# Phylogenomic analyses of malaria parasites and evolution of their exported proteins

**DOI:** 10.1186/1471-2148-11-167

**Published:** 2011-06-15

**Authors:** Christian Pick, Ingo Ebersberger, Tobias Spielmann, Iris Bruchhaus, Thorsten Burmester

**Affiliations:** 1 Institute of Zoology and Zoological Museum, University of Hamburg, Martin-Luther-King-Platz 3, D-20146 Hamburg, Germany; 2 Center of Integrative Bioinformatics Vienna, University of Vienna, Medical University of Vienna, University of Veterinary Medicine Vienna, Dr.-Bohrgasse 9, 1030 Vienna, Austria; 3 Bernhard Nocht Institute for Tropical Medicine, Bernhard-Nocht-Str. 74, D-20359 Hamburg, Germany

## Abstract

**Background:**

*Plasmodium falciparum *is the most malignant agent of human malaria. It belongs to the taxon Laverania, which includes other ape-infecting *Plasmodium *species. The origin of the Laverania is still debated. *P. falciparum *exports pathogenicity-related proteins into the host cell using the *Plasmodium *export element (PEXEL). Predictions based on the presence of a PEXEL motif suggest that more than 300 proteins are exported by *P. falciparum*, while there are many fewer exported proteins in non-Laverania.

**Results:**

A whole-genome approach was applied to resolve the phylogeny of eight *Plasmodium *species and four outgroup taxa. By using 218 orthologous proteins we received unanimous support for a sister group position of Laverania and avian malaria parasites. This observation was corroborated by the analyses of 28 exported proteins with orthologs present in all *Plasmodium *species. Most interestingly, several deviations from the *P. falciparum *PEXEL motif were found to be present in the orthologous sequences of non-Laverania.

**Conclusion:**

Our phylogenomic analyses strongly support the hypotheses that the Laverania have been founded by a single *Plasmodium *species switching from birds to African great apes or *vice versa*. The deviations from the canonical PEXEL motif in orthologs may explain the comparably low number of exported proteins that have been predicted in non-Laverania.

## Background

Malaria is one of the most common infectious diseases, putting about two billion humans at risk and resulting in about one million fatalities each year [1]. Malaria is caused by protozoan parasites of the genus *Plasmodium *(Haemosporidae; Apicomplexa). Species of this genus undergo a complex life cycle including an asexual proliferation phase in the erythrocytes of vertebrate hosts.

Although hundreds of *Plasmodium *species are currently known, only few infect humans. In moderate climate zones, human malaria infection is largely due to *P. vivax*, but the life-threatening form of this disease is almost exclusively caused by *P. falciparum*. About 60 years ago, the high pathogenicity of *P. falciparum *led to the proposal that this parasite may be a rather recent acquisition from a non-human host [2]. Since then, it has become evident that *P. falciparum *indeed is closely related to other *Plasmodium *species from African great apes [3,4]. Together they constitute the subgenus Laverania and several reciprocal host switches have occurred during the evolution of this group of malaria parasites [5-9].

The evolutionary ancestry of *P. falciparum *and the other Laverania is still a matter of debate. Until now, it has not been conclusively agreed on whether this subgenus is more closely related to other mammalian malaria parasites or whether it shares a common ancestry with bird-infecting *Plasmodium *species (reviewed in [10]). Most molecular phylogenetic studies of the genus *Plasmodium *are based on the analysis of single proteins such as cytochrome b oxidase, adenylosuccinate lyase, and caseinolytic protease C [10]. While these proteins contain sufficient phylogenetic information to resolve the relationships within the Laverania, multiple substitutions per site (homoplasy) limit their utility at a deeper phylogenetic level [10].

Upon invasion by *P. falciparum*, erythrocytes are subjected to an extensive remodeling process resulting in altered mechanical and adhesive properties [11]. Prominent examples include the formation of cytoadherence knobs at the erythrocyte membrane and the associated exposure of PfEMP1 (*P. falciparum *erythrocyte membrane protein) at the surface of the infected cell [12]. *Plasmodium *proteins involved in this remodeling process have to pass the parasitophorous vacuole membrane (PVM) on their way from the parasite into the erythrocyte; most of these proteins are characterized by a hydrophobic signal sequence for targeting the protein to the endoplasmic reticulum (ER) and a sequence motif (RxLxE/Q/D) either referred to as *Plasmodium *export element (PEXEL; [13]) or vacuolar transport signal [14].

The PEXEL motif is cleaved by the aspartyl protease plasmepsin V in the ER of the parasite [15-17] and the nascent protein is released into the parasitophorous vacuole. From there it is transported through the PVM into the host cell by the *Plasmodium *translocon of exported proteins (PTEX; [18]). Predictions based on the presence of the PEXEL suggest that more than 300 *P. falciparum *proteins are exported into the host cell [13,14,19,20]. Notably, PfEMP1s are structurally different, having an export element that precedes the signal sequence (R/KxL/V/MxE/D; cf. [13]). This export element appears to be necessary for export [13] but is not cleaved *in vivo*, and therefore might be functionally distinct [21].

The conservation of plasmepsin V and the components of PTEX throughout the genus *Plasmodium *indicates that the same protein export machinery is used by all *Plasmodium *species [16-18]. In addition, PEXEL sequences from *P. falciparum *proteins proved to be functional in rodent malaria parasites [13] and *vice versa *[22]. Thus, in principle, the screens to detect exported proteins in *P. falciparum *should be extendable to other *Plasmodium *species. However, surprisingly few proteins have been detected outside of the Laverania using the *P. falciparum *PEXEL motif, and it has been suggested that these species export substantially fewer proteins into the host cell than *P. falciparum *[13,14,19].

Non-Laverania, however, also induce elaborate morphological changes in their host cells and the low number of predicted exported proteins may argue for a prominent role of PEXEL-negative exported proteins (PNEPs; reviewed in [23]). An additional, thus far unexplored, explanation could be a slightly different consensus of the PEXEL motif in *Plasmodium *taxa other than Laverania that could hamper the prediction of these proteins. This would inevitably lead to an underestimation of the respective exportomes.

Here, we took advantage of the available genomic sequences from eight *Plasmodium *species and four other apicomplexan species. Orthologous proteins were identified and used (i) to reconstruct the phylogeny of these species, (ii) to obtain a set of exported *P. falciparum *proteins that are conserved throughout *Plasmodium *evolution, and (iii) to investigate the evolutionary plasticity of the corresponding *Plasmodium *export elements.

## Methods

### Source of sequence data

The genomic sequences of *P. falciparum *3D7 [24], *P. yoelii *17XNL [25], *P. berghei *Anka [26], *P. chabaudi *AS [26], *P. knowlesi *H [27], *P. vivax *Sal-1 [28], as well as *P. reichenowi *and *P. gallinaceum *(both unpublished data produced by the Wellcome Trust Sanger Institute; used with permission) were obtained from PlasmoDB v. 6.1 [29]; sequences of *Toxoplasma gondii *ME49 were obtained from ToxoDB v. 5.2 [30], sequences of *Babesia bovis *T2Bo from Integr8 [31], sequences of *Cryptosporidium parvum *Iowa from CryptoDB [32]; sequences of *Theileria annulata *Ankara [33] were downloaded from the Sanger Institute http://www.sanger.ac.uk/resources/downloads/protozoa/.

### Collection of orthologs

The dataset of orthologous proteins for phylogeny reconstructions was compiled as described before [34]. In brief, InParanoid-TC was used with *P. falciparum, P. vivax, P. knowlesi, P. yoelii, P. berghei, P. chabaudi, T. gondii*, and *B. bovis *as primer taxa. For 921 proteins orthologs were present in all eight primer taxa. These 921 core orthologs served then as input for HaMStR to search for the corresponding proteins in *P. reichenowi, P. gallinaceum, C. parvum, *and *T. annulata*. Following search species - reference species pairs were used in the HaMStR search: *P. reichenowi *- *P. falciparum, P. gallinaceum *- *P. falciparum, C. parvum *- *T. gondii*, and *T. annulata *- *B. bovis*. HaMStR could extend 218 core orthologs with sequences from all four species such that each ortholog group consisted of twelve sequences. The amino acid sequences for each of the 218 core orthologs were aligned with MAFFT [35] using the options *--maxiterate 1000 *and *--localpair*. The 218 single alignments were concatenated to form a super-alignment with 192,102 aa positions. This super-alignment was processed twice: (i) positions for which less than half of the sequences were represented by an amino acid were removed, and (ii) Gblocks 0.91b [36] was applied using the following parameters: *--minimum number of sequences for a conserved position *was set to 7; *--minimum number of sequences for a flanking position *was set to 10; *--maximum number of contiguous nonconserved positions *was set to 4; *--minimum length of a block *was set to 10; and *--allowed gap positions *was set to none.

To obtain the collection of exported proteins that have functionally equivalent orthologs in the other *Plasmodium *species, the two most comprehensive predictions of exported *P. falciparum *proteins were used [19,20]. These predictions contain 396 and 422 proteins (not including the structurally distinct PfEMP1s), respectively; the combination of both resulted in a non-redundant set of 531 putatively exported proteins. Each protein was used as query for a tBLASTn search against the *P. falciparum *genome. Proteins, for which the *E *value of the best BLAST hit (not considering the hit against itself) was larger than 10^-10^, were considered to have no paralogs present in *P. falciparum *and were used for further analysis. For each paralog-free protein a reciprocal tBLASTn search was performed to identify candidate orthologs in the other *Plasmodium *species (*E *value cut-off: < 10^-10^). Proteins with a single ortholog present in each of the eight *Plasmodium *species were aligned with MAFFT [35] using the options *--maxiterate 1000 *and *--localpair*.

### Phylogeny reconstruction

Maximum likelihood (ML) trees were reconstructed with RAxML v. 7.2.2 [37] using the WAG model [38] of amino acid sequence evolution with empirical amino acid frequencies (option *F*). Substitution rate heterogeneity was modeled using a gamma distribution, allowing for a fraction of invariant sites (option *GAMMAI*). Bayesian tree search was performed with PhyloBayes v. 3.2 [39] using the WAG model. Four MCMC chains were run for 10,000 cycles. Every 10^th ^cycle was sampled and convergence of the chains was pair-wise checked with *bpcomp *allowing for a burn-in of 1,000 cycles. Increasing the burn-in or usage of other models of amino acid sequence evolution such as the CAT [40] or LG model [41] did not change the results (not shown).

### Testing of alternative phylogenies

The small number of taxa in our study allows the evaluation of every possible tree topology. However, we reduced the number of tested trees by imposing the following constraints: monophyly of the genus *Plasmodium*; monophyly of *B. bovis *and *T. annulata *(Piroplasmida); monophyly of *T. gondii *and *C. parvum *(Eimeriorina); monophyly of *P. yoelii *and *P. berghei*; monophyly of *P. falciparum *and *P. reichenowi*; monophyly of *P. vivax *and *P. knowlesi*. Note that all seven constraints represent accepted evolutionary relationships (see references in Table 1) except the monophyly of *T. gondii *and *C. parvum *[42], and have been confirmed by our unrestricted heuristic tree searches. We computed the likelihood of the resulting 105 alternative tree topologies with TREE-PUZZLE v. 5.2.pl21.1 [43] using the WAG model of sequence evolution. Substitution rate heterogeneity was modeled with a gamma distribution assuming four rate categories and empirical amino acid frequencies. Hypothesis testing was performed using the routines provided by TREE-PUZZLE and by CONSEL [44].

**Table 1:** Molecular phylogenetic analyses attempting to resolve the relationships among malaria parasites.

Reference	Gene*	Outgroup taxa	Number of ingroup taxa	Laverania + avian malaria parasites
Waters et al. 1991 [53]	SSU rRNA	*Acanthamoeba castellani*	10	Yes
Escalante and Ayala 1994 [3]	SSU rRNA	*Theileria annulata* *Babesia bovis* *Sarcocystis fusiformis*	11	No
Escalante and Ayala 1995 [56]	SSU rRNA	*Colpidium campylum* *Euplotes aediculatus* *Glaucoma chattoni* *Opisthonecta henneguyi* *Paramecium tetraurelia*	5	Yes
Qari et al. 1996 [68]	SSU rRNA	*Toxoplasma gondii*, *Paramecium tetraurelia*	13	No
Escalante et al. 1998 [69]	Cyt b	*Toxoplasma gondii*	17	No
Rathore et al. 2001 [70]	SSU rRNA	*Toxoplasma gondii*	8	Yes
	Cyt b	*Toxoplasma gondii*	8	No
	ClpC	*Toxoplasma gondii*	9	No
Perkins and Schall 2002 [58]	Cyt b	*Theileria annulata* *Leucocytozoon dubreuli* *Leucocytozoon simondi*	52	No
Kissinger et al. 2002 [57]	SSU rRNA	*Theileria annulata* *Babesia equis*	10	Yes
Leclerc et al. 2004 [59]	SSU rRNA	*Toxoplasma gondii* *Sarcocystis fusiformis* *Babesia bovis*	21	No
Roy and Irimia 2008 [60]	SSU rRNA	*Leucocytozoon caulleryi* *Leucocytozoon sabrazesi*	18	No
Martinsen et al. 2008 [61]	Cyt b, Cox I, ClpC, ASL (concatenated)	*Leucocytozoon *spp.	57	No
Ollomo et al. 2009 [4]	Cyt b, Cox I, Cox III (concatenated)	*Leucocytozoon caulleryi*	17	No
Krief et al. 2010 [7]	Dhfr-ts, Msp2 (concatenated)	*Leucocytozoon sabrazesi*	42	No
Silva et al. 2010 [53]	29 proteins (concatenated)	*Theileria annulata* *Theileria annulata*	8	No

Note that only analyses are listed that included non-*Plasmodium *species as an outgroup taxa

*Abbreviations: SSU rRNA, 18S small subunit ribosomal RNA; Cyt b, cytochrome b; ClpC, caseinolytic protease C; Cox I and III, cytochrome oxidase I and III; ASL, adenylosuccinate lyase; Dhfr-ts, dihydrofolate reductase-thymidylate synthase; Msp2, merozoite surface protein 2.

### Sequence analysis

Pairwise amino acid identities and similarities were calculated with GeneDoc v. 2.6 [45] using the Blosum 62 model. PEXEL sequences of the *P. falciparum *proteins were identified via a match to the published consensus sequences [13,14,19,20]. The putative PEXEL sequences of proteins from other *Plasmodium *species were extracted by aligning these proteins to their ortholog in *P. falciparum*; we then used the homologous amino acid positions to the *P. falciparum *PEXEL as candidate export elements in these species. PEXEL sequences from the individual proteins were aligned separately for each species by hand and the corresponding PEXEL motifs were generated with WebLogo [46]. Presence of hydrophobic signal sequences was assessed using SignalP v. 3.0 [47].

## Results and Discussion

### Evolutionary ancestry of *P. falciparum *and other Laverania

We extracted the genomic sequences of eight *Plasmodium *species (*P. falciparum, P. reichenowi, P. vivax, P. knowlesi, P. gallinaceum, P. chabaudi, P. yoelii*, and *P. berghei*) and four additional apicomplexan species (*T. gondii, C. parvum, T. annulata*, and *B. bovis*) from public databases. HaMStR, a Hidden Markov Model based tool [34], was used to identify 218 proteins with orthologs in all twelve species (Additional file 1). This number is similar to that used in a recent phylogenomic study of eight apicomplexan species, including two species from the genus *Plasmodium *[48].

**Figure 1:**
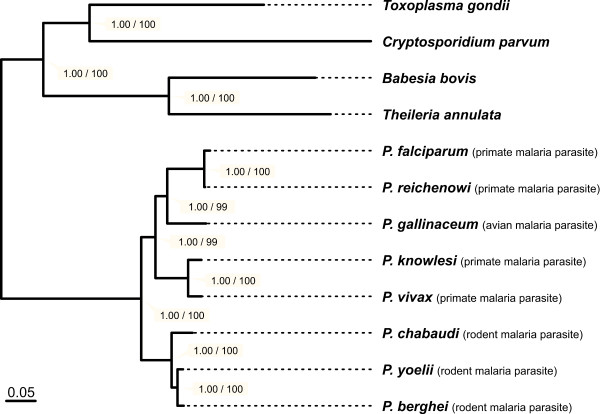
**Phylogenetic relationships of eight malaria parasites**. Phylogenetic tree reconstructions were based on 218 proteins. The single alignments were concatenated to form a super-alignment and problematic alignment regions were subsequently removed with Gblocks. This procedure resulted in an alignment with 49,521 aa positions (no missing data), which was used for ML tree reconstruction and Bayesian tree search. *T. gondii, C. parvum, T. annulata*, and *B. bovis *were used as an outgroup to root the tree. Only the ML tree is displayed, but the topology of the Bayesian tree was identical. Numbers at the nodes denote bootstrap support values (left) and Bayesian posterior probabilities (right). The scale bar equals 0.05 expected substitutions per site. See also Additional files 1, 2 and 3.

The single alignments of the 218 proteins were concatenated and positions for which less than half of the taxa were represented by an amino acid were removed. This resulted in a super-alignment with 135,360 aa positions (Additional file 2), which was used for initial maximum likelihood (ML) and Bayesian tree reconstructions. While tree topologies inferred from the ML analysis were identical with those obtained in later analyses (see below; Figure 1), MCMC chains did not converge on a single topology, indicating that the dataset includes conflicting phylogenetic information. Therefore, the 135,360 aa alignment was further processed using Gblocks [36]. This procedure has been demonstrated to improve phylogenetic analyses by reducing the impact of misaligned regions (due to very high sequence divergence) and homoplasy (due to sequence saturation) [49]. We obtained a final alignment comprising 49,521 aa positions and no missing data (Additional file 3). In Bayesian analysis, MCMC chains readily converged on the same topology (maxdiff: 0; meandiff: 0). In the resulting consensus tree all nodes received strong support and the same topology was obtained in ML analyses (Figure 1).

The phylogenetic analyses show that the eight *Plasmodium *species form a monophyletic clade (100% bootstrap support and 1.00 Bayesian posterior probabilities). The malaria parasites from rodents (*P. chabaudi, P. yoelii*, and *P. berghei*) are clearly separated from those infecting birds and primates (100% bootstrap support and 1.00 Bayesian posterior probabilities). Notably, the Laverania (*P. falciparum *and *P. reichenowi*) do not group with the other primate-infecting malaria parasites, but form a well-supported clade with *P. gallinaceum *(99% bootstrap support and 1.00 Bayesian posterior probabilities).

**Figure 2:**
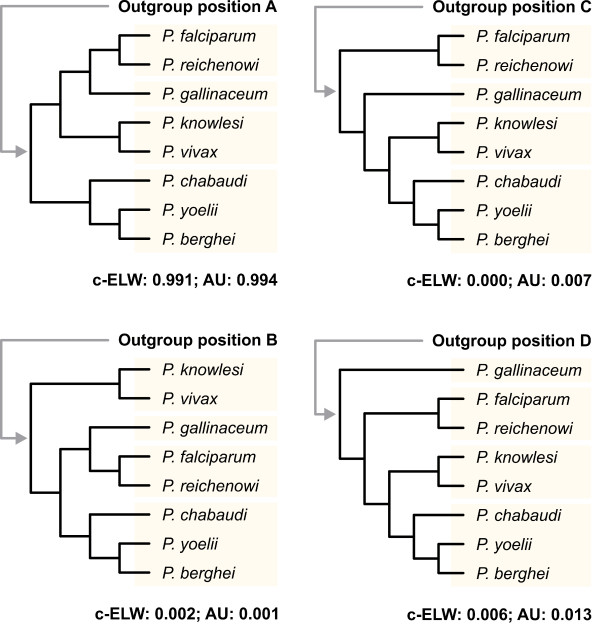
**Alternative relationships among major *Plasmodium *lineages resulting from different root placements**. The results for the individual likelihood ratio tests are given below each tree. ELW, expected likelihood weights [50]; AU, approximately unbiased test [51]. *T. gondii, C. parvum, T. annulata*, and *B. bovis *were used as an outgroup. Note that only the results from analyses with rate heterogeneity are shown; results from analyses without rate heterogeneity were essentially the same. See also Additional file 4.

ML as well as Bayesian methods return only the best tree and thus provide no information on other tree topologies with likelihoods that may not be significantly worse. To address this issue, 104 alternative tree topologies were tested by inferring their expected likelihood weights (ELW; [50]) and their probabilities in the approximately unbiased (AU) test [51]. All alternative tree topologies (including those with *P. gallinaceum *being the sister group of mammal *Plasmodium *species) were rejected with high confidence (Figure 2; Additional file 4). Thus, the position of *P. gallinaceum *as sister group to the Laverania receives unambiguous support from the data.

Until now, two other whole-genome approaches attempted to resolve the evolutionary relationships of the eight *Plasmodium *species. Dávalos and Perkins [52] based their analyses on a set of 104 proteins (~26,000 aa positions), recovering the same topology among *Plasmodium *species as displayed in Figure 1. However, no outgroup taxa were included to root the tree, and thus no information on the evolutionary ancestry of the Laverania could be provided. Silva et al. [53], on the other hand, based their analyses on a set of 29 proteins (~12,000 aa positions) and used two species from the genus *Theileria *to root the tree. While they proposed the monophyly of mammalian *Plasmodium *species, some of their results supported a grouping of *P. gallinaceum *and the Laverania.

**Figure 3:**
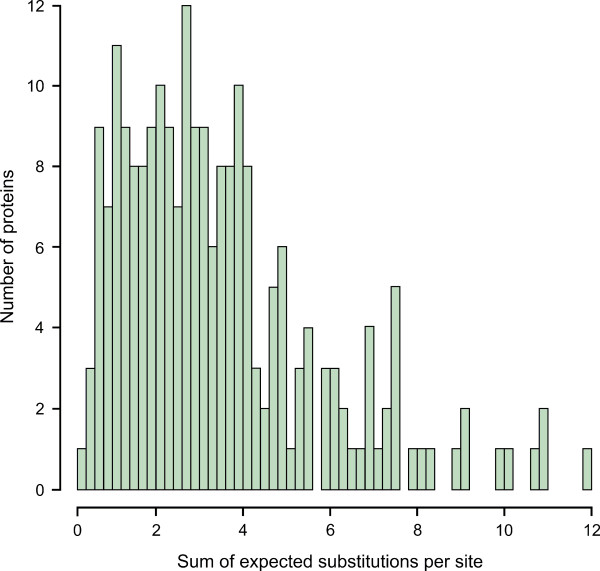
**Evolutionary rates of the 218 proteins used for phylogenetic inferences**. The length of the individual protein trees, *i.e*. the sum of its branch lengths, served as an approximation for the evolutionary rate. Note that only the proteins of primer taxa (*P. falciparum, P. vivax, P. knowlesi, P. yoelii, P. berghei, P. chabaudi, T. gondii*, and *B. bovis*) were considered. See also Additional file 5.

**Figure 4:**
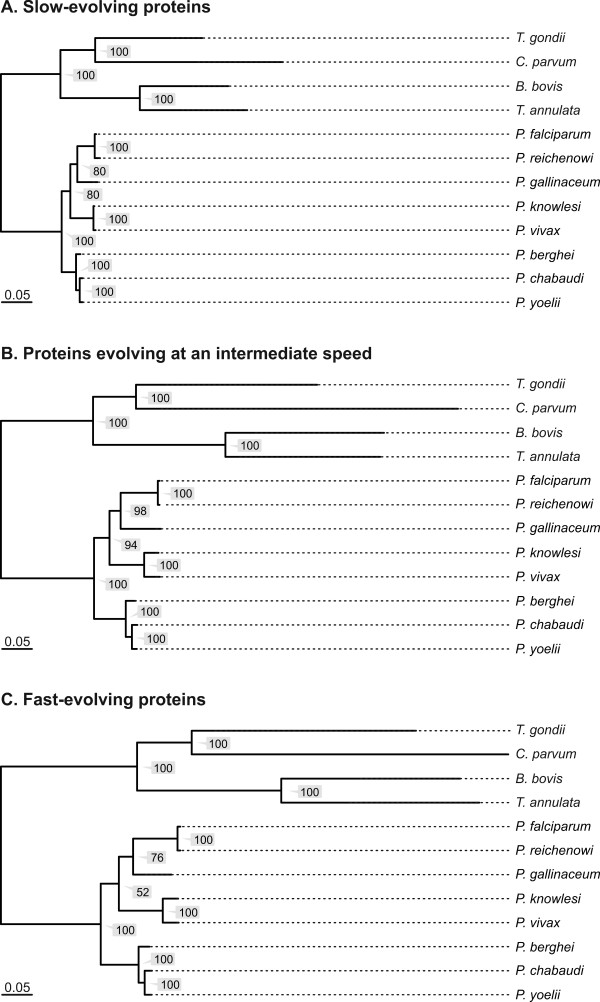
**Phylogenetic relationships inferred using three subsets of proteins with varying evolutionary rates**. The proteins used for the initial phylogenetic inference were categorized into three subsets each comprising 65 proteins: (A) Slow-evolving proteins (tree lengths of less than two expected substitutions per site); 13,670 aa of 50,332 aa (51%) were used for ML inference. (B) Proteins evolving at an intermediate speed (tree lengths of two or more but less than four expected substitutions per site); 14,967 aa of 27,063 aa (30%) were used for ML inference. (C) Fast-evolving-proteins (tree lengths of four or more expected substitutions per site); 12,021 aa of 96,774 aa (12%) were used for ML inference. *T. gondii, C. parvum, T. annulata*, and *B. bovis *were used to root the trees. Numbers at the nodes denote bootstrap support values; scale bars are equal to 0.05 expected substitutions per site.

Dávalos and Perkins [52] as well as Silva et al. [53] both used slow-evolving proteins for their phylogenetic inferences. To assess the effect of the evolutionary rate on our analysis, we partitioned the 218 proteins of our dataset. We first computed a ML tree for each protein individually. The length of this protein tree, *i.e*. the sum of its branch lengths, then served as an approximation for the evolutionary rate (Figure 3; see also Additional file 5). Subsequently, the proteins were categorized into three subsets according to their tree lengths. Dataset 1 comprised 65 slow-evolving proteins (tree lengths of less than two expected substitutions per site). Dataset 2 comprised 88 proteins evolving at an intermediate speed (tree lengths of two or more but less than four expected substitutions per site). Dataset 3 comprised the 66 fast-evolving proteins (tree length of four or more expected substitutions per site). For subsequent tree reconstruction, 65 proteins were randomly chosen from each partition such that the same number of proteins was used for each dataset. The individual alignments were concatenated, processed with Gblocks as described and used for ML tree reconstruction (Figure 4). All three datasets agree in placing *P. gallinaceum *as sister of the Laverania. The topology of the tree was thus identical with that inferred from the complete dataset (Figure 1). We conclude that our reconstruction of the *Plasmodium *phylogeny does not depend on the evolutionary rates of the proteins used for the phylogeny.

The bootstrap support for the clade consisting of *P. gallinaceum *and Laverania was maximal for the dataset comprising proteins evolving at an intermediate speed (98%) and minimal for the dataset comprising the fast-evolving proteins (76%). The branch leading to the clade consisting of *P. gallinaceum *and Laverania was short (~0.02 expected substitutions per site; cf. Figure 4). When using fast-evolving proteins, multiple substitutions in the dataset might confound the phylogenetic signal leading to artifacts due to long branch attraction [54]. On the other hand, using only slow-evolving proteins is likely to result in a dataset with a phylogenetic signal that is too weak to resolve this branch (see also Additional file 6). This may explain why proteins evolving with an intermediate rate provide the most robust tree.

The finding of a relationship between the Laverania and avian malaria parasites agrees with earlier studies by Waters et al. [55], Escalante and Ayala [56], and Kissinger et al. [57]. However, it contradicts more recent results by Perkins and Schall [58], Leclerc et al. [59], Roy and Irimia [60] and Martinsen et al. [61]. This discrepancy may be attributed to the limited phylogenetic information in the few proteins that were used in those studies [10]. While the selection of proteins may have some effect (see above), the number and choice of the outgroup taxa deserve particular attention (e.g., [62]). Alternative root placements lead to different conclusions about the order in which the individual *Plasmodium *species emerged (c.f. Figure 2). In many previous studies, only a single outgroup taxon was used (Table 1). Moreover, in some cases this outgroup was evolutionarily so distantly related that a meaningful placement of the root is unlikely (e.g., [63]). Most recent studies of *Plasmodium *phylogeny used selected species from the closely related genus *Leucocytozoon *as an outgroup (c.f. Table 1). However, the limited amount of sequence data available for this taxon - mainly a few mitochondrial genes - currently prevents its use in phylogenomic studies. Other haemosporidians (i.e., species from the genera *Haemoproteus, Parahaemoproteus*, and *Hepatocystis*) should not be considered as an outgroup since the genus *Plasmodium *has been shown to be paraphyletic with respect to these taxa (e.g., [61]). Alternative strategies for a reliable root placement employ the inclusion of multiple outgroup taxa to break the branch separating the ingroup from the outgroup, and the use of a comprehensive set of proteins [64]. Our trees include four apicomplexan species as an outgroup and are based on 218 orthologous proteins. We have obtained identical tree topologies by employing different tree reconstruction methods (ML and Bayesian inference) and different models of sequence evolution. Moreover, our findings remain unchanged when we use proteins with different evolutionary rates. Ultimately, likelihood ratio tests rejected all alternative tree topologies. Thus, we are confident that our root placement is robust and that *P. gallinaceum *and the Laverania indeed share a common ancestry.

An avian parasite as sister to the Laverania has significant implications: it suggests that a host switch from birds to African great apes or *vice versa *has occurred. Host switches have repeatedly taken place during the evolution of avian *Plasmodium *species [61]. Moreover, avian *Plasmodium *species are able to infect mammals under experimental conditions [65]. Both observations are congruent with an evolutionary scenario in which the laveranian lineage was established by a single *Plasmodium *species switching from birds to African great apes. Subsequent diversification of Laverania associated with multiple host switches within the apes eventually led to the emergence of *P. falciparum *in humans [5-9]. Note that this scenario also implies that the great diversity of malaria parasites infecting birds [61] may in fact derive from an early host switch by another mammalian *Plasmodium *species. At present, however, we cannot exclude the alternative scenario in which the avian *Plasmodium *lineage was established by a *Plasmodium *species from the laveranian lineage. Therefore, phylogenomic analyses considering additional *Plasmodium *species (and in particular those infecting birds and squamate reptiles) will be necessary to provide a more detailed picture of how the Laverania emerged.

### Evolutionary plasticity of the *Plasmodium *export element

The availability of *Plasmodium *genome sequences together with the reliable reconstruction of their phylogenetic relationships provides a robust framework to investigate the evolutionary history of exported *P. falciparum *proteins. Here, we used 531 *P. falciparum *proteins that had been predicted to be exported into the host cell [19,20] to identify functionally equivalent orthologs in the other *Plasmodium *species. BLAST searches in the *P. falciparum *genome identified 102 proteins without any recognizable paralog (Additional file 7), whereas the other 429 proteins mainly belong to large gene families such as RIFINs (repetitive interspersed family) and STEVORs (subtelomeric variable open reading frames). These gene families have a complex evolutionary history and have undergone independent lineage-specific diversifications [19]. This indicates that even if homologs of these proteins exist in the other *Plasmodium *species, they do not necessarily share the same function. These proteins were therefore excluded from further analyses.

**Figure 5:**
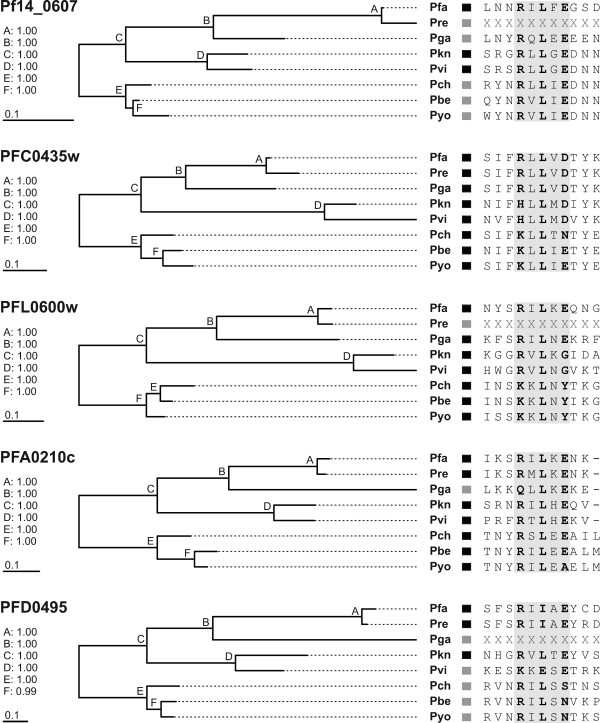
**Phylogenies of exported *P. falciparum *proteins and orthologs**. Sequence trees were inferred using a Bayesian approach; Bayesian posterior probabilities are given at the nodes and scale bars are equal to 0.1 substitutions per site. Putative PEXEL sequences are given next to each species (shaded in grey). Presence of a preceding hydrophobic signal sequence is indicated by a black box; absence (due to missing or incorrectly annotated N-terminal sequence data) is indicated by a grey box. Note that only proteins with confirmed export in *P. falciparum *are shown. Abbreviations: Pfa, *P. falciparum*; Pre, *P. reichenowi*; Pga, *P. gallinaceum*; Pkn, *P. knowlesi*; Pvi, *P. vivax*; Pch, *P. chabaudi*; Pyo, *P. yoelii*; Pbe, *P. berghei*. See also Additional file 8.

Subsequent BLAST searches for homologs of the 102 paralog-free proteins in the other *Plasmodium *species identified 33 proteins with a homolog present in each of the species (Table 2). Orthology between the members in the 33 groups of proteins was confirmed by inferring the corresponding sequence trees with a Bayesian approach as described in the Methods section (Figure 5; Additional file 7). Whereas in 27 cases this tree was congruent to the species tree, six sequence trees differed from the species tree in the position of the *P. gallinaceum *sequences. However, subsequent likelihood ratio tests revealed that superimposing the species tree did not lead to significantly worse likelihoods (Additional file 8). The pairwise similarities between the *P. falciparum *proteins and their orthologs in the other *Plasmodium *species are given in Table 2. The orthologs from *P. reichenowi *display the highest degree of similarity. This finding is expected given the sister group relationship of *P. falciparum *and *P. reichenowi*. Among the remaining six non-laveranian taxa the orthologs from *P. gallinaceum *are overall most similar to the *P. falciparum *proteins. This lends further support to our conclusion that the Laverania and *P. gallinaceum *share a common ancestry.

**Table 2:** Exported *P. falciparum *proteins with one-to-one orthologs present in all *Plasmodium *species.

Gene*	Pre	Pga	Pkn	Pvi	Pch	Pbe	Pyo
**MAL13P1.56**^2^	99.3 (99.7)	72.5 (85.8)	72.2 (84.5)	72.6 (85.2)	67.8 (81.7)	69.0 (82.7)	69.0 (82.3)
**MAL13P1.68**^2^	99.7 (99.7)	74.2 (87.4)	62.4 (81.2)	62.4 (82.2)	55.4 (76.0)	58.5 (82.5)	58.1 (79.9)
**MAL8P1.25**^2^	97.7 (98.2)	63.0 (84.3)	54.0 (75.9)	55.9 (77.3)	49.9 (72.8)	50.7 (73.6)	50.1 (74.2)
**MAL8P1.93**^2^	92.3 (95.3)	51.4 (75.0)	62.3 (84.9)	58.9 (84.2)	54.6 (82.3)	58.9 (85.1)	53.2 (82.3)
**PF08_0024**^2^	97.3 (99.1)	54.7 (79.6)	51.8 (74.1)	51.4 (74.1)	53.5 (72.5)	54.1 (72.4)	52.3 (71.7)
**PF08_0091**^2^	96.7 (98.3)	50.2 (74.6)	52.3 (75.2)	56.1 (75.8)	47.3 (71.5)	49.4 (72.6)	47.6 (71.6)
**PF10_0070**^2^	97.5 (99.0)	77.8 (91.7)	73.5 (84.7)	76.2 (85.9)	69.7 (86.7)	63.1 (77.3)	73.4 (89.8)
**PF10_0134**^2^	97.8 (99.5)	71.0 (89.4)	68.1 (87.0)	68.1 (85.3)	61.8 (80.6)	60.3 (83.6)	61.6 (84.1)
**PF10_0177**^2,3,6^	93.8 (97.7)	74.9 (89.0)	40.9 (62.6)	42.3 (68.4)	45.2 (66.5)	46.5 (68.1)	47.5 (68.9)
**PF10_0321**^2,5^	93.2 (97.2)	79.6 (92.2)	56.7 (76.3)	55.5 (77.1)	64.3 (82.1)	65.2 (82.4)	65.3 (82.0)
**PF11_0212**^2^	95.3 (97.6)	79.5 (90.4)	60.2 (77.6)	67.5 (84.2)	61.5 (78.4)	61.5 (77.9)	60.7 (78.3)
**PF11_0343**^1^	99.2 (99,8)	72.3 (89.1)	63.3 (84.1)	62.7 (83.3)	63.3 (80.6)	60.7 (81.2)	60.9 (79.7)
**PF13_0090**^2,3,6^	95.5 (97.8)	72.7 (89.5)	62.1 (79.4)	61.9 (79.4)	63.7 (81.0)	64.4 (81.9)	64.4 (81.9)
**PF13_0218**^2,3^	89.1 (94.1)	66.3 (84.8)	50.4 (69.8)	49.8 (68.0)	56.6 (78.1)	55.4 (76.7)	55.8 (76.5)
**PF13_0317**^2,3^	98.9 (99.6)	92.1 (98.9)	88.7 (98.3)	89.3 (98.3)	91.5 (98.3)	92.1 (98.3)	91.5 (98.3)
**PF14_0239**^2^	97.0 (98.0)	71.1 (88.3)	49.0 (74.0)	42.6 (71.4)	53.4 (75.8)	57.1 (78.1)	56.6 (78.2)
**PF14_0281**^1,2^	97.3 (98.7)	65.8 (80.8)	73.5 (81.6)	75.2 (83.4)	64.4 (78.4)	62.9 (78.7)	61.5 (78.4)
**PF14_0553**^1,2^	97.5 (98.1)	57.1 (79.0)	59.5 (81.2)	57.2 (80.9)	49.3 (69.7)	52.9 (73.9)	52.3 (72.6)
**PF14_0607**^2,4,5^	95.8 (97.3)	83.2 (94.6)	68.5 (83.6)	68.9 (83.3)	78.9 (91.9)	79.4 (92.9)	77.4 (86.9)
**PF14_0614**^2^	96.6 (97.8)	90.4 (94.8)	63.2 (81.5)	64.0 (81.5)	55.7 (76.9)	54.5 (77.2)	55.0 (76.8)
**PFA0200w**^2^	95.1 (98.2)	52.2 (69.6)	42.1 (60.5)	45.2 (65.5)	44.2 (62.3)	45.2 (61.9)	42.0 (62.3)
**PFA0210c**^1,2,3,4^	92.5 (96.3)	42.0 (68.6)	48.0 (68.5)	47.5 (66.6)	43.0 (64.0)	42.1 (69.3)	42.3 (67.6)
**PFC0435w**^2,3,4^	90.4 (94.9)	62.1 (82.4)	46.6 (72.9)	46.2 (74.5)	52.0 (75.1)	50.4 (74.5)	54.2 (81.3)
**PFC0555c**^2,3^	94.4 (97.9)	68.8 (82.4)	64.3 (80.2)	60.1 (79.3)	68.7 (85.1)	68.0 (82.3)	67.0 (83.7)
**PFC0935c**^2^	96.1 (99.3)	85.2 (94.4)	78.2 (89.6)	79.2 (90.4)	78.3 (90.2)	78.6 (90.2)	79.6 (90.7)
**PFD0495c**^1,2,4^	89.0 (94.0)	23.4 (51.5)	27.4 (53.6)	24.4 (50.9)	25.1 (47.3)	25.0 (47.8)	26.2 (48.9)
**PFE1190c**^2^	94.2 (94.2)	59.4 (77.2)	35.2 (57.2)	33.3 (57.8)	33.1 (52.1)	34.1 (56.1)	31.7 (53.1)
**PFI1010w**^2^	92.7 (96.2)	62.6 (84.1)	46.4 (70.8)	46.9 (70.4)	55.5 (75.6)	50.0 (70.1)	52.0 (76.1)
**PFL0535c**^2^	98.1 (99.0)	59.5 (81.0)	53.2 (74.4)	52.3 (74.4)	53.1 (74.8)	52.8 (74.6)	53.6 (74.5)
**PFL0600w**^1,2,3,4^	93.0 (99.4)	55.3 (74.4)	46.4 (69.7)	42.4 (65.1)	47.6 (66.9)	47.0 (67.2)	49.0 (68.3)
**PFL0805w**^2^	98.4 (98.9)	62.4 (80.5)	56.2 (72.5)	56.3 (73.8)	51.2 (69.7)	52.0 (70.7)	53.1 (71.2)
**PFL1630c**^1,2^	97.6 (98.5)	77.3 (91.0)	67.6 (80.1)	66.7 (83.7)	72.9 (85.9)	73.4 (88.5)	73.2 (87.4)
**PFL1660c**^1,2,6^	97.0 (98.9)	55.7 (77.6)	42.1 (65.9)	39.7 (65.7)	40.2 (61.1)	40.9 (62.9)	35.7 (57.9)

Shown are the percent amino acid identities (similarities) of the *P. falciparum *protein to their orthologs in the other *Plasmodium *species. See also Additional file 8

*Exported proteins originally predicted by ^1^Sargeant et al. [19] and ^2^van Ooij et al. [20]; ^3^found to be conserved within the genus *Plasmodium *[20]; ^4^export of these proteins has been experimentally validated in *P. falciparum *[20]; experimental approaches by ^5^Sargeant et al. [19] and ^6^van Ooij et al. [20] suggest that these proteins in fact are not exported. Abbreviations: Pre, *P. reichenowi*; Pga, *P. gallinaceum*; Pkn, *P. knowlesi*; Pvi, *P. vivax*; Pch, *P. chabaudi*; Pyo, *P. yoelii*; Pbe, *P. berghei*.

Both reciprocal best BLAST hit searches and phylogenetic tree reconstructions indicate that the proteins in the 33 groups are encoded by genes that remained single copy throughout evolution (one-to-one orthologs). Ample evidence exists that such one-to-one orthologs are functionally equivalent [66]. Therefore, we conclude that if the *P. falciparum *protein is exported, its orthologs in other *Plasmodium *species are exported as well and hence, that these proteins are suitable to assess the evolutionary plasticity of the PEXEL motif. Note that five of these 33 proteins have already been confirmed to be exported in *P. falciparum *using GFP-constructs ([20]; Table 2). However, five proteins appear not to be exported ([19,20]; Table 2); thus they were omitted from further analyses.

**Figure 6:**
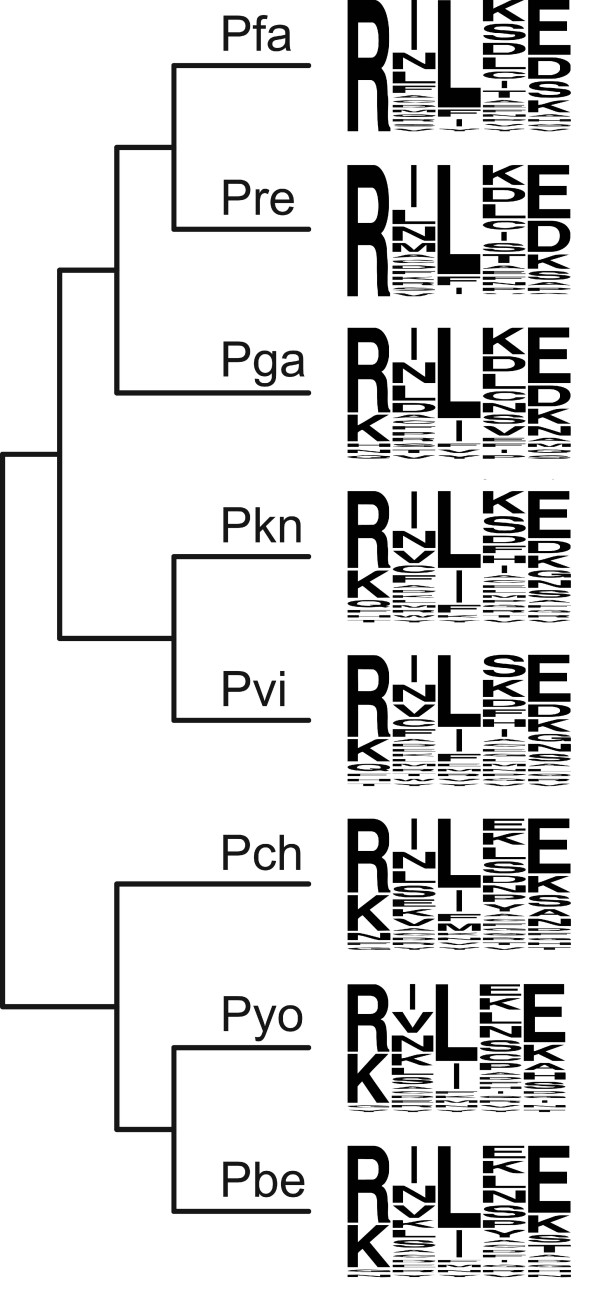
**Sequence logos representing the PEXEL motifs of eight *Plasmodium *species**. Note that only the proteins presented in Table 2 were used to draw the individual PEXEL motifs. Phylogenetic relationships were drawn according to Figure 1. For details on how the PEXEL motif mediates protein export refer to the recent review by Goldberg and Cowman [21]. Abbreviations: Pfa, *P. falciparum*; Pre, *P. reichenowi*; Pga, *P. gallinaceum*; Pkn, *P. knowlesi*; Pvi, *P. vivax*; Pch, *P. chabaudi*; Pyo, *P. yoelii*, Pbe, *P. berghei*. See also Additional file 8.

The amino acid alignments of the remaining 28 ortholog groups were used to identify the regions that correspond to the *P. falciparum *PEXEL in the sequences from the other species. Subsequently, the candidate PEXEL sequences from all proteins were extracted and aligned separately for each species (Additional file 8). From these alignments the individual PEXEL motifs were determined and compared to those of *P. falciparum *(Figure 6). The motifs were found to be largely similar across the different *Plasmodium *species. However, several deviations from the functionally important amino acids were observed; the amino acids at position 1 and 3 are crucial for an efficient cleavage by plasmepsin V, while the amino acid at position 5 affects the export rate of the nascent protein [67].

The most prominent difference between the *Plasmodium *species was found for the positively charged amino acid at position 1 of the PEXEL motif. All 28 *P. falciparum *proteins harbor an arginine (R), whereas about 20% of the proteins from non-Laverania have a lysine (K) at this position. Three lines of evidence indicate that this alternate PEXEL is nevertheless functional: (i) lysine at position 1 of the PEXEL was found in orthologs of those *P. falciparum *proteins whose export into the host cell has been confirmed (Figure 5), and thus our observation is not restricted to proteins that might have been erroneously predicted as being exported; (ii) recent experimental evidence suggests that the typical cleavage at the leucine (L) at position 3 can occur in proteins containing lysine at position 1 (PFI1780w and MAL3P8.15; [70]); and (iii) a small number of proteins with a lysine at position 1 of the PEXEL have already been predicted to be exported using a Hidden Markov Model based prediction method (21 in *P. falciparum*, three or less in each of the other *Plasmodium *species; cf. [19]). Other deviations at position 1 that are less prominent include the presence of histidine (H) in the *P. knowlesi *and *P. vivax *orthologs of PFC0435w and of glutamine (Q) in the *P. gallinaceum *protein that is orthologous to PFA0210c (Figure 5). Both PFC0435w and PFA0210c belong to the confirmed set of exported proteins in *P. falciparum *[20] and therefore these PEXEL sequences are likely to be functional as well. Position 3, which almost invariably harbors a hydrophobic leucine (L), was also found almost invariable in the orthologs of the confirmed exported *P. falciparum *proteins. However, several orthologs of *P. falciparum *proteins that have not yet been confirmed to be exported have an isoleucine (I) at this position (Figure 6). Position 5, which is considered to be the least conserved position [13,14], was found to be even more variable in the group of confirmed exported *P. falciparum *proteins.

Even though it remains to be demonstrated that these orthologous proteins are cleaved and exported with the same efficiency, these observations suggest that the PEXEL motif is more variable than previously acknowledged. This provides a possible explanation for the small number of exported proteins predicted for some *Plasmodium *species. Taking this plasticity into account will be essential to arrive at a more comprehensive set of exported proteins for all *Plasmodium *species.

## Conclusion

Our phylogenetic analyses of orthologs deduced from the *Plasmodium *genomes strongly suggests that the subgenus Laverania was established by a single host switch from birds to African great apes (or *vice versa*). However, sequences from additional bird-infecting *Plasmodium *species and the closely related Haemosporida are required to better understand the early evolution of the Laverania. Exported proteins, as identified by the PEXEL motif, play a major role in *Plasmodium *virulence and facilitate the parasite's survival in the host cell. Our results suggest that the number of exported proteins is higher in the non-laveranian *Plasmodium *species than previously assumed. Comprehensive knowledge of their diversity and evolution will help to unravel the emergence of the high pathogenicity of *P. falciparum*, and may allow the identification of novel targets for malaria therapy.

## Authors' contributions

All authors conceived the study. CP and IE analyzed the data. All authors drafted the manuscript. All authors read and approved the final version of the manuscript.

## Supplementary Material

Additional file 1 **218 *P. falciparum *proteins with orthologs present in *P. reichenowi, P. vivax, P. knowlesi, P. gallinaceum, P. chabaudi, P. yoelii*, and *P. berghei*, as well as the four outgroup taxa (*T. gondii, C. parvum, T. annulata*, and *B. bovis*)**. Click here for file

Additional file 2 **Alignment with 135,360 aa positions**. Total amount of missing data was 10.2%; amount of missing data per species was 7.1% for *T. gondii*, 14.8% for *C. parvum*, 12.2% for *T. annulata*, 12.5% for *B. bovis*, 0.5%; for *P. falciparum*, 35.4% for *P. reichenowi*; 25.4% for *P. gallinaceum*, 0.7% for *P. knowlesi*, 0.6% for *P. vivax*, 0.8% for *P. chabaudi*, 4.0% for *P. yoelii*, and 14.2% for *P. berghei*. Click here for file

Additional file 3 **Alignment with 49,521 aa positions**. Total amount of missing data was none. Click here for file

Additional file 4 **Likelihood ratio tests for 104 different tree topologies (with and without rate heterogeneity), including their expected likelihood weights (ELW) and probabilities in the approximately unbiased (AU) test**. User specified test trees, as well as TREE-PUZZLE and CONSEL output files are given. Click here for file

Additional file 5 **Sum of the expected substitutions per site for each of the 218 proteins used for phylogenetic inference**. Note that only the proteins of primer taxa (*P. falciparum, P. vivax, P. knowlesi, P. yoelii, P. berghei, P. chabaudi, T. gondii*, and *B. bovis*) were considered. Click here for file

Additional file 6 **Split network analyses of *Plasmodium *phylogeny, as reconstructed from the 65 slow-evolving proteins in our dataset**. The network was calculated with SplitsTree v. 4.11.3 [71], considering only splits with frequencies of at least 10%. Notably, a sister group position of *P. gallinaceum *to all mammalian *Plasmodium *species is not supported. Click here for file

Additional file 7 **102 paralog-free *P. falciparum *proteins that are exported into the host cell and information on the presence or absence of orthologs in other *Plamodium *species**. Click here for file

Additional file 8 **Phylogeny of exported *P. falciparum *proteins with orthologs present in all *Plasmodium *species; corresponding amino acid alignments and information on the amount of missing data; *P. falciparum *PEXEL motifs and orthologous sequences of the other *Plasmodium *species**. Click here for file
