# Supplementary material for: Phylogenomic analyses of malaria parasites and evolution of their exported proteins
Source: BMC Evol Biol. 2011 Jun 15;11:167. doi: 10.1186/1471-2148-11-167 (PMC3146879; doi:10.1186/1471-2148-11-167)
Supplement: Additional file 1 — 218 P. falciparum proteins with orthologs present in P. reichenowi, P. vivax, P. knowlesi, P. gallinaceum, P. chabaudi, P. yoelii, and P. berghei, as well as the four outgroup taxa (T. gondii, C. parvum, T. annulata, and B. bovis). [file 1471-2148-11-167-S1.PDF]

Additional file 1

218 *P. falciparum* proteins with orthologs present *P. reichenowi, P. vivax, P. knowlesi, P. gallinaceum*, *P. chabaudi*, *P. yoelii*, and *P. berghei*, as well as the four outgroup taxa (*T. gondii*, *C. parvum*, *T. parva*, and *B. bovis*).

MAL13P1.190	proteasome regulatory component
MAL13P1.205	GTP-binding protein
MAL13P1.234	hypothetical protein
MAL13P1.279	cell division control protein 2 homolog
MAL13P1.308	hypothetical protein
MAL13P1.337	Skp1 family protein
MAL13P1.344	RNAse L inhibitor protein
MAL13P1.36	hypothetical protein
MAL13P1.52	hypothetical protein
MAL13P1.76	hypothetical protein
MAL13P1.92	40S ribosomal protein S15
MAL7P1.122	conserved GTP-binding protein
MAL7P1.145	mismatch repair protein pms1 homologue
MAL7P1.162	dynein heavy chain
MAL8P1.125	tyrosyl-tRNA synthetase
MAL8P1.128	proteasome subunit alpha
MAL8P1.150	hypothetical protein
MAL8P1.65	hypothetical protein
MAL8P1.83	eukaryotic translation initiation factor
MAL8P1.96	hypothetical protein
PF07_0079	60S ribosomal protein L11a
PF07_0091	cell cycle control protein cwf15 homologue
PF07_0092	hypothetical protein
PF07_0112	proteasome subunit alpha type 5
PF07_0117	eukaryotic translation initiation factor 2 alpha subunit
PF08_0006	prohibitin
PF08_0048	ATP-dependant helicase
PF08_0069	importin beta
PF08_0113	vacuolar proton translocating ATPase subunit A, putative
PF08_0125	tubulin gamma chain
PF08_0126	DNA repair protein rad54
PF10_0086	adenylate kinase
PF10_0087	diphthine synthase
PF10_0123	GMP synthetase
PF10_0165	DNA polymerase delta catalytic subunit
PF10_0174	26s proteasome subunit p55, putative
PF10_0245	glucosamine-fructose-6-phosphate aminotransferase, putative
PF10_0264	40S ribosomal protein S2B, putative
PF10_0293	transcription factor, putative
PF10_0294	RNA helicase, putative
PF10_0306	MORN repeat protein, putative
PF11_0051	phenylalanyl-tRNA synthetase beta chain, putative
PF11_0055	hypothetical protein
PF11_0087	Rad51 homolog
PF11_0098	endoplasmic reticulum-resident calcium binding protein
PF11_0108	U5 snRNP - associated protein, putative

PF11_0112	vacuolar sorting protein 35, putative
PF11_0114	actin-like protein homolog, ALP1 homolog
PF11_0142	ubiquitin domain containing protein
PF11_0156	Ser/Thr protein kinase
PF11_0157	glycerol-3-phosphate dehydrogenase, putative
PF11_0183	GTP-binding nuclear protein ran/tc4
PF11_0187	clathrin assembly protein AP19, putative
PF11_0202	clathrin coat assembly protein, putative
PF11_0203	peptidase, putative
PF11_0225	PfGCN20
PF11_0251	endoplasmic reticulum oxidoreductin, putative
PF11_0258	co-chaperone GrpE, putative
PF11_0259	nuclear preribosomal assembly protein, putative
PF11_0265	mitochondrial inner membrane translocase subunit TIM44, putative
PF11_0270	threonine - tRNA ligase, putative
PF11_0282	deoxyuridine 5'-triphosphate nucleotidohydrolase, putative
PF11_0303	26S proteasome regulatory complex subunit, putative
PF11_0313	60S ribosomal protein P0
PF11_0331	TCP-1/cpn60 chaperonin family
PF11_0377	casein kinase 1, PfCK1
PF13_0016	methyl transferase-like protein
PF13_0063	26S proteasome regulatory subunit 7
PF13_0156	proteasome subunit beta type 7 precursor
PF13_0177	ATP-dependent RNA helicase
PF13_0178	translation initiation factor 6
PF13_0205	Tryptophan - tRNA ligase
PF13_0217	hypothetical protein
PF13_0227	vacuolar ATP synthase subunit D
PF13_0251	DNA topoisomerase III
PF13_0257	Glutamate - tRNA ligase
PF13_0305	elongation factor 1 alpha
PF13_0308	DNA helicase
PF13_0313	zinc finger protein
PF13_0315	RNA binding protein
PF13_0316	40S ribosomal protein S13
PF13_0324	vesicle transport protein
PF13_0328	proliferating cell nuclear antigen
PF13_0330	ATP-dependent DNA helicase
PF14_0064	phosphatase, putative
PF14_0067	LCCL domain-containing protein CCP3
PF14_0104	eukaryotic translation initiation factor 2 gamma subunit, putative
PF14_0127	N-myristoyltransferase
PF14_0193	conserved protein, unknown function
PF14_0324	Hsp70/Hsp90 organizing protein, putative
PF14_0328	mitochondrial import inner membrane translocase subunit Tim17, putative
PF14_0352	ribonucleoside-diphosphate reductase, large subunit
PF14_0359	HSP40, subfamily A, putative
PF14_0360	eIF2A
PF14_0361	Sec62, putative
PF14_0368	thioredoxin peroxidase 1
PF14_0370	DEAD/DEAH box helicase, putative
PF14_0378	triosephosphate isomerase
PF14_0391	60S ribosomal protein L1, putative
PF14_0393	structure specific recognition protein
PF14_0429	RNA helicase, putative
PF14_0469	transcription factor IIIb subunit, putative

PF14_0493	sortilin, putative
PF14_0517	peptidase, putative
PF14_0518	nifU protein, putative
PF14_0548	ATPase, putative
PF14_0585	40S ribosomal protein S28e, putative
PF14_0649	conserved Plasmodium protein, unknown function
PF14_0661	conserved Plasmodium protein, unknown function
PF14_0677	RNA 3'-Terminal Phosphate Cyclase-like protein, putative
PF14_0688	Pre-mRNA-splicing factor ISY1 homolog, putative
PF14_0723	LCCL domain-containing protein CCP1
PFA0145c	aspartate-tRNA ligase
PFA0400c	beta3 proteasome subunit
PFA0525w	transcription initiation factor TFIIB
PFB0275w	metabolite/drug transporter, putative
PFB0445c	DEAD box helicase, UAP56
PFB0525w	asparagine-tRNA ligase, putative
PFB0550w	peptide chain release factor subunit 1, putative
PFB0595w	heat shock 40 kDa protein, putative
PFB0640c	sec31p putative
PFB0750w	vacuolar protein-sorting protein VPS45, putative
PFB0830w	40S ribosomal protein S26e, putative
PFB0840w	replication factor C, subunit 2
PFC0160w	binding protein
PFC0185w	hypothetical protein
PFC0290w	40S ribosomal protein S23
PFC0295c	40S ribosomal protein S12
PFC0350c	T-complex protein eta subunit
PFC0365w	hypothetical protein
PFC0375c	splicing factor
PFC0475c	hypothetical protein
PFC0720w	hypothetical protein
PFC0805w	DNA-directed RNA polymerase II
PFD0180c	CGI-201 protein, short form
PFD0420c	flap exonuclease
PFD0450c	pre-mrna splicing factor
PFD0515w	exosome complex exonuclease rrp4
PFD0525w	hypothetical protein
PFD0720w	hypothetical protein
PFD0725c	arsenical pump-driving ATPase
PFD0880w	hypothetical protein
PFD1110w	hypothetical protein
PFE0165w	actin depolymerizing factor
PFE0185c	60S ribosomal subunit protein L31
PFE0465c	RNA polymerase I
PFE0485w	phosphatidylinositol 4-kinase
PFE0625w	GTPase
PFE0785c	hypothetical protein
PFE0870w	transcriptional regulator
PFE0890c	hypothetical protein
PFE0895c	zinc finger protein
PFE0965c	vacuolar ATP synthetase
PFE1005w	40S ribosomal subunit protein S9
PFE1050w	adenosylhomocysteinase(S-adenosyl-L-homocystein e hydrolase)
PFE1140c	G10 protein
PFE1155c	mitochondrial processing peptidase alpha subunit
PFE1195w	karyopherin beta

PFE1250w	long-chain fatty acid CoA ligase
PFE1340w	transmembrane protein
PFF0185c	hypothetical protein
PFF0305c	ubiquitin-conjugating enzyme E2
PFF0345w	translation initiation factor IF-2
PFF0450c	transporter protein
PFF0500c	step II splicing factor
PFF0535c	trancription factor
PFF0610c	hypothetical protein
PFF0825c	mitochondrial import receptor subunit tom40
PFF0940c	cell division cycle protein 48 homologue
PFF1155w	hexokinase
PFF1345w	transportin
PFF1350c	acetyl-CoA synthetase
PFI0200c	adapter-related protein
PFI0300w	developmental protein
PFI0415c	ribosomal RNA methyltransferase
PFI0480w	helicase with Zn-finger motif
PFI0735c	NADH dehydrogenase
PFI0880c	acid phosphatase
PFI0895c	hypothetical protein
PFI0920c	hypothetical protein
PFI0935w	DNAJ-like molecular chaperone protein
PFI1020c	Inosine-5'-monophosphate dehydrogenase
PFI1130c	DNA-directed RNA polymerase II
PFI1140w	NADPH-cytochrome p450 reductase
PFI1170c	thioredoxin reductase
PFI1260c	histone deacetylase
PFI1455c	hypothetical protein
PFI1565w	hypothetical protein
PFI1570c	aminopeptidase
PFI1625c	organelle processing peptidase
PFI1650w	DNA excision-repair helicase
PFI1685w	cAMP-dependent protein kinase catalytic subunit
PFI1700c	vesicle transport protein
PFL0095c	hypothetical protein
PFL0130c	conserved Plasmodium protein
PFL0310c	eukaryotic translation initiation factor 3 subunit 8, putative
PFL0580w	DNA replication licensing factor MCM5, putative
PFL0620c	glycerol-3-phosphate acyltransferase
PFL0660w	dynein light chain 1, putative
PFL0670c	bifunctional aminoacyl-tRNA synthetase, putative
PFL0815w	DNA-binding chaperone, putative
PFL0830w	RNA binding protein, putative
PFL0895c	conserved Plasmodium protein
PFL0930w	clathrin heavy chain, putative
PFL0950c	aminophospholipid-transporting P-ATPase
PFL1010c	conserved Plasmodium protein
PFL1110c	CAMP-dependent protein kinase regulatory subunit, putative
PFL1180w	chromatin assembly protein (ASF1), putative
PFL1245w	ubiquitin-activating enzyme E1, putative
PFL1425w	t-complex protein 1, gamma subunit, putative
PFL1680w	splicing factor 3b, subunit 3, 130kD, putative
PFL1790w	ubiquitin-activating enzyme, putative
PFL2005w	replication factor C subunit 4
PFL2060c	rabGDI protein

PFL2225w	myosin A tail domain interacting protein
PFL2310w	RNA binding protein, putative
PFL2460w	coronin
PFL2465c	thymidylate kinase, putative
