# Supplementary material for: Phylogenomic analyses of malaria parasites and evolution of their exported proteins
Source: BMC Evol Biol. 2011 Jun 15;11:167. doi: 10.1186/1471-2148-11-167 (PMC3146879; doi:10.1186/1471-2148-11-167)
Supplement: Additional file 2 — Alignment with 135,360 aa positions. Total amount of missing data was 10.2%; amount of missing data per species was 7.1% for T. gondii, 14.8% for C. parvum, 12.2% for T. annulata, 12.5% for B. bovis, 0.5%; for P. falciparum, 35.4% for P. reichenowi; 25.4% for P. gallinaceum, 0.7% for P. knowlesi, 0.6% for P. vivax, 0.8% for P. chabaudi, 4.0% for P. yoelii, and 14.2% for P. berghei. [file 1471-2148-11-167-S2.PDF]

Additional file 2

Alignment with 135,360 aa positions. Total amount of missing data was 10.2%; amount of missing data per species was 7.1% for *T. gondii*, 14.8% for *C. parvum*, 12.2% for *T. annulata*, 12.5% for *B. bovis*, 0.5%; for *P. falciparum*, 35.4% for *P. reichenowi*; 25.4% for *P. gallinaceum*, 0.7% for *P. knowlesi*, 0.6% for *P. vivax*, 0.8% for *P. chabaudi*, 4.0% for *P. yoelii*, and 14.2% for *P. berghei*.

```
> Toxoplasma gondii
-----MLRVRYSQHPPLVTLCVSALLERGLAACNSLQDSLLSVQKTESGSEEVFTDIAAA
KLLCTLSKTRLLYPAA---APAPSFADASETACIDTMLSLATSLQVSSLSPKQLGSLSSH
LQLRTFLCGFHLSLADLAVYTQLRRHANQAGAPPQGWKDKFVHVSRWYAFIHGQPQISNV
VAVALRKAGANAKGAEASKNEGKKDKRAAAAQASYEGKLEGAVQGKVVTRFPPEPSGYLH
IGHAKAALLNSYFAQKYNGKMLFRFDDTNPAKENYEFESSIAEDLRLLNVEWAAISHTSD
YFEQMQSLCERLIKEGKFYVDDTPTELMREQRAEGIESCRRNVPVEESLQRWREMLKGSA
EGQKCCVRAKIDMQSKNKCMRDPVMYRCVADCLHHRHGDKFKAYPTYDFACPVVDSIEGV
THALRTNEYADRIPQYQWVQQAAGLPPVHIYEFSRLCFVKTLLSKRKLKQFVDSGLVEGW
DDPRMPTVRGIRRRGLQVEALLEFILEQGPSKAGNLMEWDKLWTKNKQIIDPIVPRFMAV
G-KDAVPVCIKGAPETVESKKRRMHAKNESLGEADLLLFNKVFIDRDDAALCEDGEEVTL
MHWGNCIFDKVVKTASGEISEIQATLHLEGDFRKTKKKLHWLANLPAQNTELVLREYDHL
ITVDKIDQ-EEENWEKFINRETRFDTPAVGDPLLKQLKEGNERQFRQSGLVKRRGRGMNK
RFKVVSSVDRGNLRVSSEGRQL-PRHAAVGKEMAEYAVIIDNGSGYMKAGLASQEEPSAV
FPTIVGRPRGDGSGAVFVGEEAIANRHHLSFTYPIDHGHIDNWVDMEEVWNATYNMLGVQ
PNEHAVLVTEPPLCSQRHREKMAEMFFETYGAPEMNISVTGLMAIYGTGRATGFVLDIGE
GITQCVPVFDGYLEKASVKRSDFGGQELQMYLQKILCDMGYPMTTRDDYEHVRVIKETLC
FCSLNPSEDQNRDDLEKTYHLPDGLTLRDGITEITLGPERFYPPEALFNPQLCGRDSPSL
IELVWSSIMACPIESRKSLIGSIVLSGGSSMFPGFPERLEQELKNTAPPQARPHVHVLSH
PSRGSLVWQGARLYCQPEMRPLQDHIWITRQEWEEIGMKIVAKKAAFRITSMDVLERLSV
ISRITTELHNHWGVNDRDLAEFVVHLGEEASSLDEFREQLQENGAAVSTSLAVSLYTTIQ
KEKKSAASTSASSSSFREPAFPDHHELTEKEKKFPGLCMPNDFSRPELQLERPDDHAPLS
EHAQRLLNAEKLQKEQKEALKRGRENKFAPIQANSIPLGGKKREAERAGEKKAGSAPAHI
RY------AIYEGVVEKVVEFGCFVRLEFDEGTRQGLLHVADMIKTDGRPIQPQDVVHRN
MVVKVKILGIAGTKISLSMREVDQETGEDLKPRGEFEEDTAGEKKIRIDDEAEEREKQGI
GRTGIRIDNNVESLYCRKRKLMSDFDKWEAQQLLHSGLLTREEHPDEELGILPSAEVDED
---VEVEIREDEALFLRGQTTRTGMQLSPVKIVANPDGSLARAAATATALAKERREIRNA
QEAAILDSIPKDMSRPWEDPAPGPGERTIAQALKGLGQSYEMPEWKKMYIGKSVSFGQKS
NKSIAEQRQSLPIYRLREPLLKAIKENQVLIVIGETGSGKTTQMTQYLAEEGLVPPGMIG
CTQPRRVAAISVAKRVAEEFGCRVGQEVGYNIRFEDCTSPDTIIKYMTDGMLLREALVDA
SLKRYCVVMLDEAHERTISTDVLFGLLKDCCRRRPDFKLIVTSATLDAEKFSNYFFNSHI
FTIPGRTFPVEILYTKEPEADYVEASLITVLQIHLCEPPGDILLFLTGQEEIDTACQTLH
ERMQKLESTNPPPLIILPVYSALPSEMQTMIFDPAPPGCRKCVVATNIAEASLTIDGIYF
VIDPGFAKMKMYNPKTGMDSLVVAPISQANARQRAGRAGRTGPGKCYRLYTEQAYRCEML
PVAVPEIQRTNLENTVLLLKAMGVNDMLNFDFMDPPPVQTLINALESLYELGALDDEGLL
TRLGRKMAEFPMEPQLSKMLLASVDLKCSDEIITIVSMLSVQNVFYRPKDKQAMSDQRKS
CFHQPEGDHVTYLEIYRGWQRNRFSNSWCFENFIQSRAMRRAQDVRKQLITIMDRYKLDV
-----ISAGKDYNRIRRCICAGYFRHACRRDPQEGYRTLVDHTQVFLHPSSALYNRHPEW
LIYHELVLTTREYLRDCCTIEPQWLVEVAPKLFKLADQQRLSRRKMRERIEPLYDRFAEP
NAWRLSKRRGMSEDYDHLYKVILVGDATVGKTHLLSRYIRGTLPKSPKATIGVEFATRTV
PLAVGGTVKAQIWDTAGQERYRSITSAHYRRAVGALLVYDVTRKSTFLNASKWLEELRQN
SEPDIVIMMVGNKLDLVEKDPTARDVPYELAAKFAQANGLYFSEASAVTAFNVKHIFEHL
LQEIYNHRTQGEEFSGRANGADAYRDAQALGGVRLAANAN------MYGRNPQNLSCCME
QPKLAKVEKVLGRTGSRGGVLQVRVTFMDETDLAGRSLIRNVKGPVREGDILALLETERE
ARRLRMAADLENVATVELLEELKRRYACLAKPEGRYIFIGAPGSGKGTQSVKLKKSHCLC
HLSTGDMLRHAVATGTEYGKQAKAKLDAGELVSDEIVLGLIDEKLKTPECRRGFILDGFP
RNEAQAAGLDNLLKQKNQKLDGVLYFDVPDNILVERVSGRRIHLPSGRVYHVTYHPPKVA
GLDDVTGEPLHHRKDDNEATLKKRLDVFHKETVPVIEHYAKMGLLYKMDAAKDSTAVTKE
MYDFVGGKVEKKCPVCYQKLPNPADVLGPMDKELNYFMWMPGFEWRPEPKVGEYDGSPSC
REGGRPAADEDMQEALEEMVEADEMYARFNARASGGKVSTGDAMILARQLGLAPSYADKQ
AFEEKSGDNLDYASFQKFVGTSTHPEDNIEDLVEAFAYFDVSKHGYLTRKQMGNILMTYG
EPLTTEEFNALAAEYFTSDQIDYRQFCKAMLEMAAFARKSLPLCVFSLFLFSF-AFSALS
GAPSQFAEAAMPKLSGEKLAELMQMDVKDIKERMLALFDLIDTNQDNTIDTEEAKEWSAK
LKNAMHQHQVRMEFQAIDKDNDGKVSLSELEATYVDSLDQKQLEQHKKEVEQRFKTVDKD
NDGLLDLSEIRILMDPGKDEGLMKIEIEEILNAQDKNGDRKITVTEFIETEGTGSLNDVE
KTELEKEFKSYDLNADGAIDVEELQQIIKDPHSHEIRMLLEEFTKDLKDGK-VGREQWEK
EFESFAVSMLTDNGEVLRFPEDYSIEFPFKTAVP----QVDLDDEDKH------------
----------DEL-------------MVAVSTLCCLIPVYIAKYSRRGRASRLRMCQALL
SIINCFAAGAFLGLALIHVLPEAVTQLSETGILLVLDGDSSHHSYNIAYLLAAVGFTAML
GLEILLGGGHTHCCTEANYCPDEADSRTSSSVVPSSERQRESCSKVLSAFPESGDRGVTR
ESGWEKINGFLRCCSKLEGTKISLALALGVHAIFEGIILGTTQTSQNVWIATLAILGHKG
AEAVAVASTLLKMNMNTVPFVVMLAAFIIASPLGILLGAFAATAGTRVSGVFNALAVGAI
LYAANEM-LSEFSGSCSRVRRFVKFLAFVVGLGALFGLNLIHTPYCRHLHSHGH-SAHGM
QSKFWAAASDESE-DSGFSSSESEAEQEQQVPAGEASRWAAADTSSSDDEGRVVKSLKDR
RWTAMRETIRQMRNHMKIADFSELYKDYESLVRCLQKSEQEGLPSFFVRIIVELEQFLEE
RHRDKEAFKKLSKAKATAFNTLRAKVRKTNEQWQEKVDACKADPSQFASSDSDDDDD---
--FDSDDSSDLSDISDSSESEEEEKPKRKAMNEDGSEDSEDSEWSDSDASSLSSGEETDK
HKAAMAKWGLRSSRKQKKKKTKRISKTK-----DEGALSPGEAGAEFSIKDMESLFDTND
LDASVIRKRVQLVVEKRGRRGVDRLEQTRILKRLAELAARVGPQSELEVLAHLISAEFDT
TSGVFACLSSAIWLDVFHQLEAVLGHLEEGTAYVLVPTVASSDAVDAALVAPDAQVVSSG
ILTSFVERLDDELMKSLQFTDVHSEEYKERLGQSVDMIAVLCRAWVHLTDCQSKPQ-AAT
LALRINEHMHYKHDAIAASMWDLVR-------------------KRVPAEVAQHLPDEGM
KPSDFVEKLTNLVFESESSRERTRALLHLAYSRALHDDFYKARDLLHTPNMQELALQTDT
QTQILYNRDLVQLGLCAFRNGRINEAHACLSEVCP--KHKELLAQGLSNLKNVECTPEQE
RAEKRRLLPYHMHISMELIEGAHNICAMLIEVPHMAHDPFEHRKRPISKHFRRMLETYDK
QAFLGPPENARETVMAATKALQKGDWKECCRFIFSLGIWEKLMNATEIQEMLKLKVKQEA
MRTYIFTYLTLYDSFSISQLCGMFELPESTVHSIVSKMMINEEIHASWDESSQFILISRV
ERTRLQQLASTLTENVNNAVEQNELTLNMKNPKCAATTDSAGAAESAK-GTGAREARGAV
GVTFAKMRAWSCVHTFRCRNISLFVFPEAEHQPRLLETLQDVPKPFLDKARGIVEQLRAT
LDANSAYLLCFQLLKLAGLEAEIPRLVVFGQQSMGKTTLLDYIMGGPIGYSSTDTGTRQP
VVILLRPSEADSVQCWLGGEEIEVKELQARMKEIMSSQGERISSQELEVELAVPNGVHAV
```

FVDLPGVKDDSKAGATQTRSVVRTYVQNNPNDLYILVKKASDDPANWPWSLREFILSAPP
KGLGLTPRQTVVVGTRAKEFLVNEKNDIRTQSQLLERVLKRAVKDSSGAPLPLFLLELFS
LSIEEKDALDFAAKRAAMNRQMDEGGRTVRQLLETAFEPGPNPQLSRKLTEFFSPSRFKK
ELNHKFQSLLSEQMGILERRLVRKRLETQKRISDLEDQLALQSPQSCRECIKLYLRELMQ
VVTELVTGNYTIIRLPHNGDEFLKTFGGNLRDNLEDGHELACDLFPEH--YDRGFLTKII
KEAEEVFRRQDLEESLAP-----------------------------------VTGPDGR
VVMQPGHIVRYTLSKDDTNMFGLVQSPASDASSKEATVTFYFRSGQAQEQSQFKTVDKQR
LAVLLPLQTIIPSTPPPAGFRCWRRFYRPDGWVGLQPVELLSLRQRFSEKSEAVVKALAG
PDLRSAANLDELFADAATGDGE-SHAHRRTHL----SALQRISGDFADTRLLNQLALTHL
GRWLKFHICNIEPDRRFSDHVLLQMMRSVRHVIDKADWEPLVADLLQANVGAGMLQLARL
AACAAASALRRILRAASAEVNRQIKCGDLRSGLLFLATNHRFMEELEQSLEEYTRKKARD
CAQAMRDLIFEQTHAIHFEMIEDFFDGCKRFEADFLGGS----MMGEVTQHVKDSLAMRK
QRLGIADIYARQAGTPDSMIYEEVRIQFWVVKMLLSAPLTTKLYMHFIKDIKDKSMHLAS
EDKYSVTCENDLERTLQEQMLCESPAGRALARTDEELMEHFNISMNKDELINQLNAARRT
EEYTKLALEGVAKLLHQLQKGSGVDFLTRLDSPMRRAMATAGMQQLLVSTLDPRQQVREQ
AEQQLVGARDGDFSLFLISLARVLDAQLSADLAKQIAAVTFKNCISAKDVVLDSAAADKW
RAVAEAAKQAMRLQLLAAIKTE-HIQVANAVCQVLSKIGRIELPGDGFPELLPFLLTLVT
EATMTPERNALTCLSYLCEEHADIVEETGEDPVLSEAHCNNILTAVVQGMKDE-DVQLKV
AALKALYHALIFSKKNFENQTEREYIIQVVLENTKV-AHQAVQVSAFECLVKVAEEYYSM
LEPYMSGVGPLSWEALKSGDASVCIAAMELWNTIADVEI---DIQQQEEEAACHIVKQAL
PFLLPILLNTLTQQDSEETDADSWTAAMAAGTCLGLCAQVVKNDILPPVIQFVSENFASP
DWTRREAAVLAFGSVMEGPDTEALKPLVEESFASLVDVLQDSSVAVRDTAAWTLGRIAQF
HTPVVLQKLVNADESNSLLAAIVRRLLDQPRVAVNVCWLLHELADHMTA--GDGERPAST
PLDPLFQRLCDALIQVSERADADERSLRDAAFNCLGALINNAGESCKPSMLKLLDHFVQQ
LSQSFMFEANEATRQRQGLLCGVIQILCLRLGDQVQPVASQIWACLARIFSASESITNDA
LLATSALVNASGPSTAAFAEDIVCIIASGLENTDELQTVRICVELVGDVSRALGPAFAPY
SSPLLARMYQMLQDPNVERALKPCVMVAVGDAAMTMGEAFAPYLDSFMAILHQAGNTTYD
VGPSNNEEWLWYIHDLREGVLQAYMSIVYSFKEKCMQEQLKLYVNAMLDVVKAVAATSPK
MRGVENVKQAIELVGDLISTYGGDLTLHLQRAPFMEQLLQLAQVLGTVKDAGGEACLQKA
QWLRQLMARYS---MTTFVPLATDGDGTASAVAVGDWLLQIINLKNPSQTQSYYTQFLEQ
FDKDEETGEQKIRDQLFELLLSQHQLVFNYATQARQPAAAEK-GEKPQNRKTFLEAVHEV
EEFFTVLIAMVVLRIENVEQAGQAAGTLCSVFRASTDMAEFRLRLLQSLYNAFPPSFPYR
FPIFVATLEYAAETNLFSVMLPYIRYINEWMRDWNLPPSSKRQVFLILANELKKLKKADE
AYPFLKRHVQFFQNEKEEILSNGATISAAVELVEDSIRLPDVIVFDGLMDLHAVVHLRKT
-AHAPLIELLQIFVNQGPKELEAFKNKH-PQVFEEHGLNYEQCLGKIRLLAVASLVHGKK
EVSIRAIGDALQLSEAGAEEVAVQAIGQGIVDAKIDQLARVLHVRSTMQREFGRQQWEEL
LERIDHWSEGVRALMGCMQSVKNQVAARNHWCGILLLLGCVVHCSAQHDASDFYVFNDCS
ATSTE------GPEFVAQRAIQPGTGYWSSAGGLPDDEQVTWTGYLATPGKIKGVRVRWQ
YAPGEVQVAISSNGVDYHVALPWRPAGSSEAAYDEDILFGHDEEAKVVVIGMRKQIHGFY
GINEAKPLGSGEPLMMIIGGITSPAEMCLQ----------------------------EL
WYSTPQMQLVSARSTPPKCMTLQDGSTEDGGNIVLTDCLRALEEGDGRSSWTFEGNSQLR
LQRAG-AWCMTQKDVNGSSPGVGDLIRSGEATASSSSSSDAAHGANAAIDKDSDSSWRSD
PVGEA-EQTVVLTVNLGKASNVVSVRIQWEYPALSYEIYTSPDGKSYVQQAVNPANPVVD
TLDELHAGAAQFIQIRMLKPHPRLGKADDSFFYGIYEVHVYANRLGSAVSPCTQAANSDD
ARDKYFVEYVTSFNPVLADKITSMEEDVLLRQKGLNTKAKDLEALLPEMEGCRNDKIQFV
ERMKRASRRAGKIFGQFQSATGADHRHN---ARSGMKEGE--------------------
-----IMPQCSPAPLRVYCDMTTGTSIYVWNGHPPRKPGVTLDD-VVSLNDVRNACARVG
LEPMVPKSPQHFQSILSALHQMGFNLNGKGAVPLAFDYSCLYGACTGEYRDLSDGATDLT
SLVL--SQSAPDSSPVKDAAGLGLNGERTSFFDLSSAPLVAVICSTNTIEGEGSAPDIDI
DCDTTAEHEAFEGIINTNVVVECPADCADDT--SLPVYGSDGVYSASSSICRAAIHAGLI
-KTGGVVNVSIESPRASYEGSVQNGIVSSALEISPDSRALG-------------------
-SIRLSTIFKDCPV--VHHAATSFLETATEAGGS----TMQFNADMTLDADIQEALQQTI
HMIDLMHNVDPEIFAEAKEEAGIVVGASRKQLKPAEKLHHAQSAQVLEIFVGTESLAARW
LAEAGNMFQTLDQLNQKLRIAEQRHLEQTGFESFKLYPQSMAFKDYFETFDSMRAKHGPS
NWGYASAPIQGRRASIGQSRNIVGTSETEGTYAMLRGRRFYDAEIQVSFYAVGSGSVGIA
FKIRDFNNMYLLWMNQKQAVKRLLRIEDGQPTIVAERKDGGYIQGKWFNVRIETNKGVIR
VCIGEEGSAVIEVFSVLDERFMVGSVGFFSSGMEAGVFFEGLNIDAKDCTTPSKAIAPAP
PR---CSTFAETFYGNPSIYRKIDASDGSWVYK---------------------------
-VAQTRASKNGYFVFEFFPQCTGGI----VGGIFRFTSPQEYQVAELAPYELRIRAISNG
HPKTVARTPV------SMSLNEWHRMEINFEGSTVSVRLEGPGGVKNLSADDLFGGQTRD
GMVGFSAYNCGGVAFDSIQLSPYKMESIESIFTSATKAWQPCLTNVHILHRRDECQRMF-
VKEPSFRQIACAQDFCSECCNYNTSLLPRSEWAQCEKKCRRNDPLASLLASGMITRLSTC
LK-ELDDAAAHCKKGNMACQKEACELCCISWSPAGSLDQIGADL---QDATEREQEECKF
QCAKHFNLEL--MP
AQAAARAGAMPPEDGHPRPPS-----------------RSAKVPSGDTKRESDIEAGAQS
PLLVIPRIDLFPEP--

-------------------------EAGKQVDWLPPRYAEGLTPE---------------
--------------------LRRNLEERGLRPCSLREVLERRNKMWAKVQMISPEARGEK
----ERLDSLFKQQCVGWGNGWV--YGKWNPVILCPDFRQGGLQVSRSGRRQLWECL-KS
VVEGFVDPRLQICEVVDPTHPVRFATPPQETCYTVVYVGPKIPASKARVVFGEYTGVV-K
DGGLVKKRFEYVFDLSFSALAWRRAE------EFETASDSS-------------------
-----DDSLPSRDSRDEKLSESPVSSGSVTSQRQ-----------SRLPGGKDRKKSRDA
KGL-TRVELPARSQFVLDSSEACNEMSLVNHYGTIELLGDCVCKRNSEWQQVFVDGWPHI
VLTSIPGVAVEPGDEILADFGNAWFFKVQDASHEAIARELLEYRVGIGEAAAEKPVSGLC
EKLRDEPLSSGFPMCSDLLLVTQADSGDVVHCDGCDRPCHLRCLPDPEA-ILNDWRWYCA
VCRHLNYKILLSSEALASLEKEVKWTAAQ--AGNMGVHALAKTPDGEA--AADE------
ERKKERRPSCGGIEHHSR------RRESPEPVAGGRRRAERETEDE----NPGKAKKWRG
SRVSKEQETEKEDQTKSEGSPTLLSGRQRSR-------GLREPRQEEKTRAGAELVGTQN
SGVKTRSSNIEAPVGVNTDGSVSLGGEAQEKENKEPLTAVGMEFLNVPLEELGKGSGAML
WNEADEASLSPVAVSRTNEGNDVVLRPGSTEATGTEGTCSAESLPEREAHEDCSQSESSP
SRGLDRSGAQPTSVDSVRPRARCHSSERRSPPVGIRERRAVFASCNEKASLGRMTPSRVE
IVKGHLVDPKGLLG-SLQPCDVCYKALGGRASTEVCRLAKRHLSPNWDHPHSKDAILTAF
QEKVHSLQDEAKAQSVAEEIRQLK---------------------MSIAAKAQGHHAGSG
GLGAEK------SHGNGCSGLLCGSQHAGGASKKRPRGVGTAEVSVRDEKD---------
------------CRPEDNLPAP--------------------------------------

LTSGFIPLIGVHLGKTVIDRCFNVGWFEGLITEYSAVEDGKWYFQVEYEDGDTECLKVEE
LVHLLAEHGIRHTDPATTTTLDKLLSPEIKKELNCKEEKEEPEVARKRKKNGKKAKKTEN
SENETEADEAEDEGRSEKKTEAMSATEAAQALKAKGNAAFQEGKYEDAVGFFTEAIKCTP
DDAVLYSNRSGAYASLNKLEEALNDAEMCVKLRPTWGKGYSRKGLAEFRMMKYKEAEATY
HKGLQVDPTNEQLKEGLNQVQQQTDQFFMQAMLAAAQAVNKHPKLAKYQQEDPEYTHRLT
EILKQIQKNPQSLKLIMAQPDVRVKEGVIAAMGGDLEEEEELPQSRTRGSAENASATKAA
AGVKAAEQPAKELTKEEQEAEELKQKGNELYKQKKFEAALEAYDEAIEKNPNEILYLNNK
AAVYMELGDYDKCLAECQKALDKRYECKADFSKVAKVYCRMAACKTRSGDYSGAIAMYEK
ALCEDNNRMTRNALNEVKKLKEKKEKEDYINPALAEQHREKGNEYFKQGDYPAAKKEYDE
AIRRNPKDAKLYSNRAAALTKLCEYPSALRDADTSVQVDPAFVKGWSRKGNLHMLLKEYP
KALQAFDKGLALEPTNQECIQGKMAVMNKVQQLQSSGEVDPEQMAHSLADPEIQAILKDP
QMNIVLMNIQEKPELIHEYLRDPKIKDGINKLIAAGILRVAMDEEYDVVVCGTGLKECIL
SGLLSTHGKKVLHVDRNAYYGGESASLNLTHLYEKFKPGETPPQSLGFNRDWNVDLIPKF
VMACGKLVKVLLTTKVTRYLEWQVIEGTYVYQFQKAGFFSSAKYIHKVPATDTEALTSPL
MPLLEKNRCKNFLSFCAQWELDNPETWKGFDPKRHSMKQVYDYFGLQPNTIDFVGHAVAL
YTSDDYLHQPMGQTMEKIKLYMYSISRYGKSPFIYPLYGLGGLPEGFSRLCAINGGTYML
NKPIDGFVYGEDGKVCGVKSTDGEVARCKMVVCDPSYV-YDPKKVRKSGQVIRCICILGS

```
PIPNTSNASSCQIIIPQRQVNRTNDIYVMLVSSAHGVALKGKYIAIISTTVETADPLKEI
SPALELLGPIEQQFVQVSDVYEAVTDGKEDNVFVSESFDATSHFESATEDVLKIWKNMTG
EDLDLSVKAEPEDLQEKTRKAQNKSLQPVCRDYTIHLHKLIHGIQFKKRAPRALREIRRF
AQKTMHTKDVRIDTKLNKFIWSGGIRNVPRRVRVRIARRRNDDEDSKEKFYTLVQHVPVA
SFENLKTEYVNEEMEGGS----GSLYDVDDTAASAGDPSKNKSGQEEDKVVLRLRACISC
RLIMSEQQFYDEGCPNCGFLQMDGDRHRVWDCTTVNFAGFVAVMKPMSSWVARHNKLTEV
VPGCYAVSVVGELPESVKDDVHRAM------------------TFPDVYEAAEDSFLFLD
ALAEDLPFLRRRPSLVLEMGSGSGCVISFLRTLFLSSPLFIPCFFAVDCNLSATEATLET
ARRSEKSEVDAVASDLFRAFRPSGLFDVVLFNPPYVPGSPRDRPRNPADWAWWGGEDGRE
VIDQFLRQVTANLTAAGVLYLVSFPRSGSGDVSRAEEVCAF-------------------
------LEKSGFGIAHDFKGRTQEQSDFPILCETCLGDNPYVRMQRNREGKECRICTRPY
TAFRWKPGPKARYKSTVVCQTCAKLKNVCQTCLFDLQYGLPVQVRDKLLEGAVELPDHPL
NRDYMADRLEKAADQLPYGKLEDHEGRLRAIARTQPYYRRNAPRVCTFWQRGECKRGDEC
PYLHQEVHHDPALANQNLRDRYSGQDDPVAEKILRLAASKPNADSVTADTVFVGGLTKGV
TEQDLRDAFYAFGELLSIKMYRGQQFAFLCYAERSSAEEAVKQLHSNLVIKGVRLRVAWA
KPSDKKKKPDDNDVTAAQEDPGVTVIPPPSIRAPV-PLPFMLDSADANRGAPATAMYASM
NPVEAEYMRMRESRLGNILHNVSGSDTPERPPPIEETLANHGDAVSQDWRWLALRCEQRL
NAVLQQRT---TKSAKLTDTLGPSFDIRQFACFMPRTVLEAIADRRIRHSDDFDIQIEQF
TAAVVFCDASGFTALTEALDTKPNGAERLGNIINQFFDKIIKIVHYWGGDVIKFSGDAMT
IVWPVDDESPQ------------PDADGSEKDGKAAREEE----TGDDFYRIDSQVACQL
AVQCCLTLHQTLHGYVTGCDDKVLTLHIGVGFGQVHILQVGGIMDRWEYVVAGAPLEEIS
VAEPLAGSGETVVSPSVASALAGAADLEEVPNPPGRIFYKVKGLHEKEKPREQIEEVELP
VQPPPPL--APIDVESDDIDLLRRYIPPSVFRRLTTGCNVFLNELRVVSVVFICVRGLDV
STRTGSLIAHKLMKMTQKAAYTMEGSVNKFLVDDKGVLLLVMFGLPPVYHLDDPIRAIMA
ALRVIDGMKVFGLDAGIGITSGRVWCGTVGNEIRKEYTALGDYVNLAARLMAKAGPREIF
VDVNTFQAARHALEFKQLPSMHVKGKEHPVQVFMPTGVMINQ------------------
----------------------------------------KKDAVEDWSGKKQLRE----
-----MMLPSAPYLEQTSLLGKSSKYNAFRPPLDYPVPTGPLFAHEWCEPFLPELQPFAA
VGGVMVIRGKEGLGTHELAKLLGRKDLKRQTFFISNMPDSVQMNIGNPLLAWRKLCTEMV
ERWRVSDNREKKGYSKDNSVYGLTKELIHPSFHWRLEDMKPVIHGLVLPYELPENQVMLK
QRLKNLKSYQRRHGGMRHPLIMSPAINLLLKPAEWVGDAAAA------AASAARVITPVN
NWMRKLSSSQDDEVSDSDDSD------------------------DSGTGPPRLGLAN--
-------------------------------------VVRR-------------------
------------------------------------------------------------
-------------------------------------------------VSSFHSLFFWR
LFSSDVFPFHLF------------------------------------------------
-----------------------------------------------LLDAPFM------
------------------------------------------------------------
------------------------------------------------MSDRAVDVPPDY
RSSRLIVKNLPPYITTAELKQKLS----SLGGEITDVCLLRNE-------RGKSRQCAFV
GFKTQEQAMNVKDHFQSTFIHTRKVEISYALP-RTTLSPSERGEAGSNADSANRGKKVVL
PRGTGEAKGKVTVVEEESVGARKAGVSSVRTRVLFDDDSDSEESSGESDADDNEDGKKEK
HQRKKASSDTISGADDSKSGDDEIDDLAWLRQQSARTALAESADLTNADAEDTPVSRRPD
DTRDGGGAADSTEMAE------NEAADLSHGRLLIQNLPATTVDELRALCEEYGEVAETH
LV-----------VDE-------ETQR---------------------------------
---------------------PRGFGFVSFVFPEHAVASLPRLNGSIFQGRILRAFPARP
DATRERRLQLREERRLARQKKLAGSSYKLQQLVSQDEQFAAEKVWNLLYVSANSAADAVL
SELQADKAALLNLAGKNAAATVALMEAHLLTQTRAWIKAEGISLEAFERRGNTLLTATYN
QEKKEGSR-SDIARSRDTLIVKHLTAHVNEAELLRLFERVGPLARFLLAPSKTVAIVQYE
REKDAEVAFRHLAYRQYKNVPLFLEKAPVNVFVEREESKNERQKKLAGSDDSLKKRKVKE
IETIGNDVRGDEVQGVSLFVKNVNFCTSEATLNDVFAGCPGLRRTILMVKKKAVSSSAAE
AENGDAQTLLSMGYAFVEFDSAENALAACKRMQGVVVDDHVLQISISKAAGRRPRGEPGT
GVKVSGKSQAQSNKVLVRNLAFQASASDLRGLFSAYGNVTRVCIPRQHEGRSRGFGFVDF
ATKQEAQNAVEALTGSHLYGRRLVLEPAQL---EQVSKK---------------------
--------------------------ASVMFSECCQCAPCGEGEPGAAQP----------
-------------QVPGSRLVSGVVATQGEARILEPLVQERVVEVLKEEIQERVIEVPQV
QYVDRIVEVPQHVVHEKVTHVAKPIIQERVKHVHKPVYQQKIIEVPQVKVVDKIVEVPQY
VYQEKIIEVPRVVVQERVIPVPRKVVKEKIVEISQVDYRDVVVEKPLEAVEILQEEEVVE
VPKPVVVEKVVDVEKVVEVPHIQHTYRNVMTPQYRHIPKPVEVPMTHYRPIPVEKIVDRN
VPVPVELQIVQEYLCPKIEPRYKEVPVPVHVQRTIEHPVPKEAMGNPKLLPLYYQGTQEG
VEGIVT-----PGHPCYSLSCMPRREQQPIELGTGTVEVMAQQGYPTMPVPQGWDGAAPP
AGWEGSKEHIPTALRSDQH-------------SSGAMTPVSAGAAVGSAPRG---PEDNQ
GQQAPHHQAGGE----GPFVQVSVTPLGQQQVNMFSTRSEYDRGVNTFSPEGRLFQVEYA
LGAIKLGSTAVGIQTKDGVILASERRITSCLLDHRSIQKIVEIDDHIACAMSGLIADART
LIDHARVECANHFFTYNEKMSIHSCIDSVADLALDFSDVSDGRRKKMMSRPFGVALLVAG
VDDQGPSLWCADPSGTVTKYQAVAIGSAQEGAETMLQEQYSQSMSFEDAEALVLVVLRQV
MEEKLNCNNVEVACVKTSDRKYHQYSSEELQALIDRLPAPTIPTTDLSSMERQKIALSNF
LLEICPALLNCSRQSLQSLLAKEGDRTLQTFLIEQQ-QVLVVGR---EPVAKKKNGTTSG
APGDAANTERPEEPADENDEEEQFRLFVELGFAAHALTRAYSVAFLKRPAEDGEAASSPA
DAPRRKTIGQQLAVLCCGGDDTESSLDVTQLYMQRGLAPMLNVALT--------------
--QKETEGPGGNGSASGPAEEGAAEAAQKAATSSLHQTVNKKMAELLLAVQQAQQNVDIP
LIQLPVDPVIEETVKAAQAARKATVEDLGDYLHNTGYLIALQNHVNRWIKDIQKVCRMQR
DASTGSAAEEVNFWEGMERAILRVEEQLKTPEAELTLQVLKQTRKVFATMTFEQDAGLKQ
ASDMVASVNILIRDFPVNDLLGATSVEQLTQAVRTVFMHLRKLKNAAQYPLSRAYHLVEA
LSRDLSQRLVAVLSQQHLLLLGFEEFEHATGGCSELFRTWEEEVRQFKEMVRDQAKKRGL
SERPPAKLVCEHLVIQERIGELRQFRRQHHRLKEVISRVLSENAGGDLTAQKEVASAYEL
LQSVDVLDLSRAGQEAWEAARKAYDDRIDRVESQITARLRDRLGASTTSGEMFRVFSQFN
ALFFRPRVRGAIQEYQTTLLQQVKMDLRKLQESFLDGHQRVETLTMAEARDMAPVAGSIV
WAKQLERRLEGLIKRIEDVLGHGWEQHVEGQKLRQDIDAFRQRLNQNQLFENWLQVVKDE
KRLDTGDRIFLIRVLGGRGFELDVNFDLDLLTLFKEVRLLNTLQYRIPYSVKVMADEAKV
LYPFVTTLRVVLRTYTEACRLIEGSPSPPGTGGVAALLVASYQREAQQRLAEGLSLRWDS
DRLESYVRKVAEVMYSFEGKVETGLKRHQDALQDIDAIKVLPLDASKESLLEMLAKVQKH
IEEQQLQHFSNIPRWTRLLDSHVETILRSRVVDIVNEWVKQFEGWP---HAGTSLVKKAA
VLEIQLRNHVLQLFPPVEAAHQYWMGELHNTIASVCLVPRLSSRI-GASAVEDDFADSCS
SDDKKSKLCKNRTYRHLSQLVDPAVFFHAYDVINKHIQRVKEYVQTWLQYQVLWDVDVNE
VISRVSDDIETWQHLLNEIKAARSTFDTHENREEFGATIVDHQQAQSKLNTKYDGWHREI
LHAFGQKVADRTEAFYKRLHALLTDLEEQADDPTETSISAMTFLSMSQFQMDAFVNGVGP
QLLSSLVSNLTRFLLKLQEASKADVEWEVTFESLRASERLLERQRFAFPSDWLWIDRVEG
ELETFQQLLRHQALLVEQSREPIVDMVKRYNARVQFRLKHLYSEWMIHRPVKSDVSPQHA
TQVLEGYESQLNQLAEQYAFGEKAKNVLGLDDLPAGDEEQFSPDSLAEEIKDMKGVWNAL
SSLFTDVGALRETPWATVAPKAVRQRLEELLEKIKKVPPRFRQYDAFEEMRAQLTSYLKL
NLLITDLRTDALKDRHWKLILTTLKIKKTLQEVTLGTLWHADLVANESAVREILVQAQGE
TALEEFLRQVKESWQDRELTFVAYGTKTKLVKGWDDLFQLIDDQVAALQSMKLSPYFKIF
EEEALTWEEKLNRLRGLLDSWIEVQRKWVYLEGVFTGSQDIPMLLPQEHQRFRGIDQDFK
NIMKKAASVKNVMEVAGMDDLGRQLDRLSDLLSRIQKALGEYLEKQREQFARFYFVGDED
LLEMIGNARDVKVVQRHVNKLFAGIAVLDTDPENGTIIVGMSSKEGESVPFSTTIPILQY
ASLKDWLAAVEQQMVVTLAENLASAIAKLEQVNMLLAAKEETNAEVLHWVASYPLQALLL
ALQVSWTRSVETALAQEATGEA-ESHPLATGVLDYTCKLLEFLADRVVTDVGVTVRQRMV
QIITELVHQRDVCRTLIDQGVVTKDDFRWLQYMRFYFSPPAANAQDSLRIAMADATLSYG
FEYLGMAERLIQTPLTDKCFLTLTQALNMKLGGNPFGPAGTGKTESVKALGTALGRYTLV
FNCDETFDFNAMGRLFAGLCQVGAWGCFDEFNRLDEKILSAVSEQILTIQTGLREGLSSI
ELLDKNVKLSTNVGIFVTMNPGYAGRSNLPDNLKQLFREIAMIVPDKPLIAQVTLFAQGF
RSAERLASKIISLFDLCDRQLSKQPHYDFGLRSLKSALNSAGSLKRQWLQDASAAG----
----AAESEESVVQVEETLLLRSVCDTVVPKLVAQDVPLLKSLLAGVFPGADVTMLEEKL
LCEEIERLAQGRHMQCRGEWKEKVLQLYQIQKLQHGVMLVGPVGTGKSAAWKVLLDAMER
LDGVKGHAHILDPKAVPKEQLYGRLDSTTLEWQDGVFTAILRKILQAGST---PLKRHWI
```

VFDGDVDPEWAENLNSVLDDNKLLTLPNGERLQIPSNVRLLFEVDTLKHATLATVSRCGM
VWFSDSVVEVSTLFQHHLSEIQLGNLDGSQAMQKLGRL----------------------
--------------------------------PSQKEESRDQAEREVTRSGSSAWRPYFE
KDGFVCQALARAMSYDHIMVPTHVRLVESTISLLKKGIAKLAEQSEKASEGEEPQEAREF
FVKWLLLAMLWGFGGSLSLSNRLNFTKEVQRLSPIRLPSAL-------DANEDGRDVTLL
DFEPSVEDGEWHTWKEKVKQVEIEPHQVADANLVIQTVDTLRHKHVVEGWLDERRPFILC
GPPGSGKTMTLTSVLKERTDFDIAFLNFSSGTTPQVLLKTFDQYCEFTKSPKGMVMRPTQ
PGKKLIVFCDECNLPLPDKYGTQSVITFMREIVETGGFWRLMPQMAQWVWVRVERVQFAG
ACNPPTDAGRHPMSDRFLRFAPLLFVDFPGTESLRQIYGTFNRAMLRPFPQLRAHAEALT
DSMVDFYDEFSRSFTVDMQPHYIYSPRELTRWKLAMSEALDGGDELDLKTMVRVFVHEGL
RIFSDRLVHEAERQKTDQMIDQITLKHFQGVDASALQRPILLTSLVTRRYEEVSRDELRA
LLQGKLRVFNEEVFNVQLVFFNEVLEHVTRIDRVLRQPLGHLLLVGASGAGKTILTKFVA
WMNGLSVFQIKAGRNYNTAAFEQDLRVVMKRAAIKEEKIAFILDESNALGPAFLERMNAL
LASGEVPGLFEGDEFTALINECKAAYGSGEYLESGEIFARFTRLVQRNLHIIFTMNPANP
EFYNRQATSPALFNRCVIDWFGDWNECAMLEVAKAFTAPILLPPEGFDADQVGEDQAGPV
DPDEEARRMRLASSIVAFHAAVALSNKKLQRAGKKSNWMTPRDFLDFLHHFVNLVGEKAD
ATGEQQRHLQAGLQTLRVAEEQVAEMRSALTEKESVLTEKNEEAEKKMGQMVEQQAEAEE
KKRGAEQLTRKLDEQTGVIEERRQAVQKQLAEVEPLLREAAEAVTNIPKKSLDELKSMAN
PPAMAKIAVEAVAVLITDAGEKPLTWEDARKVLKNQDFITKVVNFDCSCVSVATRRCVQT
RIGGGDWDLEKINRASKAAGPLAKWVESSVAFVAISEQVDPLQKEIDVLEVEALKNKEEL
LQQQELIGQLERKLQQYKKDYAQLISEVQLIQREMEDVQKKCQRSMRLLQNLGSEKGRWL
EQSDALRRAGVTFIGDSLLAAAFCAYLGFFEYAHRQRLLDEWQDILKIECIRFCPDLSYV
DFLSLPSERLHWVASGLPPDDLSIQNAIILKRFLRYPLVIDPAGQATNFLTQLMAAKKLT
KTSFTDQNFLKALEAALRFGTTLLVQDVEKVDPILNSVLNRETHKLGGRELITVGDAEID
LSPAFMLFLATRDPTAQFTPDLCSRVTIVNFTLTPSSLVNQCLNLILKSERPDVDKRRTD
MLKLQGEFKVKIRELEDGLLQALSNVKGNILDDDAVLATMENLKQQAAEVEREAARTEDV
MAEVEKTSNMYLVWALAAGRIYFMLLNLSCISFFYQYDLRFFLNILGDTLRHPDLEKV-G
SSDYAARLDFLMERLFSTAYQRLAPGLRYRDRLVVGLEMAHIRAEIELKSSIWSAEMQLL
LAGTILKGGKEAGDEDEDRREWKEDVDGAINAEQMKALQKLSLLPFFVDLETQMRENRED
FRAMVMSQEPEKLIPAVVFV------------------------TKAKWLRHLREILLLR
ALRPDRVTLLLAALVEAVLGEHFLTVPELTQTAFYDLIQHQAGAAVPVALVSSPGFDPSS
KVTALAAAYKQHLTSIAMGSKEGFLLAEKAIGQASRQGNWVLFKNVHLSTKWLQGLEKQL
YRMQVHPNFRIFLTMEATPALPLNLMRVSYTFVFEPPSGVKASLLRSYATMNAESSASHP
LVARGRLQFLLAFLHAVVLERRRYAPVGWCKQYEFSDADQVCALKIINSWVDKGGTAEDR
VMAEYIAPERLPWDAIRTLLKQVCYGGRLDNPVDQKILSSLVDYLFQPAAFEASFPLNIA
DSLSLPQLLTAPDLFKQASAYEAWAEQLWSVDSPTWLGFSAQAERLLASRQSLAAVGSWA
AVLLRGNEEAHDFQAISVQAARGRGQLTSQMSSEMEGAAGSSWLS---------DLVPGV
QAMIGLLPQVVPDVRRTEETVKDPMFRCFEREITVARRLLATIQDALAQLLLVCKGEAKL
TNALRDLAQRISSSEVPPEWKGVFTSAELTLAEWIEDFSRRLKHLMLVAQSFGPST-IEE
TSQKIPEELSAALTRQSDGGELSQRRVWLGGLLYPSAYLTASRQAVAQAKGWSLDDLAME
VQIGVSDP---PDDQSFVITGVTVEGAAWNTQGQCLSLSMEEEEENIDILRQSSSSFYPS
GVFVLHSPLPPRLTESSAGPRRVVAVSEREARELLKTHLPDWEVAHAFLKKNSDRRADGA
VDPAATEAPSPPTPLTDETRAAGNDACGALTGAAEQATSGAQAAAGAPSRAPAPAAAPQR
RPTLFNLLDALAHDSVTTPDQRNCIRSVVTDFLNHSLPHAKVYTFIGAIVGHDVLHAMVR
KLEEQPSRLGAPDQGGLTRIEKAFGLTKTSAAGPGSAGPAGTGRLASKCEEERKHGDAEQ
GNYFQSAQSRTGGRAHAATGPGAGAGNAHARDSSLASVARQADGVARVGGQQSRDEVVKA
LRRNLEARRQVHGS------VFDRVKWLPVRRALTPVPVFGHSLVTFSNTAVLFGGSNTE
GA-VSFRHVFVVNTNDFSVRGFPMSGEPPCERDGHTTTGLTTAAGPAVILFGGCSQDAFL
NDVYTLEVDPKRWVRRHPVGKAPQPRDQHVAAVFPARTECRENSQEVLSEFLFIFGGRIG
SPESYQATNDMWTLHLATDTWAQVLLGSRLRPLPRFGCCGVWSNDVFLTLFGGETTHAPE
RMQPRHLLDDMWHFKILDVVPAHVRGAHVVGEWIQEKFEDSVAPRSHYSAIFITQRYQEP
R-AEPRTVERLMLITSGLTYEAEARRKVVETDRITVYFFTRKKWFTLKPR-YPADYVCGA
RQRHVACFFEAQPLTQRAPRPPVPCLLIHGGFRGKQVLNDAWVLSLTGEDPYRQQQLTMG
RSMLSLRMYPSYYIRETHSPGMLWALCGQQRWAFGAIAQLVENSLSPVVASRNVFVSWEE
SPEKEPMLCIQDDGQGVDYPAMNALLRLFGTFEPGD------------------------
---------------------------------RMRKS----------------------
------------------------------YEYGVGFKIAFGRLSSSCAVMSRTQGTIGV
GMLSMELMGHCDARELVAPMCMWRLPNKELINRDPNNAADHRHHQRLLMSYTPFTTPNLL
AEQINLLGTVPGTRLVFWDLRDDLDFLVFDPKEETLFLNTAPAS----------------
---QRAQRGLHAEGVRLPEK--------PDEVPAPKPRKPELLDLDEDGKPTVEMETGVS
GENSAQRDGTSP-------GAGADYPRVQEKDGEEGAEKSGEKGAEQMAEKTNEEKSGEK
KS------------------GRMQGGLPPVFPLWTSARHSLDYCLPTYLFWLHLHSPALL
HVQGRPLLPTAASF---------ARKKQG-------------------------------
-------------------------------EVREEN---------------GETRVEK-
--GCALETTTAQQS----------LEEEKE-------AKRDEEEKKREGRDKHEEL----
--AETPGPGR--------

-SLYFFLKERLYCQAELQYLFTPADHATGCFALFGFLNDPSETV--SSRVCEAGVLLYFR
QRLVRRLDAAFPDFPRALNAARDPPDDSLFGGEMFKYCLTAVVHVPEWMLPSMTKQDFRH
ENNLPYMKFKSRLLKLIDEYLRRCRNPQALQAWLDDRARRLREYHEELRRSRPKRRLGEE
FTRSPTPPVTHS-SSGAPIWTGERGSGAGASLPPVKQRAVSRPTGGPGAWQE-----REG
DERKEGLDAK-EDRTEEDQGTLDVERMQPQGEREAGSEEASGTGGSSVADSMVAETEE--
---------GAARTMEPNAVDGSMSEQPMEGVKSEGMAGIRRPQSVPAEKKEESQKKRSP
NRLIVEEAINDDNSVVALNPAKMEELQIFRGDTVLLKGKMRHDTVCVVLADQDLDEGKIR
LNKVVRKNLRVKLGDMVHVSACPDCPYGKRIHVLPLDDTIEGITGN-LFDIYLKPYFMEA
YRPVRKGDLFLVRGGFRPVEFKVVGVDPGEFCIVAPDTVIHCEGDPVKREEEERLDEVGY
DDIGGCRKQMAQIREMIELPLRHPTLFKTLGVKPPRGVLLYGPPGSGKTLIAKAVANETG
AFFFLINGPEVMSKMAGEAESNLRRAFEEAEKNAPAIIFIDEIDSIAPKREKTNGEVERR
VVSQLLTLMDGLKGRGQVVVIGATNRQNSIDPALRRFGRFDREIDIGVPDDNGRLEILRI
HTRNMKLANDVKLEELAANTHGFVGADLAQLCTEAALSCIREKMDLIDLEDDTIDAQVLN
SMAVTQEHFTSALQCCNPSSLRETVVEVPNVKWDDIGGLEDVKRNLQEMILYPIDHPEKY
EKFGMSPSRGVLFYGPPGCGKTLLAKAVASECSANFVSIKGPELLTMWFGESEANVREVF
DKARAASPCVLFFDELDSIGTQRGNSLGDAGGAGDRVMNQMLTEIDGVGPMKNLFFIGAT
NRPELLDEALLRPGRLDQLIYIPLPDLPARISILQATLRKAPVAKNVPVPFLAQKTAGFS
GADLAELCQRAAKAAIRDAIAAEELAQVNAGADEM--------DAEEEEKTDIVYEITRK
HFEEGLAGARRSVSQTDLTKYDNFRMKFDPLYKSQAAGGETQVLIEWPDDANDASAGDDA
DDDDLYSMRRKGGDRWKQVMLDAATLRECQMEGMAELEELDGGEALIFDPSVLSLDSVAE
SPESAVARVGKGKQRKTGKKKVKDSSQDSLSSDFTREPVKASEEAADPASSHKSKSRKSV
--SSSSDTRKNSTASDRSSAPARALYQSNASPTASPPSRMPPRCTLDDATEQWQFVTELR
TAL--PAWLSSSEDLLHPAVLRALQDCRFFAPTPIQRSVLLAALRDRKDIVGAAETGSGK
TLAYGVPIVCNLLAQALRRNEKSSKGAGNIECLIVVPSRELALQVQRHLEALCVYTPLAA
VCLVGGLALQKQLRLLGQRPQIVIGTPGRLFAVIQIERGQEFMASLHTLRFLVLDEADRL
TQEGCFQEMEGLLDAIYKAQSPKKRKIQTFIFSATLAMDPSSRQSVLQALMDRVHLRKKR
LAVFDLSDDPDWKFGKAGHPPGEHNHAAGPLKLPDGLSITALKTAAGEEELYLVLFLLKL
F-------PANEPLKVMIFVNAISYVYRLDPILSLVLGRDKHQACQRGG-----SKGAPL
AVVGLHSNLQQKQRLKRVERF-QSANRAVIVCTDVACRGLDLKQVKEVIHFQAPRSPSIF
VHRSGRTARAQQSGETVCVLSPTQVTEWLQVLKPVQINVGELDPPACMHKSSRSQLTKVK
RILAIATQIESAGHHKKERRATSWLKKAAQAADIMLDSDISEDEEASARSAAQARTTSIL
RTELSQRTPTIMEMYDANATICSSVHSASLTLFSQDEVRALSSCKISDTNVFLDPHASSS
SLSGGLHDSRLGSLDGREICETCGCR-SDCPGHLGHIDLALPVFQPIFLPSLVKVLKLSC
LHCERLRVSTPAASLFVKAFELLQLGCFDEVEEASRCASSALRAPTRAVLRIFRKIEEE-
------LQRRAGADAPLAASREAPASPAKG--AQASVEDLEERASPGETRRKTRETQAAV
TEKDEAKEQQRVARLLSESTDFEKEKTRLLLHSAWQVATWRKLRDLLWTRAAASCVCANC
GRSSAVTFHVAQQATGVEMRWKWTGQPPLFSFLRNA------------------------
-------TPRREEEGEGRKK---------SDAKDD--------AAALFDERCLTYG-KSG
RAIGKLHLHAFQLMPMLQNVFKNSDRRLLHCLFPMSIQLSSSCFFISSLPVSANKFRPPL

AGLHPRTATLLELLKVNEKVQFALAIMRHPDPTGALENPESLAELFAETEPEQEPARKKK
LAAEPGSDDAPAAKVEKLKEFVLVHAGNAAWLTSYAVQLQQKVNEIVDKTKAEKPLT---
APAGIRQWMERKAGTIRQKLMGKRVNYAARTVLAPDALIATNEVGVPLDFAMKLSIPEKV
TPRNVHVLSEMVINGPHKHPGALAIMDDAGNLFNLEFLSPATRRAKAHQLLLSASASNGS
FTVYRHVLDGDAVLMNRQPSLHRVSLMGHFVKILTKENVFRLNFVNCSSYNADFDGDEMN
MHLPQDHLARAEAAYILNADCHFLVPKSGEPLRGLIQDLCLAGCLLTARDTFLDIHSFQT
LVYSGLSCYLDSGPGRVILDRGRKAS-SRLHASEVSLPKSGKTDGNAEDEAGGEEGRERF
KAPGDPYRRQLCRSNCRLHVEPPAILWPKPMWTGKQVITCILKTLVDGMAASRFSKPTDE
DALNRYEGVNLTGKAKTPGDAWAEDGDKEEVVIIRNSELLQGVFDKAQFGPTAGGLVHCV
FLLFGGAAAGLLLQSLAALFCAFLKLRSATTSPADFVMRREGELQRQALVRQVIQAGTYL
QEHFTARMNALMGWDDGADNPLLSHAEAGGETGSGVAQDEQAKVASKDAALLHMAAASVA
PMNGAALEGAL--------------KLNLARLSDAAAYALRVLGTSTADTDEAAKGRKGR
VKHEELQRKD-----RSSESAYPLSLNPWVLSRLLKTPELLAASGALPTRLRRLIETLSQ
VQK--TGSPTARKELLYFLACSPSLQQQLPHLAKVLGTERLLPAHQRLRTTLAKESQTET
LDGSFHCPPPNGSAIQGDAALLQKRMYHPSWLWGGNPSASDAAYAMAA------------
-NIFETERYVLKGARNAGVGELRTFLM-PSLLTAR-------------------------
-------RIGALYTGHFRGREDTFNEMLDAFFQSAMGKVASKSTDIMKGSYFLYRFPRNG
FASMIKSGSKGSNVNFAMISVFLGQQSLEGQRVKFMTSDRSLPAFLPFDFGSRARGFITS
SFLSGLKEEEYFFHCMAGREGLVDTAVKTAKSGYLQRSVTKGLEALTVRYDGSVRDADDS
IVQFVYGDDGRDPAHRLTFEKIDMLLENPAI----------LQRVHAPPAELSNGKQ---
--CLIQGCGSPLTCLGVTAFQFENDMQLVVDKAVASYESLRAAYSRTREELEKILRVIYH
KCCMPPGEAVGCVAAQSIGEPATQMTLNTFHLAGAANVTMGIPRLRELLQQGAVTKTPIL
YIPLRTAANRLSDLEDEGRVLVRNAGMALRSFTSFGLADCVHSVGVEANVCYQPPNCPKG
CPAFHLPPYVSRNNSAQS----------------IAPSSRMYWQYEVCLQLENLAHFSQV
VKSFTPAQICTLVLRKVIRPFLKKAYRLMVAAVAHHGRTDFAEMRSDLQAFFQNAICQDN
KLTQWDHRSKVKEILRTARVGDSTT---LLSSGATVRAGNEDDRGNRAEADADEKEADGS
DPASSADEEKEEKGDDQDEGDDDEQQSEGGRASEQSDGEQESSKRQRSKS--DSARGDAT
HIKVEQTSEDESSAATSSDEDDDDPEDDGHTGKAEESTVSSRTLDAAFESMFSPLLLSFR
LVKGETFDMNTHLFFPQDKKQCRASKTPLAVPAPV-------------------------
---------------------------------------ESDGLWADGRKYKGFV--LIT
REVASILHEVFVCPRTWRIILKFGWPFERCPYRLELLPLLMGLLKKSKMQEPEGVAAPRI
VQGEGCV-TKQKVEIQCEGSNFGWIHRMKDEAIDHNGILTNDVRAVLKYYGIEAARVCLA
RELERVFSAYGIKVDYRHLALIADFMTHGGTLRPFNRLGMAASNAPFLQMSYETSFKFLT
EACERAAVDSLSTPSGSLVVGAPSVIGSQQHQVFTQLHPEAARNRLLIELREAQRMNDSQ
VRLIPNHDNLYEWVAVIVGPKESPYQNGKWKLRLICPPTYPLSPPTVTFLTKCFHPNVDF
RTGAVCLNILRGSWTPAWNLHYVCLAICALMDIPNADSPLNCDAGNLIRSGDLRGFRSMA
RMYTIEYASAEIMAPQQHWNEPLARVMDVVLNCTIRFKTAAEVGKRAVFYCRGEDLFAWL
MNNREMLQKKHADALDGQSLASETDVIEFCDKLIRFGFMYRAQYKPIDGVIEQDEEGRFK
RPKWPKRLAMTPKQNFDPQAFYVVVYEGSKSWQHFILFCIIAAVLCVCMFPAWPLKLKVA
VWYLSVVLLTLILVLVFVRLVLFVFFWFFGYQFWLLPNLFNEDAGIIDSFLPWIEWHRSQ
DDWAMFAARIFCAILTAGTLYKLSETHTPASVANFAKQSFLDVLDWGHQRLAAPPGGYSK
YASITDKQEGGQGETNEENAEGKADEDEYSCLKQCGFASFEHLMQRCLVKCSCVGDLLES
KCF-AKCPEATQAALREAARDACEEEEKRMSTLAESFLCDLQDLEEDEDDELESSADVSE
AKSSEQKVEEDDLDSPIPDAVQEYEQRQQENVGVVVSDLMLQPDFLELLDRVRALTPQD-
-----------------------------------AENDNEELHLIEKCNERVIDIDKDI
LNIHKFIKDIYSMKFPELESIVQSPLEYIGVVLRIQNQTDLTQVDLSDLLPSPTIMALTV
AASLSSGRRLPDEEFCHAIAAAKEAIALAEKRKEILQYLESRMSLIAPNVSAILGAALAA
RLLTRVGGLKMLAKMPSQNIMLVGSQKKTSFSLSSKAGATGASLLCSCEILLLTPVAFRT
RALRLLAGKVSLAARVDFFGQSKEGEKGKAMREEIVRALIKAQEPPPAPQKKALPAPDER
ARPKRGGKKYRRMKEKYELTEVHKQLNRMQFGVE--EDQNGLKAKGLGMLGKSIARLKIQ
AKQQKKRGAGARAGNETAGC--GFSSSLTFTPIQGIELCNPDAAGAAANPTKTNYFSSTG
KFTKVMSPAASPATAVAQETIPICEVAQLLHQLRLSEETE-PHAESKERLLELIKKHDML
PLYRQVCEEFRWPADQVLVQSMEEKHKEELAAIDMRLEEAKEKYGDVEVREALLTRANFY
CRIGDLKQAVKAYDVAYQKTVGAGGKIDITLSLIRLGFVFSDKELVKKYLARAEEELEKG
GDWERRNKMKVYKGLHLLTCRQFKEAAQLFLGVLATFPTCSLFSFEKFIFYATLLALLTE
DRSVLREQLLTAPPVLEG---------------------------PIARRVKCDLYLGRH
YLYFIRAIRLRAYSQFLEPYKSVTIENMATAFGVSPSFIEAEVSGFIASGKLSCRIDRVN
AVVESNRPDERSRSYLRILKQGDLLLNRIQKLSRVIAMMANINWPGLYRWSMEYHDGTLP
--RSLSKEDSDFLQNAIREAMRHQEDPAKVLAEQLAVIDGFNQGDLLAALAVMERLIDDY
SELARDFEKLGALQPCLRLLESRDRHVVKTALTILSLIVANNPDVQEAVYKQHGLALLMN
LLKEAPNSSLRVKALTALACQMRHHRPSELAFVTAGGLALLVHAMLSRDEKYQEKAASLT
RHLLQEGLLAFSQVEKYDLPGAVAGLLERTPF--TNIQFGETVVQLAIALLQQHRATMAK
GPVLAGLRQTLLDRQRGLRKVEDLLPEDFSTQAALLEEALSIAKGSALHKLRRAGRAEED
QDAGEEDSDSEEDADDQQVTDERTMKGRRRRRGDAAVADDNEEEPAAAGSPENAPTFASL
GVPPALIRTAASLHIFHPSPIQVLSLPHTLRGKNVCGLAPTGSGKTLGYCWPLLQRIGRD
GHAFMGLVLLPARELAIQVLDQFRIYGVQLGVRVCLLLGGRDLVEEGKLLDQCPHIVIAT
PGRMSDHVQNDPRMKKRLSLVDVLVLDEADRLLSDEFEDDLKTILSCVPTS-SQGRQTLL
FSATVSPALLALQRRFGDDAMPLVDAHPTDQPAPNLSHFYMFVPTRMQPIYLLYLL-EHT
SPFRGIVFAGSVRQTQQICTALEIL-KQSATPLHSLMEQRKRVACLEKFRSETSRLLICT
DVAGRGLDLPRVEFVINMQVPGKAQDYVHRTGRTARAGRKGVALTFVDPKSVRAVHRIEA
LINTQLQPLSINEQDVLKFLSNYSKVVQKSLLFLNEIGFDEKAEQTKAFKEARRKMVRTP
WVGGNWKCNGTVGSITDLCGEFGKTEFDPKTIDVVIFPPALHAPLTREKLPKKYHVGLQN
CSKTHNGAFTGEISVEMIKDFGLRWILAGHSERRQYYGESDEVVAEKVNIILQKDLNVVL
CVGEQLKDREANKTNDVVDAQLAACLPKISDWDRVVIAYEPVWAIGTGKVATPAQAQEVH
EHIREFLKAKVSEDVANKVRIVYGGSVNASNSTELILQPDLDGFLVGGASLKKDFLDIIA
SGMMAPKQKKET---EAVDDARLRIAIVNADRCKPKKCRQECKRNCPVVRTGKLCIEADA
TSKIAFISEPLCIGCGICVKKCPFEAIAIINLPRDLGKDVTHRFGANSFKLHRLPVPRPG
QVLGLVGTNGIGKSTALKILSAKLKPNLGKYSNPPDWQEILAFFRGSELQNFFTRMLEED
LKASIKPQYVDHVPKQVKGTVGQVIQLKDETGRGAELMKDMELDHLVDREIGNLSGGELQ
RFCICVTAI-QKNNVFMFDEPSSYLDVKQRLKAALVIRSCLQYDNFIIVVEHDLSVLDYL
SDYICCLWGKPGAYGVVTMPFSVREGINIFLDGFVPTENLRFRDESLNFKLAADQDILPE
EIQRLHFYKYPAMTKTLGSFKLRVESGHFSDSEILVMLGQNGTGKSTLIRMLAGLLKADE
EV---ELPNLHVSYKPQTITAKYQGTVRDLFFAKIRESFNHPQFQTDVVKPLQLETIMDQ
EVQHLSGGELQRVALIVALGKPADIYLIDEPSAYLDSEQRIMAARVIKRFILHAKKTAFV
VEHDFIMATYLADRVIVYEGEPGVECVARSPDNLVSGMNRFLKSLEITFRRDPTNFRPRI
NKMESVKDKEQKLMGNYFMLDDASLVCAISGVIPEEPVFSKTGLIYEKRLIKKHLETSGV
CPVTAQSLSEADLADVKCPKASRPRPVTAASIPGLLSLFQSEWDATMTEVFALKQHLETA
RQQLSQSLYQQDAATRVISRLLRERDASRQQVHALQQQLLQAQKRAAASAAEPGLSEELV
QEMQALAKQLLVARKKRQIDNVLPASRAASFKCTYSLPLHSSADRGVLCCEFDPKNPKTV
T---------ATGGYDGNVILFDLEKQKTLHKLTGHTKAVRSAKLHVTEPVVVSASDDKT
VRIWRASGD-SPYKTAATIRKHRGEVTCLSLHPLGNYFASCAADKTWAFSDIQEGRCLQM
QKNLPCQYKCVSFHPDGMILGGGGVDGSVHIWDMKGLAYRAALKGHSGSINQLAFSENGY
YLATASSDGTVRLWDLRKSLSFQTIDMNEAATCVTFDKSGQYIACGATNNIALYHFEARA
AAANVINLLDHTDKVTDVKIGEGAAMLLSSSMDRTVRVWT-MESCHAYHGQIKDGLFHGK
GTLIYSGNEKYEGEFVFGKREGHGRFLYADGATYEGKWVEDRIHGQGVAHFASGNRYEGQ
WEMGRINGFGKLSYSNGDEYEGEWVDGKMHGRGTYRYAEGDVYTGEWRDDKRHGKGSVTY
VSAKGSVVEKYEGDWVNGKMHGHGKYIYSDGGVYEGDWIDGKMHGKGTYVFPNGNVYEGE
WAHDMKDGYGVLTYQNGEKYEGYWKQDKVHGKGTLTYTRGDKYIGDWMDAKKDGEGELIY
ANGDRFKGQWADDRANGFGVFTYANGNRYEGEWTDDKRHGRGVFYCAEDGSAYEGEFVGG
RKEGNGILRLATGHQLEGTWSGGQLVRVTSFVFAQDSPWLNVDLIRVQEVSDVNRVERIA
AHSHIRGLGLTDALQPRKFSQGMVGQPDARKAAGLVCKLVKAGRIAGRAVLLAGQPGSGK
TAIAMAVAKELGESTPFTHISGSEIFSLEMSKTEALTQAFRRSINVLIKQEAEIIEGEVV
EIEINRQTSAKAGQPSARTGRMMLKTTEMETLYDLGAKMIDALTKEGVTAGDVITIDKST
GKVTRVGRGFSRAKDYDAVGPATRFVQCPEGELQKRKEVVHSVTLHEIDVINSRAQGFLA
LFAGDTGEIKSEVREQIDQKVADWRAEGKAEVVPGVLFIDEVHMLDIECFSFLNRALEHE
TSPIVIMATNRGITTIRGTDYKSPHGIPLDLLDRSLIIPTQPYEEKDMLKIIELRAEEED

VELEESARLLLCKIAAECSLRYALHLITVANLVCRKRRGSVVTVQDIRRVYSLFIDVKRS
TQYLVEYQQEFMFSELPGGAPSQSPSQAVRSAGADRQEGLEKTKGSDMAHLYGNDSDFWT
SDDEETEDLPSVESSRLSFASPPFSPLALA-QRCEAVWRRRRTFGILDEDNSADNNI---
ASPVVAEPSSSTGLEVQSETAKDDEEKSAVKKVAASWEAEGDPFPLEKREHADDGLAATN
AHAASAVSLFDEDHPGKPREGPNFFFFPPLDSVASSLSSKGVHEGNEDEVRLSAAAVTAA
LSLSTSSLVSPVQSPTAAQDSASSDDVEGKKQEEARTRRGSQDEAAVSAAEGGKQFIRKR
WCVEDNESDVSLFRQVPLALEFPFPLDDFQKRAILHLEKYQTVFVAAHTSAGKTVVAEYA
IALAVRRNRRCIYTSPLKALSNQKYREFRLKFPSVGIVTGDVCINPDANCLIVTTEILRS
LLYLGDALIGQVDSVIFDEAHYINDIERGVVWEEAIILLPKQVNMVLLSATLPNYRQFAE
WIGSVKQREVFTLSTDRRPTPLRHFLFFHDKAFLLMDAKGRFQAGAYNEAFKHVREKGNP
QAARKPPPSSRGGTR------------------------------------QALRESSHQ
SSKGVFQTAEAKLKTEIHRLQGLITKLEKDNELPVVVFCFSRRKCETYAQAMRRLDVVLS
HDRSKIHLFVKDCLMALSPADRDLPQIRFVCGLIHRGVGIHHGGLLPIIKEMVEILFQRG
LVRVLFATETLAIGLNMPARSVVFSALKKHDGQRSRMLLASEYTQMAGRAGRRGIDTFGH
VYIFCSDDLPEPKELTGMMVEKANPLHSRFRLTYQTLLLLAARSHMSMTSFLSQSFKEAA
RTSLLPVFKRDLRRKRKELHALPDVRCVFGE-----PAIEDLAELEDRSRGIAEEIHMPT
-NLSNAGA--------------------AAPLGDGREPG----------------RDPRS
WETSERSVSLLQVE--
-----------------------------------MLCEEIVTCPVGVSSKNIA-----N
GAVGPEDSQLLGILAQELERLASADARGEDTTNAGARKSACGALTPM-------------
----------SFSKPLKQLELMAEIAEISSQLADESLDVYPEMQARLTVMKKLKLIDDHT
GLTVKGRVACQVMSGDELTLTELLFQGGLENLQPEEIAAVLSAFVAPDGPVEQVPAPTAG
IQRVRDQAEELHVAILKLQANSGVRINAEDWWKLCNFSLSLVAYDWANGVSFGDIMHKTN
AQEGSIVRAILRLDELLRKIRQAAILIGDPDLGAKLQQTSDRIRRDIVFAMSLYLQ----

--------------------------MARGQSSDAGLSPGEKLSQMRTLMKDRNLDAFVV
YSGDAHGSEIPAPSDERRQFLTGFDGSSGVAVVTADEALLWTDGRYFVQAEQQLDASWTL
MKQNT---------LFNNSKVKRVGIDGHCTPISEYRQLLHAGFSPPSAPCLGASSSLSL
KNDGASRPSDSKELILSENLVDLARPPAPCAEIHVHPLSYAGATTREKAAQVLQQM-AAA
RCDVLLISALDDVAWFLNLRGADVPCSPVFLSYCLISGNPAQDASPLIVLYTNEARIKGA
VAEELAKSRVYVRPYASVCNDLRHVLQNKPSFVKAGQKGNAEADVVNREKTGAEMLWLDP
TANVSIFATANETRVTLTVTPAAKQKAVKNPAELEGMKEAHVQDGVALAKFLTWLEERSE
DPQAESFTEWEVAQVVDGLRALSPSFRGISFSTIASANANAAIVHYRPIREHSAPVTSSL
FLLDSGAHYVGGTTDVTRTVHTGTPSESQKRYFTLVLKGFIGLSRQVFPQGTRGPQLDVL
ARQHLWASGLDYRHGTGHGVGSYLNVHEGPIGISCQAGETLAEGNVLSVEPGFYQQGSLG
IRIENLVYVTKATNFENMRFLRFDQLTVVPIQKKLILPSLLTNEEIQWLNDYHQKVWTLV
APRLQEEAKQNNDHTLSWLEKATAPLPLHMSFSLLVSCLRSPPLWGSPRACRRVPEARSP
S-GPRFSTMASGRRAEKGTV-AALATEEKIGDPHFQVSENSPFLQKRLQVFEDLYEKQTR
RLK-EKPR--REISIELPDGSKKSGTAFETSPYDVALQISKGLAEASLVAKVLYEQPSAE
SEAITAADDEEEAECPEESPAWILYDMKRPLEGSCRLQLLKFDSDEAKHVFWHSSAHILG
QAIEATFGAQLTVGPALTNGFYYDAYMGDAKVTEESYGRLEAAAAAVIKEDQAFRRLVCS
KAEALELFADNPFKVQLIASKIPEHGLTTVYCCGSLVDLCRGPHIPSTGKVKAFQVIKHS
ASYWLGRQHLDSLQRVYGVSFPDKKLLKDYLKLLEEAKKRDHRVLGQNLHLFFFDTNVSP
GSCFWLPDGAKVYNKLCMFMREEYRFRGFQEVISPNIFSCDLWKVSGHYQNYKENMYLFD
VEGKEWGLKPMNCPGHCIMFKHLAPSYRQLPLRLADFGVLHRNELSGSLTGLTRVRRFQQ
DDAHIFCRLDQVKEEVADALNFLFFVYDQFGFSFELFLSTRPKKALGERAVWDSAEAALK
AALEETGRPWQLNPGDGAFYGPKIDIRLWDALKRPHQCGTIQLDFQLPIRFNLQYRTQDE
AVAGA------------RDKAKEDKETGEKRTDSEEEA--FGVELPLRPGMARPVIIHRA
ILGSIERMCAVVIEHTGGKLPFWLSPKQCIVLPISDKVNDYAASVRDVLHSCGYEVGLDV
SNNTVNKKIREAQLQQWNYLLVVGEKERADKTVTVRDRADPEHQKVLSMEDLLTLFEKQG
MPNSRKTLTLDAWKKS--
--MEGLEILQNRGYDSCG
ITSISPEGQLVTTKFASRTTCDSIEILRREGKHPHRGNHIGIAHTRWATHGSKTDENAHP
HHDWKDRISLVHNGTIDNFAVLKKALIDRGCTFRSSTDSEVVANLIGWYLDQPDAFEEAV
KRAVGELQGTWGLCVVHKDHPDRLVLARNGSPLLVGSVGDQLFVASEPAALARHTNQYLM
LKDGEIAVVTAQGVGQLEATRPVHRIAKETIELSPEPFAHWTLKEIFEQPQALARAMNYG
GRIAPYQNRVKLGGLDQNRESLLTVKSLLLCGCGTSLYAGIYGELLMQWLRCFDQVRAVD
ASEVDIYHLPRQDAGVLLLSQSGETLDTVRACQLADFQGLKKFSVVNQVGSLLARMTNCG
VYVNAGREVAVASTKAFTSQVAVLSLIAAWFAQNQPTQAFPDRCSALMDAIHRLPVYAGM
TLN-CRALCQNIAERLKDAKTLFVLGKGFGYPVALEGALKIKELAYLHAEGFPAGALKHG
PFALIDEKEKTPVILVLLADQHAASLLNAAQQVKARGAHLICVTDEPDIVKDLADDVLVV
PSNGPLTALLACIPLQLLAYELAIAKGINPDKPRGLAKTVTVMMKPEYDYLFKLLLIGDS
GVGKSCLLLRFADDTYTESYISTIGVDFKIRTIDLDGKTVKLQIWDTAGQERFRTITSSY
YRGAHGIIIVYDVTDRESFNNVKNWMMEIDKYAMEGVSKLLVGNKCDLTSKRTVTYEEGK
EFADSCNMRFIETSAKNAHNVEQAFHIMASEIKARVQVNQQQRPNANVRLPSQPVRSVSS
GCCMPKIRTLRNKKPPEGWELIETTLLELNRKMREAELEPHEGKRKCESAWPIFKLHHQR
SRYIYDCYYKRKAISKELYEYCLREGYADAKLIAKWKKAGYEKLCCLRCIQAGDQNFSTT
CICRVPKNCLADQNIQCVHCGCRGCASGD-FLSENCVLRCTSSVYPASHSTHRTNDLQST
LSAVVLPERLPCTPLAAAAVVAVWNSGSLLVSTPLFSVCRTETISCAPPAQRQQERAFAS
SVISTNPTNTNNGAPSCADGEFDYDLAVIGGGSGGLACAKMAAAQGAETVVFDFVQPSTQ
GSTWGLGGTCVNVGCVPKYLFHHTGLAGANAHWDGPHMGWKGKFEEQDWGVCVEKVQNYI
KSLNFGYRTGLRKAGVTYINAYAKFVSPHELAYTFRG---EDKICKARNIVVAVGGRPHI
PEEVEGAKELAITSDDIFSLKQAPNKTLCVGASYISLECAGFLRELGFDVTVAVRSILLR
GFDRQCAEQVGLCLEEAGVRILRETIPAKMVKANGKIQVTFQVGKVEEFDTVLYATGRKA
DTSNLNLQAAGVETTETG-KIVCDGDSHTSAPSVYAIGDAVENFPELTPVAIKAGEILAR
RLFANSTEHMDFTNIPTTVFTPIEYAHTGYSEEAAEAEFGRDDLEVYLFQFSPLFFSCVH
REKAPQARKSPEDVDITPPCLAKLICVKSEDEKVVGIHFVGPNAGELMQGFALAVRLGAK
KRDFDKCVGIHPTNAEAFMALTVTKASGEPFVASGGCGGGKCGMPDSRHEELLLNLARHH
PDIESLLQTFFDFLERRTDFFHVIESSG--ASAG------LESP-----NMGFREGRAEA
MVRQAFSKAQMAYRRRAQPHLLAQVGAVPSSEGHVGKPEKTQESRQMETEATEAQRSKEF
RNTGVTSSQGTAGKELTTWNGGVCENYRWTQSFTDLTLQLDKNKYRGKKDVSVSITPTAL
KVVVAGDVLLEGEWEDHVNAAESLWQVEDGPYLLLSIEKARENWWASVLKGEKKIDTTKI
ESVKRVEDFDAATQAHIRKMMFDQQQKLRGEKTSDELEKEELLRKAWDAEGSPFAGLQQS
RAASVQESEPQQQQAQQL-AEEVQSGPLKLEHLLAKGFTKRDLELLKDAGYQTVECIAFA
PVKNLVAVKGLSEQKVEKLKKASKELCNLGFCSAQEYLEARENLIRFTTGSVQLDSLLKG
GIETGNLTELFGEFRTGKTQLCHTLAVTCQLPIEQAGGEGKCLWIDTEGTFRPERIVSIA
KRFGLNANDCLDNVAYARAYNCDHQMELLMEASAMMAESRFALLIVDSATALYRSEYTGR
GELASRQTHLCRFLRCLQRIADTYGVAVVVSNQVVAKVDNMGMFSGNEKLPIGGNIMAHA
SQTRLYLRKGRGESRICKIYDSPSLAEGEAVFAIGEGGIGDYEDNMALKEVCRSACIAWG
PPTRGPAHTKCAASQEELALGTMGLDPHLELATVDFASSSTALPVV--------------
-ASVSASSPFQCIAWGSSGEHGNGVGVGERALGLLAGGMADGDVTLWDAEAILRSGSGSA
KGLLASVPVHNQRVHCIHFNPSRPSLLAAGGSSGSVSILDVENVYDVAVYEPGGEGQSGD
ECTSLAWNRVVPHVLATAFSSGTTSVWDLKQRKTAA---------------------TQL
LLAYDDDRHPVLQLWDLRNSSYPLREVYGHTRGIVNAALNPMDSRMLLTSGRDRIVCWWL
EEDGFSVYMQQQTQEPYVQLQWAPSTPGVFAAAAPI-RVCIKGLGSGGVSTDKYIPSWFR
RPRGVHVGFGEDIAFFGPD------STSVVCAALGVPPSATGSAEAEASLR----EEERE
LLGQHGEWREEEKELQQEALKLDDHLQRGDWHQACEEEVAETEQKMPEKVWNLLMRLFQQ
PRSSLVEAFTLPESEVVKRVEAFLGRPLPLKKKETESQMQSSVPQPGPMSAAAVAPPGSY
LRPSAYPQQQPLSSGSPIYGGNQVGPGGVGSVAQGN-GVEPQQAFRQEEVDPEKFFLQLS
SGPENEKRPCAGEGRESSAGREGQQGGEAERAK---------SAEEVESEKAGQSRLGSS
QSGPVDWSSEVGSLVRACLLCGCLEGAIELLQSYGSEADALLLAAAAGASLWRSACRAYI
EKIADPFMKLVGYVILEDFPALIAAVPVSAWRDALALLCCYVTDGTQLASLCRQLGDRLS
ANGSDVFGALFCYFCAGDFSGACRIWRQPTRSRSVLGRHLLATMKRMLILRAALRYGEPC
VEFEEVAVTYASYLSSDPALVPAMRLLSAGASDVRAAAATLAFRLFHAQAEQMQRGFFPP
PSPFEGKLAPGAPGLKQPARASGAEPQVGLHGDPSLSQRQAQPPMTGGSVASVGSAVDNR
FPSF-PPKGYGGVSPPAAEG--GPVHPPSSAFGATGQPAVRPPLASKPPPVSSPFYQGAP

```
STTSQPSAGPMPPAGPYPPAHPSNGVAPPCPTSQGPMPPSHGVSPLGGPAPP--------
-------AGGLSHVHP---GSSPSPP--PPQMTLPQASHGTFPPSGLQQGVP--------
--QGPPGPFEGSGNPQGGFGSLTGTQQLPLPPPSGS-------AGGVPTLQQQQAEEQPI
-----PAYSAPTVGAQPIKPGMPVPWPIPTSAQLNSRTTKSTYAANVNIHKATEKPDGAP
MPPEKQAYVQRVISQLL-----QFQTGAAANDLNQKMEELFNKMRSGELSSSASQRLVQL
CELVEQQQHIQAQKVHAELSSTEWGTSNKGWLMGLKRLLPKMATRAQFESSNEVGVFAKL
TNSYCLVALGGSEHFYSTLEAELAPHIPVVHATVGGTRVIGRVCVGNRRGLIVPSITTDQ
ELQHLRNSLPDSVEIRRVEERLSALGNNVACNDYVALLHTDMDKETEEIVQDVLGVEAFR
ATIGKQTLVGSYCHFTNQGGLVHVMTPVEDMEELSQLLQVPLTAGTVNRGSDLVGAGLIA
NDWAAFCGMDTTATELAVVERIFKIATRNKLNLVDDLTLRSSLIDTLSMLEALVERLLSR
YLALYVTGISRDLSVAVWSGDVELEDLQLKPEISDLFGLPFRVVSGRLKRIRLSIPWARL
GSAPVCLEVEGVHVLLEPKPPEQTDEELIAQLRDAKKQQIDVVEQQLLDARSQLLKATDA
AASAVFFKFANKIVNSIQIDVRDIHIAFADPSRGFKIGVLLADCSVHNTDALWRRSVGSS
AGGAPMVLYKACELNGLAVYCSLACGAGGETAVAGSELNALPRSKSSSQSSSASSLPETS
PGATPADRDDTARDRLHTETKEVRDSR------------------APSPCDTGGLDTGGG
RSAEAAGAEEREETLQAKTSGSSEFAEAFFLAPIPEQNYVLKPLHMRLLISHSTTLRELC
ARLEVKKESQGIEIGRTQLKALLDMSHEATQRRRRCESLLLRNVDAVRLDAEGLKGLTEK
EFIGLYTRIRG------ALPLSEDEKLRLQTLYDVVGVRHLAKWQAICKEALTRIAEQTQ
QRQLLRQQELAERLSWWQWATQSRRKREADEASGDAEDQPLLTEEEVNYIVNSVQSDAME
NMAVPTIYKLSFDLVHFGISLRDDLPPDE-------SSPSPGPVASF------------F
SSPLALQKRAAAAAGAGGPGEAGDADGLLSASSSLPV--------SFSSPSAFLAIELFN
WSASLNLQNKQDASGAENTQWSFSFSLSNFCVRHREQIIMHFRKDIRADGEHVRDGGSGV
HSPAGSGDSPSPAASARLELSHDVTDAGNILGVVMRLAPLVAAFRPDLLKNLLAFFVF--
-----EEPEDVRRHREREQQSRTRDALSELPARSAGDAADGLGSDDEALEQVEEMKKTEF
VEHLKARGGTVYSAAVQRFPALLQIDIFIAAPILHFSTEGRGQVALQFGTLLLRTDGTCP
YDDLRGIVELNETRLICQPP---NQEEISLLRPIPIRVHLELQREDLQISFLLEEIFLEI
TPEALAVLLAVPASVAQSLIEVKKRRAGADETARKMAKTGADRGPGAEADGASRTEKSLA
GVLLSAATVSAAKEIEAASGAGEAAETGTSDEDAETKRLNISCCIDHCGFVIRTASAASV
LRAEFCSMAVQLQADEGKYAGWAELQRVFVCDPSTASPLFLTLETEMQLTDTQLYETMAT
SVQVIPGASGAGAVPPPVLSPTGASRARPPLSPSAGSVSKEAPEGDDDSEFQDAIEEREK
SVRVDLTAVFPKRKAPEVSVAVSTATVEVHWQQESIKQILETLREYKRLIQNKLEVHLEE
LRETAKRGD-----YTFLSREAVAAMQETLHNVQQSLAYIDVQQHLNSGASANPPASAAG
KAEAGVEPQSRGAQTQKDAGRGDP----------------------RAEAWMASGLSGDM
PPLAAEREVEAVVTLDLKVKGAAIAFWKERHIFSRMGIRDFEMQYRVLANGDGTVDLKVG
GGAIIMEERCILAPRGQGSESDPRGSQE------------SLEKSGATNRKPGDPSQQST
SLFSARIRHYAGVKPYSVCMQGVLHQVCYVYFQKDVTKLLEYLNDGIFNVFITKSYHAMK
QAATRSYFLFAVEIACPVFILCEDKEIIPGYVPHAPAKTLAVTPSPSLSSSASSSGQMAS
SASSSSALAYAGSYITLDLGHLRVRNAYTWDDAMPADGDSGGCRSAARPGSEDEKCRSPE
ARRRSAKSRAPFPVAERGDDEDLSPSSCRVFTLSVDLFGLTASTVSRAGSREVGGCLLEN
VDAAVSFRSSRGLVDVELDTTPWRLRLSRQQLTFVLDVVNENLAGKGYRPAPVVLSEGGG
GPSEAGGDLRREPHSGLGRREGDAQRAEQVRQALEDSTARVRLRMTVPQLLLEASFGPEA
PLALFSLKRVVVGAEVSLFSLYFTYRVVLCAQSFAIDDIRTNSANFYKRLAECAGASWRQ
GDGPAPNET--LRPANITREAAASFGDSGKLSSVGSSGALFVAGPRPQTSTSFE------
-----------GEDSDSEALRPAIKIEFASSLEEGGMHIAIYNATIYLLMVQLMDFISYF
TSSWAFSTMRCYPKPAPPVLHRSAPEFGPAGAGDEVEPALASYETADSVAELPVETRPSE
SPEARHPSQVLLPASAVLPEMGPRDKPFGITVTVSSGRFILYTDLKKPTAPILQWTNDFY
VNMVSGADSLQMKNIDILDCRIARLDVLPPALSGRQRERTGSLGRGLRLASLSESLPKAH
ASAVLLCEAFRVQGHGSYRSVDAKEEYRKQRAEAKAEEEKVLLSDLVLLFDIPVFFLRVS
SRDMALLLAAATTIYADTPSVNPDDPSPPVRPPPPDPRLLASAQRALGGAGTVGAKTEDA
ASGGSGTVASGAGDVSVSRALKVDVKLHGVHLLLLDDLRASVVPLVRMRLVASILVEKNS
QQFLLRLKDLHCGFDYLNHRMGSWEPFVERFCITFDYRPTPPSSVLSEDPWRSFKAVKTY
LVTSTSPLWANVTPELCKLLAWFVPYLAAHLGDLGKATEEEEGSIRGEDASRPRRSGRLE
ARDRSLFATGLAPDSKDARASDEAPRRPLGRLESKVADADREEDRSGVWGRRSEGGTRER
RTEDDARSNAAFRYVNLSGNCLYAFTLIETKGRSTADQAQSVVGPSILRLASRTGLPPSR
LASRTQEMLLDRQGTTEAPMHATVGVLVLLQPTGTATPLDDLTQGLTEEASRRARRRQRV
YLARCPPVGAVARLCEMFPDVPRAAILRNLNVTQDVAATANAFLLRSQVRSGEEASGARG
SGLRPKLDI-----SPAEIGPFPRGMLAVAVDIMRNCCVTLLPPFSYA------------
------------------------------------------------------------
----------------------------------------------------GHSPQTGA
QSA-----------------------------LLDIPE----------------------
--------------------YTDLAGTAGWASLPSSG-AAGARGKGGKAGA---------
---RKGSE----------TVGP--------------------------------------
------------------------------------WADS----DDASSLKSSASFAPHT
FASRQFQMLKH--------------------------SMSSASLKALSSGDKRPGKSGSG
DAG------------ANSFEAGA--------------------GLRPTAFSPERP-----
-DSRRDSDEREATAALHAQA-----GPRSSARASTHAPAS--SKTLGG---LRRLGSTTA
AGP-DGPGSPLA---------------------------SAASVPSAASGGRLSGLSGAG
QRTTVGQ----ASSIRDSVQRSDGSFSALSPADREKSLILA----PGP------------
---SGY--------------RDFVDAAAAADDSTFA--------------------SFSE
LPGLVSSASLASAFGKATLGSEVLSGPRGASVAPDSSLLF----FGEEV---------LP
FLPSPAAKNELVCEVLS-------------------------------------------
------------------------------------------------------------
--------------------PHPSYKLLMVTSTVKVFNYSG-LPLQICFLD-SQLNPLLL
PCAEARKART-----------ETIYGASCAAPEGGSAS---------------------L
SPRQTSFHQISCSHPELRVVP-------------------PWLQEK-------ETW----
------------------------------------------------------------
-----IEKQMARDVQAQIENVFLLDAD----ATLAAASPAPAFHS---------------
------STDTCSRGR---------DEDAVASRGRDED-------------AVASRG----
-----------SADRKAETCAERGAEQGDDPRETRDLHRATYAYTFLLED-KHFMSVP--
----------QPAILGAGW---------------------------CY------------
-LCFRPAAF---------AQKGE---------EDSNPRREQDGGLGTKNEVRH-------
-----------GGLSDRSRSSFQLSDSLET-LA---------------------------
------------------------------------------------------------
------------------------------------------------------------
---------MLKRLGVRKGDVVSIYLPMGPEIVYAMLACARIGAVHNVIFAGFAASSLAE
RIHDAESHILITADQSSRGGKTIFLKDIADEAMAICPQVRHLVVFRCK------------
------------------------------------------------------------
------------------------------------------------------------
-------GEPLKNWVEGRDIWGNELMSQMQPYCPLEVMDSEDPLFILYTSGSTGKPKGLC
HSTAGYLLYAALTHKCVFDYHPGDIYACVADCGWITGHSYVVYGPLCNGATTLLFQSIPT
YPDPGRYWQMIEKWKASQFYTAPTALRALMRYGDSWPKKYELSSLRVLGTVGEPINPEAW
RWYASVIGQNRCSVVDTYWQTETGGHVLTPIPGATVTKPGSATLPFFGIEPVVLDPVSGE
EKQGNNVCGVLCIRRLWPGVARSVHGAHLRLMSTYYWPYKGYFFTGDGVFRDADGYYWIT
GRVDDTLNVSGHRLTTAEIEHALVQHDDVAEAAVVGVPHDVKGSGIFCFVILKEGVSNR-
-----------------------------------PHLQDELKRVVRKYIGPIATPDYIV
IARDLPKTKSGKIMRRLLRKIAAEIDDFGDTSTLVNSHCLESLIIEAKMARGGSLAKAMP
GRLLLLSGVALGTAAALYVLLRGRDAASPRSQTRAGASAGEAEERSADKKLSCRDTLFVY
FGSQSGTAEAFSEELASAVQDIEELHVEVVDLEDFDPDAFLAQQFKVLVLATHGEGEPTD
NARGFFEWLQRYTKDLREGRTPSCAVFGLGSSDYERFNRMGRRTFKMLEFIEKRQLCPLG
VGDAAKALDADFALWRQAFLPALKRIADPTALPVSPVSYPPLSGVSTASTPELDADPQRR
GDSEQETREDLSRFSSAASLANAQEEEAAGRAGPHGTSAKLFFQMHALPVNSVRQLRQQV
TFSSASHRVAASLTDCPAVQYRAADNMYCLHKNTAEEITWWHTFLGFKEQGLNDFVHWVP
SRPVSSPSSS---------PALSYAVPFPTPCTVEEALGFFSNLTGQLPKFAAACLSLWI
EDPEERTQWLRLFCDEAQQAYEACVRSPLLSLRELLPLLAPSSTQELGLILSLLAAHAPR
AYTIASSPKTGTRSVALCVQRESLAITTARLEEHGFKIFGSSSAVRSARRGPLFEDRLFF
GACSSFITQQLRPGDVLKALIKPSSFRLPADV-KRPIIMVAAGTGIAPFIAFLREFACLG
```

G--------WLAPVVLFFGCQRANKDFLYREELLDAAEESFLTHLFLAFSREPGQKTYVQ
HKIAEQRNLVLQLLQQQATLYICGRTAMAAGVTKTLAHAAAETLGGDNAFVHDLRKSRRI
VEEVWAQVVPWVEKYRPRRVEDMAHQVEPKKMLRRILETGNMPHLLFYGPPGTGKTSAAL
ALVRELFGREEAKNRLLELNASDDRGIKVVRERIKQYTKTNIAKGKINPETGREMPTWKI
VILDEADMMTQDAQSALRRIMEAFSRTTRFIIICNYVHRIIDPIFSRCSPHRFEPVARDA
QEARIRHICDSEGLVVTSGAVDALLRISQGDLRRAVTLLQSAASIDDNLHEDAILEVAGQ
PPARIVTDFLRACQASP-SQASSEVDNVISQGWDVCLLLQEMIRQVVVSPHLKDLQKARV
INDIAQKEFAVFQGASPYLQLLSLSLRIHDCLAAPMTVAIDGGDVKEKLDRLFSYWDSVE
GDSWHGVDAFVILVGKASEEEEGK-AEQMQMWLTGFQFPETLFVFTRTGEWWVLTSPKKL
EHLRQVESCREGIFLLSRADGLPEAMEKIHQAIKNADEASIGCLQQSASGGGFGQQVSDF
TESN--RSKFVDDGIVATMAVHTRVEIENIRSASVVCVAMVKTQIVNRIETVLDNEQQES
HAAIADLAEKLLKDGKQIEKLREKRNIDPSEVDLLYSNVQSGDVFDLRASAQPTNANLSQ
SEGSIIVSLGVKYKELCAAVARTLLLNGTKEQKEVYSFTFELLNYVISL-LKPGASFSSI
YADARAFVEEKKPAL--------ADHLLKMVGHCMGLEYRSNSLVLNAKNSKSVVERGMV
FNISVGFSHLTTAKGKNYAIWLADTVLLPKEEGAVVLTDGTSKVLRHVSYELEDAEEEPA
EKKDRVKEKEKETRKAATTGGAISATILNNAESVILKDRLRRRT-GSQAATAQQEAEERD
ERQRQLRKKKSEQLRLRFEEEKDGGGERKKKEGKKMEDIKCFSGPEGFPRDVKANKLYVD
FKSESLLVPIHGSHLPFHLSTVKNVTCSEAQGDSFYVLRINFQVPGSQTTLKGEENPLPD
LKPDTVFIKELMFKSEDGRHLQTIFRTIKEQLKRVKQKALEDDVAGEVTEQDKLILNRSG
RRVLLKDLMIRPNIAPGRKLIGALEAHTNGLRFTVNTRGQIDQVDITYSNIKHAMFQPCE
RELIVLIHFHLKSAIMVGKKRTQDVQFYTEAGTQTDDLDNRRNRSFHDPDETQDEMRERE
LKRRLNNEFKRFVQQVEDIAKVEFDLPYRELRFTGVPMKSNVEILPTANCLVHLIEWPPF
VLPLEDIELVSFERVAHGLRNFDVIFVFQDYTKPVKRIDLVPIEFLDNLKRWLNELEIVW
YEGKQNLNWNAILKQIREDPHGFVEAGGFEMFLGDDSVSGEEGDTDEDDDDEEEEDGEEG
SSEEDSS-DSDDDESLADESDEDEEYNDSDEEEGLSWDELEERAKKEDRKRRTDDSDDNE
RRKKKRK------MGIGGSKSPPLEDWVVRVG-GTQEMKTASNAVVTSKYTVWNFVVKNL
WEQFHKVSNVYFVVICCLQMIPQISTTNGVPTLALPLSIVLVVNAAKDAFEDWQRHRSDR
IENGQVTHCIAMA----PDRAACSL--RLRDAKDVAVEVR-RTSRSSGLSAEDAAAAQL-
-------GLTSAQARDLGITPKQWKDVQVGDVILCLKNDCFPADMVLLATSDTRGGAFVE
TASLDGETNLKLKQTHRVTFEWLGSFLPLAVCYLLTRAGRIRCQVPNRDLNTYEGVLELD
GSCD--------------LGETDDSELDAA----AAFANKARCGTLRAWPQ----VDAAL
SVQQLLLRGCRLRNTEWILGLVVYTGQETKIQMNSSTPPRKSSRVERLTNRLTLSIWFVQ
TLLCLSVSVGHTVLLFDPTAKARTYLT-AAGNSEGPVVFCILNFFTWMVLTCNLVPISLV
LQMGMVKALQSLFIAQDESMFFHPVPKAQSAGENGQRGSDRRDSASDRRSEQSRRNSRSE
SRENSRMNSVKEDRSSLVSASDPARSPVSFAAASSTRSSKFSLEKAEVGENAWPRTSDLN
EELGQVSYIFSDKTGTMTSNVMEFRKCCVRGLSYGQGLTEVRRQALRRLGLPVPADPLPP
DEPTTPQVQMVDSALRHQLNDPNHPMHPYLVDFFLHLAINHAVVLETDPFGMTRYSASSP
DEGALVYGARHFGIEFLGQTPSGLEVSVLGRKLHVRVLASVEFSSKRKRSSMLCEIPVSA
TQTRAPR---
------------------SKKRIVLFTKGADTVILPLLKQRREAETQMLNTMEEYAADGL
RTLCIAKREVDTDEFFTWFQAYQQAERATVGRQEQIEAVAERLEVQLELQGITGVEDKLQ
AGVADTIEKLRAAGIKVWMLTGDKVETAINIGFATSLLTREMTQRTYVWEELDRDKALL-
--RERLEAQEMSILAKHQG------------------KSGRGEAKGSPGR-----ERQAH
ALVVDGEALQQMLEPDMEQLFVSVCTNCVTVICSRVTPHQKGAVVSLIKRHLQKITLAIG
DGANDCNMIQSADIGIGLKGEEGMQAFNCSDYGLVQFRFLLPLLLTHGSWNYRRISKLVL
YMFYKNLVLVLPMFFFGYISLFSGQKFYFEFLYQMYNVVFTAIPITLYGVFDQDVDKKLA
LKYPQLYRCGQIDLYLNLRVFLKWMLNGVWQAIVIFVVPTFVFGCNAVPTTTGRTMDLWM
VGTVMFMMNMIVVNIKVLLETYYLTTIIWAGFYISLLACLLFVFLFSSWPGFAGSVLGCV
FYLFIDAAACAVIATVAVTSLARDWLWKAFRVNCAPQLYHLIQQREYNGSRSSFIPDSRY
PGGSRSPRRGPRQSTRGYAFSEADPFFSATLRKQTNALTAEKWTFHQSQRRFVAAGVSAF
AKTSQLQGFPRLQLRRYSKKVHEHFFNPKNAGAFDETRSKVGTAVVGKAACGDVIKLQVL
VEDGKIRDARFKTFGCGSAIASSSYATELIKGKTCDDALKLKNTDIAEYLNLPPVKVHCS
LLAEDAVRHAILDYQKKQKTMFPLSATQKSHGSREKGLTNERAKNVKSRNAKPYGPPQKV
VVLGTGWASVNFFRHLDPNIYDVTVISPRNYFTFTPLLPSVCAGTLSPLSCIEPVRSLTY
RNGKVADFYEAHCTDVDFKNRIVACDSRQGGHFKVKYDYLVIAVGSESNTFGIKDVAANA
FFLKEVEHAMAIRKKVMNNFELAALPQTSEKERDRLLHFVVVGGGPTGVESAAEFADFIK
EDMSKYFPQLIPHVSISLIEGGSRLLGTYPPDISAFAEKTLTELHVKLLLRSTVVGVDAT
SVYVSNEPGASKEPKELLHGFVLWASGVGEVPLVKKIIAENFPNVGKRGLPVDAQLRLLN
QP--NVYALGDCAAIAPPRLADAAQELFSKAGAAEPTPQWLGRHAPTLAQQFPQLSPLKF
NFAKLQSNEHPADQFKSFLAEIDAAYRPPAPTAQNARQEGIYLAKVFNECPHPEEKADAP
AFQETWNGSLAYVGSGQAVAHLPYFNIKGGFLSLPFWKAVYTQMQITWRSRTICLFDWLK
TFFAGRDVGHMHRSRKMMEVKNKMPAPVQITAEQLLREAVDRQLDDLSQQQRIVDEEELQ
QYRVRKRKEFEDTLRRQRHHIGTWIKYAEWEAAQKEFRRARSVFERALNVDFQNTTLWLK
YIEMESKNKFINSCRNLYDRVCLLLPRQEQFWFKYAHMEELLGNYAGARNVFERWMEWNP
SDKGWMLYIHFEERCKELDRARKVFERYLSNRPSQESFLRFCKFEERHRQIPRARAGFEK
AIELLPEDMLDEHFFLKFAQFEERQRETERAKVIYQQALEQLPKGESDLLYEKYVTFQKQ
FGDKEGIEDTVLSKRVFVYEEELHGHPLNYDCWIDYIRLEES-------RGDIDKIRNVY
ERALANVPPVLEKRFWKRYVYIWISYALFEELQAKDVERCRQVYVKMLEVIPHKKFSFAK
IWSLYASFEVRQRDLDKARLIFGRAIAECGKPKIFVAYAQLELRLGCIDRCRKIYAKFIE
LHPFNPRAWIAMIDLEVLAEEQARARALCELAIGMEEMDTPELLWKAYIDMEVGWGAVDR
ARSLYERLLEKTQHVKVFKSFADFEWRIVESLPNARKVIERGIEVCKENSWDEERASLLE
HWLSMERESGDAQSIGRVFNMLPKKVKKIRVERDSGAESTVETTAYVFPDDPGSAANLKI
LQAAKLWKRKQAAA---------MSDVESVADAPVEEEEEVKDLMTAIRKVLKNALIHDG
LVRGLHEVAKALDAKKAQVCFLSESCSEPAYKKLVQGLCKEHGIPLLDV-TDSKELGEWA
GLCKVDKDGTARKVVGASCVCVTDFGEESEALTFLQNHIKT-LS-MRRSKRDKTRPSSSS
SSASVRSRKEEGRREREDREREREDRGERERREDRERERRADRGRERERRENRERERRYER
DDKGRERRDGQRREREVREREESDDDLAVEIFDEEE-EDRFLEERRRQRELLLAKYAAQT
GDDDGSAKREEKAEGESSRNCETEEERNSPLRMRSEKREKRNGDRREETGKDGERRFDDE
SANIQEADGDSLVSVEMSSPSVSDFEESSPSEVCSVRRASPTNGKSETAGEETGFMEALQ
KRLREEKQKLRRFVIQMKES---------------AEDEESGTSSEEKNDDVDMFSTASQ
GASKRSRKKRATVSV-SATAGPAENWNDSEGYYQATVGELLDDRYRVECEAIGKGVFSNV
LKCYDLQEKRFVAIKCIRHNDMMKKAAEKETSILRLLNSTDKDDKRHIVRLLRHFEYRGH
FCLVFEWLWGNLRTALKKYGGGKGLNAPAIHAYSKQLFVALKHLSRCRIIHADLKPDNIL
LNEKFSSLKVCDLGSASDVSDNEITAYLVSRFYRAPEIILGCRYDLQIDVWSAAATIYEL
ATGQVLFPGRTNNDMLKCIMEVKGKIPTKMIKAGQLSSHHFDENLDFIYRDRDAFFKKEV
TRVLHDLRPTRNLTENLIEKQHWLKGNSPKINFLRRKMRQLGDLLEKCLALDPQKRLTPD
EALQHPFLKESIHFGAEHTQ--

--MEIDSRYQETFDFKMSNLPSLTSLLPEMLQAVPGIDEALSFAELMQNVQSMKYSVIVF
DTAPTGHTLRLLAFPDLLERGLKKLSTFKDKIQSALQMLNAVSGQQIQEQDFAAKIENLK
AVTTSVREAFQDPAHTTFVCVCIPEFLSVYETERLVQELAKQKIDCSNIVVNQVLFPVGL
ASADAETLEELLAPPAAARLRQLIRRMQIRLLALEKSYHSRRAMQSRYLQQIQDLYSFDF
HVVPIPQQPEEVRGIERLLRFGDLLSSCRPLPILPAASSRIVPSASPSESRRAIERLSQV
EEQRCASAGSRELTASAPCAFVESSPLYPYSPSYIRGSQKYEPVEGDVVVGIVSGKKNEA
YTVNIRARGDGSLPLVSSFEGATRRHKPELRRGSLILCRVERASPELG-AELTCIDPNCK
KSWTSQEKLLGELEGGFVLDVPAPLAISLSSPHCFLLESLGERLAFEIASGANGRVWIRA
KDAGDAILIGNILVACYGKERHVMQAIINTLVS------MRALTLLHRPHCLSSASSASA
RTDSREVPRTHRLLQLLRGHLHLPAPTHAARLAATVPGTSLVGALDSVMFSRPPVGFENF
SQRQAAEADANAKAEKYRAAHPKPPPPPPILAWFGLSTFFLYSLLK--SRREDMSMQEFL
SKYVANGLVDKVEVLDRGECRAYLSPPGASTPNKAVVRFRTGSAESFIEKMENFQSSLGI
HPRDFLPI-YISDQHEFHLFDFLGSLFLFFLIANMVSELIFMRRMAGGGLNRLLGNSASR
RARVKAETVKVRFSDVAGLHEAKREILEFVTFLKHPQSFRRLGAKLPKGALLVGPPGTGK
TLLAKAVAGEAGVPFFSMSGSEFVEIFVGVGASRVRELFDEARKVAPSIIFIDEIDSVGA
KRST----SFGNSERDNTLNQLLVEMDGFN-PEETVVVLAGTNRDDLLDDALKRPGRFDR
LVQIRRPDVAERKEIFKVHLKPLRLAPTIDAVALSERMAALTPGFVGADIANLCNEAAIQ
AARRRSKVGVEQRDFEAATERTIAGLPSPKDLLSSHQRRAIAYHECGHAIAGWFLKHGNP

VLKLTIIPRSSGALGFAQQMPPTVELHEKDALLDRIAVLLGGRAAEEIFIGAISSGAADD
IQKASRLARLSVMQFGMSDRLGLVDYSLQQGGEQNFYRPYSEHTAKVIDDEVSQIINDQY
ERVKTLLKEREKEVHSLCELLISRESITYSEILECIGPRPVPPDPQMAAYIQALPTRPLL
PETGDKETGN----KEPGNKETGEKETGDNRRDDDDSGGQERDLDNEAAEAVAGRKPASG
PVACKADKDED------AMPSSFTSREDARKAKELEEARKAGTAEPEKDEEGNAINPHIP
QYISKAPWYLNQQKPGLKHQRFVPGEKIRLWYERGRKAAAKKTKFEKGACANCGAKTHSE
KECVERPRSKKAKFTQSNCADEEVFEDLKLSYDGKRDRYAGYDPSEYKWVIRAYELAEIE
RKRRKALELEKKEEKERREKKKEKDSGEEKDSGSSDSGTSDSDSDSGDSSDEDFDQTSVA
CSDDRFRINTKNLRIREDTAKYLLNLDINSAFYDPKSRSMRGNPFEHLKE--EEQALFKG
DNCARKTGDVLKAQQLQLFAWEAYKHGAQVHFNAQPTQLEKLYQEHVDRKKELEEEKKNA
LLNKYGGKEHLNADP-RMLLAQTEVYVEYSRDGNIAKGRNRVLIKSKYEEDAYVGNHTSV
FGSWYNLATQKWGFKCCRQTDFAADCTGRMENNGSSGKSTAAATDGVAFDSHGETPAAHY
VTEIDPLVAQDGGSGSAVSEDGARIAERERMLMA------QQGDGVEATGVDKSIAARED
FYRRQRFQRALSPERADPFSAASRGESARTYADVMLEQHLDREKQAAVRQIRKLQEDAEL
QLQKRRRWDWGTAGAEKKKSRWDATPTIGG---TGQETPTPGQWGATPAVDQAGPTPSMG
RKRSRWDATPVLGGSADMTGGAGMTPGGSTPMTAVTG-------------MVGATPLTPG
TPISTAEQLLQLRMQQEQDERTRYLTDEELDRLLPKEGYEIVPPPADYNPPPSSAAAAAA
RARHQAIGMTPGVAAT---------------PTPIGVTPLYTMPEEG-----GMEGLMTG
GGI----GGQLTAEGGGVTMKAEDYQFFAKLFEDKDEDAMTQEEVKERKIQLLLLKIKNG
TPPLRRSALRTLTEKSKEFGAAALFNQILPLMMQSTLEDQERHLLVKVIDRVLYRLDDAV
RPYVHKILVVIEPLLIDEDYYARIEGREIISNLAKAAGLATMIATMRPDIDHPDEYVRNT
TARAFAVVASALGIPSLILFLKAVCQSKKSWQARHTGIKIIQQMSILMGCGVLPHLKQLV
EIIQHGLDDQVLKVKTVTALALAALAESAAPYGIEAFDSVLRPLWKGMGEMRGKGLAAYL
KAIGSIIPLMDAYHASYYTREVMVMLVREFETNDEEMKKIVLRVVRQCVATEGVESEYIR
TDIVPPFFAKVWLVRNALDRRTAKLLIETVVEIANKAGGAHIIQQIVEDLKDPSEPFRKV
TLEALEQIIVNNGVVDVDNRLEEQLVDGLLYAFQEQTSEDATALLNGFSTIVNALGTRIK
PYLPQICGVIRWRLNTPSAKLRQQAADLIARIAVVMQKCGEEQMLGHLGLFLYEYLGEEY
PEVLGSILGALKAIVNVIGMNKMTPPIKDLLPRLTPILKNRHEKVQENVIDLVGRIADRG
GDLVSPKEWDRICFDLLDMLKASKKAIRRATVNTFGYIARTIGPQDVLATLLNNLKVQER
QLRLCTTIAIAIVAETCLPYSVLPALMNEYRVQELNVQNGVLKTLSFMFEYIGEMAKDYI
YTVVPLLEDALMDRDLVHRQTAAWATKHLALGVHGLSCEDALLHLMNFVWPNIFEKSPHL
VQAFFDAVDGMRVSLGAGIVFRYVLLGLFHPAKKVREVYWRVYNNLYIGHQDSMVAFYPP
LPDDEKGCYSRDELLYVIPTKQRVGALDVRALVASVRPSIVGLRVTNVYDFSSSSYILKF
AGKESKVFLFIHAGFRLYTTEWKKDKGALPSPFCVRLRKGLRGKKLEDIHQHGADRVVIL
TFGKSENALHLVVELYVSGNIILTDHTNLIQAVLRRHTAPGQRIAVNEYYRLDPPEMSLL
RDAIRPIDSDVVKRQLTAI----------------------QEAACGKDTKSKTQVTWAL
ATTGDESAKKRDGLDEAVESLRRAVMECLRMLRSAEDFLASATPPVEGYIVATRRSASSA
EEKYDYEEFSPVVLRQFADLQVLVQRLQVDRNTNSQKEADEKEEEKGEKEKKGDNGGDDE
GNAETRATGDVESAVAAVH-ANVAPGTRVLLHFRDINMCVDEYFSSVDVQKSERA-EAQA
RQEALSRVEKIKSDQEQRMQLLEEEAANLLQQAQAVEANVVLVEQIIQLLRAALATGVDW
DELGRQMKLQAKEGHPLAVHVHELKLEKQRAMLLLEAPRREEAEEPG-------------
--------EASETILVPVDVALSAHGNAQLLHSQVKQLKAKTQKTSAATAAALAAADRKA
QRTLKQKDQQLQAQQQLQKVRKAFWFEKFHWFISSDHYLVLAGRDAQQNEILFRRYLRSN
DVYVHADVHGAATCIIKNSREDPPVPLTTLQQCGEFAVCRSSAWTTKSPSAAWWVYGRQV
SKSAPSGLYLSTGSFMIRGRRNFIQVHRLEMGFGLLFRLADEA---------SVARHVAA
RTRLALEEAGSEGPQGSP----SSSSSSAVLAEGESGAEGSRRGKASVGGEDESAAQSA-
--------SSTQTHTEFLRDTAIEHRAATEAPQEVV------------------------

-----------------------ESRRASGASDDGDPVLAQEKQEERQRQLL-------N
QVKSQLEEQLRMATRSPSALRGGRLSRCSVEFSATLQDEANQAGRDGQR-----IRRRHP
TGHLSVQDIAERGAAAAAEVVEVQEFVRGEQSRLEEEARSRRAAASDRVDMAEELRPLQQ
ELLQRQYLQRGTRAESARSRRPSGKSEGDDCHVTFSPDPPKVAEKTISALHVPPEVIPRR
AEP-ASLLLVQIEEELREARGEEDDEDRLRRRDTRSRFRSPQAHGGDRAPSVLVGGEEAK
TELASSSSSGSAEKGQTQSPSGGAPGGERDFL-AVENLSRTGADLENARRSPCSERSATS
THSLTPAGSRISRRMSVDEGPPPDGFTEETLP----TPRELLSHSSIDFQHSGGDEAEES
VDDQEIHIIVRRHGPTPKGFCRSEEDDGTTSAGTRRSRRAVSEGSASACFERGLSGRVSK
EDGETERNRKKEPEEE--EGEEEETEKSAEELQKAASSDTTEDEEDTDDAVKEETINAEI
PSRKRMSAAE------RRRQKKGNREAKDDPAGTAEEKEDMGGKEKAKGQPVPRGKRGKL
AKMKKKYGDQDEEEKQFKMSLIGAEEIKRGGPTATANAAAPALPGRKAAQREERRELKEL
EEEGDERLTEQCSQIDLLTASPLPEDALLCVVPVTAPYSAMSKYKFKAKLVPGSMKKGNA
GQAALRHFLQQADDDRQKQLIKSITLAEVALSMISDVRLSVPGIQNQLMGRMYGPGKGMS
ASALPWRRKPPTWLKIKPSDVEEHIAKLAKKGQTPSQIGVTLRDSFGVPQVKSVTGNKIL
RILKLQGLAPELPEDLYYLIKKAVSVRKHLERNRKDKDAKFRLILVESRIHRLARYYKRT
KQLPATWKYQSATASALVASALRKRVAYFYDPDIGSYYYGPGHPMKPQRIRMAHALVLSY
DLYKHMEVYRPHKSIEPELCLFHSSDYISFLSSVSPENYKEFSLQLKNFNVGEATDCPVF
DGLFTFQQACAGASIDAAKKLNHHQADICVNWSGGLHHAKRSEASGFCYINDIVLGILEL
LKYHARVMYIDIDIHHGDGVEEAFYVSHRVMTVSFHKFGDFFPGTGDVTDVGASQGKYYA
VNVPLNDGMDDDSFVALFKPVITKCVDVYRPGAIVLQCGADSLTGDRLGKFNLTIKGHAA
CVAFVKSLDIPLLVLGGGGYTIRNVARCWAYETGVVLDRHREMSPHVPLNDYYDYYAPDF
QLHLTPSSIPNSNSPEHLEKIKTRVLSNLSYLEHAPGVQFAYVPPDFFGEDNDDEDEFMQ
NQVDNE-GGGRAAGATAHTAAAPYRIRRKDYANDFEDMADRDQKVPIMPADANAVDTRCA
QDMFSFAEAARRRQPEQFMGLVRQLIAAAATSVLTELTVLHHACFLGDVSVVKQLISYHM
DPEARHPLNDDTPIFFAARNAHFEVVRFLVNLVGPQCLAAQSRTCMTPFLATASSTHDER
PHELLKILEFMYLHGVSLEEQNSMGYTALFLAAKHGNPNIVQWLVARGASMNHRDHTGGT
VLHAAVASAEDDPLQFLCEHGAVKLIDTRADIGIQMTVLQRCLLKRQWFSYLLLLSWRLQ
YQLFGYTRALRSSYASLYWTITLCNLPLFFNAFCQLHALDLASWDGALWIFLWGLTQLFW
WKTYSGDAGIAPGSKDLIKDQYESIHPVMTPNIVGPNHYRLLQLEREQLRLNLELQELRR
TPLPFQAGREEDTRILVTALKSLRGEVLALMSGVANERRKRVSHEYLETLLNGPPTLKVC
VTCAITKTPRIHHCADCGHCLERQDHHCVWVDTCIARNNFQPFWFFLLCLTCLLVCHEQL
LLRYCAHLFLFNQLLWMAASVGAGMLNFMALVFVVYLLVRTTRVMFTNVTYYEFLKKPEH
IRRRFMTSGWLWDFRGLSFVAMIRNAILFWRNNLEASLLNKHQPQGAFMSA-RCVDWKRY
LYNGAPVQQGEKRKAEEGEKRADQRGAPSEASLRGKRRRLIRVGSPGSSDDVPASSRLAS
PKAASLRGSLGSGDARSRLQVSSESSDEDD-----------------ALEALQDCLREST
EMTSRLVQALGGAESGTQRDVAEKIGRGRCHPEKSFALPADLREHTSSVDRLKEYQQCGV
HWLLTLHESNRNGILADEMGLGKTAQTCVFLNYLYQSGRVSSPTIIAAPASLLDNWMQEL
EAWAPFLADRVLKYHGKQMERREMALQFLDALDSEKVLVLVTSLNTLTSKWDVQYLKQIR
EVAYLVVDEAHSLKNKDSLVYRKLNRTLKCERRLLLTGSPIQNRTLELRNLLLFLMPSVF
DGDSLDLALKAF---YRQ---SRRQKAAEQRRRRLASPSAGSARDSPKTADAHTTEVSVT
EGDSASPSSPSSSSSSAAASSSVLEAGGEISAQGVAESRSSGQSLPLLDKKDKAALGKE-
EPCQQAAEVECLQRILSPFILRRLKNEVLGCLPKKKNVVLRCEMQGRQRELYIQEIKTWE
SELTRSLEKLTSELTGSLPPSPSSSASSASSSS-----SAAETARSAVSEKEKPSSVELD
SGNSEGPDSSTAEKPQDETQLVGEKRDKSRSTDRVSNGVEEGESASSCSGDSGEASEKGE
RGKFVNSLLARLRRICNHPVLMQGAYTNEQLEEITRHFWLRVDGFKGNPREKVDLEIRKW
SDYEIHQAIQQQISQGDSRLAHLSLPKEMIMDSAKIRKMIELVSEIKKKGEKALIFSQYT
TYLDVVEESL--
-----------------------TTFCGDIKCRLDGSTAVEDRQALVDDFSTNPDLTIFL
LSTKAGGQGLNLTAARTVILMDQDWNPQNDRQAEDRVHRLGQTQDVTIYRLCCRGTVEES
ILKCCQAKLDLDVAFGGNSEVLMIRPQGPVLVLKQNTKREQGRKAQLANIQASKAIADIV
RTTLGPSSMLKMLLDPLGGIVLTNDGNAILREVDVLHPAAKTMIELSRTQDEEVGDGSTS
VVVLAGEVLAGAVDLLKQQLHPSVISHGYVLALEDTLKYMSEIAIDVDVSDDAKLRQVVD
ACLNTKFSSRWEGRISQMAIDAVRKVEIKLPNGKKEIDIKRYAKVEKIPGGDLEESRVLD
GVMVNKDVTHAKMRRYIANPKVLLLDCPLEYKKGESQTYVEITKEDEWAKLLEQEEKEVR
AMCDDIIASGCNLVITEKGVSDLAQHFLVKAGISCIRRVRKTDNNRIARVTGATIVNRTE
EITKEDVGTKCGLFEVKKIGDEYFTFLTQCKEKGACTVLLRGGSKDVLNEVERNLQDAMN
VARNIMLEGKLLPGGGATEMAISALLLANAKNVESVKQYPYKAVANSLEVIPRTLAQNCG
TNVVKVMTELRAKHASCSGDKWGVDGETGAIVDMVAKQVWDSLAVKQQIVKTAIEAAAML

```
LRIDDVLSGVRKEGGADNMDADP-MDRITVLNVAEKPSVAKEASRLLS-GGYQNAPTQSR
FNPVHQFEFSLQNRSCTMLFTSVRGHLMSLDFASAYQNWNTIPPEDLYGAPVEKNISQDA
GDVRKNLVDLARKAQWLVLWLDCDREGENIAFEVLSVCREANASLNVFRAVFSALTKADL
ERACRTLQAPDRNQSDAVDTRSEIDLRIGASFTRFLTMRYRSRVKLPSPTISYGPCQSPT
LGFVVSRFVEVENFVREAYWTIRVTIS---------------------KPDDEKGAN---
---------APPLSIDFTWERQRLFDQLAVLVLYEMCLENPEATVTDVRGTERTKQRPVP
LCTVELHKMASRKLRMSSARCMSIAESLYQRGIISYPRTETEVFSPTMDLLSLIRVHIGS
TAWGTYAQKLVDGD-FLWPSDGARDDKAHPPIHPLKLMQRSEL-EEEEWKLYEFVTRHFL
ACCSEDAFGFETRVEIDIAGEGFYATGLTVLERRWLDVYPYEQWSGNRLPRFLVNERVMP
SSILMSERQTEPPPLLTEADLIDLMDKNGIGTDATMHDHIRTIQDRHYCYKNENMQFVPT
DLGVALYQGFKRLAESGVDLSLPDLRARMEADMALVARGAKTKQEVLDLHVSEMKRVFLL
LKSHAAHMDTEMRNRLGGLEDNAMSVFTFGKHAGKSFSTVAREDSSYCAWALSTPNPSGS
LLLFVDFLKRRRGEIQDKAKPSACIYGENEKKGSSSAPDSSHSSSSHALAPSSPASASSS
GGNVLRSHQKSCQVLCSPSPSLPRSSSASSSASSSASSSSSASSASSASSASSGSSPSSA
ASERRVTLDVSVAFQLCAPDAFRIVAQKTVSRRGGGWMTHLPRELWTFLKALGPSVEAGG
RYSALAFAAEKYDMVLKSLNE-CFIFTDLFPLPPFVLRSFRAFMAFATPQRLPRKTALIL
LHETSPSTKRRLATLASPAGEELIKELKPFQLEGYRFGIQRNGRVLVGDEMGLGKTLQAL
AIAAFYHKEWPFLVICPSSIRFQWRDQALRWLSELLVLDEICLVKSGRAEIPGRTKMVII
SYDMITKQKKFMVPYKVVICDESHYLKNFQAKRTQAICPLLKNAKRAILLSGTPALNRPV
ELFQQFDALLPDLCTYREFADRYSVQVWNPFTRHFEYEGHQHPEELHLLLKHTVMIRRLK
EQVHSELPEKIRSRVPIEIPAKELKAIREKLAELEAEGQPESLNADGAGGVERASDGLGH
RSSSSPLVTELFTLTGLAKRAGVCEFLSYLFDGGMKVIVFAHHRAVLDYIEEFLQAEAKR
TIRIDGRTPQDKREQLVKEFQTSPSCQVALLSITACGHGLNLTAAGTVVFAELYWVPGQM
IQAEDRSHRIGTEFSSVQIHYLIAEGTLDETVFRILQRKWRLMTSTLDGEQQQLALSAPH
PFDKYLAQIAKDEETGRGFSAERASATERRTAHRAQVRGSRDGGRQSSLARFLQRADGKR
ENPGEERKRGEKEP-RSGSLRREPKRTHRADQTEVDMAQDATAAPPA------GAAGAAE
KKASSPLLTLCIAVEQLRQGVEQQDDRQVTRMFRQFKTLRTTCTPHTLLLVPATTADAAR
STRDTLAFWKAMQRALHAMDVDIPPPPAESFPLAAKDFASCLPEVEALLALLLLLRLIDS
RRFAQAVEFGDLLVDRVLGWRRRRLDLVGAKCIFYWYRARELDRALTHVRAKLLNAYTVA
SVQHDTMTQATVLNLLLRNYIASNQYELALKLISKACFPENLRSNAQQARHLYYLGCIQA
VRLEYSAAFAKLQLALRKAPQQPRVAAGFRLAVLKKAIVVELLMGDIPERAIFSRKETRA
ALLPYKHIVLAVRSGDLHAFARVMSDFEKAFIKDGTLFLIRRLHHNVIRAGLRLISLSYS
RISLEDVAEKLGLDSAASAENIVAKAILDGVIEAAIDHEKKCVESKASVAIYSSTEPQKA
FNKRITFCLQLHTDAVKAMQYPEAEETQTRGFDDERRKALQEEMARVEDEELDDDDDMLS
RCQFCGSPRVSLLRVASRLRSCRACFVRAFEDDVSAFIRRFSLFQRGQKVAVCVSGGKDS
AVLLHVLHTLNAREDLGLSLHLLAVDEGIKGYRDHALAAVRRNSEVYNLPLHVVSYASLY
GWTMDRIAQLLGKTHSCTFCGIFRRQAFERGAQDIGADVLCTGHNADDGAETFLMNILRG
DMQRLPVSGAPLTG----------------------------------------------
----------------------------SREGPSVMRVKPLLASYQREVVLYAHFNRLDY
FATECTYSGAAYRGLVRNFLSSLQNQHQQRVLDLLHAARKLWVPSRVSSEARPCVLCGFL
TKNDLCRACALVQALNENKGNKLMRGNEKGSERRDSIKPAVVRKEGESPASPRSSALRAS
PTAASGPVDVLGAAEERATQETQWSDLLQLWSRDGPAEERALTPHDAKKDLVFFQLDIAN
ATRNVVPAAVRARHRALPVAPPATVCASYEAAEAAATANSLGPMFRVYGVTEDGRSVCAN
VHGFFPYFYCPVPVSIQESLTARLRSFLDALLKTQASAKAYDVRCLDIRMEKKESLMYYT
PGQTSLFFRITLALPNWVAPTRTLIERGISIEKVALPPATFESNVPFVLRYLVDTKTTGC
SWLLGRGGAYVVRPASLQETSAQLELDIHYADLLPLPPAERWQKLPPLRILSFDIECVKL
KGEGFPEAETDPVIQISSIVMLQSVPADPLCRVLFALKECASIAGSVVLWFDDEKEMLAK
WAEFVRQVDPDFLSGYNCVNFDLNYLITRAATLKVVGFNRLSKLKSLESKIRDSSFSSRA
LGTHEGKDIATEGRIQFDLLELVRRDYKLKSYSLNFVSFEFLKEQKEDVHYNMIGDLFRG
CPSSRRRIGVYCLKDAYLPLRLLKELLFLYNYVEMSRVTGTPLNFLLTRGQQIKVTAQLL
RKCKELNYVVPVVKRTGGDNSQYEGATVLEPRKGFYDKPIATLDFASLYPSIMIAHNICY
STLVASSAAHTMNNPDDVTVTTTSPPHKFVKKHIRRGVLPMIVEELIAARKAARKEMAAA
KDEMTRQVLNGRQLALKISANSVYGYTGTTTGGQLPCLEVATTITCFGRDMIDFTRREVE
KMFCRDNQHACNATVIYGDTDSVMVDFGDFSIAEAMKLGEEAAQALSEKFVKPIRLEFEK
VYCPFLLMNKKRYAGLLYTRPEKYDKIDSKGIETVRRDFSLLVQTMADTVLRKMLIDKDV
EAAKEYTRRKVAELLQNKIDLSLLVQTKSLGKMDYDTRLPHVELAKKLRKRDAGTAPSVG
DRVSYVVIQGAKGQAQYERAEDPLYVLENNLPIDTQHYLEGIKKPLCRIFEGVMSNPESL
FSGSHTMKRTVSISTQGALSKFVQRGVQCVGCRSVIREGALCRRCQEN-EAEIVVNKMAE
MAEKEKEHSDLWTECQRCQGSLHQDVICINRDCPIFYRRAKVKKDIGTLEERLSSLSLSS
M-----------------------------------------------------------
-----------------------------------------------VATVDSEGPGKPI
LSVAPMLAVTNTHFRNFMRCFTREAQLWTEMVTDGAILNNMDRLQQNLCLEDIEHPIVCQ
LGGSDPKTLAEAGKLIEKLGFDEINLNVGCPSNRVVSQGCFGAALMKTPETVRDIVHEIR
RHVQIPVTVKTRIGYDHCDSRDVLRNFVQTVSAGGCRHFIVHARKAWLKGVDPKKNRSVP
PLLYGRVYDLCDQFPHLDFSLNGGVRSIQQAIDLAAGKWTDGTAEMDALLRRITRDKTVK
PLRGVMLGRAASADPCILANVDTDFYGDANPPTSHSRRTVLEAYRNYIKPFAEDGEY-SH
FALLKPVLGVFNGMPGNRLFRFTLDHLMRDQSRVWTAEEILAHTMAAVDETFPGVLEYPL
RDELSSKREANESMEVESAAETAYPSVEEVLQELEGNAAEEDLYGQYAWECEAERSWDQL
IESSEGFVLLHEEGVKTRRIDRRQQHEP-QIKKGIIRSLVLLIDMSEAMREKDFRPDRLR
CVCQLAEEFIGTFLLQNPLAQLAQVALRPSGSASMQLFSSSASESIAALREKRKHPGVGT
PSLNNGLHLAKDLLAGVPPYCTREVLVLFGSLRTCDVGCIEETIAAVKKSNICCNVICLA
AELHVLKNLCQATGGRHDVPLHREHLRALLMQHTLPPAWSASMQPCLIRMGFPSLKSTST
AALCSCHQHLTFSSYVCPQCGAKLCTTPNRCRCCFLHLVSPADISRSFHSLCPPLPFDPV
PADPEAKRLCACCTTQLDRGGAQCPDCGEIFCHDCDIYSHEQLRQCAFCVMRDIAASGGL
ASHPLYYYSTHNALRAAEAENFFPVTFVELHGTRTCKINVYPFCDVQMVKRILLKKMNLS
SCMKVRDIRLLYKGSELPNWRLMNIFTD------------APLKKLHWSIRSDNMRASIR
PLVSQKLRGSLIQVIEEVKLGFRRNVAPKLTMDGTGGTYILFDARRRPVGIFKPEDEEAF
APCNPRGYEGRIGQAGFRGGVLSGEGAGREYAAHILDSLYNCPAGIPPTTMVEACHPAFC
YKSPVQLGTTAMQLKWKAGSLQQFAQAKESCGDYNPLLFSVSDVHRIALFDIRVMNLDRN
DGNILVAPLHTFQDITASLNRSESAHAATEAAHAAQSLVTPEGQQTKFRLIPIDHGLILP
DVIDVATVDLVWFDWPQCKMPFSEHDLALVYSFNAERDAERLRRKLLMRDDCLRTLRLST
RFLQQCVRHHLNLHQIATIAARDDVDQPSALELLVRTSLQRAYLTMDCASLVSTNRLGFG
LDLAELKLLDESVLASLD-------GRPRRETSSRSSRAASRGSGDL--SPSARRTCCAC
CCR----HGPPSFAKRRDDEASGRATCDENKTERASGRSESGEALCARSFSGSSAESQRG
ASGSDETKQERDSTPCGRSQCEDSGTDGRCDAQQGEGSDCGTFSSESEESEESTANRCSG
SDDNDAGDENDEDESGYDGARGRRTSCSGSEREEPRGKSEKLNTSHSHAAVTPHLRSLPS
AEAGRRRRRKKARAKKPDAHTRTKKEGKGEKETTHEKKEEFDAVRGTQRGTVRRLNTAAG
TAYRRQQPHGVGSTWLLYDTDGRSIPLDWSEPRFDRLFFDCFEELLRKYIVQQHPEWASY
PYKGSRLDAQLKKEKEAGEMEVRVGGKYRLGRKIGSGSFGDIYIGANILTGDEVAIKLES
IKSKHPQLLYESKLYKLLAGGIGIPMVHWYGIEGDYNVMVIDLLGPSLEDLFSICNRKLS
LKTVLMLADQMLNRIEFVHSKNFIHRDIKPDNFLIGRGKKMSVVYIIDFGLAKKYRDPKT
QQHIPYREGKNLTGTARYASINTHLGIEQSRRDDLEALGYVLMYFNRGSLPWQGLKATTK
KDKYDKIMEKKMSTPIEILCKHFPFEFITYLNYCRSLRFEDRPDYAYLRRLFKDLFFREG
YQYDFIFDWTFINTEKDRASRRSQQVYVEDNRQVEENQNELPMAVTTPIIAFSRRCFLDS
LSTLVGRKFLLLDEKLAGPLSLLVDAPTLQQHGVDRCYPCTFPVNSLSAGAAAAPFVLFV
CRPRLALLPLLVQHIHFIEKTYPSGRHYVVLFVPSASDFLANELRRASTLQLNLESTTVP
IYFFPLAPDVLSLELPNSFRDIHVFGDPSPCLHAAAAVQLLQQLKNAVIPHLRCLGSAAK
TVADHLIQQRKEKQAAAQQQAIWGEAAATTGSDLWGGAGGRNPGRGAFDPA---------
-------------------------------------------GGLLEDGNGEVLEQLVP
VAPPVRLG---------------------------------------RPHQVSDETPANA
RAGKDRASGPRTMP--------WNKAETNTSDKKGVDADAANR-----------------
--------------------------------------QGAGPEPLAEPAGCCAAPPSQG
DAAHSETVSTRARA-----------ASTGPAVPSVDMLVLVDRRSDLITPLCSAFTYEAL
LDAVFGIDAAAVEVPQQLLQQKQAGGAGGGGPGNGGSGPKGFPVLLPCGRRQKVPLFSDG
LFATLRDLHQSALGAHLHRVANEIQQTYKEKDELRSIQEISVFMNKFKVKQQEHSSLSLH
VRLASFLASVTKDPAFFRRLTLEDELLQTGSATSSSAGATLSAAVENMVDASAPVEDVYR
```

LLCLASVVNGGLKGKQLEGLRKGLIQQHGIREAVRMAHLQRVGLLRQADNTLGSWKTLKK
ECNLIVEEEHDIAYACSGYAPLSVRLLQFLHEQPNGWRSIPHILSLLWGPAMEVRQQPSG
QKLLELQQREQRADLSEGEGNSNVTTVMVMYLGGVTYAEIAAIRRLNEMEIAKHYIIVTT
EIINYKKLIASCGEMADVAQEEARLARTAEEDDLLQNLTEVESLCPNCEENGTTLLLLHK
VPHFKEIVLISFSCPHCHYSNREVQSAACLAPQGVRLELTVQSAADLDRQIVRSEHATLI
VKEVELEVPPKRDRGELTTVEGAIRRMIDALRAGQPV-----------------------
--RRAEAPEVAEKIDEVILR
LVKCIAGETLPWTLILDDPSGNSYIEALPEEERDFQLFVKHYERTKEQLHAMGFYEAQNE
EKE----------------PREGSKPHVWDLSK---------PLPEGDASPAEGREGEEK
EDYLFSLPVSCPHCGTEGSNNVCEIDVPGFRRCLIFSFLCQSCGGRHSEIKAAGAFGAVG
RKWILNVETAEDLNRDVLKSDTAVVEIPSLDFSMRGGVQGGEFTTVEGLLGKLATALGDS
APFACGDSAPQE---------------KREKLSELIGKLQKLERGENL-PFTLVVDDSAD
MSFIGRRRLSIDEQLKTERYKRTKEQEDDLGLTDMKV--MEIPAPSSPTALKAGSLSSSF
SEAVSPSKKTASPVHGAGRGMYVVNRRGEEEPVSFDQILKRIEKLSFGLHPLVDPARVAQ
AVINGMYAGIRTSELDDLAAQTSAYMAASHPDFSRLAARIAIDNLHKNTTDNFLTVIDQL
HGYVDKLGREAKLVSTEVYEFVRENEQALNEALNYSRDFDYDYFGFKTLERSYLLKIHDR
IVERPQHMLMRVACGIHCGDVEKAIETYELMSQKFFTHATPTLFNAGTPRPQMSSCFLLT
MQEDSIDGIFSTLKQCALISKTAGGLGLAVTDIRATNSYIRGTNGYSNGLLPMLRVFNDA
ARYVDQGGGKRKGSLAIYLEPWHFDVFDFLDIKKNHGKEERRARDLFCALWIPDLFMERV
NDNAGWTLMCPNECPGLTEVWGDEFKELYERYEREGRGRKTIPAQHLWFAILQAQIETGT
PYMLYKDACNRKSNQKNLGTIKCSNLCTEVVEYTSKDEVAVCNLASVSLPKFVDRESRTF
DYEHLKRIVKVMTRNLNRVIDRNYYPVPEAKKSNLRHRPVGLGVQGLADAFMLLRYPFDS
PEARVLNRNIFECIYFAALEASCELAAEEGPYATYEGSPVSQGILQFDMWGVTPSSGLCD
WDGLREKIKAHGVRNSLLVSPMPTASTSQILGNNEAFEPYTSNIYYRRVLSGEFFVVNPH
LLRDLLERDLWSEDVKQQLIAHNGSVQNMDVIPDDLKALYKTVWEIKQRVVLDLAIDRAP
FIDQSHSLNIHMVNPTYAKLSTMHFYGWRGGLKTGLYYLRTQAAADAIKFTVDSQLAMSA
KALSGATVGALTTTKRQDVAETE------VCRWGASPDEPCEMCSGSTYSSQNSPFFPRM
TLEEIKRDFTKHKLECPDTMWREPQPEQVQGLYSFAIESIFGLTVNDVRIEEVTGDVRSC
LPSIDSLQFLSQDGRLHAKAIGNLRFIRLCQRLNRVLGLPEFSREFASPTASGVQRFASA
A-----------------------------------QLVEQELLRFRAERQTQQPLAQRQ
KQQQSELEEELRQRHSELGALMEEFKERQAVHGRLELELGDLVLELMNLKQEREELHDQV
VHSPEKLMERRDELRVQQKHLDAQLQELENLAASQQKLLLAFAKAVKKAKKAMEILSAHR
DQVLAPHLGFRSDMRTREKLFRELGEQKEQLSKAREELARQLEDQERKKDEEETELRGHL
ARAKREVEERKKALADQQDMTAAFLREAEKLEEKLAEQKQRHKLLVDAIEEEIQKVYAAF
LTYVSQMQFIRSQMPLSLDLSQSASF-MDLFRRLIPSNKDGSGDHSQRLPPNKCVMSNFN
FGPTLGTGSFGRVFMAKRKDDPAPPVAIKRLKKAAVIRQKQVDHILSEKRILQMINHPFT
VNMLGTFKDDRYLYIVMEYVIGGEFFTLLRKTRRFENDAARFYAAQVTLIFEYLHDRNII
YRDLKPENLLVDAEGYLKLTDFGFAKVIEYRTYTLCGTPEYIAPEVLLNKGHGKPVDWWT
LGILIYEMILGYPPFFDDEPMGVYQKILGGRIAFPKFFDKNAKLLVKRLLTPDLAQRYGN
LKNGVADVKDHRWFAGFDWNACLKKSLPSPYKPPVKGMDDTSNFEAYPESTEQAPPVTGT
MDPFTSW-MLSQFYVLSPRGDCLITKDYRNDAPKGAAEIFYRHVTCWQGEASPLFCVNGI
TFAFLRRSGLYFVLTTQQNPSPAVLIELLHRLTKIIQDFCGVLNEEAIRKNFVMIYELLD
EIVDYGYPQLTSTESLKSAVYSEAILVDPPPVKTSSLSTLANLAPKTIPSNASHRPVSAN
IRRSEIFVDVLERLTVVLSSTGQVVNASLDGSIQMKSYLDGKYLLKLALNDDIVFVSQTT
GSPNTVWVDACNFHECVDLSEFDAQRLLTFVPPDGEFVLMNYRVAHCQAVPFRIFPSIDW
RCGQTGELTVKVKADIPEQTYAATVALSIPLPKGIVACSTELLPPVPLQSAEFLPAEKRL
VWNIRKFHGGAEMIMRARFTSSSPVTASRKEFGPISMTFEIPMFNVSNLQVRYLRIAEKN
GVASPFRWVRYVTQSSSYICRVASSASSTSSASAHAEGAHETHKRLRGEAMRWSAASLDD
RERKSVFSGDGVVVPGQQIVV---EEGCMRGHGTYEKGGKIFAAVCGILEKVNKLVYIRP
LRSRYQGSVGDVVVGRVTDILNGKWLVDVNSGQAACLALAAISLPE--HRRRLDEDMLEM
QNFFVVGDVICCEVQRVRADGQILLHTRSTRYGRLMNGVFLAVAPQQIQRQSHHIVQLSC
GVQVVLGLNGYIWISLPMKTSAKDTMNYAHVQT---THEKVSKEMRLAISRVRNIVLCLA
RSNFDISTQTIERMYDV-SVSRGWEAKDLADPLVMQELVEAFLVARLGKDMPVLYHLTLQ
KPTAIVHALQGNFSAPRAQEVVVSRGRVLELLRPDDQGKLQAISSTEVFGIIRSIAAFRL
TGANRDYLAIGSDSGRLAIVQFSAEKNEFERVHCETYGKTGIRRVVPGEYLAVDPKGRTL
MVAAVERQKFVYIVNRDNKAQLTISSPLEAHKSHAICHDLCGVDMGFDNPLFASLEQNVE
SSDRKPATP----GVTVPKGLCLWEMDLGLNHVIKKATLPVPASAHCLIPVPGGADGPSG
LLVCCGNFLLYKKPDHEEICCAIPRRLETGSDRGLAVVAFAVHRMKDFFFILIQTEYGDI
YKVEISHEEGVVREVVCRYFDTVPVANALCVLKSGYLFVASEFGNHLFYQFTGIGSDASD
PRCSSTHPLGREAIIAFKPRPLRNLALVDELQSLSPITDLKVLDAQGTGAPQVYVLCGKG
PRSTLRILQHGLGVEEMADNELPGRARAVWTTKLSHQNAFDGYIFVAFDGSSLVLQIGDT
VEEVTDSAFLTNVSSLLVALMYDDSFIQVHETGIRHIKSKRVNEWRAPGGRRIKAAAANE
RQLVISLAGGELVLFEVDDAHTLVETARRNINVESTCMSMQAIPKGRLRASFLAVGGLDN
MVRILSLEKDRNLRQLSTQLLPNDATPESVCLATLTGLGKSQDNGVLYLHVGLNTGVMIR
SVVDPVLGTLLDQRSRFLGGRAVRFHAVTLQGQPAILALSEKSWLCYTFQHKLHCIPLNY
DPLECVASFCSEQCTDGFVAIAGGSLRIFRCQRLGETFGQTVLPLSFTPRAMAALPHPSA
AESQGDAAGADPSRRASALAIVEADHNAYDESTKAEIRRALKGIKVNQEEEEDKEE--EE
KEQQDLPEDHYGTFKAGPGKWGSCIRIVNPLMAMTIDKVSLETDEAALSCCFCEMEGLPL
LVVGTVTAMTLKPKKVPHASIKVFSYDDKFSLSLVHSTPVEDYPMALTAFRGMLLAGVGH
KLRLYALGRKRLLKKCEYKNLPCGVAFIRVAGDRLFVGDVRESVHVMRYRLSENLFYVLA
DDVVPRWLTKGEVLDYHTFVAADKFDSVFICRVPSEAKEDELGDTTGLRLRGDTTYLTDK
CFKLQSLLHFHIGEIVTALERATLTSAASESIVYGTIMGSIGSFSPFLTKHELDLFTHLE
MVMRSEKPPLAGREHIMFRSYYHPAKNTVDGDLCESYALLPYEDQKRIAQDFEKTPADIL
KHLEDIRNRILMEDEEPKKTIPLDDADINILKSYGLSPYAFAIKRLDTDIKSLTEKITKL
CGVRESDTGLCQPSQWDLAADKQLMQE-QPLQVARCTKIIYPGDEEPKYIINVKQIAKFV
VGLGEKVAAMDIEEGMRVGVDRNKYKIQIPLPPKIDPTVTMMTVEEKPDVTYNDVGGAKE
QLEKLREVLELPLLHPERFLSLGIDPPKGVLLYGPPGTGKTLTARAVANRTDACFICVIG
SELVQKYVGEGARMVRELFQMARSRRACILFIDEVDAIGGSRGGENAHGDHEVQRTMLEI
VNQLDGFEARGNIKVLMATNRPDTLDPALLRPGRLDRKVEFGLPDLEGRTHIFKIHAKTL
SVDRNIRYELLARLCPNSTGADIRSVCTEAGILAIRARKKSISEKNFIDAINRVIKGYKK
FSATAKYMVYNMAMESHLNFLLCIDFSPV--TDDLSDLTRQNAQSMVAQLCALPHETTDD
G-LLVALPKNSTFKLPRMHPAPAAKKLTRWEAFAKEKGIQKRKRSRLVWDANTKDWVPRW
GHKGIQQMNALAEAVIEDKDGEYVVECPFEAKAKEKKLRQAKQKLRELRNTLEADAAGGE
AAAGLRHRRQTKEELKEVMRRAQTSTASFGQFDRLAKNEKREKQKTRTKGVSLSLDDERS
KYRRHLKNILSAAM---AGMCTDLVSLVSQHIRAILERVPGAKVLLLDQETTGMVSTAVS
QSDILQKEVFLVDRID---------------------ALPRGRFEHLSCVGFLRPTNENL
LLLLQLLRQARFKDIYLFFTSSVHQQLLRRLAKQDEADKVVQVEEFYVDLFALDPHVFTL
NIPAV---
----------TSLQVQDLSLWTPYEESLFQRMVDGVFSCIALLRIFPLVRFQANSVVSKR
LAAAMQVRLSEN----ADLLDKRPQTS---LPGRSADASGGS--------SSGGSRLVLL
IVDRREDPVTPLLNQWTYRAMLHELIGIRNNRVDMRRTTEDLLDIVMSPMQDKFYRENLD
SNFGDLGLNVQKYVREYQSKAKSTGQLESVDDMQRFVDAYPEVRKLAGNVSKHVAVIHAL
SKIVNDRALLDVSSLEQEVACRESRSDHFAQVADMLRNERVSSMDKLRLVLLIALRYEGD
PRIQDLTAGLRQAGIDEEEIRLVRAMTQYAGRHARSADLFSNRNFLAVAKNTIQRGLKGT
SNVYTQHKSLLWFTVESLIKGRLSTEQFPVSSPLHLLQPSREKPQTVVVFMVGGATFEEA
RDMAELSKQTGCTILLGGSTIHNSRSFLADLSQLVKEMPWKLARRDELMATRQAERPGEY
PPPTVVTPLRTSAYDFPEATFVTRPCLPAKKATGHKNVFERLTDTAYYTGSHRERFDEFG
NGRGIAGREYLYAYDGLTESPSRCHEVYSSVIKRPRKPVVTPGTLGIQRFGVQIPAPRLM
WLYRNGDKHDDGTPFFVRPYIKSMESLYQQITKEITPIAGPVRRIFDQNFRVITDLDDIV
DGAKYLCTSGEPPAAYDRLEKFLSEWVIQKMSEDDNDDSDHEEKPAKKKARGKVPKSPAT
KKTAAKGQASAARPKAKRRATKSPSTETKSRSRTTAAGTKRKKENKDEGEEEEDIP-NDA
RKYFKDGQKHITPPNGEGTRAFYESLYEENPNSLIALRYVIEYGVLTGTKLHESLPKYAL
LRELGAFKGAGGGVQPEFKDGLNEQQNKAARKAKKMDGDVDTNIEQWKIKRLIKNLESAR
GNGTSMISLIIKPKDEITRINKMLADEFGTASNIKSRVNRLSVLSAITSTQQRLKLYNKT
PPNGLVVYCGTIITDDGKEKKVSIDFEPFKAVNTSLYLCDNKFHVEALAELLESDAKFGF
IVLDGNGALFATLQGSTKEVLHRFTVDLPKKHGRGGQSAMRFARLRLEKRHNYVRKVAET

AVQMFITQDKVNVSGLILAGSADFKNDLATSGMFDQRLQAKVIKIVDVSYGGDNGFNQAI
ELSAEALTNVKFIQEKKLIGRFFDEVAQDTGKYVFGVQETLQALEMGAVELLIVFEGLPL
ERVTLKNPVTNAEKVIHITPDQARDDSLFKEDGVELEVADKISFSEWLVNNYKNFGTTLD
FVTNRSQEGAQFERGFGGLGGILRYKVDFQDYDETVDDEDEFII-------MEHDQEKLL
DEASAVVKEQARYMKRAIDSDNLREALKHASNMICELRTSLLSPKTYYELYMLVFHELQH
LSAFFSDKSRHNRKMSELYESVQHAGNIIPRLYLLITVGASYIKSREAPACDILRDMTEL
CKGVQHPMRGLFLRFYLTQMCKDKLPDVGSEYEREGAGTMNDAFAFLLTNFTEATRLWVR
LQHQG----------SARERQKREKERHDLRVLVGSTLVRMAQLDGMSVEFYREEALPRL
LEQVVGCRDGMAQQYLLDCIIQVFSDECHLQTLDPFLQACLNVQPTVDLKAIFVNLLNRL
ANFVQSEPESVPADVDVFALFRRYILELQDRYLLSLSSSSQSNGVKEGSL----------
---------------------------------VAGSLVGNLSGGKTPPSTDLTSLLELQ
MAFLSFTLTLFPDDGILASTAVLLSRCLSEKREDSPAGVEAVVELLSSPLRTLSLSVLEI
EHFPCLMGYLDFDTRKQVAVSMVSAVLGSNVALDQPSALSRFLEFISPLVLDAPDTPLDE
DEG---GASSAFSAEQQSVSKLVHLLHNP-DTDLHFALLCIAREKFGELRRLRYTLPPLV
VAALQLVPRILDRVEEHQRGD---------------------SDLPAPTVSAKKIFQFVH
GSCTQLVQCSAQTALRLFLMSAIVADSANL-------RCPGSYEAITYEYLTQALVCYEE
EISDSKSQYNLISEFVGSVVGHIHTLEKDNYENISAKITQHAAKLLKRPDQCRAILTCSH
LFWNNESVRDSRRVLECLQKCLKIADIAVQSSTAHVCLFTDILDKYIYYYERDNHEVTVD
FIQNLLALCAEHVNFALQEAGQEEAFRNTVRYLKRKKEEGAKWRGLTSVSTSMLEAKLQH
ASVLRRLFESIKDMVSDVNLDCDETGLRLQAMDSSHVALVALKLDDVGFVHFRCDRERSL
GLNLASVCKVFKLCSNADSCSIQNEEDSDTVTFVFENEADEKLSSFSLRLMAIDQDALRV
PEDETAHDVTVTMSAREFANVVRIMGEFSDSVRVEVDKLGVKFVTQGDLGVGEVLLKPKP
FANGDDSGVEIKVNSPVCQTYAVKYLNYFAKAASLSSSVTLSLTDQNPIEVRFDILEPAS
GGTASCIGHVKFFLAPKMDDDMDDDEM----SLVGGLFLANSIGSVSATEWCRAAPEDGR
PEYGVCLAAGEGSARYIIEIVPVVGPGIDLQSNVKLTVAGSDGSETKPIPIASEAFGTEK
RVTVDRIDVGDPESVGVFITG-NSAWKCKRITVWKDFRYWLFDCTGVLDPEHRDAIFTMS
GNKMYQAIMQTGNDEHAGTSGGIELTLIGSERQSSPKLLVQDVRPGAERRIRFRAADVGD
VTALVLRNTADTDPWYCEFVRIKTDDGRVFAFNVKRWIGTPYESAIRVSLKPSGDDTPAQ
DVECHTRALELYSRI------PENIGIFKVRCPMNCQASTFASVDGSSIHPMASSICAAA
IHDGVLSPSGGEVVVSVVGELPEYTGSTLNPTGTVSTSFRSSPDIPSASFFVYRTDSIDE
VDKDVRVVDAYGKLSSTGRLEIRRNGVWGSVCKQGDFTVFTIDSARKACHELGYLHGMYL
EDGCDSVEGQYVCAGAKYPVSVAGVMCMGPEKSLADCTFEEPARCADHSMDVAVRCTNTP
PAEP-PLGSLRIVDESGAPATNGVGRLQFYNEG-WGSVCSDGWTRESEKVACLQMGYTGI
KHGGYSDDGCDDVNGVNLCGPDTEKITAFGVACGGEETSLRHCPHEISSDIYCVHEEDII
VGCRGDGDPSGMGLFRTEDIPALSKRSLPPKIELNCGDRPLSQKQMG-GQAGAMFVATCP
EGCSEEPGSLKGTYIYTDDSPICKAAIHVGVV-GPLGGNIVVVLGEGQDSFMESERNGVK
SESFGRYDRTFMVSIPINSVKARTAKKYVDGATYAGRSGGGKF---------TVTLVDVL
PERFRWFAPEDFTGFRGRPEDYVDTEKLPGAKALTGFSDFTFALQMAVT-GKRKWRSILT
QSGCEGFTFAIDDKDELVFEQNCHPKFISTGIKPAIGEPLHIAVSYYSPEKVVNIYVNGR
QVVSEKTDFDFNLKSKLIIGRAADSESEYFIGHITALKVFSYALSPEQVRRVAAEAGSGL
GA---------------GGPVPRGERRTDDGRVCLSPCSSEEPLLNEILPTNPAIQLTCE
DTLRREEFNGITEQRFLVSCPSDCTSKVAQVYGS-KVYSEASSLCKAALHSGAL---ATQ
GGEVIIVTHSGLKSYSGSTGKNGVSSVGEDEPQLRSFSVLKAPNFRKLTCRDDGAFALKM
NPGDKELVICPPGCLASEGTVYGTKVYSPISSVCRAAIHSGHLTNEGGEVELQATGQHEE
FKGSDKNGIQSVNSGWYLRAITFVRGAPGNEGVASSRQLSQPEIEVTVWDGDEEKPIGKR
SRRATSSPST---------STSQRHEKIASLKGLWNKKLKGRLRTQQKLNDEATRRLRTT
ELMLFEEEGGL--------------------------------------LTEGLEKSYAI
PQADIVKEADLGTREKIFSLDLPFGPYAVDFSRNGRHMLIGGKKGSLSLLDCHTFQPLCE
INVKETVRDVQILHNHTMWAAAQKKYLYIYDQQGIELHCLRDHMMTYRMDFLPYHYLLVS
VGEFGELVYRDISTGQIAARHKTRRGPCDCMRQNPSNAVMHLGHIKGTVSLWTPNLGKPA
VELLCHKGRVTSVDVYRDYMVTSGIDGSWKIWDLRKPLHSFQYFG-SPPSSARWSQTGML
AMGFGSHVQFWKDAWSTP--YLTQHYDSKQVESLAFRPFEDLCAVGLTTGIDTIVVPQSG
IANFDTFEANPYETSAQRREREIHSLLEKLQPDMITVKSSRVGAIDSAPRAVKQREMAEK
AEMKKTKKKTKQRGRNTAAKVQKKAVLQYNQKVRTLTAKR----LEQAKAEQKASRSTKD
DRE--QAFSALDRFRNRESAEATQEDKKDKEIEFLALTKDAAELYRYE------------
--------------------------------------HGRPLEDIRKPVATFEGVEQAL
WTADGKNLVLVRKDAPNRVDIVSLDDNKNIFSVTGASAVVKCGFSPRETFLFI-CFRHEA
ANHENFCMYRM------------------------------ADGEK------------VL
SFTLKQINANTWPPLRWTSQESFCCRMVTNEVHIFRDNAFNHPHDRIRCENV--VSFSPC
NEKKKLLTCAVFVAGRKGAPSAVKLFDLENGSSCLCTKSFFQGNEVTFKWSNDGRAVLAL
VHT--DASEKSYYGSDALYFLKADGSYDCQLMPEEGPIHDVQWSPSTLEFALCKGPMPPE
ILLYDGSSAPKLSFGRRMQNTLRWDPFARMLLTAGFGNLAGDVDVWHKQQKKVIAKAQAP
FTVTCDFTADGLHFVAATTSPRLRVDNHVTIHSISGHAVCRLDFPVLYRVLVRPLATLNQ
KQSHLLQAFLADDSQSQAGPAKSVSAPPVSRGVYRPPGSTGAQATVNGSNAASASRASSK
SPPGYSPPGAAAKAASGKKRK-----------MSARKVTLVSQEGDEFDVDIEVASMSAL
IKTMVEEDSDCQESIPLPNVDTCILKKIIEYCEHHHNNPPEEIPKPLKSSNLAEVVSEWD
YQFINSDQKILFALILAANYLNIKPLLDLSVAKVATMIKAKTPEEIRRIFNIVNDFTPEE
EAQVREENKWCEDAMSSPTERVLLASSSPFPPVLSPETPLSGTSSSHLPILDDEAAGVLE
ATAAFVASPDSEDAVRSESTSASQWLRQASVKPVSASAARPLEQITDSVSTRDSRPDDNM
EGSGASETYRDCAVTAWKREAECEPGPAQTEQP-AGYASRERGETCSLSEESATPSA---
PSPLHSSSSPQHGS----------SNASEM---------SPLEDMPSPPASAGLEV-PSV
AGLDGVAGPLEQQPAAGKVLGFSERSMSNAIALGEASSKGRLPGLSSPSSSALGSFASLS
SSLGRRG-------------------DEGSLLRLFQSEYFDAYFHLYYLFHRQEAGVHEY
LVNLLYVKRTDEDIIFYLPQLVQLSLVRFKTSSLHRFLLDKASKSMHLALMASWLYQSMV
EDKVAGLEEPAQKMTQEVEMAVVNSKPLGAQGQCGREERGRNSENAFAIDLFKRRRIVQL
IRQHQQALRESRSAQPASRASAPLPPQSVLSDALDKAQR--------EPTASSPQSSSTA
SDSIGREPLPSVTDQEAASSLYRNASRWSATSSSSAAATSTACASRGSLRRPTVSAGALV
PRIPQNLRHLTGPTTVAGAKVKLPSVYGKLGNPLSLSTLQLLPFSTSREMEEELQQFIMK
QRRCDYFNTLNHFISLLIDVSNALALEPDRSLRPPLLSLFLESLNSWILCRRLYVTAMVG
TWTMTGVTIPFHEVGSSVERGPSGVAVAGATGHSLQILRIDVKECRALSSKKRVPYLLVF
EVADLDEDLTQFEDCQFGATAAAADMVSLREDGSPAEAKTA---------------CHSG
HASPRAQSPFNLSPVS-----------------RRRQLAK-----RGARGERAGTGGAED
GAPALDWSSLYVYQAIVEELKHQKLYEPGPSSEDSVTVIRRALSLVPPDPPSSADAIPGT
KPP------SSSTQPSNGSAVSVGSSVGNKVSSSQSSLLSSSLSSTLSALSSSASSVPQT
ASSAAPSAPSEPSSSSLESFSFSSSFSSSSPGLGAASTPSHPAATADHLRHDRGTKKGDD
EEYRVESEH----AQETAEGTTVWRAK-------------QKKPLWGELWADKRERLRRR
SPYGHLRSWDIRCVLVKGGDDLRQELLASQLVRQFKAIFDEARLPLWLRPYEILVTGSNS
GVMEFVPDTCSVDVLKKRHNTDSIARVFDALFADNPFEAKKNFIESHAAYSLVSYFLQVK
DRHNGNLLLDAEGHLIHIDYGYLLSNSPGNINFETSPFKLTQEFLDVMDGETSDNYEYFR
TLIIRGFLEARKHADRIILLVEMMLSATKMPCFSGGPQYTLDALRERFMIGLPEDTCIER
IVDLIETSINNFRTVQYDNFQRITNGILMPAKVTFKVSGGSQFVLELEPEWTVKQVKEKC
ATQTEIPVQAQRLIYKGRILKDADLISTHDVQDGHIIHLVKSAAAVASNNAPAGGSSSAS
QPSSDSSTNTPP----TADPFLQMFMRGGMG-AGMPGTGMPGAQDSTSGMPGMPGLP--P
DMNPDTLMQLMQSPLLQQTMQNLSQNPQMLRTMMESNPMLRQMM---PMMQNVLDNPELL
RTFLNPQMMQASLQMQQAMQNMQRAQQSTAQGDGGATAT----------PGAGLGAATGG
AGQPDLMSMMQQAQQMLQQVSGGMPGANPFMG-GFGSMGSFGSMPGNPAADTRPPEERFA
RQLESLREMGFIDRDANIQALQETGGDVNAAISRLLERGIGMKKGEENPMRKIRIEKLTL
NICVGESGDRLTRAARVLEQLTGQRPQFSKARFTIRSFGIRRNEKIACYVTVRGKKAEDI
LEKGLKVKEYELKKKNFSDSGNFGFGIQEHIDLGIKYDPSTGIYGMDFYVQLTRPGNRVA
HRKRARGRVGHSHRVTKEDSIKWFQQTYDGIVLNLCYEGSAFLRQRLLLSTLTGQPVRIS
QIRASASG------------LSLPGLRPHEASLLRLLCKVTVGTRVHIDATGTVLTYVPG
QLLGS----DEASPDRLAHTCHPGRGLSYYLEVLLLLAPFCKNPLSISLSGVTDGSVDPP
VDLTRAVTVPTLQRLLALVGLSLEVKILRRGFPPAGGGEVSVHCSGVGPLHPFALVDVGR
VKRVRGVAFAENVSPLLARRCINRVRHVFNAFLPDVWVYLDVKKKTAKKQEKRRGVGVAL
VAETITGNVRGA--------------FSCETFHESDGDKGAEAGDCARATGNCKLTRLLE
KEAREAQREQ--------SADGSEELGQSEKMGQDAARRLLLEIMQGGVVDTHHQYMAL

```
LFAAAAEEHQPCKLRLSRLTPYTTQFLRHLRDFLGVTFHFEETNADTPE--VLLRCVGVG
LRNTARKTF-MNAAGSNVSKEFFHLIKAIGEARSKQEEDRIVANEIATLKVKLNEPNISQ
HKMQELLIRSLYVELLGHDASFAHFHAVKMTNQNNIKAKRLGYLACNLFLSEDNELMLLL
INTIQKDLASPNILNVQSALHCVARLLTPEMLPALLPSLSSLLQHSHAAVRRKAVMAVHK
VLDLESEVMEGLREKMRRALCDSDPSVMAVSLHVIHRLAAKNVAAWRDLVSSLVSILKQI
IDRKLPKDYEYHRVPAPWIQIKILSLLSTLAAGDQRASEEVYELLQEVMRRADAGVNVAY
AVIYECVRTIAALYPYPKLLDIAGCSISRFISAENNNLRYVGVTGLAAVVQVSPAYATQH
QLVVVDCLEDSDDTLKRKTLDLLVKITNPVNVAVVVEKLLGHSRATVDAHLRANLIQKII
MLAERYSPDPRWFLETILCVLEVSGPSLPYSTAYSLLQLVA-------------------
-----------------------------------EGPTDDEESDK--AFREYAVNDMVA
LLERKKVIPDVLMQVISWVLGEFGCY----YHAGESVIDLLASCLERSYEDP----AWIL
TAILKLCVFYGRCASVSTVHLLQKQQRQLVTEVQERCFRGLLLLQSWQVLREIFPFDAST
EDVHVDRNLPFLNAFVEESVAAGGKRYVSRHQRVKEEGDRAVETVAPSLNFAPYAPPR--
--------APSAAVVGAQARGAEGMSPSGMAAAVRGPEEAEKEGGFQVHGPRRWGPTGYR
SAASSVVSPSADPADSS-----------SRASSSPQAAASVETAGRQPLASHQ-------
------------------------------------------------------------
---------------------------------PRAMTARELTAAALFSGTAAAPVTRGT
SGGASFATSSLPRSPVSSSSGGRRAAEREAQAGSEDARGEASEATSSVDLLELESPGGGT
EPQPHGDRGRKSEKRETACLSLDFLSNGEHADKGRRTVASLLPTARWASLPGEASLSIPC
VSASGVTRSCQEFLTNVLQQAIALHVVEAAQTPLMDAYNGSAVVAMAGKDCVGIASDTRL
GVNQFGTVSADFQKVFKMNNHTFVGLAGLATDVQTVHKELVFRSNLYQLREETEMPPEIM
SNVVSSMLYGRRFAPYFVSPVVAGLHPE-----------THQPFLSAFDYIGAACYAKDF
VCNGTSAEQLVGVCESLWKPDMNEDELMETLSQSLLAAVDRDCVAGWGAVVHVLTPNTIT
TRRLKCRMDPASSVSAIFSSSAPLRLSNVRVLVVGAGGIGCEVCKDLLLSGFRRLCVVDL
DTIDVSNLNRQFFFRNAHVGLSKAFVLAAACSALLTVWGVVGQKMNILDFTIQLLQTYDV
VISALDNQKARRHLNGLCIAADLPLIEAGSTGYSGQVMPILKNETLCYDCEAKPRDQQFP
VCTLRQRPERPEHCIAWAKMIYELVFGVEDNENLLDLKRTFLDVSSTAEAEGREMSRKMM
KELFHDQIVDLLRLSKKQEVLPTPLCVQGLTDKETFEASHASEDAKTTNKGPKQNAAPTE
N--------TQGEGSGLESQRTWSVQECQEVFERSFLGLLERQKTTERREAGIPFDKDDD
LAMDFVAAAANLRMHNFHIALKSRWFIQAVAGSIIPAIATNAVVAALQ------------
---------------------VWVKPFVTGVRPDAAGRLILPEVVDPPRASCFLCQQQTV
TIELASLSAWNIETFVERIVKGELGLAHPYLDSESRNLYDVDEVDEQREDGTGEGKQPLT
NFGIVSGTILTATDFSRGDFQCNLLILERSEEKNKDDPHIFRLVRESGASSTAEQVSQGD
KATAEQKTQEKRPRDETSQATSTEEMGKLSKDRRDIYYRRAKEEGYRARSAYKLLQLDDE
LHFLSPPETEERETRNDGKASRKNAESLCVRAVDLCAAPGSWSQVLRRRLRDNFRRKLAR
EKQVSEQARDSENSPSSTSSSSLSSPSLPAPPLIVAVDLQEMAPIPGVHALQADITHEST
VKAILDFFAQ-------------------QPADLVVCDGAPDVTGMHDIDEFIQAQLLFA
ALRVACKVLKPGGVFVCKAFRGEQIPLVYVQLKTLFAEVRCCKPAASRNSSIEAFLVCKG
FELPVGLDACIAAESGD-------------------------------------------
----------------------------NNREQDSEQEAAQGCLVPFLSCGDLAG-----
--YDADRNYPVDDRHVFLPPTQPPVHPPYEQALHLKRGSA--AAEFDGGRVLVLDFGSQY
SHLIVRRLREIGVYSELRRCDIGLQEIKGFSPSAVILSGGPASVYEAGSPHLCPSFFAWA
QEAKVAVLGICYGMQEICHALGGKVEGGEKREFGSTWVELHLEEAAANGG---------A
VALFAGIEERKEMLVWMSHGDKVTAIPEGFTTFASTAACPYAAIGDPERHFYGLQFHPEV
THTPQGTQLLKSFVLDIAKLKPSWTMKNFLQAEIRKIQTLVGDAHVLGALSGGVDSTVAA
ALVHKAIGDRFHGVLIDTGLLRKDEAQNTLAKLKACFPSLSIECVDASEAFFSKLAGVRD
PEKKRKIIGNLFVDEFVRVVNEKKIHTHNTFLLQGTLYPDVIESCSFKGPSHTIKTHHNV
GGLPEKMHLKVLEPLRELFKDEVRALGLELGLPHERVFRHPFPGPGLAVRIVGEVTPERA
RVLQEADAIFIEEIRKANLYDKIAQAFVVLLPAPSVGVMGDCRTYEWTCVLRAVETTDFM
TADWVRLPYDLVARCSTRIINEVKGINRVTMDVSSKPPATIEWELGSSSPSKRRCDDSKD
GETPSFGGRTSCVCLVRAPIMPLLSVEKG-PSILFRPFKSPLKGHQPGVSESLRKTLGVR
LKRSSA--AVASKQFKSPPPA----PTSQALPEANPLVLYIPPPDKVEVDPMLTRWLREH
QRQGVKFMFDCLMGLKEFQGEGCILADDMGLGKTLQSITILWTLLEQNIEGQPAVRRAVV
VCPASLVNNWAAEIQKWLQGRCGCTPVADNCKEKVVSKFEGFKYDRQSRILIASYETFRM
HVHRLEGVPIDMVVCDEAHRLKNDKTKTSLAIIELPAKKRLLLSGTPIQNDLDEFFALVS
LCNPNVVGDAHTFRRRYANPILVGREPDATEDQQQLAAERLTELSSLTNLFILRRTNSLL
AKVLPPKVVLNVFCRLTPLQKEFYRSFLSSKSCRKMFTAEATGRVLSSIQGLMKLCNHPS
LVKSQALQGFEKC------EKYFSELD---------------------------------
-----------------LEGQKARSRMVRTEISGKLLLLARLLDVIRTTTNDKIVLISNY
TQTLDLFDRMCRDCGYPVMRLDGQTSIKKRHAMITKFNDNPHSFVFLLSSKAGGCGVNLI
GANRLVLFDPDWNPANDKQALARVWRDGQKKSCYIYRFFSTGTIEEKIYQRQICKDGLSA
MLVSDENQIKDSLSTELVKDLFRLREDTLSDTHDMLECRRCGCRAKRRET--SVPQLEDF
DEDDLVTWAHHRDLASVPDPCLRQAALACVDPLACDREDE----EMISSEISFVMSCRIE
FKDEGKPQSPAAAAPVCGKSSAEASARAPASPVALSSKKRKLADSEEDVDETSSEEE---
----------------------------FPPSFFLPRLRDFQPTEGDSSVSCPPQVLCSL
CT--AQRRVEAAGRAFLERFERPSRKAGA-------------------------------
-----GVSPGRAKDKDAKRGDESDEDFSAVDESKRKGKTAACFASSTRLKRLLGEAQTLY
EVLGVHEGTTTEEIKKQYRRLVLEHHPDKAVTRRSPGSSEEGASGRSSPH-------AKE
KREELTDGQATSEASADAGHARFLKIQEAYEALTDTEFRRQYDSALP-------------
-----------------WSSRRPVPSLGDAQTPMSRVRSFYDFWFDFQSWRDFGVHDEYD
LNEAECREERRWMERENLKIRKKHVKAERARIQKLVETAYSVDPRVLMEKKKREEEKAAR
QRVAEEQRRQREEEEQEAEERERREARTRELQEQRRQRECHKKWRQRTRQFCRDSHPQFL
DSLQLQDLCQKLDLHQLRDLCGEIQKVACIETPLVDES----------------------
---------------------------GESPAPLPEGLGEA--SPETCQKVGDIFARRMH
QMK-------------------------------------EADRIKEEEQMKEN----AR
RQAERKAQEEAKRLARQSSWTPDELSLLAKGLQKFPGGTARRWKLIADLIGTKTQEEVVE
KTKEMSEGASLKAMGSKISQVAFDQFRVHNQGAFKKIDADPDRKDVGETRPQTAASPAKE
P-QETAESTDWTPAQQMALEKALAKHPATMPANERWTAIAAEVPGKTKKECVERFRQIRA
AILAKKSS-MPTKRRNGGRAKHGRGHSAVVRCSNCGRSCPKDKAIKRFNVRNIVDASSQR
DLREASVYNTYTLPKLYIKQCYCVSCAIHSRVVRVRSVEQRKVRENPQRRPQGGQQMAHS
FARGRP------------------PAARTLAVFLAFLAVLFFSSSVLVANAASYANKKVS
VSEVSFDSHIEDIQWCGTDHRTILLKTRRGRLYRSQDGGKSWTEITDLLK---------S
SEAATGTVAVDSIIVSPVDKRVVLIVGSKRNHFISEDSAATFRRLKYKNTIHNFHFHPTR
PKYAILSTWTDACYSGSGTASDCNHQLFYTRDLGRSFKLVADYVVQFSWGDKKLGNTDHI
FFTQHRGRSGDQPRYGGWSKNVDLMYTPDFGATITRLVYRGNKFLLSNGYFFVAKVKDAA
KQTVSLLVSTDGGKSFQMAKLPVEIEERSYTVLDTSEDAIMLHVNHG-HDNKGDTGNVYI
SDAKGVRYSLSLPNNIRTSTGECEFDKVLSLEGVYLANFKDSVDSSASVDGDLEKLEEEI
EEEAEGVQVDLEKKHKSVTRSRQEEVIRTVISFDKGGVWSYLKAPKVDSRGQKIDCPDRC
WLHLNGITRFSDFAPFYSVENAVGIIMGTGNVGSYLRPEKDEANTYLSRDGGVSWIEAHK
GAFIYEMGDHGGLLVMADDTKKTNQVVFSWNEGQSWYDFELGAAPLFVDNIVIEPNASSV
EFLLYGKRDSAGVLFHLDFNALNQQQCKGIWAADSVSSDYETWSPSDGAGGERCILGKHI
TYTRRKQTSECFNGRDFDRPKVSKVCPCTMEDYECEFGFTRAIGSTQCVATDAAAAAAAC
TSSSFFYTSAYRKVPGDVCEGGWMPEKVAVPCPAHSPVSRGGKTVLLLLLFIVVVMVVIN
YLAKTGRLKKFFRNAGFDSFANVSYGLVGASAGGPERSKYEPELGFIEAEQDENEEDAPT
LMNYGNAAGGQRTDFELDDSRPLFPSHVSSRELAPPRFDEDNVELLGMVAMADRKAVIKN
ADMPEDLQQDAIDCANQALEKYNIEKDIAAFIKKEFDRKHNPTWHCVVGRNFGSYVTHET
HHFIYFYIGQVAVLLFKSGALLYSSALTLRPPDQAYLSRQPQGESKSFTPGHLEDQVPTS
CKQAIPVFGCSTRVRLAALLDGGEASVEK------FVNRETPVTVCGWSRSVRKQGGGSL
CFVVLSDGSTSSNLQVVVEAGIGGFPQLLKCGAGCSFRFTGDIVKSPAKGQ--------A
VELAVRDSKGHRFEILGMT-DAAKYPLAKKEHTREYLREIAHLRPRSYLIGAVMRVRSNL
AMATHRFFQDRGFLYIHTPIVTASDCEGAGEMFQVSTLL---------PPPPPETRENEK
KTDG----PL-IPLTKDKKGVDYSRDFFGRPAFLTVSGQLAVEPYCCSLSDVYTFGPTFR
AENSHTSRHLAEFWMVEPEIAFATLEDNMVVAEAYVKFCVQWVLDNCRADIEWFQKNQEE
GLIARLENILAEPFARVSYTEAIEVLKAEEAQFKEKVEWGMDMGSEHERYLTENVYKKPC
IVYNYPKDIKAFYMKLNEDGKTVRAMDVLVPKIGELVGGSQREDDRDRLAAMIAAKDLDP
KPYWWYMELREYGTIPHAGFGLGFERLVMLVTGIENIRDTIPYPRYPGHAEFM-ATNSDV
```

SVSAEEQRREAKALQKLEELGIGYQLHRHAPVATVDAMVKASFRDKGVIAANLFLKEK-Q
RFFLLTVTHDLPIPLKGIAKLLSAPRMRLADEELLGPMLDVSKGSVTPLAAMCDEKKEVT
LVFDSAMKKNPDVLVHPLHNKATVALKASDLVKFVEACGHSVMWLDVDEAVKLA------
---AAPAGGANKPNAPAGKAPGAKEAVTDSSMLGVTAKKDENFSEWYTQAIVRSEMIEYY
DISGCYIMRPWAFHIWEKVQRFFDDEIKKMGVENSYFPMFVSRHKLEKEKDHVEGFSPEV
AWVTHYGDSPLPEKIAIRPTSETIMYPAYAKWIRSHRDLPLKLNQWCSVVRWEFKQPTPF
LRTREFLWQEGHTAHATEEEAWELVLDILELYRRWYEECLAVPVIKGEKSEGEKFAGGKK
TTTVEAFIPENGRGIQAATSHLLGTNFAKMFEIEFEDEEGHKRLVHQTSWGCTTRSLGVM
IMTHGDDKGLVIPPRVASVQVVIIPILFKDENTGEILGKCRELKTMLEKADIRVRIDDRS
NYTPGWKYNHWEVKGVPLRLELGPKDLAKGTARVVRRDTGEAYQISWADLAPKLLELMEG
IQRSLFEKAKARLHEGIEKISTFDEVMPALNRKHLVLAPWCEDPESEEQIKKETQKLSEE
AGDSEQVMTGAMKTLCIPFDQPPMPEGTKCFYTGKPAKRWTLWGRSYMAGPGKSDKRKTY
FSRLFALLEKYPRVLVVEADHVGSKQMADIRLALRGKAVVLMGKNTMIRTALKQKMSEMP
QLEKLLPLVRLNVGFIFCIEDPAEVRRIVAENKVPAPARQGVFAPIDVFIPAGPTGMDPG
STSFFQALGIATKIVKGQIEIQNEVHLIKEGDKVTASAATLLQKLNIKPFEYGLAIQHVY
DDGSVYKASVLDITDEVILEKFRAGTMNVAALSREVGFPTTASAPHSILEAFKFCTSLVL
ESDYSFPQMQRIKDILENPEAFAAAAPAAGAAAAAEAPKE------EEPEEEEDDMGFSL
FDMPPRHDLTREPCPGRILEDLGGAFGMGALGGFLWHFAKGWRNSPKYEKFAGGMLSGSM
KSPLVGSSFAVWGGLYATFDCSLIYLRGKEDSWNPVLSGALTGGVLSMRSGWRSCMKNAA
IGGVLLGIIEVVQLAFQRSTGPTPRQQYRQYLEMEQRRAAEQAGMDSSELWGVPDSRKEE
EEDDDEEEPNVTEDDKAVRLFDFDAQGKKISDRVYRAGTTSSESSSTSESEVSDESYEFD
DEDFAELQKLRRNNR--PGAPMDPMSRLRMWESSFFINSFMNLENKQQYYFYTVTFGHVK
SRRIQGTLRRRTLYTPGYMLRPGEFKALSAPLVIWPFKVFLLPYNQLKSFCVTIEMWKIN
ELSFNTLYASAKLTLQEIVDSEQEFQILLRRKIAGKKRSYEVHKVRVSLMLSEVFDIDLS
FDSWWFLPDKAMPQKIREVPKQLLFSVPVRSGDRNRRKTPISQMNFWPSAGLFRYRGSLQ
SISNNYITVTLLYSRPKEWYRPPAHLGLCIMALKSVLQYPLFRGIVKKLTTDPRKFQQGE
LVGNIRCFIRSVGIHEYEEIPNRPAQPLAGAALVTQLNLREQYLVVRLFKCEHLPAANVD
SYSSDPIVKIKWDAMVNTSNKRENTLRPVYNQNFYFPVRLLDPRERSNPTLKQTALPLDL
YTKGPISFEVWDNDELTSDFLGGAEVHLNAVWREGQWEERCLAEGMEDDKTRLPDGRFAT
KRHEGEDDDDEEEEDPYEWPYRTLVLRKILELKGSALPPSQQGKSTLWFEMFFIPPMPDN
VELPDPPQVQSSNDIWSQVTKKWNSDFSKYQKMYSEWFPDAPTDRRFLCTAEHVQTRSTF
PLPSFLAPIAVPSQISPEGELLHWINSITFLSPPKQMASWQVPSSILITRKG--------
----------------GTTNDGQDEHVWVMTRHAGGWVMMWEVTTKKRYSLPFRWGYPP-
---GGPPKGFAAALEHQQENAEKYWEGLYLQEWYQWSMQQAQLAHAEAVA-----VANAV
PDVELYGDQVVADELVDPEVLLDEEAQNIDPEAQRTTGAVKQKIDKDKMMKLLRSQIEHL
PITPVKTLTDPNKTLSYLPYSSVEVVFNNFQLWGNLQNHHPACITYDMEDEWKWRPLLME
PADPIDSDVLLAPPIRDKKCQLLAEDLASSVLEHIRLHRMKKGLEVFFEHREDLLFRLTF
YLDLLEYRMHLDDLHKPDPGPNHMGWSSFLQETDCQFYQTISQQTTLTSEEK--AMPGTQ
EALHMYAEYGGDAY--CMFANVPP---------------------------PGGFFDPFA
AGEAANGWSVQ------------VRKPPAPELAMNP------------------------
--------------------------------------VEAYEQQVRKKLCNFMRCSRAV
Q---

--------------------------------MALAIFGDRQSGQDVRTANAAAVQSIAN
ILRSSLGPQGLDKMLVDDIGDMTITNDGATILKQLEVQHPAAKVLVELSDLQDKEVGDGT
TSVVLLAAEFLRVGNQLVKEGVHPTAVIAGFKLAMKESVKFIQEHLTSRVDANNREVLMN
VATTTISSKLIGTETAHFADLVVRAILSVKMITERGDVKYPVSSINIIKTHGKSMRESSL
VEGYALKAGRAAQGMPQCVKNAKVALLDFNLRQHRMQLGVQIQVDNPEELEKIRQKEKDI
TAAKIQKILASGANVILTTQGIDDMAMKYFVEAGAIAVRRVDRKDLRRIAKITEGTVVLT
MATLDGDEKFDASCLGTCEEVYEERIGDWDHLMFKGCKGGKAATVILRGANEYMLDEVDR
SVHDALCAVSRALEYTHVCPGGGAVETSLSVYLENFARTLGSREQLAIAAFAEALLIIPK
TLAVNAALDATELVARLRAVHAKAQGAAGNGDEELKWHGLDLTSGKTRNNMAAGVIEAAV
SKTKALRFATEAAVTILRIDDLIKIAPEPERGQQDMDVPLIKNLYAEAWKQQYSDLRLST
KQTESCGLAANTEYIAAPWDVGGGGVLGILRLADIGRNPAVAKIKGHTASIQDTNFSPFH
RDILATACEDTIVRIWQLPEEVTGTTELKEPIATLTGALKKVLSAEWNPAVSGILASGCF
DGTVAFWNVEKNENFASVKFQESLLSAKWSWKGDLLACTTKDKALNIVDPRAAQVVGSVA
CHDGSKACKCTWIDGLAGRDGHVFTTGFGKMQEREMAIWDTRKFDKPVYHAEIDRGSSPL
YPIFDETTGMLYVCGKGDSSCRYYQYHGGTLRSVDAYRSSVPIKNFCFIPKLAVDQMRAE
IGRMLKQENGNVLQPISFIVPRKNQDVFQADLYPPAPDVEPSMTAEEWFKGENKAIRRRS
VKRMTVDTACVAAVAQAHGAAADSQALQE-LQSEVASLKAQLTELDRLRKENEELKANG-
GDTAALLQENQELKANELETLRKENAELIKELSAQSAMAVQLKMRVSELAEALSNEKSTT
AQLEARLRDLEGRFISAAKSQKAAEQEAMRLHAMSSLVAEAVCSQQVVLGLKSICKELVE
NAIDAGATTVEVRFVDGGMASVEVRDNGSGIAPQDFPMLGRRHATSKINKFTDLYSALDT
MGFRGEALASLCALSDVEILTRTASEPFATRLRFDHHGKIIHQEPAAREVGTSVTVSNLF
ASLPLRRRALRQQRQKHLQESLAFLQKFALLHADCRILVTDFTPARNGGSSVTLLNTRGA
TRLLDAAVTVYGERQMQQCTQVSLAGAEWSVEALLSRPPLGVRTSALQLFFVNKRVVEFP
PLLQKLINKKYREVCSHCFPIVIAFATVAPHLLEVNLRKDKQEVLLAVEKEITEALLVGF
YFFLSFPASLLADSRGGPLHCICSFAVPPSYSFSMFFQKTITDFVAPTVASFQTVRGFSQ
LPRAAAAKRETDRQEEDGTSRGESTRQRAGVDEA-------------------RLRLAQL
GEQSGE--TRDLKLSGDLDRSTREPTSVPEKENVADE---TFISFSSVSSPPFLSASSFP
ALDSTVPSNLSALEGETRDVHDDHDE-EGDRTRRRRATSP--QTQCSSDEENLPLSVRLE
KPTDQFHSSSSTACFSSSSSLVSQESSSASKKHPSKPKDGGAEREREEQEKADAKGKRSE
EDADEFVSESKLLKETVASAVAEC-------HCTEEDLEALYGASDAETGDETKHEAV--
----SSSSSSPASSSSSSFSSSSASSSASPCASSFRFSDCPAA--FSFASSSDTQIGAFD
PGTSSMFLDKRAFREMKIVGQFNQGFIIGCLQVPQTPDHVSPSASSSSSSSSLQSALPAS
KREVRSLFIVDQHASDEKKRFEDLNEGFKPATQPLLIPLRLHLPVDMARAVSDFDREIRE
NGFRVTIAREPAREERDACAGEDAADASVEDELVISLSALPVVEGRQLKEEDFIEFLAAL
RRRRKSEKRADEDDSDEGEEESEEDDAHAFHHRLCRPKKVWEILASRACRSAIMIGDSLT
VNQMQTVLKNLATLHLPFNCPHGRPTVRHLFDVEQSTRIGKDLSAEQHRELTKAQAHRQE
EKDHQAIFAEEKGLFDEDDPDVLSFDGHKMGTVEERRNIKREVYMIYPGLEQQLEMAFTC
HDLQHEGKLPYTTLEPIIRHLLMQYGLIEYVTRFSDSEGCLDANQIRAELNEFGVNTGGL
CCGSS-LTLEDFKSLAVIWLRKILDCHADDQAVWMAKLKAEQEEQAEAYTRAMREFQDTF
TQQHALYQQGLQEQQKQINDWNKLLEDAQKTQQEVRRMEEARLKEATAAEEAMKEQIDLI
SQYKEKLEKIAAAD--TSGKCFVYPAAATPYGACASAGAQEPTRRRRVKKEHPSR-ACCM
TTLEQNPADELVDYEEDE---------QN------DAKEKGVEDVVVGRGNYVSIHASGF
RDFFLKPELLRAIGDAGFEHPSEVQHETIPHAITGVDVLCQAKSGMGKTAVFVLSILQQL
NLDTSGE-------------EGNTQGVVCLGIAHTRELAFQIKNEFDRFSKYLKNVKCEV
VYGGISIQKNIDMLKDKTTPHILIGTPGRVLALIKGKHLNAEKVAHFVLDECDKCLEKLD
MRKDVQNIFMATPKKKQVMFFSATMNKEIRDVCKRFMQSPVEVFIDDESKLTLHGLLQYY
VKLQESEKNRKLNDLLDTLEFNQVIIFVKSVSRAQALDRLLTECNFPSIAIHAGLDQEER
INRYQQFKNFEKRIMVATDLFGRGIDIERVNIVINYDMPDSSDSYLHRVGRAGRFGTKGL
AITFVASQDDTNVLNDVQTRFEVHIAEMPQSIDASQYINQM-------------------
------------------------PQPSEAYMSRAKNRQSVSAEAYGEWNKRK-KFVAPV
YPKTAEQKERITKVIESSFLFSSLDIEDLETVINAFQEVSVKKGTVIIRQGDDGDRLYLI
ETGEVDVMKKFGEKEKFLCKMHPGDAFGELALMYNAPRAATVIAADDMLLWALDRDSFTN
IVRDAAAKKREIFEESLKEVRILEDMDPYERSKLSDALRTATYEDGDVIIKEGETGDTFY
ILLEGAAEAIKNDKVVMEYKKGGFFGELALLKDQPRAATVVAKSHVQVAYMDRKSFKRLL
GPVEQILMRNQDNYRKAMKQLGLDTKYLDK-MLLRVQALTPEVRAMYAAHGHYHEGDSGV
DLFVVQDQEIQPGETAFVKLGIKAAAFTP----------------AEGGEQEKNVSWLIM
PRSSISKTPLRLANSVGLIDAGYRGEIMAAVDNIKTVPHTLKKGDRIVQAVAFSGEGITL
ELVDELNKTARGEGGFGSTTCKMLQCDPNSTFFGFMGITAAMVFSNLGAAYGTAKSGVGI
SSMGVMRPDLVMRSIIPVVMAGILGIYGLIISIVINGSMDTPDTYSSYAGYGHLAAGLTV
GLSAMAAGLAIGIVGDAGVRANAQQPKLFVGMMLILIFAEALGLYGLIIGLVVATKKVDG
LCSSYKAMMDSCMGDEDQSRKDFQETGSASRGDSVAAPSEEEESPPAASAASDRPSSSQP
RTTSSAAAKK----QSPTVRFEVRRLLVPPHRMSPLRKQWTEILEPLVTHLKLQVRMNLR

RVELRTDSVQANSSLLQKGSDFVRAFLLGFEVRDAIALLRLDDLFIESFEIKDVKRLNGD
HLSRCIARLNGREGKTKYAIENATRTRLVFADSRIHILGSFENIKLARHSICSLVLGAPP
GKVYNHLRTVTRRLAERLMGNQCCAGRDNAYKRKAQENEYGNRGSFRHMLSFVGDDVVRF
DRAYDNNDIAEFVRLCSSTCEIEKLEERMHPWAADPETIGALAATQLAIFSSREQEPHMK
DEIREAGGIEALVKLLASKELDRKHAAVVALSFLSVDNVENCIAMYNAGALPYLIQGMKG
DIEGMRAACAQTARNIYVLDVKYRREFMKNGGVTQLVRFLDINPE--ATNVYTQLEAIYH
LEDLIGDENDEIPEFVQAVKAAGAIPKLKKLQDCKDQDVADAANLLLVRLSEMADGWDAE
KIFNTVFGFTYDDLILMPGHIDFGVNDVDLSTRITRNLHVRTPIVSSPMDTVTEHRMAIG
CALMGGMGVIHNNMETARQVAEVQKVKRYENGFILDPFVLRPSDSVADVYRIKEKYGYSS
VPITDTGMLGGKLLGIVTSRDIDFLTDVHTPLSEVMTSDLVVGHEPVQLAEANELLRESK
KGKLPIVNDNFELVALISRNDLKKNREFPLASKDSNKQLLVGAAVSTKPHDIERAKALQE
AGADVLVVDSSQGDSIYQVDLVKRLKAAFPELQIIGGNVVTARQAKSLIDAGVDGLRIGM
GSGSICTTQVVCAVGRAQATAVYHVCKYAHG-DLPCIADGGIQNSGHVMKALALGANAVM
MGSMLAGTEEAPGEYYFHNGVRVKTYRGMGSLDAMSEASRTSAARYFAEN----------
QTIRVAQGVSGCVVDKGTVMQLIPYVIQGVKHGMQDIGARTLRDLHAQLVGGELRFDVRS
GAAQREGDVHDLHSFERKLMLRPSSVSSASASSHASAPSSFASSPTLATSSSSRSSHHSS
SRTHRSRRSGSSFIKSVLAQVKQDMERNHELKKAMEDLK-ASSLSQKSEKLSSRVQEAAA
TASALASQMANQTQTLWGDAATQASRIQEATEQNAALKGVAQAVGRARQR-LNHALSKVA
SATEIFDDDDAEKKAAKWRQDMAVKRFKEQEAREQELAQRPSDSEAADEGAKKPQGARRT
EEEEPREDALVLSQRTSWDRFSTKFKEMSFLQNFFENPLVAQLFGETEIAASIREMKILD
PKFKLADMHNMMERVIAAHIVQAFLLGDEGTLAVHCAEGAFAAMRASIIERRAQKVRLDS
EILQLGNVELVGARRSADECPWFVYTFTCQQVNCLRSEDGRVVEGREDDIRRVVYSIAVS
KHPKPETEGLLYPWMIREIAIIGSEAVWMPELACKMCGVDDSGNLLHPGCVSLGGLYGLN
QEEEKTVQEELQSLQERLQKLLVKMGRGGPVSQREPARVPEVVFQNILEKEIRSFQDLVI
DEHKEKRKLFRQLAGGCRRHVETVEKKKQMKAEEEERRLRAVAKSTCGPVEVFWRRIERL
VWEREKRQLQRQLHEKKKQRLDRLVSEAMQAAQHTSETMSSRRNSFVSDEEAKTERQGPR
SESTEGGEEEWTASMFAKDQEEEDDRLEAEMEREDEQEDLQSELQGLQDEASIPVEELLK
RTYGVEGGKEKAHRADKDEEDGEEEGEEGNMEKEEEDEQEDTEASGGRGQADRQEEDGGA
FGKQKEEEELDAAMEAEEEEE--EEDELKRLQEDAELPIEELSSEDEVVIPVQRSLRRRR
QRGDSSCEVKQEASVKPSKAEVVCVCSTEEENLTGAPALVRATLRTYQSEGVQWLFALHD
KGLNGILADEMGLGKTLQTIVLLARLALERGVWGPHLIVVPTSVMLNWEREFFKFCPGFK
VLVYFGSAQERAKKRTGWSRPYAFHVCIASYSTVVKDAQIFRRKKWYSLVLDEAQNIKNF
HSRRWQTLLTFNTQHRLLLTGTPLQNNLAELWSLMHFLMPTVFQSHDDFKEWFGDPLTAA
IEQEQVSEHQQLLEKLHALLRPYLLRRLKKDVEKQMPRKYEHVVRCSLTKRQKCLYDEFM
QRRQVQQTMAAGNYRGMMNILMQLRKVCNHPDLFEPRPIETPVGGVNALSYDIPAMICLE
ERFRRVTLPIISLIHYEFSSLQGLAQNLSPLRLLAPA-----------SSSSLLQFSPF-
---DLLSAATPLEECDLLSLPQPVYTRRLHADNSRNSSASSSVAAGFPALSPHVCVDMRV
QGCPGSLAPCASPAAATASGVSPNSTFQSHSSSHCSLLSGSREAVPPLRSAERTFSWVHP
DADRTPSGVHASSVALGEAALAGHDVK----NEGKQPFLSDASGSAMPPSSVLPLSATKR
RRLMTAAMSPLLRNCFPVPPAFVSHLMRQQEDSEKDGKVMEDEDLVLNDAEAAALSSGLV
DATGGESFLDSFTPGESEFTAGEDRGMHKDTQTTFLARPAEPFSAAVDANEFWRSEEMRC
EESLEFLQGSAAELHEAVERQRRIFPHKQTLQDDCGKLIVLAELLTKLRADGHRCLLFTQ
FSKMLDVLESWINHQGFTYVRLDGSTKVDQRQRVVTRFNANPRIFLFISSTRAGGVGLNL
TGADTVIFYDTDWNPAMDRQAMDRCHRIGQTRDVHVYRLVTEHSIEENIWRKQLQKRLLD
EVVVDRGLFTMENTTRHLGQQTQDKAREWFANAETLKDLLASPEESGFKDDIYADRILHD
SAEDPRSGKREFEAAILEVEDVEDVAAMQQTTREEKQAKQELQQDFRGTVDLNEMPALAA
YCVRLINENKPPSLLAQIAQLKTQVRAEGDEDEEKPNDEDRQSESEEPQRSSEDDGPALW
EMVLIIVGLGLSDEKDITIKGLEEVKNADFVYLEAYTAVLGVGPPKLEEFFGKKIIEADR
TFVEQGSDEMLERALSSNVAFLVVGDPFCATTHADLYLRARKKNVTVRVVHNASIMNAIG
SCGLQLYRFGETVSIPFFEESWRPDSFYMKIKKNKEAGFHTLCLLDIKTKEQSVENMMRG
RQIYEPPRFMSVEAAVRQLLEVEDKLGGKVCPRDAKAFGLARIGAPSQQITSGTLEELLS
VDFGPPLHSLVICAPTLHEMEREFFELFSAKV--MGKPNGIRTARKLRSRRRVQKWADKT
YKKAHLGTRWKANPFGGSSHAKGIVVEKLGIEAKQPNSAIRKCVRVQLIKNGKKITAFVP
RDGSLNYIDENDEVLVAGFGRSGHAVGDIPGVRFKVVKVAGASLLALFKEKKEKPRTMGI
KGLGKFVGDFAPRAIKRQEPGSFTGRVIAIDASMSLYQFMVAIRDGNSFGNFTNDAGDCT
SHIAGMLNRAIRLLEQGVRPVYVFDGKPPELKSGELAKRRELRESAQEAAEKAREEGNVE
ELRKQIVRSVRVSKQHNEDVKRLLRLMGLPVVEAPCEAEAQCAELTKNRKVWATATEDAD
ALTFGATRLIRNLTFGERA---SGSGASATASGILVIDLPTLLEELQFSQEQFIDFCILC
GCDYCGTLKGVGAKTAYSLVKEHGSIEKILEVVDPEK--VPDGFCFQEAREFFRHPEVTP
ADRVHVAWGEVDVDGLKAFLVQENQFNEQRVENYITRLKKARGKTAQTRLESFFGATVTK
SSSLMHKEKQKELEKKKNKGKTAGARPESKKECERERLEKT---ETANAAKGEEKDAKGE
PTAEKEKVEVKEETADAAQDSGGQEKNSETTSTDKGKRMSLLFSENASVPSPRG------
ETN-----LFAEGMYFGSFPFDDMRGPRRGGSKEKNNTKFYEILEVDRTASVADIKKSYR
KLAIKHHPDKGGDPEKFKEISRAYEVLSDPEKRRIYDDHGEEGLENGGAGADPTDIFDLF
FGGGRPSKKKGEDIVSAMKVTLEQMYSGATKRMAINKDVLCKQCNGVGGPADALTTCHDC
DGHGVRVQTRQIGPMIQQTQSVCPACKGAGKSMDPSKRCKSCTGKGVVKERKILEIYIEK
GAKNHHKVIFRGDADERPNEIPGDVIFILEQQEHAVFKRRGNDLFMTKKISLLESLCGFK
FVLTHLDGRQLLIQSPPGTVTKPDAVQIIKGEGMPQQKNPFLKGDLFIVFEVEFP--EHV
SDADAKSLSILPKPTEAV-MVSEDDPHVEVHVAEPVDPDELRNRQQTQRSGEAYEEDDED
EHPGQQRVQCRQQMSSFSSLSSDVTHWQPNAEHTAEIVALFEKAGSTDNAVQQQLAQAFQ
TLNAMV-DAPCYLTEILSSAQFSSDVRQLAGLTLKSNLQQKQPHALPAFVSYIRPRLLAA
IEENEKTVRSAAGSAITCLLS-LEGVGAWPEALQRLFQLLDDAREDVVDGAFSALSKIVE
DAFPERGADEEILAQFCNSHLLPKLFALALPPHKPSVRKHAVACLGHFAQNRAFAPQELF
EAFFPQYWQLLGQLAQESDAEMTRFVVQGMVQVVEVRPDVVFNSGEAVLSFVVRCCGHED
YRVRLDAVEFWPVLLRDTGY-------------------------HQPLHDYRDHALELL
RHHLPTLLPLLVKNTTYHEYDYLCMDPSQLEDDNAEVADEARDIKPRFHRQSGPEGAS--
---EEEEEEDSRGT
WGDGWSVRKGSALALDHIASVYREAVLPEVLPLIEASLVDANWERREAAVLALGALAQGC
QDSLEPYLPNVLQFLLNLCDDPKPLLRSISCWCVSRYAAWICRHE--EQFLKPVLVQILK
HVLDRNKRVQEAACSAFATIEEEASLHLVPYLPDILSTLKQAFCFYQTKNLLILYDAVGT
LADSVGSALATEAYSREIMEPLFGKFQNINLQDPGLIGLFECVTNCATALGAYFVPYAQA
VTERCVWILMESLTQVD---
-RFEKGEQAERP-SRDLIESCLDLLSGVTEALGPEMCELLAHQNLNFIPLLLR-------
--CCQDPAAGMLQSSFALVGDL
SKHCVKFLQPHLSVLMPTLSEHLLHHATSVQNNAGWAIGELALRAEPQFIEPHVDSIASK
LIGIVNCPELHRSLLQNVSISLGRLGIVCPAKLAPHLGDFLQQWCIIMRHAKNDEEKANG
FDGTAVRRYSP----------------------RKFHIFREKERDKFK------------
-----------------LGIPTHAMLLADAHLIWERALYVHSNEDSKPGWRAACRSVDAK
SFVDKHSQDTALQDGSVSTSATREKSERSFLPLSSSAFHPLAAEATPEGESESPSASPNL
THSLRPLAWLRRVCWSATEGDEDA----ACREEERHDSREGETLVLMAAVLAVLSSLCPG
KRESVSSELFNLLGAKCIHLLDMLVANASLFLRELHRLRRARVTARQLVGASVFIQRKSE
MLFQRQQKVEGKQRRQALEESILDVLLEEDFEDAPRADPPTQRQKIEPSTLPAGATWRQE
QKIEEVFIPPPETKTVPQSSLIPIAALPAWARVCFNVTHLNTLQSRVYAAAFLSGKSMLV
SAPTGAGKTNVAVLSILQQVFEHIAPRGRARPRVSHGRSGVDGETNRSAAAQDLHAPSLS
CRGIHLE-GSNTEVASSSSAASASVCSDGPPSARLFKVVYIAPMKSLVVEVVDKLAAALG
KVGLVVKEMTGDVSLSPHEMQSVHVIVTVPEKWDILTRNARNSNFGADDSLMTSVKCIII
DEIHLLDDERGPVLESIVARVLRHVEETQVHTRLIGISATLPNWQDVAAFLRVEPSRAFF
FGADTRPIPLEQTLVGALESDAQRRRQKVNDVCYAKVVEAVKNGHQALVFVHSRRETVAT
AEFLVQAAQAQGHLGLFVSQSSSYALLASQAHKSRCREVASLFSNGVAIHHAGLLRSDRL
LAEKLFR---------TATLAWGVNLPARTVIIKGTSVYDSKSGGFRDISVLDVLQIFGR
AGRPQYDTRGSAVLITEHERLMRYVGQLTHSLPVESKFLENLENALNAEVAIGTVSSVDE
AVDWLRYTFCFVRMCRNPRVYGADETDDPELCALRRKLIVDAAETLHKHRLIRFNSRTQR
LDPTNLGRMACRYYVDYETASLFRQDVELGEDRVILRLLGLAKEFASLKVRDDEESELSN
LRAICRVPIVGDFDAEAKVQTLVQAALAQAPIKAFSLCADSNYVQA--------------
----GDASSAEKILEWTKAVERGLWPTSHVLMHFCNPNCFDPDVQKRRQPYVPRANE--H
PGKQNRLVLREGMVSRLEKHQFALGRLRDLGASEIASLVASKADGQDVALAIRMVPDLEL

D--VNPITAAILRVSIALRFTEELWSAWWHGNGELFHLWVADVDTQRLLHTEEVTMQKEN
IREAREVSFALPLHEPTSTQFQVLVISDRWVGVSFQHLFSVRHCLLPDKRQAHTELLDLH
PLPRTALNNPEFEALYNFLYFNPIQTQTFHVCYHTNYNVLLGAPTGSGKTIVAELAMLRL
FATSPKQKIVYIAPLKALAAERLEDWKARFEGKLKKRVAEFTADAEAEARDFWKADIFVC
TPEKWD------------------------------GPVLEAIVSRMRYVSSQTDQPVRF
VGLSTALANASDVAAWLGIGKIGLFNFKPAVRPVPCSVHIQGFPQKHYCPRMNAMNKPVF
EALLTHASPRPSLVFVSSRRQTRRTAQELVSLLHTRHEHATDLFLDVRPEEADE----FS
QTVESVQDASLRTTLHHGVAIHHAGLSPHDRAVSARLFEKGFVRVLVATATLAWGMNLPA
RLVVVKGTEYYDAETNRYKDFPITDLLQMIGRAGRPQFDSQAVAVIFCHEPKKNFYKRFL
YQPFPVESCLLNVLAEHLNAEIVGGTIQTKQQAIEYLTWTYFFRRLTSNPSYYDPSLMIQ
DFTSSDRLQASAVRAIAAFVDKAVCEALDELLEASALRLRAEEKLQVLESTPLGRIACVN
YISPKSAKMLSDALREEENRRLSFVDIVKLLADVPEYKQMPVRHNEDNLNADFSAICPYP
ITVNSPHTKTFLLFQAQMFQLPVPIADYNTDLKSALDNAMRILQAMLDICTEEAQLRYAL
DVILLFQCLIQATHPARSSLRALKHLRSAEPSRLRRLSCLGIHSLHKCPAAVLLKAGFTD
KHTCEELKKFPRLRVSTRLFVKEAEGAS---EHSPPQPLRQGDGREGETLVHTVRPGADL
HLEVSLKYSNLPPQVAFTPNFHKQKTAGWFLIMATKRGEKEKYDSIVNGAQPRSSLPLDE
RVQLCLSIAQECIQPDELRTLLEKKPFPLCYDGFEPSGRMHVAQGLLKVTNVNKLTKAGC
VFVFWVADWFAMLNNKMNGDLKKIQKVGEYFIEVWKAAGMDMQNVRFLWASEEINRNSDQ
YWVQVMQIARSFTITRVKRCSQIMGRQEGDEQPAAQIMYPCMQCADIFYLGADICQLGMD
QRKVNMLAREYCDEIKRKLKPVILSHQMLPGLLEGQEKMSKSNPDSAIFMEDTETEVNRK
IKKAFCPAGVIDGNPCVTYVEQLVFPAFGEFAVKRSEANGGDVTYATVEEFRNAYIKGNL
HPGDLKESLARELNRRLEPIRKHFTENEHAKQLLQQIKAYKVTKEPRRPSLFQVLQSGPL
KVSVVGSGNWGSVIAKIVALNAQKSYVFHNEVRMWVYEEMVDGEKLTDIINKTHENRKYL
PGHKLPENLVALPDVVEACRDSDLLIFIMPHQFATKVCEQLAEARVVPGHARAITLLKGL
HVENGKPTLFSDIFKTKLDIDCCVLSGANVANDVAREEFAEATIGYEETDAALIWQQLFD
TPYFKVNALPDVAGVQVCGAVKNVIAIAAGFCDGLGLGTNAKTAIIRLGVEEMKQFAMIF
FDNIMAETFFDSAGYADVITTVFGGRNARCAAEFLRRGGKATWDQIEADLLKGQKLQGTL
TTKEVYEVIAAYEVERLFPLFSVTYEIAFNGRDPNDLIRVFKTSEVRQHKTSLAERLLTT
IGRAGVLLGSAGFVASSCLYDVDGGQRAVMFNRFGGVAKKPIGEGMHLYFPWFQVPFLYD
VRIRPKVINTTTGTRDLQMVSVGLRLLYRPMEDRLPIIHQTLGPDYDERVLPSIGNEVLK
AVVARYDAESLLTQRDKVSHDIRDAITNRARQFDLVLDDVAITHLSYGKEFSKAIEEKQV
AQQESERTKFIVARTEQEKKAAVVRAEGEAEAATLISEAIKQHGTGLIEVRRLDAAKEIA
DTMAKSRNVMYLPSGVNMLLSQQ--STMDSISQLALQRGGYDFSNHQRNVRLLMEAAKLP
PARKTGTTICGVVCKDGVVLGADTRATEGTIVADKNCSKLHRIADNMYAAGAGTSADLDH
MCDWLAVQVELHRLNTNAKPRVSMAVSVLSQELFKYQGYKGCAVVLGGVDFKGPQIYKIH
PHGSTDCSNFAAMGSGSLNAMAVLEAGYKDGMTLEEGKNLVRDAIKAGVLNDLGSGGNID
LCIITREGAQHLRKFETPTQRPFQATHP-VFPKGTTPVLLEKIEQLKSRLEFSQAVGRGR
GELSSSARTAAELFGRPPPGYIPGRGRGATGFAGGVSRDDTDNVHDKSDLSDANFDPFTG
YSEALFNDAEYDEEDKEADRIYDTVDMRMDARRKSRRENRLKEEIAKMRAEKPTIHQQFA
DLKRSLATVTKEEWEAIPSVGDYLKRKQ-KKPQMFSMAPDSLLLQGRN-STSYSNSIASA
----GSATPIGFGMQ----TPLM-GMATPL-----GLQTP--------------------
-LGLRTPLLGSGSGTGSS----GAGTPSLNDLGEARGTVLSVKLDKVMDNLSGQTVIDPK
GYLTDLNSMQLQSDADVADIKKARTLLKSVTATNPHHAPGWIAAARLEELAGKLQAAREL
IATGCQQCPKSEDVWLEAARLE-KPANAKAVLAKAVSVLPHSVRLWFDAYAREKDLDQRK
RVLRKALEFIPNSVRLWKEAVSLEEEKNARIMLTRAVECVPQSVEIWLALARLSSYEEAQ
KVLNEARKKCPTSPEIWVAACKLEETQGNLKMVDTIIARARDNLIARGVAQTRDVWLRLA
EEAEASGFMATCQAIVRATMKVGVEGMNAKRIWKEDAEEALSRGSVATARALYTCAIERL
KTKKSLWLALADLETKHGTTQDLEKLLAKAVVCCPQAEVLWLMLAKQHWLQGDVQAARKV
LAEAFVHNENNEAISLAAVKLERENHEFARARKILKRTRAHVNTQKVWIQSVQLERQVGD
YDAAIALCEEALKSHAECPKLWMIGGQLHREHPT------KKDEEKAAEVFQRGTVVCCR
SVPLWLCAVDCQREQGKWSVARAILEKAKLR---------------NPKNPD--------
--LWHAAIRIEVEANKQM
AQHVASKAVQECPNSGLVWAEAIFLEEKSAQTHKAVDALTKCENDVHLVLAVACLFWKEG
KISKARKWLNRSVTLDASFGDAWAAFLAFELENGGEKECRNIINKASLAQPNRGQIC---
--RQSMPLSSA
RCSSLAGHSAEVDRRMPSTTPSLLRAFPPMPEETPKREEAAPSVAEVAEAPVSKKAQKKA
EKEAAKEAAKAAKREALEAEQRAAQAVMNKQVEDLEKDAFGYLPVSRMRTNREWI-----
-------------------PVDQLRNH---VGKTVWVRGRIQESRGKGSVGFLMLRERQE
TVQGVLDAKR-GNTKDMIKWTMSLPLESVVDLQGTVVEPEVEIQSTSQKGELLVTKIFCV
SKAAQELPFQLRDAMRPEDGSEGGIRVNMDTRLDNRILDLRTPVNQAIFRIQSETLQLFR
EFLLSRNFVELHSPKLIGGASEGGASCFTLKYFNRDACLAQSPQLYKQMAMCADFERVFE
IGPVFRAENSNTHRHLCEFTGLDLEMTFKEHYSEVLDLLDDLFKFIFKGLSERCKKEIDL
--FHQQHPAEPFTWIEETPRLSFEEGVQMLREAGCPN-IPEDLSEFDLSTEQEKMLGRLV
KEKYHTDFYMLLQYPLKVRPFYTMPDPHNKMYSNSYDFFMRNEEITSGAQRVHDADLLTQ
RCLECGVPPSSIQTYIDSFRLGTAPHGGAGIGLERVVMLFLGAKNIRQVSLFPRDPKRLT
PMKTSSRHEEQQHREKEEKSFLSGLFDKNSSKDAAAKSSSSVGDTDTMEGSSAALAPGTS
MLGSSEAGESTSNRLIPSESKEL-DKTRAPIPVPYEQFSREWMAVAGQDNFDGFRLEATK
QVNKVLTANHTFMLGTQSKEGGCSYSFGPTLVIGEPNFFGMARMNSDGFLQARFIKAISK
TFDIKFNSNSSISEDAKDMYEVSFDKMGSDWAANLKLAWQGTWILNGLFSQVITPKLQLG
GELTWVAATGISMGSVGARYFNENNTVTCQIGVGPDFSSPMGFANDVYSTKAQYVRKVTD
RLSMGTELEFTHPDMSSAMRVGWQYLFRQARVQGLVDTAGRVSMFAQDYNGFGLSGMIDY
WHGDYKFGFQMNVVPPPPQAEQPPAAAAQAVPEFKLILVGDGGVGKTTLVKRHLTGEFEK
KYIPTLGVEVHPLKFQTNFGMITFNVWDTAGQEKFGGLRDGYYIKGQCAIMMFDVTARIT
YKNIPNWYRDITRVCENIPIVLVGNKVDVKDRQVKARQIQFHRKRNLQYYDISARSNYNF
EKPFLWLARRLTNQPALSFVGEHAKAPEIQIDPNLVQEAERELQAAVSTAIDDDDEDLMA
SRT-PFQDAALSIVKGAACIALSLKTARTTQNVKFSKDNRCSITLGPIEVEPTAAEEALL
KTLTKQKIEENCPFRVFTIPRDLATRMYQESYLDEFGIPANVKEVRLVVLPEWNINANMY
PVLKSTGQIADIQFESVKFNEEKKQLCLSLHVVPGPDEQSPAL-----------------
----------------AEEEKLGPEEIPSRAQVLPPSGVEGDDFDDDQTITPWDVQAAEG
GIDYNKLLRKFGCSSITPELISRIEALTGKRAHHLLRRGIFFSHRDLNLLLDSLEKGPGF
YLYTGRGPSSEALHIGHLVPFMFTKYLQDVFDVPLVIQLTDDEKFLFKDSLTLEETHRLA
FENAKDIIACGFDPDKTFIFSDLSYIQHLYPVILEIQKKVTYNQVRGLFGFVESDNVGKS
AFPAVQAAPSFPTAFPMIFKKDVRCLIPQAIDQDPYFRMTRDVAPRLGLLKPALIHSRFI
PALQGFKTKMSGSVE------------TSSIYVSDTPEQIKNKINKYAFSGGQPTEAEQR
VKGADLDIDIPFQYLTFILNDDDQLKEIGEKYQKGEMLTGEVKAILIKELQALVLGHQER
RAKVTDDMVRQFMDPFRPCFKKYAQ-MDSGGSMAYEPSIVVKEVSQNKVKFLLENCDVSI
ANGLRRVMISEVPSLAIDLVTVYENTSVLHDEYISHRLGLLPIDSTRVAEFVNRDDCDCA
DHCSRCSVQYALDVVCER-DSLLVTHRDIVADNADLGCPMPVPLLREMQ-EKEMDGIPIV
KLRKNQSVNMRMTATKGIGKVHAKWSVATASYKFEPEIAFEEDLLARAPAEVKRQIAASC
PREVFSFIDDTTGGTGVLRVENKMNCIFCDQCKVKAQELGFRRLVRVEPNERKFHFTVES
TGVMPAEQIVEMAFDILLNKVTELEGLVQASARATGASTFASAAEARPS-GGSLDLDMA-
EQSKVKDLTLSAFGRKEIELAEGEMPGLMALRKEFGPRQPLKGAKISGSLHMTVQTAVLI
ETLKALGAELRWCSCNIFSTQDHAAAAIVKNNSAAVFAWKGETLEEYWWCTVMALTWGEG
AGPDLLVDDGGDATLLIHEGVKAEKEFARSGALPDPAATDNLEMKVVYKILAEGLQKDPQ
KWSRMAANLKGVSEETTTGVHRLLQMAKKKELLFPAINVNDCVTKSKFDNVYGCRHSLPD
GIMRATDVMLGGKRVVICGFGDVGKGCAAAMKGAGARVVVTEVDPICALQAAMEGYSVST
LESQLATADIFISATGNKDIITARHMSQMKNNAIVGNIGHFDNEVDMAGLLAWPGIQKVN
IKPQVDRFIFPEDNHVIVLAEGRLLNLGCATGHPSFVMSCSFTNQTLAQLDLWDNVATKR
YQNDVYLLPKELDEKVARLHLPALGCELTTMSQEQAAYVGIKPEGPYKSASYRYMYSLGV
EGDETLPVQQAFVSSASEGYGQNSTGPTPRGENHSPFIYFFTSALLRTGSCLQAACLLLM
CLFYVGFGGGGLFVFDEFAGPESVRMSDAFHLITAILMIGYLIGTFAIALFQLFVADSSK
WTRGFRSGSKTISCAVTLDVVSGCLRVTQYIHAYFFSSVQWWAKYQRTQSDWGLYHFSST
LHAFALVTYGVGFFLMESYHDQGTYEEYAWSMLGLYTTAGLAELFMVYTGYGAFFTLLLV
AALMVTVSWAFQFEPLLEKWSPDLHSRNLNENMLPFSETSGYTHQATMGTAAYGSGRCLT
GEFRVEEMPSMQRSASVLGRQSTSMPGANDMPTTYDYSTLQQQIATSGLEGLVRSL----
-MADANQPKRRTFRTFSYRGVELDKLLDMKMEDLLELFRARQRRKFQRGIKRKATTLMKK

LRISKKNCAFGEKPEPVNTHLRDLVVVPEMIGSVVGVYNGKQFINVEIKPEMVGYYLGEF
SISYKPVRHGKPGIGATHSSRFIPLKMDKQAKLSLQEDSIAKMLICKAHIGTKNVEHKMR
PYVFKSTSEGIHLINLAKTWEKILMAARVIVAIENPADVLVISARPYGSRAVLKFSQYVG
AQAIAGRWTPGMLTNQITQKFMEPRLLIVTDPRTDAQAVRESAYANVPVIALCDTDSPLE
HVDICIPCNNKGKESIALMYWLLAREVLYLRGELPRSVPWDVMVDMFLWRDPEEFERKEL
EDEEAAPHTAQADATQWGADAAAADWKQAGDWTGGAGGEWDSAVAAEEWM-IRSVSRSVG
TSSQQHNVPKHFDILPYPPLKVRVHPVVVLTILDAYLRREEGQMNVIGTLLGTVSEGNVV
DISDCFVDRHSLTDEGLLQIIKDHHETMYELKQQVSTKDIVVGWFCTGSEMTELTCAVHG
WFKQFNSVSKFHPQPPLTEPIHLMVNTTMDRDNLSIKAYMQVPMNMAKDACFQFQELPLE
LFASSSDRAGLSLLLKVREANRRHRQQHDGSRASALSKQGLGAALEKLSDQLNKCGAYVR
SVLDGSEKADPEIGRFLSKALCVEAVQDLEVFEQMCQNALQDNLMVVHLTSLARLQFAVA
EKLNTS-FFMEAFVSLLRAVISPDPALRKEAERQIKEGKEKQPAALVSLVLQTVQTHSDD
EVRLQAAVLFRSFFRGVID--------SEGHVWRQLGDAERTQVKQILLHCLDTEKNKLV
RNNICDTISDLASDLIPVDQWNDLGQVLLAMIQSGVPVKQQTGLKILSEIAPVLTEQLAA
AAPLVCRIISACMASQDVNTRVEAFALLVAVVEDMNKRVYKKFLPVQLLVIDTLEATLRA
GAEDDYAL--AERMMVSVIQLCEGGVSLLRPHLSQFCDYLLAISLKTNDEETPEGLQNCR
KYAIEALLCCVEQKSQVMLRVPNFLNRLLEALLMCMLDIRDSSYAKWLEE--GEEEDEQR
FFDVGEEGLDRICRAYSSDEDSILLPALFNYVTTFLQRPEWEYRFVALMAISQTIEYVQE
DQ-EEELDYIAKTLMRYLGDGDFRVRFAAAQAIGQMSLDQTPYVQEQFASEMLPLLIARM
DDEVPRVQGHACAAFVNFSEEVEKAEMLKVASQVMEKLLTKIRPGTPKTVREHAVTCIAV
VAGVLEESFVPYYSAVVPSLLDVITNSTALELRSLRGKAIECISIVGLSVSREQFAEDGK
VAMEAMLQIAESTAEEGDAVREYLTEALGRMCRAMGADFLVYLPRILPRLLEVLTVKPKE
LAEDDEDMTYVILDSNTSLGLKTSLLEEQSRALDLLCTITTVLQDPLTSFLQPLAEAVFP
LLTHLLSEDIKQKALETMASLIGTCKQLVRSGASVKEMLRAMTLRTCGEVFKSLQDEDD-
------VDSMVAEAAGLNDCLSKAGGEVFTDEEVAQQALNVFSALEKSFERRIDISKNRQ
DEEVDEDDMLRLEEEDQQEQTLRSSLLEIVGTLMATHPKEFLRSASEAAAAFVQQFLRED
APDDKSVALYVCDDILQHLKEDGLSLWPLFMPRLIQCLLSSDARVLQAAAYGVQQGALLQ
AFQPFVQEAAKNLLTAVNRSQKTKN-KMEQAATDNTAAALGDLLRCYGGSMHEEEQLLSA
WLGNLPLKQDETEGLRMHKFLMEAVLQNNAVVLGPNASNLPRLLQILCAVYRTDFSDSDL
NEEIKKLFAQINASPLAALC-QNFTAKEQKKLQKILK----MPTVSVPRDELFRRLGRTY
SVHEFEELCFEFGIELDEVKDGSTETIYKIEVPANRYDLLCTEGISRALYAFNNDAPLPA
YRLEP------ATPQFTMTVKPAVNQVRPFVVCAILRNVTLTKAGLASFIEFQDKLHHTL
CRRRSLVAIGTHDLSKIQPPFVYDARPPKNFEFVPLGCDSQMNGEQVMAHFSSHLQLKAY
LPLIQNSPVYPLILDAKDRILSLPPIINSEFSKVTEDTRDIFIECTAVDITKAQIVLNTL
VAMFSEYCKEPYTVEPIRVVYEDVKSLQNGNGWVFPRVNSRSMPFSLDYVRQLTGIPDLT
ADACANLLKRMMIHTSKATQAGILEASIPITRSDILHERDIVEDVAIAYSFNRLPVTRSY
MLTGDALNCLSEKIRNFCTVCGYTEALNFSLSSAAENSSSLGRTPGDGKSSLFNPLEYQV
NAQPVRLANSKTREFDQVRTTLISGLLKTIVANKRRELPIKLFEIGDVCLLD-STTEVGA
RNLRYCCLAFADEHSSGLEEVHGVLDALLQSLQFVGEYAIAEMEAAVAAAASRITFKLVP
SHEETFLPGRQVQVVASLKSVLLGVMGTLHPHTLKAFGLTIPVSIFELNVEAFVQWLPAM
AESAPGGSAGPAIALGNIRGFGRNDIGLFKMSGDLFGWKNRKTGSVHQYKAADIVSASWI
MTGFDAYQLRILLGPHKNDLMVRFDGFHEKNFADLSRHFDAHFKVKLQRGQQAYRGWHWG
DVKMEGNNLQLTVDGCAAFDIHAQEIAQVTTPSKNDLAIELIQDDTRDQQEDQLLEVRFY
QPFADDAEGPLQQLKQKLVKKSGVAETKMDSVALLNDVPLLVPRGRYEIDIGRRALKFHG
KSYDYTIQYSSINRMFLVPRPNSPHVNFILSLENAMRQGQTSYPFVVMQFDSESVHSVDV
NLEPAELQQRGLEKLIEGKTFHVVTRLFRALVGKSVIVPGDFKSVKQQFGIACSYRAQSG
HLYPLNRSFLFIVKPVIFIRYDDVVSVEFSRTGASTTNRFFAFTVSVRGGGEYEFTSIDR
NEYKPLVDFLMEKGIRIKNMETP---EPSRGGALEAADLPSDDEDDDYDEDDSDSEDEDF
QEDDEDDNASESPSEEEEEDSKMGAAVPKADLGDCRFYEERFPDVEDLVMVKVNRIADLG
AYVSLLEYNNMEGMILMSELSKRRFRSVNKLIRVGRHEVVMVLRVDPKKGYIDLSKRRVS
PEDIVKCEEKFSKSKKVHQTVRHAAQKHGMKVDDLNRSVIWPLYRKYGHALDALKEAAMR
PDEVFAGLEVDEEVRKSLIQDIQLRLAPQALKLRARVDVWCFGKQGIDAVKAALQAGQEV
GDDEVTINIKLIAPPQYVVVTSCYDKELGMRKIEQAMKAISDKIKSFSGGDFKQQGEIVV
MGGDEEKRLEELLEEANEDDSSDD--EEEEEDEGMGEDDIPEDEVEDDEEQDFLGSALSP
SSAASVTDQRFQFSFEPSLEQQKQEEGRLLNEQGISRADCVSGRGKHTVVCRHWWKGMCM
KGEFCDFLHQLIYHRMPACRN---QLCCPDTRRGCCPFRHEDGGPAGAPKAVPGTDYVSG
GAGASGNQQECVNYFLGFCKHGPKCRRKHTAKNRRDIPPILPTWFLDQILANPQVFPTEL
AESEEQAEVIGRCRQLSQASRGS-VPAPVSSHANLSSSSSCGNLSQAGGKDGSAFRGPFP
SSMAGSACFSQREGIGSLS----PSTTCVVPLLVDPFGHVSPQSTKRFFIIKSNRMSNIY
TSIQHGVWATSKGNSRKLSNAFTSTDHVLLLFSANESGGFQGFGRMMSLPDPQLFPGIWG
PVQLRLGSNFRVMWLKQCKIEFEELGKVTNPWNDDLPLRKSRDGTEVPPALGSLLCTWMS
QRPSEDLLAGTGIDPATRIDHSAFFAMLLQNKLLSVPPSGTV-------EGTLQPTAPID
LRHCEPSMATSVGVRLSEKDLGAVLRDSDEWAFLEYLLQMSVRTSRVRLLQAWHIATPHL
VSAFERRTQGRLLTYSFVDTASLDEDNSLQDIARRGLKIPMTGMRFAVGNFSLPGFPLMR
GGPAEAEDPFVLPSSASTRQEEAKKDRSKFLDAARSQLLTGERRIFEYLLCQVGVGRSVV
VEDEKEACGSRFSLLWEYDSAYLEKGAL-APEESLTVHYQAVDDSKRPASLPSIFGDGKK
VTPGVLPQYTFRHDYVVYDSSQVLPRFFLLFEMDPSLEELFAVPLCDNCQDNPACIWCPA
DAARLCASCDELIHQQNRLVSRHIRVPLNEMPRAFGTCRRHPGEVYELFCSICHVAVCRL
CRGNHLHAAPSKALSLSIVAGGRSLMPLNKPYQAVLHMAKKPHFVLAARRTELEKRLEEV
QKMLDGVRRNCREEEQRCYAILEEAITQLHSCTEDKMGAVLSRQLELQREIDTMDWSESF
LQYLRTVLPPADFLHAWLRHCRLRDELDALAGDPSMLSRNVFPDMRLQGRVDILTDSALR
RRDYVAVEGRGMAPKKKEQAEEKILLGRPKNTLKMGLVGLPNVGKSTTFNLLCKQAVPAE
NYPFCTIDPHEARMNVPDDRFKWLCTHFHPKSEVSATLAIFDIAGLVPGAHKGEGLGNAF
LSHIQAVDGIYHVVRAFEEADIVHSEGEVNPVKDLETISDELRMKDVERCEKLIEELDRL
VGKGQKDKQKKVHLDVLKVVLEHLNNKRWVSQVDWKASEVEVLNEYQFLTAKQVVYLVNM
SEKDFIRQKNKWLAKIHGWVEANVSGPIIPYSAQFESKLAELDDAAREKYIKDVGASRSQ
LDKIVTTGYHALHLIHFFTCGEDEVKCWTIRQGTKAPQAAGVIHTDFERGFICAEVYKYE
DLVAAGSENAVKAAGKYLQKGKDYVVEDGDIIFFKFNVTNSGKK----------------
--MQPRQPGDEAKQAELEVVRKMMTPTREVLLELHESFLKELQRGLEMHKRHGITWVPEE
CSMKMLDSCVSNLPTGAEVGEAYAIDFGGSTCRAVRCSLLGKGKMEIIQDKICLRS-AEH
RCTKGFMDKKAGGKELFDQFAMCIRGLMDRSGDLKKAEEVPVGFTFSFPCAQAALNSSFL
IEWTKGFETGRENPDRVEGKDVAVLLADALQRHNVPAVCKAIVNDTVGTLVSCAYQRVPG
TPECRVGLIIGTGFNACYVEPEASNYGYTGTVVNMEAGNFHKDLPRNEIDVEVDEKTHNR
GKQQFEKLVSGYYIGEIVRVAAVRVFGARAPEKASVRHSIHGETASTIRDDHSQDKAASI
QAIKECWGVTMDLDDIKCIWEICRLVFDRSAAFAATLAVALCYRTGRLDTGS-TVGIDGA
LYVKNQWYREAVEYYTKLVAGDAAKNIHYCIADDGSGKGAALIADVN-------------
MYFLVRWLCIGIVRSFFKEVAVLHPERIPLYGPAIFVGNHNNQFMDASMLVANIPRQVHF
LVALKSMKRRVIGFLSRLAGCIPVDRQDDRAFKGPGLVTREDGPLSILGNSTRFLSDVRP
GYKLRLDGKDFLVLRVLSDTHVVVRQPPEAPCDGREEGEEYKILPKIEQREVYEAVTHSL
VDGDCIGIFPEGGSHDRTTLLPLKPGVAIMAIGGAAAGA-DVMIVPVGLVYHNPHKTQSR
ATIHIGEPIPISREAVVEYQQDRRAATARILTDVEHGMRSCIITAQDHETMTLIHLCCSL
YPPERLRLSPEKLFNLNQLLSKLFWRCADSPELSNLRQDLAQYQGALQRAGIPDHDVWML
KQSTAGASLCFAEKLIALLFAIGLGVPLLPLWGPLRVIAYFLAERHRAQALAASSVKVKG
MDVVASYKVIVLLVCVPLFNLVYGAIFGLVFRRTLAETLATMLLCICLLPVAYYFSMRQA
EKILPLIRQMRTLIIVVVGKVNIWRENERELITQRMNLQFSVRETLLKLGPQTSPAFMEE
LYSILPKAVLVADIKRLIRKKEDFAPLQMKSLMNNAEEILMAEKTGKDRGSLMREMRVSN
KGVSPPPHTFWDTQPVPKLNEPKEGKE-GPIE-TKTVDQVRSEPYKLPDGFVWCECDVRD
PEELKEVYDLLSQHYVEDDDNLFRFNYSADFLDWALTAPGCHRDWVIGVRVSSTNKLVGF
ITATPSQIRVFSDSVPMAEVNFLCVHKKLRSKRLAPVLIKEITRRVNLRSIWQAVYTAGV
VLPTPVAQCRYWHRSLNPKKLIEVGFSGLSERMTISRSIKLYRVKESPSTPGLRPAKPED
VPHIHKLLSNYLRNFKLHCEFTQEEVAHWLLPREGVVHVYVRSTKGTVTDLISFYELPSS
VIGNQKYKEIKAAYSFYNVATTVPLKQLIEDALCLAKQLDFDVFNALDVMENKSFVEVSR
FPEREGK----------------------MSVVNVTNIRLGNNPSPISSPFVFEICFEA
LTPLKEDIEWRVVYVGSAECEEEGGGDYLLDSVMLGPIERGVLAFEFAVNAPDYTKMDPS
SVVGMQAVLVCALYKQQEFMRIGYYLNNAYSDTVLRENPPDVPIYDKLVRCI-VDEPRVT
RFPIVWDEDSGVG----TPEGAQAGGST----AAGQAVESPAATEAGKVSVEAPD---GE

SKERKKDARTETQETSGEARNAGAEEEEKEGTDERETEEEEEEEEEEE------------
MSHYTCGAY---------------DCVVGGGPTSTPPVNNAVFLFAL----------LVL
VCLHFPLTQLSEDARIVQPILHRLKQRTFFRIFKLSLLADTCLRDNADKCTEPQRCGLCQ
CSDDEIPQTWRQKPDEEFVDRRHSASFTRPFSMFKTPERLQGGTFPKTQATGVYVDLLLN
PPCFTKYDGGAVWRLLYDQVSGASSAWACSNNANLRRFLSGMQANIAALAAANFNTRDDA
PPPMPYPTTSMGNGTPAPPVFISNLDFFRERLAVHPARIQNLYFTFGVLLRAACGLTDIL
QECSCETGNNREDLSTRAELLHMLNSSLHACSSDHMNESFFDSSRLHFLQTTEGIDKLLR
CVPCEKCRLHGKIKLTALHIAARMLSPLHAMSLERNQVAALINALYYFAESIRVVEHMQD
RIYMHNVSVCAFFIALLLLVLLFIVYFVWTSVRSRRKRRQKLMMARNAERANAVLNKWLA
VKDVKGAASRERRPRDVNSCQDLKQAEKWRSEVMREIGKLITQVQDASLGEHRIRDLNDD
INRMIRVKKAWEFRIKELGGPDYSASATALE-AMSKMSAGADGYMYFGAAKELKGVRELL
EREASEQQK----PRKTRAMLFKNITPDYYGWRDEEDGEILLAEKVREEELRQEALGASR
GSSATMTTLRSEPMLRGTLVLPSPLAHGCLEALGRQGSVHFLDMNAHSLTRQFNTYIQRI
DEMERIIRFLEEEVNRLQDAGAKPEGAQNFLDNDKSYQLDKVEEALNRLHAQFVRFKSNN
ADLIQQKNAALEEKCVMTTAVQQLQGSAMK-DGFSAGAQNSPADGTAAGNSEEERQDDNM
TPNADDPEAGLTSSVSTVAGMIATSAIGRFQRMLFRTTRGNAFCFFQSTAEKLVDS----
--------------------NTGNEVEKGVFVIYYQGAAHSLLREKIVKVCAAFDAKPYE
WPHSAEEAATRLAGLQSLLDDKERALAAYEKYFLSEISLLLEVTRPGGSSLLEEWKMFCQ
KEKAVYATLNQFQGKDMTLRCDCWIPRDKEEEIRSILKDVSTDEQASAFLLIEK-GQPTA
MPPTYFKTTEFTEPSQVMVDTYGVPRYQEANPAVLTTITFPFLFGVMYGDIGHGLCVMLM
GLWLLFRANALKQ-DRTAALHGAVKYRYMVFLMGFFAFFGGFMYNDWFALGLDIFGSRWT
LKGA----EAGSSSITMRKKDG----EFPYPFGFDPAWKGATNELLFTNSFKMKFSVIVG
FAQMFAGVLLKGSNAIFFREPLDFVFEFIPQVMFICSLVGYMDFLILYKWATP---ADQN
KPNLINTIINMCMLAEVKSEDEMFSNQQTVERILLVFMVISIPLMLIPKPLILCSRLKKN
HPPGAHKNSSTIELEHAPS-----------GGANGAGAAAAVG----DGNENSAIEAGLR
KQGTTEEREGNVLSVIKEEI--------GEE--HEGPGDIFIHQMIETIEFILGTISNTA
SYLRLWALSLAHQQLALVFYTQTVVRAIELTDTTFVALALFVIFAAYACITFAVILCMDF
LEVSLHALRLQWVEFQNKFFKGDGYKFAPLYFIKLLQGED---MEETPVYFNTSRGFSSS
SPSALMLPSSLPSVQQMKFNFRQYLNHGGA------ELKRRLLAHAKNERCLLHVDLNDL
KVYQHHVSDLKCVDTPYCKKKHAAPPDFARQLVTGLRQRPLVYLPTCERVCEEVIEKSAI
LPPRQLAASLVQINLMDTSATHTPIRHLLSRQQEQFVVTTGIIISSRPPASRLQTVTIQC
RYCNHKMIISLPEWREQLQLPRFCLYSQQRAAVASTAGPASADLGCRNKPDPYFMVTNEC
SFVDVQSLKLQELPEDVPTGDMPRHLLLNCTRLLTDQAFPGDRLIIHGVLTTNVATPASK
ETDQPHSSYLHVLGLQKVSFLARTRLSFQTEQRDVFYKLAQSQNILDKIAKSLAPALYGM
EEVKRACACLLFGGTQKVVADGSRLRGDINVLLLGDPGVAKSQILKFVDKIAPISVYTSG
KGSSAAGLTAAVLRDRTGVFTLEGGCMCLADGGVVCVDEFDKMDERDVVAMHEAMEQQTI
SISKAGINTVLNSRCAVLAAANPSFGSFDDTQDSSEQHEFKATILSRFDLIFLLRDKENY
ETDSALCTHILNLHAQKNVQDDEIIPFELLKNYIQFAKSLPPPLLGADARDALKNFYVQT
RQDVRDDKRSKTRKIPITLRQLESLVRISESFAKMELSPVASSKHVQMAIELFSVSTAET
ARHSLVFEGLSPAEQKLIKQAEEAVLGRLQKGQRAQRKNLLRDLQMQGFDKNVLNRMTKS
YRTYSKTARSPKRPFEKERLDQEMKLLGEYGLKNKREVWRVQYALAKIRSAARELLTLEE
KDPRRVFQGTALLRRMVRLGLLGESEQKLDYVLGLTVAKFLERRLQTKVFKLGLAKSIHH
ARVLIRQRHIRVGKHIVDIPSFMVRVDSEKHVDYAITSPFGGGRPGRVKRRSLKGGNAEG
GDDE-MGRSTQAMYDRHITIFSPDGNLYQVEYAMKAVRNCNLTCVAIKGDDSACLVVQKK
VAAHQLTQDKHLDSSFVSSLYTLSPTIGSCLVGVFPDCRSIAFRARQEAGEFAYKNGYDM
PVYALAKRIADINQVYTQFAYMRLHACTGIMISYDEEAGPSIYKFDPAGFFAGYKACASG
TKEQEATNILEKMVKKRSRTTPKEVIE--------CAISAMQHVLAMDFKASDIEVGVVT
KDQPAFRILSEHEVEDHLTSIAEREM----------------------------------
--------EGAIPQAGEMENTVSGV-------------PRPAHMAPRVEIKLFVGRVPQS
MEDAALRPIFEEFGEVKEAVIIRDKATGKHKNSAFIKMDSIAAADAAIRGLNSTRILEQS
MGPITVKYATGEAEKLGFATSSEPGQDQAKLFIGSIPRTMTEDEVRQFFSTYGTVEEVFV
MKDNVQNTGKGCCFVKFAFKEEALHAVRTLSGKHTFEGCTRPVEVRFAESKAARQQQMAS
NMGGMGGPAMGMGSSTQMGTPN-----------NSNPRAAGQWKEYFTQDGRAYYHNEYT
NVTTWDRPQEFYQLPPAMAAGSGQSSNAEGPPGANIFVFHVPNEWTYYDLMQNFGGFGNV
VSARVATDRTTGRNRGFAFVSYDNVESAATAVNNMNGFMAGGKRLKVSVKKGEEQYVPQN
NPQGPRGNAGGMQQQWGSGYSQG----------------GGVYGGSGGQQRFTPYMTTAH
RPTWHQALGGEHQGGNRLVA-TQKVSAKDQPGNLTLKTRKAAQQTSDRSEFR---VQLEA
KEKKALADR----KAEATLTALPPDYENPFPEDADDV-----------------------
------------------DAPGPPSEASSDEDDDSD-EEEALLRELEKIKKERMEQAKQA
EEEKKRQAEQQERMLRANPLLMADG-------AVKRRWDDEVVFRNQAKNQKAKRQFVND
PVRSEFHKKFLSKYIRMDAEEKLDRAIELSRQATERDKAGAFAEAFELYKAALDSWHLLC
RCQTNALLKAKLYRKMGEYVARAEVLKNFLEKQKQHAFAAACRADDSEEDKIREKLLTAI
VTEKPEVRWHHIAGLEAAKEALQEAVILPSRFPSLFTGMHRGEKNVDLLQPPGTGKTFLA
KAVAAEAQATFLSVSSADLVSKWQGESEKLVRSLFAMARERRPSIIFIDEIDSMCGARSE
GDSDSSRRIKTEFLVQMQGLQKDAPGVLVLGATNVPWALDSAIRRRFERRVYIPLPDLRA
RLQLVSLSLGTTP--HQLGDAEFDTLARQTEGFSGADISVVVRDALFQPLRKCRAATHFK
RVFLDGTHFLSPCPPGDSDPSKVEMRLMEVPPNRLLPPELSMEDFIAVLRNARPSVSEED
IRRHEEWTRRFGVEGQMNASILFRRNAPGVWTPAFQRTEKRFLSGAALQPKAGPAPEYRR
VPFVKEDMEKVMEEVEFKYYYVENTKGNVYEGIPLDQSILEPADLRDYVPPHSNIQYSKL
DNGLRIASMDRGGLTASLGLFVHAGTRFE----DVTNFGVTHMIQNLAFASTAHLSLLRT
VKTIEVLGANAGCVVGREHLVYSAECLRSHMPLLVPMLTGNVLFPRFLPWELKACKEKLI
MARKRLEHMPDQMVSELLHTTAWHNNTLGHKLHCTERSLGHYNPDVIRHYMLQHFSPENM
VFVGVNVNHDELCTWLMRAFVDYNAIPPSKRTVASPVYTGGDVRLET-PSPHAHMAIAFE
TPGGWNGGDLVAYSVLQTILGGGGAFSTGGPGKGMYTRLYLNVLNQNEWVESAMAFNTQY
TDSGIFGLYMLADPTKSANAVKVMAEQFGKMVSVTKEELQRAKNSLKSSIFMNLECRGIV
MEDVGRQLLMSNRVISPQEFCTAIDAVTEADIKRVVDAMYKKPPTVVAYGDVSTVPHYEE
VRAALRMASGMGVDENCVARFNELKIRKTVKWIVFKIENTKIVVEKDGKGNA-DEFRGAL
PAND---CRFGVYDCGNKIQFVLWCPDNAPVKPRMTYASSKDALLKKLDGATAVALEAHE
MGDLAALATQP----------------NLPGGTPSYAGASQ--APTQQPFYGYTSQPSPS
DMRQSYSNGMPAYPSDSASVPSYPAPPLASGNPQPAAGVYASQIP--------SSPFHAA
TPANPGAAPPPRTAYYPSGQSSGYAGYGAAASGVSHSGAYASTGSAAQQVLQEIQQFNSP
RYFTRASVARVPHSSSLQQKLHLPIGLFIRPLAPIRPGSPTVPSVNFGNCGVVRCKRCRT
YINPFVHWEAHGRRWSCNLCGYVNDTPQFYMRGLDEKGKREDRFERAELSMGSVEFVAPA
DYMIRPPQPPAYLFLIDVSATAVASGLVESACAGIRAALTSGKLPG-----CTDSGAGRG
DSRTMVGIVTYDSAVHFYALGSGKRRPQVLVMPEIDDVFLPLSDDLFVNFAENRDAVLEA
LDTIPSLWRSNGCIDNCMGSAVKAALLVMKHVGGKILLFASFPPTVGALVTKRS------
GGLAGADGAGTDGGSRQQPD-REVELLKPASEAYQQFAQQLTQSQVSVDLFIAPAPYCDL
ATILPLAELTGGEVRYYSAFSRLQHSQQLENDIVHIITRTTGWEAVMRIRVSRGWKISAR
HGRFFLRGTDLFVLPTVNADSTFSILMELDEQAGADNVVAVQAALLYTNSDGERRIRVHT
FCMPVSQNIQDIVSSMDPEVVTCLLIQQAIDQSLRSKIADGRAFLQTSCSQLLSTQLAAL
EAGRLVALFILGVLKSNAFRPVKEVSTDKRVFSWSRLLALPVDRICAYVHPRMLPLHNLS
NFGGPSDRQEEVILPPALRLTAEEMTQDGAYLLENGEEMLLWIGRSISPQWLASVFGAHS
LDCVHPDVAAASIGDSQDPVGLRVRAVVQSVQGEHLCNAMRLLVIRQGDPSEQKFFAQLM
EDRVPSLLLTFAEFLQKVDMRSQLQAPAPPPQSQSGKDETSSTTLDIWIEKYRPETLDDV
VGNDQVMRRLRIIAREGNMPHLMLAGPPGTGKTSSVLCLCKQLLGSRWRAYTLELNASDE
RTIDVIREKVKHFAKEKRDLPAGRHKIVILDEVDAMTEAAQQALRRIMEQFSDTTRFALA
CNSSASVIEPLQSRCAILRFRKLDDSQLVRRLRQVCAMEALQVTDDGIEAIVFCADGDMR
SALNNLQSTVSAFGVVNRENVEKVCDNPPPEAVRSMLMECLAGKWREAHDIAAELLRRGY
TPMDVVLTTRSVLSRFENEKEHILLEYLKYVGLAHMTMSAGLSTPLQLE-----------
------MASEPFIDFKPSKAGLPSQSSAEDEPLVKSD--------------KKAKKERLN
SMK-TKSLMMVNASAALDGCDDQLLPATFRALERDLSFHPSLLGYITLAQTMCLSLLCPV
WGYLSDRHSRKWLLAFGTFAWGLTTTLLGLVSDFWQVTALRALNGVFLGSVGPISQSILA
DTAASKSLGFSFGLIQLCSCIGRLVGGVVTTSVALLDVGMLRGWRVCFFCVGGASMVLGL
LIAFFLEEIPRKK-SRRRAKFVDES--GGTGGPTPPAQEEQNWLSF--FKDVFSQSLSTP
SIVIILAEGLLGTVPWSAFSFNTMYFQYCAMSDLEAAVLTGSLLMGAAAGGVLGGLLGDR
LFYWSRGHGRPLVGQVAMMCRIPLLVLAYVVVPKEEEYFYAYFLIALFVGFTSMSGVAVN

RPILSDVVRPDHKGTVFAVTVALEGSSAAILGAPLVGVLAESAFGYERTSLLVKDMPDSL
RLGNASALAKSLFLLTVIPWSISFVLYGMLHFTYERDQIALAKIVHEEYEHAEDDDDVAP
TDASSKRNPDAGVATAVQSVIQHQTFKRMLLFGLRSLADFCSPSNQLYQENALDALDRGV
LSAIQTAVTTFSDDDDLMLCASRVLWAMSVAIKEEMDPAHIARVHSEGS-PVIVAVVNSS
PTDPQTIEDSMNFVDNLKRAGAPVDGASLAGGMLSIFTKTALDMKTAKRVTAALAIAAET
AEGSTALYNAGGTSVLLTYCLDQGDLSDAGVEMVEGAFDTVRYMAGYQCTDATTLPQCIA
LMDKYRGRKSASAKGSSALAAMIGPEQLQKCLNTLKTAEAGSAEYDEALVTLGSMSYISS
FTDEIVRAGGVPLLIELINSGLPQMEGNPEKIASMISGAAKMLARIASNPVNVDAIVQAG
GVATLCTAVSYCTESMEALGALCMALVPLASRESLAHEIVQYQTFATVLPILYQNVESPE
IAALAMELVATGSQHEEIQEHMLQNQAAEICSLCCQYHTADASYQQHAISALNRLVPRLT
TLHGVSEYGGIQGVIASLNANVNNEQVALLAVQLLDNFSEVSDAKTYMSDGTCVDAVLAA
MLEHEGNDLLISAGVHCLARIATEDDCARHLNVLDTAIQTARGNPDGVYRVLAAISGLSR
VPSLRQIFEEKNASDTILAGISSWIECSRFEGQNRIIKAALKTVKNMKISGDGDLTSCFA
AMCDVACLPQVKRVVELEEPDNNILVADTAAFRDLAATMRITGAENLERCIESVLRVMRK
YPDSRRAQLNCLETLNYLAQCDGGEGVAILSRTGGLNAVVQYLTRAPMYLDAQIAGFTVL
ATSAKIDSNVGETLRKCNCLQALKVAMRTHAKSKELKRTIAPLVALLMPTDALETEIQEL
LNECASACEKNNFPHLHENLAALNELLISSEGAKIAARLGIGAHMCKYQEYISAHEQ--D
ALAVTDYDILGKDLFDATVSECAHAMEQVASTRSGRNALIKAGNVATLISLYESLKAPQS
QYSEEAAIHCLEALRILLKSDKRSAELAFERNFVSTLCVGIDSFPHSAPVLGATCACLAA
MATTPERVQMLTAQPAFESLLQKLVFVIQND-PSKDNKLVAMRALQELVEITNDATMANK
IAEAGAVTALFRIIDEYGDDEQLTVQAAEVLALLGAFEDLRRFYDNDVRFPAQVLTAALT
KQKNNETAVVHLLDVLNKLATSEDRAVLRELGVMEQVADAMRVHSESEAVTRLGGELFAK
MGADEQIKSLMLQIIEVESGAEDTAQTVDILCGRLAVFLAAPLEDPRDALQHTEKCLGSL
VATLQTYPGSERLEGNVALVCRRLCDRCFDDADDPYGAWAVAASGMLAQFAGMVAGETVL
ANKKFLGPAYRTFTACCANAYCMPTMVEVAPSFLPQTYTLLEMHKNDAETVARVLEFLRY
FAEDPTACGLIVQNMSGSSGDVVALTVLLMQQHQNNDAVVCAGMEFLGALAYTLSQAGYL
PTLADGSVLRDCDALMGSNSSSARQLAHMHMIEKMLLSKAYDALIQEQALKKLTSLKAED
DKKRFSDEER--LYAAMACVLLAAGGAGLTGEMEKFNGFEVV--LQAIEEFGENPTVIKE
VNRALQGLSMADVNMTARTVKEAVPKLCTEATTAIQTDAECADTFCDLMLQLVSQEGNGR
QLLQVYGLEETLQ----GVENLAAYYGEDFGTQLSEKVAMIRQAMEDDQPREKTCKDVYD
LLNSRVQQGLSVAISEVAILQEEVEFLVSQMGMYNQEQLDHQTAMGADHQYGNMAFELLA
ATSANVKLLQANEFSKMELALIKGQADPEIVLYAVKALTAFCKFPPAAQDT-ARIQGCPA
LVTEACSKINK-SGLPNERKEEHLCARYFLVERTAINRNLYNKTPIMTELINSWNDYDKG
AYTTTLLRFVFRAMRRVVSDAHVEELLKANVLQRLIGIISDVNADMALLPDVLFLLGSLA
VVPEIKTKIGELNGIAACTDLLQRALPKPNTAPVVTNVCLAFANICIGHKKNTEIFSKLG
GPALNVKVLNDRGHEYDVCNAASVLLCNLLYKNESMKKLLGTNGAPAALVKGLSNYDGSE
EKTAIRCLESVFKAISNLSLYTPNIQPFLDAGIENAYSTWLSNLSETFPDAQLETGCRTL
VNLVMENEENNMRKFGVCLLPCMAVAKQGRTDTKALLLLLDIEASLCRLKENAEAFAANG
GIETTIRLIHQFDYDVGLLTLGIHLLGIQSAVKDSIQRMMDADVFSILVGCVEVDAEGNE
VTDLVVGGLRCTRRIVRSEELAFEYCNAGGIATIANVICKSINQPMVMLEACRVLLGLLF
YTTRSQGVVSEPPPEEDELANAAYGGWYQMGMDEVMIDAILQAVCACAAVEAHAKQLRLQ
RVCLGLAAYFASEQMGTSSLVGSGIEQVLTQIMTNFAGEGTTMQLSCVIINSIAMTSGDM
YEEIKTSALLSALKTSVGKMATKKEEKALKETCAATLEAASSGEDPFDAFSKTVTELDFK
FTEWNVDPYPNGVHDLPSNVKEALRKGGKLKVFLPEKEKEEIRWRSSQDLNVFEWCMGND
QDYNNRIPIVRIRNVAKGLVHPALKAAAKKEPRKVAAKFTMCLFGPPNDDFPEGVELPMV
AKSQKERDAFVEMMVQWRDAATYNFMAEGGRRYGANFLSFVNETGSPYHSVLAVQQRLIA
CGFSQLDERDKWDLRLGGKYFVTRNHSCIAAFVIGEKFKATSGGFTVVAAHTDSPCLRLR
PNSNVKKEGVQQVGVECYGGGLWHTWFDRGLGVAGKVVVKQDGSLAEKLIRVDRPILVLP
NLAIHLQSAEEIS-AFKINKETHLQPVLCTEVYTQLLAANDSTAREE-KRTEEADSSCSH
LAAAPLLSLIAQELRVENEDIVEWDLCLMDATPGRFCGVHEEFVESPRLDNLGSTWAAFS
ALMECPSPHP----------EEISMAVGFDHEEIGSESYTGAGSNVLMVWMERVAQALSA
A----------EFYPQILSRSFLVSSDMAHGVHPNYAERHQGQNKPQMQQGVVIKENANQ
RYATNATSMALTRAVAE-----KGQVPMQMFTVKNDSRCGSTVGPILSARLGVRTIDIGI
PQWAMHSCRETCGILDLYALQLLLKEFFASFRSIDNSYKGM-MAAASSFFSVFFSVFLAT
PTAVVAQLKFVGLGNWGSGSYGQKTVADTLKKVAANEHISFIASPGSNFLGGVSSLNDTR
WQSEFENVYSDANGALKMPFFTVLGVDDWSRNYTSEALRTELTYAVKDGKLAPEAAAENH
GYPKWTLPNWWYHYLMHFPANTGGAFINSGHKDMSVGMIFIDTWVLSSSFPFSNVTSRAW
ADLEKTLELAPKILDYIIVVADRAVYSSGASKGDSMLQYYLQPLLKKANVDAYISGYDFS
LEVISDDNISHVSCGAGSKAAGSPIVKHSGSLYYAGETGFCLFELTAEGLVTRLVSGTTG
ETLYTHKQPLKNRPERKSIDAFNFVSQLPEVRYYPVPEMGKMPGRDVFVRVVGTIGLCIA
TIFLSLSVANGLSRYMKMAAFPKRGCLVVFEGIDRSGKSTQARLAYERLTKEGHAAELIR
FPDRTTPIGQVLDRFLANHQVPPPQLTHLLFAANRWEAQARILRALGEGRTVLVDRYSFS
GIAYTVGAAPVDATFCRESERGLLAPDAVFFLDVAPQETQKRGGYGDEVYEKLATQERVY
QVYRQFDNLPYWRRIAASSSIEELHEKLFEQLKSVIETQPDQLLPLGLMRLLRLGLLNL-
-----------------CGTVLEDLTLVDGLQFAESSSGGVSMVGQFVSSCTGAGRGLRA
GVGDSREQTLQRGFNNIQSIAERLRLSSQHISSAHRLYLMATQRNFTLGRRSVLVASACL
YAICRRERTPHLLIDFCDVLRTNVRALGQVFMKLLRVLHLQVPHVDPSLFLERFACQMQL
GDKTHTVAQTGVRLIQAMNRDWISTGRRPMGLCGAALLIAARYHNFQMNAEDIAHIVRVS
GPTVNRRLQEFKQTATAQLAVADFESTDLLSLPSQAQPPCRIKAYRRKLKLMQKKEEETL
ALTDSPAVPALPSTVPARMRSEEESDEPAEANGAEQGENGESNRRETEEGEDVSLATVCG
GPKRFAEFAAVGNLHLDTELLCKEAPSPEDILMLAGRMASALDGRVQSNLSQSTSASCED
FGTQTDVSLDASVAGESR-----------------------------DTRTAGESGAEEG
RTLSLDASGTGQAGSEDLPPPGTHGCRLRC--------------------SDVPASASSS
ASSSVSSSSASSSILSGVSRASCEDSEESRSVRSGFGDTETVGSTERGEEDDDSAEEEIR
AMFLSESEQRAKALIWDELTKDVMPLVHRRLKEKKLREEMMLNQSKRKRLQSTGRSDAGS
APAASAAESVRLALQMSSRSIVGKINDDALNSLFGNM--------ATPQESSGAAAHIDT
DLYSRQIGAFGLETMGKLITLRVLISGMRGVGAECAKNLILAGPNTVVLHDPAPCEMRDL
GSNFCLTEEHVKKGVSRAEASKNYLAELNQYVTVDVLPDEKLTQVVSRFDVVIVTEAGNE
ELKKTNAFCRSAS-KPVGFIAANVFGLAASVFVDLGERFVCLDSDGEEPREVIVAGITHE
RAATVHTHTDKLLPFQDGDFVVFREVQGM-EINDQPMQIRVTGKHSFQIGDTTAFSPYVS
GGIARQVKMPQTIRFKSYEASCRAPVAAGE------------------------AMLIVP
DLGKFGQSEQLHLAFQAVLNFRDRNGHALPPHSQQAAVAACVAEAQRLNGEAKQLAEVVF
VDQVDEKLVANVAAYAQCQISPMAAFVGGVIAQEVVKFTGKFSPLRGFLYMDAFEALALG
ETGKHREKYSIDSRYADQVALFGSEFQHALGRTHAFVVGAGALGCELLKSLALMGCCGPE
KEGKVTVTDMDRIEVSNLNRQFLFRREHVGKAKSVTAAASVQTMNPDLQIVALEDRVGVE
TETVFTDDFWRSQHIIVNALDNIQARQYVDGRCVWFGLPLLESGTLGTKGNVQVVLPFMT
QCYSDSADPPEESIPLCTLRHFPHAIEHTIEWARDCFQGVFCDAVSEPNKFRENPQKYLE
RLRGEGILSVQKDRLEKIRDLVSQWQDKESFERCVEKAVFLFQDLFFNQISQLLYSFPLD
HRTSEGTLFWAPPKRPPTPISFDANDPASLDFVVAASNLFAFNFGLPAVRDVSKIQAIAA
RVAIPQFTPKRLHINTDDAEKPNLSLSAEAEEEVVAGLEKHLLATDLEKMVFVPVEFEKD
DDTNFHIDLVHAASTLRAMNYKIPCCDRNKTKIIAGRIIPAIATTTAMITGLVSLELLKT
VTY---------------KQRK--LEDFKNAFANLALPLWLFSEPMPPNRVVDKDFDPVA
CGPIRAMPKGFSCWDKIQVDIPGCTVQQLCEFLEEKFDVEVNILSVGNFCLYNSFLPVHK
QQRFKRSIVELIEEVTKTS---GQKSVAVESSCSASDGVDVLLPTICVLNKMSKLSTDGL
KKAIGEILEGSREKKRKFVETVELQIGLKDYDTQRDKRFSGSVRLPNVPRPRMRVCVMGD
AVHCEQAKELGLEFMDVEAMKKLNKNKKLVKKLARKYDAFLASQVLIPQIPRLLGPGLNK
AGKFPTLITHNDKLEDKIQEIKSSIKFQLKKVLCMGVAVGNVEMTEEQLRVNLTLAINFL
VSLLKKNWNNVKTLHIKSTMGKPQQIYGM--------AAERKWADFELDDDYELGDLVNT
ATGFEEGVKTVTTYTQTLRGETLKVTKRIKVIRKCQRINKDVYARKNIVPFGLDSLSATT
VRSHEEIIIEVPRSSKQRKFKEEEEEDDFYMNPSKTSRDLRQKFRALREDDEAGAAGGDG
DEPRARREGEENRRREECTIRVTNLSEDVKDEDLTELFGKIGKIDRIYLAKHKEKKCSKG
FAFITYQRREDAVRAIRQLNRHGYDNLLLNVEWAKPSNRM-DEVLQRSFVYGLCTTTILS
PTLQYLRLFKKPVLTAMLLYLFYAAIPTDRRDLYTILDVDVMASKNDILQAYRTVSKKFH
PDKVAAAEASGGFMEIKKAQEVLMSDTRRSNYDRFGDYKYGDIDEKTTMIVVCLALVSHL
LCFCVGFLVSYPKHVTFARQIYVVYSLAMFCNELQLRFVENGDSLAFLPYVSSLVPFERV
HFFRSLFPCVLCASLCLSKWNFSDNPAKRLALLKALLSTNRVLTERTQELIRVTAYLKTM

GTPSTAAIKTQQQQLKSARARFSGQEMPVVPTAEAGKEKDKASSQRPSQSEQKDKKDVAE
WEDFTNTLNEEQKELFKRMVQYRKTEAEEQARESSRRRFDINWGQVLMWVCILYVWIYYK
MGKDYYRILGVGKDASEADLKKAYRKLAMKWHPDKHADADAKKKAEAQFKDIAEAYDVLS
DKEKRQIYDQFGEEGLKSG-GGGSANFVYREVDPSELFSRFFGSDRMF-FGG--DDDFGP
FGS-VGMGSHSNFPFRMHHAGSGSFPSKPKTYEVDLSLSLEELYTGTKKKLKITRTRYRN
GQMLKEDNVLSIDVKPGWKEGTKITFAGEGDQDSPTSPPGDVVFVVKTKPNSRFVRDGNH
LIHKVAIPLVKALTGFTVPIESLDGRSFKVKVDTVVTPKSRKIVPNEGMPVSKRPGEKGD
LILEFDIHFPKTLTDDQKTKLKELLPNV-MASTACRFFVAFFWASFAGVASAAYFYVQES
QDKCFVESVPVGVALTVTYKNPENPGVTCSIIFKDPSGRSVYSKEVLPTEQGKISHMTAT
AGEYKVCISCASSKWFNTQLLKWSISIELGDTEINLDELAKKDQVDSLQLKLQAIAKRLE
AMQAENEYERVQEERFQRTHESINSRVLWFSVLQLLLLCATTLVSVFYLIRYFHSQKIIA
SKGKKGKQDDDLDAVLEEVGASVPGKAESPAVAEKGKK-----GKKDKKEADDLDDLLAE
LESPAEKPAVSDEEAEGRGAPGGLTKNQLKKQKKKQAAQKKKDDSDDEKGPLSAAARAAQ
ERLRLLKEEEERKKEEEERKRQEEEQRKREEEEEKALREQRRAERKQRLKEEGKLLSSKE
KKERERQQAFLRQLQEKGVVL---GEDQSETSHKKKSSRAVDGKKKKKKEKT---ETPED
AEEAESSRRREEEPREEEKKKEEEPAHENWEELLDLDDDGDSPDAKTNTEKEQQSQKVKK
GGANARQRAEAAAAVEAAAAAEKKAQEEAAEKKKKEELRSPIVCILGHVDTGKTKLLDKI
RHTNVQDNEAGGITQQIGATYFPPEALVEQCRKFS-KAELRLPGLLIIDTPGHSSFTNLR
ARGSSLCDLAIVVVDIMHGMEQQTRESLELLKQKKCPFIIALNKIDRLYGWNSIPWQDIP
KHLASQGPSTRDEFDRRAMEAITQLQEEGFNCRLYWENEDVRKNVSVVPTSAVTGEGVPD
LLNLVVQVNQTLMQRTIAKERQLQCTILEVKAIDGLGTTIDVILVHGTLYEGDKIVVCGM
SGPIVTTIRALLTPQPLKELRVKGEYIQHAFIQAAMGVKISAPNLEEAVAGTSVFVVEED
DDIEDLKEEVMSDMGSIFKSVDRTGSGVYVMASTLGSLEALLVFLQESKIPVFGVNIGTV
QKKDVKKASIMREKGRPDLSVILAFNVKVDPEAEKEAKTLGVKIMTAEIIYHLFDEFTAY
FQSMKEEKKKAVAQEAIFPCILGVLPQYIFNKKDPLILGVVVEEGILKVGTPLCVPDKGL
RIGRVVSLEVNKKPCDKATKGQEVCVKIAGEPTVMIGRHFDAKNKLVSRLTRDSIDCLKE
HFRDEMSKDDWKTVIHLKKILGIQMANATTDHLRPQDLETLDISKLTPLSPDVISRQATI
NFGTIGHVAHGKSTIVRAVSGVQTVRFKHEKERNITIKLGYANAKIYKCSNPECPEPECY
KSYGSSKEDEPPCPRPGCGHKMKLLRHVSFVDCPGHDILMATMLNGAAVMDAALLLIAGN
EPCPQPQTSEHLAAVEIMRLQHIIILQNKVELIKESQAQQQQEEIRAFVAGTAADKAPII
PVSAVLKFNIDILCQYICTLVPVPVRDFTSPPQMIIIRSFDVNKPGEEATNLQGGVAGGS
ISQGVLKVGDDIEVRPGIISKDSAGNIQCRPIVSTIMSMFAEENPLKYAVPGGLIGVGTN
IDPTLTRADRLVGQVLGHPGNLPDCFGEMEVSYYLLRRLLGVRSQEGDKSTKVSKLKKGE
FLMVNIASTSVGARVAGLKPEMAKLELTGPVCTRVGDKIALSRRVDKHWRLIGWGQIHKG
KTLPLLESLMIHFLLLVSRQGKVRLSRWYLPLPMKERSELLREAASRVLQRSAKQCNVVE
WRDTKLVFKRYASLFFIACVDSNENALLTLEVIHHFVEILDRYFGNVCELDLIFNFHKAY
YLLDEIICGGELQETSKKAVLRVMNAQDALMEESNTK-------LERARLARQTLSSASH
EHLCACSPSAFRASLGSTVAAPFSPLALAARALSTASAGPSNAAP-RGGASVERMEDEEG
S-GERRKRSPAESADREQPE----NEEGEMETALEDKYRQCVEEVESLKKKNRELQDKAL
RAFADMENARMRHQKEMASLKDYAVSDFAKAMLEVADAMAYATNSLKEDALIGPEELQQI
YDGVKLTENLLHKTLDRFGVEQYNPEGEKFNPALHEALFELEHPEKAKGEVAQVIQRGYK
IKERVLRAAKVGVSKGMSESIIDALRTVTKVDDGTLEYMAGMLEEE--GVARLSVDTACD
LIGDFLHDRAVCEKLLSLLRKDAAPRNAAGEQNKRDGEGSLSKPRDFATPMKLGDMAGHS
DPFLGLHQNSAKVNYNAPVAAAAQQQQRLQQQRERHLRQLKEWEKNKPPLPPPKRKHGD-
--------KQLKKMGEGILVDSFSIAVAGRELLIDAQLKLVKGRRYGLVGRNGIGKSTLL
SALVRQEIHGVDPDIAIGMVEQEHLWGSETVLDAVLAVDEDRLRLLEEEQVLLKQEDQ--
--------------------SEKVGRRLGVIYERLQEIDAANAEKTAATILRGLGFTESM
QHMKVTALSGGWRMRVLLARCLFSDPDVLLLDEPTNHLDLEAVQWLTNYLSGKDKIVIVV
SHAREFLNDVCTDMIHFTNQNLTYYKGDFDTFESVRAAQLLQQQRQAEAQQAKIKHVQSF
IDKFRYNAKRASLVQSRIKLLSKLPMLDMVAEDPSLHFNFKEPEVLAAPLLQAEEVSFSY
APRKSDEESEGA----AEDAETDDAEPTAGASPSE------KADENARGKEGGKLIVRGL
NLNVDMDSRIALCGVNGSGKSTILKLLVGSEQPTKGMVHRNGKLRIGYFTQQHVDQLDLT
LNAVQSLQMRYPEAGLKDEAARTYLGQFGISGLLALEPLYILSGGQKSRVAIALMAFNNP
HILILDEPTNHLDLDAVQALIAALNNFKGGVLLVSHDSHLLSCVVEEIFYMDEQAHKLQK
YHGDFLKYRKELLKRAKMPREIVTLQVGQCGNQIGMEFWKQLCMEHGIDQEGLLVKQHAY
AEDRKDVFFYQADDEHYIPRALLFDLEPRVVNAIQTSEYKNLYNPENFFISKEGGGAGNN
WGSGYAQAERVQEELLEMIDREADGSESLEGFVLCHSIAGGTGSGMGSYLLEALCDRYPK
KLLQTFSVFPLLTTETSDVVVQPYNSVLTLKRLALNADCVVVLDNTALNNIAVERLKIHN
PSFQQTNALVSTVMAASTSTLRYPSYMNTDMLSLISSLVPTPRCHFLMTGYTPLTLDACI
SSVQKTTVMDVMRRLLQTKNIMVSASLRRGMYISMLNIIRGEADPTQVHKSLQRIKDRRL
VNFIRWNPASIQVALSKQSPFISSPHKVSALMMANHTSIASLFERCIVQYDRLFKRKAFL
DNYKKEPMFSSADGVGNFDEMECSKEVCVNLIDEYRRAEGDDYLSSFRHMEKYQKLEKIG
EGTYGVVYKAQDHSGEISALKKIRLEAEDEGIPSTAIREISLLKELHHPNIVRLRDVIHT
DRRLTLVFEYLDQDLKKLLDVCDGGLEPSTTKSFLFQLLCGIAYCHEHRVLHRDLKPQNL
LINREGALKLADFGLARAFGIPVRSYTHEVVTLWYRAPDVLMGSKTYSTPVDIWSVGCIF
AEMVNGRPLFPGTGNEDQLMKIFKVLGTPQVSEHPQLAELPHWNRDFPQFPPLPWDQVVP
KLDPLGTDLLSRMLRFDSNQRISARQAMQHPYFSDLAMGNKHSVIPLIFDMKIKARELKR
QSERCYRESEGEKGKVKAALQKNNSEGARVFAQNFIRKQQEGLHCLQLSSKLDAVASRLD
AAHRSQQMSKQMRSAAYGLSNALRTLDGGTSLRQMEEFAKLFDDLDVRSDSVSTLLDAST
STSIPLEQVDDVLKHVATAAKIHMMLELNRDFSKSVETFERLLIFVMGKEKTHINLVVIG
HVDSGKSTTTGHLIYKLGGIDKRTIEKFEKESSEMGKGSFKYAWVLDKLKAERERGITID
IALWQFETPKYHYTVIDAPGHRDFIKNMITGTSQADVALLVVPAEAGGFEGAFSKEGQTR
EHALLAFTLGVKQMIVGINKMDSCNYSEDRFNEIQKEVAMYLKKVGYNPEKVPFVAISGF
VGDNMVEKSTNMSWYKGKTLVEALDTMEAPKRPSDKPLRLPLQDVYKIGGIGTVPVGRVE
TGILKAGMVLTFAPVGLTTECKSVEMHHEVMEQAVPGDNVGFNVKNVSVKELKRGYVASD
SKNDPAKGCATFLAQVIVLNHPGEIKNGYSPVIDCHTAHIACKFAEIKTKMDKRSGKTLE
EAPKCIKSGDAAMVNMEPSKPMVVEAFTDYPPLGRFAVRDMKQTVAVGVIKSVEKKEPGA
GSAVKAAKKMSHLLNAPIILLKDGVDTSQGRGQIISNINACQVIADIVRSTLGPRGMDKL
IHSENGVTISNDGATVVSLLNVVHPAAALLVDIAKAQDDEVGDGTTSVVLLAGEFLESAK
TFIEGGMAPQILINGYRTACQLAIEKIRELKVDLSSTPPHEKRVLLERCAQTTLNSKLVS
GHKDFFAKMVVDAVSMLDDSLDKEMIGVKKVTGGSYTDSFVVQGVAFAKTFSYAGFEQQP
KRFENAKILLLNLELELKAEKENAEVRLKTPDEYQAIIDAEWDIIYEKLDKISATGAQVV
LSRLPIGDLATQYFADRDIFCAGRVDEGDMKRTARAIGAKIQTTVNSITPDVLGTCGVFE
ERQIGGERFNLFMHCPQTQSATIVLRGGAPQFLDEADRSLNDAVMIVRRALQTQTIVGGA
GAIEMELSKYIRDVSQGISGKQQLVIRAFARALECIPRALATNAGYDATDILNKLRHKHA
QGKGEQWFGVDCMNGGVCDAMKEFIWEPALVKENALAAATEATCILLSIDETIKQPTPND
KRGGPPRRMGANFTEFGDLVLLIGDFHIPQRAVDLPPCFRELLNTDKIRHVLCTGNVGCA
SVVDSLRSISSSLHIVKGDADAGFDFPEYKVLQFGQFKVGLIHGHQIVPYGDGGSLLHWQ
RKLDCDILVYGHLHKDSVVELEGKFFVNPGSATGAYQPWLTEKVPSFMLMAVQGSSVVLY
VYEEKNGKAEVVMSEFKKDNTQSGPQIVYNVPIEDPAGKKRTSIYRSVFSPDAIVDNFAD
RPCQSAWDLFQRGVAISGEKRCLGTRVKADGTLGPYQWKTYREVEQLALEVGSGILSLDA
APKLHFEEETFQQMRFLAFYSKNREEWAICEQACNAYGITIVPLYDTLGPESTAFILAQT
RLRSVVASEECARRLLDSIEAAKSVNASRVTPETEDAAEQTTEGAGKEATETALLDQATK
LGIQGKENLIPAKLSTVSTVCYTSGTTSRPKGVLMSHGNFVATIAGAVRGPLTVPSMALH
PEDTYLSYLPLAHVYERSLQNILFSLGGSVGFYGGNTLKLLDDMQTLEPAVFSSVPRLFN
RIHDRVLDSVRDKSTVAQTLFNQGMTTKIRKIRATGSPVHAMWDKLVFNKTKVLLGSKLR
YMLVGSAPLDVSVHEKIEALFSTPVVEGYGMTETMAASFISIAGENTAGHIGGCCPCVEF
CLFDISEMPQHRIDDPPAGELCLRGPTITP-GYFRNREETEKAFDQDGWLHSGDVAVIVP
SNAVKIIDRKKNIFKLAQGEYVSPEKIENVYIQAPLVAQAFVTGYSSQSCLVAIIVPDAE
KAEKWAVQRKLDTSLETVCTLPEFHRAVTESMAAVAKEHQLKGFEVVKHFRLVSEPFSIE
NELLTPTMKIKRYVAKEFFAKEIDALYSEMETEMSDWDPVVKEWLVDTGYCCAGGIANAE
DGVVFAAAADDD--DGWSKLYKDDHEEDTIGEDGNACKVSINEASTIKAAVDDGSAPNGV
WIGGQKYKVVRPEKGFEYNDCTFDITMCARSKGGAHLIKTPNGSIVIALYDEEKEQDKGN
SRTSALAFAEYLHQSGYQSSQHTASVSFSASSSRPAAAPLVSFNQPSRAAPVLLPTVSTR
QAKTCPYCNGAQTIVFDSSSGDQLCRECGLIVEEKVLSEEQEWRNFSAESSSGGADRNRV
GDALDAWLEDGIGTTMLVASKRLQQLEAATSVGSGDRQLKVAFNYIRLIGEAFALRDNVL

ERAKEITKDLMQDGQLRTRSNTTTMLAITYLACREAGVTRTVKELVVYDRAISEKELGKA
INRIKKLLPQRGGVNSESATQLLPRYCSRLQLSMHVADVAEHVAKRATQVIISSHRPNSV
AAAAIWLVVKLLNS-STNPNLPKASEIASVTGAGEHTLRSIYKDMLDVAEHLLPREFQPT
VEGGLDGLRARYSSRKRKAGISTLQDIRLPTWQQIRRFVQDPEIVTAEVLFLILTLSNVF
VMYRLFLDVVPYPVFVTWWQLAQGLFMAWCLGETGKEFPKFAYFPRVEFDKKLLKKLVVP
SIVNAFMLVLANVVLYRTNCVATLPVTVSFAVVLHHVTRFIGCGEEYMPMRWQAVGLLML
AFVLGCTDSMTVGAQVLPWAVLYSIFSAAFRAAYLQKVMHEVDGRGNLLYNHQHIIGVAL
LPILCLVCGESRVITSMPMNFTLLHTWQVWGCLVTVGALPFLKNIVSNRLIRRTGQAPWR
FLEIVSIALVFLIGLGFGVPGWQGFICILLVLAGRTFCAYDVIMNAADVEAARETRGGSR
APQELGDANEGSQSSKPFLQAIQEDGGENYADVCDDEA----------------------
---HSSM--P
QDIRPAPNRMNLAIMKQKRKGAQQGYQLLKKKSDALTARFRGMLKQIVQLKQAVGADMNT
AAFSLAKATWAAGDFKSQLLERVRRPATFLNVAADNVAGVTLPIFHICTDPSVDVLKNVG
VAAGGQVIMAAREMFLKVFSELVKLASLQTAFFTLDEEIKMTNRRVNALSNVVLPRIDGG
INYIVRELDEMEREEFFRLKKIQEKKRM--RKEEEDRLHENAAGAANGHAPQSLLNDDDD
DLLFC--AFLPLVCFAFLSLAAGAKIRIDPMTHDVALVTQQNWDGKVQKFRAHQVFAVLF
YKDGCSECQSLLDEFNTLAKKMKGMVGVLAVDCGESAKLCTDQ--------NVSSYPTIL
IFPPLPLPPYKFEGTPNYASLSKVGPLIPSSVIIPTDMKDFEKAMATHVQVPKVLVFSDK
PKASVLLKALSTAFKDKLHLVMINAKLPEVVKKYRVTKFPHIVVTRKDKKDEAYKGEFNF
QVLFDWLNVFSETFVLGGGFSDHTPKAETQPWKFQVIPELTKRSNVDLCFKKSLKALCVI
YLKQGRELTAEETDMLEALKYQYD---SGKGPDFRFMFMDVDTETGFRDLFAMESYPSVV
VFNPHLKTRFTKLDADEHLSKESIASLLDKISSGNARFKVVPSSKMPSFVETEKKEKADK
KDELSLSSSAALYSSAPLKRVDQLQFGVFSSEQLRKMSVCEVTTSELYEHGMPKANGLND
LRLGTLDYRQQCRTCNMDVKNCPGHFGHLNLVKPVYHYGFLGAVLRVLRCVCYACGKLLV
DRRDPKMQHILKIRSPSRRLKHVLDACAGRKRCEGYLPADGMPV----------------
-----------------PLAEEGEGGCGCVQPRYFKEGPNIMVLFPDNDVTE---DIRRI
FAAEEAYAVLRRISEEDLKMMGFDPERAHPASFILSTLPIPPLAVRPSVQYGSARSEDDL
TLKLVDIVKTNLSLKRQGD-SVPGAVLQEMVMLLQYHVTTLFDNDIPGMPVATTRGKKPI
KSIRARLKGKEGRLRGNLMGKRVDFSARTVITGDPMLPIDTVGVPKSIAMTLTYPEFVTP
LNIGQLRQLVKTGPFDWPGAKYVIRDDGSRFDLRHAKKGGEVVLEVGYRVERHMRDGDFV
LFNRQPSLHKMSIMGHQVKILPYSTFRLNLSVTSPYNADFDGDEMNLHLAQSEETRAEIK
HLMKVPKQIVSPQGNKPVMGIVQDSLLAVSKFTRRDTFLTKPMVYNLLLQIPYWSGVVPP
PAILHPVPLWTGKQLFSLLLFFDSS-----------------------------------
----------------------------------VSGGNTKTRINMQRDVGAGLKKENLF
LSERDERVIIRQGELLAGKICKKIVGSASGSLIHLLWLEAGPERTKDFLSTLQKLTNYWL
LHQGFTVGCKDIIANEETNEKVRDILDQAKKEVDKLIRLAHRGRLESQPGKSLRESFEAR
VNKELNSARERSGKVAAESLDESNNIMAMVLAGSKGSTINISQIMACVGQQNVEGKRIPF
GFNERSLPHFHKFDYSPQSRGFVENSYLSGLEPHELFFHAMGGREGIIDTACKTSETGYI
QRRLMKAMEDVMVYYDRTVRNSICEVLQFLYGEDGMSGEFVEDQTVELMTLDNEKLKRLY
RHDVDQESYGKGWLS--DELRNEILTDYELQQPLEEEFEAIREDKNRLCRHIFKDGETKQ
HIPINILRLLEFAKAQFPS---

-------------GDSSRK-QSPIEIARKVNELLEKLVVVKQTTNADAISAEVQENATIF
MKAHLRTVLNSRRLLERECIGPKALQWLLGEVERHFHRALAHAGECVGAIAAQSIGEPAT
QMTLNTFHFAGVGSKNVTLGVPRLKELINVAKQVKTPSLTVYLQDEIAMDQERAKDVQTH
LEHTTLDRVTVVSQVIYDPDPTDTIIPQDRQWVRDYYEFPDDDELPSNLGRWLLRIQMAS
KVMIDKKLTMKEIGEKIYAEFPNEELDCIWTDDNSDDLVLRIRLKQQPGE----------
-----GEDEDKDEDD---

---VAE
AGDRFLQKLMVQCLAGITLRGITNISKVYMREEARTIYNNQLGKFERTNNWVLDTDGCNL
EDVLPIPMVDDTRTTSNDMTEIFHVLGIEAVRRALLRELRAVISFDGSYVNYRHLAILCD
TMTQKGYLMSITRHGINRIDKGPLMKCSFEETVEILMEAAVFAEADHLRGVTENIMMGQL
CPLGTGYFDVLIDEEKLKDASHNLDGLGEFDGGVGTSPTS---ITPDGSFTPSPLNSVFS
PMPFSTVYSSFAASPANP--------------------LSPTTGTNAGVDTQQLGGKFSP
TQTPRSPTSPL-SPLSCFSPRNLSPLSPSGVFSPTSPALETSSPSYSPSSPQYFQNDSSA
VDMISPGGVGSSYSPTSPTL----SPSYSPSAPSY-------SPTSP-----------AY
SPTSAPLSPFSPSNNPMSPSYSPTSPTSPYSPAYSPTSPRYSPTSPMYSPTFAPYSPQSP
SYGGPLSPS---FTVQSPRYSPSSPAYSPTSPMPNVAGSPSYEDSHAPGGDTYSPT----
--------SPMYEA-------NDPFSPDPFAAELESPTEDMVVFQIDEVTVYFPYDFIYP
EQYAYIRALKHTLDAKGHAVLEMPTGTGKTVALLSLLTSYQLAHPRLGKILYCTRTVPEM
EKALLELKGVIDYRIEEIRKARETKARQEQSPCEEKEQTTSASGDSLGRKRSKTTESEDY
IGKDMARDGYILGVGLSARRNMCINPAVYNQPDRERIDEACRQLTAPWVRQQHLTSYNPP
SHGDAPTGDIENLGEGGEREDGEEGKEERERRRRRAEPHYSPSLCAWYENLDRYFAPQFF
PAGVYTIEELKIAAGNWRHPLHRNLPFCPYFLARRLLHIANVVVLNYQYILDPKVSQAAL
LTPLPDGMSSSHTGSVVVFDEAHNIDNVCIEALSVNINRSVMEAALRNLSQLAEKIEEVK
KEDAERLREEYENLVRGIKRRRATEEESASSSSAPSSSAATSSSS---------------
-------------------------EAGGGSACVRPEDEGERIR-EKRKESRDPGRIVVA
NETLDLLASPLLPDDALLEQAVPGSIRKAEHFVALMRRIVSYLKSYIKIYELKSEGPLSF
LHMFEKETHIDASLLKFFYDRLKSLFNTLQITEVESYAPLTLVADFCTLVATYCEGFILI
CDPYPEAVGLYDPLLQLSCLDASLAMQPVLKRFQSLILTSGTISPLELYPKLLNFVPVIT
ESFPMSLDRNCICPLIVARGSDQIPLTSKFEYRHDMNVLRNYSNLLIDLCKHVPDGLVCF
FTSYSYMESVLSSWYHSGVLAQVLDYKLIFIETKDVVATTLALHNFRRACECGRGAVFFS
IARGKVAEGIDFDRHFGRCVVLFGVPFQYTLSRVLKARLDFIREHYQIPDNEFLTFDAMR
QAAQCVGRVIRSKNDYGLMIFADARYSRIDKRSKLPPWILKHLDNAHLALNTETAISVAR
TFLRHMSQPPPPPSASRLDARQLQQQQCWELVRRTLHLSDGQLQSRGFFSAAPAA-ATA
RSV--DAAIPEEAFNQPPTLTTTLPNGIRVATQRLPHQTATVGVWIDSGSRYDTKETNGA
AHFLEHMTFKGTKRRSRIQLEQEIENMGAHLNAYTSREQTVYYAKAFKKDIPQCVDILSD
ILLNSTIDEEAVQMEKHVILREMEEVERQTEEVIFDRLHTTAFRDSPLGYTILGPEENIR
NMTREHILEYINRNYTSDRMVVAAAGDVDHKELTALVEKHFAGLPQPKRSII----LPTE
KPFFCGSELLHRNDDMGPTAHVAVGFEGVPWKSPDAVTFMLMQAIVGSYRKHDEGIVPGK
VSANATVRNVCNKMTVGCADMFSAFNTCYSDTGLFGFYAQCDEVALEHCVMEIMFGITSL
SYAVTDEEVERAKAQLKTQLLGHLDSTTAVAEDIGRQMLAYGRRMPLAEFLKRLEVIDAE
EVKRVAWKYLHDARPKVGALGSLEGESGEKKKRDSTRQMRSMASLPKMLWKLEALLIFPS
ETPHLPPSAQKKLFDEATWVWREGEDVAQE-EDNFTFIVELATFVQLLKRCHYRSVLFGA
SPLACLLLCCHEADPPSLSALDTALQESCAAFVSSPSSGEGENGRNKVDEALLQSLRLRH
VLLASVAAAQLFLQVNWTGPPIRAQRPAESSEAPGQPEGDAKPPPESGAAGAETRNALQR
AMEAFNADGEDVYELVRGLPYLWLACRLAAWLRGHSAR----------------LLSASS
DSAISC----------APVFPVFSSFLWSARLAMVSQRILVSAAACLAPLRVSVEE-LAA
ALKAIKVLPSSFSLPRAPEDVRNVDATEASRRLLNSLSSSSLPASEWFTDRQRALMLLEL
SLHLLMYGKVDALTEVMDAACEAANIRFSFTGAEGIRRKYQQRAIAQLVVAA--------
-----TPIRPSSSSLPLSSSSSSSSSSPSSASASAS--SSSASASSSSASASSSSASASS
EYDSVTSREEGEKRDTEEEKTEEET------------------ADAEAEAKRRDWRLQDV
HADADVLERPQLVDSVDAQAAPLDGLEQALLLARGLRILESNPARDELALEQLNAVAVRC
LTLKEEAKQGECSGEAAREAALDALEIKSADWLLHSAALWLRCKAEYHRSKTVERACLQL
NALIDQFTDRLPAPSQRLRFIYHVDYPSSWQGRRELARSMVRRLSLCQAYESFKDLAMWE
EAAECLYAADRRADAEELLLERLKVRESPPLWCTLGDLRKS--VECYEKAWTLSNKRCAR
AQRSLGRLYMGEENFSKAAEAYARALELNPLHRASWFILGCCEMRLERWEDAVQAFGRVV
AL-EPQDGDAWANLAAVHSQREAWTAARLCIGEAAKYRRESWRVWDNLLKISVRTRDIPG
VNEALRHYVDLNVTDRIPIWIYSFLTHAVLGEKESLNPGKSPAASFKSTLKTLEFLAQNM
ADQAELWKALSSLQSVKGDFLSAAETRLKHFRALSALLRRVNGPSERGAQQKEAIDALRA
AAHLLQRAVDGDDPVDIARRMRDINTTACSLTEELRQRANELATQTSAFLQMLASPPSNA
AMSDTEDRQEDAEENESSSEEDEGERRRSKKSKKSSRRSSTERRSRSRDGGRRKKRRAVN
AFLDVEAQEGDDEEEEDNDDDYSEEAAQAARLAAQATERRQRRGGASHLESAIDSLTRRY
QDQTF-----EEGEEEDDLDADGFAEDPNENNLLPDITDPKLWMVKLNKTGVEREVCISI
LNKSFQMQEKDCEIYSCYASDDLKGYVYVEAFSQYAIKEALQGLRLITYGEIKMVPLEEM

TAVFNSVRSRAYIPQRNDFVRVKRGLYANDIAQIHQVEEQGMVVTVRLIPRLDLNALLDR
EKRGD--FSR
KSDMATLKQGVRAQKRFFDRDEVDARGGQVEQGILPGTVRFAGMTFEESGYLLRRMAVRH
LLVGSAAPSLAEVTEFVQQGKDEDDLHIRRPLSSFLKRQRSAYRLGERVRVIGGELQGMR
GKIGEREEESVEVIFDDRKGPVMLKASHVVKDLQLGENVRAVGGVNAGHSGLITSIDLAK
QTATVFSPAAGLEFTCGLESLTVAPDAGLGEEGLSSVRGFHLGDFVELTSGEKGVLVFID
KNESIRILLPSNSALCATAAQLASKRSTDLLTTLDESRVTVEKGATVVITKTQEKGAHAK
VLHVCRDRLFLKVMGRSENGGIIAVDAKHVRVSGHDFRSPGFPQDRGALRGGLTRGGMHR
NPYLGKLVKIMRGRYRGLMATVRRVEQSELHVLLQMKEKELKIPKSDAQLIESSREYQSF
VGGMAAPLSTPLAGV---------SASTSLESARGASSYLLGPSGASLSSSAHGRTLTPV
SSSAAFMLASGDLKGDRTACFFDARAQGESVRASSMKIDDLFDGHLLT--------PRED
DAASNSSMSMSVANASTVGGC---------AQTPDWCCPGVEVCVIGTGGYRGKIGAIGQ
ILPQDDHLLCHVYIEEDLIAIAAESLTPVIPEQPNKRVMILDRDCSEFGKVQAISPTCAV
IRTQNAEVMCNPR--LLVNFNPPMAQQFPIQLHSVVNLADQGVSASSFRFGNVAMHGDKN
LVVKD--TETNELFIFSLSVREFTKKPTQAEAALMHPSEKVVALRAKTEGSAGHMIQVLN
LETKVRLGTAQMNEPVVYWRWVAPTLLALVTDRAVYHWTVAEPEKVCSREGRLAD-AVQI
ISYAVDKDLKWCILTGISTQDGGKTIDGSMQLYSMELKKQQQLEGHAACFNNIVIDEVGP
QPVICFTEKKRGSPDFKLHIRDIYSSRDGGQTPLRLAVDLRMPEDAPTDFPLSIHISQKF
GVVYIVTKGGYLILLDALTGTELFRHRILQDAVFLATDSPQTGGILTVNKRGLVCLCNIN
LQALIPYINLVYVPNRQQIATSLAKRYGLPGAELMQEFNQHFASGNFKTAARIAATLKSG
VLRTAQVIQQFKSVPTQPGQTSAILVYFSTLLEYDSLNAVESLELVRPVAVQGRKDFVEK
WLREGKLECTEELGDVVRTLDAQLAVRVYREAKAGAKVLQTLTELGNFDEIIAFAKETK-
LEADYAGLLRNLVNVH---------------------PENAVKFAQQL------------
-------------LSSEPPLADVTQVSEVLLQQHKYQEFTSLMLDFLKGNKPEQGPLQTR
LLEVNLLHSPQVAETIFQMEMLTHFDRAKIAALCEKVGLSQRALELYTDIADIKRVMLQS
GGKISQEFTQQFFGNLPPDASLEILTDMLRSSSQNLQAVVAVAIKFHGQIGTTKLVEMFE
KFSSYEGVFYFLGSILA---
--FSSDPEVHFKYIEAAAKLNHTQEVERVCRESKC-YEPQRVKEFLKQVKLPDPRPLIYV
CDLHGYVTELAEYLFKNSLLKYIEVYVSRVNSANAPLVIGTLIDQDAAEDFIRNLLQSVR
GGCSAQQLVEEFEKRNRLRLLLQWLEARVAEGNQEPAVHNALAKIYIDTNRDAESFLKTN
AYYDSLVVGKYCEERDPHLAFTAYKRAWGACDEQLVELTNKNGLFRLQARYLVERQAPEL
WAYVLREENPHRRQAIDQVVSSALPESSSADEVSAAVNAFINAQLPHELIELLEKIVLHN
SDFSNNKNLQNLLILTAMKADSSRIIDYVNRLHNYDGAAIAQVAMEYGLREEAFTIYKRF
GLFGEAADTLLK----------------------------------SAEEGSEADLERAV
EFAQRCNEADVWRKLGRAQLRQKRVRDAIESFLKAGDGDVYKEVVEAAAAEDAYDALVDF
LLMARKKITVKDQVIDSELVYAYAKTDRLEEMDAFLSGTNTANVQAVGDRLFAEQRYKAA
KLLYASLPNYAKLASCFVRLEDFAASVDAARKAKNPKTWKEVAFAALSKGELKCAHAAAL
SLIVHPDHLDSLIERYEQLCLFKELIELLEQGLQGERTHVGLYTELGVLYATYESSKLMD
YIRQHSGKVNIPRLIRACERQSLWKEAVYLHMNYDEYEQAANCLIMH-PAAWSHELFVQI
LQKVSNSDVFYRAISFYLEYHPLQLCLLLKSLDKKLDHSRVVQHVRKAGHLAVVEKYLRE
TQHLNITAVNEAVNELLVEGEDVDGLRESILEYDNFDQLALAQTLENHPRVEMRRLAALL
FKKNRKFKQAIELSKRDRQYQDAIDAARDSGNTQLVGDLLRFFLDLEDPAGFAACLYTCY
PLVKPDVALELAWRHKCMDYCMPFLIQVVREVTTRVDALDKKEETREKEEEKQKNAPNDY
VPDYTMPPTGSPLMGGLALMPPQSNAFPPTLGSAPANLGQSFA--MAPMVSQPAPAFEAE
AVMADGSFGKISLSQFKGKKYVVLFFYPFDFTFVCPSEILAFHRLHGEFEKRGCQLLGVS
VDSKFVHNAWRNVELKDGGIGKISFPLLADVSHKMAEDYGVLHPEGMAFRGLFLIDKEGV
LQHCVINNLPLGRSADEALRMLDALQHVEQYGEVCPANWKKGDKAMKPTAEGVKEYLGSK
MAQSTASAILKDDAGVNEGKMTEDFSQAASQLVDKISQLKQVGPTNPIFDELFALEKKCR
QANDGASGSRLCCFYLQNLEDLRKAGELCDQLVVLCKKRGQLKRVISDIVKLAVTWLTEM
K-KEDKVEVIETLKRITEGKIFVEVERARLVLMLAEMKEAEGKIDEAATILQEVQVETFG
AMERREKTEYILKQMSLVLRRGDFIRCQIISKKISTKLLDNDELQDLKIRYYSLMIVYYL
HEGMILDCCKAYQSIFITPSVQQKEDQWIKSLQCYILFLLLAPFDDEAKQLATVQTMEAK
KLKEIPVFAQLLKDMTTVVLLSWPLPYEATLKAHEVFQNTPHEGGEGRWALLRRRVIQHN
IRVIATYYSCIEMDRVASLLDITKDEAESEISELVCSNFIEAKIDRPAGTVEFGKRKGTF
DRLNSWAADVTSLLDRVDLCSHLIQKERMVHAARAKNAALLARNAS-GNDDGDGQETMEI
LMEISTLLDTGLDRATLQILVELVEQGVHPEALSRVVLEMRRELQAVREKEERSERNNPI

> ***Cryptosporidium parvum***

--------------MSTNNKT---KSDNKSKTGQYEGKLPNAIQGQVVTRFPPEPSGYLH
IGHAKAALLNYYYANKYEGKLLLRFDDTNPVLENEEFQESIEADLKILGITPFNVSFTSD
HFDTIMNYCEEMFKLGKAYVDDTCVEVMREERGKGIESKNRNNSVDENLRLWKEMIDGTE
HGLKCCVRAKIDMQCKNKCMRDPVLYRCVL-TPHHRTGTKYKVYPTYDFACPIVDSIEGV
THALRTNEYSDRIEQYNWVINALGLRPVEIYEFSRLNFVNTVLSKRKLTWIVNNGLVEGW
DDPRFPTVRGIVRRGLSPSALLQFVTEQGPSKNSNLMEWDKLWTINKQLMDPVVPRFFAV
G-QDAVALNLSEAPEEPLINQRDLHQKNPDLGKGQIVMYKSILIDRDDATQILSGEEITL
MKWGNAIIQDISQIDKQPTGIFEGKLNLEGDFRSTKKKIHWLANLPSVLTKCILREYDHL
I--NKRKPEDGDEIQDLVNTESLFETPAFCDPLIKDLKSGDRLQLERRGYFIVDKPKPEE
PIILIKIPDGKSKASSTISSKVDAQKLAKGKNMSSFCIVIDCGSAYMKAGTDSDKSPTCI
FPSMVGQYRDEDNNPIFVGEEAIAQRKKLSLTFPIDHGHIDDWTKFEELLNYLFRGLDID
PIDSSVIITKPPLCSNRHEEKITELMFEMFQTQSLNIALQGLMALYSAGRTTGVACDIGE
GVTQVVPVYDGYCDSSSLRRADIGGQEITMYLQKLLSDKGYIATTRDDLEHVRIIKETLC
YIAKDPAAENEREEINEVYTLPDGLTLHDEHNKIEIDKERFYAPEVIFDPKLIMRDVQPV
HELIMESIMSSPMEVRKTLMGSILLSGGTSLTTGIEERLEEELMYICPSQAKSNIRVTPA
DDRNYAIWKGAQLFS--ALRDYQENLWISRDEYLEEGVNICIKKQPFFSSKMEELKHLRI
LNKVQARIFEEWNTNDRDLAEYLCYIGKESKSLKEFNGKVTSSGDKISHEVLGDIYSIIN
D-----------------------TESKLNKVNSDSLKLQKDSNNDNLNFAIPNEKAPLS
KHALELIRKED---
--HYVDINRKNKGQSETITGFGM-------------------------KDTNLKKGFGAI
SGIKL----------------VDSCNVKSQYSG---------------------------
---------------NKQERVSNDYEKWEIMQLLNSGVISRDEIPYDICDTTGDTDFQNV
EISTEIELRNYEPLFLRGQSIKKFNFDSSIQVVVNPEGSLNKAAELASNIARERREIRDF
QEKTLIDSIPRDMNRPWEDPNPEAGERTIASALRGIGMSQTTPEWKRQYLGKSLSFGKKN
TASISEQRKNLPIYPMRDSLVDAIRNNQVIVVIGETGSGKTTQITQYLYEEGFCKDGIIG
CTQPRRVAATSIARRVAQEMGCTLGSTVGFAIRFEDITTPETKIKYMTDGMLLREALSDN
CLSQYSVIMLDEAHERTITTDVLFGLLKETCIKRPKFRLIVTSATLEADKFSAYFMNCNI
FTIPGRTFPVEILYSKEPVDDYVEATLVTVLQIHLREPPGDILVFLTGQEEIDNACQTLH
ERMKRLENMKPPPLIILPVYSSQPSEIQSLIFEDAPPGCRKCVIATNIAEASLTIDGIFF
VVDPGFSKMMVFNSKTGMDSLTVTPISQASAKQRSGRAGRTGPGKCYRLYTEAAFNTEML
PTTVPEIQRTNLANTVLLLKALGVNDLLNFDFMDPPPTTTLLIALETLFELGALDEEGFL
TRLGRKMAELPMEPKLSKMVLSSVDLGCSDEIITITSMLSVQNVFYRPKDKQAQADRKKS
KFYHPQGDHLTYLNVYNSWKKQRYSVPWCYENFLQSRALKGAQDVRKQLINIFDKYKLDI
-----ISAENDHDKIRKAICAGFFSNSCKKDSQEGYRNLVDNQHVYLHPSSTLFNKSPEW
ILYHELVFTSKEYIRDCCTIKPHWLVDFAPNLFQFADQDQLSKRKKKEKIQPLYNKYEDP
NSWRLSKRRRMPDEYDHLYKIILVGDATVGKTHLLSRYTRDALPKTPQPTIGVEFATRTV
PLSIGGTVKAQIWDTAGQERYRAITRAHYRRSVGALLVYDITRKSSFLNASKWLEDIKQN
SEPDIVVMLVGNKLDLVEKDPSKREVPFDIAANFAQENNLFFSEASAVTRCNVKHIFEHL
LQEVYNQKMKDNSSNLDMNGKFIKD---------------------------------DS
APKLAKVEKILGRTGSRGGVIQVRVQFMGESELAGRSLIRNVRGPVREGDILALLETERE
ARRLRMS----------------------NSKKHNLILIGAPGSGKGTQCEFIKKEYGLA
HLSTGDMLREAIKNGTKIGLEAKSIIESGNFVGDEIVLGLVKEKFDLGVCVNGFVLDGFP
RTIPQAEGLAKILSEIGDSLTSVIYFEIDDSEIIERISGRCTHPASGRIYHVKYNPPKQP
GIDDVTGEPLVWRDDDNAEAVKVRLDVFHKQTAPLVKFYEDLGILKRVNAKLPPKEVTEQ

IKKILENSLLNSCSGCKVVIDDYKTTGREYGVFVGYYSESEESDYDESDVVTLDK-KPSN
IQDSSSAEAVSIEDELEEG-EADIIREMFDNASSGGECSLDQTCHLAHRMGLAPSKSDLE
QLNEETGGKVTYEDFERWIMSITHPEDHIDYMVSYFRKYDLRGNGKISRQQFIWLTSIGG
DILTREEAEAILDKLSIGGDVYYEDLLRKIMDMKSLKLVLTTFLVLTLSLLAG-FVFGTN
ENP-----NFLRLVSDKKMYDTMTVSEEIILERFGSLLEIIDLDKNGLLEKDELIKWIKF
VSERSSLKEVEAEFRILDKNKDGKLSNEEFINHFVSEKDEKEITELNNFYRELFKEVDTD
KDGYLTVGEYYYLTNYYSLSKELFVKINSFLSQNDKNGDGIIDIEEIKQIQKDNAEVITN
DSGKLVVFG-VDVSGEKELSVKKIIFLLRQEIQDAITEAYNQLIDVYNTRKSIPIDFVRS
NYVIYIQSILTDYGDVFKYPHDYDLSKEKCTELFGENEKSDEENEFDTDEDTDDNNPNDD
NQEFPSLEARDELMNLILSKILSFISVIFVGGIGVYIPIYIGYLNPI-----------LL
QYINVFAGGTLLSLSLCHLIPEAEEIVRANDISLKFI----GVEIPIVAYLTLFGLTIIL
FFEKALFSPQDVACVGVSNSDVNGEKKNNNNNNNNNNNNNNSNSNSFFTCPNNQHRNSTS
QVSLKQESNIQKSKTNSTLNVYFLVSALSVHAIFEGMLVGISKNHISVLTITLVIIAHKW
VEGIAVSAGIKKHEISKTTVNQLLISFILMSPLGIIIGQLFSYNSPLINVVLTCISSGAL
LYVALEMILDEFNC---GENRKQKFILFLLGILLVSIINIIQHKLGGCTL-HHHHHGHSM
QGKFWAEGSESSSMDSSNSESESSSDSEMEMQAARNSRWAISDSDSSEDDRRVVRGAKEK
FHDELRMIIRRVNNHVKVSDFSSLSDEYDKLLKCMQKSKNYGIPKFFISVLVELEAFLDE
KFRDKEAIKKLSKAKATSFNTLRAKFRKSVEEYRDQMDDCKNNPLAYQDDNLSDDDSD--
--SNSYSDSDSDSDSDSDSSSSSDSRSVSGSDSDGNGSSDSDSSSSSSSSEWSSDEYEDR
HASALAKWGTRTVKKKEGKKANRQKQVK-----KDLQDTTVSGGANNNIPSGDLLSFEGE
VTVDMIVEKVGEIVAARGKKGTDRQEQIRLLKKAAEISRPLSLQAYADVLTHLISAQFDT
ITGAFHCITSGIWSEICDNINILLDLVLLNNKDKRVYISNTDENSSDIVDLKEQDKSTVT
FLVSFIERLDGESLKALQLTDVHSSEYKDRLVQSLHLLALLWRCYKICE-ERGYYDLVSS
LSVHLINQLHFKNDSLAIKVWEFVRQILE-----SEELSANSDSSNTDGKIPDIPVPKKF
KPSELITELVGNVYKYGDSKDKLRVLIQHVYNMALHDNYNEAVTLFQSIGVYDMALGSDV
NIQILYNRSLVQLGLSAFRLGQISEAQQLLSEICMPNRNRELLAQGMSNMKSQERTPEQE
RAEKRRLLPYHMHLSLEVIDCIYLICSMLLEVPYLAYHNSHIKLRPISKQFRRLLEQYER
QAITGPPESLRDTIIAATRSLQLGKWRECRDYVFSLSIWDNQEDLQQTQERLELNIKQEA
LRTYLFTYGHLYSSYSVNNLIEMFQLPREKIHSLLSKMMLKNELQALWDQTGEFVLLNHK
QASKIQNDSLIVADKLLQFVDCNESMISSKGNASGGKNPNVINNRNSA-GVGNPNNQGGS
G-QYNKHKSSSSVPSSSSMNLRPIRV-MIENNEREFQEFLDFDNPDIEKIMAMLKSMREN
VPTKHLYLYLSELLKLNGLGEEIPRIVILGQQSMGKTTVIDYLIGHPLGYSTNDIGTCCP
IVFHISPSEEEITECLIGGEKVTFETLPEKILERMKEIKMQISSQELRIDIRSKGAIEMI
IVDLPGLKEDTKEGSKITQKIVYEYVKNHPNDIYILVKRSIDDPANWSWRQKSFLLEE--
--LGLGKEQAIVVGTRALEYLQEEVKEISNGKQLLERIKKRELNDASGAPLPLFMLELFS
LSREERAIKKMSSRKNAMERRISAGEKAIKEIITQLCDQDHETEVKERLLSYFSRDLFEM
ELRSKFGRILLKQLNSLE-RLFKNKSCSSIYT--CEELPNH---QLWREDIILIIRKFAE
IVYEIVTGNFKILDF--NGSQFLDNFGGNLWMNLNDAKE---------TLNDENLIYRNQ
NNSLNKQK---QKYLMGS
FNLNSSSEIIYLRQKRSPYLIGKCISLVSDSSSNSDLIKVEFKKKDGLTGDHIKYLSKSQ
LV---PVKKVQSKVEQNNCY--WLVGFKEERWKKLIPVQVLSKVYYLELCGEYALYIDSN
WDQIDILNLEEKTSYPTQVINK-EFTQDINKSNINND-------PMYDLLLLNEMSFRLI
FKWFKETFKSLKLKSTYNEQILCQMLRSVSNVMSTSNWEPAVVDVLQMNVKELVIPLVNL
TSFACSEALKRIMKAAL-NVLKKESEQVTTSNLLFKST--EFSSVFKTTIDHFCLEKARI
CSESMKELIIEQASAIVMDLNYD-------------------------------------
------DLY-----TDWEKVHQASKSHFEKLKESLSSSLCTKLYLSFVNDFKE----IGI
RHKQKLNIESYLKESILDGLLSEE-----------EVIQRYKLDKAIKDNEKFKEKHKNL
KENYLFILKIIKMLKYNIQFERM--------------MD---LTQVLLNCHNSVESIRKN
AEQQLQAAQEQNIGEYLTLLAEELFNESKPELSRQLAGLLLKNAVSGIEPRIDIERRGMW
ISLPQNVTSKIKALVLESILSPVAS-VRGASCQVIAKLGRVELPCKRWPELLPYLIRLVQ
NNSDNKMRSSLTALGYLCED-SKILENEVSSLIITEDISNQILTAIVQGMNDP-DSETAL
AATKSFYYALYFARSNFSNEMERNLIFQVLCTLCGTNKRELLQTAAYECLVSIATEYYDY
LGSYLSVLTPMTIKGIKGVYEPVSICCIEFWNTIADLEI---ELSLEDEHNNMHYISQVQ
AALIPVMLETLLRQNDDD-DLESWTVSKAAGACLTLCSQLLGDNILEPTLGFIHSNFSHS
NWHNREAAVLAYGSILEGPSIQKMQPIVETSVTNLCQALNDNVVAVRDTCAWTIGRIVTF
HPTIIFPLLGLPPHSNGLLSLLLQRLADEPRVCTNICWIIHQIAES-----SQYVEGGQK
VLDIWFPYIVQSLLQAFKRENSDENNMKQACFNALSMVVANSGADNDMNLVNLAHELIQK
LMEKNPIPRSDAAVLNIQLICGALYALTTKLGKRLTLQYSQILLRLYFELLRQGGSSEES
ILSLTSLIVAMSHDFSPYVNECISIIIPLIQGYDELDTCKYSIELVGDLVRSVGKGINPS
LEIIIKTLCALLAKNDVDRKVKPLAIIALGDISMNLGEDFIPYAISVLQLFQQASITQYN
DGPVNSEDWIEYLGELREAVLQGYTGIVYGMKDAKRLEILGPYVPSIIQFIDNIVNDYSG
EFPNSNLKNATALVGDLITAFNGQLIQYLLSKDKRSILENICTVGETSRDP---DIISNI
KWVRKLCNM------MMQLVFTSNGNGRSTSILLCDWLFTILDYLDKKKSNSFYEKVCKL
VRDDISEVDMA---SVFELLVENLDGIFEILKDAEVKKEIVI---RDDVKRSFGELYKES
EEFFIPLISMLLHPKFDKSRIDTCKKIFLEKLMSKGEIPKLRINVLQVYYDIFGQDLEL-
---LVNILKYKAQYGCFNNFETMESSLDKKITNIDESKSEKRNLYLIISSESKDNKNYKK
AYDYLVGFFETFKSKSQSIKSNSRILDLGVQFLISTILLPEILFFDSLLSMPIYQYIKES
KEYKVLLELFDICYQGTVGDFLQNNNQEYQNFLDKLPINESNIVNKLQLLTISTLAKGKS
SIKLDELEKEFRLSSFDTQDAVVNAISVGLIDGNISENSNTVNINCVTKRQFGKAEWESL
DKKLNQWMGHLTSLSSILANNSNNNNTWKTFSFLLAFLSISLIVTAKDGESNYYFFKKAI
SSSTLVG-SSFNGEFDPSNALNMGNSEWRSASNLSAEQQIVYTAFISATAKAIGLKIVWS
SSPRETQIQVSPDGINYEIVVPNRRATQNEKEFTEDFMFDHSRDVRAVKIQMKAKPSQYI
GIRQLVALGSGSPLAMIISGITDEKDNCLQVIGGRVQDDFAQVDLDSCVYGISAGDGREL
WKMGPNGQIISAASNPPKCLIVANGDFSGSSPLLIQDCEIAEQAEDGRSFWEFNENSQLQ
VK--GTNACVNQINKAGFGVGTTKL----ELSGTASSTMDALHTVDAAIDSDIATYWASA
AFESTGEHKVSINLSANMTYQLKNLRIDWEFPPFTYSIAAKVDGE-MKQITKVEGNPSNT
TLNEMNGVISDFIQIVMEKPHPRYGKYSNKYLYGIREIELMTNNLRSVVSFCRDAALSKD
ARDKYFLNYVEDFNSKASEMVKSAARDVLSRTKNLGVLNDRVNTLLPSIDECFTDKKEYV
SRLEDYISNAKQLKEGLG------AVKDEISVGVGNEPGDEPNLPAEDCFSIASRNPNAI
SGFYWILPRCAPEPIRVWCDIKFGASYYIWNGNPGSYIS------VTSVSDIRKYCAHVG
MEPLVLRNIEQISGIKSALGIMGFKLSEGSGIPLAVDYGCLDGKCTGSYQDLRDGTTDLT
SLVL--SLASPESTATTDAVGLGFSENYLSFFNLKNSNIVGIVCSTNVVPTQKQINHVDI
DCDTVAYNPIFEGPINTNIIVQCPPGCAEYK---TKVYGSDGVYSEKSSICKAALQVGVI
SNTGGMFNVALEGPKNSYEGSTKNEIESEKLSGIPTR-----------------------
-SLRISTISKECPIDVIHQLSTATTLNQQAKQGQVKVSTPVIKTDLTNEPFISKILKEAS
QYIDESYGVDPSLSFATQEESKMAILEMRRKLKPAEMLTKSQSPKIMDVVDNVDHIANEL
LKVTGVYKSITKELLDKLDDVEAIHSSQTGFQSFTLDTQKMPFEQTFSVYDMPRTQRGPS
DWGYTTTAINGRTKLLGQNSPISSEHSPRGTFAQIKNRRFFDFTLTTEVYPPVSGIFGIA
FRVRDLSNYYLLEFNQEEERIRLLRLKEGITYILDTKEDFKFKEGEFQKVEITVQRDRIY
AKIGHVK------LQVVDEGLLSGTVAFYSNGVVGGVYFDNVSIKAKSCKKFFMSPAPRS
PR---CSNYKQCYLSTFTTYDIVKNNDGNWEYIKEYK---GRTNAMVVKTSGE-----SR
ILLKTHTCKNGYFSFDFNPPCSNGK----VSGIFRYTDNDNYYQVIVSNTSTTLVKKTKA
KTTLLKKVMF------GFTQNEWNRILVNFEGTTIYVYSTKYNNTKEIIKVTEKDTQVSH
GRVGLSVKNCSETAFDKVSLEPFRVDSNENSTDKTDSIYKSCTLNTHILQREQHCETMFG
SN--SQKSVTCTNNYCTECCDYYTNMIDRTYYNSCKSACSINDSYAENSTKEYINQLATC
IS-GTSKAFDICTETDQKCKIEVCDLCCNSAFDG-----------VQKDIQGIVVKACQA
QCSHRFKVLA--
--MEEVN-----

--------------------INQVLLERGISYTQILDAHAKKLKNEINTRCREFQSVDEL
INFSNSDQNRNDSNGLLSNFDW---RSSYLPHIYSATLGLKNGFIDPKLQGELYQSILKC
INQGFVSGNIGIVRISDHSHPVRLATPIDKTCYSLVWRKKDLSETDIPIVLGEYTGDVKY

```
VGDEEISDESYSFQLTFKSSAFKFTGAVVSGSHQQFGDFSA-------------------
---NNTDDQPTYFPDEFEYTEANDNSYSPNKS------------SFFSKPFSQTRNGNI
KKIGARLLLPNENEYILSADKTCNEMAFLNHYECV--FNNFNFRINVQWHSVYVDGWPHI
ILTSIPGVGIKLGDELAADFGENWFSRIRRISENNIKNELILFRLRF----GSVNFSDLD
ENLSLSNLINFYEICEVCQNSITSKSKFYLLCNGCNRAFHWYCVNRPEFRKTSSKNWFCF
QCLSLTLRILPSPGLINSIQNEDPKSIVFRPDYNLRHFNEYGGLEGLT--KLKPCYLCYS
STISNQMIGIATIDEFFN-SVQYTYNSDNNQLVSNSSLHLSLVADDYGKRKYTASTQSST
EDIIRPRYIIERSRRVICSLRQSLYQSEKHRELLYKLEKLRKIQDERNISEGNGMIPVLC
IKLLNSRYYPKERIFHIEYL---LANDIENHRINIDDLLITDNHVKSTKKTSEN---LIP
YRNSKKH---------TIFGISEEFYKSENFE-------------FNIIKMKNEKLLTE
TYKNFNIDSIYDKVKLIHKFIIQENQDNEDPKYEYIDQPEWNIICGSLNIESNLIKYFVG
VNISRISHKTTTFNITSKICI---------GNVFLIKTIPITIYNEF----SAELNEKSH
SEILNTIRFSAI-EILAWTQSRLSEIFIKETSLNNNDSLNSDFNSANLIAQALQPYPGIK
WINETHSWKV--VYSNEFGQKISKFFDTIPFDENATVESLYLKASVSQTNNKVMLSTSRK
LNKITNEISSPLKFIFNEWGNTIEDFLARYNLNINSLRPFPDNLDCDSTFSNEKRDKFEF
NIINCYSAAYSLAIGN-VNIDKEFYNRKIRKYNSKVKNNTNP-----------------
----------TQLKKQIYKKHLINNRHESSLNKSIKQKNSNETFVYSYQDKDTNTSSEES
LLGNICES---------KIEPKELINEDLKGNSFSDEDKLSIRAKSNKRKTIVRALPAPS
DEYSKQADELRPFPHSVKEAFPMT-----------------------------------
------------------------------------------------------------
------------------------------------------------------------
------------------------------------------------------------
----------------KTAEFYKNKGNELYKQKKFDEALVQYDLAIEIDPNDISFLTNK
GAVYLEMGEYQKCLEVCMQALEKRFEVKADFTKVAKAYNRMASCYIKMNELQKAKEMYEK
SLLEDNNRHTRTSLKELERLIEKAEKEAYINPELAEKHRIEGNDLFKQKNYPAAKKEYDE
AIKRNPSDSRLYSNRAACYMQLLEYPSALIDVQKALDLDPKFTKAWSRKGNIHYFLKEYH
KALHAYQEGLKCDPDNKECNEGLKNTMAKIQQVSSSDQIDEEQVAHALADPEIQSLLSDP
QFRLVLEQLKQNPATLTQVIQDPTIANGIQKLMAAGILRMGMDEKYDVLICGTGLTECII
SGLLSTSGKKVLHIDRNSYYGGEAASLNLTTLYQKFRPGTSPPANYGANRDWNVDLIPKF
VMASGDLVKILLKTKVTRYLEWQVIEGTYVYQFQKGGLLFNPKFIHKVPATEMEALKSPL
LGIMEKNRCRSFFSFVANWSDDDVSKQMGFNRDKNTMKDIYDHFGLSSTTIDFVGHALAL
YTNDDYINKPCGETLDKIRLYMMSLSRYGKSPFIYPVYGLGGLPEGFSRLCAIHGGTFML
NTNIEKFLYDEQGKVSGVVTSQGK-AECKMVICDPSYVLDSKPKVKCIGKVLRCICILNS
PINDTNDVSSCQIIIPQNELGRKNDIYVMMVSCTHGVALKGKYIAIISTTVETENPLLEI
NPAIKLLGSIEEQFFYTSDLYEPIDSGKDDNVFVSKSCDASSHFESLTQDVLRLWKNITG
EDLDLTNIPNEDEADG-MAQGNKGSMAPCTRDYTINLSKMVHKISFKKRAPRAIKGIREF
AGKVMKTEDVRIDAKLNKFIFSKGVRNLPTRVRVRISRKRSESEDSKDSLYTLVQYIPVA
TFAGLQTEKVQD------------FLIHGTRKIQIRDSKTNKSTTKENYVLRLRACLSC
RIILSDQQFYEGGCPNCTHLAMEFDRQKVNSCTSMSFKGMISMLKPNESWVARYNKLLNI
VPGCYAVSVSGDM---VSDDEGNYM---------IDLSFTKKSDWENVYEPSEDSFLMED
ALILEKNEIKAKPRLICEIGCGSGYLTACLLKIIKDAEFSLPISYLVDVNTKALEMSEKV
ISNNKINSIELIKMSLFTCLNRNGLFEIIIFNPPYVPSSNKELNQSGIDSAWSGGVNGLF
FVSYFLPSLVDVLAPKGVCYLLLEENNCPEQILK--EPRYSWNVQFIIDRKVQLEHLYIL
KLTPK-MSH----------GLSYEDCNFPIVCDRCLGESKFLRMTKSNQERSCKICNRPC
TMFRWKLHNSKKYNQTTICYSCGKIKNVCQSCVSDLNFGLSLYARDDYIKSKINIPDSVD
NRDHFMENKLNGNDETFIKRIDENQKKRKEIKKILAKDDKIEAKEVTNYSKNTTECGSSS
KTTIKSISK--------------------------------------------------
------------------------------------------------------------
------------------------------------------------------------
--------NMGQVPLIAAKSTLKEKVVPQRPPDLDIIASGERDRISDDWRWLALKSEKRL
NN-MKKMN---SSSEQLKKKLGSTFDINQFSCFVPRTVLEAIADKRIGYADDFDVIIESF
KGAIMFCDASGFTALTAALDKQLNGAERLGECINNFFTPLIQIIHYWGGDVIKFSGDAIC
VVWPVDDESINLGVNLITLARDSDDNSPATSEKRWNKEEQQYSSNAADTYSVDMFHACRL
ACECCMDIHRTLHNFPTPIPGRHLTLHIGVGYGRTTILQVGGVMDRWEYVVGGPPFEEIA
IAEPLAKSGETVISPSVFSILGKHVSVEPCEGPD---FKKLNSLL--------------
-PPPPPL--ASIHIHDEDVDLLRRYIPPPVYRCLSSGYNVFLNEVRRLTCIFVSVKGLDI
STHYGSKTAHQLMQLVQKAAYTMEGSVNKFLVDDKGVLLLIIFGLPPVYHLDDPLRAVFA
GLRILDSIRFMGLSSSIGIGTGRVWIGTVGCEIRKEYTALGDTVNLSARLMARAGENEIL
CDLNTYEACNHAMQFIQKESFYPKGKDVSIDTFSPTGILTRSRLQGSTGGKAETGNPEDS
NLSNN--KDNSNIKDGLSTDNLLTTENSNNQNTDINLNIENLQSIRTWRGWKPLKK----
--------LKKFFKPRYSYGLPDMNGPNFRVIPDYAMESGPLMPWEYYEPWDPILRPTLD
LGGVMTIYGKENCGIDEIIQNIGEKL-ERKVFLCSNMPDTPFINIGNPLLPWKTLCTDIV
ESWRLTKSREKKGFSNDNSIYGLAKELTHPSFHWTLHKLKPVLQGLVLPSELSENKAFLR
MGKYKNKESKKMKDIFRNPLL-------FVKNLNGQPESLDK------ELDGGELENDEN
LNKEVFKDEADDDITSSDEDLSDED--------------------EKGLGPIITSLVNGF
SMYENSIICLHVKSGTCFYAGMEQESWKIAKMIARIAMIRRKRKVEHDFKELKKWRRKHS
RICLFCKGFKRPIQHISKDTLYLYKTTKCTPPAFKSSPPLLFILICSSNTDSIEEQKEII
QWAKECNAFIEVPKLTLNETSKFIGHQLGV-NTQVPFQLVEYVHRASGGIPEHIKRTLLQ
LLSHKAISISTQDDQQMEDKGSLILDSDKSLNNPLSNNISRNSDDSKEWSDDSIVPIIGN
KQSNRFSSNDLSGSRHRSSFTKFGQSSYLSRNSEDNRRYIK--VTGQLRKIPFAHEIVAE
CMSKLELLEPDEQMAAKIASAFTFAFTASELHEVYPQKKSPLEFLKMIKAMVNKDVFEVC
DTIHNSKHITLYIEYYRFQNVAFQRVVSESLLSDERKRLARISRKTLEMSDSGGGTKGEA
TSSRIIIKNLPSYLSEKRLKDHIS----SIGCNITDVKIVKKRSEKNPE-VESSRKFGFV
GFYSEEDAKKVLEYFNGTFIDTCRINVQYAFPPKENTEVDEKEPITLWISQKNSNKSWLD
NNEINSVKNDSSVKSSEIVKPTKAGVSNVRKHIQFSESESEPESDSDPDPDQNSDQDSE-
------KHETFNENDDNSSTNVSESDL---------------------------------
SSESNTDSDDNQELEEVVDIGEIVTSPMETSRLMVVNISSTTEEDLNKFFSKWGEVKSVN
II------------------RSPESGV---------------------------------
---------------------SKGYGFVQYEFVEHAVSALSQAHLSLLHGRVLRVSPAFN
KPTKTITDSFNESNVIV------HSDYKLKALNKRKESSIDKKTWNLLYISGNSAVNAFI
DNEDVKKHDIVDIQAPDLASRVSLMETHVISATKEWLKKEGISVKAFEVEGSDIFT----
-AKLKFEGVKNVERSKDTIIIKHLSDQVTLSDLQKICSPFGRINRLCLSPSKTIAIVQFL
EESSAESAFKRLAFKRFKSVPLYIEWAPVNLFVSETE-------------TQKEKETES
KVVNKKEILDINTNAVHVFVKNLSFDTTNKALENLFSKVEGFRKATITMKTHSDSDGNII
K--------KSMGYGFLEFKTSENAKECIKRMQSVTLDGHTLELKISKKQVDEKTGSQLT
DIGV----KNVSNKLLIKNLPFQATKSDIMSLFNSVGTVTSIRIPKKSDGTNKGYCFIEF
LGKLEAISALEQFQHTHLYGRHLIIEVAEN---DDDSSP--------------------G
KKKA----------------------KSSM-----------------------------
-----------------------PAKKNNGGQTDRDSESGLTDRQSNKVESNIVEVPEL
HFHEKYSAVNIPVVQEKIRYETKEVIQEREVHVVKPVVKEKIVEVPKYKVVEKVVEVPQV
VVQEKLIEVERVEVTSRSRNQSRP--------ISPVEKKQLDI-----------------
-----------------------------VTQYRQVPKPVEVPIAFYSAIPVPILIDRA
VPVPMELQIIQDILCPKIEAIYKDIPIPIPVKRTIEKPVPIGLYDQPELLQKYLHDPNEP
LPNPVAALEQMAQQASMAAA---LNQQNMMQMMQGSVQQEPQE-----------------
------------------------------------------------------------
--------------------------------MFASRNEYDRGVNTFSPEGRLFQVEYA
IAAIKLGSTAVGIKTKEGVILASEKRISSPLLEPRNLEKIMIIDRHVGCCMSGLVADAKT
MIDHARVESQNYFFTYNENIPTQSVVQSISDLALDFSDIKEKGKKKSMSRPFGVAMLIAG
ADSDGSSLWMTDPSGTYTQYSAAAIGTAQEGAEAILLENYNSNMSLKEAEDLALIVLRQV
MEEKINSVNVEVAAVK--EKKFIIYDDKEIQRVLDRLPPPTHIVSQLS-SQRAKTSLINF
LLEVVPSALDCSRQQLTSLLTGKGDEQLKRFLRETDVHCLIVGK---EKVMESNVGRETN
SEGNLNDTISGENRLDYNEEVVQHQVFIETFMTSRCLKASLMV-FVKNPSVDRGAETSES
DSPHSKSLSSSLQCIQLGVGSQLTPYEVLNQYLQFAFTPLLDALGN-------------
--AKTTSGTIEIGDSVDSTESG---------SHLGLENIQRKVNELCLALQQGQDDSMIP
MVKLNLDPKVAEYAKEYKNT---KINNMDEIIEDSTFLSSLQVSITQWIREIQSLARFQR
DIG-LSVTSEVKFWSSYERSLQQILKQVQSPEVEWSLSVLRQSKKFLAAISLEVDTGLKQ
```

SIEKVQNINTLIHDIPINDLLVATSIDEITSAVTLFFQQLRKIKGAASYPISRTFQLVEV
YSNDMTKQMYKVLQNNSLMLLEFPVFEHLVNGCNELGQVWEDEWRELKDVIRDLIKKRGL
SERAHPKMDFAHVPLIQRLNDIVIFRKQHQKLKDTLLELLSQEQNSGIDAKKDLQQAYAC
VANINILDLSAIGLEAWEAGKQAYNNKVDASETLLIKQLREQLSGCGNTLEMFRILGRYN
PLFFRPRIRSAVEEYQSILLEKVQSDFQLLQLRYQNPYQRSAASQFSCLRDIPNIGGMLV
WSHLLQERIQSIYFKLEDIFGPNWEFESQGHKVKTDGDHITSKIHPNQIVDFWLQQRKDD
KTYDLNKPVLGLMKLSTTSFILASTIDSGILSLFKDLRIIQSYAYRVPYSVKVLADELKL
LYPYISHLNSLCINWMRIATQLEVEPGK-----RISPLLTPFRQEVYNIIQEGISLSWSS
DRLEGFIRRFYDSVDTLEQKFEAVMTLDEQLSVLVSQIRNIQVSKGLGELVSHLSKIGSK
CQEFQSLLLAYRIDYLFYWDQVIQSFLLPKLKEFVQLWINQFISIGDNPNHTILGSLSST
IITLQLKNQKITLNPSLEDVKSLWLRRFHQEITKITNLPRVTNISQDISKAKEELATLIS
GKNSVKHIPLDHTYRHLFLFLPKSLLEWSYKSIDDVFSSIKVSIDYWTRFEALWFVELSS
VTGKLNNLLQ-WQQLVKEMRNLRTTFSSNEDLRWFSKIALDTSAIQAKIMSKYDGLAREI
LHLYASKLNEQLSSYWTSISTIKQTLDAQWLIWIDKNSSSISTLVLKSENIPSVMTNLPP
G-GEALHSSLIDLVSKMVSAQDQDQKIGSDLPLFSDGQKFLEVQRFTFPNDWIWVEQFEG
EYDKSHQRLEHCANVIFPSRRKIENYLKLCLQLIFMSYQELLSEWTRLRGITFVKNPFLM
LDQIGVIENKVNVLRQDIESFNMIFKAFKFDDTELNKVTSETFDTILRDLKDFKEIWNQL
NVFFVMISEYKRMLWTEVNVKTLKTNLESLISNLKSIPVKYRQYDAFEQLQHDVEGYNKN
MGYISRLKAEYMKERHWKLLLSKVSMKFSFSTLTLGDVWSINFDASSAIMSEILAKAQGE
HGLESYIQGMKELWNGFEIEFTGLPNNTKVIKNWDIMLTAIDDHLSALQNMSLSPFYEIF
QEESQLWTEKLTKLRFTLDLWMETQRRWIYLQGIFLASTDIANLLPQEYKRFQTVDAEVQ
GLLKKSQSRPKVIDLLTFEGLTKSLERISDYLNKIQKALGEYLEKQRSMFPRFYFIGDED
LLEMIGNGKDITVAQRHFNKIFAGITFLKFQEQIESMIIGMGSKEGEVIHFKDPIPILKD
TSLVEWLGKVVQAMQITLNDLIGKAIEDIISN--FGMSSFTEDDHIRSNFEKYPTQVLLV
AWMSWWTKLTEESFSSGTSTKQ---------LQEFIQRILSRLSDIVATLEENTCHKKYN
QLIVEFVHERDVLSHLIEQNVTSSQSFHWLQYMRMYWMSKDCQKKDDIIVRTANSAFIYG
YEYLGIPEKLVQTPLTDRTYLTLTQALHMRLGGNPFGPAGTGKTETVKALGNQLGRFVLV
FNCDEQFDFTAMGRIFVGLCQVGAWGCFDEFNRLQARILSAVSEQILTIQTALIKNSNTV
ELLNKTIPMSQDVGIFVTMNPGYAGRSELPDNLKHLLREIAMVVPDRQRIAEVTLFAQGF
QFGEMISRQIVTLFELCQSQMTSQPHYDFGLRSMKSVLRSAGKLKKSAISENKE------
----ALDDPQKLVILEQQLIIRSISSTLLPKLVSTDVPLLTTLFQGVFPQVPFESLNDSL
MVEQVKLICKRNSLEATSQWLDKTLQLFEIQKLNHGIMLVGSTGTGKTTVRKTLLEAMDT
VQGSKTISYVIDPKTIDKESLFGKLNPVTLEWTDGVFTAILRKIINSSDNNNNINKKYWI
IFDGDVDPEWAENLNSVLDDNKLLTLPNGERLELPPWVRVVFEVHSLATATLATVSRCGM
IWFNDEIISDEMYFTSFLNKIKMGNNT-----------INNVNTGSSNTGGGGGGSSKIS
EATSDQLGEGSDNIGNNTTGMEE--------FESAEGTGIENLETLLRNKAYSIWQSILF
KDSFGSKCLSFASKRPHTMVFTRIRVLEAAFSLLNSSIKLLLDSNSTMGNGKLPPMLTEF
FSRWLIWSLIWGFSSSMNLSDRISYTKEVISIVTFTLPP------------MEDENISIL
DFKVVIPTGEWRKWSECKETEDLPLNKVLDSNVIIETVDTLRHFQVLHAWLHAHLPAILC
GPPGSGKTMTLSSVLRSMTDVDIVSLNFSSATTPEILLKTLEHYCEFIKAPRGWICRPMV
PNKWLVVFCDECNLPEPDRYGTQRVIMFIRQLIECKGFWRRES--SQWSFVTLERVQFIG
ACNPPTDTGRHPLSDRFLRHSPILFVDFPGKSSLNQIYSVFNRAILKPFPTLSNHADALT
KAMVDIYDASAKTLTVDLQPHYIYSPRELTRWKISIYSGLHSSKELTLTQLIRLVLYEGE
RIFQDRLVEQSEKNWSQEMMNEMIIKHFPNLSEKDLYRPLLFTNIVTSICKEIPRSIVSE
YLQDRLTSYYEEQGTSKLVFFDEFLDNINRVDRVLRQPFGHLLLIGPPGCGKTLLADMVS
WLNGLNVFTIKPGRKYDIFAFEADLRSVMKRAAIKGEKLTFIFEESHALGPAFIERMNAL
LASGEVPGLFEGDEYNQLLNECRTAFSNSSSVDGNELFARFTKLVQENLHIVFTLNPANP
NFKETQSLSPALFNRCVVNWMGQLNNQALSQIARSFLHQVLIQSEETTESAIPTNCMPSV
EQPEERVALTIDCIISLFHAEVGEDGGIKKTSTCNTNSQTPRDFFDFLKHIIKIYKEKNE
TLLEQQQHLSSGLETLRSTEQEVATLQQELGEKEKILIAKNVEAEQKMQQMIKEQGEAEE
KKKTTETLAKSLDEQQKVIAERSSEVEIQLKDVEPILREAENAVSNIPKKNLDELRSMAN
PPGLVKKTIDAVAILLTNNSTKPQAWEESRKLLKSSDFITKVLNFDSNTITLKTMQRLQK
ELESPEWDTEKINRASHAAGPLSSWVSSILQYSLISEKVQPLKTEISQLEKSKLENEKGL
EAAQKLVGELQERIDVYKKEYAELISQVQLIKREKDLVTNKVERSIRLLGNLTTEQDRWR
EAKEGFKTEFSNIMGDCLLSAAFICFAGGLDQILRSHYIKLWQEILDEFQLSHTNANTFV
DYLSKPNERLLWQSYGLSNDDLSVENAIIIKRHIRYPFIIDPSGYATSFLLEMKGGGKLQ
TSTFSDSNFPKLLESSLRFGSSLLIQDVSSLDPLIYNVLNQEIHLHGGRSLITVGDSEVD
FSPHFRVYLTTQDPTIQYGPDLTSRVTMVNFTVTPTSLLEQSRNIILKELRPDIDKKRTD
LLRLHGEYRVQLRECEDNLLLALSNVKGNILEDETIINTLEVLKKKAQDIQIETAKMENT
MEHIDQVLTHLLPLSLTATRIYFTLQHLSSISPIYQYDLEFFNRILYGVLERNKSKINSN
SSDFDHKIKIIILDLLHEAYLRVAQGLLHNDRIVFGLHLIYIESYCNL--SLDN------
LMGKEIDLLTKSASASNNNILDSIPKSFGLDREQMNQLSVMISSKSFSKLCESFNSNKNE
WEQLMISTEPEVILDTLGT-------------------NNDD--EKREILKNFKKCLLLK
IIRPDRIVTLFNKLISDTLGSEFSNISEFSKDLLKEIVLKQITEITPILFVTSPGFDASL
VVSQLANEQNSNLLSIAIGSAESLEKAESSIKTAMRKGTWVMLKNVHLSSGNLDYLEQLL
QNRQPNKGFRLFLATELPKKISFNLIRISMTIVLESPVGLKAALQRASEITIKTVDDSQS
STIRIKLYFLLAFLHSVILERKRYTPLGWTKFYDFSEADLQCCINIVNQWTI-------T
NISG--DPEQIPWEAVRKLVSQVAYGGRLDNIVDKQILYILIDEILSSKSFEDGSELSCR
NDQIKPNILLAPDTSKKVQNYLDWVEKLSSTNWPTWLGLSPLAENVISAQKGQKMSLNWS
VLIIRSRNETPDLIPKDKNEELMGDKIEQEEALKSDTTGIPSWLLKISLTKMENSLRIFS
EYIIKNSRNII---EYSENNSLDPILRYYVSEISKFYRGFKNIWENIHELLNILGGESKL
TNNLRSIGLSIQSDLVPDLWLKLLPILLYSLDIWLSDLIRKLSQNCLLISSICKKLEGGE
DSSKMDIFLSNVLISSPGIKKIGLKTIWVGGVCDPGAFFTASKQYAAKRFGCSYNDIVIS
IGEMVENENEDGDEAKFVLGGLTAVALEWKQESKLFELSMKQLKEQMKDQEENKGDIHNT
---------------LENGTLMLIKDDALRRLDLENCNAMQIPSADFKLYYRT-------
---SEDTIVAISKKLNNEEEKGDLNLIKEKLNHWNKELELTEIAYIQKNIRIGNEYAVTK
LPTFLSLLNALCSDKVTTGEQRKRIRDTVNLFLMGEIRYDDIFLIFSKILGDQVLHHLIA
SLEGPLENDGSLDFSWMAR---SFEFKKEISPFEKLLSYT--------------------
---DIQNF
LKDSIKLFPLNGFDPLDIPN-------------------VGYSSVKCGQYLIFLGGIKSS
KREYSFDKINVLNTETLKVSSFICPGTTPKSTIHHSSNLLMSQSGPKILLSGGSKEFKFS
DTTSVFDMKTRGW--KILDGCANTPRFLHTSLTYPQ---HSTGKSS---QFIIVFGGLTK
SLTEISPSNDLWVFSTKFNSWTQVAVTQTELPMPRFGHSMAWISEKTFLIYGGETRTKED
DNYVGALLNDIWSFTINSN-DHDIENLNIRGHWTKISSTGSPRPASSHACISLSFLKND-
--SRIEKVTDMILGSTDDSNNTLNDQEPASEDQMGIFDINSKKWTNLKPSPTSKSMLIEQ
DDFSTSLIFETTSI----NKNTIPCILLQSNLKAKGSQRRFFALSLLGLEPYFITEEPTI
SESVSKYLKPKANKNFSISASVFSPFSSSQSWIFGAIAHLTDNSFSTEVNSSIFEISICK
-----NYISVIDNGSGLCYEDLNRLFKHFGTDSCGSMDECLTDSK---------------
---------------------------------NSSPL----------------------
------------------------------KMYGLGFKHAFSRLSDTCMVFTKTSNYIGV
GLLCKSIMKSENLIEYWTPICYWYSDTMKPLIPKGSSISEYEENQRLILKYGFVKDPSLF
CDHFNSIDSCSGTKMLF---SLDEKYIKLHPTQ---YLEVSDKGMNLLNQGNS-------
---DSNHLNSCVFSIPEEEM--
-----------------------------NTQSSESTN----------------------
---------------------------STISPFWKSERYSIDYSLSTYLSWLYLNKTQKI
FCQGRLI---

---SHDDKSESL----

-SLYDFLTQNLNQPVELSRIYKNGSND-GAFALIGKLCSSSNTTMTSEKIFEAGILLYYN
GRLIRRLEHTFPCT---------KPK------ELNEFQLTALINVPHWLKPASNKQEFLL
ERTGIFEEFQDHIRELISVYINIHHDQSKLKAWEAEFNNSSSED-SSLIRPNKIPKLEG-

------------------------------------YRMTNGDSSANNTSPGQIEKKRAP

```
NRLLVDDAINDDNSVVCLSPAKMEELKLFRGDTILLKGKKRRDTICIVLVDPDLEEGKIR
MNKVVRKNLRVKLGDTVSVLECGDVPYGKRIHVLPFDDCLEGITGN-LFETYLKPYFLEA
YRPVKKGDAFLVRGGFRPIEFKVVGVDPEEYCIVAPDTVIHCEGDPIKREDEEKMDDIGY
DDIGGCRKQMAQIREMIELPLRHPGLFKALGVKPPRGVLLYGPPGSGKTLIAKAVANETG
AFFFLINGPEVMSKMAGEAEGNLRRAFEEAEKNSPAIIFIDEIDSIAPKREKTNGEVERR
VVSQLLTLMDGLKGRGQVVVIAATNRPNSIDPALRRFGRFDREIDIGVPDDNGRLEIIRI
HTRNMKLAKDVKIDDIAANTHGFVGADLAQLCTEAALCCIREKMDVIDMEDETIDAVILD
SMAVSQDHFNSALGVCNPSSLRETVVEVPNIKWDDIGGLEEVKRNLQEMILYPIEHPEKF
ERFGMSPSRGVLFYGPPGCGKTLLAKAVASECSANFISVKGPELLTLWFGESEANVREVF
DKARAAAPCVLFFDELDSIGTQRGSSMGDAGGAGDRVMNQLLTEIDGVGVKKNLFFIGAT
NRPEILDEALLRPGRLDQLIYIPLPDLPARVSVLQAILRKSPLSKNVPISFIAQKTEGFS
GADLAELCQRAAKAAIRDAIAAEELKKASGDDSAM---------KIEDEVDSHIYEIGRK
HFEEAFAGARRSVSITDLAKYDQFRMKFDPVYVTQSGG--EGFTIDWPDSTHAQYSAPID
DDDDLYSMGGAKGLKWKEVELGSDLLSEFTKYGVSMIEE-------ISDPTDISFYELGK
DNNLTSNSDNKSKMGVEKPSKVKKSKKRANKDIN-----KGEVELSDSITKKQKKAKKSL
--ENKEINNKTQKISSKQN------------------------DQILDPEEYWEEYYDLK
QKL--SNWTNSTKLVIHPSILKGLSELGFLNPTPIQAACLVPAIRDRKDIVGAAETGSGK
TLAYGIPIIANIMFSAQRRREKRMKSDQALQALIVLPSRELAIQVRDHLRALGKYTGLGI
HAFVGGLSLEKQERLIATRVQVAVGTPGRLSALIFSRKQVNTTLSIDELRFLVLDEADRL
IEQGHFRELKQILQLIYSDNESKTRKIQNYLFSATLTLPNHLHNKAMQSIMQYVKLRENQ
LFIIDLSFKTKSLLDENGEQVQTMSQPNGIIQLPKGLKISMIKCESDELEMRLVLYLLKY
FAPIWDTKSYIENGKILVFVNSISYVYRLAPLLSLVLCKDTHEKELPRKKCSKACNEKSL
KIIGIHGNLSQKQRIQAIESF-KSSNSAILICTDVLARGLDIPEVDVVVHLQAPRNISLM
IHRSGRTARASRQGECVLFCTPKDVPAYSKHLKAISLSFESVGIPDELQGLLSSQVSHVQ
QRLELANEIEGLGHSIRKKRNNSWMLNKAIEADLELSDQ-----EEFSLSPDQMKEIQNL
NLKRNYFFDFVKNMN--SAPICSDIHSASFDLLRASEVKKLSVCHVTSTTT-FESTGESK
V--GGLHDLRMGPLSNRDFCGTCGSR-NECPGHIGHIELELPVFHPLFLGNLVRILKCCC
WHCCCIRLSKKVCNGYVKRFE---------------------------------SIGKNS
NVGSKDL--------EIY-----NVNDAQS------------------------------
---------------------------------SYSLEIWEGIKREFLDKASKHSSCSSC
GNAFKGRVKESQLGIGITLSWPEGSKPPWSSSLNQEDTRSEYSDSGNSGDSDNQEEGSIS
SSQQSNESKKRKKNTETKNK---------SSAKLFKRSYYDD-FSMNIPQEAKSHT-KQG
RGMITQHFQAFQIVPIIKNIWEN-NRKIFEFIFPICKSLGWEAFFMFTVPVSANKFRPLG
MALHPRTSGLLEILTANDRLKFYVDLQKENTNESSIEGEDS-------------------
-SKTGKGTKTIKEEIEVNSNNLISY----------VSSLQEKVNMYLDQSKSFKPSS---
APPGIRQWLEKKAGVIRQKMMGKRVNYSARTVIGPDPYLDTNEIGVPLMFALNLTIPERV
GTHNVSLMANLVENGPNLYPGANKLL-LGGILYDLSRLDQNQRKAKARLLLSSVLSANHH
PIVYRHLIDGDIVLMNRQPTLHRPSIMAHYIRIQPKDNIMRLNYVNCGTYNADFDGDEMN
LHLPQSLFSRSEAKYIMDASRQYSVPKSGEPLRGLIQDSCIGGAFLTSKSTFLNREEYFH
LLYTSLSFIFDKKSSALIFGSNNRFSLGKVKIDECILRNQSNLNSNVQNNFEIEFSLDSL
DTCIAPKNIPKCKTDFKIHLEPPTIIKPIHLWTGKQVFTSLLKTLLYNLVSTEP------
---SVYAGINLISKSRTPGDSWKEDGEKEAVVIIRNSELLQGVIDKNQIGSNSYCLVHLC
TELVGPLIGGKLLSAIFNLSQVYLQMRGFTCSISDMLLTPQAESERIELMKKYNHASVFI
QEAFQYYIFKEIGGNDLN---------------NQTYFSEKFDIYK--------------
----KTKKAIL--------------DLKKDKYS---------------------------
-----GQKND-----------NYLEINPSELVDFSDNKNLTSTV------LDVLIDTIRN
FSE--NSNNKLEKKLLL-LELVKSLKQQRLSIDGLFKVEENPDKFTHDDSGILFPKDVST
NNSNKDSDP------NHDAIL-----YDGSWLSIGSIEDEENEFNNHG------------
-----------------------DFFAYPNELTRR-------------------------
-------KLESLIGNKLNTKKDSLLKIMDAISKIGLGDITSKLGNLLKGSSSKFPFPYNG
FSSMVLTGAKGSRVNYNMICVMLGQQELEGRRVPIMPSMKTLPSFATYDLGSRAGGLITD
RYLDGLHAQEFFFHCMSGREGLVDTAVKTARSGYLQRCILKGLESMIVNYDGTVRSEDDT
IVQFIYGDDGIDVSKSSYLSKLEDLVRNKTLSFEKVFKYENNQNILSDEKSFKKVKKSVE
RDMTVNSLLPTILNGGSVSELFLNSLSETNIQKLTRISSDSKYYSNNNYNMEKLLRLKYM
KTQISPGEAVGCLAAQSIGEPATQMTLNTFHLAGAANVTLGIPRLKELLQTGGDSKTPYI
FIPLLKDINSKKSSKNDNKDFINYAEKVLDCFKSIPLSDIIENVGVESSIVYDEDKFKS-
----------------------------------SHKKSAYCWNYEISIQFSDLEVFCQS
LPHYNMSSLMNLTLSKCIKPFLRNVSLIL--------GDILNNYYWKLLLFI--------
------------------------------------------------------------
------------------------------------------------------------
------------------------------------------------------------
------------------------------------------------------------
---------------------------------------------------------IIR
RHSLIFFK----------------------------------------------LNSKSI
LL----------------------------------------------------------
-------------TNFTQLTLIRLNSSRY-------------------------------
----------------------------------------II-----------------N
TYFFSRENDLMYWDGEIVGPDETPYEGFVFKIEIIVSPQYPILPPSVKFVTPIFHPNVNF
FTGEVCIDVIKDNWTPAWTLHAVCRAIISILCDPNPNSPLNCDAGNILRCGDKKGFNNMA
RMYSLEYASKYKMQVDQELMYTKFVRFLLGSKTGVRTKGAIEVGRRAVDYFRGIEFIRWM
RKNEDHIKESYPEVVKNISFESINDLNQVGTELIRRSFIVRAEYKPVG-----TNTGDLK
IPKWPKRLCVTSNQNFDEHSFYIITFDSNKTMSNILMISILVSVVALFMFPAWPLAIKIS
VWYISVVFLSAMLIVFFGRLIVFAFLWFFGFDFWILPNLFDEDTGVIDSFKPLYSIIRRG
DDWFMLSIRVFCAIFLAGAIYQLSKTHTASDVGQFARQSFLDVLDWGHRKIS-PPEDFT-
-------PTLHNSGSNEHIGERNLSNEEHICITRCRFESKDAILDECATDCHCLSELLSL
PCLKSRCPSEFEEELNNAMAKNCQSNQEKQSSLQELLLKDLEDLDYSASSNINN------
KGQISEDGKYSDD---------EIISKIGNL-NDRLFGKMIDSSTNVMNSNYQL------
-----------------------------------KDFGNEIYGTFQEIIDIVGIIDSKV
SEKYECVKALYKDHFGELASIIINKIDYLEVVKRIITHENFEDVKLNDILPNSTIMTITI
SSS-TSKRNIPTLEKKESILKCIDFVQINESKSKILEFLQFQIEAITPNLSALIGPEIAA
NLLCVSGGLKNLAEMPSQNIMVLGSKKNGHFSTGPVRLELLQSIISQSDIVRNIQDKYKK
KAIRLVSLKCGLCARIDFSSTDKSPDHGVNYRNYILNILEKAQEPPQKPMKKPLPIPKDF
PKSRRGGKRIRKIKEKFKQTKIKKEMNRMKFGEEEEEYTVDGKTIGLGLLSA--GGRRIR
GLQVGSSSSSKIETLSGSDSKLGSSTSISFTPYQGMIF----------------------
-----M--------EELLQSVPDLKLSEKICQLDSKYCKFGEKESMKNEILSYIKKNSMA
PLFKILSKDIKIDNDEKFLDVLIEDNNSELNEINEKIKDARENYGDVEVRNCHYKKLVFY
SRIGSKEKSLQELEVAIEKTIG-GFKLELLFLGIRTGFFWNDLNLISKYIKRAEDLFKTT
SDWERKNRFKVYKAIYYLITRNFSAASELFLDSLTTFTAAELISFDRLIFYTIVSSIISI
DRRTIKSKLLTSPDILKIALQPDNLLEFIESFYYGRYSQFFERLVNITYILQKDYYFNRH
HKYYLRIVRSKAYIQYLEPYESVSILSMAESFGITQEFLEKDIVTFISSSKLSCTIDKVK
QVIICSRNDKKINQYNELVQKGDLLLNRLQRLTRIIQVM---------------------
---------------------KNVVDPNKLILEAKDKLSLSNDEEVLASLAVIDRCV-EL
PDCALNFEKLGIVQPLLSCL-SRSEEVRSITYQILSKSMQNNLPVQNSFAKLGALSLLKQ
SVQGEDSETNKSKGITAISSLVRHNKTLEGSFISDNGIPLIALWLHSENVGVRERALSLL
RHLLIQGVVKSEYIIGNNIIDTILTLSKNNSISNQNIQYGETISETLLELINAFNPKLSS
SS-KDKIREEVNKRMIFLDYCKLHPDDDISPEYSTLIQCEKLLV----------------
------------------------------------------------------MEFLNL
GLHKWVQDTCDSLKIRTPTAIQSKSIPYILKGRNVVGNAPTGSGKTLCYCLPILQILAED
PFSVFGLVLVPSRELSYQVLDQFQVFGNKVNANCQVLTGGFDESEQIHILNQRPHILIGT
PGRLSSIISYPGNISDLLRNLRFLVLDEADRLLSESLEDDMLPILSILPKS-CTGRQTLL
FSATLTNAIKEIVNNYSTAPMIIVNENPDDSPVEKIRQMYLFLNHRVRLVYLHYILQEQV
IDQQGIIFTATKQQCQMLTSCLEIM-GYSVTGLHSLMNQRRRLASLGKFRSKTSKLLVAT
GVAARGLDIPDVEFVINYDFPRSFEDYIHRIGRVGRANKTGISLTFVTEQDVPYVYEFES
KMKKEMELLKLDEDEVLKNMNRVTVAQQKALLMLEEIGFNEKNQEKLKVLMSKNKMSRKY
FVGGNFKCNGTKESLKTLIDSFK--QVESSNSEVYVFPTSLHISLVKEFFGNDFKIGSQN
ISCTGNGAFTGEVSCEMLKDMDVDCSLVGHSERRQYYSETDQIVNNKVKKGLENGLKIVL
```

CIGESLSERETGKTNDVIQKQLTEALKDVSDLSNLVIAYEPIWAIGTGVVATPGQAQEAH
AFIREYVTRMYNPQVSSNLRIIYGGSVTPDNCNELIKCADIDGFLVGGASLKPTFAKIIE
SAQMPPKKSTKSGG-GDENESRLRIAIVEADRCKPKNCRQECKSFCPVVRTGKLCVEVDS
SSKIASISEPLCIGCGICVKKCPYSAITIINLPKNMSKDTTHRYGKNSFKLHRLPVPRPG
QVLGLVGTNGIGKSTALQILSGKLRPNLGDYTKELEWKEVIAYFRGSELQTYFNKMQNGE
LKTVIKPQYVDHIPKRVKGRVGDIINAKDEKSIAETLIDQLELRHLLDRQVGELSGGELQ
RFALCVSTS-VASMVYMYDEPSSYLDIKQRINAAKVIRNVLNHENYVIVVEHDLSVLDYL
SDLVCCLWGSPGVYGVVTTPFSVREGINIFLDGFVPTENMRFREEGLNFKI-VDQDE--I
AMQRSNFVKYPGFTKTMGSFKLSAEAGDFGNSEIIVMLGQNGCGKTTFIKILAGVSKPDD
SDVIDQMPEFNVSYKPQTISPKFEGSVRDLFLMKIRDSFMDVQFTSEVVKPFNIERISDQ
QVKLLSGGELQRVALILALGKKADIYLIDEPSAYLDSEQRITASKIIKRFILHSQKTAFV
VEHDFIMATYLADRVILFTGTPGVDCFAHRPENLVTGMNKFLKILEISFRRDPMNFRPRI
NKLDSVKDKEQKMSGNYFLLEEMSLICSISGTTPEDPVISKTGYIFEKRLIEEYIRCNNS
CPITKSELSLDDLIQVKSKSNLKPRLIKNTSIPGILDSLRTEWDAMAMEMFQLRSELEQT
KSQLTHSLYQHDAACRVIARITREKDQAISRLAEIQNSILEKDEDNITSLQSVGIPEDVI
DLFTEYSDKMRPIRKKQSFPDLIPAEQVKEFSLKSEIKLKENCNIEVGVAFNDEIY----
----------VSGMENGEILVSSIENCEQAVSIFAIEKYSGSQYSSKNTSQLLSFQNSNK
IHIFSRNQDESDFNSSF---ESNIIINSLEKHPLGMHIIANSSNGLFSMFDLESRKQLFL
HQEQNNSYSQLKPHPDGLILGGIVPNGNIDIWDIRNLEKISSLEHPISRNSSLCFSNNGY
YLLSTSLDNKIHLWDLRKSAILDSLELSTLPNNLQIDESGKYASCFSKNHFSIFSLFNKH
KLQLISSLSNFSDNIHHIHFNQNLNSFSSLCHSGIIRMWTMETSSHSYSGDIKGGLFHGR
GVLIYSKNEKYEGDFVMGKREGFGKFTYADGASYEGEWVDDKIHGQGKASFSSGNTYEGQ
WENGKINGYGKLTFSNGDVYEGEWVDGKMHGRGVYKYVDGDIYSGEWRDDKRHGKGTVTY
VSSTGQIIEKYEGDWVNGKMHGHGKYVYVDSAVYEGDWFEGSMHGKGTYIFPCGNVYEGE
WVNDVKEGYGVLTYQNGEKYEGYWKDGKVNGKGTLTYSRGDKYVGDWLDAKKHGEGELFY
SNNDRFKGNWVADKACGFGVYTYANGNRYEGYWENDRRHGKGIFYCAEDNNVYEGEWANG
RKDGKGILRFAMGHSIQGVWKDGVLSQFHSLQFPPESQWSNPNFPKIAEIKEFQRLERIG
AHSHIRGLGLNDALDARYSSDGMVGQKLARRAAGIIVRMVKQGKIAGRAILLSGQPGTGK
TAIAMAIAKAIGSDTPFTHISASEVFSLEMNKTEALRQALRRSIGVRIKEEIDVIEGEVA
ELEIDR-----SNTTGVKVGRMALRSTDMETVYDIGSKMIESLQAENIVAGDVISINKSS
GKITKLGRSFTRSKDYDAVGYQTRFIACPEGELQKKREVVHNVTLHDIDVINSRTQGFLA
LFAGDTGEIKPEVRAQIDEKVAEWKEESRAEIVHGVLFIDEVHMLDVECFSFLNKALEEE
TSPILIMASNRGITKIRGTDYKSPHGIPIDLLDRCLIIPTVPYSEEDVKKIIQERASEED
LKLTDSAYQILTRIAMDTSLRYSLHLLTVSQVLANRKKKEEIDLDEIKKAYSLFIDVKRS
TQYLIDYQQEYLFSEIEHSNNDVKDKDNGNDTMRDTAVQEKMTLI-----MFANQDDFWT
SDEEEEEEEGVEEEGVEE----------------------EEVEGEEDKDEEKND-----
----------------QKNDLDMV-----------EEKVSRISLKE--------------

-----------------------------GKEGEENGEEE--------ERKKKKGFIRKK
WSIED-ERDVSDFRLENVVLKYPYELDDFQKRAVINIHNGDHVLVAAHTSAGKTAVAEYA
IELANKNGRKAIYTSPIKALSSQKYREFLNRFNRIGIITGDVSINPDAQCVIMTTEILRT
MLYRNDPCIEQIQTVIFDEVHYINDLERGVVWEEVLILLDPKVQLVLLSATIPNYIEFAN
WIGRIKQNTVYCIRTLHRPVPLKHYLYIYEKCFQIMDENNKFNINGYKEMLDFIKTSKSK
KSISSKSKVSISKANQEIQSQEDPNQEDPSQEDPNQE---DPSQEDPTQEVQSQEEPIQE
DQSQELSSAETKFKTEVYRLQVFLKLLEKNDQLPVILFGFSRRKVEQLATNLPNLNFLYN
HEKSNIITFIKESTSKLNELDQKIPQLLQCKELALRGIGIHHSGMLPIIKEMTEILFTRG
LIKVLFATETISMGINCPARSIVFTSIKKYDGRKNRILLSSEYTQMSGRAGRRGIDTFGN
VFIFNSETIPECIDIVKMMLNTYLPVQSKFRLTYQMILQLSCRHSLKIEDMMTKSFKEMF
RSINLPIFHRNLNRKLKRHQIISKLHSILES-YNSNLVISNYNQRNTNQKLISGQLFQKL
CNSSISQKLNPGRLILFNSFSLTGKSVYSIMDGNDNNNNNNNKGIGDSNNDIQFYQNYQN
SNKNRQISVVQNLIDL-DRSKKNNNQ-RNSSSSYSSKNSTTINLIHTFNSISTSLYNNTN
LFKNINNYYIFQNVPLELFMFIF-DFNASATNNQTNLLKSINFKDISILHNIAILLKNSQ
NQQLNTNRGNITEYSSSSSSNIPENWPKLLPISKTLKSIEIEYYELLQRYNDLFPEFVNN
NCYSFIIEENNIQLIVELNKLDHEISIYKHFINDESLDDYPEMKLKIQLLIEKGFLNENL
TITTKGRIASELLTSDELTLIEILLNGMLHKLNNHEITAILSCFVFPEKGIDRPSLPSVE
LLNAHDELINIHTDYEKTHYKHQINLDTEHFWSLCNDKFMLIAYKWSNKESLKEIMEEMN
LHEGTIVRTILRLDELVRKLIIAAKMMGDKILEEKLCLIHENIARDIIFMTSLYFN----
ALHLIKITLP--
--------------------------------IMSNISKLKKLEELRSIMSQHGVDAYII
SSSDPHMSEYTPDKYKRREFMTGFSGSQGICLVTQSSAHLIVDGRYIVEAKKTATPE-YQ
VHLLKKGFYADIVDILKEESFDTLGIDVEVTSWMSFKALANYI--ELSDLHLNTNFR---
------------IKLLNLNFVDVEEIEQARSEIFVHGIEYAGESSKSKVSKVLLEM-KKL
NAKILFLSSLTQISWLLNLRGSDVHCTPVFLSYLIVEILDDKVGIDKLKVFVNVESIKEE
VLKNEFQDKISIIQIENIMDELFHSFSKLNSH----------------TKNELNKIWLEN
FCNLAAMDTLFKKLLITSESPIIMLRAIKNKIELKGMRECHIYDGLALTKFLYYLYKAGR
DKTLFNVSEWDLSQKLLEFRKQQPKFVYPSFDTISSIGENGAIIHYRPEKENSSIIKPDL
YLCDSGGQYHTGTTDVTRTLFFGRPTIEQIESFTRVLIGFIRLHKLVFPIGTNATAIDVL
ARASLWEAGLDYLHGTGHGVGSFLSVHEEPWSICVGRDGALAAGAVVSIEPGYYEEGKYG
IRIENLAEIIEVDGYRKNKFLKFSPLTFAPIQKEMIDISILSDDELDWLNWYHSKTLENL
EPLVDDDPE-----FLKWLVQACSPINRYM------------------------------
----------DPKRIQKGTE----KTLSSVGKGEFKVEKKPEFINRRLSLFKTLYEKQCA
LLQ-EKASKNEPIKITLQNGDQRDGVKFQTSPMEIARQISKKLAESCIVAKIRYNKSLFI
EDDEVRGIEEDEEETCNQSDGWRLWDATRPLEGDCELEILTFDSLEGSGVFWHSSSHILG
QCLENEYGAQVTIGPALNPGFYYDSYMGTHSVSNTEYSDIENCAKTIISEKQQFERLQCN
KEEALELFKDNPFKVSLIMSKIPDGAQTTIYRCGSFVDLCTGPHIPHTGIVKAFKVTKNS
GCNWLGNTENDALQRVYGVSFPDKKRLDEYLNMLEEAKKRDHRLLGSNLQLFFFDSNVSP
GSCFWLPAGARLYNKLMDFIRNEYRIREFTEVITPNIFSCDLWKTSGHYFAYKENMFIFD
VEEKEWGLKPMNCPGHCVMFKHMNPSYRQLPIRLADFGVLHRNEFSGALNGLTRVRRFQQ
DDAHIFCTPEQIQEEVFKALDFLFFIYGQLGFTFDLFLSTMPKEHLGTEEQWKEAENALK
SALDKTGRDWKLNPGDGAFYGPKIDIMLWDALKRQHQCGTIQLDFQLPIRFNLQYRTDE-
-------------------------NISEESTNQHQNAD-QNPDNSLKQGYRRPVIIHRA
ILGSVERMSAVILEHTGGKLPFWLSPRQAIVLSISEKTVEYAKSVERELCRRGFDVSGDY
SAATINKKIRESQLLQWNYMLVIGENEARDKKVTLRCRDTTIPQELLTLDQLILKFSSMG
FPSSIDSNSSNQ-----------------------------SKYKFLVPIVNRINDDLKE
KNEKSKFRLTKTMKDEQDFLRVPNKAYCCGIIGYIGSGDAQKVLMQGIEILQNRGYDSCG
MSTIDDQGELITTKYSSKESGDSIERLKNDSELLHGNHHIGIAHTRWATHGGKTDFNAHP
HQDYKKRISIVHNGTIDNYCSLKSELMEKGIKFQSETDTEVIANLIGSYLDDGEDFQNAV
QKALSRLQGTWGIAVLHKDYKDLMILARHGSPLLVGVQSGHIYIASETSALANYTNQYVA
LQDGEIALLSHEGINKLITPSRLLSIDHEKVESSPSPYLHWTLKEIYDQPHALARSLNFG
GRISPYNNMVKLGGLDQRLDELKNVQNMILLGCGTSFHAALFAQLLMEHISGFNTVSAKD
ASEIFVTGFPREHAGAIAISQSGETADTVKAINIADKLGIPKISVVNVVGSMLARTTGCG
VYLNAGREVAVASTKAFSTQVLVLSLIAAWFAQNRD-SVISQRCQELLEAIHRVPISVGV
SLQ-AKDQCEQIAEMIKDNNSIFVLGKGYGYPVALEGALKIKEISYIHSEGYSAGALKHG
PFALIDKDSQTPVILVILSDENQSLMMNVAQQVKARGARVICITDDENLCKDIDCEKVLI
PSNGPLTALNAVIPLQLIAYYLAIKRGINPDKPRGLAKAVTVFMNPEYDYLFKLLLIGDS
GVGKSCLLLRFADDTYTDSYISTIGVDFKIRTISLENKTVKLQIWDTAGQERFRTITSSY
YRGAHGIIIVYDVTDRDSFDNVKQWIQEIDRYAMENVNKLLVGNKCDLVSKRVVTSDEGR
ELADSHGIKFIETSAKNAYNVEQAFHTMAGEIKKRVQVNSQNRGSAQQKLGAQPIR--QG
GCCM--VKQFTNVPIPQGWDQIKDELEKYNEMMREAENSSTKGKKKNEYLWPIYRINHLR
SRFIYTKYYLDKEISRDLYEYCLDHGYADKDLIAKWKKQGYEYLCCINCISTSNTNYGTT
CICRVPRDQLQDEDIECVNCGCNGCST---FFVRVMKFTLVTILFLIF------------

---------MKKAKIVMADLTFMYDLVVIGGGSGGMAAAKEAAKYGKKVALFDFVKPSTQ
GTKWGLGGTCVNVGCVPKKLMHYSALIASSIHHDAQMFGHKTSS-SFEWGKLVETLRNHI
RMLNFSYRTGLRVGNVEYINALAKLIDPHSVEYEDNG---QKKTITSRYILLATGGRPSI
PETVPGAIQYSITSDDIFFLSKSPGKTLVIGASYIGLETAGFLNELGFDTTVAMRSIPLR

```
GFDRQCSEKIVEYMKATGTKFLVGVVPINIEKVNENIKVSFSDGSVEEFETVLYATGRNP
DVKGLNLNAIGVEVSDSG-KIIAPK-DATSVPSIFAVGDIVEGRPELTPVAVKAGILLAR
RLFAGSNEFIDYDFVPTTVFTPIEYGHVGLSSEAAIAKYGEDDIEEYLSEFSTLEIAAAH
REKPEHLRENEMDFALPLNCLAKLVVVKSQGEKVVGFHFVGPNAGEITQGFSLAVKLGAT
KKDFDDMIGIHPTDAEVFGILEVTKRSGESFVSSGGCGGGKCG-----------------
------------------------MSELDL------------------------------
------------------------------------------------------------
--------------------GKCGDGYKWTQTEEEIYCELNKEARLIKNSLKIVIDMKSI
KIFLKGICVFTKELSSNIDSETATWYLEECKDLVILLEKKKKCWWDSCFLGESKLDLSQV
QGRKNFRDCDERTKSEIIKLINNNNNNNNNNNNNNNNFDIHQVLKESWNKENSPFQG--MS
TINPNEIVNENNENQNNL-NETVYNGPLKLEHLLPSGLTKRDLEILRENGYHTIECLAYA
PKKALLSVKGISEQKCDKIKSACKELVAMGFCSGTEYLEARTNLIKFTTGSSQLDRLLQG
GIETGSITEIFGEFRTGKTQLCHTLAVTCQLPVEHKGGEGKCLWIDTEGTFRPERIVQIA
DRFNLNASDCLDNIAYARGFNTEHQMDLLQSAVAMMTESRFALMIVDSATALYRSEYNGR
GELATRQSHLGQFLRALQKIADTFGVAVVITNQVMSKVDAMAMF-QNDKVPIGGNIIAHA
SQTRLFLKKGRGETRICKIYDSPNLPEGDATFSITEGGINDPKDEMLLKEIASSAKISWC
PISTK-------NKLSLLALGGCGLNPCLSVGLIDITNNGKDIQNI--------------
-GTSSLNSRCTSIAWGSF-------NPEENALGLICTGMEDGNMSLFKPLSTYDSNRKDG
EIMLERTNIYPSAVSCLEFNKTDHQLLASGGNDGKVYVLDLSNYYEPGKENKHGDSD---
-VTALKWNPKVPHIMSSSSSNGTTAIWDLKMKKSAISFRDPAQRSRPSTLAWVPNQPTQI
VVGYDDDRNPSLQLWDLRNVSYPFKEVSAHQKGVMSIEFSPIDPNLLLSSGKDKTICWTL
LNNQPEIFTEIHSQQWSVQNQWSPNIPGIFATASHNDKVSIYSLSSHSST-TTYIPSWYH
KPCGVNFTFSGKLVSFSSK------------STGGVPQ----------------------
---FNLYRVPTNPEITAHADMFDQLLNQGDFIGYCQKKCQESESSNEKLAWSLVENMFSE
DSVEIASLLGFDFNNASTTANQFLGRKCGVISQQEELQ--KELNQQNNISIQD--PASTS
FTGMSYQNSPTNMNGNNISGFQTLSG----------------QMVSNQSLDPEKFFQQLG
ESESPNNNKDKETEFNQKDDEPYGTRFCCEGADNEMIIPQGQENQTSHDLFNSSSNLSVH
NLRQNEWTKQEEDVINELLVTGNIDSAIDVCVQRGFFAEALLLAKFGGIYLDYVQKQYLK
KHECQYTQKVLGHVIMDDLEDFVKSSNLDRWNETLAIISIYTQPNGTMENLSELLAKRLQ
EEKFDVRSALICYLCARNFRNSIEIWGNMASCQSSQIIGLQDFVEKVAILQAATRFNGND
ENITKQVVQYAEILANSGRIVAAMKFLSLASNDYSINSSTLRDRIYNAAPELMSSGFNKP
NFPFTIIDVAPFVNANAN-IYQHNQQ----QHGMFGVNNSFQSNLNINSYPMMNNNNNNT
GMGMPPPLSSHGVPPSGPSSSNQMHPSPPPPPPSSIHSTINNGMPGGIQTQCQAFRPGIP
SSSGLPPPPPPPSTMMRPSSSLPSGNG--MMSNQLPPPPSQNMAALTRPSSPNVAYSNNV
AGVGSGLSSTNQGVKQFLPQSAQPPPVNPPNSFMNSLNKNNFGTNNVPPAIP--------
--TSIPAP------------SQIPSSVPPVAPPPPNSNNSSIIGGGMQGVLNQGISQVAP
PSNRSSITTAPHSGATPPIPGMPVPWPLPTATQQLNSTTSTTANANKQIQEASKKNNSQP
LSGQLLDMVTNVVNSNLNSIFANDP--RKKMDAQKRFDELFENLRVGNISETVSSKVVNL
CQALQTGDYLTANKIHIDLSSTEWE-NNKNWLMAFKRIIPKMALRVAYESSSEIGVFANL
TNRYCLLAHGSSAAFTSVFEAELMDHIPIINTLIGGTRLVGRCTVGNRNGLLVSNMATDQ
ELQHLRNSLPDNVKVQRIEERLSALGNCIACNDYVALIHTDMDKESEEIIQDVLGVEVFR
TTIAGHVLVGSYAKFTNQGGIVHTLASEDEMNELSALLQIPITSGTVNRGSDVVSAGCVV
NDWAGFVGMETTAAEMAVIERIFKLGTYNEEMREKDIKLKSSIIDTLAMLETILQRLLGR
YLEPYVYNLSRELRLGVLSGNLVLENLKLKENLGDILHLPLSIVSGQIGHVSITIPWTSL
GYKPLVIKLKELHVVVRPKDGNVDEDTLRKELREAKEKLIEFKEKRLSVGHLSKDERKSA
NGTNLLWRIIKKIVANVQLEIQDIHIHSRNTRSDIIMGAMIKSGFVVTTDSLGNRDTSSL
-NSESQKLDKTIEINEVGIYIGQYFPNTFNFNSID------EGRLKDFNSESSI------
---GNHHRNHNHRNSHPHNHQSPSNSPNRIK----------NDKASYEMVNIVNVRNK--
------------------------------------------------------------
---------------------------------------LLETSDEKSMNDKKTVQALES
KYLLIYDE----------------------------PHKSLGAWRLEXXXXXXXXX----
------------------------------------------------------------
------------------------------------------------------------
------------------------------------------------------------
------------------------------------------------------------
------------------------------------------------------------
------------------------------------------------------------
------------------------------------------------------------
------------------------------------------------------------
------------------------------------------------------------
------------------------------------------------------------
------------------------------------------------------------
------------------------------------------------------------
------------------------------------------------------------
------------------------------------------------------------
------------------------------------------------------------
------------------------------------------------------------
------------------------------------------------------------
------------------------------------------------------------
------------------------------------------------------------
------------------------------------------------------------
------------------------------------------------------------
------------------------------------------------------------
------------------------------------------------------------
------------------------------------------------------------
------------------------------------------------------------
------------------------------------------------------------
------------------------------------------------------------
------------------------------------------------------------
------------------------------------------------------------
------------------------------------------------------------
------------------------------------------------------------
------------------------------------------------------------
------------------------------------------------------------
------------------------------------------------------------
------------------------------------------------------------
------------------------------------------------------------
------------------------------------------------------------
------------------------------------------------------------
------------------------------------------------------------
------------------------------------------------------------
------------------------------------------------------------
------------------------------------------------------------
------------------------------------------------------------
------------------------------------------------------------
------------------------------------------------------------
------------------------------------------------------------
------------------------------------------------------------
------------------------------------------------------------
------------------------------------------------------------
------------------------------------------------------------
------------------------------------------------------------
------------------------------------------------------------
------------------------------------------------------------
------------------------------------------------------------
------------------------------------------------------------
```

```
------------------------------------------------------------
------------------------------------------------------------
------------------------------------------------------------
------------------------------------------------------------
------------------------------------------------------------
------------------------------------------------------------
------------------------------------------------------------
------------------------------------------------------------
--------------------------------AMRSPCSNNNDELNDSSVLTENM--DTV
ATRSKNLPFHETYKSKPTPADKPYRVDGIDKYKELYEQSIRDPEGFWSQMARKELRWLRD
FTKVSSGTCLQDRLAWFLNGKLNVCDNCVDRWAEIQPNTTALIWEGDDPSSIRHISYIEL
FRNVCKMANVLKRFGIKKGDSVGIYMPMIPETIYTMLACARIGAVHMVVFAGFAAQNLLE
RLVNARCKIVVTADQGSRGKKIINLKDVVDKALEKIPEIKTCIVFRHL------------
------------------------------------------------------------
------------------------------------------------------------
-------NGPIE-FVKGRDFDGETLMRSEKPYCPLEDMDSEDPLFYLYTSGSTGTPKGVQ
HSTAGYLLYAAVTQKYLFNIHPGDIFGCAGDIGWITGHSYLVYAPLCNGITTLIFEGVPT
YPNAGRYWEMVERHRITHFYAAPTAIRTLKRLGDDFVKKHDRSSLRVLGSVGEPINPSAW
RWYHSVVGEERCSIVDTYWQTETGGIVIAPIPGCFDTKPGSATFPFFGIEPAILDPDTGK
EIDGPG-SGVLCIKNSWPGMFRGIFGAHYLHEDLYTKPFPGYYFTGDGVLRDQDGYLWIT
GRIDDTINVSGHRLSSKEIEDALTNHFGIAEAAAVAIDHDVKGNALVCFVVLKDSGNRTF
DLNNS----------------------------SPHPFEYELRMCVRTQIGPVATPDHII
VVENIPKTRSGKVVRRLLRKIATGCNDYGDISTVANPECIKSIESSWAQYLKR-----MA
LNYLSICNISI-------------------GFILVLKKLAQLSSEY--IENNVPSNFSIF
YSTETGNSRKIS-ELFKKLLDEISIEANVREINSILEENYSNNSVFVFVVSTCGNGSFPA
SSRKFIRYLKMIKSGNEIFLGIKYTIIGLGSSLYEYFNSAALKLDKLISSLKGEKYCEIA
LLDEVNGNEIDFKTWWNNFLNRLKEHLNKHF-SIAVTRKKDEFVCKLRSINEISLY----
---NHSLSGNCH-------------------------VSEIYFKLIEFCPINRELLCETK
TIDGIERQIFNLKLELPKVQYKTFDIIDILPPNLDENITFFSSKLGINSIEKNITVEFVP
I---NNMTRN-------------ISVPFPNNRSLMHILKYYFDLMTLPPHSVMLQFVPYL
NSIEG-----ELISNE---SFFNENKSYEFSFHLFINRFMKSIPIPIEKFVKFTIRQYPR
SYSISSSSLASPSMIDLTI------STCIKGQISAPLNEIISEGANKKN-----SKKIIK
GLCSSFLF-EFDLNLPVLGMIRSSSLNIDN--VTSSALMFSHGSGIAPIRALLHERKYLI
NEKK-----IIKPAYLFYGCRT-ENEIIYKDELKDFKRIGALTEVFFALSKTQ--KKYVK
DIIPFYKHIILKVVDNDSIIYICGKKEFVSGIKNEVASIISNNRS--RNIIKKMFVEGRI
FIESWMSSNLWVEKYRPGNVLDISHHKDVVSMLSHVLKNGNMPHLLFHGPPGTGKTSAVL
ALSRELFGPNEYKNRILELNASDERGISVVRDKIKSWTRQVVQCNKTHEITGNLLPSWKI
VILDEAEMMTADAQSALRRIIEVSSKNTRFVIICNYISKIIEPLASRCAKFRFQPISANS
QIERLKYICSQEDVSYEDGVLETIVNLSQGDLRRGINILQSASELDKRISMSSILDVSGV
PPIKIIERIINSCKILGVESILIETAKLINEGWSVELIFKGLAEFIIMCDKIDDSKKAFL
MLRISEADASVIDGSNEYLTLLNVCSSVQTIFAN-MQVKLDVASFGRRIQFLYSVWLEKY
DKNLREVDLLYVLCGKGSSREDGVIYKSMTLWLFGFEFSDTLILFSRKKKIVIVTSQKKV
SILQQLLDNFPNIELIDRKGDLKESFENHKELVSNIAEATTTIGRIEPADGQFASQCEEL
FSGSNKETTMVTASIDYLLCHKDEVELGLCKKAAVLSVQMLKGVLLQRIETILDKELKES
HKNIGRRAEAALDDKTIHNAWKTKYGLDPEDIDLVYSLVQSGSNFELKA-VENSDENLCL
TSGCIILSIGSKYREYCANITRTYFLNSTEEQKSLYNYCLELMETLISR-IKEGTSFNDL
YSGIYQKIVEDKGTE-------LAQKFVKIMGHCIGIEFRDPSLIISPRSSDVLVQKGMT
FNLSIGFNNLDSN-GKKYAIWICDTVFLSQEGNVEVLTQGCSKKLEHVSYELDDEEPEEQ
KPVVDSKEIKKEK---------------KSKEHLIIEDRLRRTARRVNNSEHSEELKEIE
NRQKELRKRKLVELQKRFG-GKKEEKNDQKGDISDSEKLSSYKSVKEYPKERSSSRIYVD
TAKESILVPIYGLLVPFHVRLLKNVVCTQEEGRKSFILRINFLLPTGIS-----LEQLPS
TLKTPVFIKELMIRSEDGKTLNSIFRSIKELIKRFKQKEEEMAEQDMIKNQQPIDFNRSK
QRVVLKDVGIRPTIGQGRRQHGILEAHNNGFRFSS-SKGET--IDILYTSIKHAIFQPVE
NDLIVILHLHLKHSIWLGKKKTQDIQFYSEVGNQIDDLEQRRGRNVYDPDEIMEEQRERE
TKKRYNLEYKKFIQGIEELSKFEAEIPYRDLGFYGVPGRSNVQLFPTASCLVHLLEFPPF
VLSLDEIEVVSFERVEQGLRNFDMIFVTKDYSKPVKRVDSIPIEYLDLIKRWLNEMEIVY
YEGRQNLNWNAVLKTILSDIEDFVQNGGFNGFLGEDSDDEEGGSDDDDDDEDEEYSESDE
DDEDDDDDDDDEDLSDLEEESSDDSFKESDEEEGLSWDELEKQAIKEDRKRGRVDSGYSR
----KRR------LYDITSSRP----RLVVIN-NRQVRNFSGNFIRTSRYTVLNFIPKYL
FEQFCRPVNFYFLVISLLQIFPSISSTNGIPTLALPLVFVLFVGAVKDGWEDLNRHQNDR
IENEREVIVIKRFPFINHGRTLACDLEEAEKPKNKNPSLK-HVISFKSENDKQ-------
-----SNTSHSWFLGIEGSERSSSSELKVGDIVLLRNHETIPADIVLLSTSSENGDVFID
TSSLDGESNLKRRFSHKESTKMLGNNIHDVIKRARYLEGLIECSPPGKDLHNFDGTAIIR
PPIS--------------EGLVTQG---------------------------NTITQFPI
NLDNIVLRGCILRSTEWAIGCIVYAGHESKIQMNSLKPTKKMSNVDKFTNKMVLVVLVIL
FCLCMTGSLLTYKYIFDGVFDHLDYLGVSTGQAIPISFVPVVRFCTWIVLLANIIPIALV
VSMKIVKAIQGQFISRDRAMYD--------------------------------------
---------------------------------------------AVNNTYAVARNSDLN
EDLGQVRYIFSDKTGTLTRNIMEFKSLSVGGVHYGSTETSSSKEDNLIREIEIPQENILD
IKSRNSQSIKIGNLLIGTLNPDNKPKSRLGSNFMTSIASNNSSNTKQKKSNFPLYSAQFP
DEAALCYGAQYLGVSLLNPKTKHLILDVFGELLDVEILAKIPFTSDRKRSSTVVRIKRLN
------------------------------------------------------------
----SEHNMKDLRIMSELYRERIMVFSKGADSAMIPLLRQSNEKFQKDLEIGNKMANQAL
RVLCITEKEISEDEFADWNQKYQLAVNNVENRESSLQEAASMIEKDLLLQGVTGVEDRLQ
DEVPETIKCLRDAGIKIWMLTGDKVETAREIAASAGLLSPGHEILELTESTCSNKS----
EIKKRVLSICKSLGI------DLESDKSERFLRKVKASKSEK-------------KETNF
SILIDGAVLGEIFSGSLAKLFVQMCCKCRTVVFARFAPSQKGNIVRIVRKHMDEITLAVG
DGANDCNMIQTANVGVGIRGLEGNQAFLTADYGITSFKDLKVLLLVHGRLAYRRICKLAL
YMFYKNLTVGIPVFIYGFFTLWSGTRLYFDYWYQVYNVILSSVPIVVVSVFDFDVTKSES
LSKPHLYSFGPENKFLNTKICLIYLLNSAWHIFVVFTIPYILFR---VNSTNLFGSNQSI
VGAAIYYLVILVVNIKVLLMADHIHYLLGFAVAFSLFSWICTLFVCAASFSIGSDVYTIW
IPLYNNVLLWITLISGLSISLWPDYFFKVLFSEWLK-----VDDK---------------
------------------------------------------------------QLRQLI
DKRILIKKCVPIQRLFYSDTVHDHFRNPRNVGSLPSDDKNVGTAVVGKASCGDVVKLQVD
IRDGIIKDAKFKTFGCGSAIASTSYATELIIGKTTEEALKINNKTIADHLNLPPIKLHCS
LLAEDAIKHAIKNYQDKQLKMVFAEDEKKNVSNTFSTITNKATRLCSPFKTDASLGRPKV
LILGTGWGFMKLAKGLDVNSNDIKVISPNKYFCFTPLLTQIVSNRLPREVCEIPINELTY
RGNEVIKYIQGLALDIDKENKEVIYFDEKKKQERIPYDYLIINVGNEDSNI-VPGIKEYA
LYLRNVEDSIKMRDAVVNCIKEVNWDKMSDDEKRKKLTFIVAGGGPTGVEVSGAFAELTK
NFLKNEYKKLAPFINIKIIEMANKLLPTAGDKVSGYTKYVLSLAGIEVLLETKLKSVSKD
YVVIQK---EGGDEELVPYGVFVWASGASPNSLTKQICDKVEEQSFKKAIVVDERLQVHG
IP--NAYALGDCALVRPRKLAERSKEIYQNALKSSFGVKYLRDNFSI--KAFPQMFNLSK
VADLPKNEEITEEDFKNLLEKLDSMYHSPPPTAQGASQQGEYLVKLFNDYPSDKEKQECP
AFIYYWNGSTCYIYDDNIAFYTPFGSMLGGIHTKYIWRLAYTTLNPSSRSRSLLSKNWFN
PFFAGKNDEHMIANEQESNVKNKSFAPIQITVEQILKESYSGSANSVGGLSRLHESDELE
DYRIRKRKEFEDSIRRKRWKISLYLSYAKWESLQNNIKNSRSIFERGILVNYENVRIWRE
YIKLEITNGNINNARNLFERVTHLLPRIDEFWIKYIQMELILKNYINVRHIYRKWIDWKP
DPSIYIQYSKFEEECGEIKSARGVMKDLIISYPDESNFIEYIKFEQRHKNLFSS----EQ
IINILSETLIDINFFSSISDIFVEEKKIEEAIKLCNEGIKILTNEHKNLKDKLFQLFKMR
IYEKPDENIEWIKHKLQNYRDKLLHNPQDFDILFDYIIFITQYL-------ELDSVLQEY
ENLIFN-HNISDVVSWEKYLHSYLLLIYFFEINQDNSPNIYKLYNSFIQSIKNNQISFAK
VFIYFSNHQLRIGDLNKARKILGIGLGRVPCINLFDHYIDIEFKLGNFDRCRVLFTKYIE
YDPVSTNSWIKYMQFEYNLCEIKRVISIAESAISMPELDSPEIIWQYYIELMINEKNIEF
ADIIYKRLLEKTQHIQVVINYSTFIISKLHDNKLNREFILGILNKYKERQFDYQRSILLK
YWLSLEEKLASKNIIEIVNRLLPRTILDVNSKNKVFKEENYDFAHLISTNKDNEAQTLQL
IDAAKEWKKAKILD---------MSDVE-IDQQVQVEQEVVNDLTTAIRYVLKNSFVRGG
```

```
LLRGLNEVVLALDSKKAQVCFLAESCEEDCYKQLVEALCRERGIPLIMVP-DSKELGEMA
GLCKVDREGNPRKVVGAASVAIVDYGVESEAYHYLQKHIAENCRSMRR------------
------------------------------------------------------------
------------------------------------------------------------
-SSFSRSPSPEV------------------------------------------------
---------------RSPMDSLKKE---SISEITSLTKADQENKILETKEQKNSLVLASS
QDNLECKDEIKQEIMKK------------------DNKIEQNIDFEED----DIFASDSK
SKYIKPS--------GNPGYNKRLECDDDDGYYVSYIGEIIGNRYKVSSNSTGKGMFSSV
VKCVDMETQNEVAIKIIRINDMMRSTGEKEYSFVKKFK-----GAPNIVQVQGTFMHQNH
LCIVFEWLHGSLKNCIHHFSKH---NIRKTQDLAFQIFQGLKIIHEKGLVHADLKPENIL
IDSERKTIRISDFGSMHFTTDASPACYLVSRFYRAPEIILGCSYGQPIDIWSIGCVLYEC
FTGDIMFKGRTNNDMIKLIMEYRGNFPKRLLNQGVFTKSHFSECLQFKWIN-----TQGM
LQIIKNYTANKNIYNNLMDSVK--SSNIVYSETEQTLIRRLSNLIEKCLIIDPKKRISAK
EALEHSFFQLK---------MS--------TAYFDADC----DLEPSLKSLFSLKTLKWI
FVGGKGGVGKTTTSCSIASRLAEERESVLILSTDPAHNLSDAFVQKFSNAPTLVNGYKNL
YAMELDASYQQAVEFKLKEENLFSKFLPDLISALPGIDEALGFATLMQSVKSMSYSVIVF
DTAPTGHTLRLLSFPSLLEKGLSKLFSIKQNMSGALQLINSVSGNAIEEETLNSKLEDLK
AITTSVKETFQDPSKTTFVCVCIPEFLSVYETERLIQELAKQSISCSHIVVNQVMFPIDL
PSGN-DQGESVLKDSSEEKIVCSYNKLLSYSKLLYSKYYSKRNMQMKYLEQIRDLYSYDF
HVAYIPTLNNEVSKIRVLIS---------------KIN----------------------
-------------------------------------STLYISK----------------
--VSLNCSHDGILPDM-AFEGATKRNKPNLVPGNYVCSRINYVDLESGEIELTCITPEEK
KTWSNNENYLGVLKDGMTVTVPQSVAQILLADQSYVLEALSKYFAYEICVGQNGIIWFSA
SNIKEMLLIMSALKVIPNCTKPQVS-LNNFFWYIYLEISMLNAQLVN-------------
--------------FMIKKGFSQSNTYSNTLFKLLSQKSSSLLKLSPTYQNGA---LSRF
AVNYWMKNKLNINNTNFNSQNDRLKFDKFLISIFGISSLGLFSKMKYDEYKNTITLQEFV
NLYLSKGYVDRIQV-NNERGKAYLKE-NVNNPKLKIIYFSIGDFSSFESKMKQVQDSMGL
NTLNFVPIEYSHLISFKKIVNDLLPRAIGITIALLLLRSFS-KSISNSASDRLIKSNYSS
FSQVKNMNKNIKFSDIAGMKEAKQEIYELVEFLKDPKRFQDLGAKIPKGALLVGPPGTGK
TLLAKAVAGEANVPFFYISGSDFIEIFVGMGASRVRELFSQARKLSPSIVFIDEIDAVGR
KRAKGGFAASSNDERESTLNQILVEMDGF-TENNGVIVLAGTNRSDVLDPALTRPGRFDR
IINIERPNLEERKEIFKIHLKPLKLNEKLNKDELIKYLACLSPGFVGSEIRNLCNEAAIH
AARRTSNSGVDLIDFDKASDRIIGGLKKLDGYLSPKEKKIVSLHESGHAIAGWYLKHADP
ILKVSIVPRTGGALGFAQMVPNELRLLSKEALLDKIAVLLAGRASEELYSESITTGAYDD
LQKATMIANSMITLYGMDPQIGLTTFNSNNTSSYSLYKPYSEATSQAIDNCIRKMINDQY
SRVKELLILKKEQVHKLSDLLLNKETVTNQDINECIG--PMPSK----------------
------------------------------------------------------------
-----------------LM--------------NLDERDDSATEEIGRDDSDDALNPHIP
QFIAKAPWYINQQESSLEHQRLRADEAIDLDSGIKKGVLGEHVKYRKGACTNCGSMTHQA
RDCLERPRKVGAKWNNLDCPDEIIPERKTTNLDEKRDRWRGFRPEDYKPIIEQFEAVEEL
ARQKRAKKVEE-----------------------SLSNNKNNPEDKNEELDEDFDETTFG
ASSDKTRTNVRNLRIREDTAKYLRNLDLNSAFYDPKSRSMREDPFHKSSN---IGNTYRG
DNAIRNSGEVSKILLMEAFAYNKHKQGESIHLQALPTRSEMLYKSSISNKDNDNLKKLEE
ISRKYERNNQ----PSISKLELTERPTTVGDLSSMNTKTTKKFVSSIYVEDEYISNHTQV
WGSYYDLEAKKWGFRCCKQTCRFSKCTNIM------------------------------
----------------------------------------SDEVGKL-------------
--------------------DWTAMEDGRGEGLVILEQEKAPTRPQ--------------
----------------RRKKRWDIVPDEDQ------------------------ECGKLD
QKKSRWDNGEVETLR-----------------------------------EAKETVETNS
S--EQSSEVCSAQRTTSEEGRQKEFTDEELDKILPSEGYEIVKPPEGYEKLRRANLENKR
KLL-------------------------------EPKITLYDIPEPTKSFQEGESKDVPQ
GFMRQV----FHSELGELSLRIEDFHFFGKLFSNISDDDLSPEEVNERLVLTLLLKIKNG
APILRRKAMKQIVETARDHGPGIILNHLLPLLMQSTLEEQERHMLVKALDRILQRLGEKV
KPYVHKILVVIEPMLIDQDYYARQEGREIISNLAKAVGLATMIATMRPDIDHPDEYVRNT
TAKAFAILASAMGIPSLVIFLQAVCQSKKSWQARHTGIRIVQQIAILHGSSVLPHLKSLV
QIISHGLSDENQKVRVITALSLASLAEASSPYGIEAFEPILGQIWKGISEYRSRNLASYL
KAMGQMISLMETNQACYYIKEISPVLVREFGSQDDEMKRIVLRVLEQCVSVEEIGSEFVK
QKLLGPFFGQFWTSRNSLDKRTSKLVINTTVSLSKQVGLEPILDGLLIFLRDGSETFRIQ
ALETVRNVMEIVPVIHLEQRLEKLLVDGILYIFQESSTDEDSSVVENVGRILTLLGTRSK
QYLPQISSIIRWRLNTPSPRARQTAADLVAGIIGVMKQCEEEQMIAHIGLFLYEYLGEEY
PEVLGSIIGALHAIVTQVRVEKLSPPIKELVPRLTPILKNRHEKVQENIIQLLGCCAKKG
GDFVSPKEWDRICFDLLDSLKANKKSIRRASVKTFGHIAKTIGPQDVLVTLLNNLRVQER
QLRVCTTIAIAIISEICMPYTVLPAIMNEYRIPDLNVQNGVLKTLSFMFEYIGTMSKDYI
YALTPLLEVALTDRDQVHRQTAAWACKHLALGVAGTGCNDALIHLLNFLWPNVFENSPHL
VQAVYEALDAFRVALGPGVILNYLLQGLFHPAKKVRSVYWRIYNNLYIGSQDSLVPFFPP
IPQIGNRNFDINEFYYFIMVKSRMTSVDICAMVHGISKDLKGQKLINIYDINSRTYLFKF
GGEEKK-FLLVESGIRFHTTQWKRENEHKTSFFNSKLRRYIRNKKLDDISQMGMDRIVKL
TFGFGDNTFYLIFEFFVAGNIILTDCNYKILVILRDTNSIGKYYNWKDCEAVLSPSRSIS
---------SPIKSSFTKNF--DPLRLLSNYNNMKEELKIFNELEHIDSNKEESEISYSK
KRKQAEKNNSGDVFSRIMKSFHLSILEKLLESEANEPFTFSMIDEISEKFINCINKASDA
LSHFNHNYKGILIIEVPSE----KEILQGDTILNNQTSFTGNNE----------------
HESKEKVYISYSPYIEGHSAQTIPKGIIISRVLDNFCKCVDEFYSSIDIVKESKF-ATQE
HKTIYSKVDKVKIDQERRLEGLSSEKEACIVRAKFMESHQEILEKILQLIRHLIATGAQW
QDIWNEIQQQKKNNHPLARHIKSLNLKDDKVKILFSQRDLGSETTPVVD-----------
--------QIGKSIEFDLIISKSIQSNIRFQYMESKALAEKFEKTQLAYKIALKKVTNIA
KKDAEKASKGVSNVPRIKKLRAQYWFEKFYWFISSDGYLIIGGHDASQNELLFRRYLEKN
DRYIHADIHGATTCIVKNTNNVQDIPLNTLCEAGQMSICYSKAWVNKTVISAWWVYPDQV
SKNAPSGEYLSTGSFVIRGKKNFLPPLKLEMGCALYFIKSKQNFNQVDSNCNKITPLSIQ
PNNNTEESKADDSNQDDS------------------------------------------
------------------------------------------------------------
------------------------------------------------------------
------------------------------------------------------------
-------------------------------------AEISELKVEGDD------ADEES
DFESNLLERFAEGKHQNSAHVRF---SVGDVSDIIPPIKIEHRVQFDELLLSRPFNPLSI
DEASNQTSSVNSKLASKVSIDTSSSDSESEKSVTTLLRVNNSCADESNHLNLNSSRRSSV
DEGPLGFSASTLPTPAELLSHKVESSSS----SEEVLDNTGFTNKANRDSSEADSNKNTS
IESNIDWMKNLSSPQQSFNR--------ENVIKATDAHDQ--------------------
---FNNKLRENYRRRSIDGGPTPRGFIPDHVPSARELLQKIRFD-PVDLELEHLSHMRE-
---------------------RGRFISDAVPYLPEELQRLIM-----------VSNQNKK
HRNLPELDLNKSIPKN----------------------------RDFDEESQ--------
----------------RIQQTSLERLPKTSEEATSTKNNINSTNNKQKNSALPRGKKSKL
KKVADKYGEQDDEERKIKMMLFGSKEMKKANDDRSSNKTKDSNQNRQHISQEKRRKEQEM
EKVYKNRIVDNSTEDSLLPTNKDEDSEIIAVIPTFAPFTCIKDFKYCARLTPGGVIKRSK
AAQDINHFSNISYKEKDQEYIKALKIDDLIKNLMNPVKVQFSNEKSKLMGRVYGRGKGIS
SSAKPYRRRPPTWIKLKPSEAEDLITKLARKGLTPSQIGVTLRDSHGIPMVQSLTGSKIL
RILKKNGLAPDIPEDLYFLIKRAVSIRKHLEKFRKDTAAKYRLILVESRIHRLARYFKRS
KQLPANWKYQAATASTLVALKMAKRVSYFYDGDIGSYYYGPGHPMKPQRIRMAHNLILSY
DLYKHMEIYKPHKSPQSELVYFHEEDYINFLSSINPDNSKDFGLQLKRFNLGETTDCPVF
DGLFEFQQICAGGSIDGAYKLNNEQSDICINWSGGLHHAKRSEASGFCYINDIVLGILEL
LKYHARVMYIDIDVHHGDGVEEAFYLSHRVLTVSFHKFGEFFPGTGDITDIGVAQGKYYS
VNVPLNDGIDDDSFLSLFKPIISKCIEVYRPGAIVLQCGADSVRGDRLGRFNLSIKGHAE
CVEFCKKFNIPLLILGGGGYTIRNVARTWAYETATILDRTDLISDNIPLNDYYDYFAPDF
KLHIPPLNLPNMNSPEHLEKIKAKVIDNLRYLEHAPGVEFAYVPSDFFDREASNEDEERE
EELSSWQGGGRAAGSTESQGNKSSRKLQKEHASEFY-----------M-----SKRSNCY
EAEYTLAIAAQRRDKNTLHNIIQQLIETGNTGILNESNALHWSVYYRWDDVSIHLLSLGC
DPFMGD-INGDTTVHYCIKSGNFSMLKYLYEKHGNKIFYVKNRNQFGLMLTAASEVAEGR
```

```
VQDLLRVMEWLYLQGYSLEEQDILGQTPIFWATKRGNYGIIQWLISHGANIGHRDHKGNT
LLHAACSDVDDETVSFLCDLGLIHLISHSNYENPANTAFQICWTRKNYWLALMLNFWYYH
NKLFGRISLLRNPYAVYYWFISILNLLVVFIMVSTLKSYREYLDGFYTWMILFAAAQLFW
VLSNIGNDNYVKHQKIIPKLPRDLYLPLLTPQAKNSSIYQLMLKENEQIQLNNQLIDMNK
LLFNGDSKYREKYNNHISRQIELSNEIIGLMPFVAKERQSNNSKIYIDIVEGKPINSTIC
VTCRTERPFRGHHCSDCGYCIQRFDHHCVWIDSCVGYGNQRAFFFFLKFLTISFFLYYYL
FQQYCSFRFKDSNNNWFTMLIMFNSINIPWMIFVLYLFIRHVKSIMSNVTFYEYYKKPEA
IKKRFATEGFCYDMQGRTFSTCIRSAFAYFTKNNSWDNLDFFTHIMYNMKQ---------
-----DLTVVKRIKIEEKCKKVTDIENKAGYM---NSEKYTSLFDGSDRDDNRGFKRLVR
RKRSARNLSDSSSVSSSSIESDLENSSQDDQTFEEKEAKRLRELMQREEDALSFCLDISS
KMKEGIVDFFVGNDNETKKQITDNIKNGHCNISKLTEELKKSEEYINSFDRLKEYQIVGV
SWLLALHQNSYNGILADEMGLGKTAQTCVWLQYLFDSGKLVKPVIISCPASLLDNWTKEV
SIWAPRIR--AVKYHGSQKERRQIADQLFEEYEESRVDVIITTFQMLSNKMDINMAFKHF
EFSYMIVDEAHNIKNSASQRYKSMSRKIQSERKLLLTGTPISNSISELSNMLIFLMPEVF
NSDLLEDAYNS----YKRKISNRE------------------------------------
------------------------------------------------------------
KLDEANSEVLFLQQIIAPFVLRRSKQDVLSCLPKKHTFIEFCELTKIQKKQYDNEIDKDS
NQESSDEKKLINLIERKIQDKVNSHFGDSA--------SAENEKES--------------
--------------------------DDNLNSNDLKTGLQDYDQDQAEQVKSREGHHYDP
--KYVNSIIFRMRRICNHTLLHQGHYTSKQIEELVDYLSDNVDEFKGYSRIKVEQYITQL
CDYEIHQLVSRLIVKNSPLLERFRIDDELIINSCKLKKMNEIIQSVIENKEKCLIFCHHT
MLLDIIEEYI--KIKYNIPDF---------------------------------------
------------------------------YLRLDGTTPILERQNMIEKFQTT-QVPLFL
LSTKAAGQGLNLTVASSVIMMDLDYNPQIEKQAEDRVHRIGQSKQVKIFKLVCKDTIEEN
IFNCCQSKLTLDNAFGGNQRQFLKMMPNQVLVVQQPSQRDQGRKAQLRNIQAGKTVADVV
RTTLGPKAMLKMLLDPLGGIVLTNDGNSILREVDVAHPGAKSIIELSRTIDEEVGDGTTS
VVVLAGEFLACAEPLLQKNIHPTIIAKGYLRALDDSIKFMEEMSVKLDVNNKESLVSVVD
SCLKTKFSGRWGSLISSMALKAAETVSIANSGSPKEIDIKRYVRIEKIPGGEIEDSYVLD
GVVVNKDVVHPRMKRLIINPKVLLLDCTLEYKKGESQTNVEITKEADWEALLRQEEEEVE
AMCKDIIATGCNVVFTEKGVSDLAQHFLVKAGISVIRRVRKSDNNRIARVTGATIASRTE
ELTPNDVGTCCGRFEVKKIGDEYFCFLTESKTPKACSILLRGGSKDVLNELERNLHDALA
VARNILLDPALLPGGGGTEMAISCYLNEKSFSIDDTQVWAYKAFAQALEIIPKTLAQNCG
ANVMKTLTLLKSHYSATRGQVHGINGCTGAITNVTELGIWDTLAVKQQVYKTAVEATLML
LRIDDVLSSVSKKKDQNNIEQNPNIDRKTILCVAEKNSVAKEIANILCPQSPNKLKSKSK
TNVALYFPYEFCGDECNMIVTSVRGHLKQLGFASKYSDWGSVNPVVLLDTPVISSILPDC
MGIADNLTYFSKISSYLILWLDCDREGENIAFEVLSICLNSNKNLITFRAHFSAITKFEI
NNAMKKLSFPNRALSDAVEARKEIDLRVGSSFTRFLTLRYSRLFPIPERTLSYGTCQFPT
LGFVVSRYKKIINFREEDEWTITLEV----------------------KMKIYEGNN---
---------KEMSNVIFEWERGKLFDNLFTFIIYETCIENSNARIKEIVNNKILKRKPLP
LNTVEMTKIASDKLKFSPIKCINIAENLYRKGYISYPRTETNSFSDSIDISKYIKEQEKS
SIFGSFANELLYLNSFSVPRKGCKDDGSHPPIHPVKCLNKEQASSNEEWLLYELITRHFL
SSCSEDAVIMETIVKVDISGETFKTKGTVIIEENWLKIYPFERIKTKILPAFNLGNLAFP
YKLFLRKSKTIPPTLLSEAELIDLMDKNGIGTDATMHEHIEKIQVRQYVKKNSKSLLSPT
ALGVALYNGFELISNPACNLMHFRIRQIIERSIEKITSGEYSRSYVVEQIAAFMKNIYEK
MLEFISYLDSALHAYFPKWDAKCMNVLDVGKYKGRTFEYIEKNDPGYSNWVLSLESPTNR
LEAFSKYLRNKISNG------NTNEYGFLDKDNIINKSKIGNSCSYLEDERKFGHIG-NG
LGTIQSDSDELEDILQKCEKELVKNTTV-------------------------IANGLNT
ILSCKMKLDCGIVFSLVSPTMFRISSEKNNK--QLSLPKILGDYLKEYKSKENITSKKMR
TYSTFTFPGIYYEKLIKNISDR---FEPIIPIPDFILDRFPDFKKYGDFERISNLTPKYN
PDTVCIYDSICYNSLNHLKCNYLYSSLRPFQKVGILVGLKKHGRVLIGDEMGLGKTLQAL
SIITYFRQEWPVLVICPSSIRFQWYQQALDWLSPEINKSNITLIRTSNDTYSRKSNIIII
SYDLITRNEHFRSDFQVVIADESHFLKNSTAKRTQMIVPLLHKARRAILLSGTPALNNPT
ELYEQINAIVPKFPSYLDFAQRYSDTRINKFSHRKEFYGSRNTEELHLFIRESVMIRRLK
KQVLHELPPKQRSKIPLEIKDKVGIKMI---KELLADPN------CQVELSNFD------
EDDSSSSMCNLHKLTCEIKINPVQEYIEYLLENDEKYVIFGHHHVMLDAIESVLLKKRKT
FIRIDGKTPGNKREEYVKEFQNNENCKVALLSITACGQGLNLTSAGTVIFAELYWVPGTM
LQAEDRCHRIGTQYSCINIHYLIAEETLDDKMWGTLCRKQKIMASTLDGIDQRKN-----
-------DIQHFHFK---------------------------------------------
----------------------------------QGNMEKEKNQDNVEKTQEATTKQNVE
NTVDKFVLELISSMDLIKRGVESADQRLIGRALRLCSLSRISLKRKYVLEVDNIEKIIST
EESENITILKSLRSNLDTE-AEMKKTINAIEKMDWNSMKPCLKECITLLGLLILIRMLRR
NNFQECLEFSKILFDYCRRSTSQTMDQLISKVLYFYSRIHEICGEFTNIRNDVLESYRNA
VLNHNNMTQAACINLILRNYVLTKRYDLGLKALEKMVYPENLSSGIHQARYLYYSGRIYS
AQLEYQSAFNSFTQSLRKTPQTKRGSLNFALSVQKFAIVVQMLMGEVPDRSIFNSTDLRK
GLVPYLELVKAVRSGDMKEFDLNLQKRGKIYERDGTLSLIKRLAHNVIRSGLKTICSSYN
RIYLDDIAKYFGWDNSHDVEGVVSKAILDKVVDAKINDDIKCLESQHKCETYGSESMLNN
LHSRIAFSLLLRSNAIKAMEYPQNMPINNENNDDARRLSQEEIEAAV--NSVDDG--MI-
--------------------------------------MKYSMFKRGNRVGVCISGGKDS
SVLLNVLYELNKRKDYGIELELIAVDEGIKGYRDDSLEVVKYQQEYYNCPLTILSFKDMF
NTTMDEIQSKSSKSNSCTYCGVFRRKALDIGSYKVNADVICTGHSCDDTCETLLLNILRG
DFNRLRRCINPITNNEITTT--KDQMQNHDSQNEAFLNI---------------------
----------------------------------KPRVKPLMYCYEKEIVLYAHYLNLKY
FSTECTYSVDAYRGVSREFIRKIQSFDYKYSFNMILAAQELNLEQSNSSPNRKCTICGYI
SSSTICNGCNLINALKHDNPNLILKN----QRQKKKILLQES------------------
-MMEKGEEIISFTQENAEASRKLFENCLDRWRRNKVSLDPQCGNISKDDNLDFCILDAKF
ST------GKFPSQNPSPEEKYNS--SSYNFSNKTNQDNIETPIIQLFGSTSTGITVLIN
IHCFFPYLYCEVPKST-PPNPEKIKTQIESGLGATE-----GKNVLNVQVLKKESIMHYK
GDSESQFYKITLQLPNLVPTCRSMIENG----NLDCIPQAYEANIPFILRYLIDRDLSTG
SWVSIPKQKSYIRSPFQRISSCQIEMDLFYDDLIVLGSSGKWATLPPIRILSFDIECITE
SGVGFPEPHKDSVIQISSVVTLLDSP-ESICNVIFTLKECASVAEAFVFWFESEKDMLLA
WKDFLLALDPDVITGYNCINFDMNYLLERAKLFQLNDFFFFTRLAKTKITSKDTRFSSRA
FGTHESKLINIEGRILWDILETIRREHKLKSYSLNYVSTNFLKEQKEDVHYSMIRGLQDG
NPETRKRIAIYCLKDSILPLRLMKHLKLFPNIIEMARVTGTTIDILLSRGQQIKVTSQIL
RKCKSTNFLMPTVKNQSDGDNQYEGATVLEPLKGFYKDPISTLDFASLYPSIMIAHNICY
STLIPPLN-LKNV-PEDLRKT-SPTGHSFVLSSVRKGILPLIVEELIAARKKAKKEMEEA
TDPTLISILNGRQLALKTSANSVYGYTGAVAGGQLPCLELSTSITSYGRAMIDITKNEVE
KIYRKENGYSADAKVVYGDTDSVMIQFGVSDIGEAMKLGLEAATSISKLFVKPIKLEFEK
VYCPFLLMNKKRYAGVLFKNPQFHERIDCKGIETVRRDNCLLVQKVVDTVLKKILVDKDV
EAAKQYTRNVISDLLKNKIDLSLLVVSKSLGKDDYTAKLAHVELAKRLKLRDPGSAPNIG
DRVSYVVIKGAKGQPQYDRAEDPLYVLENNLAIDTQHYVDTLKSSVIRVFEGVMKDPEKL
FSGSHTRSVTVLSVTGGALAGFVKKGLQCMNCKTIIKEGPLCKDCQENMECSVLINKLHE
FREKEIEYNSLWTQCQRCQGSAFQDIICTSRDCPIFYRRTKVRKDVNSIGEQIKRLDLNW
MK----------------------------------------------------------
--------------------------------DDQSYNEEEQKQKLLTTYNN---LSNSI
ISVAPMLDVTNSHFRMLCRIISKKTELWTEMVVDDTIIHCYNDENRHFKKNDIENPLVLQ
LGGNNPQKMEKAIEIAYKYGFQNFNLNVGCPSCKVASKGSFGASLFKNPLRVAKIVDTCN
KKVNKRISVKTRIGVDQYDTYQHLYNFISLVSGCGANVFIIHARKAWLNGINPSKNRTIP
KLKYYWVYMLTLDFPNLTFILNGGVTSIQECISILTGDWYINYKDDIKDNELEINNYTIN
KIKGVMIGREVMNNPFILSKVDSMIYGGNNSSIHLTRRIVLERYVEYLSKDQGQFTIGEL
NMYLKPVFGIFHGFSGTKFWRRTLSEFIQKSLKINSPADILIQSMNLFSKDYSNLLDSIV
-------------M------------------DFEKLNSLDEQEVVYSWEQGLIKNWERL
VETESGLVVIDNNADESYNIELLKRGPNTNIRRGLLRNIVIILDMTSNMLELDYKPDRLQ
CMVKCNEIFIKQLLEDNPLTQISVISIYDGIGEVIISYNSNFLEIMTSILNYLKKGCKGS
MSIQNGLEKAKYLLVSIPPYGTKEIIFFLGSMRSVDNSFLFNWVEGFSSNNIIINAILFI
PELYIIKTITKMTGGVCLCAMNNDHLLKLTLENFIKPIASTPLNINLVTMGFPEYVSNQT
HPFCSCHRSLTHRGYSCPKCKSIVCYLPTKCPVCLIYLISPNHLAKSFAYLFQHPDITPI
```

KNESSIKTKCELCESQFSNNVYLCTNCSTQFCNECCKFIFNTLHQCPICCSLRIMNQNNF
PVAQQVYFSTHNALRAG-ENEIQKFVLYELDGTKRILLSIYPFYDCQMVKRLVIKYLDLP
EGTSIRDIQLFYRGVEIPNGRFMHTF-------------EKQRHPLHYSLRMNKSDFGIR
STGLK-WSSKIQKLVVEVKLAMQRNVHPKLTLDGTGATYRMYNAKGQVVAMFKPLDEEAF
SPNNPRGYQGKLGQQGFRSGVLSGEGASREVATAIWDAYYHNFAGVPDTTLLEACHQAFN
YDSWNKITLEEITVDWKLGAFQEFISTTETVGNFNPSVFCIRDVHRIGILDICLFNLDRN
DSNILVVPNYSIK-----FNISNSNNPSSATSPYEHPLSTPDGKKTKYKLIPIDHGLCLP
DVLDVAQFDWVWFDWPHSKIPFSRSELRVIKYMDPDADAERLKRKLLIRSECLRSMRVSV
RWLRLASSMHLNLYQIASFLCREDLEIPSSIELLIQRSLQHCYRAFDATALISSNRLGNI
IDLATTSSAPNKKGQNTNTVDNFIDDCELSDNSQSESEFSSDNSWKQKSSQSQKSDIHSC
PTE----QLTPSSFQKSCLNGAS-------------------------------------

--RS
TAYRTLGLAPQNATWMLLDRENNIIPIQWGDKHFEHVFFEVLEQEMKHLIIDKHPKWEEY
PYFGEEVFDEGKNKDISAKMEVRVGGKYRLGRKIGSGSFGDIYLGTNVTNSEEVAIKLES
VKSRHPQLLYESKLYKILAGGIGVPTVHWYGIEGDYNVMILDLLGPSLEDLFTICNRKFS
LKTVLMLADQMLNRIEFVHSKNFIHRDIKPDNFLIGRGKKLNVVYIIDFGLAKKYRDPKS
QAHIPYREGKNLTGTARYASINTHLGIEQSRRDDLEALGYVLMYFNRGTLPWQGLKATSK
KDKYDKIMERKIATPIETLCKHHPFEFITFLNYCRALRFEDRPDYAYLRRLFKDLFFREG
YQYDFIFDWSFLQPEKDRRRTGNSVVAGEGGQNVHESQQDEQM---DLIRLNCRKTLLSF
LEL-VSEKQIFIETSISSFLSLITDIVDYNNFGFKRIQPIISEDERLICDFEFDSNVVFL
CHPSLSSTCTIVKHIELISALLKIDVCFYIGFVPFYSKVILRLLHQ-ASLKVTPECYSVN
LFWAPIDETSLSMEFRNIFFDFHVYKEESSLQLVAYSLYWLLKFTNSTNVPIYSIGSAGV
SVLEHLIRCFK---

--------------------------------------ENNSLSNILKPFDWNTLFETND
DRNYKYPEFKRSDDLLTNTLEFTQYDLTNEFPMGFDQVIIIDRRCDLVTPFSTPFSYHAL
LDFLFGVQKTYVDIPTKKVASDV----------------------YEESPHWKLPLFGDP
LFAILKDLKLKDVGIYLHQKANELQSLYQEKEKLKDISAIGDFIRKLKGKQREQGTLAKH
VNIATYLNEYFTKDQTLRRLELEDSIMSDSHQSVTGVVKELSTKFDLLDQEDIQIEEIYR
LLCLSCIIENGFKNKKVEQIKKHILSVFGFEELYRMNILERVGLFKFDPNKKSYWQLIKR
LLNLFVDESNDISCVYSGYAPISTRLIEILCREMNNSKSLKEALNYVWGPSVELTPS---
-----------------MQSMIKHNSCLVVFVGGVTLGEIATLRKLQEIINKE-IIVATT
EVINHKSFFESCKKMG-----------GNSEYSRDDDTTIIQSLCMNCHKEGETKLLLTS
IPQFRDVILMSFECPHCGFKNNEIQSGGVLQDKGECIELVVTNVSDLDRQIVKSEFATIS
ILEQELDIPPSTQKGVISTIEGIITKTIQGLSLNQEA-----------------------
--RIAQDQEVGGKIEKIINN
LKGYLEGKGLPFTIKLDDPSGNSFIQNPIAPLVDHNMKRKLYDRTKEQLEEMGYYGVQNL
KDDESYQPKKDNSNVTFSKP-EHLIPHYIDLNK---------SIED------QGELGSNE
DRI--KFDVPCPNCGNNGESDVCEIDIPGFRRCLIMAFVCNFCGIKTNELKPSGAYGELA
KKWILTVESELDLNRDILKSDTASIEIPEIELEMGMGSLGSLFTTVEGMIVKITDSLKDC
FTFQ-GDSATSE---------------QKKGFQRVIEKLENL---EKKEKFTLIIDDAAD
HSFIGKRIVQDDQQLKTEKYQRTDYQNETLGINDIKVEY---------------------
-------------------IMYVVNRKGEEEPVSFDQILSRITKLSYGLHPLVDPARVTQ
AVINGLYSGIKTSELDELASQTCAYMAATHNDFSKLAARISTSNLHKNTSSDIGDVASQL
YNFKDNQGCPAPLISKPVYDFIMENRERINSKIDFSKDFEYDYFAFKTLERSYLLKIDNK
VVERPQHLLMRVSCGIHCGDIEAALETYELLSQKYFTHATPTLFNSGTPRPQMSSCFLLR
IPEDSINGIFDTLTKCANISKTAGGLGVAVSNIRGTGSYIRGTNGRSNGLIPMLRVYNDT
ARYIDQGGGKRKGAIAIYLEPWHVDVVEFIEIRKNHGKEEMRCRDLFPALWVPDLFMERV
EKDQDWTLMCPDECRGLQDVWGDDFKKLYEEYEKQGRGRKTMKAQKLWFLILQAQIETGT
PFICYKDAANSKSNQKNLGTIVSSNLCTEIIEYTSTDEVAVCNLASIGLPKFVDKNNKTF
DFDKLKEVTKVITRNLNKLIDVGYYSLKECKKSNLRHRPLGIGIQGLADCFMMLRMPYES
EGAKKLNKQIFEVIYYAALDASCELAEKYGPYETYSGSPASKGILQFDMWGVTPDSGLCD
WDLLKDRISKHGIRNSLLISPMPTASTSQILGNNESFEPFTSNIYHRRVLSGEFFVVNPH
LLNDLLELGLWDDRLKQNIIANNGSIQNILTIPEDIRELYKTVWEIKQKTVIDMAADRGP
YVCQSQSLNIHMENANFAKLSSMHFYGWKKGLKTGIYYLRTQSATRPIQFTVDQQLLKSE
TK-------------EKDSLETNEAQKLIACPLNMKDDEECMMCSGMRLQSKQKYQFPDL
EMGELMNELDMLGFEVGSNFWESINHEIAVELYMNCLSIALEIDTEDIRPEELIGQLPSS
AAG-----IISENGKSQIKPIGNLRFLRYCKILWVMIGIDDFSMNIYRPTPDRIYSFLCG
FVNLMRFKEDRWMTYKNEFYEIEEILDSVDKSNEQIKQKKEDLNNIRVRYNEQSGEIANR
RRDNQEYQEKMRSLHGEFLQNQQELKRLTQSEHDLKEQLKDVEFRITTGNQDIQDLKDQV
VQSPERLRNTLEELNKSLENERKQIDQISIKNNELKERQNLLQKTEKRLGKAKTFLEQTI
SG-IKDANNIKQSIKEIEHHIEKDKWTIEQTTEEQQHYESLIEEAQTLLNQEKQKLDVQS
SEAERQAELMEKEIQNLKVSINALNIQHSQLLNVLTNYKKHLISKLNDISKYNLKLLDET
SKENSTINSIENTIVMQHN-------MDGIWKRLVSRYPATSNMNKHDNNQKKYSIDDFQ
LIRTLGTGSFGRVFLSKHKEDN-SIYAIKRLKKSVVIRQKQVDHITNEKAILSRIKHPFL
VRMFGTFKDDRYLYIMMEFVIGGEFFTYLRRCRHFDNETSRFYAAQVVLMFEYLHGKNII
YRDLKPENILIDKDGYLKLTDFGFAKAIEYRTFTLCGTPEYIAPEVLLNKGHGKPVDWWT
LGILIYEMVVGFPPFYDDEPMGIYQKILAGKIFFPKYFDKNCKSLVKRLLTPDLTKRYGN
LKGGVSDIKLHKWFYNYDFNSLISRKVDPPYIPKVNSYDDSSNFEEYPDSHEQPTTVTGN
ADPFVDWGMISQFLILNVRGDTIIFRDFRGEKSLSESDVFYKKIKQGNSDEPTVIYFEEQ
IYIYLRQSSLFFVLTSYYDVSPTYIIELLYRIIKLVRDFCGTVNEDSIRRNFILVYELID
EIIDYGYPQIVSTNQLKYCVYNETKVLIFPSSSTQNHGNISNSGPKTISSNASQRPITCV
DRNNEVFVDIFERISLVLNHLGEISRFNIEGGILMKSYLIGQPELTLGFSNSIVLKEDDE
LSPSSTIIDDCNFHESVNVNEFLNDKVLTLKPPEGEIIVMNYRISKTLNIPFKFTTLIEA
SGNSKFDFVIKLKVDIPESSFATNLTMLCPLPEKTNTVSLETIHPPVQQTSQYDDKNQRI
IWKIKKIHGGTEIILKSKICLSFETDLNRKKIGPLFLNFEIPMFNLSNIQVKYLKISEKY
GQQNNYRWVRYVTQSNSYIYRL--------------------------------------

---M
KSILDEGDVICTEVQRVQSEGICHLHTRSAKYGKLANGMVVKVPNKLIQRQAQHIVKLKY
GIQLILGLNGYVWISLPFEHSHTDTLNYSSSSV---KPEIVNSNIRKSIVMLAFIIKLFG
LKNMQITPERICKVFDRFTEL-----------HLSYK-------------MPHFYSLTLE
SHGSILSAIQGSYSAAKAHEIVVNRGRSLELLRLDNAAQIQSICLMDTFSLVRSISNLRL
IGSGKDLIVVTSDSGNIVILDFNKDKNQFERIHSEPYGKSGCRRIVPGHYLAVDPMGRSI
MIAAIERQKLVYTLTRKNKDALDISSPMEAHKSHMVCFALVAMDVGFDNPMFATIEQSYS
TENEELQQT--------KKHLIFWEVDLGLNYVSRKSSQVITESSHTLISVPGGNDGPSG
VLVCDYKGIIYCKMGHSNIFCPYPFRFGDSSEYGTMIVASSLHKLKGFFLILVQTELGDI
YRINLIHNEGIVKEMRIYYYDTIPVCNSLLLLRSGFLFASHEFGNHTNYQIVSLGDDKTD
PYTSSLPDSNDLKRVYFRPRNCQCIRKSEEILSLSPITDIKVIDTNNDGTPQIVATCGRG
PRSTLRVCSYGKNVEEIAENPLPGRPRCIWTLKNGIDPSLSGYIIISFIDRSLVLTIGEH
VEETNDTLFTLNETTMYAASMFYNSFLQVLETHVKLIIQDRIYDWKTPDSRKIIAADSNG
RQVSLALEGGLIVILELNVNGVLVEVCRREITCEIICIGIQQLSSGQMRSDYVVVGSTEN
ALRLYKIDSEKRLKQTCTQILPNNSIPENVQLYH------SNKYGHLILFVGLTTGVILS
CKVDASNGSISDPRSKYLGNRGVNICRIDFGGEMSLVCMSSRPWLVDSQTSGVNFTPLQY
RCIDSIAPLNTHQVNNGYVAVSGSTLLIFQVTQFGESFSQSSINLSYTPRKLLTLPSPQL
FTGLLMSSGTLDIPKDQMIAIVETDHNSFDFGTKKEIILALQDVKMEEVEEDEEKQ----
ESGVLLAESEVGGFFAGEGKWGGCVRIVNLKSMETIQLIPLDTNEGCISACVCKFDELDC
LVLGTVYGMKLENHDSFGAAIKIFKYDSNYNFELVHITPIENSATALTGWRGRLLVGINK
TLRVYSLGKKRLLRKSEYRNIPQGLTWIKVVNDRIFAGDISNGVLVFKFNNTSNQFILVA
KDPMPRWLTSAEVLDYHTIAVSDKFDNIIVSRVPAEASDDFSFVTSFTDNNNSQSSALMR
THQINTVAQFHLGDIVTCLQKSQLTPTSAEAIIYGTVLGSIGSLSPILNNEDIELLSKLE
ILLRKQKSTLLSRDHLMFRSYYSPVHNVIDGDFCQTFTILDSQIQSEIASKLDVTVEEIY

KKLDDYKTRLFMGKEEEDQSRPLDQVDIDIMRKYGMGPYVSKIKQVETEVKDLMSAISKL
NSIRESDTGLAPPSQWDLAADRQNMQMEQPLQVARCTKIIDAGTEEAKYMINIKQMAKYV
VGLGDKVAPTDIEEGMRVGVDRTKYKIQIPLPPKIDPSVAMMTVEDKPDVTYNDIGGAKE
QLERLREVVEMPLLHPERFVQLGIDPPKGVLLYGPPGTGKTLTARAVANRTDACFICVIG
SELVQKYVGEGARMVRELFKLARSKKACILFIDEVDAIGGARGDESAHGDHEVQRTMLEI
VNQLDGFDARGNIKVLMATNRPDTLDPALLRPGRLDRKVEFGLPDLEGRTQIFRIHAKVM
SMERDIRFELLSRLCPNCTGADIRSVCTEAGMFAIRARRKSISEKDLLDAINKVVKGYKK
FSATAKYMVYNMELEVCLSNLIGIDISSIN-ETEINKSGANNLQLMLNSIWSLSKENTKD
G-TVVSLPSNQEILLPRAYPLPTKKEKTRWEKFAELKGIKKRKRSRMVYDPITDDFVPRW
GRNSIKKQKKHNEAIIEIKGNMDENKDPREAINLKRDMMINKQRLRELKNKMNSSGIGNL
KDT---NVKRSKTQVKELFDRVSLSTASYGRRDKALPDEDRTTNKVKKVKKILDTKNENE
KYKSIYSKILKQNM--

--------------YNLPSSWTTYEETLFSRMIEGIYSASLQLGEIPVIRYLANSPLCRN
IAFAVERRLLD-----SNLIDLVSGEF---VNTRSESYD-----------DKRNESTILL
ILDRREDPVTPLLTQWTYHAMIHELLEIKNNRLCLDNSNKEKEEYVLSEQYDDFFRDHKY
DNFGDIGFSIRDLVNNHHESSKTNHRLETIDDISRFVQMYPDFKKEYNNIYKHVNILHEL
SRIVQERDLMRISALEQDLTVCDNVDEHSRQIGNLLSDTRISQFDKLRLALLYSLKYEKE
EQINNFKYHLG------TQANYIDKLLQVFGENFRSGDLFLNKTLLNIAKNTINK--SNN
NNIYIQHKTLLYYILENLVKGKLKNSRFPST---TDDYNSSKKPLKIMVFVVGGVTLEES
RDVNVIRNLYDVDIILGGTNLLNSKLFIKDLELLINSMSKSLESKEKSNLEHRHSSKAKK
NSNNLKLQTKLISSPDTEKQTKLVNKFHTIPEDSKTSVFDRLLDPKLYTGMHKYRFDKDG
NGLGKAGREYLFREDGYTESTKRKHEVVSSSIKRHSYANISSNDF--------LQKAKVI
WLYKNGDKYDKGTIYYVKPYIRNMTQLLNEINRNVRLLGGPVRALYDQGLKQINGVSEIV
DGAKYLCTSGEQPTSIEKLEKFLSYWIINKMSKRISKRSSKSCANKNEELDSDFSDNIRI
KKKAQRPKKTAANTKNTKKASKKPTKKAKSASKPPSKPRKRQSIKSENDSEDDDIN-KDI
KKQFRDGQKYITPPNGDATRAFYESLYNENPYSIIALKYCVENGILMGAKHTSAYKRLEH
LRESGLLKRSLGGIQNEAIESLKRFDSKNGGFP--RMSDNDTNVKQWKVRRLIATLEAAR
GNGTSMISLVIKPKDEISRISKMLADEYGTASNIKSRVNRLSVLSAITSTQQRLKLYNRT
PQNGLVVYCGTLITEDGKEKKVNIDFEPFKPINTSLYLCDNKFHVDALKELLETDDKFGF
IIVDGNGALYGVVQGSSREVLLRFNVDLPKKHGRGGQSALRFARLRMEKRHNYLRKVAET
ATTMFITNDQVNVAALILAGSADFKNELAQSDIFDQRLASKILKIVDVSYGGDNGFNQAI
ELSSDALQNVKFVQEKKLITKFFDEVAQDTGKYVYGINETLQALEMGAIELLIVWENLET
KRMVVKNPSTGEEKVFLNSPTEQHDESKFKETGAELDVIEILPLTEWLVNTYQNYGAQLE
FVTNKSQEGNQFQKGFGGFGGILRYKVDFQDYAVVEDDLDEFIDRIYKV--ENYGQNTLL
AEASNVVKEQAYYMKRAIDQDGLRDALRHASNMLCELRTSSLSPKHYYELYMQIFQEMRD
LSHFFDDKSRHGRKMSDLYDSVQHAGNIVPRLFLLITAGACYIRSLEAPAKDILKDMSEL
CKGVQHPMRGLFLRYFLIQTCKDVLPDTGSIYEENGGGTVMDTWDFLYSNFCESTRLWIR
LQNHG----------TPKDKLRRERERHDLRILVGANLVRISHLEGLTQQLYIQEILPKL
LNVVLSCEDVLAQQYLLDCIIQVFSDENHLKTLELLLSACMKTLPGVDLKPILTNLMNRL
SNYLQSNDKSLINDIDIFELFRKNLSELHERPTPNIQ-----------------------
--KQISNNLERDLSSLLELH
AAFLAFTLTLYPDDLILGSTVTLLTNALG-VRVDDSRCIDTIVEILSLPFQSMPLSIMEM
NHFPNLLYFLNKQAGKKVALSLINTIVENNTPFDDADALQRFCSFILPMLDEK-GTHTGE
EVDLSITENNEFIYQQMKVSKLVHQIKHE-DVNQIFSMYGILFDLFSRSSRFKYTFPTLG
YCAINLIETTLSKEK---------------------------TDNEPSKLSVKKIFQFIH
KIAIILVTCAPELALDLFLQGSIMADKAND---------SDGYEAICYEFLTQSLVCFEE
ELAESKRQFQGLMSIIGTLVGRIQCLSKDNYELLAAKLAQYSAKLLRKPDQCRAILMCSH
LFWNNEENRDATRVLECLQKCLKIADSAVQVAPSNSVLFIDILEKYMYYLEQGNQNITTD
FISKLVALCHEQIQFSGSEIQQGPKLNNLMTHIKNNHD---LYKGVKLTSMDMFEARLSN
GGILRKVFEAITNLVSDVNLECNESGVTIQAMDNSHVSLVGLYLKDTAFERYRCDKNRTL
GLNTQNVVKLLKLCSNDDQVLLRHDDDSESLIFIFETPNGDRVSEFELTLISIDQDSLQI
P--ETSFSSVVTMPSNEFQRLCRDMAQFSDSLSIDVTSKNVRFSTKGSLGSGSIILRPKE
GA--DSEAVALDVSEPCQLVFSLRYLNNFAKATPLSNSVKLSMSENQPLELEYPLEGSGS
-------GHLRFYLARKITED-DEQEMQGKATIIKALAWASSEGLSISKDYCRAVPLDGF
ADFAECAPP-SNEAEYTIEVVPVVASGIDLRSEVDIVLYGGDDTQTSPIPISSSITGTVR
SLSTKQLDIGAPQQVGIFLKS-NTRWKCLKITVVKDYRYWVFSCNGELTPQHRSLTYGVT
GNKLYTASIMTGKSGEAGTKGGVEISISGEAGQSDKILLKQGFYSSTLRRMLFRAADVGK
IKEISLTNTALDDPWYCEYVRIQSEDGESIDFTVRRWIGRPFSQMVTLTNSISGSNSPAQ
DIDCNTRAIDIVPRA------PVTTITFKVKCPVNCGSEKFATIYGTGIHAASSSICKSA
IFDNILTPSGGELVLTIVPSLPKYFGGKGA-YSIESSDFFPKPGSEIFSFYTYLSDSIDF
IQSEVRVVDSTGNLASSGRLEVRRNGIWGSVSLKGESTVTNELAATLACKQLGYSFG-SI
LPSCTYVNGGNVCTPKGYPISVAGLQCNGNEIDIAQCMFEEP-KTTNSMTTVAIQCSSIA
PKDTM-HGALRIVDSTGVPSRTGAGRLQVYMDG-WGSVCSDGFNSVSERVACTQMGYSGV
KNGGASTKSCKDFGGEDVCGSEQEQILMVDVACKGDEEKLVNCQHYSSNNIYCVHEEDVV
VSCSGSGDPTGNGAYLSEKSPSILPSIPYNKYQFDCNDSPVSIK----GAVGTGAIIACP
SKCSKASSHIKGSYIYSDDTPVCKAAIHAGIITDEVGGEVVITFVSGQKQYFGSFNNGIQ
SRSIETSDLASIRSFTISKLSATINRRENDFSQQIVNEA-------------------IS
PPVLSWVCPTDYPGFRGGKNDFIDATNLPGGDRISSLDDFTISARVLITGGQGTWRTIMA
HTECLGISLYIDRDNTIHFDQLCNSDTVSTSYAPAINEWLFLTVAYNFNEKRVVMYINGN
EVANKVTGFVFNLKTKLIIGRSSDSDSEYFLGQISNVNIWDKFFTPSEVKSAMEKSWHQK
GIS--------------GGGELKGKRHTIDGRQCITSCQSEFPVAYKMYATNPSIQIKCD
TNLDNARFSSK-ANKFRVECPAGCSKSRYPVKGH-KVYSPKSSICQAAMHMGII---SDT
GGEFVVALYPGLKQYTGAAGKNNIVSTDENTPQTRSFVVGAAPKPRQLTCSDTASFIFQL
PVGERELVSCPSNCGESDAKVYGMDIYSPISSICRAAIHASKLSSTAGEIQIEVGPEQFH
FEQSNRNGIQSQSTGYYIRSFKIV---------------MMAEVKDD-------FKFGN-
-------------------SSKRRHSSLSSLKGLKDKKLKSKLSLQQKIFHTASERLANS
EILLPETPGFL--------------------------------------ETEGLEKSFSI
TQNELGENISIGAINKRFSLDLSSGPYSINYSRNGNQLLMGGRKGHISMLDISNGAPLLE
LPLAEQINDVHFLHDHTLFAIAQKKYIYIYDREGIEIHCIRDVTSPYKLDFLPYHFLLCS
IGEFGELRYQDVSTGQVAALHKTGRGPCHIMKHNQFNGVIHLGHSDGVVSLWTPNVSTPV
MEIFAQKGGLTALDISNNSLITAGNDNSWKVWDIRTPLYIYKSFG-SSVRSVSISGTGLV
ALGFGSHIQIWKDLHSKP--YITHNHPAVRVSEVEFQPWNDVLCIGHTHGIETIIAPGAG
YANFDSRECNIFETSKQRRNREVRMLLEKLPASTITLYPDLIGKVNKTPRAVEDLEENTQ
GENKTKKKKNKKRGRSKITNVLKNKQIAYASTVRSKASKIATQGKQNSQKAGFNSPTRTA
KASKKVTRNALERFKS--F--MSRAVEYDDE--IVCLGKGG--VYRLR------------
-----------------------GMTKNDVNSEELTVNNPIPFLEGVEIYAQ--------
-EPQGRGYCFVPKDSKNSVILITKGEGEVELKIENDFPVRKLQYSPLGSYLLVLTVFEQG
KNENNLQVYKL------------------------------KENVK-----------LLF
SFPFKTNSASTWPPYRWSNNEIFVFKVQDTSVSIFKSDTESNPIGTLNFSKS--IQLSVS
NEIDGGSDLG--EEGSKLSSLNFTVVSAQNMDVYVYHAEMDSKKEIQLSWNNSGDSILVF
AQIEGETLGKSYFGSSSLHLLRIQNSKHQVVVPEEGPVNDVAWSPKSNEFLLCKGIIPPE
LTLNSGIGSPKVSFGKSRRNTIRWNPSGKWFAYGGFGNLAGDLDIWDLDKQKLIGQTNAS
CCITLEWSVCGRFLLASTTSPRLRVDNAIRIFRYNGDLLKRVNFDTLYSAYWSPKRSVKY
RSVSPGREFNSK--------------PQPEKQIYRPKWASSGARDSNNVPTVVNQQKNEY
VLPTLEASAAAVASRQKQKQKPKK--------MIFPKVRLLSSEGEEFAVDVRVATASTL
VRNIIEADVGIDDPVPLPNVRGDVLRKVLDYCEYHVDNPSKEIPKPLRSNSLSNIVCEWD
EEFVNIQQEFLFELMLAANYLDIKPLLDLSCAKVATMIKGKKAEEIRQIFNIENDFTPEE
ESAIREENKWCDEPM---

-------VEGWLTTSKSMSGLFGKSLISKQ---------STSGSINFNDDELTDNIVVDN
NNKSDNL------YSRKDSKGGEFDKSKGSLLRLFQSDVFDAHLHLYYIYHHKEFGVHEY

LVNLLYER--SDEILWYLPQLCEISLNRFEKSSLWKYFLDKSSENMNFSLILSWMYQAMN
EDNIPLISEYAQNMLQEIEMAMVNSKLKPD---IRYIEKRLYFWRRSNLSVFQQNMKLND
IEDEFKDNDNTMLMNRENTNTQSLAIYSTLPSSETINLNESDINTNTIPNNNSDIWIYRI
INDDFIEYIQTKLGNRQFKNLYSNATCAAYYN-----PNPGTSLNYKSGVSSSAFGSLNS
GFLPTSIISGSSSFGTFSSKVSIPYFFSKLGSPFDLKIINDIQFRSKDNYISEIEKYLLK
QHRYNYFNRVNQLVSQFLEISNYLQSNINKEHRCEILEFIIEELNLWLFYSRFSISISRT
TFLLNSISLPMPSFNNEFDGNSKGIIGFSHSDNNCQFLRIIPNECKIYNSRKRVHFLLAI
EIADLDDQDYELGKNS--

-----------------QDLISKDILNY----------------------LKNHGIKL--
--------------------------------------TDNNDENQNRSQDILLNFLNIL
SVGISESIDMGLSQEDFNQITFIDNKDSDEI-----------------------------
-------------EDSSIHDNELSDED-----------IFINKDWPQETWSSKIKRYRKE
SPYSKLKSWGIKTLLLKSSDDLRQEYLASQLLQQFDWIFKINKLPLWLHPYEILVIGSHG
GFIEYIQDTFSIDSLKRRFQTDNLLNCFDQLFKDEELKARKCFVESHAAYSLVSYFLQVK
DRHNGNFLIDKHGHIIQIDYGFMLSNSPGNVNFEQSPFKLTQEFLDVMMGENSSDFQYFQ
QLIIKGFLASRKHVDQIVLTIESMTSASKLPCFSTNKEYFIQDIRDRFFLHLTEEQCVVK
VTELIQSSINNWRSIQYDAFQRITNGILIEISIVFKVSGGTQFNISVPRNTIVKDLKDKI
SEPSNIPSSQQRLIYKGRILKDSDSLDGMKVESGHTMHLVKSGVQV--NQKPTNILDQAA
DYNSSTNNNNSNTNHNSNDPFAAMMMLNGSD-LGFQTQNLRNFQNSYNGFGGIPNNN--N
FGNIPDLNSLMNSPIFQQSINELANNPQLVRNILHSNPMFAQLSANNPMLDQMLNNPEMM
RMMLNPQMIQSVL---NSNNMNSSAANSNPFSSLG-------------------------
GPNVAQINGLLNDPSIASMLSGVANGANRSVNSNVPN----------------PAPQMYA
TQLSQLRDMGFIDTDASLSALQESGGDINAAINKLLERGVGM-TAEVNPMKNIKIEKLVI
NISVGQSGDRLTRAAKVLEQLTDQKPVFGQARFTIRSFSIRRAEKISCYVTVRGDKAEEI
LEKGLKVKEYELRKRNFSATGNFGFGIDEHIDLGIKYDPSTGIYGMDFFVQLTRPGNRVS
LRRKCRSKVGKHGRVTKDEAMQWFQSKYDGIILNLIFKGGNFLRQRLALSTITGKPIIIK
EIRA--------------DDTLNPGLNDSETSLLHLLDYVTDGSKIKINDTGTTLNYTPG
IITGG-----GTSDKPLIHECHPSRSLSYYIEFLLMFAQFGRDPLCIKLIGITDNSTDHS
SDLIRMVTIPLIKKMIPQVG-EISLSIIKRSVSQNSPGQVQLNIPTIKKIEPILMDGIGR
IKRIRGSWYTGSASAQIAIKMISKARGILNKFIPDVWIYFDKPKKDSLNDEEITGFGMTL
VAESIKGITIGSNCISSSIDVQKMLNIPMESFEEEQVFNSDNINEYQDLSANGEYSNESR
NETSSSIQEIQ--------NNEEFKSLTEWEKIGWLTASRLLLEIDSQSNVDTSHQIYPL
LFMSLSQDTATSKLKLSKLAPMTIQFMRDLKTFTNIEFTIKEDTENGLF--IILSCTGIG
FSNFARKAAKMQYVDSFSSRQLFDLVKSIGECRSKHEEDNIILNESSMLKIKLIQNSLSS
NKLREYMIRAIYIEMLGHDASFAYIHAIKMTNDKNAFVKRIGYLACSIFLNKNHELLVLL
VNTLQRDLNSRNQLDVASALSSLPYLLNFEIFSSIENSLLMLLSHQTPAIRRKAYLALIC
ALEIKPAIIEENADILNRGLCDSDISVKNSVLYLIDKISSYNPKLCIPLIPHLTAIMKQI
LESNIPKEYDYYFISAPWTQINILRTLSKVA-SFEKNTNQIYEILYNLIKKVEYTANISY
AVLESCIDTIASINPNNELLDKADEIISRFLNSDLNYLKYIGIKSLSKIALIDPSYAIPH
QIVVVDCLEDKDETIRRCTLELLCNMSNPQNIQVVISKLINNLKISTDIHFCKELVKNIL
LLSEKFAPSYNWYLNTMVSLFELSGEFVGKDKVNNIAQIIA-------------------
-----------------------------------EGPTGNDISDH--EFRVHASTLFLQ
LLKEKVILPEILYNLGIWVIGEYGSCVENDVNEINRVCQSDIKCSNKSLANSFRTNSMII
TALLK-SYSYTIISSKKLSNLINQRIKEFISHLSDQTNSKCINMEYVELLSCILPFDASC
EEIYVDRELSFLDELVTEYKLS--EKYMNRKGRKNERDLIEVEREIEQRNIEVQVINH--
--------KTQQLANPFTETNIEKIHGSNKTKTVRNNSQIMDSSSNKGYNTKKWSPEGFG
KHEK----LQTRGRDLG-----------SKLTNNPKSILVESKKDCQ-------------

-----------------------------------NFSSKQREANALFSGI---------
------SNTQKKKSPSIVS---

----------------------------------IMTYNGGAILAMKGKECVAFATDMRL
GSNGYRTISTDFDKVIRPSSKTLMGFSGLATDIHTLTNLIKFKTNLYHLREEREIGVKAL
SHMTASILYSKRFSPYFVEPIVAGLDNN------------NVPFIAGYDLIGCLSVCSEF
AISGTADNQLFGICESYYRPDLEPDELMEIVSQCMLSGIDRDAFSGWGCKVYLLTPEKLV
TKTLKSRMDLFENVKKVLGEELFFKIQLAKILVVGAGGIGCELVKDLILSGFSNITIIDM
DGIDISNLNRQFFFRRKHVGMNKSTVVALEAKKLFKVSNIVGIVGNIMDYNTEFFSQFDV
VLNALDNISARSYVNKICIASNIELIDSGSAGYNGQVHPIIPRVSRCYECYPPPTQKTFP
VCTIRSVPDKPQHSIAWSKYLFDIVFGVEDSDNILDISKVQIDLDSLKQLEKNEYIVNMF
NFLFYSEITLLANNQEMYISNNKKIPIPWDDIQRKNYIDRNSEDDLVN------------
-----------------SEQKVFSIKENAELFFNSARKII-INRMNEIGTASLCFDKDNK
DAMDFVSAASNLRSYNFHIPLQSRWSCQSIAGSIVPAVASTNAIVSGVQIAQLLLMLKSK
LSSLTNPEAGNNNKLLFVNKFVWIRSIPMGR------FIICPESLEKCNPKCLICSQVLV
KIKIVSFDKWNLMEFVKGIICQHLKLSEPSVELNGKCIWDPDLL--EDDHFIKYSQRSLI
NWKFSDGCIVSITDFSCGEFQCDAIINICDEIKSEKD---FQTYEKNGELFTITSKTSSN
DSCSDYLSKRSFNASENVENTPKKRMGKLSRDRRDIYYRRAKQEGFRARSAYKLIQIDEK
YNIFDKV----------------------TRAVDLCAAPGSWSQVLSTKLLNN-------
-------SEYNEGQPKETDQIDSN----GEAPLIVAVDLQEMAPIYGVNIIKGDITSQLT
VSRILEYFQG-------------------KKADLVLCDGSPDVTGLHDIDEYIQNQLLVS
SLSITSKIMRKGGTFVAKIFRGENISRIYQQMFCYFELVDCCKPESSRNSSLEAFIVCRY
FK-------------FD---
----------------------------DQDSTNVNFEIPDPLTVPFISCGDLSE-----
--YDPDKTYETEYTSS-LEPIQPPINAPYH------------MIN---SKIIILDFGSQY
SHLIAKRFRALGYYSEIALPSTNLNTFN--NAKGIVFSGGPSSVYDDNIPEFNNDILNL-
---NIPILGLCYGHYIVNIGYGGQVHKAPIGEFGYATLNINNNI---------------Y
SPIFQNLDNNSQQQVWMSHQDEIIIPGNDFQLIASTINCKYAALQNLEKKRFSLQFHCEV
NDTPCGNIIFNNFA-QYCNMEKNWSQDFVLNHILNDIKIQKNNKNVLLFLSGGVDSTVTF
ALLNKALGDKVLGLHIDNGFMRKNESKNI-ELLYHKFGFKNFIIKDYSNSFLNIIKNITD
PQKKRMAIGEHFINIKNIFIQEQNFDPNNWLLAQGTLYPDIIESGGTKN-SNTIKTHHNR
VDMIYDLKGLIIEPLRELYKDEVRMIGKKIGLNDELIMRHPFPGPGLSINVIGDFDKYRL
DQLREVDNIVINELNNFGWYNQIFQHLTIHLPASN--------PEKASFVLRPVCSEDVM
TARFAFFPKDLLEIIIQKI-SLLNFVDAIYFDVTNKPPATFGWEMIRINTNK-----SEG
GLDDTNAVRQTEIALIRSPIMSMLSVESG-KPVMFQSFRSPMSGHVPGVSESRKMTLGVR
IPRDSTCNSIKQPGFKLPPKADPKGEPEPQV-NVNPLILWISSEDVIEVDSMLTKWLREH
QRQGVTFIFECLMGLRDFDGNGCILADDMGLGKTLQSITILWTLLNQGFDGKPSVRKAVV
VCPASLVKNWASEIEKWLQGKCKCTPVAERDREKVVSAFAGFKYDTMSRILIASYETFRM
HVEQLDGVPIDLVICDEAHRLKNDKTKTAMAINNLPAKKRLLLSGTPIQNDLVEFYSLVS
LANPQVLGDVSQFKKIYANPILEGREPDASEYQQELATQRLQELSNITNHFILRRANTLL
AKVLPPKIILNIFCNLTPIQNYLYRRFLRSSACKKLLDSDSTGQVLSSIQSLMKLCNHPT
LIRPKGGKGFEGS------EKYLEMIH-----------------------GRSVSGESGG
EYKKRV------TIGSSIRNSNRTGFSSKPNLSGKLYLLSRLLFHIRSNTKDRVVLVSNY
TQTLDVFECLCRDLQVPCVRLDGSTSITRRHNLVKTFNDNSNSFAFLLSSKAGGCGINLI
GANRLVMFDPDWNPANDKQALARVWRDGQKKNCYIYRLFSTGTIEEKIYQRQLCKDGLSA
MLVTSNNELKDSISADLVRDLFTLKEDTISDTHDMIQCNRCHDSNGQPLD--MVPQTTG-
LEDDLNTWGHYHSFSEIPDEILSLTLNECTNKMAVDMEGEPHPLELPSDFVSFVMACRIE
LQDQEDEQTKESNGEKPMEVDKENNSISPVNPVINIAKRIKDSNGSED--------DYDM
DEEMEYYSSSDSGME-------------RSILFALPPIP----VGLHGELASGPVIQSSV
AEFAFKKKIEPVGEAFFYRYEVK----------------------YSNKINFLKEKIKIE
SSIPINNIYSQEECKKKSKKISG---------NKSGNKLAKGVLSLARLKELVEEKETLY
EKLGLDENVCVKEIKQAYRRLVLSHHPDKNK-----------------------------
------------ENSSDARSEEFLKIQEAYEILSDKNLRHAYDSALPFDDSIPSVY-VSE
NNDFYEFFSPIFRRNSRWSIVKPVPEIGNIDDNIEVIESFYEFWRGFQSNRDFSIHEEHE
LNHAECREEKRWMERQNFKIRSKYIRNEISRINRLVDLAYKNDPRIKQHNKRKEEEKRKK
LEQKKIEEEKKR------------LMDEQQKKKAIELKSAIKSLRVSIRN----KMKNIS
DVNIFFDKFQKEDFSVCLKTIEDIHIFLKNMHLTEAAQSIEKINL--------KLWEEWL

```
LKLDR-----------------------EQLDNFLNKWIEVNCSDDYLKKLYLIFTIKIL
NIN-------------------------------------NFDANKSLQPTKKINESNMS
NLSNDYVTVNT--ENLSSDWTVSEMSLLAKALQKYPGGYKNRWDMISEYLKTKTKEQILT
KVKELSESEKLAKLSNEVKESAFDTFIQSNKGVLKKFDNIPDVRDYSGTSIINNSAKNDA
T-QQKKEIDLWTRDQQCSLERALKQYPSSLPSNERWELISSCIPGKDSSQCLARYKLIRE
RLLKKQK--MPSKRRNNGRSKKGRGHVVAIRCSNCGRCVSKDKAIKRFVVRNIVDASSQR
DIRDASVYSTYALPKLYNKMCYCVSCAIHSRTVRVRSVTDRKIRANPNSQRFKAQVMKLR
IA------------------------------AFKVLVVVFGT-NYGLVN----ADKKVS
VSEIVFESPIETLEWAGTDHNVVIAKTQKGHVYRSTNRGKNWRDITDVFAKMTSHSSS-S
SHSNLNGFSVKSITINPVDKNVILIVGSKHTHFISSNAGETFRRISHSGTIHTWIFHPNK
AKLALFSSWTEGCNKSSSAGNKCIHQLFVTKDLGATYHKVIDYVVQFHWDGNNKSNQNRI
FFTHHRKKQGDQPRYGGWMKTIDFSYTDDFGSHIETPVKGGNKFLASNGYIFVARILDYE
RQTVSLLVSTNDANSFTQVQLPHTLTEKSYTILDTSEKTVMLHVNHG-EDSLKGTGNIYI
SNYLGTRFALSLLNNVRTATGECEFDRVMSLEGVYIANVKDDELE-NDLDHISTTLHEFT
ESETEDEDVETLKTEHKTKIGKVDQPVRTVITFDKGGEWNYLKAPTIDSRGNRINCSDGC
YLHLHGVSNYQSLAPFYSVENAVGIILGTGNVGSYLSFDHDDINTYMSRDGGLTWQEVHK
GAFIYELGDFGGLIVMANDLKHTNQVIFSWNEGLSWYDFELGSKTLQVDNILIEPNSSSM
EFLLYGSRGKSGVLYHLDFNTLGQVQCVGATTPDRPDSDYETWSPYDGRNSEKCMLGKQL
VYTRKKQTAECYNGQDFRRPVEKKRCACSEEDFTCEFGFSRQIGSFECRPESLDVKFGQC
TSSGIFYVTAYRKLPGDDCIGGWLPPPVAIPCPSHAPTSSHARIVLVIALFIIIFMLKQG
MFGDWTRFGEL----GYDAYRNVQYKVLGVAKSAITLNNFNTDNMFMEDDEDEDDHAIGN
MTKISGNINNIKSSNLSQRQRSNSNNYISAGQITPPQDNSSGIELL----MSEKKAVIKN
ADMSDEMQQDAISCAAAAIERHNVEKDIAAYIKKEFDRKYNPTWHCVVGRNFGSYVTHET
RHFIYFYMGQIAVLLFKSG-----------------------------------MIKPEK
KF--VTEYSVTGRLRIASILDGEDGGVSY---------IGKKVTVGGWARTVRKQCSDTL
LFISLNDGSTSSNLQCVVEKTVKGFEEGLKATAGCSFKITGTIVKSPAQGQ--------S
VELLLN-TGDDELKICGL-CDASKYPLAKKHHSKEFLREVAHLRPRSQFFSSVMRIRNSL
AIAIHEYFQKNGFMYIHTPIITAADCEGAGEMFQVTTVL---------PPESKNNISNIS
KIEG-----------SQDMAVDYKKDFFGKASYLTVSGQLALENFACSMSDVYTFGPTFR
AENSHTTRHLAEFWMIEPEMAFADLSDNMKLGEGLLKYTVEYVLINNMPDLLYLDKNIEN
GLVERLKVICKEEFARISYTEAIEMLKPHDKEFTVPVSWGMDLGSEHEKYITDVLEKRPC
IIYNYPKDIKSFYMKLNEDGNTVAAMDILVPKIGEVIGGSQREDDIEKLENAIKSRNMDP
APYWWYNELRKYGSVPHSGFGLGFERLIMMVTGVENVRDVIPFPRYPNHCEFLPSKNKEF
KIMSVDEKLYSEVLDQLSKLSINYSKFDHDATPNMESMVEVAEKHNTDFAKNLLIKSKNE
GLFFVLAHHETDTKMKNLGQIFGVNKLRLADEDVLNDTLKVKRGCLTPLSLIFEKSGGIQ
VYFDECLKDKK-VFVHPLTNTESFSIHINDIVKFAESCGKKVKWFSMDNL----------
-----------EEQKPAGKPEESKE---NESLLGITADKITSFADWYSQVIVKSEMIEYY
DISGCYILRPWSYFIWETIQSVFDQKIKQHDVQNAYFPIFVTQKKLETEKDHVEGFSPEV
AWVTKSGKSDLAEPIAIRPTSETIMYPYFAKWIRSHRDLPLKINQWTSIVRWEFKHPTPF
IRTREFLWQEGHTAHSTRKEALEMVDIILNEYASIYEDLLATPVVKGTKSENEKFPGGDI
TKSIEGFIPEIGRAVQAATSHLLGQNFSKMFGVEFEDEKGNKEYAHQTSWGLTTRAIGVM
IMTHGDNKGLVLPPKVAPVQVIIIPIIFKTVITEEQKKICNEVECILKKAGVRVKIDDRS
NYTPGWKYNHWEVKGVCLRFEVGPRDIEKRSVRVVVRDNMEKMDIPISELESKIPKLLEE
FQNRLLFKAKQRQNESIIRVDTFDKVMDTLNQKKMVIAPWCEDVSCEEEIKKETARLSLD
NEDNQ-SMTGAMKSLCIPNDQIKIEEGTKCFFCDKLAKKFTLFGRSYMPSPEKAAKKKQY
FERLSEYATSYPRILVANADHVGSKQMADIRLALRGKAAVLMGKNTMIRTALKQMLGSHP
ELEKLIELVRLNVGLIFCIDEPSEVRKIIEEYRVPAPARQGVIAPCNVVVPAGATGLDPS
QTSFFQALGIATKIVKGQVEIQSDVNLIDEGKKVTASQAVLLQKLNIKPFSYGLKVNNIY
DHGSVYSSSVLDITSEDLISRVAEATKYVAAFSKETAIPTLPSARDGIISSFRNCVALGL
DVDFDFPEMQAIKNALANPSSFVAASTAADNTTAVGASAPV-----EEEEEEEGDLGFSL
FDILSNSDFTREPFPGRVFEDLGGAFSMGCIGGFITSFFKGVKSSTKREMFSSGMLFARK
AAPSLGTSFAIWGGTFSCFDCLFAKLRGKEDHWNAIFSGTATGGLLAIRGGLKTSLKSAM
VGGILLTIIESVSIGINRKTIQTPRQQFKEQMSEERDKTSKI--M---------------
------------------------------------------------------------
------------------------------------------------------------
------------------------------------------------------------
------------------------------------------------------------
-------------------------------------ITPYSTGGYWKSVCEFVFTGTRC
QLRNAYIIVEVLA----YLKFGPSVIGAAMISLASVESYPLVKTIIKRITIDIYKIVGGE
IIGNVFCYSKSTG-QPNENIRSRPSQVASGAALVSQLNINERYLVFRLFKCDNLAAADGV
TGTSDPIVKVTWDGVVNTSSVREKTRRPVYNQNLYFPVRILDQRELLIPSFRRKCLPIDL
LCKGPLKIEVWNRSDTSSILLGSGEVNLNKIYTLGKEDFRCLAEGLIDKRSRRADGKNFD
QEGDRAENDDNIFVNLYTLPYNTLVLNMNMLLQSKGF-GKSVIKPSIHFEMFFIPPFPAD
VVIPAPPSTKAISNIWKQLGNRWNRDFHKWKGIYKQWFSSAPDKRNFNVLQRHPQTEEIF
PLCSYISPIAVPNLISNEGSLLHWINNFMYITLESQIPELTPPNLFLLSKKGSIGDHVLM
FCSCLLGIGFDAYVCKGTIENGTLEHMWVMTRHRFGHVQFWEVTNKQRYVLPCRYGIDPY
YSMRNPFQQNAEVMPSVNDNNDEYYNGLYLQQWFEWLKQTGQV-------KYVETHANEN
MGIDIWGEFLVPDIKIDPKEVEYNNEEDEEDIGNRKSDKIKQNTSLDIIKKVITEYIKNI
PIIPDKRFLTP-ELISYVPYSTIELVFNNRQVWGNLGNHHPALITYDLNNPYKWRTFCGS
KPENIFQDLIIEPPLQGRAVEKLEESISVSVEENIVLNRQKLGKDTLFDRSSELEERINA
YLTLLEFKLSLDPLYDPGPPDNHTAWSALKEEKEMKEKQEKEQKKILKMKKKTRKITLGS
EGNYSGQDPQVDFFNANFYGNLEN--KAESNPNHNKSNDNLVPQCYTNMAINASYNSNNQ
NAVLQYDQNSQATNNLNNQNINMNNNNAQQFVKNPVNKSANIQTNENKTQALTKSLKGDQ
KFNVSNIPPNIANIISKSNENNSSSFFLGEIPSK----KNNLETDIKQNMENEIIDLKLL
E-------NEKDLEKIENDMK-------YWDEMADNLEIEPELPDEGVNLSKAKISNWKK
PEKEKDKANKDSQKENIPEQESESKTTTREDEKSKNNTQANSES-QDIDEDED-------
------IEPPPWRKLNRPLKYINDQISKWNWYYRMEQLYYDWQANFPTFPMSTFTGFPIH
FSTADPNDVRSFIVGSKRFRIFIELPQDEVSYMLASIHGERQHGSDVRSNNLTAVMSIAN
ILKSSLGPQGLDKMLVDEVGEVIVTNDGATILSQLEVKHPAGRVLVDLSELQDKEVGDGT
TSVVLLAAELLRRGTSLVSNGSHPSNVISGYKLALKECVRYISGSLSIN-ETISEECCLN
VAKTVLSSKLAGADSEFFGKLVVDSIMTVKATDPTGAVKYPVKSLNILKTHGKGLSESFL
VEGYALHTGRACQGMPTSVKNVKIACIDFPLKQYRMQMGIRVELEDPKELARIRLEEKEV
IHKRIEKILATGCNVVLTSGGIDDQCMKYFVSAGCIAVRRVEKVDLRRIAKATGATICLT
MAQLDSEESFDPAYLGECQEVREERIGDTDYMMFLGCKTHRAASIVLRGANEVLLDELER
SLNDALCSVSKLLESNGYVAGGGAVEAALSIYLEDFARTLGSREQLAIAEFAEALLVIPK
TLAINSAKDATDLVARLRAYHASAQQVKSQDQDKYKFFGLDLINGTVRDNVQAGVLEPTI
SKLKSLRFATEAAITLLRIDDYIKVKPVENQQGN-MHDSKFRNLYGECMQDRFSGLHIVT
HTVDGCGIAASTEFIAFPAECSGGGSVGVIDNSSFGYKPNVVYFKGHKAAVSDVEFSPFY
SCLLVSASQDKTIKLWEIPEH-AAQKSLKDPLAVFRGHTKKVSLVKFNPSAEWILASASR
DNTIKIWNCETVQDEINIGLPGLPTSIKWSYDGSLLAVSCMDKVTRIIDPRSERITYQWQ
AHNGSRKSRCEWMGGTMGNPNWLLTTGFSDKGERQIGVWDVRYLDKSVGVEFVDIEQPSL
IPFWDEGTGLFYLAGKGDTNIKVFEYSDGCVRRLEEYRSVNSLKGYCLTPKQNVEVMKCE
IDRILRLESGGVIQPVSFIVPRKTNE-FYSDLYPDAIGNEAAMGPEEWISGHTGEPMRMS
LRDIVQTQSNMFKRMRSVVKNARVILKKD-----LAKAEMQLSTMQDELYSIESLK----
----------YQLKEKEIAQLKAKTSLV--------------------------------
----------------------------IMLKKLSKEDQDRICSQQVITELQDCIKELVD
NAIDAGCTEILISLTDFGSSTIEVLDNGKGIEDLN--KIGERGVTSKLENFDNIHEDLST
LGFRGEGLNSIINSSEIEIDTKYDNKENK--IVFEKGKCLIHKYHFSSKSGTRIRVIGLF
LPYISRRTQFLRNIRLQLKSLIVLIEEYAICYPMIRFLLSNRTLSQSNQIQSELIMTRGV
SSQKEVAQYIWGKSVLGNSLDFKLEGEKWIISGFISSLDKGRPSPDHQIFTVNNRPVDPI
KRISRVISSIHSTLSSKLYPAFVINIHLPQSLLDINVTPNKRIVMLPAKVETVLAENIQ-
--------------------------LQDSYQNNIPIKREEFMENRNSILNYPSTSQDHL
TTEIS---------------QSQISSSQKPKDKV-------------------VLSYNHL
TDNLSNSGKAPELVFSEE--------SSRLGEKKINEDEKNESEKDEISNTSFS-ESKME
SIEL-SHQTIGTKRHRSDEVSLREFY----------------PNDFLTNQEKDSNIRKIV
LPSFNENMDEGMDVDMDENKDKSKNKD---------------------------------
```

```
-----------------------------KSKTKDKNKENEEKEEKEKYKEKYKEKDTVV
LNQVVPRNLTKIRKNCIPVESIMELRYNKTFGGRFNKKKVNRD-EKKFLPNEDITTENDQ
SSNRCFNFKKHLFNQLQVIGQFNKGFILTKLSIKEEQNYINH---------KNNEKNGEN
MMESLHIFIIDQHASDEKARFEKLNSDLNIQTQKLISPLSVSLTPSQEQLVISYKDIFEQ
NGFRFIF------------------NSNSEIGSRIQLTQLPVILGIPLKQIDFLDLLSQI
NKYKVEEEEKEDSISVSTGDQENTNVTLWCPSGIPRPRRIWSILASK-------------
------------------------------------------------------------
-----------------------------MGRAQDAQDVREWAFATFPGLETNLEAIFAY
HCTDGVGLLDYETIEKISRHLIMNFGYTDLLLRFTTPNGTLDNRHVSNGLA------ILD
VDIRKPITLDTFKNFVVCWLDRLIEAQDKDVENLNSALNNEQEKQKARILHAVAQWRERF
KTIDDFHVFCDDVRDAKKVELDEVVADVYDMQQQLRLLQRQKQAEEAAIQEALSNAPTAT
QIISEEIAKTFKNNIQGDVTQIAYSSELTPFGAPVSAGASTSFKRNIPKARKSTKLGMCV
VRMSVQNTEELVDYEEEETRI--------------EEESRADEGSKVGRGNYVAIHASGF
RDFFLKPELIRAIGDAGFEHPSEVQHETIPHAITGVDILCQAKSGMGKTAVFVLSILQQL
NPDEE------------------SKNVECICIGHTRELAFQVKNEFDRFSKYLKNVKPQV
VYGGIPIQKDIDMLS-NSTPNILIGTPGRIIALIRQKKLVTEGIAHFVLDECDKCLESLD
MRKDVQEIFMSTPRKKQVMMFSATMTKEIRDVCRKFMQNPVEIFVDDETKLTLHGLLQYY
VKLGESEKNRKLNDLLDQLEFNQVIIFVKSVSRAQALHKLLTECSFPSICIHAALSQQER
ISRYQQFKNFEKRIMVATDLFGRGIDIERVNIVINYDMPENTDSYLHRVGRAGRFGTKGL
AITMVSSQTDSQVLNDVQSRFEVNIAEMPNQIDTSSYINQNVDAVHPKKSSGKEDKR-KK
TSGTSSGSESESDSDDVRDNIE-----IPKNFLARGPRTSVSAEAYGAWNKMK-DFTPPS
YPKTKEQEKRIREKLLESFMFTSLDDDELKTVILACVETSVKKDTEIITQGDNGDKLYII
DQGVVECYKKTTEPRKHLCDLNPGDAFGELALLYNCPRAASVVAKTDCLLWALDRETFNH
IVKGSASKRISTYETFLKEVEILKTMDVYELNKLTMVLKSSIFEDGQEIIKQGEQGDTFY
LIITGNAVALKDNVEVMSYKRGDYFGELALLRNAPRAATVKARGRCKVAYLDRKAFKRVL
GPIEDILKRNTDKYKTVIKKITTKV------MHLSLLPLCPIAEELYKNHKTFHDGDSGL
DLFIIEDQVIKAGETAYIKLGFKAAAH---------------------SDDGKPVSYLLF
ARSSISKSPIRLCNSVGLIDAGYRGELLAPVDNIKDFDFQVKKGERYFQLVSFNGEKITF
SIVDELDKTTRGEGGFGSTDKQMITCDPNSLFFGFLGIAGCLIFANLGAAYGIAKSGVGI
SSMAVMRPDLIMRSIIPAVMAGILGIYGLIGSLVIFFQMGEPNLYSAYTAYAQMSAGLVI
GLSSLAAGLAIGIVGDAGVRAAAQQPRLLTGMILILVFGEALAIYGVIIGIIMGTTKPTG
LCASYI-M-------------------------------GETKSMDMDMVEEEREGPFKP
LTGTEVNRSN-----------EIRRVMVPENRMTPLKNQWINIVTPLVEHMGLHVRMNTK
RVELKYGPDCTDIGSLQKGVDFIKAFLLGFELQDAIALLRLDDLYIESFEIKDVKRLNGA
HLSRCIGRISGRDGKTKYAIENSTRTRIVLAGQKLHLMGAFHNIKLARDALCSLILGTPP
GKVYNHLRIVSKRVQEKLMGSGCCSGRESEEAKKLAQEKMN----RSEAL---GDDISRF
DLAYDKNDIQEFINLLSSTQPIDKLDEPMHPWAADPKTVGALAATQLAILAARDSQPELK
DEIRKKGGIQGLLELLKSKEEDRIDGAIVALSFLSVNNVECCNVMFDCGVLPYLVKCMSS
EIDGLRAASAQTARNIFILGLNQRKEFMRLGGITVLVSLLNPPTKDKPESWYTPLEAVYH
IEDLIIDQNEELLEYTRAIRKCGVVEKLQILTKSNNRDVSEAAEILLARVAE--SNMGTK
NIGK---GLTFEDILLVPNYSEVLPREVSLETKLTKNVSLKIPLISSAMDTVTEHLMAVG
MARLGGIGIIHKNMDMESQVNEVLKVKNWISN-------LEKNESTPD------------
----------------------------------------------------QNLDKEST
DGKDTKSNNNID--------------AYSNENLDNKGRLRVGAAIGV--NEIERAKLLVE
AGVDVIVLDSAHGHSLNIIRTLKEIKSKM-NIDVIVGNVVTEEATKELIENGADGIKVGI
GPGSICTTRIVAGVGVPQITAIEKCSSVAF--GIPIIADGGIRYSGDIGKALAVGASSVM
IGSILAGTEESPGEKELIGDTVYKYYRGMGSVGAM------SGDRYFQEKRP--------
ENKMVPEGIEGRVKYKGEMEGVVYQLVGGLRSCMGYLGSASIEELWKKSSYVEITTSGLR
ESHVHDVEIVKEVMNYSK-M--------EGLSSNALTKVCINSLRQDISNKYYMHFTQLN
NLNFRKRRCYSSYFNKFVSQLKNEIKSDKILQDDIALLKKKFD------NLYFKFYSNLP
FINFYSSVYFINIAELYK-INRFLSHFFTAKISSIFSRFLSSNLFSRHI-------EIIK
VLF--YDENRNVIELSSWKSDCNKESNSEIKTSNKKIINKNI---------------EGK
TIVPIVENSVVLHNYSLRNRLGVKLKDMLLLKNIFESEYFKTIYNGNKISKVVKEMKAIN
PNFKLTDFMSFFEEYILPKFMESYLKCDEHKLRLHCGDVAYRQLCSNIEKLKKMGLCLNT
KILQLGNVELIGAEKIQTNQPEFMFTFKTQQINCLQDSNGRIISGSIDDIKELQYSIKVT
PHPNANVPGLEYPYLITNLEIIGSIPIFM-------------------------------
--------SKIRFQKSQLSI------------EPEPPKEIDEDHRDFLIKESKWMYGCFI
DEHKWKIKSFKAVAQCAIRYLQTKNQRLKKRKEEEDKRLRIVSKNISVNINKFWNNISKI
VRHRKLSELNKLLRIKQFEKLDKLVSETEKGKKT-------QPNDKYCKIDLKSKSDS--
---------DDLETDNWDYIEKVDNKLDLEMESSDDSDEVNAELDELNNDASLNLEELYL
KYYGNPYDKKNN------------------LKRDKSDHAYIG----------------DA
IKRKKNDEYVETTISKHIEEELGTEKEMNDTDKKNDFSLKDETLSNVANTPIDKAIANLE
EKNNPQNCLETNNALAKVSIDQ-----------IKIPFLLKNNMREYQVAGLEWMVKLYK
KGLNGILADEMGLGKTIQTISLLAYLACYMKNWGPHLIVVPTSVMLNWEMEFKRWLPCFK
VITYFGTPKERQKKRIGWNDPNAFNVCIASYTLILQDAHIFKRKQWQYLILDEAQNIKNF
KSQKWQVMLSFNTERRLLLTGTPLQNNLMELWSLLHFLMPHIFTSHHDFKTWFSDPLTTA
IENQQVENERNLLSRLHSVLRPFLLRRLKKDVEKEMPSKIEHVIKCPLSKRQKELYDEFL
ESKTTQNTIAGGDYIGLMNVLMQLRKVCNHPDLFEPRTIKTPI-VKLMINYSFSSLIFF-
---------------PNITMSSGLGKNISTNNYLYIG-----------KKTNKSKYTTLF
---DKLTNHRFVNIPNIFILYNEIHMSKFQAQSQCELTYKYNIENSKRTIGTENNRFIRI
GLDRLASVEFNSPLIGMDEYTKDVNKYILDHISNNKVKNK----SNSVINSTESVSLFYK
EDNR--KIASEWRDYPVQIF---HSIKNTNCDSKPCIKFGSPFNVVYGKDCRNFIFDQIQ
NKLKMVGEKTFSFSTFPNLFSNRKKSYKNTAISYKSIDYNTLNEM---------------
---NAKLINLEMFPSKLLFVKKQLPMLSQFNMILERKVISTNFSIPIEGKYELFCNSYIS
LQVDNLINSSNAYLHNISFFKKCIVPPRRIIEDDCGKFQILSRLLHKLFNEGHRCIIFTQ
MSKMLDVLESFINYRGYNYLRLDGSTKVDDRQKLVNRFNRDQRIYLFISSTRSGGVGLNL
TGADTVIFYDSDWNPAMDRQAMDRCHRIGQTRDVNIYRLVSEWTIEESIFKKQLQKRLLD
DVVVDQGRFTSE----------------FFSKNDIQKMIGSRNQNNSDNNSIYVTRVLHE
SSTANDNQKKEFEDVLAAVEDLDDINALKKSSREIATENDDFINEFENQYDITTLNNLLK
YCIEFFESSVPLDIQNEVDLLEFQIN-NINSDSSIENSLSEYSQGELPYD----------
-MVLYLIGTGLNDERDMTLGGIELMKVADKIYLESYTSILSQRADLMKYTDGKPLIEADR
KMVEENCDEIIEEAKDKSIVLLVVGDPFCATTHSDLVLRAHEKDVKVEVRHNASIISAIG
CTGLQVYRFGETVSIPFFDGSWQPSSFYDKIKANIERGLHTLCLLDIKVKEQTIENMMRN
RPIFEPPRFMTVNQAISQLFILEDKLKQNVISPNSLAIGVARIGSSDQKIVSGTLSELSD
TDFGNPLHSLVICHPDLHLIEQRFFEIYR-KKLKIGKPKGINTARELRTRRRTQKWADKQ
YKRANLGTRYKSNPFGGASHAKGIVVERFGIEAKQPNSAVRKCVRVQLIKNGKKITAFVP
RDGCLNYIDENDEVLVAGFGRKGHSVGDIPGVRFKAVKVSGVSLLALFKEKKEKPRSMGI
KGLTKFLADNAPKSIQQQGIGSLLGKRVAIDASMWIYQFLAAIREGSQWGNLTNSSGEST
SHINGMLSRTTRLLEAGIKPVFVFDGAPPEMKKDELTKRDERREKALAELEKAQEIGDEE
LIKKQSVRTIHVTKKQVEDVKKLLGFLGMPCIDAPSEAEAQCAELCKDGLVYGVVTEDAD
SLTFGTPIQIKQLNFSESSNKITDKSPSKQKNGMQIIKLSLILSELDINMDQFIDLCILS
GCDYCGTIRGIGTSTAYKLLKKYHNIESILKNIDQTKNPIPGNFDFSKVRELFKNPLVSK
NNQIKIKWSNPKYEELMEWLIKEQNFNEARVNSYCERIKKSKNKTSQTCLDGFFKTASNE
RKNTH--ETPRPPLSEKQKSETRKEV-DSSLSCDKKKVKIE---ETKIISEWGAPVSKNL
SSQAEDLAENSSESSEIKVNKIEENKDSESSTVENTPSLQTKSPEPTMRPVKRK------
-----LNRLISESMFFSSMPF-DMGGGGGRMSREVDNKKLYEILEVSQEATLSEIKKAYR
RLAIKHHPDKGGDQEKFKEVSRAYEVLSDPEKRKIYDEYGEEGLEGGGGGADPVDLFDVI
FGGGRGGKRRGEDLVTHLKVTLEQIYNGAVRKMAINKDTICADCEGVGGPKDAIQYCELC
QGQGVRVQIRQIGPMVQQTQSPCNPCKGTGKTIPVTKQCKKCSGSGSVKERKVLEVNIDK
GIPNHHKVTFHGEADEKQGEIPGDVVFVLDEQEHSVFKRKGGDLFIEKDITLVEALTGFK
FIITHLDGRKLLVKSNPGDITKPSDIKCVNNEGMPTYKNPFVKGHLFVIINIIFP--DKL
DSKTQDLVKLLPAPKALN--VDEDDPSIEIHYTSNTKPSEVKDRIQK----EAYQEDDED
HHGGAERVSCRQQR----EGSQMLSSWKYDVTKCQEVLSILRQADSSESSVQLQVTNALN
SFVINSPDAPCYFALIFSGNSEGLDVRQRAGLLLKNYLVQYGIPSSSEYIEYLKVTSLNA
LNDSQRLIRSTAGTIVTTFVNTEQGKPLLVESLRHLSQLLDMATNDSIDGAFDCLIKICE
DELEYLEHTHPRLMSFLDASILPKLFQISQGEHLILSKNETAKCITLYAQYHLFSSGNAL
```

```
HDFFTRYWQVIGILAKQESRQIRLLSIMGIITILEDDPGVILDGVNIIIDFVLCCCSGDS
YNLRLESFEFWPLYLRNE------------------------------------KGINIL
RPFLPRLLICLLENSIFTDFDYIEMDPSHFEDKTE---DDLHSIGPRFHQGRTDNS----
-------------------------------------------------ESNDDEVELGA
WGNQWTVRKASALALDHISVIYGDEILGELLPKIEATLQDPNWEKQESAILVLGAIARGC
IKGLSPFLPRVLSYLVKLTSNSKPLIRSISCWCISRFTPWLALQQG-QPILNSAFGALLA
RMLDPNKRVEEAACSATATFIEDSAQSLSPFLDDIVNTISGALTVYQYRNLLILCDTIST
LCFSVGPNVFSQTFENNLVPLLITKWKSFPIDHPCLVASMDAIAKIFAVVGNKASGFADS
ILQHCIQNILMSALN---------------------------------------------
-NLKNSEEVSYS-VPDTAECALDLVSSIVEAVREPT-----------IPTLKRYGF----
---------------------------ANYILI------FCQDESYPNIKQSVFACVGDI
AKFGGDFLKPLLPTLLQLLVLNLSSPNIGIANNAAWAIGEIAMYGNPEFLEHIIDMLVSR
ITTSCNSSSHIDNLAINACITIGRVAVVAPSRVGGRLGVCADRFFLVLTNVRNDQEKMNA
VQGICFAIQTNTSLSASSINSFFDLLNSMQPALDSKSSFSENILPILQ----STLKSIYL
GLADK-----HNGYVQTLVTSFLSMP----------------------------------
------------------------------------------------------------
-AKHDDICSLYPYTGIIWKELSRIQLSQESLIDETNCNNEEIKFNIMADLIRLLSKFLEE
SMNNFMIETMDLFGNDGIFLTSIVLENLENLILEIEQLLITKEECINLVGIIFSYVDISN
-----SNSLQDEHRLEYQNNTSIYKFVSDDFQN--KIDFSVEEPKVV---------TTKT
ELFEKVFISPLIIPASVESQLISTEKLNKKLRSAFGLKMFNFVQSKVFSSIYLSNRNVLV
AAPTGSGKTNIALLAILRSISDF-------------------------------------
-------VGINTLDSDSVSE---------EPDPIKFKIVFIAPMKSLVSEITRKYSVALQ
ELRIKVVEVTSDAAVPKEVIDRNHIIVTVPEKLDIMTRTYTFDDNTGQVNLFNSLQCVIL
DEIHMLGDERGPSVEAIVSRILYNVEISQRPIRLVGLSATLPNWEDFATFLNVNKNDAFF
FSQALRPTPLEKTIIGVNEKDISSISDLYNSIAFKIVLDCLEKNEQALVFVHSRNETLST
ALYFKRMLNISDEMNKVNKKEFSGNYLLKALRDCDNSSIKDLFNFGLGIHHAGLISSQRK
LSEALFSQGLIRVLITTATLAWGVNLPARHVIIKGTNVYDSKKGSFKDLGILDILQIFGR
AGRPQFEKLGSAYMITSSDKVQSYVKKLTFQAPIESQLESNLCNLLNAEIARGSILNAKD
ASRWLKYTFLVTRVKKSPIAYGFKAENDPNLAQFCYNNISKCLDLLYQSKLIRYNIINEE
VSPTHYGRLASKYYIDFNTANIFRKLILEDSDFNILEIVGKAKEFSSMAPREEEIEELEN
IVVASIVKKRIDVTNSSKVALLLIAYSLRIEITTPTLVMDSIYISQNGARILRFIFELIQ
LSTFGVSERAQRVLEWSKMLEMRIFYTQSVLRHFVYFSSLDKTLNPNETF-------ASN
RNTKFKGPLKIGSIKKLEDYA-SWEMIKDLAICELKHIVYS--DAEKISEYIKYIPKIDF
KALVSPVTLKIVRLGIKLYPNWK-WSQRWHGIREKFYLWVTNPNDGAILYTNQVQVTQKS
VNSTISITDLIPIPDEDPPFFNIRIISDKWVNLDFEIDFNLRPALILNSIPDITELLNIP
PIPIKSLKYPEIIDYYNNKFLNPVQSQLFHILFYSDENIFLGAPTGSGKTMVAEIAIFRA
LESKKKSKIVYIAPLKSLANERFNDWKFLFSNTLGLNVVLITGSSQTSLLELEKASIIIS
TPEKWESFTRRWKSRSFVQDIKLIIFDEIHLIGEPRGSVVETLVCKTRFISHFVNKKIRS
LSLSTSLSNAKELSSWLEVGASGYYNFPPEIRPVPCTVYISGFQEKNYCPRMATMNRPIY
NKILTHSPKKPVIIFVASRRQTRITAMSLSHMCYCEG--QPNRFIN--TEQKDSFGLSLA
GSIRMAKDKSLKQTLESGIGIHHAGLSESDRNLVENLFLNGMIQIVVATSTLAWGVNFPA
HFAIIKGTEYFDAKLGQYIDYPITDVLQMVGRSGRPQYDSHSVACIMTLEAKKPFYKRFL
YDSLPLESCFVSPLIEIFNAEVSSLSIKSIPDAICFLSNSFFFKRVVINPAFYDPNVFQV
EIAQASLLDVPRVRLIVYILEKLINDTLRALIELKCIRISQDDH--SIFPTLLGQISSFF
YIKCCTISKMNKFLNVLKNHFVSWVEILSLISQAQEFETYPVRHNEDKICTKMLKYLPFK
LPMTSPHQKVFILLQANIFSIPVTVVDFINDINSILDQVPRILHAFIQLNKLYLSPSAFS
STVLLLESLQQKCHPFVSPFYQIPQTKNVSFDIFEKFKSKSLYEIVSKEINIRKELNDAN
LNILNFLYEIPLFQLKSTIVSVQANNLTIEDRKANKLVFLERLSKSRFKLMENERHYLHY
NFNVSLTLTNLSKIQNYKVS-----------EM---------PKYILKGSEKRSKLTLEE
RHKLCLSVGEECIQEAELLELLKRKEHPICYDGFEPSGRMHIAQCILKTINVNKLTECGC
VFVFYVADWFALLNNKMGGDLEKIKIVGEYFVHIWKAAGMDMTNVRFVWASDFINEDSNE
YWLRVFDISRKFNITRIKRCCQIMGRQENDEQPCASVFYPCMQCADIFQLKADICQLGMD
QRKVNMLAREYCDAAGIKHKPVILSHKMLPGLLEGQEKMSKSDTSSAIFVEDTPEAVVKK
IKKAFCPPGIIEGNPCIEYINTLVFPKFGHFHVSRKEEYGGDITFTNKEDFHKAYLSGDL
HPGDLKKGLSDALNLMLQPIRDHFNTDPRAKELLQLVQSFKVTK----MCLRQHLRCSSL
KVTIFGAGSFGSAISCVVGYNTERTLIFNSEVKLWLYDERLEGEYLADVINRDHVNVKYL
PDFKLPNNIRAVTDLKEACEDCNLMIFVIPSQFIRSVASQIRKLDIFSRAVRAVSLTKGF
LVENGHPFLISKIIEEELGIDCCVLSGANVASGLAAKEFGEATLACSDYDDAYIWQYLFD
TPWFKIDCVPDVICTELFGGLKNIIALLVGMIQGLGCGTNTVAAVMRLGVLEMILYGSIF
FSSIMTRVFFESCGIADLVTTCLGGRNVRGGKAFTLSNGQKPWEEIEAEVTGGQHLAGLV
TLKEINETLESIDVDKHFPLFRSCFKIAYTGAPPRSLIDILGRNELRELRFYEVERILTR
IARGGLLLGAIGTIPMSFMFNVDGGEKAIMFNRFGGVSPKAISEGTHFFLPWFQVPFIYD
VRVKPKVINTTTGTKDLQMVNLSLRLLFKPCTEFLPRLHQNLGPDYDEKVLPSVGNEILK
AVVAKYDAESLLTQREKVSREIRESIMQRTKQFDIIMEDVAITHLTYGKEFEKAIEEKQV
AQQDAERVKFVVQKAEYEKQAAIIRASGEAQAAEMISKAVSNSGWGIVDVRRLDGARDII
ENLSKSDRVTLIQGDQQHL--HFRVMN-AYLNAYFSEKSGFLFENNIATAPCLSDSTE-L
KTVKTGTTIVGVACNDCVVLGADTRATNGPIVADKDCEKIHRLSDNIFAAGAGTAADLDH
VTSLIEGNLELQKLQMNRKPRVAHAVSMLSDHLYKYQGYIGAHLIVAGSDSTGNFVFQVS
ANGCIMQLPFTSMGSGSLCARSILEARYRDGLTESECVELVSDAIRAGIYNDLYSGSNVN
ILIIKNNYVKHFRHFTKASERIYRQPKPISFPVGTTPIIAEKTEDLSSFVVEVSDINS--
SDLQISK--KHNIFGEPPPDYVPGKGRGAIGFASGVSRDDQTIEADIGDYSDTKFDKFSG
FNEHLFNDIKYDDDDRQADSIYEMIEEKLSTRRKKQKEKKIREEILKVREHRPTLQEQFS
GLKKSLGDVKIEEWDQIPEPGDYIKN---KKPKLFLPVPDEIIQS----SHKNLFETLTQ
----KNCSNSELNTEL--------------------------------------------
-------------------------TTELNELGTAKGNILSLKLDKAMGSVSGQSVIDPS
KYLSSLNTAGIKLNGDLSDIKKARLLLKSVVNTNPKHSPGWIAAARFEEFVGRLSHAREI
IAKGCEMCPKNEDIWLEAIRLG-KPEQIDKIIVKSIKFIPNSTKVWMVAANRETNKNKKL
LIIKKALEFIPNSIKLWKEAISLVDNESEKALLSKAVKCVPQSEELWLRYARLSEYCDAQ
KILNEARKVLPTFPGIWVEAAKLEEQNGKVEKVELIVKRCISNLSAKRFVHSRDDWLNRA
GECEKEGYSNTCISIIKNTWNLGIDDDAINDQVFSYIDNFIKSNNIISARAMFESSADMF
KSKEYFWIKWANFEEKYGNFEKVDHVLQKSLKNCPDKQILWLKAAQNQSANGNAEIARLI
LSKGYSSSLNKEEIVLEAARLELSQGEIERAKIILERERTNSPSVQIWVESIKLENDQKN
YDLCILYCSESVKEYPSSPNLWLLYGFIYRKAF-------PDRINEALKIYEEGLNFCSD
SIELWFSTIELLMLLQNWKKARTFLDLARSK---------------NKNQPE--------
--------------------------------------------LWMQTIKLEKNANNEF
IPQILSKALKECPKSGLLYAESIFTEQKQKQKSKFLIALEQCGNDPYVLVAIAISFWKEN
DFHKSRKWFKSALEIDNKIGDTWIHYIAFELLNGDFQSQRDALNDFINATPNKGFEWNNI
RRTHFFWDQKSNEILIICLELIYGIKKSFIVSNEIKQLL------------GLIM-----
------------------------------------------TTEPEVKEQVLSKKALKKQ
EKQ-ALKEAKKAEAKSNQQQQQQSVHWSSVIVHSTEASPYGVIPFNCKIENRTFS-----
-------------------KIKELNSN--QKGEKIWLRGRITESRCKGSLGFVLLRQTFY
RLQLVVDANN-GSSKEMIKWLGCLPIESMIDVYGTIVVPETPVVSSTQDIEVLVERVYCV
SSACSELPFQLKDANRVETEDSTIIKVLQDVRLDNRVLDLRTYLSQAIFRIQSEVCRLLR
EFLIEREFIEIHTPKLLPGASESGATVFKVDYFSNTACLAQSPQLHKQMSICGDLERVFE
IGPVFRAENSNTHRHLCEFVGVDIEMNIENTYHELVDVFDAMFRHIFQGINTHCKNELLI
--VSEYNPFTPFVISEKTPRLTFEEGCNLLKEAG-AE-IPEDLSDFDISTEQERLLGSIV
KEKYNSDFYMMLKYPLKVRPFYTMPDFEPEKWSNSFDFFMRGEEILSGAQRVHDYDLLVK
RCQECGVSEHSLRDYLNSFKLGAPPHGGCGIGLERVIMLFLNLGNIRKSSMFPRDPKRLS
PM----------------------------------------------------------
----SRLQETDG------------GNERTNKSLQYENFSLESQSIFQRESFDGFRAEIAK
SVTSNLQTSYSLFLGTS-NIKGYSYQLGPSYQSSSKNTLILARVNDEGTVSGRFSRCFGN
NIEGRISINSSLSDENKNMSEVSIDYNGEESSYSLKVAYQGIFLLNGLFSQLITNKLQLG
GELTWIAANNTSIMSLGSRYCHGKNIFFNQITRQPDFTSPGRIFANIHSLRSSFYRKLSD
RLSLASELEVSIPNFESTLRFGYEYLFKTARIQGMIDTCGKISLQCLDNKGFGISAAIDY
LRNDYKFGFMMQFFPNEKDDKLDD--MAAQAPEFKLALVGDGGVGKTTLVKRHLTGEFEK
KYIPTIGVEVHPLKFNTDFGPLIFNVWDTAGQEKFGGLRDGYYIKGQCAIIMFDVTSRIT
```

```
YKNVPNWHRDIVRVCENIPIVLVGNKVDVKDRQVKARQIQYHRKRNLQYYDISARSNYNF
EKPFLWLARRLTNQPALQLVGQHAKAPEVNIDPTLVAQAERELSEAANAPIEDDDEDLMS
TKN-PILFSALCVVKGSISTLFGLSGKVSKLKFKN-EKVSFNYSLSKEIEMNDDTLEE-L
NNIISYKIKENSFFQVFKILSKEAASIYGEHLESDQAIPDDI-ELRIVTLRNFYLSATRN
PVLRNTKDIGNVLIENISLDHENSALLVNFKVESEENFKDLCC-----------------
----------------EEYSIQDIKDVPSLEDSLPIS-INLDII-GDELVNPWEVKANAY
GIDYNKLIDKFGCKLITKDMIERMERLTGQKAHHFFRRNIFLSHRDFEKILDVYEKGELF
YLYTGRGPSSESLHVGHLVPFLFTKYLQDTFKVPLVIQLTDDEKFIFKSNLTLEETHNYA
YENMKDIIACGFDPELTFIFTNLEYIAELYPDILRIEKKISCSQIKSIFGFKDSCNVGKF
AFPAVQAAPAFSSSFPHIFRTDIHCLVPHAIDQDPYFRMVRDVAPRLGYLKPSSIHSIFL
PSLQGSQTKMSASVQ------------NSSIFVNDNEESIRNKIMKYAFSGGQATEEEQR
RLGANLDVDVSWQYLRFLMEDDEKLEEIGKKYSSGEMLSGEIKSILVQELVKLTKNHQKN
REAINDDVIAKFTNKSREQLLKLFKKMM-LGKGMRSPKAEITELTPNSIRFTLSNTDLSM
ANTLRRIILAEIPTLAIDLVTMIDNTSVLHDEFIVHRMGLIPLDSTNIRDFNFKDRCECQ
ERCNKCSVEYLLDVTCEGGATRNVTHYDIQPVATSTLVPMPVPKKD-DSVDGMNNGILIA
KLGPGQRIAMRMTACKGIGKFHAKWIVSVATFTNEADVRINYSLSSNLTLKQRKEIVNCC
PKDVFALGKNSYSTKEELEVVSSKSCIFCNECVNLTKNYGYINLIRVDTKPDKFHFLVES
TGALPVESIVELAFEILQEKLNTLSRGVRSETAQSGLDTNGSG--GVNITADELDLS---
MESRIKDISLAEFGLQDMEIAKTDMMGLVELQRKYRDSKPLKGARITGSLHLTIETSVLV
ETLYELGAEIRWCSCNIYSTQDHAAAALVKKNIATVFAWKNETIEDYWVCLNDAMTWRNP
NGPNLIVDDGGDATLILHEGVKAEIEYEKYNKIPEYLETESMDLKCMYKVLKMELLKNPF
RWRGMLKDLYGVSEETTTGVLRLKIMESEGKLLLPAINVNDSVTKSKFDNTYGCRQSLLH
GLFNGCIQMLAGKKIVVLGYGEVGKGCAQGLSGVGARVIVTEIDPICALQASMEGYQVSV
LEDVVSEADIFITATGNKDVITVEHMRKMKENAYIANIGHFDDEIDVYGLENYPGIKVIE
VKQNVHKFTFPDTQKVILLCKGRLVNLGCATGHPPLVMSMSFTNQVLAQMDLWKSRESKN
TRFFVKKLSKELDEYVARLHLDVLGIKLTKLTETQAKYINVSINGPYKSEDYRYMYSQPY
SGEEPMLYQSRGPNKSSNQFIMDDEALNERGEEITPLISNFSCITLRTGTLLQCASLLVL
IILYNVFGNKGLFTFDLYGNGGTLAEDLSYYAGVVSMLCVYFIGVLFLAGFQEFIADNSK
APLGFRAGSRLLNTANIIQIIVVALRVTQFSFTYSYFNQKWYGKFSQTKGDWCLYNFGIV
CEAVSLIMYGISFFYVESYADVGVGEQYAYWMLTLFTLAGISELMMLFTGFGSFFILFFA
AALIVTCTWAFQFEPLLESNSPLLFSRDINADVLPKNQTVNYQPNFTYQLYAGASG----
---------------------------VSSVPNPYGVEMQQ-------------------
-MAD-TEQKKRTFRTYSYRGVDLDKLLTMKLDEVVELLPARKRRKIARGLNRRTAAFIAK
LRKSKAECPMGEKPVAVRTHLRNMVILPEMVGSVAGVYNGKTYVTVEIKPEMIGMYLGEF
SITYKPVRHGKPGVGSTSSSRFIPLKLNMPPAETRKLDDIAKMIVCKTHIGTKNVEDKML
SYVYKRTHEGIFLINLAKTWEKIQIAARIIATIDNLADVVVVSQRPYGSRPALKFAQHTG
AHAMVGRFTPGTLTNQITQKFMEPRLLIVTDPRVDSQAVIESSYANIPVIALCDTDSPLQ
YVDVAIPCNNKGKESIALMYYLLAREVNFLRG---KTDKWDVMVDFFWRDPEEYENSAI
GAELNDGMGVDIEGAAVDSAAAANEWGGVSNWGGSAADEWRNAP-----MSIYCAQNNII
SESHLSDTNLTFDSLSKPSISCRVNPLVILSILDSHLRRQSGHQYVIGTLLGYINEGGNV
IVTDSFVDRHSFTDDGMLSIMIDTHETMFELKQKTNSRLQVIGWYSTCSGINSVSCAVNN
WFKTDIGSSKFQQTPLLSEPIHIVVDPSFSNGKLSINGYIQIQSTWTNSIVSIFRPIPLD
IVASPCERLHISRILRPLLE--KHHLNAVGIPPKSLPQSGVSQLLTKLILLVQRCQNYVK
KVLSGEVSMDPMIGRQIDYAIHSIYDFEYNIYNIIKSNSTQDALIIGCLTELTRVQLSLS
EKLQSLIITMDALCKLYEGFSSPDGSIRQESETQFNNLKQSDPNTFLQLTLGILQQQPNS
QYRVQAAISLRNVFREFVLT-------PDNCIWNKVSAENQAICLATLLKCLETEQVNVV
SLNLSDTVSLIAMELFPCGKWPDLLPFLFRLISNSVSIPARHAFRIIGEIMPVLDDVVTS
HRDNIVSTINTALQFPDVEIRFEAIGLISSIVESNDKKNWSPLVPLIPSILETLQNLI--
SAQHALVID----VLYRLTTISESEPAFYRQHFSIFFPQILNIAK------NIQLSTDIR
QAAMECLLCIVETRPMMCVKHPSFVNDMVSTLLSFMLEFDDDSPEQEENDLESDIDDDDC
LYPIGEEGLDRLARALDADS---FIPIFYQFVTIYMQETSWKYRYAAIMAIAQTIEYLPE
DDYQDRIGQIISRVLGFLQDQFPRVRYACCQTIGQISLDHSPFIQEAFHSSVIPQLIQAI
DDPISKVSSHALSALINFTEEVVAEDLQPYVRPLMEKLLGILHPQPPRIVREQCITMVAV
IAGVIENDFTPYYSTVIPYLKKTMNEA-SPQLRTLKGKCIECITIIGFSIDYSIFKNDAQ
EIMLVFLQLLGSGLKGDDPLKEYLQEALQRMCRIMKQDFVPYLPHLLPGIFNILETREDS
LVDKSDGGSLGMLSAHDFIGMRTSLVLDMESSLDILNTFIEVLGPSYHEYIAGTIKVIHP
LIRFSLSDEIKEKTYEVLSGLLKIMRELAERDSNIRTQFSQIMNELITLFLSVMDEECTG
T-----IDSQVIVINGVQECLDSFGNNILNNEQVAIIASRCFDMLQQSFVRRRKLDSVGE
NGEIDDDDKRCIEEEKEQEQELRLNILGILGVLMKYYPNVYWERVGNITIQLVSQHIVKN
NVEDRVLGFHLSADVFQYLCPTAYQHCLPWIQHILEGINDPIASIQQNCAYSLAQAAKLE
QFSGVLNDALQVLL--LRLQSKTKG-KSFNLAKDNCISVLSNIIVHHQGSINNINEIVNL
WISLLPIKYDTDEAQNSHTALMNLVDSKNPLILGQNLENFPRILAIFIDIYGTSMSNDSL
NSRIKLLIAQTGPNNLQSFA-ANFSKKQIDKLQKLCQ----MPVISVSTRLLSSYLNKEV
TSEWLDEVCFNFGLELDCIEFDEEIKVAKIEIPANRPDILCLEGLVIALGCFIGNSNIPL
FNLKP------KGNDQKIIVKRNVARVRPFILCAILRDLSFNEDVYKSFIDYQEKLHNNI
CRKRSLVSIGTHDLDMIEGPFYYDAKPHSEIKFVPLIGSTEVDGNQLLDLLNKHQQLKKY
VSLVENEIFLPVVTDSNGIVLSVPPLINGSQSKITLNTKNVFIEVTATDYSRASIVLNQI
VSSFSMYCKNKFEIEPVKVEYEHYEVLANGYHVVTPNLERLEFVVKASEASNLLGINPLD
PIVTQSLLRKMMIDSEIIKDSESLKCFVPINRSDILHPVDIIEDIGISFGFNNIELKKSR
FYELDKSSLMAEQVKRELSLLGISESLNWALCKHSDCFESLFRTENIKLKNLFESEQYNL
NCPPVVVKDAKTSEFEILRTTLIQSLLKTIASNKSLPLPQKIFEVGDVVILD-NNTPSGS
RNDKRVSIAYCNSSGSGLEEIHGFLDQLLSKLGLVAEYSLNEPNSIHPNII---MYSLSE
VNDPSFLPTRSVKITIQKVEVVIGIMGVIHPKVLNNFSLTLPTSVLELRLEPIMNLWPDM
------ATTAGVLSYGNIRGLGYQDQGIFKASKELFGWKNRRTNATYHYKPEEVMGVEWI
QTSCEDSSCQLRVFIREKKDCIHFTGFKTEDYSVIKSHFETYYGINLETKELNTKGINWG
DLTIHNDTICIGNEGKVMMYVPSININQIAMPSKSELVLEFNEGVNAGEDCDELMEIRLF
VPNQGNSLSSAEKLRSDLLKLTGIGSSGSDKVCRWNDIHLLVPRGRYEIEVLVNCLKLHG
KSFDYTILFQSISRLFLLPRPGTSLVNLVVALETPMRQGNTKYPFVVMQFDTQQDIEMPL
NLSEKEIQRFTLSPIMTGKFWDIVTRILKSLTGHSIIVPGDFRSASMYHCIRCSYKAQDG
LLYPLNRSFIFITKPVILIRFDDILNIEFSRMGG-NQTRFFELTITIRGGGDYSFTSIDK
AEYNPLIKFLQEKNIRIKNLQESSSKKESSTKSILEQDLPSDDEDEDFENDEESYSSSSG
SSDGDEEGGSEDEDEADDDDDDLKMETKQQDVVDCRFYENKFPEPEEFVMARVKRITEVG
AFVSLLEYNDIEGMILMSELSKRRIRSVNKLTRVGRLEVVVVLRVDKNKGYIDLSKRRVN
PEDVERCEEKYSKMKKVYQTVRHIAQKEGISVEELSEKLIWPLHRKYGHALDALKEAAVN
PEAVLGEFDLPASVKEALIQDIKFRLSPQQLKLRARIHVCCCGYDGIDAVRHAIIAGQEV
TKDDVEIQVKFIAPPQYLVTAVSYGKAAGVEAIEKAMETIKNVITQYKGGDFKRQGEIEV
VGGDDEKN---DLNSASESNESDYDSEDEEEDETMGEDMDENDNVEDDDE----------
--------MSARLSIEDVIERMEDEKLRLLQNQSVTRYD-PNVRGRHSVVCRHWIRNMCM
KGDFCDFLHQYNYERMPPCFIYQKYGVCVDEALGNCPFKHKADDT---------------
--------PLCAQYFLGFCKYGPKCKRRHEPKARHEIPDFLPDSFLLSIIQDKNLIP---
----KMPETSSIIKVLDDICKESYAQAVSEVSSNI-------------------------
------------------------------------------------------------
------------------------------------------------------------
------------------------------------------------------------
------------------------------------------------------------
-------MQKTLVTHISHQDLDTVQRGTEEWNLLEHYLQVCCRSSRSLIQQIWHVNIGSI
STSFDRRTQDKLVLYCWVDTNELDQNNSIQDVSRRGFKIPSSGMKFTSGNIRLPGVPQAS
HNGGTSVN--------SRDLQQANNTRSN--SMFESDYTPATRRLFEFFLCKVGVGCSVV
KNDESEADGDRFPIPPEYDTVFLRNKQI-NPPSMI--------NSK--NEFN--LSSALA
LSAGVLPQHTFKHEYIIYDSFQVVPEYLIQFEYDSSSPELFAVPWCDECQDSPAILWCQA
DTARLCDSCDERTHRHNKLVTRHIRIPINQMPRNSGNCPVHTMDSLEEFCTLCHVPMCRL
CRPSHTHAD---------EKTPSMIMPISRAYRAQLERHNKPHPCLNSRKMELLEKLESQ
QLIFNDVRANMFEVEQRIYSILESSITQLQIATESKMNKILCEQLILNQLLQQVEWSEGF
LQYLQSILPPADFLFAWLKHCHYREEVFQLCDILTS-NEQFFPDSRLVGRVDILSESALK
HNK-HHKHAKTMPPKKNEQKEVKVLLGRPRGNLKMGLVGLPNVGKSTTFNLLCKQAVPAE
NFPFCTIEPHEARMNVPDDRFRALCKHFSPKSEVPATLTIFDIAGLVPGAHKGEGLGNAF
```

LSNIQAVDGIYHVVRAFESDEIVHTEGEVNPVKDLETISNELRMKDLERVDKLISELNRL
-TKNGTDRTKKEQLQILKDVYAHLEAGNWISSKDWKSSEVELLNEHQFLTAKPVVYLVNL
SEKDYIRQKNKFLPKIAEWVKEHIDGVIIPYSAEFEAALAELTEEEKMAYIKEKGASKSM
IDKIINTGYSALNLLHYFTAGEDEVKCWTIRSGTKAPQAAGIIHTDFERGFICAEVYKFT
DLMELGSEAAIKAAGKYLQKGKDYVVEDGDIIFFKFNVTNSGKK----------------
--------MEEENQKRLDLYEEYFFLSNLKLLELVDDFHKSLEDGLENHRLKIDKYHSNY
KPFKMLDSCVDRLPTGKEKGVYYAIDMGGTNLRCVRVNLLGNGQSETKFKKVKLNKISKV
DQEVNIFDKTVSSETMFNSIAVFFNEFLDECGDLNDLTNLEVGFTFSFPIVQSKIASAKL
VIWTKEIETGRLTDDPVEGKDIGDLLNLAFKRNGVPAQCKCVLNDTVGTLISAMYDNNIS
ENQPLIGIVVGTGVNACYLEPNSSNFGYKGVIINTECGDFSTKLPITDCDYSMDWFSDNR
GEQIFEKMISGTYLGEISRLLIINFLKNKTPEIFFQKNSLKTEHIAKIINDNHQDLKSIE
NYLKETFSSNLDHNSTYIIAKISQMVLMRAASLVSAIIAAFFKRFNK-PRNQITIAIDGS
VWTKIPKFQKYVKDSLSSLIQESGGSIHFYESDDGSGRGAAILASTTVNTH------LPN
MYFLVKLLAQIITRTFFKNVTVLGKERIPLYGPVLFVGNHMNQFVDAAMLIAIFPRQIRF
LIAEASFKRPIIGRLAQSAGCIPVQRPQDLRYRGIGGLIWSSSSVRIRGIKTRFKIDVKF
QDTLIIDDLGVSVLEVVSDDEIIIDKGIGTESATYTDGFPFFIMPKIDQSTVYEEVSDAM
RNGHSIGIFPEGGSHDRTTLLPLKPGVAVMALSSVLEGAEDLLIVPVGLNYYEPHKTLSS
AVVEIGQPIPVTLELAQKYEESPSDAVKELLSMVEKGMNSVLLSARDYTTLTCIRLCVQL
YPPDRTALSQDNYYLLHQLFSQFFWALHDDPELEHLRKDLCEYEDTINHYGVPDREVWQL
KQPVYECIKLIITKFLLLILVSIVGGSFFPLWAPIRYIPAFMAERHRKKALANSKVKIRG
TDVLASYRILVIIALLPMLTVFYGIFFSLAFMQNLNSKIATVIGVFIILPPIFYISRVSF
EMIMPLTRNIRTLFYVVVSNINYLRGTERTLLHMRIKLQQKIRDLVYTKGPQVSPNFIQS
FTTVVPDAVIQADNKRINYSLGEYVPVVTRAKYDAREEILLIRKTNLLKEMSLGSFMNTA
ANAIK-PHKFWNTQPVVQNDDSSSEYSFGPIEIEP--DSFRKEIYKLPDGFSWFDCNLWD
SQDFEDTYQLLKDHYVEDDDSQFRFNYSKEFLRWALCVPGQKKNWLVGVRVNETKKMVGF
ISAIPIKVRIHNCIMNTSVVNFLCVHKKLRSKRLAPVLIKEITRRIRCEKIFQSIYTCGK
NITKPFTIGTYWHRIINVKKLLEAGFIGIPRNMTMSSLIKYHRIPADKRIEGFRPSVDSD
AEQICKLFENYFMKYKIHQCFNVEDVKHYFTNIDKVIVTYVRNKNKEITDLFSFFIIEST
VINNERFPTINIAYSYFNIANTCSLKELFNEMLITAKNNNCDAFNTLDLMQNLQVIQDSK
FIIGTGRLRYYVFNWKIPQISPSNVGIILFMSLVNVTSVKVFKNPTEITDPFEFEISFEC
LQNLEEDLEWKIVYISCAESK---DMDQELDCIALGPITRGALKFIFKAPSPDFTKIPPE
DIHGMAVVLILGSYRNEEFIRIGYYVHNVYTDPLLEDNPPDIPILDKLQRIILSESPRLT
RFNIGWDTKQ---------EDQNLSSCCDEDQDSQDSINSQISSNSSPKPSQISN-----
------------------LNSQKLSSSQGPTSQSSNPNNNENLDANMSTISSN-------
MRLIAAHILSFLTVLELI------------------GDGNVIF---IFKSLQRNGFRPDL
RLNGLPTNELFQDAQILQEKMEKIRLMNSIRIFALDLSQNCIFEEKRNSCGDTGKCQVMG
NESDLSSTLFKANDINIADNGNCNEKKTK-------------------------VDMLKN
PPSNTQYKGGEVWRHIYYEI-------EKVDFPLLKKLISGVQSNIAIHASERYRKIRD-
------------------DDYDYSLLNFINKFVLFEDRGVNLLVTFHYIVQSICILGPSF
DKFMKDLDNSAENTKLKEEVKDIFNSHYYSCRPEYKRNIIPPQFLPKLEKFFKTFISTLG
CVECEKCILHGTIKGNTLDLTVKALGGSKNIILNPTYFVTYLNGLYIFSSSITIIDLFTF
RVR---------IFLAFSLFIAIVFFFCLKRVSSYLNKRREITFFFS-------------
-----------------------------RFEIMEEISNLAKKLRDCTMSENEVRHLNSD
INSLLKEKYKWECRIVELGGPNYRSRHGQYIESLGGISMPNSSLKIFGVAISLPEYKELL
NYDDKTELSS---KIVSEPPKVTICDAYYEEINKKEEDRIKTLEREKEIEFKKKKRFLEK
EI--NMGILRSESMSHGTLVLPNDRAREYIDILGREVNLQFVDMNSITMNRQYKKYIQRI
DEMERILRVLFSEIEKLPDVKVLKGNYENFLDHDHVYQLDKVEESLQSLYGQFISFRDNN
ADLIHQKSSAIEECAVAKAASLSFAPISMYSDFYMTNAVERGEG----------MHGGNP
TPSSPLMNPGIMMMFSSIAGVVKHEDQEKFARALFRATRGNTFTHFQSIAENIMDP----
--------------------KTSKDVQKVVFVIYFQGATTSAVYDKISRICDAFNVSIYP
WPSSYEHAIQRISELNTLIQDKEKALQAYEQYITLEIETLLQPVNSNGNSLIEEWRLFCI
KEKSIYATLNLFEGSDITLRADCWYPTEEEEKIRKILIAESSTQHVGAFLLTDDEANISN
TPPTYIKTNDFTVAFQDFVNSYGIPRYQEVNPALFTLVSFPFLFGIMYGDVGHGFIVFLI
GLVLVLNYGKLKK-INDENMKILVSGRYMITMMGFFATYCGLIYNDFFAAGLDIFGSRYT
LSHD----KLPDGSHVFLPNNNSTSASFPYPFGFDPVWKGAVNEMSFLNSFKMKFSVIIA
FFQMTLGVILKGFNNLYFKNYVDFFMEFIPQFIFMVGFIGYLNFLIFFKWLTPI--EGYN
KPSILNALIGLLFGADIPLSDRFYLSQPVVQKYITLALLISVPWMFFPKPLYLIYKSRKQ
KKASEEE-----------------------SRIRQQHLSSYSS----VSSRFTSFTNSSK
KI---SRSKSNLLS----EDDHNLIGHEVEESSHSDPTEIFIHQLIETVEFLIGSISNTA
SYLRLWALSLAHNMLALVALQFTIMKALNSKLLIVKVVQLFNLFFMFFAFTSFIMILMDS
LECFLHGLRLQWVEFQNKFYKGDGILFAPLNHMRIILETE---MQNGTIYYHPTRDLEKG
DWNNDTVNSSLLSLEECRSKFSNFIKNFNQGG--EYIYREMLINNISIGEYTLRLELHHL
SIDEVSCSNDSDNFVN-----GVRTSNELCCLIQCFKNAPLKYIPICEGVLKEV--YLEL
VGKHKKEISEIQLQIVSN-QEPTLIRDLRSNVMEKLVTVPGIVIQSSKPQKKASKLKILC
RQCKGTRNINIPIWRQGTMLPRVCNT-----------TPIGDAPKC--PLDPYFTLCDES
EYIDIQSMKFQELPEHVPTGDIPRNISLHMTRGLIDKVIPGNRLYVVGVLSSTDKESSSS
RNGSLRTSYLYVIGVMNYGSIKNSSISNQYNEIEEFRRISSLPNIHELIVNSIAPAIYGN
ETIKQAIACLLFSGSSKCLPDGNRIRGDLNVLLLGDPSTAKSQLLKFVEQVAPICIYTSG
KGSSAAGLTAAIVKDANGVYALEGGAMVLADGGVVCIDEFDKMRDDDRVAIHEAMEQQTI
SIAKAGITTILKARCSILAAANPTFGSYDDSKDLTQQHDFESTILSRFDLIFLLKDEKNV
ERDKLIASHIVELHSGIKGKDCSSLQFEQLQKYINYCREFIHPRLSLDAAAILENFYVKI
REDNREDTNKASKRIPITVRQLEAITRIAESFAKMEMQNIASEKHVEMAIKLFKNATIEA
IKSNLLLDNLSPAEQSAIIDAEVAIKNRIPIKARAGKATIVKDLALIGYDPYYLTK----

---------------MERLGLLSEEERKLDYVLGLTISKFLERRLQTRVFKLGLAKSIHH
ARVLIRQNHIRVGRQIVDVPSFMVRLDSEKHIDFALTSPFGGGRAGRVRRRTLKNQSGGG
EDEE-MSRLSQSLHDRHITIFSPEGKLYQIEYTFRAVKNSNITAIAIKGKDTVCIVCEKK
VPNQQGQQDKLLDPAYVTSLYKVRKHIGAVMLGLAPDCRSIISKCREIAGKFAFEKGVEI
PVSYLSHKIADVNQLYTQHASMRLLGASGMFISIDDEDGPSLYKIDPAGYFASYRACAVG
TKEREGNNALEKIIKNEPLNSCEEVIS--------ASIDCLKTLLGVDFKAEDIEVGVVT
KDMPEFRLLSVEEIDNYLTNIAERDMVDRGSLNSSKISHEGSGRSIIQDNNSNTIKGIAR
VSESDRLDHRFCSDGSATSH-TGSNSNVDPIVCTPHNFTVPNYPAPNVEIKLFVGRVPRN
IEEDDLRDLFKLYGRVVNVSVIREKSTGIHRGAALVTMESVAQADFALRELNSIKVLDEL
RGPLKVQYSTGEPERLGFESESIPGVDQVKLFVGALPRNIIEDEIRELFSPYGQINEIFI
MREPHSGVGKGCAFVKYAFKEQGLFAIKSLHGALTLADVNRPIEVRFASKNHQSSTSSQS
GQHSQSSSHHSLTEQLQIGPFTGVSATGANAVQNLMPRCIGMWKEYFTSDGKPYYHNELT
QVTQWEVPPEFLSFRPN------FSREVVGPPGANIFIFNVPYEWDKKSLVGLFCRFGNI
LSAHLMVDKTSGRNKGVAFVSYDNIHSAAEAVNHMNGFITQGRKLKVSIKQGQEHFVKNN
LPQGGCSQTGYMENSQNNIQSDSSNNNNDVNVNINNNNSNNSCNNNNHNFSSGPSMN-GH
-------------SVNRYTIESRKLHKSDQRGHLTLKYRDNNETKKNESVIR---IDRDI
KQYSGT----------NFASNEKSDSSN-FEVSDDNQIVKETTHLSPTKDELLE------
------------------EGELELELESGSEKEELD-DDEELLKELEKIKKERVEREKEK
K------NNDIKLMMQNNPLLEEYNDKVDEEYILKKRWTEDTVFRNQNRVSQPKKRFIND
ILHSDQHREFMRKYIL---FEMINNILDLIKQGTEKEKIGNLEEALNIYISALQKWDHIC
KYQNDERVKKVLLTRMEQLVSRAEQIKNLINNNGK------IQNPSNINDPLKDAIRSCI
LMESPNISWDDIIGLEQAKTSLKEAVILPAKFPELFQGKLKPWKGILLYGPPGTGKTFLA
KACATEMKGTFLSISSADLTSKWQGESEKLIKALFDVARERAPSIIFIDEIDSLCSSRNE
QENEATRRIKTEFLVQMDGVNSNSNNILVLGTTNIPWEIDSGIRRRFERRIYIPLPDEES
RVLLIKNGLKSIN--HSLIDDDINYIAKMTHGYSSSDVSILIKDALFEPIRKCSESNWFK
KVVENFKIYWTPCSPSNIDHYDKETSLYDIPNNQLLPPKLTKSDLIHVLSKTKSSITNLD
IDKFTEWTNKFGLSGEM---------------------------------------IQQK
IRLFRHVLRNISTQVRTKKPIFINGLRSSYKIAPQN----------GIGPI-----FSEL
SNGMRVITLENSNKIASLGIIIKMGSRFE----SKSSFGSSRVLFNMILSQEGKTSQNCL
PNKLALNGLMLAGGFNREYTSFLLEYLKDQIENTQEFFDGIFKFYKFSDEELELAKKNIK
EELLFELENPSIMLNELLHSTAWKENSLGNNQSTSFDQVSDLNIQNLTDFRNSNFLSRNT
IIVGTGISHDHLIKKILNSSRKFNNLKNDEQTMKIPKYVGGLVKNKLPHYGFTDILIAFE

TNLNWKGRELVALSVLQAYLGGGSSFSVGGPGKGIHSKLFLDVLNKFDWVESCNCFVNQY
SDTGLFGIHITSYPGYSLESIKVIAKQLGKMKNISERELERAKNLVLSTICTAYENRSHY
MEEISKQILSYSEFIELDEIINCIKSIGIEDIKKVADLILSKRPTVVAVGDMNQVPNYNE
IISIISMSSGVKIHQDCIDAFQKQKIRKQHRYLLYKMDSTYILFKTSGPEETYEDFLKSI
PETE---CFYATIDLGQKLIFLMFTPENAKVKDRMVFASSKDGFVKKLEGVHGKLLQASE
RSDVADYFGHP--------------FGNVGGGAMQYSQQTQIPAPKTSP-----------
---FGMQGQQAQSQTIGQNQGYSQSGGYYGSS---------------------NDNTM--
----QGQNRVRPGMVIPTGMGQQVQGQNQTQTGFYNSGNNTTIGGL---SMSEIQQLNAP
SYFVRPSVSKVPSSASLKQKAHIPVGLVFQPLASPPPGYPEVPTVSFGSSGVVRCKPCRT
YINPFVRWEAGGRRWICNMCGYSNETLSFYYCGLDDQGRRTDRFERPELSVGSTEFIASG
EYMVRPPQPPVYFIVLDVSMPSVSSGLVETVCLAVKNAILSDNIPG--------------
GGRAMIGIITFDSSIHFYDLNSNLSQPHMFVVSDLNDLFLPLSEGVLVNIADSTEQITNL
LDNLPNLWRNNRVSENCMGSAIKAAYMAIRHIGGKVLLFSSGAPTVGDQTIKLNREQ---
--------MRNSGKESKDID-REVELLKPENDGYSNFVHALVRAYISVDLFMCSQPYVDL
PTISPIVKKTSGDLTYIFGFNSYLHGQKLREDIFSTLTRNSAWEACIRFRVSRGWKISNW
YGNFYFSGVDLLLAPNCHRDQAFSIVIDMDENVAPDPFVYIQAALLHTNSDGERRIRVHT
MALPVTQNYVDLVSSMDVQATVSIICQNAMELSLKSKLLDGRNYLQTICSQIILPQSNQI
EAAKQLPLYILGVLKCPAFRDYKEVSSDTRIYHWIRLSSLRLESQMILFYPRMFCLSSWN
LQQQETDQ--SLVLPPALNLTAEKMTQADAYLLEDGESMYLWLGRAIPTTFIQQVFGVAT
LDQLHPDYAETVIGSTGEKLGVKVAILINNIREQRKPPFMKLYTIRQGDPLENKFFLSLI
EDKTQGFMLSYNDFLNKILPRSNPNMLQRYGNKLVKSKQILEMSTQIWIEKYRPKILDEM
VGNEEVLTRLKVLAKHGNMPNLLLSGPPGTGKTTSIHCLASEMLGSKYGRAVLELNASDD
RGIDVVRDKIKSFAREKIDLPEGRHKIVILDEVDSMTDSAQQALRRLMEVYSESTRFALA
CNQSTKIIEPIQSRCAIIRYSKLTDAQIRKRLFEIIKMENIPYVDSGIDTLVFTADGDMR
IVINNLQATYHGFSMVSRDNVLKVSDIPSPEKIKSILDSCVKCNWRLAHSIVEELFIGGY
SPLDIVITMRNVLKRYQL-PERAILEYLKEVGRCHFVMLDGCATPLQLDKLLGQLCMISA
RCGLNSMDKQPIIDQKNSKKKLKSTLSNNSGSSVELY--------------EASELQRLH
SIA-LRSIAIVCLAASLDGCDEQLLPASLRALEVDLKLSPKDLGNIILCQILCLALSCPI
WGLLADRYSRKYILATGVTVWGFVTILLAFSSSYWEVLITRAVNGAFLGSVGPLAQSVLA
DTLNSKNRGLGFGMIQLSSCVGRVFGAVLTTSISQKLIVGFQGWRFAFLLVGVLSAILGG
IIVFLMDEIPHLH-LHRFRSIRSDP--NSESEQEPLEQQEQSEFSLQFMKNVITQSLIVK
SVILMILEGISGTIPWSSLTFMTMYLQYCDLSNFQAALVVATMLAGSMIGGPMGGLLGDC
LNRISADHGRPLVGQISMAIRIPIMCILFLVIPKESSSFYYFMVLSFLMGFFAIAGAAAS
RPILSDVVRASHRATVFSIAVLFEGISAATFGAPVVGILSENVFGYKTTAENVSQMNADS
RLINANALANALVFLTVFPWCISLLLYSLLHFTYGNDKRSLKIELSEISGY----TRTIR
SRLESQRAPDNGVATAISSANQHKKFKRMLLFSLRSLSDLCVPPTLLYKENSLDAMQRGI
LEIMSEALQQFPDDEDISTNCVRIMFGISEFMKESQDAELTNMFISRGGSNTVAMAVNGL
SNDSAILASICTTIENLFCIGAL-DSTTAASCLGKFYESKYLGSSELVKVTGAICAIAHQ
ESGIAALVQAGIGSKTLNFAHNWTKSDNESAALIENCLEIVRLCASYGDVPEDALKAVVH
IIDLYRSRRGITEKGGACLELLMNPQKLMECLVVIKSPNSDITQKDDSLTMLSAMAYVSS
FADEIVRQGGISMVVEALNQGVSEYEEHTSKSSRIIIGSLRVLARVASNPSNVDNLIEIG
GIDAICSAFTTCINNLEISTAVCQSLYPLFVRE--TTAVAAASIMGDLLQLFYSNVENKD
FTKCAADLLSIASQHAPLAEALISVQSVEILATCLNYYCDDAAYQSASLAALNHLAPFIK
TLQPISEFGGISGIEKSIIENVKDEVLVTTAIQLVEKLSTVSDSSIYLSQGEMVSAVLEA
MLVHNSNDYIKEAGLSILEIIATDKDVTLHTTNLPKV---ALSDPNAAYKDLAAIAGLCK
ISSLAPLFSSLNICDDILGYVRKWISESPFEGQEKLISAAFQTTSTLKISAQGDLGPTIV
QLLDISTLPQMKTFAE-KNTNDNPVVENMKAVRSLIEVQRIGSDETVVNVVNSLVSTIRK
YIENRVVQLQCIKSFCIIAETES--GAKAEIDNGVIKVCLAFLQRTKVLLECQIAGFTLL
SLLVKYSPESIEILRKSGAVEIVQGAMRLHNRSSELRLIIAPLALALVPVGEVKKIISEK
VGEIKISLDSGDLKTILQAIITCNELAVTPEGCKFTISEGIPALIPRIEELASS------
KINSSEHGVVAQNI----LSACSTLCSLLTASRPGKVAIVKNNVTVSLLNMYRNLNCSLN
EEVETGLVDNLVAVANIVKFDVKAAETCFENGAVQLICTSLDNFQDNEKILGSACSAIAA
MATSPKRVEILLAEPSFNNLLTRLVNTVNES-SKAEVRIKCLNAINDLISSHDTSIIQIT
TDVGSIV-AAFGVVDKYPSESSQIRAACRVLNSIGNYVDIRAYFEQDLHRCVEVVLRALD
AQKNNEEVVEDLLILLNNLTNSMDNTILRECAIIELMQNVMMVHNDNPAVISKCGEVLSK
CGADETIKSLMVSIINQQENGPDCARDLDQLCRQLAVFVASQPENPEDALQYTEACLQSL
VAAAAEYPTDIRLLSSIAVLTQRITDRAFDNSEDSFGSWAVATSGMMQHIEDNLEDTSGV
SSKRYVCSGIRVLSGCLNNPYTRNYVLERCGTFLPHIGEILEKYQSDSDVAAETYEFMRI
LAEDPTGAQYVAQTPMGD----ISTIISTIKKHRKNDTVAVEGMKLIGNLVINAGASSYI
CSVNGLNELQGLTEGSNKADIRDA--ALCDLMTKLISAG--CQFEDKSLVSRQIRCRAYD
DDQSLAEARREGVAVAISKLIASASESSENGKIQGIDSPAEI--LSMIQTYPTSPDVVRE
ATKALTPMLKVNENAASLTYNSVLPILATSALPVVTADPYAADAVADLMLNLAQIQGMGV
EMSQNQCTDDILN----AISQLGDYYGDEFGQALKAKVEEITNTMASDIPAELSLKVVYD
MLKRREAEGLSLSITESTAVAEKLEYLVQTMSQLVTQKMD--GPTSIDFKFGCMAAQVIS
SIPEDVEYLVDNQWPKTMLLNIQNQ-DCDTKQAIASGLIDIAKSDKAAIQC-ATAPGTAQ
ICCDFVQEIHA-SPMDEAKKEELLIVRIQLVEKIAVSRQLFVGTSMLDILLSIWEAYDNK
KYSLNILRHVFRALRRIVSDDFVSALLNANVLKRLIAIIKA-KNDIGVLPDVLYLLGSLA
IIPNIKSEIGENGGIDSICELLRASIPPEQISPTVTNGCLALANLTIQHTNNKQIFTKSK
GPEIIKSLFNSYIGYWDVINSLGVLIVNLGYKKDETKKELGSSGIPALIVQFLNSYNGEQ
ERIATRAFMSVLKAVANMCLYTPNIAVFASSHIEKVFNHLL-EVSSNLPKETILMELRTL
CNIASENEASQLASFNILIQPLLNVLNAPGDDPDIKRLCFDVLSMLCRSPSNAADFFKAN
GTDIVIKQLLKNDYDTGLMTSAIHLLSYQTSVPEQMDYLVSAGIYRVIISIVEGKEGS--
-SDLKIAAFRLLRRCMSDPSNALDFLSIGGAQSICESIKNSSDQTLVLVEAIRVLLGLLY
SGSPDS----------EYSAADAPKGYQVCQLDVNDCNAIVKSVNHAIHKEENGRHLRLM
RAGFGIMAYLLSENLCIESIANSETVNVMSKVMTIFASDMDSTALICQYISFLSKYALDL
VPGIVNDDFRNALENSASKAKGAR-----KDFVTGVSTAIMSGD--YSALSVLCGEFDFD
ITHWNVEPYPNGVQDLPKETKDFLRNGGKLKIVLDGKSRDEFTWRASQDLYKLEWKVGKD
NDFNNSLPIGKIRNIWKGLQSTVLKAANMVEPRKITGPTCFVVVGPPSEDQPQGMELSLK
AKSKSERDGIIENFVMWREAATYH-VSKMTKEFSDKFLEFLNNTGSPYHSVDETIKILKG
SGFVKLDD--NEEIKKGGKYYITLNYSTILAFKVGEEFDLEDSMVVITGSHTDSPCLRVR
PSSITCNEGYTQLSVSTYGGGLWHTWFDRGLGIAGKVV---DNACNEYLIKIKRPICAIP
NLAIHLTTSEERS-SFVYDKEKHLQPIISKTDTNDKYGE---------------------
-LPRSLLDEICREINVQPQNISSFDLCLMDSVDSRYVGINDEFIDSPRLDNLGGVFSCFT
ALIDASESNS----------SDLLISVAFDHEEVGSVSFSGAHSDFLKQSLKLILAGLEN
SNVVNANRNSDLEFCKIMKRSIFLSVDMAHSIHPNYPERHQSKHKPSPNNGIVIKTNFNQ
AYTTDCTTRTFLKTIAN-----NFNIKTQDFLVKNSSPCGSTIGPIVASNLGIRTADIGA
CMLAMHSCREFMYYSDIYDLQNFIKVFYMTWSKVKLESKLLEM---LLVFGI-VLCIFVS
SNRVNGELYFASLSNYGC-SGNQKKVASVLKAQAEKTPFSLLVSPGDNFPGGID------
FKHCFENIYSEK--SLQIPLFAAMGQADWDNGNANLLLRNNVTY-----------DSNND
IFPRFSFPNYFYHYVSHYTDTSNV----LSRRDGTVLFVFIDTFILSSSFPDHKVSEQAF
QNLNATLHYGHKHHDFTVVVGNKYLASS--YSIDSSLK-KVQQLILDTHVELVISGQNRG
TYNSTIEGTTFLNCDS-----------------------YCSFKVDGSKMVPYIINGNQV
EL-----SPINTQLPSPSL-SFQGIAELPALEMIELKKGAKHLSREAFLKIVGTVGLMIL
ATCI---GSLIINKK--M--
-----------LDGYLGK-IKMAPQVSHLLFTANRWELNNYIKENLENGIDVVCDRYSFS
GIAYSSGAINLDFEWCKSREQGLISPDIVVFLEVNLDLSSTRSGFGVEIYENISDQISVR
KVYQKFSNYSFWRNINASQ-SPDVSLNIYSTYYSFNSIIFDSTYLFQNEKMGPRICPQCS
SASIEVHEGRGETICTNCGTVLEENTMVEGLQFSECSNGSMQMVGHFVPSSGVRGFAMVY
GNRESREHVLQRGYHNLQRIADQLRLSSSHIESAQRVFLMAVQRSFTIGRNNMHVASACL
YAICRREKTPHMLIDFSDVLQTPVKVLGQVFMKLLRLLRLHVPNIDPSMFMERFAAQMKL
GEKTHAVAATGVRIVQALTRNWITTGRRPTGLCGAALLISARYHGIPVSSSEIAQIVRIS
SPTLLKRLAEFKHTSTAQLTADEFENTDLLSLPIVRGPPCFE---KNRLKD---EANKAL
AISNGEDKLAISNYDKLGNDEDRRKDDPEDAQEEEQGDEE----EEESETIDVEADDFEG
KSLSFPKFLGGIELDINQDKLCNDEPTSQDITTIASKLINAFDKAKCASENSQSGTST--

---------------------CADSTSAEDLVMDPLHFLFKDFGD-----PQVETEDKQT

AKGSIQEETSIDNLIEPVNKLNIANSDLSTTIGSSLEDRDDISSS---DDLSDISDSEIN
DLLLDEDEREAKRLLWDEITKDTLPPSWLRKINKESMNNNNTGQPKKRKTEQSEKSKAQK
VQPENAIESVLMALQKAGPGAAKHVNSDTLAKLFTCL--------EQLTMNADKRDEIDT
NLYSRQIGTLGLEAMGKLIKLRVLIVGLRGLGVEIAKNIILAGPKSITLVDDEICSFSDM
GANFYITENDVKKGAKRSDACLNKLASLNEYVQVTVFHGEITSQVIFNHDVIVCADVPLS
LQIKYNELCRDHT-PNIGFISANSLGLCGSIFVDFGDSFNVFDGNGEEPKSAIIAKISRG
KETTITCLAEKLLPFQEGDYVMFREVQGMTELNGGPHKIISTGKHQFTIQDSSMFREYER
EGLVTQVKVPINYSFRSLKDALEYPICDEQ-----------------------GILIVP
DLNKFGRSEQLFFSINSVLKYSDIKG-SRPEHTDLQAINECHSLAVEMNENSKKRQDVIS
VSSIDRDILEKVCKYSRCCISPMAAFLGGIAAQEIVKFVGKYTPLRQFFFFDAFEQLDLI
SNEIHEEFMPLGSRYDDQIIIFGRSFQNRLSEKNIFIVGAGALGCEFLKSMALLG-VGCG
PNGTVTITDMDNIEVSNLNRQFLFRQEHVGSPKSAIAAQVIRTINKDINIISLQTRVGTD
TEDVFDDIFWNKTSFVINALDNVPSRMYINDRCLWYEKPLLESGTLGTKANSETYLPHKT
QSYSDNRDPAEESIPLCTLKHFPHAIEHTIEWARDAFQGIFTSDPQEAITFLNSPCEYIQ
NLKQRGNPNVILEKSQKIFELINWISEKDTHEDCIRMAIHLFHDYFYCQIKQLLTNFPPD
HINSDGLPFWSGPKRCPTPIKLNIQDKLHFDFILSASNLYSNMVRLPEISDSSIIFKVSN
ETILPEFNAKTTIIKIDDSNENSSSSSIILDTSIAEEYTNKLLSFTENRIKIQPIEFEKD
DDSNFHIDFMNSCANLRARNYSIKECDRHKCKMIAGRIIPAMATTTAMITGLVSFEALKV
SSL---------------GEYK--IELFKNSFINLSLPLYVITEPLPAPKTISKEFDPIV
EGPLRARPEGFTAWDKLVIEQKDGTVQNIIDYLTNKMNLETQIISFGNICLYNAYIPNH-
QERKCIPIALLIEQITKKKLHVTKNSIALEVSCCDDDGVDTIIPSIKFIFKSGKVSSETI
RRAISEILEGSKAKPRKFVETVELQIGLKDYDTQRDKRFAGTVRLPNVPRPNARVCVMGD
AADCERAQKLGFDVMDIEEMKKINKNKKVVKKLCKKYDLFLASQVLLPQIPRLLGPGLNK
AGKFPTVITPSDKIDEKANELKASIKFQLKKVLCLGVAIGNVNMTEEEIRQNLTLAINFL
VSLLKKNWHNIKSLTVKSTMGKSVRIYGM--STDFMNVRRSAWADEDIDDFEPSFD--EP
LKGFEDGIKHVISYIKDSKGSTIKITKKIKEVRNIIKINKNVVNRRLIMERNLAFNSDTD
VKLGDEISIDIPKNTDSMNFQDKDNDDDYYFNNNKLSKSGNNYPFILGDEQIEKITYGQQ
NNPPFLRTTEQSSHRDDCTVRVANLSEDATEEDLQELFKTAGRVVKVFLAKNKNSKNTKG
FAFITYSKREEAQNAIRKLNRHGYDNLLLNVEWAKTKEKMMESILIQGLVGSIIVYMVFS
RGKNNLNKFRYIISILLCSSIIAYTIVSKPQTYYDIIQVDRWAPRTELVQAIRIAKKKYH
PDKNKLDEKSN-FYEIQKIETVFSSETKRNNYNKYGDFRDGNIEDRNIILCLILGAAFHV
YSCLFGLFLSFPSIFRKSRGLFPVYSIAVFCAELHMRLSDDPNPLSFLPVIGGLLPFEKI
RVLRSLFTCVLIISFVYASNYYRDYDEILNGMLKGNLITNKRIIDLTQSLI---VQLQTH
GIGSTSTSSGQNKNSNST---SSSNATPLPPKIK-----DSDNEQEDPQLE-VIQQLSEP
LKELASTMNESQKKQLQTVLNIAFTAKGKENKKEGGGFLSRIFGSSFFWIILVIIKNYLS
---DYYKILGISKDASDQDIKRAYRKLAIKYHPDKQANPEGKKKAEEMFKELGEAYEVLS
DKEKRNIYNQYGSEGLQAGFGMGGGIF----IDPNEIFARFFASDRAGSFG---DEEGSS
FFF-----SGPSGMFRQVHMSSTHNRHAPRSHEVPLLVTLEELYLGKRKKIKVTRKRFIE
HKVRNEENIVEVEIKPGWKDGTKLTYSGEGDQESPGTSPGDLVLIIQTKTHPRFTRDDCH
LIMKVTIPLVRALTGFTCPVTTLDNRNLQIPIKEIVNPKTRKIVPNEGMPIKNQPGQKGD
LILEFDICFPKSLTPEQKKLIKEALD---QA------IILFLIIKCIRVYSAIYFYVQEG
TEKCFIQEVPKSVPIHVKYENVNNLGIDCTVIFKNTENIEVFSRHVNENDKGSVAYLPEV
DGDHKVCIRCDSSNWFKSEQMKWILSIDTGNEHLDVDSIASKDEANYVEEFINSLISKVN
NQVSEGEYEFEKQEMFLKQALSVNKRIVIYSIIQLTLVSVISYFSIIHMRNFLRKQRII-
-----------VLIFIVTLGILL------------------LFQKQDQDLEDLDALLAE
FGTVD------KEVKAEETVSQTL-KNRLKKQKKKQAKSH--ALATEDPKPISAAAKAAA
ERLRQIQENEQEKRREEEEKKKEEERRRLEEEEALLQKQKQRKERREQLKAEGKLLSAKE
KAVKQKREQFVEYLKQQGVV----STSEGSLANSSFSSGLATRKKKNNKSNL---EDILS
TDIIENSERNQDMETKT---VIQEFVLDSWEKAVDYEAGSKSPNVSTKNIRDLVPPKKVD
GIADT-----------------NCIEHIAEESCEDLGFRSPVCCILGHVDTGKTKLLDKM
RKTNVQDNEAGGITQQIGATYFPPEMLSEQVKKVEADFELQIPGLLFIDTPGHESFNNLR
SRGSSLCDIAVLVVDIMHGLEPQTRESIGLLRSRKCPFIIALNKIDRLYGWIEQNWSSSR
STLSIQNESTRDEFDTRLNRVLLELSEEGLNCDIYWKNDDFRGNVSIVPTSAVTGEGVPD
LIYLIAQLTQNYMGLHLQLNRELSCTILEVKAIDGLGVTIDVILVSGILREGDTIIVCGL
SAPIVTTIRALLTPQPMHEMRVKGEYIHHRFIKASMGVKICANGLDDAVAGTQLLVQSKN
EEIESLKEEVMKDMGDIFSSVDRTGNGVYVMASTLGSLEALLVFLKSSNIPVVALNIGTV
HKSDVRRASIMHERGFPEMAVILAFDIKVDAEAEVEAKKLNVRIMKANIIYHLCDMFTKY
YSDVQEEKKKEKSQKVVFPCILKIIPQYIFNARDPIICGVYVEEGILKPGTPLCIPEKDL
MIGRVTSVEFNKKPVNEGKKGQEVAVKIASDTNITYGRHFDHNDKLVSRITRDSIDILKQ
HFRDDLSKDDWKLVIQLKKTFGIPFIKMSSDHLSKQDADNLDLTKLTPITPEVISRQATI
NLGTIGHVAHGKSTVVRAVSGVQTVRFKDEKERNITIKLGYANAKIYKCTNPNCPPPKCY
RSYGSNKEDEPMCEVPSCNHKMQLLRHVSFVDCPGHDILMSTMLNGAAVMDAALLLVAGN
ETCPQPQTSEHLAAVEIMKLKHIIILQNKVELIKEAQAQEQYKQIKDFVAGTSAQDAPII
PISAVLKFNIDVLCEYICTQIPIPIRDFTSSPRMIIIRSFDVNKPGEDAQNMRGGVAGGS
IIKGVLKIGDEIEVRPGIYNKDADGEFTCRPIRSKIVSLFAEHNDLKYAVSGGLIGVGTK
IDPTLTRANRLSGQVLGHPGFLPEIYDSINITLYRMRRLLGVRAHDGSKQAKVSKLREGE
LLMVNIGSTTTGGRVTKIRDDLATFQLSSPVCCSVGDKLAISRRVDKHWRLIGFGDIISG
DTVRIINDYMFRFFLLISRQGKTRLEKWYSSYQQSERKRFIKEVTQMIINRQGKLCNFID
WKGHTLVVKRYASLYFVACIDKNDNELLALEIIHHYVEVLDRYFGNVCELDLIFNFHKAY
FILDEIILAGEIEESSKKAALRVISTQDSMMDENKDHKRAGALF-------------MLP
FLTLALGAGSVRVGLSNFSKGQLFFV---------------------GKNLLKNLGTRAI
S-SKSKPTSVCEEEERAILE------AEVAKIGLQERIKTLEKDASGYIHKIEESKEKLL
RSLAENENLRQRHRKDLEAAREYSISGFAKSLLDVSDSLSRALLSVDIENVDKNS-IKSL
YNGISMTYSSLEKVFEAHGIKRFQSLGKQFNPKEHEAVFEVKDTSKPKGQVCEELLPGYK
IHDRVLRAAKVATIKNMQEDVAKHIEQACKLDKVVLEYLVKVVYAELENKGRMSKDRLFE
IMDTFLRQNSVVNNIYEELGIEPEESELAG-------VLKVIEPTKILKSEVETDKLKY-
DPLLDLDGGSGNFNKNIPIGESIKLQKAAAKEKERQLRLLKQWEKQKLEVPVPVCQHPI-
--------DRSRRKLTDIQIPNITIFISGRALLSDASLNLTLKHKYGLIGRNGIGKSTLL
TYIVRREIPGIPADVSIACVEQEHYRPEETVLEAVLSIDTERFELLEEEKRLLAGNDEES
GNSELL--------------KEENTARLSQIYERLTEIDAYTAENRASVILVGLGFTQEM
LKEKVIRLSGGWRMRVALARAIYANPDILLLDEPTNHLDILAVTWLENFLKEWDKICVIV
SHSRDLLNQVCSDIIHFNDNKLTYYKGNYDTFEEVRSIDLILKQKHLEQQAAEKERIQRF
IDRFRCNASRASLVQSRIKYLERLPILEEVRKDPTVVFDF-----------NAMDISGST
ITGKSDDTYVSL-------------IECCGVSFYY------SQESSNTTKQ----IVHDF
SMNIQSNSKIAICGGNGSGKTTVLKLIMGQLNPTKGMIKRDPKIRIGYFAQHHIESLDLT
LNSIQQLQARYPVADISDEKARNFFGRFGITGSLALEPLYVLSGGQKSRVAIAIMAYLNP
HLLILDEPTNHLDLDSIQALIVALNSFNGGVIIVSHDAHLISCVANSIWHIDHVNKTLKE
FKGDFDLYRKTVVRTSVMPRELVTLQIGQCGNQIGMEFWKQLCTEHGLDANGTVIKDSGP
LEDRKDVFFYLSDDSHYVPRALLFDLEPRVLNSIKSSEFKHLYNPENFFIGKEGGGAGNN
WGCGYDTGEEVYDEILDMIDREVEGCESMEGFVLCHSIAGGTGSGMGSYILELLNEHYPR
KLIKTFSVFPMLNNESSDVVVQPYNSVLTLKRLALNADCVVVLDNTALSGIAEDRLNLTN
PTFAQTNALVSTVMAASTTTLRYPGYMHNNLVSLFSTLVPTPRCHFLVTSYTPITISQNI
QNVRKTTTLDVMRRLLQPSNIMVSTNMRKGTYISILNIIRGETDPMEVHKSLQRIRDRNM
VRFIQWGPAAIQVALSKYNPY-STQHKVNGLMMANNTAIAGLFQRCVAQFDRLMKRGAFL
DNYKKQSVF-----QDGLDEFENAREVSEMLIEEYKRAERNDYMLAGYCMEKYQKLEKVG
EGTYGVVYKAKDSQGRIVALKRIRLDAEDEGIPSTAIREISLLKELHHPNIVSLIDVIHS
ERCLTLVFEFMEKDLKKVLDENKTGLQDSQIKIYLYQLLRGVAHCHQHRILHRDLKPQNL
LINSDGALKLADFGLARAFGIPVRSYTHEVVTLWYRAPDVLMGSKKYSTSVDIWSIGCIF
AEMITGKPLFPGVTDDDQLPKIFSILGTPNPREWPQVQELPLWQRTFQVFEKKPWSSIIP
GFCQEGIDLLSNMLCFDPNKRISARDAMNHPYFKDLDMGNRISIDDSIFELKLQKKELER
QYNKRDRDSKSERKKAKDAIISGKADLSKIYAENSLRMQEECKELLIMISKLDRLCARLE
KASSKEKISTQLLDIMPKLKKQLSEEGVMGYSNKIKEIGQLLNTIEDSQETVGENVGKEK
CMHA-EDSVERLLDELLNEHAMEIAISSKTEINELLEEKLE-----MGKEKTHINLVVIG
HVDSGKSTTTGHLIYKLGGIDKRTIEKFEKESSEMGKGSFKYAWVLDKLKAERERGITID
IALWQFETPKYHYTVIDAPGHRDFIKNMITGTSQADVALLVVPADR--FEGAFSKEGQTR
EHALLAFTLGVRQMIVGINKMDTCEYKQSRFDEIFNEVDGYLKKVGYNTEKIPFVAISGF

VGDNMVERSDKMPWYKGKTLVEALDTMEPPKRPTDKPLRLPLQDVYKIGGVGTVPVGRVE
TGIIRPGMNVTFAPAGVTTEVKSVEMHHEQMPEAVPGDNVGFNVKNVSIKDIKRGFVASD
AKNDPAKGCEDFTAQVIVLNHPGEIKNGYSPVVDCHTAHISCKFQTITAKMDKRSGKVLE
ENPKLIKSGDAALVVMQPLKPLCVEAFTDYPPLGRFAVRDMKQTVAVGVIKSVTKKEATS
K--------IEEMLRAPIILLKEGSDTSQGKGQILSNITACQAIVDIVKTTLGPYGMDKL
IHSGNDVTITNDGATVLNLLGIVHPAAKLLVEIAKAQDDEVGDGTTSVVILAGEFLKEAK
GFIEDGMSPQVIISGFRKASQIAIDKVNSMKISLSEETPEKRRSMLIKCAETTLNSKLLA
HYKTHFAEVVVDAVSYLDNEMDKDLIGIKKINGGSVMDSFLVKGVAFKKTFSYAGFEQQP
KHFVNPRILLLNLELELKAEKDNAEVRISDPTAYQSIVDAEWKIIFEKLDLIANSGVNVV
LSRLAIGDLATQYFADRNIFCAGRVEEQDLKRTSLATGAIVQTTVYGLNKDVFGTCSEFE
EVQIGAERYNIFRDCAKTKSSTMILRGGAQQFIDEAERSLNDAIMIVRRAMKSSYIVPGG
GAIEMAVSKTIRDHARTILGKEQLVMNSYARALEAIPRSLATNSGFDSIDILNRLRQKHS
QNTEDSNYGVDCSNGGICDTFNSFIWEPAVNKLSAYSSATEAACSILSIDETVKNKSSED
RMQGPPRRMSS--TDFGDLVLLIGDLKIPYGAKELPSNFRELLATDKINYVLCTGNVCSQ
EYVEMLKNITKNVYIVSGDLDSNGVFPEYVVVQIGEFKIGLMHGNQVLPWDDPGSLEQWQ
RRLDCDILVTGHTHKLRVFEKNGKLFLNPGTATGAFSALTPDAPPSFMLMALQGNKVVLY
VYDLRDGKTNVAMSEFSK-VFYQHDDFIYSCPIEGTGDSNSTEIRRCKETINSELSNFHE
-KLENCWGILLHGKEASNNGDYMGVRVPKNGELSDYKFVKYSYVIRKAKEVGSGLLHIGA
VQERSFEDCNIK-LKCVALFAKNSLNWSITEQACNAYGISTIPLYDVLGNSGLTYILNST
LPKTVFCSVSCCKKLIPLLESMKS--VKFLIMLDSKLIQESATDYIKENVTIMDFDDLIK
IGKSNLREVSPGNLESIHSIHYTSGTTGNPKGAVLTNRAWVSCTAAFVYGQLGREGTKLG
SNDRHISYLPLAHIFERIVHMVITYLGGKIGFYSGDVQKIVDDIQLFKPTIFITVPRVLN
RIHDEVMMSIEEKPKPLQMLFHFALKQK----ERSNSPFHFIWDSIIFNKTKKILGGNVK
AILSGAAPLDETVLTRIRCFSCSYCFEGYGMTELLA-ACMSEINDNSKNIIGGPPGCYEF
KLVSIPEMDYSVKNDPPTGELLMRGPSSFS-GYFRNEEETKAVCEKEGWIRTGDICQLLP
NGSIRIVDRRKNIFKLSQGEYVAPEKLENIFVCCELISQALVIGRSTESFLVAIFVLDEE
FTMKYVRTNQLDLKFKDAVTHPKIIEKIKLDIEKAELQHKILGYEKIRAFKCISTPFSVE
NELLTPTFKVVRHKAIKFYEESINEMYKDGYSKMSEWDDMVKEWLIDTGSVCAGGLCSI-
DGAFYAASADQG--DAWKTLVREDHEENVIQSDGVSEAELINDQTTLCQAISEGKAPNGV
WVGGNKYKIIRVEKDFQQNDATVHVTFCNRPQGGCFLVDTQNGTVVVAVYDESKDQSSGN
CKKVALQLAEYLVSQGYQRSQLGNSTDGLTGPIRSSDKLLSSNPYPERKIQQTIGELSFR
ATKFCKRCAGNSIVVYDSKSGSEICTRCGVVVEDRVINEEQEWRSFSND--GDGNSKSRI
GSVNDVWLDDGTDSQLLVGDKKLMKTLKHNTQASSDRMIKEVFNQLRQIANSFSLHDNII
ERCKEIVKEQSNLNILKS--GKKHVLAIVYLACREEGVSRTVKELLSFDRTISERELVRS
INKLKKDLPRRGPTLSSSAAELMPRFCHYLQLSHEIVGIAEYVCRTAEQYINKSHRPNSL
AAGAIYFVCNLCNIQ------IEMKSVAVAAKSGETTVRGVYKELLLVGEKLLPSDFTPK
LVGGIDTLKRRSKIFE-----DNQHSETL--LGTLREKYENSAILSLSSLFIFITLCNMF
IMYRLLLDVIPYPIGVTFSQLLVGMMLAYVLG--GCDLRKVK-------DETLFSEYAVP
VGVYLLMFTIANILLKSTPVIAVYPCILALAVVLHHIFRYLFCDQRNL-LSFKTVLITAL
SYIVACFDTNLVPPTLLGLSILYAISSAIFRAVCLEKALHVTERDANKLYNVQVFCGVFA
LLFATIIEPEFFLSQSKSFSEFILSI----GCLVTVGTIPFVKNIIANKLVTIGQQAPWR
FSEIVSVALFFVFGVIFFEITITIIISFILVISGRTMSMVDIINNDNKRHSGSHHKKRHA
PKNTLAFDVHAAQIADPPMRDMSMQDSDHESEMDHEAINEQGIIDQDDVDPESGIVQNE-
---NEELLIY
R---------ALQAIKLKSKGAKQGYDLLKRKSDALSNKFRGMLKEIVETKRSIGNDIKE
ASFALAKATWAAGDFKDRIIESCKRPTVTMEVGTENIAGVRLPIFEMNVDNNSSTTCHIG
VASGGQVIQSTREIYMKVLRDLVKLASLQTAFFSLDEEIKMTNRRVNALQNVVLPKLEDG
MNYILRELDEIEREEFFRLKKIQEKKKEWAEAELQEKKKDKSNSKENDSSSDSILEQKNE
GILF---YVLSFLFSTFVSFCASSKVILDPLQHDVQILTAPNFESLLSKYRKSGVTSVFF
YNGDDKAVSELLGWYNDAARELKGMAKVAAINCKEFKQFCS----------KIGNSGKII
IYPVIPIPTFEFNGEKSVRNLKNLLRYIPDNVSVISKVIKIEDFLTRHISVPKVLVFSEK
EIPPTIIHSLANEFNKKLLFGFIPNKKNDITKKFQISSFPTIMVYKTASKPEFYKGEIKF
LPLFEFLNVYAETFVMGGGFDNESQDSGSKPWLLQRIPELTGLSYNDVCGKH--KNLCFI
YLKNG-EISLEEQSMLEELQDLFTPHIAGRGTNFKWMWMDISMEDEFMKLFNDGALPSAV
VLGTSKRLKFTVLPRNQTANKDTIKDFLDKVIGGDARFTNIKGQKLPKFIQNKNTKLEKS
RDELTALELNSPFSSKELKRVFSIEFGVLSPELIRKYSVLEIKVSDIYENGRPKSGGLND
PLLGTTDYHILCETCHMDIKTCPGHFAHIELGKPMFHIGFITTVLKLLRCVCYACSKLFV
DTLDPKFKQIKRIKNPRLRLKKILEMRSSSSRC---------------------------
------------------IISSDGSGCGFVQPTYSKEGYNLYINQNQD--DDESVDSKRL
LAAEEVLTIFKRISHDDMKILGFDPVKCNPSWFINTVIPIPPPAVRPYVQFGSDRSEDDL
TLKLQQIIKLNENLKKQIRIGSPEHIISEMSTVLQYHLITLINNDLPGLPQSRTRSNRPI
KSLRARLKGKDGRIRGNLMGKRVDFSARTVITGDPNLAVDQVGVPYSIAMTLTYPEVVTP
YNIEELKELVQRGPHEWPGATSIIQEDGTKVDLRNTSNNSNNILQYGWKVERHMKNDDLV
LFNRQPSLHKMSIMGHRVKILPYSTFRLNLSVTSPYNADFDGDEMNLHLAQSHETRSEIK
NLMMVPRQIVSPQGNKPVIGIVQDSLLGIFLLTRKSTFLTRDKFLQLLLCIPYWNGNIPP
PAILRPQPLWTGKQIITVLLAFTTD-----------------------------------
-------------------------------------LGEPIELNLMRDGSIVSSNNNPW
ISELDNQVIIQKGEHICGVLTKKIAGSSSGSLIHILWNEIGPEKTGLFLTYTQMVVNTWL
LEHGFTVGCQDIQPQNYTLEKIMNSLEGSKLQVQQIIRRAQKGKLDCQPGKSLIESFEAQ
VNQELNSARELSGTIATENLDSKNNIVAMVQCGSKGSTINISQIMACVGQQNVEGKRIPF
GFRDRSLPHFLKYDYGPESRGFVSNSYLSGLTPQEVFFHAMGGREGIIDTACKTSETGYI
QRRLIKAMEDCMVQYDRTVRNSNGDIIQFLYGEDGMAGEFIEDQVIELLQMEAAVFQHKY
RHNLEHPRWGSSWAGVQPSLIKNLQFDLESQNILNSEYKQLVTDREQLGKDIFPDGETRQ
HLPININRIIQMAQQKFPQ---

---------RVSDTSSSKD-WGPVYIIKKVESLLDKLLLWKNVGSNDSIIAEVQKNATTL
LGIHIRSSLASKKILEVDRLGPTAFEWILGEIEHQFYRSLAHSGEMVGTIAAQSIGEPAT
QMTLNTFHFAGVGSKNVTLGVPRLRELINVAKNVRTPTLTVYLESGIANDQEQAKDVLTL
LEHTMLQNITTIAHILYDPEQDKTIVEADKSWVSDYYEFPDDADITSRLGTWLLRIQLNN
KLVTDKKLSMKEIGDKILMEFSKDELDCIWTDDNSDELVLRLRIKNIEPT--------NN
IRDNIHSSEENDSIS---

---PSITSGV
EEHKFLEKLMTECLSQITLRGINNIKKVYMKEEQVSRYDSVSQKMVRDNQWVLDTDGCNL
EAVLCHTAIDSTRTISNDITEVFSVLGIEAVRRALLRELRTVISFDGSYVNYRHLALLCD
NMTQKGHLMSITRHGINRVDKGPLQKCSFEETVEVLMDAAMFGETDYLNGVSENVMLGQL
APYGTACFDVLIDPDKLRDTATHLEIHGEFAKLGKFSTLSQRSFESDSFESPM------S
PLPMSPLYQSY--EP-----------------------MSP-----------MISGQFSP
VMTPRSPMSPV-SPGGIFSP-VYSPMSPSMDYTPTSPSVNNTLSSYSPSSPLNSDNKNIS
PDLSPTSPHYSPTSPHYSPTSPHYSPHYSPTSPHY-------SPTSP-----------HY
SPTSPHYSPTSPHYSPTSPHYSPTSPYSPTSPHYSPTSPHYSPTSPHYSPTSPHYSPSSP
HYNNPASPNYSPYSPTSPNYNPTSPNYSPSSPNY---------ISNNSGSPGYSPS----
--------SPQYDP---IAVASVPRSPSYSVGKPLDETKNMVRFFIEELEVFFPYDNVYP
EQLEYMKYLKQILDAHSHGVLEMPTGTGKTVTLLSFITSYQLVHPNMGKLIYCTRTVAEM
EKALQELKTVVDYCKKEIENDKIK--LEQEIKSENNSSLASVSESRFSA-----------
--------ASILGIGMTARRNMCINPRVSVHADRDKIDSMCRSMTAPWVRAKHQMEARER
SEGDANSSKMTEI-------ADIEEMLE----------SGCTTLCPYYEAYERVWSSDLV
PTGIYTIDEFKDFSKNWEHPLGKKIQFCPYFASKRLIQTAKVVVLNYQYILDPKVAQASL
LGGGTVSQGFSHANSVVVFDEAHNIDNVCIEALSVNMNRQILSGAARNLRTLKSEIESLS
SLDEQRLQDEYTRLIQGLRNSGQVQDEAV-------------------------------

--LEDLERFPVLPEEMIKKGLIPGSIRRAEHFITIMKKLILYLQEYIRVYSTRIEGPLTF
VKHIEASCYIQSGLLKFCDERLRSLLNTLRIVDSDQYSSLELVCTFFTILGSYSKGFIVI
VDPYPEVSGLYDPVIQLSCLDSSIAMRPILKRYQSIVLTSGTLSPLDLYPKLLGFIPVVS
QSLTMTLDRTCICPLIVTRGSDQTPLSSKFESRADVSIQQNYGKLILEITKKVPDGVVCF
FSSYLYMEQMLSQWYESGLLAQIMEHKLVFVETKDIVSTTLALHHYRKACDIGRGAIFFS

```
IARGKVAEGIDFDRHYGRCVVMVGIPYQYTLSKILQSRLSFLKENYGIQENEFLTFDAMR
QASQCVGRVIRSKADYGLMIFADLRYNKKDKREKIPPWILKHLKPEYSTLSTDMAVSISS
NFLKQMSQPYAVSNSIKLGS-----------------------ILKLGCSNLLRVL---P
TRYARFNSTLSFKRNDPDLKISKLSNGMRVATMKFGPNSLTFGLWVDSGSRNEDPGKNGI
AHFLEHLIFKGTYNRSRKEIESQIEDLGAHLNAYTTREQTVYQIRCFNQDLPKCMDLLSD
IIKNSKFCKSAIEQEKGVVLREMEEVSKSEEEIIFDDLHKEMYKNHPLGNTILGPKENIL
GFKREDLINYIRTNYIPEKMMILGVGNIDHNSFKNIAETYFGNDSNNSRNLGGYKNINLS
NSQYLNEVLVHKKNNSDGKTLLAMAYNGTSWNSKDFLKVMFLQSMLGEYGTNNINRVTG-
-----YKNQIIERILSGIKDFFETFNTCYKDTGKL-------------------------
------------------------------------------------------------
--------YLHSY---------------------------SMSNHKDLVFGWEERLVDTS
SKLKSKDSLAPELFEKLLKSL---------------EIAESYSCLKLINDPHFKLLIS--
------PLYKEYHSNLETKSLEKISQNVLDYYMSYILK---------DNDQVSQKIAQFE
ILCLSILLLNIYMQSNWTGPPFKIDDGVSKDWKPEKKEADYNNNELSGLISLDNDEFGNN
CTCCLEIDGEYLYQRCNSAPFLTWSIFLIEFLSNRSTD------NKDEFNSESNILKLSK
YIVIGD-----------EIPKLSFINFWKQRFYRIWQRSLEGGIKFAATLESTILEKYSN
WLKNWNIIPDDFP-------IDRFDNLLRSFRFDEELSSNQALFSNKLDKNVSSIFLLDL
SLSLSTFSRANPCAILLKQISLMNGFKYSFTGALGQKRMNQRESTAQLVVEV--------
--------------------YRSSDNLE-------------------------------N
EIDRNVVESEEPTEDSINNETEEKI------------------LDRQYP---ENVLLKSV
DPNIDIYENVKLDDDKNSHLTSLAIIEQCVLLIHGVAIYETSPTNDVIAYEQLNALTNRI
LKFDITKIKDE------------DLRKKIHNWLCFSTALWYRCYAEHHRGRTADRACLQL
QSLVDQFNDAEPGPEERLRLVFSVNYPNIWEAKKELGIRMMRIGSVLSAYNMFVEMCMWE
DAVDCLIVADRKTEAIELVKEQLKVRETPRLYCSLGDLTQD--LSFYEKSWELSRHRFAR
AQRSLGNAYFKKSQFELALDAYTKASLVNSTNVNCWFSLGCVALRLERWEIAQQAFSRVV
SL-DPQQGEAWANLAAALSKKELWDEAQSAINEGLKHSRDNWMMWDSSLKIAIKREDLSR
IIECLSGIMKLASYKERFPWSLPNIINLLKSEKYAHEINNSDRPYINKSLNLLNQISQY-
SSKPVVYYVLSEIQLLKNDFSSAYSSKMKELRSMEVIFNEKISSDDKEKYLQDIPNIYSD
IKEIIYKCNDSDPNEERVDEVNMVITTIIKRLKVTKPVWSSALEQKLSSSL---------
-----------------------------SGKKKSSKRSY----------------NNVN
AFLDVEALVGEDDEEDEYEDIYHEESEYASNAAEIGARRRRHLGGAGHLEEAIAQLERRY
EQKEG--GEVSEAERETEFGSSEVTSSVQACDVLPTSRDPKLWLVKVDRAGLEKDICIAL
VQKAAECQKKELPILSAYVASSYRGYIYVEAEAPNFVNEALQGFTGVRLSSIKIIPVKEM
TRVFSVDMQEKEQLMRESWVRVRSGIYGGDLAQIYEVDEHEANVILRLVPRLDVPALIRK
SQ---------------------------------------------------------N
SQDVSFSKSRIRPPAKLFDRDKVESLGGVVELTHLRGTVKFANQLFEEKGYLLKKMKANR
LVVGDAQPTIEEIKRFFGVSLSEVNID---SKTLLKTQKSTSFFVGDTVTITRGELIGIK
AKVVAVINKSLEVLPIDKSEPILTQVDLVCKSFDIGDSVQIVEGVNEGESGLVTSFDQNY
TVAIIYPLNGAQPIRCPTNFLKKVSQDVVVTSGLSTVDGFSLDDLVQLYNGKVGVIVFVG
RNKNLRLLTSTGESITIKSSEISSKRTSLMHRIPDHNGNIFGVKSTVQILEGANSGKSGK
VEHIWKSTCFIKLPSKLDDGGYFTCEGRQ-LLTLKTAESSRMDAAGNSGGGYGLRRGGPD
DAFINQKVRILRGKHKALLGSIRGFKGNNVEVLLDIGPHTVLLRREDIVLVGATIQASSF
------------------------------------------------------------
--------------------------------------------------------GGQA
RQMNTQPASQVKQPNWTGGKELSQHTDENSNNHPLFCRRGVEVTVISQGEFFNQKGIISD
ILEVTCYIILIIQIPDEMIAIAPQSITPNKPV-PGENSISVSPQTGVFGIIDSVEGDDFL
ILDSNRQSTTKVSGQFVFKYHTPMQNKIPITTNVLANLEELGINSSCFRFGSLTLEGDKY
VGVKESVDGGSQIVVIDTQSKGINRKPMKAESALIHPIENILVVRGRYED-NGCTVQIFN
LDSKEKLGAFLFPESVVFWRWLTPRILAIVGDKGIYHWTISIPVRIFERAGKLAEQSTQI
VGYQTDSGQRWCMLMGLNESTGSISVKGQLQLFSVEKRQQQLIEGFSGNFGELIVDDHSP
ASIVCFVEKKQEHSNARLHVMDISGQRAGSPTPFKAVIELPTVDEGSADFPIYTYVSSYF
GVIFIITRGGILYIVEPTSNTLLYCNKVCQDSVFLGSPSRFH-GICMANKKGLVLHITLN
SSSVLTYIQ-SNPELSNSNLLRWTQRYGYQGTDSIRMFNESIKRQDYQNACRVVSLNKNG
SLRTPATLNHFKMVDSSN---KLLFQYFTTVFKFHTLNQFESTEFCRLLLITQPLAFLQV
LINEEKLAFSEELGDLLQNGEKKLALKIFKKCTPQNKILQTLIEIGNFSHVSEFIREQRA
ITTDIRSLLNSLLIAN----NIDATVEFVKTVILPPAPSSQTDLANSTS-----------
-------------AESLDLNIDKTSVVEIFVSHSRYKEITSILLDHLKANKPEDSALQTK
LLEVNLLHAPQVAEALFQMDLFTHYDKHAIAALCEKAGLYERALENFSDMRDIRRILGVA
CGSLNTDWLANYLSKLSPRTRFDCLKELLSNNNTVLQSVIQVCIKNVDNIGIENIITLFE
QQGIWEGIYYVVGSNLTQYLSGNSLSGSNLSDSGTQNRGGAGVVLAGSGRLPSSNGSVNG
GTGSFSTFIVFKYIEASVHLGQIQEAERICRDFPQSYEPEQVIEYFKSIKMSDLRPLIWV
CDLHHRVEELISYLYHMSLYKYIQVYTLKINPSQTPLVIGTLIDLDGSEDLVKSLLQEIK
TLGSFGELIQQAENRNRLKLLLSWLEERVQEGYQDPALHNALAKIYIDMNKDSENFLKTN
PHYDAKTIGDYCEDRDPYLAYIAYRKAWGQCDDEIIQVTYKNDLYRLLARYLVERQDLDL
WNKVLGNSSNCRQAIIDQVTSSILPEYNKSEEISCVIRAFINAEVPNSLLEVLEKIIFHN
TEFSQNKNLQNLLLLTSIKIDVRRLDDYVLRLDNYDAKEVAKVAIDHGLYSHAFQIYKKF
SFNNEAVETLLMISKLLKNEGIEIDGQG-------SEFMKNLDSMISIEKLANMDLSKVQ
DFASYCNDNSVWDVLGQQYLKISRTKDAVDCFIKSENTRDYRLIIEHCLSVKAYRELLGY
LQMVRRLKTSKDPIVDTELAYCMSKLELLQDLQSFLQGINTVQLQKIGDRLMDEQDYRYS
IIFYQAIPNYSRLTSCYIQLGEYNNALETAKKANSPKTWKELLQICMQIGESELAHQAGL
NIIVYPDYCEDVVSEYEKKGLTAELLTLLEGAIQNDRANGSLFTELGILYAKYTPEKLMD
YCSSYSGRINIPKLTRICEQRQLWNEVVYLYLQYQEFDQAVLTVISHPKEAWKNDQFLSI
LQNVTNVDILYKSMTFYLQEHPELLNSLLMTLKPKKSSTRFIQQYFSQGDISIIQEFLES
ISDENIQVVNEALINLYIEKVEVEKLMKLILTCDNYDQAKLSARLEKHPMNDLRKLAVKI
LDKNSNYQQALSICQKEMLIDEAILVVYKSGNVALIEELLEFLLSNNKKENFVACLYTCY
EFLRPDTVMEMAWKHNCLDATMPFFIQSLRDMTNRIDVLEKKLETLQITDIATTASGGHP
SV--SNSQNISSCGPNSSISPGSSHHHVNNNNSNSSNTINNNYFHMSTLVRKLAPNFTAE
AVMADGSFKKVSLSDYRG-KYVVLFFYPLNFTFVCPSEILAFNQAQKDFEKLGVQLLAVS
VDSQYSHAAWRRTPLEQGGIGPVNFPLISDSSHSISKNYGVLLEEGIALRGLFIIDKEGV
VRSEVIYDLPLGRSVEETLRVIDALQFTETYGEVCPANWKKGQKGMSATHEGVSSYLKDS
MQDETVNPISELESLVKDPKCTQDLSAETDSLITKLENAGVEVNFQNAVEELLVLEKKCR
QVSDSNSSCKIIKIILWLLKYSKNIDEPLSVIQKICKKRSQLKKVISYIIQLSRKHIETV
TNLEESYKIITILSEITQGKIYLELERARLMLILSNIKQEDNDLKEASKLLEDITVETIG
NMDLREKTQYVLEQMRLSLLCKDFVRLQIFAKKINPKIIEK--FIDLKVIYYQYLIILWH
YEQSPKEISMCFLNLLNSIANFENDPTTASCIEGYVIYLILGPYSTNIRDELKFNKDQKH
IERNVQYISDLLNDYINNELIFLESTKNNQLSNCFFFSNNDYENKKDRFTLFMQRIQERN
ISIISCYYKTISFQRVQDLLNLDGQELQLVVNHLVERGIFSAKINQPAGIITFTSNNN-N
GQFNKFHNNVGEILNKLDLLKDLISNDMMIHQFNSKINNK-------LSGNAINQEVLET
LADISSLLNTGLDKETILILIKLLELGVQPETLSELVIEIRREIESYQIY----------
```

> ***Babesia bovis***

```
NGSKCAVTVQHAKDVPFAAIVIDAIA-------KQLSRSDDDVVKLEQNKAITPGTYVLD
KVSMQI--SQLVRELCLRVSTCKRMLYIDSTASLDKWLSYISEHKTEEQLWQHYTKINQH
LTMRTFLVGYRMTAVDVLQCSLL-----ANSQGIRKRLTELKHLERWFNYIMAIPGVNSN
PGTATTNRAENKVFTKNDKKP---FTKAEQFENSYKGALKNVEHGKLVVRFAPEPSGYLH
IGHTKAALLNHYFAKQHGGKMLLRFDDTNPLKEKIEYEETIKEDLEALGVPFASVSYTSD
YFELIQEYAIKMIKAGLAYCDDTDTNTMRYQRNEGIASAARDLPVETNLANFEEMLKGTE
KGTRFCLRAKIDMTHTNKCLRDPVMYRCIADAQHNRHGDRYKAFPTYDFACPIVDSMEGV
THALRSNEYSARIPQYQWFIQQCNLRPVEIYEFSRLNFVKTVLSKRKLQWFVDEGIVTGW
DDPRMPTVRGIMRRGLTKKALFDFILEQGPSKAVNLMEWDKLWAKNKQIIDPIVPRYAAV
E-IDAVELKLDNFSVELPPSKRMLHPKDPNMGECDIWFTPTVLLDRVDADEIVDGEEVTL
MRWGNVFVSK---------PAFTGQINPGGDFKKTKKKLHWLPKDDDKLVKCTLKLYSDL
LAVDKIDPKEPEDMRKFLEPVTEWTTECLADKQLASLSKGTIVQLERRGYYIIDKPASEG
HLVMVSIPDGKMKKK-----------------MSNYSVIIDTGSLYLKAGLSDQAEPLLH
CPSIYGDVRKEMGNERYYGNECQQRMSYMSTTNVVDHGHIFDYDIAAGLWKYAFDTMDVE
```

```
PKGASVLMIEPVLCSAEHHRKTGEILLEGMGVEEIHTSMSGVLSLYGVGKSSGMVVDIGD
GMIQVVPMEEGHLEKKAIRRIDFGGLELTMYLQKLLCELGYPMTSRDDFDTCRKIKEELC
FTSLDPRADENNAGLEKQYVLPDGQTLRDGENVVGLSVERFYVCEILFNPTIIGFSHPGL
SQLLWQSIQDSGLTQRKLLMENIYLCGASSKFENLGERLSFELQNLAPAAARSNIMVNTS
RDQHLLSWKGACFLGTPEVRQTYKEQWITKADYEEEGPRVFLRQMV----KMEELQSLSV
NAKVAQILKNHLDTDHPDLVDFIIHLAKRARNATEFNKLLDENDAEMPLALGEQLFNTVM
A---------------RGGTWDDIISSNDDRLNFPALCMKNATQTQDIYKLEPKKDGELS
EAAKKLLEQETVAQRDRGDDVRSRSDRYARDYRDRYSDRHRHRTREDRSRRSASSSPES-
--YLDDKGAIFKGRVTKVVEFGGFVRFKSKNGIHSGLVHVSEILPGNRRLSEASEALKED
MVVYVKKIGMKNDKISLSMKSVDQKTGRDLCSGHQDSYYTMGAP---------------M
LQTASSSLDYSNGMDGRKRRMMTDLERWEHQQLVNSGVLPKSERVAMDLAAQHEPELDEE
---IDIEINDACPTFLKGQTRRSGIELSPIKIVSNPEGSLARTIATSSTIAKERRETERM
QEDTIQRSAGAG----------SMTNNSTSQFMEELRR-MNMKQRREGALHDKRDPGTRN
IKTIQEQRESLPIFALRDELLQAVQENDILIVVGETGSGKSTQIPQYLAESGYTSESVIG
CTQPRRVAAMSVAKRVSEEVGCRLGQEVGYCIRFEDCTTKDTVIKFMTDGMLLREVLQDP
LLEQYACIMLDEAHERTIATDVLFALLKNCCSKRENFKLIVTSATLEAEKFSTYFNDASI
FSIPGRMFPVEILHTTDQESDYMEASLITVLNIHLNEPAGDILLFLTGQEEIDVACRTLH
ERMKRLESMSPPPLIILPVYAALPGEMQGAIFEPTPPGCRKCVIATNIAEASLTIDGIFY
VIDPGFAKVKRYNPRTGMESLVVVPISQASAKQRAGRAGRTGPGKCYRLYTEDAYRSEML
PTAVPEIQRTNLANVVILLKAMGINDFLNFDFMDKPPVETLIDALDNLYHLGALDDEGLL
TRLGRKMAEFPMDPNLAKMLLTSVDLECSDEVITIVSMLSIQNIFYRPQDKQAEADRAKS
RFTQAEGDHLTLLYVYNQWRKNKFSSVWCHENFLQSRALLRAQDVRKQLISIMDRYRFKV
-----VSCGNNAEVISKSVCAGYFHHSARRDPQEGYRTIVDQQNVFIHPSSALYNRSPEY
VVYHELVMTTKEYMRDLTIVKAQWLLELAPSMFK--RSEGVSKSKMGQKIEPLHNKFEEK
DGWRLSKRRGMQDDYDNVYKIILLGDATVGKSHLLSHYIRGTLPKQAKATIGVEFATRTV
PLASGGTVKAQIWDTAGQERYRSITSAHYRRAVGALLVYDITNRQSFYNCHKWLDELRMA
AEPDIVVVLVGNKIDLAIQDPSVRQVHREQATMFANEWNLHLFEASAVSGYNVKDVFEFL
LQEIHNLKSRADATHGND------EATINTMESSRVDPMYSARRTSNEGQDGCRGGSCME
QPKLAKVNKVLGRTGSRGGVTQVRVDFMGEGGWEGRVLIRNVKGPVREGDILALLETERE
ARRLRMS-GLHGYETQTLVEELRRRYDCLSKPQGNFIFMGAPGSGKGTQSLLLRDSHCYC
HLSTGDILRSAIRSGDPIGMEAKTYMDQGKLVPDDVVVKLIEGNINSPRCSRGFILDGFP
RTETQADRLKTLLSNLGKRLNAVFLFECPDDEIQRRITGRLVHEPSGRVYHMTSKPPKVP
MRDDITNEPLTQRKDDTLEVIRTRLDAYHKQTAPLIKYYENMHLLHRIDANRPEMKVNEE
INKIVENMMVSECGSCYNQLPQPEVVLSPEDSELNYYLWMPGVQYQPRLQR-----KLSE
LRSITTESEVVVDEVVEEVIAKDELFVKFNNAASNGMMDLKTAAEFIRQLGATPSQAELV
EYSATCGNSLNFDQLKELLALSMYPKEDAAYLQKVLVSLS-KDQEKIEFPHFEYIMANYG
EPMTAEELAAVKSIVFGNNNV---LTCGELAAMQSLS-----YVFMVIALASR-ATAWMF
DEKDGNGADEHPE----------GLTREQVDARMVKLFNIIDENKDGEVTSTELEKFNSR
NLQRVQNMQLEQEMQMMDKNKDGFVDFEEISISFPPEA--GTPEDFMEGLQRRFNVADKD
GNGKLNKTEVYILLNPAHDESMLDLEVKDIMLTHDKNGDGLISIEEYLSSKPEE---EQD
DEFLEAEFKPFDLNNDGLLSILEIIAAFKEEARDTLETNLEDVIAIIGEGP-IDFATWNK
HALELSTSSITDHGELLRHPEDYDIDLAADTR-----ERKGQDT----------------
-----AGYSNTELMGPGIAKLLSALLIAISGLVGCLVPYLVYYFSRSGEDLRQDKIDSRL
CLCNCLGSGIIMGMAYLHILPEAVSQWEVESLLWNIG----TGHFNPAYFLAMIAFCIML
LIERVLSSGRTPCSAAFNICNEIPEKEEATGCCKESEAQQICCESEMPSCARARFRHHHS
RLMKRFMEIICPLCE---CNGLCITFALFLHSLFEGIVIGLVESQWNIWLMTVGIALHKW
AAGMALSSFISSN--SRKSALVMQTIFCLSSPLGILVGAITAGRSPTAEAVLNCFAVGTL
IYIGMEIITHELFCHIHRKTAFWKWLCVVIGLMVIFITAIIQVYITGGCA-HSHLHQHAV
QSKFWGEDSDDSYSDSSGYSSEVSERDAQKRPGA-AAAWVASDSSSDDDENRVVKSARAK
ALEAIQNIGKALDHHKTISDYSEVLKDYDNLARLVEKQSRGRIPKLIVQIIVELQEFIDE
KQKDKDSLKKMSKARSISFNTLKSRLRKFNEQYAAVVDEYNKDPSSYVDILKESSDEDDD
SDEESSEESSVEEESASEDESEVDAEDDDGGDEDSEEDSDYSDWSDSDASSISDADAGDK
HKSALAKWGVKKVQKPKASKKPKGKVVKKK-EEEKADLGAADNPFSLSTPEINAVISQVM
VSADSVRSYVKSIVERRGKRGGNTAETIRHLKVLPYIAKGISHSLYIFVVETLLHIEFDS
YSNAYGAMTPKQWIDSYRIVSHLIDELRRHPHALLSSETNERDPANAGVSREERIQRSMT
ILESVVRKLNDELYKGLLYIEVGSDDYNTMLVYNVNMLYLLHKTLLYCL--SKGKEHAAN
IAIIMLEHLHYKDDVVSGKIWELVR-------------------QKVTEQFKVYFPDDNK
KASDIVEELAYFVFEHGSPREKIRACLYLAFNKSLHGLYYEAKDLLLTPNIHDLAMETSI
TTQILLNRNIAQLGICAFRKGLISEAHSYLMDLCSQNRHKELLAQGLSMVKGHEKPPEQE
RAEKRRLLPYHMHLNIELIETVNNICACLLESANLAKSSLNS-REIISRQFRRMYEMHEK
QVFMGPPENNRDVVMSTFRHLQNGNWKECYDLLAGLTIWNRLPDREEVLQILKDRIKVEA
FNTYIFKYVPVYDSFSVDQLSSMFDLDNNVIHSLLSKMIITGDIHATWDDSSKYCLINHT
EPTDLQKCAIKLAENLTTAVEQNEMTLNMKNPKFALSQDRRFQTRENKFSYGRSRDDRHH
APTFNRNR-RPIQGARLHR---------M-----SIEDMTTLRPEALVKAQDYLKLIKKT
GDPNLIFIHCYHIMKVGGLDAEVPRLVVFGQQSMGKTTVLDFIMGGPIGYSSTDTGTKQP
VVIIMRPMEGGKIWCLFNGKLMDIRSVQDAMRQHMQNIGDSIIAEELEVEVFVPNGLNAI
FVDLPGIKDDSKVGAEFTRNVVRNYVKSNPNDLYILVKKSSDDPANWPWSLREFITTAPP
TGLGLSPHQTVVVGTRAREFLLNEKTDIRTAEQLLERVLKRSVIDSRGNMLPLHLLELFS
LSIQAKESGDFIANKAEMKRQIAMGQREVEDLIKTSFESKEGMTVVERLLQMFSINGFLT
TLDSKYQTLVANTFRNLERKLVRKKMELERTLQMLEQRMNRLNPQSLRESIALFIRQFVE
VVQKMMTGNYTIMKLPVPPEEFLKYYGGSLRDNLEDGNELALNLFPQGDMYEPDFYDNIS
TRTETLFN----------------------------------------------KKLTMM
DSVKPGRYVRYFTSKINAHMFGLIEPPISNDYKSDEMINVEFQINNGQENSLHKNIDRSR
ISLLTPLPSVSDQMQVP--LYAWHKTQSSTGWILVRPIVIDRLDKGAKTDHDKSGR--DG
DKSNR-AS-DGSVVVDNDAKNNEIFLEDPKVVPYYNSALEMVSGEHAEAHLLNQLAVTNI
CNWLKFQIKHMEPDKHFTSEVIYQMLRSVHHVVDRADWEPLIADLVQSNVRGALLHAARL
SACAAAAALRRVLKASLAEVFRCIQQTECDQTLYCLSESLHFQEQIDQLCEEYCRQKATE
CANAMMNLILEQTYSIQFDVAVDIFDYCYQFEKYFVSRAGHRSLMGEALYRVRENFAMRK
RRLAMTDIFEKSAKTSLELIYEEVKVQFWATKMLLASPLATKIYTHFIKDVVDRMS--SE
MQDPSASGDDELENFLQKNILYETIDGNWVARSNTRMAQDYDVNSKYDRFAQHFHDTKNL
LEYVNIALESIALMKRADGGPEFVANLDKLGA-----MDTN-LLNILAACLMPSAPTFAE
AQRQLGEMRERHLPDFVRALCDVIANPHVDRDIRQLAGVLLKNCFQ-RDPKCHDASRNVL
LQVPEDCLRYIKVQLLNVMKSGGETQSSLASCYVISRIAELELRRNGWPEFFDIIIGMID
PNDVEACRNSLTCLRYLIEDLASVFYNQGVA-FLSKAESDRLLTSIVKGAFMS-DTQSRR
TVMICMQHILPFVDSNMAIPNERDTIVQAICYNSGPSNPSNLRAAANDCLVQLVTDYYEV
IGPCLQYIVPLLWEGIDTRIEEVAIPAFEFWNTICETEI---ALSYENSESNQHIIQQVI
SYLLPKILFTMTLHEFEDFDSDTWTLPMAAGVCLSLCSQAVKNDIVPSVLQFINENFHHA
QWNCREAAVLAYGYIMEGPDADTLRLLVRDSFDRLCDVLDDPSIAVQDTAAWTIGRIASF
HCAVILPHLGSLEDPGSNICKIMRALFKPARVAANICWFFHELAEGL----GQI-EASRY
MLDAMFPKICDALVHRANMDDCMERNLFSSAYSSLANVITNVSDQCRPQMSGMLEHFERV
LAQSVASDHSAEGRSRQETVCGVIQVLLTRVE--FLPNTQSLWLNLFAIL--QDDLSEDA
LLTASALLNRMGSEFTPYMQRLVDIVITGLQRTDLTNACKACIELASDMARVMDRHIQPY
VPQLMELLLGTLSDINTHQKLKPAIVTALGDIAMASGSGFNNFVEPSMRLLLQAAGTSFD
LGPVDNEEWIWYINDLREGTLLSFTGILYGQKDINQVDSLRGYVSSILYFIQQVVETPAE
YFSAGNFRLAVALTGDLITAFGGDLSVHLVNSPLVEKIYERQKALEAEEHPMASECREKV
NWLYKLLNVK--------------------------------------------------
------------------------------------------------------------
-------MYMLQLRFTSNAQIDKAGTLLLKAISGGDTFVELRLKLMQMLYNSVDVTLPLR
VVVYVEILEFASRHDLFHTLLPVIQKLDEWMKDWVIDKKTRINIYRIISEQLDKMGNSEL
SFHYWAKCIECCD---QDALYTSDNLKVIADFCVRSINADGVLYFDRLRHKPAIDHLSKT
-EFALVVDILDLLIQGSEDDFDVFLSKHGDSALAAIGISPETVRAKLKLLTIASICQNEP
EVPIARIQECLKLSKDESEELVVTAITKGVLDGLIDQRSEKVIIRSVMQRQFRKEQLQQL
HSNLLQWKSCVSNLISVLGNQP-----INQLARLILIIAVQIVTVYARNANEFYTFKDAR
ATSTYVTHTDDLQKFGPARAFQIGASYWCSAGNHASTDFVSWTGELWDVAKISQIDVIWE
YAPNEVEISTSMAQDSFNVVMPFRKTFESKPSYKEVFKLDAPVEAKFVRLTLRGPINEYF
GIREVHIVGAGNPLFVIKSGISSPLEMCLQLEEGRRDN-NTRVILDLCTYAIAAADGRDL
```

```
WRHDSRQRLVSAVTKPPKCLTSVNPNKI--GPLVIADC---KDDGDDQCRWEFLGNGQIS
LKNAT-DLCMTQSDVYSDKAGMGDLLQIMKKKVKLIASTADRHKVESIMDENVKTYWASN
LFLDSGVHTVTITIDFGEITRAAKVRIDWEYQPVTYTVEGSTDNIVFKELARNMSNADHV
TIDTLEDRDFKQLRLVMLRPHHTYGKVEGGYVYGIRQLQVLSSNLETVVGDCRAAANTPD
ARDKYFVSYVSAFEPALAHEIKNMENEIHGITGEVIDDMATLGDTLDETDTCMQEKKEYD
KTLEQIHTRETAMWKQIQMSSLC-SKQNSVDVTYSTI-GETMKTPAEDCYSIKQQSKGVT
SGFFWIQPQCSKHPLRVYCDMGSQTSMLIWDGKNGGSGPQALHN-LTSPQAIRYQCAAYG
LEPLIIKSKHQVDGLREALFLMGFERKADHYIPLAYKFGNAH-----KFRDLMNIYTFMT
ESSIVGTKTPPEQPAMQNAAGLSLATGEIEVFDLETANIAAIVCSTNVT--EDNVTPIPI
KCDDRIDNEKLVGNTNTNIVVSCSEHCADKT--ELPVYGTDGVYSDRSSICRAAIHAGVI
-ANKGTFTVSIETGLPFYSGSTENGIQSYAFNKS-WQGSRDLLDPGMPEKEIEHTGPPSR
FSIRILPRKRVCPI---VQTHGSFLQVPSNTPGKDATDIAMSPSDGNLDPTTKEAAVKVL
SDMNAMYGLDIKTVINTIENIAAIVSRAKKYIKPLESVSMHQEKKMTTLYDRLESAATYL
KESSESQKSNYQRLLH-----EQQAKGVESEAPLVMDYTTMAFSRTFKIHDTSMTSGGQS
RWGYSDSPFDGHMSYIVQSSDIDSSLLGEGAYAMLKDRRYFDFDITVDLLAKNEGSVGIA
FRSQGHFNYYLLLLNSRQSNKQLLKVQEGVTHVLATNPDDGYKKNTWIKVNISATGNLIE
VT-----CDGKRILKVLDTSFLHGGVGLYSCGSNGSFYFDNFTVTPKPILMESPEVRLGR
LKAVKCSTYNETYTGDFDAYKVVNPSHTSWRFKDIIG---GKHKAHQYHISQDPRGIGSL
AILNDRTCTAGFIGFRFLPMCEGGS----IGAVVRFANVQNMILIEMTASELSIRLIQPG
GSKVLGTVPA------SYAISKWNAMQVGISSDRISVKVKKNVDHGSLFSGLFGNTDYEC
KAIGLKSAGCDSCYFDQFHVSPEESTSSSMFLQSSVNIWRPCAESVHVLNRIALCKQMFR
NRGEAS---ACAESFCFPCCSYHTRLLGEVHREACMTTCQKNHHVSNLYLEKFLSYVNSC
VS-LEGPAFKHC-EGDRQCLKRACSLCCSSGHDEKGP--------LDKALVALETSSCLM
QCNTLV----MSDDLVEDTLGPLLGVSDWL-----DWLKDRNEVYYYAQNGQYGDFSGLI
FNFSPDSSSLPYRGGKGQ---LSKRCANENECSRDRSVARSG--VNPVTFAKDDERPDYA
SLIRVETHEGFRSQQTQELVDKSSTRHDVTDIL---------------------------
------------------------------------------------------------
------------------------------------------------------------
------------------------------------------------------------
-----------SDEAHTNLDAQTFLNTFGMVDDIHLYHWNKIDPR---------------
--------------------ILKLARNRGLEPTTLGHVLRRREKLWAKIQCIDKSARSDF
----EKHLDLYQQQFEHYSKGWVRGRYNWNNVIFTPNIGDSSDLIRRYGKKRLYKNLYDC
LVHGWVHPGLVVCEVKDATHPIRFATPPDQDCYTVIYAGPTILDTTQRVVFGEYTGVVYR
EESLEDSLFEYAFELNFSTAAWINA-------DFDYYEESA-------------------
----------------------------------------------------------DI
TNRGDQVLLPNTSKYVLDSSRACNELSLVNHYQSIKAYGEKWPFPNCEWQQVFLDGWPHV
VLTSKLGVSIKPGDELVADFGALWFSKVEETAHKAIRNEIIQYRLGTQQLE-QRPLSSID
IGLKSNTLAAAAAVCSICYISSDNDGDYSVSCDGCDRFFHIRCIRSLNKAVSDTYKFYCS
YCRHLAKKIYMHDTGYDYMPLNKPHDITKKCSNTVSLNTISGNVNSLA--PISCRMR---
TLANPTYVDLNMVDHDKP-SGSEEIQQTLTDSNTSESVISSSSQSEYETLHKNGAKENLT
DDLQQY----SHQQLNSPATNRLGGLHDTIRCVTCMRPLKQNVSDTNQLTNTHDSVSKDK
VLLNN----VKQSLVSSG--SGASHQSTATLQNNEAELKL--NSAK--ESSSSEPHRVAD
TVNESQ-------------HSFECLISERHT-------ENISGDKDTVQLDLFD-----
--DEDPMKSIKLVNRNKKPAIIVNTSQWIPIHFFKAIE-------KHHNNTTLLSSFDGA
VNSRWQVEPFAHFGDSVKLCTECYNDKGPKANVYVCRLLKRHLGGNFDHPMNRELVLMAY
EQHVQMLLRLLVNVAINFLCR-VCCHFIAK-------LPFPSYYMGKKLGTPEGLLTPMG
MISKEKIEGKEPSHLVSFVTSVKNAVVEVPLSSTSDPSISFEMLALSDEFTRLMNCKD--
ICEYLNDLMSHKVLDGDNIPGQLQQMLTGDELS-HLSERFPGPFVSQTAYT---------
---------------------------AKKRQRGHADSS---------------------
-------LFNIQPGFSYIYRQFKDGYYKGIITNLV---DGSEYFQVGYSDGDDEVITHDD
LIVEIIDNLQHMESVIRKLSPNDMRNEDILKAVSEKQTTISVISRKVRRDMMQHLIPSKN
TEISKKLDRCN-NSYGSKRKLLMAD------HKQLGNEAFKAGRFLDAVQHFTAAIQANP
SDGILYSNRSGAYASLQRFQEALDDANQCVSLKPDWPKGYSRKGLALYKLGRLQEARTAY
QEGLKIDPANEPLMSGLREVESASDPEFMYLSAAMSQLVATNPKLQQYQQQDPSYVMNLC
RMISGLKTNPQSLQHVMMDPNPAIREGIMAYIQMASG-----------MPQPEETPEPP
QKKEEPKPKEEPLSEDQQKAKEYKEEGNKLYKQKRFEEALEMYKKAIEHDPDNLLLENNK
AAVYLEMGDYAKCIATCNAAIDRRYEVKADFLVISKIYNRLGSCYTKMEDYDAALAAYQK
SLLEDNNRNTRCAMNEVERLKEKKEREAYIDPQKAEEHREKGNAFFKKFQFPEAKKEYDE
AIRRNPSDIKLYTNRAAALTKLGEYPSALADCNKAVEMDPTFVKAWARKGNLHVLLKEYS
KALEAYDKGLALDPNNQECITGKYDCMAKIQAMSQSGTVDEEQYRQAMADPEVQQMLGDP
QFQIILKRLSENPAAMNEYLSDPKIAKGIQKLMACGILRTAMDAVYDVCVCGTGLKESIL
SGLLSQSGKKVLVMDKNGYYGGEAASLNLTNLYKRFRPGEERPDSYGPNRDWNVDLIPKF
VLAGGTLVKILKATETSHYLEWQVLDGSYVYQHQKATLFYDEKFIHKVPATDKEALQSPL
MGFFEKTRCHNFYRFVAQFDHTNPETWKGLNPFKDSIRAYYDKYGLEENTVDFLGHAVAL
HTCDDYMNEPAYFSIMKMKLYMNSLMRFGSSPFIYPVYGLGGIPEAFSRRCAIYNGTYML
NKPINGFEFDDEGKVCAVKTAEGEVARCSMVVCDPTYVVTLPHKVKSVGKVIRCICILSQ
PIPGTNDASSCQVIIPQKQLNRKHDIYITLVSHSHGVAAKGKYIALISTTVETANPEREI
QPALKLIGNIDEKFVQVSEIYVPTEDVVKDNIFVSESYDATSHFESASNDVLKMYRAITG
KELDLNSVDNGNDSE-AKDKVKKRALQPITRDYTIHLHKLVHRVSFKRKAPTAIKKIKEF
ASKAMKTKDVRIDTTLNRYIWSNGIRNLPRRVRVRVSRKRNDDDDAKEPMFTLVQHVPVE
DFTGLQTEVVASEMSQGGTLIYTDMFSSDDREDEIDDNERRTRRGDSDHAIFKLRACLSC
RLIMSED------------QMDGDRRRTLDCTTANFSGLLSIMDPQKSWAARYNSLVDV
IPGCYAISVVGELPESVHDDIGR-M-GSSDYHIRLDCTHVEFGYRDSVYCPGEDTFLFIE
ALENDIDYISKNMLCVMEIGCGSGYISTYFIKLLQGSHRTIPFVITVDINPAATMATVET
LERNKVVEADTMTADMFLPLLPQEVLDMVMFNPPYVPSEEIGNPKSAIDRAWEGGFMGRE
IIDRFLETVQQYLSNNGIFYLLLEKRNDIPDVIQ--SIRQRFH-PEVKLK----------
------MDVSGFGIARDVRKQTSEKSEFPTLCETCLGPNPLIRMLKERCGKECKICERPF
TMFRWKPGPKARYKQTIVCQSCSKMKNVCQTCLFDLEYGLPVQVRDEYLKNGLELSEVKA
NLNHQLGKLEAGTLELP---KSESNPMLEKLARVAPYYRRNKPRICTFWLRNACNRGEEC
PYSHDNKHHDPSLAKQNIKDRFRGENDPVANKIFKRIEEAKQKDEESSRTLVKEGV----
------------------------------------------------------------
--------------------------------------------------------YPSM
LPHEAQRRRMLTMNL-------RGKNVAEPPEESDIDLADEEEQMHEEWEWLSIRCERRL
NEHMYFYK-----KEDIYDTLGSTSAPDKYTSYVPKLVRRFIHR-RIGKHEMPSRTIEEC
YAVTVFCDASGFTALAEAIERSPEAAATLGKSLNDFFDPLISIINNWGGDIMKFAGDAAL
VAWFIPKNDGH-----------DNHSGTQDSGRKLASGR----GAECFSHEDAWKQCAL
ALKCCEELHKTLHDFPTNIEGKTLTLHIGVGFGKVCVIHVGGLLERWEVVVAGKPLEEIA
VAEPLASSGQTVISQSVYDVMCSHIEVDTIDNPDYHLFRRFVEDVDV------------
--DPGDI--ESFDFPIESLSEFRHYMPNYIFNKLLSGYSTFNNEMRRLSTLFISVPGLDV
ASKSGRERAHQFMKICQRATYALEGSVNKFLVDDKGVVLLIFIGFPPVYHTDDPVRAVFV
GLKIAHDCAKLGLKPGIGITSGSVWCGTLGNDIRREYTCLGDYVNLSARLMGKAARYEIL
VDEATVKEAGSILEFIELESVKLKGKEAWVKIFTPTGKVRKLDPQ--------------
-------------------------------------ALQNMSPLETWR-----------
-------------------RWESYNEVRRKLLTN----------------------GLLE
RGGVMLVNGHNGCGIGDMQSSIVKQEGFTNCFSVSTMEDSPVISIASLHYAWRNLCTSLV
STWATCPARKRMMGAYKKAVSDIVKELLDPEYHWRVAGIKSLILGLEVEPL---------
------------RTLFGHSLIPAPQ------STPGVMGKLQR------WMSEGELRD---
--------EADTGSTTESEDGYDSDSLFPEEDQRYDTTLLDQRMLEESIAPFVASMVEGF
SLKECICIVLNIRMGSSVYATMADDSWVTVCHLARLAMQRRKELMDGT-----------
----------------------------------STAKPFLFIIMSSPQHIFIRWHMELV
AVANECNALIKLPKLTQDQLADFIAHALDLESNSVPEELVDYIYQNTSGMPRFAYRTLDK
LVASGAIVLEDVETGSAET-----------------------SDEADTFYKTGL------
-----------------------EQMQDLGIYLDDPKRVRKRLVSWNLDNYGLIDETLSF
AMSAVDRLQPDELFVAKVASVLPSPFTRTDLFGKNPQGFTDEQFDTILRNLLANEIVEPI
SNIIDAASPGD--LSLRLASAAIAKVLTQRVMEDERAWFAEHFAVAQ-M--------KVG
SNTRLLIKNLPLSLDEAQTQRLISQKCKDIGLEFTDCKLLTKSGKGKTKSETVSRGLCFV
```

```
GFADEADATKFKDFYNGTYFRAAKVTIEYSRPFYATEAPTESSER--IIKRSKESLD--D
GEVATTL-------VKHVSKYTKAGVAGERKHTVFDDAGS--------------------
-------------QDDELLPEDEGDSVAMLPN----------------------------
----------------------EDVDPESLDRVVIFNLPNVTESAIRELCRPFGPITEVH
LP-----------LNKSLDTDSTQDKL---------------------------------
---------------------TKGYCYVTWVFPSDAIKFRDAKNRSIFCGRIIHVDLAKP
RSSASDGSTYDILNRRIRERHAEKSSYKRQLQKKRQSTSGESSIWNTLHIDINATVAAVS
RELELQKKEVM--DENSAAVNVALTETLVLNELTRWLDEQGIDYKRFQVQKDT-------
-DDAGSEAVTEVQRSDDTIIIKNLSKESCEHELVELFSQYGTLMRLSISPYQVMGIVQYI
DPKCASTAFKKMAYRPYKGLPIYVEWAPVKLFHEDAP--------------TLDPKPTEE
VDYFTDTVGDPSLSNTSVYIKNLHFKTRNDALQHHFGSCKGYITSKVLLKDNDT------
---------LSRGFGFVEFDSLVNAKAAIRAKTGLIIDGKVIEMSIAK-RVDKPVAEVPE
HKLL-----KATSKIIVKNLAFQATKQDLYKLFSFYGNVKSVRIPKSLKSNNRGFAFVEY
SSKQESARAVESLQHSHLYGRHLVLEFAEE---DENSEQ---------------------
----------------------------EM------------------------------
-STSPLPHSYISKTPSAMSDSAGLKTPPADAIILEPIVRERIIEVERPEIQERIVDVPEI
QYVQRVTEVPEPVIQENIVRVAKPVLQERIKKVVKPIIQEKVVEVPVVEIVEKVVEVPQY
VYQEKVIEIPKVVVQERVVNIPKKVTKERIVEVPKIQYKEVIKERIVEVEQEIPEIVHKD
VRVVHYIDRPVEVEKIVEVPQIQHKYKDVITPQYRNVPTPVEVPVKQIRNVPVTKIIERD
VPVPVEIDVVQEFTCRNIEARYHEIPVPVHVQRIIEHPLPQEAFQNTNLVPMYYCAVQDN
LRQMQQNMPIQNIKNQF-------------------------------------------
------------------------------------------------------------
---------------------------------MFTARNEYDRGVNTFSPEGRIFQVEYA
LGAIKLGSTALAIATKEGVIFASEHRVNSPLMESVSLEKIMEIDTHIGCTMSGLIADART
LIDHGRLECANHRFVYNEPLAIRSCVESIADMALDFSDIFDTRKKKTMSRPFGVALLVGG
IDIDGPSIWCVDPSGTSIKYKASAIGSAQEGAESVLQERYRDDMSFRDAELLVLEVLRQV
MKDQMTTKNIEMARIKIHDRQFREYTEDEIGEIIQDLPQ----MENM--GHVKYTDYVKF
ISQVASLYLGVSQHSVESLLNNQTF--VKNALLDSKGGKLLVSA-DALSTENEKWDIRTH
RGWDLFGTDRKAVEKVIQEDDGLSKTMLPLFWNCNLSPCKLSLQLLED-IINPILMNEKY
GDNRHDLVNDALNSIMIFQIDSKSSIEPPYFVIGESLSRVVTYGGP--------------
-NAKRWDIQI--------------------PSEAMLEDIKQNVVQLYHKMLNNELSSATS
SVNDVVDG---DIISSETITSDSSSDTYSSFSDSDQFENSQRIDMFDGDANVEDSTKLSM
GIEKINVEEETTFWLHYEMALMDLHEQLCSPLVENTISLLEYNNVDVKSISFDDAMETTS
RMDWVENINIIMRSFPVGYLKGVGDVGVLKDGIYIVLKHLSKARFLKHYSSNKLSDIILG
LTVEMLASCAAIIGDGINKAIDVELSRDLFKSIEEIMTIWNHGVEALK---------ADL
DIDGNSTVTFFTGECNNRISEARDVLQAIEQSIHRYLSIYA---DAAIIVFNGNLQHENI
KYCEKIVDVINQSNSLCKSMLDSYSLNINKIQVNESFDFNDILPCNTIMTTFRSCISDIS
NLDSIPQVLYYVTQFSSNVVNIIADEVEALETITTMPYYITISDSGAEAHNIARIKGKLM
VRNYILQVLEKIVKRLGLVVGS------------ETIKNLINKMGKHENVNTLILQCSTE
CGLQMCRKMIDITKVEEMQCVILYPFDNSLDNLGKWM-YMAYFGMSVDYE---STAEMHT
ILSIVLIVRHIFRSFNEIGEL----------KANALYLPEPFSLAIQNMTWENLMI---P
DTLETVYNIVSEFLDTSRSVALIGF------LEDIKPIISTLTATQLLTILSYVKSI-KS
SEETHVDY----------EESCDTKYIIDMVLLSIKTWNET-----TMQLEHELYNNSRI
TVTFKEEDSLLHMDPTIETLKGDLYNDIAPKINAPMYLLNELSALVLFDCLIDTIKRK--
-------------------------IDSILTNLDNDFQRHKNFINHWETH----------
-FIMLSNENATTRRLVEELDKLEQGLLTND-----NPVGPANMLIPERI--KYQQVKRLE
MNLFQIVCNHALKESRNILQKTETNLDWQDLIKSDEFKKLLACMTEQPFEPTSILNTYVD
YRLDPFLISLYNAFAKNDKLTSQQAAWTSHADAISTLENICKEKSLDPPNGWIYSATIKE
NVELLFDNADQHDEVMLEDLKPISQLPVVYEKL--EHLLDLTWQKTKDKIDQGYIDHNDI
LCTIEHIAETENLLGRQDLAVRELLVQFSFE-------LHLTTNTERIQLSDYKTQWGNA
KVF---LNEYDKILEQDTKTTTKQNIVDRLLNSVCKV-MKNKVNKIYSLLNGTIGTYLNN
DDFILRLKDYLSNFKSPDIKLEES---------ITINQWLLIHDTCSEAVNFVLKSYEFN
KMVRDWIETLSEKYDTMELPCIEVPHGSIEIPHWKNIMYAIDDSLAILNTYISSIYAEEL
KGDINEWITTLSGAKIEIERWKNTETQLQYLYNLFRSST-VRKKLANEAQLLNCILKEYN
MITV------SLTYVNDLSRCEEKLSEIANSIKDLEDRLGIYLDEQRFICPRYFFLRDDE
LFHIIGMVN-IDEMKSNISKMFPGIFALECN---DGSITGIKSKDGDSLPLDENIIYETV
EPYK-VLMDMHTSIKNSIRSQILRCHEEFTP---IYCNEKMNPDAFWGCFSRYVSQALVV
SLSVSWTRCMESTKSS------------ATNLHILLNKMIDILSKP--PQTYISQRKKME
KISIVLIYQLQKSKLL---PLQNLESWDWQRCIRYYINRKN-----DVELHIGHKVHIYG
YEFMGVGPPMIITPLTETCLISISEAMDNCLIPNPQGPAGTGKTESIKVLAELCGHPFWI
FNCSEGFDSISMERIFAGLCQMGAWGIFDEFNRLIDGVLSSIAEKIQQMIKCKKGNIGDI
TLVNRKILLDKNVGIFITINPGYISRRQFPLNLRKLCRPIIMENVDLKQIIHAMLMLNGI
SDSSLVSKTLWDILH-CCRICFGELIYDFGLRCSKSILHISMNLHRDNNKNP--------
----------YSVYSIIDYLKTALSTVILPRLLSNEKCVLNTVVVGCLPDLIHQTSDDKL
FDSQYETLSEVPNL------KEKCSTLFSLMKLTKGIILYGPSGSGKTLCLSATVNIMRQ
INGGNYDVIRFDPNAIDPNELYGNDN--NGSWQEGLFSYTLR-------QYSCSPRNLII
IFDGDIYSSWVENMNSLLDDNLVLTLTNGHRIPLTPNVTILFETHSLQHVTLATTSRCSL
IRFEIKESYLCIPEYKSYDFL---------------------------------------
-------------------------------------------DTDIRLHRYHLFKAYLS
SHDYVNFIFSFALSYGSYM-----------------------------------------
--------------------GKLEWQSFLDKID---------------------------
---------------DISKPNEQRIVYTAAAGQIVGESNNSELHIIVKTLLLHGNSAVFT
STQNSDLSVIIGNIVSSIDGWFLVTMYLSKNAGRKAIMKVLQKHTVITLIGSNLTMLPID
GASRMVLLIEDIDCLTTEPTDLQ-CWSLIRELVEFKALFTKNETDN-WTKVHLKDISIFL
ATKQPSP---NLIPQRLRKSLPVINIEL--------------------------------
--LLDCRNYTQLTITVD----------DTESIEF----------DASQRDG---------
--FRDKLIY---------------------------------------------------
-------------LNIDLSTIEHFSESFQTLRQFLQQKCNRLIIMGCDMHLRKILCEATS
SFNGYKITCL------DHTSWNSTMVNAFQNAVFKCEKICIYIDWDLLVSPEVACEMKHM
IITGDYSSILSTDVTDICENKAHLT-------DVESSGQDIQRSVFGHIKYIIS------
--TKTQNVDASLISKSMLLTLPHLSSNFKASVQNALMP----------------------
---ADFKF-----DMRKIYDI---YVGKFRQKS------SLSDFLVYIISTKDNIIDNKT
AHMTEYKHFETGIKRINNAKSEIASMQTILDSQRTKLVEKNEEAKIKVDQITKLKNEAKI
KQEKANEMKISLEKEKGVLIDRNKEIQHQLEAVAPLIEESQKEIESINRKSLDELRSMSN
PPSIIKDTMEMVVLLLTNSTSSNIAWDICRKVIKSADFITKIVQFNTQALNPVTVSIVKE
RLKNPSWDKDRISKASKAAGPLARWVESILRYGEIALNVAPLLKEVELLKESNAKNEELL
NAQSELIMNLENDIDQYQVEYSDLVQSIADVKTEIENASLRLVRSEKIMSNLSTEVGHWN
NSIATLERNNDCIIGNAILVSSLLNLCGMMKSHDRKKFYKMVTDVLKQEEINYSVDPSYL
D---------------------DCTSRMISNMKYRHCIILDATDMMYIKYID----GDYK
KVSACDEHFLSILYASKKCNLTLVITDIYAHTNIKHVILDEVLNK--GKGLKILFEISTD
DIRRCEQLKTEDDHYIQNIFDLCYK---LRLKMTEEHFNSFCLDILMKEINPTLYNAHTD
VIITQHEIKGKLHGKEDELLGILSTTE-DILADN-LSDYLDEYRNERDKLHDSLSECEKV
VDRFNKFIMEHSDFTSLISSLYNTIKKLESINSMYAFDVYMLLHAMKNCHSRPLRNQV--
--------------LIKVVYSWVCQSIFTDDLLNWTVDIIYYNQVDRC------------
--------------------------------------------ADINNLLEEISQPQSE
SSKLYQKIE---------------------------------------------------
------------HMRQIYLQYDDSLLFGIQTDIFDYY--------NLIIVVTKSFDDPTE
FLYSYATSKGHELKTLAMSTLADMSCIISNIEVLLSEGYWVVLKNAHLMPSWFERFECQF
PNKS--DSPKLFVTWDHTMSINRNIMLRRYRLIYQGADSFQSTFHRLYQLYSHLFIDCD-
-LIKSHLMMKAVLVHAIIICRQSYIPYGWTQMYLFDLNDLSLALNATTRFLENELNSYNG
SILEFQQSSLLLKDWKTRIYE--IYQPKISHDLDCKILYDIICFTDIGKYIIPLPSL---
-------------------NILEWIEMIPNDTSSSLIGLPDFTETLMLESKFRKL-----
-------------------------RNFRMSSAAHITQ-DTTCSNIASSLNIFDGQMPLL
DAALQRQWRKLPMKHDERASEYNKLKTMFEQQVPVTVNF--DVFAEPLEILDILRIQASI
SSGFDLDNLILTAGLFPVVSNLD-----------ITNIAKRTCNCKPIN-----------
------------------GKLLIDHLELMGASYDCKTHRICIPTETSVESREPTQVLV--
```

```
---------------------------EWIRHTCNIKQ-MDH------------------
------------------------------------------------------------
----------------------------------------------RESAEGDAEARGAN
RPTLFNLLEVLASDAYTTPQQRHSIRGVVTEFLNNKFEHSKVYSYIGAIVGHDVLYSVIK
QLESDPDRVPLPDQNGLMRIESAFNLSRMYNAAHNDTNMGMT------------------
-------QNGPVPAVQTMHPPAPPAHFAGG----------------------SKEDILRT
IRRNLGTRKGIGVPAASAGLLLQKACWMPIRRSISAGALYGHSLICIGSRAYLIGGSNGN
PHGVNFAKAHVVSLVNFSSKQMNFSGEVPPPREGNSANVAVIRQTHSIIIFGGFNGERFF
NDIHILDLEKRKWNRRHATGRVPLPRDEHCAVVYPARCEASQKP-KGGAEFLFIFGGKTG
VRSQLESLNDMWAYHIESATWAQVEYAEGKQPLPRFGLCALWADDDTLCVFGGETNGPQV
DRSERTLLDDLWMFKFSSPT---------TGTWYQEIYEGNIGPRSHYSSIFIAQRCKEL
HTGVPRTIERLMLLTGGLTYAPGSRRTVVASDKLYFYFFSQRRWYILKPN-YPRNYLFEP
RQLHVACFFEKRNILFPGSKPSVPCIFFHGGFHRKQVLNDAWVLSLTGEDLFLISPTDSN
NLKTKHNVFPPWFYRESHSPDLFWALCTMQRWAFGAISQLVSNCLKESTSSTKISIKWEA
SPQGDPMLCIQDDGNGLDYTSMNKMLKLFGQSKLG-------------------------
---------------------------------ERGPA----------------------
------------------------------YEYGVGFKMAFARTAFGCAVMSRTIDSIGI
GMLSMELMSQCESREMSVPLCMWRLPSKELINKEGSRMVDQRHHQRLLMTYSPFNSATLL
AEQINKLGTAPGTRIMFWQLRDDMDNIWYSRKDHTLFLKNSTF-----------------
------------------------------------------------------------
----------------------DELDTVDDLVAPAERPE---------------------
---------------------------LNAFPLWKSSTYSMDYCLPVYLYWLHLRTSCAI
TVQDYDLVPY---------------DMKVE------------------------QQKT--
------------------------ENETPDITMVDAT---------------NPETIEQ-
--QE--------------------NVKAEE------------ETKETTTPSITTQG----
---------------------------DVYNNRTGCSYCSVAPVDRGLMEDR--------
------------------------------------------------------------
-SLWSFLNRRLYCKVQLPFLFNPADHKHGCYAMIGFLNRPSNTEVTDERVCETGILLYFK
ERLITRLEGPFPAPHTEIESAHQPPKDSLFKGELYRFALTAVVNVPDWLIPSASKQEFVQ
ENNTAYVEFKAKLMALLSEYSKVCYNDAARKEWEAQKMHQLVEH-DRLQEELRIERTTSD
SHTIPTWR----NDAERDVAAGEMSDSSATHGQGGSPPVE--------------------
------------------------------------------------------------
------------------------------------MADIEGRGVASDGDGLDAAKKKYL
NRLLVEEAINDDNSVVSLHPNRIEELGLFRGDTVMLKGKKRHTTVCIVLADKDLDEGKVR
MNKIVRKNLRVMLGDFVRIAPCSDVPYGKKIQVLPLDDTVEGLSRDALFDVYLKPYFLES
YRPVKKGDLFLVRGAFKAVEFKVVEVDPGEYCIVAPDTVIYHEGDPIKRDEEEKLDDVGY
DDIGGCRRQMAQIREMIELPLRHPGLFKTLGVKPPRGVLLYGPPGSGKTLIARAVANETG
AYFFLINGPEVMSKMAGEAESNLRRAFAEAEKNAPAIIFIDEVDSIAPKREKTNGEVERR
VVSQLLTLMDGLKGRGQVVVIAATNRQNSIDPALRRFGRFDKEIDIGVPDDTGRLEILKI
HTRNMKLAPEVKLEELAANSHGFVGADLAQLCTEAALGCIREKMGAIDLEEDTIDTAILD
SMAVTQEHFNAAIATCNPSSLRETVVEIPNVKWDDIGGLESVKNSLREMILYPIEHPEKF
EKFGMSPSRGVLFYGPPGCGKTLLAKAVASECSANFISIKGPELLTMWFGESEANVREVF
DKARTSAPCVLFFDELDSIGAARSGGAGEGTVAGDRVMNQLLTEIDGVSAKKNIFFIGAT
NRPNLLDEALLRPGRLDQLIYIPLPDLPARVSILNALLRKSPVADNVPISYLAQKTAGFS
GADLAEMCQIAARSAIRDAIAYEEKHGKTPTEGT----------------PDFTYEIQRK
HFQEGLANARHSVTSTDLAKFDNFRNKFDPLYKTRGAGG-DEIDIDWPDDNETNNHMDVV
DDDDLYAMDDAPMFMWTPVEYDASLQA----EGLLGLEVL--------------------
-----------------------------------------QDDSVEPVPKKQRKSKKAS
--GTAQESPSLPEVSD---------------------------DQVNEATRGWNNILFDQ
WAI-------------PIGIRRNLYGANMTSPTVIQRLVFKPSLTAGMHIVACAETGSGK
TLAFSLPIALSLKCSD----------EKPVSALAILPTRELALQVKDTMSMLIKGTQHSV
FAIIGGMSIQKQERVLKHQPEVIVATPGRLWDIMQ------DVSITFKLQYLVLDEADRL
VSEQSFKELGNIVDKVRE------RDTQCFIYSATIL------EKDLQALFKLLKLSNPM
VCTASHKVLPYDQFIKKRPFKKTVEEDTSNTTLPPNLRFRIIKCLDDERELKLIAYMMEH
H-------VNNDSAHIIIFVNSISYAYRLEPLLSLIFWRDKHELRIAKKHCMTLNKEPKF
NITSIHSRLKQKQRLKRLEQYVTKNKKAVLICTDVAARGVDIPNITEVIHFQVPRNASTF
VHRSGRTARCEAAGTAVCICESSEVDTWNDLFKAINKNMATEEAMACIPP---KQYQRYK
RLLALASDIEEQEHKIKKEKTKAWLQMAAKQADIELSDEESDTDAMKARTYRSLKAGKRT
LMEANSNPKCHGHMMKYETTVSTEVCGLSFEFLSSDAIRALSASEIGNPSHHGQECAGP-
---SCIHDSALGPSDLCNICSTCQDV-AACDGHLGHVNFPVPIYHPLLMPRLVKLLKSTC
LYCRKLKLTRQRIVKLIKLFDLLHVGLLSDMLLFDEWCPNRSKQCSLEEKDELPKFRSD-
------ILVSEGSQNDLV-----EVISAKE--DYLDSVKLENRKRTANAPITTSLSDNDL
NITTTGTPNAKLDAMNKNMSKAKKPKSVVAPSSHFDLTFWNNLRNRFLAEAGAVNQCPHC
ERKDLFNIAAASDLSSIDISWPLDADAPINTF-----------------DFNFNDEHVGN
ND-----K-------NVSSKE----------------------LQSLIPGVINRLS-ATG
RSIRQVQLQAFQVVPYLKELFMS-NEVILGHVFPQTRKMGWKMFVMFCMGVPANRFRPVL
LGLHARTSMLLDIVVARETLCVLLKVGDNSNLKKCMRDPN--------------------
-YMESLFGKVEDRQIQAFRDYIVACSDFNSAVLNTIQNLQRKVSLYTDSSKTGSTINSKL
QRPGIKQSLEHKEGAVRHNMLGKRVNYAARTVIAPDCFLDSNQMGIPLMFATELTVPENV
TSYNVNMLRKLLINGPRIYPGANFYRDEYGKLYNLGTLSINERKAKAKLLLTDISDGRQP
RVVYRHVLDGDVVLMNRQPTLHKPGIMAHFVRVLTNQKIFRLNYVNCNTYNADFDGDEMN
LHLPQDPLAQSEAQIIANADCQFTVPKDGQPIRGLIQDHCLGGAYLTSKDTFLTCEQYFN
LVYVALQEFFKWHKTIYIRSESGLRK----------MDNGIQVDVQLHQ------LAKRL
KLLSDPYARRCAHGNTEMHVEEPAILFPQRLWTGKQVITTLLKTIVDGIAKGLH--MTDV
NFLRTYKGINLYSKSKTPGDAWANDGCKESIIIIRNSELLQGVLDKSQFGATPYGLTHLV
YELLGPRASGSLLNAFSYLFTSFLQMHGATCSPQDFILTSEAERHRERILRRIKHCGIHL
QEVFISALS-----NTRSDTPML---ENGRGRSPEVILSDSLSVFKDILIELKTFPR---
------------------------------------------------------------
------------------------------LKSLLQGIDIKAISDV-----RDMLKIVSQ
YLS--KNDATPKI--------QSKLADIVSRAIEALC------SVPELRGYLLGQLGNLN
AESSMNMP-------KNDGILVK---YFPSWQ---KEYD---------------------
------ADMKMSISK-----VDRSYILGDSIGTCS-------------------------
-------SMYRLINEKFKGNEKIFYKMFDRFFQGNITGASNDASTVV--DNTLLKFPQNG
FASMVLTGAKGSKVNFSMICSILAQQSLEGRRVPVMPSVRTLPSFAFGDLGARAGGFITD
RFLTGLRPQEYFFHCMSGREGLVDTCVKTAKSGYLQRCILKAMEDVICCYDATVRSSDGT
IIQFSYGEDGIDVQKSAYLKRPNDVIEN---------------------AALISLGQ---
-----------------------SDLPLKLEEGFVNLLGNKFTTDDVKA--------HYK
RSQCEPGEAVGCIAGQSIGEPATQMTLNTFHLAGATNVTLGIPRLVELMQTTGSASTPYF
SAPLLG---------DSDDNIAHNAQLAINALRNVPLTDVIHSVAVEDSTY---------
----------------------------------MDAKGDKYCEYEAIVQFEDLVAFTNV
VPSISIFDILKITAQCLLNGFLKKITGLMIVTMDSHVPYEITEYSDYLQECWRRLVLQ--
-------ETIQRKSKLADRIRRMAL---GGAQGAILKISAKNLADGLTD-----------
---------VTSTIGPRETPSEDNDVNEDANDSKETQVNDDA------------------
-----DDDPD----KSSDHEDSDPESEVDEDGTTTVDSCVD----------SD-------
--DDNSHNPD----SDAEETITSDSPKPMTAAAIA-------------------------
-------------------------LKLKKGTVNLDRKAKTFLEFENEVKMGKQAMSSIN
TKVFHFAKSLRYCETTGRMVLKFGWPFTKCPYHLNLLQLLRQETSSQILRNSPGVKQPRV
VC--HTDNNGEVYKLHCDGTNLQRLFLLRDGLVDFNRLHVNDIATVYRYYGIEAARASIV
SELQNVFSVYGINVDYRHLSLIADFMTNKGDIRTFNRYGMARHASPLLQMSFESTLKFIM
DACERGGYDDLGSPAGAIVAGRPIRLGGGLCKVLPQIDLTLARNRLLKELKESSRTDDPN
IRLEPVNSNIHHWHAYIRGLKNSPYEKGIFKLNILCPPNYPINPPAVKFITKCFHPNINF
ETGELCMDILKSNWSPAWTLQYLCKGITYILDDPNADSPLNCDAGNLIRSGDLIGYRSMA
EMYTVDCALDKFMNED-TLRKETVALMEALLNGGIKVKSAAEVGKRAVEYTRGDEISKWI
EANKETVFKLCPNILRDLDITHDDNVLDICNVLIEAGFIYRAQYQPIEGTIEKTSSGSFR
RPMWPKRLIKTAKQEFNPVGFYIISYEGNQRWNYLMLTAMIVGIFSICMFQAWPLALKLA
VWYASVVLLSILFTLIILRLILFLLFWFFGYDFWLFPNLFDEDLGVIDSFKPLHSICYRN
```

```
DATYMLCCRVLCSIVLAASINELRKTHDLKDVGDFAKQSFMDIIEWGHNKLTAVPEEPSL
YKSIGMDLETEFNERTEEAGEDNLDDDDYKCLLACGYKSLHQLMKECMLSCECMRELLEK
PCL-AGCPEETIRVLTESKTDICRKTRKGMASLVDSFLDDLEELEREEEESERAEN----
NRTQISELCESDDDAPIIDAVEEYFSSVSNP-HFVFSKKVNDPKLNGLLEKVRQLALE--
-----------------------------------KDWSHSELTLIEECNQAVQEIDNEI
INIYNYVRDIYSKRFPKLESIVYSPLDYIAVVRRAQNEMDFTKVTLSDILPNTMVMAITV
AATTSSGSYLSSHVLKEVLAACNEGMILADFRNDILVYLETRMALLAPNVSAIIGTALAA
RLITQAGGLTTLAKMPSQNIMLVGGNRKGTVV---------PGVIYSCDIIQNAPSAVKH
RAVKLVSGKLSLAAKIDMFKEATDGSMGAEYRNMIEQALQKAQEPPPAPLKKSLPVPEER
KSTKRGGKRLRKAKERLAVSEFRKYANRLKFGEEAEEEYGLESGDGFGMLGKHTGKLRLQ
HKQQKLAAKKRQIAIQSSGATNGMSSSLVFTPLQGIELCNPEAAKPAPKK-KNAILDNSG
GFFKVMFNRKENAATATLKTLPNFEIDRLRHLLTLPEDVGIDTASVKQQLLDHIVQNDMY
PYLERMREQLPIFRDVVSMDNLKENNERVISELDEKIDFAEKNYGTSEVKDAILEKANYY
LKIGDMDNAVFQYEVALKQTVGVNSKLEIVLCLLRIAFFFHDIPLLMKYMEKAKTDIDNR
GDWEMRNRLHVYEALELIICRKFKPAAEILMNSLSTFTATELITLEDVVMYTVVLSLVTM
DRPTLRTKVLESPEVSQVATEGSLLHQLLYDFYHCNYKNYMFNLARTYDLILRDKYMSRH
CKYILRQARLPAYRQFLRPYKSVTIENMSHAFQLPPDFIEDELVSYISGMRLDCKIDLVN
GIIENNIMDERNTNYIEIVKEGDMLLNRIQKLSRIVDMMLSDRWKQLKKNLNTSVNGKTG
NKKPISKEDVEFLQKAVHDHDKKVKSAAQLIHDMAEKIDNVDE--LLDAFDTMEQFYEEH
PGNASSVHRTGMLDAIAQHIKQGNTKILPAALSLLITTISNNEKVQEEATKGPLMQNLLD
LREKVDNTNLEPKLITAIAAVTRHCTTAEKHFVKVGGMRYIAQCTAKHNLKVKEKAALLI
YHFVNLQKLDKREANNVQLLTTVRNLMP-LDVKNHGIQYAEVCVNLFAAIVSKYPNSINK
ND-ALTLWNQLAKAIENT----EAAK-DTLKEVRQAI--NKLRSG----------TTVND
ATSIDKDKWTLENVNDMQFKLIKKQAKKRKVENDAKECDENKEVLLVHDEKTVGMSFKDL
GVPEWLIQLASAVSIKAPTKIQELCLPAAFSGHNVIGCAQTGTGKTICFCWPILVALAKN
PYGVFGLVLTGSRELAFQIGDQFNVFGVQMNIRICVCVGGDDFVEQSLQLEARPHVVIAT
PGRLAYQVDIKENIANVFKKVKYLVFDEADKLLHSEFEEPLQQILKCLPTT-NDGRITYL
FSATITKAIQELSKSFKGTQFHMFDVTKGQEEQLDLKQNYLFLPENVHLPYLVHLLQTQL
LERQGIIFTATKKKCQLTAVALEYL-DFKVTCIHSLMKQRKRNACLAKFRTGVSKILVAT
DLIARGIDIQAVSFVVNLDFPRTTEDYIHRVGRTARSKSTGIAISFVDEFDIEKLKTVEK
AANIKLEKLEVDDAQAVKLLNKVSMATQRAQIYLQENN------------YFQNKAIRKR
WVGGNWKCNGSFELVSTLAKAFDAFKFDGNAMDVVLFPSNLYITKALECFDKQFKIGVQN
FSQAKCGAFTGEIAIPMLNELGLEWTLIGHSERRTLFGETDAIVAQKVNTAQQGKLRAAV
CIGENLTERESGRVEAVLTTQLDAFMGMVTDWDIVVIAYEPVWAIGTGKVATCEEVREAH
QMIRDYMTSKLG-SVAETIRIVYGGSVNEQNCQELLHVPNMDGFLVGGKSITPGFADIME
AAYMKGQGKGHGGSGASMSESKLRVAIVSTDKCKPKKCRQECKRNCPVAKAGKQCIIADP
TSKIAFISETLCIGCGICVKKCPFEAITIINLPRDLGKDTTHRFGPNTFKLHRLPVPRPG
QILGLVGTNGIGKSTALKILSGKLKPNLGRFDNEPDWPEIIQYFRGSELQSYFTRILENS
MKTAVKPQYVDNIPRQVSGRVGEILEAKDKKGRAEDLIITLELAHLLNRQVSELSGGELQ
RFAICVAVL-CDSDVTMFDEPSSYLDIRQRIIAARVIRETVEHDKYVIVVEHDLSVLDYM
SDYICCLWGRPSVYGVVTSPFSVREGINIFLDGFVPTENLRFREESLCFRMSTDVDM--E
EIERLHNYKYPEMKKTLGTFSLTVSAGDFCDSEILVMLGENGTGKTTFIKMLAGILAADN
AEAGEAMPKLSISYKPQIITAKFDGTLRQLLMMKIKEAFGSPMFQTDVIKPLQIEDLYDQ
QLKNLSGGELQRVALILVLGKPADIYLIDEPSAYLDSEQRIMASRVIKRFILHQKKTAFI
VEHDFIMATYLADRVIVFEGKPGVHATALAPEPLVVGFNRFLKSLDVTFRRDQANFRPRI
NKYDSVKDKEQKASGDYFSVETMSLICSISGVQPEEPCISKTGYVFERKLIEKHLQESQT
CPATGKPLTVDDLIPIQCDKTVIPRPATAMSIPGLLSLMQSEWDALALETYNLRKHTNTV
RKQLCQSLYEHDAATRVIARLIKERDAALQQVESLEKLLLEFRTNYNAGAIEVGLDDSSV
SRIEDLAKALMAERKKRDVARYSTTESIAKYTLKGDYRAHSSTSPGILSVTLDYNA----
---ARGGHNLFTGGADGAVVYFDLDAGRTVTRMTSHLKPVNTVVSHPYANVVISGSDDKT
VRVWKGPEDASDFKCSHVLKSSKAPVVSMSLHPSNEYFLAGASDGLWHLVDLESGQIIKI
CRDIPSPCSKVQFHPDGLLAAGSGTDGAVHIWDIRTQSLASTLKHDTSPLSSLSFSENGY
HLATVSQEGHLRLWDLRKSVVFANADCNMSPTAVSFDGSGSTIAVA-STKVELYRLFDKT
QVELMGTLEGHSGYLTDLEFGPDSKFVLTTAKDKSLRLFSTDLRANSYTGQVKDGLFHGV
GIFYYGDNERYEGNFVYGKREGKGKFFYTDGAVYDGDWVDDKIKGHGVAHFASGNVYEGH
WDNGRINGYGTLKYVNGDVYEGEWMDGAMHGQGTYKYAEGDIYVGEWRNDKRHGKGILNY
MSPKGEVLESYDGDWVDNAMSGKGKYQYSDGAVYEGDWYNGKMHGSGQYVFPNGNKYDGE
WVNDHKEGYGTLTYATGEKYDGYWVNDKAHGHGSFIYPSNDKYIGEWQNSKKHGTGELIY
VNGDRFKGTWVDDDATGFGVFEYANGNRYEGEWLMNKRHGRATFYCQEDGSTYNGEYANN
RKEGFGTLKLGLGHVIHGLWTLGSLATIEKFEISPSSPWSDPDLTQTIELSDVVKIERVG
IHSHIRGLGVDDNLNVEYQADGLVGQVQARRAAALVVKMMKTGCITGRGILLAGQPGSGK
TALAIAISKALGPDTPFTHLNASEVYSMELSKTECLLQAFRRSVGIRVEEEAEIIEGEVT
EIEIDKFANRQFNSAPTRVGKMTIKTTDMETLYDVGHKLIEALRKESVTAGDVIRIDKST
GSVRKLGRVYSRARDYDAVGPHIKYVQCPSGELQKRQKVVHTVTLHDVDVVNSRSEGFLA
LFAGDTGEIDNNIRKQIDEKVREWQADNRAELLPGVLFIDEAHMLDVECFSFLCRHLETE
MCPFLILATNRGITNVRGTFYKSPHGIPLDLLDRLLIIPTYPFQPEDTEKIIQERCNEED
VELDEESLHLLCKVASETSLRYALQLINAADLIRRRRGTKVVTSLDIRRAFGLFLDTRRS
TKYLVEFQHDFMFSELEPEDEQPSDNMQLEDTKESSSTMDIDKDKQ-MARLYDNISDFWT
SDEEGTEESHRVTSGLGHPM--------------------RDVDNHRNADHPLDF-----
-------------SLAFRNPLDSVGWIPTIENTQEKYSVFDILDREDYDKRFDIDIDVLE
RQLFGESMHYDEE----------------------------------------------E
FHLNVSEIPDCLDTSYDVDTSIGERDATTKIRHLENVDESLSNDMVVPKPEEGKQYIRTK
WSVVD---DSSTPELEDLVIEYPFELDDFQKRAIYHLHKMKHVFVAAHTSSGKTVVAEYA
IALALSRGKKAVYTSPIKALSNQKFREFTKRYETVGIITGDVSCNPNAPCLIVTTEILRN
LLYRGDPIIGQLGVVIFDEVHYINDFQRGVVWEEVFIMLPKSIQLVMLSATVPNYAEFAD
WIGAIMEREVITIVTTRRPVPLVHFMYIYNRIFLLLDNKGVFNKDAYHNMYKI-------
------------------------------------------------------------
SSQNKGSSKRTTFKGQVQKLQRLIRHLEMTQKLPVVLFCFSRAKCESYAREMPNLNLNSN
HQRSKIHIFLKESLSSISEDDRDLMQVKSIIKLLYRGIGVHHSGLLPLMKEIVEILFSRG
LIKVLFATETFAMGVNMPARSVIFTSIHKHDGQKTRHLTASEYTQMAGRAGRRGLDSFGS
VYIFCPDDPPDLQDLTTMMFEKSTKLESKFRITYNMLLQVHSREHMNITEMMLKSFKETY
KMKNIPIFKRDNIRKRQELSTIPKVDCIYGE-----PSIEEYHKLDGCSRTIADNLNESL
WNHRENAQFKAGRVIMVHSLAVCNTSCYGSIVVSPKGKS---------------------
----STLKVLLLLPDLYDEGKAYDKV----------------------------------
----------------------HTTISSNHDQIHFSVCESVRISNISFIYNNVINMKTMQ
GVTEDLSITLLSSVASELHKLLLTSKIELLAFNKQLKQTSIQFYDVMLKQRDIYHEISLN
PCNKCHLREKHYSIQERVESCRNELERITSLLKEESLSSYDEMVAKVEVLKQLDFLDENG
KPTVKGRIATYLTTGDEITLTETITQNVLNDLEPEECAAILSAFVHNDRPEKEVPSPTAA
IQKARDMVLDLHSKVDVVQRALNVVVSREDHSALCNFSLSYVIYQWAIGTPFSEIMQYTD
LQEGHIVRAITRLDELCRKIGQVANINGDQALQSKIEKVSNSIKRGIVFMPSLYLS----
------------------------------------------------------------
-----------------------------------MADNLTPTHKLMKALVTHNLDALII
DHDDPHATEIPHEAFGGLEFVSKFTGSWGQALVSTEGAWLWTDSRYYIQAARELQQPWEL
MPYGM----------IKTKGYKKIGIDAHTTPQKVLEHYESVADTAF-------------
------------FVELYKNPIYESRPTLPVDHIFIHPEKYTGMSTIAKLTEIRGAL-KKE
KADAVVFSVLDEIAYVLNLRGSDCDTSPLFYSYLVV------GEID-AVLFIDERKVPDS
VRDELASWGVQIMPYEELFLFLRHLPQKMTKK----------------NDVKTYTLWASH
SASVAICDSFMSKRLIQKPTPACWMKAIKNKVELEGMTEAHIQDAIALAEFFAKVENMKQ
DGTLFTADELILGSMSSQCRADMPDNRGISFHPISSIGSNCAVVHYRATEEIKAKIEPKI
YLLDSGGQYPGGTTDVTRTIHFGTPSDEEKEAYTQVLKGHLALGHAIFPEHTSGATLDIL
ARQYLWASGRNYYHGTGHGVGSYLNVHEGPMSISPRMGDYLEPGMVLSNEPGFYKEGHYG
IRIENMIYVKPVESKDKTEFLTFETLTLVPYCKELMNIAMLSQQEIDWINQYHARIADIL
LPRMEASPTKY-ADAIKYIKAAAEPISV-M------------------------------
-------------------GTSVASADDRVVKASFQLQKNPEFIKSRLAIFEELYNRQQK
RID-ECEKVDIIINLEIPDKHVETGKSWLTCPADLLKHLSKSESQGIIVAEIRPSTKLVE
HVADIEGDGE-------YTDEWLMWDLYRPLECDCTVRFHTFESPKGQHVFWHSSAHLLG
```

SALETLYGAYITIGPALSQGFYYDCYLGNNTFKPDDMALITEQVSTVTKTKSPFQRLVCS
KEEALELMKHNPFKVELIRKKVPDGSSTVCYRCGDFVDLCRGPHLPFTTSVKAMTVTKFS
SCYWLSKNTADSLQRVYGISFPKEAQLKAYERRIEDAKANDHRLLGTTLNLFHFDNIHSP
GSCFWLPNGAKIYNRLVDFMRENYRLRGYQEVITPNIYSCELWKTSGHYDNYKENMYMFT
VDEAEWGMKGMNCPGHCLMFKHMNPSYRQLPLRFADFGVLHRNELTGSLSGLTRVRRFQQ
DDAHIFCTPEQISAEVLSMLKFIDDVYSLFNFEYNFKLSTKPAKALGDDELWNKAEAGLQ
DALEKCGRPWTLNPGDGAFYGPKIDVILLDSLEREHQCGTVQLDFNLPIRFNLGYRDKD-
--SEDNTDCKRGYSRPVIVHRA
IFGSLERFIAIVIEHMKGKLPFWLSPTQVAVLPISDKHLDYANKIYRKLLHLGYNCYVDS
SANTMNKKIKLNQAQRYNYMLILGDREVETETVSVRAMDS-QDNEVLTLEQLYAKLADEA
ESYKRNISCFHLEE---------------------------------MSFVANIRDIL--
YRGDNNSVKPRSRAARLGIRRLRRAAECCGVVAYVGTQECRDILLDDIQSIKHRGYDSCG
IGTMRSDGSIEVTKCSSYAPANCFDRLRERLVDRHLGSTIGIAHTRWATMGPPTDENAHP
HCDPKGRVALVHNGTVTNTVELFNELRENGLEPHPDSDSSAIAYLIGLNLDLGADPFTAM
KTVVSRLEGSWAICLITANNPHSLYVARSGCPLLLMKDDDSVYIASEAVAFMHRTDYFIV
LEDGDVMELNYPVVEKLFTTREVCRITKQIIRSTPEPHQFWIQKEVFDQPLVARVALQH-
--FKLVNASQKVRLLKQELEGLMDVHKINLVAAGSSHNAAKYVASLFQRSAMFDHIETDD
PTELKMYRYTNPDSTFIYVSQSGETLDTVNACNQLAELNPSKIALLNNLNTLLDRSCDLT
MLVNIGREISLASTKTFTVQIMLLMALIGYIVQAQDLEKHHEELRQMKKSIIGYGLALKK
VLC-TEDICQRVAERLCNVDSMYVIGSGEGYAIAQEAALKFKEITYIHAEGIASGTMKHG
SLASIDPKKHTPVICIMTPDE-PEVTVNATKQLKARGAFIIMLASNPAMGEGM-DEFIQI
PECGMLTAACAVVPVQMMAYKIAMLRGWNPDTPRGLAKTVTVVMKE-YDYLFKIIIIGDS
GAGKSSLLLRFADDTYSESYMSTIGVDFKIKTVKIDNVTIKLQIWDTAGQERFRTITSTY
YRGAHGIITVYDVTSRVSFESVKTWLVDIEKYSSANISKLLIGNKVDLEDSRAVTYDEAR
EFAEQNNMDYIEASAKTAQNVEKAFESIARALKDKATRCTTPAAANTFNLSTTRVSTNRD
KCCMPRIRTLNSRPPPEGWDVISDTLDSFDERMKAAERESGEGKRRSEVQWPIFRIHHQR
SRYIYDLFYVQKAISRELYDYCVREGYADPNLISKWRKPGYERLCCLRCIQTANQNFGTT
CVCRVPKRDLEPKIIECVLCGCHGCASCDF------------------------------
-----MRRRTPFVAFLSAAPRVLVTWSYMLLRPGFV------------------SGSYST
SVVNENMSNTNNSAPSSVEPEL-YDLAVIGGGCSGLAAAKEAARLGAKTVLFDYVRPSPR
GTKWGLGGTCVNVGCIPKKLMHYAGILGHA-EHDREMLGWSDASPKHDWSKMIQTIQNYV
KMLNFSYRSGLLTTGVKYINAFATLEKDHQVSYLGPN---GPERIKAKHILIAIGTRPII
PEEVKGAYEYSITSDDLMSLSHPVGKTLIVGGSFVALECAGFLTALGYDVTVAVRSIILR
GFDRQCAEKVGDLMENMGTRFIRGSSPTSVTKADGKLEVTFDNGHVETYDTLMYATGRKL
HGIYKYLSDLGIKFSKSG--LIETDNGMTGYPNVYAVGDVAEGNPALATVAVKDGEMLAR
RLFGNSNKLMDLNYVPMCVFTPIEYGKCGLSEEEAVKKYG--DVDIYLKEFTSLEFSAVH
RHKVEWMQTDEMDVDMPPTCLSKMICKK--DGTIVGIHFVGPNAGEIIQGLCVAVRLGAK
KSDFDDTIGVHPTDAESFMNLTVTKASGESWVQSAGCGGGRCGMKE--FNNVMHMAARNF
NNIEELLDAFFGFLCTQTDFYHTQISDEQMKKYG------IDKTV---NQRGFKPGYIKE
LIMHINDKYLDIYRQRNQPYLLQQEESIMTKPTSVISQSTEQAPRSVKPCVIDEDLPKVP
NVSGTALELLTNKYTLNTWNGGVNNVYCWAQTGTDLTIEIVTKEHLAPKEVNLQLKRNSM
ILYIGNETIIEGEFPYQINADESMWSVEDRTRLIISIEKTEERWWDTVIVGHPKIDATQI
ESVKRFDEFSEPQQHEMVKLMKQHKEKDLPLEFLKN------------------------
-MSCQELQRVENSIVVPY-TDTYASGLQVVECLLSKGFLQRDIDVLKAAGYVTLDSIAQV
ASKTLLEVKGLSEQKVAKIKEIVKELCPPDICTAAEYLECRLNLIKFTTGSTALDALLQG
GIESGSITEIIGDFSTGKTQLCHTLAITSQLPIEQNGGEGKCLWIDTQNSFRPERLGPIA
NRFGLSHAECVANIVYVKVSNTEQQFDMLVEAAHYMAQSRFAMLIVDSATALYRTDYTGR
GELAARQMSLGKYFRALKRLADIYGVAVVVTNQVMARVDNMSFMGGNDKVPVGGHVVAQN
TQTRLFLRKARGNSRVCKVYNSPSLPEGEAVFAIAEGGIVDYDDS-------MKGYGLFS
PFASTTGSILLAPCYDEIGSPHLSSNEKLKLLGFEIKEQKETTGNIFDLHFDSSINYNAF
GHSNSFDGHLSAIVWMEM----------TGAHGMIAIGTTEGDVYLLDGNAIAEGD---A
ARVISKTKVCNTPIKRLSYN-AKTNMLAVASVDGQISVCELTNPSEPKVIDTSYGKWRVG
LVTGLSWNHRLGHILATSSDSSGLVVWDLKARKPASSFRDPSGRTNPIAVEWMAEQMTQL
VVGYGDDRSPALQLWDLRNCSVPLKEVRGHTMGLTSLAICPQDPNILITSGRDHTRCWTL
DATPFHAISSMQTGALSHHKRWHPHVPGLFLAQNTDDDISVHNVMCMTQE-ESYMPSWVR
NTSGVISGFAASVTTWNAAAIKYTLSTNVDE-----------------------------
---------ETSKALDDSLQLFCNLADSKDLESVCEQRISSAENEFERLTWSMMHAIHKG
NAEAIVTTLGFKMPEP---------------------------QLESNIS----------
------------AASQQIASGQ-------------TGFNDPFSSTPLNDDDGEAFFNSLS
SKTNENS-----------------VEFVGQSPS---------TEEDTLTIQGEP----ME
PSSYLNWG---EDDLCSKVVVGDFEGAASLCLERGRTTEALFLAYVGGIDLWLKVAADIT
NKMDNPLLRTLHLIMKDETHMIVEQCPLDRWREALVYIITNSMDVHTYSELCRILGNRLQ
GNREHVLPASILFMCSGDVSRVLECWEHLEKGKNA-LHILGESVIRTTALSLSIPGNTST
DCVGRKAVMLAETFVEFGDLNKAVQCLSLPLISGCPQVISLLGRIKGMGSYV------PP
DQPVAN--
----------------------IHRQIPSSPAQPA-------------------------

PRTTGDYKTAPGAVAGAMYPGMPVPWPLPTATQQKVSRTRSTEDANRKIIAKSAAALPQR
MKPADLEFATKVLGSLI----SQADTTRAAQDNRRRIAELMSSLENGEQSVEANNLILTM
CRAIHSGDHVNANIILSTISTKLWNTANKNWIMCLKRIVPRMAIRTQYENSNEVGVFATL
TNSYALVSLGSSCNFASVFEAELMPQIPVVQTTIGGTRVVGSVTVGNRKGLLVSSICTDT
ELRHLRNSLPDSVEIRRIDDRLSALGNVITCNDYVGLIHVDIDRETEEIVEDVLGIEVFR
ASIAGNVLIGSYCRFQNKGGLVHVKTTTDEMEELSQLLQIPLTSGTVNRGSDVIGGGLIA
NDWVAFCGMSTTATEIATIERIFQLSRPKDPSVSNSHTLRNALIDTLIMFEGLVKRLLDT
YLAPYVDGITQNLQLAVWSGNIVLENLTLKTDITEKLSLPFDVHFGKIGRLKVTIPWASL
GATPVKVLVDSVYLCISGIASNKTDDELLRYVRESKEKLTNMLENEYSAFVNSLNIKTDV
N-SSYLLRLSQKVLNNIQIDLNSIHLHCADQRHAF--GFIIDSISIRKYRPEDGNGITTD
DSTREEAVTHVCSLLGFSIYERPGDGDDEEFSRSSSYHGLNEDVTTDAVADIEDIATQQH
SGLIEAPIEQETVELSAEAKRRIKRAETTLMKFSEGSLATNLEKLSFSLLLAINVKNKSI
FASLRVGTE-------------------------K---------------TAGVERES--
---------LRIILSLESLRSFVELWTCCVIEAERSEKMLMERAHTVDLSPEGIKGTTRE
EFITLYTKFNGDP-------LSIMEDDRLTVLTDVVPARHLARWRFAARKLTDAVAASEQ
SKKQALANNAVATTTWFGWLKGVATSVRNIFKSGVPKEDAVLAVADIPAIEAGDVENES-
----PTS---STPVDKIELE--DNATMEYTDTLDPVSMDNADEFMEYTKFRTIGTQVECL
PGGLVLTGEEIDLIRDVLALGDMFEDSMATNNCALPHFEFIIDVGHTTATADTLCMSTGN
LNLQIYCNAVVDTNDRDTYNAYFSVNLDSFDVRYKDTTIMHF----------IKSVSQDG
DSEAKSIEDDPESGDIDLNMTHKVTNQGNVMMVNGELKPMETHMIPAVMSEIVEILLYIL
PNFTSRPPSGNMGH-----------------------VMEAYKNNGLALN----------
KINLDDADRNTVRMAAEHMPALFSFDIKFAAPILLLYNDVGDCINLRFGTLNLLSNGPCP
LSAMSGTIELKETQLSCHQHGNTHNSWYSFLRPLPVKIHYDCDIKSLNFDILFEELFMQA
TPMDTALLYSVPSEILSCVM----------GARASLRRGRAS------------------
---------------------KPLVDPNATKTTLENIKYNVKVIFCHSGFAVMNAEGCEV
FKLDMHNVSLEIAFTNRNLRVLLGFEKIMMSNPAMDMPLF--------FTHLDP------
--------------------------------APMSRGS---LLDEDDNEFEDALEDTAK
SLQIEIKRTHDAQV----SIAANITDMEGNWQYSSIKLIIDTMEEYKKCCNL---KGAEE
SSDGAPKSDPAEAHTDSAKESLVNSRTITESDIQPMSSEPAAQPDLPQSFTGLPVRSIQT
NISHVTTPRSDCGYSIQ-------DSIRHEGTTFIRGTLPEELEDC-------NIILDEL
PTLLSEQNSNYITKVTVNVKGAAIVFWNAQQVEARLSVSKIAYYLHKFGSKESSSQLELG
TAKLTLGTRCLLSY----------------------TDTKSLEEDGVT--------AMDK
PLMSLKWKCYDSIEPYSMCLTGVLERIVIVYFHQDMTRLLDYLDDGILSVFISKSYHKVV
EAAASTHMFYSFSVASPIFLIPENKAILPQT---WDHAPNHMTKSSVNLDTGDPFRQMAQ
KLSGIVDTWYIGSYLLFELGHLYIRNGYT-------------------------------
----------------------QRDGNDKQSAIYLKMLGTRASIIETHGSPEVMGSVLQS
TDLGVCYIG-GGMLDLGIDSEAWVLKLTRMQLSFIIDVLNENICGSSY------------

```
--------------------KTRTQGVVQ--Q--QKPQFHCFIRMSLKRFEFDTWFAPEK
PLARLRFNAITVCLNYASVRMTSCYQFALCGKTLTVDDTRKDSINAHRRLINCFVQE--S
P--------PDEEEVQCMEP------------SVNGLLQNWLRNYRNRTGHDLF------
-------------SSEDAADQYGVKVVLVNENNQTNVDAMVNNAAISLLCIHAMDLLRYF
TLSYAMSSMATCPKQY--------------------------------------------
---------------QITQPIVSE-KTYLFNFKTNSGKFIAFTQMDSTTSPQLELSTDFI
LEMTMQNGSFNFMKVDVIGCKLERV-------------------------------YPYS
GKRQELCSCLEVFGSGQYVS--------------EQQGYKMF-----FNFTVPPSNLTLY
TKDLSVILAAFTSVLTDGPS---------AAPPREDDGTMVKGNNGDG------------
------------------KFLSVSFNIKGIKVTFFDDMRKCLVPMMRLSISDNIEYMGLP
IEKRYTIVRCSTRLEYFNAVIGDWEPCLERCNCSLEYRQTKPARSSTEDDWKDFVPNKVL
KLSSSHNILINITPSLCQLLLWFIPMLTDNIQ----------------------------
----------RGLVS-----------------MDTDG------EEPDVQI----------
-------ENSAYRYVNLTEYEYRVFTIGSAGNT----------AKGINGLR---------
----------------------------TLQQTNIPKELDS-IVSTVGVD---DTNSSNI
YVVGRPSPEIVGNICDTMGIQSRETVERDLMVTRSITKTMENLNLSKS------------
---------Y---IPSTYKTPFENGTCAAMVPLARNCCVLLNAPVEDI------------
-------------------------------------------------KDTTICEVKTP
HPSHKLLLFTSTVRVYNRSGMPLLLTFLDQTFNVVHVSNLKTRGAPISILDTSSGDDFGE
TTTVQFPDSSLDYLREQQADA-GYTMLLEHNHFASVPECAFQGPAQAVISFLPAPLAMNR
TFMDAFNDRAMKKEQMQSNTWLKLSNTTGWSKIVDSSKHNGTRVRQCYCPGKSSFLYFVV
SVIKKRSAFPANVDMRDVVIYPALSIMNTLPMELDVCLSAND--RVPSDEAAATRKNDRC
IQEVLTLLRESITHIYSLPPNESLTFMAKIC--SNIWSERVTRIYGTSETHTMLAIPIRG
MAPVELELIRYPGGLPVSSTSFQGHLSLILNAPWRFIDRTGLGISFQKRLPRA-VICGLS
FFSSEDEDDALHLCVNGKIS------------------DRMEA-------SVRMPAVGGY
SYALVGSGGKHHSVCLITEKIAIGGLSHVACRITSAIPSFLFTNNLGTDLYIRKDNRMTP
ICV--NSGKSVA-----------------------IPWVSSKAI-A-DPATLPSAVATI-
-EFNMAEDGIWSNPILLSESHSGQTYMSLKHDKNNKPIVFCVSVVPKGGAKYCSIS-LPQ
NLNEGYVLINQCPYIKAAMVRTFHQGGEVGTKGNGV--------------------YFTA
KYGQSVHLGWQQPFLNKTRLCQVMLWLDKNTVAPIKPLVINIGSFRYRQVTTPEHAGNYT
VIVTAENRGDYISITTS--------PSRYLEFESKLGEATSSTRTSISSIRVPS----VA
YSNASDCGDSTPERREKETRNVLDL--------------------------------NTE
LLNSEGLVDTNEAEDEVLNEVVRSLQMQVQLSQIGLSLVSHNLHEELIFLEMSTVSFVCL
WNGENQRLELRISDIQIDNQTDEEQCSTILVNR-----RKTIGNDHQRHVLQVYVDRPFA
SSKDLCLKKVFVSLDDLEVDISDNLLSRVYNFYKECMKCMDFASQKKVDLRLIDTWVQEE
AREKMGSPLPPRMLVLDFLFIERFNLVLWCSFDLEKLHMLGDLMRMGLRIICVSRHFELM
GAPLQFQQEYFCNCRGSIQSFYEQIKDKYLHAALGCIGSLLGYSSLLNIPKIPINVGRNT
IEFAAEAVDSVSSGIGSLLSKFTFDNEYINKRQRDRMAVPSGNMRDGILSAGKSIGEGFM
SLTNIVTKPIEGAQKGGMGGFIRGLGKGLAGSIVKPIDKVGQAVSHVSRTIKVNMSKQLE
GQRWCAEPCRRPRMLWGEYSQLKPYSLSDAEIRQQLGHKFAKNIVHCETVSKRSHPQSHI
ALLFYPSKVYYVDL------KPRPTIIWKVPIADIQECRASCYGVIIRYSDGTLQVPCTN
AGLIYSIFTAMQQAKRQSKSSIVIGPELFESYNMTDASTAGNNADTTSGSTDELYAEING
TFPFDPSLVDVTYPPIRHE-GRKYHISSLDEYKEMHRRSLEDPEGFWGDMAKKELRWIHP
FSRVSEGDFTAADYMWFVGGKINACENCVDRWAEERPNDIAIIAEGDDPTKVTTVTYREL
RHNVCRFANVLIDKGVRKGDVVTLYMPSIPELAYAMLACARIGAIHSVVFGGYSAASIAE
RIRDADSHIIITVDESYRGGKTIKMKSIVDEALLSCAGVTTCLVLRYA------------
------------------------------------------------------------
------------------------------------------------------------
-------GVKVN-MKEGRDFWLDDLLEHVRPYCPIEVMDSEDSLFLLYTSGSTGRPKGVS
HTTGGYLVYAHATTKYIFDAHVGDIFGCVADLGWITGHTYVVYGPLLNGLTTFMFSSLPN
YPDPGRYWRMIEQHRITQFYTAPTAIRSLMRHGDDYPRQYDISSVRVLGSVGEPINPEAW
RWYYEVVGRGRTNVVDTYWQTENGGIVVAPLAGVTPMKPGSATLPFFGIDVALVDSATGK
EILQNDVGGLLVVRRPWPGIFRTLYNSHKRGIVTYFSKVPGSYLTGDAAYRDKDGYIWIN
GRVDDTLNISGHRIGSADIEHALVEVSYVAEAAAVAFPHPIKGNGIFCFVSLKDGFDI--
---------------------------------VHDVLERELKLAVRRIVGPFATPDIII
SSPNLPKTRSGKIMRRILRKLVSQAKDLGDTSTLADQTVLEGLTVLCHDVLERFNHIHM-
----IITSTVL-----FYVAYYTDDANKKENDNETNNNTTEKASNTNIDNADVSKKIVVY
YGSQTGTAERFAKTLAHRLADWNSIQRSSVNLEEFDEHDLRPNTIAIFLIATHDDGHFPD
NAERFVRWLRLMEDESKRLECLEYCIFGLGSTEYPQFNNASKNLNNILINLGAKALLPIK
LGDDATDLKSDFEEWSRDVCNALAKRLVIEPVSETFYEKPLLVSLTWRDC--VPLELRYL
S--KDQIKSDCTPI-------------------SATVVCKQQWQCVDHVVIDNINMTPES
EETTN-------MLTIEYGEFNAAETINVLYANPPKVVSYFMDKLNIKEQDMEKLITFVP
RY--VDTQMN-------------FEAPFPIPCTIGDALRYYLDLTGLPDEETLRNLGTFL
QSNHSCQYLNKLFKRK---ALMKIMKELHLTLQEFIEIFMHDAIFNIGGFLQIIPKKVTK
AYTVSSHPK--TNKIDLTIKKRNFTFKTFKSRIQKEIGYEVRPGTEKMFA----LHRMYK
GACTHYLC-SLQKGDVVKLYKRPSAFSLISDIFDKPLVMIANGSGIAPFRALWQQGN---
---------TTHRRIIFFGFRD-EQHILYKEEI-EGLKNMPNYSVNIALSRTGKH-EYVQ
HILRNHIIQVQDILNSSGLIYVCGSKAMGAQVKAMLQHFL-------KIDIAVLKTQQKY
VEELWKGNVPWVEKYRPASLDDIVFHTNAMTTMRHIVESYDMPHMIFHGPPGTGKTSAAL
AIARQIYGPEGMKERVLELNASDERGINVVRERIKTYTRLNISSNRVNTQTGRVMPNFKM
IILDEADMITPDAQAALRRIIENFSNISRFILICNYVHKIIGPIYSRCSAFHFKPISQDA
QIERLRYICTAESLEYEDHALDFLTQVSQGDMRRSVTILQSTASLFNKVTEEAVRNVSGY
PPKEIVNEIFATCKGTT-QDVEELCKKIIYDGWEVATLFQQISEYVVNSASLTDVQKATI
TIELSGRELALIQGGLQYFQLASLCFHIRSIIASG-------------------------
------------------------------------------------------------
-------------------------------------------------MGAFAENCMEI
IGSH--QTFDVTFQLTNIMAIRTKVDMEIQRKAAQVSCAVMKSQLINQIENILDSEEKKT
HANIVGEAFNVYNDTKFLDKLQRKYNVNPSEIEVAYSNVQSGNTFNLSVGVPPNDMILSH
ASGTIIVSVCSKYSELFACLTRTLLLDGTAKHKEAYTFALSALDFALTK-LKPGVTFGSI
YDDVHNFVASEKPNF--------ADRLLRSVGHIMGIEFTDPNFTLVSGNDKCIVATGMV
FHISLGFVHLDAD-GKEFAIWIGDTVEVTDEGAV-VLTSTVSKSLENISYELEDEEETPM
HTNE----------TKPQKQPSVSSELLRDADSVILKERLRKRDRPGQNQVSESEIKARM
ERQLQLRKQKVEAIARRVKEEGGLAGTAKQRNVVKMDKLRAFSSPNTFPRDLIPNQIYVD
VVNEVIMLPVNGYHLPFSILTVKNASCNSED-NQTYNLRINFQVPGSHTTSKNDVNPLPE
VAENSIFVKEVMYRSSDSKHIQNVFRAIKDLIKQVKQRETDADANRVIAEQEKLMLKKEG
RRIVLKDLMVRPNVHGARRIIGFLEAHHNGLRYVVNTRDRVDHIDITYANIRHAIFQPCE
RELIVLLHFHLKSPIMVGKRKSMDVQFYCEVGTQIDDLDNRRGRSYNDPDETLEEMRDRE
MKRRLNAEFKQFVTQLQEMSNLVFDMPYRELMFSGVPSKSNVEILPTAHCLVNLVEWPPF
VLTLEDVEMVSLERVQHGLRNFDMVLVNKDYSKAVRRIDLIPVEYLDVLKSWLNELDMVW
YEGKNNLQWTNILKTILDDVDAFVENGGFDGFLGESEGEEES---LDDEDEEYEHDSEEE
VEEDSDEEYGDDDESLADEDEEEEEYEDEDEEEGLSWDEMEAFARKEDDKHRYEDDNSNQ
RSKKRRR-----------------------------------------------------
--------------------------------MLLPLSIVMLGNAAKDAYEDYRRYLNDN
EVNYSIANVVMNPKLTKHDETI-----KYEKPANADADIV--------------------
-----------------NIVNAKWQALEVGEIVVLQNGDTIPADMVLIGSSEINGVAFVE
TSCLDGESNLKKKEAVLKLAEYLTRDINTTLNKVQNITGRLTCEPPNQNLITFDGSLHYK
QD----------------PEYTNQSKSKTKAEV-APSAKRSTGDKLDQFGY----TETSI
SMLQLLLRGCKLRNTKWAIGIIVYSGHETKIYKNIPSTPHKVSNLSKIMVRLTFIVWVVQ
IVFCSIAAIWTIYRHYYQMDQQMPYLT-SKGDDHSMLYVFTVSFFSWIAISATFVPISTI
VSMNVARIAQAFFINADADMYY--------------------------------------
---------------------------------------------DELNMYAAARTTSLN
EDLGQVHYLFSDKTGTLTCNKMVFRKFAAAGRSYGKGYTDIRRFVLSRQGAILEPEPENP
YYTKASHVNLVDDELFKQLKTPSDPRHTHLVEFFMHLVCNNAVLTDIQESGDVQYNSQSP
DELCFVHAAKFADFKLMDKTSNSITLSVFGRKMHVRTLANIEFDYIRRCSSAIIAFPKDP
THLEDPD-----------------------------------------------------
-----------------LNKFRIILFCKGGDNVMLNKLKNKSEMDKDTISHCEQYCRDGL
```

```
RTLVFAKRELTTQEFNDWNQKYIEAQGNILNREESVARCATLIEQQLELQGITGIEDKLQ
DGVGEAIELLGYAGINVWMLTGDNLETAINIGIATNLLRNFSQRIDLHSGVCPKEQMGA-
KVREWLNKVKSGSD-----------------------------------------NAIHR
CVVIDGIATNELTKEHIVDDFIELCTHCHSAICCRMTPAHKGLFVSLFKKKLGTVMMAIG
DGGNDCNMIQTADVGIGIKGKEGLQAYNVSDYGIGQFRFLVPLILDHGRNCYRRIAKTVA
YMFYKNITLIMPIFFYGYLSLFSGQRILLEVLVALYNVLFTGISVILVGSIDRDIDRSLS
YQYPHVYQLGQRNYYLNPKVFLGWLFNSFIHAVVIFFVVTFGLSRYTLPGGSGMPLNSQQ
LGVGMMLIVMVIVSSKLIMETWFYTRLTTATYLFSFFNFLLCIYVVSLSSKLGSALLGGA
MILMSNGRFWIVLLTSTMAALYRDYLTKVIHYSFMPHYYQYVQRKEYLATELPSFTE---
--------------------------------------------------------MQYI
NVISDIRGIRRLCRRFYSPEVKDHFYKPRNVGSFDKNDPNVGTAVVGKAACGDVIKFQVR
VEDGVIKDACFKTFGCGSAIASSSYVTELIKGKTCAEAESIKNTDISEVLKLPPVKVHCS
LLAEDAVKMALKDYNSKQNKMLMASSVCCMKGRSLNTYTYSLLRRTFSSTTEQRDHKQRI
VVLGTGWSSLFFVKNLDLSKFDLQVVSPRNYFTFTPLLPKLVSGRISTKTCTVPFSSFVQ
KHRGSFNFVHASCVNVDPHSKLVYCVSDPNTRVNLPYDRLVIAVGAESNTFGIPGVAEHA
YFMKEVEHANIIYQKIISNFEQASLPGISEEEKRRLLHLVIVGGGPTGVETTGEIAILLN
K-MAQSFPAVASYVKVTIVEGGQRLLGTFSLGNSQYADRVLSAKDVNILLGKQVCAVGEN
DCTVKDA--TTGETVTMPCGIVLWASGLKQLELVDKVRAHFKVQNNPRALLVDQHLALRG
TGDHSIFAVGDCCKILPDKLSEHFDEVSKAIGGT--TPDALLRNLKTLSWRFPQVSSNKL
NPKDPAFVEFPKEQLLELMDYIDSRYMPPFPTAQNAKQESVYLANLFNKGFNIQGT---A
AFNDVWKGSLASIGGNHVVGNFPHFSLNGGIKAFVLWLGVYLTMFPSGKMRFCYLGDSLV
QTLYGRHLIKMAFDPAKLQVKNKMPAAVQITAEQILRDAVEWQSRENKQNRTFVDQDELV
YYKAQRRKEFEDKLRRQRHHMGTWIKYALWEANQQDFRRARSVFERALQVDPNNVNLWLR
YIETEMKNKNVNAARNLFDRVVSLLPRVDQFWFKYAHFEELLGNYAGARTVFERWMEWNP
DDRSWMLYIKFEERCGELDRCRQIFERFLESRPSCASFLKFAKFEQRQKNYPLARAAYVK
CLEIIPPELLTEEFFLKFAAFETQQGNLSGAEKVYEQGLGILPRESSEQLYRSFVSFQKQ
HRDRETIDNLVVTKRRNEYEEQLIDSPCNYDIWFDYIRMEEQQLGPHATDAQRARVCELY
ERAISNLPQVDDRRLWRRYSYLWVGYAIFSELTLQQLDRAVAVYRKALQVLPKD---FAK
FYILLAELYLRQGDLDSMRKTFGLGLGQCKKPKLFETYAQIELKLGNLDRCRHIHAKYIE
TWPFKPESWLSFIELELMLNERKRVRGLCEAAIAMDQMDMPETVWNRYIEIEREWQQYAH
VRNIYERLLLKTTHIKVFLSYCEFEFTSGFP-DNARAIAERALEYYKASNHQVERAGMLA
HLLKFERTYGNDETISKTKDRQPKKVRRKRKMAD---GTVTEDIVYIFPEDG--VQQNKL
LQAAMRWKSQANVT---------MSDDERTDAAPVAEE--------PLQRLLHLAMANGC
LLRGIHQVTKAIEAKNARACVVSTQTSEEAYLKLIRALCKEHGVPCIETEFDSEKIGEWA
GLCKYDIEGVARKIVGATSVAITNFDEKSESAEEMISLIKS-LA-MGGHRRDSSSESLSS
VSDRSRSRAES----RSHHDRKSRSGRVDRYSDIHSERSESSEDRQSNRTILVGARRSFS
HSSVTEGSDEGMTLYFIVNMLEEGEITELVLDDDEMDVDKFLEQRRLERQKLLEKHSSAS
RSASSETESPNTLPGDNTDPVQSSPLPHMDSTPSEASHSRSRNPFATATPNNVTSNNSEE
SISNTEVLPDKPLS-GSNLDDVQESAASDDGQISELTASDIDDDDVPVPTKSTSIVNALQ
SEILGEKIKLRNMMLKLREDHKSEVDETVVTPVQESGAVSASDEYSDSDDDVDIFAEASS
KKERPSSKRRKTQRRETTVRGLSDEWNDAEGYYQATIGELLGDRYKVISESAGKGVFASV
ARCLDIDTNTTVAVKVIRNHDIMVRAAEKEISILQRLNNSDPEDRRHVVRLLGRFDYRGH
VCMVFPWLWGNLRNALRLHGKGKGFSLPYIHSYTRQLFIALRHLARNGVMHADLKPDNIL
VNDDFTRITVCDLGSASDVSENEITAYLVSRFYRAPEIILGLRYDCKIDIWSAAATIFEL
ATGDILFPGRTNNHMLKLMMEYKGKVPNRVIRAGQLSSQHFDDNLDFIYVSRDSFSHKDS
VKLVSDLRAKRSITDVLLERQPWIKGTSPKKDAMVRRMRQLGDLLERCLAIDPAKRLSAD
EALQHPFIRG----------MV------EITEVIEPEW----QTRNDIQNLVNQKTLQWV
FVGGKGGVGKTTISSSIATALAETRESVLLLSTDPAHSLSDAFGQKFTHEPRLVNGFTNL
YAMELNTSQIIDGLDGLRETHSFLKNVPDILMMLPGIDEALSFVELMQSVQSRRFSVTIF
DTAPTGHTLKFLKLPDVLEKILDSLLKLENTMGGLLQLFSSMTKAQMSQNELFDKIKLLG
DMINTTHEQMKNPDLTTFICVCIPEFLSVYETERLIQDLAKSEIDCSYIIVNQVLKHIQL
GGL----IEDAWDGLT-EEQQRIMTPFFEKVREHHSTHNSRVDVQRKYLSDIKDLYQEDF
NIVAVHQNKQEVRGKDALVAFAKKLMQHSPLPI--SRNN---------------------
---------------------------YFDAPSFVSDSQLYRPKIGDHVIGVVVAKNSDF
YSLDIGGLTEAILPAVDGFRGATKRNRPNINEGDVIFCQITKQYPNELPVEVSCLDVDDM
KQWTTKETYFGSLEGGFMFSVPIPYSYCLSGARCHVLEKCAGRFKYEIAVGLNGRVWIKS
TNEVDTILIARYIRMCFGLSLAQTEALFLYLD-------MTILS---RQALRRVFGASGH
RFLRNNGPEHH------HLGSSLPRQYGTTVVSAIQPKSKRFVTSYQVLAAKPPTGFGKF
TRKDDATEDTNNKNDD-----DRKGFEPYTLIIIGIGSMLLLDVLDSGGLRNEITLQEFI
GKYLMKGLVERIQIVNKEFCRCSLVT-GVDHTMPRVVSFRIGSLEAFEQKLDDIQASMGI
HPQDYIGIHYVNEVNVLGELKHYIPFMVVMMLLGLGLRKLTVR--STGGMDRFFRMGKMN
IVDAKDVKVDVKFKDVAGMHEAKKEISEFVDFLKNPKAYEHYGAKIPKGALLCGAPGTGK
TLLAKAVAGEANVPFYSISGSDFIEVFVGVGPSRVRDLFEKARKNAPAIVFIDEIDAVGK
KRAKGGFSAGANDERENTLNQILVEMDGFKSSS-GVIVLAGTNRADILDPALVRPGRFDR
TITINKPDLDERFEIFKVHLSPIKLNKNLDMDDVARRLAALTPSFVGAEIANVSNEAAIQ
AVRRKSTDGVSLADFDAAIERVMAGLRRSNALLSPAQKLAVAYHEVGHALIGWWLEHADP
VLKVSIIPRSSGALGFSQQLPDEAMLFSREALLDKVAVMLGGRAAEDIFIGRITTGATDD
LNKVTRMCYAFVSQWGMNPALGLVSYQRGSGDEPEFYRTYSENTAQLIDTEVRTMIESQY
ARVKSMLREKAELVHKLSKLLYQRETITYHDIASCIGEREFPVEEKLRPYVLSGIEGRVE
PIKLPETTGE------AINEIKGTEKSDDNQETNSEVS----------------------
-----------------KMTNNSSAVPDSNCVQHTNGPSVEGASTVEKVVTKDETNPYIP
RFIAKAPWYLNATS-GLSHQRVQQHQKSALDEPVLRGVTSRAVRYRKNACENCGASTHDV
KSCVERPRKKGAKYTNANCPDEYIVENKATGYEAVRDRWSGFDASTHQLVLNAHQVVEDE
LYRRRMEDLAS---------------------------TLHDTNDKSCSIGN-------V
ASDDRSRNTMPNLRIREDTAKYLINLNIDSAFYDPKSRCLRDDPLLGMSN--SDHHTFRG
DNALFTSGEASRPGEIEQFAWEAQQKGSNVSFMAQPTELEFMFKDSSLKREEAKASKRQS
LLDRFGGSAYIRKD---------------GDEDLIDTNLPVVSHTTEKETDVMLLGHTSV
WGSYYDRSTGLWGYKCCLSTSNTSRCTAGMGASNRFHNSGDPDDITSSAEVT--------
-----EVIDDDLDVDSVSTEDFVEP---------------SEDEGEV-LLNKKSVLARED
SYRRQRFDRKLSPERYDPFA-KEADPDERTYADIMKETEIMRTRKEIEHFMEREGMTLD-
---DVRKLD-------SRRSGFDDDSD----------IPATPQFPDTPQFAETPDLSEPR
KRASRWDKTPQMEAQ--------------TPSAGLGE-------------YGMSTPFTPA
G--LQPE---QYAFST---DRNRYLTDEELDEMLPSEGYEIIEPPEGYVASKHISYHSHR
----------------------------------GPETPSFVIKDEN------------L
RKPYDI--PGTPSLLLDVEVKAEDQGFFGKLFDDKTEEDLTADEITERRILALLLKIKNG
TPPLRRQALRLLTQKAREFGPGPLFNQILPLMMQTTLEDQERHLMVKVIDRILFKLEDQV
RPYVHKILVVIEPLLIDEDYYARVEGREIISNLSKAAGLATMIGTMRPDIDHPDEYVRNT
TARAFAVVAHATGIQSLILFLKAVCQSKKSWQARHTGIKIVQQIAILVGCGVLPHLKQLV
SIIASGLEDEVLKVRTMTALALASLAEASAPFGIEAFDIVLRPLWKGITEHRGKGLAAFL
KAIGMIVPLMDPYYASYYTKEVMNILVKEFATPDEEMKHIVLKVVRQCISTEGIQADYIR
HELLDPFFKSFWIVRNSMDKKNLDLIIETTVEISNKVG-LEVINRLVDDLKDPSESFRVM
VAQCIEAVISSLQKVELDQRMEELLIDGMIYAFQQQASDDCTVLLDAFGTLLHYLGDRSL
PYLTQIVGVIRWRLGTQSPRTRQQAADMIAKIAPIMRLCGRQDMLASLGQHLYEYLGEEY
PEVLGSILGALKAIVSAIGPAAMSPPIKDLLPRLTPILKNRHEKVQENVIELVGRIADRG
GDLVSPKEWDRICFDLLELLKANKKAIRRATVNTFGYIARTIGPNDVVATLLNHLRVQER
QLRLCTTIAIAIVAETCLPYSVLPALMTEYRVPEINVQTGVLKALCFLFEYIGEMAKDYI
YAITPLLENALMDRNLVHRQTAAWTCKHLALGVAGLNCEDALLHLLNYVWPNIFETSPHL
TQSCFDAIDGFRVALGPGVIFNYILQGLFHPATKVREVYWRLYNNLYVGNQDALVPLFPL
VREGVENCHQATELLYTIMVRERLNAVDVAAVVGNLRSQILDYNLVNIYDVTSRVYVLKF
SRNEDKRFVLFEIGHRIHTTQFLRTTDKLPSNFNVKLRKHLRTRKLRGIYQIAQDRVVDF
TFSSGEYAYHLIVQLFLPGNVYLTDYSYKVLTVLRPQNAGDSFFRVGETYGIPEASVPWN
---------IPVSPAVIDGILS----------GMGHGNDASNSQKKVTNSRGKPETGDSS
KQSIVNGSDQG-DIGSEFKDRSVSMLLKLIFPS-VTLRMYALVKAIGADICDSDVSAVES
----------STIYTAVEALRSTLDSLSNPVNLNLGYLYKKGTE----------------
-------YEDFGCFDYGDG----------WERFDDFNMALDAYFTKSELRKIERK-EQPK
KP---IKLQKIKDDQNRRELEREREVHRLGVSIALVEGHRDTFDTVLDLMRSLVASGASW
```

QEITDQLSRQRDSGHLLARHIRSVNIPDRRVDVCLPNDDPGYYTNVTSMGDK-----NKR
GSKKSQSSDQFDDTSVTLDYGLTCFQNLEIMYSQKKRMAEKLERTRAGHQFALKRVDREK
EKQVKSRGDR---NVSLVKVRKRMWFEKFHWFITSDGFLVLGGRDSTQNELLVKRYLTKG
DLYFHADVHGAASCILKNPSGNAESFPNTIDEAACFSLCLSSAWSQKMVVPAWWVHHHQV
SRSAPSGEYLPHGSFMIRGKKNYVQPQRLEMAIGVVFHIE----------------VPDI
DEEEVEAPAGPD--

-------------------------------------TEDAPQD----------VESDES
DASLTVDDLIGHGEEPV------VNDD----------VVMSDESPSSD---DDMLENKRV
VRFNLDN------------------DTEPKE------RVGNFHLL-------------RK
GTGCTGFNPDDLAEKLSALGL---------------------------------------
----------------------------------IDPDDT--------------------
----------------DSPESHVRFIEPDKPIHIPEAVERLRKRLPTGI-----------

--IAPKKPRGPSRLARV---KA
AKARKKYGDDDEEIQQLRCQLTGSRLLKSGIDTPVVEPVPE------ESLPKPVFQRQAI
QPLDDRELSSHMRQLRALSKSPSEGDVILSAIPMCAPYGALKSHPYHLKLVPGNNKKGAI
ASQALSHFLKVDESKS--PFIKLITTDQFALTLIENCKVPGLSGNSKRMGRMYGKGKGIS
SSSIPYRRRPASWVKTKPLDVEQQIVKLAKKGMTPSQIGVVLRDSNAIPLVKTITNNKIL
RILRGHGWAPDIPEDLYFLVKKAVVMRKHMEHNLNDKDSKFRLILVESRIHRLARYFKKK
RRLPANWKYKSETASALLG--MEKRVSYFYDPDVGSYYYGPGHPMKPQRIRMAHALVLSY
DLYRHMEVFRPHKAVEPELLAFHDHEYLQFLSGVSPDNYRDFAYQLKRFNVGEATDCPVF
DGLYVFQQSCSGASIDGAHRLNNQQADISINWSGGLHHAKRSEASGFCYLNDIVLAILEL
LKYHARVMYIDIDVHHGDGVEEAFYVTHRVMTVSFHKFGNFFPGTGDVTDVGVASGKYYS
VNVPLNDGMDDESFVDMFRTVVGKCVEVYEPGAIVLQCGADSLTGDRLGRFNLTNKGHAG
CVAFCRSLNIPLLVLGGGGYTIRNVARCWAYETGVVLDKHNEMAEQISLNEYYDYYAPDF
NLHLQPTNMPNYNTSEHLDRIKMKIIENLRHVERAPGVQFAHVPNDFFQYD-DDEDEAAQ
LEVFDE-GGGVAPAIVPHRKTVNHRLRRKDYKNDYYHLPDRDQHIPI-------------
-----MLSAATNKDHATLCAILRPLLDSNDVSQLDSICALHWACYCGHEGLVDSLLTAGC
DPHYPDDINLETPIYFAIRGSNVHIVQMLLKRFGTEILCHENRKRLTPFLLAASEFIEEN
VISTLHMLEFLYLSGVSLEEQDSYGRTALMLASRRGCQFVVQWLLSRGANLAHRDHLGNS
VLHHACHSSDLDTLRFLCRHGAIGLIHAKSLAVTSSSAFGICCVKRRFLQYSVLKLWSWQ
YGLTGRIITFSSPYPVYYWLLAFINLLLYYKMYATMGQMTPLQASVDIWILGWLMNQCFW
FITFASDPGKARKTPVISQLHRTKDGPMLGGP----CERQMHLLQKQQELINFELYRINC
DTFRRNDGLAQRMQVCKEESQLLQTDMHALYPQVGVERRDHCVFGYADAVMGNAKDHGVC
ITCNMIRAPRTHHCGSCGVCIVRQDHHCAWVDNCVGKGNQRSFCVFIASIFISLCHTYYV
LYLHFAKEIAKFDWIWDCFNILFGVVNAAWLIFVGYLSLRMVRNMTTDVTFYEFLRKPEY
IRKRFKVQGSWWDLNGLTPLKIIANCGDFWLP---------------KMVTSFFLNGLRN
RNNVGSIDDIGQYSLAEGEAKVSAQSNDAMANVVKRKRKLKRQRDDSDSDD---------
---------------------ADEAAAAMAERIA-REEQKKRRIILDNLETLASFLKISK
NMMKAAVDGFGGGDPAMETQIRETIRRGGCAFIHTRADLSKFE-IAEKFRNLKLYQQCGV
HWLSLIHSESANGILADEMGLGKTAQASVFLSYLYEAGGCRRSTLILVPLSVLDNWCNEL
KRWAPNLKDRIVKYHGAQSARFQIACDMLDDVSKGSFAILVSTMATVSSKEDIRMLRGLR
EFEYMIVDEAHSLKNSETIAYRRLNGSFNIRHRLLITGTPIQNRESELGNLIQFAMPELF
DQFKVNAGINYLIEHYKDAVNAQRQQSQHKLKSRKYTYVKESDPVATEITAVDAEESTPS
SSEPEMDRDTESVEPTNEAELNSELSGGDLGCQGSTQ-----------------------
TPETIDPSIRVLQRLIAPFILRRKKRTVMHELPEKRTLLVRCKMTGIQLDMYMTEVETKI
KQNRDTL---
---------------------------------VQKCKVTEGEVLSMCNGRSRDYSRRDD
--FLVKSMVFLMRRICNHPLLVRGYYKEDLLQKLIKYYWSKVEGYKGNPLERVEKELRSW
SDFEIHRSLQSLVPV-EPRLERFLIPKEQFLESAKVQEMFKIIDRVEQAGKKALIFSQFT
MYLDLLETCL--
-----------------------GLHKPQLYLRLDGGHNPSTRTDIVERFTNDPNITLLL
ISTKAGGTGLNLTVASTVILMDLDWNPHNDAQAENRSHRIGQTEPVDVYKLMCEDTIEEY
IWECCQRKLLLDDAFSGNLEEDM-KPQQTVLVFKPTLKKESDRKAQLATIQASRALSEVI
RTTLGPRAMLKMMLDPMGGIVITNDGNAILREIDVANPAAKSLIELSRSQDEEVGDGTTS
CVILSGQMLANAEPLLKREIHPSKIVEGYIEALDDALAVMESIATKIDLNDDDAVKDVIQ
SCLETKFSSKWGNLIASLALSAVQKVKVDRPDGRTAIDIKRYAKVEKLPGGMPEDSIVLD
GILINKDVTHPSMSRRIENPRVLILDCTLEYKKGESQTIVDISDEAGWAKLLEQEETEIF
NMCQNIIQTGCNVCVTEKGVSDLAQHFLSKAGISCIRRIRKTDANRLARVTGATIVNRTE
ELLPSDVGTGCGLFEVKKIGDEYFSYFINCKDPKACSIVLRGSSKDVLNEIERNLQDALN
VCRNIMLEGKLLPGGGATEIEVSCRLAEKLDSKSGLKRWSYRAAAKAFEVIPKTLAQNCG
ANPVRVITELTSLH-VSGNVNAGLDGETGEVCDTMKRKIFDTFAVKAQIFKSAIEAACML
LRIDMIVSGIGKRDESQKLAVNE-M--ESVLNVAEKPSVARSISDILSGGRMRSEMSNAR
TNPVYSFTAPLEGRHCKMYFTSVRGHLMEIDFDARYRNWQRTPIESLFCAPIQAAVSSNC
KDIEKNLLHYARMCSKLVLWLDCDREGEAIAYEVMAVCQRVNSAIAIRRAIFSAVTRHEI
ETACATLREPNPNLAQAVEARQEIDLRIGSSMTRFMTTRYRERIDTESKVLSYGPCQLPT
LGFVVDRFFKIESFQPESFWSIQVFIE---------------------------------
---------SGGKDYLFRWDRVKLFDRLAVLTLYEACLENPMAKVTHVSNRSTKRYPPLP
LDTVEMHKDAAMHLKISSHESMRLAESLYNKGYVSYPRTETNVFPSSMNLRDLVGMFSSH
PLFGEYANRLLQGE--IIPRKGNKNDEAHPPIHPVKPLRREEAESPQSWDLYVYITRRFL
ACCSPAAVGDESIVFLDISGEGFHAKGLVVRERNWLDIYPYASWNGTPIPAMEEGQELMP
SRILLSDGCTRPPGLLTETDLIDLMNKHGIGTDATMHEHIQKVQDRRYVKKESNFTLIPT
NLGKALYNAFKSYAHQQIDLTKPNLRALMESDICAIAEGSQDKGDVVNKYSQQMRQIYLH
IEGSIGAFDESMNGLQDVS--IRMEVFIIGKYRGKTFEEVYRTDAGYVSWAKQIDTPTGQ
LFRFRLYIHNRESNTSGNPSFTTAVKPFNPSQPSSTAPKESLGRCLDALSDTSPESV-ST
MVSIFDGSDNSSTELLEDIKDSGAFKDFPSSFITEATGLTASSGDTEATSSSGIPDSNDT
NDSSTLRIEGMLALVLYSDSEFYIAYQKPSA--LPSWTSVVPPELFQFLASLQCRSVKDQ
RHSYLIFHANQYDVVLKALRKALSKKKPVDPIPNFILRSFSEFDRYARKINLPEKTQDSL
SGHLCPYTSNNLDNVHTLVGDELYNQLKPFQREGVNYGIRRNGRVLIGDEMGLGKTLQAL
AISAFYRINWPLLIICPSSLRFQWREQCLRWLPHLVKSDDICMIMTGKTEYTDETKIVIT
SYDLCVSNRRLRHGFETIICDESHYLKNQNAKRTQFITPLLKEATRVILLSGTPSLNNPA
ELYEQLSCLIPSFCSSSTFVERYCEKRLHWFTKRMTYSGSQHASELHMFLVKTVMIRRLK
ENVLNELPPKIRSKVPIYIAPDILRGLS---KTGPAIIN-------------------SK
PNFNAPNTQEVFRKTGEAKVKGVCDYVLHLIKSSVKFIIFAHHMFMMDAIEQVLKAQHCC
YMRIDGSTNAQQRESRVTEFQNNSKCRVALLSLTACGVGLNLTSSSTVVFAELHWVPGQM
IQAEDRAHRMGTKHRIINIHYLIAEGSIEETMWRVVSRKWETVTATLNGEVSNLA-----
--------MTKESEKAFINEMAAQYKITEL------------------------------
----------------------------------LKMVEKENKINEA--------PASTT
TNGDAFRELLQHCTALIEAAVAKVDGRFMVRLIHHIKTLRSMLKKHDATTLPILKQSIAI
YISGNCPVSAMALEYLPQEGVKVPYPVAPMLGVEEFLAKNSLGETKILMLTLALIYLIDL
KKFEEAMQFAEHLAKYVLEFKSKIMDHLAAKVYFYYARAFELGGRFNQTRTFLMNVYRKA
CLHHEPMTEAVTFNCVVRNLVHHKLYSSAATLLMKTTFPESLSASTQYARYLYYCGNILA
VQLEYSEAHNKLMQALRKAPQSDNAAFGFKLATTKMAVIVSLLMGDVPSKSTFTNPIMKR
NLEPYEEVVITVMNGDLIEFSKVCGKYKTLFEKDDTMFLISRLRHNVIKGGLRKINLAYS
RIPIEKVAQKLGINSVEETECIIAKAISDGIIEATIHHEEKYMESKANVNLYTSEQPMMA
FNKRINFCLKLHSNAIQAMRYPDEPDITRHQTPKEEQIAAKKDLVMEEESSLKEN----M
KCARCNERPPTLRRCASRLPYCRQCFINEFEKEVYDLIEEHKLIVDGDVVCIGVSGGKDS
SVLAHVLATLKERYHRKWTLYLLAADEGIKGYRDDSLGVVMGMSNPNYDGLKILNFKERF
GFDMDEVVSLIGQKNNCTVCGTFRRQILEIGARMLGANKLCTGHNIDDNAETVLLNICRN
DLFKLARNLNSLDSVGSTAKMGREVTASTTSGMHINNEATPVGDSYHS------TDQTKS
TRFMPLLYDTKMDECDTQTAEMSQKLHLSDKGHELLRIKPLMHCYEKEIVMYARYLNLEY
FSTECIYAPEAYRGYMRTFIKQLEAVDPRIIQNITYSSEQFYASYLSNRKVNVCKKCGIE

GINDLCKACILVEKLTALRSNA-----------NEHIKVAHSS-----------------
-MVAETQLQEP-VTDTYTLNGIDYGELFRRCCRPTL-------DTTQLEDIVMFQTDADY
TS----HYEKVGDEPLPS---------------SNNTNNVELPIIRLYGVTRESHSVLVN
VHNFMSYFYVEKPVNFKKEHIPLLIDFFNKHLMELPPFKKTCRYVIDIDIVQITPLMLYK
PNAKCDYLKVITTLPRMVSSLRSFIESGISLPRIPFPRQVCEANIPYVLRFLLDGEITGG
CWIRLPKGTYSIVA--DKTSHCSIEVSMDYKDLDPMPIEGEWQSIGPIRTLSFDIECVKF
TGPGFPNANNDPVIQIASVLHTHGDSLDSAQKFVFTLDTCDNLHGAHVLSFANEAALLMA
WKQFFIAVDPDFLTGYNIINFDIPYLINRANALKLPEFTQLPRITRSKSSVRDIISSNNT
MGTFENKEINIEGRILFDVYDLVRRDHKLKSYTLNYVSFEFLKQQKEDVHHSTMSKLQLG
SAADRRRIASYCLKDSVLPLLLIDKLLLLFNYVEMARVTSTPIKMLISRGQQIRVTMQIY
RQCKLMGYAVPSVNRAANSEASYEGATVLEPKKGYHRNAIAVLDFQSLYPSIMIAHNLCY
STLVPPAD-ASKYPAEDLTTVPGHPGLYFIKAHVRKGMLPLIVERLIEARKKAKEEMKVC
TDPMLRSVLDGRQLALKITTNSVYGYTGASTSGFLPCVEVATAITSFGRYMIVDTKEKIE
SHFTKANGYDTDASVVYGDTDSVMINFGTKDISRAIELGVQAAAMITNQAIKPITLLFEK
VYLPLLLLAKKRYAGLYYTKADKYDKIDCKGIETVRRDFCLLVQQMMERILHMLLVDLDL
NGAIEFVKSRISQLLRNEIDISLLVVTKSLGKVDYDARLPHVELAKKLRQRDPGKAPSVG
DRVSYLIVKGTKNQPQFDRAEEPLYVVENNIPVDTQHYLDAIKSTLMRIFEVIMPNPESL
FSGEHTRFVTLTSSSEGALSMFMKKVERCLGCKTVIKVPPFCDHCNLEKRQQVMLQKLEE
RRQKEADYFDLWSQCQRCQGSLHNEVICDNRDCPIFYKRVRTGKVLTSMEATYQSLHLEY
M---
--SMEDADISGRSHDRPL
VQIAPMLDITYYEFRQFMRLITKRAQLWTEMMADGSLIYDSDKVGSMLHCDANEHPIVLQ
LGGNNLDSLSKAGNIALGYGYTEFNLNVGCPSTRVSGKGCFGAALMNDAPLVGRIVKHLR
SELGTPVTVKHRLGVDHNDSYEFVRDFISTVADQGCTDFIVHARKAWLNGINPRKNRSIP
PLDHERVYRLCQEFPNLNISINGGIKSLEEIKYALS-----------------------R
GVYGVMVGRMAYENPCGLVRVDTDIYGATNPATCKTRRILLETYANFIDSQASRTSDMNV
CMLVKPILGTFHGEVGTKIFRQALSDMNVYETLGTKCGHYIRRAIAIMDEVNPEALDRPL
H------------M--TQNEATKTFIVEELITEFEQQQRDEEIYAQYAWEKDFDKSWEQL
VDKDGVLQFVQTDVTSDGTVALSEADSS-IQKRGVVRNIVIMFDTSDGMREVDFKPDRLH
CAAGAVKGLIQGLFHQGPMTQLAIITMRNKRSTMVSKLGTPPSEHIALLDEKIKEGTDGV
PSLQNGLEMAISILTDMPPYTTREILVIFGATKTFDPGNILTTLQKLKDEHVSVSAVSIA
PEMYILKHICTETGGSYAVCKDSVHLKSLLNDHTMPPKWRPWMEPVLTKVGFPPLEKANT
ASLCICHSTLTYKAYICPQCHSKSCAIPTKCKCCRLYLVSPPDISRMFHQLIPPKPFINV
SW---------------------------------DIVSHSV------------MSSGGL
RISQQNYFSTHNALRVSPGDKGVRYVLHELHGERHGALRLYPFYDVQMVKRMLIKKLNLS
GGVSVADLRILYKGVELPNYRTMETYMS----------KGKTDHRLFWSLREKNPSAGIR
RTGIK-TTARMDAIINEIALSLKSNIKPKLTMDGTGGTYLMYNKHRKCCAVFKPIDEEAF
APCNPRGYEGTLNHQGFRSGVLSGEGASREVAAYLLDSAYGGVCGVPDTTMVEASHPCFK
NSCDERFVKDASGPKWKPGSLQEFIDCKESSGNYNPALFSVGDVHRIGIFDIRVVNLDRN
DGNILVMDMRQCNHECVPG----------------------VPSSARYKLIPIDHGLILP
DVIDVADMDLVWFEWPQSEIPFSKNELRLIFAYNPDKDAERLRKRLLIRPECLRTMRVSV
RLLQIGAAMHLNLKQIARIMCRSDMDDPSDLECMIKRAVEQAYKATEATSVISTRRLGHT
LDLISHSVKFASMADDIDHGDSISGDRHADHSNHESSETSSDESFAHHVVPAHHSTTHEL
KYR----DIVRTTYRRRRPMQEE-------------------------------------

-----------RSIWVLEDSKGHQIHFEW-DQQFEHIFYAIAERMFAKFIQKEHPEWSTY
PYNGEGDENFPWSAESGFSLEIRVGGKYRLGRKIGSGSFGDIFMGTQIQTGEDVAIKLES
HRSRHPQLLYESKLYRLLAGGVGIPTIHWYGIEGEYNILIMDLLGPSLEDLFTICNRKLS
LKSVLMLADQMLNRIEYCHSKNFIHRDIKPDNFLIGRGKKISIVYIIDFGLAKKYRDPKT
GQHICYREGKNLTGTARYASINTHLGIEQSRRDDLEALGYVLLYFMRGSLPWQGLKANSK
KDKYDKISEKKIAIPVDLLCKQLPFEFVTFINYARSLRFEDRPDYPYLRRILKDLFFRQG
YQYDFIFDWTFLHTAQLMHTMVNEDIERFERMNRERDAEEPAMSLVDQVAADGRVEFLSS
IKKVLIRSCLQCTSDVKELVDHLFLNGSLLDIGLKSIDLIQTEPRRERSEDTTKSQQIYL
IRPTFEDAGILKAVMSY-----DRSECF-VLCLPGASEMFPDVLQGGIGKQTGNRIVHLN
VHMAPLESCALSMFISRSMESFYMEGDPMASWFFAKAVEHLESILDGAINLLTCLGHLAR
FSGEILIKSRRDCAA---ELIVNSTKHSS-------------------------------

--------------------------------------VSSDMPFLTRELRNQRLLTDMP
TSGTDEDPSATVND-TQRRMK------LLKQSGTIDEAVIIDRRVDMVTPMCLNVTYEGL
LDNVFGITNGSLQCPLGVVDGSVGDASGILE-----QYRSNFGTLFPDTTVVPLRSS---
LYREIRWLNYSEVGKHLHQRALQVHKGY-ERGDLATLDEMGAFVKKFKNLQKEHSELSIH
VNMMSWMNSLISGDCMQLLHQLEDSILQSATDIKPSKIASLTAKILDLIYWNVDVTQVYR
LLILLSQTRDGVKSSELQSIKRAIVDQYGFCQLMTLHKLETMGLIRINDPDGLRWARLCK
KLNLLVDREADYASIFGGYAPISVRLFQLIIL-ARNVSSVEADLRLLDCPVAVLRQKSVL
----------------PGNPTGRSHCKLLGFLGGVTLGEIAAIAALNQKRGEQ-TLILAT
NVISMKSMLGPSN-MA---DSAISNVVTDAAVTMASDRASVESLCVQCGAMGITMVLMHM
IPHFKEVILMSFECPSCGYRNSELQDAAPLQDYGLRLKAHVAYEGALRNQVVLSGTTSCR
IEEIDFEFQPTMEKGTVTTIEGYLMRLAGGLGDHISSIADAMRENPGIIIEM--------
---------------------------------------ADGKQHTAAEYLYSLNSIKQS
LIEFSEGDK-PFTLILDDPAGNTYIE----ETQHLSVESTRYNRSPEQQEMLGYIAKEDD
KKE-------------------------IDLTT---------PIADDDDVGKEGL-----
-----SLPVDCPHCGKVGNNKICEVLVPGFGPCVIMAFTCENCGAKSNEIKPGGGYKEHA
RKWTLKVQDVTDLNRDVIISETATIHIPLLELDMTAGTIGAVYTTVEGMLIKHADSLETA
YPFLLGDSA----------------DPSNTTLKDKVRQLRALASGEFKQPYEIIIDDPAD
HSFVGARNSGDDSNLRSETYQRTAEQNDLLGLTDMVTEYMDS------------------
GDVVPPSSPDAHSTELAPKAMFVINRKGESEEVHFDRILERIRKLSFGLHSLVDAPRVTQ
SVINGMYSGIRTSQLDELAAQTCAYMAATHPDYSKLAARIVVDNLHKNTLDNYADVITAL
FKYKYIYDSNASLISEEVYDFIMANIDRINAEIDYSRDFQYDYFGLKTLERSYLLRINDK
IVERPQHMLMRVSAGIHTGDIERTIQTYHLMSQKYFTHATPTLFFSGTPRPQMSSCFLLD
MKDDSLAGIFETLTQCAFISKCAGGIGLACHKIRASGAYIRGTNGKSNGLVPMLRIFNST
ARYVDQGGGKRKGSFAIYLEPWHADIMDFLDLRKNHGAEEARARDLFYALWIPDLFMKRV
ESNGDWTLMCPDECRGLYDVWGDEFEALYTKYEMEGLGRKTIPAQTLWFAILQSQIETGN
PFMLYKDACNAKSNQQNLGTIKSSNLCCEIVQYTSPEEVAVCNLASIALPMYVDKENKTY
DFKKLYDVARVITYNLNKVIDRNYYPVPEAKVSNHRHRPIGVGVQGLADTFMLMRYPFES
EEAKELNRRIFETIYYGCLDESISLAEKYGTYESYPGSPASQGKLQFDLWGATVDNKLWD
WDGLKQRMAKHGLRNSLFLAPMPTASTSQILGNNESFEPYTSNIYYRRVLSGEFFVVNPH
LLRDLVDLGLWDDAMKEKLIAYNGSLRHIEGIPQHIKDLYKTVWEIKQKSIIDMAADRGI
FIDQSQSLNIYLDQPTFGKLTSMHFYGWKKGLKTGTYYLRTQAATDAIKFTVDTSVSQMA
KATSQQQRAKATDG-----VSTTSTEDPSVCRLDPNSNEPCVMCSGTEEPQILYELFRKV
RVDEVVEDLKELGVDVREETLKNPTPEAALCFYGLAIQVVFGKTRTDIRPEDVYSHIHVY
DSGVEGLNITSDNLEFLKRGIGNLRFWRYCQKLHETLGLQKIERYIFNPTPESFHRFISA
FVVYRRFREALKSLFEDSVARLNFCVEQDAQLDESINKVRRELQNLQRLKDENLTNIESS
VEERAILEHSLLQAKNVFNDSKDEKAKLDGEIEHIKLSINEVQLKKTKSRHLNETLSEQV
VVEPDALYNQKNDLEAEDSVATATLNNLEERFEDVIGRIKELEESSKFLVDMKTKLSQHV
DEVLKPLLENNSTSQLLQNRNETLREQISQQKCKLEEAKLSLTNTQQQYEDKMNKADAEI
KQAHKFAEEIERDVQGIKNTTAEQNDELSKLSNELVNRERYMIATFASIEEYFSKVKEAA
EAYKSSMDAVTERLHSTVTKSHPYGALIAMLKRFFLRYAKANDSGNLKNPPAKLRKEDFE
FVRTLGTGGFGRVILAIPKGIDTEPCAIKRLKKHSLIVQKQVDHVVSEKKLLSSVNHPFI
VNMLGTFKDPHYLYIVMECVFGGDFFGYLRSVDKLESESAMFYAAQITCIFDYMHSHNII
YRDLKPENLLVGSDGYLKLTDFGFAKVVELRTYTLCGTPEYLAPEILLNKGHGKAVDWWT
LGILIYEMLVGYPPFYNEDPMGIYKKILDCRLVFPRNYDPDAKALTKQLLTFDPSKRIGN
LHKGAKDIIASQWFAKMDFDKLLQKKLTPPYVPELKGKNDTTKFGTYPDSVEVPRAVEGK

ADPFDNWMALSRFFVISSGGDRILLRCLRGEGEGGSAEEFYSAVTEHHEGNLPLIRIGDV
FYYSLKRNGLYFVATTSFAVPPSYMLELLNRIIGTFKDFCGILTEESLRQNFILAYELLD
ELLDFGYVQCTNTSQLKQKVYNVALVPKIHARSMARLSLGTNPNPKTVPSSVSQRPIKEG
ARSNEIFVDVLEKVSAILGADDTYKSVTVEGQIRMKSFLSGNPMVRVALNEDIVINNRRC
KVPNVAVLDFCNFHECVDTREFEKARLLSLTPLEGEFTLMSYRISGNAVIPFRIKAAVDI
DGDA-ATITVNVFSTMPEHIN-AFVKLHCPLPSCTTGATLSTVPHDNGQATEYRPKDQSI
SWEVRKYRGCTGYTLRASVNLGSHGSKIKREFGPLNLTFEAPLFSVSNVRVRYLGVLQPP
SSGPSYRWVRYVTSSQSYIYRFLDS-----------------------------------
----------KITLPGDVVNT--RASSLLQGRGTYVDNGVLRASFLGTFKQVNQLAYVEP
LHGKYFAQVGDVVVGVVQRIGGNCWYIDIGSSMRVQLSIFQVNTEELATRRKLDEDVYEM
RNLFGIGHVISCEVQRVSPNGTVLLQTRTSKYGRLNNGILVKVKPNLMLRQSKHMQELSC
GISMILGCNGFIWLG-PIQKSGDAT---------------IHAQVYHDICTFRRIILSLA
GLSICINFALLCEIFEFFKEK-----------EQAPELLERFFSEKRA--MPVFFHLTLQ
RPTGITQAVQGNFSAPKAQEIVVVRSHTIELLSPDDSGKLRSLCVSEVFGIVRVVSTFRL
TGTQRDYLVVCSDSGRLVILEYCNVSATFKRVHCETYGKTGIRRIVPGQYLAVDPKGRAL
IVGAVEKEKFVYILNRDSKANLTISSPLEAHKSRSICHALVGLDVGFENPIFVSIEESYE
AVDAAQVDE-EVTDDIIKKGLSFWEMDLGLNHVVRKMTLPVDISAHHLIPIPGG-DGPGG
VIVCCENFLLYRKIDHPEVLCAYPRRLEMAQDKPLMMVSSALHKMRDFFFVLVQSEYGDI
YKIELFHEEGKVNEIVCRYFDTVPLGNSICILRAGYLFVAAEFGDHQLYQFTGIGTNEND
PLCSSIHPQGKDAIVAFKPRVNQNLQLVDELSSLSAITDLKVIDVQGLGQQQIFLGCGKG
ERSTLRVLRHGLSVEELADNELPGRPKQVWTVPTGVDSIYDGFILVGFEGNTLVLSVGEA
VEEVTDSCFLTSITTLHVSMMGDGSYIQVHDGGVRHVYDMKVREWKTPTAKRVKVAASNQ
HQLVLALSGGELVYFELDESHTLVEVAKRSLNVEITCLSLQPTAKGRLMANFMSVGALDN
LVRVLSL--DRQLKQYSTQLLPNNSTPESVCIAEFQ----IGTHPVLMLVVGLNTGVMIR
ATIDAVSGALSDQYTRFLGSRAVKFKYI----KNQVIGTSDRPWLLYEYQGVVQCLPLSY
DTLESVASFSSPLCNDGYVAISGSNLRIFRCCRLGETFSEHRLPLDYTPRKLVMMPNPNV
-------------GLNYMVAVVESDHNAYGPENVAEISKALGDIKLDN------------
-EVGDLL--PLANYKAGTGRWASCVRIVNPLNLTTAAKLLFETNEAATAAAVVVLDGMQC
LCIGTTVGYDLKNTDDVESYIRVYCYGANFEIRLLHVTRVGGVVRAFTGYEGRLLASVGK
RIRLYALGKKQLLLKAEHRTCSDGFIWLNAVGSRIFAGDIREGIQILFYSEEAAEFEWVG
GATGPRWLTSCAQLDYSTVIAGDKFDSIFVTRVPQEE--------------------STR
HIQLENVCQFHLGDLPTAMDKAALS-QSTHVVLYGTVMGSIGALVPFQSKDELDFLQHLE
MLMATEAPPLCGREHSFYRSYYVPVQQVVDGDLCEQFRHLTEAQQRKVAQQLDTTVNNVL
RKLDDIKNRIMMDGDPQSVSTPLNDAEISILKSFGIGPYTESISESEKGIKTVVQRINKL
SGIKESDTGLNPLHMWDLMYDQQALQEGVPLQVARCTTIINPGTPQAKYVINVKQIAKFV
VGLGEKAAATDIEEGMRVGVDRNKYKIQIALPPRIDPSVTMMTVEEKPDITYNDVGGCKV
QLEKLREVVEMPLLYPERFVELGIDPPKGVLLYGPPGTGKTLTARAVANRTDACFICVIG
SELVQKYVGEGARLVRELFQMARSKKACILFIDEVDAIGGSRGDESAHGDHEVQRTMLEI
VNQLDGFDARGNIKVIMATNRPDTLDPALLRPGRIDRKIEFGLPDLDGRKHIFKIHAKTM
SVEKNIRYELLARLCPNSTGADLRSVCTEAGMFAIRSRRKTITEKDFIDAITKVIQGYKK
FSATGRYMVYNMGLEYCLRNLIAIDATPVDAAESLTNLSRDNTQLLINRIFSLGRTTTED
G-IFATLPKENGIVMPRMYPLPKPKPKTRWQIFAEARGIKKHKRSRLVFDKSVNDWVPRW
GYKSIKKGPLHAPPIVEVTGSKVPDVDPFEAASRKKSERKTRQKIREIRNKVEGD-----
----------SLNRAHTALERAKTSTRSCGKFDKK-KKGVNDKKTIKRKAVSRPLNEERS
GHLATLRKLNIS-MDVNPYHFGSITALSRDNYASMLDRVKGLKVLILDNATAGSMSLVQT
HSYLLEGGVLLTTNIGDDFYKNDMR--------------SLSNLRHLRAVYIIQPSHSNI
LRLCDQLRGGYFKEYYLYFTSTPLEGQLEMLAKNDVLELVCGVYAYHTDFFAICRHFFLL
DAGTS---
------------------DLYTSVHSGEAGRVSQGLFNVFRVIKQVPAIVHVNNSSEARN
LGQKVQALLDNDSLNSEAILKSYTKFG-----------------------TSDSHGCCLL
IYDRKFDCITPLMHQWSYQSMIYEMLNVTRNSVRIG-----EEDFVLNPDFDDFYGSHLF
KEFSDVESALTTMIQESK---------KTFSGAVDILQSLPQQTKICNETKRHVAILHEL
STQIQRKNLLNTGLLEQDMGTHSKSADAFEAVVEIINTATVDSFEKLRLALIFCLEYRRH
EKINMIKDNLRLNGLE-SMVPKLSALLDYA---VRPDNASDSTALFSKAKNTINKSLSGA
SSPYMQYTSRLAHIVQSLLKGRLDTHSFSMIATSDSGFNLGTRPSSVVVYVIGGATFNEY
RDLQGVSAATGVPILLGGSRLLNSQNFTNNF------MPSPVKVKQRAVKTQQHRTDVYN
PKSSLKERRVSIYDNDIVETFPRTHRHEHAPFTKPRDVFERLTDYRFFTGSHRERFDENG
YGRGLAGREDVYIFDGNTESVSRPHEVYSTVLRGPPKTVVPRGVLGLQKFGVQIATPKLM
WLYRNGDKYHDGIAFYVRPFIKNMDILYQHISRDLELIAGPVRRIYDQNLHLITSLDEIV
DGAKYLCTSGEPPAPAHRLQKFMDEWVIQKM-------GKHEEKNPDSEATGDKKKR---
RSADKESEASKKKPKKSVENEKAKDKTSKKNVDKRNSKEVKKEGPKDKGKKKVSKSLSDI
SIFAKLGQKHVTPPKGDGTRGFYESLLEAHPNSVLAVVYCVEYGLFGGQKHHELYERYQE
MRQQGLLKGAAGGVRPAAIQVLQKMNRKSAKKSGDM-ADNNQNIEQWKIKQLIRSLEAAK
GNGTSMISLIIRPKDEIPRINKMLADEYGTASNIKSRVNRLSVLGAITSTQQKLKLYNRT
PPNGLVLYCGTVITDDGKEKRVSLDFEPFKPINTSLYLCDNKFHVEALKELLECDDKFGF
IVMDGNGALYGTIQGSSKEILHTFSVDLPKKHGRGGQSALRFARLRMERRHNYVRKVAEN
AVLMFITNDKVNVSGLILAGSADFKNDLMGSDMFDQRLAAKVLKIVDVSYGGENGFQQAI
ELSAECLSNVKFIQEKKLITRFFEEISTDTGKYIFGAKETIEALENGTVETLLVYEALEV
VRVVLHNPVTGEDSVIFLNDANERRDEEFKKNNVDLETVERKLLTEWIVENYSNYGAALH
FVSNKSQEGAQFQMGFGGIGGFLRYRLDMTEYYDDQLEDDDFM-MSRRGYGHELDQCKLL
EESLFYVKEHAYYMRQALDANDLGEALKRGINVISELRTSSLTPTSYYELYMKVFNELQI
LSDFMGNEEKSGVKLNQLYETVQQSCFILPRLYLLIMAASHCIREGKVSSNEILSDVTEL
CRGVQHPVRGLFLRYFLIQICKDKLPDSDANNPNG----TLESFNFLMSNFKESVRLWIR
LNNGC---------HSLLEQKRCDKQRLELGLLVGTNLVRMAQLEHLDCEFYTQTALPAI
LEEIESTKDVAAKKYLLDCLIQAFSDEYHLKSLPNLLKVIVNSISTNDCVKVVCTLMNRL
STYFQSS-ESAGDDVHVFEVFHDHLSTINI------------------------------
---RDGITLKCFLELQ
ASFVEFTSTVYPGEVILTHVVNVLSSCGTENMIHEPEACESIVKLLTLPLHTLGLRSLDM
QHNEPLLGFLPKHLHRNVARAMIDALIDSKLKIESCEVFESICRYLKSLF----------
EKAEYEPSGHILMENQNHVSRFIHTIETY-DPKDQFDIYQRLSKRMISPMHYRYSISTLI
CRSLMLVFKPFEPAGESTP-------------------VHRSPDTRDAADMALSIFNFVN
DLLARVKPMIPEESLKLSMMSAITVNELCGMQEESNYMKFGN---VCHNFIANCCIIFEE
EVVESDSQHRCLLYLISAFCSKITILDPEHQSSMAMRLAKYAIGMLKLEQQCSALAHVAS
SFANSG---DHFKVKWALQRSLTAVQQHQYLPLGDVSQALAPV--------KVVAANLQN
RFD----------------------LSDLMEAINQY----------------MLELKLNH
AVVLRRIFDCMRDIISDGNIDFDATGMSLQALDGNHVALVHLKLHESGFSLYRCDRPRAL
GINLNSVTKAFKSCSNHDSVLIQSEEEKDYISFIFENNVDDRVMSFSLKLMSIEQDALSI
PENTEGYDAEITLGSKELANICKQMNEFSDTLKLDVSINSVSFATQGDLGFGEIVLKNRP
PTNESDCGVSVKVRRPIKQSYATKYLLMFAKSCCLSDVVTLGLCQNRPIEVKYDVKDAIG
DADSPILGELKFYLAPKVDDAMDTDAM---------------------------------
--------------------------------VLTLGMFT------------SNHSEISC
RLSVERIDIGDPRAIELDSG--SSDWSCERITVFRGSKYWIFDCIGSAEVNGDLPRYLLS
GNKIYTISIQTGSHKGSGTSGKVSVMLLGPLGKSNTKILGTNFYSGSYLTFVVEAADVGD
VNGILLFNSAESDPWYCEDVRITSANDSVKSFQVKRWVGAPYESSVEIATTEGTSETTPM
DIQCHTRAIDIYSMS------VRKPFNITVRCPMNCQVSPLAHVMGSSLHHSSSSICASA
MFDGVLTPSGGEVVLSIVGPLKQYFGITNPDTHVESHDYEPSFEKPYYSFYTFLNNSIDS
IDSSVRLVDAFGKLSSFGRLEVLKNGKWGTVCNKGKFGAFNEAAANLVCRKLGFKRGIHI
LENCSNVNDQNLCVPRGYPVSYAGLMCMGTEDDISSCIIEEAVDCLAHRDDVVVKCTNSM
SHDGVGFGTLRLVDSSGSPTSTGTGRLEFYNKG-FGSVCNESWDKTAAMIACQEMGYTGL
KNGGMVGHDCSDVHGVNLCAPLTSKISAVDFRCTGLERALGQCPHESTDDIYCTHEQDVI
LSCSGNGDPSEFRNKRRVEPYSPEMKKLPRTLNLTCYDTLSSHAELQ-MKHGEFAIAMCP
SGCKGDPSSLKGTYIYTEDSSICKAAIHVGVI-DDNGGEIVVIRAITQPTFYGSVMNGVT
SLGAHKEAGAFLVSRATRHIMAMQLKPSRDSATPYDLKSSDAT---------------PT
PPKVRWFPELGWKGFNGSALDFVSLANFPNAEKMKTLRDFTFSVQMNPTELSGKWSTIFS
FQGCGGLTCAIDNTGEVIIEENCRPELFKTSYFPKIGRRTNLTIVYYSLTRDLGFFADGN
LIASRNTDFNFNLQGDLILGKSAETDSDFFVGQILSVEVFDYLMSPNQVQHHNRMLQSIE

```
GNR--------------IYSRHSSTRMTVEGNLCLSKCVKMRSPGRESGPTNPAIHLGCH
DTLEDERFNGATGDQFLVSCSRSCTDPLLTLKGS-KVYTSDSSICKAAIHAGAI---PHE
GGEAIVTIMHGKESYDYCMGHYDRDCKYKVEP-SRDAIILSIPRSKGALFNMYGYWYIHA
ENAHAKVSSWLPGTAYDSVWYSLKGIYNPISSVCQAAIHSGHLGELGGEVEIEILGSQDS
FNGSTAHGMTSLESGQYLKSFRILQGTA-----------MSTEIRNTKAF----KSFGST
AEKARHALSK------------------------------RNVERYKKLKHDALSELEAT
SVLHINEPGYL--------------------------------------EPEEGERTYHI
SQSELLKSVDVGTKRKVFNLQLSLGPYFANYTRDGRHMLLGGSKGQLALFDIIDMKPFFD
ISVKQTIRAVQFLDNHELMAVAQKKYVHIYDNHGMEVYVLRDLGLTYQLDYMAPFWLLTS
IGEFGELAWQDVSSGQVVARYKTRKGPCRLMRHNKDNGVVHLGHGNGVVTLWTPNQGRPA
VEMLAHRGPVVSMAIHQNYMATSGFDGYWSTWDLRSKSIHVNFIGKTPPQAMTVSQTGIL
GMALGGRVEFYRDVFTAPGLYLRHMYHGDQVNDIQFQPFEDICAVGTGTGFSTMLVPGAG
IANFDAYEPNPYESSSK---RQVQRLLDKIPYDTMTLKHVDVGDYNRDHEAVVDDKVDSG
GTALMNRRKRRKPLKASTAQKLTKYERTFQRRQQAAKDRLKRLRAPEEAKRRVVDALTYK
DSDKGVDGAALSRFKRASDMNLRGDTSDNADWYIIAHGKKT-QLYQFT------------
-----------------------PRNAATQSNDE------------CTLIWEEVDISHCC
PTHGGDRICINRKGS-NILEIIDLFSRKITIQLPPDCVISGILFSTKDTYLVVHTAWS-E
ANPNNLVIYRI------------------------------NGPDV----------TPVL
AIPYKSYTVKRYP--IWTPCETYCTIRVNNDLFVFKDGQYSTPLSKISLTFGDDISISPV
T-KNGACYLAAFTPEQKGTSGLVRIYNLQDATKPCYENVFKYAEEGEFFWSTRGTTAVLR
TFINNVKGLSSYYGGNGLFLLQPHKANHETIMETEGQAHEVSWSRTANDILIIKGTRPAE
LDMYDGNGNKILTFGRHHRNTIRRDAFDRLVLIGGFGNLSGDIDIWDLKKRCKIAQTKSD
CAVFCDFAPDGRYFVTATTCPRMRVNNCFKVFSYSGKLVSQIDFDELYHLYICS-PGRTF
VARDPSPTACTV-------------APTVKKSVYRAPGSRPDLRLEAQTTVATPARVSIP
RPPGADPTLLAQAARISRKKKDQANKRNN---MI---VKLVSAEGDTFTVNSEVLTPSVL
LTNMLQGYDEETEPIELKNIPTRTLGKILDYCKYHYNNPAKPIPKPLKSTRLADVVCPWD
LEFVNVDKEALFELMLAENFLDIKPLLDLTCAKVASMIKGKTTDEIRDEFNIVNDFTPEE
EAMIREENEWCKDLMDKEDKGKTEMISSPDEDSISEHNDMSLTHESVDPVVGNTSYAYEV
STPDDVCSPYRASDAEEHFYSVANEMTTSDTTRIKHTLSGPIDEHLMDQLHRDVYPDLSA
KDTPKSKTYT--VHENILNLDHVQPSDSSNDVPDTTPVEPTSSDVVIASDESVTSNDDTD
NDDLQASCSSDPKE----------NSSPED---------SSIDSQPNDDTADGPLPDEG-
SATDNAINSGPPQPDES--SSFWKDSDVEPRSADDSNTSSKSVSIHSDDQDTIRDAEVKR
KSSKKRLDKVVAGTSENPIDITDLTVDSGSLLQLYQNDYFDAYMHMHHLYHRKEPGVHEY
LVNLLYSKRTDSEVMFYLPQLCQLSISKYGKSSLHRFLLDRASTSMHFALKLSWFYQAII
SDQASKIGHLAQKMTQETEMAVVNCKLISPLSQSAAPTVDKE-LNLCSKGLLIRRAAITA
IRAG-------------------------------------------------HEGIFNT
---------------------------------------------------ATHIDALLR
PTMPKQMIYNATTLLCPFSKVKVPTPYSKIGDPLDLET--PPYVECPDEIQYELERLMMK
QRRLDYFNMLSNFVKLCMEVSKLLTTIAKRDARLPLLTLFAKSMNEWMLVQRCVVAAYEE
SFAYRGLSLPMNKMGRDK-----------QTNIPFQFLRIVEDEIKIFLSKKRAPFVLYF
EIANLDEDVRVISRGE--------------------------------------------
---------------------------------------------------------QGK
SIESITFRDMYVYEAIVKDLVASGLLDVADVSMNPLECIRYTMGMLPPEVLQSHNERR--
-----------------------------------EQFPLDSWRGEDDSVSSLLSAIHGV
AIGSSIKSMIQHRQEN---YNSNSPHSAS-------------------------------
-------------ETKFPILERMSPEE-------------AKAYIFPELFEEKKKKLRKY
SPYGNLESWNVRAVIIKGGDDLRQEYMVNQLLLLFKEIFANAHLPLWIRPVEILVTGPNC
GIIEFLHDTCSVDVVKRKFNVDSIARAFERMYAKTIYEARKNFIESHAAYSIISYLLQVR
DRHNGNILLDSDGHVIHIDYGFCLSNTPGNISFETSPFKLTKEYVDVMGGETSDNFGYFK
ALVIRGLLEARKHMDRIVLLVEMMTDAHKMPCFAAGTTYTMDMLRERFMLNLSEDVCIDR
IVAMIEESLYNFTTVQYDNFQRLTNGIKMAINVTIKISGGETFVVEVELSMTILELKGKC
ADRAGATPENQRLIFKGRIVKDEDTLESLKVEDGNTIHLVRSGVKRTSPPPSVSGTQSES
CPLSATSNVTDASGQFNPEMLNQMM--QGAG--------------GFGGMPGMGGIPGVD
DFNPQTAAALFNNPMIQDMMQQIANNPQLFKDIVSSNPMLQPMVQQNPMLSYMMNNPELL
RNMMRPGVLQAGLQLHQSMQQQPQSAGAN-------------------------------
VGNPLMSAGGYPGFAFP----------------------------TSMPADTRPPEERYA
FQLQTLQEMGFTNRDENISVLNLTNGDISAAISRLLEARGNM--TQENVMRDIQIAKLVL
NVGVGESGDRLTRAGKVLEQLTDQKPVFSKCRFTIRSLGVRRNEKIACHVTVRGKKALEL
LERGLKVKEYELKSENFSNTGNFGFGIQEHIDLGLKYDPSTGIYGMDFYVQLIRPGYRVT
KRRKCKSKIGKQHKVTKEDAMKWFQEKFDGIIFNIELDSLSHLRIVAVLGFISNTSVIIR
DKRS---------------------LLTHESQLLYLLAKVTQGGRIEVDK--DMVKIYPG
RIVGG----------KFRFECSPIAPLSYYLEPLVYLSVFSSEPFHITLIRQGGD-----
---------AVEEDFVNNYSLVLRIGCTSVDFKFKEPFEVVFTNSPLVQIAPMDFSTLSK
VKKIRGSVMVRCLQPSLGTNVIIGCKHVLSQVCDNIYIDLVSPKSAT-----QPYISVSL
IAEGT-NVVYTADH---------TISLKNQATEPAVPAISGNT----------SLSKILE
IAQRKSSQPEP--SATPNDVTPSIHE--HYEAIGASVARRLLGEIQLKGVFDTTHQHLPL
IFMAMSGDYQISVLRMGRMNDYSVQMLRKIKQFLGVTFIFEEEEDS-----ILLKCVGSS
FHNSGITTFMLNLAAPHLSRDFYRFIKLLCEARSKDEEERLVLAEISALKRLFLSKDVDR
DQLKEYLVRAVYVEMLGFEASFAHIHAINLAQERNIVRKKAGYWACRQLLQPDSELMLLL
INTIQKDLQSPHFMDIACALQCVCDLINRDMVPTILPSVIRCLDSENEHVRKHAIMAIRR
FHEFDNSCVENLTDIIERGICDPRPSVMGCTLSLLHDVIATKPRAYRHLVPSLVHILNQI
VDRRLNRGYDYHRVPAPWIQISIISIFGRMGRGDRRVSEQIYGCLQNVLQQAESCVVIAN
AIIFECVKTIAAITPRDSLTTMCSIAVSRMLSSENNNLRYAGISGLGTLVGINMSYAVEN
QLVVVSCLEDRDETIRRRTLDLLYRMTNSKNVVTIVNCFLVQLRSKCERYWSAELVSKIS
LLCEKFAPSALWYFETVLELMLLAPDLLKDELLFSTVHVLR-------------------
----------------------------------------ENMTDT--SFRSSVMSQVSD
LMKRSDNLPEMVVKMISWIYANFTTV----GYIGDTYIDTLLQFLQRYRQDS----SWVL
GCIRTLIIANDYNVSPSVESVLSQFECSNCTEVTQRCKEIRSLCT---LRPKLRFMDAST
LDI------SFLSDYVQQSLLNGARRYERPTESVSRPTETDIANSVPELRFEPYAMQA--
----------TQMAEYKETLTVDDLVNEEI---------------ICADVPRSWGPSGYV
DKGSVDHDPESTTTRDDEPPAVQHELDLSILSGGVRAPRVAEPQGYVWRKSDK-------
------------------------------------------------------------
---------------------------------PSPSRQHVEMARALFQGIGTSDDGS--
------------------------------------------------------------
------------------------------------------------------------
----------------------------------IESYNGGAVVAMVGNGCVAIACDKRL
GMNGQHTISSNFPKAFKVTDTAYFAACGLATDIQTMKSEIEFKTNMYALRCEKNMGVKTL
AHMVGSLLYSRRFGPWFVSSVVAGLDKD-------------TPHIYCFDLIGAPCNAKDF
VVVGTCSEQLYGICESLYRPNMEPSELFETISQCLMAAIDRDCLSGWGAEVHMITPEKIT
VSTLKTRMDRKDIVVNSNVDNYYDLLRNVSLLVVGAGGIGCELIKNLVLCGVRNLVIVDI
DTIDVSNLNRQFLYRAEDVGRYKAEVARDALLKWVPKCKVTAEVCDVLKWRPIDLSKYDV
VLNALDNIRARSHINYCCMRAGIPLIEAGSTGYNGQVYPIVHGITACYDCHEKPRNKDIP
VCSVRQIPEKAEHCVAWARQLYELIFGPDNDNNMLDLDPQIPDVDSITDSTAQKWVRDIF
EYLFDTQITQLLTLDKVWAERQPPRPIKLHDESTSSFVKNNPEDLPPTKMRKTGNQMVSH
CKPTVHEE-THNHSFGLKTMEVKTMDELVKQFRSALLGFI---SHRKNILGSAIFDKEDP
ICVDFVSSAANLRMINFNIPHLSTWDVQSIAGSITPAIAATNAIVAATQVMQLIHLLTTR
HISPGHSAD--YSLLRDKCKFVWIKSAVAGSAPLTRGALSSPEPLDEPNPKCAVCQQKVI
CVELRSLDDWTLESFASTICKMHMGMTMVNIDFDGRNLLDAEFM-EEDESYSAKMNTPLK
QYGIVYGSILMVTCLDSGR-QEEMQILEGLSTSADKFRLI--------------------
------------------------DMAHTTKENRDVYYRKAKEDGYRARSVYKLIQIFQA
YGIFHPIGDNLANVRKFDILEQRGNSYDCRNVVDLCAAPGSWTQCIRDFVYNEYFVYK--
-DAAKNLAENNTLCVKKVESCNL-------KPVIIAVDLQEMAPVKGVQILKGDITNEQV
MEKIKNLFVENVSKRLLETTEDNSELADSSLAQIITCDGAPDVSGVHQTDAYVQSCLIRA
AISVCSAILDPNGLFVCKAFCNDSDAPIYRHVNIFFDDFSIHKPAASRLTSAECFVIARG
FK-PMGKITRGLNG----------------------------------------------
-----------------------------YVHTAVSQDVKHEWLIPLMCCGDLGG-----
```

```
--YDKAINS---------------------------------MTT-DKNIIFIFDFGSHQ
SGSMVRFLRGLGITCELFPVEKAVETLSGRVPAAVILSGGSESVKDADSIKIDKKILEIC
ASKHVPVLALAYAMYALCETLGGKVTKLGPQESEYRKENVHVEAG--------------S
DSILEGVPS--SFVAHGCHLDKIDSIPTGFKVSMKDAHGNVAGIVNSQSNVRAFCFHPQE
VEEGKADMIMKKFCFELSRCEKTWDMRSYHEQTKKDVIKQGSKKFVVAGLSGGVDSTVCA
AIVHEAIKERFHGIMINTGLMRYQETQKCYDRLKAEIPGIQLTIRDSSAVFFKELKGVHD
PENKRKVIGRVYIEEFEKAMKELGYNHDNCLLLQGTIYPDILESDLNRRSKLPVKSHHNV
GGLPENLKFELIEPVRLLFKEEVRDLGRLLGLSETTFKRHPFPGPGLGARVLGELTPERV
EIARQADRYMFEELEARGLVDKVSQCACVLLPTRSTGLRNSARVYGMVVVVRIIMTVDFV
TAKFAHIDMECLAAISRKITENIPEVNRVCYDITDKPPATIEWEM---------MRINYG
DLEKSERQNQPQVCLIRVPFFSMLHVDSG-PVTVFQPFKSPVEGHLPGVSLAKRKTLGCR
IRNLGRLNLLHSPVGAAKPTE----ADDSSLPPVNPLVLYTSPEDKIEVDPMLSRFLRDH
QRQGVQFVFDCLMGLKEFNGQGCILADDMGLGKTLQSITVMWTLLNNGLNGKPAARKCAI
VCPASLVNNWESEIKKWLKGKCPCTAVAEGVKEKVVSKFTGFKYDRQSNVIISSYETFRL
HAKKLEGVPIDLVICDEAHRLKNDKTLTSVAIQNLPAKMRLMLSGTPIQNDLNEFYALVS
LCNPNVLGDISNFRKHYANPILLGREPDATKAQQEIAAERLADLSYITNQFVLRRTNTLL
SKVLPPKINMNVFCNLTETQKIIYTSYTNSASCRKLINSGEMTKSLGVILSLMKVCNHPG
LIKPSPSKKPTKA------DELIKEIH---------------------------------
----------------ANYNTLQKNRSCYPELSAKTLVLFRLLHNIRRTTSDRIVIISNY
TQTLDVFERMCKQCNYPCVRLDGTLSIKKRHKLVTTFNDNSHSFAFLLSSKAGGCGINLI
GANRLVLFDPDWNPANDKQALARVWRDGQRKTCYIYRFFSTGTIEEKIYQRQICKDGLSA
MLVTDENEIKDSLSGEYLRNLFEFKEDTISDTHDSLSCTRCNETTG------HVPQTADF
VEDDLLTWAHHTDLNTLPDPCLKSAIFE--NNPPVQTD---------EGYVSFVMSCLVE
FKESQTPQQRPVNT----EPAVKPSQENKRNATTNNSQKRKIDTSADD-EESMDSDDGDM
EDEEEEFQDEMSAEE-------------ERGLLALCNAPFNDDQPLVFKSEK--------
----AVRKIEPAGFAFFLTKEWELDVSYHHPVTNVSRM-------TMDIVKSYVS-CTNK
SLFSLKTQWAQ-LLTDAKCAEHLVDEVDKESVDSPKLTKANSKVCLGLVRDFVSKNQNAY
ELLDVCDSDDLSKIKANYKRIVLLLHPDKAGNTR-----------------------VPE
DMEHYVNKYRIRHLGDEQLKQQFILLQDAFTIMSDPQLRHEYDCSLPFDETIPTKEEAKL
ADDFYGLFAPVFELNARWSRTKPVPSLGAANSSDDDIDFFYDFWRNFETTRTFSHAAPHL
LDDAESREEKRWMERENLKVQRKLIKKELVRIQKLVDLAQAFDPRLKARAERRLQEKVER
KRLQEEQRLREQRE-LEEALARQRTELELSQNREKFEKQIVKKLRQHVRA-IGNKVPNGA
TLSQHFDRLSELDYAFMKETCESIYALLGHPTCLEGTD--ESL-----------------
----------------------------QFVKKMDSAVKAATIDDK-----VDVFESIL-
----------------------------------------LSVSSKIVPPAA------GA
PQDTHTVKRAEETEEQPVQWTTEELSRLSKGVEMHVAGVTDRWSLIAKHVKTKTAAQCIQ
MAREIASGKRL--------------------------DENTPAVNI--------------
--ANGVHSDSWSVEQQSEFEAALVKYPSSLDPASRWRLIASEVRGKTPKECLSRFKMIKA
TIAASSGKNMPTKRRNGGRSKHSRGRVRPIRCSNCGRSVPKDKAIKRFNVRNIVDASSQR
DIKEACAFSTFNLPKLYIKQCYCVSCAIHSRVVRVRSVEARKIRTFVQPRSMPMRIMS--
--------------------------------SLSILAVIIADHSHLDPTPPSGGGKKVS
VTEISFDSLIDDLVWCGNDHKTVLLKTQSGRLYRSADGGKQWSEITHLFQGTARN-----
-----GNYRVDSIVLCETDRNVVVIVGDSRNHFISGNAGQSFQPIAYEGPVNMWVFHPSK
PSWALLSSWEGACFAS-DNDEDCVHSVFVTRDMGRSFQRVSKYVAQFSWGDVASRSEDRI
YYSRYALESGDQPKQDGWNNNISFMYTDDFGNENVLIMEGGNKFLVSGIYVFVARVSDPI
RQTVNLYVSTDNAETFDRAQLPVELEERSYTILDTSQGAVIIHVGHEYEGGDVEVGNVYI
SDASGLRYSLSLPNNIRAASGECEFDKVYSLEGVYLANFRDDSGGVLNPRNKFKTHIDG-
------TKTQLDQKRRHVAHTKIEPDIRTVVSFNKGAEWHYLQPPKLDSEGKPYDCEGKC
FLHLHGITQYKNFAPFYSVEHATGLVLATGNVGARLRFDPSQVNTFLSRDGGLTWVEAHK
GAFIYEFGDYGGLIVMAEDQRKTKEVVFSWNEGATWFDFNLSKHELNVNNVVIEPNCSSL
NFILYGNRNGIGVAFHLDFSALGQPLCKGIWSIDSTSSDYETWRPTD-PHGNQCLLGRKM
AYKRRKQASECFNGKEFKATVEREVCPCTPDDYECEIGFTRSIGSNTCRVDGTWLMREGC
TSSSFFWTDAYRKIPGDVCTSGWIPHQVAVPCPPHSPLSNGSKMVLTTILVLSFIMLAIV
YISHNENLKHLFHNYGFKQFSYVAYTPVNSKKPMYAGGRFEPELGFIDAEQE--HDE-PT
LLNY------------LSGNRMGGNSH-----QQPTKQDQQQIELLDATAKIRPETVVKH
VDMDEPTKKFALELASDAIEKFKIEKDIAAYMKREFDKRFDPTWHCVVGRNFGSYVTHEK
HCFIYFYIGSIAILLFKNGALVKAALEKSKETKQPKYERIGYLLSSLLKGVKRETISAHL
TKL-------YGQTDLGDARDVHDGPIPFSEVKSSGKADIPMISVAGWCKTVRMQNAGKL
GFVILNDGSCKADLQVVVHEGAIGHQAVDKCTSGTTIAIRGTLVERPSKPGSFEGKEKEE
YEVHVAPLEGHYLEVIGENFDAAKYPIAKKYITQEFLREVAHLRPRSYFISSVMRVRSAL
AMATHIYFQRLGCIYLHTPLITTTDCEGAGELFQVTTMLEDCKTLGDLAK---KMTAHGK
KAKI-----------LEEMEMDYKKDFFKRKAYLTCSGQLSAENYACSMGKVYTFGPTFR
AEHSHTSRHLAEFWMVEPEIDFCDLAKNMDIAEEYVQFAIKYALDHCLDDLLYLESIGDK
GLVDSLRSFATEKFVRITYTDVIDILMQHETAFENHVEWGIDLSSEHERFIAEAVFKKPT
IIYNYPAKIKAFYMRRNDDGKTCAAMDVIVPRFGELVGGSQREERRDVLEDSIKQNGLCM
DDYTWYMDLRTYGSIPHSGFGLGFERLVMLVTGVSNIRDVIPFPRYVGKADFMLSRIASA
HFKMENTMKTEELYKMFQRLGVMYTEYVHPPLIAVKQSLEIDFKGKYDIVKNLWLKDESK
RYYLLVALHDTKIDFKYLSKQVKVKHLRMGPEECMEAMVGMKRGHLNPFAVVNDKNNEVK
VLLDERIKEQKEIMAHALHNTTSVCISTEDLLRFLRENNHEPTFVATCGE----------
---ETEVQPSA--AQPAVSQPATANADKEGHILGITVKKDTNFPEWYTQAIIRGGMVEYY
DISGCYIYLPSSYFIWEVFQQWFNENIKKEGVENCYFPMFVSKQKLETEKNHIEGFSPEV
AWVTKYGDSDFVEPIAIRPTSETIMYPEFSKWIRSHRDLPLKLNQWCSVVRWEFKQPTPF
LRSREFLWQEGHTAHKSEEDAMNTVMTMLRLYQRFYEEFLAVPVIPGEKSVEERFAGGKS
TMTIEAYIPGSGRGIQAATSHLLGTNFAKMFDIVFEDENGVKQLAHQTSWGFTTRSIGVS
IMIHGDDQGLVIPPRVSKVQIVIVPIIAKKELQKDIMAVAEDMYARLKKAGFRTHLDDRV
GYTPGFKFNHWELRGVPLRIEIGARDIQTQSCRVCYRYNGHKTDVKLETLEQSIGEALED
IQRKMYERAKAQMDESVVKVMEFDGVMPALNSQKLVLVPWCEDPETESEIKAETQRLSQE
GSDGK---TGSMKCLCLPLDQPEMPPGTKCFWTGKPATRWALFGRSYMARMSKQEKKKAY
FERLTHLVKTYPQILIVSVDYVGSRQMAHVRHSLRGKAEILIGKNTMIRMVLNTSFPNSE
AISKLLSCVKLNVGFVFCMGDPLEVRRIILDNKVPAPAKQGVIAPCDVFISAGATGMDPS
QTSFFQALGISTKIVKGQIEIQNDVHLIKVNDRVTASSATLLQKLNMKPFAYGLKIEKFY
DSGHLVEASALDITEDDSLDSVKTAVTNVNAFALAIGFPTSLSITHSLIGAFKNCVALAL
ENDYCFKEMQGIKDRLDNPELFAAAAPTAGAVEAVQEAAPEAA---EEPEEEEDDMGFSL
FD-MEGRDVSREPCPDRIVEDMGGAFGMGCVGGFLWHFVKGARNAPRGIIMQNAFYNARS
RAPVLGGNFAVWGGTFSTFDCTFQYLRGKEDHWNAIASGFATGGTLALRGGMGHAARNAV
IGGLLLSIIEVVSVVVNRRMTPTPRQQFERQMEYERQLK-----MTQIEIWEQ------R
SSADDTSSSDVERTENRLPLFGKILSATKIRPPSPKESSEAVTVESESDYTTAEESSSDY
DDDWIRLQSIRANNKQSPASYADWKLLPRAWDSVFYISAMHNMENREQRFFYCIKVQHVE
RISRASGLGNITMYTPKFTLKAGEDMKLPTPLFIWRKRRLTLPYGQLQHYSIEIELWKTH
RMRINSLHASQRITFQEIIERNSNFNITLNMHIELSERRYPVHKISVFLLLEEVFDFFFV
FENWWFTMSPELPDAVKTLPKTLKISVPTAGSYNQSSTTEASDSAYWSAPGTFKFQGTLR
QLKYASFTTKVYCHKTRFFVKPPALLGTCVLSLKSVQELPLVRGVVKKLTLGSRNMFSGT
IQGNIRCCIKSASISFFEDLKVRPAQPITGSALITQLDCRCHYLVVRVLRCASLPASNTD
SNTSDPMVKVKWDGIVNCTGVVESTVSPIYNQNMYFPIHLVDHRELIDPALIKHSLPVDM
SSKGPVMVEVWDHDETSSEFLGSVEVSLSKLYTNGVMQRRSLVDGIFSAGSY--------
---EPELDDDDDLSNVPYRRHMTRVYEATLPLTGATV-ASRGRKPTVSMEMYILPPMPTD
LYIPDEGKKVMRTDIYRDLSRRWNRDFDAWQVAYCDKYTAAVSSRRFTCVTKEDMQSDLI
PLCCFVKPIQVYIQLSPPGELMHWISNFTFKEDSMVNESWQMPSRFVLTRKGGLHDRALL
LCSCLLGLGYDAYVCKGTLDNGKREHCWVMTRHSDGTVTFWETANKRMWHLPQRWKTQ--
-------------IPVEKKEDKVMETG----SYQEINPKQAENVNNQPKHMTKRKQAKRL
MDCEIYGSDYVADVKVDLHSIFSGNEFIADGKVSLPKHT--TFLVDKQQY---EKAARRR
TFDPKAHLLVEGKTLVHLPYSTIEVIFNDKQLWGNLQFQHPGCITYDLELNDQWKPFLQA
PPNTFMPDVQITAPAPSSVCGATAKEIYGDIVEMIELMYAQKGRVANVAKDQQMDERLES
LIDLLEFRQRLDPQFDPGMPPHLKGWSTK----------NQAKPKYVKPEIK--------
----KKAALKVDA-NQDMEENDAKTGHVGGGGSQEKIEQMRSMMETANVLAEGVKKQDKG
QPTASTDDSVDNTAIVTVHNKERTKVSPAQPRTMPPRRN--VGLVARLKAIVFPAPKHNA
```

MYNEDAIVLFKNNRPWTISPSGFR---LKKPMPK----IKEPEFDIFKVLSNASQHLNVQ
ESEYSYKLDSSDCIQLEDDMEVPTPTSVIELPWNHPLSDYRQCINLAVHLKASKPEPQGF
NRRVQKRKMKL----------SERKGGFMKRIIGKFKLQETPDAHAMPQFMVDMQSVIKE
PRLTQNYADHPFPIPKEYLIHQSKQLSKWNWYYNMEARQFAWRRHLPIPHNHTFVGVPIH
FSTSDINDIRHLMHYSKRCKKMLVPNVDRCVNMSLGILGERITGKDVRMRNVTAVQAIAN
ILRSSLGPKGLDKMLVDDVGDVTISNDGATILKQLEIQHPAAKLLVDLSELQDQEVGDGT
TSVVLLAVELLRRANDLANSGIHATSIIAGYKMAIKECVKYIKDNLSKRMSDLGDEMAVN
IAKTTLSSKMVCVNLEYFASMVVKAIKAIETCDDMGNRKFPVEAVNILKTHGKSLKDSFL
VNGYSIMMGRAAQGMPIDISNAKIAFLDFPLKHYRLHFGVQVQITDPVELEQIRLKEKDV
TKERVQKILATGANVVLTSQGIDDMSLKYFTEAGVMAYRRVPRKDLRRIARLTGGKLVLT
LSTFEGEEAFPEDSLGTCGKVYEQRVGDVDFTFFEQCSSSRAATIILRGANDYMVEEADR
SIHDALCAVSRALEKDSLVPGGGCVETALSLHLEAYSRTLASREQMAIAEFAESLLVIPK
TLALNAALDATELVSKLRALHAKAQSSATPEDKECKWYGISLADGELRNNLKAGILEATV
SKIKSIKFATEAAVTILRIDELVTLEPEKEHDDH-MSTVKLKNLFGEPFKQVYCDLKINP
KPTASGGMAASPTYVAFPWEVGGGGLVSLIGLDKLGRNSGAEKLRGHAGSLQDMVFNDFD
YSVLATGSDDCSVRVWRVGNNEG------SALCNLAGHTKKTTNVVWNASTDYVLLSGSM
DNTVKVWDVKHGSAVSTIPIEGNYSYCNWSYDGNTVLVSTKESYVAFADPRDGKVKLAFK
AHDSNKATSVQWLGGNYGGD-YLATTGYVGNQTRQIRVWDARNTDKPVVSKDIDSAPGPL
IPYWDSDTGLLTVVGKGDLTVRIFQYLEGDLNRAGEFKCNGTIKSFCFLPNSACDKSRCE
LGRLLYNCTSKEINPISIVVLRRNSQAAMGEIYGNVEQR-RRTLAEEW-HGCDLGAPQKS
ISMQSSFNSQVSDSAKARTMLSVASPSADWESTATGKAFIEIVGHVNHLSIRYQPA----
FNSADMLEHLENLEKETMINLVKKEHGISMTSSGRNASQVMNSSATPNMAAVNTAAKVTS
VIPKPKEETTSNNASDGQAAVESATKGLMSIVGLSADRSASHRSLQVISEVKCVVRELVE
NAIDAGATDIVIKLVDQGLTSISVSDNASGIEAFNFEQLAKRSSTSKIKQFEDIFTSLSS
HGFRGEALNSIANVSTLEVETRVAKEEVGWYLKFDRDGSLIEKTPIAKKVGTVVTSSKLF
EPYPVRRNLLIKGAKSQATGAVGIVQQYALIYPEIRFLLTNMSST--NHQISSLFSSTGS
KSIREVSGEIFGSNFIKNVLDIKLTRDTWSVEGIISTPQTGRQNNDIQILFINRRPVDGM
KKLKRCIKDVHKQFSSKYNVAYVLNINIDSKHVDVNLAPDKRRLFLMQEDTITRQLKEGI
LELYMMRMAKD-------------------SITNDPLRMKQLQFSRSSTASSPDMQSSRT
ESTDSCPGNEVNGDHRDPGPRGNAKCKQRSLQSF-------------------MVTSTRD
NSVLKNSNTIDQWFADDH--------KVKAVVNRMPSSSRAETASSSVSTLSIH-EEVLD
AYAMSTQQSLTTVYNSSNDMNIAKDS-TDSGGIDITELTY--NTDRIVKQEPLAHTPRDV
TPDPQFNGDSNSSTTLEYNSTPEEDGV---------------------------------
-------------------------------PIKLDPHLSVVKQEVVARDSLTDAQEISN
VDRPSPRTEDAVNSDTAQTNEVLNLKLQRVYDDNFRKSEVYRM----FGSSSEPGFHPID
TET----IDPKIFLRMQVCGQFNNGFIIAKLESKY----------------------SES
NKVKYAVYLIDPHAADEKTKFEKYNSSVKIQRQPLVCERKVDLSPFHQQVVQANLDLLYE
NGFAATVVRQVVNDEGGY-----------NREPGIYLSSFPQVLGQILGEEDFVSFVHDL
AQSGS-----SSQPDPTNTSASQV---LWGANTIPRPKRIWNILANRACKDAVKLGDPLT
MKQMIVIKDRLAGLVHPWNCPHGRPTMKCLITTEQINSIITQ------------------
-----------------------------MVSTIPLEELKKTVLQSYPGIEQRLRSVFVQ
HDIVGDDRIPYEIVELLLRNFFYECGLEDYIREVTNSEGLMDHNRVAPYLIGSSMEDLSD
SNPERMVCCDEMATLAIVWLKVLSDVYKQEVSMMCGTFSSSTVKQETDVVHTVA------
-------------NANSPSELQSQTAE-FDASQQY--LEHINDSEDEAQLNQKN-----V
ENYIHTLVSEASNNRQKGVKCFVYSSEMTPNGGCISAGAALPQRRERTQGRRKPTAGCCM
AKKTPVPAEDLVDYEEQEGQVTSHNVIKS------GASSKGREDGTMGRGSYVAIHASGF
RDFFLKPEILRAIGDAGFEHPSEVQHETIPHAITGVDILCQAKSGMGKTAVFVLSVLQQL
DVQEDGTLAGGVKRDAGEAVAPSADRVACLGISHTRELAYQIKNEFDRFSKYMNGVRCEV
VYGGVPISRDIEMLKDEKCPHILVGTPGRLLALIKGKHLNMDGIRHFVLDECDKCLEKLD
MRADVQSIFMSTPKKKQVMFFSATMNNDVRDVCKRFVRSPVEVFVDDESKLTLHGLLQYY
VKLSESDKNRKLNDLLDNLEFNQVIIFVKSVSRAQTLDNLLNECNFPSIAIHAGLDQDER
IARYTQFKNFDKRIMVSTDLFGRGIDVERVNIVINYDMPDSTDSYLHRVGRAGRFGTKGL
AITFVATEADSTALADVQKRFEVDIPEMPESIDTSLYVNQM-------------------
------------------STDDIDAISAEAIFARARNRVSISAEVFGAYNNPD-LFVAPV
HEKTPEQATRIKETVLKCFLFSSVDAKDIDTLVKAFESSDVSAGTKVIQQGDPGDKLYLI
ESGTARFTKTSATEQHDLGTAGEGGCFGELALMYNAPRACSVVAETDMKLWSLDRSTFNH
IVRNAVIKKREKYDSLLQSVALLSNLDPYDRCRLADALTEKTFVD-EDIIVEGDKGTSVF
MILEGNAEAYCQGKLVKSYSEGGYFGEIALIAQTPRASTVKAKGKCVVAELERESCVTLL
GPMEECFRDNLKEYQKVLAELNIENKNLESVMHIKILPRSPEVAERYKTHKSYYPGDCGL
DLFCPDTITLAPKKTTDVVLGVKIAAYRVHDKS------------EIGSSSMRNVGWILA
PRSSISKTPLRLANSIGIIDAAYRGDIKVAFDNISDEPYTIQSGDRLVQVISYDGEEISY
ELVNELDQTERGEKGFGSTGR-MIPCDPHSIFFGLMGAVSSMVFSSLGAAYGTARSGVGI
SSMGVMRPDLVMRSIIPVIMAGVLGIYGLIMAVIIVLNMGHPGSYSAYAGYSHLSAGLIV
GFSGLASGLAIGIVGDAGVRANAQQTRLFVGMVLTLVFAETLALYGLIVGLIVALKPVAG
LCLPYASM---------------------------------------AAPTKKTIKRRNK
RTDKVVVVVKDKDGSNDAYNFVNLRRVSVPANRLTPLRNNWEVIVRTVVEHLKLQIRMCTK
NVEVRPGSEDTDLSSLQKAQDYLRAFMLGFELKDAEALIRLEDIFIESFDIKDVKRLNGD
HLSRCIGRISGKDGKTKHAIENMTRTRIVLAQDRIHIMGSFNSIKMARHSISSLILGNQP
GKVYNNLCNISKRLREKLMGNYCCFGRLREYQEKLESIEAQSYAPISNALGLPENDIYRF
DLAHDKGDIAEIVSLCESSQAIDPFTCQIHQWAENPTTVGALAATQLAIYASDEKNPEYK
DAIRKASGIPMLVKLLKSDEVDRYHAAIVALAFLSVGNHENCIEMYNAGAMPDLIRGMRS
SITGMSGACAHTCRNIFILDMDYRRFFVKCGGLKDLINLLSSSDDDDSETRATQLEAIYH
LEDFIMDGVEEIPEFVSLVKASGALSKLKLLEKSNDEDLRKAAKGMSVRLADMADGSTAA
EIFESAVGYTYDDLILLPGYISGSCNDVDVSSRLTRTLRLNTPVVSSPMDTVTEAKMAIE
IALQGGIGIIHNNLTMEESVEEVRKVKRYENGFIVDPYTLTPNHTVEDWMAIRDKYGYRS
IPITTDGRCGSKLEGIVTSGDVCFVQDKCTKIEEIMTRDPIVGHHPLTLQDANNILYKSR
KGILPIVNASGELVSIVSRSDIKRNRRFPKASHNENMQLLVGVAISTQPGSIEKAKKLMD
AGADVLVIDSSQGNSVYQIDLIKQLRQSYPNVQIIGGNVVTGSQAKNLIDAGVDALRVGM
GSGSICSTQGVVGVGRPQATAVYHVAKYAYGNGCPIIADGGIRSSGDIMKALALGASCCM
LGGAIAGTNESPGDFFYHNGIRVKQYRGMGSKAAFMTARTKSLRRYHMEE----------
DQPMVSQGVAGYTADKGSIHVLIPTMMQAVKHGMQNIGCNDIKSLHSGLYNGDVRFQIRS
YNALVEGNVSTKLMMINQSMFQMALAFRRKVSLDIFSTSGYRTS----DLTRTLHRNSQP
GRVFNGFRLFSSFVQSVINQVKRDLEKDEKFKEAMKSLE-EAQIKEKVNRLGEIYTRSKD
ACSHYVNKITETSS-----NSSAVKFVFTS------AKSLASGM------------TRVA
ELLH--DESESKALKAKWKQRVASQRAQSNINVDGSDVTAVD----TNVPDSNPQG----
---NTQEYALVLAKESVWERFGTRLRDMPFLTNFFENPVFDQLFGNSTLAKAVKEMKRLD
SSFDLPEFIESVEHVVAPHIVQCYLDGDSKSLEAHCGELAFNVLNASIRERDLQKLYLDP
NILILKDVELKGGMTMEEGYPWFIFNFKTQQINCLRDSRGHVVAGEIDDIRQVVYSMAVS
RHPDISRDGLEYPYMVHEVAIIGNTQCWMRLLHSRIEALDELVAMSKPNVESLGGLYKEF
KVATKEITIKLETHQKELEELYSQMGRNIPTKQTEPHKSSDASLWNSALKEMDDFQGLVV
GEHREKRRKYKQVVASAIKHTSKIQSRRATLEQEELRNKRLSCKNVCNMVAVYWKKIEKF
AWERMKRDLQATLLEKKRLRLDKFVEDAIKRIKTKETDTHKR--------GKTNDGAAVV
KNDDVPADDEFIVSEEMKQMERDDAELEVAMERENEAQDYKQEIGALEDDLNMPIEEILK
RYQ-----EDAAKYAAEYQETSTPGTDSDCLDSTESDSQDPNGNFNVDTKEPQSLEGEDM
YKKQEMEEALDQDMSSDSDDDTKQQQEIHALQNDAEMPIEQLDHMDIQELPSPSSTHEVA
GSNSDPQELSAVSS------------ASDDPDEVQVPCLIRAVLRPYQLDGLRWLASLYR
NKSNGILADEMGLGKTLQTIALLAHLACDHGNWGPHLIVVPTSVLLNWEMEFKKFCPGFT
ILSYYGTPAERAKKRVGWNKEYAFNVCIVSYATVVQDAHILKRKSWVYMVLDEAQNIKNF
HSKRWQTLLTFNTQGRLLLTGTPLQNSLQELWSLMHFILPDIFTSHSEFKEWFSDPLTES
IEKEQTSQTAQLVKKLHTVLRPYLLRRLKKDVEKQMPSKYEHVIKCYLSRRQRILYDEFI
TSRSTVDAMSNPSYRSMLFVLMQLRKICNHPDQLQPRPVESPY-YGMMQDVVIPSMMLLE
DRKHDQRLYIISFYRNPPNSLPGLTGKIERVATPVSF------RSYQGLKVPFNRLYSEN
---DNGYHKKLVHLP----VVDRLPISKCLSSKRSGGRRKQLINRMRSYDPVNYRRYVSS
GMEPVKSRVITDTGSINH--PADPMKLGDGAPGQTATEN--------AINGGSNIGVLGG
LREDTPDRMSMTRTIGVQV---
----------LDIADLGGTTK-LNEYIKQAKSKNKDTLVNRRELLEVNQGILNLFKVDKR

```
PKPKVELFADLVQPSADDIAEKYWWIMSRFVCTTGRPVQCVPRKVLISGTNELRARKAQK
KLSRPFIRSKTVTMEMTPGLQRILFPPRNLLHDDCGKFLVLGNLLNKLKNEGHRCLLYTQ
FSKMLDILENWINLMGFTYIRLDGSTKVDMRQRIVTRFNENQKIFLFISSTRAGGVGLTL
TGADTVIFYDTDWNPAMDRQAMDRCHRIGQTREVNVYRLISEHTVEENIWRKQLQKRRLD
DIVVDKGNFDTE-------------THTWFSNVDTLLNILKEQGSSSNNDDIYGRKVLHE
SEAPTEKTKSRVVNMLAEAEDEDDANALKSHHNELGSGTKDFQD---FQSDIISMPALVA
YSIKLLLRFNTPTLIAQRDEMQVKIKVASIDSDYVSSDSGDSSYSGTDSDSSDPEVEE--
-MTLTLVGLGLGAVEDITLRGLKAIQNADAVLLEIYTSALIDSLHDLESFIGKSIEQADR
ISVEESADKILEEARAKNVVLLVAGDPLSATTHCDLCLRAENAGVDVEVIHNASIINAIG
RTGMQLYRFGEIVSIPFFETNWSPDSFYDKIVKNMEANLHTLCLLDIKVRERSIENLMNN
RMIFEPPRYMSVNIAIDQIFRID--HTKHRLPSNTRAIGVARLGSKTAKIAAGTLKELKD
IDFGEPLHSMVICAPQLHDIEEEYFKHYR--IMGSGVPRGMLAARKLKSRRRSQRWADKA
YKKAHLGTRWKCNPFKGSSHAKGIVVEKIAIEAKQPNSAYRKSVRVQLIKNGKKITAFVP
RDGCLNYIDENDEVLVAGFGRSGHSVGDLPGVRFKVVKVAGVSLLALYKEKKEKPRSMGI
KGLIGFLSDAAPGCISEVTLESLSGTSIAIDASTALYQFTIAIREGSYLSSLTNSKGEST
SHIAGLLNRCIRLLELGIRPVFVFDSTPPEAKSQTLAKRKLLREEAESSLEKAIEEDDKE
AIRKYVGRTVRITQKENESAKKLLRLVGVPVIEAAEEAEAQCAYLCQRGFVTAVGSEDAD
ALVFRCGVLLKNLTASNKP--------------VVRVDLAKALELLELTHEQFTDFCILC
GCDYCGTLKGVGPKTAYNLIKKHGSISRILEVRSETLE------GYEAAQEYFRDPKV--
RDITTIDRCEANIDGLREFLISENDFSEERVDKLIERLQKARSKKTQLSLKSFFGYPPRA
ANIT------RNYTVPIKESAVDSTSDKDEVPSDDKVPSVN---EVPTVDK--VPSVNEV
PTVEEDTSEPPSEKKRNKRVPIEVDDSLVPDNLKRFICVASELGSDSVDRLLRRLLYSRW
RERYLGPRMLSRYMFFGDFGFGGMPGGHHHRSREVDNEKFYKVLGLSRDCSESEIKKAYR
KLAIKHHPDKGGDSEMFKEITRAYEVLSDPEKRRIYDEAGEDGLEGNMPHGDPSDIFDLF
FGGGRKGKKRGEDVVTQLKVTLEQIYNGAMRKLAINKDVVCDTCDGLGGPSDAFVSCDLC
NGRGIRVQIRQMGAMIQQSQSMCHACNGQGRSINESKKCKSCSGKGVKQMKKILEVNIDR
GVPDQHKVTFHGEADERPNEIPGNVVFIICQAPHDQFKRSGSDLIIVKQIQLYEALTGAV
FYIKHLDGRVLRIQTPANEVIRPSSIFVIENEGMPVYQSAFSKGNLYVNFEVQFPVSRKF
SAAEKDQLKLFPYKPESK---PSGTTAAEDVDAREVDPQEIHDRAHAQSQQQA-DSDRED
HHHEGRSVQCNQQM------------FQADRATYIQLLEVLQKSDKADTQTQKDVSDFIT
QFELRERCSVLYFLEAALT-APELHMRQMAAICLKRAINLKWASLDNDVKMQLKNGLVRG
IQLNDSEVRTMFGSAFVALFA-VEGYERWPDAPGLLLTLLSESPNEIVQTAGSTLVMLVE
DMAASGEAAWAHFMTFVTNQLVPRILELASTIPGSL--PFFCKMLCALIDTGCFN-TVIF
ETHFPAFWSLMGSIAQHQDPWVRKCVLKGMTETWNRRPLAILDSSAAVFAFVICSTNDAD
NTVQLEALQFWAQLLKSRLE-----------------------------ESVNSRLISQL
RTHLPQLIPVLIEHTRYSSWDYMSMDESHFEEDNAAVPDRVEDVPPRPEGE---------
-------------------------------------------------MTADEDEESAT
WGNNWTPRKGAALALDYISQVYGQEIVQFLLEHIEKRLADSDWEMKESAVLVLGAIASGC
MLAMAPYLPKVVEYLIELTRHPKPLMRSIACWCLARYAGWACHENPNENWLYRVLTAVLA
RVLDRSKRVQEAACSALASFIEEGGSQLKPHLEPIVETIVKAFSSYQARNLMFLYDTVGT
MGQVFGESLVQTPCCEYLLQSVLQRLGSTETHAPQYLALMDCISYLVQSWQQLYARYAEV
TIARAMNAVFEVLYDAK-------------------------------------------
-CYEITDGGTEPPRWDIIGCSLDMIATVIGVLQEHSRQLVATVCVTLDPDVIK-ELKLDK
---------------------------PTGYIP--DMINLCCQCADATVLQNVFALLGDV
AWQCADLVA--TETVIASLNLNLLNPSKIVSNNVCWALGVISHTDHKKRIESVVHEFYPK
LVSIL-VTETESMILQNVCITIGYFAAGYPAYVGANLQQFLEPWLRNISRSSSEHDKANA
LVSMAQVVLNTAQVPQGALAAITRVILECPPWCKELDITLHALAQRLSLN-PVEWNFLQD
SEKAK------------LRERTNI------------------------------------
------------------------------------------------------------
--------------------------------------------------------MLNC
DENVIAANLVDKLGFEYAEFITQVIQNRKNLLRQWEVQLRGRIAQDGIAEMQALLLRYTG
-----KGIDYHRHNMLAISPDELM----KSMEGQQRINTALGIPIYIPPKKDLNIEREYH
ECYDKAVIPPLSNPFVAENDLIRIDSLPQWAQKAFGIEKLNTIQSMVYNTAFKTSQNMLI
SAPTGCGKTNVALLCALQNFESY-------------------------------------
-----------------------------NGGEKNTKVVYVAPMKALASEVTGKFSKSLV
DLGLRVREVTGDTQVPTSELGSIDVLITTPEKLDVITRNSYSTGTQSDDSFLTKVSCLII
DEVHLLNDTRGIVLETVVARILRLIESTQETRRIVGISATLPNWKDVAEFLRVAPEHAYH
FGPEYRHVPLSQVFYGVKGKDITG---TMYEICFDHIIQTLENGKQCIIFVHSRNETSMT
ANKLIEMIQESSHQKLFQPNRDIYQRFHKQLKKSKHDNVERFAEYCMSIHHAGMVRRDRD
VVENMFKEGLIKVLVSTSTLAWGVNLPANCVIIKGT--FIGGLGVDRNINYLELTQIMGR
AGRPQFDTSGTGVLITEHKNLNDYIKMQTEQLPIESHLHRHLENALNAEIVLGTVVDEAD
AVTWLRYTFLYVRMRKNALKYGIKSSNDGEIFNQLHKIVRDAAINLDRSKLIRYHEPSGE
FASTDLGRIAARYYVDYETIYNFAVSLNADTDEYILERVCECKEFENLMYRNDELEELSD
LMRHSIFKPTRGLNHTTKISLLIEAHINRTYIKSSSLISDMNYIIQNIGRLLLAYFEVSM
SETVCAPPIGNLIYKWALMFERQIWDVKNVLYHFCRPYHAMYDRAKMQSS----------
----KLPTLSEGTATRLSTYNL--ESLMDLTHSEFSQLVKSRSEASAVESFLGFVPYPQI
IPSSRPITSCITEVNVKITLKNN-WSTRWNGKNEIFYIWLCS--EECILNKSKVMLNCN-
-KTSATVDMFVPQREDDTYF-ILKVFSSKWLGLSFEQQLRTKRLA--YVEEGYTKLLKLW
PMPTKALCDKF---DYQHKYFNPLQTQMLSYCLYHDDNLLVGAPTGSGKTVVAELAMFRL
WRTQVCKKVVYIAPLKALAYERLKDWNKKFG--MFKKVVEVTGDSRTSVKEIVNSDVIVT
TPEKWDGISRHWKTRKYVRSVGLIVIDEVHLLGESRGAVLEAIVSRLCFISKFTQSNTRL
VCLSTALANPGEIADWISVSSTKVFNFSPAVRPVKCHLYIDGFPLKAYCPRMNSMNRPAF
STIMRHDISAPVLVFVSSRRQTRTTARDFVSLLQVKSLRWTN----------------ID
ISARPFIDENLNVFVEHGIGIHHAGLHDSDRIRIEEMYLKGEIKVLIATATLAWGVNLPA
KIVIVKGTEYYDGKTKKYADYSVTDILQMVGRAGRRVFDKEAYAYVYTESRKVDFYKAFM
FSPFPAESSFHERLLDSMNSEIASGTIANKAQGLQYLKNTFFFKRLKKNPQYY-------
-------LNIDLFNAIEDLTNWVISKCVEKLNELGCISTKNDDN--VFIPSIIGILASQY
YISCETMANIMSSLS-DNTYYDSVSKILRIISNAKEFGEVPLRHNEDVYNMQLSADAVMP
IEASNPHAKTFLLLQARLFKLKMPIFDYNNDLKSVMDQLPRIFQAFIDLMACYRNFKNIE
YAMMIYKHLVIQSNLFDPIL----DFNDRSTIEARVVNVRSNSTLSKRGMTFVLTTHTVE
MGDIDIVVTVDNLTNTNDMYYLTLSNIYSNALYGFKKITLPGDFSFRYGIYECEKNLQ--
-------------------------------NMAIKNGLREELENHFRGMQIESQLSLKD
AIDICNQMSVECIKQEELEELLSLKGYPICYDGFEPSGRMHIAQGICKAYKVNQLKRMGI
SSVMWIADWFAMLNDKLNGNMENIRLVGEYFKHVWRASGMDMNAVKFLWASEEINKNPDL
YWRLVMDISRSFNITRLKRCSEALGRADGDNRPGASLLYPAMQCADIFYIGADICQLGLD
QRKINMLAREYCELKSVGARPIILSHHMMPSLAQQGGKMSKSIPNSAIFMEDSAEEVNAK
IKSAWCPEKEVCDNPCIAYFEHIVFPMFNTVTIPRKEKNGGDVIYQCHSELKQDYLSGNL
HPADLKPALASYINRLLEPVRIYFRDNPEANELARRVAELQQLK-----------MATGK
KVCVVGCGNWGSAVAKLVAENTPRYPEFDDTVIIYVLEEVFEGRNLSEIINTDHENKKYL
PGIKLPHNILAVPDLKQCIQDSDIFIIVIPHQFVNSTVAKIKSFNVMKPGSLAINLVKGI
ELTEKVVNCFTDTIEKELGIPCLALSGANVAKNVAMEEFSEATIGYKNKEHAVLFQRLFD
RPYFKINCVPGVSAVQVFGAIKNAVAIAAGFCDGLGLGSNTKAAIMRIGLNEIYRFACKF
FKDINTDVVFESAGVADLITTCIGGRNVRCAAEFAKHGGKKSWHDIENEMLGGQKLQGTS
TCEEVYKVLVAHNMENDFPLFVVTYNIAFQGAEPAELIRKFSNETLNPF----MEKLVRR
LGKLSVLAGSVALVPSTCLVDVDGGQRVVMFNRFGGVSEKTLGEGSHFYLPWFQMPHIYD
IRTKPKVINTTTGTRDLQMVSISLRLLYRPITENLPRIHQKLGPDYDERVLPSISNEVLK
AVVARYNAESLLTQRDQVSSDIRMAITARAKQFDIKLDDVAITHLSYGKDFSKAIEQKQV
AQQESERVKFIVQKSEQEKIAAIVKAEGEAEAANLISRAIQEHGTGMLEIRKLEAAKEIA
ETLASSKNIAYVPNTTNILLNASNMMEFGYVREYMSHQGGWDFSNHSRNARIKDNIKG-F
KVLKTGTTICGVLVKDGVVLAADTRATEGPIVADKNCSKLHRISDFIYCAGAGVAADLEH
TTLWLENNIELLRLNLKQKPKVQMCVSMLVHELFKYQGYKQCALILGGYDSKGPHLFSIS
PRGSSDSLPFCTMGSGSLNAMSVLESEYRDGMSISEAVALATKAISAGILNDLGSGGNVD
VCVINRDGATHTRAHAVVGTRTYAPI-PRSIPPGASCVLREKILSMKEYIVVDSDAGRGR
ADFGYSSVSSADPFGKAPEGYIPGRGRGATSFAGGVSRDDVSEAVDLTVVG--GEDSLNL
ENEQLFKDAEYDDEDREADLIYDFIDNRMDERRRSRRESQIRTEVNKHRADKPTIHQQLA
PLKRDLKNLSLEEWESIPSIGDYFKRKQQKRQHQYTAAPDSLLYS----AKVHMQSESSI
```

----GTSTPLGFSTPL----GIMGGSATPS-----GVRS---------------------
-SLISAT---------------SGDTSSLNDLGEARGAVLSITLDKVMDNISGQTVVDPK
GYLTDLNSMNIKSDSDIADIKKARKLLKSVIATNPNHAPGWIAAARIEELAGKISSAREI
IAQACEKCGDREDVWLEAARLE-KPEYAKAVLAKAVRMVPQSVKIWVEAARRESNVNDKR
RILRKALEFIPNSVRLWKDAISLEDETDAYVMLKRAVECVPDSVDLWLALARLCSYQEAQ
KVLNEARKHLPTNADIWITAAKLEESNGNQQMVEKIISRGLDNLSKKGVIHVRSNWLKQA
EQCEENNFVQTAQAIIKCTMNIGLDPALLKETWLEDGERMEEKKLFACARAIYRSALEQM
KTKKSLWLALAELETRHGKPEDVDDVLSQATKYCPNSDILWLMAAKHKWIQGDVESARAI
LADAYSKNMDVESISLAAVKLEREHDEFERARALLERSRKQCGTRKIWMQSIQLERQLGN
YSVAIDLCDQALEIHPYFDKLWMIAGQLRLELP-------EPDVATAINIFKDGADQCPW
SVGLWLLALESLVRDNEHAKARALVDAAKTKIRCIPRLKHTEQVATNTKKLSPTELL---
------RLARAGDIPAATTTSMQEEEE--LIESLCQNILKSCDLLWLRAVDIELESNAGN
AYFMMSSSLQEFPDSGNLWARAIFLEERNAQNSKAVDALNQCSNSPLVVMAAAKLFWRDG
KVLKTRKWFKRALAIEESNGVIWGTFLAFELDSGDNDAIKDVINGCTKAEPSTGYDWCRV
VKRVVNWRLTWPHKMYKFVEEHYNDILTEKLPDDIKAVLVPDQSKAGI---DGSMYTARS
SCTSGRFHS-------PGSSPSRLHIM--------------DKREDNLDQVISPAAQ---
----AKLDAKQQRLKAREEQERQKQLLLETTVSDLDSASFGYLPTISCLPTRQWV-----
-------------------EIKDIDSA---EGQVVWLRGRINDVRGKGTMTFIILRQQHE
SLQCIVDSRQEMVSKDMAKWAASLSMESIVDVLGKVVRPEVPVTSTTAKCELQVHKLICI
SKANPTMPFLIRDANNTDDKNPSVIKVNQDTRLDNRPLDLRATLNLAIFKIQSEICQQFR
NFLLKRDFIEIHSPKLLGGTSEGGCNVFKLKYFDNDACLAQSPQLYKQLAVVGDFRRVFE
IGPVFRAENSNTHRHLCEFVGLDMEMEIRDNYMEVVDLIDDMLKHVFHGINTECKTEVEI
--FQKHQPVEPFKWLDQTPRIRFTEAVEMLRGTELAESIPDDISSYDFSTEQEKRLGRLV
KEKYNTDYYIVYEYPLNARPFYTMPKEDGIMFTHSYDFFMRGEEILSGAQRIHNPDLLLQ
RAKECGIDPKTISCYIEAFKMGASPHAG-------------------------------Y
WMSFFRVFGSRCGAVGRSTGYLQNALVSWRKRVPDGKPLLSA----------PTQLVLFS
PAHCDEQPMPEAPPKAPQPPSPY-------ELLVFENLTREFKNVITQDNYDGFRLEADR
QLTNNLQLSHSLYLGTVMSETGYLYQIGTNYASSDGNCLAMGKIGLDGMLTGRVFAKYAD
-CEFKVSGNSFLRYDTRNAYEAGVDYFGKGWTASLKGAWQGTWILNAAYTHQLLPCLTLG
SELTYIVANGTSIGAIAARYVRGDHVFTGQWSKQPNFKGMDYNLQDTDTCRLQYVRRVNE
RLSLCTELEVTPATKESALRVGWDYLFRHARVQGNIDTAGRISMQAQDYSGFGISGCIDY
WSNIYRFGFMMHLLPPPEQKPEAM--MAEEMPQFKLLLVGDGGVGKTTLVKRHLTGEFEK
KYIPTLGVEVHPLKFRTNCGGIQFNAWDTAGQEKYGGLRDGYYIKGECAIIMFDVTSRIT
YRNVPNWHRDIVRVCDNIPMVLCGNKADVKERQVKAGHIQFHRKRNLQYYDLSARSNFNF
ERPFLWLARRLLNQPQLVFVGECAKEPEFRIDPQLAQECERNLEAAANVAIDDDG-DLMT
KYE-CYFDSALHIIKGAACIAFSIPTGRTTKSIVRKSTTAGVITLTSVLNQPTDSQYTLF
KQLIAQKIDENSKFRAFLVDRQIAESVYGESIYDARVVPIDVQRLRLVALEEWNINASTR
AVVRTTGQVGHIDIEKLTYKHDSQTLLISFVIQPETDIPDILT-----------------
----------------DTENISSPDQCPPKFMVLPPGEVDLQNDELFGMERSANATSATN
EIDYDKLIREFGCQRITQEQIERMERLIKQPVHPYLKRGLFFSHRGLDQLLDAYEQGVPF
FIYTGRGPSTGALHLGHLVPFLFTKWLQEVFQVPVVIMMSDDEKFLFREQLEYDAVRDMA
RENAKDIISCGFDPDLTFIYRNTDYIQDLYGIALKMQKKTTFNQVKGIFGFNMSSNIGCI
AYPAIEGAAAFCQAYPKIFRNDMLCLVPQGIDQDPFFRMTRDLAPRLGYLKPISIHSKFI
PSLLGVTQKMSSSIE------------GSAIFVTDTPKMIRDKVHKYAFSGGRDTAEEHR
KLGANLEVDVSYHYLRFLMEDEAKLEDIGARYKAGEIMSSTVKDMLVDVVCGIINDYKTR
REKVTDDVLDTFMDPNRECFKRFRKNME-WSSRQLRPSIDIVSLTSTKMIFILLNADVAT
ANSLRRIMLSEIPSLAIEVVTVLENTSVLHDEYIAHRLGLLPIESSSARNFEYRDKCTCS
DKCSRCTVEYSLDVKCMD-DTRVVTHYDI-NLDSNVNAPMPVPRPSADALGNATGGIPIV
KLKRGQCLSLKMTATKGLGKFHAKWMVANVAYKMEPVITINRQVAEKMNLNEKTQIVQSC
PRKVFKLSAYSTATKAEIVIDNNLNCIYCDECLNQAREMGYRDLIRVQPDETKFHFTIES
NGAISPEKILEICLDTLEEKLGSLHQHFEAQARSIGAKVHTTSKSGVTTMGGSIDLDMSD
TEGVCKDITQSEFGKILLKLRMEESPGLNSLLEVEEASKPLKGVRVSGVLHLTGEVGCLI
RTLNRLGATVRWASSNPFSAHDGICAALKAHDETTIFAWKGETVEEYWWCVYQSLRWPNA
DGPQLIVDDGCSAYTMIKYGLDLERRHAKDGSLLNPKDFDDRDIQCLVTFLNTLVTKEGY
FWEKLANQVVGLSEETTSGVTHFRKFWRAEGLLFPVMSTNDCVTKQKYDNIYGCRHSGIH
GFFNGGDFLIGGKTVVVIGYGNVGKGVAQGFRGQGAKVKITEIDPICALQAAMEGFDVVL
LEDVLETADIFISCTGGIDNITVEHMKRMKNNAILGNIGQGDQEIQMAELHKVPGLEITN
IKPGSDYFKFPDTGKVIILAFGRLYNLGCANGHPAFVMSASFTDQALCLLELWKNRDNDT
YGKCINKLPKTLDETVARYHLKALNVKLTVPTAKQCAYLGIKPEGPFKEDGYHYMYPYVG
EHQDHYGMNYNHTGQMDADYIMENEVISPRGEQHTPMVGFFSSKLLRSGLTFQCASLTLM
FIFYWAFGGNGIFVFDLYAAPESVKISRPFHLTVSSLQAIYLLGTFYVAMFQVFVSDNSK
AVRGFRAGSKILSAAVTLDLISSMLRLVQYIYAYFYMSMKWWTRYQQTKADWIFFQFGSF
TNSFALVMYGAAFFYLEAYHDEGTSEEVAWTNLVLFCLAGLTELLMVFTGYGALFSLFLL
AAIMSATVWSFSFEPLLEKWSPSLHSRDVNADFAEDAYEQGQQASYNQEQPEYTHIPNQI
NE--------------------GMYYQQQ-------------------------------
-MADAQGNRKRTFRTFKFRGYELEKLLEMPMENFVGLLKSRQRRRFSRGIKTRIRTLLKK
LKAAKKDLAYGEKPEPVKTHLRDTIIIPEMIGSIVGVYNGKQYINVEIKPEMVGHYVGEF
SITYKPVMHGKPGIGSTHSSRFIPLKMPAQTELTPDEDSIRMMLTCKVHIGTKNVENKMK
NYVFTRTQEGVHLIDLSWTLKKIKLAARAIVAVTNPAEVVVVSARPYGSRAILKFSHYVG
SQPIAGSWVPGTLTNQITQKFIEPRLMIATDPRTDAQALKESSYVNVPVIALCDTDSPLQ
FVDIAIPCNNKGKKSIALMYWLLAREVLYLRNQIQRHLPWEIMVDTFFWRDAEQMELKAE
QKEAMSSLRPPMDWNQMTGARANADWKVMA----GGNDEWGNFVDGRNWMADNTLPISLV
SESSDKLGSKCFHLSSSSPLKWKLHPVVVFTILDSYMRREEGQHNIIGTLLGVICEGNVV
EITDCFVDRHSLTEEGLLQIIKDHHENMYDLKQKVNPKEQVVGWFSTGGEMTELTCAVHG
WFKQFNTVSKFYPQPNLYEPIHLLVDATCDSGSMSIKAYVQLPLNLTKEACFQFHEVDLE
LMISPSESVGVSTLLKSIES-AKHRKFDRMRK-----TSTFDLSLSKLSELIDLCIERVE
AAIAGNKDADPELGRFLLKATTTDSIISQAKLEKLSALALQDNLMIAYLSNLANLQFALA
EHLNAS--FMEAFSTLLEGLASANAATRADADAKITSLKNENINTALELTLQVLLNDANE
ERRLQSAILLRLLL------DLSKSGDAPRNMWRAVNPEVKILLKQSLLQSIHGESKGII
RNNTCDTIADLCIACLEVGEWPELTRCVIQLMQSDNPVYKKSGFKLLGECFNFFADELSP
HLDSVIQLTKNALADGNPSVRTETICAVSEILEDDILEVASKLGDTTPYMIEHIKQLV--
ISNETSSRDELERSMAGIIMIVENNAKILRRHIQLFFNSMWEIATAHSAHIN--LDSDIK
CLAIEALITLVEKKPKMALSIPNFGMKMVKCLMEAMLDIEHDSYAEWLET-GEDDDDTQR
LYDVGEEGLDRMGRALEEIDNCPFMDWVLSHASEFIQQNNWQHIFVGIMAISQTVEYLTD
EEVEERMPSIIKIMVEKLKDQDFRIRFAACQTIGQIALDHQPYVQMTFFDEVIPALIAMF
EDRSPRVQSHAMSAFINFAEEVQKEDLLPLSDMIVKQLLTKVNPAANKSVREQAITSLAV
VAGVLEEHFIKYYSTVVPLMKEAIAKCVGPEERTCRGKAIECISIIGMTIGKDVFLNDGI
ECMNALIVIMQEPSEPDDPVKEYIDEALGRLCTALGVNFCHFLPTIVPLLIRSLENNVKS
FGEGGEDMTLMMGTEGA-AGLRTSLVEEMERTLNLVSNIVEELKENYDDYVVPTANALMP
ILNYVLTSEMKQRALYAVAHLINAKKLAIQKHGGSNELLYEIVLSTLNNVINNLQKARDD
TQMSLPVDILTANADGLYKCLDYAGPGILNVGIINAVGQKLLELIEESSKFKKLYSKYRS
NRDLDPDEILSIEIDEENEQRYRTSLLELFGVIMKHHPDEFMQTCHNACVQFVLAHLEKT
QADDIAVGLYLCDNMIEHLKSRTVPIWPQILPYIFKYVESKNANVRQSACFGVSLLARLP
EFSGMENEAAAKVASALRLTFSTSK-QEQQSATDNAIAALGEIIRHHGDKLNEAASYLNL
WLKSLPLKADEEEAKRVHKNLMDLIIANNPTILGPDNCNMAQIAKIFITIYETDFSTEEL
NKQIIQLMKHLGEAFLQQLA-PTLPKRLQAQLKNIARAM--MPTITVPKVELLSRLGGNL
SLEELDELCFSFGLEYEECIDEKGVEVVKIEIPANRYDLLCLEGLVTALLAYKNDSGFPE
VKYSV----DTPRDTAYIEAVPESKFTRPYIFAACLRGVKLTQQRYDSLIELQTKLHQNL
CRKRTLAAIGTHDMDCISEPLVYSLEKPDDIVFAPLSDNREYSASELMRIYEGNSHLKPY
LKILENCDRYPVLRDSSGKVCSWPPITNSDATKVTLDTRNILIEVTATDRVKGSICLNMV
VYCFSQYCEHPFTVEPFYTKYID-------NTVISPDLTERVLHGDMSYFRSLVGVPSLD
GQQVCSLLKRMMVKGRHCPETDSVEAVVSINRPDIQHACDLGEDIAIAYGYNRIAKESFN
CGGLRPLTYLKRTVRDVLSSCGYKETLMPILDSLVNMYDRMGWIKPDKLGS---------
-KRPVLLKNAKVSDLEACRVSLLPGILNTISNLRSASLPIQVFEVGDVVWQS-DESDVQA
VNNCNIACGYANSSTAGLEEVQGASETLLNELGFYSEYQRWELGESKIPIPEKWMYRLEP
FDDNAFMPGRCVKLVSSAVSATLGVMGVVHPDVVHRFHIYVPVTVLELDLSLIRSIS--M

------TTASAVLVYAGIRRPDSIDSGAFKVSTELFGWKNKRTGEVIQHKRSDLNSISII
NIGGGMYQVRFDMNASKGYEILRFSGFSEKAVDELKQHFDEHFKISPEVGSVAHTGWHWG
VYGFENDTFKLTIDDNAGIDIDAKDVTQVTVPTKTDLAVEFKQNK-GYINGDELMEIRFC
IPNKDDNELALEDLKQTFLLKAGLDELKSETLAFLTDVPLIVPRGRFEIEFSRKHIKYHG
KSYDYTMFFTNISRMFLVPKPNSPHINFIIGLHQPMRQGQTRYPFVVMQFDAEEDIELEI
NMPEEDLESMKLEKVMTGKTFNVVTKLFGTLVNKPIVVPGEFKSEKEEAGFSCTYKATSG
YMFPLNRSLLFIVKPVIFIRFDEIISVEFSRTGVSTQNRFFAFSISTKNGQEYEFTNVDR
AEFEPLSKYLASRDVKIKRLDEQ--------DASAMYRASQLEEEDDDDEED-----EDF
EDDGESDDDDE--SEEEDEGSN--MSTKKTTLGDCRFYEQKFPNPGDLLMVKVNRIEAQG
VYVSLLEYDDREGLILLSELSKRRYRSINKLVKVGRHEVVLVLRVDPVKGYIDLSKRRVS
PEDIVKCEEKFSKAKKVHQTVRHIAQKHGMSVEELNRICIWPLYQRYPNALDALKEAAIN
KTNIFKDLPIAPEVIDSLIADIQLRLTPQALKLRCMIDVWCFGPDGIEAVKSALMLAK--
ANEEHNIQVKLIAPPQYEIMTTCHDKDKGMDIITTALEEISNKIRSYAGGEFKQRGDIII
VGDEDERHLYSLLEGQESSEEEDSSSESEEEDEGMGDESM--DAVEPDADMDFYARVEAP
----KVRKCNVLMSLALSVVKRESEKAALLKLKGYERADIERNRGKHSVVCRHWLKGMCM
KGEFCDFLHQLVYSRMPPCRLFEKNGFCIDNQRGNCIFQHIVEQPESITAQDPRIANGIN
FAEISHETD----------------------------PDGFATAF---ILAVASVFPKIT
AIMEEAPPSPQPVDMLDPVEA-------VATEASIPESDDA-------------------
---------ASIPGLLKFDDRRL-------------HSSFVNSKNTKCFMIKSNNMMNIY
FSICYGIWATGINNTAKLINAFQSCEHVILIFSGNESGGFQGYARMMTLPISGLYKGIWG
SFQSRLGDNFRVKWIKQCSVEFEVLRHVTNQYNQNLPLKKSRDGTELPLDVAEIICNTLW
NAPDDDLLKGTPMATWERIDHKTYFEELESKNLLYT--------------------TFGL
SFSPINRM------------RNPVARDGDEWNVLEFALQLRCRTSRVNLLQAWNITKPDA
ISVFSRWAQTAPIVEAFINAETLDRNNSVQDVCTRGMDIGSNGFKVSIGNIKCSTLPLTR
STLRDA----QQAETQSGALDNQGKILPPK-NATTTNMMYGDKRIYEYFVCDVALGKSIS
VADEVEAQRVRLSMPVEYDSVYIESSEN-NNFLDVTLVPRGSD-----DTLSVEHLQDDV
FPQGVLPQYAFRKDYIVYGASQILPKYLIQFECDPSAEETFALPLCDSCQNDAATLYCAS
DTAKICKKCDEKLHS-HKVVSRHIRVPLNKMPRPVAKCRLHPSKVYTMYCTVCHLPVCQL
CTSGHIHGQGSQ------GSGSTRFIPIANAYDSAIEMQQHSSETVTRRKEYLNKLLEQL
KSIEKTVEDNCERVEVSCYENLEASLNDLHTSIQSSVEIIVAEQTENQRHLNQLKWAEHF
AAYLKNTLLPADYLRAWLRHCRFRAEVSSTA--CKEPLQEVFPNIDLKGELSIMHEQSVD
HAY-------HMAPKKAPQEEPRVLLGRPRNNLKMGLVGLPNVGKSTTFNLLSKQMVPAE
NFPFCTINPHEAVINVPDERFKHLCKVFQPKKEIAASLSIFDIAGLVRGAHKGEGLGNAF
LSHIDAVDGIYHVVRGFEDDDIIHTDGEVNPINDLETINQELILKDLDKCTKALVEINKV
YQRNMKIKSKKEELDTMTKAKEVLEKNQWISQASWKASEVPILNEYNFLTAKPVVYLVNL
SEKDFVRQKNKWLPKIAKWVADNNPGPIVPYSAQFESALEAFTDDAREAYLKDKNGATSK
IDKIIASGYNCLNLIHYFTCGPDEVRCWTIRKGTKAPQAAGVIHTDFERGFICAETYNYT
DIVEFGSESDVKGNGRYLQKGKDYVVQDGDIIFFKFNVTNKK--IGEISVIRQAEAVEVP
YKVTQVLDCDEA--VRLDQIIGQLDIPIRYLKDVAQAFYAELVNGLMAHRRHRNLWLPNE
CSFKMLDSYITHLPTGNEKGCYYAIDFGGSNLRAVRINVTGTGTMERMQSTFSLRH-ATA
LRPKGLLDRTATATELFDHFAKNIGNLMEEAGDVSDPGHYPVGFTFSFPCTMLSRRNAIL
LDWTKGFETGRDTTEQVEGRDIGMLMDEAFKRNHVNARVSIILNDTVGTLMSVAYQKPPG
YPECRMGLILGTGFNICYVEHDYLHYGYIGKVVNIECGNFDKLLPTTPVDFEIDWYTSNS
GRGMMEKLIAGAYLGDIIRRNMLLYLREKAPAKMWNIGTFTSIDAAEILNDQSETFEKAK
EIVKENWDAVLEHHHLAGLRRICEAAFSRSAGLAAAITATARKTRSYVTNKTTCAVDGS
LYVKNQWYRDRLAYYLKVSRSDLIGSVVMYACDDGSGKGAAIAAAMMAED-------VFN
VYMFIKWLTIVVIRTFFMKVTVINEERLPLYGPVILVGNHNNQFIDAATLIYAVPRQISF
LMAAKSLARRMIGSLARLAGCIPVHRQEDLKYAGIGKITWEDNSTTIRGVDTHFTMDVGV
GDKLFFIDEKIGVENVTSDTELTLQRPISRPCKDKKNGEEFVILPKVDLSDTYDAVSTAL
RFGNSIAIFPEGGSHDRTNLLPLKPGVVLMAIYSLLDGAEDVVILPVGLAYGDSHGLQSN
ATVYYGTGITISKRDVEEFQVDRHTVVNRILGIIEKGLSSCMITAPNKDIKGWIDLCGSL
YPPERSMVPTNKAFELRKILARIFWDHGEDHKTKELIKKLASYKQMLKNSFLHDDEVWLL
RQSLHSATLLFVEQGIMFFCYCFLALSFFPLWFPMYIISKILAEQHRQKALKASVVKLEG
ADVVASYKILVLMGITPLFNLGYGLLLGLYFGRNPKDIALIVIGSMVVLPLLYYINLRYF
NELPMLLRQLHIFPLILMGKINVWRENERELITTRTELQLLVREFIHEVGPKVCDNFMDD
LNAIMPKVMIDADTSRLKRSKSQWVPIFAKNYYENGEEILMSL-TSSLSAKSLLDIISDA
TNKPQVNHVFWNTQPVLRFSEDVSSDEIGPIDATSSVSKIPTRPYLLPDAFEWVDIDIND
ETHLTQLYTLLNENYVEDGECMFRFDYKPAFLQWAMTPPGYKKNWHVGVRVRSSKRLVGF
ISGVAANIKVLGTSLKAAEINFLCVHKQLRSKRLAPVLIKEVTRRINLCDIWQAVYTAGV
LIPRPVATCRYWHRPLDIRKLVTAQFSTIGNRMTISRAQRLYKLPVINDDFSMRPMEPRD
IEGVTKLLNSYLTSYKIHQVFTDEEVNHAFLPKKDIVYTYVKSEEGMVTDILSFYCLESS
VINNPRVSHIRAAYSYYNVATTVSFKNLMQKALHFAHEHSFDVFNALDLMENSSILEDLK
FGEGDGGLHYYIYNWRVTNVSFQYYDISFLMSLVNVTNITVGNNVCPVTAPLVFQIEFEC
LEDLKHDVEWKIIYVTSESNDQNSEGEIVLDAVCLGPIYKGILEFEFRVAPPDFNKLDPN
GILGMQAVLVTASYCDQEFIRIGYYTNNCYDDPELRECPPDTPIIEKMVRCI-IDQPRVT
RFPIKWDSDELL-----DPEGNDISRVI----NDHDEASSEDESDYDH-SLDKPD---SS
NAAQMDN----TGSTSSTLNISEGNTQDKTTSRKRPLDDDLELSMESRKVHRATGLFTIS
MDGVKTDAI---------------------TRIVVPAHAEGIMGVNVSPLKHTARFVGIH
DDDKFSIEHLLSDAELVHSKLAPLMRSLYFRILKVNLDTPCPLRERNDICSNIPKCYVGR
CQPQEVSPEPTLDGLEHFVDKLAEMDPKDLYVDNPWYKDFLGIYSH-NRDKAVYVDLMHN
PPSYTGYRGGEDWNSIYDLQSDCGDEVPCDQTEHLFRLISGMQSSVAAWSAWNYKCVNSV
---AAYQLKSE------LPKYESNPQFYFKMLGNHPERIENMYYTFQAMLKTVCRLSPFL
KGFAKNLGQHPEWAHLQRSIFDFLSPAYEQRSSEHCDTASPQLRHPALLKKFNSIADIVD
CVGCEKCRLHGKLKLTALQIAVRAFGQTERLVLERNEIAALLHALDYFAESIIFVQRFEE
LKKRQLILYPLRIMLAIFVILVVAYRHVIAQLCLRWPDDDQDSMARNSEKANAMLNKWLR
IKSAAHDTQLTRKPRHTSEVTDYRTAEHWRNLLVKDVMISISRIQNASLGEFAIRDLNDE
INRLIGLRKRWDERVIELGGPDQRALSSAIENAHGAELKGGGGYRYFGAAKNLPGVQELF
EKQEMDERRVD--TYVTRAELYRKINPDYYGFRDDEDGMLSAAEAELERSLHTKGLSEVS
HC--LMGIFRSVTMSHGTLVIPQERARDCIDLLCRNTNIQFVDMNERRLDRPYKKYIQRI
LNMERMIRVLTEEVTSLPGTMIVKDRIDDFLRYDKVYRLDQVEESLVKLYEQFEKFKQND
LMLKTELEEVMNEYSVMLVALKQLNASKKQRVQKSSGMDESAESNAQLLSESDETEMVNI
SPSPESDSSGSTLAFSNIAGVISAEDKDAFSRAIFRAMRGNVYTFFQDIKEAILSRGLIT
EEEA----------------SIRGNEEKIVFVIYCQSASGSSTFQKLQKLCNGFQAKTFA
WSKSHSHINQRLQELEEIIRDRQKALNAFKRYFREEIACLLECPRPDGNSVIEEWSLFCR
KEKYIYYILNHFEGSDITLRADCWFPEEEEETIRTCLQAEKSEGRVSALLLIDHQFKPAT
MPPTYNKNDVFTSAFQGVVDTYGVPRYKEMNPTPFTIVTFPFLFGIMFGDIGHGMCVILA
GLFLIIRYPQLRKKYNDEMALMILNGRYMILLMGIFATYTGFIYNDFLSLPNNFFGSCWV
RERAALAHGAAAAGEVT-ETLVKSTESFPVSFGLDVAWIHAVNEQPMLHSFKMKLSIIVG
FLQMMMGILLKGMNAIYFRQPLDFFFEFIPQLVLMCCFVGYITFLIFYKWLTPVT-ADYP
KPSIIITLIDMCLFKELAEHDVMYPGQRHVQKVLVSMMMLCIPLMLLPKPLYMWYQQRR-
-RIVIGD-DHPKDHEMVYR-------GYDVSEIANAEII--------DREENAVETSPFK
RVATDSTRYEGEFAVTIH---NDGTI--SEDHSGHSMTDIFIHQLIETIEFSLGIISNTA
SYLRLWALSLSHQQLSAVFFNQTVLRTLSGESVVGTTISLFFTSTLFAVITAAVMLGMDT
LECYLHAMRLQWVEFQNKFYKADGKPFKPFNVKVLLENPEMLGLQEGAVY--SSHGGLAG
TDEVLNIPSSLPSLQNVYTYFSIFLNSYSADSMQQGKLKRMLLENIKNNQYLLEVKLDDL
FQFQNIVENNKEGAIAEVDFANALPASYAGDLVRVLTERPLIYLSTIERACFDVCKLHHK
FDEVDESFNFIQINLLNTFCRPTPIRALLAAKQERFVVVPGIVVQAYRPQHKMKIMTIQC
RYCEHKMKLDVPLWISKPQIPRTCRYAATLKATGSETANMENQLGCFNAHNPYVVLPNEC
QFVDVQTLKLQELAEDVPTGDMPRHLQLNVTRYLCDKMIPGDRVMVHGVLTNYNVNARGN
DSTAIGSSYLHVLGIEKLTQGKGEAISFDLEETNDLVLLATQPDIHDKIFRSIAPAIYGM
ENVKKAVACALFGGSRKEVGKDNRVRGDINILMLGDPSVAKSQILKFVDHVAPISVYTSG
KGSSAAGLTAAVVRDKMGVFSLEGGAMVLADGGVVCIDEFDKMREDDAVAIHEAMEQQTI
SISKAGITTMLNTRCAVIAAANPTFGSYSDDTDTSEQHEFKTTILSRFDLIFLLRDKENV
RRDSTLCKHILSLHANQSQTEICPIPMMKLRRLIQYAKQAVSPMLSSDAKDTLRNFYVQK
RREYREDKRNATKKIPITLRQLESLVRIAESFARMELSPISTEKHIQMAIELFMVATGET
MKQSLAVEVMSQHEQLMVKQVEDYILHRLPNGHRLSRRYLIRDLENRGFALQYISKMSGS

LRNYSKTFRNPKRPFEKERLDQELKLIGEYGLKNKREVWRVQYVLSKIRSAARYLLTLDD
KDVKRQFQGDALLRRMVRYGLMGENERKLDFVLGLTLNKMMERRLQTKVFKLGLAKSIHH
ARCMIRQRHIRVGKQIVDIPSFMVRVDSEKHIDFALTSPFGGGRPGRVHRKALRAAAE--
-----MSRASNAAYDRHITIFSPEGKLFQLEYALKAVKNSNITGLAIKDNDAIAVVCQKK
LSVQQGNQDVLLDQTCVTHLYHITDDIMALLIGLPGDCMSILYKSREIALEYQYKYGCSI
PAKVLCEKIADINQVHTQHAYMRLRACTGLIAAIDEELKPVIYKFDASGWYSGYKACGIG
TKDQESENALERILKQRETVDISDKIKSDLQKETHVTVEGLKALIAIDNGAKGVEVAVCT
IDNPTFRQLSEQEIETYLTYIAETDM----------------------------------
---------------------------PDEDGDHTTEVTYPCAPAPPVEIKLFVARIPKT
YEEAEIRKMFEEFGDVKDVVIIRDKATNAHKCCAFVRMVSISQADAAIKRLNNNCVVDTA
LGAVLVKYASGETERLGFTSLVEPGVNDAKLFVGSIPKNAEEDLIREIFGPYGTLEDIFI
MKDQ-NGAGKGCAFVKMAYKEQGLYAIRSLDGMKQLEGCPRPMEVRFAESKANKQQNM--
-MMQHQMPGRGIIVQPPYPIPT-----------PTHVRQIGVWREYLSPEGKPYFYNEQT
GHTQWERPPEFDNAASS-------AQVPAGPPGANLFIFHIPNEWTHHDLVHTFSQFGKI
LSSRIASDRSTGRHKGYAFVSYDTPESAAQAIQHLNGFTVLGKRLKVTIKKGDESTVPVA
SSVPGASMQARLATPAYQAYQHTSAHGGQSFAQQTYPQQPYYGSYQR--------MSTAH
RPTWHNAIG----RSPGANFGTFKVSSRDLPSHSELKRRDSTETADHKRKLSEHLENKER
EHREKNELERNFIPLEDAVKLLNQD-VNEFPEDSDDC-----------------------
--------------------------PANEGSDSEDDDEAMLMRELEKIKAEKEEARQRE
IKATLESEEGRERILSQNPLLA--------KTTQTRRWDEDVVFKNPNREVEKKE-----
----------------MNDDERQQRAVTLSQEAIELDKAGRYSEAFDRYLRALDQWTIVC
KYQQNPVLQDRFYAKMREYVERAEALKQMLKAGNANETKAPC-TDDSQSTGISEQLEALL
EVKRPHVKWSDIAGLETAKQSLQEAVVFPMRFPNLFTGSLKPWRGILLYGPPGTGKTYLA
KACATELDASFIAISSSDVLSKWLGESEKFVKSLFQAARERAPCVIFIDEIDSLCSSRSE
SDSECGRRVKTEFLVQMQGVSEDSDGVLVLAATNLPWALDSAIIRRFDRRIYIPLPDLQA
RRQLLELSLKSCE--HELTSDDLDELAQCTEGYSGSDVNVVVRDARMQPLRKCRDASFFK
KVIRNGEEFYTPCAAGLC--
---------------EM----------------------------------KKAPISYEK
VPFVDEDLERVMEEVDFRFYYIDDGGRNPYSSIPMNEEIYAEGTEGRFEAVDNSMKFAKL
ENGLRIASVDRGGMDSLLGLYVGAGSRYE----GADELGVSSMIENMAFHSTAHLSHLRT
IKTVETLGGNASCNAFREHIAYHGECLRRDVPIMVNLLIGNVLFPRFLPWEMKASKSRLD
DRRKQIMSSPDQYITELLHSVAWHNNTLGLPNYCSESSVSNFKPEVMRNFMLRHFAPNNC
IIVGVNTDIAELSKWVMRAYNEYNAIEPVARNVEKPVYTGGVRYHED-NSPMLHLAVAYQ
IPGGWDSSELVVFTVLQSLLGGGGAFSTGGPGKGMHSRLFLNVLNKHEFVESCMAFSTVY
SDAGMFGMYMVVAPQASRGAIDVMSNEFRNMLSVTPKELERAKNSLKSFLHMSLEHKAVQ
MEDIARQLLLCDRVLTVPELERAIDSVTALDIQRCVQSMLKGKPSVVALGNLAFMPHPEE
LLKHFHMESGIKVPQETIQVFNQMKLKKSCRYLILGISGDVVTVVNQGSGEV-DELYDAL
PKDD---CAFVLYDTGRYVVLFMYASPSAPTNSRTIYSTTKQTVEKSLEGSKVYKHLVED
KDEITELKGQ------------TDPFGNRAKHAQQSFQNQNVRPPMTAP----------G
QVQPMSSGLEPRNDIA-------------------------------------SDNVIKG
SLP---------GQVFHDNLTFNHVGPTTASSGFTEN---------ITESLENMKSMNAP
SQFIRSTVGVLPSSVKLQQKTNIPLAVVLRPMAPLSDSDPEIPFVNTNSETISRCKRCRT
YINPFITLDGSRRYWTCNICGVSNELPNRY---SDIPSIGMDDSVPVELRKGLIEYMASA
DYMARSPQAPTIMFVIDVSVSAVNSGMLEVVCQTISDLIKTRELPG--------------
GPRTLVGIMTFDTSVHIYQMNSGGSSPNILMLSDLNDLFLPLPNGILLNLYESESEILDL
LSLLPSTWRNTNVAGSCMGSAMRVAHFAMQKIGGKMCIFMATPSYFGDFSLNADALS---
--------QKSSGGSN----------LHPV-EKCKDFTTVLGKDNVSVELFVCP-QSVNL
KQLYHLASLTAANVHHIP-LRTHVGNAKLSDELTRVLTRETGWESVMRVRASKGWKITNW
YGHCHVRGSDLMVLANCHADQTYTVTFEHEENVVTDKIAYIQSALLHTTSNGERRIRVCT
YAIPISDNVSQVLCSVDPEAVVLTVAHLGINSVLGGKLSDARAQVQTHCSRIANSSLNAQ
SALSQIVVYTLGLLKSPCFSE-GNVPDDTRVYHCMRLMSLPLDHLAVYCYPRLMCISDL-
--GSDYNDKGLGTLPPSLKLTHSTLSQDSAYLIENGECMILWVGKGVSSQWLQSVFDVPS
VDALNCDLAESFMASCRSPAAIRLSALIRNLRHMYLP-YMHLYVSKQGDDSEMKFFAWLI
EDKTPGMMLTLAEFTNAISLRSPLNFTLPS------------MSNDIWIEKYRPGILKDV
IGNPEVTRRLEVIAREGNMPNLLLCGPPGTGKTTSILCLAHEMLGNHFKNAVLELNASDD
RGVDVVRGAIKNFAKKSVVLPPNKHKIIILDEVDSMTEAAQQALRRIMEIYSKTTRFALA
CNQSTKIIEPIQSRCAVIRYEPLQDEMILTRLIHICKEENVQYTNDGMEALLFTANGDMR
RAVNNLQNVSSGYNLITSQNVYKVCDVPSPELIRKLLQDCLDGQWRPAHEKAEDLLALGH
SPFDILMTVRSVLKTLKA-PEHILCEFLKTISLSHMTMISGLSSPLQLE-----------
------MASK----------------SDQPSQLVKYG-----------------------
----LTAKILYHLLAFMDGYDIQSLSVCMRAFEISLGLSPSSLAWMASVEIVSLVACGTI
WGYLADFYKIRYLLCIAMNLVGLSAIGIGCASNYALIMFLRVVHGAAMGCTAPAIQQIVT
GATDKDSYGTAFGIIHAVSCFGRLVSAILITSVAIKVFGKIYGWRICYIAVGIVWILLGA
LMAIYLDSADESN---------------------ITIEKEPNNIW-----ETLRAIFRTW
TSIILLFAIFISDAPFAAFTYMILYLQYLGLSDLEAGVACALTLLGGLLGGGFGGFAVDM
CHKKSTRYGRLIAGNAIMLLRLSVTLAFFLPLPQNGLSWYHY-VEIILLGSSLMTVSAID
RPIMGAVVEKKYQASATGINRCIAGILSSLTFLPLAGLLTEMAFGYQKSQLPIDQLEENV
RSTNSDALRKAMMFIIGIGTVINTMCYIAFFFTYPKDSAETEERENEVVSI---------
-----RRAADGGVAQAVASVKEHQKFKKMLMFGMRSLSDFCNPQLQLYVENALDALNRNV
LPSLVTALSNFGDDEDIIFSCSQILQAMACGCVEEENQEISQKFAKDGGITAVELILNQC
PQDDVVLAYCYTVLECLGKIQRLPDGGAGLAGTISVNGAPSMGADVAVKALACLSSICAT
SSGPAKMKQHNGINVMLQLCLALPS-EKSKVASVESAMKAGASLAKAGILDKTCLDPVIR
VIEKYKTSKAVVGYGSDIVKSVVSTAALKQSLNDIQKCQAGTPEHQAAVDTLRSLSYISS
VGEQLAKDGALGLIVDLVKNATAQLETHAEVMLSVIAGAARILGTVAVTKSYCDEVVQKG
GVDNLVAALPLCTSDGACVASIADALAQLLQSG--PDAFIKTDAVATSLPILYQMADQEN
VAISMLGFVSAASQQSELQPAFVANKVVEILCTCSQYHLESLAIHMNIMHVFNRFSSHVT
DLSVVYEYGGLQGIAASMSAHYKDDKYCLEVLKLLLTFTCTPGSDQYMKTGDIIDTILEL
MLEHQKNDAVNGAAMKVLELLATEDDVRRTMKDLSRALQVSKEDVDGAYSKIAAVTGLAR
IARLRPIIVKSDMLKEIMQTVSTWVEGSNFTGRSKLTKAAMQCAQA-TVEDAAQCENTIS
MICDLACMPQVRRVIDLEGGEDNFLLHCTGALAHLCSVDRGYSSEQCKAVVEHINRVMRK
HMDIKNAETQCIDALSHFLQVTGNDGMDALLSTNTIVAVVGYLSKIPMYLPCQIVGVGFL
LACAKMDYRALECLKQCNTYQLLRALNRTHKKSRKLKTMVGELLSMIMPPDAFEAELIQL
LADLEKGMEVNDILAVHTALSSINQLLVSNECIRIAVRLHVPEILVKVCDWTSKSKALAD
KVIVEPDDVAGKDLIDSINLEIAQSIYNMGQNRSGLIAMTKLGTYATALKVWESVQGPVT
PILEDEACASLDAMTQLFVHDMANVDAALKNDVLSKVCRGFTLFQSSPNVIRSICRCLAA
MCTTDARAKALVESPEFNKLTAMLVQLLDGD---ENVSLGSVKAIAELLKCNNKLVIDHF
AKKTNLVSGLLRNIEMYPENSKLVALSATCLNYFGKGVVAGR---NDIPAVLTNITKALN
ENKNVATTVLPVLKLLLNMCTPETKQALKSSGVMEVVSDVMMIHIDDEAITSVGGELFGY
LGAEAQIRALMRQVIEVQMRQDTMAQEVDSLCTRLAMFLLSPLENRAEALADTEEFLGAL
NTCMAYSADNKNLIANATLVSRRLGDAVFNDFEDQFGAWAIANSSNLQQIIAILNSDYGA
SNVKFLCHAYRVFSCCAVNVYVTDMMQAECQTVMPRTMELLERYRNNPDVANAILDFLSN
LSKSQSGLRLFVDACNQRGTDPLTLLTDIMITHKQLDNLVIPAMQLMGDIVASGMF---D
NSASVKAALAAAERICVGAVSEERKLAFMDMVCKMLGSL--EKSECQRVVENVVMLKA--
---GVIARKSPDVLKAYARILAAAASCDLMQHLNKIGAMKML---AELLEDGSDKSALLS
VFQSLQKCVKSCPACAVPLLREALPKMLVSGADLITNDSECSHGFCDMLLDAVAIEGVGR
VLGGMEPLMNMLH----NLQMKAKENGD---KSLGDKILQILKGIANDQPKIRNCQSIYL
MLEERKSEEKTLSIVQAMELCDDVNFVLQTMAKYNEKVLAYESDEGKDIIYGGMAVDLLF
SLEENAQYLMDNGVLDVLMESLAKQNHLNITNSLASSLCTGAMRPNVVAKM-LPRKDFMP
VIGGYVKRVHQGKGMSDNIVEVVLINAMLLVERTAINRRIYLNADVINVLVDIWDAYDKQ
KYTMSLLRQTFRTLRKIVAEAHVDIMLKCNMVSRIKNILAT-SEDYNLIPDALFLVGSMA
VIKQIKTQICEVGLLDGIVQLMNRHVNPDSPNAIITNTCLALANACIGHKQATDRFMQLK
GPEINVRILRDYSEIHEVTNGASILLCNMLYKNDAFKDVYGKLGAPEALVRCLRAYAGSN
QAYAIRCIESLFKAISNLALYAANVTLFLDAGIEQSFCAWLSNLDPSFSDVQLKIGLQTL
SNLVMENKEGNMRKFSVLLMPVLHVMSQERQDSKVALLLFDILSSLCRSDENSELFLQNG
GCELCIQTMRRVSYDLVTLTLGINLLASQTTSPAGVQKLLELDVFSLLLANLTFEEYTPE
LTDILIATLRCLRKLVNSPEMVYLFCSQDGLSTAISVSRKTQQSPAIVIESLRLILGMLA

LTETKE--------------EGAAPAWENIGMEKEDIENLLNITFVCGGNEQAQKMIRLQ
KIVFSVIGYFMSQGLGSEVLIMNNFSSLGHIYLTNFPGTVEMVVLMTVVLENTFTVPAEV
RNNILTKEIMKKYRDVASSLPNKKEDKALYNRCHALVTALASSEN---KTLESTGHFNFE
LSGWNVDPYPHGTHDLPEAVKQGLRTGGRVKGYIRDNKRVGIRWRSSQDLNYLEW--GEE
EDYPYRIAVRRIRNIARGLRHPILEAANAKEPRKVTNNTCFCIMGSATEDFPDGFALPIK
CKNIKERDAVVELLVQWREAATYNYMSNSAQRFVRSILPYLDNTGSPAHSVVELEKFLKD
NFAKKLNRYEKWSLVKGGTYYLYDHNATMMAFHVGKGYNVENKGIVIAAGHTDSPALKLE
YKSENVAHAFNQPGV-----------------------------LEERLIRIEKPIIVVP
NLSVHLQTSEERQ-VLKLNKEKHLRGVVATEAVHNLHSN---------------------
-GSHPVLGFIAKELGVKVEDIVDMDLCMFDITKSSLSGLYEEFLSSARLDNLASCFSVLG
GFVDFVKSNG-------DYSNYITCVIFYNYEEMGSLMASGANSDITIEWIKKIFNSMDS
S------------FEENKDRAMVLNVDMSHAVHPNHSERHSDTHQPHFHEGLVMKRNING
RYATELRAAAVVIETAR-----EAGIPIQDFRVPNDSPCGSTVGPFLSSRLCVPVVDVGI
PQLAMHSIREICSTVDMWHLKEVVRVSGSVC-----------MNI--LTQAALFAILASH
VAIVKAQLRFASVGNWGTGSKYQKRVAETLKKSIANDRVTFIVSPGSNFEYGVTGSNDTK
WDTHFQSVYRSEDGSMEIPMFTVLGAGDWLGDFNSQINRNQQAYFT----SQVDEKNGAK
GLPRWTMPNWWYHYYTHFATTASMSLLKSGHKDMSVGFIFIDTWILSTAFPYKDVSNAAW
ADLKKVLEIAPKILDYIIVVGDKPIQSSGPSKGDAQLSYYLLPLLRDAQVDAYIAGYDHN
MEVIDSNGIAMIVTGNAGTGGRKPIMKTTNSAFFSEKAGFCIHELGADGMETKFINGETG
DVMYTHKQAIKKRPQRQYGNEVQHVSALPTVSLYPIGEMASPTQMDAFVKIVGTIGLIIA
GLHLTLLSGTTLGKAASMREKLLLGKLLVFEGIDRSGKSTQVKLLSQKLTDKGIDHKLLK
FPCYETECGKMLATHLASHAVRSRRCIHLLFSANRWEMMKEIVATLVSGTHIIVDRYAFS
GVAYSVGAENLSYEWCITADDGLLSPDLVVYLDNPAAVSAARSNFGDERYEQEGKLEAVR
RVYEEFSSLPYWHKYDATLPQEVLSKAIYERVASVLQSEPRRLHDAEFQKSVESTCKYCG
SDQIESCKQQGELVCRNCGAVLQENNVLEAVQYAENPAGNSTLIGRFVPTGGGGMGSLKY
SSSQTLDQLVKRGEQNIQRTACHLNISSELVTKATRIYSLAVQRNFTMGRNNKHVACCCL
YTACRRFKAPYLLIDFADVLQVPVKIIGQVFMKLVRMLHLEVPNVDPSIFFERFANELQL
KDKVDQIITTGVRLIQAMRRDWLCTGRRPTGLCGAALVVAARIHGVPLNAEAVASVVRIS
HPTIMKRLSEFRGTSTARLLTTEIDTVDLEKLPANPYPPCMVTKMEKKRRL---------
------------------------ESDTVSTCSDNARPSE---TSEELSPLLSPKQL---
------PALGDIDLKLPNELLCSDEPTANDINRIANSIIEATPQLGWVVSNQSTEIIP--
-------YEDKQTQE--
---------------------TATCTTIA---------------------IPEING----
-----------EGFLS---------------------------------SDDEDDEREFS
KMILSPEEKEAKTLLWDEVTKDIMPEVWRRQAERKRKEALGKTVKKRKYCRK---TYSDY
PEAQNAAESTRMALERHAKHFSNRMNQEFLDSILA-M------------DIDDTASEVDT
DLYSRQIGTFGIETMGKIQKLKVLILGMKGVGVEIAKNLALMGVEAICITDDNIVERRDL
GVNFFIRSSDVEVKTV-SDACLHHLQDLNRNVQITVHHGPIVEELITRHDVVVCCDQQYE
MLINVNRACRNNKNKRVGFIVADTFGMVGAVFVDFGNEFVCVDPSGKEINTAIVSGISNE
EAGLVYIHTEGSMPFQSGDFVTFSEVEGMDELNNGPIEITIKDKESFTIGDTRGFGQYVT
GGIVKEIRRSKQIDFISLEDAIQNPSKN--------------------------GCMITM
DLSLIGRAEQLHWISMAYR-------------ISGQSADAVLATAKTLNTKAQ----SCA
VEKIDEDVLNSFVKNARYRISPICSFVGGVVAHEVVKFTGKYHPIDQWLYCDFTLPTEIT
SGN--NSDIGYDSRYSDHIAIWGREIQSKIQSAKIFTVGSGALGCEFMKHFALLG-CGTQ
NGGIVKITDNDRIEVSNISRQFLFRKKHVGMSKSKVAAISAKEINEHMKIDALELAVGAD
SENMFNDSFWEELTVVVNALDNIKARTYVDGRCVWYEKPLLESGTLGTMGNVQVIIPHMT
QCYSESQDPQENSIPLCTLKHFPYQVDHTIQWARDLFEGIFTQTAHDLKRIQQNSPD-VD
DISD--------EKISLIAKLLK-INDTNVKTELLQIAAELVNKYFINDINQLLYSFPKD
HRTSDGHKFWSPPKRMPTPLTFNPSEKYVSMFLIATANILATVIGKKVLVNQDDVAMM-P
PMQFEPFKPKILKLSQDK-----LNVVVETPAECTISRSKSMQEIMNSRNVFESVEFEKD
DDTNYHIEFIWATANLRCQNYDIDQCDRMKAKMISGKIIPAIATTTSMIAGLVMLEFVKT
ICY---------------QKLK--IEHFRNSFCCLATPLWLQSEPMPPTTTSDKEYDPVV
GGAIRALPPNFTVWDKVKINIPNGTVGDVIEAIRVKFNVEAIILSAGNTCIYNSFMPAHQ
RERRSQPIAQLLEKLTKAPLLPSCSYLVIEASCTDDDDVDVVIPTIQFGFRMSKLTVETL
NEAISAILTQSQEKKRNFVETVELQISLKDYDTQRDKRFSGTVVLQNVPRERMKICVFGD
QVHCDQAKSLGIDYIDLEGLKKFNRNKTLVKKLANKYGAFLASQTLLPQIPRFLGPGLNK
AGKFPTQLTHNDNMEEKVREIKSSVKFQLKKVLCMGVAVGNVEMTHEQLRANIVLAINYL
VSLLKKNWHNVRGLTVKSTMGKPFRIYGM---VDGSMISETKWADIEADEDYDVGIDSHR
LTAFEQGIKVVTSYTKNRQGQTVKITKRIKESRIPRRIHKEAKARADGSIFKVDMEAGIT
MASQEEILIEQPVGRRSKVNVEDNLDLVYAPNLTRATRELKMKFKSLRDDSDVVDMMDSK
DMPPSRKEGGDRRNFDENTVRVTNLSEDVREKDLVELFSRVGRIHRAYLAKHKETQYSKG
FAFITYATRQDALNAINKLNRQGYDNLLLNVEWAKPPNRMINKLLCHHLCAVTATHTLLV
EKNPSSRRCRFAIVILIISVLFWHGLPNTGNTLYSTVGLPSNATQYDVVTHHSRMKTRFQ
KEG---------AEDIKNAFQILLNDEKRKMYRRFGDLDDVIGESNVALVAAALGLAYHA
LSSIICFALYGTSQMAFTRYVMMLYSSIAFALEMECRFVTANSIFKNIFYVNKLLPFQQI
ALLRGAAPAIALFLNAICARLFVDIERLSYFLWHSSVTTNRVILEKMVDVVDATNYIRSM
GPKSTAHVKLNKSVLNVS------------------------SEDETSEEKEDDSHASDD
TGKIVKILESMDVSQRKKVAKLLEKSEGDNETEEAASWFERLKTPAIYFALFVLFKYYWK
MGKDYYSILGVSRGSNDAELKKAYRKLAMQWHPDKHPDPVAKQKAEDMFKNVSEAYDVLS
DPEKRKIYDQFGEEGLKGTAGAGTTQYVYTGVDPSELFKRFFSTDRGFMFNGNFGDDMGG
FGD----------AFQMHHTTHSR-PSKSVNYELDLPVTLEELYTGTTKKMKITRKRFSG
NTEYKEEQILKVDVKAGWKDGTKLTFAHEGDQASPTSPPGDLIFIIRSKPHPRFTRDGNN
LIYKFTVPLVKALTGFQATLTTLDNRRVTTRIVDVVSPSYRKVIPNEGMPISKSPSHRGD
LILEFDITFPRTLTPEQKKQMIAVFNELGMK------PALSVVAVLTAYAAGFFFNIEYN
DRRCFYENVPQMAMISVNYEMLNQEARACVVRISDEEKKVLQNTNLKEATRGRVTYVAKN
EGTYYICIDCPGQLWYTAQMAKIALSIEIADDDVDRETTAKKDEMKNLSEEMRKFSINII
NIKNHQRLENNTAKELHDTYKSMYNYLLYFYLLQLFIIAATALFSVYHITRFFKAYRIVM
APKKKQVIDDDELALLDEFNADRGVVIEAPVEDSKTKKKKSKSAKKTQEDDAELDELLDE
LNALD-----EDDAAESEEQAKAL-KNKLKKEKKKQAKLAKKDGEDQDKKPMSLAARLAA
ERQRQLQEMEEKRKEEEERRRIEEEERKRVEEEEREARRQQRKERRERMKKEGKPMTMKE
KMAAEIRRKFLEQVEKDGLIDVDRDN---EPKKLDLSS---FRKKKVVKQTG---TEGQG
AADAAGPGMGTEESHSS---TESEDVIENWEEL---DQESAKE------------PVVQS
EPVETKAAP-------------KRVKHKAQDSDEPVEYRSPICCVLGHVDTGKTKLLDKI
RHSNVQNAEAGGITQQIGATFFPKKMLDKHCELINPEFKLKSPGLLIIDTPGHESFNNLR
ARGSSLCDIAILVVDIMHGLEPQTIESIGLLRGRKCYFVIALNKIDRLYKWKTTPWATFH
KTFENQLEDTRGEFFERARNIMTELSEQGLNSELYWENDDIRRNISICPTSAITGEGISD
LICLILQLTQKIMVKNITHKEEFRCSVLEVKAIEGLGTTVDVILLSGTINEGDKIVLCGL
SGPIVTTIRTLLTPQPLAELRVKGEYVKHTSIKAAMGVKLVAQGLEETVAGTELLLVEDD
DDIEQLCEDVMQDMSSIFGNVNRTGVGVYVMASTLGSLEALLQFLTDKKIPVFSVNIGTV
QKKDVKKASIMREKGYPEYSVILAFDVKCATEAEKEAQVLGVKIMSADIIYHLLDSFVKY
LEETQEQKKQSRISEVVFPCELTILPHCVFNKKDPFVFGVHVDNGILKPNTPLVAMAKGL
MLGRVASMEHNKKPVDKAVKGQEICIKVVGEPNIAYGRHFDCNDRVYSRITRDSIDVLKE
YFRDEMTNDAWKVVIHLKKVFGIILSTKDTSHLREQDLSKLDVSTLTSLSPEVISRQATI
NIGTIGHVAHGKSTVVHALSGVHTVRFKHEKERNITIKLGYANAKIYKCTNPECPPPECY
KSYGSSKEDDPLCLRPGCGHKMELKRHVSFVDCPGHDILMATMLNGAAVMDAALLLIAGN
EPCPQPQTSEHLAAVEIMRLRNIIILQNKVELIKESQALQRQEEIKKFVSGTAADSAPII
PISAVLNYNIDVICEYLVTQVAVPKRDFKLAPQMIVIRSFDVNKPGEEVENLQGGVAGGS
ILHGVLKVGDQIEIRPGIISKDSNGNIQCKPIISRIVSLFAEQNELQFAVPGGLVGVGTS
MDPTLTRADRLVGQVVGHVGQLPDCFVEIEVSYYLLRRLLGIKAPDGDRSTKVSKLKKGE
FLMINIGSTSVGGRVTGIKPDMAKFELTGPVCTRVGDKVALSRRVDKHWRLIGWGQINKG
KALTLQKSLMIKFFMAISRQCKLRLVKWFVPVDNKDRTAIMRELSHLVVNRNSKQCNFIE
WRDDKLVFRRYASLYFVLCVDRDANELLMLEIIQHYVELLDRYFCNVCELDMVFNVTKAY
HILDEMLIDGNLYECSKKAVLRNVSAQDALCEKTKGILSGNSSY------------MIAH
QSVFFIALSAFRGAFKCVRSRVLYSTIRPLHCNSAYSHFKSRSPV--ESNLPQRLYFSSD
ATEAKKDADGENVEELDAQVPEEKTAED-DSVDLTEKVTELEGKLAELTNTLKELQLKYR
ISLDNCEQIERISANKLQNAKLYAITQFAKDMLDVADAFELAFKALGSQHNVDLDS--KF

IEGIKMTESQLHKTFEKYGIKRFESLNQMFNPEVHEAMYEIQDDSVEKNTILQVVFNGYT
IKDRILRAAKVGVSRKM--LFH
CQSSYFAYFLFTSTVIAINGAHKGAQ----------------GCFSLARYSTIQQG--YI
------------SNTCVPPTRLTPLYNNHWNDGNNYVNEASESQISGGERNLALSLP-SI
EKNIVKHFDTVKESGTILDVKDLTIWIGDRVLFDNIAFRINRGDCIGIVGNNGTGKTSLL
DTLYYRL-SGIEPPITTLYASMDHFLRPDNVDSRLLNTYQRLHYLAVRGHSLYMRQNSQL
DNSKVVNAPNRSSDSDSNRSKFEWVKELLFLYHSFVQETCKNVDIMISNLVDMFGLRAAL
-PLRVGELSGGFKMRLHLLTQLISKPKLLLLDEPTNNLDMPSVLFLSSTLKTIGLSVILI
SHNPQFLNELCSSIFQAGDGTLSVYSGGFDDFVHKGSNVMGIKTSRLQNLELTLKKLQQQ
YNAQLES-TKGSQKQKRVLLSQKRRLITETENLVERIRGAKKSSYSDAYEKLLLS-----
-----DAQVNPGNLSHLKLVKR--GMYVFPDKPAF------DLDNVTVLNKEGEVLLSNV
SLTIQNGDRILLLGNNGAGKSTLISLL------------GRGNGIVNTFSQNCSDLLNSK
L-TVGALAMKFGDLDMSDEQLSKYLASFHLI-DFMDVKVSDLSFGERSRLLLALQFLRNS
TFLFLDEPTNHLDVYMQATLSTLLNNVKGGIIVATHDMELIKNLVTGVVYIHERDR-MYT
FNGDFEAYMKLRSENPHMPKEIITLQVGQCGNQIGIEFWKQLCAEHGIDQEGHVIGNQNY
HHDRKDVFFYQADDEHYIPRAVLFDLEPRVVHGIMTSEYQRLYNPENVFLSKDGGGAGNN
WARGYATADRVQDELFDIIDREADGSDSLEGFVLCHSISGGTGSGMGSYLLESLNEKYPK
RLIQTYSVFPHLTTETSDVVVQPYNSILTLKRLTLNADSVVVLDNAALNRILVEKLKVTT
PSIQETNTLVSNVMAASTATLRYPGPINNDLLGLMASLIAVPRCHFLITSYTPLTLQKHV
SSIQKTTVLDVMRRLFQTQNVMMSAPMKDGKYMSALNVIMGDVDPTEIHKSLQRIRERKL
VEFIKWNPASIQVALSKHSPYVPQQHKVSGLLLANHTSIAGLFQRCIQQFDKLYSRRAFL
DNYKKEAMFSSPDGQGNFEEMEHSRDISQLLIDEYKRAEQDDFFDLGQLMKGYHKLEKIG
EGTYGVVYKAQNDHGEIFALKKIRVEEEDEGIPSTAIREISLLKELHHPNIVCLRDVIHS
EKCLTLVFEYLDQDLKKLLDVCDGGLETSTAKSFLYQLLKGVAYCHEHRILHRDLKPQNL
LINRKGILKLADFGLARAFAIPVRSYTHEVVTLWYRAPDVLMGSKKYSTEVDIWSVGCIF
AEMINGVPLFPGVSEQDQLKRIFKVLGSPNVGTWPGVVDLPAYNPDMDQFEKQPWNVIVP
KLGGAGVDLISKMLQLDPFQRISARDALCHEYFNDVSMGINQSSMPTSLDLRLQAREALK
LQSQCEREEVNEKEKVKRSLEKGNMEAARIHAGNAIRKHNEALRYLQYHSKLEILRSQVE
SAERTNALNEQLKEALPRLSKMTK-YKSNGNTNLMQELEKIFDDLDVSEAYGDMTMNSVN
AHLAPQNEVDNLISKVADEYALDIMLNASRVIGSSLSNRNIGRLTQMPKEKTHINLVVIG
HVDSGKSTTTGHLIYKLGGIDKRTIEKFEKESTDMGKGSFKYAWVLDKLKSERERGITID
ITLWKFETTKYYYTVIDAPGHRDFIKNMITGTSQADVAMLVVPAEAGGFEAAFSKEGQTR
EHALLAFTLGVKQIICAINKMDKCDYKEDRYSEIQKEVQGYLKKVGYNIEKVPFVAISGF
MGDNMVERSTNMPWYKGKTLVEALDQMEPPKRPVDKPLRLPLQGVYKIGGIGTVPVGRVE
TGMLKAGMILTFAPNPITTECKSVEMHHETVEVAYPGDNVGFNVKNVSTSDIRSGHVASD
SKNDPAKAAVSFTAQVIVLNHPGTIKAGYCPVVDCHTAHISCKFEEITSRMDKRTGKSLE
ENPKTIKNGDAAMVVLKPMKPMVVESFTEYAPLGRFAVRDMKQTVAVGVIKSVEKKEPGS
SSAQKAAKKMSHLLNLPVLLLKEGTDTSQGRAQIISNINACQVVVDCIKTTLGPRGMDKL
IHSANGVTITNDGATVLKLLDVAHPAAAVLVDIAKSQDDEVGDGTTSVTILAGELLTEAK
QFIIDGISPQVIIKYFRIACERALERIESLSIDIDSKDEATKRSLLIKCAETSLNSKLLS
GHKNFFAQMVVDAVMLLDSDLDQDMIGIKKVTGGSCSDSMLIKGVAFKKTFTYAGAEQQP
KKFIDPKILLINIELELKAEKENAEILIKDPNQYQSIIDAEWTILHDKLEKIAKMGTNVV
LSKLPIGDIATQFFADRNIFAAGRVEQADMIRTSKATGALIQNTVNGISTDVLGTCGIFE
ERQIGNDRFNIFEGCPKTTTATLILRGGAQQFVEESERSLNDAICIVRRTTRTQKIVGGG
GAIEMELSKALREYSLSVAGKQQLIISAFARALEVIPKTLAQNAGFNATDVISKLRRDHA
LSKDVNWFGVNCLNGDIVDAFQDCIWEPAMVKKNAIYAATEAACQVLSIDETVKHASQSQ
QMQ-----MSNGGGDLGELLMLVGDLHVPQRALDLPQCFRDLLNTDKIKQVLCTGNVGSQ
QMKDLLLGISPNLHMVKGDFDQDTTLPEELIIHVGNFKIGLINGYQLPSWGDKNAVYEYA
KNRDVDVLVYGHTHISDVSKISGKILVNPGSATGAFQPWAPNAIPTFMLMAVQGSKIVIY
VYEEHEGQANVVMSEVDQD--------MVSQSYPNSCGSGYSPIYRCPKYMDKLLKDVHD
GKIATGWDIFQHGLSLNPDAPCLGSREKADGSLGEYVFKSYKEVESLVQRFGSGLRSLKG
VNKVEVAPEPVE-ATMIGIYASNCVEWLICEQTCNGYGYTIVPIYDTIGEESIIHILENS
DINIVVCDPACAEKLARVLPKAMSHTIKVIIVIGKEMIQTT-------DIPVYLFSEILD
MGDKALIPFEPATPAMINTISYTSGTSGIPKGVILTQGQMASLIVVVNHVVGELNGISVD
AVKCYLSYLPLAHMYERLYIDSSLFVGGKIGVYSGDVRNILDDLETLKPTVFVSVPRLFF
RIHDKVFANVSRKPWFIRWLFNTALNSKVNRLRNTGNCKHRFWDKIVFKRFPALFGGNVR
WMMTGSAPLAPRTYDRIRAIFGTELLSGYGLTETAAGAVMNRQGETDTTHVGGIIPTLEF
RLKSLPEFEYSVKDENPRGEIMFRGEHVTC-GYFRNPEATAEAF-VDGWLLTGDIAELLP
NGAIKIIDRRKNLFKLVQGEYISPEKLEAVLIGCALISQAFVTGKSTEVYPVAIVVPDET
EAQYWAESQGHDMTLQDICQHPVFKEAIMEQMAQAYDEANVKGFERCKQIYIEAEPFSIG
NNMLTTTNKLRRHHAKKRYEDIIDSLYRMSHTEMADWVPTIKQLALADNACYGCGIANAE
DGELFSAADIDHDDLCWDSVYRDPYEFEATDENGQPIKHQITEKATIMEVFEKRRSSIGI
FIGGNKYTFANYDDDCPVGDYTFKCVSAAKNKGGAHLVKTPGGYIVICVFDENRGQNKTA
SRMAAFALAEYMAANGY----------------------------------STLRDRAKR
KRLRCVDCGDAGMIVVDQTEGSQICVNCGRVAENVLISEQQEWRSFSAESSSKNNDRNRV
GEANDVWMETTSGTTFIGASRKMQNTNMVINDNDNKRHLKSAFVILRQVDDTMNLSDLVI
ERSKEMLKELDNAGHLKGRCNMLNVLSIVYMASREVGVCRSLDELTIYEAKISQRDLSRA
IGRMKKLLPQRGNATVEDSAQIIPSE----QSSNATKDITPYIASRRYHIF---HYTSSM

---------------EKQCQ-DFEELKIFKSWDQVKPYFTNPSLITVEALFVLLTLCNVY
VLHTLFVTVIAAPVFITWWQLTQGLVTAYLLGDFGTIYPKLAYFPAVKIDVNLLKTLAMP
TVAYVAMLCSTNIMLCKAPSTAAFPILASGAVAAHHAARFIACGEEYMPMRWKAIGFLLL
AFVIGATDKHIAPGNIITVAFIYAFLAAVFRAGFMERALHIVGGRGNALHNHQHFLGAMI
LPFVFLVNGELKVLNTLPWDITAARTWQVWGCFVAVGALPFVKNVVSNRLIRQTGQAPWR
MLELIAVALLFIIGSVKQSPSWQVVLATIFVIVGRFLGAVDVIRNLQYARDA--------
-------ASLGPNSSEPFLQK------DEAALVQE-------------------------
---MESM---
-----------LQILKQKRTNAHLGYSLLKRKSDALASKFRKLLKDTIQGKEKVIEGFNE
ASYALSNAVWSAGDFKSLVVESVGRSAVTLRVRTENVAGVIIPHFELKIDPTVDVIANIG
LTTGGHVIHSVKTAHLEFLETLAELASLQVSFMMLEQEIKMTNRRVNALDNLVIPTIDNN
LEYIKRELDELEREEFYRLKMVRNMNQD--DDIPKRATDSQDVHETGQSEPQTIFESVDE
DIVVM--HFCSTLAALLQPFTANATLLVDPMQHELQVGNETTFVAKVKVARQTTSTAAFF
YTPSDSGIKKLIDEIDVVSRDLKGIFTIVAIDCSNRTSICEAELG------SGYKTPILR
VYPKLPVPAYNYSGELTSAKVKRLVKHVASQVEII-NSGKLPDFMGKFETMPKALLFSDK
TGPNYMYKALSLAMDKKLLLGFVNVENPELKTRYKIKSLPSLIVIKPDTKVDRFEGTFDY
QSMFDWLNVYAETFLLGSGYDVGNTKAKSKPWLHDQLPQLTIESHMDICFNKS-HGFCVI
YMVNG-KISNEDRQMMIDLSSRYTGQFTG-----KWMWMDLESEKGFAEMFNIGELPSVV
IFNPKKRLRYMLFDGQEPVTRKGIEEMLEKVLGGDARFTLVKGDKLPNFAPIDSA----R
HDELTVAELNKPFSPCDLKKVRGIEFGMFDPEMIKRLSVCEVTQTELYREGVPHTGGLND
LRMGTTDARHNCLTCFMDVKYCTGHFGHITLAKEMYHCCLLTSVLKVLRCVCFNCSSLLC
DKEDPRVKNLMKLRSTGTRLLKMAEICTSSPRCQQA------------------------
-----------------EVSGSKMKGCGYPQPKYTKEGTSLMIQFSDKEVDEDCEEIKRS
FTAEEAYKVLKNISHDDMRYLGFNPERSQPAWMILKVLPVPPPAVRPYVAYGSDRSEDDL
TLKLLDIVKANNMLRKHEERATAPHIAEEISQLLQYHLTTLFDNELPGMPVASTRSKKPI
KSLRARLKGKGGRLRGNLMGKRVDFCARTVITGDPNLPVDVIGVPKSIAMTLTFSETVTP
LNLENLRKKVEMGPHEWPGAKYIVRADGSRFDLRHVKRASDLQLEYGYRVERHLQDGDYI
LFNRQPSLHKMSIMGHRCKVLPYSTFRLNLSVTSPYNADFDGDEMNLHLAQTHETRAEVK
HLMLVPKQIVSPQGNRPVMGIVQDSLLGISKFTRRDCFLTKDMLMNLLMWIPYWDGKLPQ
PCIFHPVPLWTGKQVVSIMLTFDQM-----------------------------------
--------------------------------------NSLTCINLMRNSAVALENDNPY
CSANDSKVIISKNEHLSGIICKKTVGTSSGSLIHVLWHEAGPDRCKDFLTTLQKVVNNWL
VYNGFTVSCSDIMASESTLTQVAEILERSKKEVQRLVGLAQRGKLKCQPGKSLFESFEAR
VNKELNEAREQSGTIAAKSLDERNNILAMVNSGSKGSTINISQIIACVGQQNVEGKRVPF
GFRDRSLPHFIKHDYGPESRGFVSNSYLSGLTPQEMFFHAMGGREGVIDTACKTSETGYV
QRRLMKAMEDIMVHYDKTVRSGGGDILQFLYGEDGMGAEYVEDQTLDLMKLDFASLNRLY
AHDFRNENYGVGWILD-ESVRTNILTDFSKQVILVEEYQRILDMKAILCKQVFPDGEARQ

```
HLPINISRLLEYAKTQFPT-----------------------------------------
------------------------------------------------------------
-------------TTESRKLMNPVDIAQRVQQLLDSLTIVVTSGPHDILAAEAQENATIL
IKAHLSTALNSRRLMEREKIGNLAFDWLLGEVKRIFYKSICHPGECVGAIAAQSIGEPAT
QMTLNTFHFAGVSSKNVTLGLPRLKELINVVRNVKTPSLTIHLDRGVAHDQERAKDMQTR
LEYTTLDKVVALSQVIYDPNVSQTIVPKDYAWVREYYEFPDED--MNRLGPWVLRIQLSN
KVMTDKRLTMKEIVDRIYQEFSNDEIDCIHTDDNNDELVLRIRVKYSNMD----------
----------VDQQN---------------------------------------------
------------------------------------------------------------
---------------------------------------------------------MGE
NEGEFLQRFMSQVLVNVKLRGVSKITKVYMREEARTKYNETNGRFERVSQWVLDTDGCNL
EDVLSIPCVDAQKSISNDISEIFHVLGIEAARMALLRELRAVISFDGSYVNYRHLSLLCD
VMTQKGHIMSITRHGLNRADRGPLVKCSFEETLEALVDAAVFAELDLLKGVTENVMLGQL
CPMGTGSFDIMIDDEKLRDANQNLQIMPDSALSGFTSPES---TSPDSM-SPAKINSMLS
PLPFSPSYAALMMSPIVNVS-------------------SPSFVTSPAVDTKALGGSFSP
TMSPQSPVSPSYAPLSP----NLSP-NPLGMMSPTSP--------YSPTSPVYGQ-----
-----------VYSPA--------SPAYSPTSPLY-------SPTSP-----------VY
SPTSPAYSPTSPAYSPTSPAYSPTSPYSPTSPAYSPTSPAYSPTSPAYSPTSPAYSPTSP
NLGLPTSPA---YRPTSPAYSPTSPAYSPTSPAY------------SPTSPVYSPT----
--------SPEYSP-------EDPFSPNPFEEEEN-----MVRFWLDGIEVFFPYPQIYP
EQLAYLRSLKSTLDAQGHAVLEMPTGTGKTVALFSLITSYQLARPEMGRLIYCTRTIPEM
EKSLLELKEVIKYRDAELEKDRVA--MEASSTSDSTGEPSSQSGSSNTKNR---------
------------------------------------------------------------
-HDLTSVWRRIEYPSSDEHVLRFDGTS-VQGVPTAIEHYTTMGLCGHYEYLERVWNPTMI
PSGVYTLEGLKEYCSNFRHPSGVSTPICPYFAARRALDIANVVVLNYQYLLDPKVSEAAF
SNLYSTLPSSEKEKIVVVFDEAHNIDNVCIEAMSVEINDDTLDEAYANLDILAGHVARQR
EKDERQLLEEYRRLAERIVDSSI-------------------------------------
------------------------------------------------------------
--DIEGYMSPVLPDD-VIQKAIPGNIRRAEHFISFLRTVVGYLKQYLKVQEPRSEGPLMF
LHRFEQETGIAYSTLQYTYNRMKSLLNTLSITALGDLSSIQLVADFCTLVGTYTTGFIVI
VEPYPQGS-LYEPVIQFSCLDASIAMQPVVENFQSVILTSGTISPLEMYPKILNFTPVLT
QSLPMSLDRDCLCPLIVAKGANQLQMSTRYELRNDVTVLRNYGTLLIELCKHIPDGVVCF
FPSYAYMELIVSHWYECGIIASIMEHKLIFMETKDVVTTTMALHNYRKACDVGRGALFLS
ICRGKVAEGIDFDRHYGRCVILIGVPFQYTLSRVLKARLDFMRTKYGIMENEFLTFDAMR
QAAQCVGRIIRNKSDFGLMVFADSRYSRADKRSKLPPWILKNLEPGNMSLTTESAVTAAK
VLLRNIAQDYVSSRLTRFDQDMLNDEAKWWSSVQNVLRLSKY-TTAANARNFRTTLIRRA
ADLLKHSKIITNANNQPPCEITTLKNGLRVASVWMPGNSTTVGVWIDSGSRFETKETNGA
AHFLEHMIFKGTKNRSRLELEEEIEQKGAHLNAYTAREQTGYYARCFNKDVPWCTELLSD
ILQNSLIEPSQMEAEKHVILREMEEVEKSTEEVIFDRLHMTAFRDSSLGFTILGPVENIQ
NMKREYLVDYIQKNYTADRMVFCCVGNVEHDKVVELAEKHLCTVSQCCATMTGTGKVQLE
KPYFVGSELLNRNDDMGPHAYLAVAFEGVSWTNPDSVCFMLMQSIIGSYKKNQEGIVPGK
VSGNKTVHAIANRMTVGCAEAFSAFNTCYKDTGLFGFYAQCDEVAVDHCVGELMFGVTSM
SYSITDEEVERAKRQLMLQFLSMNDSTSTVAEEVARQIIVYGRRMPVTEFLLRLEQIDAE
EVKRVAWKYLHDHEVAVTAMGPLHGMPSLIDIRQKTYWLRYMYCNGAMIPQLELILLAPC
KERAISYIKDDVIASGHSDLL-----------DLVTFAAEVYRGVTLLDRCMGQSVFLDV
DPSVAI-----------------SCQTLYDHLVSRVHE-FISNGSKQ--------LERIY
VLAGLVLLLNTFIRLNWLGPPCGCSSESDKHLASRKAIEDPSDNDVTLDTSVTGYTLVAS
ILDALEIDGETVYSGVYGSPYFVAALAFLGCLNGSIVG-----------------QGLSS
NSVLTK-----------ESSTLNTVGIWQGRVAFIWQRIVRNSTLNPCPFRTCVID-FGE
SLKSCGILPQDFNLLLKEVNIASVDVPNTVSTGMTFLANPDLVSRFECPGYLRPLLILEL
GVRLPYYNMSRLFDSLLNIASSTLNFGYTFTGKLGIRRKHQVRETAQLVLVT--------
--------------------DRSNDNGPSG------------------------------
-------------------TKDEHG----------------------NASVPENIGLISV
NDDSDILERPRLSEEADES--QLSLAEQCLLLCHALHMLKSTPESDELNLEFLNAIVVRC
L----------------------DNVSATTSWLLTSVALWVRCKTEYHRTKTVERATLQL
YKLSDAYYEPSAAPGARLEYIWNVWYPSAWGIKREIARRMSSIGSFLTAFEIYKQLHMWE
DAIQCLIIVGRKKDALELVNQQLKTAPSALLWCFLGDIEGD--ISHYKTAWEVSKHRCAR
AQRTLGSYYFNKGDLDQAIASLELALSINPMRESSQFMLGCCYLKKGSLERAISVFARVV
SM-NPSCHDAWANMCSAHLNIGNMKEATICIEQAVKHNGNKWEFWDIRMRIALRSRDIQN
VCFAMEKLISLGKKSAIDPLMVAFLVDASTKFDRNHATQR-------IIARTLDTITKHI
TDNGDIWSQCARYFGFQKCYLEALECTFREYRALESEIVTSLTEAKGDLQIKRVTSCLGA
MVSLLKRMSVSDKRATVIETLRSVRERIHSRIEAVNAQWQMEMDSLIENAEMARHKVDVD
DL--------------SFPESDASSDNDKPTRKKLKQSAE------------AKRRAIVS
AFLDTEAQVGDEEDEENYEEFLPDESTEAENLENRSRIRRRRIGGAGLLENAIDKLTKRY
QDMTFEEDFIDDGEAEAEQSYGVIE---DANVLIPDINDPKLWMLKLNKSQSSKFVAISL
LNKFLKLQGRKLGIYSCFAPDEIKSFIYVEADTKSAIIDAFSDLRNLNLSKLKMVPINEV
STIYAMESRQQTVPLIGEYVRIKTGRYAGDLAQVHESDELNGTVVVKVIPRLQEDSKNFE
G------LNVDDK---------------------------------------GSEFNFNK
ANMVKKTKS-KIFVKRLFDREAVELKGGLIEQGFTPGTFRYNNMTFLDAGFLLLRISVRR
IVVGSADATLSELKEFNLEDNYGNVAN------VINTSNLHLFRLGEKIRVTRGELINVI
GHIASMHNDEIEIQPEDESPNFKIQPSCVMKYFQEGDNVRIIDGINRGESGLISIVDFEK
KTAVVFSPQKSEQFKVKLDYLVKVKET-VSFESLGSLNGYFLGDLIQSSKGEVGIIVSIS
KS-CFTVLLDTNVEAKWVMSDILCKRSSFGYSSKDVNNATLYTHNKVLIVNGPYKNKQGV
VKHLWKNKCFVQLEKNV----YLVVDS-GVVLNMSISETPNFAEQREAPERGLSNKIRVP
NKFIGKTVKILVGRHKGLLGDVISVDQSEFTIILKVKPMVVRIKKVDVTILDNRDSKMRY
------------------------ANTLFDKSSLKSKQ----------------------
------------------------------------------------------------
------------------------LSNQTGKQVNKWIKRGVVIRIIGNGEYRNKIGIIDE
VVEDTDLQIVHVFVDEDYIAIAAESVEVLRPTK-ENQVVTLLRENDVIGTVVSVGETHVT
VRLENGDIEEAVENLALYGSLNIMAG-APVKINTLLRLNSLGFKDGCFRFGALTLGGDRF
VCIRESDESSHSVSIIDLYNGNVSRRPIKAESTIMNPHKPIIALKASIQN--GHFIQVFH
LETKEKIGTHQFTESVVFWNWISPTKLGIVTDNSVYHWNIEEPVLIFNRSGKLAEPSTKL
VDYASDAENKWCILTGVYSTDQGATVEGAIQLYSTERRQQQLLEGYAGTFGKLRISTPDA
TGLLVFCEHKRGGQTSKLHCMDVYSQRTEGPVPLKVSKDIEGLHSNTGDFPRFVHILDSC
GFVCIITKCGFAYYHDVATATPLYSCKISDSPIFAAAAKYVDGGSIAVNQSGDIIEILID
ENRILSTLK-------EEVRISLATRFGYPGSEMMRSFEEYFSRREYKQAALLVATLKNG
ALRTNETMERFLNAPVLAGETSPALHYFSVMLEHGKLTYQESLGLVRRVVAQGRKELVKK
WLDEGKLTESEELGDLLRTMDPLLAFKMFVSLCCHMKAILCLLDAGHATKVVPYIRKIAS
TTGSTGDKVA--------QVDGLPSMNVVVEHMLSTQPTDIISFINDLI-----------
-----------SGLGPDEPLCDVGSVAEILIKHNKLQELTKILLEYLKPNRVEHAALQTR
LLEVNLQQQPRVADMILQLNVLTHFDRAYIARLCEDAGMFDMAIQHYNSFFDVKRLIIKA
GGNMNRAVLEKSMKNMSPENALEVLREMLDSAEISNDHVVSCALTMHNHIGTMQVVQLFE
RSASSDVLFSFLRALPV-------------------------------------IQGSAA
ESTEQNATIVYTFIKCCIDRNEMEDLERICKESNV-YDGVRVKDLLKQSALPNPKSLLIV
CHKLGELAELTEYLYRNGMEKAIEVYVNTINPGGVATVVSTLFDLSASEHVIHSILENLH
DPNGMKALIQIADERHQLLMLRDWLEKRVEEGHKETEIHTALAKIRVSSQKDAEQFLSTN
KIYDRSIIGRFCEDRDPMLAYLVYSEA--QLDSDVLRLCIGNGFYKMLAAYALKRSSPSL
WHDIFSETSGNRKHVCEELVI-LAPDSSNASEISCALKALLDAGMNEEVIALLEQLLLKQ
TQFSSSSNLQNLLLATAVKTNPSKLEEYLSKLDNYDVAALSKLSDSLGQSRSSFAILKSA
GRSLDALEALL-------------------------------------STTDADVLEEAH
EYVQSLDKSELWFRLGRAYLGEKKVAQAIDAYVRSGDLSDHQRIKHACSNEP--ELFLQW
LSNGRALKKSRD--LDTDYLLCLADRGDIDKFKEVLNGQHSADVGYVGSKLMESRKYREA
VLIYSSIPNFAKLALCHLHLGEFYQAADAALNSRNPQVLRQVVEECVGKNQLGTAHKVAI
ELLTYPDFLPGIVTLYETTGNTNELIKLLEKSAPS----VAVSTELAIAIAKYKPEELMN
HFKTNTEEINTARVARECCNLWLWQEAVYLY-SLDTPDTALISMIAHYGLAWDEKLFFET
AATANNPEALYKAIHFCIQCKPLLLSRLLACAKGRVDAVRVVKILRNAGCLGLARNYLEQ
```

VADKTSGAVNDALFEIYVEEEECELLERSLEKLTTFDQAKLCAMLQEHRLPKMRSIAAQL
YIQSRDFGKAAVIYRRNGDYVDAIMGVQTSRSESLAMDMVRYFVDQGLLEEFLVCLIVNY
SLLDPADVLEYAWLHKVMDILVPFLSHTIRTVSKAVS-----------------------
-------------RPAAVQRPPPQLSLPFH---------------M-IAVGQPAPNFRCE
AVMPDNSFKEISLSDYAGKKYVCLFFYPLDFTFVCPTEIVAFNDAMAQFEARNVQILACS
VDSKFAHVTWRNTPRDKGGIGNVMFPVLTDITKTVCDAYEVLIEEGVALRGLFLIDKKGI
VQHLQINNLPLGRSVTEVLRIIDALQFYEKHGEVCPANWKAGDKGMAATTEGVIAHLTSK
MEDNTAEFL-STEATFRDEPMVEDLSDLSSKTLAIVESMLKIEFLKTVLLELMLVEKRCR
IARDGVSNSRMCNFILQLLYDIGDYPNVIYYLVLLSRKRGQLKATITSMVNYAKKWISEI
FDMEVKMNLINTLIHITQGKMFLEVQRADLAYTLAKIKEESGQIEEAANIMHNTEVETFG
ILPKKEKVRYLLEQMRLHLLNNDYLRFYIASNKIDDRVLDNDGFEEHKMTYYEYMVHYHL
HSKDYFEVAKAYRQRLDCTIKLDL-NDWLSDLESVVIFLMISAISEETIKYRDFLASEEK
RLRETPVLSSLFKELLSDNMIPFPLAADTVINSHVIFTDQRYPGGAERLSTLADRVIQHN
IMVASKFYTTLQVTRLSELTNTTCDKLEEEISAMVHAKTIYAKIDRPAGLIRFGERKDSD
TLLLSWSTDIANLMGLVDQCSRLVQKEKMIHEARLKQVELEKNLTEGDSDEGIKEEGLEI
IYEISKILNTGLDRETLAILVGLCEKGVDPTVLAHLVKNLREAKKKFLSEITAPKPGK--

> ***Theileria annulata***
NKGSEQLVVNYSQNPPYGAVILSSIS-------KYLQKSDSSKVVFNLDNKLQNNEFSLN
GDKFNE--VDLLKNLCSVLPYCDKLLDVVNNSELEYWFKLAENKGNFTENDSYLENMNKH
LAQRTYLIGHRLSLSDILHFSLLRRTCN-----VNSLKNKYPHLARWYNFISNVPGTSGC
L------NGYETPSSATYKKP---FTKQEQQDNSYKGVLKGAKEGCVVTRFPPEPSGYLH
IGHAKAALLNFYFAQKYRGKMLVRFDDTNPSKEKDEYVDSIMEDLESLGIKYDELSYTSD
YFDTFQEYAVKLIKKGCCYCDDTDVETMRKQRGEGVESLARNNSVEKNLELFSEMLKGSE
VGVKNCLRAKMDMTSKNKCLRDPVFYRCVTNVPHHRTGDKYKAYPTYEFACPIVDSLQGV
SHSLRTNEYSDRIPLYYWVLEKCELRHVEVYEFSRMNFVRTTLSKRKLRWFVENKLVTGW
DDPRMPTVKGILRRGLSVKALFEFILDQGPSKSVNLMEWDKLWAKNKQIIDPESPRYTAV
V-SDHVVLKVVNFQPAV--KTRPLHPKNPELGEIELVFGDQVMIEREDFNLIDQSEEVTL
MKWGNAFVDK---------ANLQLKLNLQGDFKLTKKKIHWLPVKTKTAVDCDLVEYGHL
LKVDKVDSDDDNNMKEFLEPQTEWVTKALGEWALSNLKKGTVLQLERKGYYIVDQPHDSG
RLVLVQIPDGKAAK---------PKQKKCFNIMADDCVVIDNGSFSLKIGLSGSYSPSFI
VRSVVGNPRQNFTEEFYYSEDVYSSLEFMSLDHLIDHGHISDFDKMEGLWQYCFSKLEVH
SDCRPVLLTEAPFTSPKHRIKSSEIFFERFNIDDLNISVAGLLSMYGLGKLTGTVVEIGD
GVTQVLPVIEGYSERSSIKRVDFGGIELTMYLQKMLCTRGYCLTSRPDFELVRELKEKFS
FCSLDPFTDENRSDLVEVYELPDGNVLRDGENEIDLSLERFYVCEPLFNPSIVNSDAPSI
VNTVWNAITSSPIQDRTTLTNSIFVCGGTSLFPNFEKRLQMELQDIAPPGGRSKVRVTAP
QDRHTLAWRGGSILSEPKLKDISANMWVSREEWDESGPEIIMRKFGLD--NMDELQSLSV
ISKVNEMLKNYLEIDQSELAEFIIYLAKKSKDLDEFNKLLNENDANMSFDFINQLYRIII
T---------------QMDEWDNIIKSNNDETNFPALSLKNNPNRKELL--LPSPTVELS
EWAKELINKENLQKSNKDKDNRDRDKHRRSLSRDRDRSRYRERDRDDRHKRSRSRDRDR-
-----DRDRDRRSRSREI-----------------------------NRSRDSSRDFEPD
TV----------------MEELEE------------------------------------
----------ENDILNNKKRYINDIERWENQQLLKSGILTNEEKFKLKSELELQEEIQ--
---PEVTINLNPPNFLKGQTIRSGIVLSPIKLVAKPEGSLQRTITTSLQIQNELKSIHHT
HNTT-----------------HNTHSTHTTTHSTNTTHTHTTASTKRVY-----------
-KNILEERKNLPIYKLREEIINEIIHNQILIVIGETGSGKTTQIPQYLYESKFTEKKMIG
ITQPRRISCINIAKRVSDEMYCIIGNEVGYCIRFSDVTSDKTIIKYMTDGMLLREILHDP
LLNNYITIMLDEAHERTIATDVLFSLLKETCMKRKDFRLIVTSATLESEKFSKYFFNSKI
FKIPGRSFPVEIFHSKEQEFDYLETSLITILNIHLNEKPGDILLFLTGEEDIETGIKILE
ERLNKLKNMNIPKLLLFPVYSALPQDQQQQIFQPAPPGTRKCILATNIAEASITIDGILY
VIDPGLCKIKSYNPKTGMESLIITPISQANARQRAGRAGRTAPGKCFRLYTEKTFHEEML
PTPIPEIQRVNLTNVVIILKSMGINDFLHFDFMDKPCNEMLIDALDILYHLGALDDEGLL
THLGRKMAQFPIDPTLSKILLYSIEMDCYNEIITIISMLSVQNIFYRPSDKREKADQSRR
KFFQSEGDHLTYLYIYNQWSNNQFSNYYCYNNFLQYRALIKVQDIKKQLISIIDKYKFMK
KKMK-IDNLNKTERIQKCICSGFFHHSAKRD-EDSYRTLLDEQKVYIHPSSSLFQRNPEY
VLYHELILTSKEYMRDLTIIKSKWLLELAPTMFISSNN---TFKHSSNKIIKLKLKY---
----------MAEEYEHVYKIILLGDATVGKSHLLCRYIKGNLPIQSKATIGVEFATKTV
PLASGGSIKAQIWDTAGQERYRSITSAHYRRAVGALLVYDVTNRISFYNCKKWLNELRAS
SYDDIVILLIANKIDLINKYNSNEKVLMIEGMEFANENNLYFFEASAVTGYNVKEIFEFL
IQQIYNLKSRLPSIRFTNKNFNTNLSSKNFTGTENFTGTKNLEKNSRQFQN-CLTNNCME
QPKLAKVNKILGRTGSRGGVTQVRVDFMGESGWEGRTLIRNVKGPVREGDILALLETERE
ARRLRMA-NLEDYETTDLLSELKRRYNCLGKPQGNFVFLGPPGSGKGTQSHILKNSHCYC
HVSTGDLFREAIKSGTPLGLKAKEFIDKGLLVPDDLTLSLVQERINSPKCRRGFLLDGYP
RNISQAKDLGKLLKSVGKKLNGVFSFNASDEVIEKRVVGRLVHPGSNRVYHKVFKPPKEE
GKDDLTGEPLITRKDDSPDIIRKRLEVYKKETAPLVEYYNNENLHHSVNANNSVESITKV
IFLGFNVTLVQSCKDCYNDLPQPEFVLSPEDSELNYFLWMPGFKYQPSFQE-----KLTK
LKSLSTNSDSTVHEALEEMIEVDVLEESFKKRARGGILDVHLAGVLARELGASPSQADLL
EFEATCGKSVNFENFKDFLAVSMYSNENREYLQDLLANFE-NMSGGISLSKFENLMKNYG
EPLTDSELNELYKLVRVEKNV---VLSNDLLNMEF-------KLILVISIYGT-FKCWCS
ATPESFFKSDLSEENDKNKVD--------YTHHMLQLFDKIDLNSDGVLSKSELDTFSTT
LSKVISDRQLANEMETIDKDKDGKVSLEELLAAFSIEVGEEDALNNKEPLIQRFKVADKN
KDGHLDLPELGDLINPSRSPELLKLEVDDVLKAHDSDGDGKISYDEYKKYRNED---GED
ETQSSNDFKQFDKDGDGYLTRSELEDVYKEEEEFDSFTMYDDVTSIVGSTSDLTRELWEK
HSDELSRSSVTDFQEVLEHPADYGLTFEVPSETPGT------------------------
--------VHVELMNIILSKFLASSCLFGSAIIGCFIPSIIRKSIKNGVSSKTSKKEGIM
CLCNCLAAGFIMGMSFLHMFPETVEQCSSNGLIIMIK----ENKLNLAFVIMLSSFSIML
FLERVLSFGRTPCCSVFNDCKDVSIDEESLVSCGIKNEENISHAEHCPCNS-PRYKHKHS
QILATIKNLLCPICD---CNGLCITLALFLHSVFEGLVVGLEDHEIHMWLITLAIVLHKW
AAGMALASFLVGN--TKSTVYALFSIFCFGSPLGVLIGSLILDSNLKVIGVLNSIALGTL
VYVGFEIIVHELFCEIKKRTALYKWISFIIGIAFIFSTLILEFLLSN----HDH-----M
QSKFWGDDSDDSYSDSSEYSSKSDDKD--NRHGVDANKWT-LDSSDDEGESRIVKSAKAK
ALETIQNHVKTIEHLKKINDYSELLKDYDILAKFVEKQSSTRLPKLVVKLIVELSQFMEA
QQKDKESYKKLSKAKTISFNTLRSRLRKFNEQHSEIIEEYNKDPDNFYDVLQQSEDE---
--EEESTESSYSEWDEDEEEEE---EEVEAAEEKSPRSGDTSGWSDSGSHPASEAEDGDK
HNKAMDKWGVKKAEMAEKTKKTKSKTKKREVLEKEVKLGNDDNPFSVMIPEVQEMLNTVH
LSEEALRLFVKSIIEKRGKRGTNNFENLKILQSLPYIAKTISQSLYLEVLETLVHVLFDT
YSHAYGAMTPTEWINTYRIASHLVSELISNPKSYLSSEMAEKVNNDKVKAFDDRVKTSLG
VLSTIVQKLNDELYKGLLYTEVHNPDYKTMLAYTIDMLYLLHRTLVYYLKFEKGNEFAAS
TALMILDHCHYKDDEISAKIWELVR-------------------NKIKKQTEKFFPNENK
KPSDLVTELVNFVFVYGTQRDKIRACLHLAYNKSLHGHYYEAKDLLAASNLTEVASETDI
STQILVNRNLAQLGICAFRAGLISEAHSYLMDMCLQNRHKELLAQGLSNVKNMEKTPEQE
RAEKRRLLPYHMHISIELIESVNYICALLLESANYARYPLKA-KEVISRQFRRMYDAYER
QVFVGPPESNREVILTAFKHLQNGDWKKCYNFILSLNTWNNMPDREKVQETLKELIKVEG
FRTYIFKYVNIYDSFSVEQLSSMFSLDENVVHSLISKMIVNGEILGSWDYSSKCCLINHS
EPTELQKLAVKLAENLSTAVEQNELTLNMKNSKFALSQDRRFQQRDTRYNYGSRHDDGRI
SFNFNRNR-RFMQQPRQTKQFPVPTR--MLNRRPKLEDLTDISVECIAFAKQHLEVIKSL
TDVKSMYIHCYQLMKLGGLEAEVPRLVVFGQQSMGKTTLLDFIMGGPIGYSSTDTGTKQP
VSIIMKPLTANSIVCRFNGRFMTIHEVQDAMRVHMQSLGQTILSDELEVEVYVPNALYAI
FVDLPGIKDDSKVGAELTRNVVRNYVSNNPNDLYILVKKASDDPSNWPWSLKEFITSSAP
AGLGLTPQQTMVVGTRAKEFLINEKTDIKTHEELIERVYKRGVIDSKGQMLPLHLLELFS
LSIQAKESGDFLSNRDEMKAQISSSQRQIYDLLLNSFNGTEANRLRDELFNIFSIDSFLK
TLNYKFQHLMNNQLNNLERRLVRKKIELERQIAVMEGKLNRFSPQTLRESIKQFIRQLIE
VVHNMITGNYTIMKLPIPAEQFLKTYGGNLQDNLQDGHELALNLFPMPEMYDPEFYSKIT
KRTEILYK--KKLTMM
DSIKPGRYVRYFTSKINSHMFGLIEPPLNKDYSSDELINVEFQINNGVETSLHKNIDRNK

```
LILLTPLPSVSERLQVP--LYAWHKTQASTGWVLVRPIVIDRLEKKFKTKLDKIDRMEKF
DKIDRVSSLDKSVDKPDRTTNDEIYLEDRTSMPYYNSALEMVSGEHADSNLLNQLAFTNV
SHWLKFHIKHMEPEHVYTSGVLYQMLRSIHHVVDRADWEPLVADLVQSNVRGTLLHASRL
SACACAAALRRVLRASLEEVFRCIRINDLDHSLLGLSESLHFQEQIDQLSEEFCRQKALE
CSNDMKNLILEQTYSIQFDVAVDIFDSCSEFEKYFINTNKYRGHMQEFLNNVKENMAIRK
RRLAMNDLFEKGTKNSIELIYEEVKVQFWATKMLLSSPLTSKLYSHFIKNVLERKQ--VI
NEDPAIKLEAEFEEFLQSNILYELVDGNLVPKNNAKLSNDYDLNVKFDRFVEKYNKTKRI
LEYVTYALEGITKFKHHAHNEEVVNFLSHLTL-----MDPS-FIKVLESSLDPSSKYFIE
AQQKLQMAKESNLPEFIKALSEVIANHEAGSGARHLAGILLKNCFEFK---TEEEKMNFY
KNTSADVLYYLKIRMINVMKTGKESQAVLAACTVVARIAQIELSNKSWPEFFDIILAMVD
SNQFNQTRSSLICLSYLIEDLSNIYENQNVN-LLSEVEVNRLLTSVIKGVYIE-DPQSCR
MALRSLQNLLFFIGNNMEVDAERDVIVEAICRRCSDTNDIEIRTAAFDCLVQLVSEYYSR
LIPSLPVIVPFLWQAIDSKIEQIAIPAFEFWNTICEIEM---QNSLNSNDGNGGIIKQVI
PYLLPKILFTMTLHKFEDMDVDTWTLPMAAGICLSLCSQTVKNDIVHSVLEFVKENFKSA
EWNRREAAVLAYGYIMEGPDSETLRILVSESFDNLCDVLVDSSIAVRDTAAWTIGRIATF
HCEVVLNHLGSPDVPNSNLSKIVRALFDVPRVAVNVCYFINELAEHI----NDYNKGPTN
LLDCMFATLCEMLVNRSTMNDTLERNLYVSIYSSLCALIAGVSNNCMSELVALLDHFVVL
VSQMTSADSSYEQRLKTQSVFGAIQVLVSRVG--YVKNLNQLMSSIFEFL--AVDLDEDA
LLTLSALVNVIEAQILPFIPKIVDAVLTGLQS--EVGICKISIGLTSDVSRCMESPFSTY
LERFMTILIKILQDVNGDKTLKPLIIVAIGDIAMAVGGAFSSYVQNTMTLLLQAATTTYE
MGPVDNEDWIYFVNQLQEASLQCFTGIVYGLKEGGALHLLRPYVSSLLQFAQQVVETPDP
FFDTNLYKLAVSLIGDLSSSFGSDLSRHLVDSNLIRGIENRLKQLELAQDPGIKDCRDRV
CWLHSTLVIK----MTTFVPLSQDTEGAVSSASLGDWVLGILKVRCPSKTSTYYAQLLDQ
FQEVEGEQIIKESFQLFELLLSEHLTVFEFLEESKNNGTTVMLKAESEVRVKYSDALKQV
EEYFTVLMYMLQLRFTSSGQIEKAGHLLMKAIQGGESFLELRLRLLQMLYNSVESTLPLR
VDVYIAILEFASKHNIFHTLVPIVKEVDEWMVDWSIDKKTKIKIYHIIAEELDKLNRADL
AYKFWKKRVECLN--EPDLFTTDENVMATVTFVVRSLRSEDILYFDQLLLMPAVAHLKET
-RYAPLISLLEIFIRGNLEDLDLFLKENAD-FVSELKLDRKPLVEKLTLLTISTMCQQQS
EIPIEMIEKNLQLPPEEAEQMIVNAINKGVMEALIDQNSKKVIINHVVHREFGNEELKQL
YNNLKQWRNCINTLINIVDNKSLNSA---MRLYNIILFLIFVKKIGCRNANEFYTFKDSK
ATSVFIAPNNDSEKHGPNRAFQIGSSYWSSQGHHSDTDFVSWTGELYEKATISQINVFWE
YAPKEVEISLSLTGDDFNVVMPFRPTFENKRSYKENFKLEMPYETKFVRLTLRGAINTYF
GIRHIHLVGYGTPLFMIKSGISSKEEMCLQVEEGTVEN-QPRVVLDLCVNAIAASDGREL
WRQNSRMQIVSAVSYPEMCLTTVESSKV--GPVIIDNC------NEDSCTWEFMGNSQLS
VKNAN-ELCLTQRDENGSAAGMGNLVEKYKSELKVTSTSSEKHEEKYAVDLDKKTYWASL
IFPDDGYHVTSLTLDFDKMVHASRVLIDWEYQPLSYTIEASSDKITFKEISRNLSNSYHT
TTDSFPGTDFKQLRIVMMKPHYLHGKVSGGYVYGIRDLSVLTNNLLTVVGDCRAAANSAD
ARDKYFVFYVSVFEPLLSKKVRGMESDLNSVITEVSNELSHLKESLEESANCMDEKKDYD
NQLNLASKKSQDLWKLFNSSTI--TKCDPYTLELSVI-GENKNNPAEDCYNIYNLNPNVK
SGFYWIQPPCSKAPLRVYCDMDSHTSMYIWNGKDNSISIQPLNN-IRTPTSLRFQCASYG
LEPLVIKSRRQIDHLKKAMKLMPLSNKLDSYLPLAYKFGSEP-----KFKDFMNIFSFIS
GNSGVENSSSDESNHDKNAVGISMSTQEIERFDLETSDISGIACSTNAT--DEITAPLEL
DCSDSLGSKKIFGSLNTSVIVRCDEDCSQKE--NLFIYGTNGLYSDHSSVCLAAIHAGII
-SSHGSFVVSIENGRKYYDGDKQNGVQSYFYNKT-WHGLKDIIHENETENKFEHNGPDRK
YSIRILPREKRCPI---VETKGSFLQLNNNTNSTDNVENGETIGDTVYDPNTVKYANKVI
EIMDSLYGVDNKTVTNVVEQISVVVSQAKKHLRSLEPIHNKQNDTITSLFDNGGSLVHSY
YDTLDSSKMLYSNLLT-----DEKNKTIDVSSDFELDYHKMPFSKTFTVHDSSM-ANGKS
NWGYSNSPVEGHDSYVAQSGDLDSELIAEGSFAFLNHFKYFDFEISLNVLATNEGSIGIA
FRVHDQFNFYLFVMSYFKSNKQLIKVEDGVAYVLATNPDEGYNKMHWYNLKVNCTHYYTE
VH-----CDNRLIFRVIDTSFLYGSFGLYSSGSNGSFYFDNLSIKAKNVKLLESPLRIER
LKHIKCCNFSETCTGSFENYNIINPKYTDWSFEKLFN---GEHKVHQVKACYDPHDIGSL
AILKDRYCSGGFFRFRVLPQCESGT----VGAVVHYLDEKNMCLVEINSKEFRIRQFSSG
VVKVLASEEA------SISISKWNVIEIELTATEIKARAKTNVPHNFILKSLLSSYDFKV
SALGLKSSACSSCYFDSLQISHSEPKELNTNFMSVSTAWVPCLAS-NLLERRRLCGLITR
NNALDE----CMSDFCNQCCSHHTRLLGDHVTLKCTKKCDKNNLLFKINSGKFNSYLSAC
TS-FQGPAFNHC-NLDPNCLFHSCKLCCNSGYYNQ----------FDDKRKLIESTYCEN
KCKILK-IHAMNYCSNEPVLGSLLSYADWCKESEEEKLHKRTKVKVKRSNGTSKPDSVEN
LRNSSENNGEISNGKQSK---TNKEPEKRKRYGVNGSESKGARDQNPESMTSD----SYI
DLLELANYSSDSSKNTQ-------FKSNTTTSM---------------------------
------------------------------------------------------------
------------------------------------------------------------
------------------------------------------------------------
-----------NNNGIGNMSTTSMNNLNNGVNNNGKSCRKRMDLREVSLERLSTLDSLGS
RSDSGGVNFNVDSEEFKYESLLSELRRRGFEPKTLGDVLRSRNKQWAKTQCIHSSAKEDF
----ERHLNMYKNHFRSYRKGWIFEKLTWSNIIFCPNFRPGGEIIYRFSRKKTYNTLYDT
INKGWVHPDLLVCLVRDASHPVRFATPPEQDCYTAIYTGPEISENDTKVIFGEYTGIVYR
EDCVPDSIFEYAFELNFTSASWVDA-------ELEFRHDKQ-------------------
----------------------------------------------------------YI
RDVNGTIFLPNNSKYVLDSTHAFNELSMVNHCQSIAAYGEYFLQANCEWQQVIFDGWPHV
ILTNKQGVKIQTGDELIADFGALWFQKVEENCHRQLKRELISYRLLSLVQENDKSLNNLD
YFVVNNNTSYVCAICSTQTLSSDEDDEDCIVCDGCDRIFHIKCLEKITTTNFQAYKWYCM
YCRYLCKQIIQHDLNQDFMPLDNTPETSQQDSSSVNVKSEESNEKKEA--EADPESDESE
ELVNNNLIDLNKITNYIN-KLIYNTSNSPDNSATSQKENTPTPKDNYNFENHKNADNVEG
GKLEKEKEYPKHKRKKIKSCLSLNYLKRKNR----RKMMVKNVKTKSKLFKGNKTKTLKR
IKLINSKYLVKQKILSNTTNTGSPRSDTNEERNNTAKNKIGPNSVETITSSSQNPNYMVD
QNNKDKEADDELTINSLNMNETSELLEAENKTASTEETCSTTSGNESELLRKCNDTINSR
NSGSEKVNSYDTLTKAVNSPVVKDNQSSDDPTFNNATYQQIINKVKDNPDEVLNVSYDNS
IMNNWQVEPFSIFGEAVRVCVDCNKKCGPKANVYVCRVLKRHLSGNFDHPKSKELILISF
EQHINYLLRLLVNIIIDFLIK-LSCHFLTSNNHNQSNSCFKDIRVGSEVTKNSELVETQK
KLTSRSNVKKDSNNVNEFIKCISNNIKRIP---QGDILFNYELLGFEEDFVRIINCDD--
LFKYINDLSSYMCYNSDQVPSSIQQILSKDELMVNTSSNIPHNNVGEKAYQ----DTSEY
NSEVCMKSELDQVIKS-TNCELEYWDPASRRFSDEVDEDTVKINFRYNKRILKLRPEDV-
QSQEFIPMIGVKPGKTIIYRKFSDGYYQGLVNKYT---NSSTYFKIEYSDGDDELMDPFD
LMNEIILNLSNIENILLQLSNNDVNNGRYIQQIQVKNNNSNDKAKTVNEDGTNSGSEING
VETSEKLKEVDITGHSKRRKCQMLD------LKNLGNEAFKAGKFKEAAEFFTKAIELNP
NDHVLYSNRSGAYASMYMYNEALADANKCIELKPDWPKGYSRKGLCEYKLGSPEKAKETY
NLGLTYDPNNEALKKALYEVENDKSDTYIQSLLMVSQMIQQNPKLRKYQEQDPEYSSKLA
RLISHMNTDPAVLQQILTDPNPALRDGLMACIGIN----------------EPTEKREAP
PKEPEKTEPKEPLTPSQAESKKYKEEGNNLYKQKKFAEALEMYNKAIELDPNNLLLENNK
AAVYLEMGDYEKCIKTCNDAIDRRYDVMADFTLVSKIYNRLAACYTKMEKYDDAISCYQK
SLIENNTRQTRSLLSDLERLKERKEKEAYINPELAEQHREKGNEYFKEFKFPEAKKEYDE
AIKRNPSDAKLYSNRAAALLKLCEYPSALADCNKAIELDPTFVKAWARKGNLHVLMKEYH
KAMDAYDKGLKVDPNNNECLQGRYNCINKIQEMNK-GNIDEEQYKHAMSDPEVQEIICDP
QFQLILKKISENPTTMGEYLKDPKISHGIQKLMAAGLLTTLMDEIYDVCVLGTGLKESIL
SGLLSQSGKKVLVMDRNPYYGGESASLNLTNLYKYFKRESTPPESFGVNRDWNVDLIPKF
VLAGGKLVKILRATETSQYLEWQVLDGSYVYQHQKGNFLYSEKFIHKVPASDKEALSSPL
MGFLEKNRCHNFYKFVFNFNERDKSTWKNHNPFLESITAYYKHYGLEENTIDFLGHAVAL
YTNDDYLKLPACEPIKKMKLYMESLMRFGSSPFIYPVYGLGGIPEAFSRKCAIHRGTFML
NKPVKEFKFDEDGKVCGVVTAEGELARCSMVVCDPTYCLLAPEKVKSTGKVIRCICILSN
PIPETNNASSCQIIIPQKQLNRKHDVYVTLVSYSHGVTSKGKFVCIISTTVETNDPVSEI
TPALGLIGKVEEHFINISDIYVPTSKECKDNIFVTESYDATSHFESASNDVLKLWREMTG
SEYDLSKVDVRLDL--ILDKHKRKTLAPLTRDYTIHLHKMVHRVTFKRKAPTAVKKIKEF
ASRAMKTKDVRLDTRLNEFLWSNGIKNLPRRVRVRVSRRRNDDEDAKEPMYTLVQHIPVD
DFSGLQTEVVANEMNN----------PVNDDEEEDIKNNVIRKHTEGDKPNPKLRACISC
RLIMSENQFYENGCGNCSFLQMDGDHRRILDCTSSNFNGFISIIDPQKSWSARYNNLSDL
IPGCYAISVNGTLPESIKDELLQ-MMIVCQLLINYSFVY-SEDYKNNVYIPSEDTFFFVD
```

```
VISKDFKNIKSKPILILEIGSGSGYISTYILNLFKAQEHNIPLCISTDINKIGTLSTSMM
IKSNKLESSECVCMDLFNNLRPV-EFDFIFFNPPYVVGTDDDTSDM-IDKAWNGGINGSE
TIIRFINSVDKYISSGGFVYLLVEKRNKINEILE--LIKENFHYKIIGTRKVIGEELSII
KFYKSTMES-----ARDITILKDEKSEFPILCETCLGPNPLIRMMKQNMGKECKVCERPF
TIFRWKPGPKSRYKQTIICQICAKIKNLCQTCLFDLQYGLPIQVRDKFMNNPIELPDSNK
NLIYKLNNIQTNNEPQI---DPHNEEILKRISRVAPYYRRNKPRLCTFWIRGICNRGEEC
PYSHEQDSFDPSLSKQNILGRYKGKNDPLALKIFKQLELERDNERDKENEKGSEME----
------------------------------------------------------------
--------------------------------------------------------YPSM
NPTEAIKRNMKIFRSCSWFFNQTTDETDASVDDFDEFEEDFYERVRNDWEWLSLRYERRL
NELLKLIK---NNKTNINNPNNVNVNLSPFLSFIPNIMQSFLVNYSLDSDFYGNIIIFEG
DVPIVFCDISGFTNLTQRIENSPQSVANLGRFLNDFFDPIINLIYHFNGDIIKFSGDAIL
AIFCGSDKSTL----------------STNDSLEASSNGT-------DVEQEKDLERCLN
SIKCCIELHNILNNYPIHYSNDNITIHISCIYGNIKIKLVGGIFERYEYVVIGDVLSELK
VLSNLSKSNETVLSPKLYNKVRNYISTQQIIF-DNNIYYKLLSL-DETSDSHTTIDETDF
FDDNFSINIGPFHFNVDNIKLLKFFMPRYIYNRLLINFNICNNEMRKMTIIFINFRINTT
NTLISSKLPYEIMLICQKCTYSYEGTINKLIVDDKGISILIFMGFPPLFHEDDAFRSILL
SLKVFKMSKKIGFNVGIGICTGKVWCGLLGNKLRNEFTSIGNIVNISNRLMMNSFNIQIL
IDENTYNECSSMIELLELEKIKVKGRDEKLTVYTPTGKLKIHNND-------------DM
NLSDNNIVNDTIIKHLYHNTELISLFSTDKDNKRLSILEDSED-----------------
------------------------------------------------------------
-NHIIVINDETTMNNRLIHKYINNTDDDHKVLYVNYDSNRL---IYNLEHTYKLLIHKII
SLYNNSKVQRNSFDKFDLNVEETLKMLLNPKFHWYINILNKLIDN-----RNSDNKLQHL
LSSNYNNDHVNA----VQLLLNSNKRNLNIGNTEPRDVNLQNIINNEV------------
----------------------------------------------DNLESYIYSLIKGI
NLYYNIILIINIVKASSVNDSRDNKVELNGEQLVHIDMARIDKLLKKLIRYNKKQRTN--
----------------------------------PDKNRFMLVVLNNDQTDY-----GIK
DPVGISSKVIDIKRLKKTELKNVLSNIMNDSNTTEDSELVDNVYNITNGVHSITYEFIRY
LIDNNF------------------------------------------------------
-------------NYDKFSNHLYKFFHTTNKSVDDNVDSKS--DSVGLEEKQIIRLVHNY
YEYKLNVIKSDELFILKILTILNKPIEMNQLAYILSSHKNDYNLDKEAQMCKKVDVEEHV
NKLNNLIESGDEKGVIFVNNKILKGIISNIILPDEKLRIIDNLAVHTYM-----SSEPYG
DKSRLIIKNIPNSLDNKSLDKLITKKCKDLGVTKSDIRLLTKEKKVNNELKRISRGICYV
GFLSENDATKFLNHYNNSYFNSCKVTIEYSK--RANTNSNNSLNCINGITNGINGIK--G
GSVGEYGREDEDVKVITEVKPVKAGISSKRVHITFNNSDS--------------------
-------------DDESQIADNEPDADPAHA-----------------------------
IEEDSSGIGFKTENFS------TDSSNIDMNRVVIFNLPNVTEEMIRKLVKPFGKVEQIH
IP-----------LNKTTGNNVSESAI---------------------------------
---------------------TRGMCFVTFCFESDALKFIEEKNNSIFSGRIITISHAKS
NNLKFFQKYIPKGKKY-RNKDTDETSYSEFKAKKKRSEIENRTIWNILHIDINSAIKSIA
SDLRISPEEIL--KGEQAGVNAAISESFILNKVKKWLSDQGIN-----------------
------SEVTNYNEEDDVLLIKNLPHDTEDRELIRLFSSCGKIIKFTTSPFKLLGLVQFS
SKTESEKAFRTLSYKMFQNLPLYLQKVPKSLLPNLNTNSDHTDDSINELND--KVDDTDS
DDQLDDKIESDRIGHVSVYVSNIDGNVSEEEFEKHFSSLKGFVISKIIKPHGS---KLEE
K--------GGVRYGFIEFDNVNNAKEAIKRLCGTVIGSKIITLELSKNKYDRNK-----
----EEGPKEENDVIIVKNLPFQATKKELLELFKYYANVKTVRIPKSAGNTHRGFGFVVF
MSKNDAKLAMENLKNVHLYGRRLVLQYVEN------------------------------
--------------------------SNQM------------------------------
---------------LDSSPVSVLKSPPSDAIILDPVVHENIIRIPKPEIKERIVEVPEI
EYVEKIIEVPDPVYEEKIVHIPKPVVCERVKQVKRPVIQEKIVEVPVVQVVEKVVEVPQY
VYQEKVVEVPKVVVQERIVSIPRKVTKERVVEVPRVEYREVITEKVVEVEEEVPEIVEKE
VHVTHYIDRPYEVEKVIEVPQVQHLYKDVYKTQYRNVPQPVEVPVTHYRRVPVEQVIDRN
VPVPVELEVVKKIQCQKFEPRYREVPVPVHVQRVIDHPLPEKVLRNPETAPEYYKDVQKD
LERLTK------------------------------------------------------
------------------------------------------------------------
---------------------------------MFATRSEYDRGVNTFSPEGRLFQVEYA
LGAMKLGSTAIAVATKEGVIFASERRSNSPLLEFVSLEKIMEIDDHIACAMSGLIADAKT
LVDHARSECVNHTFVYNERMGIRSCVESIADLALEFSDVFDTKKKKTMSRPFGVALLVGG
IDSEGPVIWCVDPSGTIIKYKAAAIGSAQEGAESILLQKYDENMEFSDAEVLVLEILRQV
MEEKMSPKNVEMARIRVDDVKYREYDEESLEKLISSLPE----LENLGAVLIGIPDCVDF
ISSICALYFNISDVNINDILNSFYFDIVSNFINQKDPEILTIYFKDDLSCEN---GVDFS
NKIVSYGKREDVAKLSSSNKFTKGFILLPLVSTSEFPLNLTILSHLKS-LLTFYSASNTH
GNTENKQISEKLRQILLTDLEKEFVTDDPKFPFCKSLIQYIDPDTK--------------
-TLKVYEIKI--------------------NDEFVLEDIKSDVRERYLSMFSEDEEYDTS
DISDYISEKSSETTSSSFDTSDTSSDTSDTSSDTSDSSDTSVIDLTLKTSKIQRLTGLVN
DCSNIELFEEIEFWNNYQIEINELRKKMESPNFVNTLLLLEYNGISVENISFKESKGLVS
KIEEVKEINSFMAELKADNFINCEKLSDFPLLIEKVFTTLRKYKHLRYMKSDRLLLLINS
IILNSIYSVSRIISIELNQTME-ENHKVLMKSIRIVYDLKNRTVDLVK------------
------------------------------------------------------------
-YFEKNNQLLTLNNNMI-IILDLYRDILSVCKIHEKF--------STVISELSEYLEDNS
ELDNCPKIITELNEINSSVISGYSNLNEGMENVLKLD-----------------------
---VIFETKYKV-----------------------------------ENYKNEFLKCVNS
Y---------------------LYTLD---------------------------------
----------LFEAF---------------------------------------------
------------------------------------PVIEMINSLD--------------
------------------ESECKSMLVFNV------------------------------
------------------------------------------------DVYIDQITDE--
-------------------------YDKIIRDIDDN--------------------DYEA
VLIPFQYDNIPYK----------------------------DMVIQERI-----------
MNLVHIVDVLNTISSKSILKTTKNNLNFRDFNYSDEHISL-----------SEYREKYLP
YHLDR-----YHKLLKNHIFTSTNT----------ILDEILIIRQLDVE---VYDKNIYD
DIILI-------EKIYKEYMYFVQESSELSDKL--LHIIG----------DKSYLNHIN-
------------------------------------------------------------
------------------------------ISTLVRV-----------------------
------------------------------------------YKLKRKEIQSFLEFEKNN
YKIRSYFTKLKQDSDDVTIEYKHIYGNQIYIYSIDNIIDYINDNITKLLVIKSSKCDLKI
LNNCDLWICAIEKSKSFVENFKMFNDKFQKLLNIVSC-------------KFMDFKDHGN
IICSLKEDIDKLSEVNKLSNIEQEFQSMIKRVDDIEKVAKKRLNDIRYKCPRLYFINDAD
LVELVGGGD----MDLFLSRIFPGIARVITN---ENIVSGIESSQGEIIIFDSKIDYCSG
DNIK-FINELDSIIKCTIKRLFTLGLEELKV---CYSHFNFDDSFFSKWISKYPTQILIL
TLCICWTESVECC---------------FTKILNFLSKIINFIISNTIDVFDNL---KYY
QLFLLLNYQINKTREI---EMGDKSKLGWLKCVRYYHDND------DVIIKIMYKEYKYS
FNYLFNCQKMITTKVTEHFYSIASMTLSCNLLPSAQGPAGTGKTETIKSLSYIAGSNVMV
FNLSELYEVEDMEKILSGLYQLGFWGIFDEFNRLSECVLSSVTEKL---------SNKNI
TLLDRNIQVNGNSAIFITMNPGYSGRSELPLNCLNLCQQFFMEKIDLHSILNINLKIFSF
KFCSKLSDRIIFLLN-SLDLVFTSAKFDFGLRFVKNLLNIIKNLIITSIEWT--------
--------------DEYDIFLQSLNRLLLPRLTNEEKELYKHFIKN---NAELKYSNDEL
EFVELIKLSNVDDI-----ILNKSLEIYQMSKISSLVILYGESGTGKSLSFDKFIECIEK
TKEVE--VVKFDPNSLDTCELYGYMD--VDDWVEGLIPKILK---------SNPDKDMYI
VFDGDLKQEWVENLNSLLDDNRILTLSNGDRISLRNNVKIFLETDSLKDITPATVSRSTI
VFFDCKIGTNSLIGSDTQDSL--GN-----------------------------------
------------------------------------------------------------
------------------------------------------------------------
-----------------------ESESFIIKCN---------------------------
---------------DSCESKLLKR---------------IEWHCVIN------------
------------------------TIYLSQGLGSRDIINYL-----YSLLGENIQENAID
```

```
GSKRTTILVKDIHILN-DTSKWSSLCSIFRQMIKYKFFYRYFPEESEWIKVHLDGIVFVF
CST--YECGNKCRLLSMLKEV------------------------------------VLT
NSSIEFDEFSQLKTFINHKYGRQMSHYDLTAIKL----------LLSKKHGGWLILKHLQ
SRFNDNSLQLMEQVKCICKVD---------------------------------------
--------INPETINNSVITCKSVEDTYNFIYFTLLYNKSFLYISGPSVSGKSFLSRIAS
TNLGFEFIE-------AHDMSKDDIVSIIISCGLNNKKCVIFINIDILV-DMKLYFIVKN
MISKEYSVVL----YDALIRDVYDK--NSVSIPYDDFINEFKMKINDNIRFILTGN----
------NIENSLMNRLIKYNMPCISYDEYVLMSHGMI-----------------------
---EDSKE-----LIPDIYNVCKEYVGD-----------GFINYIFILKMTKHWYQISFE
INVKLLKHLNRGIDKIKLFKNNVDKMESSLNQTRCKLDQMNIESETKIKMLEIKRKDCEE
KKKLAMELASGLKERKSILDHKKQMIKSQINNIDPLIKKAKEDVENINKRSLEELKSMNN
PPFIVKYTIETVSMILKN--GRKIQWDDAKKLLKSSDFITKIILYDIENMQENVYNMLKE
RLEISEWDVNRIFKASKAAGPLAKWANSILICYEIYKQVIPLKNEIIQIEEEYIRNENIL
AEQNMLISQSQNEIEQNQLDYEKNIQVSSKIQNEIEINQNELMVSKKVIDDLTNELVRWN
KRVSHIENHNKYLLCNSTIEAIMPIYVS-LDYNTRTTFIQKCIETLKKKFENYSFDIKSI
---------------------FNIEKYIIMYKRLRHFVVLDSSESEYTSLIQ----EPYT
QVSCCDVNFIKVLNSVKSLSGSLLVKDICCDVNIKLKILNEFKEK-------------VD
VETDFTMYIISTNEYFGMIESIYNRAIVLNMKLNSECFLEYTKNKIIDVLEPEIYSLYIN
SNKNIDELNFRINDNETRLLDKL--VDSDVFDPD-FVKFIDEYKQVNEQLDSSLNDLKKS
YEIYEQAIDSKLPLIELLYYIYKEVEELGNLNKLYCFDYNALFYFTKVLYNRDNYITD--
--------------IIILTYNWISEGILETDGIIWGTKMIL-------------------
--------------------------------------------SKYNNELKEINDSSNN
TSNSIQ------------------------------------------------------
------------LLINNYLKSQ-----DYNDIILDYY--------DTIVLLTDGFEDPVY
ISESISNKREKSLDAYAVGSLSEMTRVTNSMLKSLNMGNYIILKNIHLSKTWINKIETDF
LNKY--KSSKIFLTCDMNVELSKNMLLSCHKILSE-LTELKSLISHLFNIFANNSYLKC-
---SRFILLKCVVIHSIIILRQVYIPFGWSKKYNFNTNDLKIILTFLSESLN-------K
SDLDQVKTSCLINDIIKE-----VYTAKITCEIDLKLLDDIIQVSDQR------------
--------------VQESDDIGTWIENNSLDDGFAFIGFTKYTQNEILNSKTYNN-----
-------------------------KSCIEYDGEYKTELNTDCKVFQNKDEI-------A
EATVKKSVN--PLEH---TKELENITRFINGENMGTINLNNIILAN--RLINLVKF--CI
PNNIDPEEMELTLT--------------------IDNLGELL------------------
------------------GNWFKVRVKLWNGEYDPDDNLILSGTDCGREH----ELFF--
---------------------------CWARKVENTSEHMTD------------------
------------------------------------------------------------
-------------------------------------------KSKSAPAKGETTPPKNS
RPTLFNLLEVLANYSMTTPSQRQCIRDIVTEFLANKFEHSKVYSYIGAVVGHDRLHAVIK
QLEADPDRSPLPDQDGLMKIESAFNLTRNYKTS--GTNGDST------------------
---------------------------------------------------ANKDEILRS
IRRNLSKKCELTRSTASAGVLLQKACWLPIRRTLTAGPLYGHSLICIGNRAYILGGSNGR
NQGFNLSKAHLINLLDFSSKQIMLSGEVPTHREGNSCNVAVLNQSHSVVLFGGFNGEVFF
NDVYLLDLEKRRWSKRNPTGPLPTPRDEHSALIYPPRCDSSQRSKEGPVVYLFVFGGKTG
LLNKFKCLNDMWAYNVIGNYWTMVE-TGESKPCPRFGVCALWADDETVCVFGGETSSPNV
DRSERVLLDDLWMFHLNPLSSDRNGEVKLSGVWVQDYYERNIGPRSHYSSIFIAQRCKEA
NTGAPRTVERLMFLTGGLTYPPGNKKVVVASDKLYFYFFSQKRWYSLKPN-YPRNYIFEP
RQLHVACFFEKKNILFPGTKPSVPCIFFHGGFHKKQILSDSWVLSLTGEDLFFLSTETNN
NPKMELRMYPPWYYRESHSPSLLWGMCSVQKWVFGALAHLVDNSLKDTVSSTNLSIKFEP
SPKGELMLSVQDDGNGLDYNSMNRLLKLFGRTYNSYNTSDDPDSRG--------------
---------------------------------KE-------------------------
-------------------------------EYGLGFKLAYGRLGNSVAVMSRTHDSIGI
GMLSLDLMCQCESREMAAPMCMWKLPSKELISRDGPCLIDQRHHQRLLMSYSPFNSAALL
AEQINVLGVNPGTRLLFWQLRDDLDSLVL--EDGTLLSSNHTH-----------------
-----------TFGNRHDQN--------EDETNN---------DHHEVGSVQVME--GGV
VMDSNNPSGTKP-KPNGMTSPPPELVSEENNVSDEQRNEENAVPKEEKDAGYNEQEAGFI
DKLEQLKSLMGYTGAW-----------YEAFPLWKTAKYSIDYCLPVYLYWLHLHSSCTL
SVQDVQLKPNPVHDHLGSKSVDLDIDLRMEAAQLNDLDRNQNGENFKRVREPFSSAQE--
------------------------EEEAVNAKVTKFN---------------NAVDVEQ-
--NSSQSDNNMDKL----------NQKKKEPQCLGTMAMQNAQNKEQHGKDMSVEE----
-------------CFLDVLGSASGLSMDSYSGKNTCGYCGVSPIGRSLSGGE--------
------------------------------------------------------------
-TLYSFLRAKLHKSVQLPFLFHPEDHAQGAFALIGFLNYPTNSSVSEDRVCEAGVLLYYK
GRLIRRLEGNFPAAMDELESAKLPPKASLFRGNMYRFALTAIINVPNWLVPSITKQEFIH
ENNRVFLTFKAKLIRLLSEYCKVCMDEHKRHSWYLEKLKQIVDYQTTLNNYMNRQDEVEG
LDVVPSWKLANT-ESSQEITTREESLNEEVAVP---------------------------
----------NDDEVNMSSKNLNLNHLTT-------------------------------
------------------------------------MTNETNQNAVPEED-LEQKERKYL
NRLLVEDALNDDNSVVALNPKRIDELGLFRGDTILLRGKKRRSTVCIVLADNDLDETKAR
MNKIVRKNLRVMLGDFVRVSPCPDVPYGKKIQVLPIDDTVEGLSKESLFNVYLKPYFLES
YRPVKKGDLFLVRGAFKAVEFKVVEVDPGEYCIVAPDTIIFHEGDPIKREDEEKLDDVGY
DDIGGCRRQMAQIREMIELPLRHPGLFKTLGVKPPRGVLLYGPPGSGKTLIARAVANETG
AFFFLINGPEVMSKMAGEAESNLRRAFAEAEKNAPSIIFIDEIDSIAPKREKTNGEVERR
VVSQLLTLMDGLKGRGQVVVIAATNRQNSIDPALRRFGRFDKEIDIGVPDDQGRLEILKI
HTRNMKLDPQVKLEELAANSHGFVGADLAQLCTESALSCIREKMGVIDLEDDTIDSSILD
SLAVTQEHFNNAMNTCNPSSLRETVVEIPNVKWEDIGGLEQVKASLREMILYPIEHPEKF
EKFGMSPSRGVLFYGPPGCGKTLLAKAVASECSANFISVKGPELLTMWFGESEANVREVF
DKARTSAPCVLFFDELDSIGTSRGNNVGDAGGAGDRVMNQLLTEIDGVGAKKNIFFIGAT
NRPNLLDEALLRPGRLDQLIYIPLPDLPARVSILNAILKKSPVADNVPISYLAQKTNGFS
GADLAEMCQIAARAAIRDAIKHEEMMNNTTDQNNN----------AMPNGTEFKYEITRK
HFQEGLANARHSVTSSDITKYDAFRTKFDPLYKNRNAATPNDIDFDWPENDEV--IMDPI
EEDDLYSMSDS---KWKDLDIPGDLFS----EGLISLEVL--------------------
-----------------------------------------DNTQNHQGSKLNSIKSSSK
--RNDKPPPDNDKISQ-------------------EKEVTIDYEEVNKKTNGWTNLSEEN
YTI-------------PQQILVNLYKNNFNSPTPIQRLTLVPSIIKKTHVLISSETGSGK
TLCFVLPIVISLLSEKI---------DKKIESLVILPTRELAVQVKKIFFMILEGIDIRV
LSIIGGISVQKQERLLKKDPSIVVATPGRLHDFVN----DGKLRGLFQLRHLVLDEVDKF
FEDNSYKEVQLIVKYVKR------AKIQCFLSSATIL------KENLISLFKLLNISNPT
VCICSKDSIPYDQLSNKLLYKKLFTKSLTQVSIPENLTFKLIDSEDKYKEVRLIGYLVDY
L-------CNVESKKCIIFVNTITYVYRLESLLSLIFWKDVHEHRLKRKYCTTFDVNTKL
DVSGIHSRLKQKQRLNRLEKF-SSNKKSILICTDVASRGLDIPNIDIVIHFHPPKDKSLF
LHRSGRTARLKSDGVSVCFCSPNNRELWKKLFTEINKNIDKIDQIEEIPRDKSHDDTGLK
DLLQLAETIEKSEFQMKEKSVQSWFQNAARKADIMLSDDETQEETKRQRSYKAMKSEKKK
LLKVF--------MKNYDSTVSTEVNGLSLLFLSSAEIKSLSCSEIKNAGRIDHESAGK-
---SCIYDPSMGSTEIYRLCSTCNEM-TNCDGHLGHISFSIPLFHPMMINTLCKLLKTVC
FYCGRFKFKSNTWFDFLLLFETAQRGDLEDLIILDQ------------------------
------ILTLESDDHEIK-----DVNPAQQLRDKIITEERFNRKR---------------
---------------NLLMTEIKEKKSEFLDYFDHDSNMWNEIRNRFFNKTSQKFFCNEC
SQKGKIMIKPSSDLSYIEVSWPQNHDRPFKRFIEED----------YGGDVNYNTSETVN
SK-----NFEEEKMIELTNKV----------------------NEDIISCSLKSLN-QTG
KSVNTVHLQSFHLYPYLDHLFRV-YSRLLNHVFPQSIVLGHKIFFMDCMGVSANRFRPPL
VTLHSRSTALLDIVMANEFVKLFLQFKDEANYNDNGSDVSEQLNGGNQEVKSKTLKLSLV
EYLSKMNYKIDEKQLEQFESYLKDYKDLNEAIRDQILNLQKKLSSYMDNGN---------
PRPGVKQTMEHKTGTVRQNMLGKRVNYSARTVIAPDCFIDTNQMGMPLKFALELTVPEYV
TKYNVNFLRKLVINGPKVYPGAKMLRDTNGKIYNLSALSYNERVAKAKLLLVGLSEGNAT
KIVYRHILDGDVVLMNRQPTLHKPGIMAHFVKILTNQKIFRLNYVNCSTYNADFDGDEMN
LHLPQDYLSQSEAQLIANADCQFVVPKNGQPIRGLIQDHCQGGALLTSRDTFFTKSEFFN
LVYLSFNSFVSTNSLMY-LSNQDLHF----------VEEDIKLDVQLHQ------LVKRL
```

KVLSDPFSRKITNRNIKIYLDQPAILYPKQLYTGKQVITCVLKTLIDNISVGLG--DTDN
---NKYNGINLQSKSQTPGDAWSYDGDKESTIIIRNSELLQGVLDKSQFGASSYGLTHLI
YELLGPRVCGMLLNSFSYLFTSFLQMRGATCSPKDFFLTETAESERNKILKRIKYAGIHL
QELFISLSR-----NTNNIPPNTNGHDNGNYNENKKLLNELVSDFKLFYDNVKNY-----

-------------------------------MSEMKFEDLDDLGSF-----RSFVKALLN
VFSSISNSNNRNV--------NEAIRKYMCKLIERCS------KVTRLRNLLLQYLLRLP
PDSNLKLV----------------QLKFPNWL---AQYSTEPKQNPIE------------
-NFMEYAELSNSLNKNGLKNNGASYKLGDSYTTCM-------------------------
-------KFYKMVCN--SPRENHLLSTFDRFFQNNIVSVSSSINELV--DSTVVKFPKNG
FATMVSTGAKGSKVNFAMISCALSQQTLEGKRVPVMPSVRTLPCFAFGDFGSRAGGFISD
RFLTGLRPQEYFFHCMSGREGLVDTCVKTAKSGYLQRCVLKAMEDIIVCYDSTVRDSNSN
IIQFKYGEDGIDVCKSSYLSQLHDLQVNEIY------------QWSNDPKNLKSSDS---
-----------------------DSMDLDEQEGDLD-NGSRFYLRNSKTETDSNFKAYFE
RCKCDPGEAVGCIAGQSIGEPATQMTLNTFHLAGAANVTLGIPRLVEILQNTSHCSTPYF
SVPILG---------KNEDEIAENAENAINALRKIYLSDIVQSVGMETNVY---------
----------------------------------VNKNSEKEWEYSATIQFDDFNLFRKV
IGHFDTSDIIKVCSHSLIKSFMKRVLGQMIVTMDINVPFELSDKTDQLEEFWNMFVME--
-------KQIVKRDKLSTRIRKMIL---GSSSDIPKGVGGQDITDTFPS-----------
---------LEDSVCDSHGGDEDED-SEGGTESEESEVDEEV------------------
-----GEEEDEGHGEEQEKEDDLDEEEEGEEEFEVLDASQS----------SSATVEDIM
DLDKETGDPNLMNLDEQDDTLSVDDFNSPTLSPTR-------------------------
---MRNESSGIKEGKQKIFTIN
RKVFHFAKSLEYSEETSTMVLKFGWPVIKCPYFLDLLPLLKQEISQLVLRDSYGIRQSRI
VF--QTVDDKEEYTLHCDGTNLKRLFMLRENIVDFNRIKMNDVATVFKYYGIEAARSCIV
SELQKVFSVYGIKVDYRHLTLIADFMTQKGDVRTFTRYGMARHTSPLLQMSFESTMKFLM
DASERGAYDNLKSPAGGLMTGKPVHVGSSLCRLMHVVDL-LARGRLIKEMKEASKLDDPN
IKLYVSNSNIFNWTAYIRGPEGTPFESGIFKLLIHCPNSYPIQPPTVHFVTKCFHPNINF
QTGELCIDILKSNWSPAWTIQYLCRGVIYILSSPNPDSPLNCDAGNLVRYGDLIGYKSMA
KMYTHEYSLKEFMNKDYKTNNELSSLMDVMLKGGVKVKSAAEVGKRAVQFTRGDEILKWL
MNNKELVYNKCPSFFQNSKLEDNQDVSNFVDNLIENGFMYRAQYQPLEGTLEKSETGSYK
RPMWPKRLIRTQKQRFDTVGFYIISYEGSQKWNYLKLCGIIFGIIVSNVY----------
VPSVATVFKALVMASIILRLILFLIMWFCGYDFWLFPNLFDEDLGVVDSFKPLYSFTYRK
DNLTMMACRLLCSILIGVSIYQLGKTHDINDIYKFTKQSFLDVLDWGHQKLAAPPEETSF
YKSLGVDLTTEFKEKGSEEPTEEGEEDDYNCLLGCGFKSLDDLVKNCMTDCDCMSNLLEN
SCL-RHCPNETVSSLTEVKIDICKRYKRKMGTLADSFLADLEDLENEDNEQENSDL---N
KGLNATENFESDEETPIVDAVAEYFNTSGSS-ENSFSTLIKDPEINSIVEKAKLLSLS--
-----------------------------------KDVKATEISFIDECNKTVIKIDKEI
INIFNYVRDIYSKRFPKLESIVYSPLDYIAVVKRAQNESDFTKIDLTDLLPNSMIMAVTV
ASTVASGTCLSTQFLNKVVSACNEGLLLAEFRNDLLVYLEGRMILIAPNTSALIGSALTA
RIIARVGSVENLSKIPSQNLMMIGADKNQNYILNGLHLNIILGILNNCDLVLNSEPSLRI
KALRLVCSKVSLASRIDLFKQHKDGKMGHEYRKSILQSLAKAVELPPAPMKKSLPVPEEK
GGRKRGGRRHRKTKEKYSLGEFQKYRNRLKFGVDAEDDFGLEMGNTIYII----------
------LTKKRVVSMQSSGATNGMSSSLIFTPLQGIELCNPNMNREVKRK---SVLDNQ-
DFLKVMMFRNDNATVTTLKSLPNFEIEKLRHLFSVHEDLGIDPEKIKKPLLDLIKQNHMF
PYYERIKPKLGIIEDDFEIESFKELNAKTLAELDEKIEFAEKNFGSSEIKDSILDKGNYY
FKIGDHENTVRVYEQALEKTVGINSKLEIMLTILRAAFFFNDLPLLVKYMEKAKSDIEKG
GDWELRNRLHIYEAVQLMLCRKFKEATELFLESLSTFTATELISLEELVLYSIVLSLITM
GRNVINKKVLLSSEVAQVASPGSSLYQLISDYYNCNYKNYMKHLVDVSKLILKDRYLGRH
CRYFVRQARLPAYKQFLRPYKSVTLKNMADAFQVSTEFIEEELVSYISGMRLDCKIDKVN
GIIENNVGDERNNNYVKTIKQVSCFIHKILR----AIFM--PNWKGLLKWSLSK------
--KQMSKEDLEFLEGAVNEHEKQVANSVSEVNRISENGENKNKEVLLESMQKLEEYFEEH
PSNATSLARQGLLESFTLLLKSDDMEVLSSTLSIISCSFSNNESVLEEASKTQLVPNLLK
LKNKLKDTQLEPRLITAISSSIRNCRRAEQLFVTLGGLSYLKDSLESTNLKTRERAILLF
NHFISLDKASRLIMATLNPYKILNLLLP-LDPENNGIQFTELSCTLVFLILQKHSNAFTG
EE-LNEVSKVLDRELQSL----NAVDYEKAKQIEHLLICTVLEISESSVSLAIANESYSS
ENSLSTNKWDLHDFRSKSFKLISKNKFKRE--KLGSETSNHTSDIQKNNKKENLETFEGL
GVPNWIIEICKSLQIKKPTKIQQLCLPSAFKGKNLIGCSETGTGKTICFCWPILTSLAKN
PYGVYSLVLTPTRELAFQISDQFRIFGVNMNILVLSCVGGVDIVSQSIEMEKRPHVIIAT
PGRLAYQVSNAENLSSIFSNVKYLVFDESDRLLDITFQEHLKEILKCIPKS-SEGRITFM
FSATITDAIRTLASKISNTNFEFYDATEESSKVRKIEHEYLFLPQ-----------HERF
LKDKGIIFTCTKKRCQLVSLTLDQL-DFKVTCIHSLMKQSKRTDSLSKFRSGYSNLLVAT
DLVSRGIDVPEVAFVINLDFPTTPSDYIHRVGRTGRGGRQGIAFSFIDEFDVDKVKNVEN
SVEIRLKKYEIKDKEAVKLLNKVTVATQKAHIFLQERE------------Y---KYIKRK
WLGGNWKCNGTKQSISDLLLHFNRHQTKNNNLDVVLFPPSLYVEHTRNELKSEFELGVQN
VSQSKSGAFTGELSLTMFTDFGLKWSLVGHSERRQLFNEDDSYVCEKVMMLQENGVNAVV
CFGETLSEREQGQTENVLKRQLDAFVKHVKDWDKVVLAYEPVWAIGTGKVATVDQVKEAH
KFVRDYVRGLVG-DVADKVRLVYGGSVNEKNCLELSKCSDVDGFLVGGASLKKEFLDILK
SLEMKRQDKAKPNE-SGMGENKLRIAIVSSDKCKPKKCRQECKRTCPVTKTGKQCIEVDP
TSKIAFISEHLCIGCGICVKKCPFEAITIINLPRDLGKDTTHRFGPNSFKLHRLPVPRPG
QVLGLVGTNGIGKSTALKVLSGKLKPNLGKFDSPPEWSEILQYFRGSELQGYFTKMLEDN
LTTAVKPQYVDNIPKQVGGLVGDILEAKDKRGIGQDLIVTLELSHLLSRKVSELSGGELQ
RFAICVAIL-CDADVLMFDEPSSYLDIKQRIIAARVIRQCIHHERYIIVVEHDLSVLDYL
SDYVCCLWGKPSVYGVVTSPFSVREGINIFLDGFVPTENLRFREDSLSFKVSTDVDL--E
EVESIHCYKYPQLDKKLGSFSLTVMPGDFNDSEIIVLLGENGTGKTTFIKMLAGKLQPDN
ADYEDLMPKLSVSYKPQKLSVKFDGTLRQLFHSKIRESFLSPIFQADVVKPMQIDNILDQ
QLKNLSGGELQRAAIILVLGTPADIYLIDEPSAYLDSEQRIVASRVIKRFTLYKKKTTFI
VEHDFIMATYLANRVIVFEGQPGITATALSPEPLATGFNRFLKSLDVTFRRDPTNFRPRI
NKYDSVKDKEQKACGLYFTMDTMTFLCTISGVQPQEPCLSKTGYIFERRLIEKHLEESPV
CPATGEPLTPQDLINIKTDVVTKPRPVTASSIPGLLSLLQSEWDALALETHNMRSHVDEV
RKQLSYSLYQHDAATRVIARLIKQRDSALQEVEALKQQLLLFRTNYDVNSLETEFDKDTM
VRLQDLAKVLLSERKKRDLSGYLDAEAFSKFKCAGEFRLHSSTKPGVLCVALDKSKNA--
---QSLEESFFTGGNDGSVVYFDLFNQKTVHTLNGHMKPVNTVVTHPLDNIALSGSDDST
IRVWREFE--TEFKCTYVLKHHKTSIKNLAMHPSGEYLLSLSSDGVWGLCNIDSGKVIKM
HRNVPK-CNALKIHPDGLVCIGAATNGTLQVWDIRDSTLKDPITTSSSAWVDLDFNENGY
YLVSVSEAGELVLWDLRKQTVINTFSCNVNPTRVKFDQSGLYMGVS-STKVEVLYMKEKS
KFELVHTLEGHNANVTDLEFGPYSKFLLTTCLDKSLRLYNSSTPKNTYAGQVFDGLFHGS
GTFYYNDFERYEGDFVLGKREGRGKFYYADGSIYDGEWLNDKINGHGVAYFSSGNFYDGN
WENGRINGYGTLKYANGDVYEGDWLDGAMHGHGTYKYSEGDIYVGQWRQDKRHGKGTMTY
VDKLGKPCEKYEGDWVDNIMNGKGIYKYSDGSYYDGDWCNGKMHGTGKYVYADGNKYEGE
WVEDTKQGFGILIYSNGEKYEGFWQNDKCHGSGILFYSTNDKYNGEWVDGKKNGPGEIIY
VNGDRFRGNWEDDHANGHGIYEYSNGNRYEGDWVMDKRHGTGTFFCKQDSSTYRGGFVNG
KKEGYGTLTLGCGHIVHGVWHYGSLVSIDNFEISPTSPWNNPDLSKTIEVSDVTKIERIG
IHSHITGLGLDEYLNPKYQKDGLVGQLQARRAAGVVVNMLKEGKIGGRAILLAGQPGSGK
TAIAMAISKALGTDVPFTHINASEVYSMEMSKTESLTQAFRKSIGLKVREECEVIEGEVT
EIEVDKFTNAAWGTPRDKVGKMTMKTTDMETLYDIGGKLIDALKRENVSVGDIIQIDKSS
GRVTKLGRAYSYSHDYDAMSPNVNFIPCPSGELQRRKEVVHTVTLHDVDVINSRSQGFLS
LFTGDTGEIKSEIRDQIDLKVQEWQDDGRAELIQGVLFIDEVHMLDIECFSYLSRALEAD
NCPIVIMATNRGITRIRGTDYKSPHGIPLDVLDRVLIIPTFPYQPEDTKLIITERCTEED
VDVEEDSLELLVKVATDISLRYALQLITASSLIRKRKGGGSVTCDDIKRSFNLFLDSKRS
TKYLINFQHDYMFSELKPNDEKMDED---------------------MTNYYENPFEFWT
SDEEDTEEGNFLIKERFKPQSESK----------------AEVKWHQNED----------
-------------SNNLETTLDFLSWIPLISEPTNEHSIFRVFDKE-FENFKQLNYEYIE
KELYNS---I
FNDNIKENDENLESSAYTPLGVGE---NKILEQESAIVEPPPFKLIVPKPDENKRYIRKK
WAIVD---DKEAPELSDLIVKYPFELDDFQKKSIYHLINGKHVFVSAHTSAGKTVVAEYS

IALAISRGQKAIYTSPIKALSNQKYREFKVKFENVGIITGDVLCNPGASCLIVTTEILRN
LLYRGDAVIGQISVVIFDEIHYINDLSRGVVWEEVIILLPRNIQLVMLSATVPNYLEFAE
WIGNVMQKEVLIIMTNHRPVPLKHYLYIYDRFFLIHGAKG-FNKEAYHIMYKY-------

TSTLKINDKKSTFKGQVQKLQRLLKQLESEDKMPVVLFCFSRQKCEQYAKDMPNLNLVYN
KQASKIHLFLKESLDGLSESDRNLPQLRKMVNLLTRGIGVHHSGLLPIIKEMVEILFSRG
LIKVLFATETFAMGVNMPARSVVFTSIYKHDGINYRYLTSSEYTQMAGRAGRRGLDTFGN
VYIFCCDEPPDVQDLTNMMIERSTRLESRFRITYNMLLQIQSRDHMNITEMMLKSFRERE
KMMKIPLLKKQINKKKHELMSLPPISCIYGD-----PTIENYYKTLNYSMNVSHELHQHL
WNHKESRVFKFGRVLMLHSTKISRTLSYSFITEIVDEKN---------------------
----HTFKVATIITESVSD---LDEA----------------------------------
----------------------NIKTVENGELRHYYNHE-VNLSSVSFIFDHVF------
-LDTDLDRNVI-----ELCKLIEKNDFKLMSFSKKFKQISLQFYEILLKQRDLYQLFKGN
PCTDCLLREQHFKTQDKIHNYELEIEDINKQLKDESLYFYEDMSNKLEVLKQLDFLDENN
RPTLKGRIATFITTSDEITLTEVLTQGILSELTPPECAAILSAFIYNDKPEKEVPSPTLA
LQQAKNQVVSIHKKIDVVQRALGVRVSHEDFNSLCNFSLSYVIYQWASGTPFQEIMELTD
LQEGHIVRVILRLDELCRKLLQTANIFGHQKLAEKIDLVCNAIRRDIVFKQSLYLSMIFH
KFHSYCSKINFLIPTSSFYLR---------------------------------------
--------------------------------FSTMSLHQSRLSSLVNLLTEKKLDSFIV
DRVDPHNTEVPHSTFDRLSFISGFTGSYGFALVTHDQCYLWTDSRYFIQAERQLSKPWVL
MKLLESSTKESKFPLIYFISVKTVGFDLYSTTYKSYENMLKKAPEKE-------------
------------FVGLTENPVDVERPPFPLNPLKLHPLKYSGVSVSDKLVEVRKEM-TTN
KVNVLALTNLDEVAYMLNLRGSDVETSPLFYSYLVV-------EMDKIILFVDHRKLNEE
VTSYLKSLSVETRDYNDVFSYLETVGTDQKGSA---------------DPVPAFKMWSST
FSSVHLCNSFLKRELFLETTPVCDLKACKNETELKCMAEAHIADGIAMAKFFATVYEMKE
NGTLFDKDEYELGQLSSECRFEQENNVGLSFEPISSISENGAVVHYRALKESCSKIGPHM
YLLDSGGQYLTGTTDVTRTVHFGTPTEEEKLAYTLVLKGHLALRHAKFPEGTPGESLDVL
AKLPLWERGMNYYHGTGHGVGSYLNVHEGPCNITPRIGKPLKPGMVLSNEPGFYEAGKFG
VRIENMFYVKELDSKDNRKFYEFDDLTLVPYCKDLMDHSLLTKQEVEWVNEYHKRISDTL
VPLMSSRPGY--EKAVEFLKKSAQPLTHNM------------------------------
-------------------GNSSDNCSEKVVKNSFNLQTNPKFIKDRLELFNALYQKQQQ
KLK-ERENLDISLVLVTDDGEVSSGKSFLTTPYNVLYSLDKKRAKKAVVAKVLYENRPSE
VFADVDEDNSD-----KSASEWVLWDMHRPFEDSCKVEFLDFNSEQGQHVYWHSSAHILG
SALETCFGGFLTIGPALSSGFYYDVYLGNNSVKPEDVKPLLSHVEALTNLNSPFERLVCT
KEEALELMKYNPFKVQLIKNKVPDGENTVCYRCGDFVDLCRGPHIPTTSMVNSFDVTKIS
SSYWLSNAKSDTLQRVYGISFPEKEQLKMYKNRIEEAKQRDHRTIGTDLKLFYFDTVHSP
GSCFWLPNGAKIYNRLVEFMRDNYRVRGYQEVITPNIFSCDLWKQSGHYDNYKENMYLFN
LEDTEWGMKPMNCPGHCLMFKHLFLSYKQLPLRLADFGVLHRNELTGSLSGLTRVRRFQQ
DDAHIFCTREQIMDEVLNILTFIGKVYSLFDFRYEFKLSTKPAKALGEDELWEIAEKSLE
EALNKTGTKWTMNPGDGAFYGPKIDVVLFDCLDREHQCGTIQLDFNLPIRFNLEYRDKS-
--ESSAENVRCGFNRPVIIHRA
IFGSIERFVAIVLEQTKGKLPFWLSPNQLLFIPITDNHLDYALELYEKINGLGYNVDVDK
SNNTINKKIKFGQQQRWNYMGIVGDREVETRTVSLRPRDS-DEQNVISLDDLVKLFESQQ
LKYV---MNFATKFLDFLSI
KKSKNDNIKKKG-----------FYTYCCGIVGYLGNEDTNEILLHGINAMKSRGYDSCG
VCTLHK-GKLKVTKCCSVTPADSFNILKDKVLSSHPPSTVGIGHTRWATVGKLSNKNSHP
HIDLAKNIALVHNGTISNIEDLYHDLISQKNKFSPDSDSESVAIFVGLEYENTGDLLLAF
KNTIKKLKGTWALCMMSQHYPNSLFVAAHEAPLLVARSERGVYVGSEPNVFMKYVKDCIV
LNDGDILELSLENVESYYSQYNLLKLESEVVEETCEPYPNWYTKEILEQIYILSEQSNFL
YQFSFQNQNQVMNEEANELLSLRDKKKLLFVACGSSLHAATYVAKILQKIHHFDLVEVDD
ASDLTLYRYHDKDVTVVHISNSGETLDCILALNFIKRINPDSISIINTVHTSLERSSDAT
IHLRIGREKSVPSTKAFTAQVTVLLIFSLYIISNNEEGSCHNYIASLYKSLSIFPSAIAK
LLK-NDEQYDSLAQWLLKEKIVYILGRGCGHVVALEASLKMKEVAYIQAEGVLSGAMKHG
IYAMIKEEENTTTISIITSED-KEMTINSTLQIKARGGYIIVITDLEDEVD-FADVLIRI
PSIGALTPALAIIPIQIITSKIAILSNRNPDIPRGLAKTVTTLMKE-YDYLFKIIVIGDS
GTGKSSLLLRFADNTYSESYMSTIGVDFKIKTVKIDNTTIKLQIWDTAGQERFRTITSTY
YRGAHGIICVYDVTNKLSFDHIETWLQDIDKYATSNVCKLLIGNKIDLVDSRVVLADEAK
HVAEQNNMNYIEASAKTDSNVEKAFTTIAKALKDKVTQYPSNAPTSTVNLNASKVTTNRD
SCCMPRVRTLNTKPPPEGWELISETLESLDDKMKQAQLESGEGKRKTEILWPIFRIHHQR
SRYIYDMFYQKKLISRELYDYCIREGYADANLISKWRKQGYEYLCCLRCIQTSGQNFETT
CICRVPKRDLEPKVIECVLCGCRGCASCDFFKKRNLITITVPKIFLSSYLIFRQSDFLSY
SFCYNFNHLTDYRNFFKINSNNSYSNSSN-------------SYSINTSLQSSQSNSYST
QSNHTNNTNTNFEKNSFRKNMIHYDLIVLGGGPAGMAAAKEASRLGKRTVLFDYVTPSAR
GTSWGVGGTCVNVGCIPKKLMHYASLLRSS-NYDKFQYGLTNTQLTPNWNKLIQTIQNYI
KMLNFSYRSSLLTSGVDYINAFGILKHNKIIEYNLNN---EIKYVSGDKIIIAIGERPYI
PSDVEGANEYAITSDDLFQLNTNPGKTLIVGASYVALECAGFLTGLGYNVDVSVRSILLR
GFDRQCVKKVEELMEASGVLFLYHKLPIKIEKHNQQLKVTFNDQSVNYYDTVLYAIGRIP
SQYTQHLKEVGIEFDGNGNILVTN--EETNIKDIYAVGDIVSKVPKLAPVAIKSSELLIQ
RLYSNNNTQMNYENVPKCVYTPFEYSSCGLTEEEAIEKYGEDNLEIYLKEYNNLEISPVH
RIN----KKTNDEFDYPMTCLSKVICLK--DGKIIGMHFVGPNAGEIMQGFSVLLTLNAK
KSDLDKTVGIHPTDAESFVNLTVTKSSGKSWIATGGCAGGKCGMTAN-FDEFMFMALKEC
KGIEDVLEKFFSFLRRRTDFCHTVLTPEQLKEFG------LDDSV---NSRGFRPNQMRD
LVNKIIEDNILLYRRTNQPYLL---------PSNCRSDSSTQNFSSDRPTNEAENRTPNP
HKS-------DKKYTVNTWNGAVTEKYAWSQTFRDVTLEILSPKKITTKDVSVLITKDRL
TINLQGQVLIDGEFCNKVNSFDSFWSIEDGFRILLNIEKAEELWWDCVIKGHETIDTQEI
ESVKRLDEFSSSEQNAILKLIKDHKEKKFQ------------------------------
-MSSDELSLVKEENSMGV-SESGSQNPQRLECLLSKGLLQRDLDLLREAGYSTLECVAYA
PQKNLLVIKGLSEQKVLKIKAACRELCHLGFCSGQDYLEARGNLIKFTTGSSQLDKLLQG
GVETGSITEIIGEFKTGKSQLCHTLAVTCQLPVEQSGGEGKCLWVDSEGTFRPERIVSIA
KRFGLSPSDCLDNVAYARAYNTDHQLELLVEASAMMAQTRFALLIVDSATSLYRSDYSGR
GELASRQMHLCKFLRALQRIADTFGVAVVITNQVVARVDAMSFFGGNDKLPVGGHIIAHA
SQTRLFLRQSKGESRICKVYDSPVLPEGEAVFAITDGGINDYHDR-------MKGFGVFS
PCSATSRYVLAASGLHENSTPREGTQPLLNLLDFKVTENSETSSDFFSNTFEN--NFNQY
ATSRSLNAHLTTMKWIKL------GVPYTDEKTLVVVGSSSGDLVFYDAKALTENK----
--TLVSTNVCSVPIKCLGY--CGKNMLGVAGVDGQVSVVDLGDASNYAVVDVSYGKWKVG
QVTSLSWNYRLPHILASASDTSGVVVWDLKVRKPASTFRDPMGRVNPVALDWVPDQATQL
VVAYADDNAPSVQLWDLRNPSGPLFESKAHTRGLTDVKFSPHDPNVLLTSSKDTTKCWHL
TPETFTLLSTFQTEALSHHSRWHPNVPGLFLSQSNDGQLNVHHLYSGTVE-GSYVPVWTR
KRGGIASGFAGQVTSWNNVELKFTLTSQLDG-----------------------------
---------DSLKLLDGVLEVMRMLNDESMFTEFCESKVNSSTSEYEKLTWSVLKCQYKG
QLKDLLSTLGYSNFEPVTNLS-------------------PDSNVGGNMN----------
------------LPAQSIGTLA----------TQSNRTNSLSEVTSQDDKEAEDFFNSLT
LKSNLLDPNSNL------------LDTQNNLMN---------TNNNLLDLNAGSSVTGVR
SLSGLDWCSDMK--LKNKLVNADFKEAAKRCLDLNKVVEGLLLAYVGGQEVFLELVEKVV
ESKKDPFLNLLHLLMQGDVETVLLNSNLNDWKETLCIIYTYGGDLTLFRTLTHKFAKRLY
EQGY-YQEATISYILACDYYQVANLWKLQQSLSNSRLQGLGDMMLKLSVLSFSMWSNSQN
ESFDTVLMELSEVFIDAGMLDKALETLNVTGASNSEQIRQMKSKVENQLTTQYTGSVVQP
TAQYVT---QATPQYAGQSLLQQTGTQSMVQQQAQYATQPATQYTPQTMVHPVQPVVQGV
GTGLRPAVSVSQTMAST-----TTRTVPLTALQPTTHRTTQQTYPAQSTGYPSTTYQGST
SVSGMSQSFTTASS----TNSTAVSVSTGFLTSQLPSNASNLSSYNSLPHSTNL------
-------TTTQANLNPVPANTVPTNLNPVPSHLNTSLTKASNAATMSQTSPG--------
-----------------YNKTNASHPVNPLTSRP------------------QTHTMGAS
GQSYSVGSSAPGAVAGAVKPGMPVPWPNPTATQQLSSKTVATQESNKLIIAST-QNVPKM
MPKADLDMVISTLNVMT----GRLGETKMDLDTKKNVNDLINMLKQGSLNAEANSLLATL
CRAVSQDDNFNSNLILNNIISKLWNNQNKSWIMCLKRLVPKMAIRAQYENSNEVGVFSTL
TNSYALVSLGSSTNFSSLFEAELTPHIPVVHTTIGGTRVIGRVSVGNKKGLLVSSICTDK

ELRHLRNSLPDSVEIRRIDERLSALGNCISANDYVGLIHVDMDKETEEIVEDVLGIEVFR
ASIAGDVLIGSYTRFQNKGGLVHVKTTTSEMEELSQLLQIPLTSGTINRGSDVIGAGIVV
NDWVAFCGMSTTATEIATVERIFNLARPSNSSIIDSYSLKSALIDTLIMFEGLVKRMLDT
YLAPYVEGITQNLQMAVWSGNISLENLTLKNDIVSRLALPFHDVSGKIGSMNIRIPWTSL
GTTPIRIVIDSVYICIDNRSNEKTDEEILAHLRKKKNNLISILEHEYFELAN-LQSEGKL
S-SSYIIKLSQKILNNIQIDFTNIHLQFFDEHSSF--AFKIDSIFVRKSKADETKKSKTN
EEFREEPVTHVCSLLGLSIYETNFNLKKNEISSNN-------------------------
-------ILQHSEEKNSENGEVVNDYIGLLM----------LEPLSFRLYLAINARNKSI
YASLCIGSDPGHSKISETVINYTNYIKSNISSLTN---------------KVSTNRESVV
STRDDKVDRLKIVLTSDVIRTFTKMWVDINMVRKRGKRILLSKSNEVKLDPESLQTSTKA
EFIKLYTKGLGTPNDTHNTEMDKEESERLQDIIDLVPARYIARWKFACKRRSSATVMTKE
NK------------SWFKWAKNIVTTTYNNYQSGKQNQETSDSNKDEPQ------QEKE-
----PRK---SFRQSLFSLN--NSFIPGKTEQQNPVQNEEKNEILEKV-------IVENL
PGGLVLTEDEIKMIQETISLEELFDDSFTTSSSVLPSFQFIVQLINSSAEEEIIVVDIQN
LQSRLYLHAVMDMKDKDMYEGYFDLNLDSFDVIMRDKKIMTFSQQNIPFQSIVTNTSKLE
LPESNHKLELCQSISMNLRIYHRIMEKGNVVVVNGELRPIETNLFP----ELIPVFFDLV
SLFNPIKQIFNEDF-----------------------SVDAYNTNLAGPNTDKEVEYDEL
TSELNPSDRE-----LEHLPSFVNFDIKFSAPILIIYFE-DKRIDFYFGTLLLKSNGDCP
IGHIDGTLELKQTQITC----NYMDKSYNFLRPLPVKIHYDVDMEQINLNVIFEEIFAQL
EPMATNILFKVPNEIIKILL----------NNMDGSQKSNVN------------------
-------------------GIDEITDTNTSQVRNKLKQYKISVLIRHSGFSVSNLNLHEI
FKLDMYNVAVKMSYTLLNFKFSLSMESFIVSNPTTKIPLFKTKNTLPSYTDVEP------
------TLGSLNITQYPSQSEKQEKDGAEDLLASVKRMS----LERVEDEYVDAIEDVTK
SLDLEISTNIDENQIHN-AVNAQIIEMEGNWEYSTIKLIVDCFQEYK-------------
-----------DIFSNHISVNTITSLTTTFKNFKDKFIFPNTSSFVNQTDDAKP------
NPENLDNSSSKCFENIE-------G----------------------------FLIDDEL
PMLGSELKRHIVTKCKLIIKGASVLFNNSNDVIAKLSVGKIRFKLYKFQNDEKIIKLSIQ
TGRLYFGGRCILSHLTN-----------YEPKSDANLDSISDNQSDEV--------EVQN
NLLELKLKCYNTKEPYSICFQGKVDRVVFVYFQHDINRFLEYFDDGILSVFLSKSYHRVV
QRADEIYFFYHFSITSPIFILPENKAAIPDCKVHYKQTPLYDTDKREYENEGVSY---FM
NISNVVDLWYFGSYILFELGHLEFRNSYS-------------------------------
----------------------KLFESYK-STIFLKMLGTKAQIMEKNGTPNISGVILDP
TDLKVCTRG-RDLMEIGIDSKALVTNLTRNQMTFVIDVFNENIGGASYMVN---------
--------------------KSETNNFTQ--NFPTKSESRYLIRLSINKFQFYCYNNSSN
PLGLFEFEDITCCLDYASVKLVSYYQFGFISKSLTITDLRVNSFNKYKTLLKCNNEGEFS
A--------LLEEETNIVK-------------SVNGLLFEWLKNYKTKTGNDFFFTKAKT
HQSGVSVDNSYLEDSDTRLQYHGIKCVIVTDPEESFIDVIVSKSEIPLLFVYFFDIVRFF
SLSYGTSSMALFPKIS--
---------------QVN----IE-RVYILNLLVSKSKFISFSKMDKLDSPRLELVTDFI
LQMHVHGNSFKFTKLDIIDCLLSRV-------------------------------YPNT
NIKQVLCGNFLMYGKGHYLS--------------EVSS-KMF-----FQFTIPQINLILY
TRDLSIIIYCFKSMFTDGPS---------VIPIKLVNNQLIDDNT--S------------
------------------KFLTLSFDIKGIEMMFYDDLKKSIVPLVKFTVSEKLEIINLP
IEKRYSLIRCNCHLYYFNTNVGDWEPFIEKFIFNLEYQINKPNRVTDDNTWKDFVAEYQL
KITCNNNLLVNITPNLCQLLQYFLPILKSNLQSPCLNNPNNINQLENEVIN---------
---VNEETKLNDLDN-----------------LETVGGSKKMVEEDDIEM----------
-------YN--YRYINLTNYEYYGFIMDLNKSR----------TEAFSDVM---------
----------------------------NIVTTTNPKQLDS-LVNQLHKE--VSSNDYCI
HLITKPPKYLLKHLSKELNC-SEQLIETDLLTTTSVFRTISNLSSISS------------
---------HGFNKHNAYKMNFNNGTVLSNIPLSKNCTVLLSVPVKDIKMYNLAEKNMGE
LEKVSDVLNSTNTSNESTTSVETNTTGMGTEVMGMSRSNRVTNVANTAPKNNLICQVLTP
HPSHKLLLFTSTVRIYNKSGMPIVMCFLDRNYNQQYMYNLNHRTIPCNLLNQSNMDNFKQ
YTTNNIYTGLSEVQKHTQNKGIGYCVVIPNDYMLSVPENVFITDSQTIFSFKPLYRHSFD
IFSPSKADRSKSGENLLKGDYSKLVNKSGWSKLIDTSHHVGTRLRQCYCPEKNGFIYFVV
SVNHKRSSLPANVNVSEVVIYPSISVMNTLPLDLDLKLLYKDYNLNSSKYGGKDEHNTRK
EEVVYKLNNKSILHIYSVPPNESLSLYLKLCKTSTDWCTRIDNIYGNSDTKTTFNMMVNS
-SLLELELIRFPGALPVNNLCIQGHLSLIVNAPWLFIDRTGLSLVPQHFNRIY-TTNNLS
FLYDNNNNNYKLISLKNPVQSTGPSGPRYSSTRSEDFIDENEY-------DIKMPIIGGY
VYTTIDIKGKNHCVCLITEKVYIPGLSHISSKVTSALPEYLFTNNLQEPVHLRKDSRTQS
MQILPNSTQSISHLTNF------------------INGASPASVNR-EGAKEDTSENYI-
-EIKVGMETCWSNVIYLNEIYSGETFMSLPKENSSKSLVYNITIIPKNGIKYISINPTPT
MLTRGYLLYNNCSVIKVVMIRTFHK----SDIRNGS--------------------CFTA
KYGQVVNFGWPNPFVHKTKLIQVLLWLDKNTVAPSKPLIFNFSTFRYRRLTTPEYHTNYS
ITILAENRIDYYIIIIN--------PCE-------------------------N----LN
ITNPLNSSDSLSKKETEKTDMEEID--------------------------------DSI
LLQDNNLKDSKEEDLQVFEEVIKSYQVILQINQIGVSIVSQSLHEELFFLEMGGIMSLFM
CKEDNQRLELKIFDVQLDNQSDSEIGRTILVNR-----SKVETKD--TSFLQVYIDRPFS
NCKDISIKKMFISLDDLQVDFNDLLFTKIFNYYRECMINLDFSNDKKINIDMIEKWLNME
NDSPTSSVKLPRSISIDYLYIEAFNLVVWCCFELDKLHMLGDLLRVGIRILSVSRNFQLM
GAPLSFQREYISVNRTSIFTFYEQMKEKYLQACLSSIVSILGYSNLLNIPKLPITVGKCT
IELAVDAVDSFSSGLSMFLSKFTFDKEYIKRRTTTKDVT---SIKDGIFSAGKSIGEGLF
SLTNIVTKPIEGAQKEGVGGFIKGLGKGIVGSIVKPIDKVGLAVSQVSRGIKANINKE--
-EKYAVEPCRKPRMLWGEFSQIRTYSTVDAEIKYVLGSKYSKYIMDCLVILKQVNNGKYI
ALLFYPSKIYLVDLINNPNQGKKPYTMWKLLISNITDVRASSHGVIIKCGTEQYQVPCTR
AEMINNIYSCFQRAIKHSSSQITIGPELFA--NM---ESNKRFSRFVLNRTSTDAANDDF
EVVTSSYDIDSLYEPKPIP-GHKFNISTFDQYERMYGESISNSDEFWGKIAKSSLHWISP
YTKVSEGDFVDKNYAWFLNGKLNACYNCVDRWAEVRPDAVAIIYEGDEPEDQRKVTFNEL
KMNVCKVANVLKMLGVRKGDCVTIYMPTIPELCYSILACARLGAVHSVVFGGFSATSLAE
RIHDSNSHIVITADGGLRATKHIKTKDIMDDALTRCAFVTHCLVFRNI------------

-------GSDVK-MTPGRDLWMHEAMESVRPYCPVETMDSEDVLFILYTSGSTGKPKGLA
HTTGGYLVYAYATVRYIFDSHEGDVFGCMADVGWITGHTYVIYGPLLNGITTVMFGSLPN
YPTPERYWNIVQEHGLTQFYTAPTAIRSLMKFGDDPLKGHNLSSLRVLGSVGEPINPEAW
KWYYNVVGNGKVPVVDSYWQTETGGIIISPIPGVTFTKPGSATYPFFGIELAIIDANTGE
ELEGNNCSGLLCIKKPWPGMFRTIFGDHSRIHETYFPTKYPYYFTGDGAFRDKHGYIWIS
GRIDDTINVSGHRLCSAEIEYALTQVDIVSEAAVVGYPHALKGQGIFCFVSLKEASLRL-
---------------------------------PKEEVIGRLKMSIRHYVGPFATPDVVL
ITPNLPKTRSGKIMRRILRRLASKFHDFGDVSTLANPEVVHQLIEDVKQVQ-------MI
EDLSVIINLGL-------------------GISCIITQLYILKYKNKIKKLDFDKKCKIF
YASQTGTSERFSRILYDKLLQ-FNISNKPIDLEDFKEEEYIEDSIFIFLISTHYDGLFPD
NTKNFLKILKKLEINNTNLK-INYCIFGLGNSDYEYFNQAAKLLQSSLNSLNANEFIPIY
LSDELNGINKEFDNWFKILIKSFNKIYNKNFI---ITNQEPNYYKSWRHL--CELEMRYE
---NIIMKNNFVPI-------------------PKDIICKQQYQCIEAKIIQNYNLIPNS
DQSVHEHLFQD-MVILIKLLFHHHFLYQILLRYPVTVLAHTATR-------------ILP
ER--SSLSLS-------------YGAWFYGTSTSTTTVKLYCDLTSLPNDEIILNFCTFI
KDKKEIERIEQIINSK----LMKLIREVKLLFNEFLILFFPNVKFNLSGFLQLIPKQIPK
AYTISSIPN---KNIKIIVKKVEYNLHSLKTFYKINMNKNIFNEVTKTELY---KRRIYK
GNCSNYLC-NLIVGDTIKFFIRSSIFSNIN--FNNNFLLIANGTG-TPLRCLQQQLQSLI
EEKK-----LNNKVIIYLGFRS-KNHILYHEEL-NHLQNLNNFHISYAFSREQVNSK---

-----TKDVPWVEKYRPKKISDVIFQTQAVSIMEQIIETFNMPHMIFHGPPGTGKTSAAL
AMARQIYGLEGMRERVLELNASDERGIDVVRDRIKTYTRINISNNRVNPETNRVMPNYKM
IILDEADMITADAQAALRRVIENYSSISRFILICNYLHKIIGPIYSRCSVFHFKPIETNS
QIDRLKYICNQEGITFDP---KFLTTISSGDMRKSITILQSTACLYNEITENAIYSVSGK
PPKRVVESIFEVCRRPE-GDVESVCKQIVHDGWDISSIFQQICEYVVESDSIGDIEKSKI

SLELANRDFALLQGGSQYFQLASACFHIKNTITGGMSVSINFEEASLKLKKLSSIFKRPE
DD---KIDLLFVCTGKSRSESNSTTSELLQLWLTGFQFPETVMVFASDGTLSILTSPKKV
NYLEPLKNHYEKVKFYNRPGQNDESLTKIFESF----NGVVGMLNDPKPLGDFSDFCLDF
VKDF--TRKDVTVEVSTIMAVRTEVDLEIQKQSSQLSCGVMKTMLINQIEEVLDSESKKT
HSSLVAHALNIQKDQKFIEKMEKKFNMVGSDMEVIYGNVQSGSNYLLSIGAKPTDDDLSH
DPGTIIVSVCSKYNEMCSCLTRTLILDGTQYMKDAYKLALKVFEYALTV-LKPGVTFGSV
YSSVYDFVAKEKPGH--------EDYLTKSVGHTIGLEFKDSNFLLTSNNT-NLVLDNMV
FHLSVGFLEIHE--GKKFAVWIADTVHVSSSGNT-VLTSFVSKGLENVSYELEEEEEEVK
YEEE------------EEKKPVVSSQILKDAESVILKERLRNRG-----GVSKEEMENLL
AHQKKLRELKIEEITRRVKDGSGLAGDSKQKQVVKMDKIKVFQSPDYFSNELTPNKIFVD
WRNEVVMLPVNGYHLPFSVMMIKNVTCNPEN-NNLYMLRINFQVPGSHTTSRNDQNPLPD
LQENSIFIKEVLYKSKDVKHLQNVFKSLKELIKQMKQREND-DMGLTLADQEKLNLNRTG
KRIVLKDLMIRPSVHGSRRVLGFLEAHHNGLRYLVNSRDRVDSVDISYANVRHAIFQPCQ
RELIVLLHFHLKSPILVGKKKTLDVQFFSEVGTQIDDLDNRRGRSYNDPDETLEEMRERE
LKRKFNTDFKQFVSQLKDLTSMKVDLPIRELMFTGVPLKSNVELLPTVNCLVHLVEWPPF
VLPLTDIEIVSLERVQHGLRNFDIVFVNRDYSKPIKRVDLVPIEYLDTIKRWLNELDIVW
YEGKNNLQWTNILKTILEDVEAFVESGGFDGFLGEGEDEEDSGEEEDDEDEEYKDDESDE
EEEESEEEY---SESLADEDEDDEEYE-EEEDEGLSWDELEERAKKADAGKVYDD-----
RKAKRRKMRIYEYVYNKYFKKPDEEIRNIKLNI--KQIFPCSNRVKTTKYTPYNFIFKNL
YEQLCIPSNLYFVFIAVLQSTPQVSSTQGYPIVLLPLIIVLLFSALKDGYEDYQRYLSDN
QLNNNIVQIVNLPYVTDDNNTDSNFDSNFDTPISSNFDISKNATTTNGTCTNGGTATKKS
KVDEFVGT--ERYDFVEGLVNKYWKDLKVGEYVFLQNKDIAPADLVLLATSEENGFAYVD
ASSLDGETNIKKKESIYDVYNELGSDFEHVITEVQRLLGHFKCEGPNKNLISFDGHLKYL
SDKITPIPLNKKLITNYKPNSTTNSTKDTGTVVNTNTNTKDTNSKEDPLGGINESKETQV
NLNNLLLRECKLVNTQWIIGFVVYTGHDTKIYKNISKAPYKVSNLQKKMMKTTLLICIIQ
FILCLVATFYNLYIHTNKVYEKYSYLS--LETRASGFYIFIIYYLSWMALTANFVPISAI
VTLNAVKLIQGFFIQFDDSMYC--------------------------------------
---HELKMNAKARNTMLN
EELGQIKYLFSDKTGTLTCNKMEFRKFSIMGHSYGKGYTDVIRFVYAKKGIFLESEVANP
NYDKESHVNLVDDVLFKELNDPNHPRHEYLIDFFLHLAINNWAVPD--SSNNRIYMCPSP
DELCFVNAAAFCGFRLLQRNSNFVLISIFNQIYKIKIIAQADFDYKRKCSTTIISIPLKP
GTINDPSNRV------------GVNPLNTVNSSINT-----------------------K
GVPEETGTVGASTVTEVTENNRIILYCKGGDNIMIKKLKEIKEVDVVTLRNMKKYSVGGL
RTLVFAKREIELKEFNEWLKEYNRIKLTIESRDEKLAECVSKLECDLELQGVTGIEDKLQ
TGVSECIEQLLMAGIRIWMLTGDNLDTSINIGIATNLVNMLSDRIMLDSNTVPNDKLFL-
EMKKHINRIDQENNITKHRCLILDSISIEYIFSSITGTTANNSTKST--------KDTNN
TKSTEGNNNTKETPLGVKEIFIEILKRVHSVICCRMTPYLKGAVVTFVKNKLGGITLAVG
DGANDCNMIQIAHVGIGIKGREGSQAFNASDFGIGEFRFLSPLILHHGRLCYRNLSKCIS
YMFYKNVILIIPLFFYAYISLFSGQKIYYSLFVAIYNVVFTSIPVGIFGIVDQDYNREFS
VKYPHVYQLGQINHYFNVIKFSGWILNAIIQSAVIFFMMTVGLGEFSIPFPYGLIADAPT
LGIMLLSSVFIIVSCKLVLETWYFTKITLLSHLISIFFFIITVCSFSSSPIYSANSIGSA
FVLFTSYRFWIVILGTLMLSMYRDYFYKVFKYSFCPQYYHHVQKVEYLKIEQPVTF----
--------------------------------------KSAEFLFGLLQGNFISSGTQVP
EKSSLLLGINRILVRRYSPEVNDHFNNPRNVGSFDKDDPSVGTAIVGKAACGDVIKLQVK
IKDEVIEDACFKTFGCGSAIASSSYVTEMVKGKTCKEALAIKNTDISGTFLLYSY-----
----------ITNYLQGDT-MNSISRFFPSKDDKFGIIKSAFWHRKFASTSSSYSKKPKV
LFLGSGWSSVFFIKNLNPKLFDLTVISPRNYFTFTPLLPKILSGTVETNTSTEPIIEYMR
RNFNNPQFIHAKCVDVDSDAKSVTCDPSGEPSFSVPYDFLVIGVGAQTNTFGTKGVEEYA
YFLKEIEHAEVAFQKIVDNFRAASMPSLSDSERRRLLHFLVVGGGPTGVECTGELSVLMS
RHLGKCYPELMPFVKVSIVEAGQRLLPSLSQSTSKFVLNVFNKSNVNMYFGKVVSEVKQK
SCVLKEI--KTGNTEEIECGLVLWASGLKETDLVTKLKRKWNIPESSRALLVDQYLRLQG
LD--NIFCLGDCCKITPTKLSENVELVLEKVGSP--TLEALVNARKTLAKDFPQLNDSKW
NHKDEKFQKSTKEHFVEVLKLVDHGYCPPFPTAQNAKQAAIYLSRLFNSGAVLTGRYVDS
AFCEKWKGTLASLGGMKVVMNSPYFNVNGGLFPFFLWNGVYMLMFSSFKMRLSFFFDLLK
NFFFSRHLINMPFDPSKLQVKNKMPAAVQITAEQILRDAVEWQTKEVKTKQTIADEEELN
FYKAQKRKEFEDTLRRQRHHIGTWIKYAVWEANQQEFRRARSIFERALLVDPNNPSLWLR
YIETEMKNKNINSARNLFDRVVCLLPRIDQFWFKYAHFEELLGNYAGARSIYERWMEWNP
EDKAWMLYIKFEERCGELDRCRSIFNRYIENRPSCMSFLKLVKFEEKYKKVSRARSAFVK
CVEVLDPELLDEDFFIKFANFEQRQNNIEGANSVYEQGLKLLDKTKSEKLYDNFISFQKQ
FKN-EFIDDLISVKKRNEYEGDIALNPDNYDTWFNYIKLEESILENMLLEAQKDRIVQVY
ERAIANLPKDNNRKLWRRYSYLWIFYAFFSELQLDSKERAEEIYLKSLQILPRD---FSK
IYIYLSQLYLRMGDLKKMRSVMGNAIGLCKKEKIFETYSDIELKLGNIDRCRIIFTKYVE
IYPYNYKSWLSYINFELLLNEINRVRKLCEYAIEMEQMNNPEAIWNKYISIEKNY-SYSN
VISLYKKLLQKTQHIKIYKEYSKYEYENGNN-EKGREVIEEGIKLYKDSS--VERSKLLY
HLVEMEKKYGNEQTVQNAKKRLPKKILRKRKLEN---DQEVDDIIYVFPDDK---TNTKI
LENALKWKKQQK-----------MA--EDGSPNYMDEDEHVTDLSTAIQKVLLFSLSHGG
LVRGLHEVAKALDSKTAQVCFLSKGCSEPAYVKLVQALCKEHSIPLIETDVDSKTLGQWS
GLCKYDIEGKPRKIVGATSVAVKDFGEESEALVFLQKHISK-LKKMSDSNRDRYTHRDRN
SYRDRSIHRDRHYRDRSYRNRSQRDKSRRSNSIVNRSRRSNSNHKYSQKIVENKHYRSYH
PSHTDEEKDTKKRKYYYEKKPEEGEIEEVVLEEEDEDLDKFLESRRKERHKLILKHSIQT
DNSSDTANTTDLLDNNPQDSLKNENKENKEIEPEEVKDKKCINPFSTPTPNTEPEDNKEL
NSVNSETPGQTASA-ETGIDSVKEELEDDISPISQTVSESPNKDMTDETTSSTNVYSDLQ
KKLLLEKQKLRSFIINMKRS---------------EQDEVEEEVLEEEEDDMDMFSTNSA
VKKRKVVRRVITN--KLENRSLAENWNDSEGYYQAMIGEVMNDRYSVISELAGKGVFSSV
LKCYDSVENRNVAIKVIRNNDMMIKAAEKEMDILRRLNETDKEDKKHIVQLLTSFRYRGH
LCMVFNWYWGNLRSHLKMNGKGYGLNISYIHSYTRQLFIALRHMKKNKIMHADLKPDNIL
VNDDYNKVKICDLGSASDESENDITSYLVSRFYRAPEIILGCRYNCKIDVWSAAATIYEL
ATGDILFPGRNNNHMLKLMMEFKGKIPSKMIRAGQFSTNHFDENLDFVYTSTDPLTKTTV
TRVIQDLRPTRNITDAIFERQPWTKANSPKKDVLIKKIRQLGELLEKCLTLDPNKRFSPD
DALQHPFIRS----------MN----------ESSMESVNGLNLRNDVKNLVEQESYKWI
FVGGKGGVGKTTISCSLSSILSERRESVLLLSTDPAHSLSDAFNQKFTDTPTLVNGYENL
YAMELDVTRVSDTVFYLKFYLSFINANPN------------------RSVQSMKYSVIVF
DTAPTGHTLKFLNLPDTLDKLLESFLKVESLCGVAMKLFSALN-NSLPKEEIFQKIKRFK
NNLTLIMNQMKDPNKTTFVCVCIPEFLSVYETERLIQSLAKTDIDCSYIIVNQVLSYINL
EEH-VNNTKKSLENLT-PENKKVLEDFFELVLEQQNNLNGRLNIQRKYIEDIKQLYEGFF
NIG-----KNQTH---
---------------------------------MISDNNIYIPKVGDHIIGIITNKNNDY
YTVNINNLFNGYVMCIDGFRGTTKKYKPALNIGTFIFIQLDNVNYNNNLIELSCITTDDN
KNWSTNETYFGQLSGGFLFPVPLNYIKILYGEDNLVTQLL-SNLKYEIVLGFNGRYDENS
TLS------------------------------------MVHPCASHSSRFLKCFPLSLV
RFSKNH-PKLHRLLFCRNCHTLLNRKFITNNSNNFNFTKGEFSTQSSSNTNKTTDPNKQL
NDQNKESDDGNKNNDEEFKEEPKSRFDPSTMFAVGLGAIMVLELIDTGVLKNEITFQEFL
SKYFIKGYVDRIQVVNKDFCRCYLSDLSPIK-TPKFVSFRLGSIDAFEQKIDDIQGSMGL
HPQNYIPIHYVNEVNFLAEVKKITPFVLVTLLLAMGVRKISVK--SSSGMDRFLKMGKAS
PLEGRDVKVNVKFEDVAGMREAKCEITEFVDFLRSPKTYESYGAKIPKGVLLCGAPGTGK
TLLAKAVAGEANVPFYSMSGSDFIEVFVGVGPSRVRDLFEKARKNAPSIVFIDEIDAIGR
KRSKSGFNAGSNDERENTLNQLLVEMDGFKSSS-GVIVLAGTNRADILDPALTRPGRFDR
TVNISRPDLEERYEIFKVHLKPIKINENVDMDEFARKLAALTPNFVGAEIANVCNEAAIQ
AARRKSANGVEMVDFDNAIERVMAGMKKSGDILTPQQKLAVAYHEVGHALVGWFLENADP
VLKVSIIPRSSGALGFNQQMPDDSMLFTRDALLDKIAVILGGRAAEDIFIGKITTGATDD
LSKVTKMCYAFVSQWGMNKEIGLVSFQRDNTDDPYFYRNYSENTAQLIDQQVRTIIEDQY
LRVKKMLLGKAELVHKLSKLLYDKETITYQDIVQCVGEREFPIKDKYKPYIEVNCNDNVK
N---
-----------------DMLLKSKSRDDLKRDRELDEARKAGTAPALKDELGNDINPHIP
QYISKAPWYLDQGEPSLRHQRVSEVQKAPIDYVTLRGVKNKALKFRKGACENCGAMTHDS
KSCVERPRKKGAKYTNENCPDEYIVENTDKGYDATRDRWSGFDPSTHLQLVEEYRDLEQE

```
RALNKIMNISK-------------------------EDELSDEESKEHSVMN------FD
CKDDKTRTTTRNLRIREDTAKYLINLDVNSAFYDPKSRSMREDPLLGV------NCCFKG
DNYYFNSEETYKPKELEMFAWESKSKGVDVDFIANPTKLEKLFNETKERKEKETKESKQK
LIERFKASEYVNN------------YEELKPLSSVTKEDIITFKESEYEDEAKMLGHSQI
WGSFYDVEKGLWGYKCCKITNRSQRCNI-MADSDS-------------------------
----------------------------------------DEETTESRVTKGKSIYERED
DYRRQRLRQRLSPERYDPFSGKTPLPEERTFADVMKETEISRQRNEISKHISKHGISKEV
EIEETRRS--------SQRDRWDSTPDAGE--------AQTPAFDSTPMDSTPFDEMLEK
KKVSRWDKTPMMEQQ--------------TPMAHTGM-------------YGMATPMTPQ
I--VVPESMLKFNITTSYEDRNRYLTDEELDELLPVEGYEIVLPPPDYQPYRKPSSYTY-
----------------------------------GTVTPHFTIPDDV-------------
RKPYDI--PGTPSILQDVEIKAEDQHFFSKLFDDSTEDDLTSDEITERRILALLLKVKNG
TPPHRRQALRLLASKAKEFGPGPLFNQILPLMMQSTLQDQERHLMVKVIDRILFKLRDSV
RPYVHKILVVIEPLLIDEDYYARVEGREIISNLSKAAGLATMIGVMRPDIDHPDEYVRNT
TARAFAVVASSMGVSSLILFLKAVCQSKKSWQARHTGIKIIQQISILIGCGVLPYLKQLI
DIIKHGLNDEHQKVRTITALALAALAESSAPYGIEAFDPVLRPLWKGITEYKGKNLASFL
KAIGNIIPLMDPYYANYYTREVMLILINEFNTPDEEMKSIVLKVVRQCVSTEGVTAEYIK
SDLLSPFFSKFWIVRNSLDKKNSDLLIETTVEIAQKVGTCAILEKLVEDLKDPSEPFRRM
VAQCIEAIITNIDILEISTRLEELLIDGMLYAFQEQVNEDSGVLLDSFGTLIHVLGARVK
PYLPQITGLIRWRLGTQSARTRQQAADLISKIAPVMKVCDELQMLNHLSLYLYEYLGEEY
PEVLGSILCALKSIVNVVGTTEITPPIKDLLPRLTPILKNRHEKVQENVIELIGRIADRG
GDLVSPKEWDRICFDLIDLLRANKKSIRRATVNTFGYIARCIGPHDVLSTLLNHLKVQER
QLRICTTIAIAIVAETCLPYSVLPAMMNEYKIPDQNIQTGILKSLCFMFEYIGEMSKDYI
YSIVPLLEDALMCRDLVHRQTAAWTCKYLALGVFGLNCEDALIHLLNYVWPNIFETSPHL
TQSVFDALDGFRVSLGPSIIFNYTLQGLFHPARRVREAYWRVYNNLYLGHQDALVPLYPL
ITEGVERKHQSNELLYMIMAKERLNAVDVAVTVSNLKKLITNLTLVNIYDITNRVFILKF
SKNENKIYILIEIGCRIHSTQFLRSVDHLPSNFNAKLRKHLRNRRLRDISQMSQDRVIDF
TFSSEEYAHHLIVQLFLPGNIYLTDSEYKVLTVLRPQNTGDKFFKVGTNYVYDMDYNSWF
---------EVVKKPLVEELIV--------------------------------------
-------------------GRKLTEIIKSIFPSAFILSVFKLKNTRGESVDSKELDRV--
---YNLDDL-DTVYQYVECVRMYLSELLS-GNKIPGYLYKNPRG----------------
-------MEDFGLFEQENS-----------EYFEDFNDAVDTFFTKHELAKQEKK-SVDK
RP---TKINKIKIDQNKRELNLMEDIQKIDSKIKLLEEHVDVAENCLNLTKALIASGASW
NDIYEQLQIQRKQNHPLVHYIKEIHIPTQTLIFYSNQNQDQHNEQNKQN---QFQQNQQK
NENKQNKKNTRDEVVVELDYRLNSHQNLKKLYNERKRLENKLERTRIGKEYALKKVTKSL
KKEENKKTDKKGRDVKISSVRRRFWFEKFYWFITSQGYLVLAGRDALQNELLVKKYLTNG
DLYFHADIHGASSVILKNENSNVEDVKESIDEAGNFAVCLSTAWNEKFSVQSWWVYWHQV
SKTPPTGEYVPQGSFVIRGKKNYLPPQKLEMGITYLFQVQPFHSLD----------EPIQ
DNENVEGDVVEGDEPGDH------------------------------------------
------------------------------------------------------------
------------------------------------------------------------
------------------------------------------------------------
-------------------------------------SEDEEDDQEGED---TVVSDDED
DEEGDTEEEDGEGEEEDTVEDEDVNDDEEDTEDPDNTLEGEDEEDTED-DADDTLETDDP
EELDVQE------------------DSETEDTVEKSVRIGHVEMLEEYEANINKRTRGRL
PTA-LKFKLLKLKNQLQKLNL------------------TKHSSKSSKSSSNQLSTKSST
DHSTELPTK--SSTDQTDQQ--------DELIDSVDPNTK--------------------
----------------STLRKSVRILEPEKLEHTEEAVRKIRSRKLTCL-NPNLSKLNDF
IPFET-------------------------------------------------------
--------------------------------------------QDSDDKINTDTVKCDV
K---------------LKNTNTELKNPKTEPKNMNTEVKNVKEDKKRGSNRMMRFINQKV
NKIKKKYGQDDEETQELRRLLTGSKKIQQKSQKTQITTTSITKFSHGNSVSSQGGKFKEI
ETISDKELEYYMKQLSCLTKDLKEDDDVINVIPMCAPYSAIKHYKNALKLVPGNSKKGTI
ATQSLQHFIKNDPERA--NYLKLITTDQLTLTLIGNCKFTSSKM----MGRMYGKGKGIS
SSSIPYGRKPPSWLKTKPFEVEEQIAKLAKKGQTPSQIGVSLRDSMAIPQVKAVTNNKIL
RILKAQGLAPEIPEDLYFLIKKAVSMRKHMEQNLNDKDSKFRLILVESRIHRLARYYKKK
RQLPATWKYQASTAGTLVA--MEKRVSYFYDPDVGSFYYGPGHPMKPQRIRMAHALVLSY
DLYRHMEIFRPHKAVEPELLSFHDSEYVHFLSGVSPENYRDFTYQLKRFNVGEATDCPVF
DGLYVFQQSCSGASIDAAHRLNNQQADICVNWSGGLHHAKRSEASGFCYLNDIVLGILEL
LKYHARVMYIDIDVHHGDGVEEAFYVTHRVMTISFHKFGNFFPGTGDVTDVGVSSGKYYS
VNVPLNDGIDDESFVDLFKVVVGKCVEVYCPGAIVLQCGADSLTGDRLGRFNLTIKGHAA
CVQYVRSLNIPLLVLGGGGYTIRNVARCWAYETGVILNKHTDMSNQISLNDYYDYYAPDF
QLHLTPSQMTNYNTKEHLDKIKVKILDNLRYVEKSPGVQFAHVPADFLTRD-DDVDEDLQ
KQIFDE-GGGITTLSTRKRVSTTHRLRRRDNKGEFYDLPDRDESIPLM-----TVSSLGA
ENNYKLMNSSNNRDFYTMNQMLDPLIDSRRVGFLDSVSALHWCCYAGHVDLVNRLLDVGC
DPFLADPVNYETPVYYAIKSSNSYIISILLKRFGPLILCHENVKCKTPFLVAVSEFTCDN
LVAVLHVLEFLYLSGVSVDEQDNNGTSALMYACKRGQLFVVQWLLRRGADISHRDHYGAT
VLHYAALSPNLDVLLFLAQNGLAPLTWIKSISNNDQTPMDLCWEKANHLRYMLLWLFWLQ
WKVFGKVFRFNMFYPILYWIMNLFNLALVIPMYRSLKDSTNFPNSFYSCLAFFLATNALW
IINKLSDPGVAPN----NKNDRTAFSPTNDD--VGGFEGVLESLHRRQQMVNFEFYCVNI
NSTAGNNPFVSRVTACNDEINNITTEMRSVYPQVADERCNKNSINYNKAVLNFDTTNKVC
FTCGKTKAPREHHCSLCNTCLLRQDHHCGWIDNCVGAGNQREFFVFLTLLVFVFWHFYLI
LCKYLAHRFQGGFSLFDVFLFLYGFINGLMSIFVLYLWTRIVRCMITDVTYYEFLKKPSH
IRVRFKVHDRFWDFADLNFKNVLNNIVKFWTN-------------NLVMEE---------
-----------------------------------NEKFLYIINFFLESDD---------
------------------------------EIIETKDEIRIKKQVLSSLESFYSFYQLSQ
VRYGASSSNILTAERGSLKMCNEIYDIGCLKITRTFGNLSKFK-SIDSFHKLKNYQQCGV
HWLSIIHNHSSNAILADEMGLGKTVQVCIFLQYLYTDTEATNINIIIVPLNIMYNWYNEL
KLWTNI--KNIIIYHGTQSQRINIANDTFDTPGKEGFIIIITTFGMI--NDDIKLMKKLQ
PFEYLIIDEAHLIKNSNSNIYKKLS-NYQFNHKLLITGTPIQNNMNELCNLLQFSMNN-F
NSYDINNSINSFIK-YKSFIQNNLTTTMGKGADTFDTPGKGANFMGTECTIGKGANSTLC
TSEKNI-NEIAVVTNFGESDTYEGKGANSMGMEGTGAVGPDTVTEKLKKKINNIIEFNN-
KKYKISKELKILQKLTTPFILRRLKKNVINELPIKYSNFIPCHMINYQYYIYQSFITTEV
TNTKSTINNLTTTKETTTEETTTKDIGTVGA-------STVTEKNTAVVTNSKDTNTRIE
DTEDEGVPNTKEVGEEDEGTPLRAKEDHLRA---------KEDPLR---------AKEDP
FGEEINNKIYKLRRICNHPLLIRKIYEDEMLPKISKIIKKLHEEFHEYNLNKIIEYLFTL
SDFNIHQLLLQCLQYCDSNLNEIKINKKLYLESTKIRKMLELISNIIKKKEKILIFSQFT
NYLDIIEYIM--------------------------------------------------
-----------------------KLENMKPILRLDGTVTLIEREKIIKKFN-NEDVYILL
ISIKVGNVGLNLSIANHVILMDQSWNPYNDIQAEDRCHRIGQQKIVHVYKLFVKNTIEEY
IINQSYNKLQLNSLFNH-----M--DKGMYLVFKPSLKKESDRKAQLATIQASKALSDIV
RTTLGPRSMLKMLLDPMGGIVITNDGNSILREIDVNNPGAKSLIELSRSLDEEVGDGTTS
CVILCGELLSNCATLIKKEIHPTEIIQGLMEALDDTLVALDHISIPININNHDKLLNIIQ
SSLSTKFSNRWGNLISKLALDSIFKLYNSNKSNQTVLDIKRLIKIEKIIGGYIEDSIVLD
GVVVNKDVVHSNMRRRIENPRILILDCTLEYKKGESQTMVDIYDETVWNKLLLQEETEIK
QMCQYIINSNCNLIITEKGVSDLAQHYLVKANITCLRRVRKSDTNRIAKACGATIVNRPE
EITESDIGYNCKLFHVDKIGDEYYSFFDQCINTKACSILLRGSSKDVLNEIERNLYDALS
ICRNIIYNCKLLPGGGATEVYISNYLNQQISKKVGLKRLSYECASKAFQVIPKTLAQNCG
INPVKLMSELLMLH-NNGEIHMGINGETGEIINVINHNIYDIYLVKSQVYKSSFESVSSI
TNTQY-------------------M--LNVLNVAEKPSVAKNITEILSCGNATRELTHSK
TNPVYSFPHWFEGNMCKMYFTSVKGHLMNLDFDQPYRSWHRTAIIDLFTGKFYQIMSSTH
SPIHHERLQGYR------------KKHIKIRKNVLEVCFRVNKNMIVKRAIFSSVTSSDI
EHACVNLKQPNKNLANAVETRQEIDLRIGSAITRYLTLKYKTQIDTKAAILSYGTCQLPT
LGFVVERFILIENFVSEPFWTIQVEVT---------------------------------
--------------------------QLAVLTLYENCIQNPSGIIRKVLKKEVRKHPPLP
LDTIEMNKDVSRYLRISSHKCMQLAEGLYNKGFISYPRTETNVFPSSIDLKSIIGKLSSV
```

```
PEFSEYTNTLLNEDGFHEPTKGNNNDEAHPPIHPVNSLTRDRAESEEHWLLYEYITRRFL
ACCSKDSIGHQSNVILEISGELFNLKGLIIQERNWLNIYKYTTWEAKLIPNFSENQEILP
NEIILKDGATQPPDLLSESNLIDLMNKNAIGTDATMHEHIQKIQDRFYCIKDDKLRFVPT
NLGKAIYYGFKEYNYQNIDLTKPILRANMERDMSDISIGIKDKDQVVLNYVNLLRSIFQL
ISNNSNKFDGYITCLSRIV---CMSVFSFGKHKGRTFMYVYNNDSSYVNWIRSLPEPSGK
LLEFLDFAKKYEMCKS--PENNTDSMQIQHNSPSPNANRASLESNFFSLEEKTGAEI-SK
KRKIAN----------NPTPDITRDKDFEVEKALLSPSLKHMLDTSEQKKSNMDSDDCDG
VNADGVEIEGLLSLVLYSEDEFYLSYNRNRG--YGHWSCYVPNEITEIMNKMGLNSRMLG
KERCVTYKASDYSTVLKGLRSAMRDKNSVEAIPNFVLRVFPSFIPFSRDFTLEDKTRQIM
SGVQDEYTKENMDNLSKLIGEELWSQLMPFQRQGVFFGLSKNGRILIGDEMGLGKTLQAL
AIAAFYQKDWPLLIICPSSLRFQWMDQCLTWLPHLVDEYQILMVMSSKPDLLDMYKVVII
SYDLMVRIKELK-EFNAVICDESHYLKNKSSQRSKRVVPVLKSAKRAILLSGTPALNFPS
ELFEQIAAIIPGFSSSHLFIDRYCKKRTNWFTKRIEYVDSKHTNELHLFLISTVMIRRLK
NDVLTQLPPKIRSKIPIEIPEKLIKTTKVMLEKFPSRKGIENYNVVMEHLSNFSDKDSDV
KRAHLSLMSKLFQLTGESKTKGVCKYIEEILENNNKFIIFAHHMFMMDAIEDTLKSKKVG
YIRIDGSTKINDRARLVNLFQNNNGVRVALLSLTSCGVGLNLTSSSTVIFAELYWVPGVL
LQAEDRVHRIGTKFNKININYLIAQNSVEEVMWKVINKKYKTVTSTLDGETGTLSL----
--------LTNNKKKGVQTTLNLQPK----------------------------------
-----------------------------------EMVA----------------VDNVG
TQNDAFKDLVSQCISLLEAAVAKSESRFVIRLMRHYKSLRSLLKAHPATSVPFLRSLLKE
YAFGPCPVAEKAYNFLPFDLPKFHYTTSTYLEIQEYVSQDSLTETKVMLSTLALIYLIDT
RNYEKAMDLADSLAHLMLSLNKRIMDYLGAKVYFYYSRSFELGGRFKDARK---------
-LTFDCFAQ--------KSMPASRPNDTGSYFQLHTEEPDSPQAVLLSIQ----IRKVLS
VQLEYSEAYTKLIQSLRKAPQNDKTAYGFKLLATKMSVIVGLLMCDIPSKSVFTNPSMRK
DLAPYEAVVVAVRNGDLNSFLQLCDKYAHCFEKDDTMFLISRLRDNVIKGGLRKINLAYS
KINLANVAHKLGLESVEHTENIIAKAIHDGIIEAVIDHENQCVNSKVNVDLYKSYEPMRA
FHKRIQFCLKLHSNAIQAMRYPEDPESTKENKTTKDQLESVRK----------------V
KCEYCFEGSAVLRRSFSGLPSCKTCFIESFENDIYEYIMSEKLISDGDSVCIGVSGGKDS
SVLAHVLSKIKEKYKMDWNLYLLGVDEGIKGYRDDSLKVVKSI------------FKDRF
GFSMDQVVKLIGKRGNCTVCGSFRRQMLEIGARIFGANVLCTGHNADDMAETVLLNLFRG
DLLKLTNSNITTYSINSVGELNKEDLDNQIDGKNVNLEI---------------------
------------------KSKLNQS--TNNKLRKIKRVKPLKYSFEKEIVLYARYLSLDY
FSTECIYSPEAYRGHMRSFIKNLELINPKIILNIIKSGDNFHSEHTSNGEANFCQICGID
SINNICKPCKIVQQLHNLETNEFCDSGAGKNNIRKGVKLRYDG-----------------
-MSSNDLNWHNTMYSNFYGTQNDYGKIFSKLRRPTF-------------DFVFFLTDADY
TF----KVVKCTSDGTLS---------------ETGDHHTEVPVVRLYGVTKEQQSVLVC
VDDFQPYFYIEKPPELLEENFEDLKQLFNKHLSEQNQFKKSLRHVLDIQKTRLTSLMMYD
ENGEKDFLRIVVSSPRMVSNLRSYIESGVELE-IPLYRQTYEANLPYVLRFLLDNNVICG
SWLMIPQNCYTLNNPKQLVSTCNIEVNCGYSDIITLPLENEYETIGPIKILSFDIECIKL
NGTGFPNASNDPVIQISSVIHTHGNDVNNTKNFVFTLKECDSLANCTILSFDNEEQLLLA
WNDFVIFVDPDFLTGYNIILFDLPYLLTRSSVLNIERFKKLTRIKSVNCNFKDSISNNNI
LGLYENKEINIEGRILFDVYDLVRRDYKLKSYSLNYVSFEFLKQQKEDVHYSTILKLFNG
NNNDRRRIASYCLKDSILPLLLINKLLLLYNYIEMSRVTTTPIKLLITRGQQIRVTMQIY
KQCKKMNYVIPVITKNSSNENSYEGATVLEPLKGYHKNPISVLDFQSLYPSIMIAHNICY
STLLNYNN-INNYRPEDVVRVPGYNDICFISVNKRKGILPIIVENLINERKKAKKMMNEC
KDEMLKKVYDGRQLALKITTNSVYGYTGATSGGFLPCIDVATAITSFGRNMIVNTKNLIE
EHFTVKNGFKFDSKVIYGDTDSVMINFGTDDIQEAIDLGKNAADLITSKSVKPITLLFEK
VYKPLLLLSKKRYAGLYYVNSEKYEKIDCKGIESVRRDFCLLIQQMLEQILYLLLVKLDL
DGAIEFVKKKVSELLKNEIDVSLLVITKSLGKVDYEQRLPHVELAKKLRKRDPGKAPGVG
DRISYIIVKGTKGEPLFDRAEEPLYVTENNLPIDTSYYLDSLKNVLLRIFEVVMTNPNSL
FNGEHTRVININSSTKGLMNKFLTKIRRCLSCNIVLSTEMFCSNCNTSKKQQILLDKLTA
CRIKEDLYHKLWTHCQRCQGNLHNAVMCDNRDCPIFYRRVKVSKDLTQLQSTLSTLQLQY
M---LYEMIYVPYLYTLLYHIIINLFIRIVSGICLNK-----------------------
--------------NSYAFLHLNLTNSHLITSKQQFRVVENHKTNLNTMRNGIVSHSKPS
LQIAPMLDVTYLQFRQFMRLLTRKTQLWTEMFVASSLINSEESVSRWLKFEENEHPIVAQ
LGGNCPETLVEAGRILKKFGYDEINLNVGCPSPRVSGKGCFGASLMREKELVRDIVHNMI
KELEIPVTVKTRLGVDESDSYEFVKDFVSTVSQSGCEHFIIHSRKAWLKGINPKKNRTVP
PLQYEKVFRLQRDFPHLKFTINGGFKTMESILDALN-----SENVDTDNN--------PH
KLNGVMIGRLAYENPCILSNVDKIIYGVENPDTCYTRRILLEVYANYIDENVEETANMNI
SLIVKPILGVFHGEQGNKIFRRKF------------------------------------
-------------M--------KKYILEELLTEYEQNNNEDEAYQQYSWELDVDKSWEQL
IEKDGILQFIKQEFDIDYNTNLKQHNVDIIYKRGIIRSIMILFDMSEQMHEMDFKPDRLY
CAFNSLKEFMNVLYGSGPITQVGIIVMRNKICNVITQFGTNPDEQMELLSNILKDGPEGS
SSLQNGLEMCLKIMCELPYYMTREILIIFGSNKTLDPGNILITLDKLKQNFITVNCISLS
PELYILKG----------------------------------------------------
---------------------------------CGIFMVSPPDISRAFHHLIPPKSFHKI
ERN----INCSGCNLNIEIG-YECQNCQGIFCEYCDKYIHQDLHQCPICLFQH-MSSGCT
RISQQNYYSTHNALVANVDDQSVRFVLHELEGTRHGALRIFPFYDIQMIKRLLIKKLSLP
LTTRVKDLRILYKGQELPNYRTMDCYINNS----------KRDDKLYWCLKQSPTGSGIR
PLGMK-MNSKMQDLFNEIAISMKNNIKPKLTLDGTGGTYEIHNKNGKCVGIFKPCDEEAF
TPYNPRGYTGTMNQQGFRPGVLSGEGATREVAAYLLDSTYKNFSNVPTTIMVEIAHQSLN
NCNTNNLTNNGSRLKWKIGSLQEFVVSRGTSGNYNYNFFSVEDVHKIAILDIRLLNLDRN
DCNILVTNVPQSTGETNPYRYHVNGDGAENCSPNHQKI-----KDEKYKLVPIDHGLILP
DIIDICDLDWVWYEWPQCKVPFSAQELDLIFSFNVDKDIELLRKYLHIREECLRTIKVTT
KFLQIAASMNLNLYQIATIIVRHDIDIPSEIEIIIKKAIEQAYKMSDNTSIISRNRLGNI
IDLMENSLNPILSAKDQSENPAIQPNDTEDELSPDLSELPSSTF--FHYPTYTESTSERD
KVR----THSRSTIRRIKRSTVT-------------------------------------
------------------------------------------------------------
------------------------------------------------------------
------------------------------------------------------------
-----------NNTWSITDSGGHAILIEW-DYKFNNLFFKILEEMLVGNIQRLHPNWASY
PFNGNKKHLYQKNQQNNTLLEIRVGGKYRLGRKIGSGSFGDIFIGTHITTGEDVAIKLES
HRSRHPQLLYESKLYKLLAGGVGIPNIHWYGIEGEYNILIMDLLGPSLEDLFTICNRKLS
LKTVLMLADQMLNRIEYCHSKNFIHRDIKPDNFLIGRGKKMSIVYIIDFGLAKKYRDSKT
AQHICYREGKNLTGTARYASINTHLGIEQSRRDDMEALGYVLLYFMRGSLPWQGLKATSK
KDKYDKISEKKIAIPVDLLCKQLPFEFVTFINYARSLRFEDRPDYSYLRRILKDLFFRQG
YQYDFIFDWTFLHTAQLMHTMMTEDMDRFERITKEREHEDTNMILLEQVSLDSKNNFYRT
FIDIITLSTLICSDDVYDMLNVLF-NDSILELGFLSFNRIEDGIQSKEDYNVSKVNEVFV
IRPTFRDSQRLATLLQT-----RHTNVIRVVCLPEICELYPEVLSNGSNTPYTNNLVELP
IHMVPVDG-ILSMFMPNSFSDFYLNGDPTTAWFFAKALDYLQRHLGGSILNVTGVGTLSK
YVIELMLKNRRDLAA---NMIVEGMDKIQN------------------------------
------------------------------------------------------------
------------------------------------------------------------
------------------------------------------------------------
--------------------------------------EKCFIPIKLLQYYHDNMLTDVS
NFNIYESKKVNKLD-LDSRI-------LLTNSASFKSSIIIDRKVDLITPMCTNFTYEGL
LDSVFGLKCNTVNVDSQALDGKLVSSLDLVV-----DFQKSKQKNNFNKKPVSLKSK---
LYEEIRWLDFSKVGSYLHEKALRVKKGY-EGGGMQTIGEMGEFVKKFKSLQQEHVTLSTH
VNIMGYLNSFVKSERFHLVQNIEDCILQGSTDGNKSKLANISKLWLELIFWNIQVFTIFR
LLILLSQTMDGLKQSDFDTIKKAIIYQYGFQYLKTIQNFIKSGLIKINNVDSSRWQKIYG
KFNLLVDSESDCSGIFGGYAPLSIRIVQLLSV-SNDSAPLSSEFGLLNCQIVSTKQKPVT
LNRLSKDSRSLNDSLSSEYIDDPTEHCLICYIGGITIGEVASVSLINSFNKSK-FVVLTT
DIVNSIKLTNM---M----ENESRIELENPGDYVNDDEAVVESVCMSCGENGTTRILARK
IPHFNDILVMSFECSFCDNKNNEILNISKLQNLGVSYNIHVNNPEGLNNQIVITNTSAVK
LIDLEFEIPKLDRKGIVTTIEGLLTNIINNLTDHISSFESLGVDNAETDAIL--------
---------------------------NKELTNSDVSIKVDENVYKLAEYLQKLDKIKNR
```

```
LVSYSTGLE-SFTLFIDDPSGNSYVE---NDNNKLELTVNKYERTNEHLEKMGYACQPED
EGS----------------PNESNGPHQSDHED---------GLND--------------
-----FFFVNCTNCGFKGKNQICEIAIPGFDKCIIMSFVCDNCNYRTNELKPGGGIKEYG
KVWHLKINSVDDIKRDIILSNTCEISINELELTISPGSLSSLFTTIEGLINKIIENLHST
FPFLIGDSSLNSDEMSHNNGDSVNGYINKSKIKSLINKLKEICRNGSKEGINIAFNDPLD
NTFIFTYTIVVDENLYYINYNRTDEQNEEFGLI------MDAHQQDHNNTTSGESSRGYE
DHDLPSNKSRAHNSEAESKIMYVINRHGEKEDVSFDKILNRIRKLSLGLHSLVDAPRVTQ
SVINGMYTGIRTSELDELAAQTCAYMAVTHPDYSRLAANITIDNLHKNTLNDFSEVIRVL
HSYKHVYNTNASLISDDVFEFVMENKDRLNAEIDYSRDFQYDYFGFKTLERSYLLKTNGK
IVERPQHMIMRVSAGIHCGDLERTIQTYHLMSQRYFTHATPTLFNAGTKHPQMSSCFLLD
MQDDSLAGIFNTLSQCAFISKSAGGIGLAIHKIRASGSYIRGTNGISNGIVPMLKIFNST
AKYVDQGGGKRKGSFAIYLEPWHADIFKLLDLRKNHGSEDQRARDLFYALWIPDLFMKRV
EANKNWTLMCPDECRGLYEVWGDEFERLYTQYEQQGMGRKTIPAQKLWFAILQSQIETGT
PYMLYKDACNSKSNQQNLGTIKSSNLCCEIVQFTSKDEVAVCNLASVALPKFVNTQTRTF
DFKKLYEICRVITYNLNKVIDRNYYPVKQARASNFRHRPMGVGVQGLADTFMLMRYPFES
DEARELNKRIFETMYYACLSESIDLARQYGTYESYEGSPASKGLLQFDLWGAKVDNSLWD
WDKLKADLREHGLRNSLFIAPMPTASTSQILGNNESFEPYTSNIYYRRVLSGEFFVVNPH
LLNDLIDLGLWNETMKQKLIAYNGSLKHIDEIPSHIKELYKTVWEIKQKHIIDMAADRGI
FIDQSQSLNIHMEQPTFSKLTSMHFYGWKKGLKTGVYYLRTQPATDAIKFTVDASISQLA
KSRVKPANSIMSDGMSDGTMSTTSADEPQMCSLNPNPNEPCFMCSSSSHLVLIHDLFKSV
RPDEILEDLQKFGVSVSASAFRNPKPEESLGLYSLAIQVVFGKTIHDIKPEELSGKLYET
KTEVQGVDFSSENVDFLKNAIGNLRLWRYSQRLHKVLGLGEIERYLFNPTAKSFNELISA
FVVYLRFRQALYSLYESSIQKLDNLAELDLRLDENLKNLVNENDKFKNSLLEKVVEQESL
LKLKNELENRMIVANEEFNNARETKKKLEIEKEKINSLVNDTMLSKSKARFTFDDLSQHF
STNIETANRLYSELNAEYISQSQRLEALVSDVERLNTLHGSLVETLETFELLKSNLEEHY
NEVIIPHIKSLELSKRLSSQTESLKLKINQLTRTRDSISKNIELQKTLQSEKLKKLQAKV
DEAHRFAQESSNSESGLKVKIYDMRNLASQLNTKAQDVRKNSQELFSNLSQRIQTLYDSI
DSYNETIKLLNKSLTNS---------LIRSFKNLFSRKARYTVIKSV-IPKNTFLPKSLK
FIKVLGQGGFGKVFLAQKVEENFELCAVKRLQKHPLIVQKQVDHIISENKLLASVNHPFI
VKHLGSYKDNHYLYLVMEYVSSGDFFTYLRKENYLESHDAMFYAAQVTAMFEYLHDNNII
YRDLKPENLLLCFDGYLKLTDFGFAKVVEFRTYTLCGTPEYMSPEIILHLGYGKAVDWWT
LGILIYEMLAGYPPFYDSNPQSLYDKILKCKLKFPIHYDEDAKYLTSRLLVTEPSQRFGN
LHKGIDDIKKCKWFESMDFDALVEKELSPPHIPERKEDLPAEEF---IDSYRMPKEVTGE
DDPFVDWMTISQFFIISHSGDVLLSRNFRNETTKNV-DNFYNYLKEN-SNIGPIFELEGM
LYFYIRRSNLYFVMSTRYITSPSYVMELLNKITNYLKDFIGILNEETIKSNFVLAYEILD
EILDYGYIQCISINQLKQKIYNTSTVTTDNIKP-------MMSNRNMLPSVVSNKSLINP
NNKNEIFVDVIEKVTAKLGSD---VKTTVEGQIQIKSYLKGSPSIQMYISNNVQFSNNTS
RTSNKIVIEDYNLESDVEMV----NNVMKFIPKEGEYTILNYKIK-NVKMPFDIKTQLVN
NTENTVGLSIRVACNLPINVH-SFFLLKCKLPNNVNTINMSVNPKFFQQVSEYKLENNTI
SWNIKNIQGSSEVVLNSEIVFNNKVNSN--QFGPINLIFEVPLYNITNLKVSLTNSQFPN
SDKIPQR---------------VDT-----------------------------------
----------NVVVPGDVVSS--DSSFLLQGHGTYVSDKQLRASYLGSVKHVNQLVYVEP
FGGKYVGQIGDIVIGVVDSIQGNKWLLDVNSVELAQLSILQVNTEELANRRKIDEDIYEM
SNLFNVNDIVSCEIQRISSTGTIMLQTRTSKYGKLENGILVKIRPNLVIRKGKHIYDMVC
------GFSGYIWLTSQLKRQDDSL---------------EKQNFITRVCTIRSILLMFS
SKLIKVNFEVIEKAFNIYSHLFPDSCNCRMKPYKTLELLYIYSKSRRLLNMPVLYNLTLK
KPTGVTASVQGSFSAPKAQEFVVARSHILELYSLNSLGKLQQLVSVEAFGIVRALAAFRL
TGALKDYLAVTSDSGRLVILEYSTQTNSFKRIHSETYGKTGVRRIVPGQYLAVDPKGRAI
MIGAIERQKFVYILNRDTKANLTISSPLEAHKSHSICFDLVALEVGYENPMFASLEQSYE
NVDAMQIDIMVLNSELLRKGLSFWEMDLGLNHVVKKVTLPVDLSAHLLVPVPG---GPGG
VLVCCENYLVYKNLEHPDVFCSYPRRLEMSESQTLLITNYSVHKMKDFFFILLLSEYGDL
YKIELSHDDNTVSEVVIRYFDTVDVGISMCILRSGYLFIGSESGDHKLYQFTSLDNGDKD
VICTSLHPDAKNAIIAFKPRVLQNLVVVDRMSSMGLVVDMKVADVMGLNNYDIFVACGRW
YNSRLKCLRYGFNTEELAFNELPGRPKHVFTIKS-LESNFDEYIIISFQGNTLVLSIGEA
VEEVTDSFFLTSITTLHCCYMYRNGYVQIHDGGYRYLSGDIIKEWKVQSTKRVKLADNND
TQLILVLTGGEIIYFQLTDTEVLVEVGRRNLSTEITCLAIQH-PNSGTKAEFCCCGSIDN
IVRIMKL--DKNLKLCSSQILGNNSLPESVTLLT---------NDEIYLYVGLNNGVLIR
NTLDMI-GNLIDQESRFMGTKPLKLKLLQYMEKQCLILMSIKTYIIYPNNNNLDILPLYI
NTVDSIDTFNSLLCLNGFVCILGNNLKIFRCIINGDVFSEITIPLEYTPRKLLLLPSPST
MGSQVQSIGSQGVKLNVLILIVESDYNSYNIEQVEEINKEMMSIKLEG------------
-DNFEVM--ELKNYRAGVGKWSSCIRIINPINLETIAKLLFTENEAATTAYTCILNSIQL
LIVGTIKNAHLYPTHMVESCIRVYEYDSNYNIKLLHITNTKGWIRCFNNYENKLLCAIGS
KLRMYSLGKKQMLLKGEHRSLTSGFMDIKVIGSRIYCGDIRESVQLLFYGEDLGEFELTT
TSTGPRWLSSMELLDYSTVIAGDKFDSIFVSRVPHNE-------------------DVVR
SNYFEYHNQFHLGDIVTSFQRVRINPIHSEVVLYTTLMGSIGVLVPFVSKDELDFLQHLE
MLMCNQIDTVTGREVQMFRSYYFPVQNIVDGDLCEMYMTL--DDKYNIANQLNLKVNEII
KKLKNIRNRIFMDSEEKKKTVPLSETEVKILKSYGVGPYAAPIRAADNDIKEVINRINKL
SGVKESDTGLNPPHMWDLVLDQRSLQEGAPLQVARCTNIINPGTPQAKYIINVKQIAKFV
VGLGEKAAATDIEEGIRVGVDRNKYKIQITLPPKIDPSVTMMTVEEKPNITYNDIGGCKD
QLEKLREVVEMPLLQPERFVQLGIDPPNGVLLYGPPGTGKTLTARAVANRTDACFICVIG
SELVQRYVGEGARLVRELFQMARSKKACILFIDEVDAIGGSRGEDASNGDHEVQRTMLEI
VNQLDGFDARGNIKVLMATNRPDTLDSALLRPGRIDRRIEFGLPDLEGRKHIFKIHSRTM
SVDKNIRYELLARLCPNSTGADLRSVCTEAGMFAIRARRKSISEKDLIDAISKVIKGYKK
FSATGRYMVYNMALDYCLKNLIAIDATPTSTDENLLKLSCENAQLLVNKIFGLERTITSD
G-VFADLPSNDEIVMPRVFPLPKPREKTRWEKFAEMKGIKKRKRSRKVFDPTVNDWVPRW
GYKSIKKNKVNRPPIMEVKPGDDD--NVLDVESAKRSLVKMKQKMRELRNKMEQN-----
------SPKDSKSQLKKTLQRVKESTKGCGKHGKV---KGKTKVSIKRKPVIQSLKSEKD
SYLSSIKKLNL--MEKSPYEFDDLVSLMQENFSSIIEKVKGLKILVLDNTTSRIISLVLT
HSYLLQNEVLLTLNINSLNLDSGGIVDDG---PDRLISGSDPNLRHLKSVFVIEPNVDNV
NKLCSELKCPTFKSYHLFFTNKLDEGFLEILARADQFNIINGVYEYFIDINILHTNMFTI
NNTPPAININ--------------------------------------------------
----------TNNTANMLSVNMAETDKLDNRMVNSLFSVCCLLNQIPTIVYRRNDVISQT
LSNKLQMLFNNNNLNLQSILQQYNKYNN----------------------GVEGVGCVLL
ILDRKDDLVVPLMNQWTYRAMIHELIGINNNKVVVD-----DSEFVLN---DQFFNSHIF
HEFIHVEEDLNTLISHNKNTTNKVDGVNTTSMVENVLENLPEKNRMINDVMKHVKILHEL
SKIIQKNKLLDSGLLEQDIATNRR--NTLNDVIEYITDKNNSYYEKVRIALLFVLTCNDS
VKIKKVKDYLIMGKMN-ELVALVDRAADLIKGKRRMN---KEEFTLSTLKDKITKVSLDT
QSPYLQFKSNLYTTTYNLIRGKLDTEMYTMVPSADLGYTLKHKPASVTHY-------NNY
TNY--------LTILMTNTNINKSNNMVDDIDLGNNIMNKNDLSKEKVCYTPELGEVAHL
SPKSLKQPEARVKFPDSEVPRYHNKLFKNPEGLKERCVFERLTDHRFFTGSHRERFDENG
RGRGLAGRENLYFFDGNTESYSRVHEVYSSVLPRQRRPMVPPNTLGVKKFGVQIEPPKLV
WLYRNGDKHHDGVSFYIRPYIRTMKTLLIEIGKELTLIAGPVRKIYDQNLRPVTQLDDFV
DGAKYLCTSGEPPASPEKLEKFMSEWVIQKMPQDDSEEEFDLNEGAKKKANKKTKKP---
KTNSAKSEKTPRKKKNEENNKKSSKKESKSKAGKSDLKKVKTRKSKPKAPKEKTVKIELN
SSFSKPGQRYMTPPQGDGTRGFYESLYEENPNSLIAIKFCVEYGIFGGSKHDEVLHKYTQ
LQKHGHFKGTSGAIKPSAITFLQKLKTN-------M---SDTNIEQWKIKRLIQKLESAK
GNGTSMISLIIRPKDDVNRISKMLSDEYGTASNIKSRVNRLSVLSAITSTMQRLKLYRNT
PPNGLVVYCGTVITDDGKEKKVSIDFEPFKPINTSLYLCDNKFHVESLNELLESDDKFGF
IVMDGNGALYGTIQGSTKEVLHSFTVDLPKKHGRGGQSALRFARLRMEKRHNYIRKVAET
AVNMFITNDKVNVTGLILAGNADFKNDLANSDIFDQRLSSKVLKIVDVCYGGENGFQQAI
ELSSECLSNVKFIHEKKLIKRFFDEIAHDTGKYVYGVYDTINALENGMIEVLIIYEQLEI
MRVLVKNPSTNTESVLLLNQEQERDEANFKDNNVDLEVLDKIPLIEWIINNYHNYGSTLD
FVTNKSQEGSQFQSGFGGIGGILRYKMEAGYETNDDNDDDDFMLSDSVVDNRPMDQGKML
EEAIFFVKEQSYYMKKAIEMEDVSNSLKHGSNIISELRTSSLSPIHYYELYMKVFNELEY
LADFIGDHAKKTNIIPELYVSVQQATFILPRLYLLVMVGAHYIKSKKVTAKEILDDITEL
CKGIQHPMRGLFLRYYLVQICKDKLPDSDPDNENG----FIDSFDFLMNNFCESIRLWIR
```

LNTAG------------NDKKKLDKERLELGLLVGANLVRITQLEGVDINFYSSTALPRI
LSEIKSIDDNVAQKYLLDCLIQAFSDEFHIQTIDDILSACVSSVKSGNFTSILMTMMNRL
SVFLTSNPESLPEGVDIFSTFQKHLSTINVVYNLSVQ-----------------------
---GNQEPEGPQVGIKGYLDLQ
AAFLEFITTLYPGEFVLNKVVEVLSNILGDVVI-EGPAANSIVKLLTVPIKALSLKALEL
SYNEKLISFLSWEMRKEMSYNLIDELVTTNILMDELSSFEIFFNLVSPLFLPF-----DE
EKGEYISDHEKIKLEQYQICKLIQAIKCS-DVCDQFSIYKDLTERILKSLRMKHTLPCLV
NCSLSLLFSSSNREFSQTQSTQFQT----------PNVQFKNMKISFNHDFSMEILKYIH
HLMELLQPISPKKTLKLLMLVSISVDEFARFGENTKFMT--DMKMMCLDFLMKACNCYED
EISGGENQVYCIKYMCSAVSSRITILDSEDYLNVAMLLAKYALNLIRLTQRCEVLCCASH
LF-NSPQYYNEQRLVWCLEKCVTLVETLFYTPSKLVESLMFPLETSKYFKLKNTTKLVQN
KLDKLSQLLP------------KDEYEEKVKALQEYTN----------LLTKMLELKLNN
AVVLRRIFDCIRDLITDGNIDFDATGMTLQALDGNHIALVHLKLHESGFVLYRCDRPRAL
GININSVTKAFKSTTNNDSVLIQSEEDKDLINFVFENNVEDRVSSFSLKLMTIEQDALSI
PENTEGFDAEITLSSKEMTNICKQMNEFSDTIKMDISGNSITFSTEGDLGHGEIVLRNRA
PSSEGDCGVTIRVKNPIKQSYATKYLLMFTKSGCLSDSVTFGLSQNRPIEVKYEVRDSM-
-EDSRVLGELKFYLAPKIDDDIETE-MTFYSNIVNGCKLLFSYNFAYSEEWCKVASNDSE
PRYEQCLPGTEQSLVYWNKSFYFV---------ISLTHFQPGG---------NNFQNSRR
RQLVDRVDVGDPRSLELKLN--DTNWSCEKIVVYKGSRFWTFDCIKSRNKEEHVTTYLLS
GNKTYTVSVQTGGQKDAGSNGTISLTLLGSSGRTNEKTLSNSFYPASYQTFQVKGADVGD
INGLIISNDAVNDPWYCDNIRIASN-EVVNSFPVKRWIGYPFEPSVEISTEYLTSETSPL
DVMCNTRAVDIYSGT------FVKPFNVTVRCPMNCNNDPLMSIEGSSIHPSSTSICGAA
IYDGVTTPSGGQVVISIVQSMAKYFGGTGS-NGLKSEDYEPPADKNNFSFFMFLDESIDE
IDKSVRLVDGFGKLTSFGRLEIFRNGKWGTVCKRGKHSVFNNQSATFVCNTLGFKYGINI
EENCTNVNNQNFCAPPGYHVSCSGLKCKGDETDINNCTLEEPIDCKNHKEDVVVKCTNFS
PTTNLEYGTLRIVDSTGATSSTGTGRLEIYNNG--------KFVKPNANRRFRD------
------DKECSDFNGLNLCAQVHTKIAANNFKCNGGESKLKDCEHESGDDIYCTHDQDVI
VSCIGNGDPSEFRNLKKVEPFVPPLKKLSKIINLSCYDTLTSHSQFQ-GEYGSFKVASCP
MGCMDEPASIKGTYIYTRDSSICKAAIHSGVL-DNSGGEIVVIISYAQEHFYGTSMNGVE
SLSLSRREKAFMVSKPTKYIMSKVEKEVLETSKA--------------------------
------------NGFNGNHADYINTKDLPGSKLIKTFRDATFIFEIVPTGGVGNWSTIFS
FQSCGGFLCSIDNFGELVLQENCKPELVKTSYFPTMGKRAHISILYYVLTKDISVFVDGV
PVTNQLTTFDLNFQGDLILGKMADTDSDYFKGHILGFQAFDYVMSPEQIRQQYASSQNSE
SLT----------------SNLTKVRLTTEGNVCLTKCMKKFFGKYDVNATNPAIKLDCS
DTIESENFNGPNGKSFLISCTKSCIDPDLTLKGT-KVYTSDSSICKAALHSGAI---PKD
GGEAILTVLNGLSEYGNCHGHFGITSQKSNVPFMRSFSLHRAPKLIHLSCQDTAVFVLKM
HIGTRVLLDCPPGCKAKPYNVFGTGVYSPTSSLCQSAIHSGKLDNDGGEVEIEVEGERNT
FDSNESNGILSQSSGHYLKSFKIIKVRK-------------MNFKDTDFI----KFFGSK
NEKRKQSISN------------------------------KKVQKNRNILEESRNAVDLT
SILLPNEPGYI--------------------------------------IPDENEKTYEL
TQTKLLSLVDQGTKNKALKLNLPYGPYFVDFSANGRYLLLGGEKGQLSLICTQTYKDFFD
ISVRSSEDLCSHLRQHGVRSVYLKRPTIIYLFLSYSKIQITNFQLTYKLEYLYYHYLLVT
VGEFGDLCYQDISTGEVVAKHNTKKGPCKVMCQNKNNAVIHLGHNDGLVSLYVPNMEK-V
TDC--------------SYDDCLGFDGYWKVWDLRKEAVIRQYVGSNPPTCATVSQTGIL
SLNIGSRVEFYNNVFDGPNLYLKHHFNSQEIKSVAYQPYEDVCAVGTTFGMSNLIIPGSG
YPNFDALEHNPYETGKIRKDREVQRLLEKLPADSITLNAQPIGSYSRDLSQAQFSTEEPE
KVEKNKETTRKRPIKSSAKYKAERYNKVFMRRQQAVEDKIKSLKKQEEAKEEILTKMTKN
DVVKGVMGAALSRFFKKK-VSLRDHSMASDHWFLVALSKKTLHIYKFY------------
-----------------------PKTATNPGKQNSVILLRFLVEK-LELFREYSDISKSF
VSNKGNLVCLLKESS-NSIQLTELESSKVSLNLPNDSVLKEVVFSPLDTFLTVLTSWS-Q
SNPNNLVVYNL------------------------------KDPNY----------SVVL
SLPYKSYVVSRFP--TWTQDESFCILRVMDEIKVWKNNDFSTDKTNSLLSRGNNISLSPP
N-DKGFCYIGIFASNQKFDRGVLRIYSTTQLEKPIFNKEFDASEEGELFWNSKGTSVLFR
TFANSVKGLASYYGANSLYLINLNNSKFKTISTNDGIIHDISWSRNGKDFLLLKGPMPAE
IDLYDGVGLKTLSFGKNNRNTVKRDPFDRLVLMGGFGNLRGEIDIWDMKTKKKISQSKSE
CSVSCEFSPDGMYFVTATTVPRMRVDCCFKIFSYSGKLVQRVDFEELYHVYVRN-PKFKF
RQRDPSPSVTLE-------------NSTTSTPLYRPPGSLSGPKIIKQTNIPANAVINKP
KPPGADAALLSAASKIKKKNRNKK--------MTKKVITLVSAEGVSCTVNRDVICMSNV
IKNILNDIDDESEPIPLPNIKTNVLNKIIEYCKHHYNNPPSQIPQPLKSAQLNEVVSEWD
YEFVNVDKEFLFELILAENFLDIKPLLDLTCAKVASMIKGKTPEQIRREFDIVNDFTPEE
EAKVRIMN------M---

-----------------------EEEENKDINPDSPDVNPESLSTANSSDPE-HKDDYLE
QPALKEEISEDVEEENLEVHEIDHNKESEL---------NHMNSDESKQHEESVEPDEKV
TEPNNQLNHNQMEVDDSLLNDFERSLHLKDKTND----STKSLSQRTESSKSKHSSKSHR
KSSRRSQQ---FGTSENPLDITEITVDCGSLLELYQNDYFDAFMHMYHLYHKKEPGVHEY
LVNMLYTKRTDEEISYYLPQLCQLSISKYGKSSLHRFLLDKASVSMHFALQLSWFYQAAI
DDHIPSIDRLTQKMTQDTEMAVVNSKLITPVSFSRPSSEMSISENTTFPCLLDRRLLLES
FSRRTQQ--KSDLYNL
---FTNVNSLLG
AKIPTSLVHNTFMLSTPTSKVKLVGVHPKLGNAIVLET--HPILDYTQDLHLELQKLMMK
QRRLNYFNTLNNFVELCMQNSSLLTTESKRDARDPLLRVFASALNEWMLLRRCIVAAYEE
SFAYTGLSLPFDFINKPQSNKLNGYSTVHNNDALLQILRILENETKIFFSRKRAPFVFYV
EMGNLDEDIELISYNTN---

KDRETGMKDLYVFDAIVQDLLNYELLTPEQVEKNPLECIRYALDMLPQESLDRYNILSEE
KMN-----------------TDSSDNYSAKTQTSDDSFRHSSYSNTDENMVTELDSLSET
ESKSNENTEYSRVDPD---SQENSPNIVNSPQVGDTNTPNQNVEESSNRDVSPVDKEESD
PTKETPRDG----ETKTLNVKNLSPQE-------------VRKIVFAETFDQKKEKIRRK
SPYGNLKSWDLHAFVIKGNDDLRQEKMANQVLESFRRIFEKANLPLWLRPIEILVTGSNS
GIMEFLHDTYSVDVIKRKFNAESLAPVFEKLFYSNIYEARKNFIESHAAYSIVSYLLQVK
DRHNGNILLDPYGHVIHIDYGFCLSNFPGKISFETSPFKLTKEYLDVIEGGNPDNFVYFK
TLITKGLLEVRKHVDEVVLLVEMMTTANKMPCFLAGTSYTIEMFKERFMLNQSEEACIRK
ITEIIDASVNSFSTVQYDTYQRLTNGILMGLNITVKVSGGETFTLDVEPEMTVLQLKEKC
SDKANAPADKQRLIFKGRIIKDEEVLSALNVEDGNTIHLVRSGLKPASSPPTTTPTATTT
T----AQNTTENVPGFNQDFMSQMF--QG----------------------GMGNLPGMP
ELNPQSAAALLNSPVVQEMLTQISSNPELFRTLVESSPFLQPMMQQNPMFGQMLNNPELL
RTLMRPGMLQAGLQMHQAMQQSNANNQGNGNTA---------------------------
TQNPPNTENPFANFQMPANMAGTMAGTTPFQPTTMPF--------QAPPVDTRPPEERFS
SQLQSLQEMGFTDQAANLQALVQTNGDISAAIARLLNRTQ-M--PETNPMRDIRINKLVL
NIGVGESGDRLTRAGKVLEQLTDQKPVFSKCRFTIRSLGVRRNEKIACHVTVRGQKALDI
LERGLKVKEYELRKKNFSDTGNFGFGIQEHIDLGLKYDPSTGIYGMDFYVQLVRPGYRVC
KRRKCKTRVGKSHKVTKEEAMKWFQDKFDGLIFNLEDISRDYLRLSVVFSFLTNKPLQIQ
LVEP--------------------IRSYEVSLLKLVTKVTDGTRTEVDR--NTIKLMPG
RFIGG----------DFTFQCDNSIPLTYYLEPLVLLFPFSSNTTTITLSHSINNNSNST
ETNNSNRSPAHVEDLNEYYSIILQIG-SEVVFRYKEPYEVYFSCGPVKKVEPIILWKSPK
IKRIRGTVVARNLQPSLGKNAIIAAKRVLDQVCDNTWIALNTPPGKY-----KPTLIISL
IAEGTKNCIFTSNTLSNPLD----ISSTTGSSKNTGTVTSNNTSNMNKVGMGRSLLKILN
LAKNTNNTDGD--KNTECDNKPSVNQIGECERVGEKCAYRLCSEIALDSVIDTTHQHLLL
YFMALSGDHQVSQIRLAKLNRYSVHLLRLIKIHLGIIFKFQEIQTESGHNVLLVKCVGSN
YQNINLKSFMLNL---EISNDFHKFIKSINETNSKYGLEKLIYNEITKLKLSFQNKNITK
NEIYKNLLKCLHINMFGFNIKFAYIHAINLAQDKDLKYKSLGYLCCTLMLQNNNDLIILL
INTIQKDLNSNNVMNKIIVLNNLNYLINEEMLEIILPMILNCLIHENELVRKKTIILLTN
IFQQFPTHLNNIHSIIERGIYDINPSVMNVTLILIRISIINGIKLNKELINLLINIWKQI
LDNKLNKTYYYNKIPAPFIQINIIKLLTILC-NNIYTSNLIYNILYKFTQSIESEKNVNI
MLIYEFIKLLSNIHINNILLSISSIYISKLLNSEGIIMCYMSIICINKLIDKKLIINIED

```
QLKLLKLLTIDDEIIQINILKLLFKLINKNNFKLIFNTIFKHYNS---LNFKSFIEFNII
NIFSSDTPDSISTTNNLTTTVETTTEDIGEEKNPNEIAVVTKTGESDTFMDTGADSTANS
TAMECTSNNTLNNLNINWLINKCIKLLAENNIILLTHKTQELTNELNKLMEEEILEEKLE
MLEKNIEILEKNMKFLFWIFNNLRYNITSDTVLGHTAIECTTTTKDSTTKDI--------
GKGAKVAVGPSTVTGDTVTELYNKLMEKNCKKMKQIIIEIKNLRK---YKPKI------T
NNKFYDIKLSFLNNWIEKD----ERRYQRYE----RNEKMEI-IEEQKLNYKPYQLIN--
-----------------TISNIENIENIDK----------EEEKLIINIKNKSWGPKGYI
NNKEQDNNTKDSTTEEKEIAVVTNTGESEVEGKGANSTATECTKDISSTSKDTGTVG---
------------------------------------------------------------
---------------------------------ASTVMEKE-LAKKLFQNIS--------
------------------------------------------------------------
------------------------------------------------------------
----------------------------------ITSYNGGALVAMMGEGCVAIACDKRL
GLNQQVTVSSNFPKAFKVTESCFFAASGLATDVQTLKDEIMFKVNMYKLRGDKEMSVKTL
SNMVGSMLYSRRFGPWFVDSVIAGLDTD------------SSPYITCFDLVGAPCTPTDF
VVAGTCSEQLYGVCEALFKPGMDPEQLFETVSQCLMAGIDRDCLSGWGAEVHVITPDRVI
SRSLKTRMD---------LDEYYEYLNNASILLVGAGGIGCEVIKNLMLNGVKKLTIVDM
DTIDVSNLNRQFLYLPEHVNKYKAEVARMRALEINPKSEVKSLVCDVNSWEPNDLLQYDV
VLNALDNIKARSHINYCCIQSGVPLIESGSTGYNGQVYPIVKDMTKCYECDPLPKTSSIP
VCSIRQIPEKPTHCIAWARMLYQLLFGTPDNNNLLDLSPTLPDLNNLDEPVVVDYLNRIF
DFLFNSEVKSLLKMEEVWINRDPPKPLEFTLKRKANQIEKEDEKEPPNSKR---------
---------------------VLELEELYEQFSTSVKEIL---LNNSDMVGSLIFSKNDE
VCVDFVSSAANLRMINFGIKPLSTWDVQSIAGSIVPAIASTNAIVASFQVVQLLHLLKFL
KSN--------DKSLDTYCRKVWIKSSVMGSNPLVKGKLSQPELLEPPNPKCTTCQQKSF
KVKIKSLD-LTLHDLVQSVLSKSMGLAMVSLDFNLKNIYDGEEF-EEDPEYSKAVKNSLK
FYGLSDNSILTVTDLN-GDSQFELVLQLDDGLKSNFLLLYYSIFKFY-------------
------------------------HMAHTTKENRDIYYRKAKEEGFRARSAYKLLQIFES
FHILYPKNINKNDYIDKIVISERTKENLCKNVVDLCSAPGSWSQLLSKMVHEDYETLK--
-NACKKLQTQQEVCKNLVEYVNI-------KPVIVAIDIQQMAPIEGVRFLKGDITDPEI
LKEVLQLFIENVSRNINQAYGGEYDEKLRRNAQLITCDGAPDISGLHETDSFLQSYLIKS
ALSVCFSLLDPDGCFICKTFFSSENTPIFTQVSSFFDYCTIFKPSASRSSSFEHFIVAVG
YK-PFGHI----------------------------------------------------
------------------------------LNTPKSQEIDDEIDSELKEIIKAAGKAIS-
--WD--------------------------------------MKE-SKGWVLVLDFGCTF
SSTLVRAVRELGVRCELESLEKFKGLDKKDLPSGVFLVGGNESVFDQDVLSVEKSLLDTL
KGHKVPVLSLSFGMMSVAKSFGAKLRKTDNKDYVVTKCTLK------------------N
SELFDSVPN--SLTVYLNTLDMVESVPSGFEVIAKHSEVPV-GLYNSDYNLFCLYFHPES
VDTEKGNTILHNFLYKVCKCDRTWTLSEYLERELKNIEEQGSDKYVVAALSGGVDSTVSA
KMVQSVIGDRFHGVMVDTGLMRHNEIAECEKRVRKEVPGIKLTVRYSQNVFFKELAGVVD
PEMKRKIIGRVYIEEFEKAIKELGFNEKNCLLLQGTIYPDIIESELNRRSKLPVKSHHNV
GGLPERMKFELIEPVRYLFKEEVRELGRLLKLSEETFKRQPFPGPGLGVRVLGALTPENV
QKVRMADKIVREEVEKSKA--DVSQYFCIYLPLKSTGLVKGQRVYGTTVVVRCVKTKDYV
TAKWVHFDHEVLDRISQRITSEVPGVNRVLLDITNKEPATIEWEMRTLFPHEKRLRLTNG
SEEDSNGYYGSNICLIRAPLFNFLSVEAG-TPIILKPFKSPLEGHQPGVSESQRKTLGCR
IKSDTKLKDFRTGIYLEKPES------LEDLPPDNPLVLYTSDPEEIKVDSILSRFLRDH
QRQGVQFIFDCLMGLKGFNGRGCILADDMGLGKTLQSITVMWTLLNQGLDNKPAARKCAI
ICPASLVNNWESEIKKWLRGKCPCTAVAESSKEKVISSFQGFKYDRTSKVIISSYETYRL
HCSYLEGVNIDLLICDEAHRLKNDKTRTSQSISTSSAQMRLMLSGTPIQNDLNEFYSLVS
LCNPDVLGDVNNFRRNFANPILIGREPYATPAEQQKASERLAELSNITNQFVLRRTNALL
AKVLPPKIILNVFCNLTDVQKDIYKSFVNSKRWKNIMNQDRESRALSAIQSLMKLCNHPY
LIKRGGLMSSPDV------DSLLLDIE---------------------------------
-----------------NATKSSKYKCCRCDLSGKFLVLFRLLYQIRKNSNDRVVIISNY
TQTLDLFERLCKECSYPFERLDGGTSIKKRHKLVTTFNDNSNSFVFLLSSKAGGCGINLI
GANRLVLFDPDWNPANDKQALARVWRDGQTKVCYIYRFFSTGTIEEKIYQRQICKDGLSS
MLVTDINELKDSLSGEYLKNLFEYKEEVLSDTHDLIECKRCSHEDGMFLKVMHVPQLKEF
LEDDLNTWAHHADLTTIPDQYLVNAATK--NEPQIQYEDM----RLSDDFVSFVMACRIE
FQDTEESVDVENMSVKK-------------------AKSDTVSTTFDSTQETIESDE---
--------------------------------MRLCNHSHNEGLKISPKSSK--------
----GTKTIEPAGFTFFLLKEWKLNINAKE-VDNSTKKDFEFLDLNLEPVRAFSDFCQKY
EFYKL--IWTRIILSDFPSS---ESESGDEEVSRKRDKKFKSIKTLGFVRNFIAKEKNAY
ELLNCTDSDETAKIKANYRRLVLLLHPDKGVVQK-----------------------IPD
DLKEYSDKYKVSNISKEEKSELFLLLQDAFTILSDPDMRLEYDSNLPFDEYIPTHEEAKR
-KDFFLLFGPVFQMNSRWSRVKPVPLLGTNESDDDYVEEFYEFWRCFETLRTFSHAAPHL
LEDAESREEKRWMERENLKVQKKLIKKEQLRIQKLIDITQQYDPRLKRRQDRIRNEKLIK
QKQAQEAKLLELKR-IELEKEQQRLELEKLTEKIKFQKQITKKLRQHMRM-IYQKCISLG
EIGSTLEKLVTLDYDEMKKFSTQLYDILKLEFDFERCPDNEYL-----------------
----------------------------EDLKLLDPKLKELALSNNEEIELSQLFQ----
-----------------------------------------QISLKINPNQSYNNTTTVE
PTVTSTVGQSTGSSDNMGEWTKEDLKRLSKGVEINPAGTPGRWNLIAKYVKTKTAPQCIE
MSKLIANNSDI---------TPYLYNSSNTN------DGNTDSGNTSNVNGKCVASTSDT
GVNNGVNMSGWSESQQTVMIRRLKLHLRNIP----------------------RISTL--
---------MPFKRRNAGRSKHGRGHVNPVRCSNCGRSVPKDKSIKRFNVRNIVDASAQR
DIREACAYSLFNVPKLYIKQCYCVSCAIHSRVVRVRSAQGRKVRTYVQQRQMQVKLMNIT
F-------------------------------YKYITLNIWCVLQYTNVNVNAIGKKKVS
VTEISFDSLVEDLVWCGPENHVVLLKTASGRLYRSTNSGKQWSEITNTLSNSSSSTVNTN
SSNSVDKFHVDSMFVCESDKNVVAVLGTGNTHYISSDGALTFKQLGFKGEINMFFFHPTK
ASWSLLSTWEGDCLSK-GTGGDCKHVVYASRDLGSSFRRVAEYVGQFSWGDPTHGSADRI
YFSRYDMAFGDQPKQDGWNDAISFRYTDDYGATQHQLLSGGNKFLVSNGYIFVARVADSS
RQTVKLCVSTDNGVTFNEARISTELDEKSYTILDTSEGAVIIHVGHHYEGSDVEVGNVYI
SDASGLNYSLSLPNNVRASSGECEFDKVLSMEGIYLANFRDDSGGYLDPSQQFKTHVNGN
VATKSDNMTQIEKKRRNVGHKKIESNVRTVISFNKGAQWNYLTPPKVDSYGQQYQCPDRC
YLHLHGITQFKNFAPFYSVENAVGLILGTGNVGDHLSFDAANVNTFLSRDGGLTWREVHK
GAFIYEFGDHGGLLVMAQDQSRTREVVFSWNEGASWFDFSLSKHDLSVNNIVIEPSSSAT
EFLLYGNRNGVGVVFHLDFATLNQPACRGIWAINSSSSDYETWVPRD-MAGNECLLGKKV
TYKRRKQASECFNGRDFKRSVDRELCQCTRQDYECEVGFTRSIGSDVCKVEGHFLEREGC
TSASYFYTNAYRKVPGDVCVGGWVPDLVAVPCPPHSPLSKESKLLLAFMLVLALFMAAVV
YVSNDERFKHLFHNYGFKTFQYVPYSTLSQKK---LGGRFEPELGFIDAEQE--HEEIPT
LLSYLHDNDK--------------------------HQDSKTIELLDAKPTI-EDAVIKN
VDMDETTKAFALKVAFDAITKFEVEKDIAGHIKKEFDKTYEPTWHCIVGKNFGSFVTHEK
HCFIYFYLGKMAFLIFKNGDLLKKAQEMAGVSDSTKVTKSKKTLDYKNSDETKSSECPEV
KNVQVSNTGGSGDQSFENFENELELEQKF-------SKEKCLVKVFGWCKSMRSQDGGRL
LFVIVNDGSCKPNLQIVVHNDSKGYKEALKCKSGTSIQANGFLVNRVTQPKPAKEVSKEN
YELHITSDDSNYIQVLGLTNCPGTYPIAKKDLTMEFLRENAHLRPRTYLISAVMRIRSSL
SIAIHLFFQSKNFHYLNSPVITTADCEGAGELFQVTTMFENVKNVKELSKPSNDAENNCK
PEDSSGFNPVDTTVDKTNKAVDFKKDFFKKKSFLTCSGQLSAENYCCSMGSVYTFGPTFR
AENSHTNRHLSEFWMIEPEMTLVDLPGLMELTEEFIKFLVNYILKHNYDDLVFFNNTVDK
ELLSRLYNIVNKEFVHLSYTGVIDILQEYIQPFENDVHWGIDLQSEHERFISEKVFNGPV
IIYNYPKQIKAFYMRRNDDDKTVAAMDLIIPKIGELIGGSQREERFDYLEKSIKENKLNM
QDYWWYLDLRRYGTIVHSGFGLGFERLIMMVTGVQNIKDVIPFPRYSGHSLFM-------
-----SETQVDKLFKLFHTLGLRYKVIKHPNLVTVKDMLEESLAGFRDCIRNLFMLDSSK
RFYLISALYDTEVNWKVLRKGLNVNKLHMGDENDMLSLLGVGRGNLTPFSAMNDTENKVR
VLFDIRLKSKVDVVAHPLSNDQSVVMKMEDMLKFLTQINHFPTFLPLNDLDSTT------
---NIDKNSVNSNNTVNSVNTNEKNKKGEMNILGITVKKHENFAEWYSQVIIRSEMIEYY
DISGCYIFLPSSYFIWETFNEWFNKQIKQRGVENCYFPMFVTKEKLEAEKTHLEGFSPEV
AWVTRNGDVDLPDPIAIRPTSETIMYPEFARWIRSHRDLPLKLNQWNSVVRWEFKQPTPF
IRSREFLWQEGHTAHTTEQEALDTVYEMLNLYSRFYEEYLSVPVIKGVKSENEKFAGSKM
```

TTSLESFIPANGRGVQAATSHLLGTTFSDMFDIRYEDEEGVKQKVHQTSWGFTTRSIGIM
IMVHGDDKGLVLPPRVAKVQVVIVPIMSKTAEQ--VMLRVNELFQLLTEAGFRVKLDDRR
GYTPGFKFNHWELRGVPLRLEVGAKDVENNTVRLVRRDNFAKSDVPLENLTETIHQLLDE
IQNSLLTSAREKMEQSIIKAYDFEEVMVALNNDKLILAPWCEEPETEDEIKLETQKRSEN
------------------------------------VYQHNLY--SYMAKLSKSEKKKLY
FERLTNLMKTYSKILIVSVDHVGSRQMASVRHSLRGMATILMGKNTVIRTALQKNFPDSP
DVEKVTQCVKLNTGFVFCEADPMEVREVILNNRVPAPARQGVIAPSDVFIPAGSTGLDPS
QTSFFQALGISTKIVKGQIEIQNEVHLIKKDDKVSASGATLLQKLNIKPFSYGLKVEKIY
DSGAISDASVLDVTDEDILAVVKLGVSYANALSRQLGYPTTLSVDHAMLEGFKNCVGLVL
DSDYTFPQMAAVKQFLENPEAFAVATPSAPVTSEVKEEKK------VEEEEEDDDLGFSL
FD-MQGRDISRQPCPDRIVEDMGGAFGMGSVGGFLWHFIAGAKNSPRGLILKNALYTASS
KSPVLGGNFAIWGGTFSTFDCTFQALRNKEDHWNAIFSGFVTGGVLALRGGLKNASRNAF
IGGVLLSIIETVSIVVNKRIAVTPRQHFQRQMEYEKQLK-----MGKVEVWELQESSENS
SYVESQETPDEERENRAVDLFDNDYTKRRLRKHSDSEDTEEEESEFESEHSTSKESLSDN
DYKEFLLQYVRHYGQEVNQTLYNFECLPRHWNGVFYITSAHNLESVPQNYFYYIEIGRIP
NAQSR--------------YRPGERLTLGNPIFICNKLKMEISYGDLKDYFVKIDLWKKH
RLSLNTLHATRFLTFQDVIENKSNLTLALDMPVEDSERKIPLHKLNVVMLLEEIFDFMFV
FENWSFTPSPHLPDHLKNSPKNIKVSVIREGKGWDSKWAGVSNDDYWPSAGVFNYRGTIR
HMKISYFQVKVYCTNKKFWRRTTTLLGTCVMSLRSVRDYPLVRGAVKKLAKGADQMQSGT
IQGNIVCYMSSAGLKHFYENKKRPAQPISGSSLLTHLDKSYRYLVIRLVRCESLPSTNTD
TNSSDPMVKVRWDGIVNCTPTLECTTSPVYNHNMYFPIHLVDEEETKNIFLIMNCLPVDL
LSKGPVVMEVWDGDDTSTEFLGGVEVPLSKLFLNGIETRRSLVDGIYNEGSK--------
---QGLDDEDGHEVG---VEHLTPVYRDTLTLTGYTV-PHRGSKPTITFEMYILPPMPQG
LTVPNDQKRQVKSDTYRDLARRWDKEFETWQKMYKDQTLSNFPQRRFLTTTTEISPTEVI
ILSCFVTPIQIPSQLSRPGTLMHWISNFALKEDSIDTDNWQTPSRFLTTRKGGLHDRAVL
LCSCLLGINFDAYVCKGTIKNGTVEHCWVMTRHSDSTVSFWETGNKKIWHLPKRWRVS--
-------------KQDSDYEDLNVTSGPILDNYKNITFEPSSNLQ----QQVPNKNVHGL
TKYEMYGNDYVPDQKVDLDALLHKHDEVNFDTSSLRDNLSKTLLDNPQRK---QQFAKTV
QLNPKQDLIIPFETLVHLPYSSIETVFNNKQLWGNIQNHHPSCILYDLEDPLQWKPFLKV
PLDELSQYIQITPPPTMNKCIETSNDISNDICDMIEVMRAHRGLKCSISKEDEMKEKLGN
FLDLMEYRESLDPQFDPGMPIHLLGWSNR--KKLKKLSKTRNVQEVVNNMKK--------
----STKQPQFDSYYSTESTDVSP--------NQQVNEQSEKVSE---------KSQKSA
RDLVEAEQNADENPEV---NTEPERKTTSRGRKRRHSRR--EKRTKPLEKRTVEDPKHQS
L------IRYRLNLEKKLVKNGFEISLKKKSKPKNVKNLKQPK--IHKSFYDVT-HVEFQ
ISRF-------------NPINTPSSSGVMSSLEKFSFNKHSDIIHSAVHRMREKSRNHNL
GRREDSKNVKLTNYMNQQNYLVKNRKSFLKKLRTAVN-KRGSRFERNPRFIEDMNLVVQM
PEFRKEYMYADYNIPYEFTIHKSVETSNWNHYYHYEALFYSWQKNIPVMANHTFVGIPIH
FSTSDVNEIRRLLCCNKRFKKLFVNKLDDLVYMTVGILGHRTTGKEVRAGNVNAVQAIAN
ILKSSLGPKGLDKMLVDDLGDVTITNDGATMLKQLEVQHPAAKLLVDLSELQDQEVGDGT
TSVVLIAAELLKRANALANSGIHPTSIITGYKMALRESVKFIRDHMSLSLDSMGTEVLMN
IAKTTLSSKLVGFDSEYFAQLVVKAIKTVKTLSDDGDYKYPVGRINVIKVHGKSAKESYV
VNGYAVLMGRASQGMPLAVKNAKIAFLDFPLKQYRLHLGIQVNVTDPQELENIRLKEKDI
TKERVKKILDSGCNVVLSSQGIDDMSMKYFVEAGVIAARRVPKKDLKNISKITNGKLLLT
LSNLDGEESFSSEYLGTCESVEEKRIGDWDALFFNTSNNSSSCTLVLRGANDFFINELER
SVHDALCALSTALEQNSLVPGGGSVETSLSIHLHNYSKSMSSREQLAIDEFAEALLVIPK
TLSLNAALDATEHVSLLKSYHAKYHS-DREKYKEYKWYGLSLSNGKVGNNLEMGVLEATM
SKVKSVKFATEAAITILRIDDLITLEPEKQPMGEDMGCIKLKNIFGEHWKQSYRDLKISA
KPTQSCGLASSSKNIAVGFIIFYGGIVSIIDINNYERNPPVYKLLGHTGSILDIDFNGFN
ENILCSSSDDCSIKIWDISNLES--KELNESTFTLQGHRMKVTNLKWNPTVDYALLTTSF
DCTAKVWDASNGKEIFSTSICEHPSSCSWTPNGDKILVSTKEANVSLIDPRSGNCCTKFK
AHDSNKLTNALWLGGHYGDD-HLFTSGFVDNKTRQIRVWDTRKLDKHLISNDIDSSPSPL
IPHWDPQIGLIVLASKGDLTVRIFQYIDKELNRAGEFKATGSMKSFCLVPTDICDRTKCE
LGRFLFNTDCKQINLTSMFIIRRNSATTMNELYGEEYDT-ARYSVRDWEAGIEKNLGNLT
IS-----------KSKCTQMI---------KDKDAGCKFREISKTIQECNAKYGTN----
FNNPELIPVLEQLKDNDLIKTIKT------------------------------------
----------------------------MSLQSLDHQHSCISRSFQVINGISCVIRELVE
NAIDAHSTNIEIKLYNAGVDLIKVTDNGTGISEINFDNLGSKHSTSKIRKFEDLFTSLTT
FGFRGEALYSMCNLSNVEIETRTPNSDNGWLLKFDSLGNIINKSPIASNIGTCVKVRELF
GEYPVRRKLLVKNSKSQISKSVSQIQQYALINPHISFNFTTVT---NNKKFSNIFATSGK
DEIRGVVEEIF--------------------EGLVSNPYNERGYREMEYFYINKRPITKV
QKFKNAISAIFSHFSSRAKPSFILNLTMNCDNVDVNISPDKRSAYIYSEDYIIRTFKENL
YEILKPKTNLL-------------------QINSEI----LTQFISNSINIKQEKRQLDT
IDTNSQTITHTNGDTN--EEHI
SDTMST-----GMVTDSM--------GINAITDTVDN---TITTTSAITYGALNLPEILD
TKDTTVNTTSISTKSISTDIITTESF----------------STENISNRISTNSITDCM
-----------SDSITDYMSDSITNC----------------------------------
-------------------------------------MSDGINLDRINTLEEY-------
--------LELVEECCSSISRRIKICERRLFERNMKKLLPEESIMKCKLTNEDLM-----
---EHNFLNPQVFEKMELIGQFNKSFIITKLTFP-----------------------DIK
TKYNFSLYVIDQHAADEKAKYSEINRIDTIFNTSKFFKIN------NVQVAENSMNVLLS
NGFDIKVCRELVFNDGDV---CMSSVLTEKIGRGVYVNTLPQILGKVLGEDDFIDFLNEL
----------STIDYIENNQQSDY---IWGLGNIPRPHKIWSILASKACKSSVRAGDGLT
NGQMKNIIKKMGTLIHPWNCPHGRPSIKCLVSHQQLQELLSN------------------
-----------------------------ME---ELEELRLRVITAYPNVEKDIEILFNT
NDISNENLLPYDIVEALLRHYLLQQGFQDYIFTFLNSNGMLDLKYIRNYLHENNMLVV--
-NEETMLNIKEMKLLAIIWLKLLSDSYFSKQPNYLTHMTNNNTNNNT-------------
-------------NNQFIHITTNTLTNSNGTDEGTMGTNGTNGTNGTGMGNDMEEEEEEI
MKYMKNIKDEIEKNKMCGIKCYTYPASLIPNGSCASAGAIIPHRRLKNNKPIKNIFGCCM
A-KKNVVTEDLVDYEEEESKVSTKDVIKS------AAGKKSKSDGTMGRGSYVAIHASGF
RDFFLKPEILRAISDAGFEHPSEVQHETIPHAITGVDILCQAKSGMGKTAVFVLSILQQL
DVEAEEG-----KRDADDNVKP-VSRVSCVGISHTRELAFQIKNEFDRFSKYLPQVRCEV
VYGGVPIQKDVAMLKDAKTPHILVGTPGRLLALVKAKHLNMDSVKHFVLDECDKCLEKLD
MRQDVQSIFLSTPKKKQVMFFSATMNNDIRELCKRFMQSPVEVFVDDESKLTLHGLLQYY
VKLAESDKNRKLNDLLDTLEFNQVIIFVKSVSRAVTLNNLLTECNFPSIAIHAGLDQSER
INRYTQFKNFDKRIMVATDLFGRGIDVERVNIVINYDMPDSTDSYLHRVGRAGRFGTKGL
AITFVSSPEDSSQLEDVQKRFEVNISEIPATIDTSLYLNQM-------------------
------------------ASSENFEFD-EAMLARARPRLSVSAEVFGDNHSKS-KVVIPN
YEKTPQEQAKIMEMIKRCFLFSGVNSSGLDLLVKAFDFKTANPGDVLIKQGDDGDKLYLI
ESGTVEVTRKNTGQEEFLCNLTAGDYFGELALMYNSPRAATVVAKTEMHLWTLDRTTFNH
VVRMAVIKKREKYDSILSKLDLFKKVNPYDRCRLADALVERTFED-ETVIKQGEPGSSLF
MVLEGQAESFVENKLVKSYNPGDYFGEIGFILKKPRASTVKAKGKCLFVELERENFINLL
GPMEDVLNKNIKNYKKVLEELKLENKHLKSLMHVLLKPNNDEVKALYQNHKSFYEGDCGL
DLFCVEDQTVEAHDTSIINLGIRVSAFKK----------------QEDGRPTKSVGWLMF
PRSSMAKTPLRLSNSVGVIDPQFRGELRLSLDNIKDFPYTVKKGDRLVQMVSYDGEPITF
EVVDELDETQRGSKGFGSTGR-MVQCDPHSIFFGMMGVVCAMVFSNLGAAYGTARSGVGI
SSMGVMRPDLVMKSIIPVIMAGVLGIYGLIISIVITGNYGEPGEYSHFLGYSHLAAGLVV
GLCSLAAGLAIGIVGDAGVRAHAQQTRLFVGMVLTLVFAETLALYGLIIGLVVAMKAPKG
LCTSFLKMISKHL--------------------------LKKSTKNKSNQVKSTKKTRSK
KIIKVYDPKPIKKGLKSYAFVNLRRIPVPPNRMTPLQNNWENIVRTVVERLELQIRMCTK
TVEVRPSREGMDLSLLQKAQDYIRAFMLGFELKDAEAILRLEDIFIESFEINDVKRLHGD
HLSRCIGRISGKDGRTKHAIENMTKTRVILANNKIHIMGSFNSIKLARHSICSLILGSQP
GKVYNNLCNSAKRLRERIM-------------------ESDSYKPISDILGLDECHIYKF
NKAFQNGDIEKLVELCYSTTPIQKLENKLHTWAENPKTIGALSATQLSVYASKEDEPEFK
DDILAANGIEALADLLKSEV-----------------NSKCCEEMYEQDVFPYLIKGMKS
SLDPFRAACAQTCRNIFKLDIKYRKEFMRLGGLVALVSLLNLSD-DSDEMYLTQLEAIYH
LEDFCMDGIEEIPELVEYVKASNALSKLQSLEKVHF---------------TMADGYSAA

```
EFFNTKLSLSYEDLIILPGYIRDSVDKVDLSSNVTRNIKLRIPILSSPMDTVTESKMATA
MALLGGLGVIHNNLSIDNLIKEVKAVKRFENGFVHNPVCLKPTSTVSDWVEIRDKLGFTS
VPITSDGNPGSKLLGIVTKTDMYFVESKNVSLEEIMSTNLVVGKHPMKLNDANELLFMSK
KGVLPIVNEDYELMSIVTRSDFYKSKLYPYASKDDNKQLLVGAAISTRVNGLEVAKKLID
AKVDVILVDSSQGNSVFQIDLIKQLKSAYPNVQIIGGNVVSAQQAKNVLEAGCDSIKVGM
GIGSICTTQNICGVGRGQATSVYYVSRYTHWNGVPVIADGGIKTSGDIVKALSLGASCVM
GGSIFAGSKEAPGEYYFNNGVRMKSYRGMGSKDAINDSLQNSLSRYHLVD----------
DQKIISQGVSGLVIDKGSVNNILPNLTQGVKHGLQNIGAFSVKELHEALYSGQLRLEQRT
AQSIVDANVCRTINNPK--M--FRRLFNPRFSNNILTRYRNNTF----SSEFAKPSNPVN
SSVCLSSRFFSSFMQTVLKQVKKDLEKDKSLQEAMEKLESESQISEKISRISEYFEKTRD
FSKDCINKISETK------NSKIVRNCLDS------AKSIAVGF------------DRVS
SYLY--DEGHAKRIRTKMK--LNKRKPESSKPENESVVTEPNDSGN--------------
---DTPDNSLVLAKESVWDRFGSKIRDMPFLYEFFENPIISKLFGDTSLASALREMKRLD
PSFNLPDLVELVEHVIAPHVVESYLKGDGEALKLHCGEVAFNILNTSIKERNLQKLVLDP
SILILKNVELKGGMKVKEGDPWLIFNFTTQQINCLRDTKGKVVLGQIDDIREVVYSIAIS
RHPNP-FENLEYPYMVIQYIYTKQITILMSLLRKD---ISELRKLGLDNVQELGGNYEQF
KDEIKDINKLINSLQSKLNELYSEMGKSIPNKINEPSKDKHILKFEEILEEMKDFQDLVN
DEHKEKRKIYKNLVTNTSKYIIKMEQDLEKNKTERNKQVLLIYRNLSNSIDLFWKKIEKF
AWEHLKKDLQQELIKKKKQNLDKFIQDAIKKIKTTTQINHSEPQNHIPKPKNKNQQDKVS
PKPNVSNDEEYVLNKDEERMELDDLKLEDEM----DSDKEENELNDLQKEAEMPLEELLK
-MYQNQTDEQ--SEEPDYDEIEQ--TDSDAQASDRDEAEKVSLSMSDDPQSPNSDRNKDE
ERMELDDLKLEDEMDSDKEEN-----ELNDLQKEAEMPLEELNQSDEADVPVPKKKYKYK
KDDSNKVEIKTNKTLTEEQNNRVQVNQEEDDVDIEVPFLIKGVLRPYQKEGLRWLVSLYE
RNINGILADEMGLGKTLQTICLLAYLACNKGNWGPHIIIVPTSILLNWVMEFNKFCPGFK
VLAYYGTPAERAKKRTGWNKPYSFNVLISSYTIVVQDSYILKRRAWEYMILDEAQNIKNF
TSKRWQTLLTFNTKFRLLLTGTPLQNSLQELWSLMHFILPNIFTSHTQFNIWFTDPLNQA
LDNEKKKNNMELVEKLHAIFRPYLLRRLKKDVEKQMPSKYEHVLKCTLTKRQQVLYDEYI
SSNKEASKEERLSYRSMLNILIQLRKICNHPDQLKSRDAQIPIEFN---TLQLPYLFQIS
DKLKHN-----------FDNR-NLTNKINNSGKSF-------------LEVCSKRKRTER
LIVDKRIKKRRVDLN---SQLSFLSLNNLVSSTFDDNKEVLNQNFVDLEKPNNYNHYNGY
GINSEKLKSEPQNNSEVT------------------------------------------
---------------------------------------------------KRNMINKLS
REQLLQGQK-INLGPMNDNFK-ARKLTVNQNPSDELSNKMLSQKLLNRDNILNL-----N
LKNNLEI---------NKIIESYLILILNFLLISV-KVVSKPVKLYFSGTQDWYMNEVKT
KLLKRDDKKNKVTVEAVNSNYKLLFPSRRSINDDCGKFKVLGPLLLKLKSEDHRCIIYTQ
FSKMLDILENWINFMGFTYIRLDGSTKIDMRQKIINRFNENTKIFLFISSTRTGGVGITL
TGADTVIFYDTDWNPAIDRQAMDRCHRIGQTKDVNVYRLITEHTVEENIWRKQLQKRKLD
DLIVDQGQFDVQ-------------HNNWFSNLDTLINIFQNKRDEQDEEDIYGKKILHE
SNVDFQTKGVQNIKMLIEVEDADDSMALKKLKKENENINKQD-----FETDLINIPGLVT
YCIQFLLKYQTVTLERQVENMKLKIQIEDYQNQQNNEQEEDEDDIYTDYTSSENEELSQE
DMVLSIVGLGLGDVEDITIKGFKAIKDADIVYLEIYTSFLINSKHKLEEYYGKEIKEVDR
IFVEEQNDTLLNEAKDKNVALLIAGDPFYATTHVEIYYKAMNSGINVNVIHNASILNSVG
ITGLQLYRFGETVSIPFFEENWKPFSFYDKIMQNYNNNLHTLCLLDIKVRERSVENIMKN
KLIFEEPSYLTVTHYHNYYYNFTIILRFKIGKIDFMVIGIARLSSEDQIIKSGKLEDLLN
FDFGPPLHSLIVCSPHLHHYEQLFFNHYS---MGSGAPRGIRAARKLRNRRRQQRWADKA
YKKANLGTRWKCNPFKGSSHAKGIVVEKIAIEAKQPNSAYRKSVRVQLIKNGKKITAFVP
RDGCLNFIDENDEVLVSGFGRSGHSVGDLPGVRFKVVKVSGVSLLALYKEKKEKPRSMGI
KGLIPFLSEKVPSSISELSLECLSGESLAIDASAALYQFTIAIRDSSYFSSLVNSKGEST
SHIYGLMNRCSKLLEYGIKPVFVFDSKPPELKSKTLDKRRQKREEAKTDFKKAISEGDKE
SAKKLVGRTVKVTKDMNDSAKKLLRLMGIPVIEALEEAEAQCAYLVTKNLCHFVASEDTD
TLVFGGWFLLRNVTSSANK-------------KIVKVDLQKVLDGLEFNFDQFVDFCILC
GCDYCDTLEGVGPKTAYSLVKKYQSLEEIVRFKG------GDYDEFKEAKDYFLSPKVNE
YDENSVKMGTIDPEGLTEFLVQENNFSKERVEKFIEKLLKFKTKKIQTSLLSFLTNPQPT
NKSKSLDEGPEDYKVNTNTTDTNSTKDTTNNNLENKVKIEN---EENDTGR--RDSIDDL
FKEFEDEFEPKSKEKQHERNVILIDDDDDVLTTANSEKMNCEIGKVKEEPNKKG------
-----KFSLISIYMFFGGFPFDGMPGGHSRSKEPVDTEKLYKLLDLSKDCSDSEIKKAYR
KLAIKHHPDKGGDPEKFKEISKAYEILSDPDKRRIYDEHGEEGLDGSYTATDASDIFDLF
FGGSRKGKKRGEDIVSHLKVSLEQIYNGTMRKLAINKDIICNGCDGHGGPKDSFVTCTSC
NGQGIRVQIRQMGSMIHQTQTTCSSCNGQGKSLPESKRCKNCNGKGVKQTKKILEVFVEK
GVPDQHKITFHGEADERPNEIPGSVIFIINQNPHDTFKRNGNDLFMTKSIPLYQALTGCT
FYLTHLDDRILKINTPAGEVVKPGSCKVITGEGMPIYKSAYGKGNLYVTFDVIFPVGRTF
SPSEKEMLLLFPFTPETP---AKPDTQVDEYTAQHFDLDDYKSSDNS------REYEEEE
GH--GDRVQCRQQM-------------PLDRALYSQLLEILTLSEKGDTETQKYVHESLA
SFQNNRADVPLYYLEASLS-GPTFHTKQMALLLLKNSVLQNWNKTDPAIQSVIKNEIINI
VNLREVKLRNAVASCYVSIFN-VQGYDQWPTGLYNLLSIISNSADEVVETAVVTLVMILE
DTISN-GAMTPNYLNYLKSDFIVKLFQITSKSPALT--EITSRILLTLLDSNVIL-EYLV
SDLFGQFWNLLGVMATLDNYNVKKCVLKAMHNLWDLVPMSILQSSDALFPFISKLCSDDA
YTIQIDALDFYTHILQSQLY-------------------------TSSNNQIRCLLLGKM
ANEFGTLLKTLVDNTKYSSWDYMSMDRTHLEDDNANIPDDMQDVP---------------
-------------------------------------------------IKTREDEETNT
WGNTWTVRKGSALLLDTISQLYAQEVIKILLSYIQEKLDSTDWELKESGVLTLGAISKGS
LYTLYPYLPKVIDYLIVVATDPKPLLRIISCWCLSRFVEWMFLPNNTNTYLSKTLSVILR
GMLDRNKRVQESACSSFTSFEECGTTLLLPYAGQILHVILSCIELYQSRNFMILYDVIGT
LYQSLGESITQQAEHNQLIDVLLNRLEIVGLGDVQYIGLIECLSSIISVLGSKLPQFVQK
ITKHCVSSLCELVGDIT-------------------------------------------
-ELEYFYQSVQV-LTDTISILLTSTQGSVNC--NEACNVIN-------------GLRISN
---------------------------GIDLVI---VINELCSSKISVILQSCIALMGDL
SNSSIQLNQDSLKVLVPNEQSSINSSSTGVVNNCVWVFGVLCDNQLGIYNSSNIDLVFLL
VVKVINLCSNNFCILQNCCVTLGKFSNHFPNVAIKYLNSFLNPLCKHLIHSKNDKEKFNT
TLSISNLILLHQSNNTQSVNSLGNVIGKEELETCNVLLLFKLYISCTK----GTLDNFGD
SFGTNLVQLIDPDTKNQLNSLTSLMV----------------------------------
---------------NVGDSFLLKYQLRYNLFFKGSLRINNYVSGKKKKVSDDDELKYIE
TEDAGTFTWCLRNCKRICETSENL-----------------SPASLMNQILTLLKE---D
NSEIIASVLCDTLGFNNLNFISKLITFRQNLTRQWQSMLYNKLDDANYERLNRLVLQKTG
-----RDLRSQKKRLEIIPPQDLFCLIGLDFKSEKKFNTAVTF----PKQLSLNLIKEDN
EIYEKLVIPPSENRIVPEDELIPISTLPEWVQRAFGVEKLNLIQSKVFNSAFNTQQNLLI
SAPTGCGKTNVGLLCLLQNYREY-------------------------------------
-----------------------------EQGKKCGKVIYISPMKALASEIVEKYSKSLA
HSGLVVREVTGDFQVPKSELEEIDILVTTPEKCDVVTRNSFSTATQSDDSFLTRVNLIIF
DEIHLLNDERGPVIETIAARFFRLIEWTQVTRRVVGMSATLPNYEDIATFLRVPPEHTYY
FGREYRHVPLQQIFYGIKNDDIYKNNML--MICFDHIVETLESGKQCMVFVHSRNETFTT
ASRIVELVNKSEKSELFEPDLSQVKRFSAQL--MRRNNLKLLSDYSISIHHAGLSKSDRD
LVEEMFKSGLIKVLVCTSTLAWGVNLPAHSVIIKGT--FIGGVGVDRNINNLELNQIMGR
AGRPQFDVEGKGILLTDHKNLYNYVRMQTERVPIESQLHMHLENFLNAEIAIGSINNDTD
ALLWLQYTYLFVRMVKNPLFYGINGDDDDTLLKYRHEIIKNAAKNLNKSKLIRYSSKTGD
FSSTDLGRIAARYYVDYETTHNFASSINMDNHEFILDKLSECREFESILYRNEEYDELLD
LMHLVIYKPKGGINHKNKVSVLIQAYIAKLFIKTSSLAMDLNFIVQNVPRLARAYFEISM
CETVCGPPV-EQIHDWVIILERQIFNR-NILSNFTSPMN-----NTTPSK----------
----DLVLLSPSVVDRFTRFKL--EDIVNFSYQEVLDIVRSKQEALTIYKYIKYIPYPEV
KLYNQPITDKITKLTVSVEIKNE-WSKRWNGSNESFYVWVCT--SSRLLSHSQVNFTS--
-KGVQFVEFFVPIHNRNEPF-CVKIFSSNWLGLSFEISTKLQTTGEFNSADKYTRLLKLN
PLPTSVLNQYN---VYKFPYFNPLQTQVFHKAFRTDESLVVAAPTGSGKTLVAELGLFRL
FDKHPDKIAVYIAPLKALAHERFKDWCKKL---HFKKILQLTGDTSSNRDELDRYDIVIT
TPEKWDGISRHWRRKKLVTKVALIILDELHLLGESRGAIIESIISRQYTINHSTGAQVRY
ICLSTSLSNLNEISEWIGIPN--VYNFSPAVRPVKCNLYIDGFSIKAYCPRMNSMNKPCF
DTIIKHDHSSNVLIFVSSRRQTRMTAQDLVGLLQFHNISFSN-----------------S
```

```
CDNYFFDDEWLNTFVPHGIGIHHAGLSTKDRELVQDLFLNGKLKVLIATSTLAWGVNLPA
KIVIIKGTEFYDGRVKKYIDYSATDIIQMVGRAGRNIYDGEAYAYVFTETRKVGFYKAFM
FTPFPTESFFLEKINDCLNSEIATGSVTTKKSALEYLSRTFLYKRLKSNPKYYAPNMLYE
DKGDVNLLNVVKLNKLEDICEAIVNNSISSLVKLGCIALEPEDELKLLVPTLNGILASQY
YVNCKTIHEFSSI---DFSENLGFYEIARTLSNATEFNLVPLRHNEDVYNVQLSNLCPSK
IEASDPNAKTFLLFQARLFNLKLPVFDYNNDTKSILDQLPRIIQCLLDVFIINRNFKNVE
YLLLLYKCLHLGLNPLNLQL----VFEVNYEISVKV---------SKKGGKYTISSDVV-
----NLAVMLDEINHTTEFHYLFLVNQSTNVIYGYKKVYKQSTHSFKYVLIIFYLLRLRI
DIGSNLTLLLILSCPTLLSYQQIILHINNKSKMSIKCGLREELDRYFRGSRSQMDLTAEE
KVKICLSLSEECIKPDELLSLLQKKDYPLAYDGFEPSGRMHIAQGLCKVDTVNIMSKIGI
KSVIWIADWHAMLNNKFGGDLKKIRIVGEYFIHIWKAAGMNPDSVKFLWASDEVDKNPDL
YWRLSMDISRSFNITRMKRCSQALGRTEGDDQPSAQLLYPAIQCADIFFIGADICQLGMD
QRKINVLAREYSELRKIPRSPVVLSHRMLPGLVEGQEKMSKSNPNSAIFMDDSAEEVSSK
IKKAFCPPGVVDGNPVIAYFCFIVFKRFNSVTIERKEKDGGDVTFNSPEELKDAFLKGSL
HPADVKNALVKYLNLMMQPIRDYFEV------ILTFILISLAQSGIERGISSLLLKMVGK
KVTVVGCGNWGTAAAKVISENTPKFNLFNPTVRMWVLEEKVDGVNLSELINTTHENKKYL
PGIKLPDNLLAVPDLNECVKDADLFIFVIPHQFVKSTAMKIKDSGLLKKEAVALTLVKGI
MILDNKPVLVSDVIERELGIPCSALSGANVANCIAREEFSEATVAYTTKEEGKVWQRLFD
RPYFKIRCIKDVAGIQVYGAIKNVVALSAGFCDGLGLGSNTKAAVMRIGLVEIHKFAKLF
FPTVSEEVVFESAGVADLITTCIGGRNVRCAAEFAAKHGSRSWNEIEQEFLNGQKLQGVS
TCHEVYEVLKTHNLLEQFPLFHVTYKVAFESTHPSELINSLSTEELESFE---MSQFMGR
VSKLAGLGAASVVVPYLCLFDVDGGERAVMFNRFGGVSKKTFGEGSHFYLPWFQVPYLYD
IRAKPKVINTTTGTQDLQMVSISLRLLYRPLAEHLPRIHQKLGPDFDERVLPSIGNEVLK
AVVAKYNAESLLTQRDKVSKDIREAITARAMQFDIKLDDVAITHLSYGKDFSKAIEEKQV
AQQESERVKFIVAKSEQEKIAAIIRAEGEAEAANLISKAVQTHGSGMLEVRKLEAAKEIA
ETLSNSKNVVYVPNNLNMLINPTNLMNYDYLNTYMSQKGGWDFSNYSRNSRIQNSISG-H
NYLKTGTTICGVMGRDSVVLAADTRATQGIIVADKNCSKLHKISDNIYCAGAGVAADLEH
TTLWLANNIELHRLNTKKAPRVQMCISMLVHELFKYQGYKQCALILGGFDYTGPHLFSVS
PHGSSDSLPFCTMGSGSLNAMTVLEQNYFDGMSVEEATELAVKAISAGITNDLGSGGNVD
VVVMDKNGSKHSRTFKKVCSRTYAAP-DNKLTAGTTEYLREHIEHIKKHIIVDQDVAKGR
SELGYNNATPSDPFGKPPPGYVPGKGRGATSFAGGVSRDDTHDDSDLNDLG--GPYQVHC
ENEQLFKDAEYDDDDREADLVYEAIDAKMDERRKSRREQSLKSEITKLRSEKPTIHEQLA
QYKRNLSTLTKEDWESIPYIGDYLKRKQQKKQQTYVPAPDSLIYS----SRASMQHTSSI
----GTETPLGFSTPL----GIM-GAKTPL-----GIQTP--------------------
-GGFTTP---------------SGRTSSLNLLGEARGEVLSSTLDKVTDNLSGQTVVDPK
GYLTDLNSM--KTEFEEADVQKARTLLKSLINTNQKHAQGWIAAARMEELAGKIEAAREL
IAQGCENCPDKEDVWLEAARLE-KPEYAKSILAKAIKIIPTSVKLWLEAADKETSNDNRK
RVLRKALEFIPNSIRLWKEAISLENETNAYILLKRAVECVPESLDMWLALARLCPYEEAQ
KVLNEARKKLPTNVDIWITAAKLEESNKNYEMVDRIIVRAIDNLSKKGVVHIRSNWLKQA
ETAEANSFIKTAQSIIKNTMTIGVDDNNRKSTWLEDGETFVEHGSYECARTLYKTALEHM
KTRTSLWLALVELESKHGTPDQVEEHLKSAVTYCPNSEILWLMYAKHKWVGGDVESSRAI
LSKALTMNENNEAISLAAVKLDRETHEYDRARKLLEKARTRCNTPKVWMKSVQLERQLKN
YEKALELVEKALEIHPYFDKLWMISGQLKLEKQ-------PKDIEGATLTYKQGVETCPW
SVNLWLLSIELQIELKEFTKARALVETAKNKIRTISSIKKNTDITKQTKVLTNAELA---
------RMAR---------LSMESDDPG-SVKEMIEKITSQCDLIWLKGVEIELETVREN
AHFAMSKALQELPDSGLLWAHSIFLEEPNAQKTKAAEALKRNQNSPHIVLAAAKIFWNCK
MIDKARRWFQTCITLDDSNGVSWGTFIAFELDCGTEESMKQAINKFIEAEPNRGYEWCRV
TKKVENWNLSLPQKLYKFIEQHYPEVLTDKVPEDVLNVLNPPQVKKES---D--M-----
------------------------------------------------------------
-----------------------KNPMIEKLVMNIE-DKYGYVPFGYELTRREFK-----
-------------------DIKDLDNS---INKNIWIRGRIHELRGKGGICFLILRQQRE
LLQCVIDSKSENNTKDMVKWTSTLSFESIVDVYGKVVVPNMPILSTTSSSELNVSKIFCI
SKSSTNLPFLLRDANNTDNNNPNVIKVNQDTRLDNRALDLRCFMNNIIFKIQSIVCQLYR
EFLLSNDFIEIHTPKLLSGSSEGGSSVFKFKYFEQDACLAQSPQLYKQMAICGDLKRVFE
IGPVFRAENSNTHRHLCEYVGLDLEMELKNNYMEVVNLIDEMLKFVFNGLKN--SNEINY
--FYKLNPIQPFQFLNQTPKITFQQAVEMLNEI--VE-IPKDLNEYDFTTEHEKLLGKII
KNKYNTDYYIIYQYPLNVRPFYTMPLDERTIWSRSYDFFMRGEEILSGAQRIHDSEELEK
RAKECGIDVNTIKDYIQVFKYASFPHAGAGIGLERVVMLFLGLTNIRKTSMFPRDPKPVV
SMSWIRSFGASSSLENVFKTYFQNFKNTLKSKALDAKSINGF----------ERLLPLLS
VAHCSEQKQESKEEQQHQQQSPF-------DLLVYENLAREYKNVITQDNYDGFRLEADR
QLTKNLQASHSLYLGTLLKDVGYIYQIGANYASDDGKKFLMFKVGLDGNIALKAFAKLGD
RLELKAVTNSRLKIDNQSTFEVGADYLSDYWTATLKCCWQGTFIWNICYTHQILPCFTVG
SEVTYIDANGASIGSLSARYVRGDNIFTCQLTRQPDFKRMDFSKKEINCARVQYTRKVND
RLSLATELELSPSIKESALRVGWEYLFRHARVQGNIDSCGRIAMQTQDYNGFGVSGCIDY
WNNIYRFGFMMHLLPQPEQNQETP--MAEEVPQFKLLLVGDGGVGKTTLVKRHLTGEFEK
KYIPTLGVEVHPLKFRTNCGTVQFNAWDTAGQEKYGGLRDGYYIKGECAIIMFDVTSRIT
YRNVPNWHRDIVRVCENIPMVLVGNKADVKERQVKAAHIQFHRKRNLQYYDLSARSNYNF
ERPFLWLARRLLNKPQLVFVGECAKAPEIAIDPLLVQQSERELEAAANVAIDDDG-DMMV
EKACSFVESSFHIIKGAACTAFSIPTARSTKSVKRLANYNGIMTLSKLNNIPSDGDYNLF
KKLIEDKIAENARFRVINVDRSMAESLYGESIYDDFVVPATVKTLRLVVLDEWNINANIH
NVLYSTGLIREICINKFKYKPETNTLDIHFNVIPASE--ELLC-----------------
----------------KEEDINKVEDCPLKENVLPPGEVEYNSA---QYLNIIDSS-KTD
VVDYNKLTFQFGCSLITNHILNRVKALVGN-VHPFLRRKMFFCHRDFDQLLDNYEKGNKF
FIYTGRGPSSEALHLGHLIPFIFTCWLQKSFNVPVVIMLSDDEKFVFREELELENVRNLA
LENAKDIIAVGFDPELTFIFRNTDYIQHLYKTTLKLQKKTTFNQLRGSFGFDYSTNVGMI
SYPLLEGAASFSESFPNLFKEDIMCLVPQGIDQDPFFRLTRSLAPKLGLKKPALIHSKFI
PSLLGVNQKMSSSIE------------GSAIFVTDDAETIKRKIHKYAFSGGRDTAKEHM
ELGPNLDVDVSYQYLNFLVESDEELEEITQKYKSGQMLTNELKNKLVDILVPIVENHQRL
RSQVDDEMLRKFMDPNRESFRQFFK-MD-WAPRQIKPSIEVVDLRKDRMDFILLNSDVST
ANAIRRVILSEIPSLAIEIVTVLENTSVLHDEYISHRLGLLPIDSTLASEFEFRDRCQCT
DKCAKCTVDYTLDVSCNDSNSRLVTHFDIVPDDTGKNLPMPIPRADVTNFNGATDGIPIV
KLKRGQSINMKLTASKGLGKFHAKWIVANVNYKMEPRFSFNSALMEQLSSDEKAGIAASC
PRNVFKYTHSDKTFKLDLQVVNKLDCIYCDECINYCRELGHKDLIRIQPDESKFHFTIES
TGSIPPEKILEIALIVLEKKLQDLQSNFEAQSRTLGSTTTAHHREGPRAPQPSIDLDMLE
DKTYYKGDDYGDTGVFVLDLCLHECPGLVNVMKHHAHLKPFKGVQISGCLHMTKETMTFA
RTLVELGAVVRWCSSNPNSSNNQAIFTTDKKGKITLFGYKGESIQEYFWCMYQSLNWADT
SMPLLLVDDGCCSYNMIHYGKKLEDMYRDTGKLYDLSVLDDNDTRALLTFLNHLVTKKPN
FFTNLVKRTVGLSEETTSGLTEMRKLYRNHGLLFPIYSTNDVVCKTKFDNNYGARYSSVD
GMHRGVEVLLGGRQVVVIGYGNTGKGVCMGFRGAGAIVKVCEADPICALQCVMDGYQVVL
LEDVVETADIFVTATGCIEVIRLHHVRRMKEGAILGNIGQGDREILIHEILTDPNLEVTE
VRKNVHYYKFKDLNKVIILSYGRLYNLGCANGHPSLVMSMSFTTQFFALSQLLENKG--K
FDNKIHKMPKKLDEMVARYHLEFINAKLTVLTDRQCEFLGIDKDGPYKHEDYKYMYPYSS
TPKTYDSMQFNPSGPVDPDYMA--EVASPRGESHTPFVGFFSSKLLRSGFTLQTVTLSLM
FVFYWAFGGTGIFVFDLYAAPESVKVSKPFHLTVSTLMALYLLGTLYIAMFQVFVTDNSK
SVRGFRAGSKILSAAVTLDLLSNMLRLVQYVYSYFFMSMKWWTRYQQTKADWIFFQFGSF
SNSFALVMYGAAFFYLEAYHDEGTSEEVAWINLTLFSLAGLAELFMVFTGYGALFSLFLL
LANFSGTFWAFSFEPLLDKWSPSLHSRDVNADFVTDCPNQNNDPNLNYENPNQPGMDYHN
SA--------------------GMNYQNDSNPTNTGYQYQEGGFSYPFDQGYNEFNPFNT
YMDGRSILKKRTFRTFKYRGYELEKLLEMPIEKLTELLPARQRRRFSRGVKRQSLTLLNK
LRAAKKDLPYGQKPEPVKTHLRNMVVIPEMIGSIVGVHNGKQYINVEIKPEMVGYYLGEF
SITYKPVRHGKPGIGATHSSRFIPLKMAPTSKLTPDEDSIRMMLTAKVHIGTKNVENKMR
KYVYSRTQEGVHLINLAHTLEKLKVAARAIVTVSNPEEVVVVSARPYGSRAVLKFSHYVG
SHPIAGRWIPGTLTNQITQKFIEPRLLVATDPRTDAQSLKESSYVSLPVIALCDTDSPLN
YVDIAIPCNNKGKESIALMYWLLAREVLYLRDQLKRWMPWDVLVDTFFWRDPEQFEQKPE
NTEDLMASRPNVDWSTVDPSHANSDWKVMA----AGHEEWGSFVDTRQWMSRSKLSDFNA
GESYQHLGSKCFHSTPLTNIKWKVHPMVIFTILDSYMRREDGQYNVIGTLLGVVCEANTV
```

EITDSFVDRHSLTDEGLLQIIKDHHENMYELKQKINPKEQVVGWFCTGSEMTELTCAVHG
WFKQFNSVSKFYPNPNLYEPIHLLVDASCDSASMMIKAYVQLPLTITKDACFQFHEVDLE
LLVSPSDTAGISYLLKSLDN-SKSRNPHNSDT-----PNTFENSLLKLKDLIDKCIKLVE
NAMEGNSKIYPEVGRFLLKTVSTEKLMNLKKIEKICELSLQDNLMVTVKIHIQTWQTYNS
RSLST----MEVFVALLEALSSSDNSLRSDADAKITTLKNHDLNGILRLTLNVMLTEPKD
ERRLQSVVLIRILL------DVSRSGDAPHNTWQLVSNDVKSLLKSSLLKSIESETHQSI
RRNVCDTIADLVSRSMSPGEWPELSSITIRLIQNDNPLYRKSGLKLLGECFSYFAEDLVS
KSKEVAALIKTSLMSVDTGVRTEAICVVGVAVDYEEVGVSSHLRDTAPLILDSLRRLL--
GCTEPGARDELESSLTGVLMILENNAKFFKPHIELFFTRMLELALAEGPARG--LDGELR
ALALELLLVLPEKKPQTALSVPNFGLRMVNCLMTCMLDIQDESYAEWLET--GNEDDTHT
LYSASEEGLDRLGRALESIDNCPFMDWILSTASQYLQTPQWQYKFVAIMAISQTVEFLTD
DQ-IDRLSSIISIMLEKLTDGDYRIRFAVCQTIGQIALDHQPYVQLNFHEEVLPPLIKAF
EDPSPRVQSHALSAFINFAEEVQKDHLLPYSDVVVQRLLAKISANTSRSVTEQAVTSLAV
TAGVLEEHFIKYYNTIIPLMKEIITKCITTEERTCRGKAIECISIIGMSIGKDVFRNDGI
ECMNALIQIMEQPSESDDPVREYINEALGRLCTALGNNFVPFLPKIVPLLLGELTKSSKS
V--GDQDVTLMMGLDGG-AGLRTSLVDELEQTLSLIAIIVDELKELYEDYIPSTAQAVLP
LLTCVLTAELKQKALSAMANMIEAKRIAIEKRNSGKEMLLELLLNIMNAVLTDLEKSRLD
SEYAVPVDILSVSANGLYRCLDCAGPGILNQNVLNLLTTKLLLITEKSSKIKAIYRKCRA
SKDLDPDEILALEEDEEAEQTFRSSLLDIFGVVMKHHPDEFMKTCHPQCLQFVCMNLEKN
VPDDVAIALYFCGDMIEFLQSRVVSFWDKFLPHVLNNIESKNASVRQYACYGVSRLSKLP
EFAHLANESAIKISSALKMRFPSSQ-KDQQNATDNAVAALGDLIRYQGNNLADANNYLTL
WLKNLPLKQDETEGKRVHKELMELVLSNNQTILGADNSNLGQLAKIFISIYETDFSTEEL
NTLILHLMKHLGQDFLKQLS-PTLSKRLQMQLKVIAKSLRIMPTVSVLKDEFFHQLGTKL
SLEELESLCFDFGVEYDGTTDENGRELIRIDLPANRYDLLSLEGLVTAFLCFKWNLQPPN
FTLAP----NKPPNYSRIDVQKENSSIRPFVFCAVLRGVVLNENRYKSLIDMQEKLHQNL
CRKRTIAAIGTHDMDTVTPPFTYTFERPEEIIFSPLTDSREYNALELMEVYDSHQQLKNY
SKLLKGAPYYPVIRDSDRNVCSLPPVINSHRTRITVNTRNIFIEVTSTDFNKGSIVLNQL
VSSFSKYCDEPYTIEPVLVQYDK--------PFTTPDLSRRSLRASVSYLSKLVGIKELT
AEYSCDLLGRMMVKSLPV-DSETIEAMVPITRSDIQHPCDLGEDIAIAYGYKNIKRNRFT
MGNLLKKTLLADKVRFVFTSCSFKETLMPVLDSFKSSYEMMCKPFPKDNDK---------
-RAPVVIKNGQLSENETVRTSLLPGLLKTVHYKKGSNLPIRLFEVGEVVWRS-EDSDVGA
KNNTNCGCVYANT-SSGLEEVQGVSELLLNNLGFISEYQVWEYNEFDKPIPESWRYKLEE
IEDPSFLPGRCVSFVTTSEPQTFGTMGIIHPNVLKNFHIPFPGNISPYKYLFLTI----M
------ATLGTVSSFGNIKGPDVPDFGAFKVSNELFGWKNKRTGEVLQHRSSDVSSITFV
KTNSNLYQLRIELNESKQFKVLRFDGFTEKNVLDLSKHFEENYKMSCDKDEVSCTGWHWG
TYEFDNTTFRLRINNNSGLEIDAQSIIQATIPSKTDLAIELKNAN-PLNNSDDLVEIRFC
VPSKEDAEIKLEDLKQTFLVKSGLDEMKSEKIALLMDIPLIVPRGRYEIEFTKRSIKLHG
KSYDYTLLFTNIIRMFLLPKPNSPYINFILGLSQSMRQGQTRYAYIVMQFESDHETKVDL
NLQDNDLKQYKLDKVLEGKTYNVVSRLFGSLVNRSIVVPGDFKSEKGDSAISCTYKATSG
HLFPLNRSLLFIVKPVIFIRFEDIVSVEFSRTGVVTQNRFFAILVSMRGGIEYEFTNIDK
TEFKNLNEYLMTKDIKVKTSEET--------ERVEQTNYEQEEEEEEDDEED-----EDF
QDESEE-------SEEEE------MPVSKLNLGDCRFYEQKYPEPEDLVMVKVNRIEAQG
VYVSLLEYDDREGLILLNELSKRRYRSINKLVKIGRHEVVLVLRVDPLKGYIDLSKRRVT
PEDIIKCEERFSKSKKVHQTVRHIAQKHGISVEELNRECIWPLYKSYPHALDALKEAAAN
KDNVFKNISISQEVIDSLLQDIQLRLVPQALKLKCSIDVWCFGPEGINAVKMSLGKAKLL
---DPQISIRLIAPPQYEILTSCFDKEAGLALMNQTLEVIEKNIKSFIGGDFKQKCDVVV
I-GDDEKHLEDLLELHETTDEEDEDEEEEEEDEGMGDESMLSNEMEEEEEMDFSTLFKNP
EDDQKNDKEDPLITLALDVLKRDSEKVALLRLKGFERVDIERSSGKHSVVCRHWLKGMCM
KGEFCDFLHQLVYSRMPPCKSVEKNSFCTDRLKGCCIFKHTGDDESTDFLGSKELDHSDD
L---

---------SYKNDLEKFTHAFNSA----LLFA---------FPRIV-------------

-------M------------ENVVIRDSDEWDSIEFALQLRCRSSRVRLLQAWNIIRPES
LSMFNRRSKNMLVLEAFVDTESLDRSNSVQDVCTRGFDIGAPGFLVTFGNLSLSGFPLLS
TDSKGK----TLLDNETCALDPNGNPLPSKTPPEVSSSIYGEKRVFEYFVCDVGVGKSLE
VPGVVEAQTSRVSMPVEYDSYFLTNSEN-LRLDSLTVFAGSNE-----IENGALDVSEEV
QSKGVLPQNTFKHNYIIYDSSQILPRYLIQFEFDPSADESFALPLCDNCQSDVSTIYCPS
DSARICTKCDVRLHSNNKVVSRHIRVPLSEMPRPYTKCKIHQTKSYHLYCTVCETPICQL
CTVNHIHE----------LEGSTSFIPISTAYEAVVNLNSTTDVSLRERKNQLLEILNKV
DEVKNKVSENCQNVEAQCYEKLESALSDLNRMVECSLEVVSTEQTENKRQLNEINWSESF
VDHMRSTLLPADYLRSWLRHCRLRDEFAQNS--SQQPLKELFPDISLEAELSIIPKDTHA
HLQ-------TMAPKKNQPSEPKVLLGRPKNNLKLGLVGLPNVGKSTTFNLLSKQCVPAE
NFPFCTINPHEAVVSVPDDRFDHLCKVFAPKKEISATVTIFDIAGLVRGAHKGEGLGNAF
LSHIDAVDGILHVVRAFEDDEIVHTDGEVNPVNDLDTINQELILKDLDKCNKAISEVDKV
YQRNMKIKSKKEELDTLIKVKEHLESNKWISQGNWKSSEVPYINEYNFLTAKPIVYLVNL
SENDFVRQKNKWLSKIAKWVQENNPGPIIPYSAQFEQSLEAFTEEALKSYLKDKNNAVSK
IGKIITSGYHELQLIHYFTCGPDEVRCWTIRNGTKAPQAAGMSNMNITRGFICAEVYNYQ
DIVQFGTESKLELMGDICRRGKDYVVNDGDIIFFKFNVTASKK-SSVSTLIREVEIYRGD
SAIPKSLVPDPE--VRLQQIVDLLTVSLNDLKDVSHNFYSELMHGLKAHRRHRNLWLPNE
CSFKMLDSFIPNIPTGKEKGSYFALDFGGSNFRAVRIVIDGDGKMERNQSTFSLRY-SSA
LGPKGLLDQKATATELFDHFAKKIEHVMRESGVDPNPSVHKVGFTFSFPCTMLSPCNAIL
LDWTKDFETGRATNDQVEGKDVGLLMNEAFKRNNINAEVSIVLNDTVGTLLSCAYQKPKD
YPPCRVGVILGTGFNICYEEDEFRRFGYVGRVINIECGNFDTELPLNPVDFEIDFYTSNR
GRGKLEKLVAGAYLGEIIRRFMILYLREQAPPKMWEVGTFTSVDASEILNDNSDDLSLSR
QVAMRAWDVELPKKSLIALRKISEAAFGRSAGFAAASICATARKAKAYVTSKTTVAIDGS
LYVKNEWYRNKLQYYINVTRPDLVGNVVLLSSDDGSGKGAAIAAAMFA---------AFN
CYLFIKWLCRVVISSIFTNITIIHEERLPLYGPLIVVSNHNNQFVDAALLIYAIPRQMCF
LVATKTLKRKLIGTLCSLAGCISVYRPDDFKYTGVGKIHWKKGTNLIHGRDTKFTLDLNL
GDKLQFDLEKIVITEIISDTQLKLETPLQLECPD-KNGVQFSVIPKMDQSETYEQVSASL
KHGNAIVIFPEGGSHDRTNLLPLKPGVALMAFFSILDGAEDVVILPVGLVYNNVKKDQSD
ATIYYGNVISITREECAEFERDRRSVVTKLLGKIEQEINNCMITAPDIKIKKWIDLCASL
YPPERSKVPVNIAFDLRKFISKIFWKYGDSPETLELVDKLSVYKKRLDNGYLHDDEIWLL
KQSMYSAFLSFLEDTIRLICYSIIGLSFAPLWVPLYLLSNYLAERHRVKALKNSSVKLVG
TDVFVSYKCLVLIVVVPLLNFSLGLFIGLYFYRLVN------------------------

--MTEDSSSIRERILRKLSISH
TSSYIPEHKFWDTQLVTKLTDVVNSNDYGPIDSNEDISKVKKNPIALPNGFEWVSLDIND
EEDRNQVYKLLSENYVEDGDALFRFDYKPEFLIWALTVPNYNKEWQIGVQVSSCKTLIGY
ITAVPVNVNVIGNTLKLAEVNFLCIHKKFRSKRLAPVLIKEITRRVNLSGIWQAIYTAGI
VIPKPIAKCRYWHRPLDIKRLISARFSGVGRRMTISRAIRIYKVNDIPNVE-MRPMEQKD
VLSVHKLLRKYLQKYKLYQEFDVHEVEHQFMPREDIIQTFVKTNEDEVTDMVSYYSLPST
VINNRKVHTIRAAYSFYNIATTMPFKSLMEHAIFFAKSQGYDVYNALDLMENSLVFKDLK
FGMGDGDLHYYMFNYRVPDLKSTDVGMVLLMSLINVTNIKIGNNVCNIKLPLIFQIEFEC
LEHLKHDVEWKVIYITSNSNNSNNKGEIILDAVCLGPLYKGILEFEFRVNPPNFHRLNPE
CILGMQAILISGNYCEQEFIRIGYYTNNVYDEESLVENPPDLPILDKIVRCI-IDQPRVT
RFPIKWDNDYLV-----DFEGNNLNFIIQTDSNNTEDTTSPTNSNNNS-SNSSSELTTSS
NSNEVDT----TSHTTEDSTNSSGTSEDSSTVGPSTVTEEENINTAAVTTTRESGTFSED
MLFYIM-----FYRIILFTLSIVCDCTVYNNYNISEKNSNIIVSLNSSNFSQISPIEVDN
VENIVKFTRLVSEAETIHLKLSNILSDYYFRIFLVNLDSNCKSKPRINSCPSVAKCHVDR
CNSREVPLSIMGNRKENYVLGRGTD----GFGFN---REFLNRSNL-VQDNCVFVDLLRN
PPSYTGYSGQDDWRLMHEEI-DKCDNMSCKESLHFYKLISGMRGTISAFSALNYECTNPY
---EAYEDQTE------LPSYQSNYHYYYNKLSKHRDRLENIYYTYNYILSAVVRLKGYL
DNFNSNLQLVNNDLYLH--LKDFLNISTDSNGANHNN--LQTECGKEMVEKFDRLIELVN

```
CVECEKCKLHGKLKLSAIQTSIKILDKLGELDLNRNDLVALFHGLDYFAQSILIIERFEQ
HKKRRIMLYPLRFLLGCLLFIVVIYKQEILSPIFKL-------MARNSEKANAMLNKWLR
IKSEQEQTLI--RPRHTAEVTNLKEAEKWRSATIKEIMFNINKIQDASLGEFVVRDLNDE
INRLIGIRKHWDDRIIELGGTDYRRLSANLESNYGSELKGGTGYKYFGAAKNLPGVRELF
EKQQKEVEQSL--KDVSRAELYQMINPDYYGFKDELDEDLLIQEYNKEQELLTNS-----
----IMGIFRSETMVHGTLVIPHERARSCIDLLSRHTNIQYIDMNERRMDRPYKKYVQRI
DHMERMIRVLYEEIAKLPNSKIVRHNIDNFLEHDNMYRLDQVEESLVKLYDQFQMFKEND
SLLRLERDEALSEYYVLLVASKQLSPDRSFISLPVTNLDESSDSREHLLNDNTQTEMINL
SPYDLSSRTSSSISFTNIAGLISSQEKEAFSRAIFRAMRGNVFTLLHDLRAMVLSKGLVD
QE------------------ELDADNDKTVFVIYCQSSNNNATYNKIKKLCTGFQAKLFN
WCKTQSELAPRLKTLEDVIKDKKRALEAYKEYFRSEIACLLEVIRPGGNSVIEEWFLFCK
KEKYLYYILNHFEGSDITLRADCWFPADEEEKIREHLLAEKASGSVSALLLVDIQAPLSH
IPPTYNKTNKISKSFQNVVDTYGISRYKEVNPAPFTVMTFPFLFGLMFGDIAHGFCVILF
ALFLILYYRKLKRKFSGDIANMILEGRYMILLMGIMATYAGFIYNDFLSLPNSFFGTGWV
SNGTPPEGGSESDGTYV-ETLVKSAKNFPVVFGLDSAWIGAVNEQSVLHSFKMKFSVIFG
FFQMTLGIVLKGFNAIYFSSVLDFFFEFVPQLAMMCSFVGYMNFLIFHKWLTPVD-SGYA
KPSIITTLIDMCMMKTLEPHEIMYEGQQTVQRVLMIILILSVPMMLIPKPLILYFTIKKQ
GRTRTNN-NSTRDYEMVYC-------GPELEAIARENVPNYPH----RRSSLDLGVDKFK
KV--DAKNKDNQFSVTIQKDENEAVP--SEPHHAPKLSELFIHQFIETIEFTLGTISNTA
SYLRLWALSLSHQQLSLVLFKQLILNCLDSSTLFVMIFGLFIRSIFFSVFTFFIMLCMDS
LECYLHALRLQWVEFQNKFFKADGRFFRPFNIKLLLDDPTMLGLQEGRVY--NTNVSRGL
GDEYLKLPNKLPSLDNVYTYFSSFLNAYSSTSLQQGKLKRTLLENLKNNNYLLELKLEDL
FHFQNVMDANNEDVNSNNRNSEKLPPFYVHELAKVLTQRPLIYLSTIEKVCYDVCKMN--
MDYEDEKFNFIQINLLNNYASPTPIRSLLSDKQETFVVVPGIIVQANRTQHKMRVCTIQC
RYCGHKMIIEVPLWISRPQIPKTCRYSSTMK-TMGENIHVDQQLGCHSVQNPYVIIVNEC
QFVDVQLLKMQELAEDVPTGDMPRHLQLNVTRYLCDKVIPGDRIYAHGVLTSYNNS--PN
SHTDINSSYLHVLGIQKLN--VNETYEFDIDEGNDLLLLASQPDIHTKIFNSIAPSIYGL
DDVKKACACALFGGTRKEVGNGAKIRGDINILILGDPSIAKSQILKFIDFIAPISIYTSG
KGSSAAGLTAAVVRDSMGVFSLEGGAMVLADGGVVCIDEFDKMRPDDAVAIHEAMEQQTI
SISKAGITTILNTRCSVIAAANPNLGSYNNYQDNNEQHDFKTTILSRFDLIFMLKDNEDI
NHDKLLCKHILSLHNNQNKQNVVPISNNKLRRFIQYSKQVVSPILSNEAKDSLRNFYVQK
RKEYREDKRSSTKKIPITLRQLESLVRVSESLARMELSPIASEKHVQMAIQLFIVSTGEA
MKSTLNVDNMSLDDQHKIKLSEELILNIVKKGQRTTRRFIIKELQKQYINMVYIQQMVGN
YRNYSRTSRNPKRPFEKERLDQELKLIGEYGLKNKREVWRVQYVLSKIRSAARYLLTLDP
KDNKRLFQGEALLRRMVRYGLMNENEQKLDFVLGLTLSKLMERRLQTKVFKLGLAKSIHH
ARCLIRQRHICVHRQLVDIPSFLVRVDSEKHIELALTSPYAGARPGRVRRKTLRLQKGGE
QSEE-MSRASHSGYDRHITIFSPEGKLFQLEYALKAVKNCNLTGLAIKDDSAIAVVAQKK
LPAQQGNQDVLLDTSSVTSLYHITDEIFALLVGLPGDCLSILYKARQVALDYSYKYGINI
PASVLCQKISDLNQVYTQHAYMRLHACTGLILSIEPDVGPKIYKFDSSGWFAGYKACGIG
AKEQESENALEKTLKRREAMSLQDAMKSNLHQETKVTIEALRCMIDIDLGASSIEVAVAS
KDNPFFRQLSEEEIETYLTHITESDMEEQILAELESSQNDHKTTHSSNTIHNDTAETSDA
DAMDANTIEN--SIEHEMDNDVNSSEPVDYEQIECDKQDLPCSPAPPVEIKLFVARIPKT
HEESDLRRLFEEFGVVKDVIVIRDKTSNAHKNCAFVKMASICQADAAVRRLNNQRVIDSS
LGAVQIRYATGEVERLGFTQMAEPGVDEAKLFVGSLPKSLTEEDLSSLFKEFGEAMEVFV
LKDLACGGNKGCGFVKMKYKEQALHAIKELNGKKMLEGSVRPLEVRFAMNKTGLSQDFGM
NHPPPSNPPSNSPGQKHVRKDKNFGYVN---YNNGNPRMAGPWKEYISPDGRFYYYNIDN
GTTQWEVPKEFLNLNSNYSNSGGNNYNNSVGGDSSLFIFHIPPQWNNNDLFRTFSPFGRV
VQARIAVDRSTNRSKGYAFVSYDNPESATQAVANMNGFTIMGKKLRVNYKTTNNRSNPY-
-------------------------------------------------------MSTAH
RPTWHNAIG----VGLEERGSTVSVSSKDLPSHTELKRRTLSELSETKKLLRERLEETET
AHKTKNLIEKNFLSLEDATKLLNCE-VNAFPEDEDDF-----------------------
---------------------------VEVDEETVDDEEAALLRELEKIKKEKEEIKERE
REEQLKSEENREKLLSRNPLMN--------PSKQARRWDEDVVFKNPQSQSQPTKYVFLN
SRKSPFLGSSYPFYI-M--KKEVEKAIELTKEAIDLDNRKEYKRSLDMYIRALQQWSMIC
KCETDTNLRDKYFNKMKQYLERAENIKSYLNTTSS--NQVPADSTTTHDNEYFNEFESLI
NKNSNEIQWDDIVGHDGVKIILKESILLPMKFPKLFSNNIINYNCILLYGPPGTGKTYLA
NALSNEFKYHFLSISSSNILSKYYGESERYIRNLFNFCILKSPCVLFIDEIDSICNTRNV
NQHEATNRIKTEFMIQINRKMTASNVVLLLGSTNLPWLLDNAIIRRFEKRIYIPLPNQNN
RFDLIKKLFTNVK--HTLTDSDFLYMAENTNNFNCYDINILVKEIVLYALKK--------
---YNNN-----------------ESLLNISENDLIIPSINIEDAMEVLKDFRPSVVVDD
IKLYEQWTQHHGTQ-LMRGLLGNIGGLI---------RSNRIRRFTTLDKIHKDPPDYKK
VPFLKEDLDSVLSEVEFNFYYFTTDKDYPYKNVPMNEPIYVTGSEGKFTPLDHKFQYAKL
ENGLRIATLDKGGLDTHLALYVNAGSAHE----NDQNQGVTSMIENMAFHSTAHLSHLRT
IKTVETLGANVSCNAFREHTVYQAEFLRQDLPFLVNLLVGNVLFPRFLTWELAANKHRLS
EKRNKVLENPDQLVTEHLHSVAWHNNTLGNFNFCLEPSEDKYTPELMRDFMLNHFYPQNC
VLVSVNSGLDELSKWAMRAFSEYNPIPNPSGEVLEPKYTGGVKYVEG-NTPFTHVTVAYP
VK-GWDSKQVVVTTLLQSILGGGGSFSTGGPGKGLTTSLYNNVLNRYEFVESCMAFNTVH
STSGLFGIYLVVNGAYASQVFTLVKDEFERMKRITNHELSGGKNSLKSFLHMSMEHKAVL
CEDVGRQLLFCNRVLDASDLENLIDEVTLDDLKSVVNELRVNNPSVVVYGKLSKVPHPDT
VLQLLHMESGIKVSEETVAKFNQMKLKKKTRYMVLKVKGNFVDVENDGEGDV-EELLTVL
PNDE---CTFVVYDKGQNLVLFMFAPSGATTQSRTVYSTTKQTVENALPGVRLHRNLVED
HDEVRDLKSNWPNPKQGLTSRASNPFPNVQNSVHQTFANTT--PPVNPPYGYNQPQTFPG
QQFPYNPNQGLVNRSGLGNATYSNPPSVHFSSSLDYPPAFSEPPPPKLEEIN-QSQTPQG
VTPPKSTSKTLTGQVFHDN------FSSTSTLGYKTD----------EDVLDSVVLANTT
KNFVSLSVGTLPATDQLHQKSGFTLSYTISPLNPIARLVETVPLVNHGNESITRCKQCRA
YINPFVRTDASKRFWICNLCETSNELQTRYLSSFNQY-PGSNQANELELNCGVIEFMASA
DYTVRPPQPPSYLFLIDVSSNAVNSRMLEVVCKTIKELILDNEFAS--------------
DNRTLVGLMTFDSSVHFYQISRGSENYQLLVVADLEDLFLPLPGEVLLNLQESSEDFLKL
LDTLPSLWKNTTTTGSALGSAIRSAHYSMKHVGGKLIVFAASPCTFGDFSITSAQKSE--
--------SGTQSSNVLNMNKKPTTKIHPL-EKCKDFSCMMCQTQTTLDLFVCP-QSLNL
DKLQYMSTMTSGNIYYHP-FKNHAENFKLVNELKHLVRRTTVWESVMRIRLSKGWKVTNW
HGNCFVRGSDLMVLPTTSEDHSYTITFSNNTSSAGKKVMYIQTALLHTNSSGERRIRVFN
TAVGVSNDLGTVLNSVNVETLVFNMLLGAVKVYQSGKMADARNHLTTHCSRVMNSILGSA
DSARVLSLYVLGLLKSTIFTDEHN--QDYRVYLSTKFRSCKIDQVILYAYPQLYNLSN--
------ELNSQELLLQGLPLSIESVLQECFYLLFNGEYLLLWVGKNVPNNLLQTTFDAPS
FEHLNVALVPHALEQSTSNNAHKVKVCLETLRA-RVPPYVPLMVLKQGES-DSLFYSSLV
QDKTHGMMVTFQEFYNSMQPTSALGIKR------------MDAKIDIWIEKYRPSTLEEI
IGNPEITKRLQFIAKEGNMPNLLLCGPPGTGKTTSVLCLAREMLGSHFKSAVVELNASDD
RGVDVVRENIKNFAKKSLILPANKHKIVILDEVDSMTEPAQQALRRIMEIYSSTTRFALA
CNQSNKIIEPIQSRCAVIRYSKLKDEQILKRLVTICDLENLTYTDEGMEALLFSADGDLR
RAVNNLQIVSAGFKIVTKDNVFKVCDIPSPDLIQKMLENCLNGNWRLAHEKVNQLLELGH
SPVDIIVTMRSLLKTMDA-PEHVLLEYIKVF-------LNNFSRSCI-------------
------M----------------------DEQIEDSM--------------YLKVKRRFF
ERI-GVIFSVFNIATFLEYFNLQILPSSMRGLEMSLYFTSKDNSMLSMAENLGLVSFIPI
WGALCDKIELKYILLFGVIATGLINIWLSTISNYSLILILRIFNGGLIGSVTPSAQKFIA
TNMQNK-LPFGFGILHAVMCLGRMISGLIATSFSTEVYRDIYGWRIVIFIFGATSLALSP
ILLLVVRRIDNNK-ITEQIRIGD------------KIKRLVYYLL-----FITKESLNNA
TSLLLALLNFFSDGPFVAFNYVTLLIQYMRLSNVVSAVTTGLTILGGIIGGIIGAFISGY
FDRKWPKHGLLNFGILNVGIRITTFMISFLVIDVTNIDYGLLAVCLIINGMTFMTVSCVD
RTLLANVVMPSVHSSSISIIRCIGGVLSAVIFNPVLAKLNESVFHFQSSSLAVKHMPFDL
IKKNSDALRYSISIISLGTTGVVLLLYIIIHFTYGKDCEKIKKRIEKESGLFEI------
-----RRAPDSGVSNAVSTIIEHQKFKKMLVFGLRSLSDLCNPSNQLYVENALEALERNV
VKGILTAVENFGDDEDLVLCSTTIMSTMSQGCVEYEDEKLLKKLVNEGGVDVVQKALERA
PQDEDVLENCLKFLENLSNLGGLGKRGPEFVPHVCKCMEVVKSYRVANRLVVCLSNLTDQ
NESCLALKNSQGVQPLLDLCLKFTN-QDKSTGLVESCFKTLVALSQLKLVDESNLQSVIN
LVECCKDSKVVLSRASEVIKSVVDTEKLQNSLKVLEKNQYDSPEYKVAVNTLRSLSYIST
```

LSDELAKKGVIPILLKLLSSGDAKPGASAEQLAEVVFGTSRMLASISSNSEYGQQVLSNK
GLDVLVRALSQSTQHPRSVVGLSFALVQLLKHE--PKSF--GSAVSVALPILYQLSEDEA
VSQALVEFLLACSQYSELESVFIQNKVLEILSTCCQYHTSNLAYQFNVVSILNRFSRFIE
NLKLIHEYGGLQGITFALEQNYKDLKYCLEVLNFISALASTSNSQKYLTTGDIADVILEL
MLHYRDNDAVIDLSVKILDLVLEEKDVEKYAKRLNTAMPNSVKDPEGTFKALAALTGVHK
ISRLRPLLSKYNPLNGVLSAVTGWLDHNGVRQRTNLTRAALSLVVVCQ--TKEEIQETLF
TVSELACVYQVKKVIDLEQSDDNFLLHCTGAIVVLCGIDRTYDEAEMQESVECVSKVMKR
HQDVRQAQASLLEALNRLMEQSDKLFLSAMLNTGCLGLIVKYLSTVPVYLNLQILGIGLI
HKCSQLDPQVVEFLKTSNCFQLLRTVNRTHTKNKKLKAIVGSLLSLLMPADALESELDQL
LKDLVNYVAEKDAEGVNTCLVSINQLLVSKEAVKAAVMLNIYSPVKKALDWTLANPNQYN
GE---------ENIFEPTLAEFSLLGLNMLVSRMGLVYATKNSFTKFFLTLFTCLTANSK
VMMEDGVVSNLDGLVLLFRHDVTNIDEALKEDLLAKLSGCFTRILNSPAVVSSVCRCLGS
MCATPGKLDKLLNNSNFSKFCNYLVDNLSSPQEKEKVVETTLVALNELLSTKYQILIDYF
DKNTKIVSSLVGLLEEYYKTYEIVVLASNLLSYFDKRVVLSRL--SNLSSFLETVSKTLL
YNKADVGTVIALLTLLVHLLDDTNKEEFKKTSVVENISTVMLMHLENDEVSRLGGILFSL
LGAECQISALMKNVIQVQAKEENMGQKVDKLCLMLAMYLSSELKERSEALKHTEPFLSSL
NDCVSFLADNSNLLSSTCCVSRRLCDSAFEDHEDPFGAWAVASSSNMAQISSLVKTEYGY
SNLKFLVHAFRVFTACVYNGYTSDTMLGEAPGLMVRTVQVLERYKENSELVFNVLEYCMY
LANAPASTTPGVQVLLENFGNPVDLITSALTVNKNNEFLLVIGFNLLAKLVSSTKT---L
DANQVSSSVETCLKLTKTVSTKGDTVDSSHLGKNLMDSNGYDNYMSGDVVSNMVMFEG--
---SNFEGLSP---VQFALFVLNSATAGEMAQLERLGVVQKLIGLEGLLQNNKNKDELLQ
LFSGLSDAVKRDAIKAVLVFKEIFPKL--KGNEVLTSDAEVVSLLLNTLIAAAEVKGVGS
LLHSIDQFVKYLD----LVSNLS-----------PDLVTKLKTLIKKDLPKKKTCETVYE
NLTESVSSDYSSVTLENALMMEDFRFVSDLLLKYNGKTQKSGDPECRDFGFGFKCVDVWS
RCEGNPNVMAKSGLSALLISALAKQSDLVLTDALLVSICSCCRQKDLLLSF-VSNKDLVP
NADAFLAKAYQKRLVIEAAYENVLFNAMLLIDLTAVNRKVYLKSNVIPSLMEVWSLHDQN
KYSSTILRQVFRTLRKIVSDQNVQLMVKCKLVQRVNKVFSQLSVNEGLIPDALFLIGSMA
IILEIKEEICNVQLLEKIVTVLVRYVKSGSSSAIVTNCCLALANICVDFKTASDKFCSLN
GPRLNLDVFVNFKSNFEVANGSSILLCNLLYKNDKLKELYGKNGTPSALVECLMNFVGST
EPTSLRCLQSLFKAISNLGLYTKNLQYFMEAHIESPFKKWLNKVSHTKDDVELKVGLNCL
SNLVVENNGDYMRSFGVILEDLVGMLNLEFGDGKVVFLVLDVLNNLCRSKENAGRFVDLE
GVFATVKNMRRLSYDVEVLVTGIHLFRHMAY-GNGAVKLLEVDIFSFLLDIL---VQANE
LNDVVISSLRCLRRLVHSPEMVYLLCSQDGLDTVIKCSTRMKDQSSVLVESIRLILGMLY
YTDPYTGVSSTKQSDSDESTGLSDGGWENIGMTATSIVEVIKFSCYVASLESCLKMSRLQ
SSVVSLCVYFMSCGLCGEELAMNGFSLVLENFISSFCLTAPNLALLAIALEASFNYPPDL
RNSILTKPIQKKLRDLTLVVTDKQ----SKAKLTKLLEHVSNNTSP-----SVIGKFDLG
LSEWNVDPYPNGVHDLPESMKEMLRNGGKFQLITEGEKEFEYSWRSSQDLLTLEWMH-DG
LQEKNKVAFMRVRNIARGLKHDLLVKANQKDYKRVSNTNTLVLLGSSTEEFPQGFALPMV
FKNNHEREAVAEAFIQWRDASSFN-MSKEAQVLARKFVDFLNATGSPFHTVKQLSLYLTN
HLIKHLNESENWKLENGQSYYVTNNNGTMMAFNIGKKFDPSKGGLILVCSHTDSPCLKLD
FKCHVNNKGFNQLSVTTYGGGLWHTWMDRDLGLAGKVVVKSNGKLEEKLLHVQKPLILLP
NLAIHLQNSTERE-ALKLNKDNHLKPLISTEVVHNLNST---------------------
-QTEPLLKLVSSELKCEVEDLVDFELCLMDSNPSCLSGVYEEFVSSGRLDNLGSCFGSIS
AFTDFVLNQG-------EDNDAVVVTVSYNYEEIGSSLSYGADSNVTFLWLEKLFGALGC
S------------LMETRDRALVVSADMTHGVHPNYSEKHISTHSPAFHAGVVLKWNVNG
RYATETHSSSLLRTAAA-----SAGVPLQEFRVGNETPCGSTVGPILGSRLCVPVADVGF
PQLAMHSCREMCSTVDLLNFKLLLQVLLFMV-----------MSISHLVLYSLPALIVIY
SSSVKASLRFASLGNWGTGSKTQKLVAEKLKEYVKNERLTYLLSPGFNFNNGVNGLNDEK
WKKYFESVYNDDSGLMDLPMFTVLGSEDWLGDYNAQYNRYHQFYLNQD--LSSSDSNNSS
HNPRLIMPNWWYHFFTSFSTNASVSLLKSGHKDLSVGFIFVDTWVLSNQFPYKDVTNDAW
NELKKTLEIAPKVVDYIVVVGDKPVLSSGKSKGDTFLSYKLLPLLKQAQVDAYVAGYDQD
MELLDYEGTALVVCGSSGNKGRKSVLKSPHSKFYTEEPGFCVHELNAEGFTTKFVNGNTG
EVMYTYVQPKKKRKQRQQGNELKLINKLPDVMFHPVGDLDVASYSDAFTKIIGTLGLLIL
GFHLLLTAGTTVAKT--MESEVLRGKLIVFEGIDRSGKSTQINLLSQKLSEENIKNEVLS
FPYKTTPTGKLLREYLRNRESIPKQALHLLFSANRWEVMNRIIKLLKSGTHVITDRYAFS
GIAYSVGAEGLDFNWCLIPDTGIVEPDVVFYMDLAPECSSARGNFGSEIYENLEHMENVY
EVFKRFGNLSYWNIIKAHDDQKQIHHKIYSIAKKCQF----------F--MGMRKCEYCG
SSEIEDYTHLGELVCQDCGAVLQENTILEQVEYSDNNSGNTQVLGRFVSNLSSGRQALTH
TTWHSREQVINRGNENIKKIAEALRLSPHHIDAAKRIYLLAVQRNFTMGRNNLHVASCCL
YTICRRERTPHLLIDFSDVLLTPVKTIGQIFMKLVRMLHISVPNIDPSIFFERFATQLQL
KD-IHKIINTGNRIIQAMNRDWLCTGRRPTGLCGAALLVAARFHGISLSAESVSSVVRIS
HPTILKRLSEFKVTSTAHIKVSEFDNTDIDSLPKLTLPPCLLNKLARKKKY---------
-------------------EGSDYTSECVSTSSDYDRVST----DYERTSVRSGINS---
-------PMNEINTGLSTDLLCTDEPTPNQINTIAQSILSSINSMS--TPTKYSQTTL--
-------HSNTIDSI---
---------------------NSDSTIVD---------------------ANEDNGADSP
LNAADSNLNPVESVLS---------------------------------EDDEDDIAMFK
EMILPENERKMKMILWDEVTKDIMPKVYRRQMERKRREQLGQNVKKRKYVRR---VYSEY
PEAQDAVESARMALERHAKGFMNHVNKDVFKSLLSTM---------------SFEEKIDT
NLYSRQIGTFGFDMMGKLQKLNVLIIGMKSTGIEIAKNLALMGVESIKILDNDVVQRRDL
GVNYFVRASSVGKESI-ASACLHNLKDLNRNVDIKVINN-VNEELVVGNDVVVCCDQNVE
VLKNLNRICRANSGKRIGFIACDTFGMIGSVFVDFGDNFISFDPTGTELKTGIIESITND
KEGLVTLITDGVIDFQTGDYVRFSEIEGMTELNNEPVQIKVNSKNSFLIGDLSHYTPHTS
GGLVTEVRYPKRIEFRSYEDCVLNPSST--------------------------GCLYTI
DYSLVNRAEQLHWITMGYK-------------HGSGDPKSTLTNAQMMNSNAK----SCG
VESVDEELFKSFFSQVNFKVPPLASFIGGIVAHEVIKFTGKYHPINQWLYVDFSLPKEML
SGD--FSGRGFDERYFDQVSLWGSDLQNKLQNSKIFIVGAGALGCEFLKNFALLG-CGSQ
QEGLLTITDNDRIEVSNISRQFLFRTRHVGLSKSSVACESALEINPSIKVKPLEIRVGEE
TEDIFDEHFWSSLNVVVNALDNIQARQYVDGICVWYEKPLVESGTLGTLGNVQVVVPHMT
QSYSESQDPPETSIPLCTLKHFPYQVEHTIEWARDVFEGLFTQIPLDIKKIRQNDEVNSS
NI-DVGVTEIPYERLELISKLLN-CTPKNAKEQLLRISSELYNLHFVNNIQQLLNSFPKD
HVLSDGQKFWSPPKRPPTPLTFDLSDKIVQLFILSTTKIFASMMNLDLDVVESDILSL-R
GLRLPEFQPRVLKLSQDK-----LN--VEVQSDTSADSNPLLNEITNSNRTLNAVEFEKD
DESNYHIEFIWSASVLRCRNYAIKECNKMKAKLISGKIIPAIATTTAMIGGLVTIEFLKA
LCY---------------RSLK--ISHFRNAFACLATPIWLQSEPLPPIPTKDKDYDPVT
CGPVRALPPNFTVWNKLIVLIPNGTVKQLIDWIRSKFNIEVIILSAGNLCIYNSFLPQHR
NERLNAVITELVERLGKKKIGVRCSHLVIDASCTDSDDVDVVIPTIKFQFR---------
--------------------MMLLQ---QFWKVQRPKKETGTVVLQNAPKKNMKVCVFGD
AVHCDEAKALGVDYIDLEGLKKFNRNKTLVKKLANKYSAFLASQSLLPQIPRFLGPGLNK
AGKFPTQLLHTDKMEDKINELRSSVKFQLKKVLCMGVAVGNVEMSPEQLRANIVLSVNYL
VSLLKKNWNNVKGLTIKSTMGKPQRIYGM----------------EPEDNEVENLIELDH
LKEFEQGIKSLVSYSRNSRGHSVKITKRVKEIVVSKRIHKSVFERKNLVPFNLNNDAGAN
FVSNEEIVIELSRNER-RLNQDDDSDLIYSPNFLKLSRDLKLKFKSLKEEDNVPEEVEEE
P-PPSRKEGGERRSFDDNTIRITNLSEDIREKDLTELFGRVGRIHRAYLAKYKETQNPKG
FAFVTYVNKEDAREAINKFNRWGYNNLLLNVEWARPSKDMLNVLLCHTVISAVANNLLLV
GDHFVNSIFRFTLHALIASFLFYHELPKLGDTYFSYLGLPITATHSDVLKSYDRIQEFLK
VSWKINNLIQTGVAKLKKAYQTLSNQESRMLYSAYGDVDDAKNPQDFTIIVLTVSLSYYC
VSAILCCALNRSNRLKFAKYITVLYNAVGFFLEVELRFLY-ACFMKNIFYLNKLLPYQHV
EFLRAIPPFVMMVASLLTHSFVTDMDKLNMLLWQSAVATNGIILEKMANVVNATDYLKSL
GRDNLSLPEDLFKFGSG---
---FGEFLDSLDESQKKKLVDLLNNGQNGKAKTPKSRIIEFLKPALIYIVMFLVIRVFLK
MGKDYYSILGVKRGCNDAELKKAYRKLAMQWHPDKHQDPNSKLKAEEMFKNVSEAYDVLS
DPEKRKIYDQFGEEGLKGTAPGGSHTYVNTGVDPSELFRKIFGNDRSFMFGG--GDEMGG
FGD-------------VFHVT-SS-SMKSTNYELELPLTLEELYTGTVKKMKVTRKRFNG
NKQYKEEHTLKIDIKPGWKDGTKLTFTGEGDQQSPMATPGDLIFIIKTKKHMRFVRDGNN
LIYKFTVPLVKALTGFNAVLTTLDNRRLTIRVTEVVSHKSRKVIAREGMPLSKNPNQRGD

LILEFDVVFPETLTNEQKASISNIFN---MD-------LIFLFALFNLFACSLYFLVPQN
GERCFFENVPEKALLSVSYDLISEEGRDCVLSISDNDRRVLKNMKLDQDQHKRLSFVSPA
TDNYNICVHCPGRLWYMSQMCKISLNFEIADSDVNYETTAKKHQVESLTNHLHHFIASAN
VIQEYQISENRNSTKLYDNYKSMNNWILAFYLLEILVVVLTSAFSVYHITRFFKTQCFIG
D---------------------------TLPSKQKGKKKKGKSVKQES-DDDEFDKLLAD
LGTLESKDPNSEEQKETQEISKTL-KNKLKKEKKKQAKLQKKESQAEEKKPLSQAARMAA
EVQRKLREMEEQKREEEERLRIEEEERRKEEEEEREKKRQQRKEKRERKKKEGKPTSAKE
KAAAEMSKRFLEQFGK-AIL----DENQNEQPKKTLPQ----KKPKRVKSVQ---EETED
NPNEQQEPTSDDLNEED---TVSESDIDNWEDL---DYEKETN------------QTIAT
KPV-------------------KSVKYVVNEVDDDVDFRSPICCVLGHVDTGKTKLLDKI
RHSNVQNAEAGGITQQIGATFFPKDLLDMHCHKIDEEMYVKSPGLLIIDTPGHESFNNLR
ARGSSLCDIAILVVDIMHGLEPQTIESINLLKARKCYFVIALNKIDRIYNWSSTPWLTFR
ESLEKQPKESQLEFSDRTKQIMLELSENGLNSSLYWENDNIKKNVSICPTSAITGEGISD
LLYLLVQLTQLLMSKRLTFSQKLKCTVLEVKTIEGLGVTIDVILLDGILREGDKIVLCGL
SGPIVTTIRTLLTPQPLSELRVKGEYVKHSYIKAAMSVKIVANGLDDTVAGTELFVVGEG
DDVDELCNEVMTDISSIFDCIDRTGIGVYVMASTLGSLEALLHFLNDKKIKIYSVNIGPV
QKKDVKKASIMREKGHPEYSTILAFDIKVTQDAEKEAEILGVKLLSADIIYHLLDSFLAY
MDQVQEERKQQQIQNVVFPCELTILPHCVFNKKDPFVFGVHVDAGVLKSNTPLVAITKTL
FLGRVASLEHNKKPVEQALKGQEVCIKVVGEPNVAYGRHFDHTNKVYSKITRESIDILKE
YFREEVAMDGWKLVAQLKKVFNIFMTTNAEDHLLKQDLSTLDVAKLTSLTPEVISRQATI
NIGTIGHVAHGKSTVVKALSGVHTVRFKHEKERNITIKLGYANAKIYKCTNPEHEPPSCY
KSYGSSKIDDPLCEKPGCGHKMELKRHVSFVDCPGHDILMATMLNGAAVMDAALLLIAGN
ESCPQPQTSEHLAAVEIMRLKNILILQNKVELIKESQALLRQQEIKKFISGTAADGAPII
PISAVLNYNIDVISEYLVTQIAVPKRNFTVPPQMIIIRSFDVNKPGEEIENLQGGVAGGS
ILYGVLKVNDEIEVRPGIISKDQNGQITCKSIKSRVISLFAEQNNLQYAIPGGLIGVGTT
MDPTLTRADRLVGQVIGYINTLPDCFIEIEVTYYLLRRLLGIKVTDNDKNVKVSKLKKNE
FLMVNIGSTSVGGRVTGIKPDMAKFELTGPVCTRIGDKVAISRRVDKHWRLIGWGQINKG
KSLQLI---MIKFFISLNRQSKVRLVRWFIPVTSKEKSSIIQDLSHMVVNRSLKQCNFLE
WREYKVVFKRFASLYFIACVDKDANELLILEMIQRYVEILDSYFCNVCELDLVFNFTKAY
HLLDEILIDGDIYDTNKKGILRNMAAQDAMSEKTKTFSSKS---MKSS------LISPVC
VNIL-IKYPPFRKNI-----RYYTTTLMTKYARFHEFQTFRNP----NLRLYSTVEDSKS
S-EQKSTKDSNEEPDLDEENREETNLSPEEL--LNQENELLKQKLSTLETKLKELELKYK
MSLSNCDNLCKIHKKELENTKVYAVTEFAKGLLEVADTFELALKHLGESDPKKSTE--DF
VDGIKMTEAMLHQTFEKFGIKKYESMMEDFDPQIHEAMFEVKDNDS-HNKVVQVVKNGYT
ISGRVLRPAKVGVSKRM--------------------------------------KLVLH
LINCIILYFCFRSHI---------AQ----------------PSINFTKNYNISDSCDHE
------------LNNVIPY----SSYRLLLSPSEDVLTETKEWILTKDEDDLASSAHFDV
NFNDLYSFKGRSKQGVLLEVKDASICLVDRILFDKVSFSVNTGECVGIIGNNGVGKSSMF
DSIYERLKSGLDKP--QMSVSMDFNIKDEETANNISN-NKYLYSFVSKLMELYMRQNYDL
NPESTVSSVFESNGSTGTETANKIAGDILSLYRNLITQKFREMKSLIKKLVSIFGMESVI
-GSTVNTLSGGFKMRLYLFILLLHSPSLLLLDEPTNNLDTTTVKFLIDTLKNSNLSVLVV
SHDTFLLNSICTSIFQMGDGTITPFKGNFDEFVQKGTNEKNVRNARIERLRKNIAILNRD
IQELKAN-KKGSLNALKVTISQKQEMLNKYQEELDELVGSPISKYTKTYNRVLFNINENA
RNIKEDSPENTFNAIFQKILRKNTGQMLNTFGPLF------KLINVTLDTHTGKRIFDNL
NLTIRPSDRIVLLGENGIGKTTLLKLLFKSQATFKNNLMSVNTAKLSYYSQNCSNILNYD
K-TVYNLLRDYVGDEVEQNALAEYLGCFHLA-DYMDSCVSNLSFGERSRLLLSLLLINKA
HFLLMDEPTNHLDLFMKKLLKLIVNGIKGGFMIATHDLDFIRDLVNSFIYIYSKDK-VFR
FSDKFPEYLCFKSQFPDMTKEIVTLHVGQCGNNIGNEFWNQICLEHGINKDGFLL-DKTP
IGDDKDVFFFQTGTNRYYPRALLIDLEPRVISSILNSENKNLFNPENVFLSKDSMGAGNN
WGVGYTYGNQFNDELSEIVDREVDNADNLEGFVLSHSIGGGTGSGLGSYLLEMINENYPK
KLIKTFSVFPQL-KKSSDVVVQPYNTILSLKRLILNADLVNVIDNNVVNNHSFNSLKENS
TSFEENNIQIGNIMSSVTSCIRFPGPINNDLISLVSSLVIIPRCHFLISS---------I
MDHENITLLNLVKKLYYPNNFLVYTSTKNGKYLSALNIIRGNNNPSDVYKCIEKIKEKRL
VEFIKWNPANIQVNLIKQSPH--KQDKTNGILVANHTSINQVFEDCILQFDKLYSRRAFL
DNYRKAEFFSREDGKGDFEEMEHSREVVELVKEEYIRSQQDDYYDLSCKMRRYHKMEKIG
EGTYGVVYKAQNNHGEICALKKIRVEEEDEGIPSTAIREISLLKELHHPNIVWLRDVIHS
EKCLTLVFEYLDQDLKKLLDACDGGLEPTTAKSFLYQILRGISYCHDHRILHRDLKPQNL
LINREGVLKLADFGLARAFAIPVRSYTHEVVTLWYRAPDVLMGSKKYSTAVDIWSVGCIF
AEMINGVPLFPGISEQDQLKRIFKILGTPNVDSWPQVVNLPAYNPDFCYYEKQAWSSIVP
KLNESGIDLISRMLQLDPVQRISAKEALKHDYFKDLHMGGSQSLMDTKFNLTLQSREAQK
LHYKCLKEEEDERIKVKSAIESNNPESARIHATNSIFKRNEAMRFLEFKSRLDILSAQID
SAMRTQQLTEELKKVLPQLNKFLK-YNKLDSVDVITDFKKIFENMDGNESYTGENVNRES
SFKVPPREVEALISRVAQEYDLDMMTKKMETQNESKPQSYNKPGLSMGKEKTHINLVVIG
HVDSGKSTTTGHLIYKLGGIDKRTIEKFEKESADMGKGSFKYAWVLDKLKNERERGITID
ITLWKFETGKYYYTVIDAPGHRDFIKNMITGTSQADVAMLVVPAESGGFEAAFSKEGQTR
EHALLAFTLGVKQMICAINKMDKCDYKEDRYNEIQKEVCGYLKKIGYNVEKVPFVPISGF
LGDNMIDKSDKMPWYKGKILVEALDLMEPPKRPVDKPLRLPIQAVYKIGGIGTVPVGRVE
TGQLKPGMIVTFAPSQITTECKSVEMHHESVEVASPGDNVGFNVKNVSTSDIRPGHVASD
SKNDPAKEANKFDAQVIVLNHPGTIKEGYSPVVDCHTAHISCKFEQIQSRMDKRTGKTLE
ENPKTIKNGDAAMVTLKPNKPMVVETFTEYPPLGRFAVRDMKQTVAVGVIKTVDKKEPGS
SSAQKAAKKMSHLMNLPILVLKEGTDTSQGQAQIISNINACQAIVDCVKTTLGPRGMDKL
IHTENDVTITNDGATVLKLLDITHPAASVLVDIAKSQDDEVGDGTTSVTVLAGELLNEAK
AFILDGINPQVIIKYYREACQVALNLIDKVAINLSNKSSSDKRELLVKCAETTFNSKLLS
GYKTFFAEMVVEAVATLDEDLDEDMIGVKKVTGGSCEDSLLVKGVAFKKTFSYAGAEQQP
KKFINPKILLLNLELELKSEKENAEIVINNPQEYQKIIDAEYQIIFEKLENAVKLGANVV
LSKLPIGDLATQYFADKNVFCAGRVDENDLIRTSKATGASIQTTLNNLSVDVLGTCGVFE
EVQIGSERYNMFTDCKSASTCTIVLRGGGQQFIDESERSLHDAIMIVRRATKCNTILPGA
GAIEMLLSTYLLHYSKSLVGKRHIIMNGFAKALECIPRNLATNSGYNSNDLLSLLRNKYN
KNNEENWYGIDCYKGSVCNAYVECIWEPSLVKKNSIYSATEAACLVLSVDETVKNQSRQQ
LQGALPPRMGNDEHDLGELLMVLGDLHVPQRSLFLPPCFKRLLKTDKIKRVICTGNVGSK
EMLEVLNDISPSLHIVQGDYDDDFDHPDTLTLSVGDLKIGVINGYQIPTWNNKDLLLKVA
VDMNVDILVYGHSHVSDISKHGGKIFVNPGSATGCYQPWQPNSIPTFMLMAIQGSKVVIY
VYEEHDGEAQVIMTELDNL------NHVYSVPLEGSEEEGYTPVYRRPDYKDKLLEDYFD
GKIQTGWDVFNRGLSLSRDKPFLGKRVKEDGTLGEFQFLTYGEGETQIKRFGSGLLKLNK
FQEVFVKEENQT-VRMVGIYSQNTVEWLITEQVCNAYNLTLVPLYDTLGEESLLYIVNVT
KLNVIVCDYKCSLKLMELLPKSNG-TVSLLVVTGDELPPELVKGSESLSVTFKTYTEMVN
LGKENELEFTPCTKDSIGTISYTSGVSGIPKGVIIKHFQHVSLIVIVNRIVCDEKS-KLE
NPKVHLSYLPLSHMFERLYIGTSIIDGSAIGLFSGDIKNVLEDIKALKPNVFPSVPRVYM
RIHDKIFSTVSQKSFLIKSLFGLGLHRKLKKIKKTGVVTHRFWDKILFSKFNMLLGGRVN
WMLTGSAPLTPKIFDNIRALFSIPLVSGYGLTETCAGAFHTERYEPDSTHVGGPVPCMEF
RLKSLPDYNYYTTDKIPKGELLLRGHNIVS-SYFNDEVTNKESFDENKWFLTGDIAELLP
NGAIRIIDRRKNIFKLSQGEYISPEKIESILNTVPIICQSYVTGKSHFLKPVAVVVPDEF
ELELWSKKYGFNLDRKEQCNLKELQEYMSKEIEKVFHNSDVKGFEKIKNFYIEHEMFTIE
NNLLTTTSKLKRYVLSQVGQIGLILLLEILRTNMAEWVPVLKETALANNSCYGAGIANGE
DGELFVASDVDHEGLHWDSVYKEGYEYETMDDAGNPLKVKVDEKFTIREVFEKKMSSEGI
FLGGEKYTFASYDPDMESGSFKFECVCGAKNKGGCHLIKTPGNYIVVVVYDETRGQDKTV
SRMAAFNLAEYLASNGY----------------------------------VVLNR-SQS
LGLTCTTCKDSSTVVVDHVEGNQLCLNCGRVLENVLISEQQEWRNFNTESLGAGAEKSRV
GELNDVWLDGTSSTTFIGGSKKMQHLNLMTNYDSSDRALKTSFTLLRTIADSLNMRDQVV
ERSKEILKELNQMGHLRSRSNSLNTLAVLYLACRESSVSRSLRELVIYDRSLTVKELGRA
INRLKKVLPNRGNAPTEDVSQLMPRFCSRLNLSNEFMTTCEAIAQKSFVLLTSAHRTTSL
AGGIIYLVTRLFFADDSP---ISISDISQVCGTSTGTIKTTFKELCLYLDKLIPPKHRDK
MHNITNIPNTSGKVESSQPGRDNYKGQF--RWENVKTYFTDPTLLTLEVLFIVLTLSNVY
VVHTLFVTVLPAPLFVTWWQLAQGLWTAWVLGDFGTSYPKLAYFPPVTIDSKLLKELFIP
TVSYVAMLSSANVLLSKAPSTAAFPILASGAVAAHHAARFVACGEEYMPLRWKAVGFLLM
AFVLGATDSKVAPGNVVTVACLYALLAAVFRAGCMERALHVVNGKGNALHNHQHLIGTLL

LPLAIVFSGELTVLRDLPFDPFSTRTWQVWGCFVTVGALPFLKNVVSNRLIRQTGQAPWR
CLELLSVALLFVIGSAQLSPSWRSVLSTGFVLAGRFLGAMDVVKNLNPDSEP--------
-------TEMYNQASKPFLVE------EHSSTH---------------------------
---ELAMSSL
S-VLLIPSRMNLQNLKQRRHNAHLGYSLLKRKSDALTSKFHRLLRATVQGKERLVEGLKD
ATYSLANAVWSAEDFKSLVIESVGRPSVTLKLRGENIAGVLLPVFSLHTDPTVDLFANLS
LSSGGSAIQSVKTTHLAALDILVELASLQISFIILNEEIRMTNRRINALDNVLIPSIDRN
LEYIRRELDEMEREEFYRLKMIKKHKER--EESEK---------ETEPRQYQSFFDPVDD
DIVVMPTLFSSINLTL-------ARQYVDPMQHDLQVVNSKTFTTNVQLARQTNVVGAFF
YSTGDTNLTSTINELNSVAKDLKGMITIVAVNCTDQSNLCKSELG------EEYLTPAFK
IYPKLPMPAFDFKKELLKEDVKKLLKHLPSNVERV-EVGMLAHFMTKHELMPKVLLFSDK
EHPSYVYKALSNAFNKKLLLGFVDAKHPDLKKEYGVKSLPTMLVIKPNGKPQKYKGEFKY
LPMFEWLNVNAEAFLLGGGYSDKGDPAKAKPWKFDKVPKLNFFSHKDICFNKT-QGLCII
YLTDS-DLKPEEKEMLVHLSEKYTGQITG-----KWMWMNLNEETEFAQLFEVKVLPSAV
VFNPKKRLRYFLHDGNTSVTKSSMERMLEKILGGDARFTLVNGE-LPKFKIDDLL----T
TEELTVIDLNRPYSPCELKRIRAIEFGLLDPELVKRMSVCEITLTELYREGIPQTGGLND
LRMGTTDPRHICLTCSMDLKYCPGHFGHIVLAKPMYHYGFMMTVLKILRCVCFNCSKFLL
NKEDPRVQLILKSPSSSFRLNHSLELCSASRTCRID------------------------
-----------------TQSNSKIEGCGYPQPSYTKEGPNLMIQFSQKEADEQCNDIKRP
LTPEEAFKVLRGITLEDMKCLGFVPERSQPCWLILQVLPVPPPAVRPYVQYGSDRSEDDL
TLKLLDVLKTNNLIKRHDKRATAPHIIQEMCQLLQFHITTLFDNDIPGMPIASTRSKKPI
KSIRARLKGKEGRLRGNLMGKRVDFSARTVITGDPNIPIDTIGVPKSIAMTLTFCETVNS
LNYESLRKKVETGPHDWPGAKYIIRDDGTRFDLRHVKKSSELQLEYGYKVERHMQDGDYI
LFNRQPSLHKMSIMGHRAKILPYSTFRLNLSVTTPYNADFDGDEMNLHLVQTHETRAEVK
HLMLVPKQIVSPQGNRPVMGIVQDSLLAVSKFTKRDTFLTRDELMSLLIWIPYWDGKLPQ
PAIFFPKNLWTGKQVISVLLTFNQS-----------------------------------
--------------------------------------QGITNINLIRDGTIKLDKDNLN
CSINDSRVIIRKNEHLSGIICKKTVGCSSGSLIHILWHEAGPEKCRDFLTTLQKVVNNWF
LQIGFTVSCSDIYCSESTLNKVYRILDKSKKEVQKLVLQAQKGKLKCQPGKSLFESFEAR
VNKELNDAREQSGSVVASSLNLTNNILSMVNSGSKGSTINISQIIACVGQQNVEGKRIPF
GFRDRSLPHFIKHDYGPESRGFVSNSYLSGLTPQEMFFHAMGGREGIIDTACKTSETGYV
QRRLIKAMEDVMVQYDRTARNGNGEILQFLYGEDGMGAEYIEDQFIDLMRLDDEEIHRRF
SHDFRSESYGLGWIS--EDIRNAILSDFSQQVILVEEFQKLLDLKKMICEEIFPGGDYSQ
HLPINIKRILEYATTQFPA---

-------------SSTSK--LNPVEIAQRTSKLLESLIIINTSGPTDILSEEAQQNATIL
IKAHLRCHLNSRYLMEHVQISSLALDWVYGEVERCFFRAIANPGECVGAIAAQSIGEPAT
QMTLNTFHFAGVSSKNVTLGLPRLKELINVVTNVRTPSLTIYLDPSINKDQERAKEMQTL
LEYTSFEKIVLGYTVVYDPVVDRTIIKEDYEWVRDYYEFPDEE--MGALGQFVLRIVLNS
KIMTDKRLTMKEVGEIIYSEFSNGEIDAIYTDDNSELLVMRIRVKYG-GE----------
----------GENSG---

---PSD
TEADFLNKFMTDVLCGIKLRGVKGITKVYMREENCVRYNSTVGSFDRVSQWVLDTDGCNL
ESVLPITCVDYTKTFSNDVSEIFHVFGIEAARRALLREIRAVISFDGAYVNYRHLSLLCD
IMTQKGYLMSITRHGINRADRGPLIKCSFEETLETLLTAAVFGEVDHLKGVTENVIVGQL
SPYGTGAFDIMIDEVKLRDANQT-NAINVADTLGLVSPDS----SPLSPSTPATKQTM-S
PMAFSPTYTSLMMSPVGGIG------------------MGPST-PGLMVDTYALGGSFSP
T-SPTSPMSPT-SPMSP-----TSP------MSPTSA------------SPVY-------
------------------------SPAYSPTSPM--------SPTSP-------------
---TSALSPTSPVYSP---AYSPTSP----TSAMSPTSPVY-----------SPAYSPTSP
NLGAPTSPV---Y---SPAYSPTTPNY-----GY------------SPTSPL-SPT----
--------SPAYSP-------TDPFSPNPFEDID------MVRFWIEGIEVYFPYPKIYP
EQIAYMKSLKNVLDSKGHAVLEMPTGTGKTVALFSFVSSYQLAKPELGKLVYCTRTIHEM
EKALNELSVVISYRNSQLNIDHSS--TYEGSNSYKTSNNMNETNQNYIKNIKEEKNVKIE
QDVETPQNDHFLAVGLCSRRNLCIHPEVSSQADRTKIDEKCIFHFHTPVEYYRYLVMPIL
ICDLTSIWRRMQFTKRNEHTLEYEGSKPIKIKPSAIEEFNSMGLCGYYETIERIWNPTFM
PSGVYTLEGLKEYCLNFKDPTGRPSPICPYFAARRAIDDANIVVLNYQYLIDPKVSDAVF
YHLCTESYLKEKDDIVVVFDEAHNIDNVCIEALSVELSTETLDNAYSDLSRLENSVRELR
LRDEELLLEEYRRLVETTDFGSA-------------------------------------

--DIEGYMNPLLRQD-IVDRVMPGSIRKAEHFISFLKVVIGYLKKYIKVKEPKSEGPLMF
LYRFQNETGIISDVFQHTYNRFKSLLNTLKMT-VGNLTALHLVIDFCSLVGTYYKGFIII
VDPFPKSA-AYDPVIQFSCLDASVAMKPVLENFQSVILTSGTMSPLEFYPKILNFSPILT
QSLPMSLDRECLCPIIVSKGDNQVHMTTKFDLRKDITLLRNYGSLVIELCKSIPDGVVCF
FPSYAYMELILSHWYETGILSSIMSHKLVFIETKESISTSLALHNYRRACDAGRGGLFLS
VCRGKVAEGIDFDMHYGRCVILIGIPFQYTLSRTLKARLDFMRCNYGILESEFITFDAMR
QAAQCIGRVIRNKGDYGLMVLADSRYTRVGKRSKLPVWILKRLDLGNFYLTCESASSIGK
AFIRRMSQEYISTKRTKFGQTTLNNEKLYWSTVKSILDL----YNHSPNPKFNLTL-KRA
TDLFKDSKLHPNALNQPPCHVSTLKNGLRVATVWMPGSSSTVGVWIDSGSRFETPETNGS
AHFLEHMIFKGTKSRSRQQLEEQIEHKGAHLNAYTSREQTAYYARCFNNDIPWCTELLSD
ILQNSQIDPDHMENEKHVILREMEEVEKSHDEVIFDRLHMTAFRDCSLGFTILGPVENIK
NMQREYLLDYINHNYTADRMVLCAVGNFDHDKFVTLAEKHFSTIPK------PVTKVELE
KPYFVGSELLNRNDEMGPYAHMAVAFEGVPWNSPDSVAFMLMQSIIGTYNKSNEGVVPGK
VSGNKTIHAVANRMTVGCAEFFSAFNTFYKDTGLFGFYAKCDEVAVDHCVGELLFGITSL
SYSVTDEEVERAKRQLMLQFLSMTESTSSVAEEVARQVLVYGRRMPVAEFLLRLEKIDAE
EVKRVAWKYLHDSEIAVTAMGPLHGMPSLIDLRQKTYWLRYM---ECIIRELELLLLDPC
NDDKIKQLSENNVVDKLSKII-----------ELVLSVKNLYSSIILLEELFEKPILIND
NISLWD------------------KSNYFNYISQNFTE-FLENSNDFDFQ-----LNSFI
LLCTIVLCINVVIRANWIGPKFSL-QTSQKTLSNKSNTLDENDNELNLKTTWEKSEFLKQ
MINEFVIDGETIYEGTLGLQYLFNYQNTSNVNTNTDVI-----------------SSENT
EDSINN-----------VKIEMKSKEIWRSRIGYIYQLMIEDSGLILCPYHCSIIN-FLH
YLRLSNVL--------EEYDVYNIDVNDGSDSILKSVLMPDLGME--FDDYFKSLLLIEL
LQRLPIYNLNRLYKPVSENLTKYLNFSFEFTGKLGVRRKFQSFSIPQLVLSY--------
-------SKPFP--------TNSSPNQK-------------------------------L
KCDPN--------------LLDENL------------------ENPQEPKDEITVGLNEI
NEENDIYETPRFDEDEKTE--ELSDLEQLFLLSKGLNILSTTSKHDQLSLQFLNTISNTI
L------------------------QQSKKRRLIMLMSLWLRCKTEYNRTKTIERATIQL
NNILNAYYVTTMQSGNRNEYFFQMSYPAIWNIKREIGKYMFMIGSITTACNMYKELHMWE
DVIKCLVLTQQKQQANELINERIKIMATPSLYCYLGDISND--IQHYHTAWEMSNERCGR
AVRSIGVKYYNSGDFEKALEFLEKSIQLNPMNENVQFIVGCCYLKLLKFENAITPFSRVV
SI-NPDNSDAWANISSAHFKVSNYQSGKIAITQALKSNSTRWQFWDILLRISANLNDVKC
ACNCIQTLINLGMKDKVEVWAVKYLVDSSINELNSQVVDE-----------TLKLITSNI
TESAGVWSEYSRYLSNKEDYVGALESKFKQYRKVEQNIQINLNNPKNELLELELFEVLKS
MVELANKVDEDDKCESVKKLVDKNNKMALSLYESKASDFAGRIEEVYNVVGMNEESPSNS
DLFGDD---EDTSTLLELTEEAPVEETKKKRSRKLKRDSS------------KKRKIDVS
AFLDTEAQVGDDEDEN-YEYLLPDESTEAERLEHRSRLKRRRIGGTQYLETAIDKLAKRY
QDQTFEEDEIQESQP---IEYDGIE---ETSALIPDLGDPKLWIVRTNRQTPDRNLAISI
MNKSLRYQSKNLGIYSCFVPDGVTGYLYIEADNKTTVIEALSEFRQISLNTLKLVPINEM
SNVFTTGFDNVYIPRVGEFVRVKFGRYAGDLGQVYESDENTSTVTLKLIPRIDPSILSNN
DNFNDGFDGIDSI---------------------------------------NSIGTVGT
AAPVNGVKKNIKYNKMLFDRDAVELNGGLIEHGIIPGTFRYQGMTFLDSGHILQKFSSKR
LVTQDANPTLAELREFSTDSSLTEVLS-HVTRSA--GSKSALYKLGDRVKVVKGELMSVT
GKIIGVEAEEVEVQPDDKEPNFRINLNSVIKDLVEGDNVRAIGGSNEGKTGLVVMVNAKN
RSALVFSPQTGEEFKSTLEHLALIPREGLGDEGVGGINGFVVTDLVQTTSGEVGVIISVE
RSGSVTILTDGNNRLKVSPGQITCKRTSVGSSSRDFNNQPIEPRQRVMVVRGNYRNKTGQ

VLHLWRNTLFLSVENDV-----IVVSSTDCLRAGNATSTATKPVTTNAPIGNV--KLVRK
NPLIGKTVKILQGRYKGLLGDIIHVEPTQFTILLKVKPKTVRYPKSECVILDNWHHNIRY
------------------------ANTYSNNSRINTTSTVLGSTGINSNM----------
--DTYS
TVTTTTDINALANTNTTVPTNTTTVSGIVENKMSPWSVEGVVVKVIGPGGYYGKMGVVGE
VFENTSLQVLHVMVEEDYIAIAAESVEPVPPSKPGEQALVFSMGKDKFGTVVNVSGDKVT
VKLTTGVVTVDKEGVALMSQQ--MNN-YPIITNTILNLRNLGFVDNNFKFDVLSLE-DRY
ISIKEQDGDNLTVAIIDLYNNNIIRKPMKAEAAIMNPNKPIIALRAKLDN--NYSIQVYL
IYT--NFSYYQFDQRIIYWKWLNNMELVIITETLVYHWMISNPKYMFELTGKLLDSNTKI
VGYSSDITNKWCLVFGIYSNDQGVSIDGVIQLYSVDKRQQQLFEGYAGTFTQLRTNNLKK
SNLLIFCEHKKNTHNIKLHLMDIGSTTTGGTDILKINCIMERNEEFPNDFPISIHVFDSN
GIILILTKNGFAHFYFSNTLTHLFTVRISMYSLFVSCNKKENNGALTVNRIGEVINIIID
ENNLLSYLTVGTMEMRKEVCNDIATCYGIKGSDLIELFEKYFNNEQYKQAAQIVATLKSN
KLRTYDIIQRFKNKSTETG--NALSYYFSILLEVDKLNEIESIELIKPVILQNRKELIKK
WLENNKLTESETLGDLIYEIDYTIAFKIYNKLHLYQKAINCLLYPITSVTVTGTPNSITT
TTSTTTTHTTTTTHTTTTQIDIIINYIYKIINMVGMDGITIIKFINNLLDTNTNDNGTSF
GGVVGASTVTGTVTVENVLLCNIIEIMEKLIEMKKLKEMNEILLDYLKEDLEIHEKLQTR
LLQVNIENDKRIGEEILKLDILTKFNKNYIAKLCEDNELYEYSLKYYNDITNIKRILIKG
IYFLSQNIINQTLLTFNIQDSLYFLQQLHEKVDRLENIIINICLILYNKINLKELTNILT
N----KLFYKFFKKL--SKQ
ELDEDINEIIIRYMKLCIEYNDIIELENILLYNNN-FNLFDAKELIKNSQLSTTKPFIII
CYRLNNIQELLLFLFNELILKDIKI-LLEMNPKLLTEVIIILLEL--SEELINKILINIT
NITLLKEIIKLIEERHQLIILKPFLEMKCKE-INEPILHTALMKIYITLGENPEDYLINN
KYYLKEEIANYCQDIDLQLSLLIYKHN--SMYEHFIKFSLKHQLYKSLYQFLLKESNIKF
YQYYYSNCND---------IIKLCIENCNSIEISVLIKFLLSENLNEDLIILLEGLLLNQ
TEFTNNSNLQNLLLATTIKTDSTKLNDYLNKLNNYDVNSLAKLANDLKLHYSAFLIYNKS
NKYNEAFNQLI---LLKSTEGTKVEVKGARSEEVKSESTKDSEDTVGPSTVTEKILKLMY
KYSEEINNEMIWFKLGKIYLENNKLKESINCYLKSKNFTHHHEIKLKLMNNQMNELLLFW
LLETNKIKPTSELIID--LLIHLAKLRFLQHFNFLINSSVTVDLNIVGNELIKLGLYMEA
IQVFIKMNNYNKLAICYIEINKYNEACEAALMSKNPKILKQTFDYLIQNVDNKLLNRVGI
ELLNYPEFLVSVVASYESMGLFDDLIELLRNTTKT----VATSTELAICIAKYHPEELME
HLRNVSNSLNISKTARECSNLWLWREAVFLY-TIDDSDKAILSMILH-PECFEEQLFFRT
LANVSNTEVIYKALYFYIQQYPTAVNKLLSCVKFKLDTGRVIKILRNNNCLQLAKEYFQQ
-SDRNSQQINDTLYEIYVEEHDFESLEQDVSKYINYDYMKLCTLLEEHPLARMREIGAKI
LSRHNHYSRASIIYMKNNNYIKAIDCARLSKSTQLVHETINNLILKEELNYLLVALVINF
HLLDLPMVLESVWLNNVLDVIMPLIIHLQRFINYK-------------------------
-------------KPNSH---------------------------MSPKVGLQAPNFKCE
AVMPDGSFKEISLGDYLGKKYVVLFFYPLDFTFVCPTEIVAFNDAVAQFEQRNVQLLACS
VDSKYCHLAWRNTPRDKAGVGQVKFPMLSDMTKEVATSYGVLVDAGLALRGLFLIDKKGV
LQHSLVNNLPLGRSVNEVLRLVDALQVFETKGEVCPANWKLGDKGMPPTTEGVVAHLTTK
MDDESYGII-DKDSYYKDPPREEDLSELVNNTLESVKPRLPEEVLKELLLELMLVEKKCR
QKFDGESNSSICKFILTVLYDFNDFNNMFYYLVLLCKKRGQLKTTIVSMVDLAEHWLDTI
PSLEVRLELFNTLDKITLGKIYLEKQRAQIIFKLAKLKEDEGNIKESASILQNIEVETYG
SLNKLEKIRYILEQMRVNLLNGDYIRFFMTSKKITESVLDN--YVPEKLQFYDFMIQYYH
HDFDIENITKSLYTIYSTKKKLFLEEDYLTVLEKLLLYLILLSLNEENITYMKVNEDEKK
FMKQLLTISPFFQQFLNNFLIQHQLDSDEKINS---LLDERCS------KLLYDRIIQHN
VKIISKYYNKITLERLSTLLNIDSEKLENEISNMVEMGIIEAKINRITGIIKFQKKLQTE
IILNNWVNNITK-----------------------------------GGNNTIEDTDFDL
ICKLSDYLDTGIDRKLLRILVNLCLLGVNPNSLVNILKRLIKFRNTLLNQQ---------

> ***Plasmodium falciparum***
MIENTKVNIFYGNKYPFICKSILNVYKNKNKKITETTVNVDENIKFIKDANICSSDKIFA
KILDHLLKTDLYNISEKIDPKNNKVVNIYVQTQYEEWLDFFRSRNVNKDIVFICEHLNKH
LHLNTFVVSEYLTLSDIFIYFQMYKYFHVSSTQYAKYSKMYKNINRWHKLIDSLIYFEDA
EWKKNLKCLICDNKMNFDDRKDNLKCKKKTVTTSYSGKLENAVIGNVVTRFPPEPSGYLH
VGHAKAAFLNNYYAQMYEGKMLLRFDDTNPVLEDIKYEKSIIEDLENLGLKYEKISYSSD
HFDLLEKYCIDMIKMNKAYADDTGVEDMRNQRGEGIESINRNNSIEKNLELFNEMRKGTE
IGQKNCIRAKINMQSKNKCMRDPVMYRCIVDVPHHKHQFKYKCYPTYDFACPIIDSIEGV
THALRTNEYSDRIEQYNWFISTLNLRKVYIYEFSRLAFVKTVMSKRKLKWFVENNVVDSW
VDPRFPTIKGILRRGLTKEALFQFILEQGPSKAGNLMQWDKLWSINKQIIDPIIPRYAAV
DKNSSILLILTDLTDQVIQKERDLHMKNKSLGTCNMYYNNKYLIELEDAQTLLENEEITL
IKLGNIIIKNIEKENGK-IKQINALSNFHGDFKTTKKKIHWLPYLPQQLITCTLYEYDHL
ITVDKFENDNKDDWTNFINFNSKHETLVYAEPSISSLKVSDKFQFERRGYFILDKIDPHH
HLHLIKIPDGKSKNMSIITTKVDPKQLAGTKDMENKTIVIDNGSGYIKAGINSSEEPTIV
FPTIVGIEKNDETKRIYTGDEAFFHESNLNIYRPIDHGHISDWDKAQKVWDYTLNCVDPS
KSIKDILLTEPPLCSISHRKKMGEIFFEYFDTLNLNLSVSGLMSLYASGLTTGLVLDIGE
GVTQCLPVFDGYIEKNSIIRSDFGGEELSMFLQKLICDIGYSMTTTKSLEYVKNIKETIC
FCSLNPSEDQLRNDLAATYTLPDGDVLRDGYDSIEIAHERFYVAEALFNPQLCHRDNLSI
IDIIWKSILSCPMENRKILSSSIVLSGGSSLFPNLVERIETEVRNNAPESARSMVKVHAL
ENRAIMAWCGAQIFSQAELRESQTGIWISKEEYEEIGSNIFLTKARKKKKHMDCLSKINL
IRKVNEELYNSIGVEDDNLSEFLIYLCEKSTSLEEFCKDVFENGGVIEQAVLKYIYNMIK
KKKKDHDNEDDVNSKENNLEYKKIEMKNEKMKKYHCLTLKN-EDITNLVNDKKEKSIKGT
KRHSKEHHHSNEHSKEHLHSKHHHSNKHHHSNHHHSNDHHSNKHSSYYNNKKKKEKTHRK
NEMALKLNNIFNGTINKITDFGLFVSFKTNEGYKEGLVHATDILPNRKRVVNMNEKFKRN
MKVKVKVKGIFNQKISLNMSEVDQKTGKNLVNDDM-NKEEDNYISLFDDINEDFNDLKKK
NANVFKDDPKETLKYESVIKIQSDYSKWEIQQLIKSGVVFDENIRNEYKNLKIDEKIEDE
EDIIEIEVNEKEPAFLKGQTTKAGAKLSPIQIIVNAEGSLAKAITTTSALAKERKEQKQN
EQNAIYDNIPKDISRPWEDPKPNLGERTIAEALKNIGKNYDIPEWKKNYNNNNISVGVKN
TLPINEQRSKLPIYNLKNDLMKAIEKNNVLIVIGETGSGKTTQIPQYLHEANYTEKGIVG
CTQPRRVAAMSIAKRVSEEFGCILGQEVGYSIRFDDCTSNDTIIKYLTDGMLLRETLSDT
LLTKYSFIILDEAHERTISTDILFCLLKDVVRKRADFKLIVTSATLDAEKFSTYFFNSPI
FTIPGKIFPVEILHSKEPESDYVEASLITVLNIHLNEHPGDILVFLTGQDEINTACEILH
ERMKKLESMSPPPLIILPIYSSLPSEMQSVIFEPAPPGCRKCILATNIAEASLTIDGIFF
VIDPGFCKIKKYDSKRDMDSLIVAPISKANAKQRAGRAGRTGPGKCYRLYTEEAYKNEMS
EMSVPEIQRINLGSIVLLLKALGINDFLHFDFMDSPSVETLIHSLENLYYLGALDDNGYL
TKLGKKMANFPMEPNLSKILLTSLNFNCTDDVVTIVSMLSVQNIFYRPQNKALLADKKKN
KFIMPQGDLITYLNIYNKWKENSFSNYWCHENFIQSRALKRAQDVRKQMLSIFEKYNYQV
KKSTSKNDATKYVNICKSICSGYFNHVCKRDTQQGYTTLLTNQQVFIHPSSTLFNKNPLF
VVYHELVLTNKEYIRDCTIIQPQWLIQLAPNLFIPADEKKISKIKLREKIEPLHNYYEEP
NAWRLSRRKG--------------------KTHLLSRYIRGSLPSVAKATIGVEFATRTI
PLAVGGTVKAQIWDTAGQERYRSITSAHYRRSAGAILVYDITKKKTFLSISKWLEEIRQN
ADKDIVIMLVGNKVDLTEEDETKRKVTYEQGANFARENNLFFAEASAVSKLNVKHIFENL
LQEIYNNRLKNNNRSFSNRSVATCESAIQLTKARSVIKLNEVYDNQSEDNNMNKVKCCME
KSKLAKVEKVLGRTGSRGGVIQVRAQFMGDSELAGRFLIRNVKGPVREGDILALLETERE
ARRLRMNENLENFSTIDLLNELKRRYACLSKPDGRYIFLGAPGSGKGTQSLNLKKSHCYC
HLSTGDLLREAAEKKTELGLKIKNIINEGKLVDDQMVLSLVDEKLKTPQCKKGFILDGYP
RNVKQAEDLNKLLQKNQTKLDGVFYFNVPDEVLVNRISGRLIHKPSGRIYHKIFNPPKVP
FRDDVTNEPLIQREDDNEDVLKKRLTVFKSETSPLISYYKNKNLLINLDATQPANDLEKK
ISQHIDG-MKQECNVCYFNLPDPESTLGPYDNELNYFTWGPGFEYEPEPQRKPLSIEESF
ENSEESEESVADIQQLEEKVDESDVRIYFNEKSSGGKISIDNASYNARKLGLAPSSIDEK
KIKELYGDNLTYEQYLEYLSICVHDKDNVEELIKMFAHFDNNCTGYLTKSQMKNILTTWG
DALTDQEAIDALNAFSSEDNIDYKLFCEDILQMKINLYKLLCFICVIFLLHKNVRSGDNM
KYNDMKGLDDLSKLNDDQVKDILGLKIDGAKERIEKLFHLIDKNNDKEITEEELNTWSSF
LKNEIFLKQVQAEMGQIDSDKDGFISLNELNDAFAQNLDAKEVEKHSEGLLKRFQIVDKD
KDGKLSINEVGLLIDPMKDEELKELEINEILEHHDVNKDGKISLDEFKQTRSDESSVKKD

DEMALDDFNFFDANKDGFIDKEEIIKVYFDPAHESGAINVNEIKENIFEGKKITYDLWNE
KALKIAVTSLTDYGDVIRYPEDFKLDIGKNVILPTARSRAFEDDDMDADN-TEDDKDEAD
DASQQKSPAIDELMDLLFAKIICIGIFLVVTTFGCFIPHLMGLYKEKENEEKNKRVKNIL
SNLNCFGSGFIFSIIMFHLLPETIHIISDHGNIRIFNTSDSQMKILYIFFFVFIGFCMQL
GLEYVLPVDTNICCVSNLDSKKKLEDTLS-QHITKNASTTVNIEMQNVSIDDHIHEHSCE
GVHTHDEKSIGKFLEILTLQSFFLTISLAIHSCIEGMIIGTSTDVNYVFISSFCILLHKW
IAGVTVSLSLNSNNMNKTLKAILLLTFVFASPLGIVLGHMAKSAGQKVTCLINAVSIGTL
LFIGCEILLNEIKQNISRKVRLCKWLSFCFSCLIAFALISFTTSMAPHTHGHHHDHDHDM
QSKFWAKAIDEDSGDNVTESSESEVEEKKPIVSAQAERWAAIDSSSSEEEERVIKTYEGK
RLHFYETTGNSLNENMYNNDFNLLLKDYENLYKFMIKESADCIPNFAIIYLDKLSKYVEK
TFQNNVEKKNLSKNKAQTLNKLRAKIRKCSEFYQEKLNLYNENPDDFKDDRKKDMDDDED
DEEEEEEDEEEQEDQDDDNEDEKKEEKEDYDDDNDEEKDDSDDWSYSEDDNYASDEEDDK
TKNAMSKWGLKTSTKVEKKKTVKQKKVKKESTKKEEKANRVEDSQSAKNKGYAELLSTKN
LTEDVIRERVKLVIEKRGRKGLDKHEHINILSKLCEIAKTISTQSYIEVLEHLINLEFDV
VSSVYTYMSFNIWNKVFKYVELILDILIQNENFYLVSINIAEEITEETTNEKEKIIKSCK
TLISFLAKLDDELLKALLYIDVQTEEYRKRLGKTIHMIGLLKKGYNYVKCLKNMPDLAIH
ISSRILEHMYYKPEMLFKQIWTYLMNGKEMLSDSSKEDGQGKKKKRIDLFDEKLINNDSI
NPPKIIEEYVYEIFEFGTKQQKVKALLQLSYNKSLYDEYLEAKELLNVANVHELAMNSDI
QTQILYNRNLIQLGLCAFRHGKIYEAHCCLMEICSQNKHKELIAQGISNLKNQEKTLEQE
RAEKRRLLSYHMHISIELIECVNNICAMLLEVPNLAKNTYESKKDIISRQFRRFLDIYDK
QIFNNPPENNKEIIILATKHLQKGNWKLCCEKIFSLSIWPKFPDKEKVQNILKEKIKQEA
MRTYIFRYISIYESFSIDQLCVMFDLNQNVVHSILSKMMINQEIPAFWNESSKFILISKV
NPTTLQNLALKLAENVNEVMEQNELALNMKNPKFMFMQERRTQMKEEKSNWTQKKGDQKY
QKNYNQKKNAHYKKNYKDNNANKNYKMEHGNGSDKIFDLNSISREKLELVEVFYDKMKDT
TNANALYLYALQIFKICNIHNELPRLVVFGQQSMGKTTLLDFIMGGPMGYTSTDTGTKQP
IVIILKPSDTNKIECYLNKKKVNIDDLHEKMKAIMLNLNESIILKELEVEISIPGGIYAT
FVDLPGIKDDSKAGSELTRKIVRNYVQNFPNDIYILVKKASDDPANWPYNLREFILKPRP
LGLGLQPKQCIVVGTRALEFLNNELTNIKTLSELHERVKKRSITDNNDNVLPLYLLELFS
IPIEQKEKNDFLTNRISMYSKILNGRKNVLDLLLNKFENDCNDSIKKELIDCFDVEKFKQ
EVNSKFMNILIQQLRKVEVKLEKKKAKMDFYNKKLEELYNKGNILSVREQVKLYIRELVN
IVSNLLTGNYPILNLPKNGENFLKKYGGTLMDNLKDGNELAVELFEKQGLYDENFLNYLN
EYLSRYSNDMDLSKSASSEFNNMNDGLNNNNNSIDDNNSNNRTDSYENHMDLDKIKIENK
PLLVEGVPVRFMLAKEESNMFGVVQNAQSDAKGKNILVNFYFRNNNTDEQVQVKSVEKDR
LTVIKAVETLNGDPSFLNGLQVWYKMVRDDGWVGFDKAEIIKVFNNNSKIKDVLIRNLSK
NDEIISVKIIDLYMDYVNNHNHDDENKNDENNNNDDDILKRIAGPHTDLKILNQLAITYI
CKWLKYNISKLEPEKEFSDEVLLQMMRSIHNIVDQSDWKPLIVDLLQANISGNILHLTKL
ASCSAAVALKRVFKAGLGEINRKIKNNYIDENIYLLSTNQKFIEELNQALHNFCKERAIV
CAGEMKDIVFEQTYAVHFEIIEEIFDGCKLFEDNFLTPNGVKPTMSLINKNVKQNLAYRN
HQLSLTDVKINKKSRSKELIQEEVKLQFWAIKMLISVPLATKIYAHFLNNILPKNKHLAN
VLDYSIDCESYLEKYIQSKLLNKEVNGVLVPIDDKELMTHYSIIDNRDNILRKIENQKRL
NQYFTIVANSIKLLKNNLSKESTLDFVTKLDFSQDKKMESSNIGQILYATVDPNINIRSE
AENKLKLAKESNFVQYINQLSNEFCKSENDPYLRQIAGLLIKNAFTSKDNYESEEKARTW
LNFPEDIKMELKNNLLVLLSQQSDKIVIGTACQIISIIAKIELSHNKSSELLHKLVNNII
EKNAYTKKSSTVCLAYLTEDIADVCNESKSKYAFTQPDLDLILTAIINSLCEPAEESTHC
ANMKVLYNLMSFIEHNFKTQVERDIIMKTVIDGCKDTERQSVQIAAYECLINIVSYFYSY
LDAYMYAIGPLTWVAIESENERIAISAIEFWNTVCEEETFIDQYELQEGKKNHNIVKQAM
VFLLPKIFNAMITQESEDIDIDAWTLSMASATFLALSAQLLKNDIVEPVISFVEENFIHE
DWRRRDAAVLAYGSIMEGPDTEKLKPLVEESVGQLSEVLRDPSVAVRDTAAWTIGKITTY
HSEIIYNVLGSYNDSNSLYGILLERLNDYPRVAANVCWVFNQLAVNKRTSYNKMTNSYVT
DLDDSFCVLCKKLIDVSSREDSDIRNLREAAFNALNVVILSVSDNCLKYMIELLSHMMYL
LTNTYLNPLTEEVKSLQGYYCGTMQFIINRLGNQCKPFLKPIYLSIFRLFEIRTDICEDA
LLACSAIINVMAEDFREHLKTFLNVIFKGLKNVSETSTCKICIEMISDICIPWTSEYEKE
MEAILECLWEALKTFGVHDSIKISILTVLGDIATALNRSFSKYLNFFANILSETSKITIT
SGSPDSDDWVNYVFELRDAILLTYSNIIYALIDGNEINKLKPYIPNILDFIELILIKEIN
HFNAQNFQNSVSLLGDLVHAYGYELIENSKLTDLIISVYGKIDILSSQGDEKCETCVSKI
KWLKRICNARLSQKMSIYIQLTHDLSGTVTAVTIGDWILGILKEKNIQEYEVFFKKFLHI
YQKQDKDSECNNRSEIFSLLLSASDFVFETLVETKKNNKIKVIIDNKESVKSYNEFYKEV
EEYFVILISILQLEFKSVEELNNATNNFIKAIKNYNEFPELRLKILQLLYNSFNVNFSFR
FPTFIAILQFCSQNNIFHNMLPYIKYIDDWIKEWNISTREKRHIYLIISQELKKLKKYEE
SYEHLKKHIYYYDNEHKDILNHPNSINASIELIVDAININNNIYFHEIINLHAIQNLQFI
PQHQPLFDLLLIFYKYTINEFLVFKNKHGSDFFNKYNIDIQTCESKIYLLSIISLFNDHK
VQNIQFISKQLNISVVQIENILVAAIGSGVIDAKIDQINQTVHMKTTILRNFDDENWKQL
NNQITKYINNVQKILELTKHANK----MHHLLFIIWYIILNYYVSGQESATNFYKFIDSF
ASSTYMSEESGSSAYDAKRAIQNNPNYWCSSGNHSNDEEITWTGYLNTKGFIKGVKVSWA
YSPEFVKISVSSDGEKYRTIIPYKKISSNEASFDEIYFFKRLEEAMSIKIGLKNARHKYF
GIREVKLIGGGNPYFLLLSGISSEEEMCLQVEEGLINNDNTSIILDSCTNALASGDGREL
WKTNSNNQVISAFSDPPKCLSVVNLDDLENNKIVLYDCLRALEDGDGKSNWIFESNSQIR
LQKSGDAFCISQKNIYGNIPGIHDILLNLDVSIYSNSTLDDDHNPDNTIDGNLNSYWASA
TFTDNYDHLVYLVLDLNKITDLSRIKIYWEYPPLHYNISVSTDNQNFTVVSENLANPSYI
TVDSLKNMETRYIKISMIKTHPKHGELGDNFLYGIRSIEVQANNLETVINHCRDAANSDD
ARDKYFVEYITEFDKDLTNKLINLEDDVTKNVSSISDNLSKLEELLPNIETCLEEKKTYD
EELKESKEKANDLNNKLSLLTSVNNTLDSDILKLGILPGDSYNFPANDCAVIKNVQENPL
SGFYWIKPKCSPEPLRVYCDMDSSTSIYIWNGNPPKSPDHLITNMINSVNDIRQHCAEVG
LQPLILRSKNQLNSLIISLKKIGYSLNGKVNIPLAYDYSCDHGSCSGRFHDLLNGNIDIS
TLIYLKASESPDSTKVRQTAGISYDDGSFKFFNLETSDISAIVCSTNSTENDSALQYLSI
NCETTGMEDSFHSIVNTNIVVLCPLGCDDEKYHDASIYGSRGTYSDNSSICRAAIHSDII
DNKGGLVNVTIESGMDHYVGSINNNIESISLNKN-EKGLLDIIPEEKENNIREESSIFHH
KTIRVSSLIEDCPLDLFLFNQTSFLEKGNNIRNNKGTELKYNDDENMTVKNFHELISNLM
ENIDAIHGVDSSVISIVQEETIRIIEKTKKELKPADMLSKKQIEDAMNLYNLTENLAIYL
YDLSSKYIQDLEKLKNTLEELKGAQKVAHNFGTFKLNYETMNFSTHFSLFDSNLIKNKES
VWGYSDTNILGHENSIGQMNSVSSQEIGEGYYAKLKGLNFYDFDFNISVLSRGTGCLGVV
FRAKDDFNFYLFDICDKDGTKRLSKVENGQVHILKKVVSDVTLNNQWNKYKIITKHANID
IYEVDKDNNMIKILSSLDERFLSGTVGLYSQIYGLGTFFDDLEVIALPCTQLSELNTLNK
NVKSNCPYYKENYLNNLMSYDIIYNPNFNWNVEKENENYLLCSKNEEVKNAKDEKDIYTI
VLLKLRECTDGTFNFDIQVSDETGNKLSYIYILFHYKDENNFNALEMKDGKLAFLTNKNG
KSFILSERNEEENDNNTFVQNEWIHVNLHFDKSTFKVIIITNNNEDKFVLSAKSRNDVPL
GKVGFLVHNFDEVKFDSILLSPTITKVDENFLQVKSKTWANCEDSVHVLHRRFSCETDIY
PNETKEKHIKCIKNFCKECCLYHTQLLDSNEKNECEKHCKQNDNLAAKMQTLFEKFINRC
VSLNENEDYETCDKNDKKCKNKVCVLCCKKHDPTTSKELKVLPMNQFKKIQENEIIECQL
QCNMIHSI--MSISPLKYESKILSNLANMLKLSEKEECDKKKFVYNYNIETYGVDFFLDI
CNALSRSNYKPYLSEEGKKEILSKERMNKERMDDGKKVKTALYLKNNNSITEQIENKIEN
DILNEQNSSLEKVNHRKGVISVSKYKSELTSITRMSNEIIMQTILEHTKDMEDDHRTNIK
KSKTYESRNNYYYNDDHDTKRNIPKRTVVQKKYVQRKTNITNKKRISQNKPVTRNSYKKN
DRINTTIYEKKKKKVRRSNANRRKRNDNKIDDNTLNNNKIKIDGNINKINKIDGNISDDK
TSDNNKIKIDGNTPNNNKIDDNTSNNTPNNNKTDNITNMDNTNINPEKEYIKENEIYKMH
PNKALLSNNTNEKDYDYNESDNASYSTDDSKYVKYLKKKKKLQEQGKIIIDLNLFNGNSV
KTTNKRDKEEKLQEKLRMKQIKKLLDEKQMVICKIEDIAKKRNKKYVKAQVLSKDSKIER
AYFSHKMKKFYNSKSSYFGCGWSSNKKEWTPFIHAPFFENQHNAIYKNRNKKLYEEIYDT
LLHGRIHPDIKVVELKDHKHPIRLCTPYNEDCYSVVYTGKKINATDDRVIFGEYTGYVAN
NKELSQEKHQYMFALTFNKKVFNDRKHVVFINEVELDEEQNSHVVSNVMNKVKAHEKNNI
NNTNIYNSPHILNGNNNNNINSNTTEETMSNRKIDKIKPVSNNLNSYINKCKTNEHNKKS
RDINNLIILPDNYTYAVDSSYMFNEMSLVNHYKTCSVFNNFDFRINSEWQLVYLDGWPHI
ILTSIPGVEINPGEEIFADFGFEWFEKVNDICLNEFIKNNYFYRLYKLNTSREILFNGME
DIVEKYNLLKNHITCNICMHNVNTDGNNFILCSGCNHVYHLKCVHKFNTEVNENYEWFCS
SCLQFCFNIIKQKEFVDYIEKENQKRFIQLLD-DLNGDNLDTVNNEKKSDALDKYNCVGN
INNSNSIDNQKYLEVLENIRKLLQCKENIDDLFKNKEYVNSFLQKIDDEMNMFENKRSED

ESVKLD--DLKHIFENSFELHLLIDYKKKLEDLLESTEKVDSFLNDKNVLNTMKIRKRTL
SFLMENKKYIDNFIQMNEEKNECEKKKEEDIIHNTTCRKVEKLEDRIIQNSHNNQLANPE
YCSTLKGEEDSVSLI----DNKSMCERVDNHSICNEVNSSISSTYEKTLYNKSNNIIRNL
KEMKKYERECQNN-----------------NSYSNMYCSYMMKLSKKILGFPLIKDFSKG
LSTLEPSLPLNDHLKKLSVCSNCYSKHHDLAKAIICRVTKMHFEANYNDYLTDEDLFKTS
SEFIQSVIRELANTVKEYRKKELNRVYLQHATENDSM-ILDRVNKDMSLELNYNNVTTRT
SIHNCSCTKTNKEYNESFYKIVSPSFNNDTVEKSNIIIQENNHINYECQNKEVEKCHDDF
YSKDMHILYKNTFLPREEIDNCSIEKINDVEEKVINDTLNDMSNDVPNDMSNDVPNGMSN
DVPNGMSNDVPNDISNDNDVSNNVPNDVSNDVPNDVPNDVPNDVNLYEQKNDLNSYEKSC
KLNNFIPLIGVELGKTKFQREFTNGTFVGRVTEQIKDENNNNFFVVTYEDGDVEWITPSF
LFQEILKQSTNNTTYPLASTFKDIFYEDFKKDIKLNNHSYELKIEKKKRKSMFEYVPNNN
-VTKRQRHAFEENVLKKKNTHSMVNKEEAQRLKELGNKCFQEGKYEEAVKYFSDAITNDP
LDHVLYSNLSGAFASLGRFYEALESANKCISIKKDWPKGYIRKGCAEHGLRQLSNAEKTY
LEGLKIDPNNKSLQDALSKVRNENMLENAQLIAHLNNIIENDPQLKSYKEENSNYPHELL
NTIKSINSNPMNIRIILSTCHPKISEGVEKFFGFKFTGEGNDAEERQRQQREEEEERRKKK
EKKEEEEMKKQNRTPEQIQGDEHKLKGNEFYKQKKFDEALKEYEEAIQINPNDIMYHYNK
AAVHIEMKNYDKAVETCLYAIENRYNFKAEFIQVAKLYNRLAISYINMKKYDLAIEAYRK
SLVEDNNRATRNALKELERRKEKEEKEAYIDPDKAEEHKNKGNEYFKNNDFPNAKKEYDE
AIRRNPNDAKLYSNRAAALTKLIEYPSALEDVMKAIELDPTFVKAYSRKGNLHFFMKDYY
KALQAYNKGLELDPNNKECLEGYQRCAFKIDEMSKSEKVDEEQFKKSMADPEIQQIISDP
QFQIILQKLNENPNSISEYIKDPKIFNGLQKLIAAGILKVRMNEHYDVIILGTGLKECIL
SGLLSHYGKKILVLDRNPYYGGETASLNLTNLYNTFKPKENIPSKYGENRHWNVDLIPKF
ILVGGNLVKILKKTRVTNYLEWLVVEGSYVYQHQKKGFLTSEKFIHKVPATDMEALVSPL
LSLMEKNRCKNFYQYVSEWDANKRNTWDNLDPYKLTMLEIYKHFNLCQLTIDFLGHAVAL
YLNDDYLKQPAYLTLERIKLYMQSISAFGKSPFIYPLYGLGGIPEGFSRMCAINGGTFML
NKNVVDFVFDDDNKVCGIKSSDGEIAYCDKVICDPSYVMHLKNKIKKIGQVIRCICILSN
PIPETNQTNSCQIIIPQNQLNRKSDIYINLVSFQHGVTLKGKYIAIVSATVETNNPIKEI
EKPLELLGTIEEKFVKISDLYVSTSKKPADNIFVTSSYDATSHFETATNDLLQIWENLWG
QKLNFDDLNTNADGEAVTVKKQKKTLKPVTKFITINLSKLTHKVCYKRKAPRAIKEIRSI
AGKLMHTKDVRLDVKLNKFIWSKGVRNPPKRVRVKLERKRNEDEDSKEKMYTIVEHVMVD
SYKGLVNECEANEMSASKGRKKSDLKKIDDSFVENKDSPKKSRSGLQEDKALKLRACLSC
RMLKSESEFYQNGCINCKFLQLAGDRHRIHDCTTENFNGFMAITTPNKSWMAQYNDLSKY
APGFYALQVIGELPESIRDLKPNYMKNSEQVRINFNYIYSNKEIRNDVYLPSSDTFTFVE
ALEEEVDKISQDINIVLEMGSGSGYIILSLYEMLLSRNKKIDMLYCVDINKKACECIKNL
TYENKIFNVEIIRNNLFNNIRRCELFDIVLFNPPYVITGPDEMNKTDLTASYAGGKYGRE
IIMKFLLDIHNYLSNKGVIYLLLEKSNIPQEILNCEHVKNIYIYEEIKKKKTLNETIFIY
KLMKKKMDRYGHNVRSDIKKQGYEDSNLPILCETCLGENPYVRIIREENGKECKICKNVF
THFRWKPGENSRYKQTVICMKCAKVKNVCQTCLFDLQYNLPVQVRDKFLENSIVLPENET
NRNFFLEQMENDMSS-TYDKMNRINMDLSKLKRRDPYFKRNMARVCSFWRKNSCNRGDEC
PYLHKEIHLDKSLSNQNIKNRYTGENDILAEKILLKHNEKNNDDKNMSNKICIQGISESV
SQANIKECFKKFGDIKSIKVIPKDSKMFISYSNSQAAKKASDKYKDGLLLNGCNLTVHLQ
DNPTYNNKNQQPVINYMNNNMYQNNMSPQ---MMYNPMYYPY--YNMNNMHPSSAPYSSM
LPSEAEQRKMLKNIFSEYLKSYDNKDENYER--INNLSRNYSCDYTSDWRWFSIKCERFL
ENELYKKYEEREKKKKNKNIIKENENLAIFRSFFPKILLNIYSKCENPAQFFSNLVIQKF
NSVVFFCDASGFSNLAEQLDKRINGTELLGNCLNKFFNILIKIIDYWGGDIIKFSGDAVL
VIWPLHNVLKNKKKKNGTGDHQNKNNKISKSNIAQANNSDDYNKKKKNQNAVEIRKICLL
ALGCCMDIHKLLNKFPTPIENKYLKVHIAITYGKVSFLQIGNVLRKREYLLSGKPLEEIG
AGESLAKNGESVLSYSFYKNIKDKVMVQGTCKKNFFLFVKMKEEIDMKKLKKNYEEEEEQ
QQNEREESRQSTIIYENFDLLLKTFIPDIVYRKLSLGCNIFFNEIRKVTIIFVSVKDIDA
STMTGVHSAHGIMKLTQKAVFTMEGTINKFILDDKGILILIMFGLPPLYHCDDTIRALLT
CFRLIDALKSLKLNGSIGISTGKIWCGIIGNKIRKEYTALGDSVNVAARLCFKAGNKEIY
VDENTYNNCKHFISFQKLISIKVKGKNKLIKIYSPIGTINKKSIDYVYNTSNDNNYFTDE
ELLVNKTNYQQIFDFSFLNNNFLFIYYKSFFKNKIYHLFKEKNSFYYLQIKKDMD-NNHQ
NLNQNNNTYNNLLTYDMIYSLSRFNKIKTSLHVDYKSYTGPFLLHEYYDPFHFKFKELSR
IGGVLFVEGNENLGIFEFIKLITNGLYNFKLFNVSNMPNSLYINITNPLLPWKILCNDIL
NTWKLSHMRKKNLLLNKNDNFNMLREITHPSYHWFFKSMIHVIDDLDIPFFKTKKKKKKK
EKEKEKEYYNHKTNKKNEHNLIQNKINTNTQTQNASNNSNNNIFCQKIHNSRREKKKNKK
KSKEKIKKDDDDDIHDDNHDDNHDDDDISHAYSRNDIQTKMKNELKENRIGIISSMIYYF
TLHENMFIIFNYRSGTSLNINIEDDAWKISNNIAKLAMLKRKKISKHLQKNLKDWRKNHS
RKCDFCKGFYKTLCPNIKDNILLYKSPLCNPFFMHKHKPLIFLFINGMKNNSIKQLKKIK
KYAQQCEASIELKPFNKYDLYEFVSLCLNIHKDKISTQLIEYLNKTCFGIPKFVQYTLFY
LLTNHYIELYKYEKDKNDKENNDELHPSVDARKNNEKNNEKNENQQNNQHNNDEQNYDKN
NYINHFSKQIISVQNETVKSSVFPSNSTTSCNDNSNNDDINLVVVKDLNEAPLVPRLTAY
CMSLIDSLNQEEQLLAKLCSFFNNTFNIKKMECIYPKYISRCELKKIIVKLVEKNVFCLY
EDPKSVTLPKDEEIYFCITNFSLKKVLNDLLENEEKEYIKKIYKKYIEMTFSSKNEEDGY
DKTRLIIKNIPKYMNEIDLKKHFFKMKDNINFQITDIKIMKRKKIIKNKEHYESRRICFI
GFLNNYHCQCFKKFFNNTYINTSKIIIEDAISPDLKSNNSICNNSHFKIKQKDKNKDKNK
GKSIQIVKEQNCINKIVQVKKTKAGMNTTRTHVIYMDEDNLQNDNSEIKKKKRKKKKKKK
--EKHNNEDNNTQDASNTHSNNEDNALEWLKEISKKKKKKDESSMVSNIKNDNIEHVKNV
DKSDMTINMDTQKNDTTYELDISESEEMNTGKLIIFNLPPINEQDIKSLCERYGPIVDVK
VFKNLIKGDTQIYLNNKSKTKSSDDFFIKLLKGNTSNNNHNNNKKNKDDDYNKKDDDQSK
VEQHSNNQKCNKNILYNDLTNVNVYAFVNFMFPSSCEKAKIHLDNKIYRGKILIAKYSKE
KITNYEEENINEKNIFIKLSNDCKTSYKKIVEIQKKKSCQNENMWNILYTDINTSINNFC
KENNCSVESVLNIKDKNIAVNVSLTETYIINKIKTWIKNEGIYLDAFEQIYVKKKKNEDN
EVDKDDKEIVKYRRSDDTIIIKNLSIYTNQNDIINLFKQYGILKRVSFSPYNNICIIQYE
NADNAKKAFISNSYIRYKKLPLYLEWAPLNLFAKQKDDKRTIKREETNEDDSHNVNVNDD
GDDDED---DDEGTHASIYIKNINFNTKEEDLKNLFDKMEGFITCNIVKSKKVILQKDKE
SKISNQYNTVSSGYGFAEFKNKELAMEAIKRLTGTRLNDHLLEMSLSHNRIRKKKKKNED
EKLVLKNKKQVTKKLVVKNLAFQVNKEELRKLFSAFGNVKSVRIPKNVYNRSRGYAFIEF
MSKKESCNAIESLQHTHLYGRHLIIDFADDFMFDKNVDEYDKLKELNNQQNKDIITSEQA
KRKSIYESQKSKQVSESKKRRLTSNLKSI-MVKCNDNQSKNCTRNFCNVNEEDRKEEGMN
NNNYSVEKNKFSQSMNQENYENFVDCPPQNARILKPLIQEKIVEIMKPEIEEKIIEVPQV
QYIEKLVEVPHVILQEKLIHIPKPVIHERIKKCSKTIFQEKIVEVPQIKVVDKIVEVPQY
VYQEKIIEVPKIMVQERIIPVPKKIVKEKIVEIPQIELKNIDIEKVQEIPEYIPEVVQKD
IPYTQIVDRPYHVEKIVEVPHVQHIYRNIVSPQYRHIPKPVEIPMAHYRTFPVEKIVDRN
VPVPVELQIVQEFLCPKIEARYKEIPVPVHVQRIIEHPIPKDAMNNPHLLPLYYQEDN-N
IEMSTNTMEQNKNKNCFIFNLNRNDSRNSS-HASPSVELLLHNDKSQKQFYNNHTNKKTS
IISNPVFNNY--NQNGTLNQFNPNDNINNNMEHIIHHNNNNIPSNVNTHNKINNNPYENN
TVLKNNYSVNIPTKEFANNIYSSPSPVLSSHTEMFSTRSEYDRGVNTFSPEGRLFQVEYA
LGAIKLGSTAVGICVNDGVILASERRISSTLIEKDSVEKLLSIDDHIGCAMSGLMADART
LIDYARVECNHYKFIYNENINIKSCVELISELALDFSNLSDSKRKKIMSRPFGVALLIGG
VDKNGPCLWYTEPSGTNTRFSAASIGSAQEGAELLLQENYKKDMTFEQAEILALTVLRQV
MEDKLSTSNVEICAIKKSDQTFYKYNTDDISRIIDVLPSPVYPTIDMTAMELEKTHLINY
FLEICPTVLDCSRKELQSVLIKKEEEKIRKFLLDKNINLIVIGKEGNDNVEREMDDKEND
NVDINMNEEYNSMRTSNLDNKYNNFLFVELMINYKCVTKSISIAFMKRNKENFLSLNDKI
DNRNKINLSNELLMFVCGQNDCNTPLDLVYLYLSQGFNNIFDAASGYGQNISGPDSSHFN
YGTKK-----DVENMFSINNKYIGYEGENKVSNILMNNVSKKLNELLISMKNAQIDLNIP
IINLHVDKRIKKLLEENPNVDDMKPDKLKQLCESQEFINQLQKDVTKWIEDIQKLTRLNG
EFKSGSALSEINFWIGYENALYQLESQLKNPEVILTLHILKNAKRYFATMSFDSDIQLKQ
SKEYVLNVNILMKDFPIEDLLGATSIQQIIQAVRNIFNHLKKLKNTTKYPLSRSYNFVES
LSRDLNNTMKKVLCTQSLMNMDYEEFDVLISGCVEIFRLWNEEMRIFKDMVRELIKKRSF
NERAPAKMVFEHIHLQERLDEIKKFRKQHEKFKSVISTVFGNNKSLGINLYKDINTAYNI
FLSLDPLDLSKNGEDNWEKAKLSYESKVNRVESQITFKLRDQLGGSKTSAEMFHAFSKFN
PLFFRPKIRGAIQEYQNTLIQIVVDDLRKLQMIYINGYLKSDSQKVSTIRDIPLVAGSII
WAKQIERKLEDSLKRIENVLGRGWEQHSEGKILRQNIDNFKNLLSQNKTFEKWLKNIKSA
DKFDMYDNIINIKKLGGNNYEILANYDFQFFNIFKEVRYLQSINLRVPYSIKVKADETKL

IYPYALTLQKTFRTYMKICISMDNQAKDVPFNQTIKKLVAAIHNTVQNKIKEGIYLHWDS
DIIETYVRKLSETINTFEFMVDEAMNKNKIVLDSLEKMKTCEVCFDCNELKSLIEIIQKK
ADELYLEHYRNVHIWIEELNIHINKILTERLEEIIKTWTCEFVNWP---NNGKRFICKEN
IHEFKIKNQKFYLHPSIDSMRQIWFSKLSDAINIICGITRIKNIYQKKEKTNEKIQIKSK
GKNVINDVILDNTYKYIIYFIDKKIYDNAIKSINDMVDKAQKYESIWLQYKTLWQIEIGD
IISSFGEDIETWKIFMNEIKQTENTFDTLDTEKYFGPIVIDYRILQSKVSSKFEIWQKEI
VSEFSKKLGEKTLYLKEEIEKALYDLNTQSELKNETEITAMHILSMTPKDIVGIMNTYDM
DILSKTCNSIYNFIEKINEINKKEEEWSKKCDLLKQSEVLLEKQRYTFPTNWLYIDNIIG
KLETVKQICKYQIKLIKDYLPYIQSMVLDFDRKVQNNIKELFEEWNKNKPSHGNANSTKA
LQIITTFEERIDIINEQYEISEKIRKLLELENSESEIGFHVSPNILKEEINCVKGIWDEL
KIIYSNICDMKKMLWSNVDPKDVKHRLNNLLESIKKIPAKYRQYEIFDNVQNEIQQYLKT
YSLLLDLKSESLKERHWKLILQKLNIKIYYNKLTLGNLWSLHLCIHENVLSEILNQAQGE
MALEQFLRGLKDTWNEYELELVQYQNKCKLIKGWNDIFSTIDDHLNAIQSMKISSYIKIF
EEETFTWDDKLNRLRNLLDVWMNVQRKWVYLEGVLKGSSDIKSLLPQEYNRFKIIDSDFI
NIMKKTSDKPKLLELFQMEGFQKQLDRLSDSLSKIQKALGEYLEKQRNKFPRFYFVGDED
LLEMIGNSKDAKIIQRNVNKMFAGINSFILKENTNDIILGMSSREGEEVLFLEALNISSF
NTLKEWLIVLEKSMKSSLEFYLDEAAKEILEMDMIECTKIE-NNKILLWSEKYPNQIILL
CLQILWTTNIENELINFSKNPPDESNTLFHKSEKICLNLLEFLAVNVVKQKDHRTRQKFV
QMITELVHQRDVIRILIDKNVNNVNSFIWLQYMRYYWDSKKKENKINLIIKMADATFEYG
YEYLGMCEKLVQTELTDACFLTLTQALKMKLGGNPFGPAGTGKTESVKALGAQLGRYVLV
FNCDESFDFTAMGRIFVGLCQVGAWGCFDEFNRLEERILSAVSEQILTIQTSLVQRKNEI
EILNKKIGLNKNVGIFVTMNPGYAGRSNLPDNLKQLFRSFAMIEPNKQLIVEVTLFSQGF
ISAEHLSSKIVSLFDLCSEQLSKQPHYDFGLRSLKSVLNSAGNLKRLTLLKDESKYNNNN
ERKTTTNTNENIISMEQTLLLKSVCDTVYPKLVSSDIILIQSLLKGVFPNVNVGDLEEKG
LINEIHRLCKLRHFTPEEKWITKICQIYQIMKLQHGVMLVGDVGTGKSSAWKILLDSLEA
LDNIKGVSYVIDAKSLDKEEIYGKLDNINLEWTDGVFTGILRKIIYNSSQSGNTNKRHWI
VFDGDVDPEWAENLNSVLDDNKLLTLPNGERLPIPESVRILFEVDTLKHATLATVSRCGM
IWFSRDILSPIILFKHKLNMLKYGDNDYPRKMDKFKLLLINNNEREKNQNGNENGNENEK
KNSNNIYSMNHFDNIDSENIMDNIRMNSRIFFEENEQETSSYIIRTIPYRAVNIISDYFE
ENEFVHQCLVEAENYEHVMDYEYIRVIESTCLLLQKGFDNLVKKNEKINNTLSDDDIEKY
ISKWLVVSILWGIGGSLNLETREKFSMFVQSICSIPLPNDLLSKGMPNMDNTNKISNTLL
DYQPNIEDGEWINWKELVQIIDVDRTEISDATLVIETMDTIRHETILEGWLHLKKPFILC
GPPGSGKTMTLTSVLKKSSEFDIASLNFSSGSLPNLLLQTFDHYCEYVKTTSELVLRPLQ
PGKWLIIFADEINLPTPDKYDTQRIIMFMRQIYESQGFWKYDVNNNSWNWVKIERITFAG
ACNPPTDAGRNPLSNRFLRHTSVLYVDFPGYESLKQIYGTFNRAILRKFPQSSHMADNLT
QAMVDFYTKFSETFTIDMQPHYIYSPRELTRWKLALYETLESCDELKTKDLVRLCICEGL
RIFQDRLIYKKEKKETDKIIDDIFKYSFPDITKEDLLRPILFNSYMKNYYTEIDKKDLKV
LILSKLKIFNEEEINVQLVLFDDVLDHITRIDRVLRLPLGHLLLVGASGAGKTILSRFVS
WINGLSVFQIRAGRNYTTESFEADLRHIMKRAGIKEEKITFIFDESNVLGPAFLERMNAL
LASGEVPGLFEGDNYITLINECKSAYRSNIGLDESDIFKKFTKQVQQNLHIVFTMNPANP
DFANRQATSPALFNRCVIDWFGDWPYSALLQVASEFIFNLILPDNNFYMDYVEDGPIKGK
IQYKNNKAYFLSRAIVEIHNSVVHINNVLMKKGNRYNYMTPRDFLDFIKHFLKIIDEKKE
EVSSQKNHLNSGLNKLKDTEIQVAELRNSLAIKKKTLAEKDLEAEEKMKLMIEQQTETED
KKKKAEILSKKLDEQFIIIDQRKEVVRKELSEVEPKFREAEEAVKNIPKKNFDELRAMAN
PPILVRNAVEAVAILIMNEGDKNVTWEDARKIMKGQDFINKVLYLDKKAVKPQTSSQIKK
RINNNDWDVERINKASRAAGPLAKWVESVITFLNILETVQPLEKEIEKLQEETKVAEDQY
NEQRDIICELEKKLVQYKNDYAQLISQVQNIKQEMEMVENKIKRSINLIDNLKSEKERWS
ETFINLEEASETFVGDCLIAAAFCAYIGFFEHYERQRLKRTWGEIIKMHYIKYRNDLSFI
EFLSRPSERLQWIGNELPSDDLSIENAIIINNYIRYPMIIDPSDQATTFLLNQYSDKKIL
KTSFSDKNFIKNLESALRFGSTLLVYDVEKIDAILNSVLNQETHKQGGRLLITIGDSEVD
FSPSFNLFLTSRDAHFQFTPDLCSRVTFVNFTLTPSSLQNQCLNMILKNERPDIDKKRCD
LLKLQGEYKVKIRELEESLLLELSNVKGNILDDDNVISTMEKLKVQGAEASKEVNIAEEV
MVEVENVSNQYLFLAQGSARIYFILQHLCNINFLYQYDLNFFFNIMKDMFNNDHLLSIVK
KKDYKERLKVLEDLLFSLTYNRVARGLLQEDRYVFGLQLCYVKSIINPNIDMDQSYLHYL
LKDHYSNQEIDEF----EHKKIEKNLLPEYNDEQINALNNLIKHKSFSNLKKCILNNKQK
WIELLHSAEPEELVCSILNKSDEMLNKNKNLNILNNVENNDNTNNKNDISSCLKESLIIK
AIRPDKLENCFNKIINHILGRDFLWIPELSMNDFEKYVKENANGNIPIVLISSPGFDPSN
KVQQLSEKCKIPLFSIAMGSEEGYISAERVIFTAQSNGGWVLLKNIHISTKWLHELEKNI
HKATTNKNFRLFLTMEFNPRIPQSLMRISLTFMFEPPVGIKFSILRSFSLFLENRELCEP
KIARLRLYFIVSYLHAILLERRRYTPIGWTKKYEFSDSDLMCALSVVDSWLDKASTKIGK
NVSEHIDPCNIPWEAIKKILNEAIYGGRLDNMVDQKILDTFIDHLMNSNSFETDFKLNIC
NSTSNKDFLVSPDLFRNINDYINWTNNMSNTDLPAWLGFGQQAEGLLTTRTNFSIISKWN
ILYSKSRSDVYEPLPHSPDKDKNKDVISSSERTESETSESSCTVSRSIHVYSNNENILFI
NKILENLPQNIPCLEKNEEKLRNAVFRCFERENNLFSDLLKLIKTNLNQLKNVLEEKVKY
TNKIRALAKDLNSFNVPSNWLLDGNTTNLNLTNWLKELINRLYQIIVITLEFNEKSCIDI
NEKK-QKNINIENNEDHNNKKLSINFIWLGGLFYPRAFITATRQLSAFKFKNSLDDLELS
VLIGNNNNMKYDDMIHFTITCLSIEGAEWSNKDNVN---MSKNKKGNKNNNQHNCNINHN
NNNNNNFVGNHNLPSQMQKQNRLNLVNHSNKSNTMNFKNMNFDEKNSKLFNNISLLMNKN
ILSNVNAAAQISLGASSEFMKNNKLGVNNSQKNNTYNLHNQNQNASNQQINQSYNDNFTY
RPTLFNLLEVLANHNITTPDQRVCIRGIVTDFLNNELPHGKIYAYIGAVVGHDILHDIIK
KLEKDPNRNVVPDASGLARIEAAFGLSKSSYDFINNNSQNSTGNLSNVLFNNRNLNSALL
NNQFYSNSSKAAADISNLLKYAKNEKMSMSNNNVMNFNRKKGNNLHKNSFSDEEEELMKV
IKKNIESYKMNNIKNILISSVFGRIKWLKIMNESPHAYLYGHSIVKFGNKLYMFGGSNGK
NKKIPFTHTLTFSLIYYNYKLLPLSGNCPEEREGHTTHLVSLHNGLSVFLFGGSNENIYY
NDIYLLDMETRKWTRRSVKGKIPLPRDQHSSLVYPAKCEHVRGEKPNLTEGVIIFGGKCL
YNNSIVSLNDMWIFSFDSILWIRINYLCDDIPMGRFGMNLVWSDTNTICLFGGEYCEISK
THKERTLLDDMWIFKVHNN-NN-NKKFTMVGEWYRENYEGDIGCRSNYSSVFITQRHQDF
KGAEPKTIERLMILCSGITYVYDNKQKIVSTDEVFVYFFSQKKWYLLKGKLCNDEYLYNG
RQRHVGCFFESKNVLGRANRNPVPCVFIQGGFKKNSVFGDAWLLSLTGENPLRIQEYDTS
RERISTTQMPLYYFRDTHSISLLYSFCTLQKWLFGAFANLVDNCVHAHNPAENVFIKYEL
TPEHDGMLSIQDDGEGLDFNAMNRVLRMYGNYKYQDNSSVVLYNSGTNIKKHALPNNDFI
NNKGDDYNDYLLDASSALLPKKQKNTENDNNEEKDNITGDHNKNGEDENKVGSHNAYDND
QNNGYVSDDYYTKNYDQELFYNENVNNIFDVKYGVGFKMSFARISSSCAIMSRTFNTIGI
GLLSLELMNHCEAKELATPLCMWKLPNKELINRNIANKSEHRHHQKLLMSYTPFNSPSLL
AEQINILGTYSGTRLLYWDFRDDMDFIIFSPLNNNIYLSSSPLSVDEIKYNKKKKNNNKN
NKNNNNKQVSCVEDVKLNNRDIKRKGNF-DEENVKKVKVQHDESCANVYAK--NDEEGSD
KDASTKDENMKKEHPNEKDDDNNN-----NNNNNNENGDDNN-KSYNDQIFKENEQYQYY
NSTIKKLNLFEYNSHLNTSISKDKYKASQIFPLWDHPKDSIDYCLSTYLYWLYLRRNTNI
FLQNTLLIPTCMRKNDNDVNSEKKKKKKKKKKGKKKNLQEESNTTEQTSSLSKDSNSNKD
SEENVNNDEERNSKRRNNDDESNESSSTSNR---DKSDADNMNNDVIKNNKKGKEEMHKI
KFEEALEYGISQESSRKNSMKYKEEDDEEEEEDDDDEEDDDDDDEEDEEEEGEDEEEEDQ
ENQDENQDENRDESQNE---NENQDDNENEEPNEGSNENEEKPNDGSIENNEDKEAKDSD
ENNENECKQKKKKQEDKNEKNLSTDSKNNDSEKTRQGSYVNTYMDGLKIKDEYYINNTSK
YTLYNFLRKKLHKMVEFHYLFTPSDYEYGSFIMMGFLNDNNSPSIEVNRVCETGILLYYK
NRLIKRLDAPFIDTAYNLALAKYPPNPSLYEGNLYKYALTVIVNVPYWLKPSISKQEFIH
ENNYAFLVFKKKLIGLIKHYLFICQDNVKLRKWRESRDLKLKRYLDKSSFSFDKSKNESD
NDDENDYKITNQ--EKENAQSEKEGKGKNAQSPNNDNDQNEVVGANEREDDSTNRNNSSN
EEENEGTPADNEDENDDSNNSNNSNQEQKEKILENDEEEATDDNEEKNEEEDDNEEEEEY
ENNDNEKYNSDDVNEDNKSLEDAEEEDTI-------MEDNTDKKALVDENNGKVPKKKNL
SRLIVEEATNDDNSVVALNTKRMEELNFFRGDTIIIKGKKRHSTICIILNDNDLDEGKIR
INKVARKNLRVCLGDVVYVKSCPEIPYGKKIQVLPIDDTIEGLAKDTLFEIFLKPYFNES
YRPVKKGDLFLVRGGFMSVEFKVVEVDPDDFCIVSPDTVIYYEGDPIKRDDEEKLDEIGY
DDIGGCKKQLAQIREMIELPLRHPGLFKTLGVKPPRGVLLYGPPGSGKTCIARAVANETG
AFFFLINGPEVMSKMAGEAEANLRRAFEEAEKNSPAIIFIDEIDSIAPKREKTNGEVERR
VVSQLLTLMDGIKSRGQVVVIAATNRQNSIDPALRRFGRFDREIDIGVPDDNGRFEILRI
HTKNMKLSPDVKLEELASNTHGFVGADLAQLCTEAALTCIREKMDVIDLEDEIIDKEVLE

SMCVTQDHFNMALGTCNPSSLRETVVEVPNVKWDDIGGLDEVKSTLREMILYPIDHPDKF
EKFGMSPSRGVLFYGPPGCGKTLLAKAVASECSANFVSIKGPELLTMWFGESEANVREVF
DKARAAAPCVLFFDELDSIGTQRGSSLGDGSGAGDRVMNQLLTEIDGVGPKKNLFFIGAT
NRPELLDEALLRPGRLDQLIYIPLPDLGARISILTAILRKCPVAENVPIDFLAQKTAGFS
GADLAELCQRAARAAIRDAIDAEEMNKKSKLELSNKKEETNENDQQKNDDDNIKYEITRH
HFKEGLAGARRSVSQADLIKYDNFRIKFDPLYKTKTGGTGDDFIIDWPDEDNNDDTPAYV
VDEDLYSMKQTSNMNFKPLKIKPSLLKELKKEGLISIEEAEGNNVKILSADNLNSHNIHY
GNKCKNVSKNNESKKKLSLKRKKDSTKRENQDDTTIKIEDNNFIEK--RKRKRKRGKKKK
NMREQEREQEREQENEEKRGEKKSTENVNISNISNKKKELFDIERIIKNEEELEKDNNIK
YKIHCNNWNNGGKIFILHSVMKSLFDNAFFKPTEIQSKTLEKSINDKNDIVVISKTGTGK
TLTFCLPILNNILINKLKEYKKKLKNIQKFRCLILVPTRELALQILKHFNYINKYINLFI
STIIGGLNLNKQKRILMKKPEILICTPGRLKYFLHLENPIKYIYQMKNIRYLVCDEIDKM
IEISFMKDISYIAKHIYKSVDKKKKLIQTFLLSATLSLTVQLQNDNMTKLLNSIIIRKDK
SFIINLSNEQN-------------VYNDNSNILPELLTLYIVKLNERDIVCKLFYLIKSY
FSYDINNNQNDEINKIIIFVNTIKSAKQLNAIFKHLFLHNNLESSIPKKYRSNLYIKNKV
NIYSIHSKQKLKERLENINKFSQQNHKAILFCTDVLSRGIDLDKCDLIIQLNCPISDITF
VHRSGRTARNFKKGKCICFITETEIYKWKTSLEKVGINIQDLQELDYLKSINEEDYAKIN
KAIECANKMIEFQDKLILKKKDSLLKKLAREAELDDEDEYSDSENEYEHLNNQTIYKNIL
HLKKELYNTLYKQMYDINKSICAEIKSAELDVLNSGELRRISMGKYENEKYEYEKTHKSG
VVTKMYYDPKYGTIDHNQICSVCFERHENCTGHIGHIEFVLPLFNPLFYKELQELLNLIC
YHCYNFCCSEDVIFLLKHIFQLKSLNQIESTNKIKYKKNCDYEYIIDQVEKLYKKICKDS
NISLDDIVEAIYDDYENVDEKNKNNNNKNNNNNNNNNNNNYNNDVDIDVDNNNNNVDINV
DNNDNKENKPDYNKLKERIKKYKFDANKLAFQSNYNYEEYLILSKTIKLHMKKSSKCCFC
KFQRNISAKVSQRRDTINFIGVHTNNPFFKKYMPKKKQKKNEKDDQNIDDINYNDDVDNM
QHMNDDESQLNSIQSRNENNKNVNDKQMDRESSYPHNNNNNNNNKNSTFADMVNHYNRQAP
KEKCTIRLFSFQVIDILKKIFENNNKDIINLLYPFTKRDGYKKFFLYDMGVSGNRFRSQS
KGIHVRTKITNTLCRFNNFIKLCINAKKKVDFDYLLEHNKKDLKLYKDMFSVFSLKMKYK
FNNNIMDDDTDITKMDFLKYYTLVYNNKSAYINILFSTLQLGVNTFYDSTLTEKVNNKHL
KNISIRQILDKKEGILRKNIMGKRVNNCARTVISPDTFIETNQIGMPIEFAKVLTIDEYI
TQNNFTYIKKLIENGPNIYPGALSYRDSNGRVFKLSHKYEDRLKVIEKIEKLNFQT-ENS
YILHRHARDGDVVIMNRQPTLHKFSIMAHYLKIFEKEKVFRLNYVNCSSYNADFDGDEMN
LHLLQTPLARAEATHLMNCDFLFTSFKDGSPLRGLAQDFILGGLHLTSLETFLNYDEYCN
LLQCSLNSLISQKNSFFIKKKNNNNNTNRTNSIMKNTRNSQYNDEARNNQFNGNVLKITN
YNYLDPYAIILNRTSFTIITEEPAILYPKKLWTGKQLITSILKTVIDKVAIETYNKYNDN
EFMNTYKGINYVAKAKTSPDLWSFDPLKENEVIIKNSELLQGVLDKLHFGASSNSLVHLC
HELFGPKTSAVMLDCFGRLFINFLQLRGTSLSLYDFILNKNAKKEKSLIKKRISFTGFYL
QNLFAYSIANSVNADITEMNSYTKKKMNTGNQRKKLILNKMYDLYEKQNELIN-------
----KENYLNKRNIKKDSVKDEHILSISENSYLLALLNKELQNVIYHNKEKNEKENEKEN
KIANTDDEQNYEKYLIDERNNHFLNSVTNKLYKALKEPTFFNSTSFTIKKITKVIEKVLT
FLKAARCNKRLKIYIIFELFQNKNIMKYFPSLVNYINSFHFDVSNEEIIKNVINNIYFKN
EFIDDEKIKEKTIQEIKKYKLKKFLHFYAELHKKCEGRGEQNDYNIDENDYNDNNNNNND
MNHTSDDVYDEKIIRNCLMSSLPSFTLNDEITNLSYNGKYQYYEDYLKKKDEEDLNFKYY
NMDDVFYKMEYLIHNYFYLKRSDFDNLIDSLFQPYLCRVSSGTNNLINMNNLMNKFLNNG
FSNMIFTGAKGSKVNYSMICGMLDQQYLEGKRVPRMRSGKTLPSFHRYDYGARSCGLITD
CFLEGLRPQEYFFHCMSGREGLIDTAVKTAKSGYIQRCLIKCMESVILHYDGTVRNEDNS
IIQFLYGEDGIDPSKTAYLDLSKDLLHNYHLSFSKYLNNNMHSKILNNQELFLKHEKTKD
NEEPLISKYNPYTYIGSVSDKFNNKLNNTLLWNDLNFPEYENVPMNFDTLSSSLLKSKYY
NSLCNPGESIGILVAQSFGEPATQMTLNTFHLAGTENVTMGIPRLKEIFLTSKLTSKPMI
YVPIKLQVDNLNDANKMKNYITSIADKILSSYKSIFLTDVIYGIGVDRKIILKEN-QNEQ
KHTINLSDIINMNDEELSGDTQLAKINNVLKNCNINFDMKKEWNYEIIIQFENLYHFCNI
NKHLTISFILYKVVNALLNSVLNKVHETFVFHNLINMNSLNHEYYDELYDFISKKMSEEF
NFNMDKYEYKDDEHQINAKLKFMEKENENNISGEYQNASHTIASDSMKSYNDISNKENMS
PYDDGKGKDSDIELKDNSEKNSNSDQTDEEEEDDELKSEKEK-EEDEKEYDEEG--ESDT
NSKIDSDHKSDSDNKSDSD-HKSDSDHKSDSDHKSDSDHKSNDSDIDSDSESEMVSSNKE
IININNKDSNESKLSECSNTKGTVNYFSSETSATHKNKKLNIEDDEHINNIHFIEKNKYN
EMDNKEEITKSFTRLSYDYKNNINLISSDDDQENIEENEEGQKMKKSEQRYVKNIIDKIK
SSLLCFIKNIHFSPVTWILKLNIGWDINFFPYPIDFLNHIKSEINKEVLFKVKDLNNPKI
LKGKDITHSGAEYELQIEGKNIYKLYNIKDKYIDKTKLYCNDIYAIVKTYGIEAGRLCIT
KELKKVFDAYGIQIDFRHLSFISDFMTHTGDLKAFNRHGLGYFRNVFHKMSFECATNFLI
QGCIHNSIDYLSTASSSLFFGKHIKVGTNLADIVTCIDK-MTKNRLLIESREAKKQNDPD
ISLTHSEYNLYEWQAVIRGPKDSPYEGGKWKLNIKCKSTYPIDPPLITFVTKFFHPNVNF
VTGELCMDILKANWSPAWTIQSLCRAILFLFNEPNADSPLNCDAGNLIRSGDIKGFQSMA
RMYTVEYAMEDDMNRMEELDPDMLGVLKCAFNAGVKVKSAAEVGKRAVEYFRGDDFVNFL
STNGEMLKKKFPNLFINRNLSDMKEIEEFADMFIQKGYIYKAQYKPIKGINEKDENGVYK
RPKWPKRLIMTSKQNFDKTSFYILVHERNKKLQYFMLISLISIVLICCMFPVWPLRLKLA
LWHISVAFLTLISIIIVGRLVAFIFFWFFGVDYWIFPNLFDEECNVVESFLPLQSWVYRN
DTWFLFVARMCTAVLLAIAIQQLGKTHSIADIRNFATQSYIDIIEWGNKKLAASPENVSM
YKSIDSKATFEGN-VDDDDTVYDENEENYDCLKKCGFTSFEELVRKCFLKCECMEKVIKS
ECYKKKCSKVTKAVLDEAHKEACFDKSDNMATLADSFLKDLEDLEFED--EEEENNPFIK
RSNNRMEDEDNDDYEEIVDAIEEFLNEKKKNKERKISELLYDEEFLNMMNDIKTYMFEEE
EKKKEKEKDDENVDGDEEIEVLLKEKDNNNNNNNSDEDNFYDDVMIEKCMETILKIDTEI
LNIHKYVKDIYSTKFPELDSIVYSPVEYISVVNKIRNEVDLKNIDFSDILPNTTVMAITV
ASSMTTGICLPDNLLKNCISFCNEGIQLNEYRNIILLYLESKMFYLAPNVTMLLGSSLTA
RLISAVGSLKNLSIISSQNLIVIASTKKSLFGLSNVHKTLGIGILCCSEIVQSVPDAYKK
KAISLLASKCSLAARIDYFKKYKEGQYGLLLRQYIISHLIKLQEPPPLKQKKILPMPDEK
RKRKRGGKRYRKLKEKTQITELTKQINRLPFGPETTDDFYNFNDQNTMLLNSNITKLKYT
NKQKNLITKKRNLNVHSSGATGGLSSSLIFTPLQGIELYNPSLINAKNKQTENKYFSNTA
EFRKIMEDNKEENCELNKNSYPNFHLADLFYLLQLPNISINERNDLLQSLFDEIKKNHMY
PYYNYICQELNLNFDEEYFKSLKEKADEELNQIENKLSESAENFDSLDNKNDVLLKANFF
CKISDKENAFKEYEETYKKGIGMGMKLDILLTMIRICIFYNDVKNLKKYLEQARTQMEKG
GDWERKNKLKIYEALNYIMIRNFAEASKILIDAASTFTATEIISYEKIIFYVVILGIMTE
ERTVLDKKILNSSVILQITSSDEDLHTYLHSFYHCDYRTFMEKTIKIAMRVKRDRYLGRH
YRYFIRNTRVRAYKQFLEPFKNVTLKNMAFAFGVSEEFIENEISSFIANGKLNCKIDKVN
GSIESNQPNERNTMYQNTIKKGDILLNRIQKLSRVIDMMVNINWPGLLKWSTKYADGTID
TNKRLSKEDIEFLQGAIKDALSQVEDPYEAINEAVRNFENKDEGIILASAKIVERLVDEY
PEVARNLDKIKAIDPLLKLLDNSNNHILESVLQILSLALSNNPELQDSVFKKNALKTLLI
KLQESQKTIIDKKLITAISALIRHHDQGENKFIDYGGVGFLVYGMQTNIFKYQEKSALLL
KHLIHQNKITFDIFIKNDIMKGLVALVNNKNIDETGIQYGETTAELFLALIQNHRHKLAK
SGYLHTIKKLIEDRLSYLRIVQDSASYDVSQEIELFTDCLKLTK----MIICARTKKPSL
RFCKLFRRYYIRKFLKNKIKEDKKKRYKYKERNVDGTRLYNEEQINSYSDQSNNITFEEL
GVEDWLIKISKSVHILYPTKIQQLCLPLIIQGKNVIGSSETGSGKTICYCWSILQELNKN
VYGIFSLILLPTRELVFQIIEQFHLYGSKIGVMILSCIGGFSLIEQRKSVMTKPHIIVGT
PGRISDILESSIDIQNCFKRLRFLVLDEADLLLQKCFEDKLQNILNNLPKNYANERKTLF
FSSTITNSLQLLIDTFPYNNLILVNVNKKQKPPKNLDQRYIYVEEIAHITYLIYILKNKV
NNLSGIIFTANSYKCELVYTVLNMLGIDNVDAMHSSKDQKNRFATLAKFKNGLCKILVAT
DIISRGIDIPKISFVINFDFPNDTVQYIHRVGRTARANRKGLAISFIDKKDVNSFNQVKN
IMKDKLKPYTLNKKEVLENMFKIGRVIKKAEIMLEEKKDIKRENERLKHYIFHNEMARKY
FVAANWKCNGTLESIKSLTNSFNNLDFDPSKLDVVVFPVSVHYDHTRKLLQSKFSTGIQN
VSKFGNGSYTGEVSAEIAKDLNIEYVIIGHFERRKYFHETDEDVREKLQASLKNNLKAVV
CFGESLEQREQNKTIEVITKQVKAFVDLIDNFDNVILAYEPLWAIGTGKTATPEQAQLVH
KEIRKIVKDTCGEKQANQIRILYGGSVNTENCSSLIQQEDIDGFLVGNASLKESFVDIIK
SAMMKKKNKEDLYKENKLEASKLRIAIVSSDKCKPKKCHLECKKNCPIVKTGKFCIEVDH
ASKIAYISETLCIGCGICVKKCPFTSISIINLPKDINKDVVHRYGPNTFKLHRLPIPKLG
QILGLVGTNGIGKSTALKILSSKLKPNLGKFNNPPEWRDILSFFRGNELQIFFTKLLEEK
LSPIIKPQNVDLIPKQIKGNILEIINKKDKFNQKDKYIAELDLEHLLDRNVEDLSGGELQ
RFALLMSIIGQSTNVYMFDEPSSYLDIKQRISMAKIIHKLVKHDNYIIVVEHDLSILDYL

SDYVCCLWGKAGAYGVVTCPFSVREGINIFLDGFVPTDNLRIREESLNFKLATDQDATDE
DKKRLHFYNYPTMVKTLNSFSLTIDKGHFSESEIFVLLGQNGSGKSTFIRLFAGLIKPDN
LESLSFLESLSVSYKPQQIQAKFTGTVRQLLMSKLKGLYNDPYFNNEIIKPLKIESILDN
QVLTLSGGELQKVAIIVTLAKNTNIYLIDEPSAYLDSEQRIIVSKIIKRFILNTNKTAFI
VEHDFIMATYLADHVIVFDGQAGVNTVANTPQTLAAGMNKFLKIIDVTFRRDPSNYRPRI
NKYDSVKDKEQKLNGTYFIIDEMSIICTISGQTPEEPVISKTGYIFEKRLIEKHIINYGI
CPVSGEVLTLEDLYPIKNEKIVKPRPITASSIPGLLSIFQTEWDSIISEMFSLRTHVNDI
RNELSHSLYQYDAATRVIAKLLKEKNGYKEEIENLKKQIFQLKSSNDLDIYEIGLNEELL
EKMQNVAKDLLINRKKRKIDNVCSVEQWKDFKNTNEFNIHSSTIPGVTCITLDVNKYKYN
YNDDHMKHNFFSGGNDGNVYYVSLNDNKILSKLQGHLKKVNSIISHPSNFICITASNDKT
IRIWKGDDDNYQFVSAHVITKHKDHVTSLALHPLENYFISSSKDSMWILHDLETAKTIKT
SKDNPSSFKHLAIHPDGMMFGIAAQDSNIHIYDIKSQEYKATLNGHTKSLNCLSFSENGY
YLASSSKDNTVKLWDLRKAQSFQTITLNETPNFISFDYSGKYLSIAVENDIQIYNFETKN
QANLIKTLSSHTDIVTQTCFGSTTSYILSSSMDKTIKLWNMTEVTHCYNGNIKDGLFHGF
GILIYSQHEKYEGDFVYGKREGRGKFTYADGATYEGEWVDDKIHGKGIANFVSGNIYEGE
WENGKINGFGMLCYNNGDKYEGEWLDGKMHGRGTYTYEDGDVYIGEWKNDKRHGKGCVKY
KGNENKIAETYEGDWVDGKMQGRGTYFFADGGIYEGDWVDGKMEGKGVYKYLNGNKYEGE
WINDMKNGYGTLAYVNGELYEGYWKNDKVHGKGTLTYSKGDKYIGEWKYAKKCGEGELIY
ASGDKFKGQWKNDKANGYGILLYNNGNKYEGEWLDDHRHGMGTFTCKEDGTIYSGHFQFN
RKHGKGTLTFVNGHILQGIWNSGLLEKVINYELTPSSPWNDPDLMKLEEVKDIQKIERIG
AHSHIRGLGLNDCLDARYCSEGMIGQMSARKAAGIVLRMIKEGRISGRAILLAGQPGTGK
TAIAMGIAKALGEDTPFTHISGSEVYSLEMSKTEALTQAFRRSIGVRVKEESEVIEGEVV
EIEIEKFNERDINNKNKKLGKMILKTTEMETLYDLGSKMIEALQKENITAGDVICIDKGT
GKITKIGKSFARSKDYDAMDPNTLFVQCPEGELQKRKEVVHTVTLHDIDAINSRTQGFLA
LFSGDTGEIKNEIREHIDMKINEWQEDEKAEIVPGVLFIDEVHMLDIECFSYLNRALESE
QSPIVIMATNRGITHIRGTDYKAPHGIPLDLLDRTLIIPTYPYKHQDILKILEQRAEEED
VDIDEYAKELLCKIASESSLRYALHLITLANLVSKKRKATEVTVQDVRRVYNLFIDVKRS
TQYLIEYQNEFMFSELPKEELSVKEEDSSEEKRELHEKNSENSSTSNMAYIYSNLSDFWT
SDDDEGDEGSNEEEEEKSVSNDKE------NNLKNETYGYNEYNIYKREDMNMMYNDDNS
FNKTIDERLHFNINDIMNECSNEISENFTINNLLEKYDINNFIYNDNLLKYCETGVAIND
EKPNNEKLILNEDFEFNVKCSFNYVNPPSYGLKINRDVKNEVPQENIKNNNLEMVIYNKE
EMLVEKKVLDDNENVDMEKIDHPNDDNNNKKYDDDNNNNNEAQILNNNIENTKIKYIRKK
WVIEDNESDISNFNKNDLLLNYDFELDDFQKRSIKHLNNFKHVFVAAHTSAGKTLIAEHA
IALSIKLQKKAIYTSPIKALSNQKYYEFKNIFKDVGIITGDVKMNVNANCIIMTTEILRN
LLYLNDNIINNIHCVIFDEVHYVNDEDRGVIWEESIIMLPHHVQILLLSATVPNYLEFAD
WVGFTKQKEVISISTKKRPVPLLHYIYVYDSVYLVMDEKNKFYSSAFKEIYVKIREKQEA
NNKNTKQITSSSNLKKNNNYYDSKNKYLTTTNNKENDNTQINNNNNNNNNVIGYYEYCKQ
KRKQKLFANEASMKTEIQKLQTLIKKLDQDNKLPVVLFCFSRIKCETYAKCMPHLNFLDT
NKKSKVHLFIKESISKLPKQDRELNQIQSLSKLLEKGIGVHHSGLLPILKEIVEILFSKG
LIKVLFATETFAMGINMPTKSVVFTSIYKHDHLRKRILTSSEYTQMSGRAGRRSSDKYGY
VYICCCDNIPDQVQLTEMMMQKAVSLKSKFKVTYNMILKLLINKQINIEKMLFSSFLESC
RALQIPLFKKDLKRKRKLLQNIKEVQCIYEQNKNAYPPIEQYVQINYRLKYIGLNLHKKL
LNTKSSNCFVIGRVMLLNNIHILHSSVYAIYLGCDKSNNKKNDKVDFAQNSIFFQNNYED
DRSNERFFFLFILPDFMAFEDLPDYILTSKKKKTTSNNNNNDNNNNNNKSLSNSVSENIN
LYENYKNVFSKKNNKSDIKIIYHSSFDTDMNKKHFVVCSNVCIENISIITNTVIKLPNVN
NAGILNNPKNLLLYTFELDRLIEKNNFEPFVLTKMLKSLKCEFYSVLVNQADYLENLKKS
KCYNCNLKEKHYQLICKKNDCLDDIENIERNINAKSLNLYEDLEGKLNVLKHFGFIDDQN
NLTVKGKIASYITLTDEITLTQVIFENVLNKLNPAEIAAVLSCFVAPEKKVEESPDLTVN
LQEVKAALTNIHSSFEEFYKVIRLRISSEDHWKLCNFKIMFIAYKWTLGVSFAELLEQCE
LEEGLIVRSILRLDDLCRKVKIAFLYLGNIDLAQKVEKTSHLLRRDIIFTTSLYLQ---M
VFHLNIFNKGKRQNLVSAYLNHSGSDCVNKSEVSSDNNNNNNNNNNKIAHNFFSKKYQRN
FENNNQENNKNIIYSGSNIFTEMMSNNNTVDVNMMDNNPAARLEELRTIMKKNKIDVYIL
INSDEHNSEIINEKDKKIVKITNYSGADGILIVTKDKPILYVNALYELQAMNELDQNLFT
LRISRIDNRDEIFETISSLEFNTIAFDGKNTSVVFYEKLRKALLNAYPKKKIVEKIIYNN
NFDDVNKKDDLNFLVLEKSLVEIKDYPVNNKTLYIHDRKYNGACAGEKIDKLKQSLYDIK
NVDNLLLSELDEIAYLLNLRGYDYQYSPLFYSYLLFQFDREEQDFSKIVFFTTVKNLPAD
VKNLLEINKVIVKEYEEIVPYLRDVV------------------IPSIPDFKKYDISLSP
YINLMIYKLFDRKNVLLQNSPVVKMKAVKNDVEIDNMKQAHILDGLALLQFFHWCEQKRK
TKELFNETEMSLRHKVDYFRSTKKNFIFPSFSTISASGPNAAVIHYECTDKTNATIKPAI
YLLDSGGQYLHGTTDVTRTTHFGEPTAEEKRIYTLVLKGHLRLRKVIFASYTNSSALDFI
ARENLFNNFMDYNHGTGHGVGLTLNVHEGGCSIGPVGGAPLKKNMVLSNEPGYYMKDKFG
VRIENMQYVISKEITDTTEYLSFDDLTMYPYEKKLLDFSLLTNQEIKELNEYHTTIRNTL
LPLVKQSPQEYGESVEKYLIEITEPIAIHMIFFLLSKICKNNAILKSLKKNNKIVLKNII
TFLKLFISKNNYIYNTKMFSSNCNTNDVEVMKKKLVVGENPEFIKKRLEKFNEIKEKRKQ
ELEENFEELRKPITIELLDGSIKSGESYVTTPFDIALSISKRLAEDSIVCKVTYLEKVDV
ELCDIEEGDDHNEEANEKSKESILWDLNVPLLGNCRVEFINIQSEEGQKIFWHSSAHILG
SSLEKLFGGFLTIGPALKEGFYYDIFLNNFSINNEDYKRIEDEFNKLVKDNVPFEKVICT
KEEALELFDYNPFKLELIRSKIPDNKKTSVYRCGNFIDLCLGPHIKNTGKVKTFKVLKNS
SAYWLGQKENDSLQRVYGISFQKKSELVEYLKFLEEAKKRDHRNVGKILNLFFFEKETSP
GSCFWLPHGSKIYNKLIEFIRKEYRIRKYEEVISPNVFSCDLWKTSGHYQNYKDCMFLFN
VENKEWGMKPMNCPGHCLMFKQLNVSYRSLPVRLADFGVLHRNEISGSLSGLTRVRRFQQ
DDSHIFCSMEHIKQEVLNTLNFLFYVYNLFGFKYELFLSTRPKKFIGQISTWNLAEQHLK
DALYAANVQWKINEGDGAFYGPKIDILVKDSLNRTHQCGTIQLDFQLPVRFNLQYKNKEY
VTNNEKNDNDDNVHNNNNNEKKEFKDSQNHNEDKHDNHSPINEEGLLKKGFERPIIIHRA
ILGSVERFVAILIEHTAGKLPFWLSPRQAIVLPIGDKYNDYANYVYETLHNNMFDVDLDT
SVNTLNKKIREAQLKQFNYILVVGEKELTTNTVTLRDRDDQNNQHVYTIQELINKFNKLL
DVNSKKFNQIKEFNSNNNISSHKNTYNNISHKNTYNNIYYAKLYIFFMAICLALNKFLNT
QEENNENKKKKKKKIMNFFGLSNETASCCGIMAYMGNRDASKILIDGIEILQNRGYDSCG
MSTISNKNVLKTTKYASNTTCDAIEKLKSNYLNSHKNDHIGIAHTRWATHGCKTDENAHP
HVDYGERISIVHNGIIENYREIKTFLLKNNIPFKSNTDTEVVANLIGYFLDKKQSFQDAV
LSAITQLEGTWSFCIIHKNHPDEMILASNGSPLHIGFKDDEIFIASEHTALFMFTNEYIS
LKNGEILSISKDKINDLKLLKKVENIPEIAIQKTPHPYPHWTIKEIHEQSATLSKSLNNG
GRFSSGDHLVKLGGLDPYIQDLNKIENLVLVGCGTSYYAALFAKYLMNYLNCFNTVQVMD
PIDFNISVIPKEKEGVIFISQSGETRDVIKACKLAEDLNVRKLSVVNSVGSTIANMTGRG
VYLNAGREVGVASTKCFTSEVSVLTLIALWFFQHKKNNQSSNKATSLINSLHRLPLYTGV
TIKSCENTCKTLSEKFKNTKSMLIIGNGLSYPIAQEGALKIKELAYIHCEGFTGASLKHG
PYALLGGEDNIPVIMLLFNDNTKNAMINTGEQIKSRGAHIVCLTDDENLVKHFADDIILI
PNNGILTPLLAVIPLQMLAYYTSVNKGINPDKPRCLAKTVTVSMNDSYDSLFKILLIGDS
GVGKSCLLLRFADDTYTDSYISTIGVDFKIKTIEIEDKIIKLQIWDTAGQERFRTITSSY
YRGAQGIIIVYDVTDRDSFNNVKNWIIEIEKYASEDVQKILIGNKIDLKNDRNVSYEEGK
ELADSCNIQFLETSAKIAHNVEQAFKTMAYEIKNKSQHETINKGKTNINLNARPIKDTKK
KCCMPRIRTMNSRKPPEGWHKVESFLEEMNKKMRSLENEDTSKKRKNEILWPIFQINHKT
SRYIYELYYKRKEISRELYDYLVQEKYVDGALISKWRKQGYENLCCLKCIQVSDSNFSNT
CICRVPKSDLGNKVLQCVNCGCRGCASGDNMNNVISFIGNSSNKYFQINQLHFIRIIIHS
KNNLINSNSSYNVFYNKYFIKNTFQNKNKLSSIYSKLNFSIKNMCKDKNEKKNYEHVNAN
KNGYLASEKNELTKNKVEEHTYDYDYVVIGGGPGGMASAKEAAAHGARVLLFDYVKPSSQ
GTKWGIGGTCVNVGCVPKKLMHYAGHMGSIFKLDSKAYGWKFDNLKHDWKKLVTTVQSHI
RSLNFSYMTGLRSSKVKYINGLAKLKDKNTVSYYLKGDLSKEETVTGKYILIATGCRPHI
PDDVEGAKELSITSDDIFSLKKDPGKTLVVGASYVALECSGFLNSLGYDVTVAVRSIVLR
GFDQQCAVKVKLYMEEQGVMFKNGILPKKLTKMDDKILVEFSDKTSELYDTVLYAIGRKG
DIDGLNLESLNMNVNKSNNKIIADHLSCTNIPSIFAVGDVAENVPELAPVAIKAGEILAR
RLFKDSDEIMDYSYIPTSIYTPIEYGACGYSEEKAYELYGKSNVEVFLQEFNNLEISAVH
RQKHIRAQKDEYDLDVSSTCLAKLVCLKNEDNRVIGFHYVGPNAGEVTQGMALALRLKVK
KKDFDNCIGIHPTDAESFMNLFVTISSGLSYAAKGGCGGGKCGMEREKHENMLMAIARDF
NSVDDLIETFLTFLENKTDYFHVMLNDKDVETLSEKYDGAILKNLLNNNNCGFKAHSREQ
LLIKSFRKHQINYIMRKQPYIIENEEIKNKYLTSCDELKKIKYMPTTKDMNKEKQENSTK

VNSDVHSLNVTQENHISIWNGGKTDKYYWNQALKEINLEMPFNEEIKPNDVNVQITNTNI
KIYHCGVLKLEGMFYEEVDKQECVWSIEDKKNIIIYLEKKRENWWPCVIKGDTEIDTKNI
ESKKNLTDFDEKTQGQIRKFLLEQRMKNEGIPTPEDLRKQNIINNVLSSKGEPFGRMKQA
NTKEDKSQKISNSSTIDEIEEQLYTGPLKIEQLLAKGFVKRDLELLKEGGLQTVECVAYA
PMRTLCAIKGISEQKAEKLKKACKELCNSGFCNAIDYHDARQNLIKFTTGSKQLDALLKG
GIETGGITELFGEFRTGKSQLCHTLAITCQLPIEQSGGEGKCLWIDTEGTFRPERIVAIA
KRYGLHPTDCLNNIAYAKAYNCDHQTELLIDASAMMADARFALLIVDSATALYRSEYIGR
GELANRQSHLCRFLRGLQRIADIYGVAVIITNQVVAKVDAMSMFGGHEKIPIGGNIIAHA
SQTRLYLRKGRGESRICKIYDSPVLPEGEAVFAITEGGIADYEEKMALKSINISGNFEWC
PFEEYKNYLLCFNSHNLLYSNNNSLNNYIYLLDINLNSEIRNLEIVNKYNFEDALKYDND
VNNNSVNEYVTCFEWMNSNNFVDINNNEELSKGIIVGGLTNGDIVLLNAKNLFETNRNYD
NFILSKTNIHDNGINCLEYNRHKNNLIATGGNDGQLFITDIENLYSPTSYDPYLDKNNLQ
KITCLNWNKKVSHILATSSNNGNTVIWDLKIKKSAVSFRDPHSRTKTSSLSWLSNQPTQV
LISYDDDKNPCLQLWDLRNSNYPIKEIIGHSKGINNICFSPIDTNLLLSSGKDVTKCWYL
DNNNFDIFNEINNSANNIYSKWSPYIPDLFASSTNMDTIQINSINNGNKMTSKYIPTFYK
KEAGICIGFGGKICTFDNSTNNSNVNNMNNVNNMNNINNNDNSCDGEYDSNKGKNKSTQK
KFLIKYHIYPTDMELISEADNFEKYITSGNYKEFCESKINKCDDDHEKLTWQILQLLCTS
QRGDIVKYLGHDINNIVDKIMQTIGKQPGFIFKTLIDEENNNNNNSTNQMQNDVLLHNDP
NLMNNYLLKDNMNPNIMLNNNNNNINNRNGQNLLGDTNHNEENFNGNFDIDPEKFFRELG
EKTENEKIKQNEEDISGNDEHLLSIKGKENKTKNKKSGLGTDNNDNGDHNKNEGEHILNE
KNNTNNWNLGIEAIIKECVLIGNIETAVELCLHKNRMADALLLSSFGGEQLWHKTKTIYI
KKQNDNFLKNINYVLDDKLENLINNVDLNSWEEALSILCTYAINNPNFNSLCEMLAKRLQ
NEKFDIRAASICYLCACNFSETVEIWNNMPSKKTSLLNVLQDIVEKMTILKMIIKYENFN
SIMNQKISQYAELLANSGRLKAAMTFLCLIQHDQSIESLILRDRIYNSANHVLCQQIKPP
ISPFQIVDIKPSPNVYQNNMYNNNNNNININSSSNNNNNNNNNKVLSSMHHPMQQFNQCN
VNKMPPPLPMNTQMNNSTSSIQPPPSVPPTKFHTQIINNTMNSRSSI-ATTTKNYPTSNL
NSVIPTSMNNMNTNVTPPVAVPNMNNNNNNNTMNPTYPSLPKFPNYNLNSQVQQNSIPEK
QTSPMFSSNSYGNINKTHTNAVPPPPPPMPSNQLNN-TRSSFADIQNVVSPPRNKNQSIS
STANLNYQHDNQFNKRECMEQPVYPMTNQSSMFSNNTMQKKNVPGGFTSQMNYGMQPTGS
PPPSSLSTTSPIAGALTVTPGMPVPWPIPTTTQQLGSTTQSTANENKKIQTATKEQNGVL
MNRNHIENIKKTISNLLNIYTSQESVKKKADDVSSKVYELFEKLDCGAFNEQINDSLLNL
VNCINANDFKTTNKIIVDLSRNLWDGSNKAWIMGVKHIIPKMAIRVQFENSNEVGVFSRL
TNSYALVALGGSENFSSVFESELSQHIPLVYTTIGGTRVIGRVCVGNRKGLLVSSICTDQ
ELLHLRNCLPENVKIKRIEERLSALGNCITTNDYVGLIHTDIDKETEEIIQDVLDIEVFR
TSIAGNLLVGTYSYFTNNGGLLHAMTSSQEIEELSELLQIPLITGTINRGSDLIGSGLVA
NDWSAFCGMDTTAIELSIIEKVFKLNNITDNNMEDTFKYKSSIIQTMIMFESLVEKLLNK
FLAPYVEGIERNLHLGVWSGNIVLENLKLKPQITEILDLSFKIIHGNIGRINIQIPWSSL
GKNPVCVLIKNVHIYIKPRCYKKSEEVIIEELRKAKMHRLQLLEEEISLIKLQKNNEKSS
EKSTLIFKLLNKIINNIQIDIQDILIHFEDPDKNFSIGFILKSSSVKNCQNNDEMSTQAN
SNAENKTLNHIIEFKGLCIYSNSDIKSRTKKKIRKKNKKRSSNGKNISEMKYEKSELSLS
NENMNKTDTENNNNNLNNNDNNLNNNNNNLNNNDNNLNNNDNNLNNNDQNKDTHDYSKLS
SCDS----STQDDYLKTTLNRISSFLKEEENIILHNYNYLIKPFDLVLPVEQSSNKKELK
AKLEISDKWEGITLTKTQITKIIEIMNEANKSRNQTNKLLLKHAWTVRLDIESLRNETKN
EFINLYNKVLGEEYNISNTELSAQEINRLQILYDVVGVRHLAKWRLHCRTTLEKFIEEKN
LKKKFLYDSIYKQQSWWSWVTGNKKEIENKVQNILNSEQDIINENELFMIQEAMTNDDNY
DVVMPSKYDFQFKLANFSINVYDDCKK--RIIDNRNHHNNHNNNNNNIRGVSNKSVTNNR
QHIKEDKLNFNDIIKDELSINSSCETLLTSSINLSSTDSKLNKRRVRYERVNILSINFYQ
IYSSLSLQSVVDHNDHDNFQWKFIIELQNFIAKHKNKVFMEFRNNKNSFPYNMNTSGSLY
KNSIFYSLLYSQTVCAYLEINHLVTEKGNTLSTILRLNPLELYLSPLLIKSILSFTFPLL
DIINKSHKTAMKKYKSKNVSLVEKQKQNDNEDNNNDDEDDDDEDDEEALLQIKKMEQSEL
LEGLKERGENVYNRAVQHLPELFEFYIHICGPILHFDNLTNGIVELHLGNLVAKTEHPCT
YNKFNLIFEFNETQITCLKIGSGTGEKFYILQPIPVKIYVEYDLKILKTNIILDGIFFQI
NPDAISIILAVPTSITRYLTGVYSKNKNENEFNKNKKKTSVEKKGIMKGDDITRASMSNI
DSGVQKRNSITDIIKGNKDNDNVVEMNNGMPKEEESFLYDIDFLIKNSSFSIKNKKNCEI
LKYEACGIFYKNYLQKKRKIIKMEIEQLWICDPSNKQPIFFTLTKDINSKDIFLFRSLST
YLMGNNNNINNNSSHIRKNSFNIGSNAFGSLFESAKVMDDENKVEEEDDDFMDAIEEKQL
SISLQILQEHNEKNIEETHINFIISDIELHWKYKTIKQIFKTMKEYKKTLQYGIEKDMTY
IKNKLKNEKDLKNYKMFISENTLRSVQETLKNVKDSLNILDMESAKDNNVQEKEKDNVKM
NAEGIHLMNPYYARDEDLNMIADNKNNEEININNANVNDNNNNNDDLNDNILSKGLNDTL
PKLHGQQNSYPKYFFNCFIKSASLAFWKKKKIFSRIQVSNIFYENKIYINFDQKMFLNIE
KGIMSMNNKNIISNNINDYKYDLFISKDMQNDDDKEKEEYLDNLSIEIKKKKTMHKESKE
DLFIGKIKVYNDKRNYNICLLCEIPRMYYIFYLKDLRLFLEYLDDGILNVFISKSYKKVV
QAAQTKYFLFNFTFMDPIVIIPEDKNMIYNYKGEVKNVSRYFKSSTSNNEDTNDYNSIGR
NTYNSNNIPYIESYLQFHLSKLKLKNSYTLKCTEEIRVAQRRQMILKKKKYKGRSEKEKI
NKNKYEESFIRSDIVSKEQLRPNNDLVKLDFTLYIDILDIESKACENGGSSNIEGEILHK
VNLGFCLINARNGIFIYLNGNDLCLDLTVFQLAFLLDIINENFCYKGYFPMCFICDKDVS
LDSMINSEWFKKGYMNFNNFNNNNNNNNKKENVTKKTGLKLYVYINFESLKIKTAFDTNT
PVAIITFQYISMSFRLVLLDFYYVYFFDLHGNSLYIDDARKNSINYYKRVAYCCIENEKK
KFKRENKKANEEAYNDTLNKSNVYEYNSYQINDVQDEYDEKVQKLHNNSSNNISDIDYKN
LLIYMFENNVFLNEKKKRKKQKGIKIKINSFIEDLLLNIELDDAYICFFFLIFIDIYKFL
TTGFNLSTLHLYPKPSPYIVSYKNENKKKKIKSINIEENVFKRERIHYDEEIIVQKKKII
RRSSVLEQSKYIKGKNINEVLKLDNKPFHVSFKVNNGNFIIFTDVEKVSHPIIMWSNNFV
FSFSLFNKCIVFRKIYAIDSKVKRINYVSSYKYDTIKKRNNNNNKIFDPKNISNPLHNYH
KKKILLCDNLNVMGEAVYESVDTRTEDYKLKSSVRKDNNSPFITVFELDINIGNFDIRLS
NDDVEILLKASSTLFGDVPSSFTNIVVGPVIPPDARNRKEMERNDHNKKISDVSSSTNLS
YSNTSLSNNNNNNQTNSYRDIKINIKLHNVMCTFIDNIRNSIVPILRMIFSMNISINLYT
DECSYNISDLNSKLEYFNNCIGEWEPFLEKCNISLDIHNIFPNDEYDEDKEANKSPISII
KINSIKALWFNITPQLINLLFLFVPVFSEKVSNGLRKNANNKLNVRNTSINKDIESEQMN
SLNSRCDSGSVELEYKNMSVKSKSIFEDCSSVFNSFPCDTDGKEANDFHLDDFLKKEENR
STYYNRIDNSVIYYVNLTSEYFYAFLMPESKIEEIMKRKKSVTMKNVYKSVNEENIMMEH
TLISSDDNSTHNEGNEDYISEEIPDVYAKIITTNELISLDRLLINEIENNSLIEKKCLYL
YLIPIPPTNVVNIVHDMFSNISKRDIVLDLMITKNPKKTIENLLIYNQHKEHIRIDDNNK
MRMKAEENVFKYNKSNKFVGPFLRNTECVIINLIKNSCTSLTTSLKNSNVIHLLKPGDFV
FDEKSSKKNGSNNNNNNNMSSILNKENIEWERRNEEMIKQTYIACKYRLKNNNVCEIISP
MPNYKILFMSSTVRIINKCGIPLEFCFFDGSRNPILLTSLENRTIPINTLYPNHSDSFKN
YKVSNSLNINPNIKLRQKNNNLSFTVILNHEYLLSAPECVFCGPSHVYMSFKPINVITPT
TNIANNNNNNNNNSSSSSHDPMSINIENGWSDIFSSDISQGTYVKKCKYKDGNNFFYFLV
KIENKISALPAEKNLKIITIYPHVSVVNAIPAFVDIIITSDNSEHVYMQELNKLEREIYH
IDEEIKYKKKSIFYIYEIKKYTCLNLKMKIGNSQCEWSEKLFLVEDDEESVTRFSLHFKK
YASVEVEIIKNFSGYFNSLSSILGNKQLIFSLPRCFIDRTGLGIKAINSNKYYPVINGIT
LLGDHSQIDLLLPHKTQPNNNVNNKNDSRYVSDYNDIFNDFNYNNSVILFKATLPPIGSY
TETNVICKNFFYTFCLNTEKIKTTNIPYIISRIITVVPQFIISNKLNFPLLIKQYQNDQM
QGVRANDTSPLYFTKKSSILLFQFKCLDPCRTNNEKNHMLLDPKNVIKSNG--------N
KYYKLNKNIYWSSVIYPSENFVGTNYMVINTGNHEKSDVYAITCIPDRGTKNIIIEKLEN
KK-KGFIAYNNSSIAKYLKIRTFHDDTRHMKIHDEENYMEKNMFLSNFLKPTDMEHYFNI
EPNKYSYLGWVNPFIYVTRNVQIEIVLEDLKIIPKSPFVLKFAIYNYSQKTYYINYYNIT
FVICIEYIEDLITIKLSHKLNISSNSSHNIMFSKHMNSCDKSVTSNNDIKLGLNKKKKYM
EMENQVNIEKYNIINDISNERNIIDKNSENSLYAQKQIIDHDEYHYVKYIDEKGSYKNNS
ISSSNNYY--KNESVKMISNTYKNVHIIINVTQIGVSIISNILKEEVFFIELSKLCALFY
MKNEEEVIDIKITDVQIDCQLESCEKCVLLANRGISSSNKNMNANEEKIFLNIYVERSFI
SHNDVIFKKIQVSLDDVEIEMDAETLNGINLLTAEYIESISIVQKKNLLYEEIQKWTILP
VYVNYKSPEIPLAINIQYMQIDKFTLIVWCSFLLDKMHMMSDLLRIGLRILMVSGKLELL
GAPVTLNQEIFNNIRVSIKSFYALLKDKYSHSILACLGFIVGYSSLINIPKIPLEIGRNT
IGLAVYAVDNVSVGIGSFLSNLTFDSEYINRRQKERTFKTNTNMKEGLISAVKNIGEGVL
SLSNIVTKPIEGAQKEGFGGFFKGIGKGVAGSLVKPLDKVGQAVSDVTRGIKAEVSKPIG
GHKYKTKRHRKPRMLWGEYGKLKEYNINEAELRECLGLKFSKNIMKCLTVHKQENPPSHY

ALLLYPKVIIYANLYANTSEKKTDIVIWSIKIEDITEIRASSHGLIVRTNTSTYKIPCNN
AILINKIYRELHNSKNSINSTVILGANNSAKYLMNNLKSYGSLSKVDSVQINGMDIDTNI
FLNMNSYPTKEIYDDENNKNDGNHNIKNFQAYKAMYEESINNPEAFWGNLAKNNLIWYKL
FTKVFLGNFKKGNINWFVNGKINACENCVDRWVEKDPNKIALIWEQDCPDQYKKITYQKL
LEKTCKVANLLKLIGVKKQDTVTIYLPMIPELIYSMLACVRIGAIHNVVFAGYSAASLSD
RIIDSRSTVLITSDFGLRGGKLTKLKQIADVAMDMCGMIQTCIVFKNKTKMKDKNIQIKT
PMENPIYNFTSFDSNSAQNFIQKSTHSHQSDHQNDFSLQQEDNNYTNKGKTTLHKKTKNN
PINHKISYSEKSYKELINTDMVNNDNQFGDLKKKFININNIITCNQNGYSTNKQYFPEPD
KNSTSNFDETLCVLKKGRDIDGTALLKNMRSYCPIEYVDSEDFLFLLYTSGSTGKPKGVA
HTTAGYLLYAYTTCKYIFDVKENDIFGCVADIGWVTGHTYVLYGPLLNGITTVIFSSIPT
YPDCGRYWSLIQTHKVTQFYTAPTALRALMKYGDEWIQKYDLSSCRILGSVGEPINPETW
RWYYNVVGKKKCTIVDTYWQTETGGIVIAPIPHLFSMKPGCASLPFLGVQLEILDSKTLQ
PLSGNNVCGLLCIKSPWPGMLRTVYGNHQRLIKTYFTMCPNYYFTGDGAFRDEDGYYWIS
GRIDDTLNVAGHRLGAAEIEHALVQHFYIAEAAVVSFHHNVKGEGILCFVVKKKGDIKNY
SKGITNRNELHNNSPFLICSNNITDIEEFKKNFTDEKLIEQLKLYVRQVIGPIATPDLIC
VVPDLPKTRSGKIVRRILRCIANGITDFGDISTVSNYEVIETINNTLIECKKKLKIEIMF
MRWNKISRYTLLMVVSFWLFYRSENFNLLRRLISKLRSLFPLFIKNNFLNNEIKNSVKIY
FGSQSGTAEEFAKELKANLNDLFHIQANIIDLEYFNKEEIKSFGIRIFIVATYGDGEPTD
NAVEFFKWLKSLNNDNDYFRNTKYSIMGLGSKQYKHFNKIAKKLDTFLLNFKAHQISETI
YGDDDDNIYHDFEVWKNKFFMQLPKLLNMKNIPIYVPKEDIIELTSWRDMAEIKLDIQYY
DHLEEDNKKEKNVVTENIINESVTNNQQLLNHNSTDIIGKFYFNHLTGKVISNTKLLKNV
DLSNNGDKVNHINISIENIIYKAADNLSILTKNTKEVITWWLKRLNIDEKEKTKKFTFVK
RNTMNDPKDDNNNNNYNEYDDNHIYVPFPTPCSVEDALSYYCDLTTIPRLNILKKFKCFI
KDIEELKMFNFILSNNQRNTFFNICKECDMTFIEFVDMFMQSAVFELSPFLQLIPRNTPK
SYTISSSPKESKDILSLTVKKKQYCIHSLRRALKNLKTNDMFPKLNEQKLRELCSRRWFK
GSSSYYLTEELNVNDIVKFNIKPSKFVLPENIQSSHIIMIATGAGIAPFKAFLSEFIYYD
QQIVKDNFVRKGKRILFYGCRKREVDFLYEMEIMDALDKKHIDETYFAFSRDQESKIYVQ
DLILQKKELVWNLLQKGAYIYVCGNSNMSKDVNKTINSLPLHFKQNDKKFTKKLKKSGRY
IYEIWMENIPWVEKYRPKRLDDIVHQNNAVMMLKEVVRTKNMPHLIFHGPPGTGKTSAIN
ALAHELFGKENISERVLELNASDDRGINVVREKIKAYTRISISKNKIHSETKEVLPSWKL
VVLDEADMMTEDAQSALRRIIEIYSNVTRFILICNYIHKISDPIFSRCSCYRFQSIPINI
KKEKLLYICQNENIDIVDDALEKIIETTEGDLRRAVSILQLCSCINTKITLNSVLDVSGL
PSDNIVYKIIDACKMKDLKLVEKTVQDIIEDGFDVAYIFKSFNNYFVTNTEYEDSLKYQI
LLELSRHDYRLHCGATQYIQLLSFASSVHSLLNSVMSEALDIDNAKAKIGLVFSYWKKVA
NNDFSKCSVFCVLSGKSSKDENATIQEQFQMWLTGYQLTETFFVFLKNERILILTSDKKK
RFLQPLLDNIKNVDVLERSNDNTSNFENIKSTIESTNCDEIALLKDKDATGSFFENCYDF
IKTLNKKEMDVNNNIKELLNFRSDTDMKIQKSGSDIACIILKSILITTIENALDNEEFES
HDKIKEKALKFMDNKKCVMKLKDKLKVDIEEIDVIYSNVQSGNNFTLTYKNSNDKNYLSQ
NEGTILVGVGLKYKELCCNITRTLLLNARTQHKELYNFTISIEKYIIKECLKVGTNFSSV
YKKTLEYVKEHKKEYKTLSNIQIENYFVKCIGHIIGIEFIDKEYLIIESNHQGKIQKNTS
YNLSVGFENVQGLEKNKFAIWISDTICIDDNEDVIVLTDAISKEINTISYELEDTKSDDE
EDDDNNNNNNNNGLHKDKKKTGISASILNNAASVIVSDRLRRRNKNSLAHNNEQEMEELN
KRQHELKEKKINDIKIRFSKGTNDYKDLNKKNIKKLEDLKTYNDPDLLPKDLRPNIICVD
NKHECILLPINGLHIPFHVSTIKNLSSNYEDNNDIFVLRINFLVPGNQGVVKGELNTFPT
LQQNQMYIRELIFKSPNEKHFQMVVKQVKELIKQVKQKEVEADVNESKTSQDRLVLNKSG
RRIVLRDLMTRPNIFTGRKILGTLELHMNGLRYAANSRGTTEFIDILFDDIKHAFYQPCD
GQLIILIHFHLKRYIMVGKKKTLDVQFYCEAGTQIDDLDRAKARNVYDPDEMHDEMKERE
QKNKLNLIFKNFVQQMQDISKIEFEIPYPELTFSGVPNKSNVEIFVTANTINHLVEWPPF
ILSVEDIEIASLERVHHGLRNFDMIFVFKDYTKPVKRIDVIPTEYIDTIKKWLTTIDIVY
YEGKNNLQWGNILKTILSDIDSFVNSKGFDGFLGEDDDEEEETADDEDEDDEYEVDESEL
SAEEDSEYDDSEDESLATESDGDEEVEEDSEDEGLSWDELEERAKKDDKKRFADDEGYNK
RKKKKKNMSLVYRKTLNFLKGKNEDDKDVKININGENKRTCNNSVITSKYTVFNFIFLNM
YEQFHKISNVYFFFIGILQVIPQFTATNGIPTVFFPLLIVLTANAIKDAFEDWNRHKTDK
IENNRMCYGIVSEEEKKIYQEKSNKKNIFKKLKRYFFGN-RKICNTENYYDEDDMCDEIT
DINDYINNYEDNLENIEGTVKKRWKDIKAGDIILCRRSEFFCADILLLCTSHKNGIAFVE
TSSLDGETNLKVKEANTFLFNILGNDRNSAIDNVKNLKGFILSDKPNKDLSTMYGTIYFE
KDKKIDVENIQELLKKTTEEIEYRKKRLSSVDLSGSNLCNNNNKSDSKIKNNDNYIRIPF
DEKQFVLRGCKLKNTDWIMGIVIYVGRETKIQMNSSKSIKKTSKLEILTNKMTIIIWVIQ
MIICLISAYYNAIIVSSSRKNRFRYLPFNLEKAKKPYIVGIISFFSWVVITGNFVPISLI
VTMSFVKVVQAYFISCDKNMIHKVQADVPSFGEQKEIPNIKDDISSDADVIKMRTKKIAD
SSLLHIDENQTEEDNQNINGNNGIISNAPKREISRVISFKDSKEKNYIYFNAVPRTSSLI
EELGQIEYIFSDKTGTLTCNIMEFRKCAINGISYGKGLTEIKRNILKKKNLEIPVEPTMK
FKKKTPHVNIIDNDIINHLKDPNHFNHVNLINFFLHLAINHAVICEKDKEGVTTYSSSSP
DEEALVNAAKHFDITFLYRREGKYGISIFGKIYEIDTLATIEFTSKRKMSSVICRIPVIN
PDYNHPTDAKSSNMDKKKNNMDGKNEEKEETDDLNVKKEAHSKLNNNNNNNIRIDNLYDD
KNNHNEGRTPEVITCKNSKNSKIMLFCKGAGSIILKKLAKRTDVDEITIEHMETYADEGL
RTLCIAQRELSEESFAEWYHLYKEASLSIKDREEKLESVAEYIENDLILQGITGIEDKLQ
EGVSSTIEDLRMAGIHIWMLTGDKIETAMNIGIAANLIDNYSEQFIYTEEYIESEEALIK
KIDDDILMVEKSLNIPHYNFDDENNNVRNNFIKNFFCAKNKSGLLLNPDKYNMLINTLNY
VLVVDGSVIDLLLSEKMERKFFYLADKCSSVICGRVSPYQKGAIVSSANRLLNKITLAIG
DGANDRNMINTANIGIGIRGQEGVQAFNSSDYGISQFRFLKNLLLVHGRLSYRRISKLVV
YMFYKNMVLIFPLFIFGSISLYSGQKIYFEFLLHLFNVLFTAIPVVIHAVLDQDISLNTA
MEKPNLYKLGIHHYYFNIRTFISWVMNSLFHGSVVFLIPLYFLSYYNIPTSDGIPYDIWT
VGCATYFLTVLIVNFKILFETYYLNILPISGIALSIFSFVLLVTAFSFMCVGSIHLLGTI
VYLVQSLRFWLVVILGLFTALLRDYVFKVYKRNFNPEIYHLLLDQENAKIGMNDVIDKLN
EFDKDDDIRIEKSKSLGYAFSEADPACIQLIRKQDNMIMKSKILCNLFKGKSCICLYTNC
LNENNVNKCYYNLVRNYSDHVKDHFNKPRNVGSFDKNEKNIGTSIVGKASCGDVIKLQLK
IENDVIKDARFMAFGCGSAIASSSYATELIKGKTIDEALKIKNNDIASHLSLPPVKIHCS
LLAEDAIKHAIKNYREKVLT-MLVKFRKCGQANIFRSISNVRKKNNLKNNKDI-ERKEKI
IILGSGWGGFNFLLNIDFKKYDVTLISPRNYFTFTPLLPCLCSGTLSVNVCTESIRNFLR
KKNYCGNYLQLECTDVFYEDKYINCIDIENNKVKLFYDYLIIAVGAKTNTFNINGVDKYA
YFVKDIDDALKIRKKFLDILEKCTLPNISNEEKKKMLHVAVVGGGPTGVEVTAEFADFIN
KEVKINYKDIFNFISISIIEGGNNLLPTFTQNISDFTKENFHNLNINVLTNYYVIDVDKH
SFHIQSSLNKNEK-KKLSYGLLIWASGLAQTTLIQKFLKTIPVQANNAILKVDEKLRVIG
IPSNNIYAIGDCKKIQPKLLHEHTNEIIKILTGNKLTSEALKLKQSELTKTFPQLSISKW
DYEKNKKGEMTPQQFHDYLFEIDKNYKSPTPTAQNAKQEAYYLSNVFNNFIHTNQKFNIP
SFIEKWKGSLAYIGNHQVVADLPYYELKGGRFSSTFWKVVYIQLLLSWKSRFHFFIDFIK
TKWYGRPFIKMNFNSKKLQVKNKNAAEVQITAEQLINEALELEEVEHKVNYNLIDEEELN
EYRINKRKEYEDKIRKRRYMISTYIKYGLWEIKQKDIERCRSIFERALNIDYTNKNLWLK
YIEVELINKNINSARNLLERVVLLLPLENIFWKKYAHLEEILNNYVNARNIYERWIKFKI
DESSFLCYIYFEERCNEINKCREIFERLIVSIPKLECFYKFIKFEKKYKNIVRARAAYEK
CIELLPSCYIDENFYIHFCNFEEEQNEYERCKKIYIEALKILPKNKSELLYKNFLQFQKK
YANKDELHESLLIKERIFYEDELKKNKNDYDIWFNYIKLEESNINNINKEKCIIRIRDLY
ERAISIIPIISSKKFWKRYIYLWINYSIFEELYAQNIQRARDVYNNIIKILSSYEFTFKK
IFILYATFELRQLNVNKARSIFNNALQTIPNEKIFEKFCEFELKLGNIRECRNVYAKYVE
AFPFNSKAWISMINFELSLDEVERARQIAEIAINLDDMKLPELIWKNYIDMEINLQEYDN
ARKLYDRLLNITQHYKVYKSYAEFTYIYLDDIEMCRKILEEGIEFCKKNELINERCILLN
FLCDIEKDYGDKEIIDKTLKRLPKKVKKRKIIKNNDDEIIEEYITYVFPDDGNQSQNMKI
LEKALEWKKKMEDMKKEVNDDNVMSDVESADNNVVVEEKAVFDNVTAIQKVIKNAHVHDG
LKIGIREVIKSIESQEAKVCFLSDVCSEPAYKKLITTLCAEKNIPLFMVQNDSKDLGHWA
GLFKLDNEGNARKIIGASSVAVVDFGEDSAEKDFLLSQNQTVTA-MSKDKRNSFASNSFD
SSNDEKKSKNGNKIYKSKHEENSPDGDSYKINNNEKEKSKEKLKKDQKKKSKEIYNSFNS
PNSTSSDSDGNGLHLNFSNASSSSSENGFKILRTQENEDKLLEERRRKREALKEKLKNMV
KENEQNNDANEILQND-----------------QINKDYNNETFLLSENKNDNDIITNEI
PSNPSYIDQNDAACIFAPNNDVIEDTCSSLSSDHEIIEEKQNKEKPEAVKECSDLYNDLK
KKIDEEKAKIRSFIIKQKELHERLKDSLYVNKSKGNEEDEMQEEYDEDNDDFDMFSCVQA

NKKRKVEKVHITDYYTGNNANLSDNWNDSEGYYKAMVGEVIDKRYSVVCELVGKGVFSNV
LKCYDMVNKIPVAVKVIRDNDMMKKAAEKEISILKKLNQYDKDNKRHIIRLLSSIKYKNH
LCLVFEWMWGNLRIALKKYGNGHGLNATAVHCYTKQLFIALRHMRKCRIMHADLKPDNIL
INEKFNALKVCDLGSASDISENEITSYLVSRFYRAPEIILGFRYDAQIDVWSAAATVFEL
ATGKILFPGKSNNHMIKLMMEYKGKFSHKMIKGGQFYSQHFNENLDFLYVDRDHYSKKEV
VRVISDLRPTKNITCDLLEHQYWLKGNSPKMQFLKKKIKQLGDLLEKCLILDPSKRYTPD
QALQHPYLRESIHFSKSQNEM-DESNSVSCSLSLESDGYSDEEYDTNLNKLIENESLNWI
FVGGKGGVGKTTTSCSIAVQLSKRRESVLLLSTDPAHNTSDAFNQKFTNQPTLINSFDNL
YCMEIDTNYSENTAFKLNKKEMFDNILPELLHSFPGIDEALCFAELMQSIKNMKYSVIVF
DTAPTGHTLRLLAFPDLLKKALGYLINIREKLKGTLNVLKNFTNNEMEFDSLYEKINHLN
AMSSSIQANFQNPMKTTFVCVCIPEFLSVYETERLIQELTKKNISCYNIVVNQVVFPLDS
PNVNLENCKNLLSQIKNEQIQSYFNDLISKTEELEDVYISRRKLQSKYLTQIKNLYSNDF
HIVCMPQLKNEIRGLNNISSFSEMLLQSKDIPIYKMEDDVIISGTHMTIEKDKLEKMNEN
NFEKIYDNEYENIYRSKCSGILLKTPYYPYKYDIMNTSYKYIPKVGDLVIGIVKSKKLDY
YQMDINCNCECIIHKIDSFKYATKSSFPNLLNGTLLYMVVEKMNLENNSVVTSCINSSDV
KSWINYENYLGELVDGFVFSVNIASAKSLIGDRCYILDLIGQDIKYEIAVGHNGRIWIKA
NDPLEINLIHSALKHSFGKTRAQMNVLWKSIYNLNKKTTMYRADIIRDNLLDYNNTQKEC
LHSLSYLNNNNNNVIKKHMSFTNSRYIGFLNNNVSKYKLNFFQFLKNMYLSKAPKGFERF
ENKSPNS------NNFKPEEEKPRKYDNYFFYIFFILLLFFLLFVDSNGLYNEITQNDFF
YKYLSKGYVEKIKLINKDYVKAYLNSHGIKKYHMKYVSFRVGNSDSFEKKVEAVQREMNI
KRDELIEVQYVNEANILNEVKGYIPSILFFLLLIFLFQKITLKNVTNSGMDKLFKFSKIS
PINKNSLKTDVKFSSVAGMKQAKEEIMEFVDFLKNPTKYEILGAKIPKGALLCGAPGTGK
TLLAKAVAGEANVPFFNISGSDFIEVFVGIGPSRVRELFAQARKHAPSIIFIDEIDAVGR
KRSKGGFSGGGNDERENTLNQMLVEMDGFHTSNDKVVVLAGTNRVDILDPAITRPGRFDR
IVNISKPDINERSEIFQVHLKNLKLHESLDIKNISYILASLTPGFVGADIANVVNEGAIQ
CARRSNLLGVQIKDFELAIERVIGGLPKSSSLISPLEKKIISYHETGHALIGWFLEFADP
VLKVSIIPRNNGALGYSQHLSEEIMLFSRDAILDKIAVILGGRAAEELFIGKITTGAIDD
LNKVTQLAYSYVSQYGMNQEIGLVSFQPNSNSEYNLYRPHSECLAHLIDNEVRSLIETQY
KRVKSILMKNEKHVHNLANLLYEKETISYHDIVKCVGERPYPVKSAYEKFVKANPYKAIS
SEPLLEDKKETNDFEKGHIKENTKECTKENTKENTKDNTKENTKENNSSKDDTKQTSCKE
KLGDSKKKDNKISNVKLRMNTKNATREEKKKELELNEARKAGKVEALKDEEGNDINPHMP
QYIIKAPWYLNQTKPGLKHQRYKGSDKIKIEEERNKKLFLNNNKG-QDFCKNCGSVAHKE
KDCLERTRKKKVNFMKND-NEDFICVTQDLGYDGNRDRWVGYNVNNFDYIYKEYEKIVEE
KKKRKAEDLKKQYEKKSVKKKKTHNNNDDDEKSSSESENLDESSNEDNKLKPDIMNNQTI
NKNEKNRNIARNLRIREDTAKYLYNLSLNSAFYDPKSRSMREDPFANIRKHLDDDNYYKG
ENYYNNTDDAIESKKLEVFAWESYKRGENVHFNAQPTQLELMYKEYLEKKKKIIKKKQED
ILKTYKCQNNEEQQPNQEELLQSEVYTEYKPIEQIHNMKKNIKVPSKYEEDIYLFDHSSV
FGSYYDKHTKKWGYKCCSSTNKYDKCFQTMVNIRKNGAKSDASNKNMSYDEKIGRSEIRY
VKEIDPDMSDQENQNDDDEKENININKFRNEIIECDLINNHMNDIDLGLTKDKSIKKREN
AFQKKKYDYTLSPQRADPFADKSPSPGERTYTDIMLENKKKSKMKEASRKNNNYNNTDDA
NDSDADILRGMKNEKNNLKSKWDVVKHEKNLNMSNMPTHATEKWTESSFVVNNN----KK
KKNSRWDKISKEQEDKLSNNMNTPYISMNTPYIISNNMNTPYNMNTPYIPNNIKTPFTPM
VTSLSTDDLIKIKIKNEIDIRNRPLTDEDLDELLPSEGYEIVQAPEEYEAIRNNKLKTMF
KNT--IINTPLFPQKPKIEDTYEKKTNRNNNSSFI-HTPFYELPNTSNNLTDEEAMCQIK
YKQLEMNNPQLLNELKYIQLKNEDYIYFSKLFESINEEELSQEELKERKFMILLLKIKNG
TPSIRRTALRTITEKVKELGPETLFNLILPLMMQNTLEDQERHLLVKVIDRILFKLDDLV
RPYVHKILVVIEPLLIDEDYYARVEGREIISNLAKAAGLATMIGIMRPDIDHPDEYVRNT
TARAFAVVASALGIPSLILFLKAVCQSKKNWEARHTGIKIVQQIAILMGCAVLPHLKDLV
QIIAHGLHDEQQKVRTITALAVAALAEAAAPYGIEAFDSVLRPLWKGITEYRGKVLASFL
KAIGLIIPLMDSYHANYYTKEVMIILINEFNSPDDEMKKIVLKCVKQCIQTEGVDKDYIN
EEIVNPFFEKFWVMRNSNDKKSFNLIVDTTVEIAKKIGAYSVIYRIVDDLKDPSEQYRKM
VMQTIQNVVNELGVDDIDQKLEEQLIDGMLYAFQEQTSEDYYILLNSFDIICNKLNIRMK
PYLPQIAGILRWRLNTPLPKVRQQSADLISRITNLIKICDEKQMLGHLSLYLYEYLGEEY
PEVLANIIRALKSILLVLGVQNMTPPIKDLLPRITPILKNRHEKVQENVIDLIGIIADKG
GDLVSPKEWDRICFDLIELLKSNKKLIRRATIQTFGYIARTIGPFEVLTVLLNNLKVQER
QLRVCTTVAIAIVADTCLPYSVLAALMNEYKTQDMNVQNGVLKALSFMFEYIGEIAKDYV
YSVVTLLEHALMDRDLVHRQIATWACKHLALGCFGLNRQDALIHLLNYVWPNIFETSPHL
IQAVIDSIDGFRVALGPAIIFQYLVQGIFHPSRKVREIYWKIYNNVYIGHQDSLVPIYPP
FELLNDSTFVRDELRYTIMAKQRLTALDIRAIVTLCKKNIVGCIVTNIYNISNKIYVIKC
SRKEQKLFFLVEAEKRIHITEWKREKDVMPSSFTMKLRKHLRSRKISNIKQLGADRVIDI
QFGYDEKASHLIVELYIAGNIILTDENYKILSILKSNDTVGKKYNVNDIYNVEEHMSVLL
YKNLKGPDDEYLKKCIMEILLYDIEEYNKLNSGTVPKKNGNNNDNKMLGTKGKEKCKNEK
SNKNNKSKNNNNANCSDNEKMKKVKKLKTLCDLASKLILFAHNDLIIHSFLECEVNPLDL
LENYDINRLCEIFCHVIEECLSILNKLSDETCTIKGYGFYNI--ENDTNKKINNNNNNNN
DKKEEYNFTEFSPIILKNHEMKLNEGKIKYISFDDYNLCVDTYFSKLELSKYDKQQEITK
SKNAITKVDKIKLDHERRIEQLEKEVLLLKKKITLIQLNDVLIEEGIKLMRSALSTSANW
EKIWEHIKIFKKQEHPIAVRIKSVNFKNCEMDYLLSDCDDRKGNKMGDDGDDDDDDDDDD
NNNNNKSCVKPKTFA----------------------AEEKIRKTKMATDFAVKKVEKKK
KNKDNKQKGKKSSVGQIQKLRKVYWFEKFHWFISSENYLVIAGRDALQNEILFRRYFQKN
DIYVHADIHGAASCIIKNPYKDTPIPDKTLSEAGQLAICRSSAWNNKIITSAWWVYYNQV
SKSAPSGEYLKTGSFVIRGKKNYLPHVKLEMGFCVLFQIEKNEDLNVENLPLEENTID--
IDDIKREDDKGDDMAHNNDNNDKNHVDNKNNVHNYVNYLNNISV----------HYTNEE
HGIEIKENDSVKGTNENEKNKSRDMYIKILYNILSDDIFKTDFFHKSVLANKKIYNMICI
LYPLVIYNILRKMNNPTYIYKSKYVSNIYVNKFYHTFCKIIKYFFHIYNIRKFKRESFFI
NVFFKGLDSIYNFLIFLDSTLYE-RCKSSQVSLSCYNKDVEKDNCGNVYKNMSIDFVNKK
MLNYIINYKENSNIENYDSKEKALFVACEVYGRPVTFKEDSDEEIVKKNKLLKEIESDEN
SNIGNINSDNSNEENKKEKCVSFVNYDNRSNDNVSRPFRSRKGTGYVKMDMNKLLKEMEE
DETKDVNFKRKN-IDSDVV----------KRTVSFSSEEEHICIEQPASLYMPRPVRTRK
ATGFVKMDMKKLLDEIGVDDMYEEKNEDQNV--KKVTF-ESYKNNDVIKKSVSFSSEEEF
IPVDEPVSLKVTRPVRSRKATGFVKMDVSKLLQQIDSDERNEE-----MKSDDKKE--KK
VTFKNKDISSELEKKSTTYTSEDEYNISSKNSESSPMVRPVRTRKPTGFVKMDVSKLLRE
VEYEEEGDDGSGRSNDVIKKTRTRKATGFVQVDMSKLLKEVEQEEEEEEEGNDN---NDD
DDDIKGEEENKNNDDDNDKDFKDEGEDESEEKSENENV------DDNDDDEEEENTSEGY
SGEKKNTNYDNHNVNDNKNNMKRNKENDTLKKNVSFEYNENENKNENKNVHLLRGARTKK
KRMKKKYKEDDEESRLLHMKIIGSKMMKHEIEMPKKKEEELKPFEMQNKYKGPKEVITNY
EKINEDDMDIKLNEIHKLTNSPNEGDNLSFAIPMCAPYSAIQTHKYKIKLVPGNTKKGKV
ADSCISYFLKNATNEKEKELIKNISMDELGNCIIANCTPDLKELKELSMGRMYGKGKGIS
SSTLPYKRKQPSWLKQKPSEIEDAIIKLAKKGQTPSQIGATLRDNYGIPQVKSVTGNKIL
RILRAQGIATTIPEDLYFLIKKAVSMRKHLEKNKKDKDCKFRLILTESKIHRISRYYKRK
KLLPSNWKYQSSTASALIAMSNRKKVAYFHDPDIGSYYYGAGHPMKPQRIRMTHSLIVSY
NLYKYMEVYRPHKSDVNELTLFHDYEYIDFLSSISLENYREFTYQLKRFNVGEATDCPVF
DGLFQFQQSCAGASIDGASKLNHHCADICVNWSGGLHHAKMSEASGFCYINDIVLGILEL
LKYHARVMYIDIDVHHGDGVEEAFYVTHRVMTVSFHKFGDYFPGTGDITDVGVNHGKYYS
VNVPLNDGMTDDAFVDLFKVVIDKCVQTYRPGAIIIQCGADSLTGDRLGRFNLTIKGHAR
CVEHVRSYNIPLLVLGGGGYTIRNVSRCWAYETGVVLNKHHEMPDQISLNDYYDYYAPDF
QLHLQPSNIPNYNSPEHLSRIKMKIAENLRHIEHAPGVQFSYVPPDFFNSDIDDESDKNQ
YELKDDSGGGRAPGTRAKEHSTTHHLRRKNYDDDFFDLSDRDQSIVY-MNDNESLDSEVL
EKQYEIIKYAKYQDFIRLQILIQPYLLNNDIEMLNSINILHWACYCGFTELVKKLISFNC
DIEKEDLVNNDTPIYYAIKNSNYEIVLLLIKCFGISILFHKNRRRMSPFLTAICEFNEDK
ILEALHILELLYMNGVSLEEQNEHGQTALFLSVKKNNISTLQWLLTKEVNINHRDFYGNT
VLHIAVRHCDIDILRLLCDYGCLNMVYYSSIENKNTNVFQLCIKNRYFLVYILLKKWVLQ
NKICSKLKICKTIYAFYFWFFAILNLIVYFNIAHSFSIINKYHFKSLIWITIWFFQQFLW
CMLYFKSPGFYKENHMFNKNKKNNSNPMYNGTFKTNAEYQLNNIEREIFQINKKLISTNF
NTLTPINHDQALSNKYNDILINLEYQKLSLYSQVSQERINSLDEDYRNAILYNQNPRNIC
VTCNIIKPPRVHHCAECFHCIVHQDHHCVWVDNCIGIKNQRCFYMFIFCIFVLLLYNYYY
VYLYFHLFQATINYAFGSLVILCNFINVTLFAFITYLFARNTRTILTNVTFYEHYKKPNH

ITDKYNTDLRCWDFQNLNLKGILKNIYYFWSLNYDEPYIRQCTKTNT-MDVFEFVDPLRK
RKINNNILDFNKYVYKDKMEVGEEKTKDDEIIKKKKKKKLRRLLDSSSSDDNNENEEISS
KKIKIRKLNSDNEDNKLKNNNKVEDEKQSNDEILDEDDENQKAEYEYNLNTLYQCLYISI
QIKNRIIDHFTSEKKDKKEELIKEFVKGSFRVSNFQANYEDFEKYVDTFHKLKCYQKCGV
LWLYVLYKQNKNGILADEMGLGKTAQTCVFLDYMYKTGTIKNKTIIVAPTSLLKNWDNEI
NMWCPYLRNHKIIYYGSQSERRYLAYDIFSSKGSNNIHLIITSINMLMGKNDVSYFRQIK
KYDYLIFDEAHFLKNKNSLIYKKLQKKIVFNNKILLTGSPIQNKTQELTNLLLFLMPHIF
TETNINNAMQAFIAMYEEEVQTRNKKKKMDEGTTPKLTTFVEEIMKKNETQSNNNNNNNN
NSNNNSNSNNNSNNNNNNSNNNNNSSNNIMMDKDNNVIVSDTNKSIIKNYLIQTIRDDLK
HVELKNKEIILLQLIIEPYILRRSKKHVFIDMPKKHSLIIKLPLNNTQLNLYKDEIFSKM
QKTFKHLEFLETHSSKKELQKIYAILNKKEIKEKKEIQQNVDKENYNGHCNNNTNNMESA
DHNVDVHDDDDDDREMDEETINIEKTEENKNSNIVIHKRDDEDNIKNSNSHNNNNNNKEV
RGKMINASIFILRRICNHPLLHKYYYTVEDIKKISKYFYYNTDQYVDLDLKTVENEFMKI
SDFDIHLSIKHLISQGDNKLNKYLITKEHILNSSKINHMLSLIKDIRKKKEKVLIFSQFT
TFLDIIEEALLYEFIYDDSDYMDHIQTIKSSQSINKNDETKRHDDNNNNNINNTKDTNCE
YNKDDEEICLTSSTLSTSSVGTQNDAGHQIYVRLDGSTNTIERQKIIKRFSKDENIFVFL
LSTKAGGVGLNLIAANHVILMDQDWNPHNDRQAEDRVHRLGQKNEVFIYRLCCKNTIEEA
ILKCNKAKLHLDQAFGGNNELLMLKNPGTVLVFKPNTKREEGRKTQLSNIQASRAVSDIV
KTTLGPMAMLKMMLDPLGGIVITNDGNCILREVDVAHPAAKSLIELSRSQDEEVGDGTTS
VVILSGELLSVAETFLRQNIHPTIIVNCYMNALDSSLKFLEEISIDIDVNSESDLLKAID
SCLSTKFVNRYNKIVSKLALEATRCVKMDNLMGRKEIDIKRYAKVEKIPGGDITDSYVLK
GVMINKDITHPKMRRYIKNPRILLLDCTLEYKKAESQTNVEILDEKTWNELLLQEEIEVK
KMCEYIIDSKCDIVITEKGVSDLAQHFLVKKNISVIRRVRKTDLNRLERISGATIVNRCE
EIVEGDIGTKCGLFEIKKIGDDYYSFFVECKDPHACTILLRGSTKDVLNEIERNLHDGMN
VAKNILMEGKLLYGGGCTEIRVGQYLIKEAAKFNDSRKSITEAVASALEIIPKILAQNSG
VNVVKTMNELRIKHEQEGGQEFGIDGITGDIIKVTTKNIWDLLSVKKQIYKSAIEAASMI
LRIDDVVSGVGKDEKVQKTIKNEFMARLKVLNVAEKPSVASSIAEILSKGRPNKIKSCSK
YNPVFTFDYKIKNDIWYMYVTSVTGHLTDQKFDDRYKNWHNTDPQELFDAEITVYVEKDK
KNIENNLKKYSKECNMLILWLDCDREGEHICFEVINACRITNRKLIIHRAQFSAVTEKDI
IHAINNLKEPNKNLAYSVDVRREIDLRMGSIFTRFMTIRYIELVKHETSIISYGPCQFPT
LGFVVNRYLDIKNFKNEYYWSIKMKYVYNDDSNNYSDETDDYYGKKKTKKKKKKKNSKKK
KKKNNNKKNKHNNVVDFTWSRIRLFDHLAVILIYEELLKNPLCKITNVYESETRKYKPYP
LNTLQMTKLVSIYFKISSKECMMLAEKLYNKGYISYPRTETNYFPDSMNLHKIINELRKN
DNFGWYANKLCEEHKYQKPRKGKMNDKAHPPIHPVKNMNKSLKVEEKEWKLYEFICKHFL
AVCSNDAIGYNTKVTAKIQEEQFFCKGLKIKEKNYLEIYTYEKWNDKIIPSFQVDDEFYP
TSLLIEEGITQPPKYLSESNLLTLMDKFSIGTDATMHEHIENIQKRNYVIKNSKSLFIPT
NLGIALVQSYKKFKDIGIDLTDPSLRAKMEKDMSLVASGVKQKNEIIRNYIDIMKYIYQE
IYNRIDVLDKNIHYYLNNPDQYTMEVFNFGKYNGKTFEEVFEKHKSYVTWVKSLESPSGS
LLDFKNYVLQKEGKK---NSSNMNNNHYNNEKN---SNNNYSNNMYENDRNANMNNNYNN
EKNINNNYSENRNSYNNFEKSSVDTNYTNEEKKKFHNMNSSEYRNYEEKVMSDINNAYNQ
PE-EKKEFDIIVAFEIFSDDSFKIVQKDNNNKKFVSFRNFISKDLFKIISELNPTLKKIN
NYTCITFEAEKYEYVLNNLKEKCTILGGVHTIPNFLLKCFKNYTKFSEPQRISEITANIL
TNTMCSYTKQHYDKLDHLLGEKLSVELRNFQREGVFFGLKKNGRVLIGDEMGLGKTLQAL
ALMAFYKDDWPFIVVCPSSIRFQWKDQALRWLSHLIREEHICVVKNGKTDIPSNTKMIII
SYELITKNDKYQDKYKSIICDESHYLKNSLSKRTKVITPIIKNAKRCVLLSGTPALNKPS
ELYEQISSIMPNFFNYHEFCDRYCFKDKNLYTKKIEYVGCKHTEELHLFLTNTIMIRRLK
KDVLKELPDKLRSKIPVEIPQKELSEILNYYKKLESKKNINFHDIDNIHLSNWSNSKSED
GDDENLSISHLFKITGYAKVKAIKEYISYLIDADIKFLLFCHHKLVMDEVETFLKEQKCS
YIRVDGLTPMEKREIYIKNFQNDDNVKIALLSITACGMGLNLTAANTVVFGELFWVPGQI
IQAEDRAHRIGTAHDVVNIHYLIAQNTIDEIVWKIINRKWNTLTTALNGIEDSLNVKEVN
KFDKFMVDLTNDTNKSYPTSLVTTPKVRRKSSEHGTFHNSSPKKKNRDIRDFFTANKESV
EKSWNNNKRSYHDSPMNDSPNTTLKFLSKKYKTEINMKDKHKKVEYAEKDVEIKEIEKDD
GDNKVFVNNLLTSVNYINNAIVHKDNRHMLRMLKYIKNIRLSIKNDKNMSMPIIIYLIKR
IFKENYPIYNILNKYMNIYEEGLYKEISDFYNINEKTYMNCLPELEVFFYLLILLYLLDE
KCYDEGMELSTIIINRINKINRRSLDYINAKVYFYYSWIHELGGKLSQVRQKLLFIYRNA
CLHRDIMTQTVVVNLILRDYIKHNLYDLAVRFISKTSFPENLSSNAQYARYLYYIGKILA
VQLDYSEAHRKITQALRKAPQHTQSAKGFKLAATKMEIVVELLMGDIPDRSIFSNKIMYK
KLIPYKHVVSAVRNGDINKFAQVMNNYTDLFIHDGVYLLIKRIHHNVIKTALRIINLSYS
RISINDIGKKIGIESSLDVVGITAKAIHDGVIEGTIDYENQYVESKSNSDIYITGDPMKT
FHKRIAFCLQLYSDAIKAMQYPDENEKKENEEAKERKMRQQEELAQAEEGDLGDDNDLLM
LCEKCNEKNVCMMRPSNKEKLCKECFIESFEEDVHDTILKKRMFEDNDKICIAVSGGKDS
SVLAHVLVNLKKKYNYNWELFLLAIDEGIKGYRDDSLKVVYKLEKLYNLPLKILKFENLF
SYTMDDVVKFIGKKNNCTVCGVFRRQSFEKGALLFNATKLVTGHNADDLAETILMNMCRG
DIDKLAKNINDLSNADHMNKKYKNYENVDNNDNNDNNDNYDNNDNYDDNNNNNDNNNDNN
NDNNNDNNNNNNNDNNNNNNNNNSNNNNNNNSFFLPRLKPLMWCYEKEIVLYAFHLKLDY
FSTECTYSPNSFRGNLRCFIKDIELINAQFILNIIHSAEFFYFNSINQKTLNVCIKCGAY
TSNKICKACLIIDGLNNYTDNSFLYSNKKK--AKKKISIHFDNKKMEEKTCPFTNVIPYG
LLYDKLKKEKNNDVPENY-VIEEFDKLLKNYERPNVYDEIGNATFKNDEDLITFQIDLDY
TVENIFKNMIYNESGSNN-SILNDIYMPYRILLSKDKNYVSVPIIRIYSLRKDGCSVLIN
VHNFFPYFYVEKPDDFDNEDLIKLEMLMNENLNLNSQYKIYEKKILKIEIVKTESLMYFK
KNGKKDFLKITVLLPKMVPSLKKYFEGIVHVNNKSIGGIVYEANLPFILRYIIDHKITGS
SWINCKKGHYYIRNKNKKISNCTFEIDISYEHVEPITLENEYQQIPKLRILSFDIECIKL
DGKGFPEAKNDPIIQISSILYFQGEPIDNCTKFIFTLLECASIPGSNVIWFNDEKTLLEA
WNEFIIRIDPDFLTGYNIINFDLPYILNRGTALNLKKLKFLGRIKNVASTVKDSSFSSKQ
FGTHETKEINIFGRIQFDVYDLIKRDYKLKSYTLNYVSFEFLKEQKEDVHYSIMNDLQNE
SPESRKRIATYCIKDGVLPLRLIDKLLFIYNYVEMARVTGTPFVYLLTRGQQIKVTSQLY
RKCKELNYVIPSTYMKVNTNEKYEGATVLEPIKGYYIEPISTLDFASLYPSIMIAHNLCY
STLIKSNHEVSDLQNDDITTIQGKNNLKFVKKNVKKGILPLIVEELIEARKKVKLLIKNE
KNNITKMVLNGRQLALKISANSVYGYTGASSGGQLPCLEVAVSITTLGRSMIEKTKERVE
SFYCKSNGYEHNSTVIYGDTDSVMVKFGTNNIEEAMTLGKDAAERISKEFLSPIKLEFEK
VYCPYLLLNKKRYAGLLYTNPNKHDKMDCKGIETVRRDFCILIQQMMETVLNKLLIEKNL
NSAIEYTKSKIKELLTNNIDMSLLVVTKSLGKTDYETRLPHVELAKKLKQRDSATAPNVG
DRVSYIIVKGVKGQAQYERAEDPLYVLDNNLAIDYNHYLDAIKSPLSRIFEVIMQNSDSL
FSGDHTRHKTILTSSQTALSKFLKKSVRCIGCNSSIKKPPLCNHCKENKEFSIYMQKIKD
FKNKQNEFFQLWTECQRCQGNLHVDVICMNRDCPIFYRRAKIKKDIANLQEQVTSLRMDW
MQIKLYVIIYILLIIIKLNHGNSKLNNKTYTHGRVYNHTVNQYNKKRKQYNNTKKYIYCF
NKINIQNYIYKKILSNMSILSEKSIENVISPC-EKKYKLEDISNGINYDYRKNEKSTTPF
IQVAPMINVTNRHFRAMVRIITKRAQLWTEMIVDNTLLYNLNNLEEHLGFDNNEHPIVCQ
LGGCDMNSMSEAAILVEQAGYDEININVGCPSTKVANKGAFGASLMKNPEQVRNIVYEIK
KKVQIPVTVKIRTGVDNYDSFDFLKTFIETVSSVGCNHFIVHARKAWLKGLDPKQNRKIP
PLEYYKVYDLCKLYPHLKFTLNGGIQTIQEAIALLNGYMPENNNNNDTSNFIQIDNYNIN
PLNGIMIGRACMENITVLSQTDKLVYNQDIPSTAYSRRTILEAYKKYLEKNSLFYNLSSS
FELLKPVLGILKGMPGHRIFRNKLDTYIRNYTSTLPCSEILEKAIADVDHIAPGCLDLPL
HDYNLQKEYIKNYMQNPQNSENKVIFVEDVVRDFDENEIFEEITGKFTWEQDVERSWNLL
VENNGVLQHVSQENNENENKEKYRKNQGSSLRKGIFRHIIILFDMSSSMKERDLKPDRIN
VALECVESFLKNFFFKNPVGHVGVVALKNSSAKLIQPFTSNVDDILNSILKERTAGLQGS
PSLEEGLQIAHDLLIDMPLYGTKEVLIMYGSIRTCDKKNILNVLELLVKSNIYVNCISIA
PEMHILKHICEKSNGFYKICSSKNSLMNEINNNAETPLWMQGMEPQLIHICFPTKKKIST
QIMCSCHGKLNTDTYVCNFCNSYTCKIPSKCKVCGIHLISMHDLSHITNNLQGSPLFIEI
KNEQGNYKVCSSCNQQLYNKIYQCTKCQHIFCLECDIFIHEELNQCPFCLINDTMGSSSL
PMSQQMYFSTHNALRINEENDVINTLFYEINGSRHISLLIFPFYDVQMLKRLLIKKLNLP
GGVKVNDIIIFYKGIKLPNYRIISTYIDNNNNRNDKKKKKKKINKLYWAIKDTNPNASIR
VIDSKNYPEFFEDILNEIKLSFKKNIAPKLTMDGTGGTYLLYNAKKKICSVFKPLDEEAF
APFNPRGYEGKMYQEGFRAGVLSGEGASREIAAYLLDNCYNNFSNVPCTIMVEACNPHFN
NKSKLKYVDKETNLKWKCGSLQEFIDSRESVGNYDYKQFSIRDIHKIAILDIRVMNLDRN
DGNILVSPLKTLKDSCNQFVYRNNKFTSSNNEDMLKRIITIDKKPSRYTLIPIDHGLILP

HIMDVAEIDLVWFEWPQIKIPLDDEELEVVFSFDPDKDAEKVRNKLLIREDCIRTMRVCT
RLLQIGAKMHLTLYEIAKISTRKNIDEESILEHLVKDSITQAYQMMDYTSLMSTNRLGYI
LDLAEIKMNKKKKNSKIKTVEINDDQLYEKNQGNNEKGETTGGEGSLKSPPSIKKDNDII
KSNYNKNVSNNNLIKDVDDMISINSSSNTTNSIKNVSLINESYKNIYSNNVIKNNNSNKN
IENNIDLMNKKEDTNGLKQNNLKTHLNSRSDFSKECENTTESFSNNSPDNLLDKPLDTPL
DNMLENISGEVSDMSNIFCNSPNDDTKVKKKKKKKDSDLDGYPKDQKNYTNCDDGDKINS
DDNGINKENEMINQVKNNLNDD-EEEEEEEEEEEEEENDDYDVTFKKPSGTIKRISENTG
TAYRSMEMNKINSIWMIRDKNNKIINVKWENKIFEKLFFETFENYIKKYINDYHRDWRNY
PYNGSKITTTKHAYLNNIKMEIRVANKYALGKKLGSGSFGDIYVAKDIVTMEEFAVKLES
TRSKHPQLLYESKLYKILGGGIGVPKVYWYGIEGDFTIMVLDLLGPSLEDLFTLCNRKFS
LKTVLMTADQMLNRIEYVHSKNFIHRDIKPDNFLIGRGKKVTLIHIIDFGLAKKYRDSRS
HTHIPYKEGKNLTGTARYASINTHLGIEQSRRDDIEALGYVLMYFLRGSLPWQGLKAISK
KDKYDKIMEKKISTSVEVLCRNASFEFVTYLNYCRSLRFEDRPDYTYLRRLLKDLFIREG
FTYDFLFDWTCVYASEKDKKKMLENKNRFDQTADQEGRVKQNMDSLEHLKEQERIVLLNI
LHNFEGKKCLFFEKSLHVMLSVILNDDDINKEHIENIFFH----KVNVNAINNISNVLFF
LRPYFYEIKNIFEIIDKIEKSKKRKNNYVFIFVPYMSYLCKQEILKYNVLDIPLKIILFP
LYFFPLYNDVYSLEIKNLFKEYYVDNDFSNLIFCSFSLMFLQFLFNGVFKNIKSIGNLAQ
FSSEQLIQLRKEIMADFQDDFYDILTNIQNFQDLKNFKNANDTTVVPLKYALHKIFISEK
KTKKKEEPSGNYSQDEDDEDDDDEDDEEDDDDDEDDDDEDNDDDHHDDDHDDHINQLNKP
VSPNEPENISKPYFKSPKKKTSCSENNDDDEIEEYEKEKQHILQRISKKNE--KDGIIHE
EIKPEELQKCQEKEEENYQDEYKNEYIHKNEIKKKKKKSLSSNESDIISENNQEEKNKQN
NNNN-----NNDNNYNNKVNRVTSKKANKKKKKNGHLSSNNIAHDFMINCDNTKYLMKKK
DRDNLEKKNKKEKNYLLEKIGISNFNFLLNVCDHIDSCIIIDRRVDMITPFCTPFTYEGL
IDHFFGIDNLQIEIPRYIIFNEENKHVKYDMMENKDNNMNNYDNSKTMKVRVKLKSSVDV
LYNDIKDLSQNEIGSFLHKKASDIQKTYKEKDSLKDIEEINQYMRIFKEKHYEHNSLSTH
VNIASFILNNIKKEYNFNKLKLEDEIIQLEINTNKNILLSIVKQIQLLIYTNDDIYEVYR
LLCLFSILTNGINQNYINELKKDIIEQYGIKELTRLNKLYTCNILKFNNKQKFLWTQLKN
NFHLLSNDENDISYVCNGYAPLSVRLIEYMAAFKNNLQVFPEIFNLLNGPTLDIIQNPIG
YETLPIQNKTKNNHINLNNTKKKKKNVLLFYIGGISYAEIASIRALNKKNQHYNYLIFTT
EIVSSKKFLQSME--------MSNEYTNKEKLDVMDDTIEVRSMCINCEKEGLNKIVKIN
IPYFKNVLIHSFECEFCNYKNNVIQDLNQIKDKGVKISMKINNKELLDRQLIKSEYGVLK
IPEIDFEIPKETQKGSINTIEGFLHTALNNLTIYLKEIKNMYNEANTITANEANETNEAN
EANKSNLTNEANKSNVTSIDEEKNNNNDENHNENGANTNGTCQQITIENYIKMIENTVQN
LSKFVILKEFPFTIQIIDPSGLSSLEHYEDDLKYKIVHIEYYNRTKEELNELGFYEEYFE
ENEKTNDINSNMIGTNTNE-QQNIKKENFDFIKKYIHMNDNSNNNTNTTMKYKTLSNEEE
AKLIESFASNCPCCNHMGMNNFCEINIPGFKKCLILSFVCPNCNFKTSEIKSSGEINPKG
KKITLTVNNKNDLNRFVIKSETASINIPVVELTSDYGTLGGTLTTVEGLIMKIIESLEEK
FKFLLGDSNINTHQYENENTPNNNNDTTSYKIRELIKKLYKLCKTEEFCPYDLIIDDIAS
NSYISSDVVGEDQNLNEEEYERTYEQNDMLGITSMQTEYMGDNVKRLPLPSENGEIKKST
SGRLSDDGIKRTPSGKPIQTMYVLNRKGEEEDISFDQILKRIQRLSYGLHELVDPARVTQ
GVINGMYSGIKTCELDELAAQTCAYMATTHPDFSILAARITTDNLHKNTSDDVAEVAEAL
YTYKDVRGRPASLISKEVYDFILLHKDRLNKEIDYTRDFNYDYFGFKTLERSYLLRINNK
IIERPQHLLMRVSIGIHIDDIDKALETYHLMSQKYFTHATPTLFNSGTPRPQMSSCFLLS
MKADSIEGIFETLKQCALISKTAGGIGVAVQDIRGQNSYIRGTNGISNGLVPMLRVFNDT
ARYVDQGGGKRKGSFAVYIEPWHSDIFEFLDLRKNHGKEELRARDLFYAVWVPDLFMKRV
KENKNWTLMCPNECPGLSETWGEEFEKLYTKYEEENMGKKTVLAQDLWFAILQSQIETGV
PYMLYKDSCNAKSNQKNLGTIKCSNLCCEIIEYTSPDEVAVCNLASIALCKFVDLEKKEF
NFKKLYEITKIITRNLDKIIERNYYPVKEAKTSNTRHRPIGIGVQGLADTFMLLRYPYES
DAAKELNKRIFETMYYAALEMSVELASIHGPYESYQGSPASQGILQFDMWNAKVDNKYWD
WDELKAKIRKHGLRNSLLLAPMPTASTSQILGNNESFEPYTSNIYYRRVLSGEFFVVNPH
LLKDLFDRGLWDEDMKQQLIAHNGSIQYISEIPDDLKELYKTVWEIKQKNIIDMAADRGI
FIDQSQSLNIYIQKPTFAKLSSMHFYGWEKGLKTGAYYLRTQAATDAIKFTVDTHVAKNA
VKLKNADGVQITREVSRETISTESTVTQNVCPLRRNNDEQCLMCSGNKMASTHNEIIPRL
GFEEMRNEMNKYGVEINQSTLKNPSTEDIQGIYSLCIKYILNKDIQNIRIEEYTGDLKSS
LPTVDGLQILPNEGKNHLQAIGNLRFLRHCEKINKILNLDNILSYIFKPVGSHMTKLINA
FIHFMKYRDQLYNENGEKIKSIQEKKNEYDVLENEYDALENELNKLLLKHEDIRNNIINE
KNIKRNYEEDIIKNQNLLNSQQSLIISLNSTKDKIVNETNELIFQYSRYRQKKEDLEDQI
VPSPEKLQKYNEELKDHLYEHIAQFEDDRKKNEDIKNKINIADICIKKLVDLLTALNEHI
EHTIKLHIEKKNNLQTIEKQYKSLTNEKQNFITKNTEQDKIIKETKEFLQQEQTKWNQKI
KQEQHNTILIQQKVKDIYQNVDDLNIKTNREINQINNIIKHIQDIINHYNKNILLITELI
QNTKNSHSILTHKVLNNIQKDISANM-MQFIKNLQLNKKKDSDSSEQTNKKNKMKYEDFN
FIRTLGTGSFGRVILATYKNGNYPPVAIKRFEKCKIIRQKQVDHVFSERKILNYINHPFC
VNLHGSFKDDSYLYLVLEFVIGGEFFTFLRRNKRFPNDVGCFYAAQIVLIFEYLQSLNIV
YRDLKPENLLLDKDGFIKMTDFGFAKIVETRTYTLCGTPEYIAPEILLNVGHGKAADWWT
LGIFIYEILVGCPPFYANEPLLIYQKILEGIIYFPKFLDNNCKHLMKKLLSHDLTKRYGN
LKKGAQNVKEHPWFSNIDWVNLLNKNVEVPYKPKYKNIFDSSNFERVQEDLTIADKITNE
NDPFYDWMVISQFYILSPRGDTIINRDFRGDIIKGSAEVFFRNVKLYKGDAPPVFYLNGI
NFTYLKSNSLYFVVTSLFNISPSYLIELLHRLLKIFKDFCGQITEELIRTNFILIYEIID
EIIDYGYLQNSNTEYIKNLIHNEIATNNNTVKKFANLPNFSIKNTNTLPSNASQKPIQIN
DKKNEIFIDIVEKINLIMNSNGEIVYSYIDGVIQIKSYLLGNPFIKIALNDDLYIKNIHH
DNSNNIIIDDCNFNHLVNLSQFEKDKILSLYQPDGECVLMNYRINNNFKAPFKIYANVIY
NQNHTVELCIRIRLDIPSQYTCTNVFVYCNLCKHITNVHLDLNTNSDLFSAQYISNENKL
LWTIKKFKGEHEYSIRSKITLSPHYAFSKRDFGPIYILFEIPMFNLSKLRIKYLRIIENY
KTSNTHRWVRYITQSSSYVYRLMDILITTPYDKEVCEIGEITNKIISLETVKHTKSSLHK
NRDIQCPNKKKLVLPGECVLDKNDKDRFLKGGGLYEEDDMFYACILGRVNYINKLIYVEP
LKGKYTGSVGDLLVGKIKDISNDKWIVEIGSYCRAYLSISQTNISIFSQRIRLYNDVINM
IHIYKPDDIIACEIQRISTDGTIILHTRSSIYGKLSNGVLIIVPQTLIHNQKKHIFVFPC
NVQIILGMNGYIWISSPIKKSKDTNPNSVDQNIEENKFEDVDHTTRKNITIITNIIKLLV
KYHININYDTINKIYVHYTSNTNNTPSYILKPYVADSYLFNYIDKFVSSNMPVLYHLTLQ
KPTAITKTVYGNFSGPRFHEIIVAKGQVLELLRSDKQGKLNVIISKDIFGIIRSISTFRL
TGSNKDYIVIGSDSGRLVILEYNNEKNDFVRVHCETYGKTGIRRIIPGEYIAVDPKGRAL
MICAVEKQKFVYILNRDNKENLTISSPLEAHKSHSICHDVVGLNVGFENPMFVSIEQNYE
SLDKQINEENDFSLDYAKKVLCFWELDLGLNHVIKKHILPIDITAHLLIPLPGGQQGPSG
VLICCENFLVYKKVDHEDIYCAYPRRLEIGQDKNISIICWTMHRIKKFFFILIQSEYGDL
YKIEVDHEDGIVKEIVCKYFDTVPIGNSISVLKSGSLFVAAEFGNHYFYQFSGIGDDNKQ
FMCTSNHPLGKNAIIAFKTNKLKNLYLVDQIYSLSPILDMKIIDAKNTHTPQIYTLCGRG
PRSSLRILQHGLSIEELADNELPGKPKYIWTIKKDNLSEYDGYIVVSFEGNTLILEIGES
VEEVSDTLLLNNVTTLHINILYDNSFIQVYDTGIRHINGKVVQEWVAPKNKQIKAASSNS
SQIVISLSGGELIYFEIDESHTLVEIFRKNLNVEVLCLSIQQIPPNRVRANFLAVGCLDN
VVRLLSIEKDKYFKQLSTHLLPNNSSPQDICISEMNDNGNMKERNIIFLNIGLNTGVLLR
SIIDPVAGTLSNHYSKYLGAKSIKICPVNVNKNPALLVLCEKTYLCYMHQGKFLYSPLNY
DMLEYASSFYSPQCSDGYVAISSNSLRIFRFYRLGEVFSQNILHLTFTPRKIVPLPFPSL
FYDHDSSLELERKKKIRMLAIIEADHNSYDENTQREIQKALKDIKLSDTERRKENDSNDE
ENEDEYYYDRIGTFKAGQGKWGSCIKIINPVNLQILDKISLDMEEAALSVCACELEALHC
LIVGTTTNLSLKTKSLTSASLRVYTYDIQYKLNLLHITPIEEQPYCFCSYNGKLIASIGN
KLRIYALGKKKLLKKCEYKDIPEAIVSIKISGNRIFACDIRESVLIFFYDPNQNTLRLIS
DDIIPRWITCSEILDHHTIMAADKFDSVFILRVPEEAKQDEYGITNKCWYGGEIMNSSTK
NRKLEHMMSFHIGEIVTSMQKVRLSPTSSECIIYSTIMGTIGAFIPYDNKEELELTQHLE
IILRTEKPPLCGREHIFFRSYYHPVQNVVDGDLCEQFSSLSYDAQKKIANDLERTPEDIL
RKLEDIRNKILMEEETNTQSKPLDDEDINILKSYGSGPYSKTIKKVEGDISGLLVNINKL
CGVRESDTGLCLPNQWDLQLDKQMLNEEQPLQVARCTKIINADTDQTKYIINVKQIAKFV
VGLGDKVAPSDIEEGMRVGVDRTKYKIQILLPPKIDPSVTMMTVEEKPDITYNDIGGCKE
QLEKLREVVEMPLLQPERFVTLGIDPPKGVLLYGPPGTGKTLTARAIANRTDACFICVIG
SELVQKYVGEGARMVRELFQMAKSKKACILFIDEVDAIGGSRGDESAHGDHEVQRTMLEI
VNQLDGFDNRGNIKVLMATNRPDTLDSALVRPGRIDRRIEFSLPDLEGRTHIFKIHANTM
NMSRDVRFELLARLCPNSTGSDIRSVCTEAGMFAIRARRKTITEKDLLLAINKVIHGCKQ

FSATGKYMVYNMDIDFCSHHLLAYDNSIINDENEIKAKVEQNLPLIFSKINKLQKEKEDE
EDIIYSVTNDNIFNMPRYSRIPKEKKKTKWQLFAENKLRMKKNKSGLIYDKVSKGWVRRF
QKKQIKLNEQKNNFVHEYKNKEDIYEDPFEKEQEEKDIKKMKQKMRELKNKFDQKGISTE
DIKYIQRQKRKRENLIDNLKMAQISSSTFGRKDKKLKKEKKLKLQTNQKYEKRDLKDEVK
QNNRLAALVLKSVMENNPYVFKSLVNIYEEYLNLIIHRVKGYKVLVLDDETKSIISLIFS
HSYILEKEIFLTLNFNDKNIFEDIYNNNDKKENFDFMNYKIKNLKHLKVIFLLRPTYTNI
LRLMSELKKPLFSEYYIFFTNTINDIYIEKLAKADEFDVIKNIIEYYIDTYVLHDYLFHL
NIDYTSFLYKNDHKFIKEKKKKENNNNINSNNNYSSDGRYEKLTIEEFNKNNMIYDNNNN
NNNNINSGNIINNDNRNNSNITLYMNQIVQRIIDGLFSFLCCIRQVPDVIYNRHSKICKH
IIDMLKEKMLRHQSVFNNILDIYEKYNDKIETNNEPNYQFNHLINQNIHEITEGDACYFL
ILDRNEDPITPLLTQWTYQSMLHELIGIENNKINLNCNNKEEEQIVMSCNYDDFYNEHLF
DNFGDLGQAVKNYVDIYQEETSKKTNLESIDDIQKFIDIYPNYKKLSGNVTKHVNILHKF
SDIVQKRQLFYISELEQSIACYHTKNDHFKQVIDTIKNYTYTNYDVLRLSLLYSLKYADE
QHINVIKNELAKRNIQKDQILLIDALLLYSSQQTKYNQLFKEQTFLNLAKTTITRTIKGT
SNVFTLHKSYLYYLLEDIIKYKINTQLYTTTNLLHTEPTLNKKINSIVVFFIGGATYEEY
RDVQHLSKKYNISIVLGSTHMHNSQSFLADVLQLIKKMTHKINSCKHPCCEEDNLKSVKN
KVYHKKYIPSIIQKKKKERNEHAPPLLKDIQDKRCTNVFERLNDKQFYTGVQKTKFMELK
NNKNKSS----YCYNNI--------KVFSTMLKKPCNYVVTPGTLGIQKYGIQTGRPKTI
FLFNNEKKYDKGVYFLVKSYIKNIKSLCYEITKILQPSIGPTRKIYDQNFSLVRNVNDLI
NGGKYLCTSGDPPAPIRNLSHFLT------MKNHEDIKKKGLKKIENKKISKHLKRRSDS
SSKDSEMNSYSSKEEDDIDEKKIKIKMSDSICGTPTKDGFG---ELDVEGENNSNEINDA
RKYFKEGQKIITPPNGDGTRAFYESLLEENPNSIIAIKYCIEHGILSGTKHHEALYKYYV
LKKNNAFKSNFGGVRIDFKKLLEESCKDNKCSYTKM-EDHDANVEQWKIKRLIKKLENAK
GNGTSMISLIIKNKDEVSRINKMLADELGTASNIKSRVNRLSVLSAITSTQQKLKLYNKT
PPKGLVVYCGTVITEDGKEKKMSIDFEPFRPINTSLYLCDNKFHVEALKELLESDDKFGF
IIVDGNGALFGTIQGNTREVIRRFTVDLPKKHGRGGQSALRFARLRLEKRHNYVRKVAEV
ATSVFITNDKVNVTGIVLAGSADFKNDLLNSDMFDQRLFAKVIKIVDISYGGDNGFNQAI
ELSSEALQNVKFIQEKKLIGKFFEEIAQDTGKVVYGIDDTLKALEIGAVELLILYEGLDI
IRLTTKNPVTNQTKTMHISPCDEKQESLYKENNVELEVVEKISLTDWVIGNYKKYGASLD
FVTNKSQEGAQFQKGFGGFGGMLRYKIDLNLYDEDVESDVELFMSTYKDSNNTIDQKKFL
DECIFVVKEQSFYMKQALENGSLRDTLKYASNMLCELRTSHLSPKYYYELYMLIFNELQH
LDNFISDKKKHKKKFIDIYESVQHAGNIIPRLYLLIIVGRNYIKNKDIKAKYILKDMTEL
CKGVQHPLRGLFLRYFLIQMCKDRIPDTGSEYEEAGGGDINDAFEFLLTNFYESLKLWSR
MNDKVLKVPMIQDDNTMNSKIKILKEKMDVKMLVGSILVRMSQLEGMTKQYYIENCLPKI
LLYLSNINDCLIQQYIFESIVQVFSDECHIYSLEILLNAILKMNTSIDFKSILITLLKRL
RSFIANNKCDLPKDIDIFNLFYDHLVVYVNRTLDTYTKVNYNDSINRSHHLSKDIINNND
NNND-DNNNKDNDDNNNNNNNNNNNNNNNNYYNKNNTGKNCTIVNTDEFVNNVVKMLQVI
YEFIFLCIRIYDDDIIISKLFGLPYTIVSNVNMNNDTICEEIISIIVLPFNYLGLSALNA
RNMQTLLNSITEKHKKKLSLDIIDAIIECKKKYITYEDVEKILKYISYIFHEKDKKNKNN
DEDIFNLENNNSAYTCEKICKFFHIITNTKNIEEKYNICMLFYKYISNSTYLVHLLPTII
FTLLHVVTQITKLGQEDHFNQNDDKNSSDSYNNDKNNFILDEKKINQYNIYVKNIFKFIH
TNLLTVASQMPILTFKLFLYSAIVVNNYNSFVQTHEFLTFDNLEAICYEFITQPLIIYEE
DINISAQQFDCIVWIVGILCTHINLLDNENYNNIALKLTQHANKLLKKKDQCIGVLKCSH
LYWENKKYRNSNKVIECLQKSIKNAEIAIQSNNDNIILFTYMLDKYLYYYEAQNIDVSEE
TLHYLIDICQDYYNKTNDDTNFKQEYKKVIKYVHDKQKNSNVFQKINSILRSMLEAKLNN
ASILKKLFECIKDLVNDANVDADESGLKLQALDGNHVSLVSLHLLDSGFSHYRCDRERVL
GVNIASLNKVFKLCGANESVVISSKDDEDNLNFVFENNKEDKVTNFSLKLMSIELDSLNI
PDCEEGFDAEVELSSKELTNIFRNLSEFSDTVFIEIDSNCIKFTTKGIVGDAEVALKPRD
STSEDDIGVTIKSKKKIKQSFAIKYLNLFSKSNILADVVVLGLSDSRPIEFKYEIKDTSP
DSDTLKIGFVKFFLAPKMDDDMDNKDMIKFKDILIFINIYILILNAYCKEWCKATFEYGK
SDYAECISEGDSISRYMIETIPILSKDVDLYSNVSLVLSNGYGSKTKEIIIGNENDGLLN
KIFTVRSDIGNPEYIHIKLNSQNKNWKCKKITVWKDYKYWVFDCIGSLNDKKPESTYFLS
GNKIYIAYVQTGKDIEAATTGTIEIILLGNNKRSNTKVLHEGFSAGGLKKIKFQASDVGE
LEDIILINNSLNDPWYCDFVKIKSDNNKIYIFNVKSWIGAPYDKNVKVNIKSDTIEGTSK
DIDCHIRAIDLINTNNINKLLQNKVQIFKVRCPQNCHNSEFAIIEGSSIHPSSTSICAAA
IHDGSISPSGGEIIVTVASELNHYYTIKEKFNELEALDFSAKADEKNFTFFTYHLDSIDD
IISNVRIVDSFGKLSSLGRLEIRNKNSWGTVCVKGPNFEFNDDAAKRACKDLGFPNGIHI
KENCANLNGQNYCAGYKYPFASSGILCTGHEKDISKCNADDFSHCVDHHDDVIIQCLHYS
SNELINDGAIRLVDINGAPSNNGIGRLQIYYHGVFGSVCSEGWVKEAENIVCHELGYNGL
KGNGFSHHSCTDISGENLCGPDTEKINAVNIKCKGDEKLLRNCPHETHDDIYCSHDEDVI
VGCVGGDNNSHGEEQNKKHLINLEKKKFHPKIELTCFDKMLSKSNLSVATTGDVFLASCP
EKCDEEVGIVKGTFLYTYDSPICKSAIHSGVLPNNIAEDIVLSIAHTHNNFIGTKRNNIE
SHDFKGTSKSFTISIPTMSLLREERKSNPKSEDEMINKNEIDLTYDHSVNKLDHHITSSM
KPTFQWIAPTGFVGFNGKENDYIDCTNLPNEKYIRSLSNFTFIVYFTLNGGEGTWRTILS
HSLCEGISISINEDNELIIEQNCNPHLLKSKFKPKFGQTYHISLVFNKINKTLYLYINGK
KVITEKNTYNFTLSGDLIIGRSNQTTKDYFIGNIHLVEIYKYTLSEQEIKESLNSSLSLE
YLNMNILEQMSNTKKKKNQKNKKGVQKTIDGRDCVTPCKSKNMINKDLQINTQQINLKCQ
DNLLSEQFNGKIGSQFLVSCIENCTKSKYFIKGTNNYYTPDSSICKAAIHAGIYKPNGNK
NNTFVIRIVEGLLEYKSSRGHFGILSKSEKQSQLRSFSVLSENEENIFTCSTDGQFILNL
SVGEKRTINCPSNCNIIKDKIYGTNIYSPTSVLCKAAIHSGALSNQGGLVEIIVGTGQEE
FKGSTQNNVESFSSNNHSRSITFNKHME-MSSLYVEQKVEPEKIKEEKINVNVNKILYKA
HKNIKKEKIKSKRIDYLCVDKNDLPKNLPNTKNIKNRKLKNQLNKDVKLAILSSKKLLAN
TKFMPLKEGYIKYNSVNEPKVHIAPSKDEEDKKKKKKKKKNELIDSKEPSDEEDNKNRRA
SQKYIYDNADVGTQKKVFDLKLNMGPYSCTYTRNGKYLLMTGVKGHVSLIDTHNLESLCE
FQVDEMIRCNTTLHNYKLFAVSQKKYMYIYDNTGMEINCIKDILYTYNMVFLPYHFLLTS
IGEFGELVYQDISVGNIVTRKKTKRGPCSIMKQNKKDAIIYLGHKNGHVTLWSPNIDKSI
CDIFCHHTPISSIGVHENYLITASVDSTYKLWDIRKMEYIKSYKSHNIINNIDISDTSLV
AFSMNTHFRTYKNLFTNPQLYITHNIYGDQINSISFQPFEDICSLGLKHSIKTLLVPGAG
IANIDTFFNNPYETKKQVRENEVKLLLDKLPADTIKFNTDQIGNINPYLINHNEQKN---
DTNVYNKNNMKKKNKKNI----------------KNKKNLKN--NKNEENINRMHKQSEH
ETD-----------------MIPDNNENNNNLYFLAFSKNKVTIYEYDVDLSKNIVDDIN
DEIKRCEEEDMNHIRNNNIINNNTNNTNQIKKSTNKFNNDFVLNRGIKIYKEYDDVELAT
CTNDYKKIILVRKSDLNVAEIIDIKSNKNVTCIKTKTQIKRIITSPRDNYIVLHCQYKPD
ITNTNLYVYKLNVKSSKKKKKKDKKNSECENN------NNNSDNNESTNDNIIYNENLII
ELTLKSYSNKNWPFFKWSDSESICTCLINNQVYIYKDNNFNTTVNKLKLDNIEFFDISPE
LNNKKGILIATYEQGSKGNPSIFKIFHIDNLNKHIYSKNFFNSDEIKLKWNKNGSSLLLQ
IHTQVDKEKQSYYGSSNLYFIDTVTLKDVNVMTNKGLIYDTIWSNNQNKFYVCKGEIPAE
IVLHDKNGNVSHSYGRHKFNTLKLNYNEKLLLTGGFGNLSGDISIWNTSTKKEITKTKSS
CAVICEFFNDGKHFLTATTHPRLRVDNNLKIFKYNGLIVSRINFEELYNVIILPFNKIKF
QQTEEPVDINLEHTTLQPYINKQLGIDSKKTGIYRAPRSTGLVNLNGYLSSKLPDREKSS
LPPGCNFVNENKNYKKKKKKRSSKSKQKRETFMKNDKIKLVSFEGDEFIVDKNTASMSTV
IMNILEVMTAEEDTIPLPNIKTPILKKIIEYMEYHINNPADEIPKPLITSNLQDVVSSWD
FDFVNTDKETLYELIEASNYLDIKPLLDLTCGKIASMMKDKTTEEIRAEFDIVNDFTREE
EKQIREENRWCGDIMLNEVDIENSQHPIIEEKNLKEEGKVFLKGEPIHKHLYKHSYNTQV
YKDEVTHMRLRNRENKNLNENN---------SDDNKKVNKKNGCVDINMNEHTNKKEDGD
DDDGD-----DDDGDDDDDDDDDDDGDDDGDDDG-----DDDGDDGDDDSNSNDDIKNNHK
KNMIKLHCTSPKKKEHIEENLKDHTNDEEKNIKIIEMYEKKLENEEIKKQNK--------
---EDANENNKSQIRESISKIFKECPTSKPKIINEINNEKYDNNNNNNNNNNN------N
NNNSNNNNKNNSDTEKNKLKLNKNTLKEGSLLRLFRCEYFDTHLHIRYLYDRKEVGVHEY
LVNSLYTQRKYEDILFYLPQLSQISLVRYESSSLYRFLLEKASKSMHFALKLSWLYHSIV
EDNSSKYKDLAQRMIQEIEMAVVNCKPLNNECKSFEENKQSYLLDLAYPLLFKRKFILKK
IKTNEKLNKIKMYNKCLNNSYNSYTLKNLHVSNPIEMDTTSFLRNHKNSNDSTNDDNCNV
LKREDHQDVINISSEENEERDINKNLKEE-VTTLNKISRENNLQNLQSEKKLKQAHTTPC
PKLPPCYIINSGSLSIARAKVKLPSTYSKLGNPLSFSKFSLPDFNYSYDMIEELQQFFMK
QRRCDYFSLLNNFINLLISVSNLLAAEPDIDLRNELLRRFIYSLNSWMNMRRCIVACCEN
IFAMTGLCIPLESMSSST---FNHDTNNRLSYNNLQILHFNNEECKIFFSKKRAPYLLMF

EVADLDEDISHISDNIFYTKKSLFFQQDNNNNNNNNNNNNYNYNSVREEYSRKSLILSRN
FCCSKNYGYYEENLNTFDYENRLSLEDKKKKNSKKKKYAKEQEQTNNEKKEKERKKKNIF
DLNSLKMDDIYVYNCIVNDLRRENLISFTSKEDENLALIKKCLGLKMVEEEKNENDDDDN
DDDSSSSFCVKKNYLLNSRSVSMPSYLNNNLEKCDTSSYDNTSNNIKTSIETISNGIDDI
QVEKNIEKNETFEEMKNEFIDYNEHMVKLSPYYTTTNHRGKNKNDDTDDNSDDDNINNYN
NNSGNNNQDSNFPNNILDLSEYFKPENYLNEEFKKKNCKIIKRLLWGELFEEKKKRIRKV
SPYGKLKTWDLKCVIIKGGDDLRQELLASQLIKQFKIIFENAGLPLWLRPYEILVTGSNS
GIIEYVNDTCSVDSLKRKFGADSISTIFNIVFSDYIFEAKKNFIESHAAYSLISYLLQVK
DRHNGNLLLDSDGHLIHIDYGFMLTNSPGNVNFETSPFKLTQEYLDIMDGEKSDNYEYFR
RLIVSGFLEARKHSEEIILFVELMMPALKIPCFANGTQFCIESLKERFMTNLTVDVCIQR
INALIEASINNFRSVQYDYFQRITNGIMMVINVSFKVTGGKEFTVAIEPDITVLDLKKIC
AEHVDIPVEAQRIIFKGKILKDKESLTLYGVADGNTMHLVRSAMATKDAQEEKESNKNEN
ATNNDSRPSNENNNEMGDNPLVQMLMQSGAGDMGNFNSALGGANFNLGNFANMLNANGAG
DFNRDTISSLLNNPLARSVLNELSNNPEMLTNLVSNNPILRNTFSQSPLMQPVLENPNLL
REFMRPEILQAGLQIENALNMNNNNNNNNNNNSNGAFRMEDILNNLNNLSNPNNTTNNNN
ANPGNNLNSFLQSPELLQAFQQVMSSNRNLGNFNFPNANMNLDFNTTNVTDNRPPEERYA
SQLSSLQEMGFLDNAANIQALQETGGDVNSAVTRLLERGFNMENKEQNVMREIKVNKLVL
NICVGESGDRLTRAARVLEQLTEQKPIFGKCRFTIRSFGVRRNEKISCFVTVRGKKALEI
LEKGLKVKEYELRRKNFSDTGNFGFGIQEHIDLGIKYDPSTGIYGMDFYVHLSRSGYRVT
RRTRRRSKISKTHKVTKEDAMKWFQTKFDGILLKMEFYGSNFFRLRLALSLISGKAITIK
NIRKKRKSIKNGMNNEMDDNYNKYGLKEYEAKILKLIDKLCDNTTIKINEEGDELYFEPG
YLIGNTNEEDSRNMYNMTFHCGKERSITYYLEFLIMIVLFFKNPVNILLKGITDDYIDNN
VYTCKIICEHFFKEILKLNEDFLYINILKRSTKPECSGEVHFFMRNIKKINPFDITNVGV
VNKISGSILSNNISLMFRNKIMNFAKKQLYHFTPYINIDVQKEDDKKNKNINSHFISFSL
FAHTKCNCIYAADICVDELFLKKLKEGINHRDKHEHMQDSSNILEDKQHYMNGNYHTNDE
NIVPSYTQKDQQNNIITKHINNNLHDVDIYERLGFFISLKMINQIKGLSSVDTNYQWLPL
LYMALATDTSVSKITLSIIKPYSITLIRLLRDFFNVVFKIEKVQKSPIHYSYLIQCVGIG
YQNIFKKTFMLGLGGSCLSKEFFDLAKSIGEARSKQEEDRIICNEIILLKSRFSDPNTSV
KQIKEYLIRAIYIEMLGHDASFAHIHAVKLAHEKNILCKRTGYLSCNLFLHKDHELMLLL
INTIQKDLKSDNYLEIWAALSCVCKLLNNEMIPAIFPVIQDLLNHKNELIRKKVCMLLHK
MYIIEPSLIKDIDIYLKKLLCDVDPSVMGASLNLIHSIAKNDMIYSIKLVPYLVSILKQI
CENKLPKDYDYHRIPAPWIQIKILSIFRILGYSNKKLSEQMYEVLQKTMQRADFGINVGY
AIIYECVKTIATIYPSHHLLELASLSISRFISSDNHNLKYVGVTGLALIVKINPLYATEH
QLAVVDCLEDKDETLKIKTLDLLYEMTNPLNVQVIVEKLIFHMKNSVDIHFKHDLACKII
ELIERYTPDDIWFLNKINTLFLSVGELLDESYSYSLIKLLKKYKSASNGNINMECLNYMK
EQKEGTDDVNDNNGNNNNNNNNNNNNHSNYNNMSSQKKKKNKMNDDIYNLRKYAVNTYIR
LLESNENIPFILMQIICWVIGEYSHLCDIENYSTEDIIDLLCECLEKNFSNPDRVKSCLI
TAIFKLCSRNNIRDHVVAKKIIDKYKNSKITDLQQRCYEYDLILNNPILMNNVFSLRNTK
KKIVIDETLSFLNPYIEEHLRSGGKSYISKDLRKNEQHDESIKSTNPVLNFTPYELPLNN
KLSSDNYNISSNNPIYEKNYNYQEDAVVPMDEQASTMKDKGKIYKLNVMGPKKWKKEVYN
IDQGQNDDEKRDMEKKKKKKKSGKKNNKEKNQDNNNNNNNNNNNNNNNNNHIDGNQNNSE
VINKDKVKSNYYDEEDEEDEEYEDDSDDDEDENINEENENEKRTDNVNDYQNYERFDDKD
IHYSKEFNRYHNNNKTNVYNWKIKGNINTVANNKKELTEKEKMAAALFNGLISNNSPLKD
IKSSYVSTISMKKNMSILSNKNYFTSKNNNDLHNEQVENRDVTKNEKGNNNNNNDGCTNK
LVDINVDNSDHLNEITKNKDQFDMIDLNELPKKIDPTKSSDINIELWDKITTKKKAVFIN
STNLNILQTLKKIENNINTNVIEVSKMDSLISCF-----------MSGSNCVAIACDLRL
GANTFTTVSTKFSKIFKMNNNVYVGLSGLATDIQTLYEILRYRVNLYEVRQDAEMDVECF
ANMLSSILYSNRFSPYFVNPIVVGFKLKHYVDEEGEKKVNYEPYLTAYDLIGAKCETRDF
VVNGVTSEQLFGMCESLYVKDQDENGLFETISQCLLSALDRDCISGWGAEVLVLTPEKII
KKKLKARMDMHKTIRKLFSDEVCDKIENMKILLVGAGGIGSEFLKNIITIGCKNIDIIDI
DTIDITNLNRQFLFKKKDVKKYKSLVAKERALMHKKDLNINAYTFDVCTMKSSDIKKYDY
VINALDNIKARKYVNKLCIMEKKVLIEAGSTGYNGQVYPIYYNHTKCYSCEEKPKNKTYA
ICTIRQTPSLPEHCVAWGRLIFETFFCKNDNETLIDIKNHIEEESKKRNMDKKEIIIFIF
NYLFNDTIKELIYLKKDYTTIPIPIHFEENINQNEINIDKLND-NKNN---MGDNKYCNN
FKCEEKDREKDDNVLMLSSQNIWDKKKCIEMYIKTFLKLYKYLNINKKEEEYLIFDKDDD
ECINFITSISNIRMLNFCISQKSKFDIQSIAGNIIPAISSTNAIVASLQAFQLIHVIEYF
ETLKNKN----FNIRNSKAKHVWVKSIVNGNKIFSRGNLVNAEPLEIPNPNCYICQQPTI
HIYIKNFEKMTLYNFVKDICMNELSFLYPFLDKDDRNIFDYDLFQENDDDYIQNLYASLS
YWNIKHDEILLLTDFQNNNDQLEMHLKEDPSLETEYFIQQKNVPSKKRKINYMQNMDTES
VKKKRHILDEHKETNDEKQPTKKRDMGKLSKDRRDIYYRKAKESGYRARSSFKLIQINEK
FGILKLFKPDIYNCNNEKDLINIYNEHYCYNIVDLCAAPGSWSQVLKNICLYNYYQILYM
NNENIKNIQHEEFLNKFSLYINYNKIKCIKEPNIVAVDLQEIGNMNYVKIIQGDITKMST
IDKILKCMNNKINSNVFYNMEQQKKENNFSYAHTVVSDGAPDITGMNDIDEFIQSQLILS
SLKVCCSVLKVGGNFISKIFRGEHTGLLILHLNKFFQRVYICKPQSSRNKSLESFLVCLN
FSLPRSNIISMNNHNAENKINDKSEEQMRKFHAQLITYKDKDDQHTTKHNKNISDEIETK
ENNVTPNSDDKNNLDIFNFYCSDSDEEIKYFNSEDEEVVNNYISSESFMSNKLFSFIATD
NYYDSDKSYLLPENYVRHEPQLMPLKPPYMLSLQKKRQEVKKMEGEEYDKILVLNFGSQY
FHLIVKRLNNIKIFSETKDYGVELKDIKDMNIKGVILSGGPYSVTEAGSPHLKKEVFEYF
LEKKIPIFGICYGMQEIAVQMNGEVKKSKTSEYGCTDVNILRNDNINNDLYSNYKLMNET
CCLFENIK-SDITTVWMNHNDEVTKIPENFYLVSSSENCLICSIYNKEYNIYGVQYHPEV
YESLDGELMFYNFAYNICKCKKQFDPIRYHELELKNIEKYKHDHYVIAAMSGGIDSTVAA
AYTHKIFKERFFGIFIDNGLLRKNEAENVYTFLKSTFPDMNITKIDASENFLSNLQGVTD
PEQKRKIIGKLFIEEFEKAVNNIDIDINKTFLLQGTLYPDIIESKCSKNLSDTIKTHHNV
GGLPKNLKFKLFEPFKYLFKDDVKTLSRELNLPEEITNRHPFPGPGLAIRVIGEINKHKL
NILREVDDIFINDLKQYGLYNQISQAFAVLLSSKSVGVRGDARSYDYVCVLRAVKTSSFM
TANWYQIPYDILDKITTRILSEVKGVNRILYDVSSKPPATIEFEMRKYNGNVKRSRDDEY
EENENVNGERTSITLVRATLLPILSIGKNEKTTLFHPFKNPLPGFVSDKTLYLKKTLGVK
VRRTGWINLMKNSIPRRSDETEEQEKIEEVIKKHEPLILYKDENDKIEVDPILAQYLREH
QREGVQFVFECLMNIKDDKISGCILADDMGLGKTLQSITVLYTLLKQGFHKKCAVRRCLI
LCPASLINNWNDEISKWIPNRCNVTCVNDNAKEKIVSKLEGFKYDIQSTVLICSYECFRI
NNEFLDKSSIDMIICDEAHRLKNDKTKTYTSIYNLTAKKRLLLSGTPIQNDLGEFYALIS
LCNPDLFDDINLFRKKFANPILIGRDKDATEKEQEIASERLTELSNITNKFILRRTNNLL
SKVLPVKYLINIFIKLNPIQEALYVLFLKDKKILKNDNTNNKVNVLINIKKLEKICNHPL
LLNVNDIKEIGQVTLWKLIEDVIFEMQCNRGNKRDNKNDMSSISNNNMKNNQNDASGYVG
NKRKPIELDYNKPVKRIIEECKRDIYRSYYNFSCKFLLLHFLLKNIKQNTNDKVVIVSNY
TQTLDYMEILCKENMYKFVRLDGGINIKKRHKVINDFTHSADIFIFLLSSKSGGCGINLI
SSNRLILLDPDWNPANDKQALARVWREGQKKICYIYRLFCTGTIDEKVYQRQISKDGLSN
MIVTTTNLSKDQMSDENVKKLFNYKMNTVSETHDNIECNRCNLNDNNNNNEHFAEQLEDF
EEDDVNTWAHHQNIDTVPDNILIKAVKDAEEYKKNDMNSMPLLQKLTHDFVTFTMSCKIE
YRDDLIKQKSEQAKMREKGASQTNKEMKLESQDGKKREEDEDEDEFDGDEEDNDEEDEDD
EEEEENDDDNDEEDECNLIWLDKATKKRSKDILCLEDIPYKDEIKYLNELKVLPYVSCIH
CVNIRKKKVENAGFMFYIKYEVPLLLNEGKKSKENKRVDIEEIFLYKDKIDYMLNLEKSI
NKNISNDKENKRKKANEKDEEMNKDDYDNKENNGADNIKGQFKKGSNLIKKCIDQNIDVY
DVLGVEETDDLETIKSCYKKLILLFHPDKNKGTAYLNEKEKEKEKKKGKNNNNNNNNEK
DFLYFIEKYNIEKLTNDEKKNIFLKIQDSYTILSDKILRKQYDSSIPFDERIPTLTQLEE
AKNFYNFLRPVFKRNAKWSAIKPVPDIGDENTDIKEVKYFYDFWYNFNNWRDFSYQNEYD
YEQAECREERRWMERENKKIQKKASKTENLRIIKLVDLAYNNDPRIIAENKRIKLEKLKK
KEQAMIEKKNQQNENIQH----NNNNNIPSHKKNID-KASVKLWKHHIKSLCLTKLSNLV
NTEDIQQKISIMSFDDLCEFIYDIYVILNFTVPKNNTDTTSTL--TNIKNNTIKKVYNPN
INDPKGSTSTTNNNHVNNNNNNNISTDGKTSTGFIGHLKNVHLDYKQIQTLIDTFKKYIQ
DHSFILQNNDHQLNDQHNYNENEIEKQTHANNYSNVNTQDHSNNEKIYEHVKKENDSTQI
NINESNEQNNQENELQSNKWSAQEVSLLAKALKLYPGGTRNRWVLISNSIKTKTVKEVIK
KTKEMFENDTLKNLGRNFDETPFDHFKNQNKGVMKKIDDNLDKREYKLTKENNNQVETDN
LNGDVEKKKPWTHEEQHLLEQALIKYPTSIPIKERLKLVSHELKTRTVDEVILRMKTLRA
QIMAQKSSKMPKKRRNGGRSKHNRGHVNPLRCSNCGRCVPKDKAIKRFNIRNIVDTSAQR
DIKEASVYSTFQLPKLYIKQCYCVSCAIHSRFVRVRSREQRRVRKETAKHVNPSQLMKKK

LEKKLLEYEKGYYSNKVKMRNMLFKSINKNHLFLVFLLFLFFSFSCINFTQCQITKKKVS
VSEINFDSAVDDVQWCGNNHMTVLVKTVKGRLYRSSDGGKIWTNITGNLSDKENNKSDPS
SNHMNEMTTVDLIMVNSVNKNIVLIIGNQKNHYISEDSGETFRLLNYQNKINFWQFHSTK
THWALVSSWTEACYSNDDNSGECMQTLSLTKDLGRTFQLIDIYVVQFNWGDKESHLEDTI
YYTRHKNRNGHQQRFSGWSKDVDFVATHNFGKSVDVLVKQGNKFLISNGYIFVAKLNDVI
KQTVNMMVSTDGGKTFNKANLPENIHEKSYTVLDTSEGSIMLHVNHGTSSEKINTGNVYI
SDASGLNYTLSLPNNIRTSSGECEFDRVLSLDGVYIANFLDVNNEIKDEDLRFQNFKSAI
EEDIAPFETNTEKRKKQYNKGKNEDAVRTVISFNKGGEWSYLKAPKVDSRGNKYECGDNC
YLHLHGITNYHQYAPFYSIENAVGIIMGTGNVGSHLRYESDEVNTFLSRDGGVTWIEAHK
GPYIYEYGDYGGLIVMADDLRKTNQIVFSWNEGQSWFDFELGQFSIDVDNIVAEPNSASV
EFLVYGTRNDVGVLYHLDFNALGQPLCKGLWAADSVSSDYETWSPTSGNFNDKCILGRKI
TYTRRKQTSECFNGKDLKRTVDKKPCECTPEDYECETGFTRKIGSYECKPNDPTLTIEGC
TSSSYFYANAYRKVPGDICVNGWVPEKVPVPCPSYAPFNKTAKSILFIIFIMGIVMLIIT
YICRNPKFKNLFYNYGFDTFENVKYSVIKTKRGNVNNNVFEPEMEFIDAEQDDNEEDVPT
LLPYSSDRNRSTNDFTLARNRTNQNNNITSRNISPSKNYADNIELLMADRKSNKNAVVKN
VDMTEEMQIDAIDCANQALQKYNVEKDIAAHIKKEFDRKYDPTWHCVVGRNFGSYVTHET
KNFIYFYIGQVAILLFKSGDDITVNEEILQKAQEFQVENEKDIKMKKLKPITEGLLKPEV
DLLQISERGSRGRVKICNVLNVPRSEKEYNSDKVENKYIGKIITVCGWSKAIRKQGGGRF
CFVNLNDGSCHLNLQIVVNQCIENYEKLLKCGAGCCFRFTGELIISPVQNDNKKGLLKEN
VELALNNNDIHNFEIYGENLDPQKYPLSKKNHGKEFLREVAHLRPRSYFISSVIRIRNSL
SIATHLFFQSRGFLYIHTPLITTSDCEGGGEMFTVTTLLNENGDIRSIPRINLKNKKKEK
REDINTC-IVSSPQYEDNYLIDYKKDFFSKQAFLTVSGQLSLENLCSSMGDVYTFGPTFR
AENSHTSRHLAEFWMIEPEIAFADLYDNMELAEAYIKYCIDYVLNNNFHDIYYFEENVET
NLIKRLKNILNEDFAKITYTNAIEILQNYSDSFEVKVEWGMDLQSEHERFIAEKIFKKPV
IVYNYPKDLKAFYMKLNEDNKTVAAMDVLVPKIGEVIGGSQREDNLERLDKMIKEKKLNI
DSYWWYRQLRQYGSHPHAGFGLGFERLIMLVTGVDNIKDTIPFPRYPGHAEFNNNTNGEI
IIPQEYLEKSECLFKELKELNINFKEVKHGLAATIKDLLEMNLENSTNILKNLFLKDKKK
NYFLICTLNNKTVDLKNLSNILKTNNLRFVDENNLNNILNIQPGCLSPLAIKNDKENIVK
LYFDEEIKNMQEVIIHPLHNYSSLYIKTQDVIKFCESFNHAPEYVQIKEDTTSKKKEDVQ
EEMEELQNNNNLKGKLLSNNEVEDNKSKDSNILGITSKKIENFSDWYTQVIVKSELIEYY
DISGCYILRPAAYYIWECVQAFFNKEIKKLNVENSYFPLFVTKNKLEKEKNHIEGFSPEV
AWVTKYGDSNLPEEIAIRPTSETIMYSVFPKWIRSYRDLPLKLNQWNTVVRWEFKQPTPF
IRTREFLWQEGHTAHKNEEEAVKLVFDILDLYRRWYEEYLAVPIIKGIKSEGEKFGGANF
TSTAEAFISENGRAIQAATSHYLGTNFAKMFKIEFEDENEVKQYVHQTSWGCTTRSIGIM
IMTHGDDKGLVLPPNVSKYKVVIVPIFYKTTDENAIHSYCKDIEKILKNAQINCVYDDRA
SYSPGYKFNHWELRGIPIRIEVGPKDLQNNSCVIVRRDNNEKCNVKKESVLLETQQMLVD
IHKNLFLKAKKKLDDSIVQVTSFSEVMNALNKKKMVLAPWCEDIATEEEIKKETQRLSLN
QTNSETTLSGAMKPLCIPLDQPPMPPNMKCFWSGKPAKRWCLFGRSYMAKLSKQQKKQMY
IEKLSSLIQQYSKILIVHVDNVGSNQMASVRKSLRGKATILMGKNTRIRTALKKNLQAVP
QIEKLLPLVKLNMGFVFCKDDLSEIRNIILDNKSPAPARLGVIAPIDVFIPPGPTGMDPS
HTSFFQSLGISTKIVKGQIEIQEHVHLIKQGEKVTASSATLLQKFNMKPFSYGVDVRTVY
DDGVIYDAKVLDITDEDILEKFSKGVSNVAALSRATGVITEASYPHVFVEAFKNIVALII
DSDYTFPLMENIKKMVENPEAFAAVAAPASAAKADEPKKEEAKKEEEEEEEEEDGFMGFGM
FDMLQERDLAREPCPDRIIEDMGGAFGMGCIGGYIWHFLKGARNSPKGDVLSGALYSSRM
RAPILGGNFAVWGGTFSCFDCAFQYMRKKEDHWNAIGSGFCTGGVLAMRGGWRSASRNAI
VGGVLLAIIEIVSIVLTRKTTPTPRQQFQQQMELEKRMATKNNRMLNDEIWLYKEEYKEK
DYDDDGNERKPKDHFLEVPLFDEDYNGRDINKKLNYLSSKMTKPKIKEEEVMSEESISTT
DDEEFLDLYEELKREQSMKEFYNPMLKPRLWRLEFFIKYIHNLESQKQKNFYLVSFGN-K
KLKLYGSLKNEVLYTPGYTFEAGEVKYLRVPLPIWTEKEMSISYNDLENFELGIEMWQIK
GLVFNELYACAKKTLKDIIEAEPDMNIILKRKIEKKNIIFETQRLGVYMQLSEIFEFHMA
LDSWWFISTAEMPTYLKKLPKFLRFKFPLSERDWIIHSSYNSSNNFWLYPGYFCFIGTYQ
QLANAFFILTVLCYNSNYRYKPPILLGSCIISLKSVTEYPFFKGIVKKLTLDKSRFKQGE
IVGNIKCFINSYGMEENDINIQRPSQPLSDATLVNQLILNDHYLVIRVIKCENLAISSVD
LNNVNINVWVKWDGMVNKTDTVTKTTSPFFYQNLYYPIRLVDKKELTNEHLIRNVLPIDL
ISKGDICFEVHNNNEIYSTILGIFELPFADIFNYGTEDFRSLAEENTKSTSQYNDYKYNQ
FGSGRMDPSVDNYDDYYVKKYKTIVYKNTLELMYSKVNIHTKKESTISVEAFVIPPLPEN
LVFMKEEKVQNSSIIYKSMSKRWEKDFSKFNDTYNQWFPKAIKNRKFPCISRNDFDNNYY
PLCSFVTSINLPAQVSTPGPLFHWLNNIEYIENEDESSIFTSPYFFLSYKKGTIQDHVLL
LCCCLKGLEYDAYVCKGTINNGKKEHYWVMTRHEEGWVCFWEVTNKCIIHLKNRWNNNNF
LKNSDIIMENEMLRKINMQKDNKYYSGEYLMNFVKYGLEELKKKEREIREEYDMKEENKL
YSMDMYGEKKMKDEKVNVDEILYNDEEFFNNMFEIKYEKNDTYKCNKTLKYLLENFSKYI
PISPKMFLLDYENTLTYVPYSSIEIVFNDQQLYGNMQNLHPSCILYDLENNYHWRPFLNH
APSQIKNEITISTPLSDKLSVKYTKDLEEEIREMILFMRNKEGLETNFNESSEIRYFLEM
YIDLCEYKLNLDNNYNTKPENYEYSEEKKKKKNEGENFETYETKGNIQEGNETNQMVGRV
EGKNTWSQYKNDFYDLNFYNSYHPVENIEMMNKEKKQHLTNIPNDYIYGMNDEIYNNGKE
QYIKNYVFNKDSANVLNNYDENVKRKNIKRFSMSAVHRKENEYCDDEYCEEIYKEMKEKY
YYDKDENIEEEEGYYKH--MENIPNKFIKKYNKKLQKRLEKKSKRIKDELRGKKLWMNN-
NGKVNKEMFANFIDTNNYLMKIPSKEIYERKKKKNSYNDDNNNNNNYCNVLYKNNNYHLK
LFSKRKFKKKIKLRRTKEGHRYSTKRPIYLIPSNREIINEHITNNCDISNDEELVASRNK
KKFVTQYPPKTWSRLNPTSKYVAHQISQWNWYYSLEEQYFNWQYKFPVPPNHTFVGFPIH
FSTIDFTEVKSFLLHSKRFENIMKLSIYKISFMSLSIYGNRESGQDVRTANVTAVQAISN
ILKSSLGPQGLDKMLVDNIGDVTITNDGATILKQLEVQHPAAKILVNLSELQDQEVGDGT
TSVVLLASELLRRGNELIKMDIHPTTVICGYKLAMKESVKYIKEKLSERVSNLGKDVIIN
IAKTTLSSKFISYESDYFAKMVANAIQSVKIINESGKTKYPVSSVNVIKVHGMSSLDSKL
IEGYAIMSGRASQAMPTVIKNAKIAFLDFPLKQYRLHLGVQVNINDPKELEKIRQKEKDI
TKERVNKILESGANVILTTQGIDDMPLKYFVEAGAIAVRRVNKDDLRRIAKLTNGQIRLT
LSSIDGTEKFEASSLGYCDEVYEDKVGDWDLMFFKGCRTSKSNTILLRGANDFVLDEMQR
SIHDALCSVSRALESNYVVVGGGCVEVALSVYLEDFAKTLGSREQLAIAEFAESLLVIPK
ILALNASYDSIDLVCKLRAYHTKSQVMNIEDSKDYKWYGLDLVNGKVANNLKNGVLEAMI
SKIKSIRFATEATITILRIDDLIKLVPEERKMEEPMNVPLIKNLYPDPSNNLYGDLRICS
RATETCGIACSAGYIAVPWQVEGGGMIGVIRLENQVRNPPVIKLKSHTSPILDLSFNPCY
SEILASCSEDMSIRIWEIRHEDENVNEVKDPLCILNGHKKKVNILSWNPMNYFILSSTSF
DSSVNIWDIENEKKAFEINMPKKLSSLQWDIGGNLLSGTCQNKQIHIIDPRKQEICNSFL
IHDGGKSTKCIWIDGFGGEDKCILTTGFSKNNMRELKLWSLKNTTSPLTTITLDNAASPL
LPHYDESVGMIYLIGKGDGNCRYYQYSQGSIRKVDEYKSCLPFRSFGFLPKRMCDVYKCE
IGRVYKNENNTDIRPISFYVPRKNSSIFQEDLYPPIIMRDPERSTNKWIDGINLDIKRVS
IKDLTEDDLLITKKFKQLPKESKSILIQDNNNPKKGSVMRQFTKKFTFRKKKETTEIQGE
IMGETKSSIEADFEPQECKENKKGNKLNLKDNVDDDDYLITNENENYKENNDSSIQSIRS
NSKSNTLQSEENNVLDNILDMKMCKSTAMKIKNIGEESIHNICSSQVIFTLSSVVKELVE
NSIDADASEIKIKLVESGIKLIEVNDNGVGIKKINFENICARHATSKIKDFNDIHSSLNT
LGFRGEALNSLCMLSNVNITTKNEENDHAYLLKFDKLGRLYHEEPIARLRGTTVSCENIF
HNIPIRKKDFIKNIKTQVSDLLLLMQQYAIIYHNIKFVIYNIVTSKGCTKNMNLLITNGT
DSIKKNFYSIYGKRNIGNLIEFNIDGNEWNIRGYISDSNSGRRDKDLQFYYINSRPIHIL
KNVNKLINTIYREFNSRLYPIIICNILSDTKNIDINVTPDKREVFFTFEQEMCEHMKTAL
VKLFTPKTSNLIDTQIDDYFLKANNIIKMEPNYNNEVTHHQIKNDESSSLFQNDDYNFSN
IYRANTSTQESMHCDLNKKQYENSEKEKNEKNVENKKNNVYEYKLNNDDTKNNIYEYKLN
SDDTKN-NIYEYKLNNDDTKNNIYEYKLNNDDTKNNIYEYKLNNDSNNTIYEYKLNNDDS
-----------------NNTIYEYKLDDSNNTIYIYEYKLNNDSNNTIYEYKLNNDDSNN
IYEYKLNNDDSNNTIYEYKLNNDDSNNIYEYKNNDDSNNSDDSNNTPYLNGQENEETEEG
NLKDCTNYSFEDLKENIKSNCIKKIPIDINMYINREELKSGFDYDQIHVINLTNSEKIKN
IIFQKMKEETPINNYLCLTDDQEEKEYKNLFDGNLSLKESNNNNTNNVNNNEDINFSNID
ETQKDLYFQSSLFNKLKICGQFNKGFVISKIDLLYFEKKKKKHDNNSINSN------DNK
HKSNYALFIIDQHAADEKSNFEKYNKIFTMKSQKLISKIHVQVSPAQVHIIQKYMSIFLQ
NGFEVQILEEPIHKRRKTNNNPIDDEEEMLMELNVYLLSLPVFNGKILEVVDFMSLLHHL
TEHPVASYNES-EVSVKTTIDLNNKTDTWFNYNFPRPQKVWRILASKACRNAIMVGKALN
IYEMIKIKKKLSFLKNPWNCPHGRPTIKYLINNVDIKNCFKNYYLKLYDEITNLILSQNY

DAYKYLFHNHVFFLIISTKPFLGPVLKFQMGSSKEDEIEKQNIYATYPGLEQQLDMVFAC
HDISKQGKLSYKTVEMILRHFLMQCGFMEYVCRFVDENGTLDLKHVSNYLSIKKLMYKLK
CCGESMLTLDEMKELVIIFLKKISDTYTEDQTKWLEQMKSSQEQQDKALEEAMYKYEKNI
LFHHAVKEQQILQNDKKLNEWNENVENAYEAQQEILRQFESSRKKNIDISLEKNNELIIA
KDYIDKIKEAATDNKYDNSKCFIYPASSAPCGACTSAGAIIHHRRYKEKRRKKEYSLCLM
ASMDHNAQDELVDYEDDENILDSKDVKGNLGNNILNNNNK----GGAMRGSYATVHTGGF
KDFFLKPELLRAISESGFEHPSEVQQETIPAAITGTDILCQAKSGMGKTAVFVLSILQQL
DTNENQDMQDTKEMNNDNNNNGDNKFVRCLGLAHTRELAYQIKNEFDRFSKYLKNVRCEV
VYGGISMNKHIKLFKEDNIPHIIIGTPGRILALIREKYLITDKIQHFVLDECDKCLEKLD
MRSDVQKIFISTPLKKQVMFFSATMAKEMRDVCKKFLQNPVEIFIDDEAKLKLHGLLQHY
VKLQEKDKTRKLIEILDALEFNQVIIFVKSVTRAITLDKLLTECNFPSIAIHGGLEQQER
IERYDKFKKFENRILVSTDLFGRGIDIERVNIVINYDMPENSDSYLHRVGRAGRFGTKGL
AVTFVSSQEDTLALNEVQTRFEVAISEMPNKIDCNEYINQMSEILDGLDYSEMSKQVILN
DDGSSDGNDTDVHSMFDRKEIERKVLDLESIHFIQKKRLSVSAEAYGDWNKKIDNFIPKV
YKKDEKEKAKIREALNESFLFNHLNKKEFEIIVNAFFDKNVEKGVNIINEGDYGDLLYVI
DQGEVEIYKTKNNKKEVLTVLKSKDVFGELALLYNSKRAATATALTKCHLWALDRESFTY
IIKDMVAKKRKMYEDILSHVNILKDMDPYERCKVADCLKSKSYNDGEIIIKEGEEGDTFF
ILIDGNAVASKDNKVIKTYTKGDYFGELALLKNKPRAATIKAQNFCQVVYLDRKSFKRLL
GPIEDILHRNVENYKKVLNELGLDTTCIDENMHLKIVCLSDEVREMYKNHKTHHEGDSGL
DLFIVKDEVLKPKSTTFVKLGIKAIALQYKSNYYYKCEKSENKKKDDDKSNIVNTSFLLF
PRSSISKTPLRLANSIGLIDAGYRGEIIAALDNTSDQEYHIKKNDKLVQLVSFTGEPLSF
ELVEELDETSRGEGGFGSTSNNMRQCDPNSAFFGFMGIAASSIFSNLGAAFGTAKSGVGV
CSVGVMRPDLIMKSILPVVMAGVLGIYGIIMSILIYGKMTPAEGYSTFAGYAHLSSGLIV
GLSSLAAGLAIGIVGDAGVRANAQQNRLFIGMILILVFSETLALYGLIIGIYISIAETPK
LCTPYNVMTKRIIKNDIT-SKGIVRSSKSSFKVEDKESDNKGQILTIQNIIENDDSTNDK
KINKKLIIKNKKKNNTNMNTHEMRIITIPKQRRTSVIKNWLELIKPIVTHLKLEIRMNKD
KIEVRTCKLTEDKNNLQKSSDYIKAYLLGFTLEDSLALLRIEDLYIESFQIQDVKILKGD
HLSRCIGRICGSNGSTKYAIENATKTRIVIANDKIHILGSFNNIKMARHSICSLILGSTQ
GKIFNKLNILAKRMKERFMGNNCCAGRDLLYKNKLQEFGIEGSKTIRKLLSFTSNDILRF
DKAYDENDVQEFVNLCSSTCEIEKLEDRMHPWAADPKTIGALSATQLAILASKENEPHYK
DAIREANGIAVFINLLKSHELDRVHAAVVALSFLSVDNVKNCICMFESGALPYLISGMKS
NIDGMKAACAQTCRNIFVLDKKYKKEFLKLGGITQLVNLLELPSNDDSQPLYTQLEAIYH
LEDFILNDGDEIPEFLEAVKNSNSIKNLKTLQQCPEQDLAEASNVLLLRLTDMASGWKAD
EVFGGVMSYTYDDIICMPGYIDFALSDIDLTNNMTDNITLKTPVISSPMDTVTGHKMSIA
LALSGGLGVIHNNMSIEKQIEEVKKVKRFENGFIFDPYTFSPEHTVADVLETKNRVGYKS
YPITVDGKVGSKLVGIITGVDYLYLTNKSMKIGDIMTTDVVTGSYPINLSDANKVLCDEK
KSVLPIVNKNNELIALVCRNDMHKNRIFPHASKSQNKQLIVGASISTREHDLERANQLIK
NMIDVICIDSSQGNSIYQIDTIKKIKSAHPDIPIIGGNVVTSQQAKNLIDAGADVLRIGM
GSGSICTTQDVCAVGRAQGTAVYHVSKYAHTRNIKTIADGGIKNSGNIVKALSLGADFVM
LGNLLAATEESCSEYYFENNVRLKIYRGMGSMEAMYNKGFNSKSRYLVDERKNEYTDENI
DEIKVSQGVSASLVDKGSVLNLIPHLFKAVKHGFQSMGIRNIPELHSKLYSGDIRFDVRS
FNTIKEGKVSDNLIFNNKKMFQKNLKYNKFISSKC-IQNIYVTNTLCV-------YNQHL
CSPTQLRKNFSTFMRNVIEQVKKDMKENKQYQEALKELKEKTEIDDKRIKLKKRIEENID
FFQKLKEKNVEAVKILYNDVNNFVTYCFTKYYALKLSKNLCIQFFVTLKNVFIKSTNKFT
ELSEKWDENSAFVKLDKWRQDMAIKRYKKKKEKENQHINNNINEENNDEPSNNIININET
ENDKSQNYELILAQESAWDKFGSKLKDMPFLNNFFENPILGKLFGETELAAALREMKMID
KNFKLSELMYLFEYVISKHIVESYLIGDEETLRLHCGSSAFNSLNASITERKKKKVFLDT
NVLIYKNHELKGAQRMEESSPWFIFTFHTQQINCLKNKNDEIIEGKIDDIREVVYTIALS
KHPEPEKEGLLYPYIVREFAIIGNTPSWMNEIKSESLLQTRPFKLGIEDIQNLGSSYIEN
NEKLKKYNNEISSLKKELDILNEKMGKTTTTKIVEPAKTPEFTFWYYELKEMKGFQDLVM
YEVKKKKKHFKVLSHSCLKYLSNREKMKIKKQEEEEKRLKLYSKNISSYMDVFWKKIEKL
VWEEKKRELQQTLNKKKEMRFKKFVKEAIKKIKDARHNAHELFENKYVSMSSNNNSEIVN
NASSVDNGDKELKEDDLTDQEEEDYLLDEQMSSTDESENKEEEINMLDDEANLPIEELLK
RMYGFKSGEDYINFMENEDDANEENNDEKDNNIDEDDNNNDENNSDHKSEDNNIDETGED
YKSDKENTRFHNKLKKEKYDEEKREEENKNYEKDEHEYESDNDIENDSDETSEHIKRDSR
SSCQKQNCEKKRRINDSDDNDDILTCNMDEKHLTKIPPIIKATLRDYQHAGLHWLLYLYK
NNINGILADEMGLGKTLQCISLLSYLAYYFNIWGPHLVIVPTSILINWEIELKRFCPCFK
ILSYYGNQNERYKKRVGWFNKDSFHICISSYSTVVKDHLVFKRKRWKYIILDEAHNIKNF
NTKRWNIILSLKRDNCLLITGTPLQNSLEELWSLLHFLMPNIFTSHLDFKEWFSDPLNLA
IEKSKIHHSKELIDRLHTVIRPYILRRLKKNVEKEMPNKYEHIIKCKLTRRQQILYDEFI
NNKNVQNTLNTGNYIGLMNILIQLRKVCNHCDLFTNKYIQTPYYYMLSIRYFVPRFFILF
EKNYYADFYLILFLHNEFTSLGGRDVTKETSPSSKSFYDNELYDNNHISEL-YDNNHISE
LYDNNHISELYDNPMPSGFTKTSEQFGQIVSHDYNKLCADNEEKRSNSKDNNNNNAFLSI
LKLLNQSNPLNNDNNNNNINIEDVHSYIYNSIYKEYIPKNSDEFLTELNNNYDILSLYID
PYNRINNKHIYHKSTSNENTIYKYNFKVINNDTQYQNIFTDDTNNSYYNSLEHNLWIKRN
QIDERKKEEELRNERIPIFGKNFLDLIKKEFTKDKNIVYNYTNNVPIDYSSVKEVWVEDN
PMMNMSYIIEFLFPNMEQFLKRHEKMIHNFTLINNPSVICKSHDIRINNNLLNYSN-DKM
NPIILQIKNATRVYHDAFLKQSIIFPLNKDISLGSGKLCALEKLLSKCKREGNKCLLFTQ
FIKMLDILEIFLNHLNYSFIRLDGSTKVEQRQKIVTKFNNDKSIFIFISSTRSGSIGINL
TAANVVIFYDTDWNPSIDKQAMDRCHRIGQTKDVHVFRFVCEYTVEENIWKKQLQKRKLD
NICINMGNFNNSNTHSKITDTDPTHNKDWFTNVDTIKEVFINKKNNDDDDDMYKDRLLHE
QVENKDKMNVRFEKTLEHVEDKDDIRALNETKKETQNEISQNMQEFTTRNDFQDSYNLTS
YCFNFLNENLTDSLKQQIDEMRMKIEIEMMNTG-DENMSLSDLSNKSHNSE---------
-MVLYIIGLGLGDEKDITIKGKELIEKSDVVYLETYTSILFVSKDVLEETYKKSIEEVDR
DFAEENCDKILDEAKNKKVSFLVVGDPLCATTHHDIILRAKKKNIDVEIIHNTSIISAIG
ECGMQLYNFGQIVSIPYFEDNYKPTSYYDKIYINLKNNFHTLCLLDIKVKERTVENIMRN
KKIYEPPRFMTINDSIEQLLYCEHIHKKNIITKNTLGIAIIQIGTDNQQIISGDLLTLKD
ISYNKPLHSLIICAPTLHDIEKEYFDLYHMKKMGSGKPSGLRAARKLRIRRRTQRWADKS
YKKSHLGTRWKSNPFRGSSHAKGIVVEKVAIEAKQPNSAYRKCVRVQLIKNGKKITAFVP
GDGCLNFIDENDEVLVSGFGRSGHSVGDLPGVKFKVVKVARVSLLALFKEKKEKPRSMGI
KGLTKFIADAAPNAIKEIKIESLMGRIIAIDASMSLYQFIIAIRDSEQYGNLTNESGETT
SHISGLMSRSIRLMENGLKPIYVFDGAPPELKGSELEKRGEKRQKAEELLKKAKEEGNLE
EIKKQSGRTVRVTRKQNEEAKKLLTLMGIPIIEAPCEAESQCAFLTKYNLAHATATEDAD
ALVFGTKILIRNLNANATSNQNKNKNNSKRGYILTEINLEQVLKGLNLTMDEFIDFCILC
GCDYCDTIKGIGSKTAYNLIKEYNCIEKIIENIDQNKYQVPSNFRFQEARKSFINPNVLP
KEDIKIDWNEPQIEELKHFLIKDYNFNELRVTNYINRLLKARKVTTQRRLDNFFTACTKK
STKLIVEETKKEQTLPARKGKKRPTAGDKNKQKAVKRKEENVDNEKNKSVDNEDGIKSVD
DEKNLDDEKNKDDLDNFSSNLFDSDKESESGNIIKNEKQHTENKDNISDEIKKKFLLPFC
PKDVTKKKYTQRCMFFSSFPFDSMGGQQARRKREVNNNKFYEVLNLKKNCTTDEVKKAYR
KLAIIHHPDKGGDPEKFKEISRAYEVLSDEEKRKLYDEYGEEGLENGEQPADATDLFDFI
LNAGKGKKKRGEDIVSEVKVTLEQLYNGATKKLAISKDIICTNCEGHGGPKDAKVDCKQC
NGRGTKTYMRYHSSVLHQTEVTCNTCRGKGKIFNEKDKCANCKGMCVLKTRKIIEVYIPK
GAPNKHKIVFNGEADEKPNVITGNLVVILNEKQHPVFRREGIDLFMNYKISLYESLTGFV
AEVTHLDERKILVNCTNSGFIRHGDIREVLDEGMPTYKDPFKKGNLYITFEVEYPMDLII
TNENKEVLKILKKQNEVEKKYDLENSELEVVSCSPVDKEYIKVRVTKQQQQEAYDDEDHQ
PEMEGGRVACAQQMNSYFENNNTYMEWKLDEKIYKTIVGALGSCNSSNNSVQVEVTKVLK
DLNENVADAALYLLHIFMNKQENNDVRQVGGLLLKNYINSKNKFLSNDILKIIKNEIFKL
VEDEVKEIRNTSGSVITTILTKYEGIEQWPEALYNLLLLIERGNNDVVDGAFRAILIIIE
DELMNRKNRDSLFFQFCKSQLLQKLFQFCSPQEKNIKKKYAAECLDLFITSSCFTTNGVF
NDMFAQLWECLGYLASEEDPQILKIVVSCMTIITDTRYSSIFSNLDAIIQFMVNATNSND
RKVQLEALEFWPIFIKDRSYVAYASYNNPKSDINKSDSNYIDENVYKNINELRTEALKRL
KNYLPYLCKILIDNTVYTKWDYLAMDESHFQNDNANVPDLIQDISPELYNN--KNNDIAQ
EEIKMNKMNNINNNNNNINNSSNNNINNNNLLNKNDDINNNNNTEDFSDDEKNDEMSSRT
WGNDWTVRKGAALCLDYLSNVYNDEILEFVLPHIEEKLMSDKWNIRESAVLTLGAIAKGC
MYSLSPFIPKVLEYLIKLLNDEKPLARSISCWCVTRFSSWICHPDNCEKWFEPVLLNLLK
RILDSNKRVQEAACSSFANLEEDALELLNNYLHEIVHTIQQAFQIYQAKNYFILFDVVGT

LIDSVNIVKENNELAHEVVYAILSKWVNIRISSPYIIALMECMSCITSAYGKEFLKYAKD
VIRTCIKFLVILYIDLEEEVKYFYSKKLFNSTSFISNRNNISSTANELLTYYKITNDDYF
ICMKDIDTISPSKKKDLIECSFDLLSRILSVINSSIMDILNESEYNFIPLVHKYCLKLQN
IKFDDFNNKIMSNHEELRNIINPDGTLQNGYIMKNEYSELAKQFLNFGILQSNFALIGDI
SRFCPQYLITYLNDIIPFLIAHITHPSTPVSNNASWAIGEISIHINSEYMEIYVDEITKQ
LIYICQNSKYHGCLLQNICITFGRLTSTYPKKLIFYFPQFLKTWLKIMAHGTQENEKINS
LKAVLETLYINLDLAAEHLKDIVYIILKYKYVSQNVNIFFHQFLATMKEKYPNHWKEIYS
QTGDSLSSPIPNDMLSDLATRFNLMKERINEKFKINSYQDYSDKNNEWNIDDAINYIKRN
AFFKKYKDINDVNDDDHINDDDKFYQNFENILIEEKKYLKKLLRENNLKEYVDGKVLENE
ENEESGLMYVYNTCYNILSSKNEINYNEGYIIINKNNTMKKIYINEINELLIEIIYITLT
R-ENLENSLVDLLGDRFLDFIIQIIKNKEQIEKDIKLLSKQIVIKENTLVSNFFITNKSS
KKIKNQQILYKKENIEKIIDHFLYLLTDDSYNQMKKIYIPNDQDKLEEIHVPKNTIYSYT
DNITKVKISRLENLQFHKKELVSVNVLPFWHKYIFDFEYFNYVQSKVFNSAFRSNKNLLV
CAPTGCGKTNIALLVILQQIILFCEQNKIKLEKIVKIKNGLLSDIKKRNDDNERTRDELK
SGYNENNNGDKNFDDDNNFDDDVDGDGENYINSKEFKIIYIAPMKSLVFEITNLFRKKLK
IFNLNVCEYTKEYSLSSKELEQVHIIVTVPEKLDILLRNSSYSTTVSDESLIKYIKCLIL
DEVHLLNTDRGDVIETIVSRFLQYSETSQSIRRIMAMSATLPNYKDVRDFLKVENDMCYY
FNEKYRSIQLDKTLYGIHEENNNKLYIAKNIYTYNEIINSLKKDKQCIIFVCSRNETNKT
IEFLINHALKNNEIEYFVNNVYTDNDIKKKIKKSNNLYIKQFYEYGCTIHHAGMSRSDKI
LVESLFKKKVFNVLCCTSTLAWGVNLPVHTVIIKGTNYFSSESGKLEDMDILDINQIFGR
CGRPQYESHGHAILITERTKLYKYIKLLTNNTVIESNFLKNIENHLNAEISIGTIKNIED
GIKWLEYTYLFIRMKKNPYLYDVDINNDLNLYNKRKDIIMKAIQNLSENKLVRRVLLTND
FIGTFYGQIAAKYYVDYKTIGMFAENVQNNNYIEIIQVISKAKEFENIQIRNEDMKDFLY
LKDKCDIKEEYDESKNMTLRILIEVYLRRLQINNFSIICEINYIVQNIIRILYAYYDICL
NILKNISNLIMNTHNLIVAILRRLPINCGIFRHFCYKNEMLEKNKKAILTNKDRLKNNNR
RNQNYTVYLKEAAVNILEKKNLTYESIEHLSKKELLFFMRNEIYTNQILYYRNIIPNLDI
DGYIQPITQTIMKINLNVKLTNTIWSDQWNDIIENFHIFLLNTLNNDILYFQKFSIHKKD
RKKIHDISFEFPLSNQIPPQITVQFLSMNWCNLSFVHIFNTNNLFINQKINVFSEILPLT
PLSTNVLNIPNYIKFFSFKYFNPIQTQMFHATFHTDENILLGAPTGSGKTVIGELCILRN
LLRCEGQKSVYICPMKAIVNERYKSWKSKFKSLFNKNVIELTGDKNENKENIAESNIIIC
TPEKLDVITRNWKNKKFVKNINLIIFDEIHLLGENRGGVIEILVNRFKNMQNELNKKIRL
IGLTTVITSVDDLILWLDVKENYLFNFPSSCRIVPCKTHILGFTQKAYCNRMSVMNKNVF
DAINQYAQTKNVLIFVSSRRQTRLTAYDIISLLHVENLLNDKNHIDSEEERKNVANILFQ
NYLNLIENEHLKEILKYGIGIHHAGLNENDKTIVEYLFLNKIIQILICTSTLAWGINLPA
YLVIIKGNEFYDAKTKKYKDIPYTDLLQMIGRAGRPQFDDKALAILLVQEKRKNAIKNFL
YHPMNIESNIMENFNEHINAEICSNVINNKEDIFNYLTKSYYFKRLFSNPSYYIKEVQYV
QFFENSKLSTHAKKIIYDHLNDVIENGIKFLVQNKCLEVVQENYVLNYYATPLGHIASMY
YIKCETVYFFYTSIQTLKNDSLEFYDIFELVVQAKEFDDIPLRHNEDKYNVKLRNQIPLD
IDMNMPNVKTYLLLLSRFYECTYETVDYHIDLKLVMDQIARVINAFIDICLLFHNYNYIK
KLILIFNCINQKVKPNTNSLYIIKDITEYQIYKLKQLDIYNINQLIKFDKSYLYSLNIFD
ITQINFILQLPVFNMNVKLFYKDIHSENRNTTNSYVNIPYAQHFRNQHKYSFKINSYYSK
EIVIKLFFNFMNRTGKEASS----QNVQWFAEMETTDTKREEQEIEEKKAQEEKIEDVDK
ILNDILSISSECIQPDELRVKLLLKRKLICYDGFEPSGRMHIAQGLLKSIIVNKLTSNGC
TFIFWIADWFAHLNNKMSGDLKKIKKVGSYFIEVWKSCGMNMENVQFLWASEEINKKPNE
YWSLVLDISRSFNINRMKRCLKIMGRSEGEENYCSQILYPCMQCADIFFLNVDICQLGID
QRKVNMLAREYCDIKKIKKKPVILSHGMLPGLLEGQEKMSKSDENSAIFMDDSESDVNRK
IKKAYCPPNVIENNPIYAYAKSIIFPSYNEFNLVRKEKNGGDKTYYTLQELEHDYVNGFI
HPLDLKDNVAMYINKLLQPVRDHFQNNIEAKNLLNEIKKYKVTKGNKYSLIRNPQSNVPL
KVSIIGSGNWGTVVSKIIGMNAQKLKIFHPIVKMYVKEEIVEEEKLSDIINKKKENIKYM
KGMKIPENILAISNLKEVIDDADLLIFVLPHQYLDNVLDEIVQNNNLKKNARAISLIKGI
KMVRSKPQLLSDIIEKKLDIECLALSGSNIAEELSREHFSESTIGFEKKGNEVIWQNLFD
RTYFKVNCIQDKPGVEICGALKNVIALGVGFIDGLTASYNTKSAIIRIGLEEMKKFAKMF
FPNVLDETFLDSCGLADLITTCLGGRNFKCAKEFAKRKGRDSWELIEAELLNGQKLQGID
TTKEVYDVLEYHQLKTEFPLICTIYEISFQKKNPCSIIDVLSTKKLRNIKYK-MERILSS
IGKLSVVAGGLSLIPYTFIYDVDGGERCVMFNRFGGVSENTFGEGSHFYVPWFQTPYIYD
IKMKPKVINTTTGTRDLQIVTISLRLLFRPHTQHLPYLHSTLGPDYDERVLPSIGNEVLK
AVVAKYNAESLLTQRDKISKEIRESITARAKHFNILLDDVAITHLSYGKEFAKAIEDKQV
AQQESERVKFIVAKTEQEKIAAVIKAQGEAEAAKLISSAVKEYGKSLIEIRKLEAAKEIA
ENLSKSKNVTYFPSNSNILLNPRDFMKLEYINILKEENGGYNFDNLKRNEILKEKGVKFP
QFRKTGTTICGLVCQNAVILGADTRATEGPIVADKNCSKLHYISKNIWCAGAGVAGDLEH
TTLWLQHNVELHRLNTNTQPRVSMCVSRLTQELFKYQGYKVCAIVLGGVDVNGPQLYGIH
PHGSSCLLPFTALGSGSLNAMAVLEAKYRDNMTIEEGKNLVCEAICAGIFNDLGSGGNVD
ICVITKDSYQHIRPYKEPNMRLYHLPHPTIYPKGTTPILSEKIEYIKKFISVEDAMTKNK
NMSINNNKVKYEPFGKPPPGYIPGKGRGVTGFSGGVSRDDTTDDKDKNDYSDFNYDEFHG
YSESLFKDTEYDEEDKEADEIYDKIDSLMDIRRKSRRENKLKEEISKMRATKPTITQQFG
DLKKNLANVTIEEWESIPTVLQYSKQKQKKVQKNYLPAPDSLIMSRINESNIHLNFNNSA
SSGHGLKTPLGLKTPLGLKTPLS-GLQTPYMRNPSGMDTPLINNNIKSNMSISGLNTPFT
LSGYNTPLSASNVSGYNTPLFNNTHKLSLNDLGEARGTVLSVKLDELIDNVEGQTVIDPK
GYLTNLNASSLINDADIADINKARSLLKSVISTNPKHGPGWIAAARIEELAQRKDKAKEI
IMKGCVVCSKNEDIWLEAVRLEEKLSEVKIILAKAIKHIPTSVKLWLEAYKKEKNVDDKR
KVLRKAIECIPNSVKLWKEAISLENENNAYILLKRAVECIPQSIEMWIALARLCTYTEAQ
KVLNEARKKIPTSAEIWINASQLEEKQGNIKMVDIIIKRCIENLSSKNVIFDRDKWIKFA
EECEQSKFTHTCESIIRNTMHIGVETLNKKRIYKQDAQNCIHNKSIHTARTLYNEALKIF
KTKKSLWLALANLELTHGKREDVDEVLHRAVQSCPHSSVLWLMLAKQKWLNNEIDKAREI
LAESFIHNQNTEEISLAAIKLERENNEFDRARFLLKKSRVQCNTPKIWMQSVQLERLLRN
YKEAKMLAHEALKIHKHFDKLYMIAGQIELEMYKYSNDNSNDPYKNAQNIYEEGLKYCAS
SINLWICAIDLQIEKKNYTGARALTEKAKIKIKYLNSFNNNSHILKSKEIIETNEQNYDT
QLNKNMDGSKSNNTTASNISKSKNELEKKVNNNAYIKIIENYDLLWLKLIEIELCCNNKN
LNPIISEALKECPSSGILWSKAIELENKNLQNSKSVSAFNHCGNNAYVILTVAKLFWVNF
KIQKARKWFYRVINLNPHFGDGWATFLAFEIDQQNEINQKDIINKCIKAEPNRGYLWNKI
TKRVENWRLKYPQKLYKYIKDIYPHVLNKKISDPIWNIITDENADTSFRKKKKK-INCLL
QLNGRLNYLKKYLLLINLFNLKNKDINLFVCGLRKMEKDDVVSSTPAATEEMNDKKKEKK
AKKLAEKELKLAKKLERENLKNEAAKVLDYVCEDINKDNYGYIKVSTLQKYADSIELYNL
EDIYNFFVKSEDCENEKREDVKDAYNENLLGKKKIWVRGRIHDIRSKGSIAFIILRHKLY
SLQCILDIKNNNNDKNMMKWVSNLSLECIVDIYGEIKKPEIPIDSTNIKYEIHINKIFCL
SKTMKELPFLLKDANMKETNDEITIKVNQDNRLNNRCFDLRTYANYSIFSLQSVICHIFR
TFLLQHNFVEIHTPKLLGESSEGGANAFKINYFNQNGYLAQSPQLYKQMCINSGFDKVFE
VGPVFRAENSNTYRHLCEYVSLDIEMTYKFDYMENVHFYDSMFKHIFKELTNNEKNKTFI
KTIKNQYPSDDFVWLDKTPIFTYEEAIKILIKNGKLFLKEEDILTYDLTTDLEKELGKLI
KLSHNTDYYIIINFPSSLRPFYTMYKEDDPKISNSYDFFMRGEEILSGSQRISDMKLLLE
NIKLFNLDPNKLNFYIDSFAYSSYPHSGCGIGLERVLMLFLGLNNIRKTSLFPRDPKRLI
PMEITNIFKKLLCRQNVVHTENNSFFDVFKDPNEEGLKRK--NEVDNEMKNK----SMVK
ENNDDTIKTSEEDKLKEQNLLGYANSFDAPNALLFENLNKEYKFITTQDNFDGFRFEVDK
NINKFLQSTHTLFLGTTLREVGYLYQFGANFTNLDNSLLMISRINIDGSVNGRFCKKINN
NIDCKLNFNTYAKNDTRNMYEMSLEVNKPLYTYNFKSIWQGAWIFNTSYTQLLTKKLQAG
VDLTYIASNCASIGSFGLRYNHKNNVLTMQIVRQPNFKSPEFMLNQTHLYKIQYAKKISD
RLSLGTELEITPQTKESAMRLGWDYSFRHAKVQGSIDTSGKISVFTQDYSGFGVSGYIDY
LNNDYKFGFMMHISPSQEQPQTAAMDSQEYIPQYKLILVGDGGVGKTTFVKRHLTGEFEK
KYIPTLGVEVHPLKFQTNFGKTQFNVWDTAGQEKFGGLRDGYYIKSDCAIIMFDVSSRIT
YKNVPNWYRDITRVCETIPMVLVGNKVDVKDRQVKSRQIQFHRKRNLQYYDLSARSNYNF
EKPFLWLARRLSNQPNLVFVGEHAKAPEFQIDLNIVREAEKELEQAAAVAIDEEDIENMD
KLKTIYLDSALSIIKGALCIILQIPTSRTTESVKKKANNVGVITVKSILSEPTIHQYDDI
KKLIKNKLQECVPFYNYNMNRSFAEKIYGDCIYDNYGLSKEINEINLIILEEWNINCNKN
RVLKNTGLIKEITINQFKYSTNKESLEVHFAVSPKYTFEELSTMYKNEKGLYEFLLSPII
KIICNENDKKLLDNMNEECTYLNAEDILPKNKVLPPSGIENIDYERSKDVTPWDVNINEE
GINYNKLIKEFGCSKITENHIKRIEKLTNSKAHHFIRRGIFFSHRDLDFLLNYYEQHKCF

YIYTGRGPSSLSMHLGHLIPFYFCKYLQEAFNVPLVIQLSDDEKYLFNQNYSLEYINTLT
NENVKDIISVGLNPELTFIFKNTEYAGYLYPTVLSIHKKTTLNQSMNVFGFNHSDNIGKI
SYPSFQIAPCFSQCFPNFLGKNIPCLVPQGIDQDPYFRLSRDIAVKMALHKPVVVHSVFM
PGLQGVNSKMSSTKKKKDKSNSTFDHNNSVIFLTDTPEQIKNKINKYAFSGGGTTIQEHR
EKGGNLDKDISYQYLRYLLEDDNKLNEIGEKYKKGEMLSGEIKKILIDVLTELVLKHQEK
KKSLTDEEISYFFDPNKPSLQKFKNMMTTYNDNTNKHQINITKLTKDELYFTLYNSNSGI
ANALRRIMLSEIPTLAIDVVNVYENTSAFHDEFIAHRLGLIPIDSRNVNNYEFREKCKCK
ETCSKCTIQYIIEVKCNHSNKIDVSHYDIESLEHEPNVPMPIPHGNKNSV--SENAIPIV
TLSKNQTLHMKLIATKGIGKMHAKWIPANVSYRIDHKVLIKHNLIDKLSNEHKLLLANNL
NKDCYILNKDTHDDDIQLKLKENMSVVMAESSIDLLSELGYKDIIKIVYDETMFHFHVES
VGSIPPEQIVQMAIDILENKLKVLEPQIKSSFYSIDEVAKQLKEQGVSLYGIQLDLEMVE
NKSKVKDISLAPFGKMQMEISENEMPGLMRIREEYGKDQPLKNAKITGCLHMTVECALLI
ETLQKLGAQIRWCSCNIYSTADYAAAAVSTLENVTVFAWKNETLEEYWWCVESALTWGDD
NGPDMIVDDGGDATLLVHKGVEYEKLYEEKNILPDPEKAKNEEERCFLTLLKNSILKNPK
KWTNIAKKIIGVSEETTTGVLRLKKMDKQNELLFTAINVNDAVTKQKYDNVYGCRHSLPD
GLMRATDFLISGKIVVICGYGDVGKGCASSMKGLGARVYITEIDPICAIQAVMEGFNVVT
LDEIVDKGDFFITCTGNVDVIKLEHLLKMKNNAVVGNIGHFDDEIQVNELFNYKGIHIEN
VKPQVDRITLPNGNKIIVLARGRLLNLGCATGHPAFVMSFSFCNQTFAQLDLWQNKDTNK
YENKVYLLPKHLDEKVALYHLKKLNASLTELDDNQCQFLGVNKSGPFKSNEYRYMGSYGM
DVDDAYLHQGQYAAPYDNQYQQDDDTPSPRGENHTPFVGYFSSHLLRTGFFLQCVSLVLM
FVFYWAFGGTGIFIFDLYAGPECVKVSSTFHLTISILMALYLLGTLYIAMFQVFVADNSK
WCRGFRAGSKLLSAAVTLDLLSSILRLVQYLYAYFYMSMRWWARYQQTKSDWTLLHFGSI
VHSFALFIYGAAFFYMEAYHDEGTYEELAWSNLTLFKLAGLAELIMVFSGFGAFFSILLL
GAIVCATVWAFSFEPLLEKWSPELHSRDINADVLPEIKHEDEQCAYNEENIYEPYSVNPD
NVEYGEEMVAKNMNEKYIDGNTNIYSANNGIPTTYDYTQIEGQLKKNVEMGVTENNYTAQ
YMEDANKPKKRTFRTFQYRGVDLDKLLDLSQDELIKLFKARQRRKFQRGISKKAKSLLKK
IRKSKKNCEPGEKPNPVPTHLRNMTIIPEMVGSIVAVHNGKQYTNVEIKPEMIGYYLGEF
SITYKHTRHGKPGIGATHSSRFIPLKMSNKKGQSPKEESIAKMLICKVHIGTKNLENKMK
RYVYTRAKDGVHIINLAKTYEKLQLAARIIVAISNPADVVVVSARPFGSRAVLKFAQYTG
AQAIAGRWTPGMLTNQIIQKFTEPRLLIVTDPRTDAQSVKESAYANIPVIALCDSDSPLE
HVDIAIPCNNKGKESIALMYWLLAQEVLYLKGVIPRSEPWNVMVDMFLWRDPEQFELKNL
ANEENTPTAPHLIENQYAAEAPYDEWTKKEEWNDNTNEDWKNPIAAEEWMSINRYNYKTM
NSLLKDKIPQHFDILPHTTIKCIIHPSVIFTILDAYLRRDEDQTHVIGTLMGSVIDTNLV
EISDCFVDKHSLNEGGFLQIIKDHHETMYELKQKIRPRDQVVGWFCSGSELSELSCAVHG
WFKEHNSISKFYPHTPLNEPIHLLVDASLESGFLNIKAYVQLPISLVKDYFVHFHEIQTE
LLPCNVERADVSLVKGNIHAHKDNHTNNLEHNHNNNQIDMNELSLKKLLIMLKQCKSYVQ
DVIDQKKKGNISVGRYLHKVLSNDTFLTLEKFDSINESVLQDNLMISYLSNLANLQFLIA
EKLNSSSLQMDKIVEVIEGLSSSESHIRNECENTLNYYKKNDLNNTVLSILKLLKTHKDS
QVRLQCAILIRNIFRVYIKSEEKEKNEEEENYWVLLPDNLKNIVKSELISNIGTETDKMV
RSNICNNIIDLSSKLLLHNQWPELLSVTFEFCNSNNVDVLISGYKILGGILSCIPDELDG
KQEIISSICMKGLNSSNVQVRGECINLISCIVEDNSSSLVKSVHGCIPLILQSLSLMAKN
SSSDIAVLEECEKVLQSIGKMIDYNAKFFTKHITSLCDILFSICMKDENELNYDFDNSLK
SLSIEALVTIPERRPKMALSVPHFVDKIIHLSMLFMLDINNDCFNEWMNSIKEGKDDSQE
LYDIGEESLDRVGKAFSELEEAEFIHILFNKVSEFLMKNTWEHKYVGIMAIAQTIEYLPE
DEIEEQLEHVIKMLLQILVDQDVRVRYAACQAIGQISLDHQPYVQKEFFSEILSALINTM
NDVHLRVQSHATAAFVNYAEELDKMALLPYADIIIDILLQKLNSSNYLLVREQAVTAIAV
IAGVIEEDFLKYYSTVVPMMKDIIQKAVSEEERTCRGKAIECISIIGLSVGKDIFIEDAK
ECMNALLQISSTKMDPDDTVKEYIQEAIGRICRALGNDFYPYLSSIVPTILSVLSVLPKP
LTDDEEDLTITMVSNGQYVGLKTSLLEDQEKALDLLIIIIEVLKENYKDYIQATATAVLP
MLNYELSDEIKQKALTAVSELIESARILSEKTDNDKSMLLAILTAAAEKVLKSLLETKLD
DNYEYILDVMIIESHGLYMCLQKAGSNVLPENTLKLFFNQIFALLQYSTDRRVVYNQKKN
NDDVDEDELLIIDREEELEQNYRTNLLDILGVLIKYHPTQFLNTCCELCIGFINNYMNSP
NSEDVALALYVCDDLLEFLQEKSVNLWDFFMNPLLLNINHADDKVKQAACYGVIQATKIE
AFGKYANIAVEYLLKLVHESTSNKKPKEYISAIDNAIAALGDVVLMHTSKFNNAEDLIKV
WLNHLPIKEDDAEGRRVHKNLIDLVSQNHPLLFGKDNSNTAKIIEIFLTIYETDFSDTDC
NKKISTLINSLDKSYLNNLASSTLSHKQAKKLNNILNPNRKMPTISVYEDDLFEKLGEEI
IEEKLLDVCFDFGLEVDDIEYKNDKKIYKIEVPANRYDLICVEGLCRALKNFMCKFDDIK
YDISMNNYDICIKGNQYIKVDGSVDDRRGYVVCCVLKNMNINDSVYNNIIEIQEKLHHNL
GKKRSVLAIGIHDYDKIKFPLKYKFEKKEKINFIPLNEKTNLNGMNLIDFYSKNLNLKPY
LKIIKDFDKYPIIVDSNEQILSLPPIINCDHTKISLNTKNVFIECTAIDRNKAQIALNIL
CSMLSEYCVPKYSIQSFVVIYDDQNLKKKETQFLYPIFENKSLTCNIDYVRKLSGISHIT
VHEVNNLLKRMMLS-CDIMDNNTFKVTIPFYRSDIMHCCDIIEDIAIAYGYGNIKYEPPQ
ICKKHSLNNCSELFRNVLVECGYTEVMTNALLSRDENYNCMLRTHKSYDDPNINLDEYNP
LAAPIQIKNSKTSEYEIIRTSLIVNLLKFVSANKHRELPLRFFEIGDVSYATYNQTDTNA
VNKKYLSIIFSDKFTAGLEELHGVLEAILKEYQLFSDYKIEEKKKENISIRSDMYYKLIP
KEDPSFLNERIVDIVLFPHNLKFGVLGIIHPKVLENFSLDIPVSAIEINVETLLNVLMMM
GDNNTSSGSSPVLSVGNIRGFGGSDFGSFRMSNEFLGWKNKKTNNVYQYKCSDIDEGCWI
KTSYNNNRLHLKLGESKENIIIYFDGFPDRNVNEITQHFQKYFNIRLNNRKIATKGWNWG
EFKLENSNLCFDIDNKYAFNLPTNNINQLNVQIKTDIAMEFKNDENNKGNEDFLAEIRFY
YPHENDENQNFQNLKNDLLEKVNIGDTKSESIASLSNIPLLVPRGRYDIEMYSSTFKLHG
KSYDFNIQYTNINKMILVPKSNSNQYVLIFSLSNKMKQGQTEYPFILIQLNNDDDMELDI
SASDEVMTKYKLEKTISGKAHDVVTKLFTALVNKNVIVPGDYRTSKNQHGITCSYRAASG
QLYPLNKYFLFIVKPVILISFDDIVTLSFQRTGNINQHRFFSLIIKHKRGMSYEYTNIDK
SEYNPLLTFLKSKNINIQDDANDLEKKQDFHNELDESDEEEYVADDDDDEEDYVAEEEDE
DDDDDDDDEEEEEEEEEEDDDKMTEMRVKADLGDCRFYKKKFPEVDDLIMVKVNRIEDMG
AYVSILEYNDMEGMILMSELSKRRFRSVNKLIRVGRHEVVLVLRVDSQKGYIDLSKRRVS
PKDIIKCEEKFSKSKKVHQTVRHVAKEHGITVEELNEKAIWPLYERYGHALDALKEATMN
PENVFKGLDISEEIKNSLLKDIKLRLTPQALKLRGRIDVWCFGYEGIDAVKEALKKGKEI
SNDKVSINIKLIAPPQYVIVTSCHDKDLGMAKIQEAMKVISDKIKEYKGGDFKQQGEILV
IGGDEEKRLEELLDKHDGISSDDDYNTSDEDDENSSEEDENTSEDEEEEDDLYIEMQKEN
YEDRISYIDDGTITLEKHVKMRLEEQTNLLSIKGIDRVDIKQKKGKHSIICIHYIKNMCM
KNLFCNYLHQLIYSRIPTCKNYMKYNYCADRVRGCCMFRHNLDNSNNNYYNESKDEYLDD
ALKFLHEKNICVNYLLGFCSLGYGCRKIHKTKSRRNIISTLPKFFLDSILVNKHLYTHLY
NNPKKLNDMNKLKDALIILSGEKYIDKNLSTNKNDNILKDVIQNNNIFSNNIDIGIENNI
SHGDNKSSHTNNEKGYFHNDNNNQENNINIPNVYDLNNNLIVAEKMKVFIIKCNQISHLY
LSILYGVWATGKNNTRKFVNLFKDNYTIIFLFSVNESGGFQGYAKMVTMPIKNLYENLWG
PITSRLGGNFRIQWIKMAKIDFDNFKHIVNPYNDNLPLKKSRDGTELPLNVASILCNKMN
DMPNEDFLTGTIYEFKRRINHSSFFINLHKQNILNTNTRWDILIFNLNQKSDCTNITLID
GTEQNITMSNPIGINILDNNLIQIFRDSDEWLTLEYFLQVNARTSRVKLVQAWHISPVQV
INKFQKKNSEKLVLKTFIDTSALDTDNTIQDICARGFCISSKGLKLSIGNFNIPGFPLTS
LNKNKTFNQCNELTYENDPQNSRKKKLRKIMEHSQNSLLSGERGIFEFLLCDVGVGLSLS
VYEKDVSTYNRDVLPIEYDSIFIKKQKEESLDDALTIHYKNKENENNNNEINLALGGDIM
PTYGVLPYYTFHHEYIIYDNSQILPRYLIQFECDPNDDEHFSLPLCDYCSDAPAILYCES
DEVKLCEKCDTLIHSQNKIVKKHIRKALNEAQKISGKCKRHMTNDVNMFCTICHIPICNL
CISSHVHTDLTTNQLKNSNNNQDTIISLNMAYKAILHHSNNPSNFIKQKKKNLNDLLAKI
DTLHEQVRINMNDAEKSVYTILEDLVKQLHIITDQKMCSVLSEEYELKRQFNEIIWNESF
LYYLQTILPPADFMNAWLKHCQFREQIEKNSKVVEELHSLIFPDMRIKGNINIVTESSIQ
HDKIYHPNDKLMAPKKKEEEPPKLLLGRPKNTLKMGLVGLPNVGKSTTFNVLTKLNIPAE
NYPFCTIDPHEAKVTVEDERFEWLVKHFNPKSNVHAYLSIFDIAGLVKNAHLGEGLGNNF
LSNIAAVDGIYHVVRAFENEDIIHTEGNINPVRDLEIINSELIYKDISHCEKNLEEVTKV
LNRNKKDKVKQNEHDVLTSVLNYLKEHKWIKDGTWKSNEIEVLNEYNFLTAKPVVYLVNM
SEADFIRQKNKYLAKIYNWVQEKNKGTIIPYSAEVEQKILSMDEEEKKQYFETNNIKQSM
LNKIIKTGYYEINLIHFFTCGHDEVKCWTIRKGTKAPQAAGVIHTDFEKGFICAEVYKYT
DLVEYKSEGEVKANGKYLQKGKDYVVEDGDIIFFKFNVSSGGKKMSEYDIAKNDVTYTKL
DTIECDIPINEELSWRINKFVNQLRISYSTLEEFVDNFVYELKKGLEAHRKHPNLWIPHE
CSFKMLDSCIANIPTGQEKGTYYAIDFGGTNFRAVRASLDGKGKIKRDQETYSLKFTGSY

SHEKGLLDKHATASQLFDHFAERIKYIMGEFNDLDNKEVKSVGFTFSFPCTSPSINCSIL
IDWTKGFETGRATNDPVEGRDVCKLMNDAFVRAAIPAKVCCVLNDAVGTLMSCAYQKGRG
TPPCYIGIILGTGSNGCYYEPEWKKYKYAGKIINIEFGNFDKDLPTSPIDLVMDWYSANR
SRQLFEKMISGAYLGEIVRRFMVNVLQSACSKKMWISDSFNSESGSVVLNDTSKNFEDSR
KVAKAAWDMDFTDEQIYVLRKICEAVYNRSAALAAGTIAAIAKRIKIIEHSKFTCGVDGS
LFVKNAWYCKRLQEHLKVILADKAENLIIIPADDGSGKGAAITAAVIALNADIPQLPMPD
FYFLIRWLCKVIVKSVFRDVNVINPENVPLYGSVIFVGNHNNQFIDACVLIANIPRQVKF
IVAEKSMRRAVIGKLASVIGCISVKRPQDLKFKGIGHICWNEGDVKITGINTRFRLDVQI
GDKLLIQNKMFPVVKIESETELLIQEVINIECEDKMNGVPFKIIPKINQTEVYNLVTNSL
KNGDTIGIFPEGGSHDRTNLLPLKPGVAIMTLCALADGIEDVSIIPVGLSYSKLYQLQGC
ATLFYGNAIIISQDLCKEYNNNNREAISKLLSKIEEGMRSCMLTSKDHETSRCIELCVSL
YTPERMTISKNKIYNNLQLFCKMFWKFGNSKVIENLSYELKCYEKLLQANKIKDDEVWML
KQSTSAATLKFIEHICTFIFCVIFGMTFSLLWLPLVLISIYLAERHRKAALRNSTIKIQG
GDVVSSYKVLVLIVLLPTFNIVYGLLFSIYLYHSWLKRILFVFLSMCILPICYYINLNYA
VQIPSLLRQMKILLKVICGKINVWRDNERELISTRHELQLKVRDLVSTLGPDVSDDFLEQ
LYRNIPKFVVDVDTKRLIRGKDEFLPILQRSQLEYKEEILMNDDKKDFVGRDLYQLIRNA
KDKIKIDYKFWYTQPVPKINDEFDENVNEPFISDNKVEDVRKEEYKLPSGYAWCVCDITK
ENDRSDIYNLLTDNYVEDDDNVFRFNYSSEFLLWALSSPNYVKNWHIGVKYESTNKLVGF
ISAIPIDMCVNKNIIKMAEVNFLCVHKSLRSKRLAPVLIKEITRRINLESIWQAIYTAGV
YLPKPISTARYFHRSINVKKLIEIGFSCLNTRLTMSRAIKLYRIDDTLNIKNLRLMKKKD
IDGLQKLLNEHLKQYNLHAIFSKEDVAHWFTPIDQVIYTYVNEENGEIKDLISFYSLPSK
VLGNNKYNILNAAFSFYNITTTTTFKNLIQDAICLAKRNNFDVFNALEVMDNYSVFQDLK
FGEGDGSLKYYLYNWKCASCHPSKIGIVLLMSEVNVTKVIVNNPICDILDPFVFTIEFEA
LNKLEADLEWKIFYISAVNNEGESNQDIELDNIFLGPIERGVMMFDYAVNPPDYKNMDID
SVLGLQAILISANYKEKEFIRIAYYMNSFYKDMELRENPPVVPQYDKICRHIFVENPRIV
KFSIGWDSEEKDEFKEFDKEIEKIELLNCTQIKDENENNSNITNQSTQGNFVLSNNDPNN
NNNNNNNPSITSSMIFNVNNNFNQNNFLKNNELSSDLDKCEIFNANINGISRITIDNKNS
MKICLIPVFLSIIYVILFLKGKKIG-DLYITEHLRNKFENILLYFNIFEVQYPESYLNNE
KLLGLHVRDIEKDAYRAYPILKELKQKDYFRIFKVNLHLSCKFLKGNEKCKEIKKCSVCE
CEQDEIPYNFRTNEIEIIENKMTNEDLKKTFIESKLYKDILGIYAPSNEGFISYVDLVYN
SPSFTAYEGRNIWNMIYKENCFQNEKSECEEMNSFYKIISGMQSNIAVLSSEYFYLKNDF
LFGEIQINNKFNNDYFKNLNYDYSVTFFKEKIGLYPERIENLYFTFAILLRSMCRLKSLF
KQCKCNSGYEQNDKEAVRLLDDLLENFYHSCSSKEFLEPLFPQHGKDILSKFMNITNILD
CVPCVKCRLHGKLKTTALQIALVEGSDEHIGSLERNEITALINALYYFADSILIINKFED
RLKLKKTIFIFYVLSFSLFLFLVIYSIIFLTIRNYKKKKKKVAMARNVEKGKSMLNQWIK
AKEISDKREFFKIPKNIDEVENLDDALKYRIYIIKEMCKKIKEIQNHSLSDQHIRELNDQ
INKLIFIKNKWEARIVELGGRDYSKESNLLINAHSSELRGSSNYKYFGAAKNLKGVRELL
FKENEDKKQLNIKKKKDARNFEKVINIHYFGYCDEANEHLLQQEVKIQKKLEKMDLKILK
KYKH-MGIFRSEIMKHGTLVLPSDRAREYLDCLGKEVDIQFIDMNEKTMKRQYKKYIQRI
DDMERILRFLEENINKLPNVKIKKSKIDNFLEHDNIYELDQVEESLNRLHVQFVRFCNNN
KDLIDEKNNAIEEKHVILTALNQLSPGFIRIGGDQQQQHHNNNNNNNIVSPFDEIEENNI
SLSTHIMKDGINMMFTNISGVIKTKDQESFSRTIFRAFRGNTYTYFQNIDEDADDNDLLK
NVEELESLKAGDTNNMYKNNKKKKKDLKSVFVVYCQGSAQSNIYDKIMKICKAYDVKTYD
WPRTYEHAKKRLKELREIINDKEKALKAYEEYFINEIFVLINVVEPNKNSLIEEWKLFCK
KERHIYNNLNYFEGSDITLRCDCWYSANDEEKIRHILINKSSNDLVSALLLSDKILRPNV
SPPTYIKTNEFTKSYQSMVDTYGVPRYGEINPAISTIITFPFLFGIMYGDVGHGLCIFLF
ALFLIIMNNKVKNKNNNEMVTMLFDGRYMLLLMGFFAVYAGFLYNDFFSMPLNLFSSMFM
LDKQVDNMEYYKRREITDSATGEVQYAYPYIFGFDCKWLGAENELTYINSFKMKFSIIIG
FIHMTFGVLMKGFNALHFKRKMDFFFEFLPQLVMMLSMIGYLVFLIIYKWVTPVGYGGFQ
KQGIINTIINMYLMKEINSTNQFYPYQSIIQILLLSLFVLCIPFMFICKPAIRTYHIMKE
KKKAMAKFNEAGAKEMINAFHHNAKDG--NHNNNLLSMHKRNTKISSNYDENEY-LNKKK
KKRTDDDIEAHLLSSTYEE-KGLQSSSAEENHHEENISEIWIEQLIETIEFILGLISNTA
SYLRLWALSLAHQQLSFVFFEQTILNSLKRNSFMSVLINLILFSQLFSILTIAVILCMDT
LECFLHSLRLQWVEFQNKFYKGDGIPFKPFNIKKLLNENEMIGIQEGRAFFNPTRNPNNR
PENNIRIEENLPSLNEVYNYFQDFLSRYSSNTLKEGNLKRTLFENVKNNNFTLDLKLDDI
YKQQTVFENNEISATPRSERKMKKPENYSAILARALSERPLTYLPTIERVCYEVCETANI
LNDEDEHLNYIQINLLNTFIRPTPIRGLLAATQERFVVVPGIIVQASKPQHKMRKITLQC
RYCDHKMSIDVPLWRDKPQLPPYCRYSSTMKSSMGLGNAMDSQLGCNGVLEPYVILPNEC
TFVDIQSLKMQELPEAVPTGDMPRHLQLNVTRYLCEKMIPGDRVYVHGVLTSYNPNPRPT
RADGTNFSYLHVLGFQKYDDMSGNDLNFDVEERNELTLLAAEHNIHEKIFKSIAPELYGM
DEVKKACACLLFGGTRKRIGEETKIRGDINMLMLGDPSVAKSQILKFVNRCAPVSVYTSG
KGSSAAGLTAAVMRDSQGVFSLEGGAMVLADGGVVCIDEFDKMRDDDVVAIHEAMEQQTI
SISKAGITTMLNTRCSVIAAANPSFGSYDDSQDTTYQHDFKTTILSRFDIIFLLRNKQDV
EKDTLLCNHIVALHASKHKSQEGEIPLSKLTRYIQYAKREIAPLLSKEARDSLRNFYVQT
RAEYRGDRRSVTKKIPITLRQLESLIRLAESFAKMELSQFATDKHVQMSIDLFSASTAET
AKQCLIFETMSPLEQKAVKQAEDAILGRLGKGQRASRVNLFRELQLRGFDRSALSKMPKS
YRNYSKTARNPKRPFEKERLDQELKLIGEYGLKNKREIWRVQYLLAKIRSAARYLLTLDE
KSSKRIFQGEALLRRMVRQGLLGENEEKLDYVLGLTLPKLLERRLQTKVFKLGLAKSVHH
ARVLIRQRHIRVGKQMVDIPSFLVRVDSEKHIDFATTSPFGGARPGRVKRKSLKKQKEKT
EAEAEMVRPSQSMYDRHLTIFSPDGNLYQIEYAIKAVKNTNITSVGVKGENCAVIISQKK
MATQYISQDKLLDYNNITNIYNITDEIGCSMVGMPGDCLSMVYKARSEASEFLYSNGYNV
NAETLCRNICDKIQVYTQHAYMRLHACSGMIIGIDENNKPELFKFDPSGFCAGYRACVIG
NKEQESISVLERLLEKRKKKIQQETIDEDIRNTTILAIEALQTILAFDLKASEIEVAIVS
TKNRNFTQISEKEIDNYLTYIAERDMDDHENIDINDNSSDANTANYNQDKNENTSEVNDY
SKEDIEGDENLKSSKGKQENNTKSSSDKDENTIDYGNHDFPCNPAPPVPIKLFIGRVPKN
IEEDQLRPIFEEYGIVNEVVIIRDKITNVHKSSAFVKMASISEADNAIRLLNNQKTLDAQ
LGSLQVKYASGELNKLGFPQNIESGVDQAKLFIGSLPKNITEDNIKEMFSPYGTVEEVFI
MKDNSTGLGKGCSFVKFSYKEQALYAIKSLNGKKTLEGCTRPVEVRFAEPKSSKQPQIPL
TLQPMQNPPHAMAPQPSISSPNNINFGNNFSVNNNYPRQVGPWKEYYSGEGRPYYYNEQT
NTTQWEMPKEFETLFMNNSANMHNLSESSGPPGANLFIFHVPNEWQQTDLIQAFSPFGEL
LSARIATEKNTGRNRGFAFVSYDSLESAAAAISQMNGFMALNKKLKVTVKKGEEDEMKKY
VNQNGVNSFQQVPRVQKAIPAQPASM-QPNLAYHQNPQAQNFFYSNSNSYRCGPYMTTAH
RPTWYNAIGGDNQGGNRKVSQTLKVCSRDLPGHLKMKTRDLSDYVEDKNMIKNNLIQLEN
KNNKETSKGNKLLAIENIKKLTNHDFKNPFPEDEDDIIGDEWKKKSHKKNKKKKDMNDYN
SSDNNSSDNNSSDNNSSDSSDYNSSDNNSSDDDESDEEEKELLRELENLKRERMEKLKKE
KEEQELLKKKKNNVLTNNPLINLEDSDNDEVSTKKRKWTDDAIFRNTCEKKKKTPSYIND
TVRSAFHKKFLFKYIHMDSEETINLAVKYAKEAVVEDEKKNYKEALNLYIQSLQYFNFFC
KYEKNSNIRDLILKKMEVYMTRAENLKEMLNKKDSIENKEKITNTEETKENMKKQIKQFI
LNKNNNIKWSDVCGLETAKEVLKEAIIFPLKFPKLFNSSTLPYKGILLYGPPGTGKTFLA
LACSNECNMNFFNVSSSDLVSKYQGESEKYIKCLFETAKEHSPAIIFIDEIDSLCGSRTD
GENESTRRIKTEFLINMSGLTNYKNNIIVMGATNTPWSLDSGFRRRFEKRIYIPLPNIYA
RAKIFEKYINQNENN-NISKEDIKQFATLTENYTGADIDILCRDAVYMPVKKCLLSKFFK
QVKKNNKICYTPCSPGDSDPTKVEKNVMSLSENELSLPPLTVQDFKTAISNAKPSLSVDD
IKKYEEWTHQYGMNGTMKKNIQCLNIFYTKWRNIHNSSIVRSKENLNLQKITGEKQNFNK
VSFKKEKIEDVIKEVKFDYYYFNEGKKNKYKDIPLNISIINESDFPPFKAVDEKLHFSVL
ENDLKIISTNRNNSVCSIGLYVKCGSRYEEINDKVNEQGMSVMLENMAFHSTAHLSHLRT
IKSLEKIGATVSCNAFREHMVYSCECLKEYLPIVTNLIIGNVLFPRFLSWEMKNNVNRLN
LMREKLFENNELYITELLHNTAWYNNTLGNKLYVYESSIENYTSENLRNFMLKHFSPKNM
TLIGVNVEHDELTKWTSRAFQDYVPIPYTNQKEVTPKYTGGFISVEDKNVKKTNIAIAYE
TQGGWKSSDMITLTVLQTLMGGGGSFSTGGPGKGMYSRLFLNVLNSYNFIESCMAFSTQH
SDTGLFGLYFTGEPSNTSDIIKAMALEFQKMNRVTDEELNRAKKSLKSFMWMSLEYKSIL
MEDLARQMMILNRILTGKQLSDAIDSITKEDIQRVVHNFLKTKPTVVVYGNINYSPHYDE
ICNILGMISGIRVNDNCVTEFNNMKIRKTCGWIIFVIQNCEIIIHSKGASTTLTELVQSI
DKNNEIQCAYVVFDAVSKIHFFMYARESSNSRDRMTYASSKQAILKKIEGVNVLTSVIES
AQDVADLKMQPYDYNRGLNN----------NNTANYNNQNNANAPINNPFLHNNDIGN--
------------NNNMKTNEGPYNAPTYYNPGQHQQSGVYGMNTNNIGENMYNDGHNNTS

YINQGQPYRNVTSQFIPVS-SNNTLKAGGNMLGYDNMGNINHVQPIINDTYQEFLQFNAF
SHFVKSSVSYMPANTTLKQKAYVPLGFVIQPLAPIPDGYPELASVNFGNSTVVRCKKCRT
YINPFVRFEAGGKKWNCNMCYNINDTPQFYFVPLDEKGKRKDLFQRPELCTGSVEFIAPS
DYMIRPPQPSVYLFLIDVTVTSVNSGLLDVVCSTIKSLLPKNNDSTNNNNNNNSKNLKSF
DSRTLIGIMTFDSTIHFYNLNSNLKQTQMMVVPDIQDIFIPLPEDILVNVHECQNVIDVL
LDNLPGMWRNNKISDCCAGNALKAAFMVLKKVGGKLLFFLSSVPNIGDLTVSVNRDNKDK
SKYKNIYSSSSSGNNVVDSKLREVELLNPCNNLYAELAQNITQYQIAVDLFACPLYNLDL
ASIYPLIKNSGGSLYYYPQFNVHQYNDKLRQELLFALTTETAWESVMRIRISRGWKITNW
YGNYQFRGADLLALPNCHSGQNFSIIVDLEENVVQDSIVYVQSALLYTNSNGERRIRLHT
YALPITQNIKTITDSINPQVVVSLLAHQSIDISKKGKIADGRNLIQNLCSQVLSSQLLQS
ECARLLSLYILGMLKSIAFRDSGDVPPDLRIYHWYRLENIPVESVEANFYPRMFSLHNLE
KHHGHLDENNNIVFPDALNLTCENMTQDGCYIVEDGETIVMWIGRSINPQWIYAVFGVQT
IDQLNTEYAENHLGSTGNPFGVQILNIINALRKIRTPCYMKLLVVKQGDPLEYKFFSYLI
EDRSQHMMLSLKEFLAKICPKFPQFTPSMTMEEDSFKNRLLKRNIDIWIEKYRPEFLDEV
VGNPFVINTLKSIITSGNMPNLLLAGAPGTGKTTSILCLASEMLGNQAKKAVLELNASDD
RGINVIRDRIKSFAKEIISLPPGKHKIIILDEVDSMTTAAQQSLRRIMELYSDTTRFALA
CNQSEKIIDALQSRCAIIRYFKLSDDQVLKRILKICDLENIKYTDDGLDALTFIADGDLR
KAVNCLQSTYAGLEVINKENVLHICDIPSPERIENLLKHCVNSEWKKAHDIAYSMIKEGH
TPYDISLTSSNVLRRFNIGSEVIQIEFLKIGAMACNTMATGLTSVIQLDKLLADWCMAAK
ILRSKAMAKNQYMEDRNIREP-NTLLGEETEQLVDSFHYENNSSSIYKKVNSNRSKNGKH
SMAFHKSLAVVNVAAGLDGCDDQLLPASFRALEADLNLHPSLLGYITLAQTLMLSLFSPI
WGFLSDKYSRKWMLVFGTALWGVATILLANINDFAHILFFRAINGLALGSIGPISQSILA
DAAKNESLGLSFGLVQLSSSLGRLIGGVVTTTVALKYFGGIRGWRLCFIVVGILSVLLSI
IVALFVEDAPRQVRKNKKMDYLDNSLDNNNSFTGLSHQSTRTYILYQNIVELLKDSLSKK
SIIIILLEGFTGTIPWLALSFNTMFFQYCGLSDLQAAIITGFLLIGSAIGGVVGGHFGDI
MHDISNKHGRPLLGQLAMFGRVPLVLLIYLVIPKRKESFELFALSCFCIGLSSIAGVAVN
RPIVSDIIRPDYRGTVFSLTIAIEGVGSSLIGAPLFGYLAEKIFKYQNNNLLISDMPEDI
RINNAQALSKTLFYLTIIPWILSFIFYSLLHFTYGKEYLKMNEIIQNEYKYDDEDEETIP
EKKML---------------MQHENFKRMLMFGLRSLSDFCNPTSKAYKENASDALDRGV
VVSIKNAVINYKDDDDILFCSSRVLLSMSDYCMSEKDTNALKKLITDGGVDGIVEIVKSF
PSDPDTLKNCMAFIKNMNDSNYQIEGREVGIALLNVFTSKTYTNKLTNGIVLALCIISKS
TSGSKGLNDEGAHHKLLDYCLNINSLNDDTAEIVESVFDIIKNMSSNGYVDPTIIEKSVI
ILDKFKSYPRVISKGSDAMKCAVGPEELSKCLNVLKKSAQGSKEQDAALELLSSLSYISS
ITDKVVESGGIPVLIELINSGLQQYESNPEKISRLVAGASRMLGRISNNPPHAAIVVEYG
GIATLCTAISYFPNDVECSKAICNALTPFVSRSNYVSEINNYSLFASLLPILYASLESVE
LAKASMECIASASMINEFHEQMVNNQAIEILSTCVQYHLTEMDYLLNCFTAYFRLSDYIT
TVEPINQYGGVDGIANALLAVSSNSKIVEIGLKLINKMLTTSDSVNYLSNKQIVDSVLTV
MLENENKEVIIQEGTKIMEKLATESDCQRHITNLETIFNSSETNQEEAYKTLAAISGLSR
IESLKNILESKGADTSIFNGIKIWIESARFIEQTKLIKAGLKTIKTLKLNASATLHDVLG
SIVDLMCLSQVKRIAESDEPDENILITSAECINYLTEVNKINSAEIVEACLENIFKLMKK
YSESRLTQINLISAMNNILLSSNKIGVDILINKGYIKHIITYLQKVPMYVDVQIIGFTVL
ANLVKISPDSVEGIKKLNALIPLQTALRTHAKNMKLKTTCAPLLSVLMPLDSLTNEIQDI
IKLCNKSMNDKNLSKLHEYLVALNELLLTPEACKIASRSNIGEFFNDLTAWLKKNPTVYD
SSSKDDYEISGRSLFDAVISEIAHASTNISQTRLGLVHLTKCNMVSSLVQLHDILKLPGD
NYTEEAVSNILEALSLLLKYDITNAEIGFNSGLIKKLCAGINYFSHSDSVINKTFGCLAC
MCTSKNRVGQLISCPEYEGLIKLIVELIGDSEKNKLSRGSAIKAVYELLKTEDEDIIKDI
SCKTSIVDNLYKIMGEYQADLSIVQDCSRCLAIIVDYVNIEEIMKVDKYTPMKVLLECLN
KSKNDELTVLEMLTVLVKLCNSDDKMKLKELGAIDVISDITMIHSENEEISRLGGVMYSY
MGADEQVKKLMKLILNVKKEDSDAVQKIDNFTSKLEMFLRAPLENPSDALQYTEVTLQVL
NSYLASEVDNSSLQTNIALVTKRLVDRVKHDSEDPLGSWAVASAGTLNQYIDMISNKIGL
SNFKFVSPVYGVLAACVMNPYTKQLVMDKLPLLIDNTYEILEQNKNRPSVVQGVFEFLEQ
VVNDEEGSKLLYKKYNGSMGNLIDQTITVMNLNRANDSVYLPGIKLLSAICQTASVSGYG
NDMNTSNIIESCELLLNLGSEKDRTVEFLNLIDTMILGNLLDEKVASEALKKINSLVAEE
NLSKYTEENRVEIIKSYAGLVKDCAATGLFAHIRGIEKVEILNNLGKCMEYEQNDEVTIA
VLDALSQISESDPSIATKILTSSLPIIMENNRDNILNDAATAEAFLQALQNLVLHEGVGR
QLINNTELQNLLKDLEQALDERKEELGNDYVEDMKLKISNICNAINGDKPKEKKCKDVYD
VLTDYKKSNLIIDVLQEPSLEEDMDFVLERLKVYNQDNLLPTTDTGTDNSYGHMAIEMFC
ENEVNINELIKRNFHVSAFYALSKQSNQNVIHYSCRSICAFTKNPKGLE-AVKDIKDFAN
IISKSVGDLSKENTLDKEIKEDFLIHRVLLIDRTAHNRNVYDKTNAVHYLIDIWNQYDNG
DYSVLLLRHVFRSMRKIVSDAHVQTLLKAGVLVRLINIINDIETDKIIFPDVLFLIGSLS
VVKEIKIQIGELKGIDACVNLLLRSINVEKMEPTITNCCLALANMCIDHKGNSSIFCNLK
GPDVNVKILKLYRSNFDVTNGASVLLCNILFRNEEMKKQYGINGAPAELVECLKSYDGSD
DKNAVRCIESLFKAISNLSLYTVNVKYFLEAQIQVSYESWLRNLNESFPDAQLETGLRTL
SNLVMENDEENMKNFGVTLIPVLNVLKQNREDTKVIFLLLDILCSLCRLNANAKIFAENN
GIETTINVIQLYDYDINLLSLAIHLLSNQCKIESSLPLLVNADAFSILISCMEAEADEFE
MTELVVSSLRCVRRLIQSEELAYEFCNCGGVPSMANIICKSIKKSIVMLEVLRVLLCVLY
YTQNVEGVTNEYPEEEEELYNARLGGWYNISMDKEMIDSIIQAVLTCAYDVNHQKQLRLQ
KVSLGLLAYFAYHRLGIISMTASGFDSLTRELLNNFGGDAVIMQLLAICIDNIAMYSVEV
YDTTITRDIIKCFKSALSKMNNKKEDKQLWQKVQLTLEAMNSADDPLEAFKNTLLIFDFN
LSEFDKDPYINGVHDLASNIKDCLRKGGHSKIYYQSDQRLLFKWKASQDLNTLEWTIGDD
TERVFKISVVRIKNISKGLSHPILISANKREPRKVSAKVTLCIYGPPTEDFPEGLELPIK
TKTQKERDAFVDLIVLWRDAASYNYMDKKAREYAQDALKFIQRSGSNFLACKNLKERLEN
NGFINLSEGETWNLNKNEGYVLCKENRNICGFFVGKNFNIDTGSILISIGHIDSCALKIS
PNNNVIKKKIHQINVECYGSGLWHTWFDRSLGLSGQVLYKKGNKLVEKLIQINKSVLFLP
SLAIHLQNRTRYDFSVKINYENHIKPIISTTLFNQLNKCKRN-NVHHNKRDDEKNPNSKD
INSYPLLYLLSKELNCKEEDILDFELCLMDTQEPCFTGVYEEFIEGARFDNLLGSFCVFE
GFIELVNSIKNHTSNENNIHNNLYISIGYDHEEIGSLSEVGARSYCTKNFIDRIISSVFK
KEIHEKNLSVQEIYGNLVNRSFILNVDMAHCSHPNYPETVQDNHQLFFHEGIAIKYNTNK
NYVTSPLHASLIKRTFELYYNKKQQIKYQNFMVKNDTPCGSTVGSMVAANLSMPGIDIGI
PQLAMHSIREIAAVHDVFFLIKGVFAFYTYYNQVLSTCVHDKMNYCKTTFHIFFVLFFIT
IYEIKCQLRFASLGDWGKDTKGQILNAKYFKQFIKNERVTFIVSPGSNFIDGVKGLNDPA
WKNLYEDVYSEEKGDMYMPFFTVLGTRDWTGNYNAQLLKGQGIYIEKNGETSIEKDADAT
NYPKWIMPNYWYHYFTHFTVSSGPSIVKTGHKDLAAAFIFIDTWVLSSNFPYKKIHEKAW
NDLKSQLSVAKKIADFIIVVGDQPIYSSGYSRGSSYLAYYLLPLLKDAEVDLYISGHDNN
MEVIEDNDMAHITCGSGSMSQGKSGMKNSKSLFFSSDIGFCVHELSNNGIVTKFVSSKKG
EVIYTHKLNIKKKKTLDKVNALQHFAALPNVELTDVPSSGPMGNKDTFVRVVGTIGILIG
SVIVFIGASSFLSKNMKMTDDKKKGKFIVFEGLDRSGKSTQSKLLVEYLKNNNVEVKHLY
FPNRETGIGQIISKYLKMENSMSNETIHLLFSANRWEHMNEIKSLLLKGIWVVCDRYAYS
GVAYSSGALNLNKTWCMNPDQGLIKPDVVFYLNVPPNYAQNRSDYGEEIYEKVETQKKIY
ETYKHFAHEDYWINIDATRKIEDIHNDIVKEVTKIK-VEPEEFNFLWSITLNQVVCKNCL
SSDVETNEGQGEVICLRCGSVLEENKIVESLEFVENNNGAISMVGQFIPSSGNKSFILSW
GIRESREISLQKGYVNIQKIADHLHLSSQHIEAAQRIYLMALQRNFTMGRNNSYVAASCL
YTICRREKSPIMLIDFSDILQTPVKPLGKTFLKLLRLLHINVPNIDPSLFLERFAHKLNL
KNDIYKVTYTGIKLIQAMTRDWISTGRRPTGLCGAALLISTRMHGICINSNTIADIVRIS
NPTIIKRLAEFKNTSTAQIKASEFDKISINDIPSNTIPPCVIYYNKKKFKDNISEKNKTL
SLCDDVDNL--SEDMSCTLINNEENKMDSDMLNDNFPSSK---NEENKTTLLSSTHSNID
--KNHSDHLSNSDYSIHVDEICNENPKGNDIHNLAQKIMNTINLDINSNILKNVDTKINL
HNNLMDTSVNTDVSTNTQKYMCEKKKKIDNNNNNNDDNNKEEDNNNDDNKEEDNNQSSIS
NNICSNNICSNNLSNESND--NTYSTTLKERVTCEINNILNGMDEYNNLLKEQKKSDNNN
LSNDLNGMNNINNILTDFNYFFDNNSNNANDQNEALDLHSDCSSKPSCNSLSDVYDSEIE
NIILSEKEREMKMLIWDDMMKNHLPHLSKQLKKHKKRNHNDNNKNKDTNIKKKQNTFEDY
SQVQSTGESVLKALGKSDKNLPNKINYDVLQSLFTSMFDSSESWKRSDCLEKMTENKIDT
DLYSRQLGTYGFDLMNKLVKLNILIINVKGVGLECAKNLILSGPQSVCIYDNDICDISDI
GVNFYINEKDVEDKSCRSDAVLKELQELNNYVHIYNYKGTIEKNWLENFDVVICCDINKE
DLIKYNNMIRGIDKKRIAFLSCNIYGLCGYIFVDFNKEFICYDSNGEQVKSCNVSKISKE
LEGKVSFDFDKTSPFEEGDYVQFSNVEGMTEINNKIYKIKNLKKYTFEIGDTSLYSEYIK

GGICTQVKKHLKLNFYPYEYICVNPLNNENISNNQNDNHLDTCNNIIYENIPQPNSFIIS
DYAKFDMSNHLHYSIQALKWYELQNEKGLPENSDEDALEKIYNYAVTLNNKDKEEKKSYA
VEQLKKDVVYNVCRYSKSHIAPVASFFGGLLAQEVIKFTGKYMPIYQLLYLDFFECISLN
EKVDINEIKKMNCKNDNIITVFGKSFQKKLNNLNVFLVGSGALGCEYAKLFSLLDMCTRN
SEGKLTITDNDNIEVSNLNRQFLFRREHVGKSKSLVSSEIIKKKNNNMHVQSLETKVGAE
NEHIFNEEFWTKQNIIVNALDNIQARQYVDNKCVWYSKPLFESGTLGTKGNVQVIIPYLT
QSYNDSYDPPEDSIPLCTLKHFPYDIVHTIEYARDIFQGLFYNTPLSIKQFLNDKEEYIN
KIQEEGNNASLLENLQNVINSLKEISSQCNFDFCIKKSVELFHNNFINQINQLLYSFPLD
YKLSSGEYFWVGQKKPPQPIVFDVNNEMIQEFLLSTSNLLAQVYNIPPCFDINYIINVAK
KIEVKPFEPKKVKINMDEKNLNNISISFAEEEKIIDDFCKELLNIPTNNIKINPIEFDKD
EQTNLHVNFIYAFSNLRAINYKINTCDKLKAKIVAGKIIPALATTTSIITGLVGIELLKY
VNYYDNIQAYVKLSDEQRKKEKDVLSYFKNAFINSALPLFLFSEPMPPLRMMDKEYDELM
KGPVKAIPNGFSSWDKIVISIKNGTIKDLIDHINEKYSIDVNLISVGNACLYNCYLPAHN
KERLNKPIHELYKQISKQDLLEDKNYIIVEASCSDQDLVDVLIPSIQFIYKMSKLNQDLL
KKAISDVFEGTKQKKRKFVETIELQIGLKDYDTQRDKRFSGTVRLSNEVRKKLKVCILGD
AVHVEEAQKLELDYMDIEAMKKLNKDKTLVKKLAKKYDAFLASQVILPQIPKLLGPGLNK
AGKFPSLITHNDKINDKILELKSSIKFQLKKVLCMGVPVGHANLKEEELRSNIVHAINFL
VSLLKKNWQNIRTLHIKSTMGKPQRIYGMIQESINDNEIKKKWEDIDADDDLEANKKLSY
FTNYENGIKVVTKYSENLKKQT--VTKKIQEVVIKKKLNKEINNRLNLKNFNIDIHSSVT
VEPTDVVNIEVPKNNPLDFLKDTEYDYLFAEQTNKTAKDLKNRFKILKDEETEDVKAEDP
SKAAGRREGLYYRPHDECTVRVTNLSEDVNENELSNLFGKVGNIVRMFLAKHKETQNSKG
FAFITYSKREEAKRAIEKLNRHGFENLLLSVEWAKPSNRMLNDIILQVIVAAFGVAIVNS
DKIKFLQKFKYATYILILSFLLYKGIPWKRENYYTYLNITPNATKQEIQTAYRQAAKIYH
PDKNPDESANSSFIKLKQAYDVLTDDVRRSNYNRFGDYKNGEIDDNTATLLICLSLVQHT
MFFIIGYFLSYPKKLEFSRQIFLVYNIASFCFELQFRFIEDDTTFDWLPALGYLLPYEKI
KFLRTFFPIVFFISICASAYSYTDKNATLIFLMRSILSTNRIIVERSNDVIESTNYLKKN
GEKLVTKLQQIRKGEASSQLNLKDDENLDKEYLEGDKKN------------TVDSKLLSN
VKEFALTLDSQQISLLEKCFDLMKNKDANE-KNKKKSWFEFFSIQVIFGVIFVYIWLTSK
MGKDYYSILGVSRDCTTNDLKKAYRKLAMMWHPDKHNDEKSKKEAEEKFKNIAEAYDVLA
DEEKRKIYDTYGEEGLKGSIPTGGNTYVYSGVDPSELFSRIFGSDGQFSFTSTFDEDFSP
FSTFVNMTSRKSRPSTTTNINTNNYN-KPATYEVPLSLSLEELYSGCKKKLKITRKRFMG
TKSYEDDNYVTIDVKAGWKDGTKITFYGEGDQLSPMAQPGDLVFKVKTKTHDRFLRDANH
LIYKCPVPLDKALTGFQFIVKSLDNRDINVRVDDIVTPKSRKIVAKEGMPSSKYPSMKGD
LIVEFDIVFPKSLTSEKKKIIRETLANTFIKEVRAVVLLLFVLLLNSSNVKAAYFFVKEG
VDKCFVESVANNVVITATYDNYGLKDVKCLINIKDQQGKVLYSHDTSKIRKGKISYLSTK
DGLYHICISCPSTNWFKSTAIKWSLSIEVGGSDIDPENLAKKSELSETLTILNNLKKKFN
SMKLQQIYQKQMASNLYEYNKSVHNKMFYCYIVEIIILVVITVYSIIHLKNYFKAHKLMM
SKNKKGKKKEDLDAILAELGIEEKREDDEPTKMSKSKKKKEKLKQKKEQKNKEDDEKDDQ
KGIDGDTDQKKDQDDQKDDDNQNEADNQNEQKKKKNKKKKDK---EKKTGGMSEIAKAAA
ERLRLLKEYEEKKKEEERLKKEEEEERIRKEEEEKEKKRIAKLEKKMQLKKEGKLLSAKA
KEEKKKNEIYLKQLKESGMLIEPREKSKSEYINLDINKTKLLLKKKKTKSIMDDDKNNKQ
MLSNSSLKLNEEDELQGSQKDEKDIVLDNWEDFLHIDEAKKKEQEEIERKEKERKEKEKE
EREKERKSVKSFHIKNDILMRKKKKIDNELDDDDNNEYRSAIVCILGHVDTGKTKLLDKL
RHTNVQDNEAGGITQQIGATFFPKDVLDKEIKKIDDTIKCLSKGIMIIDTPGHESFYNLR
KRGSSLCDIAILVIDLMHGLEQQTKESIQILKQRNCPFVIALNKIDRLYMWNKNDWSPFN
NTFKKQKPDTQEEFHDRLKNIINELSEQGLNCQLYWENMNPRKYVSIVPTSAITGEGIAD
LIMVLVKLTQTFMLKNIQYHDKLECTVLEVKNIEGLGTTIDVILTNGILRESDTIVLCGI
NGPIVTVIRALLTPQPLKELRIKNEYIHHKYIKACIGVKISANNLEEVLCGTSLFVVNNI
EEEEEYKKKVMTDVSDVFNHVDKTGVGLYVMASTLGSLEALLIFLNDSKIPVFGVNIGTI
QKKDVKKASIMREKGRPEYAVILAFDVKIDPEAEKEAALLGVEIMQRDIIYHLFDSFTAY
LKKIEEEKKQSKITDAIFPCELSVINDCVFNKKDPIVIGVRVECGQLKIGTPLFVPEKNI
KIGNVVSVQSNKKNFDKARKGDEVCIKICGEPHVTYGKHFDSTQKIYSKITRESIDVLKE
YFRSELTMEDWKLVVQLKKIFNIVMNINEKDKLAEQNLETLDVTKLTPLSEDVISRQATI
NLGTIGHVAHGKSTLVHAISGVHTVRFKHEKERNITIKLGYANAKIYKCTNPDCLPPECY
KSYESSKEDNPICPRKDCNHEMKLLRHVSFVDCPGHDILMATMLNGAAVMDAALLLVAGN
ESCPQPQTSEHLAAVEIMRLKHILILQNKVELIKEEQALKQQEEIRNFVSGTAADSAPII
PISAVLKYNIDVVCEYIVTQISIPKRDFISSPHMIVIRSFDVNKPGEDIETLQGGVAGGS
ILHGVLKVGDKIEIRPGIISKDDKGEITCRPIISQILSMFAENNNLKYAVPGGLIGVGTK
IDPILTRADRLVGQVIGHLNKLPDCFAEIEISYYLLRRLLGVKSQDGEKNTKVAKLKNGE
FLMINIGSTSIGCRVTGIKTELAKLELTGPVCTKIGDKIALSRRVDKHWRLIGWGQINKG
KPLELQEPIMIHFVLLISRQGKTRLAKWYIPLSQKEKAKIIRETSQITLNRTPKLCNFVE
WREYKLVFKRYASLFFIACIDKGDNELITLEIIHHYVEILDKYFGNVCELDLIFNFHKAY
YLLDEILVTGEMQESSKKTILRIVAAQDSLMEDNKTTKKLGALIMRYSNIIKKGILSRYC
SNIYGMQYIP-RARYLTFCKNEKCNTMLKKYHPFSTSKMSDNAHYNETNETNETIENSAS
STEGKKKKNSLNESEEANEKKEDINYEDFNKIDLINEIKKTKRDMEEKMVDNKVLKEKYL
SVLAENENLRNRYMKEIETSKLYCISNFAKSLLDVADNLSLAIKNINEESLKTNEEINNI
YKGIEMTETILHNIFNKYGIDKYNPINEKFNPQLHEAIFEINDSTKEKGTVATVIQHGYK
IKDRILRAAKVGVVKNMMEELFKILFELVKIDEEIQNYLKERIIDEKYEIEKNGVDYFYD
LVCSYADDKIKRSKIANIFNKHFKNDIQNGNPNQNKNLEKLEEVFNFKEYWKDKDMSNYS
DPFLGVYEKQINYNTSVPISESLKISKEKEKQKQKQLNLFKEWVKNKPKIPQPVRVHNLL
HCSDVGKNKTKIDKLYDIRIDNFNISIGQRSLLSDTTLKINVMNKYGLIGKNGIGKSTLL
AKLARYEIEEIKKDISIACIEQELCLENVTVLESVLMVDKLRHDLLLELEQLEARKIKLD
PNEEILNDTKNVENMEPIETVESIEQKILDIYEKLNSISYLEAEKEASKILCGLGFDSNL
QKKKVNSLSGGMKMRLCLSRILFSNNDIILLDEPTNHLDIYTIQFLIDYIKKLNKTCIIV
SHDRDFLNEVCTDIIHFHNHQLTYYSGNYDQFEKTRIEHLLQQQREHDSLELKKKHVQKF
IDRFRYNSKRAALVQSRIKLLNKLPVVNLEKEDTPFSFSFLEPFYMSNVLIKLKNVSFRN
KMFTNLRVRKNKNILIGDHENETNEETYEDENELDNNGQPVSKTYEDKYKFKHEYLFKNA
SFEVDMDSRIAICGVNGSGKTTLLKIILNLITVYEGELHVSNKANIGYYSQYHVDCLNPI
YNSIQQLQYTYSNKNIKEEEAIKYFNKFNIPTNILYEPIYVLSGGQKSKLALAILAYKNP
NVLILDEPSNHLDIESVQALIVALNMYKGGLIIISHDTYLIKHVADEIYHINNITKELVK
IDYEFDKYTQLLLNNKIMPREIITLQCGQCGNQIGVEFWKQLCNEHNIDQEGILKNNNFL
NEDRKDIFFYQADDEHFIPRALLFDLEPRVINSIQTSEYRNLYNPENMFISKEGGGAGNN
WGCGYSQGHKVEEEIIDMIDREVDNSDNLEGFILSHSIAGGTGSGMGSYLLELLNDNYSK
KMIQTFSVFPLLTNESSDVVVQPYNSILTLKRLILSTDSVVVIDNTSLNRIFVERLKLNN
PTFQQTNTIISNVMSASTTTLRYPGSMNNDMISLISSLIINPKCHFLITSYTPITIDKHI
SNVQKTTVLDVMKRLLHTKNIMVSAPVRRGMYISILNIIRGETDPTQVHKGLQRIRDRKL
VNFIKWNPASIQVTLAKQSPHVVSQHKVCGLMMANHTSISTLFERCVTQFDRLYKRRAFL
ENYKKESMFSSADGQGNFEEMESSKEITQNLIDEYKSAERDDYFTNTYIMEKYHGLEKIG
EGTYGVVYKAQNNYGETFALKKIRLEKEDEGIPSTTIREISILKELKHSNIVKLYDVIHT
KKRLVLVFEHLDQDLKKLLDVCEGGLESVTAKSFLLQLLNGIAYCHDRRVLHRDLKPQNL
LINREGELKIADFGLARAFGIPVRKYTHEVVTLWYRAPDVLMGSKKYSTTIDIWSVGCIF
AEMVNGTPLFPGVSEADQLMRIFRILGTPNSKNWPNVTELPKYDPNFTVYEPLPWESFLK
GLDESGIDLLSKMLKLDPNQRITAKQALEHAYFKENNMGNKISTEDHIFRLKLKTKELEK
LSQRSELEEKKLIGDVKKAIQAGKIELARLYAEKCIRKKNEKVNYLNLSNKLDVLVSRLE
GAHRCASLVKDVGVMIPLIQKINAETNAIKIGNDVTKLENIFDEISISSELINDTVQTSS
AISAPTEEVDELISKIADEHALKLQIGSVHPINKHLEEISNMSERIMGKEKTHINLVVIG
HVDSGKSTTTGHIIYKLGGIDRRTIEKFEKESAEMGKGSFKYAWVLDKLKAERERGITID
IALWKFETPRYFFTVIDAPGHKDFIKNMITGTSQADVALLVVPAEVGGFEGAFSKEGQTK
EHALLAFTLGVKQIVVGVNKMDTVKYSEDRYEEIKKEVKDYLKKVGYQADKVDFIPISGF
EGDNLIEKSDKTPWYKGRTLIEALDTMEPPKRPYDKPLRIPLQGVYKIGGIGTVPVGRVE
TGILKAGMVLNFAPSAVVSECKSVEMHKEVLEEARPGDNIGFNVKNVSVKEIKRGYVASD
TKNEPAKGCSKFTAQVIILNHPGEIKNGYTPVLDCHTSHISCKFLNIDSKIDKRSGKVVE
ENPKAIKSGDSALVSLEPKKPMVVETFTEYPPLGRFAIRDMRQTIAVGIIKSVEKKEPGA
VTAKAPAKKMSHLMSLPIVLLKEGTDTAQGRSQIIRNINACQIIVDIVKTTLGPRGMDKL
IYTERDVTITNDGATVMNLLNISHPAASILVDIAKSQDDEVGDGTTSVVVVAGELLNEAK
GLLNDGIEPNMIIDGFRNACNVAINKLNELSLNFSNKNEEEKRSILLKCAQTALNSKLVS

NHKEFFGELVVNAVYKLGDNLDKSNIGIKKVTGGSCLDTQLIYGVAFKKTFSYAGFEQQP
KKFINPKILLLNVELELKAEKENAEVRIENPNEYNSIVQAEWDIIFKKLNLIKDCGANIV
LSKLPIGDIATQFFADHDIFCAGRVEDADLKRTANATGALVQTSLFNLNDDVLGTCGVFE
EVQIGNERYNIFKECLKTKSVTIILRGGAKQFIEEVERSINDAIMIVLRCITNSEIVPGA
GSIEMQLSKYLRIYSRSICNKEQIVLFSFAKALESIPRHLSHNAGYDSTDILNKLRKKHS
EQTSDIWYGVDCMEGDIINAYDNCIFEVTKIKRNVIYSATEAACLILSIDETIKNPSSAA
GTQRSPYSMSGKLEDIGELVLLIGDFHSPIRNLGLPDCFKELLKTDKIKHVLCTGNVGCN
ENLELLKNIADSVHITKGDMDDNFDFPEDITLCIGDFKISLIHGHQIIPWGDMNALLQWQ
KKYDSDIIISGHTHKNSIVQYEGKYFINPGSVTGAFQPWLSEPTPTFILMAVAKSNIVLY
VYEEKNGKTNVEMSELHKSMDNGNNEILYSVKISEPRDKNSTGIYRHPEYKDKLCENFDD
KKFNNMWEVFDRVSTKFKERDCMGVREKEDNKRGAYQWKNFGEVKELIMKVGSGLLNMNA
CPLIMCDDQRIPKARFLGLYMPNCPEWNICDLGCNAYNIITVPLYDSLGPQSSRFILDQT
QMETIVCDKTCARNLFKSLETCEEIYLKTLILVD-EIDDEIKKECSKYNLKIILWEELIK
QGEKKIVKVPQGALNNIFSICYTSGTTGYPKGVIMTNRNFIGILAAAYIGPSRLPDLCIN
ENDIHISYLPLAHIYERLMIYLFMAHGVKVGYYSGNVQTLLEDIQELKPTLFISVPRLYN
RIHERIFNSLKKKSGLVQSLFNKGLQNKIKRLSSSGSTTHVLWDKLLFNKAKKILGGHVR
AMLNGSAPISVDVVKKLRTIFCVPMFEGYGMTESLGASFITHSQDRNIGHIGGPVPCIEF
KLVSVPEMNYLVTDNPPKGELYLRGPSTCNLGYFKLEKETNELLEKDGFIRTGDIALLSP
NGSLTIIDRKKNIFKLAQGEYVAVEKVEASYKQSLFISQIFVFGYSYESVLVCVICPSTD
SIDIWRTQKKIKATDEEVIKLPEFKADVINDLTSIGKKDGLKGFEQIKDIHFTLEAFTIE
NDLMTPTGKIKRHEAKKRFKKEIDEMYEKLKQ-M-SWDSYLNDRLLATNQVSGAGLASEE
DGVVYACVAQGEESDKWSLFYKEDYDIEVEDENGTKTTKTINEGQTILVVFNEGYAPDGV
WLGGTKYQFINIERDLEFEGYNFDVATCAKLKGGLHLVKVPGGNILVVLYDEEKEQDRGN
SKIAALTFAKELAESSQSSISPNKKFALTSADKPITLIRTNVSNENNNVRRKMLRNFRNS
RNTTCPDCKEKGIIICDTSEGTQICNGCGMVVESKILSEEQEWRNFQNDGQSKNTDRNRV
GEVSDIWLENNTSTTFIKSSKKLQHLNMMTQINKNDQTLISAFNILKLICDTFFLRSNVI
ERAKEITKELQDMEQLKNRINNLNMLAVVYLACREAGHIKSIKELITFDRSYKEKDLGKT
INKLKKVLPSRAFVYNENISHLIYSLSNRLQLSIDLIEAIEYVVKKASTLITTSHRLNSL
CGGSIHLIVELNTNEEKNMKLPNLSQIATVCGVTTNTLKTTFKELLNAAEYIIPKYYLSE
DNPKLSMLKQKYLSEDKRKKNMKFEKFNIPEWSDIERYFKDPELLTAEILFVALTLCNVF
VMYRLFLDVIPYPIFVTWWQLAQGLLVAYVCGELGREFPKFAYFPKVEINENMLKVLFVP
SIFYCLMLVLSNYLLYKTPCIASYPVLVSFTVVFHHLTRFIGCGEEYMPLRWKSIVFLLA
AFIIGCFDSKTTGKGVIVWALLYALFSAIFRAGFMQKIMHLVDGKGNTLHNNQHLLGVLI
LPVLILLSGELSVFVHMPYDITSLHTWQMWGCLITVGTLPFIKNVISNRLVRRTGQGPWR
FLEIISIILVFFIGMTYNAPSFKGYLAILCVIIGRSLGAFDVLLNASDYMHAEDERKRKN
PRASYAKSRQGTQASKPFLSSADNEEDEESSFSSNDSKSYQGSQDYDDKESVNSSSQQYS
VHKGTSHMDSSSNQTSHGVISADEGRGEHSRAATFKSKSKLSRNYSSKQEMLDSQAMGAL
DESTPVPSRITLQLMKQKKKSAFQGYSLLKKKSDALFIHFRDVLKDIVKTKTKVGEEMRN
ASFSLAKSVWAAGDFKGQIIEGIKRPVVTLSLSTNNVAGVKLPIFQVNIDPTVDVLGNLG
VAAGGQVINNTRENYLQCLNMLVKLASMQVAFFSLDEEIKMTNRRVNALNNIVLPRLDGG
INYIIKELDEIEREEFYRLKKIKEKKSDKLKDSNIDTDADGD-YNASKRQYNYACTQKDD
DIIFM--CVISFFILLLFCKAYNTSSLLETIKHDLQIVNNSNFNTVVNKFRNEKVFAVLF
FKKSNKNIKNVIKNYNDVASKFKGILTLCVVDCDENASLCENELSLYVPDYKSSNTHHFL
IYPINPMPKFVFKDEITESNIKKYTYLIPSKIDIIKDTQSFNVFLSKHENMPKVLIFSNK
KKPNYVLNALSNSFNKKLMFAYINNENEELVAKYNVKNLPTILLLKKNKVVDTYKGKQNY
INIFDWLNVYSETFVLGGGFDISPDKTNEKPWKFELIPKFTKLSHGDICFKKADKGLCLI
YLKEGEKLEKSEIDMLLSLKEKFKPHIDGRGINFRFMWIDIAMETHFRSLLEFKKYPSVV
VFNPYKRIRYAKLNEDLTATKENIEKLLEKISGGDAKFTMLKGQTLPEFIQDPNAAKANE
KDELMTVDLNIPYSACELKRVKRLELGVLDPEIIKKISVCEIVNVDIYKDGFPREGGLND
IRMGTIDYRTLCGTCNMNVKYCPGHFGHIELAKPMYHYGFMNVVLNVLRCVCYHCGRLLC
NVNSSKVKYIEKIKVNSLRLRKLAELCLGIRACDHSVEEEGLNINDNSLNNFYNNDLSNL
NMNQQMLLNKSNYTNIFEMVSKEDVDCGCVQPKYSREGPNMYIQFLH-SSEEDIDESKRK
LSAEEALEILKKIRKEEMSILGFNSDRCVPASLILTCIPIPPPCARPYVQYGNQRSEDDL
TLKLLDIVKTNIQLKRQTDRGAKSHVLQDLCSLLQFHITTLFDNDIPGMPIATTRSKKPI
KAIRTRLKGKEGRLRGNLMGKRVDFSARTVITGDPNLNIDYIGVPKSVAMTLTFCETVTP
LNYDNLKKLVERGPYEWPGAKYIIRDNGTKYDLRHVRRNSEKELEYGYKVERHMTDEDYI
LFNRQPSLHKMSIMGHKAKILPYSTFRLNLSVTSPYNADFDGDEMNLHLAQSHETRSEIK
HLMIVQRQIVSPQGNKPVMGIVQDSLLAIRKFTRRDNFLTKEEVMSLLIWIPYWNHVIPT
PAIIKPRALWTGKQIFSMLLQFDDMDVNTNVNKDSSYYYGNSTNDNNDDYLEKRSGNNHP
NSPLSIGDNINGNVSSPNNNNNNNNSNNNNNGINSFKRFNMVKINLMRDSSTSSKDDNPY
CSINDGKVIIKNNELLSGIICKRTVGSSSGSLIHVLWHEMGPDKTKDFLSALQKVTNNWL
EYVGFTVSCSDIIASNKVLGKVREILDKSKSEVSKLVEKAQKGELECQPGKSLYESFETR
VNNELNCAREMAGKVASESLDERNNIFSMVASGSKGSIINISQIISCVGQQNVEGKRIPF
GFNHRSLPHFIKFDYGPESRGFVSNSYLSGLTPQEVFFHAMGGREGIIDTACKTSETGYI
QRRLIKAMEDVMVQYDRTVRNSYGDIIQFLYGEDGMAGEYIEDQIIDLMKLDNKEINKLY
KYNFDEEPFGKDYYIGNDGSRNTSYIDYNKQNILNQEFEELYKCKNYLCKEIFPDGDIRQ
HLPINMNRLIEYAKSQFPCIPFVSNNNSTNISNSRKLGNLSSTHNHKESKKRRKRRRRKF
DKFKNNNELMSEIKKEYENNDLNNMMISQSPFKGMNEFHMGVADNDDDDYDDDDDDYDDD
DDYDDDDLDKNNYDE--NSMLNPIDVVHKVNNFLEKLVIIKQINSNDTLSVEAQNNATIL
LKAHLRTYLNSKLLTQTHKVSVKGLDWLLQEIEKIFYKSLCHPGECVGALAAQSIGEPAT
QMTLNTFHFAGVGSKNVTLGVPRLKELINIVKNVKTPSTTIYLDDMVSNDQQKAKDILTK
LEYTTLKQLTSHAQIIYDPNTTTTILEEDKSWVNEFYEFPDEDDTQYSLGEWVLRIQLTN
IHVNEKKLTMKEIVYIIYSVFSSDELDIIYTDDNSEDLVLRIRVKYLNGEYNFMNYDVVD
NANEQVDEQEEDEEHNNNTTNIFKVKNNISSDINTKNEDSISINSSNNEQVKNINSSPVS
NNMHNNNNNIKKEDGNEGALRGGNNDDDEEEEEEEEDFLFGKDGKNKNTNNKSNNNENKN
KKSGNNNSNNSNTDDNDDNKSDITIKEDNDVAFMTSKNAEEDLELKNKNHIEHN-ISRED
TEDTFLKKLMEQCLSTLKLRGIENITKVYMREESKITYDSDNGKFVRSSHWVLDTDGCNL
ENIFCAPQVDFKKTVSNDIVEIFEVLGIEAVRRALLKELRTVISFDSSYVNYRHLSILCD
VMTQKGYLMSITRHGINRVDKGPLIKCSFEETVEILLEAAAFAQVDNLRGITENIMLGQL
CKIGTGSFDIIIDNQKLNDANQNLETIQDLTSAGFTTPDSLHVITPDGLQSPVAINTINS
PLPFSPTYNANLLSPTAPIDNVNNLLSPQNLQNYGDNVMSPTSKDINNLDTLKLGGKFSP
TQSPKSPTSVMHSPFSPFDHQNQQPVDATLLFSPKNN----NIMNYNVFSPKPNINNIQS
PNIYSPNPMLDIFSPKPQINHNIYSPSYSPTSPTYNANNAYYSPTSPKNQNDQMNVNSQY
NVMSPVYSVTSPKYSPTSPKYSPTSPYSPTSPKYSPTSPKYSPTSPKYSPTSPKYSPTSP
VAQNIASPNYSPYSITSPKFSPTSPAYSISSPVYDKSGVVNAHQPMSPAYILQSPVQIKQ
NVQDANMFSPIQQAHVDEAKNDDPFSPMPYNIDEDEMKENMVVFNLDDVEIFFPYDYIYP
EQYAYMKYLKKTLDSEGHCVLEMPTGTGKTVAIFSLITSYQYHKKDEGKFIFCTRTVAEM
EKSLIELKKVIQYRINVMKQRKVEKLKNEKDDVNDVIKNDDV--NDVIKNDKEYNKHDDN
NLKEFGENSEILAIGISARRCMCINDKVLLKHEREKIDEECRKLTATFIREKKYINNKID
NIYHPNVDKISDFILRNRHHLDIEDYFDIYNSRNSLEEYDNIGLCGYYENYKKEFLYDLI
KPGVYTIEDLKVLCKNYKNKENVNVPICPYFCAKKIIEISKVIILNYQYVIDPKVSKALF
SWKDMNKNVHLKKNDIIVFDEAHNIDSVCLEALSVNIDRNILNKASMNITKLMKKIEQSK
MLNEQKLKEECNKILEKIKLQKCNQSLNVTNNVINIDNHMDNINKTNEQEKKKRKVESSA
YFDEDMNLMFSDFFDESFGDNEKKKDIKINDDIKNNDDINNDDRNGRNNNNDRNNNNEDE
DHLNDLCYSPLLMEDIIKNVVIPGNIRKSEHFLNLMRIVVVYLKKYINIYDITSEGPLSF
LYKFEKDTKLDTSFFKYCFDRLKSLLNNLQIVNIEDYSSLNIVCNFCTLLGNYFKGFIII
CEPYPEATGIYDPLIQFACLDSSIAMKTVINKYKSIILTSGTITPLELYPKLLNFKTVLT
ASFPMSFDRNCVCPLIVTKGSDLIPLSSQFSLRNDLSVIKNYGILLVDMCKCIPDGIVAY
FPSYIYMEQVISSWYELGVIANILEYKLIFIETKDIVSTTIALHNFKKACDLGKGAVFLS
ICRGKIAEGIDFDKHYGKCVILFGIPYQYTLSKILKSRLDFLKETYNIQENEFLTFDAMR
QASQCVGRIIRNKKDYGIMIFSDIRYAKHDKKNKLPPWIIKCMDISNINLTVTTAVDISK
QFLLNMSQEYRETGQTKISPLMLKNQVKCWTMVKSILNMDDFIMWKRKVVNVVSCIRKNY
RSNYSTYNLPQEIINQPITRVTELSNKLKVATVHTNCEIPTIGLWISSGSKYENKKNNGV
AHFLEHMIFKGTKKRNRIQLEKEIENMGAHLNAYTAREQTGYYCKCFKNDIKWCIELLSD
ILSNSIFDDNLIELEKHVILREMEEVEKCKDEVIFDKLHMTAFRDHPLGFTILGPEENIK
NMKRKDIIDYINKNYTSDRMVLCAVGDVQHEEIVKLAELNFNHLKTQEQKNNSIIHNNND

```
KPFFCGSEIIIRDDDSGPNAHVAVAFEGVPWNSPDSITFMLMQCIIGTYKKNEEGILPGK
LSANRTVNNICNKMTVGCADYFTSFNTCYNNTGLFGFYVQCDEIAVEHALGELMFGVTSL
SYSITDEEVELAKIHLKTQLISMFESSSTLAEEVSRQLLVYGRKISLAEFILRLNEIDTE
EVKRVAWKYLHDRDIAVAAIGALHGMPQYIDLRQKTYWLRYMEKLQEIFWSVEELIYIHY
QDEHIYEIKEKEIIESLSILYYNNNNNNNNNENLIEEFMDLFIVIKLIKNAKHKECIRHR
FIHNIFDYIKDIFSNKCINNIEDIKKEICNYIFMEKKK--MEYDNNIFLHTCYIFLYEIK
LLLCCYILLNIFVQYNWTGPPLNYDIK----DKQIIHDEDIYFKDFIHAIHIKNNKFLNS
SLSFLSLEGEEIYEYCELINSFSLSIIFLGLINNYNNQDNHIFSFCEYDHFFSSEYDGNR
NKSMTSNEINKLFDHLKLLYFVKSKYLWKARIFFIWQRLFTSSNDYYYSLKINIIDKPIH
IFKNIKIIPPDFELIEQDININEIDIVDCVKENLKHVKYCNLFYNNYMSTQFKIYVLSNF
SIYLAFYNYTSAYQRILDLLSQISHFQYFFTGRMGIKRMYQKIPATILVLKTKLDQQEDN
TSTILKEIEPLSEYDILRSDYRIIDHDEHNVDSNNNNICNKQNASLKREETNLLSNNNTN
ELERQRMSNGCCNNNINEHKTDEHDFDLCVDDKNESKEIVKISNEVEKNNSKVTWKLKDF
DPDTDILEEPHFVDSQNNIFKVLSFQEQICLINYCFSMIRFNPHYDEIKFEKLNAVISRC
LKCYDVNQKDSNNKSNNNNNNNNSQQLKYQNWLLHSCMLWFKCKGETFRFKTVDRAAAQL
NELHKECYDIKPESVERLKFIYDVYYPTTWEMKKEIGSVMIKIGSVVTAFNIFKDLKLWE
EAISCLIQADRKEEARELLDDLLKKKKSPCLLCLYGLIEKQRALNYYIDAWEISNFKYAK
AARLIGKYYYEKEMYEKCSEYLEKALELSPLFPEIWFILGCSYMKIQNFDESIKAFTRMI
SMTNDNSCKSYGNLAYLYMKKGTYKAAKICINQAVKMNNNEWKYWDTYLKLSIIQNDIDS
FCLALRMICQVNQVKQIQPWVFDYISDVIVKDKPTLIPNKNGLAYLDKIITTMEHLSQYI
TELDTFWNAHSFFLFIKGKFQDSFEAKIKEIRSIESIIQKCNNEKLVDALISKIVVAIKF
LYHLIKSHDVGE-KGTFLYQLKSLIESTLNHYKNLGSENLQELSQIKNILNMAEKEKEQN
NIFSDDESDEMIKNNKDISEHDAKNIEMSRKRKKINNTNTKDMIESGSKKRRIGKNKYLS
TFLDTEAQVGDDDEEEEYESSYIDEFEEAKRLEKKKLYEQKLKSGTNHLAQAINKLSKRY
ENEKEVKDILTDGETLTDGEILGDDYSDERRERLQTIDSPKMWLIKLFKNNVERNLAIGI
YHKFMKLKDNDFNIKGIYVSDNLKGYIYIEADSLYMLKKFLLGFKFINLNEISIVPIQEL
TSIFTMCHSKINIPKVNEYVRIKRGLYINDIGQIYEIHEKGIYAIVRLIPRITYEHFMNA
KKSKYYNQ--KSSNKLTSIIDKNNKSNSYFNDKLDMNRSMSNGYMSNGYASNKKKDSLDE
ALQL-RKKKKERPLQKLFDRDEIERIGGVIETGPYPRTIKYQNNIFEENGYILKKMNIKY
LITENANITLTEIREFNKNNMAGEKATLGITKSFINKNSLHLFKKDERVKILKGELCNLI
GTITAVNDNVLTINPDNLAKQFKFLPSDVTKYFIEGDNVTVINGLHKGKSGLISLIDYKE
NVALIFSPSLNTELRCSIQDLSLSINNSEGLGGVHTLNGFSVGDLIELNDRQIGVLTYID
KNKHIRVLTNNNTTLHTTIGCITSKRSAVGQVGRDENNNIVQSRDNIQIVRGAHRNKVAV
IMYIWKNKIFGKINKKIEDNGFVVLECQNCSLVGNPAEKKKIITNNNLFRSNT-RKNNNF
NSFIGKTVKILTGVYKGLLADVIDAERDEFTLLLKIKPKTVRQKRIECAISDSYKDQNAC
EENDEMDKLKPLQMFNNNNNNNNNHNHYDNRYNHHDNRYNHHDNRYNHHDNRYNHHDNRY
NHHDNRYDNPKDYKSNNNHYEDDYLKKKHSINDSSNNIKHEEHKNYVTHIGEENKKNKYE
TKKEY---------------NNIINNNDQKEESPIWLHIDIMVKVITPGPFYNEIGKIID
VITKNAYTILKIQTDKTTFNIVADAVVPLKPQKKNDHILIFDKGEKIEGTVQDIKINEVH
ANTASGLLTCHLKNTFLYKNYIPMSQNNPLSVCVADNLINYDIQNESFRLGNVSVEGDKY
ICVKENVNENTQVVVINLHNKNSTRKHMKAESVIIHPNDPILALKGTIKNMNTIFLQVFN
IETKEKICSLNLNEYMNYWKWINNDTIAIVCEKNVYHWNINTLTKVFEKAQIFIDNNSQI
LYYSTDKDMKWCILCGISTQDQGKSIDGYMQLYSCEKKLHQTIEGFIGCFGSFVFDNLDM
KPLFCFVEKKKNSTISRLHLMDIYTNKTETTIPYKIVKEINLINENLNDFPIYISLNTLQ
GVIYIVTKCSYVYVFDEGTLSLIVKEKISEDNIFICCDSKNGEGIYAVNKKGIIFYITIN
YFHLINHLKLSNLEFKDKIIKNLCVKYGYPGCDYISAYKKCINDMDFKKASKIICLMKNT
KLRTQQVLNSFKSFKNTSGQLSPLLLYFSVLLEYDKLNTYESIELVKPVVLQKKKEYLEK
WIKDDKLTCSEELGDLVKVLDLRLALNIYLRCSSHNKILSTYCLLNMFNNVLSYINNFKQ
INFDYVGIFITIVNYEFPQGNADNKDSAFVGDFNSGSKNETMDFFNDGASNDQNNSKLNK
GHINEVAIQYIKFLCENNISFDINKIIDYLLSKKKLQEATSILLDYLKENKAEHKNLQTK
LFEFNLYNNVQVAETLFQMDIFTYYDKNKIAYLCEEKGLYQRALENYTNINDIKRVITKS
TGKISIEWIKNYFSTLSDSVCEELLFDFMKGSKINMEVVISICVQYYDKIGIKKIINKFE
ENKNYEGIFYFVSNILNDLPNLIVKGNDNLSYNNMNDEDASILLTSDICSTPQYSYETAN
LKLEDVHYIMFKYIEACVKINNIQELDRICKDKNAKYNPEQIKNFLKDCKLSDPRPLIYV
CDIHNYIEELAEYLYKNSLLKYIEVYVIKVNPNNAHKVIGVLLDLDASEDFLLNLLNNIK
NISNIGNLIEIAEKRNRLKLLLPWLESRANEGYENIELHNALAKIYIDLNKDPENFLKNN
NFYDKKMIGKYCEDLDPHLAYTAYERSNGECDEELIYITSKNGLFKLQARYLISRQSIEL
WKVVLDETNKYRKNVIDQVIGSTLIESNNADEITVTVKAFIEKKLSSELIELLEKIVLHN
SEFSDNKNLQNLLILTAIKSDSKKVMEYINRLDNFSGPQIAAVAYEYKLREEAFVIYKKF
NCYTSAISVLLDKILYKNQKNKNFHEFSSHDSTVPNYEDIDSTESTCDLSNYEDDLNRAI
EFAQKCNNNDVWFILGKAQLKLNKIIDSIDSFVKSNNPEAYKEVIEKCKENNFYEHLITY
LNTLREQNSLKDVLVDSELLYAYAKLKKTNEMNKFINTTNSANLQLIGDRLYKEEEYEVA
KILYSNIPNNQKLTACYLKLKEYALAIEAAKKAKSLKTWKEVNFICVKYKQLKYAHTAGL
QLIMHADHLDEIIKIYEKKKYINELMNLLENGLNNERAHVGIYTELGILYAKYKPEKLME
FIRNYTNKMNTRKLIDVCENEYLLKEAVYLYISYDEYNLAVDTIIKHSPTAYTADTFMQV
IHKVTNSDIIHKVIDFYIEEHPLNLYNLLKILENKIDNNRLVQTLKKSNNLPLIQKYLED
IQAQNITAVNETLNEIYLQNDDYISLRNSIDEYDNFNQTNLLNKLENHKLPEMRRIAALL
YKKNKKYKEAINLSKKEKQYKDAIEIARVSKNNVYIEDLINYFIESKNKEAFCACLIVCY
DLLKPDYILEIVWTSGFKDQAMLYFIQIISDYTNQIEHMKKQIEDMEKEKKMNKSAPNDY
SA--MNNQFNYSLNNNLSIMPPQNNFMSSNS-FDKYDMFNNNTHFMASYVGREAPYFKAE
AVFADNTFGEVNLHDFIGKKYVLLYFYPLDFTFVCPSEIIALDKALDAFKERNVELIGCS
VDSKYTHLAWKKTPLTKGGIGNIQHTLISDITKSISRSYNVLFGDSVSLRAFVLIDKQGV
VQHLLVNNLAIGRSVEEVLRIIDAVQHHEQHGDVCPANWKKGKVAMKPSEEGVSEYLSKL
MDDNTANIIGSHDNILTDPRIAQDCSAETNELLNKAEQYYKVGELDVMVEELISLEKKCR
QSYDGISTSKICCFILNKYKEVGNYSKVNEYLIFFNKKRGQLKRTIIDIINLCKGWIEDI
QNKEEKLNLINTLCLVSEGKIFVEVERSEVIRMLSKIKEEDGNIEEAANILQDVQVETFI
SMNKRDKTEYILEQMRLVLLRKDFIRCHVISRKINPSLLNTEEFADLKLKYYMYMIQYYI
NEESYLDVAKCYEERFHTDIVLNDRNLWIDEMKCYIIFLILSPYDEQQNKLSNLLKMQKK
KLKEIPIYQNLVQDFIEQDLIQWPLPYQQELFNFFIFDDSIFLGGQNRKDLFKKKVMHHN
IHVISNCYDQISLNRLAQLLNASIEDSENLLSELVSAKFINSKIDRLNGIIKFGQKKNPE
NLLNSWSLQINDILDLLEESSHLIQKERMLHEAKLKRIQLENKKMTLMNDKEAKKEAIEI
IYEISNILNVNLDKETIVILIQLCEYGVSPKILSHIIIQLKKEREKFLDNLNN-MNNLNK
```

> ***Plasmodium reichenowi***

```
------------------------------------------------------------
------------------------------------------------------------
------------------------------------------------------------
------------------------------------------------------------
------------------------------------------------------------
------------------------------------------------------------
------------------------------------------------------------
------------------------------------------------------------
------------------------------------------------------------
------------------------------------------------------------
---------------GK-IKQINALSNFHGDFKTTKKKIHWLPYLPQQLITCTLYEYDHL
ITVDKFENDNKEDWTNFINFNSKHETLVYAEPSISSLKISDKFQFERRGYFILDKIDPHQ
HLHLIKIPDGKSKNMSIITTKVDPKQLAGTKDKKNTIYIFLFIIGYIKAGINSSAEPSIV
FPTIVGIEKNDETKKIYTGDEAFFHESNLNIYRPIDHGHISDWDKAQKVWDYTLNCVDPS
KSIKDILLTEPPLCSISHRKKMGEIFFEYFDTLNLNLSVSGLMSLYASGLTTGLVLDIGE
GVTQCLPVFDGYIEKNSIIRSDFGGEELSMFLQKLICDIGYSMTTTKSLEYVKNIKETIC
FCSLNPSEDQLRNDLAATYTLPDGDVLRDGYDSIEISHERFYVAEALFNPQLCHRDNLSI
IDIIWKSILSCPMENRKILSSSIVLSGGSSLFPNLVERIETEVRNNAPESARSMVKVI--
-----------------NFKYMITHTYIYRNKMYAINLYILL------------------
------------------------------------------------------------
------------------------------------------------------------
```

```
------------------------------------------------------------
------------------------------------------------------------
------------------------------------------------------------
------------------------------------------------------------
------------------------------------------------------------
------------------------------------------------------------
------------------------------------------------------------
------------------------------------------------------------
------------------------------------------------------------
-----------ILHSKKPKSDYVKASLITVLNIHLNEHPENILVFLTGQNEINTACEILH
ERMKKLKSMSPPPLIILPIYSSLPSKMQSVIFEPAPPGCQKCILATNIAKASLTIDGIFF
VIDPGFCKIKKYDSKRDMDSLIVAPISKANAKQRAGRAGRTGPGKCYRLYTEEAYKNEMS
EMSVPEIQRINLGSIVLLLKALGINDFLHFDFMDSPSVETLIHSLENLYYLGALDDNGYL
TKLGKKMANFPMEPNLSKILLTSLNFNCTDDVVTIVSMLSVQNIFYRPQNKALLADKKKN
KFIMPQGDLITYLNIYNKWKENSFSNYWCHENFIQSRALKRAQDVRKQMLSIFEKYNYQV
KKSTSKNDATKYVNICKSICSGYFNHVCKRDTQQGYTTLLTNQQVFIHPSSTLFNKNPLF
VVYHELVLTNKEYIRDCTIIQPQWLIQLAPNLFIPADEKKISKIKLREKIEPLHNYYEEP
NAWRLSRRKG--------------------------------------------------
------------------ENKKDMHMYMYSFFFIIIIIIFFFFFFFFF------------
----FFFFLV--------------KVTYEQGANFARENNLFFAEASAVSKLNVKHIFENL
LQEIYNNRLKNNNRSFSNRSVATCESAIQLTKARSVIKLNEVYDNQSEDNNMNKVKCCME
KSKLAKVEKVLGRTGSRGGVIQVRAQFMGDSELAGRFLIRNVKGPVREGDILALLETERE
ARRLR-----------------------------------------GTQSLNLKKSHCYC
HLSTGDLLREAAEKKTELGLKIKNIINEGKLVDDQMVLSLVDEKLKTPQCKKGFILDGYP
RNVKQAEDLNKLLQKNQTKLDGVFYFNVPDEVLVNRISGRLIHKPSGRIYHKIFNPPKVP
FRDDVTNEPLIQREDDNEDVLKKRLTVFKSETSPVISYYKNKNLLIN-------------
-------KMKQECNVCYFNLPDPESTLGPYDNELNYFTWGPGFEYEPEPQRKPLSIEESS
ENSEESEESVADIEQLEEKVDESDVRIYFNEKSSGGKISIDNASYNARKLGLAPSSIDEK
KIKELYGDNLTYEQYLEYLSICVHDKDNVEELIKMFAHFDNNCTGYLTKSQMKNILTTWG
DALTDQEATDALNAFSSEDNIDYKLFCEDILQMKINLYKLLCFIWVIFFLHKNVKSGDNM
KYNDMKGLNDLSKLNDDQGKDILGLKIDGAKERIEKLFHLIDKNNDKEITEKKLNTWSSF
LKNEIFLKQVQAEMGQIDSDKDGFISLNELNDAFAQNLDAKEVEKHSEGLLKRFQIVDKD
KDGKLSINEVGLLIDPMKDEELKELEINEILEHHDVNKDGKISLDEFKQTRSDESSVKKD
DEMALDDFNFFDTNKDGFIDKEEIIKVYFDPAHESGAINVNEIKENIFEGKKITYDLWNE
KALKIAVTSLTDYGDVIRYPEDFKLDIGKNVILPTARSRAFEDDDIDADN-TEDDKDETD
DASQQKSPAIDELMDLLFAKIICISIFMVVTTFGCFIPHLMGLYKEKENEEKNERVKNIL
SNLNCFGSGFIFSIIMFHLLPETIHIISDHGNIRIFNTSDSQMKILYIFFFVFIGFCMQL
GLEYVLPVDTNICCVSNIDSKKKLEDTLS-------------------------------
------------------------------------------------------------
------------------------------------------------------------
-----QILLNEIKQNISRKVRLCKWLSFCFSCLIAFALISFTTSMAPHTHGHHHDHGHN-
--------------------------EKKPIVSAQAERWAAIDSSSSEEEERVIKTYEGK
RLHFYETTGNSLNENMDNNDFNLLLKDYENLYKFMIKESADCIPNFAIIYLDKLSKYVEK
TFQNNVEKKNLSKNKAQTLNKLRAKIRKCSEFYQEKLNLYNENPDDFKDDRKKDMDDDED
DEEEEEEDEEQQQEQEDDSEDEKKEEKEDYDDDNDEEKDDSDDWSYSEDDNYASDEEDDK
TKNAMSKWGLKTSTKVEKKKTVKQKKVKKESTKKEEKANRVEDSQSAKNKGYAELLSTKN
LTEDVIRERVKLVIEKRGRKGLDKHEHINILSKLCEIAKTISTQSYIEVLEHLINLEFDV
VSSVYTYMSFNIWNKVFKYVELILDILIQNENFYLVSINIAEEITEETTNEKEKIIKSCK
TLISFLAKLDDELLKALLYIDVQTEEYRKRLGKTIHMIGLLKKGYNYVKCLKNMPDLAIH
ISSRILEHMYYKPEMLFKQIWTYLMNGKEMLSDPSKEKWTRLEKKK--------------
------------------KIEKIK------------------------------------
-----------------------------------------------------KKPLGQE
RAEKRRLLSYHMHISIELIECVNNICAMLLEVPNLAKNTYESKKDIISRQFRRFLDIYDK
QIFNNPPENNKEIIILATKHLQKGNWKLCCEKIFSLSIWPKFPDKEKVQNILKEKIKQEA
MRTYIFRYISIYESFSIDQLCVMFDLNQNVVHSILSKMMINQEIPAFWNESSKFILISKV
NPTTLQNLALKLAENVNEVMEQNELALNMKNPK---------------------------
------------------------------------------------------------
-NETYIYIY---------------------------------------------------
----------------------------------------------IYIYIYIYDNIFFC
IYDH-----------------VHIY-------IYIYMQII----------LFCFIL----
LGLGLQPKQCIVVGTRALEFLNNELTNIKTLSELYERVKKRSITDNNDNVLPLYLLELFS
IPIEQKEKNDFLTNRISMYSKILNGRKNVLDLLLNKFENDCNDSIKKELIDCFDVEKFKQ
EVNSKFMNILIQQLRKVEVKLEKKKAKMDFYNKKLEELYNKGNILSVREQVKLYIRELVN
IVSNLLTGNYPILNLPKNGENFLKKYGGTLMDNLKDGNELAVELFEKQGLYDENFLNYLN
EYLSRYSNDMDLSKSASSEFNNMNDGLNNNNNSIDDNNSNNRTDSYENHMDLDKIKIENK
PLLVEGVPVRFMLAKEESNMFGVVQNAQSDTKGKNILVNFYFRNNNTDEQVQVKSVEKDR
LTVIKAVETLNGDPSFLNGLQVWYKMVRDDGWVGFDKAEIIKVFNNNSKIKDVLIRNLSK
NDEIISVKIIDLYMDYVNNHNHDDENKNDENNNNDDDILKRIAGPHTDLKILNQLAITL-
------------------------------------------------------------
---------------------------YMGRHIYIY----------------------IM
CM----------------------------------------------------------
-------------------------YMYIYIYIYIYISYVSYIYIYFI------------
--------------------------GVLVPIDDKELMTHYSIIDNRDNILRKIENQKRL
NQYFTIVANSIKLLKNNVSTHNLLHYIQGIGFCYTRF-----------------------
------------------------------------------------------------
------------------------------------------------------------
------------------------------------------------------------
------------------------------------------------------------
------------------------------------------------------------
------------------------------------------------------------
------------------------------------------------------------
------------------------------------------------------------
------------------------------------------------------------
------------------------------------------------------------
------------------------------------------------------------
------------KTFGVHDSIKISILTVLGDIATALNRSFSKYLNFFANILSETSKITIT
SGSPDSDDWVNYVFELRDAILLTYSNIIYALIDGNEINKLKPYIPNILDFIELILIKEIN
HFNAQNFQNSVSLLGDLVHAYGYELIENSKLTDLIISVYGKIDILSSQGDEKCETCVSKI
KWLKRICNARLSQKMSVYIQLTHDLSGTVTAVTIGDWILGILKEKNIQEYEVFFKKFLHI
YQKQDKDSECNNRSEIFSLLLSASDFVFETLVETKKNNKIKVIIDNKENVKSYNEFYKEV
EEYFVILISILQLEFKSVEELNNATNNFIKAIKNYNEFPELRLKILQLLYNSFNVNFSFR
FPTFIAILQFCSQNNIFHNMLPYIKFIDDWIKEWNISTREKRHIYLIISQELKKLKKYEE
SYEHLKKHIYYYDNEHKDILNHPNCINASIELIVDAININNNIYFHEIINLHAIQNLQYI
PQHQPLFDLLLIFYKYTINEFLAFKNKHGSDFFNKYNIDLQTCEKKIYLLSIISLFNDHK
VQNIQFISKQLNISVVQIENILVAAIGSGVIDAKIDQINQTVHMKTTILRNFDEENWKQL
NNQITKYINNVQKILDLTKHANK----MHHLLFIIWYIILNYYVSGQESATNFYKFIDSF
ASSTYISEESGSSAYDAKRAIQNNPNYWCSSGNHSNDEEITWTGYLNTKGFIKGVKVSWT
YSPEFVKISVSSDGEKYRTIIPYKKISSNEASFDEIYFFKRLEEAMSIKIGLKNATHKYF
GIREVKLIGGGNPYFLLLSGISSEEEMCLQVEEGLINNDNTSIILDSCTNALASGDGREL
WKTNSNNQIISAFSDPPKCLSVVNLDDLENNKIVLYDCLRALEDGDGKSNWIFESNSQIR
LQKSGDAFCISQKNIYGNIPGIHDILLNLDVSIYSNSTLDDDHNPDNTIDGNLNSYWASA
TFTDNYDHLVYLILDLNKITDLSRIKIYWEYPPLHYNISVSTDNQNFTVVSENLANPSYV
TVDSLRNMETRYIKISMIKTHPKHGELGDNFLYGIRSIEVQANNLETVINHCRDAANSDD
ARDKYFVEYITEFDKDLTNKLINLEDDVTKNVSSISDNLSKLEELLPNIETCLEEKKTYD
EELKESKEKANDLNNKLSLLTSVNNTLDSDILKLGILPGDSYNFPANDCAVIKNVQENPL
SGFYWIKPKCSPEPLRVYCDMDSSTSIYIWNGNPPKSPDHLITNMINSVNDIRQHCAEVG
```

```
LQPLILRSKNQLNSLIISLKKIGYSLNGKVNIPLAYDYSCDHGSCSGRFHDLLNGNIDIS
TLIYLKASESPDSTKVRQTA----------------------------------------
----------------RNII----------------------------------------
------------------------------------------------------------
------------------------------------------------------------
------------------------------------------------------------
------------------------------------------------------------
------------------------------------------------------------
------------------------------------------------------------
------------------------------------------------------------
------------------------------------------------------------
------------------------------------------------------------
------------------------------------------------------------
------------------------------------------------------------
------------------------------------------------------------
------------------------------------------------------------
------------------------------------------------------------
---------------------------------------------KNNSITEQIENKIEN
DILNEQNSSLEKVNHRKGVISVSKYKSELTSITRMSNEIIMQTILEHTKDMDDDHRSNIK
KSKTYESRNNYYYNDDHDTKRNIPKRTVVQKKYVQRKTNITNKKRISQNKPVTRNSYKKN
DRINTTLYEKKKKKVRRSNANRRKRNDNKIDGKISDNNKIKIDGNINKINKIDGNILDGN
TLDNNKIKIDGNTPSNNKIDDNTSNSTLNNNKTDNITNMDDTNINPEKEYIKENEIYKMY
PNKALLSNNTNEKDYDYNESDNASYSTDDSKYVKYLKKKKKLQEQGKIIIDLNLFHGNSV
KTTNKRDKEEKLQEKLRMKQIKKLLDEKQMVICKIEDIAKKRNKKYVKAQVLSKDSKIER
AYFSHKMKKFYNSKSSYFGCGWSSNKKEWTPFIHAPFFENQHNAIYKNRNKKLYEEIYDT
LLHGRIHPDIKVVELKDHKHPIRLCTPYNEDCYSVVYTGKKINATDDRVIFGEYTGYVAN
NKELSQEKHQYMFALTFNKKVFNDRKHVVFINEVELDEEQNSHVESNVMNKVKIHEKNNI
NNTNIYNSPHILNG--NNNINSNTTEETMSNRKIDKIKPVSNNINSYINKCKTNEHNKKS
RDINNLIILPDNYTYAVDSSYMFNEMSLVNHYKTCSVFNNFDFRINSEWQLVYLDGWPHI
ILTSIPGVEINPGEELFADFGFEWFEKVNDICLNEFIKNNYFYRLYKLNTSREILFNGMD
DIVEKYNLLKNHITCNICMHNVNTDGNNFILCSGCNHVYHLKCVHKFNTEVNENYEWFCS
SCLQFCFNIIKQKEFVDYIEKENQKRFIQLLG-DLNGDNLDSVNNRKKSDAVDNYNRVGN
INNCNSIDN---------------------------------------------------
------------------------------------------------------------
------------------------------------------------------------
---------------------------------------------------------RSF
K-----------------------------------------------------------
--------------KIWSVCSNCYSKHHDLAKAIICRVTKMHFEANYNDYLTDEDLFKTS
SEFIQSVIRELANTVKEYRKKELNRVYLQHATENDNM-ILDRVNKDMSLELNYNNVTTRT
SIHNCSCTKTNKEYNESFYKIVSPSFNNDTVEKSNIIIQENNHINYECQNKEVEKCHDDF
YSKDMHILYKNTFLPREEIDNCSIEKINDVEEKVINDTLNDMSNGISNDVPNDVP----N
DVPNDVPNDVPNDVPNDNDVPNDVPNDVSNDVPNDVPNDVPNDVNLCEKKNDLNSYEKSC
KLNNFIPLIGVELGKTKFQREFTNGTFVGTVTEQIKDENNNNFFVVTYEDGDVEWITPSF
LFQEILKQSTNNTTYPLASTFKDIFYEDFKKDIKLNNHSYELKIEKKKRKSMFEYVPNNN
-VTKRQRHAFEENVLKKKNTHSMVNKEEAQRLKELGNKCFQEGKYEEAIKYFSDAITNDP
LDHVLYSNLSGSFASLGRFYEALESANKCISIKKDWPKGYIRKGCAEHGLRQLANAEKTY
LEGLKIDPNNKSLQDALSKVRNENMLENAQLIAHLNNIIENDPQLKSYKEENSNYPNELL
NTIKSINSNPMNIRIILSTCHPKISEGVEKFFGFKFTGEGNDAEERQRQQREEEERRKKK
EKKEEEEMKKQNRTPEQIQGDEHKLKGNEFYKQKKFDEALKEYEEAILINPNDIMYHYNK
AAVYIEMKNYDKAIETCLYAIENRYNFKAEFIQVAKLYNRLAISYINMKKYDLAIEAYRK
SLVEDNNRATRNALKELERRKEKEEKEAYIDPDKAEEHKNKGNEYFKNNDFPNAKKEYDE
AIRRNPNDAKLYSNRAAALTKLIEYPSALEDVMKAIELDPTFVKAYSRKGNLHFFMKDYY
KALQAYNKGLELDPNNKECLEGYQRCAFKIDEMSKSEKVDEEQFKKSMADPEIQQIISDP
QFQIILQKLNENPNSISEYIKDPKIFNGLQKLIAAGILKVRHIYIYIYIYIYIYIYICIY
FFFCTCYKDIIIILQCFIY------------LYPLFPSEENIPSKYGENRHWNVDLIPKF
ILVGGNLVKILKKTRVTNYLEWLVVEGSYVYQHQKKGFLSSEKFIHKVPATDMEALVSPL
LSLMEKNRCKNFYQYVSEWDANKRNTWDNLDPYKLTMLEIYKHFNLCQLTIDFLGHAVAL
YLNDDYLKQPAYLTLERIKLYMQSISAFGKSPFIYPLYGLGGIPEGFSRMCAINGGTFML
NKNVVDFVFDDDNKVCGIKSSDGEIAYCDKVICDPSYVMHLKNKIKKIGQVIRCICILSN
PIPETNQTNSCQIIIPQNQLNRKSDIYINLVSFQHGVTLKGKYIAIVSATVETNNPIKEI
EKPLELLGTIEEKFVKISDLYVSTSKKPDDNIFVTSSYDATSHFETATNDLLQIWENLWG
QKLNFDDLNTNADGEA--------------------------------------------
--------EVRLDVKLNKFFWFKGVRNPPKRVWVKFERKRNEDGGSKGKMYSFVGQVMVD
SYKGFVKEWEANG-----------------------------------------------
--------FYQKGCINCKFLQLARDRPRIHDCPTENFNGFMAITTPNKSWMAQYNDLSKY
APGFYALQVIGELPESIRDLKPNYN---------------KSKCVYNIYIFIDTYIFFIY
GFI---------------HRSGSGYIILSLFEMLLSRNKKIDMLYCVDINKKACECIKNL
TYENKIFNVEIIRNNLFNNIRRCELFDIVLFNPPYVITGPDEMKKTDLTASYAGGKYGRE
IIMKFLLDIHNYLSNKGVIYLLLEKSNIPQEILNYEHVKNIYIYEEIKKKKTLNETIFIY
KLMKKKMDRYGHNVRSDIKKQGYEDSNLPILCETCLGENPYVRIIREENGKECKICKNVF
THFRWKPGENSRYKQTVICMKCAKVKNVCQTCLFDLQYNLPVQVRDKFLENSIVLPENET
NRNFFLEQMENDMSS-TYDKMNRINMDLSKLKRRDPYFKRNMARVCSFWRKNSCNRGDEC
PYLHKEIHLDKSLSNQNIKNRYTGENDILAEKILLKHNEKNNDDKNMSNKICIQGISESV
SQANIKECFKKFGDIKSIKVIPKDSKMFISYSNSQAAKKASDKYKDGLLLNGCNLTVHLQ
DNPTYNNKNQHPVINYMNNNMYQNNMSPQ---IMYNPMYYPY--YNMNNMHPSSAPYSSM
LPSEAEQRK---------------------------------------------------
------------------------------------------------------------
------------------------------------------------------------
------------------------------------------------------------
------------------------------------------------------------
------------------------------------------------------------
------------------------------------------------------------
--------------------------------------------LRPV------------
------------------------------------------------------------
------------------------------------------------------------
----------------------------------------RKNSFYYLEIKKEMDNNNHE
NLNQNNNIYNNPLTYDMIYSLSRFNKIKTSLHVDYKSYTGPFLLHEYYDPFHFKFKELSR
IGGVLFVEGNENLGIFEFIKLITNSLYNFKLFNVSNMPNSLYINITNPLLPWKILCNDIL
NTWKLSHMRKKNLLPNRNDNFNMLREITHPSYHWFFKSMIHVIDDLDIPFFKTKKKKKKK
EKEKEKEYYNHKTNKKNEHNLIQNKINTNTQPQSASNNSNNNIFCQKIHNSRREKKKNKK
KSKEKNKKDDDDDIHDDIHDDIHDDDNISHTYSRNDIQTKMKNELKENRIGIISSMIYYF
TLHENMFIIFNYRSGTSLNINIEDDAWKISNNIAKLAMLKRKKISKHLQKNLKDWRKNHS
RKCDFCKGFYKTLCPNIKDNILLYKSPLCNPFFMHKHKPLIFLFINGMKNNNIKQLKKIK
KYAQQCEASIELKPFNKYDLYEFVSLCLNIHKDKISTQLIEYLNKTCFGIPKFVQYTLFY
LLTNHYLELYKYEQDKNDKENNDELHPSVDARKNNEQNNEQNQNQQNNQHNNDEQNYDKN
NYINHFSKQIISVQNETIKSSVFPSNSTTSCNDNSNNDDINLVIVKDLNEAPLVPRLV--
----------------------KKKKKKKNMSCSCKMFI---------------------
------------------------------------------------------------
------------------------------------------------------------
----------YRRCF----------------SPALKSNNLKFNNSHFKIKQKDNDNDKDK
GKFVQIVKEQNCINKIVQVKKTKAGMNSTRTHVIYMDEDNLQNHNSEIEKKKKKKKKKKK
QEEKHNNEDNNTQDASNTHSNNEDNALEWLKEISKKEKKKDESLIVPNIKKDNIEHVENV
DKNDRTINMDTTKNDTTYELDISESEEMNTGKLIIFNLPPVDVQDIKSLCERYGPIVDVN
VFKKSTKADTQIYLNEKSKTKSSDDFFIKLLKGNTSNNNSNNNNKNKDEDYKKKNDDQSK
DEEHSNNHKYNKNMLYNDLTNVNVYAFVNFMFPSSCEKAKIHLDNKIYRGKILTAKYCKE
KITNYKEENINEKNIFIKLSNDCKSSYKKILEIQKKKSCQNENMWNILYTDINTSINNFC
```

KENNCSVESVLNIRDKNIAVNVSLTETYIINKIKTWIKKEGIYLDAFEQIYVKTKKNEDN
EINKDDKEIVKYRRSDDTIIIKNLSIYTNQNDIINLFKQYGTLKRVSFSPYNNICIIQYE
NADNAKKAFISNSYIRYKKLPLYLEWAPLNLFVKQKDDKRTIKREETNEHDNKNGDSRDD
GDDDDDGDGDEEGTHASIYIKNINFNTKEENLKDLFEKMEGFITCNIVKSKKVILQKDKE
SKISNQYNTVSSGYGFAEFKNKELAIEAIKRLTGTRLNDHVLEMSLSHNRIRKKKTKNED
EKLVLKNKKQVTKKLVVKNLAFQVNKEELRKLFSAFGNVKSVRIPKNVYNRSRGYAFIEF
MSKKECCNAIESLQHTHLYGRHLIIDFADDFIFDKNVDEYDKLKELNNQQNKDIITSEQA
KRKSIYESQKSKQVSESKKRRLTSNLKSIYMVKCNDSQSKNCGYNFCNVNEEDRKEEVMK
NNNYSVEKNMFSQSMNQENYENFVDCPPQNARILKPLIQEKIVEIMKPEIEEKIIEVPQV
QYIEKLVEVPHVILQEKLIHIPKPVIHERIKKCSKTIFQEKIVEVPQIKVVDKIVEVPQY
VYQEKIIEVPKIMVQERIIPVPKKIVKEKIVEIPQIELKNIDIEKVQEIPEYIPEVVQKD
IPYTQIVDRPYHVEKIVEVPHVQHIYRNIVSPQYRHIPKPVEIPMAHYRTFPVEKIVDRN
VPVPVELQIVQEFLCPKIEARYKEIPVPVHVQRIIEHPIPKDAMNNPHLLPLYYQEDN-N
IEMSTNTMEQNKNKNCFIFNLNRNDSRNSS-HASPSVELLLHNDKSQKQFYNNHTNKKNS
IISNPVFNNY--NQNGTLNQFNLNDNINNNMDHIIHH-NNNIPSNVNTYNKINNNPYENN
TVLKNNYSVNIPTKEFASNLYSSPSPVLSSHTE---------------------------
-----LGSTAVGICVNDGVILASERRISSTLIEKDSVEKLLSIDDHIGCAMSGLMADART
LIDYARVECNHYKFIYNENINIKSCVELISELALDFSNLSDSKRKKIMSRPFGVALLIGG
VDKNGPCLWYTEPSGTNTRFSAASIGSAQEGAELLLQENYKKDMTFEEAEILALTVLRQV
MEDKLSTSNVEICAIKKSNQTFYKYNTDDISRIIDVLPSPVYPTIDLAA-----------

----------------------------------LKGSSDIKSLLPQEYNRFKIIDSDFI
NIMKKTSDKPKLLELFQMEGFQKQLDRLSDSLSKIQKALGEYLEKQRNKFPRFYFVGDED
LLEMIGNSKDAKIIQRNVNKMFAGINSFILKENTNDIILGMSSREGEEVLFLEPLNISSF
NTLKEWLIVLEKSMKSSLEFYLDEAAKEILEMDMMECTKIE-NNKILLWSEKYPNQIILL
CLQILWTTNIENELINFSKNSSDESNTLFHKSEKICLNLLEFLAVNVVKQKDHRTRQKFV
QMITELVHQRDVIRILIDKNVNNVNSFIWLQYMRYYWDSKKKDNKINLIIKMADATFEYG
YEYLGMCEKLVQTELTDACFLTLTQALKMKLGGNPFGPAGTGKTESVKALGAQLGRYVLV
FNCDESFDFTAMGRIFVGLCQVGAWGCFDEFNRLEERILSAVSEQILTIQTSLVQRKNEI
EILNKKIGLNKNVGIFVTMNPGYAGRSNLP------------------------------

------------------------------RIYVSRG-----------------------

------KHL---

----------------------------VNFTFNCHSLQNQCLNMILKNERPDIDKKRCD
LLKLQGEYKVKIRELEESLLLELSNVKGNILDDDNVISTMEKLKVQGAEASKEVNIAEEV
MVEVENVSNQYLFLAQGSARIYFILQHLCNINFLYQYDLNFFFNIMKDMFNNDHLLSIVK
KKDYKERLKVLEYLLFSLTYNRVARGLLQEDRYVFGLQLCYVKSIINPNIDMDQSYLHYL
LKDHYSNQEIDEF----EHKKIEKSLLPEYNDEQINALNNLIKHKSFSNLKECILNNKQK
WIELLHSAEPEALVCSILNKSDEMLNKNKNSNILNNVENNDNTNNKNDISSCLKESLIIK
AIRPDKLETCFNKIINHILGRDFLWIPELSMNDFEKYVKENANGNIPIVLISSPGFDPSN
KVQQLSEKCKIPLFSIAMGSEEGYISAERVIFTAQSNGGWVLLKNIHISTKWLHELEKNI
HKATTNKNFRLFLTMEFNPRIPQSLMRISLTFMFEPPVGIKFSILRSFSLFLENRELCEP
KIARLRLYFIVSYLHAIIWKEE--------------------------------------

--DILL--


```
------------------------------------------------------------
------------------------------------------------------------
------------------------------------------------------------
--------DD-------------------------------------Y------------
---------------------------------IYERF----------------------
------------------------------------------------------------
------------------------------------------------------------
------------------------------------------------------------
-----------------------------NNEEKDNITGDHNNNGEDENKVGSHNAYDND
QNNGYVSDDYYTKNYDQELFYNENVNNIFDVKYGVGFKMSFARISSSCAIMSRTFNTIGI
GLLSLELMNHCEAKELATPLCMWKLPNKELINRNIANKSEHRHHQKLLMSYTPFNSPSLL
AEQINILGTYSGTRLLYWDFRDDMDFIIFSPLNNNIYLSSSPLSVDEIKYNKKKK-NNKN
NKNNSNKQVSCVEDVKLSNRDIKRKGNF-DEENVKKVKVQHDESCANVYAK--NDEEGSD
KDASTKEENMKKEHPNEKDDDDNN-----NNNNNNENGDNNN---YKDQIFKENEQYQYY
NSTIKKLNLFEYNSHLNTSISKDKYKANQIFPLWDHPKDSIDYCLSTYLYWLYLRRNTNI
FLQNTLLIPTCMRKNDNDDNSEKKKKKKKKKKGKKKNLQEESNTTEQTSSLSKDSNSNKD
SEENVNNDEERNSKRRNNDDESNESSSTSNR---DKSDADNVNNDVIXNYKKGXEEMHXI
NLKKHWNMVXHRKVQE--------------------------------------------
------------------------------------------------------------
------------------------------------------------------------
-XWYEIXRRR--------------------------------------------------
------------------------------------------------------------
------------------------------------------------------------
------------------------------------------------------------
------------------------------------------------------------
------------------------------------------------------------
------------------------------------------------------------
------------------------------------------------------------
------------------------------------------------------------
------------------------------------------------------------
------------------------------------------------------------
--------------RGQLVVIAATNRQNSIDPALRRFGRFDREIDIGVPDDNGRFEILRI
HTKNMKLSPDVKLEELASNTHGFVGADLAQLCTEAALTCIREKMDVIDLEDEIIDKEVLE
SMCVTQDHFNMALGTCNPSSLRETVVEVPNVKWDDIGGLDEVKSTLREMILYPIDHPDKF
EKFGMSPSRGVLFYGPPGCGKTLLAKAVASECSANFVSIKGPELLTMWFGESEANVREVF
DKARAAAPCVLFFDELDSIGTQRGSSLGDGSGAGDRVMNQLLTEIDGVGPKKNLFFIGAT
NRPELLDEALLRPGRLDQLIYIPLPDLGARISILTAILRKCPVAENVPIDFLAQKTAGFS
GADLAELCQRAARAAIRDAIDAEEMNKKSKLELSNKKEETNENDQQKNDDDNIKYEITRH
HFKEGLAGARRSVSQADLIKYDNFRIKFDPLYKTKTGGTGDDFIIDWPDEDNNDDTPAYV
VDEDLYSI-FIISIIFILFYFILFLFYRLKKEGLISIEEAEGSNVKILSTDNLNSHNLHY
GNKCKNDSKNNES-KKLSLKRKKDSTKSENQNDTTIKIEDNNFIEK--RKRKRKRGKKKK
NM-EGEGEEEREQKNGKKRGQKKSTENVNISNISSKKKELFNIEKIIKTEEELEKDNNIK
YKIHCNNWNNGGKIFILHSVMKSLFDNAFFKPTEIQSKTLEKSINDKNDIVVISKTGTGK
TLTFCLPILNNILINKLKEYKKKFKNIQKFRCLILVPTRELALQILKHFNYINKYINLFI
STIIGGLNLNKQKRILRKKPEILICTPGRLKYFLHLENPIKYIYEMKNIRYLVCDEIDKM
IEISFMKDISYIAKHIYKSVDKKKKLIQTFLLSATLSLTIQLQNDNMTKLLNSIIIRKDK
SFIINLSNEQN-------------VYNDNSNILPELLTLYIVKLNERDIVCKLFYLIKSY
FSYDTNNKQNDEINKIIIFVNTIKSAKQLNAIFKHLFLHNNLESSIPKKYRSNLYIKNKL
NIYSIHSKQKLKERLENINKFSQQNHKAILFCTDVLSRGIDLDKCDLIIQLNCPISDITF
VHRSGRTARNFKKGNKSNIKNIIYIYIY------IYVYI---------------------
-----------------------------------------------------YIYFFFF
YHFRKVYLFYYRNMYDINKSICAEIKSAELDVLNSGELRRISMGKYENEKYEYEKTHKSG
VVTKMYYDPKYGTIDHNQICSVCFERHENCTGHIGHIEFVLPLFNPLFYKELQELLNLIC
YHCYNFCCSEDVIFLLKHIFQLKSLNQIESTNKIKYKKNCDYEYIIDQVEKLYKKICKDS
NISLDDIVEAIYDDYENVDKKNKNNNNNNNNNNNN---------VDINVD-NNNNVHIDV
DNNDNKENKPNYNKLKERIKKYKFDPNKLAFQSNYNYEEYLILSKTIKLHMKKSSKCCFC
KFQRNISAKVSQRRDTINFIGVHTNNPFFKKYMPKKKQKKNEKDDQNIDDINYNDDADNM
QHMNDDENQLNSIQSRNENNKNINDKQMDRVGSYPYNNNNNNNNKSSTFTDMVNHYNRQPP
KEKCTIRLFSFQVIDILKKIFENNNKDIINLLYPFTKRDGYKKFFLYDMGVSGNRFRSQS
KGIHVRTKITNTLCRFNNFIKLCINAKKKVDFDYLLEHNKKDLKLYKDMFSVFSLKMKYK
FNNNIMDDDTDITKMDFLKYYTLVYNNKSAYINILFSTLQLGVNTFYDSTLTEKLNNKHL
KNISIRQILDKKEGILRKNIMGKRVNNCARTVISPDTFIETNXIGMPIEFAKVLTIDEYI
TQNNFSYXXKV-------------------------------------------------
------------------------------------------------------------
------------------------------------------------------------
------------------------------------------------------------
------------------------------------------------------------
----KYKWTPYISXC---------------------------------------------
----------------------------TXLNLGIWVLVPGGKR----------------
------------------------------------------------------------
------------------------------------------------------------
------------------------------------------------------------
------------------------------------------------------------
----------------------------------------KMDYNI--------------
------------------------------------------------------------
------------------------------------------------------------
------------------------------------------------------------
------------------------------------------------------------
------------------------------------------------------------
------------------------------------------------------------
------------------------------------------------------------
------------------------------------------------------------
------------------------------------------------------------
------------------------------------------------------------
------------------------------------------------------------
------------------------------------KSEKEK-EGDEIEYGERG--ESDA
SSKIDSDH------KSDSD-HKSDSDHKSNSDHKNDSDMDSNDINMDSDSESEMVSSNKE
IININNKDSNESKLSECSNTRGTVNYFSSETSATHKNKKLNIEDDEHINNIHFIEKNKYN
EMDNKEEITKSFTKLSYDYKNNINLISSDDDQENIEQNEEGQKMKKSEQRYVKNIIDKIK
SSLLCFIKNIHFSPVTWILKLNIGWDINFFPYPIDFLNHIKSEINKEVLFKVKDLNNPKI
LKGKDITHSGAEYELQIEGKNIYKLYNIKDKYIDKTKLYCNDIYAIVKTYGIEAGRLCIT
KELKKVFDAYGIQIDFRHLSFISDFMTHTGDLKAFNRHGLGYFRNVFHKMSFECATNFLI
QGCIHNSI------------GKEKKYIINK------------------------------
----------------------------------------TVQVKLMLRTLHIFTCTCSY
CSGELCMDILKANWSPAWTIQSLCRAILFLFNEPNADSPLNCDAGNLIRSGDIKGFQSMA
RMYTVEYAMEND-----ELDPDMLGVLKCAFNGGVKVKSAAEVGKRAVEYFRGDDFVNFL
STNGEMLKKKFPNLFINRNLSDMKEIEEFADMFIQKGYIYKAQYKPIKGINEKDENGVYK
RPKWPKRLIMTSKQNFDKTSFYILVHERNKKLQYFMLISLISIVLICCMFPVWPLRLKLA
LWHISVAFLTLISIIIVGRLVAFIFFWFFGVDYWIFPNLFDEECNVVESFLPLQSWVYRN
DTWFLFVARMCTAVLLAIAIQQLGKTHSIADIRNFATQSYIDIIEWGNKKLAASPENVSM
YKSIDSKATFEGN-VDDDDTVYDENEENYDCLKKCGFTSFEELVRKCFLKCECMEVII--
------------------------------ATLADSFLKDLEDLEFEEEEEEEENNPFIK
RSNNRVDDEDNDDYEEIVDAIEEFLNEKKKNKERKISELLYDEEFLNMMNDIKTYMFEEE
QKKKEKEKDDEHVDGDEKIEGLLKERESDKFNNNSDEDNSYDDVMIEKCMETILRIDTEI
LNIHKYVKDIYSTKFPELDSIVYSPVEYISVVNKIRNEVDLKNIDFSDILPNTTVMAITV
ASSMTTGICLPDHLLKNCISFCNEGIQLNEYRNIILLYLESKMFYLAPNVTMLLGSSLTA
```

RLISAVGSLKNLSIISSQNLIVIASTKKSLFGLSNVHKTLGIGILCCSEIVQSVPDAYKK
KAISLLASKCSLAARIDYFKKYKEGQYGLLLRQYIISHLIKLQEPPPLKQKKILPMPDEK
RKRKRGGKRYRKLKEKTQITELTKQINRLPFGPETTDDFYNFNDQNTILLNSNITKLKYT
NKQKNLITKKRNLNVHSSGATGGLSSSLIFTPLQGIELYNPSLINKKNKQTENKYFSNTA
EFRKI---

---MTE
ERTVLDKKILNSSVILQITSSDEDLHTYLHSFYHCDYRTFMEKTIKIAMRVKRDRYLGRH
YRYFIRNTRVRAYKQFLEPFKNVTLKNMAFAFGVSEEFIENEISSFIANGKLNCKIDKVN
GSIESNQPNERNTMYQNTIKK-------------VYNVSVYICRYIFLILYISFIDGTID
TNKRLSKEDIEFLQGAIKDALSQVEDPYEAINEAVRNFENKDEGIILASAKIVERLVDEY
PEVARNLDKIKAIDPLLKLLDNSNNHILESVLQILSLALSNNPELQDSVFKKNALKTLLI
KLQESQKTIIDKKLITAISALIRHHDQGENKFIDYGGVGFLVYGMQTNIFKYQEKSALLL
KHLIHQNKITFDIFIKNDIMKGLVALVNNKNIDETGIQYGETTAELFLALIQNHRHKLAK
SGYLHTIKKLIEDRLSYLRIVQDSA-------------------ESLSMLICARRKKPSL
RFCKLFRRYYIRKFLKNKIKEDKKKRYKYKECNVEGTPLYNGEQINSYSDQSNNITFEEL
GVEDWLIKISKSVHILYPTKIQQLCLPLIIQGKNVIGSSETGSGKTICYCWSILQELNKN
VYGIFSLILLPTRELVFQIIEQFLLYGSKIGVTILSCIGGFSLIEQRKSVMTKPHIIVGT
PGRISDILESSIDIQNCFKRLRFLVLDEADLLLQKCFEDKLQNILNNLPKNYTNERKTLF
FSSTITNSLQLLIDTFPYNNLILVNVNKKQKPPKNLDQRYIYVEEIAHITYLIYILKNKV
NNLSGIIFTANSYKCELIYTVLNMLGIDNVDAMHSSKDQKNRFATLAKFKNGLCKILVAT
DIISRGIDIPKISFVINFDFPNDIVQYIHRVGRTARANRKGLAISFIDKKDVNSFNQVKN
IMKDKLKPYTLNKKEVLENMFKIGRVIKKAEILLEEKKDIKRENERLKHYIFHNEYTSTY
IYIYMFI------FVKYLFVCF-------YSLDVVVFPVSVHYEHTRKLLQSKFSTGIQN
VSKFGNGSYTGEVSAEIAKDLNIEYVIIGHFERRKYFHETDEDVREKLQASLKNNLKAVV
CFGESLEQREQNKTIEVITKQVKAFVDLIDNFDNVILAYEPLWAIGTGKTATPEQAQLVH
KEIRKIVKDTCGEKQANQIRILYGGSVNTENCSSLIQQEDIDGFLVGNASLKESFVDIIK
SAMMKKKNKEDLYKENKLEASKLRIAIVSSDKCKPKKCHLECKKNCPIVKTGKFCIEVDH
ASKIAYISETLCIGCGICVKKCPFTSISIINLPKDINKDVVHRYGPNTFKLHRLPIPKLG
QILGLVGTNGIGKSTALKILSSKLKPNLGKFNNPPEWRDILSFFRGNELQIFFTKLLEEK
LSPIIKPQNVDLIPKQIKGNILEIINKKDKFNQKDKYIAELDLEHLLDRNVEDLSGGELQ
RFALLMSIIGQSTNVYMFDEPSSYLDIKQRISMAKIIHKLVKHDNYIIVVEHDLSILDYL
SDYVCCLWGKAGAYGVVTCPFSVREGINIFLDGFVPTDNLRIREESLNFKLATDQDATDE
DKKRLHFYNYPTMVKTLNSFSLTIDKGHFSESEIFVLLGQNGSGKSTFIRLFAGLIKPHN
LESLSFLESLSVSYKPQQIQAKFTGTVRQLLMSKLKGLYNDPYFNNEIIKPLKIESILDN
QVLTLSGGELQKVAIIVTLAKNTNIYLIDEPSAYLDSEQRIIVSKIIKRFILNTNKTAFI
VEHDFIMATYLADHVIVFDGQAGVNTVANTPQTLAAGMNKFLKIIDVTFRRDPTNYRPRI
NKYDSVKDKEQKLNGTYIYIYIMSIICTISGQTPEEPVISKTGYIFEKRLIEKHIINYGI
CPVSGEVLTLEDLYPIKNEKIVKPRPITASSIPGLLSIFQTEWDSIISEMFSLRTHVNDI
RNELSHSLYQYDAATRVIAKLLKEKNSYKEEIENLKKQIFELKSSNDLDIYEIGLNEELL
EKMQNVAKDLLINRKKRKIDNVCSVEQWKDFKNTNEFNIHSSTIPGVTCITLDVNKYKYN
YYDDHMKHNFFSGGNDGNVYYVSLNDNKILSKLQGHLKKVNSIISHPSNFICITASNDKT
IRIWKGDDDNYQFVSAHVITKHKDHVTSLALHPLENYFISSSKDSMWILHDLETAKTIKT
SKDNPSSFKHLAIHPDGMMFGIAAQDSNIHIYDIKSQEYKATLNGHTKSLNCLSFSENGY
YLASSSKDNTVKLWDLRKAQSFQTITLNETPTFYFF------------------------
--MTEVTHCYNGNIKDGLFHGF
GILIYSQHEKYEGDFVYGKREGRGKFTYADGATYEGEWVDDKIHGKGIANFVSGNIYEGE
WENGKINGFGILCYNNGDKYEGEWLDGKMHGRGTYTYEDGDVYIGEWKNDKRHGKGCVKY
KGNENKIAETYEGDWVDGKMQGRGTYFFADGGIYEGDWVDGKMEGKGVYKYLNGNKYEGE
WINDMKNGYGTLTYVNGELYEGYWKNDKVHGKGTLTYSKGDKYIGEWKYAKKCGEGELIY
ASGDKFKGQWKNDKANGYGILLYNNGNKYEGEWLDDHRHGMGTFTCKEDGTIYSGLFQFN
RKHGKGTLTFVNGHILQGIWNSGLLEKVINYELTPSSPWNDPDMMKLEEVKDIQKIERIG
AHSHIRGLGLNDCLDARYCSEGMIGQMSARKAAGIVLRMIKEGRISGRAILLAGQPGTGK
TAIAMGIAKALGEDTPFTHISGSEVYSLEMSKTEALTQAFRRSIGVRVKEESEVIEGEVV
EIEIEKFNERDINNKNKKLGKMILKTTEMETLYDLGSKMIEALQKENITAGDVICIDKGT
GKITKIGKSFARSKDYDAMDPNTLFVQCPEGELQKRKEVVHTVTLHDIDAINSRTQGFLA
LFSGDTGEIKNEIREHIDMKINEWQEDEKAEIVPGVLFIDEVHMLDIECFSYLNRALESE
QSPIVIMATNRGITHIRGTDYKAPHGIPLDLLDRTLIIPTYPYKHQDILKILEQRAEEED
VDIDEYAKELLCKIASESSLRYALHLITLANLVSKKRKATEVTVQDVRRVYNLFIDVKRS
TQYLIEYQNEFMFSELPKEEPSIKEEDSSEEKRELHEKNSENSSTSNMAYIYSNLSDFWT
SDDDEGDEGSNEEGEEKSASNDKE------NNLKNETYGYNEYNIYKRED--MMYNDDNS
FNKTIDQRLNFNINDIMNECSNDISENFTINNLLEKYDINNFIFNDNLLKYCETGVAIND
EKPNNEKLTLNEDFEFNVKCSFNYVNPPSYGLKINRDVKNEVPQENIKSNNLEMVIHNKE
EMLVNKKVLDDNKNDDDDDNNNKNDDDNNNKNDDD----NEQQILHNHIENTKIKYIRKK
WVIEDNESDISNFNKNDLLLNYDFELDDFQKRSIKHLNNFKHVFVAAHTSAGKTLIAEHA
IALSIKLQKKAIYTSPIKALSNQKYYEFKNIFKDVGIITGDVKMNVNANCIIMTTEILRN
LLYLNDNIINNIHCVIFDEVHYVNDEDRGVIWEESIIMLPHHVQILLLSATVPNYLEFAD
WVGFTKQKEVISISTKKRPVPLLHYIYAYDSVYLVMDEKNKFYSSAFKEIYVKIREKQEA
NNKNTKQITSSSNLKKNNNYYDSKNKYLTTINNKENDTTQNNNNNNNNNNVKGYYEYCKQ
KRKQKLFANEASMKTEIQKLQTLIKKLDQDNKLPVVLFCFSRIKCETYAKCMPHLNFLDT
NKKSKVHLFIKESISKLPKQDRELNQIQSLSKLLEKGIGVHHSGLLPILKEIVEILFSKG
LIKVLFATETFAMGINMPTKSVVFTSIYKHDHLRKRILTSSEYTQMSGRAGRRSSDKYGY
VYICCCDNIPDQVQLTEMMMQKAVSLKSKFKVTYNMILKLLINKQINIEKMLFSSFLESC
RALQIPLFKKDLKRKRKLLQNIKEVQCIYEQNKNAYPPIEQYVQINYRLKYIGLNLHKKL
LNTKSSNCFVIGRVMLLNNIHILHSSVYAIYLGCDKSNNKKNDKVDFAQNSIFFQNNYED
DRSNERFFFLFILPDFMTFENLPDYILTSKKKKTTSNNNNNDNINNNNNSLSNSVSENIN
LYENYKNVFSKKNNKSDIKIIYHSSFDTDMNKKHFVVCSNVCIENISIITNTVIKLPNVN
NAGILNNPKNLLLYTFELDRLIEKNNFEPFVLTKMLKSLKCEFYSVLVNQADYLENLKKS
KCYNCNLKEKHYQLICKKNDCLDDIENIERNINAKSLNLYEDLEGKLNVLKHFGFIDDQN
NLTVKGKIASYITLTDEITLTQVIFENVLNKLNPAEIAAVLSCFVAPEKKVEESPDLTVN
LQEVKAALTNIHSSFEEFYKVIRLRISSEDHWKLCNFKIMFIAYKWTLGVSFAELLEQCE
LEEGLIVRSILRLDDLCRKVKIAFLYLGNIDLAQKVEKTSHLLRRDIIFTTSLYLQ----

--LDGLALLQFFHWCEQKRK
TKELFNETEMSLRHKVDYFRSTKKNFIFPSFSTISASGPNAAVIHYECTDKTNATIKPAI
YLLDSGGQYLHGTTDVTRTTHFGEPTAEEKRIYTLVLKGHLRLRKVIFASYTNSSALDFI
ARENLFNNFMDYNHGTGHGVGLTLNVHEGGCSIGPVGGAPLKKNMVLSNEPGYYMKDKFG
VRIENMQYVISKEITDTTEYLSFDDLTMYPYEKKLLDFSLLTNQEIKELNEYHTTIRNTL
LPLVKQSPQEYGESVEKYLIEITEPIAIHMIFFLLSKICKYNAILKSLKKKNNKIVLNII
TFLKLFISKNNYIYNTKMFSSNSNTNDVEVMKKKLVVGENPQFIKKRLEKFNEIKEKRKQ
ELEENFEQLRKPITIELLDGSIKSGESYVTTPFDIALSISKRLAEDSIVCKVTYLEKVDV
ELCDIEEGDDHNEQTNENSKESILWDLNVPLLGNCKLEFINIQSEEGQKIFWHSSAHILG
SSLEKLFGGFLTIGPALKEGFYYDIFLNNFSINNEDYKRIEDEFNKLVKENVPFEKVICT
KEEALELFEYNPFKLELIRSKIPDNKKTSVYKCGNFIDLCLGPHIKNTGKVKTFKVLKNS
SAYWLGQKENDSLQRVYGISFQKKSELVEYLKFLEEAKKRDHRNVGKILNLFFFEKETSP
GSCFWLPHGSKIYNKLIEFIRKEYRIRKYEEVISPNVFSCDLWKTSGHYQNYKDCMFLFN
VENKEWGMKPMNCPGHCLMFKQLNVSYRSLPVRLADFGVLHRNEISGSLSGLTRVRRFQQ
DDSHIFCSMEHIKQEVLNTLNFLFYVYNLFGFKYELFLSTRPKKFIGQISTWNLAEQHLK
DALDAANIQWKINEGDGAFYGPKIDILVKDSLNRTHQCGTIQLDFQLPVRFNLQYKNKEY

VNNNEKNDNDDNIHNNNNNEKKEFKDPQNHNEDKHDNNSPINEEALLKKGFERPIIIHRA
ILGSVERFVAILIEHTAGKLPFWLSPRQAIVLPISDKYNDYANYVYETLHNNMFDVDVDT
SVNTLNKKIREAQLKQFNYILVVGEKELTTNTVTLRDRDDQNNQHVHTIQELIDKFNKLL
DVNSRKFNQIKEFNSNNNISSHKGTYNNISHKNTYNNLYYAKLYIFFMAICLALNKFLNT
QEENKEN-KKKKKKIMNFFGLSNETASCCGIMAYIGNRDASKILIDGIEILQNRGYDSCG
MSTISNKNVLKTTKYASNTTCDAIEKLKSNYLNSHKNDHIGIAHTRWATHGCKTDENAHP
HVDYGERISIVHNGIIENYREIKTFLLKNNIPFKSNTDTEVVANLIGYFLDKKQSFQDAV
LSAITQLEGTWSFCIIHKNHPDEMILASNGSPLHIGFKDDEIFIASEHTALFMFTNEYIS
LKNGEILSISKDKINDLKLLKKVENIPEIAIQKTPHPYPHWTIKEIHEQSATLSKSLNNG
GRFSSGDHLVKLGGLDPYIQDLNKIENLVLVGCGTSYYAALFAKYLMNYLNCFNTVQVMD
PIDFNISVIPKEKEGVIFISQSGETRDVIKACKLAEDLNVRKLSVVNSVGSTIANMTGRG
VYLNAGREVGVASTKCFTSEVSVLTLIALWFFQHKKNNQSSNKATSLINSLHRLPLYAGV
TIKSCENTCKTLSEKFKNTKSMLIIGNGLSYPIAQEGALKIKELAYIHCEGFTGASLKHG
PYALLGGEDNIPVIMLLFNDNTKNAMINTGEQIKSRGAHIVCLTDDENLVKHFADDIILI
PNNGILTPLLAVIPLQMLAYYTSVNKGINPDKPRCLAKTVTVSKEI-HKWIHIYIYI---
-----YIYIFFF-------YI---------------------------------------
------------------------YIFLFLRYASEDVQKILIGNKIDLKNDRNVSYEEGK
ELADSCNIQFLETSAKIAHNVEQAFKTMAYEIKNKSQHETINKGKTNINLNARPIKDTKK
KCCMPRIRTMNSRKPPEGWHKVESFLEEMNKKMRSLENEDTSKKRKNEILWPIFQINHKT
SRYIYELYYKRKEISSK---
------------------------------MNNVISFIGNSSNKYFQINQVHFIRIIIHS
KNNLINSNSSYNIFYNKYFIENTFQSKNKLSSIYSKLNFSIKNMCKDKNEKKNYEHVNAN
KNGYLASEKNELTKNKVEEHTYDYDYVVIGGGPGGMASAKEAAAHGARVLLFDYVKPSSQ
GTKWGIGGTCVNVGCVPKKLMHYAGHMGSIFKLDSKAYGWKFDNLKHDWKKLVTTVQSHI
RSLNFSYMTGLRSSKVKYINGLAKLKGKNTVSYYLKGDLSKEETVTGKYILIATGCRPHI
PDDVEGAKELSITSDDIFSLKKDPGKTLVVGASYVALECSGFLNSLGYDVTVAVRSIVLR
GFDQQCAVKVKLYMEEQGVMFKNGILPKKLTKMDDKILVEFSDKTSELYDTVLYAIGRKG
DIDGLNLESLNMNVNKSNNKIIADHLSCTNIPSIFAVGDVAENVPELAPVAIKAGEILAR
RLFKDSDEIMDYSYIPTSIYTPIEYGACGYSEEKAYELYGKSNVEVFLQEFNNLEISAVH
RQKHIRAQKDEYDLDVSSTCLAKLVCLKNEDNRVIGFHYVGPNAGEVTQGMALALRLKVK
KKDFDNCIGIHPTDAESFMNLFVTISSGLSYAAKGGCGGGKCG-----------------
-------------------YFFILMT----------------------------------
-------------------YFISHFKHLFLY-----------------------------
---KISYIFVIEENHISIWNGGKTDKYYWNQALKEINLEMPLNEEIKPNDVNVQITNTNI
KIYHCGVLKLEGMFYEEVDKQECVWSIEDKKKIIIYLEKKRENWWPCVIKGDTEIDTKNI
ESKKNLTDFDEKTQGQIRKFLLEQRM------------------------------MKQA
NTKEDKSQKISNSSTIDEIEEQLYTGPLKIEQLLAKGFVKRDLELLKEGGLQTVECVAYA
PMRTLCAIKGISEQKAEKLKKACKELCNSGFCNAIDYHDARQNLIKFTTGSKQLDALLKG
GIETGGITELFGEFRTGKSQLCHTLAITCQLPIEQSGGEGKCLWIDTEGTFRPERIVAIA
KRYGLHPTDCLNNIAYAKAYNCDHQTELLIDASAMMADARFALLIVDSATALYRSEYIGR
GELANRQSHLCRFLRGLQRIADIYGVAVIITNQVVAKVDAMSMFGGHEKIPIGGNIIAHA
SQTRLYLRKGRGESRICKIYDSPVLPEGEAVFAITEGGIADYEEK---------------

------------------VAVPNMNNNNNNNTMNPTYPSLPKFPNYNLNSQVQQNSTPEK
QTSPMFSSNSYGNINKTQTNAVPPPPPPMPSNQLNN-SRSSFADIQHVVPPPRNKNQSIS
STANLNYQHDNQFNKRECMEQPVYPMTNQSSMFSNNTMQKKNVPGGFTSQMNYGMQPTGS
PPPSSTKLIS--
----------------IYVYIFK-------------------------------------
---MAIRVQFENSNEVGVFSRL
TNSYALVALGGSENFSSVFESELSQHIPLVYTTIGGTRVIGRVCVGNRKGLLVSSICTDQ
ELLHLRNCLPENVKIKRIEERLSALGNCITTNDYVGLIHTDIDKETEEIIQDVLDIEVFR
TSIAGNLLVGTYSYFTNNGGLLHAMTSSQEIEELSELLQIPLITGTINRGSDLIGSGLVA
NDWSAFCGMDTTAIELSIIEKVFKLNNITDNNVEDTFKYKSSIIQTMI------------


```
------------------------------------------------------------
------------------------------------------------------------
------------------------------------------------------------
------------------------------------------------------------
------------------------------------------------------------
------------------------------------------------------------
------------------------------------------------------------
------------------------------------------------------------
------------------------------------------------------------
------------------------------------------------------------
------------------------------------------------------------
------------------------------------------------------------
------------------------------------------------------------
------------------------------------------------------------
------------------------------------------------------------
------------------------------------------------------------
------------------------------------------------------------
-----------------------------------------------------------Y
TETNVIGKNFFYTFCLNSRKLXPXQFXIYISRIITVVPQFIISNKLNFPLLIKQYQNDQI
QGVRANDTSPLYFTKKSSILLFQFKCLDPCRTNNEKNHMLLDPKNVIKSNG--------N
KYYKLNKNIYWSSVVYPSENFVGTNYMVINTGNHEKSDVYAITCIPDRGTKNIIIEKLEN
KK-KGFIAYNNSSIAKYLKIRTFHDDTRHMKIHDEENYIEKNMFLSNFLKPTDMEHYFNI
EPNKYSYLGWVNPFIYVTRNVQIEIVLEDLKIIPKSPFVLKFAIYNYSQKTYYINYYNIT
FVICIEYIEDLITIKLSHKLNISSNSSHNIMLSKHMNSCDKSVTSNNDIKLGLNKKKKHM
EMENQVNIEKYNIINDISNERNIIDKNSENSLYAQKQIIDHDEYHYVKYIDEKGSYKNNS
ISSSNNYY--KNESVKVISNTYKNVHIIINVTQIGVSIISNILKEEVFFIELSKLCALFY
MKNEEEVIDIKITDVQIDCQLESCEKCVLLANRGISSSNKNMNANEEKIFLNIYVERSFI
SHNDVIFKKIQVSLDDVEIEMDAETLNGINLLTAEYIESISIVQKKNLLYEEIQKWTILP
VYVNYKSPEIPLAINIQYMQIDKFTLIVWCSFLLDKMHMMSDLLRIGLRILMVSGKLELL
GAPVTLNQEIFNNIRVSIKSFYALLKDKYSHSILACLGFIVGYSSLINIPKIPLEIGRNT
IGLAVYAVDNVSVGIGSFLSNLTFDSEYINRRQKERTFKTNTNMKEGLISAVKNIGEGVL
SLSNIVTKPIEGAQKEGFGGFFKGIGKGVAGSLVKPLDKVGQAVSDVTRGIKAEVSKPIG
GHKYKTKRHRKPRMLWGEYGKLKEYNINEAELRECLGLKFSKNIMKCLTVHKQENPPSHY
ALLLYPKVIIYANLYANTSEKKTDIVIWSIKIEDITEIRASSHGLIVRTNTSTYKIPCNN
AILINKIYRELHNSKNSINSTVILGANNSSKYL---------------------------
------------------------------------------------------------
------------------------------------------------------------
------------------------------------------------------------
------------------------------------------------------------
------------------------------------------------------------
------------------------------------------------------------
------------------------------------------------------------
--------YA------------ND------------------------------------
------------------------------------------------------------
-------------IVDTYWQTETGGIVIAPIPHLFSMKPGCASLPFLGVQLEILDSKNLQ
PLSGNNVCGLLCIKSPWPGMLRTVYGNHQRLIKTYFTMCPNYYFTGDGAFRDEDGYYWIS
GRIDDTLNVAGHRLGAAEIEHALVQHFYIAEAAVVSFHHNVKGEGILCFVVKKKGDIKNY
GKGNTNTNELHNNNPFLICSNNITDIEEFKKNFTDEKLIEQLKLYVRQVIGPIATPDLIC
VVPDLPKTRSGKIIRRILRCIANGITDLGDISTVSNYEVIETINNILIECKKKLKIEIMF
MRWNKISRYTLLMVVSFWLFYKSENFNLLRRLISKLRSLFPLFIKNNFLNNEIKNSVKIY
FGSQSGTAEEFAKELKANLNDLFHIQANIIDLEYFNKEEIKSFGIRIFIVATYGDGEPTD
NAVEFFKWLKSLNNDNDYFRNTKYSIMGLGSKQYKHFNKIAKKLDTFLLNFKAHQISETI
YGDDDDNIYHDFEVWKNKFFMQLPKLLNMKNIPLYVPKEDIIEFTSWRDMSEIKLDIQYY
DHLEEDNKKEKHVVTENIINESVTNNQQLLNHKSTDIIGKFYFNHLTGKVISNKKLLKNV
DLSNNGDKVNHINISIENIIYKAADNLSILTKNTKEVITWWLKRLNIDEKEKTKKFTFVN
RNTMNDPKNDNNNNNYNEYDDNHIYVPFPTPCSVEDALSYYCDLTTIPRLNILKKFKCFI
KDIEELKMFNYILSNNKRNTFFNICKECDMTFIEFVDMFMQSAVFELSPFLQLIPRNTPK
SYTISSSPKESKDILSLTVKKKQYCIHSLRRALKNLKTNDMFPKLNEQKLRELCSRRWFK
GSSSYYLTEELNVNDIVKFNIKPSKFVLPENIQSSHIIMIATGAGIAPFKAFLSEFIYYD
QQIVKDNFVRKGKRILFYGCRKREVDFLYEMEIMDALDKKHIDETYFAFSRDQESKIYVQ
DLILQKKELVWNLLQKGAYIYVCGNSNMSKDVNKTINSLPLHFKQNDKKFTKKLKKSGRY
IYEIWMENIPWVEKYRPKRLDDIVHQNNAVMMLKEVVRTKNMPHLIFHGPPGTGKTSAIN
ALAHELFGKENISERVLELNASDDRGINVVREKIKAYTRISISKNKIHSETKEVLPSWKL
VVLDEADMMTEDAQSALRRIIEIYSNVTRFILICNYIHKISDPIFSRCSCYRFQSIPINI
KKEKLLYICQNENIDIVDDALEKIIETTEGDLRRAVSILQLCSCINTKITLNSVLDVSGL
PSDNIVYKIIDACKMKDLKLVEKTVQDIIEDGFDVAYIFKSFNNYFVTNTEYEDSLKYQI
LLELSRHDYRLHCGATQYIQLLSFASSVHSLLNSV-------------------------
------------------------------------------------------------
------------------------------------------------------------
------------------------------------------------------------
------------------------------------------------------------
------------------------------------------------------------
------------------------------------------------------------
------------------------------------------------------------
------------------------------------------------------------
------------------------------------------------------------
------------------------------------ALTIHF-----------------D
ITQNQMYIRELIFKSPNEKHFQMVVKQVKELIKQVKQKEVEADVNESKTSQDRLVLNKSG
RRIVLRDLMTRPNIFTGRKILGTLELHMNGLRYAANSRGTTEFIDILFDDIKHAFYQPCD
GQLIILIHFHLKRYIMVGKKKTLDVQFYCEAGTQIDDLDRAKARNVYDPDEMHDEMKERE
QKNKLNLIFKNFVQQMQDISKIEFEIPYPELTFSGVPNKSNVEIFVTANTINHLVEWPPF
ILSVEDIEIASLERVHHGLRNFDMIFVFKDYTKPVKRIDVIPTEYIDTIKKWLTTIDIVY
YEGKNNLQWGNILKTILSDIDSFVNSKGFDGFLGEDDDEEEETADDEDEDDEYEVDESEL
VNI---------------------------------------------------------
-----NN-----------------------------------------------------
------------------------------------------------------------
------------------------------------------------------------
------------------------------------------------------------
------------------------------------------------------------
------------------------------------------------------------
------------------------------------------------------------
------------------------------------------------------------
------------------------------------------------------------
------------------------------------------------------------
------------------------------------------------------------
------------------------------------------------------------
--------------------------------YEIDTLATIEFTSKRKMSSVICRIPVIN
PDYNYPRDVKSSNMDKNKNSMDGKNEEKEETDDLNVKKEAHSKLNNNNNNNISIDNLYDD
KNNHNEGRTPEVITCKNSKNSKIMLFCKGAGSIILKKLAKRTDVDEITIEHMETYADEGL
RTLCIAQRELSEESFAEWYHLYKEASLSIKDREEKLESVAEYIENDLILQGITGIEDKLQ
EGVSSTIEDLRMAGIHVWMLTGDKIETAMNIGIAANLIDNYSEQFIYTEEYIESEEALIK
KIDDDILMVEKSLNIPHYNFDDENNNVRNNFIKNFFCAKNKSGLLLNPDKYNMLINTLNY
VLVVDGSVIDILLSEKMERKFFYLADKCSSVICGRVSPYQKGAIVSSANRLLNKITLAIG
DGANDRNMINTANIGVGIRGQEGVQAFNSSDYGISQFRFLKNLLLVHGRLSYRRISKLVV
YMFYKNMVLIFPLFIFGSISLYSGQKIYFEFLLHLFNVLFTAIPVVIHAVLDQDISLNTA
MEKPNLYKLGIHHYYFNIRTFISWVMNSLFHGSVVFLIPLYFLSYYNIPTSDGIPYDIWT
```

VGCATYFLTVLIVNFKILFETYYLNILPISGIALSIFSFVLLVTAFSFMCVGSIHLLGTI
VYLVQSLRFWLVVILGLFTALLRDYVFKVYKRNFNPEIYHLLLDQVIKKIK-NYLI----
-----------KSFNIIYSFS-----------------MKSKILCNLFKGKSCICLYTNC
LNENNVNKCYYNLVRNYSDHVKDHFNKPRNVGSFDKNEKNIGTSIVGKASCGDVIKLQLK
IENDVIKDARFMAFGCGSAIASSSYATELIKGKTIDEALKIKNNDIASHLSLPPVKIHCS
LLAEDAIKHAIKNYREKILTYMLVKFRKCGQANIFRSISNVRKKNNLKNNKDI-ERKEKI
IILGSGWGGFNFLLNIDFKKYDVTLISPRNYFTFTPLLPCLCSGTLSVNVCTESIRNFLR
KKNYSGNYLQLECTDVFYEDKYINCIDIENNKVKLFYDYLIIAVGAKTNTFNINGVDKYA
YFVKDIDDALKIRKKFLDILEKCTLPNISNEEKKKMLHVAVVGGGPTGVEVTAEFADFIN
KEVKINYKEIFNFISISIIEGGNNLLPTFTQNISDFTKKNFHNLNINVLTNYYVIDVDKH
SFYIQSSLNKNEK-KKLSYGLLIWASGLAQTTLIQKFLKTIPVQANNAILKVDEKLRVIG
IPSNNIYAIGDCKKIQPKLLHEHTNEIIKILTGNKLTSEALKLKQIELTKTFPQLSVSKW
DYEKNKKGEMTPQQFHDYLFEIDKNYKSPTPTAQNAKQEAYYLSNVFNNFIHTNKKFNIP
SFIEKWKGSLAYIGNHQVVADLPYYELKGGRFSSTFWKVVYIQLLLSWKSRFHFFIDFIK
TKWYGRPFIKIYIYIYIYQVKNKNAAEVQITAEQLINEALELEEVEHKVNYNLIDEEELN
EYKINKRKEYEDKIRKRRYMISTYIKYALWEIKQKDIERCRSIFERALNIDYTNKNLWLK
YIEVELINKNINSARNLLERVVLLLPLENIFWKKYAHLEEILNNYINARNIYERWIKFKI
DESSFLCYIYFEERCNEINKCREIFERLIVSIPKLECFYKFIKFEKKYKNIDRARAGYEK
CIELLPSSYLDENFYIHFCNFEQEQNEYERCKKIYIEALKILPKSKSELLYKHFLQFQKK
YANKDELNESLLIKERIFYEDELKKHRNDYDIWFNYIKLEESNINNINKEKCIIRIRDLY
ERAISIIPIISSKKFWKRYIYLWINYSIFEELYAENIQRARDVYINIIKILSTYEFTFKK
IFILYATFELRQLNVHKARSIFNNALQTIPNEKIFEKFCEFELKLGNIRECRNVYAKYVE
AFPFNSK---
VRHSYDQ---
----------------------KKKNKKKK--------------------------NMKI
LEKALEWKKKMEDMKKEVNDDNV---------------------------VIKNAHVHDG
LKIGIREVIKSIESQEAKVCFLSDVCSEPAYKKLITTLCAEKNIPLFMVQNDSKDLGHWA
GLFKLDNEGNARKIIGASSVAVVDFGEDSAEKDFLLSQNQTVTA-M--------------

--FL------------------

---KIY--LY
ITICLCKYNIYIYI--
-----YIYIYMY-IF--------------LFFLKAMVGEVIDKRYSVVCELVGKGVFSNV
LKCYDMVNKIPVAVKVIRDNDMMKKAAEKEISILKKLNQYDKDNKRHIIRLLSSIKYKNH
LCLVFEWMWGNLRIALKKH---
--------KLCCLSS-----------------------------------W---------
---------------------------HKMVIYIYIY----------IYI----------
---------------------FYFLGNSPKMQFLKKKIKQLGDLLEKCLILDPSKRYTPD
QALQHPYLRESIHFSKSQNE--

-----ENCKNLLSQIKNEQIQSYFNDFISKTEELEDVYISRRKFQSKYLTQIKNLYQQ--
-----------------------------------MEDDVIISGTHMTIEKDKFEKMNEN
NFEKIYDNEYENIYRSKCSGILLKTPYYPYKYDIMNTSYKYIPKVGDLVIGIVKSKKLDY
YQMDINCNCECIIHKIDSFKYATKSSFPNLLNGTLLYMVVEKINLENNSVVTSCINSSDV
KSWINYENYLGELVDGFVFSVNIASAKSLIGDRCYILDLIGQDIKYEIAVGHNGRF----
----EIYKIKNILIYIYICEKYNKNICICAIYSLILYIF---------------------

-----------------------RLH----------------------------------

ILKY-------GALGYSQHLSEEIMLFSRDAILDKIAVILGGRAAEELFIGKITTGAIDD
LNKVTQLAYSYVSQYGMNQEIGLVSFQPNSNSEYNLYRPHSECLAHLIDNEVRSLIETQY
KRVKSILMKNEKHVHNLANLLYEKETISYHDIVKCVGERPYPVKSAYEKFVKANPYKAIS
SEPLLEDKKETKDFEKGHIKENTKECKKENTKECTKENT-------NSSKDDTKQTSCKE
KLGDSQKKDNKMSNVKLRF---
-YFY---FYFDLFVFGLKHQRYKGSDKIKIEEERNKKLFLNNNKG-QDFCKNCGSVAHKE
KDCLERTRKKKVNYMKND-NEDFICVTQDLGYDGNRDRWVGYNVNNFDYIYKEYEKIVEE
KKKRKAEDLKKQYEKKSVKKKKTHNNDDDDDKSSSESENLDESSNEDNKLKPDVMNNQTI
NKNEKNRNIARNLRIREDTAKYLYNLSLNSAFYDPKSRSMREDPFANIRKHLDDDNYYKG
ENYYNNTDDAIESKKLEVFAWESYKRGENVHFNAQPTQLELMYKEYLEKKKKIIKKKQED
ILKTYKCQNNEEQQPNQEELLQSEVYTEYKPIEQIHNMKKYIKVPSKYEEDIYLFDHSSV
FGSYYDKHTKKWGYKCCSSTNKYDKCFQT-------------------------------

-----IIPLMDSYHANYYTKEVMIILINEFNSPDDEMKKIVLKCVKQCIQTEGVDKEYIN
EEIVNPFFEKFWVMRNSNDKKSFTLIVDTTVEIAKKIGAYSVISRIVDDLKDPSEQYRKM
VMQTIQNVVNELGVDDIDQKLEEQLIDGMLYAFQEQTSEDYYILLNSFDIICNQLNIRMK
PYLPQIAGILRWRLNTPLPKVRQQSADLISRITKLIKICDEKQMLGHLSLYLYEYLGEEY
PEVLANIIRALKSILLVLGVQNMTPPIKDLLPRITPILKNRHEKVQENVIDLIGIIADKG
GDLVSPKEWDRICFDLIELLKSNKKLIRRATIQTFGYIARTIGPFEVLTVLLNNLKVQER
QLRVCTTVAIAIVADTCLPYSVLAALMNEYKTQDMNVQNGVLKALSFMFEYIGEIAKDYV
YSVVTLLEHALMDRDLVHRQIATWACKHLALGCFGLNRQDALIHLLNYVWPNIFETSPHL
IQAVIDSIDGFRVALGPAIIFQYLVQGIFHPSRKVREIYWKIYNNVYIGHQDSLVPIYPP
FELLNDSTFVRDELRYTI--
--------------KRMSI---


```
------------------------------------------------------------
------------------------------------------------------------
-------------------------------------------------KLLKEIESEEN
SNIGNINSDNSNEENKKERCVSFVNYDKRSNDNISRPFRSRKGTGYVKMDMNKLLKEIEE
DETKDINFKRKN-IDSDVV----------KRTVSFSSEEEHICIEQPASLYMPRPVRTRK
ATGFVKMDMKKLLDEIGVDDMYEEKNEDQNVQ-KKVAF-ESYKNNDVIKKSVSFSSEEEY
IPVDEPVSLKVTRPVRSRKATGFVKMDVSKLLQQIDSDERNEE-----MKNDDKKE--KR
VPFKNKDISSELEKKSTTYTSEDDYNISSKNSESSPMVRPVRTRKPTGFVKMDVSKLLRE
VEYEEEGDDGTGRSNDIIKKTRTRKATGFVKVDMSKLLKEVEQEEEEEENDNDNDDDNDD
DDDIKGEEENKNNDDDNDKDFKDEDEEKSEEKSEEKSEESEEKSEDDDDDEEEENTSEGY
SGEKKNTNYDNHNVNDNKNNMKRNKENDKLKKNVSFEYNENENKNKNKN-----------
------------------------------------------------------------
---------------------------------MCL------------------------
------------------------------------------------MGRMYGKGKGIS
SSTLPYKRKQPSWLKQKPSEIEDAIIKLAKKGQTPSQIGATLRDNYGIPQVKSVTGNKIL
RILRAQGIATTIPEDLYFLIKKAVSMRKHLEKNKKDKDCKFRLILTESKIHRISRYYKRK
KLLPSNWKYQSSTASALIA-----------------------------------------
------------------------------------------------------------
----------------------------------------ASE-----------------
------------------------------------------------------------
------------------------------------------------------------
------------------------------YXT---------------------------
-----------------------------RHIEHAPGVQFSYVPPDFFNSDIDDESDKNQ
YELKDDSGGGRAPGTRAKEHSTTHHLRRKNYDDDFFDLSDRDQSIVYFMNDNESLDSEVL
EKQYEIIKYAKYQDFIRLQILIQPYLLNNDIETLNSINILHWACYCGFTELVKKLISFNC
DIEKEDLVNNDTPIYYAIKNSNYEIVLLLIKCFGISILFHKNRRRMSPFLTAICEFNEDK
ILEALHILELLYMNGVSLEEQNEHGQTALFLSVKKNNISTLQWLLTKEVNINHRDFYGNT
VLHIAVRHCDIDILRLLCDYGCLNMVYYSSIENKNTNVFQLCIKNRYFLVYILLKKWVLQ
NKICSKLKICKTIYAFYFWFFAILNLIVYFNIAHSFSIINKYHFKSLIWITIWLFQQFLW
CMLYFKSPGFYKENYMFNKNKKNNSNPTYNGTFKTNAEYQLNNIEREIFQINKKLISTNF
NTLTPINHDQALSNKYNDILINLEYQKLSLYSQVSQERINSLDEDYRNAILYNQNPR---
---------------------VKSNHKYLYIST--------------------VLHIYIY
IYIYF-------------------------------------------------------
----------CFSFSRIFV-----------------------------------------
------------------------------------------------------------
------------------------------------------------------------
--------------------------------------------------KLKCYQKCGV
LWLYVLYKQNKNGILADEMGLGKTAQTWVFLDYMDKTGTIKNKTIIVAPTSLLKNWDNEI
NMWCPYLRNHKIIYYGSQSERRYLAYDIFSSKGNNNIHLIITSVNMLMGKNDVSYFRQIK
KYDYLIFDEAHFLKNKNSLIYKKLQKKIVFNNKILLTGSPIQNKTQELTNLLLFLMPHIF
TETNINNAMQAFIAMYEEEVQARNKKKKMDEGTTPKLKTFVEEIMKKNETQNNNNNNNN-
-----------NNNNSNSNNNNNSSSNNIIVDKDNNVIVSDTNKNIIKNYLIQTIRDDLK
HVELKNKEIILLQLIIEPYILRRSKKHVFIDMPKKHSLIIKLPLNNTQLNLYKDEIFSKM
QKTFKHLEFLETHSSKKELQKIYSILNKKEIKEKKEIQQNEDKEKYNGHCNNNTNDMEFA
DHNVDGHDDDDDDREMDEETINIEKTVENKNSNIVIHKRDDEDNIKNSNSHNNNNNNNKEV
RGKMINASIFILRRICNHPLLHKYYYTVEDIKKISKYFYYNTDQYVDLDLKTVENEFMKI
SDFDIHLSIKHLISQGDNKLNKYLITKEHILNSSKINHMLSLIKDIRKKKEKVLIFSQFT
TFLDIIEEALLYEFIYDDSDYMDHIQTIKSSQSINKNDETKRHDDNNNNNINNTKDTNCE
YNKDDEEICLTSSTLSTSSVGTQNDAGHQIYVRLDGSTNTIERQKIIKRFSKDENIFVFL
LSTKAGGVGLNLIAANHVILMDQV--------------------KIFFLRRITKNKK---
---------------------------------------------------ASRAVSDIV
KTTLGPMAMLKMMLDPLGGIVITNDGNCILREVDVAHPAAKSLIELSRSQDEEVGDGTTS
VVILSGELLSVAETFLRQNIHPTIIVNCYMNALNSSLKFLEEISIAIDVNSESDLLKAID
SCLSTKFVNRYNKIVSKLALEATRCVKMDNLMGKKEIDIKRFAKVEKIPGGDITDSYVLK
GVMINKDITHPKMRRYIKNPRILLLDCTLEYKKAESQTNVEILDEKTWNELLLQEEIEVK
KMCEYIIDSKCDIVITEKGVSDLAQHFLVKKNISVIRRVRKTDLNRLERISGATIVNRCE
EIVESDIGTKCGLFEIKKIGDDYYSFFVECKDPHACTILLRGSTKDVLNEIERNLHDGMN
VAKNILMEGKLLYGGGCTEIRVGQHLIKEASKFNDSRKSITEAVASALEIIPKILAQNSG
VNVVKTMNELRIKHEQEGGQEFGIDGITGDIINVTTKNIWDLLSVKKQIYKSAIEAASMI
LRIDDVVSGVGKDEKVQKTIKNEFI-----------------------------------
--PV--------------------------------------------------------
-------------------------EGE--------------------------------
------------------------------------------------------------
----------------------------NDDSNNYSDETDDYYGKKKKKKKKKNKNSKKK
KNKNNNKKNKHNNVVDFTWSRIRLFDHLAVILIYEELLKNPLCKITNVYESETRKYKPYP
LNTLQMTKLVSIYFKISSKECMMLAEKLYNKGYISYPRTETNYFPDSMNLHKIINELRKN
DNFGWYANKLCEQNKYQKPRKGKMNDKAHPPIHPVKNMNKSLKVEEKEWKLYEFICKHFL
AVCSNDAIGYNTKVTAKIQEEQFFCKGLKIKEKNYLEIYTYEKWNDKIIPSFQVDDEFYP
TSLLIEEGITQPPKYLSESNLLTLMDKFSIGTDATMHEHIENIQKRNYVIKNSKSLFIPT
NLGIALVQSYKKFKDIGIDLTDPSLRAKMEKDMSLVASG---------------------
-----------------------Y------------------------------------
------------------------------------------------------------
------------------------------------------------------------
------------------------------------------------------------
------------------------------------------------------------
------------------------------------------------------------
-------------------------------------EKNIVLLRMEKLDIPSNTKMIII
SYELITKNDKYQDKYKSIICDESHYLKNSLSKRTKVITPIIKNAKRCVLLSGTPALNKPS
ELYEQISSIMPNFFNYHEFCDRYCFKDKNLYTKKIEYVGCKHTEELHLFLTNTIMIRRLK
KDVLKELPDKLRSKIPVEIPQKELSEILNYYKKLESKKNINFHDIDNIHLSNWSNSKSED
GDDENLSISHLFKITGYAKVKAIKEYISYLIDADIKFLLFCHHKLVMDEVESFLKEQKCS
YIRVDGLTPMEKREIYIKNFQNDDNVKIALLSITACGMGLNLTAANTVVFGELFWVPGQI
IQAEDRAHRIGTAHDVVNIHYLIAQNTIDEIVWKIINRKWNTLTTALNGIEDSLNVKEVN
KFDKFMVDLTNDTNKSYPTSLVTTPKVRRKSSEHGTFHNSSPKKKNRDIRDFFPANKESV
EKSLNNNKRSYHDSPMEDSPNKTLKFLSKKYKTEINMKDKHKKVEYAEKDVEIKEIEKDD
GDNKVFVNNLLTSVNYINNAIVHKDNRHMLRMLKYIKNIRLSIRNDKNMSMSIIIYLIKR
IFKENYPIYNILNKYMNIYEEGLYKEISDFYNINEKTYMNCLPELEVFFYLLILLYLLDE
KCYDEGMELSTIIINRINKINRRSLDYINAKVYFYYSWIHELGGKLSQVRQKLLFIYRNA
CLHRDIMTQTVVVNLILRDYIKHNLYDLAVRFISKTSFPENLSSNAQYARYLYYIGKILA
VQLDYSEAHRKITQALRKAPQHTQSAKGFKLAATKMEIVVELLMGDIPDRSIFSNKIMYK
KLIPYKHVVSAVRNGDINKFAQVMNNYTDLFIHDGVYLLIKRIHHNVIKTALRIINLSYS
RISIVRKKK---------------------------------------------------
-----------------------------------------------------------M
LCEKCNEKNVCMMRPSNKEKLCKECFIESFEEDVHDTILKKRMFEDNDKICIAVSGGKDS
SVLAHVLVNLKKKYNYKWELFLLAIDEGIKGYRDDSLKVVYKLEKLYNLPLKILKFENLF
SYTMDDVVKFIGKKNNCTVCGVFRRQSFEKGALLFNATKLVTGHNADDLAETILMNMCRG
DIDKLAKNVNDLSNADHMNKKYKNYENIDNNDNNNNNDNNDYNDNNDNNNDYNDNNDYND
NNDYNDNNDYNDNNDNYDDIILNDIMSER-------------------------------
LNEKCSCINNNLNNDGGKKNKEMQKCEQDY--------EEKYNNDDNNNNDDNNNNNDRY
NDNNN------NNDNNNNNDNNSNNSN---NNNNSNISNNSNNSNMEEKTCPFTNVIPYG
LLYDKLKKEKNNDVPENY-VIEEFDKLLKNYERPNVYDEIGNATFKNDEDLITFQIDLDY
TVENIFKNMIYNEIGSNN-SILNDIYMPYRILLSKDKNYVSVPIIRIYSLRKDGCSVLIN
VHNFFPYFYVEKPDDFDNEDLIKLEMLMNENLNLNSQYKIYEKKILKIEIVKTESLMYFK
KNGKKDFLKITVLLPKMVPSLKKYFEGIVHVNNKSIGGIVYEANLPFILRYIIDHKITGS
SWINCKKGHYYIRNKNKKISNCTFEIDISYEHVEPITLENEYQQIPKLRILSFDIECIKL
DGKGFPEAKNDPIIQISSILYFQGEPIDNCTKFIFTLLECASIPGSNVIWFNDEKTLLEA
```

```
WNEFIIRIDPDFLTGYNIINFDLPYILNRGTALNLKKLKFLGRIKNVASTVKDSSFSSKQ
FGTHETKEINIFGRIQFDVYDLIKRDYKLKSYTLNYVSFEFLKEQKEDVHYSIMNDLQNE
SPESRKRIATYCIKDGVLPLRLIDKLLFIYNYVEMARVTGTPFVYLLTRGQQIKVTSQLY
RKCKELNYVIPSTYMKVNTNEKYEGATVLEPIKGYYIEPISTLDFASLYPSIMIAHNLCY
STLIKSNNEVSDLQNDDITTIQGKNNLKFVKKNVKKGILPLIVEELIEARKKVKLLIKNE
KNNITKMVLNGRQLALKISANSVYGYTGASSGGQLPCLEVAVSITTLGRSMIEKTKERVE
SFYSKSNGYEHNSTVIYGDTDSVMVKFGTNNIEEAMTLGKDAAERISKEFLSPIKLEFEK
VYCPYLLLNKKRYAGLLYTNPNKHDKMDCKGIETVRRDFCILIQQMMETVLNKLLMKK--
------------------------------------------------------------
-----------------------------------------IK-----------------
------------------------------------------------------------
------------------------------------------------------------
MQIKIYGIIYILLLIIKLNHGNSKLNNKTYTLGRVYNHTVNQYNKKRKQYNNTKKYIYCF
NKINIHNYRYKKILSNMSVLSEKSIENVISPC-EKKYKLEDISNGINYDYRKNEKNTTPF
IQVAPMINVTNRHFRAMVRIITKRAQLWTEMIVDNTLLYNLNNLEEHLGFDNNEHPIVCQ
LGGCDMNSMSEAAILVEQAGYDEININVGCPSTKVANKGAFGASLMKNPEQVRNIVYEIK
KKVQIPVTVKIRTGVDNYDSFDFLKTFIETVSSVGCNHFIVHARKAWLKGLDPKQNRKIP
PLEYYKVYDLCKLYPHLKFTLNGGIQTIQEAIALLNGYMPENNNNNDTSNFIRIDNYNIN
PLNGVMIGRACMENITVLSQTDKLVYNQDIPSTAYSRRTILEAYKKYLEKNSLFYNLSSS
FELLKPVLGILKGMPGHRIFRNKLDTYIRNYTSTLPCSEILEKAIADVDDIAPGCLDLPL
HDYNQQKEYIKNY------KYMRIIY----------------------------------
----------------------------------IYMYIYIFIYLFIYM---------IY
FILFCLKSFLKNFFFKNPVGHVGVVALKNSSAKLIQPFTSNVDDILSSILKERTAGLQGS
PSLEEGLQIAHDLLIDMPLYGTKEVLIMYGSIRTCDKKNILNVLELLVKSNIYVNCISIA
PEMHILKHICEKSNGFYKICSSKNSLMNEINNNAETPLWMQGMEPQLIHICFPTKKKIST
QIMCSCHGKLNTDTYVCNFCNSYTCKIPSKCKVCGIHLISMHDLSHITNNLQGSPLFIEI
KNEQGNYKVCSSCNQQLYNKIYQCTKCQHIFCLECDIFIHEELNQCPFCLINDTMGSSSL
PMSQQMYFSTHNALRINEENDVINTLFYEINGSRHISLLIFPFYDVQMLKRLLIKKLNLP
GGVKVNDIIIFYKGIKLPNYRIISTYIDNNNNRNDKKKKKKKINKLYWAIKDTNPNASIR
VIDSKNYPEFFEDILNEIKLSFKKNIAPKLTMDGTGGTYLLYNAKKKICSVFKPLDEEAF
APFNPRGYEGKMYQEGFRAGVLSGEGASREIAAYLLDNCYNNFSNVPCTIMVEACNPHFN
NKSKLKYVDKETNLKWKCGSLQEFIDSRESVGNYDYKQFSIRDIHKIAILDIRVMNLDRN
DGNILVSPLKTLKDSCNQFVYRNNKFTSSNNEDMLKRIITIDKKPSRES-----------
------------------------------------------------------------
------------------------------------------------------------
----------------------------------YEKGETTGGGGSVKSPPSIKKDNDII
KSNYNKNVSSNNLIKDVDDMISINSSSNTTNSIKNVSFRNQSYKNIYSNNVI-TNNSNKN
IENNIDLMNKKEDTIGLKQNNLNSHSNSRTGASKECENTTERFSNNSPNNLLDKPLDTPL
DNMLENISGEVSDMSNIFCNSPNDDTKVKKKKKKKDSDLDSYPKDQKNYTNYDDGDKINS
DDNVINKENEMINQVKNNLNDDEEEEEEEEEEEEEEENDDYDVTFKKPSGTIKRISENTG
TAYRSMEMNKINSIWMIRDKNNKIINVKWENKIFEKLFFETFENYIKKYINDYHPDWRNY
PYNGSKITTTKHAYLNNIK-----------------------------------------
-------------------------FIYVY--------------------LF--------
--------------------------------FCIGRGKKVTLIHIIDFGLAKKYRDSRS
HTHIPYKEGKNLTGTARYASINTHLGIEQSRRDDIEALGYVLMYFLRGSLPWQGLKAISK
KDKYDKIMEKKISTSVEVLCRNASCK----------------------------------
------------------------------------------------------------
------------------------------------------------------------
------------------------------------------------------------
------------------------------------------------------------
------------------------------------------------------------
------------------------------------------------------------
------------------------------------------------------------
------------------------------------------------THNNNNNNNKNH
NNNNNHNNINNINNYNNKVNRVTSQKASKKKKKNAHLSNNNIAHDFMINCDNTKYLMKKK
DRDNLEKKKKKEKNYLLEKIGISNFNFLLNVCDHIDSCIIIDRRVDMITPFCTPFTYEGL
IDHFFGIDNLQIEIPRYIIFNEENKNVKYDMMENKDNNMNNYDNAKTMKVRVKLKSSVDV
LYNDIKDLSQNEIGSFLHKKASDIQKTYKEKDSLKDIEEINQYMRIFKEKHYEHNSLSTH
VNIASFILNNIKKEYNFNKLKLEDEIIQLETNTNKNILLSIVKQIQLLIYTNEDIYEVYR
LLCLFSILTNGINQNYINELKKDIIEQYGIKELTRLNKLYTCNILKFNNKQKFLWAQLKN
NFHLLSNDENDISYVCNGYAPLSVRLIEYMAALKNNLQVFPEIFNLLNGPTLDIIQNPIG
YETLPMQNKTKNNHINLNST--KKKNVLLFYIGGISYAEIASIRALNKKNQHYNYLIFTT
EIVSSKKFLQSME-IYIEIKNMSGEYTDKEKLDVMDDTIEVRSMCINCEKEGLNKIVKIN
IPYFKNVLIHSFECEFCNYKNNVIQDLNQIKDKGVKISMKINNEELLDRQLIKSEYGVLK
IPEIDFEIPKETQKGSINTIEGFLHTALNNLTIYLKEIKNMYNEANTITANETNEANETN
EANEKNVTNEANEENVTTLDEEKNNNNDENHNENGANTNGTCQQITIENYIKMIENTVQN
LSKFVILKEFPFTIQIIDPSGLSSLEHYEDDLKYKIVDIEYYNRTKEELNELGFYEEYFE
ENEKTNDINSNMIGTNTNE-QQNIKKENFDFIKKYIHMNDNSNNNTNTTMKYKTLSNEEE
AKLIESFASNCPCCNHMGMNNFCEINIPGFKKCLILSFVCPNCNFKTSEIKSSGEINPKG
KKITLTVNNKNDLNRFVIKSETASINIPVVELTSDYGTLGGTLTTVEGLIMKIIESLEEK
FKFLLGDSNINTHQYENENTPNNNNDTTYFQ-----------------------------
--------------------------------------NMSDNVKRLPLPSENGEIKKTT
SGRLSDDGIKRTPSGKPIQTMYVLNRKGEEEDISFDQILKRIQRLSYGLHELVDPARVTQ
GVINGMYSGIKTCELDELAAQTCAYMATTHPDFSILAARITTDNLHKNTSDDVAEVAEAL
YTYKDVRGRPASLISKEVYDFILLHKDRLNKEIDYTRDFNYDYFGFKTLERSYLLRINNK
IIERPQHLLMRVSIGIHIDDIDKALETYHLMSQKYFTHATPTLFNSGTPRPQMSSCFLLS
MKADSIEGIFETLKQCALISKTAGGIGVAVQDIRGQNSYIRGTNGISNGLVPMLRVFNDT
ARYVDQGGGKRKGSFAVYIEPWHSDIFEFLDLRKNHGKEELRARDLFYAVWVPDLFMKRV
KENKNWTLMCPNECPGLSETWGEEFEKLYTKYEEENMGKKTVLAQDLWFAILQSQIETGV
PYMLYKDSCNAKSNQKNLGTIKCSNLCCEIIEYTSPDEVAVCNLASIALCKFVDLEKKEF
NFKKLYEITKIITRNLDKIIERNYYPVKEAKTSNTRHRPIGIGVQGLADTFMLLRYPYES
DAAKELNKRIFETMYYAALEMSVELASIHGPYESYQGSPASQGILQFDMWNAKVDNKYWD
WDELKAKIRKHGLRNSLLLAPMPTASTSQILGNNESFEPYTSNIYYRRVLSGEFFVVNPH
LLKDLFDRGLWDEDMKQQLIAHNGSIQYISEIPDDLKELYKTVWEIKQKNIIDMAADRGI
FIDQVK-------------------------LK----YKR--------------------
----------------------------------------------NKMASTHNDIVPRL
CFEEMRNEMNKYGVEINQSTLKNPSTEDIQGVYSICIKYILNKDIQNIRIEEYTGDLKSS
LPTVDGLQILPNEGKNHLQAIGNLRFLRHCEKINKILNLDNILSYIFKPVGSHMTKLINA
FIHFMKYRDQLYNENDEKIRSIQEKKNEYDVLANEYDVLENELNKLLLKHEDIRNNIINE
KNIKRNYEEEIIKNQNLLNSQQSLIISLNSTKDKIINETNELIFQYSRYRQKKEDLEDQI
VPSPEKLQKYNEELKDHLYEHIAQFDDDKKKNEDIKNKINIADICIKKLVDLLTALNDHI
EHTIKLHIEKKNNLQTIEKQYKSLTNEKQDFITKNTEQDKIIKETKECLQREQTKWNQKI
KQEQHNTILIQQKVKDIYQNVDDLNIKTNREINQINNIIKHIQDIINHYNKNILLITELI
QNTKNSHNILTHKVLNNIQKDISANM----------------------------------
------------------------------------------------------------
--------INKYIYIYI-------------------------------------------
----------------------------------------YFYIYMFISIGLSKQADWWT
LGIFIYEILVGCPPFYANEPLLIYQKILEGIIYFPKFLDNNCKHLMKKLLSHDLTKRYGN
LKKGAQNVKEHPWFSNIDWVNLLNKNVEVPYKPKYKNIFDSSNFERVQEDLTIADKITNE
NDPFYDWRGISQFYILSPRGDTIINRDFRGDIIKGRAEVFFRNGKLYKGDAPPVFYLNGI
NFTYLKSNSLYFGVTSLFNISPSYLIELLHRLLKIFKDFWGQITEELIRTNFILIYEIID
EIIDYGYLQNSNTEYIKNLIHNEITTNNNTVKKFTNLPNFSIKNTNTLPSNASQKPIQIN
DKKNEIFIDIVEKINLIMNSNGEIVYSYIDGVIQIKSYLLGNPFIKIALNDDLYIKNIHH
DNSNNIIIDDCNFNHLVNLSQFEKDKILSLYQPDGECVLMNYRINNNFKAPFKIYANVIY
NQNHT-------------------------------------------------------
------------------------------------------------------------
```

```
----------------------MDILITTPYDKEVCEIGEITNKIISLETIKHTKSSLHK
NRDIQCPNKKKLVLPGECVLDKNDKDRFLKGGGLYEEDDMFHACILGRVNYINKLIYVEP
LKGKYTGSVGDLLVGKIKDISNDKWIVEIGSYCRAFLSISQTNISIFSQRIRLYNDVINM
IHIYKPDDIIACEIQRISTDGTIILHTRSSIYGKLSNGVLIIVPQTLIHNQKKHIFVFPC
NVQIILGMNGYIWISSPIKKSKDTNPNSVDQNIEENKFEDVDHTTRKNISIITNIIKLLV
KYHININYDTINKIYVHYTSNTNNTPSYILKPYVADSYLFNYIDKFVSSNMPVLYHLTLQ
KPTAITKTVYGNFSGPRFHEIIVAKGQVLELLRSDKQGKLNVIISKDIFGIIRSISTFRL
TGSNKDYIVIGSDSGRLVILEYNNEKNDFVRVHCETYGKTGIRRIIPGEYIAVDPKGRAL
MICAVEKQKFVYILNRDNKENLTISSPLEAHKSHSICHDVVGLNVGFENPMFVSIEQNYE
SLDKQINEENDFSLDYAKKVLCFWELDLGLNHVIKKHILPIDITAHLLIPLPGGQQGPSG
VLICCENFLVYKKVDHEDIYCAYPRRLEIGQDKNISIICWTMHRIKKFFFILIQSEYGDL
YKIEVDHEDGIVKEIVCKYFDTVPIANSISVLKSGSLFVAAEFGNHYFYQFSGIGDDNKQ
FMCTSNHPLGKNAIIAFKTNKLKNLYLVDQIYSLSPILDMKIIDAKNTHTPQIYTLCGRG
PRSSLRILQHGLSIEELADNELPGKPKYIWTIKKDNLSEYDGYIVVSFEGNTLILEIGES
VEEVSDTLLLNNVTTLHINILYDNSFIQVYDTGIRHINGKIVQEWVAPKNKQIKAASSNS
SQIVISLSGGELIYFEIDESHTLVEIFRKNLNVEVLCLSIQQIPPNRVRANFLAVGCLDN
VVRLLSIEKDKYFKQLSTHLLPNNSSPQDICISEMNDNGNTKERNIIFLNIGLNTGVLLR
SIIDPVAGTLSNHYSKYLGAKSIKICPVNVNKNPALLVLCEKTYLCYMHQGKFLYSPLNY
DMLEYASSFYSPQCSDGYVAISSNSLRIFRFYRLGEVFSQNILHLTFTPRKIVPLPFPSL
FYDHDSSLELERKKKISMLAIIEADHNSYDENTQREIQKALKDIKLSDTERRKENNSNDE
ENEDEYYYDRIGTFKAGQGKWGSCIXIINPVNLQILDKISLDMEEAALSVCACELHALHC
LIVGTTTNLSLKTKALTSASLRVYTYDIQYKLNLLHITPIEEQPYCFCSYNGKLIASIGN
KLRIYALGKKKLLKKCEYKDIPEAIVSIKISGNRIFACDIRESVLIFFYDPNQNTLRLIS
DDIIPRWITCSEILDHHTIMAADKFDSVFILRV---------------------------
---------------------------------------------------RLELTQHLE
IILRTEKPPLCGREHIFFRSYYHPVQYIYNWDIL----YFSFNLKKK-------------
------KKKILL------------------------------------------------
------------------------------------------------------------
-------------------------------------------LKKKPDITYNDIGGCKK
QLEKFREVVEMPLLQPERFVTLRIDPPKGVLLYGPPGTGKTLTARAIANRTDACFICVIG
SELVQKYVGEGARMVRELFQMAKSKKACILFIDEVDAIGGSRGDESAHGDHEVQRTMLEI
VNQLDGFDNRGNIKVLMATNRPDTLDSALVRPGRIDRRIEFSLPDLEGRTHIFKIHANTM
NMSRDVRFELLARLCPNSTGSDIRSVCTEAGMFAIRARRKTITEKDLLLAINKVIHGCKQ
FSATGKYMVYNI---FC----------------RIKAKVEQNVPLIFSKINKLQKEKEDD
EDIIYSVTNDNIFNMPRYSRIPKEKKKTKWQLFAENKLRMKKNKSGLIYDKASKGWVRRF
QKKQIKLNEQKNNFVHEYKNKEDIYEDPFEKEQEEKDIKKMKQKMRELKNKFDQKGISTE
DIKYIQRQKRKRENLIDNLKM---------------------------------------
--------------------XHXMRNIKXNY-----SSCEGYKVLVLDDETKSIISLIFS
HSYIXEKEIFLTLNFNDKNIFEDIYNNNDKKEDFDFMNYKIKNLKHLKVIFLLRPTYTNI
LRLMSELKKPLFSEYYIFFTNIVNDIYIEKLAKADEFDVIKNIIEYYIDTYVLHDYLFHL
NIDYTSFLYKNDHIFIKEKKKKENNNNINSSNNYSSDGRYEKLTIEEFNKNNMIYDNNNN
NINNINSNNIINNDNRNNSNIRLYGNQIVQRIIDGLFSFLCCIRQVPDVIYNKHSKICKH
IIDMLKEKMLRHQSVFNNILDIYEKYNDKIETNNEPNYQFNHLINQNIHEITEGDACYFL
ILDRNEDPITPLLTQWTYQSMLHELIGIENNKINLNSNNKEEEQIVMSCNYDDFYNEHLF
DNFGDLGQAVKNYVDMYQVETSKKTNLESIDDIQKFIDIYPNYKKLSGNVTKHVNILHKF
SDIVQKRQLFYISELEQSIACYHTKNDHFKQVIDTIKNYTYTNYDVLRLSLLYSLKYGDE
QHINIIKNELAKRNIQKDQICLIDALLLYSSQQTKYNQLFKEQTFLNLAKTTITRTIKGT
SNVFTLHKSYLYYLLEDIIKYKINTQLYTTTNLLHTEPTLNKKINSIVVFFIGGATYEEY
RDVQYLSKKYNISIVLGSTHLHNSQSFLADVLQLIKK----INSCKHPCCEEDNLKSVKN
KVYHKKYIPSIIQKKKKERNEHAPPLLKDIQDKRCTNVFERLNDKQFYTGVQKNKFMELK
NNKNKSS----YCYNDI--------KVFSTMLKKPCNYVVTPGTLGIQKYGIQTGRTKTI
FLFNNEKKYDKGVYFLVKSYIKNIKSLCYEITKILQPSIGPTRKIYDQNFSIVRNVNDLI
NGGKYLCTSGDPPAPIRNLSHFLT-------KNHEDIKKKGLKKIENKKISKHLKRRSNS
SSKVSEMNSYSSKEEDDKEEKKIKIKMSDSICGTPTKEGFG---ELDVEGENNSNEINDA
RKYFKEGQKIITPPNGDGTRAFYESLLEENPNSVIAIKYCIEHGILSGTKHHEALYKYYV
LKKNNAFKSNFGGVRIDFKKLLEETCKDNKCSYSK-------------------------
------------------------------------------------------------
------------------------------------------------------------
------------------------------------------------------------
--------------------------------------------------------PQAI
ELSSEALQNVKFIQEKKLIGKFFEEIAQDTGKVVYGIDDTLKALEIGAVELLILYEGLDI
IRLTTKNPVTNQTKTMHISPCDEKQESLYKENNVELEVVEKISLTDWVIGNYKKYGASLD
FVTNKSQEGAQFQKGFGGFGGMLRYKIDLNLYDEDVESDVELF-----------------
------------------------------------------------------------
------------------------------------------------------------
------------------------------------------------------------
------------------------------------------------------------
------------------------------------------------------------
-----------------------SLVVYVNRTLDTYTKVNYNDSINRSHHLSKDIINNND
NNNDGDNNNNNNKNNNNNNNNNNNNNNNNNNKNKNNTGKNCTIVNTDEFVNNVVKMLQVI
YEFIFLCIRIYDDDIIISKLFALPYTIVSNVNMNNDTICEEIISIIVLPFNYLGLSALNA
RNMQTLLNSITEKHKKKLSLDIIDAIIECKKKYITYEDVEKILKYISYIFHEKEKKNKNN
DEDIFNLENNNSAYTCEKICKFFHIITNTKNIEEKYNICMLFYKYISNSTYLVHLLPTII
FTLLHVVTQITKLGQEDHFNQNDDKNSSDSYNNDKNNFILDEKKVNQYNMYVKNIFKFIH
TNLLTVASQMPIVTFKLFLYSAIVVNNYNSFVQTHEFLTFDNLEAICYEFITQPLIIYEE
DINISAQQFDCIVWIVGILCTHINLLDNENYNNIALKLTQHANKLLKKKDQCIGVLKCSH
LYWENKKYRNNNKVIECLQKSIKNAEIAIQSNTDNIILFTYMLDKYLYYYEAQNIDVSEE
TLHYLIDICQDYYNKTNDDTNFKQEYKKVIKYVHDKQKNSNVFQKINSILRSMLEAKLNN
ASILKKLFECIKDLVNDANVDADESGLKLQALDGNHVSLVSLHLLDSGFSHYRCDRERVL
GVNIASLNKVFKLCGANESVVISSKDDEDNLNFVFENNKEDKVTNFSLKLMSIELDSLNI
PDCEEGFDAEVELSSKELTNIFRNLSEFSDTVFIEIDSNCIKFTTKGIVGDAEVALKPRD
STSEDDIGVTIKSKKKIKQSFAIKYLNLFSKSNILADVVVLGLSDSRPIEFKYEIKDTSP
DSDTLKIGFVKFFLAPKMDDDMDNKDV---------------------------------
------------------------------------------------------------
------------------------------------------------------------
------------------------------------------------------------
------------------------------------------------------------
------------------------------------------------------------
------------------------------------------------------------
------------------------------------------------------------
------LQHQEMC-----------------------------------------------
------------------------------------------------------------
------------------------------------------------------------
------------------------------------------------------XLASCP
EKCDEEVGIVKGTFLYTYDSPICKSAIHSGVLPNNVAEDIVLSIAHTHNNFIGTKRNNIE
SHDFKGTSKSFTISIPTMSLLREERKSNPKSEDEMINKNEIGLTYDHSFNKLDHHITSSM
KPTFQWIAPTGFVGFNGKENDYIDCTNLPNEKYIKSLSNFTFIVYFTLSGGEGTWRTILS
HSLCEGISISINEDNELIIEQNCNPHLLKSKFKPKFGQTYHISLVFNKINKTLYLYINGK
KVITEKNTYNFTLSGDLIIGRSNQTTKDYFIGNIHLVEIYKYTLSEQEIKESFNSSLSLE
YLNMNILEQMSNTKKKKNQKNKKGVQKTIDGRDCLTPCKSKNMINKDLQINTQQINLKCQ
DNLLSEQFNGKIGSQFLVSCIENCTKSKYFIKGTNNYYTPDSSICKAAIHAGIYKPNGNK
NNTFVIRIVEGLLEYKSSRGHFGILSKSEKQSQLRSFSVLSENEQNIFTCSTDGQFILNL
SVGEKRTINCPSNCNIIKNKIYGTNIYSPISVLCKAAIHSGALSNQGGIVEIIVGTGQQE
FKGSTQNNVESFSSNNHSRSITFNKHMEKMSSLFVEQKVEPEKIKEEKIKVNVNKILYKA
HKNIKKEKIKSKRIDYLCVDKNDLPKNLPNTKNIKNRKLKNQLNKDVKLAILSSKKLLAN
TKFMPLKEGYIKYNSVNEPNVQIAPSKEEEEKKKKKKKKKSELIDSTELSDEEDNKNKRA
```

SQKYIYDNADVGTQKKVFDLKLNMGPYTCTYTRNGKYLLMTGVKGHVSLIDTHNLESLCE
FQVDEMIRCNTTLHNYKLFAVSQKKYMYIYDNTGMEINCIKDILYTYNMVFLPYHFLLTS
IGEFGELVYQDISVGNIVTRKKTKRGPCSIMKQNKKDAIIYLGHKNGHVTLWSPNIDKSI
CDIFCHHTPISALGVHDNYLITASVDSTYKLWDIRKMEYIKSYKSHNIINNIDISDTSLV
AFSMNTHFRTYKDFFTNPQLYITHNIYGDQINSISFQPFEDICSLGLKHSIKTLLVPGAG
IANIDTFFNNPYETKKQVRENEVKLLLDKLPADTIKFNTDQIGNINPYLINHNEQKN---
HTNLYNKNYMKKKNKKNK----------------KNKKNKKNKKNKNEENINLMHKQSEH
ETD----------------IMISDNNENNNNLYFLAFSKNKVTIYEYDVDLSKNIVDDIN
DEIKRCEEEDMNHIRNNNIINNNTNNTNQIKKSTNKFNNDFVLNRGIKIYKEYDDVELAT
CTNDYKKIILVRKSDLNVAEIIDIKSNKNVTCIKTKTQIKRIITSPRDNYIVLHCQYKPD
ITNTNLYVYKINVKSSKKKKKKDKKNSECENNNNKNNSNNNSNNNESTNDNIIYNENLII
ELTLKSYSNKNWPFFKWSDSESICTCLINNQVYIYKDNNFNTTVNKLKLDNIEFFDISPE
LNNKKGILIATYEQGSKGNPSIFKIFHIDNLNKHIYSKNFFNSDEIKLKWNKNGSSLLLQ
IHTQVDKEKQSYYGSSNLYFIDTVTLKDVNVMTNKGLIYDTIWSNNQNKFYVCKGEIPAE
IVLHDKNGNVSHSYGRHKFNTLKLNYNEKLLLTGGFGNLSGDISIWNTSTKKEITKTKSS
CAVICEFFNDGKHFLTATTHPRLRVDNNLKIFKYNGLIVSRINFEELYNVIILPFNKIKF
QQTEEPVDINLEHTTLQPYINKQLGIDSKKTGIYRAPRSTGLVNLNGYLSSKLPDREKSS
LPPGCNFVNENKNYKKKKKKRSSKSKQKRETFKYNNEYIGRYAKQYEIIKKKKKKKLKYF
LYFYFIVMTAEEDTIPLPNIKTPILKKIIEYMEYHINNPADEIPKPLITSNLQDVTIKRK
EMFI-------FFIWM---YINICLYIYIYIYMCSSCFYFLFFSFLFFFFFICNNN----
----RLYLHGILISN---

--YHGY
I---HSIV
EDNSSKYKDLAQRMIQEIEMAVVNCKPLNNECKSFEENKQSYLLDLAYPLLFKRKFILKK
IKTNEKLNKIKMYNKCLNNSYNSYTLKNLHVSNPIERDTTSFLRNHKNSNDSTNDDNCNV
VKREDHQDIINISSEENEERDINKNLKEEVVTTLNKISRENNLQNLQSEKKLKQAHTTPC
PKLPPCYIINSGSLSIARAKVKLPSTYSKLGNPLSFSKFSLPDFNYSYDMIEELQQFFMK
QRRCDYFSLLNNFINLLISVSNLLAAEPDIDLRNELLRRFIYSLNSWMNMRRCIVACCEN
IFAMTGLCIPLESMSSST---YNNDTNNRLSYNNLQILHFNNEECKIFFSKKRAPYLLMF
EVADLDEDISHISDNIFYTKKSLFFQQDNNNNNNNNNCYNYNYNSVREEYSRKSLILSRN
FCCSKNYGYYEENLNTFDYENRISLEDKKKKNSKKKKYAKEQEQTNNEKKEKDRKKKNIF
DLNSLKMDDIYVYNCIVNDLRRENLISFTSKEDENLALIKKCLGLKMVEEEEEKN---EN
DDDSSSSFCVKKNYLLNSRSVSMPSYLNNNLEKCDTSSYDNTSNNIKTSIETISNGIDDI
LVEKNIEKNETFEEMKNEFIDYNEHMVKLSPYYTTTNHRGKNKNDDTDDNSDDDNINSYN
NNTGNNNQDSNFPNNILDLSEYFKPENYLNEEFKKKNCKIIKRLLWGELFEEKKKRIRKI
SPYGKLKTWDLKCVIIKGGDDLRQELLASQLIKQFKIIFENAGLPLWLRPYEILVTGSNS
GIIEYVNDTCSVDSLKRKFGADSISTIFNIVFSDYIFEAKK-------------------

--------VTNKRTI---
-KYIYIHFLHQIYIY---------------------IYI---------------------
------------------NICLYFFLPLLPSDMGNFNSALGGANFNLGNFANMLNANGGG
DFNRDTISSLLNNPLARSVLNELSNNPEMLTNLVSNNPILRNTFSQSPLMQPVLENPNLL
REFMRPEILQAGLQIENALNMNNNNNNNNNNNSNGGFRMEDILNNLNNLSNPNNTTNNNN
ANPGNNLNSFLQSPELLQVFQQVMSSNRNLGNLNFPNANMNLDFNTTNVTDNRPPEERYA
SQLSSLQEMGFLDNAANIQALQETGGDVNSAVTRLLERGFN-------------------
------SGDRLTRAARVLEQLTEQKPIFGKCRFTIRSFGVRRNEKISCFVTVRGKKALEI
LEKGLKVKEYELRRKNFSDTGNFGFGIQEHIDLGIKYDPSTGIYGMDFYVHLSRSGYRVT
RRTRRRSKISKTHKVTKEDAMKWFQTKFDGILLKMEFCGSNFFRLRLALSLISGKAITIK
NIRMKRKSIKNGMNNEMDDNYNNYGLQEYEAKILKLIDKLCDNTTIKINEEGDELYFEPG
YLIGNTNEEDSRNMYNMTFHCGKERSITYYLEFLIMIVLFFKNPVNILLKGITDDYIDNN
VYTCKIICEHFFKEILKLNEDFLYINILKRSTKPECSGEVHFFMRNIKKINPFDITNVGV
VNKISGSILSNNISLMFRNKIMNCAKKQLYHFTPYINIDVQKEDDKKNKNINSHFISFSL
FAHTKYNCIYAADICVDELFLKKLKEGINNRDKHEHMHDRSNILEHKQYYMNDNYHNNDE
NILPSYTQKNQQNHLLTKHINNNLHDVDIYERLGFFISLKMINQIKGLSSVDTNYQWLPL
LYMALAADTSVSKITLSVIKPYSITLIRLLRDFFNVVFKIEKVQKSPIHYSYLSQCVGIG
YQNM----V---
KQIKEYLIRAIYIEMLGHDASFAHIHAVKLAHEKNILCKRTGYLSCNLFLHKDHELMLLL
INTIQKDLKSDNYLEIWAALSCVCKLLNNEMIPAIFPVIQDLLNHKNELIRKKVCMLLHK
MYIIEPSLIKDIDIYLKRLLCDVDPSVMGASLNLIHSIAKNDMIYSIKLVPYLVSILKQI
CENKLPKDYDYHRIPAPWIQIKILSIFRILGYSNKKLSEQMYEVLQKTMQRADFGINVGY
AIIYECVKTIATIYPSHHLLELASLSISRFISSDNHNLKYVGVTGLALIVKINPLYATEH
QLAVVDCLEDKDETLKIKTLDLLYEMTNPLNVQVIVEKLIFHMKNSVDIHFKHDLACKII
ELIERYTPDDIWFLNKINTLFLSVGELLDESYSYSLIKLLKKYKSASNDNINMECLNYMK
EQKEGTDDVNDNNGNNNQ--

----------------------------------IYNYNGGCVLGMSGLNCVAIACDLRL
GANTFTTVSTKFSKIFKMNNNVYVGLSGLATDIQTLYEILRYRVNLYEVRQDAEMDVECF
ANMLSSILYSNRFSPYFVNPIVVGFKLKHYVDEEGEKKVNYEPYLTAYDLIGAKCETRDF
VVNGVTSEQLFGMCESLYVKDQVKE-----------------------------------
---------MHKTIRKLFSDEVCDKIENMRILLVGAGGIGSEFLKNIITIGCKNIDIIDI
DTIDITNLNRQFLFKKKDVKEYKSHVAKERALEHKKDLNINAYTFDVCTMKSSDIRKYDY
VINALDNIKARKYVNKLCIMEKKVLIEAGSTGYNGQVYPIYYNQTKCYSCEEKPKNKTYA
ICTIRQTPSLPEHCVAWGRLIFETFFCKNDNETLIDIKNHIEEESKKRNMDKKEIIIFIF
NYLFNDTIKELIYLKKDYTTIPIPIHFEENIKKNEKNIDKLNDNNKNN---MVDNKYSNN
FNCEETNREKDDNVLMLSSQNIWDKKKCIQMYIKTFLKLYKYLNINKKEQQYLIFDKDDD
ECINFITSISNIRMLNFSINQKSKFDIQSIAGNIIPAISSTNAIVASLQAFQLIHVIEYF
ETLKKKNKKKIFNIRNSKAKHVWVKSIVNGNKIFSRGNLVNAEPLEIPNPNCYICQQPTI
HIYIKNFDKMTLYNFVKDICMNELSFLYPFLDKDDRNIFDYDLFQENDDDYIQNLYASLN
YWNIKHDEILLLTDFQNNNDQLEMHLKEDPSLETEYFIQQKKVPSKKRNITYMHNMDTE-
------------------------RMGKLSKDRRDIYYRKAKESGYRARSSFKLIQINEK
FGILKLFKPDIYNCNNEKDLINIYNEHYCYNIVDLCAAPGSWSQVLKNICLYNYYQILYM
NDENIKNIQHEEFLNKFSLYINYNKIKCIKKPNIVAVDLQEIGNMNYVKIIQGDITKMST
IDKILKCMNNKINSNVFYNMEQQKNENNFSYAHTVVSDGAPDITGMNDIDEFIQSQLILS
SLKVCCSVLKVGGNFISKIFRGEHTGLLILHLNKFFQRVYICKPQSSRNKSLESFLVCLN
FSLPRSNIISMNNHNAENKINDKSEEQMRKFHAQLITYKDKDDQHTTKHNKDISEEIETK
ENNVTPNSDDKKNLDIFNFYCSDSDEEIKYFNSEDEEVVNNYISSECFMSNKLFSFIATD
NYYDSDKSYLLPENYVRHEPQLMPLKPPYMLSLQKKRQEVKKMEGEEYDKILVLNFGSQY
FHLIVKRLNNIKIFSETKDYGVELKDIKDMNIKGVILSGGPYSVTESGSPHLKKEVFEYF
LEKKIPIFGICYGMQEIAIQMNGEVKKSKTSEYGCTDVNILRNDNINNDLYSNYKLMNET
CCLFENIK-SDITTVWMNHNDEVTKIPENFYLVSSSENCLICSIYNKEYNIYGVQYHPEV
YESLDGELMFYNFAYNICKCKKQFDPIRYHELELKNIEKYKHDHYVIAAMSGGIDSTVAA
AYTHKIFKERFFGIFIDNGLLRKNEAENVYTFLKSTFPDMNITKIDASENFLSNLQGVTD
PEQKRKIIGKLFIQEFEKAVNNIDIDINKTFLLQGTLYPDIIESKCSKNLSDTIKTHHNV

```
GGLPKNLKFKLFEPFKYLFKDDVKTLSRELNLPDEITNRHPFPGPGLAIRVIGEINKHKL
NILREVDDIFINDLKQYGLYNQISQAFAVLLSSKSVGVRGDARSYDYVCVLRAVKTSSFM
TANWYQIPYDILDKITTRILSEVKGVNRILYDVSSKPPATIEFEMRKYNGNVKRSRDDEY
EENEIVNGERTSITLVRATLLPILSIGKNEKTTLFHPFKNPLPGFVSDKTLYLKKTLGVK
VRRTGWINLMKNSIPRRSDETEEQEKIEEVIKKHEPLILYKDENDKIEVDPILAQYLREH
QREGVQFVFECLMNIKDDKISGCILADDMGLGKTLQSITVLYTLLKQGFHKKSAVRRCLI
LCPASLINNWNDEISKWIPNRCNVTCVNDNAKEKIVSKLEGFKYDIQSTVLICSYECFRI
NNEFLDKSSIDMIICDEAHRLKNDKTKTYTSIYNLTAKKRLLLSGTPIQNDLGEFYALIS
LCNPDLFDDINFFRKKFANPILIGRDKDATEKEQEIASERLTELSNITNKFILRRTNNLL
SKVLPVKYLINIFIKLNPIQEALYVLFLKDKKILKNDNTNNKVNVLINIKKLEKICNHPL
LLNVNDIKEIGQVTLWKLIEDVICEMQSNRGNKRDNKNDISSISNNNMNNNQNDGSGYIG
NKRKPIELDYNKPVKRIIEECKRDIYRSYYNFSCKFLLLHFLLKNIKQNTNDKVVIVSNY
TQTLDYMEILCKENMYKFVRLDGGINIKKRHKVINDFTHTADIFIFLLSSKSGGCGINLI
SSNRLILLDPDWNPANDKQALARVWREGQKKICYIYRLFCTGTIDEKVYQRQISKDGLSN
MIVTTTNLSKDQMSDENVKKLFNYKMNTVSETHDNIECNRCSLNDNNNNN----------
------------------------------------------------------------
------------------------------------------------------------
---------------CNLIWSDKATKKRSKDILCLEDIPYKDEIKYLNELKILPYVSCIH
CVNIRKRKVENAGFMFYIKYEVPLLLNEAKKSKENKRVDIEEIFLYKDKIDYMLNLEKSI
NKNISNDKQDKRKKGNEKNEELNKDDYDNKE-NGADNIKGQFKKGSNLIKKCIDQNIDVY
DVLGVEETDDLETIKSCYKKLILLFHPDKNKGTTYLNEKEKEKNKKKGKNNNNNNNNNEK
DFLYFIEKYNIEKLTNDEKKNIFLKIQDSYTILSDKILRKQYDSSIPFDESIPTLTQLEE
AKNFYNFLRPVFKRNAKWSAIKPVPDIGDENTEIKEVKYFYDFWYNFNNWRDFSYQNEYD
YEQAECREERRWMERENKKIQKKASKTENLRIIKLVDLAYNNDPRIIAENKRIKLEKLKK
KEQAMNEKKNQQNENLQHNNNNNNNNNVTSHKKNID-KASVKLWKHHIKSLCLTKLSNLV
NTEDIQQKLSIMSFDDLCEFIYDIYVILNFTVPKNNTDTASTL--TNIKNNTVKKVYNPN
INDPK----------------YNISADGKTSTGFIGHLKNVHLDYKQIQTLLDTFKKYIQ
DHSFILQNSDHPLSNQHNYKENEIEKQTHANNYSNVNTQDHSNNEKIYEHVKKENDSTQI
NINESNEQNNQENELQNNKWSAQEVSLLAKALKLYPGGTRNRWVLISNSIKTKTVKEVIK
KTKEMFENDTLKNLGKNFDETPFDHFKNQNKGVMKKIDDNLDKREYKLTKENNNQVQTDN
LNGDVEKKKPWTHEEQHLLEQALIKYPTSIPIKERLKLVSLELKTRTVDEVILRMKTLRA
QIMAQKSSKMPKKRRNGGRSKHNRGHVNPLRCSNCGRCVPKDKAIKRFNIRNIVDTSAQR
DIKEASVYSTFQLPKLYIKQCYCVSCAIHSRFVRVRSREQRRVRKETAKHVNPSQLMKKN
LEKKLLEYEKGYYSNKVKMRNMLFKSINKNHLFLVFLLFLFFSFSCINFTQCQITKKKVS
VSEINFDSAVDDVQWCGNNHMTVLVKTVKGRLYRSSDGGKIWTNITGNLSDKENNKSEPS
SNHMNEMTTVDLIMVNSVNKNIVLIIGNQKNHYISEDSGETFRLLNYQNKINFWQFHSTK
THWALVSSWTEACYSNDDNSGECMQTLSLTKDLGRTFQLIDIYVVQFNWGDKESHLEDTI
YYTRHKNRNGHQQRFSGWSKDVDFVATHNFGKSVDVLVKQGNKFLISNGYIFVAKLNDVI
KQTVNMMVSTDGGKTFNKANLPENIHEKSYTVLDTSEGSIMLHVNHGTSSEKINTGNVYI
SDASGLNYTLSLPNNIRTSSGECEFDRVLSLDGVYIANFLDVNDEIKDEDLRFQNFKSAI
EEDIAPFETNTEKRKKQYNKGKNEDAVRTVISFNKGGEWSYLKAPKVDSRGNKYECGDNC
YLHLHGITNYHQYAPFYSIENAVGIIMGTGNVGSHLRYESDEVNTFLSRDGGVTWIEAHK
GPYIYEYGDYGGLIVMADDLRKTNQIVFSWNEGQSWFDFELGQFSIDVDNIVAEPNSASV
EFLVYGTRNDVGVLYHLDFNALGQPLCKGLWAADSVSSDYETWSPTSGNFNDKCILGRKI
TYTRRKQTSECFNGKDLKRTVDKKPCECTPEDYECETGFTRKIGSYECKPNDPTLTIEGC
TSSSYFYANAYRKVPGDICVNGWVPEKVPVPCPSYAPFNKSKKNQIYIL-----------
----------------------SKY-----------------------------------
----------------------------------------------MADRKSNKNAVVKN
VDMTEEMQIDAIDCANQALQKYNVEKDIAAHIKKEFDRKYDPTWHCVVGRNFGSYVTHET
KNFIYFYIGQVAILLFKSGDDIIVNEEILQKAQEFQVENEKDIEMKKLKPITEGLLKPEV
DLLQISERGSRGRVKICNVLNVPRSEKEYNSDKVENNYIGKIITVCGWSKAIRKQGGGRF
CFVNLNDGSCHLNLQVVVNQCIENYEKLLKCGAGCCFRFTGELIISPVQNDNKKGLLKEN
VELALNNNDIHNFEIYGENLDPQKYPLSKKNHGKEFLREVAHLRPRSYFISSVIRIRNSL
SIATHLFFQSRGFLYIHTPLITTSDCEGGGEMFTVTTLLNENGDIRSIPRINLKNKKKEK
REDTNACNIIASPQYEDNYLIDYKKDFFSKQAFLTVSGQLSLENLCSSMGDVYTFGPTFR
AENSHTSRHLAEFWMIEPEIAFADLYDNMELAEAYIKYCIEYVLNNNFHDIYYFEENVET
NLIKRLKNILNEDFAKITYTNAIEILKNYSDSFEVKVEWGMDLQSEHERFIAEKIFKKPV
IVYNYPKDLKAFYMKLNEDNKTVAAMDVLVPKIGEVIGGSQREDNLERLDKMIKEKKLNI
DSYWWYRQLRQYGSHPHAGFGLGFERLIMLVTGVDNIKDTIPFPRYPGHAEF--------
-------------------------GVKHGLAATIKDLLEMDLENSTNILKNLFLKDKKK
NYFLICTLNNKIVDLKNLSNILKTNNLRFVDENNLNNILNIQPGCLSPLAIKNDKENVVK
LYFDEEIKNMQEVIIHPLHNYSSLYIKTEDVIKFCESFNHAPEYIQIKEDTTSKKKEDVQ
EEMEELQNNKNLKGKLLSNNEVEDNKSKDSNILGITSKKIENFSDWYTQVIVKSELIEYY
DISGCYILRPAAYYIWECVQAFFNKEIKKLNVENSYFPLFVTKNKLEKEKHHIEGFSPEV
AWVTKYGDSNLPEEIAIRPTSETIMYSVFPKWIRSYRDLPLKLNQWNTVVRWEFKQPTPF
IRTREFLWQEGHTAHKNEEEAVKLVFDILDLYRRWYEEYLAVPIIKGIKSEGEKFGGANF
TSTAEAFISENGRAIQAATSHYLGTNFAKMFKIEFEDENEVKQYVHQTSWGCTTRSIGIM
IMTHGDDKGLVLPPNVSKYKVVIVPIFYKTTDENAIHSYCKDIEKILKNAQINCVYDDRA
SYSPGYKFNHWELRGIPIRIEVGPKDLQSNSCVIVRRDNNEKCNVKKESVLLETQQMLVD
IHKNLFLKAKKKLDDSIVQVTSFSEVMNALNKKKMVLAPWCEDIATEEEIKKETQRLSLN
QTNSETTLSGAMKPLCIPLDQPPMPPNMKCFWSGKPAKRWCLFGRSYMGKLSKQQKKQMY
IEKLSSLIQQYSKILIGHVDNGGSNQMARVRKSLRGKATILMGKNTRIRTALKKNLQAVP
QIEKLLPLVKLNMGFVFCKDDLSEIRNIILENKSPAPARLGVIAPIDVFIPPGPTGMDPS
HTSFFQSLGISTKIVKGQIEIQEHVHLIKQGEKVTASSATLLQKFNMKPFSYGVDVRTVY
DDGVIYDAKVLDITDEDILEKFSKGVSNVAALSRATGVITEASYPHVFVEAFKNIVALII
DSDYTFPLMENIKKMVENPEAFAAVAAPASAAKADEPKKEEAKKEEEEEEEEDGFMGFGM
FDMLQERDLAREPCPDRIIEDMGGAFGMGCIGGYIWHFLKGARNSPKGDVLSGALYSSRM
RAPILGGNFAVWGGTFSCFDCAFQYMRKKEDHWNAIGSGFCTGGVLAMRGGWRSASRNAI
VGGVLLAIIEIVSIVLTRKTTPTPRQQFQQQMELEKRMATKNNRY---------------
------------------------------------------------------------
----------------------------------------------LQINNYLI------
------------------------------------------------------------
------------------------------------------------------------
---------------------FLHFYFILF---------------FYFF--YFFF-----
------------------------------------------------------------
------------------------------------------------------------
------------------------------------------------------------
------------------------------------------------------------
------------------------------------------------------------
------------------------------------------------------------
--------------------LFHF--------------------FFLC------------
------------------------------------------------------------
------------------------------------------------------------
------------------------------------------------------------
------------------------------------------------------------
------------------VRVKYTKDLEEEIREMILFMRNKEGLETNFNESSEIRYFLEM
YIDLCEYKLNLDNNYNTKPENYEYSEEKKKKKNEGENFETYETKGNIQEENENNQMVDRV
EGKNTWSQYKNDFYDLNFYNSYHPVKNIEMMNKEKKQHLTNIPNDYIYGMNDEIYNNGKE
QYIKNYVFNKDSTNVLNNYDENVKRKNIKRFSMSAVHRKENEYCDDEYCEEIYKELKEKY
YYDKDENIEEEEGYYKH--MENIPNKFIKKYNKKLQKRLEKKSKRIKDELRGKKLWMNN-
NGKVNKEMFANFIDTNNYLMNIPSKEIYERKKKKNPYNDD--NNNNYCNIPYKNNNYHLK
LFSKRKFKKKIKFRTTKEGHRYSTKRPIYLIPSNREIINEHITNNSDISHDEELVASRNK
KKFVKQ------------------------------------------------VG----
--------------------------------LFV------CSFVYLFVSLVTAVQAISN
ILKSSLGPQGLDKMLVDNIGDVTITNDGATILKQLEVQHPAAKILVNLSELQDQEVGDGT
TSVVLLASELLRRGNELIKMDIHPTTVICGYKLAMKESVKYIKEKLSERVSNLGKDVIIN
```

```
IAKTTLSSKFISYESDYFAKMVANAIQSVKIINESGKTKYPVSSVNVIKVHGMSSLDSKL
IEGYAIMSGRASQAMPTVIKNAKIAFLDFPLKQYRLHLGVQVNINDPKELEKIRQKEKDI
TKERVNKILESGANVILTTQGIDDMPLKYFVEAGAIAVRRVNKDDLRRIAKLTNGQIRLT
LSSIDGTEKFEASSLGYCDEVYEDKVGDWDLMFFKGCRTSKSNTILLRGANDFVLDEMQR
SIHDALCSVSRALESNYVVVGGGCVEVALSVYLEDFAKTLGSREQLAIAEFAESLLVIPK
ILALKASYDSIDLVCKLRGFYTKFPGVNFEDLKGFKRYGFGLVKGKVANHFKNGVFEALI
SKNKFFRFGSGATITILGKDVFFKVVPEGGKMEEP-------------------------
---------------------------------NQVRNPPVIKLKSHTSSIEFVHVNPAY
SEILASCSEDMSIRIWEIRHEDENVNEVKDPLCILNGHKKKVNILSWNPMNYFILSSTSF
DSSVNIWDIENEKKAFEINMPKKLSSLQWDIGGNLLSGTCQNKKIHIIDPRKQEICNSFL
IHDGGKSTKCIWIDGFGGEDKCILTTGFSKNNMRELKLWSLKNTELPLTTITLDNAAAPL
LPHYDESVGMIYLIGKGDGNCRYYQYSQGSIRKVDEYKSCLPFRSFGFLPKRMCDVYKCE
IGRVYKNENNTDIRPISFYVPRKNSSIFQEDLYPPIIMRDPERNTNKWIDGINQDIKRIS
IKDLTEDDILITKKFKELPKESKSIVIQDNNNAKKGSIMRQFTKKFTFRKKKETTDIQDE
IMGETKSSIEADFQPQECKENKKGNKLNLKDNVDDDDYLITNENEHYKENNDSSIQSIRS
NSKSNILQSEEKNVLDNILDMKMCKSTA--------------------------------
------------------------------------------------------------
------------------------------------------------------------
------------------------------------------------------------
------------------------------------------------------------
------------------------------------------------------------
------------------------------------------------------------
------------------------------------------------------------
------------------------------------------------------------
------------------------------------------------------------
------------------------------------------------------------
-----------------------------------------------------------N
IIFQKMKEETPINNYLCLTDDQEEKEYKNLFDGNLSLKESNNN-TNNVNNNEDINFSNID
ETQKDLYFQSSLFNKLKICGQFNKGFVISKIDLLYFEKKKKKHDNNSINSNDNNLISDNK
HKSNYALFIIDQHAADEKSNFEKYNKIFTMKSQKLISKIHVQVSPAQVHIIQKYMSIFLQ
NGFEVQILEEPIHKRRKTNNNPIDDEEEMLMELNVYLLSLPVFNGKILEVVDFMSLLHHL
TEHPVASYNES-EVSVKTTVDLNNKTDTWFNYNFPRPQKVWRILASK-------------
------------------------------------------------------------
-----------------------------L------------------------------
--------------------FL----------------GTLDLKHVSNYLSIKKLMYKLK
CCGESMLTLDEMKELVIIFLKKISDTYTEDQTKWLEQMKSSQEQQGKALEEAMYKYEKNI
LFHHAVKEQQILQNDKKLNEWNENVENAYEAQQEILRQFESSRKRNIDISLEKNNELIIA
KDYIDKIKEAATDNKYDNSKCFIYPASSAPCGACTSAGAIIHHRRYKEPRRKKEYSLCLL
-----------------------------------------------------------F
TYFCFIP--------------------------LGTDILCQAKSGMGKTAVFVLSILQQL
DTNENQDMQDTKDMNNDNNNNGDNKFVRCLGLAHTRELAYQIKNEFDRFSKYLKNVRCEV
VYGGISMNKHIKLFKEDNIPHIIIGTPGRILALIREKYLITDKIQHFVLDECDKCLEKLD
MRSDVQKIFISTPLKKQVMFFSATMAKEMRDVCKKFLQNPVEIFIDDEAKLKLHGLLQHY
VKLQEKDKTRKLIEILDALEFNQVIIFVKSVTRAITLDKLLTECNFPSIAIHGGLEQQER
IERYDKFKKFENRILVSTDLFGRGIDIERVNIVINYDMPENSDSYLHRVGRAGRFGTKGL
AVTFVSSQEDTLALNEVQTRFEVAISEMPNKIDCNEYINQY--ICFTLIYCPFFQLPIPD
DDGSSDGNDTDVHSMFDRKEIERKVLDLESIHFIQKKRLSVSAEAYGDWNKKIDNFIPKV
YKKDEKEKAKIREALNESFLFNHLNKKEFEIIVNAFFDKNVEKGVNIINEGDYGDLLYVI
DQGEVEIYKTKNNKKEVLTVLKSKDVFGELALLYNSKRAATATALTKCHLWALDRESFTY
IIKDMVAKKRKMYEDILSHVNILKDMDPYERCKVADCLKSKSYNDGEIIIKEGEEGDTFF
ILIDGNAVASKDNKVIKTYTKGDYFGELALLKNKPRAATIKAQNFCQVVYLDRKSFKRLL
GPIEDILHRNVENYKKVLNELGLDTTCIDENMHLKIVCLSDEVREMYKNHKTHHEGDSGL
DLFIVKDEVLKPKSTTFVKLGIKAIALQYKSNYYYKCEKSENKKKDDDKSNIVNTSFLLF
PRSSISKTPLRLANSIGLIDAGYRGEIIAALDNTSDQEYHIKKNDKLVQLVSFTGEPLSF
ELVEELDETSRGEGGFGSTSNNIIYKVPNNLFFFFLK-------KKLGAAFGTAKSGVGV
CSVGVMRPDLIMKSILPVVMAGVLGIYGIIMSILIYGKSKKIF-----------------
------------------------------------------------------------
------------------------------------------------------------
------------------------------------------------------------
-------------------SDYIKAYLLGFTLEDSLALLRIEDLYIESFQIQDVKILKGD
HLSRCIGRICGSNGSTKYAIENATKTRIVIANDKIHILGSFNNIKMARHSICSLILGSTQ
GKIFNKLNILAKRMKERF------------------------------------------
---------------------KKKKKIYMHIF------------TKV-------------
----------------------RIYANVSYFIFFV--DVKNCICMFESGALPYLISGMKS
NIDGMKAACAQTCRNIYVLGNKKKN-----------------------------------
------------------IKN-----KICTF--------------------IMASGWKAD
EVFGGVMSYTYDDIICMPGYIDFALSDIDLTNNMTDNITLKTPVISSPMDTVTGHKMSIA
LALSGGLGVIHNNMSIEKQIEEVKKVKRFENGFIFDPYTFSPEHTVADVLETKNRVGYKS
YPITVDGKVGSKLVGIITGVDYLYLTNKSMKIGDIMTTDVVTGSYPINLSDANKVLCDEK
KSVLPIVNKNNELIALVCRNDMHKNRIFPHASKSQNKQLIVGASISTREHDLERANQLIK
NMIDVICIDSSQGNSIYQIDTIKKIKSAHPDIPIIAGNVVTSQQAKNLIDAGADVLRIGM
GSGSICTTQDVCAVGRAQGTAVYHVSKYAHTRNVKTIADGGIKNSGNIVKALSLGADFVM
LGNLLAATEESCSEYYFENNVRLKIYRGMGSMEAMYNKGFNSKSRYLVDERKNEYTDENI
DEIKVSQGVSASLVDKGSVLNLIPHLFKAVKHGFQSMGIRKIPELHSKLYSGDIRL----
------------------------------------------------------------
--------------------VKKDMKENKQYQEALKELKEKTEIDDKRIKLKKRIEENID
FFQKLKEKNVEAVKILYNDVNNFVTYCFTKYYALKLSKNLCIQFFVTLKNVLNKSTNKFT
ELSEKWDENSAFVKLDKWRQDMAIXRYXKKKEKENQHINNNINEENNDEPSNNIININET
ENDKSQNYELILAQESAWDKFGSKLKDMPFLNNFFENPILGKLFGETELAAALREMKMID
KNFKLSELMYLFEYVISKHIVESYLIGDEETLRLHCGSSAFNSLNASITERKKKKVFLDT
NVLIYKNHELKGAQRMEESSPWFIFTFHTQQINCLKNKNDEIIEGKIDDIREVVYTIALS
KHPEPEKEGLLYPYIVREFAIIGNTPSWKNEIKSESLLQTRPFKLGIEDIQNLGSSYIEN
NEKLKKYNNEISSLKKE-------------------------------------------
------------------------------------------------------------
------------------------------------------------------------
------------------------------------------------------------
------------------------------------------------------------
------------------------------------------------------------
------------------------------------------------------------
------------------------------------------------------------
------------------------------------------------------------
------------------------------------------------------------
------------------------------------------------------------
------------------------------------------------------------
------------------------------------------------------------
------------------------------------------------------------
------------------------------------------------------------
------------------------------------------------------------
------------------------------------------------------------
------------------------------------------------------------
------------------------------------------------------------
------------------------------------------------------------
------------------------------------------------------------
------------------------------------------------------------
------------------------------------------------------------
------------------------------------------------------------
```

```
-MVLYIIGLGLGDEKDITIKGKELIEKSDVVYLETYTSILFVSKDVLEETYKKSIEEVDR
DFAEENCDKILDEAKNKKVSFLVVGDPLCATTHHDIILRAKKKNIDVEIIHNTSIISAIG
ECGMQLYNFGPIVSIPYFEDNYKPTSYYDKIYINLKNNFHTLCLLDIKVKERTVENIMRN
KKIYEPPRFMTINDSIEQLLYCEHIHKKNIITKNTLGIAIIQIGTDNQQIISGDLLTLKD
ISYNKPLHSLIICAPTLHDIEKEYFDLYLMKK----------------------------
-----------------------------------PNSAYRKCVRVQLIKNGKKITAFVP
GDGCLNFIDENDEVLVSGFGRSGHSVGDLPGVKFKVVKVARVSLLALFKEKKEKPRSMGI
KGLTKFIADAAPNAIKEIKIESLMGRIIAIDASMSLYQFIIAIRDSEQYGNLTNESGETT
SHISGLMSRSIRLMENGLKPIYVFDGAPPELKGSELEKRGEKRQKAEELLKKAKEEGNLE
EIKKQSGRTVRVTRKQNEEAKKLLTLMGIPVIEAPCEAESQCAFLTKYNLAHATATEDAD
ALVFGTKILIRNLNANATSNQNKNKNNSKRGYILTEINLEQVLKGLNLTMDEFIDFCILC
GCDYCDTIKGIGSKTAYNLIKEYNCIEKIIENIDQNKYQVPSNFRFQEARKSFINPNVLP
KEDIKIDWNEPQIEELKHFLIKDYNFNELRVTNYINRLLKARKVTTQRRLDNFFTACTKK
STKLIVEETKKQQTLPARKGKKRPTSGDKNKQKGVKRKEENVDDEKNKNEDDIDDIKNMD
DIKNEDDIKNEDDLDNFSSNLFDSDKESESGNIIKKEKQHTENKDTISDEIKKKFLLPFC
PKDVTKKKYTQRC---------------------VNNNKFYEVLNLKKNCTTDEVKKAYR
KLAIIHHPDKGGDPQKFKEISRAYEVLSDEEKRKLYDEYGEEGLENGEQPADATDLFDFI
LNAGKGKKKRGEDIVSEVKVTLEQLYNGATKKLAISKDIICTNCEGHGGPKDAKVDCKQC
NGRGTKTYMRYHSSVLHQTEVTCNTCRGKGKIFNEKDKCANCKGMCVLKTRKIIEVYIPK
GAPNKHKIVFNGEADEKPNVITGNLVVILNEKQHPVFRREGIDLFMNYKISLYESLTGFV
AEVTHLDERKILVNCTNSGFIRHGDIREVLDEGMPTYKDPFKKGNLYITFEVEYPMDLII
TNENKEVLKILKKQNEVEKKYDLENSELEVVSCSPVDKEYIKVRVTKQQQQEAYDDEDHQ
PEMEGGRVACAQQ-----------------------------------------------
------------------------------------------------------------
------------------------------------------------------------
------------------------------------------------------------
------------------------------------------------------------
--------KFWPIFIKDRSYVAYASYNNPKSDINKSDSNYIDESVYKNINELRTEALKRL
KNYLPYLCKILIDNTVYTKWDYLTMDESHFQNDNANVPDLIQDISPELYNN--KNNDIAQ
EEIKMNKMNNNNNNNKNNNNSNNNNINNNNLLNKNDDINNNNNTEDFSDDEKNDEMSSRT
WGNDWTVRKGAALCLDYLSNVYNDEILEFVLPHIEEKLMSDKWNIRESAVLTLGAIAKGC
MYSLSPFIPKVLEYLIKLLNDEKPLARSISCWCVTRFSSWICHPDNCEKWFEPVLLNLLK
RILDSNKRVQEAACSSFANLEEDALELLNNYLHEIVHTIQQAFQIYQAKNYFILFDVVGT
LIDSVNIVKENNELAHEIVYAILSKWVNIRISSPYIIALMECMSCITSAYGKEFLKYAKD
VIRTCIKFLVILYIDLEEEVKYFYSKKLFNSTSFISNRNNISSTANELLTYYKITNDDYF
ICMKDIDTISPSKKKDLIECSFDLLSRILSVINSSIMDILNESEYNFIPLVHKYCLKLQN
IKFDDFNNKIMNNHEELRNIINPDGTLQNGYIMKNEYSELAKQFLNFGILQSNFALIGDI
SRFCPQYLITYLNDIIPFLIAHITHPSTPVSNNASWAIGEISIHINSEYMEIYVDEITKQ
LIYICQNSKYHGCLLQNICITFGRLTSTYPKKLIFYFPQFLKTWLKIMAHGTQENEKINS
LKAVLETLYINLDLAAEHLKDIVYIILKYKYVSQNVNIFFHQFLATMKEKYPNHWKQIYS
QTGDSLSSPIPSDMLSDLATRFNLMKERINEKFKINSYQDYSDKNNEWNIDDAINYIKRN
AFFKKYKDINDVNDDDHINDDDKFYQNFENILIEEKKYLKKLLRESNLKEYMDGKVLENE
ENEESGLMYVYNTCYNILSSKNEINYNEGYIIINKNNTMKKIYINEINELLIEIIYITLT
T-ENLENSLVDLLGDRFLDFIIQIIKNKEQIEKDIKLLSKQIVIKENTLVSNFFITNKSS
KKIKNQHILYKKENIEKIIDHFLYLLTDDSYNQMKKIYIPNDQDKLEEIHVPKNTIYSYT
XHITKGXISRLENYIS-XXELI--------------------------------------
------------------------------------------------------------
------------------------------------------------------------
------------------------------------------------------------
------------------------------------------------------------
------------------------------------------------------------
------------------------------------------------------------
------------------------------------------------------------
------------------------------------------------------------
------------------------------------------------------------
------------------------------------------------------------
------------------------------------------------------------
------------------------------------------------------------
------------------------------------------------------------
------------------------------------------------------------
------------------------------------------------------------
------------------------------------------------------------
------------------------------------------------------------
------------------------------------------------------------
------------------------------------------------------------
------------------------------------------------------------
------------------------------------------------------------
------------------------------------------------------------
------------------------------------------------------------
------------------------------------------------------------
------------------------------------------------------------
------------------------------------------------------------
------------------------------------------------------------
------------------------------------------------------------
-------------------------------QL---------VQVFIFIISSHGYLLYIF
IIIIIILFPYTCIYMPHIYIYIYL-----------------FFIGLLKSIIVNKLTSNGC
TFIFWIADWFAHLNNKMSGDLKKIKKVGSYFIEVWKSCGMNMENVQFLWASEEINKKPNE
YWSLVLDISRSFNINRMKRCLKIMGRSEGEENYCSQILYPCMQCADIFFLNVDICQLGID
QRKVNMLAREYCDIKKIKKKPVILSHGMLPGLLEGQEKMSKSDENSAIFMDDSESDVNRK
IKKAFCPPNVIENNPIYAYAKSIIFPSYNQFNLVRKEKNGGTKRQK-----ENTHTHTHI
Y---------IYIYVYMYTFA---------------------------------------
-------------------------------VKMYVKEEIVEEEKLSDIINKKKENIKYM
KGMKIPENILAISNLKEVIDDADLLIFVLPHQYLD-----VIQKNKIKK-----------
--------------------------------------------------------FIYI
YIYIFFSCI-------------------------------------------FIIYSLLI
LP------------------------------------------------------QGID
TTKEVYDVLEYHQLKTEFPLICTIYEISFQKKNPCSIIDVLSTKKLRNIKYK-MERILSS
IGKLSVVAGGLSLIPYTFIYDVDGGERCVMFNRFGGVSENTFGEGSHFYVPWFQTPYIYD
IKMKPKVINTTTGTRDLQIVTISLRLLFRPHTQHLPYLHSTLGPDYDERVLPSIGNEVLK
AVVAKYNAESLLTQRDKISKEIRENITTRAKHFNILLDDVAITHLSYGKEFAKAIEDKQV
AQQESERVKFIVAKTEQEKIAAVIKAQGEAEAAKLISSAVKEYGKSLIEIRKLEAAKEIA
ENLSKSKNVTYFPSNSNILLNPTDFMKLEYINILKEENGGYNFDNLKRNEILKEKGVKFP
QFRKTGTTICGLVCQNAVILGADTRATEGPIVADKNCSKLHYISKNIWCAGAGVAGDLEH
TTLWLQHNVELHRLNTNTQPRVSMCVSRLTQELFKYQGYKVCAIVLGGVDVNGPQLYGIH
PHGSSCLLPFTALGSGSLNAMAVLEAKYRDNMTIEEGKNLVCEAICAGIFNDLGSGGNVD
ICVITKDSYQHIRPYKEPNMRLYHLPHPTIYPKGTTPILSEKIEYIKKFISVEDA-----
---------------------------------------DT-------------------
------------------------------------------------------------
------------YW----------------------------------------------
----GLKTPLGLKTPLGLKTPLS-GLQTPYMRNSSGMDTPLINNNIKSNMSISGLNTPFT
LSGYNTPLSASNVSGYNTPLFNNTHKLSLNDLGEARGTVLSVKLDELIDNVEGQTVIDPK
GYLTNLNASSLINDADIADINKARSLLKSVISTNPKHGPGWIAAARIEELAQRKDKAKEI
IMKGCVVCSKNEDIWLEAVRLEEKLSEVKIILAKAIKHIPTSVKLWLEAYKKEKNVDDKR
KVLRKAIECIPNSVKLWKEAISLENENNAYIL----------------------------
------------------------------------------------------------
------------------------------------------------------------
```

```
------------------------------------------------------------
--------------------------------FIMVKINRNCITP---------------
------------------------------------------------------------
------------------------------------------------------------
--------------------------------------------------------HNKN
VNPIISEALKECPSSGILWSKAIELENKNLQNSKSVSAFNHCGNNAYVILTVAKLFWVNF
KIQKARKWFYRVINLNPHFGDGWATFLAFEIDQQNEINQKDIINKCIKAEPNRG---NNR
YIHIYIY-IYIYLFMYYY------NVVY----------------------SSSFYINCLF
QLNGRLNYLKKYLLLINLFKLQNKDINLFVCGLRKMEKDDVVSSTPVATEEMNDKKKEKK
AKKQAEKELKLAKKLERENLKNEAAKVLDYVCEDIHKDNYGYIKVSTLQKYGDSIELYNL
EDIYNFFVKSEDCENEKREDVKDAYNKNLLGKKKIWVRGRIHDIRSKGSIAFIILRHKLY
SLQCILDIKNNNNDKNMMKWVSNLSLECIVDIYGEIKKPEIPIDSTNIKYEIHINKIFCL
SKTMKELPFLLKDANMKETNDEITIKVNQDNRLNNRCFDLRTYANYSIFSLQSVICHIFR
TFLLQHNFVEIHTPKLLGESSEGGANAFKINYFNQNGYLAQSPQLYKQMCINSGFDKVFE
VGPVFRAENSNTYRHLCEYVSLDIEMTYKFDYMENVHFYDCMFKHIFKELTNNEKNQTFI
KTIKNQYPSDDFVWLDKTPIFTYEEAIKILIKNGKLFLKEEDILTYDLTTDLEKELGKLI
KLSHNTDYYIIINFPSSLRPFYTMYKEDDPKISNSYDFFMRGEEILSGSQRISDMKLLLE
NIKLFNLDPNKLNFYIDSFAYSSYPHSGCGIGLERVLMLFLGLNNIRKTSLFPRDPKRLI
PMEITNIFKKLLCRQNVVHTENNSFFDVFKDPNEEGLKRK--NEVDNEMKNK----SVMK
ENKDDTLKTSEEDKLKEQNLLGYANTFDAPNALLFENLNKEYKFITTQDNFDGFRFEVDK
NINKFLQSTHTLFLGTTLREVGYLYQFGANFTNLDNTLLMISRINIDGSVNGRFCKKINN
TIDCKLNFNTYAKSDTRNMYEMSLEVNKPLYTYNFKSIWQGAWIFNTSYTQLLTKKLQAG
VDLTYIASNCASIGSFGLRYNHKNNVLTMQIVRQPNFKSPEFMLNQTHLYKIQYAKKISD
RLSLGTELEITPQTKESAMRLGWDYSFRHAKVQGSIDTSGKISVFTQDYSGFGVSGYIDY
LNNDYKFGFMMHISPSQEQPQPAA--LENLKKNIYVIKRRNNKMGIYLYVYIYIYIFFFF
FFLATLGVEVHPLKFQTNFGKTQFNVWDTAGQEKFGGLRDGYYIKSDCAIIMFDVSSRIT
YKNVPNWYRDITRVCETIPMVLVGNKVDVKDRQVKSRQIQFHRKRNLQYYDLSARSNYNF
EKPFLWLARRLSNQPNLVFVGEHAKAPEFQIDLNIVREAEKELEQAAAVAIDEEDIEN--
----PYI---------LFLFLFQFFFFFST----FKANNVGVITVKSILSEPTIHQYDDI
KKLIKNKLQECVPFYNYNMNRSFAEKIYGDCIYDNYGLSKEINEINLIILEEWNINCNKN
RVLKNTGLIKEITINQFKYSTNKESLEVHFAVSPKYTFEELSTMYKNEKGLYEFLLSPII
KIICNENDKILLDNMNEECTYLNVEDILPKNKVLPPSGIENIDYERSKDVTPWDVNINEE
GINYNKLIKEFGCSKITENHIKRIEKLTNSKAHHFIRRGIFFSHRDLDFLLNYYEQHKCF
YIYTGRGPSSLSMHLGHLIPFYFCKYLQEAFNVPLVIQLSDDEKYLFNQNYSLEYINTLT
NENVKDIISVGLNPELTFIFKNTEYAGYLYPTVLSIHKKTTLNQSMNVFGFNHSDNIGKI
SYPSFQIAPCFSQCFPNFLGKNIPCLVPQGIDQDPYFRLSRDIAVKMALHKPVVVHSVFM
PGLQGVNSKMSSTKKKKDKSNSTFDHNNSVIFLTDTPEQIKNKINKYAFSGGGTTIQEHR
EKGGNLDKDISYQYLRYFLEDDNKLNEIGEKYKKGEMFSGEIKKILIDVFTELVLKPQEK
KKFFTDEEIFFFFDPNKPFF------MTTYNDNTNKHQINITKLTKDELDFTLYNSNSGI
ANALRRIMLSEIPTLAIDVVNVYENTSAFHDEFIAHRLGLIPIDSRNVNNYEFREKCKCK
ETCSKCTIQYIIEVKCNHSNKIDVSHYDIESLEHEPNVPMPIPHGNKNSV--SENAIPIV
TLSKNQTLHMKLIATKGIGKMHAKWIPANVSYRIDHKVLIKHNLIDKLSNEHKLLLANNL
NNDCYILNKDTHDDDIQLKLKENMSVVMAESSIDLLSELGYKDIIKIVYDETMFHFHVES
VGSIPPEQIVQMAIDILENKLKVLEPQIKSSFYSIDEVAKQLKEQGVSLYGIQLDLEMVE
NKSKVKDISLAPFGKMQMEISENEMPGLMRIREEYRKDQPLKNAKITGCLHMTVECALLI
ETLQKLGAQIRWCSCNIYSTADYAAAAVSTLENVTVFAWKNETLEEYWWCVESALTWGDD
NGPDMIVDDGGDATLLVHKGVEYEKLYEEKNILPDPEKAKNEEERCFLTLLKNSILKNPK
KWTNIAKKIIGVSEETTTGVLRLKKMDKQNELLFTAINVNDAVTKQKYDNVYGCRHSLPD
GLMRATDFLISGKIVVICGYGDVGKGCASSMKGLGARVYVTEIDPICAIQAVMEGFNVVT
LDEIVDKGDFFITCTGNVDVIKLEHLLKMKNNAVVGNIGHFDDEIQVNELFNYKGIHIEN
VKPQVDRITLPNGNKIIVLARGRLLNLGCATGHPAFVMSFSFCNQTFAQLDLWQNKDTNK
YENKVYLLPKHLDEKVALYHLKKLNASLTELDDNQCQFLGVNKSGPFKSNEYRYMGSYGM
DVDDAYLHQGQYAAPYDNQYQPDDDTPSPRGENHTPFVGYFSSHLLRTGFFLQCVSLVLM
FVFYWAFGGTGIFIFDLYAGPECVKVSSTFHLTISILMALYLLGTLYIAMFQVFVADNSK
WCRGFRAGSKLLSAAVTLDLLSSILRLVQYLYAYFYMSMRWWARYQQTKSDWTLLHFGSI
VHSFALFIYGAAFFYMEAYHDEGTYEELAWSNLTLFKLAGLAGAYIYIYIYIYIYIYILI
YFMYNNVYWIIRAKFFY-------------------------------------------
----------------------------------YLYKVM--------------------
-QEDANKPKKRTFRTFQYRGVDLDKLLDLSQDELIKLFKARQRRKFQRGISKKAKSLLKK
IRKSKKNCEPGEKPNPVPTHLRNMTIIPEMVGSIVAVHNGKQYTNVEIKPEMIGYYLGEF
SITYKHTRHGKPGIGATHSSRFIPLK----------------------------------
------------------------------------------------------------
------------------------------------------------------------
----------------------------------------KLMVNVFLGEAPEQFELKNL
GNEENTPTAPHFLENQYAGEAPFDEWAKKEEWNGYSNGGWKNPIAAEEW-----------
------------------------------------------------------------
---------------GFLQIIKDHHETMYELKQKIRPRDQVVGWFCSGSELSELSCAVHG
WFKEHNSISKFYPHTPLNEPIHLLVDASLESGFLNIKAYVQLPISLVKDYFVHFHEIQTE
LLPCNVERADVLLVKENIHSHKDNHTNNLEHNHNNNQIDMNELSLKKLLIMLKQCKSYVQ
DVIDQKKKGNISVGRYLHKVLSNDTFLTLEKFDSINESVLQDNLMISYLSNLANLQFLIA
EKLNSSSLQ---------------------------------------------------
------------------------------------------------------------
------------------------------------------------------------
------------------------------------------------------------
------------------------------------------------------------
------------------------------------------------------------
------------------------------------------------------------
------------------------------------------------------------
------------------------------------------------------------
----------------IPM-----------------------------------------
------------------------------------------------------------
------------------------------------------------------------
------------------------------------------------------------
----------------IIYVLQKAGLNVCPENTLKIFFNQIFAXLQYSTDRRVVYNQKKN
NDDVDEDELLIIDREEELEQNYRTNLLDILGVLIKYHPTQFLNTCCELCIGFINNYMNSP
NSEDVALALYVCDDLLEFLQEKSVNLWDFFMNPLLLNINHADDKVKQAACYGVIQATKIE
AFGKYANIAVEYLLKLVHESTSNKKPKEYISAIDNAIAALGDVVLMHTSKFNNAEDLIKV
WLNHLPIKEDDAEGRRVHKNLIDLVSQNHPLLFGKDNSNTSKIIEIFLTIYETDFSDTDC
NKKISTLINSLDKSYLNNLASSTLSHKQAKKLNNILNPNRKMPTISVYEDDLFEKLGEEI
IEEKLLDVCFDFGLEVDDIEYKNDKKIYKIEVPANRYDLICVEGLCRALKNFMCKFDDIK
YDISMNNYDICIKGNQYIKVDGSVDDRRGYVVCCVLKNMNINDSVYNNIIEIQEKLHHNL
GKKRSVLAIGIHDYDKIKFPLKYKFENKEKINFIPLNEKINLNGMNLMDFYSKNLNLKPY
LKIIKDFDKYPIIVDSSEQILSLPPIINCDHTKISLNTKNVFIECTAIDRNKAQIALNIL
CSMLSEYCVPKYSIQSFVVIYDDKNIKKKETQFLYPIFENKSLTCNIDYVRKLSGISHIT
VHEVNNLLKRMMLS-CDIMDNNTFKVTIPFYRSDIMHCCDIIEDIAIAYGYGNIKYEPPQ
ICKKHSLNNCSELFRNVLVECGYTEVMTNALLSRDENYNCMLRTHKSYDDPNINLDEYNP
LAAPIQIKNSKTSEYEIIRTSLIVNLLKFVSANKHRELPLRFFEIGDVSYATYNKTDTNA
VNKKYLSIIFSDKFTAGLEELHGVLEAILKEYQLFSDYKIEEKKKENISIRSDVYYKLIP
KEGNK-------------------------------------------------NNEMKM
GDNNTSSGSSPVLSVGNIRGFGGSDFGSFRMSNEFLGWKNKKTNNVYQYKCSDIDEGCWI
KTSYNNNRLHLKLGESKENIIIYFDGFPDRNVNEITQHFQKYFNIRLNNRKIATKGWNWG
EFKLENSNLCFDIDNKYAFNLPTNNINQLNVQIKTDIAMEFKNEENNKGNEDFLAEIRFY
YPHENDENQNFQNLKNDLLEKVNIGDTKSESIASLSNIPLLVPRGRYDIEMYSSTFKLHG
KSYDFNIQYTNINKMILVPKSNSNQYVLIFSLSNKMKQGQTEYPFILIQLNNDDDMELDI
SASDEVMRKYKLEKTISGKAHDVVTKLFTALVNKNVIVPGDYRTSKNQHGITCSYRAASG
QLYPLNKYFLFIVKPVILISFDDIVTLSFQRTGNINQHRFFSLIIKHKRGMSYEYTNIDK
```

SEYNPLLTFLKSKNINIQDDANDLEKKQDFHNELDESDEEEYVADDDDDEEDYVAEEEDE
DDDDDDDDEEEEEEEEEEDDDK--------------------------------------
--PKKDISIYQKEEYP
QKIIIKCEEKFSKSKKVHQTVRHVAKEHGITVEELNEKAIWPLYERYGHALDALKEATMN
PENVFKGLDIFEEIKNSLLKDIKLRLTPQALKLRGRIDVWCFGYEGIDAVKEALKKGKEI
SNDKVSINIKLIAPPQYVIVTSCHDKDLGMAKIQEAMKVISDKIKEYKGGDFKQQGEILV
IGGDEEKRLEELLDKHDGISSDDDYNTSDEDDENSSEEDENTSEDEEEED----------

--ILY----------------------IIFLFSVNESGGFQGYAKMVTMPIKNLYENLWG
PITSRLGGNFRIQWIKMAKIDFDNFKHIVNPYNDNLPLKKSRDGTELPLNVASILCNKMN
DMPNEDFLTGTIYEFKRRINHSSFFINLHKQNILNTNTMWDILIFNLNQKSDCTNITLID
GTEQNITI-----------------------NIYIYIYVHIIYSMYTIIYYSFFF----F
ISPFFFFFFFYLSFFFFF------------------FFIPPFFLFFYFFFFSIPGFPLTS
LNKSKTFNQCNELTYENDPQNSRKKKLKKIIEHSQNSLLSGERGIFEFLLCDVGVGLSLS
VNEKDVSTYNRDVLPIEYDSIFIKKQKEESLDDALTIHYKNKENENNNNEINLALGGDIM
ATYGVLPYYTFHHEYIIYDNSQILPRYLIQFECDPNDDEHFSLPLCDYCSDAPAILYCES
DEVKLCEKCDTLIHSQNKIVKKHIRKALNEAQKISGKCKRHMTNDVNMFCTICHIPICNL
CISSHVHTDLTTNQLKNSNNNQDTIISLNMAYKAILHHSNNPSNFIKQKKKNLNDLLAKI
DTLHEQVRINMNDTEKSVYTILEDLVKQLHIITDQKMCSVLSEEYELKRQFNEIIWNESF
LYYLQTILPPADFMNAWLKHCQFREQIEKNSKVVEELHSLIFPDMRIKGNINIVTESSMH
HDKIYHPSDKL-------------------------FFFLSLLGKSTTFNVLTKLNIPAE
NYPFCTIDPHEAKVTVEDERFEWLVKHFNPKSNVHAYLSIFDIAGLVKNAHLGEGLGNNF
LSNISAVDGIYHVVRAFENEDIIHTEGNINPVRDLEIINSELIYKDISHCEKNLEEVTKV
LNRNKKDKVKQNEHDVLTSVLNYLKEHKWIKDGTWKSNEIEVLNEYNFLTAKPVVYLVNM
SEADFIRQKNKYLAKIYNWVQEKNKGTIIPYSAEVEQKILSMDEEEKKQYFQTNNIKQSM
LNKIIKTGYYEINLIHFFTCGHDEVKCWTIRKGTKAPQAAGVIHTDFEKGFICAEVYKYT
DLVEYKSEGEVKANGKYLQKGKDYVVEDGDIIFFKFNVSSGGKKMSEYDIAKNDVTYTKL
DTIECDIPINEELSWRINKFVNQLRISYSTLEEFVDNFVYELKKGLEAHRKHPNLWIPHE
CSFKMLDSCIANIPTGQEKGTYYAIDFGGTNFRAVRASLDGKGKIKRDQETYSLKFTGSY
SHEKGLLDKHATASQLFDHFAERIKYIMGEFNDLDNKEVKSVGFTFSFPCTSPSINCSIL
IDWTKGFETGRATNDPVEGRDVCKLMNDAFVRAAIPAKVCCVLNDAVGTLMSCAYQKGRG
TPPCYIGIILGTGSNGCYYEPEWKKYKYAGKIINIEFGNFDKDLPTSPIDLVMDWYSANR
SRQLFEKMISGAYLGEIVRRFMVNVLQSACSKKMWISDSFNSESGSVVLNDTSKNFEDSR
KVAKAAWDMDFTDEQIYALRKICEAVYNRSAALAAGTIAAIAKRIKIIEHSKFTCGVDGS
LFVKNAWYCKRLQEHLKVILADKAENLIIIPADDGSGKGAAITAAVIALNADIPQLPMPD
FYFLIRWLCKVIVKSVFRDVNVINPENVPLYGSVIFVGNHNNQFIDACVLIANIPRQVKF
IVAEKSMRRAVIGKLASVIGCISVKRPQDLKFKGIGHICWNEGDVKITGINTRFRLDVQM
GDKLLIQNKMFPVVKIESETELLIQEVINIECEDKINGVPFKILPKINQTEVYNLVTNSL
KNGDTIGIFPEGGSHDRTNLLPLKPGVAIMTLCALADGIEDVSIIPVGLSYSKLYQLQGC
ATLFYGNAIIISQDLCKEYNNNNREAISKLLSKIEEGMRSCMLTSKDHETSRCIELCVSL
YTPERMTISKNKIYNNLQLFCKMFWKFGNSKVIENLSYELKCYEKLLQANKIKDDEVWML
KQSTSAATLKFIEHICTFIFCVIFGMTFSLLWLPLVLISIYLAERHRKDALRNSTIKIQG
GDVVSSYKVLVLIVLLPTFNIVYGLLFSIYLYHSWLKRILFVFLSMCILPICYYINLNYA
VQIPSLLRQMKILLKVICGKINVWRDNERELISTRHELQLRVRDLVSTLGPDVSDDFLEQ
LYRNIPKFVVDADTKRLIRGKDEFLPILQRSQLEYKEEIL--------------------
--EEYKLPSGYAWCVCDITK
ENDRSDIYNLLTDNYVEDDDNVFRFNYSSEFLLWALSSPNYVKNWHIGVKYESTNKLVGF
ISAIPIDMCVNKNIIKMAEVNFLCVHKSLRSKRLAPVLIKEITRRINLESIWQAIYTAGV
YLPKPVSTARYFHRSINVKKLIEIGFSCLNTRLTMSRAIKLYRIDDTLNIKNLRLMKKKD
IDGLQKLLNEHLKQYNLHAIFSKEDVAHWFTPIDQVIYTYVNEENGEIKDLISFYSLPSK
VLGNNKYNILHAAFSFYNISTTTTFKNLIQDAICLAKRNNFDVFNALEVMDNYSVFQDLK
FGEGDGSLKYYLYNWKCASCHPSKIGIVLLF-----------------------------
---IYMLKYEFLYFSISQMDID
SVLGLQAILISANYKEKEFIRIAYYMNSFYKDMELRENPPVVPQYDKICRHIFVENPRIV
KFSIGWDSEEKDEFKEFDKEIEKIELLNCTQIKDENENNSNITNQSTQGNFVLSNNDPNN
NNNNNNNPSITSSMIFNVNNNFNQNNFLKNNELSSDLDKCEIFNANINGISRITIDNKNS
MKICLIPVFLSIIYVILFLKGKKIG-DLYITEHLRNKFENILLYFNIFEVQYPRSYINNE
KLLGLHVRDIEKDAYRVYPILKELKQKDYFRVFKVNLHLSCKFLKGNEKCKEIKKCSVCE
CEQDEIPYNFRTNEIEIIENKMTNEDLKKTFIESKLYKDILGIYAPSNEGFISYVDLVYN
SPSFTAYEGRNIW---

---HTP--------------
----SDMLIYIYIYIYI--------------YIFIHIC----------------------
-----------------------------IFFFLASELRGSSNYKYFGAAKNLKGVRELL
FKENEDKKQLNIKKKKDARNFEKVINIHYFGYCDETNEHLLQQEVKIQKKLEKMDLKILK
KYKP-MGIFRSEIMKHGTLVLPSDRAREYLDCLGKEVDIQFIDMNEKTMKRQYKKYIQRI
DDMERILRFLEENINKLPNVKIKKSKIDNFLEHDNIYELDQVEESLNRLHVQFVRFCNNN
KDLIDEKNNAIEEKHVILTALNQLSPGVIRIGADQQHHHH---NNNNIVSPFDEVEENNI
SLSTHIMKDGINMMFTNISGVIKTKDQESFSRTIFRAFRGNTYTYFQNIDEDADDNDLLK
NVEELESLKVGDTNNMYKNNKKKKKDLKSVFVVYCQGSAQSNIYDKIMKICKAYDVKTYD
WPRTYEHAKKRLKELREIINDKEKALKAYEEYFINEIFVLINVVEPNKNSLIEEWKLFCK
KERHIYNNLNYFEGSDITLRCDCWYSANDEEKIRHILINKSSNDLVSALLLSDKILRPNV
SPPTYIKTNEFTKSYQSMVDTYGVPRYGEINPAISTIITFPFLFGIMYGDVGHGLCIFLF
ALFLIIMNNKVKNKNNNEMVTMLFDGRYMLLLMGFFAVYAGFLYNDFFSMPLNLFSSMFM
LDKQVDNMEYYKRREITDSATGEVQYAYPYIFGFDCKWLGAENELTYINSFKMKFSIIIG
FIHMTFGVLMKGFNALHFKRKMDFFFEFLPQLIMMLSMIGYLVFLIIYKWVTPVGYGGFQ
KQGIINTIINMYLMKEINSTNQFYPYQNIIQILLLSLFVLCIPFMFICKPAIRTYHIMKE
KKKTMAKYTEAGAKEMINAFHHNAKDGNLNNNNNLLSMHKRNTKISSNYDENEY-LNKKK
KKRTDDDIEAHLLSSTYEE-KGLQSSSAEENHHEENISEIWIEQLIETIEFILGLISNTA
SYLRLWALSLAHQQLSFVFFEQTILNSLKRNSFMSVLINLILFSQLFSILTIAVILCMDT
LECFLHSLRLQWVEFQNKFYKGDGIPFKPFNIKKLLNENEMIGIQEGRAFFNPTRNPNNR
PENNIRIEENLPSLNEVYNYFQDFLSRYSSNTLKEGNLKRTLFENVKNNNFTLDLKLDDI
YKQQTVFENNEISATPRSERKMKKPENYSAILARALSERPLTYLPTIERVCYEVCETANI
LNDEDEHLNYIQINLLNTFIRPTPIRGLLAATQERFVVVPGIIVQASKPQHKMRKITLQC
RYCDHKMSIDVPLWRDKPQLPPYCRYSSTMKSSMGLGNAMDSQLGCNGVLEPYVILPNEC
TFVDIQSLKMQELPEAVPTGDMPRHLQLNVTRYLCEKMIPGDRVYVHGVLTSYNPNPRPT
RADGTNFSYLHVLGFQKYDDMSGNDLNFDVEERNELTLLAAEHNIHEKIFKSIAPELYGM
DEVKKACACLLFGGTRKRIGEETKIRGDINMLMLGDPSVAKSQILKFVNRCAPVSVYTSG
KGSSAAG---

--RRSS
IKRKNNIYIY-IYIYIYIYIYIFIYLFIYLLIYIFVLCFRVQYLLAKIRSAARYLLTLDE
KSSKRIFQGEALLRRMVRQGLLGENEEKLDYVLGLTLPKLLERRLQTKVFKLGLAKSVHH
ARVLIRQRHIRVGKQMVDIPSFLVRVDSEKHIDFATTSPFGGARPGRVKRKSLKKQKEKT
EADAEISSTVQIIIVIIISYYNYYPFLKFLEYAIKAVKNTNITSVGVKGENCAVIISQKK
MATQYISQDKLLDYNNITNIYNITDEIGCSMVGMPGDCLSMVYKARSEASEFLYSNGYNV
NAETLCRNICDKIQVYTQHAYMRLHACSNIYIYI----------------------YI--

```
-------------------------MDDHENIDINDNSSDANTANYNQDKNGNTSEVNDY
SKEDIEGDENLKSSKGKQENNTKSSSDKDENTIDYGNHDFPCNPAPPVPIKLFIGRVPKN
IEEDQLRPIFEEYGIVNEVVIIRDKITNVHKSSAFVKMASISEADNAIRLLNNQKTLDAQ
LGSLQVKYASGELNKLGFPQNIESGVDQAKLFIGSLPKNITEDNIKEMFSPYGTVEEVFI
MKDNSTGLGKGCSFVKFSYKEQALYAIKSLNGKKTLEGCTRPVEVRFAEPKSSKQPQIPL
TLQPMQNPPHAMAPQPSISSPNNINFGNNFSVNN--------------------------
-------------------------------------IIHVKLDLGKN-IIQ------EK
VDHIIITNKQT----------------------QHNG----------KCQKNSKRYL---
-------------------------------------------------------YSYRI
QYICYSFFYSSKKGGNRKVSQTLKVCSRDLPGHLKMKTRDLSDHVEDKNMIKNNLIQLEN
KNNKETIKGNKLLAIENIKKLTNHDFKNPFPEDEDDIIGDEWKKNCHKKNKKKKDMNDYN
SSDNNSSDNNSSDNNSSDSSDNNSSDNNSSDDDESDEEEKELLRELENLKRERMEKLKKE
KEEQELLKKKKNNVLTNNPLINLEDSDNDEVSTKKRKWTDDAIFRNTCEKKKKTPSYIND
TVRSAFHKKFLFKYIHMDSEETINLAVKYAKEAVVEDEKKNYKEALNLYIQSLQYFNFFC
KYEKNSNIRDLILKKMEVYMTRAENLKEMLNKKDSIENKEKITNTEETKENMKKQIKQFI
LNKNNNIKWPDVCGLETAKEVLKEAIIFPLKFPKLFNSSTLPYKGILLYGPPGTGKTFLA
LACSNECNMNFFNVSSSDLVSKYQGESEKYIKCLFETAKEHSPAIIFIDEIDSLCGSRTD
GENESTRRIKTEFLINMSGLTNYKNNIIVMGATNTPWSLDSGFRRRFEKRIYIPLPNIYA
RSKIFEKYINQNENN-NISKEDIKQFATLTENYTGADIDILCRDAVYMPVKKCLLSKFFK
QVKKNNKICYTPCSPGDSDPTKVEKNVMSLSENELSLPPLTVQDFKTAISNAKPSLSVDD
IKKYEEWTHQYGMNGTKYIYIYIYMYIC--------------------------------
----------IYVNIYIFFFFF--------------------------------------
---LKILHTCYIFYFFVTGLYVKCGSRYEEINDKVNEQGMSVMLENMAFHSTAHLSHLRT
IKSLEKIGATVSCNAFREHMVYSCECLKEYLPIVTNLIIGNVLFPRFLSWEMKNNVNRLN
LMREKLFENNELYITELLHNTAWYNNTLGNKLYVYESSIENYTSENLRNFMLKHFSPKNM
TLIGVNVEHDELTKWTSRAFQDYVPIPYTNQKEVTPKYTGGFISVEDKNVKKTNIAIAYE
TQGGWKSSDMITLTVLQTLMGGGGSFSTGGPGKGMYSRLFLNVLNSYNFIESCMAFSTQH
SDTGLFGLYFTGEPSNTSDIINAMALEFQKMNRVTDEELNRAKKSLKSFMWMSLEYKSIL
MEDLARQMMILNRILTGKQLSDAIDSITKEDIQRVVHNFLKTKPTVVVYGNINYSPHYDE
ICNILGMISGIRVNDNCVTEFNNMKIRKTCGWIIFVIQNCEIIIHSKGASTTLTELVQSI
DKNNEIQCAYVVFDAIKWIIYLMHKH---------------------INKIN--------
-------N----------------------------------------------------
------------------------------------------------------------
------------------------------------------------------------
-----------------------------------PDGYPELASVNFGNSTVVRCKKCRT
YINPFVRFEAGGKKWNCNMCYNINDTPQFYFVPLDEKGKRKDLFQRPELCTGSVEFIAPS
DYMIRPPQPPVYLFLIDVTVTSVNSGLLDVVCSTIKSLLPKNNDSTNNNNNNNSKNLKSF
DSRTLIGIMTFDSTIHFYNLNSNLKQTQMMVVPDIQDIFIPLPEDILVNVHECQNVIDVL
LDNLPGMWRNNKISDCCAGNALKAAFMVLKKVGGKLLFFLSSVPNIGDLTVSVNRDNKDK
SKYKNIYSSSSSANNVVDSKLREVELLNPCNNLYAELAQNVTQYQIAVDLFACPLYNLDL
ASIYPLIKNSGGSLYYYPQFNVHQYNDKLRQELLFALTTETAWESVMRIRISRGWTITNW
YGNYQFRGADLLALPNCHSGQNFSIIVDLEENVVQDSIVYVQSALLYTNSNGERRIRLHT
YALPITQNIKTITDSINPQVVVSLLAHQAIDISKKGKIADGRNLIQNLCSQVLSSQLLQS
ECARLLSLYILGMLKSIAFRDSGDVPPDLRIYHWYRLENIPVESVEANFYPRMFSLHNLE
KHHGHLDENNNIVFPDALNLTCENMTQDGCYIVEDGETIVMWIGRYIKAKMI--------
------------------------------------PRYCKCGCM---------------
------------------------------------------------------------
-------------------------GAPGTGKTTSILCLASEMLGNQAKKAVLELNASDD
RGINVIRDRIKSFAKEIISLPPGKHKIIILDEVDSMTTAAQQSLRRIMELYSDTTRFALA
CNQSEKIIDALQSRCAIIRYFKLSDDQVLKRILKICDLENIKYTDDGLDALTFIADGDLR
KAVNCLQSTYAGLEVINKENVLHICDIPSPERIENLLKHCVNSEWKKAHDIAYSMIKEGH
TPYDISLTSSNVLRRFNIGSEVIQIEFLKIGAMACNTMATGLTSVIQLDKLLADWCMAAK
ILRSKAMAKNQYMEDRNIREP-NSLLGEETEQLVDSFHYENNSSSIYKRVNSNRSKNGKH
SMAFHKSLAVVNVAAGLDGCDDQLLPASFRALEADLNLHPSLLGYITLAQTLMLSLFSPI
WGFLSDKYSRKWMLVFGTALWGVATILLANINDFAHILFFRAINGLALGSIGPISQSILA
DAAKNESLGLSFGLVQLSSSLGRLIGGVVTTTVALKYFGGIRGWRLCFIVVGILSVLLSI
IVALFVEDAPRQVRKNKKMDYLDSSLDNNNSFTGLSHQSTRTYILYQNIVELLKDSLSKK
SIIIILLEGFTGTIPWLALSFNTMFFQYCGLSDLQAAIITGFLLIGSAIGGVVGGHFGDI
MHDISNKHGRPLLGQLAMFGRVPLVLLIYLVIPKRKESFELFALSCFCLGLSSIAGVAVN
RPIVSDIIRPDYRGTVFSLTIAIEGVGSSLIGAPLFGYLAEKIFKYQNNNLLIADMPEDI
RINNAQALSKTLFYLTIIPWILSFIFYSLLHFTYGKEYLKMNEIIQNEYKYDDEDEETIP
EKKMLKRGGDPGVSTAVSSIMQHENFKRMLMFGLRSLSDFCNPTSKAYKENASDALDRGV
VVSIKNAVINYKDDDDILFCSSRVLLSMSDYCMSEKDTNALKKLITDGGVDGIVEIVKCF
PSDQDTLKNCMAFIKNMNDFNYQIEGREVGIALLNVFTSKTYTNKLTNGIVLALCIISKS
TSGSKGLNDEGAHHKLLDYCLNINSLNDDTAEIVESVFDIIKNMSSNGYVDPTIIEKSVI
ILDKFKSYPRVISKGSDAMKCAVGPEELTKCLNVLKKSAQGSKEQDAALELLSSLSYISS
ITDKVVESGGIPVLIELINSGLQQYESNPEKISRLVAGASRMLGRISNNPPHAAIVVEYG
GIATLCTAISYFPNDVECSKAICNALTPFVSRSNYVSEINNYSLFASLLPILYGSLESVE
LAKASMECIASASMINEFHEQMVNNQAIEILSTCVQYHLTEMDYLLNCFTAYFRLSDYIT
TVEPINQYGGVDGIANALLAVSSNSKVVEIGLKLINKMLTTSDSVNYLSNKQIVDSVLTV
MLENENKEVIIQEGTKIMEKLATESDCQRHITNLETIFNSSETNQEEAYKTLAAISGLSR
IESLKNILESKGADTSIFNGIKIWIESARFIEQTKLIKAGLKTIKTLKLNASATLHDVLG
SIVDLMCLSQVKRIAESDEPDENILITSSECINYLTEVNKIHSAEIVEASLENIFKLMKK
YSESRLTQINLISAMNNILLSSNKIGVDILINKGYIKHIITYLQKVPMYVDVQIIGFTVL
ANLVKISPDSVESIKKLNALIPLQTALRTHAKNMKLKTTCAPLLSVLMPLDSLTNEIQDI
IKLCNKSMNDKNLSKLHEYLVALNELLLTPEACKIASRSNIGEFFNDLTAWLKKNPTVYD
SSSKDDYEISGRSLFDAVISEIAHASTNISQTRLGLVHLTKCNMVSSLVQLHDILKLPGD
NYTEEAVSNILEALSLLLKYDITNAEIGFNSGLIKKLCAGINYFSHSDSVINKTFGCLAC
MCTSKNRVGQLISCPEYEGLIKLIVELIGDSEKNKLSRGSAIKAVYELLKTEDEDIIKDI
SCKTSIVDNLYKIMGEYQADLSIVQDCSRCLAIIVDYVNIEEIMKVDKYTPMKVLLECLN
KSKNDELTVLEMLTVLVKLCNSDDKMKLKELGAIDVISDITMIHSENEEISRLGGVMYSY
MGADEQVKKLMKLILNVKKEDSDAVQKIDNFTSKLEMFLRAPLENPSDALQYTEATLQVL
NSYLASEVDNSSLQTNIALVTKRLVDRVKHDSEDPLGSWAVASAGTLNQYIDMISNKIGL
SNFKFVSPVYGVLAACVMNPYTKQLVMDKFPLLIDNTYEILEQNKNRPSVVQGVFEFLEQ
VVNDEEGSKLLYKKYNGSMGNLIDQTITVMNLNRANDSVYLPGIKLLSGICQTASVSGYG
DDMNTSNIIESCELLLNLGSEKDRTVEFLNLIDTMILGNLLDEKVASEALKKINSLVSEE
NLSKYTEENRVEIIKSYAGLVKDCAATGLFAHIRGIEKVEILNNLGKCMEYEQNEEVIIA
VLDALSQISESDPSIATKILTSSLPIIMENNRDNILNDAATGEAFLQSLENLVLHEGVGR
QLINNTELQNLLKDLEQALDERKEELGNDYVEDMKLKISNIFNAINGDKPKEKKCKDVYD
VLTDYKKSNIIIDVLQEPSLEEDMDFVLERLKVYNQDNLLPTTATGTDNSYGHMAIEMFC
ENEVNINELIKRNFHVSAFHSLSKQNNENVIHYSCRSICAFTKNPKGLE-AVKDIKDFAN
IISKSVGDLSKENTLDKEIKEDFLIHRVLLIDRTAHNRNVYDKTNAVHYLIDIWNQYDNG
DYSVLLLRHVFRSMRKIVSDAHVQTL----------------------------------
------------------------------------------------HSFQSSLFLH--
------------------------------------------------------------
--------------------FSSSIRYYI-------------------------------
------ND----------------------------------------------------
------------------------------------------------------------
--------------------------------------------------------CLWF
Y-----------------------------------------------------------
--------------------------SLTRELLNNFGGDAVIMQLLAICIDNIAMYSVEV
YDTTITRDIIKCFKSALSKMNNKKEDKQLWQKVELTLEAMNSADDPLEAFKNTLLIFDFN
LSEFDKDPYVNGVHDLASNIKDCLRKGGQSKIYYQSDQRLLFKWKASQDLNTLEWTIGDD
TERVFKISVVRIKNISKGLSHPILISANKREPRKVSAKVTLCIYGPPTEDFPEGLELPIK
TKTQKERDAFVDLIVLWRDAASYNYMDKKAREYAQDALKFIQRSGSNFLACKNLKERLEN
NGFINLSEGETWNLNKNEGYVLCKENRNICGFFVGKNFNIDTGSILISIGHIDSCALKIS
```

PNNNVIKKKIHQINVECYGSGLWHTWFDRSLGLSGQVLYKKENKLVEKLIQINKSVLFLP
SLAIHLQNRTRYDFSVKINYENHIKPIISTTLFNQLNKCKRN-NLQHNKRDDEKNPNSKD
INSYPLLYLLSKELNCKEEDILDFELCLMDTQEPCFTGVYEEFIEGARFDNLLGSFCVFE
GFIELVNSIKNQTSNENNIHNNLYISIGYDHEEIGSLSEVGARSYCTKNFIDRIISSLFK
KEIHENNLSVQEIYGNLVNRSFILNVDMAHCSHPNYPETVQDNHQLFFHEGIAIKYNTNK
NYVTSPLHASLIKRTFELYYNKKQQIKYQNFMVKNDTPCGSTVGSMVAANLSMPGIDIGI
PQLAMHSIREIAAVHDVFFLIKGVFAFYTYYNQVLSTCVHDKMNYCKTTFHIFFVLFFIT
IYEIKCQLRFASLGDWGKDTKGQILNAKYFKQFIKNERVTFIVSPGSNFIDGVKGLNDPA
WKNLYEDVYSEEKGDMYMPFFTVLGTRDWTGNYNAQLLKGQGIYIEKNGETSIEKDADAT
NYPKWIMPNYWYHYFTHFTVSSGPSIVKTGHKDLAAAFLFIDTWVLSSNFPYKKIHEKAW
NDLKSQLSVAKKIADFIIVVGDQPIYSSGSSRGSSYLAYYLLPLLKDAEVDLYISGHDNN
MEIIEDNDMAHITCGSGSMSQGKSGMKNSKSLFFSSDIGFCVHELSNNGIVTKFVSSKKG
EVIYTHKLNIKKKKTLDKVNALQHFAALPNVELTDVPSSGPMGNKDTFVRVVGTIGILIG
SVIVFIGASSFLSKNMKN--
-------------IYIYT--------YIYFFS-----------------------LYVYI
LINYNDIIMNLNKTWCMNPDQGLIKPDVVFYLNVPPNYAQNRSDYGNMCLII--------
---LILVNQVVCKNCL
SSDVETNEGQGEVICLRCGSVLEENKIVESLFEVENNNGAISMVKKI-------------

-----------------------------------K-FDSSESWKRIDCLEKMTENKIDT
DLYSRQLGTYGFDLMNKLVKLNILIINVKGVGLECAKNLILSGPQSVCIYDNDICDISDV
GVNFYINEKDVEDKSCRSDAVLKELQELNNYVHIYNYKGTIEKKWLENFDVVICCDINKQ
DLIKYNNMIRGIDKKRIAFLSCNIYGLCGYIFVDFNKEFICYDSNGEQVKSCNVSKISKE
LEGKVSFDFDKTSPFEEGDYVQFSNVEGMTEINNKIYKIKNLKKYTFEIGDTSTYSDYIK
GGICTQVKKHLKLNFYPYEYICVNPLNNENISNNQNDNHLDTCNNIIYENVPLPNSFIIS
DYAKFDMSNHLHYSIQALKWYELQNEKGLPENSDEDALEKIYNYAVTLNNKDKEEKKSYA
VEQLKKDVVYNVCRYSKSHIAPIASFFGGLLAQEVIKFTGKYMPIYQLLYLDFFECISLN
EKVDINEIKKMNCKNDNIITVFGKSFQKKLNDLNVFLVGSGALGCEYAKLFSLLDMCTRN
SEGKLTITDNDNIEVSNLNRQFLFRREHVGKSKSLVSSEIIKKKNNNMHVQSLETKVGAE
NEHIFNEAFWTKQNIIVNALDNIQARQYVDNKCVWYSKPLFESGTLGTKGNVQVIIPYLT
QSYNDSYDPPEDSIPLCTLKHFPYDIVHTIEYARDIFQGLFYNTPLSIKQFLNDKEEYIN
KIQEEGNNASLLENLQNVINSLKEISSECNFDFCIKKSVELFHNNFINQINQLLYSFPLD
YKLSSGEYFWVGQKKPPQPIVFDVNNEMIQEFLLSTSNLLAQVYNIPPCFDINYIINVAK
KIEVKPFEPKKVKINMDEKNLNNISISFAEEEKIIDDFCKELLNIPTNNIKINPIEFDKD
EQTNLHVNFIYAFANLRAINYKINTCDKLKAKIVAGKIIPALATTTSIITGLVGIELLKY
VNYYDNIQAYVKLSDEQRKKQKDVLSYFKNAFINSALPLFLFSEPMPPLRMMDKEYDELM
KGPVKAIPNGFSSWDKIVISIS----------------------KIIEMCIYNIYI----
--LYVYSKLNQDLL
KKAISDVFEGTKQKKRKFVETIELQIGLKDYDTQRDKRFSGTVRLSNEVRKKLKVCILGD
AVHVEEAQKLELDYMDIEAMKKLNKDKTLVKKLAKKYDAFLASQVILPQIPKLLGPGLNK
AGKFPSLITHNDK---
----------------------------KLQ-----------------------------
---HSSVT
VEPTDVVNIEVPKNNPLDFLKDTEYDYLFAEQTNKTAKDLKNRFKILKDEETEVI-----
-------------------LRF----------------------------------NIKI
YNFF--

--------------------IFLVYNIASFCFELQFRFIEDDTTFDWLPALGYLLPYEKI
KFLRTFFPIVFFISICASAYSYTDKNATLIFLMRSILSTNRIIVERSNDVIESTNYLKKN
GEKLVTKLQQIRKGEASSLLNLKDDENLDKEYLEGDKKN------------TVDSKLLSN
VKEFALTLDSQQISLLEKCFDLMKNKDANE-KNKKKSWFEFFSIQVIFGVIFVYIWLTSK

---YVLVYMIFF
FFFFFFFF-------FWTDINTNNYN-KPATYEVPLSLSLEELYSGCKKKLKITRKRFMG
TKSYEDDNYVTIDVKAGWKDGTKITFYGEGDQLSPMAQPGDLVFKVKTKTHDRFLRDANH
LIYKCPVPLDKALTGFQFIVKSLDNRDINVRVDDIVTPKSRKIVAKEGMPSSKYPSMKGD
LIVEFDIVFPKSLTSEKKKIIRETLANTFVXYIYIYIYILFI------------------
-------------------------DVKCLINVKDQQGKVLYSHDTSKIRKGKVSYLSTK
DGLYHICISCPSTNWFKSTAIKWSLSIEVGGSDIDPENLAKKSELSETLTILNNLKKKFN
SMKLQQIYQKQMVQK----NK--------------------------------------V

---HVDTGKTKLLDKL
RHTNVQDNEAGGITQQIGATFFPKDVLDKEIKKIDDTIKCLSKGIMIIDTPGHESFYNLR
KRGSSLCDIAILVIDLMHGLEQQTKESIQILKQRNCPFVIALNKIDRLYMWNKNDWSPFN
NTFKKQKPDTQEEFHDRLKNIINELSEQGLNCQLYWENMNPRKYVSIVPTSAITGEGIAD
LIMVLVKLTQTFMLKNIQYHDKLECTVLEVKNIEGLGTTIDVILTNGILRESDTIVLCGI
NGPIVTVIRALLTPQPLKELRIKNEYIHHKYIKACIGVKISANNLEEVLCGTSLFVVNNI
EEEEEYKKKVMTDVSDVFNHVDKTGVGLYVMASTLGSLEALLIFLNDSKIPVFGVNIGTI
QKKDVKKASIMREKGRPEYAVILAFDVKIDPEAEKEAALLGVEIMQRDIIYHLFDSFTAY
LKKIEEEKKQSKITDAIFPCELSVINDCVFNKKDPIVIGVRVECGQLKIGTPLFVPEKNI
KIGNVVSVQSNKKNFDKARKGDEVCIKICGEPHVTYGKHFDSTQKIYSKITRESIDVLKE
YFRSELTMEDWKLVVQLKKIFNIVMNINEKDKLAEQNLETLDVTKLTPLSEDVISRQATI
NLGTIGHVAHGKSTLVHAISGVHTVRFKHEKERNITIKLGYANAKIYKCTNPDCLPPECY
KSYESSKEDNPICPRKDCNHEMKLLRHVSFVDCPGHDILMATMLNGAAVMDAALLLVAGN
ESCPQPQTSEHLAAVEIMRLKHILILQNKVELIKEEQALKQQEEIRNFVSGTAADSAPII
PISAVLKYNIDVVCEYIVTQISIPKKGIXXSTXMXLV-----------------------

--QSLYIYIYIYIFIYLFIY
LFIYIFYFGRYASLFFIACIDKGDNELITLEIIHHYVEILDKYFGNVCELDLIFNFHKAY
YLLDEILVTGEMQESSKKTILRIVAAQDSLMEDNKTTKKLGALIMKYSNIIKKGILSRYC
SNIYGMQYIP-RARYLTFCKNEKCNTMLKKYHPFSTSKMSDNAHYNEANETNETIENSAS
SPEGKKKKNSLNESEEANEKKEDINYEDFNKIDLINEIKKTKRDMEEKMVDNKVLKEKYL
SVLAENENLRNRYMKEIETSKLYCISNFAKSLLDVADNLSLAIKNINEESLKTNEEINNI
YKGIEMTETILHNIFNKYGIDKYNPINEKFNPQLHEAIFEINDSTKEKGTVATVIQHGYK
IKDRILRYFKCHIISYTXRQI---------------------------------------

----------SLDIESVQALIVALNMYKGGLIIISHDTYLIKHVADEIYHINNITKELVK
IDYEFDKYTQLFLNNKIMPREIITLQCGQCGNQIGVEFWKQLCNEHNIDQEGILKNNNFL
NEDRKDIFFYQADDEHFIPRALLFDLEPRVINSIQTSEYRNLYNPENMFISKEGGGAGNN
WGCGYSQGHKVEEEIIDMIDREVDNSDNLEGFILSHSIAGGTGSGMGSYLLELLNDNYSK
KMIQTFSVFPLLTNESSDVVVQPYNSILTLKRLILSTDSVVVIDNTSLNRIFVERLKLNN
PTFQQTNTIISNVMSASTTTLRYPGSMNNDMISLISSLIINPKCHFLITSYTPITIDKHI
SNVQKTTVLDVMKRLLHTKNIMVSAPVRRGMYISILNIIRGETDPTQVHKGLQRIRDRKL
VNFIKWNPASIQVTLAKQSPHVVSQHKVCGLMMANHTSISALFERCVTQFDRLYKRRAFL
ENYKKESMFSSADGQGNFEEMESSKEITQNLIDEYKSAERDDYFTNTYI-----------

------------LNISRAFGIPVRKYTHEVVTLWYRAPDVLMGSKKYSTTIDIWSVGCIF
AEMVNGTPLFPGVSEADQLMRIFRILGTPNSKNWPNVTELPKYDPNFTVYEPLPWESFVK
KN---KIVHIYIYIYLYLYLYLCAYTEMILIFVYTNI---------------------EK
LSQRSELEEKKLIGDVKKAIQAGKIELARLYAEKCIRKKNEKVNYLNLSNKLDVLVSRLE
GAHRCASVSLQ----------------------------KIL------------------
--MGKEKTHINLVVIG
HVDSGKSTTTGHIIYKLGGIDRRTIEKFEKESAEMGKGSFKYAWVLDKLKAERERGITID
IALWKFETPRYFFTVIDAPGHKDFIKNMITGTSQADVALLVVPAEVGGFEGAFSKEGQTK
EHALLAFTLGVKQIVVGVNKMDTVKYSEDRYEEIKKEVKDYLKKVGYQADKVDFIPISGF
EGDNLIEKSDKTPWYKGRTLIEALDTMEPPKRPYDKPLRIPLQGVYKIGGIGTVPVGRVE
TGILKAGMVLNFAPSAVVSECKSVEMHKEVLEEARPGDNIGFNVKNVSVKEIKRGYVASD
TKNEPAKGCSKFTAQVIILNHPGEIKNGYTPVLDCHTSHISCKFLNIDSKIDKRSGKVVE
ENPKAIKSGDSALVSLEPKKPMVVETFTEYPPLGRFAIRDMRQTIAVGIIKSVEKKEPGA
VTAKAPAKKFFFFKPLPIVLLKEGTDTAQGRSQIIKNINACQIIVDIVKTTLGPRGMDKL
IYTERDVTITNDGATVMNLLNISHPAASILVDIAKSQDDEVGDGTTSVVVVAGELLNEAK
GLLNDGIEPNMIIDGFRNACNVAINKLNELSLNFSNKNEEEKRSILLKCAQTALNSKLVS
NHKEFFGELVVNAVYKLGDNLDKSNIGIKKVTGGSCLDTQLIYGVAFKKTFSYAGFEQQP
KKFINPKILLLNVELELKAEKENAEVRIENPNEYNSIVQAEWDIIFKKLNLIKDSGANIV
LSKLPIGDIATQFFADHDIFCAGRVEDADLKRTANATGAIVQTSLFNLNNDVLGTCGVFE
EVQIGNERYNIFKECLKTKSVTIILRGGAKQFIEEVERSINDAIMIVLRCITNSEIVPGA
GSIEMQLSKYLRIYSRSICNKEQIVLFSFAKALESIPRHLSHNAGYDSTDILNKLRKKHS
EQTSDIWYGVDCMEGDIINAYDNCIFEVTKIKRNVIYSATEAACLILSIDETIKNPSSAA
GTQRSPYSMSGKLEDIGELVLLIGDFHSPIRNLGLPDCFKELLKTDKIKHVLCTGNVGCN
ENLELLKNIADSVHITKGDMDDNFDFPEDITLCIGDFKISLIHGHQIIPWGDMNALLQWQ
KKYDSDIIISGHTHKNSIVQYEGKYFINPGSVTGAFQPWLSEPTPTFILMAIAKSNIVLY
VYEEKNGKTNVEMSELHK-MDNGNNEILYSVKISEPRDKNSTGIYRHPEYKDKLCENFDD
KKYNNMWELFDRVSTKFKDKECMGVREKEDNKRGAYQWKNFGEIKELIIKVGSGLLNMNA
CPLIMCDDQRIPKARFLGLYMPNCPEWNICDLGCNAYNIITVPLYDSLGPQSSRFILDQT
QMETIVCNKTCARNLFKSLETCEEIYLKTLILVD-EIDDEIKEECSKYNMKIILWEELIK
QGEKKIVKVPQGSLNNIFSICYTSGTTGYPKGVIMTNRNFIGILAAAYIGPSKFPDLCFN
ENDIHISYLPLAHIYERLMMYLFIAHGVKVGYYSGNIQTLLEDIQELKPTLFISVPRLYN
RIHERIFNSLKKKSGIVQSLFNKGLQNKIKRLNSSGSTTHVLWDKLLFNKAKKILGGRIR
AMLNGSAPISVDVVKKLRTIFCVNILEGYGMTESLGASFITHSRDRNIGHIGGPVPCIEF
KLVSVPEMNYLVTDNPPKGELYLRGPSTCNLGYFKLEKETNELLEKDGFIRTGDIVVLNP
NGSLTIIDRKKNIFKLAQGEYVAVEKVEASYKQSLFISQIFVFGYSYESVLVCVICPSTD
SIDIWRTQKKIKATDEEVIKLPEFKADVINDLTSLGKKDGLKGFEQIKDIHFTLEAFTIE
NDLMTPTGKIKRHEAKKRFKKEIDEMYEKLKQ-TF-------------------------
----------------TFIFYLFVCIFFFILKNGTKTTKTINEGQTILVVFNEGYAPDGV
WLGGTKYQFINIERDLEFEGYNFDVATCAKLKGGLHLVKVPGGNILVVLYDEEKEQDRGK
QKFMSL--

-----------DMEQLKNRINNLNMLAVVYLACREAGHIKSIKELITFDRSYKEKDLGKT
INKLKKVLPSRAFVYNENISHLIYSLSNRLQLSIDLIEAIEYVVKKASTLITTSHRLNSL
CGGSIHLIVELNTNEEKNMKLPNLSQIATVCGVTTNTLKTTFKELLNAAEYIIPKYYLSE
DNPKLSMLKQKYLSEDKRKKNMKFEKFNIPKWSDIERYFKDPELLTAEILFVALTLCNVF
VMYRLFLDVIPYPIFVTWWQLAQGLLVAYVCGELGREFPKFAYFPKVEINENMLKVLFVP
SIFYCLMLVLSNYLLYKTPCIASYPVLVSFTVVFHHLTRFIGCGEEYMPLRWKSIVFLLA
AFIIGCFDSKTTGKGVIVWALLYALFSAIFRAGFMQKIMHLVDGKGNTLHNNQHLLGVLI
LPVLILLTGELSVFVHMPYDITSLHTWQMWGCLITVGTLPFIKNVISNRLVRRTGQGPWR
FLEIISIILVFFIGMTYNAPSFKGYLAILCVIIGRSLGAFDVLLNASDYMHAEDERKRKN
PRASYAKSRQGTQASKPFLSSADNEEDEESSFSSNDSKSYQGSQDYDDKESVNSSSQQYS
VHKGTSHMDSSSNQTSHGVISADEGRGEHSRAATFKSKSKLSRNYSSKQEMLDSQA----
---TKTKVGEEMRN
ASFSLAKSVWAAGDFKGQIIEGIKRPVVTLSLSTNNVAGVKLPIFQVNIDPTVDVLGNLG
VAAGGQVINNTRENYLQCLNMLVKLASMQV-------IHTHKN-----------------

--II---------------------------RH--------------------YVFSFL-
-----------------------CIIVICIY-----------------------------

----------------KLILIYI----------------------------DVYM----Y
VYFFFFFFFFFFFF--
-----------------------------RGINFRFMWIDIAMETHFRSLLEFKKYPSVV
VFNPYKRIRYAKLNEDLTATKENIEKLLEKISGGDAKFTMLKGQTLPEFIQDPNAAKENE
KDEL--

-------------------------DVNTNVSKDSTYYYGNSINDNTDDYVEKRSGNNHP
NSPLSIGDNINGNMSSPNNNNNSNNNNNNNNGINSFKRFNMVKINLMRDSSTSSKDDNPY
CSINDGKVIIKNNELLSGIICKRTVGSSSGSLIHVLWHEMGPDKTKDFLSALQKVTNNWL
EYVGFTVSCSDIIASNKVLGKVREILDKSKSEVSKLVEKAQKGELECQPGKSLYESFETR
VNNELNCAREMAGKVASESLDERNNIFSMVASGSKGSIINISQIISCVGQQNVEGKRIPF
GFNHRSLPHFIKFDYGPESRGFVSNSYLSGLTPQEVFFHAMGGREGIIDTACKTSETGYI
QRRLIKAMEDVMVQYDRTVRNSYGDIIQFLYGEDGMAGEYIEDQIIDLMKLDNKEINKLY
KYNFDEEPFGKDFYIGNDGSRNTSYIDYNKQNILNQEFEELYKCKNYLCKEIFPDGDIRQ
HLPINMNRLIEYAKSQFPCIPFVSNNNSTNISNSRKLGNLSSTHNHKESKKRRKRRRRKF
DKYKNNNELMSEIKKEYENNDLNNMMISQSPFKGMNEFHMGVADNDDDDDDYDDDDDDDN
DDYDDDNDDKNNYDE--NSMLNPIDVVHKVNNFLEKLVIIKQINSNDTLSVEAQNNATIL
LKAHLRTYLNSKLLTQTHKVSVKGLDWLLQEIEKIFYKSLCHPGECVGALAAQSIGEPAT
QMTLNTFHFAGVGSKNVTLGVPRLKELINIVKNVKTPSTTIYLDDMVSNDQQKAKDILTK
LEYTTLKQLTSHAQIIYDPNTTTTILEEDKSWVNEFYEFPDEDDTQYSLGEWVLRIQLTN
IHVNEKKLTMKEIVYIIYSVFSSDELDIIYTDDNSEDLVLRIRVKYLNGEYNFMNYDVVD

```
NANEQVDEQEEDEEHNNNSTNIFKVKNNISSDINTKNEDSISINSSNNGQVKNMNSSPVS
NNMHNNNNNIKKEDGNEGVLRGGNNDDDDEEEEEEEDFLFGKDGKNKNTNNKSNNNENKN
KKSGNNNNNNSNTDDDDENKSDITIKEDDNIAFMTSKNAEEDLELKNKNHIEHN-ISRED
TEDTFLKKLMEQCLSTLKLRGIENITKVYMREESKITYDSDNGKFVRSSHWVLDTDGCNL
ENIFCAPQVDFKKTVSNDIVEIFEVLGIEAVRRALLKELRTVISFDSSYVNYRHLSILCD
VMTQKGYLMSITRHGINRVDKGPLIKCSFEETVEILLEAAAFAQVDNLKGITENIMLGQL
CKIGTGSFDIIIDNQKLNDANQNLETIQDLTSAGFTTPDSLHVITPDGLQSPVAINTINS
PLPFSPTYNANLLSPTAPIDNVNNLLSPQNLQNYGDNVMSPSSKDINNLDTLKLGGKFSP
TQSPKSPTSIMHSPFSPFDHQNQQPVDATLLFSPKNN----NMMNYNVFSPKPNINNIQS
PNIYSPNPMLDIFSPKPQINHNIYSPSYSPTSPTYNANNAYYSPTSPKNQNDQMNVNSQY
NVMSPVYSVTSPKYSPTSPKYSPTSPYSPTSPKYSPTSPKYSPTSPKYSPTSPKYSPTSP
VAQNIASPNYSPYSITSPKFSPTSPAYSISSPVYDKSGVVNAHQPMSPAYILQSPVQIKQ
NVQDANMFSPIQQAHVDEAKNDDPFSPMPYNIDEDEMKENMVVFKLDDVEIFFPYDYIYP
EQYAYMKYLKKTLDSEGHCVLEMPTGTGKTVAIFSLITSYQYHKKDEGKFIFCTRTVAEM
EKSLIELKKVIEYRIDVMKQRKVEKLKNEKDDVNDDVNNDDVNNNDVINNDKEYNNHDDN
YLKEFGENSEILAIGISARRCMCINDKVLLKHEREKIDEECRKLTATFIREKKYINNKID
NIYHPNVDKISDFILKNRHHLDIEDYFDIYNSRNSLEEYDNIGLCGYYENYKKEFLYDLI
KPGVYTIEDLKVLCKNYKNKENVSVPICPYFCAKKIIEISKVIILNYQYVIDPKVSKALF
NWKDMNKNVHLKKNDIIVFDEAHNIDSVCLEALSVNIDRNILNKASMNITKLMKKIEQSK
MLNEQKLKEECNKILEKIKLQKCNQSLSVTNNVINVDNHRDNLNKTNEQEKKKRKVESSA
YFDEDMNLMFSDFLDESFGDNEKKKGISNNNNDINNNNNNDINNNIEKNMNNNNNNNEDE
DHLNDLCYSPLLMEDIIRNVVIPGNIRKSEHFLNLMRIVVVYLKKYINIYDITSEGPLSF
LYKFEKDTKLDTSFFKYCFDRLKSLLNNLQIVNIEDYSSLNIVCNFCTLLGNYFKGFIII
CEPYPEATGIYDPLIQFACLDSSIAMKTVINKYKSIILTSGTITPLELYPKLLNFKTVLT
ASFPMSFDRNCVCPLIVTKGSDLIPLSSQFSLRNDLSVIKNYGILLVDMCKCIPDGIVAY
FPSYIYMEQVISSWYELGVIANILDYKLIFIETKDIVSTTIALHNFKKACDLGKGAVFLS
ICRGKIAEGIDFDKHYGKCVILFGIPYQYTLSKILKSRLDFLKETYNIQENEFLTFDAMR
QASQCVGRIIRNKKDYGIMIFSDIRYAKHDKKNKLPPWIIKCMDISNINLTVTTAVDISK
QFLLNMSQEYRETGQTKISQLMLKNQAKCWTMVKSILNMDDFIMWTRKVINVVSCIRKNY
RSNYSTFNLPQEIINQPITRVTELSNKLKVATVHTNCEIPTIGLWISSGSKYENKKNNGV
AHFLEHMIFKGTKKRNRIQLEKEIENMGAHLNAYTAREQTGYYCKCFKSDIKWCIELLSD
ILSNSIFDDNLIELEKHVILREMEEVEKCKDEVIFDKLHMTAFRDHPLGFTILGPEENIK
NMKRKDIIDYIDKNYTSDRMVLCAVGDVQHEEIVKLAELNFNHLKTQEQKNNSVIHNNND
KPFFCGSEIIIRDDDSGPNAHVAVAFEGVPWNSPDSITFMLMQCIIGTYKKNEEGILPGK
LSANRTVNNICNKMTVGCADYFTSFNTCYNNTGLFGFYVQCDEIAVEHALGELMFGVTSL
SYSITDEEVELAKIHLKTQLISMFESSSTLAEEVSRQLLVYGRKISLAEFILRLNEIDTE
EVKRVAWKYLHDRDIAVAAIGALHGMPQYIDLRQKTYWLRYY------------------
------------------------------------------------------------
------------------------------------------------------------
------------------------------------------------------------
-------------------NSFSLCVIFLGLINNYNNKDNHIFSFCEYDHFFGSQCDGNM
NKSMGSNENNKLYDNLKLLYFVKSKYLWKARIFFIWQRLFTSSNDYYYSLKINIIDKPIE
IFKTIKIIPPDFELIEQDININEIDIVDCVKENLKHVKYCNLFYNNYMSTQFKIYVLSNF
SMYLAFYNYTSAYQRILDLLSQISHFQYTFTGRMGIKRMYQKIPATILVLKTKLNEQEDN
TSTILKEIEPLSEYDILRSDYRIIDHDEHNVDDDTNNICNKQNVSLKREEINLLSNNNTN
ELEHQRMSNACCNNNINEHKTDEHNFDLCNDDKNESKEIVKISNEVQKNNSKVTWKLKDF
DPDTDILEEPHFVDSQNNIFKVLSFQEQICLINYCFSMIRFNPHYDEIKFEKLNAVISRC
LKCYDVNQKDSNNKNNNNKNNNNSQQLKYQNWLLHSCMLWFKCKGETFRFKTVDRAAAQL
NELHKECYDIKPESVERLKFIYDVYYPTTWEMKKEIGSVMIKIGSVVTAFNIFKDLKLWE
EAISCLIQADRKEEARELLDDLLKKKKSPCLLCLYGLIEKQRALNYYIDAWEVSNFKYAK
AARLIGKYYYEKEMYEKCSEYLEKALELSPLFPEIWFILGCSYMKIQNFDESIKAFTRMI
SMTNDNSCKSYGNLAYLYMKKGTYKAAKICINQAVKMNNNEWKYWDTYLKLSIIQNDIDS
FCLALRMICQVNQVKQIQPWVFDYISDVIVKDKPTLIPNKNGLAYLDKIITTMEHLSQYI
TELDTFWNAHSFFLFIKGKFPDSFEAKIKEIRSIEVQHKKYIYIYK---NIYIYIYIYIY
IYIYIFIHYILG------------------------------------------------
------------------------------------------------------------
------------------------------------------------------------
------------------------------------------------------------
-----------------------------------------------------IVPI---
------------------------------------------------------------
------------------------------------------------------------
-------------------------IHGII------------------------------
IITE--------------------------------------------------------
-----------------------------------------ING----------------
GXAII-------------------------------------------------------
---------------------------------------------------------III
IIIIWMEMVIPLLHK---------------------------------------------
------------------------------------------------------------
---------------HNDRHNHNDYNHHDNRYNHHDNRYNHHDNRYNHHDNRYNHHDNRY
NHHDNRYDNPKDYKSNNNHYEDDYLKKKHSINDSSNNIKHEEHKNYVTHIGEENKKNKYE
TKKEYNN-------------NNIINNNDQKEESPIWLHIDIMVKVITPGPFYNEIGKIID
VITKNAYTILKIQTDKTTFNIVADAVVPLKPQKKNDHILIFDKGEKIEGTVQDIKINEVQ
ANTASGLLTCHLKNTFLYKNYIPMSQNNPLSVSVADNLINYDIQNESFRLGNVSVEGDKY
ICVKENVNENTQVVVINLHNKNSTRKHMKAESVIIHPNDPILALKGTIKNMNTIFLQVFN
IETKEKICSLNLNEYMNYWKWINNDTIAIVCEKNVYHWNINTLTKVFEKAQIFIDNNSQI
LYYSTDKDMKWCILCGISTQDQGKSIDGYMQLYSCEKKLHQTIEGFIGCFGSFIFDNLDM
KPLFCFVEKKKNSTISRLHLMDIYTNKTETTIPYKIVKEINLINENLNDFPIYISLNTLQ
GVIYIVTKCSYVYVFDEGTLSLIVKEKISEDNIFICCDSKNGEGIYAVNKKGIIFYITLN
YFHLINHLKLSNLEFKDKIIKNLCVKYGYPGCDYISAYKKCINDMDFKKASKIICLMKNT
KLRTQQVLNSFKSFKNTSGQLSPLLLYFSVLLEYDKLNTYESIELVKPVVLQKKKEYLEK
WIKDDKLTCSEELGDLVKVLDLRLALNIYLRCSAHNKILSTYCLLNMFNNVLSYINNFKQ
INFDYVSIFITIVNYEFPHGNADNKETAYVGDFNSGSKNETMDFFNDGASNDQNNSKLNK
GNINEVAIQYIKFLCENNISFDINKIIDYLLSKKKLQEATSNLLDYLKEIKQNIKIYKPK
LFEI--------------------------------------------------------
------------------------------------------------------------
------------------------------------------------------------
------------------------------------------------------------
------------------------------------------------------------
------------------------------------------------------------
------------------------------------------------------------
------------------------------------------------------------
------------------------------------------------------------
------------------------------------------------------------
------------------------------------------------------------
------------------------------------------------------------
------------------------------------------------------------
------------------------------------------------------------
------------------------------------------------------------
------------------------------------------------------------
------------------------------------------------------------
------------------------------------------------------------
---------------------------------------------MASYVGREAPYFKAE
AVFADNTFGEVNLHDFIGKKYVLLYFYPLDFTFVCPSEIIALDKALDAFKERNVELIGCS
VDSKYTHLAWKKTPLSKGGIGNIQHTLISDITKSISRSYNVLFGDSVSLRAFVLIDKQGV
VQHLLVNNLAIGRSVEEVLRIIDAVQHHEQHGDVCPANWKKGKVAMKPSEEGVSEYLSKL
```

```
MDDNTANIIGSHDNILTDPRITQDCSAETNELLNRAEQHFK-------------------
--------------------------------VFKKKKKKKKK-----------------
------------------------------------------------------------
---------------------------------------------KKKIYMYTYK-----
------------------------------------------------------------
------------------------------------------------------------
------------------------------------------------------------
-----------------------------------------------MNDKEAKKEAIEI
IYEISNILNVNLDKETIVILIQLCEYGVSPKILSHIIIQLKKEREKFLDNLNN-MNNLNK
```

> ***Plasmodium gallinaceum***

```
MKEFNKLNIYYGKKYPFACKTVYNIYINNIRKHKLNIENIEEKIQFLNDEKILSRDKIFA
KNLDHILKTNLYYILC--ENKKNKVLNAYVQTQYDEWIDFFRYKNLQKDIVSICEYIDRH
LHLNTFISSHFLTLSDLYVFYEMYKYFNVNSCYFIKYSKQYKNINRWFKLINCLLNFEDN
ELRNHLKEESELKISDTTKDNGNQGIKQKNISTSYSGKLENAEVGKVVTRFPPEPSGYLH
IGHAKAAFLNSYYANMYNGKMLLRFDDTNPILEDIKYEKSIMEDLQNLGLKYEKISYTSD
YFDLLEDYCVKLIRMNKAYADDTNVEDMRNQRGEGIESVNRENSIEKNLELFEEMKKGTE
IGQKNCIRAKINMQSKNKCMRDPVLYRCVVDVPHHKHHFKYKCYPTYDFACPIIDSIEGV
THALRTNEYSDRIEQYNWVISTLQLRKVSIYEFSRISFVRTVMSKRKLKWFVENNVVDSW
LDPRMPTIKGILRRGLTKEALFQFILEQGPSKSGNLMQWDKLWSINKQIIDPIVPRYAAV
DKKKAVLLTLSDLKSDIIEKQRDLHMKNKSLGTCKMYYTNKFLIELEDATTLCEKEEITL
IKLGNVIITNIIKNEEN-IKEIIATSNFQGDFKTTKKKIHWLPYIIEKLITCTLYEYDHL
ITVDKFESDDKNDWKNFINYNSKFETTVYADPAITSLKVSDKLQFERRGYFIVDQV-CNN
QLHLIKIPDGKSKNMSIIGTKVDPKSLAGTKDKI--LIIFFFFLGYIKAGLNAYEEPSII
FPTLIGNLRHNENPETFVGDDAIYHESELSIYRPIDHGHISDWDMAQKIWDYTLNCIDKN
KNAKDILLTEPPLCSTSHRTKMGEMFFEYFDFLNLNISVSGLMSIYAAGLTTGLVLDVGE
GVTQCLPIFDGYIEKNSVIRSDFGGEELSMFLQKLICDIGYSMTTRKNFEYVKSIKENLC
FCSLNPSQDQLRDDLSVTYTLPDGDVLRDGYDTIEISHERFYVPEALFNPQLCHRDSLSI
VDIIWKSILLCPIENRKSLSSCIVLSGGSTLFPNLVERIETEVKNNAPANARSAVKV---
--------------------------------------------------T---------
------------------------------------------------------------
------------------------------------------------------------
------------------------------------------------------------
------------------------------------------------------------
------------------------------------------------------------
------------------------------------------------------------
------------------------------------------------------------
------------------------------------------------------------
------------------------------------------------------------
------------------------------------------------------------
------------------------------------------------------------
-----------ILHSKEPESDYVEASLITVLNIHLNEHPGDILVFLTGQEEINTACEILH
ERMKKLESMCPPPLIILPIYSSLPSEMQSVIFEPAPPGCRKCILATNIAEASLTIDGIFF
VIDPGFCKVKKYDSKRDMDSLIVAPISKANAKQRAGRAGRTGPGKCYRLYTEEAYKNEMA
ETSVPEIQRINLGSIVLLLKALGVNDFLHFDFMDSPSIETLIHSLENLYYLGALDDNGYL
TKLGKKMANFPMEPNLSKILLTSINFNCADDVVTIVSMLSVQNIFYRPQNKALLADKKKN
KFVMPQGDLITYLNIYNKWKENSLSNYWCHENFIHSRALKRAQDVRKQLLSIFEKYNYEV
KKNTSKNDATRYVNICKSICSGYFNHVCKRDSQQGYTTLLTNQQVFIHPSSTLFNKNPLF
VVYHELVLTNKEYIRDCTIIQPQWLIQLAPNLFIPADEKKISKIKLREKIEPLHNYYEEP
NAWRLSRRKG--------------------------------------------------
------------------------------------------------------------
-------------------------VTYEQGANFAKENNLFFAETSAVSKLNVKHIFESL
LQEIYNNRLKNNNGSFSNRSNATCESAIQLTKAQSVIKLNDKNENEIEEDNKNKVKCCME
KSKLAKVEKVLGRTGSRGSVIQVRAQFMGDTELSGRFLIRNVKGPVREGDILALLETERE
ARRLR--------------------------------FL------KGTQSLNLKKSHCYC
HLSTGDLLREAIEKKSEIGNKVKDIMSEGKLVDDDIVLSLVDEKLKSPQCKKGFILDGYP
RNVKQAEDLNKLLQKNQTKLNGVFYFNVPDDVLVKRISGRLIHKPSGRIYHKIFNPPKTP
FKDDLTDEPLIQREDDNEEVLKKRLHIFKNETTPLVNYYENKNLLINLDATQPPQDLEKK
ISQHIDNKMEKNCPVCYFNLPDPESTLAPYDTELNYFMWGPGFEWQPEPQEKASSVEETY
---EESEESDADLENLDEKVDKNDAKTFFNEKSKNGKISVENASVNARKLGLAPSSTDEE
KIKEKYGDNLTYDQYLEYLSLCIHDRDNTEELVKMFAHFDINATGYLSKKQMKNILTTWG
DALTEEEATDALNAFSKEDKIDYKLFCEEILQLKRIAYIFFLEKSWVFYLKQN-ATPNLL
KFPDLKGLDDLSHLSNEKVMDILGMNIQGAKERLEKIFHIIDKNNDMILSDAELEIWSNF
VKNEVFLKQVQVEMKQIDSDKDGFISLAELNDAFSQNLDEKEVEKHSEGLLKRFQIVDKD
KDSKLNINEVGLLIDPVKDEELKELEISEILDHHDINKDGKISLEEFKQTRTDDPNSKKD
DDVALDDFNFFDSNKDGFIDKEEIIKVYFDPSHETGSININEIKDGIFEGKEISYDLWNE
KALKIAVTSLTDYGDIIRFPEDFKLDIGKNVVLPSSKGHVSEEEDLDQDEETEDNKNEEQ
DETEEKEPTTDELMDTFLAKTICIFIFIIVTTTGCLIPYLIGIYGKKHESQNENKIKNIL
SNLNCFGSGFIFSIIMFHLLPETIFVAIEHDNMKIFNSYDKEIKILYIFFFVFIGFCMQL
ALEYVLPTDRHICCVLHENENVIPNENYIK------------------------------
------------------------------------------------------------
---------------SNSLKIILLMIFIISSPLGIILGYVVQAAGITILKFVNLFG---Q
IYFNLIFFLQEKK-----------------------------------------------
--------------------------NKKPLVSAQAERWAAIDSSSSEEEERVIKSYEGK
RLDFYENMGNSINENMNNNEFNQLLKDYENLYKFMIKESSENIPKFAIVYLDKLSKYVEK
IFQNNVEKKNLSKNKAQTLNKLRAKIRKCSEFYQERLNAYNENPDDFKDDLEENEEEE--
--EEEEEEEEEEEKEEGEDEKEKKAEKSKKDSDKNVEEDNSDEWSYSDEEEYASNEEDDK
TKSAISKWGLKTSEKVEKKKTIKVKKSKKETNKKEEKTVHIDEVQSSKNKGYLDLLNTKN
LTEDVIRKRVKLVIEKRGRKGLDKHEHINILSKLCEIAKTISTQSYIEVLEHLINLEFDV
VSSVYTYMSFNIWNKAFKYIELILDLLIQNDNFYLVSINLAEEITEESTNEKEKIIKSCK
TLISFLAKLDDELLKALLYIDVQTEEYRKRLGKTIHVIGLLHKGYNYVKYTKKMPELAIY
ISSRILEHMYYKSETIFKQIWNFINYGKEYLESPNKENNINEKSKEKINEENSVKTNIEI
CPKKIVENYVYEIFEYGTKQQKIRALLQLSYNKSLYDDFLEAKELLNVANVHELALNSDI
QTQILYNRNLIQLGLCAFRHGRIYEAHCCLMEICSQNKHKELIAQGMSNLKNQEKTLEQE
RAEKRRLLSFHMHISIELIECVNNICAMLLEVPNLAKNSYESKKDIISRQFRRFLDMYDK
QVFNSPPENNKEIIILATKYLQKGNWKLCCEKIFGLSIWSKFTDKEKVQDILKEKIKQEA
MRTYIFRYIFIYDSFSIDQLCLMFDLSQNTVHSILSKMMINQEIPACWNESSQFILINKI
NPTILQSMALKLAENVNDVMEQNELALNMKNPK---------------------------
------------------------------------------------------------
------HIY---------------------------------------------------
IVI-----------------------------------------------------IYMC
--------------------------------IYIF--------------MYNFYIS---
IGLGLQPKQCIVVGTRALEFLNNELNNIKTLSELYERVKKRSIMDNNDNILSLYLLELFS
IPIEQKEKNDFMTNRIAMYSKILNGRKNVLDLLLNKFENDCNDSIKKELIDCFDVEKFKQ
EVNNKFMNILIQQLRKVEVKLEKKKAKMEFYNKKLEELYNKGNILSIREQIKLYIRELVN
IISNLLTGNYPILNLPKNGESFLKKYGGTLMDNLRDGNDLAIELFEKKGLYDENFLNALH
ENLNKYYSMNDLNKS-SSEFNNINDGINN-NNSLDGSNHSNR-EIYDNFNDIEKKNNENK
SLLVEGQLVRFILAKEESNMFGIIQNSSTDVKSKNILVTFYFRNSNTEEQVQIKSVEKDR
LTVIKPVETLNCDASFLNGQLVWYKMVRDDGWVGFDKAEIIKVFNNNSKIKDVLIRNISK
NDEIISIKISDLYMDYVN-----DETKNDSKKENDEDIIKKIAGPHSDLKILNQLAITYI
CKWLKHNIAKIEPEKEFSDEVLLQMMRSIHNIVDQSDWKPLVVDLLQANISGNILHLTKL
ASCSTAVALKRVFKAALGEINRKIKNNYIDENIYLLSTNTKFLEELNQALHNFCNERAII
CSNEMKDIVFEQTYAVHFEIIEEIFDGCKLFEDNFLTPNGVKPTMSIINKNVKQNLAYRN
HQLSLTDIKINKKSKSKELIQEEVKLQFWAIKMLISVPLATKIYAHFLNNVLPKNKHLAN
IFDYSIDCESFLEKYIQNKLLNKEVNGILKSI----------------------------
```

```
------LYNSI--------------------------MESNNIAQVLYATVDPNIDIRSE
AESKLKHAKETNFVQYINQLSNEFCKSQNDPYLRQIAGLLIKNAFTAKDNFESEERARTW
INFPEEIKNELKNSLLLLLSQPCDKIVIGTACQIISIITKIELSHNKSSELLHKLVNNII
EKNAYTKKSSTVCLAYLTEDIADICNESKTKYVFTQPDLDLILTAIINSLCEPAEESIHC
ANMKVLYNLMSFIDQNFKTQVERDIIMKTVIDGCKDTERLSVQIAAYECLINIVSYFYSY
LDAYMYAIGPLTWTAIESDNERIAISAIEFWNTVCEEETFINQYELHEGKKNHNIVKQAM
VFLLPKIFNAMITQESEDIDIDAWTLSMASATFLALSAQLLKNDIVEPVISFVEENFIHE
DWRRREAAVLAYGSIMEGPDTEKLKPLVEESVGQLSEVLRDPSVSVRDTAAWTIGKITTF
HSEIIYNVLGNYNDSNSLYGILLERLNDYPRVAANVCWVINQLAVNKKSSFDKITNSYIT
HLDDSFCVLCKKLIDVTSREDADTRNLREAAFNALNVVILNVSDNCLKYMIELLSHMMYL
LTNTYINPLTEEVKSLQGYYCGTMQFIINRLGSQCKPFLKPIYLSIFRLFEIRNDICEDA
LLACSAIINVMGEDFREHLKTFLNVIFKGLRNVSETSTCKICIEMVSDICIPWTVEFEKE
MELILECLWDALKTFGVHDSIKISILTVLGDIATALNRCFSRYLNFFANILTETSKITIS
SGPPESDDWVNYVFELRDAILLTYSNIIYALIDGNEIAKLKTYIPNILDLIELILVKEIN
HFNAQNFQNAVSLLGDLVHAYGYELIENSKLTDLIISVYGKIDILSSQGDEKCESCVLKI
KWLKRICNARLAQKMSVYIQLTHDISGTVTAVTIGDWILGILKQKNIQEYEVFFKKFLNI
YQKQDKESESNNRSEIFSLLLSASDFVFETLVETKKNNKIKVIIDNKESVKSFNEFYKEV
EEYFVILISILQLEFKSVEDLNNATNNFIKAIKNYNVFPELRLKILQLLYNSFSVNFSFR
FPTFIAILQFSSQNNIFHNILPYIKFIDQWIKEWNISSREKRQIYLIIAQELKKLKKYED
SFKHLKKHIYYFQKESEEILNHPNTINASVELIVDSINLNSNIYFHEILNLDAIQNLQNI
KEHQPLFELLVIFYKYGIHEFLTFKNTYGVDFFEKYNINLEACENKIYLLSIISLFKDIK
IQNIQYISEKLNITPLKIEQILVAAIGSGVIDAKIDQINKTIQMKTTILRHFDDTHWELL
NNQINKYINNVQKILDLTSQTKI----MQYLLYFFLCVILKCFVSGQESATSFYKFIDST
ASSTYISEESGSSLYDAKRAIQNNPNYWCSSGNHSKDEEITWIGYLNTKGFIKGVKISWE
YCPELVKISVSADGENFKTVIPYKRISGNEASFEEIYFFKKLEEVISIKIGLKNPIHKYF
GIREVKIIGGGNPYFLLLSGITSENEMCLQVEEGLINNDNTSVILDSCINALSSGDGREL
WKTNSNNQIISAFSDPPKCLSVINLDNLENNKIVLYDCLRALEDGDGKSNWIFESNSQIR
LQKSGESLCISQKNIYGNVPGIHDILLNLDVSIDSNSILDNDHNPDNTIDGNLNSYWASA
TFADNYEHLVYLNLDLNKLVEISRIKIYWEYPPLHYNIEVSSDNQNFKVIVENLANPSHI
TIDTLKNIETRYIKISMIKPHPNHGKMEDQFLYGIRSIEVQANNLETVIEYCRDAANSDD
ARDKYFVEYITEFDKDLTNKLINIEDDVSKNVNSISNNLSKLEELLPSIETCLQEKKGYD
EELKEFKEKANELNDKLSSLVSVNSVHDNDLLKLGIFPGDSSSYPASDCSVIKNLQENPQ
SGFYWIKAKCSSEPLRVYCDMTSSTSIYIWNGNPPKSPDHLITNIINSVDDIRYHCAEVG
LEPLILRSRSQLNSLILALKKIGFVLNGKINIPLAYDYSCDHGSCSGKFHDLLNGNIDLT
TLIYLKTSESPDSTKIRQTAGISYDDGSFKFFNLETSDISAIACSTNSTENDSALQYLSI
NCETTALEDYFNTIINTNIVVLCPVGCDNEKFQNAAIYGSKGVYSDNSSICRAAIHSDII
DTKGGLVNITIESGLDHYDGSINNNIESISLNKN-KNGLLDIITEENNESIKEESSIFHH
RTIRINELTEDCPMDLFQFNQTSFLQKENLME---RKEIRYNDEENVETVNLHELISVLL
SNIDSIHGVDPSIISNIQDETVRILEKTKRELKPADVLSKKQIEDAINLYNVTENLTLYL
YELSEKYIYDLEKLKERLEELKRAQKVAYNFGTFKLNYETMNFSTHFRIFDSKSMKNKPS
TWGYTDTNILGHKNSIGQTKSVSNTEIGEGSYAKLKGLNFYDSEIIVSMLSRGTGCLGVV
FRAKDDFNFYLFDICNR-GVKRLSKVENGNVHILRESFGEVNINNQWNKFKIITSHGNID
IYEVNEDLEEVKILSSLDERFLSGTVGLYSQIHGQGSFFDDLEIIAHPCLDLLKKNKNSE
NSMNNCPFYTENYMNTKFPYIIINDIDYSWRFETDNDNHLLCSRN--IKDDVLDDNRYTI
GLLKQRKCTEGYFTFDINIPNKDNNNESYIYVLFHYENELNYNALEIRYDSIRFFTRNNN
KIEVLSELKDNEKIERLKMENEWINVKLNFLKSKFHVIISSNDYHLELDAGSFDKNIIRP
GNVGFLVYNLIEVKFDSILLSPPISKQYENFIQVKSKAWASCEESIHVLNRRSSCETDIY
PNETKEKHINCIKNFCEECCLHHTKLLDSNEKKQCEKHCKKNDHLAAKMQKVFEKFLNKC
VSLEENKDYEKCNSNDGICRNKTCMLCCKKNDPTTSKELKGLSANESKDIQEKEIIECQL
QCNQIYTTNKMSASPIKYESKILSNLANMLKLSEKEGYTGKKFEYYYNVETYGVDFFLDI
CSALCRSNYKPFLTEEGKNEILSKERLNRDRLNDGKTPNKKMYLKD-KYITEEIEDKIEN
EILREKGNFQKKEDIREGIISVGKYKSELTSLSRRSNEKIMKDIFEHTKNMEYSLNKDIK
KSNTFENVNNYDFYDRSQLIKNDSILSFSNEKIDKRYQSVNKNKKLSQNKVLKRNSYKKN
NNASAYFYDQKKEEKQVTPKNKKNKNDINLFLNKNENNKMSTDKNVMLIKTVKKKIEKNN
SQQKEKSKMHNETVNSIVVSNEVQKENYNNSVENDNSSINKNEISIKKEIKINNNTYEIE
NEKSISESYSNEEEYYYNESDGASYSTDDSSYIKYLKKKKKLQEQGKIVIDLDLLHGNSI
ISANKQNKEEKLQDKLRIGELKKILEEKKMVFCKIEDIAKKRNKRYVKTQVLSEDSKIER
VYFSHKMKKFYNSKSGYFGCGWSNNKMEWTPFIHAPFFDNQHNAIYKNRNKKLYEEIYDT
LLHGRIHPDIRIVELKDHKHPVRLCTPHNEDCYSIVYIGKKINATDDRIIFGEYTGFVAN
NRELSQEKHQYMFALTFSKKVFNDKKNVVFINEIESDEEEN---------DNSTESYINS
NTINN---------------EISEKLTNRMNKVKKTFCNLNNSQNNSIHKVKRPKVGKKS
KNLNNLIILPDNYTYAVDSSYMFNEMSLVNHYKTCSIFNNYDFRINAEWQLVYLDGWPHI
ILTSIPGIEIYPGEEIFADFGFEWFERINDTCLNEFIKNNFEHRLNKLNLSKDKLFNGLD
DIVEKYNLLKNNTTCNICMHNVNIDGNNFITCSGCNNIYHLKCVHKLNEEVNENYDWFCS
SCIQFSLNLINQKEFLNYIEKKNQKKFINFFS-DVHIDNLKKYNKSLYNNTLDKTICVEF
----QNSDTYNELEGYEKANKLLKSNDNINDIEKNREIVEDLLEKKEIENNLLEIKENEE
NNLLL----------NDFELQLLVYYKKKIDELLIIREKIDFFLKNDNYKNTLKRRKGTL
SFLTQSKKCIEDFLRINENINLQKKMKDSNFTKNENEQKLQ-------KKKSNDNLVSNE
NYKSPKEEKEEKEGKEENTLKKEELPFEKPSNDTSEIDSNFSNTYEKILYKKSNNIVKNL
KEMRKKD-----------------------KNNKGTCCNYMMKLSKKYIGFPLIKDFEKG
INTKQPSLPLNDYLKKLSVCSSCYNKHHDLAKAIICRITKMHFHSNYNDCLTDEDLFKSS
SECIQSVIRELANTIKEYRKKELDGAYLNIIKNNNS--LIEND--NMNLEVNFSNVT--H
KINNNNFGKIKK--DDNFFYEKRESLSKENHNTNDKDFKKHLDSSNTGKNENIDNYDD--
GKKEKHTLHNDL---SETVNNKESYKINNFNK-FKEDSNTELNRSNEEYLKNEKNGLYEN
KKY--FQNDINNSSTKNNNDQNKIPEN--EDLKNET---LKNEKILDKEINHFNSIVNNN
KVSNFIPLFGIELGKTKFQREFTNGTFVGTVTKHIKDEDNNNFFVITYEDGDVEWITPCF
LFQELLKXSTNNIEYPLPTNFKESIYAALDPDTKPVNCSPPTQ-----------------
-CSHPTPH-------------PMINKEEAQRLKELGNKCFQEGKYEDSVKHFSDAIKNDP
LDHVLYSNLSGAYSSLGRFYEALENANKCINLKKDWPKGYIRKGCAEHGLRQLENSEKTY
LEGLKIDPNNKSLKDGLEKVRQEKLLENMEFMNHINKLIDNDENLKSYKQENENYPNELL
DTIKKINSNPMNIRLILSSCNKKISEGVEKFFGIKFNDDSSEAEERERQRKKEEEEKERE
RKRKEEEERKKNRSPEEILGEEHKLKGNEFYKQKKFEEALKEYDEAIRVNPNDIMYYYNK
AAVYIEMKNYEKSIETCLYAIENRYNFKADFNKVAKLYNRLAICYTNLKNYDKAIEAYQK
SLVEDNNRVTRNALKELERIKEKEEKEAYIDPVKAEEHKNKGNEYFKNGDFPNAKKEYDE
AIRRNPNDAKLYSNRAAALTKLIEYPSALEDVMKAIELDPKFVKAYSRKGNLHFFMKDYY
KALQAYNKGLEIDPNNKECMEGYQRCVYKIDEMSKSEKVDEEQFKKSMSDPEIQQIISDP
QFQIILQRLNENPNSISEYLKDPKIFNGLQKLIAAGILKVRF------------------
----------------------------------FFFLDEKIPSKYGENRHWNVDLIPKF
ILVGGNLVKILKKTRVTNYLEWLVVEGSYVYQHQKKGLLYSEKFIHKVPATDMEALVSPL
LSLMEKNRCKNFYQYVSEWDSNNKSTWKNLDPFKLTMMDIYKYFNLCQLTIDFLGHAVAL
YLNDDYLKEPAYVTLERIKLYMQSISAFGKSPFIYPLYGLGGIPEGFSRMCAINGGTFML
NKNAVDFIFND-NKVCGIKSSDGEVAYCDKVICDPSYVMHLENKIKKIGQVIRCICILSN
PIPETNQANSCQIIIPQNQLNRKSDIYINLVSFQHGVTLKGSI-----------------
------------------------------------------------------------
------------------------------------------------------------
--------DVRLDVKLNKFIWSKGIRNPPKRVRVKLERKRNEDEDSKEKMYTIVQHVMVD
SFKGLVNECEANE-----------------------------------------------
--------FYQNGCNNCKFLQMTGDRHRIHDCTTENFHGFIAITNPNKSWMAQYNDLSKY
VPGFYALQIVGELPESIRDLKANYI---------------------KFY-----------
-------------------RAGTGYLILSLYEMLLKRNKKLELLYCIDINEAACNCIKNL
SNINKVSNVEILNNNLFNNMRKCNQFDLVLFNPPYVPTEEDEMNKTDIVASYSGGKLGRE
IILKFLLNVYDYVSNKGVIYLLLEKNNISHEIMNNVDIKKRFNYIELKKKKTLNETIFIY
KLSKKLMDRYGHNVRTDVKKQGYENSDLPILCETCLGENPYVRLIREENGKECKICKNAF
TLFRWKPGQKARYKQTVICGMCAKVKNVCQTCLFDLEYNLPVQVRDKFIENAITLPENET
NRNFFLEQMEKNMST-SYNKANHINIDLSKLKRYDPYFKRNMARVCSFWRNNSCNRGSEC
PYLHKEIHLDKSLYNQNIKNRYTGENDVLAEKILKKYNKENEGENFMANTICIHGISESV
```

```
NQVNIKECFKKYGEIKSIKMIPKDCKMFISYKNYSSAKKASEEYKDGLELNGCNLTVLLQ
EEIYNSNIKNQGNMNFLSN----------YQNINVPYMYYPFYSNNLNTLSPNATPYSSM
IPSEAEQRK---------------------------------NDYTSDWRWFSIKCERLI
ENELFKKSNKENTTKDYKNLIKANESMMNFKSFFPKILLNLYSKCENPNKFFSNLVIQNF
NAVVVI------------------------------------------------------
------------------------------------------------------------
------------------------------------------------------------
------------------------------------------------------------
------------------------------------------------------------
----------------------------------------------PLY-----------
------------------------------------------------------------
------------------------------------------------------------
------------------------------------------------------------
------------------------------------------------------------
------------------VKII--------------------------------------
------------------------------------------------------------
------------------------------------------------------------
------------------------------------------------------------
------------------------------------------------------------
------------------------------------------------------------
------NTFIK-------------------------------------------------
------------------------------------------------------------
------------------------------------------------------------
------------------------------------------------------------
------------------------------------------------------------
-------------------KNIFFKMKGNYEFKITDIKIMKRKKIINGKELYESRKICFI
GFINNEDCENFKKSFNNTYINTSKITIEDAFSPIISENVKQKKTY--DLKKKEEINKING
KKSVKIIKNENFINKLIPIKKTKAGMNTTRSHIIYLDDETDINKSAKPN--NSKKKKNTV
IEKINKENNENIENIENNKENAEDNALEWLKKISKNNENDKNVEEKKNLNEEEIIKEDNN
KNADNTSSIKN-----------DECENMNTGKIIIFNLPPTNEHDIKDLCEKYGPVVDVK
IYKNIKKGKFEINLNE--TNKSNEDFFFKLLKNSEDNS----KEKEKKKKENNSNNEENK
NDEKGENIVGNKYIIENDLTNIKVYAFVSFMFPSACEKAKENLNNAIYKGKILYVKYAKE
KLGDFEISEKSKNNIIIKLSNDSKSSYKKILEIQRKRNCQNENIWNILYTDINSNIYNFC
KENKCSRESLLNIKDKNIAVNVSLTETYIINKMKEWIKKEGIYLEAFEKIYKKNEEREEN
DNSYNTEKFIKYKRSNDIIIIKNLSMNTNQNDIINLFKKYGTLKKVSFSPYNNLAIIQFE
NPENAKKAFISNSYIRYKKLPLYLEWAPINLYEKKIE-ENDMEKNNSNSNDNTNEIKKNE
DEILENETSDEEITHASIYIKNINFNTKEEDLKNLFKNLEGFLTCNIVKSKKVISQKEYE
KNNSVEPKYLSLGYGFAEFKSKEFAIEAIKKLTGTSLDDHILELSLSHNRVKKKMNKKDE
EKKIIKEQKKISKKLVVKNLAFQVTKEELRKLFSAFGNVKSVRIPKNAYNRSRGYAFIEF
MSKNECLSAIDSLQHTHLYGRHLIIDFANDFIFEKDMDEFDRLKENNNKNNENSITSEQA
KRKAVYESKKNNEISESKKRKALNNLKNIMLNKCYQSKCRSYGYCCCEQKDENIMKNELN
EHFYSLDEKKFSQGISQENVENYLENPPKNARILKPLIQEKIVEIMKPEIEEKIIEVPQV
QYVEKVVEVPHVILQEKLIHIPKPVIHERIKKCPKTIFQEKIVEVPQIKVVDKIVEVPQY
VYQEKIIEVPKVMVQERIIPVPKKIIKEKIVEIPQIELKNINIEKIQEIPEYIPEVVKKD
VPYTQIVDRPFHVEKVVEVPHVQHIYRNIVSPQYRHIPKPVEVPMAHYRTFPVEKLVDRN
VPVPVELQIVQEFLCPKIEARYKEIPVPVHVQRIIEHPIPKDAMNNPHLLPLYYQEDNID
IPRNKS-----NQNNCFVFNCNKNNTRTS--KMNPSVELLLHNDRSTDQLYS-QDPNMKC
NFS--SNNMYMNQNLNDMNRYTYNNGINKSMPANINLNYPNGNQNLQNMNRMSNNPYDNN
NIFKNNNSINYSNNTGLNNTYNTISPTIPPNNE---------------------------
-----LGSTAVGICVNDGVILASERRISSALIEKDSVEKLLAIDDHIGCAMSGLMADART
LIDYARVECNHYKFIYNENINIKSCVELISELALDFSNLSDNKRKKIMSRPFGVALLIGG
VDKNGPCLWYTEPSGTNTRFLAASIGSAQEGAELLLQENYNKNMSFQEAEILALTVLRQV
MEDKLSSTNVEIAAIKKSEKTFYKYNSEDISRIIDILPSPTYPTIDMTAMELEKTHLINY
FLEICPTVLDCSRTELQSILAKKEEDKIRRFMVDKNINLIVIGKETKEVIENEGTERN--
---MKLENEQED-KKLNLDKEPCSFLFVELNINYRCVTKSISIAFMKRNKEKFSTLNEEL
DKGNKINLSNELLMFVCGQNDCNTPLDLVYLYLSQGFNTIFDAASNTTQQSSSNEFPQIG
YSRKD-------VDTVSGTNKAICYEGENKISNILMANVSKKLNELLISMKNAQIDLNIP
IINLSVDKRIKKIVDETPNINEIKPEILKGLCESQEFLNQLQKDVTKWVEEIQKLTRLNG
DFKGGSALSEINFWIGYENALSHLENQLKNPEVLLTLQILKNAKRYFATMSFDSDIQLRQ
SKEYVLNVNILMKDFPINDLLGATSIQQIIQAIRNIFNHLKKLKNTTKYPLSRSYNFVEA
LSRDLANTMRKVLSPQPLLGMDYQEFEVLISGCMEAFRVWNEEMRIFKDMVRELIKKRSF
NERAPAKMTFEHSYLQERLDEVRKFRKQHQKFKSVITKVFGNDRTIGINLNKDINLAYSI
FLSLDALDLSKTGEDLWEKAKISYESKINRVESQITFKLRDQLGGAKTSSEMFNAFSKFN
PLFFRPKISGAIQEYQNTLIQIVVDDLRKLQMIYINGYLKSDSEKVSTIRDIPLVAGSII
WAKQIERKLENSLKRIENVLGRGWEQHSEGKNLRQNIDNFKNLLSQNKTFEKWLKNIKNE
DKFDMYEKIIKIRKLGAKNYEILANYDPEFFNLFKEVRYLQSTNLRVPYSIKVKADETKL
IYPYALTLQKTFRTYMKICLFMENEAKLIPFNSTISKLVASIHNDVQNKIKEGINLHWDS
DILETYVRKLSEIMNTFESMVDEAANKNYFVLDTLEKLKTCEITNDGQEIRKLIECIQEK
ADELYLEHYKNVHIWIDELNILLNKILSKRLEEIIKSWTNEFINWP---NNGKRFIEKET
IHEFKIKNQKFYLYPSIDSMRQIWYYKLSEAINIICGITRVKNIFQKKDKTKETIQIKIE
DGNIINDVILDNTYRYIMYFIDKKIYDNAVRAINDTVEKASKYESIWLQYKSLWKIEIDD
IITSFGEDIQTWKNFMDEIKQTENTFDTLDTEKYFGPIVIDYRILQSKVSSKFEIWQKEI
VSEFSKKLSEKTLHLKEEIENALNDLNMQATLTNESEITAMHILSMTPKDIIGIINSYDM
DVLSKACNSIHNFMEKINEIDAKESEWDNKCDILKQSEVLLEKQRYVFPSNWLYIDNIIG
KLETVKQILSYQIKLVKDYFPYLQSLVLEFDVKVQNNIKVLFEEWNKSKPSHGNANSTKA
LQIINAFESRIRIINKQYEISEKTKTLMKISNDESEVSFHVSPDVLKEEIICVKGIWDEL
KIIYSSISDMKKTLWSNVEPKNIKNILNNLLDSIKKIPAKYRQYEIFDSVQNEIQQYLKT
YFLLLELKSESLKERHWRSILQKFQIKVYYNKLTLGDLWSLNLNAHENVLREILNQSQGE
MALEEFLRGLKDTWNEYELELVQYQNKCKLIKGWNDIFSTIDDHLNALQSMKISSYVKIF
EEETLTWDDKLNRLRNLLDVWMNVQRKWVYLEGVLKGSSDIKSLLPQEYNRFKIIDKDFI
SIMKKTSEKPKLLELFQIDGFQKQLDRLSDSLSKIQKALGEYLEKKEANFQDF-------
------------------------------------------------------------
---------------------------------------------------------ILL
EMKI--------------------------------------------------------
----------------------------------------------------------YI
FRYV---RKVSQTQLTDACFLTLTQALKMKLGGNPFGPAGTGKTESVKALGAQLGRYVLV
FNCDESFDFTAMGRIFVGLCQVGAWGCFDEFNRLEERILSAVSEQILTIQTSLAQRKNEI
ELLNKKIGLNKNVGIFVTMNPGYAGRSNLPDNLKQLFRSFAMIEPNKQLIVQVTLFSQGF
ISAEHLSSKIVSLFDLCSEQLSKQPHYDFGLRSLKSVLNSAGNLKRLALLESVNKNDENK
NDKNLNNSIENIVAVEQTLLLKSVCDTVYPKLVSSDILLIKNLLTGVFPNANTAAFEEKA
LINEIYRMCKLRYYTPEEKWITKICQIYQIMKLQHGVMLVGDVGTGKSSAWKILLDSLEA
LDNIKGLSYVIDAKSLDKEEIYGKLDNINLEWTDGVFTAILRKILYNSAQSGNPPKRHWI
VFDGDVDPEWAENLNSVLDDNKLLTLPNGERLPIPESVRILFEVDTLKHATLATVSRCGM
IWFSRDILSPTILFKHRLHKLKYGDNDYLRKMDNFKAFIMNNSENVENESTRMNDSTILE
N--SKSFENNSED-KTKEKLMDNLRINSRIFFDENEENLNSYKVKTIAYRAIDLISDYFE
ENEFVHQCLLGAKNFEHVMDYEYIRVIESTCSLLQKGIDNLVQKNEKLNDTLTDSEIEKY
ISKWLVVSVLWGIGGSLNLESRQKFSQFVQSICFVPLPPDLLSVDTLD-ENSSKLFKTLL
DYEPSIEDGEWHSWKKRVEIVDVDRTEISDATLVIETMDTIRHATVLEGWLNLKKPFILC
GPPGSGKTMTLTSVLKKSSEFDIAALNFSSGSLPNLLLQTFDHYCEYVKTTSELVLRPLQ
PGKWLIVFADEINLPTPDKYDTQRIIMFMRQLYESQGFWKYESSNNQWNWVKIERITFAG
ACNPPTDAGRNPLSNRFLRHTPVLYVDFPGYESLKQIYGTFNRAILRKFPESSHMANNLT
LAMVDFYTKFSETFTIDMQPHYIYSPRELTRWKLALYETLEHYDELKKRDLVRLCISEGL
RIFQDRLIYKKEKKQTDKMIDDIFKYSFPDITDNDLQRPILFSCYMQNQYTEIDKNDLKE
LIQGKLKIFNEEEINVQLVLFDEVLDHITRIDRVLRLPLGHLLLVGASGAGKTILSRFVS
WINGLSVFQIRAGRNYSTESFEADLRYIMKRAGIKEEKITFIFDESNVLGPAFLERMNAL
LASGEVPGLFEGDNYTALINECKAAYGSKVGLDESDIFKKFTKQVQQNLHIVFTMNPANP
```

```
DFSNRQATSPALFNRCVIDWFGDWSYSALLQVASEFIFNLDLPNKAFYMEYIEDAHIKGK
IQYKDNKSYFLSKAIVEIHNSVGKINNILMKKGNKYNYMTPRDFLDFIKHFLKIIDEKKE
EVTDQKKHLNSGLNKLKDTEEQVAELRNSLAIKQKTLAEKDFEAEEKMKLMIEQQAETED
KKKKAEILAKKLDEQFIIIEQRKEVVRKELSEVEPKFRDAEEAVKNIPKKNFDELRAMAN
PPILVRNAIEAVAILIINEGDKSVTWEDARKVMKGQDFINKVLYLDKKTVKPQTSAQIKK
RINNSDWDVERINKASRAAGPLAKWVESVITFLNILETVKPLENEIEKLQEETRIAEDQY
IEQKEIIAELEKKLLQYKNDYAQLISQVQNIKQEMEKVENKIKRSINLIENLKSEKERWS
ETFINLEAASETFIGDCLIAAAFCAYIGFFEHYERQKLKKTWGEIIKFHHIKHRYDLSFI
EFLSRPSERLQWIAHRLPSDELSMENAIIINRYI--------------------------
------------------------------------------------------------
------------------------------------------------------------
------------------------------------------------------------
------------------------------------------------------------
------------------------------------------------------------
------------------------------------------------------------
------------------------------------------------------------
------------------------------------------------------------
------------------------------------------------------------
------------------------------------------------------------
------------------------------------------------------------
------------------------------------------------------------
------------------------------------------------------------
------------------------------------------------------------
------------------------------------------------------------
------------------------------------------------------------
-------------------------------------------------------DIQ--
------------------------------------------NKKANKTANQQNCNLNHS
NNIMSHTVAAANLNSQHAKQMRLHQLNHSHKPHLLSYHNVNYDDKNNKLYNNINNLNNKN
VSNGNSTAPQVSSTSNNEMIKNNKLGCANRQKNINHHIHNNHIY-NHKSQQQSYNENYGY
RPTLFNLLEVLANHNITTPDQRVCIRGIVTDFLNNELPHGKIYAYIGAVVGHDILHDIIR
KLEKDPNRNIVPDASGLARIEAAFGLSKSSYDFMNSNSNNSSNNISNVLFNHRNLSSNIL
NNHIYSSNSKTSNDINNLLKYTKNEKYSVSCNNFLNLNKKNNNNMNKNNFSDDEEELMKA
IKKNIETYKMNNIKNILISSVFGRIKWLKIMNESPHAYLYGHSIVKFGNKLYMFGGSNNK
NKKVPFNHTLTFSLIYYNYKLLPLGGNYPEERDGHTTHLISLKNGLSIFLFGGSNENIYY
NDIYILDMETRKWSLREVKGKLPLPRDQHSSLVYPAKSEHLRGEKSNLTEGVIIFGGKCL
YNNSIINLNDMWIFLFHLNIWVRINYLSEETPIGRHGMNLVWSDTNTLCLFGGECYDNSK
NFKERKLLDDMWIFKLHTN-KKFTKKIRVTGEWQREVYEGKIGCRSNYSSIFITQRHQDF
KGAEPKTIERLMLLCSGITYVTDNKKKIVSSDEIYVYFFSQKRWYLLKGKLYNEELIYNG
RQRHVGCFFESKNVLGRANKNPIPCIFIQGGFKKNSVFGDAWLLSLTGENPLRIHEYDSS
REKISTTQMPLYYFRDTHSISLLYSFCTLQKWLFGAFANLVDNCIQSSNPAENVFIKYEL
TPEHDGMLSIQDDGEGLDFNAMNRILRMYGNYRNYDKNLLYNNNN-TSVKKHNLPNSDFV
NNKVDEFNDDSAKNSQEIESKQKQKNYEEIDESDKERENEEVPKNKNLNEKNNDYNEMNN
KGGNSNNDDYDYKTHDEDSFYNENANNTFDLKYGVGFKMAYARISSSCAIMSRTVNTIGI
GLLSLELMNHCDAKELATPLCMWKLPNKELINRNIANKSEHRHHQKLLMSYTPFNSPSLL
AEQINILGTYSGTRLLYWDFRDDMDFIVFCSSNNNIYLSSSPLSLEEIKYGKKRKNITNV
SNN--SNKYSCVEDIKLNNRDIKRKGNF-EDEKMKKAKTEKDSSIGNNNRDLVKEEKGNN
STYSKNDDNNQDDNDNQSKTNKNNVKREKNTNSCDENK-EKNNGIYNKARNNDYDPYQYY
NSSLKKLNLFEYNAHLHPSISKEKYNISSIFPLWDHPKDSIDYCLSTYLYWLYLRRNTNI
FLQNTLLIPTCMRNNDKSNLKKKKKKSKKGKNRKKLKKKKNNNNNNSDFNKNIIKNRKEKE
SDNQYHATEENKDDRDNNNTTNFESNENVNKEIEEDSKNEDIEKNNDK----EQDQIEKK
KLQEALEYGIKQEKKEGNEVHKKEKEDEDEEDDDVDNEEDDDDDDDDDDDDNDDDDDDDD
DDDDEEEEEDDIDGDQEEEKEQKESGLQKTEKDENDGHNVKEEYDNENTEGKKIKKIKDN
DNEKEENEKKAKKKLEKNENYVNNDIKNEENEKVKKENYVNLYMENLKIKDEYYINNTSK
YTLYSFLKRKLYKQVEFHYLFTPSDYEYGSFIMMGFLNDNDNTLIEVKRVCEAGILLYYK
NRLIKRLDAPFIDSAYNLSLSKYPPNPSLYEGNLYKYALTVIVNVPNWLKPSISKQEFIH
ENNYAFLVFKKKLISLIKYYLNICQDNLKLKKWKESRDLKLKNYLEKVQYKLRKSKNESE
NEYEKAYKIVYNEDIE-----KEEELEREEN--RQIVKEIE-KAQEEKRQRENDEDEKDG
EHVEKGNNGHNDDEIKKEDNELEEEEEEEDEDEPEDDNEQDDEGKEDEQE-EYDEEKEDE
DDADDDDDEQEEDEQKDEGHDNDGDDDDQERDDDEQKH----------------------
-----------------ISLFMLYFLNIYIYFFLFI-------YIFFILYMHML------
--------IFLYFLDVVYVKPCPEIPYGKKIQVLPIDDTIEGLAKDTLFEIFLKPYFNES
YRPVKKGDLFLVRGGFMSVEFKVVEVDPDDYCIVSPDTVIYYEGDPIKRDDEEKLDEIGY
DDIGGCKKQLAQIREMIELPLRHPGLFKTLGVKPPRGVLLYGPPGSGKTCIARAVANETG
AFFFLINGPEVMSKMAGEAEANLRRAFEEAEKNSPAIIFIDEIDSIAPKREKTNGEVERR
VVSQLLTLMDGIKSRGQVVVIAATNRQNSIDPALRRFGRFDREIDIGVPDDNGRFEILRI
HTKNMKLSPDVKLEELASNTHGFVGADLAQLCTEAALTCIREKMDVIDLEDEIIDKEVLE
SMCVTQDHFNMALGTCNPSSLRETVVEVPNVKWDDIGGLDEVKNTLREMILYPIDHPDKF
EKFGMSPSRGVLFYGPPGCGKTLLAKAVASECSANFVSIKGPELLTMWFGESEANVREVF
DKARAAAPCVLFFDELDSIGTQRGTTLGDGSGAGDRVMNQLLTEIDGVGPKKNLFFIGAT
NRPELLDEALLRPGRLDQLIYIPLPDLAARISILNAILRKCPVADNVPIDFLAQKTAGFS
GADLAELCQRAARAAIRDAIDAEEMNKKSKLQLNPNENQNNENQNQDNEENAVKYEITRH
HFKEGLAGARRSVSQADLIKYDNFRLKFDPLYKTKTGGANEDFIIDWPDEENNEEPQDYN
VDDDLYST---------------SLLK---------------------------------
---------------------------------------HNEDEKKNVKSKCSTVINKKI
-----KKKEKRKLKNYKNNGNLKNIKEKSIKII--KKKEIFDINTIIENEEKFKNIDDVK
YIIQYRKWTLNGKINVLYSIMKSLYDSNFFKPTEIQFKTLEHSINYKKDVIVISKTGTGK
TLTFCLAILNNIVINKLNEYKK--KCKQKFRCLILVPTRELALQILNHFSFINKYINLYI
TTIIGGLNVNKQKRIISKKPEVIICTPGRLRFFLELEEKIDYIYEMKNIRYLACDEVDKM
IESSFINDINFIVKRLYKCVNDKKKYVQTFLLSATLGLTVQLQNENLSKLLNSIIIRKEK
SFFINLSGDHL--------------NNDNFNILPDGLTLNIVKCEKKMVLIKLFYLLKLY
FFNEMDN--EEEIKKIIIFVNTINSTKELKMIFRFLFFDPSLESAIPKKFRSNLFLKSKI
NIYSIHSKLKLKERIANITKFSESNNKSVLFCTDVLSRGIDLDKCDLIVQLNCPITDITF
VHRSGRTARNFKKGINIYIYIYIYIYIY------IYIYI---------------------
------------------------------------------------------------
-------------MYDINNARCVEIKSAELDVLNSDELKRISLGKYENEHYEFEKVQKSG
VTTKLYYDPRYGTIDNKQICSVCFERHENCFGHVGHLEFVLPLFNPLFYKELQELLNLVC
YNCYNFCCSEDVIFLLKQVFQLKTLNEIESTNKLIYKENCDYEYLISEIEKLYKKICKDS
DISLEDIIESISDE----DESNTDNNLHNNDNDNLTTK---------GKEKNYLEELSEI
D---NKEN----NKLKEKINKYKFDANKLAYQSNYNYEEYLILSKTIKMHMKKSSKCYYC
KFHRNISAKISQKRDTINFVGIYTKNSFFKKYFPKMKKKDDDSFDIENEEKKKKRKLDES
DHFVDINEK------------NFKKKEMNSDSSIEHSLNNNDNRNTSFSEMLDNYKKQNI
KEKCTIRLFSFQVIDILKKIFVKNNKEIIDLLYPFTKKDGYKIFFLYDMGISANRFRSRY
RGMHKRTKMAGTLCKINNFLNLCLNAKKEIDFDSLLEHNKKELTLYKQMFSLLSLKIRYK
LNNNIMDDDTEITKMEFLKCYSLVYHNKSTYINILFSSLQIGVNTFYDSNLADKINNRNM
TNIGIREILDKKEGILRKNIMGKRVNHCARTVISPDTFIETNQVGMPLEFAKVLTVDEYV
THHNLEYIKKLIENGPHIYPGAISYKDSKGRVFKISHDYEQRLKLLKQLDNINFRN-ENC
YIVHRHARDGDIVIMNRQPTLHKFSIMAHYIKIFEKEKIFRLNYVNCSSYNADFDGDEMN
MHLLQTPLARAEASHLMNCDFLFTSFKDGSPLRGLAQDFILGGLHLTSLETFLNYDEYCN
LLQCSLNCLISQKNSFFFKKKKNMHKTTTNNNNNNNNQNTPYNNKQNFNSFSGNNVKITN
YNYLDPYANILNQINFSIVTDEPAILFPKKIWTGKQLITSILKTIIDKVAVETFNKNNDN
EFMKNYKGINYTSKAKTSAELWSFDPVKECEVIIKNSELLQGILDKFHFGASSNSLIHLC
FELFGPKTAGVLLDCFGRLFISFLQLRGSSLSLYDFILNKDAKREKSLIKKRISFTGFYL
QNLFVHSIAKSLNADINDMNSYAKRKKECNNEKNDIIINKMYDSYKKKEEIISYFEQKKG
KLKLEEKEANYKNYGTDDILKEKYIDLKEEKYFIALLNKKIEDICAQNNENTSNLDDKKR
REMDSSDSDNYEKYIDEKKDSIILNTVIHKLYNSIKEPSFFSNPTFTGKKIIKIIEKILD
FLKEAKCNKRLKIFIIFELFQNKNIMKYFPSLMNYLHDFNYDLSNEEIIKNVVNNIYFE-
```

EYCPTSPFKDNIIKNKDEYKLNKFINFYESSIHKK----EDDAYTKKETNSVDNISNNDD
YNIKEISLHDKKLIKNCLISSFPSFLLNDDICNLSYNDKYQYFEEYLVKEEKEDGYFKFY
DMKDIFYKMEYLINNFFSLNRFDFDTLIDSLFQPYLCRVSSSTDHLINMNNLINKFLNNG
FSNMIFTGAKGSKVNYSMICGILDQQYLEGKRVPRMKSGKTLPSFHRYDYGARSCGLITD
CFLEGLRPQEYFFHCMSGREGLIDTAVKTAKSGYIQRCLIKCMESVVLHYDGTVRNEDNS
IIQFLYGEDGIDPSKTAYLDSSNDLLYNYNLSFSKYLKNNVHEKILNNEDIFQNHIRKWK
DEEPVICHYNPYTFIGSVSDKFNKKLSKTF-FNNFNFPNYRDLPKDFDTLSTLLLKSKYF
NSLCNPGESIGILVAQSFGEPATQMTLNTFHLAGTENVTMGIPRLKEIFLTSKLTSKPMI
YVPIKIELKT-NDINKMKLYITNIADKILNSYKSICLSDVVYGVGVERKLILKEN--KNN
VHYINGS---EFDDEKVSVNVQVAKIMNVLKNVNISFDCKKEWNYEIIIQFENLYHFCKI
YKQISIPFILHKVTSAILTSVLNKIQESLIFHNINSISSFNHEYYDELYDFISKKLVTEF
NFNVEKYEYKDDEGQINAKLKYMANENKTDKSGDKQNDDQDDVNYSLQNKNAYNNNVGDS
SYNGKKNKESDIEDNDMSEKEENIYKTESEAEKEEEESESEEGKGDENEYDEDDVIDSEV
ESEKDEDYNEENKNYGNDD-DDDDDDDEDEDEEKVEEENKQNVCRYEKDMSKLKNSKNTS
DKNKNEKNSDELKLSESSNDSGTVNYFSSEASVTH-------------------------
-----------------DDSNNIK-----------------DKICGENSHHKKNILDKLK
HQQLRFVKKINFSPITWTLKFEVGWDINFFPYPIDFLTYLKSEISKETLYKVKDLNNPKI
LKGKDITHSGSEYELQIEGKNIYKLYNIKDKYIDKTKLYCNDIYAIVKTYGIEAGRLCIT
KELKKVFDAYGIKIDFRHLSFISDFMTHTGKKKKKKK-----------------------
--------------------KKKIIITK--------------------------------

--GELCMDILKANWSPAWTIQSLCRAILFLFTEPNAESPLNCDAGLKLQR-KILFF----
------------LYTIQELDPDMLGLLKCAFNGGVKVKSAAEVGRRAVEYFRGDDFFNFI
NTNKDMLKKKFPKLFVNRNLIDSKDIEDFADIFIQKGYIYKAQYKPIKGVIEKDENGIYK
RPKWPKRLIMTSKQNFDKTSFYILVHERNKKLQYVMLIGLITIVLICCMFPVWPLKLKLA
LWHLSVVFITIISVIIVGRLCTFVFFWFFGVDYWIFPNLFDEDCNVIESFYPIQSWVYRK
DTWLLFVARIFTAILLSIGIHQLGKTHSISDIRNFATQSFIDILEWGNKKLAASPENVSM
YKSIDAKATFEGEKEEEEEVIYDENEENYDCLKKCGFQTFEELVRRCFLKCECMEVLILM
ISYKKKGEKNKNTYFKKIPSFLCFIFILY-ATLADSFLKDLEDLELEDD-NFSDFN---K
KDNEEEKIEDNDDYEEIVDAIEEFLNERKKKKERKISELLYNEEFLKMMNDIKDYIREEE
EQHEQMNDN----------KALRERRE------KNEKEKDEEEVLIEKCIELIIKIDTEI
LNIHKYVKDIYSTKFPELDSIVYSPLEYISVVSKIKNEIDLTNIDFSDILPNTTVMAITV
ASSMTTGIKLSDHLLKNCLSFCSEALELNENRKMILLYLESKMFLLAPNLTMLLGSALTA
RLISSVGSLKNLSVTSSQNIIVVGGSKKSVLGLSNVNKTFGIGILSSSEIVQSVPDSYKK
KAIKLLAGKCSLAARIELL---

---MTE
ERTVLNKKILNNSVILQVTSSDDDLQSYISSFYHCDYRTFMEKIIKIAIRVKRDKYIGRH
YRYFIRNTRVRAYKQFLEPFKNVTLKNMAYAFGVSEEFIESEVSSFIANGKLNCKIDKVN
GSIESNQPNERNTMYLNTIKK---VLVKKNKLNFYISIYMNININPRLTFFIKFLDGTID
TNKRLSKEDIEFLQGAIKEALSQVEDPYEAINEAVLNFENKDEGIILASAKIVERLVDEY
PEVARNLDKIKAIDPLLKLLDHSNNHILESVLQILSLALSNNPDLQESVFKKNALKTLLI
KLQESQKTLIDKKLITAISALIRHHDEAENKFIDYGGVGFLVYGMQTNIYKYQEKSALLL
KHLVHQNKITFEIFIKNEIMKGLICLANNKNIDETGIQYGETTAELFLAIIQNHRHKLAK
SGNLHNLKKLIEDRLIYLRVIQNSTSYDVSHEMELFNECLKLTKFFFPKKLCTMSKKLLL
RYSKLNRLSQIKKFLKKLLLEKKKKKKKEKEK----------DEENPNERENNNITFENL
GIEDWLIKISKSVQINYPTKIQQLCLPLIIQGKNVIGTSETGSGKTICYCWSILQELNKN
FFAIFALILLPTRELVFQIIEQFLLYGSKIGVKVLSCIGGYSLIEQRRNILSKPHIVIGT
PGRVSDILNNCEDIKSCFKRLKFLVLDEADLLLQKCFESKLKIILDNLQK--SNERRTLF
FSSTITHSIQLLIKSFPNDNLMLVNANKNQKPVKNLDQRYIYVDDIAQITYLVYILKNKL
TDLSGIIFTANSYKCQLIYSVLNILGNFSVECIHSSKEQKKRISSLSKFKNGLCKIMVAT
DIISRGIDIPRVAFVINFDFPNDTIQYIHRIGRTARANRKGLAISFIDKKDIKNFKDVKK
IMKDKLKPYILDKNEVLKDMFKIGKAIKKAEIMLEEQKDIRKENEQLKYFIYHNE-----
-----------------------------NFLDVVIFPVSVHYEYTRKLLDKKFSMGIQN
TSKYGNGSYTGEISAEIAKDMNVEYVIIGHFERRKYFFEKDEDVREKLQQCLKNNLKAVV
CFGESLEEREQNKTIDVIKTQVKAFIDLIQNFDNVILAYEPLWAIGTGKTATPEQAQNVH
KEIRNIVKENCGEKQSKQIRILYGGSVNTENCASLIKQEDIDGFLVGNASLKDTFVDIIK
SALMGKKNKEDLYKENKLEASKLRIAIVSNDKCKPKKCHLECKKNCPIVKTGKFCIEVEH
SSKIAYISETLCIGCGICVKKCPFSAITIINLPKDINKDVVHRYGPNTFKLHRLPIPKLG
QILGLVGTNGIGKSTALKILSSKLKPNLGKFNNPPEWRDILSFFRGNELQIFFTKLLEEK
LSPIIKPQNVDLIPKQIKGNILDIIKKKDKLNQKDKYISELDLEHLLDRNVEDLSGGELQ
RFALLISIIGQSTNVYMFDEPSSYLDIKQRISMAKIIHKLVKHDNYIIVVEHDLSILDYL
SDYVCCLWGKAGAYGVVTCPFSVREGINIFLDGFVPTDNLRIREESLNFKLATDQDATDE
EKKRLHFYNYPSIVKTLNSFTLTIEKGIFSESEIFVLLGQNGSGKSTFIRLFAGLIKPDN
LESLSFLESLSVSYKPQQIQAKFTGTVRQLLMSKLKGLYTDPYFNNEIIKPLKIDSILDN
QVLTLSGGELQKVAIIVTLAKNTNIYLIDEPSAYLDSEQRIIVSKIIKRFILNTNKTAFI
VEHDFIMATYLADHVIVFDGQAGVNTVANTPQTLTAGMNKFLKIIDVTFRRDPTNYRPRI
NKYDSVKDKEQKLNGMLKIQNCMSILCTISGQTPEEPVVSKTGYIFEKRLIEKHIINYGI
CPVSGELLTLQDLYPIKNEKIVKPRPISASSIPGLLSIFQTEWDSIISEMFSLRTHVNDI
RNQLSHSLYQYDAATRVIAKLLKEKNSYKEEIDNLRNQIFQLKSGNDINEFEIGITDDLL
NEMQTTAKNLLMKRKKRKIENVCSVDLWKEFKNSNEFNIHSSVVAGVTCLSLDINKYKYN
YYDDHSNHNFFSGGKDGNIYYVSLSDNKILSKLQGHLKKINSIVSHPSNFICISGSNDKT
IRIWKGDSDTNEYVTSHLIKKHKDNITSLSLHPLENCFISSSKDNTWILHDLETSKTIKI
CKNNPSSFKNVSIHPDGMMLGVASDDSNIHIYDIKSQEYKASLTGHTKAVECISFSENGY
YLASCSKDNTVKLWDLRKALSFQTLELEHTPKHITFDYSGKYLSVSIGNDIQIYNFETKN
EVTLIKTLMSHTDAVTQTCFGSRTSYLLSSSMDKTIKLWN-------YHGNIKDGLFHGY
GVLIYSKNEKYEGDFVYGKREGKGKFTYADGATYEGEWIDDKIHGKGIAKFISGNVYEGE
WENGKINGFGILSYNNGDIYEGEWLDGKMHGRGTYTYEDGDVYIGEWKNDKRHGKGCVKY
KGNENKIAETYEGDWVEGKMQGKGIYTFADGGIYEGDWIDGKMEGKGIYKYLNGNVYEGE
WSNDMKNGYGVLTYVNGEKYEGYWKNDKVHGKGTLTYSKGDKYIGGWKYAKKCGEGELIY
ASGDRFKGEWKNDQANGFGILLYANGNKYEGEWIDDQRHGYGTFSCKEDGSVYSGYFAYN
RREGKGTLTFVNGNILEGIWNLGVLTKVTNFKLSPSSPWNDADLMKLEEVKDIQKIERIG
AHSHIRGLGLNDCLDARYCSEGMIGQMSARKAAGIVLRMIKEGRISGRAILLAGQPGTGK
TAIAMGIAKALGEDTPFTHISGSEVYSLEMSKTEALTQAFRRSIGVRVKEESEVIEGEVV
EIEIEKYNESDINNSNRKVGKMILKTTEMETLYDLGNKMIDALQKENITAGDVISIDKST
GKITKIGKSFARSKDYDAMDPNTNFVQCPEGELQKRKEVVHTVTLHDIDAINSRTQGFLA
LFSGDTGEIKNEIREHIDMKISEWQEDEKAEIVPGVLFIDEVHMLDIECFSYLNRALESE
QSPIVIMATNRGITHIRGTDYKAPHGIPLDLLDRTLIIPTYPYKHQDILKILEQRAEEED
VEIDEYAKELLCKIASESSLRYALHLITLANLVSKKRKATEVTVQDVRRVYNLFIDVKRS
TQYLIEYQNEFMFSELPKEDECVK-EEVCEEKREIQEKQSVNSNISNMAYLYSNLSDFWT
SDEDDEDEENEDEKDEEEESEEEEKKKNKINEEKNEFYNYNENIFEKRENLKENDLYTNG
TFNVVNEGLNFNINDLMNDSVNDLSDKFSISNILQNYDVNDFIFNDSLLNYCETGVAIND
EAPCNEKLSLNENFNFKHSNSFDYVNPPNFKSKITNEIKSDINESNNNNINEETNKLNEI
NLYNENFTKSDNYNLDSKDICQLNDEKNLKSSKEDNFNDKDDINIKINEEKTKVKYIRKK
WVIEDNHSDVSNFSKNDMLLNYDFELDDFQKRSINHLNNFKHVFVAAHTSAGKTLIAEHA
IALSIKLQKKAIYTSPIKALSNQKYNEFKNIFKNVGIITGDVKMNVNANCIIMTTEILRN
LLYLNDKIINNIHCVIFDEVHYVNDEDRGVIWEESIIMLPDHVQILLLSATVPNYLEFAD
WVGFTKKKEVISISTKKRPVPLLHYIYAYDSIFLIMDEKNKFYSSSFKEIYIKIREKEEA
SKNQK----------------QLQYKKKSNYDPKSK----PVQNKSETDNTKGYYEYCKQ
KRKQKTFFNEASMKTEIQKLQTLIKKLEQDNKLPVVLFCFSRIKCETYAKCMPHLNFLNN
KEKSKVHLFIKESISKLCTRDRELNQIKNLSKLLEKGIGIHHSGLLPILKEIVEILFSKG
LIKVLFATETFAMGINMPTKSVVFTSIYKHDHLKKRILTSSEYTQMSGRAGRRSSDKYGY

VYIFCCDNIPDQVQLTEMMMQKAVSLKSKFKVTYNMILKLLINKQINIEKMLFSSFLESC
RALQIPLFKKDLKRKKRLLQNIKEVECIYMQ-GDIIPPIENYVQIDYKLKLIGLDLHKKL
YNMKNSNCFVIGRVMLLNNISILHSSVYAIYMGCDKKSNKKNDKVDFAQNSIFFQNNKEK
DESNERFFFLFILPDFMTYEDMLLQMDCDKEKDNNSYNHENKNNSNNNASYSNSISENIN
LYENYKNIFSERRNKNDIKIIYHSSFDTDGNKKHFVLCSNICIENISIITNTVIKLPNVK
TTSLLNNPKNLLLYTLELDRLIEKENFEPIVLSKVLKALKCEFYSVLINQTDYLESLKKS
KCYNCNLKEKHYELVCKKNDCLNDIENIEKNINAKSLNLYEDMEGKLNVLRHFSFIDDQN
NLTIKGKIASYITLTDEITLTQVIFENLLNKLNPPEIAAVLSCFVAPEKKIEESPDLTVN
LQDVKASLTNIHSKFEEFYKVIRLRVSSEDHWKLCNFKIMFIAYKWALGASFAELLEQTE
LEEGLIVRSILRLDDLCRKVKIAFLYLGNIELAQKVEETSNLLRRDIIFTTSLYLQFFLM
VFHLSFINRRKGKGFIAAYYLNSKIKSQNNINEYPQNKINEFCLNNKVCNVLYAKYFKKV
FENLANQEDKKGLPISNNLNSYINTINETMAEDHNIKPHKTRLEELKTVMKRNNIDVYIL
MNNDEHNSEIINDKDKKIYFLSNFSGADGTLIITQDEQILYVNALYELQANKELDKDIFT
LKIIRINNREEIYDTISSLQFNNIAFDGKNTGVRFFEKLRKSISQKYTNRKIEEKVIYGN
NFDYLKQNNNINLLILENSLVEIKNFEVNTKSVFIHDLTFNGSSIAEKIAKMKYSFEEIK
DVNNILISELDEIAYFLNLRGYDYKFSPLFYSYLLFQFNRQKNDFDKIILFTIVNNLTEE
VKDYLNKNNIIIKDYESVIEYLRDNISTKLQDIGSKTVIDLTEDNYNNTELISYDISLSP
FINLIIYMLFNKKNVLLQNSPIVNMKAIKNDIEIENMKEAHILDALALLQFFHWVNEKKK
SKELFNETEISLKNKIDYFRSTKKNFLFPSFATISAIGPNAAIIHYESTESSNTKISPNI
YLLDSGGQYLHGTTDVTRTTHFGEPTEEEKKAYTLVLKGHLHLRKVIFASYTNSLALDFM
ARSYLFKNYMDYSHGTGHGVGLSLNVHEGGCSISPVSGTPLKENMVLSNEPGYYLENKFG
IRIENMQYVIVKKKKDNTEYLSFNDLTVYPYEKKLLDFSLLNQEEIRDINEYHDNIRKTL
LPLLKENPTEYDEGIIDYLMEITEPILI-MIIFFLSNICFGINFLASAKKNK--------
-IKKKNFIKLTFHSFKKMTH-NINNSHKENEKKKVVIEKDPSFIKERLEKYNELKEKKKE
QLL-KNEHLKRQINIELLDGSIRNGECYVTTPFQIASSISKKLAEDSIVSKVTYKEKVEL
ELCDIEDAEENNSDLKNSNCNSLLWDMNEPLIGNCKIEFLNIENEEAKKVFWHSSAHILG
SSLEKLFGGYLTIGPALNEGFYYDIFLGDFSISNEHYKKIEEEFNNLVKQNVEFEKLICT
KDEVLELFKYNPFKLELIKSKIPDNKKTSVYKCGNFIDLCLGPHIKNAGKVKAFKVLKNS
AAYWLGNKNNESLQRVYGITFQKKNELMEYIKFLEEAKKRDHRNLGKKLNFFFFEKDTSP
GSCFWLPHGAKIYNKLIDFIKKEYRLRKYDEVISPNVYSCDLWKTSGHYQNYKECMFIFN
VENKEWGIKPMNCPGHCLMFKQLNLSYRSLPIRLADFGVLHRNEITGSLSGLTRVRRFQQ
DDSHIFCSYEHIKKEVLDTLNFIFFVYIHLVLSMNY------------------------
-------------------------ICLLD-------------------QANISVRNQH-

---------GIMLKKN--

----------------NVIGDNTIKIGNVCEKNVLQSLKYGEKNFFMLIIFFALYEILAN
EE-----KKKKKKKYRNFFNFSNKPADCCGIMGYLGNRDASKILIEGIEILQNRGYDSCG
MSTISSNNVLKTTKFSSTSTSDAIDKLKCNYMTNHVNDNIGIAHTRWATHGSKTDENAHP
HTDYGERISLVHNGIIENYRELKTFLLKNNIPFKSNTDTEVVANLIGYFLDKNEKFEDAV
LSAIRQLEGTWSFCIIHKNYPDQIILAANGSPLHIGFKENEIFIASEHSALFMFTNEYIS
LKNGEILVVNKEKINDLKILKKLESIPEIAIQKTPHPFPHWTIKEIHEQSQSLSKSLNNG
GRFSIANNCVKLGGLDPYSEDLSKIDNLILIGCGTSYYAALYGKYIMNYLNCFNTVQVMD
PVDFNISVIPKEKEGVIFVSQSGETRDVIKACKLAHDLNLKKMSVVNSVGSTIANMTGCG
VYLNAGREVAVASTKCFTSQVSVLILIALWFFQNKKNYSTSNKISSLIYSLHRLPLYADM
TIKSCESLCKLLSHKLSNYKSMLIIGNGLSYPIALEGALKIKELTYIHCEGFTGSSLKHG
PYALLGGDDNIPVIMLVFNDSTKNVMINTGEQIKSRGAHIICLTDDENLVKHFADDIILI
PNNGLLTPLLAVIPLQMLAYYISVSKGINPDKPRCLAKTVTVSIRILQFFFYKLILL---
---FHCIFFFFF-FYYYLSYICMLIHFF------------------------F-------
------------------------FVFIFKRYASEDVQKILIGNKIDLKNDRNVSYEEGK
ELADSCNIQFLETSAKIAHNVEQAFKTMAYEIKNKSHLENQQKGKANINLNAKPVKDNKK
KCCMPRIRTMNSRKPPEGWDKVESFLDEMNKKMRSLENEDTSKKRKNEILWPIFQINHQT
SRYIYELYYKRKEISSNVYIYIFIFIYLLFYLFIHFF-----------------------
------------------------------MSNALFKLRKSSVKYLQNYQYHFTRNILFF
KKSFFTNTVSNNLNKNKIVLNNILYKKRN-------LNKPTETMCSYQNQEKKHEHVNIT
GDKFRN-SEQVHVENGINEKSYDYDYIVIGGGPGGMASAKEAAANGAKVLLFDFVKPSIQ
GTKWGIGGTCVNVGCVPKKLMHYAGNMGCLFNLDSNEYGWKFDNLKHDWNKLVTTVQSHI
RSLNFSYMTSLRSAKVKYINGLAKLKNENTVSYYLKGDLSKEETVTGKYILIATGCRPYI
PEDVEGAKELSITSDDIFSLKRDPGKTLVVGASYVALECAGFLNSLGYDVSVSVRSIVLR
GFDQQCANKVKTYMEEQGVVFLN-VLPKKLTKQNEKILVEFSNQTKELYDTVLYATGRKG
DTKHLNLEGINININKNNNKIIANNFSCTNIPNIFAVGDVVENIPELAPVAIKAGEILAR
RLFKQSDEIMDYSYIPTSIYTPIEYGSCGYSEEKAYEIFGKSNVEVFLQEFNNLEISAVH
RKKHSKVRKDEYDIDISSTCFAKLVCLKNEDNRVIGFHYVGPNAGEVTQGMALALKLKAK
KKDFDNCIGIHPTDAESFMNLHITLSSGLSYAAKGGCGGGKCGMAYERHENMLMAIARDF
SSIDDLIEMFLSFLENKTDYFHLMINEEDIKTLSKKYEGSMLKNILNNNHCGFKANNRED
CLMKLFRKHQLNYIIRKQPYIIENEEIKKKYFPLCDELIKLNNIKINEKIKDKNENKQSK
EDTKTTINVPCNKKYI--

--MKPA
NAKEDKLQKVINSNAIDEVEEQLYTGPLKIEQLLAKGFVKRDLELLKEGGLQTVECVAYA
PMRTLCSIKGISEQKAEKLKKACKELCNSGFCNAIDYHDARQNLIKFTTGSKQLDALLKG
GIETGGITELFGEFRTGKSQLCHTLAITCQLPIEQSGGEGKCLWIDTEGTFRPERIVAIA
KRYGLHPTDCLNNIAYAKAYNCDHQTELLIDASAMMADTRFALLIVDSATALYRSEYIGR
GELANRQSHLCRFLRGLQRIADIYGVAVIITNQVVAKVDAMSMFGGHEKIPIGGNIIAHA
SQTRLYLRKGRGESRICKIYDSPVLPEGEAVFAITEGGIADYEEKKGLKSININGNFDWC
PFEEYKNYLICFNSHNLLYSNNNNINNYIYLLDINLNNDSRNIEIVNKLNFEDALKNESD
GSSN-ISEYVTCFEWINSNNFVETENEDELNKGIIVGGLTNGDIVLLNPQNLFEKNLDYE
NFIINKVNVHDNSINCLEFNRHKNHLIATGGNDGQLFITDIENIYSPTSYDPYLDKNNLQ
KINCLNWNRKVSHILATSSNNGNTVIWDLKIKKSAVSFRDPHSRTKTSSLCWLANQPTQI
LVSYDDDRNPCLQLWDLRNSNYPIKEIIGHSKGINNICFSNIDTNLLLSSGKDITKCWYL
NNNNFDIFNEINNSANNIYSKWSPFIPDIFASSTNMDTIQINSINNGNKMTTKYIPNFYI
KDSGICFGFGGKICCFNNS----SISSSSTSLHTSAIQHKNINNNEDNEINKMKNKKLEN
KFLIKCHIYPTEMELISEADNFEKYITNGNYKEFCESKIMKSDDDHEKLTWKILQLLYTS
QKSEIVKELGYDISNIIQKIMYNIGKQPGFIFKNILDK-----DISINDISNN-------
------LDENNDRDYNNITCTSNLMN--DNNLSSSNMGDNIQKMNGSFDVDPEKFFRELG
EKTEHEKSKEKL---------------------------SIQNSENEDDYNSNSDDNLDN
KKTYSDWNTGIESIIKECVLVGNIETAVELCLHKDRMADALLLASFGGEQLWHKTKTIYI
NKQNDNFLRNINYILDNKLDLLVNNIDLSSWGEALSILCTYAINNPNFNNLCETLAKRLQ
NEKFDIRAASICYLCACNFSETVEIWNNMPSKKASLLNVLQDLVEKMTVLKMVIKYDQFN
PIMNQKINQYAELLANSGRLKAAMTFLCLIQDDQSLESLILRDRIFNSANHILGQQIEPP
ILPFQVVDIKPSPTIHQNLLYNQGNQNQQFNQSFQYNQSQQYKQNLSNNPNQPKLYNQQN
LQRLPPPISVSQISSTHINQ----NQLVSNNYNSQVINNTVN-RSSFLNTTNQQFS-SNV
NTIQSP---TNIPS-YMPPNIPS--NTSPVNT--PPFSPPSKFSNFNTTQKIQD---QEK
QQSSMF-NQSYTNINKSLQNNM-PSSVVPPSNTLNQINRPSFSRFQNITAPPRNNNPPTS
SSPNINQPQENQYSKRECFDQQIFDPNNISSIPPNNNMQTKVFQNSFTNKVNFQLNSN--
--------TSSLSGK---

---MAIRVQFENSNEVGVFSRL
TNSYALIALGSSENFSSVFESELSQHIPLVYTTIGGTKVIGRVCVGNRKGLLVSSICTDQ
ELLHLRNSLPENVKIKRIEERLSALGNCITANDYVGLIHTDIDRETEEIIQDVLDIEVFR
TSIAGNLLVGTYSYFTNNGGLLHAMTSSQEIEELSELLQIPLITGTINRGSDLIGSGLVA
NDWSAFCGMDTTAIELSIIEKVFKLNNINDNNITNNFKYKSSIIKTMIMFESLVEKLLNK
FLAPYVEGIEHNLHLGVWSGNIVLENLKLKPQITEILDLSFKVIYGNIGRINIQIPWSSL
GKNPVCVLIKNVHIYVKPRCYKKSEDVIIEELRKAKMHRLEMLEEEISIIKLQKNNEKSS
EKSTLIFKLLNKIINNIQIDIQDILIHFEDTEKNFSIGFILKSSSVKNCHSKEENSTQTN
STIENKKLNHIIEFKGLCIYSNSNIKKKKKKEKEEKEKKKKKGSCKDSNEEKEQ------

```
--EVNKHMKENIENKTLMNNDKTNDISDKMENYNFSSNLKNNYLNTNDINEDNNIFSKLK
SNE-----HNKDDYLHDMLNCLNSFLKYDDK-----------------------------
------------------------------------------------------------
------------------------------------------------------------
------------------------------------------------------------
------------------------------------------------------------
------------------------------------------------------------
------------------------------------------------------------
------------------------------------------------------------
------------------------------------------------------------
------------------------------------------------------------
------------------------------------------------------------
------------------------------------------------------------
------------------------------------------------------------
------------------------------------------------------------
------------------------------------------------------------
------------------------------------------------------------
------------------------------------------------------------
------------------------------------------------------------
------------------------------------------------------------
------------------------------------------------------------
------------------------------------------------------------
------------------------------------------------------------
------------------------------------------------------------
------------------------------------------------------------
------------------------------------------------------------
------------------------------------------------------------
------------------------------------------------------------
------------------------------------------------------------
------------------------------------------------------------
------------------------------------------------------------
------------------------------------------------------------
------------------------------------------------------------
------------------------------------------------------------
------------------------------------------------------------
------------------------------------------------------------
------------------------------------------------------------
------------------------------------------------------------
------------------------------------------------------------
------------------------------------------------------------
------------------------------------------------------------
------------------------------------------------------------
------------------------------------------------------------
------------------------------------------------------------
------------------------------------------------------------
------------------------------------------------------------
------------------------------------------------------------
------------------------------------------------------------
------------------------------------------------------------
------------------------------------------------------------
------------------------------------------------------------
------------------------------------------------------------
------------------------------------------------------------
------------------------------------------------------------
------------------------------------------------------------
------------------------------------------------------------
------------------------------------------------------------
------------------------------------------------------------
------------------------------------------------------------
------------------------------------------------------------
------------------------------------------------------------
------------------------------------------------------------
------------------------------------------------------------
------------------------------------------------------------
------------------------------------------------------------
------------------------------------------------------------
------------------------------------------------------------
--------------------------------IMNNINSHGNISKIDSGQISSIDIDESI
FINRKSYPVNELYEDENKK--GNNQIENFKSYKEMYEESIKNPELFWGNIGRTSLRWFKL
FTKVYSGDFRKGNVSWFVNGKINACENCVDRWAEIHPNKTAIIWEKDNPNEYKKISYQKL
LENTCRVANLLKIYGVKKQDTVTIYLPMIPELVYSMLACARLGAIHNVVFAGYSAGSLSD
RIIDSKSKILITCDFGLRGGKLTKLKKIADAAMDLCGTIEKCIVFKNKMKINESNIYLKT
PMQNQFYHSASIDSQNIKDSIKSGSSSKIKNNGSDINENVSDQINNYMDIYAMNNKTPDK
TILKPNSICSKTYQNVCTNGKTNNSGDIINNGEELCSYNNDIKCKSIASPEEKQFQNKTM
ENIPSNFDENICTLKEGRDVDGSSLMKNMRPYCPIEYVDSEDFLCLLYTSGSTGKPKGVA
HTTSGYLLYAYTTCKYIFDVTENDIFGCVADIGWVTGHSYVLYGPLLNGITTVLFSSIPT
YPDCGRYWNMIETHKVTQFYTAPTALRALMKHGDHWVENYDLSSCRILGSVGEPINPETW
RWYYNVVGKKKCVIVDTYWQTETGGIVIAPIPNLFKLKPGCATLPFFGVELEILDSKTLN
PLKGPNVCGLLCIKSPWPGMLRTVYGNHNRLIKTYFATCPNYYFTGDGAYRDEDGYYWIS
GRIDDTLNVSGHRLGAAEIEHALVQHFYIAEAAVVSFFHKVKGEGILCFVVKKKGDLKNY
GKVTN------EKNVILSSTHNIEDLEDFKKTYTDEKLIEELKLQVRQVIGPIATPDLIC
IVPDLPKTRSGKIIRRILRCIANGLNDYGDITTVANYEIIDVINNKFNECKENFHFTI--
------------------------------------------------------------
------------------------------------------------------------
------------------------------------------------------------
------------------------------------------------------------
------------------------------------------------------------
------------------------------------------------------------
-----------EKELLKNMNDNSIRIPFPTPCTVEKALEYYCDLTTIPRVNVLKKFKCFI
KDKEELKMFNFILSSNQRNTFFNICKESDMTLIEFVDIYMSSSQFEISPFLQLIPKNNPK
NYTISSSPKESENTISLTIKKKQYPIHSLRRTLKNLKKNDMLPKISEQKLRELCGRRWFK
GSCSFYLTEELNIHDTIMFNLKPSKFILPDNMESSNIIMIATGTGIAPFKAFITEFKHFD
QMFTQNGLVRRGKRILFYGCRKKEVDFLYEKEILDAKEKKYIDEVYFAFSRDQEKKIYVQ
DIILEKKELVWNLIQKGAYIYVCGNNDMSKDVKKTINNLPLYNKHNDKKITKKLKKLGRY
IEEMWMDNLPWVEKYRPKKLDDIVHQSNAVRMLKEVVKTKNMPHLIFHGPPGTGKTSAIN
ALAHELFGKDNIKDRVLELNASDDRGINIVREKIKAYTRISISKNKINNETNETLPSWKL
VVLDEADMMTDDAQSALRRIIEIYSNVTRFILICNYIHKISDPIFSRCSCYRFQSIPINI
KKEKLLYICKSENINITDDALEKIIETTQGDLRRAVSILQLCSCINPQITLESVLDVSGL
PNDNIILKIIDACKMKDLKNVEKTVQDIIEDGFDVSYIFKAFNNYFVTNSDYQDSLKYQI
LLELSRHDYRLHSGATQYIQLLSFAVKIHSLLNSIMNESLDVENAKAKIKLVFSYWVNIA
NNEFSKTNIFCVLSGKSSKDENATIQEQFQMWLTGYQLTETFFIFLKNEKLIILTSDKKR
KFLQPLLDNMDNVEILERSNDNKENFEKIKNSIESANSSEIAILRDTDATGTFFENCYDF
IKKLDKRELDVSSSIKHLLNFRSESDMKIQKSGSDIACIILKSTLITTIENALDNEEFES
HEKIKDNALKFHENKKCLMKLKDKLKVDIDEIDIIYSNVQSGNKFTLTYKNSNDKNYLSQ
NEGTIIVGLGLKYKELCCNITRTLLLNAKTHHKELYNFTLNIEKYIIKECLKVNSTFSSV
YKKAFDYIKQNKKECDSLQNINLEEYFVKCIGHVIGIEFMEKEFLITESNENGRIEKNTS
```

```
YNLSVGFENVQGDDKSNFAIWLSDTIYINDKEEVIVLTDTLSKEINTISYELEDSSSNDE
EKKDVKKEKK--NGDVDKKKIGISASILNNAASVIVSDRLRRRNKNSIAHNNEQEMEELN
RRQNELKEKKINEIKFRFSKGTNDYKDMNKKNIKKLEDIKAYNDPDLLPKDLKPNVICVD
NKHECILLPINGLHIPFHVSTIKNLSSNYEDNNDIFVLRINFLVPGNQGVVKGDFNTFPT
LQAKEMYIKELIFKSKDEKHFQMVVKQVKELIKQVKQKEVEADVNDSKHTQDKLILNKSG
RRIILRDLMTRPNIFTGRKILGTLELHTNGLRYSANSRGTTEYIDILFDDIKHAFYQPCD
GQLIILIHFHLKRYIMVGKKKTLDVQFYCEAGTQIDDLDRAKARNVYDPDEMHDEMKERE
QKNKLNLIFKNFVQQMQDASKIEFEIPYPELTFSGVPNKSNVEIFVTANTINHLVEWPPF
ILSVEDIEIASLERVHHGLRNFDMIFVFKDYTKPVKRIDVIPTEYIDTIKKWLTTIDIVY
-----------XKLXLSDIDSFVNSKGFEGFLGEDDDEEEHSAEDEDEDDEYELDESEL
VII---------------------------------------------------------
-------LYIFHIFYFIFYRGP----DEIKININSANKKHCNNSVITSKYTIFNFIFLNI
YEQFHKISNVYFFFIGILQAIPQFTATNGIPTVFFPLLIVLIANAIKDAYEDWNRHKTDK
IENNRICYVITNDETNVDYNNKKNEKKKSCKLWNFLTRKKKKVRSTESSIDEGAFTEDRS
DVNDFMKNYEDSFENIEGTIKKRWKDITVGDIILCRKSEFFCADILLLCTSDKNGIAFVE
TSSLDGETNLKVKEANGFLFNILSSDRTEAIQKVKQIKGFILSDIPNKDLSTMYGTIYFL
KDEKVNVLDNNEILQSNTEQIESRKKRISSSDVSGSKVSNMNSKTISRLKKTDNYLKIPF
DSKQFVLRGCKLKNTDWIIGIVIYTGKETKIQMNSTKPIRKTSKLERLTNRMTIIIWFIQ
MIICFISAYYNAMIVSSSRKKKFPYLPFNLEKEKTPIVVGVISFFSWIVITGNFVPISLI
VTMSFVKVVQAYFISCDKNMIHKVQTDNP-FGEQKEISSVKEENSYDLNEKQ---KNKVT
NNLLKISDNQSKQDKKDLESSNSLMTNVQKNELSKINKSQEIKEKNYIYFNAVPRTSSLI
EELGQIEYIFSDKTGTLTCNIMEFKKCAVNGISYGKGLTEIKKNILKKKNLEIPDEPVMK
SKKKTSNVNIVDSNLVDHLHDINHFNHAHLISFFLHLAINHSVICERGKDGVNTYSSSSP
DEEALVNAAKHFDITFLYRKDGKYGISIFGKIYEIDTLASIEFTSQRKMSSIICRIPIFS
CNHGNNNDFNSSNNNNNNNNSNEKNIHKRENNDFIIKNDATSKL----------------
---ADNNNVSETCNCKNSKKSKIMLFCKGAGSVILKKLAKKTKVDDITIEHMEAYADEGL
RTLCIAQRELSEETFAKWYELYKEASLSIKDRERNLEHVASFIENDLILQGVTGIEDKLQ
EGVSSTIEDLRIAGIHIWMLTGDKIETAMNIGIAANLIDNYSVQYVYTEDYISSENELMK
KLDEDILKAEKSLNLPHYDFDDNDDNTKNNFCKNIFFRKKKNSLSIKPDNYKTLTSSFSH
VLVVDGNVIDILLSKKFEKKFFYLADKCTSVICGRVSPYQKGAIVSSANRLLKKITLAIG
DGANDRNMINRANIGIGIRGQEGVQAFNSSDYGISQFRFLKNLLLVHGRLSYRRISKLVV
YMFYKNIVLIFPLFIFGSLSLYSGQKIYYEFLLQVYNVFFTSIPVIIFAILDQDINLNTA
LEKPVLYKLGIYHYYFNINTFVSWVMNSLFHGSVVFLIPLYFLSYYNIPSLDGIPYDIWT
VGCVTFFLTVLVVNLKILLETYYLNMLPIIAVSLSIFSFLLLVTSFSFMYFGSSHILGTV
IYLVRSLRFWLVSLLSLFVALSRDYIFKVYKRNFNPEVYHILQDHVKKKKKKKKFHILR
FFTNFYFFKIHRKVHLTFWNK-----LSQIILKIKHM-MVGKIIEICKQKE---ILYKFF
LRGKYMNN--YMYTRNYSDNVKDHFNKPRNVGSFDKNEKNIGTSIVGKASCGDVIKLQLK
IEDNVIKDARFMAFGCGSAIASSSYATELIKGKTIDEALKIKNDDIASHLNLPPVKIHCS
LLAEDAIKHAIKNYREKVINYMLIKSGKNGISNLIRKIRNFQENNEIKKINS--KNKEKV
VILGSGWGGMHFLINIDFKKYDVTLISPRNYFTFTPLLPCLCSGTLSINVCTESVRNFLK
KNGSVGNYLQLECTDIIYKDNYITCKDNDNNEMKINYDYLIIAVGAKTNSFNIKGVDKFA
FYVKDIDDVLKIRRKFFENLEKSTLPNTTNEEKKNLLHIVIVGGGPTGVEVAGEFADFVN
REVKQNYEDIFNFISISIIEGGKNLLPTFTQNISDFTKYNFRNLNINVLTNYYVTEVNEN
YFYIQSSIDKSDK-KKFPYGVLIWASGLAQTTLVNNLLKKIPQQVNNKILNVDSQLKVIG
VESNNVYAIGDCKKIFPLQLQKYTSELIDSLHCSKLTSDTLKSKSEELSEVFPQASKYKW
DYDKNKKGEMNEQEFHEYLCKMDENYKSPAPTAXNAKQEAYYLSNVFNNFSTRDKKLTVP
FFL---------------------------------------------------------
-------------------------DIQITAEQLINEALDLEEVEKKVNYNLIDEDELN
EYKISKRKEFEDKIRKRRYLISTYIKYALWEIKQKDIRRGRSIFERALNIDYTNKNLWLK
YIEVELTNKNINSARNLLERVVLLLPLENIFWKKYAHLEEILNNFINARNIYERWIKWNI
DETSFLCYINFEERCKEINKCREIFEKLIVKIPKLECFYKFIKFEKKYKNIERARACFEK
CIELLPSSFIDENFYIYFCHFEEENNEYERCRKIYIEALKRLPKNKSTLLYKNFLQFQKK
YANKDELDQTLLLKERIYYEDELKKTPNDYDIWFNYIXLEEIYINVINKEKSLIRNRELY
ERAISGIPLXTNKNFG--------------------------------------------
------------------------------------------------------------
-----------------------------------------------XDIXI-------
-------------XGVYKSYAEFEYIYYDDIKKCREILENGIEFCKKNELINERCVLLN
FLYDIEKDYGDNEIIEKTLKRLPKKVKKRKIIKNKDDETVEEFITYVFPDDGTQSQVTII
TIFFF-----------------MK---------------FFFFILIFKVIKNAHVHDG
LKVGIREVIKAIESKEAKVCFLSSVCSEPAYKKLITALCTEKNIPLFLVQNDSKDLGHWA
GLYKLDKEGNARKIIGASSVAIVDFGEDSAEREFLMSQKQTAVA----------------
------------------------------------------------------------
------------------------------------------------------------
------------------------------------------------------------
------------------------------------------------------GLY----
---------LFIFLLKL-------------------------------------------
-----ILILYIT-YF-------------SLFLKAIVGEVIDNRYSVVCELVGKGVFSNV
LKCYDMVNKMPVAIKVIRDNDMMKKAAEKEISILKKLNQYDKDNKRHIVRLLRSIKYKNH
LCLVFEWMWGNLRIALKK------------------------------------------
------------------------------------------------------------
--------GKSNNHMIKLMMEYKGKFSHKMIKGGQFYSQHFNENLDFIYVDRDSYTKKEI
VRVISDLRPTKNITCDLLEHQYWLKG-----KFFKKKIKN--------------------
--------------------FKDDSSALSCSLSLESDTCNEDEYDTNLSNLIENKTLNWI
FVGGKGGVGKTTTSCSIAVQLAKRRESVLLLSTDPAHNTSDAFNQKFTNKPTLVKSFNNL
YCMEIDTTFSENTAFKLNKKDIFENIIPELLQSFPGIDEALCFAELMQSIKNMKYSVIVF
DTAPTGHTLRLLAFPDLLKKALGYLINIREKLKGTFNVLKNLTNNEMELENLYEKINHLN
AMSSSIQSNFQNPLKTTFVCVCIPEFLSVYETERLIQELTKKNISCYNIVVNQVVFPLES
PEIDVNECANLLQKINNKEIEKCFNSLIIKTRELEDVYISRRKLQSKYLMQIKNLYGNDF
NIVCMPQLKNEIRGLDNISSFSEMLLESKDIPIYNAEDEVIISGDYVKIEKDKLNKINEN
NFEKIHNSEHENFYRSKCSGILLKTPYYPYKYEIMNTSYKYIPKVGDLVIGIVKSKKLDY
YQMDINCNCDCIIHKIEGFKYATKSSFPNLVNGTLLYMIVEKINLDNNSVVASCINSSDV
KSWINYENYLGELIDGFLFSVNISFSKSLIGDRCYILDLIGQDINYEIAIGHNGRF----
------------------------------------------------------------
------------------------------------------------------------
------------------------------------------------------------
------------------------------------------------------------
------------------------------------------------------------
------------------------------------------------------------
--------------------------------------------------------CCWK
KKIKRWICRGGNDERENTLNQMLVEMDGFHTSNDKVVVLAGTNRVDILDPAITRPGRFDR
IVNISKPDINERSEIFQVHLKNLKLHETLDIKNISYILASLTPGFVGADIANVVNEGAIQ
CARRSNLLGVQIKDFELAIERVIGGLPKSSSLISPLEKKIISYHETGHALIGWFLEFADP
VLKVSIIPRNNGALGYSQHLSEEIMLFSREAILDKIAVILGGRAAEELFIGKITTGAIDD
LNKVTQLSYSFVSQYGMNKEIGLVSFQPNSNSEYNLYRPHSECLAHLIDNEVRNLIETQY
KRVKSILLKNEQHVHNLANLLYQKETISYHDIVKCVGERPHPIKSNYEKFVKANPYKLIP
NETETNVINKKDEIMSENDSENKEKISNENDKCNNNETKRKNDVNNNNDQDKNDKQMDIN
KKVNKNKKDNKISNLNIRI-----------------------------------------
--------FI-----GLKHQRYKGTDKVKIEEERNKKIFLKNNK-IQEFCKNCGSAAHKE
KYCLERTRKKKINFMKKDNDDDFICVTQDLGYDGNRDRWVGYNSDNFEQIYREYEKIVEE
KKKRKAEELKKKYEKKSIKKKKNLENDDTKNDKSS------SESETLEKCNDDLKDDK--
------------------------------------------------------------
----------------------------------------FIKEF-------------
----------------NDELIHSEIYTEYKPSDQIDPKRKKIKVLSKYEEDIYAFDHSSV
FGSYYDKEKKKWGYKCCLSTNKFEKCLHTMGTNREFDRKDLYKNNNVEKLN--GGDIEY
VKEIDTTLNDDYPDNFLNNKNRINIDKLKNEIIENDLLNNDINDISLGLVKDKTIKKREN
EFQKKKYDYILSPERVDPFSEKSPSPGERTYTDIMLEINNEGKKSKIINKNKKIMNKN--
```

--EEKYKMN-ENSKKESVKTKWDYVEEENNLNFGSIPTPAPNKWVDTPFILNEGN--LKK
KKVSRWDKIGNSENV-----------SLKTPKISILDTNNNVIINTPFVNTNIYTPLTPG
ISTLQTDTFIKMKIKNEMDIRNRPLTDEDLNELLPSEGYEIVKAPEEYETIRKNKLKALF
KNINN---TPLLNNNNSGLSTNNMEIKENKESTFI-NTPFYDIPTVNNTLKDEEANYLMQ
NKQLEINNPHLLNELKYIQLKNEDYIYFNKLFQNVNEEDLSQDELKERKFMLLLLKIKNG
TPSIRRTALRTITEKAKELGPNTLFNLILPLMMQNTLEDQERHLLVKVIDRILFKLDDLV
RPYVHKILVVIEPLLIDEDYYARVEGREIISNLAKAAGLATMIGIMRPDIDHPDEYVRNT
TARAFAVVASALGIPSLILFLKAVCQSKKNWEARHTGIKIVQQIAILMGCAVLPHLKQLV
NIIAHGLHDEQQKVRTITALALAALAEASAPYGIEAFDSVLRPLWKGITEYRGKVLAAFL
KAIGLIIPLMDSYHANYYTREVMIILINEFSSPDEEMKKIVLKCVKQCIQTEGIEKDYIN
QEIVNPFFEKFWVMRNCNDKKNFTLIVETTVEIANKIGGSVVISRIVDDLKDPSEQYRKM
VMQTIQNIINNLGVDDIDQKLEEQLIDGILYAFQEQVSDDYYVLLNAFDVIVNKLQLRMK
PYLPQIAGIIRWRLNTPLPKIRQQSADLISRIAMLIKICDEQQMLGHLALYLYEYLGEEY
PEVLGNIIRALKSIVVVLGVQNMTPPIKDLLPRITPILKNRHEKVQENVIDLIGIIADKG
GDLVSPKEWDRICFDLIELLKSNKKLIRRATIQTFGYIARTIGPFEVLTVLLNNLRVQER
QLRVCTTVAIAIVADTCLPYSVLAALMNEYRTQDLNVQNGVLKALSFMFEYIGEIAKDYV
YSVVSLLEHALIDRDLVHRQIATWACKHLALGCFGLNREDALIHLLNYVWPNIFETSPHL
IQAVIDSIDGFRVALGPAIIFQYLIQGLFHPSRKVREIYWRIYNNVYIGHQDSLVPIYPP
FEKLNDSNFSRDELKYTL------RALDIRAIITLCKKNIIGCIVTNIYNISNKIYVLKC
SKKEQKYFLLVEAEKRLHITEWKREKDVMPSSFTMKLRKHLRSRKITNITQLGGDRVIDI
KFGFDDKAYHLIVELYISGNIVLTDHDYKIINILKNIDTFGKSYSINEIYNIEEHSSILL
YKNLISTNKENIKKCISEILLYKIPNVNDNGNCINNKNEKYININDETNKKKKDIKKNNS
LNDVKNNSNIKDIVNNSNDKSKKIKKIKTLADLASKLVVFAHNDLIIHALMVNDINPFDL
LDNYQLIDITEIMYKVINECLNLLNILSNEENIIKGYGFILKNEEQGGNSTENENNSINE
EEKKEFEICEFSPIILSNHKSKINENKMDIIEFDDFNTCVDTYFSRMELSKYDKQQEINK
NKNMISKMDKIKLDHERRINQLEKEVSILKKKITLIQMNDRFVEESILLMRAAISTSANW
EKIWDHIKIYKKQCHPIASRINSVNFNNCEMELLLTDFESDSNRDNEDNCDLKMDNL-KK
LDMNKKMV------SVTINLNNSVYGNIEDYQKSRKKTEEKIRKTRTATDFAVKKVEKKK
KDKENKQKVKQKTVCQIQKLRKVYWFEKFHWFTSSENYLVIAGRDALQNEILFRRYFQKN
DIYVHADIHGAASCIIKNPYKDKPIPEKTLAEAGQLAICRSSAWNNKIITSAWWVYYHQV
SKSAPTGEYIKTGSFVIRGKKNYLPHAKLEMGLCILFHIDKILDENIEENNLDITEKVIE
KDEINTNKSSSYKDQSKGNEANIHYNSTTTTTTTTTTTNNNNNNNTLKMQKDNTNSYNFN
NNLNISLKSSYKKKNKYTYRKFFNFILGRSYIMNILNFIHILILQKLGIFYYYEYNTIKI
LCKFFNLNLFSNKKNFLLYHKNYHQNICNNCKGFKKYYKRYFCFFLTYKKNLTPIKSVKI
RKFCIIFNALTFFFVHIIASNINSSKNTPDINHNVFKNIQNNAKSNNVIQI-NNTLLRYY
MKKTLIENLGRKTDINFD-NRKHSFMQCEVYGTPVTFNDDDLHECSKEKRLFKDVVDEEV
DYKNNEKRNNKIGEEEEKKNVSFYDFEKRESLNLSRPVRSRKPTGFVKMDVSKLLEEIGD
EEKNDEN-----KVKIKVDFDNNESIEKNKKKISLTTEEEDIYIEKQESLNLPRPVRSRK
PTGFVKMDVSKLLKNIEDEENYEK--------HIKQTFNNN-SIDKEKKKDVSFTSEKDN
IYVEKQESLNLPRPVRSRKPTGYVKMDVSKLLKNIEDEEKYDENLKNIEDEEENEGDLNK
QESFNSSENNDKKKVSVTFISEDESSCVEKQESLN-IPRPARSRKPTGFVKMDVSKLLKN
IEDEEKNEK---ISNEKKKKSRSRKPTGLVKIDVNKLLEDIEEKKDYQNVDNEDDEKKNK
DGDNEDEEEEEKNDDDEEKEEEEEEEEEEEEKEKEEEENENENEEDNDEDENEEDNDEDE
DKEEEEEDENENEEDEEDEDEEDDEDDEDENEEDNDEDENVEDNDEDED----------K
EEEEEEEEEDDEDENE---------EEEEEEEEDDEDEDEDKEEEEEEEDEDENEEEEDD
EDKNEEEDDGEDED-----------------EGGCGGENKAEYSEYKSNENRKKKNKGKK
DKNEQDKDKTNTEEKHQKNTTEYIENKKKKNTKLKQKSSSNNELNEDLNGSYVRKRKRYI
KFNCSIKRKQPSWLKQKPSEIEDTIIKLAKKGQTPSQIGATLRDNYGIPQVKSVTGNKIL
RILRAPGVATTIPEDLYFLIKKAVSMGKHFEKNKKDKDCKFRLILTESKIHRISRYYKRK
KFFPSNWKYQSSPASAFFAMSNRKKVAYFHDPDIGSYYYGAGHPMKPQRIRMTHSLIVSY
NLYKYMEVYRPHKSDVHEMTLFHDYEYVDFLSSITLENYREFTYQLKRFNVGEATDCPVF
DGLFQFQQSCAGASIDGASKLNHHCADICINWSGGLHHAKMSEASGFCYINDIVLGILEL
LKYHARVMYIDIDVHHGDGVEEAFYVTHRVMTVSFHKFGDYFPGTGDITDIGVHHGKYYS
VNVPLNDGITDEAFVDLFKVVIDKCVQTYKPGAIIIQCGADSLTGDRLGRFNLTIKGHAK
CVEHVRSYNLPLLVLGGGGYTIRNVARCWTYETGVVLNKHHEMPDQISLNDYYDYYAPDF
QLHLQPSNMPNYNSPEHLNRIKMKITENLRNIEHAPGVQFSYVPPDFFDSEMDDESDKNQ
YELDDDSGGGRAAGTRIKEHSSSHHLRRKNYEDDFFDLSDRDQKMILKNDDNERINSEIL
EKQYEIIKCAKYQDFIRMQILIQPYILNNDIEMLNSINILHWSCYCGFTELVKKLIDLNC
DVEKEDLVNSDTSIYYAIKNSNYEIVLLLIKKFGASILFHKNRRKMSPFLTAISEFNEDK
ILETLHILELLYMNGISLEEQNEHGQTALFLSVKKNNISTLQWLLSKNVNINHRDFYGNT
ILHIAVRYSDIDIIRLLCDYGCLNLVYCSSIENKNTNVFQLCIKNRYFLIYILLKKWILQ
NKICKNLKICKTIYAFYFWFFSLLNLIIYFNISYSFSLITKYQTLSIVWLSLWIFQKFLW
CLLYFRSPGFYKENNVFNKNKK-YSNYIYDGSFKRKAEYHLNSIEKEIYQINKKIISTNI
NSSNVPNFNDSLFNKYNDLLLNLEYQKLSLYSHVSQERINSLDINYRNAILNNQNPRVI-
--------------YKFLYFYIFKDFYIFYI-------------IFIFRMFVLLVK----

--MDVFEFVDPLRK
RKITKNLFDFDKYIYKDKNEKSEEEKKSSENNLKKKKKKLRRILNSSSSDE---NFENIK
KKNSKLKKEKEIIDKKEFNDE--------DCVNDNISIESDKEEYESSLNHLFECLLISI
KIKNKIIEYFASDVREKRQEIIKEFVKGSFRVSNCDANFQNFEKNVDTFHKLKSYQKCGV
FWLYILYKEKKNGILADEMGLGKTAQTCVFLDYMYRTKELQNKTIIVAPTSLLKNWNNEI
NIWCPYLKNHKIIYYGNQNERKYLAYDIFTNQS-NNIHLIVTSINMLIGKNDVSYFRQIK
KYDYLIFDEAHFLKNKNSLIYKKLQKKIVFNNKILLTGSPIQNKTEELMNLLLFLMPEIF
TEKNINSAMDAFVKLYQQIVNKKEDYELEEN-------IQVNKTMEIHDINSEEEIAN--
---------------------------------------SESAKNMIKNYLIQTIKNDVK
HVELKNKEIILLQLIIEPYILRRSKKHVFIDMPKKHSVIIKLPLNNTQLNLYKNEIFSKL
QHTHKHLEFIQKHSSKKELEKLYSILEKKENKEIQNYKENVEEKNE--------------
------KNDDYDDKEIDEETINIEKTEENKDSNIIINKREDEQVI-----ENINNNSKDV
RGKMINASIFILRRICNHPLLHKYYYSIEDIKNISKYFYNNTDQYLDLDLKTVETEFMKI
SDFDIHLSIKHLISQGDNNLNKYLITKEHILNSTKIHHMISLIKEIRQKKEKVLIFSQFT
TFLDIIEEALLYEFIYDEQDFSDHLQHIKSSQSINKNEKSEQEDLENNNESNFNKNINND
ENKNEKDFYISSSTFSTSTLSTEEKTGNEIYVRLDGTTNTIERQKIIKRFSKNENIFIFL
LSTKAGGVGLNLIAANHVILMDQVIQYYN-------------------FYIVKKNIL---
---ASRAVSEII
KTTLGPMAMLKMMLDPLGGIVITNDGNSILREVDVAHPAAKSLIELSRSQDEEVGDGTTS
VVILSGELLNIAETFLKQNIHPTIIVNCYMNALNCCLKYLEEIAIEVNINDESNLLKAID
SCLNTKFVSRYNKIISKLALEATLCVKIDNIMGKKEIDIKRYAKVEKIPGGEITDSYVLK
GVMINKDITHPKMRRYIKNPRILLLDCTLEYKKAESQTNVEILDENTWNQLLLQEEIEVK
KLCEYIIDSKCDVVVTEKGVSDLAQHFLVKKNISVIRRVRKTDLNRLERISGATIVNRCD
EIVESDIGTKCGLFEVKKIGDDYYSFFIDCEDPHACTILLRGSTKDVLNEIERNLHDGMN
VAKNIILEGKLLCGGGCTEMRVGQNLIKQAKNFDDSRKSITEAVASALEIIPKILAQNCG
VNVVKTINELRIKHENPGGEKYGIDGITGEIIDVSTKNIWDLLSVKKQIYKSAIEAAAMI
LRIDDVVSGIGKDDKNQKHIQNEYMARIKVLNVAEKPSVASSIADILSKGKQHKKKSYSK
YNPVFTFEYKIKDDIWLMNVTSVTGHLTEQKFEDKYKNWSNTDPQELFDAKIITYVENDK
KAIENNLKKYSKDCHMLVLWLDCDREGEHICFEVINTCYKTNKKLKIQRAQFSAVTEKDI
IYAINNLKEPNRNLAYSVDVRREIDLRMGSIFTRFMTIRYIELVKNETNIISYGPCQFPT
LGFVVNRYLDIKNFKNEYYWYIKMQYLYDRDDNIDDD---NSITNNKKKRKKKEKNI---
---------KSDNIIDFTWSRIKLFDHLYVVLIYEKLLKNPLCKVTNIYEKETKKYRPLP
LNTLQMTKQVSKFFHISSKECMNIAEKLYNKGYISYPRTETNYFTDSMDLQKIVNELSKN
NLFGWYASKLTKNNNFKEPRKGKLNDKAHPPIHPVKNMNRSDKIDEKEWILYEFICRHFL
AVCSDDAIGFNSKVIVRIGDEQFFCKGLKIIKKNYLEIYTYEKWNDKILPTFHINDEFFP
YSLLIEEGITQPPKFLSESNLLSLMDKFSIGTDATMHEHIENIQKRNYVTKNSKSLFIPT
NLGIALILSYKKFKDIGVDLTEPSLRAKMEKDMSLVASGAKEKNEIIRNYIDIMKYIYQE
IYNRIELLDKNIHFYLKNTEITYFEVFNFGKYNGKSFEEVYEKHKSYVSWVKKLDNPTGS
LILFKNYILQKEGEK---NDNNM----------SQKSNNENIYNMFEK-KKTNENNNFSD
KGNIYDSYDKNKNSYIN-----------NDEKNIIDKMNSSEYKNYEQELMNQINNSNNR

```
KE-EKLELDIIVAFEIFDDDHFKIVQKDNNTRKFVNFKNFLSKELFKIISEFNPTIKKIN
NYSCVMFEADKYEYVLNNLKEKCNILGGVHAIPNFLLKCFKNYAKFSEPKKICEITANIL
TNTMCSYTREHFDKLDILLGEKLSTELKNFQREGVYFGLKKNGRVLIGDEMGLGKTLQAL
ALMAFYHDDWPFIVVCPSSIRFQWKDQALRWLSHLISEKHICVVKSGKTDIPSNCKMIII
SYELITKNDKYQNKYKSIICDESHYLKNSFSKRTKAITPIIKSAKRCVLLSGTPALNKPS
ELYEQVSSVIPNLFNYHEFCDRYCFKDKNIYTRKIEYVGCKHTEELHLFLTNTIMIRRLK
KDVLKELPEKLRSKIPVEIPPKELSEILTYYKKLEMKKNINIDEFDEVHFSQMNNSKSED
NEEENISISHLFKMTGYAKVKAIKEYITYLIDADIKFLLFCHHKLVMDEIEEFLKEKKTQ
FIRVDGLTSMDKRELYIKNFQENENIKIALLSLTACGIGLNLTAANTVVFGELYWVPGQI
IQAEDRAHRIGTTHENVNIHYLIAQNTIDEIVWKIINRKWNTLTTALNGMEDTLNVKEVN
KFDQFMVDLTNNHNKSYPSSLVNTPKIRRRSSEFRSFSNINNNKKDRDIRDFFKPINKSV
EKSNNKKKRSYNNSPIIDSSNSSPNIFNKKLKNEII------------------------
------------------------------------------------------------
------------------------------------------------------------
------------------------------------------------------------
------------------------------------------------------------
------------------------------------------------------------
------------------------------------------------------------
----KDIGKKIGVESSLDVEGITAKAIHDGVIEATIDYENQYLESKSNSDIYITGEPMKT
FHKRIAFCLQLYSDAIKAMQYPDENEKKENEEAKERKIRQQEELAQAEEGDLGDDNDLLM
LCEKCNEKSICLMKPSNREKLCKECFIKNFEDEVHMTIIKKRMFEANDKICIAVSGGKDS
SVLTHVLVNIKKKYNYKWDLFLLAIDEGIKGYRDDSLKVVYKLEKLYNLPLKILKFNDIF
SYTMDDVVKFIGKKNNCTVCGVFRRQAMEKGALLFNATKLVTGHNADDMAETILMNLCRG
DIDKLARNINDYTFTSPLNNSMKEILTV-------NKKHIENNYDYMNLCQVKTKDNIEK
SPIQNKINDHFNNEKNISNNIINPKESEKRKNFFLPRLKPLMWCYEKEIVLYAFHLKLDY
FSTECTYSPNSFRGNLRSFIKDIETINPQFILNIIHSAEFFYFNANNKKMLNVCIKCGAY
TSNQVCKACLIVTGLNNYKDNSFLYSNKKK--NKKKISINYEKK----------------
------------------------------------------------------------
------------------------------------------------------------
------------------------------------------------------------
------------------------------------------------------------
------KNNYYIRSKNRKISNCTFEIDASYESIEPLLIENEYQQIPKLRILSFDIECIKL
DGKGFPDAQNDPIIQISSILYLQGDPIDHSIKFIFTLQECASIPGSNVIWFNDEKSLLNA
WNEFLVRLDPDFLTGYNIINFDLPYILNRGTALNLKKLKYLGRIKNISSSVKDSSFSSKQ
FGMHETKEINITGRIQFDVYDLIKRDYKLKSYTLNYVSFEFLKEQKEDVHYSIMNDLQRE
SPESRKRIATYCIKDGVLPLRLIDKLLFIYNYVEMARVTGTPFVYLLTRGQQIKVTSQLY
RKCKELNYVIPSTYIKVNTNEKYEGATVLEPIKGYYIEPISTLDFASLYPSIMIAHNLCY
STLIKNNNEISELNDDDITVIQGKGNLKFVKKNVKKGILPLIVEELIEARKKVKLLMKKE
ENRITQMVLNGRQLALKISANSVYGYTGASSGGQLPCLEVAVSITTLGRCMIEKTKERVE
NYYNKNNGFEYNSTVIYGDTDSVMVKFGTNNIEEAMRLGKDAAERISKEFLPPIKLEFEK
VYCPYLLLNKKRYAGLLYTNPLKHDKMDCKGIETVRRDFCILIQQMMETVLNKLLIEKNL
NSAIEYTKSKIKELLTNNIDMSLLVVTKSLGKTDYETRLPHVELAKKLKQRDSATAPNVG
DRVSYIIVKGSKGQAQYERAEDPLYVLDNNLSIDYQHYLDSIKNTLSRIFEVIMQNSDSL
FCGEHTRHKTILTSSQTALSKFLQKSIRCIGCNSSIKKPPLCNHCKSNKEFSIYMQKIKE
FKNKQNEFFQLWSECQRCQGNLHVDVICMNRDCPIFYRRAKIKKDVDNLQKQVSSLRIDW
MQVKLIGFILCIYFLLLKNHGKTKLKRKTYTLVRVYIEKVKLYKKKKKLWNISKKYIYCF
NKVTDFTKIKNK--NNMLTLKENNTGEMLSSC-ENIFKFENISTYLNSSYWKNKNEVIPF
IQVAPMINVTNRHFRALVRIFTKKAQLWTEMIVDNTLLYNLNNLEEYLGFNDNEHPIVCQ
LGGCDITSMSEAAILVEQAGYDEININVGCPSTKVANKGAFGASLMKKPEHVRNIVYEIK
KKVQIPVTVKLRTGVDDYDSFSFLKSFIETVSSAGCNHFIVHARKAWLKGLDPKKNRKIP
PLEYKKVYDLCKLYPNLKFTLNGGVKTIEEATALLNGYMPQ-NNDLNQNNYVFVENYNIN
PLNGVMIGRACMENTTILYQTDKLIYNEKVPDTAYSRRTALNAYKSYLEENSIFYNVASA
FELLKPILGILKGMPGHRLFRNKLDVYIRNYASTLQCSEILEKAMIDVDKIAPGCLDLPL
NDYKMHQEYLKNY-----------------------------------------------
------------------------------------------------------------
-------TFLKNFFFKNPVGHIGIVALKNSSAKLIQPLTSSIDDILNSLLKERSLGLQGS
PSLQEGLEIAHDLLIDMPLYGTKEILIMYGSIRTCDKKNILKILELLIKSNIYVNCISIA
PEMHILKHICEKTNGIYKICTDKNSLINEINNCAETPLWMNGMEPQLIHICFPIKKKINT
QIMCSCHNNLNTDTYICNFCNSYTCKIPSKCKVCGIHLISMHDLSHITNNLQGSPLFTEI
TNENKQYNLCVSCNQQLDDKVSQCTKCKNIFCIDCDVFIHEDLNQCPFCLINDIMGRSSL
AMAQQMYFSTHNALRINEENDVISTLFYEINGNRHISLLIFPFYDVQMLKRLLIKKLNLP
GGIKVNDIIIFYKGIKLPNYRIISTYLDNNNNE---KKKRKTVNKLYWAIKDTNPNASIR
VIDNKSYPPFFENILNEIKLAFKKNISPKLTMDGTGGTYLLFNGKKKVCSVFKPLDEEAF
APFNPRGYEGKMYQEGFRSGVLSGEGASREIAAYILDNNYNNFSNVPCTIMVEACHPHFN
NRSKLKYIDNESNLKWKCGSLQEFIDSKESVGNYDYKQFSIRDIHKIAILDIRVMNLDRN
DGNILVSPLKSLKDSYNQFLYRNNQFFSTNNEDILKRIVTIDKKPSRY------------
------------------------------------------------------------
------------------------------------------------------------
------------------------------------------------------------
------------------------------------------------------------
------------------------------------------------------------
------------------------------------------------------------
------------------------------------------------------------
------------------------------------------------------------
------------------------------------------------------------
--------FYFSCL------------------------------------LF--------
------------------------------------------------------------
----------------------SHLGIEQSRRDDIEALGYVLMYFLRGSLPWQGLKAISK
KDKYDKIMEKKISTSVEVLCRNASCN---------------------IKYILKDIYV---
FLYIFWKNKIYIMKEKKKKKNILSSLYIFGVLYK--------I-----------------
------------------------------------------------------------
------------------------------------------------------------
------------------------------------------------------------
-----------------------------------ILKNLMNHVVVPIKYALHKIFLSEE
THKKKEDEEADEEEEEDEEEEDEEEEEEDENENENENEDEEEEEEEEKEDENEKEEGEEGE
GEEEEKENEKDEEDKEKEKKKEEEEEEKENEKKEEKEESVENMKKNYMEEDEEEEEEEEKE
EEKEEEEEEEKEEEEEEEEKEDENENENENEEEEEEEEEEEEEEDEEEDENENENEEEEEEEEE
EEEDEEEEGENENENENEDEDEDEEEEKKKQYKRKKKKSATITHNFMNKIDNTKYFMKKK
DRKKMEKEYKKEKNYLLEKIGITNFNFLLNVCYNIDSCIIIDRKIDMITPFCTPFTYEGL
IDHLFGIDNLQIEIPRYIIFNDEKSVSK--------KNEKNFDDLNSMKVRVKLKSSVDV
LYNDIKDLSQNEIGRFLHKKASEIQKTYEEKDKLKDIEQINEYMRKFKAKHYEHNSLSTH
VNIASFILNTIKREKNFDKLKLEDEIIQLNSNTNKSKLLNILKKIQLLIYTGEDIYEVYR
ILCLFSIITNGFNENYINDIKKDIVEQYGINELTRLNKLHTCNILKYQSKQKFIWNNLKN
HFNLLSNEINDISYVCNGYAPLSVRLIEYMNVLKNNLQIIPEVLNFLNGPTLDVIQNSVG
YENFEIKQKKKKFN--------KKKNIILFYIGGISYAEIASIRKLNEKCENYNYLIFTT
EIISSKKILQSMNLMENI-PKDIQINNTHQQGNMLDDTIEVKSMCINCEKEGINKIVKIE
IPYFKNVLIHSFECEFCNYKNNVIQDLNTIKDKGIKIIFKVTKDEHLDRQLIKSEYGILK
IPEIDFEIPKETQKGSINTIEGFIQTALNNLSVYLENIKSMYNEANEGNEENEKEGKEEK
EKEKIDEKDIKKEKQENNVEEEKKSSNKVNKNET-SNENNACKQITIENYIKMIESTIYN
LSKYVISKEFPFTIEIIDPSGLSSLEYYDEDIKNNIVTIEHYERTKKELNELGFYEEDFE
EKGVDKESYMNHSENKLNE-EHNIKKENFDFIKKYIN-----NNDKNATMNYKTASDDEE
-KLIESFTSNCPCCNYLGSNNFCEIVIPGFKKCLILSYVCANCNFKTSEIKSSGEINPKG
KKITLTVKNKSDLDRFVIKSETASIHIPIIDLTSDYGTLGGSLTTVEGLIIKIIESLEDK
FKFLLGDSNIYNHKNKNENTEDNDDDSMTKKIKDLITNLYKLCKSEELFPFDLIIDDIAS
NSYISSDKI-DDENLKEEEYERTFEQNDMLGLTSMKTEYMSDNIKRLPLQSENMEVKKTL
SGRVADDGIKRTPSGKPIQTMYVLNRKGEEEDISFDQILKRIQRLSYGLHELVDPARVTQ
```

```
GVINGMYSGIKTCELDELAAQTCAYMATTHPDFSILAARITTDNLHKNTSDDIAEVAEAL
YSYKDIRGRPASLISKEVYDFIMLHKDRLNKEIDYTRDFNYDYFGFKTLERSYLLRINKK
IIERPQHLLMRVSIGIHIDDIEKALETYHLMSQKYFTHATPTLFNSGTPRPQMSSCFLLS
IKADSIEGIFETLKQCALISKTAGGIGVAVQDVRGQNSYIRGTNGISNGLVPMLRVFNDT
ARYVDQGGGKRKGSFAVYIEPWHSDIFEFLDLRKNHGKEELRARDLFYAIWVPDLFMRRV
KENKNWTLMCPNECPGLSDTWGEEFEKLYTKYEEQNLGKKTVLAQDLWFAILQSQIETGV
PYMLYKDSCNAKSNQKNLGTIKCSNLCCEIIEYTSPDEVAVCNLASIALCKFVDVEKKQF
DFKKLYEITKIITRNLDKIIERNYYPVKEARTSNIRHRPIGIGVQGLADTFMLLRYPYES
EPAKELNKRIFETMYYAALEMSVELAQIYGPYESFQGSPASQGILQFDMWNAKVDNKYWN
WDELKAKIRKHGLRNSLLLAPMPTASTSQILGNNESFEPYTSNIYYRRVLSGEFFVVNPH
LLKDLFDRGLWDEDMKQQLIAHNGSVQYISEIPDDLKELYKTVWEIKQKNIIDMAADRGI
FIDQVK------------------------------------------------------
----------------------------------------------YSKMNIGNEIVPKY
GFEEMKNEISKYGVEITQSTLKNPTTEDVQGVYSICIKYILNKDINNIRIEEFTGDLKSS
MPSIDGIQILPNEGKNHLQAIGNLRFIRHCEKINKILNLDNILSYIFKPVSSHITKLISA
FIHFMKYKEEIYNENDAKIKKIQEKKNEDNILESELKTVDSELNMLLGRHDEVKTSIINE
KSTKRNYEEEIIENQNLLNAQQSIIISLKSTKDKIINETNELIFQFSRLRQKKEDLEDQI
VPSPEKLQQYNEELKDLLSEHMSYFESDKKKNEEIKSKINVADLSIKKLVDLLTILTNHV
ENTIKQHIDKKNELKNLEKNLKSLKIEKENLENKKEEQDNILSNTEQCFEEEKNKWNKKI
HEEEKKITQVEKNVKTIYENIDNINFKTNREIKEINNIVKHIHDTINSYSRNCDIITDLI
ENTRNSQKILSDKVQNNIQKYIKANV----------------------------------
------------------------------------------------------------
------------------------------------------------------------
-------------------------------------------------------ADWWT
LGIFIYEILVGCPPFYANEPLLIYQKILEGIIYFPKFLDSNCKHLMKKLLSHDLTKRYGN
LKKGAENVKEHPWFRNIDWNNLLNKNVEVPYKPKYKNMFDASNFEKVEEDLTTADKITNE
SDPFVEWMVISQFYILSPRGDTIINRDFRGDITKSSAEVFFRNVKLYKGDAPPLFYLNGI
NFTYLKNNSLYFVLTSLFNISPSYLIELLNRLLKIFKDFCGQITEEIIRMNFILIYEIID
EVIDYGYLQNSNTEYIRNLIHNEITTT-NANKKFANLSNFSIKNSNTLPSNASQKPIQIN
DKKNEIFIDIVEKINLIMNSKSEIIYSYIDGVIQIKSYLLGNPYIKIAFNDDLYIKNIHN
DTSNNIIIDDCNFNHLVNLSQFEKEKILSLYQPDGECVLMNYRINNNFKAPFKIFANIIY
NQNHTIYIYIQ-------------------------------------------------
------------------------------------------------------------
----------------------MDICITTPYDNEIDEIEEFNNRILAVETIKHNKSNLNK
NIDSTCPNIKKLVLPGEVILEKKDKDRFLKGSGLYEENEKFYACILGTINYINKLVYVEP
LKGKYTGAVGDLLVGKVKDINNDKWVVEIGSYSRALLSISQTNISLFSQRIRLYNDVINM
INIYKPNDIIACEVQRILTDGCIILHTRSSIYGKLSNGILITVPQTLIQNQKKHIFVFPF
NVQIILGMNGFIWISSPIKKSKDTNPNSIDEDIEGNKFEEVDDTTRRNISIISNIIKLLA
KYHININYDIITKIYMQYTTNKNNNPCYILKPYVSDSFLFNYIDQINKKN----------
-----------------------------------------------IF-----------
----------------------------FMTIHL--------------------------
---------FKYMIQ---------------------------------------------
------------------------ELD---------------------------------
--------------------------------------------------ILME------
------------------------------------------------------------
------------------------------------------------------------
------------------------------------------------------------
---------------------------------------KXVQEWVTPKNKQIKAASSNN
AQIVIALSGGELIYFEIDESHTLVEIFRKNLNVEVLCLSMQQISENRLRANFLAVGCLDN
VVRLLSIEKDKYFKQLSTHLLPNNSSPQDICISEMKELGNDKERNVLFLNIGLNTGVLLR
SIIDPIAGTLSNHYSKYLGAKSVKICHVNVNKNPALLVLCEKTYLCYVHQGKYVYSPLNY
DMLEYASSFYSEQCSDGYVAISGNSLRIFRFYRLGEVFSQNILHLTFTPRKIVPLPFPSL
FYDHDSSVEVERKKKIRMLAIIEADHNSYDENTQKEIQKALKDIKLDIEDKENKKNTNEE
ENEEELLYDRIGTFKAGAGKWGSCIKIINPINLQIVDKISLEMEEAALSVCACELEALHC
LIVGTTSNMSLKSRSVPAAALRVYTYDIKYKLNLLHITPIEDQPFCFCPFNGRVIASIGN
KLRIYALGKKKLLKKCEYKDIPEAIVSIKVSGDRIFASDIRESVLIFFYDSNQNTIRLVS
DDIIPRWITCSEILDHHTIIAADKFDSVFILRVFTKI-----------------------
--YLEHIMSFHIGEIVTSLQKVKLSPTSSECIIYSTIMGTIGAFIPYDNKEEV-------
---------------------YNKIKEN--------------------------------
-NLKQF-NKL------------------------GSGPYSKSIKKVEGDISGLLTNINKL
CGVRESDTGLCLPNQWDLQLDKQMLNEEQPLQVARCTKIINADTDQTKYIINVKQIAKFV
VGLGDKVAPTDIEEGMRVGVDRTKYKIQILLPPKIDPTVTMMTVEEKPDITYNDIGGCKE
QLEKLREVVEMPLLQPERFVTLGIDPPKGVLLYGPPGTGKTLTARAIANRTDACFICVIG
SELVQKYVGEGARMVRELFQMAKSKKACILFIDEVDAIGGSRGDESAHGDHEVQRTMLEI
VNQLDGFDNRGNIKVLMATNRPDTLDSALVRPGRIDRKIEFSLPDLEGRTHIFKIHANTM
NMSRDVRFELLARLCPNSTGSDIRSVCTEAGMFAIRARRKTITEKDLLLAINKVIHGCKQ
FSATGKYMVYN----YIYLYIFIYIYIFIPSSFSIKIKVEENLSSIIKKINELKNEKHND
GELIYFLEKNNFFNIPRYSKIPKTKKSTKWEMFAEKKLMKKKNKSGLIFDKNSKGWVRRY
QKKHIKENEEKLKFVHEYKDNENLYEDPFEKKEEEKEIKKMKQKMREMKNKFDQKGISNE
DIKYIQRQKRKRENLIDNLKM---------------------------------------
------------------------------------------------------------
---ILKKGNFLTLNFKEKKIFGDLI----KVENFVFKNLKIKNLKLLKAIFLLEPTHTNI
LKLVKELKKPFFLKNYLFFTNFLGENFLEKLAKAEGF-----------------------
------------------------------------------------------------
------------------------------------------------------------
------------------------------------------------------------
------------------------------------------------------------
------------------------------------------------------------
------------------------------------------------LSF---------
------------------------------------------------------------
------------------------------------------------------------
-------------------------------------IKYVGISKDVYNSDEDALKPIKH
FDSDEDFQSVLLEKSKKKKNCNHTNLLDIKTNRKYKNVFDRLTDTNFYTGIHKERFDELG
NGRGKAGTTDIYIHDGWTQAKTRNHEIYSSDIKKSKKPVVTPGTLGIQKYGIQIASPKNI
WIFRNGDKHHNGILFLVKPHINNLKTLFFEITKVLSPTIGPIRKIYDQNFRLVRSVEHLV
EGAKYLCTSGDPPAPIDRLQLFLSKWVIQN--KKDEYLKNSNKKIENSNSTKHVKKNCNN
ISKVNKGLNQNNKVKNEKNNKKKKIKKGNSSSKTTVKEMI----DEDEEEEDKDNICADA
RKYFKEGQKIITPPNGDGTRAFYESLFEENPNSVIAIKYCIEHGVLSGTKHHEALYKYFI
LKKNNAFKSNFGGIKSEFLKLLEDSCKNEKCSYSK-------------------------
------------------------------------------------------------
------------------------------------------------------------
--------------------------------GR--------------KRY---------
------------------------------------------------------------
-------KMLNFFKKKNLLESFLKKLPKILGKVVYGIDDPLKALEIGAVEFLIVFEGLDF
FGLTPGNNFSNPAKTLHFSPNGEKQEFFFKENNVEFEVFEKIFLTDWVFNNYKKFGASLD
FVTNKSQEGAQF------------------------------------------------
------------------ENGSLRDTLKYASNMLCELRTSLLSPKYYYELYMIIFNELQH
LDNFISDKKKHKKKFIDIYESVQHAGNIIPRLYLLIIVGRNYIKNKDIKAKYILKDMTEL
CKGIQHPLRGLFLRYFLIQMCKDRIPDTGSEYEEAGGGNIDDAFEFLLTNFYESLKLWSR
MNDKTLRIPNLPDENVVNNRIKVLKDKMDVKMLVGSILVRMSQLEGMTTQYYIEKCLPKL
LLYLSNINDSLIQQYIFESIVQVFSDECHIYSLETLLNAVLKINSSLDFKSILITLLKRL
RSFIESNKYEFPKNVDIFNLFYDHLILYVNKTLDIYAKTKYNSSYE-------NAKKCKL
NNGNIEKNSDNDDVSQNNNNFNDINSNINNLSNKYNVNDNASIINSEECVQNVVKMLQVI
YEFIFLCIRIYND-ISISKLFVLPYTVVSNVCLDNDLICEQVISIIVLPFNYLGLNALNS
KDMQKLLSSVNEKHKKKLSLDIIDAIIECKNKSIVYEDVEEVLKYISSIFHDE-----EN
KDDVFNLENNS-AYTSEKLCKFFHIITNTNDIEQKYNICMLFYKYIANSGYLIHLLPTII
```

FSMLNLVTSITNLVTNQSEKKED--TYDDLIEDKNQNDILDEKRVNEYNIYVKNIFKFIH
TNLLSVSSKIPILAFKLFLYSAVVVNNYNSFLQAYHYISFDNLXAICYEFITPPLIIYED
DINISSPTI------------RLDYLGDWNSENTP-------------------------
---PTPKYRNSSKVFGRLPKSIKKADIAIPSKRGKKYLFGKKVPKYVYFFGAGNFGIPGG
Q------------------------FALFNRYM-------------------MLEAKLNN
ASILKKLFECIKDLVNDANVDADETGLKLQALDGNHVSLVSLHLLDSGFSHYRCDRERVL
GVNIASLNKVFKLCAANESVVISSKDDEDNLNFVFENNKEDKVTNFSLKLMSIELDSLNI
PDCEEGFDAEVELSSKELTNIFRNLSEFSDTVFIEIDSNSIKFTTKGLVGDAEVALKPRD
STSEDDIGVNIKSKKKIKQSFAIKYLNLFSKSSILSDVVTLGLSDSRPIEFKYEIKDTSP
DADTLKIGFVKFFLAPKMDDDMDNKDIVNYKYIIVFLDFLFFLLKIYGKEWCKAKFEYGK
SDYAECLNEGDNITKYMIETVPIISSDVNLYSNVSIVLSNGYGLKTKEIIIGNENEGLLN
KIFSVRKDIGNPEYIHIKMNSPNKKWKCKKITIWKDFKYWIFDCIDHLDDRKPEATYFLS
GNKLYTAYVQTGKDIEAGTTGTIEIILLGNEKRSNTKILHEGFNSGSLKKIKFQASDVGT
LEDIILSNNSKNDPWYCDFVKIKSD-NKVYVFNVKNWIGHPYDKNVKVNIKNDNVEGTAK
DIDCHIRANDLINTNNLPKNLANKVQIFKVRCPQNCQNAEFASIEGSSIHPSSSSICASA
IHDGSLSPSGGELIVTVGSELKYYHALNEKYNGLEAMDFSTKADEKNFSFYSYHLDSIDD
IKSDIRIVNSFGKLSSLGRLEIRTKNMWGSVCKKGPNFTFNDDTAKRACKDLGFPNGIYI
KENCGNVNGQNYCADYGYPFSSAGIMCSGNEKSLVNCNGDDASHCIDHHDDVIIQCLHYS
SNELVSDGTIRIVDVNGSPSNNGVGRLQIYYNGTFGSVCSEGWAKESEKIVCQELGYTGL
KGSGFSHHLCANISGENLCGHDTEKINAVNIKCKGNEKYLKNCPHETHEDIYCSHDEDIV
IGCSGEGDASGLGISGKKNLMNLEKKNFHPKIELTCFDKIVSKSDLSSANVGDIFIVNCP
EKCDEEMGIIKGTFLYTFDSPICKAAIHAGVLTNNVTEDIVVIIAHKHKNFIGTRRNNIE
SHTFSGISKSYSVSIPTTSLLNKERRSNPKAEDEIL-KKEDDFSYQNVFDKNTKHLSTSI
QPTFQWISPTGFLGFNGKENDFIDCSNLPNEKYIKGLYNFSFIIYFTLNGGEGTWRTILS
HSLCEGISISIDEENELIIEQNCNPYIIKSRFKPKFGETYHIAIIFNKTNKTIALYINGK
KAIFEKAKYDFTLNGDLIIGRSNQSTTDYFIGNIHLVEVYKYVLSDEEIKESLNSVLSLD
YVNISTTENI-------KKKKKKGARKTIDGRECITPCKSRSIINKDLQVNAEKINLKCE
DDLLSEQFNGKIGSQFLVNCLEDCTKSKFLVKGSNNFYTPDSSICKAAIHAGIYIPNNVN
KGSFIVRIVEGLLEYKSSRGHFGIVSKSEKQSQLRSFSVFLENEDNIFTCSTNGQFILNL
SVGEKIIISCPSNCDKVNNKIFGTNIYSPLSSVCKAAIHSGALSNQGGQVEIIVGPKQEE
FKGSKQNNIESYDSTSQNLSLTFMRYTGKMSDLFVEQNINTEKIEEEKVDFDENKIIYKI
KKKIKKKGIS--RINYLPLKDEKLHKTLPNTKNIKNKKLKNKLKKDVKLAIISSKRILAN
SKFKPVEEGYIKFDSCEEKNVKTNVNKKEEVNKNKEVNKNKEVNENKE--NEKKEVGKYL
SQNYIYNNADIGTQKKVFDLSLNLGPYSCNYSRNGKYLLATGEKGHISLIDTQNLESLCE
FEVDETVRCNTILHNHKLFAVSQKKYIYIYDNTGIEINCIKDILYTYKMEFLPYHFLLVS
IGEFGELVYQDISVGNIVTRKKTKRGSCSIMKQNKSDAIIYLGHKNGHVTLWSPNVDKSV
CDIFCHYTPISSIGVHDNYLITASIDCTYKLWDMRKLQFIKSYRSHNVINNIEISDTSIV
GFSMNSHFRTYKNFFTYPEVYLTHNTYGDKINSLSFQPFEDICCAGLKYSIKTLLVPGAG
LANIDTFVNNPYETKKQVKENEIRLLLDKLPPDTIRFKKNEIGKLNPYINVEDNKMSNYK
NNSKIRNMNNHRHSKKNINEADVSYNDQNENYDSTNDDFANEEKKNNNNKIDIMRKKKKK
KKKKNTLNSNIPKFQRKSSIEIMLQNNECNNLYFLALSRNKATIYEYDVNLSKNIIDEIN
EEFKKNEEQN-ENKDNNKISFLNEPRSNILKKSTSKFNNDFLLNRGIRVYKEYNDVELAT
CTNDYKNIILVRKSSLNVAEIIEIKNDKNITYIKTKTQIKRIMTSPRDNYIVLHCLYKPD
IVNTNLYVYKIGNKSNKKKKNKEKKNIEENSEKINENDIENKNNNNNTNDNIIYNESLVI
ELTLKSYSNHNWPFFKWSDSESICTCLINNQINIYKGNNLQTPFSKLKLDNIEFFELSPE
FNNKKDILLATYEKGSKGNPSIFKIFNLDNLNKHIYSKSFFNSDEIKIKWNKNGTALLLQ
IHTQVDKEKHSYYGSSSLYFIDTVTLKEVNIMTNKGLIYDTIWSNNQNKFYVCKGEIPAE
IVLHDKNGNVSHSFGRHKYNTLKLNCNEKLLLTGGFGNLSGDISIWNTVSKKEITKTKSS
CAVICEFFNDGKHFLTATTNPRLRVDNNLKIYKYNGIIVSRINFEELYNVIILPFNKIKF
NE-KESIDFNLDNSDMQIYINKQLGIDERKTGIYRAPRSSGLLNMNGNLTSKD-NKVKSS
LPPGSNFVNENKSYKKKKKKKSSKNKQKRETFN---------------------------
----FLVMTEEEDTIPLPNIKTQILKKIIEYMEYHIQNPADEIPKPLSTSNLQDVIN---
----------------------IKILL---------------------------------
-----------LKIN------KIMEHKSNIEKNLNEEKINSLKNELKYKKVYKHNFNTHI
YKDELNYIHLKNIEDEYILNENPFMNLSQQDFSDHKKKLKSYDSEILESNNMTNEKENKI
VKKIEPYFKKEEDNICNNEDYNNEKNNDNTSNSDSINSINRNNNKDNNVNSNNDNFNNTD
NNIDNNNCNCNNESNEDKEKREDKKYEEEKKIKIIEIYEKKLENEEKKKNKE--------
------LKNNKVQIRENISKIFKESPSSKPKIINEIRKKKSDNNVNETVVIEK------N
NSDANNNNNNNIDIEKKKIKMNKNTLKEGSLLRLFRCEYFDTHLHIRYLYDRKEVGVHEY
LVNSLYTQRKYEDILFYLPQLSQISLVRYETSSLYRFLLDKASKSMHFALKLCWIYHSIV
EDNSSKYKDLAQKMIQEIEMAVVNCKPLNNECKTFKENKQSYLLDLAYPLLFKRKFILIK
IRTNEKFNKMKMYNRSSKHSYHSLNLKDFIERSSFKENTITECDNNNNINNSSKSDLNNS
KKEEE--EIEKVKEKRKEEEEKEGEGEGEGEGKEKEIEKKEKIENIKNEEKIKEENNTL-
PKLPPCYIINSGSLSIARAKVKLPSTYSKLGNPLSLSKFSLPDLNYSFDMIEELQQFFMK
QRRCDYFSLLNNFINLIISVSNLLSSEPDIDLRNELLKRFIYSLNSWMNMRRCIVASCEN
TFAMTGLCIPLESLSS------NNKNGNNFSYNNLQILHFNHEECKIFFSKKRAPYLLVF
EVADLDEDISDIPDNIFYTKSSVLNDENNKNYEKERESS------KMEEINKKNHILSKN
FCCSKSYSVYVDNNTSFEYEKRKNFEDKKKK-----KYTKENVQNNEEK-----RKKYIL
DLNELKMDDLYVYNCIVNDLRRENLISFNTKEEENLSLIRKCIGLKADEEEEEEKKKKDN
QTTISTMMCAKKNQ-LNSRSVSMPNYLSNSTTKVDSKYNNNTNSNIKVSRENINSGIDDI
LIEKNIEQIETYEELKKNFIDYNNNN-------------------IDKSHNDSEEPKEN
NNISKNNNPIEYPNNLLDINEYFKPENYLNEEFKKKNCRIIKRLLWGELFEEKKKKIRKI
SPYGKLKSWDLKCVIVKGGDDLRQELLASQLIRQFKIIFDNAGLPLWLRPYEILVTGSNS
GIIEYVNDTCSVDSLKRKFATDNISTIFNIVFADYIFEAKKVNIYYIYIYYIYIYI----
--------------YIYI------------------------------------YIYIY
MFIIF---
-------------------------FHF-SLHLSFFF----WFIILLYFFVYV-------
--YIFIIFFYLFFFF----------FFLYDVL----------------------------
-------------------------------DVNNFNSGTGSGNLNFGNFANLLNSNG-G
EFNRDTISSLLNNPLARSLLNELSNNPEMLTNLVSNNPILRNTFSQSPIMQPVLENPNLL
REFMRPEVLQAGLQIENALNMNNNNNNNNNSSQR-GIRMEDLLNNLNNFNTSANNTENNN
NSNINNINSLFQSQELLQTFQQVMRANRNLANLNFPNSSQNLDFNSSNVTDNRSPEEKYA
SQLLSLQEMGFIDNAANIQALQETGGDVNSAVTRLLEKGFN-------------------
------SGDRLTRAARVLEQLTEQKPIFGKCRFTIRSFGVRRNEKISCYVTVRGKKALEI
LEKGLKVKEYELRRKNFSDTGNFGFGIQEHIDLGIKYDPSTGIYGMDFYVHLSRPGYRVT
RRRRK-------------------------ILIV--------------------------

----------------------------------LIIYIY--------------------

IYKCHGII--
-----------------------------NGNSIQNYENKLECKKIEENFLINNNDP
SSKEENSCIDY--NNFSNGITHNFHQVDIYERLGFFISLKMINEIKGLSSIDTNYQWLPI
LYMALANDTSVSKITLNILKPYSIVLIRLLSDFFSVAFDIKKVEKSQIDYSYIIKCVGVG
YRNFSKKTF---

---GKNFYPCKWINSCII
TAIFKLCCFNNITDHVVAKKLIEKYKNSQLTDMQQRCYEYDLILKNPTLMKNLFSV-DSK
QNIVVDENLSFLNPFIEKHLRNGGKSYIEKDLRKNAIQFETSKNNIPVLNFTPYEVPVNN
NFHLDNLNVCCNNSTYEKNYNYDPSLNMSKTDSIDNIKEKEKPFKLNVIGPKKWRKKVVK

NEEKKNNK-------KKKKKKKMNENNYNKY--------NNIMESKKRLEDIEVENKLPK
NINTEEDEDNYYDEENEDEEEIEEEEDEDDDDDTKEGIENEKKMDSIENYKNYERFDEKN
IDHLKEYEYQIDNHESNIYEYKNSFDYTNNKAHNHELTEKEKMAAALFNGLISNNSSESN
TRNYFVSSFSAKKGISGLSNRNYFKNKKDGESTKRILENIQSK-GDAINQNEQMKNSSNI
MD----NNINKSEDIKRKPFSFDMLDLNESSQNMDETYESKTD------IEEVIYNLLLL
FSYFKIKEKKKKK---------------------IFNYNGGCVLGMSGTDCVAIACDLRL
GSNGFTTVSTNFTKIFKMNDYVYVGLSGLATDIQTLYELLRFRVNLYQIRQEKLMDVDCF
SNMLSSILYSNRFSPYFVNPIVVGFKIKHSIDEKGNKTSTYEPYLTAFDLIGAKCETNDF
VINGITSEQLYGMCESLYIKDQVI------------------------------------
---------MHKTIRKIFDSKICDKIENMKIFLVGAGGIGSEFLKNIITIGCKNIDIIDI
DTIDITNLNRQFLFKKKDVKKYKSVVAKERALKYNKNLNINAYTFDVCIMKSSDIKKYDY
IINALDNIKARKYVNKLCVMEKKVLIEAGSTGYNGQVYPIYPNETKCYNCEEKPKNKTYA
ICTIRQTPTLPEHCVAWGKLIFEMFFCKNDNETLIDIKNHIEEESKKRNMEKMEIIIFIF
NYLFHDTICELISLKKDFAIMPIPILFEENSNDNTCNIEKKSYDKSEKKNEFEKNKTKNN
VNIDENIKIKEENTIQLCAQNIWDKKKCIEIYVKTFLKLYSYLNINKNVEEYLIFDKDDD
DCINFITAISNLRMMNFSINQKSKFDIQSIAGNIIPAISSTNAIVASLQAFQLIHVIEYF
EILKYKNDEKI---

------------------------N-----------------------------------

-----------GG---------------FYQFNVIFDEVVINDKESHINDT---------
--KKDEKNENISSITESK
-VDKNIINNRNKNIDIFNFYCSDSDEEIKYFNSEDEEIVNDYISSENFRNSKLFSFVATK
NYYDSDKSYLLPQNYIRHEPQLMPLKPPYLLSLQKKRQETKKMEG-DYDKILVINFGTQY
FHLIIKRLNNIKIFSETKDFNIDLKDIIDLNIKGVILSGGPKSTIEPGSPHLKDEVFEYF
LKNKIPILGICYGMQEIALKMKGEVKKSKNCEYGSTDVQIITTENAHDDRYKNYKLMD-N
CDLYDNIN-DKKITVWMNHTEEVSKIPDSFYLTNSSENCLITSFYDKEHNIHGVQYHPEV
YESLVGEQLFFNFAYKVCKCKKQFDPIRYHELEFKKIEKHAHDHYVIAAMSGGIDSTVAA
AITYKIFKNRFFGIFIDNGLLRKNEGENVFSFLKKLFPDMNLTRINASDNFLKNLKGVTE
PEKKRKIIGKLFIEEFEKAVTNMNIDVNYTYLLQGTLYPDVIESKCSKNISDTIKTHHNV
GGLPTNLKFKLFEPFRYLFKDDVKKLAQELNLPKEIINRHPFPGPGLAIRVVGEIDEHKL
NILREIDDIFIKDLIHYGLYDEINQAFAVLLTTKSVGVRGDARSYEYVCVLRAVKTSSFM
TANWFKIPYDILEKISTRILSEVKGINRVLYDISSKPPSTIEFEMRKYHGNVKRNRDDPI
EENDNITSDKTCITLVRATLLPILSIGKNEKTTMFHPFKNPLPGFVSDKTLYLKKTLGAK
VRRTGWINLMKNSIPRRSDEADEKDQIEEIIQKHNPLILYEDENDKIQVDPILAQHLREH
QREGVQFIFECLMNLRDEKISGCILADDMGLGKTLQSISVLYTLLKQGFHNKSAIRRCLI
LCPASLINNWNDEISKWLPGRCNVTCVNDNVKEKIVSKLEGFKYDLKSNVLICSYECFRI
NNEFLDKSSIDMIICDEAHRLKNDKTKTYTSIYNLSAKKRLLLSGTPIQNDLGEFFALIS
LCNPDLFDDTNLFRKKYANPILIGRDKDATEKEQEIASERLSELSNITNKFILRRTNNLL
SKVLPVKYLINIFIKLNPLQEALYLLFLKDKKILKNDQSNNKVNVLINIKKLEKICNHPL
LLNANDIKDIGQIPLSKLIEDLTCEIEKSKNEKINTNDKNSNIK---NEKNLHNSSENCK
LEKKKFDIDYEKPVKRILEECKRDIHRSYYNLSCKFLLLHFLLKNIKQNTTDKVVIVSNY
TQTLDYMEILCKENFYKFVRLDGGISIKKRHKVISDFTHSPDIFIFLLSSKSGGCGINLI
SSNRLILLDPDWNPANDKQALARVWREGQKKICYIYRLFCTGTIDEKVYQRQISKDGLSS
MIVTNTNLSKDQLSDENVKKLFNYKINTICETHDNIECNRCNSKNST---ENFTEQLEDF
EEEDVNTWAHHLDIETVPDEILIKATKDATDFKKNDINNCPILQKLTPDFVTFTMSCKIE
YRDDLIKQKLNQAKMCAEKL----KKQNIIEKNSKKQKKNKKIDGKYEENDESEDEFEDE
FEDEFEEDEEEDEIKMNDKL--NNEHRRNEELFCITDINVKSCDEYEKEISSLPYISTKY
CVNIKKKKIESAGFMFYIKYEIPILKEKKKKIKDNKNVIIDELFLYKDKLNYLKDIEEDN
N----NKNENAKIEQEEKKKKKNDDDYDDTKDYNKKQEKASYKKSSNLVKKCIDENINVY
EILGVDETDDIEAIKASYKKLILIFHPDKNKGTAYLNAKEKKKKKEKEKNDLDEKNNDTK
DLMYYMEKFNIDKLTPEEKKLMFLKIQDSYTILSDKTLRKQYDSSVPFDEYIPTLKELEE
ASNFYDFLRPVFKRNAKWSAKKPVPDIGDENTNIKNVKYFYDFWYNFVNWRDFSYHNAYD
YEQAECREERRWMERENKKIQKKVSKLENLRILKLVDLAYNNDPRIIAENKRIKTEKQKK
KELAILEKQKKMDT-LDDTKKDNNTTNNSNEKKNKE-KAAVKIWKHHIKSVCLTKLSNTV
NYDRLQEELLLMPFETICDFIYDIYVLLNFNFYKSDILPTEKMSSNNLKENNSKKNDNKN
LNHLKNNNNNNNNNNNNNMNNSNDGNNDNKSTGFIGHLKSIELGENDIESLIQIFKKYVN
ESVFIIEKKEEINREDLNL-------SNNMKDDNCIEEHRDHNDMEKYDEKEEKNNIEEK
KKTNENTEKNEEDVEKNDKWTAHEVSLLAKALKFYPGGTKNRWVLISSYIKTKSIKEVIK
KTKEMFENETLKNLSKNFDESPFDHFKNQNKGVMKKIDDNLDKRELKIEKETSNLNNT--
-NGYINQKKPWTQEEQNLLEKALMKYPSSIPLKERLELVSSEIKTRTTDEVILRMKKLRA
QILAKKSAKMPKKRRNGGRSKHNRGHVNPLRCSNCGRCVPKDKAIKRFNIRNIVDASAQR
DIKEASVYSTFQLPKLYIKQCYCVSCAIHSRFVRVRSREQRRVRKETAKHINPSQL----
--QLKDAQ
AVRIFMQMLTE---

--KFLV--------------
----------------------------IYALMDGFLKKFLFLVQHT-------------

-LHL----------------TEVRII----------------------------------
--YIYIY------------------IIFS-------FSFHL-------------------
------------IYFFILFISF--------------------------------------

---------------------------------------LGAKSILFIIFIMAMIMLIVT
YICRNPKFKNLFYNYGII--
--YIMADRKANKNAVVKN
VDMTEEMQIDAIDCANQALQKYNVEKDIAAHIKKEFDRKYDPTWHCVVGRNFGSYVTHET
KNFIYFYIGQVAILLFKSGMNVEIEEETLQMAKDFQKSNQEEILLKKLKPFSEGLLKPNS
NLLYISEKGTRNRVKICNVLNIPKSEKEFNEGVKKNVFIDQIITVCGWSKAVRKQGGGRF
CFVNLNDGSCHLNLQVIVDQHIDNYDKLLKCGVGCCFRFTGKLILSPVQNESNKGILKEN
VELTLNDNSAHSFEIYGENLDPQKYPLSKKNHSKEFLREVAHLRPRSYFISSVMRIRNAL
SIATHLFFQSRGFLYIHTPLITASDCEGGGEMFTVTTLLNENGDIDSIPKIKCKELKKEK
REDN------NIQSDGDKYMIDFKKDFFNRQAFLTVSGQLSVENFCSSMGDVYTFGPTFR
AENSHTSRHLAEFWMIEPEIAFSDLYDNMELAESYIKYCIEYVLNNNYHDIVYFEENVEK
DLIKRLKNVLEEDFAKITYTNAIDLLMKYSESFEVPVHWGMDLQSEHERFIAERIFKKPV
IVYNYPKDLKAFYMKLNEDKKTVAAMDVLAPKIGEIVGGSQREDNLELLDKMIQEKKLNL
DNYWWYRQLRKYGSHPHSGFGLGFERLIMLVTGVDNIKDTIPFPRYSGHAEFM-NTSCEV
NIPEEELKKSEVLFKELKELNINYREVKHGVAHTIKDLLEMNLDNSTNIIKNLFLKDKKK
KFFFICTLNSKTVDLKYLANALNTSNLRFVDEKNLKNILNLLPGCLTPLAMKYDKDNVVK
LYFDAELKNMDEIIIHPLHNYSSLYMKKIDVIKFCDIHNHSPEYIKIEEEKI--KKEDGQ
EEKDNQKNNE-ETCLNLKVNNKFDNQSKEYNLLGITSKKLENFSDWYTQVIVKSELIEYY
DISGCYILRPGAYYIWECVQSFFDKEIKKRGVENSYFPLFVTKGKLEKEKNHIEGFSPEV
AWVTKYGDSNLPEEIAIRPTSETIMYSAFSKWIRSHRDLPLKLNQWNTVVRWEFKQPTPF
IRTREFLWQEGHTAHKNEEEAVKFVFDILDLYRRWYEEYLAVPIIKGLKSEGEKFAGANF
TSTAEAFISENGRAIQAATSHYLGSNFAKMFKIEFEDENEVKKYVHQTSWGCTTRSIGIM
IMTHGDDKGLVLPPNVSKYKAVIVPILYKNTDENLVHSYCKDIEKVLKNAQINCIFDDRS
LYSPGYKFNHWELRGIPIRIEVGPKDIQNNSCVFVRRDNNEKTNVKKESVLLEAQQMLVD
IHKNLFLKAKKKLDESIVKVTNFSEVMDALNKKKMILAPWCEDITTEDEIKKETQRLSLN
QTNTETTLSGAMKPLCIPLDQPPILPNTKCFWSGKPAKRWCLFGRSYMAKLSKAQKKQMY
IDKLSTLIQQYTKILIVHVDNVGSDQMATVRQSLRGKAVILMGKNTRIRTALKKNLQAVP
QIEKLLPLVKLNMGFVFCNDDLSEVRKIILENRSPAPARLGVIAPIDVFIPPGPTGMDPS

```
HTSFFQSLGISTKIVKGQIEIQEKVHLIKQGEKVTASSATLLQKFNMKPFSYGVEVKTVY
DDGVIYDANVLDITEEDILKKFSKGVSNVAALSRAVGIITEASYPHVFVEAFKNIVSLVI
DSDYTFPLMQNIKNMVENPQAYVAAAPAASATKTEEPKKEEAKKEEEEEDDEDGFVGFGM
FDMLQERDLAREPCPDRIIEDMGGAFGMGCVGGYIWHFLKGARNSPKGDILSGALYSSRM
RAPILGGNFAVWGGTFSCFDCAFQYVRKKEDHWNAIGSGFCTGGVLAMRGGWRSASRNAI
VGGVLLAIIEVVSIVLTRKTTPTPRQQFQQQMEMEKRMASKNNK----------------
------------------------------------------------------------
------------------------------------------------------------
------------------------------------------------------------
------------------------------------------------------------
------------------------------------------------------------
------------------------------------------------------------
------------------------------------------------------------
-NMMHMFVKVK--------------------KNLH-------------------------
------------------------------------------------------------
------------------------------------------------------------
------------------------------------------------------------
--------------------IYMYLNKYIYIN------------IYIYLNK---------
--------YLNIFFFQGTINNGKKVHYWVMTRHENGWICFWEVTNKSIIHLKNRWNNKNF
LKNSEIIKQNEMLNKIKENNEEKYYNGEYLMNFVRYGLEELKRIEKEIKEDYDLKEENEL
YTMDICGEYNTKDEKVDIEEILCNDEDFFNNMFEIKYEKTDSYNCNKALKYLLENFSKYI
PISPKMFLIDYENTLAYVPYTSIEIVFNDEQLYGNMQNHHPACILYDFENNNHWRPLLNH
SPVSIKNEITISTPLSDKLSYIFLYILIYICVLFFFF----------FFFLKE-----EQ
YFNWQYYKF---------------------------------------------------
------------------------------------------------------------
------------------------------------------------------------
------------------------------------------------------------
------------------------------------------------------------
------------------------------------------------------------
----------------------------------------------PVPPNHTFVGFPIH
FSTIG---------------------------HLLYLIYFLQCMYIYFFFIVTAVQALSN
ILKSSLGPQGLDKMLVDNIGDVTITNDGATILKQLEVQHPAAKILVNLSELQDQEVGDGT
TSVVLLASELLRRGNELIKMDIHPTTVICGYKLAMKESVKYIKEKLSEKVNNLGKDVIIN
IAKTTLSSKFISYESEYFAKMVANAIQSVKIINEAGKVKYPVSSVNVIKVHGLSSLDSKL
IDGYAIMSGRASQSMPSAIKNAKIAFLDFPLKQYRLHLGVQVNINDPTELEKIRQREKDI
TKERVNKILESGANVILTTQGIDDMPLKYFVEAGAIAVRRVNKDDLRRIAKLTNGQIRLT
LSSIDGTEKFEASSLGYCDEVYEEKVGDWDLMFFKGCKTSKCNTILLRGANDFVLDEMKR
SIHDSLCSVSRALESNYVVVGGGCVEVALSVYLEDFAKTLGSREQLAIAEFAESLLVIPK
ILALNASYDSIDLVCKLRAYHTKSQVMNTEDSKDYKWYGLDLVNGKVANNLKNGVLEAMI
SKIKSIRFATEATITILRIDDLIKLSPEERKEEQNF---------------LYGYIQIFF
----------FFFFLKTPWEVEGGGMLGVIRLKDQIKNPPVIKMKGHTANILDLSFNPCY
SKIIASSSEDLTVRIWELPYEDENITEITNPACILKGHKKKVSIIDWNPMSYYILASTAF
DSFLNIWDIENEKKAFQINMPKKLTSLKWNIRGNLLSGACLNKNLHIIDPRKKEICFSFG
VHNGGKSVKSTWIDGLCGDDNCVLSTGFSKDNMREIKLWDLKNTSTPLTSISLDNASAPL
LPFYDETLGIVYLIGKGDGNCRYYQYLQGNIHKVEEYKSNLPFRSFGFLPKQMCDIYKCE
LGRVYKNENNNSIRPISFIVPRKNSSVFQKDLYPPIIVHDPEYDSKNWVDGVDLDIKRIG
INELTNDDLKITKKFKSIPQSFKSIIIQDPIAPKKSSIIRQFTKRLTFFKKNNEHD--NS
SLNGDIIICNNSFKEDEISKIEKDNKFYYEEYEKNKKYIKYLENTINNLKKQIKNKEDST
KTNENKIKDNLNKFFDNVTHMKLCKKRNY-------------------------------
------------------------SQNGKCLEK-------------------DCH-----
------------------------------------------------------------
----------------------------------IK------------IKKVSVVMTH--
--LLKMYYYYFIKKEMINTNDYYNNDN--NKSNYNEENILKIEDKNTEIYYNNQIPMEFQ
KNSNNNNNNNSNFENS--YNFYKNEISSEDKNVNHNIYENKTNEYNYPTFEEKEKKQQR-
----ILKNDIYSTYEKEDYYY----------------------------------DSIST
KDKGN----ITHSYNSDYTEIKNININDKKIYNENKIINSSEYILEYDMVKQEYYEYEMK
NIADNS-DIYEFKIKNNE-NKKMYECETENIHNNLDTYENKLENDKNNTVYEYKLENDTN
EN-----MNENKSKKSEKENVYEYKLENDENNMYIYEYKLENDKNNTVYEYKLENDQDEN
IYEYKLEN-DKYNTVYEYKLGNNENDNVCEYKKSDEDENQNKLNNSPVYEHKMTKEVEQD
NNYDSIDYSIDDLKENIRGSCIKSIPIDINMYINREKLKSGYDYDQIHVINLTNSEKIKN
NVFHKMKDESKLNNYLCLTDNDQENDYENLFNNSLNIKESISTTKNSNYDNEDIDFSNID
ENQKDLYFKSNLFDKLKICGQFNRGFVISKINLLYFKNQEEKNNSSDNNSNNNCSNNNCN
NKNDYALFIIDQHAADEKSNFEKYNKTFTMKSQKLISKINLQLSPAQIHLIQKYFQIFLE
NGFEVEIVEEPIQKKRKLN--NINEEEEILMEVKVYLLSLPVFNGKILEIVDFMSLLHHL
TEHPLTFDKKSFEIFKENKNHSNQKVDTWFNYNFPRPQKVWRILASK-------------
------------------------------------------------------------
-----------------------------F------------------------------
----------------LLNIFLI---------------GRLDLNHVENYLSSKKLVHKLK
CCGEIMLTLEEMKALVIIFLKKISDTYIEDQAKWMQNMKVSQEEQGKALEAAMSEYEKNV
LFSHALKEQRIFQNNKKLGEWNDTIENAYEAQQEILRQFETKKREKMELISEKNKEIEIA
KDYIQKIKEAAVDNRYDNSKCFVYPASSAPCGACTSAGAVLPYRRYKEPRQKKEYSMCIL
SSLSASVRATLLLPPPSSNPLESVDALHPLIVSSLFLTTSSENAVPLVRRGYIYIYRGGF
FFFFF---------------------------FLGTDILCQAKSGMGKTAVFVLSILQQL
DTNENKEIKEEKEINNKSNNKSQNKYVRCLGLAHTRELAYQIKNEFDRFSKYLKNVRCEV
VYGGISMSKHIKLFKEENIPHIIIGTPGRILALIREKYLVTDKIQHFVLDECDKCLEKLD
MRSDVQKIFISTPLKKQVMFFSATMAKEMRDVCKKFLQNPVEIFIDDEAKLKLHGLLQHY
VKLQEKDKTRKLIEILDALEFNQVIIFVKSVTRAITLDKLLTECNFPSIAIHGGLDQQER
IERYDKFKKFENRILVSTDLFGRGIDIERVNIVINYDMPENSDSYLHRVGRAGRFGTKGL
AITFVSSQEDTLALNEVQTRFEVAISEMPNKIDCNEYINQ--------------KISFLD
EEYSSDGDETEYHSEMDKNEIEKKESDIANIEGRKNKRLSVSAEAYGEWNKKKENFVAKI
YEKDEEEKLKIREALNESFLFNHLNEKEFETIVDAFFDKNVNEGMNIINEGDDGDLLYVI
DEGEVEIYKNKGDKKEILTVLKSKDVFGELALLYNSKRAATATALTNCHLWALDRESFTY
IIKDNVAKKRQMYEDILKQVTILQDMDPYERSKVADCLKSKNYTDEEIIIKEGEEGDTFY
MLIDGNAIALKNDKIIKTYKKGDYFGELALLKNQPRAATVKCQKFCQVVYLDRKSFKRLL
GPIEEILYRNVENYKDVLKQLGLDTTCIEGNMHLKIKCLSDEVRELYKNHKTYHEGDSGL
DLFIIKDEVIKAKSTTFVKLGIKVVALQYKCDCYNKCCE---KNKKDDTKNVINTSFLLF
PRSSISKTPLRLANSIGLIDAGYRGEIIAALDNISEDDYHIKKNDKLVQLVSFTGEPLSF
ELVDELDETSRGEGGFGSTSNN------------FFNDSFCVLISDLGAAFGTAKSGVGV
CSVGVMRPDLVMKSILPVIMSGVLGIYGIIMSILIKGRSTYI------------------
------------------------------------------------------------
-------MTKRIIKDSLSEKNDKKNIYEEKIKENNN-----RNILTLNKILEEKDTENKK
KVKNKLICKK----KISTNTNEMRIITIPRQRKSSVLKNWLELIKPIVTNLKLEIRMNKD
KIEVRTCKLTEDKNNLQKSSDYIKAYLLGFSIEDALALLRIEDLYIESFQIQDVKMLKGD
HLSRCIGRICGSNGSTKYAIENATKTRIVIAGDKIHILGSYNNIKMARYSICSLILGSTQ
GKIFNKLNILAKRLKERFIY---------------------------------------I
NMKWNFIVILYLVIFLFSLNQIEKLEDRMHPWAADPKTIGALSATQLAILASKESKEKLK
E----------------------------------------------------------N
KIYIKKSKC-----NIFFFLSHF-------------------------------------
---LIQNDGDEIPEFLEAVKNSNAIDNLKNLQQVLKKI-------YML---LMANGWNAE
QIFGGVMSYTYDDIICMPGYIDFSLSEIDLCNNLTSNISLKTPIISSPMDTVTEHKMSIS
LALSGGLGLIHNNMSIENQIEEVKKVKRFENGFIFDPYTFSPENTVADVLETKKRVGYKS
YPITVDGKVGSKLVGIITSVDYLYLTNKSKKIRDIMTTDVVTGNYPINLTDANKVLCEEK
KSVLPIVNNNYELIALVCRNDMHKNKIFPYASKSKNKQLIVGAAISTREEDLERANQLIK
NMIDVICIDSSQGNSIYQIDTIKKIKSAYPEVPIIGGNVVTCDQAKNLIDAGADILRIGM
GSGSICTTQDVCAVGRAQGTAVYHVSNYAHTRNIKTIADGGIKNSGNIVKALSLGADFVM
LGNLLAATEESCSDYYFENNVRLKIYRGMGSMEAMYNKNFNSKSRYLVEDKKISSFDDQN
```

EEIKVSQGVSASLVDKGSVLNLIPHLVKAVKHGFQSMGIKNIQELHKKLYSGDLKFDVRS
FNAIKEGKISDNLIFSNNK---
--------------------VKKDMKENKQYQEALKELKEKTEINDKTMKLKKKIEENIN
FYKNIKEKNVEAIKILYNDVHNFVKYCFENNYILKHSKNISIQFFSILKKFIINYSDKFI
QLKERWNDNSAFIQLDKWRQEMAIKRYKKKNKQENEKINESDLSENSEQKIENDMNINEK
NSEQKENNELILAYESAWDKFGSKLKDMPFLNNFFENPFIGKLFGETELAAALREMKMYD
KNFKLSELMYLFEYVISKHIVESYLIGDEETLRLHCGSSAFNSLNASINERKKKKVYLDT
NVLIYKNHELKGAQRMEESSPWFIFTFHTQQINCLKNKNDEIIEGKIDDIREVVYTIALS
KHPEPEKEGLLYPYIVREFAIIGNTPSW--------------------------------
--KEFQDLVL
YEIKRKKRHFKILSQSWVKYLSNKEKLKLKKQEEEEKRLKLYSKNISSYMDLFWKKIEKL
VWEEKKKELQKTLNKKKEMRFKKFVKGAIKKIKCARHNAHELFENKSFSVSSYNNSEQLN
NSSNLDNLDKELKEEDLTDQEEEDILLDEQMESSDELEDKKKEISLLDDEANIPIEELLK
NIYGFKSGEEYLNLMQKKEEDYEEGNDEK----DEEDEKDEEKNEEKKGEKIKKEKTKEE
KKEEKKEEKKEENLKKEKYEEEKNEENLKKEEYEEYSKEEKDDVENEKNKSILR-----K
ASNENKNNEKKRKINSSSKKDEELVCNMDEKHLTKIPPIIKATLRDYQHAGLHWLLYLYK
NNINGILADEMGLGKTLQCISLLSYLAFYFNIWGPHLIIVPTSILINWEIELKRFCPCFK
ILSYYGSQNERYKKRIGWFNNDSFHICISSYSTVVKDHIIFKRKKWKYIILDEAHNIKNF
NTKRWNIILSLKRENCLLITGTPLQNSLEELWSLLHFLMPNIFTSHLDFKEWFSDPLNLA
IQKSKIHDSKELIDKLHTVIRPYILRRLKKNVEKEMPNKYEHIIKCKLTRRQKILYDEFI
NNKNVQNTLNAGNYIGLMNILIQLRKVCNHCDLFTNKYIQTPFYYNLPINFYIPRFFILF
NKNYYLDFYLIIFLHNEFTSMGGKCVNKKKKEEDKEKNEVPNINNCEQTSF-CDNFQNTN
IILNDSCNESNINL--
--SCNFQHVQNNN---------
--------------------IFDNNSQIMQNSSNFTNFQNIQSNNNIDNFQKMN------
-----------------------------NFNIDNFQNMHNINN----------------
--------------------------INNFQGTNN-------------------------

----------------------------------ISNCNN--------------------
-------YYNTQKN--
-------NFDNFPNSSSNKNFCNFQPNNFFISKDLQNENYIN------NDSMIKNNIPHS
YLFLTNINNSDISGNLNTFNEKENISVNFKNTSEN---FITHILEFTNKNDFQEVYNLTS
YCFNFLNDNLTDSLKQQIDEMKMKIEIEMMNGGGDENISISDSSSQSEASKHENDSKDIE
EMVLYIIGLGLGDEKDITVKGKELIEKSDIVYLESYTSILFISKEDLEKFYKKKIYEVDR
NFAEENCEEILNEAKNKSVSFLVVGDPLCATTHHDIILRAKKENIDVHIIHNTSIISAIG
ESGMQLYNFGQIVSIPFFEKDYKPTSFYDKIKINLDNNFHTLCLLDIKIKERTIENIMKN
RNIFEPPRYMTINEAIDQLLYCEDIHKKNVITKNTLGIAMVRIGSNNQQIISGNLLTLKS
LNYDKPLHSLIICASTLHDIEKEYFNIYLHKN----------------------------
-----------------------------------PNSAYRKCVRVQLIKNGKKITAFVP
GDGCLNFIDENDEVLVSGFGRSGHSVGDLPGVKFKVVKVARVSLLALFKEKKEKPRSMGI
KGLTKFIADAAPNAIKEIKIENLMGRIIAIDASMSLYQFIIAIRDSDQYGNLTNESGETT
SHISGLMSRSIKLMENGLKPIYVFDGAPPELKGSELEKRGEKRQKAEELLQKAKEEGNLE
EIKKQSGRTVRVTKKQNEEAKKLLTLMGIPVVEAPCEAESQCAFLTKYNIAHATATEDAD
ALVFGTKILIRNLNANASSNQNKNKNSSKRGYILTEINLQQVLEGLNLTMNQFIDFCILC
GCDYCDTIKGIGSKTAYNLIKEYNCIENIIQNIDKNKYQIPANFRFEEARNSFLNPKVLT
KEEIKIDWNEPKIEELKDLLIKEYNFNEVRVTNYINRLLKARKATTQRRLDNFFTACTKK
STKLVIEESQKE-LKKKLKGTKRDITN-INTKLNKKQKTENIKKESEKNEQIKKEKYEIS
KEPEE---KSQENNDSQDKNSLDYQTNSESGNLKNDNKEHSLNINNICDNIKKAFLLPFC
PKNVTKRKLVQRC---------------------VNNSKYYEVLNLKKNCTTEEVKKAYR
KLAIIHHPDKGGDPEKFKEISRAYEVLADEEKRKLYDEYGEEGLENGEQPADATDLFDFI
LNAGKGKKKRGEDIVSEVKVTLEQLYNGATKKLAISKDVICSNCEGHGGPKDAKVDCKQC
NGRGTKTYMRYHSSVLHQTEVTCNGCRGKGKIFNEKDKCTNCKGGCVLKTRKIIEVYIPK
GAPNKHKIVFNGEADEKPNVITGNLVVVLNEKQHPVFRREGVDLFMNYKISLYQSLTGFV
AEVTHLDERKILVNCTNTGFIRHGDIREIKEEGMPTYKDPFKKGNLYITFEVEYPMDLVI
TNEKKEVLKFLKKQNESEKNYDLENSEFEVVTCQAVDKEYLKQRVSKQQQQEAYDDDDHQ
PEMEGGRVACAQQAYCCFENSNSYMEWKPNEKIYKTIIEALTSCNSSNNTVQTEVTKVLK
DLNENVSDAALYLLHIFMNKEEKNDVRQVGGLLLKNYINSKNKFLTNDILKIIKNEIFKL
VEDEVKEIRNTSGSVITTILTKYEGIEKWPEALYNLLVLIDRGNNDVVDGAFRAIIIIIE
DELMNRKSTDSLFFQFCKTQLLQKLFSFCSPQEKSIKKKYAAECLDLFITSSCFTLNGVF
DDYFPQLWECLGYLASEEDTQILKIVVTCMTIITDTRYSSIFSNLDAIIQFMVNATNSND
RKVQLEALEFWPVFIKDRSYIAYSNCNNNKNDVNK-SENYIDENVYKNINDLRNEALKTL
KNYLPYLCKILVDNTVYTKWDYLTMDESHFQNDNANVPDLIQDITPELYNN--KNNDINQ
DEIKMNKLNNNDNNS-------NNNMNDTS------DI-NNENLDDYSDDEKNDDMTART
WGNDWTVRKGAALCLDYLSNVYNDDILEYILPHIEEKLMSDKWNIRESAVLTLGAIAKGC
MYSLSPFIPKVLEYLIKLLNDEKPLARSISCWCVTRFSSWICHPDNCDKWFEPVLLNLLK
RILDSNKRVQEAACSSFANLEEDALELLNNYLHEIVHTIQQAFQIYQAKNYFILFDVVGT
LIDSVNIVKENNELAHQIVNSILSKWNNIRISSPYIIALMECMSCITSAYGKEFLKYSQN
VIRTCIKFLVLLYIDLEEEIKYYYSKKSGNSISYIVNRNNISPTASELLSYYKITNEEYF
TSIKDIDSISPSKKKDLIECSFDLLSRILSVVNSNIMNIISENEYNFIPLVHRYCLKLEN
IKFDGMNK---INHDFKIL--NCDTSLKNGIIGKEESTELAKQFLNFGILQSNFALIGDI
SRFCTQYLISYLNDIIPFLIAHITHPSTPVSNNASWAIGEISIHINSQYMEMYVDEIIKQ
LIFICQNSKYHGCLLQNICITLGRLSSTYPKKIIFYFPQFLKTWLKIMSHGTQENEKINA
LKAVLDTLYLNLDIAAENLKDIVYIILKYKYVSQNLNIFFHQFLSTMKQKYPNQWKEIYQ
PTNDSHSSPIPNDMLNDLNVRFNL------------------------------------

------------FILWLDVKENYLFNFPSSCRIVPCKTHVLGFTQKAYCNRMSVMNKNVF
DSINQYAQSKNVLIFVSSRRQTRLTAYDIISLLHVENLLNDKNHINSEQDKTDIGGLLFQ
NYLNVIEDKHLKELLKYGIGIHHAGLNENDKSIVEYFFLNKIIQILICTSTLAWGINLPA
YLVIIKGNEYYDAKTKKYKDISYTDLLQMIGRAGRPQFDDKALAILLVQEKKKNAIKNFL
YHPMNIESNILENLNEFINAEICSNVINNKEDMFNYLTKSYYFKRLFSNPSYYIKDIQYV
QLYENNKLSSQAKKTIYDHLNQIIDNTINFLVENNCINVIQEDYDSKYYSTPLGHIASMY
YIKCETAYFFYKSIENLK-KVLDFYDLFELIAQAKEFKDIPLRHNEEKYNVKLRNQIPLD
IDMNMPNIKTYLLLLSCLYECSFESVDYNIDLKLVLDQIARVINGFIDICLLFHKYSYIK
NIILIFQCINQKIIPTKNSLYTIKNINEYQIKKLRELNIYNIKELIKFDKEFIYSMNIFD

KNQLDYILQIPVFNVNIKLYDKNTE---EKDKKYLSNIQFMTYIKNENKFCYKMNYYESN
KIVIKIFFNFFKKVEKNINSSSSIGNIQW---------------------------DLTI
KIFLFFFIFYYFLFFFIFYYFLLF-----------------FFIGLLKCLIVNKLTSNGC
KFIFWIADWFAQLNNKMSGDLKKIRKMGNYFIEVWKSCGMNMENVEFLWASDEINKKPND
YWSLVIDISRSFNINRIKRCLKIMGRSEGEDNYCSQILYPCMQCADIFFLNVDICQLGID
QRKVNMLAREYCDIKKMKKKPIILSHEMLPGLLEGQEKMSKSDENSAIFMDDLEADVNRK
IKKAYCPPNIIENNPIFSYAKSIIYPYYNKFHLVRKEKNGGLDKNKL-------------

-------------------------------VKMFVKEEIIDTEKLSDIINKKKENVKYM
KGMKIPDNIVAVSDLNEATEEADLLVFVLPHQYLN--------------------VIKKI
K---

------ETFLDSCGLADLITTCLGGRNLKCAKEFAIRKGKTTWEKIEEELLNGQKLQ-VF
TLK--MEKVLSS
IGKLSVVAGGLSLIPYTFIYDVDGGERCVMFNRFGGVSEKTYGEGSHFYIPWFQTPYIYD
IKMKPKVINTTTGTRDLQIVTISLRLLFRPHTQHLPYLHSTLGPDYDERVLPSIGNEVLK
AVVAKYNAESLLTQRDKISKEIRESITARAKHFNIMLDDVAITHLSYGKEFAKAIEDKQV
AQQESERVKFIVAKTEQEKIAAVIKAQGEAEAAKLISTAVKEYGNSLLEIRKLEAAKEIA
ENLSKSKNITYFPSSSNILLNTKNLMKLEYINVLKEENGGYNFENLNRNEILKEKGITFP
QFRKTGTTICGLVCQNAVILGADTRATEGPIVADKNCSKLHYISKNIYCAGAGVAGDLEH
TTLWLQHNVELHRLNTNSQPRVAMCVSRLTQELFKYQGYKVCAIVLGGVDVTGPQLYGIH
PHGSSCLLPFTALGSGSLNAMAVLEAKYRDNMTIEEGKNLVCEAICAGIFNDLGSGGNVD
ICVITKDGTQHIRSYKQPNVRLYHLPHPTIYPKGTTPVLCEKIENIKKYITVEDAIKNNK
NEMYNFNPIKIEPFGKPPVGYIPGKGRGVTGFSGGVSRDDTTDDKDKNDYSDFNYDEFHG
YSESLFKDTEYDEEDKEADEIYENIDARMDIRRKSRRENKLKEEISKMRAQKPTIQEQFS
DLKKNLANVTLEEWESIPSVFQYSKQRQKKAPKNYLPPPDSLIMSRINESNMHLNYNNSS
SSGLGLQTPLGLRTPLGAQTPIGLGFQTPFMKSSTGYETPMYNRNPKNNMAYSGLNTPFT
LSGYNTPLNASNVSGYNTPLLNNVNKLSLNDLGEARGTVLSVKLDELIDNVEGQTVIDPK
GYLTNLNAKSLINDADVADINKARSLLKSVISTNPKHGPGWIAAARVEELSQRKDKAKEI
IMKGCIECSKNEDVWLEAVRLEDKISEAKIIIAKAIKHIPTSVKLWLEAYKKEKNIDDKR
KVLRKAIECIPNSVRLWKEAISLENENNAYILLKRAVECIPQCIEMWIALARLCNYNEAQ
KVLNEARKKIPTSAEIWINASKLEEKQGNINMVDTIIKRCIENLSSKNVIFERDKWIKFA
EECEKSNFPHTCESIIRNTMNIGVETLNKKSIYKQDAQNCINNKSFHTARILYNEALKIF
KTKKSLWLALANLELNYGKKESVDEVLQRAVHNCPHASVLWLMLAKQKWLNNEIDKAREI
LAESFMHNQNTEVISLAAIKLERENNEFDRARFLLKKSRVQCNTPKIWMQSVQLERLLRN
YKEAKYLVYEALKIHKYFDKLYMIAGQIELEYDKFNKENVEDPYINAQKIYEQGLKYCPS
SINLWICAIDLQIEKKNYTSARALVEKAKIKIKYMHSLNNNNHVLKNKEIIETSDLAFEE
ELSKNFDDIKN-PNSNSNISNNKNDLEKNSTRSASVKVIENYDLLWLKLIEIELCCNNKN
INPFISEALKECPSSGILWSKAIELENKNLQNSKSVSAFNNCGNNAYVVLTVAKLFWFNF
KIHKARKWFYRVITLNPYFGDGWATFLAFEIDQQNEINQKDIINKCIKAEPNRGII---I
YMHIYTF-LSIHISMYIYICFFFFFFIF--------------------FRLYVE------

--------------------------------------NYGYLNVINMKLN--DIKLYIL
ETLYNSLL-NECTEEFKREPIEKKRNEEDILKNNIWIRGRIHDIRGKGSLSFIILRHKIY
SLQCILDINNNNNDKNMIKWVNNLSLESIVDIYGKLIKPEIPIDSTNIKYEIHINKIFCI
SKTVKELPFLLKDANMKETSEESTIKVNQDNRLNNRCIDLRTYANFSIFYLQSQICQIFR
NFLIQNNFIEIHTPKLLGESSEGGANAFQINYFNQNGYLAQSPQLYKQMCINSGFDRVFE
IGPVFRAENSNTYRHLCEYISLDIEMTYKYDYMENVYFYDSLFKHIFKELTTLGKNDMFI
KTIKNQYPSEDFKWLDVTPIFTYEEAIRLLIKHNKLDLKEEDILNYDLTTDMEKELGKII
KDSHNTHYYIIINFPSSLRPFYTMYDEKNPKISNSYDFFMRGEEVLSGAQRINDVNLLLN
NIKSLNLDSQKLNFYIDSFSYSSYPHSGCGIGLERVLMLFLGLNNIRKTSLFPRDPKRLI
PMEITNIFKKLL-RQNVVYTESNSLFSIFKKQNEETLKEK--TE---ETPNV----ESLS
ELKNDSLKKTEEEKLKEQNFLPYTNNFDSSNALLFENLNKEYKFITTQDNFDGFRFEVDK
NVNKYLQSTHTLFLGTSLRDIGYLYQFGANFTNSDNSLLMISRINIDGSVNGRFCKKINP
NIDCKLNFNTFAKSDTRNMYEMSLEVNKPVYTYNIKTIWQGAWIFNASYTQLLTKKFQAG
VDLTYIASNCASIGSFGLRYNHKNNVITMQVIRQPNFKSPEFMLNQTHLYKIQYAKKISD
RLSLGTELELTPQTKESAMRLGWDYSFRHAKVQGTIDTSGKISVFTQDYSGFGVSGYIDY
LNNEYKFGFMMHISPSQEQPQQQP----------------NTSIRIYIYIYIFVYIYFFF
FFLATLGVEVHPLKFQTNFGKTQFNVWDTAGQEKFGGLRDGYYIKSDCAIIMFDVSSRIT
YKNVPNWYRDITRVCETIPMVLVGNKVDVKDRQVKSRQIQFHRKRNLQYYDLSARSNYNF
EKPFLWLARRLSNQPNLVFVGEHAKAPEFQIDLNVVREAEKELEQAAAVAIDEEDIEN--
------------------------------------QNNVGIITVKSIFKEPTISQYNDI
KQLIKTKIEENCPFYNYQINRTIAEKIYGDTIYDNYGLSKEINEVNLIILEEWNINCNRN
RVLKHSGLIKNIEINKFKYLNNKESLEVHFLVNPKYTFEELNTIYKNEEELNNFLLSPII
KVTNKKIYEIE--DKKSEFSYLYEEDILPKNKVLPPSGIENVNYESSKVVTPWDVNIGEE
GINYNKLIKEFGCSKISDEHIRKIEKLTNRKAHHFIRRGIFFSHRDLDFLLNYYEQNGYF
YIYTGRGPSSLSMHLGHLIPFYFCKYLQDAFNVPLIIQLSDDEKFLFNQNYSLDDINRFT
KENVKDIIAVGFNPELTFIFKNTEYANHLYPTVLAIHKKTTLNQSMNVFGFNNSDNIGKI
SYPSFQIAPCFSQCFPNFLKKNIPCLVPQGIDQDPYFRLSRDIAVKLALYKPVVIHSVFM
PGLQGVNTKMSSTKKKDNNMDSKQDINNSVIFLTDSPEQIKNKINKYAFSGGGATIAEHK
EKGADLEKDISYQYLRYFLVDDEKLNEIGEKYKKGEMLSGEIKKILIDILTDLVQKHQEK
RNSLTDEDILYFFNDNKSSLKKFKDMMIASNDISNKHKIEITKLTKDEMSFILYNSNSGL
ANALRRIILSEIPTLAIDVVNVYENTSPFHDEYLAHRLGLIPIDSRNVNNYEFREKCKCK
ETCSKCTIQYVIEVKCNNANKIDISHYDIESLDHEPNIPMPIPHGKKNTK--RENAIPIV
TLSKNQTLHMKLIATKGIGKMHAKWIPANVSYRIDHKIKIKHHLIDKLPQSHKLMLANNL
NKDCYVLKNIDEDRDVQLKLNENMSVVMAENCIEILNELGYKDVIKIIYDDTKFHFHIES
VGSLPPEQIVEMALEVLENKLKNLEPQIKSSFYSIDEVAKQLKEQGVSLYGIQLDLE---

----------------------------RKKYFTRSSVSKKREEKCFLNLLKKSILKNPK
KWTNISKKIIGVSEETTTGVLRLKKMDKNKELLFPAINVNDSVTKQKYDNVYGCRHSLPD
GLMRSTDFLISGKIVVICGYGDVGKGCASSMKGLGARVYVTEIDPICAIQAIMEGFNVVT
LDEIVDKGDFFITCTGNVDVIKLEHLLKMKNNAVVGNIGHFDDEIQVNELFNYEGVHIEN
VKPQVDRVTLPNGNKIIVLAKGRLLNLGCATGHPAFVMSFSFCNQVFAQLNLWENRNNSK
YENKVYLLPKELDEKVALYHLKKLNASLTELDDNQCEFLGVSKNGPYKSNEYRYMYTYGV
EGDDGYLQQAQYPSPYENQYQQDEESPSPRGESHTPFIGYFSSHLLRTGFFLQCVSLILM
FIFYWAFGGTGIFVFDLYAGPECVKVSSAFHLTISILMAIYLLGTLYIAMFQVFVADNSK
WCRGFRAGSKLLSAAVTLDLLSSILRLVQYLYAYFYMNMRWWARYQQTKSDWTLLHFGSI
VHSFALFIYGAAFFYMEAYHDEGTYEELAWSNLTLFKLAGLAGIF---------------

--ADANKPKKRTFRTFQYRGVDLDKLLDISQDELIKLFRARQRRKFKRGISKKAKSLLKK
LRKAKKECEPGEKPKPIPTHLRNMTIIPEMVGSIVAVHNGKQYTNVEIKPEMIGYYLGEF
SITYKHTRHGKPGIGATHSSRFIPLK---------------------VHIGTKNLENKMK
RYVYTRAKDGVHIINLAKTYEKLQLAARIIVAISNPADVVVVSARPFGSRAVLKFAQYTG
AQAIAGRWTPGMLTNQIIQKFTEPRLLIVTDPRTDAQPVKESSYANIPVIALCDSDSPLE
HVDIAIPCNNKGKESIALMYWLLAQEVLYLKGTLKRSEPWNVMVDMFLWRDPEQFELKNL
ANEENAATAPHLVENQFATEAPYEEWNKKEEWNDNTNEDWKNPITADEW-----------

---------------GFLQIIKDHHETMYELKQKIRPRDQVVGWFCSGSELSELSCAVHG
WFKEHNSISKFYPHSPLNEPIHLLVDAALESGFLNIKAFVQLPISLVKEYFVHFHEIQTE
LLPCNVERAEVLPYKEKLTVNKDKDNNNNANNAEIITNEMNEISLKKLLIMLKQCKSYVQ
DVIDKKKKGNLSVGRYLHKVLSNDTFLSLEKFDSLNENILQDNLMISYLSNLANLQFLIA
EKLNASSLQC--


```
------------------------------------------------------------
------------------------------------------------------------
------------------------------------------------------------
------------------------------------------------------------
------------------------------------------------------------
------------------------------------------------------------
--------------------------------RSC-------------------------
------------------------------------------------------------
------------------------------------------------------------
------------------------------------------------------------
------------------------------------------------------------
----------------------------------------FRRFCFNAPL----------
------------------------------------------------------------
------------------------------------------------ESFIMPEELIKI
WVKNFPIKKNYPKGRGVFKNLIGLVSQNLPLFFGKGGLKPSKKIEIFFTIFETDFLDRGC
KKKIFSQKNFLDQFFLKNLANFPLTHKQGKKLDFFFNTPRKMPTISVYEEELIEKIGENI
SETKLKDLCFDFGLEIDEVEYKNDKKIYKIEVPANRYDLICVEGLGRALKNFLGNYERIK
YELLINDKDLCLKENQIIRVHESVDERRGYVVSCALKNMKINENVYNNIIELQEKLHHNI
GKKRSILAIGIHDYDKIKFPLEYKFEEKEKINFTPLNEKNNLNGSNLLNFYENNLNLKPY
LKIIKDFERYPIIVDADKKIISLPPIINCDHTKITLNTKNIFIECTAIDKNKAEIALNIL
CSMLSEYCTPKYSIYSFLVKYDEKHELEKGSSYLYPLFQKKMLKCDIDYVRKLSGISDIT
IDSIEKLLKKMMIS-INVIDEFCFNIEVPFYRSDIMHCCDIIEDIAIAYGYGNILPQNSK
FSKKNILNTCSDLFKNALVECGYTEVMTNTLVSKKENYDYMLRNHMSYDTSEINMDEYNP
LAPPVQILNSKTSEYEIVRTSLIVNLLKFVSTNKHRELPLRFFEIGDVAYTSYNMTDTNA
VNKRHLSVIFADKFTAGLEELHGILEAILKEFNLFSDYKIEEKKKENIYVRSDIFYKLIP
KE---------------------GIYIYIYIYIYIYIYIYIYIYIY------IYIYIYIM
GDNNTNSNNNSVISIGNIRGFGGGDYGSFRMSNEFLGWKNKKTNNVYQYKFSDISEGEWI
KTSYNNNRLHLKLIESKDNLIVYFDGFPDRNINEITQHFQKYFNIRLSNRKITTKGWNWG
EFKFENSNLLFDIDKKYAFNIPTNNINQLNVQIKTDIAMELKNEE-NQTNEDSLAEIRFY
YPHENDENQTFQNLKNDLLEKVNIGDSKSESIASLTNIPLLVPRGRYEIEMYIRTFKLHG
KSYDFTIQYTNINKMILVPKSNSNQYVLIFSLNNKMKQGQTEYPFILIQLNNDDDMDINI
NANEEIIKKYKLEKTISGKPYDVVTRLFTSLVKKSVIIPGDFRTAKNQYGITCSYRAASG
QLYPLNKYFLFIIKPVILISFEDIVTLSFQRTGNINQHRFFSLIIKHKRGISYEYTNIDK
NEYVPLLNFLKSKNLNIQDDANNTENKQDFRDELSESDEEEYV--EDEDEEDYVAEEEEE
EEEEEEEEEEEEEEEEEEDDEN--------------FF----------------------
-----------LGMILMSELSKRRFRSVNKLIRVGRHEVVLVLRVDSQKGYIDLSKRRVS
PKDIIKCEEKFSKSKKVHQTVRHVAQKHGMTVEELNRRAIWPLYKKYDHALDALKEATMN
PEGVFKGLDIDEEIKKSLLADIQLRLTPQALKLRGRIDVWCFSYEGIDAVKEALKKGKEI
SNEEVSINIKLIAPPQYVIVTSCHDKELGMAKIQEAMKVISDKIKEYKGGDFKQQGDILV
IGGDEEKRLEELLDKHDELSSEEEDNSSEEDEENSSEEDDNSSEEGEDEE----------
--------------------------------------------GGH-------------
------------------------------------------------------------
------------------------------------------------------------
------------------------------------------------------------
------------------------------------------------------------
------------------------------------------------------------
------------------------------------------------------------
------------FGNKRGISPLPFFSNLPKQNFLKPPPLGGMFIFFLNQKSGFSQISFFG
GAGPNF-F----------------------------------------------------
---------------------------------------------LCMFNFSLPGFPLTS
LNKKNIINDSNELIYEQDVTYLGKKKKKKIIEHSETVLLSGERSLFEFFLCDVGVGLSLA
VSENEIAAYNRDTLPIEYDSIFIKKKKDKTLDDSLTIHYKCKKNENNNNEINLVLGGDTM
ATYGVLPYYTFHHEYIIYDNSQLLPRYLIQFECDPSADEHFAIPLCDYCQDAPSAFYCES
DEVRLCEKCDNIIHSQNKLVMKHIRKSLNEAQKIPGKCKIHLHNDINMFCTVCHVPICEL
CMSIHSHTDLSSENLININYSSDTIMSLNMAYKAIIQYSSKPSKLIKERKRNLNDLLNKI
EKLYEQVKLNIHDAEENVYTILEDLVKQLHMITEKKMCSVLSEEYELKRQFNEIIWNESF
LHYLQTILPPADFMNAWLKHCKRREEIEKNSLIVEKIYSLVFPDMRIKGNINIVTEGSIE
HENLFHSTENL----------MMIISFRPKNTLKMGLVGLPNVGKSTTFNVLTKLNIPAE
NYPFCTIDPHEAKVTVEDDRFDWLVNHFKPKSSVHAYLSIFDIAGLVKNAHLGEGLGNNF
LSNIAAVDGIYHVVRAFENEDIIHTEGNINPVRDMEIINSELIYKDISHCEKNLEEINKV
LNRNKKDKIKQNEHDVLTGVLKHLKENKWIKDGNWKNSEVEVINDFNFLTAKPVVYLVNM
SEEDFIRQKNKYLAKIYNWVQENNKGTIIPYCAEFEQKILNMTEEEKIDYFKQKNIKQSM
LNKIIKTGYYEINLIHFFTCGQDEVKCWTIRKGTKAPQAAGVIHTDFEKGFICAEVYKYT
DLVEHKSESEVKANGKYLQKGKDYIVEDGDIVFFKFNVSSSGKKMGEYNLVQDNVVYSKL
DTIECDIPINEELLERINKFVDHLRLPYYLLEEFVDNFVYELKKGLEAHRKHPNLWIPHE
CSFKMLDSCISEIPSGKEKGTYYAIDFGGTNFRAVRASLDGNGKIKRDQETYSLKFTGTF
SHEKGLLDKNATASQLFDHFAERIKYIMGEFNDLDSKEVKSVGFTFSFPCTSPSINCAIL
IDWTKGFETGRATNDPVEGRDVCKLMNEAFTRSSVPAKVCCVVNDAVGTLMSCAYQKGKS
TPPCYIGIILGTGSNGCYYEPEWKKYKYSGKIINIEFGNFDKDLPLAPIDLVMDWYSSNR
SRQLFEKMISGAYLGEMVRRYMVNVLQSASSKKMWITDTFNSESGSVVLNDTSKNFEETR
KVAKDAWDIDLTDEQVYALRKICEAVYNRSAGLAAGAIAAIAKRIKIIEHSKFSCGVDGS
LFVKNAWYCKRLQEHLKVILADKSENLIIIPADDGSGKGAAITAAVVASCSNV----MPN
LYFVIRWLCKAIVRSLFRDVNVINPENVPLYGSVIFVGNHNNQFIDACVLVANIPRQVKF
IVAEKSMKRAVIGKLASIIGCISVKRPQDLKFKGIGNIFWEIGDIRIKGVNTRFRLDVQI
GDKLMTQNKIFCVTKIESETELLIQEAINIECDDKVNGVPFKIIPKVNQSDVYNLVTNSL
KNGDTIGIFPEGGSHDRTNLLPLKPGVAIMTLCALADGIEDVSIIPVGLSYSKLYQLQGC
VTLFYGNAIMISQDLCKDYNNNHRETISKLLTKIEEGMRSCMLTSKDHETSRCIELCVSL
YTPERMTISKNKIYNNLQLFCKMFWKFGNTKVIKNLCYQLQCYEKLLQANKIKDDEVWML
KQSTSAATLKFIEHICSVIFCVIFGMTFSLLWLPLVLISIHLAEKHREDALKNSTVKIQG
CDVVSSYKVLVLIVLLPTFNLFYGLIFSLYLYETWIKRIIFTFLSICILPICYYINLNYS
VQIPTLLRQMKILLKVICGKINVWRDNERELISTRHELQLKVRELVSTLGPNVSDDFLEQ
LYRNIPKFVVDADTKRLIRGKDEWVPILQRSQLEYKEEIL--------------------
------------------------------------------------------------
------------------------------------------------------------
------------------------------------------------------------
-------------------------------------------------FKNLRKMKKKD
IDGLHNLLNNYLEQFKLHAVFSKEDIAHWFLPIDNVIYTYVNEENGEIKDLISFYSLPSQ
ILGNEKYNILNAAYSFYNIATTTSIKNLMQDAIYLAKKNSFDVFNALEIMHNKSIFEDLK
FGEGDGSLKYYLYNWKCASFDPSNVGIVLL------------------------------
--------------------------------------------------------MDID
SVLGLQAILISANYKEKEFIRIAYYMNAFYKDIELREKPPLIPQYDKICRHIFVENPRIV
KFNIGWDSGEKDEFKEFDKENEKIEFFKCTKVKDENECSSNITNQSTQENFNISN-----
--NNNGNSYISSNFNINNVNNYNQNNFLKNNEQSNDLDKCEIFNANLNGISRITIDNKNN
MKIPIFSFFFTLIFFGIFLKAKKFDVFLSYHKRISKKINEILLYFNIFEVQYPLSYINNE
KILGLHMKDIEKDAYKVYPILKELKQKDYFRIFKVNLHISCKFLKISEKCKEMKKCSVCE
CDDDKIPYNFRTNEIEIIEDKLSNEDLKKTFTESKLYNDILGIYAPSNEGFLSYVDLVYN
SPCFTAYEGKNIWLYKF-------------------------------------------
------------------------------------------------------------
------------------------------------------------------------
--------------------------------------------LFIYIHVKIYIIKYTS
YIHLY--------------------------------------L----------------
------NVCFLYFYVYVTLIFYLNVIILIYDFILFFYY----------------------
----------------------------VFFYIIGSELKGNNNYKYFGAAKNLKGVKELL
FRENEERKKLALKKKKDKRNLDKFVNIHYFGYCDDENEILLNEELKIQKKLEKNDLKILK
KINNN-------TMKHGTLVLPSDRAREYLDCLGKEVDIQFVDMNEKTMKRQYKKYIQRI
DDMERILRFLEENINKLPNVKIKKSKIDNFLEHDNIYELDQVEESLNRLYVQFVRFCNNN
```

KDLVDERNNAIEEKHVILAAINQLKPDTSKNGNINMNIHENSI-------NVDDSNEENI
SLSTHIMKDGINMMFTNISGVIKTKDQESFSRTIFRALRGNAYTYFQNIDDEMEESVSIN
E---NESLNPLLNKENEKNNKKKKNELKSVFVVYCQGSAQSSIYEKIMKICKAYDVKTYD
WPKTYEQSKKRLRELKEIINDKDKALKAYEEYFINEIFVLINVVEPNKNSLIEEWKLFCK
KERHIYNNLNYFEGSDITLRCDCWYSASDEEKIRHILINKSSNDLVSALLLSDKVLTPNI
SPPTYIKTNEFTKSYQVIIDTYGVPRYGEINPAISTIITFPFLFGIMYGDVGHGFCIFLF
AIFMILIHNKVKNKKKNEMIAMFFDGRYMLLLMGFFSVYAGFLYNDFFSMPLNLFSSMFE
KNNEIDNIKYYKRKEIV-TKDGKTEFRHPYIFGFDSNWLGAENELIFINSFKMKFSVIIG
FLHMTFGTIIKGFNTLYFNKKLDFFFEFIPQLVMMICIIGYLVFLIIYKWVTPAGYGGYE
KQGIINTIINMYLHKDIDKTNQFYSHQSIIETLLLSLFIISIPVMLICKPAIRTYKIMKD
KKKNLSRYNENLEKEMTNTFNHNYK----SNHIESLSMHKRIGNHPYEDNENDY-LFKKK
NKKTDHEMEAHLLSSSPDHISENVC-------HEENISEIWIEQLIETIEFVLGLISNTA
SYLRLWALSLAHQQLSYVFFEQTILNSLKQDKFITVLISLIIFVQLFSILTILIILCMDT
LECFLHSLRLQWVEFQNKFYKGDGIPFRPFNIKKILSENDMIYI----------------

-NNKLYFENNEISATPRSERKMKKPENYSAILARALSERPLTYLPTIERVCYEVCETANI
LNDEDEHLNYIQINLLNTFIRPTPIRGLLAATQERFVVVPGIIVQASKPQHKMRKITLQC
RYCDHKMSIDVPLWKDKPQLPPYCRYSSTMKSSMGAANPMDSQLGCNGVLEPYVILPNEC
TFVDIQSLKMQELPEAVPTGDMPRHLQLNATRYLCEKMIPGDRVYVHGVLTSYNPNPKPT
RADGTNFSYLHVLGFQKYDDMTGNDLNFDVEERNELTLLAAEHDIHEKIFKSVAPELYGM
DEVKKACACLLFGGTRKRIGEETKIRGDINMLMLGDPSVAKSQILKFVNRCAPVSVYTSG
KGSSAAGLTAAVMRDSQGVFSLEGGAMVLADGGVVCIDEFDKMRDDDVVAIHEAMEQQTI
SISKAGITTMLNTRCAVIAAANPSFGSYDDSQDTTDQHDFKTTILSRFDIIFLLRNKQDV
EKDTLLCNHIVALHASKHKSQEGEIPLSKLTRYIQYAKREIAPLLSKEARDSLRNFYVQT
RAEYRGDRRSVTKKIPITLRQLESLIRLAERYI---------------------------
---CTII--------------------------------ILYNIKLHL--------FFIN
YIFLFFIFFYYLYKFSV----CEIHLYKFIFFFISCSYFRVQYLLAKIRSAARYLLTLDE
KSPKRIFQGEALLRRMVRQGLLGENEEKLDYVLGLTLPKLLERRLQTKVFKLGLAKSVHH
ARVLIRQRHIRVGKQMVDIPSFLVRVDSEKHIDFATTSPFGGAKPGRVKRKTLRNQKEKN
ETEENI---HNIFLFYMKDFPHFFIFFFSSEYAIKAVKNTNITSIGVKGENCAVIISQKK
MATQYISQDKLLDYNNITNIYNITDEIGCSMVGMPGDCLSMVYKARSEASEFLYLNGYNV
NVETLCRNICDKIQVYTQHAYMRLHACSKICTYV----------------------CI--

-------------------------MDNNENIDSNDNSSDTNTVNYNNGKSENLSEGNDF
AKENNHLNGNAKNSKTKQENKTKNSSDKDDNN-DYNNHKFPCHPAPPVPIKLFIGRIPKN
VEEDQLRPIFEEYGIVNEVVIIRDKITNVHKSSAFVKMACIAEADNAIRSLNNQKTLDPQ
LGSLQVKYASGEVIKLGFPQNIESGVDQAKLFIGSLPKNITEDNIKEMFSPYGSVEEVFI
MKDNSTGLGKGCSFVKFSYKEQALYAINSLNGKKTLEGCTRPIEVRFAEPKSAKQPQIPL
TLQPMQNSTHGISPQPHINNPNNINYANNFSINNNYPRQIGPWKEYFSGEGRPYYYNEQT
NTTQWEMPKEFETLFMNSSPNMHNLSDSSGLKKKNIYLTF------KNVFIIVFYYFFFF
LFNRPSRCK-------FIYFSCS-------------------------------------
---VEKIK
NNIFFNAIFLNNLGGNRKVSQTVKVCSRDLPSHTKMKTRDLSDYVEDKTLIKNNLLKLEN
NNNNDTKT-NKLLAIENIKKYTNQEYTNPFPQDEDDEIDDDFKSYKKKDKKKKKNNNDTN
KKDTDNEKDEEYEDENDEDEYEDDDDDDDDDDDDDDDEEKELLRELENLKREKLEKLKKE
KEEQEIMKNKKNNVLTNNPLINLEDSDNEEKNVKKRKWTEEAIFRNTCERKEKKVSFIND
TVRSAFHKKFLFKYIHMDSEETINLAVKYAKDAVIEDEKKNYKEALNLYIQSLQYFHFFC
KYEKNANIRELILKKMEIYMARAESLKKIINKKEVIETKEKVGNSEETKENMKKQVKEFI
MNKNNNVKWSDVCGLELAKEVLKEAIIFPLKFPKLFNSSSLPYKGILLYGPPGTGKTFLA
LACANECSMNFFNVSSSDLISKYQGESEKYIKCLFETAKENSPSIIFIDEIDSLCGSRTD
GENESTRRIKTEFLINMSGLQNYENNIIVMGATNTPWSLDSGFRRRFEKRIYIPLPNLYA
RMKIFEKYIKNSESS-DIQKEDIKYFATLTENYTGADIDIICRDAVYMPIKKCLLSKFFK
KVNKNNKIYYTPCSPGDTDKTKVEKNVMNINENELLLPTLSVQDFKTAITNAKPSLSLDD
LKRYEEWTKQYGMSGTI---
-------------------YFF--------------------------------------
-----------------IGLYVKCGSRYEEINEKVNEQGMSVMIENMAFHSTAHLSHLRT
IKSLEKIGATVSCNAFREHIVYSCECLKEYLPIVINLLIGNVLFPRFLSWEMKNNVNRLN
LLREKLFENNELYITELLHNTAWYNNTLGNKLYVCESSIENYTSENLRNFMLKHFSPKNM
ALVGVNVDHDELTKWTARAFQDYVSIPFTNQKEVTPKYTGGFVSVEDKNVKKTNIAIAYE
TKGGWKSSDMITLTVLQTLMGGGGSFSTGGPGKGMYSRLFLNVLNNYNFIESCMAFSTQH
TDTGLFGLYFTGEPTNTVDIINAMALEFHKMNKVTEEELNRAKKSLKSFMWMSLEYKSIL
MEDLARQMMILNRILSGKQLCDAIDAITKEDIYKVVNKFLKSKPTVVVYGNINHSPHYDE
ICKILA-----------IYNFS--------------------------------------
--------FFFLFFLVNKIHFFMYARGSSNSRDRMTYASSKQALLKKIEGVNVLTSVIEG
VQDVADFKMQPFDY-RGMNNYYNNNNNN---NNTNYNNQNNVNKPINNPFSYNNNN----
---------EITNNNIQGNENIY-AQNYYQPQQL--QGIYAQNSNNLNENIYGDGTNQNS
YL-NQPQYRGIANQFIPNA-INNNFKQPSNVIGFDNTSNVNNVQPIINESYQELLQFNAF
SHFIKSSVSYMPANTTLKQKSYVPLGFVIQPLAPIPDGYPELSSVNFGNSTVVRCKKCRT
YINPFVRFESGGKKWNCNMCYHINDTPQFYFVPLDEKGKRKDLFQRPELCTGSVEFIAPS
DYMIRPPQPPVYLFLIDVTVTSVNSGLLDVICNTIKKLLPKNND---------MNNKKCF
DSRTLIGIITFDSTIHFYNLNSNLKQTQMMIVPDIQDIFIPLPEDILVNAHECQNIIDNL
LDNLPTMWRNNKVSDCCAGNALKAAFMVLKKIGGKLLFFLSSVPNIGELTVNLNRETKEK
SKYKNIYSSNNSGNNVVDAKLREVELLTPYNNLYAELAQNITQYQIAVDLFACPLYNLDL
ATIYPLIKNSGGSLYYYPQFNVHQYNEKLREELLFALTTETAWESVMRIRISRGWKITNW
YGNFQFRGVDLLALPNCHSSQSFSIIVDLEENVVQDSVVYVQSALLYTNSNGERRIRLHT
YALPVTQNIKTITDSINPQVVVSLLSHQAIDISKKGKIADGRNLIQTLCSQVLSSQLLPS
ESAKLLSIYILGMLKSVSFRDSGDVPSDLRIYHWSRIENIPVESREVYFYPRMFSLHNLE
KHHGNYDENNTVMLPDTLNLTCDNMTQDGCYIIEDGETITMWIGRYY-------------
--FEYKYLYNNL
------------------------------MEEESFKNRLLKRNIDIWIEKYRPEYLDEV
VGNPFVINTLKSIISSGNMPNLLLA-----------------------------------
-------------------------VIVFL------------------------------
------------------------------------YMENI-------------------

------MNIDPCIKE-KENEETNNILCEETETLVDSGIYDKALKDGLKKRSSSLKRDIKH
DLAFHKSLAVVNIAAGLDGCDDQLLPASFRALEADLDLHPSLLGYITLVQTLMLSLFSPI
WGFLSDKYSRKWMLVFGTALWGIATILLANINDFAHILFFRAINGLALGSIGPISQSILA
DAAKNESLGLSFGLVQLSSSTGRLIGGVITTTVALKYFGGIRGWRLCFIVVGILSILLSI
TVALFVDDAPRQIRKKEKVEYINDDIDAGTGGIRIINNNSHSYILYKNIVDILKDSLSKK
SIIIILLEGFTGTIPWLALSFNTMFFQYCGLTDLQAAVITGFLLVGAALGGVIGGLFGDV
MHDISNDHGRPFLGQLAMFGRVPLVILTYIVIPKRQESFELFALSCFCLGMSSIAGVAVN
RPIVSDIIRPDYRGTVFSLTIAIEGVGSSLIGAPLFGYLAENVFNYQNNNLLISDMSEDL
RKNNAEALSKTLLYLTLIPWILSFVFYSLLHFTYGKEYRKMNKIIENEYKYDDEDEETVA
EKLEKKRGSDPGVSTAVSSIMQHQNFKRMLMFGLRSLSDFCNPTSKIYKENAYDALDRGA
VGSIKNAVVNYKDDDDILFCSSKILCAMSDYCCSEKDQEALQKLITDGGVDAIVEIIKSS
PSDQETVKNCMSFIQNMNESNVHIEGGDLGIGLLNVFTSNSYNTKLGKEIANALSVAAKS
SSGAKALNDEGAHHKLIDQCLSFQTLNDDVASMVEGAFNTIKNMASNGYIDATIIEKSVL
ILDKFKSYPRVVSKGSDAMKCAVGPEQLTDCLNVLKKSEQGSKEQDSALELLSSLSYISS
ITDKVVQSGGIPVLIELINSGLQQYESNPEKISRLVAGASRMLGRISANPPHAAIVDEYG
GIATLCTALSYFPNDPECASAICNALTPFVSRSNYVSEINNYTLFASLLPILYGSLESIE
LAKASMECIASASMINEFHEHMVNNQAIEILSTCIQYHLTETDYLLNCLTAYFRLSDYIT
SIESINQYGGVTGIADALSAVSNDKKIAEIGLKLINKMLTASDALNYMSNQYVVDAVLTI
MLENENEEVIIQEGTKIMEKLATESDCQRHISNLESVINSAQSNPESAYKTLAAISGLSR
IESLRKILESKKADYSIFNGIKVWIEAPRFNDQTKLIKAGLKTIKILKLNSSSTIHEVIE
SIVDIMCSANVKRIIESEEPDENILITSVVCINDLTKVSKISSTEIVEANIDSILKLMKK

```
YSESRLTQTNLLSAINNILLSSDRIGADVLVNKGYIKQITNYLQKVPMYVDVQIIGFTVL
ANLIKINPESIEAIRKANALNPLQIALRTHVRNNKLKMTCAPLLALLMPLDSLFKEIEEL
LKLCDNSMKEKNIAKLHECLVSLNELLLTPEASKISSRCNIGEYLFNIEKWLKANPNAAD
SITNKDYDIIGRSLYDATISEIGHACINISQTRIGLVHLTKNKMASALIEAYDLLQLPGN
DYTEEAVSNILESLSLLLKHDIINADTAFNSGLVKKLCVGINYFAESDVVIKSTCGCLAC
MCTNGKRVNQLISHPEYEELISLIVKLIGNLEKNKHSRANAIKALYELLKTQKEEIIVHL
SNKTHVIDNLFKIMEEYQDYLPIVQESSKCLAIIADYVTLEEKMKIDKHSAIKILIESLN
KNKNDEETALEILRVLVKLCNANDKMQLKELGAIDVVSDVTMIHNDNEEISKLGGVLFSY
MGADEQVKKLMKLILNVKRDDPDSVQKIDNLTGKLEMFLRAPLENPQDALQYTEATLQVL
NEYLASEVDNVSLQANIALVTKRLVDRVKYDYEDQLGSWVVASAGTLNQYMDMISNKIGL
SNAKFVSPVYSVLAGCVLNP----------------------------------------
------------------------------------------------------------
------------------------------------------------------------
------------------------------------------------------------
------------------------------------------------------------
------------------------------------------------------------
------------------------------------------------------------
------------------------------------------------------------
------------------------------------------------------------
------------------------------------------------------------
------------------------------------------------------------
------------------------------------------------------------
------------------------------------------------------------
------------------------------------------------------------
------------------------------------------------------------
------------------------------------------------------------
------------------------------------------------------------
------------------------------------------------------------
------------------------------------------------------------
------------------------------------------------------------
------------------------------------------------------------
---------------------TRNNMDRKAREYAQDLLEFIRNSGSSFLACKNLREKLKK
NNFIHLKEGEKWNLKTNEAYVLSKENRNICSFFIGKNFNMENGSILISIGHIDSCSLKIS
PNNNIIKSKINQINVECYGAGMWNTWFDRGLGLSGQVVYKKEDKLIEKLIQINKSLFFLP
SLAIHLQKRTRFDFSPNINYEDHLKPILSTTLFDNMVKKDTMKNS--LKKNDSLSTEEKE
INSSPLLYILSKELNCKEEDILDFELCLIDAQDPCFTGAYEEFIEGPRFDNLVGSYCIFE
AFIELVNNIKNNEKNNN-YDNNLYICIGYDHEEIGSLSEVGAKSYFTKNIIERIIRCVFK
NELNNNETSIDEIYGSLVTRSILLSVDMSHCSHPNYPETIQNNHQLYFHEGIAIKYNTNK
NYVTSPFYASLVKRTFELFSEKNELIKHQNFMVKNDTPCGSTVGSMLAASLSMPGIDIGI
PQLAMHSIREIAGVHDIYYLIKGTLAFYTYYNKVLSCCEIDSMNVCKTVFNVFFVLFFIT
IYQIQCQLRFASLGDWGKESKSQLLNAKYLKQYIKNERVTFIVSPGSNFLDGVKGLNDPA
WKNLYEDVYAEESGNMYMPFFTVLGARDWTGNYTSEVLKGQGIYVEKDGETAIEKKGDAT
EYPKWIIPNYWDHYFTHFTGSTGPSIVKTGHKDMAAAFIFIDTWILSPNFPYKNIPERAW
DDLKFSINCLQKK---LQISGDQPIYSSGLSEGISYLAYYLLPLLKDAQVDLYIAGHDNN
MEVIEDNDITHVTCGSGAIASGKPAIKSSNSLFFSNDIGFCLHELNNNGIITKFISSKSG
EVIYTHKTNLKTKKGLDKLNSLQYVASLPKVELMDIPASGPMGNKDTFIKIVGTVGLLIG
SLIVFIGASSFLSKNMKT------------------------------------------
-------------LYIVN----FKFIFIFLFSSFSFYIRNEIKSLLLRGVWVVCDRYAYS
GVAYSAGALNLTKNWCMNPDQGLIKPDIVFYLDVPPNYAQNRSEYGYI------------
-----------------------------------------------K------------
-------------------------------------------VGQFIPSSGNKSFILSW
GIRESREISLQKGYINIQKIADHLHLSSQHIEAAQRIYLMALQRNFTMGRNNSYVAASCL
YTICRREKSPIMLIDFSDILQTPVKPLGKTFLKLLRLLHINVPNIDPSLFLERFAHKLNL
KNDIYKVTYTGIKLIQAMTRDWISTGRRPTGLCGAALLISTRIHGISINSNTIADIVRIS
NPTIIKRLSEFKNTNTAKIKASEFDKISITDIPSNSIPPCVIYDNKKKLKHNFIEKNKTL
SLCDSEEQDNRSRKSISYGETFSDDI---FSQKSNDENNLNTSNDNNLSHIDGPSFNNNN
TESEKEKNINHSNNGINLEEICNENPEGNDIDNLALKIINTINIENNPNILKNVNNNFSI
N---CD-YTSNLEKCEFKKMKSTTSMCSINKNSSNSSNLSLSRYYNETTLETNNIIESNN
NTVSSNILEQNNLDKEKQKIDSSYCTTLKETVSCELNSILNEVEDYNFLECNIQNKDRNN
K----KNMNNV-NILTDFNYFFENNSNSVNDQNTSFCGNSDTSIDQNDETLSDIYDSEIE
SMILTEKEKEVKMLIWDDMMKNYLPHICKQLKRQKKRSNEENNNEKK-KIRNKKKKVEDY
SETQSTGDSVLKALEKSDKNLPNKINYDVLKSLFSS-VEKRENFTNINSLDKMEDNKIDT
DLYSRQLGTYGYELMNKLIKLNILIINVKSVGLECAKNLILSGPKSVCIYDNDICEISDV
GVNFYISESNVHNKNCRSDAVLKHLQELNNYVHVYNFKGEIDKNMLEKFDVVVACDINNN
DIIKYNNLVRSIKTKKIAFLCCNIYGLCGYIFVDFNNEFVCYDQTGENVKSCNVSKISKE
SEGKVSFDFDKSAPFQNGDYVKFTDVEGMTEINNKIYKINNLQKYTFTIGDTSNFSNYIK
GGICTQLKIPLKLNFHSYEYISKYPLSKKVERK------EIIINDTIHEEIGIPDSFIVS
DYAKIDMSNQLHYAIQALKWYEAQNNNMLPENYEEEVFEKIYKYAVELNEKDKQQELNQS
VEELKKEIVYNVSRYCKSHIAPVASFFGGLLAQEVIKFTGKFMPIYQILYLDFFECIT-S
TEPDMNEIKKLNCKNDNIISIFGKSFQKKLNELNVFLVGSGALGCEYSKLFCLLDMCESN
NNGVLTITDNDNIEISNLNRQFLFRREHVGKSKSHIASEIIKNKNKNINVQSLETKICAE
NEHIFNESFWERQNIIVNALDNIQARQYVDNKCVWYSKPLFESGTLGTKGNVQIIIPFLT
QSYNDSYDPPEDSIPLCTLKHFPYDIVHTIEYARDIFQGLFYNTPLNIQQFLNNKKDYIQ
KIEEEGNNATLLETLQNVINTLKEVSNQCNFEFCIRKSVELFHTNFINQINQLLHSFPID
YKLSSGEYFWIGQKKPPQPISFDLNNEYVTEFLLSTSNLYAQVYNIPSCYDINFIKEVAN
KIEVKPFQPKSVKVNIDEKNLNNISFSHTSEAKLIEDYCEELLKIETSNIKVTPIEFDKD
EETNLHVNFIYSFANLRAKNYKIETCDKLKAKIVAGKIIPALATTTSMITGLVGIELLKY
VNYYDYIQKYVKLSEEERKKEKDILSYFKNAFINSALPLFLFSEPMPPLKTVDKEYDELM
KGPIRAIPNGFTSWDKIQISISNNK-KYMIIYILAKFIISIYINSFFFILLLLFF-----
-----------------------------------------------FFFLFSKLNQDLL
KKALADVFEGTRQKKRKFVETIELQIGLKDYDTQRDKRFSGTVKLSNEVRKKLKVCILGD
AVHVEEAQKLQLDYMDIEAMKKLNKDKTLVKKLAKKYDAFLASQVILPQIPKLLGPGLNK
AGKFPSLITHNDKINDKINELKSSIKFQLKKVLCMGVPVGHANLKEDELRSNIVHAINFL
VSLLKKNWQNIKTLHIKSTMGKPQRIYGF--------FKEKKWEDIDADDDLENNKKLSY
FTNYENGIKVVTKYSENLKKQTVKVIINISQ-----------------------------
-------------------------------NFLSIC-----------------------
-----------------------------------------------------------C
LIFILY---------------------MLNIFYR-----Y--------------------
------------IFFLIF------------------------------------------
------------------------------------------------------------
-------------------KIFLVYNIASFCFELQFRFIEDDTTFDWLPFLGYLLPYEKI
KLLRMLFPIVFFISICASAHIYTDKNATMIFLMRSILSTNRIIVERSNDVIESTNYLKKH
GTDIISKLQHIRKNELSSTLNLKDASKIGNENLEVNKYDTENSGKKSSKDEVIDSKLLDN
IQKFSLTLDSQQMNLLEKCFELMKNKSSKD-KNKKKSWFEFFSLQMIFGIIFVYIWLTSK
------------------------------------------------------------
-------------------------------------------SSNEYNFLYIYYNHFYI
FFFFFFFI-------FFIDINSNNYN-KPATYEVPLSLTLEELYSGCKKKLKITRKRFMG
TKSYEDDNFVTIDVKAGWKDGTKITFYGEGDQLSPMAQPGDLVFKVKTKAHDRFLRDSNH
LIYKCPVPLDKALTGFQFIVKSLDNRDINVRIDDIVTPKTRKIVANEGMPSSKHPGMKGD
LIVEFDIVFPKNLTSEKKRIIREVLANTFFKKKKKI-LLLFI------------------
-------------------------DVKCLINIKDQNGRVLYSHETSKITKGKISYLTNK
DGLYYICISCPSTNWFKSTSIKWTLSIDVGGTDIDLENLAKKSELSETLSILKSLKKKFN
SMKLQQGYQKHMVDTYTFINIVIEKNIY--------IFSYIYIFSYIYIFSYI------M
SKNKKGKKKDDLDAILAEFGIDEKREDEEMTKMSKSKKKKEKLKQKKEQKSKENEEKKEE
NLENLNIEMEKSLEDKTKEENVAEVDDTIKKNKKKKKKEKEK---EKKSTTVSEIAKAAT
ERLRLIKEYEEKKKEEERLKREEEEARIKKEEEEKERKRLAKLEKKMQLKKEGKLLSAKA
```

KEEKKKKELYLKTLKESGVLVEPKEKVKAEVINFDINKTKLLLKKKKNKPAINAEEKNKQ
LLNNE----KESKEESKENLEEKEIVLDDWEDFLNMDEENKKDKEENEKKEKDKEKKENK
TAANTKKNTKKGKKK------------IENDADDNSEYRSAIVCILGHVDTGKTKLLDKL
RHTNVQDNEAGGITQQIGATFFPKDVLEKEIKKIDESIKCLSKGIMIIDTPGHESFYNLR
KRGSSLCDIAILVIDLMHGLEQQTKESIQILKQRNCPFVIALNKIDRLYMWNKNDWSPFN
NTFKKQKQDTQDEFYDRLRQILNQLAEQGLNCQLYWENTNPRKTVSIVPTSAITGEGIAD
LIMILVKLTQSFMLKNIQYHDKLECTVLEVKNIEGLGTTIDVILTNGILRESDTIVLCGI
NGPIVTVIRALLTPQPLKELRIKNEYIHHKYIKACIGVKISANNLEEVLCGTSLFVANNT
EEIEEYKKKVMTDISDVFSHVDKTGVGLYVMASTLGSLEALLIFLNDSKIPVFAVNIGTV
QKKDVKKASIMREKGRPEYSVILAFDVKIDPEAEKEAAILGVEIMQKDIIYHLFDAFTNY
LKKIEEEKKQSKMTDAIFPCELSIINDCVFNKKDPIVMGVKIESGILKIGTPLYIPEKNL
KIGNVVSLESNKKSCDKARKGEEVCIKICGEPHVTYGRHFDFNQKIYSKITRESIDVLKE
YFRNELTMEDWKLVVHLKKIFNIIMNINEKDKLAEQNLENLDVTKLTPLSEDVISRQATI
NLGTIGHVAHGKSTLVHAISGVHTVRFKHEKERNITIKLGYANAKIYKCTNPECRPPECY
KSYESSKEDDPMCPRENCNHKMKLLRHVSFVDCPGHDILMATMLNGAAVMDAALLLVAGN
ESCPQPQTSEHLAAVEIMRLKHILILQNKVELIKEEQALKQQEEIRNFVSGTAADSAPII
PISAVLKYNIDVVCEYIVTQISIPKRDFISPPHMIVIRSFDVNKPGEDIETLQGGVAGGS
ILHGVLKVGDKIEIRPGIISKDEKGEITCRPIISQILTMFAENNNLKYAVPGGLIGVGTR
IDPILTRADRLVGQVIGHLNKLPDCFAEIEISYYLLRRLLGVKSQDGEKNTKVAKLKNGE
FLMINIGSTSIGCRVTGIKTELAKLELTGPVCTKIGDKIALSRRVDKHWRLIGWGQINKG
KPLELQEPI---NFMRINNFIG
IFIYITFSFRYASLFFITCIDKNDNELITLEIIHHYVEILDKYFGNVCELDLIFNFHKAY
YLLDEILVTGELQESSKKTVLRVVAAQDALMEDNKSSKKLGAII----------------

---------------------------FAKSYXDVADNLSLAIQNINEESLKHNEEISNI
HKGIQMTETILHNIFNKYGINKYNPINEKFNPSLHEAIFEINDNTKEKGTVATVIQQGYK
IKDRILR---------NMKKLLENLFETVEFDEETESYLKERIIDEKKKILENGVDYFYD
LISSFSDKKIKKSKITEIFNKHIKNCNLV-DENKNINNEKLSKSLNLKEYWKNNNVIGYN
DPFLGIHEKQINYNTNIPLSESIKINKEKQKQKQKQLNLFNEWIKNKIKIPSPVRVHNLL
HCDDFNKNKTKIEKMYDIKIDNFNLSIGQRNLLNDTTLKINVMNKYGLIGKNGIGKSTLL
AKLARHEIEEIKKDISIACIEQDLFLEDVTVLESVLMVDKLRHDLLKELEYLESIKSKRN
DNVENLKGEKKKMNNTKESDTENVDEKILDIYEKLNSISYLETEKEASKILCGLGFDSNL
QKKKVNSLSGGMRMRLCLSRILFSNNDIILLDEPTNHLDIYTIQFLIDYIKKLNKTCIIV
SHDRDFLNEVCTDIIHFHNKKLTYYSGNYDQFEKTRIEHLLQQQREFDSIEMKKKHVQKF
IDRFRCNSKRASLVQSRIKLLNKLPIINLEKEETPFSFSFLEPFYISNVLIRLKNVSFKN
SMFKNIQIKKNESIIIGDNFNETNIK-MNAESPNLTTTEEISTINNNDYQFKHEFLFKNA
SFEVDMDSRIAICGVNGSGKTTLIKIILNLIDVFEGELHVSNKANIGYYSQYHVDSLNPI
FNSIQQLQYNYSSKNIKEEEAIKYFNKFNIPTNILYEPIYVLSGGQKSKLALAILAYKNP
NILILDEPSNHLDIESVQALIVALNLYKGGIIIISHDTYLIKHVADEIYHINNCTKELVK
INYDFDKYTKLILENKIMPREIITLQCGQCGNQIGVEFWKQLCNEHNIDQEGILKNNNFL
NEDRKDIFFYQADDEHFIPRALLFDLEPRVINSIQTSEYRNLYNPENMFISKEGGGAGNN
WGCGYSQGHKVEEEIIDMIDREVDNSDNLEGFILSHSIAGGTGSGMGSYLLELLNDNYSK
KMIQTFSVFPLLTNESSDVVVQPYNSILTLKRLILSTDSVVVIDNTSLNRIFVDKLKLNN
PTFQQTNTIISNVMSASTTTLRYPGSMNNDMISLISSLIINPKCHFLVTSYTPITIEKHI
SNVQKTTVLDVMKRLLHTKNIMVSVPVRRGMYISILNIIRGETDPTQVHKGLQRIRDRKL
VNFIKWNPASIQVTLAKQSPHVVSTHKVCGLMMANHTSISTLFERCVTQFDRLFKRRAFL
ENYKKEPMFSSADGQGNFEEMESSKEITQNLIDEYKSAERDDYFNHTYL-----------

--------KFISNCIYRAFGIPVRKYTHEVVTLWYRAPDVLMGSKKYSTTIDIWSVGCIF
AEMVNGRPLFPGVSETDQLMRIFKILGTPNSQNWPSVTELPKYDPNFTVYEPLPWESFVT
KYIFCVLSIVYNSIFFSFFIYVLNNSIFIIFLVKRIRF----------------------

-----LQLVKEVNVMIPLIQKINSETNAVKIGNDVTKLENIFDEIVLSIRL---------
--------------KIYKEKI-------------------------MGKEKTHINLVVIG
HVDSGKSTTTGHIIYKLGGIDRRTIEKFEKESAEMGKGSFKYAWVLDKLKAERERGITID
IALWKFETPRYYFTVIDAPGHKDFIKNMITGTSQADVALLIVPAEVGGFEGAFSKEGQTK
EHALLAFTLGVKQIVVGVNKMDTVKYSEERYEEIKKEVRDYLKKVGYAADKVDFIPISGF
EGDNLIEKSDKTPWYKGRTLIEALDTMEPPKRPYDKPLRIPLQGVYKIGGIGTVPVGRVE
TGILKAGMVLNFAPSAVVSECKSVEMHKEVLEEARPGDNIGFNVKNVSVKEIKRGYVASD
TKNEPAKGCAKFTAQVIILNHPGEIKNGYTPVLDCHTSHISCKFVNIDSKIDKRSGKVVE
ENPKAIKSGDSALVSLEPKKPMVVETFTEYPPLGRFAIRDMRQTIAVGIIKSVEKKEPGA
VSSNKPAKK-----SLPIVLLKEGTDKSQGKSQIIRNINACQIIVDIVKTTLGPRGMDKL
IYTDKEVTITNDGATVMNLLNITHPAASILVDIAKSQDDEVGDGTTSVVIVAGELLNEAK
GLLNDGIEPNMIIDGFRNACNVAINKLNELSLNFSSKNEEEKRNILLKCAQTALNSKLVS
NHKAFFAELVVNAAYKLGDNLDKSNIGIKKVTGGSCLDTQLIYGVAFKKTFSYAGFEQQP
KKFNNPKILLLNVELELKAEKENAEVRIENPNEYNSIVQAEWDIIFKKLNLIKDSGANIV
LSKLPIGDIATQFFADHNIFCAGRVEDADLKRTANATGAVIQTSLFNLNENILGNCGIFE
EVQIGNERYNIFKECLKTKSVTIILRGGAKQFIEEVERSINDAIMIVLRCMSNSEIVPGA
GSIEMQLSKYLRIYSRSICNKEQIVLYSFAKALESIPRHLSHNAGYDSTDILNKLRKKHS
EGTNDIWYGVDCLEGDIINAYEHCIFEVTKIKRNVIYSATEAACLILSIDETIKNPSSSE
KAPRNPYAMSGKLEDIGELVLLIGDFHSPIRNLGLPDCFKDLLKTDKIKHVLCTGNVGSR
ENLELLKNIADSVHITKGDMDDNYDFPEEISLNIGDFKISLIHGHQIIPWGNMNSLLQWQ
KKYDSDIIISGHTHKNSIVQYEGKYFINPGSATGAFQPWLAETVPSFILMAVAKNSIVVY
VYEEKYGKTNVEMSELRK-MYNEKNEIIYSVKISEDVGNECTGVYRSPMCKDRLMENFED
KPFNSVWEMFSDVADKFKDNDCLGSRIKENNKLGEYKWKTYGEVKELVMLIGSGLLNCDA
CPLIDCGEDKVIKARFLGFYMPNNPEWTICDLSCNAYNIITVPLYDSLGIESSKFILEQT
LMKTIICNKSCALNLFKSLETCDEIYLKKLIFVD-EIDDEIEKMSKKFNLELIVWNDLIN
EGKKNILEISPANPDAIASICYTSGTTGFPKGVIMVNRNMMATLNASMLELLKMSDLDVN
DKDTHISYLPLAHIYERMILFLCFVFGIRIGYYSGNIQALIEDIQVLKPSIFISVPRLYN
RIHERIYNSLKKKSHLIQALFNKGIDHKVKKLNGSGSYTHFLWDKFLFNKAKKIMGGNIK
VMLNASAPISCDVVKKLKSIFCAPILEGYGMTETMGPSFLSHMYDPLMGHIGGPIACMEF
KIVSVPEMNYFINDKPPKGELYLRGPSICKLGYFKLEKETKELINEDGWVCTGDIVVLND
NGSVSIIDRKKNIFKLSQGEYVAVEKIESSYRQSLFINQIFVFGYSSESFLVSVIFPSTD
TIEIWKKNKKIDKNDEEIIILPDFKNDVINDLIKIGKNDGLKGYEQIKDIYFTLEPFTIE
NDLLTPTGKIKRHVALKKFKSQIDQMYQKAKNS---------------------------
----------------------YIYIFFFFLKNGDKIKKTINEGQTLLTVFKEGYAPDGV
WLGGTKFQFINIDRDLDFEGYTFDVATCAKLKGGLHLIKIPGGNILVALYDEEKEHDRGI

-----------DLEQLKNRINNLNMLAVVYLACREAGHIKSIKELITFDRSYKEKDLGKT
INKLKKVLPTRAFVYNENISHLIYTLSNRLQLSTDLIEAIEYVVKKATTLITTSHRLNSL
CGGSIHLIVELNTNEEKNIKLPNLSQIASVCGVTTNTLKTTFKELLNASEYILPKYYLTE
NNQKLSLLKQKYISEEKKKKKNKMAKLSLPEWSDVERYFKDPELITAEILFVALTLCNVF
VMYRLFLDVIPFPIFVTWWQLAQGLLVAYVCGELGREFPKLAYFPKVEINENMLKVLFIP
SIFYCLMLVLSNYLLYKTPCIASYPVLVSFTVVFHHLTRFVGCGEEYMPLRWQSIFFLLV
AFIIGCFDSKIMGKGVILWALLYALSSAIFRAGFMQKIMHLVDGRGNTLHNNQHLLGVLI
LPILIILSGEWTVFQYMPYDFTSLHTWQMWGCLITVGTMPFIKNVISNRLVRRTGQGPWR
FLEILSIILVFLIGMTFNAPTYKGYIAILCVIIGRSIGAFDVLLNASDYMLAEDERRRKN
PRSNYAKNRQSTQASKPFLSSADNEEDEESSYSSNDSKSYQGSQDYDDKESVNSSSQQYS
VNKGTSRMGSSSNQTSNAGISGDESRRTSGRGSGFQSKSKLSRNYSSKEEMLDSQA----
---TKVKVGEEMRN
ASFALAKAVWAAGDFKGQIIEGIKRPAVTLSLSTNNVAGVKLPIFQVQIDPTVDVLGNLG
VASGGQVINNTRENYLQCLNMLVKLASMQV-------RKKKKRKNKNLCNNNYQLVVYSY

FIYLKCYIFILFFTHLY---
--YILKKVYFLFFLFLLFCNTF-YASILESIKHDLQIVNNQNFNTIVNKFKNEKVFVILF
FKKTNKNIKNIIKDYNDVAIKYKGIITFCIADCDTNSSLCENELSLYVPNYKTSDSHYFL
VYPVNPMPKFLFNEEVTESNIKKYTYLIPSKIEIIKDEKDLNVFISKHENMPKVLIFSNK
KKPNYVLNALSNSFNKKLMFCYINNEMQDLVKKYNIKNFPSIIILKKNKVIDTYKGKHTY
INIFDWLNIHSETFVLGGGFDISPDKSDEKPWKFELIPKFTKLSHGDICFKKADKGLCLI
YLKEGEKLEKSEIDMLLSLKEKYKPHIDGR------------------------------
---------YTKKKKKKKKKKKN-------------------------------------
----MTVDLNVPYSACDLKRVKRLELGVLDPEIIKKISVCEIVNVDIYKDGLPREGGLND
IRMGTIDYKTLCGTCNMNVKYCPGHFGHIQLAKPMYHYGFMNVVLNVLRCVCYHCGRLLC
NMNNSKVKYIEKIKVNSLRLRKLSELCSSIKVCDHSVHEDSLNINDNSIDNFYNNDLSNL
NVNQKMLLNPNTYSNIFEMVSKEDVDCGCVQPKYTREGPNMFIQFLH-SSEEDIDESKRK
LSAEEALEILKKIRKEEMGILGFNSDRCIPSSLILTYIPIPPPCARPYVQYGNQRSEDDL
TLKLLDIVKTNIQLKRQTDRGAKSHVLQDLCSLLQFHITTLFDNDIPGMPIATTRSKKPI
KAIRTRLKGKEGRLRGNLMGKRVDFSARTVITGDPNLDIDYIGVPKSVAMTLTFCETVTP
LNYEDLKRRVERGPYEWPGAKYIIRDNGTKYDLRHVRKSSEKELEYGYKVERHMTDEDYI
LFNRQPSLHKMSIMGHKAKILPYSTFRLNLAVTSPYNADFDGDEMNLHLAQSHETRSEIK
HLMIVQKQIVSPQGNKPVMGIVQDSLLAIRKFTRRDNFLCKEEVMSLLIWIPYWNHVIPT
PAIIKPKALWTGKQIFSMLLQFDDVNRNNDLLNNKM----NDNNLKNEEEVKRKIGNNQP
NSPCSIVDNIN-HVSNTFNSNIKHQVNNNDN-YNSFKNPNMVKINLIRDSSTSCKDDNPY
CSVNDGKVIIKNNELLSGIICKRTVGSSSGSLIHILWHEMGPDKTKDFISALQKVTNNWL
EYIGFTVSCSDIIASNKILDKVKEILNKSKKEVTKLVKKAQRGELECQPGKSLYESFETR
VNNELNCAREMAGKVASESLDEKNNIFSMVASGSKGSIINISQIISCVGQQNVEGKRIPF
GFNQRSLPHFIKFDYGPESRGFVSNSYLSGLSPQEVFFHAMGGREGIIDTACKTSETGYI
QRRLIKAMEDVMVQYDRTVRNSYGDIIQFLYGEDGMAGEYVEDQIIDLMKLDNKEINKLY
KYNFDEESYGKDFYLCKSEERKESYIDYNKQNILNQEFEELYKCKNYLCKEIFPDGDIRQ
HLPINMNRLIEYAKSQFPFIPLINSKSKN--------SSDRIRSKIKLKKKKKKKKVKTN
DYTQNNNELLSEIKKEYENNDLSNIIKCQKSYNEMESFDEEANNEIETDDDNEEEDDNDD
NSYYYSDSSKSNIEQEDNSMVNPIDIVHKVNNFLENLVIIKQINSNDTLSLEAQNNATIL
LKAHLRTYLNSKLLTQTHKISLKGIDWLLQEIEKNFYKSLCHPGECVGALAAQSIGEPAT
QMTLNTFHFAGVGSKNVTLGVPRLKELINIVKNVKTPSTTIYLDDLVSNDQQKAKDILTK
LEYTTLKQLTSHAQIIYDPYTTTTILEEDKSWVNEFYEFPDEDDTQYTLGEWVLRIQLTN
IHVNEKKLTMKEIVYIIYSVFSSDELDIIYTDDNSEDLILRIRVKYLNGEYNFINDDDDD
DHGDEEEEEDD----YNNISNSFKTKKNASPDLNEKKDDNLSVNSYNTQKNNNSNENKIR
NNTKEGEND--------------------YENDNEDDTLFGNNVKN--NNDNSSNSKNMK
ELGSDDMYNKSTS-----------------------KNMDDDATLKNRSKTNNPLLSKED
TEDTFLKKLMEQCLSTLKLRGIENITKVYMREEAKINYDTESGKFIRSSHWVLDTDGCNL
ENIFCAPLVDYKKTISNDIVEIFEVLGIEAVRRALLKELRTVISFDSSYVNYRHLSILCD
VMTQKGYLMSITRHGINRVDKGPLIKCSFEETVEILLEAAAFAQVDHLKGITENIMLGQL
CKIGTGAFDIIIDNQKLNDANQNLETIQDITSAGFTTPDTYHGITPDGLQSPIPINTLNS
PLPFSPTYNSNLLSPTAPIDNVNNILSPQCLQNYPDNIMSPSKNDFNNLDTLKLGGKFSP
THSPKSPTSIVHSPFSPFDHHNQQPIDPNLLFSPKSN-----LMNYNVFSPKANMNNIQS
PIMYSPNPMLDIFSPKPQIQSNIYSPSYSPTSPSYNADNVYYSPTSPKNQMDDVKSNIKY
NVMSPVYSVTSPKYSPMSL---
--N--IHQ
HLL---

-------------KRFIVFDEAHNIDSVCLEALSVNIDRNILNKASLNIKKLLKKIENSK
AINEEKLKEECNKILQKIKSQKLNNKKKKSDDEANIKSDYEKNIFSNNKDFKRRRIENEV
YFDDDMNLVFGDMNEEKICEEENIVEKKKDTSESLVEDLKELIRDGDKTNENRNNLLIEE
GDISDLHFNPLLAEDTIKNIIIPGNIRKSEHFLNLMRIVVVYLKKYINIYEITSEGPLSF
LYKCEKDTKLDTSFFKYCFDRLKSLLNTLQIVNIDDYSSLNIVCNFCTLLGNYFKGFIII
CEPYPEATGIYDPVIQFACLDSSIAMKSVINKYKSIILTSGTITPLELYPKLLNFKTVLT
ASFPMSFDRNCVCPLIVTKSSDLVPLSSQFSLRNDINVIKNYGILLVEMCKNIPDGIVAY
FPSYIYMEQVISTWYELGVIANILEYKLIFIETKDIVSTTIALHNFKKACDFGKGAVFLS
ICRGKIAEGIDFDKHYGKCVILFGIPYQYTLSKILKSRLDFLKETYNIQENEFLTFDAMR
QASQCVGRIIRNKKDYGIMIFSDIRYSRNDKKNKLPPWIIKCMDISNVNLTIGTAVNISK
NFLLNMSQQYKETGQTKISQDLLKSQKKCWQTVKSILNMDDFIMFKKKVQNILSVINKKF
SRNYNSYNLAQEIINQPITRISELSNKLKIATVKNNCEIPTIGIWISSGSKYENKFNNGV
AHFLEHMIFKGTKKRNRIQLEKEIENMGAHLNAYTAREQTGYYCKCFKEDVKWCIELLSD
ILTNSVFDENLIEMEKHVILREMEEVEKSKDEVIFDKLHMTAFRDHPLGYTILGPVENIK
NMKRQDIIKYIQKNYTSDRMVLCAVGDVDHEEIVKLAEKNFSHLKPQPKS---ILKSLDE
KPFFCGSEIIVRDDDSGPNAHVAVAFEGVEWKSPDSITFMLMQCIIGSYKKNEEGVLPGK
LSANRTVNNICNKMTVGCADYFTSFNTCYNNTGLFGFYVQCDEIAVEHALGELMFGITSL
SYSITDEEVELAKIQLKTQLISMFESSSTLAEEVSRQILVYGRKITLAEFILRLNEIDTE
EVKRVAWKYLHDRDIAVAAIGALHGMPQYTDLRQKTYWLRY----KRLFVNRRTNLY-YF
ENEDLYNNKEKEIIKNISILFKNSNENSTENKEIILEITNFFNVLKLVKTVNHKNCITHP
WVYDIFKFIKEIAKNDSVVNINQVKKEVFEFFFKEKKKQIIKYRNNFFLNTCFIFINEIK
ILLCCCILLNIFVQYNWTGPPLNCDFKKLKEQKIQINDSDNYFKEFMDAINIKNDEFLNS
CLEFLSLEGEYIYEYSELIYSFCMCIILLGIINNFNDEDQHMFSLNEYNYFKINLNDPSN
NKIVHSESNEKKYDELKLLYFIKSKYLWKARIYFIWQRLFVSSNDYFYFLKIHIIDKPFN
IFKNIKLLPEEFELIEQEVNLRAIDIFDHLNENMKDIEHCDLFFENYMSEKLKIYLLSNF
SIYLAFYNYTSGYKKIMDIISELSQFHYSFTGRMGIKRKYQKIPATILVLKTKLKDEEDN
TSTILKEIDPFSEYDILKSDYKIIENET-NEEEETNDLRSEDHKSLKREEIDKKQNEQQN
ELNNKKLGGTCCDNNNADKKLEITNYENCSNNNDSEKESSNRKKCLEEPNGKVTWKLKDL
DPDTDILEEPFFVDSQNNYFKILSFQEQIILINYCFSLIRFNPHYDEIKFEKLSAVISRC
LKCYDVNAT--VEKENEGKNNNYLLQIKYQNWLLHSCILWFKCKYETFKFKTVDRAAAQL
NELLKECYELEPKSIERLKFIYDVYYPTTWEMKKEIGKVMIKIGSMISAFNIFKDLKLWE
EAITCLIEAGRKEEAKELLDDIIQKKRTPALLCLYGLIDRNNALNYYEDAWNLSNFKYSK
AARFIGKYYYNKEMYKECCEYLEKSLEISPLFSDIWFILGCSYMKIDKFDEAIKSFTRMI
SMTNENCAKSYGNLAYLYMKKGTYKAAKICINQAVKVNNNEWKFWDTYLKLSILQNDVDS
FCLSIRTLCQLNQVKQIQPWVFDYISDLIVKDKPTFIQNKNGLSYLDKMITTMDNLSIYI
TEYDSFWNAYSFFLFIKGKFTDSFDTKIKEIRSIEVG-----------------------

--KSGTNHLAQAINKLSQRY
ENEKEIKDTLTDGETLTDEEMSGE-EYFDEGECLSTFDSPKMWLIKLFKNNIERNLAIGI
YYKYMKQKDNDYNIKGIYVSDNLKGYIYIEADSLYMLKKFLLGFKFINLNEISIVPVQEL
TSIFAMCHSKVIIPKVNEYVRIKRGVYMNDIGQIFEVHEKGIYAIVRLIPRISYDKYNNF
KKDNYNNM---SSNRVSSTLDENNKNDSYLNDKLDINRNAILSKSHNENIINEKNDILDE
ALQFRRKKKKERPLKKFFDREEIEQIGGVIEHGPYPRTIKYQNNIFEENGYLLKKMNIKY
LISENANITLTEIRDFNKNNTNEEDINLHLSKSFINKNSLHLFKKNERVKILKGELYNLI
GTITSVNENVLTVNPDNLAKEFKFLPSDVTKYFNEGDNVTVINGIHKGKSGLISLLDYKE
NVALIFSPSLNTELRSSIQDLALSTNNSEGLGGVNTLNGFSIGDLIELSDRQIGVLTYID
KNKHIRVLTNNNKILHTTIGAITSKRSAIGQVCKDENNNVIQAKDTIQIIRGIHKNKVAV
VNYIWKNKIFAKINRKIEDNGFVVVDCENCILSGNKNEKKKVITHNNLFRNNNFFKKNNF
QSFIGKTVKILTGVYKGLLGDVIDAEKDEFTLLLKIKPKTVRQKRIECAIADAYKDQNIF
DEKFNGNSQKGLEKMKKNYETNKNHNVNSTISNKNENTYIKEDNHTSY-NKNYDNNYNKH
SGNSKKFNEEDRYKKNKNEESKNYNKKKDHMS-KEYNLKYEEDKNYVCNFDEEIKKKQED
EKKKQKNAQDIEKKEDTSKQHSIIDDNDNNQKDPIWLIEGVMVKVITPGQFFNEIGRIMS
ITKKNSYVILKIQTEKTSFSIVSDAVVPLKPEKKNDQVIILDNGKTIEGLVIDIHNKEVQ
VNTIHGNITSHLENMFLYKKYFS-------------------------------------

```
------------------------------------------------------------
------------------------------------------------------------
------------------------------------------------------------
------------------------------------------------------------
------------------------------------------------------------
------------------------------------------------------------
------------------------------------------------------------
------------------------------------------------------------
------------------------------------------------------------
------------------------------------------------------------
------------------------------------------------------------
------------------------------------------------------------
------------------------------------------------------------
------------------------------------------------------------
------------------------------------------------------------
------------------------------------------------------------
------------------------------------------------------------
------------------------------------------------------------
------------------------------------------------------------
------------------------------------------------------------
------------------------------------------------------------
------------------------------------------------------------
------------------------------------------------------------
------------------------------------------------------------
------------------------------------------------------------
-----------------------NLY------------------------LPL-YKILED
IQAQNITAVNETLNEIYLQNDDYISLRNSIDEYDNFNQMNLINKLENHKLPEMRRIASLL
YKKNKKYKEAINLSKKEEQYKDAIEIARVSKNNTYIEDLINYFIEINNKEAFCACLIVCY
DLLKPDYILEIIWSSGFTDQAMLYFIQIVSDYTNQIDIMKKQIEDMQKEKKMNKSAPNDY
SATSISNQFNYSLNNNLSIMPPQNNYMSNNSQFDKYDMFNNNTNFMSMYVGKEAPFFKAE
AVFGDNSFGEVSLTDFIGKKYVLLYFYPLDFTFVCPSEIIALDRALHKFKERDVELLGCS
VDSKFSHLAWKKTSLSNGGIGNIKHTLLSDITKSISKDYNVLFDDSVSLRAFFLIDKKGI
IQHALINNLAIGRSVDEILRIIDALQHHEKYGDVCPANWAKGKEAMKPSAEGVSDYLSKI
EEDNTAKIIGSHDNLLTDPRIAQDFSNETDELLKKAEKYYK-------------------
--------------------------------VNF-------------------------
------------------------------------------------------------
------------------------------------------------------------
------------------------------------------------------------
------------------------------------------------------------
------------------------------------------------------------
-----------------------------------------------MSDKEAKKEAIEI
IYEISNILNVNLDKETIVILIQLCEYGVSPKILSHIIIQLKKEKEKFLQNLNNNMSSLK-
```

> ***Plasmodium knowlesi***

```
MIGGDKIIIYYGKKYPFVCRTILNVHKILSQKEGRSNHFPNDKVKFVKDENINTTDKIFA
KTLDEILQTKLYHSFCENNKKEDKQTDVYVQAQYDEWVDYFRNKHMQKEILIICEYVNKH
LHLNTFICGEYLTLSDLYVYYEMYRYFNVANCYNVKYTKQFRNINRWFKLINCLVNYPDE
ELSTMVKKELEKNQVNITKDANSKLLKQKTVCTSYGGKLENAEKGNVITRFPPEPSGYLH
IGHAKAAFLNSYYANMYEGKMLLRFDDTNPVLEDIKFETSIMEDLENLGLKYEKISYTSD
YFADLEDYCIKLIKMGKAYADDTNVEEMRNQRGEGIESRNRENSVEQNLQLFEEMRKGTE
IGQKNCIRAKIDMQSKNKCMRDPVLYRCIVDTPHHRHGFKFKCYPTYDFACPIIDSIEGV
THALRTNEYSDRIEQYNWIITTFQLRKVYIYEFSRISFVRTVMSKRKLKWFVENKVVDGW
LDPRMPTIKGILRRGLTKEALFQFILEQGPSKSGNLMQWDKLWSINKQIIDPIIPRFAAV
DKKKGVILKLTDLTEDIVEKTRDLHMKNKSLGTCSMLYTNRFFIELEDAQTLGEQEEITL
IKLGNVIITSIVKGDDNVIKEVIGKSNFDGDFKTTKKKIHWVPYIPEKLITCTLYEYDHL
ITVDKFENDNKEDWTKFINSNSKYETVVYSEPAITSLKVSDRFQFERRGYFIVDKV-VDN
HLHLIKIPDGKSKNMSIISSKVNPQNLAGTKTMDKQTVVIDNGSGYIKAGLNAYDEPSIV
FPTIVGLHRNSEDKASYVGDEAIFREADLSFYRPIDHGHISDWDLARIVWEYAIKCVDKN
RSVESILLTEPPLCSTSHRTKMGEIFFEDFDYKNINISVSGLMSIYATGLTTGLVLDIGD
GVTQCIPVFDGYIEKNSIIRSDFGGEELSMFLQKLICDIGYSMTTRKNFEYIKTMKETLC
FCSLNPPKDQLREDLSVTYTLPDGDVLRDGYNSVELSHERFYVPEALFNPLICQRDSLSI
VDIVWKSILLCPIENRKTLTSYIVLSGGSSLFPNLVERLEREVKNNAPESARSAVKVHAH
ENRAIMAWCGARIFSQPEIRQAQEGLWISKEEYEEIGSNIFLIKVSRKDENMECLSKINL
IQKVNAELYKSLGVEDDNLAEYLIYLCKKAKSLEEFCKEVFENGGEIEQSVLKYLYDIIK
ITDDTEEEEEDGVDKEKRLEMKMIEKKNEKMKRFHCLTIKNDEQLTRLSLEGGSDTATGG
GRSRKRSHHRDDRSQDGSHIHKDRHDRRSRYEKSRHEDRSRHRDEHHRRDRSHSREGDHR
RRFALRVNNIFSGKVSKIMDFGMFVSFRTEGGYKEGLVHCTDILPNRKRVVNMSEKFQKG
MKVKVKVKAIFGEKINLNMSEVDQKTGKDLVSDYEEKEGQKNVMSIFDDLHEDYKELKKH
NSDVFKEDVKDTMKYESVIKMQSDYAKWEIQQLIKGGIMYDEEIKKEYKNLRNDEKIEDE
EEIIEIEVNEKEPSFLKGQTTKAGAKLSPIQVIVNAEGSLARAITTTCALAKERKEQKQN
EQNAIYDSIPKDISRPWEDPKPELGERTIAEALKNIGKNYDLPEWRKNYLHNNISIGVKN
PMPVNEQREKLPIYHLKKDLMKAIAKNNVLIVIGETGSGKTTQIPQYLHEANYTDKGIVG
CTQPRRVAAMSIAKRVSEEFGCILGQEVGYSIRFDDCTSNDTIIKYLTDGMLLRETLSDT
MLSKYSFIILDEAHERTISTDILFCLLKDVVKRRPDFKLIVTSATLDAEKFSTYFFNSPI
FTIPGKIFPVEILHSKEPESDYVEACLITVLNIHLNEHPGDILVFLTGQDEINTACEILH
ERMKKLESMSPPPLIILPIYSSLPSEMQSVIFDPAPQGCRKCVLATNIAEASLTIDGIFF
VIDPGFCKIRKYDSKRDMDSLVVAPISKANAKQRAGRAGRTGPGKCYRLYTEDAYKNEMA
ETSIPEIQRINLGSTVLLLKALGVNDFLHFDFMDSPSVDTLIHSLENLYYLGALDDNGYL
TKLGKKMSNFPMEPTLSKILLTSINFNCADDVVTIVSMLSVQNIFYRPQNKALLADKKKN
KFIMPQGDLITYLNIYNRWRENNYSNYWCHENFIHSRALRRSQDVRKQILSIFERYNYEV
EKNKSRNDSAKYVSICKSICSGYFSHVCKRDAQQGYTTLLTNQQVFIHPSSTLFNKNPLF
VVYHELVLTNKEYIRDCTIIQPQWLIQLAPNLFIPADEKKISKIKLREKIEPLHNYYEEP
NAWRLSRRKGMSEEYDHLYKIILVGDATVGKTHLLSRYIRGSLPSVAKATIGVEFATRTI
PLAIGGTVKAQIWDTAGQERYRSITSAHYRRSAGAILVYDVTKKKTFLSISKWLEEIRQN
ADKDIVIMLVGNKVDLVEHDEMKRKVTYEQGASFARENNLFFAEASAVSKLNVKHVFENL
LQEIYNNRMKNNSCSLSTRSSATCESAIQLTRARSTIKLSDLNENETEDEHRARSTCCME
KSKLAKVEKVLGRTGSRGGVIQVRAQFMGDTELSGRFLIRNVKGPVREGDILALLETERE
ARRLRMSDNLEKFSTIDLLNELKRRYSCLNKPDGRYIFLGAPGSGKGTQSLNLKKSHCYC
HLSTGDLLREAAEKKNDLGNKIRNIINEGKLVDDDVVLTLVDDKLKSPQCKKGFILDGYP
RNVKQAEDLNKLLQTNQMKLNGVFYFNVPDDVLVKRISGRLIHKPSGRIYHKTFNPPKTP
FKDDITNEPLIQREDDNEEVLKKRLNVFKSETTPLINYYKNKNLLINLDATQPANDIEKK
ISQHIGG-MEKQCPVCYYNLPDPESAIAPYDTELNYFMWGPGFEWQPEPEVKNILVEENY
EESEQSEESVAGLEELDEKVNRNEAKDMFNTKSSNGKLRIEDASHNARKLGLAPSSTDEK
KIRDLYGDSLTYEQYLEYLTMCVHDRDNMEELIKMFSHFDNNSSGFLTKNQMKNILTTWG
DALTEQEANDALNAFSSEDRINYKLFCEDILSMKRYVYN-ILVACLVFYVKEDLSADMPM
KYADMRALDDLTNLNDDQVKDILGLDIKGAKERIEKLFAVIDKNNDKVISEEELNAWSIY
VKNEVFLKQVQVEMKQIDADKDGFISLPELNEAFSQNLDAKEVEKHAEGLLKRFQIVDKD
KDNKLNINEVGLLIDPMKDEDLKELEINEILEHHDVNKDGRISVDEFKQTRTDDPHVKKD
DDIALDDFNFFDTNKDGFIDKEEIVKVYFDPSNEAGSINIAEVKDSIFEGKPITYDLWNE
KALKLAVTSLTDYGDILRYPEDFKLDIGKNVILPSTRSRAGMEDDLDQEDSLGDKEDGAQ
DDSTQKGTTPDELMDLLLAKAICIFTFVCVATFGCSIPYLIGLLGNKKNAEHENRIKGVL
SNLNCFGSGFIFSIVMFHLLPETIMTVTSHSHIVLFNSSDPEAKTLFIFLFVFIGFAMQL
ALEYVLPVDGNMCCVVNDNVKSMLEDNFAKNKYQSKKSDSINIEMHSVTVNEHQYHHACD
SQHFKKQQNIVKFLHVLTLQSFFLTISLAVHSCIEGMIVGTSNDVNFVFINSFCILSHKW
IAGVTVALSLNQNNISKNLKIILLIIFIFSSPLGIIIGHLIKSSGEKVTCVINAVSIGTL
```

LFIGCEILLNEIKQKFSRKIRLAKWLSFCSSCVIAFLLIMMTQHLAPHAHAHSLDHGHSM
QSKFWAKGADDDSGDNITESSDDEVDEKKPLVSAQAERWAAIDSSSSEEEERVLKSYEGK
RIDFYKNIGSSLNESMESSDFNQMLKDYESLYKFMVKESSERIPNFAIVYLDKLSKYVDT
TFQNNVEKKVLSKSKAQTLNKLKAKIRKCSAYYQSKLDLYHENPEEFKLAMEDDLDDDDD
EDEDDQTDEEEEDDEEP--ADQEEEKKEKAKDEKTPGEEDSDDWSNSEEEEYVSDDGDDK
TKSAMSKWGLKTSEKVEKKKVVKT-KVKKEGTKKEEKVVHVDENQSAKNKAYAELLSTKN
LSEEVIRNRVKFVIEKRGRKGLDKHEHINILSKLCELAKTVSTQSYIEVLEQLINLEFDV
VSSLYTYMSFNIWNKAFKYIEIILDLLIQNESFYLVSINITEEITEEVINEKEKISRSCK
TLISFLAKLDDELLKALIYIDAQTEEYRRRLGKTIHMIALLYKGYNYVKFTKKMPELAIF
ISTRILEHMYYKPEALFMQVWKFVTNGKE-----NAENSGNNNVNGKVAEKMPTKGEDQI
SPKQIVEKYVYEIFEHGTKQQKVKALLQLSFHRSLHDEFLEAKELLNVSNVHELALSSDT
QTQILYNRNLIQLGLCAFRHGKIFEAHCCLGEICSQNKHRELIAQGVSNLKNQEKTLEQE
RAEKRRLLSFHMHISIELIECVNNICAMLLEVPNLARHSFESKKDIISRQFRRFLDIYDK
QVFNSPPENNKEIILLATKYLQKGNWKMCCEKIFSLSIWSKFNDTEKVQNILREKIKQEA
MRTYIFRYISIYDSFSVDQLCIMFDLNQNVVHSILSKMMINHEIPACWNESSSHILINKV
NPTALQTVAIKLAENINEIMEQNELTLNMRNPKFMFMQEKRTQMKDDKSNWSHKKGDGKY
GKGYHQHKNVHYKKNYKEKSMPKGFAMEYGNGGDKIFDLNSISREKLELVEVFYDKMKDT
TNANALYLYALQIFKICNIHNELPRLVVFGQQSMGKTTLLDFIMGGPMGYTSSDTGTKQP
IVIILKPSDTNKIECYLNKKKVSIDDLHEKMKAIMVNLNESIIPKELEVEISIPGGIYAT
FVDLPGIKDDSKAGSELTRKIVRNYVQNFPNDIYILVKKASDDPANWPYHLREFFMKPKP
MGLGLQNKQCIVVGTRALEFLNNELSTIKTLTELYDRVKKRGIMDHNDNILSLYLLELFS
IPIEQKEKNDFLTNRISMYSKILNGRKNVLDLLLNKFENDCNDSIKKELLDCFDVEKFKQ
EVNSKFMNILIQQLRKVEVKLEKKKSKMEFYNKKLEELYNKGNILSIREQVKLYIRELVN
IVSNLLTGNYPILNLPNNGEDFLKKYGGTLMENLKDGNELAVELFEKQGLYDENFLNYLN
EYLCKYASENDINQSISSESNDINNILNNNNNGNNNNSSNKRADAYDTLNDMEKKKLNSV
PALVEGQPVRFILAKEESSMFGVVQNTPTDAKSKNILVSFYFRNSNTEEQVQIKSVEKDR
LTVIKAVETLNGDPSFLNGLQVWYKMIRDDGWVGFDKAEIIKVFNNNSKIKDVLIRNLSK
NDEVISIKINDLYMDYVIDENKEENEENEEMKENDDDILKRIAGAHTDLKILNQLAITYI
CKWLKYNIAKIEPEKDFSDEVLLQMMRSIHNIVDQSDWKPLVVDLLQANISGNILHLTKL
ASCSAAVALNRVFKAGLGEINRKIKNNYMDENIYLLSTNPKFLDELNQALHNFCKERAIT
CASEMKDIVFEQTYAVHFEIIEEIFDGCKLFEDNFLTPSGVKPTMSIITKNVKQNLAYRN
HQLSLTDIKINKKSKSKELIQEEVKLQFWAIKMLISVPFATKIYAHFLNNILPKNKHLAN
VLDYSIDCESTLEKYIQSKLLNREVNGVMVPIDDKELMSHYNIIDNRDNLLRKLENQKRL
NQYFTIVSNSIKLLKNNLSKESTLDFVTKLDFGQNKKMEASNIAQVLYATVDPNISIRSE
AESKLKHAKETDFVQYINQLSNEFCKTQNDPYLRQIAGLLIKNAFASKDNYENEEKARTW
VNFPENIKNELKNSMLHLLSQQGEKVVIGTACQIISLIAKIELSHNKSSELLHKLVNNII
EKNAYTKKSSTVCLAYLTEDIADICNESKSKYVFTQPDLDLILTAIINSLCEPGEESVHC
ASMKVLYNLMSFIDQNFKTQVERDIIMKTVIDGCKDSERSTVQVAAYECLINIVSYFYSY
LDAYMYAIGPLTWTAIESDNERIAISAIEFWNTVCEEETFIDQYELQEGKKNHNIVKQAM
VFLLPKIFNAMINQESEDIDIDAWTISMASATFLALSAQLLKNDIVEPVISFVEENFTHE
DWRRRDAAVLAYGSIMEGPDTEKLKPLVEESVGQLSEVLRDPSVSVRDTAAWTIGKITTY
HSEIIYNVLGNYNDSNSLYGILLERLNDYPRVAANVCWVFNQLASNKRSSYNKMTNSCTT
ELDDSFCVLCKKLIDVTSREDADTRNLREAAFNALNVVIDNVSDNCLKYMIELLSHMMYL
LTNTYLNPLTEEVKSLQGYYCGTMQFIINRLGNQCKPFLKPIYLSIFRLFEIRTDICEDA
LLACSAIINVMGEDFREHLKTFLNVIFKGLRNVSETSTCRICIEMISDICIPWTFEFEKE
MELILECLWDALKTFGVHDSIKISILTVLGDIATALNRSFSRYLNFFANILAETSKITIA
SGSAESDDWVNYVFELRDAILLTYSNIIYALIDGNEIAKLKIYITNILDLIELILIKEIN
HFNAQNFQNAVSLLGDLVHAYGYELIENSKLTDLIISVYGKIDILSSQGDEKCDSCVSKI
KWLKRICNARLGQKMSVYIQLTHDLSGTVTAVTIGDWILGILKQKNIQEYEVFFKKFLNI
YQKQDKDSESNNRSEIFSLLLSASDLVFETLVETKKNNKIKVIIDNKEIVKTYNEFYREV
EEYFVILISILQLEFKSVEDLNNATNNFIMAIKNFNVFPELRLKILQLLYNSFSVNFSFR
FPTFIAILQFSSQNNIFHFMLPYIKFIDEWIKEWNISSREKRQIYLIIAQELKKLKKYEE
SFKHLNKHVYYFQKEAPEVLNHPTTVNATIELIADAINLNNNIYFHQLLTLDAVQNLQNI
EEHKPIFHLLNIFYQYSIHEFLAFKNNYGEELFTKYNIDLQVAENKIYLLSIISLFKDTK
VQNIQYISEELNISALKIEQILVAAIGSGVIDAKIDQINKSVQMKTTILRHFDEAHWEIL
NAQINKYINNVQKILDITTSRSRPMQKMNYLIPVLWCIFLSFLARGQESATNFYKFVDSS
ASSTYISEESGSSLYDAKRAIQNNPGYWCSSGNHGKDEEISWTGYLNTKGFIKGVKISWE
YSPESVSISVSSDGENYKNVIPYRRISGNEASFDEIYFFKKLEEVISIRIGLKNAIHKYF
GIREVKIIGGGNPYFLLLSGITSDNEMCLQVEEGLVNNDNTSVILDSCINALASGDGREL
WKTNSNNQIISAFSNPPKCLSVINLDNLEKNKIVLYDCLRALEDGDGKSNWIFESNSQIR
LQRSGEPLCISQKNIYGNVPGIHDILLNMDITVDANSTLDDDHNADNTIDGNLNSYWASA
TFADNYEHVVNIILDLNKYVEISRVKISWEYPPLHYSISASIDNQEYKVIAENLANPSFV
TIDSLKNLETRYIKITMMKPHPKHGEMGDQFLYGVRSIEVQANNLETTIGYCRDAANSDD
ARDKYFVEYITEFDEDLTSKLINLEDDVSKNVNSISDNLSKLEELLPNIETCIQEKKEYD
EELKESKEKANELNEKLSSLVSVNSIHDNDLLRLDILPGDSSSYPATDCSVIKNVQDISQ
SGFYWIKPKCAPEPLRVYCDMESSTSLYVWNGNPPKHSDHLISNIINSVDDIRQHCAEIG
LEPLILRSKSQLNSLILSLKKMGYTLNGKNNIPLAYDYSCDHGSCSGKFHDLVNGNIDLT
TLIYLKASESPDSTKVRQTAGISYDDGSFKFFNLETSDISAIVCSTNSTENDDALQYLRI
NCETTALEDYFNSIVNTNIVVLCPLGCASDNFKSNAVYGSRGIYADNSSICRAAIHAGVV
DNKGGLVNVTIESGMDRYEGSISNNVESISLNKDPAGGLLDIITKDEEENIKEERSIFHH
WTIRIGNLSAECPMDLFQYKHTSFFQKGETTK-E-RKEMAYSEDENRDSIIFHELITDLL
RNIDAIHGVDPSVISIVQDETVRVIEKSKKELRPADVLSKKQIDDAINLYNITENLALYL
YDLSGKYMYDLERLKERLDELKKEQKVALNFGTFKLNYETMNFSSYFQIFDSKLTKNVSS
NWGYADTDIAGHKNSIGQTSSISNREIGEAYYAKLKGLNFYDFEINVSILSRGNGCSGIV
FRAKDDFNFYLFDVCDRGGMKRLCKVENGNVEVLKEKLTGVNTNNKWNKYKIVTSHANID
IYEVDENSNETNILRSLDERFLSGTVGLYSQIHGQGSFFDNLEVIPRPCSELSKRGAQKK
QRESSCPYYKENYLSETLPYSFINDVDYEWSFARGGDEHLLCSKMVQISDKSSEQSSNTI
ALLKQRKCSDGYFTLDMNFSKDEGNNDELVKILFNFVNEQNYNALEMTNDGIRIVTHRNS
KSETLSELTDREKIKKVLVPNEWINIKLHFQKSKITVVISNNEEELQLNTDIADVDMIRS
GQVGFWVQNFSEVKFASIILSSAMSHNDGNFIQTKSKAWAACEDSVHVLNRRSSCQTNIF
PNETKEKHINCIKNFCEECCLYHTQMLDSNEKKQCEKHCKKNDHLAAKMQTLFEKFLNRC
VSLEENKDYKQCADNDTECRNRVCVLCCKRNDPENSKELKGLSLQKSKDIQQKEVIECQF
QCNRAH---AMNISPIKYESKILNNLASMLKLSEKEEYKGKKFEYYYNVETYGVDFFLDI
CNALGRSNYKPFLSEEGKKEVLSKERLNKDRLNDGKTPKRKMYLQDNIYITEEMEEKMEN
DILREKNNNEDPPDLRDGIITVSRYKAELASITRKSNEKIIKTILDHTKNMDTSISGDLN
GSNTFEENNNYDSFEHNELKHSDSANGFSNDGTGSTDNNAHSKKRTLKNNPVANNLIQMN
GSAGNIVLRKKRERQRRSQTITKNKLSTNSSRKSSSVKKKSVDGGVALPDSAPCDEAQTD
APTLELILQGNETPRALIQSGVVQQAEWDKKLGSNVTEVQTKISTGEEELNSKGYQYDVY
QKTSLVKNASNEESYDLEGSDDASYSTDDSNYKRYMRKKTKLLQEGKIVIDLNILNGNAK
NASEKKEKEEKNQEKIRMDNIRKKLEEKKMFFCKIEEVAKRRNKRHVKTQVLSKDCKIER
AYFSHKMKKFYNSKSGYFGCGWSNNKTQWTPFIHAPFFDNNYNTIYKNRNKKMYEEIYDT
LLHGRIHPAVRIVELKDHKHPVRLCTPYSEDCYSVVYTGEKILASDDRVIFGEYTGYVAN
NSELSQDKHQYMFALSFSKKVFNDRKNVVFINEIESDEEGVGDNHHGEEANDIITHRDSS
NHSEYATTEGIYLSDEKICKEASVKKGDINTNRGSTVPNVDTIQCQNFIREK-----KKY
KGMYNKIILPDNYTYAVDSSYMFNEMSLVNHYKTCSIFNNYDFRINAEWQLVYLDGWPHI
ILTSIPGVEINTGEEIFADFGFEWFERVNDICLNDFIKNSYACRLSNLNLGIEKTFSGID
DIVEKYNLIKNHTTCNICMHSVNTDGSNFIACSGCNHIYHLTCVQKLNAEVNENYDWFCA
SCVKFCINMLNQEEFVANLEMENHKLLIQLFSDDMCVEKLNRLPTLLALEANDTPLCIPD
KDAKEVTNNSNCIEQYDRIRKLLRCRKNIIDLVKNKEIINSYLENTEKEMNSLDNIQ-EV
GNLSFKKDKMKSLLQNSFELHLLIEYKKKIDDLLLSREKVDTFFNDEKAQRTMHLRKQTI
NYLLENRKCIEKLVDSMEKGTSIVFAHK------------------EVNRSDRD--TTLM
GLKEEFPQEHNLAIEKWDPSFNRGESP--GSVIKEHTNGSISNSYEKILYRKSNDIMNNL
REIKNGTPRGRIKSDIVNCPTSSNGNSNMNGSNRNLCFNNMMKLSKKILGFPLLTDFERG
MSTNQPCLPLSDHLKRLSVCTVCYSKHGDLAKAIICRVTKMHFEPNYNDGLSDEDMFKTS
SECVQSIIRELANTIKEYRKRELSGAYLQEVELSASSGEINANSAGAPLAGHHNHMGAEA
HSSSSVPSRTEIQPRQPFYEMMSNPACIGGHQRGRNTPVENNTPPLLLENR--MSYTDDY

TFKREQMNSTKNKSSYEENDGGSSKKKSK----LRTKSSNKVIDENDDSLKTNKAGSHGG
RCR--VHGEIGQ-----DPCFQGHAEEHVHPRKEGNQEGEKNHMAQSEQKGKDVTLENIS
KTSNFVPLMGVQLGKTKIQREFTNGTYVGTVMEQIKNENGIPFFVVTYDDGDTEWMTPYF
LFQELLKQSTNSVEYPLATTFKEVFNPEFKKDLKLNNCSLELKIERRKRKSNCESASNSN
SVSKRQKYAQEENSTKKKKQQFMINKEEAQRLKGIGNKCFQEGKFDEAVTHFTNAIKNDP
QDHVLHSNLSGAYASLGRFYEALESANKCISLKKDWAKGYIRKGCAEHGLRQLDNAEKSY
LEGLQIDPNNKSLNDALENVRKEKLAENMEYINHINTIIQNDATLKSYQEENANYSIELL
NTIKAINANPMNIRYILSSSDRKINEGVEKFFGIKFNEDAGYDEERQRKIEEEEMKKKKQ
KKEEEEERKKKNRSPEEIQADEHKQKGNEFYKQKKFQDALHEYDEAIKINPNQIMYHYNK
AAVYIEMKEFDKAVETCLNAIENRYNFKADFSQVAKMYNRLAISYTNMKNYDKAIEAYRK
SLVEDNNRATRNALKELERKKEKEEREAYIDPEKAEEHKNKGNEYFKNNDYPNAKKEYDE
AIRRNPNDAKLYSNRAAALTKLLEYPSALEDVMKALELDPNFVKAYSRKGNLHFFMKDYY
KALQAYNKGLELDPNNKECTEGYQRCVYKIDEMSKSEKVDEEQLKKSMADPEIQQIISDP
QFQIILQKLNENPNSISEYIKDPKIFNGLQKLIAAGILKVRMNEHYDVIILGTGLKECIL
SGLLSHYGKKILVLDRNPYYGGETASLNLTNLYSTFKPSEKIPSKYGENRHWNVDLIPKF
ILVGGNLVKILKKTRVTNYLEWLVVEGSYVYQHQKKGLLYSEKFIHKVPATDMEALVSPL
LSLMEKNRCKNFYKYVSEWDANNRNTWDGLDPYRLTMMDIYKYFNLCQLTIDFLGHAVAL
YLNDDYLNEPAYKTLERIKLYMQSISAFGKSPFIYPLYGLGGIPEGFSRMCAINGGTFML
NKNVVDFVFNENKKVCGIKSSDGEIAYCDKVICDPSYVTHLKDKVKKIGQVIRCICILSN
PIPETNDINSCQIIIPQNQLNRKSDIYINLVSFQHGVSLKGKYIAIVSATVETNNPIKEI
EKPLELLGPIEDKFVKISDLYVSTTNKPDDNIFVTSSYDATSHFETATNDLLQIWENLFG
EKLNFDDLNNKADNE-MAVKKQKKTLKPVTKVITINLSKLTHDVCYKRKAPRAIKEIRNI
AGKLMHTKDVRLDVKLNKFIWSKGIRNPPKRVRVKIERKRNEDEDSKEKMYTIVQHVMVD
TYKGLLNECEVNEMATPKGRKKSDKEKIDESFSEHKESPKKSRNNMQEEKALKMRACLSC
RLLRTEAEFYQNGCSNCKFLQMAGDRHRIHDCTTENFNGFMAITTPTKSWMAQYNDLSKY
APGFYALQVVGELPESIRDLRPNYMESCPEVKINFHHIYSNQELRKNVYLPSSDTFTFLE
ALEEDVETISPTVHAALEMGTGSGYLILSLYELLLKRKKKINLLYCLDINEKACKCVRKL
TLENKISNVEIINTDLFSNLKHCKQFDLVLFNPPYVVTEEEEMNKTDIVASYAGGKYGRE
IILKFLLSVYDYVSDKGVIYLLMEKNNRPNEILNDGNISSRFNYTELKKKKTLNETIFIY
KLSKKGMDRYGHNVRADVKKQGAESAELPILCETCLGENPYVRLIKEENGKECKICNNPF
TLFRWKPGQKSRYKQTIICNMCAKVKNVCQTCLFDLQYNLPVQVRDKFLETSISMPENET
NRNFFLEQVEKNLDTNTYNKIDRGNMDLSKLRRRDPYFKRNMARVCSFWRKNECNRGAEC
PYLHKEIHMDKSLSNQNIKSRYTGENDVLAEKILTRYEKQNEDNRFMANNICIHGISEAV
SQVNVKDCFKKFGEIKSIKMIPKDSKMFISYANSQSAKKAAETYKDGLELNGSNLTVILQ
EEVMPRGSQNQRNVNFMKKNNKADAPPPPGMMMPARPMFFPYQNYNFNPKAPNSAPYLSM
RPSEAEQRKMIKNIFNEYLKSHEVRRTDHEG--LRKVARDYKNNYTSDWRWFSIKCERII
ENELQKKSLREEDKKAYGSWVKEAQNLSTFRSFFPKILLKVYSRCDDPHKFFSKLVIQKF
DAVVFFCDASGFSNLAEQLDKRINGTELLGNCLNKFFNILIKIIDCWGGDIIKFSGDAVL
VIWPLR----RGHRGANSTPNGTASNGTSNGASNGIPNGIPNGAKKPNQKEKNVKKICML
ALGCCVDIHKLLNKFPTPIENKYLKVHIAITYGKVSFLQLGNILNKRDYLLSGKPLEEIG
VAESLARNGESVISHRFYEKVKDRIVVKETAKKKFFLFVKMKEEIDMRELKREYEEGDWE
LP---TGQSETPLVHEHFCHLLKSFIPDIVYRKIATGCNVFINEIRKVTIIFLSVKDIDT
STMIGVYSAHGIMKLTQKAVFTMEGTINKFIYDDKGILILIMFGLPPLYHCDDSIRALLT
CFRLIDALKSLKLNGSIGISTGRIWCGLIGNKIRKEYTALGDSVNVAARLCCKAGNKEIY
VDENTFNGCKHFISFQKLISIKVKGKKKLIRIYSPIGTINRSCPQFGLEQGGAHGYFTDE
ELQAANAEMNEVTDFSFLNKNYLLRYYQRTYRRGIKRLLGKKDCLYYLRRCEGGDDAFSQ
GEDTWRNPWRD--TWQAAYRDDPYSEIKANTPLDYITHTGPLLLHEYYDPLYCDFFQMYN
TGGVLFLQGNEHLGIFEIITLVSRKLSNYKTFKISNMPHSLYINVTNPLLPWKMLCNEML
QLWSSCKMRKANTLIRNEDNYNLLREITYPSYHWFFKCMSSVVDDLAIPYWG--DSGRKV
QTE---KKGRKTRSKQTGAVLPSTQQSTDKDTDVGTGDPTDELTAPDTYARQHEDSKSKK
KTKKKKKKYGKDGSKQRTRMKKQKGKLCSGSYPFAEAHPLLKRDAKGNRMGIISSMLYYF
TLHEYTFVVFNYRAGTSLNIMGDESALGICKNIAKLAMYKRNKILNNIHRGIKHWRRHHC
RGCNYCKGFYKTLFPNINDNILLYKSPLCDPIFKGKHKPLIFLFVNGMKDDAIGAIRKIK
KCAEQCNGYLKLEELNRERLCDFVCLCLRVKKDELNKELVNYLHKTCFGIPKFVQYTLFY
LLTKKYIKLYRYACGVRESEPVSEAPNQRDTSIGGTEEHSRNSSSTINAGSTSLHDPTSY
TYINEETKQIITQQNGFQNFQFCEET----EYDDNRSDDNVIKVVRDLNSAPLVPRLTAH
CMSLIDSLNQEELLLAKLCSFFKQNFNIKKMECIFPRYISRSELKRIIKELIRKRVFQVC
EENRENQVPTEEALFFCITNFSLKKVLNDLLENEEKKYIQKIYKKYLDMDNRPFQNESNF
DKTRLIIKNIPKYMNEMDLKKHFFKMKGNYEFKITDIKIMKRKKIVKNKEIFESRKICFI
GFINNTDCENFKKSFNNTYINTSKIVIEDAFSPLISKNAQQSRNATSALKRIGDIQKMKK
DKSVKIVKSNNFVNKTIPIKKTKAGMSTTRSHMIFLDDQPVQGEVKEEEKKKKKKKKKKE
KKKIKMEGDNPSESNAENEEAEEEGALDWLKKISKREESEINNH--KGAQRNRLFEDEMS
TASNLSSDEKEVKKKEDNGMDDENCENMNTGKIIIFNLPPVSEQDVKNLCERFGPVVDVK
VFRNVKKGAVVLNLNEKKNNKSSDDFFIKLLKENHDSFANEKKEKKKKKK--VDEDGEA
EDWKNKNRSNNESILNSDLTNVKVYAFVNFMFPSACERAKQFLNHAIFRGKVLSVKYAKE
KIGDYEYTEKEKNNVFIKLSHDSKTSYKKILEIQKKRNCQNENIWNILYTDINSSIHSFC
KENKCSPNSILNIKDRNIAVNVSLTETYIINKMKEWIKKEGIYLEAFEQIYRKRDEGGEG
EEPSHTNHVVKYKRSDDTIIVKNLSIQTNQKEVISLFKKYGVLSKVSFSPYNNIAILQFE
KAENAKKAFISNSYIRYKKLPLYLEWAPMNLFQKKENNKGETKSGEIPAKEEQEGAPKEE
QHYSESESSDEEITHASIYVKNLNFNTKEEDLKKLFEKLEGFITCNIVKSKKAISKKNDE
KGKEPEQKLLSQGYGFVEFKSKELALEAIKKLTATTLDGHVLELSLSRNRVKKKKNKNNE
EKEVVKEKKKITKKLLVKNLAFQVTKEELRKLFSAFGNIKSVRIPKNAYNRSRGYAFVEF
MSKNECLTAIESLQHTHLYGRHLIIDFADDFIFDKNVNEFDKMKEEGVNNNNKCITSEQA
KRKATYEKEKKNQVTESKRRKVMNNMQDIMLNNCGESNCRGYAQNLCDQQKKGTMQRDPN
DQYYQVASNLFSNSMGGTRVLNSGDAPPQNARILKPLVQEKVVEIMKPEIEEKIIEVPQV
QYIEKLVEVPHVILQEKLIHVPKPVIHERIKKCPKTIFQEKIVEVPQIKVVDKIVEVPQY
VYQEKIIQVPKIMVQERIIPVPKKVIQEKIVEIPQIELKNINIEKVQEIPEYIPEVVKKD
IPYTQIVDRPFHVEKIVEVPHVQHIYRNIVSPQYRHIPKPVEVPMAHYRTFPIEKLVDRN
VPVPVELQIVQEFLCPKIEARYKEIPVPVHVQRIIEHPIPKDAMNNPFLLPLYYQEDNID
TTSSKG-----SSKNCFPFNWNNKQNATKINKGTQSVELLLHNDKYHNKVNINHDANMRA
KFVPPSGNVYPSTHLRDDNTYHYPNGQHISAPGNASLNHLYKAQHVNSHTNFNTAPQDQN
DMFNGSNSMGFTSDTEMGNAYSPISALVSPHSEMFSTRSEYDRGVNTFSPEGRLFQVEYA
LGAIKLGSTAVGICVNDGVILASERRIASTLIEKSSVEKLLPIDDHIGCAMSGLMADART
LIDYARVECNHYKFIYNENINIKSCVELISELALDFSNLSDNKRKKIMSRPFGVALLIGG
VDKNGPCLWYTEPSGTNTRFLAASIGSAQEGAELLLQENYNKNMTFEEAEILALTVLRQV
MEDKLSSSNVEIAAIKKSDQTFYKYNTDDISRIIEALPSPIYPTIDMTAMELEKTHLINY
FLEICPTVLDCSRKELQSVLAKKEEEKIRRFMADKNVNLIVIGKEKKEDADNEGSDRNGH
SGSTQEGAEQEESRGAKLEKEPGHVLFVELNINYKCVTKSVAIAFMKRKKENFLSMHDRI
EKGNKTNLSSELLMFVCGQHDCDTPLDLVYLYLSQGFNTIFDAASSATHNADAKDSIPNG
YSRKELESSGSAMNTNPGGNKYLTYEGENKLSNVLMTNVSKKLNELLISMKNAQIDLNIP
IINLSVDRRIKELVREYPNVDEIKPDKLKALCESQEFLNHLQKDVTKWVEEIQKLTRLNG
DFKSGSALAEINFWIGYENALLQLENQLRTPEVLLTLQILKSAKRYFATMSFDSDIQLKQ
SKEYVLNVNILMKDFPIDDLLGAQTVQQIIQAVRIIFTHLKKLKNTTKYPLSRSYNFVEA
LSRDLGNTLKKVLLPQALLSMDYQEFDVLISGCMEVFRLWNDEMRVFKDMVRELIKKRSF
NERAPAKMVFEHINLQERLDEIRKFRKQHQKFKMVITKVFGDDRTVGVNLLKDINSAYDI
FLPLDALDLSKTGEELWEKSKISYESRINKVESQITFKLRDQLGGAKTSTEMFNAFSKFN
PLFFRPKIRGAIQEYQNSLIQIVVDDLRKLQMVYINGYVKSPSEKVSTIRDIPLVAGSII
WAKQIERKLEDSLKRIENVLGRGWEQHSEGKNLRQSIDNFKTLLSQNKTFEKWLKGVKSA
DKFDIYEKIIKIKKLGVKNFEIVANYDFEFFNLFKEVRYLQSINLRVPYSIKVKADETKL
IYPYALTLQKTFRSYMKICLFMENEAKAVPFNSTITKLVASIHNNVQAKIKEGIGLHWDS
DVLETYVRKLTEVVNTFESMVDEAVRKNSFVLDTLDRIKVCEITMQGDEVTKLVKSIQEK
ADELYLEHYRNVHIWIDELNILLNKILTSRLEEVIKAWTGEFVNWTSGFQKGKKFIMKET
THEFKIINQKFYLHPSIDTMRQMWYTRLAQAINIICGIPRVKNIYQKKDRTKETIQIRKE
KDSIASDIILDNTYRHIIYFIDKKVYDDAIECINDTVEKARKYESMWLQYNTLWKIEIGD
IITSFGEDIEAWKGFMNEIKETEYTFDTLNTEKNFGPIVIDYRILQSKVSSKFENWQKEI
VSEFSKKLSEKIVLLKAEIEKALDDLTLQAEVTNESDITAMHILSMAPKDIIAIINTHDM

DVLSKICNSVYKFIEKINEISTKGAEWDSRCDVLKQSEVLLEKHRYVFPISWVHIDNVIG
KVETVNQICSYQIKLVKDYYPYIQSLVLEFDTNVQNSIKVLFEEWNKNKPSHGNTNSTKA
LQIISAFESRIDVSNGQYEISEKIKKLMKIRSEESENGFHVSPNILKEEIACVKGIWDEL
KSIYSTISEMKKMLWCNVEPKNVKNTLNGLLDCIKRIPAKYRQYEIFDNVQEEIQQYLKT
YGLLLDLKSESLKDRHWRLILHKLQIKIYYNKLTLGNLWSLNLSGHENALREILNQSQGE
MALEEFLRGLKDTWNEYELELVQYQNKCKLIKGWNDIFSTIDDHLNALQSMKISSYVKIF
EEETLTWDEKLNRLRNLLDVWMNVQRKWVYLEGVLKGSSDIKSLLPQEYNRFKIIDTDFI
NIMRKTSEKPKLMELFQMEGFQKQLDRLSDSLSKIQKALGEYLEKQRNQFPRFYFVGDED
LLEMIGNSKDAKVIQRNVNKMFAGISSFIIKENTSDVILGMSSREGEEIFFEEPICLSAY
KTLKEWLITLESYMKATLQNSLDKAAKEILQMDVLECANCRGNNKILEWASKYPNQIVLL
CLQILWTSNIEGDMQNTSSNEQNPGKNIFQRSEKICVSLLEFLAVSVVKQRDHRTRQKIV
QMITELVHQRDVIRVLIEKNVRSVHNFTWLQYMRFYWDSSKKNSDVNLLIKMADATFEYG
YEYLGMCEKLVQTKLTDACFLTLTQALKMKLGGNPFGPAGTGKTESVKALGAQLGRYVLV
FNCDESFDFTAMGRIFVGLCQVGAWGCFDEFNRLEERILSAVSEQILTIQMSLAQRKKEI
EILNKKIGLNKNVGIFVTMNPGYAGRSNLPDNLKQLFRSFAMIEPNKQLIVEVTLFSQGF
ISAEHLSSKIVSLFELCSEQLSKQPHYDFGLRSLKSVLNSAGNLKRQALLEGAHGGNKEG
ENAKTDSSTESIVEMEQNLLLKSVCDTVYPKLVSSDILLIRSLLTGVFPNINVAPFEEKA
LVNEIHRICKSRFYTPEEKWVTKICQIYQIMKLQHGVMLVGDVGTGKSSAWKVLLDAMEA
LDNIKGVSYIIDAKSLDKEEIYGKLDNINLEWTDGVFTAILRKILYNSAQSGNATRRHWI
IFDGDVDPEWAENLNSVLDDNKLLTLPNGERLPIPESVRILFEVDTLKHATLATVSRCGM
IWFSRDILSPTLLFKHKLAKLKYGNHDYPRQMDRFKAFIMNNNESIRNDSAHMNDSTTMQ
RTSNDSLSGSHENIRMSDNLMDHMKKNSHIFFDENEDELNVCSPNSIASRSIKLISDYFE
ENEFVHQCLLGANNFDHVMDYEYIRAIESTFLLLEKGIDNLVVKNEKTNNAITDGDIEKY
ISKWLVMSILWGIGGSLNLEAREKFSIYVESICSIPLPTDLSRSRQMDDTDSSGAPRTLL
DYEPSIEDGEWHCWKERVEMIDVDRGEVSDATLVIETMDTIRHATVLEGWLNLKKPFILC
GPPGSGKTMTLTSVLKKSSEFDIAALNFSSGSLPNLLLQTFDHYCEYVKTTSELVLRPVQ
PGKWLIIFADEINLPTPDKYDTQRIIMFMRQIYESQGFWKYDSNNNQWNWVKIERITFAG
ACNPPTDAGRNPLSNRFLRHTAILYVDFPGYESLKQIYGTFNRAILRKFGDASHMADNLT
LAMVNFYTKFSETFTIDMQPHYIYSPRELTRWKLALYETLEHCDHLEKRDLVRLCICEGL
RIFQDRLIYKKEKKQTDKIIDDIFKFCFPGIGEADLQRPILFTSYVQNEYKEIDKRDLKE
LIKAKLKVFNEEEINVQLVLFDDVLDHITRIDRVLRLPLGHLLLVGASGAGKTILSRFVS
WINGLSVFQIRAGRNYCTESFEADLRNVMKRSGIKEEKITFIFDESNVLGPAFLERMNAL
LASGEVPGLFEGDNYTTLINECKAAYGSNVGLDESDIFKKFTKQVQKNLHIVFTMNPANP
DFANRQATSPALFNRCVIDWFGDWPYSALLQVASEFIYNLDLPDKNFHMDSVEEGPIKGK
IQYKDNKSYYLSRAIVEIHNSVVRINGVLLKKGSKYNYMTPRDFLDFIKHFLKIIEEKKE
EVSDQKKHLNLGLSKLKDTEVQVAELRNSLAINKKTLAEKDTEAEEKMKLMIEQQAETED
KKKKAEILAKKLDEQFIIIEQRKEIVRKELSEVEPKFREAEEAVKNIPKKNFDELRAMAN
PPILVRNAVEAVAILIMNEGDKSVTWEDARKIMKGQDFINKVLYLDKKMVKPQTSGQIKK
RLSHSDWDVDRINKASRAAGPLAKWVESVITFLNILETVQPLENEIEKLQEETRIAEDQY
NEQKDIISELEKKLVQYKNDYAQLISQVQSIKQEMENVENKIKRSINLIENLKSEKERWS
ETFINLEAASETFVGDCLIAAAFCAYIGFFEHYERQKLKKTWGEIIKMHHIKHRHDLSFI
EFLSNPSERLQWIANELPSDELSIENAIIIKSYIRYPMIIDPSDQASTFLLNQYREKKIV
KTSFSDKNFIKNLESALRFGSTLLVYDVEKVDAILNSVLNQETHKQGGRLLITIGDSEID
YSPAFNLFLTSRDAHFQFTPDLCSRVTFVNFTLTPSSLQNQCLNMILKNERPDIDKKRCD
LLKLQGEYKVKIRELEESLLLELSNVKGNILDDDNVISTMEKLKVQAAEASREVNIAEEV
MVEIENVSNQYLFLAQGSARIYFILQHLGSINFLYQYDLNFFFNLMKDMLRKEELPSNVK
KNNYQERLMCLEHLLFSLTYNRVARGLLQEDRYVFALQLCYVKGVINPGIYIDPNHLHFL
LKDHYGSHHADTAVSSEEGNKIEKGLLPDYTDEQISTLNNLAKHKSFANLRKSITNDKEK
WIQLIHSTEPEQIISSIMNESREVFNRNTDPQYVHNLRSSDNINKEEQILACLKESLIIK
AIRPDKLDKCFNKLINVILGKDFLWIPELSTNEFEKYVKENASGNIPIVLISAPGFDPSN
KVQQLSEKCRVPLFSIAMGSEEGYISAEKIISSAQKNGGWVLLKNIHLSPKWLHQLEKNI
HKATTNQNFRLFLTMEINPKIPPNLLRISLTFMFEPPVGIKSSILRTFSLFLENRNLDDP
KIARMRLYFLVSFLHAIILERRRYTPIGWTKGYEFGDSDLSCALSVVDNWLDRASTKIGK
SVIEHIDPSNIPWDAIKKILNEVVYGGRLDNLVDSKILDTFIEHLMNSNSFEADFKLNIC
KSSSEKDLLNSPDLFRHLNDYLKWTTNMSNTDLPAWLGFGKQAEGLLTTRTNFSILSKWN
ILYSKSGSDVYEPLPHAPDVISPDDSSS-----EEGELFESSIARTVTHRYSQNENVLFI
NQILDSLPEYIPELERNEENMSNAVFRCFERENHLFRNLLKVIRGNLNQLKNVLEEKLKY
TNKLRALAKDLNSFNVPPSWLVDGNATNLNLTNWIRELINRFYQIIVITTELNESRRANL
NKQKSGRSSSRTFSNDLTDKKLSIHFIWLGGLFYPRAFITATRQLCASKFKNSLDDLELS
VAIGPSSPEMLDDTVHFTITCLSIEGAQWSDLDEVSADSMNKNKKINKMVNQSSCNLNQN
NNVVTHIVPSTNLSSQHAKQIRLNQMNHVHKSHLLPYHGSNYEEKSSKLYNNGSSVNNKN
VLNGAAANAQGSSNLSSELIKNNKLGCTNRQKNVNHHVHSSHAYNNNKSQQHSYHENLGY
RPTLFNLLEVLANHNITTPDQRVCIRGIVTDFLNNELPHGKIYAYIGAVVGHDILHDIIR
KLEKDPNRNVVPDASGLARIEAAFGLSKSSYDFMNHSSNSTSNNIPNVLFNQRNLNSALL
NNHIYSNNVKTQADLNNLLKYAKNDKYALSCNNLINVGKKKGNNMSRGNASDDEEELMKV
IKKNIETYKMNNIKNMLISSVFGRIKWLKIMNESPHAYLYGHSIVKFGNKLYMFGGTNSK
HKKVPFNHTLTFSLIYYNYKLLPLGGNYPEERDGHTTHLISLKTGLAVFLFGGSNENTYF
NDIYTLDMETRKWSQRITKGNIPLPRDQHCSLVYPAKSEHVRGEKTNLTEGVIIFGGKCL
YNNNIVNLNDMWIFLFQINMWVRINYSSEESPIGRYGMNFVWSDTNTLCLFGGEYFDSNK
NCRERKLLDDMWTFRLNAATNNMLKRIKITGEWKREIYEGKIGCRSNYSSIFITQRHQDF
KSAEPKTIEKLMLLCSGITYMNDNQLKIVSSDEIYVYFFSQKRWYLLKGKLYNEEFIYNA
RQRHVGCFFESKNVLGRANKNPVPCIFIQGGFKKNSIFGDAWLLSLTGDNPLRIHEYDTS
REKISTTQMPLYYFRDTHSISLLYSFCTLQKWLFGAFANLVDNCVHALNPAENVFIKYEL
TPEHDGMLSIQDDGEGLDFNAMNRILRLYGNYRNYDNNNSYNENDGASVKKHNFPNSHFT
GSSRIDDSDENGENSKESGSNTTRDGQEGSGENS-NPTGEEVQTVGNKNEKSTEGVTQRR
KKKCSFNDQYYHKNYDQEYFYNENANSIFDIKYGVGFKMSFARISHSCAIMSRTINTIGI
GLLSLELMNHCDAKELATPLCMWKLPNKELINRNIANKSEHRHHQKLLMSYTPFNSPSLL
AEQINILGTYSGTRLLYWDFRDDMDFIVFSPANNNIYLSSSPLSIDEIKCSKKKRGITDG
ASSDDSRKLHCIEDIKLSNREVKRKAHYEETEKYKRSKVEKDEQVSQVGEKLVKDEQGDI
GEGTATDDIKKDEGSSNDVDSAPQGGADNDGNSAGNQKDEGAKPKSNKSRYYEKEEFKHS
NDILKKMNLFEYNSHLNPSISKEKYNVSPIFPLWDHPKDSIDYCLSTYLYWLYLRRSTNI
FLQNTLLIPTCMRKDESSTGAHKGKRTKKGKKIKKGAEAENTTNAEDGNSTVAVK-GEKN
GDENTHMNDLNKDGMPSEVHVKEESNSSINKEASSNGDINGCKGNSGKGNEGGFTKLEKK
KLEEALEYGISQEVKKRKHVHRNDSDDEDDDEENDNDDEEEDDDDDEEEDDEDDDDDDEGK
ENANAAENSNTVEGEEAEINGE-ATPNGEYVGDAENTEKAQETKDAELNDSTLVKNPDAA
ENQKEKRMKKTKNKREKNESGVNNDSKNEENEKARKENYVNVYMDTLKIKDEYYINNTHK
YTLYNFLRRKLYRMVEFHYLFTPSDYEYGSFIMMGFLNDNTSSSIEVNRVCEAGILLYYK
NRLIKRLDAPFIDTAYNLSLSKYPPNPSLYEGNLYKYALTVIVNVPNWLKPSISKQEFVH
ENNYAFLLFKKKLVSLIKYYLCICEDTAKLTKWRESRDFKLKRYLENMQYSNKPSKNYSD
NEYEKAYKIVHTDQ-EEVVSHKEGESQRRERGTAHLPRCGESRTHEGKRRRENDMPEKSK
EETKQHSQGEDDEEDDHDDEEGEDDEEEEDEEQRHADAEEEEHNEEEDQNDEYAEEDEGD
DEADDEVDEVEEEEEEEEGHVEEEQEEDEEYDPEEEMENSADVKAVADENNGKVTKKKNL
CRLIVEEATNDDNSVVALNTKRMEELNFFRGDTILIKGKKRHSTICIILNDNELDEGKIR
INKVARKNLRVCLGDIVYVKPCPEIPYGKKIQVLPLDDTIEGLAKDTLFEIFLKPYFNES
YRPVKKGDLFLVRGGFMSVEFKVVEVDPDDFCIVSPDTVIYYEGDPIKRDDEEKLDEIGY
DDIGGCKKQLAQIREMIELPLRHPGLFKTLGVKPPRGVLLYGPPGSGKTCIARAVANETG
AFFFLINGPEVMSKMAGEAEANLRRAFEEAEKNSPAIIFIDEIDSIAPKREKTNGEVERR
VVSQLLTLMDGIKSRGQVVVIAATNRQNSIDPALRRFGRFDREIDIGVPDDNGRFEILRI
HTKNMKLSPDVKLEELASSTHGFVGADLAQLCTEAALTCIREKMDVIDLEDEIIDKEVLE
SMCVTQDHFNMALGTCNPSSLRETVVEVPNVKWDDIGGLDEVKNTLREMILYPIDHPDKF
EKFGMSPSRGVLFYGPPGCGKTLLAKAVASECSANFVSIKGPELLTMWFGESEANVREVF
DKARAAAPCVLFFDELDSIGTQRGSTLGDGSGAGDRVMNQLLTEIDGVGPKKNLFFIGAT
NRPELLDEALLRPGRLDQLIYIPLPDLAARISILSAILRKCPVADNVPIDFLAQKTAGFS
GADLAELCQRAARAAIRDAIDAEEMNKKSKLQMYPNEKENAQNTVQNNEENTIKYEITRH
HFKEGLAGARRSVSQADLIKYDNFRIKFDPLYKTKTGGGNDDFIIDWPDEENNEEPQEYN
VDDDLYSMRPESKMIFKPFAVKTPLLKELRKKGLVSIEVADAGSVKVLTEDDLEGSPQEG

ETSATNKERGNKNGSKLSPSKRSKKRKRENVDQTNKVMKQGEDTHRGLKRRKKKRGRKKK
ST-----GCNEEGVSIDQAACSI-------DSTEAPKRERFDIQKVIQNEENFKGTDQVK
YKVHYVRWNMNGKLHLLYSIMKSLHDAKFWKPTEIQKQTLEHSINLKNDIIVVSKTGTGK
TLTFCIPILNNIVKNKLREYQKKGSCISKLRCLILVPTRELAIQILSHFSYINKYTHIYI
ATIIGGLNLNKQKRIISNKPEILVCTPGRLKYFLLLSDKINYLDKMKHVRYFACDEIDKM
IETSFMRDINFIAKHLYKSVAQKKKFIQTFLLSATLGLSVHLQNENLAKLLNYVTIRKEK
SCIINLADDHL---------VDGPSRGEAGSILPDGLSLNVVKCERKNIIHKLFYLLKMY
FLTDSMGQEHGEVKKIVIFVNTIKETKDLNSIFRFLFFDQGLESSVPKKYRCGMSLKERI
NIFSIHSKQSLKERMQSISKFSQSTNHSVMFCTDVLSRGIDLDKCDVIIQLSCPVSDITF
VHRSGRTARNFKTGTCICFITDDEIARWKSSLKKIGLIFENLQELEMVKRISNHEQTKIN
SAILCCNKMIELQNKIKDNKNKSLLSKLAREAELEDEEGSSDSDNPSGKITNETILKQLL
RLKKELYSTLYGKMYDINKVVSVEVRSAELDVLNSEELRKISLGKYENELLEYEKTNSSG
MTSKIYFDPKYGTIDYRQICSVCFEKHENCVGHIGHLEFTLPLFNPLFYKDVYDLLGLVC
LNCYHVCCSEDTIFLLKHIYQLRALNEIESSSGISCKTNYDRQYVVGKIEELYRKICEQ-
--------EGLISGKEF-EGTNYANQADGQDYINLSDA------------PNTKDEDEKT
HQKD--DVQLNVKKIKEKIKKFNFDASKLAFESNYNYEEYLKLIKTLKVLMKRNGRCPIC
KFKRNISARMSQKRDTINFVGMYLQNSFLKKYMKEEKKRGKGE------DPSVGGEEELE
EMVEREEEGENIYGSRNEMMNAPDQVGQAYDASEASPSLADGGTRDNFPGTTFNYRKQNV
KEKTTVRLFSFQVIDLLGKIFKKNDKDIMNLLYPFTKRDGHKVFFLHDMGISANRFRVKL
RGIHKKSKMLNVLNKFNALLNLCIRAKKEIDFEELMEKNKKDLQLYKEMFSLFSIRMKYK
FNVEIMDDDTEITKVDFLKGYDLIYRNKSAYINVLFSNLQVAMNTFFDNSFSAYLSNAKM
SNQGLKEILDKKEGILRKNIMGKRVNNCARTVISPDTFIETNQIGVPLEFAKKLTMDECI
TESNLDYVKRLILNGPDVYPGALSYRDARGNVFKLPSEYEKREHIVRLLEKMDLKKQCAT
MVLHRHARDGDIVIMNRQPTLHKFSIMAHYIKIFQKEKVFRLNYVNCSSYNADFDGDEMN
LHLLQTPHARSEASHLMNSDFLFTSFKDGSPLRGLAQDFILGGLHLTSLETFLDYDEYCN
LLQCALNCLLSRKDCFFFEKSRSSNS-----------------------------WKIRN
HSYLDPYAVVLNRTHFTIVTEEPTILFPRKLWTGKQLITSILKTIIDKVAMETYNQRKDN
EFMQNYKGINYYAKAKTDPSLWSFDPINECEVIIKNSELLQGVLDKLHFGASSNSFVHLC
YELFGPKIAAVILDCFGRLFISFLQLRGTSLSLFDFILKKDAREEKINIRKRISFTGFYL
QNLFTHSIAKALNADVNEMSAYSRGKLNSYRQNNDLFANNLYELYKRRSDFINDFVGGEF
GLTGRETHQREEKLPSEEIPSEEQLDIKEEAYFSALLHKEIQNLLAAPNSSPSSLVECAL
ERMASDQEDEYAKGVKEEVPQNMRSALVKKLFRSLSEPHFMDSPNFIGDKATRVIEKVVR
FLKKANCSRRLKVYILFELFQNKSVMKHFPALASCVNDLSHNVSNEEILHHVLSSVSAD-
-RAQVQPDQENGVVAQE--MVSKFLRLLQDME---NGRGSEEDQEEREYDGEGYDGEGYD
P---PMNQHDEEMIRDSLKKSFPSFLLNEEIGNLSFNGKDHYYESYVQKGTEEDTIFQFY
NMKDIFNKLEFLIHTYFLQMRGDFDGLIDSLFQPYLCRVSSNTNNLISMENIMNKFLNNG
FSNMIFTGAKGSKVNYAMICGMLDQQYLDGKRVPRMRSGKTLPSFHRYDYGARACGLITD
CFLEGLRPQEYFFHCMSGREGLIDTAVKTAKSGYIQRCLVKCMESVILHYDGTVRNEDNA
IIQFLYGEDGIDPSRISYLDTPKDLISNYHVAFSKYLLHDVVPKMERNERLFEGRDRWG-
--DPIETQFSPYAYMGATSERFREKMHHTI-ANDLNLADCYNLPDDFDTQSASLLKSKYF
RSLCDPGESIGILVAQSFGEPATQMTLNTFHLAGTENVTMGIPRLKEIFLNSKLTSKPMI
YVPIKMDERGSNDAKAVKSYINEIAEKILSTYKSICLSDVIYGVGVDRKLILRESAKDAQ
MHSIQVVTRQGEDEQMEGGDVQATKVMNALQNTQLSFEIQKEWNYEIVIQFENLYHFCQI
NNHLTVQSILSKAVSCVLYSVLNKIQESLLNYNMRYVHSFNHEHYDELFEFVCAKMQDEF
NFSMQKLEYKNDEDKINAKLKFMGSENHGGIGGPFGRNSNGEDTYGEEEEDGLSDHMDRN
NMGMENDNEDDGQAEEKNDSNSRS--SESGEDGDQDSPSEEDKDYDESEREEEEVEAEEV
EAEEVEGEEEENVQ-AEDEEQLEEDDDQVKDSTAFKGRAKR------------------
--------DNE------SDVSNRDKFLSSVQRMTKRSPKLSTSAERLGEEVYTEESNEAN
ENEEWEKMAEDFANLPYDYKNNVKLKNDSEEEDNHEENASTEKIKSENKKWVRNIIEKLK
CSLLCFVKNISFSPVTWVLKFEIGWDINFFPHAIDFLSYVQAELSKEILFKVKDLNNPKV
LKGKDITHSGAEYELQIEGKNIFQLYNIKDTFIDKTKLYCNDIYTIVKTYGIEAGRLCIT
KELKKVFEAYGIKIDFRHLSFISDFMTHTGDLKSFSRHGLGCFRNVFHKMSFECATNFLT
QGCVHNSVDYLSTASSSLFFGKPIKVGTNVADIVTHIEGEMTKNRLLIESREAKKQNDPD
ITLTHSDYNLYEWQAIIRGPKDSPYEGGKWKLSIKCKGTYPIDPPVITFVTKFFHPNVNF
VTGELCMDILKANWSPAWTIQSLCRAILFLFTEPNADSPLNCDAGNLLRSGDIKGFQSMA
RMYTHEFAMQNDMSRSEDLDPEIHALLTCAFNGGVKVKGAVEVGKRAVEYFRGDEFVHFL
YTKRDMLKKKFPVLLQNRTLEDLKDIEEFADTLIQKGFIYKAQYKPIKGISEVDENGMYR
RPKWPRRLIMSSKQNFDRTSFYILVHERNKKLQYLMLITLISVVLICCMFPIWPLKLKLA
LWHLSVVFVSLMSVIVFVRLFLFIFFWFFGVDYWLFPNLFDEECSIIESFTPVNDWYYRN
DSWVFVIARMFTAVLLAIGIHQLGKTHSISDISNFATQSFIDIIEWGNKKLAASPENVSI
YKSLDSKATFEIE-FQDDGVVYDENEENYDCLKGCGFQTFEDLVRKCFLKCECMEKVIKS
ECYKKKCSRVTKEVLDEAHKEACFGKKDKQATLADTFLQDLEDLEFEEESNFINFNEGDK
ISHVGEGKFEEDDYEEIVDAIEEFLNEKIKKKERKISELLYDEEFLKIMNNIRNYVMEE-
GSGADDEDNEEGGDGEARRAKKRRKKGEDDVDEDAAASKESDEVLIEKCIELIIQIDTEI
LNIHKYVKDIYSTKFPELDSIVYTPLEYISVVSKIKNESDLKNIDFSDILPNTTVMAITV
ASSMTTGINLSDHSLKNCLSFCNEALELNENRRMILLYLESKMFLLAPNLTMLLGSALTA
RLISSVGSLKNLSITSSQNLIVVGSSKKSVLGLSNVRKTFGIGILSTSEIVQSVPDAYKK
KAISLLAGKCSLASRVDYFKKYPEGQYGLLLRENLISHLIKLQEPPPMKQKKILPMPDEK
RKRKRGGKRYRKLKEKTEITELRKQINRLPFGPNSNEDFYTFTDQNAALLNSNITKLKYQ
SKQKVNVAKRKNLSVQSSGVTGGLSSSLIFTPLQGIELFNPSVINPRPDPVENKYFSSKA
QFRKVMEGNKEDICELNKNSYPNFYLADLFYILQLTHISPDEKNETWMKLQDEIKKNNMY
PYYNYVCEELNIPVDQELYNSLKKNADEEINEIEKKIQEASENFDSVDTKNDVLLKANFF
CKIGDKENALKEYEEAYKKDIGIGVKLDILLTIIRISIFFNDLKNTKKYLEQARTQMEKG
GDWERKNKLKIYEALNYIMIRNFPEASKILIDAASTFTATEIISYDDIIFYVVILGIMTE
ERTVLDKKILNSSVILQVTSSDEDLHSYISSFYHCEYRAFMEKTIKIAMRVKRDRYFGRH
YRYFIRNTRVRAYRQFLEPFKSVTLKNMAYAFGVSEEFIESEISSFIANGKLNCKIDKVN
GSIESNQPNERNTLYLNTIKKGDILLNRIQKLSRVIDMMVNINWPGLLKWSTKYADGTID
TNKRLSKEDIEFLQGAIKEALSQVEDPYEAIKEAVRNFENADEGIILASAKIVERLVDEY
PEVSRNLHKINAIEPLLKLLNQTNNHILESVLQIFSLALSNNPDLQECVFKKNGLKTLLL
KLQESQKTVIDKKLITAISALIRHHDEAENKFIDYGGVGFLVYGMQTNIYQYQEKSALLL
KHLVHQNKITFEIFLKNEIMKGLICLAKNKNIDETGIQYGETTAELFLALIQNHRHKLAK
SGYLHNMKILIEDRLAYLRVVQDSASYDVSQEIDLFTDCLKLTSMTESRNIKRKGRPPLL
RHSKLRKIFYIRMYLERKRREA---RSTRRGGGRKSGRLGDDEGNSPGEHSAPPVTFEDL
GVEDWLIKISKTVQITHPTKIQQLCLPLIMRGHNVIGTSETGTGKTICYCWGILQELNKN
AFGVFALVLLPTRELVVQVVEQFLLYGYKIGIKILSCIGGFSIIEQRRGVLAKPHVVVGT
PGRTCDIMENCEDVTNCFKRLSFFVLDEADLLLQKCYESKLEVILRSLPKQGTSRRRTLL
FSSTITDTLELLANSFPHENLILVNVNKKQKPLKNLDQRYIYVDEIAQMTYLVYLLRKEL
PDQSGIIFTDNSYRCELVYTVLCMLGGFSVESIHSSKDPKKRLAALSKIKNGGCKILVAT
DIISRGIDIPKTSFVINYDFPNDTVLYVHRVGRTARANRKGVAISFVDKRDVNSFNNVMA
IMKSALKPLRLKKKEVLQDMFQVGRVLKKAELLLEEREDARREHQRMQRFVHRAMMTRKY
FVSANWKCNGTQESIKALAASFNELEFDPAKLDVVVFPVSVHYELAKSLLKPKFHTGIQN
VSKYGNGSYTGEISAEIAKDLNIEYVIIGHFERRKFFNETDEDVKEKLQQCLKNNLKVVV
CFGESLEQREKNQTIDVIKKQVNSFVHLINNFDNVVLAYEPIWAIGTGKTATPEQAQEVH
KEIRNIVKEKCGEKNAQQIRILYGGSVNTENCASLIKQEDIDGFLVGNASLKPSFVEIIK
SAMMKKKNKEDLYKENKLEASKLRIAIVSTDKCKPKKCHLECKKNCPIVKTGKFCIEVEH
ASKIAYISETLCIGCGICVKKCPFAAITIINLPKDINKDVVHRYGPNTFKLHRLPVPKLG
QILGLVGTNGIGKSTALKILSSKLKPNLGKFSNPPEWRDILSFFRGNELQIFFTKLLEEQ
LTPIIKPQNVDLIPKQIKGNILEIINKKDKLNRKDQYMSALELDHLLDRNVEDLSGGELQ
RFALLLSIIGQTTNVYMFDEPSSYLDIKQRISMAKIIHSLVRHDNYIIVVEHDLSILDYL
SDYVCCLWGKAGAYGVVTSPFSVREGINVFLDGFVPTDNLRIREESLNFKLATDQDVTDE
DKKRLHFYNYPTIKKTLNSFTLTIDKGIFSESEIFVLLGQNGSGKSTFIRLFAGLIKPDN
VESLSFLESLSVSYKPQQIQAKYTGTVRQLLMSKLKGLYTDPYFNNEIIKPLKIEGILDN
QVLTLSGGELQKVAIIITLAKNTNIYLIDEPSAYLDSEQRIIVSKIIKRFILNTNKTAFV
VEHDFIMATYLADHVIVFDGQAGVNTIANTPQTLVAGMNKFLKIIDVTFRRDPTNYRPRI
NKYDSVKDKEQKLNGTYFIIDEMSILCTISGQTPDEPVVSKTGYIFEKRLIEKHILNYGI
CPVSGEVLTLQDLYPLKNEKVVKPRPITASSIPGLLSILQTEWDSLIAEMFTLRTHVNDI

RNQLTHSLYQYDAATRVIAKLLKEKNNYTEEIKNLRNQILSIRSGNDLSELEVGLSEELL
NQMQDVAKNLLITRKKRNIEHVTSTDKWKEVTNTNEFDVHSAALPGVTCLSLDINKMKYN
YYDDHMNHNFFSGGVDGNVYYISLEESKILAKLQGHLKKVNAIVSHPTYPVCISGANDKT
VRIWKGDVDSNEFTTSHVITKHKDQINSLALHPMENYFVSSSKDNVWILHDLETSRTIKI
CKTNPSPFKHLAIHPDGIMIGIGSEDSNIHIYDIKSEEYKASLSGHTSEVQYISFSENGY
YLASCSKDKTAKLWDLRKAQCFQTIDVNEHPKSIEFDFSGKYLSLAAGNDIHIYNFETKT
HANLVKTLSSHTDTVTQTCFGSRTAYLLSSSMDKTVKVWNLTEETHCYNGNIKDGLFHGR
GILMYSRNEKYEGDFVYGKREGKGKFTYADGATYEGDWVDDKIHGKGTAKFVSGNVYEGE
WDNGRINGFGILKYNNGDIYEGEWLDGKMHGRGTYTYEDGDVYVGEWKNDKRHGKGCVKY
KGSENKIAETYEGDWFEGKMQGKGTYFFADGGIYEGDWVDGKMEGKGVYKFLNGNKYDGD
WSNDMKNGYGVLTYVNGEMYEGYWKDDKVHGKGTLTYSRGDKYIGEWKFAKKSGQGELIY
ASGDKFKGEWKNDKANGFGVLVYSNGNKYEGEWVDDQRHGFGTFTCKEDGSIYAGHFAFN
RKEGRGTLTFVGGNVLEGLWTMGVLTKVSKFQLSPASPWHDPDLMKLEEVKDIQKIERIG
AHSHIRGLGLNDCLDARYCSEGMIGQMSARKAAGIVLRMIKEGRISGRAILLAGQPGTGK
TAIAMGIAKALGEDTPFTHISGSEVYSLEMSKTEALTQAFRRSIGVRVKEESEVIEGEVV
EIEIEKFNDKDINNINKKVGKMILKTTEMETLYDLGNKMIEALQKENITAGDVICIDKGT
GKITKIGKSFARSKDYDAMDPNTNFVQCPEGELQKRKEVVHTVTLHDIDAINSRTQGFLA
LFSGDTGEIKNEIREHIDMKINEWQEDEKAEIVPGVLFIDEVHMLDIECFSYLNRALESE
QSPIVIMATNRGITHIRGTDYKAPHGIPLDLLDRTLIIPTYPYKHQDIMKILEQRAEEED
VEIDQYAKELLCKIASESSLRYALHLITLANLVSKKRKATEVTVQDVRRVYNLFIDVKRS
TQYLIEYQNEFMFSELPKEDEGINPEDSYDEKREIQEKKSVNDNADSMAYIYSNLSDFWT
SDDEDDEEVDGTEEDAPSS--GANGVKKLS-DQNHELYNYDENAFEKRENVEINDTGNSP
FANVMDSGLNFNINEIMSGLTNNVVDNFSIKSMIEKYDISNFIYNDTLLKYCEAGVAIND
DSACNEKLSLNENFHFSCFGPVDYVTPPKGNVKGDAETDS--AVENIIDEPLEPDFEGDI
TGQGGELHKSGDATMEGVVPGQTDDKVDDK--VGDKVDDKDKDDDKA-DDRANVKYIRKK
WVIEDNHSDVTDFNQNDLLLSYDFELDDFQKRSIKHLNNFKHVFVAAHTSAGKTLIAEHA
IAMSIKLNKKAIYTSPIKALSNQKYHEFKNIFKSVGIITGDIKMNVNANCLIMTTEILRN
LLYINDNIINNIHCVIFDEVHYVNDNERGFIWEESIIMLPPHVQILLLSATVPNYLEFAD
WVGFTKKKEIVSISTKKRPVPLLHYIYAYDSIFQIMDENNKIYSSAFKEIYTKVREKEQA
SGGHGGHHGGHHGGHHGGQSKQAPSAKWSGHDGKNKQPSGPGSTPTNNNPPIAYNEYCKQ
KRRQKLFANEANMKTEIQKLQALIKKLEQDNKLPVVLFCFSRIKCETYAKSMPHLNFLDN
KHKSKVHLFIKESIAKLCTQDRELNQIKILSKLLEKGIGIHHSGLLPILKEIVEILFSKG
LIKILFATETFAMGINMPAKSVVFTSIYKHDQLKKRILTSSEYTQMSGRAGRRSSDKYGY
VYIYCPDKIPDQVQLTEMLMQKAVSLKSKFKVTYNMILKLLINKQINIEKMLFSSFLESC
RAVQIPLFKKDLKRKKKLLQSIKQVQCIYANDVKYVSPIENYVQIDCKLKHIGLSLHRKL
FSTKSSNCFVIGRVMMLNNIATLHSSIYGVYLGCDKSSNKKKEKVDFAPNSIFFQHSGAE
EESNERFFFLFILPDFMTYEEFLQKMNTNGVKQPTQMNRTSVMQR------NRFPHENIN
IFENYKNLFSKQSTPTGVQIIHHNSFDTDMHKKHFVVCSNVPLDHISIITNTVIVLPNVK
TTAILNNPSNMLLYSLELDRLIEKGNFEPFVLTKMLKSLKCEFYSVLIKQADYLEALKKS
QCYSCNMKEKHYEIVCKRNKCINDIENIEQNINAKSLNLYEDLEGRLDVLRHFSFIDEDH
NLTVKGKIASYITMTDEITLTQVIFENVLNNLNPPEIAAVLSCFVAPEKKIEESPDLTVN
LQDVKMALTNIHSQFEEFYKIIRLKISSEDHWKLCSFKIMFIAYKWALGVSFAELLEQCE
LEEGLIVRSILRLDDLCRKVKIAFLYLGNVDLAERLETTCTLLRRDIIFMTSLYLQFCLM
VFHLNIFYKGTRVNYVSAYISSGNNGGHNNPNKIDNHKISANETSNRSAASYFKKKFPQI
YQQNTQTSEKKEFERTSQLQQLQESDNLEEDKDNMAAPIEERLANLKKVMQENNIDVYIL
INSDEHNSEIINDKDKKIYFLSNYSGADGILILTKDKQILYVNALYELQANKELNHDIFT
LRVSRITNRDEIYETIASLEFNNIAVDGKNTSVNFYEKLKGKIESTYPEKKVEEKVIYGK
NMNQIEKNENINFLILEKSLVELKQNEVNNKQVFIHDRLYNGACAGQKLEKFRQAFFDKK
NVDKILLSELDEIAYILNLRGFDYTFSPLFYGYLYFEFNREKDEFGKMILFTVSKNLSPS
SIRHLNTIKVDVKEYETVVEYLRDNVSSKTMALKAGNETSAVHTNLSEDSQKKYEISLSP
YINLMIYMLFNKEKILLEKSPVVDLKAVKNDVEIDNMKEAHVLDALALLQFFHWCDEKRK
TKELFNETEMSLKNKVDYFRSTKSNFISPSFATISASGPNAAVIHYEVTESTNSKITPSI
YLLDSGGQYLHGTTDVTRTTHFGEPTAEEKKIYTLVLKGHLHLRKVIFASYTNSMALDFI
ARENLFKHFLDYNHGTGHGVGLFLNVHEGGCSIGPTAGTPLKPYMVLSNEPGFYLENKFG
VRIENMQFVISKKKTDNTEFYSFEDLTLYPYEKKLLDYSILTTEEIKDINEYHDNIRKTL
LPRLKKNPSEYGEGVVKYLMDITQPISINMLCVLLTNTYNNFNYKPGTRSVGNVVHWNDE
QLWKKNIIKVNKLPFKRMASIKSQGDLLEELKKNLVIGENPAFIQERLAKYNELKEKKRI
EREKSDPKFTRSINVQLLDGSVKVGQCNVTTPFHIATSISKRLAEDSIVARVTYVEKVEL
ELCDIEEELEEQDEVNETLGCPLLWDMNVPLLGSCKIEFLNLENEEAKKTFWHSSAHILG
SSLEKLFGGYLTIGPPLKEGFYYDIYLGDFSITNEDYKKIEEEFNKLIKQNAEFEKLICT
KDEVMELFQYNPFKLELIKSKIPENKKTSVYKCGNFIDLCLGPHIKSTGKAKAFKVLKNS
AAYWLGNKNNDSLQRIYGITFQKKTELNDYLNFLEEAKKRDHRNVGKKLHFFFFDKDTSP
GSCFWLPHGAKIYNKLVDFIRREYRIRMYDEVITPNVFSCDLWRTSGHYQNYKDCMFIFN
VEQKEWGMKPMNCPGHCIMFKQLNASYRSLPIRLADFGVLHRNEISGSLSGLTRVRRFQQ
DDSHIFCTFDQIKEEVLNTLHFIFFIYDLFGFKYELFLSTRPKKFIGNISTWDFAEQGLK
DALNSANISWKINEGDGAFYGPKIDILLRDSINRTHQCGTIQLDFQLPCRFNLQYKNKDF
GALNEGDNAESGTMKRE---EVAEKGEGNQPSQNKGSDTPTDTAEQLKKGFDRPVIIHRA
ILGSVERFVAILIEHTAGKLPFWISPRQAIVLPVSDKFNDYANYVYQTLNNHFFDVEIDT
SLNTLNKKIREAQLNQFNFILVVGEKELTTNTVTVRNRDDQNNHEVCSLEELIARFRKLL
DVNSMPFNQIKPFNQVLCAGDRGRKGANNRTWENQRGRTYGRMALLVAATIGGLNLLLKN
AEQGEKRWKKKTRNIIPSPNMRNEIANCCGIMAYLGNRDASKILFDGIEVLQNRGYDSCG
MSTISRTDTLKTTKYASTSTSDAIEKLRGNYSTSHKNDKIGIAHTRWATHGCKVDENAHP
HMDYKERISIVHNGIIENYRELKSFLLGKNIPFKSNTDTEVVANLIGYFLDQKENFQNAV
LSAIRQLEGTWSFCIIHKDHPDEMILAANGSPLHIGFKDNEIFVASEHSALFMFTNEYIS
LKNGEILSINKDKINDLKTEKKVESIPEVVIQKTPHPFPHWTLKEIHEQSITLSKTLNNG
GRFSILNSSVKLGGLDPYVDELSQIENIILIGCGTSYYAALFAKYVMHYLNCFNTVQVMD
PMDFNVSVIPKEKEGVIFISQSGETRDVIKACKLADDLNLKKLSVVNSVGSTIANMTGRG
VYLNAGREVGVASTKCFTAEVSVLTLIAIWFFQNKKEHSSHSKVSSLINSMHRLPLYAGT
TVKSCEAKCKTLANKLINAKSMLIVGNGLSYPIALEGALKIKELSYIHCEGFTGSALKHG
PYALLGGDENMPVIMLIFNDPSKNVMINTGEQIKSRGAHIICLTDDEDLCKHFADDIILI
PNNGLLTPLLAVIPLQMLAYYTSVARGNNPDRPRCLAKTVTVSMNDSYDSLFKILLIGDS
GVGKSCLLLRFADDTYTDSYISTIGVDFKIKTIEIEDKIIKLQIWDTAGQERFRTITSSY
YRGAQGIIIVYDVTDRDSFNNVKNWIIEIEKYASEDVQKILIGNKIDLKNDRNVSYEEGK
ELADSCNIQFLETSAKIAHNVEQAFKTMAHEIKNKSQLENQQKGRANINLNAKPIKDNRK
KCCMPRIRTMNSRKPPEGWSKVESFLDEMNKKMRSLENEDTSKKRKSEILWPIFQINHQT
SRYIYELYYKRKEISRELYDYLVQEKYVDGALISKWRKQGYENLCCLKCIQVSDSNFSNT
CICRVPKSNLGDRVLQCVNCGCRGCSSGDKMNKRLASLAISSIK-LEASQHLFVREVLHS
SGFARASIPPRKIIEQRHPVKSPRWHIESLQRRNFFQVFLKETMSNAKSENPKYGHAQME
GKNALEPNQSNNHECKSQECNYDYDYVVIGGGPGGMASAKEAASHGAKVLLFDYVKPSNK
GTKWGIGGTCVNVGCVPKKLMHYAGNMGSLFKMDSNPYGWSYNNLIHNWGKLVTTVQSHI
RSLNFSYMTGLRSSKVKYMNGLASLKDEHTVSYYLKGDMSKEELVTAKYILIATGCRPSI
PDDVEGAKELSITSDDIFSMKKDPGKTLIVGASYVALECAGFLNSLGYDVTVAVRSIVLR
GFDSQCALKVKIYMEEQGVLFKHGVLPKKLSKEGEKISVLFNDGTTELFDTVLYATGRKG
DIDMLNLDQVKIQVNKNTNKIITNEVSCTNISNIFAVGDVAVDVPELAPVAIKAGEILAR
RLFNQSEEIMDYTFIPTSIYTPIEYGTCGYSEEKAYEIFGTSNVEVFLQEFNNLEISAVH
REKHVRAQKDQYDTDVSSTCLSKLVCLKNEDNRVVGFHYVGPNAGEITQGMALALRLKAK
KSDFDKCIGIHPTDAESFMNLTITRSSGLSFAAKGGCGGGKCGMENEKHENMLLAIARDF
QSIDNLIETFLSFLEHKTDYFHIMLNEDDIGTLAEKYDGAVGKDILNNNKCGFKAHSREE
NLMKLFRKHQLNYIIKKQPYLIENEDMRNKYLPSCDELKKLNTSKFCEVKNKQNDMKKKV
ETGTTPITVTTSETHISTWNGGQTEKYLWNQALNEINIEMPLHTEIKTSEINVQITNKSI
KVYHKNEIKLEGTFYEEVNKQECMWNIEDKKKLIIFLEKKRENWWPCVLMGDPEIDTSKI
ESKKNLTDFDEKTQGEIRKFLHQQKIKNEGAQSPEELKAQNIMKNAISSKGYPFMKMKAA
NARPDEIQTISHNNAVEEVEEPLYSGPLKIEQLLAKGFVKRDLELLKEGGLQTVECVAYA
PMRTLCAIKGISEQKAEKLKKACKELCNSGFCNAVDYHDARQNLIKFTTGSKQLDSLLKG
GIETGGITELFGEFRTGKSQLCHTLAITCQLPIEQSGGEGKCLWIDTEGTFRPERIVAIA
KRYGLHPTDCLNNIAYAKAYNCDHQTELLIDASAMMADARFALLIVDSATALYRSEYIGR

GELANRQSHLCRFLRGLQRIADIYGVAVIITNQVVAKVDAMNVFGGNDKIPIGGNIIAHA
SQTRLYLRKSRGESRICKIYDSPVLPEAEAVFAITEGGIADYEEKMALKSINISGNFDWC
PFKEHKNYLTCFTSHNLLYSNNNNLNNYVYLLDINLNSDGRNLEIVSKLNFEEALKSEKS
GKKKGTNEYVTSFEWINSSNFIESEGNYELQKGIIVGGLTNGDVILLNAQNLFAEKPTHE
QFILSRANVHEGSINCLECNKHKNYLVATGGNDGQLFITDIENIYSPTSYDPYLDKNNLQ
KITCLNWNQKVSHILATSSNNGNTVIWDLKIKKAAVSFRDPHSRTKTSSLAWLANQPTQI
LVAYDDEKSPCLQLWDLRNANYPIKEIIGHSKGINDISFSSIDTNLLISSGKDTTKCWYL
CNNNFDVYNEVNNSANNIYSKWSPFIPDMFASATNMDTIQINSINNGMQMTSKYIPNFYR
KDAGITFGFGGKICCFDNTALETKQGASEGTDVGNVG-NAGNAGNAGNANAVVGGSAPEN
KFLIKCHIYPTEMELISEADKFEKYIASGNYFEFCESKISKTEDEYEKLTWRILQLLCTS
QKEGIVKHLGYDINEIIQKIEDTTGKKPGFIFNKLAEEEKEKMNSSSNVLAPDMMDPSMA
GLMTGTANYNHVNGGLMASGPGGIDPNNAAAGASSMLVDNMQTFNSSFDVDPEKFFRELG
EKKENEETNEGEEEKHQKGDREAQQKGKDKGKKKGMGIGGGMADE----EYDDESKDINE
TNDKTNWNSGIESIIKECVLVGNIETAVELCLHKNRMADALFLSSFGGEQLWHKTKTLYI
RKQKSTFMRSLNYILDDQLELLVQQIDLSSWGEALSILCTYALNKGNFNNLCETLAKRLQ
NEKFDIRAASICYLCASNFDQTVEIWNDMPSTQNSLLNVLQDMVEKMTVLKMATKHEMFN
AIMNKKINQYAELLANSGRLKAAMTFLCLTQEDQTEESLILRDRIFNSGANMLCHQVKPP
MSPFQVLHVKSTFGGMLHQNYQQ------------CNQSPQFKSNIMGNSGQSKVFPTKS
ATSMPPPMPGQMPMQSSPSHHTAYPSMPPNKFNTHVIGAPPPHRASFTSTSSKNFPLGNV
NPVNPPSLSSMSPSYMMPPSGPSPSSTCPISTSPPSYATMSNISNFSAPQGIKSEQDNHK
QGGPMFSSQSYAN-QRRIQGANAPPPTPLQTNQINN---RNFPSMQNIAPPPVNRNQSIS
SNNTMSFQKDNQFLKREYMDQQG--PYGGAVSPP--VMQPNNFGTSFTNKVGIGGQSSG-
PPSSGVSTTSPIAGALTVTPGMPVPWPIPTTTQQLGSTTTSTANENKKIQTATKEQKGVF
MGRSNVDNVKKTISNFLNGYMSQEPIKKKAEDISIKVHELFDKLDTGSFNEQINDSIVNM
VNALNANDFRVANKIIVELSRNLWDGSNKSWIMGLKCIVPKMAIRVQFENSNEVGVFARL
TNSYALIAMGGSENFSSVFESELSQHIPLVYTTIGGTKVIGRVCVGNRKGLLVSSICTDQ
ELLHLRNALPENVKIKRIEERLSALGNCITANDYVGLVHTDIDRETEEIIQDVLDIEVFR
TSIAGNLLVGTYSYFTNNGGLVHAMTSSQEIEELSELLQIPLITGTVNRGSDLIGAGLVA
NDWSAFCGMDTTAIELNIIEKVFKLNSIEDANIEDNFKYKSSIIQTMIMLESLVEKLLNK
FLAPYVEGIERNLHLGVWSGNIVLENLKLKPQITEILDLSFKIIHGNIGRINIQIPWSKL
GKSPVCVLIKNVHIYIKPRSYKKSENVIIEELRRAKMHRLELLEEEISLIKQQKNKEKSS
EKSTLIFKLLNKIINNIHVDIQDILIHFEDPERNFSIGFILKSSSVKNCLVKEGNAASAN
ASEENKKLNHIIEFKGLCIYSNSNIRKKRRRKKGEKGKTGGENDRNISAGGPKRQATSAS
SGSLTRGDPD-NRSSLSDDEDEGNDVSGRVSNLDSSSKLKNDLSISYDKNDDLAFESKLG
SANPEEDQDSKDNYLKRTLTQISNFLKDDDSRILNYYNYLIKPFDLVLPVEQSSNKKELK
AKLEIGEKWEGITLTRTQITKIIEIMNEANKSRNQTNKLLLKYAYTVKLDIESLRNETKN
EFMNLYNKVLAEKYNISTTELTSQEMDRLQILYDVVGVRHLAKWRLQCKNTLEKIIEEKN
LKKKYLYDSIYKQQSWWSWVTGNKKDIENKVQSILNSEQDIINEKELYILQEAMTNEDNY
DVVLPTKYDFQFKLANFSINVYDDCKKGKRVLLQRGELAERGRINGNTEGTSNPS-----
-----------NPLVEEISLSGSEDTQLNSSPAISPSNSNYIKKGMKYEEINILSINFYQ
IYSSLSLQSVVDHNDNDNFQWKFIIELQNFVAKHKNKIFMEFRTNKNLFPYNVNSTGSLY
QNAIYYSLLHSQSLCAYVEINHLVTEKGNTLSTLLRLNPLELYVSPLLIKNILSFAFPLI
DIVNKKHKSVFRRGKEKDSLLDGATETGETPKSTAGDTSEDDEEDEEALEHIKKMEQTEL
IEGLKEKGENVYNRAVQHLPELFEFYIHICGPILHFDNLTNGIVELHLGNLIAKTEFPCT
YHKFNLIFEFNETQITCLKASLQKSEKFYILQPIPVKVYVEYDLKILRTNIILDGIFFQI
NPDAVSIILAVPTSITRYLTRFYSKNKKEKDFLKGRKKTSVEKKVSSKGEGNICLKIPSG
DGASKENNSVSVHAPNDDESKGNLTDVNTNKLEEESFLYDIDFLIKNSSFSIKNGKNREV
LKYEACGISYKNYLQKKKKIVKVEIEQLWICDPSNRQPIFFTLTKNVNSKDIFLFRSLST
YLSDKHVIGHKGDDNTVSRDASNEGNGSGDIFQSAERIEEEPFEDEEDDDFMDAIEEKQL
SINLQILQEQNQMNRAKTHVNLIISDIELHWKYKTIKQIFKTMKEYKKKLQYGIEKDMKY
IKNKLKNEEELKNYKMFISEHTLRSVQETLNNVKNSLNILDLESARK-NEEDLK-EEMSS
AGDGTHPGSAQMGSSPVHPRGSNFGKTQHMGGRSTQRGAQNDGDDDYEEDILSNGLNNTL
PKLYGQKNSYVKYFFNCYIKSASLAFWQKRKIFSKIQVSSIYYENKVYLNFDQKMFVNIE
KGIISMNSKNIISNNINDYTYDLFISRRVKPSEDSKGENVVDNLSEEIKKKKKKSKQKRQ
DLFVGKIKVYKDKRNYNICLLCDISSIYYIFYLRDLKLFLEYLDDGILNVFISKSYKKVV
QVAQAKYFLFHCTILDPVVIIPEDKNIIYNYRGEVKDVSKISKASING-------GANGN
RGGNS--IPYIESYLKFHLGTLKFKNSYTVNYTQGIVENQRRALRERRKRKRQARARATM
QGRSTSKRQMGVHCSDKGASSTEKVGGKYDFTLYVDLLDMESRACENGGSINIQGNILQK
VNIGLCLVSTKDGVFIYMNGNDFCLDLTVFQLAFLLDIINENFCYKGYFPMCFICDKGVN
IDSMLNAEWFRKGNINLGNLETNSANVEKGSEPVRKNGLKLYLYINLESLKIKTSFDENT
PVAIITFQNISISFHLVLLDFYYIYFFDLYSNSLYIDDVRKNSINYYKRVAYCCIENEAS
RVKRKRGEKEEKMRVNFVKENAEAKISSVSVNSAAVGSNPVGGSRHPCVDRRVPSTDYKG
LLRYMFESNVFLSEEKKKKRQKGIKIRINSFIEDIILSIELDDAYICCFFIIFMDIYKFL
STGFNLSTLHLYPKPSPYIVSAKEPGRKKKGKNIRIEEDVFKSEAKSEAENIPPQDKVNI
SSGSSANSSKFIRERNINEILKLDNKPIHISFKVNNGNFIVFTNLENVAHPILLWSNNFV
FSFSLLNKCIIFRKIYAINSKIKRINYVSNFSSDLSRKRKTGGVKEGDPKDVSNPLHNYH
KKKILLCDNLNVKGEAIYESVDTKKENYILKANVRKKDYPEFISVFELDIHIGNFDIRLS
NDDVEILLRASSTLFGDVPSSYTNIAVGAVIPP--RKRSVVMGDLNNEALSEISTDSNTI
YSDSSSTLTNGNEQVKTYRDIKIVIKLHNVKCTFIDDIRNSIVPIIRTIMSMSICLNFYS
DECSYNINDLNSKVEYFNNCIGDWEPFLEKCNISLDIHKILPNDSFSEDTESSKSPISII
KINSVNALWFNITPQLVNLLFLFLPVFTDKVLKGLKKKENNRFMASNTLEKEENQESHLS
LFNKNCESVCAELDEKKLSIKSKSIYEDCSSVFESVPGDIDQTKDTDIHLDEFLNKEENR
NSYDCMRDNSVVYYVNLTSDFFYAFVMPEGSVEEVKKKRPCAGAKGGRGYRIVTTIGSGN
TGDRVKKNNYHSEGSEADDSSTAPELYAKIVTTNELVSLDRLLVNEIANNSLIEKKCLYL
YLIPIPPTNVVNVIHDMFANVSKRDIVLDLMITKNPKKTIENLLVYTQGRVDQKENINLM
SNCKAKLSHFKYSQSNKFVGPFLRNCECVIINLIKNSCTTLTTSLKSSNVIHMMKPLDYI
FDEAEKWTPPKDISGAIRGDAALNKSSVKWEMLNEEMDKQTNMACKYRLKNNTVCEIISP
MPNYKILFMSSTVRIVNKCGIPLEFCFFDGSRNPILLTSLENRTIPISTLYPNHSDSFKN
YKVSNSLNINPNIKLRQKNNNLSFTVILNHEYLLSTPECAFCGPSHVYLSFKPINLVSSS
KGIPDFSRNGLDSPKGEGHDTSSISSEKGWSDIFSSDMNQGTYVKKCKMKDSNNYLYFIV
KIENKISALPAEKNLKIITIYPHVSVVNAIPALVDIIITSENNERVYIHELKRLEKEISN
IDEEIKIKKRSIFYIYEIKKYSCLNMKMKIGNSQCEWSDKLFLLEDDEESVMRFSLNFKR
FASVEVELIKNFSGYFNSLNSILGNKQLIFSLPRCFIDRTGLGIKAINSNKYYPIINGIT
LLGDNSQIDLLLPHKHNEYLRSCNGVRSLEVGDYSDIFNDFNYDNSLILFKATLPPIGSY
TETSVVCKNFFYTFCLNTEKIKTTNIPYIISRIITVVPQFIISNKLNHPILVKQFQTDKI
QGVRQNDTSPLYFPKKQNILLFRFRALEEEAACVGDGTADDDGVNSVDGHGNRKREDYVS
KQYKISKNPFWSSVIFPSENFVGTNYMVVNNSKEEKSEVYVITCIPDMGTKNIIIEKLDN
NMNKGFIAYNCSSLARYLKIRTFHDDTKHLKMDEEEYKFEKNMFVKNFLSTADMEHYFNI
EYDQSSYLGWVNPFIYVTRNVQIEIVLHDMKTIPRTPFILKFAQYNYAEKLFQVYYYDVV
FIISIEYIQDLIAIKLTHRISISRG---ALAGGGGFIATTSYTNDTREGGRVSKNSVDMQ
EYHKIEVAEGGYELRDKGGHEMGVVRSAMGGSYIQKKMIGHSEYNYVKYGTEKGGSRYQG
QANGGNYYGRREEAVKMISNTYKNVHVIINVTQIGVSIISNILKEEVFFIELSKLCALFY
MKNEEEVIDIKITDVQVDCQLESCEKCVLLANRGISTNGKGMNTNDEKIFLNIYVERSFI
SHKDVIFKKIQISLDDVEIEMDAETLNGINLLIAEYIEGISIVQKKNLLYEEIQKWTILP
VYVNYKSPDIPLAINIQYMQIDKFTLIVWCSFLLDKMHMMSDLLRIGLRILMVSGKLELL
GAPVTLNQEIFNNIRVSLKSFYALLKDKYSHSILACLGFIVGYSSLINIPKIPLEIGRNT
IGLAVYAVDNVSVGIGSFLSNLTFDAEYINRRQKERNFKTNTNMKEGLLSAVKNIGEGVL
SLSNIVTKPIEGAQKEGVGGFFKGIGKGVAGSLVKPLDKVGQAVSDVTRGIKAEVSKPIG
GHKYKIKRHRKPRMLWGEYGKIKEYNLHEAELRECLGLKFSKNIMKCLTIHKQENPPAHY
SLLLYPKVIIYANLYANQGEKKGDIVIWSLRIEDISEIRASSHGVIIRTGNSNYKIPCNN
ALLINKIYRELHSSKSSINSTIILGDAPSSPCVM--------------------------
----------------IIKRAQMEHIKDVSAYKEMYEKSINNPELFWGSIAKNSVRWFKL
FTKVYSGNFKKGNTSWFVNGKINACDNCVDRWAEKHPMKTAIIWEKDTPNDFKKITYQKL
LEKTCKIANLLKMHGVKKQDVVTIYLPMIPEIVYSMLACARIGAIHNVVFAGYSAGSLCD
RILDSKSSILITSDFGMRGGKLTKLKQIADAAMDMCGAIKVCIVFKNKTKIGDTNRALDS
TSQCKLQDSTSVNLKGAENFLAQDATRLINSNQNEMNQNEYEQMNHAKGIHTLHKKCKNS

SLTKKNPTSDKTHKEIANEEATKNMADRNSHGKESCDYMNSVTCKSMMEATFEGKGYPSN
DCRKSNFDENICTLKEGRDVDGSALMKNMRAYCPIEYVDSEDFLCLLYTSGSTGKPKGVA
HTTAGYLLYAYTTCKYIFDVNPDDIFGCVADIGWVTGHTYVLYGPLLNGITTTLFSSIPT
YPDCGRYWSLIETHKITQFYTAPTALRALMKHGNSYIDKYDLSSCRILGSVGEPINPETW
RWYYNIVGKKKCAIVDTYWQTETGGIVIAPIPNLFKMKPGCATLPFFGVELEILDSKTLE
PLKGPNVCGLLCIKSPWPGMLRTVYGNHSRLVKTYFSSCPNYYFTGDGAYRDEDGYYWIS
GRIDDTLNVSGHRLGAAEIEHALVQHSCIAEAAVVSFRHVVKGEGILCFVVKKVCVSKKW
GEVEGRNGNTESVNAHLTSTSNLEKMDTMHVNYPDEKIIEELKLYVRRAIGPIATPDLIC
IVPDLPKTRSGKIIRRILRCIANGINDFGDMTTVANYEVIDIIVNKFKDSKSKFQSKMMR
RSSRLLLQGSIFCACALFLAWKTRHATLFQKVTNIVRRCLRLKSQRDITNEQIRNNVKIY
FGSQGGTAEQFSKELSENLRDIFNIKAEVIDLEYFNKDEISKFGVRIFIVATYGDGEPTD
NACAFFKWLKNLNDDNEYFRNTIYSIMGLGSKQYKHFNKVAKKLANYLTKFKATQISENV
FGDDDDNIYQDFEIWKREFFKELVKMLHMKEIPVQFFSENVVELVDWTTLPEINLRIRYG
EEADEGALPHGMEQ----------TETIILPHQTTDITGKFYFNHHTGRVMSNENLLKHV
NHTSDDDKVNRIVISVEKVSFKAADTLVVLAKNPNEIISWWLKRLRIDDTDRKKRFTFVS
KDTTENRSQESTLQSSILQENTPVCVPFPTPCTVEDALAYYCDLTTIPRVNILKKFKCFI
KDVEELKMFNYILSNKQRSTFFNICKESDMTLIEFVDIFMPKAEFELTPFLQLIPRNVPK
SYTISSSPKEGEDTISLTVKKKQYPIHSLRKALKEFKNNDMLPPISEQKLRELCSRRWFK
GSSSFYLTEELTPHDSVKFNLKSSKFCLPPYLESTNIIMVATGTGIAPFKAFITEFKHFD
QTCVYNGVAKKAKRILFFGCRKREIDFLYEREISEAVEGKHIDEVFLAFSRDQHEKVYVQ
DLILERKDLVWSLIQKGAYIYVCGNNNMSKDVNKTINSLPMHYKQNNKKFTKSLKKAGRY
VEEMWMDNLPWVEKYRPKKLDDIVHQTNAVSMLKEVVRTKNMPHLIFHGPPGTGKTSAIN
ALAHELFGRDNISERVLELNASDDRGINVVREKIKAYTRISISKNKINSETNETLPPWKL
VVLDEADMMTEDAQSALRRIIEIYSNVTRFILICNYIHKISDPIYSRCSCYRFQGIPIDV
KKEKLLYICKSEGINILDDALDKIIETTQGDLRRAVSILQLCSCIDPMITLESVLDVSGL
PADDIISKIIDACKMKDLKNVEKAVQDIIEDGYDVAYIFKSLNNYFVMNTEYQDSVKSQI
LLELSRHDYRLHSGATKYIQLMSFASSVHSLLNAAMNESLDIENAKAKLKLVFSFWESSE
NKSFSQSNAFCVLSGKSSKEENATTQEQFQMWLMGYQLTETFFLFLSKEKLIILTSDKKK
KFLQPLLDSVQNVHVMERSNDNSDNFAKIKEMINDTGTDEIAILKDKDATGNFFESCYGF
IKSLEIPQIDVNSELKFLLNFRSESDMKIQKSGSDIACIILKNILITTIENALDSEEFQS
HDKIKDKALKFHENKKCVLKLKEKLKVDIDDIDVIYSNVQSGNQFTLNYKNSSNKNYLSQ
NEGTILVGVGVKYKELCSNVNRTLLLNAKTQHKELYNFTLAIEKYIIKECLQVNSTYSDV
YKKAISFIKKNKQDYSTLSQIDLEDYFVKCIGHVIGIEFMEKEFLITENNNTGIIQKNTS
YNLSVGFENVPGNDKNNFAIWISDTVCINDNGEVNILTDSISKEINTISYELEDSKSEEE
MDNNVKSEKKEQNAEYQKKKTGISASILNNAASVIVSDRLRRRNKNSLAHNNEQEMEELN
KRQSELKEKKMNEIKIRFSKGTSDYKDPNKKNIKKLEDVKAYNDVDLLPRDLRPNIISID
NKHECILLPINGAHVPFHVSTIKNLSSNYEDNNDIYVLRINFQVPGGQGVLKADFNTFPT
LQEKEMYIKELIFKSNDERHFQNIVKQVKDLIKHVRQKEVEADVNDPEHAQEKLVLNKSG
RRIILRDLMTRPNIFTGRKILGTLELHTNGLRYSANSRGTTEHIDILFDDIKYAFYQPSD
GQLIILIHFHLKRYIMVGKKKTLDVQFYCEAGTQIDDLDRAKARNVYDPDEMHDEMKERE
QKNRLNLIFKNFVQQMQDASKIEFEIPYPELTFSGVPNKSNVEIFVTANTINHLVEWPPF
ILSVEDIEIASLERIHHGLRNFDMIFVFKDYTKPVKRIDVIPTEYIDTIKKWLTTIDIVF
YEGKNNLQWGNILKTILADIDSFVESKGFDGFLGDDDDEEEQSAEDEDDDDEYELDESEM
SAEDDSDYDDSEDESLATESDGEGEVEEDSEDEGLSWDELEERAKKDDKKRFAEDEGYNK
RKKKKKNMSLLYKKTLDLLKGNNNDANGIRININSQNKRGHNNSVITSKYTIWNFIFLNA
YEQFHKISNIYFLIIGILQLVPEFTATNRLPTILFPLTIVLVANAIKDAYEDWNRHKTDK
IENNRMCYVVADTEINEEEESTSFFKSIFRKIRKFLIRK-KKICSMDNSFDEEIYVDEIT
DVDTFMSSFEGNLGHVEGTVKKRWKDVNAGDIILCRRSEFFCADILLLSTSDKNGIAFVE
TSSLDGETNLKVKEANGFVFNILTSDRSEAIDKVKNLKGFIISEKPNKDLTTMYGTIYFE
KDETTDLKFNSEILKGTTELVEYRRRRVSSADFSSSSIGNTQEEWIGELNDEDKHVRIPF
DEKHFVLRGCKLKNTDWVIGIVIYIGKETKIQMNSTKPIIKTSKLEILTNKLTIIVWLIQ
MIICFISAYYNAVIVSMSKKSKFKYLPFNLEESKKPFIVGVISFFSWVVITANFIPISLI
VTMSFVKVVQAYFISCDKNMIHKVVADIPTFGMQKETASLRDENSVELDIKESTHTDKSK
INLAPQENTESSEATSNDNATVNATSDEPKKTLTRVITFKDVKEKNYIYFNAVPRTSSLI
EELGQIEYIFSDKTGTLTCNVMEFRKCAINGISYGNGLTEIKKHILKKRNMAIPEEPVLK
STERTPNVNIVDEDLVKHLHDVTHFNHAAVIGFFVHLAINHSVICDYGKEPTTTYSSSSP
DEEALVYAAKHFGITFLYRRDGKYGISIFGTVYEIETLAIVEFTSKRKMSSVICRIPVRA
HTDGAGSNGAYGEVGRGKGGTGP----KVEEHSALVRNEMISPLNNPLDENIAHGGKKLK
KGN-KQNQKDEIITCKNCKESKIMLFCKGAGSVILNKLANKTEIDDITIEHMETYADEGL
RTLCIAQRELTEEEFAEWYRLYKEATVSLKDREENLENVAEFIENNLTLQGVTGIEDKLQ
EGVSSTIEDLRLAGIHIWMLTGDKIETAMNIGIAANLIDNYSEQFIYTADLISCEEALMN
KIDEDIINIEKTLNLPHYDFNAKFNDKNPGFLRRCFTVKEESNLSLSPDNYKKIISSFNH
VLVVDGGLLDTLLSKKFERKFFYLADKCSSVICGRVSPYQKGAIVSSANRLLKKITLAIG
DGANDRNMINTANIGVGIRGQEGVQAFNSSDYGISQFRFLKNLLLVHGRLSYARISKLVV
YMFYKNIVLIFPLFMFGSISLYSGQKIYYEFLLHLYNIVFTSIPVVAHAILDKDVSLKTA
LVTPSLYKLGIYHYYFNISTFVSWVINSLFHGLVVFLLPLYFLSYYNIPSADGTPFDMWT
VGSVTYFLTVLVVNFKVLLETYCLNVLPLTAVFMSILSFVILVSSCSFMCFGTNNFLGTA
VILAKSLRFWLVVLLGLFTTLSRDFIFKVFKRNFNPEVYHFLLDQEDKPKGKNNVINSSD
THKKDEEIKIEKCKSLGYAFSEVDPACVKLIRKQDKMIMMATFLSLLCKGRGGACVNRNI
LE----NGCYPCAARSYSDHVKDHFNKPRNVGSFDKNEKNIGTSIVGKASCGDVIKLQLK
IEDNVIKDARFMAFGCGSAIASSSYATELIKGKTIDEALKIKNNDIASHLSLPPVKIHCS
LLAEDAIKHAIKNYREKVIN-MSMKVRRNGVHSVLKNVKYVYESSRDISTSRIYDRKEKV
VILGSGWGGIHFFINIDFKKYDVTLISPRSYFTFTPLLPCLCSGTLSAKVCTENVSTFLK
KKGSSGKYLQMECTDISPEERQVICRDNKNNEVKIAYDHLVISVGAKTNSFNIKGVDKHA
FFVKDIEGVINIRKRFLDVLDICCTDKISNEEKKKLLHVVVVGGGPTGVEVAGEFADFIN
KDVKKKYKNIFPLISVSIIEGGKNLLPTFTQNISDFTKRTFHTANINVLTNYYVKEVDED
TICVQSSLDQNEKKKQIPYGLLIWASGLAQTPLITNFLKKIPEQVNNRILNVNGHLAVIG
IKEQNIYAIGDCKKIQPLQLHQNFHEVLDYFSSSSTTSDLLKSKANELSKKFPQVSQSKW
DYKKNKKTQMDKHQFCEYLKEIDENYKSPIPTAQNAKQEAYFLSNLFNTLMDKKADHQFP
SFVEKWKGSIAYIGSHQVVAHLPFFEITGGLFSFTFWKMVYIQLLLTWRSRFAFIMDFLR
IKFFGRPFSKMNVNSKKLQVKNKNAAEVQITAEQLINEALDLEEVEQKVNYNLIDEDELN
EYKISKRKEYEDKIRKRRYLISTYIKYALWEVKQKDIRRARSIFERALNIDYTNINLWLK
YIEVELLNKNINSARNLFERAVLLLPMENIFWKKYAHLEEILNNFVNARNIYERWIKWKI
DETSFLCYINFEERCREIDNCRNIFERLIVTLPKMECFYRFIKFERKYRNVDRARACFEK
CIELLPPSFLDEHFYINFCNFEEENNEYERCRKIYIEALKILPKNKSEFLYKSFLQFQKK
YADKEELDETLMIKERITYEEEIKKNPSDYDTWFNYIKLEESNINLVNKDRCVVRIRELY
ERAISIIPPVANKKFWKRYIYLWINYAIFEELHAENVQRARDVYRNVLKILKNQNFTFKK
IYLLYANFEIRQMDIPKVRSIFNRAIESVKKEEIFEEYCEMELRLGNIKECRDIYAKYVE
AFPFNSKAWISMINFELSLDEVERARQIAEIAIHLDDMKLPELIWKNYIDLEINLQEYEN
AKKLYERLLNITQHYKVYKSYAEFQYIYFDDIAKCREILENGIEFCKKNELVNERCILLN
FLYEIEKDYGDKDVIDKTQKRLPKKIKKRKIIKKDDDEVVEEFITYVFPDDGNQSQSGQG
KRRSRRRKKTRRKRKKKTRRKAMMSDVESVENNVVVEEKTVFDHTTAIQKVLKNALVHDG
LKIGIREVIKSIESKEAKACFLSDVCSEPAYKKLITALCTEKNIPLFMVENDSKDLGQWV
GLFKLDKEGNARKIIGASSVSIIDFGEESPERDYLMQQNQPAATAMAKDKRGRVISNSYE
SDEDKYSKRIKKHHHKINFAEKDPDNGSYKKKNYENDKSKLNLKKDQKKNSKENLNSFSS
HHSISSSSDANNLGLNISGGSTSNSEDEFKILKEEENEDKFLEERRRKREAIKERLKDLV
SENEKGNDVVSGDLGDSVVSNGNDRSGKEEPSENV-KKEEGQDAFSSCNKNDLGESLNEI
PPMLDDVDHDDAACIFAPNKEVMGETCSSLSSDHEMIDEKPTKEKNDSFKESSDLYSDLK
KKIMEEKAKIRAFIIKQKELHERTKDGPLTNKNTDPHGVEEYEEEDNDNDDVDMFSSVQP
SKKEKIEKIRITNYYASDNVNLSDNWNDSEGYYKAIVGEVIDNRYSVVCELVGKGVFSNV
LKCYDKVGKIPVAIKVIRDNDMMRKAAEKEISILKKLNEYDKDNKRHIVRLLRSLKYKNH
LCLVFEWMWGNLRIALKKYGNGYGLNATAVHCYTKQLFIALRHMRKCRIMHADLKPDNIL
INEKFNALKVCDLGSASDISENEITSYLVSRFYRAPEIILGFRYDAQIDVWSAAATVFEL
ATGKILFPGKSNNHMIKLMMEYKGKFSHKMIKGGQFYSQHFNDNLDFIYVDRDYYTKKEV
VRIISDLRPTKNITCDLLEHQYWLKGNSPKMQFLKKKIKQLGDLLEKCLMLDPTKRYTPD
QALQHPYLRESIHFSKTQNEM-SDADSLSCSLTLESDEYDEEEYDTNLSKLLENKTLNWI

FVGGKGGVGKTTTSCSIAVQLAKRRESVLLLSTDPAHNTSDAFNQKFTNQPTLINSFDNL
YCMEIDTTYSENTAFKLNKTEFFDSIIPELLQSFPGIDEALCFAELMQSIKNMKYSVIVF
DTAPTGHTLRLLAFPELLKKALGYLISLREKLKGTLNMLKSFTNNEVELEGIYEKINHLN
AMSISIQSNFQNPLKTTFVCVCIPEFLSVYETERLIQELTKKNISCYNIVVNQVVFPLDC
PTVNVSHCEGLLKQIKDKKIQESFSSLVQKTKELEDVYISRRKLQSKYLTQIKNLYGNDF
HIVCMPQLKSEIRGLENISNFSEMLLESKDIPIYRMEDGVIISGDYIQMGKDKVKGINEH
NFEQVHDVESENFYRSKCSGILLKAPYYPYKYDIMNTSYKYMPKVGDLVIGIVKSKKLDY
YQMDINCNCECIIHKIESFKYATKSSFPNLANGTLLYMIVEKINLDNNSVVASCINSSDV
KSWINYENYLGELVDGYMFPVSIAYAKSLIGDKCYILDLIGTDISYEVAVGHNGRVWIKT
DDPRETNMIHTALRYSYGKTKAQMDVLWKSIYNLGKKDTMSRSQIFLNGMMGRAGPVEQF
LSCRKYLPHIGQNPFLAQSEFFNPRKYEKVAI-GLQENRRKFHQMVQMYLSKAPKGFENF
ERTNKKKEEG--SSFKPDEEKNNKKFDNFFFFFFFMLLLFFFLFVDSNGLYNEVTQNDFF
MNYLSKGYVEKIKLVNKDYVKAYLNVHGMSKYHQKYVSFRIGNSDAFERKVEHIQREMNI
QREEIIEVQYTNETNMLSEVKGYIPTILFFLLFAFIFQKITLKNVANSGMDRLFKMNKMN
PINKHQLKTDVKFSNVAGMKQAKEEIMEFVDFLRAPSKYENLGAKMPKGALLCGAPGTGK
TLLAKAVAGEANVPFFNISGSDFIEVFVGIGPSRVRELFAQARKHAPSIIFIDEIDAVGR
KRSKGGFAGGGNDERENTLNQMLVEMDGFHTSNDKVVVLAGTNRVDILDPAITRPGRFDR
IVHISKPDINERSEIFQVHLKNLKLHHTLDIENISYLLASLTPGFVGADIANVVNEGAIQ
CARRSNMVGVQVKDFELAIERVIGGLPKSSSLISPLEKKIISYHETGHALIGWLLEHADP
VLKVSILPRSNGALGYSQHLSEEVMLFSRDAILDKVAVILGGRAAEELFIGKITTGAIDD
LNKVTQLSYSYVSQYGMNKEIGLVSFQPNTSSEYSFYRPHSECLAHLIDNEVRCLIETQY
NRVKSILLKHEKQVHKLADLLFQKETISYQDIVECIGERPYPVKSTYEKFVKANPYKLIR
PGETPSEQAIGNGVATAGESEKGIQGTHAMPQMGEPNEASAEPLVKREERHITGKKETSE
ENSDDGKKNNKVSHVGTRMNTKNVTREEKKKEKELNEARKAGKIEALKDEEGNDINPHMP
QYIIKAPWYLNQTAPGLKHQRYKGTDKVKIEEERNKKVYVKNLKNVSDFCKNCGSTAHKE
KDCLERTRKKKLNFANRDNEDDFVCLTQDLGYDGNRDRWVGYNPDNFEHVYKEYEKIVEE
KKKRKAEELKQKYEKKATSGKKQRANDQSGSDSDNEEDEIDDDARKATTGAEDTQKSS-G
NTNEKHRNVARNLRIREDTAKYLYNLSLNSAFYDPKSRSMREDPFAGTGKNLDDNNHYKG
ENYYNNTDEAIESKKLEIFAWETYKRGENVHFNAQPTQLELLYREFLSKKKKLIKKKEED
ILKTYKCENVSKDATAGEELTQSEVYTEYKPVDQVDPKVKRIKIMSRYEEDIHLFDHSSV
FGSYYDRDKKKWGYRCCRSTNKFEKCSSVMGAKRSEYDEEGKKHKYGKEEMNQLSGNVEY
DLEIETRLEDEDSANHVGNHNHISIDTLKEEVVQNDFNNDDLDDKTLGLVKDKSIKRREN
EFQRRKYDYVMSPGRADPFGEKSPSPGERTYADVMMDINNESYKMKLSSGGTK-AGAKDI
GGGGNRKMRWGVTEGDQGKSKWGALSEEENMSFGNMPTPAPSKWVDTPFVLNDGG-AVKK
KKISRWDKVGDGNTTADGTGGATPGGMMKTPYLSGNFVNTPYVLNQLGKTPTMLTPMTPG
MTSVSIDSIIKMKIKNEMEMRNRPLTDEDLDELLPSEGYEIVQPPEEYEAIRRNKLKVFF
KTMATTVGTPLLMGSAARVTTEDDKTGDHHSGTMPSGTPFYEIPSTTTNQMKGDAQTILQ
SSQFQINNPQLLSELKYVQLKNEDFIYFNKLFETVDESDLSQEELKERKLMILLLKIKNG
TPSVRRAALRAITDKVKELGPETLFNLILPLMMQNTLEDQERHLLVKVIDRILFKLDDLV
RPYVHKILVVIEPLLIDEDYYARVEGREIISNLAKAAGLATMIGIMRPDIDHPDEYVRNT
TARAFAVVASALGIPSLILFLKAVCQSKKNWEARHTGIKIVQQIAILMGCAVLPHLRQLV
SIVAHGLHDEQQKVKTITALAIAALAEAAAPYGIEAFDSVLRPLWKGIAEHRGKVLGAFL
KAIGLIIPLMDPYHASYYTREVMVILINEFNSPDEEMKKVVLKCVKQCIQTEGIEKDYIN
EEVVNPFFEKFWVLRSSHDKRNLHLIVETTVEISNKIGGAAVIARIVDDLKDPSEQFRKM
VMQTIQSIVNNQGVDDIDQTLEEQLIDGILYAFQEQASEDYYILLNSFDAIVNKLQVRMK
PYLPQIAGIIRWRLNTPLPKIRQQSAELISRIANLMHLCGEHQMLGHLALYLYEYLGEEY
PEVLGNIIGALKSIVVVLGVQHMTPPIKDLLPRITPILKNRHEKVQENVIDLIGIIADKG
GDLVSPKEWDRICFDLIELLKSNKKLIRRATIQTFGYIARTIGPFEVLTVLLNNLRVQER
QLRVCTTVAIAIVADTCLPYSVLAALMNEYRTQDLNVQNGVLKALSFMFEYIGEIAKDYV
YAVVPLLEHALMDRDLVHRQIATWACKHLALGCFGLNREDALIHLLNYVWPNIFETSPHL
IQAVIDSIDGFRVALGPAIIFQYLVQGIFHPSRKVREIYWKIYNNVYIGHQDSLVPIYPP
FEHLDDSNFARDELRYTLMAKQRLTALDIRAIITLCKNIIVGCVVTNIYNISNKIYVLKC
SKKEQKYFFLVEAEKRIHITEWKREKDVMPSAFTMKLRKHLRSRKITNISQLGGDRVVDI
QFGFDDKACHLIVELYIAGNIILTDNNHKILSILKSNDAIGKKYNVNDVYNVEEHTSILL
HKNLHVVDMENVKKTINEIMVLAISGERDNENATVNKQGSDNNNNNNGGRTGKGNVKNDA
SGSLNKKLNKKTPICSDNEKGKKMKKLKTLTDLASKLIIFAHSDLIVHSLIACDVNPSDS
LEKYNLSDLSCILLKAINEALSVLNSFSIEGSCVRGYGFALKGGEKGTRAEKNKTDGGYS
KSGEEITFTEFSPIILNNHKNKVEENKLEIIHFDDFNKCVDSYFSRMELSKYDKQQEVIK
IKKSLTKMDKIKLDHERRIEQLEKEVSSLRKKISLIQMNDELVEQAIQLMRAAVATNANW
EKIWEHIKLFKKQNHPIALRISSVNFNNCEMELLL---DDGEENEEGSDDSSEADEE---
--SPKRATGRESKLAVTINLNNSVYGNVEDYQKMRKKAEEKIRKTKISTNFAVKKVEKKK
KEKENKQKGKNKTVGQIQKLRKVYWFEKFHWFISSENYLVIAGRDALQNEILFRRYFQKN
DVYVHADIHGASTCIIKNPYKDIPIPEKTLSEAGQLAICRSSAWNNKIITSAWWVHYHQV
SKSAPTGEYLKTGSFVIRGKKNYLPHVKLEMGLCIIFQVDNAAVENDEENNLDDTQKSFE
NDDEKKNSDGDQEVVEDADNGNGSYIPETVLTKGRDLHNNDSTSVCTSFSVSNTNAKCVS
NCVAIPSRGSLKRKRRYLYKRFVGFFFSVRNRTNLLNFEKKPLLEKLGITYNYDYDIVKG
LLNWIGSNLWSDEDNSLIRSRNGHLSECAICTGLRKTYKKYMYSFVYYHKKHTTLRDLTL
RPFFRRLDAITFFVSHKVFAEKGYLKGIKYMYDDSFEGLGDTRMVNDGMELFSSRFMNGG
LSEQVSNICVKDYLCKREKRAPFIFVDCMVYGAPVTFKDEDSNDSDADKKVNVRILAPPS
GGIHDEKRECYDEGEGKAKKVSFVRDDG-EGSKMVRPARSRKGTGFVKMDVSKLLQEIEE
EEEEEKELSEETGISTKVAFFKAGERRSEKKVVTFTSGGESVDDEKQSSLNAPRPVRSRK
PTGFVKMDVTKLLEEIENEQSFEDAGEAGEAKQKNVMFSESPHRFSTRKKSVSFSSEDEH
IYVERQESMKVPRPVRSRKPTGFVKMDVTKLLEEIENEEKVDEALNDLNGEEEHKEDTPK
VTFAEGTLGTDLTKKKVSLKYEDEYVQGEKNELMNKAPRPVRSRKPTGFVKMDVAKLLEE
IENEEKLDEGKEPSNDKKGKKRPRKATGFVKMDLSKLLEDVEEDEDNDDNDNDNDSNENN
DDDNSDDNNDDENNDDNDMDGEGCYEESNESDGSNESDQTDRDEWDSDSDDGHDGNVQSS
AKESKEKANNEKGSHENSNMQSENKAKKNDIKKQNVSINHGTVKDQKKD----------K
KRMKKKYREDDEEERQLHMKIIGARKMKHEMEKEKVDKVESKATEQENKYKSPKEVTVNF
EEINEEEMKDKMSELKKLVCTPKEGDNLVFAIPMCAPYSAIQNQKYKVKLVPGNAKKGKV
AESCISYFLKMSTEEKEKELIKNISVDELGNCIITNSTPDLKELKPATMGRMYGKGKGIS
SSTIPYKRKQPSWLKQKPSEIEDAIIKLAKKGQTPSQIGATLRDNYGIPQVKAVTGNKIL
RILRAHGVATTIPEDLYFLIKKAVSMRKHLEKNKKDKDCKFRLILTESKIHRISRYYKRK
KLLPSNWKYQSSTASALIAMSNRKKVAYFHDPDVGSYYYGAGHPMKPQRIRMTHSLIVSY
NLYKYMEVYRPHKSDVNELTLFHDYEYVDFLSSISMENYRDFTCQLKRFNVGEATDCPVF
DGLFQFQQSCAGASIDGASKLNHHCADICVNWSGGLHHAKMSEASGFCYINDIVLGILEL
LKYHARVMYIDIDVHHGDGVEEAFYVTHRVMTVSFHKFGDYFPGTGDITDIGVHHGKYYS
VNVPLNDGITDDAFVDLFKAVIDKCVQTYRPGAIILQCGADSLTGDRLGRFNLTIKGHAR
CVEHVRSYNIPLLVLGGGGYTIRNVSRCWAYETGVVLNKHHEMPDQISLNDYYDYYAPDF
QLHLQPSSIPNYNSPEHLSRIKIKIAENLRNIEHAPGVQFAYVPPDFFDSEIDDECDKNQ
YELKDDSGGGRAPGTRSKEHSTTHHLRRKNYEDDFFDLSDRDQNIVLMYSDNEPIDREIL
EKQYEIIKCAKYQDFIRLQIIIQPYILNNDIEMLNGINVLHWACYCGFTELIKKLISLNL
DIEKEDLVNNDTAIYYAIKNSHYEIVLLLIEHFGPSILFHKNRRKMSPFLTAISEFNEDK
ILEALHILELLYLNGASLEEQNEYGQTALFLCVKRNNISTLQWLLSKSVNINHRDFYGNT
ILHIAVKYCDIDILRLLCDYGSLNLVHHTSMQNDSINVFQLCLRNRYFLVYILLKKWIIQ
DKLCKGIKICKTIYAFYFWFFAMLNLIVYLNISRSFWIHRKYHSLSITWIVIWLFQQFLW
FILYFKSPGFYKQNETLTKRHSRPFSYTYDSSFKRKTEYQLNSIEMELFKINKIISSSHM
NPQIMDYQIEAQQAKYDELIVNLECQKLALYEKVSKERINSLDVDYRNAILYNRNPRNVC
VTCNIVKPPRVHHCAECFHCIVHQDHHCVWVDNCIGIKNQRAFYLFIFSMFVLLLYNYYY
VFLYFSLFHKTVDYAFALLVILCNFINITLFAFITYLFARNTRTILTNITFYEHFKKPSH
ITDKYNTELRCWDFQNLSLIKMLKNVYSFWSLNYDEPYLRHGKKADRIMDVFEFVEPLRK
RKITNNLFDFDQYVYKEKVDESEEKKSEHHVV-KKKKKKLRRVLDSSNSEGNKSNEDTSP
RSRKKRNGPKDESPKKEYEDEHQQNESNTN-SDDHDSSLSNQEEYEQNLKDLFECILGSI
KIKNKIIEYFTSNVKEKRQEIIKDFVKGSFRVSNFDANFTNFEKDVDTFHKLKKYQKCGV
FWLYVLYKENKNGILADEMGLGKTAQTCVFLDYMYRTKELKNKTIIVAPTSLLKNWNNEI
NMWCPYLKNHKIIYYGSQNERKYLAYDIFTNKS-SNIHIIVTSVNMLIGKNDVSYFRQIK
KYDYLIFDEAHFLKNKNSLVYKKLQKKISFNNKILLTGSPIQNKTQELMNLLLFLMPDIF

TEKSINSAMNAFVKMYQDILESKEN--DDHNNDCCGTPRNSAKNMDIYDIKSVEKKEP-M
EMNDSTG---------------------------NAITTEDTRNIIKNYLIETIKDDVK
YAQLKNKEIILLQLIIEPYILRRSKKHVFIDMPKKHSIIIKLPMNTTQLNLYKDEIMSKI
QHTHKHLEFLSKHSNKKELEKLAVIFEKKDIKDGGDKESVTEAATEGGTHNGDTSDHVNT
NEKGGINEYDDEDDKIDEKTINIEKAEENKDGNIVINKRQDEPMEK----ENANNISKEV
RGRMINASIFILRRICNHPLLHKYYYSVEDIKKISKYFYTNTDQYLDLDLKTVENEFMKI
SDFDIHLSIKHLISQGDENLNRYLISKDHILNSSKIHHMISLIKDIRKKKEKVLIFSQFT
TFLDIIEEALLYEFVYDEQDFSHHQQCVTEGGKDGQIDPNVDATEEGETRQDNEEDTNSY
LNKNDKDVYLSSSTSSTSSLASDHKGNNQIYVRLDGSTNTIERQKIIKRFSNNDNIFIFL
LTTKAGGVGLNLIAANHVILMDQDWNPHNDRQAEDRVHRLGQKNEVYIYRLCCKNTIEET
ILRCCKAKLHLDQAFGGNSDLLMLKNPGAVLVFKPNTKREEGHKTQLSNIQASRAVSEIV
KTTLGPMAMLKMMLDPLGGIVITNDGNSILREIDVAHPAAKSLIELSRSQDEEVGDGTTS
VVILSGELLSIAELFLKQKIHPTIIVNCYMDSLSKVVKFLESIAVQVDVNDEKSLLKAID
SCLSTKFVNRYNKMVSKLALQAVQCVKIENVMGKKEIDIKRYAKVEKIPGGDITDSYVLK
GVMLNKDIVHPKMRRRIKNPRILLLDCTLEYKKAESQTNVEILDEQTWNQLLLQEEIEVK
KLCEHIIDSRCDIVVTEKGVSDLAQHFLVKKNISVIRRVRKTDLNRLERISGATIVNRCD
EIVESDIGTKCGLFEVKKIGDDYYSHFIECENPRACTILLRGSTKDVLNEVERNLHDGMN
VAKNIILEGKLLYGGGCTEMRVGQHLISQASQYDDSRKSIMEAVGSALEIIPKILAQNSG
ANVVKTINELRIKHETPGGEKFGVDGITGEIIDVSTKNIWDLLAVKKQIYKSAIEAAAMI
LRIDDVVSGIGKEDKLQKPVQNQFMAPIHVLNVAEKPSVASAIANILSRGKMERKKSCSK
YNPVFTFNYQRDNETWSMYVTSVTGHLTEQKFDDKYRNWLNTDPQVLFDAEITIYVENDK
KTIENNLKRYSKFCNLLILWLDCDREGEHICFEVINTCFVMNRDLRIKRAQFSAVTEKDI
IHAINNLKYPNRNLANSVDVRREIDLRMGSIFTRFLTIRYVHLIKTRTNIISYGPCQFPT
LGFVVNRYMDIKNFVNEYYWSIKMQYLWEDAEKSGDESDGDSSAWRKKKTKRKKKNEKEK
KNKGKKEDTNQNTIADFTWSRSRLYDHLAVVLIYEELLKNPLCRITNIYQNEVKKYRPLP
LNTLQMTKLVSKHFHISSKQCMNIAEKLYNKGFISYPRTETNSFPISMNLRSIVSELKKN
NIFGSYANKLCQGNNFKEPRKGKLNDKAHPPIHPVKNMQKTDGVEEKEWIIYEFICRHFL
AVCSEDAIGFNSKVVATIGKEEFFCKGLKIVKKNYLEIYIYEKWNNKNIPPFEVNQEFHP
YSLLVEEGITQPPKYLSESDLLSLMDKYGIGTDATMHEHIENIQKRNYVFKNSKNLFIPT
NLGIALVLSYKKFKDIGIDLTDPSLRAKMEKDMTLVASGTKNKEETIRNYIDIMKYIYQE
MFNRIDFLDEQIKYYLHNAAEHCMGTFNFGKHNGKTYEEVFEKDKSYVTWVKNLETPTGS
LIHFKEYVLEREKEEQGRQVPSPGHLGHHNTLGRKSNGNWSRGQGWEG--ASCNGRRESG
GADTYRSFDQSPSAVLKGMDTSYKNEGYQKTDKSVNKMNSREYKMYEQGIMNEINKSYNK
SEGEKVEFDIIVAFEIFSVDTFKIVQMDNNSRKYASFKNYLPKELFKILSEFNPTLKKLN
NYSCTTFEADKYEYVLSNLKEKCTILGGVQSIPNFLLKCFKLYSRFSEPQKVSEMTANIL
TNTLCPYTEKNYDKMDILVGEKLSAELKNFQREGVYFGLKKNGRVLIGDEMGLGKTLQAL
ALMAFYQEDWPFIVVCPSSIRFQWKDQALRWLSHLLSENEICVVKSGKTDIPRNCKMIII
SYELMTKNDKYQNKYKSIVCDESHYLKNSFSKRTKAITPIIRSAKRCVLLSGTPALNKPS
ELYEQVSSIIPDLFNYNEFCERYCFRDKNMYTRKFEYVGCKHTEELHLFLTNTIMIRRLK
KDVLKELPEKLRSKIPVEIPPKELSEILTYQRMLEGKKNINLDDLDGFPLTGGANDTPNR
IDEENVSISHLFKITGYAKVKAIKEYITYLIDADIKFLLFCHHKLVMDEIDKFLTEKKCM
FIRIDGLTPIDKRELYIKSFQNDDKIKIALLSLTACGLGLNLTAANTVVFGELYWVPGQI
IQAEDRAHRIGTTHEVINIHYLIAQKTIDETVWRIINRKWNTLTTALNGMEDSLNVKEVN
KFDRFMLDLTNDANKSYPTSLVTTPKVRRKSFEHNKPFESSNTKRERDIRDFFKPSERSA
EKSDCVKKRSYDTPTKDFSSNASPNIFSKRYKTELHMKEKHKKVEYAEKEAEAPADGTTD
TTSSVFVNNLLTSINNINNAIVYKDNRYILRMLKYIKCMRLSIKNESSTLMPIMTNLISK
TFKEGYPIYEILFKYINEFKE-LPKEVPEFSNINDKTYTHAAPEIEVFFYIMILLYLLDK
KCYNEAMDLSTVIVNRITKLNRRSLDCINAKVYFYFSWVHELGGKLSHVRQKLLYIYRNA
CLHRDIMTQTVVLNLILRDYIKHNLYDLAVKFVSKTSFPENSSSNAQHARYLYYIGKILA
IQLDYSEAHSKITQAIRKAPQNNQSAKGFKLEATKMEIIVELLMGDIPDRSLFSNKIMRN
KLIPYKHVVTAVRNGDINKFSKVMNDYNQLFMRDGVYLLIKRIHHNVIKTALRIINLSYS
RISISDIGKKIGVESPLDIVGITAKAIHDGVIEATIDYDNQYVESKSNSDVYVTSDPMKT
FHKRIAFCLQLYSDAVKAMQYPDENEKKENEEAKERKIRQQEELAQAEEGDLGDDNDLL-
------------MKPSNRRKLCRECFTECFEEDVHATIVKKNMFHDKDKICIAVSGGKDS
SVLAHVLVHIKRKYNYKWDLFLLAIDEGIKGYRDDSLKIVFKLQEKYNLPLKVLKFEDIF
TYTMDTVVSFIGKKNNCTVCGVFRRQAMERGALLFNATKLVTGHNADDLAETILMNMCRG
DLEKLAKGVDSMSEMKM------------GTSEMEKAKFGACCGG---ECTGGEATAVEP
GHRSPSSTGNPDALIGGPSTHVESAPPEEEHTFFLPRLKPLMWCYEKEIVLYAFYKKLDY
FSTECTYSPNSFRGNLRSFIKDLEMINPQFILNIIHSSEFFYHNSSRRKVLQVCTRCGVY
TSNPVCKACLIVEGLRNYKDNSFLYANTKKNGEKRRIPIRYDPGTMESQKCPFTSVIPYG
MLYEKLKKEKNNQLPENV-VIEQFDQLLTTYERPSPYDANGQICISNQSDLFLFQIDIEY
TVDNIFKNMVSMNDGTPNGSALADIFSTYRMLQNSEKNYISVPVIHIYTVTNDGYSALVS
VHNFFPYFYVEMPSNFQKEDLLKLECMMNDNLNMNNQYKMYEQKIMNIEIVKTESLMYYK
REGKKNFLKITVLLPKMVPTLKKYFEGIVNVNGKNIGGIVYEANLPFILRYIIDKKITGS
SWLLCKKENFYIRPRHKKISNCSFEIDISFEHLEPMPLENEFQQIPKLRILSFDIECIKL
DGKGFPEAKNDPIIQISSILYFQGDPIGKCSKFIFTLKECASIPGSNVIWFHDEKTLLDA
WNEFITRLDPDFLTGYNIINFDLPYILNRGTALNLKKLKMLGRIKNISSVVKDSNFSSKQ
FGNHETKEININGRIQFDVYDLIRRDYKLKSYTLNYVSFEFLKEQKEDVHYSIMNDLQNE
SPESRKRIATYCIKDGILPLRLIDKLLFIYNYVEMARVTGTPFVYLLTRGQQIKVTSQLY
RKCKELNYVIPSTYIKSGSNEKYEGATVLEPIKGYYIEPISTLDFASLYPSIMIAHNLCY
STLVKNNSEIEGLEQEDITSIQGKSNIKFVKKSVKKGILPMIVEELIDARKKVKLLIKNE
KNKITKMVLNGRQLALKISANSVYGYTGAASGGQLPCLEVAVSITTLGRSMIDKTKESVE
KYYNKSNGFEHNCTVVYGDTDSVMIKFGTSSIAEAMALGKDAAQRISKEFLHPIKLEFEK
VYCPYLLLNKKRYAGLLYTTPEKHDKMDCKGIETVRRDFCILIQQMMETVLNKLLIEKNL
DSAIEYTKSKIKDLLTNNIDMSLLVVTKSLGKTDYETRLPHVELAKKLKQRDSATAPNVG
DRVSYIIIKGVKGQAQYERAEDPLYVLDNNLAIDYNHYLDAIKNTLSRIFEVIMNNSDSL
FCGEHTRHKTILTSSQTALSKFLQKAVRCIGCNSSIKKPPLCNHCKSNKELSIYMQKMNN
FKKKQNEFFQLWTECQRCQGNLHAEVICMNRDCPIFYRRAKIKKDMANVQEQITALRADW
MRVALRDFAACLYLARPLNNGQPKLNQRRHPLVGAYYNRENLHKRGKKLWGQSKRNVYCF
SSIRRHQKILKQNKISMSVAAERNVEESIHLFGEEEFKLEDISTCLSDIFEKEKCENIPF
IQVAPMINVTNRHFRAFVRTITRRAQLWTEMIVDNTLLYNLNNLEEYLGFNSNEHPVVCQ
LGGSDPTSLAEAAVLVEQAGYDEINLNVGCPSTKVANKGAFGAYLMKKPEHVRNIVYEIK
RKVQIPVSVKIRTGVDDCDSFPFLRSFIECVSSVGCTHFIVHARKAWLKGLDPKQNRSVP
PLQYSKVYTLCQLYPHLKFTLNGGIKTIEEAVALLNGYLPREDKCDGEKTYVQVQNYHVN
PLNGVMLGRACMENTTVLSQTDQLVYNEKPPHTAFSRRTVLDAYKSYLEENSSLCSLSSA
FELLKPILGILKGMPGHRIFRNKVDMYIRNYSSTLPCSGILEKAMVDVDAVAPGCLDLPL
ADYKLQQEYIKNYMQKSQNFENETVLVEEVVRDFDKNEVFEEITAKFTWEQDVERSWNLL
VENNGVLQHVSQENFEEKSKQKYKKNQVCALRKGIFRHIIILFDMSSSMKERDFKPDRIN
VVLECVENFLTNFFFKNPVGHVGVVALKNSSAKLIQPLTSNMEDIMNALVKERSMGLQGS
PSLEQGLEIAHDLLVDIPLYGTKEILIMYGSIRTCDKKNILNILNLIVKNNMHVNCVSIS
PEMHILKHICEETNGVYKICMTKNALMNEMNNVTETPLWMIGMEPQLIHICFPIKKKIST
QIMCSCHNHLNTDTYICNFCNSYTCKIPSKCKVCGMHLISMHDLSHITNNLQGSPLFIEI
KNEKNGPSACVSCNKPLYDKVSQCSKCKNIFCLGCDLYIHEDLNQCPFCLILDTMGSNSL
PMSQQMYFSTHNALRVNDENDVISTLFYEINGNRHISLLIFPFYDVQMLKRLLIKKLNLP
GGVKVNDIIIFYKGIKLPNYRIISTYLECTGNEKNKKKKKKINKLYWAIKDINPNASIR
VIDQKSYPPFFENILHDIKLAFKKNISPKLTMDGTGGTYLLFNSKKKVCSVFKPLDEEAF
APFNPRGYEGKMYQEGFRSGVLSGEGASREIAAYILDNSYNNFSSVPCTIMVEACNPHFN
NKSKLKYVDHENNLKWKCGSLQEFVDSRESVGNYDHKQFSIRDIHKIAILDIRVMNLDRN
DGNILVSPLKSLKDSCNQFLYRNNRSLGTSDEDTLKRIVTIDNKPSRYSLIPIDHGLIMP
HIMDVAEIDLVWFEWPQTKVPFDDEELEVIFTFDPDKDAEKIRNKLLIREDCIRTMRVCT
RLLQIGARMHLNLHEIAKISTRKNIDEESVLEHLVRDSIVQAYQMMDYTSLMSTNRLGYI
LDLAEIKINKKKKNSRMKSIENIDEALKERNETTDKTGINKMEGGAFHFKPLLEDKSKSL
DCSYGKSLPSGNTFQDVGGMETAESGSNSRRMEKEDSAEGSGKLQIISESVSCAPGGKGN
LSNAFEVTEKKEKYDGLYRGTLDVDLNIRSEQKEENKTSTNSFSNKSDYTLVTASSSSEN
SPSSDG-----------PHSASDQDTRVKGKKRKKKKKKKYFDKDDNEKREDEEGKGLPS
DSEELQNETTKGDLLKGGVQDDEEEGDDESSSEEDEETDDDEVPFRKPTGTIKRITESSG

TAYRGMEMNKVNSVWMIRDKNNKIINVKWENKIFEKLFFETFENYVKRYISDYHPEWKQY
PYKGSHISGIKHGYLKSIEMEIRVANKYALGKKLGSGSFGDIYVAKDIVTMEEYAVKLES
TRSKHPQLLYESKLYKILGGGIGVPKVYWYGIEGDFTIMVLDLLGPSLEDLFTLCNRKFS
LKTVLMTADQMLNRIEYVHSKNFIHRDIKPDNFLIGRGKKVTLIHIIDFGLAKKYRDSRS
HTHIPYKEGKNLTGTARYASINTHLGIEQSRRDDIEALGYVLMYFLRGSLPWQGLKAISK
KDKYDKIMEKKISTSVEVLCRNSSFEFVTYLNYCRSLRFEDRPDYTYLRRLLKDLFIREG
FSYDFLFDWTCVYASEKDKKKMLENKNRFDQTADQEGRVKQNMNLTDELRQKGRTQLLSI
FKRFKGDKCLFFERSLHIILNVVLKDDDIKKERIEHVFLLDKETEVNVSKVDKVNNILFF
LRPNFYEVENVFKIIERVAKNEGGTRKYALVFIPYMTPMCEAEILKHNLLDIHIRVIVFP
LYFFPLYNDVFSLEIKGLYKEYYVDNDFTNLLMCSFSFMFLQHLFNGVFKNIKSLGQLSH
SIVEQLIQLRKEIVAKFDEDFLDVITNLQNVQDMRHFRDSGQPLAIPLKYALHKIFLSER
SSRGKFQTRSCAEGKEEDEDGSDDGSGDDGDEDNRDGEVSSDHNGDKSDHLGAPEGDSPG
GMEDPPNGVEGKAHKEKEHAPRESTGMHNEKSVKREMLSQEMFVTGASPSEQVNIDSASE
RSRNSTSRIKRRNPSNNATDGNKSSSSF-------EEGLPPSRGRKAIKGAIHRERRKGE
SRGRPSKGK----TKKVIESVRMKKTDSDVTEEDYESSSASTGPNFMATEDNTNFLLKKK
DKQKMERKNKREKSYLQEKLGIANFNFLLNVSNKIDSCIIIDRKIDMVTPFSTPFTYEGL
LDHLFGISNLQIEVPRYIIFNDMNNPGGDMK-----HSQQNKDSLKNMMVRIKLKNSVDV
LYNDIKDLSPNQVGLYLHNKASEIQKTYKEKDTLKDIEEINKFLKKIKMKHFEHNSLSTH
VNLASFILTTMKKEPNFNKLKLEDEIIQLNNTSNRTTLHNIVQQIQLLIYSNEDIHEVYR
LLCLFSVVTNGFSENYTNEVKKDILEHYGIDELSRINKLHFCNILKHQPKQKFIWSHLRN
HFNLLSNEHNDISYVCNGYAPLSVRLIEYMGILKNNMQAFPEIFNLLSGPTLDIVQNPVG
YGKFALDKGKKDTPWGENNPDGEKDVVLLFYVGGISYAEIAAIRNLNKHSQTYHYLIFTT
EIISSRRLLQSLAAMETTTNNQTSPLKEEADISPLDETIQVKSMCINCEQEGLNKIAKLH
IPYFKNVLIHSFECGFCNYRNNVIQDLNTIKEKGVKIIFQISQREHMDRQLIKSEYGVLK
IPQIDFEIPKDTQKGSINTIEGFLQTALSNLTEYLRNLKHMYCEANGLPEGAPNGEPGVE
AHNVEGEIINKSERGENAHGEDARATDQMSQGDKSADSNLQGQQMTIENYIKLIESTVHK
LSTYVVSKEPMFTIEIIDPSGLSSLEHYDEDLQKGTVTVEHYKRSKQELNEMGFYEEDFE
SKNEGANLETNGGKEKEDHVMEKVKKENFDFIKKYVHMNDASSVNSGTAVRYENISEDEE
GKLIESFTSNCPCCNYMGSNNFCEINIPGFKKCLIMSYVCANCNFKTSEIKSSGEINPKG
KKITLTVRSKSDLNRFVIKSDTASIHIPIVDLTSDYGTLGGTLTTIEGLILKIIESLEDK
FKFLLGDSSTNTHQTNDNVLPGNNDESVTNKIKTLIANLYKLCKTEELCPFDLVIDDIAS
NSYISSDDIMHDENLKEEEYERTFEQNDVLGLTSMNTEYMADSAKRLPLTVDNADMKKIP
SGRVSDDGIKRTPSGKPIQTMYVLNRKGEEEDISFDQILKRIQRLSYGLHELVDPARVTQ
GVINGMYSGIKTCELDELAAQTCAYMATTHPDFSILAARITTDNLHKNTSDDIAEVAEAL
YMYKDVRGRPASLISKEVYDFMIQHKDRLNKEIDYTRDFNYDYFGFKTLERSYLLRINGK
IIERPQHLLMRVSIGIHIGDLEKALETYHLMSQKYFTHATPTLFNSGTPRPQMSSCFLLC
MKSDSIEGIFETLKQCALISKTAGGIGVAVQDIRGQNSYIRGTNGISNGLVPMLRVFNDT
ARYVDQGGGKRKGSFAVYIEPWHSDIFEFLDLRKNHGKEELRARDLFYAVWVPDLFMKRV
KENQNWTLMCPNECPGLSESWGAEFEKLYLKYEEEGMGKKTVLAQDLWFAILQSQIETGV
PYMLYKDSCNAKSNQKNLGTIKCSNLCCEIIEYTSPDEVAVCNLASIALCKFVDVDKREF
NFKKLYEITKIITRNLDQIIERNYYPVQEAERSNKRHRPIGIGVQGLADTFMLLRYPYES
DSAKELNKRIFETMYYAALEMSMELAQIYGPYETYQGSPASQGILQFDMWNVKVDNKYWD
WDKLKEKIKKHGLRNSLLLAPMPTASTSQILGNNESFEPYTSNIYYRRVLSGEFFVVNPH
LLKDLFDRGLWDEDMKQQLIAHNGSVQYISEIPNDLKELYKTVWEIKQKNIIDMAADRGA
FIDQSQSLNIYIQKPTFAKLSSMHFYGWEKGLKTGAYYLRTQAATDAIKFTVDTQVAKNA
AKMKNEEGVAITREVSRETISTESTVTQNVCPLRRNKDDQCLMCSG---MSGGGEVVPRL
SFEEMRSEMSKYGVDITQGTLKNPTTEDMQGVYSMCIKHILNKDINNIRIEEFTGDLKSS
MPSIDGIQILPNEGKNHLQAIGNLRFIRHCEQINRILCVENTLSYLFKPVSSHITRLINA
FIHFTKYKEQIYLDNDFKIRKIEEGKSEDLALDTELKAVRNELQSLLDNYEQIKNSVLTE
KNKKRDYEEEIIENQNLLNAQQSTIISLRSTKDKIVNETNELIFQFSRFRQKKEDLEDQI
VPSPEKLQEYNHELKNLLLEHVSYYESDKKKNEEIKNKINVADLCLKKLVELLTCLTGHL
NDTIKHHIEKKDQLKDLEKDLKTLKTDKDNLTLKKKDQEKILRDTEQYYAEQKDKWNAKV
EGEEKNVVVVEKKVNQMYEKIDELNREADREAQEIDSIVKLIQDTLDNYSRNFAAIDDLT
ERTRNSHALLAGKVRTLVLPCAGKP-MIQFLKNLHLHKKKDSASTKEPKRENKMKYEDFN
FIRTLGTGSFGRVILARYKNEDFPPVAIKRFEKSKIIKQKQVDHVFSERKILNYIKHPFC
VNLYGSFKDESYLYLVLEFVIGGEFFTFLRRNKRFPNDVGCFYAAQIVLIFEYLQSLNIV
YRDLKPENLLLDKDGFIKMTDFGFAKVVDTRTYTLCGTPEYIAPEILLNVGHGKAADWWT
LGIFIYEMLVGCPPFYANEPLLIYQKILEGIIYFPKFLDTNCKHLMKKLLSHDLTKRYGN
LKKGAQNVKEHPWFGNIDWVSLLHKNVEVPYKPKYKNVFDTSNFERVQEDLTIADKITNE
NDPFFDWMVVSQFYILSPRGDTIINRDFRGDVSKGSGEMFFRNVKLHKGDAPPLFYLNGI
HFTYLKYNNLYFVFTSLFNTSPSYILELLYRLVKIVKDFCGHISEEVIRANFILIYEIVD
EIIDYGYIQNSNTESIRHLIHNEISATSKSTKKLANLSNFSMKNSNTLPSNASQKPIQLN
EKKNEIFIDIVEKINLIMNFKGEIIYSYVDGVIQVKSYLQGTPYIKIALNEDLYIKNLHS
DNTNNVIIDDCNFNHLVNLSQFEREKILSLYQPDGECILMNYRINNNFKAPFRIYASVTY
GPNHTVELCIRIRLDIPSQYTCTNVFVNCNLCKHITNVHLDQSCASDLFSAQYIANENRL
LWTIKKFKGESEHSIRSKITLSPGYTFCKRDFGPIYMLFEIPMFNLSKLRIKYLRIIENY
KSSNTHRWVRYITQSSSYVYRLMDVRVTTPYDEELEDIEEFNNRILATETLKHKEENVQR
SGDEVCPNIKKLVLPGEAILSKKDKGRFLKGSGLYEEDEKYFACIVGTVNYINKLVYVEP
LRGKYTGAVGDLLVGKIKDISNDKWVVEIGSYCKALLSISQTNISLFCQRIRLYNDVINM
INIYKPNDIIACEVQRILPDGCIILHTRSSIYGKLSNGILITVPQTLIQNQKKHIFVFPC
NVQIILGMNGFIWISSPIKKSKETNPNSVDEDIEDNKFEEVDDTTRRNISIISNIIKLLA
KYHININYDIITKIYMHYTSNRNNSASYILKPYVSDSYLFSYIDKFAQWGMPVLYHLTLQ
KPTAITKIAYGNFSGPKVHEIVVSKGQVLELLRADKQGKLNLIVSKDIFGIIRCLQTFRL
TGSNKDYVVIGSDSGRLVILQFSNEKNDFVRVHCETYGKSGLRRIIPGEYIAVDPKGRAL
MICAIERQKFVYILNRDNKEQLTISSPLDAHKSHTICHDVVGMDVGFENPMFASIEQNYE
MYDKQVTNTTEIDACTRKTLLCLWEMDLGLNHVIRKHTLPIDMSAHLLIPIPGGQQGPSG
VIVCCDNYLVYKKVEHVDVYCAYPRRLETGQEKNISIVCSTVHRIRKFFFILIQSEYGDL
YKIEMDHQDGVVKEITCKYFDTVPVANAICVMKSGSLFVAAEFGNHFFYQFSGIGDDDNE
AMCTSKHPSGRNAIIAFRTKKLTNLFLIDQVYSLSPILDMKILDAKNANSPQIYALCGRG
PRSSLRILQHGLSIEELADNELPGRPKYIWTIKKDNASDYDGYIIVSFEGSTLILEIGET
VEEVVDTLLLTNVTTIHVNILYDNSLIQVHDTGIRHINGKVINEWVPPKNKQVKAATSNA
TQIVISLSGGELIYFEIDESHSLVEIFRKSLNVEILCLSIQEVEENKVRANFLAVGCLDN
VVRLLSIEKEKYFNQLSTFILPNNSSAQDICISEMCELGNDKERKLLFLNIGLNNGVLLR
SVVDPITGTLTNHYSKYLGAKNVKICPVHVKKNPALLVLCEKTYLCYVHQGKYIYSPLNY
DILEYASSFHSEQCSDGYVAISGSSLRIFRFYRLGEVFSQNILHLSFTPRKIVPLPFPSL
FYDHDTSIEIERQKNIRMLAIIEADHNAYDENTLREIQRALKGIQLEGEEHTGSNGEA-D
DEEEELLYDRIGTVKAGPGKWGSCIKIIHPVSLQTIDKISLEMEEAALSVCACELEALHC
LIVGTTTNLSLKNRTATTAALRVYTYDINYKLNLLHITPVEDQPFCFSPFNGRLLASVGN
KLRIYALGKKKLLKKCEYKDIPEAIISIKVSGDRIFASDIRESVLVFFYDANMNALRLIS
DDIIPRWITCSEILDHHTIMAADKFDSVFVLRVPEEAKQEEYGISNKCWYGGEMMAGSNK
NRRLEHIMNFHVGEIVTSLQKVKLSPTSSECIIYSTIMGTIGAFIPYDNKEELELTQHLE
IILRTENPPLCGREHIFFRSYYHPVQHVIDGDLCEQFSSLPYDIQRKVAADLERTPDDIL
RKLEDIRNKILMDEDAPTQSKPLDDEDINILKSYGSGPYSTTIKKVESDISGLVSNINKL
CGVRESDTGLCLPNQWDLQLDKQMLNEEQPLQVARCTKIINGDTDQTKYIINVKQIAKFV
VGLGDKVAPSDIEEGMRVGVDRTKYKIQILLPPKIDPTVTMMTVEEKPDITYNDIGGCKE
QLERLREVVEMPLLQPERFVTLGIDPPKGVLLYGPPGTGKTLTARAIANRTDACFICVIG
SELVQKYVGEGARMVRELFQMAKSKKACILFIDEVDAIGGSRGDESAHGDHEVQRTMLEI
VNQLDGFDNRGNIKVLMATNRPDTLDSALVRPGRIDRKIEFSLPDLEGRTHIFKIHANTM
NMSRDVRFELLARLCPNSTGSDIRSVCTEAGMFAIRARRKTITEKDLLLAINKVIHGCKQ
FSATGKIYGVQMSIEFCSQHLLAYDNSIIPSENDIKSRAQENLKYIVQCMEALKSGKDDF
GDPSYQMTKINHFNIPRYSKIPKEKKNTKWENFAKKKM-MKKNKSGLIYDKNTKGWVRRF
QKKQIKINEENASFVHEYKPSDNIYEDPFERMEEEKEIKKMKQKMREMKNKFQQEGISTE
DIKYIARQKKKRDNLIDNLKTAQISSSTFGRQDKKLKKEKKMKVKMKQKCEKRLVKDEIK
QNNKLASIVLKSLMETNPYVFKSLVQIYEEYLNLMIHRVKGYKVLILDDETKTIISLIFS
HSYILEKEIFLTLNFNDSNIFEDATNGSGKNDKFDFKNYKIKNLKHLKAIFLLRPTHTNI
LKLMKELRRPIFLEYYLFFTNVLSDKYTEKLAKADEFEVVKNIMEYYIDAYVLHDNLFSL

NIDYTSFFYKNDHNNYSRRRKKKGMHWTDGNYNLGDDGKYDKLTIDEFEEGNMDSFGQFQ
NNNDYNVNTIVDDDVANYSNFDLFENQVVQRIVDGVFSLLCSVKQVPDIIYNRKSPICKH
IIDLLKIKMLRNESVFSGVLDSYERYNGASQQGEHSNYQFNSIGNQPPNMGTEGNCCYML
ILDRREDPITPLLTQWTYQAMLHELIGIENNKINMG-INPEESQIVMSCIYDDFYNEHLF
DNFGDLGKAVKTYVDVYQEETSRKSNLESIDDIQKFIEIYPNYKKLSGNVTKHVNILHKF
SEIVEKRQLFYMSELEQSIAIYDKKSEHFKQVIETIRNGMYSNYDVLRLSLLYSLKYEDE
EEVEMIKTELTKRNIDKDQVLLIDALLMYANEEARNNQLFKEQTFLDFAKTTITRTIKGT
SNVFTLHKSYIYYLIEDLLKSKLDSQTYTTTNLLNIEPNVNKRVNSMIIFFIGGATYEEY
RDLQYLSKRYNISFLLGATQIHNSQSFLADALQLVKDM-------DYTNLYDGALSGSSH
FYPDGDLQSVKKH-CTNLSFPLEVGIPKEKERRRHENVFDRLTDENFYTGMHKQKFDALR
RRAASKGICQNLKLKHLQVEGGGGHPPRTIFQKKCKTSVVTPGTLGMQKYGIQIAQPKNI
WLFRNGDEHHNGLLFLVKPHVNNWKSLLSEITKVLSPTIGPVRIIYNANFRLVRTVKELN
DGEKYLCTSGEPPARVDRLTRFLSPWVMHAM---------------------------CS
DIKGEKNERDERGEQGKMSNMKKQAKKSESASSESVKESFEDDEDEDAGGEDKDEDFADA
RRYFKEGQKCITPPNGDGTRAFYESLLEENPNSIIAIKYCIEHGVLNGTKHHQAIYKYKV
LKKNNAFRNNFGGIRGEFIKMLEEPPKSEDGSYSKMDEDRDANIEQWKIKRLIKKLENAK
GNGTSMISLIIKNKDEVSRINKMLADELGTASNIKSRVNRLSVLSAITSTQQKLKLYSKT
PPKGLVVFCGTVITEDGKEKKMSIDFEPFRPINTSLYLCDNKFHVEALKELLESDDKFGF
IIVDGNGALFGTIQGNTREVIRRFTVDLPKKHGRGGQSALRFARLRLEKRHNYLRKVAEV
ATSVFITNDKINVSGIVLAGSADFKNDLMHSDMFDQRLFTKVIKIVDISYGGDNGFNQAI
ELSSEALQNVKFIQEKKLIGKFFEEIAQDTGKVVYGIEDTLKALEVGAVELLILYEGLDI
IRLTTRNAITNTTRTIHISPQDEKQESLYKENNVELEVVEKISLTDWVINNYKKYGASLD
FVTNKSQEGAQFQKGFGGFGGMLRYKLDLNLYDEDVESDAELFMNSYRDANTTLDQKKFL
DECIFIVKEQSFYMKQAVENGSLRDTLKHASNMLCELRTSQLSPKYYYELYMLIFNELQH
LDTFISDKKKHKKRFIDIYESVQHAGNIIPRLYLLIIVGRNYIKNKDIKAKYILKDMTEL
CKGIQHPLRGLFLRYFLIQMCKDRIPDTGSEYEEAGGGNIDDAFEFLLSNFYESIKLWSR
MSDKVIKLAGQDEQVMHNNRNKVLREKMDVKMLVGSNLVRMSQLEGMTRQYYIEKCLPKL
LQNLSTINDSLIQQYIFESIVQVFSDECHIYTLDILLNAILKTNSSLDFKGILITLLKRL
RFFIESNKYEVPKEVDIFSLFYEHLVLYVNRTLDSYEKGSYRSSFQQEGQTVVEEKKKKA
VSTCGTTTSTNVITTNNVTTMGTHHINENDLPNYSNEKNNQENDEDDEFVEDIVKMLQVI
YEFIFLCIRIYDDVITISKLFDLPYTIACNVNLNNDVLCEQIINIIVLPFNYLGISALKG
KNMQALLRSISQKHKKKLSLDIIDAIIDCKNKAIVYKDVEEILNYISPIFNEDSHQRETH
QADFFNLENSTITYAAKKMCKFFHIITNTHDIDERYNICILFYKHIENGPYLVHLLPTIV
FTMLDLVMTITNLAPSSGTKTLSQHTYDDLNLGDRADDPYLEDKIKQYNIYVKNILKFIH
TNLLCVSSQIPMLALKLFLYSAVVVNNYDRFVQAHEFLSFENLEAICYEFITQPLIIYEE
DINISSQQYNCIIWITGILCSHITLLQNENYENIALKLTQHANKLLKKKDQCLGILACSH
IYWENKKYRNSAKVLECLQKCIRNAEIAVQSNNDNVVLFLFLLQKYVYYYEAQNIEVTED
SVHYLLHICQEEYSRESCDANFKKEFLQTVKYIHDKKNSSNAFAKIGSVLR-MLEAKLNN
ASILKKLFECIKDLVNDANVDADESGLKLQALDGNHVSLVSLHLLDSGFSHYRCDRERVL
GVNIASLNKVFKLCGANESVVISSKDDEDNLNFVFENNKEDKVTNFSLKLMSIELDSLNI
PDCEEGFDAEVELSSKELTNIFRNLSEFSDTVFIEIDSNIIKFTTKGLVGDAEVALKPRE
STSEDDVGVTIKSRKKIKQSFAIKYLNLFSKSTILCDVVTLGLSDNRPIEFKYEIKDTSP
DADTLKVGFVKFFLAPKMDDEMDNKDMVNTKHLLIQLLIQIAPLTTHAKEWCKVKFEFGK
ADYAECQSETENIAKYMIHTTPVISNDVDLYANVSLVMSNGYGFKTKEINIGSEEQGLLS
KTFAVATDIGSPEYVHVKINSVKKNWKCKKITIWKNYKYWVFDCAASLNDKNPQGTYFLS
GNKMYTAFVKTGNEVQAGTSGTVDIVLVGNNRRSNTKVLHEGFQSGVLKKIKFQASDVGK
IEDIILTNNARDDPWYCDFVKIKSD-NKLYIFNVKSWIGHPYEKTVKINIRADQSGGATK
DIDCHIRGNDLINMNNLPHALQSKVQIFKVRCPQNCQNAEFASVEGSSIHPSSSSICTSA
IHDGSLTPSGGSIIITVGSDLHQYHSVKEKINNIEAIDVLTKVDEPNFSFYTYRLESIDD
VKSNVRIVDAFGKLSSLGRLEIRSDNTWGTVCKKGPNFTFSDDSAKRACADLGFANGVYI
KEMCFNLNGQNYCAGYKYPFSSAGMVCSGNEKSLLNCNADDSSHCVDHHDDVIIQCLNEQ
TNESLSDGMIRLVDITGAPTTNGIGRLEMFYNGSFGSVCSEGWVKEGEKIACRELGYTGL
KGNGFSHHLCANIAGENLCGHDADKINAVNIKCKGDEKSLQNCAHETHEDIYCSHDEDIV
LGCSGGEGQGGSQENAKKHFLTLEKKNFPRKIELTCFDKIVSIADLSAAQVGEVFLASCP
EKCDEEIGIIKGTFLYTFDSPICKAAIHAGVLSSNVADDIVLIISHKHHNFVGTKRNNVE
SHEFAGTSKGFSISIPTRSIIQEERKSNHKYAEENSDKDDDGLEDQHAEYSLNGRLTTGT
QPTFQWIPPMGSPGFNGKENDFVNCINLPNEKYIKSMSNFTFIIYFTVSGGAGNWRTLLS
HSLCDGISISVNEENELIIEQNCNPHLLKSKFKPVIGQTYHLAVAFNKTNKSVTLYVNGK
KLPTEKVKYDFTLNGDLVIGRSNQSTTDYFIGSIHLVEVYKFVLADDEIKQLANAALSLD
YLSGGSDSNYNDIRRKRGKKTSGMNRKTVDGRECMTPCKPQSIINKELQINAEQINLSCK
DDLLSHQFNSKIGSHFLVHCSDNCSKSNFIVKGSNNYYTPDSSICKAAIHAGVYRPKGDE
NNSFILKIVNGLFEYKSARGHMGIVSKAERQSQLRSFSIFPENDDNILSCSSNGHLFLNL
PVGEKRTIICPSGCDKMEGKIWGTNVYTPSSTLCKAAIHSGALSNQGGLVDMSIGSGVDK
FTGATQNGIESHSSAQHSRSLIFSTHSQRDDGILVLHEVTPQAVEEECINVDVEKLLNKL
KKKNREKILQKNRIDYLPVDPKELPQNLPITKKIKNKKIKMKLKRDVRLAILSSKKLLAN
RKFKRVEEGFIRAAPPSKGEVEKMMDNGEMMNKGEVMNKGEVMNKGEVTVGASAGARSLL
TQKELYAQADMGIKKKIIDLRLNLGAYQCRYSRNGKYLLSTGEKGHITLMDTQNLEPMCE
LQVEESVRCSTILHNHKLFAVAQKKYIYFYDNTGIEVNCIKDILYTYQLEFLPYHFLLTS
IGEFGELVYQDISMGNIITRKKTKRGPCNIMKQSKHDATIYLGHQNGHVTVWTPNIDKPV
CDIYCHATPISAIALHKNYLITSSVDCTYKLWDLRKLQFIESYRSHNIINEMDISDTGIV
GMAINSHFRTYKDFFNKPQLYLTHNNYGDKINSLAFQPFEDICCVGSRYSIKSLLIPGAG
LANIDTYVNNPYETKKQVRENEIRSLLDKLPPDTITFGKNKLGLINPFASNVKQVNGKTQ
TNHKNPRNHNTIHSKQQMGDSSDDESSDDATGGLRRGRKGSLHQKPPGMKKKK---KKKN
KNK--FLNSNVPKHLRKSSMKSTETSNSNKNLFFLALSKNKATIYEYDVRFSGCIINDIE
EEIKRSEENGIDMKETTESNETRNPSFTQVKKNGNKFNNDFVLNRGIRIYKEFDDVELAT
CTNDYKKIILVRKSNLHVAEIIDLRQDVDSFQIKAKTPIKAICSSPKDSHLVLYCQYKPE
VSPHNLFMYKIGKAADPKKKKRKKKGNDDHKMSHHAEEHSDHEDGARGHCDDHSDEIPVR
VDTLKSYSSKLWPFYKWSENESICVIRINNTIYIYKENNFTSYVNKMTLENLEFLDVSPE
QGNKKGVFIATYERGSKGKPSVFKLFKSSDFGNHVYSKTFFNSDEMKLKWNKNASAVLLN
VHTQVDKEKQYYYGLSNLFFIETNKFSEVNIMTDRGQIYDYIWSPNQNKFYVCKGDIPAE
IVSYDKCANVAHSFGRHKFNTLRLNCSEKLLLTGGFGNLSGDITLWNTSTEKEVSKTKAS
CAVVCEFFNDGKHFLTATTHPRLRVDNHLKIFTHDGFIVSRINFEELYKVIILPFNKINF
SESDASLGIYMENSPQELYIHKQLGIDSKKTGVYRAPGTSATLTLNGFMNAKKPQRAIKT
KTPGANFVQQKEKKSKKKKNKKKKNTQTEEESMKNDKIKLVSFEGDEFIVDKYTASMSTV
ILNILEVMTAEEDTIPLPNIKTPILKKIIEYMEYHINNPADEIPKPLITSNLQDVVSTWD
YDFVNTDKETLYELIEASNYLDIKPLLDLTCGKIASMMKDKTTEEIRAEFDIVNDFTREE
EKQIREENRWCGDIMDNKTDHAKGQTSKEDADNVTEGATSTPGESTSYRSLYKHNYKAHA
YVDDFVCLRLKGRGGEVHDDRSPMGGCTHDDSPDDRLGDKPVGEPSGNSVNLEEVPAPGR
SNVGRGSVHRDETSSPREDDQYVEVEKRMEDRQDEGTYSKTGAQHSNQNNEEKRENSNLC
SDNHMKEENSPHESNHHKMQEKDQPAEKQKKIKIIEMFKMKLENKEMNRSRHCDDVDHSG
VDSPSTSSNRRTQIRESISRIFSESPSSKPKIINEIKCSDSHVGEGDDGGGSHHGGSSND
RESHHGNSSNDRDKADNKANGAKSALKEGSLLRLFRCEYFDTHLHIRYLYDRREVGVHEY
LVNSLYTQRKYEDILFYLPQLSLISLVRYDSSSLYRFLLYKASKSMHFALKLSWIYHSIV
EDNTSKYKDLAHKMTQEIEMAVVNCKPFNSKCTSGNENKQSYLLNLAHPLLFKRKYIIKR
IKDNARFRQNRLFKNCLQRPCNSYLLKGSEPEGQGKVEPGG------QSKVE--------
---PE------------------GQGKVEPGGQSTVGQGKVKMASPKKCRKRETTSTAPP
PKLPHCYIINSGALSIARAKVKLPSTYAKLGNPLSASKFFLPEFSCSFDMIEDLQQFFMK
QRRCDYFSLLNNFVNLTISVSNLLSTEPDIEIRNELLNRFIYSLNSWMLMRRCIVAACTN
VFSMTGLCIPLECLSS--SSHLNHRREQRTNKSSLQILHFNYDECKIFFSKKRAPYLLMF
EVADLDEDISHIPDNLFYPTR--GENQPDRETSHSGVKGPIRHISPQPQPRRKGHRIEKN
FCSSKNYGIFNENSSSYDYEPRSSMYDGKRKNSDVEQGNDVLKESNNMGKDKVKGKKTHA
NLDDLKMEHLYVYNCIVNDLRKENLISFPSVEEDNLSIIRKCIGMKVDEDDEEDNEENGE
EDDEEGIGEGAKNFLVTPRSASMPNYLSSSMEKCNISSNDNDSNEIKTSVESISSELSGL
SPGGLYENKMGAE------------------DGECNLSRGKDPDDGA-QPSDPSLVTTH-
-TRNSLHSSGEKTIDPLDISEYFKPENYTNEEFKKKNCRIIKRLLWGELFEEKKKKIRKT
SPYGKLKTWDLKCVIVKGGDDLRQELLASQLIRQFKVIFENAGLPLWLRPYEILVTGANS

GIIEYVNDTCSVDSLKRKFGVESISTIFNVVFADYIFEAKKNFIESHAAYSLISYLLQVK
DRHNGNLLLDSDGHLIHIDYGFMLTNSPGNVNFETSPFKLTQEYLDIMDGEKSENYEYFR
RLIVSGFLEARKHSEEIILFVELMMPALKIPCFANGTQFCIDSLKERFMTNLAVDVCIQR
INALIESSINNFRSVQYDYFQRITNGIMMAISVSFKVTGGKEFTISVEPEITVLELKQKC
AEHVEIPVECQRLIFKGKILKDKEPLTTYGVSDGITMHLVRSAAPVKEPEAEKETNKNEG
AANNANVGANEHMNDFSDNPLVQMFLQSGAGNM-NMNAGLGAGNFNLGAFANFLNPGGNG
EINRDSISSLLNNPLARSLMNEISNNPEMLANIVSNNPLLRNTFSQSPIMQPVLENPNLL
RELMRPEFLQAGLQFENALNMSNGGNTSGANNAGQGLRMEDLLSNLNNLANANADANNNT
NNNLNNMSSLFQSPELLQTFQQVMRANRNLGNFNFPGAGQNMDFNTGNAADNRPPEERYA
SQLLSLQEMGFIDNDANIQALQETGGDVNSAVTRLLEKGFNMEKKEDNVMREIKVNKLVL
NICVGESGDRLTRAARVLEQLTEQKPIFGKCRFTIRSFGVRRNEKISCFVTVRGKKALEI
LEKGLKVKEYELRKKNFSETGNFGFGIQEHIDLGIKYDPSTGIYGMDFYVHLSRPGYRVT
RRRRKRSTISKTHKVTKDEAMKWFQTKFDGILLKMEFKGSNFLRFRLALSMISGKAITIK
NIRKKKKKKKSGRDNQGDGDQ-KDGLREYEAKLLKLIDKLCDNTTIKINEEGDELYFKPG
FLMGNVNDEVRISDLENTFHCGKERSITYFLEFLLMVTPFFKNPVKLTLKGITDDSTDSS
VYTCKIVSEHFFKNILKLDDYFLNITILKRGVRSDCSGEVLFFMNNLKTVEPFDMNDAGV
VKKINGTIVCNKISAVFRNKLMNFAKRNLLNFTPYVSIEAEDAKVKNF-KAQNHFISFSL
FAHTKNKCIYSTDLCVDEFFLKHARGVLNSGAKSGEIDDQDDAMSDENDEEMNDEEMSDR
AKDDGATNKLQQRNAEEDKQNITLHDADIYERLGFFIALKMMNEIKGLPSVDSNYQWLPL
FYMAMANDMAVSKISLSMVKPYSIALIRLLRDFFSVVFDIKKVEKSPVDHSYLIKCVGIG
YRNISKKTFMLGFTGSCLSKEFFDLAKSIGEARSKQEEDRIICNEIVLLKSRFADPSASV
KQIKEYLIRAIYIEMLGHDASFAYIHAVKLAHEKNILCKRTGYLSCNLFLNKDHELMLLL
INTIQKDLKSDNHLEIWAALNCVCKLLNSEMIPAIFPIIKNLLNHKNELIRKKVCMLLHK
MYLIDPSLIKEIDLFLKKLLCDVDPSVMGASLNLIFCIAKNDITYCIKLVPYLVSILKQI
CENKLPKDYDYHRIPAPWIQIKILAIFRILGYSNKKISEQMYEVLQKTMQRADFGINVGY
AIIYECVKTIATIYPSHHLLELASLSISRFISSDNHNLKYVGVTGLALIVKINPMYASKH
QLAVVDCLEDKDETLKMKTLDLLYQMTNPLNVKVIVDKLLFHVENSLDIHFKHDLACKII
QLIERYTPDDIWFLNTINSLFLSVGELLDESYSYSLIKLLKLNEEVNCGDDNLDNSNYAK
EGERANKDVSDDSGDGNSGGYGRDDNHTGENHISGELKGKKKINDDVYNLRKYAVNTYIT
MLENNENIPFILMQIICWVLGEYSYLCDIENYTTEDIIDLLCECLEKTFNNPDRVKSCII
TAIFKLCCFNNVTDHVVAKKLIEKYKNSKLTDMQQRCYEYSSILNNPTLIKNVFSI-SSK
QKMIMDENLSFLNPFVDKYLASGGKAYIKKELRETETNFESAKNSIPVLNFTPYELPINN
RIHIDTFHTSNSGSAYERNYKYDSPEHISLEDRTSNTKERERTFKLNVVGPKKWTKEACK
VGGKNNNEKNAQKNGKKKKKKKKKKDNQATYQLEG----SHENANRNKMSKEEGQKGLHK
FSNMQGEIDSKYDDEDDDGEDDNDDDDSDDDDDASDGREDEKRMDGLGDYQNYERFDERD
IHNLGDYKNDRENNNSPFYKQERAHSNNNATVTHNELTEKEKMAAALFNGLISNNSSVDY
SRNNYSSSFSSKKNHSLLSNRNYFYSKSRTDEKGIHFLERRNSS---------VESSSQQ
MEKKNSSSSNKLDEMRKSKYAFDMLDLNEPSAKRDFMEEEEEEKELWNSISSQKKAVFLN
STTLSIFQIIKKIENNLNANVVEVSKKEALMSCI-----------MSGAQCVAIACDLRL
GANNFTTISTNFTKVFKMNDYVYVGLSGLATDIQTLYELLRYRVNLYEIRQETPMDIDCF
ANMLSSILYANRFSPYFVNPIVVGFRVKHTFDDEGKKIRTFEPYLTAYDLIGAKCETKDF
VVNGVTSEQLYGMCESLYIKDQDKDGLFETISQCLLSALDRDCLSGWGAEVYVLTPDSIM
KKKLKARMDMHRTLRKIFDRQTCDKIESMKILLVGAGGIGSEFLKNIITIGCRNIDIVDI
DTIDITNLNRQFLFKKDDVKKYKSFVAKQRALQHKKDLNINAYTFDVCTMKGSDIAKYDY
VVNALDNIKARKYVNKLCVMEKKVLIEAGSTGYNGQVYPILANETKCYNCEEKPKNKTYA
ICTIRQTPSLPEHCVAWGRLIFETFFCKSDNETLIDIKNHIEEESKKRNMEQYEIITFIF
NYLFYDTIKELATLKKDYGTEPIPILFKDNAKKE-DKCDEMEKEESNT-LKEGEPPHGGG
QNEAHKHKETEPASITLCSQNIWKQDECIKMYTEAFEKLYSYLNINKKTEEYLVFDKDDD
DCINFITAISNLRMMNFSIKQKSKFDVQSIAGNIIPAISSTNAIVASLQASQLIHVIEHL
ERVKGGDEAVEISLRDSKAKHVWVKSIVSGNKMFSRGNIVNAENLEAPNPRCYICQQPMI
DIYIKSFSEMTLYDFVKNVCTNELAFLYPFLDKQDRNIFDYDSFLEEDEEYIKSLYNSLS
EWDIKNDEILILTDFQNDKDQLEIHLKEDPTLEVPYDIKQKVVKKTKADELNGPEAPPPS
SKKRKHINEEEEPLNDKKKARTQNDMGKLSKDRRDIYYRKAKENGYRARSSFKLIQINEK
FGIFKLFDPKNCGERDVEKIRNIYNENFCYNIVDLCAAPGSWSQVLKNICLYNYYQMLHW
NGTLSNCDEHENFLNNFSLYINFNKKEFVKKPKIIAVDLQEIGNMKYVQIIQGDITKAST
VQEILRCMKEGGAGTDQEDGQKAERKNVPTYAHAVVSDGAPDITGMNDIDEFIQSQLILS
SLKVCCSVLKIGGNFISKIFRGEHTGLLILHLNKFFERVYVCKPQSSRNKSLESFLVCLN
FSLPLSKITALSHHTGGKEFNLQPEEELRKMHAKLITEKDNEGEEDSQVETDVNKEGDGK
HINQCATDNTHKNVDIFNFYCSDSDEEIKYFNSDDEEIVNEYIASEVFMNDKLFSFVATE
NYYDSDKSYLLPQNYVRHEPQLMPLQPPYLLSLQKKRQEGKKMEGGDYDKILVLNFGSQY
FHLIVKRLNNIKIFSETRDYGIELKEVKELNIKGVILSGGPHSVTEENAPHLNKEVLEYF
LEKKIPIFAICYGMQEIAVQMNGEVNKSKNSEYGCTDVNIITSKNKSEEKYKNYNLVDKK
CLLFDGIKDPEKSTVWMNHTDEVTKIPENFFLVNSTDDCLICAMYNEEYNIYGVQYHPEV
YESLDGEQMFYNFAYKICKCTKKFDPIRYHEIELNNIKKYAKDHYVIAAMSGGIDSTVAA
AFTHQIFKERFYGIFIDNGLLRKNEGEQVYSFLKGLFPDMNLTKIDASEIFLNNLKGVTD
PEQKRKIIGKLFIEEFEKAVKNINIDIEKTYLLQGTLYPDIIESKCSKKLSDTIKTHHNV
GGLPENLKFKLFEPFKYLFKDDVKKLSKELNLPDEITNRHPFPGPGLAIRVIGEIDKHKL
SILREVDDIFIKDLKAYNLYNDISQAFAVLLPTKSVGVRGDARSYDYVCSLRAVKTSSFM
TASWYKIPYDILEKISTRILSEVKGVNRILYDISSKPPATIEFEMRKFTGNVKRNRDD--
------SPERTCITLVRATLLPILSIDKNEKTTLFHPFKNPLPGFVSDKSLYVKKTLGAK
VRRTGWINL-TNCIPRRSEGAEPDEQKENEIKDFVPLILYEDENNKIEVDPILAQFLREH
QREGVTFVFECLMNLRDEKISGCILADDMGLGKTLQSISVLYTLLKQGINKKPAVRRCLI
LCPASLINNWNDEINKWLPGRCNVTCVNDNAKEKILSKLEGFKYDYKSTVIICSYECFRI
NNESLDKSSIDMIICDEAHRLKNDKTKTYMSIYNLSARKRLLLSGTPIQNDLSEFFALIS
LCNPDLFDDTILFRKKYANPILIGRDKDATEKEQQIASERLAELSTITNKFILRRTNNLL
SKVLPVKYLINIFIKLNPIQEALYMLFLKDKRILKNDQSTNRVNVLINIKKLEKICNHPL
LLNANDIKDVGQVSFAKLIEEAAKELEKKRGAKRANARSGGTSPENNTQGDRDRDRERDR
DRRRAVEMDYDKPVKKLLEECRKDTHRSYYNMSCKFQLLHFLLKTIKQNTTDKVVIVSNY
TQTLDYMEILCRENSYKFVRLDGGINIKKRHKVINDFTHSNDIFIFLLSSKSGGCGINLI
SSNRLVLLDPDWNPANDKQALARVWREGQKKICYIYRFFCTGTIDEKVYQRQISKDGLSS
MIVTNTNLCKDQLSDENVKKLFNYRMNTLCETHDNIECTRCTIKNCTEKNENFSEQLEDF
EEEDVNTWAHHQSVETVPDEILVKAVKAATDYKINDMNNFPVLKKLSDLFVTFAMSCKIE
FRDDLIKQKAQKQAELKSL-----TGHSAAAEG---RDVRGESGADDDEDDEADVEDDDE
EDEEEEDEDDEADGEMKDIILPKNACTQSEEQFCLQDIKAKNEEDYASQLSSLPYISTHH
GVSIRKKKVEPAGFMFYVKYEIALLKGKKKKTKEGKQIPIEEIFLYKEKIDYLNELKGKN
QEGDHGGDYNKEKSTKNNKDKNYEDDYDDTNNNKKKQEKANNKKSSNLVKKCINNNINVY
EVLSVEEADDLETIKASYKKLILLFHPDKNKGTTFLNQKEKERKRKKEREGQHGSQETQK
DFMYYMEKYNIEKLTPEEKKIMFLKIQDSYAVLSDKTLRKQYDSSIPFDEYIPTLTELEE
APNFYEFLRPVFKRNAKWSAKKPVPDIGDEHTDIKNVKYFYDFWYNFINWRDFSYQNEYN
YEEAECREERRWMERENKKIQKKASKAENLRIIKLVDLAYNNDPRIIAENKRVKLEKQRK
KELSILEKQKKIDGATNEMEKESTTNQQSNDKKNKDNKAAVKIWRHHIKSLCVTKLAKYV
DSDLVQERLSLMPFETLCEFIDDIYVFLNFNLQKGNSLSVEKTNVNSVYENGSKKEENNT
LNGHIVNAEKVTNGVNSGEPKVSDGDDGNRTTGFIGHLKNIELAEKEIELLILIFKKYIN
QFTLVVEKEQTKNLEETANTKNKNEKGAQITEEETINRSPSPVQEQVSDNVKLMNELGSG
HVSENDKKKNEESSNTCGKWTAQEVSLLAKALKSYPGGTRNRWEQISNFIKTKSVKEVIK
KTKEMFENETLKNLSRNFEETAFDNFKNQNKGVMKKIDDNLDKRDVKMTTDGEENLTNNS
ANGHAEVKRPWTHQEQHLLEQALMKHPASLPKKERLQLVASEIKTRTLEEVILRMKTLRA
QILAKKAAKMPKKRRNGGRSKHNRGHVNPLRCSNCGRCVPKDKAIKRFNIRNIVDTSAQR
DIKEASVYSTFQLPKLYIKQCYCVSCAIHSRFVRVRSRQQRRVRKETSKHVNPSQLMKKK
VERKVPRFKCHNQSDERKTQTASYHADNKERRALLFFNFLFLFFSLTDFAQCQVAKKKVS
VSEINFDSAVEDVQWCGNNHMTVLVKTVKGRLYRSADGGKIWTNITSSLSENPVNKNEP-
ANHTNEVTTVDLIMVNPINKNIVLIIGNNKNHFVSEDSGESFRIVNYKNKINFWQFHSTK
THWALVSSWTSACYSTDEKSGECMQTLSVTKDLGRTFQLMDIYVVQFNWGDKDTHSEDTV
YYTRHKNRNGHQQRFSGWSKDVDFVKTDNFGKNVDVLVKQGNKFLISNGYIFVARLNDVI
KQTVNMMVSTDGGKTFNKANLPENIHEKSYTVLDTSEGAIMLHVNHGTSSDRINTGNVYI
SDASGLNYTLSLPNNIRTSSGECEFDRILSLDGVYIANFLDENDDIKDEDLRFRNFNAQL

EEDISPFQTNTEKRKKQAAKGKNEDLVRTVISFNKGGHWSYLKAPKVDSRGNKYDCGDNC
FLHLHGITNYHQYAPFYSIENAVGIIMGTGNVGSHLRYESDEVNTFLSRDGGVTWMEAHK
GPYIYEYGDHGGLIVMADDLRKTNQIVFSWNEGQSWFDFELGQFSIEVDNIVAEPNSSSV
EFLVYGTRNDVGVLYHLDFNALGQPLCKGLWAADSVSSDYETWSPTSGSFSDKCILGRKI
TYTRRKQTSECFNGKDLKRTVDKKACECTPEDYECETGFTRKVGSYECKPNDPTLTIEGC
TSSSYFYANAYRKVPGDICVNGWVPEKVPVPCPAHAPFNRSAKSILFILFILGLIMLIVT
YLCRNPKFKHLFYNYGFDTFEHVKYSVIKTKRGNVNTNVFEPEMEFIDAEQDDNEEDVPT
LLSYSNDRNRTRNDFKLTRNRSNQNNYLTSRNVSPPQKYPENIELLMADRKSNKNAVVKN
VDMTEEMQIDAIDCANQALQKYNVEKDIAAHIKKEFDRKYDPTWHCVVGRNFGSYVTHET
KNFIYFYIGQVAILLFKSGMDIQLDDGVIQKAKALQEANEEEIKMKKLKPQSKGLLSPKS
NLLEVTDTGCRSRIRICNVLNVPKSEGEFNDTSRKNKYIDQIITVCGWSKAVRKQGGGRF
CFVNLNDGSCHLNLQIIVDQSIQNYEKLLKCGVGCCFRFTGKLIVSPVQNEGEKGLIKEN
VELSLKDNSIHNFEIYGENLDPQKYPLSKKNHGREFLREVAHLRPRSYFISSVIRIRNAL
AIATHLFFQSRGFLYIHTPLITTSDCEGGGEMFTVTTLLGEDADYSAIPRVKKLAKEGKK
REDIQATHAHESSAQPTQYLIDYKKDFFSKQAFLTVSGQLSLENLCSSMGDVYTFGPTFR
AENSHTSRHLAEFWMIEPEIAFADLYDNMELAESYIKYCIGYVLSNHFDDIYYFEKNVEN
GLISRLKNVLDEKFAKITYTNVIDLLLPYSEKFEVPVKWGMDLQSEHERFVAEQIFKKPV
IVYNYPKDLKAFYMKLNEDQKTVAAMDVLVPKIGEVIGGSQREDNLERLDKMILEKKLNM
ESYWWYRQLRKFGSHPHAGFGLGFERLIMLVTGVDNIKDTIPFPRYPGHAEFMSSPNCEE
KVPEEELKKAEILCRELDELKIQYKEVKHAKANSIKDLLDMNLENSKNIIKNLFLKDKKK
NYFFLCTVNWKTVDLKYLSTIFKTSNLRFVDEGNLKSMLNLLPGCLTPLALKCDQENLVK
LYFDEELKNMENIIVHPMHNYSSLYMKQEDVVKFCELHNHAPEYIHIEEGQNKMKKEDMH
LEEEKQKG---TNAGGSGGNAGKDDNAKDANILGIVAKKTVNFSEWYTQVIVKSELIEYY
DISGCYILRPASYYIWECLQTFFNKEIKKLDVENSYFPLFVTKNKLEKEKNHIEGFSPEV
AWVTKYGDSTLPEEIAIRPTSETIMYSVFSKWIRSHRDLPLKLNQWNTVVRWEFKQPTPF
IRTREFLWQEGHTAHKNEEEAVKMVFDILDLYRRWYEECLAVPIIKGIKSEGEKFGGANF
TSTAEAFISENGRAIQAATSHYLGTNFAKMFKIEFEDENEKKQYVHQTSWGCTTRSIGIM
IMTHGDDKGLVLPPKVAKFKVVIVPILYKNTDENVIFNYCKDIEKVLKNAQINCILDDRE
LYSPGYKFNHWELRGVPIRIEVGPKDIQNNSCLFVRRDTNEKFNVKKESVLLETQQMLVD
IHKKLFLKAKKKLDESIVQVTSFSEVMDALNRKKMVLAPWCEDISTEDEIKKETQRLSQN
QANTETSLSGAMKPLCIPLDQPPLPPNTKCFWSGKPAKRWCLFGRSYMAKLSKAQKKQIY
MDKLSSLIQQYTKILIVHVDNVGSNQMATVRQSLRGKAIILMGKNTRIRTALKKNLQTVP
QIEKLLPLVKLNMGFVFCKDDLTEVRNIILQNKSPAPARLGVIAPIDVFIPPGPTGMDPS
HTSFFQSLGISTKIVKGQIEIQENVHLIKQGEKVTASSATLLQKFNMKPFSYGVDVRTVY
DDGVIYDAKVLDITEEDILAKFSKGVANVAALSRSIGVITEASYPHVFVEAFKNIVALVI
DTDYTFPLMKKIKDMVENPEAYAASAPVA-ETKADAP-KEEAKKEEEEEEEEDGFMGFGM
FDMLQERDLAREPCPDRIIEDMGGAFGMGCIGGYIWHFLKGARNSPKGDVLSGALYSGRM
RAPILGGNFAVWGGTFSCFDCTFQYIRKKEDHWNAIGSGFFTGGVLAMRGGWRSASRNAI
VGGVLLAIIEVVSLVLTRKTTPTPRQQFQQQMELEKKMASKNSKMLKDEVWLYKNEDKEG
SIYNEEGGEKPKEHFLEVPLFDEDECGRIIQKNLRHLPSRKIKAKKNEESEVSEESLSTT
DEEEFLDLYEELKREQSVKEFYNPMLRPRLWRFEFFIKYIHNLENQLHRNFYLVSFGNTK
KLKLYGDMQKEVLYTPGYSIEPGEIKYLKVPLPIWAEKELSISYEDLQNFEVVIEMWCIK
GFTFNELCASSKKTLKEVIENDPDTNVILKRKIEKKNITFETQSLGVYMQLSEIFEFHMA
LDSWWFIANSEMPNYLKTLPKFLRFKFPLSEDDWVIHSSHKSQNNFWLYPGYFCFIGTYH
QLANAFFILTVLSHNTSYRYKPPILLGSCIISLKSVTEHPFFKGTVKKLTLDKTKFRQGE
IVGNIKCFVNSYGIEEGDIPVQRPVQPLSDATLVNQLLLNEHYLVIRVIKCENLAISSID
LNNVNINVWVKWDGIVNKTDTVSKSTSPFFYQNLYFPIRLVDKKELTHESLIKNVLPVDL
ISKGEICFEVHNNNEIHSTILGIFELPFADIFNYGTPDYRSLAQEASQSISPYNDYKNYD
ATNEGMDLSSDNYDDYYVRKYKTIVYKNTLELMYSTLSVQTKKESTISVEAFVIPPLPSG
LLFIEKEKIQNASMIYKSMSKRWERDFSKFKDTYMQWFPRADKNRSFPCVSRNEFDSNYY
PLCSFVTSINLPAQVSTPGPLFHWLNNIEYIENEDESSIFTPPYFFLSYKKGTIQDHVLL
LCCCLKGLEYDAYVCKGTINNGKKNHYWVMTRHEDGWVCFWEVTNKCIIHLKRRWNNNKS
SMDPEVNIQKEIMNKVVDSNREKYYTGEYLMNFVRYGLEELKKREKEIKEEYDMKEENKL
YTMDLYGENHMKDEKVNVNEILFNDDELFNNMFEVKFERNETYSSTKALKYLLENFSKNI
PIAPKMFLLDYDKTLAYVPYSSIEVVFNDEQLYGNMQNHHPACILYDLENNYHWRPLLNH
SPVPIKSEITISTPLSDRLSEKYTKDLEEEIQEMILFMRTKEGLETSFEHSKEIRHFLEM
YIDLCEYKLNLDNNYNTKPENYESTGGNDVQKNSGAEAAYASPTTRVGCVEKGENEGDNN
QLRRTWSQYKNDYYNLNFYQTYHPSKEIGMINEEQKKHLVNIPEDYIYGMNDEVYNNGKE
QYIKNYVFCRDNADILRNYDDSVKRKNIKRFSNAPVHYQERPSSEANEEEELYKEIKENY
HYDEDETEEEDESERRHIDREGVLNKYIKKYNKKLQKSLKQKEKKIKEEMVNVKLGGEFF
QSRLGRESFLSYVDTKAFLKSIPSKGDHTHDNWNGAVTQWSRRGGDGVGRTYRLNKPYIV
LRRSHTSRKKIRISGKGERRRGDSRFDRHPFVIGKNKVEERKRNFTSSPDREEIGTLHRR
RLFICQIPPKTWSRLDSTSKYVSHQISQWNWYYSLEEQYFNWQYKFPVPPNHTFVGFPIH
FSTIDFYEVKSFLLHSKRFENIMKLSIDNISFMSLSIYGNRESGQDVRTANVTAVQALSN
ILKSSLGPQGLDKMLVDNIGDVTITNDGATILKQLEVQHPAAKILVNLSELQDQEVGDGT
TSVVLLASELLRRGNELIKMDIHPTTVICGYKLAMKESVKYIKEKLSERVSNLGKDVIIN
VAKTTLSSKFIGYESDYFAKMVANAIQSVKIVNDAGKTKYPVSSVNVIKVHGMSSLDSKL
IDGYAIMSGRASQSMPTGVKNAKIAFLDFPLKQYRLHLGVQVNINDPTELEKIRQREKDI
TKERVNKILESGANVILTTQGIDDMPLKYFVESGAIAVRRVNKDDLRRIAKLTNGQIRLT
MSSLDGTEKFEPSSLGYCDEVYEERVGDWDVMFFKGCKTSKSNTILLRGANDFVLDEMQR
SIHDALCSVSRALESNYVVVGGGCVEVALSVYLEDFAKTLGSREQLAIAEFAESLLVIPK
ILALNASYDSIDLVCKLRAYHTKSQVMNTEDPKDYRWYGLDLVNGKVANNLKNGVLEAMI
SKIKSIRFATEATITILRIDDLIKLTPEERREEEPMSIPYLKNLYPDPSNKLFDDLRICS
RVIDSCGIACSSGFVAVPWEVEGGGLIGAIRLENQMRKPPVIKLKGHTSSILDLQFNPCF
SEILASGSEDLTVRVWEIPHNDESVKEIKDPQCILKGHKKKISIIDWNPMNYYIMCSSGF
DSFVNIWDIENEKRAFQIVMPKKLSSLKWNIKGNLLSGTCVGKHMHIIDPRKREIASSFH
IHSGGKNTKNIWIDGLGGDDNYILSTGFSKNNFREMKLWDLRNTTSALVTMSIDNASAPL
IPHYDESTGLIYIIGKGDGNCRYYQHSLGSIRKVNEYKSCSPFRSFGFLPKQICDVYKCE
IGRVYKNENNSSIRPISFYVPRKNPTKFQEDLYPPILLHDPENSSRNWIKGKDNKMSRIN
IKDLTEDDLRITKKYKFVPQSFNSIIIGEDYTSKRTSIIRQLTKKFTFFKKGIHNDGFSS
SFKESVFIYPKSFKEKWLLTEKGGAQFSSNNSLERGAAEEQDELEEFPLESEQPCDGESR
GTSEKTQRAGGSNCFNGLRCARLCRGK-MKIQNIGEESIHNICSSQVIFTLSSVVKELVE
NSIDADATEIKIKLVENGIKLIQVNDNGAGIKKSNFENVCARHATSKITEFEDIHTSLNT
LGFRGEALNSLCMLSDLHIVTKHDESSHGYLLKFDGLGRLSHEEPIARLRGTTVSCENIF
KNIPIRKKDFIKNIKSQLSDLLLLMQQYAIIYSHIKFCIQNIVTVKGNVKNMNMLLTNGK
ESIKNSVHTIYGKRNIGNLIDFNIDEEEWKLRAYISDNNSGRRDRDIQFYYINNRPIHVL
KNVNKIINSIYREFNSRLYPIIICNILSDTKNFDINVTPDKREVFFIYENELCEKIKTSL
VSLLTPKTSQLVDTHIGDYFLKANNI-KEEDATEQGGSMTQLLVSEAPQGGFSASETIGI
GEKGKKTTPSWEGSSTGESFSEGGRDSTGGVKEEVPIKSPPEHANMWPLQKEEYPDEEYT
PPVNSGDLFFRSPVANRGRARQGWQVEGSVLKKGAQGDGLTEEEAPRVQAYEYQLEEGCE
MEDHP------------SAHAFEYKLEDSPKL--VQEYKLEASPKFFVQENKLEDSPKLC
VQEYKLED-SPKLCVQEYKLEDSPKLYVQEYKEESPKVHEGELNPKSQPEGENNRSADSP
D--DALDYTFEQLKENIKSRCIKSIPIDINMYINREQMKSGFDYDQVHVVNLTNSEKIKN
IIFPKGKEEEKANSFFCLTDKKQVSEYADLFNGSLSIKEAGGMSTSGGGSGEDISFNNID
ERQRDLYFKSNLFEKLQICGQFNKGFVISKIDLLYFQRGTEGKVVDP--GGSGGEEAEPK
GNSNYALFIIDQHAADEKSNFEKYNKIFTMKSQKLISKIDLELSPAQIYIIEKNLEIFLH
NGFDVEIVEEPAQKRRRLKADATDTGEGVLMQVKVYLLSLPVFNGKILEVEDFMSLLHHL
TEHPITYDKAKFQMFIRNKGQPNKKTDTWFNHNFPRPQKVWRILASKACRNAIMVGKPLN
VTEMIKIKKKLSVLKNPWNCPHGRPTIKYIINDMDIKSCFENYYVKLYDEITNLVVTKNY
DAYKYLFHNHAFFLIMSTKPMLGPLLKFQM--------------------EWVNVCVYLR
AHAQSSS-------------YLFSMCCQFYTVPFFLHSGQLDLKHVESYMTNKKWLSKLK
CCGDNPLTVDEMKALVLMFLKKISDTYVDDQAKWMDKMRSSQEEQGKALEEAMNEYEKNI
LFTHALKEQQLLHNNKKLTEWNETIENAYEAQQEVLRQFEAKKREDKKMALEKNNELIIA
KDYIDKIKEAATDSRYANSKCFVYPASSAPCGACTSAGAIAPYRRFKEPRRKKQYSLCLM
ATMDHSVQDELVDYEDDENILDAKDVKGNLDNSLLNNNNKGVNENGAMRGSYATVHTGGF
KDFFLKPELLRAISESGFEHPSEVQQETIPAAITGTDILCQAKSGMGKTAVFVLSILQQL

ETNDEKDIKDEKEMNSNGDGASKNKFVRCLGLAHTRELAYQIKNEFDRFSKYLKGVRCEV
VYGGISMSKHIKMFKEESIPHIIIGTPGRILALIREKYLLTDKIQHFVLDECDKCLEKLD
MRSDVQKIFISTPLKKQVMFFSATMAKEMRDVCKKFLQNPVEIFIDDEAKLKLHGLLQHY
VKLQEKDKTRKLIEILDALEFNQVIIFVKSVTRAITLDKLLTECNFPSIAIHGGLDQQER
IERYDKFKKFENRILVSTDLFGRGIDIERINIVINYDMPENSDSYLHRVGRAGRFGTKGL
AVTFVSSQEDTLALNEVQTRFEVAISEMPNKIDCNEYINQMNDAQEESQLSGANQLPLIS
EDYSSDGDETDCLSEVDKKEMELKESDIVNLSVSQGKRMSVSAEAYGEWNKKKLNFVPKV
YKKDENEKEKIREALNDSFLFNHLNKNEMETIVNAFFDEHVEKNVNIINEGEEGDLLYVI
DEGEVEIYKMKENKKEVLTILKSKDVFGELALLYNSKRAATAKALTKCHLWALDRESFTY
IIKDNIAKKRKMYEDFLTHISILKDMDPYERSKVADSLKTKTFSDEEVIIKEGEPGDTFY
IIVDGSALAIKDKTVIKTYSKGDYFGELALLKNQPRAATVKAKDSCQVVYLDRKSFKRLL
GPIEEILHRNVENYKKVLKELGLDTACIEGNMHLQILCLNDEVREMYKNHKTHHEGDSGL
DLFIIKDEVLKPKSTTFVKLGIKAIALQYKYNYYYKSNANTGAQKDQNSPEVVNTSYLLF
PRSSISKTPLRLANSIGLIDAGYRGELIAALDNTSEEEYVIKKNDKLVQLVSFTGEPLSF
ELVDELDETSRGEGGFGSTSNKMRPCDPNSAFFGFMGIAASSIFSNLGAAYGTAKSGVGV
CSVGVMRPDLIMKSILPVVMAGVLGIYGIIMSILIYGKMSPAAEYSTFSGYTHLSSGLIV
GLSSLAAGLAIGIVGDAGVRANAQQNRLFIGMILILVFSETLALYGLIIGIYISISDTPK
LCAPYATMTKRMPNKASSSARGGKIEKKEKHEEVDHEENNTRNILTMENILEEKDRDNNK
KVKNKIILKNRKNTSSTFNNNEMRILTIPKHRISSIKTNWMELIKPIVTHLKLEIRMVGD
KIQVRTCKLTEDKNNLQKSSDYIKAYLLGFTIEDSLALLRIDDLYIESFQVKDVKILKGD
HLSRCIGRICGSNGATKYAIENATKTRIVIAGDKIHILGSFNNIKMARYSICSLILGSTQ
GKIFNKLNILAKRMKERFMGNHCCAGRDLLYKNKLQEFGIEGSKTIRKLLSFTSNDILRF
DKAYDENDVQEFVNLCSSTCEIEKLEDRMHPWAADPKTIGALSATQLAILASKENEPHYK
DAIREADGIPVFINLLKSHELDRVHAAVVALSFLSVDNVKNCITMFENGALPYLISGMKS
NIDGMKAACAQTCRNIFVLDKKYKKEFLKLGGITQLVNLLEMPRKDDSQPLYTQLEAIYH
LEDFILNDGDEVPEFLEAVKNSNAIKSLKNLQQCPEQDVAEASNVLLLRLTDMANGWPAE
KVFGDVMSYTYDDIICLPGYINFPMSEIDLSNNLTPNICLKTPIISSPMDTVTEHKMSIS
LALCGGLGIIHNNMSIENQIEEVKKVKRFENGFIFDPYTFSPEHTVADVIATKNKVGYKS
YPITVDGKVGSKLVGIITGVDYLYLTDKTRKIKDIMTTDVVTGKYPINLSDANKVLCEEK
KSVLPIVNNNYELIALVCRNDMHKNKIFPHASKSQNKQLIVGASISTREHDLERADQLIK
NMIDIICIDSSQGNSIYQIDTIKKIKGAHPHIPIIAGNVVTCDQAKNLIDAGADVLRIGM
GSGSICTTQDVCAIGRAQGTAVYHVSNYAHTRNIKTIADGGIKNSGNIVKALSIGADFVM
MGNLLAATEESCSDYYFENNVRLKIYRGMGSMEAMYNKGFNSKSRYLVEERKNDNLCDQN
EEIKVSQGVSASLVDKGSVLNLIPHLVKAVKHGFQSMGIKSIPELHSKLYSGDLKFDVRS
FNTIKEGRISDNLIFTNKKMVGKNAKCRRFISGSSKVKSIFDR-KYVFYFLWSDVRNAQR
GAVHLTKKFFSTFMKNVIDQVKKDMKENKQYQEALKELKEKTEIDHKAVKLKKKIRENIN
LLKHIKEKNEEAVKLVCNDVNNFIHYCFENYTIFRFSKNASVKFFLILQKFLLCTSNKLT
ELADKWNENNAFVKLDKWRQEMAIRRYKKSAHAGSNVDENTDKSND----HPNQSNTEVM
EEEKAGSSELVLAQESAWDKFGSKLKDMPFLNNFFENPILGKLFGETELAAALREMKMYD
QNFKLSELMYLFEFVISKHIVESYLIGDEETLRVHCGQSAFNSLNASINERKKKKVYLDT
NVLIYKNHELKGAQRMEESSPWFIFTFHTQQINCLKNANDEIIEGNIDDIREVVYTIALS
KHPEPEREGLLYPYIVREFAIIGNTPSWMNSTTFDNVMNARSYRLSLDDIQNLGSLYSEN
NKKIAKYNEEINLLKQQLNYLNEKMGKGYVHKVIEPAKPSELTFWCYELKEMKEFQDLVM
YEIKKKKKHFKILSQGCVKYLSNREKIKQKKQEEEEKRLKTHSKHISSYMDMFWKKIEKL
VWEEKKRELQKTLNKKKEMRFKRFVKDAIKKIKRARQNMHELFINRHASISSFNPSENV-
TFSVQNVGDDELKEEDLTNQEEEDVLLDEEMESSDESEEKGKEISLLDDEATMPIEELLK
RMYGFKSGEEYLDLMREGDEPEEED-----PSAKEGA---EEEAPNGEGSAQEGEPDNPE
QGEETAH--------SVNSESKTNEKKRKFEDSEEETLPAEGNLTKKSESHIERRRKRRK
RNKKGRKSYDGGSHSSTSSNDDMLMCNMDEKHLTKIPPFIKATLRDYQHAGLHWLLYLYK
NNINGILADEMGLGKTLQCISLLSYLAYNFDIWGPHLIIVPTSILINWEIELKRFSPCFK
ILSYFGNQNERYKKRVGWFNKDSFHVCISSYSTIVKDHLIFKRKRWKYIILDEAHNIKNF
NTKRWNIILSLKRENCLLVTGTPLQNSLEELWSLLHFLMPNIFTSHLDFKEWFSDPLNLA
IQKSKINDSRELIDRLHTVIRPYILRRLKKNVEKEMPNKYEHIIKCKLTRRQQVLYDEFI
QNKQVQNTLTSGNYIGLMNILIQLRKVCNHCDLFTNKDIQTPYYYLLPISFYIPRFFILF
EKTYHLDFQLILFLHREFTSLGGVPLHRQADGKKLSKQTDFSSPLEQLSGGENKEYTGSY
STKDRIDVIPLDTFPHARNILDEPQHGEIVGNMDEETTSGAFRQRS-----------SST
GSTTGKKRPLPPSG----VNVEDVNTYLSNHLKRRSLPRNSDEFVKEMNNHYDTLSLFID
PANKHLNKTTLSKHASNEMVLYKYNMKVINSDTQYRNFFSDESNQSYLNSLEHNLWIKRQ
RKFDEEDRRKLKNNRTPLFGTNLLSLLRREFSRDGFVPYHTSNNLPVNDSLMRGLCAADT
TGASGSTILEKLFPTMELFLKTHQREIHNFTVMNTPAVISSSHRIAVNNNL--LQNCSHL
EPILHRIKIATRVYHEPFHKQSIIFPLNKDITLGSGKLFALEKLLNKCKREGNKCLLFTQ
FIKMLDILEIFLNHLNYTFIRLDGSTKVEQRQKIVTKFNNDKSIFLFISSTRSGSIGINL
TAANVVIFYDTDWNPSIDKQAMDRCHRIGQTKDVHVFRFVCEYTVEENIWKKQLQKRKLD
TICISMGNFSNLNNESRGNPIPNNDSKDWFANADTIKEIFINKRNNDEDDDIYKDRLLHE
HMDETDKTNVRFEKTLEHVEDKDDINALHENKKEKLHAISQDLQEFTNRNDFQETYTLTS
YCFNFLNDNLTDSLKQQIEEMKMKIEIEMMNVGADDNDNDDDNNDDDNNDDGNDDDSDSP
SMALYIIGLGLGDERDVSVRGKELIEMSDVVYLESYTSVLFVSKNALEEFYKKNIKEVDR
NFAEENCEEILEEAVNKKVSFLVVGDPLCATTHHDIILRAKKKNINVQVIHNASIMSAIG
ESGMQLYNFGQTVSIPYFEETYKPTSFYDKIKVNLDNNFHTLCLLDIKVKERTIENLMKN
KNIYEPPRYMTINEAIEQLLYCEEVHKKNVITDNTRGIAIVRIGSNSQQIVSGNLLTLKT
IKYNDPLHSLIICAPTLHDVEREYFDMYMHK-MGSGKPSGLRAARKLRIRRRTQRWADKG
YKKSHLGTRWKSNPFRGSSHAKGIVVEKVAIEAKQPNSAYRKCVRVQLIKNGKKITAFVP
GDGCLNFIDENDEVLVSGFGRSGHSVGDLPGVKFKVVKVARVSLLALFKEKKEKPRSMGI
KGLTKFIADAAPNAIKEIKIENLMGRVVAIDASMSLYQFIIAIRDSEQYGNLTNESGETT
SHISGLMSRSIKLMENGLKPIYVFDGAPPELKGSELEKRGEKRQKAEELLKKAKEEGNLE
EIKKQSGRTVRVTKKQNEEAKKLLTLMGIPVVEAPCEAESQCAFLTKYNLAHATATEDAD
ALVFGTKILIRNLNANASSSQNKNKNSSKRGYILTEINLEQVLKGLNLNMNEFIDFCILC
GCDYCDTIKGIGSKTAYNLIKEYNSIEKIIENIDKNKYQIPSNFRFVEARDSFINPKVLS
KEEIKIDWGEPKIEELKNFLIKDYNFNEVRVTNYINRLLKARKVTTQRRLDNFFTACTKK
STKLVNEESQKKEVKPKRKGKKRDAPNDSSTKLNSKQNKKPKGEKESKTEKDDGDTHNGN
DNEEEDPFDTEEDEDNPSVNFFHHKSDSESGNVKKESTEQEANATPTGDEMKKKFLLPYC
PKNVTKRKSVQRCMFFSSFPFDSMGGQQTRRKREVNNSKYYEVLNLKKNCTTDEVKKAYR
KLAIIHHPDKGGDPEKFKEISRAYEVLSDEEKRKLYDEYGEEGLENGEQPTEATDLFDFI
LNAGKGKKKRGEDIVSEVKVTLEQLYNGATKKLAISKDVICANCEGHGGPKDAKVDCKQC
NGRGTKTYMRYHSSVLHQTEVTCNGCRGKGKIFNEKDKCANCKGGCVLKTRKIIEVYIPK
GAPNKHKIVFNGEADEKPNVITGNLVVILNEKPHQLFRREGVDLFISHKISLYESLTGFV
AEIVHLDERKILVDCTNSGFVRHGDIREIAEEGMPTYKDPFKKGNLYITFEVEYPMDLII
TNEKKEILKILKKQNEIEKKYDLENSECEVVTCQTVDKEYLKQRLSKQQQQDAYDDEDHQ
PEMEGQRVACAQQMNNCFDNNNSYMEWKPNEKIYKTIIQALSSCNSSNNSVQIEVTKVLK
DLNENVTDAALYQLHIFLNKQEKNDVRQVAGLLLKNYINSKNKFLNNEILKIIKNEIFKL
VEDEVKEIRNTAGSVITTILTKYEGIDKWPEALYNLLLLIERGNNDVVDGAFRAIIIIIE
DELMNRKSADSFFFQFCKTQLLQKLFAYCAPQEKSIKKKYAAECLDLFITASCFTTNGVF
NDYFPQLWECLGFLASEEDTQILKIVVTCMTVITDTRYSSIFNNLDAVIQFMVNATNSCD
RKVQLEALEFWPVFIKDRSYIAYSNYNNNSNDINKVENNYIDENVYKNINELRNEGLKIL
KNYLPYLCKILVDNTVYTKWDYLTMDESHFQNDNANVPDLIQDISPEMYNNSSKNNDMNQ
EEMKINKMNNNNDNHHMGSNSHNENMKSNNMGQAGDEGNNDNHMDDYTDDEKNDEMTART
WGNDWTVRKGAALCLDYLSNVYNDEILEFILPHIEEKLMSDKWNIRESAVLTLGAIAKGC
MYSLSPFIPKVLEYLIKLLNDEKPLARSISCWCVTRFSSWICHPDNCDKWFEPVLLNLLK
RILDTNKRVQEAACSSFANLEEDALDLLNNYLHEIVHTIQQAFQIYQAKNYFILFDVVGT
LIDSVNIVKENNDLAHEIVNSILSKWNTIRINSPYIIALMECMSCITSAYGKDFLKYAKN
VIRTCIKFLVLLYIDLEEEIKYYYSKKAGNTNTYMVNRNNISMTASELLAYYKISNDDYF
TTLKDIDSISPSKKKDLIECSFDLLSRILSVINSNIMNIISENEFNFIPLVHKYCLKFDN
IKFDGILNKLIMNQGFNKMVFSSSSVVKNGRIVNDELTELAKNFLNFGILQSNFALIGDI
SRFCAQYLIPYLNDIIPFLIAHIAHPSTPVSNNASWAIGEISIHINPQYMEVYVDEIIKQ
LIYICQNSKYHGCLLQNICITLGRLSSIYPKKIIFYFPQFLKTWLKIMSHGTQENEKINS
LKAILETLYLNLDIAAENLQDIVYIILKYKYVCQNMNNFFHQFLATMKQKYPTQWKGIYS

STGDSLSSPIPNEMLSDLSTRFNLMKPRLNNRVKGNSYYEYSIKHGRYDIDQAVAYIERS
SFFRSSLNSNSDSSHNNNRKEPTLQRKLLRLMREEKKYMRNGTHKEKSKGRSKDHDRDHH
EGGESGLMYVHDTCEQILNSGKAFQNTDRYIVMNGRNQIKKISLNEMNELITEIIYTTMT
R-DNLENSLLDLLGEEHIDFIFNIIKNKESIRRDIKLLSKYVDVKEKVLASNFFITDKTT
DGINNKNILIKKENIEKIVDFFLYTLKENNFGDVKKIYIPNEENKLEEIQVPRNTSYSYT
DNVTKVRINRLENFQFSKDELVPVTVLPFWHRYIFEFEHFNYVQSKVFKAAFQTNKNLLV
SAPTGCGKTNIALLVILQQICLFCEQNGISLERIAQVRTGEGGEVRRPDVHHAERKGQMI
GDPPEEFMNEDPPSDEGAS-DHAPPRGGNSISAKEFKIIYIAPMKSLVFEITNLFQRKLK
IFNLKVCEYTKEHSLTSKQLEGVHIIVTVPEKLDILLRNSSYSTTVSDESLIKHIKCLIL
DEVHLLNTDRGDVIETIVARFLRYSETSQSVRRIMAMSATLPNYNDVRDFLKVEKDMCFY
FNESYRSIQLDKTLYGIHEKNMNKLNIAKNIYAYGEIINALKKDKQCIIFVCSRNDTNKT
IQFLIDYAVKNGEINYFVNNLYTDSDINKRIKKSNNMYVKQFYEFGCSIHHAGMSRFDKI
LVEDLFKKKAFNVLCCTSTLAWGVNLPVHTVIIKGTNFFSSESGKMEDMDILNINQIFGR
CGRPQYEDHGHAILITERTKLYKYIKLLTNNTIIESTFLKNIENHLNAEISIGTTKNVED
GIKWLEYTYLYVRMQKNPNLYDADLTTDIHLYNKRKEIILKAIQNLSENKLVRRVFLTND
FIGTFYGHIAAKYYVDYQTIGIFAANIDRSNYVEIIDVISKSKEFENIQIRNEDMKDFMW
LKSKCEISEQYDESKCMTLRILIESYLRRIQINNFSLICEINYVIQNIIRILYAYYEICL
NILKNISNLIMNTHNLIVSILRRLPINCCVFRHFCYKNELLEKKNLTFSSHNAKDKKPQR
KNQNYTVYLKESVVNILEKKKLTHESIDTLTKSELLFFLRNEVYTNQILYYKNVIPNLHI
DGYIQPITQTIMKINLQVQLINTIWSDQWNDLQENFHLFLLNTLNNDILYFQKFSIHKKD
RKKIHDISFEFPISNQMPPQITVQFLSMNWCNLSYVHIFNTNNLFINQKINIFSEIFPVV
PLSTQILRIPSYIKFFSFRYFNPIQTQMFHATFHTDENILLGAPTGSGKTVIGELCILRN
LLHHEREKSVYICPMKAIVNERYKSWASKFKNLLNKNVIELTGDKNENKENIVDSDIIIC
TPEKLDVISRNWKNKKFIKNVSLIIFDEIHLLGENRGGVIEILINRFKNMEQYLNKKIRL
VGLTTVITSVDDLVLWLDVKENYLFNFPSSCRIVPCKTHILGFTQKAYCARMSVMNKNVF
DAINQYAQSKNVLIFVSSRRQTRVTGYDIISLLHTENLLNDKSHIQSGEEKTQVANMLFQ
NYLNIIENEHLKDLLRYGIGIHHAGLNENDKNIVEYFFLNKIIQILICTSTLAWGINLPA
YLVIIKGNEFYDAKTKKYKDISYTDLLQMIGRAGRPQFDDKALAILLVQEKRKNAIKNFL
YHPMNIESNIIENLNEHINAEICSKVINNKEDIFNYLTKSYYFKRLFSNPSYYIKDVQYV
QLFDNNILSNQAKKVIYEHVNKIIDSTISFLENNKCIQVTMEDYMQSYYSTPLGHIASVY
YLKCETVSFFYKMVERAQEKELDFYGLFALIAQAREFDDVPLRHNEDQYNVKLRNQIPLD
IDMNMKNIKTYLLLLSRLYECTYETVDYHIDLKLVMDQIARVINGFIDICLLFGKYNYIK
NLILIYQCINQKIQPTQNSLYQIKDINNSQLAKLEELQIKNLNDLLKFDKSFLYSLNIFH
TSQLDFIFKIPSLTSSVKLFYKNVNPSNAEKKNNFVSIPCVNHLKNENKFVFKMKHHDPN
EIIIRVFFCFFNKVSRDSNSASSGANVQWYAEMESDGVKREEVEAQETAPQENPQEDVSR
RMTEILSIASECIQPEELKARLLLRRRLICYDGFEPSGRMHIAQGLLKCQIVNKLTSNGC
TFIFWIADWFAQLNNKMSGDLKKIRKVGNYFIEVWKSCGMNMENVKFLWASEEINKKPNE
YWSLVIDISKSFNINRIKRCLKIMGRSEGEENYCSQIMYPCMQCADIFFLNVDICQLGID
QRKVNMLAREYCDIKKIKKKPIILSHEMLPGLLEGQEKMSKSDENSAIFMDDSEADVNRK
IKKGYCPPGVIENNPIFAYARNIIFPHYNEFALLRKEKNGGNKTYTTIAELEADYLSGAL
HPLDLKDNVALYLNKMLQPVRDHFQNNAEAKSLLNEIRKYKVTKVSRNTLVSNTTNNLPL
KVSVIGSGSWGTVVSKIVAENTHKSKIFHPLVKMYVKEEIVDNDKLSNIINKKKENVKYM
KGMKVPDNVLATSNLKDAVEGADLLIFVVPHQYLESVLNEIVKNENLKKDAKAISLMKGI
KIDNCKPMLLSSVIEEKLNIGCAALSGSNIANELSTENFSESTIGFEDAQEAGIWQELFD
RTYFKINCVEDKPGVETCGALKNVVALGVGFLDASSHSYNTKSAIIRIGLDEMKRFTRFF
FPDVLDETFLDSCGLADLITTCLGGRNLKCAREFATRNGADTWDQIEMELLNGQKLQGIH
TAKEVYSVLEHHKLKNEFPLFTTIYEIAFLHKNPSSIIDVLSTKKLRHIKYKGMEKLLSS
VGRLSVVAGGLSLIPYTFIYDVDGGERCVMFNRFGGVSENTYGEGSHFYIPWFQTPYIYD
IKMKPKVINTTTGTRDLQIVTLSLRLLFRPHTKQLPYLHSTLGPDYDERVLPSIGNEVLK
AVVAKYNAESLLTQRDKISKEIRESITARAKHFNILLDDVAITHLSYGKEFAKAIEDKQV
AQQESERVKFIVAKTEQEKIAAVIKAQGEAEAAKLISSAVKEYGNSLLEIRKLEAAKEIA
ENLSKSKNVTYFPASSNILLNPKNLMKLEYMNALKEESGGFNFENIKRNEILKEKGITFP
NFRKTGTTICGLVCQNAVILGADTRATEGPIVADKNCSKLHYISKNIYCAGAGVAGDLEH
TTLWLQHNVELHRLNTNTQPRVAMCVSRLTQELFKYQGYKVCAIVLGGVDVTGPQLYGIH
PHGSSCLLPFTALGSGSLSAMAVLEAKYRDNMTIEEGKELVCEAICAGIFNDLGSGGNVD
ICVITKDGSQHIRPYKQPNVRLYHLAQPTVFPKGTTPVLCQKIENIKKYITVEDAMRSNK
NDMYTFNAGKVEPFGKAPVGYIPGKGRGVTGFSGGVSRDDTTDEKDKNDYSDFNYDEFHG
YSESLFKDAEYDEDDKEADAIYENIDARMDVRRKSRREIKLKEEIQKIRAQKPTIQEQFS
DLKKGLASVTAEEWESIPTVMNYSRQKQKKVPKNYLPTPDSLIMSRLNDANMHLNYSSSS
SNGLGIQTPLGLRTPLGASTPIGLGMQTPFMKGGGGLETPFLSRHLASSSTYSGLNTPFT
LSGYNTPLSASTAGGYNTPLMNGVNKLSLNDVGEARGTVLSVKLDELIDSVEGQTVIDPK
GYLTNLNAKSLVNDADIADINKARSLLKSVISTNPKHGPGWIAAARVEELAQRKDKAKEI
ITKGCIECSKNEDIWLEAVRLEDKLSEVKIILTKGIKEIPTSVKLWLEAYRKESNIDDKR
KVLRKAIECIPNSVRLWKEAISLESENNAYILLKRAVECIPQCIEMWIALARLCPYTEAQ
KVLNEARKKIPTSAEIWINASKLEEKQGNNNMVDIIIKRCIENLSSKNVVFERDKWLKFA
EESEKSDFPLTCESIIKNTMNIGVESLNKKRIYKQDAENCIKNKSIHTARAIYNEALKIF
KTKKSLWLALANLELAYGNKESVEQVLQRAVKSCPHSSVLWLMYAKQKWLNNEIDKAREI
LAESFMHNQNTEVISLAAIKLERENNEFDRARFLLKKSRVQCNTPKIWMQSIQLERLLRN
YKDAKELAQEALQIHKRFDKLYMIAGQIELEMMRAEMDHGNEHYTTAQKIYDEGLKHCPS
SINLWLCAIDLQIEKKNYTSARALVEKAKIKIKNIHATNTNNYVLKNKEIIESNEFALDD
ELNRNDEEDTKSGTNVSNLTNTKNDLSKNAAMNASIRVIENYDLLWIKLIEIESLCNSNN
ITPVISEALKECPSSGILWSKAIEFENKNLQNSKSVTAFNNCGNNAYVILTVAKLFWQHF
KTQKARKWFYRVISLNPNFGDGWATFLAFEIDQQNEVNQKDIINKCIKAEPNRGYMWNKI
TKRVENWRLKYPQKLYKYIKELFPEVLKKKISEQILEIMSDENVSLPVRRKRKKMQKYCF
SLLRKFYRVQKYVSAKEGRIRTKNRQSSIMEGGTKEMNDKEGNHVVNAADEVSDKKKEKK
AKKLAEKEKKLAKKAERENLKNEATKILEHVCEDIEKDNYGFVKVSKIKENKDDIKLLNL
EEIYYTLMKNATVDNGKRESVVAESGKEHLLQGDIWVRGRIHDIRSKGSLAFIILRQKLY
SMQCILDIKNNDNDKNMMKWVSNLPLESIVDIYGKLTKPEVPIDSTNIKYEVHIKKIFCI
SKTTKELPFLLKDANMKETNEEGSIKVNQDNRLNNRCIDLRTYANYSIFCLQSQICTLFK
NFLLKNNFIEIHTPKLLGESSEGGANAFQINYFNQKGFLAQSPQLYKQMCINSGFDRVFE
VAPVFRAENSNTYRHLCEYVSLDIEMTYKYDFLENVFFYDSLFKHIFTELTKGEKSEMLI
KTVKGQYPCEDFQWLEVTPIFTYEEAIKMLIQHNKLDLKDEDILSYDMSTDMEKELGKIV
KASHHTDYYIIINFPSALRPFYTMYKEDNPAISNSYDFFMRGEEILSGSQRISDVNLLLE
NIKRFNLDASKLNFYIDSFAYSSYPHSGCGIGLERVLMLFLGLNNIRKTSLFPRDPKRLI
PMEIPGF--GFLHRWRRERGEKFGFLDALKKGNASLW-RRGRSNELPKEGEKSTLLDNDG
DGKKNKMNEDDTNALKEQANSPMPNSFDAPEALLFENLNKEYKFITTQDNFDGFRFEVDK
SVNKYLQSTHTLFLGTTLRDVGYLYQFGANFTNSDNSLLMISRVNIDGSVNGRFCKKIKN
DIDCKLNFNTYAKSDTRNMYEMAIEVNKPIYTYNVKTIWQGTWIFNASYTQMLSRKFQAG
VDLTYIASNCASIGSFGLRYQHKNNVLSMQVVRQPNFKSPEFMLNQTHLYKIQYAKKISD
RLSLGTELEVTPQTKESAMRLGWDYSFRHAKVQGSIDTSGKIAVFTQDYSGFGVSGYIDY
PNNEYKFGFMMHIAPAQEQPVQA-MDAQEFIPQYKLILVGDGGVGKTTFVKRHLTGEFEK
KYIPTLGVEVHPLKFQTNFGKTQFNVWDTAGQEKFGGLRDGYYIKSDCAIIMFDVSSRIT
YKNVPNWYRDITRVCETIPMVLVGNKVDVKDRQVKSRQIQFHRKRNLQYYDLSARSNYNF
EKPFLWLARRLSNQPNLVFVGEHAKAPEFQIDLNIVREAEKELEQAAAVAIDEEDIENMD
KLKTVYLDSALSIIKGALCVILQIPTSRTTESVKRKQNNNGVLTVKSILMEPTISQYDDI
KKLIRNKIQEQVPFYNYQMKRALAEKIYGDCIYDNFGLSKDINEVNLIALEEWNINCNRN
RVLQNTGLIKDIQINEFKYLTTKESLEVHFAVNPKYTFEELSGMYKSEKNLINFLLAPIV
KVYTSEEESVLKGPTNDDFVYVHVEDILQKNKVLPPSGVENVNYERSKEVTPWDVNITEE
GINYNKLIKEFGCSKITENHIKRIEQLTNRRAHHFIRREIFFSHRDLDFLLNYYEQNKCF
YIYTGRGPSSLSMHLGHLIPFYFCKYLQDAFNVPLVIQMSDDEKFLFNQNYSLEYINTLT
EENVKDIIAVGLNPELTFIFKNTKYAGYLYPTVLSIHKKTTLNQSMNVFGFNHSDNIGKI
SYPSFQIAPCFSQCFEQFLPPNIPCLVPQGIDQDPYFRLSRDIAVKMALHKPVVVHSVFM
PGLQGVNTKMSSTKKKDEAGNSAKDHNNSVIFLTDTPEEIKNKINKYAFSGGGATIEEHR
KKGGNLEKDISYQYLRYFMEDDDKLHEIGEKYRRGEMLSGELKKILIEILTELIQKHQQR
KQAITEAEIAYFFDASKPGLKKFRGTMNAAKDAAKRHEVEITKLTKDEMTFVLHNSNTGM
ANALRRIMLSEIPTLAIDVVNVYENTSPFHDEFLAHRLGLIPIDSRNVKNFEFREKCKCK

ETCSKCTIQYLIQVKCNNVSKIDVTHYDIESVEHEPNVPMPVPFEDRNNKMAQSNAIPIV
TLSKNQTLHMKLIATKGIGKMHAKWIPANVSYRIDHKVSIKHHLINSLSPEHKLLLANSL
NKDCYVLKNAEHDRDMSLRLNENMSVVMAESCIEVLNELGYKDVVKIIYDETKFHFKVES
VGSMPPEQVVEMAIEILENKLKTLEPQIKSSFYSIDEVAKQLKEQGVSLYGIQLDLEMYE
NKSKVKDLSLAPFGKLQMQISENEMPGIMSIREEYEKLKPLKGAKITGCLHMTVECALLI
ETLQKLGAQIRWCSCNIYSTVDYAAAAVSTLENVVVFAWKGETLEEYWWCVESALTWENE
EGPDLIVDDGGDAALLVHKGVEYEKLYEEKKILPDPESAKNEEEKCFLSLLKKSILKNPK
KWTNIAKKIVGVSEETTTGVLRLKKMEKNNELLFSAINVNDAVTKQKYDNIYGCRHSLPD
GLMRATDFLISGKIVVICGYGDVGKGCASSMKGLGARVYVTEIDPICAIQAVMEGFNVVT
LEEIVEKGDFFITCTGNVDVIKLEHLMKMKNNAVVGNIGHFDDEIQVNELFNSEGIHIEN
VKPQVDRVTLPNGNKIIVLAKGRLLNLGCATGHPAFVMSFSFCNQVFAQLDLWENRNSSK
YQNKVYLLPKQLDEKVALYHLKKLNVSLTQLDDKQCEFLGVTKDGPYKGDSYRYMYTYGV
EGDDTYLPQPQYPSPYENQYGQDDESPSPRGENHTPFVGYFSSHLLRTGFFLQCVSLMLM
FIFYWAFGGTGIFVFDLYAGPECVKVSSAFHLTISILMAIYLLGTLYIAMFQVFVADNSK
WCRGFRAGSKLLSAAVTLDLLSSILRLVQYLYAYFYMSMRWWARYQQTKSDWTLLHFGSI
VHSFALFIYGAAFFYMEAYHDEGTYEELAWSNLTLFKLAGLAELLMVFSGFGAFFSILLL
GAIMCATVWAFSFEPLLEKWSPELHSRDINADVLPEIKHENEQGGYNEENVYEPYNVNPE
NMEYGQEMVPQNMSEKYVNGNSNIYAQNNGMPTTYDYSQIEGQVKQNAEMGVSENYTNGQ
FMEDANKPKKRTFRTFHYRGVELDKLLDLSQEELVKLFRARQRRKFKRGISKKEKSLLKK
LRKAKKECEVGEKPRAIPTHLRNMTIIPEMVGSIVAVHNGKQYNNVEIKPEMIGYYLGEF
SITYKHTRHGKPGIGATHSSRFIPLKMTNKKVQSPKEESIAKMLICKVHIGTKNLENKMK
RYVYTRAKDGVHILNLAKTYEKLQLAARIIVAINNPADVVVVSARPFGSRAVLKFAQYTG
AQAIAGRWTPGMLTNQIIQKFTEPRLLIVTDPRTDAQPVKESAYANIPVIALCDSDSPLE
HVDIAIPCNNKGKESIALMYWLLAQEVLYLKGTLPRSKPWNVMVDMFLWRDPEQFELKNL
AIEETAPAAPHLTENQFATEAPYEEWNKKEEWNDNANEEWKNPIAAEEWMSVNRYNFKTM
SSLLKDKTPQHFDILPHTSIKCVVHPSVIFTILDAYLRRDEDQTHVIGTLMGCIVDSNLV
EISDCFVDKHSLNEGGFLQIIKDHHETMYELKQKVRPRDQVVGWFCSGSELSELSCAVHG
WFKEHNSISKFYPHSPLNEPIHLLVDAALESGFLNIKAYVQLPITLVKEYFVHFHEIQTE
LLPSNVERAEVLQYQEKGSSGKDKDGHMHGNKNSVLANDMNEMSLKKLLIMLKQCKSYVQ
DVIDKKKKGNLEVGRYLHKVFSNDSFSTLEKFDSINESILQDNLMISYLSNLAHLQFLIA
EKLNATPMQMEKIVEVIEGLSSSDSHVRNECENTLNFYKKNDLNNTVLSILKLLKSHKDS
QVRLQCAILIRNLFRAYIKSGEKEKGDEEENYWDLLPDNLKNIVKSELISNISSETDKMV
RSNLCNNIIDLSSKLLVNKQWPELLSVTLDFCNSNNNDVLISGFKILGGILSCIPYQLEL
KREVVSSVCMKGLNSSDVQVRGECINLISCIVEDNNSVLMKCVQPCIPHILQSLSLMVKN
SSSDISVLEECEKVLQAIGKMIDYNAKFFAKHISNLCDILFSICMKGENELNYDFDSSLK
SLSIEALITIPERRPKMALSVPHFVDKIVHLSMLFMLDINNDSFNEWMNSIKEGKDDNQE
LYDIGEESLDRVGKAFSELEEAEFIHILYNKVSEFLMKNTWEHKYVAIMAIAQTIEYLPE
DEIEDQLEHVIKMLLQVLLDQDVRVRYAACQAIGQISLDHQPYVQKEYPRQIITALITTM
NDVHLRVQSHATAAFVNYAEELDKMALLPFADMIIDILLQKLNSSNYLLVREQAVTAIAV
IAGVIEEDFLKYYSTVVPMMKDIIQKAVSEEERTCRGKAIECISIIGLSVGKEIFLEDAK
ECMNALLQISSTKMDPDDTVKEYIQEAIGRICRALGNDFFPYLSSIVPTILSLLSIMPKP
LTDDEEDLTITMVSNGQYVGLKTSLLEDQEKALDLLIIIIEVLKENYKDYIEATATAVLP
MLNYELSDEIKQKALTAVSELIEAARILSEKTDNNKTMLHAILTAAAEKVLKSLSETKLD
DNYEYILDVMIIESNGLYMCLQKAGANVLPDGTLKLFFNQIFKLLQCSTDRRLVYNQKKN
NDDVDEDELLIIDREEELEQNYRTNLLDILGVLIKYHSTQFLNTCCDICITFINTYMNSP
NAEDVALALYVCDDLLEFLQENSVCLWEYFMNPLLLNINHTDDKVKQAACYGVIQATKIE
AFSKYANVAVEYLLKLVHQNTSSKKPKEFISAIDNAVAALGDIVLMHTSKFNNAEELIKL
WLNNLPLKEDDAEGRRVHKNLIDLVSQNHPLLFGKDNSNTAKIIEIFLTIYETDFSDADC
NKKIASLINSLDQAYLSNLASSALTNKQAKKLNHIVNSNRKMPTISVYEEDIIEKLGEKI
EEEKLNDICFEFGIEIDDIEYKGGKKIYKIEVPANRYDLVCVEGLCRALKSFIGKYENIK
YALLTNSEEACMKEKHFMRVDESVDERRSYVVSAVLKNVKINESVYNNIIELQEKLHHNL
GKKRVLLAIGIHDYDKINFPVTYKFEEKEKINFIPLNETKNVNGNNFLKFYEENINLKSY
LKIIKDFDKFPVIVDADNQILSLPPIINCDHTKITYDTKNLFIECTAIDKNKAEIAVNII
CSMLSEYCTPKYAIHSFFVQYDRKHKAEKGNGYLYPVFKNKSLTCHIDYVRKLSGILDLS
VKDVEPLLKKMMIS-SKVIDSSTFTVDVPFYRSDIMHCCDIVEDIAIAYGYGNIVSEKIE
IAKKNSLSAYTELFRNVLSECTYTEVMTNALLSKRENYDCMLRKPRDYNDDRINLDEYNP
LAPPVQIMNSKTSEYEIVRTSLIVNMLKFVSANKHRELPLRFFEIGDVSYTTYNKTDTNA
VNKRYLSVIFADKFTAGLEEAHGMLETVLKEFQLFSDYKIEEKRKENVAIRSDVFYKLVP
KEDPSFLNERVVDIVLCPHNLKFGIMGIIHPKVLENFSIDIPVSVIEINIETIMDVLMIM
GDNAGSSGSNPVISIGNIRGFGGCDYGSFRMSNEFLGWKNKQTNSVYQYKCSDISEAEWI
KTGYNNNRLHIKFNKQKDNLIIFFDGFPDRNISEITQHFQKYFNLRLASRKIATKGWNWG
EFKLENTNINFDIDNKYAFSIPTNSINQLNVQIKTDIAMELKNEDYKKTNEDFLSEIRFC
YPHENDENKHFQNFKNDLLEKVNIGDSKSECIASLANIPLLVPRGRYEIEMYPKSFKLHG
KSYDFTVQYTNINKMLLVPKSNSNQYVLIFSLNNKMKQGQTEYPFILVQLNNDDDMELDI
NASEEDLKKYKLEKSLCGRAYEVIPRLFSALVKKNAIIPGDFRTAKNEHGITCSYRAASG
QLYPLNKYFLFIVKPVILISFDDIVTLTFQRTGNINQHRFFSVIIKHKRGMSYEYTNIDK
SEYLPLLEFLKSKNIHIQDDANVADKKQDFGDELSESDEEEYVADDDDDEEDYVAEEEED
DDDDGSDDEEEEEEEEEEDDDKMGDLRSKADLGDCRFYEKKFPEVDDLIMVKVNRIEDMG
AYVSILEYNDMEGMILMSELSKRRFRSVNKLIRVGRHEVVLVLRVDSQKGYIDLSKRRVS
PKDILKCEEHFSKSKKVHQTVRHVAQKHNMTVEELNRIAIWPLYKKYGHALDALKEATVN
PEAVFKGIELNEDVKNSLMADIQLRLAAQALKLRGRIDVWCFSYEGIDAVKEALKKGKEV
SNDEVSINIKLIAPPQYVIVTSCQDKDLGMAKIQEAMKVISDKIKEYKGGDFKQQGEILV
IGGDDEKRLEELLDKQDDLSSDNEYNSSDDDDENSSDDEENSSDIEEEEDQLYLSMQDDV
YVDVHSRENAHIMHLEKQVKIKLEEQSSLLSIKGIDRVDIQQKKGKHSIICIHYIKNMCM
KNLFCNYLHQLIYAKIPACKNFLKNNYCADKVRGSCMFRHTQDNVNSGGFGESKDEHLDD
VLKLLYDKNICVNYLLGFCNLGYNCKRVHKCKSRKNLISILPKFYLDNILVNHSLYGQLY
SNQRKLSDMNKLKDALIILSGEKYQEKTLSATSSNLKDKEIFPTDQRGNSDTQRYRGGGL
SLQKTDYVHRDNEKGFTNNEKRNQENVMTIPNIYDINNNIIPSEKIKIFIIKCNQISHLY
LSILYGVWATGKNNTRKFINLFKENYTIVFLFSVNESGGFQGYAKMVTVPIKNLYENLWG
PISNRLGGNFRIQWIKIAKIDFDAFRNMRNPCNDNLPLKKSRDGTELPLNLASIICNRIY
ALPSEDFLAGTIYEYKRRINHAAFFLNLHKQGLLNSNTVWDMRIYCLNKQSDCERITFID
GTEQAIGMLDPLRISFLDNNLTQIFRDSEEWLTLEYLLQMNVRTSRVRLVQAWHVSPVNL
INKFEKRNSNKLVLKSFIDVSSLDMDNCIQDICTRGFSVSRRGLRLSVGNFNIPGFPLTS
LDAGYEMVNPNEQDAGTGATHKAKKTLRNILKHSETVLLSGERSVFEFFLCDVGVGLSLS
VSEKDAEIYNRDTVPIEYDSVFIEKKKVSALDHSLTVHYSDRDEYQNSERINLALGGDTM
ASCGVLPHYTFHHEYIIFDNSQILPRYLIQFECDPNGEEHFSLPLCDYCGNAPSVFYCES
DEVKLCAKCDHMIHTQNKLVRKHIRKTLNEAQTISGKCKIHVEERVSMFCTICHMPICNK
CISSHEHTDLCGENSPWLSKKNDAIISLNLAYKAIIKHSAIPSNLVKEEKKKLNDMLKKV
DKLYEQVRSNIKDAEKHVYTILEDVIKQLHVVTDQKMCAVLSEEYELKRQFCEIAWNENF
LYYLQTVLPPADFMNAWLKHCLVREEIEKNSGYAERPNALVFPDMCIRGNINVVTEESAH
HSE----RKRHMAPKKKEEE-QKVLLGRPKNTLKMGLVGLPNVGKSTTFNVLTKLNIPAE
NYPFCTIDPHEAKVTVEDERFDWLVSHFKPKSNVHAYLSIFDIAGLVKNAHLGEGLGNNF
LSNIAAVDGIYHVVRAFENEDIIHTEGNINPVRDMEIINSELIYKDISHCEKNLEEVTKV
LNRNKKDKVKQNEHDVLTTVLSFLKEHKWIKDGNWKSSEVEVINEYNFLTAKPVVYLVNM
SETDFIRQKNKHLAKIYNWVQEKNKGTIIPYCADMELKLLSMSEDEKKTYFEENKIKQSM
LSKIVKTGYYEINLIHFFTCGADEVKCWTIRKGTKAPQAAGVIHTDFEKGFICAEVYKYT
DLVEFKSESEVKANGRYLQKGKDYVVEDGDIIFFKFNVSSSGKKMSYYNLEKDDTVYYKL
DTIKCDIPINEELQARINKHVNQLRITYSTLEEFVDNFVYELKKGLEAHRRHPNLWIPHE
CSFKMLDSCIADIPTGQEKGTYYAIDFGGTNFRAVRASLDGNGKIKRDQETYSLKFTGTF
SHEKGLLDKHATASQLFDHFAERIKYIMGEFKDLDNREGKNVGFTFSFPCTSPSINCSIL
IDWTKGFETGRATNDPVEGRDVCKLMNDAFVRSEVPAKVCCVVNDAVGTLMSCAYQKGKT
TPPCYIGIILGTGSNGCYYEPEWKKYKYAGKIINIELGNFDKDLPLTPIDLVMDWYSANR
SRQLFEKMISGAYLGEIVRRFMVNVLQSASSEKMWKSDSFNSESGSVVLNDTSPNFEDSR
KVAKAAWDMDFTDEQIYALRKICESVYNRSAALAAAAIAAIAKRIKIIEHSKFSCGVDGS
LFVKNAWYCKRLQEHLKVILADKAENLIIIPADDGSGKGAAITAAVVSQSSSIKRLPMPD
LYFLIRWLCKAIVSSLFGDVNVINPENVPLYGSVIFVGNHNNQFIDACVLVANIPRQVKF

IVAEKSMRRAVIGKLASIIGCISVKRPEDLKFKGIGHVCWVKGDNKIKGINTRFKLDVQI
GDKLLTQNKVFSVTKIESETELIIQDAINIECEDKKNGVPFKIIPKINQTEVYNLVTSSL
KNGDTIGIFPEGGSHDRTNLLPLKPGVAIMTLCALADGVEDVSIIPVGLSYSKLYQLQGC
VTLFYGNAIIVSQDHCKDYNNNHRETISKVLSKIEEGMRSCMLTSKDHETSRCIELCVSL
YTPERMTISKNKIYNNLQLFCKMFWKFGNSKEIENLSYELKCYEKFLQANKIKDDEVWML
KQSTSAATLKFIEHICSLIFCVIFGMTFSLLWLPLVAISIYLAEKHRESALKNSTVKIQG
CDVVASYKVLVLLVLLPTFNIMYGLVFSLYLYQSWLKRIAFVILSMCILPICYYINLNYS
AQIPSLLRQMKILLKVICGKINVWRDNERELISTRHELQLKVRALVSNLGPKVSDDFLEQ
LYRNIPKFVVDADTKRLIRGKDEWVPILQRSQLEYKEEILMNDDNKEFSGRDIYQLIKNA
KDKIKIDYKFWYTQPVPKINDEFNESVNEPFISDNKVEDVRKDEYKLPPGYSWYVCDVKD
EKDRSEIYTLLTDNYVEDDDNIFRFNYSAEFLLWALTSPNYLKSWHIGVKYDASNKLIGF
ISAIPTDICIHKRTIKMAEVNFLCVHKTLRSKRLAPVLIKEITRRINLENIWQAIYTAGV
YLPKPVSDARYYHRSINVKKLIEIGFSSLNSRLTMSRAIKLYRVDDTLNLKNMRLMKKKD
IEGVHKLLSSYLEQFNLYAVFTKEEIAHWFLPIENVIYTYVNEEAGKIKDMISFYSLPSQ
ILGNDKYSTLNAAYSFYNVTTTTTFKNLMQDAILLAKRNNFDVFNALEVMQNKSVFEDLK
FGEGDGSLKYYLYNWKCASFAPAHVGIVLLMSEVNVTKVIVNNPICDILDPFVFTIEFEA
LNKLEADLEWKIFYISAVNNDGEGNQDIELDNIFLGPIERGVMMFDYAVNPPDYKNMDVD
SVLGLQAILISANYKEKEFIRIAYYMNSFYKDMELREKPPAVPQYDKICRHIFVENPRIV
KFCITWDAEEKDDFKEFDKENEQIELMNSIKMNADQNSNSNITNESTQENIFVSSDIHNV
GTNGKMSTHMEAHENANSNADLNERGVLSSNGASTDLDKCELFNANINGISRITIDNNNN
MKVAVVSVLLPLLFLGLFLKSKEQELSLSYGKYLKNTFKEILLYFNIFDIEFPRSYVLNQ
KLLGLHVKDIEKDAYTAFPILKELKQKDYFRIFKVNLHIPCKYLKVNEKCKEMKKCSVCE
CHDDEIPYNFRTNEIEIIQNKMSTEDLKKTFIESKLYKDILGIYAPSDEGFLSYVDLVYN
SPSFTAYEGRNIWDKIYRENCFQKEENKCHEMKSFYKIISGMQSNIAILSAEYHYLQNDF
VFGDIHTEDVMKSDYFKKQNYAYNMEFFKEKIALYPDRIENLYFTFAILLRAMCRLKALV
SQCKCNSGHAQNDQEALKLLNDLLGSFYHSCSSEEFLEPIFPTQGKEILAKFMNITNILD
CVPCVKCRLHGKLKTTALQIALVEGSYEHIGSLERNEITALINSLYYFADSIIILNKFQE
RQKLKRATFIFYLLFFCFFLILVIYSVILITVCNC-KAKKKKKMARNVEKGRSMLNQWLK
AKELSEQKSFFKIPKRVNEVEDLETAVSCRRHIIKEICNKIKEIQNYSLSDQHIRELNDQ
INKLISIKNKWEIRIIELGGPDYQTESNTLINAHCSELKGNNNYKYFGAAKNLKGVKELL
LKENDDRKKFILKKKKENRFFDKYVNIHYFGYCDDQNEMLLREELKMQDQLEKKDLKTLK
RMNYNARSLPPQTMKHGTLVLPSDRAREYLDCLGKNVDIQFIDMNEKTMKRQYKKYIQRI
DDMERILRFLEENIRKLPNVKIKKSKIDSFLEHDNVYELDQVEESLNRLHVQFVRFCNNN
KELVDERNNAIEEKHVILTAMNQLNPGATKNGNVSKRLPESTL-------PYDEVNDESI
SLQTNMMKDGISMMFTNISGVIKTKDQESFSRTIFRALRGNTYTYFQSIDESGDSSSGGV
MDGVNDMDEGGGATTCM-NNKEKKNEPKSVFVVYCQGSSQSNIYQKILKICKAYDVKTYD
WPKTYEQARQRLKELKEIITDKQKALKAYEEYFINEIFVLINVVEPNKNSLIEEWKLFCK
KERYIYNSLNCFEGSDITLRCDCWFSANDEEKIRHMLITKSSNDLVSALLLSDKVLTPNI
SPPTYIKTNKFTKSYQSMVDTYGIPRYGEINPAISTIITFPFLFGIMYGDVGHGVCIFLF
ALFLILINNRIKNKSKNEMVSMLLDGRYMLLLMGFFAIYAGVLYNDFFSMPLNLFTSMFE
VDRQVDSVTYYKRRQVMNEASGQMEDADPYIFGFDAKWLGAENELTYINSFKMKFSIIIG
FLHMTFGVLMKGLNALHYRRKMDFFFEFLPQLMMMLSIIGYLVFLIIYKWVTPIGYGGYK
KQGIINTVINMYLMKDLTPDNQFYAHQGLVQAFLIAIFVLCIPLMFVCKPAIRTYHIMKE
KKSRGSIYSHHEEKEMTHTFNHLVGNGVNNGTSSSPSLHKRSPNMTFNNREDDYLSPRRR
NKPTDDEMEAHLLSPTAPEDQEGMV--EGEAHHEENISEIWIEQLIETIEFILGLISNTA
SYLRLWALSLAHQQLSFVFFEQTILNSLRKDSFMSVLINLIVFSQLFSILTIAVILCMDT
LECFLHSLRLQWVEFQNKFYKGDGIPFRPFNIKKVLSESDMLGIQEGRAFFNAARNPNNR
PENNIRIEENLPSLNEVYNYFQDFLSRYSSNTLKEGNLKRTLFENVKNNNFTLDLKLDDI
YKQQTVFENNEISATPISERKMKKPENYSAILARALSERPLTYLPTVERVCYEVCETANI
LNDEDEHLNYIQINLLNTFIRPTPIRGLLAATQERFVVVPGIIVQASKPQHKMRKITLQC
RYCDHKMSIDVPLWKDKPQLPPYCRYSSTMKSSMGVANPMDNQLGCNGVLEPYVILPNEC
TFVDIQSLKMQELPEAVPTGDMPRHLQLNATRYLCEKMIPGDRVYVHGVLTSYNPNPKPT
RADGTNFSYLHVLGFQKYDDMTGNDLNFDVEERNELTLLAAEHDIHEKIFKSIAPELYGM
DEVKKACACLLFGGTRKRIGEETKIRGDINMLMLGDPSVAKSQILKFVNRCAPVSVYTSG
KGSSAAGLTAAVMRDSQGVFSLEGGAMVLADGGVVCIDEFDKMRDDDVVAIHEAMEQQTI
SISKAGITTMLNSRCSVIAAANPSFGSYDDSQDTTDQHDFKTTILSRFDIIFLLRNKQDI
EKDTLLCNHIVALHASKHKSQEGEIPLSKLTRFIQYAKKEIAPLLSKEARDSLRNFYVQT
RAEYRGDRRSVTKKIPITLRQLESLIRLAERLESIRESN-------------------PS
MCQCSI--MPKS
YRNYSKTARNPKRPFEKERLDQELKLIGEYGLKNKREIWRVQYLLAKIRSAARYLLTLDE
KSSKRIFQGEALLRRMVRQGLLGENEEKLDYVLGLTLPKLLERRLQTKVFKLGLAKSVHH
ARVLIRQRHIRVGKQMVDIPSFLVRVDSEKHIDFATTSPFGGSRPGRVKRKSLKNQKEKN
EEENNMVRQSQSMYDRHLTIFSPDGNLYQIEYAIKAVKNTNITSIGVKGENCAVIISQKK
MATQYISQDKLLDYNNITNIYNITDEIGCSMVGMPGDCLSMVYKARMEAAEFLYSNGHNV
NVETLCRNICDKIQIFTQHAYMRLHACSGMIIGMGEDNKPSLFKFDPSGFCAGYRACVIG
NKEQESISILERLLEKRRRKVQQETLEEDIQNTIILAIEALQTILAFDLKANEIEMGIVS
KANPNFTQIGEKEIDNYLTFIAERDMDNNENIDLNDNSSDTNTGNYNHGKNENISEEMDF
SKEDNEMDGNSKSSKSKQDNKTKNASDKDDMNMDYSDHHFPCHPAPPVSIKLFIGRVPKN
MEEEQVRPIFEEFGIVKEVVIIRDKITNIHKSSAFVKMASISEADNAIRSLNNQRTLDPQ
LGSLQVKYASGEIMKLGFPQNIESGVDQAKLFIGSLPKSISEESVKEMFSPYGSVEEVFI
MKDNSTGLGKGCSFVKFAYKEQALYAINSLNGKKTLEGCARPVEVRFAEPKSAKQAQIPM
NMQSMQNSAHGISSQPHVTSPNNINFGNNFGVNNNYPRQVGAWKEYYSGEGRPYYYNEQT
NTTQWEMPKEFETLFMGTTHNMHNLSDSSGPPGANLFIFHVPNEWHQTDLIQAFSPFGEL
LSARIATEKSTGRNRGFAFVSYENIESAAAAISQMNGFMALNKKLKVTVKKGEEEEMKKF
VSQNGINSFQQMSRPQKNIPAQPNAVAQPNFPPHQNAQPQNFFYSNSNSYRCGPYMTTAH
RPTWYNAIGGENQGGNRKVSQTAKVCSRDLPGHMKMKTRDLSDYVEDKEEIRKNLIQLEG
GATDKDNK-HRLLAIENIKKLTNHEYKNPFPEDEDDEVEEEWTSRKRKKGRKAKKGNSPE
GSQSENPSDGSSDGSAED--RSDDESDDSSDDESEDEEEKELLRELENLKREKMEKERQE
KQEQELLQSRKNNVLTNNPLINLEDSDNEEGMSRKRKWTEEAIFRNTCEKKEKKITFIND
TVRTAFHKKFLFKYIHMDSEETINQAVKLAKDAVIEDEKKNYKSALNLYIQSLQYFNFFC
KYEKNSNIRDLILKKMEIYMTRAEILKELINKKESMETKEKVASTEESKENMKKQIKEFI
LNKDKNVKWSDVCGLETAKEVLKEAIIFPLKFPKLFNSSALPYKGILLYGPPGTGKTFLA
LACSNECNMNFFNVSSSDLVSKYQGESEKYIKCLFDTAKEHAPAIIFIDEIDSLCGSRTD
GENESTRRIKTEFLINMSGLNNYKNNIIVMGATNTPWSLDSGFRRRFEKRIYIPLPNVYA
RMKIFEKYINGNESNSNIGKEDIKYFATVTENYTGADIDIICRDAVYMPVKKCLLSKFFK
QVKRNGQIFYTPCSPGDPDPTKVEKNVMSLNENELLLPPLSVQDFKTAISNAKPSLSVDD
LKKYEEWTQQYGMNGTMNANVQKFKIVLKTGKQLFSSEALKVKRNPNLEKLSGEKQNLTK
VTFKKEKIEDIIKEVKFDYYYFNEGKKNIYKDIPLNIAVIKESELPAFKQVDEKLHFSVL
ENDLRIISTNRNNSVCSIGLYVKCGSRYEEINDQVNEQGMSVMLENMAFHSTAHLSHLRT
IKSLEKIGANVSCNAFREHIVYTCECLKEYLPVVTNLLIGNVLFPRFLSWEMKNNVNRLN
TMRSKLFENNELYITELLHNTAWYNNTLGNKLYVCESSVENYNATNLRNFMLKHFSPKNM
TLVGVNVDHEELTKWTSRAFQDYVSIPYTNQKEVTPKYTGGFVSVEDKNVKKTNIAIAYE
TKGGWKTSDMITLTVLQTLMGGGGSFSTGGPGKGMYSRLFLNVLNNYNFIESCMAFSTQH
SDTGLFGLYFTGEPANTMDIINAMAVEFQKMNKVTDEELNRAKKSLKSFMWMSLEYKSIL
MEDLARQMMILNRVLSGKQLCDAIDAVTKEDINRIVGHFLKTKPTVVVYGNINHSPHYDE
ICKILGMISGIRVNDTCITEFNNMKIRKTCRWIIFVIENCEIIIHSKGDTTTLTELVKSI
DQNDKIQCAYVVFDAVNKIHFFMYARESSNSRDRMTYASSKQALLKKIEGVNVLTSVIES
AQDVADFKMQPYGYSGGGNHYNSNNNNN-GGNGGNYNNPGNVNKPMNSPFFYNNGSGTNA
DMV------NRGSGNMKGNEGAY-PPNYYSPPQAMQPGMYGMNGPPLSNNIYGDGSNPNS
YINNQGQYRGVASQFVPVMNNNNALKPGGNVLGYDNTGHVNSVQPIMNDSYQEFLQFNAF
SHFVKSSVSYMPANTTLKQKAYVPLGFVIQPLAPIPDGYPELASVNFGNSTIVRCKKCRT
YINPFVRFEGGGKKWNCNMCYHVNDTPQFYFTPLDEKGKRKDLFQRPELCTGSVEFIAPS
DYMIRPPQPPVYLFLIDVTVTSVNSGLLDVVCSTIKKLLPKNRTASNDNRGVDMNNKEPF
DPRTLIGIMTFDSTVHFYNLNSNLKQTQMMVVPDIQDIFIPLPEDILVNVHECQNVIDTL
LDNLPTMWRNNKMADCCAGNALKAAIMVLKKVGGKLVFVLSSAPNIGDLTVSVNRETKEK
SSYKNLYGSGSSSNNVVDSKVREIEMLTPFNNSYTELAQSITQYQIAVDLFACPLYSLDL

ATIYPLVKNSGGSLYYYPQFNVHQYSDKLREELLFALTTETAWESVMRIRISRGWKITNW
YGNFQFRGVDLLALPNCHSSQNFSIVVDLEENVVQDSVVYVQSALLYTNSNGERRIRLHT
YALPVTQNIKTITDSINPQVVVSLLSHQAIDICKKGKIADGRNLIQTLCSQVLSSQLLLS
ETARLLSIYVLGMLKSIAFRDSGDVPPDLRIYHWSRVQNIPVDCVEAYFYPRMFSLHNLE
KHHGNYDDNNAMMFPDTISLTCENMTQDGCYIVEDGENIIMWIGRSISPQWIYEVFGVQS
LDQLNSEYAENHLGTSGSPSGVQILNIINALRKIRTPSYMRLLVVKQGDPLEYKFFACLI
EDRSQHMMMSLKEFLAKICKYLKGVAII--MEEDSFKNRLLKRNIDIWIEKYRPENLDDV
VGNPFVINTLKSIIVSGNMPNLLLAGAPGTGKTTSILCLASEMLGPQAKKAVLELNASDD
RGINVIRDRIKSFAKEIISLPPGKHKIIILDEVDSMTTAAQQSLRRIMELYSDTTRFALA
CNQSEKIIDALQSRCAIIRYFKLSDDQVLKRIVKICQMENIKYTDDGLETLTFIADGDLR
KAVNCLQSTYAGLEVINKENVLHICDIPSPERIENLLKFCINSEWKKAHDIAYDMIKEGH
TPFDVALTSSNVLRRYDLGSESVQIEFLKIGAMACNTMASGLASVIQLDRLLADWCIAAK
ALRTKCMTKHTYMEDRKMGEPIGSILVDETEKLVDTYRFDSTFTDGLKKSSSSQKKDGKH
GMAFHKSLAVVNVAAGLDGCDDQLLPASFRALEADLNLHPSLLGYITLAQTLMLSLFSPI
WGFLSDKYSRKWMLVFGTALWGVATILLANINDFAHIIFFRAINGLALGSIGPISQSILA
DAAKNESLGLSFGLVQLSSSVGRLIGGVVTTTVALKYFGGIRGWRLCFIVVGILSILLSI
IVALFVDDAPRQVRKKKKMEYLDDDIDAGSNNVRIVTQYTQSYLLYQNIMELLRDSLSKK
SIIIILLEGFTGTIPWLALSFNTMFFQYCGLRDLQAAIITGFLLIGSALGGVIGGHFGDI
MHDISNKHGRPFLGQLAMFGRVPLVILTYMVIPQRKESFELFALSCFFLGLSSIAGVAVN
RPIVSDIIRPDYRGTVFSLTIAIEGVGASLIGAPLFGYLAEKVFNYQNNNLLISEMPEEL
RRNNAEALSKTLLYLTMVPWLLSFVFYSLLHFTYGSEYKKMNEIIESEYKYDDEDEETVA
EKAHMRRGSDPGVSTAVTSIMQHQNFKRMLMFGLRSLSDFCNPTSKTYKENAFDALDRGA
VESIKNAVINYKDDDDILFCSSRVLCAMSDYCSSEKDTDALQKLITDGGVNAVVEIIKTV
PSDQDTLKNCMLFIQNMNDSNVQIEGGDLGVALLNVFTSNAYNVKLGNIIVSALSVAAKS
ASGAKILNTEGAHHKLIDHCLSLQAVNDDTAEIVEGVFDAIKNLSSNGYVVPTIIEKSVV
ILDKFKSYPRIVSKGSDAMKCAVGPEQLTNCLNILKTSKQGSKEQDSALELLSSLSYISS
ITDKVVESGGIPVLVELINSGLQQYESNPEKISRLVAGASRMLGRISNNPPHAVIVYEYG
GIATLCTALSYFPNDADCASAVCNALIPFVSRSNYVKEINNYSLFASLFPILYASLESIE
LAKASMSCIASASMINEFHEQMVNNQAIEILSTCIQYHLNETKYLLNCFTAYFRLSDYIT
TVEPIHQYGGVTGIANALSAVSNDSTISEIGLKLINKMLTASDALSYLSNQQVVDAVLTV
MLENETKEVIIQEGTKIMEKLATESDCQRHISNLESIINLAQSNPEAAYKTLAAISGLSR
IQSLKSILESKGADSSIYNGMKVWIESPRFNEQPKLIKAALKTIKILKLDAENTIHEVIG
SIVDVMCISQVKRLAEIEEPDDNILITASECINYLTEINKINNKEIVESSLDSIFKLMKK
YSESRVTQTNLLAAINNILLSSNLIGADVLVNKGYVKQIVNYIHKVPMYVDVQIIGFSVL
GNILKISPESIDSIRKANALVPLQNSLRTHEKNAKLKTTCAPLLAMLMPLDSLTKEIEDL
LKLCNDAMTENDLIKLHEYLISLNELLLNAEASKISSRCNVGEHLNHMIQWLKNNPTIYK
STVNKDYSIVGHSLYDATISEIAHACTNISQTRIGLVHLTKSDMTSALMEGYNLLELPGN
DYTEEAASNTLESLSLLLKHDITNADIGFNKGLVKKLCTGINYFSESDPVIKNTFACLAC
MCTSEKRVNQLIKHPEYDKLIALIVDLIGNSEKYKNSRGNAIKALYELLKTEDEQIITNI
ASMTPIVDNLFKIMGEYQADLPIVQDSAKCLAIIADHINIQEKMKIDQYTAIKILLETLD
KNKNDELTAEDILTVLIKLCDSNDKMKLRELGAVDVISDVTMIHSENENISRLGGVLFSY
MGADEQVKKLMKLILNVKSSDKDAVQKIDNLSSKLEMFLRAPLENPLDALEYTEATLQQL
NGYLTSQLDNASVQANIALVTKRLVDRVKYDHEDQLGAWAVASSGVLNQYTDMIANKVGL
ANSKFVSPVYSVLAGCVLNPYTKQLVLDKLGPILDNTYEILDEYKNKPYAVQGIFELLEQ
VAKDEEGAKLLYMRWNGSMGNIVDQTLSIMNLNRTNDCVFVPGIKLLGAIAETSSASGYV
NDMDNARIIESCGMLLNLGSSKERTVEFINFVDTMVVSSLLDEKVASETFKKISTLVSEE
NLSNYTQQDRVEIIKAYANLLRDSASTGLFTHIRPTDKTEALNNLARLMEYEPCEEVNVA
ILEALSEIATCDSSTSLKIVTSALPSIVQSNHDNLVNDNATAESFLQTLEKLVVKEGIGR
QIANNNEMYKLLEELEINLEGKKKELGEEYVDNIKMRISNVRTAINDDMPKEKTCKDIYD
LLTELKLKSIEINVLVDASLEEDMNYVMERLKVYNKDNLLPTTEAGTDNSYGYMAIEIFC
ENSDNVKELVKKGFHTSAFHSLIKQANENVKHYSCRALCALTKNPIGLEHVIKEIKDYAN
VISKSVGDLCMEKTLEKEDKEDFLINRVLLIDRTAHNRNVYDRTNAVNHLIEIWNQYDHG
DYSVSLLRHVFRAMRKIVSDAHVEMLLKADVLVRLTGIINNVETDKIIYPDVLFLIGSLS
VVKEIKTQIGEIKGIDACVNLLLRSLNVEKMEPTITNCCLALANMCIDHKGNSKIFCALK
GPELNVRILREYRSNFDVTNGASVLLCNILFRNEDMKRTYGTNGAPAELVQCLRSYDGSD
NKNAVRCIESLFKAISNLSLYTANVRYFLDAQIEISYESWLNKLNESFPDAELETGLRTL
SNLVMENEERNMKNFGVTLIPVLNVLKQNRENTKVIFFLLDILCSLCRHNDNAKMFAEND
GIATTINVIQLYDYDISLLTLAIHLLSNQCKIESSLPLLVKSDTFGILISCIEAETEEFE
ITELVVSSLRCIRRLIQSEELAYEFCNSGGVPSIANLICNSVKKSIVMLEALRVLLCILY
YTQNVEGVTNEYPEEEEELYNAKLGGWYNISMDKEMIDTIIQSVLTCANDNNHLKQLRLQ
KVSLGLLAYFAYHNLGIISMTASGFDTLTRENLNNFGGDGVIMQLLSICIDNIAMYSAEV
YDMTISRDIIKSFKSSVSKMSNKKEDKPIIQKVELTLEAMNSAEDPLDAFKNTILIFDFS
LSEFDKDPYVNGVHDLSSNIKDALRKGGVHKIYHNSDVRKPFKWKASQDLATLEWIVGDD
AEHIFKISVVRIKNISKGLTHPLLKGANKREPRKVNAKVTVCIYGPPTEDFPEGLELPIK
TKTQKERDAFVDLLVLWRDAASYNYMEKKVRDYAQGAVKFIKKSGSNFLACKNLREKLEE
KGFKRIQEGEKWDLRKNEGYVFSKQNRNICGFFIGKDFNMEKGSILISIGHVDTCCLKIS
PNNNTVKSKVNQLNVECYGSGLWHTWFDRSLGLSGQVVYKKDDKLVEKLIQINRSIIFLP
SLAIHLQNRTRFEFSVKVNFENHLKPIISTVLYDQLIKGKEKQNTDADKCLNGDDSHQEN
PNSSPLLYTLAKELQCEEKDILDFELCLMDVNEPCFTGAYEEFIEGARFDNLLGSYCVFE
AFAEMIDMLKGKTPPSDEAHANLYICIGYDHEEIGSLSEVGAQSYFTQNFIKRILTAISS
SQVGQNTTSIDELYGSLMSRSLILNVDMAHCSHPNYPETVQANHQLFFHEGIAIKYNTNR
NYATSPYYTCLLKRTFELFTSKNEKIKYQNFMVKNDTPCGSTVGSMVASNLSMPGVDIGI
PQLAMHSIREIAAVRDIYYLVKGILAFYTYYSHVHASCVPDEMNFCKTVFNVFFVLLFIS
SYQIKCQLRFASLGDWGKESKSQLLNAKYFKQYIKNERVTFIVSPGSNFLDGVKGLDDPS
WKSLYEDVYAEENGDMYMPFFTVLGTRDWAGNYNSELLKGQGMYLNEDGQTTIEKDIDKT
AYPKWIMPNYWYHYFTHFTVSTGPSIVKTGHKDMAAAFIFIDTWILSSNFPYKKIHERAW
ADLKAQLNVAKKIADYIIVVGDQPIYSSGSSRGNSYLAYYLLPLLKDAQVDLYISGHDHN
MEVLEDSNIAHITCGSGTMSGGKGNMKNGKSLFYSNDIGFCIHELSSNGIITKFISGKSG
DVIHTHKINLKVKKSLDKVNSLQYFASLPKVEQVDVPASGPMGNKDSFVRIVGTIGILIG
SVIVFMGASSFLSKNIKMNDGKKKGKFIVFEGLDRSGKSTQSKLLVEHLRRKNVEVNHLC
FPNRETPTGQIIAKYLKMQNDMTNETIHLLFSANRWELMGEIKRLLSKGVWVVCDRYAYS
GVAYSSGALKLSKTWCMNPDQGLIKPDVVFYLNVPPNYAQQRSEYGEEIYEKVEVQKRIY
EAYKNFSQEDYWINLDGTKNINDIHQDVVNEITKLEPVHEEPFTFLWSMKVNQIICKNCH
STDVETNEGQGEVICLRCGSVLEESKIVESLEFVENNNGAISMVGQFVPASGNKSFILSW
GVRESRELSLQKGYINIQKIAEHLHLSTQHVESAQRIYLMALQRNFTMGRNNSYVAASCL
YTICRREKSPVMLIDFSDILQTPVKPLGKTFLKLLRLLHISVPNIDPSLYLERFAHKLNL
KNDIYKVTYTGIKLIQAMTRDWICTGRRPTGLCGAALLISTRMHGIFVHSNTIANIVRIS
NPTIIKRLSEFKNTSTAKMKVSDFDKIRLNDLPSNSLPPCVIASKRRKFREDMLRNNKAV
SLCDSEEISSTVASAKCGKDSEQHLTNDTYSPNCNEDTFSNALSYNNLTLLDSRSSCNGM
GSQTGDDVENLTTNGINVEEICNENPEGNDLDELAKKIINTIDVEKQSSILKVNVSSLHM
NNSHCDGFSHSNKQWRVKKIQSTVSIATINHTNGKCTNGSLSSVQSEKQMSKAIQSDNKN
GALSKKVLHNDRLDSLNNDPETTYSSTLKETFNSELSHLLNEVDDLNILEAKENKLLHEP
NDMHKDDVDHVSSIMSDFNYFFENNSNTVESQPPNTDIPSDASVEQFNESLSDFYDSEIE
NIILSEKERKRKMLIWDDMMKSYFPQYYKQFKKQKKKRSSGADKSKKKKKKEQEQNDHHP
LDEQTTGESVIMALEKSDKSMSTKMNYDVLKSLFSSMMEKRENLEGCNLVPKKENGEIDA
DLYSRQLGTYGFELMNKLIKLNILIVNVKSVGLECAKNLVLSGPKSVCIYDNELCQVSDV
GVNFYIDEEDVANKVTRSDAVIKKLKELNRYVHIYNYKGELDENFLQSFDVVVCCDVAHS
HLVKYSKMVRNISNRKIAFLCCNIYGLCGYLFVDFGKGFVCYDKDGENTKSCNISKISKA
TEGVVSFDFDKGAPFQKGDYIKFANVEGMTQINNKIYKINDMQKYTFTIGDTSQFDEYLK
GGECTQVKSHLRMNFQPYDIVCAKPLAWD----EVSTEQTVFEGETIYEEVPPPQSFLIS
DYAKCDMSNQLHYAIQALKKYEEENNNVLPQNSEEEAFEKVFQIAVHLNQADKELKKIYT
VDEVKKDIVLKVAKYCTAHLAPVASFFGGLLAQEVIKYTGKYMPIYQLLYVDFFECISLG
GESDSDDIAKENSKNDNVITVFGKAFQKRLNELNVFLVGSGALGCEYAKLFSLLDMCSVE
HSGKLTITDNDNIEVSNLNRQFLFRRENVGKSKSLVASGIIKQKNPNINVQSLETKVGPE
NEHIFNETFWEKQHIIVNALDNIQARQYVDNKCVWYSKPLFESGTLGTKGNVQVILPFLT
QSYNDSYDPPEDSIPLCTLKHFPYDIVHTIEYARDIFQGLFYNTPLSLQEFLKDKKEYVR

KVEEEGNNASLLETLENVLSTLREVSKECNFNFCVKKAVDLFHTNFINQIDQLLYSFPLD
YKLASGEFFWVGQKKAPQVISFDINNEFVKEFLFCTSNLFAQVYNIPQCYDLKYILDVAS
QIEVKPFQPKRVKVNMDEKNLNNISISFVDDEKLIHDFCKELLNIDCQHVKVSPIEFDKD
EETNMHVNFIYSFANLRAINYKIETCDKLKAKLVAGKIIPALATTTSIITGLVGIELLKY
VNYYGYVQMYVKSTEDKRKQMKDLLSYFKNAFINTALPLILFSEPMPPIKMRDKEYDDLM
KGPIKAIPNGFTSWDKIEIHIVNGTIKNLIDHINEKFNIEVNLISVGNACLYNCYLPAHN
KERLNRPIHEIYSDITKQKLLDDKNYIVVEASCSDQDLVDVLIPSIKFIYKMSKLNQDLL
KKAIADVFEGTKTKKRKFVETIELQIGLKDYDTQRDKRFSGTVKLSNEVRKKLKVCILGD
AVHVEEAQKLELDYMDIEAMKKLNKDKTLVKKLAKKYDAFLASQVILPQIPKLLGPGLNK
AGKFPSLITHNDKINDKILELKSSIKFQLKKVLCMGVPVGHANLKEEELRSNIVHAINFL
VSLLKKNWQNIRTLHIKSTMGKPQRIYGMIQESQNENENKQKWEDIDAEDDLESNKKLSY
FTNYENGIKVVTKYSENLKKQTVKVTKKIKEVVIKKKLNKEINNRLNLKNFNIDTCSSVI
VEPTDVVNIEVPKNNLLDFLKGTEYDYLFTEQADKTAKDLKNRFKIFKDEDVEEVKPEDP
NKLGAKREMLFYRSHEECTVRVTNLSEEVNESELSNLFGRVGQISRMFLAKHKETQNSKG
FAFITYSNREEAKRAIEKLNRHGFENLLLSVEWAKPSNRMLNDIIFQVIIASFGVTIVNS
DKIKFLQKFRYAIYVLILSFLLYKGVPWKRENYYTYLNITPNATKQEIQTAYRQAAKIYH
PDKNPDESADSAFIKLKQAYDVLTDDTRRSNYNRFGDYKNGEVDDNTATLLICLSLVQHT
MFFIIGYFLSYPRKLVFARQIFLVYNIASFCFELQFRFIEDDTTFDWLPSIGYLLPYEKI
KLLRMLFPIVFFISICAAAYTYTDRNATLIYLMRSILATNRIVFERSNDVVDSTNYLKKN
GEQLVSKLQEMRKAAKYAQAKLKDKEQ-------KDKSECLEDSKKDPKDENADDKLREN
VQEFALTLDSHQMGLLEKCFEMMKNKNTKDKKNQKKSWFEFISMQMIFGIIFVYIWFTSK
MGKDYYSILGVSKDCTTNDLKKAYRKLAMMWHPDKHKDVKSKKEAEEKFKNIAEAYDVLS
DEEKRKIYDAYGEEGLKGSIPTGGGTYVYSGVDPSELFSRIFGSDGHFSFSTGFDDDFSP
FSTFVNMTSRKSRPSTSTNVNNNNYNSKPATFEVPLSLTLEELYSGCKKKLKITRKRFMG
SKSYEEDNFVTIDVKAGWKDGTKITFYGEGDQLSPMSQPGDLVFKVKTKTHDRFVREANN
LIYKCPVPLDKALTGFQFIVKTLDNREINVRVDEIVTPQTKKIVSKEGMPSSKIPNTKGD
LIVEFDIIFPKNLTSEKKKIIREALVNTFVKGIKKSLCLLVLLLLSYTYVQGAYFYVKEG
MEKCFVESAASNVIITSSYDNFGLKELKCHIIIKNQQGKVVYSHETSEISKGKVSYMAKK
NGLYYICISCPSSNWFKSTAIKWSLSIEVGGSDVDIENVAKKSELSETLAILLNLKKKFS
SMKLQQIYQKQMATNLYEHNKSVHKKMFYCYILEIIILVAITVYSIVHLKNYFKAQKLMM
SKNKKGKKKDDLDAILAEFGFDEKREDEEATKMSKSKKKKEKMKQKKEQKGKENVEEGDE
GEAKKNDDANDAANDGANDAANDGANDAAKKNKKKKKKDKEKGKEQKGTSGMSEMAKAAA
ERLRLLKEYEEKKREEEKRKQEEEEERIRKEEEEKEKKRLAKLEKKMQLKKEGKLLSAKA
KEEKKKKELYLKTLKESGVLIEPKEKAKAEIINIDLNKTKAFLKKKKNKTNANNADGAGG
TTPQGNKLVEEQAKD-----EPKEIVLDDWEDFLNVDEESKKEKQSNENQNNAKTDKERN
KSKEAKGSEKKGKKKNDTSKTNEKHSNTQNESDDESEYRSAIVCILGHVDTGKTKLLDKL
RHTNVQDNEAGGITQQIGATFFPKDVLDKEIKKVDESIKCMSKGIMIIDTPGHESFYNLR
KRGSSLCDIAILVIDLMHGLEQQTKESIQILKQRNCPFVIALNKIDRLYMWNKNDWSPFN
STFNKQKENTQDEFKDRLQNILNELAEQGLNCQLYWENPNPRKYVSIVPTSAITGEGIAD
LIMILVKLTQNFMLKNIEYHDKLECTVLEVKNIEGLGTTIDVILTNGVLKESDTIVLCGM
NGPIVTVIRALLTPQPLKELRIKNEYVHHKSIKACIGVKISANGLEEVLCGTSLFVANNT
NEIEDYKKKAMTDVSDVFNHVDKTGVGLYVMASTLGSLEALLIFLKDSKIPVFAVNIGTV
QKKDVKKASVMREKGKPEYSVILAFDVKIDPEAEKEAQILGVEIMQKDIIYHLFDAFTSY
IKKIEEEKKQSKLTDAIFPCELSIVNDCVFNKKDPIVIGVKVDCGILKIGTPLYIPEKSL
KIGNVVSILLNKKTCEKAKKGDEVSIKICGEPHITFGRHFDFNQKIYSKITRESIDVLKE
YFRSELTMEDWKLVVQLKKIYNIIMNINEKDKLAEQNLETLDVTKLTPLSEDVISRQATI
NLGTIGHVAHGKSTLVHAISGVHTVRFKHEKERNITIKLGYANAKIYKCTNPDCLPPECY
KSYESSKEDDPMCPRENCNHKMKLLRHVSFVDCPGHDILMATMLNGAAVMDAALLLVAGN
ESCPQPQTSEHLAAVEIMRLKHILILQNKVELIKEEQALKQQEEIRNFVSGTAADSAPII
PISAVLKYNIDVVCEYIVTQISIPKRDFISSPHMIVIRSFDVNKPGEDIETLQGGVAGGS
ILHGVLKVGDKIEIRPGIISKDEKGEITCRPIISQILSMFAENNNLKYAVPGGLIGVGTR
IDPILTRADRLVGQVIGHLNKLPDCFAEIEISYYLLRRLLGVKSQDGEKNTKVAKLKNGE
FLMINIGSTSIGCRVTGIKSELAKLELTGPVCTKIGDKIALSRRVDKHWRLIGWGQINKG
KPLELQEPIMIQFVLLISRQGKTRLAKWYIPLSQKEKAKIIRETSQITLNRTPKLCNFVE
WREYKLVFKRYASLFFIACIDKGDNELITLEIIHHYVEILDKYFGNVCELDLIFNFHKAY
YLLDEILVTGELQESSKKNILRVVSAQDSLMEDTKSSKKLGSLIMKYAFFLRRGVSCRSP
LNTCAIQRATFRRYYANVSPNEICGKVVQGRLFTSKAGMSDGGASNEG-NGEKDQDGQNS
--QSNQQSKGAEGTEESKEQMKEINYEKLSKADLINEIRKTKRDIEEKMVDNKILKEKYL
SVLAENENIRHRYVKEIENSKLYCISNFAKSLLDVADNLSLAIKNINEESLKQNEEISNI
YKGIQMTETILHNIFNKYGIDKYDPINEKFNPLFHEALFEINDSTKEKGTVATVVQQGYK
IKDRILRAAKVGVVKN-MKELIENILQLVDPDEETEAYLRGRINDEKHIIQKKGIDYLYD
LVSTFSDKKIKKSTIVDIFNKHMKRENGVGDENKNVDNQKLTEIVNLKEYWKKNDVVGYY
DPFLGIQEKQINYNTSIPISESIRISKEKEKQRQKQLNLFKEWVKNKIKIPSPVRVHNLL
HCSDSNKNKTKIEKMYDIRIDNFTLSIGQRSLLTDTTLKINVMNKYGLIGKNGIGKSTLL
AKLARYEIEEIKKDISIACIEQDLFLEDVTVLECVLMVDKLRHDLLMELEQLELKKSKQG
GGCPNGGDGKQEVNERGEEDEKDIDMKILNIYEKLNSINYLEAEKEASKILCGLGFDSNL
QKKKVNSLSGGMRMRLCLSRILFSNNDIILLDEPTNHLDIYTIQFLIDYIKKLNKTCIIV
SHDRDFLNEVCTDIIHFHQKQLTYYSGNYDQFEKTRVEHLLQQQREHDSIEMKKKHVQKF
IDRFRCNSKRAALVQSRIKLLNKLPVVNLEKEETPFSFSFLEPFYTSNVLIRLKNISFKN
EMFKNLQIKKNANIIIADTVEDTNGK-INEKLHNDTKGGDETTARSDDYQFRHEFLFKNA
TFEVDMDSRIAICGVNGSGKTTLIKIILNLIDTFEGELHVSNKANIGYYSQYHVDSLNPI
FNSIQQLQYNYSNKNIKEEEAIKYFNKFNIPTNVLYEPIYVLSGGQKSKLALAILAYKNP
NILILDEPSNHLDIESVQALIVALNLYKGGLIIISHDTYLIKHVADEIYHINNLTKELIK
IDYDFDKYAKLLLENKIMPREIITLQCGQCGNQIGVEFWKQLCNEHNIDKEGILKNNNYL
NEDRKDIFFYQADDEHFIPRALLFDLEPRVINSIQASEYRNLYNPENMFISKEGGGAGNN
WGCGYSQGHKVEEEIIDMIDREVDNSDNLEGFILSHSIAGGTGSGMGSYLLELLNDNYSK
KVIQTFSVFPLLTNESSDVVVQPYNSILTLKRLILSTDSVVVIDNTSLNRIFVDRLKLNN
PTFQQTNTIISNVMSASTTTLRYPGSMNNDMISLISSLIINPKCHFLVTSYTPITIDKHL
SNVQKTTVLDVMKRLLHTKNIMVSVPVRRGMYISILNIIRGETDPTQVHKGLQRIRDRKL
VNFIKWNPASIQVTLAKPSPHVVSTHKVSGLMMANHTSISTLFERCVTQFDRLFKRRAFL
ENYKKEPMFSSADGQGNFEEMESSKEITQNLIDEYKSAERDDYFSHAYLMEKYHGLEKIG
EGTYGVVYKAQNNYGETFALKKIRLEKEDEGIPSTAIREISILKELKHSNIVKLYDVIHT
KKRLILVFEHLDQDLKKLLDVCDGGLESVTAKSFLLQLLSGIAYCHEHRVLHRDLKPQNL
LINREGELKIADFGLARAFGIPVRKYTHEVVTLWYRAPDILMGSKKYSTPIDIWSVGCIF
AEMVNGRPLFPGVSETDQLMRIFRILGTPNSANWPSVTELPKYDPDFIVYEPLPWETFLK
GLDDTGIDLLSKMLRLDPNQRITAKEALQHAYFKESSMGNKISTEDHIFRLKLKTKELEK
LSNRSELEEKKLVTDVKKAIQAGKIDIARIYAEKCIRKKNEKINYLNLSNKLDVLVSRLE
GAHRCASLVKDVSMMIPLIQKINAETNAVKIGNDVMKLENIFDEISISSDLISDTVQTSS
AISAPTEEVDELISKIADEHAIKLQLGPVNSINKHLEEISNMSERIMGKEKTHINLVVIG
HVDSGKSTTTGHIIYKLGGIDRRTIEKFEKESAEMGKGSFKYAWVLDKLKAERERGITID
IALWKFETPRYFFTVIDAPGHKDFIKNMITGTSQADVALLVVPAEVGGFEGAFSKEGQTK
EHALLAFTLGVKQIVVGVNKMDTVKYSEDRYEEIKKEVKDYLKKVGYQADKVDFIPISGF
EGDNLIEKSDKTPWYKGRTLIEALDTMEPPKRPYDKPLRIPLQGVYKIGGIGTVPVGRVE
TGILKAGMVLNFAPSAVVSECKSVEMHKEVLEEARPGDNIGFNVKNVSVKEIKRGYVASD
TKNEPAKGCSKFTAQVIILNHPGEIKNGYSPVLDCHTAHISCKFLNIDSKIDKRSGKVVE
ENPKSIKSGDSALVSLEPKKPMVVETFTEYPPLGRFAIRDMRQTIAVGIIKAVEKKEPGA
VSAKNPAKKMSHLMSLPIVLLKDGTDKAQGKSQIIRNINACQIIVDIIKTTLGPRGMDKL
IYTDKDVTITNDGATVMNLLNISHPAACILVDIAKSQDEEVGDGTTSVVVVAGELLNEAK
QLINDGIEPNMIIDGFRNACTVAINKLNDLSLSFVSKSPEEKKEILIKCAQTALNSKLVS
NHKAFFSELVVNAVYKLGEYMDKSNIGIKKVTGGSCLDTQLIYGVAFKKTFSYAGFEQQP
KTFLNPKILLLNVELELKAEKENAEVRIDNPSDYNSIVQAEWEIIFKKLNLIKESGANIV
LSRLPIGDIATQFFADNDIFCAGRVEDADLKRTANATGAIVQTSLFNLNESILGNCGVFE
EVQIGNERYNIFKECLKTKSVTIILRGGANQFIEEVERSINDAIMIVLRCMGNSEIVPGA
GSIEMQLSKHLRIYSRSICNKEQIVLYSFAKALESIPRHLSHNAGYDSTDILNKLRKKHS
EETSDIWYGVDCQQGDIINAYEHCIFEVTKIKRNVIYSATEAACLILSIDETIKNPSSAD
KGPRNPYAMSGKLEDIGELVLLIGDFHSPMRNLGLPDCFKDLLKTDKIKHVLCTGNVGCR

ENLELLKNIADSVHITKGDMDDEYDFPEDTSLTIGDFKISLIHGHQIIPWGDTNALLQWQ
KKYDSDIVISGHTHKNSIVRYEGKYFINPGSATGAFQPWLSQPTPSFILMAVAKSSIVVY
VYEEKNGKTNVEMSELQK-MFKTTGKLLYSVKVSEPTREDATGAYRNPEYKDKLFDNFED
NPLNNMWEMFDQVAKKYKDRDCLGTRVKVDNKLGPYKWKSFTEIRELILAVGSGLVNTNA
CPVIRCDDTKVTRARFLGFYMPNCEEWNICDLSCSAFNIVTVPLYDSLGVESSKFILEQT
LMQTIICNKACAMNLFKSLDLCERIYLKKLILVENEADTEIKEACEKHQLTIILWKDLVE
SGKKKLQEPRPGNLKDIACICYTSGTTGYPKGVIMTNGNFVAQLASSITGPSRMTLLDIN
ENDTHISYLPLAHIYERIMVLVFCAQGVRTGYYSGNVQTLVEDIQELKPTLFISVPRLYN
RIHERIFNSLKKKSSVVQSLFNKGLEHKIKKLNNNGIPFHFFWDKLVFNKAKKILGGNVR
AMLNGSAPISPDVVKKLKAVFSVPIFEGYGMTETLGPAFISHSTDVNIGHIGGPVPCVEY
RVVSVPEMNYLITDNPPRGELHLRGPAITNLGYFKLEKETNDFIDNEGWISTGDIVSFSE
NGSITIIDRKKNIFKLSQGEYIAVEKIESVYRQSLYISQIFVFGYSYESVLVCIVCPSLD
TIEIWKNQKKINKTDEEVMQMPEYKRDVIDDLIKMGKKDGLKGYEQIKDVYFATEPFTIE
NDLLTPTGKIKRHAVQKKYKEQIDEMYRQLKAAMASWDSYLNDRLLATNQVSAAGLASEE
DGVVYACVAQADENDKWTLFYKEDYEIEVEDENGNKSKKTINEGQTLLTVFKEGYAPDGV
WLGGTKYQFINIERDLEFEGYTFDVATCAKLKGGLHLIKVPGGNILVVLYDEEKEHDRGN
SKVAALTFSKELAESGGMSSFHSKKLALSSHQERPTPLNLGTNNESYSGKRKLLRSLRSS
RNDTCPDCKEKGIIICDNSEGTQICNGCGRVLESRILSEEQEWRNFSSDGQMKSSDRNRV
GEVSDIWFENNTSTTFIKSSKKLQHLNMMTQINKSDQTLLAAFNILKLICETFFLRSNVI
ERAKEITKELQDMEQLKNRINNLNMLAVVYLACREAGHIKSIKELITFDRSFKEKDLGKT
INKLKKILPSRAFVYNENISHLIFTLSNRLQLSIDVIESIEYVVKKATTLITTSHRLNSL
CGGSIHLIVELNTSEEKNLKLPNIAQIAAVCGVTTNTLKTTFKELLSAADYILPSYYLGD
NNSKLATLRQKYLSEDRRRKK--MEKLQLPEWNDIERYFKDPELITAEILFVGLTLCNVF
VMYRLFLDVIPFPIFVTWWQLAQGLLVAYVCGELGKEFPKFAYFPKVEINENMLKVLFVP
SIFYCLMLVLSNYLLFKTPCIASYPVLVSFTVVFHHLTRFVGCGEEYMPLRWKSIVFLLA
AFVIGCFDSKTTGKGVIVWALLYALFSAIFRAGFMQKIMHLVDGKGNTLHNNQHLLGVLL
LPILIVLSGEWAVFGHMPYNIMSLHTWQMWGCLITVGALPFIKNVISNRLVRRTGQGPWR
FLEIISIVLVFFIGMTYNTPSFLGYVAIVCVIIGRSLGAFDVMLNASDYMMAEDERKRKT
SKANYAKSRQGTQASKPFLYSGDNEDDEESSFSSNDSRSYQGSQDYDDKDSVNSSSQQYS
VHKGTSRIDSSSNQTSHAGLSGDESRKASGRGASFQSKAKLSRNYSSKKELVDSQAMGAL
DESTPVPSRITLQLMKQKKKSAFQGYSLLKKKSDALFIHFRDVLKDIVKTKTKVGEEMGN
ASFSLAKAVWAAGDFKGQIIEGIKRPVVTLSLSTNNVAGVKLPIFQVHIDPTVDVLGNLG
IASGGQVINNTRENYLQCLNMLVKLASMQVAFFSLDEEIKMTNRRVNALNNIVLPRLDGG
INYIIKELDEIEREEFYRLKKIKEKKIDKLKDENVEHIPDEDHHESAKRHDNFATSQKDE
DVIFMKRVPILLLLVLFLYKTCNAVSLLDSIKHDLQIVNNHNFNTVVNKFRSEKVFSILF
FQKTNKNIKDVIKNYNDVASKFKGIFTLCIADCDENPTLCESELSLYVPDYKTKNNHHLL
IYPINPMPKFLFNEEMNEANFKKYTYLIPSKIDIIKDEKDFTVFLSKHENMPKVLVFSNK
KKPNYVLNALSNSFNKKLMFCYINNELNGLVQKYNIKTFPTIVILKKGKLVDTYKGKPNF
ISMFDWLNVHSETFVLGGGFDISPDKTVDKPWKFELVPKFTKMSHGDICFKKADKGLCVI
YLKEGDKLEKNEIDMLLSLKEKFKPHIDGRGVNFRYMWIDIATETNFRTLFEVKNYPSVV
VFNPYKRIRYAKMNEDLVATKENVEKLLEKISGGDAKFTMLKGQTLPEFVQDDSDPKANE
KDELMTVDLNVPYSACELKRVKRLELGVLDPEIIKKIGVCEIVNVDLYKDGLPREGGLND
IRMGTIDYKTLCGTCNMNVKYCPGHFGYIELAKPMYHYGFMNVVLNVLRCVCYHCGRLLC
NMNNSKVKYIEKIKVNSLRLKRLSDVCQGIKVCDHSSEEDSLHLNDNAVDNFYNNNLSNL
NVNQQMLLNPNSYSNIFEMVNKEDVDCGCVQPKYSREGPNMFIQFLH-SSEEDIDESKRK
LSAEEALEILKKIRKEEMPILGFDSDRCIPASLILTFIPIPPPCARPYVQYGNQRSEDDL
TLKLLDIVKTNIQLKRQTDRGAKSHVLQDLCALLQFHITTLFDNDIPGMPIATTRSKKPI
KAIRTRLKGKEGRLRGNLMGKRVDFSARTVITGDPNLNIDYIGVPKSVAMTLTFCETVTP
LNYDDMKKLVERGPYEWPGAKYIIRDNGTKYDLRHVRKNSERELEYGYKVERHMTDEDYI
LFNRQPSLHKMSIMGHKAKILPYSTFRLNLAVTSPYNADFDGDEMNLHLAQSHETRSEIK
HLMIVQKQIVSPQGNKPVMGIVQDSLLAIRKFTRRDNFLTKEEVMSLLIWIPYWNHVIPT
PAIMKPKALWTGKQIFSMLLQFEDLERLNDPLGNSKMGATHGSGFRVEDEMSRSGAHLHP
HSPLAIGDTVNSNVKNQFTGNEGTGTNSADGGISGWGR--NVKINLMRDSSTSCKDDNPY
CSVNDGKVIIKNNELLCGIICKRTVGSSSGSLIHILWHEMGPDKTKDFISALQKVTNNWL
EYIGFTVSCSDIIASNKVLDKVKDILNKSKREVSKIVKKAQRGELECQPGKSLYESFETR
VNNELNCAREMAGKVASESLDERNNIFSMVASGSKGSIINISQIISCVGQQNVEGKRIPF
GFNHRSLPHFIKFDYGPESRGFVSNSYLSGLTPQEVFFHAMGGREGIIDTACKTSETGYI
QRRLIKAMEDVMVQYDRTVRNSYGDIIQFLYGEDGMAGEYIEDQIIDLMKLDNKEVKKLY
KYNFDEDSYGKDFYLNNGQSRKNSYIDYNKQNVLNQEFEELLKCKNNICKEIFPDGDVRQ
HLPINMNRLIEFAKSQFPFVPVVGSKVKDPISASRMRGTTKRKLKKGKKLPPRNETKAI-
-----NSEFMSEIKKEYETNDLASAMKGQGAYSGFEPFDENLSEGP-GRNGSEDEEDDDD
EDNDDSDDE---SDSKDQSFINPVDIVHKVNRFLEKLVIIKQINSNDTLSLEAQNNATVL
LKAHLRTYLNSKLLTQTHKISMKGIDWLLQEIEKNFYKSLCHPGECVGALAAQSIGEPAT
QMTLNTFHFAGVGSKNVTLGVPRLKELINIVKNVKTPSTTIYLDDIVSNDQQKAKDILTK
LEYTTLKQLTSHAQIIYDPNTTSTILEEDKTWVDEFYEFPDEDDTQYTLGEWVLRIQLTN
IHVNEKKLTMKEIVCIIYSVFSSDELDIIYTDDNSEDLILRIRVKYLNGEYNFLNDDADQ
AQEDEYEEEEEEEENYNKIGNTFKTKKDVEEEKENDKDDNESVSSNHSRQNSINSENRFG
SASGSASGTDFEDDDGKGNHNRDRPDNNHVQGYGETDLFGKEVGKSGESEIGSEKGS---
--GTEDMFGKSNLKRNSISGTNGGVGTTGGNVPNNNTGSLDMNNMRNINNLSNPIHMKED
TEDTFLKKLMEQCLSTLKLRGIENITKVYMREESKITYDSNNGKFVRSSHWVLDTDGCNL
EHIFCAPHVDYKKTISNDIVEIFEVLGIEAVRRALLKELRTVISFDSSYVNYRHLSILCD
VMTQRGYLMSITRHGINRVDRGPLVKCSFEETVEILLEAAAFAQVDNLKGITENIMLGQL
CKIGTGVFDIIIDNQKLSDANQNLETMMDITSAGFTTPDSHHGITPDGLQSPVAINTLNS
PLPFSPTYNVNLLSPTAPLDSVNGILSPQCLQNYADNIMSPSKNDFNNLDTLKLGGKFSP
TQSPRSPTSVIHSPFSPFDNNNQQPLDPGMLFSPKNNNSSMAMMNYNVFSPKANLDNIHS
PAMYSPNRPLDIFSPKQQTQNNIYSPSYSPTSPTYHLSKAFYSPTSPKNQMDEMNPNKYH
SVLSPAYAVTSPKYSPTSPKYSPTSPYSPTSPKYSPTSPKYSPTSPKYSPTSPQYSPTSP
VPNSPSSPHYSPYAIASPKFSPTSPAYSISSPVYDQSSNARNRHPLSPAYILQSPVQVKQ
HIQDVSVFSPIQQANADEAQNDDPFSPVPYNMEEDEMEQ-MVVFHLDDLEVFFPYDYIYP
EQYAYMRYLKKTLDSEGHCVLEMPTGTGKTVAIFSLITSYQYKKNDDGKFIFCTRTVAEM
EKSLIELKKVIQYRVDTLRKRAQGSVEETGGGKVQVQSTANTTTTQPTNNQNANNGNNAG
NANSGAPPGDILAIGISARRCMCVNERVLEKHEREKIDEECRKLTATFVREKKYINQKIN
QTAHRDRDRISEFIVKNRQHIDIEDYFDIYNTRNSMEEYSEIGLCGYYENYKKEFLFDFI
EPGVYTIEDLKVICKGYKNEKNENIPMCPYFCAKKIIEVAKVIVLNYQYIIDPKVSKSLF
IGRDMNNRVNLYKKDIIVFDEAHNIDSVCLEALSVNIDRNILNKATGNIKKLLEKIETSK
IMNEEKLREDCRRILQELRAQGGEQAQRGGGGK-TYETGAEEGGKTGAEGGGKTGAEGQV
YFDEDLNLIFPRVTNPLGVEDHPSSDEKKAKEDNLIDGLAKLLGNAPSNGV---------
DDPTDLHYSPILMEDIVRNAVIPGHIRKSEHFLNLMRIVVMYLKKYVNIYEITSEGPLSF
LYKCERETKLDTSFFKFCFDRLKSILNSLQIVNVDEYSALNIVCNFCTLLGSYFQGFIII
CEPYPEATGIYDPVIQFACLDSSIAMKSVINKYKSVVLTSGTITPLELYPKLLNFKTVLT
ASFPISFDRNCVCPLIVTKSSDLVPLSSQFSLRSDITVIKNYGMLLVEMCKTIPDGIVAY
FPSYIYMEEVISSWYELGIITSILEYKLIFIETKDIVSTTIALHNFKRACDLGKGAIFLS
ICRGKIAEGIDFDKHYGKCVILFGIPYQYTLSRILKARLDFLKETYNIQENEFLTFDAMR
QASQCVGRIIRNKKDYGIMIFADIRYARNDKKSKLPPWIIKCMDISNVNLTVSTAVNISR
KFLLNMSQEYKETGQTKLSQDIICNQPKCWSLVKSVLNMDDFIMLGRT-----------L
RRYSSLKSLPKEILNQPVTRVTELPNKLKIATVKSTCEVPTIGIWISSGSKYENKHNNGV
AHFLEHMIFKGTKKRNRIQLEKEIENMGAHLNAYTAREQTSYYCRCFKDDIKWCIELLSD
ILSNSIFDENLINMEKHVILREMEEVEKSKDEVIFDKLHMTAFRDHPLGYTILGPVENIK
NMNRENIINYINTNYTSDRMVLCAVGDVEHEQVVKLAEQHFSHLKPQATNMGSASNIDNV
KPYFCGSEIIMRDDDSGPSAHVAVAFEGVDWKSPDSITFMLMQCIIGTYKKSEEGILPGK
LSANRTVNNICNKMTIGCADYFSAFNTCYNNTGLFGFYVQCDELAVEHALGELMFGVTSL
SYSITDEEVELAKIQLKTQLINMFESSSTLAEEISRQILVYGRNIPLAEFLLRLEKIDTE
EVKRVAWKHLHDREIAVAAMGALHGMPQYYDLRQKTFWLRYMEDLKSILWSVEELIYLSP
ENDAFYEKEETEITEKLIALCKSACEINCQAEELISQVRNLFHFLKLIKAGNHKECIVDP
WVRDIFQFILDTLKNDCISSISLVKKELFYYISREKIDEQSGNGNEVFFQVCFTFLHEIK
ILLCCYAFLNMFVQYNWTGPPPPTHSEKERVQTSQVCDEDKNFCQFIQTMKIKNEEFLNS

CLEFLSLEGEIIYAHCELLNAFCLSVILLDLVNNFNQIDQPIMYSNEYSYFEEGTPKGVR
ENSQDDNSTKKKYNETKLLHFVKSKYLWKARIYFIWQRLFASSNDCFYSLKIHVVDIPLR
IFKNVKLLTSDFELIDQEINVEQVEIFAHLRENEKDLQNCDLFCENYMSQNFQICALSNF
SIYLAFYNYVYAYQRLLDVLAEMSQFSYSFTGRMGIKRKYQKIPATILVLKAKRGQEEDN
TSVVLKEIEPLSEYDILRSDYRIIDDKEGDNDTEGEDGHKEE--SEKREDGTGEEPPSSK
KVNEIKSEVEKIGEENIEKTGEAQNGEEIDQKPDECSSNRDEQ-GKTNQSGKVMWKLKDL
DPDTDILEEPYFMDSQNNYFKILTFDEQITLINFCYSMIRFNPHYDEIKFEKISAVISRC
LKCYDVNVESSKEQNKIKNIENNLLQIKYQNWLLHSCILWYKCKCETFRLKTVDRAAAQL
NELLKETYDMKPHGGERVKFLFDVYYPTTWELKKEIGNVMVKTGSVVSAFNLFKDLKLWE
EAITCLIQADRKEEAKELLDDLLEKKKTPSLLCLYGLVDTKNALNYYIDAWKLSNYKYAK
AARFIGNFYYRKEMYNPCCEYLEKALEISPLFPDIWFILGCSYMKIEKIDESVKAFTRMV
SMSNEDSGKAYGNLAYLYMKKEMYRAAKICINQAVKVDNNEWKYWDTYLKLSIMQNDVDS
FCLALITLCQKNKVKQIQPWVYEYISDLIVNDKQTLIPDKNGLSYLDKVITTMGTISAYI
SECDSYWNAFSFFLFIKGRFVDSYEAKVKEIRSLESVAQKCTTKDVVDMLISKQVAAVKF
LYHIIKTHYVEEKRGYFEYQLRNIIESILRQYKDMNQQEVTELSQIMQIIKMSESSVEGN
ELFDGDDEEEEGEKTADDEVNDPDKGEVRRKRKKDDHTSPTKGGSSGLKKRKLSKKRYLS
TFLDTEAQVG-DDEEEEYASSYVDEFEEAKKLEKKKMYEMKLKSGTNHLAQTINKLSQRY
ENEKDIKDTLTDGETLTDEDMSGDEEYFDEGECLTTFDSPKMWLIKLFKNGVERNIAMGI
YYKYMKLKDNDFNIKGVYVSDNLKGYIYVEADSLYMLKRFLLGFKFINLNEMTIVPVQEL
TSIFSMCHSKVIIPKVNEYVRIKRGVYMNDIGQIFEVHEKGIYAIVRLIPRIEYDKYNHS
KKGNNTHLSLTNPMSKGASSQSGKNESYYGDNKLELNNHMVGGDTRNAGMINGQLEMLDE
ALQIKRKKKKERPLKKLFDREEIEQIGGVIEHGPYPRTIKYQNNIFEENGYLLKKMNIKY
LISENANITLTEIRDFNKNNSNEEDINLHVSKSFINKNSLHLFKKGERVKIMKGELYNLI
GTITNVSDNVLTINPDNLAKNFKFLPSDVTKYFLEGDNVTVINGIHKGKSGLISLLDYKE
NVALIFSPSLNTEFRSSIQDLSACEHSSEGLGGINSLNGFSIGDLIELSDRQIGILTYID
KNKHIRVLTSHNTTLHTTIGTITSKRSAIGQVCKDENGNIIQAKDIIQVVRGIHKNKVAV
VNYIWKNKVFAKINKKIEDNGFVVMDSHNCILTGNPNEKKRIVTHNNLFRANTSRRNNTY
QSFIGKTVKILSGVYKGLLGDVIDAERDEFTLLLKIKPKTIRQKKNECAIADSFKDHYTI
TDDRYYKSSRWRNISAKGANGSND--SYDQKNFNGNWSKTSHENHSNSNNTSYNYHHDGR
ASHPTHFNEPHSRGEGMNEGSIKNATRKNNMAEEFMNGQYEGPGSHNLNTDQRYNDGKWD
DVQSTGGINGGVSNTGFTSASNFLDKNNEDKASPLWLVEGIMIKVITPGPFYNEIGRVTE
VMKKSLYTILKIATEKTSFSIVSDAVIPLNPNKINEEVLVVDEGKVVEGIVVDIQNDDIQ
VNTAQGIFTYNVKNIFLFKKQFPMNPNNPLSVAVADSLSAYDIQNESFRLGNVSIEGDKY
ICVKENVNENTQVVVINLQNKISTRKYMKAESVIIHPNDPILALRGTIKNVNTIFLQVFN
IETKEKICSLNLNEYMNYWKWINNDTIAIVCEKNVYHWNIYTLTKVFEKAQVFIDNNSQI
LYYGTDKDMKWCILCGISTQDQGKSIDGYMQLYSCDKKLHQTIEGFIGCFGSFIFDNWEM
KPLFCFVEKKKNSSISRIHLMDIYTNKTETTMPYKIVKEINLINEHISDFPIYMSLNTLQ
GVIYIVTKCSYVYIFDEGTLTQLVKEKISEDNIFICCDRKNGEGIYAVNKKGKIYYITIN
YVNLINHIKLSNFESKDKIIQNLCVKYGYPGCDYISSYKKCINEMDFKKASKLICLLKNT
KLRTQQVLNSFKSMKNASGQLSPLLLYFSVLLEYDKLNTYESVELVKPVVLQKKKEYLEK
WIKDDKLTCSEELGDLVKVLDLRLALNIYLRCGAHNKIISTYCLLNMFNNVLSYLNNFKN
FSFDFVSIFIVIVNYESQYSNEKDGSST-MTDISGSSKNEAMDFFNDS-SNSTNDSRQGK
GSNNEVAVQYVKFLCENNIPFDINNIIDYLLSKKKLQEATSILLDFLKDNKPEHKNLQTK
LFEFNLYNNVQVAETLFQMDIFTYYDKNRIAYLCEEKGLYQRALENYTNLNDIKRVITKS
SRAISIDWIKKYFSTLSDSVCQDILFDFMKGNKINMEVVISICVQYYNKIGIKKIINKFE
ENKNYEGIFYFVSSILTHLPNLTSKSTDHLSYGNMNDDASSILLASDIGSQYSNAENSSN
LKIEDVHFIMFKYIEACVKINNIQELDRICKDKNAMYNPEQIKNFLKDCKLSDPRPLIYV
CDIHNYIEELAEYLYKNSLLKYIEVYVIKVNPNNAHKVIGVLLDLDASEDFLLNLLNNIK
NISNIGNLIEIAEKRNRLKLLLPWLESRSNEGYENIELHNALAKIYIDLNKDPENFLKNN
NFYDKKLIGKYCEDLDPHLAYTVYEKSNGECDEELIHITSKNGLFKLQAKYLVSRQSMEL
WIKVLDESNKYRKNVIDQVIGSTLIESNNAEEITVTVKAFIEKKLSSELIELLEKIVLHN
SEFSDNKNLQNLLILTAIKSDSKKVMEYINRLDNFSAPQIASVAYQYKLREEAFVIYKKF
NYYKLAISVLLDKILHVNHKSAYPCEFSGASSSVYESDSHDAQEFLLDAKLYEDDLNRAI
EYAQKCNNNDVWFILGKAQLKINKIIDAIDSFVKSNNPEAYKEVITKCKENNFYEHLITY
LNTLRDQNLLKDVLVDSELLYAYAKLKKTTEMTKFIGCTNSANLQLIGDRLFKEQEYEVA
KILYSNIPNNQKLTFCYLKLKEYSLAIEAAKKAKSLKTWKEVNFICVKYKQLKHAHTAGL
QLIMHADHLDEIIKIYEKKKYINELMSLLENGLNNERAHVGIYTELGILYAKYKPEKLME
YIRNYSNKMNTRKLIDVCQNEYLLKEAVYLYISYDEYNLAVDTIIKHSPTAYTPDTFMQV
IHKVTNSDIIHKVIDFYIEEHPLNLYNLLKILENKIDNNRLVQTLKKSNNLPLIQKYLED
IQGQNITAVNETLNEIYLQNDDYISLRNSIDEYDNFNQTNLINKLENHKLPEMRRIAALL
YKKNKKYKEAINLSKKEEQYKDAIEIARVSKNNSYVEDLINFFIEIKNKEALCACLIVCY
DILKPDYILEIVWMSGYKDQAMLYFIQVISDYTNQIDVMKKQIEDMQKEKKMNKSAPNDF
AANSMPGQFNYSLNNHLSIMPPQNTYMSSSSQYDKYDMFNNNSHFMVTYVGKEAPFFRAE
AVFGDNSFGEVNLSQFIGKKYVLLYFYPLDFTFVCPSEIIALDKALDAFHERNVELLGCS
VDSKYTHLAWKKTPREKGGIGNIKHTLLSDISKSISRDYNVLFDDSVSLRAFVLIDKNGI
VQHLLVNNLALGRSVDEILRIIDALQHHEKYGDVCPANWKKGKVSMKPSEEGVAQYLSTL
MEDSAAKIIGCHDSLLTDPRIAQDFSAETDDLLTEAENLFKIGDGELAMEKLIAMEKKCR
QAYDGSSTSKIVQFILNKYKLAGDYKKINEYLVFFNKKRGQLKKTIIDMINLCKLWIPQV
ESKEDKLNLINTLCTISEGKIFVEVERSEIVRVLSKIKEDDGNIEEAANILQEVQVETFI
SMDKRNKTEYILEQMRLVLLRKDFIRCHVISRKINPALLKAPEFADLKLKYFMYMIEYHI
NEEAYCEVAKCYEERFNTEHVLADPNLWVEELKCYIIFLALSPFEDQQTKLPNLLKTEKK
KLKEIPVFQNIVEDFINMDLIQWPLPYQDELLNFYIFNDSKFVGGQNRWNLFKKKVMHHN
IHVISNCYCQISLLRLSQLLNASVDDSESFLSELVSNKILNAKIDRLHGIIKFGQKKTPE
VLLNNWSSQIHQILNLLEESSHLIQKERMLHEAKLKRMQLESKNMSLMAEKEAKKEAIEI
IYEISNILNVNLDKETIVILIQLCEYGVSPKVLSHIIIQLKKEKQKFLQNLNNNMGNLKQ

> ***Plasmodium vivax***

MVESDGINIYYGKKYPFVCRTILNAHKSLRQKEG-GDHSAEEKVKFVKDENINTTDKIFA
KSLDQILQTKLYHSFRENNKKEDKQTDVYVQAQYDEWVDYFRSKQLQKEVLIICDHLNRH
LHLNTFICGDYLTLSDLYVYYEMHRYFNVANCYNVKYSKQFRNVNRWFKLINCLVNYSDA
ELSTMLKKEVETNQVDITKDANTMLLKQKIVSTSYVGKLENAEKGKVVTRFPPEPSGYLH
IGHAKAAFLNSYYANMYEGKMLLRFDDTNPTLEDIKYETSIMDDLENLGLKYEKISYSSD
YFATLEEYCIRLIKMEKAYADDTNVEEMRNQRGEGIESVNRQNSVEQNLQLFEEMRKGTE
IGQKNCIRAKIDMQSKNKCMRDPVLYRCIVDTPHHRHGFKFKCYPTYDFACPIIDSIEGV
THALRTNEYSDRIEQYNWFITTFQLRKVYIYEFSRISFVKTVMSKRKLKWFVENKLVDGW
LDPRMPTIKGILRRGLTKEALFQFILEQGPSKSGNLMQWDKLWSINKQIIDPIIPRFAAV
DKKKGVILKLTDLTEDVVEKTRDLHMKNKSLGTCSMFYTNRFFIELEDAQTLAEQEEITL
IKLGNVIITSIVKGDDDTVKELIGTSNFDGDFKTTKKKIHWLPYIPEKLITCTLYEYDHL
ITVDKFENDNKDDWTKFINPSSKYETVVYSEPAISSLKVCDKFQFERRGYFIVDKV-VSN
HLHLIKIPDGKSKNMSIISSKVNPKNLAGTKNMDKQTIVIDNGSGYIKAGLNAYDEPSIV
FPTIVGLPRNSEIKTSYVGDEAIFRESELSFYRPIDHGHISDWDLAQIAWEYAIKCVDQN
RSVENILLTEPPLCSTSHRTKMGEIFFEDFNFQNINISVSGLMSIYATGLTTGLVLDIGD
GVTQCIPVFDGYIEKNSIIRSDFGGEELSMFLQKLICDIGYSMTTRKNFEYVKTIKETLC
FCSLNPPKDQLRDDLNVTYTLPDGDVLRDGYDSVEISHERFYVPEALFNPLICHRDSLSI
VDIVWKSLLLCPIENRKTLTSYIVLSGGSSLFPNLVERLEKEVKNNAPESARSAVKVHAH
ENRAIMAWCGARIFSQPEMREAQEGLWISKEEYEEIGSNIFLIKVRRKDENMESLSKINL
IHKVNEELCKSLGVEDDNLAEYLIYLCKKAKSLEAFCKDVFENGGEIEQSVLKYLYDIIK
PRDGAEEEEEDGVDKEHRMEIKMIEKKNEKMKRFQCLSIKNDEQLTRLSAEGGSDTATGG
GRHRERGRPRDDRHRDRSRSRRSRSRERGRHRRSRQRDRSRNRD--HHREESRQLEGERR
RRRELRVNNIFSGTVSKVMDFGIFVSFRSEGGYKEGLVHCTDILPNRKRVANMREQFQRG
MKVKVKVKALFGEKISLNMAEVDQKTGKNLVSDDEGKPRDKNIASIFDDLHEDYKELKRH
NSDVFKEDPKDTIKYESVIKMQSDYSKWEIQQLIKSGLVYDEQLKREYLNLRNDEKIEDE
EEIIEIEVNEREPAFLKGQTTKAGAKLSPIQVIVNAEGSLARAITTTSALAKERKEQKQN
EQNAIYDSIPKDISRPWEDPKPELGERTIAEALKNIGKNFDLPEWRKNYLHNNISIGVKN
PMPVNEQRAKLPIYNLKKDLMKAIAKNNVLIVIGETGSGKTTQIPQYLHEANYTDKGIVG

CTQPRRVAAMSIAKRVSEEFGCILGQEVGYSIRFDDCTSNDTIIKYLTDGMLLRETLSDT
MLSKYSFIILDEAHERTISTDILFCLLKDVVKRRPDFKLIVTSATLDAEKFSTYFFNSPI
FTIPGKIFPVEILHSKEPESDYVEACLITVLNIHLNEHPGDILVFLTGQDEINTACEILH
ERMKKLESMSPPPLIILPIYSSLPSEMQSVIFDPAPQGCRKCILATNIAEASLTIDGIFF
VIDPGFCKIRKYDSKRDMDSLVVAPISKANAKQRAGRAGRTGPGKCYRLYTEDAYKNEMA
ETSIPEIQRINLGSTVLLLKALGVNDFLHFDFMDSPSVDTLIHSLENLYYLGALDDNGYL
TKLGKKMSNFPMEPNLSKILLTSINFNCADDVVTIVSMLSVQNIFYRPQNKALLADKKKN
KFLMPQGDLITYLNIYNRWRENNYSNYWCHENFIHSRALKRSQDVRKQILSIFERYNYEV
QKNRSRSDSAKYVSICKSICSGYFNHVCKRDAQQGYTTLLTNQQVFIHPSSTLFNKNPLF
VVYHELVLTNKEYIRDCTIIQPQWLIQLAPNLFIPADEKKISKIKLREKIEPLHNYYEEP
NAWRLSRRKGMSEEYDHLYKIILVGDATVGKTHLLSRYIRGSLPSVAKATIGVEFATRTI
PLAVGGTVKAQIWDTAGQERYRSITSAHYRRSAGAILVYDVTKKKTFLSISKWLEEIRQN
ADKDIVIMLVGNKVDLVEHDESKRKVTYEQGASFARENDLFFAEASAVSKLNVKHVFENL
LQEIYNNRMKNNSCSFSTRSSATCESAIQLTKAKSIIKLNDLNENESEDERRTRATCCME
KSKLAKVEKVLGRTGSRGGVIQVRAQFMGDTELSGRFLIRNVKGPVREGDILALLETERE
ARRLRMSDNLEKFSTVDLLNELKRRYSCLSKPDGRYIFLGAPGSGKGTQSLNLKKSHCYC
HLSTGDLLREAAEKKNDLGNKIRNIINEGKLVDDDVVLTLVDDKLKSPQCKKGFILDGYP
RNVKQAEDLNKLLQTNQMKLNGVFYFNVPDDVLVKRISGRLIHKPSGRIYHKIFNPPKTP
FKDDITNEPLIQREDDNEEVLKKRLNVFKSETTPLINYYKNKNLLINLDATQPANDLEKK
ISQHIGGKMEKQCPVCYFNLPDPESAIAPYDTELNYFMWGPGFEWQPEPEVKNILVEENF
EESEQSEESVAGLEELDEKVDRSEAKTMFNEKSSNGKVSVEDASQNARKLGLAPSSLDEK
KVRDLYGDSLTYEQYLEYLAMCVHDRDNMEELIKMFSHFDNNASGFLTKNQMRNILTTWG
DALTEQEANDALNAFSSEDRIDYKLFCEDILQMKRHVYS-ILVACLVFYLKEDLGADMPM
KYADMRALDDLTNLNDEQVNDILGLDIKGAKERISKLFAVIDKNNDKVITEEELTAWSNY
VKNEVFLKQVQVEMKQIDADKDGFISLPELNEAFSQNLDAKEVEKHAEGLLKRFQIVDKD
KDNKLNINEVGLLIDPMKDEELKELEINEILEHHDVNKDGRISMDEFKQTRTDDPHAKKD
DDVALDDFNFFDTNKDGFIDKEEIVKVYFDPSNETGSINLTEVKDTIFEGKPITLDLWNE
KALKLAVTSLTDYGDILRYPEDFKLDIGKNVVLPSTRSRAGVDDDLDQDDASGDKDDGAE
DDTTQKTPTPDELMELLLAKAICIFTFLCVATFGCSIPYLIGLLGKRKNAEHEDKIKGIL
SNLNCFGSGFIFSIVMFHLLPETIMIVSSHKHIVLFNSSDPEIKTLFIFFFVFVGFAMQL
ALEYVLPVDGNMCCVVNDNVKSMLDDNFAKNKPKSKKGEAVNIEMHNVTVNEHQYHHACD
SENFKKKQNIVKFLHVLTLQSFFLTISLAVHSCIEGMIVGTSDDVNFVFINSFCILSHKW
IAGVTVALSLNQNNISKNLKIILLIIFIFSSPLGIIIGHLIKSSGEKVTCVINAISIGTL
LFIGCEILLNEIKQKFSRKIRLAKWLSFCSSCVIAFCLIMVTQHFAPHTHAHSHDHDHGM
QSKFWAKGADDDSGDNVTESSDNEVDDKKPLVSAQAERWAAIDSSSSEEEERVIKSYEGK
RIDFYKNIGNSLNESMGSSDFNQLLKDYESVYKFMVKESSERIPNFAIVYLDKLTKYVET
TFQNNVEKKVLSKNKAQTLNKLKAKIRKCSEFYQNKLNLYHENPEEFKAAMQDEEEDEEE
EEEEDDEEEEEADEEEADEGDEADEEEGGKKKEKTPGEEDSDDWSNSEEEGYVSDDADDK
TKSAMSKWGLKTSEKVEKKKAVKTKKVKKEGTKKEEKATHVDENQSAKNKAYAELLSTKN
LSEEVIRNRVKFVIEKRGRKGLDKHEHINILSKLCELAKTISTQSYIEVLEQMINLEFDV
VSSVYTYMSFNIWNKAFKYIELILDLLIQNENFYLVSINITEEITEEVINEKEKISRSCK
TLISFLAKLDDELLKALLYIDAQTEEYRRRLGKTVHMIGLLYKGYNYVKFAKKMPDLAIF
ISTRILEHMYYKPEALFMQIWNFVTSGKDPLVDANQSDHTNQNGDAKTAEKEPAKGDDHI
SPKQIVEKYVYEIFEHGTKQQKVKALLQLSFHRSLHDEFLEAKELLNVANVHELALSSDM
QTQILYNRNLIQLGLCAFRHGKIFEAHCCLGEICSQNKHRELIAQGVSNLKNQEKTLEQE
RAEKRRLLSFHMHISIELIECVNNICAMLLEVPNLARHTYESKKDIISRQFRRFLDIYDK
QIFNSPPENNKEIILLATKYLQKGNWKLCCEKIFSLSIWSKFNEREKVQNILQEKIKQEA
MRTYIFRYISIYDSFSVEQLCIMFDLNQNVVHSILSKMMINHEIPACWNESSSHILINKV
NPTALQTVAIKLAENINEIMEQNELTLNMRNPKFMFMQERRTQMKDEKSNWSHKKGEGKY
GKGYHQHKNVHYKKNYKEKGMPKGFAMEYGNGGDKIFDLNSISREKLELVEVFYDKMKDT
TNANALYLYALQIFKICNIHNELPRLVVFGQQSMGKTTLLDFIMGGPMGYTSSDTGTKQP
IVIILKPSDTNKIECYLNKKKVSIDDLHEKMKAIMVNLSESIIPKELEVEISIPGGIYAT
FVDLPGIKDDSKSGSELTRKIVRNYVQNFPNDIYILVKKASDDPANWPYHLREFFMKPKP
MGLGLQNKQCIVVGTRALEFLNNELSTIKTLTELHDRVKKRGITDHNDNILSLYLLELFS
IPIEQKEKNDFLTNRISMYSKILNGRKNVLDLLLNKFENDCNDSIKKELLDCFDVEKFKQ
EVNSKFMNILIQQLRKVEVKLEKKKSKMEFYNKKLEELYNKGNILSIREQVKLYIRELVN
IVSNLLTGNYPILNLPNNGEDFLKKYGGTLMENLKDGNELAVELFEKQGLYDENFLNYLN
EYLYKYANENELNKSISSESNDINNILNNNSNSNNNNSS-KRADTYDTLNDMEKKNFDNV
PALVEGQPVRFILAKEESSMFGVVQNTPTDAKSKNILVSFYFRNSNTEEQVQIKSVEKDR
LTVIKAVETLNGDPSFLNGLQVWYKMIRDDGWVGFDKAEIIKVFNNNSKIKDVLIRNLSK
NDEVISIKINDLYMDYVIDENKEENEENEELKENDDDILKRIAGPHTDLKILNQLAITYI
CKWLKYNIAKIEPEKDFSDEVLLQMMRSIHNIVDQSDWKPLVVDLLQANISGNILHLTKL
ASCSAAVALNRVFKAGLGEINRKIKNNYMDENIYLLSTNPKFLDELNQALHNFCKERAIT
CASEMKDIVFEQTYAVHFEIIEEIFDGCKLFEDNFLTPSGVKPTMSIITKNVKQNLAYRN
HQLSLTDIKINKKSKSKELIQEEVKLQFWAIKMLISVPFATKIYAHFLNNILPKNKHLAN
VLDYSIDCESTLEKYIQSKLLNREVNGVLVPIDDKELMSHYNIIDNRDNLLRKLENQKRL
NQYFTIVANSIKLLKNNLSKESTLDFVTKLDFGQNKKMEASNIAQVLYATVDPNINIRSE
AESKLKHAKETNFVQYINQLSNEFCKTQNDPYLRQIAGLLIKNAFASKDNYENEEKARTW
VNFPEDIKNELKNSMLHLLSQQGEKVVIGTACQIISLIAKIELSHNKSSELLHKLVNNII
EKNAYTKKSSTVCLAYLTEDIADICNESKTKYVFTQPDLDLILTAIINSLCEPGEESIHC
ASMKVLYNLMSFIDQNFKTQVERDIIMKTVIDGCKDSERSTVQIAAYECLINIVSYFYSY
LDAYMYAIGPLTWVAIESENERIAISAIEFWNTVCEEETFIDQYELQEGKKNHNIVKQAM
VFLLPKIFNAMITQESEDIDIDAWTLSMASATFLALSAQLLKNDIVEPVISFVEENFIHE
DWRRRDAAVLAYGSIMEGPDTEKLKPLVEESVGQLSEVLRDPSVSVRDTAAWTIGKITTY
HSEIIYNVLGNYNDSNSLYGILLERLNDYPRVAANVCWVFNQLAANKRSSYNKMTNSCTT
ELDDSFCVLCKKLIDVTSREDADTRNLREAAFNALNVVIDNVSDNCLKYMIELLSHMMYL
LTNTYLNPLTEEVKSLQGYYCGTMQFIINRLGNQCKPFLKPIYLSIFRLFEIRTDICEDA
LLACSAIINVMGEDFREHLKTFLNVIFKGLRNVSETSTCRICIEMISDICIPWTFEFEKE
MELILECLWDALKTFGVHDSIKISILTVLGDIATALNRSFSRYLNFFANILAETSKITIT
SGPPESDDWVNYVFELRDAILLTYSNIIYALIDGNEIVKLKVYITNILDLIELILIKEIN
HFNAQNFQNAVSLLGDLVHAYGYELIENSKLTDLIISVYGKIDILSSQGDEKCESCVSKI
KWLKRICNARLGQKMSVYIQLTHDLSGTVTAVTIGDWILGILKQKNIQEYEVFFKKFLNI
YQKQDKDSESNNRSEIFSLLLSASDLVFETLVETKKNNKIKVIIDNKESVKTFNEFYREV
EEYFVILISILQLEFKTVEDLNNATNNFIMAIKNFNVFPELRLKILQLLYNSFSVNFSFR
FPTFIAILQFSSQNNIFHFILPYIKFIDQWIKEWNISSREKRQIYLIIAQELKKLKKYEE
SFKHLNKHVYYFQKEAPEVLNHPTTVNASVELIADAINLNNNIYFHQLLTLDAIQNLQNI
PEHQPIFHLLTIFYQYSIHEFLAFKNNYGEEFFSKYSIDLEVAENKIYLLSIISLFKETK
VQNIQYISEKLNISTLKIEKILVAAIGSGVIDAKIDQINKSVQMKTTILRHFDEAHWEIL
NAQITKYINNVQKILDITTSRSRPMQKMNYLIPVLWCIILSCFARGQESATNFYKFVDSS
ASSTYISEESGSSLYDAKRAIQNNPSYWCSSGNHSKDEEINWTGYLNTKGFIKGVKISWE
YSPELVSISVSSDGENYKNVIPYRRISGNEASFDEIYFFKKLEEVVSVKIGLKNAIHKYF
GIREVKIIGGGNPYFLLLSGITSDNEMCLQVEEGLVNNDNTSVILDSCINALASGDGREL
WKTNSNNQIISAFSDPPKCLSVINLDDLEKNKIVLYDCLRALEDGDGKSNWIFESNSQIR
LQRSGEPLCISQKNIYGNVPGIHDILLNMDVTVDANSTLDDDHNADNTVDGNLNSYWASA
TFADNYEHLVYLILDLNKYVEISRVKVSWEYPPLHYSISASVDNQEYKVIAENLANPSFV
TMDSLKNVETRYIKIVMMKPHPKHGEMGDQFLYGIRSIEVQANNLESIISYCRDAANSDD
ARDKYFVEYITEFDQDLTNKLINLEDDVSKNVNSISDNLSKLEELLPNIETCVEEKKEYD
EELKESKEKANELNDKLSSLVSVNSIHDNDLLRLGMLPGDSSSYPASDCSVIKNVQEIPQ
SGFYWIKPKCAPEPLRVYCDMVSSTSLYVWNGNPPKHSDHLISSIINSVDDIRQHCAEIG
LEPLILRSKSQLNSLILSLKKMGYTLNGKNNIPLAYDYSCDHGSCSGKFHDLVNGNIDLT
TLIYLKASESPDSTKVRQTAGISYDDGSFKFFNLETSDISAIVCSTNSTENDSALQYLNI
NCETTALEDYFNSIVNTNVVVLCPLGCASENFKNKAVYGSRGIYADSSSICRAAIHAGVV
DNKGGLVNVTIESGMDRYEGSVSNNVESISLNKDPAGGLLDIITKDREEDIKEESSIFHH
RTIRIGNLSADCPMDLFQYKQTSFLQKGDTTKGM-RKELPFSEDENRDSIIFHELITDLL
SNVDAIHGVDPSVISIVQDETVRVIEKLKKQLRPADLLSKKQIDDAINLYNITENLALYL
YDLSGKYMYDLERLKERLEELKKEQKVALNFGTFKLNYETMNFSSHFETFDSKLTKNVSS

KWGYADTDIAGHTNSVGQTSSISNREIGEGYYAKLKGLNFYDFEINVSMLSMGSGCSGIV
FRAKDDFNFYLFDVCDREGMKRLSMVENGNVVILKENLAEVNTNNKWNKYKIVTSHANID
IYEVDENSNETNILRSLDERFLSGTVGLYSQIHGQGSFFDNLEVIARPCSELSTGGASKK
QRESSCPYYKENYLGETLPYSFINDVDYEWTFARGGDEHLLCSKNVKVSAAPGEETYNTI
ALLKQRKCSDGYFTLDVNFSNDESNNDEFVKILFNFVNEQNYNALEMTNDGVRIVTHRNG
KSVTLSELTDRERIKKVLAPNEWINIKIHFHKLKVTAVISNNEEELQLDTDIADVDMIRS
GQVGFWVQNFSEVKFASIVLSGAMSNNDGHFIQTQSKAWGTCEDSVHVLNRRSSCQTDIY
PNETKEKHINCIKNFCEECCLHHTQMLDSNEKKQCEKHCKRNDHLAAKMQTLFEKFLNKC
VSLEENKDYEQCAENDAECRNKVCVLCCKRNDPGSSKALKGLSLQKSKDIQQKEVIECQF
QCNRAH---AMSISPIKYESKILSNLASMLKLSEKEEYKGKKFEYYYNVETYGVDFFLDI
CNALGRSNYKPFLSEQGKKEVLSKERLNKDRLNDGKTPKRKMYLQDNKYITEEMEEKMEN
DLLSEKNSNVNPPNFRDGIIAVSRYKAELASISRKSNERIIKTILGHSKDMDSSINGDLS
RSNSFEDNKRYGFFDKHELKHSDSANSFSIDGAKSTHKNAHSKKTTLKINPVTNNLIQKN
APAGNLFLSKNRDRQRRTQSSRKSRHSAGSSKKSGSVKKKGVDAGVAVPGGGHFEEPQTD
APALEVIAQGNEPPRKLSQSETVQHTEWDKKLGGGLTEANTKLTTGEDEPNSKDHQYDVY
QKTSLVKNASNEEDYDFNASDDASYSTDDSNYKRYMKKKTKLQQEGKIVIDLNILNGNAI
NASEKKEKEKKHQERIRMDGVRKSLEEKKMIFCKIEEVAKRRNKRHVKTQVLSEDSKIER
AYFSHKMKKFYNSKSGYFGCGWSNNRTQWTPFIHAPFFDNHHNTIYKNRNKKMYEEIYDT
LLHGRIHPSVRIVELKDHKHPVRLCTPYYEDCYSVVYTGKKILASDDRVIFGEYTGYVAN
NRELSQEKHQYTFALSFNKKVFNDRKSVVFINEIESDEEGAGGSPHGGETNGRIANYDGG
DHCEYAASEEAASNDEQNCQEGAPPSGGVNAK--GCTPNLATPPCHSYSKEK-----KKS
KGMNNKIILPDNYTYAVDSSYMFNEMSLVNHYKTCAVFNNYDFRINAEWQLVYLDGWPHI
ILTSIPGVEINTGEEIFADFGFEWFEKVNDICLNEFIKNSYAYRLSKLNLCKEKNFTGID
DIVEKYNLIKNHLTCNICMYSVNTDSSHFIACSGCNHIYHLACVQKLNAEVNENYDWFCA
SCVKFCINVLNQEEFLAYLEKENHKQLIDLFS-DMCVENSNKLSQLLAPGASDTPLCGSD
KESKGVTGNPNCAKQYDKIRKLLQCRENIIDLVRNKEIINSYLESTEKEMNSLENAE-EG
EDLSFKKDEMKLLLQNSFELHLLVEYKKKIDDLLASREKVDTFVNDEKAKNTMQLRKKTI
NYLLENRKYIEKLVDSMEQGTPIVFPHKEGTILTKGTNNLAVAHKEEVHRSEEE--TTLK
GLKEELPHEHTLAIQKYDPSFGRGGSP--GSGSTEHTNGSFSNSYETILYNKSNDVVKNL
KEIKKGAPFGGVISDAVSCPASSSS----NGGNKNLCFSNMMKLSKKILGFPLLTDFERG
MSTNQPCLPLSDHLKRLSVCTVCYSKHNDLAKAIICRVTKMHFEANYNDGLGDEDMFKTS
SECIQSVIRELANTIKEYRKRELSGAYVQELARSGSSGEIHAVIAGPPLTDDHNDIGAEA
HSPSSS------PPQKPFYGMMSDPPCSDRRPGDTNNPFENNTPPLLWDNK--VNYTDDY
TCKRGEVNSTLGKRPHEEDNKGSSQKKSK----LRTKPSNDTIGGNGDSLKTHEGGGNVG
SCT--AQGGADQLPRSDDPCVDPLPEQHAHRSKDENQKGDKNDIAPGDQKGNYVTLENIS
KASNFIPLLGVELGSTKIQREFTNGTYVGTVTEQIKDEHGNPFFVVTYEDGDAEWMTPCF
LFQELLKQSTNSVDYPLATTFKEVFNPEFKKDLKLSNCSLELKIERRKRKSNCESASNNN
SVSKRQKHAQEENSSRKKKQRFMTNKEEALRLKEIGNKCFQEGKFDEAVTHFSNAIKNDP
QDHVLHSNLSGAYASMGRFYEALESANKCISLKKDWPKGYIRKGCAEHGLRQLDSSEKTY
LEGLQIDPNNKSLKDALENVRKEKAAENMEYINHINTIIQNDANLKAYKEENQNYPNELL
NTIKAINANPMNIRYILSSCDRKISEGVEKFFGIRFNEDAAYNEERQRRMEEEEMKKKKQ
QEKEEEERKKKNRSPEEIQGDEHKQKGNEFYKQKKFEEALNEYDQAIQINPNDIMYHYNK
AAVYIEMKQLDKAIETCLYAIENRYNFKADFAQVAKVYNRLAISYANLKNYDKAIEAYRK
SLVEDNNRATRNALKELERKKEKEEREAYIDPEKAEEHKNKGNEYFKNNDYPNAKKEYDE
AIRRNPNDAKLYSNRAAALTKLIEYPSALEDVMKALELDPNFVKAYSRKGNLHFFMKDYY
KALQAYNKGLELDPNNKECLEGYQRCVYKIDEMSKSEKVDEEQFKKSMADPEIQQIISDP
QFQIILQRLNENPNSISEYIKDPKIFNGLQKLIAAGILKVRMNEHYDVIILGTGLKECIL
SGLLSHYGKKILVLDRNPYYGGETASLNLTNLYSTFKPNEKIPSKYGENRHWNVDLIPKF
ILVGGNLVKILKKTRVTNYLEWLVVEGSYVYQHQKKGLLYSEKFIHKVPATDMEALVSPL
LSLMEKNRCKNFYKYVSEWDANNRNTWDDLDPYRLTMMDIYKYFNLCQLTIDFLGHAVAL
YLNDDYLNEPAYKTLERIKLYMQSISAFGKSPFIYPLYGLGGIPEGFSRMCAINGGTFML
NKNVVDFVFNENKKVCGIKSSDGEVAYCDKVICDPSYVTHLKNKVKKIGQVIRCICILSN
PIPETNDINSCQIIIPQNQLNRKSDIYVNLVSFQHGVSLKGKYIAIVSATVETSNPLKEI
EKPLELLGPIEDKFVKISDLYVSTTSKPADNIFVTSSYDATSHFETATNDLLQIWENLFG
EKLNFDDLN-KADNE-MAVKKQKKTLKPVTKVITINLSKLTHDVCYKKKAPRAIKEIRNI
AGKLMHTKDVRLDVKLNKFIWSKGIRNPPNRVRVKIERKRNEDEDSKEKMYTIVQHVMVD
TYKGLLNECEVNEMATPKGRKKSDKEKIDEPFSEHKDSSKKSRNTLQEDKVLKMRACLSC
RLLRTEAEFYQNGCSNCKFLQMAGDRHRIHDCTTENFNGFMAITTPTKSWMAQYNDLSKY
APGFYALQVVGELPESIRDLKSNYMASSPEVKINFDHIYNNEGVRKDVYLPSSDTFTFLE
ALEEDVETISPTVRVALEMGTGSGYLILSLRELLLKKKKSLDLLYCLDINEKACNCVRKV
TWDNKISNVEIINTDLFSNLRQCKQFDLVLFNPPYVVTEEDEMNKTDIVASYAGGKLGRE
IILKFLLSVYDYVSDEGVIYLLMEKNNRPDEILRDENISGRFSYTQLKKKKTLNETIFIY
KLSKKGMDRYGHNVRADVKKQGVEGAELPILCETCLGENPYVRLIKEENGKECKICNNPF
TLFRWKPGQKSRYKQTIICNMCAKVKNVCQTCLFDLQYNLPVQVRDKFLETSISMPENET
NRNFFLEQVEKNLDTNTYDKINRGNMDLSKLRRRDPYFKRNMARVCSFWRKNECNRGAEC
PYLHKEIHLDKSLSNQNIKSRYTGENDVLAEKILTRYEQQNEGNRFMANNICIHGISEAV
SQVNVKDCFKKFGEIKSIKMIPKDSKMFISYANSQSAKNAAEAYKDGLELNGSNLTVILQ
EEVMPRGANNHRNVNFVKKNNKADAPAPPGMMVPAPPMFFHYQNYNFNPKAPNSAPYSSM
RPSEAEQRKMIKNIFSEYLKSHEGRRTDHEA--LSKVARDCKNNYTSDWRWFSIKCERII
EHELQKKSLREEDRKAYSSWVKEAQNLATFRSFFPKILLRVYSRCDDPNKFFSKLVIQKF
DAVVFFCDASGFSNLAEQLDKRINGTELLGNCLNKFFNILIKIIDCWGGDIIKFSGDAVL
VIWPLRCRRPRGQKSAGSGHNNA-----------------RSAAKRQSQKERDAKKICLL
ALGCCVDIHKLLNKFPTPIENKYLRVHIAITYGKVSFLQLGNVLNKRDYLLSGKPLEEIG
VGESLARNGESVLSHRFYNKVKDKVVVKETAKKKFLLFVKMREEIDMRELKRGYEREDWP
ASRGRSGEPPPPLAHDHFCSLLKSFIPDIVYRKMSIGCNVFLNEIRKVSIIFLSVKDIDT
STMIGVYSAHGIMKLTQKAVFTMEGTINKFIYDDKGILILIMFGLPPLYHCDDSVRALLT
CFRLVDALKSLKLNGSIGISTGRIWCGIIGNKIRKEYTALGDSVNVAARLCCKAGNKEIY
VDENTFNGCKHFISFQKLISIKVKGKNKLIRIYSPIGTINRRSPQLGLEQGGAHGYFTDE
ELQAGAAEF-EVTDFSFLNRNYLLRYYERTFRRRLKRLLGKKDCLYYLRQCAGEDAEKLT
GKDTGKDTWKD--AFHAAYEATPYGQLKAGTPLDYNTHTGPLLLHEYYDPLYCDFTQMHN
AGGVLFLQGNEHLGIFEMITLISRKLSNFKSFKISNMPHSLYINITNPLLPWKMLCNDML
HLWSACNMRKANALMGDEGNYNLLREITHPSYHWYFGCMSSVVDELVIPQLGHSDGGKKQ
RRERRKKEKKKTRSKQNGAVPPPTQQPTDKDTDVGTGDPTDELTCADTQARQPGEGTSKR
KMKRKKKKGGRDRSKERTRRKKQKSNLCTGSCRSAEVHPVFEADAKDNRIGIISSMVYYF
SLHEYTFIVFNYRAGTSLNITGDESAWGICKNIAKLAMYKRAKILSSLQRGVKHWRRHHC
RGCNYCKGFYKTLFPNINDNILLYKSPLCDPTFKGKHKPLIFLFVNGMKNDSIRAIRKIK
EFAEQCNGYLKLEELGREGLYHFVSLCLRVKKEELNKELIDYLHKTCFGIPKFVQYTLFY
LLSKRYIRLYRYACRVEASESAGKAPVQRGTSTNSTNGASTN-GASTNGVSTNERDPTAY
TYINEATKQIITQQNGFRNFQFCDETGSANGSDDRSSDDNEISVVRDLNSAPLVPRLTAH
CMSLIDSLNQEELLLAKLCSFFKQKFNIKKMECIFPRYISRSELKRTIKELIKKGVFQLC
EESRASQPPTDEALFFCIMNFSLKKVLNDLLENEEKKYIQKIYKKYLDMDYRPFQNESNF
DKTRLIIKNVPKYMNEMDLKKHFFKMKGNYEFKITDIKIMKRKKIVKNKEVFESRKICFI
GFISNTDCENFKKSFNNTYINTSKIVVEDAFSPLISKNAQQSRNATSGLRRIGEMQKMKK
DKSVQIVKSEKFVNKTIPIKKTKAGMSATRSHVIFLDDQPLQGEVQEEEKKQKKKQKKKE
KKTTKMEGEKPSESGEEKEEAAEEKALDWLKKISKKEESDKNDRNGQGAQRNRLFEDEMS
TASTQSCDEKEGKKKADNDVEEDDCENMNTGKIIIFNMPPVNEQDVKSLCERFGPVVDVK
VFKNVKKGAAVLNLNEKKNNKSSDDFFIKLLKENHDSFANEKREKQKKKKKKNSDEDGEA
SDWKNKNGSNNESILNSDLTNVKVYAFVNFMFPSACERAKNILNNAIFRGKVLSVKYAKD
KCGDYEYTEKGKNNVFIKLSHDSKTSYKKILEIQKKRNCQNENIWNLLYTDINSSIHSFC
KENKCSPQSILNIRDRNIAVNVSLTETYIINKMKEWIRKEGIYLEAFEQIYKKRSEGEEG
EEPSRANHVVKYKRSDDTIIVKNLSVQTNQKEVISLFKKFGVLSKVSFSPYNNIAILQFE
KAENAKKAFISNSYIRYKKLPLYLEWAPMNLFERKGDSKGETNEGDTPANEEQGAAPKEE
QHHSESESSDEEITHASIYIKNLNFNTKEEDLKKLFEKLDGFITCNIVKSKKAISKKNLE
KGKAPEQKLMSQGYGFVEFKSKELAVEAIKKLTATTLDGHVLELSLSRNRVKKKKNKNNE
EKEVVKEKKKITKKLLVKNLAFQVTKEELRKLFSAFGNIKSVRIPKNAYNRSRGYAFVEF
MSKNECLTAIESLQHTHLYGRHLIIDFADDFIFENNVNEFDKLKEGGVNNNHKCITSEQA

KRKATYEKEKRNQVTESKKRKLMNDMQNIMLNNCGESKCRGYAQHLCDQQEKRTKQRDAN
EQYYQVASNLFASLMSGSPMMSGGGSPPQNARILKPLVQEKVVEIMKPEIEEKIIEVPQV
QYIEKLVEVPHVILQEKLIHVPKPVIHERIKKCPKTIFQEKIVEVPQIKVVDKIVEVPQY
VYQEKIIQVPKIMVQERIIPVPKKVIQEKIVEIPQIELKNINIEKVQEIPEYIPEVVKKD
IPYTQIVDRPFHVEKIVEVPHVQHIYRNIVSPQYRHIPKPVEVPMAHYRTFPIEKLVDRN
VPVPVELQIVQEFLCPKIEARYKEIPVPVHVQRIIEHPIPKDAMNNPFLLPLYYQEDNID
TASTKG-----SSKNCFSFNWNNKQNATKINKGSPSVELLLHNDKHHSKVNPNQDQHMRA
KFVSPSESVLHTTHLRDDDTYHYPNGMHNGASGNAALNQMYRTQHVTSQANFNTAPQDQN
DMFNGSNSMGFTCDSELGGAYPPISALVSPHGEMFSTRSEYDRGVNTFSPEGRLFQVEYA
LGAIKLGSTAVGICVNDGVILASERRIASALIEKDSVEKLLPIDDHIGCAMSGLMADART
LIDYARVECNHYKFIYNENINIKSCVELISELALDFSNLSDNKRKKIMSRPFGVALLIGG
VDKNGPCLWYTEPSGTNTRFLAASIGSAQEGAELLLQENYNKDMTFEEAEILALTVLRQV
MEDKLSSSNVEIAAVKKSDQTFYKYKTEDISRIIEALPSPIYPTIDMTAMELEKTHLINY
FLEICPTVLDCSRKELQSVLAKKEEEKIRRFMGDKNVNLIVIGKEKKEDADNEGSDRNGH
SRSSQEGAEREEEGGAKLEKEPGHVLFVELNINYKCVTKSVAIAFMKRNKENFLAMHDQT
DKGNKTNLSNELLMFVCGQHDCNTPLDLVYLYLSQGFNTIFDAASSAAHSAAAKDFTPNG
YGRKELEGSGSASSTTAGGNKYLTYEGENKLSNVLMTNVSKKLNELLISMKNAQIDLNIP
IINLSVDRRIKELVREYPNVDEIKPEKLKALCESQEFLNQLQKDVTKWVEEIQKLTRLNG
DFKSGSALAEINFWIGYENALSQLENQLRTPEVLLTLQILKSAKRYFATMSFDSDIQLKQ
SKEYVLNVNILMKDFPIDDLLGAQTVQQIIQAVRIIFTHLKKLKNTTKYPLSRSYNFVEA
LSRDLGNTLKKVLSPQALLSMDYQEFDVLISGCMEVFRLWNDEMRVFKDMVRELIKKRSF
NERAPAKMVFEHINLQERLDEIRKFRKQHQKFKAVITKVFGDDRTVGVNLLKDINSAYDI
FLPLDALDLSKTGEELWEKSKLSYESRVNKVESQITFKLRDQLGGAKTSTEMFNAFSKFN
PLFFRPKIRGAIQEYQNSLIQIVVDDLRKLQMVYINGYVKSPSEKVSTIRDIPLVAGSII
WAKQIERKLEDSLKRIENVLGRGWEQHSEGKNLRQSIDNFKTLLSQNKTFEKWLKGVKNA
DKFDMYEKIVKIKKLGVKNFEIMANYDFEFFNLFKEVRYLQSINLRVPYSIKVKADETKL
IYPYALTLQKTFRSYMKICLFMENEAKVVPFNSTITKLVASIHNNVQEKIKEGIGLHWDS
DVLETYVRKLTEVVNTFESMVDEAVRKNSFVLDTLDRIKACEISIEGNEVTKLVKSIQEK
ADELYLEHYRNVHIWIEELNKLLNKILTARLEEVIKAWTGEFVNWSGGNQKGKKFIVKET
THEFKIINQKFYLHPSIDAMRQMWYSRLAEAINTICGIPRVKNIYQKKDRTTESIQIGSE
TGGPTSDVVLDNTYRHIIHFIDKQVYEDAIDSIHTTVEKARKYESMWLQYNTLWKIEIGD
IITSFGEDIQTWKGFMNEIRETEYTFDTLDTEKNFGPIVIDYRILQSKVSSKFENWQKEI
VSEFSKKLSEKIVVLKEEIEKALNDLTLQAEVTSETEITAMHILSMAPKDIIAIINTHDM
EVLSRICNSVYTFIEKINQISTKGAEWDSRCDVLKQSEVLLEKQRYAFPTSWVYIDNVIG
KVETVNQICSYQIKLVKDYFPYIQSLVLEFDANVQNSIQVLFDEWNKNKPSHGNTNSAKA
LQIISAFESRIDVTNGQYEISERIKKLMKIRSEESENGFHVSPNILKEEIACVKGIWDEL
KAIYATISEVKKMLWCNVEPKSVRSTLNGLLDCIKRIPAKYRQYEIFDSVQGEIQQYLKT
YGLLLDLKSESLKDRHWRVILHKLQIKIYYNKLTLGNLWSLNLSSHENALREILNQSQGE
MALEEFLRGLKDTWNEYELELVQYQNKCKLIKGWNDIFSTIDDHLNALQSMKISSYVKIF
EEETLTWDEKLNRLRNLLDVWMNVQRKWVYLEGVLKGSSDIKSLLPQEYNRFKIIDADFI
NIMRKTSEKPKLMEVFQMEGFQKQLDRLSDSLSKIQKALGEYLEKQRNQFPRFYFVGDED
LLEMIGNSKDAKVIQRNVNKMFAGISSFIIRENTSDVILGMSSREGEEVFFEEPICLSAY
KTLKEWLITLEIYMKATLQNCLDKAAKEVLQMDVLECANSSGNNKILEWASKYPNQIVLL
CLQILWTSNIEGDMQGASS-EQNPGGCTFQRSEQICVSLLKFLAVSVVKQRDHRTRQKIV
QMITELVHQRDVIRALIEKNVRSVHNFTWLQYMRFYWDPSKTNSGVNLVIKMADATFEYG
YEYLGMCEKLVQTKLTDACFLTLTQALKMKLGGNPFGPAGTGKTESVKALGAQLGRYVLV
FNCDESFDFTAMGRIFVGLCQVGAWGCFDEFNRLEERILSAVSEQILTIQTSLAQRKNEI
EILNKKIGLNKNVGIFVTMNPGYAGRSNLPDNLKQLFRSFAMIEPNKQLIVEVTLFSQGF
ISAEHLSSKIVSLFELCSEQLSKQPHYDFGLRSLKSVLNSAGNLKRQALLEGAHGGDNRG
SSAKGESPTESVVEMEQNLLLKSVCDTVYPKLVSSDILLIRSLLTGVFPNINIAPFEEKA
LVNEIHRICKSRFYTPEEKWVTKICQIYQIMKLQHGVMLVGDVGTGKSSAWKVLLDALEA
LDNIKGVSYIIDAKSLDKEEIYGKLDNINLEWTDGVFTAILRKILYNSAQSGNATKRQWI
IFDGDVDPEWAENLNSVLDDNKLLTLPNGERLPIPESVRILFEVDTLKHATLATVSRCGM
IWFSRDILSPTLLFKHKLAKLKYGNHDYPRKMDRFRAFIMNSSESIQNDGVRLDDSTTAQ
RTSNDSLSSSHENIRISDHLMDHMKKNSHIFFDENEDELNVCPLRSIASRSINLISDYFE
ENEFVHQCLLGASNFDHVMDYEYIRAIESTCLLLQKGIDNLVVKNEKANNSLTDGDIEKY
ISKWLVMSILWGIGGSLNLESREKFSIYVESICSIPLPAELSRRRQMDDTDSSDAPRTLL
DYEPNIEDGEWHCWKERVEMIDVDRGEVSDATLVIETMDTIRHATVLEGWLNLKKPFILC
GPPGSGKTMTLTSVLKKSSEFDIAALNFSSGSLPNLLLQTFDHYCEYVKTTSELVLRPVQ
PGKWLIIFADEINLPTPDKYDTQRIIMFMRQIYESQGFWKYDSNNNQWNWVKIERITFAG
ACNPPTDAGRNPLSNRFLRHTAILYVDFPGYESLKQIYGTFNRAILRKFGEASHMADNLT
LAMVNFYTKFSETFTIDMQPHYIYSPRELTRWKLALFETLEHCDQLEKRDLVRLCICEGL
RIFQDRLIHKKEKKQTDKIIDDIFKFCFPGIGEADLERPILFTSYVQNEYKEIDKKDLKE
LIKAKLKVFNEEEINVQLVLFDDVLDHITRIDRVLRLPLGHLLLVGASGAGKTILSRFVS
WINGLSVFQIRAGRNYCTESFESDLRSVMKRSGIKEEKITFIFDESNVLGPAFLERMNAL
LASGEVPGLFEGDNYTALINECKAAYGSNVGLDESDIFKKFTKQVQKNLHIVFTMNPANP
DFANRQATSPALFNRCVIDWFGDWPYSALLQVASEFIYNLDLPDKNFHMDSVEEGPISGK
IQYKDNKSYYLSRAIVEIHNSVVRINGVLLKKGSKYNYMTPRDFLDFIKHFLKIIEEKKE
EVSDQKKHLNLGLNKLKDTEVQVAELRNSLAINKKTLAEKDTEAEEKMKLMIEQQAETED
KKKKAEILAKKLDEQFIIIEQRKEIVRKELSEVEPKFREAEEAVKNIPKKNFDELRAMAN
PPILVRNAVEAVAILIMNEGDKSVTWEDARKIMKGQDFINKVLYLDKKMVKPQTSAQIKK
RISHTDWDVERINKASRAAGPLAKWVESVITFLNILETVQPLENEIEKLQEETRIAEDQY
NEQKDIISELEKKLVQYKNDYAQLISQVQSIKQEMENVENKIKRSISLIENLKSEKERWS
ETFINLEAASETFVGDCLLAAAFCAYIGFFEHYERQKLKKTWGEIIKMHHIKHRHDLSFI
EFLSKPSERLQWIANELPSDELSIENAIIINSYIRYPMIIDPSDQASTFLLNQYREKKIV
KTSFSDKNFIKHLESALRFGSTLLVYDVEKVDAILNSVLNQETHKQGGRLLITIGDSEID
YSPAFNLFLTSRDAHFQFTPDLCSRVTFVNFTLTPSSLQNQCLNMILKNERPDIDKKRCD
LLKLQGEYKVKIRELEESLLLELSNVKGNILDDDNVISTMEKLKVQAAEASREVSIAEEV
MVEIENVSNQYLSLAQGAARIYFILQHLGSINFLYQYDLNFFFNLMKDMLRKEELPSSEK
KSNYEERLKCLENLLFSLTYNRVARGLLQEDRYVFALQLCFVKGVINPGIYIDPNHLHFL
LKDHFANNKVEATVASEE-HKIEKGLLPDYTDEQISTLNNLMKHKSFANLKKSIQNDKEK
WVHLIHSTEPEQLISSIISESGVVPNRGADRQYVHSVSNPDNANKEDQILACLRESLIIK
AVRPDKLDKCFNKLINVILGKDFLWIPELSTNEFEKYVKENASGNIPIVLISAPGFDPSN
KVQQLSEKCRVSLSSIAMGSEEGYISAEKIISSAQKNGGWVLLKNIHLSPKWLHQLEKNI
HKATTNHNFRLFLTMEINPKIPPNLLRISLTFMFEPPVGIKSSILRTFSLFLENRNLEDP
KIARMRLYFLVSFLHAIILERRRYTPIGWTKGYEFGDSDLACALSVVDNWLDRASTKIGK
SVIEHIDPSNIPWDAIKKILNEVVYGGRLDNLVDSKILDTFIEHLMNSNSFEADFKLNIC
NSSSAKDLLTSPDLFRHLNDYLKWTSNMSNTDLPAWLGFGKQAEGLLTTRTNFSIISKWN
ILYSKSCSDVYEPLPHAPPVVSPGESSSSSEEEDDGGLSESSTASRVTHLYSQNENVLLI
NQILDSLPQYIPDLERNEENMSNAVFRCFERENHLFRDLLKVIRGNLNQLKNVLEEKLKY
TNKLRALAKDLNSFNVPPGWLVNGDATNLNLTNWVRELISRFYQIIVITTELNEKGKVNG
SKQKASRSRNRNTSDDLTDKKLSIHFIWLGGLFYPRAFITATRQLSASKFRNSLDDLELS
VAIGPSPAELLDDTVHFTITCLSIEGAQWSDAEGVSADHMNKNKKSNKMVNQSSCNLNQS
NNVVTHIVPSTNLSAQHAKQIRLNQMNHTHKSHLLPYHGANYEDKNSKLYNSVSGVGNKN
VLNGASANAHGSASPNSELMKNNKLGCTSRQKSVNHHVHGNHSYNNHKLQQQSYNENFGY
RPTLFNLLEVLANHNITTPDQRVCIRGIVTDFLNNELPHGKIYAYIGAVVGHDILHDIIR
KLEKDPNRNVVPDASGLARIEAAFGLSKSSYDFMNHNSNSTSNNIPNVLFNHRNLNSALL
NNHIYSSNVKTQADLNNLLKYAKSDKYALSCNNLINMNKKKGNNLNRGSASDDEEELMKV
IKKNIETYKMNNIKNMLISSVFGRIKWLKIMNESPHAYLYGHSIVKFGNKLYMFGGTNSK
HKKVPFNHTLTFSLIYYNYKLLPLGGNYPEERDGHTAHLISLKSGLAVFLFGGSNENTYF
NDIYTLDMETRKWSQRITKGKIPLPRDQHCSLVYPAKSEHVRGEKNNLTEGVIIFGGKCL
YNNNIVNLNDMWIFLFQLNMWVRINYSSQESPIGRYGMNFVWSDTNTLCLFGGEYFDSNK
NCRERKLLDDMWTFRLNSATNSILRRVKITGEWKREVYDGKIGCRSNYSSIFITQRHQDF
KSAEPKTIEKLMLLCSGITYMNEGKLKIVSSDEIYVYFFSQKRWYLLRGKLYNEEFIYNA
RQRHVGCFFESKNVLGRANKNPVPCIFIQGGFKKNSIFGDAWLLSLTGDNPLRIHEYDTS
REKISTTQMPLYYFRDTHSISLLYSFCTLQKWLFGAFANLVDNCVHALNPAENVFIKYEL

TPEHDGMLSIQDDGEGLDFNAMNRILRLYGNYRNYESNSLYNETGGASVKRHNFPNTDFV
SSNRIEDYDEGGGGGASGGSGATAEGQEGAGESSNNPAGEEAQATKNNSEAASEGDNQRR
KKKCSFNDEYYHKNYDQEYFYNENANGIFDIKYGVGFKMSFARISSSCAIMSRTINTIGI
GLLSLELMNHCDAKELATPLCMWKLPNKELINRNIANKSEHRHHQKLLMSYTPFNSPSLL
AEQINILGTYSGTRLLYWDFRDDMDFIVFSPANNNIYLSSSPLSMDEIKCGKKKRSAANG
TANDAARKLPCVEDIKLSNREVKRKAQFEEKEKCKRSKVEKDEQAAQVGEKLIKDEQGDP
GEGAAKD------GQSVGKDGGQNGEKCPDASSGAASPPEGAAPKGSKRGQHETEQFQHS
NDMLKKMNLFEYNCHLHPSISKEKYNVSPIFPLWDHPKDSIDYCLSTYLYWLYLRRSTNI
FLQNTLLIPTCMRKDDSSSGANKGKKPRRGKKFKKVEKKERVEKAEDPNSAVTVKEEEKK
GDENANTNDLSLDGVPSEVHVKNESNASSSKEASSNGDVQGGKGNNGKGNEGGFTKLQKK
KLEEALEYGIAQEVKKRKHVHRNETDDSAQEDDYNEDGEEEEEEEDDEEDEEEPDEEEGK
KNANVTEKANGVEGDEAEANGEAATPNGEYTADVENPEKAQETNDPQVSGAADAKNLDGG
ENQKEKRMKKTKNKREKGENAVNNDAKNEENEKARKENYVNVYMETLKIKDECYINNTNK
YTLYNFLRRKLFRMVEFHYLFTPSDYEYGSFIMMGFLNDNTSTCIEVNRICEAGILLYYK
NRLIKRLDAPFIDTAYNLSLSKYPPNPSLYEGNLYKYALTVIVNVPNWLKPSISKQEFVH
ENNYAFLLFKKKLVSLIKYYLCICEDTAKLTKWRESRDLKLKRYLENMQYSNRPSKNDSD
NEYEKAYKIMYTDQGEEGLPHKEEEPQRRDRAAAQIAKGAESRAHEGRRRRENDAPAEVE
EE----------------EEEGEDDEEEEDEEQRDADAEEEEENAEEDQNDEYVEEEEEE
VEEEEEEEEVEEEEEEEEEEEEEEKEEDEEYDPEEDMENSADVKAVADENNGKVTKKKNL
SRLIVEEATNDDNSVVALNTKRMEELNFFRGDTILIKGKKRHSTICIILNDNELDEGKIR
INKVARKNLRVCLGDIVYVKPCPEIPYGKKIQVLPLDDTIEGLAKDTLFEIFLKPYFNES
YRPVKKGDLFLVRGGFMSVEFKVVEVDPDDFCIVSPDTVIYYEGDPIKRDDEEKLDEIGY
DDIGGCKKQLAQIREMIELPLRHPGLFKTLGVKPPRGVLLYGPPGSGKTCIARAVANETG
AFFFLINGPEVMSKMAGEAEANLRRAFEEAEKNSPAIIFIDEIDSIAPKREKTNGEVERR
VVSQLLTLMDGIKTRGQVVVIAATNRQNSIDPALRRFGRFDREIDIGVPDDNGRFEILRI
HTKNMKLSPDVKLEELASSTHGFVGADLAQLCTEAALTCIREKMDVIDLEDEIIDKEVLE
SMCVTQDHFNMALGTCNPSSLRETVVEVPNVKWDDIGGLDEVKNTLREMILYPIDHPDKF
EKFGLSPSRGVLFYGPPGCGKTLLAKAVASECSANFVSIKGPELLTMWFGESEANVREVF
DKARAAAPCVLFFDELDSIGTQRGSTLGDGSGAGDRVMNQLLTEIDGVGPKKNLFFIGAT
NRPELLDEALLRPGRLDQLIYIPLPDLAARISILSAVLRKSPIADNVPIDFLAQKTAGFS
GADLAELCQRAARAAIRDSIDSEEMNKKSKLQMYPNVKENTQSPVQNNEENTVKYEITRH
HFKEGLAGARRSVSQADLIKYDNFRIKFDPLYKTKAGGGNEDFIIDWPDEENNEEPQEYN
VDDDLYSMRPQSKMIFKPLAVKTPLLKEFRKKGLVSIEVADAGSVKILTKDDLEGSPQGG
ETSGTNKQRGNKNGRKLSPSKGSKKRKRENVEQTTKVMKRGEDTHKGFKRRKKKRGRKKK
PT-----GGNEEGVSNDQAVRSA---NPTASPTESPKREKFDIQKVIQNEEHFQRADEVK
YKVHYARWNLRGKINLLYSIMKSLHDANFWKPTEIQKQTLEHSINFKKDVIVVSKTGTGK
TLTFCIPILNNIVKNKLREYRKRGSCVPKLRCIILVPTRELAIQILSHFSNINKYTHIYI
ATIIGGLNLNKQKRIISNKPEILVCTPGRLKYFLLLNDKISYLDKMKHVRYFACDEIDKM
IETSFMRDINLIAKHLYKSVGEKKKFIQTFLLSATLGLSVHLQNEHLAKLLNCVTIRKEQ
SCIINLADAHL---------EGDSSREGGGSILPDGLSLSMVKCERKKVLQKLFYLLKLY
FLSGSAGHEHGQVKKIVIFVNTIKETKELNSIFRFLFFDQGVESSVPKKYRCDLSLKERI
NIFSIHSKQSLKERMQSISKFSQSINHSVLFCTDVLSRGIDLDKCDVIIQLNCPVSDITF
VHRSGRTARNFKTGTCICFITDDEIARWKNSLKKIGLSLENLHELEMVKRISDHEDAKMN
AAILCCNKMVQLQNQIKDNKSKSLLSKLAREAELEDEEGSSDSDTPSGKITNEAILKQLL
RLKKELYNTLYREMYDVNKAVSVEVRSAELGVLTSEELRRVSLGKYQSELLEYEKMNSSG
VTSKMYFDPKYGTIDYRQICSVCFERHENCVGHIGHLEFSLPLFNPLFYKDLCELLGLVC
LDCFQVCCSEDTIFLLKHIYQLRALNEIESSNRIVCKRSYDREHVVGEIERLYRRISEQQ
HCGSEEEVDAMDYGSEA-EGQNCGSEAEGQKYASEAEG------------QHHSSEADGQ
HRGETGPAPAKLQKLKEKISMFKFDPTKLAFQSNYNYEEYLQLTKTLKLLMKKSGRCPAC
KGRRNISARMSQKRDTINFVGVYLQNSFLKKYMQVGRKREQA-------GPAAPGEEDLE
DLVERDEEEAADYGDAADHDDAANPANAASAASAASAA------RESFPQMVRSYKKQNA
KEKSTVRLFSFQVIDLLEKVFRKNDREVMHLLYPFTKRDGHKVFFLYDMGISANRFRVKL
RGVHKKTKMISVLSKFNALLNLCISAKKEIDFEELLERNKRELHLYKEMFGLFSIRMKYK
FNADLMDEDTEITKIDFLRSYDLIYRNKSAYINILFSNLQVALNTFFDSTFAAHLPNAKM
SNQGVREILDKKEGILRKNIMGKRVNNCARTVISPDTFIETNQIGVPLEFAKKLTMDECI
TENNFDYVKRLILNGPDIYPGALSYRDSRGNVFKLSTDYEKRVLLIKQLERMNLKKQCTT
LVLHRHARDGDIVIMNRQPTLHKFSIMAHFIKIFEREKVFRLNYVNCSSYNADFDGDEMN
LHLLQTAHARSEATHLMNSDFLFTSFKDGSPLRGLAQDFILGGLHLTSLETFLNYDEYCN
LLQCALNCLLSRKDCFFFEAGPRARRGNAVKSSKGGDPTNEGNAANTANTAAPPTWKLRN
HSYLDPYAVVLNRTHFTIVTEEPAILFPRRLWTGKQLITSILKTIIDQVAVETYNHRRDN
HFMKNYKGINYYAKAKTDPSLWSFDPKNECEVIIKNSELLQGVLDKLHFGASSNSFVHLC
YELFGPKTAAVMLDCFGRLFISFLQLRGTSLSLYDFILKKDAREEKLKIRKRISFTGFYL
QNLFTHSIAKALNVNVSQMNAYSRGKMNSHSENNEVYVNGLFGLYRRRGALLRDFPKGSA
PLGGSEKLPPSEKLPSEELPSEEQLDVKEEAYFSALLDKEMQHLLARPTSSPASRVERAL
NRMVG-----VAGIAEERPPPRLRAAPVRQLFRSLSEPSFMESPNFAGDKATKVIERIVR
FLKRANCSRRLKVYILFELFQSKSVLKHFPALASCVNDLSQEVSNEEILHHVLSSVSAE-
-RAQ-----GGGAATQE--MLSKFLRLLEDMEGCTQGSGQESGQESGQESGEPYGGDDQK
P---PVNEHDERMIRNCLKKSFPSFLLNDEIGNLSFNGKEHYYEDCVQKGTDEDTTFQFY
NMKDVFNKLEYLIHTYFLQMRGDFDGLIDSLFQPYLCRVSSNTNNLISMENLMNKFLNNG
FSNMIFTGAKGSKVNYAMICGMLDQQYLEGKRVPRMRSGKTLPSFHRYDYGARACGLITD
CFLEGLRPQEYFFHCMSGREGLIDTAVKTAKSGYIQRCLIKCMESVILHYDGTVRNEDNA
IVQFLYGEDGIDPSRISYLDSSRDLLSNYQVAFSKYLLHDEVPRMQRNERLFWGSGESGG
GSDPLETRFSPYAYIGATSERFREKLRHTI-ANDLNLADCYNLPEDFDSLSASLLKSKYF
RSLCDPGESIGILVAQSFGEPATQMTLNTFHLAGTENVTMGIPRLKEIFLTSKLTSKPMI
YVPIKMEARGANDASEMKNYINEIADKILSTYKSICLSDVIYGVGVDRKLILREPAKNTQ
MHTLRVVSNQAEEDQHMHGDVQAAKVMNALQNTQLSFKIKKEWSYEIVIQFENLHHFCKV
NSHITLQAILSKAVNCVLCSVLSKIQESLHSYNIRHVHSFNHEHYDELFSFVCAKMQEEF
NFSMEKFEYKNDEDKINAKLKFMASENY---GGAAGRSGGGASGYADRGEDALSDEVD--
----GNDDRDDRHGGAKRSSNSDSDGNEGDNDGDPDSVSEEDNDYDESEREEEEVGIEEV
GSEEVGSEEVGSEKGATSEEELLDEGDAAPLRSA-RERRKR-------------------
--------ERESQLSDASDVPSTDKGFSSAQSVTQRGRKASTSAERLGEDAYTEESNEPN
RDDEWERMARDFANLAYDYKNNVNLRNESDEED--DQD-GTSKLQSENKKCVNNIIDKLK
CSLLCFVKNISFSPVTWTLKFEIGWDINFFPHAIDFLSYVQTELGKEILFKVKDLNNPKI
LKGKDITHTGAEYELQIEGKNIYKLYNIKDKYIDKTKLYCNDIYTIVKTYGIEAGRLCIT
KELKKVFEAYGIKIDFRHLSFISDFMTHTGDLKSFSRHGLGCFRNVFHKMSFECATNFLT
QGCVHNSVDYLSTASSSLFFGKPIKVGTNVADIVTHIEGGMTKNRLLIESREAKKQNDPD
ITLTHSDYNLYEWQAVIRGPKDSPYEGGRWKLNIKCKSTYPIDPPVITFVTKFFHPNVNF
VTGELCMDILKANWSPAWTIQSLCRAILFLFTEPNADSPLNCDAGWFCGACG--------
------------MSRSEDLDPDMLALLTCAFDEGVKVKGAVEVGKRAVEYFRGDEFVHFL
YTRRDMLKKKFPTLLQNRTLADLKDIEEFSDTFIQKGFIYKAQYKPIKGINEIDENGMYR
RPKWPRRLIMSSKQNFDRTSFYILVHERNKKLQYLMLITLISIVLICCMFPIWPLRLKLA
LWHLSVVFISLMSVIVLIRLFLFIFFWFFGVDYWLFPNLFDEECNVVESFTPVHQWIYRN
DTWLLVIARMFTAVLLAIGIHQLGKTHSISDIGNFATQSFIDIIEWGNKKLAASPENVSM
YKSLDSKATFEVE-FQDDGVVYDENEENYDCLKGCGFQTFEELVRKCFLKCECMEKVIKS
ECYKKKCSRVTKEVLDEAHKEACFGKKDKMATLADTFLQDLEDLEFEEESSFANFGEAEK
GSHVEEAKFQEGDYEEIVDAIEEFLNEKIKKKERKVSELLYDEDFLKMMSSIRDYVMEEE
GSGEDGEADEADEGGVTRRAKKRRRNGEDAASPNATPSKKADEVLIEKCIELIIQIDTEI
LNIHKYVRDIYSTKFPELDSIVYTPLEYISVVSKIKNESDLKSIDFSDILPNTTVMAITV
ASSMTTGINLSDHSLKNCLSFCNEALELNENRRMILLYLESKMFLLAPNLTMLLGSALTA
RLISSVGSLKNLSITSSQNLIVVGSSKKSVLGLSNVRKTFGIGILSTSEIVQSVPDAFKK
KAISLLAGKCSLASRVDYFKKYSEGQYGLLLRENLINHLIKLQEPPPMKQKKILPMPDEK
RKRKRGGKRYRKLKEKTEITELRKQINRLPFGPESNEDFYTFTDQNAALLNSNITKLKYQ
SKQKVNVGRKKNLAVHSSGATGGLSSSLIFTPLQGIELFNPSVANPRADPLENKYFSSKA
QFRKVMEVNKEDICELNKNSYPNFYLADLFYILQLAHISPDEKNETWVKLQDEIKKNNMY
PYYSYVCEELNIPVDQDLYNSLKKNADEEINEIEKKIQEASENFDSVDTKNDVLLKANFF
CKIGDKENALKEYEEVYKKGIGIGVKLDILLTIIRISIFFNDLKNTKKYLEQARTQMEKG

GDWERKNKLKIYEALNYIMIRNFPEASKILIDAASTFTATEIISYDEVIFYVVILGIMTE
ERTVLDKKILNSSVILQVTSSDDDLQSYISSFYHCDYRTFMEKTIKIAMRVKRDKYLGRH
YRYFIRNTRVRAYRQFLEPFKSVTLKNMAYAFGVSEEFIESEISSFIANGKLNCKIDKVN
GSIESNQPNERNTLYLNTIKKGDILLNRIQKLSRVIDMMVNINWPGLLKWSTKYADGTID
TNKRLSKDDIEFLQGAIKDALSQVEDPYEAINEAVKNFEHADEGMVLASAKIVERLVDEY
PEVSRNLHKINAIDPLLKLLNQTNHHILESVLQIFSLALSNNPELQESVFKKNALKTLLL
KLQESQKSVIDKKLITAISALIRHHDEAENKFIDYGGVGFLVYGMQTNMYQYQEKSALLL
KHLVLQNKITFEIFLKNEIMKGLICLANNKNIDETGIQYGETTAELFLALMQNHRHRLAK
SGCLHNLKLLIEDRLAYLRVVQDSASYDVSQEIELFSDCLKLTSMTDGRKIKRKGKPPLL
RHSKLRKLFYIRMYLQRKHREA---RATRR----------QTDGNSAGGAASPPVTFEEL
GVEDWLVKISKTVQISHPTKIQQLCLPLIMRGHNVIGTSETGTGKTICYCWGILQELNKN
PFGVFALVLLPTRELVVQVVEQFLLYGHKIGIKIISCIGGSSLIEQRRGVLAKPHVVVGT
PGRTSDVMDNCEDVRSCFRRLRFLVLDEADLLLRSCYEAKLEGILRGVRGGAVGGRRTLL
FSSTITDSLELLANSFPQEDLLLVNVNRKQKPLKNLDQRYVYVDEIAQMTYLVYLLRKKL
PDQSGIIFTDNSYRCELVYTVLSMLGGFSVESIHSSKEPKKRLAALSKIKNGGCNILVAT
DIISRGIDIPKTSFVINFDFPNDSILYVHRVGRTARANRKGVAISFVDKRDVNSFNSVMQ
TMRGALKPWRLRRREVLQDMFHVGRVLKKAELLLEERADARRDHRRVQRFVHRAVMTRKY
FVSANWKCNGTQESVKALAASFNELDFDPAKLDVVVFPVSVHYELAKSLLKPKFHTGIQN
VSKFGSGSYTGEISAEIAKDLNIEYVIIGHFERRKYFNETDEDVKDKLQQCLKNNLKVVV
CFGESLEQREKNQTIDVITKQVNSFVHLIDNFDNVVLAYEPIWAIGTGKTATPEQAQEVH
KEIRNIVKEKCGEKNANQIRILYGGSVNTDNCASLIKQEDIDGFLVGNASLKSSFVEIIK
SAMMKKKNKEDLYKENKLEASKLRIAIVSTDKCKPKKCHLECKKNCPIVKTGKFCIEVEH
TSKIAYISETLCIGCGICVKKCPFSAITIINLPKDINKDVVHRYGPNTFKLHRLPIPKLG
QILGLVGTNGIGKSTALKILSSKLKPNLGKFSNPPEWRDILSFFRGNELQIFFTKLLEEQ
LTPIIKPQNVDLIPKQIKGNILEIINKKDKLNKKDQYMSALELDHLLDRNVEDLSGGELQ
RFALLISIIGQTTNVYMFDEPSSYLDIKQRISMAKIIHSLVRHDNYIIVVEHDLSILDYL
SDYVCCLWGKAGAYGVVTSPFSVREGINVFLDGFVPTDNLRIREESLNFKLATDQDVTDE
DKKRLHFYNYPTIKKTLNSFTLTINKGIFSESEIFVLLGQNGSGKSTFIRLFAGLIKPDN
VESLGFLESLSVSYKPQQIQAKYTGTVRQLLMSKLKGLYTDPYFNNEIIKPLKIEAILDN
QVLTLSGGELQKVAIIITLAKNTNIYLIDEPSAYLDSEQRIIVSKIIKRFILNTNKTAFV
VEHDFIMATYLADHVIVFDGQAGVNTVANTPQTLVAGMNKFLKIIDVTFRRDPTNYRPRI
NKYDSVKDKEQKLNGTYFIIDEMSILCTISGQTPEEPVVSKTGYVFEKRLIEKHILNYGI
CPVSGEVLTLQDLYPLKNEQVVKPRPITASSIPGLLSILQTEWDAIISEMFTLRTHVNDI
RNQLTHSLYQYDAATRVIAKLLKEKNDYKEEVKKLRNQILSIKSGNDLNEFEVGLSEDLL
NEMQEVAKNLLMTRKKRNIEHVSSAEQWKEVTNTNEFDVHSAAIPGVTCLSLDINELKYS
YYDDHKNHSFFSGGMDGNVYYVSLEQSKVLAKLQGHLKKVTAIVAHPKYSLCISGANDKT
VRIWKGDVETNEFATAHVIAKHKDQITSLALHPMENYFVSSSKDNVWILNDLETSRSIKI
CKDTPSPFRQLAIHPDGIMLGIGCEDSNIHIFDMKSQEYKASLSGHTSHVSYISFSENGY
YLASCSKDKTVKLWDLRKAQCFQTIDVEELPRSICFDFSGKYLSLAAGNDVHVFNFEAKT
KAVLVNTLSAHTDAVTQTCFGSRTAYLLSSSMDKTVKVWSLAEETHCYNGNIKDGLFHGH
GILMYSRNEKYEGDFVYGKREGKGKFTYADGATYEGDWVDDKIHGKGTAKFVSGNVYEGE
WDNGKINGFGILKYNNGDIYEGEWLDGKMHGRGTYTYEDGDIYVGEWKNDKRHGKGCVKY
KGSENKIAETYEGDWFEGKMQGKGTYFFADGGIYEGDWIDGKMEGKGVYKFLNGNKYDGD
WSNDMKNGYGILTYVNGEMYEGYWKDDKVHGKGTLTYSRGDKYIGEWKFAKKSGQGELIY
ASGDKFKGEWKNDKANGFGVLLYSNGNKYEGEWVDDQRHGFGTFTCKEDGSVYAGHFAFN
RKEGRGTLTFVDGNVLEGLWTMGVLTKVSKFQLAPTSPWHDPDLMKLEEVKDIQKIERIG
AHSHIRGLGLNDCLDARYCSEGMIGQMSARKAAGIVLRMIKEGRISGRAILLAGQPGTGK
TAIAMGIAKALGEDTPFTHISGSEVYSLEMSKTEALTQAFRRSIGVRVKEESEVIEGEVV
EIEIEKFNEKDMNNVSKKVGKMILKTTEMETLYDLGNKMIEALQKENITAGDVICIDKST
GKITKIGKSFARSKDYDAMDPNTNFVQCPEGELQKRKEVVHTVTLHDIDAINSRTQGFLA
LFSGDTGEIKNEIREHIDMKINEWQEDEKAEIVPGVLFIDEVHMLDIECFSYLNRALESE
QSPIVIMATNRGITHIRGTDYKAPHGIPLDLLDRTLIIPTYPYKHQDIMKILEQRAEEED
VEIDEYAKELLCKIASESSLRYALHLITLANLVSKKRKATEVTVQDVRRVYNLFIDVKRS
TQYLIEYQNEFMFSELPKEDEGANAEDSYEEKREIQEKKSSNDSADCMAYIYSNLSDFWT
SDDEDDEEVDGTEEDAPSAANAANAVKRSGDDQNYQLSRYDEDPFEKREDVGPNDTGSSP
FTNVMESGLNFNINEVMNGLTNNVVDNFSIKSMIEKYDISSFMYNDNLLKYCETGVAIND
DSACGEKLSLNENFHFSCFGPVEYVSPPKCGVRGGADAKG--AVEGAVEGAVEATIEATI
-----------EATPPGAAPTQPEVPLTEEPLTGEPLAELPTGEPRAGDDKANVKYIRKK
WVIEDNHSDVSNFSQNDLLLSYDFELDDFQKRSIKHLNNFKHVFVAAHTSAGKTLIAEHA
IAMSIKLNKKAIYTSPIKALSNQKYHEFKNLFKSVGIITGDIKMNVHANCIIMTTEILRN
LLYINDNIINNIHCVIFDEVHYVNDNDRGFIWEESIIMLPPHVQILLLSATVPNYLEFAD
WVGFTKKKEIISISTKKRPVPLLHYIYAYDTIFQIMDEKNKIYSSAFKEIYVKVREKEQG
GAHGGGGHGSGHAGHHGGQAKHAPSAKRNSHDGKNKQPGG-GAGAPSNNQPMAYNEYCKQ
KRRQKLFANEANMKTEIQKLQALIKKLEQDNKLPVVLFCFSRIKCETYAKSMPHLNFLDN
KHKSKVHLFIKESIAKLCTQDRELNQIKILTKLLEKGIGIHHSGLLPILKEIVEILFSKG
LIKILFATETFAMGINMPAKSVVFTSIYKHDQLKKRILTSSEYTQMSGRAGRRSSDKYGY
VYIYCADKIPDQVQLTEMLMQKAVSLKSKFKVTYNMILKLLINKQINIEKMLFSSFLESC
RAVQIPLFKKDLKRKKKLLQNIKQVQCIYANDVKYVSPIESYVQIDCKLKHIGLSLHRKL
FTMKSSNAFVIGRVMLLNNIGTLHSSIYGVYLGCDKSSSKKNEKVDFAQNSIFFQHSAAG
EESSERFFFLFILPDFMTYEELLRKM--NGVKQPGQANRSSVKQSSTNPSPGQANHQNVN
MFENYKNLFSKQSAPTGVQIVHHNSFDTDMQKKHFVVCSNVPIEHISIITNAVVVLPNVK
TTAILNNPKNMLLYSLELDRLIEKGNFEPFVLTKMLKSLKCEFYSVLINQADYLEALKKS
KCYSCNMREKHYELVCKRNNCINDIENIERNINAKSLNLYEDLEGRLDVLRHFSFIDDEH
NLTVKGKIASYITLTDEITLTQVIFENLLNNLNPPEIAAVLSCFVAPEKKIEESPDLTVN
LQDVKMALTNIHSQFEEFYKIIRLKISSEEHWKLCSFKIMFIAYKWALGVSFAELLEQSE
LEEGLIVRSILRLDDLCRKVKIAFLYLGNVDLAERLETTCTLLRRDIIFMTSLYLQSSLM
VFHLDFFYKGNRVNYVSAYISSSGDGGQNKPSFFGKRKIDANGVSNRSAVSYFKKKFPQV
YQQNRQASEKKDPETTSQLQQPQQSDNQGVRDTMANPPIEERLANLKKVMQENNIDVYIL
INSDEHNSEIINDKDKKIFYLSNYSGADGILILTKDKQIMYVNALYELQANKELNHDIFS
LRISKITNRDEIYETIASLEFNNIAVDGKNTSVAFYEKLKSKIESTYPGKTVEEKVIYEN
DMNQIVKNENINFLILEKSLVDLKDYQVNNKLVFIHDRKFNGACSGEKLEKLRQIFFENK
NVDKLLLSELDEIAYILNLRGFDYTFSPLFYGYLYFEFNREKDDFEKMILFTVSKNLSES
SIRHLNTVNVTVKEYETVVEYLRDNVSSKTMALKVGNEVGAVKAPPSKDSQKKYEISLSP
YINLMIYMLFNKDKILLEKSPVLHMKAVKNDVEIENMKEAHVLDALALLQFFHWCDEKRK
TKELFNETEMSLKNKVDYFRSTKPNFIFPSFATISASGPNAAVIHYEVTDSTNAKITPGI
YLLDSGGQYLHGTTDVTRTTHFGEPTAEEKKIYTLVLKGHLRLRKVIFASYTNSMALDFI
ARESLFKHFLDYNHGTGHGVGLFLNVHEGGCSIGPTAGTPLKPAMVLSNEPGYYLENKFG
VRIENMQFVISKKNTDNTEFYSFEDLTLYPYEKKLLDFSILTAEEIRDINEYHETIRKTL
LPRLKQNPSEYGEGVVKYLMDITQPIAINMLCVLLTNTCNNLHYKPSARRACSVAHSNDS
QLWRRSTIKVSKLAFKRMACTQPQGDLMERLKQKLVVGENPAFIQERLAKYNALKEKKRI
EREKSDPQLTRSITVQLLDGSVREGQCNVTTPFQIASSISKRLAEDSLVARVTYVERVDL
ELCDVEEGEQQQDQTQEQPR-PVLWDMGAPLLGSCKIEFLNVEHEEAKKAFWHSSAHILG
SSLEKLFGGYLTIGPPLKEGFYYDIYLGDFSITNEHYKKVEDEFSKLIKENAEFEKLICT
KEEVMDLFQYNPFKLELIRTKIPEGKKTSVYKCGNFIDLCLGPHIKSTGKAKAFKVLKNS
AAYWLGNKNNESLQRVYGITFQKKTELNDYLNFLEEAKKRDHRNVGKKLHFFFFDKDTSP
GSCFWLPHGAKIYNKLIDFVRREYRIRLYDEVITPNVFSCDLWRTSGHYQNYKDCMFIFN
VEQKEWGMKPMNCPGHCVMFKQLNASYRSLPIRLADFGVLHRNEISGSLSGLTRVRRFQQ
DDSHIFCTFEQIKEEVLGTLHFIFFIYDLFGFKYELFLSTRPKKFIGNISTWDFAEQALK
DALNSANIPWKLNEGDGAFYGPKIDILLKDSINRTHQCGTIQLDFQLPCRFNLQYKNRDF
GALAEGGDGEGAAAKREAGAEVAAGAAGAADATDNGANTAAPAAELLKRGFDRPVIIHRA
ILGSVERFVAILIEHTAGKLPFWISPRQAIVLPVSDKFNDYAHYVHQTLNNHLFDVEIDT
SLNTLNKKIREAQLNQFNFILVVGEKELTTNTVTVRNRDDQNNHEVCSLEELIARFGKLL
EVNSKPFNRVEPFQRSWCAGNRGRSGAGDGERGHPGDRACGRTALLAAATIGGLHLLLSG
AEEGEQTTNKKRRTLFFPQYMRNEIASCCGIMAYLGNRDASKILFDGIEVLQNRGYDSCG
MSTISRSSTLKTTKYASSSTSDAIEKLRGNYSASHKNDNIGIAHTRWATHGCKVDENAHP
HMDYNERISIVHNGIIENYRELKTFLLGKKIPFRSNTDTEVVANLIGYFLDQRESFQNAV

LSAIRQLEGTWSFCIIHRDHPDEMILAANGSPLHIGFKDNEIFVASEHSALFMFTNEYIS
LKNGEILSINKDKINDLKMEKKVGSIPEVVIQKTPHPFPHWTLKEIHEQSITLSKTLNNG
GRFSILNSSVKLGGLDPYVEELSKIENIILIGCGTSYYAALFAKYVMQYLSCFNTVQVMD
PIDFNVSVVPKEKEGVIFISQSGETRDVIKACKLADDLNLKKLSVVNSVGSTIANMTGRG
VYLNAGREVGVASTKCFTAEVSVLTLIAIWFFQNKKGHSSHSKVSSLINSMHRLPLYAGT
TVKSCEAKCKQLATKLTNAKSMLIVGNGLSYPIALEGALKIKELTYIHCEGFTGSALKHG
PYALLGGEDNLPVIMLVFNDPSKNVMMNTGEQIKSRGAHIICLTDDEDLCKHFADDIILI
PNNGLLTPLLAVIPLQMLAYYTAVARGNNPDRPRCLAKTVTVAMNDSYDSLFKILLIGDS
GVGKSCLLLRFADDTYTDSYISTIGVDFKIKTIEIEDKIIKLQIWDTAGQERFRTITSSY
YRGAQGIIIVYDVTDRDSFNNVKNWIIEIEKYASEDVQKVLIGNKIDLKNDRNVSYEEGK
ELADSCNIQFLETSAKIAHNVEQAFKTMAHEIKNKSQLENQQKGRVNINLNAKPIKDNKK
KCCMPRIRTMNSRKPPEGWSKVESFLDEMNKKMRSLENEDTSKKRKSEILWPIFQINHQT
SRYIYELYYKRKEISRELYDYLVQEKYVDGALISKWRKQGYENLCCLKCIQVSDSNFSNT
CICRVPKSNLGDRVLQCVNCGCRGCSSGDK------------------------------
---MSGTES-----GHVQAE
GKSALESKESNNSECKSDHCDYDYDYVVIGGGPGGMASAKEAAAHGAKVLLFDYVKPSSK
GTKWGIGGTCVNVGCVPKKLMHYAGNMGSLFKLDSTQYGWTCKDLSHNWGKLVSTVQSHI
RSLNFSYLTGLRSSQVKYINGLASLKDEHTVAYYLKGDMSQEETITAKYILIATGCRPHI
PEDVEGARELSITSDDIFSMKKVPGKTLIVGASYVALECAGFLNSLGFDVTVAVRSIVLR
GFDSQCALKVKNYMEEQGVLFKEGVLPKKLSKEEDKVAVLFTDGTTELYDTVLYATGRKG
DIAMLHLERLNIHVDKSANKIITNEGSCTNVPNIFAVGDVAVDVPELAPVAIKAGEILAR
RLFKQSQEIMDYTFIPTAIYTPIEYGACGYSEEKAYEAFGTSNVEVFLQEFNNLEISAVH
REKHVRAQKDQYDTDVSSTCLSKLVCLKSEDNRVVGFHYVGPNAGEVTQGMALALRLKAR
KSDFDSCVGIHPTDAESFMNLSVTRASGLSFAAKGGCGGGKCGMENEKHENMLLAIARDF
QSIDNLIETFFSFLEHKTDYFHVMLNEDDIGTLAKKYEGTLEKDILNNNKCGFKAHSREQ
NLMKSFRKHQLNYIIKKQPYLIENEDMKNKYLPSCDELKKLNTSKFCEVKNKQNDVQKKV
EPGAMPVTVTASETHISTWNGGQTEKYLWNQALNEINIEMPLHTEIKTSEINVQISNKSI
KVYHKHELKLEGTFYEEVNKQECMWNIEDKKKLIIFLEKKRENWWPCVIIGDPEIDTSKI
ESKKNLTDFDEKTQGEIRKFLHQQKM------------------------------MKPA
NTREDATHKFSNNNAVDEIEEHLYSGPLKIEQLLAKGFVKRDLELLKEGGLQTVECVAYA
PMRTLCAIKGISEQKAEKLKKACKELCNSGFCNAIDYHDARQNLIKFTTGSKQLDSLLKG
GIETGGITELFGEFRTGKSQLCHTLAITCQLPIEQSGGEGKCLWIDTEGTFRPERIVAIA
KRYGLHPTDCLNNIAYAKAYNCDHQTELLIDASAMMADARFALLIVDSATALYRSEYIGR
GELASRQSHLCRFLRGLQRIADIYGVAVIITNQVVAKVDAMSMFGGHEKIPIGGNIIAHA
SQTRLYLRKGRGESRICKIYDSPVLPEGEAVFAITEGGIADYEEKMALKSINISGNFDWC
PFEEHKNYLACFTSHNLLYSNGSNLNNYVYLLDISLNSEGRNLEIVSKLNFEEALKSGQS
GKKKGTNEYVTSFEWVNSSSFVESEGEGALRKGIIVGGLTNGDVILLNAQNLFVEKPSHE
QFILSKANVHEGAINCLECNKHKSHLIATGGNDGQLFITDIESICSPTSYDPYLDKNNLQ
KITCLNWNRKVSHILATSSNNGNTVIWDLKIKKAAVSFRDPHSRTKTSSLAWLANQPTQI
LVAYDDEKSPCMQLWDLRNANYPIKEIIGHSKGINNITFSTIDTNLLISSGKDTTKCWYL
SSGNLDVFNEVNNSANNVYSKWSPFIPDLFASATSMDTVQINSINNGVKMTSKYVPSFYG
RAAGITFGFGGKICCFGNA----------AEDSGGVS-GGGISGVSSAATAATAGDPPES
KFPIKCHIYSTEMELISEADKFEKYIASGNYFEFCESKIAKTEDQHEKLTWRILQSLCAS
QKEGIVKHLGYDMSEIVQKIEGSTGKQPGFIFDKLAEEEKEKLSASCTQLGGDMMGGSAA
GLVPGAPNYEHMSGAYMTGGAVGMDPNLAASTAAAMVIDNTQGFNSSFDVDPEKFFRELG
EKKENEETTQEEEEKQQKGDKTAKKKGKDKGKKKGKGGVTGAADEEGEGEGEEESKEKTQ
MSENTNWDSGIESIIKECVLVGNIETAVELCLHKNRMADALFLSSFGGEQLWYKTKTLYI
RKQKSTFMRSLNYILDDQLELLVQQIDLSSWGEALSILCTYALNKANFNTLCETLAKRLQ
NEKFDIRAASICYLCASNFDQTVEIWNDMPSTQNCLLSVLQDMVEKMTVLKMAIKHDVFN
AIMNRKINQYAELLANSGRLKAAMTFLCLTQEDQTEESLILRDRIFNSGAHMLCHQVKPP
MSPFQVLHVKSAAGGMMHQGYQP------------YNQNAQFKPNVMGNHGPSKLFPTKS
ATSMPPPM--QPSMQSAPPHHMGYSAMPPNKFNTQVINAPPPQRASFASTSAKNFPLGNV
SAVKPPSMSTMSPSFMMPPSGPPPS-----SASPPSYASMSNLSNFSSPPGMKAEQDDQK
QGGPMFPPQSYANNQRRMQGGSAPPA---QTNQMSN---RSFPSMQNVSPPPGNRNVPVA
PNNSMTFQQDNPFLKRECIDQQA--PYGGAVSPP--GMQPSSFGASFTNKVGLGGQPGG-
PPSSGLSTTSPIAGALTVTPGMPVPWPIPTTTQQLGSTTTSTANENKKIQTATKEQNGVF
MGRGNVDNVKKTISSFLNGYISQEPMKKKAEDVSIKVHELFDKLDTGSFNEQINESIISM
VSALNANDFRAANKVIVDLSRNLWDGSNKSWIMGLKCIIPKMAIRVQFENSNEVGVFSRL
TNSYALIAMGGSENFSSVFESELSQHIPLVYTTIGGTKVIGRVCVGNRKGLLVSSICTDQ
ELLHLRNALPENVKIKRIEERLSALGNCITANDYVGLIHTDIDRETEEIVQDVLDIEVFR
TSIAGNLLVGTYSYFTNNGGLLHAMTSSQEIEELSELLQIPLITGTVNRGSDLIGAGLVA
NDWSAFCGMDTTAIELSIIEKVFKLNSIEDANIEDNFKYKSSIIQTMIMLESLVEKLLNK
FLAPYVEGIERNLHLGVWSGNIVLENLKLKPQITEILDLSFKIIHGNIGRINIQIPWSKL
GKSPVCVLIKNVHIYIKPRSYKKSENVIIEELRRAKMHRLEMLEEEISLIKQQKNKEKSL
EKSTLIFKLLNKIINNIHVDIQDILIHFEDPEKNFSIGFILKSSSVKNCLVKDKNAAGAN
ASEEYKKLNHIIEFKGLCIYSNSNIRKKRRRRKGEKGTTAGQTCRSASEGPPKRQATSAS
SDSLTRGDPDNNRSSLSDDEDEANDVSGRVDDMDLSSKVKDDASFSYDQNEDLTFESKLG
SADAEEDQDSKDNYLKRTLTQISNFLKDDDSKILNYYNYLIKPFDLVLPVEQSSNKKELK
AKLEIGEKWEGITLTRTQITKIIEIMNEANKSRNQTNKLLLKYACTVKLDIESLRNETKN
EFMNLYNKVLAEKYNISTTELTSQEMNRLQILYDVVGVRHLAKWRLQCKNTLEKIIEEKN
LKKKYLYDSIYKQQSWWSWVTGNKKDIENKVQSILNSEQDIINEKELYILQEAMTNEDNY
DVVLPTKYDFQFKLANFSINVYDDCRKGRRVQRLRGGLPERGRVNADTKGTSNPSPLGED
ASNKCYGNHFGDPLGEEISLSGSEDTLLNSSPGVSSSNSNYMKKGTNYQEINILSINFYQ
IYSSLSLQSVVDHNDNDNFQWKFIIELQNFVAKHKSKVFMEFRTNKNLFPYNVSSTGSLY
QNPIYYSLLHSQSLCAYVEINHLVTEKGNTLSTLLRLNPLELYVSPLLIKNILSFAFPLI
DIVNKKHKSVLRRGKGRGSLADAAGEASEEPPRSAADTSEDDEEDDEALLHIKKMEQTEL
IEGLKEKGENVYNRAVQHLPELFEFYIHICGPILHFDNLTNGIVELHLGNLIAKTECPCT
YHKFNLIFEFNETQITCLKASLRESQKFYILQPIPVKVYVEYDLKILKTNIILDGIFFQI
NPDAVSIILAVPTSITRYLTRFYSKNKKEKDLLKGRKKTSVEKKVSSKGEGNICLQIPSA
EGASKEGDHANDHAPNEVESKANLTEANINKPEEESFLYDIDFLIKNSAFSIKNGKNREV
LKYEACGISYKNYLQKKKKIVKVEIEQLWICDPSNRQPIFFTLTKNVNSKDIFLFRSLST
YLTNDKLVGTLRGDNTVSREASNEENGSRGIFQSAQRIEDGGVGDGDDDDFMDAIEEKQL
SINLQILQKQNQINRAETHVNLIISDIELHWKYKTIKQIFKTMKEYKKKLQYGIEKDMKY
IKNKLKNEEELKNYKMFISEHTLRSVQETLNNVKNSLNILDIESAKK-SEAELENDETAC
AGDGTHVGSAQMGRPPVHPGGGNLGRDVHLDGSSVRSGAQSHSGDDSEGDILSNGLKNAL
PKLHGQKNSYVKYFFNCYVKNASLAFWQKRKIFSKIQVSSIYYENKVYLNFDQKMFVNIE
KGIISMNSKNIISNNINDYTYDLFISRRVKPSEDGKGENVVDNLSEEIKKKKKKSRQKRQ
DLFVGKIKVYKDRRNYNICLLCDISSIYYIFYLRDLKLFLEYLDDGILNVFISKSYKKVV
QVAQAKYFLFHCTISDPVVIIPEDKNIIYNYRGEVKDVSKCSKASING-------GGTAS
RGGNS--IPYIESYLKFHLGTVKFKNSYTVNYTQGIVQEQRRALRERRRGRRRARGGAAT
QGGATPTRRKGAPCSDKGGSSTASGGGKYDFTLYVDLLDMESRACENGGSTNIEGNILQK
VNIGLCLVSSKEGVFIYFSGSDFCLDLTVFQLAFLLDIINENFCYKGYFPMCFICDRGVN
IDSMLNAEWFRRGNINLGNLAAEEGKAAEASEPARKHGLKLYLYINLESLKIKTSFDGNT
PVAIITFQNISISFHLVLLDFYYIYFFDLYSNSLYIDDVRKNSINYYKRVAYCCIENEAS
RGKRKRGGKEEKMRVNFVRENAEAKISSMSVNSAAGGSNPAVGSSAAMGSNQVAAPDYKS
LLQYMFESNVFLSEEKKKKRQKGIKIRINSFIEDILLSIELDDAYICCFFIIFMDIYKFL
STGFNLSTLHLYPKPSPYIVSAKEPGRKKRGKNIRIEENVFKSEAKGETEITPPGDDKNI
SRGSSMNSSKIIRERNINEILKLDNKPIHISFKVNNGNFIVFTDLENVAHPILMWSNNFV
FSFSLLNKCIIFRKIYAVNSKIKRINYVSSFSSDESRRRKENRVRGESPKDVSNPLHNYH
KKKILLCDNLNVKGEAIYESVDTKKENYILKANVRKREYPEFISVFELDIHIGNFDIRLS
NDDVEILLRASSTLFGDVPSSYTNIAVGAVIPP--RRRAVAGADLTNGALSEISTDSNTI
LSDSSSTLSSGNEQTNSYRDIKIMIRLHNVKCTFIDDIRNSIVPIIRTIMSMSICLNFYT
DECSYNINDLNSKVEYFNNCIGDWEPFLEKCNISLDIHKILPNDAYSEDDESSKSPISII
KINSVNALWFNITPQLVNLLFLFLPVFTEKVLNGLKKKENNRCMPSNTLEKEEKESSHLS
LFNNNCDSVSAELDEKNFGIKSKSIYEDCSSVFESVPGDMDAGKETDFHLDDFLKKEENR
NSYDCMRDNSVVYYYVNLTSDFFYAFVMPESTLEEAKKRRPCAAARGGFPTGVVTPMGSAN

GRHRAKTDNAHREGSDDDGSASAPELYAKIVTTNELVSLDRLLVNEIANNSLIEKKCLYL
YLIPIPPTNVVNIIHDMFAEVSRRDIVLDLMITKNPKKTIENLLAYSQGRGRAKEGVNAT
SSCKGATSQFKYSQSNKFVGPFLKNCECVIINLIKNSCTTLTTSLKSSSVIHMMNPRDYI
FDEADASAQCGKPHGGKRHKLLVDNLLAKWERLNAEMERQTNLACKYRLKNNTVCEIISP
MPNYKILFMSSTVRIVNKCGIPLEFCFFDGTRNPILLTSLENRTIPISTLYPNHSDSFKN
YKVSNSLNVNPNIKLRQKNNNLSFTVILNHEYLLSTPECAFCGPSHVYLSFKPINLVSSV
GGIPNFSRNQLDCSKAEGSDTSSISSEKGWSDIFSSDINQGTYVKKCKIKDSNSYLYFLV
KIENKISALPAEKNMKIITIYPHVSVVNAIPALVDIIITSENNERVYVHELKRLEKEISN
IDEEIKMKKKSIFYIYEIKKYSCLNMKMKIGNSQCEWSDKLFLLEDDEESVMRFSLNFKR
FASVEVELIKNFSGYFTSLSSILGNKQLIFSLPRCFIDRTGLGIKAINSNKYYPIINGIT
LLGDNSQIDLLLPHKHNEFLRSCNGGRSYEVGDYSDIFNDFNYDNSLILFKATLPPIGSY
TETSVACKNFFYTFCLNTEKIKTTNIPYIISRIITVVPQFIISNKLNHPILVKQFQTDKI
QGVRQNDTSPLYFPKKQGLLLFRFKALEGGSGVGGSGGCGVSDVSVVDERASRKREDQAC
KQYKVSKNPFWSSVIFPSENFVGTNYMVVSNSKEEKSDVYVITCIPDMGTKNIIIERLNN
NMKRGFIAYNCSSLARYFKIRTFHDDTKHLKMDEEEYKFEKNIFLKNFLSTADMEHYFNI
EYDQYSYLGWVNPFIYVTRNVQIEIVLHDMKTIPRTPFILKFAQYNYAQKLFQVYYYDVI
FSVSIEYIQDLITIRLSHRIDISRG---VSQVGGGASGTTSYAYDSREGGRASKNSLDMQ
EYHKIEPAEGGYALRDRSGHEVVVARSAKGGGYVQKRMIGHSEYNYVKCGAEQSGGSGSG
HGRGGNYYGRREEAVKMISNTYRNVHVIINVTQIGVSIISNILKEEVFFIELSKLCALFY
MKNEEEVIDIKITDVQVDCQLESCEKCVLLANRGISTNGKGMNTNDEKIFLNIYVERSFI
SHKDVIFKKIQVSLDDVEIEMDAETLNGINLLIAEYIEGISVVQKKNLLYEEIQKWTILP
VYVNYKSPDIPLAINIQYMQIDKFTLIVWCSFLLDKMHMMSDLLRIGLRILMVSGKLELL
GAPVTLNQEIFNNIRVSLKSFYALLKDKYSHSILACLGFIVGYSSLINIPKIPLEIGRNT
IGLAVYAVDNVSVGIGSFLSNLTFDAEYINRRQKERNFKTNTNMKEGLLSAVKNIGEGVL
SLSNIVTKPIEGAQKEGVGGFFKGIGKGVAGSLVKPLDKVGQAVSDVTRGIKAEVSKPIG
GHKYKIKRHRKPRMLWGEYGKIKEYNLHEAELRECLGLKFSKNIMKCLTIHKQENPPAHY
SLLLYPKVIIYANLYASMGEKKGDIVIWSLRIEDISEIRASSHGVIIRTGNSSYKIPCNN
ALLINKIYRELHSSRSSINSTLILGDAPSSPRVMNNPESNGNITKIDSGQLNVIDIDQNI
FLNKNSFPVNESYSQNDNKTGTNGNIKDVSIYKEMYEESINNPELFWGNIAKNSVRWFKL
FTKVYIGNFKKGNISWFVNGKINACDNCVDRWVEKHPNKIAIIWEKDTPNDFKKITYQKL
LERTCKIANLLKMYGVKKQDVVTIYLPMIPEIVYTMLACARIGAIHNVVFAGYSAGSLCD
RILDSNSSVLITSDFGMRGGKLTKLKQIADAAMEMCGAIKVCIVFKNKTKISDASRALES
ASQSQLYHSTSADLKGADNFLAQGAARVINSQQNGVNQSEYQQMNNAKGVHTLHKKCKNN
NLGKKNPASDKTYNEIGNGEATNNMGNRSNHEKESCDYINSVMCKSFVEAPFEGKGYLRS
DSRKSNFDENICTLKEGRDVDGSALIKNMRAYCPIEYVDSEDFLCLLYTSGSTGKPKGVA
HTTAGYLLYAYTTCKYIFDVTADDIFGCVADIGWVTGHTYVLYGPLLNGITTVLFSSIPT
YPDCGRYWSLIETHKVTQFYTAPTALRALMKHGDSYIEKYDLSSCRILGSVGEPINPETW
RWYYNTVGKRKCVIVDTYWQTETGGIVIAPIPNLFKMKPGCASLPFFGVQLEILDSKTLE
PLNGPNVCGLLCIKSPWPGMLRTVYGNHNRLVKTYFSSCPNYYFTGDGAYRDEDGYYWIS
GRIDDTLNVSGHRLGAAEIEHALVQHSCIAEAAVVSFRHVVKGEGILCFVVKKVGVSKKW
GELEGRNGNAPNANAHLSPASNLAEMDTMHVSHTDENIIEELKLYVRRAIGPIATPDLIC
IVPDLPKTRSGKIIRRILRCIANELNDFGDITTVANHEVIDIIVNKFRDSKLKCQLGKMP
RASRLLLQGSLFCACAVFLAWKGRHSPLLGKVTNILRRILGLKSERSIENDQIRDNVKIY
FGSQGGTAEQFSKELSTNLRDIFKIKAEVIDLEYFKKEEISKFGVRIFIVATYGDGEPTD
NACAFFKWLKSLSDDNGYFRNTLYSIMGLGSKQYKHFNKVAKKLANYLAKFKARQISENV
FGDDDDNIYQDFEIWKKKFFKELVKLLHLKEIPVHIVAENAVELVDWTALPEVNLGIQYG
EEVAVGGAANGEAAKGEAADGAVGNEPTILPHQTTDITGKFFFNHHRGRVISNENLLKNV
NDSADDDKVNRIVIAVEKVSFKAADTFVVLAKNPRRVTSWWLKRLGLDDTDGKKRFTFVP
RNVMEKPPTGSALPSSRPQDPPPVCVPFPTPCSVEDALAYYCDLTTIPRVNILKKFKCFI
KDVEERKMFNHILSNKQRSTFFNICKEADMTFIEFVDIFMAKAEFELTPFLQLIPRNVPK
SYTISSSPKEAEDTISLTVKKKQYPIHSLRKALKGFKSNDMLPPISEQKLRELCSRRWFK
GASSFYLTEELSPNDSVKFNLKSSKFCFPPYLESTNIIMIATGTGIAPFKAFITEFKHFD
QMCVQNGIAKRAKRILFFGCRKREIDFLYEREIADAEQGKHIDQVFLAFSRDQHEKVYVQ
DLILERKDLVWSLIQKGAYVYVCGNSNMSRDVNKTINSLPMHYKQNNKKFTKKLKKEGRY
VEEMWMDNLPWVEKYRPKKLDDIVHQTNAVSMLKEVVRTKNMPHLIFHGPPGTGKTSAIN
ALAHELFGRDNISERVLELNASDDRGINVVREKIKAYTRISISKNKINSETNETLPPWKL
VVLDEADMMTEDAQSALRRIIEIYSNVTRFILICNYIHKISDPIYSRCSCYRFQGIPINI
KKDKLLYICKSEGINILDDALDKIIETTQGDLRRAVSILQLCSCIDPMITLESVLDVSGL
PADDIISKIIDACKMKDLKNVEKAVQDVIEDGYDVAYIFKSLNNYFVMNTEYQDSVKSQI
LLELSRHDYRLHSGATKYIQLMSFASSVHSLLNAAMSESLDIENAKAKLKLVFSFWGSSA
NKSFAQSNAFCVLSGKSSKEENATTQEQFQMWLMGYQLTETFFLFLKKERLVILTSDKKK
KFLQPLLDSLDNVQVMERSSDNTENFTQIKKMINGAETEEIAILKDKDATGNFFENCYSF
IKTLDLPQVDVNAELKFLMNFRSESDMKIQKSGSDIACIILKNILITTIENALDSEEFQS
HDKIKDKALKFHENKKCVLKLKEKLKVDIDDIDVIYSNVQSGNQFTLNYKNSNNKSYLSQ
NEGTILVGVGVKYKELCSNVNRTLLLNAKTQHKELYSFTLAIEKYIIKECLQVNNTYGEV
YKQAVSFIKKNKKDYPTLSQINVESYFVKCLGHVIGIEFMEKDFLITESNNSGMIQKNTS
YNLSVGFENVPGNDKNNFAIWISDTVCINDQGEVNILTDSISKEINTISYELEDSKSEEE
LDTNVKSEKKEQNGDLSRKKTGISASILNNAASVIVSDRLRRRNKNSLAHNNEQEMEELN
KRQSELKEKKINEIKFRFSKGTSDYKDPNKKNVKKLEDVKAYNDADLLPRDLRPNIICVD
NKHECILLPINGAHIPFHVSTIKNLSSNYEDNNDIFVLRINFQVPGNQGVLKADFNTFPT
LQEKEMYIKELIFKSNDERHFQNVVKQVKDLIKHVKQKEVEADVNDPKHAQEKLVLNKSG
RRIILRDLMTRPNIFTGRKILGTLELHTNGVRYSANSRGTTEHIDILFDDIKYAFYQPSD
GQLIILIHFHLKRYIMVGKKKTLDVQFYCEAGTQIDDLDRAKARNVYDPDEMHDEMKERE
QKNRLNLIFKNFVQQMQDISKIEFEIPYPELTFSGVPNKSNVEIFVTANTINHLVEWPPF
ILSVEDIEIASLERIHHGLRNFDMIFVFKDYTKPVKRIDVIPTEYIDTIKKWLTTIDIVY
YEGKNNLQWGNILKTILADIDSFVNSKGFDGFLGDDEEEEEQSAEDEDEDDEYEVDESEL
SAEDDSDYDDSGDESLATESDGEGEVEEDSEDEGLSWDELEERAKKDDKKRFADEEGYNK
RKKKKKNMSLLYRKTLNLLKGNNDEVNGIRININSQNKRGHNNSVITSKYTIWNFIFLNA
YEQFHKISNVYFLIIGILQLVPEFTATNRLPTILFPLTIVLVANAIKDAYEDWNRHKTDK
IENNRMCYVVANEEANEETQSTSFCKTIFRKIRNFLIRK-KKICSTNNSFDEEIYVEEIT
DVDTFMSSFEGNLGHIEGTIKKRWKDVNAGDIILCRRSEFFCADILLLSTSDKNGIAFVE
TSSLDGETNLKVKEANGFVFNILTSDRGEAIEKVKNLKGFIISEKPNKDLTTMYGTIYFE
KDEKIDLQSNSEILKETTELIENRRRRVSSADFSSSSIGNIQEDWIDELNDEDKFVRIPF
DEKHFVLRGCKLKNTDWVIGIVIYIGKETKIQMNSTKPIIKTSKLEILTNRLTIIIWLIQ
MIICFISAYYNAVIVSLSKKSKFKYLPFNLEESKKPFIVGVISFFSWVVITANFIPISLI
VTMSFVKVVQAYFISCDKNMIHKVLADIPTFGKQKETASLRDENSVELDIKEGDQTDKSK
MNLAPQESTERGEATGNSNSNGNATPIERKKTLTRVITFKDVKEKNYIYFNAVPRTSSLI
EELGQIEYIFSDKTGTLTCNVMEFRKCAINGISYGNGLTEIKKHILKKKNMAIPEEPVLK
STEKTPNVNIVDADLVNHLHDVTHFNHAALIGFFLHLAINHSVICDYAKEPTTTYSSSSP
DEEALVYAAKHFGITFLYRRDGKYGISIFGTVYEIETLAIVEFTSKRKMSSVICRIPVRA
HTGGAGGATA-AAAATATATTAP-----TGEHPALARNEVNSRLKDPLEENLPPGGERHK
KGN-NHHQKKDIITCKNCKESKIMLFCKGAGSVILNKLAKKTEVDDITIEHMETYADEGL
RTLCIAQRELTEEEFAEWYRLYKEATVSIKDREENLENVAEFIENKLTLQGVTGIEDKLQ
EGVSSTIEDLRLAGVHIWMLTGDKIETAMNIGIAANLIDNYSEQFIYTADLISCEEALMN
KIDEDIINIEKTLNLPHYDFNAKCNDKKQGFLRSCFTFKEESNLSLSPDNYKMLISSFNH
VLVVDGGLLDILLSKKFERKFFYLADKCSSVICGRVSPYQKGAIVSSANRLLKKITLAIG
DGANDRNMINTANIGVGIRGQEGVQAFNSSDYGISQFRFLKNLLLVHGRLSYTRISKLVV
YMFYKNIVLIFPLFIFGSLSLYSGQKIYYEFLLHLHNILFTAVPVVAHAILDKDVSLNTA
LVTPSLYKLGIYHYYFNISTFVSWVINSLFHGLVVFLIPLYFLSYYNIPSSDGTPYDMWT
VGSVTYFLTVLVVNLKVLLETYCLNVLPLTAVFMSILAFVILVVSCSFMCFGSNNFLGTA
VILAKSLRFWLVVLLGLFTALSRDFIFKVFKRNFNPEVYHFLLDQEDKPKGENNVINSSD
PCQKEEEIKIEKCKSLGYAFSEVDPACVKLIRKQDKLIMMATFLSLLCKGRGAARVNGNI
LQ----NGCYPCAARSYSDHVKDHFNKPRNVGSFDKNEKNIGTSIVGKASCGDVIKLQLK
IEDNVIKDARFMAFGCGSAIASSSYATELIKGKTIDEALKIKNDDIASHLSLPPVKIHCS
LLAEDAIKHAIKNYREKVIN-MSLKVRQNGVQSAVKNVKCLCENKREISTSRIYGGKEKV
VILGSGWGGIHFFISIDFKKYDVTLISPRSYFTFTPLLPCLCSGTLSAKVCTENISTFLR

KKGSSGSYLQMECTDIVPEERQVICRDNQNNEVKISYDYLIISVGAKTNSFNIKGVEKYA
FFVKDIQGVINIRRRFLDILSICSTERISNEEKKKLLHIVVVGGGPTGVEVAGEFADFIN
KDVKRKYKSIFPFISVSIIEGGNNLLPTFTQNISDFTRKTFRRSNINVLTNYYVTEVDEH
NICVQSSVDTNEERKHIPYGILIWASGLAQTPLITNFLKKIPEQVNNKILNVNGHLAVIG
IRQKNIYAIGDCKKIQPLQLHEHLNDVLHHFSSSSTTSDLLKSKASELSKKFPQVSQSKW
DYRKNKRAQMDKQQFWEYLKQIDQNYKSPTPTAQNAKQEAYFLSNLFNTLVEKKADNHFP
SFVEKWKGSIAYIGNHQVVAHLPFFEIRGGLFSFTFWKMVYIQLLLTWRSRFAFILDFLR
TKICGRPFAKMNVNSRKLQVKNKNAAEVQITAEQLINEALDLEEVEQKVNYNLIDEDELN
EYKISKRKEYEDKIRKRRYLISTYIKYALWEVKQKDMRRARSILERALNIDYTNVNLWLK
YIEVELTNKNINSARNLFERAVLLLPMENIFWKKYAHLEEILNNFLNCRNIYERWVKWKI
DETAFLCYINFEERCREINKCRDIFERLIVTLPKMECFYRFIKFERKYKNVDRARACFEK
CIQLLPPSFLDEHFYIHFCNFEEENNEYERCRKIYIEALKILPKSKSEFLYKSFLQFQKK
YADKDELDETLMIKERITYEEEIKKNPSDYDTWFNYIKLEESNINLVNKDKCIFRIRELY
ERAISVIPPVANKKFWKRYIYLWINYAIFEELHAENVQRARDVYKNALKILKKQNFTFKK
IYLLYANFEVRQMDIPKVRSIFNRAIESVKKEEIFEEYCEMELRLGNIKECRDIYAKYVE
AFPFNSKAWISMINFELSLDEVERARQIAEIAIHLDDMKLPELIWKNYIDLEINLQEYEN
AKKLYERLLNITQHYKVYKSYAEFQYIYFDDIAKCREILENGIEFCKKSELVNERCILLN
FLYEIEKDYGDKDVIDKTQKRLPKKVKKRKIIKKDDDEVVEEFITYVFPDDGNQSQNMKI
FQKALEWKKKMEEQQKREEEEAKMTDVESVDNNVVVEEKTVFDHTTAIQKVLKNALVHDG
LKIGIREVIKSIESKEAKACFLSDVCSEPAYKKLITALCTEKNIPLFMVENDSKDLGQWV
GLFKLDKEGNARKIIGASSVSIIDFGEESAERDYLMQQQNQPAAAMAKDKRGRMISSSHE
SDEDKNSKRIKK-HHKLHFAEKAPDNGSYKKKNYENDKSKIILKKDQKKNSKENLNSFSS
HHSTSSKSDSNNLGLDLSGGSTSNSEDEFKILKEEENEDKFLEERRRKREAIKERLKDLV
SDNEKGNDVVSGELGDG----DSGGDGKEDPSGNV-KKEEGEGAFSSCNKNDLAESLTEI
PPMLDEVDHDDAACIFAPNKEVMEETCSSLSSDHEMVEDKPAKEKSESVKESNDLYSDLK
RKITEEKAKIRAFIIKQKELHERTKEGPPTNKNKDTEGVEEYEEEDNENDEVDMFSSVQP
KKKKKIEKIRITNYYTSDNVNLADNWNDSEGYYKAIVGEVIDNRYSVVCELVGKGVFSNV
LKCYDKVGKIPVAIKVIRDNDMMRKAAEKEISILKKLNEYDKDNKRHIVRLLRSLKYKNH
LCLVFEWMWGNLRIALKKYGNGYGLNATAVHCYTKQLFIALRHMRKCRIMHADLKPDNIL
INEKFNALKVCDLGSASDITENEITSYLVSRFYRAPEIILGFRYDAQIDVWSAAATVFEL
ATGKILFPGKSNNHMIKLMMEYKGKFSHKMIKGGQFYSQHFNDNLDFIYVDRDYYTKKEV
VRIISDLRPTKNITCDLLEHQYWLKGNSPKMQFLKKKIKQLGDLLEKCLMLDPTKRYTPD
QALQHPYLRESIHFTKTQNEM-SDADSLSCSLTLESDEYDEEEYDTNLSKLLENKTLNWI
FVGGKGGVGKTTTSCSIAVQLAKRRESVLLLSTDPAHNTSDAFNQKFTNQPTLINSFDNL
YCMEIDTTYSENTAFKLNKTEFFDNIIPELLQSFPGIDEALCFAELMQSIKNMKYSVIVF
DTAPTGHTLRLLAFPELLKKALGYLINLREKLKGTLNMLKSFTNNEMELEGIYEKINHLN
AMSISIQSNFQNPLKTTFVCVCIPEFLSVYETERLIQELTKKNISCYNIVVNQVVFPLDS
MTVDVAHCEGLLKQIKDKQVQESFSSLVQKTKELEDVYISRRKLQSKYLTQIKNLYGNDF
HIVCMPQLKSEIRGLQNISNFSEMLLESKEIPIYRMEDGVIISGDYIQMEKDKVKRINEH
NFEQVHDVESDNLYRSKCSGILLKTPYYPYKYDIMNTSYKYMPKVGDLVIGIVKSKKLDY
YQMDINCNCECIIHKIESFKYATKNSFPNLANGTLLYMIVEKINLDNNSVVASCINSSDV
KSWINYENYLGELVDGYMFPVSISYAKSLIGDKCYLLDLIGADVSYEVAVGHNGRVWVKT
DDPRETNMIHTALKYSFGKTKAQMDVLWKSIYNLGKRDTMNKPHILLCGVTGRGGLVEEV
LSCRKYLPQVGHNWPFTEGGFPPPKRYEQAAI-GLQQKRRQFHQMVRVYLSKAPKGFESF
EKTDRKKGDGVSSSPKPDEERNHKRLDNFLFFVLFLLLVSLFLFVDSNGLYNEVTQNDFF
INYLAKGYVERIKLVNKDYVKAYLNVHGMSKYHQKYVSFRIGNSDAFERRVEIIQREMNI
QRDQLIEVQYTNEANVLHEVKSYIPTILFFLLFAFIFQKITLKNVANSGMDRLFKMNKMN
PISKQHLKTDVRFSNVAGMKQAKEEIMEFVDFLKTPSKYEALGAKMPKGALLCGAPGTGK
TLLAKAVAGEANVPFFNISGSDFIEVFVGIGPSRVRELFAQARKHAPSIIFIDEIDAVGR
KRSKGGFAAGGNDERENTLNQMLVEMDGFHTSNDKVVVLAGTNRVDILDPAITRPGRFDR
IVNISKPDINERSEIFQVHLKNLKLHHSLDIQSISYLLASLTPGFVGADIANVVNEGAIQ
CARRSNLVGVQVKDFELAIERVIGGLPKSSSLISPFEKKIISYHETGHALIGWLLEFADP
VLKVSILPRSNGALGYSQHLSEEVFLFSREALLDKVAVILGGRAAEELFIGKITTGAIDD
LNKVTQLSYSYVSQYGMNKEIGLVSFQPNSSSDYSFYRPHSECLAHLIDNEVRCLIETQY
NRVKSILRTHEEQVHKLADLLFRKETISYQDIVQCIGERPFPVKSAYEKFVKANPYRLGG
QESGGGSGE-GNGVAAIGEAEKATQGAHAAPQLGSPQLAAPNET--LAEQHPAGKKGPPG
EGSDDGNKGSKLSHVGTRMNTKNVTREEKKKEKELNEARKAGKIEALKDEEGNDINPHMP
QYIIKAPWYLNQTKPGLKHQRYRGTEKVKIEEERNKKVYVKNLKHISDFCKNCGSATHKE
KDCLERTRKKKLNFANRQNEEDFVCLTQDLGYDGNRDRWVGYDPENFEQVYREYEKIVEE
KKKRKAEELKQKYERKASAGKKRRANDHSRSDSDNEEDEADDDAPKGATGADDPQKSSNL
NASEKHRNVARNLRIREDTAKYLYNLSLNSAFYDPKSRSMREDPFATTRGNLEDSNHYKG
ENYYNNTDEAIESKKLEIFAWETYKRGENVHFNAQPTQLELLYKEFLAKKKKLIKKKEEG
ILKKYKCEDAGTDGPVGEELSQSEVYTEYKPVDQIDSKVKRVKILSRYEEDVHLFEHSSV
FGSYYDRERGKWGYRCCRSTDRFQKCFSAMGAKRSEYDEGGKKHNYGHEEMNRLGGEVQY
DLEIETHLEEEDSANRVGSRNHINVGTLKDEVVQNDLNNDHLDDEALGLVKDKSIKRREN
EFQRRKYDYMLSPGRADPFGEKSPSPGERTYADVMMGINNESHKRKLASSASSSGAAKEA
DAGGNRKMRWGVAEGDQGKSKWDVLSEEESTSFGNMPTPAPAKWVDTPLVLNDGG-AVKR
KKISRWDKVGEGTTAADAAGGATPGGLLKTPYLSGNFVNTPYVVNQLGNAPAMFTPMTPG
VASLATDSIIRMKIKNEMEIRNRPLTDEDLDELLPSEGYEIVQPPEEYEAIRRNKLKAFF
KTVASAAGTPLLVGSGAGVATQGDKTGEHPPGTLLSGTPFYELPTATATQLKGDAQTLLQ
SRQLEITNPQLLSELKYVQLKSEDFIYFSKLFQTVDESDLSQDELKERKLMILLLKIKNG
TPSVRRTALRAITDKVKELGPETLFNLILPLMMHNTLEDQERHLLVKVIDRILFKLDDLV
RPYVHKILVVIEPLLIDEDYYARVEGREIISNLAKAAGLATMIGIMRPDIDHPDEYVRNT
TARAFAVVASALGIPSLILFLKAVCQSKKNWEARHTGIKIVQQIAILMGCAVLPHLRQLV
SIVAHGLHDEQQKVKTITALAIAALAEAAAPYGIEAFDSVLRPLWKGITEHRGKVLAAFL
KAIGLIIPLMDPYHASYYTREVMVILINEFNSPDEEMKKVVLKCVKQCIQTEGIERDYIN
QEVVNPFFEKFWVLRSSHDKRNLHLIVETTVEISNKIGGAVVIARIVDDLKDPSEQFRKM
VMQTIQSIVNNQGVDDIDQTLEEQLIDGILYAFQEQASEDYYVLLNSFDAIVNKLQIRMK
PYLPQIAGIIRWRLNTPLPKIRQQSAELIARIANLMHLCEEHQMLGHLALYLYEYLGEEY
PEVLGNIIGALKSIVVVLGVQNMTPPIKDLLPRVTPILKNRHEKVQENVIDLIGIIADKG
GDLVSPKEWDRICFDLIELLKSNKKLIRRATIQTFGYIARTIGPFEVLTVLLNNLRVQER
QLRVCTTVAIAIVADTCLPYSVLAALMNEYRTQDLNVQNGVLKALSFMFEYIGEIAKDYV
YAVVPLLEHALMDRDLVHRQIATWACKHLALGCFGLNREDALIHLLNYVWPNIFETSPHL
IQAVIDSIDGFRVALGPAIIFQYLVQGIFHPSRKVREIYWKIYNNVYIGHQDSLVPVYPP
FERLADSNFARDELRYTLMAKQRLTALDIRAIITLCRNIIVGCVVTNIYNISNKIYVLKC
SKKEQKYFFLVEAEKRIHITEWKREKDVMPSAFTMKLRKHLRSRKITNISQLGGDRVVDI
QFGFDDKACHLIVELYIAGNIILTDNNHKILSILKTNDAIGKKYNINDVYNVEEHTSIML
HRSLDVVDLENVKKSISEMMLLAIFGERTNEKATVSKKGSD---NNGGGRIQKGAAKNGT
SANLNNSSNKKTPICSDNEKGKKMKKLKTLTDLASKLIVFAHSDLIVHSLIASDVNPSDL
LEKYSLSDLGQILLKAINEALCVLNSFSSEGGCVKGYGFAPKGGEKGTKAEKNKTDGVGG
RSGEEIAFTEFSPIILNNHKNKVEENKLEVVHFDDFNKCVDTYFSRMELSKYDKQQEVIK
IKKSLTKMDKIKLDHERRIDQLEKEVSTLRKKISLIQMNDELVEQAIQLMRAAVATNANW
EKIWEHIKLFKKQNHPIALRISSVNFNNCEMELLL---DDGEENGLGSDDSSEANEQSKG
KKSSNKKAATNNRFAVTINLNNSVYGNVEDYQKLRKKAEEKIRKTKISTNFAVKKVEKKK
KEKENKQKGKNKTVGQIQKIRKVYWFEKFHWFISSENYLVIAGRDALQNEILFRRYFQKN
DVYVHADIHGASTCIIKNPHKDIPIPEKTLSEAGQLAICRSSAWNNKIITSAWWVHYHQV
SKSAPTGEYLKTGSFVIRGKKNYLPHVKLEMGLCIIFQVDNAALDNNEENNLDDTQKSFE
NDGERRSSDGDQAVVGGVDAGNGTSFPATVLTRGRHQQNGGSTSKCTYPSASNENAKGVN
SCVAVPCRGHLKRKKRHMYNRFVGFFLPDRNRTNLLNFEKKPLLEKLGIAYNHDCAIVRG
LLSWVASNLWSDERNPLMRGRNGHLSKCGMCAALKRTHKRFVYFFLYYPKEHIPLGDPTL
RPFLRRLEAITFFVSHRGFAQKGYLEGIPDMHDGPFEGLEDTRMVSNAVELFPSSFTNGG
SVEQLGSNCVKDYLCEGEKRSPFIFVECAVYGAPVTFKDEDSNDSEADKKVKAELGDHPS
VSSHDEKRECGEE-EEKAKRVSFVSDHGEEGPKLVRPARSRKCTGFVKMDVSKLLQEIEE
EEEEEHHATEETGTSTKVTLCEAGEGRGEKKSVTFTSEDESVGDAKQPSLNVPRPVRTRK
PTGFVKMDVSKLLQQIGDEQSLEDVGEDNAVREVSATFGEGPRRPSIRKKSVSFSSEDEH
IYVERQESLNVPRPVRSRKPTGYVKMDVRKLLEEIENEEKSDEGLNGSNAEDEHKEETPK

VTFAEGALSPDLKKKSVSFSYEDVYMQGEKNESLN-APRPVRSRKPTGFVKMDVSKLLEE
IDLEENSDEGEEPSDNQKEKKRPRKATGFVKMDLSKLLEDVEGGDGSSDGGGAPEGKKNF
DGDGNDDDEEDEDDDDDDDGDGGSYQESEESDESDESDQTDGDGEDSDSDDSHGGNAQPS
GNKRGGKGNNEKGSHENSNKQSENEVKKNAAKEQNGSVNQGTVKDQKKDVHLLRGARTKK
KRMKKKYREDDEEERQLHMKIIGARKMKHETEKAKVEKLESKAAEQENKYKSPKEVTINF
EEINEEEMKHTSGEIKEVGVHPQGRRQLVCNSDVCA---LLSHSESKVQGEAG-ARKCQK
GEGCIVHFVENVNRRKGKGAHQKYIGGRTGKLHYNKFDPRSEGVKAGYMGRMYGKGKGIS
SSTIPYKRKQPSWLKQKPSEIEDAIIKLAKKGQTPSQIGATLRDNYGIPQVKAVTGNKIL
RILRAHGVATTIPEDLYFLIKKAVSMRKHLEKNKKDKDCKFRLILTESKIHRISRYYKRK
KLLPSNWKYQSSTASALIAMSNRKKVAYFHDPDIGSYYYGAGHPMKPQRIRMTHSLIVSY
NLYKYMEVYRPHKSDVNELTLFHDYEYVDFLSSISMENYREFTYQLKRFNVGEATDCPVF
DGLFQFQQSCAGASIDGAAKLNHHCADICVNWSGGLHHAKMSEASGFCYINDIVLGILEL
LKYHARVMYIDIDVHHGDGVEEAFYVTHRVMTVSFHKFGDYFPGTGDITDIGVHHGKYYS
VNVPLNDGITDDAFVDLFKVVIDKCVQTYKPGAIILQCGADSLTGDRLGRFNLTIKGHAR
CVEHVRSYNLPLLVLGGGGYTIRNVSRCWAYETGVVLNKHHEMPDQISLNDYYDYYAPDF
QLHLQPSSIPNYNSPEHLSRIKMKITENLRNIEHAPGVQFAYVPPDFFDSEIDDECEKNQ
YELKDDGGGGRAAGTRAKDHATSHHLRRKNYEDDFFDLSDRDQNVALMHRDDEHVDREIL
EKQQEIIKCAKQQDFIRLQIIIQPYILRNDVEMLNEINILHWACYCGFTELIKRLINLAV
DIDKEDLVNNDTAIYYAIKNSHYEIVLLLIKHFGPSILFHKNRRKMSPFLTAISEFNEGK
ILEALHILELLYLNGASLEEQNEHGQTALFLSVKRNNISTLQWLLSKGVNINHRDFYGNT
ILHIAVKHCDVDILRLLCDYGSLPLVHQTSLQNDNTNVLQLCLRNRYFLVFILLNKWMLQ
DKLCKGIKICKTVYAFYFWFFAILNLIVFANISRSFWAFRKYHALSITWLAIWLFQQFLW
CLLYFKSPGFYKQNETLTKRHGKPFAYTYDGSFKKKAEYQLNGLEMELYKLNKSISSSLM
HPQKMN---KDDHAKYDQLVAHLEGQKMPLYAQVSQERVNSLDADYRNAILYNRNPRNVC
VTCNIVKPPRVHHCAECFHCIVHQDHHCVWVDNCIGIKNQRAFYLFIFSIFALLLYNYYY
VFLYFSLFHKTVDYAFALLVILCNFINITLFAFITYLFARNTRTILTNITFYEHFKKPSH
ITDKYNTELRCWDFQNLSLKKAFRNVYSFWSLNYDEPYLRHGKKADRIMDVFEFVDPLRK
RKITNNLFDFDQYVYKEKLNESEEKKSEESVVKKKKKKKLRRVLDSSNSEGNSKEDASPP
QSGRKRNGPTDESPKKEHEDEQHPNGSNNNLSDDNYSLSNEREEHEKNLKDLFECLLASI
KIKNKIIEYFTSNVKEKRQEIIKEFVKGSFRVSNFDANFSHFEREVDTFHKLKKYQKCGV
FWLYALYREKKNGILADEMGLGKTAQTCVFLDYMYRTKELQNKTIIVAPTSLLKNWNNEI
NMWCPYLRNNKIIYYGNQNERKYLAYDIFTNKA-NNIHLIVTSINMLIGKNDVSYFRQIK
KYDYLIFDEAHFLKNKNSLIYKKLQKKIVFNNKILLTGSPIQNKTQELMNLLLFLMPEIF
TEKNINNAMSAFVKMYQEILNSKEGSDDQGEGDSAATPLKSAKTLEIYDIKSVERKEP--
--SDSTGKVTSPGGNTPSSDGNTSSPGGNTPSSGSNSTCTEETKNIIKNYLIETIKNDVK
YVKLKNKEIILLQLIIEPYILRRSKKHVFIDMPKKHSIIIKLPLNSTQLNLYKDEIMSKM
QHTHKHLEFLQKHSNRKELERLAAVFEKRDIKEVGGVAEVADAADEGATPNGRS----HA
AREVPTNDYDDEDDKIDEETINIEKAEENKDSNIVINKRQDEPADT----ESVNNTSKEV
RGKMINASIFILRRICNHPLLHKYYYSVEDIKKISKYFYANTDQYLDLDLKTVENEFMKI
SDFDIHLSIKHLISQGDENLNKYLISKEHILNSSKIHHMISLIKEIRKKKEKVLIFSQFT
TFLDIIEEALLYEFIYDEQDFADHRQGGKGGNPDEKGEQADQADQVDQADDDNEEDPNGD
FNKSEKDLYLSSSTTSTSSFTSDRKGSSQIYVRLDGSTNTIERQKIIKRFSKNDNVFIFL
LSTKAGGVGLNLIAANHVILMDQDWNPHNDRQAEDRVHRLGQKNEVYIYRLCCKNTIEET
ILRCCKAKLHLDQAFGGNSDMLMLKNPGAVLVFKPNTKREEGRKTQLSNIQASRAVSEIV
KTTLGPMAMLKMMLDPLGGIVITNDGNSILREIDVAHPAAKSLIELSRSQDEEVGDGTTS
VVILSGELLNIAELFLKQKIHPTIIVNCYMDALNKVVKFLESIAIEVDVNDEESLLKAID
SCLSTKFVNRYNKMVSTLALEAVQCVKIENVMGRKEIDIKRFAKVEKIPGGDITDSYVLK
GVMLNKDIVHPKMRRRIKNPRILLLDCTLEYKKAESQTNVEILNEETWNQLLLQEEIEVK
KLCEHIINSQCDVVVTEKGVSDLAQHFLVKRNISVIRRVRKTDLNRLERISGATIVSRCD
EIVESDIGTKCGLFDVKKIGDDYYAHFVECENPRACTILLRGSTKDVLNEVERNLHDGMN
VAKNIIMEGKLLYGGGCTEMRVGQHLISQANQYDDSRKSIMEAVGSALEIIPKILAQNSG
ANVVKTINELRIRHETPGGEKFGVNGVTGEIIDVSTENIWDLLAVKKQIYKSAIEAAAMI
LRIDDVVSGIGKEDKLQKPVQGEFMARIHVLNVAEKPSVASAIADILSRGKMEKKKSCSK
YNPLFTFNYKRGHETWSMYVTSVTGHLTEQKFDDKYRNWVNTDPQELFDAEITIYVENDK
KKIENNLKNYSKFCSLLILWLDCDREGEHICFEVINTCFAMNREIKIKRAQFSAVTQKDI
IHAINNLKYPNRNLAYSVDVRREIDLRMGSIFTRFLTIRYVHLIKTETSIISYGPCQFPT
LGFVVNRYMDIKNFVNEYYWSIKMQYWCEGGGHSGDESEGGASLGRKKR-KRNGKNSAVM
KNGKKMAGASQNTVVDFTWSRLRLYDHLAVVLIYEELLKNPLCRITNIYENQVKKYRPLP
LNTLQMTKLVSRHFHISSKQCMNIAEKLYNRGFISYPRTETNYFPSSMNLRSIVAELKKS
KVFGSYAKKLCEGNNFREPRKGRLNDQAHPPIHPVKNMHRTDKVEEREWAIYELICRHFL
AVCSDDAIGFNSKVVATIGKEEFFCKGLKIVKKNYLEIYIYEKWNDKILPPFQVNQEFYP
YSLLVDEGMTQPPKYLSECDLLSLMDKYGIGTDATMHEHIENIQKRNYVFKNKKNLFIPT
NLGIALVLSYKKFKDIGVDLTEPSLRAKMEKDMTLVASGTKEKDETIRNYIDIMKYIYQE
IYNRIDLLDEQIKHYLNSAEGLCMGTFNFGKYNGKTFEEVFEKHKSYVTWVKNLENPSGS
LIQFKEYVLEREKGERGQEKGQPGRLDHLDQFGGKSPANCSRGQGWEG--NTWDGPQERG
GGDTYRSYDRSPSAVLKGVGTSYKKEGYQEGAKSVNKMNSREYKTYEQSIMNEISNSYNK
SEGEKAEFDIIVAFEIFSDDTFKIVQKDNNSRKFASFKNFVPKELFKILSEFNPTLKKTN
NYSCITFEADKYEYVLSNLKEKCTILGGIQSIPNFLLKCFKQYSRFSEPQKVSEMTASIL
TSTLCPYTKKNYDKMDILVGDKLSAELKNFQREGVFFGLKKNGRVLIGDEMGLGKTLQAL
ALMAFYQEDWPFIVVCPSSIRFQWKDQALRWLSHLLTEDQICVVKSGKTDVPRNCKMIII
SYELMTKNDKYQNKYKSIVCDESHYLKNSFSKRTKAITPIIRSAKRCVLLSGTPALNKPS
ELYEQVSSIIPNLFNYHEFCERYCFKDKNIYTRKIEYVGCKHTEELHLFLTNTIMIRRLK
KDVLKELPEKLRSKIPVEIPPKELSEIITYHRKLESKKNINIDDLDELPFPSGGNGIPNR
GDEENVSISHLFKMTGYAKVKAIKEYITYLIDADIKFLLFCHHKLVMDEIDDFLREKKTM
FIRVDGLTPIEKREVYIKSFQNDDHVKIALLSLTACGIGLNLTAANTVVFGELYWVPGQI
IQAEDRAHRIGTTHEVVNIHYLIAQNTIDEIVWKIINRKWNTLTTALNGMEDSLNVKEVN
KFDKFMIDLTNDTNKSYPTSLVTTPKVRRKSSEHNKSFESSNTKRDRDIRDFFKSSEKSA
EKSDCAKKRSYGTPTNDCSSSTSPIIVSKRYKTELHMKEKHKKVEYADKETAAKETAAKE
DGSTIFVNNLLTSINNINSAIVYKDNRYILRMLKYIKCMRLSIKNESATLMPIMISLISK
TFKEGYPIYEILAKYIDEFRE-LPKEVPDFLNINEKTYTHAAPEIEVFFYIMVLLYLLDN
KCYNEAMDLSTVIVNRITKLNRRSLDCMNAKVYFYFSWVHELGGKLSQVRQKLLYIYRNA
CLHRDIMTQTVVLNLILRDYLKHNLYDLAVKFVSKTSFPENSSSNAQHARYLYYIGKILA
IQLDYSEAHSKITQAIRKAPQNIQSAKGFKLEATKMEIIVELLMGDIPDRSLFSNKIMRN
KLIPYKHVVTAVRNGDINRFAKVMNDYNQLFMRDGVYLLIKRIHHNVIKTALRIINLSYS
RISISDIGKKIGVESPLDIVGITAKAIHDGVIEATIDYDNLYVESKSNSDVYITSDPMKT
FHKRIAFCLQLYSDAVKAMQYPDENEKKENEEAKERKIRQQEELAQAEEGDLGDDNDLLA
PCEECGEKNIRMVKPSDRKKLCRDCFTEGFEEHVHETILKKRMFEEKDKICIAVSGGKDS
SVLAHVLVQIRRKHNYKWDLFLLAIDEGIKGYRDDSLKIVFKLQEKYHLPLKVLKFEDIF
TYTMDAVVSFIGKKNNCTVCGVFRRQAMERGALLFNATKLVTGHNADDLAETILMNMCRG
DLEKLAKGVEGAAAVEREEVAKKEEVAKEEVAKEEAAEVATCCGG---ECARGEA-STER
GQRPPSGMDTRGARMEASPTHGEPAPPEEEHPFFLPRLKPLMWCYEKEIVLYAFYKQLDY
FSTECTYSPNSFRGNLRSFIKDLEMINPQFILNIIHSSEFFYHNSSRRKVLQVCASCGAY
TSNPVCKACLIVEGLRNYKDNSFLYANKKKKGQRRRIPIRYDGGAMESKKCPFTSVIPYG
MLHEKLKREKNNQLPENV-VIEEFNQLLATYERPSPYDANGVVQISNQSDLILFQIDIEY
TVESIFKSMVFTNDGTPNGSALTSIFSPYKLLQSNEKNYVSVPVIRIYTVTNDGYSVLVN
VHNFFPYFYVEMPSGFDQEDLQKLEVMMNDSLNANSQYKIYDQKILHIEIVQTESLMYYK
RDGKRDFLKITVLLPKMVPSLKKFFEGIVKVNNKSIGGIVYEANLPFILRYLIDKKITGS
SWLLCKKKLFHIRPRHKKVSNCSLEIDISYEHVEPMLLEGEYQQIPRLRVLSFDIECIKL
DGKGFPEAKNDPIIQISSILYFQGDPIGKCSKFIFTLKECASIPGSNVIWFHDEKTLLDA
WSEFITRLDPDFLTGYNIINFDLPYILNRGTALNLKKLKMLGRIKSISSVVKESSFSSKQ
FGTHETKEININGRIQFDVYDLIKRDYKLKSYTLNYVSFEFLKEQKEDVHYSIMNDLQNE
SPESRKRIATYCIKDGILPLRLIDKLLFIYNYVEMARVTGTPFVYLLTRGQQIKVTSQLY
RKCKELNYVIPSTYIKSGSNEKYEGATVLEPIKGYYIEPISTLDFASLYPSIMIAHNLCY
STLVKNNAEIEGLKQEDVTSIQGKSNIKFVKRSVKKGILPLIVEELIDARKKVKLLIKNE
QNKITKMVLNGRQLALKISANSVYGYTGAASGGQLPCLEVAVSITTLGRCMIDKTKETVE
KYYSKSNGFEHNSTVVYGDTDSVMVKFGTNNIAEAMALGKDAAQRISKEFLHPIKLEFEK

VYCPYLLLNKKRYAGLLYTTPERHDKMDCKGIETVRRDFCILIQQMMETVLNKLLIEKNL
QSAIEYTKCKIKDLLTNNIDMSLLVVTKSLGKTDYETRLPHVELAKKLKQRDSATAPNVG
DRVSYIIVKGVKGQAQYERAEDPLYVLDNNLAIDYNHYLDAIKNTLSRIFEVIMQNSDSL
FCGEHTRHKTILTSSQTALSKFLQKAVRCIGCNSSIKKPPLCNHCKANKEFSIYMQKMNH
FKTKQNEFFQLWTECQRCQGNLHAEVICMNRDCPIFYRRAKIKKDMANVQEQISALRADW
MRVALRGLTACLYLARPPNHGQPKLNQKRHPLVGAYYNRANLHKRGKKLWGKAKRNVYSF
SSIRRHQKVLKQNRTNMSVATEMKVEEGIHLCGDQEFKLEDISSCVSEIFEKKKYENIPF
IQVAPMINVTNRHFRALVRTITRRAQVWTEMIVDNTLLYNLNNLEEHLGFNSNEHPIVCQ
LGGSDATSLAEAAVLVEQAGYDEINLNVGCPSTKVANKGAFGAYLMKKPEHVRNIVYEIK
RKVHIPVSVKIRTGVDDCDSFPFLRSFVECISSVGCSHFIVHARKAWLKGLDPKQNRSVP
PLEYPKVYSLCQLYPHLKFTLNGGVKTVEEAVALLNGYLPKKGNVDSEKTYVQVKNYQVN
PLHGVMLGRACMENTTVLSQTDQLVYNEKPPHTAFSRRTVLDAYKSYLEENSSLCSLSSA
FELLKPVLGILKGMPGHRIFRNKLDMYIRNYASTLPCSGILEKAMVDVDAVAPGCLDLPL
ADYKLQQEYIKNYMHKSQNVENKTVLVEEVVRDFDKNEVFEEITAKFTWEQDVERSWNLL
VENNGILQHVSQENLEEKGKQKYKKNQVCALRKGIFRHIIILFDMSSSMKERDFKPDRIN
VVLECVENFLTHFFFKNPVGHVGVVALKNSSAKLIQPLTSNMEDITNALLKERSMGLQGS
PSLQQGLEIAHDLLIDIPLYGTKEILIMYGSIRTCDKKNILNILNLIVKNNMHVNCVSIA
PEMHILKHICEQTNGSYKICMTKNSLMNEMNNITETPLWMMGMEPQLIHICFPIKKKIST
QIMCSCHNNLNTDTYICNFCNSYTCKIPSKCKVCGMHLISMHDLSHITNNLQGSPLFLEI
KNEEKGPSVCVSCNKRLYDKVSQCSKCGNLFCLACDLYIHEDLNQCPFCLIQDTMGSNSL
PMSQQMYFSTHNALRINDENDVISTLFYEINGNRHISLLIFPFYDVQMLKRLLIKKLNLP
GGVKVNDIIIFYKGIKLPNYRIISTYLESTSNEKKKKKKKKKLNKLYWAIKDTNPNASIR
VIDQKSYPPFFENILHDIKLAFKKNISPKLTMDGTGGTYLLFNSKKKVCSVFKPLDEEAF
APFNPRGYEGKMYQEGFRSGVLSGEGASREIAAYILDNSYNNFSSVPCTIMVEACNPHFN
NKSKLKYVDNEATLKWKCGSLQEFVDSRESVGNYDYKQFSIRDIHKIAILDIRVMNLDRN
DGNILVSPLKSLKDSCNQFLYRNNRSLGTTDEDILKRIVTIEKKPSRYSLIPIDHGLIMP
HIMDVAEIDLVWFEWPQTKVPFDDEELEVIFTFDPDKDAEKIRNKLLIREDCIRTMRVCT
RLLQIGARMHLNLHEIAKISTRKSIDEESVLEHLVRDSIVQAYQMMDYTSLMSTNRLGHI
LDLAEIKINKKKKNSKMKTIEHIDEALKGANETAD---MKRIENATCRFSPLSKDKCRSL
DYSFGKCLPSGYTCQIVKGVINAESGGSSKEVQSKDGAVEESALESASRGSGVTPPGKGS
LSNEFEALKRKEKYDGLY-GTADVDLNAGSNQNEQNKTSTNSLSNRSDYTLVTASSSSHS
DNQQDSQQDSQQDSQQVAYSASDQDVRVKRKKKKKKKKKKHFDKDGSDKREDEEGKELPS
DPEEGQTGTAQGGLLKSGLQDDDDDDDDDDDDDDDDDDDDNDVPFKKPSGTIKRISEGGG
TTYRSIEMNKVNSVWVIRDKNNKVINVKWENKIFEKLFFETFENYVKRYINEYHPEWRQY
PYNGSQISGIKHGYLKGIQMEIRVANKYALGKKLGSGSFGDIYVAKDIVTMEEYAVKLES
TRSKHPQLLYESKLYKILGGGIGVPKVYWYGIEGDFTIMVLDLLGPSLEDLFTLCNRKFS
LKTVLMTADQMLNRIEYVHSKNFIHRDIKPDNFLIGRGKKVTLIHIIDFGLAKKYRDSRS
HTHIPYKEGKNLTGTARYASINTHLGIEQSRRDDIEALGYVLMYFLRGSLPWQGLKAISK
KDKYDKIMEKKISTSVEVLCRNSSFEFVTYLNYCRSLRFEDRPDYTYLRRLLKDLFIREG
FSYDFLFDWTCVYASEKDKKKMLENKNRFDQTADQEGRVKQNMNVTDALRQQERTQLLNI
FKRFKGDKCLFFDKSLHIILNVVLNDDDINKERIQHVFLLDDETEVNVSKLDSVGNILFF
LRPQFYEVENVFKVIERVAKCEGGGRKYALVFIPYMTPMCEGEILKHNSLDIHIRVIVFP
LYFFPLYDDVFSLEMKGLYKEYYVDSDITNLLMCSFSLMFLQHLFNGVFKSIKSLGHLSH
LIVEQLINLRKEIVAKFDEDLLDILTNLQNMQDVRQFGDSGQPPAIPVKYALHKIFLSER
ARRRKKRSGGAAPGEEEEESGGEDGSGEDGHNRDHLEEDHPDEDHPDGDHPDEDHPDEDH
PDESPPDAAEGDPPGGGASPPWERTKGRDEQSIKREAPSQGTLVTGGGPNEGGSTDSASD
RSRHSRGGLKRGGTPNSGRDGSKSSSPVGVSGGKGEEGMTPNRGCSAVGG-----RRKGA
PRGRASKEAPKPGEKKRIELGEKKRTESEPGEEYPESDSSGPGPNFMAKEDNTSFLMKKN
EKQKMERKSKRERSHLQEKLGIGDFNFLLNVSPKIDCCVIIDRRIDMVTPFCTPFTYEGL
LDHLFGISNLQIEIPRYIIFNEASKASKASTPSTPLGSQHHSDVLKNMMVRVKLKNSVDV
LYNDIKDLNPNQVGLYLHNKASEIKETYKEKDTLKDIEEINTFLKKIKVKHYEHNSLSTH
VNLASFILTTMKKEANFNKLKLEDEIIQLNSTSDRTTLLNIVQQIKLLIYTNEDIYEVYR
LLCLFSVVTNGFSETHINELKKDILESYGIRELSRINKLHLCNIIKHQPKQKFIWGNLRN
HFNLLSNDHNDISYVCNGYAPLSVRLIEYMGILKNNMQAFPEIFNLLSGPTLDIVQNAVG
YGKFGINKGKEQAPWG-GDPEGQKDVVLLFYVGGISYAEIAAIRNLNRHSRDYHYLIFTT
EVISSRRLLQGLAAMETSANSETAPPKEEADGSPMDETIQVRSMCINCEQEGINQIAKLH
IPYFKNVLIHSFECGFCNYRNNVIQDLNTIKEKGVKIIFNINKREHMDRQLIKSEYGVLK
IPQIDFEIPKETQKGSINTIEGFMQTALSSLTEYLKNLKHMYCEANGLPEGA--------
-----GEVSSKSERGEGALEENAHAIDQTNQGDKPADTNLQGQQMTIESYIKLIESTVHK
LSTYLLSKEPIFTIEIVDPSGLSSLEHYDEDVQKGIVTVEHYKRSKQELNEMGFYEEDFE
GKNEAVDLGPDGAKEKENHVEEKVKKENFDFIKKYVHMGGPGSVNGSAYVRYENISAEEE
GKLIESFTSNCPCCNYMGANNFCEINIPGFKKCLIMSYVCGNCNFKTSEIKSSGEINPKG
KKITLTVRSKSDLDRFVIKSDTASIHIPIVDLTSDYGTLGGSLTTVEGLILKIIESLEDK
FKFLLGDSSTNTKQPNDDALKENNDESITSKVKSLIANLYKLCKTEELCPFDFVIDDIAS
NSYISSEDFSHDENLKEEEYERTFEQNDVLGLTSMNTEYMADGAKRLPLAAENPEMKKSP
SGRVSDDGIKRTPSGKPIQTMYVLNRKGEEEDISFDQILKRIQRLSYGLHELVDPARVTQ
GVINGMYSGIKTCELDELAAQTCAYMATTHPDFSILAARITTDNLHKNTSDDIGEVAEAL
YKYTDVRGRPASLISKEVYEFMIEHKDRLNKEIDYTRDFNYDYFGFKTLERSYLLRINGK
IIERPQHLLMRVSIGIHIGDLEKALETYHLMSQKYFTHATPTLFNSGTPRPQMSSCFLLC
MKSDSIEGIFETLKQCALISKTAGGIGVAVQDIRGQNSYIRGTNGISNGLVPMLRVFNDT
ARYVDQGGGKRKGSFAVYIEPWHSDIFEFLDLRKNHGKEELRARDLFYAVWVPDLFMKRV
KENKNWTLMCPNECPGLSESWGEEFEKLYTKYEEENMGKKTVLAQDLWFAILQSQIETGV
PYMLYKDSCNAKSNQKNLGTIKCSNLCCEIIEYTSPDEVAVCNLASIALCKFVDVEKREF
NFKKLYEITKIITRNLDQIIERNYYPVEEAKRSNKRHRPIGIGVQGLADTFMLLRYPYES
DSAKELNKRIFETMYYGALEMSMELAQLYGPYETYQGSPASQGILQFDMWNVKVDKKYWD
WDELKAKIKKHGLRNSLLLAPMPTASTSQILGNNESFEPYTSNIYYRRVLSGEFFVVNPH
LLKDLFDRGLWDEDMKQQLIAHNGSVQYISEIPADLKELYKTVWEIKQKNIIDMAADRGA
FIDQSQSLNIYIQKPTFAKLSSMHFYGWEKGLKTGAYYLRTQAATDAIKFTVDTQVAKNA
AKMKNAEPVAITREVSRETISTDSTVTQNVCPLRRNNDDQCLMCSG---MSGGGEVVPRL
SFEEMRGEMSKYGVEITQGTLKNPTTEDMQGVYSMCIKHILNKDINNIRIEEFTGDLKSS
MPSIDGIQILPNEGKNHLQAIGNLRFIRHCEQVNKILCVENTLSYLFKPVSSHMARLISA
FVHFTKYKEQIYVDNDMKIRRIEEGKSEDSALGAELKAVKNELQSLQENYEQVKNSVLSE
KNKKRDYEEEIIENQNMLNAQQSTIISLRAAKDKIVNETNEIIFQFSRFRQKKEDLEDQI
VPSPEKLQEYNQELKNLLLEHVSYFEKDKKKNEEIKNKINISDLCLKKLVDLVTILTGHF
NETIKLHIGKKEELKGLEKHLKNLKSEKEHLTMKRKQQEKILLETEQYFAQQKDKWNAKV
QAEEKNVAVVEEKASQLYGQMDELKRQADREAREIDSIVKLIQETLNNYRRNFALIDDLT
ARTRSSHALLAAQVRGQAAGRIG---MIQFLKNLQLHKKKDSGSTKEPKRKNKMKYEDFN
FIRTLGTGSFGRVILATYKNEDFPPVAIKRFEKSKIIKQKQVDHVFSERKILNYINHPFC
VNLYGSFKDESYLYLVLEFVIGGEFFTFLRRNKRFPNDVGCFYAAQIVLIFEYLQSLNIV
YRDLKPENLLLDKDGFIKMTDFGFAKVVDTRTYTLCGTPEYIAPEILLNIGHGKAADWWT
LGIFIYEILVGCPPFYANEPLLIYQKILEGIIYFPKFLDANCKHLMKKLLSHDLTKRYGN
LKKGAQNVKEHPWFGNIDWVSLLHKNVDVPYKPKYKNVFDSSNFERVQEDLTIADKITNE
NDPFFDWMVVSQFYILSPRGDTIINRDFRGDVSKGSGEMFFRNVKLHKGDAPPLFYLNGI
HFTYLKNNSLYFVFTSLLNSSPSYVLELLYRVVKIVKDFCGQINEEVIRANFILIYEIVD
EVIDYGYIQNSSTESIRHLIHNEISASSNSTKRLANLSTFTMKNSNTLPSNASQKPIQLN
EKKNEIFLDIVERINLVMNSKGEIAYSYVDGVILIKSYLQGNPFIKIALNEDLYIKNVHS
DSTNNIIIDDCNFNHLVNLSQFEREKILSLYQPDGECVLMNYRINNNFKAPFRLYATVTY
GPNHTVELCIRIRLDIPAQYTCTNVFVNCNLCKHITNVHLDLNSASDLFSAQYIANEHRL
LWTIKKFKGEHEHSIRSKITLSPGYTFAKRDFGPIYILFEIPMFNLSKLRIKYLRIIESY
KSSNTHRWVRYITQSSSYVYRLMDVRVTTPYDEELDDIEEFNNRILATETLKHKGESVQR
SGDEACPNIKKLVLPGEAILSKKDKGRFLKGSGLYEEDEKYFACIVGTVNYINKLVYVEP
LRGKYTGAVGDLLVGKIKDISNDKWVVEIGSYCKALLSISQTNISVFCQRIRLYNDVINM
INIYKPNDIIACEVQRILADGCIVLHTRSSIYGKLSNGILITVPQTLIQNQKKHIFVFPC
NVQVILGMNGFIWVSSPIKKTKDTNPNSVDQDIEDNKFEEVDDTTRRNISIISNIIKLLA
KYHININYDIVTKIYVHYTSNRNNSATYILKPYVSDSYLFSYIDKFAPWGMPVLYHLTLQ
KPTAITKIAYGNFSGPKVHEIVVSKGQVLELLRADKQGKLNLIASKDIFGIIRCLQTFRL

TGSNKDYVVIGSDSGRLTILQFSNEKNDFVRVHCETYGKSGLRRIIPGEYIAVDPKGRAL
MICAIERQKFVYILNRDTKEQLTISSPLDAHKSHTICHDVVGMDVGFENPMFASIEQNYE
ALDKQVTNTSEIDSYTRKTLLSLWEMDLGLNHVIRKYTFPIDASAHLLIPIPGGQQGPSG
VIVCCDNFLVYKKVDHADVYCAYPRRLETGQEKNLSIVCSTLHRIRKFFFILIQSELGDL
YKIEMEHEDGVVKEITCKYFDTVPVANAICVMKSGSLFVAAEFGNHFFYQFSGIGDEDNE
AMCTSKHPSGRNAIIAFRTKKLTNLFLIDQVYSLSPILDMKVIDAKNASSPQIYALCGRG
PRSSLRILQHGLSIEELADNELPGRPKFIWTIKKDNASDYDGYIIVSFEGSTLILEIGET
VEEVVDSLLLTNVTTIHVNILYDNSLIQVHDAGIRHINGKVIHEWVPPKNKQIKAATSNC
AQIVISLSGGELLYFEIDESHTLVETFRKNLNVETLCLSIQQVQENKLRANFLAVGCLDN
VVRLLSIEKEKYFNQLSTFILPNNSSAQDICITEMSELGNDKERKLLFLNIGLNNGVLLR
SVVDPITGTLTNHYSKYLGAKNVKICPVHVKKNAALLVLCEKTYLCYVHQGKYIYSPLNY
DILEYASSFHSEQCSDGYVAISGSSLRIFRFYRLGEVFSQNILHLTFTPRKIVPLPFPSL
FYDHDTSLEIERQKNIRMLAIIEADHNSYDENTLSEIQRALKGIQLDGEDAQGGSADAEE
EEEEELLYDRIGTVKAGPGKWGSCIKIIHPVNLQTIDKISLEMEEAALSVCACELEALHC
LIVGTTTSLSLKNRSAPAAALRVYTYDINYKLNLLHITPVEDQPFCFCPFNGRLLASIGN
KLRIYALGKKKLLKKCEYKDIPEAIISIKVSGDRIFASDIRESVLIFFYDANMNTLRLIS
DDIIPRWITCSEILDHHTIMAADKFDSVFVLRVPEEAKQEEYGISNKCWYGGEIMAGSNK
NRRLEHIMSFHVGEIVTSLQKVKLSPTSSECIIYSTIMGTIGAFIPYDNKEELELTQHLE
IILRTENPPLCGREHIFFRSYYHPVQHVIDGDLCEQFSSLPYDVQRKVAADLERTPDDIL
RKLEDIRNKILMDEDAPTQSKPLDDEDINILKSYGSGPYSKSIKKVESDISGLVTSINKL
CGVRESDTGLCLPNQWDLQLDKQMLNEEQPLQVARCTKIINGDTDQTKYIINVKQIAKFV
VGLGDKVAPSDIEEGMRVGVDRTKYKIQILLPPKIDPTVTMMTVEEKPDITYNDIGGCKE
QLEKLREVVEMPLLQPERFVTLGIDPPKGVLLYGPPGTGKTLTARAIANRTDACFICVIG
SELVQKYVGEGARMVRELFQMAKSKKACILFIDEVDAIGGSRGDESAHGDHEVQRTMLEI
VNQLDGFDNRGNIKVLMATNRPDTLDSALVRPGRIDRKIEFSLPDLEGRTHIFKIHANTM
NMSRNVRFELLARLCPNSTGSDIRSVCTEAGMFAIRARRKTITEKDLLLAINKVIHGCKQ
FSATGKYMVYNMSIEFCSQHLLAYDNSIITSENDIKSKAEENLRRIIQCMDALKNGKDDL
GEPAYQMTKTNHFNLPRYSRIPKEKKNTRWEIFAKKKL-MKKNKSGLIYDQNSKGWVRRF
QKKQMKINEDKANFVHEYKPSDNIYEDPFERMEEEKEIKKMKQKMREMKNKFHQEGISTE
DIKYIARQKKKRENLIDNLKTAQISSSTFGRKDKQLKKEKKMKVKTKQKCEKRLVKDEIK
QNNKLASIVLKSLMESNPYVFKSLVQIYEEYLNLIIHRVKGYKVLILDDETKTIISLIFS
HSYILEKEIFLTLNFNDSNIFEDATSSGGKSDKFDFKNYKIKNLKHLKAIFLLRPTHTNI
LKLMKELRRPIFLEYYLFFTNVLSDKYTEKLAKADEFEVVKNIMEYYIDAYVLHDNLFSL
NIDYTSFLYKNDEHSLSRRRKKKGVNPTDGGYDFGGARNYDKLTIDEFGLGNEDAFVQLQ
NSSDYNASTIADDDVSNYSSFALFENQVVQRIVDGVFSLLCSVKQVPDIIYNRNSPICKH
IIDLLKIKMLKNESVFSAVLDSYERYNGATHQGEHSTYQFNTMGSQHGNMGTEGNCCYMI
ILDRREDPITPLLTQWTYQAMLHELIGIENNKINLG-INSEESQIVMSCMYDDFYNEHLF
DNFGDLGKAVKTYVDMYQEETSRKSNLESIDDIQKFIEIYPNYKKLSGNVTKHVNILHKF
AEVVEKRQLFYMSELEQSIAIYHKKGEHFKQVIDTIRNETYTNYDVLRLSLLYTLKYEDE
EEVEVIKTELTKRNIDKDQVLLIDALLMYASEEARNNQLFKEQTFLDFAKTTITRTIKGT
SNVFTLHKSYIYYLIEDLMKAKLDSHTYTTTNLLNIEPNVNKRVNSMIVFFIGGATYEEY
RDLQYLSKRYNISFLLGATQLHNSQSFLADALQLVKDM-------DCTHLHAGGLIGSPH
LRSDGDLQCVKKHFCCFAQFPLEVGTPKEKQ-RRHRNVFDRLTDANFYTGMHKQKFDTLR
SRAASEGTSQKCKLNNLLTRGGAEQPPRGIFQKKGKTPCVVP--LGVQKYGIQIARPKNI
WLFRNGDEHHNGLLFLVKPHVHNWTSLLSEITKVLGPTIGPVRTIYNANFRQVHTVEALN
DGEKYLCTSGEPPARVDRLSRFLSRWVMHGMKKVCVINKNNAVKSEKKERTNEKVKICCS
GVKGEEN---ETGEQGEKSDMKKQGKKSDSASSESVKESFEDEEDEDAGGDDKDEAFADA
RKYFKEGQKCITPPNGDGTRAFYESLLEENPNSVIAIKYCIEHGVLSGTKHHQTIYKYNV
LKKNNAFRNNFGGLRGEFIKLLEQPPKSEEGSCSKMDEDRDANIEQWKIKRLIKKLENAK
GNGTSMISLIIKNKDEVSRINKMLADELGTASNIKSRVNRLSVLSAITSTQQKLKLYSKT
PPKGLVVFCGTVITEDGKEKKMSIDFEPFRPINTSLYLCDNKFHVEALKELLESDDKFGF
IIVDGNGALFGTIQGNTREVIRRFTVDLPKKHGRGGQSALRFARLRLEKRHNYLRKVAEV
ATSVFITNDKINVSGIVLAGSADFKNDLLHSDMFDQRLFTKVIKIVDISYGGDNGFNQAI
ELSSEALQNVKFIQEKKLIGKFFEEIAQDTGKVVYGIEDTLKALEIGAVELLILYEGLDV
IRLTTRNAITNTTRTMHISPQDEKQESLYKENNVELEVVEKISLTDWVINNYKKFGASLD
FVTNKSQEGAQFQKGFGGFGGMLRYKLDLNLYDEDVESDAELFMHSYRDANTAQDQKKFL
DECIFIVKEQSFYMKQALENGSLRDTLKHASNMLCELRTSQLSPKYYYELYMLIFQELQH
LDTFINDKKKHKKRFIDIYESVQHAGNIIPRLYLLIIVGRNYIKNKDIKAKYILKDMTEL
CKGIQNPLRGLFLRYFLIQMCKDRIPDTGSEYEEAGGGNIDDAFEFLLSNFYESIKLWSR
MSDKVMKLSGQDEQILHNNRNKVLREKMDVKMLVGSNLVRMSQLEGMTRQYYIEKCLPKL
LQNLSTINDSLIQQYIFESIVQVFSDECHIYTLDILLNAIQKINSSLDFKGILITLLKRL
RCFIESNRFEVPKEVDIFSLFYEHLVLYVHRTLDSYEKGTYSLAFQQDGQAAAGEKRAAN
SGHTANNTA-----------------NAANTPNCSHEEKNQRNDEDEEFVENVVKMLQVL
YEFIFLCIRIYDDVITISKLFELPYMIASNVNLSNDVLCEQIISIIVLPFNYLGLSALKG
KNMQALLASVSQKHKKKLSLDIIDAIIECKNKAIVYRDVEEILSYISPIFNEDGHQRDSH
QADLFNPENGAITYAAKKMCKFFHIITNTDDIDERYNICMLFYKHIENGPYLVHLLPTIV
FTMLHLVTTITNLAPSSRRKSQSRQTFDDLHSDDRSGDPFLEEKLKQYNLYVKNILKFIH
TNLLCVSSQIPMLALKLFLHSAVVVNNYERFVQAHPFLSFENLEAICYEFITQPLIIYEE
DINISSQQYSCIIWITGILCSHITLLQNENYENIALKLTQHANKLLKKKDQCLAILACSH
IYWENRKYRNSAKVLECLQKCIKNAEVAVQSNNDNVILFLFLLQKYVYYYEAENIEITED
SVHYLLHICQEEFSRETCDAGFKQEFLQTVKYVHAKKESSNAFAKIGSLLR-MLEAKLNN
ASILKKLFECIKDLVNDANVDADESGLKLQALDGNHVSLVSLHLVDSGFSHYRCDRERVL
GVNIASLNKVFKLCGANESVVISSKDDEDNLNFVFENNKEDKVTNFSLKLMSIELDSLNI
PDCEEGFDAEVELSSKELTNIFRNLSEFSDTVFIEIDSNIIKFTTKGLVGDAEVALKPRE
STSEDDVGVTIKSRKKIKQSFAIKYLNLFSKSTILSDVVTLGLSDSRPIEFKYEIKDTSP
DADTLKVGFVKFFLAPKMDDEMDNKEMVTPKQLLFQLL---PPLTTHAKEWCKAKFEFGK
ADYAECQGETENIAKYMIHTTPVISSDVDLYANVSLVMSNGYGLKTKEIIIGSEEEGLLS
KTFAVTTDVGSPEHVHVKLNSAKKNWKCKKITIWKDYKYWVFDCTGLLNEKNSEGTYFLS
GNKIYTAFVQTGKDVQAGTSGTVEIVLLGNDKRSNTKVLHEGFTSGVLKKIKFQASDVGT
LEDIILTNNARDDPWYCDFVKIKSD-NKLYVFNVKSWIGHPYEKTVRVNIRADQSGGAAK
DIDCHIRGNDLINMANLPQALQSKVQIFKVRCPQNCQNAELSSVEGSSIHPSSTAICTSA
IHDGALTPSGGSIIVTVGNDLNQYHAVREKTNQIEAIDFITKADEPNFSFYTYRLESIDD
VKSNVRIVDSFGKLSSLGRLEIRSDNTWGTVCKKGPNFTFSDDSAKRACADLGFPNGLYI
KQMCFNLNGRNYCAGYKYPFSSAGMVCSGNEKNLLQCNADDSSHCVDHHDDVIIQCLNQG
SNELVTDGMIRLVDATGAPTTNGIGRLEMFYNGSFGSVCSEGWVKEGEKIACRELGYTGL
KGNGFSHHLCTNIAGENLCGHDADKINAVNVKCKGDEKSLQSCPHETHEDIYCSHDEDLV
IGCSGGEDGGGSQVGGKKNFLNLEKKDFPRKIELTCFDKVVSIADLSSAQVGDVFLASCP
EKCNEEVGVIKGTFLYTFDSPICKAAIHAGVLSSNVADDLVLIISHKHHSFLGTKRNDVE
SHGFTGTSKSFSVSIPTRSIIQEERKSNRKYEGGNAAGNGDDLSDERGNDSLNGHLPAGT
QPTFQWTPPTGFHGFNGKETDFVNCTNLPNEKYIKSLSNFTFIIYFTLSGGGGNWRTLLS
HSLCDGISISVNEENELIIEQNCNPHLIKTKFKPVLGQTYHLAVAFNKTNKGVTLYINGK
ELTLEKAKYDFTLNGDLIIGRSNQTTTDYFIGSIHLVEVYKFVLADDEIRQSASAALSLD
YLSRGSDGNYNGGGKKRGKKKRGSNRKTVDGRQCTTPCKPKSIINKELQINGEQINLSCQ
DDLLSHQFDGKIGSQFLVHCSDDCTKSKLIVKGSNNYYTPDSSICKAAIHAGVYRPNGDE
NNSFIMRIVNGLFEYKAARGHLGIVSKSERQSQLRSFSVFPENDDNILTCSSNGHLFLNL
PVGTKRTIMCPSGCDKMEGKIWGTNVYAPSSTLCKAAIHSGVLSNQGGLVDLSLGSAVDE
FTGSTQNGVESHSSARRSRSLTFSTHS-GGEGLLVLREVTPQAVEEERIAVDVEKLLGRL
KKKVREEVLQRKRIDYLPVGANELPRNLPKTKKIKNKKIKQKLSRDVRLAILSSKKLLAN
RRFKPVEEGFIRPTPGLASAGAESPPGGPLT-----------------PTADTLSGRPLL
TQKELYARADVGTKKKVLDLRLNLGPYRCSYSRNGKYLLATGEKGHITLVDTHNLEPLCE
LEVEESVRCSTILHNHKLFAVGQKKYTYIYDNTGMEINCIKDIPYTYQLEFLPFHFLLTS
IGEFGELVYQDISMGSIITRKRTKRGPCKVMKQNKQDATIYLGHQNGHVTVWTPNIDKPV
CDLYCHATPISAVAIHKNYLITSSVDCTYKLWDMRKLQLMEDARSHNVINQMEISTTGLV
AMAINSHFRTYANFFTKSQLYLTHNMHGDRINSLSFQPFEDICCVGARHSIKSLLIPGAG
LANIDTYVNNPYETKKQVRENEVRSLLDKLPPDTITFGEGQLGRVSPFASSPAHGQGATR
SSNKHPRG-NAMQGKHQVGDSSDGSSPDEASGGRTRASGGAPQEAPPAGKKKKKSRKKKN

KNKSNFLNSNVPKHLRKSVGNSNSNSNSKSNLFFLAVSKNKATVYEYDVSFSSCIVEDIE
EQIKRSEADGADVKES---RSNGTLAATHAKKTGNKFNNDFVINRGIRIYKEFDDVELAT
CTNDYKKIILVRKGNLHVAEVIELQGETNSFQIRAKTPIKAICSSPKDSHLVLYCQYKPE
VSAHNLFVYRVGGVAGKKKKKKKKGDAAGKNEQHNQHELPNQHNQHNQHNPLNAVAPPVR
VDTLKSYSSKTWPFYKWSDSESVCALRMNSSIFVYKDNDFASYVSKAHFENLEFFDVSPE
QSKKKGVLLATYERGSKGKPSVFKIFNSSNLQSHLYTKSFFNSDEMKLSWNKNATAVLLN
VHTQVDKEKQYYYGLSNLFFIETEKYSEVNIMTDRGQIYDCIWSYNQNKFYVCKGDIPAE
IVSYDRCANVAHSFGRHKFNTLKLNGSEKLLLTGGFGNLSGDITLWNTSTKKEVTKTKSS
CAVVCEFFNDGKHFLTATTHPRLRVDNHLKIFTHNGFIVSRINFEELYNVIILPLNKIKF
CETDASLGTYVENSNAQMYIHKQLGIDSKKTGVYRAPGTSAAFTLNGFMNAKKTQRLSKP
KTPGANFVQEQKKKAPQKKKKKKKNQQTGEEPMKNDKIKLVSFEGDEFIVDKYTASMSTV
ILNILEVMTAEEDTIPLPNIKTPILKKIIEYMEYHINNPAEEIPKPLITSNLQDVVSSWD
YDFVNTDKETLYELIEASNYLDIKPLLDLTCGKIASMMKDKTTEEIRAEFDIVNDFTREE
EKQIREENRWCGDIMEGKTEGETGQTGKEDAGDTTEGATAAPEESTSYRSLYKHNYKAHV
YADDFVCLRLKGRGGEGHDDGSPVGGCPHDDT--------PVGELSTKRDNNEEVAATGR
DNLCGVAVDRDEANPPREDAQYVQVGSRMEDHQDEAIYSQAGTQHSNHANEEKRENSDLQ
SDCHLKEE-MPHESNQHKMKEKDQPPEKQNKIKIIEMFKMKLESKEMNRSRHGDDVDHSG
GASPG-ASNRCTQIRESISRIFSESPSSKPKIISELKHGEGHPNSGSNGSGSHHSSGTHH
GSGSNGSGSHERERADGKANGAKNALKEGSLLRLFRCEYFDTHLHIRYLYDRREVGVHEY
LVNSLYTQRKYEDILFYLPQLSQISLVRYESSSLYRFLLCKASNSMHFALKLSWIYHSIV
EDNSSKYKDLAHKMTQEIEMAVVNCKPFNGKGTSGNENKQSYLLNLAHPLLFKRKYLIKR
IKDNERFERTKLSRNCLNRSCNLCLLKGGEESTQSGMEVAV------QGETQRNSQSADL
SSAPN------------------AAPSAAPNAAAT---HAAAHASPKKRRKREATSTAPP
PKLPQCYIVNSGALSIARARVKLPSTYAKLGNPLSASKFFLPEFNCTFDMIGELQQFFMK
QRRCDYFSLLNNFVNLTISVSNLLSTEPDVEIRNELLNRFIYSLNSWMLMRRCIVAACTN
VFSMTGLCIPLECLSPPQSNGSSNISGSSGSSSGLQILHFNYDECKIFFSKKRAPYLLMF
EVADLDEDISHIPDGLFYPSSVGGDEAAGEEEEEAGQTASERGVTPTPPPRRKGHRIERN
FCSSKNYGLFNENSSSYDYEPRGGVHDGKRKGSEVDRGKE------ERGKGAQGKKTTGA
DLDALKMEHMYVYNCIVNDLRKENLISFPSGEEDSLSIIRKCIGMRADEEDEEGGDTEGG
DTNEEGPREATKSFLVNARSASMPNYLSSSLERCNISSNDNDSNEVKTSVESISSGLADL
PPGGLHGSEVDAQ------VDAQEDAQVEAPDGGWSPSPGKRPNDSADQPLEGAAPSPH-
---SSFLHAGEKTGDALDIDEYFKPANYTNEEFKKRYCRVIKRLLWGELFEEKKKKIRKT
SAYGKLKTWDLKCVIVKGGDDLRQELLASQLIRQFKIIFENAGLPLWLRPYEILVTGANS
GIIEYVNDTCSVDSLKRKFGADSISTIFNVVFADYIFEAKKNFIESHAAYSLVSYLLQVK
DRHNGNLLLDSDGHLIHIDYGFMLTNSPGNVNFETSPFKLTQEYLDIMDGEKSENYEYFR
RLIVSGFLEARKHSEEIILFVELMMPALKIPCFANGTQFCIDSLKERFMTNLAVDVCIQR
INALIESSINNFRSVQYDYFQRITNGIMMAINVSFKVTGGKEFTISVEPEITVLELKQKC
AEHVDIPVECQRIIFKGKILKDKEPLTTYGVSDGITMHLVRSAAPVKEPEAEKETNKNEG
AGSNANVGANEHMNDFSDNPLVQMFLQSGAGDM-NLNAGLGAGNFNLGAFANFLNPGGNG
EINRDSISSLLNNPLARSLMNEISNNPEMLANIVSNNPLLRNTFSQSPIMQPVLENPNLL
RELMRPEFLQAGLQFENALNMSSGGNNNGANNAGQSLRMEDLLSNLNNFASANAGANSNS
NNNLNNMSSLFQSPELLQTFQQVMRANRNLGGFNFPGAGQNMDFNTPNVADNRPPEERYA
SQLLSLQEMGFIDNDANIQALQETGGDVNSAVTRLLEKGFNMEKKEDNVMREIKVNKLVL
NICVGESGDRLTRAARVLEQLTEQKPIFGKCRFTIRSFGVRRNEKISCFVTVRGKKALEI
LEKGLKVKEYELRRKNFSETGNFGFGIQEHIDLGIKYDPSTGIYGMDFYVHLSRPGYRVT
RRRRKRSTISKTHKVTKDEAMKWFQTKFDGILLKMEFTGSNFLRFRLALSMISGKAITIK
NIRKKKKKKNSWRDEQSDGVE-REGLREYEAKLLKLIDKLCDDTTIKINEEGDELYFKPG
FLMGNVNDEVRISDLDNTFHCGKERSITYFLEFLLMVTPFFKNPIKLTLKGITDDSIDAT
VHTCKIVSEHFFKNILKFDDHFLNITIVRRGVQSDCSGEVHFFMNNLKTVEPFDMNDAGV
VKKITGTIVCNKISVVFRNKLMNFAKKNLLCFTPYVNIEVEEAKVKSF-KTQNHFISFSL
FAHTKNKCVYATDLCVDEFFLRHAQGVLSSGGGSAQMDDQGGAVGDEDDEATDDETTDDA
ATNDEATNKPQHRCAEEHKQNKPLHDADIYERLGFFIALKMMNEIKGLPSVDSNYQWLPL
LYMALGNDLAVSKISLSMVKPYSIALIRLLRDFFSVVFDIKKVEKSPVEHSYLIKCVGIG
YRNISKKTFMLGFTGSCLSKEFFDLAKSIGEARSKQEEDRIICNEIVLLKTRFADPNASV
KQIKEYLIRAIYIEMLGHDASFAYIHAVKLAHEKNILCKRTGYLSCNLFLNKDHELMLLL
INTIQKDLKSDNLLEIWAALNCVCKLLNSEMIPAIFPIIKNLLNHKNELIRKKVCMLLHK
MYLIDPSLIKEIDIFLKKLLCDVDPSVMGASLNLIFCIAKNEISYCIKLVPYLVSILKQI
CENKLPKDYDYHRIPAPWIQIKILAIFRILGYSNKKISEQMYEVLQKTMQRADFGINVGY
AIIYECVKTIATIYPSHHLLELASLSISRFISSDNHNLKYVGVTGLALIVKINPMYASKH
QLAVVDCLEDKDETLKMKTLDLLYQMTNPLNVKVIVDKLLFHVENSLDIHFKHDLACKII
QLIERYTPDDIWFLNTINSLFLSVGELLDESYSYSLIKLLKANEEVCSGDDDRDDSNYEN
QGESANDDTNDDSEDGNSGEFARDNPHGGENHIREEMKEKKKINDDVYNLRKYAVNTYIT
MLENNENIPFILMQIICWVLGEYSYLCDLENYSTEDIIDLLCECLEKTFNNPDRVKSCII
TAIFKLCCFNNVTDHIVAKKLIEKYKNSKLTDLQQRCYEYESILNNPTLIKNVFSI-SSR
QKMAIDENLSFLNPLVEKHLKSGGKAYITKELRQSETNFESAKSSVPVLNFTPYELPINN
RIHIDTFHTSNSGSAYERSYRYDTQAHVSLEDNTANPKEREKTFKLNVVGPKKWTKETCK
VEGKKNNEKNSHKNGKKKKKKKKKRDNQATYQLKG----SHENVNRNKVGKKDGQKRHPQ
FGNTHGRIGSRYDEEEDEVEGEEDDEDEEEDEDGNDGRGNEKRMDNLGDYQNYERFDERD
IHNVRDYRHQSSNNSSSFYKQESSHSNSNAAAAHSELTEKEKMAAALFNGLISNNSSIDY
SRNNYSSSFSSKKSHSLLSNRNYFNSKSRTDERGKHFVERKSSSSSQPFRREPAAPSSQQ
VEKKNSSSSNKLDEMRKSKCAFDMLDLNEPAAKRDFMEEEEENKELWNSISSQKKAVFLN
TATLNIFQIIKKIENNLNANVVEVSKKEALTSCIIFNYNGGCVLGMSGAQCVAIACDLRL
GANNFTTVSTNFTKIFKMNDYVYVGLSGLATDIQTLYELLRYRVNLYEIRQETPMDIDCF
ANMLSSILYANRFSPYFVNPIVVGFRVQHTLDDEGKKVCSFEPYLTAYDLIGAKCETKDF
VVNGVTSEQLYGMCESLYIKDQDKDGLFETISQCLLSALDRDCLSGWGAEVYVLTPDTIM
KKKLKARMDMHRTLRKIFDRQTCEKIESMKILLVGAGGIGSEFLKNIITIGCKNVDIVDI
DTIDITNLNRQFLFKKEDVKKYKSFVAKERALQHSKGLNINAYTFDVCTMKSSDIAKYDY
VVNALDNIKARKYVNKLCVMERKVLIEAGSTGYNGQVYPILANETKCYNCEEKPKNKTYA
ICTIRQTPSLPEHCVAWGRLIFETFFCKSDNETLMDIKNHVEEESKKRNMDQHEIITFIF
NYLFYDTIKELAALKKDYVTEPIPILFEGTAKKE-DKLGEAAEQGSG-------PPDADP
QNESHTHKETDPAAITLCSQNIWKKDECVKMYTETFAKLYSYLNINKQKEEYLVFDKDDD
DCINFITAISNLRMINFSIKQKSKFDVQSIAGNIIPAISSTNAIVASLQASQLIHIIEHF
ERVKGSDKEVAGSLRDSKAKHVWVKSIVSGNKMFSRGNVVNAEKLEPPNPSCYICQQPMI
DIYIKSFSEMTLYDFVKNVCTNELAFLYPFLDKQDRNIFDYDSFLEEDEEYIKGLHNSLS
EWDIKNDEILILTDFQNDKDQLEIHLKEDPTLEAPYDIKQKVVKRRKAEELRGSEAAPPS
AKKRKHINQEEEPLNDKKKARTQSDMGKLSKDRRDIYYRKAKENGYRARSSFKLIQINEK
FGIFKLFDPQRCGEKEKDKIGSIYNENFCYNVVDLCAAPGSWSQVLKNICLYNYYQMLHW
SGTPPNCVEHEKFLNDFSLYINFNKNNFVKMPKIIAVDLQEIGNMKYVQIIQGDITKAST
VHQILRCMRDGTSA--QLDEQNSQRNNSPTYAHAVVSDGAPDITGMNDIDEFIQSQLILS
SLKVCCSVLKIGGNFISKIFRGEHTGLLILHLNKFFERVYVCKPQSSRNKSLESFLVCLN
FSLPLSSITALSSHTGEEKFNRQPQEELRKMHAKLIAQKDDEGGDDSQVESEGNK-GEEK
HINECASDDAHKNVDIFNFYCSDSDEEIKYFNSDDEEVVNEYIASEVFMNEKLFSFVATQ
NYYDSDKSYLLPQNYVRHEPQLMPLQPPYLLSLQKKRQEGKKMEGGDYDKILVLNFGSQY
FHLIVKRLNNIKIFSETRDYGIDVKEVEELNIKGVILSGGPHSVTEENAPHLKKEVLEYF
LEKKIPIFAICYGMQEIAVQMNGEVKKSKNSEYGCTDVNIITSKNGGEEKYKNYKLVDSK
CILFDGIKNAEKSTVWMNHTDEVTKIPDNFFLVNSTDDCLICAMYNEEHNIYGVQYHPEV
YESVDGDQMFYNFAYNICECTKKFDPIRYHEIELNNIKKYAQDHYVIAAMSGGIDSTVAA
AFTHKIFKERFYGIFIDNGLLRKNEGEKVYSFLKGIFPDMNLTKIDASEIFLNNLKGVTD
PEQKRKIIGKLFIEEFEKAVKNINIDIEKTYLLQGTLYPDIIESKCSKRLSDTIKTHHNV
GGLPENLKFKLFEPFKYLFKDDVKKLSQELNLPEEITNRHPFPGPGLAIRVIGEIDKHKL
SILREVDDIFINDLKAYNLYNDISQAFAVLLPTKSVGVSGDARSYDYVCSLRAVKTSSFM
TASWYKIPYDILEKISTRILSEVKGVNRILYDISSKPPATIEFEMRKCPGRAKRSRED--
------SPDRACITLVRATLLPILSIDKNEKTTLFHPFKSPLPGFVSDRRLYVQKTLGAK
VRRTGWLNLMKNSIPRRSDEAEEQNKADEIKNEFVPLILYESENSKVEVDPILAQFLREH
QREGVTFVFECLMNLRDDRISGCILADDMGLGKTLQSISVLYTLLKQGIDKKPAVRRCLI
LCPASLINNWNDEINKWLPGRCNVTCVNDNAKEKIVSKLEGFKYDQKSTVIICSYECFRI

NNDSLDKSSIDMIICDEAHRLKNDKTKTYMSIYKLAARKRLLLSGTPIQNDLSEFFALIS
LCNPDLFDDTNSFRKKYANPILIGRDKDATEKEQEVASERLAELSTITNKFILRRTNNLL
SKVLPVKYLINIFIKLNPIQEALYVLFLKDKRILKNEQSTNKVNVLINIKKLEKICNHPL
LLNPNDMKDVGHVSLAKLIEDAAKQHQGKRQAKQEKAERTEKPPEGHPQGDRDRDRERDR
DRRRAVEMDYDKPVKKLLEECRRDTHRCYYNMSCKFQLLHFLLKTIKQNTTDKVVIVSNY
TQTLDYMEILCKENFYKFVRLDGGISIKKRHKVISDFTHSSDIFIFLLSSKSGGCGINLI
SSNRLILLDPDWNPANDKQALARVWREGQKKICYIYRLFCTGTIDEKVYQRQISKDGLSS
MIVTNTNFSKDQLSDENVKKLFNYKMNTLCETHDNIECTRCTIRNSTEKEENFSEQLEDF
EEEDVNTWAHHQNVESVPDEILAKAVKAATDYKRNDMNNFPVLKKLSDLFVTFAMSCKIE
FRDDLIKQKAQKAQKGERT-----CRPGAAADGPVEQDEEADYEGEDEEEEEEEEEEEEE
EEEEEEEEEEEEEEEMKEIFPKNACTQGEEEQFCLQDINAKNEEDYASQVSSLPYVSPHH
GVCIRKKKVEPAGFMFYVKYEIALLKGKKKKAKEGKYIPIEEIFLYKDKIDYLNELKVKN
EEGDQGGDYNKERSTKNNKDKNYEDDYDDANNSKKKQEKANNKKSSNLVKKCINNNINVY
EVLSVEEADDLETIKASYKKLILLFHPDKNKGTSFLNQKEKEKKRKKEREGQHDSHETKK
DFMYYMEKYNIEKLTPEEKKTMFLKIQDSYAVLSDKTLRKQYDSSIPFDEYIPTLTELEE
APNFYEFLRPVFKRNAKWSAKKPVPDIGDEHTDIKNVKYFYDFWYNFINWRDFSYQNEYN
YEEAECREERRWMERENKKIQKKASKAENLRIIKLVDLAYNNDPRIIAENKRLKLEKQKK
KELAILEKQKRIDATMNEVEKEGSPNLPSSDKKNKDNKAAVKIWRHHIKSLCLTKLAKHV
DSDLVQERLSLMPFETLCEFINDIYVFLNFNLHKGNPPSVEKPGANSVYENVSKKEDNNA
LNGHIVNAEKVPNGVSSGEPKLPDSDGGNRTTGFIGHLKNVQLGDKEIELLILIFKKYIN
EFTRVMEKEQKRDSEEATITRSKTEKDARVAGGGTMNSSPSPVRQQVSENVKLMNEQGSG
NHSENDKKKSEESSNMCGKWTAQEVSLLAKALKSYPGGTKNRWNLISNFIKTKSVKEVIK
KTKEMFENETLKNLSRNFEETPFDNFKNQNKGVMKKIDDNLDKRDVKVTAAEKDGLTAKS
ANGHAEVKRPWTHQEQHLLEQALMKHPASLPKKERLQLVASEIKTRTLEEVILRMKTLRA
QILAKKAAKMPKKRRNGGRSKHNRGHVNPLRCSNCGRCVPKDKAIKRFNIRNIVDTSAQR
DIKEASVYSTFQLPKLYIKQCYCVSCAIHSRFVRVRSREQRRVRKETSKHVNPSQLMKKK
AETKLSRFKCHHHSDERETLAAASRADNKERRALL-FTFLFLFFSLTRFAQCQVTKKKVS
VSEINFDSAVEDVQWCGNNHMTVLVKTVKGRLYRSSDGGKIWTNITSSLSENPVNKNEP-
ANHTNDVTTVDLIMVNPVNKNIILIIGNQKNHFVSEDSGETFRLINYKNKINFWQFHSTK
THWALVSSWTSACYSTDEKSGECMQTLSVTKDLGRTFQLIDIYVVQFNWGDKDTHSEDTV
YYTRHKNRNGHQQRFSGWSKDVDFVRTDNFGKNVDVLVKQGNKFLISNGYIFVAKLNDVI
KQTVNMMVSTDGGKTFNKANLPENIHEKSYTVLDTSEGAIMLHVNHGTSSDRINTGNVYI
SDASGLNYTLSLPNNIRTSSGECEFDRILSLDGVYIANFLDENDDIKDEDLRFHNFKAQL
EEDVSPFQTNTEKRKKQAAKGKNEDLVRTVISFNKGGHWSYLKAPKVDSRGNKYDCGDNC
FLHLHGITNYHQYAPFYSIENAVGIIMGTGNVGSHLRYESDEVNTFLSRDGGVTWMEAHK
GPYIYEYGDHGGLIVMADDLRKTNQIVFSWNEGQSWFDFELGQFSIDVDNIVAEPNSSSV
EFLVYGTRNDVGVLYHLDFNALGQPLCKGLWAADSVSSDYETWSPTSGSFSDKCILGRKI
TYTRRKQTSECFNGKDLKRTVDKKSCECTPEDYECETGFTRKVGSYECKPNDPTLTIEGC
TSSSYFYANAYRKVPGDICVNGWVPEKVPVPCPAHAPFNRSAKSILFILFILGLIMLIVT
YLCRNPKFKHLFYNYGFDTFEHVKYSVIKTKRGNVSTNVFEPEMEFIDAEQDDNEEDVPT
LLSYSNDRNRTRNDFKLTRNRSNQNNYLTSRNVSPPKKYPENIELLMADRKSNKNAVVKN
VDMTEEMQIDAIDCANQALQKYNVEKDIAAHIKKEFDRKYDPTWHCVVGRNFGSYVTHET
KNFIYFYIGQVAILLFKSGMDVQVDEAVLQKAKALQQANEEEIQAKKLKPQSEGLLSPKS
DLLQVTDKGCRSRIRICNVLNVPKSEAEFNDSSRKNEYIDQVITVCGWSKAVRKQGGGRF
CFVNLNDGSCHLNLQVIVDQSIDNYEKLLKCGVGCCFRFTGKLIISPVQNEEKKGLLKEN
VELALKDNSIHNFEIYGENLDPQKYPLSKKNHGKEFLREVAHLRPRSYFISSVIRVRNAL
ATATHLFFQSRGFLYVHTPLITTSDCEGGGEMFTVTTLLGEDADYGAIPRVRKAVKGGQK
REDNVQSATH--GAPPAQYLIDYKKDFFNKQAFLTVSGQLSLENLCSSMGDVYTFGPTFR
AENSHTSRHLAEFWMIEPEIAFADIYDNMELAESYIKYCIGYVLKNNFDDIYYFEENVEK
GLISRLRNILDENFAKITYTNVIDLLMPFSNQFEVPVKWGMDLQSEHERFVAEQIFKKPV
VVYNYPKDLKAFYMKLNEDQKTVAAMDVLVPKIGEVIGGSQREDNLELLDKMIVEKKLNM
ESYWWYRQLRKFGSHPHAGFGLGFERLIMLVTGVDNIKDTIPFPRYPGHAEFMSSPSCEE
NVPEEELKKAEVLCKELDELKIQYKEVKHAKANSIKDLLDMNLENSKNIIKNLFLKDKKK
NYFFLCTVNWKTVDLKYLSSLFKTSNLRFVDEGNLKSMLNLLPGSLTPLALKFDQENLVK
LYFDDELKSMEDMIVHPMHNYSSLYMKQEDVVKFCQLHNHAPEYIHIEEGKDKLKKEDGQ
LEEEKQKGGSGGSGVGSGANAGKDDQAKDANILGITAKKTTSFSEWYTQVIVKSELIEYY
DISGCYILRPASYYIWECVQAFFNKEIKKLDVENSYFPLFVTKNKLEREKNHIEGFSPEV
AWVTKYGDSTLPEEIAIRPTSETIMYSVFSKWIRSHRDLPLKLNQWNTVVRWEFKQPTPF
IRTREFLWQEGHTAHKNEEEAVKMVFDILDLYRRWYEECLAVPIIKGIKSEGEKFGGANF
TSTAEAFISENGRAIQAATSHYLGTNFAKMFKIEFEDENETKQYVHQTSWGCTTRSIGIM
IMTHGDDKGLVLPPKVAKFKVVIVPILYKNTDENVIFNYCKDIEKVLKSAQINCIFDDRD
LYSPGYKFNHWELRGVPIRIEVGPKDIQNNSCLCVRRDTNEKFNVKKESVLLETQQMLVD
IHKKLFLNAKKKLDDSIVQVTSFSEVMDALNRKKMVLAPWCEDISTEEEIKKETQRLSQN
QANTETSLSGAMKPLCIPLDQPPLPPNTKCFWSGKPAKRWCLFGRSYMAKLSKAQKKQIY
IEKLSSLIQQYTKILIVHVDNVGSNQMASVRQSLRGKATILMGKNTRIRTALKKNLQAVP
QIEKLLPLVKLNMGFVFCKEDLTEVRNIILQNKSPAPARLGVIAPIDVFIPPGPTGMDPS
HTSFFQSLGISTKIVKGQIEIQEHVHLIKQGEKVTASSATLLQKFNMKPFSYGVDVRTVY
DDGVIYDAKVLDITDEDILAKFGKGVANIAALSRSIGVLTEASYPHVFVEAFKNIVALVI
DTDYTFPLMKKIKDMVENPEAYAAAAPVA-AAAADAPKKEEAKKEEEEEEEEEDGFMGFGM
FDMLQERDLAREPCPDRIIEDMGGAFGMGCIGGYIWHFLKGARNSPKGDVLSGALYSGRM
RAPILGGNFAVWGGTFSCFDCTFQYIRKKEDHWNAIGSGFFTGGVLAMRGGWRSASRNAI
VGGVLLAIIEIVSLVLTRKTTPTPRQQFQQQMELEKKMASKNSKMLKDEVWLYKSEDKEG
SFYDEEGGRKPKDHFLEVPLFDEDESGRSIQKNLRSLPSRKIKAKRSEESEVSEESLSTT
DEEEFLDLYEELKREQSVKEFYNPMLRPRLWRFEFFIKYIHNLENQLQRNFYLVSFGNIK
KLKLYGDLKKEVLYTPGYSVEPGEVKYLKVPLPIWAEKELSISYEDLQNFEVAIEMWCIK
GFIFNELYASSKKTLKEVIENDPDTNIILRRRIEKRNITFEAQRLGVYMQLSEIFEFHMA
LDSWWFIANSEMPSYLKTLPKILRFKFPLSEDDWVIHSSHKSQNNFWLYPGYFCFIGTYH
QLANAFFILTVLSHNTSYRYKPPILLGSCIISLKSVTEHPFFKGTVKKLTLDKTKFRQGE
IVGNIKCFVNSYGIEEGDIPVQRPVQPLSDATLVNQLVLNDHYLVIRVIKCENLAISSID
LNNVNINVWVKWDGIVNKTDTVSKSTSPFFYQNLYFPIRLVDKKELTHESLIKNVLPVDL
ISKGEICFEVHNNNEVNSTILGIFELPFADIFNYGTPDYRSLAQEASQSSSPYNDYKNYD
AANEGADLASDNYDDYYVRKYKTVVYKNTLELMYSTLSIQTKKESTISVEAFVIPPLPSG
LVFVEKEKIQNASMIYKSMSKRWERDFAKFKDTYTQWFPRADKSRSFPCVSRNEFDGNHY
PLCSFVTSINLPAQVSTPGPLFHWLNNIEYIENEDESSIFTPPYFFLSYKKGTIQDHVLL
LCCCLKGLEYDAYVCKGTINNGKKNHYWVMTRHEDGWVCFWEVTNKCIIHLKRRWNNSNF
SRNAEVRAQEEMISKVVENDRERYYTGDYLMSFVRYGLEELKKREKQIKEEYDLKEQNKL
YTMELYRENHMKDEQVDVNEVLYNDEELFNNMFEVKFEKHETYSSSKALKYLLENFSKHI
PIAPKMFLLDYESTLAYVPYSSVEVVFNDEQLYGNLQNHHPACILYDLENNFHWRPLLNH
SPVPIKSEITISTPLSDRLSVKYTKDLEEEIQEMILFMRTKEGLDTSFEHSKEIRHFLEM
YIDLCEYKLNLDNNYNTKPENYDRAGG-------GEAAAVGGKAAEVGQAATGGEDKAHL
HLRKTWSQYKNDYYNLNFYETYHPSGHNEMISHEQKKHLANIPEDYIYGMNDEVYTQGKE
QYIKNYVFCRDSADILNNYDSSVKRKNVKRFSNAPVHYQER---EKNQEEDIPSISGDGS
IQEGDPTSEANESKRRPTDRADVPNKYIKKYNKRLEKSLKKKEKQMKEEMMDVKLGGEFL
QGRLGRQSFFSYVDTQAFLKHIPSKGDHTEGRCNGEGTQRGRRSGDGVGPSYRVNESSTL
LRKSHPSGKKLRIRGNGKGRSG-ARFERNPFV-SEKKAEDRKDGAASSPDREVTGALQRG
RLFLCQIPPKTWSRLNPTSKYAAHQISQWNWYYSLEEQYFNWQYKFPVPPNHTFVGFPIH
FSTIDFYEVKSFLLHSKRFENIMKLSIDNISFMSLSIYGNRESGQDVRTANVTAVQALSN
ILKSSLGPQGLDKMLVDNIGDVTITNDGATILKQLEVQHPAAKILVNLSELQDQEVGDGT
TSVVLLASELLRRGNELIKMDIHPTTVICGYKLAMKESVKYIKEKLSERVSNLGKDVIMN
IAKTTLSSKFIGYESDYFAKMVANAIQSVKIVNDAGKTKYPVSSVNVIKVHGMSSLDSKL
IEGYAIMSGRASQSMPTGVKNAKIAFLDFPLKQYRLHLGVQVNINEPTELEKIRQREKDI
TKERVNKILESGANVILTTQGIDDMPLKYFVESGAIAVRRVNKDDLRRIAKLTNGQIRLT
MSSLDGTEKFEPASLGYCDEVYEERVGDWDVMFFKGCKTSKSNTILLRGANDFVLDEMQR
SIHDALCSVSRALESNYVVVGGGCVEVALSVYLEDFAKTLGSREQLAIAEFAESLLVIPK
ILALNASYDSIDLVCKLRAYHTKSQVMNTEDPKDYRWYGLDLVNGKVANNLKNGVLEAMI
SKIKSIRFATEATITILRIDDLIKLTPEERREEQPM------RSAPDPSNNLFDDLRICS

RVIDSCGLACSSGFVAVPWEVEGGGLIGAIRLENQMRKPPVIKLKGHTSSILDLQFNPCF
SEILASGSEDLTIRVWEIPHNDESVKEIKDPQCILKGHKKKISIIDWNPMNYYIMCSSGF
DSFVNIWDIENEKRAFQIIMPKKLSSLKWNVKGSLLSGTCVGKHMHIIDPRKKEIASSFH
IHNGGKNTKNIWVDGLGGDENYILSTGFSKNNLREMKLWDLKNTSSALVTMSIDNASAPL
IPHYDESTGLIYVIGKGDGNCRYYQHSLGSIRKVNEYKSCSPFRSFGFLPKQICDVYKCE
IGRVYKNENNSSIRPISFYVPRKNPTKFQEDLYPPILMHDPDSSSRNWINGKDNKMERIN
VKDLTQDDLRITKKYKFVPQSFNSIIIGEEYTSKRTSIIRQFTKKFTFFKKGLHNDGFSS
SFKESVFIYPKSFKEKGLLTEQGGAQFSSSNSLERGAEEAEHPDEQFPLEGEPPCDGTSR
GTSERTRRGSGANCFDALRCARLCRRREMKIRNIGEESIHNICSSQVIFTLSSVVKELVE
NSIDADATEIKIKLVENGIKLIQVNDNGAGIKKSNFENVCARHATSKITEFEDIHSSLNT
LGFRGEALNSLCMLSDLHIVTKHEESSHGYMLTFDNLGRLSHEEPIARLRGTTVSCENIF
KNIPIRKKDFIKNIKSQLSDLLLLMQQYAIIYCKVKFSIQNVITVKGNVKNINLLLTNGN
ETVKKSVHTIYGKKNIGNLIDFNIDAEEWKLRAYISDSNSGRRDRDIQFYYINSRPIHVL
KNVNKIINSIYREFNSRLYPIIICNILSETKNFDINVTPDKREVFFIYENELCERIKTAL
VKLLTPQTSQLVDTHIGDYFLKANNIPPEEDTFAEGGAPLQLLPSEAPLGGYSPGEATQF
GQNGKKTTPSWEGASTEGSFPERAGGSAGGVKEEVPMSRSAHHAKAWPPRKEEHPQEERT
PPFDSSEQFWGTPVANGVRARQGWQVEGSLSKKGAQG-----EEAPRVEAYEYKLEGACE
LGESP------------QVRVQEYHLEESPKV--VQEYQLEESPKMLVQEYHLGESPKML
VQEYQLED-SPQVHVQEYQLEDSPQVLVQEYQEDPPKADEGELLPRSQPEGGSERGGDSP
EVAEVAEYSFDDLKENIKRSCIKSIPIDINMYINREQMMSGFDYDPIHVVTLTNSEKIKN
IIFPKGKEQEKANSYLCLTDEKQVSQYADLFNSSLSIKEAATVGATSGGSSEHISFSNID
EGQRDLYFKSNLFEKLKICGQFNKGFVISKIDLLYFQRGGGGGAVHP--VGLGEEQAEPQ
GKGSYALFIIDQHAADEKSNFEKYNKVFTMKSQRLISKIDLELSPAQIYVIEKNLEVFLH
NGFDVEIVEEPLRKRRRGGAAAAAAGEGALVQVKVYLLSLPVFNGKILEVEDFMSLLHHL
TEHPITYDKASFQMFIRNKGQPNKQTDTWFNYNFPRPQRVWRILASKACRNAVMVGKALN
VAEMIKIKKKLSVLKNPWNCPHGRPTIKYIINDVDIKSCFQNYYAKLYDEITNLIVTKNY
DAYKYLFHNHAFFLIMSTKPMLGPLLKFQMVMEWKDEGTSLHTTYVYSYTCLYCVCVFLR
LCIPTSAWTAPPSLRTVRPIFLPLNVVANSTSSLLLHPGQLDLKHVEGYMTSKKWLSKLK
CCGDNPLTVDEMKALVLMFLKKISDTYVDDQAKWMNKMRSSQEEQGKALEEAMNEYEKNI
LFNHALKEQQLLHNNKKISEWNETIENAYEAQQEVLRQFEAKKREDKKMALEKNNELIIA
KDYIDKIKEAATDSRYANSKCFVYPASSAPCGACTSAGAIAPYRRFKEPRRKKQYSLCLM
ATMDHSVQDELVDYEDDENILDAKDVKGTLDNSLLNNNNKGVNENGAMRGSYATVHTGGF
KDFFLKPELLRAISESGFEHPSEVQQETIPAAITGTDILCQAKSGMGKTAVFVLSILQQL
ETNDGKDIKEEKEMNNNGSGASKNKFVRCLGLAHTRELAYQIKNEFDRFSKYLKGVRCEV
VYGGISMSKHIKMFKEEGIPHIIIGTPGRILALIREKYLLTDKIQHFVLDECDKCLEKLD
MRSDVQKIFISTPLKKQVMFFSATMAKEMRDVCKKFLQNPVEIFIDDEAKLKLHGLLQHY
VKLQEKDKTRKLIEILDALEFNQVIIFVKSVTRAITLDKLLTECNFPSIAIHGGLDQQER
IERYDKFKKFENRILVSTDLFGRGIDIERVNIVINYDMPENSDSYLHRVGRAGRFGTKGL
AVTFVSSQEDTLALNEVQTRFEVAISEMPNKIDCNEYINQMSDAQEESQLSGTNQSPLIS
EEYSSDGDETECLSEVDKKEMELKESDLAKLSVGQSKRMSVSAEAYGEWNKKKLNFVPKV
YKKEESEKEKIREALNDSFLFNHLNKNEMETIVDAFFDEHVEKNVNIINEGEEGDLLYVI
DQGEVEIFKTKENKKEVLTVLKSKDVFGELALLYNSKRAATAKALTKCHLWALDRESFTY
IIKDNVAKKRKMYEDFLTQISILKDMDPYERSKVADSLKTKTFADQEDIIKEGEPGDTFY
IIVEGNALAIKDKTVIKTYGKGDYFGELALLKNKPRAATVKAKDTCQVVYLDRKSFKRLL
GPIEEILHRNVENYRQVLKQLGLDTACIEGNMHLQIVCLTDEVREMYKHHKTHHEGDSGL
DLFIIKDEILKPKSTTFVKLGIKAIALQYKSSYYHKSEANANANKHQKNPEIVNTSYLLF
PRSSISKTPLRLANSIGLIDAGYRGELIAALDNTSEEEYLIKKNDKLVQLVSFTGEPLSF
ELVDELDETSRGEGGFGSTSNKMRPCDPNSAFFGFMGIAASSIFSNLGAAYGTAKSGVGV
CSVGVMRPDLIMKSILPVVMAGVLGIYGIIMSILIYGKMTPAAEYSTFSGYTHLSSGLIV
GLSSLAAGLAIGIVGDAGVRANAQQNRLFIGMILILVFSETLALYGLIIGIYISISDTPK
LCDSYATMTKRMPNKANPSSRESKNEKKQKNDEVDHGENSTRNILTMENILEEKDKDNNK
KVKNKLILKNKKNTSSTFNSNEMRILTIPKHRISSVKTNWMELIKPIVTHLKLEIRMNGD
KIEVRTCKLTEDKNNLQKSSDYIKAYLLGFTIEDSLALLRIDDLYVESFQVKDVKILKGD
HLSRCIGRICGSNGATKYAIENATKTRIVIAGDKIHILGSFNNIKMARYSICSLILGSTQ
GKIFNKLNILAKRMKERFMGNQCCAGRDLLYKNKLQEFGIEGSKTIRKLLSFTGNDILRF
DKAYDENDVQEFVNLCSSTCEIEKLEDRMHPWAADPKTIGALSATQLAILASKENEPHYK
DAIREADGIPVFINLLKSHELDRVHAAVVALSFLSVDNVKNCIAMFENGALPYLISGMKS
NIDGMKAACAQTCRNVFVLDKKYKKEFLKLGGITQLVNLLEMPRKDDSQPLYTQLEAIYH
LEDFILNDGDEVPEFLEAVKTSNAIKNLKNLQQCPEQDVAEASNVLLLRLTDMANGWPAD
KIFGDVMSYTYDDIICLPGYINFPMSEIDLCNNLTPEISLKTPIISSPMDTVTEHKMSIS
LALCGGLGIIHNNMSIENQIEEVKKVKRFENGFIFDPYTFSPEHTVADVIATKNKVGYKS
YPITVDGKVGSKLVGIITGVDYLYLTDKTRKIKDIMTTEVVTGNYPINLSDANKVLCEEK
KSVLPIVNSNYELIALVCRNDMHKNKIFPHASKSQNKQLIVGASISTREHDLERANQLIK
NMIDIICIDSSQGNSIYQIDTIKKIKGAHPDIPIIGGNVVTCDQAKNLIDAGADVLRIGM
GSGSICTTQDVCAIGRAQGTAVYHVSNYAHTRNIKTIADGGIKNSGNIVKALSIGADFVM
MGNLLAATEESCSDYYFENNVRLKIYRGMGSMEAMYNKGFNSKSRYLVEERKNDGLCDQN
EEIKVSQGVSASLVDKGSVLNLIPHLVKAVKHGFQSMGIRNIPELHSRLYSGDLKFDVRS
FNTIKEGRISDNLIFTNKKMVGKKAKGRRFVSATCRVKSIFDS-RYVFYSLWSERRNAQQ
GGVHLTKKCFSTFMKNVIEQVKKDMKENKQYQEALKELKEKTEIDHKAVKLKKKIEENIN
LLKHIKGKNEEAVRLLYNDVNKFIRYCFESYTVFRFLKHTSVKFFLIMQRLLLCSSSKLT
ELADKWNENSAFVQLDKWRQEMAIKRYKRGARGGSNTDSNTESSSNGAPPLPNQSNAGSA
SEDKPGSSDLVLAHESAWDKFGSKLKDMPFLNNFFENPILGKLFGETELAAALREMKMQD
KNFKLCELMYLFEFVISKHIVESYLIGDEETLRLHCGQSAFNSLNASINERKKKKVYLDT
NVLIYKNHELKGAQRMEESSPWFIFTFHTQQINCLKNANDEIVEGHIDDIREVVYTIALS
KHPEPEREGLLYPYLVREFAIIGNTPSWMNSTPCDNVLHARSYRLSLDDIQNLGSLYFEN
NRKVAKYNEEISLLKQQLTYLNEKMGKGYVHKVIEPAKPSELTFWCYELKEMKEFQDLVM
YEIKKKKKQFKILSQSCVKYLSNREKIKQKKQEEEEKRLKTHSKHISSYMDLFWKKIEKL
VWEEKKRELQKTLNKKKEMRFKKFVKDAIKKIKRARQNVHELFVNRHASISSCNPSENV-
TFSLHNLADDELKEEDLTNQEEEDVLLDEQMESSDESEEKGKEISLLDDEASMPIEELLK
RIYGFKSGEQYLDLMRQGEGAADEG-----EAADEGGAADEGEAADEGGAADEGEADAAA
EGDAAAEGEPPQGVDGESSEAKPKEKKRKFEEAEEAAPPAEDNLTKKSNSCSGRRKKRRK
RKKRSRSHHGKEGPVSSTSNDDMLMCNMQEKHLTKIPPFIKATLRDYQHAGLHWLLYLYK
NNINGILADEMGLGKTLQCISLLSYLAYHFDIWGPHLIIVPTSILINWEIELKRFSPCFK
ILSYFGNQNERYKKRVGWFNKDSFHVCISSYSTIVKDHIIFKRKRWKYIILDEAHNIKNF
NTKRWNIILSLKRENCLLVTGTPLQNSLEELWSLLHFLMPNIFTSHLDFKEWFSDPLNLA
IQKSKINDSRELIDRLHTVIRPYILRRLKKNVEKEMPNKYEHIIKCKLTRRQQVLYDEFI
QNKQVQNTLSSGNYIGLMNILIQLRKVCNHCDLFTNKHIQTPYYYLLPITFYIPRFCVLF
ERTYHLDFHLILFLHKEFTSLGGLPRRREAGGQSASRQRDLSSLGSPHSFAAHQRGTPSD
STQNLVDAIPLQPFPPPGDTLDGPQFGQPVGGAPNEITSESVKRSS-----------SSI
GSVTGKKRPRSPSGESLTRNIEDVNTYVSNQVKRRSIPKNSDEFVCEMNNNYDILSLFID
PFNKALNKAALSKPPPNEALLYRNNLKIINIDTQYRNFFSDESNQSYLNSLEHNLWIKRQ
RRFEEKDRRRLKNSRTPLFGANLLSLLRREFSADGFVPYHSSNNLPVDRSLMREVRSADA
VTPTGVPPLEALFPTMELFLKTREREIHNFTVLNTPAVICSSHKIAVNNTL--LQNGSYL
EPILHRIKVATRVYHQPFHKQSIIFPLNKDITLGSGKLFALEKLLNKCKREGNKCLLFTQ
FIKMLDILEVFLNHLNYTFIRLDGSTKVEQRQKIVTKFNNDKSIFLFISSTRSGSIGINL
TAANVVIFYDTDWNPSIDKQAMDRCHRIGQTKDVHVFRFVCEYTVEENIWKKQLQKRKLD
TICISMGNFSNLNSRSGRTPMPSSDNKDWFANVDTIKEIFVNKRNNDEDEDIYKDRLLHE
HVDEADKTNVRFEKTLEHVEDKDDINALHENKKETLNAISQDLQEFTNRNDFQDTYTLTS
YCFNFLSDNLTDGLRQQIDEMKMKIEIEMMNVGGGGEDDEEDGEDEDDEDE-------SP
SMALYIIGLGLGDERDVSVKGKELIEMSDVVYLESYTSVLFVSKNTLEEFYKKNIKEVDR
NLAEENCEEILKEAINKKVSFLVVGDPLCATTHHDIILRAKKKNINVQVIHNASVMSAIG
ESGMQLYNFGQTVSIPYFEGNYKPTSFYDKIKVNLDNNFHTLCLLDIKVKERTIENMMKN
KNIYEPSRFMTVNEAIEQLLYCEEVLKKNVITDNTRGIAIVRIGSDSQQIVSGSLLALKS
VSYNDPLHSLIICAPTLHDVEREYFEMYQHRCMGSGKPSGLRAARKLRIRRRTQRWADKG
YKKSHLGTRWKSNPFRGSSHAKGIVVEKVAIEAKQPNSAYRKCVRVQLIKNGKKITAFVP
GDGCLNFIDENDEVLVSGFGRSGHSVGDLPGVKFKVVKVARVSLLALFKEKKEKPRSMGI

KGLTKFIADAAPNAIKEIKIENLMGRVVAIDASMSLYQFIIAIRDSEQYGNLTNESGETT
SHISGLMSRSIKLMENGLKPIYVFDGAPPELKGSELEKRGEKRQKAEELLKKAKEEGNLE
EIKKQSGRTVRVTRKQNEEAKKLLTLMGIPVVEAPCEAESQCAFLTKYNLAHATATEDAD
ALVFGTKILIRNLNANASSNQNKNKNSSKRGYILTEINLEQVLKGLNLSMNEFIDFCILC
GCDYCDTIKGIGSKTAYNLIKEYNSIEKIIENIDKNKYQVPSNFRFVEARDSFINPKVLP
KEEVKIDWCEPKIEELKNFLIKDYNFNEVRVTNYINRLLKARKVTTQRRLDNFFTACTKK
STKLVIEESQQKEAKSKRKGKKRDAPNDGAAKLNSKQSKKTKVEKEPKREKKDGEAHNGD
NNEDEDPFDTDDEEDNPSPNFFHQKSDSESGNVKKEKTEQEGNATTAGDEMKKKFLLPYC
PKNVTKRKSVQRCMFFSSFPFDSMGGQQARRKREVNNSKYYEVLNLKKNCTTDEVKKAYR
KLAIIHHPDKGGDPEKFKEISRAYEVLSDEEKRKLYDEYGEEGLENGEQPADATDLFDFI
LNAGKGKKKRGEDIVSEVKVTLEQLYNGATKKLAISKDVICANCEGHGGPKDAKVDCKQC
NGRGTKTYMRYHSSVLHQTEVTCNGCRGKGKIFNEKDKCANCKGGCVLKTRKIIEVYIPK
GAPNKHKIVFNGEADEKPNVITGNLVVILNEKPHQLFRREGVDLFITHKISLYESLTGFV
AEIMHLDERKILVDCTNSGFVRHGDIREIAEEGMPTYKDPFKKGNLYITFEVEYPMDLVI
TNEKKEILKVLKKQNEIEKKYDLENSECEVVTCQAVDKEYLKQRLSKQQQQEAYDDEDHQ
PEMEGQRVACAQQMNSCFDNNNSYVEWKPNEKIYKTVIQALSSCNSSNNSVQIEVTKVLK
DLNENVADAALYLLHIFLNKQEKNDVRQVGGLLLKNYINSKNKFLNNEILKIIKNEIFKL
VEDEVKEIRNTAGSVITTILTKYEGIDKWPEALYNLLLLIERGNNDVVDGAFRAIIIIIE
DELMNRKNADSFFFQFCKTQLLQKLFAYCAPQEKSIKKKYAAECLDLFITSSCFTTNGVF
NDYFPQLWECLGFLASEEDTQILKIVVTCMTIITDTRYSSIFNNLDAIIQFMVNATNSCD
RKVQLEALEFWPVFIKDRSYIAYSNYNNNSNDINKV-DNYIDENVYKNINELRNEALKIL
KNYLPYLCKILVDNTVYTKWDYLTMDESHFQNDNANVPDLIQDISPEMYNNSSKNNEMNQ
EEMKINKMNSNSDSHHMGSNSRNGNMNSNNMMQPGDQANNENHMDDYTDDEKNDEMTART
WGNDWTVRKGAALCLDYLSNVYNDEILEFILPHIEEKLMSDKWNIRESAVLTLGAIAKGC
MYSLSPFIPKVLEYLIKLLNDEKPLARSISCWCVTRFSSWICHPDNCDKWFEPVLLNLLK
RILDTNKRVQEAACSSFANLEEDALDLLNNYLHEIVHTIQQAFQIYQAKNYFILFDVVGT
LIDSVNIVKENNDLAHEIVNSILSKWNNIRISSPYIIALMECMSCITSAYGKDFLKYAKN
VIRTCIKFLVLLYIDLEEEIKYYYSKKAGNTNSFIVNRNNISVTASELLAYYKINNDDYF
TTLKDIDSISPSKKKDLIECSFDLLSRILSVINSNIMNIISENEFNFIPLVHRYCLKFEN
IKFDGVLNKLIMSQGFNKLVLSSSSAVKNGRIGNDECTELAKHFLNFGILQSNFALIGDI
SRFCAQYLIPYLNDIIPFLIAHITHPSTPVSNNASWAIGEISIHINPQYMEVYVDEIIKQ
LIYICQNSKYHGCLLQNICITLGRLSSTYPKKIIFYFPQFLKTWLKIMSHGTQENEKINS
LKAILETLYLNLDIAAENLQEIAYIILKYKYVSQNVNIFFHQFLATMKQKYPTQWKGIYS
STSDSLSSPIPADMLNDLSTRFNLMKSRLNNRLKGNSYYEYSIKHGTYDIDEAVAYIERS
SFFRSSLDSSLDGSHDNNRREPTLKRKLLGLIREEKKHMRNGAHKQKEKKRTKDHEEDHP
EGNESGLMYVHDTCEKILSSGKAFQNTDRYIILNRRNQIKKISLNEMNELITEIIYTTMT
K-DHLENSLLDLLGEEHIDFIFNIIKNKESIKRDIKLLSKYVDVKEKVLASNFFITDKTT
DGINSKNVLVKKENIEKIVDFFLYTLKENNFGDVKKIYIPNEENKLEEIQVPRNTSYSYT
DNVTKVRINRLENFQFNKDELVPVKVLPFWHKHIFEFEHFNYVQSKVFKAAFQTNKNLLV
SAPTGCGKTNIALLVILQQICLFCEQNGVSLERIAQVRSGQGGETHRADVHHAQRNGQLT
GDPLEEFLNEDPPSEEAASDDYAPARGANCISAKEFKIIYIAPMKSLVFEITNLFQRKLK
IFNLKVCEYTKEHSLTSKQLEEVHIIVTVPEKLDILLRNSSYSTTVSDESLIKHIKCLIL
DEVHLLNTDRGDVIETIVARFLRYSETSQSVRRIMAMSATLPNYNDVRDFLKVEKDMCFY
FNESYRSIQLDKTLYGIHEKNLNKLNIAKNMYAYGEIINALRKDKQCIIFVCSRNDTNKT
IQFLIDHAVKNGEIEYFLTNLYTDSEINKKIKKSNNMYVKQFYEFGCSVHHAGMSRYDKI
LVEDLFKKKAFNVLCCTSTLAWGVNLPVHTVIIKGTNFFSSESGKMEDMDILDINQIFGR
CGRPQYEDHGHAILITERTKLYKYIKLLTNNTIIESSFLKNIENHLNAEISIGTTKNVED
GIKWLEYTYLYVRMKKNPNLYDADLTSDMHLYNKRKEIILKAIQNLSENKLVRRVLLTND
FIGTFYGHIAAKYYVDYQTIGIFAANIDRSNYVEVIDVISKSKEFENIQIRNEDMNDFLW
LKNRCEIKEQYDESKCMTLRILIESYLRRIQISNFSLICEINYVIQNIIRILYAYYEICL
NILKNISNLIMNTHNLIVSILRRLPINSCVFRHFCYRNELLEKRNATFSSHPARGGKPQR
RNQNYTVYLKESVVNILEKKKLTHESVDTLTKSELLFFLRNEVYTNQILYYKNVIPNLHI
EGYIQPITQTIMKINLQVQLINTIWSDQWNDIQENFHLFLLNTLNNDILYFQKFAIHKKD
RKKIHDISFEFPLSNQMPPQITVQFLSMNWCNLSFVHIFNTNNLFINQKINIFSEILPVV
PLSTQILKIPSYIKFFSFRYFNPIQTQMFHATFHTDENILLGAPTGSGKTVIGELCILRN
LLHHEREKAVYVCPMKAIVNERHKSWTSKFKNLLNKNVIELTGDKNENKESIVESDIIIC
TPEKLDVISRNWKNKKFMQNVNLIIFDEIHLLGENRGGVIEILVNRFKNMEQYLNKKIRL
VGLTTVITSVDDLILWLDVKENYLFNFPSSCRIVPCKTHILGFTQKAYCARMSVMNKNVF
DAVNQYAQTKNVLIFVSSRRQTRVTAYDIISLLHTENLLNDKSHIQSGEEKTQVANMLFQ
NYLNIIENEHLKDLLKYGIGIHHAGLNENDKNIVEYFFLNKIIQILICTSTLAWGINLPA
YLVIIKGNEFYDAKTKKYKDISYTDLLQMIGRAGRPQFDDKALAILLVQEKRKNAIKNFL
YHPMNIESNITENLNEHINAEICSKVINNKEDIFNYLTKSYYFKRLFSNPSYYIKDVQYV
QLFENNILSNQAKKVIYEHVNKMIDSTISFLEANKCVEVTMQDYMHTYCSTPLGHIASVY
YLKCETVSFFYKMVEPARAKELDFYGLFALIAQAREFDDVPLRHNEDKYNVKLRNQIPLD
IDMNMKNVKTYLLLLSRLYECTYETVDYHIDLKLVMDQIARVINGFIDICLLFGRYNYIK
NLILIYQCINQKIQPSQNSLYLIKDLNDSQLAKLAELQIKNLKDLLKFDKSFLYSLNIFH
TSQLNFIFQIPTLTSSVKLFYKSVNLRNAEKKHNLISIPCVNHQKNENKFFFQMKHHDPN
EIVIRVFFCFFNKALRDANSTPPSANVQWYAPMEGEGVKREE--PQEAASQEAPQEDVSA
KLADILSVASECIQPEELKARLLLKRRLVCYDGFEPSGRMHIAQGLLKCQIVNKLTSNGC
RFIFWIADWFAQLNNKMSGDLKKIKKVGMYFIEVWKSCGMNMQNVEFLWASEEINKKPNE
YWSLVIDISKSFNINRIKRCLKIMGRSEGEENYCSQILYPCMQCADIFFLNVDICQLGID
QRKVNMLAREYCEIKKMKKKPIILSHQMLPGLLEGQEKMSKSDENSAIFMDDSEADVNRK
IKKGYCPPGVIESNPIFAYARSIVFPHYNEFALQRKEKNGGNKTYATIAELEADYLSGAL
HPLDLKDNVAIYLNKMLQPVRDHFQNDAAAKSLLSEIKKYKVTKVSRNALMANATNSLPL
KVSVIGSGSWGTVVSKIVAENTHKSKIFHPLVRMYVKEEIVDNEKLSNIINTKKENVKYM
KGMKVPDNVVAISNLKDAVEDADLLIFVVPHQYLENVLNEIVKNENLKKGAKAISLMKGI
KIDNCKPTLLSSVIEDKLKIGCAALSGSNIANELSRENFSESTIGFEDAQVAGIWQELFD
RTYFKINCVQDKPGVETCGALKNVVALGVGFLDASRHSYNTKSAIIRIGLDEMKRFARLF
FPDVLDETFLDSCGLADLITTCLGGRNLKCAREFATRNGVDSWDQIEMELLNGQKLQGIH
TAKEVYGVLEHHKLKNEFPLFRTIYEIAFRQKNPSSIIDVLSTKKLRHIRYKGMEKLLSS
IGRLSVVAGGLSLIPYTFIYDVDGGERCVMFNRFGGVSENTYGEGSHFYIPWFQTPYIYD
IKMKPKVINTTTGTRDLQIVTLSLRLLFRPHTKQLPYLHSTLGPDYDERVLPSIGNEVLK
AVVAKYNAESLLTQRDKISKEIRESITARAKHFNILLDDVAITHLSYGKEFAKAIEDKQV
AQQESERVKFIVAKTEQEKIAAVIKAQGEAEAAKLISSAVKEYGNSLLEIRKLEAAKEIA
ENLSKSKNVTYLPASSNILLNPKNFMKLEYMNALKEESGGFNFENLKRNEILKEKGVTFP
NFRKTGTTICGLVCQNAVILGADTRATEGPIVADKNCSKLHYISKNIYCAGAGVAGDLEH
TTLWLQHNVELHRLNTNTQPRVAMCVSRLTQELFKYQGYKVCAIVLGGVDVTGPQLYGIH
PHGSSCLLPFTALGSGSLSAMAVLEAKYRDNMTIEEGKELVCEAICAGIFNDLGSGGNVD
ICVITKDGSQHIRPYKQPNVRLYHLAQPTVFPKGTTPVLCQKIENIKKYITVEDAMRSNR
NGMYAFNPVKAEPFGKAPVGYIPGKGRGVTGFSGGVSRDDTTDEKDKNDYSDFNYDEFHG
YSESLFKDAEYDEDDKEADAIYENIDARMDVRRKSRREIKLKEEIQKMRAQKPTIQEQFS
DLKKGLASVTAEEWESIPTVMNFSRQKQKKVPKNYLPTPDSLIMSRLNDANMHLNYAGAS
SNGLGIQTPLGLRTPLGASTPIGLGMQTPFMKGGGGLETPFLSRHLASSSTYSGMNTPFT
LSGYNTPLSASTVGGYNTPMMNASNKLSLNDVGEARGTVLSVKLDELIDSVEGQTVIDPK
GYLTNLNAKSLVNDADIADINKARSLLKSVISTNPKHGPGWIAAARVEELAQRKDKAKEI
ITKGCIECSKNEDVWLEAVRLEDKLSEVKIILTKAIKEIPTSVKLWLEAYRKENHIDDKR
KVLRKAIECIPNSVRLWKEAISLESENNAYILLKRAVECIPQCIEMWIALARLCPYSEAQ
KVLNEARKKIPTSAEIWINASKLEEKQGNNNMVDIIIKRCIENLSSKNVVFERDKWLKFA
EESEKSQFPLTCESIIRNTMNIGVETLNKKRIYKQDAENCIKNKSIHTARAIYNEALKIF
KTKKSLWLALANLELAYGSKESVEQVLQRAVKSCPHSSVLWLMYAKQKWLNNEIDKAREI
LAESFMHNQNTEVISLAAIKLERENNEFDRARFLLKKSRVQCNTPKIWMQSIQLERLLRN
YKDAKELAREALKIHKRFDKLYMIAGQIELEMMRAEVDQQSDHYSSAQKIYDEGLRHCPA
SINLWLCAIDLQIEKKNYTSARALVEKAKIKIKSIHAANSNSHVLKNKEIIESNEFAYEE
EFNRNGDEDAKGAANVNNLAGAKNDLGKNAPINASIRVIENYDLLWIKLIEIESHCNRNN
VSPVISEALKECPSSGILWSKAIEFENKNLQNSKSVTAFNNCGNNAYVILTVAKLFWQHF
KTQKARKWFYRVISLNPHFGDGWATFLAFEIDQQNEVNQKDIINKCTKAEPNRGYMWNKI

TKRVENWRLKYPQKLYKYIKELFPEVLKKKISEQIWHIISDENVGLPIRRKRKKMQRCCF
TLVRRFHHVQKYIPVKEVIPCIKKGESTTMEGETEQVKEKEGSQVENAADEVSDKKKEKK
AKKLAEKEKKLAKKAERENLKNEATKVLEHVCEDINKESYGFVKISKMKENEKEIRLFNL
EEIYHSLMKSGATDGGKRESVVAESNGAHLLQSDIWVRGRIHDIRSKGSLAFIILRHKLY
SMQCILDIKHNDNDKNMMKWVSNLPLESIVDIKGKLSKPEVPIDSTNIKYEAHIRKIFCI
SKTAKELPFLLKDANMKETNEEGSIKVNQDNRLNNRCVDLRTYANYSIFCLQSQICTIFK
NFLLENNFIEIHTPKLLGESSEGGANAFQINYFNQKGFLAQSPQLYKQMCINSGFDRVFE
VAPVFRAENSNTYRHLCEYVSLDVEMTYKYDYLENVHFYDSMFKHIFTELSKGGKNEMLI
KTVKGQYPCEDFQWLEETPIFTYEEAIKMLIQHGKLHLKEEEILAYDMSTDMEKELGKIV
KASHHTDYYIIINFPSALRPFYTMYKEDEPAISNSYDFFMRGEEILSGSQRISDVNLLLE
NIKRFNLDANKLNFYIDSFAYSSYPHSGCGIGLERVLMLFLGLNNIRKTSLFPRDPKRLI
PMEIAGL--GLLHRWRRERGEQAAFREALKRGNAALWGRSGRPSELPIEGEKGTLPDIDE
EGKKNKMNDDDPNALKEQANSPFTNSFDAPNALLFENLNKEYKFITTQDNFDGFRFEVDK
SVNKYLQSTHTLFLGTTLRDVGYLYQFGANFTNSDNSLLMISRVNIDGSVNGRFCKRIDN
QIDCKLNFNTYAKNDQRNMYEMAVEVNKPIYTYSVKTIWQGTWIFNSSYTQMLSKKFQAG
VDLTYIASNCASIGSFGLRYNHKNNVVSMQVVRQPNFKSPEFMLNQTHLYKIQYAKKVSD
RLSLGTELEVTPETKESAMRLGWDYSFRHAKVQGSIDTSGKIAVFTQDYSGFGVSGYIDY
PNNEYKFGFMMHIAPSQEQPVQPVMDSQEYIPQYKLILVGDGGVGKTTFVKRHLTGEFEK
KYIPTLGVEVHPLKFQTNFGKTQFNVWDTAGQEKFGGLRDGYYIKSDCAIIMFDVSSRIT
YKNVPNWYRDITRVCETIPMVLVGNKVDVKDRQVKSRQIQFHRKRNLQYYDLSARSNYNF
EKPFLWLARRLSNQPNLVFVGEHAKAPEFQIDLNIVREAEKELEQAAAVAIDEEDIENMD
KLKTVYLDSALSIIKGALCVILQIPTSRTTESVKKKQNNNGVLTVRSILMEPTISQYDDI
KKLIRNKIQEEVPFYNYQMKRTLAETIYGDCIYDNFGLSKDISEVNLIALEEWNINCNRN
RVLQNTGLIKDIQINEFKYLTTKESLEVHFAVNPKYSFEELSGMYKSEKSLTDFLLAPIV
KVYTSGEESGAKGPTSEDFAYAHVDDILQKNKVLPPSGVENVNYERSKEVTPWDVNITEE
GINYNKLIKEFGCSKITESHIKRIEQLTNRRAHHFIRREIFFSHRDLDFLLNSYEQHKCF
YIYTGRGPSSLSMHLGHLIPFYFCKYLQDAFNVPLVIQMSDDEKFLFNQNYSLEYINTLT
KENVKDIIAVGLNPELTFIFKNTQYAGYLYPTVLSIHKKTTLNQSMNVFGFNHSDNIGKI
SYPSFQIAPCFSQCFPQFLPPNIPCLVPQGIDQDPYFRLSRDIAVKMALHKPVVVHSVFM
PGLQGVNTKMSSTKKKDEASNSAKEHNNSVIFLTDTPEQIKNKINKYAFSGGGATIEEHR
EKGGNLEKDISYQYLRYLMEDDEKLHEIGEKYRRGEMLSGELKKILIEVLTELIQKHQQR
RQALTEAEIAYFFDAHKPGLSKFRGAMNAAKDAAKRHEVEITKLTKDEMTFVLHNSNTGM
ANALRRIMLSEIPTLAIDVVNVYENTSPFHDEFLAHRLGLIPIDSTNVKNFEFREKCKCK
ETCSKCTIQYLIQVKCNNVSKIEITHHDIESVEHEPNVPMPVPLEDRNNQMAESNAIPIV
TLSKNQTLHMKLIATKGIGKMHAKWIPANVSYRIDHKVAIKHHLINSLSREHKLLLANSL
NKNCYVLKNAEHDREVSLRLNENMSVVMAESCIEFLNELGYKDVVKIIYDETKFHFKVES
VGSMPPEQVVEMAIEILENKLKTLEPQIKASYYSIDEVAKQLKEQGVSLYGIQLDLEMCE
NKSKVKDLSLAPFGKLQMQISENEMPGIMTIREEYEKVKPLKGAKITGCLHMTVECALLI
ETLQKLGAQIRWCSCNIYSTVDYAAAAVSTLENVVVFAWKGETLEEYWWCVENALTWENE
EGPDLIVDDGGDATLLVHKGVEYEKLYEQKQILPDPESAKNEEEKCFLSLLKKSILKNPK
KWTNIAKKIIGVSEETTTGVLRLKKMDKNEELLFTAINVNDAVTKQKYDNIYGCRHSLPD
GLMRATDFLISGKIVVICGFGDVGKGCASSMKGLGARVCVTEIDPICAIQAVMEGFNVVT
LDEIVEKGDFFITCTGNVDVIKLEHLLKMKNNAVVGNIGHFDDEIQVNELFNCEGIHIEN
VKPQVDRVTLPNGNKIIVLAKGRLLNLGCATGHPAFVMSFSFCNQVFAQLDLWESKNSSK
YQNKVYLLPKHLDEKVALYHLKKLNASLTQLDDKQCEFLGVTKGGPYKSDSYRYMYTYGV
EGDDTYLPQPQYPSPYENQYGQDDESPSPRGENHTPFIGYFSSHLLRTGFFLQCVSLMLM
FIFYWAFGGTGIFVFDLYAGPECVKVSSAFHLTISILMAIYLLGTLYIAMFQVFVADNSK
WCRGFRAGSKLLSAAVTLDLLSSILRLVQYLYAYFYMSMRWWARYQQTKSDWTLLHFGSI
VHSFALFIYGAAFFYMEAYHDEGTYEELAWSNLTLFKLAGLAELLMVFSGFGAFFSILLL
GAIMCATVWAFSFEPLLEKWSPELHSRDINADVLPEVKHEDEQGGYNEENVYEPYNVNPE
NMEYGEEMVPQNMNEKYANGNSNIYAQNNGMPTTYDYSQIEGQVKQNAEMGVSENYSSGQ
FMEDANKPKKRTFRTFHYRGVELDKLLDLSQDELVKLFRARQRRKFKRGISKKEKSLLKK
LRKAKKECEVGEKPRAIPTHLRNMTIIPEMVGSIVAVHNGKQYNNVEIKPEMIGYYLGEF
SITYKHTRHGKPGIGATHSSRFIPLKMSSKKVQSPKEESIAKMLICKVHIGTKNLENKMK
RYVYTRAKDGVHILNLAKTYEKLQLAARIIVAINNPADVVVVSARPFGSRAVLKFAQYTG
AQAIAGRWTPGMLTNQIIQKFTEPRLLIVTDPRTDAQPVKESAYANIPVIALCDSDSPLE
HVDIAIPCNNKGKESIALMYWLLAQEVLYLKGTLPRSKPWKVMVDMFLWRDPEQFELKNL
AIEETAPAAPHLAENQFATEAPYEEWNKKEEWNDNANEEWKNPIAADEWMSVNRYNFKAL
SSLLKDKTPQHFDILPHTSIKCIVHPSVIFTILDAYLRRDEDQTHVIGTLMGSVIDANLI
EISDCFVDKHSLNEGGFLQIIKDHHETMYELKQKIRPRDQVVGWFCSGSELSELSCAVHG
WFKEHNSISKFYPHSPLNEPIHLLVDAALESGFLNIKAYVQLPITLVKEYFVHFHEIQTE
LLPSNVERAEVLQYQEKGLPGKDKDSHMHGNKNSVPPNDMNEMSLKKLLIMLKQCKSYVQ
DVIDKKKKGNLDVGRYLHKVFSNDSFSTLEKFDSINESILQDNLMISYLSNLAHLQFLIA
EKLNASSLQMEKIVEVIEGLSSSDSHVRNECENTLNFYKKNDLNNTVLSILKLLKSHKDS
QVRLQCAILIRNLFRAYIKSGEKEKGEEEENYWDLLPDNLKNIVKSELISNISSETDKMV
RSNLCNNIIDLSSKLLVNKQWPELLSVTLDFCNSNNNDVLISGFKILGGILSCIPYQLEL
KREVVSSVCMKGLNSSDVQVRGECINLISCIVEDNNSVLVKCVQPCIPQILQSLSLMVKN
SSSDISVLEECEKVLQAIGKMIDYNAKFFAKHISNLCDILFSICMKGDSELNYDFDSSLK
SLSIEALITIPERRPKMALSVPHFVDKIVHLSMLFMLDINNDSFNEWMNSIKEGKDDNQE
LYDIGEESLDRVGKAFSELEEAEFIHILYNKVSEFLMKNTWEHKYVAIMAIAQTIEYLPE
DEIEDQLEHVIKMLLQVLLDQDVRVRYAACQAIGQISLDHQPYVQKEYPRQIITALITTM
NDVHLRVQSHATAAFVNYAEELDKMALLPFADMIIDILLQKLNSSNYLLVREQAVTAIAV
IAGVIEEDFLKYYSTVVPMMKDIIQKAVSEEERTCRGKAIECISIIGLSVGKEIFLEDAK
ECMNALLQISSTKMDPDDTVKEYIQEAIGRICRALGNDFFPYLSSIVPTILSLLSVLPKP
LTDDEEDLTITMVSNGQYVGLKTSLLEDQEKALDLLIIIIEVLKENYKDYIEATATAVLP
MLNYELSDEIKQKALTAVSELIEAARILSEKTDNNKTMLHAILTAAAEKVLKSLSETKLD
DNYEYILDVMIIESNGLYMCLQKAGSNVLPDGTLKLFFNQIFKLLQCSTDRRLVYNQKKN
NEDVDEDELLIIDREEELEQNYRTNLLDILGVLIKYHSTQFLNTCCDICITFINTYMNSP
NSEDVALALYVCDDLLEFLQENSVCLWEYFMNPLLLNINHTDDKVKQAACYGVIQATKIE
AFSKYANVAVDYLLKLVHQNTSSKKPKEFISAIDNAVAALGDIVLMHTSKFNNAEELIKL
WLNNLPIKEDDAEGRRVHKNLIDLVSQNHPLLFGKDNSNTAKIIEIFLTIYETDFSDADC
NKKIASLINSLDQAYLSNLASSALTNKQAKKLNHIVNSNRKMPTISVHEEDLIEKLGEKI
EEEKLNDICFEFGIEIDDVEYKGEKKIYKIEVPANRYDLVCVEGLCRALKSFIGKYENVS
YALLTNSEEACVKEKHFMRVDESVDERRSYVVSAVLKNVKMNENVYNNIIELQEKLHHNL
GKKRILLAIGIHDYDKINFPVAYKFEEKEKINFIPLNETQNVNGNNFINFYQDNINLKSY
LKIISDFEKFPVIVDAGGQILSLPPIINCDYTKITYDTRNLFIECTAIDRNKAEIAVNII
CSMLSEYCTPKYSIHSFFVQYDKNHKAEKGNGYLYPVFKNKTLTCHMDYVRKLSGILNLS
VKDVEPLLKKMMIT-SKVIDSSTFTVDVPFYRSDIMHFCDIVEDIAIAYGYGNIVSEKIE
IAKKNSLSACTELFRNVLAECTYTEVMTNALLSKRENYDCMLRKHRSYDDRKINLDEYNP
LAPPVQIMNSKTSEYEIVRTSLIVNMLKFVSANKHRELPLRFFEIGDVSYTTYDRTDTNA
VNKRYLSVIFADKFTAGLEEAHGMLETVLKEFQLFSDYKIEEKSKENVAIRSDVFYKLVP
KEDPSFLNERVVDIVLCPHNLKFGIMGIIHPKVLENFSIDIPVSVIEINIETIMDVLMMM
GENAGSSGSNPVISIGNIRGFGGCDYGSFRMSNEFLGWKNKKTNSVYQYKCSDISEAEWI
KTSYNNNRLHLKFSEQKDNLIIFFDGFPDRNTSEITQHFQKYFNLRLASRKLATRGWNWG
EFKLENSNITFDIDNKYAFTIPTNSISQLNVQIKTDIAMELKNEENKKTNEDFLSEIRFC
YPHENDENQNFQNFKNDLLEKVNIGDSKSECIASLANIPLLVPRGRYEIEMYTKSFKLHG
KSYDFTIQYTNINKMLLVPKSNSNQYVLIFSLNNKMKQGQTEYPFILIQLNNDDDMELDI
NASEEDLKKYKLEKSLSGKAYDVVTRLFTALVKKNAIIPGDYRTAKNEHGITCSYRAASG
QLYPLNKYFLFIIKPVILISFDDIVTLSFQRTGNINQHRFFSLIIKHKRGMSYEYTNIDK
SEYLPLLEFLKSKNIHIQDDANVADKKQDFGDELSESDEEEYVADDDDDEEDYVAEDEED
DDDDEADEEEEEEEEEEEDDDKMGDLRSKADLGDCRFYEKKFPEVDDLIMVKVNRIEDMG
AYVSILEYNDMEGMILMSELSKRRFRSVNKLIRVGRHEVVLVLRVDSQKGYIDLSKRRVS
PKDIMKCEEHFSKSKKVHQTVRHVAQKHNMTVEELNRIAIWPLYKKYGHALDALKEATIN
PEAVFKGIELNEDVKNSLLADIQLRLAAQALKLRGRIDVWCFSYEGIDAVKEALKKGKEV
SNDEVSINIKLIAPPQYVIVTSCQDKDLGMSKIQEAMKLISDKIKEYKGGDFKQQGEILV
IGGDDEKRLEELLDKQDDLSSDNEYNSSEDDDENSSDDEENSSEIEEEEDQLYLSMQEDA

YGDAQPSEQPHMMMHLEKQVKMKLEEQSSLLSIKGIDRVDIQQKKGKHSIICIHYIKNMCM
KNLFCNYLHQLIYAKIPACKNFLKNNYCADRVRGSCLFRHTLENVNSGGFAEGKDEHLDD
VLKLLHEKNICVNYLLGFCNLGYNCKRVHRCKGRKNWIGILPKFYLDHILVNQRLYSHLY
ANQRKLPDMSKLKDALIVLSGEKFPEKGLSPHSNSLKDKELFPTDQRGNNDTYRHLAGGF
PLQLTDFLPRHNERGFTHKDMRNQENLMSIPNIYDANNEVVPSEKIKIFIIKCNQICHLY
LSILYGVWATGKNNTRKFTSLFKENYTIVFLFSVNESGGFQGYAKMVTVPIKNLYENLWG
PITKRLGGNFRIQWVKIAKIDFDAFKNMTNPFNDNLPLKKSRDGTELPFNLASIICHRIY
ALPSEDFLAGTIYEYKRRINHAAFFENLRKQGLLNSNTVWDMRIFSLNQQSDCQRITFVD
GTEQVIGMANPLGTSILDNNLTQIFRDSEEWLTLEYLLQMNVRTSRVRLVQAWHLSPVHL
INKFEKRNANKLVLKSFIDVSSLDMDNSIQDVCTRGFSVSRRGLRLSVGNFNIPGFPLTS
LDAGRALVNPNELAGEPGATPKAKKTLRNILKHSETVLLSGERSVFEFFLCDVGVGLSLS
VSEKDAEMYNRDTVPVEYDSLFIDKQKVSALDDSLTIHYSERDEEQNSERINLALGGDTM
ASCGVLPHYTFHHEYIIFDNSQLLPRYLIQFECDPSGEEHFSLPLCDYCGNAPSVFFCES
DEVKLCAKCDQMIHSQNKLVKKHIRKTLNEAQTISGKCKIHVQQRVSMFCTICHIPICNI
CVSSHSHMDLCGESFPWLSKKNDAIISLNLAYKAIIKHSAIPSNVVKEEKKKLNDVLKKV
DKLYEQVRSNMKDAEKHVYTILEDVIKQLHETTDQKMCAVLSEEYELKRQFCEIAWNENF
LYYLQTVLPPADFMNAWLKHCLVREEIERNSGHAERPHALVFPDMCIRGNINVATEESAR
HAG----GKQHMAPKKKEEE-QKVLLGRPKNTLKMGLVGLPNVGKSTTFNVLTKLNIPAE
NYPFCTIEPHEAKVTVEDERFDWLVSHFKPKSNVHAYLSIFDIAGLVKNAHLGEGLGNNF
LSNIAAVDGIYHVVRAFENEDIIHTEGNINPVRDMEIINSELIYKDITHCEKNLEEVTKV
LNRNKKDKIKQNEHDVLTVVLNFLKEHKWIKDGNWKASEIEVINEFNFLTAKPVVYLVNM
SEADFIRQKNKHLAKIYNWVQEKNKGTIIPYCADMELKLLSMSEEEKNAYFKENNVKQSM
LSKIIKTGYYEINLIHFFTCGPDEVKCWTIRKGTKAPQAAGVIHTDFEKGFICAEVYKYA
DLVEFKSEGEVKANGKYLQKGKDYVVEDGDIVFFKFNVSSSGKKMSYYNLEKDDTVYYKL
DTIKCDIPINEELQARINKHVNQLRITYSTLEEFVDNFVYELKKGLEAHRRHPNLWIPHE
CSFKMLDSCIADIPTGQEKGTYYAIDFGGTNFRAVRASLDGNGKIKRDQETYSLKFTGTF
SHEKGLLDKHATASQLFDHFAERIKYIMGEFKDLDNPEGKNVGFTFSFPCTSPSINCSIL
IDWTKGFETGRATNDPVEGRDVCKLMNDAFVRSEVPAKVCCVVNDAVGTLMSCAYQKGKT
TPPCYIGIILGTGSNGCYYEPEWKKYKYSGKIINIELGNFDKDLPLSPIDLVMDWHSANR
SRQLFEKMISGAYLGEIVRRFMVNVLQSASSEKMWKSDSFNSELGSVVLNDTSPNFEESR
KVAKDAWDMDFTDEQIYALRKICESVYNRSAALAAAAIAAIAKRIKIIEHSKFSCGVDGS
LFVKNAWYCKRLQEHLKVILADKAENLIIIPADDGSGKGAAITAAVVSQSSSIKQLPMPD
VYFLIRWLCKAIVSSLFGDVNVINPENVPLYGSVIFVGNHNNQFIDACVLVANIPRQVKF
IVAEKSMRRAVIGKLASIIGCISVKRPEDLKFKGIGHICWVKGDKKIKGINTRFRLDVQM
GDKLLTQNKIFLVAKIESETELIIQDAINIECEDKKNGVPFKIIPKINQTEVYNLVTSSL
KNGDTIGIFPEGGSHDRTNLLPLKPGVAIMTLCALADGVEDVSIIPVGLSYSKLYQLQGC
VTLFYGNAIIISQDLCKDYNNNHRETISKVLSKIEEGMRSCMLTSKDHETSRCIELCVSL
YTPERMTISKNKIYNNLQLFCKMFWKFGNSKEIENLSYELKCYEKLLKANKIKDDEVWML
KQSTSAATLKFIEHICSLIFCVIFGMTFSLLWLPLVAISIYLAEKHRESALKNSTVKIQG
CDVVASYKVLVLLVLLPTFNIMYGLVFSLYLYESWLSRIAFVILSMCILPICYYINLNYS
AQIPTLLRQMKILLKVICGKINVWRDNERELISTRHELQLKVRDLVSNLGPDVSDDFLEQ
LYRNIPKFVVDADTKRLIRGKDEWVPILQRSQLEYKEEILMNDDNKEFSGRDIYQLIKNA
KDKIKIDYKFWYTQPVPKINDEFNESVNEPFISDNKVEDVRKDEYKLPPGYSWYVCDVKD
EKDRSEIYTLLTDNYVEDDDNIFRFNYSAEFLLWALTSPNYLKTWHIGVKYDASNKLIGF
ISAIPTDICIHKRTIKMAEVNFLCVHKTLRSKRLAPVLIKEITRRINLENIWQAIYTAGV
YLPKPVSDARYYHRSINVKKLIEIGFSSLNSRLTMSRAIKLYRVEDTLNIKNMRLMKKKD
VEGVHKLLGSYLEQFNLYAVFTKEEIAHWFLPIENVIYTYVNEENGKIKDMISFYSLPSQ
ILGNDKYSTLNAAYSFYNVTTTATFKQLMQDAILLAKRNNFDVFNALEVMQNKSVFEDLK
FGEGDGSLKYYLYNWKCASFAPAHVGIVLLMSEVNVTKVIVNNPICDILDPFVFTIEFEA
LNKLEADLEWKIFYISAVNNDGEGNQDIELDNIFLGPIERGVMMFDYAVNPPDYKNMDVD
SVLGLQAILISANYKEKEFIRIAYYMNSFYKDTELREKPPAVPQYDKICRHIFVENPRIV
KFCITWDAEEKDEFKEFDRENEQIELINCVKMNGEDNSNSNITNESTQENICVFSDVQ--
-ANGKKSTHVGAVESANASAGLNERAVLSSNAASTDLDKCELINANINGISRITIDNKHA
MKLAVVSVLLPVLFLGLFLKSKDQEVLLSYGHFLKNTFKELLLFFNIFDIEFPPSYVQNE
KLLGLHVRDIEKDAYTAYPILKELKQKDYFRIFKVNLHIPCKLSKVNEKCKEIKKCSVCE
CHDDEIPYNFRTNEIEIIEDKMSSEDLKKTFIESKLYKDILGIYAPSDEGFLSYVDLVYN
SPSFTAYEGRNIWNKIYRENCFQKEENKCHEMKSFYKIISGMQSNIAILSAEYHYLKNDF
VFGDMHTDDVMKSDYFKKQNYEYSMQFFKEKIALYPERIENLYFTFAILLRAMCRLKSLV
SQCKCNSGHAQNDKEALKVLNDLLGSFYHSCASEQFLEPIFPTQGKEILAKFMNITNILD
CVPCVKCRLHGKLKTTALQIALVEGSYEHIGSLERNEITALINSLYYFADSIIILNKFEE
RQKLKRATFIFYLLSFCFFLILIIYSIVFITVRNCKKMKKKKKMARNVEKGRSMLNQWLK
AKELSEQKSFFKIPKRVNEVEDLETAVSCRRHIIKEICNKIKEIQNYSLSDQHIRELNDQ
INKLISIKNKWEIRIIELGGPDYQTESNTLINAHCSELKGNNNYKYFGAAKNLKGVKELL
LKESDDRKKFILKKKKENRFFDKHVNIHYFGYCDDQNEMLLREELKMQNRLEQDDLKTLK
RINYNMGIFRSETMKHGTLVLPSDRAREYLDCLGKNIDIQFIDMNEKTMKRQYKKYIQRI
DDMERILRFLEENIKKLPNVKIKKSKIDSFLEHDNVYELDQVEESLNRLHVQFVRFCNNN
KDLVDERNNAVEEKHVILTAMNQLNPEGAKNGGINKRLPENSL-------PYDEVNDESV
SLQTNMMKDGMNMMFTNISGVIKTKDQESFSRTIFRALRGNTYTYFQSIDENAAPSKGAG
AESMNDPDEGGGSSSCIHNNNAKQNEPKSVFVVYCQGSSQSNIYHKILKICKAYDVKTYD
WPKTYEQARQRLKELKEIITDKEKALKAYEEYFINEIFVLINVVEPNKNSLIEEWKLFCK
KERYIYNSLNCFEGSDITLRCDCWFSANDEEKIRHMLITKSSNDLVSALLLSDKVLTPNI
SPPTYIKTNSFTKSYQAMVDTYGIPRYGEINPAISTIITFPFLFGIMYGDVGHGVCIFLF
ALFLIMINSRVKNKSQNEMVSMLLDGRYMLLLMGFFAIYAGFLYNDFFSMPLNLFTSMFE
EDRQVDTTVYYKRRKVTNATTGQLEDADPYIFGFDAKWLGAENELTYINSFKMKFSIIIG
FLHMTFGVLMKGLNALHYRRKMDFFFEFLPQLMMMLSIIGYLVFLIIYKWVTPIGYGGYQ
KQGIINTIINMYLMKDLTPQNQFYAHQGLVQAFLIAIFVLCIPLMFVCKPAIRTYHIMKE
KKGGVGGYSPHEEKEMTHTFNHLVGTAHTTAHTAHPPLHKRSP----NNREDDYLSPRRR
NKPTDDEMEAHLLSPASPEDPDGLAEQEGGAHQEENISEIWIEQLIETIEFILGLISNTA
SYLRLWALSLAHQQLSFVFFEQTILNSLKKDSFISVLVNLIVFSQLFSILTIAVILCMDT
LECFLHSLRLQWVEFQNKFYKGDGIPFRPFNIKKLLSEGDMLGIQEGRAFFNAARNPNNR
PENNIRIEDNLPSLNEVYNYFQDFLSRYSSNTLKEGNLKRTLFENVKNNNFTLDLKLDDI
YKQQTVFENNEISATPISERKMKKPENYSAILARALSERPLTYLPTVERVCYEVCETANI
LNDEDEHLNYIQINLLNTFIRPTPIRGLLAATQERFVVVPGIIVQASKPQHKMRKITLQC
RYCDHKMSIDVPLWKDKPQLPPYCRYSSTMKSSMGVANPMDNQLGCNGVLEPYVILPNEC
TFVDIQSLKMQELPEAVPTGDMPRHLQLNATRYLCEKMIPGDRVYVHGVLTSYNPNPKPT
RADGTNFSYLHVLGFQKYDDMTGNDLNFDVEERNELTLLAAEHDIHEKIFKSVAPELYGM
DEVKKACACLLFGGTRKRIGEETKIRGDINMLMLGDPSVAKSQILKFVNRCAPVSVYTSG
KGSSAAGLTAAVMRDSQGVFSLEGGAMVLADGGVVCIDEFDKMRDDDVVAIHEAMEQQTI
SISKAGITTMLNSRCSVIAAANPSFGSYDDSQDTTDQHDFKTTILSRFDIIFLLRNKQDI
EKDTLLCNHIVALHASKHKSQEGEISLSKLTRFIQYAKKEIAPLLSKEARDSLRNFYVQT
RAEYRGDRRSVTKKIPITLRQLESLIRLAESFAKMELSQFATEKHVQMSIDLFSASTAET
AKQCLIFETMSPLEQKAVKQAEDAILGRLGKGQRASRVNLFRELQLRGFDRSALSKMPKS
YRNYSKTARNPKRPFEKERLDQELKLIGEYGLKNKREIWRVQYLLAKIRSAARYLLTLDE
KSSKRIFQGEALLRRMVRQGLLGENEEKLDYVLGLTLPKLLERRLQTKVFKLGLAKSVHH
ARVLIRQRHIRVGKQMVDIPSFLVRVDSEKHIDFSTTSPFGGSRPGRVKRKTLKNQKEKN
EEENSMVRQSQSMYDRHLTIFSPDGNLYQIEYAIKAVKNTNITSIGVKGEHCAVIISQKK
MATQYISQDKLLDYNNITNIYNITDEIGCSMVGMPGDCLSMVYKARIEAAEFLYSNGHNV
NVETLCRNICDKIQIFTQHAYMRLHACSGMIIGMDEDNKPGLFKFDPSGFCAGYRACVVG
NKEQESISILERLLEKRKKKIQQETLEEDVQNTIILAIEALQTILAFDLKANEIEMAIVS
KTNPNFTQISEKEIDNYLTFIAERDMDNNENIDSNDNSSDTNTGNYNQGKNENVSEEMDF
CKEDNELDGNNKGSKSKQDNKTKNASDKDDLNMDYSDHHFPCDPAPPVSIKLFIGRVPKT
MEEEQLRPIFEEFGIVKEVVIIRDKITNIHKSSAFVKMASISEADNAIRSLNNQRTLDPQ
LGSLQVKYASGEIMKLGFPQNIESGVDQAKLFIGSLPKSITEESVKEMFSPYGSVEEVFI
MKDNSTGLGKGCSFVKFAYKEQALYAINSLNGKKTLEGCARPVEVRFAEPKSAKQTQIPM
TLQPMQNAAHGMNSQPHVTSPNNINFGQNFGVNNNYPRQVGAWKEYYSGEGRPYYYNEQT
NTTQWEMPKEFETLFMGSTPNMHNLSDSSGPPGANLFIFHVPNEWHQTDLIQAFSPFGEL

LSARIATEKSTGRNRGFAFVSYESIESAAAAISQMNGFMALNKKLKVTVKKGEEEEMKKF
VNQNGINSFQQMSRPQKNIPSQPNAVAQPNFPPHQNAQPQNFFYSNSNSYRCGPYMTTAH
RPTWYNAIGGENQGGNRKVSQTAKVCSRDLPGHTKMKTRDLSDYVEDKEVIRNNLIQLEG
EAAEKDNS-HRLLAIENIKKLTNHDYTNPFPEDEDDEVEEEWKSRRRKGGGKAKKKDSEE
GSQSDGSSDDQSDNSAGDGHSSDDASDDSSANQSEDEEEKELLRELENLKREKMEKERQE
KLAQERLQSKKNNVLTNNPLINLEDSDNEEGASRKRKWTEEAIFRNTCEKKEKRSTFIND
TVRTAFHKKFLFKYIHMDSEETINQAVKFAKDAVIEDEKKNYKAALNLYIQSLQYFNFFC
KYEKNSNIRDLILKKMEIYMTRAENLKEIINKKETMETKEKVGSTEESKENMKKQIKEFI
LNKDKNVKWSDVCGLETAKEILKEAIIFPLKFPKLFNSSALPYKGILLYGPPGTGKTFLA
LACSNECNMNFFNVSSSDLVSKYQGESEKYIKCLFETAKEHAPAIIFIDEIDSLCGSRTD
GENESTRRIKTEFLINMSGLNNYKNNIIVMGATNTPWSLDSGFRRRFEKRIYIPLPNVYA
RMKIFE---------SNIGKEDIKYFAAVTENYTGADIDIICRDAVYMPVKKCLLSKFFK
QVKRNGQIFYTPCSPGDPDATKVEKNVMSLNENELLLPPLSVQDFKTAISNAKPSLSVDD
LKKYEEWTQQYGMNGTMNGHVQKLKVVLKPGKQLYSSEALKVKRNPNLEKLSGEKQNLAK
VTFKKEKIEDIIKEVKFDYYYFNEGKKNIYRDIPLNIAVVKESELPPFQQVDEKLHFSVL
ENDLRIISTNKNNSVCSIGLYVKCGSRYEEISEQVNEQGMSVMLENMAFHSTAHLSHLRT
IKSLEKIGANVSCNAFREHIVYTCECLKEYLPVVTNLLIGNVLFPRFLSWEMKNNVNRLN
TMRTKLFENNELYITELLHNTAWYNNTLGNKLYVCESSVENYTANNLRNFMLKHFSPKNM
TLVGVNVDHEELTKWTSRAFQDYVSIPYTSQKEVTPKYTGGFVSVEDKNVKKTNIAIAYE
TKGGWKTSDMITLTVLQTLMGGGGSFSTGGPGKGMYSRLFLNVLNNYNFIESCMAFSTQH
SDTGLFGLYFTGEPANTMDIINAMALEFQKMNKVTDEELNRAKKSLKSFMWMSLEYKSIL
MEDLARQMMILNRVLSGKQLCDAIDAVTKEDISRIVGHFLKTKPTVVVYGNINHSPHYDE
ICKILGMISGIRVNDTCITEFNNMKIRKTCRWIIFVIENCEIIIHSKGATTTLTELVESI
DKNDKIQCAYVVFDAVNKIHFFMYARESSNSRDRMTYASSKQALLKKIEGVNVLTSVIES
VQDVADFKMQPYDYNRGGNNYNSNSNNNGGGNAGNYNNPGNVNKPMNSPFFYNNNSGGNA
DMA------NSGGGNMKGNEGAY-PPNYYHPPQAMQPGMYGMNGPPLSDNMYGDGSNPNA
YLNNQAQYRGVASQFVPVMNNNNALKPGGNVLGYDSTGHVNSVQPIINDSYQEFLQFNAF
SHFVKSSVSYMPANTTLKQKAYVPLGFIIQPLAPIPDGYPELASVNFGNSTVVRCKKCRT
YINPFVRFEAGGKKWNCNMCYHVNDTPQFYFTPLDEKGKRKDLFQRPELCTGSVEFIAPS
DYMIRPPQPPVYLFLIDVTVTAVNSGLLDVVCSTIKKLLPKNSPASNDGSGGDVSGKKPF
DSRTLIGIMTFDSTVHFYNLNSNLKQTQMMVVPDIQDIFIPLPEDILVNVHECQNVIDVL
LDNLPTMWRNNKMTDCCAGNALKAAIMVLKKVGGKLVFFLSSPPNIGDLTVSVNRETKEK
SSYKNIYSSGSSNNNVVDSKVKEIEMLTPFNNLYTELAQSITQYQIAVDLFACPLYNLDL
ATIYPLVKNSGGSLYYYPQFNVHQYSDKLREELLFALTTETAWESVMRIRISRGWKITNW
YGNFQFRGVDLLALPNCHSSQNFSIIVDLEENVVQDSVVYVQSALLYTNSNGERRIRLHT
YALPVTQNIKTITDSINPQVVVSLLSHQAIDICKKGKIADGRNLIQTLCSQVLSSQLLLS
ETARLLSIYILGMLKSVAFRDSGDVPPDLRIYHWSRVQNIPVESVEAYFYPRMFCLHNLE
KHHGSYDENNAMMFPDTMSLTCENMTQDGCYIVEDGENIIMWIGRSINPQWVYAVFGVQS
LEQLNSEYAENHLGTSGNPSGMQILNIINALRKIRTPSYMRLLVVKQGDPLEYKFFACLI
EDRSQHMMMSLKEFLAKICK----------MEEDSFKNRLLKRNIDIWIEKYRPEYLDDV
VGNPFVINTLKSIIVSGNMPNLLLAGAPGTGKTTSILCLASEMLGAQAKKAVLELNASDD
RGINVIRDRIKSFAKEIISLPPGKHKIIILDEVDSMTTAAQQSLRRIMELYSDTTRFALA
CNQSEKIIDALQSRCAIIRYFKLSDDQVLKRIVKICQLENIKYTDDGLETLTFIADGDLR
KAVNCLQSTYAGLEVINKENVLNICDIPSPERIENLLKFCINSEWKKAHDIAYDMIREGH
TPFDVALTSSNVLRRYDLGSEAVQIEFLKIGAMACNTMASGLASVIQLDKLLADWCIAAK
AFRTKCMTKHTYMEDRNMAEPVGSILVEETEKLVDTYRFDSAASEGPKKSKSSQKKDGKH
GMAFHKSLAVVNVAAGLDGCDDQLLPASFRALEADLNLHPSLLGYITLAQTLMLSLFSPI
WGFLSDKYSRKWMLVFGTALWGVATILLANINDFAHIIFFRAINGLALGSIGPISQSILA
DAAKNESLGLSFGLVQLSSSVGRLIGGVVTTTVALKYFGGIRGWRLCFIVVGILSILLSI
VVALFVDDAPRQVRKKKKMEYLDDDIDAGSNNVRIVTQYTQSYLLYQNIVELLRDSLSKK
SIIIILLEGFTGTIPWLALSFNTMFFQYCGLSDLQAAIITGFLLIGSALGGVIGGHFGDI
MHDISNKHGRPFLGQLAMFGRVPLVILTYLVIPQRKESFELFALSCFFLGLSSIAGVAVN
RPIVSDIIRPDYRGTVFSLTIAIEGVGASLIGAPLFGYLAEKVFNYQNNNLLIAEMPEEL
RRNNAEALSKTLLYLTLVPWLLSFVFYSLLHFTYGKEYQKMNEIIESEYKYDDEDEETVA
EK--------------------------MLMFGLRSLSDFCNPTSKTYKENAFDALDRGA
VESIKNAVINYKDDDDILFCSSRVLFAMSDYCCSEKDTDALQKLITDGGVNAVVEIIKTV
PSDQDTLKNCMLFIQNMNDSNVQIEGGDLGVALLNVFTSSSYNVKLGNVIVSTLSVAAKS
ASGSQILNAEGAHHKLIDHCLSLQAINDDTAEIVEGVFDAIKNLSSNGYVVPTIIEKSVV
ILDKFKSYPRIVSKGSDAMKCAVGPEQLTDCLNILKTSEQGSKEQDSALELLSSLSYISS
ITDKVVQSGGIPVLVELINSGLQQYESNPEKISRLVAGASRMLGRISNNPPHAVIVYEYG
GIATLCTALSYFPNDVDCASAVCNALIPFVSRSNYVKEINSYSLFASLFPILYASLESIE
LAKASISCIASASMINEFHEQMVNNQAIEILSTCIQYHLNEREYLLNCFTAYFRLSEYIT
TVEPINQYGGVTGIANALAAVSNDSTISEIGLKLINKMLTASDALSYLSNQQVVDSVLTV
MLENETKEVIIQEGTKIMEKLATESDCQRHISNLESIINLAQSNPEAAYKTLAAISGLSR
IQSLKSILESKGADNSIYNGMKVWIESPRFTEQPKLIKAALKTIKILKLNAASTIHEVIA
SIVDVMCIPQVKRLAEIEEPDDNILITAAECINYLTEINKINNKEIVESSLDSIFKLMKK
YSESRLTQTNLLAAIDNILLSSNVIGADVLVNKGYVKQIVTYIHKVPMYVDVQIIGFSVL
GNIVKISSESVDSIKKANTLVPLQNALRTHAKNAKLKTTCAPLLAMLMPLDSLTKEIEDL
LKLCNESMSENDLIKLHEYLISLNELLLTAEASKISSRCNVGEHLHHMIQWLKSNPTIYQ
STEGKDYSILGRSLYDATISEVGHACTNISQTRIGLVHLTKSDMTSALIEAYDLLQLPVK
DYTEEAVSNILESLSLLLKHDITNADIGFNNGLVKKLCAGINHFSESDAVIKSTFGCLAC
MCTSEKRVNQLISHPEYDKLIALIVDLIGNSEKYKNSRGNAIKALYELLKTEDAQIITNI
ASMTPIVDNLFKIMGEYQADLPIVQDSTKCLAIIADYVNIEEKMKIDQYSAIKILLESLG
KNKNDELTAQDILTVLIKLCDSNDKMKLRELGAVDVISDVTMIHSENEEISKLGGVLFSY
MGADEQVKKLMKLILNVKSSDSDSVQKIDNLSSKLEMFLRAPLENPLDALEYTEATLQQL
NGYLTSQLDNASLQANIALVTKRLVDRVKYDHEDQLGAWAVASAGVLNQYTDMIANKVGL
ANCKFVSPVYSVLAGCVLNPYTKQLVLDKLAPILDNTYEILEEYKNKPYAVQGIYELLEQ
VAKDEEGAKLLYMKWNGSMGNLVEQTLAIMNLNRANDCVFVPGIKLLGAIAETSNASGYV
NDMDNARIIESCGMLLNLGSSKDRTVEFINFVDTMVVSSLIDEKVASETLKKISSLVSEE
NLSNYTEQGRVEIIKAYATLLKDSASTGLFTHIRQTDKTDALNNLARLMEYEPCDEVNVA
TLEALSEIATCDSSTSLKIITSALPSIVQSNHENLVNDNATAESFVQTLEKLVVKEGIGR
QIANNDEMYKLLEELEISLDGKKKELGEEYVDNIKMRICTVRNAINDDMPKEKTCKDIYD
MLTEYKLKSMEINVLLDGSLEDDMNYVLERLKVYNKDNLLPTTEAGTDNSYGYMAIEILC
ENNDNVKELVKKGFHTSAFHSLIKQANENVKHYSCRALCALTKNPIGLEHVIKEIKEYPN
VISKSVGDLCMEKTLEKEDKEDFLINRVLLIDRTAHNRNVYDRTNAVNHLIEIWNQYDHG
DYSVSLLRHVFRAMRKIVSDAHVETLLKADVLIRLTAIINNVETDKIIYPDVLFLIGSLS
VVKEIKTQIGKIKGIDACVNLLLRSMNVEKMEPTITNCCLALANMCIDHKGNSNIFCALK
GPDLNVRILREYRSNFDVTNGASVLLCNILFRNEDMKRTYGTNGAPAELVQCLRSYDGSD
NKNAVRCIESLFKAISNLSLYTANVRYFLDAQIEVSYESWLNKLNESFPDAELETGLRTL
SNLVMENEERNMKNFGVTLIPVLNVLKQNRENTKVIFFLLDILCSLCRHHDNAKMFSENG
GIETTINAIQLYDYDISLLTLAIHLLSNQCKIESSLPLLVKSDTFGILISCMEAETEEFE
MTELVVSALRCIRRLIQSEELAYEFCNCGGVPSIANLICKSVKKSIVMLEALRVLLCILY
YTQNVEGVTNEYPEEEEELFNAKLGGWYNISMDKEMIDTIIQSVLTCANDFNHQKQLRLQ
KVSLGLLAYFAYHRLGIISMTASGFDSLTREILSNFGGDVVIMQLLAICIDNIAMYSAEV
YDMTVSRDIIKGFKSSVSKMSSKKEDKQIVQKVELTVEAMNSAEDPLDAFKDTLLFFDFG
LSEFDKDPYVNGVHDLSSNIKDALRKGGVTKIYHNSDKRKPFKWKASQDLGTLEWTVGED
SEHIFKISVVRIKNISKGLAHPLLRASNKREPRKVNAKVTLCIYGPPTEDFPEGLELPIK
AKTQKERDAFVDLLVLWRDAASYNYMEKKAREYAQGAVRFIQKSGSNFLACKNLREKLEE
RGFKRIHEGEKWELRKNEGYVLSKQSRNICGFFIGKDFTIEKGSILISIGHIDSCCLKVS
PNNNVVKSKLHQLNVECYGSGLWHTWFDRSLGLSGQVLYKKEGKLVERLIQINKSLLFLP
SLAIHLQNRTRFEFSVKINYEAHLKPILSTLLYEHLVKGG-KPGAASEKRLDAEDSHQEN
PNSSPLLYTLAKELQCQEKDILDFELCLMDVNQPCFTGAYEEFIEGARFDNLLGSFCVFE
AFAEMVDMLRGGAEAAAGAHANLYICIGYDHEEIGSLSEVGAQSYFTQNFIKRILAAVCS
SHTSSAAPSIDELYGSLMSRSLLLNVDMAHCSHPNYPETVQASHQLFFHEGIAIKYNTNK
NYVTSPYYTCLLKRTFELFASNNEKIKYQNFMVKNDTPCGSTVGSMVAANLSMPGIDIGI
PQLAMHSIREIAAVRDVYYLVKGVLAFYAYYSHVLASCVPDAMNFCKTVFNVFFVLLFIS

SYEIKCQLRFASLGDWGKESKSQLLNAKYFKQYIKNERVTFIVSPGSNFLDGVKGLDDPS
WKSLYEDVYAEETGDMYMPFFTVLGTRDWAGNYNSELLKGQGMYLNKDGQTSIEKDIDKT
PYPKWIMPNYWYHYFTHFTVSSGPSIVKTGHKDMAAAFIFIDTWILSSNFPYKKIHQRAW
SDLKAQLNVAKKIADYIIVVGDQPIYSSGSSRGNSYLAYYLLPLLKDAQVDLYISGHDHN
MEVLEDSEIAHITCGSGTLSGAKGTMKNGKSLFYSNDIGFCIHELSSNGIITKFVSGKSG
DVIYTHKLNLKGKKSLDKVNSLQYFASLPKVQHVDIPASGPMGNKDSFVRIVGTIGILIG
SVIVFIGASSFLSKNIKMNDGKKKGKFIVFEGLDRSGKSTQSKLLVEHLRRKNVEVNHLC
FPNRETPTGQIIAKYLKMQSDMTNETIHLLFSANRWELMGEIKRLLASGVWVVCDRYAYS
GVAYSAGALKLSKTWCMNPDQGLIKPDVVFYLNVPPNYAQNRSEYGEEIYEKVEVQKRIY
ETYKNFSQEDYWINLDGTKNIDDIHQDVINEITKLEPVHEELFTFLWSMKVNQVVCKNCH
STDVETNEGQGEVICLRCGSVLEENKIVESLEFVENNNGAISMVGQFVPASGNKSFILSW
GVRESRELSLQKGYINIQKIADHLHLSTQHVEAAQRIYLMALQRNFTMGRNNSYVAASCL
YTICRREKSPVMLIDFSDILQTPVKPLGKTFLKLLRLLHISVPNIDPSLYLERFAHKLNL
KNAIYKVTYTGIKLIQAMTRDWICTGRRPTGLCGAALLISTRMHGIFIHSNTIANIVRIS
NPTIIKRLSEFKNTSTAKMKAADFDRVRLNDMPSNSLPPCVIASNKRKLRQDMLRNNQAA
SLCDSEEIFSSAASAKCGKNSEQLLTNDTYSPNCNEDSFSDVLSYNNLTLLDSRSSCNGM
ESQTGDDGETLTNSGINVEEICNENPEGNDLDQLAKKIINTIDVEKQSDILKMKVSSLHM
NSSQCDGPSNSNSQVKVKKIKSTVSIASINRTNGGCQDVSLSSLRNEKQSTKPTPADAIN
GCLAKNALPNDGLESLNNEPETTYCSTLKETLNSELSHLLNDVDDFNILQAKESNRIDQP
SGSHNEDVNHVSSILSDFNYFFENNSNTVESQPPNVDIPSDASLEQLNESLSDFYDSEIE
NIILSEKERKRKMLIWDDMMKSYFPQYYKQLKKQKKKRSSGAEKSRKKKKKE---NDDHP
LDEQTTGDSVIMALEKSDKSMSTKMNYDVLKSLFSS-MEKREAPEGCSVLGKKENEQIDA
DLYSRQLGTYGFELMNKLIKLNVLIVNVKSVGLECAKNLILSGPKSVCIYDNEVCQMPDV
GVNFFINEEDVAKQVTRSDAVIKHLQELNSYVHIYNYKGELNEQFFQSFDVIVCCDVSHS
LLVKYSKMVRSISVKKIAFLCCNIYGLCGYLFVDFGKGFICYDKDGENTKVCSISKISKA
PEGMVSFDFDKGAPFQKGDYVKFSNVEGMTQINHKIFKIKDMHKYTFTIGDTSHFDEYLK
GGECTQVKSHLRMDFQPYEFVCATPLSWEAAGEEATTGEPVPDGETIYEDVPSPQSFLIS
DYAKWDMSNQLHYAIQALKKHEEANGNVLPENQEEEAFEKVFQIAVQLNEADKQSKKTYA
VEEVKKEVVVNVAKYCTAHLAPVASFFGGLLAQEVIKFTGKYMPIYQLLYVDFFECISLG
VGETTAKDTKQNGKNDNIICVFGEAFQKRLNELHVFLVGSGALGCEYAKLFSLLDMCTAK
EEGKLTITDNDSIEVSNLNRQFLFRRENVGKSKSLVASGIIKKKNPNMNVESLETKVGPE
NEHIFNESFWTKQHMVVNALDNIQARQYVDNKCVWYSKPLFESGTLGTKGNVQIILPFLT
QSYNDSYDPPEDSIPLCTLKHFPYDIVHTIEYARDIFQGLFYNTPLSLQEFLSDKKGYVK
KVEEEGNNASLLETLQNVLTTLKEVSKETNFTFCVKKAVDLFYANFINQINQLLYSFPLD
YKLASGEFFWVGQKKPPQVIPFDLNNEFVQEFLFSTANLFAQVYNIPQCYDLKHILDVAS
QIEVKPFQPKRVKVKMDEKNLNNISISFLDDEKIMQDFCKELLHIECANVKVSPIEFDKD
EETNMHVNFIYAFANLRAINYKIETCDKLKAKLVAGKIIPALATTTSIITGLVGIELLKY
VNYYGYFQTYVKATEEERKQMKDLLSYFKNAFINSALPLFLFSEPMPPIKMRDKEYDELM
KGPIRAIPNGFTSWDKIQVQIENGTIKNLIDHIGEQFGVEVNLISVGNACLYNCYLPAHN
KERLNKPIHEIYAEITKQKLLDDKNYIVVEASCSDQDLVDVLIPSIKFIYKMSKLNQDLL
KKAIADVFEGTKTKKRKFVETIELQIGLKDYDTQRDKRFSGTVKLSNEVRKKLKVCILGD
AVHVEEAQKLELDYMDIEAMKKLNKDKTLVKKLAKKYDAFLASQVILPQIPKLLGPGLNK
AGKFPSLITHNDKINDKILELKSSIKFQLKKVLCMGVPVGHANLKEEELRSNIVHAINFL
VSLLKKNWQNIRTLHIKSTMGKPQRIYGMIQESQNENENKKKWEDIDADDDLESNKKLSY
FTNYENGIKVVTKYSENLKKQTVKVTKKIKEVVIKKKLNKEINNRLNLKNFNIDTCSSVI
VEPTDVVNIEVPKNNLLDFLKGTEYDYLFTEQADKTAKDLKNRFKIFKDEDVEEVKPEDP
NKLAAKRDMLFYRSHDECTVRVTNLSEDVNESELSNLFGRVGQISRMFLAKHKETQNSKG
FAFITYSNREEAKRAIEKLNRHGFENLLLSVEWAKPSNRMLNDIIFQVIIASFGVTIVNS
DKIKFLQKFRYAIYVLILSFLLYKGIPWKRENYYTYLNITPNATKQEIQTAYRQAAKIYH
PDKNPDESADSSFIKLKQAYDILTDDVRRSNYNRFGDYKNGEVDDNTATLLICLSLVQHT
MFFIIGYFLSYPRKLEFARQIFLVYNIASFCFELQFRFIEDDTTFDWLPSIGYLLPYEKI
KLLRMLFPIVFFISICSSAYTYTDRNATLIYLMRSILATNRIVFERSNDVIESTNYLKKN
GEQLVSKLQEMRKAGKSGQAKIKDEEQ-------KGEKECLEDAKKDPKDQDTDDKLREN
VKEFALTLDSHQMGLLEKCFELMKNKSTKDKKNQKKSWFEFFSMQMIFGIIFVYIWFTSK
MGKDYYSILGVSKDCTTNDLKKAYRKLAMMWHPDKHKDVKSKKEAEEKFKNIAEAYDVLS
DEEKRKIYDAYGEEGLKGSAPTGGNTYVYSGVDPSELFSRIFGSDGHFSFSSGFDDDFSP
FSTFVNMTSRKARPSTSTNVN-NNYSSKPATFEVPLALTLEELYSGCKKKLKITRKRFMG
SKSYEDDNYVTIDVKAGWKDGTKITFYGEGDQLSPMSQPGDLVFKVKTKTHDRFVRDSNN
LIYKCPVPLDKALTGFQFIVKSLDNRDINVRVDEIVTPKTKKVVSKEGMPSSKMPNTKGD
LIVEFDIIFPKNLTGEKKKIIREALVNTFVKGTKISLPLLLLLLLSYTCVQAAYFYVKEG
VDKCFVESVASNVVITSSYDNFGLKELKCHINIKDQQGRVVYSHDTSAISKGKVSYLTKK
NGLYYICISCPSSNWFKSTAIKWSLSIEVGGSDVDIENVAKKSELSETLSILLNLKKKFT
SMKLQQIYQKQMATNLYEHNKSVHNKMFYCYILEIIILVAITVYSIVHLKNYFRAQKLMM
SKNKKGKKKDDLDAILAEFGIDEKREDEEAAKMSKSKKKKEKLKQKKEQKGKENEEEGEG
GEAKK---PPGAAADEAVDADANDANEDAKKNKKKKKKEKEKANEKKGTSGMSEMAKAAA
ERLRLLKEYEEKKREEERRKQEEEEERIRKEEEEKEKKRLAKLEKKMQLKKEGKLLSAKA
KEEKKKKELYLKTLKESGVLVEPKEKTKAEIISIDLNKTKAFLKKKKNKTNANNAEGGGG
TTPQGGKLDESLGQDGAEKKDPKEIVLDDWEDFLNVDEEGKKEKQPSEQKSGEKADRERN
KSKEGKAAEKKGKKKTDAGKSSEKNSHAQGESDGESEYRSAIVCILGHVDTGKTKLLDKL
RHTNVQDNEAGGITQQIGATFFPKDILDKQIKKVDESIKCMSKGIMIIDTPGHESFYNLR
KRGSSLCDIAILVIDLMHGLEQQTKESIQILKQRNCPFVIALNKIDRLYMWSKSDWSPFN
YTFQNQKENTQEEFQDRLKNILNELAEQGLNCHLYWENPNPRKYVSIVPTSAITGEGIAD
LIMILVKLTQNFMLKNIEYHEKLECTVLEVKNIEGLGTTIDVILTNGVLKESDTIVLCGI
NGPIVTVIRALLTPQPLKELRIKNEYVHHKSIKACIGVKISANGLEEVLCGTSLFVANNT
NEIEDYKKKAMTDVSDVFNHVDKTGVGLYVMASTLGSLEALLIFLKDSKIPVFAVNIGTV
QKKDVKKASVMREKGKPEYSVILAFDVKIDPEAEKEAQILGVEIMQKDIIYHLFDAFTSY
IKKIEEEKKQSKLTDAIFPCELSIVNDCVFNKKDPIVIGVKVDCGILKIGTPLYIPEKSL
KIGNVVSILLNKKTCEKAKKGDEVSIKICGEPHITFGRHFDFNQKIYSKITRESIDVLKE
YFRSELTMEDWRLVVQLKKIFNIIMNINEKDKLAEQNLETLDVTKLTPLSEDVISRQATI
NLGTIGHVAHGKSTLVHAISGVHTVRFKHEKERNITIKLGYANAKIYKCTNPDCLPPECY
RSYESSKEDDPICPRENCQHKMKLLRHVSFVDCPGHDILMATMLNGAAVMDAALLLVAGN
ESCPQPQTSEHLAAVEIMRLKHILILQNKVELIKEEQALKQQEEIRNFVSGTAADSAPII
PISAVLKYNIDVVCEYIVTQISIPKRDFISSPHMIVIRSFDVNKPGEDIETLQGGVAGGS
ILHGVLKVGDKIEIRPGIISKDEKGEITCRPIISQILSMFAENNNLKYAVPGGLIGVGTR
IDPILTRADRLVGQVIGHLNKLPDCFAEIEISYYLLRRLLGVKSQDGEKNTKVAKLKNGE
FLMINIGSTSIGCRVTGIKSELAKLELTGPVCTKIGDKIALSRRVDKHWRLIGWGQINKG
KPLELQEPIMIHFVLLISRQGKTRLAKWYIPLSQKEKAKIIRETSQITLNRTPKLCNFVE
WREYKLVFKRYASLFFIACIDKGDNELITLEIIHHYVEILDKYFGNVCELDLIFNFHKAY
YLLDEILVTGELQESSKKNILRVVSAQDSLMEDTKSSKKLGSLIMKYAFFLRRGVSCRSP
LNACGVQRAAFRRYYASVCPNEICGKVVQGRLFSSRAGMSDGGAREKGMKGEKGEDGQHG
QDQFDQQGKHTEGAEQKKEQMKETNYEKLNKADLINEIKKTKRDIEEKMVDNKILKEKYL
SVLAENENLRHRYVKEIENSKLYCISNFAKSLLDVADNLSLAIKNINEESLKQNEEISNI
YKGIQMTETILHNIFNKYGIDKYDPINEKFNPLFHEALFEINDDTKEKGTVATVVQQGYK
IKDRILRAAKVGVVKN-MKELIESVLQLINPDEETEAYLRERINDEKHIIQKKGVEYLYD
LVSTFSDEKIKKSTVADIFNKHMKRENGVGDEGKNADGQKLAKMVNLKEYWKKNDVVGYY
DPFLGIQEKQINYNTSIPISESIRISKEKEKQRQKQLNLFKEWVKNKIKIPSPVRVHNLL
HCSDSSKNKTKIEKMYDIRIDNFTLSIGQRSLLTDTTLKINVMNKYGLIGKNGIGKSTLL
AKLARYEIEEIKKDISIACIEQDLFLEDVTVLECVLMVDKLRHDLLTELERLELKKSKLD
GGCPNGGDAKQAVDQEGEGDEKDIDLKILTIYEKLNSINYLEAEKEASKILCGLGFDSNL
QKKKVNSLSGGMRMRLCLSRILFSNNDIILLDEPTNHLDIYTIQFLIDYIKKLNKTCIIV
SHDRDFLNEVCTDIIHFHQKELTYYSGNYDQFEKTRVEHLLQQQREHDSIEMKKKHVQKF
IDRFRCNSKRAALVQSRIKLLNKLPVVNLEKEETPFSFSFLEPFYTSNVLIRLKNISFKN
EMFKNLQIKKNANIIIADTVEDPNGK-VNGKSHNGTKGEGETTARSDDYQFRHEFLFKNA
TFEVDMDSRIAICGVNGSGKTTLIKIILNLIDTFEGELHVSNKANIGYYSQYHVDSLNPI
FNSIQQLQYNYSNKNIKEEEAIKYFNKFNIPTNVLYEPIYVLSGGQKSKLALAILAYKNP
NILILDEPSNHLDIESVQALIVALNLYKGGLIIISHDTYLIKHVADEIYHINNLTKELIK

IDYDFDKYAKLLLENKIMPREIITLQCGQCGNQIGVEFWKQLCNEHNIDREGILKNNNHL
NEDRKDIFFYQADDEHFIPRALLFDLEPRVINSIQASEYRNLYNPENMFISKEGGGAGNN
WGCGYSQGHKVEEEIIDMIDREVDNSDNLEGFILSHSIAGGTGSGMGSYLLELLNDNYSK
KVIQTFSVFPLLTNESSDVVVQPYNSILTLKRLILSTDSVVVIDNTSLNRIFVDRLKLNN
PTFQQTNTIISNVMSASTTTLRYPGSMNNDMISLISSLIINPKCHFLVTSYTPITIDKHL
SNVQKTTVLDVMKRLLHTKNIMVSVPVRRGMYISILNIIRGETDPTQVHKGLQRIRDRKL
VNFIKWNPASIQVTLAKPSPHVVSTHKVSGLMMANHTSISTLFERCVTQFDRLFKRRAFL
ENYKKEPMFSSADGQGNFEEMESSKEITQNLIDEYKSAERDDYFSHAYLMEKYHGLEKIG
EGTYGVVYKAQNNYGETFALKKIRLEKEDEGIPSTAIREISILKELKHSNIVKLYDVIHT
KKRLILVFEHLDQDLKKLLDVCDGGLESVTAKSFLLQLLSGIAYCHEHRVLHRDLKPQNL
LINREGELKIADFGLARAFGIPVRKYTHEVVTLWYRAPDILMGSKKYSTPIDMWSVGCIF
AEMVNGRPLFPGVSETDQLMRIFRILGTPNSENWPNVTELPKYDPDFMVYEPLPWETFLK
GLDDTGIDLLSKMLRLDPNQRITAKQALEHAYFKESNMGNKISTEDHIFRLKLKTKELEK
LSNRSELEEKKLVTDVKKAIQAGKIDIARLYAEKCIRKKNEKINYLNLSNKLDVLVSRLE
GAHRCASLVKDVSMMIPLIQKINTETNAAKIGNDVMKLENIFDEISISSDLINDTVQTSS
AISAPTEEVDELISKIADEHAIKLQLGPVNSINKHLEEISNMSERIMGKEKTHINLVVIG
HVDSGKSTTTGHIIYKLGGIDRRTIEKFEKESAEMGKGSFKYAWVLDKLKAERERGITID
IALWKFETPRYFFTVIDAPGHKDFIKNMITGTSQADVALLVVPAEVGGFEGAFSKEGQTK
EHALLAFTLGVKQIVVGVNKMDTVKYSEDRYEEIKKEVRDYLKKVGYQADKVDFIPISGF
EGDNLIEKSDKTPWYKGRTLIEALDTMEPPKRPYDKPLRIPLQGVYKIGGIGTVPVGRVE
TGILKAGMVLNFAPSAVVSECKSVEMHKEVLEEARPGDNIGFNVKNVSVKEIKRGYVASD
TKNEPAKGCSKFTAQVIILNHPGEIKNGYSPVLDCHTAHISCKFLNIDSKIDKRSGKVVE
ENPKSIKSGDSALVSLEPKKPMVVETFTEYPPLGRFAIRDMRQTIAVGIIKAVEKKEPGA
VSAKNPAKKMSHLMSLPIVLLKEGTDKAQGKSQIIRNINACQIIVDIIKTTLGPRGMDKL
IYTEKDVTITNDGATVMNLLSISHPAACILVDIAKSQDEEVGDGTTSVVVVAGELLNEAK
QLLNDGIEPNMIIDGFRNACTVAINKLNDLSLRFVSKSEEEKKEILIKCAQTALNSKLVS
NHKAFFSELVVNAVFKLGDSIDKSNVGIKKVTGGSCLDTQLIYGVAFKKTFSYAGFEQQP
KTFLNPKILLLNVELELKAEKENAEVRIDNPSDYNSIVQAEWEIIFKKLNLIKESGANIV
LSRLPIGDIATQFFADNDIFCAGRVEDADLKRTANATGAIVQTSLFNLNEGILGTCGVFE
EVQIGNERYNIFKECLKTKSVTIILRGGASQFIEEVERSINDAIMIVLRCMGNSEIVPGA
GSIEMQLSKHLRIYSRSICNKEQIVLYSFAKALESIPRHLSHNAGYDSTDILNKLRKKHS
EETSDIWYGVDCHEGDIINAYEHCIFEVTKIKRNVIYSATEAACLILSIDETIKNPASAD
KGPRNPYA---------------------MRNLGLPDCFKDLLKTDKIKHVLCTGNVGCR
ENLELLKNIADSVHITKGDMDDEYDFPEDISLTIGDFKMSLIHGHQIIPWGDTNALLQWQ
KKHDSDIVISGHTHKNSIVRYEGKYFINPGSATGAFQPWLSQPTPSFILMAVAKSSIVVY
VYEEKNGKTNVEMSELQK-MFTSTGELLYSVKVSEPTRKDATGAYRNPQYKDKLFDNFED
KPLNNVWEMFDRVAKKYKDRDCLGTRVKVNNKLGPYKWKSFAEVKELIIAVGSGLVNTNA
CPVIRCDDTKVTRAKFLGFYMPNCEEWNICDLSCNAFNIVTVPLYDSLGIESSKFILDQT
LMQTIICNKTCAMNLFKSLDTCEKINLKKLILVENEADAEVKKACEKHQLEIILWKDLIA
AGKKKLHEPKPGNLKDVSCICYTSGTTGYPKGVIMTNGNFVAQLTSSVTGPSRLPILNIN
DTDTHISYLPLAHIYERMMMLVFCAQGVRTGYYSGNVQNLVEDIQELKPTLFISVPRLYN
RIHERIFNSLKKKPAVVQSLFNKGLEHKIKKLNSNGIPFHFFWDKLVFNKAKKILGGNIR
VMLNGSAPISPDVVKKLKAIFCAPIFEGYGMTETLGPAFISHTTDVNIGHIGGPVPCVEY
RVVSVPEMNYLITDNPPRGELHLRGPAITNLGYFKLEKETNEFIDKDGWISTGDIVSFSE
NGSITIIDRKKNIFKLSQGEYIAVEKIESVYRQSLYISQIFVFGYSYESVLVCIVCPSLD
TIEIWKNEKKITKTDEEVMQMPEFKKDVIDDLIKMGKKDGLKGYEQIKDVYFASEPFTIE
NDLLTPTGKIKRHAVQKKYKEQIDKMYKQLKAAMASWDSYLNDRLLATNQVSAAGLASEE
DGVVYACVAQADENDKWTLFYKEDYEIEVEDENGNKSQKTINEGQTLLTVFKEGYAPDGV
WLGGTKYQFINIERDLEFEGYTFDVATCAKLKGGLHLIKVPGGNILVVLYDEEKEHDRGN
SKIAALTFSKELAESGGMSSFHNKKLALSSHNDRPTPLNLGANSESHTGKRKLLRSFRSS
RNDTCPDCKEKGIVICDNSEGTQICNGCGLVLESRILSEEQEWRNFHSDGQMKSNDRNRV
GEVSDIWFENNTSTTFIKSSKKLQHLNMMTQINKSDQTLLAAFNILKLICETFFLRSNVI
ERAKEITKELQEMEQLKNRINNLNMLAVVYLACREAGHIKSIKELITFDRSFKEKDLGKT
INKLKKILPSRAFVYNENISHLIFTLSNRLQLSIDVIEAIEYVVKKATTLITTSHRLNSL
CGGSIHLIAELNASEEKNLSLPNIGQIAAVCGVTTNTLKTTFKELLSAADYILPAYYLVG
NNSKLASLRQKYLSDDRRRRK--MEKLQLPEWNDIERYFKDPELITAEILFVGLTLCNVF
VMYRLFLDVIPFPIFVTWWQLAQGLLVAYVCGELGKEFPKFAYFPKVEISENMLKVLFVP
SIFYCLMLVLSNYLLFKTPCIASYPVLVSFTVVFHHLTRFVGCGEEYMPLRWKSIVFLLA
AFVIGCFDSKTTGKGVLIWALLYALFSAIFRAGFMQKIMHLVDGKGNTLHNNQHLLGVLL
LPILIVLSGEWAVFGHMPYNIMSLHTWQMWGCLITVGALPFIKNVISNRLVRRTGQGPWR
FLEIISIVLVFFIGMTYNAPSFLGYVAIICVIIGRSLGAFDVMLNASDYMMAEDERKRKT
SKANYAKSRQGTQASKPFLYSGDNEDDEESSFSSNDSRSYQGSQDYDDKDSVNSSSQQYS
VHKGTSRMDSSSNKTSHAGMSGDESRKAGGRGASFQSKSKLSRNYSSKKELVDSQAMGAL
DESTPVPSRITLQLMKQKKKSAFQGYSLLKKKSDALFIHFRDVLKDIVKTKTKVGEEMRN
ASFSLAKAVWAAGDFKGQIIEGIKRPVVTLSLSTNNVAGVKLPIFQVHIDPTVDVLGNLG
IASGGQVINNTRENYLQCLNMLVKLASMQVAFFSLDEEIKMTNRRVNALNNIVLPRLDGG
INYIIKELDEIEREEFYRLKKIKEKKIDKLKDANVEHIPDEDNHESAKRHDNFATSQKDD
DVIFMKRLPLVLLLPLLLYKTCNAVSLLESIKHDLQIVNNHNFNTVVNKFRSEKVFSVLF
FQKSNKNIKDVIKNYNDVAAKFKGIFTLCIADCDENPTLCESELSLYVPDYKNTNTHHLL
LYPINPMPKFLFSEEMNEANLKKYTYLIPSKIDIIKEAKDFSVFLSKHENMPKVLVFSNK
KKPNYVLNALSNSFNKKLMFCYINNELNDLVQKYNVKSFPTILILKKGKLVDTYKGKPNF
ISMFDWLNVHSETFVLGGGFDISPDKTVDKPWKFELVPKFTKMSHGDICFKKADKGLCLI
YLKEGDKLEKTEIDMLLSLKEKFKPHIDGRGINFRYMWIDIATETNFRALFEVKNYPSVV
VFNPYKRIRYAKLNEDLVATKENVEKLLEKISGGDAKFTMLKGQTLPEFVQDDSDPKANV
KDELMTVDLNVPYSACELKRVKRLELGVLDPEIIKKIGVCEIVNVDLYKDGLPREGGLND
IRMGTIDYKTLCGTCNMNVKYCPGHFGYIELAKPMYHYGFMNVVLNVLRCVCYHCGRLLC
NTSNSKVKFIEKIKVNSLRLKRLSEVCQGIKVCDHSSEEDTLHLNDNSVDNFYNNDLSNL
NVNQQMLLNPNSYGNIFEIVNKEDVDCGCVQPKYSREGPNMFIQFLH-SSEEDIDESKRK
LSAEEALDILKKIRKEEMPILGFNSDRCIPASLILTFIPIPPPCARPYVQYGNQRSEDDL
TLKLLDIVKTNIQLKRQTDRGAKSHVLQDLCALLQFHITTLFDNDIPGMPIATTRSKKPI
KAIRTRLKGKEGRLRGNLMGKRVDFSARTVITGDPNLNIDYIGVPKSVAMTLTFCETVTP
LNYDDLKKLVERGPYEWPGAKYIIRDNGTKYDLRHVRKNSERELEYGYKVERHMTDEDYI
LFNRQPSLHKMSIMGHKAKILPYSTFRLNLAVTSPYNADFDGDEMNLHLAQSHETRSEIK
HLMIVQKQIVSPQGNKPVMGIVQDSLLAIRKFTRRDNFLTKEEVMSLLIWIPYWNHVIPT
PAIMKPKALWTGKQIFSMLLQFEDLERLNDPLGNAKMGATNASGFRVEDEMGRSGAHLHP
HSPLAIGDTLSGNVKNQATINEATRTNSTDGGISGWGR--NVKINLMRDSSTSCKDDNPY
CSVNDGKVIIKNNELLCGIICKRTVGSSSGSLIHILWHEMGPDKTKDFISALQKVTNNWL
EYIGFTVSCSDIIASNKVLDKVKDILSKSKKEVSKIVKKAQRGELECQPGKSLYESFETR
VNNELNCAREMAGKVASESLDERNNIFSMVASGSKGSIINISQIISCVGQQNVEGKRIPF
GFNHRSLPHFIKFDYGPESRGFVSNSYLSGLTPQEVFFHAMGGREGIIDTACKTSETGYI
QRRLIKAMEDVMVQYDRTVRNSYGDIIQFLYGEDGMAGEYIEDQIIDLMKLDNKEVKKLY
KYNFDEDSYGKDYYLKGGQHRKGSYMEYSKQNVLNQEFEELLKCKNNICKEIFPDGDVRQ
HLPINMNRLIEFAKSQFPFVPVLGGKVKDPIGASGMGGKLKKREEKKGKNKKKKEKKATH
DQAKANSEFVSEIKKEYEANELAGAMKGQGAYSGFEPFDEDLAEGSGGDEGSGEEDSDGN
ESGEESGDE---GSGDDRSLVNPVDIVHKVNRFLEKLVIIKQINSNDTLSLEAQNNATVL
LKAHLRTYLNSKLLTQTHKISMKGIDWLLQEIEKNFYKSLCHPGECVGALAAQSIGEPAT
QMTLNTFHFAGVGSKNVTLGVPRLKELINIVKNVKTPSTTIYLDDVVSNDQQKAKDILTK
LEYTTLKQLTSHAQIIYDPNTTSTILEEDKLWVDEFYEFPDEDDTQYTLGEWVLRIQLTN
IHVNEKKLTMKEIVYIIYSVFSSDELDIIYTDDNSEDLILRIRVKYLNGEYNFLNDDPDQ
AQEDEYEEEEEEEEEYN-IGNTFKAKKNVEADKKNDKDDNQSVSSNRSRNNSVNSENRFG
SANGSASGTESEDDDGAGNPHRDRADSNYVEGYEEADLFVKGEGRSGESAVGSGVGSNVA
PNGTEDMFGKANLKRSSLSGANAGGGTTGGNAPSNNANSLDMNSMRNITNLSNPVYMKED
TEDTFLKKLMEQCLSTLKLRGIENITKVYMREESKITYDSTNGKFVRSSHWVLDTDGCNL
EHIFCASHVDYKKTVSNDIVEIFEVLGIEAVRRALLKELRTVISFDSSYVNYRHLSILCD
VMTQRGYLMSITRHGINRVDRGPLVKCSFEETVEILLEAAAFAQVDNLKGITENIMLGQL
CKIGTGVFDIIIDNQKLSDANQNLETLMDITSAGFTTPDSHHGITPDGLQSPVAINTLNS

PLPFSPTYNVNLLSPTAPLDSVNGILSPQCLQNYGDSIMSPSKNDFNNLDTLKLGGKFSP
TQSPKSPTSVIHSPFSPFDNHNQQALDPSMLFSPKNNSNSMSMMNYNVFSPKANLNSIQS
PAMYSPNRALDLFSPKQQTQNNIYSPSYSPTSPTYHLNKAYYSPTSPTNQLDEVNPNKYH
SVVSPVYSVTSPKYSPTSPKYS----YSPTSPKYSPTSPKYSPTSPKYSPTSPQYSPSSP
VPNNPSSPHYSPYAIASPKFSPTSPAYSISSPVYDQSGNARSKHPLSPAYILQSPVQVKQ
HIQDVSVFSPIQQANAEEVQNDDPFSPMPYNMEEDEMEQ-MVVFQLDDLEVFFPYDYIYP
EQYAYMRYLKKTLDSEGHCVLEMPTGTGKTVAIFSLITSYQHGKNDEGKFIFCTRTVAEM
EKSLIELKKVIQYRVDTLRRRVVSEVKGERADERAEDEMKDQTGEQTTANDTANHANDAA
QRRSGDLQGDILAIGISARRCMCVNERVLRKHEREKIDEECRKLTATFVREKKYISQRIS
KLGSTHSDRVSEFIVKNRQHIDVEDYFDIYHTRNSLEEYNDIGLCGYYENYKKEFLFELI
QPGVYTIEDLKVMCKRYKNAQNETVPICPYFCAKKVIEVAKVIVLNYQYVIDPKVSKSLF
IGREVNSRVNLHKRDIIVFDEAHNIDSVCLEALSVNIDRNVLNKATANIKKLLSKIEASK
VLNEEKLREECTRILRRVREEKRGGEALGEAMSEAANEAVKEALTGGAPGKRRPQESKGV
YFDEDLNLIFPRAANPSGVDDDAPWGGKQAKGGGLLEGLAKFLGNGSSSSAAAANAATPR
EDPTELHYSPLLMEDAVKNVVLPGNIRKSEHFLNLMRIVVMYLRKYVNIYEVTSEGPLSF
LYKCERETKLDTSFFKFCFDRLKALLNALQIVTVDDYSALNVVCNFCTLLGSYFQGFIII
CEPYPEATGIYDPVIQFACLDSSIAMKAVIGKYRSVILTSGTITPLELYPKLLNFKTVLT
ASFPMSFDRNCVCPLIVTNSSDLVPLSSQFSLRSDLTVIKNYGMLLVEMCKTIPDGIVAY
FPSYIYMEEVISSWYELGIITSILECKLIFIETKDIVSTTIALHNFKRACDLGKGAVFLS
ICRGKIAEGIDFDKHYGKCVILFGIPYQYTLSRILKSRLDFLKETYNIQENEFLTFDAMR
QASQCVGRIIRNKKDYGIMIFADIRYARNDKKSKLPPWIIKCMELSNVNLTVSTAVNISR
KFLLNMSQEYKETGQTKLSQELTSDQPRCWALVKSALNLDDFLMLGRA-----------L
RRYGSLKGLPQEVLNQPGTRVSELPNKLKIATVKSSCEVPTIGIWVSSGSKYESKQNNGV
AHFLEHMIFKGTKKRSRIQLEKEIENMGAHLNAYTAREQTSYYCRCFKGDVKWCIELLSD
ILSNSIFDEDLIEMEKHVILREMEEVEKSKDEVIFDKLHMTAFRDHALGYTILGPIENIK
NMNRQSIINYIHTNYTSDRMVLCAVGDVEHEEIVKLAEQHFSHLKPQSSHTTSASNLDAV
KPYFCGSEIIVRDDDSGPSAHVAVAFEGVDWKSPDSITFMLMQCIIGTYKKSEEGILPGK
LSANRTVNNICNKMTVGCADYFSAFNTCYNNTGLFGFYVQCDELAVEHALGELMFGVTSL
SYSITDEEVELAKIQLKTQLINMFESSSTLAEEVSRQILVYGRNIPLAEFLLRLDKIDTE
EVKRVAWKYLHDREIAVAAMGALHGMPQYYDLRQKTFWLRYMEGLKPILWSVEELIYLSP
ENDAFYEKEETEIRRKLADLCRSSGGADCHAEELTSQVRNLFHFLKLIKAGKHEECIVDP
WVRDIFQFIFDTLKNDSTNSINQVKKELFCYVSREKADEQSENGNQVFFQACFTFFHEIK
ILLCCYAFLNMFVQYNWTGPPPPTDSEKEKVQTSQGCHEDNHFCQFIQTMKIKNEEFLNS
CLEFLSLEGETTYAHCELINAFCLSVILLNLVNNFNQTDQPIMCSNEYGYFEEGAPNGVQ
VNSQDGNSMTKQYNETKLLHFVRSKYLWKARIYFIWQRLFASSNDWFYSLKIHVVDIPLR
IFKNVKLLSSDFELIDQEIKVGQVEIFAHLRQNEKDLQNCDLFCENYMSPNFQICALSNF
SIYLAFYNYTYAYQRVLDVLAEMSQFSYSFTGRMGIKRKYQKIPATILVLKAKKGQEEDN
TSAVLNEIEPLSEYDILRSDYRIIDDVKGGEDTERGDPHKEE--SEKREDDAGEAVPSSK
KADEMSPKVENFAEEHIEKKGEAQNGEQIDQKTDECSPNREEEKSKTNPNGKVMWKLKDL
DPDTDILEEPYFMDSENNYFKILTFDEQIALINFCYSMIRFNPHYDEIKFEKLSAVIARC
LKCYDVNVESGKQQHGTKNRDNNSLQIKYQNWLLHSCILWYKCKCETFRLKTVDRAAAQL
NELLKETYDEKPQGGERVKFLFDVYYPTTWELKKEIGNVMVKTGSVVSAFNLFKDLKLWD
EAITCLIQADRKGEAKELLDDLLKKKRTPSLLCLYGLVDSNNALNYYIDAWKLSNCKYAK
AARFIGNFYYRKEMYNPCCEYLEKALEISPLFPDIWFILGCSYMKIEKVDESVKAFTRMV
SMTNEDSAKAYGNLAYLYMKKGTYKAAKICINQAVKVDNNEWKYWDTYLKLSIMQNDVDS
FCLALTTICQKNQVKQIQPWIYEYISDLIVNDKPTLIPNKNGLSYLDKVLTTMGTISVHI
SECDSYWNAFSFFLFVKGRFVDSYEAKVKEIRSLESVAQKCTTKDVVDRLISKQVAAVKF
LYHVLKAHYVEEKRGTFEYQLRNIIESILRQYKDMGQQEVTELSQIMQIIKMSEASVEGN
ELFNADDEEEQAEGNADDRADDPAEGGARRKRKKEHHTSPSKGGASGLKKRKLSKKRYLS
TFLDTEAQVGDDDEEEEYASSYVEEFEEAKKLEKKKMYETKLKSGTNHLAQTINKLSQRY
ENEKEIKDTLTDGETLTDEEMSGDEEYFDEGECLSTFDSPKMWLIKLFRNGVERNLAMGI
YYKYMKLKDNDFNIKGVYVSDNLKGYIYVEADSLYMLKRFLLGFKFINLNEITIVPVQEL
TSIFSMCHSKVIIPKVNEYVRIKRGVYMNDIGQIFEVHEKGIYAIVRLIPRIEYDKYNHF
KKGNY-HLGLTGSMSKGPSAHSGKTESYYLGDKFEMNSHAIGGDARNGQTANEQLELLDE
ALQIKRKKKKERPLKKLFDREEIEQIGGVIEHGPYPRTIKYQNNIFEENGYLLKKMNIKY
LISENANITLTEIRDFNKNNTNEEDINLHVSKSFINKNSLHLFKKGERVKIMKGELYNLI
GTITNVSENVLTINPDNLAKNFKFLPSDVTKYFLEGDNVTVINGIHKGKSGLISLLDYKE
NVALIFSPSLNTEFRSSIQDLSACEHSSEGLGGINSLNGFSIGDLIELTDRQIGILTYID
RNKHIRVLTSNNKTLHTTIGTITSKRSAIGQVCKDENGNIIQAKDVIQVVRGIHKNKVAV
VNYIWKNKVFAKINKKIEDNGFVVLDNENCILTGNQNEKKKIVTHNNLFRVNSSRRVNTY
QSFIGKTVKILSGVYKGLLGDVIDAERDEFTLLLKIKPKTIRQKRTECAIADSFRENYNL
TDDRYYNSPRWRNRSVGGANGAYSLRPHDQKGFEGNWARTSHENHPNG-NSSYSYNRNGR
AGDPISFNEPPSRGGGMNEDGKISATRGGNPEGEFPNGQYGGPGSHVLNADQRYDDGKWD
DAPGTGGINGGVNNPGATSASNFMDKNNEGKASPLWLVEGIMIKVITPGPFYNEIGRVTE
VMKKSLYTILKIATEKTSFSIVSDAVIPLKPNRVNDEVLVVDDGKVVEGTVLDIQNDDVQ
VSTAQGIFTYHVGHIFLFKRQFPMNPNNPLSVAVADSLSAYDIQNESFRLGNVSIEGDKF
ICVKENVNENTQVVVINLQNKISTRKYMKAESVIIHPNDPILALRGTIKNVNTIFLQVFN
IETKEKICSLNLNEYMNYWKWINNDTIAIVCERNVYHWNIYTLTKVFEKAQVFIDNNSQI
LYYATDKDMKWCILCGISTQDQGKSIDGHMQLYSCDKKLHQTIEGFIGCFGSFIFDNWDI
KPLFCFVEKKKNSTVSRLHLMDIYTNKTEATMPYKIVKEINLINEHISDFPIYISLNTLQ
GVIYIVTKCSYVYIFDEGTLTQLVKEKISEDNIFTCCDSKNGEGIYAVNKKGKIYYITIN
YVNLINHIKLSNFDGRDKIIKNLCVKYGYPGCDYISAYKKCINEMDFKKASKIICLLKNT
KLRTQQVLNSFKSMKNASGQLSPLLLYFSVLLEYDKLNTYESVELVKPVVLQKKKEYLEK
WIKDDKLTCSEELGDLVKVLDLRLALNIYLRCGAHNKIISTYCLLNMFNNVLSYLNNFKQ
VSFDFVSIFIVIVNYESQCGNEKDASSP-MADMSTSSKNEAMDFFNDG-SNSTNDSRQGK
GSNSEVAVQYVKFLCENNIPFDINNIVDYLLSKKKLQEATSILLDFLKDNKPEHKNLQTK
LFEFNLYNNVQVAETLFQMDIFTYYDKNRIAYLCEEKGLFQRALENYTNLNDIKRVITKS
SRGISIDWIKKYFSTLSDSVCQDILFDFMKGNKINMEVVISICVQYYNKIGIKKIINKFE
ENKNYEGIFYFVSSILTHLPNLTNKATDHLSYGSMNDETSSILLASDIGSQYNNAENPSS
LKIEDVHFIMFKYIEACVKINNIQELDRICKDKNAKYNPEQIKNFLKDCKLSDPRPLIYV
CDIHNYIEELAEYLYKNSLLKYIEVYVIKVNPNNAHKVIGVLLDLDASEDFLLNLLNNIK
NISNIGNLIEIAEKRNRLKLLLPWLESRSNEGYENIELHNALAKIYIDLNKDPENFLKNN
NFYDKKLIGKYCEDLDPHLAYTVYEKSNGECDEELINITSKNGLFKLQAKYLVSRQSMEL
WKRVLDESNKYRKNVIDQVIGSTLIESNNAEEITVTVKAFIEKKLSSELIELLEKIVLHN
SEFSDNKNLQNLLILTAIKSDSKKVMEYINRLDSFSGPQIASVAYDYKLREEAFVIYKKF
NCYTSAISVLLDKILQVNNKGAYPNEFSRASSSVYESDSLNAQETILDAKSYEDDLNRAI
EYAQKCNHNDVWFILGKAQLKINKIIDAIDSFVKSNNPEAYKEVIAKCKENNFYEHLITY
LNTLRDQNLLKDVLVDSELLYAYAKLKKTTEMTKFIGSTNSANLQLIGDRLFKEQEYEVA
KILYSNIPNNQKLTFCYLKLKEYSLAIEAAKKAKSLKTWKEVNFICVKYKQLKHAHTAGL
QLIMHADHLDEIINIYEKKKYINELMSLLENGLNSERAHVGIYTELGILYAKYKPEKLME
FIRNYSNKMNTRKLIDVCQNEYLLKEAVYLYISYDEYNLAVDTIIKHSPTAYTPDTFMQV
IHKVTNSDIIHKVIDFYIEEHPLNLYNLLKILENKIDNNRLVQTLKKSNNLPLIQKYLED
IQGQNITAVNETLNEIYLQNDDYISLRNSIDDYDNFNQTNLINKLENHKLSEMRRIAALL
YKKNKKYKEAINLSKKEEQYKDAIEIARVSKNNSYVEDLINFFIEIKNKEALCACLIVCY
DILKPDYILEIVWTSGFKDQAMLYFIQVISDYTSQIDAMKKQIEDMQKEKKMNKSAPNDF
AANSMSGQFTYSLNNHLSIMPPQNSYMSSSSQFDKYDMFNNNAHFMPTYVGKEAPFFKAE
AVFGDNSFGEVNLTQFIGKKYVLLYFYPLDFTFVCPSEIIALDKALDAFHERNVELLGCS
VDSKYTHLAWKKTPLAKGGIGNIKHTLLSDITKSISKDYNVLFDDSVSLRAFVLIDMNGI
VQHLLVNNLAIGRSVDEILRIIDAIQHHEKYGDVCPANWQKGKVSMKPSEEGVAQYLSTL
MEDNAAKIIGCHDSLLTDPRIAQDFSAETEELLAEAESLFKVGEGELAVEKLIALEKKCR
QAYDGNSTSKIVRFILNQYKQAGDYQKINEYLVFFNKKRGQLKKTIIDMINLCKLWIPEV
ESKGDKLNLINTLCAISEGKIFVEVERSEIVRVLSKMKEDDGNIEEAANILQDVQVETFI
SMEKRDKTEYILEQMRLVLLRKDFIRCHVISRKINPALLKAPEFADLKLKYFMYMIEYHI
NEEAYAEVAKCYEERFNTEPVLADANLWVDELKCYIIFLALSPFEDQQIKLPNLLKTEKK
KLKEIPVFQKIVEDFIDMDLIQWPLPYEEELLQFYIFKDSKFVGGQNRWNLFKKKVMHHN
IHVISNCYCQISLLRLAQLLNASVEDSESFLSELVSNKIMNAKIDRLHGIIKFGQKKTPE

VLLNGWSSQINQILNLLEESSHLIQKERMLHEAKLKRMQLESKNMSLMAEKEAKKEAIEI
IYEISNILNVNLDKETIVILIQLCEYGVSPKVLSHIIIQLKKEKQKFLQNLSNNMGNLKQ

> ***Plasmodium chabaudi***

MIKDSKINIYYGKNYPFLCRTVFNIYQSNLKKKNLTDQNIEEVVNFINDKKVSSSDKIFA
INLDFLLKTNLYYFTCKEIINKNIITNVFFQAQYNEWIDFLRNKDIEKDIMPICEHINKH
LYLNTFLSSHYLTLSDIYIYYEMHKYFSGSITTNLKYPKQYKNINRWFKLIKSLIHSTDA
ELIQHLKRRPGNNADKSSGDH------GKGSAASYEGKLQNAEKGKVVTRFPPEPSGYLH
IGHIKAAFLNNYYANLYKGKMLLRFDDTNPVLEDVKYEKSIMEDLETLGIKYEQVSYTSD
YFQLLEEYCIKLIKMGKAYADDTNVDEMRNQRGEGIESVNRNNPIETNLKLFEEMRSGTE
VGKKNCIRAKIDMSSKNKCMRDPVLYRCIVDIPHHRHEFKYKCYPTYDFACPIIDSIEGV
THALRTNEYSDRIEQYNWFIHTLELRNVYIYEFSRLAFVRTVMSKRKLKWFVENNIVDGW
TDPRMPTIKGILRRGLAKEALFQFILEQGPSKAGNLMQWDKLWSINKQIIDPNIPRFAAV
DMNNGVILTLADLKGKIIEKTRDLHAKNKDLGTCTMYYTQKIYIELEDAQMIGPNEEITL
IKLGNVIVNGIIKDSVDKIKEIIALSNFDGDFKTTKKKIHWVPYIPDKLIKCTLYEYDHL
ITVDKFENDNKEDWTNCVNQNSKFETVAYAEPALANLKVSDKLQFERRGYFIVDKITEDK
HFHLIKIPDGKSKNMSIISTKVNPKSLAGTKKMDNNTIIIDNGSGYMKVGLNTNTLPTIV
FPTVVGNSRDKDVNQTYVGDEAFYHESELSIYRPFDHGHISDWDLVNSIWDYAINCVDPN
RSVKNILLTEPPLCSISHRKNMGEIFFENFGFENINISVSGLMSIYAAGLTTGLVLDIGE
GVTQCIPVFDGYIEKNSVIRSDFGGEELTMFMQKLICDIGYNMTTRKCYEYVKIMKETLC
FCSLNPPKDQLREDLTATYTLPDGDVLRDGYNTIEISHERFYVPEALFNPLLCHRDNLSI
PDIVCKSILSCPIENRKILSSNIILSGGCSLFPNLAERLEREIKNNSPENARSAVKVHVH
ENRAIMAWCGGQIFSQPELREAQRGVWISKEEYDETGDNIFLIKATLKLT-MDGLKKINL
IQKINEKLYDHLGIEDENLTEFIIFLCEKSTCLEEFCKEVFENGGEIEQSVLKSIYNLIK
QNGKNQQNEKDVNNNTISKEIQEIENKNAKMKKFGCLSIKNSINLPDLTEEEKDKHKSFD
KKADHKSHRHS--------NHKKETDENDKLKKNRHKDYSRKRSRSKHDDRKRSKYDDRS
KRRALKVGNIFSGSIKKITDFGMFVSFKTREGYKEGLVHITDVEKNNKK-INLNENYKRN
MIVKVKIKGIFGEKISLNMSEVDQKTGKNLVDDNDKNDFNKNETSFFDDIEDEFKHKKNK
HHDKYSKDPNETIKYESVIKMKSDYSKWEIQQLIKSGIIYDENIKNEYKNLKYEEKIDDE
EEMIEIEVNEREPNFLKGQTTKAGANLSPIQIIVNAEGTLARAITTTSALTKERKEEKKN
EQNALFDSIPKDISRPWEDPKPNLGERTIAEALKNVGKNYDLPDWKKNYINNNISIGMKN
SLPLNEQREKLPIYNLKVDLMKAINKNNVLIVIGETGSGKTTQIPQYLHEAKYTELGIVG
CTQPRRVAAMSIAKRVSEEFGCILGQEVGYSIRFDDCTSNDTIIKYLTDGMLLREALSDT
MLSKYSFIILDEAHERTISTDILFCLLKDVVKKRSDFKLIVTSATLDAEKFSTYFFNSPI
FTIPGKIFPVEILHSKEPESDYVEACLITVLNIHLNEHPGDILVFLTGQEEINTACEILH
ERMKKLESMSPPPLIILPIYSSLPSEMQSIIFEPAPPGCRKCILATNIAEASLTIDGIFF
VIDPGFCKIKKYDSKRDMDSLIIAPISKANAKQRAGRAGRTGPGKCYRLYTEEAYKNEMA
ETSVPEIQRINLGSIVLLLKALGVNDFLHFDFMDSPSIETLIHSLESLYYLGALDDNGYL
TKLGKKMSNFPMEPNLSKILLTSINFNCTDDICTIVSMISVQNIFYRPQNKILLADKKKN
KFVMPQGDLITYLNIYNKWKENSFSNYWCHENFIQSRALKRAQDVRKQLLSIFEKYNYKV
KKREGISNSTNYVNICKSICSGYFNHVCKRDSQQGYTTLLTNQQVFIHPSSTLFSKNPLF
VVYHELVLTNKEYIRDCTIIQPQWLIQLAPNLFIPADEKKISKIKLREKIEPLHNYYEEP
NAWRLSRRKGMNEEYDHLYKIILVGDATVGKTHLLSRYIRGSLPSVAKATIGVEFATRTI
PLAVGGTVKAQIWDTAGQERYRSITSAHYRRSAGAILVYDITKKKTFLNISKWLEEIRQN
SEKDIVIMLVGNKVDLAEEDETKRKVTYEQGASFARENNLFFSEASAVSKLNVKHIFENL
LQEIYNNRLKDNNSVSSTRSHETYESAIQITNAKNIIKLNDKSNKYN-DHNANQMKCCME
KSKLAKVEKVLGRTGSRGGVIQVRAQFMGDSELSGRFLIRNVKGPVREGDILALLETERE
ARRLRMNESLDKLSTIDLLNELKRRYACLSKPDGRYVFIGAPGSGKGTQSLNLKNSHCYC
HLSTGDLLREAAEKQNELGNKIRGIINEGKLVDNEIVLSLVDDKLKSPQCKKGFILDGYP
RNVKQAEDLNKLLDKNKMKLNGVFYFNVPDEVLVERICGRLIHKPSGRIYHKTLNPPKTP
FRDDITNEPLTQRDDDNEAVLKKRLGVFKNETTPLINYYKNKNLLVSLDATKPAADIEKN
ISQHIGG-MEQQCYTCYFDLPDPKSTIGPYDNELSYFMWGPGFEWKPEPVVKQISREDTY
DEAEESEESEHGFGELDEKVDKDERRKYFDEKCIGGKISIADASYNARKLGLAPSSKDEE
KIRDLYGDNLTYDQYLEYLSMSIHDKDNAEQLVKMFAYFDTNTTGFLTKNQMKNILVTWG
DALTEDEAMNALNAFSNDDRIDYKLFCEDILQMKG--YKYSLSLLSLLYLLANYGMENAM
KFPDMKGLDDLSALNDVQIKDIFGLTGDEVKERLTKLFGVIDKNQDKVLSDEEISAWFEY
VKNEVFLKQVQIEMKQIDADKDGFISLPELNDAFSQNLDPKEVEKHADGLLKRFQIVDKD
KDNKLNLNEVGLLIDPMKDNDLKELEINEILEHHDTNKDGKISVDEFKETRSDDPNMKKD
DELALDDFNFFDVNKDGFIDREEIVKVYFDPSNDAATLNLNDVKNNIFEGKPITFDLWNE
KSLKFAVTSLTDYGDVIRYPQDFKLDIGKNVVLPSSKHRMG-DDILNLDTDLSDGKDKDD
GDASDKQNITDELMDLLLAKAICIFAFLSVATGGCAIPYILGLFGKRNRNKYENKVKNIL
SNLNCFGAGFIFSIVMFHLLPETIIIASSHNDITIFKTHDPEMKTLFIFFFVFLGFCMQL
ALEYVLPTDTSLCCVDHGAVK-FTDDNDHIINVENHTIENPNTEIRNMPMNTDAYYHPHE
GDHSNHKNRFTKFLDVLALQSFFLTISLAIHSGIEGMIVGTSDDAHFVFITTFCILSHKW
IAGVTVSLSLNRNHISKNLKIILLLIFIFSSPLGIIVGHLVQSSGEKITCLINAVSIGTL
LFIGCEILLNEMQMKFPRKVRFSKWLSFCSSCLIAFSIIYGTSHIAPHQRAH-------M
QSKFWARGADNDSGDNVSDSSENEVDE-KPLVSAQAERWAVMDSSSSEEEERVIKSSEGK
RLHFYETIEDNLNDSMENDDFNQLLKEYENLYKFMAKEGADRIPNFVIIYLDKLTKYVDT
TFQNNVEKKELSKNKAQTLNKLRAKIRKCSELYQNKLNQYHENPDKFKENLERRKKDEDD
DEDDEDDDEEDEED-DEEEDDG-KKKKTNKDDED-EDEDDDDDWSYSDDAEYASDEEDDK
TKKAMSKWGLKTSEKVEKKKVAKVKKTKKEGTKKDEKGTHADDNQSSKKKTYAELLNTKN
LSEDVIRNRVKSVIEKRGRKGLDKHEHINILSKLCELAKTISTQSYIEVLEHLINLEFDV
VSSVYTYMSFNVWNKTFKYIELILDLLIQNDHFYLVSINITEEIAEDETNEKEKITKSCK
TLISFLAKLDDELLKALLYIDVQTEEYRKRLGKTVHMISLLYKGYKYVKHTKNLPDLAIY
ISTRILDHLYYKPELPFKQIWGFVKHGKEYVEKE---------GDNQKKEETNESTDADE
SPKDVIEKFVCEVFEHGTKQQKLRALLQLSYNKSLYDEFLEAREILNVGNVHELAISSDV
QTQILYNRNLIQLGLCAFRHGRIYEAHCCLVEICSQNKHRELIAQGISTLKNQEKTIEQE
RTEKRRLLSFHMHISIELIECVNNICAMLLEVPNLAKHSYESKKDIISRQFRRFLDIYDK
QIFNSPPENNREIIILATKYLQKGNWKMCCEKIFSLSIWPKFTDKEKVQAILTEKIKQEA
MRTYIFRYISVYDSFSIDQLCVMFDLPQNTVHSILSKMMVNHEIPACWNESSKYILINKV
NPTPLQTMALKLAENINEVMEQNELALNMKNPKFMLMQERKTQMKDDKSNWNHKKGDGKY
GKNYNRNKNQNYKKNYKDKNMNKNFVMEHGNVGDKIFDLNSISREMLELVEVFYDKMKET
TNANALYLYALQIFKICNIHNELPRLVVFGQQSMGKTTLLDFIMGGPMGYTSSDTGTKQP
IVIILKPSETNKIECYLNKKKVSIDDLHEKMKAIMLNLSESIIAKELEVEISIPGGIYAT
FVDLPGIKDDSKAGSELTRKIVRNYVQNFPNDIYILVKKASDDPANWPYHLKEFFMKPKP
LGLGLQNKQCIVVGTRALEFLNNELSTIKTLTELHDRVKKRGITDNNDNMLSLYLLELFS
IPIEQKEKNDFLTNRISMYSKILNGRKNVLDLLLNKFENDCSDSIKKELIDCFDVEKFKQ
EVNSKFMNILIQQLRKVEVKIEKKKAKMEIYIKKLEELYNKGNILSIREQVKLYIRELVN
IISNLLTGNYPILNLPKNGDDFLKKYGGTLMENLKDGNDLAIELYEKQGLYDENFLTYLN
EYLIKQSNENEYNKSVISDFNNCNDLLNNSNNNSNINSLNKRSDIYDSFNDRDKKR-TND
KMLKVGQAVRFLLAKEESSMFGVVQNIPNDARSKNILVNFFFRSNNVEEQIQVKSVEKER
LIVIKPVESLNSDLFFLNGLQVWYKMTRDDGWVGFDKAEIIKVFNNNSKIKDVLIRNLSK
NNEVVSIKINDLYADYTDNM--DEDADPMEEIYDEDDALKRIAGPHTDLKILNQLAITYI
CKWLKYNIAKIEPEKIFSDEVLLQMMRSIHNIVDQSDWKPLVVDLLQANISGNILYLTKL
ASCSAAVALNRVFKAGLGEINRKIKNNYLDENIYLLSTNPKFLEELNQALHNFCKERAVS
CANEMKEIVFEQTYAVHFEIIEEIFEGCKLFEDNFLTPTGVKPTMSIINKNVKQNLAYRN
HLLSLTDVKINKKSRSKELIQEEVKLQFWAIKMLISVPFATKIYAHFLNNIVPKNKHLAN
VLDYSIDCESNLEKYIQSKLLNREVNGIQVPIDDKELLSHYNIIDNRDNIFRKLENQKRL
IQYLTIVSNSIKLLKNNLSNESTLDFVTKLDFGQNKKMELNNISQILYGTVDPNIDVRTE
AENKLKQAKETNIVQYINQLSNEFCKAQNDPYLRQIAGLLIKNAFVSKDNYESDEKARIW
LNFPENIKNDLKSSLLNLLDQQGEKIVIGTACQIISIITKIELSYNKSSELLHKLVNNII
EKNAYTKKSSTICLAYLTEDIADICNESKTKYVFTQPDLDLILTAIINSLCEPAEEAIHC
ANMKVLYNLMSFIDHNFKTQVERDIIMKTVIDGCKDNDRLTVQVAAYECLINIVSYFYSY
LDAYMYAIGPLTWEAIESDNERIAISAIEFWNTVCEEETFIDQYELDEGKKNHNIVKQAM
VFLLPKIFNAMITQESEDIDIDAWTLSMASATFLALSAQLLKNDIVEPVIAFVEENFIHE

DWRRRDAAVLAYGSIMEGPDTEKLKPLVEESVGQLSEVLRDPSVSVRDTAAWTIGKITQY
HSEIIYNVLGNYNDNNSLYGILLERLNDYPRVAANVCWVFNQLAVNKRSTYNKLENTYTT
DLDDSFCVLCKKLIDVTSREDADTRNLREAAFNALNVVILNVSDNCLKYMIELLSHMMYL
LTNTYLNPLTEEVKSIQGYYCGTMQFIINRLGAQCKPFLKPIYLCIFRLFEIRNDICEDA
LLACSAIINVMGNDFREHLKTFLNVIFKGLKNVSETSTCKICIEMISDICIPWTCDMEKE
MENILECLWEALRSFGVHDSIKISILTVLGDIATALNKSFSKYLNFFANILLETSKITIA
SGSPENDDWVSYVFELRDAILLTYSNIIYALIDGNEINKLKMYIPNILDFIELILIKEIN
HFNAQNFQNSVSLLGDLVHAYGYELIENSKLTDLIISVYGKIDILSSQRDEKCESCVSKI
KWLKKICNARLGQKMSVYIQLTHDLSGTVTAVTIGDWILGILKQKNIQEYEVFFKKFLNI
YQNQDKESETNNRSEIFSLLLSASDCVFETLVETKKNKKINVIIDNKECVKSFNEFYKEV
EEYFVILISILQLEFKSVEDLNNATNNFIKAIKNYNVFPELRLKILQLLYNSFNVNFSFR
FPTFIAILQFSSQNNIFHAILPYIKSIDDWIKEWNISNHEKRQIYLIVAEELKKLKKFEE
SYKHLKKHVYYFQKEDKEILNHASTIKASVELVVDAINLNNNIYFHEIINLDAIQNLQYV
DEHKPIYELLTIFYKYNIHEFLEFKKTHGDSFFTKYNIDLESSENKIYLLSIISLFKDNK
VQNIQYISEQLNISMLKVEQILVSAIGSDIIDAKIDQINKTVHMKTTILRHFDEQQWEHL
NTQIAKYIKNVQKVIDITTHRKSTSKAMNYLFYLVLSVILNCFVRGQESATNFYKFIDSF
ASSTYISEESGSSLYDAKRAIQNNPSYWCSAGNHLKDEEITWTGYLNTKGFVKGVKISWE
YSPELVSIFVSSDGEHYKNVIPYKKISSTESSFDEIYFFKKLEEVTSIKIGLKNAIHKYF
GIREVKIIGGGNPYFLLLSGITSEQEMCLQVEEGLINNDNTSVILDSCINALASGDGREL
WKTNSNNQIISALSDPPKCLAVINLDNLENNKLVLYDCLRALEDGDGKSNWVFESNSQIR
LQRSGEPLCISQKNIHGNIPGIHDILLNTDASVDATSILDDDHSADNTIDGDLNSFWASS
IFGDNYEHLVYFVIDLNKFVEISRVKVFWEYPPLHYIISFSTEKDNYKIVAENLANPSFT
TIDSLKNIETRYIKISMVKPHPKHGEMDGQFLYGIRSIEVQANNLESVLNFCRDAANSDD
ARDKYFIEYISEFDKNLSNKLINLEDDVSKNVSSISDKLSKLEEVLPNIETCLNEKKEYD
TKLKASMDQVIELNNKITSLESVDLIHSNDLLRLGVSPGDSSSYPANDCSIIKNAQEVPM
SGFYWIKPKCSPEPLRVYCDMDSSTSLYVWNGHSPKSPDHLITNIINSVDDIRKHCAEVG
LEPLVLKSTDQLNSLIVALKKMGFILNGKINIPLAYDYSCNYGSCSGKFHDLLNGNVDLT
TLIYFKGAESPNTTTIRQTAGISYDDGSFKFFNLETSDISAIVCSTNSSENDFSMQYLSI
DCDTTALDDDFNGIVNTNIVALCPLGCDHERFKEFHVYGSNGVYSDSSSICRASIHAGVI
DKQGGLVNVAIESGLDYYKGSISNNIESISLNKGGNEALLDIITNEKKEDIKEETSIINH
RTIRISNLSQDCPIDLFEYKQLSFIEKGNFKKNEQKVENIAEYNESNEGYKTHEIINDLL
KNVDAIHGVDSSVISIIQDETVRVIEKAKKEFAPADILSKKQIDDTINLYNLTENLALYL
YDLSGKYINDLEKVKERLEELKKVQRIVHNFGAFKLNYESMNFSSYFYIFDSKLIKNKPS
VWGYVDTEILGHKNSIGQMSSISNREIGEGYFAKLKGLNFYDFEIKVSVLSKGTGCAGIA
FRAKDDFNFYLFDICDQDGVRRLSKIENGHADILKEKYGDFSINNKWSTYKITTSHANID
IYEIDDKLNEMKILSSLDEKFLSGTVGLYSQINGQGTFFDELEIIAKPCSELSKSGKHEK
KKNFNCPYYKENYDSDLFPYTIINDVEYKWSFAKEDDDYLLCKKIEN------GTVYDTI
ALLKQRKCSDGNLNFDMNYSKENENNNQHIYILFNFVNENNFNALEIRQDSLKLTSYKDS
KPITLSEFNDHDKITKILEQDEWFHVNVKFDKLKFKVVISSNNYEIKLDANNDDTDSIDL
GNVGFMVNNFDEVKFDSITLSSNVLNSNESFIESKTKTWGTCEKSIHVLNRRSSCETDIY
PNQKKEKHINCIKNFCEECCLHHTKMLDSNEKKQCEKHCKRNDGLAEKMQKLFEKFINKC
VSLEENKDYENCDDDDLNCRSKTCILCCEQNDVVDSDDIEEVSFHKSKDIQKEETIECQF
QCKVTHGIAEMSFSAIKYESKILESLASMLNLNEKDEYKGKKFEYFYNIETYGVDFFLDI
CNALCRSNYKHFLTEEGKNEVLSKERLNKERLSDKKCPTKNMYLNQNKYITEEIEERIEN
DILNSNDTFKSQQNHQDGIISVFKYKSELSSITRKSNQKIVQTIFEHTKDFNTDSTTNMS
NSNFNNTNSNFYSMENNNTKRSLRSRQFSGKKPSNKYKNLNKKVKLSQNKSLTRNSHRND
CNSITNYCNNKKIRKRRTSNNRKIRNSTNSFMPDYLYLENQIDSIYSIFKSIDKSIEKED
SPSKFKETKETEIEENIENENYKEKEDYNPYLGLGDINNDNEGADLEEQLNKQNNEYGIY
EKHTLSKNFCNDEEYEGNQSDDTSYSTNGSNYSKYLKRKKKLEEQGKIVIDLNLLHRNTI
KPNMKQCRDEKMAERLRIEKLKKILEEKKMLLCRVEDIAKKRNRIHVKTQILSEDSKIKR
IYFSHKMKKVYNYKFGYFGCGWSNNKTEWTPFIHAPFFDNQHNTIYKSRNKKLYEEIYDT
ILHGRIHPHIKVVELKDHMHPIRLCTPSNEDCYSVIYTGEKINATDERVIFGEYTGFVAN
NKELPQEKHQYIFALTFNKKVFNDRKNVVFINEIDSYENESDENENSVEGNNSEMKYANM
KNGNNYKGNKFNLDEDKHNEEISKEKE-----KGEKNKSLGNN---------KKKENDKS
KELNNLIILPDNYTYAVDSSHMFNEMSLVNHYKTCSIFNNYDFRINAEWQIVYLDGWPHI
ILTSIPGVEIETGEEIFADFGFEWFDRVNDICLNDFIKNNYEHRLNEIGIKKEKNFNGLD
DIVDKYNLLKNYTTCNICMHSVNTDCNNYILCSGCNHVYHLKCVNRLNFQINENYDWFCS
SCIQFSMNIISQKEFLEYIKKENNKRLINYFNKNIDQGKTKSSEKILMENNLDSYNCE--
KNNEIQLDDENYINNFENIKKLLQCKENIDDLLNNQQIINAFLENVDNNINILQGEEGQD
DNIILKNPELKNLIENSYELHLLIEYKNKIDDLLLCRENINFLLNNDQAKRTFQMRIKTI
NYLMQNKKCIENLVESIEKKNQGEITKNNNTTFKTSNDKLQLLMNDDAQNGNTDNTSSLD
LFKCENGEQNCVALCEENDTTQSMIIPVHGKYPYSKDGCYFTSAYENMLYKKTNNIIKNL
KDLKNKNRKSRFKNNSIKNEMNDENSNGTDEAGLNAYYNYMMKLSKKILGLPLIREFEKG
INTHQPSLPLNANLKKLEVCSNCYKKHGNLAKAVICRVTKLHFETNYNDGLGEEELHKMS
SECIQSVIKELANTIKEYRKQELNSAFIQYTKKKKQTGLINNKENDIKTGTRLTDIITPS
DIINSAFNKTDI-ENKDFYHSLSKSLYKDTYETDDNILMQHSHISYIDKNKSVESHQDEN
STLQINTYTTLG-KKYENIGDGN--GKNCHQANLSSDTIDEKNVSDQENGKNNKRISYKN
GNISAYNNDINNVCFRSDSNNNDYKNQKKNKFKNDNELLTTNEILLDDKKKEDKSISNNN
NINNFIPLFGIELGKTKFQREFTNGTYVGTVTKQIKDDNDNNFFVVTYEDGDVEWITPFF
LFQELLKQATNNIEYPLATPFKDLMNPNFKKDIKLNNYSLELKVEKKKKKGTGEYNSNNN
STTKRQRHNQEDNSSKTKKR--MVNKEEAQRLKELGNKCFQEGKFDDSVKYFSDAIKNDP
SDHVLYSNLSGAYSSLGRFYEALETANKCISIKNDWPKGYIRKACAEHGLRQLDNSEKTY
LEGLKLDPNNKSLKDGLEKVRKEKEMENMEYINHINNIINNDPKLRSYKEENSNYSTELL
NTIKAINANPMNIRFILSSCNPKISEGVEKFFGIKFNDDASYEAERERQRKKEEEEKKEK
ERKMKEEEKKKNRTPEELLGDEHKLKGNELYKQKKFEEALKEYDEAIKVNPNDIMYYYNK
AAVYLEMKSYEKSIETCIYAIENRYNFKADFSQVAKVYNRLAIGYINIKDYDKALEAYRK
SLVEDNNRATRNALKELERKKEKEEREAYIDPVKAEEHKNKGNEYFKNNDFPNAKKEYDE
AIRRNPNDAKLYSNRAAALTKLIEYPSALEDVMKAIELDPKFVKAYTRKGNLHFFMKDYY
KAIQAYNKGLELDPNNKECTEGYQRCVYKIDEMSKSEKVDEEQIKKSMADPEIQQIISDP
QFQIILQKINENPNSISEYIKDPKIFNGLQKLIAAGILTVRMNEHYDVIILGTGLKECIL
SGLLSHYGKKILVLDRNPYYGGETASLNLTNLYNTFKPREMIPSKYGENRHWNVDLIPKF
ILVGGNLVKILKKTRVTNYLEWLVVEGSYVYQHQKKSLLFSEKFIHKVPSTDMEALVSPL
LSLMEKNRCKNFYQYVSEWNANDKSTWDNLDPYRLSMMDIYKYFNLCQLTIDFLGHAVAL
YLNDDYLKQPAYITLERIKLYMHSISAFGKSPFIYPLYGLGGIPEGFSRMCAINGGTFML
NKNVTDFIYNDNKQVCGIKSSDGEVAYCDKVICDPSYVMHLENKIQKIGQVIRCICILSN
PIPETNDINSCQIIIPQNQLNRKSDIYVNLVSFQHGVSYKGKYIAIVSATVETNNPTKEI
EKALELLGPIDEKFIKISDLYVSTNPKPKDNIFVTSSYDATSHFETATNDLLQIWENLWG
QKLNFDDLNKN-DIELMAVKKQKKTLKPSTKVITINLSKLTHDVCYKRKAPRAIKEIKNI
AGKLMHTKDVRLDVKLNKFIWSKGIRNPPKRVRVKIERVRNEDEDSKERMYTLVQHVMVD
SFKGLVNECETNEMATPKGRKKSDMKKIDDPFAEHGDSTKKSRSIAQDDRALKLRACLSC
RLLQTEAEFYQSGCSNCKFLQMTGDRHRIQDCTTENFSGFIAITTPTKSWIAQYNDLSKF
NPGFYALQVVGELPESIRDLKSNYMPFSSDGKINFDYIYNNKELKNKCYLPSSDTFVFAE
ALEEDAETISPNVNMVLEMGTGSGYLILFLYELLLKKNKKVDLLYCIDINKDACNCVQNA
ISLNAISNVEIINNNLFNNLRTCGQFDIILFNPPYVETEQDEMNKTDIVASYAGGKHGRE
VILKFLHTVYDYLSNNGILYLLLEKNNIPHEIMDSTLISEKFHYTELKKKKTLNETIFIY
KLRKKIMDRYGHNVRSDVKKQGYENSDLPILCETCLGENPYVRIIREENGKECQICKNAF
TLFRWKPGHNARYKQTIICNKCAKVKNVCQTCLFDLEYNLPVQVRDKFLETSIALPENET
NRNFFLEQLEKNISTDTYDKISHGNMDLSKLKRKDPYFKRNMARVCSFWRKNACNRGDEC
PYLHKEIHLNKSLANQSIKSRYTGENDVLAETILNRYKNNNIDEKNMANKICIQGISDSI
RVENVKECFKKFGEIKSFKMIPKDSKIFISYATLTAAKNAAEKYKDGLELNGCNLTVTLQ
QDNINNNI-NHNNKKFSKNNFKPHNPPPPMMNTVVPPMYFPYNNPKFNSMPPNAIPYASM
LPSEAEQRKMIKNIFNEYLKHYDKKNLGYERDDTNTSSKDHSNEYTSDWRWFSIKCERLI
ESELNKKYEQNNEKNEYKNAIDDIDNISTFRSFFPKILLNAYSRYSDPKKFFDNLIIQKF
NAVVFFCDASGFSSLAEQLENKINGAELLGNCLNKFFNILIKIIDSWGGDIIKFSGDAVM
VIWPLNGKKKIKKEMQSLGRSGTNSFEKRGSVQSEINNGDENMDEEKRNKLNQARRVSLL
ALGCSVNIHKLLNKFPTPIENRYLKVHIAITYGKVNFLQVGNILNKRDYILSGKPLEEIG

AGESLAKDGETVISYSFYKNIKDKVDIKETCKKRFYLLAGVNDELDMEKLKEHDNEKD--
-------DKKNETVCKNFDLLLKSFIPDIVYRKLSLGYNMFVNETRKVTIIFVSVKDVDT
STMTGIYSTHSIMKITQKAVFTMEGTINKFIFDDKGILILIMFGLPPLYHSDDSIRALLT
CFRLIDGLKSLKLNGSIGVSTGKIWCGMVGNKIRKEYTALGDSVNIAARLCCKAGNKEIY
VDENTYSNCKHFIIFQSLTSIKVKGKNKSIKIYSPIGTINKRANNLAYDDLFILDYFTDK
ELLDHSPNREEVTDFNFLNKNFLLKYYKNKFKDQISDLLNKKDCLYYLKLCKEMQNNGNK
NKNEKNTLNSNCFKCSQIYSLSPYHYVKRELCTDYKTYTGPLFIHEYYDPLFFDFSEIYN
IGGVLFLEGSEHLGMFEIIKLIKKSLNNFKIFNISNMPNSLYINITNPLLPWKILCNDIT
NMWRLCNMRKKYLYLNNDDNYNILKEITHPSYHWFLKCMSNVIDDLYIPEIPSQKEAR-I
KNILDNQGMDSD-----------SHFEAYDGSHYDENRKDGRAFCNKLFNSPLEIRDTKN
ENDAINKKNSGDSIANSS-----EGENGNGNMTSINSDVILKNDMKENRISIINCMIYNF
SLYEHTFIIFNCRSGTSLNISIEEHAWRICKNISKLAIYKRKKIIKNFHKNLKNWRKNHC
RVCNFCKGFYKTLYPNIQDNILLYRSNLCNPIFKPKNKPLIFLFVNGAQNNDVKELKKMK
KYAEECNGYIKLENFNKSDLYNFVSLCLNISVDKISEELINYLNKTCFGFPKFVQYTLFY
LLKNKFIKLYKYAIPNVREQTNEKLDETIKRSKSDYTNTEKKK-QKLTKNRKGSQNRVSY
DYVNKTSQNIIREQNEFLNFTVYREASNTLINSDLSNYDQEMEVVKNLDDATLVPRLIAY
CMFIIDSLGEEDQLLAKLCSFFKDKFNIQKMECVYPKPLSRSELKKIVVNLVKKNVFEVC
EDNKSNTTPKKENIFFRITNYSLKKVLSELMENEEKKYVQNIYKKHIEMLDRNYKHQSMH
EKTRVIIKNIPKYMNEFDLKKHFFKMKGKYDFKITDIKIMKRKKIIKNKESYESRKICFI
GFISNTDCENFKKSFNNTYIHTSKITIEDAFSPILSKNSNTFQLN--GIQQSEKTNKKDQ
NNTVKVIKNDQFINKMTTIKKTKAGMNTTRSHVIFIDDDADLEKVNELNAENSEKKKKKK
KKKKKKNASENSQMEENTVEVEEENALDWLKGISKDKGNDKENSKETDEGERNLLESDDT
ESEDEISSMDKAKPLYSEPEDIDYNDNINTGKLIIFNLPPVDEQDVKSLCERYGPVIDVH
IFKNTKKGSKYICLNEKNDNKSREDFFIKLLKENNDNTSNRNKDDTKKMTNSNNTKEGNK
NESENENNKNENNIANIDLTNVKTYAFVSFVFPSSCEKAKNNLNETIYKGKILSVKYARE
KIDNSENLEKNKNNIFIKLSNESKTSYKKILEIQKKRNCQNENIWNILYTDINSNIYNFC
KETNCDPQSILNIRDKNIAVNVSLTETFIINKMKEWIRKEGIVLEAFEQIYKKENAKPEN
DEKDETDKVIKYKRSDDTIIVKNLSMHTNENDIINLFKKHGILKKISFSPYKNIAILQYE
KPEDAKKALISNSYIRYKKLPLYLEWAPVNLFEQNNKNTDETTEINENSND---ALHQNN
VEAYDSESSDEEITHSSIYIKNINFNTKEEDFKKLFEKLDGFITCNIVKSKKAIKQKHKD
NKTEQENKYISLGYGFAEFKSKELAIEAIKKLTATKLDGHVLELSLSHNRIKKNKSKNNE
EKQVIKDKKKITKKLLVKNLAFQVTKEELRKLFSAFGNIKNVRIPKNAYNRSRGYGFVEF
MSKNECLAAINALQHTHLYGRHLIIDFANDLIFDQNVDEFDKMKESQNGANPDFITSEQA
KRRSIYENEKSTKISESKKRKIMGNLENVMKSNCGESTYKNQGYKFCETSNCATKCVDQD
GQQYNAIDNIFLKESSNGNENNYLDKIPENAKMLKPLVQEKIIEVMKPEIEEKIIEVPQV
QYIEKLVEVPHVILQEKLIHVPKPVIHERIKKCPKTIFQEKIVEVPQIKIVDKIIEVPQY
VYQEKIIQVPKVMVQERIIPVPKKVIKEKIVEIPQIELKNINIEKIEEVPEYIPEVVKRD
VPYTQIIDRPFHVEKIVEVPHVQHIYRNIVSPQYRHIPKPVEVPMAHYRTFPVEKLVDRN
VPVPVEIQIVQEFLCPKIEARYKEIPVPVHVQRIIEHPIPKDAMNNPLLLPLYYQEDNFN
ISKNGNSNTSQNNKSCFMFNWNKNNNQKTLKGRSNSVELLLHNDNKNNEFFNINEANMNP
NINFKSINQFSGNQFRDINNIPMDANMNMHALGPCQSINPIGNSNNQTMNNFNNQSYDAN
ASLKISNSMNYPANISLTKPYPVVSPIVPPLNEMFSTRSEYDRGVNTFSPEGRLFQVEYA
LGAIKLGSTAIGICVDDGVILASERRIASPLIEKDSIEKLLPIDDHIGCAMSGLMADART
LIDHARVECNHYKFVYNENINIKSCVELISELALDFSNLSDSKRKKIMSRPFGVALLIGG
VDKNGPCLWYTEPSGTNTRFLAASIGSAQEGAELLLQENYNKNMSFQEAEILALTVLRQV
MEDKLSSSNVEIAAIKKSDQTFYKYKPDDITKIIDSLPSPIYPTIDMTAMEVEKTHLINY
FLEICPTVLDCSRKELQTVLAKREEEKIRKFVVDKNVNIIVIGKGKQEKID-EGHDNI--
---KKLENERDEERNINFDKESSYSLFVELNINYKCVTKSIAIAFMKRNKDTFLGLNEKL
NNGNKINLSNELLMFVCGLNDCNTPLDLVYLYLSQGFNTIFDAASYVTHGNEHDEISQKK
YSKKRDAENGSSSYAGYGNTTCGTYDGENKISNLLMTNVSKKLNELLISMKNAQIDLNIP
IINLNVDKRIKKLVEENPNIDDIKQETIKNLCESQGFLNQLQKDVTKWVEEIQKITRLNG
DFKSGSALAEINFWIGYENALLELENRLKSPEVILTLQILKNAKRYFATISFDSDIQLKQ
SKEYVLNVNILMKDFPIDDLLGAQTVQQTMQAIRNIFNHLKKLKNTTKYPLSKSYNFVEA
LSRDLGNTMKKILSNQSLLSMDYQIFDVLISGCMEVFQLWNEEIRVFKDMVRELIKKRSF
NERAPAKMIFEHVILQERLDDIRNFRKQHQKFKTVIAKVFGNDKNVGINLQKDINAAYNV
LLSLDALDLSKSGNDLWEKTKLTYESKMNKVESQITFKLRDKLGGAKTSNEMFNVFSKFN
PLFFRPKIKGAIQEYQNSLIQIVVEDIKKLQMIYINGYVKSSSEKVSTIRDIPLVSGSII
WCKQIEKKLEDSFKKIENVLGRGWEQHAEGKNLKQNIDNFKNLLNQNKTFEKWLKSIKNG
DKFDIYEKIIKIKKLGINNYEILANYDQQLFNLFKEVRYLQSINLRVPYSIKVKADETKL
IYPYALGLEKTLRIYMKICLYLENEGKDIPFNGTISMLVASAHNSVQEKIKEGINLHWDS
DILETYVRKLTEHVNMLESMVDEAVNKNSFVLDILEKIKTCQISIDGNEIKKYVELIQEK
ADELYLQHYKNVHMWINEMNKILDAILATRLEGIIKKWTEEFVGWTRRNSAKNRFILRSS
VHEFKISKHKIFLHPPTDSMRQIWFSKLSEAINLICGITRIKNIYQKKDKSREVIEIKNK
DGEVINNVMLDNTYKYIIYYVDKKVYDNAIKCINDSVEKAREYESMWFQYKTLLKVEIGD
IITNFGDDIEIWKTFMNEIKEREDKFDILDTEKNFGPIVIDYRMLQSKVSSKFEIWQKEI
VSEFSKKLLEKILAFKEDVEKCLHDLREQSELKSESEITAMHILSMRPKDIITIMNDYDM
DVLSRICSSIHDFVEKINEINKKEQEWDSKCDILKKSELMLEKQKYFFPKNWLFIDSVIG
TVETVKQVLAYQINLVKEYFPYIQSLVIEFDSKVQNNIKELYEQWNSSKLADGNTNSVKV
LQIIKTFESKINIISQDYQVSERLRKLMKISDEESEGNFHISPKMLQEEITCIKTIWDEL
KIIYSSISDLKKILWINADLKNVKNLLNNLLGSIKKIPAKYRQYEIFDKVKDEIQEYLKT
YIILYDLKSESLKERHWKLILQKLELKIHYNKLSLGNLWSSNLCYHENAIRDILNQSQGE
MVLDEFLRGLKDTWTEYELELVQYQNRCKLIKGWNDILSNIDDHLNAIQSMKISSYVKIF
EEETLNWDDKLNRLRNLLEVWMNVQRKWVYLEGVLKSSTDIKLLLPQEYNRFKIIDADFI
NIMKKTSENPKLLELFQINGFQKQLDRLSDSLSKIQKALGEYLEKQRNQFPRFYFVGDED
LLEMIGNSKDAKIIQRNINKMFAGISSFILKENTSDSILGMCSREGEEVFFKEPIHISTY
KTLKEWLMVLETRMKTTLENYLDLAAIEFMQMDILQCTKDLSNRQIIDWCCKYPNQIILL
CLQIMWTYNIEHDMELSMEKSEMASQTIFLTSEGVCRSLLKYLSEIVVKQTNNTTRQKIV
QMITELVHQRDVIRILIEKDIKNVNDFTWLQYMRFYWDKDKRNKNVNLIIKMADASFEYG
YEYLGMCEKLVQTKLTDSCFLTLTQALKMKLGGNPFGPAGTGKTESVKALGAQLGRYVLV
FNCDESFDFTAMGRIFVGLCQVGAWGCFDEFNRLEERILSAVSEQILTIQTSLSQRKKEV
EILNKKVELNKNVGIFVTMNPGYAGRSNLPDNLKQLFRSFAMIEPNKELIVQVTLFSQGF
ISAEYLSSKIVSLFDLCSEQLSKQPHYDFGLRSLKNVLNSAGNLKRLALQEKWKEDGDNG
KNSDEVENVKKTAEMEQALLLKSVCDTVYPKLVSSDIVLIKSLLNGVFPNANISLFEDKD
LINEIKRICKLKYYMPEEKWITKICQINQIMKLQHGIMLVGGVGTGKSSAWKILLDALEA
IDNIKGMSYIIDAKSLDKEEIYGKLDNINLEWTDGVFTCILRKIIYNYTTNNNITKRHWI
IFDGDVDPEWAENLNSVLDDNKLLTLPNGERLPIPESVKILFEVDTLKHATLATVSRCGM
IWFSRDILPPIVLFKHRLNKLKYGNTDYPRKIDILKSLFVSNSNNEAAGGAIMNGDGGKK
GNDYENSEISDNSIALNENIMGNMRRNSQVFYDKSDENIDVCKLKTIEYRSIELISEYFE
ENGFVQQCLVGAANFEHVMDYEYIRVIESTCLLIQKGIDNLITKYGKFNNSVSDSDIEKY
MSKWLVVSLIWGIGGSLNLESREKFSKYVESICSSSLPSDLSKRGLLNPSNLSNTNRTVL
DYEPSVEDGEWHNWKERVEITDVDRTEISDATLIIETMDTIRHATILEGWLNLKKPFILC
GPPGSGKTMTLTSVLKKSTEFDIAALNFSSGSLPNLLLQTFDHYCEYVKTTSELVLRPIQ
PGKWLIIFADEINLPTPDKYDTQRIIMFMRQIYESQGFWKYDSNNNQWNWVKIERITFAG
ACNPPTDAGRNPLSNRFLRHTSILYVDFPGYESLKQIYGTFNRAILRKFPESLHMADNLT
LAMVDFYAKFSETFTVDMEPHYIYSPRELTRWKLSIYNTLENFEKIENRELVRLCIYEGL
RIFQDRLIYKKEKKETDKIINEIFKYSFPDVKDEDLERPIIFSSYIENKYIEINKNILKE
LILAKLKIFGEEEVNVQLVLFDEVLDHITRIDRVLKLPFGHLLLVGASGVGKTILSRFVS
WMNGLSVFQIRTGRNYSTELFEVDLRNVMKRAGIKEEKITFIFDESNVLGPAFLERMNAL
LASGEVPGLFEGDNYKALINECKGQYGTNAGLDESDIFKKFTKQVQKNLHIVFTMNPANP
DFANRQSTSPALFNRCVIDWFGDWSYKALLQVASEFIFSLNLPDNNFHMDDINSKSIKSK
LDFKDKKYYFLSKAIVEIHNSVVHINQVLMKKGSKYNYMTPRDFLDFIKHFLKIIDEKRE
EISAQKKHLNAGLKKLKDTEVQVAELRNSLANKKKTLAEKDIEAEEKMKLMIEQQAETED
KKKKAEILAKKLDEQFIIIEQRKEIVRKELSEVEPKFREAEDAVKNIPKKIFDELRAMAN
PPILVRNAIEAVAILIMNEGDKNVTWEDARKIMKGQDFINKVLYLDKKTVKAQTSAQIKK
KINSPDWDVDRINKASRAAGPLAKWVESVITFLTILETIQPLENEIEKLQEETRIAENQY
NEQKEIISALEKILVQYKNDYAQLISQVQSIKQEMEIVEKKIVRSINLIENLKSEKERWS

ETFISLEEASETFIGNCLISAAFCAYIGFFEHYERQKLKKHWGEIIKIHHIKYRYDLSFI
EFLSKPSERLQWIANKLPSDDLSIENAIIISNYIRYPLIIDPSDQASTFLLNQYADKKIV
KTSFSDKNFLKNLESAIRFGSTILLYDVEKIDAILNSVLNQETHKQGGRLLIVIGDSEID
FSPNFNLFLTSRDANLQFTPDICSRVTFCNFTLTPSSLQNQCLNMILKNERPDIDKKRRD
ILKLQGEYKVKIRELEESLLLELSSLEGNILDDDNVITTMEKLKIQASEASKEVNIAEEV
MIEIENVSNMYLFLAEGCSKIYFILQYLCNINFLYQYDLNFFFNIMKDMLLDYEYLSKIK
KTDYGERIKCLEYLLFNLTYNRVARGLLQEDRYVFALHLCYVKSIINPSCCIDRVYLHLL
LKDSYVYEESTTMMDQKTSNEIGRELLPEYNDEQIRNLNNLIKHKLFYYLKDLIENNREG
WASLLHSDEPEYVVSSLIIQNKNIKRKNSSATHLNSVEEGGEINQENPISQCLKEAIIIK
AVRADKLDRCFNKLINIILGKEFLWLPELSTNEFEKYVKENALGNTPIVLISSPGFDPSN
KVHQLSEHCKVSLSSIAMGSEEGYISAEKVIASAQKYGGWVLLKNIHLSHKWLYQLEKNM
HRATSNADFRLFLTMEINPKIPPNLLRMSLTFMFEPPVGIKSSILRTFSLFLENKCINDP
KIARLRLYFIVSVLHAIILERRRYTPIGWTKKYEFSDADLTCALAVIDNWVDRSVVKIGN
NISEHIDPCNIPWDAIKKILNETVYGGRLDNIVDSKILDTFIEHLMHSNSFETDFNLNIS
HSLSSKDFLKSPDLFRNIQDYIKWTNNMINTDIPAWLGFGQHAEGLLTTRTNFSIINKWN
ILYSKSCSDMQELLPYSQDEIKLEDQIDEMDETENADTPEYDTKIRHMRTKKQNESTIFI
DKILESLPEYIPDLEKSDENVGNAVFRCFERENQLFNNLLKDIKTNLIQLKSVLEEKIKY
TNKLRALAKDINSLNVPSGWLLQTNTSNMNLTIWLRDIIKRLYQIIVITVELNKSSAAKS
NSEKYGSAKKTKHEDDENSKKLSINFIWLGGIFYPRAFITATRQMCAHKYKKSLNDLELS
ITVDEKNEEKLMEAFHFTIICLSIEGAQWSDNDRCLILP---SKNSKNPINQGNCNLNVN
NNLSTHIIPSSNLSSQHTKQIRLNQMNNSHKSHLLQYHNSNYDDKNSKLYN--NTVINKH
ISSENPGASH--ASNNNDTVKGNKIGCNTRQKNSSHALHN-GNNNSSKGQQQAYNENLGY
RPTLFNLLEVLSNHNITTPDQRVCIRGIVTDFLNNELPHGKIYAYIGAVVGHDILHDIIK
KLEKDPNRNAVPDASGLARIEASFGLSKSSYDFMNHNSTNSSNNIPNVLFNNRNLNSALL
NNHIYSNNNKTANDINNMMMKYGNDKLGGSNNNMLNMNKKKALNMRNSNITEDEEELMKV
IKKNIETYKMNNIKNILISSVFGRIKWLKIMNESPHAYLYGHSIVKYGNKLYMFGGTNNK
HKKVPFNHTLTFSLIYYNYKLLPLSGNYPEERDGHTTHLVSLKSGLSVFLFGGANNNIYY
NDIYILDMETRKWSKRIVNGKLPLPRHQHCSLVYPAKSEHLRGEKLNLTEGVIVYGGKCL
YNNNIVNLNDMWIFLFQTNMWVRINYLGDQVPTGRYGMNLIWSDTNTLCLFGGEYLYSDK
TCKTRKLLDDMWTFRLNTPV-NLANKIKITGEWKREIYEGNIGCRSNYSSIFITQRHQDF
KGAEPKTIEKLMLLCSGITY--DNKLKIVSSDEIYVYFFSQKRWYLLKGKLYNEEYMYNA
RQRHVGCFFESKNVLGRSHRNPVPCIFIHGGFKKNSVFGDAWLLSLTGENPLRLYEYDTC
REKISTTQMPLYYFRDTHSISLLYTFCTLQKWLFGAFANLVDNSVYAINPAENVFIKYEL
TPEHDGMISIQDDGEGLDFNAMNRILRMYGNYKNYDNNSFYNNNNNNNIKKHSLPNNDDS
DSHEIDNYDDNNENNADNGTDEMQQNEEGDGDKNNNLSGQNCEGIIKKENDIRNKNGPIG
KKKCTWNDTFYYKRKNQYYFYNDLSNSIFDIKYGVGFKTAFARIASSCAIMSRTINTIGI
GLLSLELMNHCDAKELATPLCMWKLPNKELINRNIANKSEHRHHQKLLMSYTPFNSPSLL
AEQINILGTYSGTRLLYWNFRDDMDFVIFNPTNNNIYLSSAPLSIDELKSKSKKRKNAQG
EGSNINSKYQGVKDIKFANRIIKRKGVFGEDGNLKKFKAEKEEE-------VIDDENNAD
DNYNKEEENIKKEHDNMSTSSKNNNIPD---DNNDENKNGNSLNYKEKMKYEDQLKYNNN
NYFKKRMNLFEYNSHLSPSISKEKYNMNEIFPLWDHPKDSIDYCLSTYLYWLYLRRSTNI
FLQNTLLIPTSMRREKKNNNDTKGKKKKKSKRNKKPQNEEQADSTNNQENEQMASNEESE
TAAENNRSEQEHENDDDNNHSDAQNEDNINNETSEQNQ-----NDDIKNNDGGLSKLEKQ
KLEEALEYGISQDVQAQNSANKNDTDDEDEEEEEEEEEEDDDEEDDEEDEEEEEE-KNDE
DNANINETNNEVEEVEGEANEVGEDGVVKTEQNEMETENNVEEEDATDKNTKKVGKTVDD
PNEDSSRRKRRRKKDDLAADAAANGKKNGEIEKMKKENYVNVYMENFKIKDEYYINNPSK
YTLYHFLRRKLYKMVESHYLFTPADHTYGSFIMMGFLNDNNNTSMEVNRVCETGILLYYK
NRLIKRLDAPFIDTPYNLFLSKYPPSPSLYEGNLYKYALTVIVNVPNWLKPSVSKQEFVH
ENNHAFLVFKKKLVTLIKHYLSICEDVVKLNKWRESRDMKLKRYLEKMQNQSVKSRNDSD
YEKSKNYNIIYNNQNDGNKKKKEEINYSGRNKPSSYANDNDPILHKNKKTKTNNDDKRDQ
NPASENESANMDNENNQENDNLDEEKEHEDEEYDEMDQEADNHENYDDEKYEQEENDEEE
DDPDDEEYNGEMDDQKNRSIDQETDEEMAEDEEYEPMENNPDTKTLGDDNNGKLPKKKNL
CRLIVEEATNDDNSVVALNTKRMEELNFFRGDTILIKGKKRHSTICIILNDNDLDEGKIR
INKVARKNLRVCLGDIVYVKACPEIPYGKKIQVLPIDDTIEGLAKDTLFEIFLKPYFNES
YRPVKKGDLFLVRGGFMSVEFKVVEVDPDDFCIVSPDTVIYYEGDPIKRDDEEKLDEIGY
DDIGGCKKQLAQIREMIELPLRHPGLFKTLGVKPPRGVLLYGPPGSGKTCIARAVANETG
AFFFLINGPEVMSKMAGEAEANLRRAFEEAEKNSPAIIFIDEIDSIAPKREKTNGEVERR
VVSQLLTLMDGIKSRGQVVVIAATNRQNSIDPALRRFGRFDREIDIGVPDDNGRFEILRI
HTKNMKLSPDVKLEELASNTHGFVGADLAQLCTEAALTCIREKMDVIDLEDEIIDKEVLE
SMCVTQDHFNMALGTCNPSSLRETVVEVPNVKWDDIGGLDEVKNTLREMILYPIDHPDKF
EKFGMAPSRGVLFYGPPGCGKTLLAKAVASECSANFVSIKGPELLTMWFGESEANVREVF
DKARAAAPCVLFFDELDSIGTQRGSSLGDGSGAGDRVMNQLLTEIDGVGPKKNLFFIGAT
NRPELLDEALLRPGRLDQLIYIPLPDLAARISILSAILRKCPVADNVPIDFLAQKTAGFS
GADLAELCQRAARAAIRDAIDAEEMNKKSKLALNPEGNQTNENQATNNEESDIKYEITRH
HFKEGLAGARRSVSQADLIKYDNFRIKFDPLYKTKSGGANEDFIIDWPDEENNEDPQDYN
VDDDLYSM------IFKKVSIDPSLLKRLSKEGLVSIEVAKASDIKILNKDNAEKGAKKS
A-----KKSKLNKVAKKAITKKGKEKKKKNEITKSVKSNKPKIGVK--RKRKRVRGKKKK
KSASEKKENDVEPKNYDKDMSNQSNKENKIKKDEPKKKQIFDIEQIIKNEEEFKNIKEIK
YKIHCSKWNKDNELNIPHSIMKSLYDHNFFSPREIQSKTLEHSINGKKDIIVVSKTGTGK
TLTFCLPILTNILQNKLKEFHTKKKTGQKFRCLILVPTRELAVQILNHFNYVNKYVNIYI
ITIIGGLNINKQLRLISKKPEIVICTPGRLKYFLQLEDKINYLCNMKNVRYFVCDEVDKM
IETSFINDIHFISKHLYKSVDDKKKFIQTFLLSATLSLTVQLHNENLAKLLNYVSIRKDK
SYIIDLTNENDHSQNSMTISNNNDISHGKTTVLPDHLKLNIIKCEKKIILHKLYYLLKLY
ILKDLQNNKENQVKKIIIFLNTIKLVKDVSTIFKYLFFEPGLESSIPNKYKTNLCLSQKI
NIYSIHSKQTLKERIQSVSKFTESNNSSILFCTDVLSRGIDLNKCDLIIQLNCPISDITF
IHRSGRTARNLKAGECICFITDDEISRWNVSLNKIGLNLENLKEYEHVKTIENNEFTKIN
KAIRYCDEMLQLQNKIKSNKEKMNLNKLAKDAELSNDDDTSDSDNPLPKKTNDHLLKNIF
RLKKQLYTTLYQEMYDINSAKCSEIKSAELDVLSSQELKRISLGKYENQLQEFEKISKDG
TSNRITYDPRFGTIDYSQICSVCFEKHDNCLGHVGHIEFMLPVFNPLFYKDLQELLNLVC
YNCYALCYSEDVIFLLKHIYQLKSLNEIESSNKFVIKKNNENEYIVSEIERLYNKICKES
NITMDDIVESICQDYEKSGNNEKTNNENNSNEDNQTYEKKINNDMNSGDEQNEPDPSKES
EKKESQETKEKIRFLKEKIKKYNFDISKLAFQSNYNYEEYLILNKAIKIHMKKGANCSYC
KFRRSITVKTSPKKDTINFVGAYTNNSFFKRYLSKKDKNKISGENG---DDNLEDNEINY
DHVDVEKPNKQGKESKSGKRKSLDNLENGDDQLVEGTDNNKDGNDESFKDAVASYKRKTS
KEKCTVRLFSFQIVDLLKKIFVNNNKDIINLLYPFTKKDGCQVFFLYDMGISANRFRTQF
RGIHKRIKVTNYICKLNNFVKLCINSKKNIDFDYMIEYNKTELDLYNEMFSLFSLKQRSR
TVSNSMDFNVEIAKSDFLRCYSLIYNNKSAYLNILFGNLQLAVNTFFDSSLIDKRVQKNL
DNICIKEILDKKEGVVRKNIMGKRVNNCARTVISPDTFIETNQIGVPIEFAKTLTIDEHI
TENNFEYIKKLIENGPDMYPGALSYKDSQGRVFKLSRSYEGRMKLINELANLLRKEKKET
LILHRHARDGDIVIMNRQPTLHKFSIMAHFLKIFEKEKVFRLNYVNCSSYNADFDGDEMN
LHLLQTPLARAEATHLMNSDFLFTSFKDGSPLRGLAQDFILGGLHLTSLETFLNYDEYCN
LLQCSLNVLLRQKNSFFFRKPKRSN-TSGSPNRKDGNKNSGSNDKGTSNSFSGINSKISN
YSYLDPYASNLSRTHFTIKTEEPTILFPKKLWTGKQLISSILKTIINELAIETYNKNGDN
NFMENFKGINYIAKAKTDPELWSFDPIKECDIIIRNSELLQGVLDKLHFGASSNSFVHLC
YELFGPKSAAVMLDCFGRLFISFLQLRGTSLSLYDFILKKKAKDEKKKIKKRISFTGFYL
QNLFVYTIGKALNADMNKMNEYSNKKISNYEECKNLFSNKLYDIYLKKSKIINNYEKKLG
KINIENEDVNTKNISSKFLFNDEEFNMKKDLYFITLLNKEIENIFIQNKLLSDNVKNDQP
EQIETYEYSNFEKYVNETGYSEFVNNIIHKLYNSIKNPSFYKSTTFTSVQIVKVIEKIIT
FLREAKCNKRLKVFILFELFQNENITKYFPSLIEFATNLNTDISSEEIVETVINNILLKS
RDVEKSSFNGKRWETTNDIILKRYLYLYNRLVKVE----KD---KKGQNNSPLKPKNDQD
QNINEVHIYDGDAIKNCLISSLPSFALNEDINNLSYNNRYQYYEKYLNIKDEKYSRFKFY
KMEDVFHKLDFLINNFFSLKRLDFDNMLDSLFQPYLCRVSSGTNNLITMENLMNKFLDNG
FSNMIFTGAKGSKVNYSMICGMLDQQYLEGKRVPRMRSGKTLPSFHRYDYGARSCGLITD
CFLEGLRPQEYFFHCMSGREGLIDTAVKTAKSGYIQRCLIKSMESIILHYDGTVRNEDNA
IIQFLYGEDGIDPSKTAYLNSPSDLINNYNISFSKYLNYGSSQLIQKNQNLFMSNGSCSK
NEDPLICQYNPYTYIGSVSDNYDSKLNNILRKSDFNLTNNDE---NFYSLSSSLLRSKYF

HSLCSPGESIGILVAQSFGEPATQMTLNTFHLAGTENVTMGIPRLKEIFLTSKLTSKPMI
YVPIKLEENNMNNPNEIKNYITNIADKILSSYQSILLSEVIYGIGIDRKLIIRDKIDNKE
IHMIQLDKVEPKDNEKKSKTKDYSKILQVLENTNLNFDLKKEWAHEIVIQFDNLNHFCKV
NKHLSVSFILYKIVNIVLISILNKIQSSISHHNIRNINFMNHEHYDELFDFISGKMSEDF
NFNFDKYEYKNDEDEINAKLKHMPKDAGGNKSGDENDYGR-SHN--LNNSKKYNEENDDN
MSMGDKNEESGNEKNNKSDGNDDSDQNDDGRESDEYKSEQEDKEYDESEENEDDSRSSQN
EQDSDDDVKHDSMNGSESDDNKYDETEKN--LKKKKSEKKSNETDSESDASSNLTITNPG
IIDGESKESNKSKKEENMGISG-------------KKRKLYIKNEKHRKEINYDSSMSSE
EAELKKEMSKRFSKYSYDYKNRMNKIDLDSDYEDLSIGIAGNKNKLYDQKYIKHIIEKMK
SSLLCFIKRIEFSPITWVLKFELSWDVNFFPYPIDFLSYLQNDISKEILFKVKDLNNPKI
LKGKDITHSGEEYELQIEGRNVYKLFNIKDKYIDKTKLYCNDIYTIVKTYGIEAGRLCIT
KELKKVFEAYGIKIDFRHLSFISDFMTHTGDLKSFNRYGMSAYRNIFHKMSFECATSFMI
QGCIQNSVDYLSTASSSLFFGKHIKVGTNVSNIITCINGTMAKNRLLIESREAKKQNDPD
IFLSHSDYNLYEWEAVIRGPKDSPYEGGKWKLSIKCKNTYPIDPPLITFITKVFHPNVNF
TTGALCMDILKTNWSPAWTIQSLCRAILFLLNEPNAESPLNCDAGNLIRSGDIKGFKSMA
KMYTKEYAS---MDNVEEIHSDMFDLLAFAFDRGVKVKSAAEVGKRAVEYFRGDDFAAFL
SSNQDVLKKKFPKLIGNRNLSEIKDIEEFGDLFIQRGFIYKAQYKPFKGIHEMDENGVYK
RPKWPKRLIMTAKQNFDKAGFYILVYERNRKLQYFMLMTLISIVLICCMFPVWPLKLKLA
LWHLSVVFITFLSAIIVGRLVAFLYFWFFGIDYWIFPNLFDEECSIVESFIPFHSWERRN
DSWILVFARMVTAVLVAIGIHQLGKTHSISDIQNFAKQSFIDIIEWGNKKLSDGPENNFM
YKSIDSKATFEGQEDPEDQIIYDENEENYDCLKKCGFPPFEELVRRCFLKCDCMEKIIKS
HCYKKKCSKATKEVLDEAHKEACFQKIKKMATLADTLLKDLEDLEEEEEDDFNNNNDGIK
KINFEEQYAEDDDYEEIVDAIEEFLNDKKKKCERKISELLYDDDFLKTMDHIKNYIIEEE
AEKLKNEEGTKETDEDENENNIKRKKAHSHEDENNDNSQNDEEVLIEKCIELIIKIDTEI
LNIHKYLKDIYSTKFPELDSIVYTPLEYISVVSRIKNESDIKNIDFSDILPNTTVMAIVV
ASSMTTGIKLPDHLLKSCMSFCDEALELNENRQKILIYLENKMFLLAPNLTMLLGSALTA
RLISCVGSLKNLSVTSSQNLIVVGNSKKSTLGLSNVNKTFGVGILSTSEIVQSVPDAYKK
KAISLLAGKCSLASRIDYFKKYKEGQYGLLLREYVINHLIKLQEPPPMKQKKILPIPDEK
KGRKRGGKRYRKLKEKTEITELRKQINRLPFGPDTNEDFYTFTDQNTALLNSNITKLKYQ
TKQKTNIPKKKLASAQSSGATGGLSSSLIFTPLHGIELFNPSINKTTSDVRENKYFSSLS
QFRKVMEEFKEDSSEECKQKYPNFYLADLFYTLQLSHLSIDERNEALNKLLEEIKKNNMY
PYYSYVCGELNLDMDQELYNTLKQKADEEIKEIENKIQEASENFDYVDTKNDILLKANFY
CKIGDKENSFKEYEAVYEKGIGIGVKLDILLTIIRISIFFNDINSTKKYLEKARAQMEKG
GDWERKNKLKIYEALNYIMIRKFPEASKILIDAASTFTATEIISYESIIFYVIILGIMTE
ERTVLDKNILNSSVILQVTSSDEDLYNYIYSFYHCDYRTFMEKTIKIALRVKKDRYLGRH
YRYFIRNTRVRAYKQFLEPFKSVTLKNMAYAFGVSEDFIEKEISSFIANGKLNCKIDKVN
DSIESNQPNERNTVYQDTIKKGDILLNRIQKLSRVIDMMVNINWPGLLKWSTKYSDGTID
TNKRLSKEDIEFLQGAIKEALSQVEDPYEAIGEAVRNFENKDEGIILASAKIIERLVDEY
PEVAKNLDKINALDPLLKLLESNNNHILESVLQIFSLALSNNPVLQDCVFKKNGLKILLL
KLQESKQTSVDKKLITAISALIRHHDEGENKFIDYGGIAFLVYGMQTNIYKYQEKSALLL
KHLIHQNKITFEIFEKNKVMNGLIALTNNKNIDETGIQYGETTAELFLALMQNHRHKLAK
GGYLKQLKELIEGRLNYLTVIQDNASYDVSQEIDLFKDCLKLTK-MRTRKIQK--RRLPL
GFRKRYRRYYIIKYLNKKFLENKKR-------NEKEDEIDSDETSDSSSDTTGPVSFEDL
GVDDWLIKISKSVQILKPTKIQKLCLPLIIEGKNVIGSSETGTGKTICYCWSMLQELNKN
FYAIFGLILLPTRELVFQIVEQFQLYGNKIGIKILSCIGGFSLIDQRRSILSKPHIVVGT
PGRVSDIIDDCIDVKNCFKRLKFLVLDEADLLLQKSFEDKLKIILNNIPKDFGEPRRTLF
FSSTITDSINLLTKAFPNDKLVLVNANKKQKPVKNLDQRYIYIDTIAQMTYLVYILKNKL
SDLSGIIFTANSYKCELIYTVLNELGSFSIESIHSSKDQRKRMSSLLKFKNGLCKILVAT
DIISRGIDIPKVAFVINFDFPNDTIQYIHRVGRTARANRKGISISFVDKRDLKSFNNVKL
IMKEKLKPYILNKNEVLSDMLKIGKVVKKAEMMLQEKKDIKKENDDLKNSIYLNEMARKY
FVSANWKCNGTKDSIKTLADSFNTVDFDPSKLDVVVFPVSVHYDLARNLLNKKIGTGIQN
VSKFGNGSYTGEVSAEIAKNLDIEYVLIGHFERRKYFHETDEDVREKLQQAIKHNLKAVV
CFGESLEQRESNKTIEVITKQVKAFVDLIENFDNVILAYEPIWAIGTGKTATPEQAQEVH
KEIRKIVKEICGEAQANKIRILYGGSVSVENCTSLIKQEDIDGFLVGTSSLKTSFTEIIK
SAMMKKKNKEDLYKENKLEASKLRIAIVSTDKCKPKKCHLECKKNCPIVKTGKFCIEVDH
SSKIAFISEMLCIGCGICVKKCPFSSITIINLPKDINKDVVHRYGPNTFKLHRLPVPKLG
QILGLVGTNGIGKSTALKILSSKLKPNLGKFDSPPEWRDILAFFRGSELQIFFTKLLEEQ
LSPIIKPQNVDLIPKQVKGNILEIINKKDKLNQKDKYIKVLELDHLLDRNVEDLSGGELQ
RFALLISIIGQTTNVYMFDEPSSYLDIKQRISMAKIIHGLVRHDNYIIVVEHDLSILDYL
SDYVCCLWGKAGAYGVVTSPFSVREGINVFLDGFIPTDNLRIREESLNFKLATDQDVTDE
DKKRLHFYTYPKMVKTLSSFTLTVDKGNFSESEIFVLLGQNGSGKSTFIRLFAGLIKPDN
VESLSFLESLSVSYKPQQIQAKFTGTVRQLLMSKLKGLYTDPYFNNEIIKPLKIDGILDN
QVLTLSGGELQKVAIIITLAKNTNIYLIDEPSAYLDSEQRIIVSKIIKRFILNTNKTAFV
VEHDFIMATYLADHVIVFDGQAGVNTVANTPQTLVAGMNKFLKIIDVTFRRDPTNYRPRI
NKYDSVKDKEQKLNGTYFIIDEMSILCTISGQTPEEPVVSKTGYIFEKRLIEKHIKNYGI
CPVSGEVLTLDDLYPIKIEKFVKPRPITATSIPGLLSIFQTEWDSMISEMFTLRTHVNDV
RNQLSHCLYQYDAATRVIAKLLKEKNNYQEEINNLRNQILQLKNGNDIDDLEMGISEDLL
NEMQNIAKDLLMNRKKRKVENVNSPNDWKKITSTNEFNIHSSVIPGVTCLAIDINKLKYN
YDDDHKNHNFFSGGKDGNIYYVSLNNNKIISKLQGHLKKVNSIISHPSNSICISGSNDKT
IRVWKGDPDTNEFVTSHVITKHKDNVHSLSLHPLENYFISSSKDSIWILHDMETGKTIKT
CKSSPSPFKNLSIHPDGMMFGIGSEDSNIYIYDIKSQEYKASLTGHTKSVECISFSENGY
YLASISKDNTLKLWDLRKATSFQTIELEDTPKHITFDYSGKYLSLSVGNDIQIFNFETKN
QASLITTLSSHTDVVTQTCFGSRTSYLLSSSMDKTVKLWSMSEEMHCYNGNIKDGLFHGY
GVLVYSKNEKYEGDFAYGRREGKGKFTYADGATYEGEWMDDKIHGKGVAHFVSGNIYEGE
WENGKISGFGILNYSNGDKYEGEWSEGKMHGRGTYIYADGDIYVGEWKNDKRHGKGCVKY
KGSKDKIAETYDGDWYEGKMQGKGVYSFADGGIYEGDWVDGKMEGKGIYKYLNGNKYDGD
WSNDMKNGYGVLTYANGEMYEGYWKDDKVHGKGTLTYSKGDKYIGDWEFAKKCGEGELIY
SSGDKFKGKWKNDKANGFGVLNYSNGNKYKGEWVNDQRHGFGVFTCKEDGSVYSGQFSHN
RKEGHGTLTFSNGTIVEGIWNLGVLTKVTKFQLYPSSPWNDPDLMKLEEVKDIQKIERIG
AHSHIRGLGLNDCLDARYCSEGMIGQMSARKAAGIVLRMIKEGRISGRAILLAGQPGTGK
TAIAMGIAKALGEDTPFTHISGSEVYSLEMSKTEALTQAFRRSIGVRVKEESEVIEGEVV
EIEIERFQEKDATTNNKKVGKMILKTTEMETLYDLGNKMIEALQKENITAGDVICIDKST
GKITKIGKSFARSKDYDAMDPNTHFVQCPEGELQKRKEVVHTVTLHDIDAINSRTQGFLA
LFSGDTGEIKNEIREHIDMKINEWQEDEKAEIVPGVLFIDEVHMLDIECFSYLNRALESE
QSPIVIMATNRGITHIRGTDYKAPHGIPLDLLDRTLIIPTYPYKHEDIMKILEQRAEEED
VEIDEFAKELLCKIASESSLRYSLHLITLANLVAKKRKATEVTVQDVRRVYNLFIDVKRS
TQYLIEYQNEFMFSELPKDDESIKDGESVDEKRENQEKK--------MAYLYSNLSDFWT
SDDEEDGERTDEDFDEDSENNYKDTNKNYINNENNE------YEFEKRQDEEGLQTSNTN
ANAVTGGESALNLVDLVNDCRNELFDNISISTILQNYDINNFIHNDNLLKYCEAGVTINN
ESSCNEPIELNEGFNFNCYSHFDYINPPNSKPSPTTTTNALISAENGENNFIDMCIQNKD
EL--NHEIKEISVKEVSNETEKEDIQEEEKNIS----NET-MNKLEIVPEKQSVKYIRKK
WVIEDNESDVSNFSKNDLLLSYDFELDNFQKRAVKHINNFKHVFIAAHTSAGKTLIAEHA
IALSIKLNKKAIYTSPIKALSNQKYYEFKNIFKNVGIITGDVKMNVNANCLIMTTEILRN
LLYLNDNIINNIHCVIFDEVHYVNDEFRGVIWEESIIMLPPHVQIVLLSATVPNYLQFAD
WVGFTKQKEVIAISTKKRPIPLLHYIYAHDSLFLIMDEKNKFYSSAFKEIYIKIREKEEA
GKKGKELSGSSHGGKKKIYYSDAKNNKDNQMEKQNKTGTTNNTGDKQNDTVKGYYQYCKQ
KQKQRMFQNEANMKTEIQKLQALIKKLDEDNKLPVVLFCFSRIKCETYAKSMPHLNFLDN
KKKSKVHLFIKESASKLCDQDRDLNQIKILSKLLENGIGVHHSGLLPILKEIVEILFSKG
LIKVLFATETFAMGINMPAKSVIFTSIYKHDHLKKRILTSSEYTQMSGRAGRRSSDTHGY
VYIYCSDNIPDQVQLTEMMMQKAVSLKSKFKVTYNMILKLLINKQINIEKMLFSSFLESC
RALQIPLFKKDLKRKKKMLQNIKQVECVYASDLNQTAPIENYVYIDHKLKNIGLNLHKKL
FDMKNSNAFVVGRIMLLNTIDLFRSSVYAVYLGCDKSSNKKNNKVNFAQNSIFFFNDKEE
NEITERFFFLFILPDFIGYGDLTNNTDSVNANENGTNNSTAVSNTNTNPEKTQMASENIN
MYENYKNVFACTNNKKDIKITYHSTFDTDMNKKHFVVCSNVGIENISLITNTIIKIPNVS
TNALLNNPKNLLLYTLELDRLIKKETFEPFTLTKMLKSLKCEFYSVLINQSDYLESLKKS
VCYNCNLKDEHYELVCKKNDCINDIENIERNINAKSLNLYEDLEGKLNVLKHFSFIDDDN

NLTIKGKIASYITLTDEITLTQVIFENVLNNLNPPEIAAVLSCFVSPEKKVEEAPDLTLN
LQDVKLALTNIHSKFEEFYRVIRLKISTEEHWKLCNFKLMFIAYKWALGVSFSELLEQSE
FEEGLIVRSIQRLDNLCRKVRIAFLYLGNVDLAEKTEKASLLLRRDIVFTTSLYLQLLFT
AIHANFFTKGNKANYVSAYINTRHNNNDNKHNTEDSNKINVTNTSQDQLNNYFSRRFYTI
QNKNQYLREGKRSSTDDNLTYNMESSNEQNNQMMGGDSYKERLQKLKKYMGDHNIDVYIL
INSDAHNSEIINDQDKKIYYLTNYSGADGILILTKDQQIIYVNALYELQATKELDTKFFD
LKIGRITNKDEIFQTIADLEFNTIAFDGKNTSVSFYEKLKNKLKFQYPDKQIQEKFIYKD
SINKIVKDKNINLYVLESPLVTVPNDNVNKKPVFIYDREFGGACAAQKIQEASDFFDENP
DVDSMLLSELDEIAYLLNLRGYDYVYSPLFYSYVYLKYNREKGRIDEIILFAKTENIKEN
VLAHLDRIHVKLMDYDSVVSFLTKNVSTKTANITRYNENNLLLSSLQGNSNPRYDISLSP
HINLMVYMLFNKEKVLLKKSPIVDMKAVKNYVEMDSIKEAHVLDGLALLQFFHWCDEKRK
TKELFKETEISLRNKIDYFRSTKKNFIFPSFSTISAIGPNSAVIHYESTEETNAKITPSI
YLLDSGGQYLYGTTDVTRTTHFGEPNADEKKLYTLVLKGHLSLRKVIFASYTNSMALDFL
ARQPLYNHFLDYNHGTGHGVGICLNVHEGGCSISPAAGTPLKESMVLSNEPGYYWADHFG
IRIENMQFVVTKKQTDDTTFLTFNDLTLYPYEKKLLDYSLLTPQEIADINEYHLTIRNTL
LPRIKENPSEYDKGVEQYLMEITEPISH-MLCICISNILFSNLFVKSIKKNKNIYSNGLR
RNKKTYIFSVD---NQKMSTLTFDDSNLETLKKNFVIKESPDFIKYRLDKFNELKEKKKK
ELEENDPNYYKEINIELLDGSIKIGQKNVTTPYQIASQISKKLSENSIVAKVIYLDDVNL
NLCDIEDEESEEKHE-ETSQEGILWDLNVPLIGNCKINFLGIDTPEGKKVFWHSSAHILG
SSLEKIYGGYLTIGPALNEGFYYDIYLGNNSIVSDDYTKIENEYNKLVKENVEFEKMVCT
KEEVLELFKYNPFKIELIKSKINDNEKTSVYKCGNFIDLCLGPHIKNTGKSKAFQVLKNS
SAYWLGDKNNDSLQRVYGISFQKKTELTDYIKFIEEAKKRDHRNVGKNLNLFFFEKETSP
GSGFWFTHGAKIYNKLIEFMRKEYRIRKYEEVITPNIFSCDLWKTSGHYQNYKNCMFIFN
IENKEWGMKPMNCPGHCLIFKQLNASYKSLPIRLADFGVLHRNEITGSLSGLTRVRRFQQ
DDAHIFCSLDHIKTEVINVLQFIFYVYNLFGFKYDLYLSTRPPKYIGDINTWNFAEQSLK
EALELANVKWKLNEGDGAFYGPKIDIVLRDSLNRTHQCGTVQLDFQLPIRFNLQYKNKEY
GVGQETDSNLVNQSNKDANEKKEDSTMGNAGNNDDGENAGNQPGGELKKGFDRPIIIHRA
ILGSVERFVAILVEHTSGKFPFWLSPRQAIVLPISDKFNEYAKYIHDVLTNNLFDVDVDI
SVNTLNKKIREAQLKQYNFILVVGEKELSTNTVTVRDRDNPNDQKVYTIQELVNNFKKML
DINSVKLNEIPPFIQKNIVEGIGRNKTGNGYNSKHLSLTYGKDFFLAMLAVLGMYEILNS
KEE----KGKKKKNFFFDFLLPKNKASCCGIMAYLGDGDASKILIDGIEILQNRGYDSCG
MSTIDKSNSLKTTKYASSSATNAIEKLRGNYMTSHKNDNIGIAHTRWATHGSKTDENAHP
HVDYKERISLVHNGMIENYRELKKFLVQKNIPFKSNTDTEVVANLIGYFLDQKQSFQDAV
VSSIKQLEGTWSFCIIHKDFPDEMILAANGSPLHIGIKDNEMFVASEHSALFAFTNEYIS
LKNGEIMSINKNNINNLKLIKKFDNIPEIVIQKTPDPYPHWTIKEIHEQSISLSKSLNNG
GRFNLQNNTVKLGGLDPYVDELKNIENIILIGCGTSYYAALFCKYIMNYLHCFNTVQVMD
PSDFNISSIPKEKEGIIFISQSGETRDIIKACKLAEYFNLKKLSVINSVGSTIANMTGRG
VYLNAGREVGVASTKCFTSEVSVLTLIALWFFQNKQNLNSNNKVSSLINSLYRLPLYADT
TIKSCEDTCKALSHKLSNAKSMFIIGNGLSYPIALEGALKIKEIAYIHCEGSTGNALKHG
PYALLGGNDNIPVIMLIFNDGTKNSMISIGEQIKSRGAHIICLTDDANLCNHFADDIILI
PNNGMLTSLLAVIPLQMLAYYMSVNLGHDPDKPRSLAKTVTV-MNDSYDSLFKILLIGDS
GVGKSCLLLRFADDTYTDSYISTIGVDFKIKTIEIDDKIIKLQIWDTAGQERFRTITSSY
YRGAQGIIIVYDVTDRDSFNNVKNWIIEIEKYASEDVQKILIGNKIDLKNDRNVSYEEGK
ELAESCNIQFLETSAKISHNVEQAFKTMAYEIKNKSQLENQQKGRTNINLNAKPIKDNKK
KCCMPHIRTMNSKKPPEGWNKVEAFLNEMNQKMRSLENEDTSKKRKNEILWPIFQINHQT
ARYIYELYYKRKEISRELYDYLVREKYVDGALISKWRKQGYENLCCLKCIQVSDSNFNNA
CICRVPKSNIGNKVIQCVNCGCRGCASGDRMNPVLVSYTFMPKKYLKISKRYITIDSIYF
QRIYTNTFLNNNINIIHSNCEIFFCNSRSLFSRRFTKNKTIKTMSNDNKRDPIND-----
---TSQTNNSTINKQSMDSSNYDYDYIVIGGGPGGMASAKEAASHGAKVLLFDFVKPSTQ
GTKWGIGGTCVNVGCVPKKLMHYAGNMGTLFKNDSEKYGWDCDNLKHDWNKLVSTVQSHI
RSLNFSYMVGLKSSKVKYINGLAKLKDKNTVSYYLKGDTSKEECVTGKYILVATGCRPNI
PDDVIGAKELSITSDDIFSLKRCPGKTLVVGASYVALECAGFLNSLGYDVTISVRSIILR
GFDQQCANKIKLYMEEQGVTFMTGVLPKKLTKENDKILVHFNNDTTEVFDTVLYAIGRKG
DIDGLNLEKLNININNNNKKIIADQFSCTNIPNIFAVGDIAENVPELAPVAIKAGEILAR
RLFKNSNEIMKYDFIPTSIYTPIEYGSCGYSEEKAYEIFGKNNIEVFLQEFNNLEISAVH
RIKHIKAQKDEYDVDISSTCFSKLVCLKNEDNRVVGFHYVGPNAGEVTQGMALALKLNAK
KSDFDNCIGIHPTDAESFMNLTITLSSGLSYAAKGGCGGGKCGMENEKHENMLMHIARDF
RSIDDLVEVFLSFLENKTDYFHLMMNDDDIQTLSKKYDGNIAKAILNNNTCGFKANSREI
ELMKSFRKHQLKYIIKNQPYIIENEDIRNKYLPPCDDLNKLSNIQIAKAKNTQNEAQKNN
QSSHTPSYDSINEKHISTWNGGRTEKYFWNQSLNEVNLEIPLNKEIKPSEIKVEITNKHI
KVQHLNEIKLEGMLYEEVNKQECVWNIEDKKKIIIFLEKKKENWWSYVIKGDPEIDTTKI
ESKKNLTDFDEKTQEEIRKMLYKQKMMNEGIKSPEELKEQALLQNVLGNKGVPVPKMKSA
NAKEDTVSQTCDNSTIEDADEHLYAGPLKIEQLLAKGFVKRDLELLKEGGLQTVECVAYA
PMRTLCSIKGISEQKAEKLKKACKELCNSGFCNAIDYHDARQNLIKFTTGSKQLDALLKG
GIETGGITELFGEFRTGKSQLCHTLAITCQLPIEQSGGEGKCLWIDTEGTFRPERIVAIA
KRYGLHPTDCLNNIAYAKAYNCDHQTELLIDASAMMADTRFALLIVDSATALYRSEYTGR
GELANRQSHLCRFLRGLQRIADIYGVAVIITNQVVAKVDAMSMFGGHEKLPIGGNIIAHA
SQTRLYLRKGRGESRICKIYDSPVLPEGEAVFAITEGGIADYEEKMALKSINISGNFDWC
PFEEYKNYLICFNSHNLLYSNNGNLNNYTYLLDINLNSDIRTLDIVHKLNFEEALKRGND
KSKKSSNEYVTSFEWINCNNFVESENESGLSKGIIVGGLTNGNLVLLNAKNLFDTNVNYD
NFILSQASLHESGINCLECNKHKNNLIATGGNDGQLFITDIENIFAPTSYDPYLDKNNLQ
KITCLNWNKKVSHILATSSNNGNTVIWDLKIKKSAVSFRDPHSRTKTSSLCWLENQPTQI
LISYDDDKNPCLQLWDLRNSNYPIKEIIGHSKGINNICFSSVDSNLLLSSGKDVTKCWYL
NSNNFDIYNEVNNSANNIYSKWSPFIPDMFASSTNVDTIQINSINNGSKMTSKYIPNFYK
KDASICFGFGGKICLFDNISDTTHLSTSENAANKSMNPNQSSIDETNMQANKNKEGNSNS
PYMIKCHIYPTEVDLIEEADKFEKYIECGKYQEFCENKIAKCDDYHEKLTWKILQLLCTS
QKEEIVKHIGYDMNEINQKIVENIGEESGFIFKKYRNETNDNINGTGNITSNNLEMNSDI
NNKHDNTRMSNLSNGENIPGYPNYGNNMQGEFSTSQMQENEPNFNESFDLDPEKFFRELG
EKNEIEKNQENEDDKTGKDGLIGKINNSDFNTKDMINTIRGQTSENSLGEINEDSKPNSN
KTNSNNWNSGIESIIKECLLVGNIEAAVELCLYQNRMADALLLSSFGGENLWHKTKNIYI
KKQNDSFLRNINYVLDDKLEHLVKTIDLSSWDEALSILCTYAINNPNFNNLCEILAKRLQ
NEKFDVRSASICYLCASNFPETVEIWDSMPSSKSTLLNALQDIVEKITVLKMVIKYNKFN
STMNQKINQYAELLANSGRLKAAMTFLSFIDDDKTMESLTLRDRIFNSATHVMPPHIKPP
PSPFQYFDVKPFG---VPDKY---NNMMNSNAHGIYNKSQGY--------------PNKS
LTSVVPPLSMSEMRKPHGPPMPPFSHTPPTKFSTQVIGGPPGAMGSTDSASNRGYGTSNF
GSNVKYPPPATGSLYIPPPSMPS-HSTTPINPSSSPYASVPNLSHLSSHQNLKLEHDKIG
-GPTMFESQSYSNVNKAMQNSVAPPPPPPISNQIPGAPRSSFSTPQSNIPPPRNRHASVA
TPPNMPFTPESQLNKRESIDSQVYNPVTPPPISP------MSHQNTFTPPQPYSQNKMGF
APPTGVKTTSPVAGAMSITPGMPVPWPIPTTTQQLGSTTQSTASENKKIQTAAKEQNGVL
MNRNNIEHIKKVISNLLNAYIAQEPVKKKADDISIKVNELFDKIYNGAFNEQINNILISL
ANSINDNDFKMANKNLMDISRNLWDGSNKAWIMGLKCIIPKMAIRVQFENSNEVGVFSRL
TNSYGLIALGGSENFSSVFEAELSQHIPIVYATIGGTRVIGRVCVGNRKGLLVSSICTDQ
ELLHLRNSLPDDVKIKRVEERLSALGNCITCNDYVGLIHTDIDRETEEIVQDVLDIEVFR
TSIAGNLLVGTYSYFTNNGGLLHAMTTSQEIEELSELLQIPLITGTINRGSDLIGSGLVA
NDWSAFCGMDTTAIELNIIEKIFKLNNIEDTNIEDTFKYKSSIVQTMIMLESLVEKLLNK
FLAPYVEGIEKNLHLGVWSGNIVLENLNLKPQITEILDLSFKIVHGSIGQINIQIPWSSL
GKNPVCVFIKNAHIYVKPRHYKKSESVIIEELRRAKMHRLEMLEEEISLIKQQKNKEKPA
EKSTLIFKLLNKIINNIQIDIQDVFIHFEDIERNFFIGFILKSSSVKNLKKKDERSSEGN
NDSESKKLNHIIEFKGLCIYSNSSVNNKKRGSKKSKKKGKKENVMKKKNSKDITHETDMV
NNNLTNGCSK--KEICSDTESKTNDNNSKEENASSTSPMSIDDKKSSKQYSNDNIDSKFK
FSSENFDNDEENDYLRKTLANINLFYKSDEHKLLDHNNYLIKPFDLVLPVEQSSNKKELK
ARLEISDKWEGITLTRTQITKIIEIMSEANNSRNEINKLLLKHASTVKLDVESLRNETKN
EFINLYNKILGEEYNISKIELTKKEQNRLQILYDVVGVRHLAKWRLQCRDTLEKIIEEKN
CKKKILYDSIYKQQSWWSWVTGNKKDIENKVQSILKTEQDFINEHELNILQEAVVNEDNY
DVVIPTKYDFEFKLSNFSINIYDDRKKRKYIINKGINSKNDDKFTEQIKEASNKNTYNET
TNKSSDHNNTSNNLSSELCLSGTSESSLSTSSNISSPNSIYRNTKLDYNEINILSINFYQ

IYSSLSLQSVVDHNDNDNFQWKFIIELQNFVAKHKNQIFMEFRNTKNAFSYSMDNSENIY
QNPIYHSLLYSQSLCAYLEINHLVTEKGNTLSTLLRLNPLELYLSPLLINNILSFTFPLM
DIINNKDITSTRRHKDKPESTLDKGEVGEAEKTTEINDTENEEDNEEALLHIKKMEQSEL
IEGLKEKGENVYNRAVQHLPELFEFYIHICGPILYFDNLTNGIVNLHLGNLIAKTEYPCA
YNKFNLIFEFNETQITCLKTSNFEDEKFYILQPIPVKVYVEYDLKILKTNIILDGIFFQI
NPDAVSIILAVPTSITRYLTRFYSKNKKEDESLKNKRKGNAEKKIDDKEDENVDTNPQNE
NDKIKNEDLSTNLMDDNIKTGLEVSNIDNNRIEEESFLYDIDFLIKTSSFSIKNSKNCDI
LKYEACGISYKNYFQRKKKIIKVEIEQLWICDPSNKQPIFFTLAKNINSKDAYPFKYVSE
YLSNSSKMLNESNFGTNEKYEHDDNNNTGCLFQSAQRLESGKVEDNDDDDFMDAIEEKQL
SINLQIMHKHNENNIAQIHVDFIVSDIELHWKYKTIKQIFKTMKEYKNILQYGIEKDMKY
IKNKLKNEKELKNYKMFISEHTLRSVQATLKNVKDSLNILDIKAS--DNQNNINENETAE
KNETINQGSLEKDQQFEGFSDENNKILRDGDVFNEYNKEKHNVENENSDVILKNGLNGTL
PKLFGEENSYVKYFFNCYVKSASLSFWEKKKIFSKIQVSNIFYENNIYLNFDQKMFINIE
KGIISLNGKNIISNNINDYTYDLFISKKSKDNRDYSDDEDVANLSDEIKRKETMTNKKKQ
FLFVGNVNVYNDKRYYNTCMMCDISGIYYIFYLRDLRLFLEYLDDGILNVFISKSYKKVV
QVAQEKYFLFQFTIVDPIVIIPEDKNIIYNYKGQVKDTSKYDKDTKNSEHNNTNNENNDK
QGSNNKCIPYIESYLKFHLSTLKFKNAYTKNCAEEIVLRQRNEIKQKRMRKEKRRTKHKL
KKKKVIKYMSDQKVTNKGDNENFDSNKKYDFTLYVDLLDIESRACENGGSKNIEGNILHK
IDIGLCLLSRKDGIFIFFNGKDLCLDLTVYQLRFLLDIINENFCYKGYFPMCFICDKNID
IDNMLDAELFRKGNIALNNLSGNNSNFNTKTDGIRKNGTKFYAYINLESLKIKTSFDNNI
PVAIITFQNISISFDLTLLDFYYIYFFNLYSTSLYIDDVRNGSVNYYKRVAYCCIENEMN
KKRGNRNAKFCENESDFVSPTSVASRDHNSSNSVTGQENRITSNDANQNFDERYEINYKS
LLNYMFDNNVFLNERKKNIKQKGIKIKINSFIEDLLLSIELDDAYICCFFVIFIDIYKFL
STGFNLSTLHLYPKPSPYIVSNKYYNNKNNSINNNINDGMAKQKPENEEKKRSKNEKEDT
NAPKILNKENYIKELNINELLKMDNKPIHIDFKVNNGNFILFTNLENVDHPIIMWSNNFV
FSFSLSNGCILFRKIYAIDSKIKRLNYVNNSRSMISNYSRVNNNNKMDSINVSNPLHNYH
KKKILLCENLNVIGEAIYESIDTKKDNYIIKKNIRKKDHPQFISVFEIDLNISNFDIRLS
NDDVEILLKASSTLFGDVPSAYTDITIGAVIPPRIRNNSIVSGNEKNEGEYDSSADSNSA
DSNLWYFLDTNAAKANAYKDVKINIKLNNVKCTFINDVKNAIVPIIRMTFSMDIKLRFYT
DECSYIMNCLNSNIEYFNNCIGEWEPFLEKCNISLDVHKVLPSDIYSEDIESIRTPISII
KINSAKALWFNITPQLVNLLFMFVPVFWEKVLNGLKSKRNESIMENTEI-EASKKNDDKS
TYDRDSERLLYNQDDKNEDKKSKSIYEDSSSIFDVIPDGADENKSMDFHLDSFLKKEENG
EAIDPIEDNSIIYYVNLTSDYYYAFTMPERNSDDMKDKRGRTTLRSGNEMNTQESLDTNK
GKHRDSYDKMSVDFANGEKGRVLPDVYTKIITTNELIPLDELLVNEIGNNTLIEKKDLYL
YLIPIPPTNIVDTLHEMFVNISKRDIVLDLMITKNPKKTIENLLYYNNLRNNSANIENRS
YKIKGAYDNYYFNKANKFAGPFLKNSECVMINLIKNSCTSLNTALKNNNVIHVINPSDYI
FNEIGPITNNSNNISSKKMEAILNREDIKLEQLNEEMNKQTNLVYKYRHKNNTICEIISP
MPNYKILFMSSTVRIINKCGIPLEFSFFDTSKSPILLTSLKNRTIDSSILYPNHSDSFKN
YKVSNSLNINPNIKIIQKNTNLSYTVILNHEYLLSPPECAFNGQSQLYLSFKPVNLVSEK
YNIGDTNENAKRNNCTASNDCSYINIDNGWSDIFSSDISQGTYVKKCKVVDNNSYLYFLV
KIEHKISALPAEKNIRIITIYPHVSIVNTIPALVDIVMMSENNERVYVHEYNRLEKEIGN
IDHEIKVLKKSIFYIYEIKKYTCLNLKMKIGNSQCEWSDRIHLDEDDDETMTRLTLNFKK
FASIEVEIIKNFNGYFNSLNSILGNKHLIFSLPRCFIDRTGLGIKAINSNKYYPVINGIT
LLGDNSQIDLLLPHKNNNIDGV-NGKKKSQRDNFDDIFNDFNYDNSLILFKANLPPIGSY
TEANVVCNNFYYTFCLNTEKIKTTNIPYIISRIITVVPQFIISNKLHYPLLIKQFQLDQI
QGVRENDTSPLYFIKKHNVLLFQFKTFEMGKHNISKNSIESNTHSSNRTGSCDNNKNEMN
KSRRYSKNMFWSSVIFPSENFVGSNYMVLNNGKDE-SDVYVITCIPNMGTKNIIIEKLEK
KLNKGYIAYNNSSLVKYLKIRTFHDDAHNLKMGKNDKQLDKNLLLNHFLKASDMEHYFNV
EYNKYSYLGWVNPFIYISRNIQIEIVLHDMNVIPRSPLVLKFASFNYSQKTYHINYFNIT
FIISIEYIEDLISIKLTHRINSPNGGDKVLAVSGSNYIVDGKDKDENDEKKYSTNSSEMK
SYNKIESIDKYEIMNDTIGTRGMVNAARNSQMYIEKKVIEQNEYNYVMNISERGRSRYSG
E-HNNSIYSNRKESIKVINNTYKNVHVILNLTQIGASIISNILKEEVFFIELSKLCALFY
MKNEEEVIDIKITDVQVDCQLESCKKCVLLANRGLNLDSKNINNNDEKTFLNVYVERLFI
SHHDVIFKKIQVLLDDIEIEMDSETLNGINLLIAEYIEGINIIQKKNLLYEEIQKWTVLP
VYVNYKSPDIPLAINIQYMQINKFTLIVWCSFLLDKMHMLSDLLRIGLRILMVSGKLELL
GAPVTLNQEIFNDIRVSIKSFYALLKDKYSHSILACLGFIVGYSSLINIPKIPLEIGRNT
IGLAVYAVDNVSVGIGSLLSNLTFDTEYINRRQKERNFKTNTNMKEGLLSAVKNIGEGVL
SLSNIVTKPIEGAQKEGVGGFFKGIGKGVAGSLVKPLDKVGQAVSDVTRGIKAEVSKPIG
GYKYKTKRHRKPRMLWGEYGKIKEYNTQEAELRECLGLRFSENIMKCLTIHKQQNPPSHY
ALLLYPKVIIYANLYANISDKKGDIVIWSVKIDEISEIRASSHGLIIRTTNGTYKIPCNN
ASLINKIYKELHNSKNSIKSTIILGDSISSPPLMNNAEHNGVASKMDSSKIDNIDIDENI
FLNSKSFPVNEAYEVDQNKFGGNNQIKDIKEYKRMYEESIKNPELFWGDMAKNNLRWSQL
FNKTYIGNFNKGNVSWFVNGKINACDNCVDRWVEKHPNKTAIIWEKDTPNDSKKISYQKL
LEKVCKIANLLKMYGVKKQDCVTIYLPMIPELIYSMLACARIGAIHNVVFAGYSTRSLSE
RIISSGSTILITSDFGLRGGKLTKLKNIADGAMEMTGMIKTCIVFKNKTKINDQNNSILN
SKSNNLYNSSNICSQGSSHFLQYTVGNATHKNNNDPSQNGHDSVLHEKDIQTLNKLSKNT
NLNKKTQNKESGYKELYSNKLNENGAQMNGHISYDNNMINHNQVNAFEQLHNGQPNCEQN
CKSKYDFDENICTLKEGRDINGSLLMKNMRPYCPIEYVDSEDFLCILYTSGSTGKPKGVT
HTTAGYLLYAFATSKYIFDIKDDDIFGCVADIGWVTGHTYVVYGPLLNGITTTLFSSIPT
YPDCSRYWSLIQTHKITQFYTAPTALRTLMKHGDYYIKNYDLSSCRILGSVGEPINPETW
RWYYNVVGKKKCVIVDTYWQTETGGIVIAPIPHLFKMKPGSASLPFFGIQLEILNSKTLE
PLNGPNVCGILCIKSSWPGMLRTVYGNHNRLIKTYFEPCPNYYFTGDGAYRDEDGYYWIS
GRIDDTLNVSGHRLGAAEIEHALVQHSCISESAVVSFSHKVKGEGILCFVVKKIASLSKY
PECNNDNNNDELNNRSILSSNGDIVQNHTHENYTDEKLIEELKQYVRKVIGPIATPDIIC
IVPDLPKTRSGKIIRRILRAIAIGVNDYGDISTVSNYDVIEIIKNKFVESKEKRYIS-MR
RDWKSILSYSIFFTGNIYFLWKIQNTYSFKSIVACIIKCLNYSKKKKFPNNAIAQNVKIY
FGSQTGTGEQFAKELCYNLQEIFDIKAEIIDLEYFDKEEIKTLGIRIFIVSTYGNGDPPD
NATEFFKWLKGLDIDNPYFRNTKYSIMGLGSKQYSHFNKIAKKLTTYLKSFKAEQISETI
YGDDDDNIYHDFEIWKNKFFKELSKILNMEHIPINFIKEEVIKLVDWKSLPDIKLDIKFE
DLEEDNKKSETNQHTSNLLNDNTTQEGQFNKPIATSLTGKFYFNHNIGTVISNTNLLKNV
DDSTNSDKVNHIIISSKNIKYKSADTLVVLTKNSKEITDWWLKRLNINETDKNKKFIFVE
RNNQNSKKHDNNKNNGN--NNHSMNSPFPTPCTIEEALQCYCDLSTIPRVNVLNNFKCFI
KDIEELKMFNYILSNNKRNIFFNICKEFDMTFIEFVDIFMQSAIFELVPFLQLIPKIAPK
SYTISSSPKDDPDTITLTVKKKQYPIHSLRKALKSFKNNNMLPNITEKKLRNLCERRWYK
GSSSYYLTEELYPNDILKFNVKTSIFTLPENLRDASIIMIATGTGIAPFKAFLTEFKIFD
QKREKNEILKKNKRILFYGCRKKGIDFLYEKEIMDALENKYIDEVYLAFSRDQSNKIYVQ
DLIREQKELVCSLIEKGAYVYICGNTEMGKDVKQTINNLCENNKKNDKKFIKKLKKSGRF
FEETWMSNLPWVEKYRPKKLDDIVHQTNAISMLKEVIKTKNMPHLIFHGPPGTGKTSAIN
ALAHELFGKENISERVLELNASDDRGITVVREKIKAYTRISISKNKINNETNEPLPPWKL
VVLDEADMMTEDAQSALRRIIEIYSNVTRFILICNYIHKISDPIYSRCSCYRFQGIPLNI
KKEKLLYICNNENINISDDALTKIIETTQGDLRRAVSVLQLCSCIDSKITVESVLDISGL
PDNDIILKIVDSCKVKDFKILEKTIQDIIEDGFDVSYIFKALNEYFVMCQDINDSIKYQI
LMELSRHDFRLHNGATKYIQLMSFASSVHSLLNQAMSEALDVDNAKEKINFMFAYWKNNS
NKDFENSNAFCILSGKSSKDDNATIQEQFQMWLLGYQLTETFFLFCKKEKLIILTSDKKK
KFLQPLLDKTDNITILERNNDNSENFEKIKNEINMHDSKELLILKDKDSTGSFFEACYNF
IKSLNKNEIDVNNNIKNLLNLRSKSDVKLQKSASDIASIIMKSVLITTIENSLDSEEYES
HNKIKEKVLKFNENKKCVVKIKDKLKADIDDIDVIYSSVQSGNKFVLNFKNTNDNNYLSQ
NDGTIVIGLGVKYKELCANINRTLLLNAKEYHKELYNFTFSLQKYIINDCLKYNTSFSDV
YKKAIQYIKDNKQNYQTIGNINLENYFIKCLGHVIGFEFMEKEFLITANNSNATIEKNTS
YNISVGFENVQLPDKNVFSTWISDTVFVNDKEEVTILTDAIGKEINTISYELEESESENE
EEDSVKKEKNKNGDSENKKKIGISASILNNASSVIVSDRLRRRNKNSLAHNNEQEIEELN
KRQNELKNKKIEEIKNRFSEGTNEYKDLNKKNIKKLEDIKSYNDADLIPRDLRSNIIHVD
NKHESILLPVNGAHIPFHVSTIKNLSSNYEDNNDIFVLRINFQVPGNQGSQKGEFNAFPK
LNEKEMYIKELIFKSNDEKHLQFVVKQVKELIKQVKQKEVEADVNDSKTSNEKLALNKSG
RRIVLRDLMTRPNIFTGRKILGTLELHTNGLRYSANSRGTTEFIDILFDDIKHAFYQPCD
GQLIILIHFHLKRYIMVGKKKTLDVQFYCEVGTQIDDLDRAKARNVYDPDEMHDEMKERE

QKNKLNLIFKNFVQQMQDISKIEFEIPYPELTFSGVPNKSNVEIFVTANTINHLIEWPPF
ILSVEDIEIASLERVHHGLRNFDMIFVFKDYTKPVKRIDVIPIEYIDTIKKWLTTIDIVY
YEGKNNLQWGNILKTILADIESFVNSKGFDGFLGEDDDEEEQSAEDEDEDDEYEVDESEL
SAEDDSEYDDSEEESLATESDGDEEVEEDSDDEGLSWDELEERAKKDDKKRFADSEGYNK
RKKKRN-LKSHKLNTHKREIGNRCEANETQISINGVNKRRHTNSVITSKYTIFNFIFLNA
YEQFHKISNVYFFIIGVLQLVPELTATNRIPTILFPLSIVLIANAINDAYEDWNRHKTDK
IENNRICYVISDGNLSQPNTSSNAFIKFLRYIKKLFPQKTKKIDSIESYYDDEEDIDEIA
DTNYFMRNYDDKLENIDGTIQKRWKDIEVGDIIICRRSDFFCADILLLSSSDPNGICFAE
TSSLDGETNLKVKEVNKYIFNNLTYNIDEAIEKVKKLRGYILSEKPNKNLSTMNGAIYLE
SDENNDLTNNKELRKSISGGTSHGEGMK-----NNNSIENNSNDNLDSYKNDDKYVKLPF
DEKNFVLRGCKLKNIDWVIGMAIYIGKETKIQMNSLKPVLKYSKLEILTNKLTIIIWLIQ
VFMCIISAYYSAIIVSFSKKSKFKYLPFNLVEPKAPIVSGIISFFSWIVITANFIPICLI
VTMSCVKVIQAYFISCDNNMIYKVEENIPSFGESRQKSMKKKNVSIEIDEIHNLSEDKSV
FVTNKKSDTSISDSTKIIPTSEDENNDILKKRSTRMRTFKDSKEKNYINFNAIPRTSSLI
EELGQIEYIFSDKTGTLTCNVMEFRKCAINGISYGTGLTEIKRKILIKNNIPIPPEPVLD
IKNKTPNVNIIDKKLVDHLKDVNHFNHASLIYFFLHLAVNHCVMCDT-SDDVNAYSSSSP
DEEALVYAAKHFGITFLYRKDGKCGVKIFDKVYEIDILATIEFTSKRKMSTIVCRIPVMS
NEDTKTSPTNNKTMPRPPNGNGERNGNGMGDSPANLGNESNAPLDNASNSDVNKIGKKEN
KNNKKMGTNNDVISCENTKKHKIVVFCKGAGCVIIKKLANKTDVDDLTIEHMETYADEGL
RTLCIAYKELSPKEFAIWYNLYKEASLSLNGREENIEKIAGGIENDLILQGVTGIEDKLQ
EGVGSTIEDLRLAGIHVWMLTGDKIETAINIGIATNLIDNGSEQFIYTEELALSEDLLMK
KLDEDIIYIEKSLNLLHFNFDT--NKVEESFFQKVISYKSTDSLALDHDTYKEDMTLNNN
VLIVDGSVLDILLSKPFERKFFYLADKCSSVICGRVSPYQKGFIVSSANRLLRKNTLAIG
DGANDCNMIKMANIGIGIRGQEGVQAFNSSDYGISQFRFLRNLILVHGRLSYRRISKLVV
YMFYKNIVFIFPLFIYGAISLYSGQKIYFEILLHSYNVLFTSLPIIILAILDKDVSINTA
LKNPCLYKLGIHNFYFNINKFISWVLNSLFHGLLVFSIPLYFLAYYNIPSSTGEPFDLWS
IGCVTYLLSVVIVNIKILLETYYLNTSPIIGVSFSIISFIVTAIAFSFTGIGNKSFLGVA
TLLATSLRFWLVFVLGLFAALTRDYVYKVYKKNFCPEAYHLLQDEEDKIENPKNIQHNSR
SSNNIEEMKPSKSELMGYAFSEADPVCVNFIRKQDKLIMNRRLLNQCFQ-KSRSSSYINV
L-INNTNNCYQLCCRNYSDNVKDHFNKPRNVGSFDKNEKNVGTSIVGKASCGDVIKLQLK
IEDDVIKDARFMAFGCGSAIASSSYATELIKGKTIDEALKIKNNDIASHLNLPPVKIHCS
LLAEDAIKHAIKNYREKVIN-MISNVKHRKVMGVFRAMSSFCDSNKLKNVNYINKKREKV
VILGSGWGGIHFLLNIDFQKYDVTLVSPRNYFTFTPLLPCLCSGTLNVDACSERIDILLK
KNNISGKYLKLECTDIVYKDKYIKCKENSNNEIKIKYDYLIISVGAKTNSFNIKGVDKYA
FYIKDVIDALKIRKKFISNLEACNGNTTNDEFVKNMLHVVVVGGGPTGVEVAAELADFVN
NDIKNKYKQIYKYISITIVEGGNNLLPTFTQNISNFTKDNFKKLNINVYTNYHVTEIDEN
HFYIKSSINKNEEPKKIPYGMIIWASGLAQTPLINNFIKKIPEQVNNRILNVNQHLKVIG
IPTNDVYAIGDCKKIEPIKSHEHMDKIINCLGDSKVTSDTLKNKSEELSNIFPQLSNKKW
DYNKNKKTEMNQKELEDYLIMVDKNYKSPPPTAQNAKQEAFYLSNIFNNHLYYKNNNIIP
PFIEKWKGSLAYIGNHQVVAHLPFYEIKGGPLSFTFWKIIYMQMLLTWRSRLIFIFSFLR
TKIYGRPFY-MYTNSKNVQVKNKNAADVQITAEQLIKEALDFEEVEKKVNYNLIDEDELN
EYKISKRKEFEDSIRKRRYLINTYIKYALWEIKQKDIKRCRSIFERALNIDYTNKNLWLK
YIEVELTNKNINSARNLLERVVLLLPLENIFWKKYAHLEEILNNFVNARNIYERWVKWKI
DETAFLCYINFEERCKEINKCREIFERLIVSIPKLECFYRFIKFEKKYKNISRARACYEK
CIELLPSQFLDQHFYIHFSKFEEENNEYERCRKIYIEALKRLPRENSDILYKNFLQFQKK
YSEKEELDQTLLYNERINFEEALKKTPNDYDIWFNYIKLEEQNINLINKEKSIIRIRELY
ERAISIIPQICTKKYWKRYIYLWINYSVFEELYADNIDRARKVYSNIFKILSKQNFTFKK
IYILYANFEIRQMDIDKVRAIFNHAIENVKNEKIFQEYCDMELRLGNVKECRTIYSKYVE
AFPFNSKAWIAMINFELSLDEIERARQIAEIAIHIDDMKLPELIWKTYIDLEINLQEYEN
ASKLYERLLNITQHYKVYKSYAEFQYVYLDNISKCREILENGIEFCKKNELTNERSILLN
FLYEIEKDHGDNEIIEKTLKRLPKKVKKKKIIKSNDDEVVEEFITYVFPDDKAQSQNMKI
LQKAMEWKKKMEKHEQKESPQEIMADAESVENNVVVEEKAVFDNITAIQKVIKNALVHDG
LKIGIREVIKSIESKEAKVCFLSNVCSEPAYKKLVTALCAEKQIPLFMIDNDSKDLGQWS
GLFKVDKEGNARKIIGASSVAVIDFGEESAERDFLMSQKPTAAAAMSKDKRGRIFFNSHG
SDNERDNSKRSKTDHKSKYSEISPHNGSNKKKNYEKE------KNDNTKTCKENINSFSF
PNSTSSISDLNNLDFDLSNGSSSNSENEFKILKEKENEEKFLEERRRKREAIKERLKNMM
DENNDNNKQ-------HDSSNNHDKENEVSIDSNAKIDDKCENTFTPCKKNDMSESLSGM
PSMLDDMEQNEAACIFAPNNEVIEETCSSLSSDHEIIDDKAPNEKNETIKESNDLYSDLK
RKINEEKIKIRNFIIKQKELHERNKDAAYVNKKNETESIDFQEQEDDDNDDVDMFSSEQT
TKKRAIENIRITDYYPANTANLSDNWNDSEGYYKAIVGEVIDNRYSVVCELVGKGVFSNV
LKCYDMTNKMHVAIKVIRDNDMMHKAAEKEISILKKLNDYDKDNKKHIIRLLRSVKYKNH
LCLIFEWMWGNLRIALKKYGNGHGLNAAAVHCYTKQLFIALRHMRKCRIMHADLKPDNIL
INEKFNALKVCDLGSASDISENEITSYLVSRFYRAPEIILGFRYDSQIDVWSAAATVFEL
ATGKILFPGKSNNHMIKLMMEYKGKFSHKMIKGGQFYSQHFNDNLDFIYVDRDHYTKKEV
VRIISDLRPTKNITCDLLEHQYWLKGNSPKMQFLKKKIKQLGDLLEKCLMLDPSKRYTPD
QALQHPYLRESIHYSKMQNEMSSDASSISCSLSLDSDSCDDEFYETNLNKLIENTSLNWI
FVGGKGGVGKTTTSCSIAIQLAKKRESVLLLSTDPAHNTSDAFNQKFTNKPTLINSFDNL
YCMEIDTTFSEDTAFKINKSDFLNSIIPELLQSFPGIDEALCFAELMQSIRNMKYSVIVF
DTAPTGHTLRLLAFPDLLKKALGYLINLKEKLKGTLNMLQSLTSNEMEFEGMYDKINHLN
TMSISIQENFQNPLKTTFVCVCIPEFLSVYETERLIQELTKKNISCYNIVVNQVVFPLTS
QDANIESCEGLLKQIKDTNIKDSFSSLILKAKELEDVYISRRKLQSKYLTQIKNLYGNYF
HIVCMPQLKSEIRGLDKIASFSEMLLQSKDIPIYSMEDGIVLSGDYIKLSNDKIKKVNKS
NFERIYDKEYEDLYRSKCSGIMLKTPYYPYKYDIMNTSHKYIPKIGDLVIGIVKSKKLDY
YQMDINSNCECIIHKIESFKYASKSSFPNLLNGTLLYMIIEKINLDNNMVVASCINSADV
KSWINYENYLGELVDGFLFSVNISYAKSLIGDKCYILDLIGKDIAYEIAIGHNGWVWIKT
NDPQETNMIRSALKHCYGKTNIQMAVLWKSIYNLYKRGNMYRSHMLENDLFTINNDGKRC
INSLVYTPNFNSNIINSDAHHPNGLVDNKTEIKKPNFVETLFNDIVKIYLSKIPKGFERF
EKSDSNKNCNGNINNAQQEEEKPKKFDNNFFYLFILLLLCFLLFVDSNSLYNEITQNDFF
YNYLSKGYVDKIKLINKDYVKAYLNSHGINKYHLKYVSFRVGNSDSFERKVELIQKEMNI
KLDEIIEVQYVNEGNLVGEIKSYIPSILFFLFLVFIFQKITLKNVANSGMDKLFKFNKIN
PINKNNYKTDIKFSSVAGMKQAKEEIMEFVDFLKNPAKYQVLGAKIPKGALLCGAPGTGK
TLLAKAVAGEANVPFFNISGSDFIEVFVGIGPSRVRELFAQARKHAPSIIFIDEIDAVGR
KRSKGGFAGGGNDERENTLNQMLVEMDGFHTSNDQVVVLAGTNRVDILDPAITRPGRFDR
IVNINKPDINERSEIFQVHLKNLKLHDSLDIKNISYILASLTPGFVGADIANVVNEGAIQ
CARRSHIQGVQMHDFELAIERVLGGLAKSTSLISPLEKKTISYHETGHALIGWFLEFADP
VLKVSIIPRSNGALGYSQHLSEEIMLFSKEAIHDKIAVILGGRAAEELFIGKITTGAIDD
LNKVTQLAYSYVSQYGMNKEIGLVSFQQNGGSEYSFYRPHSECLAHLIDNEARNLIESQY
NRVKAILKKNEKHVHNLANLLYEKETISYHDIVKCVGERPYPIKSNYEKFVKANPYKMNL
SASIEEKTGE---KAEEADSEHAQKNESHKDATNNEHDKNSNNLKKNDSGDVNGVSHATP
NNNDRNKKDEQISNFNIRMNSKNATREEKKKEKELNEARKAGKIEALKDEEGNDINPHMP
QYILKAPWYLNQTKPGLKHQRYREADKVKIEEERNRKVFVKNTSNKQDFCKNCGSAAHTE
KYCLERTRKKKKNFMNKENDEDYLCVTQDLGYDGNRDRWVGYDPNNFDHIYREYEKIVEE
QKKRKAEKLKKKYEKQAIKNKNQ--DENEGEEGGGEDSELSSDSEEGNDLNESKQNNKKN
NKDEKNKTVARNLRIREDTAKYLYNLNLNSAFYDPKSRSMREDPLANIKNNLENSNYYKG
ENYYNNTGDAIESKKLEIFAWESYKRGENVHFNAQPTQLELMYKEFLEKKNKLIKKKQED
ILKTYKCENITKEIHNEQELIHSEVYTEYKPADKIDKKNNKIKVLSKYEEDIYISDHTSV
FGSYYDRKTNKWGYKCCQRTDKFQNCFQSMGTKRNESSKRDAKKKNIKYETDNAVNEIKY
VREIDPMLNEDESDNAFLNNNNININNLKNEIVENELDNNNVDDISLGLIKDKSIRKREN
EFQRKKYDYILSPGRADPFEEKSPSPGERTYTDIMLDISKENKKNKILSNNKKEASTSD-
NQGEKRHLRFGNETGDGTKSKWDYINEDQDIDFGNMATPAPNKWGETPFILNDANIKNKK
KKLSRWDKTGDGGGNINADNGTINSDMMKTPIVSGNRYNENMMINTPIVGTNMMTPMTPY
LGSIQNNDYIKFKIKNEMDFRNRPLTDEDLDNLLPSEGYEIVKPPEEYEAIRKNKLKILF
KNMKDTTTTPLIQGSTNQSTLDDTYIDETGKSSFISHTPFYNLPTSDGTLKEEDEQILRQ
NKIMEITNPQLLNELKYIEIKNEDYIYFNKLFQNYDEEDLSQDEIKERKIMLLLLKIKNG
SPSIRRNALRTITDKVKELGPENLFNLILPLMMQNTLEDQERHLLVKVIDRILFKLDDLV
RPFVHKILVVIEPLLIDEDYYARVEGREIISNLAKAAGLATMIGIMRPDIDHPDEYVRNT

TARAFAVVASALGIPSLILFLKAVCQSKKSWEARHTGIKIVQQMAILTGCAVLPHLKQLV
NIIAHGLHDEHQKVRTITALALAALAEAAAPYGIEAFDPVLRPLWKGITEYRGKCLASFL
KAIGLIIPLMDAYHANYYTKEVMIILINEFNSPDEEMKKIVLKCVKQCIQTEGIEKDYIN
QEIVNPFFEKFWVIRNSSDKKNFNLIVDTTVEIANKIGGAVVIAKIVDDLKDPSEPYRKM
VMQTIQNIINNLGVDDIDQKLEEQLIDGILYSFQEQTSDDYYVLLNSFDVIVNKLKLRMK
PYLPQIAGIIRWRLNTPLPKVRQQSAELIARIAKLIKICDEQQMLGHLALYLYEYLGEEY
PEVLGNILKALKSIVVVLGVNNMTPPIKDLLPRITPILKNRHEKVQENVIDLIGIIADKG
GDMVSPKEWDRICFDLIELLKSNKKLIRRATIQTFGYIARTIGPFEVLTVLLNNLRVQER
QLRVCTTVAIAIVADTCLPYSVLAALMNEYKTQDLNVQNGVLKALSFMFEYIGEIAKDYV
YSVISLLEHALTDRDLVHRQIATWACKHLALGCFGLNREDALIHLLNHVWPNIFETSPHL
IQAVIDSIDGFRVALGPAIIFQYLVQGIFHPSKKVREIYWKIYNNVYIGHQDSLVPIYPP
FETMGDSNFARDELRYVLMGKQRLTALDIRAIITSCKKTIIGSVVTNIYNISNKIYVLKC
SKKEQKYFLLLEAEKRMHITEWMREKDVMPSGFTMKLRKHLRSRKITNISQLGGDRVVDI
QFGYDDNVYHLIVELYIAGNIVLTNNEYKIIFILKSNDDNKKKLKINEIYNVEEHNAVML
YKYLDIINNENIKKSINEILMISLLNSEKNDNVKLKKKGSQNIKNSSNSRKGRGGTTFGD
SNNILNDKNKNDDTQNDNKKNKKTKKIKTLNELGSKLILFAHNDLIIHSLIEWNYNPHDP
VEKYDIDTLTEIFFKVINECMKIFNMMSNEESVIYGYGFAPID-EKLSILNKNKNNVGKN
KLENDRLFVEFSPILLKNHINKIDEKKIELIKFNDFNMCVDTYFSKMELTKYDKHQEMNK
RKNALTKIDKIKLDHERRIEALEKEVNILKKKILLIQANDEFVGEAIKLMRAAISTSANW
EKIWDHVKLFKKRNHPVALKIMSVNFNNCEIELLL---NEGDTEESSSE-----DSSEKG
MEEKNKAC------TVTINLNNSVYGNIEDYEKLRKKAEEKIRKIKMSTNVAIKKVEKKK
KDKDTKQKGKHKSVFQIQKLRKIFWFEKFNWFLSSENYLVISGRDSLQNEILFRRYFQNN
DIYVHADIHGAASCIIKNPYKDIPIPEKTLAEAGQLAMCRSSAWNNKVITSAWWVYYHQV
SKTAPTGEYIKTGSFVIRGKKNYLPYAKLEMGLSIIFQVNKNVDDNNKEDALNDDKEN--
--DETVHTGSDNDCLDKENGGNADKMTATSNTADCVNHMNDSS-NCKETRIVNK-----N
NCVGIDLRGTCKQNKKYAKNKYIRLILKCLNRIDLLNVYKKNMISKLVISDKYRKEIISM
LCKLCFSNLCINMKNFLYKIKNHDNIQCYICQTFKKNYKIFRHLFLYFDSSNTCIGGIFL
KSFDRAIDSISFYFVHKMVLLKSCDKKIREWGKDEFNTFFDINKNNNFIKNLNVDTLVCA
MKNQIDEEIKLDMMNEKDKTMKLCFTPCEVYGLPVAFKDDDINDDESKNSKKGGSSNKVA
FNSGDNDKEDG-----QKKHISFA--NVENSIKIERPTRTRKPTGFVKMDIKKLLEEIDN
EEEEEEG---DTREDMKVTFDEQKKVPVEKKGVAFNLENNSV-DNKSSTLNIPRPTRTRK
PTGFVKADLSKLLEEIDNEEEGEEKSEADGSSQKGTLNSNS-STTNVRKKSVSFSSEDDR
IYIERPITSGIPRPVRTRKPTGFIKMDISKLLEEIDNEEENGENEKDEDE---KSDDTKK
VKFSEGV------EKNTNDE-NIK-----------NFSRPVRTRKPTGFVKMDVKKLLEE
IDNEEEEDDENS--GAYVEN--
--SGNSEEEENSENSEEEEEEEEESENSEEEDESENSEENSEDEEESENSEDEGSGQNSG
YGKTDYKGNMERVNESDSNKKSPNETNK------EGRFNDKLVKNEN--VHLLRGARTKK
KRMKKKYREDDDEERQLYMKIIGSRKMKHETEKQKKEKEENKVEKEENKYKRPNELKANY
EEINEDEMNMKLSELNKLTFTPKEGDVITCAIPMCAPYSAIQNHKYKVKLVPGNAKKGTI
AKSCISHFLKCSTSEIEKKFINGISMDDLGNCIITNSTTDLLSSK---MGRMYGKGKGIS
CSTIPYKRKQPSWLKQKPSEIEDAIIKLAKKGQTPSQIGATLRDNYGVPQVKAVTGNKIL
RILRAHGVATTIPEDLYFLIKKAVSMRKHLEKNKKDKDCKFRLILTESKIHRISRYYKRK
KLLPSNWKYQSSTASALIAMSNRKKVAYFHDPDIGSYYYGAGHPMKPQRIRMTHSLIVSY
NLYKYMEVYRPHKSDVNELTLFHDYEYIDFLSSISMENYRDFTYQLKRFNVGEATDCPVF
DGLFQFQQSCAGASIDGAAKLNHHCADICVNWSGGLHHAKMSEASGFCYINDIVLGILEL
LKYHARVMYIDIDVHHGDGVEEAFYVTHRVMTVSFHKFGDYFPGTGDITDVGVNHGKYYS
VNVPLNDGITDEAFVDLFKVVIDKCVQSYKPGAIILQCGADSLTGDRLGRFNLTIKGHAR
CVEHVRSYNLPLLVLGGGGYTIRNVSRCWAYETGVVLNKHHEMSDQISLNDYYDYYAPDF
QLHLQPSSIPNYNSPEHLSKIKMKIAENLRNIEHAPGVQFSYVPPDFFDSDIDDKSDKNQ
YELKDDSGGGRAAGTRGKEHSSSHHLRRKNYEDDFFDMSDRDQGII-MQDDNESIDGDIL
EKQYEIIKCAKNQDFIKFQILIQPYILNNDIEMLNTINIMHWACYSGFTELVQKLIALNC
DIEKEDLVNSDTPIYYAIKNSNYEIVLLLIKHYGSSILFHKNRRQMSPFLTAISEFNEDK
ILEALHILEFLYLNGVSLEEQNEYGQTALFLGVKKNNISILQWLLSKNVNINHVDFYGNT
ILHIAVRYTDIDILRLLCDYGCLNLVYYSTFENNNTNVFQLCINNRYFLVYILLKKWLLQ
NKICKGLKICKTIYAFYFWFFALLNLIVYINIAHSFLQIQTHHNKSVIWISLWLFQQLLW
CVLYFKNPGFYKENKFLTNRNKNNSNYMYTSDFKNQAEYQLNNIERELFQINKKLLLANL
NPMYNV--NENEILEYNDQIINLRYSKLSLYSQVSQERINSLDVNYRNAILYNQNPRNVC
VTCNIIKPPRVHHCADCFHCVVHQDHHCVWVDNCIGINNQRSFYFFILSAFILLLFNYYY
VFLYFKLFHTTINYAFGLLVILCNFINITLFAFITYLFVRNTKTILTNVTFYEHFKRPTH
ITDKYNTELECWEFQNLNFKKIVKNVYNFWTLNYDEPYLRYDKKIDSVMDVCEFVDPIRK
RKITNNLIDFDKYVYKEKNDKCEEKQSNEYVVKKKKKKLRRVLDSSNSDSKENIKKEYN
NECEENKK----------NDDELLCKSYED-SMTNDNEIEERKEYERNLKKLYNCLLGSI
KVKNKIIKYFTQNIKEKRQDIIKEFVKGSFRINNYDADFEDFEKNVNTFHKLKKYQKCGV
YWLYILYKEKKNGILADEMGLGKTAQTCVFLDYMYKTNKLQNKTIIVAPTSLLKNWDNEI
DMWCPYLKNHKIIYYGNQNERKYLAYDIFTNKKKNTIHLIITSINMLTGKNDAAYFRQIR
KYDYLIFDEAHFLKNKNSLIYKKLQKRIVFSNKILLTGSPIQNKTQELTNLLLFLMPHIF
TEQNINAAMDAFLKLYQEVIEKKDELDDTQL-ESNTQS-RNYNIMDIYEIESEPDENKKT
NKDDDVYCVSDSYGDENENNSDEEKD---------EGYMCENTRTIIKNYLDETIKHHEK
NVDIKNKEIILLQLIIEPYILRRSKKHVFIDMPKKHSIIIKLPLNNTQLDLYKDEILSKL
QHTHKHLEFLQTNSSKEEMDKLKSILEGKTEKESKNLQ--------TNE-SNSTTDTKFD
EMKGSSDNYDEDDDKIDEETINIEKEEENK-AKIIINKREDEEMVKSTSSQNSNSTTKEE
RGKMINASIFILRRICNHPLLHKYYYSVDDIKKISKYFYNNTDQYLDLDLKTVENEFMKI
SDFDIHLSIKHLISQGDNNLNKYLIDKKHILNSTKIHHMLTLIKNIKEKKEKVLIFSQYT
TFLDIIEESLLYEFIYDENDYAEHSSFVDKTQSVSKQNNEVETDHNNEVENGNNFSKRTS
FNKDDDTYMRSPSAMSTSSQLSEKRTQNNIYVRLDGSTNTIERQQIIKEFSENDNVFIFL
LSTKAGGVGLNLIAANHVILMDQDWNPHNDRQAEDRVHRLGQKKEVYIYRLCCKNTIEET
VLKCCKAKLHLDQAFGGNSDLLMMKNPGTVLVFKPNTKREEGRKTQLSNIQASRAVSEIV
KTTLGPMAMLKMMLDPLGGIVITNDGNCILREVDVAHPAAKSLIELSRSQDEEVGDGTTS
VVILSGELLSIAESFLKDKIHPTIIVNCYMTALNLSLKYLEEIAIEVDVNNEENLLKAID
SCLSTKFVNRYNKIVSKLSLEATQCVKVENVIGKKEIDIKRYAKVEKIPGGDIMDSYVLK
GVMINKDITHPKMRRYIKNPRILLLDCTLEYKKAESQTNVEILDEHTWNQLLLQEEIEVK
KLCEYIIDSRCDIVITEKGVSDLAQHFLVKKNISVIRRVRKTDLNRLERITGATIVNRCD
EIVEKDIGTKCGLFEIKKIGDDYYSFFVECENPRACTILLRGATKDVLNEVERNLHDGMN
VAKNIMLEGKLLYGGGCTEMRVSQHLIKQAANFDDSRKSIIESVASAFEIIPKILAQNSG
VNVVKCINELRTKHEKPESEKLGIDGVTGEIIDVSSKNIWDLLSVKKQIYKSAIEAASMI
LRIDDVVSGIGKDDKIQKPIKNEFMGRIKVLNVAEKPSVASAIVSILSKGKSNKKKSYSK
YNPVFTFDYKMENETWSMFVTSVTGHLTDQKFDEKYKNWNNTDPHELFDAKITIYIDKDK
KPIENNLKKYSKDCNVLILWLDCDREGEHICFEVINACSVTNKKLKIHRAQFSAVTEKDI
KYAINNLKSPNKNLAQSVDVRREIDLRMGSIFTRFMTIRYFKLVQNDTKIISYGPCQFPT
LGFVVNRYLQIKNFNNEYYWTIKMGYLYQDKDGNNSNLFLDNIGKKKKKKGKKKKNCSDD
DENNRPDSSNTNYVVDFTWSRLKLFDHLGVVLIYEDLLKNPLCRISNIFEKEVKKYRPFP
LNTLQMTKLVSKYFHISSKECMNIAEKLYNKGYISYPRTETNYFVDSMNLRKFIHELKKN
NIFGSYAAKLAENGS-CKPRKGKLNDKAHPPIHPVKNMNKANNVDFKEWKIYEFICRHFL
AVCSDDAIGFDTKVVANIGAEQFYCKGLKIKNKNYLEIYIYEKWNDKILPPFQINDEFYP
YSLIVEEGITQPPKYLSESDLLSLMDKYGIGTDATMHEHIENIQKRNYVYKNSKNLFIPT
KLGIALILSYKKFKDIGVDLTEPSLRAKMERDMFLVASGEKGKNEIIRNYIDIMKYIYQE
IYNRIDLLDENINYYINNPEIMNMETFNFGKYKGKTFEEVFENHKSYVTWVKNLENPTGS
LIEFKNYVLRRE--KHGGGDTNAEYSEYSNKYG---------------NTNNYGSDNYYG
YGNKYK--DQSQNGSFN-----YKQNGY-DNKISVNKMNSSEHKFYEQNIMNEIANRYNN
KEEEKPELDIIVAFEIFNNDSFKIVQKDNNNKKYSNFKNFVPKELFKILSEFNPTLKKVN
NYSCVTFESDKYEYVLNNLAEKCTILGGIHSIPNFLLKCFQNYSKFSQPQKISEVTANIL
TNTMCSYTKEHYDDLNFLLGEKLAEELKNFQKEGIHFGLKKNGRVLIGDEMGLGKTLQAL
ALMAFYNKDWPFIVVCPSSIRFQWKDQALRWLPHLIEEKDICVIKSGKMDIPRNTKMIII
SYELITKNDKYQNKYKCIVCDESHYLKNSFSKRTKAIVPIIKSAKRCVLLSGTPALNKPS
ELYEQVSSIIPNLFNYNEFCDRYCYKDKNIYTRKIEYVGCKHTEELHLFLTNTIMIRRLK
KDVLKELPDKLRSKIPIEIPQNELSEILLYAKKLESKKNININDLDNISLSRFNNHD-NN

IDEENITISQLFKMTGYAKVKAIKEYITYLIDADIKFLLFCHHKLVMDEIDEFLKEKKLG
FIRVDGLTPIDKREVYIKNFQSDEKIRIAILSITACGVGLNLTAANTVVFGELYWVPGQM
IQAEDRAHRIGTTHDTINIHYLVAQNTIDEVVWKIINRKWNTLTTALNGAEDSLNVKEVS
KFDKFMLDLTNDTNKSYPTSLVNTPKIKRRSSEYKALINNANTKKYPDIRDFFKKDETDT
ERQKSMKKRANYSPPKNYDSNSTSNILTKKYKTEIIMKEKTKKAEHTEHNEETKERDEK-
--INTFVSNLLASINYINNAIVYKDNRFILRMLKYIKSMRLSIKNDSDNLMPILVSLINK
IFKDTYPIYTILYKYINQYNENNKQNITEIATLNDKTYANSQPEIEVFLYILFIVHLIDK
KLYDECIELSTRIVNRVNKLNRRSLDFINAKVYFYYSWVHELRGKISQVRQELLFIYRNA
CLHRDVMTQTVVLNLILRDYIKNNLYDMAVKFVSKTLFPENMSSNVQHARYLYYIGKILA
IQLDYSESHSKITQALRKAPQNVHTAKGFKLEVTKLEIIVELLMGDIPDRSLFTNKIMRN
KLIPYKHVVTAVRNGDINKFANVMNNYKKLFVRDGVYLLIKRIHHNVIKTALRIINLSYS
RISIADIGKKIGVESPMDIVGITAKAIHDGVIGATIDYDNLYVESKPNTDIYITGDPMKA
FHKRIAFCLQLYSDAVKAMQYPDENEKTENAEAKERKMRQQEEFAQAEEGELGDDTDLLM
LCEQCNKNNVCMLRPSNKEKLCKYCFLESFEDEVHTTILKKKMFEDNDKICIAVSGGKDS
SVLTHVLVNIKKKYNYNWNLFLLAIDEGIKGYRDDSLKVVYKLEKLYNLPLSVLKFQDIF
SYTMDDVVSYIGKKNNCTVCGVFRRQAMEKGALLFNATKLVTGHNADDLAETILMNMCRG
DIDKLAKNINDVLQKKN-----NNNSSLASYSENNNPNIFPNCNDVLDGCGCKEKTNIES
NKEEEKKNQCNNDVAKIS---NDNIKYEHKMDGFIPRLKPLMWSYEKEIVLYAYHLKLDF
FSTECTYSPNSFRGNLRSFIKDLEIINPQIILNIIHSAEFFYFNTNIKKKLNTCIKCGAY
TSNMICKACLIVDGLNNYTDNSFLYANKKKKNNKKKISIEYEIEKMEQNIFSATNVIPYG
PLYDKVKKVKNNDQDENYPVVEEYEKLLKIYERPNPYDSNGCLKFNNNDDLILFQIDLDY
TVENIFRNVIYSNEGSTNNSKCDSIYEPYKAILGKEKNFVSVPIIRIYSITNCGYSVLVN
VHNFFPYFYVEKPNGFNNDDMIKLESMLNENLSLNNQFKMYENKILKIETVKTESIMYFK
KSGKTDFLKITVLLPKMVPSLKKYFENGITVNSKHFGGIVYEANLPFILRYIIDKKITGS
SWIKCEKNSYIIRSKNKQSSNCTFEIDIHYENIEPMPLENEYQKIPKLRILSFDIECIKL
DGKGFPEAKADPIIQISSILYLQGDPIENCAKFIFTLLECASIPGSNVIWFNDEKTMLEA
WNEFIIRIDPDFLTGYNIINFDIPYILNRGTALNLKKLKYIGRIKNVPSIVKDANFSSKQ
FGTHETKEININGRIQFDVYDLIKRDYRLKSYTLNYVSFEFLKEQKEDVHYSIMNDLQNE
NSESRKRIATYCIKDGLLPLRLIDKLLFIYNYVEMARVTGTPFVYLLTRGQQIKVTSQLY
RKCKELNYIIPSTYMKVNSNDKFEGATVLEPIKGYYIEPISTLDFASLYPSIMIAHNLCY
STLIKNNDEISDLNKNDITTVPGKNNFKFVKGNVKRGVLPLIVEELIKARKNVKAMMKNE
QNPITKMVLNGRQLALKISANSVYGYTGAAAGGQLPCLEIATSITTFGRSMIEKTKETVE
AYYCKKNGFEHNATVVYGDTDSVMVKFGTNDVGEAMRLGKDAADRISKEFLNPIKLEFEK
VYCPYLLLNKKRYAGLLYTNPNKHDKMDCKGIETVRRDFCILIQQMMETVLNKLLIEKDL
NSAIEYTKSKIKDLLTNNIDMSLLVVTKSLGKTEYETRLPHVELAKKLKQRDSATAPNVG
DRVSYIIIKGTKGQAQYERAEDPLYVLDNNLSIDHNHYLDAIKNPLSRIFEVIMQNSDSL
FCGEHTRHKTILTSSQTALSKFLQKTIRCIGCNSSIKKPPLCNHCKTNKEFSIYMQKIKD
LKVKQNEFFQLWTECQRCQGNLHIDVICMNRDCPIFYRRAKIKKDVANLQEQVTSLKTEW
MRIIVKVLILYMYFTYFKNHGNAKLNKSKYTLIRVYHNPFKLYKKNKKINKNNRGVFFIA
NNINTKEKIKNLKRKNMSILSSNNIDDTLGGVNQPIFELENISQSLNDLYRKRKSEIEPL
IQVAPMINVTNRHFRALVRIISKKVQLWTEMIVDNTLLYNINNLEEHLGFNKNEHPIVCQ
LGGSDPTSLSEAAVLIEQAGYDEININVGCPSTKVANKGAFGAYLMKKPELVKNIVYEIK
KKVQIPVTVKIRTGVDDLDSFSFLKSFIETVSSVGCEHFIIHSRKAWLKGLDPKQNRSVP
PLEYNKVFDLCKLYPNLKFTLNGGIKSIEQGVALLNGYVPINHNKYTDNNYIKVEDYDIN
PLYGVMIGRACMDNITVLAKTDKLVYNHDTLATAYSRRTVLEAYKSYLEQNSSFYSLVNA
FELLKPILGVLKGMPGHRLFRNKIDAYIRTYASTLNCAQILDKAMIDVDNIAPGCLDLML
DDYKAQQEYIKNYMQNSQNIDNKAIFVEEVVRDFDQNEVFEEITTKFVWEQDVERSWNLL
VENNGILQHVNQETYEEKNKQKYKKNQTCSLRKGIFRNVIILFDMSSSMTERDFKPNRIT
VILECVEIFLKNFFFKNPVGHVGVVALKNSSAKLIQQLTSNIDDVLNSLVKEQKEGLQGS
PSLQEGLEIAHNLLMDMPLYGTKEILIMYGSIRTCDKKNILKYLDLLIKNNMYVNCISIA
PEMHILKHICEKTHGIYKICTSKNILINEINQVAETPLWMHGMEPQLIHICFPVKKKINT
QIVCSCHNTLNTDTYICNFCNSYTCKIPSKCKVCGIHLISMHDLSHITNNLQASPLFIEI
KNEQNYYTYCSSCNQQLYDKVSQCTKCKNIFCLECDVFIHEDLNQCPFCLNDDDMGSNSI
AISQQMYFSTHNALRINEENDVISTLFYEINGHRHISLLIFPFYDVQMLKRLLIKKLDLP
-DIKVNDILIFYKGIKLPNYRIISTYLDNTNVDNVKKKKKKKVNKLYWAIKDPNPNASIR
VIDNKSYPPFFENILNDIKLAFKKNIAPKLTMDGTGGTYLLFNSKKKVCSVFKPADEEAF
SPFNPRGYEGKIYQEGFRAGVLSGEGASREIAAYILDNTYNNFSNVPCTIMVEACNPHFN
NKSNLKYIYNENTLKWKCGSLQEFIDSRESVGNYDHKQFSIRDIHKIAILDIRVMNLDRN
DGNILVSPLKSLKDCCNQFLYRNNKRFSTNDEDILKRIITIDQKPSRYSLIPIDHGLIMP
HIMDVAEIDLVWFDWPQTKIPFDNEVLEVIFAFDPDKDADKIRNKLLIREDCIRTMRVCT
RLLQIGARMHLNLHEIAKISTRKNIDEESILEHLVRDSIIQAYQMMDYTSLMSTNRLGHI
LDLAEIKINKKKKNNKNKTMELIDSNKTKDENSNDVKKNNSFQKEEENYETTTYAKTKSL
DIKKIENSFTTVSFKDIKEHKSSEGKYNINMNEQKEGEGNSTYKMLLQHQSDTKDSDNKF
INTKFNNISSKENIGGYYSWSKDIEIRINDHVKEKNISETNSFSTKSEYTFVTATSNSKR
EDNVHKSSNDVEQNSDGGNFGEENGAKVEKKMKKKKKKKKISNNKAKADKKKEPCNISD
EEKNQEIDQDKAEEKQKNQQDDEEEEEEEEEEEEEEEEEENYARYKKTTGTMKRINENTG
TAYRNIEMNKINSVWMIKDKNNKTINVKWENKIFEKLFFETFENYVKKYINDYHPNWRDY
PYNGSKIITTKHSYLNNIKMEIRVANKYALGKKLGSGSFGDIYVAKDIVTMEEFAVKLES
TRSKHPQLLYESKLYKILGGGIGVPKVYWYGIEGDFTIMVLDLLGPSLEDLFTLCNRKFS
LKTVLMTADQMLNRIEYVHSKNFIHRDIKPDNFLIGRGKKVTLIHIIDFGLAKKYRDSRS
HTHIPYKEGKNLTGTARYASINTHLGIEQSRRDDIEALGYVLMYFLRGSLPWQGLKAISK
KDKYDKIMEKKISTSVEVLCRNTSFEFVTYLNYCRSLRFEDRPDYTYLRRLLKDLFIREG
FTYDFLFDWTCVYASEKDKKKMLENKNRFDQIADQEGRVKQNMEALNELRDQERLQLLTI
LKKYNGRKSLFFENSLHIIINLLLTEQDIKNEKIDNVFFMTDEINTISNKANISNNIIFF
LRPYFYEIEQIFKIIENIEKIKTGNKNYLFIFIPYMTHMCEQEIYKHNVLELTIKIIIYP
LYFFPIYNDVFSLEIKNIFKDYYVDNDFSNLIFCSYALMFLQYIFNGVFRNIKSLGHASH
FISEQLMQLRKEIVADCQDNLFQLLSNIQSLQDLTNLKNSKEPSVVPIKYALHKFFLSEN
IKKKKYVTPKNYSSDEDPNFTSNLESESIQRNEFSSSTSTYKREHVENNHFSNTRETTKP
YNNRIKDNEQASDESHKFRRTESALNYSNKGSEKDTKQSDNFKETKTEPDKISSENYNNN
ENVKEIKDTYQQNSKIMNKEEHINSSDNDSTNKNNNDSNKINQDESDINGNKFREDNKHS
SNKLLSNNLDRQNEYNNSDNDQNHKIQNKKKKKIHKKIYGTGIPNFMTEFDNSKFLMKKK
DKEEAEKHEKKEKNYLLEKIGINSFLFLLNACTKVDSCIIIDRRIDMVTPFCTPFTYEGL
IDHIFCIENLQIEIPRYIIFNEGANKTDADNSKNRTHIGHTSNDLNNKKIRVKLNSSIDV
LYNDIKDLNQNEVGLFLHKKASDIQQTYKEKDSLKDIGQINKFMIKFKEKHYEHNSLSRH
VNIASYILKEIKTEHTFNKLKLEDEIIQLNTNTNKTILSNIVKQIQILIYTGENLYEIYR
LISLFSVITNGFNDTYINELKKDIIEQYGINELTRLNKLHISNILRYQPKQKFIWNTLKD
HFNLLSNDENDISYVCNGYAPLSTRLIEYIGVFKNNMQVFPEVFSLINGPTFDIIQNAVG
YEHVQVNNHNTSEQNHYINSKKNKKFVILFYVGGISYAEIASIRKLNQTNENYNYLIFTT
EIISSKRIIDSMGNMENALSNEHTKKDIENQNEIVHDTIEVKSMCINCEQEGVNKILKFE
IPYFKNILIHSFECTLCNYRNNTIQDLNPIKEKGVKIIFSVTKNEHLDRQLIKSEYGVLK
IPEINFEIPKETQKGSINTIEGFIQTALSNLTDYFINLKNMYNEANNIADDNANNKSEDV
EKNANGNINKIEEKEELNKNDEINNNNYSAKDDEPSEVNEKCHQITIENYMSMIEKTIHE
LSRFIVSKELPFTIEIIDPSGLSSLEYYDEDINSKTVVIEHYQRSKQELNELGFYEEDFE
EKKKNEEFKENNLNVNENQSGDQIKKENFDFIKKYVHMNNNSDGSNNMSVKYKTINEGEE
SKLIESFTSNCPCCNYLGDNNFCEINIPGFKKCLILSYVCPNCNYKTSEIKSSGEINPKG
KKITLTVKNKSDLNRFVIKSETASIQIPIIDLTSDYGTLGGSLTTVEGIIMQIIESLEDK
FKFLLGDSSINTHINNDENNTSNNDESVANKIKNIISNLYKLCRTEEMFPFDLIIDDIAS
NSYISCDQVGDDTNLKEEEYERNFEQNDMLGITSMDAN-MNDNIKRLPSSNEGEELKKTD
SGRIYDDGIKRTPSGKPIQTMYVLNRRGEEEDISFDQILKRIQRLSYGLHELVDPARVTQ
GVINGMYSGIKTCELDELAAQTCAYMATTHPDFSILAARITTDNLHKNTSDDIGKVAEAL
YTYKDVRGRPASLISKEVYDFIMEHKDRLNKEIDYTRDFNYDYFGFKTLERSYLLRINNK
IIERPQHLLMRVSIGIHIDDLEKALETYHLMSQKYFTHATPTLFNSGTPRPQMSSCFLLS
MKSDSIEGIFETLKQCALISKTAGGIGVAVQDIRAQNSYIRGTNGISNGLVPMLRVFNDT
ARYVDQGGGKRKGSFAVYVEPWHSDIFEFLDLRKNHGKEELRARDLFYAIWVPDLFMKRV
KENKNWTLMCPNECPGLSETWGEEFEKLYTKYEEENLGKKTILAQDLWFAILQSQIETGV
PYMLYKDACNSKSNQKNLGTIKCSNLCCEIIEYTSPDEVAVCNLASIALCKFVDREKKEF

NFKKLYDITKIITRNLDKIIERNYYPVKEAERSNKRHRPIGIGVQGLADTFMLLRYPYES
EEAKELNRRIFETMYYAALEMSVELAQVSGPYESYQGSPASQGILQFDMWNAKVDNKYWD
WDLLKKKISKHGLRNSLLLAPMPTASTSQILGNNESFEPYTSNIYYRRVLSGEFFVVNPH
LLKDLFDRGLWDEDMKQQLIAHNGSVQYISEIPSDLKELYKTVWEIKQKNIIDMAADRGI
FIDQSQSLNIYIQKPTFAKLSSMHFYGWEKGLKTGAYYLRTQAATDAIKFTVDTQVAKNA
EKLKNADGMGITREVSRETISTESTVTQNACPLRRNNDEPCLMCSG------MNDTVPKL
PFDEIRNEMHKYGVAITPATLKHPTTEDVQGVYSICIKYILNKDINNIRIEEFTGDLKSS
MPSIDGIQILPNEGKNHLQAIGNLRFFRHCEKINKILNMENTLSYIFKPTSGHITKLINA
FMHFMRYREQIYNENDTTIKQIEERKTESSILDNELKSIHSELQVLLSKHEEVRTSILNE
KNIKRDYEEEIIENQNSLNSQQSILISLKSTKDRIVNETNELIFQFSRYRQKKEDLEDQI
VPSPEKLQQYNDELKDLLYEHMSHCETSKKKNEDIKNKINVADLCIKKLVNLLTTLTSHI
NETLKVHIDKKNRLKDLGTNLKSLKEENDNLTKKKIEQENILNETENNFLQEKNKWNEKI
QDEKKNVIIVDENVKQIYENINDITTKTNQEIQEINNIVNHIQDTINTYNKNFAIIADLI
ENTKNSQKVLTNKIQTNIQNCIKTHLMIQFLKNLQLYKKRETPDIKPTTKKSKMKYEDFN
FIRTLGTGSFGRVILATYKNEDLPPVAIKRFEKSKIIKQKQVDHVFSERKILNYISHPFC
VNLYGSFKDESYLYLVLEFVIGGEFFTFLRRNKRFPNDVGCFYAAQIVLIFEYLQSLNIV
YRDLKPENLLLDKDGFIKMTDFGFAKVVNTRTYTLCGTPEYIAPEILLNAGHGKAVDWWT
LGIFIYEILVGYPPFYANEPLLIYQKILEGIIYFPKFLDNNCKHLMKKLLSHDLTKRYGN
LKKGAQSVKEHPWFANIEWNRLLNKRVDVPYKPKYKNILDASNFEQVQEDLSIADKVINE
NDPFYDWMVISQFYILSPRGDTIINRDFRGDVLKGSAEIFFRKVKLHKGDPPPLFYLNGI
NFCFLKNNNLYYVLTSLFNISPSYLIELLYRLLKIFKDFCGQLTEEIIRTNFILIYEIVD
EVIDYGYLQNSNTEYIRYLIHNEISNINTPSTKFSNLTKFTIKHSNTLPSNASQKPIQVD
NKKNEIFIDIVEKINLIMNKKGEIIYSYIDGVIQIKSYLLGNPYIKIALNDDLYIKNIHK
DNTNNIIIDDCNFNHLVNTSNFESDRILSLYQPDGECVLMNYRINNNFKAPFHLYANLLY
NTNHTVELFIRIKLDIPSRYSCTNVLVNCNLCKHISSVHLDANTNSDLFSAQYIANEHKL
LWTIKKFKGETEYTIRSKITLNQNYEYSRRDFGPIHIMFEIPMFNLSKLRIKYLKIIENY
KSSNTHRWVRYITQSSSYVYRFMDLIVITPYDEELDDIEELNNKILALEMIKHNKSNVNK
NADNSCPNIKKLVLPGESVLEKKDKARFLKGSGLYEEEENFCACILGSVNYINKLVYVEP
LRGKYTGSVGDLLVGKIKDINNDKWVVEIGSYCRALLSITQTNISLFSQRIRLYNDVINM
INIYKPNDVIACEVQRILTDGCIVLHTRSSIYGKLSNGILITVPQTLVQNQKKHIFVFPC
NVQIILGMNGFIWISSPIKKSKDTNPNSIDENIEGNKFEEVDDTTRKNISIISNIIKLLA
KYHININYDIITKIYMQYTSNKSNTPSYILKPYVSDSYLFSYLENFTK--MPILYHLTLQ
KPTAITRTVYGNFSGPKAHEIIVAKGQVLELLRADKQGKLSVIVSKDIFGIIRSLEIFRL
TGSNKDYIAIGSDSGRLVILKYDDEKNDFIRVHCETYGKSGIRRIVPGEYIAVDPKGRAL
MLCAIEKQKFVYILNRDNKENLTISSPLEAHKSHSICHAVVGLNVGFENPMFVSIEQNYE
TLDKEVTNGNQQIMEYPKKGLCFWEMDLGLNHVIKKHTIPIDITAHLLIPLPGGQQGPSG
LIVCCENYLVYKKIDHDDVYCSYPRRLEVGEEKNISIVCWTIHRIKTFFFILIQSEYGDL
YKIEVNHEDGIVKEIICKYFDTVPIANSICVLKSGALFVAAEFGNHFFYQFSGIGNDSNE
SMCTSNHPSGKNAIIAFKTQKLKNLYLVDQIYSLSPIIDMKILDAKNSSLPQIYALCGRG
PRSSLRILQHGLSIEELANNELPGKPKYIWTIKKDNSSEYDGYIIVSFEGNTLILEIGET
VEEVYDSLLLTNVTTIHINLLYDNSFIQVYDTGIRHINGKIVQEWVPPKNKQINAATSNG
SQIVVSLSGGELIYFEIDESHTLTEIFRKNINVEILCLSIQQIQQNKLRASFLAVGCLDN
VVRLLSIEKDQYFKQLSTYILPNNSSPQDICISEMKELGNQKEHTILYLNIGLNTGVLLR
SVIDPICGTLSNHYSKYLGAKSVKICHVQVNKNPALLVLSEKTYLCYVYQGKYIYSPLNY
DVLEYASSFYSEQCSDGYVAISGNSLRIFRFYRLGEVFSQNILPLTFTPRKIVPLPFPSL
FYDNDTSLEITRIKNIQMLAVIEADHNAYDENTQREIQKALRDIKLEGKEGEEDDVDEPD
NEEEELLYDRIGTPKAGAGKWGSCIKIINPINLQVIDKVSLELEEAALSVCACELEALHC
LIVGTTTNMTLKNRNVPSASLRVYTYDINYKLNLLHITPIEDQPYCFCPFNGRVIVSVGN
KLRIYALGKKKLLKKCEYKDIPEAIVSIKVSGDRIFASDIRESVLIFFYDSNQNVIRLIS
DDIIPRWITCSEILDHHTIMAADKFDSVFILRVPEEAKQEEYGIANKCWYGGEVISSSTK
NRKMEHIMSFHIGEIVTSLQKVKLSPASSECIIYSTIMGTIGAFIPYDNKEELELTQHLE
IILRTEKHALCGREHIFFRSYYHPVQHVIDGDLCEQFSSLPFDVQRKVASDLEKTPDEIL
RKLEDIRNKILMEEEPTTQSKPLDDEDINILKSYGSGPYSKSIKKVETDITGLLTNINKL
CGVRESDTGLCLPNQWDLQLDKQMLNEEQPLQVARCTKIINSDTDQTKYIINVKQIAKFV
VGLGDKVAPSDIEEGMRVGVDRTKYKIQILLPPKIDPSVTMMTVEEKPDITYNDIGGCKE
QLEKLREVVEMPLLQPERFVTLGIDPPKGVLLYGPPGTGKTLTARAIANRTDACFICVIG
SELVQKYVGEGARLVRELFQMAKSKKACILFIDEVDAIGGSRGDESAHGDHEVQRTMLEI
VNQLDGFDNRGNIKVIMATNRPDTLDSALVRPGRIDRKIEFSLPDLEGRTHIFKIHANTM
NMSRDVRFELLARLCPNSTGSDIRSVCTEAGMFAIRARRKTITEKDLLLAINKVIHGCKQ
FSATGKYMVYNMSLDFCSQHLLAYDNSLIVSENEIKAKAEENFAIVLKKINELKNERNDD
GELFYNLTQDNVFNLPRYSKIPQAKKPTRWELFAQTKLKKKRNKHGLIYDENSKGWVRRF
QKKQIKINKEKADFVHEYKPSDNIYEDPFERMEEEKDIKKMKQKMREMKNKFEQQGVSSQ
DIKYIQKQKRKRENLIDDLKMAQISSSTFGRYDKKLKKEKKLKVKINQKPEKRLLKDEIN
QNNKLAEIVLKSLMETNPYVLKNLVHIYEEYINLIINRVKGYKVLVLDDETKVIISLIFS
HSYILEKEIFLTLNFNDINIFEDIKNGSNKLDELSFQNYKIKNLKHLKAIFLLRPTHTNI
LKLMKELKKPIFLEYYLFFTNVLNNSYIEKLAKADEFECIKSVMEYYIDIYILHDKLFSL
NIDYTSFLYKNDNKILKNTIKNASGNSMNIQRDIQFKSSNNSLTFEEFNKNGNYMNYLEN
NNDDYNSNDLGDGNFHDESKNMLYEHSLINRLIEGMFSFLCSIKQVPDIIYNKHSYVCKS
IIDALKMKMLRHENIFSNILESYENYDNYTKTNPMENYQDNNLINKNIDVNTEGNCCYMV
LLDRREDPITPLLMQWTYQAMLHELIGIDNNKIILDSNNSEESQIVMSSNYDDFYNEHLF
DNFGDLGQAVQSYVDVYQKETARKSKLESIDDIQKFIEAYPNYKKLSGNVTKHVNILHKF
SELVEKRQLFHISELEQSISIYQKKLEHYKQVIETVRNYSYTNYDALRLSLLYSLKYEDK
EHIDTIKKELQKRNIEKDQISLIDSIMTYSNEQNRKNNIFKEQTFLDFAKTTITRTIKGT
SNVFTLHKSYIYYLIEDIIKYKLDTSIYTTTNLLNIAPNINKKINSIIVFIVGGATYEEY
RDVQTLSKKYNINIILGGTQIHNSQSFLADVLQLTKKM---VFPEIFADIEENKFVPIQK
SSPRDIFLIDFINNEENNENINNNRLCEKKTTKQYTSVFDRLTDQTFYTGTHKKKFEALV
KRKLNEQDMDNCLSKNLEIHKNNQFEKGKKKKKKEKNLVVTPGILGIQKYGIQIARPKSI
WLYRNGDKHHNGLLFFIKPHINNLKLLLFEITKVLDPIIGPIRKMYDQNFKLIKNIQQLA
DGAKYLCTSGDPPASIDNLGKFMSKWVIQGMKKKEESSKTKIKKKTSINMSKSEKDECSE
GSKMHEEIIKSNSISSENSNKKTKVKKIKSSSKEFIKEECEEEYGEEDSKNEEDMKINDI
TKYFKEGQKVITPPNGDGTRAFYESLLDENPNSIIAIKYCIEHGVLSGTKHHETLNKYYM
LKKNNAFRNNFGGIKCEFVEMLENIKNDKLLSMKNMEVDRDANVEQWKIKRLIKKLENAK
GNGTSMISLIIRSKDEVSRINKMLADELGTASNIKSRVNRLSVLSAITSTQQKLKLYNKT
PPKGLVVYCGTVVTEDGKEKKMSIDFEPFRPINTSLYLCDNKFHVEALKELLESDDKFGF
IIVDGNGALFGTIQGNAREVIRRFTVDLPKKHGRGGQSALRFARLRLEKRHNYLRKVAEV
STSVFITNDKVNVLGIVLAGSADFKNDLLHSDLFDQRLYAKVIKIVDISYGGDNGFNQAI
ELSGEALQNVKFIQEKKLIGKFFEEIAQDTGKVVYGIEDTLKALEIGAVELLIVYEGLDI
IRLTTKNNVTNQTKTMHIFPHDEKQESLYKENNVELEVVEKILLTDWIINNYKKYGASLD
FVTNKSQEGAQFQKGFGGFGGMLRYKIDLNLYDEDVDSDVELFMNSYKESNHVVDQKRLL
DECIFVVKEQSFFMKQALDNGSLRDTLKHASNMLCELKTTELSPKYYYELYMLIFNEMQH
LDSFINDKKKHKKKFIDIYESVQHAGNIIPRLYLLIIVGRNYIKNKDIKAKYILKDMTEL
CKGIQHPLKGLFLRYFLIQMCKDRIPDTGSEYEEAGGGNIDDAFEFLLTNFYESLKLWNR
MNDKVVPIPNIDDTILKNNRIKILKEKMDVKMLVGSILVRMSQLEGMTKQYYIEKCLPKL
LLYLSNINDSLIQQYIFESIVQVFSDECHLYSLEILLNSILKLNNSVDFKNILITLLKRL
RSFVEHDKSEFPKEIDIFNLFYNHLVIYVNRTIEQCQIN-YNDAQDMSDKVHPDSININD
EPSTLNNTKEDADASKVIPILTDHSIHKNSI--GNNDNNKVVKKDKQEIIQNIIKMLQVL
YEFIFLCICMYDS-TTINALFELAYKIVSNININDGEIYEQVISIIVLPFNHLGLDALKA
KNIQNLLNSINDKYKKKLSLNIIDAIIECKHKEMVYENVEEILKFISCIFYEDNQKKNIN
KKDPFNFENNSIVYTSEKISKFFHIITNTNNIEQKYNTTMLFYNYIYDSIYFSQLLPTII
FTLLNIVTKIIVVGTSIPDNNETTHNYYDSNFSNKETTILSENTVNQYNKYAKNIFKFIH
TNLLAISSEIPILAFKIFLYSAIVVDKYEKFVNDYSFISFDNIEAICLEFITQPLIIYEE
DINISAQQFECIIWAVGILSSHINILDNENYNNVALKLCQHANKLLKKKDQCVGLLMCSH
LYWENKKYRNSTKTYECLQKALKNAEIAMQSNSDNIFLFVHTLKKYLYYYESLNIEVTEK
NINYLIDICQEYYSDLSKDNSANQEYLQIIKDIQAKKQNPNLFENINAYLPSMLEAKLNN
ASILKKLFECIKDLVNDANIDADENGLKLQALDGNHVSLVSLHLVDSGFSHYRCDRERVL
GVNIASLNKVFKLCGINESVVISSKDDEDNLNFVFENNKEDKVTNFSLKLMSIELDSLNI

PDCDEGFDAEVELSSKELTNIFRNLSEFSDTVFIEIDSNSIKFTTKGLVGDAEVALKPRE
STSEDDVGVTIKSKKKIKQSFAIKYLNLFSKSSILSDVVILGLSDSRPIEFKYEIKDTSP
DSDALKVGFIKFFLAPKMDDDMDNKDMRIYNWVSCGFIFLQFFLSAYGKEWCKAKFEYGM
SDYAECQKEGDNLTKYMIEIIPAKANNIDLYSDVSIVLSNSQGINTKEIIIGSEKEGLFR
KIYSVRKDIDNPEYIHVKLNSKNRNWKCKKIKVWKEYKYWTFDCIGILNEEKQEATYFLS
GNKLYTAYVQTGKDIEAGTTGIIDIILLGNNKRSNTKMLHEGFTSGGLKKIKFQASDVGN
LENIILINNSYNDPWYCDFVKIKGDDNKVYVFNVKSWIGYPYNNKIKININTNNIDGNAK
DIDCHIRANDLIDTTNNSFVLQNKVHIFKVRCPQNCHSSDFSIIEGTSIHPASTSICAAA
IYDGSLTESGGEIIVTITKGLNYYYAMDGTYNNLKAIEFSTKSDENNFSFYTYHLTSIDD
IKSNVRIVDSFGKLSSLGRLEIRVNNKWGAVCKKGPNFEFSEEAAKRACKDLGFPNGIYI
KDNCSNINEQNYCAGYKYPFNASGILCSGNEQNLLSCNTDDPSYCIDHHDDVIIQCVNQL
GNDSIENGTIRLLDSTGAPTSNGIGRLQIYYNGVFGSVCSEGWTKETEKIACLELGYHNV
KANGFSKQLCSDIAGENLCGHDTERINATNFRCKGDEANLKNCPHETSEDIYCSHEEDII
IGCANAEEDGDASTSSNKHIMSMEKKQFHPKIELSCFDKISSKAELSKGNVGDIFLVSCP
EKCDEDSGVIKGTFVYTFDSYICKAAIHAGVLSSNVADDVVLIITHSRNKFIGTKRNNVE
SKEFNGESKSFSLSIPTNYIIMEERQNNSKYEDEIL-KDDNDFYYEHIFNKQNKTFFEHL
EPTFQWIAPSSFTGFNGNENEYINASNLPNEKYIRTLSNFTFITHFIPSSGKNTWRTILS
HSLCEGISISIDEENELVIEQNCNPHLIKTKFIPKFEHPYHLVLIYNKPNKSISLYINQK
KINLENMKIDFTLNGDLTIGRSNKQATDYFIGDINFVKIYKYILTEQEIKESFDSVLSNN
YLNDGMSGNGNINSRKIQNKKTKNQRKTIDGRDCISSCKSKTNVNKNIQINTEEFYLNCS
DNLLSERFSGKIGTQFLASCSEDCTSSKYIVKGSNNYYTPDTSICKAVMHSGIMRKNENN
KNSFIIKIVEGLTEYKSSRGHFGIISKAEKQSQLRSFSLFSKNEDDIFTCFTDGSFLFEL
PIGSTKNIICPENCHKIDKQIYGTNTYSPLSSVCKAAIHAGVISIKGGQIQVVVGKGQQE
FKPSTQNNVQSYIAEKQDRSFTFFKRLY-MKNLFVEQNVDTEKVPENKVNINTDKIIYKI
KKKIRKKKENNPNLNLLPHNKELLSKDFPNPKNIQNKKLKNKISTDVKLAILSSKKILTN
AQFNNIDHGYIKLTSHKDKNVQTHTQNVEKKNFSNNDTDGNLINLSKQDEDALGNSSIKV
SQKYIYENADVGTQKKVFDLHLNLGPYKCNYSRNGKYLLVTGEKGHISLLDTHNMESLCE
LDVNETVRCNTIFHNHKLFAVGQKKYIYIYDNTGIEVNCIKDILYPSQLEFLPYHFLLAS
IGDLGELVYQDISVGNIVTRKKTKRGPCSIMKQNKHDAIIYLGHKNGHVTLWSPNMDKSV
CDIFCHYTPISAIGIFDNYLITSSLDCTYKLWDIRKLEYINTFKSHNIINNIDISDTSLV
AFTMNSHFRTYKNFFTKPELYLTHNTYGDKINSIAFQPFEDICCAGLKYSIKSFIVPGAG
LANIDTFVNNPYETKKQTKENEIRQLLDKLPPETIHFKQNQIGKINPHLTLDHTQKN---
SSNQVNTIHTGRSKNKNI-------------------SLNRHTKNK-----VQFPKNVAA
SQNNG--KKN--KNKKKR---MSRSNEANDNLYFLAISKNKVTIYEYNAEFSKSIISDIE
EEIKRSETINDDTTPKGVINNTN-NHITPLKRSSNKFNNDYLINRGISIYREYEGVELAT
CTRDYKKIILVRKNDLNVAEIIDIKTDEHISYIKTKTQIKRIVTSPRDSHIVLHCQYKPD
ISNKNLYIYKIGGKNNKKKKKKDNKEAENSEQTEQNE--NKHNNVTKTDEIIYNESLVH
EMALNSYSNSNWPFFKWTDSESNCLCLINNQIYIYKDNNFNVISDKLKLEHIEFFEVSPE
QPNKRKVFLATYERGTKGNSSVFKIFNLDNLSKHIYSKNFFNSDEIKLKWNKNGTSLLLQ
IHTQVDKEKQSYYGSSNLYFIDTIQLKDVNIMTNKGLIYDTIWSYNQNKFYVCKGEIPAD
IVLHDKNGNIIHSYGKHKFNTLKLNYNEKLLLTGGFGNLSGDISIWNTINKKEITKTKSS
CAVICEFFNDDKHFLTATTHPRLRVDNNIKIYKYNGLIVSKLDFDELYNVIILPFNKIKS
QPIDISINP---NSDLQKYINKQLGIDSKKVGIYKAPGSTAKTSLNGLGIMARP--PKPS
LPPGCNFVVEEKTNKKKKKKKAPKNKDKKEPFMKNDKIKLVSFEGDEFIVDKYTASMSTV
IFNILEVMTSEEDTIPLPNIKTQILKKIIEYMEYHIHNPPDEIPKPLITSNLQDVVSVWD
YDFVNTDKETLYELIEASNYLDIKPLLDLTCGKIASMMKDKTTEEIRAEFDIVNDFTREE
EMQIREENKWCGDIMDPSITNMINDEKEDQH-------EKLPEVENKCTELPDHNCNTQT
DKSDNVCIQDPNMITSEEIQT-SKQGLSDNLNKNDYKQDTNLDEQNASSLNVQNVDDSNI
VNIKSDLNGANEDNKNTFIDTNTSKNENMHNEGQADKNIGDATIDANHIGVEKREDTN--
-ENCLEKPNHNNESNAENDCGEGKDDAAEKNRRIIEMYEKKLETEINEKEKNVDGPTESS
DRFDGSISSISAKMRESISKIFREIPSSKPKIINEIKKNDNDENYENNKN---------E
INPSNGNGSGGRDDRDNKPSFSRNTLKEGSLLRLFRCEYFDTHLHIRYLYDRKEVGVHEY
LVNSLYTQRNPEDILFYLPQLCQISLVRYESSSLYRFLLDKASKSMHFALKLNWIYNSIV
EDNIPKYKEISQKMIQEIEMAVVNCKPLNSECKIFKEDKKTDLLILAYPLLFKRKFIIKK
IRTNEKINQIKTFNKFLSNSYS---LKGF--SNPVEKANILFCSSHIKNNDQQNSDIVDG
EKVEAPQPMHPNSEHINEETENNNYLGAEQNYNDGVCPKCAHIFEAKKEKKDKNQNKPLS
PQMPSCYIINIGPLTAASGKIKLPSTYAKLGDPLSFSKFSLPDCNYSFDMIEELQQFFMK
QRRCDYFSLLNNFINLLITTSNLLANETDIDARNTLLNKFLYSLNTWMIMRRCIVASCEN
IFSMTGLCIPMESISDSKSDRCGSRRKQKKSSKHLQILHFNNDECKIFFSKKRAPYLLVF
EVADLDEDISHISDNTFYVSNRLFNIENYKNNNSIKTAQTNSYISHSKNEKSDDELKHNN
YILTKNYGGYNENGSSYECEMRPSYEEQNKKNYRDEQTSKLNSNTNSADSEVSSTNSYFK
DLNAIKMNDLYVYNAIVNDLRRENLISFTSEDEENIYLIKKCIGLAKEKSNDGYSDKEYN
NNEESSALGIKTDKFITTRSASMPNYLNNFKSAD---------NNQTGENSDICEYNEAS
ANSTDNQTAEQDENLKGEACDKK--------ETNLMKRVG-NNNNGENSGKQASVLSSHP
PNTRNCYYSVELPSMLPDISEYFKVENYLNEEFKKKNCKIIKTLLWGELFEDKKKKIRKI
SPYGKLKSWDLKSVIVKGGDDLRQELLASQLIKQFKIIFDNAGLPLWLRPYEILVTGSNS
GIIEYVHDTCSVDSLKRKFGTDSISTIFNIVFADYIFEAKKNFIESHAAYSLISYLLQVK
DRHNGNMLLDSYGHLIHIDYGFMLTNSPGNVNFETSPFKLTQEYLDIMDGENSDNYEYFR
RLIVSGFLEARKHSEEIILLVELMMPALKMPCFANGTQFCIDSLKERFMTNLTVDTCIQR
INALIEASINNFRSVQYDYFQRITNGIMMTINVSFKVTGGKEFTISIEPTITVIELKQKC
AEHVDIPVESQRIIFKGKILKDKEPLTLYNVADGNVMHLVRSSVPAKDSEAEKENNKESN
SAPDQSQGINENLNNFNDNPLIQMLMQRGAGDMNSFGQGLGGGDFNYGNLASMLNPNGNG
EFNRESISSLLNNPLARSLMNELSNNPEMLTNLISNNPLLRNTFSQSPLMQPMLDNPNLL
REFMRPEVLQAGLQIESALNNNQNNNSNNNQG---GLRMEDLLSNLSNFANPNAGLNSSN
GNNANNLNSLFQSPELLQTFQQVMRGNPNLGNLDFASLAQNLNLNTPNVTDNRPPEERYA
SQLVSLQEMGFIDNDANIQALQETGGDVNSAVTRLLERGFNMEKKEDNVMREIKVNKLIL
NICVGESGDRLTRAARVLEQLTEQKPIFGKCRFTIRSFGVRRNEKISCFVTVRGKKALEI
LEKGLKVKEYELRRKNFSDTGNFGFGIQEHIDLGIKYDPSTGIYGMDFYVHLSRPGYRVT
RRRRKRSTISKTHKVTKEDAMKWFQTKFDGILLKMEFYGSNYFRFRLALSLISGKAITIK
NIRNKNSHKKINKDDGIDDNEINEGLQEYEAKILKLIDKLCDDTTIKINEYGDELYFKPG
FLIGNVNDEIRISDLNNTFHCGNERSISYFLEFLIMIVPFFKNPVKLLLKGITDDQIDRT
VYTCKIVCENFFKTFLNVNDGFLNITILKRGTKLDATGEVSFFMNNLKMINSFDMHDAGL
VKRITGTIVCNQISMIFRNKIVNCAKKNLHNFTPYVSIEVEKEKKNSYSNSQNNFMSLSL
FAQTKNKCIYGTDLYVDKFMLQHVKDMLASRTEGEKIDDNDDEEDGEDDEEEDEGEEDDS
PFNDENNEMNSSNIDASKEEPKTLHDADIYERLGFFISLKLMNEIKGLSSIDSSYQWLPL
LYMALANDTAVSKISLSALKPYSIVLIRLLRDFFSVVFDIKKVEKSQIEYSYLIKCVGIG
YRNFSKKTFMISLGGSSLSKEFFDLAKSIGDSRSKQEEDRIICNEIVLLKSRFANPNATV
KQIKEYLIRAIYIEMLGHDASFAYIHAVKLAHEKNILCKRTGYLSCNLFLNKDHELMLLL
INTIQKDLKSDNHLEVWAALNCVCKLLNNEMIPAIFPIIKNLLNHKNELIRKKVCMLLHK
IYLIDPTLIKEIDIYLKKLLCDVDPSVMGASLNLIFAIANNDMIYCMELVPYLVSILKQI
CENKLPKDYDYHRIPAPWIQIKILSIFRILGFSNKKLSEQMYEVLQKTMQRADYGINVGY
AIIYECVKTITTIYPSHRLLELASLSISRFISSENHNLKYVGVTGLALIVKINPMYATKH
QLAVVDCLEDKDETLKMKTLDLLYEMTNPLNVQVIVDKLIFHVENSQDMHFKHDLACKII
QLIERYPPNDIWFLNKINTLFLSVGELIDEAYSYSLIKLLKENSQSNNVNKEEEHLNLLK
DSEDAGND---ENGENNKNNVSEKLNNEEDNEADLNKHEKKKSNDDTLNLRKYAVNTYIK
MLEENENIPFVLIQIICFVLGEYSYLCDLENYTAEDILDLLCECMEKNLTNPDRVKSCII
TAIFKLCCHNNITDHVVTNKIIEKYKSSQITDIQQKCYEYDSILKNRELIKNVFSKNNKT
QNIVIDENLSFLNPFIEKHLESGGKSYVSRDLRQCENTIETSKSASLTLNFTPYELPINN
NINADIYNPSSPNLLYQKNRELSSFSNSQINDDCSSQMEKKKMFKLNVVGPKKWKKETTK
VEEVEANENK-NNSTKKKKKKKKGKKNVSTYPGTNNGIINYLGEDRNKLSIEQMNKKQFN
KLDNMNQSNPYYNEERDDNEDSEDDEDEDEEDSTSENNEHEKRMDFAHDYTNYENLYGNN
IEGIKNNKYGNSNKKGTSKDEQSKFRNNGMGDTSNELSEKEKMAAALFNGLISNNTSVFD
SKNIYSQNFPNKKSLSMSSNRNDLRHNKDSSVKNNTYEESKNTNNRMTNKNEEHDPSASI
ITRSNLNNSNKLGETSKKRYSYDMIDLNETPENRSVTLENNENKEMWNDITNQKKAVFLN
KSKLSIFQTIKKFETNLDSNVVDVSKTEALVSCM-----------MSGSQCVAIACDLRL
GSNSFTTVSTNFTKIFKINDHIYVGLSGLATDIQSLYELLRYRVNLYQIRQETEMNIDCF

SNMLSNILYSNRFSPYFVNPIVVGFKVTTQVDENGNKTNVYEPYLNAYDLIGAKCETNDF
VVNGVSNEQLYGMCESMYIKDQ--NGLFETVSQCLLSALDRDCLSGWGAEVYVLTPDKII
KKKLKARMDMHKTIRKIFDSKICERLESMNILLVGAGGIGSEFLKTIITIGCKNIDIIDI
DTIDITNLNRQFLFKKKDVKKHKSLVAKERALKHRKDLNINAYTFDVCTMKGSDISKYDY
VINALDNIKARKYVNKLCVTEKKVLIEAGSTGYNGQVYPIFSNETKCYNCEEKPKNKTYA
ICTIRQTPSLPEHCVAWGKLIFETFFCKNDNETLIDIKKHIEEESKKRDMDKEEIIRFIF
NYLFHDTINELIALKKDYTIMPKPILFEENINHEPHNIDKLSQEKSGNNLKTNDN-----
-------KICENNSIQLSSQNIWDKKKCIEMYVSTFNKLYTYLNIKKETEEYLIFDKDDD
DCINFITSLSNLRMINFSIKQKSKFDIQSIAGNIIPAISSTNAIVAAFQAVQLVHVIEHF
ELLKEKETEQNITLRDSKAKHIWIKNVVSGNKIFSRGNIVNAENLETPNPNCYVCQQPVI
NVYIKDFNDITLNSFVKEICMNELSFLNPFLDNQDRNIFDYDTFLDNDDDYMKSLSNSLH
DWDIKHDDILTLTDAQDTKNQIEIHLKEDKSLENLYLISSKLTKKRKPEVAAIEERPAKS
AKKMKSAPEDDIIIVDNAETNDEKDMGKLSKDRRDIYYRKAKENGYRARSSYKLIQINDK
FEIFKLFNPSNYNNNDIGVVVNKYNDEFCYNIVDLCAAPGSWSQVLKNICLYNYYNMLYF
NNSELDTLEHEEFVKNFSLYINFNKNNNIKEPKLVAVDLQEIGNMNYINIIQGDITKMST
INQILKCMNGDEKEIENNLLNKEKDEKKFAYAHAVVSDGAPDITGMNDIDEFIQSQLILS
SLKVCCSVLKVGGNFISKIFRGEYTGLLIFHLNKFFEKIYVCKPQSSRNKSLESFLVCLN
FGLPKSAITSLFTNTNELCYDCKNENELRNVHIESINGKDQDCE-EDKLDSDLDSPENEK
GNNINITNNTKKNLDIFNFYCSDSDEDIKYFNSDEEENVNNYISSNFSSNNKLFSFVATQ
NYYDSDKSYLLPQNYIRHEPQIMPLQPPYLLSLQNRRKETKKMEGNNYDMILVLNFGSQY
FHLIVKRLNNIKIYSETKDYNVDLKDIQNLNIKGVIFSGSPHSVNAKNGPHVKKEVLNYF
IENKIPIFGICYGMQELAFQMNGKVGKSKNSEHGSTDVTLISHDYKDNASYKNYKLLETN
CLLFDDIKDTSKMNVWMNHTEEVIEIPENYYLVNSSENCFICGIYNKEYNIYGVQFHPEV
YETIDGDQMFYNFAYKICKCTKTFDPIKYHEVEFNNIKKYAHDHYVIAAMSGGIDSTVAA
AMTHKIFKDRFFGIFIDNGLLRKNEGEKVYTFIKNTFPDMNITKIDASEIFLNQLKGVTD
PEQKRKIIGKLFIEEFEKAVYSMDIDIEKTYLLQGTLYPDIIESKCSKNLSDTIKTHHNV
GGLPKNLKFKLFEPFKFLFKDDVKKLSQELNLPKELTNRHPFPGPGLAIRVIGEIDKHKL
DILREVDDIFINSLKEFNLYDDIGQAFAVIFSSKSVGVRGDARSYDHICALRAVKTTSYM
TASYYKIPHDILEKITTRILSNVKGVNRILYDISSKPPSTIEFEMRKYGGNTKRNRDDEA
DESNN--GDRKCITLVRATLLPILSIGKNEKTTLFTPFRNPLPGFVSNKSLYLKKTLGAK
VRKTGWINLMKNSIPRRSDETDENDKVEEIIRKFDPLVLYEDENSKIEVDPILAQYLREH
QREGVQFVFECLMNLKDEKISGCILADDMGLGKTLQSISVLYTLLKQGYNKKPAVRRCLI
LCPASLINNWNDEINKWLPNRCTVTCVNDNAKEKIVSKLEGFKYDLKSTILICSYECFRI
NNESIDKSAIDMIICDEAHRLKNDKTKTYTSIYKLSAKKRLLLSGTPIQNDLGEFFALIS
LCNPDLFDDTNSFRKKFANPILIGRDKDATEKEQQIASERLAELSTITNKFILRRTNNLL
SKVLPVKYLINIFVKLNPIQEALYVLFLKDKKLLKSDNSNNKVNVLINIKKLEKICNHPL
LLNANDIKDIGHIPIVKLIEDVVAESGKGRGSKISNKSSVNSSANASTENNASKDGEYLR
ERRKAVELDYDKSVKKIIEECKRDVYRCYYNLSSKFQLLHFLLKTIKQDTNDKVVIVSNY
TQTLDYMEILCKENHYKFVRLDGGISIKKRHKVISDFTNTDDIFIFLLSSKSGGCGINLI
SSNRLILLDPDWNPANDKQALARVWREGQKKICYIYRLFCTGTIDEKVYQRQISKDGLSS
MIVTNTNLSKDQLSDENVKKLFNYKQNTICETHDNIECNRCNFKDNT---EVYSEQLEDF
EEEDVNTWAHHLNIDTVPDAILIKAVKEATEYKINHMNTLPILKKLKQDFVTFSMSCKIE
FRDDLIKQKANKNNVSYQPIVVSDKKETPKSKRAKGRKADDYSDSTNDEEEEEEEEEEEE
DDEDEEDEDDEEEDEMSKYNSPNNEFEKDDEILCIEDIVNKSSTEYEKEIKNLPYISNEH
CVNIRKKKVEPAGFAFYIKYEIPILKEKEQKKNNNKCIPIDELFLYNDKIHYLEEIKKNE
T--NIDDDKNKDSSKNLKNEKYQSDNYEENNNSKKKEEKASLKKNTNLVKKCINNNINVY
EILDVEESDDLETIKSAYKKLILIFHPDKNKGTSILNEKEKVKKKKKNNKDDDKNNEIKK
DIAYYIEKYNIEKLTQEEKKSMFLKIQDSYAVLSDKILRKQYDSSIPFDETIPTKAALDE
APNFYEFLNPVFKRNAKWSSIKPVPNIGDENTSIDKVKYFYDFWYEFTSWRDFSYQNEYD
YEEAECREERRWMERENKKIQKKASKAEKLRINKLVDLAYNNDPRIIAENKRIEMEKQKK
KALAMLEKEKNKMNTLQSTNKTGSQTNSSNDKKNKDNKTTSKIWKHHIKSVCVTKLYTIF
NFNLIQDKLNDMPFDTLCQFVYEIYYFLNFNINKPDNSSAEKINI--------NEKEATF
INTNKPNTNDNNTRGRKNSLSNNTGNEGNKNTGFIAHLKGVELSEKDIELLLDIFRKYIN
NFNSILEMKENIDIQDIKNEKETPEQDGHTDEKNNITTNCSQNDKVTNEQIMEKSNSKEI
AVEEKKNKKEDDENNTNTKWTPQEISLLSKALKLYPGGTKNRWTVIANSIKTKNVKEVIK
KTKEMFENETLKNLSKNFEESAFDNFKNQNKGVMKKIDDKLDKREYKHINDQNHVSNPNS
SDNNSEQKKPWTHEEQMLLEKALMKHPATIPTKERLKLVANELKTRTVEEIVLRLKTIKA
KIMAKNAAKMPKKRRNGGRSKHNRGHVNPLRCSNCGRCVPKDKAIKRFNIRNIVDTSAQR
DIKEASVYSTFQLPKLYIKQCYCVSCAIHSRFVRVRSRQQRRVRKETTKHAHASQLMKKK
IEQNNPRHKKYSYSDENKMQNFFYKSINKNRFYLLFFLFLFS--SLINLAQCQV-KKKVS
VSEINFDSAVDDVQWCGNNHSTVLVKTVKGKLYRSSDGGKIWTNITSNLSENPSSKNDNT
TGHTPETTVVDLIMVNPINKNIVLVIGAHNSHYISDDAGETFKLINYKNKINFWQFHNKK
AHWALVSSWTAACFSTDNSSGECMQTLSLTKDLGATFQLIDIYVVQFNWGDANSHSEDTI
YYTRHRNRNGHQQRFSGWSKDVDFVSTNNFGKDVEVLVKHGNKFLISNGYIFVAKLNDVI
KQTVNMMVSTDGGKTFNKANLPKDIHEKSYTILDTSEGAIMLHVNHGSSSEKLNTGNVYI
SDASGLNYTLSLPNNIRTASGECEFDRVLSLDGVYIANFLDDQDEMKDEDLKFTNFKLQL
EEDVEPIETNTQKRKKQLIKGKNEETVRTVISFNKGGHWSYLKAPKVDSIGNKYDCGDEC
YLHLHGITNYHQYAPFYSIENAVGIIMGTGNVGSHLKYKSDEVNTFLSRDGGVTWIEAHK
GPYIYEFGDHGGLIVMSDDLRKTNQIVFSWNEGQSWFDFELGQFPIDIDNIVAEPTSSSV
EFLVYGTRNDIGVLYHLDFNALGQPLCKGLWAADSVSSDYETWSPSSGSFKDKCILGRKI
TYTRRKQTSECFNGKDLKRVVDKKLCDCTPEDYECETGFTRKVGSFECKPTDSTLTIEGC
TSSSYFYATAYRKVPGDVCVNGWVPEKVPVPCPDYSPFNNSAKSILFILFIMGLVMLIIT
YICRDPKFRSMFYNYGFDTFEHVKYSVVKTKKGNINSNVFEPEMEFIDAEQDNNEEDVPT
LMSYHNERNGQRNDFDLTRNRSNHNNYITSRTANSQKKYPENIELLMADRKPNKNAVVKN
VDMTEEMQIDAIDCANQALQKYNVEKDIAAHIKKEFDRKYDPTWHCVVGRNFGSYVTHET
KNFIYFYIGQVAILLFKSGMAIEVDEETLKIAKEFQKQNEEEIKFKKLKPETVKVNEPSS
NLLKINEAACRGRIKICNLLNVPMSENEYNDKMEKNKYIDQLVVVCGWSKAVRKQGGGRF
CFVNLNDGSCHLNLQIVVNQNIENYDKLLKCGIGCCFRFTGTLILSPVQNTEKKGLLNEN
VELTLTDNSIHSFEIYGENLDPQKYPLSKKNHGKEFLREVAHLRPRSYFISSVMRIRNAL
MVSTHLFFQSRGFICIQTPLITTSDCEGGGEMFTVTTLLNENGDISAIPKTKKKTSNNQK
REDQTANEIANTTNNTNEYVIDYKKDFFSKQAFLTVSGQLSLENLCSSMGDVYTFGPTFR
AESSHTSRHLAEFWMIEPEMAFADIYDNMEVAEAYIKYCIRYVLDNHFHDIYYFEENVEK
GLIDRLKNILNDDFAKITYTNAIDLLTKYSTNFDVPVKWGMDLQSEHERFIAEQIFKKPV
IVYNYPKDLKAFYMKLNDDQKTVAAMDVLVPKIGEVIGGSQREDNLELLDKMIVEKKLNI
ESYWWYRQLRKFGTHPHSGFGLGFERLIMLVTGVDNIKDTIPFPRYHGHAEFMCSTNSEI
DIPEADRIESQKLFDELKELNINYKEVKHGKVNSIKEILDLNLEKSENVIKNLFLKDKKK
NYFYLCVANWKKLDLKNVSTQLKTSNLRFVDDENLKNILNVNPGSLTPFSIKSDKDNIVK
LYFDEDIKNMDEVIIHPMHNYSCIYVKTSDVIKYCDLHNHTPEFINLPDSKDTEKRDDTS
IYEENPKDAANTHGKSSEKINEKDKGKDGGNILGITSKKEQNFSDWYTQVIVKSELIEYY
DISGCYILRPASYYIWECIQTFFNNEIKKLDVENSYFPLFVTKNKLEKEKNHIEGFSPEV
AWVTKYGDTNLPEEIAIRPTSETIMYSVFSKWIRSHRDLPLKLNQWNTVVRWEFKQPTPF
IRTREFLWQEGHTAHKNEEEAVKMVFDILDIYRRWYEECLAVPVIKGIKSEGEKFGGANF
TSTNETFISESGRAIQAATSHYLGTNFAKMFKIEFEDEQENKQYVHQTSWGCTTRSIGVM
IMVHSDNKGLILPPNVAKYKAVIVPILYKNTDETAIFNYCKDIEKILKNAQINCIFDDRD
LYSPGYKFNHWELRGIPIRIEVGPKDIQNNSCVFVRRDNNQKFNIKKESVLLETQQMLVD
IHKNLFLKAKKKLDDSIVQITSFDQVMDALNKKKMVLAPWCEDIATEEEIKKETQRLSMN
QTNVETSLSGAMKPLCIPLDQPPMPPNTKCFWSGKPAKRWCLFGRSYMAKLSKAQKKQIY
MDKLSSLIQQYNKILIVHVDNVGSDQMASVRQSLRGKATILMGKNTRIRTALKKNLQAVP
QIEKLLPLVKLNMGFVFCKDDLSEVRSIILQNKSPAPARLGVIAPIDVFIPPGPTGMDPS
HTSFFQSLGISTKIVKGQIEIQENVHLIKQGEKVTASSATLLQKFNMKPFSYGVDVRTVY
DDGVIYDAKVLDITEEDILAKFSKGVANVAALSRSVGIITEASYPHVFVEAFKNIVALVI
DTDYTFPMMQKIKDMVENPQAYAAAPAAASSAPKDEPKKEEAKKEEEEEDEEDGFMGFGM
FDMVQERDLAREPCPDRIIEDMGGAFGMGCIGGYIWHFLKGARNSPKGDMLSGALYSSRM
RAPILGGNFAVWGGTFSCFDCTFQYIRKKEDHWNAIGSGFFTGGVLAMRGGWRSSSRNAI
VGGVLLAIIEFVSMVLTRKTTPTPRQQFQQQMEMEKKMAAQKTKMLKDEIWLYKNEYKEN
EYYDDENDKQPKDHFLEVPLFDEDDYGKSIKRRMKAFPNKKVNSKRYEETEISEETLSTT

DEEEFLDLYEELKREQKIREFYNPMLKPRLWRLEFFIKYIHNLENNIQKNFYLISFGNTK
NVNLYGGMQKDVLYTPGYSIKPGEIQHLKIPLTIWSEKELSISYEDLKKFEVTIEMWCIK
GLIFNELYASSKINLKEIIENDPDSSIVLKRKIEKKKITFDAQRLGIYMQLSEIFEFHMA
LDSWWFIANSEMPSYLKLLPKFLRFKFPLSEGEWVIHSSNKSSNNFWLYPGYFCFIGTYR
QLANAFFILAVLCYNSNYKYKPPILLGSCIVSLKSVTEYPFFKGTVKKLTLEKRKFKQGE
IVGNIKCFVNSYGIQEDDTNIQRPVQPLSDVTLVNQLSLNDHYLVIRIIKCENLAISSLD
LNNININVWVKWDGIVNKTDTVSKTVSPFFYQNLYYPIRLVDRKELTNEKLIHNVLPIDL
ISKGDICLEVHNNNEIYSSILGIFELPFADIFNYGTDDFRALAQDNNED-SIHNENKNYE
YYDQGFDNPDDNYEDYYSRQYRTIVYKNTLDLMYSRANMQTKRKSTISIEAFVIPPLPSG
LVFFQENKSQNSSIIYKSMSKRWEQDFKGFNDIYLQWFPKAIKNR-----SKNEFDDNYY
PLCSFVTPINLPAQVSTPGPLFHWLNNIEYIENDDESVNFTPPHFFLSYKKGTIQDHALL
LCCCLKGLEYDAYVCKGTINNGKTEHYWVMTRHEKGWVCFWEVTNKSIIHLKNRWNNNNF
LKNFDILTENKMINKMNENDNDKYYNGEYLTNYIKYGLEELENRKNQILQEYDMKEENQL
YTMDLYGEHHMKDEEVNIDEVLYNDEEFFDNIFKVKYEKNNTYNCNKAIKYVLESFSKYI
PIAPKLGLLNYENTLAYVPYSSIEIIFNDQQLYGNMQNHHPACILYDLENNYHWRPLLNH
EPPQIKSEITISTPLSDKLSIKYMKELEEEIQEMIFFIRNVEGLETNFDHSTEIKYFLEM
YIDICEYKLNLDNNFNPKPENYEWKNEKEPEKNM-NGSMPGNMQDGMENINKDGN---NN
ESRKSWSQYKNDFYNINFHDSYHPAKNVEIMNSERKKDLSNIPQDYVYGMNNEIYNDGKE
EYIKNYVFNKNSTDILNNYDEKIKRKNTKRFSNAPLHYQDNGHDCEECEEEIYKEIKERY
YCYDDSECSDN------QTNKNLPSKFMKKN---LEKKLKKYEKNMKKEMIDIKLASKGV
QKENTKKLFSKFLDTQEYLINIPSKNGTTF-----EGVKKNEKISYKKKRNIKNKSKKIR
VDKNDKQCEKKKFKLGTRIHIKGKRKNKLFCKDISFFINSNIIKNSQLSVDKETVNLTKS
KQFEYEPPPKTWSRLDATSKYAAHQISQWNWYYALEEQYFNWQYKFPVPPNHTFVGFPIH
FSTPDFSEIKAFLLSSKRFDNIMKLSINNISFMSLSIYGNRENGQDVRTANVTAVQALSN
ILKSSLGPQGLDKMLVDNIGDVTITNDGATILKQLEIQHPAAKILVNLSELQDQEVGDGT
TSVVLLASELLRRGNELIKMDIHPTTVICGYKLAMKESVKYIKEKLSERVTNLGKDVIIN
IAKTTLSSKFISYESEYFAKMVANAIQSVKIINDAGKTKYPVSSVNIIKVHGLSSLDSKL
IDGYAIMSGRASQAMPSAIKNAKIAFLDFPLKQYRLHLGVQVNINDPNELEKIRQREKDI
TKERVNKILESGANVILTTQGIDDMPLKYFVEAGAIAVRRVKKDDLKRIAKLTNGQIRLT
LSSIDGTEKFEPASLGYCDEVYEEKVGDWDVMFFKGCRNSKSNTILLRGANDFVLDEMER
SIHDALCSVSRALESNYVVVGGGCVEVALSVYLEDFAKTLGSREQLAIAEFAESLLVIPK
ILALNASYDSIDLVCKLRAYHTKSQVMNTDDPKDYRWYGLDLVNGKVVNNLKNGVLEAMI
SKIKSIRFATEATITILRIDDLIKLVPEEPKQEEPM------NQNADQWNTAFDDLQICT
RITESCGIACSSEYIATPWQVQGGGVIGVIKLENMYRNPPVYKLKGHTSNILDIQFNPCY
NEVIASSSEDMSIRIWEVCNKEDKSEEIKKSLCILNGHKKKVTIIDWNPLNYYILSSSSF
DSTVNIWDIENEKKAFNISMPQKLTSLKWNNTGTLLTATCLNKKLHIIDPRQEKICTSFV
GHTGGKCAKNIWIDGYSGNENYILSTGFSKNYMREIKLWDLKNISDPISTISIDNASAPL
LPHYDESIGIIYVIGKGDGNCRYYQHSEGVLRKINEYKSCLPFKSFGFLPKQVCNIYKCE
IGRIYKNENNKSIKPISFYVPRKNPNIFQEDLYPPIIKHDPNYSSKSWINGNNLEITRIN
IKDLTDADIKIYKKFKTVPKSMDSILIGDPCLDKRSFIIRQFTKRFTFFKKNNNIEFNNN
ISSESSFNINDSEQTE----EKKKIKISIEDI--DND-----KNKSGEFSDRLKEEYVQT
ETIE----TNSNKFLDTITCKKWFGKPNMKIKSIGDESIHNICSSQVIFTLSNVVKELVE
NSIDAGATEIKVKLVENGIKLIEVSDNGNGIKKINFENVCARHATSKISEFDDIHNVLDT
LGFRGEALNSLCMLSDLYISTKHDEFEHGYLLKFDKFGKLLHEEPIARLRGTTVSCENIF
KNIPIRKKDLIKNIKNQLSDLLSLMQQYAIIYHEIKFMIFNIVTQKGSTKNMNMLITNGS
DDIKKNFYSIFGKKTIGNLIDLNIKNENWLVKGYISDSNSGRRDKDLQFYYMNSRPIHVI
KNVNKIINTIYREFNSRLYPIIICNIISDAKNIDINVTPDKREVFFTFENELCEQIKIEL
IKLLTPKTTHLVDTQIDDYFFIKNNRAIKHEEVTN-------------------------
----------EHSTNTECPDIENEDACYTSTDYKQVVVNGNEDTENFDFNRQKKSINKMD
NQKENNEDRKESYINNNIKIKKEFEKSYEDELNECPSEMKKLVKDEPVEYYEYKYEEYKN
FSDENNSNNANSNRKEENNTFFDDKELSDYSSVDFSKYGLNKGSKFDAVKTEVEEKSNES
--EEERKELGNGEVVYEYKLDDENGGGVYEYKGDGNGSEESDGGESGNGEQEPERAKVKK
EVFSENEYSFENLKENIKNSCIQNIPININTYINREDMKAGFDYDQIHLINLTNSEKVRN
AIFKKMKEVEKVNDYLCLTDETEENKYSNIFNTNLNIQKSCNNTVSGSGNEEDINFDNID
EQQKDLYFKSNLFNKLKICGQFNKGFIISKIDLLYFKN--ETEENATTDENETDENEYIE
RKNNYALFIIDQHAADEKSNFEKYNKIFTMKSQKLINKIELELSPAQIHIIEKNLVIFLR
NGFEIEIVEEPINKKRKINKDNDIPDEETLVQIKVYLLSLPVFNGKILEVVDFMSLLYHL
NNHPIIFDKGIDDSFLKDKSKLTENTGTWFNYNFPRPQKVWRILASKACRNAVMVGKPLN
ISEMITIKKKLSVLQNPWNCPHGRPTIKYVINNIEVQKCYTNYYLKLYDEVTNLRISKNY
EKYKHIFRDHIFFLIMSTKPMLGPVLKFQMGISKSEEIEKQSIYANYPGLEQQLDMVFAC
HDISKDGKLPYATVEMILRHFLMQCGFMEYVCRFVNEDGKLDLKHVENYLKCKKLLYKLQ
CCGSCMLTLDEMKNLVLVFLKKISDTYLEDQSKWMEKMKSSQEQQGKALEEAMYEYEKNI
LFNHSIKEQQLLQNNRKLDEWNECIENAYEVQQEVLRQFEAKKREQASQAVNKNNELLIA
QNYIEKIKEAASHKKQDNSKCFVYPASSAPCGACTSAGAVTPHRRYKEPRQKKEYSLCI-
--MGHNVQDELVDYEDDDNMLDNKDVKGDIGRNLLNNNNKGVNDGGAMRGSYATVHTGGF
KDFFLKPELLRAISESGFEHPSEVQQETIPAAITGTDILCQAKSGMGKTAVFVLSILQQL
ET-DSKDIKEEKDMNNAGGDASQNKYVRCLGIAHTRELAYQIKNEFDRFSKYLKNVRCEV
VYGGISMNKHVALFKGNNVPHIIIGTPGRILALIREKYLLTDKIQHFVLDECDKCLEKLD
MRGDVQKIFISTPLKKQVMFFSATMAKEMRDVCKKFLQNPVEIFIDDEAKLKLHGLLQHY
VKLQEKDKTRKLIEILDALEFNQVIIFVKSVTRAITLDKLLTECNFPSIAIHGGLNQEER
IERYDKFKKFENRILVSTDLFGRGIDIERINIVINYDMPENSDSYLHRVGRAGRFGTKGL
AVTFVSSQEDTLALNEVQTRFEVAISEMPNKIDCNEYINQMNDVSSNKDNSAN------V
DEYSSDGDETDSLSDINKKDMDSSPVDIEHINVRKSKRMSVSAEAYGEWNKKKENFVPTV
HKKDDKEKEKIRKALNESFLFNHLNHLEMETIIDAFFDEHVEEGDNIINEGDEGDLLYVI
DEGEIEIYKTKKNKKEVLTILKSKDVFGELALLYNSKRAATAKALTKCHLWALDRESFTY
IIKDNIAKKRQMYENILKQVTILKDMDPYERSRVADCLKSKTYNAGDIIINEGERGDTFY
ILTYGNATALKSDQVIKTYTKGDYFGELALLRNKPRAATVKADGVCQVVYLERKGFKRLL
GPIEKILIRNVENYKQVLKELGLDSSCIEASMHLKIMCLSDEVREMYKNHKTHHEGDSGL
DVFIIKDEVLKPKTTTFVKLGIKATALQYKCNYYYKSDKNDSNN-NNKEPEIVNTSFLLF
PRSSISKTPLRLANSIGLIDAGYRGEIILALDNTSDQEYTIKKNDKLAQIVSFSGEPLSF
ELVTELDETSRGEGGFGSTSNKMRTCDPNSAFFGFMGIAASSIFSNLGAAFGTAKSGVGV
CSVGVMRPDLIMKSILPVVMAGVLGIYGIIMSIIISGKMSPAASYSSYLGYTHLASGLIV
GLSSLAAGLAIGIVGDAGVRANAQQNRLFIGMILILVFSETLALYGLIIGIYISLSDASN
LCTPYNVMTKR-ITNSVTTRDDRKNVTKEKFEENDNGINETRNVLTLENILQDNDTDNNK
KVKNKLIFKNKKNNDSSINKNEMRIITIPRQRISSVKNNWLELIKPIVTNLKLEIRMNKD
KIEVRTCKLTEDKNNLQKSSDYIKAYLLGFSIEDALALLRIDDLYIESFQIKDVKILKGD
HLSRCIGRICGSNGSTKYAIENATKTRIVIAGDKIHILGSFNNIKMARYSICSLILGSTQ
GKIFNKLNILAKRLKERFMGNRCCTGRDLLYKSKLQELGIEGSKTIRKLLSFTNNDIIRF
DKAYDENDVQEFVNLCSSTCEIEKLEDRMHPWAADPKTIGALSATQLAILASKENEPHYK
DAIRESNGIPVFINLLKSHELDRVHAAVVALSFLSVDNVKNCICMFENGALPYLITGMKS
NIDGMKAACAQTCRNIFVLDKSYKKEFLKLGGITQLVNLLELPPTDDNLPLYTQLEAIYH
LEDFILNDGDEVPQFLEAVKKSDAIKNLKALQQCPEQDLAEASNVLLLRLTDMADGWEAE
KIFGSTISYTYDDIICMPGYIDFPLSEIDLSNNMTKDICLKTPIISSPMDTVTEHKMAIS
MALCGGLGIIHNNLSIEKQVEEVKKVKRFENGFIFDPYTFSPEHTVADVLCVKNKVGYKS
YPITSDGKVGSKLVGIITGVDYLYLPNQDVKIKEIMTTEMVTGKYPISLSDANKVLCDEK
KSILPIVNDNYELIALVCRNDMHKNKIFPHASKRENKQLIVGASISTRESDLEKVNKLAQ
NMIDIICIDSSQGNSIYQIDMIKKIKSAYPDMPIIAGNVVTSNQAKNLIDAGADVLRIGM
GSGSICTTQDVCAVGRAQGTAVYHVSNYAHTRGVKTIADGGIKNSGNIVKALSLGADFVM
LGNLLAATEESCSEYYFENNVRLKIYRGMGSMEAMYNKQFNSKSRYLVEDKLFGTVYDPT
NDIKISQGVSASLVDKGSVLNLIPHLVKAVKHGFQSIGSKSIQELHSKLYSGDLKFDIRS
INSIKEGKVSDNLIFNSKKMFCKHIKNKRILSGECEKRYIYSSTPNIITAAKFGIPNDKT
VLDGFPARNFSSFMKNVIEQVKKDMKENKQYQEALKELKEKTEIDDKAIKLKKKIEENIN
FLKNIKEKNEQAIKIIYNDINKFVTHCFENYFIFRLSKNITVKFFSGLTKVLLITTDKIT
ELGDKWNDNNAFIQLDKWRQEMAIKRYKKKTGMDEKAINNSE----TDSENGNLKNVKEN
DTNASTGGELILAYESAWDKFGSKLKDMPFLNSFFENPLLGKLFGETELAAALRVMKMED
KNFKLSELMYLFEFVISKHIVESYLIGDEETLRLHCGQAAFNSLNSSINERKKKKLFLDT

NVLIYKDHELKGAQRMEESSPWFIFTFHTQQINCLKNKNDEIVEGKIDDIREVVYTMALS
KHPEPETEGLLYPYIVREFAIIGNTPSWMNHTSFDNILQTKTFKLGIEDIQNIGSSYGEN
NKKIDRYNEEINLLKQH--DLNEKMGKGAIHKVLEPVGAPKITFWYYELKEMKEFQDLVM
YEIKKKKKHFKTLSNSCMKYLCNKEKIKLKKQEEEEKKLKIHSKNISSYMDVFWKKIEKL
VWEEKKRELQKTLNKNKEMRFKKFVKGAIKKIKNARHNAHELFENNMNDLCSNNNSEN-N
ANSNHSNEDKELGEEDLTDQEDEDILLDEEMESMDESEE--QEVNLLDDEANMPVEELLK
RIYGFKSGEEYINLMQNEEDDEDGDTDMDDQVSTQNDSTKPS--------------QNSK
KRKLPDGDGSDREPKTVKMEEAVSDAPNNDETANEADAGNNTETANDVDVPNNTEPVKEE
GNRIGE---------SPIKIDEILECNMDEKHLTKIPPFIKATLRDYQHAGLHWLLYLYK
NNINGILADEMGLGKTLQCISLLGYLAYYLNIWGPHLIIVPTSILINWEIELKRFCPCFK
ILSYYGNQNERYKKRIGWFNNDSFHVCISSYSTIVKDHIIFKRKNWKYIILDEAHNIKNF
NTKRWNIILSLKRDNCLLITGTPLQNSLEELWSLLHFLMPNIFTSHLDFKEWFSDPLNLA
IQKSKISDSKELIDRLHTVIRPYILRRLKKNVEKEMPNKYEHIIKCKLTRRQKILYDEFI
NNKKVQNTLTSGNYMGLMNILIQLRKVCNHCDLFTNKYIQTPYYYILPIQYNIPKFCLLF
ENNYYKDFYLILFLHNEFVSLGGIAKDAHPNQNQNHHYSDIPSKNNDSPFFSPKQSQNSI
LNMETLSGFPF----ECTNLNGITQTNELASKLIEEVNYG------GSFF----NYYKNI
LINLNKNKELKMGSQTVNESVQDLCTYVNNEIYKENIPKNSNEFINELNNNYDILSTFID
PQNKHAGRPSRGGHVGSQHTLYRYNMKVINREVQYKNFFTDETNQTYLNSLEHNLWIKKQ
KEEEMNKIKIIKNSRIPIFGSNLLHLLKTEFSKDKNIVYNHTNNIIINNGSMKEVHVEDK
PNDCASIVLERLFPTMEYFLKLYEKVIQNFIVINCPFVICSPPNILINRNSQNYSNISRE
EDIIKKIKKATRVYHNAFLKQSIIFPLNKDISLGSGKLFALEKLLSKCKREGNKCLLFTQ
FIKMLDILEIFLNHLNYSFIRLDGSTKVEQRQKIVTKFNNDKSYFIFISSTRSGSIGINL
TAANVVIFYDTDWNPSIDKQAMDRCHRIGQTKDVHVFRFVCEYTVEENIWKKQLQKRKLD
NICINMGNFNSQNNRNNNTSLQDHNNKDWFSNVDTIKEIFINKQNNDEDDDIYQDRLLHE
HLENPEKTNVRFEKTLEHVEDKDDINALHVTRRERQHELSQDMQEFTNKNDFQEAYTLTS
YCFNFLNENLTDSLKQQIDEMKMRIEIEMMNAKEDENNSFDSLSNQSDEVENYEQVRNEA
TMVLYIIGLGLGDEKDISVKGKELIDQSDVVYLESYTSILFISKDKLEDYYKKKIYEVDR
NFAEENCEQILDEAINKKVSFLVVGDPLCATTHHDIILRAKKKNIDVQVIHNASIMSAIG
ESGMQLYNFGQTISIPYFEGDYKPTSYYNKIKINLDNNFHTLCLLDIKVKERTIENIMKN
KNIYEPPKFMTVNEAIEQLIYCETVHNQNVITKNTLAIAIVRIGSKDQQIVSGNIFTLKT
QKYNDPLHSLIICAPNLHDIEKEYFDMYSTPIMGSGKPSGLRSARKLRIRRRTQRWADKG
YKKSHLGTRWKSNPFRGSSHAKGIVVEKVAIEAKQPNSAYRKCVRVQLIKNGKKITAFVP
GDGCLNFIDENDEVLVSGFGRSGHSVGDLPGVKFKVVKVARVSLLALFKEKKEKPRSMGI
KGLTKFIADTAPNAIKEIKIENLMGRVVAIDASMSLYQFIIAIRDGDQYGNLMNEAGETT
SHISGLMSRTIKLMENGLKPIYVFDGAPPELKGSELEKRGEKRQKAEELLLKAKEEGNLE
EIKKQSGRTVRVTKKQNEEAKKLLTLMGIPVIESPCEAEAQCAFLTKYDMAHATATEDAD
ALVFGTKILIRNLNANASSNKNKNKNSSKRGYILTEINLEQVLKGLKLTMDEFIDFCILC
GCDYCDTIKGIGSKTAYNLIKEYNCIENIIKNIDQNKYQVPDNFKYVEARQSFINPKVLE
KSEVKIDWCEPKIEELKTFLIKEHNFNEVRVTNYITRLLKARKVTTQRRLDTFFTTCTKK
STKLIIEESQKELLKAKGKGKKREL-NDNSTKLNAKKKKTNIKDEKKNTDKMDELKNKSD
ENFVK---DEENDQDDYDQNLFDEKTNSDSGNIKNENIKEDISSNDIT------MDIPKC
TNDIV-------CMFFSSFPFDSMGGQQPRRKREVNNSKYYESLNLKKNCTTDEVKKAYR
KLAIIHHPDKGGDPEKFKEISRAYEVLSDEEKRKLYDEYGEEGLEGGEQPTDATDLFDFI
LNAGKGKKKRGEDIVSEVKVTLEQLYNGATKKLAISKDVICTNCEGHGGPKDAKVDCKQC
NGRGTKTYMRYHSSVLHQTEVTCNGCRGKGKIFNEKDKCVNCKGLCVLKTRKIIEVYIPK
GAPNKHKIVFNGEADEKPNVITGNLVVILNEKQHTTFRREGVDLFMSYKISLYESLTGFV
AEITHLDERKILIDCTNAGFIKHGDIREILEEGMPTYKDPFKKGNLYITFEVEYPMDLVI
TNEKKEILKILKKQNEVEKKYDIENTDCEVVTCKAVDKEYLKQRLAMQQQQDAYDEDGHQ
PDMEGGRVACAQQMNTNFENNNLYMGWKPNEKIYKTIIEALSSCNNSNNNVQIEVTKVLK
DLNENVSDAALYLLHIFMNKQENNDVRQVGGLLLKNYINSKNKFLTNDILKIIKNEIFKL
VEDEVKEIRNTSGSVITSILTKYEGIEKWPEALYNLLLLVERGNNDVVDGAFRAIIIIIE
DELINRKNTDSLFFQFCKTQLLEKLFLYCSLQEKSIRKKYAAECLDLFINASCFATNGIF
NEYFPQLWECLGFLAAEEDPQILKTVVTCVTIITDTRYASIFNNLDGIIQFMVNATNSGD
RKVQLEALEFWPVLIKDRSYMAYSNYNSNNSNNNKTSENYIDENIYKNINELRNEALKTL
KNYLPYLCKILIDNTVYTKWDYLTMDESHFQNDNANVPDLVQDISPELYHSSNNNNFANS
NDNKNNGMLNDQDEYKGQNNSNNGNCANNNMNVNSGDIDNNDDLDDLSDEEKNDEMTSRT
WGNDWTVRKGAALCLDYLSNVYNDDILEYILPHIEEKLMSDKWNIRESAVLSLGAIAKGC
MYSLSPFIPKVLEYLIKLLNDEKPLARSISCWCVTRFSSWICHPDNCDKWFEPVLLNLLK
RVLDSNKRVQEAACSSFANLEEDALELLNNHLHEIVHTIQQAFQIYQAKNYFILFDVVGT
LIDSVNIVKENIDLAHEIVNSILIKWNSIRISSPYIIALMECMSCITSAYGKEFLKYAKI
VIRTCIKFLVLLYIDLEEEIKYYYSRKVNNSNSFIVNRNNISQTANELLNYYKISNEDYF
INLKDIDSISSSKKKDLIECSFDLLSRILSVINSDIIEIIAENEYNFIPLVHKYCLKVGC
VHIDGIMFSLLLNHNYNTKLPASSVSVKNGVIMENEYTELAKQFLNFGILQSNFALIGDI
SRFCAQYLIPFLSDIIPFLIAHISHPSIPVSNNASWAIGEISIHINSQYIEAYVDEIVKQ
HIYICQNSKYHGCLLQNICITIGRLCSTYPKKIIYYFPQFLKTWLKIMAHGTQENEKINS
LKAILEALYLNLDIAAEHLKDIAYIILKYKYVSQNVNIFFHQFLSTMKQKYPNQWKEIYS
PMMDEHSSPIPADMLNDLSTRFNLMKARINSKIKENAYQEYINKCETYDINEIVNYIKSN
TFFRKYPNSNNIQEDYKAIIDDTVRTKIADLFKKEKAYLKKYEENENNNGKTSPDETRAS
EKKEKGLIYLYNACGNLFKNQDNINGKDKFIIINSDNSMKRISENNINELLIEIIYTIFT
KNETLENSMLNLLGESNIDLIINIIKNKDEIKKDIKLLSKQVDIKENTLGANFFITSTTT
KNTKNKNILSNKENIENIIEYFINNFEDNYLDNVKKIYLPNEENQLEEINVPQNTTYSYS
NNMTKVKINRLPNMIFDQNELVSVNALPFWAKYIFNFQYFNYVQSKVFNAAFKNNKNLLV
SAPTGCGKTNIALLVILQQLVLHSEQHGINLNKIANTKKGLAPEGNGYIEKHNTTSGRLK
GETENENVAESSYDDDNNSDSSVLKKGETVINPNDFKIVYIAPMKSLVFEITTNFREKLK
IFNLNVCEYTKEHSLTSKELELVHIIVTVPEKLDILLRNSSYSSTVSDESLIKSIKCIIL
DEVHLLNTDRGDVIETIVARFLRYSETSQSMKRIMAMSATLPNYKDVSDFLKVERDMCFY
FNEKYRSIQLDKTLYGIHETNMNKLNLAKNLCAYNEVINCLKNDKQCIVFVCSRNDTNKT
IEFLIDHAIKNDEIDYFTNNLYTDYDVNKRIKKSNNIYIKKFYEYGCAIHHAGMSRYDKI
LVENLFKKKSFNVLCCTSTLAWGVNLPVHTVIIKGTNFFSSESGKMEDLDILDINQIFGR
CGRPQYESHGHAILITERTKLYKYIKLLTNNTIIESNFLKNIENHLNAEISMGTTKNAQD
GIKWLEYTYLYIRMKKNPFLYDTDIKNDRELYEKRKSIIIKAISNLSENKLVRRS-LTND
FIGTFYGHIAAKYYVDYKTIGIFASHIESNNYTEIIDVISKAKEFENIQIRNEDMNDFIY
YKNKCDIKETYDESKSMTVRILIEMYLRRIQINNFSLVCEINYIVQNIIRILYAYYEICL
NILKNISHLIINTHNLILSITRRLPMDCGLFRHFCYKNELMDKKYVHTSNNSNRGNADNR
KNQNYTVYLKETAVNILEKKNLSYETIANLSKSELFFFLRNEVYTKQILYYRNIIPNLYI
DGYIQPITQTIMKINLNVQLQNTIWSDQWNNTKEDFHIFLLNTLNNDILYFQKFTIHKKD
RKKIHDISFEFPITNIMPSQITVQFLSMNWCNLSFVHIFNTNNLFINQKINVFSQILPIV
PLSTEVMNNPNYIKFFSFKYFNPIQTQIFHAAFHTDENILLGAPTGSGKTVIGELCILRN
LLKHEDQRSVYICPMKAIVNERCIAWKNKFKTLFNKNVIELTGDKNENKDNIKNSHIIIC
TPEKLDVISRNWKNKNIIKNINLIIFDEIHLLGENRGGVIEILVNRFKNMQNYLNKKIRL
VGLTTVITSVDDLILWLDVKENYLFNFPSSCRIVPCKTHILGFNQNAYCPRMSVMNKDVF
DSINQYAQTKNVLIFVSSRRQTRLTAYDIISLLHLENLLNDKNHIKSEHEQIHVQNLIFQ
NYLNIVENEHLKDLLKYGIGIHHAGLNESDKNIVEYFFLNKIIQILICTSTLAWGINLPA
YLVIIKGNEYYDPKTKKYKDISYTDLLQMIGRAGRPQFDNEALAILLVHEKRKNAIKNFL
YHPMNIESNIMENINEHINAEICSKVIQNKEDMLTYITKSYYFKRLFSNPSYYIKDVQYI
QLFENNRLSNQAKKAIYDHINKIIENTIEFLQKNKCIEAVQEDYIQNYYSTPLGYIACIY
YTKCETAYFFYKTIEPSPKKKFDFYGLLELIAQAKEFDDVPLRHNEDKYNVALRNQIPLD
IDMNMPNVKTYLLLLSRFYECTYETVDYHIDLKLVLDQLARVINSYIDISLLFHNYTYIK
TLILIFQCVNQRIKPYTNDLYTIKGITNSQIYKLKELQIKNIKDLIKFDKSFLYSLNIFD
TSQINYILQIPNLTTNIKLYQKNGNTDTPNKINNFTNVKLGKYSKNENKYHFKISYYEKN
EILIKVFFNILNKIFKEANPSNTVSNAQWYAEMETINIKKEDNEAQVKIPQEIHDEEVEK
RYNDIMSITSECIQPEELKLKLLQKRKLICYDGFEPSGRMHIAQGLLKSHIVNTLTNNGC
TFIFWIADWFAQLNNKMSGDLNKIKKVGQYFIEVWKSCGMNMENVQFMWASDEINKNPDK
YWSTVIDISRSFNINRIKRCLTIMGRTEGEDNYCSQILYPCMQCADIFFLNVDICQLGTD
QRKVNMLAREYCDIKKIKKKPVILSHGMLPGLLEGQEKMSKSDENSAIFMDDNEADVNRK
IKKGYCPPNVIENNPIFAYAKTIIFPHYKEFNLTRKEKNGGDKLYLTIEEMEKDYISGDI

HPLDLKDNVALYINKMLQPVRDHFQNNAEAKKLLSEIKKYKVTKINKNVISPTVKHNGPL
KVSIIGSGSWGTVISKIISENTQRSKIFHPIVRMYVNEEIVDNEKLSDIINKTKENVKYM
KGMKLPNNIVAIPDINKVIEDADLLIFVVPHQYLETTLSEIMKNKNLKSTAKAISLMKGI
KICDYKPMLLSNIIEKMLNIECSVLSGSNIASELSTESFSEATIGFENLGTAEIWRELFD
RNYFKINCIQDKAGVEMCGALKNVVALGIGFSEAFKKSYNTKSAIIRIGLEEMKKFAKLF
FPSVLDETFLDSCGVADIIATCLGGRNVKCATEFAIRNGQDSWDKIESELLNGQKLQGIH
TSKEIYKILENRELKNEFPLFSIIYEIAFGYKNPSSITNVLSTKKLRHIKYRKMERILSS
IGRLSVFAGGLSLIPYTFVYDVDGGERCVMFNRFGGVSEKTYGEGSHFYFPWFQTPYIYD
IKMKPKVINTTTGTKDLQIVTLSLRLLFRPHTKHLPYLHSTLGPDYDERVLPSIGNEVLK
AVVARYNAESLLTQRDTISKEIRESITARAKQFNIVLDDVAITHLSYGKEFAKAIEDKQV
AQQESERVKFIVAKTEQEKIAAVIKAEGEAEAAKLISTAVKQYGNSLLEIRKLEAAKEIA
ENLSKSKNVTYFPSTSNILLNPKNLMKLDYINAIKEENGGYNFENLKRNEILKEKGVTFP
QFRKTGTTICGIVCQNAVILGADTRATEGPIVADKNCSKLHYISKNIYCAGAGVAGDLEH
TTLWLQHNVELHRLNTKTQPRVAMCVSRLTQELFKYQGYKVCAIVLGGVDVTGPQLYGIH
PHGSSCLLPFTALGSGSLNAMTVLEAKYRDNMTIEEGKELVCEAICAGIFNDLGSGGNVD
ICVITKDGTQHIRPYKQPNTRLYHLAHPTIFPKGTTPILYEKIESIKKHITIEDAMKSIK
NGMYNFNPSKEDPFGKAPAGYVAGKGRGVTGFSGGVSRDDITEDKDKNDYSDFNYDEFHG
YSESLFKDTEYDEEDKEADDIYEAIDSRMDVRRKSRRENKLKEEILKMRAQKPTIQEQFS
DLKKNLANVTLEEWESIPNVLNYSRQKQKKMPKSYLPAPDSLIMSKLNESNSHLNYSASS
GSGL--KTPLGMKTPLGLYTPIGLGFQTPLLKGSTGIDTPLFGKNNRSGAINSGLSTPFT
LSGYATPLNSSNISGYNTPLISNASMLSLNDLGEARGTVLSVKLDELIDNVEGQTVIDPK
GYLTNLNAKNLTSDSDIADINKARSLLKSVINTNRKHGPGWIAAARVEELAQRKDKAKEI
IMKGCIECSKNEDVWLEAVRLEDKLSEAKIILAKAIKNIPTSVKLWLEAYKKEKNVQDKR
KVLRKAIECIPNSVVLWKEAISLENENNAYILLKRAVECIPQCIEMWIALARLCKYSEAQ
KVLNEARKQIPTSAEIWINASKLEEKQGNINMVDVIIKRCIENLSQKNVVHERDKWIKFA
EECEQSDFLHTCQSIIKNTMNIGVENLNKKRIYKQDAQNCINNKSLHTARCIYNEALKIF
KTKKSLWLDLANLELTHGNQANVDDVLQRAVKNCPHSSVLWLMYAKQKWLNNEIDAARKI
LAESFMHNQNTEVISLAAVKLERENNEFERARVLLKKSRVQCNTPKIWMQSVQLERLLGN
YNDAKELVHEALKIHKKFDKLYMIAGQIELEMVELKENNLTSAYDKAQQIYQQGLKFCPD
SINLWLCAIDLQITKKSYTSARALVEKAKIKIKNIHSLSINTKILKNKEIIESNEQYHDE
EIASNLNHSKNGDSNNDNISNSKNELENKI--NASVKVIENYDLLWLKLIEIELLCNNKN
INPIISEALKECPTSGILWSKAIELENKNLQNSKSVTAFNNCGNNSYVILIVAIIFWNNY
KIVKARKWFHRAITLNPSFGDGWATFLAFEIDQENEINQKDIINKCIKAEPNRGYMWNKI
AKRRENWRLSYPQKMYKYIKERFPQVLNKPISENILKIISDENVVLPTSRK-KKMGKFSN
SLVLSEFKKLKNFFAYTKKGCLKNKDNKVDETYNEMASEQVNNNGDIIPDEVNDKKKEKK
AKKLEEKQLKLAKKLERENLKNEAAKVLEHVCEDINKENYGYVKLNMMMENGKNIHLYNP
EEIYNSLYGEKNIDNEKREDVESEVNHKLEEGSNLWVRGRIHDIRSKGSLAFIILRNKIY
SLQCILDIKNINNDKNMIKWVSNLSLESIVDIYGKLVKPEISIDSTIIKYEIHIQKIFCI
SKNSKELPFLLKDANMKETNDEATIRVNQDNRLNNRCIDLRTYANYSIFYLQSEICKIFR
NYLIDNNFTEIHTPKLLGESSEGGANAFQINYFNQNGFLAQSPQLYKQMCINSGFDRVFE
IAPVFRAENSNTYRHLCEYVSLDVEMTYKFDYMENVYFYDSMFKNIFNKLVNNEKNQLFI
KNIKNQYPSEDFKWLAVTPIFTYEQAIKILIEHKKLNLQPEEILTYDMTTDMEKELGKII
KQTHNTDYYIIINFPSGLRPFYTMYNEEDPKISNSYDFFMRGEEILSGSQRISDVKVLLD
NIKKFNLNPKKLDFYIDSFAYSSYPHSGCGIGLERVLMLFLGLNNIRKTSLFPRDPKRLT
PMEITNAFKRLF-RQKFEYIERNSIFDTRQKKDEEPNRKKGRNEIDNYQKKKYGDIDNIN
DIEKNKHND-DDKNSKDANLMPFANTFDAPNALLFENLNKEYKYITTQDNFDGFRFEVDK
NVNKYLQSTHTLFLGTSLRDVGYLYQFGANFASSDNTLLMISRVNLDGSVNGRFCKKINN
FFDCKFNFNTYNKNDPRNMYEMSLEVNNPQNTYNFKTIWQGAWIFNASYTQLLTKKLQAG
VDLTYIGSNCASIGSFGLRYNHNNHVLTMQCVRQPNFKSPEFMLNQAHSYKMQYARKISD
RLSVGTELELTPETKESAMRLGWDYSFRHAKVQGSIDTSGKIAVFTQDYSGFGVSGYIDY
LNNEYKFGFMMHIAPSQEQPQPQ-MDNQEFIPQYKLILVGDGGVGKTTFVKRHLTGEFEK
KYIPTLGVEVHPLKFQTNFGKTQFNVWDTAGQEKFGGLRDGYYIKSDCAIIMFDVSSRIT
YKNVPNWYRDITRVCETIPMVLVGNKVDVKDRQVKSRQIQFHRKRNLQYYDLSARSNYNF
EKPFLWLARRLSNQPNLVFVGEHAKAPEFQIDLNIVREAEKELEQAAAVAIDEEDIENMD
KLKTVYVDSALSIIKGALCVILQIPTGRTTESVKKKQNFIGVLTVNSIKSEPTISQYDDI
KKLVKNKILENAPFYNYQIERSFADKFYGECMYDNFGLPKNINEINLIILEEWNINCNRN
RILKHTGLIKNIEINNFKYLNNKESLEVHFSVNPMYTFEELSTLYKNEKSLHNFLLCPIR
KVRTGSESISPDGENCEEYIYINTEDLLEKNKVLPPSGVENIGYERSNEVTPWNVNINAD
GIDYNKLIKQFGCSKIKEEHIKRIEMLTNKKAHHFIRRDIFFSHRDLDFLLNYYEKNKSF
YIYTGRGPSSLSMHLGHLIPFYFCKYLQDAFNVPLIIQISDDEKFLFNKNYSLDYINKLS
KENVKDIIAVGLNPELTFIFKNTEYVHNLYNTVLSIHKKTTLNQSMNIFGFDHSDNIGKL
SYPSFQIAPCFSQCFPHFLPQNLPCLVPQGIDQDPYFRLSRDIAVKLALHKPVVIHSIFM
PSLLGVNTKMSSTTKKSENKMTSNEHNNSVIFLTDTPEQIKNKINKYAFSGGGATIQEHR
EKGGNLDTDISYQYLRYLLEDDDKLNEIGQKYKSGELLSGEIKKILIDVVVDLIQTHKKK
RDSLTDEQIDYFFNPNKPALMKFKNIMMMNGDNSKKHEIEIKSISKNEMSFILYNSNTAL
ANALRRIMLSEVPTLAVDIVNVYENTSPFHDEFIAHRIGLIPIDSRNIKSYEFRERCKCK
ETCSRCTVQYIIEVKCNNANKIDVSHYDIEALDHEPNIPMPIPNDNKHNNIDKDTAIPIV
TLSKNQTLHMKLTATKGIGKMHAKWIPANVSYIIDHKIVINHPEVDKMPKEHKLIIANNL
NPDCYILKEMDDDRDVELQLSENMSVVMAESCRDTLTELGYKDIVKVIYDETKFHFKVES
VGSMPPEQIVEMAIEILESKLKDLEPQIKASFYSIEEVAKQLKEQGVSLYGIQLDLEMYD
STSKIKDLSLAPFGKLQMEISETEMPGLMTIREEYEKLKPLKGAKITGCLHMTIETALLM
ETLQKLGAKLRWCSCNIYSTLDYAAAAVSTLENVSVFAWRGETLEEYWWCVEKALTWENG
EGPDLIVDDGADASYLVHKGAEYEKLYEEKKILPDPETGKNEEERCFLSLIKSSILKNPK
KWTNMSKKIIGMSEETTTGVLRVKKIEKNNGLLFTAINVNDSVTKQKYDNIYGCRHSLPD
GLMRATDFMISGKIVVICGYGDVGKGCASAMKGLGARVYVTEVDPICAIQAVMEGFNVVT
LEEIVEKGDFFVTCTGNVDIIKLEHLLKMKNNAVVGNIGHFDDEIQIADLFSHEGIEIEN
VKPQVDRVTLPNGNKIIVLAQGRLLNLACATGHPAFVMSFSFCNQVFAQLELWENRNTGK
YENKSYILPKELDEKVAYYHLKKLNATLTELDDNQCEFLGVSKNGPFKSEAYRYMYSYGM
EGDDTYLPQVQYPSPYENQY-QDEESPSPRGENHTPFIGYFSSHLLRTGFLLQCVSLILM
FVFYWAFGGTGIFIFDLYAGPECVKVSSAFHLTISVLMSIYLLGTLYIAMFQVFVADNSK
WCRGFRAGSKLLSAAVTLDLLSSILRLVQYLYAYFYMNMRWWARYQQTKSDWTLLHFGSI
VHSFALFMYGAAFFYMEAYHDEGTYEELAWSNLTLFKLAGLAELLMVFSGFGAFFSILLL
GAIMCATVWAFSFEPLLEKWSPELHSRDINADVLPEIKHDDENGGYNEENMYEPYNPNPE
NMEYNEEMIKHNANEKYINGNANFYDHNNGIPTTYDYSQIEGQVKQNAEMGVSENKYIAQ
YMEDPNKPKKRTFRTFHYRGIELDKLLELNQEELVKLLPARQRRKFRRGIDKKAKSLLKK
LRKAKKECEVGEKPRPIPTHLRNMTIIPEMVGSIVAVHNGKQYTNVEIKPEMIGYYLGEF
SITYKHTRHGKPGIGATHSSRFIPLKMSNKKVQSPKEESIAKMLICKVHIGTKNLENKMK
RYVYTRAKDGVHIINLAKTYEKLQLAARVIVAVSNPADVVVVSARPFGSRAVLKFAQYTG
AQAIAGRWTPGMLTNQIIQKFIEPRLLIVTDPRTDAQSVKESAYANIPVIALCDSDSPLE
HVDIAIPCNNKGKESIALMYWLLAQEVLYLKGSIPRSQAWDVMVDMFLWRDPEQFELKNL
ANEEATPTAPHLADNQYATDAPYDDWNKKDDWNDNANEEWKNPIAVDEWMSMNRYNFKTM
SSLLKNKTPQHFDIQPHTNIKCIVHPSVIFTILDSYLRRDEDQTHVIGTLMGSIIDTNLV
EISDCFVDKHSLNEGGFLQIIKDHHETMYELKQKIRPRDQVVGWFCSGSELSELSCAVHG
WFKEHNSISKFYPHSPLNEPIHLLVDASLESGFLNIKAYVQLPINLVKEYFVHFHEIQIE
LLPCNVERADVAKITDKGTKGGDNNS-----KDKLLNHEIDENSLKKLLIMLKNCKSYVQ
DIVDKKRKGNVAIGRYLHKVFSNDPFVSIEKFDSINESILQDNLMISYLSNLAHLQFLIA
EKLNASSMQMDKIVEIIEGLSNPDSRIRTECENTLNYYKKNDLNNTVLSILKLLKSHKSS
QVRLQCAILVRNLFRGYIKSDKDKADDEEENYWDLLSNNLKNIVKSELISNIGIETDKMV
RSNLCSNIIDLSSKLLLNNEWPELLSVTFEFCNSNNNDVLISGYKILGGILNCIPDELHG
KNEMISSICMKGLNSPSVQVRSECINLISCIVEDNNSPLIKCVQPCIPLILQSLSLMVKN
SVSDVAVLDECEKVLQSIGKMIDYNAKFFSKYISNLCDILFDICMKNENELNYDFDNSLK
SLSIEALITIPERRPKTALSVPRFLDKIINVSLLFMLDINNDCFNEWMNSLKESKDENQE
LYDIGEESLDRVGKAYSELSDSEFIHILYNKVSEYLMKNTWEHKYVAIMAIAQTIEYLPE
DEIEEQLENVIKMLLQVLLDQDVRVRYAACQAIGQISLDHQPYVQKEYPRQIITSLISTM
NDVHLRVQSHATAAFVNFAEELEKSALLPFSDMIIEILLQKLNTTNYLLVREQAVTAIAV
IAGVIEEDFLKYYPTVVPLMKEIIQKAVSEEERTCRGKAIECISIIGLSVGKEIFIEDAK

ECMSALLQISSTKMDPDDTVKEYIQEAIGRICRALGNDFYPYLSSIVPTILSLLSISPKP
LVDDDDDLTITMVSNGQYVGLKTSLLEDQEKALDLLIIIIEVLKENYKEYIEATASAILP
MLNYELSDEIKQKALSAVSELIEAARIISEQTDNNKSMLLAILTTSAEKVLKSLSDTKLD
DNYEYVLDIMIIESNGLYMCLQKAGANILPNNTLKMFFNEIFKLLQYSTDRRVIYNQKKN
NEDVDDDELLIIDREEELEQTYRTNLLDILGVLIKHHTNQFLSTCCDICITFINNYLNSP
HAEDIALALYVCDDLLEFLQDNSVCLWEYFMNPLLLNINHADHKVKQAACYGVIQANKIE
AFGKYANLAVEYLLKLLHQSGPSKKPKEYISAIDNAVAALGDVVLMHTSKFNNAEELIKL
WLNHLPIKEDESEGRRVHKNLIDLVSQNHPLLFGKDNSNIGKIIEIFLSVYETDFSDSDC
NKKIVSLISSLDQSYLSNLASSSLTNKQSKKLNHIMNASRKMPTISVHEEDLVEKLGKKY
NDEELINICFDFGLEIDDVEIKNGKKIYKIEVPANRYDLVCAEGLCRSLKSFIGIHEDIK
YNIIKNCSNNKLNEKHLLEVDSSVDKKRGYVVSCVLKNIKMNDQIYNNIIELQEKLHHNI
GKKRTVLAIGIHDYDKIKFPVKYKFEEKNKINFIPLNENKNLNGCDLFKFYDDNINLKPY
LKILKDFDKYPLIVDSENNILSLPPIINGDHTKITINTKNLFVECTGIDLNKLEICLNII
SSMLSEYCQPKYTIHSVLVSYNENHELEKGNKHLYPNFKNKKLTCDIEYVRKLSGITDIT
IEDIKKLLKKMMIPVTNVINNTTFEVDVPFYRSDIMHACDIVEDIAIAYGYGNIKHEPIE
ISKKHLLNTVSDMFRNSMTECGYIEVLTNALLSMKENYDCMFRKHIDYDTTNKLVDSYNP
LYPPVQIMNSKTSEYEIVRTSLIVNLLKFVAANKHRELPLRFFEIGDISYTIYNKTDTNA
FNKRNLSIIFADKVTAGLEEIHGVLESILKDFQLFSHYKIDEKRKENIHIRSDVYYELVP
INDPSFLDERVVNIVLRPHNLTFGIMGIIHPNVLENFSINIPVSVIEINLDALLNVLFVA
ADNTGTSGNSPVISIGNIRGLGGCDYGSFRMSNEFLGWKNKKTNSVHQYKCSDISEGEWI
KLSYNNNRLHLKFNESKDHLIVFFDGFPDRNLAEITQHFQKYFNIKLGNRKLATKGWNWG
EFKLENSNLIFDIDKKYAFNINTNNINQLNVQIKTDIAIELKNDEKQNTNEDVLSEIRFY
YPHENDENQNFQDLKNNLLDKVNIGDSKSECIASLSNIPLLVPRGRYEIELYSKTFKLHG
KSYDFTVQYSNINKMLLVPKTNSNQYILIFSLNNKIKQGQTEYPFILIQLNNDDDMDLDI
NAPEEDIKNYKLEKTLTGKAYDVVTRLFTALAKKNAIIPGDYRTAKNEHGITCSYRAASG
QLYPLNKYFLFVVKPVILISFDDIVTLSFQRTGNINQHRFFSLIIKHKRGMSYEYTNIDK
SEYAPLLEFLKSKNLNIQDDANVTEKKADFDDDDDDL--------SESDEEDYVAEEEDE
EDDNDDDD-----EYDDEDDDKMGEVRSKTDLGDCRFYEKKFPEVDDLIMVKVNRIEDMG
AYVSILEYNDMEGMILMSELSKRRFRSVNKLIRVGRHEVVLVLRVDNQKGYIDLSKRRVS
PKDIMKCEEHFSKSKKVHQTVRHVAQKHNMTVEELNRKVIWPLYKKYGHALDALKEATMN
PDIIFKEMDISDAVKESLLSDIKLRLTPQALKLRGRIDVWCFGYEGIDAVKEALKKGKEI
SNNEVTINIKLIAPPQYVIVTSCHDKELGMQKIQEAMKVISDKIKEYKGGDFKQQGEILV
IGGDDEKRLEELLDKHDGLSSDDE-YSSDGDDDDSSDEDDNSSDEDDD--ELYLNMYEDI
YRNKKSSNDANRIDLEKEVKLRLEEQSNLLSIKGIDRVDIKQKKGKHSIICIHYIKNMCM
KNLFCNYLHQLIYDRIPPCKNYIKYNYCSDKIRGSCMFRHTLENTNTNYYSENKEESLDE
ALKFLHEKNICVNYLLGFCNLGYNCRKIHKNRSIKNIINILPKFYLDQILINKNLYTNLY
KNQQVLNDMNKLKDALIVLSGEKYQEKNTSLTKNDRENSDMLINSRMINSGTNIHDDKNL
NRSVLNMLQNNNENMLQNNDPNNIKGVGNIPEVYNLDNTPVTSDKIKVFVIKCNQISNLY
LSILYGVWATGKNNTRKYMNFFKENYTIIFLFSVNESGGFQGYAKMVTTPIKNLYENLWG
PITNRLGGNFRVQWIKIAKIDFDVFKNITNPYNDNLPLKKSRDGTELPLNIASIICNKIH
ALPNEDFLAGTIYEYKRRINHSVYFTNLYKKNMLNTNTMWDSIIFTLNQKSDCQQITYID
GNEQNISMYNPLGINILDNNLTQIFRDSEEWLTLEYFLQMNARTSRVKLVQAWNISPVEV
VNKFKKRNFEKLVLKSFIDTSSMDMNNTIQDVCVRGFSISKKGLKLSIGNFNIPGFPLIS
LNKDNPHSNTYELTYEQNESNEGKKSLKRIMEQSENILLSGERGIFEFFVCDVGVGLSLS
VNEDEVNSYDRDILPMEYDSIFIKKKGKNNLVDSLTIHYKNKENNNNEKEINLALGGDVM
PSYGVLPYYTFNHEYVIYDSSQLLPRYLIQFECDPSAEEVFSIPLCDYCGNAPSLYYCES
DEVKLCEKCDNIIHSQNKLVKKHIRKTLNEAQSNFGNCKIHLQNEVNMFCTVCHIPICNL
CMCSHAHKDLSNVNLFNFNNNSDTIISLKMAYNAIMQHSSKPSNFIKERKKNLNNLLEKI
DKLHEQVSLNMNEAEKNVYTALEDLVKQLHTTTDKKMSSILSEEYELKRQFNEIMWNENF
LYYLQTILPPADFMNAWLKHCQYREEIEQNSEHSEKINSLIFPDIRIKGNINVITEGSIN
HENIFHSSDHLMAPKKKEEEPKVLLLGRPKNTLKMGLVGLPNVGKSTTFNVLTKLNIPAE
NYPFCTIDPHEAKVNVVDERFDWLVDHFKPKSSVHAYLSIFDIAGLVKNAHLGEGLGNNF
LSNIAAVDGIYHVVRAFENEDIIHTEGNINPVRDMEIINSELIYKDISNCERNLEEISKV
LNRNKKDKIKQNEHDVLTIVLEHLKEHKWIKDRAWKSSEIEVINEFNFLTAKPVVYLVNM
SEHDFIRQKNKYLAKIYNWVQEKNKGTIIPYCAEFEQKILSMTETEKEEYFKANNIKNSM
LNKIIKTGYYEINLIHFFTCGQDEVKCWTIRKGTKAPQAAGVIHTDFEKGFICAEVYKYT
DLVEFKSEGEVKANGKYLQKGKDYVVEDGDIIFFKFNVSSSGKKMSEYNLKKDDSQFYKL
DTIKCDVPISPEFSKRIDKFVNQLRISYGTLEEFVDNFVYELKKGLEAHRRHPNLWIPHE
CSFKMLDSCISDIPTGQERGTYYAIDFGGTNFRAVRASLDGNGKIKRDQETYSLKFTGTF
SHEKGLLDKHATASQLFDHFAERIKYIMGEFNDIDDSSEKSVGFTFSFPCTSPSINCSIL
IDWTKGFETGRATNDPVEGRDVCKLMNDAFARSSVPARVSCVVNDAVGTLMSCAYQKGKS
APPCYIGIILGTGSNGCYYEPDWKKYKYSGKIINIELGNFDKDLPLSPIDLVMDWYSANR
SRQLFEKMISGAYLGEIVRRFMVNVLQSASSKKMWQSDSFNSESGSVVLNDTTPDFSECK
KIAKQTWDMDFTDEQIYALRKICEAVYNRSAALAAAAIAAIAKRIKIIEHSKFSCGVDGS
LFVKNAWYCNRLKEHLKVILADKAENLIIIPADDGSGKGAAITAAVVSLSNSMKQLPMLD
IYIVLRWLCKAIVSSLFGDVNIINPENVPLYGSVIFVGNHNNQFIDACVLVASIPRQIKF
IVAEKSMKRPVIGELARLAGCISVKRPEDLKFKGIGRIYWNTGDTKIKGINTRFKLDVQI
GDKLMTQNKMFTVTKIESEIELILQNPININCEDKVNGVPFKIVPKINQTEVYNLVTHSL
KNGDPIGIFPEGGSHDRTNLLPLKPGVAIMTLCALADGIEDVSIIPVGLSYSKLYQLQGC
VTIFVGNAIIASQDLCNDYNNNNRETISKLLAKIEEGMRSCMLTSKNHETSRCIELCVSL
YTPERMTISKNKIYNILQLFSEMFWKFGNSKEIENLCYELQCYEKLLQANKIKDDEVWML
KQSTSAATLKFIEHICSLIFCIIFGMTFSLLWLPLVAISVYLAENHRKMSLKNSLVKIQG
GDVVASYKVLVLLVLLPTFNIIYGLLFSLYFYKSWLQRIAFTICSICILPICYYININYS
VQIPTLLRQMKILLKVICGIINVWRDNERELISTRHELQLKVRNTVSKLGHKVSDNFLEQ
LHRNIPKFVINADTKRLIRGKDEWVPILKRSQLEYREEILMDGDNKEISGRDIYQIIKNA
RDKIKIDYKFWYTQPVPKINEEFSESINEPFIADNKVENVRKDPYKLPEGYVWYVCDVND
ENDRKEVYNLLTDNYVEDDDNIFRFNYSSEFLLWALTSPNYLKEWHIGVKRVDTNKLIGF
ISAFPTDICINKKVVKMVEVNFLCVHKSLRSKRLAPVLIKEVTRRINLENIWQAVYTAGV
YLPKPISDARYYHRTINVKKLIDVGFSSLNTRLTMSRAIKLYKIDDELNLKNLRLMKKKD
VDQVHKLLNNYLSKFNIYVKFTKEEIAHWLMPIQNVIYTYVNEENGEIKDLISFYSLPSK
ILANEKYDMIYAAYSFYNVATTTSLKNLMQDAICLAKRNNFDVFNALEVMDNKSVFADLK
FGEGDGTLKYYLYNWKCASFDTSMVGIVLLMSEVNVTKVIVNNPICDILDPFVFTIEFEA
LNKLEADLEWKIFYISAVNNEGESNQDIELDNIYLGPIERGVMMFDYAVNPPDYKNMDAD
SVLGLQAILISANYKEKEFIRIAYYMNSFYKDIELREKPPVVPQYDKICRHIFVDNPRIV
KFSIPWDSEERDEFKEFDKENEKIELLNFSKMKEENQSNSNITNQSTQDNILIPNGLNYN
PNNGNFNTPINNNMQIGMQNNMNSFNYIKNNEMPVDMDRCEIINENIKGISSITIDNKNM
MKIYLISYVFPLMVYLLYTLLKDQSGFSQYNDYLISKINDALLYFNIYEIKFPSSYTNNE
KILGLHIRDIEEDSRVAYEILKELTQKNYFRIFKVNLHIPCKLQKISEKCNEKTKCSVCE
CTEDEIPYNFRTNEVEIIHDQYAQEDLKQTFIASKLYKDILGTYDPSDEGFLSYVDLIYN
SPSYTAYEGKSIWNRIYLENCFQNGENTCTEMNNFYKIISGMQSCIAALSSEYYYLKNDF
VFGDMYTDNTIKNHYIKKIDYDYNISFFKDKLALYPDRIENLYFTFAILLRALCRLKPLF
SQCKCNSGIKENDKDAFKLLNEFLEKQYHSCSSEEFLEPIFPNHGKEILSKFMNITSILD
CVPCIKCKLHGKLKTTALQIALVEGGHEHIGSLERNEATALINAIYHFADAILIIKKFEE
RLKLKKTLFMFYVVSVTLFILLVVVSVIYITIRTRHKKKIKAAMARNVEKGRSMLNQWLK
AKELNDKKTFFKIPKNVNEVDDLESAVSYRKSIIKEICSKIKEIQNLSLGDQHVRELNDQ
INKLISIKNRWEIRIIELGGPDYQSESNALINAHGSELKGNNNYKYFGAAKNLKGVKELL
FKENDDRKKLLLKRKKEKRNLDKIVNIHYFGYCDEENEILLNEELKIQKKLEKTDLEIIK
KINY-MGIFRSETMKHGTLVLPADRAREYMDCLGKQVDIQFIDMNEKTMKRQYKKYIQRI
DDMERILRFLEENINKLPNVKIKKSKIENFLEHDHIYELDQVEESLNRLHVQFVRFCNNN
KDLVDERNSAIEEKHVILTALNQLHPDMSKRSNLR-SMHDDNIDENNLMNN----SEEDA
SLSTHIMREGINMMFTNISGVIKTKDQESFSRTIFRALRGNTYTYFQNIDENMNSSGTLN
NENSSLDNKIGEDNNENKENGGNSNELKSVFVVYCHGSTHSSIYEKIMKICKAYDVRNYE
WPKTYEQATKRLSELKEIINDKEKALKAYEEYFINEIFVLINVVEPNKNSLIEEWKLFCK
KERHIYNNLNYFEGSDITLRCDCWYSANDEEKIRHILMNKSSNDLVSALLLSDKLLTPNI
SPPTYIKTNEFTSTYQSMVDTYGIPRYGEINPAISTIVTFPFLFGIMYGDVGHGICIFLF
ALFLIIVHNRMKNKNSNEMLSMLFNGRYMLLLMGFFAVYAGFLYNDFFSMPLNLFTSMFE

VDKVVDSVEYYKRKQILNAETGQMEDAPPYIFGFDSKWLGADNELTYINSFKMKFSIIIG
FCHMTFGVIIKGFNALHFKKKMDFFFEFLPQFVMMLSMIGYLVFLIIYKWVTPIGYGGYK
KQGIINTIINMYLMKEINQDNQFYEHQEIVQAIIITLFALCIPVMLFCKPAIKTYKMMKE
KKKRMAMHYQTVEKEMTNQFNGVGIYGNN-------SMHKRVTKIGYAENEDEYLLLKRR
GRKTDDEMEAHLLGPSSYHSSDDANANHGSDEHEENLSEIWIEQLIETIEFILGLISNTA
SYLRLWALSLAHQQLSLVFFEQTILSSLEKDSFMGVLISLIIFSQLFSILTIAVILCMDT
LECFLHSLRLQWVEFQNKFYKGDGIPFKPFNIKKLLSERDMLGIQEGRAFFNAARNPNNR
PENNIRIEDNLPSLNEVYNYFQDFLSRYSSNTLKEGNLKRTLFENVKNNNFTLDLKLDDI
YKQQTVFENNEISATPISERKIKKPDNYSAILARALSERPLTYLPTIERVCYEVCETANI
LSDEDEHLNYIQINLLNTFIRPTPIRGLLAATQERFVVVPGIIVQASKPQHKMRKITLQC
RYCDHKMSIDVPLWKDKPQLPPYCRYSSTMKSSMGVANPMDNQLGCNGVLEPYVILPNEC
TFVDIQSLKMQELPEAVPTGDMPRHLQLNATRYLCEKMIPGDRVYVHGVLTSYNPNPKPT
RVDGTNFSYLHVLGFQKYDDMSGNDLNFDVEERNELTLLAAEHDIHDKIFKSIAPELYGM
DEVKKACACLLFGGTRKRIGEETKIRGDINMLMLGDPSVAKSQILKFVNRCAPVSVYTSG
KGSSAAGLTAAVMRDSHGVFSLEGGAMVLADGGVVCIDEFDKMRDDDVVAIHEAMEQQTI
SISKAGITTMLNTRCSVIAAANPSFGSYDDSQDTTDQHDFKTTILSRFDIIFLLRNKQDI
EKDTLLCNHIVALHASKHKSQEGEISLSKLTRYIQYAKKEISPLLSKEARDSLRNYYVQT
RAEYRGDKRSVTKKIPITLRQLESLIRLAESFAKMELSQFATEKHVQMSIDLFSASTAET
AKQCMVFEAMSPSEQKAVKQAEDAILGKLGKGQRASRVNLFRELQLRGFDRSALSKMPKS
YRNYSKTARNPKRPFEKERLDQELKLIGEYGLKNKREIWRVQYLLAKIRSAARYLLTLDE
KSPKRIFQGEALLRRMVRQGLLGENEEKLDYVLGLTLPKLLERRLQTKVFKLGLAKSVHH
ARVLIRQRHIRVGKQMVDIPSFLVRIDSEKHIDFATASPFGGSRPGRVKRKTLRNQKEKA
EGDDNMVRQSQSMYDRHLTIFSPDGNLYQIEYAIKAVKNTNITSLGVKGENCAVIISQKK
MATQYITQDKLLDYNNITNIYNISDEIGCSMVGMPGDCLSMVYKARSEASEYLYNNGYNL
NVETLCRNICDKIQVFTQHAYMRLHACSGILIGMNEENKPELYKFDPAGFCAGYRACVIG
NKEQESISILERLLEKRKKKIQQETLEEDIQNTIILAIEALQAILAFDLKANEIEMAIVS
KTNPNFVQISEKEIDNYLTFIAERDMDNNDNIDSNDNSSDTNTANYNQGKNDIISEEIDF
TKEDNELSGSTKNSKSKNDSKGK--SDKDDINMDYSDHTFPCHPAPPVSIKLFIGRVPKN
YEEEQLRPIFEEFGIVNEVVIIRDKITNIHKSSAFVKMASISEADNAIRSLNNQRTLDQQ
LGSLQVKYASGEVMKLGFPQNVESGVDQAKLFIGSLPKNITEESIKDMFSVYGSVEEVFI
MKDNSTGLGKGCSFVKFAYKEQALYAISSLNGKKTLEGCNRPVEVRFAEPKSSKQAQSQL
GMQPMQNAPHGISPQAHPGTPNNINYAGNFGVNNNYPRQVGVWKEYYSGEGRPYYYNEQT
NTTQWEMPKEFETLFMNNNPNMHNLSDSSGPPGANLFIFHVPNEWQQTDLIQAFSPFGEL
LSARIATEKNTGRNRGFAFVSYENIESAAAAISQMNGFMALNKKLKVTVKKGEEEEMKKY
ISQNGVNTFQQMARQQKNIPSQPNAMGQPNFVAHQNPQAQNFFYSNNNSYRCGPYMTTAH
RPTWYNAIGGENQGGNRKVGQTAKVCSRDLPGHTKMKMRDLSDYTEDKEIIKNNLIELEN
KSSGREGN-NKLLAIENIKKLTNCDYNNPFPEDEDDEIQDDWKGHKTKKKKK-NNNNNYE
QSDNMSEDNSSANDSDTN---SDDDDESDEMDDESDEEEKELMRELENLKREKLEKLKRE
KEEEELMKNKKNNVLTNNPLINLEDSDNEEKQQKKRKWTDEAIFRNTCEKKPKTNTFIND
TVRTAFHKKFLFKYIHMDSEETINLAVKYAKDAVVEDEKKNYKEALNLYIQSLQYFNYFC
KYEKNDNIRELILKKMEVYITRAADLKEMLNKKETIETKEKVGTSEEAKESMKKQIKEFI
LNKDQSIKWSDVCGLETAKEILKEAVIFPLKFPKLFNSSALPYKGILLYGPPGTGKTFLA
SACANECNMNFFNVSSSDLVSKYQGESEKYIRCLFDTAKEYSPAIIFIDEIDSLCGSRTD
GENESTRRIKTEFLISMSGLNNYKNNIIVMGATNTPWSLDSGFRRRFEKRIYIPLPNLYA
RMKIFEKYINKAKSNHNITNEDIKNFANITENYTGADIDIICRDAVYMPVKKCLLSKFFK
QVKKNNKIYYMPCSPGDPDPTKIEKNVMSINENELLLPPLTLQDFKIAISNSKPSLSLDD
LKRYEEWTNLYGMNGTMNTNIQKLKLVLKFGKKSYSHDASKLKRNPNLEKLTGDKHDFSK
VIFKKEQIEDVIKEVKFDYYYFNEGKKNKYKDIPLNVSIIKESDIPPYKPVDDKLNFSIL
ENDLKIISTNKNSGVCSIGLYIKCGSRYEEISDKINEQGMSVMIENMAFHSTAHLSHLRA
IKSLEKIGANVSCNAFREHIVYTCECLNEYLPVVINLLIGNVLFPRFLSWEMKNNVNRLN
TMRAKLFENNEMYITELLHNTAWYNNTLGNKLYVSESNIENYTSENLRNFMLKHFSPKNM
TLVGVNVDHNELTKWTSRAFQDYVPIPYVKQNEVTPNYTGGFVSVEDKNIKKTNIAIAYE
TKGGWKTSDMITLTVLQTLMGGGGSFSTGGPGKGMYSRLFLNVLNNYNFIESCMAFSTQH
SDTGLFGLYFTGDPANTKDIINSMALEFHKMNKCTDEELNRAKKSLKSFMWMSLEYKSIL
MEDIARQMMILNRILSGKQLCDAIDAVTKEDINRVVSQFLKTKPTVVVYGNISHSPHYDE
ICKMLGMISGIRVNDNCVSEFNNMKIRKTCRWIIFVIENCEIIIHSKGETTSLKELVDSI
DKNNSIQCAYVVFDAVNKIHFFMYARETSNSRDRMTYASSKQALLKKIEGVNVFTSVVEN
AQDVADYKMQPHDSNRGLNNFNNSSNPNSSNNALNYNSQSNMNTPMASPFSYSGN-TNNI
NRGMGNNNDNAGYNTVKGNPNYYSGQSYYSAQNPVQHGAYAANSSNLNEGMYGEPPNSNP
YLNNQGQYMGAQNAYMPMANNASAFKDMGNNLRYDGNKPMNSVQPIVNDTYQEFLQFNAF
SHFVKSSVNYMPANATLKQKTHVPLGFTIQPLAPIPDGYPELASVNFGNSTVVRCKKCRT
YINPFARFEAGGKKWNCNMCYNINETPQFYYVPLDEKGKRKDLFQRPELCTGSVEFIAPS
DYMIRPPQPPVYLFLIDVTVTSINSGLLDVVCNTIKKLLPKNND--DNATNANSNNKKVF
DSRTLIGIITFDSTIHFYNLNSNLKQNQMMVVSDIQDIFIPLPENILVNVHECQNAIDNL
LDNLPNMWRNNKMSDCCAGNALKAAVMLIKKVGGKILFFLSSVPNIGDLTVNVNREAKDK
SSYKKIYNSGNSNNGNSDLKLREVEMLNPANNEYGEFAQSITQFQIAVDLFACPLYNLDL
ASIYPLIKNSGGSLYYYPQFNVHQYSDKLREELLFALTTETAWESVMRIRISRGWKITNW
YGNFQFRGVDLLALPNCHSSQTFSIVVDLEENVVQDSVVYVQSALLYTNSNGERRIRLHT
YALPVTQNIKTITDSINPQVAVSLLSHQAIDVIKKGKIADGRNLIQTLCSQVLSTQLSSS
ENSRLLPIYILGMLKSVAFRDSGDVPPDMRIFQWSRVENIPVESIEAYFYPKMFSLHNLE
KHHGNYDENNNFVFPPTLNLTCENMTQDGCYLVEDGENIVMWIGRSINPQWIYSVFGVQS
LEQLNSEYAENHIGSTDNPSGLQVLNIINALRKARTPSYMRLMVVKQGDPLEYKFFSYLI
EDRSQHMMISLKEFLAKFYPKYPQFTPSLSMEEDSFKNRLLKRNIDIWIEKYRPEYLEDV
VGNPFVINTLKSIIVSGNMPNLLLAGAPGTGKTTSILCLASEMLGSQAKKAVLELNASDD
RGINVIRDRIKSFAKEVISLPPGRHKIIILDEVDSMTTAAQQSLRRIMELYSDTTRFALA
CNQSEKIIDALQSRCAIIRYFKLTDDQVLKRILKICEYENIKYTDDGLETITFIADGDLR
KAVNCLQSTYAGLEVINKENVLNICDIPSPERIENLLKHCISSEWRKAHDIAYDMIKEGH
TPFDVALTSSNVLRRYDLGSEAIQIEFLKIGAMACNTMASGLSSVIQLDKLIADWCIAAK
TLRGKCMNVDSYTQNRKASFPINNVLSEETEKLVDTYKYEEEVNNIFKTGVSKFKKNGKN
SMAFHKSLAVVNVAAGLDGCDDQLLPASFRALEADLNLHPSLLGYITLAQTLMLSLFSPI
WGFLSDKYSRKWMLVFGTALWGIATIFLANINDFTHIIIFRAINGLALGSIGPISQSILA
DAAKNESLGLSFGIVQLSSSIGRLIGGVVTTTVSMKYFGTIRGWRLCFIVVGALSILLSI
IVAFFVEDAPRQVRRKETNSYMGESIIDNNNETIIEPQRSQSYMVYQNVKEMLKDSLSKK
SIIIILLEGFTGTIPWLALSFNTMFFQYCDLSDLQAAVITGFLLIGSALGGVLGGHFGDI
MHNISNKHGRPFLGQLAMFGRVPLVILTYLVIPKRKESFELFVLSCFFLGLSSIAGVAVN
RPIVSDIIRPDYRGTIFSLTIAIEGVGSSLIGAPLFGYLAEEVFHYRNNNLLISDMTTEF
RSHNAEALSKTLLYLTAVPWMLSFVFYSLLHFTYGAEYSKMNQIIESEYKYDDEDDETMV
GKGMT-----------------------MLMFGLRSLSDFCNPNSKAYKENAYDALNRDA
IPSINKAVSNYKDDDDILYCASRVLFAMSDYCCSEKDNAALNKLVNDGGVNAITEIIKTI
PKDQDTLKNCMLFIQNMKDMSGQVDG-ELSVALLNVLTSDTYNAKLGSAIVSALFVVSKS
PSGSKALNDENAHHKLIDHCLSIQNINDETAEVIEGVFDIIKNLSSNGYVVPTIIEKSVV
ILDKFKSYPRVVSKGSDTMKSAVGPEQLTNCLNILKKEQRGSKEHDSALELLSSLSYISS
ITDKIVQSGGIPVLIELINSGLQQYDSNPDKIGRLVAGASRMLGRISNNPSHAGVVYDYG
GIATLCTALSYFPNDADCVSAISIALIPFVSRSNYANEINNYLLFASLFPILYASVESID
LAKASMSCVAAASMINEFHEQMVNNQVIEILSTCIQYHLTDIDYLSNVFSVYFRLSDYIK
TIEPINQYGGIIGIANALSAVSVDSSISETGLKLLNKMLTASDALTYLSNPQIVDSVLTV
MLENENKEVIIQEGTKIMENLATESDCQRHISNLESIINLAQSNPDCAYKTLAAISGLSR
IQSLKLMLESKGADSSIYNGMKTWIESPKFNEQTKLIKAALKTIKILKLNVLNKVHEVIE
SIVELMCIAQVKRLAEGEGPDDNILITSAECINYLTEVNKISTKEIVESSLESIFKMMKK
YSESRLTQTNLLSAINNILLSSNMVCAEVLVNKGYVKQIVTYIHKVPMYVDVQIIGFSVL
ANMLKINSDSLDAIKKANTLIPLQNALRTHVKNMKLKTTCAPLLAVLMPLDTLTREIEDL
LNLCNKCIQGNNLSQLHANLVSLNELLLTAESSKISARCNIGQTLNNIVEWLKNNPDAYK
NDNVKDYSITGRSLFDATISEVAHMCTNISQSRIGLVHLTKSNMASSLIQLYNLLKIPGD
EYTEEAVANILEALSFLLKHDIVNADLAIELGLIEKLCSGINHFSESDIVIKSTFSCLAC
MCTNEKRINQLITHPEYDKLISVIVNLVGNSEKNKDSRMNAIKALHELLKTEKEEIAIDI
SSKTPIVDNLFKIMGEYQMDLPIIQNSSKCLATIADHATIEEKMKIDKYSAMKILIECLG

KNKNDESTAKEIMSVLVKLCNSSDKPHFKELGAIDIISDVTMIHGKNEEISKLGGTLFSY
MGADEQVKKLMKLILSVKDSDADAVQKIDNLTGKLELFLRAPLENPSDALQYTEATLQQL
NAYLASNLENVSLQTNIALVNKRLVDRVKYEFEDQLGAWAVASAGILNQYTDMISNKVGL
KHDKVVAPIYSVLAGCYINPYTKQLIADNLSSILVNTYELLEDNKNKPSVVQSVFELLEQ
IASDEEGSKLLHKKWNGSKGNIVDQTLSIMNINKSNDAVFIAGTRLMGALAETANTSGYI
NDMNIPRIVESCELLVNVGSSKDRVLEFISLIDKMVLANLLDERTASDALKKISSLISEE
HLSNYPEQDRATIIKAYANLLKDSAATGLFAHIRQANKLEAINNLEKLMEYEQKEDVKVA
ILEAITEISACDAFTASKLLVSLLPSVLKNDYQDILNNKNISDSFLQMLERILQTEGIGR
QLSTNNELYKVLSELEETLEKKREELGDEFVNNTKLRIANIRNAIDDDKPKEKTCKDVFD
TFTEYKNKEMPISVLEEPSIDDDMNFLLDKLRIYNKDKLSHNTERGIDNSYGYMAIEILC
ESHDNIQELIKKEFHTSAFHALVKQPEEHVKHYACRSLCAFTKDPIGIKRVITDVKDYPN
IISKSVGDLTANNALGKEDKEEFLINRVLLIDRTAHNRNVYDNTNAIRHLIEIWNQYDQG
YYSVSLLRHVFRAMRKVVSDFHVQTLLDANILVRLINIINSLETDKILYPDVLFLIGSLA
IVKEIKTQIGELKGIHACVSLLLRYISVEGMEPTITNCCLALANMCIDHKENSSIFSSLK
GPDINVRILKEYRSNFDVVNGASVLLCNILFRNEEMKKTYGVNGAPGELVECLRSYDGSD
NKNAVRCIESIFKAISNLSLYTANIKQFLDTQIEVSYESWLNKLNESFPDAELETGLRTL
SNLVMENEEINMKKFGVTLIPVLNVLKQNRENTKVIFFLLDILCSLCRLNDNAKLFAENG
GIETTINAIQLYDYDVSLLTLAIHLLSNQCKIESSLPLLIKADAFSILISCMEAETEEFE
VTELVVSSLRCTRRLIQSEELAYEFCNCGGIPTIANLITKSVKKSIVILEALRILLCVLY
YTKNVDGVVNEYSEEEEELFNARLGGWYNISMDKEMIDAILQSVLTCSNDENHQKQLRLQ
KVSLGLIAYFAYHRLGIISMTASGFDTLAKNNLNHFGGDIVIMQLLSICIDNIAINSAEI
YDMTITRDIIKSFKTSLTKMQNKKENKQIIQSVEKTLEAMSSEGDPLDTFKDTLLTFDFS
LSEFDKDPYVNGVHDLPQNIKEALRTGGQYKIYHKSEKRTMFKWKASQDLGTLEWTIGDN
ADRIFKISVVRIKNISKGLVHPLLKAANKYEPRKVHSKVVLCIYGPPTEDFPEGLELPIK
TKTNKERDAFADLLILWRDAASYNYMDKKAREYAQEAIKFIQRSGSSFMACKNLREKLES
HGLIHIKEGDQWKLQKNQGYVLCKENRNICSFFVGKNFNINNGSILISIGHIDSCALKIS
PNNKVTKDQISQLNVECYGSGLWHTWFDRSLGLSGQVVYKKDNKLVEKIIQINKSVLFLP
SLAIHLQNRTRYDFSVKVNYENHLKPILSTVLYEKLIKGNENISEKNNSSTDDEDKNSKN
INSSPLLYLLANELKCKEDDILDFELCLMDTNQPCFTGVYEEFIEGARFDNLLGTFSVFE
AYIELIKMIKSENNKNEPLENNLYICIGYDHEEIGSLSEIGAQSYFTKSFIERIIGNIFK
NELKNNETTVDEIYGSLSSRSLILNVDMAHCGHPNYPETIQQSHHLRFHDGIAIKYNTNK
NYVTSPYYACLLKRTFELYQNQNQKIKYQNFMIKNDTPCGSTVGSMVASNLSMPGMDIGI
SQLAMHSIREIAAIHDIYYLIKGVFAFYAYYNQVLSTCVHDAMNISKTLFSVFFVLFFIS
SYNVKCQLRFASLGNWGKESKSQLLNAKYLKQFIKSERVTFIVSPGSNFVDGVKGLNDPS
WKSLYEDVYEEEKGDMYMPFFTVLGTGDWTGNYNSEVLKGQGIYTEKDGVTTIENDDEKT
KYPKWIMPNYWYHYFTHFTVSSGPSIVTTGHKDMAAAFIFIDTWILSANFPYKDIHNKAW
EDLKLQLNVAKKVTDFIIVVGDKPIYSSGLSRGSSYLSYYLLPLLKEAQVDLYISGHDNN
MEVIEDSDIAFVNCGSGAISSGKSSIKSPKSLFFSSDIGFCIHELTNNGLVTKFISSNTG
DIVYTHKLGLKKRKTLDKVNSLQYFATLPKVELIDVPAAGPMGNKDTFVRIVGTIGILIG
SVIAFMGFSSFLSKNMKMNTHSKKGNFIVFEGVDRSGKSTQSKLFVDYLKNNNIQVKHLC
FPNRETTIGKIITNYLKMESTFSNETIHLLFSANRWEMMDEIKNLLINGTWVVCDRYAYS
GVAYSSGALKLPKEWCMNPDKGLIKPDAVCYLNLPPNHAQNRSEYGKEIYEKFEVQKRIY
EAYEHFSNEDYWINIDATKSIQEIHNTIVEEISKKCSSKNTQFEYLWS-MKVSLVCKNCQ
SSDIETNEGQGEIICLRCGSVLEENKIVESLEFVENNNGAISMVGQFIPSSGTKSFILSW
GIRESREISLQKGYINIQKIADNLHLSSQHIEAAQRIYLMALQRNFTMGRNNSYVAASCL
YTICRREKSPIMLIDFSDILQTPVKPLGKTFLKLLRLLHISVPNIDPSLFLERFAYKLNL
KNDIYKVTYTGIKLIQAMTRDWISTGRRPTGLCGASLLIATRIHGITINSNTIAEVVRIS
NPTIIKRLYEFKNTNIAKIKASEFDKISIDDIPSSSIPPCVISDNKKKIKYNLLQKNKTL
SLCDSEEQYALCSNSICSVENYEKNI---NSQNINASSYFELDHNKFFNTTQSGIINSSN
KNDKNEEHIRSSSNEINLDEICNDNPEGNDIDNLAFKIINTIDIEKNSEFLKISNTSLNE
LGALKNGLQNQDIQKNTQNMMDSSKLQSANLTNSETSTSALVDNENDEYNETKESTQKYK
NTSNFDIINDNNLRQNNKALDSTYCTTLKQTVNSELSNAINDINDFNLFDNNAISKNSSN
TTSGLSKMNDVNNILTDFNYFFENNSNTINDQSANFDINSDDSEQINDETISDSYDSEIE
NIILSEKERKIKMLIWDDVMKGCMPNISKNMKRQKKRQNTDINNSKN-KIPNNKQNVDNP
QDQLSTGDSVIKALEKSNKLLPKKINYDVLKSLFSSMIEKKKNEESSIGLEKMEGDKIDA
NLYSRQLGTYGFELMNKLIKMNVLIINVKGVGLECAKNLILSGPKSVCIYDNDICEISDV
GVNFYITEKHVENKVCRSNAVLSNLQELNNYVHVYNYTGNLDSKFIEKFDVVVCCDTKDS
DIIKYNNLVRGIETKNIAFLSCNVYGLCGYMFNDFGSNFICYDKDGENVKSCSISQISKE
TEGKVSFDFDKSSPFQNGDFVKFTNVEGMTEINGQIYQIQNLKKYTFTIGDTTKFSDYIK
GGECTQVKTNLKIDFKPYEYIKSKPLFCPPSEIKISIVDDNKGGKAIFEEVTLPTSFIIS
DYSKLNASNYLHYAIQGLKWYESEY-NCLPENYQMEEFEKIYKKACDLNSKDKENKLPWS
VEELDKNIIINVAKYSKAHISPITSFFGGLLAQEIVKFTGKYMPIHQLLYMDFFECINMN
DEENIDDKKKLNCKNDNIISIFGKNFQDKLNKLNIFLVGSGALGCEFAKLFSLLDMCTVE
KNGSLIITDNDNIEVSNLNRQFLFRREHIEKSKSLVASNAIKNKNKNINVISHVTKVGQE
NEHIFDEKFWTKQDFIINALDNIVARQYVDNKCVWYSKPLFESGTLGTKGNVQIIIPHMT
QSYNDSYDPPEDSIPLCTLKHFPYDIVHTIEYARDIFQGLFYNVPLSIQQFLNNKDDYIK
KIQDEGNNASSLENLENVLNTLKEIIKENNFNFCIKKAVHLFHSNFINQISQLLYSFPLD
YKLSTGEFFWVGQKKPPQVIEFDLNNTYVQEYLVSTSNLYAQVYNIPTCYDIKYIIDVAS
QIKVEPFSPKTVKVNIDEKNLNNISISYAQDNKLIQDYCNELLNIQTDSLKVSPIEFDKD
EESGLHVNFIYAFANLRAMNYKITTCDKLKTKMVAGKIIPALATTTSIITGLVGIEILKY
VNYSDSIQKYVKLTDEEKKKEKDILSYFKNAFINTALPLFIFSEPMPPLRMKDKEYDELM
KGPVKAIPNGFTTWDKIEISIKNGTIKDLVDHINEKFNIDVNLISVGNACLYNCYLPVHN
KERLNKPLHEIYEQISKQSLPSDKDYIVVEASCSDQDLVDVLIPSIKFIYKMCKLNQDLL
KKAISDVFEGTKQKKRKFVETIELQIGLKDYDTQRDKRFSGTVKLSNEVRKKLKVCILGD
AVHSEEAQKLKLDYMDIEAMKKLNKDKTLVKKLAKKYDAFLASQVILPQIPKLLGPGLNK
AGKFPSLITHNDKINDKILELRSSIKFQLKKVLCMGVPVGHANLKEDELRSNIVHAINFL
VSLLKKNWQNIRTLHIKSTMGKPQRIYGMIQESLNDNENKKKWEDIDADDDLESNKKLSY
FTNYENGVKVVTKYSENLKKQTVKVTKKIKEVIIKKRLNKEIENRLKLKNFNVDAFSSVI
VEPTDVVNIEPPKNNLLDFLKDTEYDYLFAEQTDKTAKDLKNRFKMFKDEEAEEAKPDDA
NKAGAKRDMMYYRSHDECTIRVTNLSEDVNESELSNLFGRVGQISRMFLAKHKETQNSKG
FAFITYSKREEAKRAIEKLNRHGFENLLLSVEWAKPSNRMLNDIIIQVVIASIGVTIVNS
DKIKFLHKFKYAVYALIFSFLVYKGIPWKRDNYYVYLNITPNATKQEIQTAYRQAAKIYH
PDKNSDESADSSFIKLKHAYDVLSDDVRRSNYNRFGDYKNGEVDDNTATLLICLSLVQHA
MFFIIGYFLSYRKKLEFSRQIFLVYNIASFCFELQFRFIEDDTTFDWLPVIGYLLPYEKI
KLLRMIFPIVFFISICISAYAYTDRNASLIYLMRSILSTNRIIVERSNDVVESTNYLKKN
GDQIVSKLQQMRKADGSSQLNDNNDDGDNKEYLG---------------GE--NKKLLEN
VKEFSLSLDSQQMNLLQKCYEIMKNKKIDDKKGKKKSWFEFFSMQMIFGIIFVYIWFTSK
MGKDYYSILGVSRDCTTTELKKAYRKMAMMWHPDKHKDVKSKKEAEEKFKNIAEAYDVLS
DEEKRKIYDTYGEEGLKGSIPTGANTYVYSGVDPSELFSRIFGSDGHFSFSSAFDDDFSP
FSTFVNMTSRKPRPSGNANMNHNNYNAKPATYEVPLPLSLEELYKGCKKKLKITRKRFMG
TKSYEDDNFVTIDVKAGWKDGTKITFYGEGDQVSPMAQPGDLVFKVQTKPHDRFTRDSNN
LIYKCPVPLDKALTGFQFIVKSLDNRDINVRIDEIVNPKFRKIVANEGMPSSKTPNMKGD
LIVEFDIIFPKNLTSEKKRIIREALANTFMKGIILIGYLLLIVCLNYFYVQGAFFYVKEG
TEKCFVENVAKNVIIVASYDNYGAKELKCLINVKNKKGHVLYTHDASQMSRGKISYMAKS
SGPHYVCILCPSNNWFKDTSVKWKLSIEAGGIDIDLNDAAKKSELSKTISALQSLKKKFS
SMKSQQAHQKLIADNMHEHNKNVHKNMIYCYIIEIIILIIITGYSIMHLKNYFKANKLMM
SKNKKGKKKEDLDAILAELGIEEKREDQENAQISKSKKRKEKLKQKKEQKLKENETGVVE
PEVVKEEEVKDEEKDGE-----DGENNETAKAKKKKKKKK--DKETKPETGLSAAAKAAA
ERLRLLKEYEEKQKEEERRKREEEEERIRKEEEEREKKRLARLEKKMQLKKEGKLLSAKA
KEEKKKRELYLQTLKESGMLIEPKEKAKAEIINLDLNKAKLLLKKKKNKQASNNDDKNKE
VKENENK-----DKEKEEVEEEKEIVLDDWEDFLNMDNEEKKKDDKINTDQKPKENKQNQ
IVKKGSTTDKKGKKQKSNKSGEEDKDND-KTDDEEDMYRSSIVCILGHVDTGKTKLLDKL
RHTNVQDNEAGGITQQIGATFFPKDILDKEIKKIDGTIKCLSKGIMIIDTPGHESFYNLR
KRGSSLCDIAILVIDLMHGLEQQTKESIQILKQRNCPFVIALNKIDRLYMWEQSAWEPFN
NTFKKQKEYVKEEFNNRLQTILNELSEQGLNCQLYWENKNPRKYVSIVPTSAITGEGIAD
LIMVLVKLTQSFMLKNIEYNKKLECTVLEVKNIEGLGTTIDVILTNGVLKESDTLVLCGM

NGPIVTVARALLTPQPLKELRIKNEYIHHKSIKACIGVKISANGLEEVLCGTSLFVANNN
DEIEEYKKKVMTDVSDVFNHVDKSGVGLYVMASTLGSLEALLIFLNDSKIPVFSVNIGTV
QKKDVKKASIMREKGKPEYSVILAFDVKIDPEAEKEAQILGVEIMQKDIIYHLFDAFTAY
LKKIEDEKKQSKMADAIFPCEVSIISDCVFNKKDPIVVGVKIEAGILKIGTPLYIPEKNL
KIGSVVSIESNKKSCSKAKKGEEVCVKIAGEPNVTYGRHFDFNQKIYSKITRESIDVLKQ
YFRNELTMDDWRLVVQLKKILNILMIINEKDKLAEQNLETLDVTKLTPLSEDVISRQATI
NLGTIGHVAHGKSTLVHAISGVHTVRFKHEKERNITIKLGYANAKIYKCTNPDCPPPECY
KSYESSKEDDPMCPRENCNSKMKLLRHVSFVDCPGHDILMATMLNGAAVMDAALLLVAGN
ESCPQPQTSEHLAAVEIMRLKHILILQNKVELIKEEQALKQQEEIRNFVSGTAADSAPII
PISAVLKYNIDVVCEYIVTQISIPRRDFISSPHMIVIRSFDVNKPGEDIEGLQGGVAGGS
ILHGVLKVGDQIEIRPGIISKDEKGEITCRPIISKILSMFAENNNLKYAVPGGLIGVGTR
IDPILTRADRLVGQVIGHLNKLPDCFAEIEISYYLLRRLLGVKSQDGEKNTKVAKLKNGE
FLMINIGSTSIGCRVMGIKNELAKLELTGPVCTKIGDKIALSRRVDKHWRLIGWGQINKG
KPLDLQEPIMIHFVLLISRQGKTRLAKWYMPLSQKEKAKIIRETSQITLNRTPKLCNFVE
WKEYKLVFKRYASLFFILCIDKGDNELITLEIIHHYVEVLDKYFGNVCELDLIFNFHKAY
YLLDEILVSGELQESSKKIILRVVAAQDSLMEDNKRKEHTFSQVMKYANMLKTSLICNKV
LNMHGIPSIAFRKCYTNFLDGNKSFLNVTKRRLFSSQSAQDKAYSNEQHKENMEMDSKNS
ENDNKKNMNKSENSEQSEEKTKEINYESYNKIDLINEIKKTKKDMDEKLVDNQVLKEKYL
SVLAEKENLRTRYMKEIENSKLYCISNFAKSLLDVADNLSLAIKNISEESLKSNEEINNI
YKGIEMTETILHNIFSKYGIDKYNPINEKFNPMLHEAIFEVNDTTKEKGTVATVIQPGYK
INDRILRAAKVGVVKN-MKELLEDLFKIIKPDEETENYLKERINDEKKKIMKNGVDYFYD
LICPFSDKKLKKNVIAEVYKKHVSDAINVTNENKNSNNEKLTKSLNLKEYWEKNDAIGYY
DPFLGIQEKQINYNTSIPISESIKINKEKQKQKEKQLSLFKEWVKNKIKIPSPVRVHNLL
HCSDSNKGKTKIEKMNDIRIENFNLSIGQRNLLNDTTLKINVMNKYGLIGKNGIGKSTLL
ARLARHEIENIKEDISIACIEQDLFLEDVTVLECVLMVDKVRHNLLKELEELELAKKQ--
------NDAKP------DNLKDNTDEKIINIYEKLNSINYLEAEKEASKILCGLGFDSNL
QKKKVNSLSGGMRMRLCLSRILFSNNDIILLDEPTNHLDIYTIQFLIDYIQKLNKTCIIV
SHDRNFLNEVCTDIIHFHQKQLTYYSGNYDQFEKTRIEHLLQQQREHDSIELKKKHVQKF
IDRFRYNSKRATLVQSRIKLLNKLPVINLEKDETPFKFSFLEPFYVSSVLIRLRDVSFKN
EMFKNLQIKKNANIIIADDFEDSNAKPIELENAEETKKDTLNNNIAGDYQFKHEFLFKNA
NFEVDMDSRIAICGVNGSGKTTLIKIILNLIDVYEGELYVSNKANIGYYSQYHVDSLNPV
FNSIQQLQYNYSHKNIKEDEAIKYFNKFNIPTNILYEPIYVLSGGQKSKLALAILAYKNP
NVLILDEPSNHLDIESVQALIVALSLYKGGIVLISHDTYLIKHVADEIYHINNITKEVVK
IDYEFEKYTKLLLENKIMPREIITLQCGQCGNQIGVEFWKQLCNEHNIDQEGILKNNNFL
NEDRKDIFFYQADDEHFIPRALLFDLEPRVINSIQTSEYRNLYNPENMFISKEGGGAGNN
WGSGYSQGHKVEEEIIDMIDREVDNSDNLEGFILSHSIAGGTGSGMGSYLLELLNDNYSK
KMIQTFSVFPLLTNESSDVVVQPYNSILTLKRLILSTDSVVVIDNTSLNRIFVEKLKLNN
PTFQQTNNLISNVMSASTTTLRYPGSMNNDMISLISSLIINPKCHFLVTSYTPITVDKHV
SNVQKTTVLDVMKRLLHTKNIMVSVPVRRGMYISILNIIRGETDPTQVHKGLQRIRDRKL
VNFIKWNPASIQVTLAKQSPHIVSPHKVCGLMMANHTSISTLFERCVTQFDRLFKRRAFL
ENYKKEPMFSSADGQGNFEEMESSKEITQNLIDEYKSAERDDYFSHTYMMEKYHGLEKIG
EGTYGVVYKAQNSDGESFALKKIRLEKEDEGIPSTAIREISILKELRHSNIVKLYDVIHA
KKRLILVFEHLDQDLKKLIDVCDGGLESVTAKSFLLQLLNGIAYCHEHRVLHRDLKPQNL
LINREGELKIADFGLARAFGIPARRYTHEVVTLWYRAPDILMGSKKYSTPIDIWSVGCIF
AEMVNGRPLFPGVSDTDQLMRIFKILGTPNSQNWPDVFKLPKYDPNFPVYEPLPWETFIK
GLDDTGIDLLSKMLKLDPNQRITAKQAIEHPYFKETSMGNKLSTEDHIFRLKLKTKELEK
LSNRSELEEKRFVMDVKKAIQAGKIDMARLYAEKCIRKKNEKINYLNLSNKLDVLVSRLE
GAHRSASLVKDVSVMIPLIQQINSETNAVKIGSDVMKLENIFDEISITSDLINDTVQTSS
AIVAPTEEVDELISKIADEHALKLQLGPTNTINKHLEEITNMSERIMGKEKTHINLVVIG
HVDSGKSTTTGHIIYKLGGIDRRTIEKFEKESAEMGKGSFKYAWVLDKLKAERERGITID
IALWKFETPRYFFTVIDAPGHKDFIKNMITGTSQADVALLVVPAEVGGFEGAFSKEGQTK
EHALLAFTLGVKQIVVGVNKMDTVKYSEDRYEEIKKEVKDYLKKVGYQADKVDFIPISGF
EGDNLIEKSDKTPWYKGRTLIEALDTMEPPKRPYDKPLRIPLQGVYKIGGIGTVPVGRVE
TGILKAGMVLNFAPSAVVSECKSVEMHKEVLEEARPGDNIGFNVKNVSVKEIKRGYVASD
TKNEPAKGCSKFTAQVIILNHPGEIKNGYTPVLDCHTSHISCKFLNIDSKIDKRSGKVVE
ENPKAIKSGDSALVTLEPKKPMVVETFTEYPPLGRFAIRDMRQTIAVGIIKSVEKKEPGA
VSAKAPAKKMSHLMSLPIVLLKDGTDKAQGKSQIIRNINACQVIVDIVKTTLGPRGMDKL
IYTERDVTITNDGATVMNLLNISHPAASILVDIAKSQDDEVGDGTTSVVVVAGELLNEAK
VLLNDGIEPNMIIDGFRNACNVSINKLNDLSLSFVNKNEEEKKNILLKCAQTALNSKLIS
NHKSFFAELVVNAAYQLGDNLDKSNIGIKKVTGGSCLDTQLIYGVAFKKTFSYAGFEQQP
KKFNNPKILLLNVELELKAEKENAEVRIDNPNEYNSIVQAEWDIIFQKLNLIKNSGANIV
LSRLPIGDIATQFFADHDIFCAGRVEDADLKRTATATGAVIQTSLFNLNESILGNCGLFE
EVQIGNERYNIFKECLKTKAVTIILRGGAKQFIEEVERSINDAIMIVLRCIGNSEIVPGA
GSIEMQLSKHLRIYSRSICNKEQIVLYAFAKALESIPRYLSHNAGYDSTDILNKLRKKHS
EETNDIWYGVDCLEGDIINAYSNCIFEVTKIKRNVIYSATEAACLILSIDETIRNPSSMD
KQPRNPYAMSDKLEDIGEFVLVIGDFHSPMRNLGLPDCFKELLKTDKIKHVLCTGNVGCN
ENLELLKNIADSVHITKGDMDSNFDFPEKINIKIGDFKISLVHGHQIIPWGDLNALLQWQ
KEYDSDIIISGHTHKNSINNFEGKYFINPGSATGAFQPWVSNPIPSFILMAISKSSIVVY
VYEEKDGKMNVEMSELRKQMFTSTGKLLYSVEYSPSKSKDSTGVFRNPKFKDKLVDNFDE
VPLTSVWDGFNKSAEKYKNKQCFGTRVRKDGKLGEYQWKTFQEVRELIIILVGSGLINKNV
CPVIDCEDPTVPKGRFLGLYLPNCEEWNICDFSCNAFNIITVPLYDSLGIESSKFILDQT
MMQTITCNKPCALKLLKSLGSFEQMFIKTLILVEKEIDPEIKKLSSQLNIKIVTWDDLIE
SGKKKRLEPKPGKLNDICSICYTSGTTGYPKGVIMTNHNFVAQMASSCYGPIKLPNLIID
EKDTHLSYLPLAHIYERIMLSIFIYLGIRIGYYSGNILALTDDIQALKPTLFLSVPRLYN
RIHEKICNSLKKKPSLLQSLFNKGLDQKIKKLNSTGNPSSFFWDTLLFNKAKKILGGNVR
GMLNGSAPLGVEVAKRLKCIFSVPLMEGFGMTEGLGCLFITNTYDKDVGHIGGPLPATEF
RLVSVPEMNYLVTDNPPRGELLLRGPTICSLGYFKLEKETSELIDEEGWMRTGDIACFSS
NNSLTIIDRKKNIFKLSQGEYVAVEKIESVYKQSLFIGQIFVFGYSHESFLVCIVFPSFD
TMEIWAKENKINKPNDEIIKLEKFKNDVIQDLVKIGKNNGLNGYEQIKDIHFIMDGFTIE
NDLMTPTGKIKRHAVQIKFKQDIDKMYERVKKA--SWENFLNDKLLATNQVSAAGLASEE
DGVVYECVAAPDENDKWSLFYKEDYDIEIEDENGNKSTKTISEGQTILTMFNEGYAPDGI
WLGGTKYQFINMEKGLDYEGYSFDVATCAKLKGGMHIIKVGGGHILIVLYDEEKEQDRGN
SKNAALAFSKELAESTDMSSFYNKNLSLSPSNGKAYPARISGNNEAGNINRRLFRSFRST
KNDMCPDCKEKGIIICDTSEGTQICNGCGLVVESNILSEEQEWRNFSNDGQSKGNDRNRV
GEVSDIWLENNTSTTFIKSSKKLQHLNMMTQINKNDQTLLSAFNILKLICETFFLRSNVI
ERAKEITKELQDMEQLKNRINNLNMLAVVYLACREAGHIKSIKELITFDRSYKEKDLGKT
INKLKKVLPSRAFVYNENISHLIYSLSNRLQLSTDLIEAIEYVVKKATTLITTSHRLNSL
CGGSIHLIVELNTSEEKNVKLPHLSQIASVCGVTTNTLKTTFKELLAASEYILPKNYLKD
NNTKLVILKHKYLHDDKKKRR--MEKLNLPEWTDIERYFKDPELITSELLFVGLTLCNVF
VMYRLFLDVIPFPIFVTWWQLAQGLLVAYVCGEMGKEFPKFAYFPKVEINENMLKVLFVP
SIFYCLMLVLSNYLLFKTPCISSYPVLVSFTVVFHHIIRFVGCGEEYMPLRWKSIAFLLS
AFVFGCFDSQTSGKGVIIWALLYALFSAIFRAGFMQKIMHLVEGKGNTLHNNQHMLGVLI
LPVLILLSGEWRVLGHMPYNITSLYTWQVWGCLVTVGALPFVKNVVSNRLVRRTGQGPWR
FLEIISIALVFMIGLGYNKPSFMGYLAIICVIIGRSLGAFDVLLNVSDYMISEDERKRKM
EKSSYAKSRQGTQASKPFLSSGENEDDDESSFSSNDSKSYQGSQDYDDKESINSSSNQYS
THKGTSRMVSFSNHTSHEDISIDESRDINARSSSFKSKPQYSRKYSSKQEVIDSQAMGAL
DESTPVPSRITLHLMKQKKKSAFQGYSLLKKKSDALFIHFRDVLKDIVKTKNKVGEDMRN
ASFALAKSVWAAGDFKGQIIEGIKRPVVTLSLSTNNVAGVKLPIFQVHIDPTVDVLGNLG
VAAGGQVINNTRENYLQCLNMLVKLASMQVAFFSLDEEIKMTNRRVNALNNIVLPRLEGG
INYIIKELDEIEREEFYRLKKIKEKKTENLNDATEDHLPDNGGHKHVKATNNYANIQADD
DVIFMTKLYFLFLFAIQFCSSFVNSSILESIKHNLQIVNNHNFNTVINKFRNEKVFFILF
FKDSNKDIKNVIKNYDAVAEKFKGIMTLCAIDCDANSRLCDDELSLYVPDHKTSNTHHLL
VYPISPMPKFEFKDEINEPNIKKYTYLIPSKIDVIKETKDFNIFLSKHENMPKVLIFSNK
KKPNYVLNALSNSFNKKLLFCYINNELDELVKKYNIKNFPSIIMLKKNQVVDTYKGKNNF
INMFDWLNIHSETFVMGGGFDISPGKTNYKPWKFEAVPKFTKLSHGDICFKQTDKGLCVI
YLKEGEKLDKSEEEMLITLKEKFKPQMSGRGVNFRYMWIDIATETNFRALFELKKYPSVV

VFNPHKRIRYATINEDLIATKEHIEKLLEKISGGDAKFTMLKGQTLPEFILDESEDASPG
KDELMTVDLNIPYSACELKRVKRLELGVLDPEIIKKVGVCEIVNVDIYKDGFPRDGGLND
IRMGTIDYKALCGTCNMNVKNCPGHFGYIELAKPMYHYGFMNIVLNVLRCVCYHCGRLLC
DMNSSKVKYIEKIKVNSLRLKRLSDLCQGIKVCNHSSQDDNLYINDTSIDNFYNNDLSNL
NVNQQMLLNSNNYSNLFEMISKEDVDCGCVQPKYTREGPNMYIQFLHNNNEDDVDGSKRR
LSAEEALDILKKIRKEEMSILGFNSDRCTPPSLILTCMPIPPPCARPYVQYGNQRSEDDL
TLKLLDIVKTNIQLKRQTDRGAKSHVLQDLCSLLQFHITTLFDNDIPGMPIATTRSKKPI
KAIRTRLKGKEGRLRGNLMGKRVDFSARTVITGDPNLGIDYIGVPKSVAMTLTFCETVTP
LNYDNLKKLVERGPYEWPGAKYIIRDNGSKYDLRHVRKNSEKELEYGYKVERHMTDEDYI
LFNRQPSLHKMSIMGHKAKILPYSTFRLNLAVTSPYNADFDGDEMNLHLAQSHETRSEIK
HLMIVQNQIVSPQGNKPVMGIVQDSLLAIRKFTRRDNFLTKEEVMALLIWIPYWNYKVPT
PAIIKPKPLWTGKQIFSMLLQFDDKDMNNNFDNDDRSKFGNNMGRDNENNFGKLSGSNNP
NSPLSLMDNMHGNARNEINNNVSNMA---SGGNNTYGKRYKMKINLIRDSSTSCKDDNPY
CSNNDGKVIIKNNELLSGIICKRVVGSSSGSLIHILWHEMGPDKTKDFISALQKVTNNWL
EYVGFTVSCSDIIASNKVLDKVKEILNKSKNEVTKLVKKAQSGELECQPGKSLYESFETR
VNNELNCAREMAGKVASESLDEKNNIFSMVASGSKGSIINISQIISCVGQQNVEGKRIPF
GFNHRSLPHFIKFDYGPESRGFVSNSYLSGLTPQEVFFHAMGGREGIIDTACKTSETGYI
QRRLIKAMEDVMVQYDRTVRNSYGDIIQFLYGEDGMAGEYIEDQIIDLMKLDNKEIKKLY
KYNFDDESYGKDYYLGKEGEEHPAYINYNKQNILNQEFEELYKCKNYLCKEIFSDGDIRQ
HLPINMNRLIEHAKSQFPFIPVISSKSKIQTKISRDRGNDTMGIGGNRKKKKKKRS-KKN
EKSMHNKDLMFEIKKEYENNDLSSVMKNDKSFMGFETYNKDDSDNYEDEEGSSESDDDAD
YFFSGKNENRNSYDDKINSMINPVEIVQKVNKFLDKLVIIKQINNSDTLSLEAQNNATIL
LKAHLRTYLNSKLLIHTHKISLKGIDWLLQEIEKIFYKSLCHPGECVGALAAQSIGEPAT
QMTLNTFHFAGVGSKNVTLGVPRLKELINIVKNVKTPSTTIYLDDMISNDQQKAKDILTK
LEYTTLKQLTSHAQIIYDPNTTSTILEEDKLWVNEFYEFPDEDDTQYTLGEWVLRVQLTN
IHVNEKKLTMKEIVYIIYSVFSSDELDIIYTDDNSEDLILRIRVKYLNGEYNFLSGNGDN
INDDQYEDEDDDEREYNNIANTFKIKKSMSSDMNEKNEDNASNSGNNIGSGDHIGNSSID
NIGQNMDGNVKQEGGRKGLHKSDNNEDDEDDDD-DEAFLFGSDSRNLENKEASNSSHNMN
KSPYGHDEKTSNFNDEHISGNSGGMGGSNKTMGKGDDENNQKNRKKNLNNENNTMISKED
TEDTFLKKLMEQCLSSLKLRGVENITKVYMREESKITYDSENGKFVRSSHWVLDTDGCNL
ESIFCAPSVDFKKTISNDIVEIFEVLGIEAVRRALLKELRTVISFDSSYVNYRHLSILCD
VMTQKGYLMSITRHGINRVDKGPLIKCSFEETVEILLEAAAFAQVDNLRGITENIMLGQL
CRIGTGVFDIIIDNQKLNDANQNLETIQDITSAGFTTPDSSGSITPDGLQSPLGINTISS
PLPFSPTYNSNLLSPTLPIDNMNNILSPQCLPNYSDNIMSPSKNDFNNLDTLQLGGKFSP
TQSPRSPTSVIHSPFSPFDNRSKQPLDPLLLFSPKNNAGNAGLNNYNVFSPKANMNNIQS
PLLYSPNPMIDIFSPKPQMQNNIYSPSYSPTSPTYNANNAYYSPTSPKNQADDMGGNKKY
NIMSPVYSV----------------------------------TSPKYSPTSPQYSPSSP
VPNNPSSPQYSPYSITSPKFSPTSPAYSISSPVYDKSENMAGKNPLSPAYMLQSRVQIRQ
NAQGTQVFSPIQQGGANDVQNDDPFSPIPYNMDEEEMQEEMVVFYLDDLEIFFPYDYIYP
EQYAYMKYLKKTLDSEGHCVLEMPTGTGKTVAIFSLITSYQYYKNDDSKFIFCTRTVAEM
EKSLIELKKVINYRINIIKKRNEISEQIVKTDIKEDSDMIRNE---------YKNEEDDN
AWNRFGRNSEILAMGISARRCMCVNEKVLLKHEREKIDEECRKLTATFVREKKYISKKLN
NYSNSNIDRISDFIIKNKHHIDMEDYFSIYNSKNSISEYNDLGLCGYFENYKKDFVYELI
EPGVYTIEELKTLCKNYKNRENMNAPICPYFCAKKIIEIAKVIVLNYQYIIDPKVSKSIF
LGKDINNRVNYKKNDIIVFDEAHNIDSVCLEALSVNIDRSILNKASMNITTLFKKIEKSR
IVNEDKLREECYKILKKIKSGKEEYNSMMNRNGEQMENKQFNENKDNLESQNKKNQQDEI
YFDEEMNLVFRGLLGEEPNNREGD-DVEKLTDKVIIDDLKKILNDSSDNVI--KDKDKDK
SNLEEINYSPLLMEDIIKNVVMPGNIRKSEHFLNLMRIVVVYLKKYINIYEVTSEGPLSF
LYKCEKDTKLDTSFFKYSFDRLKSLLNALQVVDTDDYSALNVVCNFCTLIGNYFKGFIII
CEPYPEATGIYDPVIQFACLDSSIAMKSVLNRYKSVVLTSGTITPLELYPKLLNFSTVLT
ASFPMSFDRNCVCPLIVTKSSDLIPLSSQYSLRNDLNVIKNYGFLLVEMCKNIPDGIISY
FPSYIYMEHVMSTWYELGIISNILEYKLIFIETKDIVSTTIALHNFKKACDLGKGAVFLS
ICRGKIAEGIDFDKHYGKCVILFGIPYQYTLSRILKARLDFLKETYNIQENEFLTFDAMR
QASQCVGRIIRNKKDYGIMIFSDIRYARNDKKGKLPPWIIKCMDVSNINLTIGAGVSISK
KFLLNMSQEYKETDQTKISQVILQNPSKCWDIVKSVLNMDDFIMLGKKIRNTLGNQLKGY
SRKYSTSNLMEEIKNQPLSKITELPNKMKVATIKNNCEVPTIGLWISSGSKYENKANNGV
AHFLEHMIFKGTNKRNRVQLEKEIENMGAHLNAYTAREQTGYYFKCFKDDVKWCIELLSD
ILTNSVFDEKLIEMEKHVILREMEEVEKSADEVIFDKLHMTAFRDHPLGYTILGPVENIK
NMKKNDILNYIQKNYTSDRMVLCAVGDVEHDNIVKLVEQNFSNIKPQDEKLILKQEFDKI
KPFFCGSEIIIRDDDSGPNAHVAVAFEGVPWTSSDSITFMLMQCIIGTYKKNEEGIVPGK
LSANRTINNISNKMTIGCADYFTSFNTCYNNTGLFGFYVQCDELAVEHAVGELMFGITSL
SYSITDEEVELAKIHLKTQLISMFESSSTLAEEISRQILVYGRPITLAEFITRLNEIDAE
EVKRVAWKYLHDRDIAVAAMGALHGMPQYFDLRQKTYWLRYMDNLKKVLWSIEELVYTNN
QDEEIYEKKEKEIKEYLLNLHKNSFSNETENEELVVENGNFFNLLKSVKECKYEKCITNP
WVSSIFKLILEKFKNENCSTIYEIKREIFNFISAEKEQGLKKEANDACIFSCFVFFYEMK
ILLCCNIFLNMFVQCNWTGPSL--------INNSEKSEEDKYFNEFIDSIKIKNDDFLNS
CLEFLSLEGEYIYEYCNMINSFCLCIIFLGFINNFSKHDEKNAHGNEYNNLMAYLRNGED
K-------NNDRGNSLDSLIFARSKYLWKARIYFIWQRLFSSSSSFMYILKRHIIDTPFN
IFKNMNILPNNFDLIERDFDLEAVDIFSCISENADDNKKCDLFCENYISEKFKIYILSNY
SVYLSFYNYTYAYDKLLEILSECSKFYYTFTGRMGIKRKYQKNPATILVLKVKLGDEEDN
TSTVFKEIEPLSEYDILRSDYKIIDNKNVNKEEKKTDLVKQITVQTKDEEFEKREDKNCT
ALENDTLNGVCN-------NPDEKKLNVCDGNNDEETNNGLSENSPMEFNGKVTWKLKDF
DPDTDILEEPYFFDSQNNYFKVLSFDEQIALINYCFSIMRFNPHYDEIKFEKLNAIISRC
LKSYDVNMESSENEENGKRVGNHLLQIKYQNNLLHSCILWFKCKCESFRLKTVDRSQAQL
NELLKEYYNDEPQNKERLKFIYDIYYPTTWEMKKEVGNIMIKTGSVVSAFNIFKDLKLWE
EAIECLIEADRKAEAKELLDTLLEKKKSPSLVCLYGLVNRESCLKYFIQAWDLSNYKYSK
AARLIGKHYYNKEMYSECCDYLEKALEISPLLPDIWFILGCAYMKIDKFDQAIKAFTRMI
SMTNENTAMAYGNLAYLYMKNNGYKAAKICINQAVKINNNEWKYWDTYLKLSIVQNDVDS
FCLALTTLCQLNQVKQIQPWVFDYISDLIVKDKPTIIPNKTGLSYLNKIIKTMNIISVHI
SEYDSFWNAYSFFLFVKGEFEDSFEAKIKEIRSIESMIQKCNVINKIEVLVSKQVNAIKF
IYHLMKTHYTEDKKGTVIYQLKNIIESILRKEKYMNHKDISELSEIMEDLKGKTSDEEGA
ELFNDEINKRDSEENE----SDEDISQSKKKRKKINHTVSITGNKTGNKKRKVGKNKYVS
TFLDTEAQVGDDEEEEEYASSYVDEFEEAKRLEKKKLYETKLKSGTNHLAQAINKLSQRY
EHEKDLKDGASDDETLTDEEMSEDEEYFDEGEGLSAFDSPKMWLIKLFRNGSERTLAMGI
YYKYMKLQSSDFNIKGIYVSDDLKGYIYIEADSLYMLKRFLLGFKFINLNDISIVPVQEL
TSIFAMSHSKVVIPKVNEYVRIKRGVYANDIGQIFEVHEKGIYAIVRLIPRIVYDKYNNS
KKDQYDNVGISASNKSHSGIANNHLDQIYLMDKMNTNNQLNNGTSAIVPLSGEKLSAIDE
ALQIRRKKKKERPLKKLFDRDEIEQIGGVIEHGPYPGTIKYQNNIFEENGYLLKKMNIKY
LISENANITLTEIRDFNKNNTNEEDINLHISKSFINKNSLHLFKKGERVKIMKGELFNLI
GTITNINDSVLSVNPDNLAKEFKFLPSDVTKYFTEGDNVTVINGLHKGKSGLISLLDYKE
NIALIFSPSLNTEFRSSIQDLTGTISSSEGLGGVNTLNGFSIGDLIELNDRQIGVLTYID
KNKHIRVLTNSNKILHTTIGSITYKRSAIGQICKDENGNIIQSKDTIQLTKGAHKNKTAI
VSYIWKNKIFAKISKKIEDNGFVVVDCENCLLSGNVNEKKKIITQHNLFRNNNMQRRNTF
QSFIGKTVKILSGVYKGLLADVIDAERDEFTLLLKIKPKTIRQKRIECAIADAYKDDKIF
DDKNIEKKRNGISSNDRRYDKNYNYDDEKKKKKHHDTSNKKYHQYDEHE-SNWKYSSHNK
DDKNYL----KNHKSTENMDDYYTSNKNGNT-NRFHNEKDNYDKRKTSPTHSEYKKHEYD
QAENQNNFNDINDEN----------RDEYNKANSDWVIAGVIIKIVTPGLFYNETGRIAE
VIKKGSYTILKIETDKTSFNIVSEAVVPLKPSKPNDEVIILDGGKINQGTVIDISNDKVE
ISTINGNINCDMKHVFLYKKV--MSSNSPLSVTLVDNLASYNIQNESFRLGNVAIEGDKF
ICVKENVNDNTQVVVINLYNQISIRKYMKADSVIIHPNDPILALRGSIKNANTIFLQVFN
IETKEKICSLNLNEPINYWKWINNDTIAIVCEKNVYHWNINVLTKVFEKAQIFIDNNSQI
LYYGTDKEMKWCILCGISTQDQGKSIDGHMQLYSCEKKLHQIIEGFIGCFGSFIFDNWDT
KPLFCFIEKKKNSSMSRLHLMDIYNSKTEGSTPYKIVKEINLINDTLNDFPIYISINTLQ
GVIYVITKCSYVYIFDESTLTEIVREKISDDNIFICCDNKNGEGVIAVNKKGKIYYITLN
YISLINSLKLSNIDVKDKIIRNLSLKYGYPGCDYISVYKKCISEMDFKKASKIICLLKNP
KLRTQQVLNSFKSFRNSQGQLSPLLLYFSVLLELDKLNTYESIELVKPVVLQKKKEYLEK

WIKDDKLACSEELGDLVKALDLRLALIIYLRCGAHNKIISTYCLLNMFNNVMTYINNYKH
TNFDFVNIFITIINYEFPSGNTNGNDSSSSLDLHNTEKNESMDFFNDNSSKDLRNANNLR
NTNIEIATQYIKLLSDNNIQLDIGKIVDYLLNNNKLQEATSILLDYLKDNKPEHKHLQTK
IFEFNLYHNVQIAETIFQMDIFTYYDKNRIAYLCEEKGLYQRALENYTNINDIKRVITKS
AGGISLEWIKNYFSTLSDSVCQDLLFDFMKGNKVNIEIIISICVQYYNKIGIKKIVNKFE
ENKNYEGIFYFISSILNELQNMGTVGNDNNISRSNNDDASSILLNSDIGIGTQFSENNSV
LTIEDLHHIMFKYIEACVKINNIQELDRICKDRNAKYNPEQIKNFLKECKLSDPRPLIYV
CDIHNFIEELAEYLYKNSLLKYIEVYVIKVNPHNAHKVIGVLLDLDASEDFLLNLLNNIK
NISNIGNLIDIAEKRNRLKLLLPWLESRSNEGYENIELHNALAKIYIDLNKDPETFLKNN
NFYDKKMIGKYCEDLDPHLAYTAYERSNGQCDEELINITSKNGLFKLQAKYLVSRQSMGL
WSMVLDESNKYRKNVIDQVIGSTLIESNNADEITVTVKAFIEKKLSSELIELLEKIVLHN
SEFRDNKNLQNLLILTAIKSDSKKVMEYINRLDNFSGPQIAAVAYEYKLREEAFVIYKKF
NCNTSAISVLLDNILIKNQKGKMSANARRKQIELAEDQEMQKDEELTNIENYEEDLNRAI
EFAQKCNVNDVWFILGKAQLKLNKIIDAIDSLIKSNNAGAYKEVIEKCKENNFYEQLIAY
LNTLREQNSLKDVLVDSELLYAYAKLKKTAEMTKFIASTNLANMQLIGDRLYKEKEYDAA
KILYGSIPNNQKLTFCHLKLKEYSLAIEAAKKTKSLKTWREVNLVCVKYKQLKFAHVAGL
QLIMHADHLDEIIKIYEKKKYINELLSLLENGLNSERAHVGIYTELGMLYAKYKPEKLME
FIRSYTNKMNTRKLIDVCHNEYLLKEAVYLYISYDEYNLAVDTIIKHSPIAYQPDIFMQV
IHKVTNSDIIHKVIDFYIEENPLNLYNLLKILENKIDNNRLVQTMKKSNNLPLIQKYLED
IQTQNITSVNETLNEIYLENDDYISLRKSIDEYDNFNQTNLINKLENHKLAEMRRIAALL
YKKNKKFKEAINLSKKEGQIKDAIDIARVSKNHLYIEDLINYFIQIKNKEALCACLIACY
DILKPDYVLEIIWLSGFKDQAMLYFIQIISDYTQQIETMKKQLEDIEKEKKMNKSAPNDY
SANTISNQFTYSLNKNLSIMPPQNNYIPNNSDFDKYDMFNSNTHFMPSIVGSQAPHFKAE
AVFGDNSFGEVSLSDYIGKKYILLYFYPLDFTFVCPSEIIALDKALESFKERNVELLGCS
VDSKFTHLAWKKTPLQQGGIGNIRHTLLSDISKSIARDYDVLFNESVALRAFVLIDKQGI
VQHLLVNNLALGRSVDEILRLIDALQHHEKVGDVCPANWQKGKESMKPSEEGVAKYLSNL
MEDTAAKIIGSSDGLLTDPRIAQDFSAETNELLAKAENYFTVGDFELIIEELILLEKKCR
QSYDGISTSKICCFILNKYKLMENYKKVNEYLIFFNKKRGQLKRTIIDIINLCKSWIVDI
QNKEEKLNLINTLCTISEGKIFVEVERSEIIRILSKIKEDDGNIEEAANILQDVHVETFI
SMDKRDKTEYILEQMRLVLLRKDFIRCHVISRKINPKLLNTDEFADLKLKYFLYMIQYYI
NEESYSDVANCYEQRFNTECVQNDPNLWIDELKCYIIFLVLSPFQEQQTKFLNLIKLQKK
KLKEIPTYEQMVNDFIKQDLIEWPLSYEQELQSFYIFNDSVFVGGENRWNLFKKKVMHHN
IHVISTCYNKISLQRLAQLINSTNEESENLLLELVSNKMLDAKIDRLYGVIKFGQKNNPQ
TLLNNWSSQIHQIVDILEESSHLIQKERMVHEAKLKRMQLENKKMALMGDKEAKKEAIEI
IYEIANILNVNLDKETIVILIQLCEYGVSPKILSHIIIQLKKEREKFLQNVSNNMENLKQ

> *Plasmodium yoelii*

MIKDSKINIYYGKNYPFLCRTVFNIYQNNIKKKNLNDKKIEEIVNFINDKTVISSDKIFA
INLDFLLKTNLYYFTSRENINKNIITNVFFQAQYNEWIDFLRNKDIEKNIIPICEHINKH
LYLNTFLSFHYLTLSDIYIYYEMHKYFSGNITTNLKYPKQYKNINRWFKLIKSLLHTHDA
ELVQNLKKKSGNNEDKSSTHPNKSTKVGGTSLSSYEGKLQNAEKGKVVTRFPPEPSGYLH
IGHIKAAFLNNYYANIYEGKMLLRFDDTNPVLEDVKFENSIMEDLKTLGIKYEKISYTSD
YFQLLEEYCIKLIKMNKAYADDTNVDEMRNQRGEGIESVNRNNSIDTNLKLFEEMRNGTD
IGKKNCIRAKIDMSSKNKCMRDPVLYRCIIDIPHHRHEFKYKCYPTYDFACPIIDSIEGV
THALRTNEYSDRIEQYNWFISTLELRNVHIYEFSRLAFVKTVMSKRKLKWFVENNIVDGW
TDPRMPTIKGILRRGLTKEALFQFILEQGPSKAGNLMQWDKLWSINKQIIDPNIPRFACV
DMNNGIILKLIDLKNEIIEKTRDLHVKNKNLGTCTMYYTQKIYIELEDAQMIEPNEEITL
IKLGNVIVKEIIKDVDNKIKEIIAVSNFDGDFKTTKKKIHWLPYIPDKLIKCTLYEYDHL
ITVDKFENDNKEDWTNCINQNSKFETVGYAEPALINLKVSDKFQFERRGYFIVDKITENN
HFHLIKIPDGKSKNMSIISTKVNPQNLSGTKK--------------MKVGLNTNTLPTIV
FPTVVGNSKNKDVSQTYVGDEAFFRERELSIYRPFDHGHISDWDLANNIWDYAINCVDPN
KSVKSVLLTEPPLCSISHRKNMGEIFFENFGFENINISVSGLMSIYAAGLTTGLVLDIGE
GVTQCIPIFDGYIEKNSVIRSDFGGEELTMFMQKLICDIGYNMTTRKSYEYVKIMKETLC
FCSLNPPKDQLREDLTTTYTLPDGDVLRDGYNTIEISHERFYVPEALFNPLLCHRDNLSI
SDIVCKSILSCPIENRKTLSSYIILSGGCSLFPNLAERLEREIKNNSPENARSAVKVHAH
ENRGIMAWCGAQVFSQPELRDAQRGVWISKDEYEEIGDNIFLIKVRKIRKKMDGLKKINL
IQKINEKLYDHLGIEDDNLTEFLIFLCEKSTCLEEFCKKVFENGGEIEQSVLKNIYNLIK
QNKQSVGNGKDSNYNHVSKEIQQIEKKNEKMKKFGCLSIKNTTNLPPLTEE--EEEEKGG
GKGEKYKNKGREEKGEKLADKRGRDRDRGKRK-------DKDRWADREDKKSRESKYGSV
KYRALKINNIFSGKIKKITDFGMFVSFKTREGYKEGLVHITDIEKNNKR-INLNDKFKRN
MIVKVKIKGIFGEKISLNMSEVDQKTGKNLANNNKEENNDENKTYFGNNDDNEFKHKKNK
NKNKYYEDPKEAIKYESVIKMKSDYSKWEIQQLIKSGIVYDENIKNEYKNLKNEEKIDDE
EEIIEIEVSEKEPNFLKGQTTKAGANLSPIQIIVNSEGTLAKAITTTSALTKERKEQKKN
EQNALFDSIPKDISRPWEDPNPNLGERTIAEALKNVGKNYDLPDWKKNYINNNISIGIKN
PLPLTEQREKLPIYNLKQDLMKAIKKNNVLIVIGETGSGKTTQIPQYLHEAKYTEHGIVG
CTQPRRVAAMSIAKRVSEEFGCILGQEVGYSIRFDDCTSTDTIIKYLTDGMLLREALSDT
MLSRYSFIILDEAHERTISTDILFCLLKDVIKKRSDFKLIVTSATLDAEKFSAYFFNSPI
FTIPGKIFPVEVRNSNK---

---MKIFKK-----
----------------------------RREAK--------------HASCIL--RNPKV
IT--
--MDSVASKAFRISLHIFTI
SMFLN-----QIWDTAGQERYRSITSAHYRRSAGAILVYDITKKKSFLNISKWLEEIRQN
SEKDIVIMLVGNKVDLAEEDETKRKVTYEQGASFARENNLFFSEASAVSKLNVKHIFENL
LQEIYNNRLKDNNSCSSTRSHETYEGGIQITNAKNIIKLNDAHDKYN-ENNTNQMKCCME
KSKLAKVEKVLGRTGSRGGVIQVRAQFMGDSELSGRFLIRNVKGPVREGDILALLETERE
ARRLRMNESLDKLSTIDLLNELKRRYACLSKPDGRYVFIGAPGSGKGTQSLNLKKSHCYC
HLSTGDLLREAAEKQNELGNKIRSIINEGKLVDNELVLSLVDDKLKSPQCKKGFILDGYP
RNVKQAEDLNKLLDKNKIKLNGVFYFNVPDEVLVERICGRLIHKPSGRIYHKTLNPPKTP
FKDDITNEPLIQRDDDNEAVLKKRLGVFKNETTPLINYYKNKNLLISLDATKPAADIEKN
ISQHISG-MEQQCHACYFELPDPKTTIGPYDSELNYFMWGPGFEWKPEPVVSQISGEDTY
DETEESEESEHGFDELDEKVNKDDRKRYFDEKSSGGKISIENASHNARRLGLAPSSKDEE
KIRELYGDNLTYDQYLEYLSMSIHDKDNAEQLVKMFAYFDTNTTGYLTKNQMKNILITWG
DALTEDEAMNALNAFSNDDKIDYKLFCEDILQMKG--YKYSLSVLSLLYLLTNYAMDNPM
KYTDMKGLEDLSTLSDVQINDIFGLTGDEVKDRLTKLFGVIDKNQDKVLSDDEITSWFEY
VKNEVFLKQVQIEMKQIDSDKDGFISLPELNDAFSQNLDPKEVEKHADGLLKRFQIVDKD
KDNKLNINEVGLLIDPMKDNDLKELEINEILEHHDTNKDGKISIEEFKETRSDDINMKKD
DELALDDFNFFDANRDGFIDRDEIVKVYFDPTNDAASVGLNDVKDNIFEGKPITFDLWNE
KALKFAVTSLTDYGDVIRYPQDFKLDIGKNVVLPSSKHRMG-DDSLNLDNDLSDDKDSDD
SDGSDKQNITDELMDLLLAKAICIFAFLSVATGGCAIPYILGLFGKRNRNKYESKVKNIL
SNLNCFGAGFIFSIVMFHLLPETIIIASSHKDITIFKTHDPEMKILFIFFFVFVGFCMQL
ALEYVLPTDAKLCCVDHGAVR-FTDDNNHILNVTNHTLDNPNPEIPNASINADIHYHPHE
GDHSSHKNRLTKILDVLALQSFFLTISLAIHSGIEGS-----------------------

---M
QSKFWARGADNDSGDNVSDSSENEVDE-KPLVSAQAERWAVMDSSSSEEEERVIKSHEGK
RLDFYENIEDNLNDSMENDDFSQLLKEYDNLYKFMVKEDPDSIPNFVIIYLDKLTKYVDT
TFQNNVEKKNLSKNKAQTLNKLRAKIRKCSELYQNKLNQYLENPDKFKDDLGRRRKDEDD
DEDDDDDDEEDEEDEDEEDEDE-KKDKTNKEDGDEEDEDEEDDWSYSEDAEYASDEEDDK
TKKAMSKWGLKTSEKVDKKKVAKVKKSKKEGTKKDDKGSHIDDNQSSKKKTYAELLNTKN
LSEDVIRNRVKSVIEKRGRKGLDKHEHINILSKLCEIAKTISTQSYIEVLEHLINLEFDV

```
VSSVYTYMSFNIWNKTFKYIELILDLLIQNEHFYLVSINITEEIAEEESNEKEKISKSCK
TLISFLAKLDDELLKALLYIDVQTEEYRKRLGKTVHMISLLYKGYKYVKYTKNLPDLAIY
ISTRILDHLYYKPELPFKQIWGFVKNGKEYVEKQT-------------KEESNESTDSDE
SPKDVVEKFVCEIFEHGTKQQRLRALLQLSYNKSLYDEFLEARELLNVGNIHELAISSDV
QTQILYNRNLIQLGLCAFRHGRIYEAHCCLVEICSQNKHRELIAQGISTLKNQEKTIEQE
RTEKRRLLSFHMHISIELIECVNNICAMLLEVPNLAKHSYESKKDIISRQFRRFLDIYDK
QIFNSPPENNREIIILATKYLQKGNWKMCCEKIFSLSIWPKFTDKEKVQAILKEKIKQEA
MRTYIFRYISVYDSFSIDQLCVMFDLPQNTVHSILSKMMVNHEIPACWNESSKYILINTI
NPTPLQTMALKLAENINEVMEQNELALNMKNPKFMLMQERKTHMKDDKSNWNNKKGDGKY
GKNYNRHKNQNYKKNYKDKNMNKNFVMEHGNVGDKIFDLNSISREMLELVEVFYDKMKET
TNANALYLYALQIFKICNIHNELPRLVVFGQQSMGKTTLLDFIMGGPMGYTSSDTGTKQP
IVIILKPSDTNKIECYLNKKKVNIDDLHEKMKAIMLNLNESIISKELEVEISIPGGIYAT
FVDLPGIKDDSKAGSELTRKIVRNYVQNFPNDIYILVKKASDDPANWPYHLKEFFMKPKP
LGLGLQTKQCIVVGTRALEFLNNELSTIKTLTELHDRVKKRGISDNNDNMLSLYLLELFS
IPIEQKEKNDFLTNRISMYSKILNGRKNVLDLLLNKFENDCSDSIKKELIDCFDVEKFKQ
EVNSKFMNILIQQLRKVEVKLEKKKAKMEIYIKKLEELYNKGNILSIREQVKLYIRELVN
IISNLLTGNYPILNLPKNGDDFLKKYGGTLMENLKDGNDLAVELYEKQGLYDENFLTYLN
EYLIKQSNENEYNKSVASDFNNCNDLLNNSNNNSNINSLNKRSDIYDNFNDRDKKR-TND
NMLKVGQTVRFLLAKEESSMFGIIQNISNDVRSKNILVNFFFRSSNIEEQIQVKSVEKER
LIVIKPVETLSGDLFFLNGLQVWYKMTRDDGWVGFDKAEIIKVFNNNSKIKDVLIRNLSK
NNEVVSIKINDLYADYTDHM--DEEADPMGETYNEDDALKKIAGPHTDLKILNQLAITYI
CKWLKYNIAKIEPEKIFSDEVLLQMMRSIHNIVDQSDWKPLVVDLLQANISGNILYLTKL
ASCSAAVALNRVFKAGLGEINRKIKNNYLDENIYLLSTNPKFLEELNQALHNFCKERAVS
CANEMKEIVFEQTYAVHFEIIEEIFEGCKLFEDNFLTPTGVKPTMAIINKNVKQNLAYRN
HQLSLTDVKINKKSRSKELIQEEVKLQFWAIKMLISVPFATKIYAHFLNNIVPKNKHLAN
ILDYSIDCESNLEKYIQSKLLNREVN----------------------------------
-------------------------------------MELNNISQILYGTVDPNIDVRTE
AENXLKQAKETNIVQYINQLFNEFCKNQNDPYLRQIAGLLIKNVFVSKDNYESEEKARIW
LNFPENIKNELKNSLLNLLDQHGEKIVIGTACQIISIITKIELSYNKSSELLHKLVNNII
EKNAYTKKSSTICLAYLTEDIADICNESKTKYVFTQPDLDLILTAIINSLCEPAEESIHC
ANMKVLYNLMSFIDHNFKTQVERDIIMKTVIDGCKDNERLTVQVAAYECLINIVSYFYSY
LDAYMYAIGPLTWEAIESDNERIAISAIEFWNTVCEEETFIDQYELDEGKKNHNIVKQAM
VFLLPKIFNAMVTQESEDIDIDAWTLSMASATFLALSAQLLKNDIVEPVISFVEENFIHE
DWRRRDAAVLAYGSIMEGPDTEKLKPLVEESVGQLYEVLRDPSVSVRDTAAWTIGKITQY
HSEIIYNVLGNYNDNNSLYGILLERLNDYPRVAANVCWVFNQLAVNKRSSYNKLENTYTT
DLDDSFCVLCKKLIDVTSREDADTRNLREAAFNALNVVILNVSDNCLKYMIELLSHMMYL
LTNTYLNPLTEEVKSLQGYYCGTMQFIINRLGNQCKPFLKPIYLCIFRLFEIRSDICEDA
LLACSAIINVMGNDFREHLKTFLNVIFKGLKNVSETSTCKICIEMISDICIPWTCDMEKE
MENILECLWEALRSFGVHDSIKISILTVLGDIATALNKSFSKYLNFFANILLETSKITIA
SGSPENDDWVSYIFELRDAILLTYSNIIYALIDGNEINKLKMYIPNILDFIELILIKEIN
HFNAQNFQNSVSLLGDLVHAYGYELIENSKLTDLIISVYGKIDILSSQRDEKCESCVSKI
KWLKKICNARLGQKMSIYIQLTHDLSGTVTAVTVGDWILGILKQKNIQEYEVFFKKFLNI
YQNQDKESETNNRSEIFSLLLSASDCVFETLIETKKNKKINVIIDNKECVKTFNEFYKEV
EEYFVILISILQLEFKSVEDLNNATNNFIKAIKNYNVFPELRLKILQLLYNSFNVNFSFR
FPTFIAILQFSSQNNLFHSILPYIKFIDEWIKEWNISNNEKRQIYLIVAEELKKLKKYED
SYKHLKKHIYYFQKESKEILNHASTIKASVELIVDSINLNNNIFFHEIINLDAIQNLQYI
EEYKPIYELLNIFYKYNIQEFLTFKKKYGDSFFTKYNIDLENSENKMYLLSIISLFKDNK
VQNIQYIADQLNITMLKVEKILVSAIGSDVIDAKIDQINKTVHMKTTILRHFDEQQWEHL
NNQIEKYIKNVQKVIDLTSHRKSTSNAMNYLFYLVLSVILNCFVKGQESATNFYKFIDSF
ASSTYISEESGSSLYDAKRAIQNNPSYWCSAGNHLKDEEITWTGYLNTKGFVKGVKISWE
YSPELVSIFVSSDGEHYKNVIPYKKISSTESSFDEIYFFKKLEEVISIKIGLKNAIHKYF
GIREVKIIGGGNPYFLLLSGITSEQEMCLQVEEGLINNDNTSVILDSCINALASGDGREL
WKTNSNNQIISALSDPPKCLAVINVDNLENNKLVLYDCLRALEDGDGKSNWVFESNSQIR
LQRSGEPLCISQKNVHGNIPGIHDILLNIDASVDATSILDDDHNADNTIDGNLNSFWASS
IFGDSYEHLVYFIIDLNKFVEISRIKVFWEYPPLHYIISFSTEKDNYKIVAENLANPSFI
TIDSLKNIETRYIKISMIKPHPKHGEMDGQFLYGIRSIEVQANNLESVLNFCRDAANSDD
ARDKYFIEYISEFDKNLSNKLINLEDDVSKNVSSISDKLSKLEEVLPNIETCLNEKKEYE
TKLKVSMEQVIELNDKITTLESVDLIHSNDLLRLGISPGDSSSYPANDCSVIKNSQEVPM
SGFYWIKPKCSPEPLRVYCDMDSSTSLYVWNGHSPKSPDHLITNIINSVDDIRKHCAEVG
LEPLVLKSTDQLNSLIFALKKMGFILNGKINIPLAYDYSCNYGSCSGKFHDLLNGNVDLT
TLIYFKGAESPNSTSIRQTAGISYDDGSFKFFNLETSDISAIVCSTNSSENDFSMQYLSI
ECDTTALDDDFNGIVNTNIVALCPLGCDNERFKEFNVYGSNGVYSDSSSICRASIHAGVI
DKQGGLVNVTIESGLDYYKGSISNNIESISLNKGGNESLLDIITNEKKEDIKEETSIINH
RTIRISNLSQDCPIDLFEYKQLSFIEKGNLKKNEQNEEKIAEYNESNESYKTHEIINDLL
KNIDAIHGVDPSVISIIQDETVRVIEKAKKEFAPADILSKKQIDDTINLYNLTENLALYL
YDLSGKYINDLEKVKERLEELKKIQRIVHNFGTFKLNYESMNFSSYFYVFDSKLIKNKPS
VWGYVDTEILGHKNSIGQMSSISNREIGEGYFAKLKGLNFYDFEIRVSVLSKGTGCAGIA
FRAKDDFNFYLFDICDQDGVRRLSKIENGHADILKEKYGEFSINNKWSTYKITTSHANID
IYEIDDKLNEMKILSSLDEKFLSGTVGLYSQINGQGTFFDGLEIIAKPCSELSKNGKHEK
KQNYNCPYYKENYDNDLFPYTIINDVEYKWGFANEDDDYLFCKKIENIDNESNDKLYDTI
ALLKQRKCSDGILNFDMNYSKENKNNKPHIYILFNFVNENNFNALEIRQDSLKLTSYKDS
KTVTLSEFNDHDKITKTLEQDEWFHVNVKFDKLKFKAVVSNNNYEIELDANNIDTDSIDL
GNVGFSVNNFDEVKFDSITLSSNVLNSNESFIESKTKTWGTCEKSIH-------------
-------------------------------------------------------FINKC
VSLEENKDYENCDDNDLNCRSKTCILCCEQNDVVDSDDIEEISFHKSKDIQKEETIECQF
QCKITHGITEMSFSAIKYESKILESLASMLNLNEKDEYKGKKFEYFYNIETYGVDFFLDI
CNALCRSNYKPFLTEEGKNEILSKERLNKERLSDKKSPKKNMYLNQNXYITEEIEEKXEN
DILNSNDTFXSQQNHQDGIISVFKYKSELSSITRKSNQKIVQTIFEHTKNFNTDSTTNMS
NSNFNNSNSNFYSIENNNTRRSLRSRQFSGKKCSNKYKNLNKKVKLSQNKSLTRNSYRND
CNSITNYCNNRKIRKRRTSNNKKNRNSTNSFMPDYLYLENQIDSIYSIFKSIDKSIEKEE
SPSKFKETKETEIEENIENENYKEKEEYNSYLGLGEISNENEDEDLEEQLNKQNSEYGIY
EKHTLSKNFCNYEEYEGNQSDDTSYSTNGSNYSKYLKKKKKLEEQGKIVIDLNLLHRKTI
KPNMKQCRDEKMAEKLRIEKLKKILEEKKMLLCRVEDIAKKRNRIHVKTQILSEDSKIKR
IYFSHKMKKVYNYKFGYFGCGWSNNKTEWTPFIHAPFFDNQHNTIYKNRNKKLYEEIYDT
ILHGRIHPHIKVVELKDHMHPIRLCTPSNEDCYSVIYTGEKINSTDERVIFGEYTGFVAN
NKELPQEKHQYIFALTFNKKVFNDRKNVVFINEIDSFENESDEKENSIEGNISEMKYANM
KNGNNYKGNQFNLDEDKHKEEINTEKEKMNDIKGEKNKSLGNSKNDIIDKSKKKKENDKS
KELNNLIIILPDNYTYAVDSSYMFNEMSLVNHYKTCSIFNNYDFRINAEWQIVYLDGWPHI
ILTSIPGVEIETGEEIFADFGFEWFDRVNDICLNDFIKNNYEHRLNEIGIKKEKNFNGLD
DIVDKYNLLKNYTTCNICIHSVNTDCNNYILCSGCNHVYHLKCVNRLNFQINENYDWFCS
SCIQFSMNIISQKEFLEYIKKENNKRLINYFNKNIEQGKTKSSEKLLMETNLDSYNCE--
KNNEIQLDDENYINNFENIKNLLQCKENIDDLLNNQQIINAFLENVDNNINILQGEEGQD
DNIILKNPELKNLIENSYELHLLIEYKNKIDDLLLCRENINFLLNNDQAKQTFQMRIKTI
NYLMQNKKCIENLVESIEKKNQDEITKNNNTTFKTTNDQLQLLMTDNAQNGNTGNLSSLD
LFKYENGEQNCVALCEENENTQSMIIPANGKYPYSKDGCYFTSAYENMLYKKTNNIIKNL
KDLKNKNRKSRFKNNSIKNEVNDENNNGTDEAGLNAYYNYMMKLSKKILGLPLIREFEKG
INTHQPALPLNANLKKLEVCSNCYKKHGNLAKAVICRVTKLHFETNYNDGLGEEELHKMS
SECIQSVIKELANTIKEYRKQELNSAFIQYTKKKKQTGLLNNKENDIKIGTRFSDIITSS
DIINSTFNKTDI-ENKDFYHSLSKSLYKDTYETDDNITIQHSHLSYIDKNRSVENQQDGN
STLQTNTYTTFG-KKYENNDDGD--GKNSHQVNLSSDTVDEKNASDQENCKNDKYISHKD
ENMYTHNSDINNVCFRSDSNNNDYKNQKTNNFKNDDELLTTNEILLDDKKKEDKPISNNN
NINNFIPLFGIELGKTKFQREFTNGTYVGTVTKQIKDDNDNHFFVVTYEDGDVEWITPFF
LFQELLKQGTNNIEYPLATPFKDLMNPNFKKDIKLNNYSLELKVEKKKKKGASEYNSNNN
SATKRQRHNQEETSSRTKKR--MINKEEAQRLKELGNKCFQEGKFEDSVKYFSDAIKNDP
SDHVLYSNLSGAYSSLGRFYEALESANKCINIKNDWPKGYIRKACAEHGLRQLDNSEKTY
LEGLKLDPNNKSLKDGLEKVRKDKEMENMEYINHINNIINNDAKLKSYKEENSNYPIELL
```

```
NTIKAINANPMNIRFILSSCNPKISEGVEKFFGIKFNDEASYEAERERQRKKEEEEKKEK
ERKMKEEEKKKNRTPEEILGEEHKLKGNDFYKQKKFEEALKEYDEAIKVNPNDIMYYYNK
AAVYLEMKSYEKCIETCIYAIENRYNFKAEFSQVAKVYNRLAIGYINIKNYDKAIEAYRK
SLVEDNNRATRNALKELERKKEKEEREAYIDPVKAEEHKNKGNEFFKNNDFPNAKKEYDE
AIRRNPNDAKLYSNRAAALTKLIEYPSALEDVMKAIELDPKFVKAYSRKGNLHFFMKDYY
KAIQAYNKGLELDPNNKECIEGYQRCVYKIDEMSKSEKVDEEQIKKSMADPEIQQIISDP
QFQIILQKINENPNSISEYIKDPKIFNGLQKLIAAGILTVRMTNKYNIFNYNITMVACI-
------YSKKILVLDRNPYYGGETASLNLTNLYNTFKPNEKIPSKYGENRHWNVDLIPKF
ILVGGNLVKILKKTRVTNYLEWLVVEGSYVYQHQKKSLLFSEKFIHKVPSTDMEALVSPL
LSLMEKNRCKNFYQYVSEWNANDRNTWDNLDPYRLTMMDIYKYFNLCQLTIDFLGHAVAL
YLNDDYLKQPAYITLERIKLYMHSISAFGKSPFIYPLYGLGGIPEGFSRMCAINGGTFML
NKNVTDFIYNDNNQVCGIKSSDGEVAYCDKVICDPSYVMHLENKIQKIGQVIRCICILSN
PIPETNDINSCQIIIPQNQLNRKSDIYVNLVSFQHGVSYKGKYIAIVSATVETNDPTKEI
EKALELLGPIDEKFIKISDLYVSTNPKPKDNIFVTSSYDATSHFETATNDLLQIWENLWG
QKLNFDDLNKS-DNELMSVKKQKKTLKPSTKVITINLSKLTHDVCYKRKAPRAIKEIKNI
AGKLMHTKDVRLDVKLNKFIWSKGIRNPPKRVRVKIERVRNEDEDSKERMYTLVQHVMVD
SYKGLVNECETNEMATPKGRKKSDMKKIDESFVEHGDSVKKSRNTGQDDKALKLRACLSC
RLLRTEAEFYQSGCTNCKFLQMTGDRHRIQDCTTENFSGFIAITTPTKSWIAQYNDLSKY
NPGFYALQVVGELPESIRDLKSSYMQYSSNGKINFDYIYNNKELKNKCYLPSSDTFVFAE
ALEDDAETISSNVNMVLEMGTGSGYLILFLYELLLKKNKRIDLLYCIDINKDACNCVQNA
INLNGISNVEIINSDLFNNLRKCGQFDIILFNPPYVETEQDEMNKTDIVASYAGGKHGRE
VILKFLHTVYDHLSNNGILYLLLEKSNIPHEIMTSTLVSEKFYYTELKKKKTLNETIFIY
RLRKKIMDRYGHNIRGDVKKQGYENSDLPILCETCLGENPYVRIIREENGKECKICKNAF
TLFRWKPGHNARYKQTIICNKCAKVKNVCQTCLFDLEYNLPVQVRDKFLETSITLPENET
NRNFFLEQLENNISTDTYDKINHGNMDLSKLKRRDPYFKRNMARVCSFWRKNACNRGDEC
PYLHKEIHLNKSLASQNIKSRYTGENDVLAETILNRYKNNNIDEKNMANKICIQGISNST
RVENIKECFKKFGEIKSFKMIPKDSKIFISYATLTAAKNAAEKYKDGLELNGSNLTVTLQ
QDNLNNNINNHNNTKFPKDNFRFHNAPPPTINNTTPTMYFPYNNSNFNSMPPNAIPYASM
LPSEAEQRKMIKNIFNEYLKHYDNKNFGYEKNDTNISSKEHYNEYISDWRWFSIKCERII
ESELNKKHLQKNEKNEYKNNITEIDNISTFRSFFPKILLNAYSRYSNPKKFFDNLIIQKF
NAIVFFCDASGFSSLAEQLDKKINGAELLGNCLNKFFNILIKIIDSWEGDIIKFSGDAVM
VIWPLNAKMKKKKKKETKVKKGINEINSSEINSSEINNSERDADEMKIKRMHQIRRMSLL
ALGCCMNIHKFLNKFPTPIENKYLKVHIAITYGKVNFLQVGNILNKRDYILSGKPLEEIG
LGESLAKDGETVISYSFYKNIKDKIEIKETCKKKYYLLIGIKEKLDINKLKENCYEQDNF
IKN----DKKNVNVCKNFDLLLKSFIPDIVYRKLSIGYNIFFNETRKVTIIFVSVKDVDT
STMTGIYSTHSIMKLTQKAVFTMEGTINKFIFDDKGILILIMFGLPPLYHSDDSIRALLT
CFRLIDGLKSLKLNGSIGVSTGKIWCGIVGNKIRKEYTALGDSVNIAARLCCKAGNKEIY
VDENTYNNCKHFIIFQSLISIKVKGKNKSIKIYSPIGTINKKGNNIDYNDLFILDYFTDK
ELLDNANGYEEVTDFNFVNNNFLLIYYKHKFKDRINDLLNRKDCLYYLKTNKETQNNSDK
NKKEENNLNSNCFKCNKIYSLSPYYFLKRELYIDYKTHTGPLFIHEYYDPLFFYFKEIYN
IGGVLFLEGNEHLGIFEFIKLIKKKLKNFKIFNIGNMPNSLYINITNSLLPWKMLCNDLT
SMWKLCNTRKKYLYFNNVDNYNILKEITHPSYHWFLKCMSNIIDDLYIPDTCLKKETKKI
NKIFDTKTINNI-----------TQIESLNGTHYYENRKNGRTFCN-------------
------------------------------------------------------------
------------------------------------------------------------
------------------------------------------------------------
------------------------------------------------------------
------------------------------------------------------------
------------------------------------------------------------
------------------------------------------------------------
------------------------------------------------------------
------------------------------------------------------------
------------------------------------------------------------
------------------------------------------------------------
------------MEENKLEVEEENALDWLKGISKDKGNDKKNDKGNDKGNSELLESDDT
ENENEISSTEKVKPLYSEPEDIDDNDNINTGKLIIFNLPPVDEQDVKSLCERYGPVIDVQ
IFKNTKKGSKYICLNEKNDQKSREDFFIKLLKENNDNTSNGNKGSTKKMANSNNTKEGNK
NESENENSKNENNITNIDLTNVKTYAFVSFVFPSSCEKAKNNLNETIYRGKVLSVKYARE
KIDNSENLEKNKNNIFIKLSNESKTSYKKILEIQKKRNCQNENIWNILYTDINSNIYNFC
KETNCDPQSILNIRDKNIAVNVSLTETFIINKMKEWIRKEGIALEAFEQIYKKENAKPEN
DENDETDKVVKYKRSDDTIIVKNLSMHTNENDIINLFKKHGILKKISFSPYKNIAILQYE
KPEDAKKALISNSYIRYKKLPLYLEWAPVNLFEQNNKNTEGTTEENENYND---TSHQNN
AEPYDSESSDEEITHSSIYIKNINFNTKEEDFKKLFEKLDGFITCNIVKSKKAIKQKHKD
NKTEEENKYISLGYGFAEFKSKELAIEAIKKLTATKLDGHVLELSLSHNRIKKNKY-NNE
EKQVIKDKKKITKKLLVKNLAFQVTKEELKKLFSAFGNIKNVRIPKNAYNRSRGYGFVEF
MSKNECLAAINALQHTHLYGRHLIIDFANDLIFDQNVDEFDKMKESQNNGNQGVITSEQA
KRKVIYENEKSAKISESKKRKIIGNLENVMKNNCGESTYNNQGYKFCETSNCGTKYVDQN
GQQYNVMDNIFLKESNNGNENNYLDKIPENARMLKPLIQEKIIEIMKPEIEEKIIEVPQV
QYIEKLVEVPHVILQEKLIHVPKPVIHERIKKCPKTIFQEKIVEVPQIKIVDKIIEVPQY
VYQEKIIQVPKVMVQERIIPVPKKVIKEKIVEIPQIELKNINIEKIEEIPEYIPEVVKRD
VPYTQIIDRPFHVEKIVEVPHVQHIYRNIVSPQYRHIPKPVEVPMAHYRTFPVEKLVDRN
VPVPVEIQIVQEFLCPKIEARYKEIPVPVHVQRIIEHPIPKDAMNNPFLLPLYYQEDNFN
ISKNGTSNTSQNNKGCFMFNWNKNNNQKPSKSRNNSVELLLHNDNKNNEFFKINENNINP
NINFKSINQYSGNTFRDINNIPMDANMNMHVLGQCQSINPIDNTNNQIMNNFNNEPYDPN
ANLRINNSMNYPATIPLKKPYPIVSPIVPPLNEMFSTRSEYDRGVNTFSPEGRLFQVEYA
LGAIKLGSTAIGICVNDGVILASERRIASPLIEKDSVEKLLPIDDHIGCAMSGLMADART
LIDHARVECNHYKFIYNENINIKSCVELISELALDFSNLSDSKRKKIMSRPFGVALLIGG
VDKNGPCLWYTEPSGTNTRFLAASIGSAQEGAELLLQENYNKNMSFEEAEILALTVLRQV
MEDKLSSSNVEIAAIKKSDQMFYKYKPDDITKIIDSLPSPIYPTIDMTSMEVEKTHLINY
FLEICPTVLDCSRKELQSVLAKREEEKIRKFVVDKNVNIIVIGKGKQEKID-EGNDNI--
---KKLENERDEERNINFDKESSYSLFVELNINYKCVTKSIAIAFMKRNKDTFLVLNEKL
NNGNKINLSNELLMFVCGLNDCNTPLDLVYLYLSQGFNTIFDAASYVTHGNEHDEISQKK
YSKKRDVENGSSSYAGYGNRKCGTYDGENKISNLLMTNVSKKLNELLISMKNAQIDLNIP
IINLNVDKRIKKLVEENPNIDDIKQETIKNLCESQDFLNQLQKDVTKWVEEIQKITRLNG
DFKSGSALAEINFWIGYENALLELENRLKSPEVILTLQILKNAKRYFATISFDSDIQLKQ
SKEYVLNVNILMKDFPIDDLLGAQTVQQTMQAIRNIFNHLKKLKNTTKYPLSKSYNFVEA
LSRDLGNTMKKILSNQSLLSMDYQIFDVLISGCMEVFQLWNEEIRVFKDMVRELIKKRSF
NERAPAKMVFEHVILQERLDDIRKFRKQHQKFKTVITKVFGNDKNVGINLHKDINAAYNV
LLSLDALDLSKSGNDLWEKTKLSYESKMNKVESQITFKLRDKLGGAKTSNEMFNVFSKFN
PLFFRPKIKGAIQEYQNSLIQIVVEDIKKLQMIYINGYVKSSSEKVSTIRDIPLVAGSII
WCKQIEKKLEDSFKKIENVLGRGWEQHAEGKNLKQNIDNFKNLLNQNKTFEKWLKSIKNG
DKFDIYEKIIKIKKIGINNYEILANYDQQLFNLFKEVRYLQSINLRVPYSIKVKADETKL
IYPYALGLEKTLRVYMKICLYLENEGKDIPFNGTISMLVASAHNSVQEKIKEGINLHWDS
DILETYVRKLTEHVNMLESMVDEAVNKNSFVLDILEKIKTCQISIDGNEIKKYVELIQEK
ADELYLQHYKNVHLWINEMNKILDAILANRLEEIIKKWTEEFVGWTRRNSTKNRFILRSS
VHEFKISKHKIFLYPPTDSMRQIWFSRLSEAINLICGITRIKNIYQKKDKSREVIEIKNK
DGEVINNVMLDNTYKYIIYYVDKNVYDNAIKCINDSVEKAKEYESMWFQYKTLLKVEIGD
IITNFGEDIEIWKTFMNEIKEREDKFDILDTEKNFGPIVIDYRILQSKVSSKFEIWQKEI
VSEFSKKLLEKILIFKEDVEKCLHDLREQSELKSESEITAMHILSMRPKDIITIMNDYDM
DVLSRICSSIHDFVEKINEINKKEQEWDSKCDILKKSELMLEKQKYFFPKNWLFIDSVIG
KVETVKQVLAYQINLVKEYFPYIQSLVIEFDSKVQNNIKELYEQWNNSKLADGNTNSVKV
LQIIKTFESKINIISQDYQVSEKLRKLMKISDEESEGNFHISPKMLKEEITCIKTIWDEL
KVIYSSISDLKKILWINADLKDVKNLLNNLLGSIKKIPAKYRQYEIFDKVKDEIQEYLKT
YVILSDLKSESLKERHWKLILQKLELKIHYNKLSLGNLWSSNLCHHENVIREILNQSQGE
MVLDEFLRGLKDTWTEYELELVQYQNRCKLIKGWNDILSNIDDHLNAIQSMKISSYVKIF
EETLNWDDKLNRLRNLLEVWMNVQRKWVYLEGVLKSSTDIKLLLPQEYNRFKIIDADFI
```

NIMKKTSENPKLLELFQINGFQKQLDRLSDSLSKIQKALGEYLEKQRNQFPRFYFVGDED
LLEMIGNSKDAKIIQRNINKMFAGISSFILKENTSDSILGMCSREGEEVFFKEPIHISTY
KTLKEWLIVLETRMKATLENYLDLAAIEFMQMDILQCTKDSTNRQIIDWCCKYPNQIILL
CLQIMWTYNIEHDMELSMEKLEMASQTIFLTSEGVCRSLLKYLSEIVVKQTNNTTRQKIV
QMITELVHQRDVIRVLIEKDIKNVNDFTWLQYMRFYWDKDKRNKNVNLIIKMADASFEYG
YEYLGMCEKLVQTKLTDACFLTLTQALKMKLGGNPFGPAGTGKTESVKALGAQLGRYVLV
FNCDESFDFTAMGRIFVGLCQVGAWGCFDEFNRLEERILSAVSEQILTIQTSLSQRKKEV
EILNKKVELNKNVGIFVTMNPGYAGRSNLPDNLKQLFRSFAMIEPNKELIVQVTLFSQGF
ISAEYLSNKIVSLFDLCSEQLSKQPHYDFGLRSLKNVLNSAGNLKRLALQEKWQGDGDNG
KSSDEMEKVKKTVEMEQTLLLKSVCDTVYPKLVSSDIVLIKSLLSGIFPNANISLFEDKD
LINEIKRICKLKYYIPEEKWITKICQINQIMKLQHGIMLVGGVGTGKSSAWKILLDALEA
IDNIKGMSYIIDAKSLDKEEIYGKLDNINLEWTDGVFTCILRKIIYNYTTNNNITKRHWI
IFDGDVDPEWAENLNSVLDDNKLLTLPNGERLPIPESVKILFEVDTLKHATLATVSRCGM
IWFSRDILPPIALFKHRLNKLKYGNSDYPRKIDILKSLFVSSSNNETTEGAIMNGDGDKK
GNDYENKGISNNSIVINENIMENMRRNSQVFYDKSEENIDVCKLQTIEYRSIELISEYFE
ENGFVQQCLVGAANFEHVMDYEYIRVIESTCLLIQKGIDNLVAKYGKFNNSVSDSDIEKY
MSKWLVVSLIWGIGGSLNLESREKFSKYVESICSVALPSDLSKRGLVNSSNLSNANRTVL
DYEPSVDDGEWHNWKERVEITDVDRTEISDATLIIETMDTIRHATILEGWLNLKKPFILC
GPPGSGKTMTLTSVLKKSTEFDIAALNFSSGSLPNLLLQTFDHYCEYVKTTSELVLRPIQ
PGKWLIIFADEINLPTPDKYDTQRIIMFMRQIYESQGFWKYDSNNNQWNWVKIERITFAG
ACNPPTDAGRNPLSNRFLRHTSILYVDFPGYESLKQIYGTFNRAILRKFPESLHMADNLT
LAMVDFYAKFSETFTVDMEPHYIYSPRELTRWKLSIYNTLENFEKIENRELVRLCIYEGL
RIFQDRLIYKKEKKETDKIINDIFKYSFPDVKDEDLERPIIFSSCIENKYIEINKNILKE
LILAKLKIFGEEEVNVQLVLFDEVLDHITRIDRVLKLPFGHLLLVGASGVGKTILSRFVS
WMNGLSVFQIRTGRNYSTELFEVDLRNVMKRAGIKEEKITFIFDESNVLGPAFLERMNAL
LASGEVPGLFEGDNYKALINECKGQYGTNAGLDESDIFKKFTKQVQKNLHIVFTMNPANP
DFANRQSTSPALFNRCVIDWFGDWSYKALLQVASEFIFSLNLPDNNFYMDNINSESIKSK
LDFKDKKYYFLSKAIVEIHNSVVHINQVLMKKGSKYNYMTPRDFLDFIKHFLKIIDEKRE
EISAQKKHLNAGLKKLKDTEVQVAELRNSLANKKKTLAEKDIEAEEKMKLMIEQQAETED
KKKKAEILAKKLDEQFIIIEQRKEIIRKELSEVEPKFREAEDAVKNIPKKIFDELRAMAN
PPILVRNAIEAVAILIMNEGDKNVTWEDARKIMKGQDFINKVLYLDKKTVKAQTSAQIKK
KISNADWDVDRINKASRAAGPLAKWVESVITFLTILETIQPLENEIEKLQEETRIAENQY
NEQKEIISALEKILVQYKNDYAQLISQVQSIKQEMEIVEKKIVRSINLIENLKSEKERWS
ETFISLEEASETFIGNCLISAAFCAYIGFFEHYERQKLKKRWGEIIKIHHIKYRYDLSFI
EFLSKPSERLQWIANKLPSDDLSIENAIIISNYIRYPLIIDPSGQASTFLLNQYADKKIV
KTSFSDKNFLKNLESAIRFGSTILLYDVEKIDAILNSVLNQETHKQGGRLLIVIGDSEID
FSPNFNLFLTSRDANLQFTPDICSRVTFCNFTLTPSSLQNQCLNMILKNERPDIDKKRRD
ILKLQGEYKVKIRELEESLLLELSSLEGNILDDDNVITTMEKLKIQASEASKEVNIAEGV
MIEIENVSNMYLFLAEGCSKIFFILQYLCNINFLYQYDLNFFFNIMKDMLLDQEYLSKIK
KNDYGERIKCLEYLLFNLTYNRVARGLLQEDRYVFALHLCYVKSIINPSCYMDKVYLHLL
LKDSYVYEEATTIMGQKTSNEIGREVLPEYNDEQIRNLNNLIKHKLFYYLKDLIENNKEG
WTSLLHSDEPECVVSSLIMQNKNIKRKNSSVTHLNSVEEGEGINQENPISQCLKEALIIK
AVRADKLDRCFNKLINIILGKEFLWLPELSTNEFEKYVKENAWGNIPIVLISSPGFDPSN
KVHQLSEHCKVSLSSIAMGSEEGYISAEKVIASAQKYGGWVLLKNIHLSHKWLYQLEKNI
HRATSNADFRLFLTMEINPKIPPNLLRMSLTFMFEPPVGIKSSILRTFSLFLENKCINDP
KIARLRLYFIVSVLHAIILERRRYTPIGWTKKYEFSDADLTCALSVIDNWVDKSAVKIGN
NISEHIDPSNIPWDAIKKILNEAVYGGRLDNIVDSKILDTFIEHLMHSNSFETDFNLNIS
NSLSSKDFLKSPDLFRNIQDYIKWTNNMINTDIPAWLGFGQHAEGLLTTRTNFSIINKWN
ILYSKSCSDMQELLPYSQDEIKLEDQVDEMDEKENTDTPEYNTKVRNIRTKEQNENIVFI
DKILESLPEYIPDLEKSDENVGNAVFRCFERENQLFNNLLKDIKTNLIQLKNVLEEKIKY
TNKLRALAKDINSLNVPSGWLLQTNTSNMNLTIWLRDIIKRLYQIIVITVELNKSSTAKS
NSEKYGSAKKTKEDDDANNKKLSINFIWLGGIFYPRAFITATRQMCAHKYKKSLNDLELS
ITVDEKNEEKLMEAFHFTIICLSIEGAQWSDKDRCLILP---TKNNKNPINQGNCNLNVN
SNLSTHIIPSSNLSSQHTKQIRLNQMNNSHKSHLMQYHNSNYDDKNSKLYNNVNTVINKH
ISSENSGAPHGSASNNNDAVKINKIGCNTRQKNAPHILHNDGNNNSCKG-QQPYNENLGY
RPTLFNLLEVLSNHNITTPDQRVCIRGIVTDFLNNELPHGKIYAYIGAVVGHDILHDIIK
KLEKDPNRNAVPDASGLARIEASFGLSKSSYDFMNHNSTNSSNNIPNVLFNNRNLNSALL
NNHIYSNNNKTANDINNMMMKYGNDKSGGSNNNMLNINKKKALNMRNNNIVEDEEELMKV
IKKNIETYKMNNIKNILISSVFGRIKWLKIMNESPHAYLYGHSIVKYGNKLYMFGGTNNK
HKKVPFNHTLTFSLIYYNYKLLPLSGNYPDERDGHTTHLVSLKSGLSVFLFGGANNNIYY
NDIYILDMETRKWSKRIVNGKLPLPRHQHCSLVYPAKSEHLRGEKLNLTEGVIIYGGKCL
YNNNIVNLNDMWIFLFQANMWVRINYLGDQIPIGRYGMNLIWSDTNTLCLFGGECLQSDK
NYKTRKLLDDMWTFRLNTPLTNLINKIKIAGEWKREIYEGNIGSRSNYSSIFITQRHQDF
KGAEPKTIEKLMLLCSGITY--DNKLKIVSSDEIYVYFFSQKRWYLLKGKLYNEEYMYSA
RQRHVGCFFESKNVLGRSNRNPVPCIFIHGGFKKNSVFGDAWLLSLTGENPLRLYEYDTC
REKISTTQMPLYYFRDTHSISLLYTFCTLQKWLFGAFANLVDNSVYAINPAENVFIKYEL
TPEHDGMISIQDDGEGLDFNAMNRILRMYGNYKNYDNNSFYNNNNNNNIKKHSLPNNDDS
DGYEIDNYDENNENNSDNNTNQTQPNEKGDGENNNNLSGSNCEGIIKKENETRNKNGVIG
KKKCTWNDSFYYKKKNQHYFYNDISNSIFDIKYGVGFKTAFARIASSCAIMSRTINTIGI
GLLSLELMNHCDAKELATPLCMWKLPNKELINRNIANKSEHRHHQKLLMSYTPFNSPSLL
AEQINILGTYSGTRLLYWNFRDDMDFIIFNPTNNNIYLSSSPLSIDELKNKNKKQKNTQG
EGSNFSSKYQGVKDIKFPNRIIKRKGVFEEDGNLKKLKTEKNED-------IIADDK---
------EENIKKEHDNISNSSKNNIILDNNNNNNDDNNNENTTNCNEKLKYEDELKYNNN
NYFKKRMNLFEYNSHLTPNISKEKYNMNQIFPLWDHPKDSIDYCLSTYLYWLYLRRSTNI
FLQNTLLIPTCMRREKKNNNDNNGKKKKSTKRNKK---------INKQNDEKIANTEESE
TVLENNKSELIQEKNDDNNHVDAQNEANVNNNISEQNQNTEINNNGIKNNVCGLTKLEKQ
KLEEALEYGISQNVQPQNSANKNGTDDEEEEDEEDEEEEEEEEEEEEEEEEEEEEEKNNE
DNGNINETNNDVEEVEGEINQVDEGGVVKTEQNEMETENNV--EDGNETNKKKVGKTIDD
TNEDSSRRKRRKKKDDLSADTTANDKKNGEIEKMKKENYVNVYMENFKIKDEYYINNPSK
YTLYHFLRRKLYKMVESHYLFTPADHTYGSFIMMGFLNDNNNTSIEVNRVCETGILLYYK
NRLIKRLDAPFIDTPYNLFLSKYPPNPSLYEGNLYKYALTVIVNVPNWLKPSVSKQEFVH
ENNHAFLVFKKKLVTLIKHYLSICEDVVKLNKWRESRDMKLKKYLEKIQNQSVKSRNDSD
YEKNKNFNIIYNDQNDGNKKKKEEINYSGKNK-TPYTNDSEPILHKNKKTKTNNDDKRDH
NPTSENESANNDNENNQENDVLEGEKEQEDDEY---DQEAENRPNFQDEKDEHEENEEEE
EDPDDEEYNAEMDDQKNRSVEQETEEEMIEDEEYEPMENNPDIKSLGDDNNGKIPKKKNL
CRLIVEEATNDDNSVVALNTKRMEELNFFRGDTILIKGKKRHSTICIILNDNDLDEGKIR
INKVARKNLRVCLGDIVYVKACPEIPYGKKIQVLPIDDTIEGLAKDTLFEIFLKPYFNES
YRPVKKGDLFLVRGGFMSVEFKVVEVDPDDFCIVSPDTVIYYEGDPIKRDDEEKLDEIGY
DDIGGCKKQLAQIREMIELPLRHPGLFKTLGVKPPRGVLLYGPPGSGKTCIARAVANETG
AFFFLINGPEVMSKMAGEAEANLRRAFEEAEKNSPAIIFIDEIDSIAPKREKTNGEVERR
VVSQLLTLMDGIKSRGQVVVIAATNRQNSIDPALRRFGRFDREIDIGVPDDNGRFEILRI
HTKNMKLSPDVKLEELASNTHGFVGADLAQLCTEAALTCIREKMDVIDLEDEIIDKEVLE
SMCVTQDHFNMALGTCNPSSLRETVVEVPNVKWDDIGGLDEVKNTLREMILYPIDHPDKF
EKFGMAPSRGVLFYGPPGCGKTLLAKAVASECSANFVSIKGPELLTMWFGESEANVREVF
DKARAAAPCVLFFDELDSIGTQRGSSLGDGSGAGDRVMNQLLTEIDGVGPKKNLFFIGAT
NRPELLDEALLRPGRLDQLIYIPLPDLAARISILSAILRKCPVADNVPIDFLAQKTAGFS
GADLAELCQRAARAAIRDAIDAEEMNKKSKLELNPEGNQTNENQDTNNEESEIKYEITRH
HFKEGLAGARRSVSQADLIKYDNFRIKFDPLYKTKSGGTNEDFIIDWPDEENNEDPQDYN
VDDDLYS---

KISSGKKENDVEPQNYDKNMSKQSNEENNIKEVEQKKKKIFDIEQIIKNEENFKNIKEIK
YKIHCSKWNKNNELNISHNIIKSLYDHNFYSPREIQSKTLQHSINEKKDIIVVSKTGTGK
TLTFCLPILSNILQNKLKEFHTKNKTGQKFRCLVLVPTRELAVQILNHFNYVNKYINIYI
ITIIGGLNINKQLRLISKKPEIIICTPGRLKYFLQLEDKINYLYNMKNVRYFVCDEVDKM
IETSFINDIHFISKHLYKCVDEKKKFIQTFLLSATLSLTVQLHNENLAKLLNYISIRKNK
SYIIDLTNEKDHDQNKILTFNNNDINYNNSTFLPDHLTLNIIKCEKKIILHKLYYLLKLY

ILKDLPTNKENKIKKIIIFLNTIKLVKDVSTIFKYLFFESGLESSLPNKYKTNLSLSKKI
NIYSIHSKQKLKERIQSVSKFTESNNSSILFCTDVMSRGIDLNKCDLIIQLNCPISDITF
IHRSGRTARNLKSGECICFITDDEISKWNISLKKIGLNLKDLKEYEHVKTIPNNEFSKIN
KSIEYCNEILQLQNKIKTNKEKSNLSKLAKDAELSNDDDTSDSDNPFPKKTNEHIFKNLF
RLKKQLYNTLYHEMYDINSANCSEIKSAELGVLSSQELKRISLGKYENQLQEFEKVSKDG
TSNRITYDPRFGTIDYTQICSVCFEKHDSCLGHIGHIEFVLPVFNPLFYKDLQELLNLVC
YNCYALCYSEDVIFLLKHIYQLKSLNEIESSNKFIIKKNNENEYIVSEIERLYNKICKES
NITMEDIVESICQDYENSGNNEKDNNENDSFEDRQTYEKKINDDIYNDDQTNEADFSKES
DNKENKETKEKIKFLKEKIKKYKFDISKLAFQSNYNYEEYVILNKAIKLHMKKGANCSNC
KFRRSIVAKTSPKKDTINFVGIYTNNSFFKKYISKKSKNEGFEENGSNDDND--------
DDAEVGKANKRGKEIKSEKIKNLNNSENGIDKFQYEGSNNKDGEDESFKDAVASYKRKTS
REKCTVRLFSFQIIDLLKKIFVENNKEIINLVYPFTKKDGYQVFFLYYMGISSNRFRTQS
RGIHKRTRITNYICKLNNYVKLCINSKKNVDFDHMIEYNKNELDLYNEMFLLFSLRLRQR
VISNSMEFNIEIAKSDFLRCYSLIYNNKIAYLNILFGNLQLSVNTFLDSSLIDKRLQKNM
DNISIREILDKKEGILRKNIMGKRVNNCARTVISPDTFIETNQIGVPIEFAKTLTIDEHI
TENNFEYVKKLIENGPDVYPGALSYKDGQGRVFKLSHSYENRMNLISRLTHILQKGKHET
LVLHRHARDGDIVIMNRQPTLHKFSIMAHFLKIFEKEKVFRLNYVNCSSYNADFDGDEMN
LHLLQTPLARAEATHLMNSDFLFTSFKDGSPLRGLAQDFILGGLHLTSLETFLNYDEYCN
LLQCSLNGLLRQKNAFFFSKTKHRN-TSNSSNTKDGNKNSGGNDKSGSNSFRGINAKISN
YSYIDPYATNLNKTNFTIKTEEPTILFPKKLWTGKQLISSILKTIINELAIETYNKNGDN
SFMENFKGINYVAKAKTDPELWSFDPKKECEIIIKNSELLQGVLDKLHFGASSNSFVHLC
YELFGPKSAAVMLDCFGRLFISFLQLRGTSLSLYDFILKKKAKVEKKKIKKRISFTGFYL
QNLFVYTIGKALNADMNKMDEYSNKKISNYEEGQNLFSNKLYDIYLNKSQIINKYERKLE
KMNIEDKHMNTKNIPNKFLFNDKELNLKKDLYFITFLNKEIEEIFTQNNMLSGNVKNEES
EETQKCKYSDFEKCLDESRYYEFVNSIINKLYNSINNPIFYKTSTFTSTQVVKVIEKIVN
FLKEAKCNKKLKVFILFELFQNENIVKYFPSLIDFASGLNTNISSEEIVETVINNILLKS
LNTEKIKFNDNKLEKTNDVILKKYLHLYSRLIKIE----RE---KKGKNNSPSKLKHDED
QNIG---IDNVDIIKNCLISSLPSFALNEDITNLSYNNRYQYYEKYLNIKDEKYSHFKFY
RMKDVFHKLDFLINNFFSLKRINFDGLLDSLFQPYLCRVSSGTNNLITMENLMNKFLTNG
FSNMIFTGAKGSKVNYSMICGMLDQQYLEGKRVPRMRSGKTLPSFHRYDYGARSCGLITD
CFLEGLRPQEYFFHCMSGREGLIDTAVKTAKSGYIQRCLIKSMESVILHYDGTVRNEDNS
IIQFLYGEDGIDPSKTAYLNLPSDLINNYNISFSKYLNYGSDQLILSNQRLFAKNDGKKK
NEDPLIFQYNPYTYIGSISDNYNAKLNNILINKKFNITNYNE---NFDSLSASLLRSKYF
HSLCSPGESIGILVAQSFGEPATQMTLNTFHLAGTENVTMGIPRLKEIFLTSKLTSKPMI
YVPIKLDEKNNNNPNQIKNYITNIADKILSSYQSILLSEVIYGIGINRKLIIRDKIENKE
IHTIKLDNLLSKNNKENEKTANYSKILQVIENTNLNFDLKKEWVHEIVIQFDNLNHFCKV
NKHLSIPFILYKMVNIVLMSILNKIQNSISHHNIKNIHFINHEHYDELFDFISEKMSEEF
NFAYEKYEYKDDEDEINAKLKYMPNKSEGNKLGDDDDH--YNTS--LKTPKKFSEDNDDN
FSMDGKNEGSDNDKNDKSDGNNDSDQN-DGRESDEYKSEQEDKEYDESEENDTDSMLSQN
AADTDDDIKHENVNSSESD-------DGDEKKKEKKSEKKENETDSESNYSSDESITDSS
IINEDPKESNKSKKEDKLNTSS-------------KKRKLNIKNEQNKKEINSDSSISSE
ETELKQKMSKKFSEYLYDYKNRMNKHNLDSDYEDPSIGISGSKNKLNDQKYIKNLIDKIK
SSLLCFIKKIDFSPITWVLKFELSWDVNFFPYPIDFLSYLQNEISKEILFKVKDLNNPKI
LKGKDITHSGEEYELQIEGRNVYKLFNIKDKYIDKTKLYCNDIYTIVKTYGIEAGRLCIT
KELKKVFEAYGIKIDFRHLSFISDFMTHTGDLKSFNRYGMAACRNVFHKMSFECATTFMI
QGCIQNSVDYLSTPSSSLFFGKHIKVGTNVSNIVTSIKAKMAKNRLLIESREAKKQNDPD
IFLSHSDYNLYEWEAIIKGPKDSPYEGGKWKLSIKCKSTYPIDPPMITFITKVFHPNVNF
TTGALCMDILKTNWSPAWTIQSLCRAILFLLNEPNAESPLNCDAGNLIRFGDIKGFNSMA
KMYTKEYAM-------------MFALLRFAFDGGVKVKSAAEVGKRAVEYFRGDDFVSFL
SSKQDILKKKFPKLIGNRNLSEMKEVEEFADLFIQKGFIYKAQYKPLKGVHEMDENGVYK
RPKWPKRLIMTSKQNFDKAGFYILVYERNKKLQYFMLMILISIVLICCMFPVWPLKLKLA
LWHLSVVFIGLLSAIIVGRIITFVFFWFFGVDYWIFPNLFDEDCSIVESFIPFHSWERRN
DSWFLVFARAVTAVLVAIGIHQLGKTHSISDIQNFAKQSFIDIIEWGNKKLSDTPENTFM
YKSIDSKATFEEQEDPEDQIVYDDNEENYDCLKKCGFPPFEELVRRCFLKCDCMEKIIKS
HCYTKKCSKATKEVLDEAHKEACFQNIKTYATLADTLLKDLEDLEEEE--NFNNNNDGIK
KINFEEQYEEEDDYEEIVDAIEEFLNDKKKKCERKISELLYDEDFLKTMNHIKNYIIEEE
AEKLKNEEGTKDTDGDENENNIKRKKAYNNEDENNDNNPNDEEVLIEKCIELIIKIDTEI
LNIHKYLKDIYSTKFPELDSIVYTPLEYISVVSRIKNESDIKNIDFSDILPNTTVMAIVV
ASSMTTGIKLPDHLLKSCMSFCNEALELNENRQKVLIYLENKMFLLAPNLTMLLGSALTA
RLISCVGSLKNLSVTSSQNLIVVGNSKKSILGLSNVNKTFGVGILSTSEIVQSVPDAYKK
KAISLLAGKCSLASRIDYFKKYKEGQYGLLLREYVINHLIKLQEPPPMKQKKILPIPDEK
KGRKRGGKRYRKLKEKTEITELRKQINRLPFGPNTNEDFYTFTDQNTALLNSNITKLKYQ
TKQKTNIPKKKNAAAQSSGATGGLSSSLIFTPLHGIELFNPSINKTTSESRENKYFSSLS
QFRKVM--
---HILFY
LYI-FKENSIKEYEEVYEKGIGIGVKLDILLTIIRISIFFNDINSTKKYLEKARAQMEKG
GDWERKNKLKIYEALNYIMIRKFPEASKILIDAASTFTATEIISYESIIFYVIILGIMTE
ERTVLDKNILNSSVILQVTSSDEDLYNYIYSFYHCDYRTFMEKTIKIALRVKKDRYLGRH
YRYFIRNTRVRAYKQFLEPFKSVTLKNMAYAFGVSEDFIEKEISSFIANGKLNCKIDKVN
DSIESNQPNERNTVYQDTIKKLIFFITNFTKKNKINIFMVNINWPGLLKWSTKYSDGTVD
TNKRLSKEDIEFLQGAIKEALSQVEDPYEAIGEAVRNFESKDEGIILASAKIIERLVDEY
PEVAKNLDKINALDPLLKLLESNNNHILESVLQIFSLALSNNPVLQDCVFKKNGLKILLL
KLQESKQTTVDKKLITAISALIRHHDEGENKFIDYGGIAFLVYGMQTNIYKYQEKSALLL
KHLIHQNKITFETFEKNKVMNGLIALANNKNIDETGIQYGETTAELFLALMQSHRHKLAK
AGYLKQLKELIEGRLNYLTVIQDNASYDVSQEIDLFKDCLKLTKKIEGRKIQK--RKLPL
GFRKRYRRYYIIKYLNKRFLKDKKRNENKNENNENENESGNEEESDCSSDTNGPESFEEL
GIDDWLIKISKSVQIIKPTKIQKLCLPLIIEGKNVIGSSETGTGKTICYCWGMLQELNKN
FYGIFGLIILPTRELVFQIVEQFHLYGNKIGIKILSCIGGFSLIEQRRNILNKPHIVVGT
PGRVSDILDDCIDVKNCFKRLRFLVLDEADLLLQKSFEDKLKIILSNIPKEFGEPRRTLF
FSSTITDSINLLNKTFPNDKLILVDANKKQKPLKNLDQRYIYIDSIAQITYLVYILKNKL
IDMSGIIFTANSYKCELIYNVLNSLGTFSVESIHSSKDQRKRMSSLLRFKNGFCKILIAT
DIISRGIDIPKVAFVINFDFPNEIIQYIHRIGRTARANRKGLSISFIDKKDLQSFNNVKI
AMKNKLKPYILNKNEVLADMLKIGKVVKKVEMMLQEKKDIKKENEDLKNSIYLDE-----
---------------------------------VVVFPVSVHYDLTRKLLNKKIGTGIQN
VSKFGNGSYTGEVSAEIAKNLNIEYVLIGHFERRKYFNETDEDVRQKLQQAIKNNLKAVV
CFGESLEQRESNKTIDVITKQVKAFVDLIENFDNVILAYEPIWAIGTGKTATPEQAQEVH
KEIRKIVKEMCGEAQANKIRILYGGSVSVENCTSLIKQEDIDGFLVGTSSLKTSFTEIIK
SAMMKKKNKEDLYKENKLESSKLRIAIVSTDKCKPKKCHLECKKNCPIVKTGKFCIEVEH
SSKIAFISEMLCIGCGICVKKCPFSSITIINLPKDINKDVVHRYGPNTFKLHRLPVPKLG
QILGLVGTNGIGKSTALKILSSKLKPNLGKFDNPPEWRDILSFFRGSELQIFFTKLLEEQ
LSPIIKPQNVDLIPKQVKGNILEIINKKDKLNQKDKYMKVLELDHLLDRNVEDLSGGELQ
RFALLISIIGQTTNVYMFDEPSSYLDIKQRISMAKIIHGLVRHDNYIIVVEHDLSILDYL
SDYVCCLWGKAGAYGVVTSPFSVREGINVFLDGFIPTDNLRIREESLNFKLATDQDVTDE
DKKRLHFYTYPKIVKTLNSFTLTIDKGNFSESEIFVLLGQNGSGKSTFIRLFAGLIKPDN
VDSLSFLESLSVSYKPQQIQAKFTGTVRQLLMSKLKGLYTDPYFNNEIIKPLKIDGILDN
QVLTLSGGELQKVAIIITLAKNTNIYLIDEPSAYLDSEQRIIVSKIIKRFILNTNKTAFV
VEHDFIMATYLADHVIVFDGQAGVNTVANTPQTLVAGMNKFLKIIDVTFRRDPTNYRPRI
NKYDSVKDKEQKLNGFIIILNSMSILCTISGQTPEEPVVSKTGYIFEKRLIEKHIKNYGI
CPISGEILTLEDLYPIKNEKYVKPRPITATSIPGLLSIFQTEWDSMISEMFNLRTHVNDV
RNQLSHSLYQYDAATRVIAKLLKEKNNYQEEINNLRNQILQLKNGNDINDLEIGINEDLL
NEMQNIAKELLMNRKKRKVENVNSPNEWKKITATNEFNIHSSIIPGVTCLTIDVNKLKYN
YDDDHKNHNFFSGGKDGNIYYVSLNNNKIISKLQGHLKKVNSIISHPSNSICISGSNDKT
IRIWKGDPDTNEYVTSHIITKHKDKINSLSLHPLENYFISSSNDSIWILHDMETGKTIKT
CKSSPSPFKNLSIHPDGMMFGIGSEDSNIYIYDIKSQEYKASLTGHTKSIESISFSENGY
YLASISKDNTLKLWDLRKATTFQTIELENTPKHITFDYSGKYLSLSVGNDIQIFNFETKN
QASLITTLSSHTDIVTQTCFGSRTSYLLSSSMDKTVKLWSMSEEMHCYNGNIKDGLFHGY

GILIYSKNEKYEGDFAYGRREGKGKFTYADGATYEGEWVDDKIHGKGVAHFVSGNVYEGE
WENGKISGFGILNYNNGDKYEGEWSEGKMHGRGTYIYADGDVYVGEWKNDKRHGKGCVKY
KGNKDKIAETYEGDWYEGKMQGKGVYSFADGGIYEGDWVDGKMEGKGIYKYLNGNKYDGD
WSNDMKNGYGILTYANGEMYEGYWKDDKVHGKGTLTYSKGDKYIGDWEFAKKSGEGELIY
SSGDKFKGKWKNDKANGFGVLNYSNGNKYKGDWVNDQRHGFGVFTCKEDGSIYSGQFSYN
RKEGQGTLTFSNGTIVEGIWNSGVLTKVTKFQLYPSSPWNDPDLMKLEEVKDIQKIERIG
AHSHIRGLGLNDCLDARYCSEGMIGQMSARKAAGIVLRMIKEGRISGRAILLAGQPGTGK
TAIAMGIAKALGEDTPFTHISGSEVYSLEMSKTEALTQAFRRSIGVRVKEESEVIEGEVV
EIEIERFQEKDATTNNKKVGKMILKTTEMETLYDLGNKMIEALQKENITAGDVICIDKGT
GKITKIGKSFARSKDYDAMDPNTHFVQCPEGELQKRKEVVHTVTLHDIDAINSRTQGFLA
LFSGDTGEIKNEIREHIDMKINEWQEDEKAEIVPGVLFIDEVHMLDIECFSYLNRALESE
QSPIVIMATNRGITHIRGTDYKAPHGIPLDLLDRTLIIPTYPYKHEDIRKILEQRAEEED
VEIDEFAKELLCKIASESSLRYSLHLITLANLVAKKRKATEVTVQDVRRVYNLFIDVKRS
TQYLIEYQNEFMFSELPKDDESVKDQESVDEKRENQEKKQIVDKGNAMAYLYSNLSDFWT
SDDEEDGERTDEDFDEDSENNYKDTNKNYINNENNE--NNKNNEFEKREDEEELQTSNMN
TNAVTGGETALDLADLLNDSRNELFDNIPISSILQNYDINNFIYNDNLLKYCEAGVTINN
ESSCNEPLTLNEEFNFNCYSHFDYINPPNIKSSYTSTSNALISIENGENNFIDMCIQNKD
EL--NHKIKDISVKEISNEMAKEEMQEDEKKISNEIINET-MKKLEVIPEKQNVKYIRKK
WVIEDNESDVSNFSKNDLLLSYNFELDDFQKRSVKHINNFKHVFVAAHTSAGKTLIAEHA
IALSIKLNKKAIYTSPIKALSNQKYYEFKNIFKNVGIITGDVKMNVNANCLIMTTEILRN
LLYLNDNIINNIHCVIFDEVHYVNDEFRGVIWEESIIMLPPHVQIVLLSATVPNYLQFAD
WVGFTKQKEVIAISTKKRPVPLLHYIYAHDSLFLIMDEKNKFYSSAFKEIYIKIREKEEA
GKKGKELMGSSHGGKKKMYYSDPKNNKDNQMEKQNKTGTTNNLGDKQNDTVKGYYQYCKQ
KQKQRMFQNEANMKTEIQKLQALIKKLDEDNKLPVVLFCFSRIKCETYAKSMPHLNFLDN
KKKSKVHLFIKESASKLCDQDRELNQIKILSKLLENGIGVHHSGLLPILKEIVEILFSKG
LIKVLFATETFAMGINMPAKSVIFTSIYKHDHLKKRILTSSEYTQMSGRAGRRSSDSYGY
VYIYCSDNIPDQVQLTEMMMQKAVSLKSKFKVTYNMILKLLINKQINIEKMLFSSFLESC
RALQIPLFKKDLKRKKKMLQNIKQVECVYTSDLNQIAPIENYVYIDHKLKNIGLNLHKKL
FDMKNSNAFVVGRIMLLNTIDLFRSSVYAVYLGCDKSSNKKNNKVNFAQNSIFFFNDKEE
NEIMERFFFLFILPDFIGYGDLTNNTDSTNVNKNGTNNSTAVSNENTNTEKTQLTSENIN
MYENYKNVFACTNNKKDINIIYHSTFDSDMNKKHFVVCSNVGIENISLITNTIIKIPNVS
TNALLNNPKNLLLYTLELDRLIKKETFEPFTLTKMLKSLKCEFYSVLINQSDYLDSLKKS
LCYNCSLKDEHYELVCKKNDCINDIENIERNINAKSLNLYEDLEGKLNVLKHFSFIDDDN
NLTIKGKIASYITLTDEITLTQIIFENVLNNLNPPEIAAVLSCFVSPEKKVEESPDLTLN
LQDVKLALTNIHSKFEEFYKVIRLKISTEEHWKLCNFKLMFIAYKWALGVSFSELLEQSE
FEEGLIVRSIQRLDNLCRKVRIAFLYLGNVDLAEKTEKASLLLRRDIVFTTSLYLQLLFT
AIHANFFSKGNKANYVSAYINSNNNNNDNKINIENNNKINDTNTHQDQLNNFFTRRFYSL
YSKNRYLREGKKNSKEVNLNYNMESSNEQNNQMMGGDSYKERLQNLKKYMGDHNIDVYII
INSDAHNSEIINDQDKKIYYLTNYSGADGILILTKDAQIVYVNSLYELQANKELDTNFFT
LKIGRITNRDEIFQTIVDLKFNTIAFDGKNTSVSFYEKLKNKIKIQFPDKKIQEKFIYKN
SINQVVKDNNINLYVLETPLVTVPNNDVNKKPIFIYDREFGGSCAAQKIQESSDFFIENP
DVDSLLLSELDEIAYLLNLRGYDYKYSPLFYSYVYLKYNRDKGRIDDIILFTKVENVQKN
VLAHLERIHVKLMDYDSVVSYLTKNVSSKSENTKNNNGKNIILGSIHENSSPRYDISLSP
HINLMIYMLFNKEKVLLKKSPIVDMKAIKNYVEMDSIKEAHVLDGLALLQFFHWCEEKRK
TKELFKETEISLRNKIDYFRSTKKNFISLSFSTISAIGPNSAIIHYESTEDTNAKITPSI
YLLDSGGQYLHGTTDVTRTTHFGEPTADEKKLYTLVLKGHLSLRKVIFASYTNSMALDFL
ARQALFNNFLDYNHGTGHGVGICLNVHEGGYSISPAAGTPLKENMVLSNEPGYYWADHFG
IRIENMQYVVTKKQTDNAKFLTFNDLTLYPYEKKLLDYSLLTPEEIADINEYHQTIRNTL
LPRIKENPSDYAKGIEQYLMDITEPIHTKMICIWISNIFFYNLFVRSVKKNKNIFSNNLR
RNQKRNIFSVE---NKKMSTLNFDNNNLEIQKKNFVIQEDPDFIQYRLNKYNELKEKKKK
ILEENDPNYYKEINIELLDGSIKTGQKNITTPYQIASQISKKLSENSIVAEIIYLDNVNL
NLCDIEDDENDETNV-DQSQEGILWDMNVPLIGNCKIKFLGIETPQGKKIFWHSSAHILG
SSLEKLYGGYLTIGPALNEGFYYDIYLGNNSIISENYNKIENEYNKLVKENVEFEKMICT
KEEVLELFKYNPFKIELIKSKINDNEKTSVYKCGNFIDLCLGPHIKNTGKVKAFKVLKNS
SAYWLGNKNNDSLQRVYGISFQKKTELTDYIKFIEEAKKRDHRNVGKNLNLFFFEKETSP
GSGFWFSHGAKIYNKLIEFMRKEYRIRKYDEVITPNIFSCDLWKTSGHYQNYKNCMFIFN
IENKEWGMKPMNCPGHCLIFKQLNASYKSLPIRLADFGVLHRNEITGSLSGLTRVRRFQQ
DDAHIFCSLDHIKTEVVNVLQFIFFVYNLFGFKYDLYLSTRPPKYIGDIETWDFAEKSLK
EALELANVKWKLNEGDGAFYGPKIDIVLRDSLNRSHQCGTVQLDFQLPIRFNLQYKNKEY
GVGQETDSNLVNNSNTDVIEKKEDSTLENIDNN---QDPINQPGGVLKKGFDRPIIIHRA
ILGSVERFVAILVEHTSGKFPFWLSPRQAIVLPISDKFNEYAKYIHTVLTNNLFDVDVDI
SVNTLNKKIREAQLKQYNFILVVGEKEISTNTVTVRDRDNPNDQKVYTVQELVNTFKKML
DINSVKLNEITPFIQKNIIEGNGWKKNNNRYSNKHLSLTYEKIFLLAMLAVLGMYEIMNS
KEE----KGKKKKNFFFDFLFPKNKASCCGIMAYLGDGDASKILIDGIEILQNRGYDSCG
MSTIDKSNSLKTTKYASSSTANAIEKLRGNYLTSHKNDNIGIAHTRWATHGSKTDENAHP
HVDYKERISLVHNGMIENYRELKKFLVQKNIPFKSNTDTEVVANLIGYFLDQKQSFQDAV
VSSIKQLEGTWSFCIIHKDFPDEMVLAANGSPLHIGIKDNEMFVASEHSALFAFTNEYIS
LKNGEIMLINKNNINNLKMIKKFDNIPEIVIQKTPDPYPHWTIKEIHEQSISLSKSLNNG
GRFNLKNNTVKLGGLDPYVEELKNIENIILIGCGTSYYAALFCKYIMNYLHCFNTVQVMD
PIEFNISAIPKEKEGIIFISQSGETRDIIKACKLAEYFNLKKLSVINSVGSTIANMTGRG
VYLNAGREVGVASTKCFTSEVSVLTLIALWFFQNKQNLNSNNKVSSLINSLYRLPLYADT
TIKSCEDTCKALSHKLSKAKSMFIIGNGLSYPIALEGALKIKEIAYIHCEGSTGNALKHG
PYALLGGDDNIPVIMLIFNDGTKNSMISIGEQIKSRGAHIICLTDDVNLCNHFADDIILI
PNNGLLTSLLAVIPLQMLAYYMSVNLGHDPDKPRSLAKTVTV-INSKFDSLFKILLIGDS
GVGKSCLLLRFADDTYTDSYISTIGVDFKIKTIEIDDKIIKLQIWDTAGQERFRTITSSY
YRGAQGIIIVYDVTDRDSFNNVKNWIIEIEKYASEDVQKILIGNKIDLKNDRSVSYEEGK
ELAESCNIQFLETSAKISHNVEQAFKTMAYEIKNKSQLENQQKGRANINLNAKPIKDNKK
KCCMPHIRTMNSKKPPEGWNKVETFLNEMNQKMRSLENEDTSKKRKNEILWPIFQINHQT
ARYIYELYYKRKEISRELYDYLVREKYVDGALISKWRKQGYENLCCLKCIQVSDSNFNNA
CICRVPKSNIGNKVIQCVNCGCRGCASGDRMNSVLVSYTFIPKKYLKISKRYITRDSIYF
QRTY--KFL-NNINSIHSNCAIFFCNCRLLFSRRFTKNKSIKTMCNDNKRNPINDNFNVN
KKNNTQTNNIAIKEENMDNSNYDYDYIVIGGGPGGMASAKEAASHGAKVLLFDFVKPSSQ
GTKWGIGGTCVNVGCVPKKLMHYAGNMGTLFKSDSDKYGWECDNLKHDWNKLVSTVQSHI
RSLNFSYMVGLKSSKVKYINGLAKLKDKNTVSYYLKGDTSKEDCVTGKYILIATGCRPNI
PDDVIGAKELSITSDDIFSLKKNPGKTLVVGASYVALECAGFLNSLGCDTTISVRSIILR
GFDQQCANKIKLYMEEQGVTFMCGVLPKKLTKENDKILVHFNNNTTELFDTVLYAIGRKG
DIDGLNLEKLNININNNNNKIITDKFSCTNIPNIFAVGDIAENVPELAPVAIKAGEILAR
RLFKNSNEIMKYNLIPTSIYTPIEYGSCGYSEEQAYEQFGKNNIEIFLQEFNNLEISAVH
RTKHLKVQKDEYDVDISSTCLSKLVCLKNEDNRVIGFHYVGPNAGEVTQGMALALKLNAK
KSDFDNCIGIHPTDAESFMNLSITLSSGLSYAAKGGCGGGKCGMENDKHENMLMHIARDF
KSIDDLVDVFLSFLENKTDYFHLMMNDDDIQTLSKKYDGDIAKAILNNNTCGFKANSRET
ELMKLFRKHQLKYIIKNQPYIIENEDIRNKYLSPCDELNKLSNIQIAKTKNTQNEAQRNN
RNPHTPAYDSINEKHISTWNGGRTEKYFWNQSLNEINLEIPLNKEIKPSEIKVEITNKHI
KVQHLNEVKLEGMFYEEVNKQECVWNIEDKKKIIIFLEKKKENWWSYVIKGDPEIDTTKI
ESKKNLTDFDEKTQGEIRKMLYKQKMMNEGLKSPEELKEQALLKNVLDNKGFPFSKMKSA
NAKEDTISQTCDNSTTEDVDEHLYTGPLKIEQLLAKGFVKRDLELLKEGGLQTVECVAYA
PMRTLCSIKGISEQKAEKLKKACKELCNSGFCNAIDYHDARQNLIKFTTGSKQLDALLKG
GIETGGITELFGEFRTGKSQLCHTLAITCQLPIEQSGGEGKCLWIDTEGTFRPERIVAIA
KRYGLHPTDCLNNIAYAKAYNCDHQTELLIDASAMMADTRFALLIVDSATALYRSEYTGR
GELANRQSHLCRFLRGLQRIADIYGVAVIITNQVVAKVDAMSMFGGHEKIPIGGNIIAHA
SQTRLYLRKGRGESRICKIYDSPVLPEGEAVFAITEGGIADYEEKMALKSINISGNFDWC
PFEEYKNYLICFNSHNLLYSNNGSLNNYTYLLDINLNNDIRSLDIVHKLNFEEALNRENN
KSKTSSNEYVTSFEWINCNNFVESQNENELNKGIIVGGLTNGNVILLNAQNLFDTSINYE
NFILSQTNIHESSINCLECNKHKNNLIATGGNDGQLFIIDIENIFSPTSYDPYLDKNNLQ
KITCLNWNKKVSHILATSSNNGNTIIWDLKIKKSAVSFRDPHSRTKTSSLCWLENQPTQI
LISYDDDKNPCLQLWDLRNSNYPIKEIIGHSKGINNICFSNIDSNLLLSSGKDVTKCWYL

```
NSNNFDIYNEVNNSGNNIYSKWSPFIPDIFASYTSMDTIQINSINNGNKMTNKYIPTFYK
KDTSICFGFGGKICLFDNISDNSNLSASENVSNKNVNQNQNNVDEGNIQINKNKSGNNNN
PYLIKCHIYPTEVDLIEEADKFEKYIACGKYQEFCENKIAKCDDYHEKLTWKILQLLCTS
QKEEIVKHIGYDMNEIHQKIVENVGEESGFIFKKYKCETNENMNISGNITSNNLEINSDV
NNKHDNTRMSNLNNGENISGYSNYGNNMHGEFVTSQMQENEQSFNESYDLDPEKFFRELG
EKNEIEKNQENEEDQNGKEKLKDSLNNVDSNTKDIINTIKCQNFENVLGEINEDSKPNSN
KTNSNNWNNGIESIIKECLLVGNIEAAVELCLYQNRMADALLLSSFGGENLWHKTKNIYI
KKQNDSFLRNINYVLDDNLECLVKTIDLSSWDEALSILCTYAINNPNFNNLCEILAKRLQ
NEKFDVRSASICYLCASNFPETVEIWDSMPSSKSTLLNALQDIVEKITVLKMVIKYNKYN
STMNQKINQYAELLANSGRLKAAMTFLSFIEDDKSIGNLTLRDRIFNSAAHIMPSHIKPP
PSPFQYFDIKPFG---LSQKYNNNNNNVNASIQGIYNKSQIH--------------PNKS
LTSIVPPLPMSEMHKQHGPSMAPFSHNSPTKFNTQMIGGSSGVMGSTDSTSNRGYSTSNF
NNNVKYPSTGMGSLYIPPPNIPS-HSTTPINASPSPYNSVQNLAHLPSHPNLKLEHEKLG
QVSNMFESQSYTNVNKAMQNSMGPPPPP--SNQIQGVSRSSFSVSQNNIPPPRNRHASVS
TPPNVPFNQEPQLNKREPIDSQIYNPVSP----PQQTQVPTQMQNTFTPQQSYSQNKIGF
APPSGMSHTNKCALTCT-------------------------------------------
------------------------------------------------------------
-----------------------------------------MAIRVQFENSNEVGVFSRL
TNSYGLIALGGSENFSSVFEAELSQHIPIIYATIGGTRVIGRVCVGNRKGLLVSSICTDQ
ELLHLRNSLPDNVKIKRVEERLSALGNCITCNDYVGLIHTDIDRETEEIVQDVLDIEVFR
TSIAGNLLVGTYSYFTNNGGLLHAMTTSQEIEELSELLQIPLVTGTINRGSDLIGSGLVA
NDWSAFCGMDTTAIELNIIEKIFKLNNIEDTNIEDTFKYKSSIVQTMIMLESLVEKLLNK
FLAPYVEGIEKNLHLGVWSGNIVLENLNLKPQITEILDLSFKIVHGSIGQINIQIPWSSL
GKNPVCVFIKNAHIYVKPRHYKKSESVLIDELRKAKMHRLELLEEEISLIKQQKNKEKPA
EKSTLIFKLLNKIINNIQIDIQDVFIHFEDIERNFFIGFILKSSSVKNLKKKDERSSETN
NDSESKKLNHIIEFKGLCIYSNSIVNKKKRGNKKSKKKGKKENTLKKKNSKDITHETDRI
NNNPTNDYSK--KETCSDTENKFNDKNSREQNGGSASPINADDKKPSKQYNNDNIDSKFK
LSNDHFDNDEENDYLKKTLANINLFYKSDDHKMLDYNNYLIKPFDLVLPVEQSSNKKELK
ARLEISDKWEGITLTRTQITKIIEIMSEANNSRNEINKLLLKHASTVKLDVESLRNETKN
EFINLYNKILGEEYNISKIELTKKEQNRLQILYDVVGVRHLAKWRLQCRDTLEKIIEEKN
CKKKILYDSIYKQQSWWSWVTGNKKDIENKVQSILKTEQDFINEHELNILQEAVVNEDNY
DVVIPTKYDFQFKLSNFSINIYDDSKKRKYIINNRINSKNDDKFNEQIKGASNKNKYNEN
THMNSDCNNTGNNLNNELCLSGTSESSLSTNSNISSLNSIYRNKKLDYNEINILSINFYQ
IYSSLSLQSVVDHNDNDNFQWKFIIELQNFVAKHRNQIFMEFRNTKNAFSHNINNSENIY
QNPIYHSLLYSQSLCAYLEINHLVTEKGNTLSTLLRLNPLELYLSPLLINNILSFTFPLM
DIINNKDITSTRRYKEKVENALVKGEIGEDEERTETNDTENDEDNEEALLHIKKMEQSEL
IEGLKEKGENVYNRAVQHLPEMFEFYIHICGPILHFDNLTNGIVNLHLGNLIARTEYPCA
YNKFNLIFEFNETQITCLKTSNFEDDKFYILQPIPVKVYVEYDLKILKTNIILDGIFFQI
NPDAVSIILAVPTSITRYLTRFYSKNKKEDESFKNKRKNNAEKKIDGKGDENVETNPQNT
NDKIKNTNVSTNLLDDNIKTQLEVSNIDKNKTEEESFLYDIDFLIKTSSFSIKNSKNCDI
LKYEACGISYKNYFQKKKKIIKIEIEQLWICDPSNKQPIFFTLAKNINSKDAYPFKYISA
YLSNSNKMLNESNFDTNEKYEHDDNNNTGYLFQSAHRLENGKLEDNDDDDFMDAIEEKQL
SINLQIIQKHNENNIAQIHVDFIVSDIELHWKYKTIKQIFKTMKEYKNILQYGIEKDMKY
IKNKLKNEKELKNYKMFISEHTLRSVQETLKNVKNSLNILDIKASTQDNQNDINENNTVD
KNETINRGSLQKDQQFEGFPGENNKNLKDLNISNEYNKEKNNVENENSDVILKNGLNDTL
PKLFGEENSYVKYFFNCYVKSASLSFWEKKKIFSKIQVSNIFYENNIYLNYDQKMFINIE
KGIISMNGKNIISNNINDYTYDLFISKKSKDNIGYNDDEYVANLSDEIKRKETMTNKKKQ
FLFVGNVNVYNDKRYYNTCMMCDISGIYYIFYLRDLRLFLEYLDDGILNVFISKSYKKVV
QVAQEKYFLFQFTIVDPIVIIPEDKNIIYNYKGQVKDTSKYDQDTKNNENNNTDNENNGK
RGSNNKCIPYIESYLKFHLSTLKFKNAYTKNCAEEIVLRQRNEIKQKRMRKEKRRTKYKL
KKKKVIKNSNDQKVISKGDNENFDSNKKYDFTLYVDLLDIESMACENGGSTNIEGNILHK
IDIGLCLLSRKDGIFIFFNGKDLCLDLTVYQLRFLLDIINENFCYKGYFPMCFICDKNID
IDNMLDAELFRKGNIALNNLSGNNSNFNTKTDGIRKNGTKFYAYINLESLKVKTSFDNNI
PVAIITFQNISISFDLILLDFYYIYFFNLYSTSLYIDDVRNGSVNYYKRVAYCCIENEMN
KKRSNMAAKFREKSSDFVSPAASADRDHNNSISVNGQGNRIICNGANQSLDERYEINYKS
LLNYMFDNNVFLNERKKNIKQKGIKIKINSFIEDLLLSIELDDAYICCFFVIFIDIYKFL
STGFNLSTLHLYPKPSPYIVSNKYYNNKNSNINNSIDDGMEKREPEDKEKKKPKNEKENI
NSTKILNKENYIKELNINELLKMDNKPIHIDFKVNNGNFILFTNLENVNHPIIMWSNNFV
FSFSLSNGCILFRKIYAIDSKIKRINYVNNNPNMISNYSRIKNNNKMDGINVSNPLHNYH
KKKILLCENLNVIGEAIYESVDTKKENYIIKKNIKKKDHPQFISVFEIDLNISNFDIRLS
NDDVEILLKASSTLFGDVPSAYTNIAIGTVTPPIFENNSIVPGKEKNEGEYDSSAYSNSI
DSNLWYFLDTNTTKTNAYKDIKINIKLNNVKCTFINDVKNAIVPIIRMTFSMDIKLRFYT
DECSYIINCLNSNIEYFNNCIGEWEPFLEKCNISLDVHKVLPSDLYSEDIESIRTPISII
KINSAKALWFNITPQLVNLLFMFVPVFWEKVLNGLKSKRNENIMENNEISEASKQNDDKS
AYDRDLERLIYNHDDKNEDKKSKSIYEDSSSIFDVIPDGADENKSMDFHLDSFLKKEETG
EAIDPIEDNSIIYYVNLTSDYYYAFAMPDRSSEYMKYDCGRTTIRNGHKMNTRETLDPHI
GKNRDNYDKMNVDSANGEKGGMLPDVYTKIITTNELISLDELLVNEIGNNTLIEKKDLYL
YLIPIPPTNIVNTLHEMFINISKRDIVLDLMITKNPQKTIENLLSYNNLRNSNPSIENRG
YKIKGAYDNYYFNKANKFVGPFLKSSECVMINLIKNSCTSLNTALKNNNVIHVISPSDYI
FNETEASNNSSNNINSKKKEVILNKEYIKLEQLNDEINKQTNLAYKYRSKNNTICEIISP
MPNYKILFMSSTVRIINKCGIPLEFSFFDTSKTPILLTSLKNRTIDSSILYPNHSDSFKN
YKVSNSLNINPNIKIIQKNTNLSYTVILNHEYLLSPPECAFYGQSQVYLSFKPLNLVSEK
YNIDDTNENTKRSSCTASNDCSYINIDNGWSDIFSSDISQGTYVKKCKVVDNNSYLYFLV
KIEHKISALPAEKNIRIITIYPHVSIVNTIPALVDIIMMSENNERVYTHEYNRLEKEIGN
VDHEIKVLKKSIFYIYEIKKYTCLNLKMKIGNSQCEWSDRIHLDEDDDETMTRLSLNFKK
FASIEVEIIKNFNGYFNSLNSILGNKHLIFSLPRCFIDRTGLGIKAINSNKYYPIINGIT
LLGDNSQIDLLLPHKNNSIDGINNGKKKKQRDNFDDIFNDFNYDNSLILFKANLPPIGSY
TETNVVCNNFYYTFCLNTEKIKTTNIPYIISRIITVVPQFIISNRLHYPLLIKQFQLDQI
QGVRENDTSPLYFIKKHNVLLFQFKVFEMGKKNKNINSIESNSYSNNGAGSYDNNKNEMS
KPSKYSKNMFWSSVIFPSENFVGSNYMVLNNGKDE-SDVYVITCIPNMGTKNIIIEKLEK
KINKGYIAYNNSSLVKYLKIRTFHDDAHNLKMGKNDKHLDKNLLLNNFLKASDMEHYFNV
EYNKYSYLGWVNPFIYISRNIQIEIVLHDMNVIPRSPLILKFASFNYSQKTYHINYFNIT
FIISIEYIEDLISIKLTHRINSPKGTDKVLAISGSNCVVDGKDRDENDEKKYSTNSSEIK
SYNKIESIDKYEIMNDNVGTRGIGNVAKNNKLYIEKKVIEHNEYNYVMNISERGRNRYSV
ENNNNSFYSNRKESIKVINNTYKNVHVILNLTQIGASIISNILKEEVFFIELSKLCALFY
MKNEEEVIDIKITDVQIDCQLESCKKCVLLANRGLNSDSKNINNNDEKTFLNVYVERLFI
SHNDVIFKKIQVLLDDIEIEMDSETLNGINLLVAEYIEGINIIQKKNLLYEEIQKWTVLP
VYVNYKSPDIPLAINIQYMQINKFTLIVWCSFLLDKMHMLSDLLRIGLRILMVSGKLELL
GAPVTLNQEIFNDIRVSIKSFYALLKDKYSHSILACLGFIVGYSSLINIPKIPLEIGRNT
IGLAVYAVDNVSVGIGSLLSNLTFDTEYINRRQKERNFKTNTNMKEGLLSAVKNIGEGVL
SLSNIVTKPIEGAQKEGVGGFFKGIGKGVAGSLVKPLDKVGQAVSDVTRGIKAEVSKPIG
GYKYKTKRHRKPRMLWGEYGKIKEYNTQEAELRECLGLRFSENIMKCLTIHKQQNPPSHY
ALLLYPKVIIYANLYANINDKKGDIVIWSVKIDEISEIRASSHGLIIRTTNGTYKIPCNN
ASLINKIYRELHNSKNSIKSTIILGNSILSPPLMNNAEHNGVASKMDSSKIDNIDIDENI
FLNSKSFPVNEAYEVDQNKFGGNNQIKDIKEYKKMYEESIKNPELFWGDMAKNNLRWSKL
FTKTYIGNFNKGNVSWFVNGKINACDNCVDRWVEKHPNKTAIIWEKDTPNDYKKISYQKL
LEKVCKIANLLKMYGVKKQDCVTIYLPMIPELIYSMLACARIGAIHNVVFAGYSTRSLSE
RIVSSGSTILITSDFGLRGGKLTKLKNIADGAMEMSGMIKTCIVFKNKTKRNDNNNNILN
SKSNNMYNTSNICSQGSSHFLQYAVGNATHKNNNDPSPNVYDSILHEKDIQTLNKLSKNT
NINKKNQNKENGYKELYNNKLTENGTQMNGHISYDNNMINCNQVNAFEHIHNGHANYEQN
CKSKYDFDENICTLKEGRDINGSMLMKNMRPYCPIEYVDSEDFLSILYTSGSTGKPKGVT
HTTAGYLLYAFATSKYIFDIKDDDIFGCVADIGWVTGHTYVVYGPLLNGITTTLFSSIPT
YPDCSRYWNLIQTHKITQFYTAPTALRTLMKHGDYYIKNYDLSSCRILGSVGEPINPETW
RWYYNVVGKKKCVIVDTYWQTETGGIVIAPIPHLFKMKPGSASLPFFGIQLEILNSKTLE
PLKGPNVCGILCIKSSWPGMLRTVYGNHNRLIKTYFEACPSYYFTGDGAYRDEDGYYWIS
GRIDDTLNVSGHRLGAAEIEHALVQHSCISESAVVSFSHKVKGEGILCFVVKKLTSFQKY
```

PECNGNNNNDELNNRSLLSSNDNIVQNHTHENYTDEKLIEELKLYVRKVIGPIATPDIIC
IVPDLPKTRSGKIIRRILRAIAIGLNDYGDISTVSNYDVIEIIKNKFMESKEKRYVASMN
SDWKTILSYSIFFTGNIYFLWKIQNAYSFKSIVACIRKCLNYSKKKKFANNAIAQNVKIY
FGSQSGTGEQFAKELCYNLQEIFDIKADIVDLEYFNKEEIKTFGIRIFIVSTYGNGDPPD
NAIEFFKWLKELDINNTYFRNTKYSIMGLGSKQYSHFNKIAKKLTTYLKNFKAEQISETI
YGDDDDNIYHDFEIWKXXFFKELSKILNMNHIPINFIKEEVIKLVDWKNLPDIKFDLKFE
DIEEDDKKSETKHHTTNLLKDNDNKEAQFNKHISTSLTGKFYFNHNIGTVISNTNLLKNV
DDSTNSDKVNHIIISAKNIKYKSADTLVVLTKNSKQTTDWWLKRLNINETDKNKKFTFVE
RNGQNSPKYDNNENNGNNGNNNSMHIPFPTPCTIEEALQCYCDLSTIPRVNVLKNFKCFI
KDIEELKMFNYILSNNKRNTFFNICKEFEMTFIEFVDIFMQSAIFELTPFLQLIPKIAPK
SYTISSSPKDDPDIITLTVKKKQYPIHSLRKALKSFKNNNMLPNITEQKLRNLCERRWYK
GSSSYYLTEELYPNDIIKFNVKTSIFTLPENLGDASIIMIATGTGIAPFKAFLTEFKLFD
QKREKNEILKKSQRILFYGCRKKGIDFLYEKEIMDSLENKYIDEVYLAFSRDQSNKIYVQ
DLIREQKERVCNLIQNGAYVYICGNTEMGKDVKQTIINLYENNKQNDKKIIKKLKKSGRF
FEETWMSNLPWVEKYRPKKLDDIVHQTNAISMLKEVIKTKNMPHLIFHGPPGTGKTSAIN
ALAHELFGKENISERVLELNASDDRGINVVREKIKAYTRISISKNKINSENNEPLPPWKL
VVLDEADMMTEDAQSALRRIIEIYSNVTRFILICNYIHKISDPIYSRCSCYRFQGIPINI
KKEKLLYICKNENIDISDNALSKIIETTQGDLRRAVSVLQLCSCIDSKITVDSVLDVSGL
PDNDVILKIVDSCKMKDFKILEKTVQDIIEDGFDVSYIFKSLNEYFVMCQDINDSIKYQI
LMELSRHDFRLHNGATKYIQLMSFASSVHSLLNQAMSEGLDVDNAKEKINFMFTYWKNNN
NKDFENSNAFCILSGKSSKDDNATIQEQFQMWLLGYQLTETFFLFCKKEKLIILTSDKKK
KFLQPLLDKMNNITILERNNDNSENFEKIKNEINMFNNKELLILKDKDSTGSFFEACYDF
IKNLNKNEIDVNNNIKSLLNLRSKSDVKLQKSASDIASIIMKSVLITTIENSLDSEEYES
HNKIKEKVLKFNENKKCVVKIKDKLKADIDDIDVIYSSVQSGNKFLLNFKNTNDNNYLSQ
NDGTIVIGIGVKYKELCANINRTLLLNAKEYHKELYNFTFSIQKYIINDCLKCNTSFSDV
YKKAIQYIKDNKQNYQTIGNINLENYFIKCLGHVIGFEFMEKEFLITINNSNATIEKNTS
YNISVGFENVQMPDKNVFSTWISDTVFVNDKDEITILTDAISKEINTISYELEESGSENE
EEDNIKKEKNKNGDNENKKKIGISASILNNASSVIVSDRLRRRNKNSLAHNNEQEIEELN
KRQNELKNKKIEEIKNRFSEGTNEYKDLNKKNIKKLEDIKSYNDADLIPRDLRSNIIHVD
NKHESILLPVNGAHIPFHVSTIKNLSSNYEDNNDIFVLRINFQVPGNQGSQKGELNSFPK
LNEKEMYIKELIFKSNDEKHLQIVVKQVKELIKQVKQKEVEADVNDSKTSNEKLALNKTG
RRIVLRDLMTRPNIFTGRKILGTLELHTNGLRYSANSRGTTEYIDILFDDIKHAFYQPCD
GQLIILIHFHLKRYIMVGKKKTLDVQFYCEVGTQIDDLDRAKARNVYDPDEMHDEMKERE
QKNKLNLIFKNFVQQMQDISKIEFEIPYPELTFSGVPNKSNVEIFVTANTINHLIEWPPF
ILSVEDIEIASLERVHHGLRNFDMIFVFKDYTKPVKRIDVIPIEYIDTIKKWLTTIDIVY
YEGKNNLQWGNILKTILADIESFVNSKGFDGFLGEDDDEEEQSAEDEDEDDEYEVDESEI
SAEEDSEYDDSEEESLATESDGDEEVEEDSDDEGLSWDELEERAKKDDKKRFADSEGYNK
RKRKKN--------MPQHIKGDNVEASETQIRINGVNKRRHTNSVITSKYTIFNFIFLNA
YEQFHKISNIYFFIIGVLQLVPELTATNRIPTILFPLSIVLIANAINDAYEDWNRHRTDK
IENNRVCYVISDGDLSQPNISSNVFIKFIQYLKNILFKKSKKIDSIESYYDDEV-IDEIA
DTNYFINSYDDRLENIDGTIQKRWKDIEVGDIIICRRSDFFCADILLLSSSDPNGICFAE
TSSLDGETNLKVKEVNKYIFNNLTYNIDEAIEKAKKLRGYILSEKPNKNLSTMNGAIYLE
SDVNTDVTNNKEIKKSISEGSSHREEIETKKDINNNFIENKSNDDLNSYKHDDKYVKLPF
DEKNFVLRGCKLKNIDWVIGMAIYIGKETKIQMNSLKPVIKYSKLEILTNKLTIIIWLIQ
VFMCIISAYYSAVIVSFSKKSKFKYLPFNLVDPRAPIVSGIVSFFSWIVITANFIPICLI
VTMSFVKVVQAYFISCDNNMVYKVEENIPSFVESRQKSMKRKNFSIEIDETHKLNEGKSM
VGTDKKNGTNPSDSTKIILNSEDDNSDIFKKKSTRMRTFKESREKSYISFNAIPRTSSLI
EELGQIEYIFSDKTGTLTCNVMEFRKCAINGISYGTGLTEIKRKILKKNNIPIPQEPVLD
PKNKTPNVNIVDKKLVNQLKDVNHFNHASLIYFFLHLAINHCVMCDT-SDNVNTYSSSSP
DEEALVYAAKHFGITFLYRKDGKCGIKIFDKVYEIDILATVEFTSKRKMSTIVCRIPVPS
NESPEPTPIDNDDMSRPRNVNG-KGGNGMGDSPVNLEKELNAAIDNVANSDVNKESNKIE
KKNKQTETNNDVISCKNTKKHKIVVFCKGAGCVIIKKLAKKTDVDDLTIEHMETYADEGL
RTLCIAYKELSQEEFAVWYNSYKEASLSLNGREENIEKIAEDIEKDLILQGVTGIEDKLQ
EGVGATIEDLRLSGIHVWMLTGDKIETAINIGIATNLIDNGSEQFIYTEELALNEDLLMK
KLDEDIIYIEKSLNLLHFNFDT--NKVEESFFKKMISYKNTDSLALDHDTYKEDMTLNNN
VLIVDGNVLDILLSKPFERKFFYLADKCSSVICGRVSPYQKGSIVSSANRLLKKNTLAIG
DGANDCNMIKMANIGVGIRGQEGVQAFNSSDYGISQFRFLRNLILIHGRLSYRRISKLVV
YMFYKNIVFIFPLFIYGSISLYSGQKIYYEFLLHLYNVMFTSLPIVILAILDKDVSLNTA
LNNPCLYKLGIHNFYFNINKFISWVLNSLFQGLLVFIIPLYFLAYYNIPSSTGEPFDIWS
IGCVTYLLAVLIVNIKILLETYYLNTSPIVAVSMSIISFIIMSIAFSFIGIGNKSFLGVA
ILLVKSLRFWLVLLLVLFTALTRDYVYKVYKKNFYPEAYHLLQDEEENNSNTKHIQYSSK
CSNNVDETKSSKSEFMGYAFSEADPVCVHFIRKQDKLIMNRRLLNQCFQ-RSRRCSYINV
LKINNTNNGYQLHCRNYSDNVKDHFNKPRNVGSFDKNEKNVGTSIVGKASCGDVIKLQLK
IEDNVIKDARFMAFGCGSAIASSSYATELIKGKTIDEALKIKNNDIASHLNLPPVKIHCS
LLAEDAIKHAIKNYREKVINYMILSVKNSKIKGIYRSVSNFCNLNKIKNVNYINKKREKV
VILGSGWGGIHFLLNIDFQKYDVTLISPRNYFTFTPLLPCLCSGTLNVDACSENIETLLK
KNKISGKYLKLECTDIVYKDKYIKCKDN-NNEIKINYDYLVISVGAKTNSFNIKGVDKYA
FYIKDIIDALKIRTKFISNLEACSSSSISDDLAKKMLHIVVVGGGPTGVEVAAELADFVN
KDIKNKYKEIYKYISISIIEGGNNLLPTFTQNISKFTEKIFKKLNINVYTNYHVIEIDEN
NFYIKSSINKNEEHKKIPYGIIIWASGLAQTPLINNFIKKIPEQENNKNLKVNQYLQIIG
IKPNNIYAIGDCKQISPINSHEHVNEIINCLGNSKITSDVLKQKSKELSNIFPQLSDTKW
DYNKNKKSEMSIKELQEYLFMIDKNYKSPPPTAQNAKQEAFYLSNIFNNYLYNSNSNTIP
PFIEKWKGSLAYVGNHQVVAHLPFYEIKGGPFSFTFWKIVYMQMLLTWKSRINFIFSFLR
TKIYGRPFI-MYINTCVIQVKNKNAADVQITAEQLIKEALDFEEVEKKVNYNLIDEDELN
EYKISKRKEFEDSIRKRRYLINTYIKYALWEIKQKDIKRCRSIFERALNIDYTNKNLWLK
YIEVELTNKNINSARNLLERVVLLLPLENIFWKKYAHLEEILNNFVNARNIYERWVKWKI
DETAFLCYINFEERCKEINKCREIFEKLIVNIPKLECFYRFIKFEKKYKNISRARACYEK
CIELLPSQFLDQHFYIHFSKFEEENNEYERCRKIYIEALKRLPRENSDILYKNFLQFQKK
YSEKEELDQTLLYNERIHFEEALKKTPNDYDIWFNYIKLEEQNINLINKEKSIIRIRELY
ERAISIIPQICTKKYWKRYIYLWINYSVFEELYADNIDRARKVYSNIFKILSKQNFTFKK
IYILYANFEIRQMDIDKVRAIFNHAIENVKNEKIFQEYCDMELRLGNVKECRTIYSKYVE
TFPFNSKAWIAMINFELSLDEIERARQIAEIAIHIDDMKLPELIWKTYIDLEINLQEYEN
ASKLYERLLNITQHYKVYKSYAEFQYVYLDNISKCREILENGIEFCKKNELTNERSILLN
FLYEIEKDHGDNEIIEKTLKRLPKKVKKKKIIKSNDDEVVEEFITYVFPDDKAQSQNMKI
LQKAMEWKKMMEQHEQKEISQEIMADAESVDNNVVIEEKAVFDNVTAIQKVIKNALVHDG
LKIGIREVIKSIESKEAKVCFLSNVCSEPAYKKLVTALCAEKQIPLFMIDNDSKDLGQWS
GLFKVDKEGNARKIIGASSVAVIDFGEESAERDFLLSQKPTTAAAMPKDKRGRIFFNSHG
SDNERDNSKRSKSGHKSNYSEISPHNGSNKKKNYEKEKSKDKLKNDNTKTSKENINSFSS
PNSTSSISDLNNLDFDLSNGSSSNSENEFKILKEKENEDKFLEERRKKREAIKERLKNMM
SENNDNNKETDVSTNKHTVSNNNDKENEISINSNAKINEKCENTFTTCKKNDMSESLSEM
PSMLDDIEQNEAACIFAPNNEVIEETCSSLSSDHEIIDDKVPNEKNETMKESNDLYTDLK
KKINEEKIKIRNFIIKQKELHERNKDPAYINKRKENESIGFQEQEDDDNEDVDMFSSEQT
NKKRAIENIRITDYYSANNANLSDNWNDSEGYYKAIVGEVIDNRYSVVCELVGKGVFSNV
LKCYDMTNKMHVAIKVIRDNHMMHKAAEKEIFILKKLNDYDKDNKKHIIRLLRSVKYKNH
LCLIFEWMWGNLRIALKKYGNGHGLNATAVHCYTKQLFIALRHMRKCRIMHADLKPDNIL
INEKFNALKVCDLGSASDISENEITSYLVSRFYRAPEIILGFRYDSQIDVWSAAATVFEL
ATGKILFPGKSNNHMIKLMMEYKGKFSHKMIKGGQFYSQHFNENLDFIYVDRDHYTKKEV
VRIISDLRPTKNITCDLLEHQYWLKGNSPKMQFLKKKIKQLGDLLEKCLMLDPSKRYTPD
QALQHPYLRESIHYSKMQNDMSSDVSSLSCSLSLDSDSCEDEFYETNLNKLIENTSLNWI
FVGGKGGVGKTTTSCSIAIQLAKKRESVLLLSTDPAHNTSDAFNQKFTNKPTLINSFDNL
YCMEIDTTFSEDTAFKINQSNFLNSIIPELLQSFPGIDEALCFAELMQSIKNMKYSVIVF
DTAPTGHTLRLLAFPDLLKKALGYLINLKEKLKGTLNMLQSLTNNEMEFEGMYDKINHLN
TMSISIQENFQNPLKTTFVCVCIPEFLSVYETERLIQELTKKNISCYNIVVNQVVFPLIC
PDANIEKCENLLKQIKDTNIQDSFNTLILKAKELEDVYISRRKLQSKYLTQIKNLYGNYF
HIVCMPQLKTEIRGLDKISNFSEMLLQSKDIPIYSMENGIVLSGDYIKLNSDQVKKVNKN
NFEQIYDKEYEDLYRSKCSGIMLKTPYYPYKYDIMNTSHKYIPKIGDLVIGIVKSKKLDY

YQMDINSNCECIIHKIESFKYASKSSFPNLLNGTLLYMIIEKINLDNNMVVASCINSADV
KSWINYENYLGELVDGFLFAVNISYAKSLIGDKCYILDLIGKDVPYEIAIGHNGWLDKNT
N-------IRAR---------------------------MYRSHIVENELFTINNDGKRC
INSLVYTPNFNDNNINSDVGHPNGLVNMKTEIGKSNFVETLLKDIVKIYLSKIPKGFERF
EKNNSNKNCNGNINNVQQEEEKPKKFDNNFFYLFILLLLCFLLFVDSNSLYNEITQNDFF
YNYLSKGYVDKIKIINKDYVKAYLNSHGINKYHLKYVSFRVGNSDSFERKVELIQKEMNI
KLDEIIEVQYVNEGNIIGEIKSYIPSILFFLFLIFIFQKITLKNVANSGMDKLFKFNKIN
PINKNNYKTDIKFSSVAGMKQAKEEIMEFVDFLKNPAKYQVLGAKIPKGALLCGAPGTGK
TLLAKAVAGEANVPFFNISGSDFIEVFVGIGPSRVRELFAQARKHAPSIIFIDEIDAVGR
KRSKGGFAGGGNDERENTLNQMLVEMDGFHTSNDQVVVLAGTNRIDILDPAITRPGRFDR
IVNINKPDINERSEIFQVHLKNLKLHDSLDIKNISYILASLTPGFVGADIANVVNEGAIQ
CARRSHIQGVQIKDFELAIERVLGGLAKSTSLISPLEKKTISYHETGHALIGWFLEFADP
VLKVSIIPRSNGALGYSQHLSEEIMLFSKEAIHDKIAVILGGRAAEELFIGKITTGAIDD
LNKVTQLAYSYVSQYGMNKEIGLVSFQQNGSGEYAFYRPHSECLAHLIDNEARSLIESQY
NRVKAILKKNEKHVHNLANLLYEKETISYHDIVKCVGERPYPIKSNYEKFVKANPYKMNL
STSMEEKSGEKNVRTQMGDSESAQKNAPQKDNNNGSDSGNNNSGSDSNDKTGNDDSPITQ
NIGDRNKKDEKISNFNIRMGGKNGG-ENGGKNGGKNGGKNGGKNRVKKQT----VWPLMF
GKIYKCSYSISTLQICLKHQRYREADKVKIEEERNRKIYVKNIKNKQDFCKNCGSAAHTE
KYCLERTRKKKKNFMNKENDEDYLCVTQDLGYDGNRDRWVGYDPNNFDHIYREYEKIVDE
QKKRKAEKLKKKYEKQSIKKKKEEENEDEEEGGDAEDSELSSDSEKDNDLNDNKQNNKKN
NKNEKNKTIARNLRIREDTAKYLYNLNLNSAFYDPKSRSMREDPLANIKNNLENSNYYKG
ENYYNNTGDAIESKKLEIFAWESYKRGENVHFNAQPTQLELMYKEFLEKKNKLIKKKQED
ILKTYKCENITKENNNQQELIHSEVYTEYKPVDQIDKKNNKIKILSKYEEDIYISDHTSI
FGSYYDRETKKWGYKCCKCTDKFQHCVQSMGTKKNESNKKDTKKKNIKYEINNVVNEIKY
VREIDPILNEDDSENVFL--NNININTLKNEIVENELNNNNLDDVSLGLIKDKSIKKREN
EFQKKKYDYILSPERADPFNEKSPSPGERTYTDIMVDINKKNIKNKILNNNKKEIATSDI
NQEEKKKMRWGNENGDEIKSKWGMINENEDVDFGNMETPAPNKWGETPFLLNDGNIKNKK
KKISRWDRTVENSTSNNGDGKMASSGMLKTPIISGNKYNENMIINTPILGTNIMTPITPY
SGSVQNNDYIKIKIKNEMDYRNRPLTDEDLDNLLPIDGYEIVKPPEEYETIRKNKLKILF
KNMNDTTTTPLIQGNKNKSTLDNTIINENGESTFISDTPFYNLPNINNSLKKEDEEILRQ
NKIMEITNPQLLNELKYIEIKNEDYIYFNKLFQNYNEDDLSQDEIKERKIMLLLLKIKNG
TPSIRRNALRTITDKVKELGPENLFNLILPMMMQNTLEDQERHLLVKVIDRILFKLDDLV
RPYVHKILVVIEPLLIDEDYYARVEGREIISNLAKAAGLATMIGIMRPDIDHPDEYVRNT
TARAFAVVASALGIPSLILFLKAVCQSKKSWEARHTGIKIVQQIAILTGCAVLPHLKQLV
NIIAHGLHDEHQKIRTITALALAALAEAAAPYGIEAFDPVLRPLWKGITEYRGKCLASFL
KAIGLIIPLMDSYHANYYTKEVMIILINEFNSPDEEMKKIVLKCVKQCIQTEGIEKDYIN
KEILNPFFEQFWIIRNSNDKRNFNLIVDTTVEIANKIGGEIVISKIVDDLKDPSESYRKM
VMQTIQNIINNLGVDYIDQKLEERLIDGILYSFQEQTSDDYYVLLNSFDIIVNKLKLRMK
PYLPQIAGIIRWRLNTPLPKVRQQSAELIARISKLIKTCDEQQMLGHLALYLYEYLGEEY
PEVLANILKALRSIVIVLGVNNMTPPIKDLLPRITPILKNRHEKVQENVINLIGIIADKG
GDMVSPKEWDRICFDLIELLKSNKKLIRRATIQTFGYIARTIGPFEVLTVLLNNLRVQER
QLRVCTTVAIAIVADTCLPYSVLAALMNEYKTQDLNVQNGVLKALSFMFEYIGEIAKDYI
YSVVSLLEHALTDRDLVHRQIATWACKHLALGCFGLNREDALIHLLNHVWPNIFETSPHL
IQAVIDSIDGFRVALGPAIIFQYLVQGIFHPSKKVREIYWKIYNNVYIGHQDSLVPIYPP
FEMIGDSNFSRDELRYVLMGKQRLTALDIRAIITSCKNTIIGSVVTNIYNISNKIYVLKC
SKKEQKYFLLLEAEKRVHITEWVREKDVMPSGFTMKLRKHLRSRKITNISQLGGDRVIDI
QFGYDDNMYHLIVELYIAGNIILTDSDYKIIFILKSNDDNKKNLKINEIYNVEEHNSVML
YKYLDIINNENVKKSINEILMIKLLKCENNDNVKLKKKGSENIKNCSNSRKNRGAISFGN
SNNVFNDKNKNDDIHDDNKKNKKIKKIKTLSELGSKLILFAHNDLIIHSLIECNLNPHDM
VDKYDINTLTEMFFKVINESMKIFNMMCNEENVICGYGFVPID-EKSLIANKNKNNGEKN
KLDNDRLFVEFSPILLKNHINKINEKKIEIIKFDNFNMCVDTYFSKIELTKYDKHQEMNK
NKNALTKMDKIKLDHEKRIEGLEKEVSMLKKKILLIELNYQFVGEAIKLMRSAISTSANW
EKIWDHIKLFKKRNHPIALKIMSVNFNNCEMELLLDDNDDDDVEESGDDNNLKNDKWEKV
IEEKNKTC------AVTINLNNSVFGNIEDYEKLRKKAEEKIRKIKMSTNIAVKKVEKKK
KDKDIKQKGKNKSVFQIKKIRKIFWFEKFNWFISSENYLVISGRDSLQNEILFRRYFQNN
DIYVHADIHGAASCIIKNPYKDIPIPEKTLSEAGQLAMCRSSAWNNKMITSAWWVYYHQV
SKTAPTGEYIKTGSFVIRGKKNYLPYAKLEMGLCIIFQINKKVNDNNEENKLTDDEPNCD
NNEENKLTDAEPNCDNNEENENLDKMSTGGDVTNCVNYMNNLNKNCQNNKIVNESLSNVK
NCVSIDLRGTCKKNKECLKNKYIKLILKCLNKIDLVNIYKKNMISKFGINKNYKNEIILI
LCKLCFSKLNINMKNFLYKIKNSECIQCYICKTFQKNCKIVKCLFLYFDSSNTFLGGFFL
KRFDKAIDSIYFYFIHKKYLLKNYSKKVVDCYKDELNKFVDVNKNNNFIKNLNGDTLMCV
MRNHVNEKINHDLVMEKDKMMKLCFRSCEVYGLPVAFKDDDITDEDSKNSKNGESSKKIA
LNSSDSDKLNGKKKEEEKRHISFV--NVENPIKIERPTRTRKATGFVKMDVKKLLEEIEN
EEEEEEDEKEETREDMKVNFYEEEKKKVEKKGVAFNFENNNVYDNKNCMVNIQRPARTRK
PTGFVKADLSKLLEEIENEEEREEKSEAEGSSQKDVLNSNS-STTNIRKKSVSFSSEDEH
IYIERPITSIIPRPVRTRKPTGFVKMDISKLLEEIDNEEESCESERDTNEREKNGDEIKK
VKFSEGG------EKNINAE-NDKTISGPENPM--KISRPFRSRKPTGFVKMNVEDLLKD
IEMDEEEEDGNGNEQDEEGEQ-------------NEEDEESEEDEENDENDENDESDE--
-NDENDESEENSDESEEENEESDESEENEESDESDESEENSDDSEDGENGQNGGGGQNE-
--KKDYKENMEKGNERYS-----NETKK------GGKFNDKLFKNE--NVHLLRGARTKK
KRMKKKYRDDDDEERQLYMKIIGSRKVRKR------------------------------

---DS---MGRMYGKGKGIS
SSTIPYKRKQPSWLKQKPSEIEDAIIKLAKKGQTPSQIGATLRDNYGIPQVKAVTGNKIL
RILRAHGVATTIPEDLYFLIKKAVSMRKHLEKNKKDKDCKFRLILTESKIHRISRYYKRK
RLLPSNWKYQSSTASALIAMSNRKKVAYFHDPDIGSYYYGAGHPMKPQRIRMTHSLIVSY
NLYKYMEVYRPHKSDVNELTLFHDYEYVDFLSSISMENYRDFTYQLKRFNVGEATDCPVF
DGLFQFQQSCAGASIDGAAKLNHHCADICVNWSGGLHHAKMSEASGFCYINDIVLGILEL
LKYHARVMYIDIDVHHGDGVEEAFYVTHRVMTVSFHKFGDYFPGTGDITDVGVNHGKYYS
VNVPLNDGITDEAFVDLFKVVIDKCVQSYKPGAIILQCGADSLTGDRLGRFNLTIKGHAR
CVEHVRSYNLPLLVLGGGGYTIRNVSRCWAYETGVVLNKHHEMSDQISLNDYYDYYAPDF
QLHLQPSSIPNYNSPEHLNKIKMKITENLRNIEHAPGVQFSYVPPDFFDSDIDDKSDKNQ
YELKDDSGGGRAAGTRGKEHSSTHHLRRKNYEDDFFDMSDRDQGII-MQDDNESIDGNIL
EKQYEIIKCAKNHDFIKFQILIQPYILNNDIEMLNAINIMHWACYSGFTELVQKLIALNC
DIEKEDLVNSDTPIYYAIKNSNYEIVLLLIKHYGSSILFHKNRRQMSPFLTAISEFNEDK
ILEALHILEFLYLNGISLEEQNEYGQTALFLGVKKNNISILQWLLSKNVNINHVDFYGNT
ILHIAVRYTDIDILRLLCDYGCLNLVYYSTFENNNTNVFQLCINNRYFLVYILLKKWLIQ
NKICKGLKICKTIYAFYFWFFALLNLFVYINITQSFLEIKKHHNKSVIWISLWLFQQLLW
CILYFKNPGFYKENKFLTNRNKNNSNYMYATDFKNQAEYQLNNIEKEIFQINKKLLQTNL
NPIHNANQNENEILEYNDKIINLKYSKLSLYSQVSQERINSLDVNYRNAILYNQNPRNVC
VTCNIIKPPRVHHCADCFHCIPSRSSLCVGRQ----------------------------
--LYRNKQPTIFLYAFALLVILCNFINITLFAFITYLFARNTKTILTNVTFYEHFKRPTH
ITDKYNTELECWEFQNLNFKKIIKNIYNFWTLNYDEPYLKYDRKI---MDVCEFVDPIRK
RKITNNLIDFDKYVYKEKSDKCEKKENTEYVVKKKKKKKLRRVLDSSNSDNQENIKKECK
NEKKKKNDNNDNDDNDNNDDDQFLGKSYDNNSIINDNEIEEKKEYERNLKKLYNCLLGSI
KIKNKIIKYFTQNVREKRQDIIKEFVKGSFRINNYDANFDDFEKNVDTFHKLKNYQKCGI
YWLYILYKEKKNGILADEMGLGKTAQTCVFLDYMYKTKKLQNKTIIVAPTSLLKNWDNEI
DMWCPYLKNYKIIYYGNQNERKYLAYDIFTNKKKNSIHLIITSINMLTGKNDAAYFRQIR
KYDYLIFDEAHFLKNKNSLIYKKLQKKITFSNKILLTGSPIQNKTQELTNLLLFLMPHIF
TEHNINSAMDAFLKLYQEVIEKKNKSDNTEN-TTNNNKLKNQQLMDIYEIESECDEEKKN
NKDDNTYSVTEANGNLNDSEKDDEDEDKNADAEDGEEYMCPNTRDIIKEYLDNTIKYHEK
NVDIKNKEIILLQLIIEPYILRRSKKHVFIDMPKKHSIIIKLPLNNTQLNLYKDEILSKL
QHTHKHLEFLETNSSKEEMEKLKSILGNKGEKEITNLK--------NNEYNNNIIDHKFE
NMK---DNYDEDDDKIDEETINIEKEEENKESKIVINKRDDEEVIKSTSLQNNKNNTKEE
RGKMINASIFILRRICNHPLLHKYYYSIDDIKKISKYFYNNTDQYLDLDLKTVENEFMKI
SDFDIHLSIKHLISQGDNNLNKYLIDKKHILNSTKIHHMLTLIKNIKQKKEKVLIFSQFT

TFLDIIEESLLYEFIYDEHDYADHSNFVDKTQNGNISESPNKYNNNDGGDNDDQFVKRTS
FNKDDDTYVRSPSAMSTSSQLSEKRFQNNVYVRLDGSTNTIERQQIIKEFSENDNVFIFL
LSTKAGGVGLNLIAANHVILMDQDWNPHNDRQAEDRVHRLGKS-------FSQKNKK---
----------------------MMKNPGTVLVFKPNTKREEGRKTQLSNIQASRAVSEIV
KTTLGPMAMLKMMLDPLGGIVITNDGNCILREVDVAHPAAKSLIELSRSQDEEVGDGTTS
VVILSGEFLNIAEAFLKNKIHPTIIVNCYMTALNLSLKYLEEIAIEVDVNNEENLLKAID
SCLSTKFVNRYNKIVSRLSLEATQCVKVENIIGKKEIDIKRYAKVEKIPGGDIMDSYVLK
GVMINKDITHPKMRRYIKNPKILLLDCTLEYKKAESQTNVEILDENTWNQLLLQEEIEVK
KMCEYIIDSKCDIVVTEKGVSDLAQHFLVKKNISVIRRVRKTDLNRLERITGATIVNRCD
EIVEKDIGTKCGLFEVKKIGDDYYSFFVECENPRACTILLRGATKDVLNEIERNLHDGMN
VAKNIMLEGKLLYGGGCTEMRVSQYLIKEAANFDDSRKSVIESVASAFEIIPKILAQNSG
VNVVKCINELRTKHEKPGSEKLGIDGVTGEIIDVSSKNIWDLLSVKKQIYKSAIEAASMI
LRIDDVVSGIGKDDKIQKPIKNEFMGRIKVLNVAEKPSVASAIVSILSKGKSNKKKSYSK
YNPVFTFDYKMENETWSMFVTSVTGHLTDQKFDDKYKNWNNTDPHELFDAKITIYIDKDK
KPIENNLKKYSKDCNVLILWLDCDREGEHICFEVINACSVTNKKLKIHRAQFSAVTEKDI
KYAINNLKSPNKNLAQSVDVRREIDLRMGSIFTRFMTIRYFKLVQNDTKIISYGPCQFPT
LGFVVNRYLQIKNFNNEYYWTIKMGYLYQDKNCNNSNLFLDNIGKKKKKKGKKKKNCSDD
DANNRPDSSNTNYVVDFTWSRLKLFDHLGVVLIYEDLLKNPLCRISNIFEKEVKKYRPFP
LNTLQMTKLVSKYFHISSKECMNIAEKLYNKGYISYPRTETNYFVDSMNLRKIIHELKKN
NIFGSYATKLAEKNS-CKPRKGKLNDKAHPPIHPVKNMNKANNVDFKEWKIYEFICRHFL
AVCSDDAIGFDTKVIANIGEEQFYCKGLKIKNKNYLEIYIYEKWNDKILPPFQINDEFYP
YSLVVEEXITQPPKYLSESDLLSLMDKYGIGTDATMHEHIENIQKRNYVYKNSKNLFIPT
KLGIALILSYKKFKDIGVDLTEPSLRAKMERDMFLVASGEKGKNEIIRNYIDIMKYIYQE
IYNRIDLLDENINYYINNPEILNMETFNFGKYKGKTFEEVFEKHKSYVTWVKNLENPTGS
LIEFKNYVLRKE--EQGGGDTNTEYTEYSNKYG---------------NSNNYGNDNYYG
NGNKYK--DKNTNGNFN-----YKQNGY-DNKNSVNKMNSSEHKLYEQNIMNEIANRYNN
KEEDKPELDIIVAFEIFNSDSFKIVQKDNNNKKYSNFKNFVPKELFKILSEFNPTLKKIN
NYSCVTFESDKYEYVLNNLAEKCTILGGIHSIPNFLLKCFQNYSKFSQPQKISEVTANIL
TNTMCSYTKVHYDNLNNLLGEKLSEELKNFQKEGVHFGLKKNGRVLIGDEMGLGKTLQAL
ALMAFYNKDWPFIVICPSSIRFQWKDQALRWLPHLIEENEICVIKSGKMDIPRNTKMIII
SYELITKNDKYQNKYKCIVCDESHYLKNSFSKRTKAIVPIIKSAKRCVLLSGTPALNKPS
ELYEQVSSIIPNLFNYNEFCDRYCYKDKNIYTRKIEYVGCKHTEELHLFLTNTIMIRRLK
KDVLKELPDKLRSKIPIEIPPNELSEILIYSKKLESKKNININDLDNINLSRFNNRD--N
NDEENITISQLFKMTGYAKVKAIKEYITYLIDADIKFLLFCHHKLVMDEIDEFLKEKKLG
FIRVDGLTPIDKREIYIKNFQSDEKIRIALLSITACGVGLNLTAANTVVFGELYWVPGQM
IQAEDRAHRIGTTHDTVNIHYLVAQNTIDEVVWKIINRKWNTLTTALNGTEDSLNVKEVS
KFDQFMLDLTNDTNKSYPTSLVNTPKIRRRSSEYKALINSGSNRKNLDIRDFFKKNKTDI
EEQKSMKKRSNYSPPKNYDSNSTSNILTKKYKTEIIMKEKGPKAESTEKNDDIKECNEK-
--INTFISNLLSSINYINNAILYRDNRFILRMLKHIKNMRLSIKNDSENLMPVLVSLINK
IFKDTYPIYIILNKYIKLYNENNKENITEIVNLNEKNYVNSLPEIEVFFYILFMVHLIDK
KLYDDCIELSNLIIKRINSLNRRSLDFINSKIYFYYSWVHELRGNISQVRQELLFIYRNA
CLHRDIMTQTVVLNLILRDYIKNNLYDMAVKFVSKTLFPENMFSNVQHARYLYYIGKILA
IQLDYSESHNKISQALRKAPQNANMAKGFKLEATKLEIVVELLMGDIPDRSLFTNKIMRK
NLIPYKHVVTAVRNGDINKFASVMNNYKKLFVKDGVYLLIKRIHHNVIKTALRIINLSYS
RISIADIGKKIGVESPMDVVGITAKAIHDGVIGATIDYDNLYVESKPNIDIYITGDPMKA
FHKRIAFCLQLYSDAVKAMQYPDENEKTENVEAKERKIRQQEEFAQAEEGELGDDTDLLM
LCEQCNKNNVCMLKPSNKEKLCKHCFLESFEDEVHTTILKKKMFEDNDKICIAVSGGKDS
SVLTHVLVNIKKKYNYNWNLFLLAIDEGIKGYRDDSLKVVYKLEKLYNLPLSVLKFQDIF
SYTMDDVVSYIGKKNNCTVCGVFRRQAMEKGALLFNATKLVTGHNADDLAETILMNMCRG
DIDKLAKNINDVIQKKNNTNNSNSNGSLASYSENSNANIFPNCNNILDSCGCKEKINIEN
NKEMEKKNECNNDVAKIS---NDNIKYENKGDGFIPRLKPLMWSYEKEIVLYAYHLKLDF
FSTECTYSPNSFRGNLRSFIKDLEIINPQIILNIIHSSEFFYFNTNIKKKLNTCIKCGAY
TSNVVCKACLIVDGLNNYTDNSFLYANKKKKNSKKKISIEYEAENMEQNIFSATNVIPYG
PLYDKVKKVKNNDQDENYPVVEEYEKLLKTYERPNPYDSNGSLKFNNNDDLILFQIDLDY
TVENIFRNVIYNNEGSTNNSKCNSIYEPYKIILGKEKNFISVPIIRIYSITNCGYSVVVN
VHNFFPYFYVEKPNGFNNDDMIKLESMLNETLSLNNQFKMYENKILKIETVKTESIMYFK
KSGKTDFLKITVLLPKMVPSLKKFFESGINVNSKHFGGIVYEANLPFILRYIIDKKITGS
SWIKCEKNSYIIRSKNKQSSNCTFEIDIHYENIEPMALENEYQKIPKLRILSFDIECIKL
DGKGFPEAKTDPIIQISSILYLQGDPIENCAKFIFTLLECASIPGSNVIWFNDEKTMLEA
WNEFVIRIDPDFLTGYNIINFDIPYILNRGTALNLKKLKYIGRIKNIPSLVKDANFSSKQ
FGTHETKEININGRIQFDVYDLIKRDYRLKSYTLNYVSFEFLKEQKEDVHYSIMNDLQNE
NSESRKRIATYCIKDGLLPLRLIDKLLFIYNYVEMARVTGTPFVYLLTRGQQIKVTSQLY
RKCKELNYIIPSTYIKVNNNDKFEGATVLEPIKGYYIEPISTLDFASLYPSIMIAHNLCY
STLIKNNDEISDLNKNDITTVPGKNNFKFVKGNVKRGVLPLIVEELIRARKNVKAMMKNE
QNPITKMVLNGRQLALKISANSVYGYTGAAAGGQLPCLEIATSITTFGRSMIEKTKETVE
AYYCKNNGFEHNATVVYGDTDSVMVKFGTNDVGEAMRLGKDAAERISKEFLHPIKLEFEK
VYCPYLLLNKKRYAGLLYTNPNKHDKMDCKGIETVRRDFCILIQQMMETVLNKLLIEKDL
NSAIEYTKSKIKDLLTNNIDMSLLVVTKSLGKAEYETRLPHVELAKKLKQRDSATAPNVG
DRVSYIIIKGTKGQAQYERAEDPLYVLDNNLSIDHNHYLDAIKNPLSRIFEVIMQNSDSL
FCGEHTRHKTILTSSQTALSKFLKKTIRCIGCNSSIKKPPLCNHCKTNKEFSIYMQKIKD
LKLKQNEFFQLWTECQRCQGNLHIDVICMNRDCPIFYRRAKIKKDVANLQEQVTSLKIEW
MRIIVKGLILYMYFTYLKNHGNAKLNENKYTLIRVYYDNFKLYKKKKKINKNNKGVFFIG
SNINIKNKITNLKKKNMSILSCNNIDETLGGGDQNIFELENISQSLNNIYRKRKNEIEPL
IQVAPMINVTNRHFRALVRIISKKVQLWTEMIVDNTLLYNINNLEEHLGFNKNEHPIVCQ
LGGSDPTSLSEAAILVEQAGYDEININVGCPSTKVANKGAFGAYLMKNPQLVKNIVYEIK
KKVQIPVTVKIRTGVDNLDSFSFLRSFIETVSSAGCDHFIIHSRKAWLKGLDPKQNRSIP
PLEYNKVFDLCKLYPHIKFTLNGGIKSIEQGVALLNGYAPIKHNNSTDNNYIKIEDYDIN
PLYGVMIGRACMENITVLAKTDKLVYNYDSPSTAHSRRTVLDAYKFYLEQNSSFYTLVNA
FELLKPILGILKGMPGHRLFRNKIDSYIRKYASTLNCAQILDKAMEDVDNIAPGCLDLIL
DDYKAQQEYIKNYMQNSQNTDNKAIFVEEVVRDFDQNEVFEEITTKFIWEQDVERSWNLL
VENNGILQHVNQETYEEKNKQKYKKNQTCSLRKGIFRNVIILFDMSSSMKERDFKPNRIT
VILECVEIFLKNFFFKNPVGHVGVVALKNSSAKLIQQLTSNIDDVLNSLVKEQKEGLNGS
PSLQEGLEIAHNLLMDMPLYGTKEILIMYGSIRTCDKKNILKYLDLLIKNNMYVNCISIA
PEMHILKHICEKTNGIYKICTSKNILINEINQVAETPLWMHGMEPQLIHICFPVKKKINT
QIVCSCHNILNTDTYICNFCNSYTCKIPSKCKICGIHLISMHDLSHITNNLQASPLFVEI
KNEQNYYTHCSSCNQQLYDKVSQCTKCKNIFCLECDVFIHEDLNQCPFCLNDEDMGSNSI
AISQQMYFSTHNALRINEENDVISTLFYEINGHRHISLLIFPFYDVQMLKRLLIKKLDLP
-DIKVNDILIFYKGIKLPNYRIISTYLDNSNVDNVKKKKKKKVNKLYWAIKDPNPNASIR
VIDNKSYPPFFENILNDIKLAFKKNIAPKLTMDGTGGTYLLFNSKKKVCSVFKPADEEAF
SPFNPRGYEGKIYQEGFRAGVLSGEGASREIAAYILDNTYNNFSNVPCTIMVEACNPHFN
NKSNLKYIYNENTLKWKCGSLQEFIDSRESVGNYDHKQFSIRDVHKIGILDIRVMNLDRN
DGNILVSPIKSLKDCCNQFLYRNNKRFSTNDEDILKRIITIDQKPSRYSLIPIDHGLIMP
HIMDVAEIDLVWFGWPQTKIPFDNEVLEVIFSFDPDKDADKIRNKLLIREDCIRTMRVCT
RLLQIGAWMHLNLHEIAKISTRKNIDEESILERLVRDSIIQAYQMMDYTSLMSTNRLGYI
LDLAEIKINKKKKNSKNKTIELIDSNKTKDENSNDIKKNTISQKEEENYETTTYAKSKSL
DIKKIENSFTIVSFKDIRDNKSSDETYNINMNDQKGGECNSTYKTLIQHRSGSKESGNKF
IHTKINNISNREHARGYYSLSKDIEVRINDQNKEKNISETNSFSNKSEYTFVTATSNSKK
DDNINKSSNDVEQNSDGGNVDEENGAKVEKKMKKKKKKKKXXSDEKQSDKKESSFNISD
DERNVENEKNEKNTIEHNQQEEEEEEEEEEEEEEEEEDDENYVRFKKTTGTMKRISENTG
TAYRNIEMNKINSVWMIKDKNNKTINVKWENKIFEKLFFETFENYVKKYINDYHPNWREY
QYNGSKVVNTKHSYLSSVKMEIRVANKYALGKKLGSGSFGDIYVAKDIVTMEEFAVKLES
TRSKHPQLLYESKLYKILGGGIGVPKVYWYGIEGDFTIMVLDLLGPSLEDLFTLCNRKFS
LKTVLMTADQMLNRIEYVHSKNFIHRDIKPDNFLIGRGKKVTLIHIIDFGLAKKYRDSRS
HTHIPYKEGKNLTGTARYASINTHLGIEQSRRDDIEALGYVLMYFLRGSLPWQGLKAISK
KDKYDKIMEKKISTSVEVLCRNTSFEFVTYLNYCRSLRFEDRPDYTYLRRLLKDLFIREG
FTYDFLFDWTCVYASEKDKKKMLENKNRFDQIADQEGRVKQNMKILDELREQERLQLLYI

LKKNNGKKTLFFEKSLHIIINLILTEQDIKNEKIDNVFFMTDEINTISEKANVSNNIIFF
LRPYFYEIEQIFKIIENIEKIKTDNKNYIFIFIPYMTYMCEQEIYKYNVLELTIKVIIYP
LYFFPLYNDVFSLEIKNIFKDYYVDNDFSNLIFCSYALMFLQYIFNGVFRNIKSLGHVSH
FISEQLMQLRKEIVADSQDNLFHILNNIQSLQDLTNFKNPKEPSVVPIKYALHKFFLSEN
IKKKKYETPKNYSYDEDLSLTSNLESEPIKRNELSSSTSTYKREHIENDHFSNTHEITNQ
YNNRSKNNEQINDDVGKIKKSETPLNDSNKDIEKDTKQSDNFKENKIEPDKINSENYKNN
ENKREIIDTYQQKYKIMNKEEHINSNDNHTTNQNNIDADKINHDESDISEPDFGEDEKNN
SNKLLSNNLDKTNEHYNSDNDEDKKIRKKKKTKKNKKIYGTGIPNFMTEFDNTKFLMKKK
DKEEAEKQEKKEKNYLLEKLGINHFLFLLNTCTKIDSCIIIDRRIDMVTPFCTPFTYEGL
IDHIFCIENLQIEIPRYIIFNTGTNKIEAENSKTTTQKGYASNDLNNKKIRVKLNSSIDV
LYNDIKNLNQNEVGIFLHKKASDIQQTYKEKDSLKDISQINKFMIKFKEKHYEHNSLSRH
VNIASYILNEIKTEHTFNKLKLEDEIIQLNTNTNKNILSSIIKKIQTLIYTGENIYEIYR
LASLFSSITNGFNDTYMNELKKDIIEQYGINELTRLNKLHISNILKYQPKQKFIWNNLKN
HFNLLSNDENDISYVCNGYAPLSTRLIEYIGFFKNNMQVFPEVFSLINGPTFDIIQNAVG
YEHVQVNNNNTSEQTHDINSKKHKKYVILFYVGGITYAEIASIRKLNQTNENYHYLIFTT
EIISSKRIFDSMGTMENSLSNENTKKDIENHNEITHDTIEVKSMCINCEQEGINKILKFE
IPYFKNILIHSFECVLCNYRNNTIQDLNPIKEKGVKILFSVTKIEHLDRQLIXSEYGVLK
XPEINFEIPKETQKGSINTIEGFIQTALSNLTDYFINLKNMYNEANNIVDDNANKESKEV
EKNVNDNVHKIEEKEQLNKDGEINNNNYSAKDDETSEVNETCYQMTIENYMSMIEKTIHK
LSKFIVSKELPFTVEIIDPSGLSSLEYYDEDINSKTVIIEHYQRSKQELNELGFYEEDFE
EKKKDEESKQNNLNINENQSGDQIKKENFDFIKKYVHMNNNSNGSNNMCVKYKTINEGEE
NKLIESFTSNCPCCNYLGDNNFCEINIPGFKKCLILSYVCPNCNYKTSEIKSSGEINPKG
KKITLTVKNKSDLNRFVIKSETASIQIPIIDLTSDYGTLGGSLTTVEGIIIQIIESLEDK
FKFLLGDSSINTHISNDKVDTSNNDDSVTNKIKNVISNLYKLCRTEEMFPFDLIIDDIAS
NSYISCDQIGDDTNLKEEEYERNFEQNDMLGITSMDAN-MNDNIKRLPSSAEEGQIKKTD
SGKIYDDGIKRTPSGKPIQTMYVLNRKGEEEDISFDQILKRIQRLSYGLHELVDPARVTQ
GVINGMYSGIKTCELDELAAQTCAYMATTHPDFSILAARITTDNLHKNTSDDIAKVAEAL
YSYKDIRGRSASLISKEVYDFIMEHKDRLNKEIDYTRDFNYDYFGFKTLERSYLLRINNK
IIERPQHLLMRVSIGIHIDDLEKALETYHLMSQKYFTHATPTLFNSGTPRPQMSSCFLLS
MKSDSIEGIFETLKQCALISKTAGGIGVAVQDIRGQNSYIRGTNGISNGLVPMLRVFNDT
ARYVDQGGGKRKGSFAVYIEPWHSDIFEFLDLRKNHGKEELRARDLFYAIWVPDLFMKRV
KENRNWTLMCPNECPGLSESWGEEFEKLYTKYEEENLGKKTVLAQDLWFAILQSQIETGV
PYMLYKDACNSKSNQKNLGTIKCSNLCCEIIEYTSPDEVAVCNLASIALCKFVDREKKEF
DFKKLYDITKIITRNLDKIIERNYYPIKEAEKSNKRHRPIGIGVQGLADTFMLLRYPYES
DEAKELNKRIFETMYYAALEMSVELAQIHGPYESYKGSPASQGILQFDMWNVKVDNKYWD
WDLLKKKISMHGLRNSLLLAPMPTASTSQILGNNESFEPYTSNIYYRRVLSGEFFVVNPH
LLKDLFDRGLWDEDMKQQLIAHNGSVQYISEIPADLKELYKTVWEIKQKNIIDMAADRGV
FIDQSQSLNIYIQKPTFAKLSSMHFYGWEKGLKTGAYYLRTQAATDAIKFTVDTQVAKNA
EKLKNADGVAITREVSRETISTESTVTQNACPLRRNNDEPCLMCSGSEEMSTMNDTLPKL
PFDEIRNEMNKYGVAITPSTLKHPTTEDVQGVYSICIKYILNKDINNIRIEEFTGDLKSS
MPSIDGIQILPNEGKNHLQAIGNLRFFRHCEKINKILNMDNTLSYIFKPTSGHITKLINA
FMHFMRYREQIYNENDATIKQIEERKNESNILDNELKSIKSELQVLLSKHEE--------
--------------------------------DRIVNETNELIFQFSRYRQKKEDLEDQI
VPSPEKLQQYNDELKDLLYEHMSHCETSKKKNEDIKNKINIADLCIKKLVNLLTILTSHI
NETLKVHIDKKNKLKDLGTNLKSLKEENENLKKKKRENENILNETENHFLQEKNKWDEKI
QDEKKNTIIVEENVKQIHESIDDITTKTNQEIQEINNIVNHIQDTVNTYNKNFAIIADLI
ENTKNSQKILTNKIQNNIQNCIKTHLMIQFLKNLQLYKKRETSDIKPNTKKSKMKYEDFN
FIRTLGTGSFGRVILATYKNEDLPPVAIKRFEKSKIIKQKQVDHVFSERKILNYINHPFC
VKLYGSFKDESYLYLVLEFVIGGEFFTFLRRNKRFPNDVGCFYAAQIVLIFEYLQSLNIV
YRDLKPENLLLDKDGFIKMTDFGFAKVVNTRTYTLCGTPEYIAPEILLNAGHGKAVDWWT
LGIFIYEILVGYPPFYANEPLLIYQKILEGIIYFPKFLDNNCKHLMKKLLSHDLTKRYGN
LKKGAQSVKEHPWFANIEWNNLLNKKVDVPYKPKYKNIFDASNFEKVQEDLSIADKVINE
NDPFFDWMVISQFYILSPRGDTIINRDFRGDVLKGSAEIFFRKVKLHKGDPPPLFYLNGI
NFCFLKNNNLYFVLTSLFNISPSYLVELLYRLLKIFKDFCGQLTEEIIRTNFILIYEIID
EVIDYGYLQNSNTEYIRYLIHNEINNINNSNTKFPNLTKFSIKHSNTLPSNASQKPIQAD
NKKNEIFIDIIEKINLIMNKKGEIIYSYIDGVIQIKSYLLGNPYIKIALNDDLYIKNIHK
DNTNNIIIDDCNFNHLVNTSNFETDRILSLYQPDGECVLMNYRINNNFKAPFHLFANVLY
NPNHTVELFIRIKLDIPSRYSCTNVLVNCNLCKHISSVHLDPNINSDLFSAHYIPNENKL
LWTIKKFK--YPYIIHHDIYLFTHFSF---------------------------------
----------------------MDLIVITPYDEELDDIEELNSKILALETIKHNKSNVNK
NADNSCPNIKKLVLPGEAVLEKKDKPRFLKGSGLYEEEENFCACLLGSVNYINKLVYVEP
LRGKYTGSVGDLLVGKIKDINNDKWVVEIGSYCRALLSITQTNISLFSQRIRLYNDVINM
INIYKPNDVIACEVQRILTDGCIILHTRSSIYGKLSNGILITVPQTLVQNQKKHIFVFPC
NVQIILGMNGFIWISSPIKKSKDTNPNSIDEDIEGNKFEEVDDTTRKNISIISNIIKLLA
KYHININYDIITKIYMQYTSNKNNTPSYILKPYVSDSYLFSYLENFTK--MPILYHLTLQ
KPTAITRTVYGNFSGPKAHEIVVAKGQVLELLRADKQGKLNVIVSKDVFGIIRSLEIFRL
LGSNKDYIAIGSDSGRLVILKYDDEKNDFIRVHCETYGKSGIRRIIPGEYIAIDPKGRAL
MLCAIEKQKFVYILNRDNKENLTISSPLEAHKSHSICHAVVALNVGFENPMFVSIEQNYE
NLDKQVLNANEQIMEYPKKGLCFWEMDLGLNHVIKKHTIPIDITAHLLIPLPGGQQGPSG
LIVCCENYLVYKKIDHDDIFCSYPRRLEVGEEKNISIVCWTIHRIKTFFFILIQSEYGDL
YKIEVNHEDGIVKEIICKYFDTVPIANSICVLKSGALFVAAEFGNHFFYQFSGIGNDSND
AMCTSNHPSGKNAIIAFKTQKLKNLYLVDQIYSLSPIVDMKILDAKNSNLPQIYALCGRG
PRSSLRILQHGLSIEELANNELPGKPRYIWTVKKDNSSEYDGYIIVSFEGNTLILEIGET
VEEVYDSLLLTNVTTIHINLLYDNSFIQVYDTGIRHINGKIVQEWIPPKNKQINAATSNG
SQIVVSLSGGELIYFEIDESHTLTEIFRKNINVEILCLSIQQIQQNKLRASFLAVGCLDN
VVRLLSIEKDQYFKQLSTYILPNNSSPQDICISEMKELGNQKEHTILYLNIGLNTGVLLR
SVIDPICGTLSNHYSKYLGAKSVKICHVQVNKNPALLVLSEKTYLCYVYQGKYIYSPLNY
DVLEYASSFYSEQCSDGYVAISGNSLRIFRFYRLGEVFSQNILHLTFTPRKIVPLPFPSL
FYDNDTSLEIKRIKNIQMLAIIEADHNAYDENTQQEIQKALRDIKLEGKGETQDGGDEQE
NDEEELLYDRIGTPKAGLGKWGSCIKIINPINLQIIDKISLELEEAALSVCACELEALHC
LIVGTTTNMTLKNRNVPSASLRVYTYDINYKLNLLHITPIEDQPYCFCPFNGRVIVSVGN
KLRIYALGKKKLLKKCEYKDIPEAIVSIKVSGDRIFASDIRESVLIFFYDSNQNLIRLIS
DDIIPRWITCSEILDHHTIIAADKFDSVFILRVPEEAKQEEYGIANKCWYGGEVINSSTK
NRKMEHIMSFHIGEIVTSLQKVKLSPTSSECIIYSTIMGTIGAFIPYDSKEELELTQHLE
IILRTEKHSLCGREHIFFRSYYHPVQHVIDGDLCEQFSSLPFEVQRKIGSDLEKTPDEIL
RKLEDIRNKILMSFFFSFNTLPMNLCLLFFLQ--GSGPYSKSIKKVETDITGLVTNINKL
CGVRESDTGLCLPNQWDLQLDKQMLNEEQPLQVARCTKIINSDTDQTKYIINVKQIAKFV
VGLGEKVAPSDIEEGMRVGVDRTKYKIQILLPPKIDPSVTMMTVEEKPDITYNDIGGCKE
QLEKLREVVEMPLLQPERFVTLGIDPPKGVLLYGPPGTGKTLTARAIANRTDACFICVIG
SELVQKYVGEGARLVRELFQMAKSKKACILFIDEVDAIGGSRGDESAHGDHEVQRTMLEI
VNQLDGFDNRGNIKVIMATNRPDTLDSALVRPGRIDRKIEFSLPDLEGRTHIFKIHANTM
NMSRDVRFELLARLCPNSTGSDIRSVCTEAGMFAIRARRKTITEKDLLLAINKVIHGCKQ
FSATGKYMVYHMSLDFCSQHLLAYDNSLITSENEIKTKTEENFALVLKKINELTNQRNDE
GELFYTLTQDNVFNLPRYSKFPEAKKPTRWELFSQTKLKKKKNKHGLIYDENSKGWVRRF
QKKQIKINKEKSDFVHEYKPSDNIYEDPFEKMEEEKDIKKMKQKMREMKNKFEQQGISSQ
DIKYIQKQKRKRENLIDNLKMAQISSSTFGRYDKKLKKEKKLKVK-------EYFKEHFG
VHFTLY-------METNPYVFKNLIHIYEEYINLIINKVKGYKVLVLDDETKMIISLIFS
HSYILEKEIFLTLNFNDINIFENIKNGNNELDELSFQNYKIKNLKHLKAIFLLRPTHTNI
LKLMKELKKPIFLEYYLFFTNTLNNSYIEKLAKADEFECIKSVMEYYIDIYVLHDKLFSF
NINYTSFLYKNDNKALKNKIKNVSGNNLNIKKNIQFKTSNNSLTFEEFDKNLNYMNYLEN
ENDEISSKDFENVGSNNESKNMLYENLLINRLIEGMFSFLCSIKQVPDIIYNKHSYVCKS
IIDALKMEMLKHESIFLNILENYENYDKYTKTNPMENYQENNLINKNIDVNTEGNCCYMV
LIDRREDPITPLLMQWTYQAMLHELIGIDNNKIILDSNNSEESQIVMSSNYDDFYNKHLF
DNFGDLGKAVQGYVDVYQKETARKSKLESIDDIQKFIEAYPNYKKLSGNVTKHVNILHKF
SELVEKRKLFHISELEQSIAIYQKKMEHFKKVIDIVKNYSYTNYDALRLSLLYSLKYEDK
EHIDVIKKELQKRNIEKDQISLIDSLMIYSNYQNRKNNLFKEQTFLDYAKTTITRTIKGT

SNVFTLHKSYIYYLIEDIIKFKLDTNIYTATNLLNIVPNMNKKINSIIVFVIGGATYKEY
RDVQDLSEKYNINIVLGGTQIHNSQSFLADVLQLTKKMNKILATEIFPDIEENKFAPIQK
SSSKDLFLIDFINNKEDNSNSNNNMLHEKKKKKIYKSVFERLTDQTFYTGTHKKKFEALV
KCKLNEQNVDNYLSKNLETHKNKNEKKNEKSEKKEKKLVVTPGILGIQKYGIQIAHPKSI
WLYRNGDKHHNGLLFFIKSHINNLKLLLFEITKVLNPIIGPIRKIYDQNFRLIKNIQQLN
DGSKYLCTSGDPPAPIDHLGKFKSKWVIQ-M-------------------SKSEKNECSE
GSKMCEELIQSNSISSENFNKKTKVKKNKRSSKEFIKEECEEEDGEEDGKNEEDMKISDI
TKYFKEGQKVITPPNGDGTRAFYESLLDENPNSIIAIKYCIEHGVLSGTKHHETLNKYYM
LKKNNAFRNNFGGIKCEFVEMLEKIKNDKLISMKNMEVDRDANVEQWKIKRLIKKLENAK
GNGTSMISLIIRSKDEVSRINKMLADELGTASNIKSRVNRLSVLSAITSTQQKLKLYNKT
PPKGLVVYCGTVVTEDGKEKKMSIDFEPFRPINTSLYLCDNKFHVEALKELLESDDKFGF
IIVDGNGALFGTIQGNAREVIRRFTVDLPKKHGRGGQSALRFARLRLEKRHNYLRKVAEV
STSVFITNDKVNVLGIVLAGSADFKNDLLHSDLFDQRLYAKVIKIVDISYGGDNGFNQAI
ELSGEALQNVKFIQEKKLIGKFFEEIAQDTGKVVYGIEDTLKALEIGAVELLIVYEGLDI
IRLTTKNNVTNQIKTMHIFPHDEKQESLYKENNVELEVVEKILLTDWIINNYKKYGASLD
FVTNKSQEGAQFQKGFGGFGGMLRYKIDLNLYDEDVDSDVELFMNSYKESNHVVDQKRLL
DECIFVVKEQSFFMKQALDNGSLRDTLKHASNMLCELKTTELSPKYYYELYMLIFNELQH
LDSFINDKKKHKKKFIDIYESVQHAGNIIPRLYLLIIVGRNYIKNKDIKAKYILKDMTEL
CKGIQHPLKGLFLRYFLIQMCKDRIPDTGSEYEESGGGNINDAFEFLLTNFYECLKLWNR
MNDKIIPVSNIDDNVLKNNKIQISKEKMDVKMLVGSILVRMSQLEGMTKQYYIEKCLPKL
LLYLSNINDSRIQQYIFESIVQVFSDECHLYSLEILLDSILKLNNSVDYKNILITLLKRL
RSFIEHNKSDFPKEIDIFNLFYNHLVIYADRTIEQCRSDGFNN-------VQNNLININD
KVCNLNKNNEDIDVSQNVTRLNDQNIQKNSIVNDNNNNNKNVKKNKEETVQIIIKMLQVL
YEFIFLCICMYDS-TRINGLFELAYKIVSNVNINDDEISEQVINIIVLPFNHLGINALKA
KNIKNLLNSINDKYKKKLSLNIIDAIIECKNKEIIYQNVEEMLKFISCIFYDDTPKKNIN
KKDPFNFENNSIIYTSEKISKFFHIITNTNDIDQKYNTSMLFYNYIYDSIYFSQLLPSII
FTFLNLVTKIIDIGTSIPENNENINTYYDTYLTNKETEILSEDKINQYNKYAKNIFKFIH
TNLLTISNDIPILAFKIFLLTSIVVNKYDKFINDYSFISFDNIEDICLEFITQALIIYEE
CINLSSQQFESIIWAIGILTSHINLLDNENYNNIALKLCQHANKLLKKKDQCIGLLMCSH
LYWENKKYRNSKKTYECLQKALKAAEIAIQSNSDNIFLFVYTLKKYIYYYELSNIEITEN
SINYLISICQEYYSDISKDSSINQEYLQICKDIQTKKQNQNLFENVNVYLPSMLEAKLNN
ASILKKLFECIKDLVNDANIDADENGLKLQALDGNHVSLVSLHLVDSGFSHYRCDRERVL
GVNIASLNKVFKLCGINESVVISSKDDEDNLNFVFENNKEDKVTNFSLKLMSIELDSLNI
PDCDEGFDAEVELSSKELTNIFRNLSEFSDTVFIEIDSNSIKFTTKGLVGDAEVALKPRE
STSEDDVGVTIKSKKKIKQSFAIKYLNLFSKSSILSDVVILGLSDSRPIEFKYEIKDTSP
DSDALKVGFIKFFLAPKMDDDMDNKDMRIYNWVSCGFIILQFFLNVYGKEWCKAKFEYGM
SDYAECQKEGDNLTKYMIEIIPAKANNINLYCDVSIVLSNSQGINTKEINIGSEKEGLFR
KIYSVRKDIDNPEYVHVKLDSKNRSWKCKKIKIWKDYKYWTFDCIGILNEEKREATYFLS
GNKLYTAYVQTGKDIEAGTTGIIDIILLGNNKRSNTKMLHEGFISGGLKKIKFQASDVGN
LENIILINNSYNDPWYCDFIKIKSDDSKIYVFNVKSWIGYPYNNKIKININTNNIDGNAK
DIDCHIRANDLIDTTNNNFVLQNKVHIFKVRCPQNCHSSEFSIIEGTSIHPASTSICAAA
IYDGSLTESGGEIIVTITKGLNYYYAMDQTYNNLKAIEFSTKSDKNNFSFYTYHLTSIDD
IKSNIRIVDSFGKLSSLGRLEIRVNNKWGAVCKKGPNFAFSEDAAKRACKDLGFPNGIYI
KENCSNINEQNYCAGYKYPFNASGIMCSGNEQNLLSCNTDDPSYCIDHHDDVIIQCINQL
GNDSIENGTIRLLDSTGSPTSNGIGRLQIYYNGVFGSICSEGWTKETEKIACLELGYHNV
KANGFSQHLCNDIAGENLCGHDTERINATNFRCKGDEPNLKNCPHETSEDIYCSHEEDII
IGCASADEEGNNSISSNKHMMSMEKKKFHPKIELSCFDKITSKAELSKGNVGDIFLVSCP
EKCDEDIGAIKGTFVYTFDSYICKAAIHAGVLSSNVTDDVVLIITHSRNKFIGTKRNNIE
SKEFIGESKSFSLSIPTNYIIMEERQNNSKYEDEIL-KEDNDFYYEHIFKKENKTFFEHL
EPTFQWIAPSSFAGFNGDENQYINANNLPNEKYIRTLSNFTFIIHFIPNNGKNKWRTILS
HSLCEGISISIDEENELIIEQNCNPHLVKTKFIPKFEHPYHLVLIYNKSNKSISLYMNQK
KINLENTKFDFTLNGDLTIGRSNKQATDYFIGDINFVKIYKYILTEHEIKESYNSVISNN
YLNDGMSGNRDINIKKTQNKKTKNNRKTIDGRDCITPCKSKTNVNKNIQINTEEFYLNCS
DNLLSERFNGKIGAQFLASCLEDCTNSKYIVKGSNNYYTPDTSICKAVMHSGIIHKTDNN
NNSFIIKIVEGLTEYKSSRGHFGIVSKSEKQSQLRSFSLFSKMEDDIFTCFTDAAFLLEL
PIGTTKNVICPENCHKIDKQIFGTNTYSPLSSVCKAAIHAGVISIKGGHIQIVVGKGQQE
FKSSTQNNIQSYIAEKQNRSFTFLKHKFMKADLFVGQNVETEKVPENKFNINTDIIIHKI
KKNIFKKKKNNLNIDLLSNNKELLSKDFPNLKNIKNKKIKKKLSTDVKLAILSSKKIITN
AQFNNIDQGYIKLATHKEKKNDVEKKDVEKKDVEKNDPNESLINLSKQNENNFQNDSIKI
SQKYIYEHADVGTQKKVFDLHLNLGPYKCNYSRNGKYLLVTGEKGHISLLDTHNMESLCE
LNVNETVRCNTTFHNHKLFAIGQKKYIYIYDNTGIEVNCIKDILYPCQLEFLPYHFLLAS
IGDLGELVYQDISVGNIITRKKTKRGPCSIMKQNKQNAIIYLGHRNGHVTLWSPNMDKSL
CDIFAHKTPISSIGVFDNYLITASIDCTYKLWDIRKLEYIKSFKSHNIINNIDISDTSMV
AFSMNSHFRTYKNFFTKPELYLTHNTGGDKINSIAFQPFEDICCAGLKYSIKSFIVPGSG
LANIDTFVNNPYETKKQIKENEIRQLLDKLPPETIQFKPNEIGKMNPYISHDNIKQN---
ILSE--HITTVRSKNKNA-------------------SLTKHQKQQNGNKSNHIPNNKKK
GKKKG--KKNIKKNDNKMS--MSRSIEANDNLYFLAISKNKVTIYEYNAEFSKTIISDIE
EEIKRSENVSNGNGQNVSSQNDVINNNTSLKKSSNKFNNDYLINRGISIYREYEGVELAT
CTRDYKKIILVRKNNLNVAEIIDIKTNEYVSYIKTKTQIKRIITSPRDSHIVLHCQYKPD
ISNKNLYIYKINGKNNKKKKKKKDIKETENPEQNEQNEHINNYNDSPKKDEIVYNESLVY
EMGLNSYSNSNWPFFKWTESESNCLCLINNQIYIYKDNNFNVISDKLKLDHIEFFEVSPE
QTNKRRVFLATYERGTKGNSSVFKIFNLDNLNKHIYSKNFFNSDEIKLKWNKNGTSLLLQ
IHTQVDKEKQSYYGSSNLYFIDTIKIKDVNIMTNKGLIYDTIWSYNQNKFYVCKGEIPAD
IVLHDKNGNIIHSYGKHKFNTLKLNNNEKLLLTGGFGNLSGDISIWNTINKKEITKTKSS
CAVICEFFNDDNHFLTATTHPRLRVDNNIKIFKYNGLIVSKLDFDELYNVIILPFNKIKS
QPIDTSINP---NSELQKYINKQLGIDSKKVGIYKAPGSTAKTSLNGLGIMARPLKPKSN
MPPGCNFVVEEKTNXKKKKKKNPKNKENKESFMKNDKIKLVSFEGDEFIVDKYTASMSTV
IFNILEVMTSEEDTIPLPNIKTQILKKIIEYMEYHIHNPPDEIPKPLITSNLQDVVSVWD
YDFVNTDKETLYELIEASNYLDIKPLLDLTCGKIASMMKDKTTEEIRAEFDIVNDFTREE
EMQIREENKWCGDIMEPSITSMVYDEKENQNEKENQNEEKLTQVENKCEALPEHNCNTHA
NKSDNICIQNSNVLKCEEIQNNSKLSLSTNLYEQNYKYNTNLYE--------QNMDDCNI
VDIKYDLNNPDEGNENSFIDTSTNKNEEINNEKQKDKNICD-IINTNNIEVEKREDTNIK
EEKCLTNFNYNNESNIEKYCEQNKDDATEKNRRIIEMYEKKLETEISQKEKNIDGLIESS
NKSDGNVSDISAKMRESISKIFRETPSSKPKIINEIKKNDNDESFENEENFENNKNEDKE
GNLSNDNGSMSRDCRDNKPSFSRNTLKEGSLLRLFRCEYFDTHLHIRYLYDRKEVGVHEY
LVNSLYTQRNPEDILFYLPQLCQISLVRYESSSLYRFLLDKASKSMHFALKLNWIYNSIV
EDNIPKYKEISQKMIQEIEMAVVNCKPLNNECKIFKENKQSDLLILAYPLLFKRKFIIKK
IRANEKINQIKKFNKFISNSYS---LKGLIESNPIEKTNTLFCSNYVNNTDQKNTDIINV
EKMDEPQSIYSNSEHINEECEKNNYLGAKEKYN-GVCPKCAHIFEIKKEKKDKKKNITLS
PQLPSCYIINSGALSVASAKIKLPSTYAKLGDPLSFSKFSLPDCNYSFDMIEELQQFFMK
QRRCDYFSLLNNFINILITTSNLLANEPDIDIRNTLLNKFIYSLNTWMVMRRCIVASCEN
IFSMTGLCIPMESISSSKSDRCGSRRKHKKNSKHLQILHFNNDECKIFFSKKRAPYLLVF
EVADLDEDISHISDNTFYVSNRLFNIENGKSEKNEKNEKSEKYLSHSNNEKSDEELKHNN
YILTKNYGGYNENSSSYECEMKSSYEEQNKKNYKKEQINKLNSNTNSVNSEISSTNSSSK
DLNVIKMNDLYVYNAIVNDLRRENLISFTSEEEENIYLIKKCIGLAEEKSNDGYSDKEYD
NNEDSSTEGIKTNKFLNTRSASMPNYLNNSKSTDITSTPVNNNDDENGHINDMCEYNEGD
GNSKNIQTTEQDKPTQGETYDKNNKKEISSINKNLIKRIGNNNNNGKSDGKHSSVLSSHS
HNTRNFYYSTELPSMLPDISEYFKVENYLNDEFKKKNCKIIKTLLWGELFEDKKKKIRKI
SPYGKLRSWDLKCVIVKGGDDLRQELLASQLIKQFKIIFDNAGLPLWLRPYEILVTGSNS
GIIEFVHDTCSVDSLKRKFGTDSISTIFNIVFADYIFEAKKNFIESHAAYSLISYLLQVK
DRHNGNMLLDSYGHLIHIDYGFMLTNSPGNVNFETSPFKLTQEYLDIMDGENSDNYEYFR
RLIVSGFLEARKHSEEIILLVELMMPALKMPCFSNGTQFCIDSLKERFMTNLTVDACIQR
INTLIETSINNFRTCAYIYIYFFLSFFFMTINVSFKVTGGKEFTISIEPTITVMELKQKC
AEHVDIPVESQRIIFKGKILKDKEPLTLYGVADGNVMHLVRSSVPAKDSEAEKENNKESN
SATDQNQGINENLNNFNDNPLVQMLMQRGAGDMNSFGQGVGDGNFNYGNLASMLNPNGNG
EFNRESISSLLNNPLARSLMNELSNNPEMLTNLISNNPLLRNTFSQSPLMQPMLDNPNLL

```
REFMRPEVLQAGLQIESALNNNQNNNNNNNNNNNPGLRMEDLLSNLSNFANPNGGLNSSN
GNNANNLNSLFQSPELLQTFQQVMRGNPNLGNFNFGNLAQNLNLNTPNVTDNRPPEERYA
SQLVSLQEMGFIDNDANIQALQETGGDVNSAVTRLLERGFNMEKKEDNVMREIKVNKLIL
NICVGESGDRLTRAARVLEQLTEQKPIFGKCRFTIRSFGVRRNEKISCFVTVRGKKALEI
LEKGLKVKEYELRRKNFSDTGNFGFGIQEHIDLGIKYDPSTGIYGMDFYVHLSRPGYRVT
RRRRKRSTISKTHKVTKEDAMKWFQTKFDGILLKMEFGGSNYFRFRLALSLISGKEITIK
NIRKKNSHKKTSKDEEIDDNEINEGLQEYEAKILKLIDKLCDDTIIKINEDGDELYFKPG
FLIGNVNDEVKISDLNNTFHCGNERSISYFLEFLIMIVPFFKNPVKLLLKGITDDQIDRT
VYTCKIVCENFFKTFLNVNDDFLNITILKRGTKLDATGEVSFFMNNLKMVNSFDMHDAGL
VKKITGTIVCNKMSMIFRNKIVNCAKKNLHNFTPYVSIEVEKEKKNNYNNSQNNFMSLSL
FAQTKNKCIYGTDLYVDKFMLQHVKDMLNSRT----VDENDEEKEEEAEVEEDEGEEKES
TFKDENKEMNNSNIDASKDALKTLHDVDIYERLGFFISLKLMNEIKGLSSIDSSYQWLPL
LYMALANDTAVSKISLSALKPYSIVLIRLLRDFFSVVFDIQKVEKSQIEYSYLIKCVGIG
YRNFSKKTFMISLGGSSLSKEFFDLAKSIGDSRSKQEEDRIICNEIVLLKSRFANPNATV
KQIKEYLIRAIYIEMLGHDASFAYIHAVKLAHEKNILCKRTGYLSCNLFLNKDHELMLLL
INTIQKDLKSDNHLEVWAALNCVCKLLNSEMIPAIFPIIKNLLNHKNELIRKKVCMLLHK
IYLIDPTLIKEIDVYLKKLLCDVDPSVMGASLNLIFAIANNDMIYCMELVPYLVSILKQI
CENKLPKDYDYHRIPAPWIQIKILSIFRILGFSNKKISEQMYEVLQKTMQRADYGINVGY
AIIYECVKTITTIYPSHRLLELASLSISRFISSENHNLKYVGVTGLALIVKINPIYATKH
QLAVVDCLEDKDETLKMKTLDLLYEMTNPLNVQVIVDKLIFHVENSQDMHFKHDLACKII
QLIERYPPNDIWFLNKINTLFLSVGELIDEAYSYSLIKLLKENSQSNNINKEEKYLNLLK
GSEDAGNDLNGESGENNKNNVGEKLKNEEDNETDLNKNEKKKSNDDTLNLRKYAVNTYIK
MLEENENIPFVLIQIICFVLGEYSYLCDLENYTAEDILDLLCECMEKNLTNPDRVKSCII
TAIFKLCCYNNITDHVVTNKIIEKYKSSQITDIQQKCYEYDLILKNSELIKNVFSKNNKT
QNIVIDENLSFLNPFIDKHLESGGKSYVAKDLRQCENNFETSKSPSLALNFTPYELPINN
NINSDIYNASSPNLLYQKNRELSSFSNSQINEDCSSQREKKKMFKLNVVGPKKWKKETSK
MEDIEINENKNNNAKKKKKKKKGKKNVNIYPGTNNGIINYLGEDRNKLSIEKTNKKQYN
NLDNVNQSNSYYNEEKEINEHSEDDEDEDEEDSTSENNEHEKRMDFMQDYTNYENLYGNS
IEGIKNNKYGNLNKKGTNKDEQSTFRNKGMEDRTNELSEKEKMAAALFNGLVSNNTSVFD
SKNIYSQNFTNKKSLSISSNKNDLKHNKDSSVKNNSFEESKNTNNRMTNKHEEHDPSASI
ITRRNLNNSNKLGDPSKKIYSYDMIDLNEMPESRDITLENNENKEMWNDIENQKKAVFLN
KSKLSIFQTIKKFETNLDSNVVDVSKTEALVSCM-----------MSGSQCVAIACDLRL
GSNSFTTVSTNFTKIFKINDHIYVGLSGLATDIQSLYELLRYRVNLYQIRQETDMNIDCF
SNMLSNILYSNRFSPYFVNPIVVGFKVNTHIDENGNKINVYEPYLNAYDLIGAKCETNDF
VVNGVSNEQLYGMCESMYIKDQDENGLFETVSQCLLSALDRDCLSGWGAEVYVLTPDKII
KKKLKARMDMHKTIRKIFDTKICERLESMHILLVGAGGIGSEFLKSIITIGCKNIDIIDI
DTIDITNLNRQFLFKKKDVKKHKSIVARERALKHRKDLNINAYTFDVCTMKGSDISKYDY
VINALDNIKARKYVNKLCITEKKVLIEAGSTGYNGQVYPIFSNETKCYNCEEKPKNKTYA
ICTIRQTPSLPEHCVAWGKLIFETFFCKNDNETLIDIKKHIEEESKKRNMDKEEIIRFIF
NYLFHDTINELISLKKDYTIMPKPILFEENSKQEPHNIEKLNQEMPN-NLKTTDNKIADK
IAGKIAGKIAENNSIQLSSQNIWDKEKCIEMYVSTFNKLYKYLNIKKTTEEYLIFDKDDD
DCINFITCLSNLRMINFSIKQKSKFDIQSIAGNIIPAISSTNAIVAAFQAAQLVHVIEHF
ELLKEKETEQNINLRDSKAKHIWIKNVVNGNKIFSRGNIVNAENLETPNPNCYVCQQPVI
NIYIKNFNDITLNSFVKEICMNELSFLYPFLDNQDRNIFDYDTFLENDDDYMKSLSNSLH
EWGIKHDDILTLTDSQDTKNQIEIHLKEDKSLENLYLINNKLTKKRKTEVATIEERPTKS
AKKMKFIPEDDIIIVEKMETNDDKD-----------------------------------
------------------------------------------------------------
------------------------------------------------------------
---------------------------------------------MNDIDEFIQSQLILS
SLKVCCSVLKIGGNFISKIFRGEYTGLLIFYLNKFFEKIYVCKPQSSRNKSLESFLVCLN
FHLPKSSITSLFINRNEVCYNSQNENELRNIHLELINGKDQDLQFDDKIDNNLVNSSDSE
ENNLNNMKKKKKNLDIFNFYCSDSDEDIKYFNSDEEENFNNYISSNFLSNNKLFSFIATQ
NYYDSDKSYLLPQNYIRHEPQIMPLQPPYLLSLQNRRKENKKMEGDNYDMILVLNFGSQY
FHLIVKRLNNIKIYSETKDYNVDLKDIKNLNIKGVILSGSPHSVNAENGPHIKKEVLNYF
IENKIPIFGICYGMQEIAFHMNGKVGKSKNSEHGSTEVTLISNDYKNNESYKNYKLLESN
CLLFDGIKNTNNMNVWMNHTEEVIEIPENYYLVNSSENCFICAFYNKENNIYGVQYHPEV
YETVDGDQMFYNFAYKICKCTKTFDPIKYHEVEFNNIKKHAHDHYVIAAMSGGIDSTVAA
AMTHKIFKDRFYGIFIDNGLLRKNEGEKVYTFIKSTFPDMNITKIDASENFLNQLKGVTD
PEQKRKIIGKLFIEEFEKAVYSMDIDIEKTYLLQGTLYPDIIESKCSKNLSDTIKTHHNV
GGLPKNLKFKLFEPFKFLFKDDVKKLSQELNLPKELTNRHPFPGPGLAIRVIGEIDKHKL
DILREVDDIFINSLKEYNLYDDIGQAFAVIFSSKSVGVRGDARSYDHICALRAVKTTSFM
TASYYKIPHDILEKITTRILSNVKGVNRILYDISSKPPSTIEFEMGKMMSYNMQSSILSM
DITND--RNYVCTYLIDNT---IKLVEKNSGTVL-----GLYKGDINNNHRRNIK-FDIK
NKKTGWINLMKNSIPRRSDETDENDKVEDIIRKFDPLVLYEDENSKIEVDPILAQYLREH
QREGVQFVFECLMNLKDEKISGCILADDMGLGKTLQSISVLYTLLKQGYNKKAAVRRCLI
LCPASLINNWNDEINKWLPNRCTVTCVNDSAKEKIVSKLEGFKYDLKSTILICSYECFRI
NNDSIDKSAIDMIICDEAHRLKNDKTKTYTSIYKLSAKKRLLLSGTPIQNDLGEFFALIS
LCNPDLFDDTNSFRKKFANPILIGRDKDATEKEQQIASERLAELSTITNKFILRRTNNLL
SKVLPVKYLINIFIKLNPIQEALYVLFLKDKKLLKPDNSNNKVNVLINIKKLEKICNHPL
LLNANDIKDIGHIPVVKLIEDVIAENVKGRGNKLSNKNSVNSSANNSTENNCSKDGDYLR
ERRKAVELDYDKSVKKLIEECKRDVYRCYYNLSSKFQLLHFLLKTIKQETNDKVVIVSNY
TQTLDYMEILCRENYYKFVRLDGGISIKKRHKVISDFTNTDDIFIFLLSSKSGGCGINLI
SSNRLILLDPDWNPANDKQALARVWREGQKKICYIYRLFCTGTIDEKVYQRQISKDGLSS
MIVTNTNLSKDQLSDENVKKLFNYKNNTICETHDNIECNRCNFKDNT---EVYSEQLEDF
EEEDVNTWAHHLNIDTVPDAVLIKAVKEATECKINHMNTLPILKKLKHEFVTFSMSCKIE
FRDDLIKQKANKNNTSYQPIAISEKKEPTKAKRAKPKEDSEYSDSTNDEEEEEEDEEEDE
EEEDEEEEENEDEYEMNKYNAPNIEFKKDDAIFCIEDIANKSSPEYEKEIKNLPLVSNEH
CVNIRKKKIEPAGFAFYTKYEIPILKEKEKNKNNNKYIPIDELFLYKDKIDYLEEIKNN-
-----EDGENKNNSKNLKNEEYQKNDYEENDNSKKKEEKPSLKKNSNIVKKCINNNINVY
EILGVEESDDFETIKLAYKKLILIFHPDKNKGTSILNQKEKAKKKKKNNNNDNKNDEEKK
DIAYYIEKYNIEKLTQEEKKSMFLKIQDSYTVLSDKVLRKQYDSSIPFDETIPTKTALDE
APNFYEFLNPIFKRNAKWSSKKPVPNIGDENTSIDKVKYFYDFWYEFTSWRDFSYQNEYD
YEDAECREERRWMERENKKIQKKASKAEKLRINKLVDLAYNNDPRIIAENKRIELEKQRK
KELAMLERQKNKMNTLQSTSKNSNQTNSSNDKKSKDNKAAYKIWKHHIKSICVTKLYNIF
NFDLIQDNINNMPFDTMCQFIYEIYLFLNFNINKSENSSVEKININQTKENKQNEKETTF
INTNKSNNNDNNKRGGKNASSNNTGNEGNKNTGFIGHLKGVELSEKDIESLLDIFRKYIN
NFNTILEMKENMNTQDIKNEKNDPEQDVHTDEKNNITTNCSQNNKVINEEIKEKSNSKEI
DVEEKKNKKEEDENNTSTKWTPQEISLLSKALKLYPGGTKDRWTVIANSIKTKNVKEVIK
KAKEMFENETLKNLSKNFEESAFDNFKNQNKGVMKKIDDKLDKREYKDINDQNNLSSAHS
SDNNSEQKKPWTHEEQMLLEKALMKHPATIPTKERLKLVSNELKTRSVEEIVLRLKTIKA
KIMAKNAAKMPKKRRNGGRSKHNRGHVNPLRCSNCGRCVPKDKAIKRFNIRNIVDTSAQR
DIKEASVYSTFQLPKLYIKQCYCVSCAIHSRFVRVRSRQQRRVRKETTKHAHASQLMKKK
IEQNNPRHKKYSYSDESKMQNFFYKSINKNRFYLLFFLFLFS--SLTHLAQCQV-KKKVS
VSEINFDSAVDDVQWCGNNHSTVLVKTIKGKLYRSSDGGKIWTNITSNLSENSSNKNDST
SGHTPETTVVDLIMVNPINKNIVLVIGAQNSHYISDDAGETFKLINYKNKINFWQFHNKK
AQWALVSSWTSACFSTDNSSGECMQTLSLTKDLGASFQLIDIYVVQFNWGDVSSHSEDTI
YYTRHRNRNGHQQRFSGWSKDVDFVSTNNFGKDVEVLVKHGNKFLISNGYIFVAKLNDVI
KQTVNMMVSTDGGKTFNKANLPKDIHEKSYTILDTSEGAIMLHVNHGSTSEKLNTGNVYI
SDASGLNYTLSLPNNIRTASGECEFDRVLSLDGVYIANFLDDQDEMKDEDLKFTNFKLQL
EEEVGPVETNTQKRKKQLTKGKNEEIVRTVISFNKGGHWSYLKAPKVDSIGNKYDCGDEC
YLHLHGITNYHQYAPFYSIENAVGIIMGTGNVGSHLKYKSDEVNTFLSRDGGVTWIEAHK
GPYIYEFGDHGGLIVMSDDLRKTNQIVFSWNEGQSWFDFELGQFPIDVDNIVAEPTSSSV
EFLVYGTRNDIGVLYHLDFNALGQPLCKGLWAADSVSSDYETWSPSSGSFTDKCILGRKI
TYTRRKQTSECFNGKDLKRIVDKKLCDCTPEDYECETGFTRKVGSFECKPTDSTLTIEGC
TSSSYFYATAYRKVPGDVCVNGWVPEKVPVPCPDYSPFNNSAKSILFILFIMGLVMLIIT
YICRNPKFRNMFYNYGFDTFEHVKYSVVKTKKGNINNNVFEPEMEFIDAEQDNNEEDVPT
```

```
LMSY-NERNGQRNDFELTRNRSNHNNYVTSRASNSQKKYPENIELLMADRKPNKNAVVKN
VDMTEEMQIDAIDCANQALQKYNVEKDIAAHIKKEFDRKYDPTWHCVVGRNFGSYVTHET
KNFIYFYIGQVAILLFKSGMAIEVDEETLKIAKEFQKKNEEEIKSKKLKPETIQLDSPSS
NLIKINEASCRGRIKISNLLNAPISETEYNDKIEKNKYIDQLVVVCGWSKAVRKQGGGRF
CFVNLNDGSCHLNLQIVVNQNIDNYDKLLKCGIGCCFRFTGTLILSPVQNTEKKGLLNQN
VELTLTDNSIHSFEIYGENLDPQKYPLSKKNHGKEFLREVAHLRPRSYFISSVMRIRNAL
MLSTHLFFQSRGFICIQTPLITTSDCEGGGEMFTVTTLFNEEGDINSIPKTKKKINNNQK
REDQNTNE-LETNNNTNQYIVDFKKDFFSKQAFLTVSGQLSLENLCSSMGDVYTFGPTFR
AENSHTSRHLAEFWMIEPEMAFADIYDNMEVAEAYIKYCIRYVLNNNFHDIYYFEENVEK
GLIDRLKNILNDNFAKITYTNAIDLLTKYSNNFEVPIKWGMDLQSEHERFISEQIFKKPV
IVYNYPKDLKAFYMKLNDDNKTVAAMDVLVPKIGEVIGGSQREDNLELLDKMINEKKLNI
ESYWWYRQLRKYGTHPHSGFGLGFERLIMLVTGVDNIKDTIPFPRYHGHAEFMCSTNSEI
DIPEADRIESQKLFDELKELNINYKEVKHGKVNSIKEILDLNLEKSENVIKNLFLKDKKK
NYFYLCVANWKKLDLKNVSTQLKTSNLRFVDDENLKNILNVNPGSLTPFSIKSDKDNIVK
LYFDEDIKNMDEVIIHPMHNYSCIYVKTTDVIKYCDLHNHTPEFINLSDSKDTEKRDDSN
INEESSKDTAKKHGKNDDKINDRDKGKDGSNILGITSKKEQNFSDWYTQVIVKSELIEYY
DISGCYILRPASYYIWECIQTFFNNEIKKLDVENSYFPLFVTKNKLEKEKNHIEGFSPEV
AWVTKYGDTNLPEEIAIRPTSETIMYSVFSKWIRSHRDLPLKLNQWNTVVRWEFKQPTPF
IRTREFLWQEGHTAHKNEEEAVKMVFDILDIYRRWYEECLAVPVIKGIKSEGEKFGGANF
TSTNETFISESGRAIQAATSHYLGTNFAKMFKIEFEDEQENKQFVHQTSWGCTTRSIGVM
IMMHSDNKGLILPPNVAKYKVVIVPILYKNTDETAIFNYCKDIEKVLKNAQINCVFDDRD
LYSPGYKFNHWELRGIPIRIEVGPKDIQNNSCVFVRRDNNQKFNIKKESVLLETQQMLVD
IHKNLFLKAKKKLDDSIVQITSFDQVMDALNKKKMVLAPWCEDITTEEEIKKETQRLSMN
QTNTETSLSGAMKPLCIPLDQPPMPPNTKCFWTGKPAKRWCLFGRSYMAKLSKAQKKQIY
MDKLSSLIQQYNKILIVHVDNVGSDQMASVRQSLRGKATILMGKNTRIRTALKKNLQAVP
QIEKLLPLVKLNMGFVFCKDDLSEVRSIILQNKSPAPARLGVIAPIDVFIPPGPTGMDPS
HTSFFQSLGISTKIVKGQIEIQENVHLIKQGEKVTASSATLLQKFNMKPFSYGVDVRTVY
DDGVIYDANVLDITEEDILKKFSKGVANVAALSRSVGIITEASYPHVFVEAFKNIVSLVI
DTDYTFPLMKKIKDMVENPQAYAAAPVAASSAQKDEPKKEAAKKEEEEEEEEEDGFMGFGM
FDMAQERDLAREPCPDRIIEDMGGAFGMGCIGGYIWHFLKGARNSPKGDMLSGALYSSRM
RAPILGGNFAVWGGTFSCFDCTFQYLRKKEDHWNAIGSGFFTGGVLAMRGGWRSSSRNAI
VGGVLLAIIEFVSMVLTRKTTPTPRQQFQQQMEMEKKMAAQKTK----------------
------------------------------------------------------------
------------------------------------------------------------
-------------------------------------------------------MWCIK
GLIFNDLYASSKINLKEIIENDPDSSIV-------------------YMQLSEIFEFHMA
LDSWWFIANSEMPSYLKLLPKFLRFKFPLSEGEWVIHSSNKSSNNFWLYPGYFCFIGTYR
QLANAFFILAVLCYNSNYRYKPPILLGSCIVSLKSVTEYPFFKGTVKKLTLEKRKFKQGE
IVGNIKCFVNSYGIQEDDTNIQRPVQPLSDVTLVNQLSINDHYLVIRIIKCENLAISSVD
LNNININVWVKWDGIVNKTDIVSKTVSPFFYQNLYYPIRLVDKKELTNEKLIHNVLPIDL
ISKGDICLEVHNNNEIYSSILGIFELPFSDIFNYGTYDYRGLVQDNNAS-SIYNENKNYD
FYDQGFDNPDENYEDYYSRQYKTIVYKNTLDLMYSRANAQTKRKSTISIEAFVIPPLPSG
LVFFKENQSQNSSIIYKAMSKRWDQDFKGFNDIYLQWFPKAIKNR---------------
---SFVTPINLPAQVSTP------------------------------------------
----------------GTINNGKTEHYWVMTRHEKGWVCFWEVTNKSIIHLKYRWNNNNF
LKNSDILAENKMINKMNENDNEKYYNGEYLTNYIKYGLEELKNRKNQIIQEYDMKEENQL
YTMDLYGEHLMKDEEVNIDEVLYNDEEFFDNIFKVKYEKNKTYSCNKAIKYVLESFSKYI
PIAPKFGLLNYENTLAYVPYTSIEVIFNDQQLYGNMQNHHPACILYDLENNYHWRPFLNH
EPPQIKSEITISTPLSDKLSIKYMNELEEELQEMIFFIRNVEGLETNFDHSKEIKYFLEM
YIDICEYKLNLDNNFNPXPENYEYKNGKEIEKNMNNGSMSGNKENHMENMNKNDNNIYNN
ESKKSWSQYKNDFYNINFHNSYHPAKNVEIMNSEKKKNLSNIPQDYVYGMNNEIYNDGKE
EYIKNYVFNKNSTDILNNYDENIKQKNIKRFSNVPLHYQYNEHNCEEYEEEIYKEIKERY
YRCDDSEFSDN------TTHKNIPKQYMKNNN--LKKKLKKYEKIMKKEMIDIKLARKGV
QDKNKKKIFSKFLDAQEYLMN---------------------------------------
------------------------------------------------------------
------------------------------------------------------------
------------------------------------------------------------
-------------MLVDNIGDVTITNDGATILKQLEIQHPAAKILVNLSELQDQEVGDGT
TSVVLLASELLRRGNELIKMDIHPTTVICGYKLAMKESVKYIKEKLSERVTNLGKDVIIN
IAKTTLSSKFISYESEYFAKMVANAIQSVKIINDSGKTKYPVSSVNILKVHGLSSLDSKL
IDGYAIMTGRASQSMPSAIKNAKIAFLDFPLKQYRLHLGVQVNINDPNELEKIRQREKDI
TKERVNKILESGANVILTTQGIDDMPLKYFVEAGAIAVRRVKKDDLKRIAKLTNGQIRLT
LSSIDGTEKFEPASLGYCDEVYEEKVGDWDVMFFKGCKNSKSNTILLRGANDFVLDEMER
SIHDALCSVSRALESNYVVVGGGCVEVALSVYLEDFAKTLGSREQLAIAEFAESLLIIPK
ILALNASYDSIDLVCKLRAYHTKSQVMNTDEPKDYRWYGLDLVNGKVVNNLKNGVLEAMI
SKIKSIRFATEATITILRIDDLIKLVPEEPKQEEP-----------DPWNTAFDDLQICT
KITESCGIACSSEYIATPWQVQGGGVIGVIKLENMCRNPPVYKLKGHTSTILDIQFNPCY
SEVIASSSEDMSIRIWEVCNKEDISEEIKNSLCVLKGHKKKVTIIDWNPLNYYILSSSGF
DSNVNIWDIENEKKAFNINMPQKLTSLKWNSIGTLLSATCLNKKLHIIDPRQEKICTTFN
GHSGGKCAKNIWIDGYSGNENYILSTGFSKNYMREMKLWDLKNISEPIYTISIDNASAPL
LPHYDESIGIIYIIGKGDGNCRYYQHSEGVLRKINEYKSCLPFKSFGFIPKQACNIYKCE
IGRIYKNENDKNIKPISFYVPRKNPNIFQKDLYPPIIMHDPNYSSKSWINGNNLEIKRIN
INELTDDDIKIKKKFKSVPKSMDSILIGDPSLDKKSFIIRQFTKRFTFFKKNNNIEFNND
NSSESSFNINDSYQND----ENKKTRFFVKDKDDDND-----ENQNCMTSGRMKEECVQT
ETSE----NNSNKCLDTITCKKLFGKTNMKIKSIGDESIHNICSSQVIFTLSNVVKELVE
NSIDAGATEIKVKLVENGIKIIEVSDNGNGIKKINFENVCARHATSKISEFDDIHNILDT
LGFRGEALNSLCMLSDLCISTKHDEFEHGYLLKFDKFGKLIHEEPIARLRGTTVSCENIF
KNIPIRKKDLIKNIKSQLNDLLTLMQQYAIIYHEIKFMIFNVVTQKGCTKNMNMLITNGS
DDIKKNFYSIFGKKNIGNLIDFNITNENWLVKGYISDSNSGRRDKDLQFYYMNNRPIHVI
KNVNKIINTIYREFNSRLYPIIIFNILSDSKNIDINVTPDKREVFFTFENELCEEIKTEL
IKLLTPKTSNFVDTQIGDYFFVKSSRLIKSEEAQNERQSERAEIERTEHGERTEYGERAE
HGERQSERAEGEYTNVGRPVFENEELHYTSIDYKKMVVSKNEDTENLYFSGQNKYDNKMA
NQKDNNEDAEKSYISNGIKIKTEFEKFDDNEHNEYTSKRTKIVKDEQVEYYEYKYEEDTN
FNDKNNSSNMDSKWKEINSSLIDSKDLSDDSSVDFSKYGLNKGSEYDAIKTKVTKEQNES
--EDEIKEIENGEIVYEYKLDDENGGEGGETGGDEKGGDEKGGGENGGVEKGGETGGGEK
GGSEQNEYLFEKLKENIKNNIIKNIPININTYINREDMKRGFDYDQINLINLTNSEKIRN
TIFKKIKDVEKANNYLCLTDEKEENKYNNLFKNDLHIKQNSNNPINGIGNEEDINFDNID
EQKKDLYFKSNLFKKLKICGQFNKGFILSKIDLLYFKNEDNKSKN--NDMNETDENENVD
RKNNYALFIIDQHAADEKSNFEKYNKIFTMKSQKLINKIELELSPAQIHIIEKNFVIFLR
NGFEIEIIEEPINKKRKINNDENIISEETLMEMKVYLLSLPVFNGKILEVVDFMSLLHHL
TNHPIIFDKQIDNSFFRNNQKLIDNTETWFNYNFPRPQKVWKILASKACRNAVMVGKTLN
ISEMIRIKKKLSVLQNPWNCPHGRPTIKYIINNIEIQKFYANYYLNLYEEITNLRKSKNY
QEYKHIFRDHIFFLIISTKPMLGPVLKFQ-------------------------------
------------------------------------------------------------
-----------MKNLVLVFLKKISDTYIEDQSKWMEKMKSSQEEQGKALEEAMYEYEKNI
LFNHSIKEQQLLQNNKKLDEWNECIENAYEVQQEVLRQFEAKKREKANQVANKNNELLIA
QNYIEKIKEAATNKKQDSSKCFVYPASSAPCGACTSAGAVTPHRRYKEPRQKKEYSLCIM
AAIDHNVQDELVDYEDDDNMLDNKDVKGDIGGNLLNNNNKGVNDSGAMRGSYATVHTGGF
KDFFLKPELLRAISESGFEHPSEVQQETIPAAITGTDILCQAKSGMGKTAVFVLSILQQL
ETNDSKDIKEEKDMNNNQNSGSQNKYVRCLGIAHTRELAYQIKNEFDRFSKYLKNVRCEV
VYGGISMNKHVVLFKGNNIPHIIIGTPGRILALIREKYMLTDKIQHFVLDECDKCLERLD
MRGDVQKIFISTPLKKQVMFFSATMAKEMRDVCKKFLQNPVEIFIDDEAKLKLHGLLQHY
VKLQEKDKTRKLIEILDALEFNQVIIFVKSVTRAITLDKLLTECNFPSISIHGGLNQEER
IERYDKFKKFENRILVSTDLFGRGIDIERINIVINYDMPENSDSYLHRVGRAGRFGTKGL
AITFVSSQEDTLALNEVQTRFEVAISEMPNKIDCNEYINQ-NDVLSNKDKSANANKDLII
EECSSDDDETDCLSETNKKDMDSNPLDIEHIEVRQSKRMSVSAEAYGEWNKKKENFVAKV
```

HKKDNKEKKKIREALNESFLFNHLNNSEMETIIDAFFDEHVEKGVNIINEGDEGDLLYVI
DEGEIEIYKTKDNKKEVLTTLKSKDVFGELALLYNSKRAATAKALTKCHLWALDRESFTY
IIKDNIAKKRQMYEDILKQVTVLKDMDPYERSKVADCLKSKTFNTGDIIINEGEQGDTFY
ILIDGKATALKNGQVIKTYTKGDYFGELALLRNQPRAATVKAESTCQVVHLERKGFKRLL
GPIEKILIRNVENYKKVLKELGIDSSCIEENMHLKIMCLSDEVREMYKNHKTHHEGDSGF
DVFIIKDEILKPKTTTFVKLGIKATALQYKCNYYYKSDKNDSNNKNNKEPEIVNTSFLLF
PRSSISKTPLRLANSIGLIDAGYRGEIILALDNTSDQEYTIKKNDKLAQIVSFTGEPLSF
ELVTELDETSRGEGGVHVITNKMIICLFRCLFFLLFP------ITDLGAAFGTAKSGVGV
CSVGVMRPDLIMKSILPVVMAGVLGIYGIIMSIIISGKMSPAASYSSFLGYTHLASGLIV
GLSSLAAGLAIGIVGDAGVRANAQQNRLFIGMILILVFSETLALYGNFFL----------
-------MTKRIIKNSTAIRDDGKNVTKEQIKENDNDVN-----------------DNNK
KVKNKLIFKNKKNNDSSINKNEMRIITIPRQRISSVKNNWLELIKPIVTNLKLEIRMNKD
KIEVRTCKLTEDKNNLQKSSDYIKAYLLGFSIEDALALLRIDDLYIXSFQIKDVKILKGD
HLSRCIGRICGSNGSTKYAIENATKTRIVIAGDKIHILGSFNNIKMARYSICSLILGSTQ
GKIFNKLNILAKRLKERFM---------------------------YFFSNIIDNDIIRF
DKAYDENDVQEFVNLCSSTCEIEKLEDRMHPWAADPKTIGALSATQLAILASKENEPHYK
DAIRESNGIPVFINLLKSHELDRVHAAVVALSFLSVDNVKNCICMFDNGALPYLITGMKS
NIDGMKAACAQTCRNIFVLDKNYKKEFLKLGGITQLVNLLELSSTDDNQPLYTQLEAIYH
LEDFILNDGDEVPQFLEATDRLLKIHKLYYFVKIKKQTLSHYTHVLFVSLKKMANGWDAE
KIFGSTISYTYDDIICMPGYIDFPLSEIDLSNNLTKDISLKTPIISSPMDTVTEHKMAIS
MALCGGLGIIHNNMSIENQIEEVKKVKRFENGFIFDPYTFSPEHTVADVLCVKNKVGYKS
YPITSDGKVGSKLVGIITGIDYLYLTNPDVKIKEIMTTELVTGKYPISLSDANKVLCDEK
KSILPIVNDNYELIALVCRNDMHKNRIFPHASKRENKQLIVGASISTRESDLEKVNKLAQ
NMIDIICIDSSQGNSIYQIDMIKKIKSAYPDMPIIAGNVVTSNQAKNLIDAGADVLRIGM
GSGSICTTQDVCAVGRAQGTAVYHVSNYAHTRNIKTIADGGIKNSGNIVKALSLGADFVM
LGNLLAATEESCSEYYFENNVRLKMYRGMGSMEAMYNKQFNSKSRYLVEDKIFGTVYDPT
NDIKISQGVSASLVDKGSVLNLIPHLVKAVKHGFQSIGIRNIQQLHSKLYSGDLKFDIRS
INSIKEGKVSDNLIFNTKKMFCKNIKYKRLFSGKCENKYIYRTNQNIITGIKFDIYNDKT
ILGNFPTRNFSSFMKNVIEQVKKDMKENKQYQEALKELKEKTEIDDKAIKLKKKIEENIN
ILKNIKEKNEQAIKLIYNDINKLITHCFENYYIFKLSKNISFKFFSGLTKILLITTDKIS
ELTDKWNDNNAFIQLDKWRQEMAIKRYKKKTGISEKDAEKRD----ADA-DAEK---ADA
GAKCGTGNELILAHESAWDKFGSKLKDMPFLNSFFENPILGKLFGETELAAALRVMKMDD
KNFKLSELMYLFEYVISKHIVESYLIGDEDTLRLHCGEAAFNSLNLSITERKKKKLFLDT
NVLIYKNHELKGAQRMEESSPWFIFTFHTQQINCLKNKDDEIIEGKIDDIREVVYTIALS
KHPEPETEGLLYPYIVREFAIIGNTPSW--------------------------------
---M
YEIKKKKKHFKTLSNSCMKYLSNKEKIKLKKQEEEEKKLKIHSKNISSYMDVFWKKIEKL
VWEEKKRELQKTLNKNKEMRFKKFVKGAIKKIKNARHNAHELFENNMNYLCSNNNSEN-N
ANSNHSNEDKELGEEDLTDQEDEDILLDEEMESMDESEE--KEVNLLDDEANVPIEELLK
KIYGFKSGEEYINLMQNEEEDEGTDTDVDDQVSTQNDSTNPT--------------QNSK
KRKLSDAGESDRELKTAKVDEAGKVEEAKVEKVDEAEVAK----ANEAEVA---------
------------------KVDEILECNMDEKHLTKIPPFIKATLRDYQHAGLHWLLYLYK
NNINGILADEMGLGKTLQCISLLGYLAYYLNIWGPHLIIVPTSILINWEIELKRFCPCFK
ILSYYGNQNERYKKRIGWFNNDSFHICISSYSTIVKDHIIFKRKNWKYIILDEAHNIKNF
NTKRWNIILSLKRDNCLLITGTPLQNSLEELWSLLHFLMPNIFTSHLDFKEWFSDPLNLA
IQKSKIYDSKELIDRLHTVIRPYILRRLKKNVEKEMPNKYEHIIKCKLTRRQKILYDEFI
NNKKVQNTLTSGNYMGLMNILIQLRKVCNHCDLFTNKYIQTPYYYILPIQYNIPKFCLLF
ENNYYKDFYLILFLHNEFVSLGGIAKDVRPKQKQNHIYTDMHTKNNDLTILDSQQFKKNM
TNIET--------------LNEINQTKEFTNKLIEEINSE------GSFNLINKNYYKNI
LTNLNKNKEIKRDSQIITETMQDLCTYVNNEIYKENIPKDSNEFVNELNNNYDILSIYID
PQNKHAGRPSRGSHVGNQSSLYRYNMKVLNREMQYKNFFTDETNQSYLNSLEHNLWIRKK
KEEEINKMKIIKNSRIPIFGSNLLHLLKTEFLKDQNIVYNHANNIIINNTSMKEVYSEDK
SNECASIVLERLFPTMEYFLKLYEKLIQNFIVINSPSVICSSPNILINSNSQNYSHISKI
EDLIKKIKKATRVYHNAFLKQSIIFPLNKDISLGSGKLFALEKLLSKCKKEGNKCLLFTQ
FIKMLDILEIFLNHLNYSFIRLDGSTKVEQRQKIVTKFNNDKSYFIFISSTRSGSIGINL
TAANVVIFYDTDWNPSIDKQAMDRCHRIGQTKDVHVFRFVCEYTVEENIWKKQLQKRKLD
NICINMGNFNSQNNRNNNTSLQDHNNKDWFSNVDTIKEIFINKQNNDDDDDIYQDRLLHE
QLENPEKTNVRFEKTLEHIEDKDDINALHVTKRERQHELSQDMHEFANKNDFQEAYTLTS
YCFNFLNENLTDSLKQQIDEMKMRIEIEMMNAKEDENNSLDNLSNPSDELENYEQVRNEP
TMVLYIIGLGLGDEKDISVKGKELIDQSDVIYLESYTSILFISKDKLEEYYKKKIYEVDR
NFAEENCEQILDEAINKKVSFLVVGDPLCATTHHDIILRAKKKNIDVQVIHNASIMSAIG
ESGMQLYNFGQTVSIPYFEGDYKPTSYYNKIKINLDNNFHTLCLLDIKVKERTIENIMKN
KNIYEPPKFMTVNEAIEQLIYCESVHNENVITKNTLAIAIVRIGSKDQQIVSGNLFTLKT
QKYNDPLHSLIICAPNLHDIEKEYFDMYSTSILLTGKPSGLRSARKLRIRRRTQRWADKG
YKKSHLGTRWKSNPFRGSSHAKGIVVEKVAIEAKQPNSAYRKCVRVQLIKNGKKITAFVP
GDGCLNFIDENDEVLVSGFGRSGHSVGDLPGVKFKVVKVARVSLLALFKEKKEKPRSMGI
KGLTKFIADTAPNAIKEIKIENLMGRVVAIDASMSLYQFIIAIRDGDQYGNLMNESGETT
SHISGLMSRTIKLMENGLKPIYVFDGAPPELKGSELEKRGEKRQKAEELLIKAKAEGNLE
EIKKQSGRTVRVTKKQNEEAKKLLTLMGIPVIESPCEAEAQCAFLTKYEMAHATATEDAD
ALVFGTKILIRNLNANASSNKNKNKNSSKRGYILTEINLEQVLKGLKLTMDEFIDFCILC
GCDYCDTIKGIGSKTAYNLIKEYNCIENIIKNIDQNKYQVPANFKYVEARQSFINPKVLE
KSEVKIDWCEPKIEELKTFLIKEHNFNEVRVTNYITRLLKARKVTTQRRLDTFFTTCTKK
STKLIIEESQKEVLKTKGKGKKREINNDSSTKLNAKKKKTNVKDEKKNNEKVDELKNKSD
ENLVK---DEEDDQDDYDQNIFDEKTNSESGNIKNENVKEDVSSNDIT------MDIPKC
TNDIV-------CMFFSSFPFDSMGGQQPRRKREVNNSKYYESLNLKKNCTTEEVKKAYR
KLAIIHHPDKGGDPEKFKEISRAYEVLSDEEKRKLYDEYGEEGLEGGEQPTDATDLFDFI
LNAGKGKKKRGEDIVSEIKVTLEQLYNGATKKLAISKDVICTNCEGHGGPKDAKVDCKQC
NGRGTKTYMRYHSSVLHQTEVTCNGCRGKGKIFNEKDKCVNCKGLCVLKTRKIIEVYIPK
GAPNKHKIIFNGEADEKPNVITGNLVVILNEKQHTTFRREGVDLFMNYKISLYESLTGFI
AEITHLDERKILIDCTNSGFIKHGDIREVLEEGMPTYKDPFKKGNLYITFEVEYPMDLVI
TKEKKEVLKILKKQNEVEKKYDIENTDCEVVTCKPVDKEYLKQRLTMQQQQEAYDEDGHQ
PEMEGGRVACAQQ-----------MGWKPNEKIYKTIIEALSSCNNSNNNVQIEVTKVLK
DLNENVSDAALYLLHIFMNKQENSDVRQVGGLLLKNYINSKNKFLTNDILKIIKNEIFKL
VEDEIKEIRNTSGSVITSILTKYEGIEKWPEALYNLLLLVERGNNDVVDGAFRAIIIIIE
DELINRKNTDSLFFQFCKTQLLEKLFLYCSLQEKSIKKKYAAECLDLFINASCFATNGIF
NEYFPQLWECLGYLAAEEDTQILKIVVTCVTIITDTRYASIFNNLDGIIQFMVNATNSGD
RKVQLEALEFWPVFIKDRSYMAYSNYNNNNSNNNKTSENYIDENIYKNINELRNEALKTL
KNYLPYLCKILIDNTVYTKWDYLTMDESHFQNDNANVPDLIQDISPELYHSSNNNNFANS
SDNKSNNMLNEQGEYKVQNNSNNGNCANNNMNVNSGDIDNNDDLDDMSDEEKNDEMTSRT
WGNDWTVRKGAALCLDYLSNVYNDDILEYILPHIEEKLMSDKWNIRESAVLSLGAIAKGC
MYSLSPFIPKVLEYLIKLLNDEKPLARSISCWCVTRFSSWICHPDNCDKWFEPVLLNLLK
RVLDSNKRVQEAACSSFANLEEDALELLNNHLHEIVHTIQQAFQIYQAKNYFILFDVVGT
LIDSVNIVKENIDLAHEIVNSILIKWNNIRISSPYIIALMECMSCITSAYGKEFLKYAKI
VIRTCIKFLVLLYIDLEEEIKYYYNRKVNNSNSFIVNRNNITQTANELLNYYKISNEDYF
INLKDIDSISSSKKKDLIECSFDLLSRILSVINSDIIEIIAENEYNFIPLVHKYCLKVGC
VHIDGIMFPLLLNHNYNTKLPVSSGSVKNGVIMENEYTELAKQFLNFGILQSNFALIGDI
SRFCAQYLIPFLNDIIPFLIAHISHPSIPVSNNASWAIGEISIHINSQYIEPYVDEIVKQ
HIYICQNSKYHGCLLQNICITVGRLCSTYPKKIIYYFPQFLKTWLKIMSHGTQENEKINS
LKAILEALYLNLDIAAEHLKDIVYIILKYKYVSQNINIFFHQFLSTMKQKYPNQWKEIYN
PMMDEHSSPIPTDMLNDLSTRFNL------------------------------------

--------------------------------------MKKISENNINELLVEIVYTIFT
KNETLENSMLTLLGESNVDLIINIIKNKDEIKKDIKLLSKQVDIKENTLGANFFITNTTT
KNTKNKNILTNKENIENIIEYFINNFEDNYLDNVKKIYLPNEENQLEEINVPQNTTYSYS
NNMTKVKINRLPNMIFDQNELVSVNALPFWGKYIFNFQYFNYVQSKVFNAAFKNNKNLLV
SAPTGCGKTNIALLVILQQLILHSEKHGINLNKIANAKNGLSSGGNGYIEKHGTTSGRLK

```
TETENENVAESSYDDDNKSDSSMPKGGENVINSNDFKIVYIAPMKSLVYEITTNFREKLK
IFNLNVCEYTKEHSLTSKELELVHIIVTVPEKLDILLRNSSYSSTVSDESLIKSIKCIIL
DEVHLLNTDRGDVIETIVARFLRYSETSQSMKRIMAMSATLPNYKDVSDFLKVERDMCFY
FNEKYRSIQLDKTLYGIHETNMNKLNLAKNLCAYNEIINSLKNDKQCIIFVCSRNDTNKT
IEFLIDHAIKNDEIDYFTNNLYTDYDVNKRIKKSNNIYIKKFYEYGCAVHHAGMSRYDKI
LVENLFKKKTFNVLCCTSTLAWGVNLPVHTVIIKGTNFFSSESGKMEDLDILDINQIFGR
CGRPQYESHGHAILITERTKLYKYIKLLTNNTIIESNFLKNIENHLNAEISMGTTKNAQD
GIKWLEYTYLYIRMKKNPFLYDTDIKNDRELYEKRKNIIIKAISNLSENKLVRRS-LTND
FIGTFYGHIAAKYYVDYKTIGIFASHIENNNYAEIIDVISKAKEFENIQIRNEDMNDFIY
YKNKCDIKENYDESKSMTVRILIEMYLRRIQINNFSLVCEINYIVQNIIRILYAYYEICL
NVLKNISHLIINTHNLILSITRRLPMDCGLFRHFCYKNELMDKKYVHTSNNKNRGNNENR
KNQNYTVYLKEAAVNILEKKNLSYETIANLSKSELFFFLRNEVYTKQILYYRNIIPNLYI
DGYIQPITQTIMKINLNVQLQNTIWSDQWNSTKEDFHIFLLNTLNNDILYFQKFTIHKKD
RKKIHDISFEFPITNIMPSQITVQFLSMNWCNLSFVHIFNTNNLFINQKINVFSEILPIV
PLSTEVMNIPNYIKFFSFKYFNPIQTQIFHAAFHTDENILLGAPTGSGKTVIGELCILRN
LLNYEDQRSVYICPMKAIVNERCIAWKNKFKTLFNKNVIELTGDKNEHKDNIKNSHIIIC
TPEKLDVISRNWKNKNIIKNINLIIFDEIHLLGENRGGVIEILVNRFKNMQHYLNKKIRL
VGLTTVITSVDDLILWLDVKENYLFNFPSSCRIVPCKTHILGFNQNAYCPRMSVMNKDVF
DSINQYAQTKNVLIFVSSRRQTRLTAYDIISLLHLENLLNDKNHIKSEQEQIHVQNLIFQ
NYLNIIENEHLKDLLKYGIGIHHAGLNESDKNIVEYFFLNKIIQILICTSTLAWGINLPA
YLVIIKGNEFYDPKTKKYKDISYTDLLQMIGRAGRPQFDNEALAILLVHEKRKNAIKNFL
YHPMNIESNIMENINEHINAEICSKVIQNKEDMLTYITKSYYFKRLFSNPSYYIKDVQYI
QLFENNRLSNQAKKAIYDHINKIIENTIEFLQKNKCIEAVQEDYIQHYYSTPLGYIACIY
YTKCETAYFFYKTIESPSKKKLDFYALLELIAQAKEFDDIPLRHNEDKYNVKLRNQIPLD
IDMNMPNVKTYLLLLSRFYECTYETVDYHIDLKLVLDQLARVINSYIDISLLFHNYTYIK
TLILIFQCVNQKIKPYTNDLYTIKGITDSQINKLKDLQIKHIKDLIKFDKNFLYSLNIFD
TSQINYILNIPNLTTNIKLYQKNDNTDTSNKINNFANIKLGKYSKNENKYHFKIRYYEKN
EILIKVFFNILSKIFKESNTSTMVSNAQWYA-METINIKKEDNEAQVKISQEIHDENVEK
RYNEIMSITSECIQPDELKLKLLQKRKLICYDGFEPSGRMHIAQGLLKSHIVNTLTNNGC
TFIFWIADWFAQLNNKMSGDLNKIKKVGQYFIEVWKSCGMNMENVQFMWASDEINKNPDK
YWSTVIDISRSFNINRIKRCLTIMGRTEGEDNYCSQILYPCMQCADIFFLNVDICQLGTD
QRKVNMLAREYCDIKKIKKKPVILSHGMLPGLLEGQEKMSKSDENSAIFMDDNEADVNRK
IKKAYCPPNVIESNPIFAYAKTIIYXHYKEFSLARKEKNGGDKLYLTIEEMEKDYINGEI
HPLDLKDNVALYINKMLQPVRDHFQNNAEAKKLLSEIKKYKITKINKNVISPTVKHNGPL
KVSIIGSGSWGTVISKIISENTQRSNIFHPIVRMYVNEEIIDNEKLSDIINKTKENVKYM
KGMKLPNNIVAIPDIDKVIEDADLLVFVVPHQYLETTLSEILKNKNLKSTAKAISLMKGI
KICDYKPMLLSNIIENMLNIECSVLSGSNIASELSTESFSEATIGFENLETAEIWRELFD
RNYFKINCIQDKSGVEMCGALKNVVALGVGFSEAFKKSYNTKSAIIRIGLEEMKKFAKLF
FPNVLDVS----------------------------------------------------
----------------------------------------------------NMERILSS
IGRLSVVAGGLSLIPYTFIYDVDGGERCVMFNRFGGVSEKTYGEGSHFYFPWFQTPYIYD
IKMKPKVINTTTGTKDLQIVTLSLRLLFRPHTKHLPYLHSTLGPDYDERVLPSIGNEVLX
AVVARYNAESLLTQRDTISKEIRESITARAKQFNIVLDDVAITHLSYGKEFAKAIEDKQV
AQQESERVKFIVAKTEQEKIAAVIKAQGEAEAAKLISSAVKEYGNSLLEIRKLEAAKEIA
ENLSKSKNVTYFPSTSNILLNPKSLMKLDYINAIKEENGGYNFENLKRNEILKEKGVIFP
KFRKTGTTICGIVCQNAVILGADTRATEGPIVADKNCSKLHYISKNIYCAGAGVAGDLEH
TTLWLQHNVELHRLNTKTQPRVAMCVSRLTQELFKYQGYKVCAIVLGGVDVTGPQLYGIH
PHGSSCLLPFTALGSGSLNAMTVLEAKYRDNMTIEEGKELVCEAICAGIFNDLGSGGNVD
ICVITKDGTQHIRPYKQPNTRLYHLSHPTVYPKGTTPILYEKIENIKKHITIEDAMKSIK
NGMYNFNTSKEDPFGKAPAGYVAGKGRGVTGFSGGVSRDDITEDRDKNDYSDFNYDEFHG
YSESLFKDTEYDEEDKEADDIYESIDSRIDVRRKSRRENKLKEEILKMRAQKPTIQEQFS
DLKKNLANVTLEEWESIPNVLNYSRQKQKKMPKNYLPAPDSLIMNKLNESNSHLNYSASS
ENGLGIKTPLGIKTPLGLYTPIGLGFQTPFLRNSKGIDTPLFGKNNKSGSINSGINTPFT
LSGYTTPLNASNISGYNTPIANNGNMLSLNDLGEARGTVLSVKLDELIDNVEGQTVIDPK
GYLTNLNAKNLTNDSDIADINKARSLLKSVINTNRKHGPGWIAAARVEELAQRKDKAKEI
IMKGCIECSKNEDVWLEAVRLEDKLSESKIILTKAIKNIPTSVKLWLEAYKKEKNVQDKR
KVLRKAIECIPNSVVLWKEAISLENENNAYILLKRAVECIPQCIEMWIALARLCKYSEAQ
KVLNEARKQIPTSAEIWINASKLEEKQGNINMVDVIIKRCIENLSQKNVIHERDKWIKFA
EECEKSDFLHTCQSIIKNTMNIGVENLNKKRIYKQDAQNCINNKSLHTARCIYNEALKIF
KTKKSLWLDLANLELTHGNQTNVDDVLQRAVKNCPHSSVLWLMYAKQKWLNNEIDAARKI
LAESFMHNQNTEVISLAAVKLERENNEFERARILLKKSRVQCNTPKIWMQSVQLERLLGN
YKDAKELIYEGLKIHKKFDKLYMIAGQIELEMANLKEKNLNNAYDKAQQIYQQGLKYCPE
SINLWLCAIDLQITKKSYTSARALVEKAKIKIKNIHSLSVNTKVLKNKEIIESNEQYHDE
EIGNNLNHTKNDDNNNDNISNSKNELENKINVNASVKVIENYDLLWLKLIEIELLCNNIN
INPIISEALKECPTSGILWSKAIELENKNLQNSKSVTAFNNCGNNSYVILIVAIIFWNNY
KIGKSRKWFYRAITLNPSFGDGWATFLAFEIDQENEINQKDIINKCIKAEPNRGYMWNKI
TKRIENWRLNYPQKMYRYIKERFPHVLNKPISDTIWKIINDENVTLPTNKKTKNMGKFSY
LSFLSEFKKFKKIYVYTKTNCLNNKNNKFEQTYNEMESEKMNNNGNIIPEEISDKKKEKK
AKKLEEKKLKLAKKLERENLKNEAAKVLEHVCEDIDKENYGYVKLNIIMENGKNINLYNP
EEIYNLLYVEKNIDNEKREDIESEMNNKLEEGSNIWVRGRIHDIRSKGSLAFIILRNKIY
SLQCILDIKNVNNDKNMIKWVSNLSLESIVDIYGKLVKPEISIDSTIIKYEIQILKIFCI
SKNSKELPFLLKDANMKETSDEATIRVNQDNRLNNRCIDLRTYANYSIFYLQSEICKIFR
NYLIDNNFTEIHTPKLLGESSEGGANAFQINYFNQNGFLAQSPQLYKQMCINSGFDRVFE
IAPVFRAENSNTYRHLCEYVSLDVEMTYKYDYMENVYFYDSMFKNIFNKLINNEKNKLFI
KNIKNQYPSEDFKWLAVTPIFTYEQAIKLLIEHKKLNLQSEEILTYDMTTDMEKELGKII
KQTHDTDYYIIINFPSGLRPFYTMYNEKDPKISNSYDFFMRGEEILSGSQRISDVKLLLD
NIKKFNLDPKKLDFYIDSFAYSSYPHSGCGIGLERVLMLFLGLNNIRKTSLFPRDPKRLT
PMEITNTLKKLF-RQNFTYIEGNSIFETQQKKDEKLNRKKGKKETENIQKPKYGDIENIN
DIEKNKQNEGDDKNSKDPNLTPFINTFDAPNALLFENLNKEYKYITTQDNFDGFRFEVDK
NVNKYLQSTHTLFLGTSLRDVGYLYQFGANFANSDNTLLMISRINLDGSVNGRFCKKINN
FVDCKFNFNTYNKSDPRNMYEMSLEVNNPQNTYNIKTIWQGAWIFNASYTQLLTKKLQAG
VDLTYIGSNCASIGSFGLRYNHKNHVLTMQCIRQPNFKSPEFMLNQAHSYKMQYARKISD
RLSVGTELEVTPETKESAMRLGWDYSFRHAKVQGSIDSSGKIAVFTQDYSGFGVSGYIDY
LNNEYKFGFMMHIAPSQEQPQPQPMDNQEFIPQYKLILVGDGGVGKTTFVKRHLTGEFEK
KYIPTLGVEVHPLKFQTNFGKTQFNVWDTAGQEKFGGLRDGYYIKSDCAIIMFDVSSRIT
YKNVPNWYRDITRVCETIPMVLVGNKVDVKDRQVKSRQIQFHRKRNLQYYDLSARSNYNF
EKPFLWLARRLSNQPNLVFVGEHAKAPEFQIDLNIVREAEKELEQAAAVAIDEEDIENME
KLKTIYVDSALSIIKGALCVILQIPTSRTTESVKKKQNFIGVLTVNSIKSEPTISQYDDI
KKLVKNKVLENAPFYNYQIERSFADKFYGDCIYDNFGVPKNINEINLIILEEWNINCNRN
RILKHTGLIKNIEINNFKYINNKESLEVHFSVNPMYTFEELSTLYKNEKSLYNFLLCPIR
KVRTNNADTITDNENNEEYIYINIEDLLEKNKVLPPSGVENIGYERSNEVTPWNVNIQGD
GIDYNKLIKQFGCSKIKEEHIKRIEMLTKKKAHHFIRRNIFFSHRDLDFLLNYYEKNKSF
YIYTGRGPSSLSMHLGHLIPFYFCKYLQDAFNVPLIIQISDDEKFLFNKNYSLDYINKLS
KENVKDIIAVGLNPELTFIFKNTQYVHNLYNTVLSIHKKTTLNQSMNIFGFDHSDNIGKI
SYPSFQIAPCFSQCFPNFLPQNLPCLVPQGIDQDPYFRLSRDIAVKLALHKPVVIHSIFM
PSLLGVNTKMSSTKKKSENKTGSNEHNNSVIFLTDTPEQIKNKINKYAFSGGGATIQEHR
EKGGNLDKDISYQYLRYLLDDDDKLNEIGEKYKSGELLSGEIKKILIDVVVDLIQTHKKK
RDSLTDDQIDYFFNPNKPGLMKFKNLMMINGDNSKKHEIEIKSISKNEMEFTLYNSNSAL
ANALRRIMLSEVPTLAIDIVNVYENTSPFHDEFIAHRIGLIPIDSRNIKNYEFRERCKCK
ETCSRCTVQYIIEVKCNNANKIDVSHYDIEALDHEPNIPMPIPNDNKNNNIEKENAIPIL
TLSKNQTIHMKLTATKGIGKMHAKWIPANVSYIIDHKIIINNHEVDKMSKEHKLLIANNL
NSDCYILKELDDDRDVELRLSENMSVVMAESCRDTLNELGYKDIVKIIYDETKFHFKIES
VGSMPPEQIVEMAIEILENKLKDLEPQIKASFYSIEEVAKQLKEQGVSLYGIQLDLEMYD
SSSKIKDLSLAPFGKLQMEISETEMPGIMTIREEYEKLKPFKGAKITGCLHMTIETALLI
ETLQKLGARIRWCSCNIFSTLDYAAAAVSTLENVSVFAWRGETLEEYWWCVEKALTWENG
EGPDLIVDDGADASYLVHKGAEYEKLYEEKKILPDPESGKNEEERCFLSLIKSSILKNPK
```

KWTNMAKKIIGMSEETTTGVLRVKKIEKNNGLLFTAINVNDSVTKQKYDNIYGCRHSLPD
GLMRATDFLISGKIVVICGYGDVGKGCASAMKGLGARVYVTEIDPICAIQAVMEGFNVVT
LEEIVEKGDFFITCTGNVDIIKLEHLLKMKNNAVVGNIGHFDDEIQVSDLFNHEGIEIEN
VKPQVDRVTLPNGNKIIVLAQGRLLNLSCATGHPAFVMSFSFCNQIFAQLELWENRNTGK
YENKSYILPKELDEKVAFYHLKKLNATLTELDDNQCEFLGVSKTGPFKSEAYRYMYSYGM
EGDDTYLPQVQYPSPYENQY-QDEESPSPRGESHTPFIGYFSSHLLRTGFLLQCLSLILM
FVFYWAFGGTGIFIFDLYAGPECVKVSSAFHLTISVLMSIYLLGTLYIAMFQVFVADNSK
WCRGFRAGSKLLSAAVTLDLLSSILRLVQYLYAYFYMNMRWWARYQQTKSDWTLLHFGSI
VHSFSLFIYGAAFFYMEAYHDEGTYEELAWSNLTLFKLAGLAELLMVFSGFGAFFSILLL
GAIMCATVWAFSFEPLLEKWSPELHSRDINADVLPEIKHDDENCGYNEENMYEPYNGNPE
SMEYNEEMIKQNGNEKYINGNSNFYEHNNGIPTTYDYSQIEGQVKQNAEMGVSENKYIGQ
YFEDANKPKKRTFRTFHYRGIELDKLLELNQEELVKLLPARQRRKFRRGIDKKAKSLLKK
LRKAKKECEVGEKPKPIPTHLRNMTIIPEMVGSIVAVHNGKQYTNVEIKPEMIGYYLGEF
SITYKHTRHGKPGIGATHSSRFIPLKMSNKKVQSPKEESIAKMLICKVHIGTKNLENKMK
RYVYTRAKDGVHIINLAKTYEKLQLAARVIVAVSNPADVVVVSARPFGSRAVLKFAQYTG
AQAIAGRWTPGMLTNQIIQKFIEPRLLIVTDPRTDAQSVKESAYANIPVIALCDSDSPLE
HVDIAIPCNNKGKESIALMYWLLAQEVLYLKGTIPRSQAWDVMVDMFLWRDPEQFELKNL
ANEEATPTAPHLADNQYATEAPYDDWNKKDDWNDNANEEWKNPITVDEW----------M
SSMLKDKTPQYFDIQPHTNIKCIVHPSVIFTILDSYLRRDEDQTHVIGTLMGSIIDTNLV
EISDCFVDKHSLNEGGFLQIIKDHHETMYELKQKIRPRDQVVGWFCSGSELSELSCAVHG
WFKEHNSISKFYPHSPLNEPIHLLVDASLESGFLNVKAYVQLPINLVKEYFVHFHEIQIE
LLPCNIERADVAKITEKITKGGD--------KEKLLNNEIDENSLKKLLIMLKNCKSYVQ
DIVDKKKKGNVAIGRYLHKVFSNDPFISVEKFDSINESILQDNLMISYLSNLAHLQFLIA
EKLNTSSMQMEKIVEIIEGLSNPDSRIRTECENTLNYYKKNDLNNTVLSILKLLKSHKNS
QVRLQCAILIRNLFRGYIKSDKDKTENEEENYWDLLSNNLKNIVKSELISNIGIETDKMV
RSNLCSNIIDLSSKLLLNNEWPELLSVTFEFCNSNNNDVLISGYKILGGILNCIPDELHG
KNEIISSICMKGLNSPSVQVRSECINLISCIVEDNNSPLIKCVQPCIPLILQSLSLMVKN
SVSDVAVLDECEKVLQSIGKMIDYNAKFFSKYISNLCDILFDICMKNENELNYDFDNSLK
SLSIEALITIPERRPKTALSVPHFLEKIINVSLLFMLDINNDCFNEWMNSLKESKDENQE
LYDIGEESLDRVGKAYSELSDPEFIHILYNKVSEYLMKNTWEHKYVAIMAIAQTIEYLPE
EEIEEQLENVIKMLLQVLLDQDVRVRYAACQAIGQISLDHQPYVQKEYPRQIITSLISTM
NDVHLRVQSHATAAFVNFAEELEKSALLPFSDMIIEILLQKLNTTNYLLVREQAVTAIAV
IAGVIEEDFLKYYPTVVPLMKEIIQKAVSEEERTCRGKAIECISIIGLSVGKEVFIEDAK
ECMSALLQISSTKMDPDDTVKEYIQEAIGRICRALGNDFYPYLSSIVPTILSLLSISPTP
LIDDDDDLTITMVSNGQYVGLKTSLLEDQEKALDLLIIIIEVLKENYKEYIEATASAILP
MLDYELSDEIKQKALSAISELIEAARIISDQTDNNKSMLLAILTTSSEKVLKSLSDTKLD
DNYEYVLDIMIIESNGLYMCLQKAGANILPNNTLKMFFNEIFKLLQYSTDRRVIYNQKKN
NEDVDDDELLIIDREEELEQTYRTNLLDILGVLIKHHTNQFLSTCCDICITFINNYLNSP
HAEDIALALYVCDDLLEFLQDNSVCLWEYFMNPLLLNINHSDNKVKQAACYGVIQANKIE
AFGKYANLAIEYILKLLHQTPPNKKPKEYISAIDNAVAALGDVVLMHTSKFNNVEELIKL
WLNNLPIKEDESEGRRVHKNLIDLVSQNHPLLFGKDNSNIGKIIEIFLSIYETEFSDSDC
NKKIVSLISSLDQSYLSNLASSSLTNKQSKKLNHIMNASRK-------------------

--NSNGCDLFNFYEDNINLKPY
LKILKDFDKYPIIVDSKNNILSLPPIINCDHTKITLDTKNIFIECTGIDLNKLEISLNII
CSMLSEYSQPKYTIHSILVLYNEHHELEKGNRYLYPNFKNKKLTCDIEYVRKLSGIKDIT
IDDVKKLLKKMMISVTDVLDNTTFEVNVPFYRSDIMHACDIVEDIAIAYGYDNIKYEPIE
ISKKHLLNTVSDMFRNSMTECSYTEVITNALLSLKENYDWMLRKHINYDTSDKLVNNYNP
LYPPVQIMNSKTSEYEIVRTSLIVNLLKFVAANKHRELPLRFFEIGDISYTMNNKTDTNA
FNKRNLSIIFADKVTAGLEEIHGVLESILKDFQLFSHYKIEEKRKEKVEIRSDVYYDLVS
IN---------------------GKIG--------------------------------P
ADNTSTSSNSPVISIGNIRGLGGCDYGSFRMSNEFLGWKNKKTNSVYQYKCNDISEGEWI
KLSYNNNRLHLKFNESKDNLIVFFDGFPDRNIAEITQHFQKYFNIKLGTRKLATKGWNWG
EFKLENSNLFFDIDKKYAFNINTNNINQLNVQIKTDIAIELKNDEKQNTNEDVLSEIRFY
YPHENDENQNFQDLKNNLLEKVNIGDSKSECIASLSNIPLLVPRGRYEIEMYSKTFKLHG
KSYDFTVQYSNINKMLLVPKTNSNQYILIFSLNNKIKQGQTEYPFILIQLSNDDDMDLDI
NASEEDIQNYKLEKTLTGKAYDVVTRLFTALAKKNAIIPGDYRTAKNEHGITCSYRAASG
QLYPLNKYFLFVVKPVILISFDDIVTLSFQRTGNINQHRFFSLIIKHKRGISYEYTNIDK
SEYAPLLEFLKSKNLNIQDDANVSEKKTDFDDDDDDL--------SESDEEDYVAEEEDE
EDDNDDDD-----EYDDEDDDKMGDIRSKTDLGDCRFYEKKFPEVDDLIMVKVNRIEDMG
AYVSILEYNDMEGMILMSELSKRRFRSVNKLIRVGRHEVVLVLRVDNQKGYIDLSKRRVS
PKDIIKCEEHFSKSKKVHQTVRHVAQKHNMTVEELNRKVIWPLYKKYGHALDALKEATMN
PDVIFKEMDISDAVKESLLSDIKLRLTPQALKLRGRIDVWCFGYEGIDAVKEALKKGKEI
SNNEVTINIKLIAPPQYVIVTSCHDKELGMQKIQEAMKVISDKIKEYKGGDFKQQGEILV
IGGDDEKRLEELLDKHDGLSSDDE-YSSDGDEDDSSDDDDNSSEEDDDD-ELYLNMYDDI
YRNKKKIDDNKIIDLEKEVKIRLEEQSNLLSIKGIDRVDIKQKKGKHSIICIHYIKNMCM
KNLFCNYLHQLIYDRIPPCKNYIKYNYCADKIRGSCMFRHTLENTNMNYYNENKEEHLDE
ALKFLHEKNICVNYLLGFCNLGYNCRKIHKNRSIKNIINILPKFYLDQILINKNLYTHLY
KSQQVLNDMNKLKDALIVLSGEKYQEKNISLTKNERENSDMFTNSRMLNSGINIHDDKNL
NRSGFNMVHNNNENKLQNNETNNIKAVGNIPEVYNLDNSPVISDKIKVFVIKCNQISHLY
LSILYGVWATGKNNTRKYVNFFKENYTIIFLFSVNESGGFQGYAKMITLPVKNLYENLWG
PITKRLGGNFRVQWIKIAKIDFDVFKNITNPYNDNLPLKKSRDGTELPLNIASIICNKIH
ALPNEDFLAGTIYEYKRRINHSVYFTNLYKKNMLNTNTMWDSIIFTLNQKSDCQQITFID
GIEQNIS---

------------------------------MEQSENILLSGERGIFEFFVCDVGVGLSLS
VNENEINSYDRDILPIEYDSIFIKKNVKNNLVDSLTIHYKNKENNNNEKEINLALGGDIM
PSYGVLPYYTFNHEYIIYDSSQLLPRYLIQFECDPSAEELFSIPLCDYCGNAPSLYYCES
DEVKLCEKCDNIIHSQNKLVKKHIRKTLNEAQSNFGNCKIHLQNEVNMFCTVCHIPICNL
CMCSHAHKDLSNVNFFNFNNNSDTIISLKMAYNAIMQHSSKPSNFIKERKKNLNNLLEKI
DKLHEQVRLNMNETEKNVYNVLEDLVKQLHTTTDKKMSSILSEEYELKRQFNEIMWNENF
LYYLQTILPPADFMNAWLKHCQYREEIEKNSEHSEKINSLIFPDIRIKGNINVITEGSIN
HENIFHSSANLMAPKKKEEEPKVLLLGRPKNTLKMGLVGLPNVGKSTTFNVLTKLNIPAE
NYPFCTIDPHEAKVTVEDERFDWLVDHFKPKSSVHAYLSIFDIAGLVKNAHLGEGLGNNF
LSNIAAVDGIYHVVRAFENEDIIHTEGNIXPVRDMEIINSELIYKDISNCERNLEEISKV
LNRNKKDKIKQNEHDVLTIVLEHLKEHKWIKDKAWKSSEIEVINEYNFLTAKPVVYLVNM
SENDFIRQKNKYLAKIYNWVQEKNKGTIIPYCAEFEQKLLSMTENEKEEYFKANNIKTSM
LNKIIKTGYYEINLIHFFTCGQDEVKCWTVRKGTKAPQAAGVIHTDFEKGFICAEVYKYT
DLVEFKSEGEVKANGKYLQKGKDYVVEDGDIVFFKFNVSSSGKKMSEYNLKKDDSPFYKL
DTIKCEVPINSEFNDRINKFVNQLRISYGTLEEFVDNFVYELKKGLEAHRRHPNLWIPHE
CSFKMLDSCISDIPTGQERGTYYAIDFGGTNFRAVRASLDGNGKIKRDQETYSLKFTGTF
SHEKGLLDKHATASQLFDHFAERIKYIMGEFNDLDDNNEKSVGFTFSFPCTSPSINCSIL
IDWTKGFETGRATNDPVEGRDVCKLMNDAFIRSSVPAKVSCVVNDAVGTLMSCAYQKGKS
APPCYIGIILGTGSNGCYYEPDWKKYKYSGKIINIELGNFDKDLPLSPIDLVMDWYSANR
SRQLFEKMISGAYLGEIVRRFMVNVLQSASSKKMWQSDSFNSESGSVVLNDTTPDFRECK
KIAKQTWDMDFTDEQIYALRKICEAVYNRSAALAAAAIAAIAKRIKIIEHSKFSCGVDGS
LFVKNAWYCNRLKEHLRVILADKAENLIIIPADDGSGKGAAITAAVVSLSSNMKQLPMPD
LYILIRWLCKAIVSSLFGDVNIINPENVPLYGSVIFVGNHNNQFIDACVLVASIPRQVKF
IVAEKSMKRAVIGDLARLAGCISVKRPEDLKFKGIGRIYWNTGDTKIKGINTRFKLDVQM
GDKLMTQNKIFSVTKIESEIELILQDPININCEDTVNGVPFKIVPKINQSEVYNLVTHSL
KNGDTIGIFPEGGSHDRTNLLPLKPGVAIMTLCALADGIEDVSIIPVGLSYSKLYQLQGC
VTIFFGNAIIASQDLCKDYNNNNRETISKLLGKIEEGMRSCMLTSKNHETSRCIELCVSL
YTPERMTISKNKIYNNLQLFSEMFWKFGNSKEIENLCYELQCYEKLLEANKIKDDEVWML
KQSTSAATLKFIEQICSLIFCTIFGMTFSLLWLPLVAISVYLAENHRKTSLKNSLVKIQG
GDVVASYKVLVLLVLLPTFNIIYGLLFSLYFYQSWLKRIAFTICSICILPICYYININYS

VQIPTLLRQMKIHLKVICGIINVWRDNERELISMRHELQLKVRNIVSKLGHKVSDSFLDQ
LHRNIPKFVINADTKRLIRGKDEWVPILKRSQLEYREEILMDGDQKEVSGRDIYQIIKNA
RDKIKIDYKFWYTQPVPKINEEFSESINEPFIADNKVENVRKDEYKLPEGYVWYVCDVND
ENDRKEVYNLLTDNYVEDDDNIFRFNYSSEFLLWALSSPNYLKEWHIGVKRVDTNKLVGF
ISAFPTDICINKKVVKMVEVNFLCVHKSLRSKRLAPVLIKEVTRRINLKNIWQAVYTAGV
YLPKPISDARYYHRTINVKKLIDVGFSSLNSRLTMSRAIKLYKIDDELNLKNLRLMKKKD
VDQVHKLLNNYLSKFNIYVKFTKEEIAHWFMPIQNVIYTYVNEVDGEIKDMISFYSLPSK
ILANEKYDMIYAAYSFYNVATTTSLKNLMQDAICLAKRNNFDVFNALEVMDNKSVFADLK
FGEGDGTLKYYLYNWKCASFDTSMVGIVLL------------------------------
--MDTD
SVLGLQAILISANYKEKEFIRIAYYMNSFYKDIELREKPPVSPQYDKICRHIFVDNPRIV
KFSIPWDSEERDEFKEFDKENEKIELLNFSKIKEENQSNSNITNQSTQDNIFVPNGVKYN
PNNGNLNITLN--------NNINALNYFKNNEMAPDMDRCEILNQNIKGISSITIDNKNM
MKIYLISYIFPTLIYLLYILLKNQKSLSKYNNYINSKINDVLLFFNIYEIKFPNSYNNNE
KILGLHIRDIEEDSRIAYKILKELTQKNYFRIFKVNLHVPCKLQKITEKCNEKTKCSVCE
CTEDEIPYNFRTNEVEIIHDKYAQEDLKKTFIASKLYKDILGTYDPSDEGFLSYVDLIYN
SPSYTAYEGKSIWNRIYLENCFQNGENSCKEMNNFYKIISGMQSCIAALSSEYYYLKNDF
VFGDMYTDNTIKNHYIKKIDYDYNLSFFKEKIALYPDRIENLYFTFAILLRALCRLKPLF
SQCKCNSGLKKKNK--
---KK---
---MARNVEKGRSMLNQWLK
AKELNDKKTFFKIPKNVNDVDDLESAVSYRKSIIKEICSKIKEIQNLSLGDQHVRELNDQ
INKLISIKNRWEIRIIELGGPDYQSESNALINAHGTELKGNNNYKYFGAAKNLKGVKELL
FKENDDRKKLLLKRRKEKRNLDKIVNIHYFGYCDEENEILLNEELKIQKKLEKTDLEIIK
KINY-M-----QTMKHGTLVLPADRAREYMDCLGKQVDIQFIDMNEKTMKRQYKKYIQRI
DDMERILRFLEENINKLPNVKIKKSKIENFLEHDNIYELDQVEESLNRLHVQFVRFCNNN
KDLIDERNSAIEEKHVILTALNQLHPDMSKRSNLR-TMHDDNIDENNLMNN----SEEDA
ALSTHIMREGINMMFTNISGVIKTKDQESFSRTIFRALRGNTYTYFQNIDENMNSTGTLN
NENNSIDSKIGENNNNNNENKENNSELKSVFVVYCHGSTHSSIYEKIMKICKAYDVRNYE
WPKTYEQANKRLNELKEIINDKEKALKAYEEYFINEIFVLINVVEPNKNSLIEEWKLFCK
KERHIYNNLNYFEGSDITLRCDCWYSANDEEKIRHILMNKSSNDLVSALLLSDKLLTPNI
SPPTYIKTNEFTSTYQSMVDTYGIPRYGEINPAISTIVTFPFLFGIMYGDVGHGICIFLF
ALFLIIVHNRMKNKNNNEMLNMLFNGRYMLLLMGFFAVYAGLLYNDFFSMPLNLFTSMFE
VDKVVDSVEYYKRKQILNAETGQMENAPPYIFGFDSKWLGADNELTYINSFKMKFSIIIG
FFHMTFGVIIKGFNALYFKKKMDFFFEFLPQLVMMLSMIGYLVFLIIYKWITPIGYGGYK
KQGIINTIINMYLLKEINQDNKFYEHQEVVQIIIITLFALCIPIMLICKPAIKTYKIIKD
KKKRMAIHYQAVEKEMTNQFVGVG-YGNN-------SMHKRVTKIGYAENEDEYLLLKRR
GKKTDDEMEAHLLGPSSYHSSSASNTNQTFDEHEENISEIWIEQLIETIEFILGLISNTA
SYLRLWALSLAHQQLSLVFFEQTILSSLEKNTFIGVVISLIIFSQLFSILTIAVILCMDT
LECFLHSLRLQWVEFQNKFYKGDGIPFKPFNIKKLLSDREMLGIQEGRAFFNAARNPNNR
PENNIRIEDNLPSLNEVYNYFQDFLSRYSSNTLKEGNLKRTLFENVKNNNFTLDLKLDDI
YKQQTVFENNEISATPISERKIKKPDNYSAILARALSERPLTYLPTVERVCYEVCETANI
LSDEDEHLNYIQINLLNTFIRPTPIRGLLAATQERFVVVPGIIVQASKPQHKMRKITLQC
RYCDHKMSIDVPLWKDKPQLPPYCRYSSTMKSSMGVANPMDNQLGCNGVLEPYVILPNEC
TFVDIQSLKMQELPEAVPTGDMPRHLQLNATRYLCEKMIPGDRVYVHGVLTSYNPNPKPT
RVDGTNFSYLHVLGFQKYDDMSGNDLNFDVEERNELTLLAAEHDIHNKIFKSVAPELYGM
DEVKKACACLLFGGTRKRIGEETKIRGDINMLMLGDPSVAKSQILKFVNRCAPVSVYTSG
KGSSAAGLTAAVMRDSHGVFSLEGGAMVLADGGVVCIDEFDKMRDDDVVAIHEAMEQQTI
SISKAGITTMLNTRCSVIAAANPSFGSYDDSQDTTDQHDFKTTILSRFDIIFLLRNKQDI
EKDTLLCNHIVALHASKHKSQEGEISLSKLTRYIQYAKKEISPLLSKEARDSLRNYYVQT
RAEYRGDKRSVTKKIPITLRQLESLIRLAESFAKMELSQFATEKHVQMSIDLFSASTAET
AKQCMVFEAMSPSEQKAVKQAEDAILGRLGKGQRASRVNLFRELQLRGFDRSALSKMPKS
YRNYSKTARNPKRPFEKERLDQELKLIGEYGLKNKREIWRVQYLLAKIRSAARYLLTLDE
KSPKRIFQGEALLRRMVRQGLLGENEEKLDYVLGLTLPKLLERRLQTKVFKLGLAKSVHH
ARVLIRQRHIRVGKQMVDIPSFLVRIDSEKHIDFATASPFGGSRPGRLKEMIINKHKSMN
WSER-MVRQSQSMYDRHLTIFSPDGNLYQIEYAIKAVKNTNITSLGVKGENCAVIISQKK
MATQYITQDKLLDYNNITNIYNISDEIGCSMVGMPGDCLSMVYKARSEASEYLYNNGYNL
NVETLCRNICDKIQVFTQHAYMRLHACSGILIGMNEDNKPELYKFDPAGFCAGYRACVIG
NKEQESISILERLLEKRKKKIQQETIEEDIQNTIILAIEALQAILAFDLKANEIEMAIVS
KKNPNFIQISEKEIDNYLTFIAERD-----------------------------------
-----------------------------------CTYFFPCHPAPPVSIKLFIGRVPKN
YEEEQLRPIFEEFGIVNEVVIIRDKITNIHKSSAFVKMASISEADNAIRSLNNQRTLDQQ
LGSLQVKYASGEVMKLGFPQNVESGVDQAKLFIGSLPKNITEESIKDMFSVYGSVEEVFI
MKDNSTGLGKGCSFVKFAYKEQALYAISSLNGKKTLEGCNRPVEVRFAEPKSSKQAQSQV
GIQPLQNAPHGISPQAHPGTPNNINYGNNFGVNNNYPRQVGVWKEYYSGEGRPYYYNEQT
NTTQWEMPKEFETLFMNNNPNIHNLSDSSGPPGANLFIFHVPNEWQQTDLIQAFSPFGEL
LSARIATEKNTGRNRGFAFVSYENIESAAAAISQMNGFMALNKKLKVTVKKGEEEEMKKY
ISQNGVNTFQQIARQQKNIPSQPNAMGQPNFAAHQNPAQNFFYSNNNSYRCGPYM----
------------SGGNRKVGQTAKVCSRDLPGHTKMKMRDLSDYTEDKEIIKNNLIELEN
KSSGREGNGNKLLAIENIKKLTNCDYANPFPEDEDDEIQDEWKAHKMKKKKKEKNNNNYE
HSDIISKENSSINDSDMNDDGSDKSDESDGSDDQSDEEEKELMRELENLKREKLEKLKKE
KEEEELMKNKKNNVLTNNPLINLEDSDNEENHQKKRKWTDEAIFKNTCEKKTKTNTFIND
TVRTAFHKKFLFKYIHMDSEETINLAVKYAKDAVIEDEKKNYKEALNLYIQSLQYFNYFC
KYEKNDNIRELILKKMEVYITRAADLKEMLNKKETIETKEKVGTSEEAKESMKKQIKDFI
LNKDQNVKWSDVCGLETAKEILKEAVIFPLKFPKLFNSSALPYKGILLYGPPGTGKTFLA
LACANECNMNFFNVSSSDLVSKYQGESEKYIRCLFDTAKEYSPAIIFIDEIDSLCGSRTD
GENESTRRIKTEFLISMSGLNNYKNNIIVMGATNTPWSLDSGFRRRFEKRIYIPLPNLYA
RMKIFEKYINKAKSNHNITNEDIKNFANITENYTGADIDIICRDAIYMPVKKCLLSKFFK
QVKKNNKIYYMPCSPGDPDPTKIEKNVMSINENELLLPPLTLQDFKIAISNSKPSLSLDD
LKRYEEWTNLYGMSGIMNTNIQKLKLVLKLGKKSYSHDVSKLKRNPNLEKLTGDKHDFSK
VIFKKEKIEDVIKEVKFDYYYFNEGKKNKYKDIPLNVSIIKESDLPPYKPVDEKLNFSIL
ENDLKIISTNKNSGVCSIGLYIKCGSRYEEINDKVNEQGMSVMIENMAFHSTAHLSHLRA
IKSLEKIGANVSCNAFREHIVYTCECLNEYLPVVINLLIGNVLFPRFLSWEMKNNVNRLN
TMRAKLFENNEMYITELLHNTAWYNNTLGNKLYVSESNIENYTSENLRNFMLKHFSPKNM
TLVGINVDHNELTKWTSRAFQDYVPIPYTKQKEVTPNYTGGFISVEDKNIKKTNIAIAYE
TKGGWKTSDMITLTVLQTLMGGGGSFSTGGPGKGMYSRLFLNVLNNYNFIESCMAFSTQH
SDTGLFGLYFTGDPANTKDIINSMALEFHKMNKCTDEELNRAKKSLKSFMWMSLEYKSIL
MEDLARQMMILNRILSGKQLCDAIDAVTKEDINRVVSQFLKTKPTVVVYGNISHSPHYDE
ICKMLGMISGIRVNDNCVTEFNNMKIRKTCRWMIFVIENCEIIIHSKGETTSLKELVDSI
DKNDTIQCAYVVFDAVNKIHFFMYARETSNSRDRMTYASSKQALLKKIEGVNVFTSVVES
ALDVADFKMQPHDSNRGLNNFNNSSNTNSSNNALNYSNQNNMNMPIASPFSYSGNTTNNI
NRGMGNNNDNAGYNTVKGNPNYYSGQSYYNSQNPVQHGAYAANSNNLNEGMYGEPPNSNP
YLNNQGQYMGAQNAYMPMSNNASAFNDVGNNLRYDANKPINSVQPIVNDTYQEFLQFNAF
SHFVKSSVNYMPANATLKQKTHVPLGFTIQPLAPIPDGYPELASVNFGNSTVVRCKKCRT
YINPFARFEAGGKKWNCNMCYNINETPQFYFVPLDEKGKRKDLFQRPELCTGSVEFIAPS
DYMIRPPQPPVYLFLIDVTVTSINSGLLDVVCNTIKKLLPKNND--DNATNANSNNKKVF
DSRTLIGIITFDSTIHFYNLNSNLKQTQMMVVSDIQDIFIPLPENILVNVHECQNVIDNL
LDNLPNMWRNNKMSDCCAGNALKAAVMLIKKVGGKILFFLSSVPNIGDLTVSVNREAKDK
SSYKKIYNSGSSNNSNSDLKLREVEMLNPANNEYGEFAQSITQFQIAVDLFACPLYNIDL
ASIYPLIKNSGGSLYYYPQFNVHQYSDKLREELLFALTTETAWESVMRIRISRGWKITNW
YGNFQFRGVDLLALPNCHSSQTFSIVVDLEENVVQDSVVYVQSALLYTNSNGERRIRLHT
YALPITQNIKTITDSINPQVVVSLLSHQAIEVIKKGKIADGRNLIQTLCSQVLSTQLSSS
ENSRLLPIYILGMLKSVAFRDSGDVPPDMRIYQWSRVENIPIESIEAYFYPRMFSLHNLE
KHHGNYDENNNFIFPATLNLTCENMTQDGCYLVEDGENMVMWIGRSINPQWIYSVFGVQS
LDQLNSEYAENHIGSSDNPSGLQVLNIINALRKARTPSYMKLMVVKQGDPLEYKFFSYLI
EDRSQHMMISLKEFLAKFYPKYPQFTPSLSMEEDSFKNRLLKRNIDIWIEKYRPEYLEDV

VGNPFVINTLKSIIVSGNMPNLLLAGAPGTGKTTSILCLASEMLGSQAKKAVLELNASDD
RGINVIRDRIKSFAKEVISLPPGRHKIIILDEVDSMTTAAQQSLRRIMELYSDTTRFALA
CNQSEKIIDALQSRCAIIRYFKLTDDQVLKRILKICEYENIKYTDDGLETITFIADGDLR
KAVNCLQSTYAGLEVINKENVLNICDIPSPERIENLLKHCISSEWRKAHDIAYDMIKEGH
TPFDVALTSSNVLRRYDLGSEAIQIEFLKIGAMACNTMASGLSSVIQLDKLIADWCIAAK
TLKGKMMNADSYTQNRKASFPINNVLAEETEKLVETYKYEDDVDDILKKGASKFKRNGKN
SMAFHKSLAVVNVAAGLDGCDDQLLPASFRALEADLNLHPSLLGYITLAQTLMLSLFSPI
WGFLSDKYSRKWMLVFGTALWGLATIFLANINDFAHIIIFRAINGLALGSIGPISQSILA
DAAKNESLGLSFGIVQLSSSIGRLIGGVVTTTVSMKYFGTIRGWRLCFIIVGALSILLSI
IVALFVEDAPRQVRKKETNSYMEESIIDNNNETIVEPKRSQSYMLYQNVREMLIDSLSKK
SIIIILLEGFTGTIPWLALSFNTMFFQYCDLSDLQAAVITGFLLIGSALGGVLGGHFGDI
MHNISNKHGRPFLGQLAMFGRVPLVILTYLVIPKRKESFELFVLSCFFLGLSSIAGVAVN
RPIVSDIIRPDYRGTIFSLTIAIEGVGSSLIGAPLFGYLAEEVFNYRNNNLLISDMTTEF
RSHNAEALSKTLLYLTAVPWILSFIFYSLLHFTYGAEYLKMNQIIESEYKYDDEDDETMA
DKVMS-MGCDPGVSTAVSSIVQHQNFKRMLMFGLRSLSDFCNPTSKAYKENAYDALNRDA
IPSINKAVNNYKDDDDILYCSSKVLFAMSDYCCSEKDNEALKKLVSDGGVNAITEIIKTI
PKDQDTLKNCMLFIQNMKDISSQIDG-ELSVALLNVFTSDTYTTKLGSSIISALSVVSKS
PSGSEALNGENAHHKLIDHCLSIQNINDETAEIIEGAFDVIKNLLSNGYVVPTIIEKSVV
ILDKFKAYPRVVSKGSDTMKSAVGPEQLTDCLNILKKEQQGSKEHDSALELLSSLSYISS
ITDKIVESGGIPVLIELINSGLQQYDSNPDKIARLVAGASRMLGRISNNPSHAGVVYEYG
GIATLCTALSYFPNDADCVSAICIALIPFVSRSNYANEINNYLLFASLFPILYASVESLD
LAKASMACVASASMINEFHEQMVNNQVIEILSTCIQYHLTDSDYLSNVFSVYFRLSDHIK
TIEPINQYGGISGIANALSAVSTDSSISETGLKLLNKMLTASDALTYLSNPQIVDSVLTV
MLENENKEVIIQEGTKIMENLATESDCQRHISNLESIINLAQSNPDRAYKTLAAISGLSR
IQSLKLMLESKGADSSIYNGMKTWIESPKFNEQTKLIKAALKTIKILKLNVLNKVHEVIA
SIVELMCVPQVKRLAEGDGPDDNILITSAECINYLTEVNKISTKEIVESSLESIFKMMKK
YSESRLTQTNLLSAINNILLSSNMICAEVLVNKGYVKQIVTYIHKVPMYVDVQIIGFSVL
ANMLKINSDSLDAIKKANTLIPLQNALRTHVKNTKLKTTCAPLLAVLMPLDTLTREIEEL
LRLCNKSIQANNLPQLHEYLVSLNELLLTAESSKISARCNIGQTLNNIVEWLKNNPDSYK
NNNAKDYSITGRSLYDATISEVAHVCSNVSQSRIGIVHLTKSNMASSLMHLYNLLKLPGD
EYTEEAVANILESLSLLLKHDIINTDIAIDLGLIEKLCTGINHFSESDTVIKSTFSCLAC
ICTTNKRINQLITHPEYDKLISVIVNLIGNSEKNKDSRKNAIKALYELLKIEKEEISVNI
STKTPIVDNLFKIMGEYQMDLPIIQDSSKCLATIAEHVTIEEKMKIDKYSAMKILIECLG
KNKNDESTAQEIMSVLVKLCNNNDKPQFKELGAIDIISDVTMIHGKNEEISRLGGILFSY
MGADEQVKKLMKMILSVKDSDADAVQKIDNLSGKLELFLRAPLENPLDALQYTDATLQKL
NAYVGSNLENVSLQTNIALVNKRLVDRVKYDFEDQLGAWAVASAGILNQYTDMITNKVGL
KHDKTVAPIYSVLSGCYLNPYTKQLITDNLSPILVSTYQILEDNKNKPSVVQSVFELLEQ
IASDEEGSKLMHMKWNGSKGNIVDQTLSIMNINKSNDAVFIAGTRLMGALADTANTSGYI
NDMDVPRIVESCELLVNVGSSKDRILEFISLIDKMILSNLLDEKIASDALKKISSLVSEE
HLSNYPEQDRTNIIKAYANLLKDAASTGLFAHVRQANKLEAINNLEKLIEYEQNEEVKLA
ILEAISEISACDPFAASKLLVSLLPSILKNDYHDILNNKNIADAFLQMLEKLVQNEGIGR
QLSTNNELYKILSELEESVEKNRGELGDDFVNNTKLRITNILNAIEDDKPKEKTCKDVFD
TFTEYKNKEMSISVLDEPSINDDINFLLDRLRIYNKDNLSHTTERGVDNSYGFMSIELLC
ENQDNVQELIKKEFHISAFHSLVKQPEEHVKHYACRSLCAFTKDPLGLQRVIADIKDYPN
VISTSVGDLAANNTLEKAEKEDFLINRVLLIDRTAHNRNIYNNTNAVRHLIEIWNQYDQG
YFSVLLLRHVFRAMRKIVSDFHVQTLLDANILVRLINIINSLETDKILYPDVLFLIGSLA
IVKEIKTQIGELKGIHACVSLLLRYINVEGMEPTITNCCLALANMCIDHKANSSIFSSLK
GPDINVRILKEYRSNFDVVNGGSVLLCNILFRNEEMKKTYGGNGAPGELVECLRSYDGSD
NKNAVRCIESIFKAISNLSLYTANIKQFLDTQIEVSYQSWLNKLNESFPDAELETGLRTL
SNLVMENEELNMRKFGVTLIPVLNVLKQNRENSKVIFFLLDILCSLCRLNENAKAFAENG
GIETTINAIQLYDYDVSLLTLAIHLLSNQCKIESSLPLLIKADAFSILISCMEAETEEFE
ITELVVSSLRCTRRLIQSEELAYEFCNCGGIPTIANLISKSVKKSIVVLEALRILLCVLY
YTKNVEGVVNEYSEEDEELFNARLGGWYNISMDKEMIDVILQSVLTCSNDENHQKQLRLQ
KVSLGLLAYFAYHRLGIISMTASGFDILAKNNLNHFGGDMVIMQLLAICIDNIAINSAEI
YDMTITRDIVKLFKSSISKIQNKKDHKQIVQSIEKTLEAMGSDGDPLDTFKDTILTFDFS
LSEFDKDPYVNGVHDLPQNVKEALRTGGQYKIYHKSDKRTLFKWKASQDLGTLEWTVGDN
LDRIFKISVVRIKNISKGLVHPLLKAANKYEPRKVNSKVVLCVYGPPTEDFPEGLELPIK
TKSNKERDAFADLLILWRDAASYNYMDKKAREYAQEALKFIQRSGSNFMACKNLREKLES
HGLIHIKEGDQWKLQKNQGYVLCKENRNICSFFIGKNFNINNGSILISIGHIDSCTLKIS
PNNKVTKDQISQLNVECYGSGLWHTWFDRGLGLSGQVVYKKDNKLIEKIIQINKSVLFLP
SLAIHLQNRTRYDFSVKVNYENHLKPIISTLLYEKLIKGNENILEKNDNNNDDDDMNSKN
LNSSPLLYLLANELKCKEEDILDFELCLMDTNKPCFTGVYEEFIEGARFDNLLGTFGVFE
AYVELIKNLKNEDNEN--LGNNLYICIGYDHEEIGSLSEIGAQSYFTKNFIERILGNIFK
NELKNNDITIDEIYGSLSNRSLILNVDMAHCGHPNYPETIQQNHHLRFHEGIAIKYNTNK
NYVTSPYYACLLKRTFELYQNQNQKIKYQNFMIKNDTPCGSTVGSMVAANLSMPGMDIGI
PQLAMHSIRELAAIHDIYYLVKGIFAFYAYYNQVLSSCVHDSMNISKTLFSVFFVLFFIS
SYNVKCQLRFASLGNWGKESKSQLLNAKYLKQFIKSERVTFIVSPGSNFMDGVKGLNDPS
WKSLYEDVYEEEKGDMYMPFFTVLGTGDWTGNYNSEVLKGQGIYVEKDGVTTIESDDEKT
KYPKWIMPNYWYHYFTHFTVSSGPSIVTTGHKDMAAAFIFIDTWILSSNFPYKDIHDKAW
DDLKLQLNVAKKVTDFIVVVGDKPIYSSGISRGSSYLSYYLLPLLKEAQVDLYISGHDNN
MEVIEDNDIAFINCGSGAISNGKSSIKNSKSLFFSSDIGFCIHELTNNGIVTKFISSKNG
DIVYTHKLGLKKRKTLDKVNSLQYFATLPKVELIDIPAVGPMGNKDTFVRIVGTIGILIG
SVIAFMGFSSFLSKNMKMNTNLKRGNFIVFEGVDRSGKSTQSKLFVEYLKNNNIQVQHLC
FPNRETTIGKIITNYLKMESTFSNETIHLLFSANRWEMMDQIKNLLINGVWVICDRXXYS
GVXYSSGALVKKFMKSSKFKNEFMKHMNIFLMRII-------------------------
---G----SLVCKNCQ
SSDIETNEGQGEIICLRCGSVLEENKIVESLEFVENNNGAISMVGQFIPSSGTKSFMLSW
GIRESREISLQKGYINIQKIADNLHLSNQHIEAAQRIYLMALQRNFTMGRNNSYVAASCL
YTICRREKSPIMLIDFSDILQTPVKPLGKTFLKLLRLLHISVPNIDPSLFLERFAYKLNL
KNDIYKVTYTGIKLIQAMTRDWISTGRRPTGLCGASLLIATRIHGINVNSNTIAEVVRIS
NPTIIKRLYEFKNTNIAKIKASEFDKISIEDIPSSSIPPCVISDNKKKNKYNLLQKNKTL
SLCGSEEQYALCSNSTCSVENYEKNK---ELQNDNISSYSDSNQNKFFNSTQNSIINSSN
KNNQIEQHIRSSSNEINLDEICNDNPEGDDIDNLAFKIINTINIEKNSEFLKISDKSLNE
LGSIKNNSQNQDIQNNTQNMIGSSKLQSTNLINHETSISTITDDDNDNNDEIKERNEKYK
NS--------NELKQNNKSVDSTYCTTLKQTVNSELSNV---INEFNLFDNNTVSKNSSN
TTSGLNRINDVNNILTDLNYFFENNSNTINDQTANFDENSDDSEQLNDETISDSYDSEIE
NIILSEKERKIKMLIWDDVMKGCMPNLSKNIKKPKKRQNTDINNSKN-KIPNNKNNVDDP
QDQLSTGDSVIKALEKSXNFCLKKL-------IMTYMIEKKRNDESSIRLENMEEYKIDA
NLYSRQLGTYGFELMNKLIKMNVLIINVKGVGLECAKNLILSGPKSVCIYDNEICEMSDV
GVNFYITENHVENKICRSNAVLSNLKELNNYVHVYNYTGNLNVKFIEQFDVVVCCDAKDS
DIIKYNNLVRSIENKNIAFLSCNIYGLCGYIFNDFGNNFICYDKDGENVKSCNISKIVKD
VEGIVSFDFDKSLPFQNGDYVKFTNVEGMNEINGKIYQIKNLKKYSFTIGDTSKFGDYIK
GGECTQIKTNLKLNFKPYEYIKNKPLFSLSDNNNINIVANQKGEQIIFEETKFPTSFIIS
DYSKLESSNYLHYAIQGLKWYETEY-NCLPENYQNDEFEKIYKKACDLNNKDKEDKQSWS
VEKLDKNIIINVAKYSKSHISPIASFFGGLLAQEIIKFTGKYMPIHQLLYMDFFECINMN
DDENINDKKKLNCKNDNIISIFGKKFQDKLNKLNIFLVGSGALGCEFAKLFSLLDMCTIE
SNGSLVITDNDNIEVSNLNRQFLFRREHIEKSKSLVASNAIKNKNKNINVISYVTKVGQE
NEHIFNEQFWSKQDFIINALDNIIARQYVDNKCVWYSKPLFESGTLGTKGNVQIIIPHMT
QSYNDSYDPPEDSIPLCTLKHFPYDIVHTIEYARDIFQGLFYNVPLSIQQFLNNKNEYIK
KIQDEGNNASLLENLENVLNTLKEIIKENNFNFCIKKAVHLFHSNFINQISQLLYSFPLD
YKLSTGEFFWVGQKKPPQIINFDINNIYVQEYLVSTSNLYAQVYNIPTCYDIKYILDVAS
QIKVEPFSPKNVKVNIDEQNLNNISISYTQDNKLIQDYCNELLNIQTDSLKVSPIEFDKD
EISGLHVNFIYAFANLRAMNYKITTCDKLKTKMVAGKIIPALSTTTSIITGLVGIEILKY
VNYSDSIQKYVKLNDQEKKNEKDILSYFKNAFINTALPLFIFSEPMPPLKIKDKEYDELM
KGPVKAIPNGFTTWDKIEISIQNGTIKDLVDHINEKFNIDVNLISVGNACLYNCYLPAHN
KERLNKPIHEIYEQISKQSLPNDKNYIVVEASCSDQDLVDVLIPSIKFIYKYSKLNQDLL

KKAISDVFEGTKQKKRKFVETIELQIGLKDYDTQRDKRFSGTVKLSNEVRKKLKVCILGD
AVHSEEAQKLKLDYMDIEAMKKLNKDKTLVKKLAKKYDAFLASQVILPQIPKLLGPGLNK
AGKFPSLITHNDKINDKILELRSSIKFQLKKVLCMGVPVGHANLKEEELRSNIVHAINFL
VSLLKKNWQNIRTLHIKSTMGKPQRIYGMFIESLNDNENKKKWEDIDADDDLESNKKLSY
FTNYENGVKVVTKYSENLKKQTVKVTKKIKEVIIKKRLNKEIENRLKLKNFNVDAFSSVI
VEPTDVVNIEPPKNNLLDFLKDTEYDYLFAEQTDKTAKDLKNRFKMFKDEEAEEAKPDDA
NKAGAKRDMMYYRSHDECTIRVTNLSEDVNESELSNLFGRVGQISRMFLAKHKETQNSKG
FAFITYSKREEAKRAIEKLNRHGFENLLLSVEWAKPSNRMLNDIIIQVVIASIGVTIVNS
DKIKFLHKFKYAVYALIFSFLIYKGIPWNRENYYIYLNITPNATKQEIQTAYRQAAKIYH
PDKNSDESADSSFIKLKHAYDVLSDDVRRSNYNRFGDYKNGEVDDNTATLLICLSLVQHA
MFFIIGYFLSYRKQLEFSRQVLFLYNIASFCFELQFRFIEDDTTFDWLPVIGYLLPYEKI
KLLRMIFPIVFFISICISAYAYTDRNASLIYLMRSILSTNRIIVERSNDVVESTNYLKKN
GDQLVSKLQQTRKSDNPMQTNNKNSENDSLDSFDSDNNNNSKDENKNPEDE--NKRLLEN
VKEFSLTLDSQQMNLLEKCFEIMKNKKTDNKKGKKKSWFEFFSLQMVFGVIFIYMWFTSK
--------------------------MAMMWHPDKHKDVKSKKEAEEKFKNIAEAYDVLS
DEEKRKIYDTYGEEGLKGSIPTGANTYVYSGVDPSELFSRIFGSDGHFSFSSAFDDDFSP
FSTFVNMTSRKPRPSANTSANHNNYNAKPATYEVPLPLSLEELYKGCKKKLKITRKRFMG
TKSYEDDNFVTIDVKAGWKDGTKITFYGEGDQISPMSQPGDLVFKVQTKPHDRFTRDSNN
LIYKCPVPLDKALTGFQFVVKSLDNRDINVRIDEIVYPKFRKIVANEGMPSSKTPNMKGD
LIVEFDIIFPKNLTSEKKRIIREALANTFMKGIILIRYLLFIIYLNYLYVQAAYFYVKEG
AEKCFVENVSKSVIIVASYDNYGT--------------------------KGKISYMTKS
SGLHYICILCPSNNWFKDSSIKWNLSIEVGGIDIDMNDTAKKSEISETLNTLKNLKKKFN
SMKTHHAHQKVIV------------RFAPLYLVQTRTIC-------MNITKMFTK---VM
SKNKKGKKKEDLDAILAELGIEEKREDQENTQISKSKKRKEKLKQKKEQKLKENDTPIAQ
TEAVKEEDAKDGEKDGEKDGEQDGENNEAAKGKKKKKKKK--EKESKAETGISAAAKAAA
ERLRLLKEFEEKQKEEERKKREEEEERIRKEEEEKEKKRLARLEKKMQLKKEGKLLSAKA
KEEKKKRELYLQTLKESGMLIEPKEKAKAEIINLDINKAKLLLKKKKNKQISNSDDKNKD
DENKEDK-----NKDNENKEEEKEIVLDDWEDFLNMDNEEKKKTEKNDIDQNTKENKQTQ
IVKKSSINDKKGKKQKNNKNGEEDKDNE-KTKDEEDIYRSSIVCILGHVDTGKTKLLDKL
RHTNVQDNEAGGITQQIGATFFPKDVLDKEIKKIDKTIKCLSKGIMIIDTPGHESFYNLR
KRGSSLCDIAILVIDLMHGLEQQTKESIQILKQRNCPFVIALNKIDRLYMWEKNDWEPFN
DTFKNQKEYVKEEFKNRLQTILNELSEQGLNCQLYWENKNPRKFVSIVPTSAITGEGIAD
LIMVLVKLTQSFMLKNIEYNNKLECTVLEVKNIEGLGTTIDVILTNGVLKESDTLVLCGM
NGPIVTVARALLTPQPLKELRIKNEYIHHKSIKACIGVKISANGLEEVLCGTSLFVANNN
DEIEEYKKKVMTDVSDVFNHVDKSGVGLYVMASTLGSLEALLIFLNDSKIPVFSVNIGTV
QKKDVKKASIMREKGKPEYAVILAFDVKIDPEAEKEAQILGVEIMQKDIIYHLFDAFTAY
LKKIEDEKKQSKMADAIFPCEVSIINDCVFNKKDPIVVGVKIEAGILKIGTPLYIPEKNL
KIGNVVSIESNKKSCNKAKKGEEVCVKIAGEPNVTYGRHFDFNQKIYSKITRESIDVLKQ
YFRNELTMDDWRLVVHLKKILNILMIINEKDKLAEQNLETLDVTKLTPLSEDVISRQATI
NLGTIGHVAHGKSTLVHAISGVHTVRFKHEKERNITIKLGYANAKIYKCTNPDCPPPECY
KSYESSKEDDPMCPRENCNSKMKLLRHVSFVDCPGHDILMATMLNGAAVMDAALLLVAGN
ESCPQPQTSEHLAAVEIMRLKHILILQNKVELIKEEQALKQQEEIRNFVSGTAADSAPII
PISAVLKYNIDVVCEYIVTQISIPRRDFISSPHMIVIRSFDVNKPGEDIETLQGGVAGGS
ILHGVLKVGDQIEIRPGIISKDEKGEITCRPIISKILSMFAENNNLKYAVPGGLIGVGTR
IDPILTRADRLVGQVIGHLNKLPDCFAEIEISYYLLRRLLGVKSQDGEKNTKVAKLKNGE
FLMINIGSTSIGCRVMGIKNELAKLELTGPVCTKIGDKIALSRRVDKHWRLIGWGQINKG
KPLDLQEPIMIHFVLLISRQGKTRLAKWYMPLSQKEKAKIIRETSQITLNRTPKLCNFVE
WKEYKLVFKRYASLFFILCIDKSDNELITLEIIHHYVEVLDKYFGNVCELDLIFNFHKAY
YLLDEILVSGELQESSKKNILRVVTSQDSLMEDNKSNKKLGSIIMKYVNVLKKTLICNRV
LNTHGMKSIVFRKYYTNLLDGNKPFLNLTKRCLFSSQSGQDKAYSNEEHNENMEINKKSS
ENDNKKNMNKSENSEHSEEKNKEINYESYNKIDLINEIKKTKKHMDEKLVDNQVLKEKYL
SVLAEKENLRTRYMKEIENNKLYCISNFAKSLLDVADNLSLAIKNISEESLKSNEEINNI
YKGIEMTETILHNIFNKYGIDKYNPINEKFNPMFHEAIFEVSDTTKEKGTVATVIQPGYK
INDRILRAAKVGVVKN-MKELLEDLFKIIELDEETESYLKERINDEKKKIVKNGVDYFYD
LICPFSDKKLKKNVIAEIYKKHVNDVIKVTNENKNNNNEKLTKSLNLKEYWEKNDVIGYY
DPFLGIQEKQVNYNTSIPLSESIKINKEKQKQKEKQLSLFKEWVKNKIKIPSPVRVHNLL
HCSDSNKSKTKIEKMNDIRIENFNLSIGQRNLLNDTTLKINVMNKYGLIGKNGIGKSTLL
AKLARHEIENIKEDISIACIEQDLFLEDVTVLECVLMVDKIRHNLLKELEELELAKKQ--
------NDTKNSNDTKCDNLKDDTDEKIINIYEKLNSINYLEAEKEASKILCGLGFDSNL
QKKKVNSLSGGMRMRLCLSRILFSNNDIILLDEPTNHLDIYTIQFLIDYIQKLNKTCIIV
SHDRNFLNEVCTDIIHFHQKQLTYYSGNYDQFEKTRVEHLLQQQREHDSIELKKKHVQKF
IDRFRYNSKRATLVQSRIKLLNKLPVVNLEKDETPFKFSFLEPFYVSSVLIRLRDVSFKN
EMFKNLQIKKNANIIIADDFEDSNAKPIELSNDDENKKDTLTNNITGDYQFKHEFLFKNA
NFEVDMDSRIAICGVNGSGKTTLIKIILNLIDVFDGELYVSNKANIGYYSQYHVDSLNPV
FNSIQQLQYNYSHKNIKEDEAIKYFNKFNIPTNILYEPIYVLSGGQKSKLALAILAYKNP
NVLILDEPSNHLDIESVQALIVALSLYKGGIVLISHDTYLIKHVADEIYHINNITKEVVK
IDYEFEKYTKLLLENKIMPREIITLQCGQCGNQIGVEFWKQLCNEHNIDQEGILKNNNFI
NEDRKDIFFYQADDEHFIPRALLFDLEPRVINSIQTSEYRNLYNPENMFISKEGGGAGNN
WGSGYSQGHKVEEEIIDMIDREVDNSDNLEGFILSHSIAGGTGSGMGSYLLELLNDNYSK
KMIQTFSVFPLLTNESSDVVVQPYNSILTLKRLILSTDSVVVIDNTSLNRIFVEKLKLNN
PTFQQTNNLISNVMSASTTTLRYPGSMNNDMISLISSLIINPKCHFLVTSYTPITVDKHI
SNVQKTTVLDVMKRLLHTKNIMVSVPVRRGMYISILNIIRGETDPTQVHKGLQRIRDRKL
VNFIKWNPASIQVTLAKQSPHVISPHKVCGLMMANHTSISTLFERCVTQFDRLFKRRAFL
ENYKKEPMFSSADGQGNFEEMESSKEITQNLIDEYKSAERDDYFSHTYMMEKYHGLEKIG
EGTYGVVYKAQNSDGESFALKKIRLEKEDEGIPSTSIREISILKELRHSNIVKLYDVIHA
KKRLILVFEHLDQDLKKLIDVCDGGLESVTAKSFLLQLLNGIAYCHEHRVLHRDLKPQNL
LINREGELKIADFGLARAFGIPARRYTHEVVTLWYRAPDILMGSKKYSTPIDIWSVGCIF
AEMVNGRPLFPGVSETDQLMRIFKILGTPNSQNWPDVFKLPKYDPNFPVYEPLPWETFIK
GLDDTGIDLLSKMLKLDPNQRITAKQAIEHPYFKETNMGNKLSTEDHIFRLKLKTKELEK
LSNRSELEEKRFVLDVKKAIQAGKIDMARLYAEKCIRKKNEKINYLNLSNKLDVLVSRLE
GAHRSASLVKDVSVMIPLIQQINSETNAVKIGSDVMKLENIFDEI---------------
--MGKEKTHINLVVIG
HVDSGKSTTTGHIIYKLGGIDRRTIEKFEKESAEMGKGSFKYAWVLDKLKAERERGITID
IALWKFETPRYFFTVIDAPGHKDFIKNMITGTSQADVALLVVPAEVGGFEGAFSKEGQTK
EHALLAFTLGVKQIVVGVNKMDTVKYSEDRYEEIKKEVKDYLKKVGYQADKVDFIPISGF
EGDNLIEKSDKTPWYKGRTLIEALDTMEPPKRPYDKPLRIPLQGVYKIGGIGTVPVGRVE
TGILKAGMVLNFAPSAVVSECKSVEMHKEVLEEARPGDNIGFNVKNVSVKEIKRGYVASD
TKNEPAKGCSKFTAQVIILNHPGEIKNGYTPVLDCHTSHISCKFLNIDSKIDKRSGKVVE
ENPKAIKSGDSALVTLEPKKPMVVETFTEYPPLGRFAIRDMRQTIAVGIIKSVEKKEPGA
VSAKAPAKKMSHLMSLPIVLLKDGTDKAQGKSQIIRNINACQVIVDIVKTTLGPRGMDKL
IYTERDVTITNDGATVMNLLNISHPAASILVDIAKSQDDEVGDGTTSVVVVAGELLNEAK
VLLNDGIEPNMIIDGFRNACNVSINKLNDLSLSFVNKSEEEKKNILLKCAQTALNSKLIS
NHKNFFAELVVNAAYQLGDNLDKTNIGIKKVTGGSCLDTQLIYGVAFKKTFSYAGFEQQP
KKFNNPKILLLNVELELKAEKENAEVRIDNPNEYNSIVQAEWDIIFQKLNLIKDCGANIV
LSRLPIGDIATQFFADHDIFCAGRVEDADLKRTATATGAVIQTSLFNLNESILGNCGLFE
EVQIGNERYNIFKDCLKTKSVTIILRGGAKQFIEEVERSINDAIMIVLRCIGNSEIVPGA
GSIEMQLSKHLRIYSRSICNKEQIVLYAFAKALESIPRYLSHNAGYDSTDILNKLRKKHS
EETNDIWYGVDCLEGDIINAYSNCIFEVTKIKRNVIYSATEAACLILSIDETVKNPSSMD
KQPRNPYAMSDKLEDIGEFVLVIGDFHSPMRNLGLPDCFKDLLKTDKIKHVLCTGNVGCN
ENLELLKNIADSVHITKGDMDNNFDFPEKINIKIGDFKISLVHGHQIIPWGDLNALLQWQ
KEYDSDIIISGHTHKNSINNFEGKYFINPGSATGAFQPWISNPTPSFILMAISKSSIVVY
VYEEKNGKMNVEMSELRKQMFTTTGKLIYAVEHSPSTSKDSTAVYRNPQFNDKLLDNFED
DPLNNVWDMFNKSAEKYKNRHCFGTRVRKNDKLGEYKWKTFKEVQELIILIGSGLINMNI
CPVIECNDTIIPKARFLGLYLPNCEEWNICDFSCNAFNIITVPLYDSLGIESSKFILDQT
MMQTIMCNRACGLKLIKSLDNFDHIYIKTLILVEKEIDPEIKKTCNKLNIKIVTWDDLIE
AGKKKKLDPKPGKLSDVCSLCYTSGTTGYPKGVIMTNQNFIAQIASSCLGPIKFPSVAIN

```
EKDTHLSYLPLAHVYERIMMCIFLHVGVRIGYYSGNILALTDDVQELKPSLFLSVPRLYN
RIHERICNSLKKKSSVIQSLFHKGLDQKLKKLNSTGNPWSLFWDTLLFNKAKKVLGGNLK
GMLNGSAPLGVEVAKKLKCIFCVPLMEGFGMTEGLGCLFITNPIDPDVGHIGGPLPAVEY
KLVSVPEMNYLVTDNPPRGELLLRGPTICSLGYFKLEKETSELLDSDGWLRTGDIASFSQ
NQSLTIIDRKKNIFKLSQGEYVAVEKIESVYKQSLFIGQIFVFGYSYESFLVCIIFPSVD
TMSIWAKENKINLPNEEIIKLEKFKNDVMQDLITIGKTDGLNGYEQIKDIHFIMEGFTIE
NDLMTPTGKLKRHAVQKKYKQEIDQMYENVKKAMYSWENFLNDKLLATNQVSAAGLASEE
DGVVYECVATPDENDKWSLFYKEDYDIEIEDENGNKTTKTITEGQTILTMFNEGYAPDGI
WLGGTKYQFINMEKGLEYEGYSFDVATCAKLKGGMHIIKVGGGHILIVLYDEEKEQDRGK
QILKALKMLKN-------------------------------------------------
------------------------------------------------------------
------------------------------------------------------------
------------MDQLKNRINNLNMLGVVYLACREAGHIKSIKELITFDRSYKEKDLGKT
INKLKKVLPSRAFVYNENISHLIYSLSNRLQLSTDLIEAIEYVVKKATTLITTSHRLNSL
CGGSIHLIVELNTSEEKNVKLPHLSQIASVCGVTTNTLKTTFKELLAASEYILPKQYLKE
NNTKLIILKHKYLHDDKKKRR--MEKSILPEWTDIERYFKDPELITSEILFVGLTLCNVF
VMYRLFLDVIPFPIFVTWWQLAQGLLVAYVCGEMGKEFPKFAYFPKVEINENMLKVLFVP
SIFYCLMLVLSNYLLFKTPCISSYPVLVSFTVVFHHIIRFIGCGEEYMPLRWKSIAFLLS
AFILGCFDSQTSGKGVIIWALLYALFSAVFRAGFMQKIMHLVEGKGNTLHNNQHMLGVLI
LPIFILLSGEWKILGHMPYNITSLYTWQVWGCLVTVGALPFVKNVVSNRLVRRTGQGPWR
FLEIISIALVFMIGLGYNRPSFMGYLAIVCVIIGRSLGAFDVLLNVSDYMISEDERKRKL
EKSSYAKSRQGTQVSKPFLSSGENEDDDESSFSSNDSKSYQGSQDYDDKESVNSSSNQYS
THKGTSRMVSFSNHTSQEDMSVDESRDINARSSSFKSKPQYSRKYSSKQEVVDSQAMGAL
DESTPVPSRITLHLMKQKKKSAFQGYSLLKKKSDALFIHFRDVLKDIVKTKNKVGEDMRN
ASFALAKSVWAAGDFKGQIIEGIKRPVVTLSLSTNNVAGVKLPIFQVHIDPTVDVLGNLG
VAAGGQVINNTRENYLQCLNMLVKLASMQVAFFSLDEEIKMTNRRVNALNNIVLPRLEGG
INYIIKELDEIEREEFYRLKKIKEKKTENLNNSIEDHLPDNGGHKHVKITNNYANIQADD
DVIFMAKLYLLFLFAIHLCSSFIRSSILESIKHNLQIVNNQNFNTVINKFRNEKVFFILF
FKNSNKDIKNVIKNYDHVAEKFKGIITLCAIDCDSNSRLCEDELSLYVPEYKSSNTHHLL
VYPINPMPKFEFKDEINEANVKKYTYLIPSKIDIIKETKDYNIFLSKHENMPKVLIFSNK
KKPNYVLNALSNSFNKKLLFCYINNELDELVKKYNIKTFPSIIMLKKNKVVDTYKGKNNF
INMFDWLNIHSETFVMGGGFDISPGKTNYKPWKFEAVPKFTKLSHGDICFKQTDKGLCVI
YLKEGDKLDKSEEEMLITLKEKFKPQMSGRGVNFRYMWIDIATETNFRSLFELKKYPSVV
VFNPHKRIRYAKINDDLIATKEHIEKLLEKISGGDAKFTMLKGQTLPEFVLNENEDTSPG
KDELMTVDLNIPYSACELKRVKRLELGVLDPEIIKKIGVCEIVNVDIYKDGFPRDGGLND
IRMGTIDYKALCGTCNMNVKNCPGHFGYIELAKPMYHYGFMNVVLNVLRCVCYHCGRLLC
DMNSSKVKYIEKIKVNSLRLKKLSELCQGIKVCNHSSQDDNIYINDTSIDNFYNNDLSNL
NVNQQMLLNSNNYSNLFEMISKEDVDCGCVQPKYTREGPNMYIQFLHNNNEDDIDGSKRR
LSAEEALDILKKIRKEEMSILGFNSDRCIPPSLILTCMPIPPPCARPYVQYGNQRSEDDL
TLKLLDIVKTNIQLKRQTDRGAKSHVLQDLCSLLQFHITTLFDNDIPGMPIATTRSKKPI
KAIRTRLKGKEGRLRGNLMGKRVDFSARTVITGDPNLNIDYIGVPKSVAMTLTFCETVTP
LNYDHLKKLVERGPYEWPGAKYIIRDNGSKYDLRHVRKNSEKELEYGYKVERHMTDEDYI
LFNRQPSLHKMSIMGHKAKILPYSTFRLNLAVTSPYNADFDGDEMNLHLAQSHETRSEIK
HLMIVQKQIVSPQGNKPVMGIVQDSLLAIRKFTRRDNFLTKEEVMGLLIWIPYWNYKVPT
PAIIKPKPLWTGKQIFSMLLQFDDKDMNNNFDNDDRFKFGNNMGRDNENNFGKLSGSNNP
NSPLSLMDNMHGNTRNEINNNVSNISNMGNGGDNGYGKRYKMKINLIRDSSTSCKDDNPY
CSNNDGKVIIKNNELLSGIICKRVVGSSSGSLIHILWHEMGPDKTKDFISALQKVTNNWL
EYVGFTVSCSDIIASNKVLDKVKEILNKSKNEVTKLVKKAQRGELECQPGKSLYESFETR
VNNELNCAREMAGKVASESLDEKNNIFSMVASGSKGSIINISQIISCVGQQNVEGKRIPF
GFNHRSLPHFIKFDYGPESRGFVSNSYLSGLTPQEVFFHAMGGREGIIDTACKTSETGYI
QRRLIKAMEDVMVQYDRTVRNSYGDIIQFLYGEDGMAGEYIEDQIIDLMKLDNKEIKKLY
KYNFDDDSYGKDYYLGKEGEENPAYIDYNKQNILNQEFEELYRCKNYLCKEIFSDGDIRQ
HLPINMNRLIEHAKSQFPFIPVISSKSKIQTKVSMDRGNDTMGIGGNRKKKKKKSKIKKN
EKSIHNKDLMFEIKKEYENNDLSTVMKNDKSFMGFETYNKEDSDNYEDEEGSSESDDDAD
YFFSGKNENRNSYDDKLNSMINPVEIVQKVNKFLDKLVIIKQINNSDTLSLEAQNNATIL
LKAHLRTYLNSKLLIHTHKISLKGIDWLLQEIEKIFYKSLCHPGECVGALAAQSIGEPAT
QMTLNTFHFAGVGSKNVTLGVPRLKELINIVKNVKTPSTTIYLDDMISNDQQKAKDILTK
LEYTTLKQLTSHAQIIYDPNTTSTILEEDKLWVNEFYEFPDEDDTQYTLGEWVLRVQLTN
IHVNEKKLTMKEIVYIIYSVFSSDELDIIYTDDNSEDLVLRIRVKYLNGEYNFLSGNGDN
INDDQYEDEDDDEREYNNIANTFKVKKSATSDMNEKNEDNASISGNNIGNVDHIVNSSID
NLGQNIDGTIKKEGGRKGIHKNDNTENDEDDDDEEEVFLFGSDSRNLENKENSNSSHNMN
KSPYGNDEKTSNFNDQHISGNNVGMGGSNKTMGKGDDENNQKNRKKNLNNENNTMISKED
TEDTFLKKLMEECLSSLKLRGVENITKVYMREESKITYDSENGKFVRSSHWVLDTDGCNL
ESIFCAPSVDFKKTISNDIVEIFEVLGIEAVRRALLKELRTVISFDSSYVNYRHLSILCD
VMTQKGYLMSITRHGINRVDKGPLIKCSFEETVEILLEAAAFAQVDNLRGITENIMLGQL
CKIGTGVFDIIIDNQKLNDANQNLETIQDITSAGFTTPDSNGSITPDGLQSPLGINTISS
PLPFSPTYNSNLLSPTLPIDNVNNILSPQCLQNYSDNIMSPSKNDFNNLDTLQLGGKFSP
TQSPRSPTSVIHSPFSPFDNRNKQPLDPLLLFSPKNNATNPGLNNYNVFSPKANMNNIQS
PLIYSPNPMIDIFSPKPQMQNNIYSPSYSPTSPTYNANNAYYSPTSPKNQADDMGGNKKY
NIMSPVYSV----------------------------------TSPKYSPTSPQYSPSSP
VPNNPSSPQYSPYSITSPKFSPTSPAYSISSPVYDKNENMAGKNPLSPAYMLQSRVQIRQ
NAQGTQVFSPIQQGGANDVQNDDPFSPIPYNMDEEEMQEEMVVFYLDNLEIFFPYDYIYP
EQYAYMKYLKKTLDSEGHCVLEMPTGTGKTVAIFSLITSYQYYKNDNSKFIFCTRTVAEM
EKSLIELKKVINYRINIIKKRNELSEKISKSNIKEDSDTIKNENSDTIKNENENNDEDDN
ISSRFGKNSEILAMGISARRCMCVNDKVLLKHEREKIDEECRKLTATFVREKKYISKKLN
NFSNSNIDRISDFIIKNKHHIDMEDYFNIYNSKNSINEYNDLGLCGYFENYKKNFVYELI
EPGVYTIEELKEICKNYKNSENINTPICPYFCAKKIIEIAKVVVLNYQYIIDPKVSKSIF
LGKDINNRVNYKKNDIIVFDEAHNIDSVCLEALSVNIDRSILNKATMNITTLFKKIEKSR
IVNENKLKEECYNILNKIKSGKEASNFKLNQNGEEVDNNQISENDENLESQNRRSKQDEV
YFDEEMNLVFKGLLEEASSEKNDEKDGEKISDKVIIDDLKEILNNNSDDNEKNDLKSKEN
FNLEEINYSPLLMEDIIKNIVMPGNIRKSEHFLNLMRIVVVYLKKYINIYEVTSEGPLSF
LYKCEKDTKLDTSFFKYSFDRLKNLLNTLQVVDTDDYAALNIVCNFCTLIGNYFKGFIII
CEPYPEATGIYDPVIQFACLDSSIAMKSVLNRYKSIILTSGTITPLELYPKLLNFSTVLT
ASFPMSFDRNCVCPLIVTKSSDLIPLSSQYSLRNDLNVIKNYGFLLVEMCKNIPDGIISY
FPSYIYMEHVMSTWYELGIISNILEYKLIFIETKDIVSTTIALHNFKKACDLGKGAVFLS
ICRGKIAEGIDFDKHYGKCVILFGIPYQYTLSRILKARLDFLKETYNIQENEFLTFDAMR
QASQCVGRIIRNKKDYGIMIFSDIRYTRNDKKGKLPPWIIKCMDVSNTNLTIGAGVNISK
KFLLNMSQEYKETDQTKISQIILQNPIKCWEIVKSILNMDDFIMIGKKINYLLGNTLKKS
SRKYSTSNLINEIKNQPLTKITELSNKMKVATIQNNCEVPTIGLWISSGSKYENKMNNGV
AHFLEHMIFKGTHKRNRIQLEKEIENMGAHLNAYTAREQTGYYFKCFKDDVKWCIELLSD
ILTNSIFDEQLIEMEKHVILREMEEVEKSTDEIIFDKLHMTAFRDHPLGYTILGPIENIK
NMKKNDILNYIQKNYTSDRMVLCAVGNVNHDNIVKLAEQHFSNIKPQDEKLIFKKEFDKI
KPFFCGSEIIMRDDDSGPNAHVAVAFEGVPWTSSDSITFMLMQCIIGTYRKNEEGIVPGK
LSANRTINNISNKMTVGCADYFTSFNTCYNNTGLFGFYVQCDELAVEHAVGELMFGITSL
SYSITDEEVELAKIHLKTQLISMFESSSTLAEEISRQILVYGRPISLAEFIIRLNEIDAE
EVKRVAWKYLHDRDIAVAAMGALHGMPQYFDLRQKTYWLRYMDNLKKVLWSIEELIYTNN
QDEDIYEKKEKEIKEYLLNLHKNSFDNETEKEELVVENGNFFNLLKLVKECKYEKCITNP
WVSSIFKLILEKFKNENYSTIYEIKKEIFNFISAEKEQGLKKGANDLPIFSCFVFFYEMK
ILLCCNIFLNMFVQCNWTGPSLII--NNEKIKNNEKSEEDKYFNEFVDSIKIKNDDFLNS
CLEFLSLEGEYIYEYCNLINAFCLCIIFLGFLNNFSKHDEKNPHGKEYNNLMAYLENTED
TKIETREENNDGENSLDSLIFARSKYLWKARIYFIWQRLFSSSSNFMYVLKVHIIDTPFN
IFKNMNILPNNFDLIERDFDLETVDIFSCISENADENKKCDLFCENYISEKFKIYILSNY
AVYLSFYNYTYAYDKLLDILSESSKFYYTFTGRMGIKRKYQKNPATILVLKVKLGDEEDN
TSTVFKEVEPLSEYDILRSDYKIIDNKSVDKEENKTDLVKQITTQTKDEAIEKREDTNCM
ALENDTLNGICN-------NVDEKNFNTCDENNNEEINNDISENTPIESNGKKTWKLKDF
DPDTDILEEPYFFDSQNNYFKILSFDEQIALINYCFSIIRFNPHYDEIKFEKLNAIISRC
```

```
LKSYDVNI---ENGENGKRGGNHLLQIKYQNYLLHSCILWFKCKCESFRLKTVDRSQAQL
NELLKEYCNDEPLNKERLKFIYDIYYPTTWEMKKEVGNIMTKTGSVVSAFNIFKDLKLWE
EAIQCLIEADRKVEAKELLDTLLEKKKSPPLVCLYGLVNRENCLKYFIKAWDLSNYKYSK
AARLIGKHYYNKEMYSECCDYLEKALEISPLLPEIWFILGCAYMKIDKFDQAIKAFTRMI
SMTNENTAMAYGNLAYLYMKNNVYKAAKICINQAVKINNNEWKYWDTYLKLSIVQNDVDS
FCLALTTLCQLNQVKQIQPWVFDYISDLIVNDKQTIIPNKTGLSYLDKIIKTMNVISAHI
SEYDSFWNAYSFFLFVKGEFEDSFEAKIKEIRSIEVIPS---------------------
----------------------------------------------FNMLLEKNSEEEEV
GLFNEEGNMKDDEEDEGVKDSDEDISQSKKKRKKTNNTVSITGNRTGNKKRKVGKNKYVS
TFLDTEAQVGDEEEEEEYASSYVDEFEEAKRLEKKKLYETKLKSGTNHLAQAINKLSQRY
ENEKDLKDGATDDETLTDEEMSEDEEYFDETEGLNAFDSPKMWLIKLFKNGAERHLAMGI
YYKYMKLQSSEFNIKGIYVSDDLKGYIYIEADSLYMLKRFLLGFKFINLNDISIVPVQEL
TSIFAMSHSKVVIPKVNEYVRIKRGVYANDIGQIFEVHEKGIYAIVRLIPRILYDKYNNS
KKDQYDNVGISTSNKNNSTITNNNLDQIYLMDKMNTNNQMHNVNNAIVPLSGEKLSAIDE
ALQIRRKKKKERPLKKLFDRDEIEQIGGVIEHGPYPGTIKYQNNIFEENGYLLKKMNIKY
LISENANITLTEIRDFNKNNTNEEDINLHISKSFINKNSLHLFKKGERVKIMKGELFNLI
GTITNINENVLSVNPDNLAKEFKFLPTDVTKYFTEGDNVTVINGLHKGKSGLISLLDYKE
NIALIFSPSLNTEFRSSIQDLTGTISSSEGLGGVNTLNGFSIGDLIELNDRQIGVLTYID
KNKHIRVLTNSNKILHTTIGSITYKRSAIGQICKDENGNIIQSKDTIQLIKGAHKNKTAI
VSYIWKNKIFAKINKKIEDNGFVVVDCENCLLSGNVNEKKKIITQHNLFRNNNMQRKNTF
QSFIGKTVKILSGVYKGLLADVIDAERDEFTLLLKIKPKTIRQKRIECAIADAYKDDKLF
DDKNTEKKKDGINKTDRKYEKNYNYDDEKKKKK-HDNSNKKYHQYDEHENNNWKYSSHNK
IHENDTYSNDKNYKSGENMDDYYTSNKNGNTENRFHNEKDKYEKKKTSPTNSEYKKHEYH
QTESQNNLNKINNENNFYKNQTDEKKNQNNKGNSDWIISGVIIKIVTPGLFYNEIGRISE
VIKKGSYIILKIETDKTSFNIVSEAVIPLKPSKPNDEVIILDGGKISQGT----------
--TIHKLIICTHIYICAYKSCLKMSSNSPISVSLVDNLASYNIQNESFRLGNVSIEGDKF
ICVKENVNDNTQVVVINLYNQVSIRKYMKADSVIIHPNDPILALRGSIKNANTIFLQVFN
IETKEKICSLNLNEHINYWKWINNDTIAIVCEKNVYHWNINVLTKVFEKAQIFIDNNSQI
LYYGTDKEMKWCILCGISTQDQGKSIDGHMQLYSCEKKLHQIIEGFIGCFGSFIFENWDT
KPLFCFIEKKKNSSMSRLHLMDIYNSKTEGSTPYKIVKEINLINDTLNDFPIYISLNTLQ
GVIYVITKCSYVYIFDESTLTEIVREKISDDNIFICCDNKNGEGIIAVNKKGKIYYITLN
YISLINSLKSSNIDVKDKIIRNLSLKYGYPGCDYISVYKKCISEMDFKKASKIICLLKNP
KLRTQQVLNSFKTFRNSQGQLSPLLLYFSVLLELDKLNTYESIELVKPVVLQKKKEYLEK
WIKDDKLACSEELGDLVKSLDLRLSLIIYLRCGAHNKIISIYCLLNMFNNVMTYINNYKH
TNFDFVNIFITIINYEFPSGNTNGNDSGSSLDLHHTEKNESLDFFNDNNSKDLKNGNNHR
NTNIEIATQYIKLLSDNNIQLDIGKIVDYLLNNNKLQEATSILLDYLKDNKPEHKHLQTK
IFEFNLHHNVQIAETIFQMDIFTYYDKNRIAYLCEEKGLYQRALENYTNINDIKRVITKS
AGGISLEWIKNYFSTLSDSVCQDLLFDFMKGNKVNIEIIISICVQYYNKIGIKKIVNKFE
ENKNYEGIFYFISSILNELQNMGTVGNDNNISRSNNDDTSSILINSDIGIGTQFPENNSV
LTIEDLHHIMFKYIEACVKINNIQELDRICKDRNAKYNPEQIKNFLKECKLSDPRPLIYV
CDIHNFIEELAEYLYKNSLLKYIEVYVIKVNPHNAHKVIGVLLDLDASEDFLLNLLNNIK
NISNIGNLIDIAEKRNRLKLLLPWLESRSNEGYENIELHNALAKIYIDLNKDPETFLKNN
NFYDKKMIGKYCEDLDPHLAYTAYERSNGQCDEELINITSKNGLFKLQAKYLVSRQSMGL
WSMVLDESNKYRKNVIDQVIGSTLIESNNADEITVTVKAFIEKKLSSELIELLEKIVLHN
SEFRDNKNLQNLLILTAIKSDSKKVMEYINRLDNFSGPQIAEVAYEYKLREEAFVIYKKF
NCNTSAISVLLDNILTKNRKGKMSANARRKKIELAEEQEMEKDETHPNIENYEEDLNRAI
EFAQKCNVNDVWFILGKAQLKLNKIIDAIDSFIKSNNAGAYKEVIEKCKENNFYEQLITY
LNTLREQNSLKDVLVDSELLYAYAKLKKTLEMTKFIASTNLANMQLIGDRLYKEKEYDAA
KILYSSIPNNQKLTFCHLKLKEYSLAIEAAKKTKSLKTWREVNLVCVKYKQLKFAHIAGL
QLIMHADHLDEIIKIYEKKKYINELLSLLENGLNSERAHVGIYTELGMLYAKYKPEKLME
FIRSYTNKMNTRKLIDVCHNEYLLKEAVYLYISYDEYNLAVDTIIKHSPIAYQPDIFMQV
IHKVTNSDIIHKVIDFYIEENPLNLYNLLKILENKIDNNRLVQTMKKSNNLPLIQKYLED
IQTQNITSVNETLNEIYLENDDYISLRKSIDEYDNFNQTNLINKLENHKLAEMRRIAALL
YKKNKKFKEAINLSKKEGQIKDAIDIARVSKNHLYVEDLINYFIQIKNKEALCACLIACY
DILKPDYVLEIIWLSGFKDHAMLYFIQIISDYTQQIETMKKQIEDIEKEKKMNKSAPNDY
SANTISNQFTYSLNKNLSIMPPQNNYIPNNSDFDKYDISYMTTYFMPSIVGNQAPSFKAE
AVFGDNTFGEVSLSDFIGKKYVLLYFYPLDFTFVCPSEIIALDKALDSFKERNVELLGCS
VDSKFTHLAWKKTPLSQGGIGNIKHTLISDISKSIARSYDVLFNESVALRAFVLIDKQGV
VQHLLVNNLALGRSVDEILRLIDALQHHEKYGDVCPANWQKGKESMKPSEEGVAKYLSNL
MEDTAAKIIGSHDGLLTDPRIAQDFSAETNELLTKAENYFKVGDFELIIEELILLEKKCR
QSYDGISTSKICCFILNKYKLMENYKKVNEYLIFFNKKRGQLKRTIIDIINLCKSWIADI
QNKEEKLNLINTLCTISEGKIFVEVERSEIIRILSKIKEEDGNIEEAANILQDVHVETFI
SMDKRDKTEYILEQMRLVLLRKDFIRCHVISRKINPTLLNTDEFADLKLKYFLYMIQYYI
NEESYSDVANCYEQRFNTDSVQNDPNLWIDELKCYIIFLILSPFQEQQTKFLNLIKLQKK
KLKEIPTYEQMVNDFIKQDLIEWPLVYEQELQSFYIFNDSVFVGGENRWHLFKKKVMHHN
IHVISTCYSKISLQRLAQLINSTNEESENLLLELVSNKMLDAKIDRLYGVIKFGQKNNPQ
TLLNNWSSQIHQIVDILEESSHLIQKERMVHEAKLKRMQLENKKMALMGDKEAKKEAIEI
IYEIANILNVNLDKETIVILIQLCEYG---------------------------------
```

> ***Plasmodium berghei***

```
---------------------------------------------------VTSSDKIFA
INLDFLLKTNLYYFTSRENINRNIITNVFFQAQYNEWIDFLRNKDIEKNIIPICEHINKH
LYLNTFLSFHYLTLSDIYIYYEMHKYFSGNITTNLKYPKQYKNINRWFRLIKALLHATDA
ELIQNLKKRLGNNGNKYSAHSDHTTKGGATSLSSYEGKLQNAEKGKVVTRFPPEPSGYLH
IGHIKAAFLNNYYANIYEGKMLLRFDDTNPVLEDVKFENSIIEDLETLSIKYEKISYTSD
YFQLLEEYCIKLIKMNKAYADDTNVDEMRNQRGEGIESVNRNNSIETNLKLFEEMRNGTD
IGKKNCIRAKINMSSKNKCMRDPVLYRCIIDIPHHRHGFKYKCYPTYDFACPIIDSIEGV
THALRTNEYSDRIEQYNWFIYTLELRNVHIYEFSRLAFVKTVMSKRKLKWFVENNIVDGW
TDPRMPTIKGILRRGLTKEALFQFILEQGPSKAGNLMQWDKLWSINKQIIDPNIPRFSSV
DMNNGVILKLTDLKNEIIEKTRDLHVKNKSLGTCTMYYTQQIYIELEDAQMIEANEEITL
IKLGNVIVKEIIKDTDNKIKEIIAVSNFDGDFKTTKKKIHWLPYIPDKLINCTLYEYDHL
ITVDKFENDNKEDWTNCINQNSKFETVAYAEPALIDLKVSDKFQFERRGYFIVDKITENK
HFHLIKIPDGKSKNMSIISTKVNPQNLSGTKKMDNNTIVIDNGSGYMKVGLNTHNLPAIV
FPTVV-EIRNKDVNQTYVGDEAFFHESELSIYRPFDHGHISDWDLANNIWDYAISCVDPN
KSVKSALLTEPPLCSISHRKNMGEIFFENFGFESINISVSGLMSIYAAGLTTGLVLDIGE
GVTQCIPIFDGYIEKNSVIRSDFGGEELTMFMQKLICDIGYNMTTRKSYEYVKIMKETLC
FCSLNPPKDQLRDDLTVTYTLPDGDVLRDGYSTIEISHERFYVPEALFNPLLCHRDNLSI
SDIVCKSILSCPIENRKILSSYIILSGGCSLFPNLVERLEREIKNNSPENARSAVKVHAH
ENRGIMAWCGAQIFSQPELRDAQRGVWVSKDEYEEIGDNIFLIKVRKKIKAMDGLKKINL
IQKINEKLYDHLGIEDDNLTEFIIFLCEKSTCLEEFCKEVFENGGEIEQSVLKNIYNLIK
KNKQAAENGNDDDYNHISKEIQEIEKKNEKMKKFGCLSIKNSTNLAPLTEEGKPKDED--
-----KGKDNS--------LNKERIDENVKLKKNRQLKYSIKK----------------A
KYRALKVNNIFSGKIKKITDFGMFISFKTRDGYKEGLVHITDIDQNNKR-INLNEKFKRN
MIVKVKIKGIFGEKISLNMSEVDQKTGQNLVSNNKEKNIDENKTYFFDNIDDEFKHKRNK
NRDKYSEDPKEAIKYESVIKMKSDYSKWEIQQLIKSGIIYDENIKNEYKNLKYEEKIDDE
EEMIEIEVNEKEPSFLKGQTTKAGANLSPIQIIVNAEGTLAKAITTTSALTKERKEQKKN
EQNALFDSIPKDISRPWEDPNPNLGERTIAEALKNVGKNYDLPDWKKNYINNNISIGIKN
SLPLTEQRKKLPIYNLKLDLMKAIKKNNVLIVIGETGSGKTTQIPQYLHEAKYTDHGIVG
CTQPRRVAAMSIAKRVSEEFGCILGQEVGYSIRFDDCTSNDTIIKYLTDGMLLREALSDT
MLSRYSFIILDXAHERTISTDILFCLLKDVVKKRSDFKLIVTSATLDAEKFSAYFFNSPI
FTIPGKIFPVEILHSKEPESDYVEACLITVLNIHLNEHPGDILVFLTGQEEINTACEILH
ERMKKLESMSPPPLIILPIYSSLPSEMQSIIFEPAPPGCRKCILATNIAEASLTIDGIFF
VIDPGFCKIKKYDSKRDMDSLIIAPISKANAKQRAGRAGRTGPGKCYRLYTEEAYKNEMA
ETSVPEIQRINLGSIVLLLKALGVNDFLHFDFMDSPSIETLIYSLENLYYLGALDDNGYL
TKLGKKMSNFPMEPNLSKILLTSINFNCTDDICTIVSMISVQNIFYRPQNKILLADKKKN
```

KFIMPQGDLITYLNIYNKWKENSFSNYWCHENFIQSRALKRAQDVRKQLLSIFEKYNYQV
KKRGDISNSTNYVNICKSICSGYFNHVCKRDSQQGYTTLLTNQQVFIHPSSTLFSKNPLF
VVYHELVLTNKEYIRDCTIIQPQWLIQLAPNLFIPADEKKISKIKLREKIEPLHNYYEEP
NAWRLSRRKGM---------------LTIGKTHLLSRYIRGSLPSVAKATIGVEFATRTI
PLAVGGTVKAQIWDTAGQERYRSITSAHYRRSAGAILVYDITKKKSFLNISKWLEEIRQN
SEKDIVIMLVGNKVDLAEEDETKRKVTYEQGASFAKENNLFFSEASAVSKLNVKHIFENL
LQ-IYNNRLKDSNSCSSTRSHETYE-XIQITNAKNIIKLNDTNEKYN-ENNPNQMKCCME
KSKLAKVEKVLGRTGSRGGVIQVRAQFMGDSELSGRFLIRNVKGPVREGDILALLETERE
ARRLRMNESLDKLSTIDLLNELKRRYACLSKPDGRYVFIGAPGSGKGTQSLNLKKSHCYC
HLSTGDLLREAAEKQNELGNKIRSIINEGKLVDNELVLSLVDDKLKSPQCKKGFILDGYP
RNVKQAEDLNKLLDKNKIKLNGVFYFNVPDEVLVERICGRLIHKPSGRIYHKTLNPPKIP
FKDDITNEPLIQRDDDNEEVLKKRLGVFKNETTPLINYYKNKNLLISLDATKPAADIEKN
ISQHISG-MEQQCHMCYFELPDAKTTIGPYDNELNYFMWGPGFEWKPEPVVKQISGEDTY
DETEESEESEHGFDELDEKVNKDDRKIYFDEKSSSGKISIENASYNARRLGLAPSSKDEE
KIRDLYGDNLTYDQYLEYLSMTIHDKDNAEQLVKMFAYFDTNTTGFLTKNQMKNILVTWG
DALTEDEAMNALNAFSNDDKIDYKLFCEDILQMKG--YKYSLSVLSLLYLLTNYAMENPM
KYTDMKGLEDLSTLSDVQINDIFGLTGDEVKDRLTKLFGVIDKNQDKVLSDDEITAWFEY
VKNEVFLKQVQIEMKQIDSDKDGFISLPELNDAFSQNLDPKEVEKHADGLLKRFQIVDKD
KDNKLNINEVGLLIDPMKDNDLKELEINEILEHHDTNKDGKISI-EFKETRSDDINMKKD
DELALDDFNFFDANRDGFIDREEIVKVYFDPSNDAASVGLNDVKDNIFEGKPITFDLWNE
KALKFAVTSLTDYGDVIRYPQDFKLDIGKNVILPSSKNRMG-DDSLNPDNDLSDDKDSDD
SDGSDKQNITDELMDLLLAKAICIFAFLSVATGGCAIPYILGLFGKRNRNKYENKVKNIL
SNLNCFGAGFIFSIVMFHLLPETIIIASSHKDITIFKTHDPEMKTLFIFFFVFVGFCMQL
ALEYVLPTDTNICCIDHGAVR-FTDDNDHILNITNHTLDNPNTEIPNVQFYKYTHYHPHE
GGHSNHKNRLTKILDVLALQSFFLTISLAIHSGIEGMIVGTSDDSHFVFITTFCILSHKW
IAGVTVSLSLNRNNISPNLKIILLLIFIFSSPLGIIVGHLVHSSGEKITCVINALSIGTL
LFIGCEILLNEIQMKYTRKVRFSKWLSFCSSCLIAFSIIYFTSHIAPHQHSHELNVKK-M
QSKFWARGADNDSGDNVSDSSENEVDE-KPLVSAQAERWAVMDSSSSEEEERVIKSSEGK
RLDFYENIEDNLNESMENDDFNQLLKEYENLYKFMVKEDSDRIPNFVIIYLDKLTKYVDT
TFQNNVEKKNLSKNKAQTLNKLRAKIRKCSELYQNKLNQYLENPEKFKDDLGRRKKDE--
DEEEDEEDEEDEEDE----EDE-EKDKINKEDG-----DEEDDWSYSEDAEYASDEEDDK
TKKAMSKWGLKTIEKVDKKKVAKIKKSKKEGTKKDDKGSHIDDNQSSKKKTYAELLNTKN
LSEDVIRNRVKSVIEKRGRKGLDKHEHINILSKLCEIAKTISTQSYIEVLEHLINLEFDV
VSSVYTYMSFNIWNKTFKYIELILDLLIQNEHFYLVSINITEEIAEEESNEKEKISKSCK
TLISFLAKLDDELLKALLYIDVQAEEYRKRLGKTVHMISLLYKGYKYVKYTKNLPDLAIY
ISTRILDHLYYKPELPFKQIWGFVKQGKEYVEKQTKEVDNTNQSDNQKKEESNESTDSDE
SPKDVVEKFVCEIFEHGTKQQRLRALLQLSYNKSLYDEFLEAREILNVGNIHELAISSDV
QTQILYNRNLIQLGLCAFRHGRIYEAHCCLVEICSQNKHRELIAQGISTLKNQEKTIEQE
RTEKRRLLSFHMHISIELIECVNNICAMLLEVPNLAKHSYESKKDIISRQFRRFLDIYDK
QIFNSPPENNREIIILATKYLQKGNWKMCCEKIFSLSIWPKFTDK-KVQAILKEKIKQEA
MRTYIFRYISVYDSFSIDQLCVMFDLPQNTVHSILSKMMVNHEIPACWNESSKYILISTI
KPTPLQTMALKLAENINEVMEQNELALNMKNPKFMLMQERKTHMKDDKSNWNSKKGDGKY
GKNYNRHKNQNYKKNYKDKNINKNFVMEHGNVGDKIFDLNSISREMLELVEVFYDKMKET
TNANALYLYALQIFKICNIHNELPRLVVFGQQSMGKTTLLDFIMGGPMGYTSSDTGTKQP
IVIILKPSDTNKIECYLNKKKVNIDDLHEKMKAIMLNLNESIISKELEVEISIPGGIYAT
FVDLPGIKDDSKAGSELTRKIVRNYVQNFPNDIYILVKKASDDPANWPYHLKEFFMKPKP
LGLGLQTKQCIVVGTRALEFLNNELSTIKTLTELHDRVKKRGISDNNDNMLSLYLLELFS
IPIEQKEKNDFLTNRISMYSKILNGRKNVLDLLLNKFENDCSDSIKKELIDCFDVEKFKQ
EVNSKFMNILIQQLRKVEVKLEKKKAKMEIYIKKLEELYNKGNILSIREQVKLYIRELVN
IISNLLTGNYPILNLPKNGDDFLKKYGGTLMENLKDGNDLAVELYEKQGLYDENFLTYLN
EYLIKQSNENEYNKSVTSDFNNCNDF---SNNNSNINSLNKRSDIYDNFNDRDKKR-TND
NMLKIGQTVRFLLAKEESSMFGIIQNISNDARSKNILVNFFFRSSNIEEQIQVKSVEKER
LIVVKPVETLSGDLFFLNGLQVWYKMTRDDGWVGFDNAEIIKVFNNSSKIKDVLIRNLSK
NNEVVSIKINDLYADYTDHM--DDEADPIDETYNEDDALKKVAGPHTDLKILNQLAITYI
CKWLKYNIAKIEPEKKFSDEVLLQMMRSIHNIVDQSDWKPLVVDLLQANISGNILYLTKL
ASCSAAVALNRVFKAGLGEINRKIKNNYIDENIYLLSTNPKFLEELNQALHNFCKERAVI
CASEMKEIVFEQTYAVHFEIIEEIFEGCKLFEDNFLTPTGVKPTMSIINKNVKQNLAYRN
HQLSLTDVKINKKSRSKELIQEEVKLQFWAIKMLISVPFATKIYAHFLNNIVPKNKHLAN
ILDYSIDCESNLEKYIQSKLLNREVNGIQVPIDDKELLSHYNIIDNRDNILRKLENQKRL
IQYISIVSNSIKLLKNNLSNESTLDFVTKLDFGQNKK-----------------------
---NYESEEKARIW
LNFPENIKNDLKNSLLNLLDQHGEKIVIGTACQIISIITKIELSYNKSSELLHKLVNNII
EKNAYTKKSSTICLAYLTEDIADICNESKTKYVFTQPDLDLILTAIINSLCEPAEESIHC
ANMKVLYNLMSFIDHNFKTQVERDIIMKTVIDGCKDNERLTVQVAAYECLINIVSYFYSY
LDAYMYAIGPLTWEAIESDNERIAISAIEFWNTVCEEETFIDQYELDEGKKNHNIVKQAM
VFLLPKIFNAMITQESEDIDIDAWTLSMASATFLALSAQLLKNDIVEPVISFVEENFIHE
DWRRRDAAVLAYGSIMEGPDTEKLKPLVEESVGQLSEVLRDPSVSVRDTAAWTIGKITQY
HSEIIYNVLGNYNDNNSLYGILLERLNDYPRVAANVCWVFNQLAVNKRSSYNKLENTYTT
DLDDSFCVLCKKLIDVTSREDADTRNLREAAFNALNVVILNVSDNCLKYMIELLSHMMYL
LTNTYLNPLTEEVKSLQGYYCGTMQFIINRLGNQCKPFLKPIYLCIFRLFEIRSDICEDA
LLACSAIINVMGNDFREHLKTFLNVIFKGLKNVSETSTCKICIEMISDICIPWTCDMEKE
MENILECLWEALRSFGVHDSIKISILTVLGDIATALNKSFSKYLNFFANILLETSKITIA
SGSPENDDWISYIFELRDAILLTYSNIIYALIDGKEINKLKMYIPNILDFIELILIKEIN
HFNAQNFQNSVSLLGDLVHAYGYELIENSKLTDLIISVYGKIDILSSQRDEKCESCVSKI
KWLKKICNARLGQKMSVYIQLTHDLSGTVTAVTIGDWILGILKQKNIQEYEVFFKKFLNI
YQNQDKESETNNRSEIFSLLLSASDCVFETLVETKKNKKINVIIDNKECVKSFNEFYKEV
EEYFVILISILQLEFKSVDDLNNATNNFIKAIKNYNVFPELRLKILQLLYNSFNVNFSFR
FPTFIAILQFSSQNNIFHAILPYIKSIDDWIKEWNISNHEKRQIYLIVAEELKKLKKYED
SYKHLKKHIYYFQKESKEILNHASTIKASVELVVDSINLNNNIFFHEIVNLDAIQNLQYI
EEHKPIYELLTIFYKYNIQEFLTFKETYGNKFFTKYNIDLENSENKIYLLSIISLFKDNK
VQNIQYISEQLNINMLKVEKILVSAIGSDIIDAKIDQINKTVHMKTTILRQFDEQQWEHL
NNQIAKYIKNVQKVLDLTTHRKSSSNAMHYLFYLVLSAILNCFVRGQESATNFYKFIDSF
ASSTYISEESGSSLYDAKRAIQNNPSYWCSAGNHLKDEEITWTGYLNTKGFVKGVKVSWE
YSPELVSIFVSSDGEHYKNVIPYKKISSTESSFDEIYFFKKLEEVISIKIGLKNAIHKYF
GIREVKIIGGGNPYFLLLSGITSEQEMCLQVEEGLINNDNTSVILDSCINALASGDGREL
WKTNSNNQIISALSDPPKCLSVINVDSLENNKLVLYDCLRALEDGDGKSNWVFESNSQIR
LQRGGEPLCISQKNIHGNIPGIHDILLNTDASVDATSILDDDHNADNTIDGNLNSFWASS
IFGDNYEHLVYFIIDLNKFVEVSRIKVFWEYPPLHYIISFSTEKDNYKIVAENLANPSFI
TIDSLKNIETRYIKISMIKPHPKHGEMDGQFLYGIRSIEVQANNLESVLNFCRDAANSDD
ARDKYFIEYVSEFDKNLSNKLINLEDDVSKNVSSISDKLSKLEEVLPNIETCLNEKKEYE
TKLKASMEQVIELNDKITSLESIDLIHSNDLLRLGISPGDSSSYPANDCSVIKNAQEVPM
SGFYWIKPKCSPEPLRVYCDMDSSTSLYVWNGHSPKSPDHLITNIINSVDDIRKHCAEVG
LEPLVLKSTDQLNSLIFALKKMGFILNGKINIPLAYDYSCNYGSCSGKFHDLLNGNVDLT
TLIYFKGAESPNSTSVRQTAGISYDDGSFKFFNLETSDISAIVCSTNSSENDFSMQYLSI
ECDTTALDDDFNGIINTNIVALCPLGCDHEKFKEFNVYGSNGVYSDSSSICRASIHAGVI
DKQGGLINVTIESGLDYYKGSISNKIESISLNKGGNESLLDIITSEKKEDIKEETSIINH
RTIRISSLSQDCPIDLFEYKQLSFIEKGNFKKNEHDVEKIAEYNESNESYKTHEIINDLL
KNIDAIHGVDSSVISIIQDETVRVIEKAKKEFAPADILSKKQIDDTINLYNLTENLALYL
YDLSGKYINDLEKVKERLKELKKIQRIVHNFGTFKLNYESMNFSSYFYVFDSKLIKNKPS
VWGYVDTEILGHKNSIGQMSSISNREIGEGYFAKLKGLTFYDFEIRVSVLSKGSGCAGIA
-GAKDDFKFYLFDICDQDGVRRLSKIENGHADILKEKYGEFSINNKWSTYKITTSHANID
IYEIDDKLNEMKILSSLDEKFLSGTVGLYSQINGQGTFFDGLEIIAKPCSELSKNGKHEK
KQNYNCPYYKENYDSDLFPYTIINDVEYKWSFAKEDDDYLLCKKIENIDNESNGKLYDTI
ALLKQRKCYDGILNFDMNYSKENKNNKPHIYILFNFVNENNFNSLEIRQDSLKLTSYKDS
KSITLSEFSDHEKITKTLEQDEWLHVSVKFDKLKFKAVVSNNNYEIELDANNIDTDLIDL
GNVGFRVNNFDEVKFDSITLSSNVLNSNESFIESTTKTWGTCEESIHVLNRRSSCETDMH

```
PNQKKEKHINCIKNFCEECCLHHTKMLDSNERKQCEKHCKRNDGLAEKMQKLFEKFINKC
VSLEENKDYENCDDNDLNCRSKTCILCCEKNGVIDSDDIEEVSFHKSKDIQKEETIECQF
QCKVTHGITE--------------------------------------------------
------------------------------------------------------------
------------------------------------------------------------
------------------------------------------------------------
------------------------------------------------------------
------------------------------------------------------------
----LSKNFCNYEEYEGNQSDDTSYSTNGSNYSKYLKRKKKLEEQGKIVIDLNLLHRNTI
KPNMKQYRDEKMAEKLRIEKLKKILEEKKMLLCRVEDIAKKRNRIHVKTQILSEDSKIKR
IYFSHKMKKVYNYKFGYFGSGWSNNKTEWTPFIHAPFFDNQHNTIYKNRNKKLYEEIYDT
ILHGRIHPHIKVVELKDHMHPIRLCTPSNEDCYSVIYTGEKIKSTDERVIFGEYTGFVAN
NKELPQEKHQYIFALTFNKKVFNDRKNVVFINEIDSYDNESDEKENSVEGNISEMKYTNM
KNGNNYKGNQFNLDEDKHKEEINTETEKMNDAKGEKNKSLGNNKNDIIDKSKKKKENDKS
KELNNLIILPDNYTYAVDSSYMFNEMSLVNHYKTCSIFNNYDFRINAEWQIVYLDGWPHI
ILTSIPGVEIETGEEIFADFGFEWFDRVNDICLNDFIKNNYEHRLNEIGIKKEKNFNGLD
DIVDKYNLLKNYTTCNICIHSVNTDCSNYIICSGCNHVYHLKCVNRLNFQINENYDWFCS
SCIQFSMNIISQKEFLEYIKKENNKRLINYFNKHIDQGKTKSSEKLLMENNLDSYNCE--
KNHEIQLDDENYINSFENIKKLLQCKENIDDLLNNQQIINAFLENVDNNINILQGEEGQN
DNIILKNPELKNLIENSYELHLLIEYKNKIDDLLLCRENINFLLNNEQAKQTFQMR-KTI
NYLMQNKKCIENLVESIEKKNRGEITKNNNATFKTTNDQLQLLMTDNAQNGNTGNLSSLD
LFKYENGEQNFVALREENETTQSTIISAHGKYPYSKDGCYFTSVYENVLYKKTNHIIKNL
KDLKNKNRKSRFKNNSIKNEVNDENNNGSDGAGLNAYYNYMMKLSKKLLGLPLIREFEKG
INTHQPSLPLNANLKKLEVCSNCYKKHGNLAKAVICRVTKLHFETNYNDGLGEEELHKMS
SECIQYVIKELANTIKEYRKQELNSAFIQYTKKKKQSGLVNNKEHDIKIGNRFSDIITSS
DIINSTFNKTDI-ENKDFYHSLSKSLYKDTYETDDNIAIQRSHLSYIDKNRSVENQQDGN
STLQTNTYTIFG-KKHENNDDGDGNGKSRHQVNISSDTIDEKNASDQENFKNDKYISYND
ENISTHNNDINNVYFRSDSNNNDYKNQKTNNFKNDDELLTTNEILLDDKKKEDKSISNNN
NINNFIPLFGIELGKTKFQREFTNGTYVGTVTKQIKDDNDNHFFVVTYEDGDVEWITPFF
LFQELLKQGTNNIEYPLATPFKDLMNPNFKKDIKLNNYFLELKVEKKKKKGTGEYNSNNN
SATKRQKHNQEGTSSKTKKR--MINKEEAQRFKELGNKCFQEGKFEDSVKYFSDAIKNDP
SDHVLYSNLSGAYSSLGRFYEALESANKCINIKNDWPKGYIRKACAEHGLRQLDNSEKTY
LEGLKLDPNNKSLKDGLEKVRKDKEMENMEYINHINNIINNDARLKSYKEENSNYPTELL
NTIKAINANPMNIRFILSSCNPKISEGVEKFFGIKFNDEASYEAERERQRKKEEEEKKEK
ERKMKEEEKKKNRTPEEILGEEHKLKGNDFYKQKKFEEALKEYDEAIKVNPNDIMYYYNK
AAVYLEMKSYEKCIETCIYAIENRYNFKAEFSQVAKVYNRLAIGYINIKNYDKAIEAYRK
SLVEDNNRATRNALKELERKKEKEEREAYIDPVKAEEHKNKGNEFFKNNDFPNAKKEYDE
AIRRNPNDAKLYSNRAAALTKLIEYPSALEDVMKAIELDPKFVKAYSRKGNLHFFMKDYY
KAIQAYNKGLELDPNNKECIEGYQRCVYKIDEMSKSEKVDEEQIKKSMADPEIQQIISDP
QFQIILQKINENPNSISEYIKDPKIFNGLQKLIAAGILTVR------VIILGTGLKECIL
SGLLSHYGKKILVLDRNPYYGGETASLNLTNLYNTFKPNEKIPSKYGENRHWNVDLIPKF
ILVGGNLVKILKKTRVTNYLEWLVVEGSYVYQHQKKSLLFSEKFIHKVPSTDMEALVSPL
LSLMEKNRCKNFYQYVSEWNANDRNTWDNLDPYRLTMMDIYKYFNLCQLTIDFLGHAVAL
YLNDDYLKQPAYITLERIKLYMHSISAFGKSPFIYPLYGLGGIPEGFSRMCAINGGTFML
NKNVTDFIYNDNNQVCGIKSSDGEVAYCDKVICDPSYVMHLENKIQKIGQVIRCICILSN
PIPETNDINSCQIIIPQNQLNRKSDIYVNLVSFQHGVSYKGKYIAIVSATVETSNPTKEI
EKALELLGPIDEKFIKISDLYVSTNPKPKDNIFVTSSYDATSHFETATNDLLQIWENLWG
HKLNFDDLNKS-DNEL--VKKQKKTLKPSTKVITINLSKLTHDVCYKRKAPRAIKEIKHI
AGKLMHTKDVRLDVKLNKFIWSKGIRNPPKRVRVKIERIRNEDEDSKERMYTLVQHVMVD
SYKGLVNECETNEMKFHNGRKKTDMKKIDEPFVEHGDSVKKSRNPGQDDKALKLRACLSC
RLLRTEAEFYQNGCSNCKFLQMTGDRHRIQDCTTENFSGFIAITTPTKSWIAQYNDLSKY
NPGFYALQVVGELPESIRDLKSSYMQFSSNGKINFDYIYNNKELKSKLYLPSSDTFVFAE
ALEDDVEAISPNVNMVLEMGTGSGYLILFLYELLLKKNKKIDLLYCIDINRDACNCVQNA
ISLNGISNVEIINSNLFNNLRTCGQFDIILFNPPYVETEQDELNKTDIVASYAGGKQGRE
VILKFLHTVYDHLSNNGILYLLLEKSNIPHEIMTSTLISEKFYYTELKKKKTLNETIFIY
KLRKKIMDRYGHNIRGDVKKQGYENSDLPILCETCLGENPYVRIIREENGKECKICKNAF
TLFRWKPGHNARYKQTIICNKCAKVKNVCQTCLFDLEYNLPVQVRDKFLETSITLPENET
NRNFFLEQLENNISTGTYDKINHGNMDLSKLKRRDPYFKRNMARVCSFWRKNACNRGDEC
PYLHKEIHLNKSLASQNIKSRYTGENDVLAETILNRYKNDNIDEKNMANKICIQGISNST
RVENIKECFKKFGEIKSFKMIPKDSKIFISYATLTAAKNAA-KYKDGLELNGSNLTVTI-
QDNPNNNFNNNNNNKFTKNNFRFHNPPPSTMNNMMPPIYFPYNNYNLNSMPPNAIPYASM
LPSEAEQRKMIKNIFNEYLKHYDNKNLGYEENDTNIPSKEYYNEYISDWRWFSIKCERII
ESELNKKHLQKNEKNEYKNNITEIDNISTFRSFFPKILLNAYSRYSNPKKFFDNLIIQKF
NAVVFFCDASGFSSLAEQLDKKINGAELLGNCLNKFFNILIKIIDSWGGDIIKFSGDAVM
VIWPLNVKMKKKKKKETKVKENIQNNEKKDRKKNEINNTNKNIDEIKIN---KIRRISLL
ALGCCMNIHKFLNKFPTPIENKYLKVHIVITYGKVNFLQVGNILNKRDYILSGKPLEEIG
FGESLAKDGETVISYSFYKNIKDKIEIKETCKKKFYLLIRMKEELDINKLKENNYEKDNI
QKNNENLDKKNINVCKNFDLLLKSFIPDIVYRKLSIGYNIFFNETRKVTVIFVSVKDVDT
STMTGIYSTHSIMKLTQKAVFTMEGTINKFIFDDKGILILIMFGLPPLYHSDDSIRALLT
CFRLIDGLKSLKLNGSIGVSTGKIWCGIVGNKIRKEYTALGDSVNIAARLCCKAGNKEIY
VDENTYNNCKHFIIFQSLISIKVKGKNKLIKIYSPIGTINKRENNIDYNDLFILDYFTDK
ELLNNLNNYEEITDFNFVNKNFLLIYYKNKFKDRINDLLNRKDCLHYLKTNKETQKNSYQ
NHKEKNTLNTNCFKCNQIYSLSPYYFLKIELYTDYKTHTGPLFIHEYYDPLFFDFKEIYN
VGGVLFLEGNEHLGIFELIKLIKKSLNNFKIFNISNMPNSLYINITNPLLPWKILCNDIT
NMWKLCNTRKKYLYFNKVDNYNILKEITHPSYHWFLKCMSNIIDDLYIPDTSMKKETKNI
NKIFDTQKINNI-----------AHIEALNGSHYDENRKNGRAFCNKLFNSTLEIRNSNN
TSGVINKKNSDNSILYSSYEEEEEEEYINVNMTNINSDYILKNDMKENRISIINCMIYNF
SLYEHTFIVFNYRCGTSLNISIEEHAWRICKNISKLAIYKRNKIVKNFHKNLKNWRKNHC
RMCNFCKGFYKTIYPNIQDNILLYQSNLCNPIFKPKNKPLIFLFVNGAKNNDVKDLKKMK
KYAKECNGYIKLENFDKNDLYNFVSLCLNVNVDKISEELINYLNKICFGFPKFVQYTLFY
LLKNKYIKLYKYNTPNVLEKTNEQLHNTVGLSTNEYTDIEKRKEQNFTKSSKTPRNFISY
YYINKASQNIIREQNKFLNFTLYRETNNTSINSDYSNYDPKMEVVKDLDNAALVPRLIAY
CMFMIDSLSEEDQLLAKLCSFFKDKFNIKKMECIYPKPLSRSELKKIIVNLVKKNVFQVC
DDNKSNTTPKEENIFFRIANYALKKVLNELMENEEKKYIQTIYKKYIEMLDKNYKHESMH
EKTRVIIKNIPKYMNEIDLKKHFFKMKGKYDFKITDIKIMKRKKIIKNKESYESRKICFI
GFISNIDCENFKKSFNNTYIHTSKIIIEDAFSPILSKNSNTFQLN--EIKQSEKTNKKDQ
NNTVKVIKNDQFINKMTTIKKTKAGMNTTRSHVIFIDKDADLEKVNELNAENGEKKKKKK
KKKKSKNANEKSEMEENKLEVEEENALDWLKGISKDKENG----KANNKGNPELLESDDT
GNENEISSMEKVKPLYSEPEDIDDNDNINTGKLIIFNLPPVDEQDVKSLCERYGPVIDVY
IFKNTKKGSKYICLNEKNDQKSREDFFIKLLKENNDNASSGNKGSTKKMANSNNTKEENK
NESENENSKNENNITNIDLTNVKTYAFVSFVFPSSCEKAKNNLNETIYRGKILSVKYARE
KIDNSEHLEKNKNNIFIKLSNESKTSYKKILEIQKKRNCQNESIWNILYTDINSNIYNFC
KETNCDPQSILNIRDKNIAVNVSLTETFIINKMKEWIRKEGIALEAFEQIYKKENSKPEN
DENDETDKVIKYKRSDDTIIVKNLSMHTNENDIINLFKKHGILKKISFSPYKNIAILQYE
QPENAKKALISNSYIRYKKLPLYLEWAPINLFEQNSKNTEGTIEENENSND---ISYQNN
AKPYDSESSDEEITHSSIYIKNINFNTKEEDFKKLFEKLDGFITCNIVKSKKVIKQKHKD
NKTEEEHKYISLGYGFAEFKSKELAIEAIKKLTATRLDGHVLELSLSHNRIKKNKYKNNE
EKQVIKDKKKITKKLLVKNLAFQVTKEELKKLFSAFGNIKNVRIPKNAYNRSRGYGFVEF
MSKNECLAAINALQHTHLYGRHLIIDFANDLIFDQNVDEFDKMKESQNNPNQDVITSEQA
KRKVIYENEKSEKISESKKRKIIGRLENV-------------------------------
---YNIMDNIFLKESNNGNDNNYLDKVPENARMLKPLVQEKIIEIMKPEIEEKIIEVPQI
QYVEKLVEVPHVILQEKLIHVPKPVIHERIKKCPKTIFQEKIVEVPQIKIVDKIIEVPQY
VYQEKIIQVPKVMVQERIIPVPKKVIKEKIVEIPQIELKNINIEKIEEIPEYIPEVVKRD
VPYTQIIDRPFHVEKIVEVPHVQHIYRNIVSPQYRHIPKPVEVPMAHYRTFPVEKLVDRN
VPVPVEIQIVQEFLCPKIEARYKEIPVPVHVQRIIEHPIPKDAMNNPFLLPLYYQEDNFN
TSKNSTSNTPQNNKGCFMFNWNKNHNQKTMKSRNNSVELLLHNDNKNNEFFNINEANINP
```

```
NINLKSINQYSGNTFRDINN----------------------------------------
-------------------------------------RSEYDRGVNTFSPEGRLFQVEYA
LGAIKLGSTAIGICVNDGVILASERRIASPLIEKDSVEKLLPIDDHIGCAMSGLMADART
LIDHARVECNHYKFIYNENINIKSCVELISELALDFSNLSDSKRKKIMSRPFGVALLIGG
VDKNGPCLWYTEPSGTNTRFLAASIGSAQEGAELLLQENYNKNMSFEEAEILALTVLRQV
MEDKLSSSNVEIASIKKSDQTFYKYKPDDITKIINSLPSPIYPTIDMTSMEVEKTHLINY
FLEICPTVLDCSRKELQSVLAKREEEKIRKFVVDKNVNIIVIGKGKQEKID-EGHDNI--
---KKLENERDEERNINFDKEPSYSLFVELNINYKCVTKSIAIAFMKRNKDTFLALNEKL
NNGNKINLSNELLMFVCGLNDCNTPLDLVYLYLSQGFNTIFDAASYVTHSAEHDEISQKK
YSKKRDVEN------GYGNRKCGTYGGDNKISNLLMTNVSKKLNELLISMKNAQIDLNIP
IICLNVDKRIEKLVEENPNIDDIKQETIKNLCESQGFLNQLQKDVTKWVEEIQKITRLNG
DFKSGSALAEINFWIGYENALLELENRLKSPEVILTLQILKNAKRYFATISFDSDIQLKQ
SKEYVLNVNILMKDFPIDDLLGAQTVQQTMQAIRNIFNHLKKLKNTTKYPLSKSYNFVEA
LSRDLGNTMKKILSNQSLLSMDYQIFDVLISGCMEVFQLWNEEIRVFKDMVRELIKKRSF
NERAPAKMIFEHVILQERLDDIRKFRKQHQKFKTVITKVFGNDKNVGINLHKDINAAYNV
LLSLDALDLSKSGNDLWEKTKLSYESKMNKVESQITFKLRDKLGGAKTSNEMFNVFSKFN
PLFFRPKIKGAIQEYQNSLIQIVVEDIKKLQMIYINGYVKSSSEKVSTIRDIPLVAGSII
WCKQIEKKLEDSFKKIENVLGRGWEQHAEGKNLKQNIDNFKNLLNQNKTFEKWLKSIKNG
DKFDIYEKIIKIKKLGINNYEILANYDQQLFNLFKEVRYLQSINLRVPYSIKVKADETKL
IYPYALGLEKTLRVYMKICLYLENEGKDIPFNGTISMLVASAHNSVQEKIKEGINLHWDS
DILETYVRKLTEHVNMLESMVDEAVNKNSFVLDILEKIKTCQISIDGNEIKKYVELIQEK
ADELYLQHYKNVHLWINEMNKILDGILATRLEEIIKKWTEEF----RRNSAKNRFILRSS
VHEFKISKHKVFLSPSTDSMRQIWFSRLSEAINLICGITRIKNIYQKKDKSREVIEIKNK
DGEVINNVMLDNTYKYIIYYVDKKVYDNAIKCINDSVEKAKEYESMWFQYKTLLKVEIGD
IITNFGEDIEIWKTFMNEIKEREDKFDILDTEKNFGPIVIDYRILQSKVSSKFEIWQKEI
VSEFSKKLLEKILIFKEDIEKCLHDLREQSELKSESEITAMHILSMRPKDIITIMNDYDM
DVLSRICSSIHDFVEKINEINKKEQEWDSKCDILKKSELMLEKQKYMFPKNWLFIDSVIG
KVETVKQILAYQINLVKEYFPYIQSLVIEFDSKVQNNIKELYEQWNRSKLADGNTNSVKV
LQIIKTFESKINIISQDYKVSERLRKLMKISDEESETNFHISPKMLKEEITCIKTIWDEL
KVIYSSISDLKKILWINSDLKNVKNLLNNLLGSIKKIPAKYRQYEIFDKVKDEIQEYLKT
YVILSDLKSESLKERHWKLILQKLELKIHYNKLSLGNLWSSNLCYHENVIREILNQSQGE
MVLDEFLRGLKDTWTEYELELVQYQNRCKLIKGWNDILSNIDDHLNAIQSMKISSYVKIF
EEETLNWDDKLNRLRNLLEVWMNVQRKWVYLEGVLKSSTDIKLLLPQEYNRFKIIDADFI
NIMKKTSENPKLLELFQINGFQKQLDRLSDSLSKIQKALGEYLEKQRNQFPRFYFVGDED
LLEMIGNSKDAKIIQRNINKMFAGINSFILKENTSDSILGMCSREGEEVFFKEPIHINAY
KTLKEWLIVLETRMKTTLENYLDLAAIEFMQMDILQCTKDSTNRQIIDWCCKYPNQIILL
CLQIMWTYNIEHDIELSMEKLEMDSQTIFLTSEEVCRSLLKYLSEVVVKQTNNTTRQKIV
QMITELVHQRDVIRILIEKDIKNVNDFTWLQYMRFYWDKDKRNKNVNLIIKMADASFEYG
YEYLGMCEKLVQTKLTDACFLTLTQALKMKLGGNPFGPAGTGKTESVKALGAQLGRYVLV
FNCDESFDFTAMGRIFVGLCQVGAWGCFDEFNRLEERILSAVSEQILTIQTSLSQRKKEV
EILNKKVELNKNVGIFVTMNPGYAGRSNLPDNLKQLFRSFAMIEPNKELIVQVTLFSQGF
ISAEYLSSKIVSLFDLCSEQLSKQPHYDFGLRSLKNVLNSAGNLKRLALQEKWQGDGDNG
KNSDEMEKVKKAVEIEQTLLLKSVCDTVYPKLVSSDIVLIKSLLSGIFPNANISLFEDKD
LINEIKRICKLKYYIPEEKWITKICQINQIMKLQHGIMLVGGVGTGKSSAWKILLDALEA
IDNIKGMSYIIDAKSLDKEEIYGKLDNINLEWTDGVFTCILRKIIYNYTTNNNITKRHWI
IFDGDVDPEWAENLNSVLDDNKLLTLPNGERLPIPESVKILFEVDTLKHATLATVSRCGM
IWFSRDILPPIVLFKHRLNKLKYGNSDYPRKIDILKSLFVSSSNNETTEGAIMSGDGSKK
GNDYENREINNNSIVMNE----NMRRNSQVFYDKSEENIDVCKLQTIEYRSIELISEYFE
ENGFVQQCLVGAAYFEHVMDYEYIRVIESTCLLIQKGIDNLVAKYGKFNNSVSDSDIEKY
MSKWLVVSLIWGIGGSLNLESREKFSKYVESICSVALPSDLSKRGLVNSSNLLNTNRTVL
DYEPSVDDGEWHNWKERVEITDVDRTEISDATLIIETMDTIRHATILEGWLNLKKPFILC
GPPGSGKTMTLTSVLKKSTEFDIAALNFSSGSLPNLLLQTFDHYCEYVKTTSELVLRPIQ
PGKWLIIFADEINLPTPDKYDTQRIIMFMRQIYESQGFWKYDTNNNQWNWVKIERITFAG
ACNPPTDAGRNPLSNRFLRHTSILYVDFPGYESLKQIYGTFNRAILRKFPESLHMADNLT
LAMVDFYAKFSETFTVDMEPHYIYSPRELTRWKLSIYKTLENFEKIENRELVRLCIYEGL
RIFQDRLIYKKEKKETDKIINDIFKYSFPDVKDEDLERPIIFSSYIENKYIEINKNILKE
LILAKLKIFGEEEVNVQLVLFDEVLDHITRIDRVLKLPFGHLLLVGASGVGKTILSRFVS
WMNGLSVFQIRTGRNYSTELFEVDLRNVMKRAGIKEEKITFIFDESNVLGPAFLERMNAL
LASGEVPGLFEGDNYKALINECKSQYGTNAGLDES-IFKKFTKQVQKNLHIVFTMNPANP
DFANRQSTSPALFNRCVIDWFGDWSYKALLQVANEFIFSLNIPDNNFYMDNISNESIKSK
LDFKDKKYYFLSKAIVEIHISVVHINQVLMKKGSKYNYMTPRDFLDFIKHFLKIIDEKRE
EISAQKKHLNAGLKKLKDTEVQVAELRNSLANKKKILAEKDIEAEEKMKLMIEQQTETED
KKKKAEILAKKLDEQFIIIEQRKEIIRKELSEVEPKFREAEDAVKNIPKKIFDELRAMAN
PPILVRNAIEAVAILIMNEGDKNVTWEDARKIMKGQDFINKVLYLDKKTVKAQTSAQIKK
KINNTDWDVDRINKASRAAGPLAKWVESVITFLTILETIQPLENEIEKLQEETRIAENQY
NEQKEIISALEKILVQYKNDYAQLISQVQSIKQEMEIVEKKIVRSINLIENLKSEKERWS
ETFISLEEASETFIGNCLISAAFCAYIGFFEHYERQKLKKRWGEIIKIHHIKYRYDLSFI
EFLSKPSERLQWIANKLPSDDLSIENAIIISNYIRYPLIIDPSDQASTFLLNQYADKKIV
KTSFSDKNFLKNLESAIRFGSTILLYDVEKIDAILNSVLNQETHKQGGRLLIVIGDSEID
FSPNFNLFLTSRDANLQFTPDICSRVTFCNFTLTPSSLQNQCLNMILKNERPDIDKKRRD
ILKLQGEYKVKIRELEESLLLELSSLEGNILDDDNVITTMEKLKIQASEASKEVNIAEGV
MIEIENVSNMYLFLAEGCSKIYFILQYLCNINFLYQYDLNFFFNIMKDMLLDQEYLSKIK
KNDYGERIKCLEYLLFNLTYNRVARGLLQEDRYVFALHLCYVKSIINPSCYMDRVYLHLL
LKDSYVYEEATTMMGQKTSNEIGREVLPDYNDEQIRNLNNLIKHKLFYYLKDLIENDREG
WTCLLHSDEPECVV--LIMRNKNIKRKNSSVTHLNSVEEGVGVNQENPISQCLKEALIIK
AVRADKLDRCFNKLINIILGKEFLWLPELSTNEFEKYVKENAWGNIPIVLISSPGFDPSN
KVHQLSEHCKISLSCIAMGSEEGYISAEKVIASAQKYGGWVLLKNIHLSHKWLYQLEKNM
HKSTSNTDFRLFLTMEINPKIPPNLLRMSLTFMFEPSVGIKSSILRTFYLFLENKCINDP
KIARLRLYFIVSVLHAIILERRRYTPIGWTKKYEFSDADLTCALAVIDNWVDKSAVKIGN
NISDHIDPCNIPWEAIKKILNETVYGGRLDNIVDSKILDTFIEHLMHSNSFETDFNLNIS
NSLSSKDFLKSPDLFRNIQDYIKWANNMINTDIPAWLGFGQHAEGLLTTRTNFSIINKWN
ILYSKSL-DMQELLPYSQDEIKLEDQVDEMDEKENTDTPEYHTNVRHIRTKEQNESIVFI
DRILESLPEYIPDLEKSDENVGNAVFRCFERENQLFNNLLKDIKTNLIQLKHVLEEKIKY
TNKLRALAKDINSLNVPSGWLLQTNTSNMNLTIWLRDIIKRLYQIIVITVELNKSSTAKS
NSEKY--------DDDENNKKLSINFIWLGGIFYPRAFITATRQMCAHKYKKSLNDLDLS
ITVDEKNEEKLMEAFHFTIICLSIEGAQWSDNDRV-------------------------
------------------------------------------------------------
------------------------------------------------------------
------------------------------------------------------------
------------------------------------------------------------
------------------------------------------------------------
------------------------------------------------------------
------------------------------------------------------------
------------------------------------------------------------
------------------------------------------------------------
-------------------------KIKITGEWKREIYEGNIGPRSNYSNIFITQRHQDF
KGAEPKTIEKLMLLCSGITY--DNKLKIVSSDEIYVYFFSQKRWYLLKGKLYNEEYMYSA
RQRHVGCFFESKNVLGRSNRNPVPCIFIHGGFKKNSVFGDAWLLSLTGENPLRLYEYDTC
REKISTTQMPLYYFRDTHSISLLYTFCTLQKWLFGAFANLVDNSVYAINPAENVFIKYEL
TPEHDGMVSIQDDGEGLDFNAMNRILRMYGNYKNYDNNSFYNNNNNNNIKKHSLPNNDDS
DSYEMDNYNENNENNADNNTDQMQQNEKGDGEKNNNLSGSNCEGMIKKENDTRNKNVIVG
KKKCTWSDSFYYKKKNQHYFYNDISNSIFDIKYGVGFKTAFARIASSCAIMSRTINTIGI
GLLSLELMNHCDAKELATPLCMWKLPNKELINRNIANKSEHRHHQKLLMSYTPFNSPSLL
AEQINILGTYSGTRLLYWNFRDDMDFIIFNPTNNNIYLSSSPLSIDEL--KNKKQKNAQG
EGSNFNSKYQGVKDIKFANRVIKRKGVFGDDENLKKLKTEKDED-------IVADDKNID
DKYNKDEDNIKKELDNMSTSSKNNIILDGDNNNNDENKNVNSTHYKEKLKYEDQLKYNND
```

NYFKKRMNLFEYNSHLTSSISKEKYNMNQIFPLWDHPKDSIDYCLSTYLYWLYLRRSTNI
FLQNTLLIPTSMRREKKNNNDNNGKKKKSTKRNKK---------TTKQNDQ---------
----------IQEKNDDNNHSNVQNEADTNNNISEQNQNTEINNNGIKNNVCGLTKLEKQ
KLEEALEYGISQDLQPQNSANKNEADGEEEEEEEEEEEEEEEEEEEEEEEEEEDE-KNNE
DNGNINETNNDVEKVEGEVNEVG-AGVVKTEPNEMETENNA---DDTEKNKKNVGKTIDN
ANEDSNRRKRRKKKDGLSTGTVANGKKNGEIEKMKKENYVNVYMENFKIKDEYYINDPSK
YTLYHFLRRKLYKMVESHYLFTPSDHTYGSFIMMGFLNDNNNTSIEVNRVCETGILLYYK
NRLIKRLDAPFIDTPYNLFLSKYPPNPSLYEGNLYKYALTVIVNVPNWLKPSVSKQEFVH
ENNHAFLVFKKKLVTLIKHYLSICEDIVKLNKWRESRDMKLKKYLEKMQNQSVKSRNDSD
YEKSKNFNIIYNDQNDGNKKKNEEIN--GKNK-TPYTNDNEPILHKNKKTKTNNDDKRDH
NPTSENESANIDNENNQENDVLEGEKEQEDDEYDEADQEAEDRPNFEDEKDEHEENEEEE
EDPDDEEYNGEMDDQKNRSVEQETEEEMIEDEEYEPMENNPDIKTLGDDNNGKIPKKKNL
CRLIVEEATNDDNSVVALNTKRMEELNFFRGDTILIKGKKRHSTICIILNDNDLDEGKIR
INKVARKNLRVCLGDIVYVKACPEIPYGKKIQVLPIDDTIEGLAKDTLFEIFLKPYFNES
YRPVKKGDLFLVRGGFMSVEFKVVEVDPDDFCIVSPDTVIYYEGDPIKRDDEEKLDEIGY
DDIGGCKKQLAQIREMIELPLRHPGLFKTLGVKPPRGVLLYGPPGSGKTCIARAVANETG
AFFFLINGPEVMSKMAGEAEANLRRAFEEAEKNSPAIIFIDEIDSIAPKREKTNGEVERR
VVSQLLTLMDGIKSRGQVVVIAATNRQNSIDPALRRFGRFDREIDIGVPDDNGRFEILRI
HTKNMKLSPDVKLEELASNTHGFVGADLAQLCTEAALTCIREKMDVIDLEDEIIDKEVLE
SMCVTQDHFNMALGTCNPSSLRETVVEVPNVKWDDIGGLDEVKNTLREMIVYPIDH----

-------------------------------EGLVSIEVAKTSDVKILNKDNVENSTNKN
SDLKKKKSNKLSKITKKTITKKSKEKKKNNKVIKSVKSNKTKIEVK--KKIKKVREKKKK
KNLCEKKGNDIEPKNYDKNITEETNEENSIKEVEQKKKKIFDIEQIIKNEENFKKIKEIK
YKIHCSKWNKNNELNISHNIIKSLYDHNFYSPREIQSKTLKHSINEKKDIIVVSKTGTGK
TLTFCLPILSNILQNKLKEFHTKNKAGQKFRCLVLVPTRELAVQILNHFNYVNKYINIYI
ITIIGGLNINKQLRLISKKPEIIICTPGRLKYFLQLEDKINYLYNMKNVRYFVCDEVDKM
IETSFINDIHFISKHLYKCVDEKKKFIQTFLLSATLSLTVQLHNENLAKLLNYISIRKDK
SYIIDLTNEKDHNQNKIAISNNNDINYNKTTFLPDHLTLNIIKCEKKIILHKLYYLLKLY
ILKDLPTNKENQVKKIIIFLNTIKLVKDVSTIFKYLFFEPGLESSLPNKYKTNLFLSKKI
NIYSIHSKQKLKERIQSISKFTESNNSSILFCTDVMSRGIDLNKCDLIIQLNCPISDITF
IHRSGTARNLKSGK--

-------------MYDINSAKCAEIKSADL-VLSSQELKRISLGKYENQLQEFEKISKDG
TSNRITYDPRFGTIDYSQICSVCFEKHDGCLGHIGHIEFVLPVFNPLFYKDLQELLNLVC
YNCYGLCYSEDVIFLLKHIYQLKSLNEIESSNKFIIKKNNENEYVVSEIERLYNKICKES
NITIEDIVESICQDYENSGNSGNGNNENGSHEDNQTYEKKINDDMYSDDQKNQGDFSKKF
AKKENKETKEKIKFLKEKIKKYKFDISKLAFQSNYNYEEYIILNKAIKLHMKKGANCSNC
KFRRSITVKTSQKKDTINFIGTYTNSSFFKKYISKNNKNGSFEEYGDEDDDNLDDNKINY
DNAEVGKGNKRGKEIKSEKRKNLNNLENGVNQFLDGVYNKKGGEDKLFKDAVASYKRKAS
NEKCTVRLFSFQIIDLLKKIFVQDNKEIINLLYPFTKKDGYQVFFLYYMGISSNRFRTQS
RGIHKRTKITNYICKLNSFVKLCINSKKNIDFDYLIEYNKNELNLYNEMFLLFSLRIRQE
Q-SSSIDFNIEIAKSDFLRCYSLIYNNKIAYLNILFSNLQLTINTFLDSTLIDKRLQKNM
DNISIKDILDKKEGILRKNIMGKRVNNCARTVISPDTFIETNQIGVPIEFAKTLTIDEHI
TENNFEYIKKLIENGPDIYPGALSYKDSQGRVFKLSHSYENRMKLINDLTNLLRREKKET
LILHRHARDGDIVIMNRQPTLHKFSIMAHFLKIFEKEKVFRLNYVNCSSYNADFDGDEMN
LHLLQTPLARAEATHLMNSDFLFTSFKDGSPLRGLAQDFILGGLHLTSLETFLNYDEYCN
LLQCSLNGLLRQKNTFFFSKAKSRN-ISGSSNTKDGNMNSGDNDKSISNSFRGINAKISN
YSYIDPYASNLNRTNFTIKTEEPTILFPKKLWTGKQLISSILKTIINELAIETYNKHGDN
SFMENFKGINYVAKAKTDPELWSFDPIKECEIIIKNSELLQGVLDKLHFGASSNSFVHLC
YELFGPKSAAVMLDCFGRLFISFLQLRGTSLSLYDFILKKKAKDEKKKIKKRISFTGFYL
QNLFVYTIGKALNADMNKMDEYSNKKISNYEECQNIFSNKLYDIYLKKSQIINKYERKLG
KINIGDKHMNTKNISKKFLFNDEEFNLKKDLYFITFLNKEIEDIFIQNRILNGNVKNEQS
EETEKCEYSNFEKYVDENVYSEFVNNIVNKLYNSIKNPSFYKTTTFTSTQIVKVIEKIVN
FLKEAKCNKKLKVFILFELFQNENITKYFPSLIELANDFNTDISSEEIVENVINNILLKS
HNVEKNKFNDSKWEKTNDVILKRYLHLYSRLIKVE----KE---KKGKNDSTSKFKHDED
QNINETIIGNPDIIKNSLISSLPSFALNEDITNLSYNNRYQYYEKYLNIKDEKYSRFKFY
RIDDVFHKLDFLINNFFSLKRLNFDGLLDSLFQPYLCRVSSGTNNLITMENLMNKFLDNG
FSNMIFTGAKGSKVNYSMICGMLDQQYLEGKRVPRMRSGKTLPSFHKYDYGARSCGLITD
CFLEGLRPQEYFFHCMSGREGLIDTAVKTAKSGYIQRCLIKAMESIVLHYDGTVRNEDNS
IIQFLYGEDGIDPSKTAYLNIPSDLI-NYNISFSKYLNYGSDKLILNNQKLFIKNENNKK
NEDPLICQYNPYTYIGSISDNYNIKLNNILKKKKFNLTNYNE---NFDSLSSSLLRSKYF
HSLCSPGESIGILVAQSFGEPATQMTLNTFHLAGTENVTMGIPRLKEIFLTSKLTSKPMI
YVPIKLEEKYINNPNKIKNYITNIADKILSSYQSILLSEVIYGIGMDRKLILRDKIENKE
IHTIVLDNLGSKNNEENGKTTDYSKILQVIENTKLNFDLKKEWVHEIVIQFDNLNHFCKV
NKHLSIPFILYKMVNIVLVSILNKIQSSISHHNIKNIHFINHEHYDELFDFISGKMSEEF
SFNHEKYEYKNDEEEINAKLKYMSKNFEGNKSGDENDYERYNGS--LKVPKKYNEENDDN
FSIDNKNEDTENEKNNKSDGNNDSDQN-DGRESDEYKSEQEDKEYDESEDNDTDSILSQN
GPDSDDDIKHESMNSSESDYNKYDEKQKNKEKKKKKSEKKKNETDSESDSSSDQSITDSL
IIDEESKGSNKSKKEDKLNTSG-------------KKKKLYIKNEQHRKEVNSDYSMSSE
EAELKKEMNKKFSEYSYDYKNRINKKNLDSDYEDPSIGISGSKNKLNDQKYIKNLTDKIK
SSLLCFIKKIDFSPITWVLKFELSWDINFFPYPIDFLSCLQNEISKEVLFKVKDLNNPKI
LKGKDITHSGEEYELQIEGRNVYKLFNIKDKYIDKTKLYCNDIYTIVKTYGIEAGRLCIT
KELKKVFEAYGIKIDFRHLSFISDFMTHTGDLKSFNRYGINAYRNVFHKMSFECATSFMI
QGCIQNSIDYLSTPSSSLFFGKHIKVGTNVSNIVTCIKGKMAKNRLLIESREAKKQNDPD
IFLSHSDYNLYEWEAIIKGPKDSPYEGGKWKLSIKCKSTYPIDPPMITFITKVFHPNVNF
TTGALCMDILKTNWSPAWTIQSLCRAILFLLNEPNAESPLNCDAGNLIRFGDIKGFKSMA
KMYTKEYAM-------------MFALLRFAFDGGVKVKSAAEVGKRAVEYFRGDDFVNFL
SSKQDILKKKFPNLIGNRNLSEMKEIEEFADLFIQKGFIYKAQYNPFKGVYEMDENGVYK
RPKWPKRLIMTSKQNFDKAGFYILVYERNKKLQYFMLMTLISIVLICCMFPVWPLKLKLA
LWHLSVVFIGLLSAIIVGRIIAFVYFWFFGVDYWIFPNLFDEECSIVESFIPFHSWERRN
DSWLLVFARVVTAVLVAIGIHQLGKTHSISDIQNFAKQSFIDIIEWGNKKLSDTPENTFM
YKSIDSKATFEDQEDPEDQIVYDENEENYDCLKKCGFPPFEELVRRCFLKCDCMEVCRKT
IYYYSKITLANKMLF---------------ATLADTLLKDLEDLEEE---NFNNNNDGIK
KIKFEEQY-EEDDYEEIVDAIEEFLNDKKKKCERKISELLYDEDFLKTMNHIKNYIIEEE
AEKLKNEEGTKDTDGDENEDNIKRKKAYNSDDENNDNNPNDEEVLIEKCIELIIKIDTEI
LNIHKYLKDIYSTKFPELDSIVYTPLEYISVVSRIKNESDIKNIDFSDILPNTTVMAIVV
ASSMTTGIKLPDHLLKSCMSFCNEALELNENRQNILIYLENKMFLLAPNLTMLLGSALTA
RLISCVGSLKNLSVTSSQNLIVVGNSKKSILGLSNVNKTFGVGILSTSEIVQSVPDAYKK
KAINLLAGKCSLASRIDYFKKYKEGQYGLLLREYVINHLIKLQEPPPMKQKKILPIPDEK
KGRKRGGKRYRKLKEKTEITELRKQINRLPFGPNTNEDFYTFTDQNTALLNSNITKLKYQ
TKQKTNIPKKKNASAQSSGATGGLSSSLIFTPLHGIELFNPSINKTTSETRENKYFSSLS
QFRKVMEEYKEDNNEECKQKYPNFYLADLFYTLQLSNLSLNERNEALLKLFEEIKKNNMY
PYYSYVCGELNLDMDQEMYTTLKQKADEEMKEIENKIQEASENFDYVDTKNDMLLKANFY
CKIGDKENSFKEYEEVYEKGIGIGVKLDILLTIIRISIFFNDINSTKKYLEKARAQMEKG
GDWERKNKLKIYEALNYIMIRKFPEASKILIDAASTFTATEIISYDSIIFYVIILGIMTE
ERTVLDKNILNSSVILQVTSSDEDLYNYIYSFYHCDYRTFMEKTIKIALRVKKDRYLGRH
YRYFIRNTRVRAYKQFLEPFKSVTLKNMAYAFGVSEDFIE--------------------
--------------------------------------MVSINWPGLLKWSTKYSDGTVD
TNKRLSKEDIEFLQGAIKEALSQVEDPYEAIGEAVRNFESKDEGIILASAKIIERLVDEY
PEVAKNLDKINALDPLLKLLESNNNHILESVLQIFSLALSNNPVLQDCVFKKNGLKILLL
KLQESKQTTVDKKLITAISALIRHHDEGENKFIDYGGIAFLVYGMQTNIYKYQEKSALLL

KHLIHQNKITFETFEKNKVMNGLIALANNKNIDETGIQYGETTAELFLALMQNHRHKLAK
VGYLKQLKELIEGRLNYLTVIQDNASYDVSQEIDLFKDCLKLTK----------------
---SFEEL
GVDDWLIKISKSVQIIKPTKIQKLCLPLIIEGKNVIGSSETGTGKTICYCWGMLQELNKN
LYAIFGLILLPTRELVFQIVEQFQLYGNKIGVKILSCIGGFSLIDQRKSILSKPHIVVGT
PGRVSDILDDCIDVKNCFKRLKFLVLDEADLLLQKSFEEKLKIILNNIPKEFGEPRRTLF
FSSTITDSINLLIKTFPNDKLILVNANKKQKPLKNLDQRYIYIDSIAQMTYLVYILKNKL
TEMSGIIFTANSYKCELIYTVLNSLGSFSIESIHSSKDQRKRMSSLLKFKNGLCKILIAT
DIISRGIDIPKVAFVINFDFPNDTIQYIHRIGRTARANRKGLSISFVDKKDLKSFNNVKI
IMKKKLKPYILNKNEVLTDMLKIGKVVKKAEMMLQEKKDIKKENADLKNSIYLDEMARKY
FVSANWKCNGTEYSIKSLADSFNTVDFDPSKLDVVVFPVSVHYDLTRKLLNKKIGTGIQN
VSKFGNGSYTGEVSAEIAKNLNIEYVLIGHFERRKYFHETDEDVRQKLQQAIKNNLKAVV
CFGESLEQRESNKTIDVITKQVKAFVDLIENFDNVILAYEPIWAIGTGKTATPEQAQEVH
KEIRKIVKEICGEAQANKIRILYGGSVSVENCTSLIKQEDIDGFLVGTSSLKTSFTEIIK
SAM---
---------------------------------KDINKDVVHRYGPNTFKLHRLPVPKLR
QILRLVGTNGIGKSTALKILSSKLKPNLRKFDNSPEWRDILSFFRGSELQIFFTKLLEEQ
LSPIIKPQNVDLIPKQVKGNILEIINKKDKLNQKDKYIKVLELDHLLDRNVEDLSGGELQ
RFALLISIIGQTTNVYMFDEPSSYLDIKQRISMAKIIHGLVRHDNYIIVVEHDLSILDYL
SDYVCCLWGKAGAYGVVTSPFSVREGINVFLDGFIPTDNLRIREESLNFKLATDQDVTDE
DKKRLHFYTYPKIVKTLNSFTLTIDKGNFSESEIFVLLGQNGSGKSTFIRLFAGLIKPDN
VDSLSFLESLSVSYKPQQIQAKFTGTVRQLLMSKLKGLYTDPYFNNEIIKPLKIDGILDN
QVLTLSGGELQKVAIIITLAKNTNIYLI--------------------------------

----------------------MSILCTISGQTPDEPVVSKTGYIFEKRLIEKHIKNYGI
CPVSGEILTLEDLYPIKIEKFVKPRPITATSIPGLLSIFQTEWDSMISEMFSLRTHVNDV
RNQLSHCLYQYDAATRVIAKLLKEKNNCQEEINNLRNQILQLKNGNDIDDLEIGISEDLL
NEMQNIAKELLMNRKKRKVENVNSPNEWKKITSTNEFNIHSSIIPGVTCLSIDINKLKYN
YDDDHKNHNFFSGGKDGNIYYVSLNNNKIISKLQGHLKKVNSIISHPSNSICISGSNDKT
IRIWKGDPDTNEYVTSHIITKHKDKINSLSLHPLENYFISSSNDSIWILHDMETGKTIKT
CKSSPSPFKNLSIHPDGMMLGIGSEDSNIYIYDIKSQEYKASLTGHTKSIESISFSENGY
YLASISKDNTLKLWDLRKATSFQTIELENTPKHITFDYSGKYLSLSVGNDIQIFNFETKN
QANLITTLSSHTDIVTQTCFGSRTSYLLSSSMDKTVKLWSMSEEMHCYNGNIKDGLFHGY
GILIYSKNEKYEGDFAYGRREGKGKFTYADGATYEGEWMDDKIHGKGMAHFVSGNIYEGE
WENGKISGFGILNYNNGDKYEGEWSEGKMHGRGTYIYADGDIYVGEWKNDKRHGKGCVKY
KGSKDKIAETYEGDWYEGKMQGKGVYSFADGGIYEGDWVDGKMEGKGIYKYLNGNKYDGD
WSNDMKNGYGILTYANGEMYEGYWKDDKVHGKGTLTYSKGDKYIGDWEFAKKSGEGELIY
SSGDKFKGKWKNDKANGFGVL-YSNGNKYKGEWVNDQRHGFGVFTCKEDGTIYSGQFSYN
RKEGQGTLTFSNGTIVEGIWNSGVLIKVTKFQLYPSSPWNDPDL----------------

---------------------------EMETLYDLRNKMIEALQKENITAGDVICIDKST
GKITKIGKSFARSKDYDAMDPNTHFVQCPEGELQKRKEVVHTVTLHDIDAINSRTQGFLA
LFSGDTGEIKNEIREHIDMKINEWQEDEKAEIVPGVLFIDEVHMLDIECFSYLNRALESE
QSPIVIMATNRGITHIRGTDYKAPHGIPLDLLDRTLIIPTYPYMHEDIMKILEQRAEEED
VEIDEFAKELLCKIASESSLRYSLHLITLANLVAKKRKATEVTVQDVRRVYNLFIDVKRS
TQYLIEYQNEFMFSELPKDDESIKDQESVGEKRE-------------MAYLYSNLSDFWT
SDDEEDGDRTDEDFDEDSENNYKDTNKNYINNKNNELSNYDANKSYMNLKRGKMK-----
YTTLIFSETALGLVDLMKDCGNELFDNIPISSILQNYDINNFIYNDNLLKYCEAGVTINN
ESSCNEPLTLNEDFNFNCYSHFDYINPPNIKSSYTSTSNALISIENGENNFIDMCIQNKD
EL--NHKIKDISVKEVSNETVKEEIQED-KKIGNEIINET-MKKLEVVPEKQNVKYIRKK
WVIEDNESDVSNFSKNDLLLSYDFELDDFQKRSVKHINNFKHVFVAAHTSAGKTLIAEHA
IALSIKLNKKAIYTSPIKALSNQKYYEFKNIFKNVGIITGDVKMNVNANCLIMTTEILRN
LLYLNDNIINNIHCVIFDEVHYVNDEFRGVIWEESIIMLPPHVQIVLLSATVPNYLQFAD
WVGFTKQKEVIAISTKKRPVPLLHYIYAHDSLFLIMDEKNKFYSSAFKEIYIKIREKEES
GKKGKELMGSSHGGKKKIYYSDAKNNKDNQMEKQNKTGTANNSGDKQNNTVKGYYQYCKQ
KQKQRMFQNEANMKTEIQKLQALIKKLDEDNKLPVVLFCFSRIKCETYAKSMPHLNFLDN
KKKSKVHLFIKESASKLCDQDRELNQIKILSKLLENGIGVHHSGLLPILKEIVEILFSKG
LIKVLFATETFAMGINMPAKSVIFTSIYKHDHLKKRILTSSEYTQMSGRAGRRSSDSYGY
VYIYCSDNIPDQVQLTEMMMQKAVSLKSKFKVTYNMILKLLINKQINIEKMLFSSFLESC
RALQIPLFKKDLKRKKKILQNIKQVECVYTSDLNHIAPIENYVYIDHKLKNVGLNLHKKL
FDMKNSNAFVVGRIMLLNTIDLFRSSVYAVYLGCDKSSNKKNNKVNFAQNSIFFFNNKEE
NEIMERFFFLFILPDFIGYGDLTNNTNSTNTHKNGTNNSTTVYNANMNTEKTQLTSENIN
MYENYKNVFACTNNKKDINIIYHSTFDTDMNKKHFVVCSNVGIENISLITNTTIKIPNVS
TNALLNNPKNLLLYTLELDRLIKKETFEPFTLTKMLKSLKCEFYSVLINQSDYLESLKKS
LCYNCSLKDEHYELVCKKNDCINDIENIERNINAKSLNLYEDLEGKLNVLKHFSFIDDDN
NLTIKGKIASYITLTDEITLTQIIFENVLNNLNPPEIAAVLSCFVSPEKKVEESPDLTLN
LQDIKLALTNIHSKFEEFYRVIRLKISTEEHWKLCNFKLMFIAYKWALGVSFSELLEQSE
FEEGLIVRSIQRLDNLCRKVRIAFLYLGNADLAEKTEKASLLLRRDIVFTTSLYLQLLLT
AIHANFFSKGNKANYVSAYINSNNNNNDNKINIEDNNKINDTNTRQDQLNNFFTRRFYTF
HNKNQYLREEKRNSKEVNLTYNMESSNEQNNQMMGGDSYKERLQNLKKYMAEHNIDVYII
INSDAHNSEIINDQDKKIYYLTNYSGADGILILTKDAQIIY-NALYELQANKELDTKFFT
LKVGRITNRDEIFQTIADLKFNTIAFDGKNTSVSFYEKLKDKIKIQFPDKKIQEKFIYKN
SINQVVKDNNINLYVLESPLVTVPNSDVNKKPIFIYSREFGGSCAAQKIQESFDFFIENP
DVDSLLLSELDEIAYLLNLRGYDYKYSPLFYSYVYLKYNRDKGIIDDIILFTKVENVQKN
VLAHLERIHVKLMDYDSVVSYLTNNVSSKSENTKNNNGKNIILGSVHENRSPRYDISLSP
HINLMIYMLFNKEKVLLKKSPIADMKAVKNYVEIDSIKEAHVLDGLALLQFFHWCEEKRK
TKELFKETEISLRDKIDYFRSTKKNF-FPSFSTISAIGPNSAVIHYESTEDTNAKITPNI
YLLDSGGQYLHGTTDVTRTTHFGEPTPEEKKLYTLVLKGHLSLRKVIFASYTNSMALDFL
ARQPLFNNFLDYNHGTGHGVGTCLNVHEGGCSISPATGTPLKENMVLSNEPGYYWADHFG
IRIENMQYVVTKKQTNDAKFLTFNDLTLYPYEKKLLDYYLLTPEEIADINEYHQTIRNTL
LPRIKENPSDYAKGIDQYLMDITEPIHTK-------------------------------
-----------------MSTLNFDDNNLEIQKKHFVIQENPDFIKYRLDKFNELKEKKKK
ILEENDPNYYKEINIELLDGSIKIGQKNVTTPYQIASQISKKLSENSIVAKVIYLDNINL
NLCDIENDHFEEKDN-DKSQAGILWDMNVPLIGNCKIKFLGIETIEGKKVFWHSSAHILG
SSLEKLYGGYLTIGPALSEGFYYDIYLGNNSILSENYNKIENEYNKLVKENVEFEKMVCT
KEEVLELFKYNPFKIELIKSKINDNEKTSVYKCGDFIDLCLGPHIKNTGKVKAFKVLKNS
SAYWLGNKNNDSLQRVYGISFQKKTELTDYIKFIEEAKKRDHRNVGKNLNLFFFEKETSP
GSGFWFAHGAKIYNKLIEFMRKEYRIRKYEEVITPNIFSCDLWKTSGHYQNYKNCMFIFN
IENKEWGMKPMNCPGHCLIFKQLNASYKSLPIRLADFGVLHRNEITGSLSGLTRVRRFQQ
DDAHIFCSLDHIKTEVVNVLQFIFFVYNLFGFKYDLYLSTRPPKYIGDIDTWNFAEKSLK
EALELANVEWKLNEGDGAFYGPKIDIVLKDSLNRSHQCGTVQLDFQLPIRFNLQYKNKEY
GVGQETDSNLVNDSNIDPIEKKEDSTLENIDNN---EDAISQSTAILKKGFDRPIIIHRA
ILGSVERFVAILVEHTSGKFPFWLSPRQAIVLPISDKFNEYAKYIHNVLTNNLFDVDVDI
SVNTLNKKIREAQLKQYNFILVVGEKEISTNTVTVRDRDNPNDQKVYTIQELVNTFKKML
DINSIKLNEITPFIQQ---MNS
KEE----KGKKKKKNFFDLLFPKNKASCCGIMAYLGDGDASKILIDGIEILQNRGYDSCG
MSTIDKSNSLKTTKYASSSASNAIEKLRGNYMTSHKNDNIGIAHTRWATHGSKTDENAHP
HADYKERISLVHNGMIENYRELKKFLVQKNIPFKSNTDTEVVANLIGYFLDQKQSFQDAV
VSSIKQLEGTWSFCIIHKDFPDEMILAANGSPLHIGIKDNEMFVASEHSALFAFTNEYIS
LKNGEIMLINKNNINNLKMIKKFDNIPEIVIQKTPDPYPHWTIKEIHEQSISLSKSLNNG
GRFNLKNNIVKLGGLDPYVDELKNIENIILIGCGTSYYAALFCKYIMNYLHCFNTVQVMD
PSDFNISSIPKEKEGIIFISQSGETRDIIKACKLAEYFNLKKLSVINSVGSTIANMTGRG
VYLNAGREVGVASTKCFTSEVSVLTLIALWFFQNKQNLSSNNKVSSLINSLYRLPRYADT
TIKSCEDTCKALSHKLSN-KSMFIIGNGISYPIALEGALKIKEIAYIHCEGSTGNALKHG
PYALLGGNDNIPVIMLIFNDGTKNSMISIGEQIKSRGAHIICLTDDVNLCNHFADDIILI

PNNGLLTSLLAVIPLQMLAYYMSVNLGHNPDKPRSLAKTVTV-MH-----IFFLFLI---
---IQ-WDTAGQERFRTITSSY
YRGAQGIIIVYDVTDRDSFNNVKNWIIEIEKYASEDVQKILIGNKIDLKNDRSVSYEEGK
ELAESCNIQFLETSAKISHNVEQAFKTMAYEIKNKSQLENQQKGRTNINLNAKPIKDNKK
KCCMPHIRTMNSKKPPEGWNKVETFLNEMNQKMRSLENEDTSKKRKNEILWPIFQINHQT
ARYIYELYYKRKEISRELYDYLVREKYVDGALISKWRKQGYENLCCLKCIQVSDSNFNNA
CICRVPKSNIGNKIIQCVNCGCRGCASGDR------------------------------
---MCNDNKRNPINDNFNVN
KTNNSQTNNIAIKEENMDSSNYDYDYIVIGGGPGGMASAKEAASHGAKVLLFDFVKPSSQ
GTKWGIGGTCVNVGCVPKKLMHYAGNMGTLFKNDSDKYGWECNNLKHDWNKLVSTVQSHI
RSLNFSYMIGLK-SKVKYINGLAKLKNKNTVSYYLKGDSSKEDCVTGKYILIATGCRPNI
PDDVIGAKELSITSDDIFSLKNDPGKTLVVGASYVALECAGFLNSLGYDTTVSVRSIILR
GFDQQCANKIKLYMEEQGVTFMCGILPKKLTKENDKILVHFNNNTTELFDTVLYAIGRKG
DIDGLNLEKLNININSNNNKIITDEFSCTNIPNIFAVGDIAENVPELAPVAIKAGEILAR
RLFKNSNEIMKYNFIPTSIYTPIEYGSCGYSEEKAYELFGKNNIEIFLQEFNNLEISAVH
RTKHIKAQKDEYDVDISSTCLSKLVCLKNEDNRVVGFHYVGPNAGEVTQGMALALKLNAK
KSDFDNCIGIHPTDAESFMNLSITLSSGLSYAAKGGCGGGKCGMENEKHENMLMHIARDF
RSIDDLVDVFLSFLENKTDYFHLMMNDDDIQTLSKKYDGDIAKVILNNNTCGFKANSRET
ELMKLFRKHQLKYIIKNQPYIIENEDMRNKYLPPCDELNKLSNIQIAKAKDTQNAAQRNN
RSIHTPAYDSINEKHISTWNGGRTEKYFWNQSLNEINLEIPLNKEIKPSEIKVEITNKHI
KVQHLNEVKLEGMFYEEVNKQECMWNIEDKKKIIIFLEKKKENWWSYVIKGDPEIDTTKI
ESKKNLTDFDEKTQGEIRKMLYKQKMMNEGLKSPEELKEQFLLKNVLDNKGLPFPKMKSA
NAKEDTISQTCDNSTTEEVDEHLYAGPLKIEQLLAKGFVKRDLELLKEGGLQTVECVAYA
PMRTLCSIKGISEQKAEKLKKACKELCNSGFCNAIDYHDARQNLIKFTTGSKQLDALLKG
GIETGGITELFGEFRTGKSQLCHTLAITCQLPIEQSGGEGKCLWIDTEGTFRPERIVAIA
KRYGLHPTDCLNNIAYAKAYNCDHQTELLIDASAMMADTRFALLIVDSATALYRSEYTGR
GELANRQSHLCRFLRGLQRIADIYGVAVIITNQVVAKVDAMSMFGGHEKIPIGGNIIAHA
SQTRLYLRKGRGESRICKIYDSPVLPEGEAVFAITEGGIADYEEKMALKSINISGNFDWC
PFEEYKNYLICFNSHNLLYSNNGSLNNYTYLLDINLNSDIRSLDIVHKLNFEEALSRENN
KNKTSSNEYVTSFEWINCNNFVESENENELNKGIIIGGLTNGNIILLNAQNLFDTNVNYD
NFILSQSNIHESSITCLECNKHKNNLIATGGNDGQLFIIHIENIFSPTSYDPYLDKNNLQ
KITCLNWNKKVSHILATSSNNGNTIIWDLKIKKSAVSFRDPHSRTKTSSLCWLENQPTQI
LISYDDDKNPCLQLWDLRNSNYPIKEIIGHSKGINNICFSSIDSNLLLSSGKDVTKCWYL
NSNNFDIYNEVNNSGNNIYSKWSPFIPDMFASSTNMDTIQINSINNGSKMTSKYIPTFYK
KDASICFGFGGKICLFDNISDNSNLSASESASNKNANQNQNIADEASMQINKNRGGNSNN
PYLIKCHIYPTEVDLIEEADKFEKYIACGKYQEFCENKIAKCDDYHEKLTWKILQLLCTS
QKEEIVKHIGYDMNEINQKIVESIGEESGFIFKKYRCETNENMNNGGNITSNNLEIDSDM
NNKHDNTRMNNLNNGENISGYSNYGNNMHGEFVTSQMQENEPNFNEPFDLDPEKFFRELG
EKTEIEKKQENEEDKNGKEDLKSSVNNVDSNTKDMISTIKCQNPEIGFGEINEDSKPNSN
KTNSNNWNTGIESIIKECLLVGNIEAAVELCLYQNRMADALLLSSFGGENLWHKTKNIYI
KKQNDSFLRNINYVLDDKLEYLVKTIDLSSWDEALSILCTYAINNPNFNNLCEILAKRLQ
NEKFDVRSASICYLCASNFPETVEIWDSMPSSKSTLLNALQDIVEKITVLKMVIKYNKYN
STMNQKINQYAELLANSGRLKAAMTFLSFTENDNAIENLTLRDRIFNSAAHIMPPHIKPP
PSPFQYFDIKPFG---LSQKYNNSNNITNTSIHGIYNKSQIH--------------PNKS
LTSIVPPLPISEMHKQHGPSMVPFSHNSPTKFNTQMISGSSDIMGSINSTSNRGYPTSNF
NNNVKYPSNGMGSLYIPPPNIPS-HSTTPINASSPPHTSVQNLAHLSSHSNLKLEHEKLP
QGLNMFESQSYTNVNKAMQNSMAPP-----SNKIPNISRSSFSVSQNNIPPPRNRHTSVS
TSQNIPFIQESQLNKRESIDSQVYNPVSPSQISPSQIQ-QTQQQNSFTPPQPYSQNKVGF
TPPSGIKTTSPVAGAMSITPGMPVPWPIPTTTQQLGSTTQSTANENKKIQTVTKEQNGVL
MNRSNIENVKRVISNLLNMYTSQELVKKKADDISVKVHELFDKIDNGAFNEQINTIIINL
ANSINENDFKTANKNLMEVSRNLWDGNNKAW----------MAIRVQFENSNEVGVFSRL
TNSYGLIALGGSENFSSVFEAELSQHIPIVYATIGGTRVIGRVCVGNRKGLLVSSICTDQ
ELLHLRNSLPDNVKIKRIEERLSALGNCITCNDYVGLIHTDIDRETEEIVQDVLDIEVFR
TSIAGNLLVGTYSYFTNNGGLLHAMTTSQEIEELSELLQIPLITGTVNRGSDLIGSGLVA
NDWSAFCGMDTTAIELNIIEKIFKLNNIEDTNIEDTFKYKSSIVQTMIMLESLVEKLLNK
FLAPYVEGIEKNLHLGVWSGNIVLENLHLKPQITEILDLSFKIVHGSIGQINIQIPWSSL
GKNPVCVFIKNAHIYVKPRHYKKSESVLIDELRKAKMHRLELLEEEISLIKQQKNKEKPA
EKSTLIFKLLNKIINNIQIDIQDVFIHFEDIERNFFIGFILKSSSVKNLKKKDERSSETN
NDSESKKLNHIIEFKGLCIYSNSNTNKKKRENKKSKKKVKKENAVKKKNSKDITHETDRI
NNNPTNDYCK--KETQSDTEKKFNDKNNGEQNGGSASPINADDKNSSKQYNNDNIDYEYK
LNNDHFDNDEGNDYLKKTLANINLFYKSDDHKMLDYNNYLIKPFDLVLPVEQSSNKKELK
ARLEISDKWEGITLTRTQITKIIEIMSEANNSRNEINKLLLKHASTVKLDVESLRNETKN
EFINLYNRILGEEYNISKIELTKKEQNRLQILYDVVGVRHLAKWRLQCRDTLEKIIEEKN
CKKKILYDSIYKQQSWWSWVTGNKKDIENKVQSILKTEQDFINEHELNILQEAVVNEDNY
DVVIPTKYDFQFKLSNFSINIYDDSKKRKYIINNRINSKNDGKFNEQIKGASNKNKYNEN
IHMNSDCNNTANNLNNELCLSVTSESSLSTNSKISSLNNMHRNKKLNYNEINILSINFYQ
IYSSLSLQSVVDHNDNDNFQWKFIIELQNFVAKHRNQIFMEFRNTKNVFSHNMNNKENIY
QNPIYHSLLYSQSLCAYLEINHLVTEKGNTLSTLLRLNPLELYLSPLLINNILSFTFPLM
DIINNKDINSTRRYKEKVENALVKGEIGEDEEITEINDTENDEDNEEALLHIKKMEQSEL
IEGLKEKGENVYNRAVQHLPEMFEFYIHICGPILHFDNLTNGIVNLHLGNLI-KTEYPCA
YNKFNLIFEFNETQITCLKTSNFEDDKFYILQPIPVKVYVEYDLKILKTNIILDGIFFQI
NPDAVSIILAVPTSITRYLTRFYSKNKKEDESFKNKRKNNAEKKIDGKGDENVETNPQNA
NDKIKNNNVSTNLLDDNIKTQLEVCNIDKNKTEEESFLYDIDFLIKTSSFSIKNSKNCDI
LKYEACGISYKNYFQKKKKIIKIEIEQLWICDPSNKQPIFFTLAKNINSKDAYPFKYISA
YLSNSNKMLNESNFDTNEKYEHGDNNNTGHLFQSAQRLENGKIEDNDDDDFMDAIEEKQL
SINLQIIQKHNENNIAQIHVDFIVSDIELHWKYKTIKQIFKTMKEYKNILQYGIEKDMKY
IKNKLKNEKELKNYKMFISEHTLRSVQETLKNVKNSLNILDIKASTQNNQNNINENNTVE
KNETINQGSLQKYQQFERFPGENNKKLKDLNIFNEYNKEKNNVENENNDVILKNGLNGTL
PKLFGEENSYVKYFFNCYVKSASLSFWEKKKIFSKIQVSNIFYENNIYLNFDQKMFINIE
KGIISMNGKNIISNNINDYTYDLFISKKSKDNIGYNDDEYVANLPDEIKRKETMTNKKKQ
FLFVGNINVYNDKRYYNICMMCDISGIYYIFYLRDLRLFLEYLDDGILNVFISKSYKKVV
QVAQEKYFLFQFTIVDPIVIIPEDKNIIYNYKGQVKDTSKYDKDTKNNENNNTDNENNDN
RGSNNKCIPYIESYLKFHLSMLKFKNAYTKNCAEEIVLRQRNEIKQRRMRKEKKRTKYKL
KKKKVIKNSNDQKVISKGDNENFDSNKKYDFTLYVDLLDIESMACENGGSKNIEGNILHK
IDIGLCLLSRKDEIFIFFNGKDLCLDLTVYQLRFLLDIINENFCYKSYFPMCFICDKNID
IDNMLDAELFRKGNIALNNLSGNNSNFNTKTDGIRKNGTKFYAYINLESLKVKTSFDNNI
PVAIITFQNISISFELILLDFYYIYFFNLYSTSLYIDDVRNGSVNYYKRVAYCCIENEMN
KKRINRTTKLRERSSDFVSHTARADRDHNNSSSINGQSNRIICNGANQNLDEIYEINYKS
LLNYVFDNNVFLNERKKNIKQKGIKIKINSFIEDLLLSIELDDAYICCFFVIFIDIYKFL
STGFNLSTLHLYPKPSPYIVSNKYYNNKNSNINNSIDNGMEKLEPENKEKKKPKNEKENI
NSTKILNKESYIKELNINELLKMDNKPIHIDFKVNNGNFILFTNLENVNHPIIMWSNNFV
FSFSLSNGCILFRKIYAIDSKIKRINYVNNSTNTISNYSSINNNNKMDSINVSNLLHNYH
KKNILLCENLNVIGEAIYESVDTKKENYIIKKNIKKKDHPQFISVFEIDLNISNFDIRLS
NDDVEILLKASSTLFGDVPSAYTNIAIGTVTPPIFENNSIVPGKEKNEGEYDSSAYSNSI
DSNLWYFLDTNTTKTNTYKDIKINIKLNNVKCTFINDVKNAIVPIIRMTFSMDIKLRFYT
DECSYIINCLNSNIEYFNNCIGEWEPFLEKCNISLDVHKVLPSDIYSEDIESIRTPISII
KINSAKALWFNITPQLVNLLFMFVPVFWEKVLMG--SKKNESIMENNEIEEASKKNYDKG
TYDRDLERLLYNQDDKNENKKSKSIYEDSSSIFDVIPDGADENKSMDFHLDSFLKKEETG
EAIYPIEDNSIIYYVNLTSDYYYAFVMPDRSSEDMKYDCGRTTIRNGHELNTRETLDTHK
GKNRDSYDKMNVDFENGEKRGVLPEVYTKIITTNELISLDELLVNEIGNNTLIEKKDLYL
YLIPIPPTNIVNTLHEMFINISKRDIVLDLMITKNTQKTIENLLSYNNLRNSNANIENRG
CKGKGAYDIYYFNKANKFVGPFLKNSECVMINLIKNSCTSLNTALKNNNVIHVISPSDYV
FNEIGENNNSSNNINSKNKEAILNKEYIKLEQLNEEINKQTNLAYKYRSKNKTICEIISP
MPNYKILFMSSTVRIINKCGIPLEFSFFDTLKTPILLTSLKNRTIDSSILYPNHSDSFKN
YKVSNSLNINPNIKIIQKNTNLSYTVILNHGYLLSPPECVFYGQSQVYLSFKPVNLVREK
CNIDDTNENTKRSSCTASNDCSYINIDNGWSDIFSSDISQGTYVKKCKVVDNNSYLYFLV

```
KIEHKISALPAEKNIRIITIYPHVSIVNTIPALVDIIMTSENNERVYIHEYNRLEKEIGN
IDHEIKILKKSIFYIYEIKKYTCLNLKMKIGNSQCEWSDRIHLDEDDDETMARLSLNFKK
FASIEVEIIKNFNGYFNSLNSILGNKNLIFSLPRCFIDRTGLGIKAINSNKYYPIINGIT
LLGDNSQIDLLLPHKNNSIDGINNGKKTKQRNNFDDIFNDFNYDNSLILFNANLPPIGSY
TETNVVCNNFYYTFCLNTEKIKTTNIPYIISRIITVVPQFIISNRLHYPLLIKQFQLDQI
QGVRENDTSPLYFIKKHNVLLFQFKMFEMGKKNKNINYIESNTYSNSGAGSYDNNKNEMN
KSSKYSKNMFWSSVIFPSENFVGSNYMVLNNGKDE-SDVYVITCIPNMGTKNIIIEKLEK
KINKGYIAYNNSSLVKYLKIRTFHDDAHNLKIGKNGKHLDKNLLLNNFLKASDMEHYFNV
EYNKYSFLGWVNPFIYISRNIQIEIVLHDMNIIPRSPLILKFASFNYSQKTYNINYFNIT
FIISIEYIEDLISIKLTHRINSPKGTDKVLAISGFNCVVDGNDRDENDEKKYSTKSSEIK
SYNKIESIDKYEIMNDNIGTQGIGNVANSNKIYIEKKVIEQNEYNYVMNISERGRSRYSV
ENNNNSFYSNRKELIKVINNTYKNVHVILNLTQIGASIISNILKEEVFFIELSKLCALFY
MKNEEEVIDI--------------------------------------------------
------------------------------------------------------------
------------------------------------------------------------
------------------------------------------------------------
------------------------------------------------------------
------------------------------------------------------------
------------------------------------------------------------
------------------------------------------------------------
---------------------------------MNNAEHNGIASKMDSSKIDNIDIDENI
FLNRKSFPVNEAYEVDQNKFGGNNQIKDIKEYKKMYEESIKNPELFWGDMAKNNLRWSKL
FTKTYIGNFNKGNVSWFINGKINACDNCVDKWVEKHPNKTAIIWEKDTPNDSKKISYQKL
LEKVCKIANLLKMYGVKKQDCVTIYLPMIPELIYSMLACVRIGAIHNVVFAGYSTRSLSE
RIVSSGSTILITSDFGLRGGKLTKLKNIADGAMDISGIIKTCIVFKNKTKINDHNNNILN
SKSNNMYNTSNICSQGSSHFLQYTVGNATHKNNNDPSPNVYDSVLREKDVQTLNKLSKNI
NTNKKNQNKENGYKELYNNKLTENGTQMNGHISYDNNMINCNQVNVFEHVHNGHANYEQT
CKSKYDFDENICTLKEGRDINGSILMKNMRPYCPIEYVDSEDFLCILYTSGSTGKPKGVT
HTTAGYLLYAFATFKYIFDIKDDDIFGCVADIGWVTGHTYVVYGPLLNGITTTLFSSIPT
YPDCSRYWNLIQTHKITQFYTAPTALRTLMKHGDYYIKNYDLSSCRILGSVGEPINPETW
RWYYNVVGKKKCVIVDTYWQTETGGIVIAPIPHLFKMKPGSASLPFFGIQLEILNSKTLE
PLNGPNVCGLLCIKGSWPGMLRTVYGNHNRLVKTYFETCPNYYFTGDGAYRDEDGYYWIS
GRIDDTLNVSGHRLGAAEIEHALVQHPCISESAVVSFSHKVKGEGILCFVVKKLTSFQKY
SECNSNNNNDELNNRSLLSSNDNIVQNYTHENYTDEKLIEELKLYVRKVIGPIATPDIIC
IVPDLPKTRSGKIIRRILRAIAIGLNDYGDISTVSNYDVIEIIKNKFMESKEKRYAS---
------------------------------------------------------------
-----GTGEQLAKELCHNLQEIFDIKADIVDLEYFNKEEIKTLGIRIFIVSTYGNGDPPD
NAIEFFKWLKELDINNTYFRNTKYSIMGLGSKQYSHFNKIAKKLTAYLKNFKAEQISETI
YGDDDDNIYHDFEIWKNNFFKELSKILNMSHIPINFIKEEVIKLVDWKNIPDIKFDLKFE
NI-EDDKKSETKCHASNLLKDNTNQEAQFNKHVSTSLTGKFYFNHNTGTVISNTNLLKNV
DDSTNSDKVNHIIISAKNIKYKSADTLVVLTKNSKQITDWWLKRLNINEIDKNKKFIFVE
RNDQNSQKYDNNDNNNNNNNNNSMHIPFPTPCTIEEALQCYCDLSTIPRVNVLKNFKCFI
KDIEELKMFNYILSNNKRNTFFNICKEFEMTFIEFVDIFMQSAIFELTPFLQLIPKISPK
SYTISSSPKDDPDMITLTVKKKQYPIHSLRKALKSFKNNNMLPNITEKKLRNLCERRWYK
GSSSYYLTEELYPNDIVKFNVKTSIFTLPENLGDASIIMIATGTGIAPFKAFLTEFKLFD
QKREKNEILKKSQRILFYGCRKKGIDFLYEKEIMDSLENKYIDEIYLAFSRDQSNKIYVQ
DLIREQKELVCNLIQKGAYVYICGNTEMGKDVKQTIVNLYENNKQNDKKIIKKLKKSGRF
FEEIWMSNLPWVEKYRPKKLDDIVHQTNAISMLKEVIKTKNMPHLIFHGPPGTGKTSAIN
ALAHELFGKENINERVLELNASDDRGINVVREKIKAYTRISISKNKINTETNEQLPPWKL
VVLDEADMMTEDAQSALRRIIEIYSNVTRFILICNYIHKISDPIYSRCSCYRFQGIPINI
KKEKLLYICKNENIDISDNALSKIIETTQGDLRRAVSVLQLCACIDSKITVDSVLDVSGL
PGNDVILKIVNSCKMKNFKILEKTIQDIIEDGFDVSYIFKALNEYFVMSQDINDSIKYQI
LMELSRHDFRLHNGATKYIQLMSFASSVHSLLNQAMSEGLDVDNAKEKINFMFTYWKNIN
NKDFENSNAFCILSGKSSKDDNATIQEQFQMWLLGYQLTETFFLFCKKEKLIILTSDKKK
KFLQPLLDKMNNITILERNNDNSENFEKIKNEINMFNDKELLILKDKDSTGSFFEACYDF
IKNLNKTEIDVNNNIKSLLNLRSKSDVKLQKSASDIASIIMKNVLITTIENSLDSEEYES
HNKIKEKVLKFNENKKCVVKIKDKLKADIDDIDVIYSSVQSGNNFLLNFKNTNDNNYLSQ
NDGTIVIGIGVKYKELCANINRTLLLNAKEYHKELYNFTFSIQKYIINDCLKYNTSFSDV
YKKAMQYIKDNKRNYQTIGNINLENYFIKCLGHVIGFEFMEKEFLITVNNSNATIEKNTS
YNISVGFENVQMPDKNVFSTWISDTVFVNDKDEITILTDAISKEINTISYELEDSGSENE
EENSVKKEKKKNEDNENKKKIGISASILNNASSVIVSDRLRRRNKNSLAHNNEQEIEELN
KRQNELKNKKIEEIKNRFSEGTNEYKDLNKKNIKKLEDIKSYNDADLLPRDLRSNIIHVD
NKHESILLPVNGAHIPFHVSTIKNLSSNYEDNNDIFVLRINFQVPGNQGSQKGEFNSFPK
LNEKEMYIKELIFKSSDEKHLQILVKQVKELIKQVKQKEVEADVNDSKTSNEKLALNKTG
RRIVLRDLMTRPNIFTGRKILGTLELHTNGLRYSANSRGTTEYIDILFDDIKHAFYQPCD
GQLIILIHFHLKRYIMVGKKKTLDVQFYCEVGTQIDDLDRAKARNVYDPDEMHDEMKERE
QKNKLNLIFKNFVQQMQDISKIEFEIPYPELTFSGVPNKSNVEIFVTANTINHLIEWPPF
ILSVEDIEIASLERVHHGLRNFDMIFVFKDYTKPVKRIDVIPVEYIDTIKKWLTTIDIVY
YEGKNNLQWGNILKTILADIESFVNSKGFDGFLGEDDDEEEQSAEDEDEDDEYEVDESEM
SAEEDSEYEDSEEESLATESDGDEEVEEDSDDEGLSWDELEERAKKGKPQYIQ-------
-------ISLLYKKMPKHIKGDNVEANATQIRINGVNKRRHTNSVITSKYTIFNFIFLNA
YEQFHKISNIYFFIIGVLQLVPELTATNRIPTILFPLSIVLIANAINDAYEDWNRHRTDK
IENNRVCYVISDGDLSQPNNSNNVFIKFIRYLKKILLKKSKKIDIIESYYDDEEDIDEMA
DTNYFMNSYDDKLENIDGTIQKRWKDIEVGDIIICRRSDFFCADILLLSSSDPNGICFAE
TSSLDGETNLKVKEVNKYIFNNLTYNMDEAIEKAKKLRGYILSEKPNKNLSTMNGAIYLE
SDINTDVTTNKEIKKSISEGSIHRDEIENSKDINNNFIENKSNDNLYSYKHDDKYIKLPF
DEKNFVLRGCKLKNIDWVIGMAIYIGKETKIQMNSLKPVIKYSKLEILTNKLTIIIWLIQ
VFMCIISAYYSAVIVSFSKKSKFKYLPFNLVEPKAPIISGIVSFFSWIVITANFIPICLI
VTMSFVKVVQAYFISCDNNMVYKVEENIPSFGESIQKSMKKKKFSIEVDEMHNSNEDKSM
VDTNKKKGTNPSDSTKIILNSEYENSDIFKKRSTRMRTFKDSKEKSYIYFNAIPRTSSLI
EELGQIEYIFSDKTGTLTCNVMEFRKCAINGISYGTGLTEIKRKILKKNNIPIPQEPVFD
PKNKTPNVNIIDKQLVDQLKDVNHFNHASLIYFFLHLSINHCVMCDT-SDNVNTYSSSSP
DEEALVYAAKHFGITFLYRKDGKCGIKIFDKVYEIDILATVEFTSKRKMSTVVCRIPIIS
NESTEPSSINNNAMPQ-------QGGNGMGDNPVNLEKELNSAIDSAGNSDMNNENNKIE
KKNKHTGTNNNVISCENTKKHKIVVFCKGAGCVIIKKLAKKTDVDDLTIEHMETYADEGL
RTLCIAYKELSQEEFAIWYNSYKEASLSLNCREQNIEKIAENIEKDLILQGVTGIEDKLQ
EGVGSTIEDLRLSGIHVWMLTGDKIETAINIGIATNLIDNGSEQFIYTEELALSEDLLMK
KLDEDIIYVEKSLSLLHFNFDT--NKVEESFFKKIISYKNTDSLALDHDTYKEDMTLNNN
VLIVDGSVLDILLSKPFERKFFYLADKCSSVICGRVSPYQKGSIVSSANRLLKKNTLAIG
DGANDCNMIKMANIGVGIRGQEGVQAFNSSDYGISQFRFLRNLILVHGRLSYRRISKLVV
YMFYKNIVFIFPLFIYGSISLYSGQKIYYEFLLHLYNVMFTSLPIVILAILDKDVSLNTA
LKNPCLYKLGIHNFYFNINKFISWVLNSLFHGLLVFIIPLYFLAYYNIPSSTGEPFDIWS
IGCVTY-LTVFIVNIKILLETYYLNTSPIVAVSMSVISFIIMSIAFSFIGIGNKSFLGVA
ILLAKSLRFWLVLLLVLF-ALTRDYVYKVYKKNFYPEAYHLLQDEENI-SNTNHIQHSSK
CSNNMDETKSSKSEFMGYAFSEADPACVHFIRKQDKLIMNRRLLNQCFQ-RSRRCSYMNV
LKINNTNNGYHLHCRNYSDNVKDHFNKPRNVGSFDKNEKNVGTSIVGKASCGDVIKLQLK
IEDNVIKDARFMAFGCGSAIASSSYATELIKGKTIDEALKIKNNDIASHLNLPPVKIHCS
LLAEDAIKHAIKNYREKVIN-MILNVKNPKIKGVFRVVSSLCNSNKLKNVNYINKKREKV
VILGSGWGGIHFLLNIDFQKYDVTLISPRNYFTFTPLLPCLCSGTLNVDACSENVETLLK
KNKISGKYLKLECIDIVYKDKYIKCKDNSNNEIKIYYDYLVISVGAKTNSFNIKGVDKYA
FYIKDIIDALKIRRKFISNLETCTNNNINDDLAKNMLHVVIVGGGPTGVEVAAELADFVN
KDIKNKYKEIYKYISISIIEGGNNLLPTFTQNISNFTKDTFKKLNINVYTNYYVIEIDEN
NFYIKSSINKNEEYQKIPYGIIIWASGLAQIPLINNFIKKIPEQVNNRILNVNQYLRVIG
IQSNNIYAIGDCKQINPIKSHEHVNEIINCLGNSKITSDMLNKNQ--------------
------------------------------------------------------------
------------------------------------------------------------
```

```
----------M-------QVKNKNAADVQITAEQLIKEALDFEEVEKKVNYNLIDEDELN
EYKISKRKEFEDSIRKRRYLINTYIKYALWEIKQKDIKRCRSVFERALNIDYTNKNLWLK
YIEVELTNKNINSARNLLERVVLLLPLENIFWKKYAHLEEILNNFVNARNIYERWVKWKI
DETAFLCYINFEERCKEINKCREIFEQLIVNIPKLECFYRFIKFEKKYKNISRARACYEK
CIELLPSQFLDQHFYIHFSKFEEENNEYERCRKIYIEALKRLPRENSDILYKNFLQFQKK
YSEKEELDQTLLYNERIHFEEALKKTPNDYDIWFNYIKLEEQNINLINKEKSIIRIRELY
ERAISIIPQIFTKKYWKRYIYLWINYSVFEELYADNIDRARQVYSNIFKILSKQNFTFKK
MYILYANFEIRQMGIDKARAIFNHAIENVKNEKIFQEYCDMELRLGNVKECRTIYSKYVE
AFPFNSKAWIAMINFELSLDEIERARQIAEIAIHIDDMKLPELIWKAYIDLEINLQEYEN
ASKLYERLLNITQHYKVYKSYAEFQYVYLDNINKCREILENGIEFCKKNELTNERSILLS
FLYEIEKDHGDNEIIEKTLERLPKKVKKKKIIKSNDDEVVEEFITYVFLDDKTQSQNMKI
LQKAMEWKKKMEQHEQKEMSQEIMADAESVDNNVVIEEKAVFDNVTAIQKVIKNALVHDG
LKIGIREVIKSIESKEAKVCFLSNVCSEPAYKKLVTALCAEKQIPLFMIDNDSKDLGQWS
GLFKVDKEGNARKIIGASSVAIIDFGEESAERDFLMSQKPTATAAMPKDKRGRIFFNSHG
SDNERDNSKRSKSDHKSNYSEISPHNGSNKKKNYEKEKSKDKLKNDNTKTCKENINSFSS
PNSTSSISDLNNLDFDLSNGSSSNSENEFKILKEKENEDKFLEERRKKREAIKERLKNMM
SENNDSNKETDVSTNKHNVSNNHDKEAEISIHSNAKINDKCENTFTTCKKNDMPESLSRI
PSI-------EAACIFAPNNEVIEETCSSLSSDHEIIDDKVPNEKNETMKEYNDLYSDLK
KKINEEKIKIRNFIIKQKELHERNKDSAYINKKKEIESIDFQEQEDGDNDDVDMFSSEQT
HKKRAIENIRITDYYSANNANLSDNWNDSEGYYKAIVGEVIDNRYSVVCELVGKGVFSNV
LKCYDMTNKIHVAIKVIRDNHMMHKAAEKEISILKKLNDYDKDNKKHIIRLLRSVKYKNH
LCLIFEWMWGNLRIALKKYGNGHGLNAAAVHCYTKQLFIALRHMRKCRIMHADLKPDNIL
INEKFNALKVCDLGSASDISENEITSYLVSRFYRAPEIILGFRYDSQIDVWSAAATVFEL
ATGKILFPGKSNNHMIKLMMEYKGKFSHKMIKGGQFYSQHFNDNLDFIYVDRDHYTKKEV
VRIISDLRPTKNITCDLLEHQYWLKGNSPKMQFLKKKIKQLGDLLEKCLMLDPSKRYTPD
QALQHPYLRESIHYSKMPNDMSSDVSSISCSLSLDSDSCEDEYYETNLNKLIENTSLNWI
FVGGKGGVGKTTTSCSIAIQLAKKRESVLLLSTDPAHNTSDAFNQKFTNKPTLINSFDNL
YCMEIDTTFSEDTAFKINKSDFFNSIIPELLQSFPGIDEALCFAELMQSIKNMKYSVIVF
DTAPTGHTLRLLAFPDLLKKALGYLINLKEKLKGTLSMLQSLTNNEMEFEGMYDKINHLN
TMSISIQENFQNPLKTTFVCVCIPEFLSVYETERLIQELTKKNISCYNIVVNQVVFPLTS
PDVNIEKCEKLLKQIKDTNIQNSFNSLILKAKELEDVYISRRKLQSKYLTQIKNLYGNYF
HIVCMPQLKTEIRGLDKISNFSEMLLQSKDIPIYSMKNGIVLSGDYIKLNNDQIKKVNKS
NFEQIYDKEYEDLYRSKCSGIMLKTPYYPYKYDIMNTSHKYIPKIGDLVIGIVKSKKLDY
YQMDINSNCECIIHKIESFKYASKSSFPNLLNGTLLYMIIEKINLDNNMVVASCINSADV
KSWINYENYLGELIDGFLFAVNISYAKSLIGDKCYILDLIGKDVPYEIAIGHNGWVWIKT
NDPQETNMIRSALKHCYGKTNIQMAVLWKSIYNLYKKGN---------------------
----------------------------------------LFKDIIKIYLSKIPKGFERC
EKNNSNKN-NGNINNAQQEEEKPKKFDNNFIYLFILLLLCFLLFVDSNSLYNEITQNDFF
YNYLSKGYVDKIKIINKDYVKAYLNSHGINKYHLKYVSFRVGNSDSFERKVELIQKEMNI
KLDEIIEVQYVNEGNLIGEIKSYIPSILFFLFLIFIFQKITLKNVANSGMDKLFKFNKIN
PINKNNYKTDIKFSSVAGMKQAKEEIMEFVDFLKNPAKYQVLGAKIPKGALLCGAPGTGK
TLLAKAVAGEANVPFFNISGSDFIEVFVGIGPSRVRELFSQARKHAPSIIFIDEIDAVGR
KRSKGGFAGGGNDERENTLNQMLVEMDGFHTSNDQVVVLAGTNRIDI-DPAITRPGRFDR
IVNINKPDINERSEIFQVHLKNLKLHDSLDIKNISYILASLTPGFVGADIANVVNEGAIQ
CARRSHIQGVQIKDFELAIERVLGGLAKSTSLISPLEKKTISYHETGHALIGWFLEFADP
VLKVSIIPRSNGALGYSQHLSEEIMLFSKEAIHDKIAVILGGRAAEELFIGKITTGAIDD
LNKVTQLAYSYVSQYGMNKEIGLVSFQQNGSGEYAFYRPHSECLAHLIDNEARNLIESQY
NRVKAILKKNEKHVHKLANLLYEKETISYHDIVKCVGERPYPIKSNYEKFVKANPYKMNL
STSMEEKTGEQNSRTQMDDSESVQKNEPQKDNNNVSDNGNDNDLKKNNEKGVNGASPVTQ
NIGNRNKKDEKISNFNIRMNSKNETREEKKKEKELNEARKAGKIEALKDEDGNDINPHMP
QYILKAPWYLNQTQPGLKHQRYREADKVKIEEERNRKIYVKNTKNKQNFCKNCGSAAHTE
KYCLERTRKKKKNFMNKENDQDYLCVTQDLGYDGNRDRWVGYDPNNFDHIYREYEKIVDE
QKKRKAEKLKKKYEKQSIKKKK---EENEEEEEEAENSELSSDSEKGNDLNDNKQNNKKN
NKNEKNKTIARNLRIREDTAKYLYNLNLNSAFYDPKSRSMREDPLANIKNNLENSNYYKG
ENYYNNTGEAIESKKLEIFAWESYKRGENVHFNAQPTQLELMYKEFLEKKNKLIKKKQED
VLKTYKCENITKEIHNEQELIHSEVYTEYKPVDQIDKKNNKIKILSKYEEDIYISDHTSV
FGSYYDRETKKWGYKCCKCTDKFQNCFQLMGTKKNEPNKKDTKKKNIKYEINNTVNDIKY
VREIDPLLNEDDSENVFLNNNNININTLKNEIVKNELDNNNIDDVSLGLIKDKSIKNREN
EFQKKKYDYILSPERADPFEEKSPSPGERTYTDIMLDINKKNIKNKILNNNKKEVSTSDI
HQEEKKKMKLGNENGDGIKSKWDFINENQDVDFGNMATPAPNKWTETPFILNDGNIKNKK
KKMSRWDKTVENSSNNNGDSKIINSDILKTPIISGNKYNENMIINTPILGTNVMTPITPY
SGSIQNNDYIKFKIKNEMDFRNRPLTDEDLDNLLPSEGYEIVKPPEEYETIRKNKLKILF
KNMKDTTTTPLIQGNTNQSTLDNTFIDENGKSTFMSHTPFYNLPNADNTLKEEDEKILRQ
NKIMEITNPQLLNELKYIEIKNEDYIYFNKLFQNYNEDDLSQDEIKERKIMLLLLKIKNG
TPSIRRNALRTITDKVKELGPENLFNLILPMMMQNTLEDQERHLLVKVIDRILFKLDDLV
RPYVHKILVVIEPLLIDEDYYARVEGREIISNLAKAAGLATMIGIMRPDIDHPDEYVRNT
TARAFAVVASALGIPSLILFLKAVCQSKKSWEARHTGIKIVQQMAILTGCAILPHLKQLV
NIIAHGLHDEHQKIRTITALALAALAEAATPYGIEAFDPVLRPLWKGITEYRGKCLASFL
KAIGLIIPLMDGYHANYYTKEVMIILINEFNSPDEEMKKIVLKCVKQCIQTEGIEKDYIN
QEIVNPFFEQFWIIRNSNDKKNFNLIVDTTVEIANKIGGAVVIAKIVDDLKDPSESYRKM
VMQTIQNIINNLGVDDIDQKLEEQLIDGILYSFQEQTSDDYYVLLNAFDVIVNKLKLRMK
PYLPQIAGIIRWRLNTPLPKVRQQSAELIARISKLIKICDEQQMLGHLALYLYEYLGEEY
PEVLGNILKALKSIVIVLGVNNMTPPIKDLLPRITPILKNRHEKVQENVIDLIGIIADKG
GDMVSPKEWDRICFDLIELLKSNKKLIRRATIQTFGYIARTIGPFEVLTVLLNNLRVQER
QLRVCTTVAIAIVADTCLPYSVLAALMNEYKTQDLNVQNGVLKALSFMFEYIGEIAKDYV
YSVVSLLEHALTDRDLVHR-IATWACKHLALGCFGLNREDALIHLLNHVWPNIFETSPHL
IQAVIDSIDGFRVALGPAIIFQYLVQGIFHPSKKVREIYWKIYNNVYIGHQDSLVPIYPP
FETIGDSNFARDELRYVL------------------------------------------
------------------------------------------------------------
------------------------------------------------------------
------------------------------------------------------------
------------------------------------------------------------
------------------------------------------------------------
-------------------------------------ICVSTYLTKMESTKYDKHQEMNK
RKNALTKIDKIKLDHERRIEGTKKQVSILKKKISLIQLNDESVGEAIKLMRSAISTSANW
EQIWDHIKLFKKRDHPIALKIMSVNFNNCEMELLL---NDDDIEENGDDNNLKNNSWEKI
ADKNSKTC------T------------------LRKKAEEKIRKIKMSTNMAVKKVEKKK
KDKDTKQKGKNKSVFQIKKLRKVFWFEKFNWFISSENYLVISGKDSLQNEILFRRYFQNN
DIYVHADVHGAATCIIKNPYKDISIPEKTLFEAGQLAMCRSSSWNNKIITSAWWVYYHQV
SKTAPTGEYIKTGSFVIRGKKNYLPYAKLEMGLCIIFQVNKQMDDNNKENALNGDKQN--
---------YESINSGDENGENVGKTSTGGDATNCVNYMNDSNNNFQDSKMINESLSNMK
NCVSIFLRGTCKGNKKYLKNKYIKLILKCLNRVDSINIYKKNMISKFGICDNYRNEIISM
LCKLCFSKLCINMKNFLHKIKNRECIQCYICRSFHKTCKIIKQLFLYFNSSNTFLGFFFL
KSFDRAIDSIFFYFIHKTYLLKNYDKKAVECCKDELNRFVGANKNNNFIKNLNRDTLMYV
MRNQINENIKRNLMMENDKTMKVCFKACEVYGLPVAFKDDDINDEDSKNSKNTGFSNKMV
LNSSDNEKLNG----EEKRHISFE--NVENSIKTERPTRTRKATGFVKMDVKKLLEEIGN
EEEEEDN----TREDIKVSFDEEKKKTVEKKGVAFNFENNNVYDNKNCMVNISRPARTRK
PTGFVKADLTKLLEEIDNEEQREEKSEAEGISQKDVLNSNS-STTNIRKKSVSFSSEDEN
IYIERPITSTIPRPVRTRKPTGFVKMDISKLLEEIYNEEESCENEKGIGE---NGDEIKK
VKFSD-V------EKNMNAE-NDKTISGPENPM--KFSRPFRSRKPTGFVKMNVDDLLKD
IEMDETEEDRNS------------------------------------------------
------------------------------------------------------------
------------------------------------------------------------
------------------------------------------------------------
------------------------------------------------------------
------------------------------------------------MGRMYGKGKGIS
```

```
SSTIPYKRKQPSWLKQKPSEIEDAIIKLAKKGQTPSQIGATLRDNYGIPQVKAVTGNKIL
RILRAHGVATTIPEDLYFLIKKAVSMRKHLEKNKKDKDCKFRLILTESKIHRISRYYKRK
RLLPSNWKYQSSTLVLLL-MSNRKKVAYFHDPDIGSYYYGAGHPMKPQRIRMTHSLIVSY
NLYKYMEVYRPHKSDVNELTLFHDYEYVDFLSSISMENYRDFTYQLKRFNVGEATDCPVF
DGLFQFQQSCAGASIDGAAKLNHHCADICVNWSGGLHHAKMSEASGFCYINDIVLGILEL
LKYHARVMYIDIDVHHGDGVEEAFYVTHRVMTVSFHKFGDYFPGTGDITDVGVNHGKYYS
VNVPLNDGITDEAFVDLFKVVIDKCVQSYKPGAIILQCGADSLTGDRLGRFNLTIKGHAR
CVEHVRSYNLPLLVLGGGGYTIRNVSRCWAYETGVVLNKHHEMSDQISLNDYYDYYAPDF
QLHLQPSSIPNYNSPEHLSKIKMKITENLRNIEHAPGVQFSYVPPDFFDSDIDDKSDKNQ
YELKDDSGGGRAAGTRGKEHSSTHHLRRKNYEDDFFDMSDRDQGII--------------
------------------------------------------------------------
------------------------------------------------------EFNEDK
ILEALHILEFLYLNGISLEEQNEYGQTALFLGVKKNNISILQWLLSKNVNINHVDFYGNT
ILHMAVRYTDIDILRLLCDYGCLNLVYYSTFENNNTNVFQLCINNRYFLVYILLKKWLIQ
NKICKGLKICKTIYAFYFWFFALLNLFVYINIAQSFLEIQKHHNKSVIWISLWLFQQLLW
CILYFKNPGFYKENKFLTNRNKNNSNCIYATDFKNQAEYKLNNIEREIFQINKKLLQTNL
NPMHDA--NENEILEYNDQIINLRYSKLSLYPQVSQERINSLDVNYRNAILYNQNPRNVC
VTCNIIKPPRVHHCADCFHCVVHQDHHCVWVDNCIGINNQRSFYMFILSAFILLLFNYYY
VFLYFKLFHTTINYAFALLVILCNFINITLFAFITYLFARNTKTILTNVTFYEHFKRPNH
ITDKYNTELECWEFQNLNFKKIIKNIYNFWTLNYDEPYLK--------------------
------------------------------------------------------------
------------------------------------------------------------
------------------------------------------------------------
------------------------------------------------------------
------------------------------------------------------------
------------------------------------------------------------
------------------------------------------------------------
-----------------NSEKEDEAE---------EGYMCANTRAIIKEYINNTIKYHEK
NIDIKNKEIILLQLIIEPYILRRSKKHVFIDMPKKHSIIIKLPLNNTQLNLYKDEILSKL
QHTHKHLEFLETNSSKEEMEKLKSILGNKGEKEIRNLK--------NNEFNNNTTDPKLE
NMKDNCDNYDEDDDKIDEETINIEKEEENKESKIIINKRDDEEIIKSTSFQNNKNNTKEE
RGKMINASIFILRRICNHPLLHKYYYSVNDIKKISKYFYNNTDQYLDLDLKTVENEFMKI
SDFDIHLSIKHLISQGDNNLNKYLIDKKHILNSTKIQHMLTLIKNIKQKKEKVLIFSQFT
TFLDIIEESLLYEFIYDEHDYADHSNFIDKTQNVNKQNNLNENDNINESPNRDQFSKRTS
FNKDDDAYIRSPSVMSTSSQLSEKKFQNNIYVRLDGSTNTIERQQIIKEFSENDNVFIFL
LSTKAGGVGLNLIAANHVILMDQDWNPHNDRQAEDRVHRLGQKKEVYIYRLCCKNTIEET
VLK---------------------------------------------------------
------------------------------------------------------------
------------------------------------------------------------
------------------------------------------------------------
---------------------------------------------KSWNQLLLQEEI-VK
KMCEYIIDSRCDILL-EKGVSDLAQHFLVKKNISVIRRVRKTDLNRLERITGATIVNRCD
EIVEKDIGTKCGLFEIKKIGDDYYSFFVECENPRACTILLRGATKDVLNEIERNLHDGMN
VAKNIMLEGKLLYGGGCTEMRVSQYLIKQAANFDDSRKSVIESVASAFEIIPKILAQNSG
VNVVKCINELRTKHEKPESEKLGIDGVTGEIIDVSSKNIWDLLSVKKQIYKSAIEAASMI
LRIDDVVSGIGKDDKIQKPIKNEFMGRIKVLNVAEKPSVASAIVSILSKGESNKKKSYSK
YNPVFTFDYKMENETWSMFVTSVTGHLTDQKFDDKYKNWNNTDPHELFDAKITIYIDKDK
KPIENNLKKYSKDCNVLILWLDCDREGE-ICFEVINACSVTNKKLKIHRAQFSAVTEKDI
KYAINNLKSPNKNLAQSVDVRREIDLRMGSIFTRFMTIRYFKLVQNDTKIISYGPCQFPT
LGFVVNRYLQIKNFNNEYYWTIKMGYLYQDKNSNNSNLFLDNIG-KKKKKGKKKKNCSDD
DANNRPYSSNTNYVVDFTWSRLKLFDHLGVVLIYEDLLKNPLCRISNIFEKEVKKYRPFP
LNTLQMTKLVSKYFHISSKECMNIAEKLYSKGYISYPRTETNYFVDSMNLRKIIHELKKN
NIFGNYATKLAEKNS-CKPIKGKLNDKAHPPIHPVKNMNKANNVDFKEWKIYEFICRHFL
AVCSDDAIGFDTKVVANIGEEQFYCKGLKIKNKNYLEIYIYEKWNDKILPPFQINDEFYP
YSLVVEEGITQPPKYLSESDLLSLMDKYGIGTDATMHEHIENIQKRNYVYKNSKNLFIPT
KLGIALILSYKKFKDIGVDLTEPSLRAKMERDMFLVASGEKGKNEIIRNYIDIMKYIYQE
IYNRIDLLDENINYYINNPKIQSMETFNFGKYKGKTFEEVFEKHKSYVTWVKNLENPTGS
LIEFKNYVLRRE--EQGGGDTNTEYTEYNNKYG---------------NNNNYGNDNYYG
NGNKYK--DKNPNGNFN-----YKQNGY-DNKSSVNKMNSSEHKLYEQNIMNEIANRYNN
KEEDKPELDIIVAFEIFNSDSFKIVQKDNNNKKYSNFKNFVPKELFKILSEFNPTLKKIN
NYSCVTFESDKYEYVLNNLAEKCTILGGIHSIPNFLLKCFQNYSKFSQPQKISEVTANIL
TNTMCSYTKVHYDNLNNLLGEKLSDELKNFQKEGVHFGLKKNGRVLIGDEMGLGKTLQAL
ALMAFYNKDWPFIVICPSSIRFQWKDQALRWLPHLIEEKDICVIKSGKMDIPRNTKMIII
SYELITKNDKYQNKYKCIVCDESHYLKNSFSKRTKAIVPIIKSAKRCVLLSGTPALNKPS
ELYEQVSSIIPNLFNYNEFCDRYCYKDKNIYTRKIEYVGCKHTEELHLFLTNTIMIRRLK
KDVLKELPDKLRSKIPIEIPPNELSEILIYSKKLESKKNININDLDNINLSRFNNHDNNN
NDEENITISQLFKMTGYAKVKAIKEYITYLIDADIKFLLFCHHKLVMDEIDEFLKEKKLG
FIRVDGLTPIDKREIYIKNFQSDEKIRIALLSITACGVGLNLTAANTVVFGELYWVPGQM
IQAEDRAHRIGTTHDTINIHYLVAQNTIDEVVWKIINRKWNTLTTALNGTEDSLNVKEVS
KFDKFMLDLTNDTNKSYPTSLVNTPKIRRRSSEYKALINSGNNKKCPDIRDFFKKNKTDI
EEQKSM------------------------------MKEKTKKNECTEQNENIKEYDEK-
--INTFVSNLLASINYINNAILYRDNRFILRMLKYIKNMRLNIKNDSENLMPILVSLINK
IFKDTYPIYTILNKYINMYNENNKQNITEITTLNDKTYANSLPEIEVFFYILFMVHLIDK
KLYDECIELSNLIVKRVNKLNRRSLDFINAKVYFYYSWVHELRGNISQVRQDLLFIYRNA
CLHRDIMTQTVVLNLILRDYIKNNLYDMAVKFVSKTIFPENMLSNVQHARYLYYIGKILA
IQLDYSESHSKITQALRKAPQNINVAKGFKLEVTKLEIIVELLMGDIPDRSLFTNKIMRN
KLIPYKHVVTAVRNGDINKFANVMNNYKKLFVKDGVYLLIKRIHHNVIKTALRIINLSYS
RISITDIGKKIGVESPMDIVGITAKAIHDGVIGATIDYDNLYVESKPNIDIYITGDPMKA
FHKRIAFCLQLYSDAVKAMQYPDENEKTENVEAKERKIRQQEEFAQAEEGELGDDTDLLM
LCEQCNKNNVCILKPSNKEKLCKYCFLESFEDEVHTTILKKKMFEDNDKICIAVSGGKDS
SVLTHVLVNIKKKYNYNWNLFLLAIDEGIKGYRDDSLKVVYKLEKLYNLPLSVLKFQDIF
SYTMDDVVSYIGKKNNCTVCGVFRRQAMEKGALLFNATKLVTGHNADDLAETILMNMCRG
DIDKLAKNINDFMQKKK-----NNNSSLTNYSENSNTNVFSNSNNVLDSCVCKEKISIQS
NKEMEKKNKCNDDIAKIS---NDNTKYENKGDGFIPRLKPLMWSYEKEIVLYAYHLKLDF
FSTECTYSPNSFRGNLRSFIKDLEIINPQIILNIIHSSEFFYFNTNIKKKLNTCIKCGAY
TSNVVCKACLIVDGLNNYTDNSFLYANKKKKNNKKKISIEYEIEKMEKNIFSATNVIPYG
PLYDKAKNVKNNDQDENYHIVEEYEKLLKIYERPNPYDSNGSLKFSSNDDLLLFQIDLDY
TVENIFRNVIYSNEGSTNNSKCDSIYESYKIILGKEKNFISVPIIRIYSITNCGYSVVVN
VHNFFPYFYVEKPNGFNNDDMIKLESMLNETLSLNNQFKMYENKILKIEAVKTESIMYFK
KSGKTDFLKITVLLPKMVPSLKKYFESGINVNSKHFGGIVYEANLPFILRYIIDKKITGS
SWIKCEKNSYIIRSKNKQSSNCTFEIDIHYENIEPMALENEYQKIPKLRILSFDIECIKL
DGKGFPEAKTDPIIQISSILYLQGDPIENCAKFIFTLLECASIPGSNVIWFNDEKTMLEA
WNEFVIRIDPDFLTSYNIINFDIPYILNRGTALNLKKLKYIGRIKNIPSLVKDANFSSKQ
FGSHETKEININGRIQFDVYDLIKRDYRLKSYTLNYVSFEFLKEQKEDVHYSIMNDLQNE
NSESRKRIATYCIKDGLLPLRLIDKLLFIYNYVEMARVTGTPFVYLLTRGQQIKVTSQLY
RKCKELNYIIPSTYIKVSNNDKFEGATVLEPIKGYYIEPISTLDFASLYPSIMIAHNLCY
STLIKNNDEISDLNKNDITTVPGKNNFKFVKGNVKRGVLPLIVEELIRARKNVKAMMKNE
QNPITKMVLNGRQLALKISANSVYGYTGAAAGGQLPCLEIATSITTFGRSMIEKTKETVE
AYYCKNNGFEHNATVVYGDTDSVMVKFGTNDVGEAMRLGKDAAERISKEFLHPIKLEFEK
VYCPYLLLNKKRYAGLLYTNPNKHDKMDCKGIETVRRDFCILIQQMMETVLNKLLIEKDL
NSAIEYTKSKIKDLLTNNIDMSLLVVTKSLGKAEYETRXAXMXLAKKLKQRDSATAPNVG
DRVSYIIIKGTKGQAQYERAEDPLYVLDNNLSIDHNHYLDAIKNPLSRIFEVIMQNSDSL
FCGEHTRHKTILTSSQTALSKFLKKTIRCIGCNSSIKKPPLCNHCKANKEFSIYMQKIKD
LKLKQNEFFQLWTECQRCQGNLHIDVICMNRDCPIFYRRAKIKKDVANLQEQVTSLKIEW
---------------------------------------------------------FIG
NNINIKNKITNLKRKTMSILSCNNINETLGGGDKNIFELENISQSLNSIYRKRKNEIEPL
```

IQVAPMINVTNRHFRALVRIISKKVQLWTEMIVDNTLLYNINNLEEHLGFNKNEHPIVCQ
LGGSDPISLSEAAILVEQAGYDELNINVGCPSTKVANKGAFGAYLMKKPQLVKNIVYEIK
KKVQIPVTVKIRTGVDDLDSFSFLRSFIETVSSAGCDHFIIHSRKAWLKGLDPKQNRSIP
PLEYNKVFDLCKLYPNIKFTLNGGIKSIEQGVALLNGYAPIKHNNCTDNNYIKIKDYDIN
PLYGVMIGRSCMENITVLAKTDKLVYNYDIPTTAYNRRTVLDAYKSYLEQNSSFYSLVNA
FELLKPILGILKGMPGHRLFRNKIDSYIRKYASILNCAQILDKAMSDVDNIAPGCLDLVL
DDYKAQQEYIKNYMQNSQNTDNNAIFVEEVVRDFDQNEVFEEITTKFIWEQDVERSWNLL
VENNGILQHVNQETYDEKNKQKYKKSQTCSLRKGIFRNVIILFDMSSCMKERDFKPNRIT
VILECVEIFLKNFFFKNPVGHVGVVALKNSSAKLIQQLTSNIDDVLNSLVKEQKEGLQGS
PSLQEGLEIAHNLLMDMPLYGTKEILIMYGSIRTCDKKNILKYLDLLIKNNMYVNCISIA
PEMHILKHICEKTNGIYKICTNKNILINEINQVAETPLWMHGMEPQLIHICFPVKKKINT
QIVCSCHNILNTDTYICNFCNSYTCKIPSKCKICGIHLISMHDLSHITNSLQASPLFVEI
RNEQNNHTHCASCNQLLYDKVSQCTKCKNIFCLECDVFIHEDLNQCPFCLNDEDMGSNSI
AISQQMYFSTHNALRINEENDVISTLFYEINGHRHISLLIFPFYDVQMLKRLLIKKLDLP
-DIKVNDILIFYKGIKLPNYRIISTYLDNSNVDNVKKKKKKKVNKLYWAIKDPNPNASIR
VIDNKSYPPFFENILNDIKLAFKKNIAPKLTMDGTGGTYLLFNSKKKVCSVFKPADEEAF
SPFNPRGYEGKIYQEGFRAGVLSGEGASREIAAYILDNTYNNFSNVPCTIMVEACNPHFN
NKSNLKYIYNENTLKWKCGSLQEFIDSRESVGNYDHKQFSIRDIHKIGILDIRVMNLDRN
DGNILVSPLKSLKDCCNQFLYRNNKSFSTNDEDILKRIITIDQKPSRYSLIPIDHGLIMP
HIMDVAEIDLVWFDWPQTKIPFDNEVLEVIFAFDPDKDADKIRNKLLIREDCIRTMRVCT
RLLQIGAWMHLNLHEIAKISTRKNIDEESILERLVRDSIIQAYQMMDYTSLMSTNRLGYI
LDLAEIKINKKKKNNKNKTMELIDSNKTKDDNSNDXKKNNIHQK-EENYETTTYAKSKSL
DIKKIENSFTIVSFKDIKENKSSEESYNTNMNEQKEGECNSTYKTLIQHQSDNKESDNKF
LYTKINNISNKEKIGGYYSLSKDIEIRINDQNKEKNISETNSFSNKSEYTFVTATSNSKK
DDDIHKTSNDAEQNSDGGNMDEENGAKVEKKVKKKKKKKKKIKSDEKQSDKKEEPFNISD
G-----------DKISSHSQEEEEEEEEEEEEEEEEEDDENYVRFKKTTGTMKRISENTG
TAYRNIEMNKINSVWMIKDKNNKTINVKWENKIFEKLFFETFENYVKKYINDYHPNWREY
PYNGSKIITTKHSYLNSVKMEIRVANKYALGKKLGSGSFGDIYVAKDIVTMEEFAVKLES
TRSKHPQLLYESKLYKILGGGIGVPKVYWYGIEGDFTIMVLDLLGPSLEDLFTLCNRKFS
LKTVLMTADQMLNRIEYVHSKNFIHRDIKPDNFLIGRGKKVTLIHIIDFGLAKKYRDSRS
HTHIPYKEGKNLTGTARYASINTHLGIEQSRRDDIEALGYVLMYFLRGSLPWQGLKAISK
KDKYDKIMEKKISTSVEVLCRNTS------------------------------------
--MKILDKLRNQERLQLLYI
LKKNNGRKSLFFEKSLHIIINLILTEQDIKNEKIDNVFFMTDEINTISSKANISNNIIFF
LRPYFYEIEQVFKIIENIEKIKTDNKNYIFIFIPYMTYMCEQEIYKYNALELTIKIIIYP
LYFFPLYNDVFSLEIKNIFKDYYVDNDFSNLIFCSYALMFLQYIFNGVFRNIKSLGHVSH
FISEQLMQLRKEIVADSQDNLFHILSNIQSLQDLTNFKNPKEPSVVPIKYALHKFFLSEN
IKKKKYETPENYSCDEDLNLTSNLESEPIQRNEFSSSTSTYKREHIENSKFSNTPEITNP
YNNRSKNNEQVNDDVDKFKKSENPANDSNKDIEKDTKKSDNFKENKMEPDKINSENYNNN
ENKREMIGTYRQNSKIMTKEEHINSNDNNSTNKNNIDTNKINHDESDINKPDFREDNKNN
SNKFLSNNLNKKNEHYNSDNDEKKKIRN-IKKKINKKFYGMGIPNFMTEFDNSKFLMKKK
DKEEAEKQEKKEKNYLLEKLGINNFLFLLNTCTKIDACIIIDRRIDMVTPFCTPFTYEGL
IDHIFCIENLQIEIPRYIIFNNGANKIEAENSKTTTPKGYPSNDLNNKKIRVKLNSSIDV
LYNDIKDLNQNEVGIFLHKKASDIQQTYKEKDSLKDIGQINKFMIKFKEKHYEHNSLSRH
VNIASYILNEIKTENTFNKLKLEDEIIQLNTNTNKTILSNIVKKIQTLIYTGENIYEIYR
LISLFSSITNGFNDASMNELKKDIIEQYGINELTRLNNLHISNILKYQPKQKFIWNTLKN
HFNLLSNDENDISYVCNGYAPLSTRLIEYIGVFKNNMQVFPEVFSLINGPTFDIIQNAVG
YEHVQVNNNNNSEHIHDINSKKHKKNIILFYVGGIS------------------------
--------------MEIALSNENIKKDIETHNEIAHDTIEVKSMCINCEQEGINKILKFE
IPYFKNILIHSFECVLCNYRNNTIQDLNPIKEKGVKILFSVTKTEHLDRQLIKSEYGVLK
IPEINFEIPKETQKGSINTIEGFIQTALSNLTDYFINLKNMYNEANNIVDDNENNESKEV
EKNVNDDIHKIEEKEQLNKDGEINNNNYSDKDDETSDVNEKCHQITIENYMSMIEKTIHK
LSRFIVSKELPFTVEIIDPSGLSSLEYYDEDINSKTVVIEHYQRSKQELNELGFYEEDFE
GKKKDENFKQNNLNINENQSGDQIKKENFDFIKKYVHMNNNSNGSNNMCVKYKTINEGEE
NKLIESFTSNCPCCNYLGDNNFCEINIPGFKKCLILSYVCPNCNYKTSEIKSSGEINPKG
KKITLTVKNKSDLNRFVIKSETASIQIPIIDLTSDYGTLGGSLTTVEGIIIQIIESLEDK
FKFLLGDSSINTHISNDEVNASNKDDSVTNKIKNVISNLYKLCRTEEMFPFDLIIDDIAS
NSYISCDQIGDDTNLKEEEYERNFEQNDMLGITSMDAN-MNDNIKRLPSSSEGDQLKKTD
SGKIFDDGIKRTPSGKPIQTMYVLNRKGEEEDVSFDQILKRIQRLSYGLHELVDPARVTQ
GVINGMYSGIKTCELDELAAQTCAYMATTHPDFSILAARITTDNLHKSTNDDIGKVAEAL
YTYKDIRGRSASLISKEVYDFIMEHKDRLNKEIDYTRDFNYDYFGFKTLERSYLLRINNK
IIERPQHLLMRVSIGIHIDDLEKALETYHLMSQKYFTHATPTLFNSGTPRPQMSSCFLLS
MKSDSIEGIFETLKQCALISKTAGGIGVAVQDIRGQNSYIRGTNGISNGLVPMLRVFNDT
ARYVDQGGGKRKGSFAVYIEPWHSDIFEFLDLRKNHGKEELRARDLFYAIWVPDLFMKRV
KENKNWTLMCPNECPGLSESWGDEFEKLYTKYEEENLGKKTVLAQDLWFAILQSQIETGV
PYMLYKDACNSKSNQKNLGTIKCSNLCCEIIEYTSPDEVAVCNLASIALCKFVDREKKEF
NFKKLYDITKIITRNLDKIIERNYYPIKEAEKSNKRHRPIGIGVQGLADTFMLLRYPYES
DEAKELNKRIFETMYYAALEMSVELAQVHGPYESYKGSPASQGILQFDMWNAKVDNKYWD
WDLLKKKISIHGLRNSLLLAPMPTASTSQILGNNESFEPYTSNIYYRRVLSGEFFVVNPH
LLKDLFDRGLWDEDMKQQLIAHNGSVQYISEIPNDLKELYKTVWEIKQKNIIDMAADRGV
FIDQSQSLNIYIQKPTFAKLSSMHFYGWEKGLKTGAYYLRTQAATDAIKFTVDTQVAKNA
EKLKNAEGATITREVSRETISTESTVTQNACPLRRNNDEPCLMCSG---MNTMNDPVPKL
PFDEIRNEMNKYGVVITPSTLKHPTTEDVQGVYSICIKYILNKDINNIRIEEFTGDLKSS
MPSIDGIQILPNEGKNHLQAIGNLRFFRHCEKINKILNMDNTLSYIFKPTSGHITKLINA
FMHFMRYREQIYNENDATIKQIEERKNESNVLDNELKSIQSELQVLLSKHEEVRTSILNE
KNIKRDYEEEIIENQNSLNSQQSILISLKSTKDRIVNETNELIFQFSRYRQKKEDLEDQI
VPSPEKLQQYNDELKDLLYEHMSHCETSKKKNEDIKNKINIADLCIKKLVNLLTILTSHI
NETLKVHIDKKNKLKDLGTNLKSLKEENENLTKKKREHENILNETENNFLQEKNKWDEKI
QDEKKNTIIVEENVKQIRESIDGITTKTNQEIKEINNIVNHIQDTVNIYNKNFAIIADLI
ENTKKSQKILKNKIQNNIQNCIKTHL----------------------------------
-------GSFGRVILATYKNEDLPPVAIKRFEKSKIIKQKQVDHVFSERKILNYISHPFC
VNLYGSFKDESYLYLVLEFVIGGEFFTFLRRNKRFPNDVGCFYAAQIVLIFEYLQSLNIV
YRDLKPENLLLDKDGFIKMTDFGFAKVVNTRTYTLCGTPEYIAPEILLNAGHGKAVDWWT
LGIFIYEILVGYPPFYANEPLLIYQKILEGIIYFPKFLDNNCKHLMKKLLSHDLTKRYGN
LKKGAQSVKEHPWFSNIEWNNLLNKRVDVPYKPKYKNIFDASNFEKVQEDLSIADKVINE
NDPFFDWMVISQFYILSPRGDTIINRDFRGDVLKGSAEIFFRKVKLHKGDPPPLFYLNGI
NFCFLKNNNLYYVLTSLFNISPSYLIELLYRLLKIFKDFCGQLTEEIIRANFILIYEIVD
EVIDYGYLQNSNTEYIRYLIHNEISNNNTSSTKFSNLTKFTIKHSNTLPSNASQKPIQVD
NKKNEIFIDIVEKINLIMNKKGEIIYSYIDGVIQIKSYLLGNPYIKIALNDDLYIKNIHK
DNTNNIIIDDCNFNHLVNTSNFETDRILSLYQPDGECVIMNYRINNNFKAPFHLFANILY
NPNHTAELFIRIKLDIPSRYSCTNVLVSCNLCKHISSVHLDGNTNSDLSSAHYIPNEHKL
LWTIKKFKGETEYTIRSKITLNQNYEYSRQDFGPIHIMFEIPMFNLSKLRIKYL-IIENY
KSSNTHRWVRYITQSSSYVYRFMDLIVITPYDEELDDIEELNSKILALEMIKHNKSNVNK
NADNSCPNIKKLVLPGEAVLEKKDKPRFLKGSGLYEEEENFCACLLGSVNYINKLVYVEP
LRGKYTGSVGDLLVGKIKDINNDKWVVEIGSYCRALLSITQTNISLFSQRIRLYNDVINM
INIYKPNDVIACEVQRILTDGCIILHTRSSIYGKLSNGILITVPQTLVQNQKKHIFVFPC
NVQIILGMNGFIWISSPIKKSKDTNPNSIDEDIEGNKFEEVDDTTRKNISIISNIIKLLA
KYHININYDIITKIYMQYTSNKSNTPSYILKPYVSDSYLFSYLENFTK--MPILYHLTLQ
KPTAITRTVYGNFSGPKAHEIVVAKGQVLELLRADKQGKLNVITSKDIFGIIRSLEIFRL
MGSNKDYIAIGSDSGRLVILKYDDEKNDFIRVHCETYGKSGIRRIIPGEYIAIDPKGRAL
MLCAIEKQKFVYILNRDNKENLTISSPLEAHKSHSICHAVVGLNVGFENPMFVSIEQNYE
ALDKQVLNTNEQIMEYPKKGLCFWEMDLGLNHVIKKHTIPIDITAHLLIPLPGGQQGPSG
LIVCCENYLVYKKIDHDDIYCSYPRRLEVGEEKNISIVCWTIHRIKTFFFILIQSEYGDL
YKIEVNHEDGIVKEIICKYFDTVPIANSICVLKSGALFVAAEFGNHFFYQFSGIGNDSNE
SMCTSNHPSGKNAIIAFKTQKLKNLYLVDQIYSL-PIVDMKILDAKNSNIPQIYALCGRG
PRSSLRILQHGLSIEELANNELPGKPRYIWTIKKDNSSEYDGYIIVSFEGNTLILEIGET

VEEVYDSLLLTNVTTIHINLLYDNSFIQVYDTGIRHINGKIVQEWVPPKNKQINAATSNG
SQIVISLSGGELIYFEIDESHTLTEIFRKNINVEILCLSIQQIQQNKLRASFLAVGCLDN
VVRLLSIEKDQYFKQLSTYILPNNSSPQDICISEMKELGSQKEHTILYLNIGLNTGVLLR
SVIDPICGTLSNHYSKYLGAKSVKICHVQVNKNPALLVLSEKTYLCYVYQGKYIYSPLNY
DVLEYASSFYSEQCSDGYVAISGNSLRIFRFYRLGEVFSQNILHLTFTPRKIVPLPFPSL
FYDNDTSLEIKRIKNIQMLAVIEADHNAYDENTQQEIQKALRDIKLEGKGEAE---DELE
NDEEELLYDRIGTPKAGLGKWGSCIKIINPINLQVIDKISLELEEAALSVCACELEALHC
LIVGTTTNMTLKSRNVPSASLRVYTYDINYKLNLLHITPIEDQPYCFCPFNGKVIVSVGN
KLRIYALGKKKLLKKCEYKDIPEAIVSIKVS-NRIFASDIRESVLIFFYDSNQNVIRLIS
DDIIPRWITCSEILDHHTIIAADKFDSVFILRVPEEAKQEEYGIANKCWYGGEVINSSTK
NRKMEHIMSFHIGEIVTSLQKVKLSPVSSECIIYSTIMGTIGAFIPYDNKEELELTQHLE
IILRTEKHALCGREHIFFRSYYHPVQHVIDGDLCEQFSSLPFEVQRKIGSDLEKTPDEIL
RKLEDIRNKILMEEEPATQSKPLDDEDINILKSYGSGPYSKSIKKVENDITGLVTNINKL
CGVRESDTGLCLPNQWDLQLDKQMLNEEQPLQVARCTKIINSDTDQTKYIINVKQIAKFV
VGLGEKVAPSDIEEGMRVGVDRTKYKIQILLPPKIDPSVTMMTVEEKPDITYNDIGGCKE
QLEKLREVVEMPLLQPERFVTLGIDPPKGVLLYGPPGTGKTLTARAIANRTDACFICVIG
SELVQKYVGEGARLVRELFQMAKSKKACILFIDEVDAIGGSRGDESAHGDHEVQRTMLEI
VNQLDGFDNRGNIKVIMATNRPDTLDSALVRPGRIDRKIEFSLPDLEGRTHIFKIHANTM
NMSRDVRFELLARLCPNSTGSDIRSVCTEAGMFAIRARRKTITEKDLLLAINKVIHGCKQ
FSATGKYMVYNMSLDFCSQHLLAYDNSLITSENEIKSKTEENFALVLKKINELTNQRNDE
GELFYNLTQDNVFNLPRYSKFPEAKKPTKWELFSQKKLKKKKNKHGLIYDENSKGWVRRF
QKKQIKINKEKSDFVHEYKPNDNIYDDPFEKMEEEKDIKKMKQKMREMKNKFEQQGISSQ
DIKYIQKQKRKRENLIDNLKMAQISSSTFGRYDKKLKKEKKLKVKINQKCEKRLLKDEIN
QNNKLAEIVLKSLMETNPYVFKNLIHIYEEYINLIINRVKGYKVLVLDDETKVIISLIFS
HSYILEKEIFLTLNFNDINIFEDIKNGN-QLEELSFQNYKIKNLKHLKAIFLLRPTHKNI
LKLIKELKKPIFLEYYLFFTNILNNSYIEKLAKADEFECIKSVMEYYIDIYVLHDKLFSL
NIDYISFLYKNDNKVLKNKIKNVSGNSLNMKRNIQFKSSNNSLTFEEFNKNVNYINYLEN
DNDDFNSKDLEDVGSNNESKNMLYEHLLVNRLIEGMFSFLCSIKQVPDIIYNKHSYVCKY
IIDALKMKMLKHENIFSNILENYENYDKYTKTNPMENYKENNLINKNIDVNTEGNCCYMV
VLDRKEDPITPLLMQWTYQAMLHELIGIDNNKIILDSNNSEESQIVMSSNYDDFYNEHLF
DNFGDLGQAVQSYVDVYQKETARKSKLESIDDIQKFIEAYPNYKKLSGNVTKHVNILHKF
SELVEKRKLFHISELEQSIAIYQKKMEHFKQVIETVKNYSYTNYDALRLSLLYSLKYEDK
EHIDIIKKELQKRNIEKDQISLIDSLMIYSNDKNRKNNIFKEQTFLDYAKTTITRTIKGA
SNVFTLHKSYIYYLIDDIIKFKLDTSIYTTTNLLNIAPNMNKQINSIIVFIIGGATYEEY
RDVQDLSKKYNINIVLGGTQIHNSQSFLADVLQLTKKM----------------------
-------------------------LHEKKRNKQYKSVFERLTDQNFYTGTHKKKFEALV
K-----------------MHTNNQFENGKKKKKKEKNLVVTPGILGIQKYGIQIARPKSI
WLYRNGDKHHNGLLFFIKSHINNFKLLLFEITKVLNPIIGPIRKIYDQNFRLIKNIQQLN
DGSKYLCTSGDPPASIDHLGKFKSKWVIQGM-----------------------------
--LKGEEDGKNEEEMKINDI
TKYFKERQKVITPPNGDGTRAFYESLLDENPNSIIAIKYCIEHGVLSGTTYHETLNKYYI
LKKNNAFRNNFGRIKCEFVDMLENIKNDKLISMKNMEVDRDANVEQWKIKRLIKKLENAK
GNGTSMISLIIRSKDEVSRINKMLADELGTASNIKSRVNRLSVLSAITSTQQKLKLYNKT
PPKGLVVYCGTVVTEDGKEKKMSIDFEPFRPINTSLYLCDNKFHVEALKELLESDDKFGF
IIVDGNGALFGTIQGNAREVIRRFTVDLPKKHGRGGQSALRFARLRLEKRHNYLRKVAEV
STSVFITNDKVNVLGIVLAGSADFKNDLLHSDLFDQRLYAKVIKIVDISYGGDNGFNQAI
ELSGEALQNVKFIQEKKLIGKFFEEIAQDTGKVVYGIEDTLKALEIGAVELLIVYEGLDI
IRLTTKNNVTNQTKTMHIFPHDEKQESLYKENNVELEVVEKILLTDWIINNYKKYGASLD
FVTNKSQEGAQFQKGFGGFGGMLRYKIDLNLYDEDVDSDVELFMNAYKEPNHVVDQKRLL
DECIFVVKEQSFFMKQALDNGSLRDTLKHASNMLCELKTTELSPKYYYELYMLIFNELQH
LDSFINDKKKHKKKFIDIYESVQHAGNIIPRLYLLIIVGRNYIKNKDIKAKYILKDMTEL
CKGIQHPLKGLFLRYFLIQMCKDRIPDTGSEYEEAGGGNIDDAFEFLLTNFYESLKLWNR
MNDKVIPIGNIDDNVLKNNKIKILKEKMDVKMLVGSILVRMSQLEGMTKQYYIEKCLPKL
LLYLSNINDSRIQQYIFESIVQVFSDECHLYSLEMLLNSILKLNNFVDFKNILITLLKRL
RSFVEHNKSEFPKEIDIFNLFYNHLVIYVNRTIEQCRFDCFNNTLDTSDKVHTNSININD
EVSNLNQSNKDIDASQNIPKFSNQNIHKNSIGNNSDNNNNNIKKDKQEIIQIIIKMLQVL
YEFIFLCICMYDS-TTINELFELAYKIVSNININDDEISEQVINIIVLPFNHLGLNALKA
KNIKNLLNSINDKYKKKLSLNIIDAIIECKNKEMAYQNVEEILKFISCIFYEDTHKKNIN
KKDPFNFENNSIIYTSEKISKFFHIITNTNDIDKKYNTSMLFYNYIYDSIYFSQLLPSII
FTLLNIVTKIIAIG--

--MLEAKLNN
AYILKKLFECIKDLVNDANIDADENGLKLQALDGNHVSLVSLHLVDSGFSHYRCDRERVL
GVNIASLNKVFKLCGINESVVISSKDDEDNLNFVFENNKEDKVTNFSLKLMSIELDSLNI
PDCDEGFDAEVELSSKELTNIFRNLSEFSDTVFIEIDSNSIKFTTKGLVGDAEVALKPRE
STSEDDVGVTIKSKKKIKQSFAIKYLNLFSKSSILSDVVILGLSDSRPIEFKYEIKDTSP
DSDALKVGFIKFFLAPKMDDDMDNKDMRIYNWVSCGFILLQFFMNVYGKEWCKAKFEYGM
SDYAECQKEGDNLTNYMIEIIPAKANNIDLYSDVSIVLSNSQGINTKEIIIGSEKEGLFR
KIYSVRKDIDNPEYVHVKLNSKNRNWKCKKIKIWKDYKYWTFDCIGILNEEKREATYFLS
GNKLYTAYVQTGKDIESGTTGIIDIILLGNNKRSNTKMLHEGFISGGLKKIKFQASDVGN
LENIILINNSYNDPWYCDFVKIKSDDSKIYIFNVKSWIGYPYNNKIKININTNNIDGNAK
DIDCHIRANDLIDTTNNNFVLQNKVHIFKVRCPQNCHNSEFSIIEGTSIHPASTSICAAA
IYDGSLTESGGEIIVTITKGLNYYYAIDEAYNNLKAIEFSTKSDENNFSFYTYHLTSIDD
IKSNIRIVDSFGKLSSLGRLEIRVNNKWGAVCKKGPNFEFSEDAAKRACKDLGFPNGIYI
KENCSNINEQNYCAGYKYPFNASGILCSGNEQNLLSCNTDDPSYCIDHHDDVIIQCVNQL
GNDSIENGTIRLLDSTGSPTSNGIGRLQIYYNGVFGSICSEGWTKETEKIACLELGYHNV
QANGFSHHLCSDIAGENLCGHDKERINATNFRCKGDEANLKNCPHETSEDIYCSHEEDII
IGCASAEEEGNDSANSNKHMMSMEKKKFHPKIELSCFDXISSKAELSKGNVGDIFLVSCP
EKCDEDIGVIKGTFVYTFDSYICKAGIHAGVLSSSVTDDMILIITHSRNKFIGTKRNNIE
SKEFNGESKSFSLSIPTNYIIMEERQNNSKYEDEIL-KEDNDFYYEHIFKKQNKTFFEHL
EPTFQWLAPSSFAGFNGDENQYINANNLPNEKYIRTLSNFTFIIHFIPNSGKNKWRTILS
HSLCEGISISIDEENELVIEQNCNPHLVKTKFIPKFEHPCHLVLIYNKPNKSISLYINQK
KINLEKMKFDFTLNGDLTIGRSNKQATDYFIGDINFVKIYKYILTEQEIKESYDSVLSNN
YLNDGMSGNRDINTKKTQNKKTKNNRKTIDGRDCITPCKSKTNVNKNVQINTEEFYLNCS
DNLLSERFNGKIGAQFLVSCLEDCTNSKYIVKGSNNYYTPDTSICKAVMHSGIMHKTKTN
NNSFIIKIVEGLTEYKSSRGHYGIVSKPEKQSQLRSFSLFSKKEDDIFTCFTDAAFLFEL
PIGTTKNIICPENCHKIDKQIFGTNTYSPLSSVCKAAIHAGVISTKGGQIQIVVGKGQQE
FKSSTQNNIQSYIAEKQNRSFTFLKRLYMKGNLFVEQNVETEKISENKLNINTDKIIHKI
KKKIRKKQENNPNINLLPNNKELLSKDFPNPKNIKNKKIKKKLSTDVKLAILSSKKIISN
AQFNNIDQGYIKLTSQKEKNIISHIQ-----NISKDATNEGLINLSKQNENFFRNDSIKI
TQKYIYENADVGTQKKVFDLHLNMGPYTCNYSRNGKYLLITGEKGHISFLDTHNMETLCE
LQVNETVKCNTIFHNHKLFAIGQKKYIYIYDNTGIEVNCIKDILYPCQLEFLPYHFLLAS
IGDLGELVYQDVSVGNIITRKKTKRGPCSIMKQNKQNAIIYLGHKNGHVTLWSPNMDKSL
CDIFSHKTAISSIGVFDNYLITAGIDCTYKLWDIRKLEYINSFKSHNIINNIDISDTSMV
AFSMNSHFRTYKNFFTKPELYLTHNTWGDRINSITFQPFEDICCAGLKYSIKSFIVPGSG
LANIDTFVNNPYETKKQTKENEIRQLLDKLPPETIQF-----------------------

---------------------MSRSSEANDNLYFLAISKNKVTIYEYNAEFSKSIISDIE
EEIKRGEHINDDNIQKGVVNNTNINNITSLKKSSNKFNNDYLINRGINIYREYEGVELAT
CTHDYKKIIIVRKNDLNVAEIIDIKTNEHVSYIKTKTQIKRIVTSPRDSHIVLHCQYKPD
ISNKNLYIYKINGKSNKKKKKKKDIKETENPEQNEQDEHINNYNNSPKKDEIIYNESLVY
EMGLNSYSNSNWPFFKWTESESNCLCLINNQIYIYKDNNFNVVSDKLKLDHIEFFEVSPE
QTNKRRIFLATYERGTKGNSSVFKIFNLDNLNKHIYSKKFFNSDEIKLKWNKNGTSLLLQ
IHTQVDKEKQSYYGSSNLYFIDTVKIKDVNIMTNKGLIYDTIWSYNQNKFYVCKGEIPAD

```
IVLHDKNGNIIHSYGKHKFNTLKLNYSEKLLLTGGFGNLSGDISIWNTINKKEITKTKSS
CAVICEFFNDDKHFLTATTHPRLRVDNNIKIFKYNGLIVSKLDFDELYNVIILPFNKIKS
KPIDSSINP---SSELQKYINKQLGIDSKKVGIYKAPGSTAKTSLNGLGIMTRSLKPKSN
MPPGCNFVVEEKTNKKKKKKKNPKNKDKKE--MKNDKIKLVSFEGDEFIVDKYTASMSTV
IFNILEVMTSEEDTIPLPNIKTQILKKVIEYMEYHIHNPPDEIPKPLITSNLQDVVSVWD
YDFVNTDKETLYELIEASNYLDIKPLLDLTCGKIASMMKDKTTEEIRAEFDIVNDFTREE
EMQIREENKWCGDI----------------------------------------------
------------------------------------------------------------
------------------------------------------------------------
------------------------DDATEKNRRIIEMYEKKLETEIDQKEKNVDRLTELS
DQIDGNASTISAKMRESISKIFRETPSSKPKIINEIKKNDNDENSEYNKS-ESISEKNEE
GNLSNDNGSMGRDDRDNKPSFSRSTLKEGSLLRLFRCEYFDTHLHIRYLYDRKEVGVHEY
LVNSLYTQRNPEDILFYLPQLCQISLVRYESSSLYRFLLDKASKSMHFALKLNWIYNSIV
EDNISKYKEISQKMIQEIEMAVV-CKPLNSECKIFKENKQSDLLILAYPLLFKRKFIIKK
IRANEKINQIKKFNKFLSNSYS---LKGLIESNPIEKTNTLFCSNHIKSIDQKNTGVMNL
EKMDPPQSIHPNSEHINEEAEKNNYLGGEQKYN-SVCPKCAHIFEIKKEKKDKKKNIALS
PQLPSCYIINSGALSVASAKIKLPSTYAKLGDPLSFSKFSLPDCNYSFDMIEELQQFFMK
QRRCDYFSLLNNFIDILITTSNLLANEPDIDIRNTILNKFIYSLNTWMVMRRCIVASCEN
IFSMTGLCIPMESISSSKSDRCGSRRKQKKNSKHLQILHFNNDECKIFFSKKRAPYLLVF
EVADLDEDISHISDNTFYVR---------------------NYFSHSNNAKSDDELKHNN
YILTKNYGGYNENSSSYECEMKTSYEEQNKKNYKEEQITKLNRNTNSADSEISSTNSYFK
DLNVIKMNDLYVYNAIVNDLRRENLISFTSEEEENIYLIKKCIGL---------------
------------------------------------------------------------
------------------------------------------------------------
------------------------------------------------------------
------------------------------------------------------------
------------------------------------------------------------
------------------------------------------------------------
------------------------------------------------------------
----------------------------MTINVSFKVTGGKEFTISIEPTITVMELKQKC
AEHVDIPVESQRIIFKGKILKDKEPLTLYSVADGNVMHLVRSSVPTKDSEAEKENNKESN
SAADQNQGVNENLNNFNDNPLVQMLMQRGAGDMNSFGQGAGDGNFNYGNLASMLNPNGNG
EFNRESISSLLNNPLARSLMNELSNNPEMLTNLISNNPLLRNTFSQSPLMQPMLDNPNLL
REFMRPEVLQAGLQIESALNNNQNNNNNNNNNQ--GLRMEDLLSNLSNFANPNGGLNSSN
GNNANNLNSLFQSPELLQTFQQVMRGNPNLGNFNFGNLAQNLNLNTPNITDNRPPEERYA
SQLVSLQEMGFIDNDANIQALQETGGDVNSAVTRLLERGFNMEKKEDNVMREIKVNKLIL
NICVGESGDRLTRAARVLEQLTEQKPIFGKCRFTIRSFGVRRNEKISCFVTVRGKKALEI
LEKGLKVKEYELRRKNFSDTGNFGFGIQEHIDLGIKYDPSTGIYGMDFYVHLSRPGYRVT
RRRRKRSTISKTHKVTKEDAMKWFQTKFDGILLKMEFEGSNYFRFRLALSLISGKAITIK
NIRKKNSHKKINKGEEIDDNEINEGLQEYEAKILKLIDKLCDDTIIKINENGDELYFKPG
FLIGNVNDEVKISDLNNTFHCGNERSISYFLEFLIMIVPFFKNPVKLLLKGITDDQIDRT
VYTCKIVCENFFKTFLNVNDDFLNITILKRGTKLDATGEVSFFMNNLKMINSFDMHDAGL
VKKITGTIVCNKISMIFRNKIVNCAKKNLHNFTPYVSIEVEKEKKNNYNNSQNNFMSLSL
FAQTKNKCIYGTDLYVDKFMLQHVKDMLN--------DHNDKE-------EEDEGEKNGS
PFKDENKETNNSNIDASKDALKTLHDADIYERLGFFISLKLMNEIKGLSSIDSSYQWLPL
LYMALANDTAVSKISLSALKPYSIVLIRLLRDFFSVVFDIQKVEKSQIEYSYLIKCVGID
YRNFSKKTF---------------------------------------------------
------------------------------------------------------------
------------------------------------------------------------
------------------------------------------------------------
------------------------------------------------------------
------------------------------------------------------------
------------------------------------------------------------
--------------------------------------------------------NLLK
GSEDARNDLNDENGENNKNHVSEKLKNEEDNETDFNKTEKKKSNDDTLNLRKYAVNTYIK
MLEENENIPFVLIQIICFVLGEYSYLCDLENYTAEDILDLLCECMEKNLTNPDRVKSCII
TAIFKLCCHNNITDHVVTNKIIEKYKNSQITDIQQKCYEYDLILKNSELIKNVFSKNNKT
QNIVIDENLLFLNPFIEKYLESGGKSYVAKDLRQCENNFETSKSPSLALNFTPYELPINN
NISSDIYNASSPNLLYPKNRELSSFSNSQINEDCSSQREKKKMFKLNVVGPKKWKKETSK
MEDVEINENKNND--KKKKKKKKGKKNVSIYPGTNNGIINYLGDDRNKLSIEKTNKKQYN
NLDNVNQSNSYYNEEREVNEHSEDDADEDEEDSTSENNEHEKRMDLVHDYTNYENLYGNS
IEDIKNNKYGNSNKKATNKDEQSKFRNNRMEDRTNELSEKEKMAAALFNGLISNNTSVFD
SKNIYSQNFPNKKSLSISSNENDLKHKKDSSVKNNSLEENKNTNNRMTNKNEERNPSASI
IAKSNLNNSNKLEASHKKIYSYDMIDLNEIPEIRGITLENNENKKMWDDIKNQKKAVFLN
KSKLSIFQTIKKFETNLDSNVVDVSKTEALVSCI-----------MTGSQCVAIACDLRL
GSNSFTTVSTNFTKIFKINDHIYVGLSGLATDIQSLYELLRYRVNLYQIRQETDMNIDCF
SNMLSNILYSNRFSPYFVNPIVVGFKVNTHIDENGNKTNVYEPYLNAYDLIGAKCETSDF
VVNGVSNEQLYGMCESMYIKDQDENGLFETVSQCLLSALDRDCLSGWGAEVYVLTPDKII
KKKLKARMDMHKTIRKIFDTKICERLESMNILLVGAGGIGSEFLKSIITIGCKNIDIIDI
DTIDITNLNRQFLFKKKDVKKHKSIVAKERALKHRKDLNINAYTFDVCTMKGSDISKYDY
VINALDNIKARKYVNKLCITEKKVLIEAGSTGYNGQVYPIFSSETKCYNCEEKPKNKTYA
ICTIRQTPSLPEHCVAWGKLIFETFFCKNDNETLIDIKKHIEEESKKRNMDKEEIIRFIF
NYLFHDTINELISLKKDYAIMPKPILFEENLKQEPHNIEKLSQEMPNSNLKTNDNKLID-
-------KIYENNSIQLSSQNIWNKEK-IEMYVSTFNKLYKYLNIKKTTEEYLIFDKDDD
DCINFITCLSNLRMINFSIKQKSKFDIQSIAGNIIPAISSTNAIVAAFQAAQLVHVIEHF
ELLKEKETEQNISLRDSRAKHIWIKNVVNGNKIFSRGNIVNAENLETPNPNCYVCQQPVI
NIYIKNFNDITLNSFVKEICMNELSFLYPFLDNQDRNIFDYDTFLENDDDYMKSLSNSLH
EWDIKHDDILTLTDAQDTKNQIEIHLKEDKSLESLYLISSKLTKKRKPKVTTIEERPTKR
Y-----------------------TMGKLSKDRRDIYYRKAKENGYRARSSYKLIQINDK
FEIFKLFNPNKYNNSDISEIIKKYNDEYCYNIVDLCAAPGSWSQVLKNICLYNYYNMLYF
NNNELD---HEEFIKNFSLYINFNKNNNIKEPKLVAVDLQEIGNMNYINIIQGDITKMST
INKILKCMNENEKDMKNNILNKEKENNKFVYAHAVVSDGAPDITGMNDIDEFIQSQLILS
SLKVCCSVLKIGGNFISKIFRGEYTSLLIFHLNKFFEKIYVCKPQSSRNKSLESFLVCLN
FHLPKSSITSLFRNTNEVCYNFQNDGELRNMHLESINGKDQEFQFDDKLNSNLDTSDSEK
ENNLNINNNKKKNLDIFNFYCSDSDEDIKYFNSDDEENFNNYISSNFSTNNKLFSFIATQ
NYYDSDKSYLLPQNYIRHEPQIMPLQPPYLLSLQNRRKENKKMEGDNYDIILVLNFGSQY
FHLIVKRLNNIKIYSETKDYNVDLKDIKNLNIKGVILSGSPHSVNAKNGPHIKKEVLNYF
IENKIPIFGICYGMQEIAFHMNGQVGKSKNSEHGSTEVTLISNDYKDNELYKNYKLLESN
CLLFDDIKNTNNMNVWMNHTEEVIEIPENYYLVNSSENCFICAFYNKENNIYGVQYHPEV
YETADGDQMFYNFAYKICKCTKTFDPIKYHEVEFNNIKKHAHDHYVIAAMSGGIDSTVAA
AMTHKIFKDRFYGIFIDNGLLRKNEGEKVYKFIKSTFPDMNITKIDASENFLNQLKGVTD
PEQKRKIIGKLFIEEFEKAVYSMDIDIEKTYLLQGTLYPDIIESKCSKNLSDTIKTHHNV
GGLPKNLKFKLFEPFKFLFKDDVKKLSQELNLPKELTNRHPFPGPGLAIRVIGEIDKHKL
DILREVDDIFINSLKEYNLYDDIGQAFAVIFSSKSVGVRGDARSYDHICALRAVKTTSFM
TASYYKIPHDILEKITTRILSNVKGVNRILYDISSKPPSTIEFEMRKYGGNTKRNRDDDA
DESNN--TDRKCITLVRATLLPILSIGKNEKTTLFTPFRNPLPGFVSDKSLYLRKTLGVK
VRKTGWINLMKNSIPRRSDETDENDKVEDIIRKFDPLVLYEDENSKIEVDPILAQYLREH
QREGVQFVFECLMNLKDEKISGCILADDMGLGKTLQSISVLYTLLKQGYNKKAAVRRCLI
LCPASLINNWNDEINKWLPNRCTVTCVNDSVKEKIVSKLEGFKYDLKSTILICSYECFRI
NNDSIDKSAIDMIICDEAHRLKNDKTKTYTSIYKLSAKKRLLLSGTPIQNDLGEFFALIS
LCNPDLFDDTNSFRKKFANPILIGRDKDATEKEQQIASERLAELSTI-NKFILRRTNNLL
SKVLPVKYLINIFIKLNPIQEGLYVLFLKDKKLLKSDNSNNKVNVLINIKKLEKICNHPL
LLNANDIKDIGHIPVVKLIEDVIAENVKGRGNKISNKNSVNLSTNNSMENNGSKDGEYLR
ERRKAVELDYDKSVKKLIEECKRDVYRCYYNLSSKFQLLHFLLKTIKHETNDKVVIVSNY
TQTLDYMEILCKENHYKFVRLDGGISIKKRHKVISDFTNTDDIFIFLLSSKSGGCGINLI
SSNRLILLDPDWNPANDKQALARVWREGQKKICYIYRLFCTGTIDEKVYQRQISKDGLSS
```

```
MIVTNTNLSKDQLSDENVKKLFNYKNNTICETHDNIECNRCNFKDNT---EVYSEQLEDF
EEEDVNTWAHHLNIDTVPDAVLIKAVKEATECKINHMNTLPILKKLKHEFVTFSMSCKIE
FRDDLIKQKANKNNISYQPIAMSEKKEPTKSKRTKQKEPSEYSDSTNDEEEDEEEDE---
---------------MNQYNAPNNEFKKDDKIFCIEDIANKSSPKYENDIKNLPLVSNEH
CVNVRKKKIEPAGFAFYTKYEIPILKEKEKIKNKNKNIPIDELFLYKDKIDYLEEIKNNK
-----DDDENKDNSKNLKNEEYQKNDYEENNNSKKKEEKASLKKNSNIVKKCINNNINVY
KVLGVEESDDFETIKLAYKKLILIFHPDKNKGTSILNEKEKAKKKKKNNNNDNKNDEAKK
DISYYIEKYNIEKLTQEEKKSMFLKVQDSYAVLSDKILRKQYDSSIPFDETIPTKTALDE
APNFYEFLSPVFKRNAKWSSKKPVPNIGDENTSIDKVKYFYDFWYEFTSWRDFSYQNEYD
YEDAECREERRWMERENKKIQKKASKAEKLRINKLVDLAYNNDPRIIAENKRIELEKQRK
KELAMLEKQKNKMNTLQSTSKNSSQTNSSNDKKNKDNKAASKIWKHHIKSICVTKLYNIF
NFNLIQDKLNDMPFDTLCQFIYEIYLFLNFNINKPENSSVEKFSISQTKENKQNEKETTF
INTSKPNNNDNNKRRGKNASSNNTGNEGNKNTGFIGHLKGVELSEKDIELLLDIFRKYIN
NFNTILEMKENINTQDIKNEKGNPEQDVHTDEKNNIKTNCYQNNKVANEEIKEKNNTKEI
DVEEKKNKKEGDENNTSTKWTPQEVSLLSKALKLYPGGTKNRWNVIANSIKTKNVKEVIK
KAKEMFENETLKNLSKNFEESAFDNFKNQNKGVMKKIDNKLDKREYKHINDQNNISSAHS
SDNNSEQKKPWTHEEQMLLEKALMKHPATIPTKERLKLVANELKTRSVEEIVLRLKTIKA
KIMAKNAAKMPKKRRNGGRSKHNRGHVNPLRCSNCGRCVPKDKAIKRFNIRNIVDTSAQR
DIKEASVYSTFQLPKLYIKQCYCVSCAIHSRFVRVRSRQQRRVRKETTKHAHVSQLMKKK
IEQNNPRHKKYSYSDESKMQNFLYKSINKNRFYLLFFLFLFS--SLTHLAQCQV-KKKVS
VSEINFDSAVDDVQWCGNNHSTVLVKTVKGKLYRSSDGGKIWTNITSNLSESFNNKNDNT
SGHTPETTAVDLIMVNPINKNIVLVIGAQNSHYISEDAGETFKLINYKNKINFWQFHNKK
AHWALVSSWTASCFSTDNTSGECMQTLSLTQDLGANFQLIDIYVVQFNWGDVSSHSEDTI
YYTRHRNRNGHQQRFSGWSKDVDFVSTNNFGKDVEVLVKHGNKFLISNGYIFVAKLNDVI
KQTVNMMVSTDGGKTFNKANLPKDIHEKSYTILDTSEGAIMLHVNHGSTSEKLNTGNVYI
SDASGLNYTLSLPNNIRTASGECEFDRVLSLDGVYIANFLDDQDEMKDDDLKFTNFKLQL
EEEVGPFETNTEKRKKQLIKGKNEEIVRTVISFNKGGHWSYLKAPKVDSIGNKYDCGDEC
YLHLHGITNYHQYAPFYSIENAVGIIMGTGNVGSHLKYKSDEVNTFLSRDGGVTWIEAHK
GPYIYEFGDHGGLIVMSDDLRKTNQIVFSWNEGQSWFDFELGQFPIDIDNIVAEPTSSSV
EFLVYGTRNDIGVLYHLDFNTLGQPLCKGLWAADSVSSDYETWSPSSGAFTDKCILGRKI
TYTRRKQTSECFNGKDLKRIVDKKFCDCTPEDYECETGFTRKVGSFECKPTDSTLTIEGC
TSSSYFYATAYRKVPGDVCVNGWVPEKVPVPCPDYSPFNNSAKSILLILFIMGLVMLIIT
YICRNPKIRNMFYNYGFDTFEHVKYSVVKTKKGNINSNAFEPEMEFIDAEQDNNEEDVPT
LMSYNNERNGQRSDFN------------------------------MADRKPNKNAVVKN
VDMTEEMQIDAIDCANQALQKYNVEKDIAAHIKKEFDRKYDPTWHCVVGRNFGSYVTHET
KNFIYFYIGQVAILLFKSGMAIEVDEETLKIAKEFQKKNEEEIKSKKLKPESIKLDNPSS
NLIKINEASCRGRIKICNLLNVPISEVEYNDKIEKNKYIDQLVVVCGWSKAVRKQ-GGRF
CFVNLNDGSCHLNLQIVVNQNIDNYDKLLKCGIGCCFRFTGTLILSPVQNTEKKGLLNEN
VELTLTDNSIHSFEIYGENLDPQKYPLSKKNHGKEFLREVAHLRPRSYFISSVMRIRNAL
MLSTHLFFQSRGFICIHTPLITASDCEGGGEMFTVTTLFNEHGDISSIPKTKKKINNNEK
REDKNTNE-LET-NNTNQYIVDFKKDFFSKQAFLTVSGQLSLENLCSSMGDVYTFGPTFR
AENSHTSRHLAEFWMIEPEMAFADIYDNMELAEAYIKYCIRYVLNNNFHDIYYFEENVEK
GLIERLKNILNDDFAKITYTNAIELLTKYSNNFDIPVKWGMDLQSEHERFISEQIFKKPV
IVYNYPKDLKAFYMKLNDDNKTVAAMDVLVPKIGEVIGGSQREDNLELLDKMIIEKKLNI
ESYWWYRQLRKYGTHPHSGFGLGFERLIMLVTGVDNIKDTIPFPRYHGHAEFMCNTNSEI
DIPEADQIESQKLFDELKELNINYKEVKHGKVNSIKDILDLNLEKSENVIKNLFLKDKKK
KYFYLCVANWKKLDLKNVSSQLKTSNLRFVDDENLKNILNVNPGSLTPFSIKSDKDNIVK
LYFDEDIKNMDEVIIHPMHNYSCIYVKTTDVIKYCDLHNHTPEFINLSDSKDTEKRDDTN
INEEISKGTTKKHGENDEKINERDKGKDEGNILGITSKKEQNFSDWYTQVIVKSELIEYY
DISGCYILRPASYYIWECIQTFFNNEIKKLDVENSYFPLFVTKNKLEKEKNHIEGFSPEV
AWVTKYGDTNLPEEIAIRLTSETIMYSVFSKWIRSHRDLPLKLNQWNTVVRWEFKQPTPF
IRT---------------------------------------------------------
------------------------------------------------------------
------------------------------------------------------------
------------------------------------------------------------
------------------------------------------------------------
-----------------------------------------------MAKLSKAQKKQIY
MDKLSSLIQQYNKILIVHVDNVGSDQMASVRQSLRGKATILMGKNTRIRTALKKNLQAVP
QIEKLLPLVKLNMGFVFCKDDLSEVRN-ILQNKSPAPARLGVIAPIDVFIPPGPTGMDPS
HTSFFQSLGISTKIVKGQIEIQENVHLIKQGEKVSASSATLLQKFNMKPFSYGVDVRTVY
DDGVIYDAKVLDITEEDILEKFSKGVANVAALSRSVGIITEASYPHVFVEAFKNIVSLVI
DTDYTFPLMKKIKDMVENPQAYAAAPVAASSAQKDEPEKEAAKKEEEEEEEEDGFMGFGM
FDMTQERDLAREPCPDRIIEDMGGAFGMGCIGGYIWHFLKGARNSPKGDMLSGALYSSRM
RAPILGGNFAVWGGTFSCFDCTFQYLRKKEDHWNAIGSGFFTGGVLAMRGGWRSSSRNAV
VGGVLLAIIEFVSMVLTRKTTPTPRQQFQQQMEMEKKMAAQKTKMLKDEIWLYKNEYKEN
EYDDDENEKQPTDHFLEVPLFDEDDYGKNIKRRI------KINSKRYDETEMSEETLSTT
YDDEFLDLYEELKREQKIREFYNPMLKPRLWRLEFFIKYIHNLENNIQKNFYLISFGNTK
NINLYGGLQNDVLYTPGYSIKPGEIQYLKIPLTIWSEKELSISYEDLKKFEVTIEMWCIK
GLIFNDLYASSKINLKEIIENDPDSSIVLKRKIEKNKMTFEAQRLGIYMQLSEIFEFHMA
LDSWWFIANSEMPNYLKLLPKFLRFKFPLSEGEWVIHSSNKSNNNFWLYPGYFCFIGTYR
QLANAFFILAVLCYNSNYRYKPPILLGSCIVSLKSVTEYPFFKGTVKKLTLEKRKFKQGE
IVGNIKCFVNSYGIQEDDTNIQRPVQPLSDVTLVNQLSLNDHYLVIRIIKCENLAISSID
LNNININVWVKWDGIVNKTDIVSKTVSPFFYQNLYYPIRLVDKKELTNEKLIHNVLPIDL
ISKGDICLEVHNNNEIYSSILGIFELPFSDIFNYGTYDYRGLVQDNNSS-SIYNENKNYD
FYDQEFDNPDENYEDYYSRQYKTIVYKNTLDLMYSRANAQTKRKSTISIEAFVIPPLPSG
LVFFKENQSQNSSIIYKSMSKRWDQDFKGFNDIYLQWFPKAIKNRSFPCISKNEFDDNYY
PLCSFVTPINLPAQVSTPGPLFHWLNNIEYIENDDESTNLTPPHFFLSYKKGTIQDHAIL
LCCCLKGLEYDAYVCKGTINNGKTEHYWVMTRHEKGWVCFWEVTNKSIIHLKDRWNNNNF
LKNFDIL-RNKMINKINENDNEKYYNGEYLVSYIKYGLEELKNRKNQIIQEYDIKEENQL
YTMDLYGEHLMKDEEVNIDELLYNDEEFFDNIFKVKYEKNKTYNCNKAIKYVLESFSKHI
PIAPKFGLLNYENTLAYVPYTSIEVIFNDQQVYGNMQNHHPACILYDLENNYHWRPFLNH
EPPQIKSEITISTPLSDKLSIKYMKELEEELQEMIFFIRNVEGLETNFDHSKEIKYFLEM
YIDICEYKLNLDNNFNLK------------------------------------------
------------------------------------------------------------
------------------------------------------------------------
------------------------------------------------------------
------------------------------------------------------------
------------------------------------------------------------
------------------------------------------------------------
--------------------------------MSLSIYGNRENGQDVRTANVTAVQALSN
ILKSSLGPQGLDKMLVDNIGDVTITNDGATILKQLEIQHPAAKILVNLSELQDQEVGDGT
TSVVLLASELLRRGNELIKMDIHPTTVICGYKLAMKESVKYIKEKLSERVTNLGKDVITN
IAKTTLSSKFISYESEYFAKMVSNAIQSVKIINDSGKTKYPVSSVNILKVHGLSSLDSKL
IDGYAIMSGRASQSMPSAIKNAKIAFLDFPLKQYRLHLGVQVNINDPNELEKIRQREKDI
TKERVNKILESGANVILTTQGIDDMPLKYFVEAGAIAVRRIKKDDLKRIAKLTNGQIRLT
LSSIDGTEKFEPASLGYCDEVYEEKVGDWDVMFFKGCKNSKSNTILLRGANDFVLDEMER
SIHDALCSVSRALESNYVVVGGGCVEVALSVYLEDFAKTLGSREQLAIAEFAESLLIIPK
ILALNASYDSIDLVCKLRAYHTKSQVMNTDEPKDYRWYGLDLVNGKVANNLKNGVLEAMI
SKIKSIRFATEATITILRIDDLIKLVPEEPKQEEPMDVPCIKNLYSDPWNSGFDDLRICT
RITESCGIACSSEYIATPWQVQ-GGVIGVIKLENMYRNPPVYKLKGHTSNILDIQFNPCY
SEVIASSSEDMSIRIWEVCNKEDKSEEIKNSLCILKGHKKKVTIIDWNPLSYYILCSSSF
DSTVNIWDIENEKKAFSINMPQKLTSLKWNSSGALLSATCLNKKLHIIDPRQEKICTTFN
AHSGSKCAKNIWIDGYSGNENYILSTGFSKNYMREMKLWDLKNISDPLCTISIDNASAPL
LPHYDESIGIIYIIGKGDGNCRYYQNSEGVLRKINEYKSCLPFKSFGFLPKQVCNIYKCE
IGRIYKNENDKSIKPISFYVPRKNPDIFQEDLYPPIIMHDRNYSSKSWINGNNLEIKRIY
I-----------------------------------------------------------
```

----------------------------MKIKSIGDESIHNICSSQVIFTLSNVVKELVE
NSIDAGATEIKVKLVENGIKLIEVNDNGNGIKKINFENVCARHATSKISEFDDIHNILNT
LGFRGEALNSLCMLSDLYISTKHDEFEHGYLLKFDKFGKLLHEEPIARLRGTTVSCENIF
KNIPIRKKDLIKNIKSQLSDLLSLMQQYAIIYHEIKFMIFNVVTQKGCTKNMNMLITNGS
DDIKKNFYSIFGKKNIGNLIDLNIKNENWSVKGYISDSNSGRRDKDLQFYYMNSRPIHVI
KNVNKIINTIYREFNSRLYPIIICNILSDSKNIDINVTPDKREVFFTFENELCEEIKTEL
IKLLTPKTSNFVDTQIDDYFFVKNNRIIKNEETKN---------------EETKNEETKN
EETKNEETKNEESTNIERPLFESDDLSYTSINYKKIVVNGNEDIENLYFNKQKKYNNKMD
NQKDKNEDTEKNYINNDIKIKKEFEKXYENEHNEYTSKIRKIVKDEEVEYYEYKYEENTN
FNDKNNSRNMDSKWKEITPSFIDDKRLSDDSGVDFSKYGLNKGSESDAIKTEVIKEKNES
--EDHIKEIENGEIVYEYKLDN--------------GSE-----------------KVKK
GIVKENEYWFEKLKENIKNNSIKNIPININTYINREDMKRGFDYDQINLINLTNSEKIRN
TIFKKMKEIEKINDYLCLTDEKEENKYNDLFKNDLNIQQNCNNSINGIENEEDINFDNID
EQKKDLYFKSNLFKKLKICGQFNKGFIISKIDLLYFKNGNSKSENVKNNRNEAEENEYIE
RKNNYALFIIDQHAADEKSNFEKYNKIFTMKSQKLINKIELELSPAQIHIIEKNFVIFLR
NGFEIEIIDEPINKKRKINNDNNIINEEALMEMKVYLLSLPVFNGKILEVVDFMSLLYHL
SNNPIIFDKQIDGSFLKNNKKLTDNTEMWFNYNFPRPQKVWKILASKACRNAVMVGKTLN
ISEMITIKKKLSVLKNPWN-PHGRPTIKYIINNIEIQKYYTHYYLKLYEEITNLRKTKNY
EEYKHIFRDHIFFLIISTKPMLGPVLKFQMGISKSEEIEKQNIYANYPGLEQQLDMVFAC
HDISKDGKLPYATVEMILRHFLMQCGFMEYVCRFVNEEGKLDLKHVENYLKCKKLLYKLQ
CCGSCMLTLDEMKNLVLVFLKKISDTYLEDQSKWMEKMKSSQEEQGKALEEAMYEYEKNI
LYNHSIKEQQLLQNNKKLDEWNECIENAYEVQQEVLRQFEAKKIEKANQVANKNNELLIA
QNYIEKIKEAATNKKQDSSKCFVYPASSAPCGACTSAGAVTPHRRYKEPRQKKEYSLCIM
AAIDHNVQDELVDYEDDDNMLDNKDVKCDIGGNLLNNNNKGVNESGAMRGSYATVHTGGF
KDFFLKPELLRAISESGFEHPSEVQQETIPAAITGTDILCQAKSGMGKTAVFVLSILQQL
ETNDSKDIKEEKDMNNNQNSGSQNKYVRCLGIAHTRELAYQIKNEFDRFSKYLKNVRCEV
VYGGISMNKHVVLFKGNNIPHIIIGTPGRILALIREKYMLTDKIQHFVLDECDKCLERLD
MRGDVQKIFISTPLKKQVMFFSATMAKEMRDVCKKFLQNPVEIFIDDEAKLKLHGLLQHY
VKLQEKDKTRKLIEILDALEFNQVIIFVKSVTRAITLDKLLTECNFPSISIHGGLNQEER
IERYDKFKKFENRILVSTDLFGRGIDIERINIVINYDMPENSDSYLHRVGRAGRFGTKGL
AITFVSSQEDTLALNEVQTRFEVAISEMPNKIDCNEYINQ-NAVLSNKDNSANVNEDLII
DECSSDDDETDCLSEVNKKDMDSNSLDVGNMEMRQSKRMSVSAEAYGEWNKKKENFVAKV
HKKDNKEKKKIREALNESFLFNHLNNSEMETIIDAFFDDHVEEGANIINEGDEGDLLYVI
DEGEIEIYKTKDNKKEVLTTLKSKDVFGELALLYNSKRAATAKALTKCHLWALDRESFTY
IIKDNIAKKRQMYEDILKHVTILKDMDPYERSKVADCLKSKTFNAGDVIINEGEQGDTFY
ILTDGNATALKNCQIIKTYTKGDYFGELALLRNQPRAATVKAESTCQVVYLERKGFKRLL
GPIEKILIRNVENYKKVLKELGIDSSCIEENMHLKIICLSDEVREMYKNHKTHHEGDSGL
DVFIIKDEILKPKTTTFVKLGIKATALQYKCNYYYKSDKNDSNNKNNNEPEIVNTSFLLF
PRSSISKTPLRLANSIGLIDAGYRGEIILALDNTSDQEYTIKKNDKLAQIVSFSGEPLSF
ELVTELDETSRGEGGFGSTSNKMRTCDPNSAFFGFMGIAASSIFSNLGAAFGTAKSGVGV
CSVGVMRPDLIMKSILPVVMAGVLGIYGIIMSIIISGKMSPAASYSSFLGYTHLASGLIV
GLSSLAAGLAIGIVGDAGVR--
-------MTKRIIKNSATIRDDRKNVTKEKIEEKDNGVNETRNVLTLENILQDNDTDNNK
KVKNKLIFKNKKNNDSSINKNEMRIITIPRQRISSVKNNWLELIKPIVTNLKLEIRMNKD
KIEVRTCKLTEDKNNLQKSSDYIKAYLLGFSIEDALALLRIDDLYIESFQIKDVKILKGD
HLSRCIGRICGSNGSTKYAIENATKTRIVIAGDKIHILGSFNNIKMARYSICSLILGSTQ
GKIFNKLNILAKRLKERFMGNRCCTGRDLLYKSKLQELGIEGSKTIRKLLSFTNNDIIRF
DKAYDENDVQEFVNLCSSTCEIEKLEDRMHPWAADPKTIGALSATQLAILASKEDEPHYK
DAIRESNGIPVFINLLKSHELDRVHAAVVALSFLSVDSVKNCICMFENGALPYLITGMKS
NIDGMKAACAQTCRNIFVLDKNYKKEFLKLGGITQLVNLLELSSTDDSQPLYTQLEAIYH
LEDFILNDGDEVPQFLEAVKKSNAIKNLKALQQCPEQDLAEASNVLLLRLTDMANGWDAE
KIFGSTISYTYDDIICMPGYIDFPLSEIDLSNNMTKDICLKTPIISSPMDTVTEHKMAIS
MALCGGLGIIHNNMSIENQIEEVKKVKRFENGFIFDPYTFSPEHTVADVLCVKNKVGYKS
YPITSDGKVGSKLVGIITGIDYLYLTNPDVKIKDIMTTELVTGKYPISLSDANKVLCNEK
KSILPIVNDNYELIALVCRNDMHKNRIFPHASKRENKQLIVGASISTRGSDLEKVNKLVQ
NMIDIICIDSSQGNSIYQIDMIKKIKSAYPDIPIIAGNVVTSNQAKNLIDAGADVLRIGM
GSGSICTTQDVCAVGRAQGTAVYHVSNYAHTRNIKTIADGGIKNSGNIVKALSLGADFVM
LGNLLAATEESCSEYYFENNVRLKMYRGMGSMEAMYNKHFNSKSRYLVEDKIFGTVYDPT
NDIKISQGVSASLVDKGSVLNLIPHLVKAVKHGFQSIGIKNIQQLHSKLYSGDLKFDIRS
INSIKEGKVSDNLIFNTKK---
------------------------MKENKQYQEALKELKEKTEIDDKAIKLKKKIEENIN
ILKNIKGKNEQAIKIIYNDIKKLVTHCFENYYIFKLSKNLSVQFFSGLTKILLITTDKIN
ELTDKWNDNNAFMKLDKWRQEMAIKRYKKKTGINEKDSDNKN----GDGKDANL-----N
DSNSGTGNELILAHESAWDKFGSKLKDMPFLNNFFENPILGKLFGETELAAALRIMKMDD
KNFKLSELMYLFEFVISKHIVESYLIGDEDTLRLHCGEAAFNSLNSSINERKKKKLFLDT
NVLIYKNHELKGAQRMEESSPWFIFTFHTQQINCLKNKDDEIVEGKIDDIREVVYTIALS
KHPEPETEGLLYPYIIREFAIIGNTPSW------------------IEDIQNIGSSYFEN
NKKIDRYNEEINLLKQQLSDLNDKMGKGVIHKSLEPVNAPKITFWYYELKEMKEFQDLVM
YEIKKKKKHFKTLSNSCMKYLSNKEKIKLKKQEEEEKKLKIHSKNISSYMDVFWKKIEKI
VWEEKKRELQKTLNKNKEMRFKKFVKGAIKKIKNSKHNAHELFENNMNYLCSNNNSEN-N
ANTNYSNEDKELGEEDLTDQEDEDILLDEEMESIDESEE--KEVNLLDDEANIPIEELLK
KIYGFKSGEEYINFMQNEEEDHGTDTDMDNQFSTQNDSINPT--------------QNSK
KRKLPNEGGSDRELKSPKIEENANIEEVKG------------------------------
---------------IPIKVDEILECNMDEKHLTKIPPFIK-TLRDYQHAGLHWLLYLYK
NNINGILADEMGLGKTLQCISLLGYLAYYLNIWGPHLIIVPTSILINWEIELKRFCPCFK
ILSYYGNQNERYKKRIGWFNNDSFHICISSYSTIVKDHIIFKRKNWKYIILDEAHNIKNF
NTKRWNIILSLKRDNCLLITGTPLQNSLEELWSLLHFLMPNIFTSHLDFKEWFSDPLNLA
IQKSKIYDSKELIDRLHTVIRPYILRRLKKNVEKEMPNKYEHIIKCKLTRRQKILYDEFI
NNKKVQNTLTSGNYMGLMNILIQLRKVCNHCDLFTNKYIQTPYYYILPIQYNIPKFCLLF
ENNYYQDFYLILFLHNEFVSLGGIAKDVHSKQKQNHNYTDMHPKNNDLTIFDSEQFQNNM
INMETLNVALSDNFHEYKNLNEINQTKDFPNKLIEEIDSK------CPFTLINKNYYKNI
LINLNKNKELKKDNQIIN--
----HVGRSSRGSHPGNQNL--

-------------------------NINSNNSNSNSNNNNSNSNSNSNN-----------
-MVLYIIGLGLGDEKDISVKGKELIDQSDVIYLESYTSILFISKDKLEAYYKKKIYEVDR
NFAEENCEQILDEAINKKVSFLVVGDPLCATTHHDIILRAKKKNIDVHVIHNASIMSAIG
ESGMQLYNFGQTVSIPYFEGEYKPTSYYNKIKINLDNNFHTLCLLDIKVKERTIENIMKN
KNIYEPPKFMTVNEAIEQLIYCESVHNENVITKNTLAIAIVRIGSNDQQIVSGNLFTLKT
QEYNDPLHSLIICAPNLHDIEKEYFDMYSTSI--TGKPSGLRSARKLRIRRRTQRWADKG
YKKSHLGTRWKSNPFRGSSHAKGIVVEKVAIEAKQPNSAYRKCVRVQLIKNGKKITAFVP
GDGCLNFIDENDEVLVSGFGRSGHSVGDLPGVKFKVVKVARVSLLALFKEKKEKPRSMGI
KGLTKFIADTAPNAIKEIKIENLMGRVVAIDASMSLYQFIIAIRDGDQYGNLMNESGETT
SHISGLMSRTIKLMENGLKPIYVFDGAPPELKGSELEKRGEKRQKAEELLIKAKEE-NLE
EIKKQSGRTVRVTKKQNEEAKKLLTLMGIPVIESPCEAEAQCAFLTKYEMAHATATEDAD
ALVFGTKILIRNLNANASSNKNKNKNSSKRGYILTEINLEQVLKGLKLTMDEFIDFCILC
GCDYCDTIKGIGSKTAYNLIKEYNCIENIIKNIDQNKYQVPANFKYVEARQSFIKPKVLE
KSEVKIDWCEPKIEELKKFLIKEHNFNEVRVTNYITRLLKARKVTTQRRLDTFFTTCTKK
STKLIIEENQKEVLKTKSKGKKREINNDNSTKLNAKKKKTNVKDEKKNNEKVDEPKNKPN

```
ENLVK---D-EDDQDDYDQNIFDERTNSESGNIKNENVKEDISSSDIT------MDIPKC
INDIV-------CMFFSSFPFDSMGGQQPRRKREVNNSKYYESLNLKKNCTIDEIKKAYR
KLAIIHHPDKGGDPEKFKEISRAYEVLSDEEKRKLYDEYGEEGLEGGEQPTDATDLFDFI
LNAGKGKKKRGEDIVSEVKVTLEQLYNGATKKLAISKDVICTNCEGHGGPKDAKVDCKQC
NGRGTKTYMRYHSSVLHQTEVTCNGCRGKGKIFNEKDKCVNCKGLCVLKTRKIIEVYIPK
GAPNKHKIIFNGEADEKPNVITGNLVVILNEKPHTTFKREGVDLFMSYKISLYESLTGFI
AEITHLDERKILIDCTNAGLIKHGDIREVLEEGMPTYKDPFKKGNLYITFEVEYPMDLVI
TKEKKEMLKILKKQNEVEKKYDIENTDCEVVTCKLVDKEYLKQRLTMQQQQEAYDEDGHQ
PEMEGGRVACAQQMNTNFE-NNLYMGWKPNEKIYKTIIEALSSCNNSNNNVQIEATKVLK
DLNENVSDAALYLLHIFMDKQENSDVRQVGGLLLKNYINSKNKFLTNDILKIIKNEIFKL
VEDEIKEIRSTSGSVITSILTKYEGIEKWPEALYNLLLLVERGNNDVVDGAFRAIIIIIE
DELINRKNTDSLFFQFCKTQLLEKLFLYCSLQEKSIKKKYAAECLDLFINASCFATNGIF
NEYFPQLWECLGYLAAEEDTQILKIVVTCVTIITDTRYASIFNNLDGIIQFMVNATNSGD
RKVQLEALEFWPVLIKDRSYMAYSNYNNNN-----TSENYIDENIYKNINELRNEALKTL
KNYLPYLCKILIDNTVYTKWDYLTMDESHFQNDNANVPDLIQDISPELYHSSNNNNFVNS
SDNKNNSMLNEQDEYKAQNNSNNGNC-TNDMNVNSGDIDNNDDLDDLSDEEKNDEMTSRT
WGNDWTVRKGAALCLDYLSNVYNDDILEYILPHIEEKLMSDKWNIRESAVLSLGAIAKGC
MYSLSPFIPKVLEYLIKLLNDEKPLARSISCWCVTRFSSWICHPDNCDKWFEPVLLNLLK
RVLDSNKRVQEAACSSFANLEEDALELLNNHLHEIVHTIQQAFQIYQAKNYFILFDVVGT
LIDSVNIVKENIDLAHEIVNSILIKWNNIRISSPYIIALMECMSCITSAYGKEFLKYAKI
VIRTCIKFLVLLYIDLEEEIKYYYNRKVNDSNSFIVNRNNITQTANELLNYYKISNEDYF
INLKDIDSISSSKKKDLIECSFDLLSRILSVINSDIIEIIAENEYNFIPLVHKYCLK---
--------PLLLNNNYNTKLPASSGSVKNGVIMENEYTELAKQFLNFGILQSNFALIGDI
SRFCAQYLIPFLNDIIPFLIAHISHPSIPVSNNASWAIGEISIHINSQCIEPYVDEIVKQ
HIYICQNSKYHGCLLQNICITVGRLCSTYPKKIIYYFPQFLKTWLKIMSHGTQENEKINS
LKAILEALYLNLDMAAEHLKDIVYIILKYKYVSQNVNIFFHQFLSTMKQKYPNQWKEIYN
PMMDEHSSPIPTDMLNDLSTRFNLMKNRINSKLKENTYQEYISKCDTYDIYEIVNYIKSN
TFFRKYTNSNNIQEDYKILIDDTVRTKIDELFKKEKAYLKKHTENENKNGKTNPDETKIS
EKKEKGLIYLYNACDNIFKNQDNINGKDKFIIINNENNMKKISENNINELLVEIVYTIFT
KNETLENNMLNLLGESNVDLIINIIKNKDEIKKDIKLISKQVDIKENTLGANFFITN-TT
KNTKNKNILTNKENIENIIEYFINNFEDNYLDNVKKIYLPNEENQLEEINVPQNTTYSYS
NNMTKVKINRLPNMIFDQNELVSVNALPFWAKYIFNFQYFNYVQSKVFNAAFKNNKNLLV
SAPTGCGKTNIALLVILQQLILHSEKHGINLNKITNTKNGLSPEGNGYIEKHGTTSGRLK
TKTENENVGESSYDDDNKSDSSTPKIGDNVINPNDFKIVYIAPMKSLVFEITTNFREKLK
IFNLNVCEYTKEHSLTSKELELVHIIVTVPEKLDILLRNSSYSSTVSDESLIKSIKCIIL
DEVHLLNTDRGDVIETIVARFLRYSETSQSMKRIMAMSATLPNYKDVSDFLKVERDMCFY
FNEKYRSIQLDKTLYGIHETNMNKLNLAKNLCAYNEIINSLKNDKQCIIFVCSRNDTNKT
IEFLIDHAIKNDEINYFTNNLYTDYDVNKRIKKSSNIYIKKFYEYGCAIHHAGMSRYDKI
LVENLFKKKTFNVLCCTSTLAWGVNLPVHTVIIKGTNFFSSESGKMEDLDILDINQIFGR
CGRPQYESHGHAIL-TERTKLYKYIKLLTNNTIIESNFLKNIENHLNAEISMGTTKNAQD
GIKWLEYTYLYIRMKKNPFLYDTDIKNDRELYEKRKSIIIKAISNLSENKLVRRS-LTND
FIGTFYGHIAAKYYVDYKTIGIFASHIENNNYAEIIDVISKAKEFENIQIRNEDMNDFIY
YKNKCDIKENYDESKSMTLRILIEMYLRRIQINNFSLVCEINYIVQNIIRILYAYYEICL
NILKNISHLIINTHNLIMSITRRLPIDCGLFRHFCYKNELMDKKYVHASNNSNRGNTENR
KNQNYTVYLKEAAVNILEKKKLSYETIANLSKSELFFFLRNEVYTKQILYYRNIIPNLYI
DGYIQPITQTIMKINLNVQLQNTIWSDQWNSTKEDFHIFLLNTLNNDILYFQKITIHKKD
RKKIHDISFEFPITNIMPSQITVQFLSMNWCNLSFVHIFNTNNLFINQKINVFSEILPIV
PLSTEVMNIPNYIKFFSFKYFNPIQTQ---------------------------------
------------------------------------------------------------
------------------------------------------------------------
------------------------------------------------------------
------------------------------------------------------------
------------------------------------------------------------
------------------------------------------------------------
------------------------------------------------------------
------------------------------------------------------------
------------------------------------------------------------
------------------------------------------------------------
------------------------------------------------------------
------------------------------------------------------------
--------------------------------METINIKKEDNEAEVKISQEIHDENVEK
RYNEIMSIASECIQPEELKLKLLQKRKLICYDGFEPSGRMHIAQGLLKSHIVNTLTNNGC
TFIFWIADWFAQLNNKMSGDLNKIKKVGQYFIEVWKSCGMNMENVQFMWASDEINKNPDK
YWSTVIDISRSFNINRIKRCLTIMGRTEGEDNYCSQILYPCMQCADIFFLNVDICQLGID
QRKVNMLAREYCDIKKIKKKPVILSHGMLPGLLEGQEKMSKSDENSAIFMDDNEADVNRK
IKKGYCPPNIIENNPIFAYAKIIIYPHYKEFNLIRKEKNGGDKLYTTIEEMEKDYICGDI
HPLDLKDNVASYINKMLQPVRNHFQNNAEAKKLLSEIKKYKITK----------------
-VSIIGSGSWGTVISKIISENTQRSKIFHPIVRMYVNEEIIDNEKLSDIINKTKENVKYM
KGMKLPNNIVAIPDIDKVIEGADLLIFVVPHQYLKTTLSEILKNKNLKSTAKAISLMKGV
KICDYKPMLLSNIIENMLNIECSVLSGSNIASELSTESFSEATIGFENLETAEIWRDLFD
RNYFKINCIQDKAGVEMCGALKNVVALGVGFSEAFKKSYNTKSAIIRIGLEEMKKFAKLF
FPNVLDETFLDSCGVA-VIATCLGGRNVKCATEFAFRNGQDSWDKIESELLNGQKLQGVY
TSKEIYKILENRDLKSEFPLFSIIYEIAFGYKHPSSITSVLSTKKLRHIKYRKMERILSS
IGRLSVVAGGLSLIPYTFIYDVDGGERCVMFNRFGGVSEKTYGEGSHFYFPWFQTPYIYD
IKMKPKVINTTTGTKDLQIVTLSLRLLFRPHTKHLPYLHSTLGPDYDERVLPSIGNEVLK
AVVARYNAESLLTQRDTISKEIRESITARAKQFNIVLDDVAITHLSYGKEFAKAIEDKQV
AQQESERVKFIVAKTEQEKIAAVIKAQGEAEAAKLISSAVKEYGNSLLEIRKLEAAKEIA
ENLSKSKNVTYFPSTSNILLNPKSFMKLDYINAIKEENGGYNFENLKRNEILKEKGVTFP
KFRKTGTTICGIVCQNAVILGADTRATEGPIVADKNCSKLHYISKNIYCAGAGVAGDLEH
TTLWLQHNVELHRLNTKTQPRVAMCVSRLTQELFKYQGYKVCAIVLGGVDVTGPQLYGIH
PHGSSCLLPFTALGSGSLNAMTVLEAKYRDNMTIEEGKELVCEAICAGIFNDLGSGGNVD
ICVITKDGTQHIRPYKQPNTRLYHLSHPTIYPKGTTPILYEKIENIKKHITIEDAMKSIK
NGMYNFNPSKEDPFGKAPVGYVAGKGRGVTGFSGGVSRDDITEDKDKNDYSDFNYDEFHG
YSESLFKDTEYDEEDKEADDIYESIDSRIDVRRKSRRENKLKEEILKIRAQKPTIQEQFS
DLKKNLANVTLEEWESIPNVLNYSRQKQKKMPKNYLPAPDSLIMNKLNESNSHLNYAGSS
GNGL--KTPLGLKTPLGLYTPMSLGFQ-PFLRNSIGIDTPLFGKNNRSGLINSGLNTPFT
LSGYSTPLNGSNINGYNTPITNNTNMLSLNDLGEARGTVLSVKLDELIDNVEGQTVIDPK
GYLTNLNAKNLTNDSDIADINKARALLKSVINTNRKHGPGWIAAARVEELAQRKDKAKEI
IIKGCIECSKNEDVWLEAIRLEDKLSESKIILAKAIKNIPTSVKLWLEAYKKEKNVQDKR
KVLRKAIECIPNSVVLWKEAISLENENNAYILLKRAVECIPQCIEMWIALARLCKYSEAQ
KVLNEARKQIPTSAEIWINASKLEEKHGNVNMVDVIIKRCIENLSQKNVIHERDKWIKFA
EECEKSDFLHTCQSIIKNTMNIGVENLNKKRIYKQDAQNCINNKSLHTARCIYNEALKIF
KTKKSLWLDLANLELTHGNQENVDDVLQRAVKNCPHSSVLWLMYAKQKWLNNEIDAARKI
LAESFMHNQNTEVISLAAVKLERENNEFQRARILLKKSRVQCNTPKIWMQSVQLERLLGN
YKDAKELVHEGLKIHKKFDKLYMIAGHIELEMANLKENNLNNAYDKAQQIYQQGLKYCPE
SINLWLCAIDLQITKKSYTSARALVEKAKIKIKNIHSLSINTKVLKNKEIIESNEQYHDE
EIGNNLNHTKNDDNNNDNISNSKNELENKINVNASVKVIENYDLLWLKLIEIELSCNNLN
INPIISEALKECPTSGILWSKAIELENKNLQNSKSVTAFNNCGNNCYVILIVAIIFWNNY
KIGKSRKWFYRAITLNPSFGDGWATFLAFEIDQENEINQKDIINKCIKAEPNRGYMWNKI
TKRIENWRLNYPQKMYRYIKERFPHVLNKPISGNIWKIINDENVALPTNKKTKS------
----------------------------------------MNNNGNIIPDEISDKKKEKK
AKKLEEKKLKLAKKLERENLKNEAAKVLEHVCEDINKESYGYVKLSIIMENGKNIHLYNP
EEIYNLLYVEKNVDNEKREDIESEMNNKLEEGSCLWVRGRIHDIRSKGSLAFIILRNKIY
SLQCILDIKNVNNDKNMIKWVNNLSLESIVDIYGKLVKPEISIDSTIIKYEIHILKIFCI
SKNSKELPFLLKDANMKEIDDEATIRVNQDNRLNNRCIDLRTYANYSIFYLQSEICKIFR
NYLIDNNFTEIHTPKLLGESSEGGANAFQINYFNQNGFLAQSPQLYKQMCINSGFDRVFE
```

```
IAPVFRAENSNTYRHLCEYVSLDVEMTYKYDYMENVYFYDSMFKNIFNKLINNEQNKLFI
KNIKNQYPSEDFKWLAVTPIFTYEQAIKLLIEHKKLNLQSEEILTYDMTTDMEKELGKII
KQTHDTDYYIIINFPSALRPFYTMYNEEDPKISNSYDFFMRGEEILSGSQRISDVKLLLD
NIKKFNLDPKKLDFYIDSFAYSSYPHSGCGIGLERVLMLFLGLNNIRKTSLFPRDPKRLT
PMEITNALKKLF-RQNFAYIERNSIFDTQQKKDEELNRKKRKNEMENLQKPKYGDIENIN
DIEKNKQNEGDDKNSKDANLAPFTNTFDAPNALLFENLNKEYKYITTQDNFDGFRFEVDK
NVNKYLQSTHTLFLGTSLRDVGYLYQFGANFANSDNTLLMISRINLDGSVNGRFCKKINH
FVDCKFNFNTYNKSDPRNMYEMSLEVNNPQNTYNFKTIWQG-----------------PG
VDLTYIGSNCASIGSFGLRYNHKNHVLTMQCVRQPNFKSPEFMLNQAHSYKMQYARKISD
RLSVGTELEVTPETKESAMRLGWDYSFRHAKVQGSIDSSGKIAVFTQDYSGFGVSGYIDY
LNNEYKFG-MMHIAPSQEQPQPQ-MDNQEFIPQYKLILVGDGGVGKTTFVKRHLTGEFEK
KYIPTLGVEVHPLKFQTNFGKTQFNVWDTAGQEKFGGLRDGYYIKSDCAIIMFDVSSRIT
YKNVPNWYRDITRVCETIPMVLVGNKVDVKDRQVKSRQIQFHRKRNLQYYDLSARSNYNF
EKPFLWLARRLSNQPNLVFVGEHAKAPEFQIDLNIVREAEKELEQAAAVAIDEEDIENME
KLKTIYVDSALSIIKGALCVILQIPTSRTTESVKKKQNFIGILTVNSIKSEPTISQYDDI
KKLVKNKILENAPFYNYQIERSFADKFYGDCIYDNFGLPKNINEINLIILEEWNINCNRN
RILKHTGLIKNIEINNFKYINNKESLEVHFSVNPMYTFEELSTLYKNEKSLYNFLLCPIR
KVCTNSEHIVTNGENNEEYIYINVDDLLEKNKVLPPSGVENIGYERSNEVTPWNVNINGD
GIDYNKLIKQFGCSKIKEEHIKRIEMLTKKKAHHFIRRNIFFSHRDLDFLLNYYEKNKSF
YIYTGRGPSSLSMHLGHLIPFYFCKYLQDVFNVPLIIQISDDEKFLFNKNYSLDYINKLS
------------------------------------------------------------
------------------------------------------------------------
------------------------------------------------------------
------------------------------------------------------------
----------------------------MNEDNSKKNKIEIKSISKNEMEFTLYNSNAAL
ANALRRIMLSEVPTLAIDIVNVYENTSPFHDEFIAHRIGLIPIDSRNIKSYEFRERCKCK
ETCSRCTVQYIIEVKCNNANKINVSHYDIEALDHEPNIPMPIPNDNKNNNIEKENAIPIV
TLSKNQTIHMKLTATKGIGKMHAKWIPANVSYIIDHKIVINNHEVDKMSKEHKLLIANNL
NPDFYILKEMDDDRNVELRLSENMSVVMAESCRDALNELGYKDIVKIIYDETKFHFKIES
VGSMPPEQIVEMAIEILESKLKDLEPQIKASFYSIEEVAKQLKEQGVSLYGIQLDLEMYD
CTSKIKDLSLAPFGKLQMEISETEMPGIMTIREEYEKLKPFKGAKITGCLHMTIETALLI
ETLQKLGARIRWCSCNIFSTLDYAAAAVSTLENVSVFAWRGETLEEYWWCVEKALTWENG
EGPDLIVDDGADASYLVHKGAEYEKLYEEKKILPDPESGKNEEERCFLSLIKSSILKNPK
KWTNMAKKIIGMSEETTTGVLRVKKIEKNNGLLFTAINVNDSVTKQKYDNIYGCRHSLPD
GLMRATDFLISGKIVVICGYGDVGKGCASAMKGLGARVYVTEIDPICAIQAVMEGFNVVT
LDEIVEKGDFFVTCTGNVDIIKLEHLLKMKNNAVVGNIGHFDDEIQVTDLFNHEGIHIEN
VKPQVDRVTLPNGNKIIVLAQGRLLNLSCATGHPAFVMSFSFCNQIFAQLELWENRNTGK
YANKSYILPKELDEKVAFYHLKKLNAT---------------------------MYSYGM
EGDDTYLPQVQYPSPYENQY-QDEESPSPRGENHTPFIGYFSSHLLRTGFLLQCLSLILM
FVFYWAFGGTGIFIFDLYAGPECVKVSSAFHLTISVLMSIYLLGTLYIAMFQVFVADNSK
WCRGFRAGSKLLSAAVTLDLLSSILRLVQYLYAYFYMNMRWWARYQQTKSDWTLLHFGSI
VHSFSLFIYGAAFFYMEAYHDEGTYEELAWSNLTLFKLAGLAELLMVFSGFGAFFSILLL
GAIMCATVWAFSFEPLLEKWSPELHSRDINADVLPEIKHDDENCGYNEENMYEAYNGNPE
SMDYNEEMIKQNGNEKYINGNSNFYEQNNGIPTTYDYSQIEGQVKQNAEMGVSENKYIGQ
Y-EDANKPKKRTFRTFHYRGIELDKLLELNQEELVKLLPARQRRKFRRGIDKKAKSLLKK
LRKAKKECEIGEKPKPIPTHLRNMTIIPEMVGSIVAVHNGKQYTNVEIKPEMIGYYLGEF
SITYKHTRHGKPGIGATHSSRFIPLKMSNKKVQSPKEESIAKMLICKVHIGTKNLENKMK
RYVYTRAKDGVHIINLAKTYEKLQLAARVIVAVSNPADVVVVSARPFGSRAVLKFAQYTG
AQAIAGRWTPGMLTNQIIQKFIEPRLLIVTDPRTDAQSVKESAYANIPVIALCDSDSPLE
HVDIAIPCNNKGKESIALMYWLLAQEVLYLKGSIPRSQAWDVMVDMFLWRDPEQFELKNL
ANEEAAPTAPHLADNQYATEAPYDDWNKKDDWNDNANEEWKNPITVDEWMSMNKYNFKTM
SNLLKDKTPQYFDIQPHTNIKCIVHPSVIFTILDSYLRRDEDQTHVIGTLMGSIIDTNLV
EISDCFVDKHSLNEGGFLQIIKDHHETMYELKQKIRPRDQVVGWFCSGSELSELSCAVHG
WFKEHNSISKFYPHSPLNEPIHLLVDASLESGFLNIKAYVQLPINLVKEYFVHFHEIQIE
ILPCNVERSDVAKITEKVTKGGDN-------KEKLLNHEIDENSLKKLLIMLKNCKSYVQ
DVVDKKKKGNVAIGRYLHKVFSNDPFISIEKFDSINESILQDNLMISYLSNLAHLQFLIA
EKLNTSSMQMEKIVEIIEGLSNPDSRIRTECENTLNYYKKNDLNNTVLSILKLLKSHKNS
QVRLQCAILVRNLFRGYIKSDKDKTDDEEENYWDLLSNNLKNIVKSELISNIGIETDKMV
RSNLCSNIIDLSSKLLLNNEWPELLSVTFEFCNSNNNDVLISGYKILGGILNCISDELHG
KNEMISSICMKGLNSPSVQVRSECINLISCIVEDNNSPLIKCVQPCIPLILQSLSLMVKN
SVSDVAVLDECEKVLQSIGKMIDYNAKFFSKYISNLCDILFDICMKNENELNYDFDNSLK
SLSIEALITIPERRPKTALSVPHFLEKIINVSLLFMLDINNDCFNEWMNSLKESKDENQE
LYDIGEESLDRVGKAYSELSDPEFIHILYNKVSEYLMKNTWEHKYVAIMAIAQTIEYLPE
EEIEEQLENVIKMLLQVLLDQDVRVRYAACQAIGQISLDHQPYVQKEYPRQVITSLISTM
NDVHLRVQSHATAAFVNFAEELEKSALLPFSDMIIEILLQKLNTTNYLLVREQAVTAIAV
IAGVIEEDFLKYYPTVVPLMKEIIQKAVSEEERTCRGKAIECISIIGLSVGKEIFIEDAK
ECMSALLQISSTKMDPDDTVKEYIQEAIGRICRALGNDFYPYLSSIVPTILSLLSISPTP
LVDDDDDLTITMVSNGQYVGLKTSLLEDQEKALDLLIIIIEVLKENYKEYIEATASAILP
MLDYELSDEIKQKALSAISELIEAARIISDQTDNNKSMLLAILTTSAEKVLKSLSDTKLD
DNYEYVLDIMIIESNGLYMCLQKAGANILPNNTLKMFFNEIFKLLQYSTDRRVIYNQKKN
NEDVDDDELLIIDREEELEQTYRTNLLDILGVLIKHHTNQFLSTCCDICITFINNYLNSP
HAEDIALALYVCDDLLEFLQDNSVCLWEYFMNPLLLNINHTDHKVKQAACYGVIQANKIE
AFGKYANLAIEYLLKLLHQTPPSKKPKEYISAIDNAVAALGDVVLMHTSKFNNSEELIKL
WLNNLPIKEDESEGRRVHKNLIDLVSQNHPLLFGKDNSNIGKIIEIFLSIYETEFSDSDC
NKKIVSLISSLDQSYLSNLASTSLTNKQSKKLNHIMNASRKMPTISVHEEDLIEKLGTRL
NEEELTNICFEFGLEIDDIEIKNGKKIYKIEVPANRYDLVCAEGLCRALKNFMGINEDIT
YNIIKDNNNNKLNEKHLLEVDASVDNKRGYVVSCILKNIKMNDQIYNNIIELQEKLHLNI
GKKRAILAIGIHDYDKIQFPVKYKFEEKKKINFIPLNENKNLNGCDLFKFYEDNINLKPY
LKILKDFDKYPIIVDSENNILSLPPIINCDYTKITLNTKNIFVECTGTDLNKLEISLNII
CSMLSEYSQPKYTIHSVLVLYNENHQLEKGNKYLYPNFKNKKLTCDIEYVRKLSGIKDIT
IDNVKKLLKKMMISVTNVINNTIFEVNVPFYRSDIMHACDIVEDIAIAYGYDNIKYEPIE
ISKKHLLNTISDMFRNSMTECSYTEVITNALLSLKENYDYMLRKHISYDTPNKFVDSYNP
LYPPVQIMNSKTSEYEIVRTSLIVNLLKFVAANKHRELPLRFFEIGDISYTIYNKTDTNA
FNKRNLSIIFADKVTAGLEEIHGVLESILKDFQLFSHYKIEEKRKEKIEIRSDVYYELVS
INDPSFLDERVVNIVLQPQNLTFGIMGVIHPNVLENFSINIPGY---------------P
ADNTSTSSNSPVISIGNIRGLGGCDYGPFRMSNEFLGWKNKKTNSVYQYKCNDISEGEWI
KLSYNNNRLHLKFNESKDNLIVFFDGFPDRNLSEITQHFQKYFNIKLGTRKLATKGWNWG
EFKLENSNLIFDIDKKYAFNINTNNINQLNVQIKTDIAIELKNDEKQNTNEDVLSEIRFY
YPHENDENQNFQDLKNNLLEKVNIGDSKSECIASLSNIPLLVPRGRYEIEMYSKTFKLHG
KSYDFTVQYSNINKMLLVPKTNSNQYILIFSLNNKIKQGQTEYPFILIQLSNDDDMDLDI
NASEEDIQNYKLEKTLTGKAYDVVTRLFTALAKKNAIIPGDYRTAKNEHGITCSYRAASG
QLYPLNKYFLFVVKPVILISFDDIVTLSFQRTGNINQHRFFSLIIKHKRGISYEYTNIDK
SEYAPLLEFLKSKNLNIQDDANVSEKKTDFDDDDDDL--------SESDEEDYVAEEEDE
DDDNDDDD-----EYDDEDDDKMGDARSKTDLGDCRFYEKKFPEVDDLIMVKVNRIEDMG
AYVSILEYNDMEGMILMSELSKRRFRSVNKLIRVGRHEVVLVLRVDNQKGYIDLSKRRVS
PKDIIKCEEHFSKSKKVHQTVRHVAQKHNMTVEELNRKVIWPLYKKYGHALDALKEATMN
PDIIFKEMDISDAVKESLLSDIKLRLTPQALKLRGRIDVWCFGYEGIDAVKEALKKGKEI
SNNEVTINIKLIAPPQYVIVTSCHDKELGMQKIQEAMKVISDKIKEYKGGDFKQQGEILV
IGGDDEKRLEELLDKHDGLSSDDE-YSSDGDEDDSSNDDDNSSDEDDDD-----------
--------DNKIIDLEKEIKLRLEEQSNLLSIKGIDRVDIKQKKGKHSIICIHYIKNMCM
KNLFCNYLHQLIYDRIPPCKNYIKYNYCADKIRGSCMFRHTLENTNMNYYNENKEEHLDE
ALKFLHEKNICVNYLLGFCNLGYNCRKTHKNKSIKNIINILPKFYLDQILINKNLYTHLY
KNQQVINDMNKLKDALIILSGEKYQEKNISLTKNEIENSDIFINNRMLNSGINIHDDKNS
NRGGFNMLHNNNENILQNNEPNNIKTVGNIPEVYNLDNIPVASDKIKVFVIKCNQISHLY
LSILYGVWATGKNNTRKYVNFFKENYTIIFLFSVNESGGFQGYAKMITLPVKNLYENLWG
PITKRLGGNFRVQWIKIAKIDFDVFKNITNPYNDNLPLKKSRDGTELPLNIASIICNKIH
```

```
ALPNEDFLAGTIYEYKRRINHSVYFTNLYKKNMLNTNTMWDSIIFTLNQKSDCQQITFID
GIEQNISMYNPLGINILDNNLTQIFRDSEEWLTLEYFLQMNARTSRVKLVQAWHISPIEV
INKFKKRNFERLVLKSFIDTSSMDMNNSIQDVCIRGFSISKKGLKLSIGNFNLPGFPLVS
LNKDNLHSNSYELTYEHNASNEDKKSLKKIMEHSENILLSGERGIFEFFVCDVGVGLSLS
VNENEINSYDRDILPIEYDSIFIKKNGKKNLIDSLTIHYKNKENNNNEKE-NLALGGDIM
PTYGVLPYYTFNHEYIIYDSSQLLPRYLIQFECDPSAEELFSIPLCDYCGNAPSLYYCES
DEVKLCEKCDDIIHSQNKLVKKHIRKTLNEAR-QIGNCKIHLQNEVNMFCTVCHIPICNL
CMCSHAHKDLSNVSFFNFNNNSDTIISLKMAYNAIMQRSSKPSNFIKERKKNLNNLLEKI
DKLHEQVSLNMNETEKNVYNVLEDLVKQLHTTTDKKMSSILSEEYELKRQFNEIMWNENF
LYYLQTILPPADFMNAWLKHCQYREEIEKNSEHSEKINSLIFPDIRIKGNINVITEESIN
HENIFHSSANLMAPKKKEEEPKVLLLGRPKNTLKMGLVGLPNVGKSTTFNVLTKLNIPAE
NYPFCTIDPHEAKVTVEDERFDWLVDHFKPKSSVHAYLSIFDIAGLVKNAHLGEGLGNNF
LSNIAAVDGIYHVVRAFENEDIIHTEGNINPVRDMEIINSELIYKDISNCERNLEEISKV
LNRNKKDKIKQNEHDVLTIVLEHLKEHKWIKDRTWKSSEIEVINEFNFLTAKPVVYLVNM
SENDFIRQKNKYLAKIYNWVQEKNKGTIIPYCAEFEQKILFMTENEKEEYFKANNIKTSM
LNKIIKTGYYEINLIHFFTCGQDEVKCWTIRKGTKAPQAAGVIHTDFEKGFICAEVYKYT
DLVEFKSEGEVKANGKYLQKGKDYVIEDGDIVFFKFNVSSSGKK------------FYKL
DTIKCEVPINSEFNDRINKFVNQLRISYGTLEEFVDNFVYELKKGLEAHRRHPNLWIPHE
CSFKMLDSCISDIPTGQERGTYYAIDFGGTNFRAVRASLDGNGKIKRDQETYSLKFTGTF
SHEKGLLDKHATASQLFDHFAERIKYIMGEFNDLDDNNEKSVGFTFSFPCTSPSINCSIL
IDWTKGFETGRATNDPVEGRDVCKLMNDAFVRSNVPAKVSCVVNDAVGTLMSCAYQKGKS
APPCYIGIILGTGSNGCYYEPDWKKYKYSGKIINIELGNFDKDLPLSPIDLVMDWYSANR
SRQLFEKMISGAYLGEIVRRFMVNVLQSASSKKMWQSDSFNSESGSVVLNDTTPDFSECK
KIAKQTWDMDFTDEQIYALRKICEAVYNRSAALAAAAIAAIAKRIKIIEHSKFSCGVDGS
LFVKNAWYCKRLKEHLRVILADKAENLIIIPADDGSGKGAAITAAVVSLSSNMKQLPMPD
LYILIRWLCKAIVSSLFGDVNIINPENVPLYGSVIFVGNHNNQFIDACVLVASIPRQVKF
IVAEKSMKRAVIGELARVAGCISVKRPEDLKFKGIGRIYWNTGDTKIKGINTRFKLDVQI
GDKLMTQNKIFSVTKIESEIELILQDPININCEDKVNGVPFKIVPKINQSEVYNLVTHSL
KNGDTIGIFPEGGSHDRTNLLPLKPGVAIMTLCALADGIEDVSIIPVGLSYSKLYQLQGC
VTIFFGNAIIASQDLCKDYNNNNRETISKLLAKIEEGMRSCMLTSKNHETSRCIELCVSL
YTPERMTISKNKIYNNLQLFSEMFWKFGNSKEIENLCYELQCYEKLLEANKIKDDEVWML
KQSTSAATLKFIEQICSLIFCTIFGMTFSLLWLPLVAISVYLAEKHRKTSLKNSLVKIQG
GDVVASYKVLVLLVLLPTFNIIYGLLFSLYFYQSWLKRIAFTICSICILPICYYININYS
VQIPTLLRQMKIHLKVICGIINVWRDNERELISMRHELQLKVRNIVSKLGHKVSDSFLDQ
LHRNIPKFVINADTKRLIRGKDEWVPILKRSQLEYREEIL----QKEISGRDIYQIIKNA
RDKIKIDYKFWYTQPVPKINEEFSESINEPFIADNKVENVRK--YKLPEGYAWYVCDVND
ENDRKEVYNLLTDNYVEDDDNIFRFNYSSEFLLWALTSPNYLKEWHIGVKRVDTNKLVSF
ISAFPTDICINKKVVKMVEVNFLCVHKSLRSKRLAPVLIKEVTRRINLKNIWQAVYTAGV
YLPKPISDARYYHRTINVKKLIDVGFSSLNSRLTMSRAIKLYKIDDELNLKNLRLMKKKD
VDQVHKLLNNYLSKFNIYVKFTKEEISHWFMPIQNVIYTYVNEENGEIKDMISFYSLPSK
ILANEKYDMIYAAYSFYNVATTTSLKNLMQDAICLAKRNNFDVFNALEVMDNKSVFADLK
FGEGDGTLKYYLYNWKCASFDTSMVGIVLLMSEVNVTKVIVNNPICDILDPFVFTIEFEA
LNKLEADLEWKIFYISAVNNEGESNQDIELDNIYLGPIERGVMMFDYAVNPPDYKNMDTD
SVLGLQAILISANYKEKEFIRIAYYMNSFYKDIELREKPPMSPQYDKICRHIFVDNPRIV
KFTIPWDSEERDEFKEFDKENEKIELLNFSKIKEENQSNSNITNQSTQDHILIPNGLNYN
PNNGNFNITLNNN----MQTNMNAFDYLKNNEVLPDMDRCEILNENIKGISSITIDNKNM
MKIYLISYVVPILVYLLYILLNNQKGLSKYNNYINSKLNDVLLFFNIYEIKFPNSYNNNE
QILGLHIRDIEEDSRVAYEILKELTQKNYFRIFKVNLHVPCKLQKISEKCNEKTKCSVCE
CTEDEIPYNFRTNEVEIIHDQYAQEDLKKTFIASKLYKDILGTYVPSDEGFLSYVDLIYN
SPSYTAYEGKSIWNRIYLENCFQNGENACKEMNNFYKIISGMQSSIAALASEYYYLKNDF
VFGDMHTDNTIKNHYIKKIDYDYNLSFFKEKIALYPDRIENLYFTFAILLRALCRLKPLF
SQCKCNSGIKENDKDAFKLLNEFLGKQYHSCSSEEFLEPIFPTHGKEILSKFMNITSILD
CVPCIKCKLHGKLKTTALQIALVEGGDEHIGSLERNEATALINAIYHFADSILIIKK---
-------------------------------------------MARNVEKGRSMLNQWLK
AKELNDKKTFFKIPKNVNDVDDLESAVSYRKSIIKEICSKIKEIQNLSLGDQHVRELNDQ
INKLISIKNRWEIRIIELGGPDYQSESNALINAHGSELKGNNNYKYFGAAKNLKGVKELL
FKENDDRKKLLLKRRKEKRNLDKIVNIHYFGYCDEENEILLNEELKIQKKLEKTDLEIIK
KMNY-MGIFRSETMKHGTLVLPADRAREYMDCLGKQVDIQFIDMNEKTMKRQYKKYIQRI
DDMERILRFLEENINKLPNVKIKKSKIENFLEHDNIYELDQVEESLNRLHVQFVRFCNNN
KDLVDERNSAIEEKHVILTALNQLHPDMSKRSNLR-TIHEDNIDENNLMNN----SEEDA
SLSTHIMREGINMMFTNISGVIKTKDQESFSRTIFRALRGNTYTYFQNIDENMSNTDTLN
NESSSMDNKIGENNNENKENGGNNNELKSVFVVYCHGSTHSSIYEKIMKICKAYDVRNYE
WPKTYEQANKRLNELKEIINDKEKALKAYEEYFINEIFVLINVVEPNKNSLIEEWKLFCK
KERHIYNNLNYFEGSDITLRCDCWYSVNDEEKIRHILMNKSTNDLVSALLLSDKLLTPNI
SPPTYIKTNEFTNTYQSMVDTYGIPRYGEINPAISTIVTFPFLFGIMYGDVGHGICIFLF
ALFLIIVHNRMKNKNNNEMLNMLFNGRYMLLLMGFFAIYAGFLYNDFFSMPLNLFTSMFE
VDKVVDSVEYYKRKQILNAETGQMENAPPYIFGFDSKWLGADNELTYINSFKMKFSIIIG
FFHMTFGVIIKGFNALYFKKKMDFFFEFLPQLVMMLSMIGYLVFLIIYKWITPIGYGGYK
KQGIINTIINMYLLKEINKDNQFYEHQEIVQIIIITLFALCIPIMLICKPAIKTYKMIKE
KKKRMAIHYQTVEKEMTNQFGGVGLYGNN-------SMHKRVTKIGYAKNEDEYLLLKRK
GKKTDDEMEAHLLGPSSYHSSNASNTNESFDEHEENISEIWIEQLIETIEFILGLISNTA
SYLRLWALSLAHQQLSLVFFEQTILSSLEKNTFMGVLISLIIFSQLFSILTIAVILCMDT
LECFLHSLRLQWVEFQNKFYKGDGIPFKPFNIKKLLSDREMLGIQEGRAFFNAARNPNNR
PENNIRIEDNLPSLNE-YNYFQDFLSRYSSNTLKEGNLKRTLFENVKNNNFTLDLKLDDI
YKQQTVFENNEISATPISERKIKKPDNYSAILARALSERPLTYLPTVERVCYEVCETANI
LSDEDEHLNYIQINLLNTFIRPTPIRGLLAATQERFVVVPGIIVQASKPQHKMRKITLQC
RYCDHKMSIDVPLWKDKPQLPPYCRYSSTMKSSMGVANPMDNQLGCNGVLEPYVILPNEC
TFVDIQSLKMQELPEAVPTGDMPRHLQLNATRYLCEKMIPGDRVYVHGVLTSYNPNPKPT
RVDGTNFSYLHVLGFQKYDDMSGNDLNFDVEERNELTLLAAEHDIHDKIFKSVAPELYGM
DEVKKACACLLFGGTRKRIGEETKIRGDINMLMLGDPSVAKSQILKFVNRCAPVSVYTSG
KGSSAAGLTAAVMRDSHGVFSLEGGAMVLADGGVVCIDEFDKMRDDDVVAIHEAMEQQTI
CICKGGITTMLNTRCSVIAAANPSFGSYDDSQDTTDQHDFKTTILSRFDIIFLLRNKQDI
EKDTLLCNHIVALHASKHKSQEGEISLSKLTRYIQYAKKEISPLLSKEARDSLRNYYVQT
RAEYRGDKRSVTKKIPITLRQLESLIRLAESFAKMELSQFATEKHVQMSIDLFSASTAET
AKQCMVFEAMSPSEQKAVKQAEDAILGRLGKGQRASRVNLFRELQL-GFDRSALSK----
-----------------ERLDQELKLIGEYGLKNKREIWRVQYLLAKIRSAARYLLTLDE
KSPKRIFQGEALLRRMVRQGLLGENEEKLDYVLGLTLPKLLERRLQTKVFKLGLAKSVHH
ARVLIRQRHIRVGKQMVDIPSFLVRIDSEKHIDFATASPFGGSRPGRVKRRTLRNQKEKA
DGDDNMVRQSQSMYDRHLTIFSPDGNLYQIEYAIKAVKNTNITSLGVKGENCAVIISQKK
MATQYITQDKLLDYNNITNIYNISDEIGCSMVGMPGDCLSMVYKARSEASEYLYNNGYNL
NVETLCRNICDKIQVFTQHAYMRLHACKGILIGMNEDNKPELYKFDPAGFCAGYRACVIG
NKEQESISILERLLEKRKKKIQQETLEEDIQNTIILAIEALQAILAFDLKANEIEMAIVS
KKNPNFIQISEKEIDNYLTFIAERD-----------------------------------
------------------------------------------------------------
------------------------------------------------------------
-------------------------------------KNITEESIKDMFSVYGSVEEVFI
MKDNSTGLGKGCSFVKFAYKEQALYAISSLNGKKTLEGCNRPVEVRFAEPKSSKQSQSQL
GIQPIQNAPHGISPQAHPGTPNNINYGNNFGVNNNYPRQVGVWKEYYSGEGRPYYYNEQT
NTTQWEMPKEFETLFMNNNPNIHNLSDSSGPPGANLFIFHVPNEWQQTDLIQAFSPFGEL
LSARIATEKNTGRNRGFAFVSYENIESAAAAISQMNGFMALNKKLKVTVKKGEEEEMKKY
ISQNGVNTFQQMARQQKNIPSQPNTIGQPNFAAHQNPQAQNFFYSNNNSYRCGPYMTTAH
RPTWYNAIGGENQGGNRKVGQTAKVCSRDLPGHTKMKMRDLNDYTEDKEIIKNNLIELEN
KSNKREGNNNKLLAIENIKKLTNCDYTNPFPEDEDDEIQDEWKAHKMKKKKKEKNNNNYE
QNDKMSEENSNIIDSDMN------------------------------------------
------------------------------------------------------------
----------------MDSEETINLAVKYAKDAVVEDEKKNYKEALNLYIQSLQYFNYFC
```

KYEKNDNIRELILKKMEVYITRAADLKEIINKKETIETKEKVGASEEAKENMKKQIKDFI
LNKDQNVKWSDVCGLETAKEILKEAVIFPLKFPKLFNSSALPYKGILLYGPPGTGKTFLA
LACANECNMNFFNVSSSDLVSKYQGESEKYIRCLFDTAKEYSPAIIFIDEIDSLCGSRTD
GENESTRRIKTEFLISMSGLNNYKNNIIVMGATNTPWSLDSGFRRRFEKRIYIPLPNLYA
RMKIFEKYINKAKSNHNITNEDIKNFANITENYTGADIDIICRDAIYMPVKKCLLSKFFK
QVKKNNKIYYMPCSPGDPDPTKIEKNVMSINENELLLPPLSLQDFKIAISNSKPSLSLDD
LKRYEEWTNLYGMSGIMNTNIQKLKLVLKLGTKSYSHDVSKLKRNPNLEKLTGDKHDFSK
VIFKKEQIEDVIKEVKFDYYYFNEGKKNKYKDIPLNVSIIKESDLPPYKPVDEKLNFSIL
ENDLKIISTNKNSGVCSIGLYIKCGSRYEEINDKINEQGMSVMIENMAFHSTAHLSHLRA
IKSLEKIGANVSCNAFREHIVYTCECLNEYLPIVINLLIGNVLFPRFLSWEMKNNVNRLN
TMRAKLFENNEMYITELLHNTAWYNNTLGNKLYVSESNIENYTSENLRNFMLKHFSPKNM
TLVGINVDHNELTKWTSRAFQDYVPIPYIKQKEVTPNYTGGFISVEDKNIKKTNIAIAYE
TKGGWKTSDMITLTVLQTLMGGGGSFSTGGPGKGMYSRLFLNVLNNYNFIESCMAFSTQH
SDTGLFGLYFTGDPANTKDIINSMALEFHKMNKCTDEELNRAKKSLKSFMWMSLEYKSIL
MEDLARQMMILNRILSGKQLCDAIDAVTKEDINRVVSQFLKTKPTVVVYGNISHSPHYDE
ICKMLGMISGIRVNDNCVTEFNNMKIRKTCRWIIFVIENCEIIIHSKGETTSLKDLVDSI
DKNNNIQCAYVVFDAVNKIHFFMYARETSNSRDRMTYASSKQALLKKIEGVNVFTSVVES
ALDVADFKMQPHDSNRGLNNFNNSSNTNSSNNALNYNNQNNMNTPIASPFSYSGNTTNNI
NRGMGNNNDNAGYNTVKGNPNYYSGQNYYNLQNPVQHDAYAANSNNLNEGMYGEPPNSNP
YLNNQGQYMGTQNAYMPMANNANTFNDIGNNLRYDANKPINSVQPIVNDTYQEFLQFNAF
SHFVKSSVNYMPANGTLKQKTHVPLGFTIQPLAPIPDGYPELASVNFGNSTVVRCKKCRT
YINPFARFESGGKKWNCNMCYNINETPQFYFVPLDEKGKRKDLFQRPELCTGSVEFIAPS
DYMIRPPQPPVYLFLIDVTVTSINSGLLDVVCNTIKKLLPKNND--DNATNANSNDKKVF
DPRTLIGIITFDSTIHFYNLNSNLKQTQMMVVSDIQDIFIPLPENILVNVHECQNVIDNL
LDNLPSMWRNNKMSDCCAGNALKAAVMLIKKVGGKILFFLSSVPNIGDLTVNVNREAKDK
SSYKKIYNSGNSGNSNTDLKLREVEMLNPANNEYGEFAQSITQFQIAVDLFACPLYNIDL
ASIYPLIKNSGGSLYYYPQFNVHQYSDKLREELLFALTTETAWESVMRIRISRGWKITNW
YGNFQFRGVDLLALPNCHSSQTFSIVVDLEENVVQDSVVYVQSALLYTNSNGERRIRLHT
YALPITQNIKTITDSINPQVVVSLLSHQAIEVIKKGKIADGRNLIQTLCSQVLSTQLSSS
ENLQLLPIYILGMLKSVAFRD-GDVPPDMRIYQWSRLENIPVESIEAYFYPRMFSLHNLE
KHHGHYDENNNFIFPATLNLTCENMTQDGCYLVEDGENIVMWIGRSINPQWIYSVFGVQS
LDQLNSEYAENHIGSSDNPSGLQVLNIINALRKARTPSYMKLTVVKQGDPLEYKFFSYLI
EDRSQHMMISLKEFLAKFYPKYPQFTPSLSMEEDSFKNRLLKRNIDIWIEKYRPEYLEDV
VGNPFVINTLKSIIVSGNMPNLLLAGAPGTGKTTSILCLASEMLGSQAKKAVLELNASDD
RGINVIRDRIKSFAKE-ISLPPGRHKIIILDEVDSMTTAAQQSLRRIMELYSDTTRFALA
CNQSEKIIDALQSRCAIIRYFKLTDDQVLKRILKICEYENIKYTDDGLETITFIADGDLR
KAVNCLQSTYAGLEVVNKENVLNICDIPSPERIENLLKHCISSEWRKAHDIAYDMIKEGH
TPFDVALTSSNVLRRYDLGSEAIQIEFLKIGAMACNTMASGLSSVIQMDKLIADWCIAAK
TLRGKCMNADSYTQNRKASFPINNVLAEETEKLVDTYKYEDEVNNILKKGTSKFKRNGKN
SMAFHKSLAVVNVAAGLDGCDDQLLPASFRALEADLNLHPSLLGYITLAQTLMLSLFSPI
WGFLSDKYSRKWMLVFGTALWGLATIFLANINDFAHIIIFRAINGLALGSIGPISQSILA
DAAKNESLGLSFGIVQLSSSIGRLIGGVVTTTVSMKYFGTIRGWRLCFIIVGALSILLSI
IVALFVEDAPRQVRRKETSSYMEGSIIDGNNEIIVEPKRSQSYMLYQNVKEMLKDSLSKK
SIIIILLEGFTGTIPWLALSFNTMFFQYCDLSDLQAAVITGFLLIGSALGGVLGGHFGDI
MHNISNKHGRPFLGQLAMFGRVPLVILTYLVIPKKKESFELFVLSCFFLGLSSIAGVAVN
RPIVSDIIRPDYRGTIFSLTIAIEGVGSSLIGAPLFGYLAEEVFNYRNNNLLISDMTTEF
RSHNAEALSKTLLYLTAVPWVLSFIFYSLLHFTYGAEYSKMNQIIESEYKYDDEDDETMA
DKVMS-MGCDPGVSTAVSSIVQHQNFKRMLMFGLRSLSDFCNPTSKAYKENAYDALNRDA
IPSINKAVNNYKDDDDILYCSSKVLFAMSDYCCSEKDNDALKKLVNDGGINAITEIIKTI
PKDQDTLKNCMLFIQNMKDISGHIDG-ELSIALLNVFTSDTHTAKLGSSIISALSVVSKS
PSGSKALNDENAHHKLIDHCLSIQNINDETAEIIEGAFDVIKNLLSNGYVVPTIIEKSVV
ILDKFKSYPRVVSKGSDTMKSAVGPEQLTDCLNILKKEQQGSKEHDSALELLSSLSYISS
ITDKIVESGGIPVLIELINSGLQQYDSNPEKIARLVAGASRMLGRISNNPSHAGVVYEYG
GIATLCTALSYFPNDVDCVSAICIALIPFVSRSNYANEINNYLLFASLFPILYASVESIE
LSKASMACIASASMINEFHEQMVNSQVIEILSTCIQYHLTDIDYLSNVFSVYFRLSDYIK
TIEPINQYGGISGIANALSAV--------------------------------IDSVLTV
MLENENKEVIIQEGTKIMENLATENDCQRHISNLESIINLAQSNPDCAYKTLAAISGLSR
IQSLKLMLESKGADSSIYNGMKTWIESPKFNEQTKLIKAALKTIKILKLNVLNKVHEVIA
SIVELMCIAQVKRLAEGEGPDDNILITSAECINYLTEVNKINTKEIVESSLESIFKMMKK
YSESRLTQTNLLSAINNILLSSNMICADVLVNKGYVKQIVTYIHKVPMYVDVQIIGFSVL
ANMLKINSDSLDAIKKANTLIPLQNALRTHVKNTKLKTTCAPLLAVLMPLDTLTREIEEL
LKLCNKSIQGNNLSQLHEYLVSLNELLLTAESSKISARCNIGQTLNNIVEWLKNNPDAYK
NDNAKDYSITGRSLYDATISETAHVCSNVSQSRIGIVHLTKNNMASSLMQLYNLLKMPGD
EYTEEAVVNILESLCLLLKHDIVNADIAIDLGLIEKLCSGINHFSEFDTVIKSTFSCLAC
ICATNKRINQLITHPEYDKLISVIVNLIGNSEKNKDSRRNAIKALYELLKVEKEEISIDI
STKTPIVDNLFKIMGEYQMDLPIIQDSSKCLSNIADHVTIEEKMKIDKYSAMKILLECLG
KNKNDESTAKEIMSVLVKLCNNNDKPQFKELGAIDVISDVTMIHGKNEEISKLGGILFSY
MGADEQVKKLMKLILSVKDNDADAVQEIDNLTGKLELFLRAPLEIHSDALQYTDATLQKL
NGYLGSNLENVSFQTNIALVNKRLVDRVKYDFEDHIGAWAVASAGILNQYTDMISNKVGL
KHDRIVAHIYSVLAGCYINPYTEQLIADNLSPILVSTYQLLEDNKNKPFVVQSIFELLEQ
----------------------------------SNDAVFISGTRLMGALAETANTSGYI
NDMDVPRIVESCELLVNVGSSKDRILEFISLIDKMVLSNLLDEKIASDALKKISSLISEE
HLSNYPDQDRTNIIKAYANLLKDSASTGLFAHIRQANKLEAINNLEKLMEYEQDEEVKLA
VLEAISEISACDPFTASKLLVSLLPSILKNDYQDILNNKNIADAFLQMLEKIVQNEGIGR
QLSTNNELYKVLHDLEEAVEKKREELGDEFVNNTKLRITNIFNAIEDDKPKEKTCKDVFD
TFTKYKNKEMSISVLDEPSINDDINFLLDRIRIYNKDNLSHNTERGIDNSYGFMSIELLC
ENQDNLQELIKKEFHIIAFHSLVKQPEEHVKHYSCRSLCAFTKDPIGIQRVIADIKDYAN
IISTSVGDLTANDTLEKEEKEDFLINRVLLIDRTANNRNIYNNTNAVRHLIEIWNQYDQG
YYSVSLLRHVFRAMRKIVSDFHVQTLLDANILVRLINIINSLETDKILYPDVLFLIGSLA
IVKEIKTQIGELKGIHACVSLLLRYINVEGMEPTITNCCLALANMCIDHKANSNIFFSLK
GPDINVRILREYRSNFDVVNGGSVLLCNILFRNEEMKKSYGGNGAPGELVECLRSYDGSD
NKNAVRCIESIFKAISNLSLYTANIKQFLDTQIEVSYQSWLNKLNESFPDAELETGLRTL
SNLVMENEELNMRKFGITLIPVLNVLKQNRENSKVIFFLLDILCSLCRLNENAKAFAENG
GIETTINAIQLYDYDVSLLTLAIHLLSNQCKIESSLPLLMKSDAFSILISCMEAETEEFE
ITELVVSSLRCTRRLIQSEELAYEFCNCGGIPTIANLISKSVKKSIVVLEALRILLCVLY
YTKNVEGVVNEYSEEDEELFNARLGGWYNISMDKEMIDVILQSVLTCSNDENHQKQLRLQ
KVSLGLLAYFAYHRLGIISMTASGFDILAKNNLNHFGGDMVIMQLLAICIDNIAMNSAEI
YDMTITRDIIKLFKSSISKIPNKKDNKQIVQSIEKTVEAMGSDGDPLDAFKDTILTFDFS
LSEFDKDPYVNGVHDLPQNIKEALRTGGQYKIYHKSDKRTLFKWKASQDLGTLEWTVGEN
VDRIFKISVVRIKNISKGLVHPLEKIS-------FISKTSICIY----------------
------------MLVY------FFFMDKKAREYAQEALKFIQRSGSNFMACKNLREKLES
HGLIHIKEGDQWTLQKNQGYVLCKENRNICSFFVGKNFNINNGSILISIGHIDSCTLKIS
PNNRVTKDQISQLNVECYGSGLWHTWFDRGLGLSGQVVYKKDNKLIEKIIQINKSVIFLP
SLAIHLQNRTRYDFSVKVNYENHLKPILSTLLYEKLIKGNENI-----SNIDDDDNNSKN
LNSSPLLYILANELKCKEEDILDFELCLMDTNKPCFTGVYGEFIEGARFDNLLGTFGVFE
AYVELIKILKNENNKNE---

---NMNISKTLFSVFFVLFFIS
SYNVKCQLRFASLGNWGKESKSQLLNAKYLKQFIKSERVTFIVSPGSNFVDGVKGLNDPS
WKSLYEDVYEEEKGDMYMPFFTVLGTGDWTGNYNSEVLKGQGIYVEKDGVTTIENDEEKT
KYPKWIMPNYWYHYFTHFTVSSGPSIVTTGHKDMAAAFIFIDTWILSSNFPYKDIHDKAW
EDLKLQLNVAKKVTDFIVVVGDKPIYSSGLSRGSSYLSYYLLPLLKEAQVDLYVSGHDNN
MEVIEDNDIAFVNCGSGAISNGKSSIKNPKSLFFSSDIGFCIHELTNNGIVTKFISSKTG
DIVYTHKLGLKKRKTLDKVNSLQYFATLPKVELIDVPAAGPMGNKDTFVRIVGTIGILIG
SVIAFMGVSSFLSKNMKMNTNFKKGNFIVFEGVDRSGKSTQSKLLVEHLKNNNIQVQYLC

```
FPNRETTIGKIIANYLKMESTFSNETIHLLFSANRWEMMDQIKNLLINGIWVICDRYAYS
GVAYSSGALKLPKEWCMNPDKGLIKPDAVCYLNVPPTYAKNRSEYGKEIYEKFEIQKRIY
EAYEHFSNEDYWINIDATKSIQEIHNTIVEEISKMCSSKKKQLEYLWS-----LVCKNCQ
SSDIETNEGQGEIICLRCGSVLEENKIVESLEFVENNNGAISMVGQFIPSSGTKSFMLSW
GIRESREISLQKGYINIQKIADNLHLSTQHIEAAQRIYLMALQRNFTMGRNNSYVAASCL
YTICRREKSPIMLIDFSDILQTP-KPLGKTFLKLLRLLHISVPNIDPSLFLERFAYKLNL
KNDIYKVTYTGIKLIQAMTRDWISTGRRPTGLCGASLLIATRIHGININSNTIAEVVRIS
NPTIIKRLYEFKNTNIAKIKASEFDKISIDDIPSSSIPPCVISDNKKKIKYNLLQKSKTL
SLCGSEEQYALCSNSTCSVENYEKNT---DLQNGNICSYSELDQSKFFNSTQN-------
------KYIQASSNEINLDEICNDNPEGNDIDNLAFKIINTINIEKNSEFLKISDSPLNE
LGSTKNNLQNQDIQNNPRNMIGSSKLQSSNLINHETSISTITDNGDDEYNEIKESNEKYK
NSNNFDMINDNNLKQNNKLLDSAYCTTLKQTVNSELSNVINDINEFDLFDNNALSKNSSN
STSSLNKINDVNNILTDFNYFFENNSNTINDQTANFDVNSDDSEQLNDETISDSYDSEIE
NIILSEKERKIKMLIWDDVMKGCMPNLSKNIKRPKKRQNTDINNSKN-KIPNNKNNDDDP
QDQLSTGDSVIKALEKSNKLLPKKINYDVLKSLFSS------------------------
----------------------------------------------VCIYDNDICEISDI
GVNFYINENHVENKICRSNAVLSNLQELNNYVHVYNYTEDLNSKFIEQFDVVVCCDTKDS
DIIKYNNLIRSIETKNIAFLSCNVYGLCGYIFNDFGNNFICYDKDGENIKSCNISKISKD
VNGIVSFDFDKSSPFQNGDFVKFTNVEGMTEINGKIYQIKNLKKYTFTIGDTSKFSDYIK
GGECTQIKTNLKLNFKPYEYIKNKPLFGLSSDNNVKIVDDKKGGKIIFEKEIFPTSFIIS
DYSKLNLSNYLHYAIQGLKWYEIEY-NCLPENNQNDEFEKIYKKACDLNSKDKENMHPWS
VEELDKNVIINVAKYSKAHISPITSFFGGLLAQEIVKFTGKYMPIHQLLYMDFFECINMN
DEENIDDKKKLNCKNDNIISIFGKKFQDKLNKLNIFLVGSGALGCEFAKLVSLLDMCTIE
SNGSLIITDNDNIEVSNLNRQFLFRKEHIEKSKSLVASNAIKNKNKNINVISYVTKVGQE
NEHIFDEQFWSKQDFIINALDNIIARQYVDNKCVWYSKPLFESGTLGTKGNVQVIIPHMT
QSYNDSYDPPEDSIPLCTLKHFPYDIVHTIEYARDIFQGLFYNVPLSIQQFLNNKNEYIK
NIQNEGNNASSLENLENVLNTLKEIIKENNFNFCIKKAVHLFHSNFINQISQLLYSFPLD
YKLSTGEFFWVGQKKPPQVIDFDINNIYVQEYLVSTSNLYAQVYNIPTCYDIKYILDVAS
QIKVEPFSPKSVKVNIDEKNLNNISISYAQDNKLIQDYCNELLNIQTDSLNVFPIEFDKD
EESGLHVNFIYAFANLRAMNYKISTCDKLKTKMVAGKIIPALSTTTSIITGLVGIEILKY
VNYSDSIQKYVKLSDQEKKNEKDILSYFKNAFINTALPLFIFSEPMPPFKIKDKEYDELM
KGPIKAIPNGFTTWDKIEISIKSGTIKDLIDHINEKFNIDVNLISVGNACLYNCYLPVHN
KERLNKPIHEIYEQISKRSLPNDKDYIVIEASCSDQDLVDVLIPSIKFIYKCSKLNQDLL
KKAINDVFEGTKQKKRKFVETIELQIGLKDYDTQRDKRFSGTVKLSNEVRKKLKVCILGD
AVHSEEAQKLKLDYMDIEAMKKLNKDKTLVKKLAKKYDAFLASQVILPQIPKLLGPGLNK
AGKFPSLITHNDKINDKILELRSSIKFQLKKVLCMGVPVGHANLKEEELRSNIVHAINFL
VSLLKKNWQNIRTLHIKSTMGKPQRIYG-IIESLNDNENKKKWEDIDADDDLESNKKLSY
FTNYENGVKVVTKYSENLKKQT--VTKKIKEVIIKKRLNKEIENRLKLKNFNVDAFSSVI
VEPTDVVNIEPPKNNLLDFLKDTEYDYLFAEQTDKTAKDLKNRFKMFKDEEAEEAKPDDA
NKVGAKRDMMYYRSHDECTIRVTNLSEDVNESELSNLFGRVGQISRMFLAKHKETQNSKG
FAFITYSKREEAKRAIEKLNRHGFENLLLSVEWAKPSNRMLNDIIIQVAIASIGVTIVNS
DKVKFLHKFKYAVYALIFSFLI-KGIPWNRENYYIYLNITPNATKQEIQTAYRQAAKIYH
PDKNSDESADSSFIKLKHAYDVLSDDVRRSNYNRFGDYKNGEVDDNTATLLICLSLVQHA
MFFIIGYFLSYRKQLEFSRQIFLVYNIASFCFELQFRFIEDDTTFDWLPVIGYLLPYEKI
KLLRMIFPIVFFISICISAYVYTDRNANLIYLMRSILSTNRIIVERSNDVVESTNYLKKN
GDQLISKLQQTRKSGNPMQINNKTSENDTLDSFESDNNNNTKDENKNAEDE--NKKLLEN
VKEFSLTLDSQQMNLLEKCFEIMKNKKTDDKKGKKKSWFEFFSLQMVFGVIFVYMWLTSK
--QDYYSILGVSRDCTTNDLKKAYRKMAMMWHPDKHKDVKSKKEAEEKFKNIAEAYDVLS
DEEKRKIYDTYGEEGLKGSIPTGANTYVYSGVDPSELFSRIFGSDGHFSFSSAFDDDFSP
FSTFVNMTSRKPRPSSNANINHNNYNAKPTTYEVPLPLSLEELYKGCKKKLKITRKRFMG
TKSYEDDNFVTIDVKAGWKDGTKITFYGEGDQISPMAQPGDLVFKVQTKPHDRFIRDSNN
LIYKCPVPLDKALTGFQFIVKSLDNRDINVRIDEIVNPKFRKIVANEGMPSSKTANMKGD
LIVEFDIIFPKNLTSEKKRIIREALANTFMKGIILIGYLLFIACLNYLYVQGAYFYVKEG
VEKCFVENVTKNVIIVSLYDNYGTKELKCLINVKDKNGLVLYTHDVSQMSKGKISYMAKS
SGLHYICILCPSNNWFKDTSIKWNFSIEVGGADIDINNTAKKSELSATLNTLQNLKKKFN
SMKSHHAHQKVIADNMHEHNKNVHKSMIYCYIIEIIILIIITGYSIMHLKNYFKANKLM-
------------------------------------------------------------
------------------------------------------------------------
----------------------------------------------MQLKKEGKLLSAKA
KEEKKKRELYLQTLKESGMLIEPKEKAKAEIINLDINKAKLLLKKKKNKQTSNADVKNKE
IKDNENK-----DKESDDIEEEKEIVLDDWEDFLNMDNEEKKKAEKNNIDQSTKENKQTQ
IVKKSSTNDKKGKKQKNNKNGEEDKDNE-KTEEEEDIYRSSIVCILGHVDTGKTKLLDKL
RHTNVQDNEAGGITQQIGATFFPKDILDKEIKKIDGTIKCLSKGIMIIDTPGHESFYNLR
KRGSSLCDIAILVIDLMHGLEQQTKESIQILKQRNCPFVIALNKIDRLYMWEKNDWEPFN
NTFKNQKEYVKEEFNNRLQTILNELSEQGLNCQLYWENKNPRKYVSIVPTSAITGEGIAD
LIMVLVKLTQSFMLKNIEYNKKLECTVLEVKNIEGLGTTIDVILTNGILKESDTLVLCGM
NGPIVTVARALLTPQPLKELRIKNEYIHHKSIKACIGVKISANGLEEVLCGTSLFVANNN
DEIEEYKKKVMTDVSDVFNHVDKSGVGLYVMASTLGSLEALLIFLNDSKIPVFSVNIGTV
QKKDVKKASIMREKGKPEYSVILAFDVKIDPEAEKEAQILGVEIMQKDIIYHLFDAFTAY
LKKIEDEKKQSKMTDAIFPCEVSIINDCVFNKKDPIVVGVKIEAGILKIGTPLYIPEKNL
KIGNVVSIESNKKSCNKAKKGEEVCVKIAGEPNVTYGRHFDFNQKIYSKITRESIDVLKQ
YFRNELTMDDWRLVVQLKKILNILMIINEKDKLAEQNLETLDVTKLTPLSEDVISRQATI
NLGTIGHVAHGKSTLVHAISGVHTVRFKHEKERNITIKLGYANAKIYKCTNPDCPPPECY
KSYESSKEDDPICPRENCNSKMKLLRHVSFVDCPGHDILMATMLNGAAVMDAALLLVAGN
ESCPQPQTSEHLAAVEIMRLKHILILQNKVELIKEEQALKQQEEIRNFVSGTAADSAPII
PISAVLKYNIDVVCEYIVTQISIPRRDFISSPHMIVIRSFDVNKPGEDIETLQGGVAGGS
ILHGVLKVGDQIEIRPGIISKDDKGEITCRPIISKILSMFAENNNLKYAVPGGLIGVGTR
IDPILTRADRLVGQVIGHLNKLPDCFAEIEISYYLLRRLLGVKSQDGEKNTKVAKLKNGE
FLMINIGSTSIGCRVMGIKNELAKLELTGPVCTKIGDKIALSRRVDKHWRLIGWGQINKG
KPLDLQEPIMIHFVLLISRQGKTRLAKWYMPLSQKEKAKIIREASQITLNRTPKLCNFVE
WKEYKLVFKRYASLFFILCIDKSDNELITLEIIHHYVEVLDKYFGNVCELDLIFNFHKAY
YLLDEILVSGELQESSKKIILRVVASQDSLMEVNKSNKKLGSII----------------
---------------------------------------------NEQHNENMEMNCKNS
EYDNKKNMCKSENSEHSEEKNKEINYECYNKIDLINEIKKTKKHMDEKLVDNQVLKEKYL
SVLAEKENLRTRYMKEIENSKLYCISNFAKSLLDVADNLSLAIKNISEESLKSNEEINNI
YKGIEMTETILHNIFNKYGIDKYNPINEKFNPMFHEAIFEVSDTTKEKGTVATVIQPGYK
INDRILR-----------------------------------------------------
------------------------------------------------------------
------------------------------------------------------------
------------------------------------------------------------
------------------------------------------------------------
------------------------------------------------------------
--------SGGMRMRLCLSRILFSNNDIILLDGPANHLDIYAIQLLIDYIPKLNITCIIV
SHDRNFLNEVCTDIIHFHRNQLTYYSGNYDQFEKTRVEHLLQQQREHDSIELKKKHVQKF
IDRFRYNSKRATLVQSRIKLLNKLPVVNLEKDETPFKFSFLEPFYVSSVLIRLRDVSFKN
EMFKNLQIKKNESIIIADDFEDSNAKPIELSNPDETKKDTLTNNIAGDYQFKHEFLFKNA
NFEVDMDSRIAICGVNGSGKTTLIKIILNLIDVFDGELYVSNKANIGYYSQYHVDNLNPV
FNSIQQLQYNYSHKNIKEDEAIKYFNKFNIPTNILYEPIYVLSGGQKSKLALAILAYKNP
NVLILDEPSNHLDIESVQALIVALSLYKGGIVLISHDTYLIKHVADEIYHINNITKEVVK
IDYEFEKYTKLLLENKIMPREIITLQCGQCGNQIGVEFWKQLCNEHNIDQEGILKNNNFL
NEDRKDIFFYQADDEHFIPRALLFDLEPRVINSIQTSEYRNLYNPENMFISKEGGGAGNN
WGSGYSQGHKVEEEIIDMIDREVDNSDNLEGFILSHSIAGGTGSGMGSYLLELLNDNYSK
KMIQTFSVFPLLTNESSDVVVQPYNSILTLKRLILSTDSVVVIDNTSLNRIFVEKLKLNN
PTFQQTNNLISNVMSASTTTLRYPGSMNNDMISLISSLIINPKCHFLVTSYTPITVDKHI
SNVQKTTVLDVMKRLLHTKNIMVSVPVRRGMYISILNIIRGETDPTQVHKGLQRIRDRKL
VNFIKWNPASIQVTLAKQSPHVISPHKVCGLMMANHTSISTLFERCVTQFDRLFKRRAFL
```

ENYKKEPMFSSADGQGNFEEMESSKEITQNLIDEYKSAERDDYFSHTYMMEKYHGLEKIG
EGTYGVVYKAQNSDGESFALKKIRLEKEDEGIPSTAIREISILKELRHSNIVKLYDVIHA
KKRLILVFEHLDQDLKKLIDVCDGGLESVTAKSFLLQLLNGIAYCHEHRVLHRDLKPQNL
LINREGELKIADFGLARAFGIPARRYTHEVVTLWYRAPDILMGSKKYSTPIDIWSVGCIF
AEMVNGRPLFPGASETDQLMRIFKILGTPNSQNWPDVFKLPKYDPNFPVYNPLPWETFIK
GLDDTGIDLLSKMLKLDPNQRITAKQAIEHPYFKETNMGNKLSTEDHIFRLKLKTKELEK
LSNRSELEEKRFVLDVKKAIQAGKIDMARLYAEKCIRKKNEKINYLNLSNKLDVLVSRLE
GAHRSASLVKDVSVMIPLIQQINSETNAVKIGSDVMKLENIFDEINITSDLINDTVQTSS
A---MGKEKTHINLVVIG
HVDSGKSTTTGHIIYKLGGIDRRTIEKFEKESAEMGKGSFKYAWVLDKLKAERERGITID
IALWKFETPRYFFTDIDAPGHKDFIKNMITGTYQADVALLVVPAEVGGFEGAFSKEGQTK
EHALLAFTLGVKQIVVGVN-MDTVKYSEDRYEEIKKEVKDYLKKVGYQADKVDFIPISGF
EGDNLIEKSDKTPWYKGRTLIEALDTMEPPKRPYDKPLRIPLQGVYKIGGIGTVPVGRVE
TGILKAGMVLNFAPSAVVSECKSVEMHKEV-EEARPGDNIGFNVKNVSVKEIKRGYVASD
TKNEPAKGCSKFTAQVIILNHPGEIKNGYTPVLDCHTSHISCKFLNIDSKIDKRSGKVVE
ENPKAIKSGDSALVTLEPKKPMVVETFTEYPPLGRFAIRDMRQTIAVGIIKSVEKKEPGA
VSAKAPAKK-----SLPIVLLKDGTDKAQGKSQIIRNINACQVIVDIVKTTLGPRGMDKL
IYTERNVTITNDGATVMN-LNISHPAASILVDIAKSQDDEVGDGTTSVVVVAGELLNEAK
VLLNDGIEPNMIIDGFRNACNVSINKLNDLSLSFVNKSEEEKKNILLKCAQTALNSKLIS
NHKSFFAELVVSAAYQLGDNLDKSNIGIKKVTGGSCLDTQLIYGVAFKKTFSYAGFEQQP
KKFNNPKILLLLNVELELKAEKENAEVRIDNPNEYNSIVQAEWDIIFQKLNLIKNSGANIV
LSRLPIGDIATQFFADHDIFCAGRVEDADLKRTATATGAVIQTSLFNLNESILGNCGLFE
EVQIGNERYNIFKDCLKTKAVTIILRGGAKQFIEEVERSINDAIMIVLRCIGNSEIVPGA
GSIEMQLSKHLRIYSRSICNKEQIVLYAFAKALESIPRYLSHNAGYDSTDILNKLRKKHS
EETNDIWYGVDCLEGDIINAYSNCIYEVTKIKRNVIYSATEAACLILSIDETIRNPSSMD
KQPRNPYAMSDKLEDIGEFVLVIGDFHSPMRNLGLPDCFKDLLKTDKIKHVLCTGNVGCN
ENLELLKNIADSVHITKGDMDNNFDFPEKISIKIGDFKISLVHGHQIIPWGDLNALLQWQ
KEYDSDIIISGHTHKNSINNFEGKYFINPGSATGAFQPWISNPTPSFILM-ISKSSIVVY
VYEEKNGKMNVEMSELRKQMFTTTGKLIYAVEHTPSTSKDSTAVYRNPKFNDKLLDNFED
NPLNNLWDMFNKSAEKYKNRHCFGTRIRKNDKLGEYKWKTFKEVQELIILIGSGLMNKNI
CPLIECNDTKIPRARFLGLYLPNCEEWNICDFSCNAFNIITVPLYDSLGIESSKFILDQT
MMQTIMCNKTCGLKLIKSLDKFDHIYIKTLILVEKEIDPEIESTCNKLNIKIVTWDDLIE
AGKKKKLDPKPGKLSDVSSICYTSGTTGYPKGVIMTNQNFIAQIASSCLGPSKFPMLNIN
EKDTHLSYLPLAHVYERIMMCLFLYLGIRVGYYSGNILALTDDIQELKPTLFLSVPRLYN
RIHERICNSLKKKSSVVQSLFHKGLDHKIKKLNNTGNPWSLFWDTLLFNKAKKILGGNLR
GMLNGSAPLGVEVAKKLKCIFCVPLMEGFGMTEGLGCLFITNPIDPDVGHIGGPLPSVEY
KLVSVPEMNYLVTDNPPRGELLLRGPTICNLGYFKLEKETNELLDSDGWMKTGDIASFSQ
NQSITIIDRKKNIFKLSQGEYVAVEKIESVYKQSLFIAQIFVFGYSYESFLVCIVFPSVD
TMRIWAKENKINKSNEEIIKLEKFKNDVMKDLIKIGKTDGLNGYEQIKDIHFIMEGFTIE
NDLMTPTGKIKRHAVQNKFKQEIDKMYENVKKA-------------------------EE
DGVVYECVATPDENDKWSLFYKEDYDIEIEDENGSKTTKTITEGQSILTMFNEGYASDGI
WLGGTKYQFINMDKGLEYEGHSFDVATCAKSKGGMHIIKVGGGHILIVLYDEEKEQDRGN
SKNAALAFSKELIESTDMCVLLLRNIYISIYYLLLFLLLSDGNHEPGNINRRLLHSFRGF
QNNICPDCKEKGIIICDTSEGTQICNGCGLVVETNILSEEQEWRNFSNDGQSKGNDRNRV
GETSDIWLENNTSTTFIKSSKKLQHLNMMTQINKNDQTLLSAFNILKLICETFFLRSNVI
ERAKEITKELQDMDQLKNRANNLNMLAVVYLACREAGHIKSIKELITFDRSYKEKDLGKT
INKLKKVLPSRAFVYNENISHLIYSLSNRLQLSTDLIEAIEYVVKKATTLITTSHRLNSL
CGGSIHLIVELNTSEEKNIKLPHLSQIASVCGVTTNTLKTTFKELLAASEYILPKNYLKD
NNTKLVILKHKYLHDDKKKRR-KMEKSILPEWTDIERYFKDPELITSEILFVGLTLCNVF
VMYRLFLDVIPFPIFVTWWQLAQGLLVAYVCGEMGKEFPKFAYFPKVEINENMLKVLFVP
SIFYCLMLVLSNYLLFKTPCISSYPVLVSFTVVFHHIIRFIGCGEEYMPLRWKSIAFLLS
AFVLGCFDSQTSGKGVIIWALLYALFSAVFRAGFMQKIMHLVEGKGNTLHNNQHMLGVLI
LPILILLSGEWKVLGHMPYNITSLYTWQVWGCLVTVGALPFVKNVVSNRLVRRTGQGPWR
FLEIISIALVFMIGLGYNRPSFMGYLAIVCVIIGRSLGAFDVLLNVSDYMISEDERKRKL
EKSSYAKSRQGTQVSKPFLSSGENEDDDESSFSSNDSKSYQGSQDYDDKESINSSSNQYS
THKGTSRMVSFSNHTSQENISIDESRDINARSSSFKSKPQYSRKYSSKQEVIDSQAMGAL
DESTPVPSRITLHLMKQKKKSAFQGYSLLKKKSDALFIHFRDVLKEIVKTKNKVGEDMRN
ASFALAKSVWAAGDFKGQIIEGIKRPVVTLSLSTNNVAGVKLPIFQVHIDPTVDVLGNLG
VAAGGQVINNTRENYLQCLNMLVKLASMQVAFFSLDEEIKMTNRRVNALNNIVLPRLEGG
INYIIKELDEIEREEFYRLKKIKEKKNENLNDSIEGHLSDNDGHKHVKITNNYANIQVDD
DVIFMAKLYLLFLFAINLCSSFIRSSILESIKHNLQIVNNQNFNTVINKFRNEKVFFILF
FKNSNKDIKNVIKNYDNVAEKFKGIITLCAIDCDSNSRLCEDELSLYVPEYKSSNTHHLL
VYPINPMPKFEFKDEINEANVKKYTYLIPSKIDIIKETKDFNIFLSKHENMPKVLIFSNK
KKPNYVLNALSNSFNKKLMFCYINNELDELVKKYNIKNFPSIIMLKKNKVVDIYKGKNNF
INMFDWLNIHSETFVMGGGFDISPGKTNYKPWKFEAVPKFTKLSHGDICFKQTDKGLCVI
YLKEGDKLDKSEEEMLITLKEKFKPQMTGRGVNFRYMWIDIATETNFRSLFELKKYPSVV
VFNPHKRIRYAKINDDLLATKEHIEKLLEKISGGDAKFTMLKGQTLPEFVLNENEDISSG
KDELMTVDLNIPYSACELKRVKRL-LGVLDPEIIKKIGVCEIVNVDIYKDGFPRDGGLND
IRMGTIDYKALCGTCNMNVKNCPGHFGYIELAKPMYHYGFMNVVLNVLRCVCYHCGRLLC
DMNSSKVKYI-KIKVNSLRLKKLSELCQGIKVCNHSSQDDNIYINDTSIDKFYNNDLSNL
NVNQQMLLNSNNYSNLFEMISKEDVDCGCVQPKYTREGPNMYIQFLHNNNEDDIDGSKRR
LSAEEALDILKKIRKEEMSILGFNSDRCIPPSLILTFMPIPPPCARPYVQYGNQRSEDDL
TLKLLDIVKTNIQLKRQTDRGAKSHVLQDLCSLLQFHITTLFDNDIPGMPIATTRSKKPI
KAIRTRLKGKEGRLRGNLMGKRVDFSARTVITGDPNLNIDYIGVPKSVAMTLTFCETVTP
LNYDNLKKLVERGPYEWPGAKYIIRDNGSKYDLRHVRKNSEKELEYGYKVERHMTDEDYI
LFNRQPSLHKMSIMGHKAKILPYSTFRLNLAVTSPYNADFDGDEMNLHLAQSHETRSEIK
HLMIVQKQIVSPQGNKPVMGIVQDSLLAIRKFTRRDNFLTKEEVMGLLIWIPYWNYKVPT
PAIIKPKPLWTGKQIFSMLLQFDDKDMNNNFDNDDRFKFGNNMGRDNENNFGKLSGSNNP
NSPLSLMDNMHGNIRNEINNNVSNISNVGNGGDNGYGKRCKMKINLIRDSSTSCKDDNPY
CSNNDGKVIIKNNELLSGIICKRVVGSSSGSLIHILWHEMGPDKTKDFISALQKVTNNWL
EYVGFTVSCSDIIASNKVLDKVKEILNKSKNEVTKLVKKAQRGELECQPGKSLYESFETR
VNNELNCAREMAGKVASESLDEKNNIFSMVASGSKGSIINISQIISCVGQQNVEGKRIPF
GFNHRSLPHFIKFDYGPESRGFVSNSYLSGLTPQEVFFHAMGGREGIIDTACKTSETGYI
QRRLIKAMEDVMVQYDRTVRNSYGDIIQFLYGEDGMAGEYIEDQIIDLMKLDNKEIKKLY
KYNFDDESYGKDYYL--GCEENPAYIDYDKQNILNQEFEELYKCKNYLCKEIFSDGDIRQ
HLPINMNRLIEHAKSQFPFIPVISSKSKIQTKISRDRGNDTMGIGGNR-KKKKKSKTKKN
EKSIHNKDLMFEIKKEYENNDLSTVMKNDKSFMGFEAYNKEDSDNYDDEEDSNESDDDAD
YYFSGNNENRNSYDDKINSMINPVEIAQKVNNFLDKLVIIKQINNSDTLSLEAQNNATIL
LKAHLRTYLNSKLLIHTHKISLKGIDWLLQEIEKIFYKSLCHPGECVGALAAQSIGEPAT
QMTLNTFHFAGVGSKNVTLGVPRLKELINIVKNVKTPSTTIYLDDMISNDQQKAKDILTK
LEHTTLKQLTSHAQIIYDPNTTSTILEEDKLWVNEFYEFPDEDDTQYTLGEWVLRVQLTN
IHVNEKKLTMKEIVYIIYSVFSSDELDIIYTDDNSEDLVLRIRVKYLNGEYNFLSGNGDN
INDDQCEDEDGDEREYNNIANTFKVKKS-TSDMNEKNEDNATISGNNIGNVDHIVNSSID
NLGQNIDGTIKKEDGRKGIHKNDNNENDE-EDDEEEVFLFGSDSRNLENKESSNSSHNMN
KIPYGNDENTSNFNDQHISGNNGGMGGSNKTMGKGDDENNQKNRKKNLNNENNTMISKED
TEDTFLKKLMEECLSSLKLRGIENITKVYMREESKITYDSENGKFVRSSHWVLDTDGCNL
ESIFCAPSVDFKKTISNDIVEIFEVLGIEAVRRALLKELRTVISFDSSYVNYRHLSILCD
VMTQKGYLMSITRHGINRVDKGPLIKCSFEETVEILLEAAAFAQVDNLRGITENIMLGQL
CKIGTGVFDIIIDNQKLNDANQNLETIQDITSAGFTTPDSNGSITPDGLQSPLGINTISS
PLPFSPTYNSNLLSPTLPIDNVNNILSPQCLLNYSDNIMSPSKSDFNNLDTLQLGGKFSP
TQSPRSPTSVIHSPFSPFDNRNKQPLDPLLLFSPKNNATNPGLNNYNVFSPKANMNNIQS
PLIYSPNPMIDIFSPKPQMQNNIYSPSYSPTSPTYNANNAYYSPTSPKNQADDMGGNKKY
NIMSPVYSV----------------------------------TSPKYSPTSPQYSPSSP
VPNNPSSPQYSPYSITSPKFSPTSPAYSISSPVYDKNENMTGKNPLSPAYMLQSRVQIRQ
NAQGTQVFSPIQQGGAHDVQNDDPFSPIPYNMDEEEMQEE--------------------

```
------------------------------------------------------------
------------------------------------------------------------
------------------------------------------------------------
------------------------------------------------------------
------------------------------------------------------------
-----------------------------------------------------------A
YFDEEMNLVFKELLEEEMNYNESNKDVEKLSDKIIIDDLKKILNNN---NEKNDLKNKDK
SNLEEINYSPLLMEDIIKNVVMPGNIRKSEHFLNLMRIIVVYLKKYINIYEVTSEGPLSF
LYKCEKDTKLDTSFFKYSFDRLKSLLNTLQVVDTDDYSALNIVCNFCTLIGNYFKGFIII
CEPYPEATGIYDPVIQFACLDSSIAMKSVLNRYKSIVLTSGTITPLELYPKLLNFSTVLT
ASFPMSFDRTCVCPLIVTKSSDLIPLSSQYSLRNDLNVIKNYGFLLVEMCKNIPDGIISY
FPSYIYMEHVMSTWYELGIISNILEYKLIFIETKDIVSTTIALHNFKKACDLGKGAVFLS
ICRGKIAEGIDFDKHYGKCVILFGIPYQYTLSRILKARLDFLKETYNIQENEFLTFDAMR
QASQCVGRIIRNKKDYGIMIFSDIRYTRNDKKSKLPPWIIKCMDISNTNLTIGASVNISK
KFLLNMSQEYKETDQTKISQVILQNPIKCWEIVKSILNMDDFIMLGKKIRNILGNTLKKY
SRKYSTSNLMKEIKNQPLTQITELSNKMKVATIQNNCEVPTIGLWISSGSKYENKTNNGV
AHFLEHMIFKGTNKRNRVQLEKEIENMGAHLNAYTAREQTGYYFKCFKDDVKWCIELLSD
ILTNSIFDEKLIEMEKHVILREMEEVEKSIDEVIFDKLHMTAFRDHPLGYTILGPIENIK
NMKKNDILNYIQKNYTSDRMVLCAVGDVDHANIVKLAEQYFSNIKPQDEKLIFKKEFDKI
KPFFCGSEIIIRDDDSGPNAHVAVAFEGVPWASSDSITFMLMQCIIGTYRKNEEGIVPGK
LSANRTINNISNKMTVGCADYFTSFNTCYNNTGLFGFYVQCDELAVEHALGELMFGITSL
SYSITDEEVELAKIHLKTQLISMFESSSTLAEEISRQILVYGRPISLAEFIIRLNEIDAE
EVKRVAWKYLHDRDIAVAAMGALHGMPQYFDLRQKTYWLRYMDNLKKVLWSIEELIYTNN
QDEDIYEKKEKKIKEYLLNLYKNCFGNETEKEELVVENGNFFNLLKLVKECKYEKCITNS
WVSSIFKLILEKFKNENYSTIYEIKREIFNFISAEKEQGLKKEANGLCIFSCFFFFYEMK
ILLCCNIFLNMFIQCNWTGPSLII--NNEKIKNNEKSEEDKYFNEFLDSIKIKNDDFLNS
CLEFLSLEGEYIYEYCNLINAFCLCIIFLGVLNNFSKHDEKSPHGKDYNNLMAYLKNNED
KKMEIREENNDEENSLDSIIFAKSKYLWKARIYFIWQRLFSSSSNFMYVLKVHIIDTPFN
IFKNMSILPNNFDLIERDFDLETVDIFSCISENADDNKACDLFCENYISEKFKIYILSNY
AVYLSFYNYTYAYDKLLDILSESSKFYYTFTGRMGIKRKYQKNPATILVLKVKLGDEEDN
TSTVFKEIEPLSEYDILRSDYKIIDNKSVDKEENKTDLIKQITDQTKDEAFENREDKNCM
ALEDDTLNGVCN-------HVDEKKFNVCDENNNSETNNSLSENSPIESNGKKTWKLKDF
DPDTDILEEPYFFDSQNNYFKILSFDEQIALINYCFSIIRFNPHYDEIKFEKLNAIISRC
LKSYDVNI---ENGENGKRGGNHLLQIKYQNYLLHSCILWFKCKCESFRLKTVDRSQAQL
NELLKEYCNDEPPNKERIKFIYDIYYPTTWEMKKEVGNVMTKTGSVVSAFNIFKDLKLWE
EAIQCLIEADRKVEAKELLDTLLEKKKSPPLVCLYGLVNRENCLKYFIEAWDLSNYKYSK
AARFIGKHYYNKEMYSECCDYLEKALEISPLLPDIWFILGCAYMKIDKFDQAIKAFTRMI
SMTNENTAMAYGNLAYLYMKNNVYKAAKICINQAVKINNNEWKYWDTYLKLSIVQNDVDS
FCLALTTLCQLNQVKQIQPWVFDYISDLIVNDKQTIIPNKTGLSYLDKIIKTMNIISAHI
SEYDSFWNAYSFFLFVKGEFEDSFEAKIKEIRSIESMIQRCNVINKIEVLVSKQVSAVKF
IYHLMKTHYTEDNKGIFMYQLKNIIESILRKEKYMNHKDISELSEIMEVLKEKNSEEEEV
ELFNEESNKKNNEEN------DEDISQSKKKRKKTNNTVSITGNKTGNKKRKVGKNKYVS
TFLDTEAQVGDEEDEEEYASSYVDEFEEAKRLEKKKLYETKLKSGTNHLAQAINKLSQRY
ENEKDLKDGATDDETLTDEEMSEDEEYFDEAEGLNAFDSPKMWLIKLFKNGAERHLAMGI
YYKYMKLQSNDFNIKGIYVSDDLKGYIYIEADSLYMLKRFLIGFKFINLNEISIVPVQEL
TSIFAMSHSKVVIPKVNEYVRIKRGVYANDIGQIFEVHEKGIYAIVRLIPRILYDKYNNS
KKDQYDNVGISTSNKSHSTITNNNLDQIYLMDKINTNNQINNGNNAIVPLSGEKLSSIDE
ALQIRRKKKKERPLKKLFDRDEIEQIGGVIEHGPYPGTIKYQNNIFEENGYLLKKMNIKY
LISENANITLTEIRDFNKNNTNEEDINLHISKSFINKNSLHLFKKGERVKIMKGELFNLI
GTITNINENVLSVNPDNLAKEFKFLPSDVTKYFTEGDNVTVINGLHKGKSGLISLLDYKE
NIALIFSPSLNTEFRSSIQDLTGTISSSEGLGGVNTLNGFSIGDLIELNDRQIGVLTYID
KNKHIRVLTNSNKILHTTIGSITYKRSAIGQICKDENGNIIQSKDTIQLIKGAHKNKMAI
VSYIWKNKIFAKINKKIEDNGFVVVDCENCLLSGNVNEKKKIITQHNLFRNNNMQRKNTF
QSFIGKTVKILSGVYKGLLADVIDAERDEFT-----------------------------
------------------------------------------------------------
------------------------------------------------------------
------------------------------------------------------------
------------------------------------------------------------
------------------------------------------------------------
------------------------------------------------------------
------------------------------------------------------------
------------------------------------------------------------
------------------------------------------------------------
------------------------------------------------------------
------------------------------------------------------------
------------------------------------------------------------
------------------------------------------------------------
------------------------------------------------------------
------------------------------------------------------------
------------------------------------------------------------
---------------LSDSVCQDLLFDFMKGNKVNIEIIISICVQYYNKIGIKKIVNKFE
ENKNYEGIFYFISSILNELQNMSTARNDNNISRSNNDDASSILLNSDIGIGTQFSENNSA
LTIEDLHHIMFKYIEACVKINNIQELDRICKDRNAKYNPEQIKNFLKECKLSDPRPLIYV
CDIHNFIEELAEYLYKNSLLKYIEVYVIKVNPHNAHKVIGVLLDLDASEDFLLNLLNNIK
NISNIGNLIDIAEKRNRLKLLLPWLESRSNECYENIELHNALAKIYIDLNKDPETFLKNN
NFYDKKMIGKYCEDLDPHLAYTAYERSNGQCDEELINITSKNGLFKLQAKYLVSRQSMGL
WSMVLDELNKYRKNVIDQVIGSTLIESNNADEITVTVKAFIEKKLSSELIELLEKIVLHN
SEFRDNKNLQNLLILTAIKSDSKKVMEYINRLDNFSGSQIAEVAYEYKLREEAFVIYKKF
NCNTSAISVLLDNILIQNRKGKMSANARRKKTEFAEEQEMEKEEKLTNVEKYEEDLNRAI
EFAQKCNVNDVWFILGKAQLKLNKIIDAIDSFIKSNNAGAYKEVIEKCKENNFYEQLITY
LNTLREQNSLKDVLVDSELLYAYAKLKKTLEMTKFIASTNLANMQLIGDRLYKEKEYDAA
KILYSSIPNNQKLTFCHLKLKEYSLAIEAAKKTKSLKTWREVNLVCVKYKQLKFAHVAGL
QLIMHADHLDEIIKIYEKKKYINELLSLLENGLNSERAHVGIYTELGMLYAKYKPEKLME
FIRSYTNKMNTRKLIDACHNEYLLKEAVYLYISYDEYNLAVDTIIKHSPIAYQPDIFMQV
IHKVTNSDIIHKVIDFYIEENPLNLYNLLKILENKIDNNRLVQTMKKSNNLPLIQKYLED
IQTQNITSVNETLNEIYLENDDYISLRKSIDEYDNFNQTNLINKLENHKLAEMRRIAALL
YKKNKKFKEAINLSKKEGQIKDAIDIARVSKNHLYVEDLINYFIQIKNKEALCACLIACY
DILKPDYVLEIIWLSGFKDHAMLYFIQIISDYTQQIETMKKQIEDIEKEKKMNKSAPNDF
SANTISNQFTYSLNKNLSIMPPQNNYIPNNSDFDKYDMFNTNTHFMPSIVGNQAPSFKAE
AVFGDNNFGEVSLSDFIGKKYILLYFYPLDFTFVCPSEIIALDKALESFKERNVELLGCS
VDSKFTHLAWKKTPLSQGGIGNIKHTLISDISKSIARSYDVLFNESVALRAFVLIDKQGV
VQHLL-NNLALGRSVDEILRLIDALQHHEKYGDVCPANWKKGKESMKPSEEGVAKYLSSL
MEDTAAKIIGSHDSLLTDPRIAQDFSAETDELLAKAENYFKVGDFELIIEELILLEKKCR
QSYDGISTSKICCFILNKYKLMENYKKVNEYLIFFNKKRGQLKRTIIDIINLCKSWIVDI
QNKEEKLNLINTLCTISEGKIFVEVERSEIIRILSKIKEDDGNIEEAANILQDVHVETFI
SMDKRDKTEYILEQMRLVLLRKDFIRCHVISRKINLTLLNTDEFADLKLKYFLYMIQYYI
NEESYSDVANCYEQRFNTDSVQNDLNLWIDELKCYIIFLILSPFQEQQTKFLNLIKLQKK
KLKEIPTYEQMVNDFIKQDLIEWPLLYEQELQSFYIFNDSVFVGGENRWHLFKKKVMHHN
IHVISTCYSKISLQRLAQLINSTNEESENLLLELVSNKMLDAKIDRLYGVIKFGQKNNPQ
TLLNNWSSQIHQIVDILEESSHLIQKERMVHEAKLKRMQLENKKMALMGDKEAKKEAIEI
IYEIANILNVNLDKETIVILIQLCEYGVSPKILSHIIIQLKKEREKFLQNVSNNMENLKQ
```
