# Supplementary material for: Phylogenomic analyses of malaria parasites and evolution of their exported proteins
Source: BMC Evol Biol. 2011 Jun 15;11:167. doi: 10.1186/1471-2148-11-167 (PMC3146879; doi:10.1186/1471-2148-11-167)
Supplement: Additional file 3 — Alignment with 49,521 aa positions. Total amount of missing data was none. [file 1471-2148-11-167-S3.PDF]

Additional file 3

Alignment with 49,521 aa positions. Amount of missing data was none.

> ***Toxoplasma gondii***

HLEGDFRKTK KKLHWLANLP AQNTELVLRE YDHLIEEENW EKFINRETRF DTPAVGDPLL
KQLKEGNERQ FRQSGLVKRR VVSSVDRGNL KAGLASQEEP SAVFPTIVFV GEEAIANRHH
LSFTYPIDHG HIDNWVDMEE VWNATYNMLG VQPNEHAVLV TEPPLCSQRH REKMAEMFFE
TYGAPEMNIS VTGLMAIYGT GRATGFVLDI GEGITQCVPV FDGYLEKASV KRSDFGGQEL
QMYLQKILCD MGYPMTTRDD YEHVRVIKET LCFCSLNPSE DQNRDDLEKT YHLPDGLTLR
DGITEITLGP ERFYPPEALF NPQLCGRDSP SLIELVWSSI MACPIESRKS LIGSIVLSGG
SSMFPGFPER LEQELKNTAP PQARPHVHVV PYELAAKFAQ ANGLYFSEAS AVTAFNVKHI
FEHLLQMEQP KLAKVEKVLG RTGSRGGVLQ VRVTFMDETD LAGRSLIRNV KGPVREGDIL
ALLETEREAR RLRGTQSVKL KKSHCLCHLS TGDMLRHAVA TGTEYGKQAK AKLDAGELVS
DEIVLGLIDE KLKTPECRRG FILDGFPRNE AQAAGLDNLL KQKNQKLDGV LYFDVPDNIL
VERVSGRRIH LPSGRVYHVT YHPPKVAGLD DVTGEPLHHR KDDNEATLKK RLDVFHKETV
PVIEHYAKMG LLCPVCYQKL PNPADVLGPM DKELNYFMWM PGFEWRPFNA RASGGKVSTG
DAMILARQLG LAPSYADKQA FEEKSGDNLD YASFQKFVGT STHPEDNIED LVEAFAYFDS
KHGYLTRKQM GNILMTYGEP LTTEEFNALA AEYFTSDQIR MLALFDLIDT NQDEAKEWSA
KLKNAMHQHQ VRMEFQAIDK DNDGKVSLSE LEATYVDSLK QLEQHKKEVE QRFKTVDKDN
DGLLDLSEIR ILMDPGKDEG LMKIEIEEIL NAQDKNGDRK ITDVEKTELE KEFKYDLNAD
GAIDVEELVG REQWEKEFES FAVSMLTDNG EVLRFPEDYS ILSIINCFAA GAFLGLALIH
VLPEYLLAAV GFTAMLGLEI LLGGGHTHCC DTSSSDDEGR VVKSLKDRRW TAMRETIRQM
RNHMKIADFS ELYKDYESLV RCLQKSEQEG LPSFFVRIIV ELEQFLEERH RDKEAFKKLS
KAKATAFNTL RAKVRKTNEQ WQEKVDACKA DPSQFWSDSD ASSLSSGEET DKHKAAMAKW
GLRSSRKQKK KKTKRDEGAL SPGEAGAEFS IKDMESLFDT NDLDASVIRK RVQLVVEKRG
RRGVDRLEQT RILKRLAELA ARVGPQSELE VLAHLISAEF DTTSGVFACL SSAIWLDVFH
QLEAVLGHLE EGTAYVLVPT VASSDAVDAA LVAPDAQVVS SGILTSFVER LDDELMKSLQ
FTDVHSEEYK ERLGQSVDMI AVLCRAWVHL TAATLALRIN EHMHYKHDAI AASMWDLVEC
TPEQERAEKR RLLPYHMHIS MELIEGAHNI CAMLIEVPHM AHDPFEHKRP ISKHFRRMLE
TYDKQAFLGP PENARETVMA ATKALQKGDW KECCRFIFSL GIWEKLMNEI QEMLKLKVKQ
EAMRTYIFTY LTLYDSFSIS QLCGMFELPE STVHSIVSKM MINEEIHASW DESSQFILIS
RVERTRLQQL ASTLTENVNN AVEQNELTLN MKNPKLGLTP RQTVVVGTRA KEFLVNEKND
IRTQSQLLER VLKRAVKDSS GAPLPLFLLE LFSLSIEEKD ALDFAAKRAA MNRQMDEGGR
TVRQLLETAF EPGPNPQLSR KLTEFFSPSR FKKELNHKFQ SLLSEQMGIL ERLVRKRLET
QKRQSCRECI KLYLRELMQV VTELVTGNYT IIRLNGDEFL KTFGGNLRDN LEDGMQPGHI
VRYTLSKDDT NMFGLVQSPA SDASSKEATV TFYFRSGQAQ EQSQFKTVDK QRLPLQTIIP
STPPPWRRFY RPDGWVGLQP VELLSLRQRF SEKSEAVVKF ADTRLLNQLA LTQFWVVKML
LSAPLTTKLY MHFVERALKP CVMVAVGDAA MTMGEAFAPY LDSFMAILHQ AGNTTYDVGP
SNNEEWLWYI HDLREGVLQA YMSIVYSFKE KCMQEQLKLY VNAMLDVVKA VAATSPKMRG
VENVKQAIEL VGDLISTYGG DLTLHLQRAP FMEQLLQLAQ VLGTVKDACL QKAQWLRQLM
AIAMVVLRIE NVEQAGQAAG TLCSVFRAST DMAEFRLRLL QSLYNAFPPS FPYFVATLEY
AAETNLFSVM LPYIRYINEW MRDWNLPPSS KRQVFLILAN ELKKLKKADE AYPFLKRHVQ
FFQEILSNGA TISAAVELVE DSIRLPDVIV FDGLMDLHAV VHLRKTAHAP LIELLQIFVN
QGPKELEAFL NYEQCLGKIR LLAVASLVHG KKEVSIRAIG DALQLSEAGA EEVAVQAIGQ
GIVDAKIDQL ARVLHVRSTM QREFGRQQWE ELLERIDHWS EGVASDFYVF NDCSATSTRA
IQPGTGYWSS AGGLPDDEQV TWTGYLATPG KIKGVRVRWQ YAPGEVQVAI SSNGVDYHVA
LPWRPAGSSE AAYDEDILFG HDEEAKVVVI GMRKQIHGFY GINEAKPLGS GEPLMMIIGG
ITSPAEMCLQ ELWYSTPQMQ LVSARSTPPK CMTLQDGSTE GDGRSSWTFE GNSQLRLQWC
MTQKDVNGSS PGVGDLEATA SSSSSSDAAH GANAAIDKDS DSSWRSDPVG EAEQTVVLTV
NLGKASNVVS VRIQWEYPAL SYEIYTSPDV QQAVNPANPV VDTLDELHAG AAQFIQIRML
KPHPRLGKAD DSFFYGIYEV HVYANRLGSA VSPCTQAANS DDARDKYFVE YVTSFNPVLA
DKITSMEEDV LLRQKGLNTK AKDLEALLPE MEGCRNDKIQ FVERMKRASR RAGKIIMPQC
SPAPLRVYCD MTTGTSIYVW NGVVSLNDVR NACARVGLEP MVPKSPQHFQ SILSALHQMG
FNLNGKGAVP LAFDYSCYRD LSDGATDLTS LVLSAPDSSP VKDAALEERG LRPCSLREVL
ERRNKMWAKV QMISPEARGE KERLDSLFKQ QCVGWGNGWY GKWNPVILCP DFRQGGLQVS
RSGRRQLWEC LKSVVEGFVD PRLQICEVVD PTHPVRFATP PQETCYTVVY VGPKIPASKA
RVVFGEYTGV VKDGGLVKKR FEYVFDLSFV ELPARSQFVL DSSEACNEMS LVNHYGTILG
DCVCKRNSEW QQVFVDGWPH IVLTSIPGVA VEPGDEILAD FGNAWFFKVQ DASHEAIARE
LLEYRVCEKL RDEPLSSGFP MCSDLLLVTQ ADSGDVVHCD GCDRPCHLRC LPDPELNDWR
WYCAVCRHLN YKIASTEVCR LAKRHLSPND AILTAFQEKV HSLQDEAKLG KTVIDRCFNV
GWFEGLITEY SDGKWYFQVE YEDGDTECLK VEELVHLELK QKGNELYKQK KFEAALEAYD
EAIEKNPNEI LYLNNKAAVY MELGDYDKCL AECQKALDKR YECKADFSKV AKVYCRMAAC
KTRSGDYSGA IAMYEKALCE DNNRMTRNAL NEVKKLKEKK EKEDYINPAL AEQHREKGNE
YFKQGDYPAA KKEYDEAIRR NPKDAKLYSN RAAALTKLCE YPSALRDADT SVQVDPAFVK
GWSRKGNLHM LLKEYPKALQ AFDKGLALEP TNQECIQGKM AVMNKVQQLQ SGEVDPEQMA
HSLADPEIQA ILKDPQMNIV LMNIQEKPEL IHEYLRDPKI KDGINKLIAA GILFKPGETP
PQSLGFNRDW NVDLIPKFVM ACGKLVKVLL TTKVTRYLEW QVIEGTYVYQ FQKAGFFSSA
KYIHKVPATD TEALTSPLMP LLEKNRCKNF LSFCAQWELD NPETWKGFDP KRHSMKQVYD
YFGLQPNTID FVGHAVALYT SDDYLHQPMG QTMEKIKLYM YSISRYGKSP FIYPLYGLGG
LPEGFSRLCA INGGTYMLNK PIDGFVYGEK VCGVKSTDGE ARCKMVVCDP SYVYDPKKVR
KSGQVIRCIC ILGSPIPNTS NASSCQIIIP QRQVNRTNDI YVMLVSSAHG VALKGKYDVR
IDTKLNKFIW SGGIRNVPRR VRVRIARRRN DDEDSKEKFY TLVQHVPVAS FENLKTEQMD
GDRHRVWDCT TVNFAGFVAV MKPMSSWVAR HNKLTEVVPG CYAVSVVGEL GSGSGCVISF
LRTLFLSSPL FIPCFFAVDC NLSASEKSEV DAVASDLFRA FRPSFDVVLF NPPYVPGSPR

DRPRNADWAW WGGEDGREVI DQFLRQVTAN LTAAGVLYLV SFPRSGSGDV EQSDFPILCE
TCLGDNPYVR MQRNREGKEC RICTRPYTAF RWKPGPKARY KSTVVCQTCA KLKNVCQTCL
FDLQYGLPVQ VRDKLLEGAV ELPDHPLNRD YMADRLELRA IARTQPYYRR NAPRVCTFWQ
RGECKRGDEC PYLHQEVHRL LIQNLPATTV DELRALCEEY GEVAETFGFV SFVFPEHAVA
SLPRLNGSIF QGRILRAFPA SYKLQQLVSQ DEQFAAEKVW NLLYVSANSA ADAVLSELQA
DKAALLGKNA AATVALMEAH LLTQTRAWIK AEGIRSRDTL IVKHLTAHVN EAELLRLFER
VGPLARFLLA PSKTVAIVQY EREKDAEVAF RHLAYRQYKN VPLFLEKAPV NVFGDEVQGV
SLFVKNVNFC TSEATLNDVF AGCPGLRRTI LSMGYAFVEF DSAENALAAC KRMQGVVVDD
HVLQISISKV LVRNLAFQAS ASDLRGLFSA YGNVTRVCIP RQHEGRSRGF GFVDFATKQE
AQNAVEALTG SHLYGRRLVL EPAQGEARIL EPLVQERVVE VLKEEIQERV IEVPQVQYVD
RIVEVPQHVV HEKVTHVAKP IIQERVKHVH KPVYQQKIIE VPQVKVVDKI VEVPQYVYQE
KIIEVPRVVV QERVIPVPRK PQYRHIPKPV EVPMTHYRPI PVEKIVDRNV PVPVELQIVQ
EYLCPKIEPR YKEVPVPVHV QRTIEHPVPK EAMGNPKLLP LYYLGSTAVG IQTKDGVILA
SERRITSCLL DHRSIQKIVE IDDHIACAMS GLIADARTLI DHARVECANH FFTYNEKMSI
HSCIDSVADL ALDFSDVSDG RRKKMMSRPF GVALLVAGVD DQGPSLWCAD PSGTVTKYQA
VAIGSAQEGA ETMLQEQYSQ SMSFEDAEAL VLVVLRQVME EKLNCNNVEV ACVKDRKYHQ
YSSEELQALI DRLPRFRGID QDFKNINVME VAGMDDLGRQ LDRLSDLLSR IQKALGEYLE
KQREQFARFE RLIQTPLTDK CFLTLTQALN MKLGGNPFGP AGTGKTESVK ALGTALGRYT
LVFNCDETFD FNAMGRLFAG LCQVGAWGCF DEFNRLDEKI LSAVSEQIIE LLDKNVKLST
NVGIFVTMNP GYAGRSNLPY GVGFKIAFGR LSSSCAVMSR TQGTIGVGML SMELMGHCDA
RELVAPMCMW RLPNKELINR DPNNAADHRH HQRLLMSYTP FTTPNLLAEQ INLLGTVPGT
RLVFFPLWTS ARHSLDYCLP TYLFWLHLHS PALLHVQGRP LRGQVVVIGA TNRQNSIDPA
LRRFGRFDRE IDIGVPDDNG RLEILRIHTR NMKLANDVKL EELAANTHGF VGADLAQLCT
EAALSCIREK MDLIDLEDDT IDAQVLNSMA VTQEHFTSAL QCCNPSSLRE TVVEVPNVKW
DDIGGLEDVK RNLQEMILYP IDHVLRALQD CRFFAPTPIQ RSVLLAALRD RKDIVGAAET
GSGKTLAYGV PIVCNLLIVV PSRELALQVQ RHLEALCVYT PLAAVCLVGG LALQKQLRLL
GQRPQIVIGT PGRLRFLVLD EADRLTQEGC FQEMEGLLDA IYIQTFIFSA TLLQALMDRV
HLRKKRLAVF DLLPDGLSIT ALKTAAGEEE LYLVLFLLKL KVMIFVNAIS YVYRLDPILS
LVLGRDKHQA CQRGGVVGLH SNLQQKQRLK RVERFSANRA VIVCTDVACR GLDLKQVKEV
IHFQAPRSPS IFVHRSGRTA RAQQSGNATI CSSVHSASLL FSQDEVRALS DSRLGSLDGR
EICETCGCDC PGHLGHIDLA LPVFQPIFLP SLVKVLKLSC LHCERLRVSW QVATWRKLRD
LLWTRAAASC VCANCGRSSA VTFHVAQQAT GVKLHLHAFQ LMPMLQNVFD RRLLHCLFPM
SIQLSSSCFF ISSLPVSANK FRPPLAGLHP RNEKVQFALA IMRHPDPTGA LELQQKVNEI
VDIRQWMERK AGTIRQKLMG KRVNYAARTV LAPDALIATN EVGVPLDFAM KLSIPEKVTP
RNVHVLSEMS PADFVMRREG DYRHLALIAD FMTGAVCLNI LRGSWTPAWN LHYVCLAICA
LMDIPNADSP LNCDAGNLIR TIRFKTAAEV GKRAVFYCRG EDLFAWLMNN REMLQKKHAD
ALDGQSLASE TDVIEFCDKL IRFGFMYRAQ YKPEEGRFKR PKWPKRLAMT PKQNFDPQAF
YVVVYEGSKS WQHFILFCII AAVLCVCMFW YLSVVLLTLI LVLVFVRLVL FVFFWFFGYQ
FWLLPNLFNE DAGIIDSFLP WIEWHRSQDD WAMFAARIFC AILTAGTLYK LSETHTPASV
ANFAKQSFLD VLDWGHQRLA EDEYSCLKQC GFASFEHLMQ RCLVKCSCVT LAESFLCDLQ
DLEEDGVVVS DLMLQIEKCN ERVIDIDKDI LNIHKFIKDI YSMKFPELES IVQSPLEYIG
VVLRIQNQTD LTQVDLSDLL PSPTIMALTV AASSSGRRLP DEEFCHAIAA AKEAIALAEK
RKEILQYLES RMSLIAPNVS AILGAALAAR LLTRVGGLKM LAKMPSQNIM LVGSQKKSLL
CSCEILLLTP VAFRTRALRL LAGKVSLAAR VDFFTEDRSV LREQLLTAPP VLEIARRVKC
DLYLGRHYLY FIRAIRLRAY SQFLEPYKSV TIENMATAFG VSPSFIEQED PAKVLAEQLA
VIDGFNQLLA ALAVMERLID YSELARDFEK LGALQPCLRL LDRHVVKTAL TILSLIVANN
PDVQEAVYKQ HGLALLMNLL KEAPNSSLRV KALTALACQM RHHRPSELAF VTAGGLALLV
HAMLSRDEKY QEKAASLTRH LLQEGLLAFS QVEKYDLPGA VAGLLETNIQ FGETVVQLAI
ALLQQHRATM AKGPLAGLRQ TLLDRQRGLF ASLGVPPALI RTAASLHIFH PSPIQVLSLP
HTLRGKNVCG LAPTGSGKTL GYCWPLLQRI GRDGHAFMGL VLLPARELAI QVLDQFRIYG
VQLGVRVCLL LGGRDLVEEP HIVIATPGRL SLVDVLVLDE ADRLLSDEFE DDLKTILSCV
PRQTLLFSAT VSPALLALQR RFGDDAMPLV DAHPTDQPAP NLSHFYGIVF AGSVRQTQQI
CTALEILQSA TPLHSLMEQR KRVACLEKFR SETSRLLICT DVAGRGLDLP RVEFVINMQV
PGKAQDYVHR TGRTARAGRK GVALTFVDPK SVRAVHRIEA LINTQLQPLS INEQDVLKFL
SNYSKVVQKS LLFLNEVVIF PPALHAPLTR EKLPKKYHVG LQNCSKTHNG AFTGEISVEM
IKDFGLRWIL AGHSERRQYY GESDEVVAEK VNIILQKDLN VVLCVGEQLK DREANKTNDV
VDAQLAACLP KISDWDRVVI AYEPVWAIGT GKVATPAQAQ EVHEHIREFL KAKVSANKVR
IVYGGSVNAS NSTELILQPD LDGFLVGGAS LKKDFLDIIA SGRDLGKDVT HRFGANSFKL
HRLPVPRPGQ VLGLVGTNGI GKSTALKILS AKLKPNLGKY SNPPDWQEIL AFFRGSELQN
FFTRMLEEDL KASIKPQYVD HVPKQVKGTV GQVIQLKDET GRGAELMKDM ELDHLVDREI
GNLSGGELQR FCICVTAIQK NNVFMFDEPS SYLDVKQRLK AALVIRSCLQ YDNFIIVVEH
DLSVLDYLSD YICCLWGKPG AYGVVTMPFS VREGINIFLD GFVPTENLRF RDESLNFKLE
EIQRLHFYKY PAMTKTLGSF KLRVESGHFS DSEILVMLGQ NGTGKSTLIR MLAGLLKADE
EVELPNLHVS YKPQTITAKY QGTVRDLFFA KIRESFNHPQ FQTDVVKPLQ LETIMDQEVQ
HLSGGELQRV ALIVALGKPA DIYLIASLVC AISGVIPEEP VFSKTGLIYE KRLIKKHLET
SGVCPVTAQS LSEADLADVK CPKASRPRPV TAASIPGLLS LFQSEWDATM TEVFALKQHL
ETARQQLSQS LYQQDAATRV ISRLLRERDA SRQQVHALQQ QLLQAQKRAA ASAAEPGLSE
ELVQEMQALA KQLLVARKKR QIDNVLPASR AASFKCTYSL PLHSSADRGV LCCEFDATGG
YDGNVILFDL EKQKTLHKLT GHTKAVRSAK LHVTEPVVVS ASDDKTVRIW RASKHRGEVT
CLSLHPLGNY FASCAADKTW AFSDIQEGRC LQMQKNLPCY KCVSFHPDGM ILGGGGVDGS
VHIWDMKGLA YRAALFSENG YYLATASSDG TVRLWDLRKS LSFQTIDMNE AATCVTFYHG
QIKDGLFHGK GTLIYSGNEK YEGEFVFGKR EGHGRFLYAD GATYEGKWVE DRIHGQGVAH
FASGNRYEGQ WEMGRINGFG KLSYSNGDEY EGEWVDGKMH GRGTYRYAEG DVYTGEWRDD
KRHGKGSVTY VSAKGSVVEK YEGDWVNGKM HGHGKYIYSD GGVYEGDWID GKMHGKGTYV
FPNGNVYEGE WAHDMKDGYG VLTYQNGEKY EGYWKQDKVH GKGTLTYTRG DKYIGDWMDA
KKDGEGELIY ANGDRFKGQW ADDRANGFGV FYANGNRYEG EWTDDKRHGR GVFYCAEDGS
AYEGEFVGGR KEGNGILRLA TGHQLEGTWS GGQLVRVTSF VFAQDSPWLN VDLEMETLYD
LGAKMIDALT KEGVTAGDVI TIDKSTGKVT RVGRGFSRAK DYDAVGPATR FVQCPEGELQ

KRKEVVHSVT LHEIDVINSR AQGFLALFAG DTGEIKSEVR EQIDQKVADW RAEGKAEVVP
GVLFIDEVHM LDIECFSFLN RALEHETSPI VIMATNRGIT TIRGTDYKSP HGIPLDLLDR
SLIIPTQPYE EKDMLKIIEL RAEEEDVELE ESARLLLCKI AAECSLRYAL HLITVANLVC
RKRRGSVVTV QDIRRVYSLF IDVKRSTQYL VEYQQEFMFS ELYGNDSDFW TSDDEETEDF
IRKRWCVEDD VSLFRQVPLA LEFPFPLDDF QKRAILHLEK YQTVFVAAHT SAGKTVVAEY
AIALAVRRNR RCIYTSPLKA LSNQKYREFR LKFPSVGIVT GDVCINPDAN CLIVTTEILR
SLLYLGDALI GQVDSVIFDE AHYINDIERG VVWEEAIILL PKQVNMVLLS ATLPNYRQFA
EWIGSVKQRE VFTLSTDRRP TPLRHFLFFH DKAFLLMDAK GFQAGAYNEA FEAKLKTEIH
RLQGLITKLE KDNELPVVVF CFSRRKCETY AQAMRRLDVV LSHDRSKIHL FVKDCLMALS
PADRDLPQIR FVCGLIHRGV GIHHGGLLPI IKEMVEILFQ RGLVRVLFAT ETLAIGLNMP
ARSVVFSALK KHDGQRSRML LASEYTQMAG RAGRRGIDTF GHVYIFCSDD LPEPKELTGM
MVEKANPLHS RFRLTYQTLL LLAARSHMSM TSFLSQSFKE AARTSLLPVF KRDLRRKRKE
LHALPDVRCV FIEDLAELED RSRGIAEEIH MPTIVTCPVG VSSKELERLA SADARGEDTT
NAGARKSACG ALTPMSFSKP LKQLELMAEI AEISSQLADE SLDVYPEMQA RLTVMKKLKL
IDDHTGLTVK GRVACQVMSG DELTLTELLF QGGLENLQPE EIAAVLSAFV APDGPVEQVP
APTAGIQRVR DQAEELHVAI LKLQANSGVR INAEDWWKLC NFSLSLVAYD WANGVSFGDI
MHKTNAQEGS IVRAILRLDE LLRKIRQAAI LIGDPDLGAK LQQTSDRIRR DIVFAMSLYL
DGVALAKFLT WLEERSEDPQ AESFTEWEVA QVVDGLRALS PSFISFSTIA SANANAAIVH
YRPIREHSAP VTSSLFLLDS GAHYVGGTTD VTRTVHTGTP SESQKRYFTL VLKGFIGLSR
QVFPQGTRGP QLDVLARQHL WASGLDYRHG TGHGVGSYLN VHEGPIGISC QAGETLAEGN
VLSVEPGFYQ QGSLGIRIEN LVYVTKATNF ENMRFLRFDQ LTVVPIQKKL ILPSLLTNEE
IQWLNDYHQK VWTLVAPRLQ EEADPHFQVS ENSPFLQKRL QVFEDLYEKI SIELPDGSKK
SGTAFETSPY DVALQISKGL AEASLVAKVL YEQPSAESEA ILYDMKRPLE GSCRLQLFWH
SSAHILGQAI EATFGAQLTV GPALTNGFYY DAYMGDAKVT EESYGRLEAA AAAVIKEDQA
FRRLVCSKAE ALELFADNPF KVQLIASKIP EHGLTTVYCC GSLVDLCRGP HIPSTGKVKA
FQVIKHSASY WLGRQHLDSL QRVYGVSFPD KKLLKDYLKL LEEAKKRDHR VLGQNLHLFF
FDTNVSPGSC FWLPDGAKVY NKLCMFMREE YRFRGFQEVI SPNIFSCDLW KVSGHYQNYK
ENMYLFDVEG KEWGLKPMNC PGHCIMFKHL APSYRQLPLR LADFGVLHRN ELSGSLTGLT
RVRRFQQDDA HIFCRLDQVK EEVADALNFL FFVYDQFGFG LEILQNRGYD SCGITSILVT
TKFASRTTCD SIEILRREGK HPHRGNHIGI AHTRWATHGS KTDENAHPHH DWKDRISLVH
NGTIDNFAVL KKALIDRGCT FRSSTDSEVV ANLIGWYLDQ PDAFEEAVKR AVGELQGTWG
LCVVHKDHPD RLVLARNGSP LLVGSVGDQL FVASEPAALA RHTNQYLMLK DGEIAVVTAQ
GVGQLEATRP VHRIAKETIE LSPEPFAHWT LKEIFEQPQA LARAMNYIAP YQNRVKLGGL
DQNRESLLTV KSLLLCGCGT SLYAGIYGEL LMQWLRCFDQ VRAVDASEVD IYHLPRQDAG
VLLLSQSGET LDTVRACQLA DFQGLKKFSV VNQVGSLLAR MTNCGVYVNA GREVAVASTK
AFTSQVAVLS LIAAWFAQNQ FPDRCSALMD AIHRLPVYAG MTLNCRALCQ NIAERLKDKT
LFVLGKGFGY PVALEGALKI KELAYLHAEG FPAGALKHGP FALIDEKEKT PVILVLLADA
ASLLNAAQQV KARGAHLICV TDEPDIVKDD VLVVPSNGPL TALLACIPLQ LLAYELAIAK
GINPDKPRGL AKTVTVWMME IDKYAMEGVS KLLVGNKCDL TSKRTVTYEE GKEFADSCNM
RFIETSAKNA HNVEQAFHIM ASEIKARVQV NQQQRPNANV RLPSQPVRIR TLRNKKPPEG
WELIETTLLE LNRKMREAEL EPHEGKRKCE SAWPIFKLHH QRSRYIYDCY YKRKAISYDL
AVIGGGSGGL ACAKMAAAQG AETVVFDFVQ PSTQGSTWGL GGTCVNVGCV PKYLFHHTGL
AGHWDGPHMG WKGKFEQDWG VCVEKVQNYI KSLNFGYRTG LRAGVTYINA YAKFVSPHEL
AYTFRGEDKI CKARNIVVAV GGRPHIPEEV EGAKELAITS DDIFSLKQAP NKTLCVGASY
ISLECAGFLR ELGFDVTVAV RSILLRGFDR QCAEQVGLCL EEAGVRIIPA KMVKANGKIQ
VTFQVGKVEE FDTVLYATGR KADTSNLNLS HTSAPSVYAI GDAVENFPEL TPVAIKAGEI
LARRLFANST EHMDFTNIPT TVFTPIEYAH TGYSEEAAEA EFGDLEVYLF QFSPLFFSCV
HREKRKSPED VDITPPCLAK LICVKDEKVV GIHFVGPNAG ELMQGFALAV RLGAKKRDFD
KCVGIHPTNA EAFMALTVTK ASGEPFVASG GCGGGKCGEE VQSGPLKLEH LLAKGFTKRD
LELLKDAGYQ TVECIAFAPV KNLVAVKGLS EQKVEKLKKA SKELCNLGFC SAQEYLEARE
NLIRFTTGSV QLDSLLKGGI ETGNLTELFG EFRTGKTQLC HTLAVTCQLP IEQAGGEGKC
LWIDTEGTFR PERIVSIAKR FGLNANDCLD NVAYARAYNC DHQMELLMEA SAMMAESRFA
LLIVDSATAL YRSEYTGRGE LASRQTHLCR FLRCLQRIAD TYGVAVVVSN QVVAKVDNMG
MFGNEKLPIG GNIMAHASQT RLYLRKGRGE SRICKIYDSP SLAEGEAVFA IGEGGIGDYM
ATRAQFESSN EVGVFAKLTN SYCLVALGGS EHFYSTLEAE LAPHIPVVHA TVGGTRVIGR
VCVGNRRGLI VPSITTDQEL QHLRNSLPDS VEIRRVEERL SALGNNVACN DYVALLHTDM
DKETEEIVQD VLGVEAFRAT IGKQTLVGSY CHFTNQGGLV HVMTPVEDME ELSQLLQVPL
TAGTVNRGSD LVGAGLIAND WAAFCGMDTT ATELAVVERI FKIATRNKLN LVDDLTLRSS
LIDTLSVVDT YWQTETGGHV LTPIPGATVT KPGSATLPFF GIEPVVLDPV SGEEKQGNNC
GVLCIRRLWP GVARSVHGAH LRLMSTYYWP YKGYFFTGDG VFRDADGYYW ITGRVDDTLN
VSGHRLTTAE IEHALVQHDD VAEAAVVGVP HDVKGSGIFC FVILKLKRVV RKYIGPIATP
DYIVIARDLP KTKSGKIMRR LLRKIAAEID DFGDTSTLVN SHCLESLPFP TPCTVEEALG
FFSNLTGQLP KFAAACLSLW IEDPEERSPL LSLRELLPLL APSSTQELGL ILSLLAAHAP
RAYTIASSPK RLFFGACSSF ITQLRPGDVL KALIKPSSFN KDFLYREELD AAEESFLTHL
FLAFSREPPW VEKYRPRRVE DMAHQVEPKK MLRRILETGN MPHLLFYGPP GTGKTSAALA
LVRELFGREE AKNRLLELNA SDDRGIKVVR ERIKQYTKTN IAKGKINPET GREMPTWKIV
ILDEADMMTQ DAQSALRRIM EAFSRTTRFI IICNYVHRII DPIFSRCSPH RFEPVARDAQ
EARIRHICDS EGLVVTSALL RISQGDLRRA VTLLQSAASI DDNLHEDAIL EVAGQPPARI
VTDFLRACQA SPSQASSEVD NVISQGWDVC LLLQEMIRQV VDLQKARVIN DIAQKEFAVF
QGASPYLQLL SLSLRIHDCL IKELMFKSED GRHLQTIFRT IKEQLKRVKQ KALEDVAGEV
TEQDKLILNR SGRRVLLKDL MIRPNIAPGR KLIGALEAHT NGLRFTVVDI TYSNIKHAMF
QPCERELIVL IHFHLKSAIM VGKKRTQDVQ FYTEAGTQTD DLDNRRNRSF HDPDETQDEM
RERELKRRLN NEFKRFVQQV EDIAKVEFDL PYRELRFTGV PMKSNVEILP TANCLVHLIE
WPPFVLPLED IELVSFERVA HGLRNFDVIF VFQDYTKPVK RIDLVPIEFL DNLKRWLNEL
EIVLKQIRED PHGFVEAGGF EMFLGDDSVS GEDEDDDDEE EEDGEEGSSE VRVLASVEFS
SKRKRSSMLC EIPRIVLFTK GADTVILPLL KQRREAETQM LNTMEEYAAD GLRTLCIAKR
EVDTDEFFTW FQAYQQAERA TVGRQEQIEA VAERLEVQLE LQGITGVEDK LQAGVADTIE
KLRAAGIKVW MLTGDKVETA INIGFATSLL TREMVDGEAL QQMLEPDMEQ LFVSVCTNCV

TVICSRVTPH QKGAVVSLIK RHLQKITLAI GDGANDCNMI QSADIGIGLK GEEGMQAFNC
SDYGLVQFRF LLPLLLTHGS WNYRRISKLV LYMFYKNLVL VLPMFFFGYI SLFSGQKFYF
EFLYQMYNVV FTAIPITLYG VFDQDVDKKL ALKYPQLYRC GQIDLYLNLR VFLKWMLNGV
WQAIVIFVVP TFVFVPTTTG RTMDLWMVGT VMFMNMIVVN IKVLLETYYL TTIIWAGFYI
SLLACLLFVF LFSSWPGFAG SVLGCVFYLF IDAAACAVIA TVAVSLARDW LWKAFRVNCA
PRRYSKKVHE HFFNPKNAGA FDETRSKVGT AVVGKAACGD VIKLQVLVED GKIRDARFKT
FGCGSAIASS SYATELIKGK TCDDALKLKN TDIAEYLNLP PVKVVVLGTG WASVNFFRHL
DPNIYDVTVI SPRNYFTFTP LLPSVCAGTL SPLSCIEPVR SLTYRNGKVA DFYEAHCTDV
DFKNRIVACD SGGHFKVKYD YLVIAVGSES NTFIKDVAAN AFFLKEVEHA MAIRKKVMNN
FELAALPQTS EKERDRLLHF VVVGGGPTGV ESAAEFADFI KESKYFPQLI PHVSISLIEG
GSRLLGTYPP DISAFAEKTL TELHVKLLLR STVVGVDATS VYVSNKELLH GFVLWASGVG
EVPLVKKIIA ENFPNVGKRG LPVDAQLRLL NQNVYALGDC AAIAPVQITA EQLLREAVDR
QLDDLSQQQR IVDEEELQQY RVRKRKEFED TLRRQRHHIG TWIKYAEWEA AQKEFRRARS
VFERALNVDF QNTTLWLKYI EMESKNKFIN SCRNLYDRVC LLLPRQEQFW FKYAHMEELL
GNYAGARNVF ERWMEWNPSD KGWMLYIHFE ERCKELDRAR KVFERYLSNR PSQESFLRFC
KFEERHRQIP RAEKAIELLP EDMLDEHFFL KFAQFEERQR ETERAKVIYQ QALEQLPKGE
SDLLYEKYVT FQKQFEGIED TVLSKRVFVY EEELHGHPLN YDCWIDYIRL EEKIRNVYER
ALAVLKNALI HDGLVRGLHE VAKALDAKKA QVCFLSESCS EPAYKKLVQG LCKEHGIPLL
DVDSKELGEW AGLCKVDKDG TARKVVGASC VCVTDFGEES EALTFLGYYQ ATVGELLDDR
YRVECEAIGK GVFSNVLKCY DLQEKRFVAI KCIRHNDMMK KAAEKETSIL RLLNKRHIVR
LLRHFEYRGH FCLVFEWLWG NLRTALKLRQ LIRRMQIRLL ALEKSYHSRR AMQSRYLQQI
QDLYIRARGD GSLPLVSFEG ATRRHKPELR RGSLILCRVE RASPELGAEL TCIDPNCKKS
WTSQEKLLGE LEGGFVLDVP APLAISLSSP HCFLLESLRL AFEIASGANG GALGFAQQMP
PTVELHEKDA LLDRIAVLLG GRAAEEIFIG AISSGAADDI QKASRLARLS VMQFGMSDRL
GLVDYSLQQG GEQNFYRPYS EHTAKVIDDE VSQIINDQYE RVKTLLKERE KEVHSLCELL
ISRESITYSE ILECIGLKHQ RFVPGEKIRL WYERGRKAAA KKTKACANCG AKTHSEKECV
ERPRSKSYDG KRDRYAGYDP SEYKWVIRAY ELAEIERKRR KALELIAKGR NRVLIKSKYE
EDAYVGNHTS VFGSWYNLAT QKWGFKCCRQ TDFAADCIIP LMDAYHASYY TREVMVMLVR
EFETNDEEMK KIVLRVVRQC VATEGVESEY IRTDIVPPFF AKVWLVRNAL DRRTAKLLIE
TVVEIANKAG IIQQIVEDLK DPSEPFRKVT LEALEQIIVN NGVVDVDNRL EEQLVDGLLY
AFQEQTSEDA TALLNGFSTI VNALGTRIKP YLPQICGVIR WRLNTPSAKL RQQAADLIAR
IAVVMQKCGE EQMLGHLGLF LYEYLGEEYP EVLGSILGAL KAIVNVIGMN KMTPPIKDLL
PRLTPILKNR HEKVQENVID LVGRIADRGG DLVSPKEWDR ICFDLLDMLK ASKKAIRRAT
VNTFGYIART IGPQDVLATL LNNLKVQERQ LRLCTTIAIA IVAETCLPYS VLPALMNEYR
VQELNVQNGV LKTLSFMFEY IGEMAKDYIY TVVPLLEDAL MDRDLVHRTA AWATKHLALG
VHGLSCEDAL LHLMNFVWPN IFEKSPHLVQ AFFDAVDGMR VSLGAGIVFR YVLLGLFHPA
KKVREVYWRV YNNLYIGHQD SMVAFYPPLP DDEKGCYSRD ELLYMGRMYG PGKGMSASAL
PWRRKPPTWL KIKPSDVEEH IAKLAKKGQT PSQIGVTLRD SFGVPQVKSV TGNKILRILK
LQGLAPELPE DLYYLIKKAV SVRKHLERNR KDKDAKFRLI LVESRIHRLA RYYKRTKQLP
ATWKYQSATA SALSYLEHAP GVQFAYVPPD FFGEDDDEDE FMQNQVDNEG GGRAAGATAH
TAAAPYRIRR KDYANDFSTH DERPHELLKI LEFMYLHGVS LEEQNSMGYT ALFLAAKHGN
PNIVQWLVAR GASMNHRDHT GGTVLHAAVA SAEDDPLQFL CEHGAVKLID TRADIGIQMT
VLQRCLLKRQ WFSYLLLLSW RLQYQLFGYT RALRSSYASL YWTITLCNLP LFFNAFCQLH
ALDLASWDGA LWIFLWGLTQ LFWWKTYSGD AGHYRLLQLE REQLRLNLRG EVLALMSGVA
NERRKRVSHE YLETLLEVEC LQRILSPFIL RRLKNEVLGC LPKKKNVVLR CEMQGRQREL
YIQEIKTWES ELTRSLVNSL LARLRRICNH PVLMQGAYTN EQLEEITRHF WLRVDGFKGN
PREKVDLEIR KWSDYEIHQA IQQQISQDSR LAHLSLPKEM IMDSAKIRKM IELVSEIKKK
GEKALIFSQY TTYLDVVEES LKCRLDGSTA VEDRQALVDD FSDLTIFLLS TKAGGQGLNL
TAARTVILMD QWAKLLEQEE KVRAMCDDII ASGCNLVEKG VSDLAQHFLV KAGISCIRRV
RKTDNNRIAR VTGATIVNRT EEITKEDVGT KCGLFEVKKI GDEYFTFLTQ CKEKGACTVL
LRGGSKDVLN EVERNLQDAM NVARNIMLEG KLLPGGGATE MAISALLLAN AKNVESVKQY
PYKAVANSLE VIPRTLAQNC GTNVVKVMTE LRAKHSGDKW GVDGETGAIV DMVAKQVWDS
LAVKQQIVKT AIEAAAMLLR IDDLAVLVLY EMCLENPEAT VTDVRGTERT KQRPVPLCTV
ELHKMASRKL RMSSARCMSI AESLYQRGII SYPRTETEVF SPTMDLLSLI RVHIGSTAWG
TYAQKLVDGD PSDGARDDKA HPPIHPLKLM EWKLYEFVTR HFLACCSEDA FGFETRVEID
IAGEGFYATG LTVLERRWLD VYPYEQWSGN RLPRFLVNER VMPSSILMSE RQTEPPPLLT
EADLIDLMDK NGIGTDATMH DHIRTIQDRH YCYKNENMQF VPTDLGVALY QGFKRLAESG
VDLSLPDLRA RMEADMALVA RGICLVKSGR AEIPGRTKMV IISYDMITKQ KKFMPYKVVI
CDESHYLKNF QAKRTQAICP LLKNAKRAIL LSGTPALNRP VELFQQFDAL LPDLCTYREF
ADRYSVQVWN PFTRHFEYEG HQHPEELHLL LKHTVMIRRL KEQVHSELPE KIRSRVPIEI
PAKELKAIRL FTLTGLAKRA GVCEFLSYLF DGGMKVIVFA HHRAVLDYIE EFLQAEAKRT
IRIDGRTPQD KREQLVKEFQ TSPSCQVALL SITACGHGLN LTAAGTVVFA ELYWVPGQMI
QAEDRSHRIG TEFSSVQIHY LIAEGTLDET VFRILQRKWR LMTSTLDGEQ QQLLFQRGQK
VAVCVSGGKD SAVLLHVLHT LNAREDLGLS LHLLAVDEGI KGYRDHALAA VYASLYGWTM
DRIAQLLGKT HSCTFCGIFR RQAFERGAQD IGADVLCTGH NADDGAETFL MNILRGDMQR
LFATECTYSG AAYRGLVRNF LSSLQCVLCG FLTKNVQALN ENKGNLQETS AQLELDIHYA
DLLPLPPAER WQKLPPLRIL SFDIECVKLK GEGFPEAETD PVIQISSIVM LQSVPLCRVL
FALKECASIA GSVVLWFDDE KEMLAKWAEF VRQVDPDFLS GYNCVNFDLN YLITRAATLK
VVGFNRLSKL KSLESKIRDS SFSSRALGTH EGKDIATEGR IQFDLLELVR RDYKLKSYSL
NFVSFEFLKE QKEDVHYNMI GDLFRGCPSS RRRIGVYCLK DAYLPLRLLK ELLFLYNYVE
MSRVTGTPLN FLLTRGQQIK VTAQLLRKCK ELNYVVPVVK RTGGDNSQYE GATVLEPRKG
FYDKPIATLD FASLYPSIMI AHNICYSTLV ASSTSPPHKF VKKHIRRGVL PMIVEELIAA
RKAARKEMAA AKDEMTRQVL NGRQLALKIS ANSVYGYTGT TTGGQLPCLE VATTITCFGR
DMIDFTRREV EKMFCRDNQH ACNATVIYGD TDSVMVDFGD FSIAEAMKLG EEAAQALSEK
FVKPIRLEFE KVYCPFLLMN KKRYAGLLYT RPEKYDKIDS KGIETVRRDF SLLVQTMADT
VLRKMLIDKP ILSVAPMLAV TNTHFRNFMR CFTREAQLWT EMVTDGAILN NMDRLQQNLC
LEDIEHPIVC QLGGSDPKTL AEAGKLIEKL GFDEINLNVG CPSNRVVSQG CFGAALMKTP
ETVRDIVHEI RRHVQIPVTV KTRIGYDHCD SRDVLRNFVQ TVSAGGCRHF IVHARKAWLK

GVDPKKNRSV PPLLYGRVYD LCDQFPHLDF SLNGGVRSIQ QAIDLALRGV MLGRAASADP
CILANVDTDF YGDANPPTSH SRRTVLEAYR NYIKPFAEDG EYFALLKPVL GVFNGMPGNR
LFRFIGTFLL QNPLAQLAQV ALRPSGSASM QLFSSSASES IAALREKRKH PGVGTPSLNN
GLHLAKDLLA GVPPYCTREV LVLFGSLRTC DVGCIEETIA AVKKSNICCN VICLAAELHV
LKCFLHLVSP ADISRSFHSL CPPLPFDPVA ASGGLASHPL YYYSTHNALR AAAENFFPVT
FVELHGTRTC KINVYPFCDV QMVKRILLKK MNLSCMKVRD IRLLYKGSEL PNWRLMNIFA
PLKKLHWSIR SDNMRASIRP LVSQLRGSLI QVIEEVKLGF RRNVAPKLTM DGTGGTYILF
DARRRPVGIF KPEDEEAFAP CNPRGYEGRI GQAGFRGGVL SGEGAGREYA AHILDSLYNC
PAGIPPTTMV EACHPAFCYK SPVQLGTTAM QLKWKAGSLQ QFAQAKESCG DYNPLLFSVS
DVHRIALFDI RVMNLDRNDG NILVAPTHLG IEQSRRDDLE ALGYVLMYFN RGSLPWQGLK
ATTKKDKYDK IMEKKMSTPI EILCTGPAVP SVDMLVLVDR RSDLITPLCS AFTYEALLDA
VFGIDAAAVE VPLFATLRDL HQSALGAHLH RVANEIQQTY EKDELRSIQE ISVFMNKFKV
KQQEHSSLSL HVRLASFLAS VTKDPAFFRR LTLEDELLQT GSATSSSAGA TLSAAVENMV
DASAPVEDVY RLLCLASVVN GGRKGLIQQH GIREAVRMAH LQRVGLLRQA DNTLGSWKTL
KKECNLIVEE EHDIAYACSG YAPLSVRLLQ FPNGWRSIPH ILSLLWGPAM EVRQNLTEVE
SLCPNCEENG TTLLLLHKVP HFKEIVLISF SCPHCHYSNR EVQSAACLAP QGVRLELTVQ
SAADLDRQIV RSEHATLIVK EVELEVPPKR DRGELTTVEG AIRRMIDALV AEKIDEVILR
LWTLILDDPS GNSYIEYERT KEQLHAMGFY SLPVSCPHCG TEGSNNVCEI DVPGFRRCLI
FSFLCQSCGG RHSEIKAAGA FGAVGRKWIL NVETAEDLNR DVLKSDTAVV EIPSLDFSMR
GGVQGGEFTT VEGLLGKLAT ALGDSAPFAM YVVNRRGEEE PVSFDQILKR IEKLSFGLHP
LVDPARVAQA VINGMYAGIR TSELDDLAAQ TSAYMAASHP DFSRLAARIA IDNLHKNTTD
NFLTVIDQLH GYVDKLGREA KLVSTEVYEF VRENEQALNE ALNYSRDFDY DYFGFKTLER
SYLLKIHDRI VERPQHMLMR VACGIHCGDV EKAIETYELM SQKFFTHATP TLFNAGTPRP
QMSSCFLLTM QEDSIDGIFS TLKQCALISK TAGGLGLAVT DIRATNSYIR GTNGYSNGLL
PMLRVFNDAA RYVDQGGGKR KGSLAIYLEP WHFDVFDFLD IKKNHGKEER RARDLFCALW
IPDLFMERVN DNAGWTLMCP NECPGLTEVW GDEFKELYER YEREGRGRKT IPAQHLWFAI
LQAQIETGTP YMLYKDACNR KSNQKNLGTI KCSNLCTEVV EYTSKDEVAV CNLASVSLPK
FVDRESRTFD YEHLKRIVKV MTRNLNRVID RNYYPVPEAK KSNLRHRPVG LGVQGLADAF
MLLRYPFDSP EARVLNRNIF ECIYFAALEA SCELAAEEGP YATYEGSPVS QGILQFDMWG
VTPSSGLCDW DGLREKIKAH GVRNSLLVSP MPTASTSQIL GNNEAFEPYT SNIYYRRVLS
GEFFVVNPHL LRDLLERDLW SEDVKQQLIA HNGSVQNMDV IPDDLKALYK TVWEIKQRVV
LDLAIDRAPF IDQSPRMTLE EIKRDFTKHK LECPDTMWRE PQPEQVQGLY SFAIESIFGL
TVNDVRIEEV TGDVRSFLSQ DGRLHAKAIG NLRFIRLCQR LNRVLGLPEF SREFASPTAS
GVQRFASAAL ELELGDLVLE LMNLKQEREE LHDQVVHSPE KLMERRDELR VQQKHLDAQL
QELENLAASQ QKLLLAFAKA VKKAKKAMEI LSAHLAPHLG FRSDMRTREK LFRELDWWTL
GILIYEMILG YPPFFDDEPM GVYQKILGGR IAFPKFFDKN AKLLVKRLLT PDLAQRYGNL
KNGVADVKDH RWFAGFDWNA CLKKSLPSPY KPPVKGMDDT SNFPESTEQA PPVTGTMDPF
TSWMLSQFYV LSPRGDCLIT KDYRNDAPKG AGEASPLFCV NGITFAFLRR SGLYFVLTTQ
QNPSPAVLIE LLHRLTKIIQ DFCGVLNEEA IRKNFVMIYE LLDEIVDYGY PQLTSTESLK
SAVYSETIPS NASHRPVSAN IRRSEIFVDV LERLDGSIQM KSYLDGKYLL KLALNDDIVF
VSQTTGSPNT VWVDACNFHE CVLTFVPPDG EFVLMNYRVA MQNFFVVGDV ICCEVQRVRA
DGQILLHTRS TRYGRLMNGV FLAVAPQQIQ RQSHHIVQLS CVSKEMRLAI SRVRNIVLCL
ARSNFDISTQ TIERMYVNEW RAPGGRRIKA AAANERQLVI SLAGGELVLF EVDDAHTLVE
TARRNINVES TCMSMQAGRL RASFLAVGGL DNMVRILSLD RNLRQLSTQL LPNDATPESV
CLLYLHVGLN TGVMIRSVVD PVGTLLDQRS RFLGGRAVRF HAVQPAILAL SEKSWLCYTF
QHKLHCIPLN YDPLECVASF CSEQCTDGFV AIAGGSLRIF RCQRLGETFG QTVLPLSFTP
RAMAALPHPS ALAIVEADHN AYDESTKAEI RRALKGIKVG TFKAGPGKWG SCIRIVNPLM
AMTIDKVSLE TDEAALSCCF CEMEGLPLLV VGTVTAMTLK ASIKVFSYDD KFSLSLVHST
PVEDYPMALT AFRGMLLAGV GHKLRLYALG RKRLLKKCEY KNLPCGVAFI RVADRLFVGD
VRESVHVMRY RLSENLFYVL ADDVVPRWLT KGEVLDYHTF VAADKFDSVF ICRVVEEKPD
VTYNDVGGAK EQLEKLREVL ELPLLHPERF LSLGIDPPKG VLLYGPPGTG KTLTARAVAN
RTDACFICVI GSELVQKYVG EGARMVRELF QMARSRRACI LFIDEVDAIG GSRGGENAHG
DHEVQRTMLE IVNQLDGFEA RGNIKVLMAT NRPDTLDPAL LRPGRLDRKV EFGLPDLEGR
THIFKIHAKT LSVDRNIRYE LLARLCPNST GADIRSVCTE AGILAIRARK KSISEKNFID
AINRVIKGYK KFSATAKYMV YNLSDLTRQN AQSMVAQLCA LVALPKNSTF KLPRMHPAPA
AKKLTRWEAF AKEKGQKRKR SRLVWDANTK DWVPRWGHKG IQQMNALAEA VIEDKDGEYV
CPFEAKAKEK KLRQAKQKLR ELRNTVFERL TDTAYYTGSH RERFKRPRKP VVTPPRLMWL
YRNGDKHDDG TPFFVRPYIK SMESLYQQIT KEITPIAGPV RRIFDQNDGA KYLCTSGEPP
AAYDRLEKFD ARKYFKDGQK HITPPNGEGT RAFYESLYEE NPNSLIALRY VIEYGVLTGT
KLHESLPKYA LLRELGAFKG AGGGVQPEFK DGLNVKFIQE KKLIGRFFDE VAQDTGKYVF
GVQETLQALE MGAVELLIVF EGLPLERVTL KNPVTNAEKV IHITPDQARD DSLFKEDGVE
LEVADKISFS EWLVNNYKNF GTTLDFVTNR SQEGAQFLEL QMAFLSFTLT LFPDLASTAV
LLSRCLSPAG VEAVVELLSS PLRTLSLSVL EIEHFPCLMG YLDFDTRKQV AVSMVSAVLG
SNVALDQPSA LSRFLEFISP LVDTDLHFAL LCIAREKFGE LRRLRYTLPP LVVAALQLVM
LEAKLQHASV LRRLFESIKD MVSDVNLDCD ETGLRLQAMD SSHVALVALK LDDVGFVHFR
CDRERSLGLN LASVCKVFKL CSNADSCSIQ NEEDSDTVTF VFENEADEKL SSFSLRLMAI
DQDALRVPET AHDVTVTMSA REFANVVRIM GEFSDSVRVE VDKLGVKFVT QGDLGVGEVL
LKPKPFADDS GVEIKVNSPV CQTYAVKYLN YFAKAASLSS SVTLSLTDQN PIEVRFDILE
GHVKFFLAPK MDDDCPEGCS EEPGSLKGTY IYTDDSPICK AAIHVGVVPL GGNIVVVLGE
GQDSFMESER NGVKSESFGR YDRTFMVSIP IGFRGRPEDY VDTEKLPGAK ALTGFSDFTF
ALQMGKRKWR SILTQSGCEG FTFAIDDKDE LVFEQNCHPK FISTGIKPAI GEPLHIAVSY
YSPEKVVNIY VNGRQVVSEK TDFDFNLKSK LIIGRAADSE SEYFIGHITA LKVFSYALSP
EQVRRVAAEA GSRRTDDGRV CLSPCSSEEP LLNEILPTNP AIQLTCEDTL RREEFNGIEQ
RFLVSCPSDC TSKVAQVYGS KVYSEASSLC KAALHSGIIV THSGLKSYSG STGKNGVSSV
GEDEPCRDDG AFALKMNPGD KELVICPPGC GTKVYSPISS VCRAAIHSGH LTNEGGEVEL
QATGQHEEFK GSDKNGIQSV NSGWYLRAQA DIVKEADLGT REKIFSLDLP FGPYAVDFSR
NGRHMLIGGK KGSLSLLDCH TFQPLCEINV KETVRDVQIL HNHTMWAAAQ KKYLYIYDQQ
GIELHCLRDH MMTYRMDFLP YHYLLVSVGE FGELVYRDIS TGQIAARHKT RRGPCDCMRQ

NPSNAVMHLG HIKGTVSLWT PNLGKDYMVT SGIDGSWKIW DLRKPLHSFQ YFGSPPSSAR
WSQTGMLAMG FGSHVQFWKD AWSTPYLTQH YDSKQVESLA FRPFEDLCAV GLTTGIDTIV
VPQSGIANFD TFEANPYETS AQREIHSLLE KLQPDMITAD GKNLVLVRKV TGASAVVKCG
FSPREVLSFT LKQINANTWP RWTSQESFCC RMVTNEVHIF RDNVAGRKGA PSAVKLFDLE
NGSSCLCTKS FFQGNEVTFK WSNDGRAVLA LVHTDASEKS YYGSDALYFL KADGSYDCQL
MPEEGPIHDV QWSPSTLEFA LCKGPMPPEI LLYDGSSAPK LSFGRRMQNT LRWDPFARML
LTAGFGNLAG DVDVWHKQQK KVIAKAQAPF TVTCDFTADG LHFVAATTSP RLRVDNHVTI
HSISGHAVCR LDFPVLYRVL VRPPGYSPPG AAAKAASGKK RKCQESIPLP NVDTCILKKI
IEYCEHHHNN PPEEIPKPLK SSNLAEVVVE DKVAGLEEPA QKMTQEVEMA VVLFKRRRIV
QLIPRIPQNL RHLTGPTTVA GAKVKLPSVY GKLGNPLSLS TLLPFSTSRE MEEELQQFIM
KQRRCDYFNT LNHFISLLID VSNALALEPD RSLRPPLLSL FLESLNSWIL CRRLYVTAMV
GTWTMTGVTI PLQILRIDVK ECRALSSKKR VPYLLVFEVA DLDEDLTQFI PVQAQRLIYP
DMNPDTLMQL MQSPLLQQTM QNLSQNPQML RTMMESNPML RQMMPMMQNV LDNPELLRTF
LNPQMMQASL PPEERFARQL ESLREMGFID RDANIQALQE TGGDVNAAIS RLLERGSGDR
LTRAARVLEQ LTGQRPQFSK ARFTIRSFGI RRNEKIACYV TVRGKKAEDI LEKGLKVKEY
ELKKKNFSDS GNFGFGIQEH IDLGIKYDPS TGIYGMDFYV QLTRPGNRVA HRKREKMGQD
AARRLLLEIM QGGVVDTHHQ YMALLFAAAA EEHQPCKLRL SRLTPYTTQF LRHLRDFLGV
TFLLRCVGVG LRNMAGKDCV GIASDTRLGV NQFGTVSADF QKVFKMNNHT FVGLAGLATD
VQTVHKELVF RSNLYQLREE TEMPPEIMSN VVSSMLYGRR FAPYFVSPVV AGPFLSAFDY
IGAACYAKDF VCNGTSAEQL VGVCESLWKP DVLVVGAGGI GCEVCKDLLL SGFRRLCVVD
LDTIDVSNLN RQFFFRNAHV GLSKAFVLAA ACSALLTVWG VVGQKMNILD FTIQLLQTYD
VVISALDNQK ARRHLNGLCI AADLPLIEAG STGYSGQVMP ILKNETLCYD CEAKPRDQQF
PVCTLRQRPE RPEHCIAWAK MIYELVFGVE DNENLLDLKR TFLDVSSTAE AEGREMSRKM
MKELFHDQIV DLLRLSKKQE VLPTPKTTER REAGIPFDKD DDLAMDFVAA AANLRMHNFH
IALKSRWFIQ AVAGSIIPAI AATNAVVAAL QFAEVRCCKP AASRNSSGRV LVLDFGSQYS
HLIVRRLREI GVYSELRRCD IGLQEIKSPS AVILSGGPAS VPHLCPSFFA WKVAVLGICY
GMQEICHALG GKVEGGEKRE FGSTVWMSHG DKVTAIPEGF TTFASTAACP YGDPERHFYG
LQFHPEVTHT PQGTQLLKSF VIAKLKPSWT MKNFLQAEIR KIQTLVGDAH VLGALSGGVD
STVAAALVHK AIGDRFHGVL IDTGLLRKDE AQNTKLKACF PSLSIECVDA SEAFFSKLAG
VRDPEKKRKI IGNLFVDEFV RVVNEKKIHT HNTFLLQGTL YPDVIESCSF KGSHTIKTHH
NVGGLPEKMH LKVLEPLREL FKDEVRALGL ELGLPHERVF RHPFPGPGLA VRIVGEVTPE
RARVLQEADA IFIEEIRKAN LIAQAFVVLL PAPSYEWTCV LRAVETTDFM TADWVRLPYD
LVARCSTRIN EVKGINRVTM DVSSKPPATI EWELPLKGHQ PGVSESLRKP LVLYIPPPDK
VEVDPMLTRW LREHQRQGVK FMFDCLMGLK EFQGEGCILA DDMGLGKTLQ SITILWTLLE
QNIEGQPAVR RAVVVCPASL VNNWAAEIQK WLQGRCGCTP VADNCKEKVV SKFEGFKYDR
QSRILIASYE TFRMHVHRLE GVPIDMVVCD EAHRLKNDKT KTSLAIIELP AKKRLLLSGT
PIQNDLDEFF ALVSLCNPNV VGDAHTFRRR YANPILVGRE PDATEDQQQL AAERLTELSS
LNLFILRRTN SLLAKVLPPK VVLNVFCRLT PLQKEFYRSF LSSKSCRKMF TAEATGRVLS
SIQGLMKLCN HPSLVKSQAL QSGKLLLLAR LLDVIRTTTN DKIVLISNYT QTLDLFDRMC
RDCGYPVMRL DGQTSIKKRH AMITKFNDNP HSFVFLLSSK AGGCGVNLIG ANRLVLFDPD
WNPANDKQAL ARVWRDGQKK SCYIYRFFST GTIEEKIYQR QICKDGLSAM LVSDENQIKD
SLSTELVKDL FRLREDTLSD THDMLECRRC RRVEAAGRAF LERFETAACF ASSTRLKRLL
GEAQTLYEVL GVHEGTTTEE IKKQYRRLVL EHHPDKFLKI QEAYEALTDT EFRRQYDSAL
PWSSRRPVPS LGDAQTPMSR VRSFYDFWFD FQSWRDFGVH DEYDLNEAEC REERRWMERE
NLKIRKKHVK AERARIQKLV ETAYSVDPRV LMEKKKREEE KAARRECHKK WRQRTRQLHQ
LRDLCGEIQK VAWTPDELSL LAKGLQKFPG GTARRWKLIA DLIGTKTQEE VVEKTKEMSE
GASLWTPAQQ MALEKALAKH PATMPMPTKR RNGGRAKHGR GHSAVVRCSN CGRSCPKDKA
IKRFNVRNIV DASSQRDLRE ASVYNTYTLP KLYIKQCYCV SCAIHSRVVR VRSVEQRKVR
ENPQRRPQGG QKKVSVSEVS FDSSYTVLDT SEDAIMLHVN HVLFHLDFNA LAVIKNADMP
EDLQQDAIDC ANQALEKYNI EKDIAAFIKK EFDRKHNPTW HCVVGRNFGS YVTHETHHFI
YFYIGQVAVL LFKSGVCGWS RSVRKQGGSL CFVVLSDGST SSNLQVVVEA GIGGFPQLLK
CGAGCSFRFT GDIVKSPAKS KGHRFEILGM DAAKYPLAKK EHTREYLREI AHLRPRSYLI
GAVMRVRSNL AMATHRFFQD RGFLYIHTPI VTASDCEGAG EMFQVSTLDY SRDFFGRPAF
LTVSGQLAVE PYCCSLSDVY TFGPTFRAEN SHTSRHLAEF WMVEPEIAFA TLEDNMVVAE
AYVKFCVQWV LDNCRADIEW FQKNQEEGLI ARLENILAEP FARVSYTEAI EVLKAEEAQF
KEKVEWGMDM GSEHERYLTE NVYKKPCIVY NYPKDIKAFY MKLNEDGKTV RAMDVLVPKI
GELVGGSQRE DDRDRLAAMI AAKDLDPKPY WWYMELREYG TIPHAGFGLG FERLVMLVTG
IENIRDTIPY PRYPGHAEFV DAMVKASFRD KGVIAANLFL KEKQRFFLLT VTHDLPIPLK
GIAKLLSAPR MRLADEELLG PMLDVSKGSV TPLAAMCDEK KEVTLVFDSA MKKNVLVHPL
HNKATVALKA SDLVKFVEAC GHSVMLGVTA KKDENFSEWY TQAIVRSEMI EYYDISGCYI
MRPWAFHIWE KVQRFFDDEI KKMGVENSYF PMFVSRHKLE KEKDHVEGFS PEVAWVTHYG
DSPLPEKIAI RPTSETIMYP AYAKWIRSHR DLPLKLNQWC SVVRWEFKQP TPFLRTMAGP
GKSDKRKTYF SRLFALLEKY PRVLVVEADH VGSKQMADIR LALRGKAVVL MGKNTMIRTA
LKQKMSEMPQ LEKLLPLVRL NVGFIFCIED PAEVRVAENK VPAPARQGVF APIDVFIPAG
PTGMDPGSTS FFQALGIATK IVKGQIEIQN EVHLIKEGDK VTASAATLLQ KLNIKPFEYG
LAIQHVYDDG SVYKASVLDI TDEVILEKFR AGTMNVAALS REVGFPTTAS APHSILEAFK
FCTSLVLESD YSFPQMQRIK DILENPEAFA AAAPAAEEPE EEEDDMGFSL FDPRHDLTRE
PCPGRILEDL GGAFGMGALG GFLWHFAKGW RNSPKYEKFA GGMLSGSMKS PLVGSSFAVW
GGLYATFDCS LIYLRGKEDS WNPVLSGALT GGVLSMRSGW RSCMKNAAIG GVLLGIIEVV
QLAFQRSTGP TPRQQYRQYL EMEQMLVDDI GDMTITNDGA TILKQLEVQH PAAKVLVELS
DLQDKEVGDG TTSVVLLAAE FLRVGNQLVK EGVHPTAVIA GFKLAMKESV KFIQEHLTSR
ANNREVLMNV ATTTISSKLI GTETAHFADL VVRAILSVKM ITERGDVKYP VSSINIIKTH
GKSMRESSLV EGYALKAGRA AQGMPQCVKN AKVALLDFNL RQHRMQLGVQ IQVDNPEELE
KIRQKEKDIT AAKIQKILAS GANVILTTQG IDDMAMKYFV EAGAIAVRRV DRKDLRRIAK
ITEGTVVLTM ATLDGDEKFD ASCLGTCEEV YEERIGDWDH LMFKGCKGGK AATVILRGAN
EYMLDEVDRS VHDALCAVSR ALEYTHVCPG GGAVETSLSV YLENFARTLG SREQLAIAAF
AEALLIIPKT LAVNAALDAT ELVARLRAVH AKAQGAGNGD EELKWHGLDL TSGKTRNNMA
AGVIEAAVSK TKALRFATEA AVTILRIDDL IKIAPERNPA VAKIKGHTAS IQDTNFSPFH

RDILATACED TIVRIWLTGA LKKVLSAEWN PAVSGILASG CFDGTVAFWN VEKNENFASV
KFQESLLSAK WSWKGDLLAC TTKDKALNIV DPRAAQVVGS VACHDGSKAC KCTWIDGLAG
RDVFTTGFGK MQEREMAIWD TRKFDKPVYH AEIDRGSSPL YPIFDETTGM LYVCGKGDSS
CRYYQYHGGT LRSVDAYRSS VPIKNFCFIP KLAVDQMRAE IGRMLKQENG NVLQPISFIV
PRKNFQADLY PPAPGENKAI RRRSVFREMK IVGQFNQGFI IGCLFIVDQH ASDEKKRFED
LNEGFKPATQ PLISLSALPV VEGRQLKEED FIEFLAALAF HHRLCRPKKV WEILASRFKS
LAVIWLRKIL DCHADDQAVW MAKLKAEQEE QQQKQINDWN KLLEDKCFVY PAAATPYGAC
ASAGAQEPTR RRRVKKEHPS RTGVDVLCQA KSGMGKTAVF VLSILQQLVV CLGIAHTREL
AFQIKNEFDR FSKYLKNVKC EVVYGGISIQ KNIDMLKTPH ILIGTPGRVL ALIKGKHLNA
EKVAHFVLDE CDKCLEKLDM RKDVQNIFMA TPKKKQVMFF SATMNKEIRD VCKRFMQSPV
EVFIDDESKL TLHGLLQYYV KLQESEKNRK LNDLLDTLEF NQVIIFVKSV SRAQALDRLL
TECNFPSIAI HAGLDQEERI NRYQQFKNFE KRIMVATDLF GRGIDIERVN IVINYDMPDS
SDSYLHRVGR AGRFGTKGLA ITFVASQDDT NVLNDVQTRF EVHIAEMPQS IDASQYINQR
QSVSAEAYGE WNKRKKFVAP VYPKTAEQKE RITKVIESSF LFSSLDIEDL ETVINAFQEV
SVKKGTVIIR QGDDGDRLYL IETGEVDVMK KFGEKEKFLC KMHPGDAFGE LALMYNAPRA
ATVIAADDML LWALDRDSFT NIVRDAAAKK REIFEESLKE VRILEDMDPY ERSKLSDALR
TATYEDDVII KEGETGDTFY ILLEGAAEAI KNDKVVMEYK KGGFFGELAL LKDQPRAATV
VAKSHVQVAY MDRKSFKRLL GPVEQILMRN QDNYRKAMKQ LMLLRVQALT PEVRAMYAAH
GHYHEGDSGV DLFVVQDQEI QPGETAFVKL GIKAAANVSW LIMPRSSISK TPLRLANSVG
LIDAGYRGEI MAAVDNIKTV PHTLKKGDRI VQAVAFSGEG ITLELVDELN KTARGEGGFG
STLGAAYGTA KSGVGISSMG VMRPDLVMRS IIPVVMAGIL GIYGLIISIV INGDFVRAFL
LGFEVRDAIA LLRLDDLFIE SFEIKDVKRL NGDHLSRCIA RLNGREGKTK YAIENATRTR
LVFADSRIHI LGSFENIKLA RHSICSLVLG APPGKVYNHL RTVTRRLAER IEKLEERMHP
WGDIEGMRAA CGFTYDDLIL MPGHIDFGVN DVDLSTRITR NLHVRTPIVS SPMDTVTEHR
MAIGCALMGG MGVIHNNMET ARQVAEVQKV KRYENGNELL RESKKGKLPI VNDNFEFPLA
SKDSNKQLLV GAAVSTDIER AKALQEAGAD VLVVDSSQGD SIYQVDLVKR LKAAQIIGGN
VVTARQAKSL IDAGVDGLRI GMGSGSICTT QVVCAVGRAQ ATAVYHVCKY AHDLPCIADG
GIQNSGHVMK ALALGANAVM MGSMLAGTEE APGEYYFHNG VRVKTYRGMG SLDAMVAQGV
SGCVVDKGTV MQLIPYVIQG VKHGMQDIGA RTLRDLHAQL VGGMERNHEL KKAMEDLKKL
SSRVQEAAAT ASALASQLVL SQRTSWDRFS TKFKEMSFLQ NFFENPLVAQ LFGETEIAAS
IREMKILDPK FKLADMHNMM ERVIAAHIVQ AFLLGDEGTL AVHCAEGAFA AMRASIIERR
AQKVRLDSEI LQLGNVELVG ARRSADECPW FVYTFTCQQV NCLRSEDGRV VEGREDDIRR
VVYSIAVSKH PKPEGLLYPW MIREIAIIGS EAVWMVLIIV GLGLSDEKDI TIKGLEEVKN
ADFVYLEAYT AVLGVGPPKL EEFFGKKIIE ADRTFVEQGS DEMLERALSS NVAFLVVGDP
FCATTHADLY LRARKKNVTV RVVHNASIMN AIGSCGLQLY RFGETVSIPF FEESWRPDSF
YMKIKKNKEA GFHTLCLLDI KTKEQSVENM MRGRQIYEPP RFMSVEAAVR QLLEVELGGK
VCPRDAKAFG LARIGAPSQQ ITSGTLEELL SVDFGPPLHS LVICAPTLHE MEREFFELFP
NSAIRKCVRV QLIKNGKKIT AFVPRDGSLN YIDENDEVLV AGFGRSGHAV GDIPGVRFKV
VKVAGASLLA LFKEKKEKPR TMGIKGLGKF VGDFAPRAIK RQEPGSFTGR VIAIDASMSL
YQFMVAIRDG NSFGNFTNDA GDCTSHIAGM LNRAIRLLEQ GVRPVYVFDG KPPELKSGEL
AKRRELRESA QEAAEKAREE NVEELRKQIV RSVRVSKQHN EDVKRLLRLM GLPVVEAPCE
AEAQCAELTK NRKVWATATE DADALTFGAT RLIRNLIDLP TLLEELQFSQ EQFIDFCILC
GCDYCGTLKG VGAKTAYSLV KEHGSIEKIL EVVDFQEARE FFRHPEVADR VHVAWGEVDV
DGLKAFLVQE NQFNEQRVEN YITRLKKARG KTAQTRLESF FKNNTKFYEI LEVDRTASVA
DIKKSYRKLA IKHHPDKGGD PEKFKEISRA YEVLSDPEKR RIYDDHGEEG LENGGAGADP
TDIFDLFFGG GRPSKKKGED IVSAMKVTLE QMYSGATKRM AINKDVLCKQ CNGVGGPADA
LTTCHDCDGH GVRVQTRQIG PMIQQTQSVC PACKGAGKSM DPSKRCKSCT GKGVVKERKI
LEIYIEKGAK NHHKVIFRGD ADERPNEIPG DVIFILEQQE HAVFKRRGND LFMTKKISLL
ESLCGFKFVL THLDGRQLLI QSPPGTVTKP DAVQIIKGEG MPQQKNPFLK GDLFIVFEVE
FPEHVSDADA KSLSILPKPT EAVSEDDPHV EVHVAEPVDP DELRNRLELL RHHLPTLLPL
LVKNTTYHEY DYLCMDPSQL EDDNAEEDSR GTWGDGWSVR KGSALALDHI ASVYREAVLP
EVLPLIEASL VDANWERREA AVLALGALAQ GCQDSLEPYL PNVLQFLLNL CDDPKPLLRS
ISCWCVSRYA AWICRHEQFL KPVLVQILKH VLDRNKRVQE AACSAFATIE EEASLHLVPY
LPDILSTLKQ AFCFYQTKNL LILYDAVGTL ADSVGSALAT EAYSREIMEP LFGKFQNINL
QDPGLIGLFE CVTNCATALG AYFVPYAQAV TERCVWILME SLTQDLIESC LDLLSGVMLQ
SSFALVGDLS KHCVKFLQLS VLMPTLSEHL LHHATSVQNN AGWAIGELAL RAEPQFIEPH
VDSIASKLIG IVCPELHRSL LQNVSISLGR LGIVCPAKLA PHLGDFLQQW CIIMRHAKND
EEKANFREKE RDKFKECIQP DELRTLLAQG LLKVTNVNKL TKAGCVFVFW VADWFAMLNN
KMNGDLKKIQ KVGEYFIEVW KAAGMDMQNV RFLWASEEIN RNSDQYWVQV MQIARSFTIT
RVKRCSQIMG RQEGDEQPAA QIMYPCMQCA DIFYLGADIC QLGMDQRKVN MLAREYCDEI
KRKLKPVILS HQMLPGLLEG QEKMSKSNPD SAIFMEDTET EVNRKIKKAF CPAGVIDGNP
CVTYVEQLVF PAFGEFAVKR SEANGGVRMW VYEEMVDGEK LTDIINKTHE NRKYLPGHKL
PENLVALPDV VEACRDSDLL IFIMPHQAER LLTTIGRAGV LLGSAGFVAS SCLYDVDGGQ
RAVMFNRFGG VAKKPIGEGM HLYFPWFQVP FLYDVRIRPK VINTTTGTRD LQMVSVGLRL
LYRPMEDRLP IIHQTLGPDY DERVLPSIGN EVLKAVVARY DAESLLTQRD KVSHDIRDAI
TNRARQFDLV LDDVAITHLS YGKEFSKAIE EKQVAQQESE RTKFIVARTE QEKKAAVVRA
EGEAEAATLI SEAIKQHGTG LIEVRRLDAA KEIADTMAKS RNVMYLPSGV NMLSISQLAL
QRGGYDFSNH QRNVRLLMEA PPARKTGTTI CGVVCKDGVV LGADTRATEG TIVADKNCSK
LHRIADNMYA AGAGTSADLD HMCDWLAVQV ELHRLNTNAK PRVSMAVSVL SQELFKYQGY
KGCAVVLGGV DFKGPQIYKI HPHGSTDCSN FAAMGSGSLN AMAVLEAGYK DGMTLEEGKN
LVRDAIKAGV LNDLGSGGNI DLCIITREGA QHLRKFETPT QRPFQAPKGT TPVLLEKIEQ
LKSRLSLNDL GEARGTVLSV KLDKVMDNLS GQTVIDPKGY LTDLNSSDAD VADIKKARTL
LKSVTATNPH HAPGWIAAAR LEELAGKLQA ARELIATGCQ QCPKSEDVWL EAARLEKPAN
AKAVLAKAVS VLPHSVRLWF DAYAREKDLD QRKRVLRKAL EFIPNSVRLW KEAVSLEEEK
NARIMILKRT RAHVNTNKQM AQHVASKAVQ ECPNSGLVWA EAIFLEEKSA QTHKAVDALT
KCENDVHLVL AVACLFWKEG KISKARKWLN RSVTLDASFG DAWAAFLAFE LENGGEKECR
NIINKASLAQ PNRGWVRGRI QESRGKGSVG FLMLRERQET VQGVLDAKRG NTKDMIKWTM
SLPLESVVDL QGTVVEPEVE IQSTSQKGEL LVTKIFCVSK AAQELPFQLR DAMRPEDGSE

GGIRVNMDTR LDNRILDLRT PVNQAIFRIQ SETLQLFREF LLSRNFVELH SPKLIGGASE
GGASCFTLKY FNRDACLAQS PQLYKQMAMC ADFERVFEIG PVFRAENSNT HRHLCEFTGL
DLEMTFKEHY SEVLDLLDDL FKFIFKGLHQ QHPAEPFTWI EETPRLSFEE GVQMLREDLS
EFDLSTEQEK MLGRLVKEKY HTDFYMLLQY PLKVRPFYTM PDPHNKMYSN SYDFFMRNEE
ITSGAQRVHD ADLLTQRCLE CGVPPSSIQT YIDSFRLGTA PHGGVPYEQF SREWMAVAGQ
DNFDGFRLEA TKQVNKVLTA NHTFMLGTKE GGCSYSFGPT LVIGEPNFFG MARMNSDGFL
QARFIKAISK DIKFNSNSSI SEDAKDMYEV SFDKMGSDWA ANLKLAWQGG GELTWVAATG
ISMGSVGARY FNENNTVTCQ IGVGPDFSSP MGFANDVYST KAQYVRKVTD RLSMGTELEF
THPDMSSAMR VGWQYLFRQA RVQGLVDTAG RVSMFAQDYN GFGLSGMIDY WHGDYKFGQM
NVVPPPPQDG GVGKTTLVKR HLTGEFEKKY IPTLGVEVHP LKFQTNFGMI TFNVWDTAGQ
EKFGGLRDGY YIKGQCAIMM FDVTARITYK NIPNWYRDIT RVCENIPIVL VGNKVDVKDR
QVKARQIQFH RKRNLQYYDI SARSNYNFEK PFLWLARRLT NQPALSFVGE HAKAPEIQID
PNLVQEAERE LQAAVSTAID DDDCSITLGP IEVEPTAAEE ALKTLTKQKI EENCPFRVFT
IPRDLATRMY QESYLDEFGI PANVEVRLVV LPEWNINANM YPVLKSTGQI ADIQFESVKF
NEEKKQLCLS LHVVPGPDEI PSRAQVLPPS GIDYNKLLRK FGCSSITPEL ISRIEALTAH
HLLRRGIFFS HRDLNLLLDS LEKGPGFYLY TGRGPSSEAL HIGHLVPFMF TKYLQDVFDV
PLVIQLTDDE KFLFKDSLTL IVVKEVSQNK VKFLLENCDV SIANGLRRVM ISEVPSLAID
LVTVYENTSV LHDEYISHRL GLLPIDSTRV AEFVNRDDCD CADHCSRCSV QYALDVVCED
SLLVTHRDID NADLGCPMPV PIPIVKLRKN QSVNMRMTAT KGIGKVHAKW SVATASYKFE
PEIAFEEDLL ARAPAEVKRQ IAASCPREVF SFLRVENKMN CIFCDQCKVK AQELGFRRLV
RVEPNERKFH FTVESTGVMP AEQIVEMAFD ILLNKVTELE GLVQASARAT GASTFASALP
DPAATDNLEM KVVYKILAEG LQKDPQKWSR MAANLKGVSE ETTTGVHRLL QMAKKKELLF
PAINVNDCVT KSKFDNVYGC RHSLPDGIMR ATDVMLGGKR VVICGFGDVG KGCAAAMKGA
GARVVVTEVD PICALQAAME GYSVSTLESQ LATADIFISA TGNKDIITAR HMSQMKNNAI
VGNIGHFDNE VDMAGLLAWP GIQKVNIKPQ VDRFIFPEDN HVIVLAEGRL LNLGCATGHP
SFVMSCSFTN QTLAQLDLWD NVRYQNDVYL LPKELDEKVA RLHLPALGMY SLGVEGDETL
PVQQAFVSSA SEGYTGPTPR GENHSPFIYF FTSALLRTGS CLQAACLLLM CLFYVGFGGG
GLFVFDEFAG PESVRMSDAF HLITAILMIG YLIGTFAIAL FQLFVADSSK WTRGFRSGSK
TISCAVTLDV VSGCLRVTQY IHAYFFSSVQ WWAKYQRTQS DWGLYHFSST LHAFALVTYG
VGFFLMESYH DQGTYEEYAW SMLGLYTTAG LAELNQPKRR TFRTFSYRGV ELDKLLDMKM
EDLLELFRAR QRRKFQRGIK RKATTLMKKL RISKKNCAFG EKPEPVNTHL RDLVVVPEMI
GSVVGVYNGK QFINVEIKPE MVGYYLGEFS ISYKPVRHGK PGIGATHSSR FIPLKVMVDM
FLWRDPEEFE RKELEDEEAA PHTAQADATQ WGADAAAADW GLLQIIKDHH ETMYELKQQV
STKDIVVGWF CTGSEMTELT CAVHGWFKQF NSVSKFHPQP PLTEPIHLMV NTTMDRDNLS
IKAYMQVPMN MAKDACFQFQ ELPLELFASS SDRAGLALEK LSDQLNKCGA YVRSVLDGSE
KADPEIGRFL SKALCVEAVQ DLEVFEQMCQ NALQDNLMVV HLTSLARLQF AVAEKLNWLG
NLPLKQDETE GLRMHKFLME AVLQNNAVVL GPNASNLPRL LQILCAVYRT DFSDSDLNEE
IKKLFAQINA SPLAALCFTA KEQKKLQKIN GEQVMAHFSS HLQLKAYLPL IQNSPVYPLI
LDAKDRILSL PPIINSEFSK VTEDTRDIFI ECTAVDITKA QIVLNTLVAM FSEYCKEPYT
VEPIRVVYPR VNSRSMPFSL DYVRQLTGIP DLTADACANL LKRMMIQAGI LEASIPITRS
DILHERDIVE DVAIAYSFNR LPVTRSYMLT GDALNCLSEK IRNFCTVCGY TEALNFSLSS
AAENSSSLGR PVRLANSKTR EFDQVRTTLI SGLLKTIVAN KRRELPIKLF EIGDVCLSTT
EVGARNLRYC CLAFADESSG LEEVHGVLDA LLQSLQFVGE YAIAEMEAAV AAAAAIALGN
IRGFGRNDIG LFKMSGDLFG WKNRKTGSVH QYKAADIVSA SWIMTGFDAY QLRILLGPHK
NDLMVRFDGF HEKNFADLSR HFDAHFKVKL QRGQQAYRGW HWGDVKMEGN NLQLTVDGCA
AFDIHAQEIA QVTTPSKNDL AIELIQDDQE DQLLEVRFYQ PFADDAEGPL QQLKQKLVKK
SGVAETKMDS VALLNDVPLL VPRGRYEIDI GRRALKFHGK SYDYTIQYSS INRMFLVPRP
NSPHVNFILS LENAMRQGQT SYPFVVMQFD SESVHSVDVN LEPAELQQRG LEKLIEGKTF
HVVTRLFRAL VGKSVIVPGD FKSVKQQFGI ACSYRAQSGH LYPLNRSFLF IVKPVIFIRY
DDVVSVEFSR TGATTNRFFA FTVSVRGGGE YEFTSIDRNE YKPLVDFLME KGIRIKGYID
LSKRRVSPED IVKCEEKFSK SKKVHQTVRH AAQKHGMKVD DLNRSVIWPL YRKYGHALDA
LKEAAMRPDE VFAGLEVDEE VRKSLIQDIQ LRLAPQALKL RARVDVWCFG KQGIDAVKAA
LQAGQVTINI KLIAPPQYVV VTSCYDKELG MRKIEQAMKA ISDKIKSFSG GDFKQQGEIV
VMLLEEANED DSSDDGERRI FEYLLCQVGV GRSVVVRFSL LWEYDSAYLG VLPQYTFRHD
YVVYDSSQVL PRFFLLFEMD PSLEELFAVP LCDNCQDNPA CIWCPADAAR LCASCDELIH
QNRLVSRHIR VPLNEMPGTC RRHPGEVYEL FCSICHVAVC RLCRGNHLHM PLNKPYQAVL
HMAKKPHFVL AARRTELEKR LEEVQKMLDG VRRNCREEEQ RCYAILEEAI TQLHSCTEDK
MGAVLSRQLE LQREIDTMDW SESFLQYLRT VLPPADFLHA WLRHCRLRDE LDALANVFPD
MRLQGRVDIL TDSALRRLVG LPNVGKSTTF NLLCKQAVPA ENYPFCTIDP HEARMNVPDD
RFKWLCTHFH PKSEVSATLA IFDIAGLVPG AHKGEGLGNA FLSHIQAVDG IYHVVRAFEE
ADIVHSEGEV NPVKDLETIS DELRMKDVER CEKLIEELDR LGKGQKDKQK KVHLDVLKVV
LEHLNNKRWV SQVDWKASEV EVLNEYQFLT AKQVVYLVNM SEKDFIRQKN KWLAKIHGWV
EANVSGPIIP YSAQFESKLA ELDDAAREKY IKDVGASRSQ LDKIVTTGYH ALHLIHFFTC
GEDEVKCWTI RQGTKAPQAA GVIHTDFERG FICAEVYKYE DLVAAGSENA VKAAGKYLQK
GKDYVVEDGD IIFFKFNVTN SGELEVVRKM MTPTREVLLE LHESFLKELQ RGLEMHKRHG
ITWVPEECSM KMLDSCVSNL PTGAEVGEAY AIDFGGSTCR AVRCSLLGKG KMEIIQDKIC
LRSAEHRCTK GFMDKKAGGK ELFDQFAMCI RGLMDRSGDL KKAEEVPVGF TFSFPCAQAA
LNSSFLIEWT KGFETGRENP DRVEGKDVAV LLADALQRHN VPAVCKAIVN DTVGTLVSCA
YQRVPGTPEC RVGLIIGTGF NACYVEPEAS NYGYTGTVVN MEAGNFHKDL PRNEIDVEVD
EKTHNRGKQQ FEKLVSGYYI GEIVRVAAVR VFGARAPEKA SVRHSIHGET ASTIRDDHSQ
DKAASIQAIK ECWGVTMDLD DIKCIWEICR LVFDRSAAFA ATLAVALCYR TGRVGIDGAL
YVKNQWYREA VEYYTKLVAG DAAKNIHYCI ADDGSGKGAA LIADYFLVRW LCIGIVRSFF
KEVAVLHPER IPLYGPAIFV GNHNNQFMDA SMLVANIPRQ VHFLVALKSM KRRVIGFLSR
LAGCIPVDRQ DDRAFKGPGL VTREDGPLSI LGNSTRFLSD VRPGYKLRLD GKDFLVLRVL
SDTHVVVRQP PEAPCDGEGE EYKILPKIEQ REVYEAVTHS LVDGDCIGIF PEGGSHDRTT
LLPLKPGVAI MAIGGAAAGD VMIVPVGLVY HNPHKTQSRA TIHIGEPIPI SREAVVEYQQ
DRRAATARIL TDVEHGMRSC IITAQDHETM TLIHLCCSLY PPERLRLSPE KLFNLNQLLS
KLFWRCADSP ELSNLRQDLA QYQGALQRAG IPDHDVWMLK QSTAGASLCF AEKLIALLFA

IGLGVPLLPL WGPLRVIAYF LAERHRAQAL AASSVKVKGM DVVASYKVIV LLVCVPLFNL
VYGAIFGLVR PAKPEDVPHI HKLLSNYLRN FKLHCEFTQE EVAHWLLPRE GVVHVYVRST
KGTVTDLISF YELPSSVIGN QKYKEIKAAY SFYNVATTVP LKQLIEDALC LAKQLDFDVF
NALDVMENKS FVEVSRFPER EGMDPSSVVG MQAVLVCALY KQQEFMRIGY YLNNAYSDTV
LRENPPDVPI YDKLVRCIVD EPRVTRFPIV WDDARIVQPI LHRLKQRTFF RIFKLSLLAD
TCLRDNADKC TEPQRCGLCQ CSDDEIPQTW RQKPDEVDLL LNPPCFTKYD GGAVWMSAGA
DGYMYFGAAK ELKGVRELLE REASEQQKPR KTRAMLFKNI TPDYYGWRDE EDGEILLAEK
VREEELMLRG TLVLPSPLAH GCLEALGRQG SVHFLDMNAH SLTRQFNTYI QRIDEMERII
RFLEEEVNRL QDAGAKPEGA QNFLDNDKSY QLDKVEEALN RLHAQFVRFK SNNADLIQQK
NAALEEKCVM TTAVQQLQGT PNADDPEAGL TSSVSTVAGM IATSAIGRFQ RMLFRTTRGN
AFCFFQSTAE KGVFVIYYQG AAHSLLREKI VKVCAAFDAK PYEWPHSAEE AATRLAGLQS
LLDDKERALA AYEKYFLSEI SLLLEVTRPG GSSLLEEWKM FCQKEKAVYA TLNQFQGKDM
TLRCDCWIPR DKEEEIRSIL KDVSTDEQAS AFLLIEKGQP TAMPPTYFKT TEFTEPSQVM
VDTYGVPRYQ EANPAVLTTI TFPFLFGVMY GDIGHGLCVM LMGLWLLFRA NALKQTAALH
GAVKYRYMVF LMGFFAFFGG FMYNDWFALG LDIFGSPYPF GFDPAWKGAT NELLFTNSFK
MKFSVIVGFA QMFAGVLLKG SNAIFFREPL DFVFEFIPQV MFICSLVGYM DFLILYKWAT
PADQNKPNLI NTIINMCMLA EVKSEDEMFS NQQTVERILL VFMVISIPLM LIPKPLILCS
RLKKDIFIHQ MIETIEFILG TISNTASYLR LWALSLAHQQ LALVFYTQTV VRAIELTDTT
FVALALFVIF AAYACITFAV ILCMDFLEVS LHALRLQWVE FQNKFFKGDG YKFAPLYFIK
LLPPDFARQL VTGLRQRPLV YLPTCERVCE EVLPPRQLAA SLVQINLMDT ATHTPIRHLL
SRQQEQFVVT TGIIISSRPP ASRLQTVTIQ CRYCNHKMII SLPEWREQLQ LPRFCLYKPD
PYFMVTNECS FVDVQSLKLQ ELPEDVPTGD MPRHLLLNCT RLLTDQAFPG DRLIIHGVLT
TNVATSKETD QPHSSYLHVL GLQKVSRTRL SFQTEQRDVF YKLAQSQNIL DKIAKSLAPA
LYGMEEVKRA CACLLFGGTQ KVVADGSRLR GDINVLLLGD PGVAKSQILK FVDKIAPISV
YTSGKGSSAA GMVRLGLLGE SEQKLDYVLG LTVAKFLERR LQTKVFKLGL AKSIHHARVL
IRQRHIRVGK HIVDIPSFMV RVDSEKHVDY AITSPFGGGR PGRVKRRSLT QAMYDRHITI
FSPDGNLYQV EYAMKAVRNC NLTCVAIKGD DSACLVVQKK VAAHQLTQDK HLDSSFVSSL
YTLSPTIGSC LVGVFPDCRS IAFRARQEAG EFAYKNGYDM PVYALAKRIA DINQVYTQFA
YMRLHACTGR TMTEDEVRQF FSTYGTVEEV FVMKDNQNTG KGCCFVKFAF KEEALHAVRT
LSGKHTFEGC TRPVEVRFAE SKAARQTQKV SAKDQPGNLT LKTRKAAQQT SDRSEFEKLD
RAIELSRQAT ERDKAGAFAE AFELYKAALD SWHLLCRCQT NALLKAKLYR KMGEYVARAE
VLKNFLEIVT EKPEVRWHHI AGLEAAKEAL QEAVILPSRF PSLFTGMHRG EKNVDLLQPP
GTGKTFLAKA VAAEAQATFL SVSSADLVSK WQGESEKLVR SLFAMARERR PSIIFIDEID
SMCGARSEGD SDSSRRIKTE FLVQMQGLQK DAPGVLVLGA TNVPWALDSA IRRRFERRVY
IPLPDLRARE FDTLARQTEG FSGADISVVV RDALFQPLRK GLFVHAGTRF EGVTHMIQNL
AFASTAHLSL LRTVKTIEVL GANAGCVVGR EHLVYSAECL RSHMPLLVPM LTGNVLFPRF
LPWELKACKE KLIMARKRLE HMPDQMVSEL LHTTAWHNNT LGHKLHCTER SLGHYNPDVI
RHYMLQHFSP ENMVFVGVNV NHDELCTWLM RAFVDYNAIP PSKRTVASPV YTGGDVRLET
PSPHAHMAIA FETPGWNGGD LVAYSVLQTI LGGGGAFSTG GPGKGMYTRL YLNVLNQNEW
VESAMAFNTQ YTDSGIFGLY MLADPTKSAN AVKVMAEQFG KMVSVTKEEL QRAKNSLKSS
IFMNLECRGI VMEDVGRQLL MSNRVISPQE FCTAIDAVTE ADIKRVVDAM YKKPPTVVAY
GDVSTVPHYE EVRAALDCGN KIQFVLGSPT VPSVNFGNCG VVRCKRCRTY INPFVHWEAH
GRRWSCNLCG YVNDTPQFYK REDRFERAEL SMGSVEFVAP ADYMIRPPQP PAYLFLIDVS
ATAVASGLVE SACAGIRAAL DSRTMVGIVT YDSAVHFYAL GSGKRRPQVL VMPEIDDVFL
PLSDDLFVNF AENRDAVLEA LDTIPSLWRS NGCIDNCMGS AVKAALLVMK HVGGKILLFA
SFPPTVGAEA YQQFAQQLTQ SQVSVDLFIA PPYCDLATIL PLAELTGGEV RYYSFSRLQH
SQQLENDIVH IITRTTGWEA VMRIRVSRGW KISARHGRFF LRGTDLFVLP TVNADSTFSI
LMELDEQAGA DNVVAVQAAL LYTNSDGERR IRVHTFCMPV SQNIQDIVSS MDPEVVTCLL
IQQAIDQSLR SKIADGRAFL QTSCSQLLST QLAALEAGRL VALFILGVLK SNAFRPTDKR
VFSWSRLLAL PVDRICAYVH PRMLPLHNPP ALRLTAEEMT QDGAYLLENG EEMLLWIGRS
ISLMMVNASA ALDGCDDQLL PATFRALERD LSFHPSLLGY ITLAQTMCLS LLCPVWGYLS
DRHSRKWLLA FGTFAWGLTT TLLGLVSDFW QVTALRALNG VFLGSVGPIS QSILADTAAS
KLGFSFGLIQ LCSCIGRLVG GVVTTSVALL DVGMLRGWRV CFFCVGGASM VLGLLIAFFL
EEIPSLSTPS IVIILAEGLL GTVPWSAFSF NTMYFQYCAM SDLEAAVLTG SLLMGAAAGG
VLGGLLGDRL FYWSRGHGRP LVGQVAMMCR IPLLVLAYVV VPKEEEYFYA YLIALFVGFT
SMSGVAVNRP ILSDVVRPDH KGTVFAVTVA LEGSSAAILG APLVGVLAES AFGYERTSLL
VKDMPDSLRL GNASALAKSL FLLTVIPWSI SFVLYGMLHF TYERDQIALA KIVHEEMLLF
GLRSLADFCS PSNQLYQENA LDALDRGVLS AIQTAVTTFS DDDDLMLCAS RVLWAMSVAI
KEEMDPAHIA RVHSEGSVIV AVVNSSPTDP QTIEDSMNFV DNLAGGMLSI FTKTALDMKT
AKRVTAALAI AAETAEGSTA LYNAGGTSVL LTYCLDAGVE MVEGAFDTVR YMAGYQCTDA
TTLPQCIALM DKYRGRKSAS AKGSSALAAM IGPEQLQKCL NTLKTAEAGS AEYDEALVTL
GSMSYISSFT DEIVRAGGVP LLIELINSGL PQMEGNPEKI ASMISGAAKM LARIASNPVN
VDAIVQAGGV ATLCTAVSYC TESMEALGAL CMALVPLASR EYQTFATVLP ILYQNVESPE
IAALAMELVA TGSQHEEIQE HMLQNQAAEI CSLCCQYHTA DASYQQHAIS ALNRLVPRLT
TLHGVSEYGG IQGVIASLNA VDAVLAAMLE HEGNDLLISA GVHCLARIAT EDDCARHLNV
LDTAARGNPD GVYRVLAAIS GLSRVPSLRQ IFEEKNASDT ILAGISSWIE CSRFEGQNRI
IKAALKTVKM CDVACLPQVK RVVEEEPDNN ILVADTAAFR DLAATMRITG AENLERCIES
VLRVMRKYPD SRRAQLNCLE TLNYLAQCDG GVAILSRTGG LNAVVQYLTR APMYLDAQIA
GFTVLATSAK IDSNVGETLR KCNCLQALKV AMRTHAKSKE LKRTIAPLVA LLMPTDALET
EIQELLNECA SACEKNNFPH LHENLAALNE LLISSEGAKI AARLGIVSEC AHAMEQVAST
RSGRNALIKA GNVATLISLY ESLKAPQSQY SEEAAIHCLE ALRILLKSDK RSAELAFERN
FVSTLCVGID SFPHSAPVLG ATCACLAAMA TTPERVQMLT AQPAFESLLQ KLVFVIQNDK
DNKLVAMRAL QELVEITNDA TMANKIAEAG AVALFRIIDE YGDDEQLTVQ AAEVLALLGA
FEDLRRDVRF PAQVLTAALT KQKNNETAVV HLLDVLNKLA TSEDRAVLRE LGVMEQVADA
MRVHSESEAV TRLGGELFAK MGADEQIKSL MLQIIEVESG AEDTAQTVDI LCGRLAVFLA
APLEDPRDAL QHTEKCLGSL VATLQTYPGS ERLEGNVALV CRRLCDRCFD DADDPYGAWA
VAASGMLAQF AGMVAGETVL ANKKFLGPAY RTFTACCANM AEGGRRYGAN FLSFVNETGS
PYHSVLAVQQ RLIACGFSQL DEWDLRLGGK YFVTRNHSCI AAFVIGEKFK ATSGGFTVVA

AHTDSPCLRL RPNSNVKKEG VQQVGVLAEK LIRVDRPILV LPNLAIHLQS AEEAFKINKE
THLQPVLCTE VYTQLPLLSL IAQELRVENE DIVEWDLCLM DATPGRFCGV HEEFVESPRL
DNLGSTWAAF SALMEFSVFL ATPTAVVAQL KFVGLGNWGS SYGQKTVADT LKKVAANEHI
SFIASPGSNF LGGVWQSEFE NVYSDAALKM PFFTVLGVDD WSRNYTSEAL RTELTYPKWT
LPNWWYHYLM HFPANTGGSG HKDMSVGMIF IDTWVLSSSF PFSNVTSRAW ADLEKTLELA
PKIIVVADRA VYSSYLQPLL KKANVDAYIS GYDFSLEVIS DDNISHVSCG AFCLFELTAE
GLVTRLVSGT TGAFNFVSQL PEVRYYPVPE MGKMPGRDVF VRVVGTIGLC IRYSFSGIAY
TVGAAPVDAT FCRESERGLL APDAVFFLVV LHDPAPCEMR DLGSNFCLTE EHVKKGAEAS
KNYLAELNQY VKPVGFIAAN VFGLAASVFV DLGERFVCLD SDGEEPREVI VAGITHERAA
TVHTHTDKLL PFQDGDFVVF REVQGMEIND QPMQIRVTGK HSFQIGDTTA FSPYVSGGIA
RQVKMPQTIR FKSYEDLGKF GQSEQLHVDQ VDEKLVANVA AYAQCQISPM AAFVGGVIAQ
EVVKFTGKFS PLRGFLYMDA FERYADQVAL FGSEFQHALG RTHAFVVGAG ALGCELLKSL
ALMGGKVTVT DMDRIEVSNL NRQFLFRREH VGKAKSVTAA ASVQTMNPDL QIVALEDRVG
VETETVFTDD FWRSQHIIVN ALDNIQARQY VDGRCVWFGL PLLESGTLGT KGNVQVVLPF
MTQCYSDSAD PPEESIPLCT LRHFPHAIEH TIEWARDCFQ GVFCDAVSEP NKFDRLEKIR
DLVSWQDKES FERCVEKAVF LFQDLFFNQI SQLLYSFPLD HRTSEGTLFW APPKRPPTPI
SFDANDPASL DFVVAASNLF AFNFGLPAVR DVSKIQAIVA IPQFTPKRLH INTDPVEFEK
DDDTNFHIDL VHAASTLRAM NYKIPCCDRN KTKIIAGRII PAIATTTAMI TGLVSLELLK
TVTYLEDFKN AFANLALPLW LFSEPMPPNR VVDKDFDPVA CGPIRAMPKG FSCWDKIQVD
IKDYDTQRDK RFSGSVRLPN VPRPRMRVCV MGDAVHCEQA KELGLEFMDV EAMKKLNKNK
KLVKKLARKY DAFLASQVLI PQIPRLLGPG LNKAGKFPTL ITHNDKIYVV YSLAMFCNEL
QLRFVEGDSL AFLPYVSSLV PFERVHFFRS LFPCVLCASL CLSKWNFSDN PAKRLALLKA
LLSTNRVLTE RTQELAYLKT MGTPSTAAIK TQQQFTNTLN EEQKELFKRR ESSRRRFDIN
WGQVLMWVCI LYVWIYYKKP KTYEVDLSLS LEELYTGTKK KLKITRTRYR NGQMLKEDNV
LSIDVKPGWK EGTKITFAGE GDQDSPTSPP GDVVFVVKTK PNSRFVRDGN HLIHKVAIPL
VKALTGFTVP IESLDGRSFK VKVDTVVTPK SRKIVPNEGM PVSKRPGEKG DLILEFDIHF
PKTLTDDQKT KLKELLGEYK VCISCASSKW FNTQLLKWSI SIELGDTEIN LDELAKKDQV
DSLQLKLHVD TGKTKLLDKI RHTNVQDNEA GGITQQIGAT YFPPEALVEQ CRKFSAELRL
PGLLIIDTPG HSSFTNLRAR GSSLCDLAIV VVDIMHGMEQ QTRESLELLK QKKCPFIIAL
NKIDRLYGWN SIPWQDIPKH LASQGPSTRD EFDRRAMEAI TQLQEEGFNC RLYWENEDVR
KNVSVVPTSA VTGEGVPDLL NLVVQVNQTL MQRTIAKERQ LQCTILEVKA IDGLGTTIDV
ILVHGTLYEG DKIVVCGMSG PIVTTIRALL TPQPLKELRV KGEYIQHAFI QAAMGVKISA
PNLEEAVAGT SVFVVEEDDD IEDLKEEVMS DMGSIFKSVD RTGSGVYVMA STLGSLEALL
VFLQESKIPV FGVNIGTVQK KDVKKASIMR EKGRPDLSVI LAFNVKVDPE AEKEAKTLGV
KIMTAEIIYH LFDEFTAYFQ SMKEEKKKAV AQEAIFPCIL GVLPQYIFNK KDPLILGVVV
EEGILKVGTP LCVPDKGLRI GRVVSLEVNK KPCDKATKGQ EVCVKIAGEP TVMIGRHFDA
KNKLVSRLTR DSIDCLKEHF RDEMSKDDWK TVIHLKKILG IQMANATTDH LRPQDLETLD
ISKLTPLSPD VISRQATINF GTIGHVAHGK STIVRAVSGV QTVRFKHEKE RNITIKLGYA
NAKIYKCSNP ECPEPECYKS YGSSKEDEPP CPRPGCGHKM KLLRHVSFVD CPGHDILMAT
MLNGAAVMDA ALLLIAGNEP CPQPQTSEHL AAVEIMRLQH IIILQNKVEL IKESQAQQQQ
EEIRAFVAGT AADKAPIIPV SAVLKFNIDI LCQYICTLVP VPVRDFTSPP QMIIIRSAKQ
CNVVEWRDTK LVFKRYASLF FIACVDSNEN ALLTLEVIHH FVEILDRYFG NVCELDLIFN
FHKAYYLLDE IICGGELQET SKKAVLRVMN AQDALMEESN FAKAMLEVAD AMAYATNSLK
EDALIGPEQI YDGVKLTENL LHKTLDRFGV EQYNPEGEKF NPALHEALFE LEHPEKKGEV
AQVIQRGYKI KERVLRHLDL DAVQALIAAL NNFKGGVLLV SHDSHLLSCV VEEIFYMDEQ
AHLQKYHGDF LKYRKELLKR AKMPREIVTL QVGQCGNQIG MEFWKQLCME HGIDQEGLLV
QHAYAEDRKD VFFYQADDEH YIPRALLFDL EPRVVNAIQT SEYKNLYNPE NFFISKEGGG
AGNNWGSGYA QAERVQEELL EMIDREADGS ESLEGFVLCH SIAGGTGSGM GSYLLEALCD
RYPKKLLQTF SVFPLLTETS DVVVQPYNSV LTLKRLALNA DCVVVLDNTA LNNIAVERLK
IHNPSFQQTN ALVSTVMAAS TSTLRYPSYM NTDMLSLISS LVPTPRCHFL MTGSSVQKTT
VMDVMRRLLQ TKNIMVSASL RRGMYISMLN IIRGEADPTQ VHKSLQRIKD RRLVNFIRWN
PASIQVALSK QSPFSPHKVS ALMMANHTSI ASLFERCIVQ YDRLFKRKAF LDNYKKEPMF
VGNFDEMECS KEVCVNLIDE YRRAEGDDYF GLARAFGIPV RSYTHEVVTL WYRAPDVLMG
SKTYSTPVDI WSVGCIFAEM VNGRPLFPGT GNEDQLMKIF KVLGTPQVSE HPQLAELPHW
NRDFPQFPPL PWDQVVPKLG TDLLSRMLRF DSNQRISARQ AMQHPYFMGK EKTHINLVVI
GHVDSGKSTT TGHLIYKLGG IDKRTIEKFE KESSEMGKGS FKYAWVLDKL KAERERGITI
DIALWQFETP KYHYTVIDAP GHRDFIKNMI TGTSQADVAL LVVPAEAFEG AFSKEGQTRE
HALLAFTLGV KQMIVGINMD SCNYSEDRFN EIQKEVAMYL KKVGYNPEKV PFVAISGFVG
DNMVEKSTNM SWYKGKTLVE ALDTMEAPKR PSDKPLRLPL QDVYKIGGIG TVPVGRVETG
ILKAGMVLTF APVGLTTECK SVEMHHEVEQ AVPGDNVGFN VKNVSVKELK RGYVASDSKN
DPAKGCATFL AQVIVLNHPG EIKNGYSPVI DCHTAHIACK FAEIKTKMDK RSGKTLEEAP
KCIKSGDAAM VNMEPSKPMV VEAFTDYPPL GRFAVRDMKQ TVAVGVIKSV EKKEPGAPII
LLKDGVDTSQ GRGQIISNIN ACQVIADIVR STLGPRGMDK LIHSENGVTI SNDGATVVSL
NVVHPAAALL VDIAKAQDDE VGDGTTSVVL LAGEFLESAK TFIEGGMAPQ ILINGYRTAC
QLAIEKIREL KVDLSSTPPH EKRVLLERCA QTTLNSKLVS GHKDFFAKMV VDAVSMLDDS
LDKEMIGVKK VTGGSYTDSF VVQGVAFAKT FSYAGFEQQP KRFENAKILL LNLELELKAE
KENAEVRLKT PDEYQAIIDA EWDIIYEKLD KISATGAQVV LSRLPIGDLA TQYFADRDIF
CAGRVDEGDM KRTARAIGAK IQTTVNSITP DVLGTCGVFE ERQIGGERFN LFMHCPQTQS
ATIVLRGGAP QFLDEADRSL NDAVMIVRRA LQTQTIVGGA GAIEMELSKY IRDVSQGISG
KQQLVIRAFA RALECIPRAL ATNAGYDATD ILNKLRHKHA QGKGEQWFGV DCMNGGVCDA
MKEFIWEPAL VKENALAAAT EATCILLSID ETIKQPTRAV DLPPCFRELL NTDKIRHVLC
TGNVGCASVV DSLRSISSSL HIVKGDADAG FDFPEYKVLQ FGQFKVGLIH GHQIVPYGDG
GSLLHWQRKL DCDILVYGHL HKDSVVELEG KFFVNPGSAT GAYQPWLTEK VPSFMLMGSS
VVLYVYEEKN GKAEVVMSEF KKYNVPIEDP AGKKRTSIYR SVFSPDAIVD NFADCLGTRV
KADGTLGPYQ WKTYREVEQL ALEVGSGIAF YSKNREEWAI CEQACNAYGI TIVPLYDTLG
PESTAFILAQ TRLRSVVASE ECARRLLDSI PAKLSTVSTV CYTSGTTSRP KGVLMSHGNF
VATIAGAVRG PLTVPHPEDT YLSYLPLAHV YERSLQNILF SLGGSVGFYG GNTLKLLDDM
QTLEPAVFSS VPRLFNRIHD RVLDSVRDKS TVAQTLFNQG MTTKRATGSP VHAMWDKLVF

NKTKVLLGSK LRYMLVGSAP LDVSVHEKIE ALFSTPVVEG YGMTETMAFI SIAGENTAGH
IGGCCPCVEF CLFDISEMPQ HRIDDPPAGE LCLRGPTIGY FRNREETEKD GWLHSGDVAV
IVPSNAVKII DRKKNIFKLA QGEYVSPEKI ENVYIQAPLV AQAFVTGYSS QSCLVAIIVP
DAEKAEKWAV QRKLDTSLET VCTLPEFHRA VTESMAAVAK EHQLKGFEVV KHFRLVSEPF
SIENELLTPT MKIKRYVAKE FFAKEIDALY GNACKVSINE ASTIKAAVDD GSAPNGVWIG
GQKYKVVRPE KGFEYNDCTF DITMCARSKG GAHLIKTPNG SIVIALYDEE KEQDKGNTTT
MLAITYLACR EAGVTRTVKE LVVYDRAISE KELGKAINRI KKLLPQRGGV NSESATQLLP
RYQLSMHVAD VAEHVAKRAT QVIWQQIRRF VQDPEIVTAE VLFLILTLSN VFVMYRLFLD
VVPYPVFVTW WQLAQGLFMA WCLGLKKLVV PSIVNAFMLV LANVVLYRTN CVATLPVTVS
FAVVLHHVTR FIGCGEEYMM RWQAVGLLML AFVLGCTDSM TVGAQVLPWA VLYSIFSAAF
RAAYLQKVMH EVDGRGNLLY NHQHIIGVAL LPILCLVCGE SRVITSMPMN FTLLHTGCLV
TVGALPFLKN IVSNRLIRRT GQAPWRFLEI VSIALVFLIG LGFGVPGWQG FICILLVLAG
RTFCAYDVIM NKQAVGADMN TAAFSLAKAT WAAGDFKSQL LERVRRPATF LNVAADNVAG
VTLPIFHICT DPSVDVLKNV GVAAGGQVIM AAREMFLKVF SELVKLASLQ FQVLFDWLNV
FSETFFTKLD ADEHLSKINM QRDVGAGLKK ENLFLSERDE RVIIRQGELL AGKICKKIVG
SASGSLIHLL WLEAGPERTK DFLSTLQKLT NYWLLHQGFT VGCKDIIANE ETNEKVRDIL
DQAKKEVDKL IRLAHRGRLE SQPGKSLRES FEARVNKELN SARERSGKVA AESLDESNNI
MAMVLAGSKG STINISQIMA CVGQQNVEGK RIPFGFNERS LPHFHKFDYS PQSRGFVENS
YLSGLEPHEL FFHAMGGREG IIDTACKTSE TGYIQRRLMK AMEDVMVYYD RTVRNSICEV
LQFLYGEDGM SGEFVEDQTV ELMTLDNEKL KRLYRHDVDQ ESYGDYELQQ PLEEEFEAIR
EDKNRLCRHI FKDGETKQHI PINILRLLEF AKAQFPSPIE IARKVNELLE KLVVVKQTTN
ADAISAEVQE NATIFMKAHL RTVLNSRRLL ERECIGPKAL QWLLGEVERH FHRALAHAGE
CVGAIAAQSI GEPATQMTLN TFHFAGVGSK NVTLGVPRLK ELINVAKQVK TPSLTVYLQD
EIAMDQERAK DVQTHLEHTT LDRVTVVSQV IYDPDPTDTI IPQDRQWVRD YYEFPDDDPS
NLGRWLLRIQ MASKVMIDKK LTMKEIGEKI YAEFPNEELD CIWTDDNSDD LVLRIRLKQQ
GDRFLQKLMV QCLAGITLRG ITNISKVYMR EEARTIYNNQ LGKFERTNNW VLDTDGCNLE
DVLPIPMVDD TRTTSNDMTE IFHVLGIEAV RRALLRELRA VISFDGSYVN YRHLAILCDT
MTQKGYLMSI TRHGINRIDK GPLMKCSFEE TVEILMEAAV FAEADHLRGV TENIMMGQLC
PLGTGYFDVL IDEEKLKDAS HNDGLGEFDG GVGTSPTSSP MPFSTVYSSF LLEQAVPGSI
RKAEHFVALM RRIVSYLKSY IKIYELKSEG PLSFLHMFEK ETHIDASLLK FFYDRLKSLF
NTLQITESYA PLTLVADFCT LVATYCEGFI LICDPYPEAV LYDPLLQLSC LDASLAMQPV
LKRFQSLILT SGTISPLELY PKLLNFVPVI TESFPMSLDR NCICPLIVAR GSDQIPLTSK
FEYRHDMNVL RNYSNLLIDL CKHVPDGLVC FFTSYSYMES VLSSWYHSGV LAQVLDYKLI
FIETKDVVAT TLALHNFRRA CECGRGAVFF SIARGKVAEG IDFDRHFGRC VVLFGVPFQY
TLSRVLKARL DFIREHYQIP DNEFLTFDAM RQAAQCVGRV IRSKNDYGLM IFADARYSRI
DKRSKLPPWI LKHLDNAHLA LNTETAISVA RTFLRHMSQP PPPPSASRNQ PPTLTTTLPN
GIRVATQRLP HQTATVGVWI DSGSRYDTKE TNGAAHFLEH MTFKGTKRRS RIQLEQEIEN
MGAHLNAYTS REQTVYYAKA FKKDIPQCVD ILSDILLNST IDEEAVQMEK HVILREMEEV
ERQTEEVIFD RLHTTAFRDS PLGYTILGPE ENIRNMTREH ILEYINRNYT SDRMVVAAAG
DVDHKELTAL VEKHFKPFFC GSELLHRNDD MGPTAHVAVG FEGVPWKSPD AVTFMLMQAI
VGSYRKHDEG IVPGTVRNVC NKMTVGCADM FSAFNTCYSD TGLFSSFLWS ARLAMVSQRI
LVSAAACLAP LRVSVEELAA ALKAIKVLTD RQRALMLLEL SLHLLMYGKV DALTEVMDAA
CEAANIRFSF TGAEGIRRKY QQRAIAQLVV LQDVHADADV LERPQLVDSV DALDGLEQAL
LLARGLRILE SNPARDELAL EQLNAVAVRC LKSADWLLHS AALWLRCKAE YHRSKTVERA
CLQLNALIDQ FTDRLPAPSQ RLRFIYHVDY PSSWQGRREL ARSMVRRLSL CQAYESFKDL
AMWEEAAECL YAADRRADAE ELLLERLKVR ESPPLWCTLG VECYEKAWTL SNKRCARAQR
SLGRLYMGEE NFSKAAEAYA RALELNPLHR ASWFILGCCE MRLERWEDAV QAFGRVVALN
LAAVHSQREA WTAARLCIGE AAKYRRESWR VWDNLLKISV RTRDIPGVNE ALRHYVDLNV
TDRIPIWIYS FLTHAVLGEK DQAELWKALS SLQSVKGDFL SAAETRLKHF RALSVLHVCR
DRLFVSQPAP AFEAEAVMAD GSFGKISLSQ FKGKYVVLFF YPFDFTFVCP SEILAFHRLH
GEFEKRGCQL LGVSVDSKFV HNAWRNVELK DGGIGKISFP LLADVSHKMA EDYGVLHPEG
MAFRGLFLID KEGVLQHCVN NLPLGRSADE ALRMLDALQH VEQYGEVCPA NWKKGDKAMK
PTAEGVKEYL GSKMAQSTAS AIKDDAGVNE GKMTEDFSQA ASQLVDKISE TMEILMEIST
LLDTGLDRAT LQILVELVEQ G

> ***Plasmodium reichenowi***
NFHGDFKTTK KKIHWLPYLP QQLITCTLYE YDHLINKEDW TNFINFNSKH ETLVYAEPSI
SSLKISDKFQ FERRGYFILD LIKIPDGKSK KAGINSSAEP SIVFPTIVYT GDEAFFHESN
LNIYRPIDHG HISDWDKAQK VWDYTLNCVD PSKSIKDILL TEPPLCSISH RKKMGEIFFE
YFDTLNLNLS VSGLMSLYAS GLTTGLVLDI GEGVTQCLPV FDGYIEKNSI IRSDFGGEEL
SMFLQKLICD IGYSMTTTKS LEYVKNIKET ICFCSLNPSE DQLRNDLAAT YTLPDGDVLR
DGYDSIEISH ERFYVAEALF NPQLCHRDNL SIIDIIWKSI LSCPMENRKI LSSSIVLSGG
SSLFPNLVER IETEVRNNAP ESARSMVKVV TYEQGANFAR ENNLFFAEAS AVSKLNVKHI
FENLLQMEKS KLAKVEKVLG RTGSRGGVIQ VRAQFMGDSE LAGRFLIRNV KGPVREGDIL
ALLETEREAR RLRGTQSLNL KKSHCYCHLS TGDLLREAAE KKTELGLKIK NIINEGKLVD
DQMVLSLVDE KLKTPQCKKG FILDGYPRNV KQAEDLNKLL QKNQTKLDGV FYFNVPDEVL
VNRISGRLIH KPSGRIYHKI FNPPKVPFRD DVTNEPLIQR EDDNEDVLKK RLTVFKSETS
PVISYYKNKN LLCNVCYFNL PDPESTLGPY DNELNYFTWG PGFEYEPFNE KSSGGKISID
NASYNARKLG LAPSSIDEKK IKELYGDNLT YEQYLEYLSI CVHDKDNVEE LIKMFAHFDN
CTGYLTKSQM KNILTTWGDA LTDQEATDAL NAFSSEDNIR IEKLFHLIDK NNDKLNTWSS
FLKNEIFLKQ VQAEMGQIDS DKDGFISLNE LNDAFAQNLK EVEKHSEGLL KRFQIVDKDK
DGKLSINEVG LLIDPMKDEE LKELEINEIL EHHDVNKDGK ISKKDDEMAL DDFNFDTNKD
GFIDKEEIIT YDLWNEKALK IAVTSLTDYG DVIRYPEDFK LLSNLNCFGS GFIFSIIMFH
LLPEFFFVFI GFCMQLGLEY VLPVDTNICC DSSSSEEEER VIKTYEGKRL HFYETTGNSL
NENMDNNDFN LLLKDYENLY KFMIKESADC IPNFAIIYLD KLSKYVEKTF QNNVEKKNLS
KNKAQTLNKL RAKIRKCSEF YQEKLNLYNE NPDDFWSYSE DDNYASDEED DKTKNAMSKW
GLKTSTKVEK KKTVKKEEKA NRVEDSQSAK NKGYAELLST KNLTEDVIRE RVKLVIEKRG
RKGLDKHEHI NILSKLCEIA KTISTQSYIE VLEHLINLEF DVVSSVYTYM SFNIWNKVFK

YVELILDILI QNENFYLVSI NIAEEITEET TNEKEKIIKS CKTLISFLAK LDDELLKALL
YIDVQTEEYR KRLGKTIHMI GLLKKGYNYV KAIHISSRIL EHMYYKPEML FKQIWTYLKK
PLGQERAEKR RLLSYHMHIS IELIECVNNI CAMLLEVPNL AKNTYESKDI ISRQFRRFLD
IYDKQIFNNP PENNKEIIIL ATKHLQKGNW KLCCEKIFSL SIWPKFPDKV QNILKEKIKQ
EAMRTYIFRY ISIYESFSID QLCVMFDLNQ NVVHSILSKM MINQEIPAFW NESSKFILIS
KVNPTTLQNL ALKLAENVNE VMEQNELALN MKNPKLGLQP KQCIVVGTRA LEFLNNELTN
IKTLSELYER VKKRSITDNN DNVLPLYLLE LFSIPIEQKE KNDFLTNRIS MYSKILNGRK
NVLDLLLNKF ENDCNDSIKK ELIDCFDVEK FKQEVNSKFM NILIQQLRKV EKLEKKKAKM
DFYLSVREQV KLYIRELVNI VSNLLTGNYP ILNLNGENFL KKYGGTLMDN LKDGLVEGVP
VRFMLAKEES NMFGVVQNAQ SDTKGKNILV NFYFRNNNTD EQVQVKSVEK DRLAVETLNG
DPSFLWYKMV RDDGWVGFDK AEIIKVFNNN SKIKDVLIRH TDLKILNQLA ITMYIYIYIY
IYISYVSYIY IYFVHDSIKI SILTVLGDIA TALNRSFSKY LNFFANILSE TSKITITSGS
PDSDDWVNYV FELRDAILLT YSNIIYALID GNEINKLKPY IPNILDFIEL ILIKEINHFN
AQNFQNSVSL LGDLVHAYGY ELIENSKLTD LIISVYGKID ILSSQGDECV SKIKWLKRIC
NISILQLEFK SVEELNNATN NFIKAIKNYN EFPELRLKIL QLLYNSFNVN FSFFIAILQF
CSQNNIFHNM LPYIKFIDDW IKEWNISTRE KRHIYLIISQ ELKKLKKYEE SYEHLKKHIY
YYDDILNHPN CINASIELIV DAININNNIY FHEIINLHAI QNLQYIQHQP LFDLLLIFYK
YTINEFLAFI DLQTCEKKIY LLSIISLFND HKVQNIQFIS KQLNISVVQI ENILVAAIGS
GVIDAKIDQI NQTVHMKTTI LRNFDEENWK QLNNQITKYI NNVATNFYKF IDSFASSTRA
IQNNPNYWCS SGNHSNDEEI TWTGYLNTKG FIKGVKVSWT YSPEFVKISV SSDGEKYRTI
IPYKKISSNE ASFDEIYFFK RLEEAMSIKI GLKNATHKYF GIREVKLIGG GNPYFLLLSG
ISSEEEMCLQ ELWKTNSNNQ IISAFSDPPK CLSVVNLDDL GDGKSNWIFE SNSQIRLQFC
ISQKNIYGNI PGIHDIDVSI YSNSTLDDDH NPDNTIDGNL NSYWASATFT DNDHLVYLIL
DLNKITDLSR IKIYWEYPPL HYNISVSTDT VVSENLANPS YVTVDSLRNM ETRYIKISMI
KTHPKHGELG DNFLYGIRSI EVQANNLETV INHCRDAANS DDARDKYFVE YITEFDKDLT
NKLINLEDDV TKNVSSISDN LSKLEELLPN IETCLEEKKT YDEELKESKE KANDLIKPKC
SPEPLRVYCD MDSSTSIYIW NGINSVNDIR QHCAEVGLQP LILRSKNQLN SLIISLKKIG
YSLNGKVNIP LAYDYSCFHD LLNGNIDIST LIYESPDSTK VRQTALDEKQ MVICKIEDIA
KKRNKKYVKA QVLSKDSKIE RHKMKKFYNS KSSYFGCGWK KEWTPFIHAP FFENQHNAIY
KNRNKKLYEE IDTLLHGRIH PDIKVVELKD HKHPIRLCTP YNEDCYSVVY TGKKINATDD
RVIFGEYTGY VNNKELSQEK HQYMFALTFI ILPDNYTYAV DSSYMFNEMS LVNHYKTCFN
NFDFRINSEW QLVYLDGWPH IILTSIPGVE INPGEELFAD FGFEWFEKVN DICLNEFIKN
NYFYRLDDIV EKYNLLKNHI TCNICMHNVN TDGNNFILCS GCNHVYHLKC VHKFNNENYE
WFCSSCLQFC FNIAKAIICR VTKMHFEANE DLFKTSSEFI QSVIRELALG KTKFQREFTN
GTFVGTVTEQ INNNNFFVVT YEDGDVEWIT PSFLFQEEHK LKGNEFYKQK KFDEALKEYE
EAILINPNDI MYHYNKAAVY IEMKNYDKAI ETCLYAIENR YNFKAEFIQV AKLYNRLAIS
YINMKKYDLA IEAYRKSLVE DNNRATRNAL KELERRKEKE EKEAYIDPDK AEEHKNKGNE
YFKNNDFPNA KKEYDEAIRR NPNDAKLYSN RAAALTKLIE YPSALEDVMK AIELDPTFVK
AYSRKGNLHF FMKDYYKALQ AYNKGLELDP NNKECLEGYQ RCAFKIDEMS KEKVDEEQFK
KSMADPEIQQ IISDPQFQII LQKLNENPNS ISEYIKDPKI FNGLQKLIAA GILFPSEENI
PSKYGENRHW NVDLIPKFIL VGGNLVKILK KTRVTNYLEW LVVEGSYVYQ HQKKGFLSSE
KFIHKVPATD MEALVSPLLS LMEKNRCKNF YQYVSEWDAN KRNTWDNLDP YKLTMLEIYK
HFNLCQLTID FLGHAVALYL NDDYLKQPAY LTLERIKLYM QSISAFGKSP FIYPLYGLGG
IPEGFSRMCA INGGTFMLNK NVVDFVFDDK VCGIKSSDGE AYCDKVICDP SYVHLKNKIK
KIGQVIRCIC ILSNPIPETN QTNSCQIIIP QNQLNRKSDI YINLVSFQHG VTLKGKYEVR
LDVKLNKFFW FKGVRNPPKR VWVKFERKRN EDGGSKGKMY SFVGQVMVDS YKGFVKEQLA
RDRPRIHDCP TENFNGFMAI TTPNKSWMAQ YNDLSKYAPG FYALQVIGEL RSGSGYIILS
LFEMLLSRNK KIDMLYCVDI NKKANKIFNV EIIRNNLFNN IRRCFDIVLF NPPYVITGPD
EMKKTLTASY AGGKYGREII MKFLLDIHNY LSNKGVIYLL LEKSNIPQEI EDSNLPILCE
TCLGENPYVR IIREENGKEC KICKNVFTHF RWKPGENSRY KQTVICMKCA KVKNVCQTCL
FDLQYNLPVQ VRDKFLENSI VLPENETNRN FFLEQMELSK LKRRDPYFKR NMARVCSFWR
KNSCNRGDEC PYLHKEIHKL IIFNLPPVDV QDIKSLCERY GPIVDVYAFV NFMFPSSCEK
AKIHLDNKIY RGKILTAKYC SYKKILEIQK KKSCQNENMW NILYTDINTS INNFCKENNC
SVESVLDKNI AVNVSLTETY IINKIKTWIK KEGIRSDDTI IIKNLSIYTN QNDIINLFKQ
YGTLKRVSFS PYNNICIIQY ENADNAKKAF ISNSYIRYKK LPLYLEWAPL NLFDEEGTHA
SIYIKNINFN TKEENLKDLF EKMEGFITCN ISSGYGFAEF KNKELAIEAI KRLTGTRLND
HVLEMSLSKL VVKNLAFQVN KEELRKLFSA FGNVKSVRIP KNVYNRSRGY AFIEFMSKKE
CCNAIESLQH THLYGRHLII DFAPQNARIL KPLIQEKIVE IMKPEIEEKI IEVPQVQYIE
KLVEVPHVIL QEKLIHIPKP VIHERIKKCS KTIFQEKIVE VPQIKVVDKI VEVPQYVYQE
KIIEVPKIMV QERIIPVPKK PQYRHIPKPV EIPMAHYRTF PVEKIVDRNV PVPVELQIVQ
EFLCPKIEAR YKEIPVPVHV QRIIEHPIPK DAMNNPHLLP LYYLGSTAVG ICVNDGVILA
SERRISSTLI EKDSVEKLLS IDDHIGCAMS GLMADARTLI DYARVECNHY KFIYNENINI
KSCVELISEL ALDFSNLSDS KRKKIMSRPF GVALLIGGVD KNGPCLWYTE PSGTNTRFSA
ASIGSAQEGA ELLLQENYKK DMTFEEAEIL ALTVLRQVME DKLSTSNVEI CAIKNQTFYK
YNTDDISRII DVLPRFKIID SDFINIKLLE LFQMEGFQKQ LDRLSDSLSK IQKALGEYLE
KQRNKFPRFE KLVQTELTDA CFLTLTQALK MKLGGNPFGP AGTGKTESVK ALGAQLGRYV
LVFNCDESFD FTAMGRIFVG LCQVGAWGCF DEFNRLEERI LSAVSEQIIE ILNKKIGLNK
NVGIFVTMNP GYAGRSNLPY GVGFKMSFAR ISSSCAIMSR TFNTIGIGLL SLELMNHCEA
KELATPLCMW KLPNKELINR NIANKSEHRH HQKLLMSYTP FNSPSLLAEQ INILGTYSGT
RLLYFPLWDH PKDSIDYCLS TYLYWLYLRR NTNIFLQNTL LRGQLVVIAA TNRQNSIDPA
LRRFGRFDRE IDIGVPDDNG RFEILRIHTK NMKLSPDVKL EELASNTHGF VGADLAQLCT
EAALTCIREK MDVIDLEDEI IDKEVLESMC VTQDHFNMAL GTCNPSSLRE TVVEVPNVKW
DDIGGLDEVK STLREMILYP IDHVMKSLFD NAFFKPTEIQ SKTLEKSIND KNDIVVISKT
GTGKTLTFCL PILNNILILV PTRELALQIL KHFNYINKYI NLFISTIIGG LNLNKQKRIL
RKKPEILICT PGRLRYLVCD EIDKMIEISF MKDISYIAKH IYIQTFLLSA TLMTKLLNSI
IIRKDKSFII NLLPELLTLY IVKLNERDIV CKLFYLIKSY KIIIFVNTIK SAKQLNAIFK
HLFLHNNLES SIPKKIYSIH SKQKLKERLE NINKFQNHKA ILFCTDVLSR GIDLDKCDLI
IQLNCPISDI TFVHRSGRTA RNFKKGNKSI CAEIKSAELV LNSGELRRIS DPKYGTIDHN

QICSVCFENC TGHIGHIEFV LPLFNPLFYK ELQELLNLIC YHCYNFCCSY NYEEYLILSK
TIKLHMKKSS KCCFCKFQRN ISAKVSQRRD TITIRLFSFQ VIDILKKIFN KDIINLLYPF
TKRDGYKKFF LYDMGVSGNR FRSQSKGIHV RNNFIKLCIN AKKKVDFDYL LELQLGVNTF
YDIRQILDKK EGILRKNIMG KRVNNCARTV ISPDTFIETN XIGMPIEFAK VLTIDEYITQ
NNFSYXXKVN LGIWVLVPGG DFRHLSFISD FMTGELCMDI LKANWSPAWT IQSLCRAILF
LFNEPNADSP LNCDAGNLIR GVKVKSAAEV GKRAVEYFRG DDFVNFLSTN GEMLKKKFPN
LFINRNLSDM KEIEEFADMF IQKGYIYKAQ YKPENGVYKR PKWPKRLIMT SKQNFDKTSF
YILVHERNKK LQYFMLISLI SIVLICCMFW HISVAFLTLI SIIIVGRLVA FIFFWFFGVD
YWIFPNLFDE ECNVVESFLP LQSWVYRNDT WFLFVARMCT AVLLAIAIQQ LGKTHSIADI
RNFATQSYID IIEWGNKKLA EENYDCLKKC GFTSFEELVR KCFLKCECMT LADSFLKDLE
DLEFEERKIS ELLYDIEKCM ETILRIDTEI LNIHKYVKDI YSTKFPELDS IVYSPVEYIS
VVNKIRNEVD LKNIDFSDIL PNTTVMAITV ASSTTGICLP DHLLKNCISF CNEGIQLNEY
RNIILLYLES KMFYLAPNVT MLLGSSLTAR LISAVGSLKN LSIISSQNLI VIASTKKGIL
CCSEIVQSVP DAYKKKAISL LASKCSLAAR IDYFTEERTV LDKKILNSSV ILQIAMRVKR
DRYLGRHYRY FIRNTRVRAY KQFLEPFKNV TLKNMAFAFG VSEEFIEVED PYEAINEAVR
NFENKDEILA SAKIVERLVE YPEVARNLDK IKAIDPLLKL LNNHILESVL QILSLALSNN
PELQDSVFKK NALKTLLIKL QESQKTIIDK KLITAISALI RHHDQGENKF IDYGGVGFLV
YGMQTNIFKY QEKSALLLKH LIHEDQNKITFD IFIKNDIMKG LVALVNTGIQ YGETTAELFL
ALIQNHRHKL AKSGLHTIKK LIEDRLSYLF EELGVEDWLI KISKSVHILY PTKIQQLCLP
LIIQGKNVIG SSETGSGKTI CYCWSILQEL NKNVYGIFSL ILLPTRELVF QIIEQFLLYG
SKIGVTILSC IGGFSLIEQP HIIVGTPGRF KRLRFLVLDE ADLLLQKCFE DKLQNILNNL
PRKTLFFSST ITNSLQLLID TFPYNNLILV NVNKKQKPPK NLDQRYGIIF TANSYKCELI
YTVLNMLDNV DAMHSSKDQK NRFATLAKFK NGLCKILVAT DIISRGIDIP KISFVINFDF
PNDIVQYIHR VGRTARANRK GLAISFIDKK DVNSFNQVKN IMKDKLKPYT LNKKEVLENM
FKIGRVIKKA EILLEEVVVF PVSVHYEHTR KLLQSKFSTG IQNVSKFGNG SYTGEVSAEI
AKDLNIEYVI IGHFERRKYF HETDEDVREK LQASLKNNLK AVVCFGESLE QREQNKTIEV
ITKQVKAFVD LIDNFDNVIL AYEPLWAIGT GKTATPEQAQ LVHKEIRKIV KDTCGANQIR
ILYGGSVNTE NCSSLIQQED IDGFLVGNAS LKESFVDIIK SAKDINKDVV HRYGPNTFKL
HRLPIPKLGQ ILGLVGTNGI GKSTALKILS SKLKPNLGKF NNPPEWRDIL SFFRGNELQI
FFTKLLEEKL SPIIKPQNVD LIPKQIKGNI LEIINKKDKF NQKDKYIAEL DLEHLLDRNV
EDLSGGELQR FALLMSIIQS TNVYMFDEPS SYLDIKQRIS MAKIIHKLVK HDNYIIVVEH
DLSILDYLSD YVCCLWGKAG AYGVVTCPFS VREGINIFLD GFVPTDNLRI REESLNFKLE
DKKRLHFYNY PTMVKTLNSF SLTIDKGHFS ESEIFVLLGQ NGSGKSTFIR LFAGLIKPHN
LEFLESLSVS YKPQQIQAKF TGTVRQLLMS KLKGLYNDPY FNNEIIKPLK IESILDNQVL
TLSGGELQKV AIIVTLAKNT NIYLIMSIIC TISGQTPEEP VISKTGYIFE KRLIEKHIIN
YGICPVSGEV LTLEDLYPIK NEKIVKPRPI TASSIPGLLS IFQTEWDSII SEMFSLRTHV
NDIRNELSHS LYQYDAATRV IAKLLKEKNS YKEEIENLKK QIFELKSSND LDIYEIGLNE
ELLEKMQNVA KDLLINRKKR KIDNVCSVEQ WKDFKNTNEF NIHSSTIPGV TCITLDFSGG
NDGNVYYVSL NDNKILSKLQ GHLKKVNSII SHPSNFICIT ASNDKTIRIW KGDKHKDHVT
SLALHPLENY FISSSKDSMW ILHDLETAKT IKTSKDNPSF KHLAIHPDGM MFGIAAQDSN
IHIYDIKSQE YKATLFSENG YYLASSSKDN TVKLWDLRKA QSFQTITLNE TPTFYFFYNG
NIKDGLFHGF GILIYSQHEK YEGDFVYGKR EGRGKFTYAD GATYEGEWVD DKIHGKGIAN
FVSGNIYEGE WENGKINGFG ILCYNNGDKY EGEWLDGKMH GRGTYTYEDG DVYIGEWKND
KRHGKGCVKY KGNENKIAET YEGDWVDGKM QGRGTYFFAD GGIYEGDWVD GKMEGKGVYK
YLNGNKYEGE WINDMKNGYG TLTYVNGELY EGYWKNDKVH GKGTLTYSKG DKYIGEWKYA
KKCGEGELIY ASGDKFKGQW KNDKANGYGI LYNNGNKYEG EWLDDHRHGM GTFTCKEDGT
IYSGLFQFNR KHGKGTLTFV NGHILQGIWN SGLLEKVINY ELTPSSPWND PDMEMETLYD
LGSKMIEALQ KENITAGDVI CIDKGTGKIT KIGKSFARSK DYDAMDPNTL FVQCPEGELQ
KRKEVVHTVT LHDIDAINSR TQGFLALFSG DTGEIKNEIR EHIDMKINEW QEDEKAEIVP
GVLFIDEVHM LDIECFSYLN RALESEQSPI VIMATNRGIT HIRGTDYKAP HGIPLDLLDR
TLIIPTYPYK HQDILKILEQ RAEEEDVDID EYAKELLCKI ASESSLRYAL HLITLANLVS
KKRKATEVTV QDVRRVYNLF IDVKRSTQYL IEYQNEFMFS ELYSNLSDFW TSDDDEGDEY
IRKKWVIEDD ISNFNKNDLL LNYDFELDDF QKRSIKHLNN FKHVFVAAHT SAGKTLIAEH
AIALSIKLQK KAIYTSPIKA LSNQKYYEFK NIFKDVGIIT GDVKMNVNAN CIIMTTEILR
NLLYLNDNII NNIHCVIFDE VHYVNDEDRG VIWEESIIML PHHVQILLLS ATVPNYLEFA
DWVGFTKQKE VISISTKKRP VPLLHYIYAY DSVYLVMDEK NFYSSAFKEI YEASMKTEIQ
KLQTLIKKLD QDNKLPVVLF CFSRIKCETY AKCMPHLNFL DTNKKSKVHL FIKESISKLP
KQDRELNQIQ SLSKLLEKGI GVHHSGLLPI LKEIVEILFS KGLIKVLFAT ETFAMGINMP
TKSVVFTSIY KHDHLRKRIL TSSEYTQMSG RAGRRSSDKY GYVYICCCDN IPDQVQLTEM
MMQKAVSLKS KFKVTYNMIL KLLINKQINI EKMLFSSFLE SCRALQIPLF KKDLKRKRKL
LQNIKEVQCI YIEQYVQINY RLKYIGLNLH KKLVCIENIS IITNELDRLI EKNNFEPFVL
TKMLKSLKCE FYSVLHYQLI CKKNDCLDDI ENIERNINAK SLNLYEDLEG KLNVLKHFGF
IDDQNNLTVK GKIASYITLT DEITLTQVIF ENVLNKLNPA EIAAVLSCFV APEKKVEESP
DLTVNLQEVK AALTNIHSSF EEFYKVIRLR ISSEDHWKLC NFKIMFIAYK WTLGVSFAEL
LEQCELEEGL IVRSILRLDD LCRKVKIAFL YLGNIDLAQK VEKTSHLLRR DIIFTTSLYL
DGLALLQFFH WCEQKRKTKE LFNETEMSLR HKVDYFRSTK KNFPSFSTIS ASGPNAAVIH
YECTDKTNAT IKPAIYLLDS GGQYLHGTTD VTRTTHFGEP TAEEKRIYTL VLKGHLRLRK
VIFASYTNSS ALDFIARENL FNNFMDYNHG TGHGVGLTLN VHEGGCSIGP VGGAPLKKNM
VLSNEPGYYM KDKFGVRIEN MQYVISKEIT DTTEYLSFDD LTMYPYEKKL LDFSLLTNQE
IKELNEYHTT IRNTLLPLVK QSPKKKLVVG ENPQFIKKRL EKFNEIKEKI TIELLDGSIK
SGESYVTTPF DIALSISKRL AEDSIVCKVT YLEKVDVELC DLWDLNVPLL GNCKLEFFWH
SSAHILGSSL EKLFGGFLTI GPALKEGFYY DIFLNNFSIN NEDYKRIEDE FNKLVKENVP
FEKVICTKEE ALELFEYNPF KLELIRSKIP DNKKTSVYKC GNFIDLCLGP HIKNTGKVKT
FKVLKNSSAY WLGQKENDSL QRVYGISFQK KSELVEYLKF LEEAKKRDHR NVGKILNLFF
FEKETSPGSC FWLPHGSKIY NKLIEFIRKE YRIRKYEEVI SPNVFSCDLW KTSGHYQNYK
DCMFLFNVEN KEWGMKPMNC PGHCLMFKQL NVSYRSLPVR LADFGVLHRN EISGSLSGLT
RVRRFQQDDS HIFCSMEHIK QEVLNTLNFL FYVYNLFGFG IEILQNRGYD SCGMSTILKT
TKYASNTTCD AIEKLKSNYL NSHKNDHIGI AHTRWATHGC KTDENAHPHV DYGERISIVH

NGIIENYREI KTFLLKNNIP FKSNTDTEVV ANLIGYFLDK KQSFQDAVLS AITQLEGTWS
FCIIHKNHPD EMILASNGSP LHIGFKDDEI FIASEHTALF MFTNEYISLK NGEILSISKD
KINDLKLLKK VENIPEIAIQ KTPHPYPHWT IKEIHEQSAT LSKSLNNFSS GDHLVKLGGL
DPYIQDLNKI ENLVLVGCGT SYYAALFAKY LMNYLNCFNT VQVMDPIDFN ISVIPKEKEG
VIFISQSGET RDVIKACKLA EDLNVRKLSV VNSVGSTIAN MTGRGVYLNA GREVGVASTK
CFTSEVSVLT LIALWFFQHK SSNKATSLIN SLHRLPLYAG VTIKCENTCK TLSEKFKNKS
MLIIGNGLSY PIAQEGALKI KELAYIHCEG FTGASLKHGP YALLGGEDNI PVIMLLFNDK
NAMINTGEQI KSRGAHIVCL TDDENLVKDD IILIPNNGIL TPLLAVIPLQ MLAYYTSVNK
GINPDKPRCL AKTVTVYIFL FLRYASEDVQ KILIGNKIDL KNDRNVSYEE GKELADSCNI
QFLETSAKIA HNVEQAFKTM AYEIKNKSQH ETINKGKTNI NLNARPIKIR TMNSRKPPEG
WHKVESFLEE MNKKMRSLEN EDTSKKRKNE ILWPIFQINH KTSRYIYELY YKRKEISYDY
VVIGGGPGGM ASAKEAAAHG ARVLLFDYVK PSSQGTKWGI GGTCVNVGCV PKKLMHYAGH
MGKLDSKAYG WKFDNKHDWK KLVTTVQSHI RSLNFSYMTG LRSKVKYING LAKLKGKNTV
SYYLKGKEET VTGKYILIAT GCRPHIPDDV EGAKELSITS DDIFSLKKDP GKTLVVGASY
VALECSGFLN SLGYDVTVAV RSIVLRGFDQ QCAVKVKLYM EEQGVMFLPK KLTKMDDKIL
VEFSDKTSEL YDTVLYAIGR KGDIDGLNLS CTNIPSIFAV GDVAENVPEL APVAIKAGEI
LARRLFKDSD EIMDYSYIPT SIYTPIEYGA CGYSEEKAYE LYGNVEVFLQ EFNNLEISAV
HRQKQKDEYD LDVSSTCLAK LVCLKDNRVI GFHYVGPNAG EVTQGMALAL RLKVKKKDFD
NCIGIHPTDA ESFMNLFVTI SSGLSYAAKG GCGGGKCGEQ LYTGPLKIEQ LLAKGFVKRD
LELLKEGGLQ TVECVAYAPM RTLCAIKGIS EQKAEKLKKA CKELCNSGFC NAIDYHDARQ
NLIKFTTGSK QLDALLKGGI ETGGITELFG EFRTGKSQLC HTLAITCQLP IEQSGGEGKC
LWIDTEGTFR PERIVAIAKR YGLHPTDCLN NIAYAKAYNC DHQTELLIDA SAMMADARFA
LLIVDSATAL YRSEYIGRGE LANRQSHLCR FLRGLQRIAD IYGVAVIITN QVVAKVDAMS
MFGHEKIPIG GNIIAHASQT RLYLRKGRGE SRICKIYDSP VLPEGEAVFA ITEGGIADYM
AIRVQFENSN EVGVFSRLTN SYALVALGGS ENFSSVFESE LSQHIPLVYT TIGGTRVIGR
VCVGNRKGLL VSSICTDQEL LHLRNCLPEN VKIKRIEERL SALGNCITTN DYVGLIHTDI
DKETEEIIQD VLDIEVFRTS IAGNLLVGTY SYFTNNGGLL HAMTSSQEIE ELSELLQIPL
ITGTINRGSD LIGSGLVAND WSAFCGMDTT AIELSIIEKV FKLNNITDNN VEDTFKYKSS
IIQTMIIVDT YWQTETGGIV IAPIPHLFSM KPGCASLPFL GVQLEILDSK NLQPLSGNNC
GLLCIKSPWP GMLRTVYGNH QRLIKTYFTM CPNYYFTGDG AFRDEDGYYW ISGRIDDTLN
VAGHRLGAAE IEHALVQHFY IAEAAVVSFH HNVKGEGILC FVVKKLKLYV RQVIGPIATP
DLICVVPDLP KTRSGKIIRR ILRCIANGIT DLGDISTVSN YEVIETIPFP TPCSVEDALS
YYCDLTTIPR LNILKKFKCF IKDIEEKECD MTFIEFVDMF MQSAVFELSP FLQLIPRNTP
KSYTISSSPK RWFKGSSSYY LTELNVNDIV KFNIKPSKFE VDFLYEMEID ALDKKHIDET
YFAFSRDQPW VEKYRPKRLD DIVHQNNAVM MLKEVVRTKN MPHLIFHGPP GTGKTSAINA
LAHELFGKEN ISERVLELNA SDDRGINVVR EKIKAYTRIS ISKNKIHSET KEVLPSWKLV
VLDEADMMTE DAQSALRRII EIYSNVTRFI LICNYIHKIS DPIFSRCSCY RFQSIPINIK
KEKLLYICQN ENIDIVDKII ETTEGDLRRA VSILQLCSCI NTKITLNSVL DVSGLPSDNI
VYKIIDACKM KDKLVEKTVQ DIIEDGFDVA YIFKSFNNYF VDSLKYQILL ELSRHDYRLH
CGATQYIQLL SFASSVHSLL IRELIFKSPN EKHFQMVVKQ VKELIKQVKQ KEVEDVNESK
TSQDRLVLNK SGRRIVLRDL MTRPNIFTGR KILGTLELHM NGLRYAAIDI LFDDIKHAFY
QPCDGQLIIL IHFHLKRYIM VGKKKTLDVQ FYCEAGTQID DLDRAKARNV YDPDEMHDEM
KEREQKNKLN LIFKNFVQQM QDISKIEFEI PYPELTFSGV PNKSNVEIFV TANTINHLVE
WPPFILSVED IEIASLERVH HGLRNFDMIF VFKDYTKPVK RIDVIPTEYI DTIKKWLTTI
DIVLKTILSD IDSFVNSKGF DGFLGEDDDE EEDEDEDDEY EVDESELVNI IDTLATIEFT
SKRKMSSVIC RIPKIMLFCK GAGSIILKKL AKRTDVDEIT IEHMETYADE GLRTLCIAQR
ELSEESFAEW YHLYKEASLS IKDREEKLES VAEYIENDLI LQGITGIEDK LQEGVSSTIE
DLRMAGIHVW MLTGDKIETA MNIGIAANLI DNYSVDGSVI DILLSEKMER KFFYLADKCS
SVICGRVSPY QKGAIVSSAN RLLNKITLAI GDGANDRNMI NTANIGVGIR GQEGVQAFNS
SDYGISQFRF LKNLLLVHGR LSYRRISKLV VYMFYKNMVL IFPLFIFGSI SLYSGQKIYF
EFLLHLFNVL FTAIPVVIHA VLDQDISLNT AMEKPNLYKL GIHHYYFNIR TFISWVMNSL
FHGSVVFLIP LYFLIPTSDG IPYDIWTVGC ATYLTVLIVN FKILFETYYL NILPISGIAL
SIFSFVLLVT AFSFMCVGSI HLLGTIVYLV QSLRFWLVVI LGLFALLRDY VFKVYKRNFN
PRNYSDHVKD HFNKPRNVGS FDKNEKNIGT SIVGKASCGD VIKLQLKIEN DVIKDARFMA
FGCGSAIASS SYATELIKGK TIDEALKIKN NDIASHLSLP PVKIIILGSG WGGFNFLLNI
DFKKYDVTLI SPRNYFTFTP LLPCLCSGTL SVNVCTESIR NFLRKKNYSG NYLQLECTDV
FYEDKYINCI DNNKVKLFYD YLIIAVGAKT NTFINGVDKY AYFVKDIDDA LKIRKKFLDI
LEKCTLPNIS NEEKKKMLHV AVVGGGPTGV EVTAEFADFI NKKINYKEIF NFISISIIEG
GNNLLPTFTQ NISDFTKKNF HNLNINVLTN YYVIDVDKHS FYIQSKKLSY GLLIWASGLA
QTTLIQKFLK TIPVQANNAI LKVDEKLRVI GINIYAIGDC KKIQPVQITA EQLINEALEL
EEVEHKVNYN LIDEEELNEY KINKRKEYED KIRKRRYMIS TYIKYALWEI KQKDIERCRS
IFERALNIDY TNKNLWLKYI EVELINKNIN SARNLLERVV LLLPLENIFW KKYAHLEEIL
NNYINARNIY ERWIKFKIDE SSFLCYIYFE ERCNEINKCR EIFERLIVSI PKLECFYKFI
KFEKKYKNID RAEKCIELLP SSYLDENFYI HFCNFEQEQN EYERCKKIYI EALKILPKSK
SELLYKHFLQ FQKKYDELNE SLLIKERIFY EDELKKHRND YDIWFNYIKL EERIRDLYER
AISVIKNAHV HDGLKIGIRE VIKSIESQEA KVCFLSDVCS EPAYKKLITT LCAEKNIPLF
MVDSKDLGHW AGLFKLDNEG NARKIIGASS VAVVDFGEDS AEKDFLFFLK AMVGEVIDKR
YSVVCELVGK GVFSNVLKCY DMVNKIPVAV KVIRDNDMMK KAAEKEISIL KKLNKRHIIR
LLSSIKYKNH LCLVFEWMWG NLRIALKIQS YFNDFISKTE ELEDVYISRR KFQSKYLTQI
KNLYINCNCE CIIHKISFKY ATKSSFPNLL NGTLLYMVVE KINLENNVVT SCINSSDVKS
WINYENYLGE LVDGFVFSVN IASAKSLIGD RCYILDLIDI KYEIAVGHNG GALGYSQHLS
EEIMLFSRDA ILDKIAVILG GRAAEELFIG KITTGAIDDL NKVTQLAYSY VSQYGMNQEI
GLVSFQPNSN SEYNLYRPHS ECLAHLIDNE VRSLIETQYK RVKSILMKNE KHVHNLANLL
YEKETISYHD IVKCVGLKHQ RYKGSDKIKI EEERNKKLFL NNNKFCKNCG SVAHKEKDCL
ERTRKKGYDG NRDRWVGYNV NNFDYIYKEY EKIVEEKKKR KAEDLIHNMK KYIKVPSKYE
EDIYLFDHSS VFGSYYDKHT KKWGYKCCSS TNKYDKCIIP LMDSYHANYY TKEVMIILIN
EFNSPDDEMK KIVLKCVKQC IQTEGVDKEY INEEIVNPFF EKFWVMRNSN DKKSFTLIVD
TTVEIAKKIG VISRIVDDLK DPSEQYRKMV MQTIQNVVNE LGVDDIDQKL EEQLIDGMLY

AFQEQTSEDY YILLNSFDII CNQLNIRMKP YLPQIAGILR WRLNTPLPKV RQQSADLISR
ITKLIKICDE KQMLGHLSLY LYEYLGEEYP EVLANIIRAL KSILLVLGVQ NMTPPIKDLL
PRITPILKNR HEKVQENVID LIGIIADKGG DLVSPKEWDR ICFDLIELLK SNKKLIRRAT
IQTFGYIART IGPFEVLTVL LNNLKVQERQ LRVCTTVAIA IVADTCLPYS VLAALMNEYK
TQDMNVQNGV LKALSFMFEY IGEIAKDYVY SVVTLLEHAL MDRDLVHRIA TWACKHLALG
CFGLNRQDAL IHLLNYVWPN IFETSPHLIQ AVIDSIDGFR VALGPAIIFQ YLVQGIFHPS
RKVREIYWKI YNNVYIGHQD SLVPIYPPFE LLNDSTFVRD ELRYMGRMYG KGKGISSSTL
PYKRKQPSWL KQKPSEIEDA IIKLAKKGQT PSQIGATLRD NYGIPQVKSV TGNKILRILR
AQGIATTIPE DLYFLIKKAV SMRKHLEKNK KDKDCKFRLI LTESKIHRIS RYYKRKKLLP
SNWKYQSSTA SALRHIEHAP GVQFSYVPPD FFNSDDDESD KNQYELKDDG GGRAPGTRAK
EHSTTHHLRR KNYDDDFEFN EDKILEALHI LELLYMNGVS LEEQNEHGQT ALFLSVKKNN
ISTLQWLLTK EVNINHRDFY GNTVLHIAVR HCDIDILRLL CDYGCLNMVY YSSIENKNTN
VFQLCIKNRY FLVYILLKKW VLQNKICSKL KICKTIYAFY FWFFAILNLI VYFNIAHSFS
IINKYHFKSL IWITIWLFQQ FLWCMLYFKS PGEYQLNNIE REIFQINLEY QKLSLYSQVS
QERINSLDED YRNAILEIIL LQLIIEPYIL RRSKKHVFID MPKKHSLIIK LPLNNTQLNL
YKDEIFSKMQ KTFKHLINAS IFILRRICNH PLLHKYYYTV EDIKKISKYF YYNTDQYVDL
DLKTVENEFM KISDFDIHLS IKHLISQDNK LNKYLITKEH ILNSSKINHM LSLIKDIRKK
KEKVLIFSQF TTFLDIIEEA LYVRLDGSTN TIERQKIIKR FSNIFVFLLS TKAGGVGLNL
IAANHVILMD QWNELLLQEE IVKKMCEYII DSKCDIVEKG VSDLAQHFLV KKNISVIRRV
RKTDLNRLER ISGATIVNRC EEIVESDIGT KCGLFEIKKI GDDYYSFFVE CKDPHACTIL
LRGSTKDVLN EIERNLHDGM NVAKNILMEG KLLYGGGCTE IRVGQHLIKE ASKFNDSRKS
ITEAVASALE IIPKILAQNS GVNVVKTMNE LRIKHGGQEF GIDGITGDII NVTTKNIWDL
LSVKKQIYKS AIEAASMILR IDDLAVILIY EELLKNPLCK ITNVYESETR KYKPYPLNTL
QMTKLVSIYF KISSKECMML AEKLYNKGYI SYPRTETNYF PDSMNLHKII NELRKNDNFG
WYANKLCEQN PRKGKMNDKA HPPIHPVKNM EWKLYEFICK HFLAVCSNDA IGYNTKVTAK
IQEEQFFCKG LKIKEKNYLE IYTYEKWNDK IIPSFQVDDE FYPTSLLIEE GITQPPKYLS
ESNLLTLMDK FSIGTDATMH EHIENIQKRN YVIKNSKSLF IPTNLGIALV QSYKKFKDIG
IDLTDPSLRA KMEKDMSLVA SGIVLLRMEK LDIPSNTKMI IISYELITKN DKYQKYKSII
CDESHYLKNS LSKRTKVITP IIKNAKRCVL LSGTPALNKP SELYEQISSI MPNFFNYHEF
CDRYCFKDKN LYTKKIEYVG CKHTEELHLF LTNTIMIRRL KKDVLKELPD KLRSKIPVEI
PQKELSEILL FKITGYAKVK AIKEYISYLI DADIKFLLFC HHKLVMDEVE SFLKEQKCSY
IRVDGLTPME KREIYIKNFQ NDDNVKIALL SITACGMGLN LTAANTVVFG ELFWVPGQII
QAEDRAHRIG TAHDVVNIHY LIAQNTIDEI VWKIINRKWN TLTTALNGIE DSLMFEDNDK
ICIAVSGGKD SSVLAHVLVN LKKKYNYKWE LFLLAIDEGI KGYRDDSLKV VFENLFSYTM
DDVVKFIGKK NNCTVCGVFR RQSFEKGALL FNATKLVTGH NADDLAETIL MNMCRGDIDK
LLNEKCSCIN NNLNNDGGKK NKEMQNNNND RYNDNNNDNN NNNDNKKISN CTFEIDISYE
HVEPITLENE YQQIPKLRIL SFDIECIKLD GKGFPEAKND PIIQISSILY FQGEPCTKFI
FTLLECASIP GSNVIWFNDE KTLLEAWNEF IIRIDPDFLT GYNIINFDLP YILNRGTALN
LKKLKFLGRI KNVASTVKDS SFSSKQFGTH ETKEINIFGR IQFDVYDLIK RDYKLKSYTL
NYVSFEFLKE QKEDVHYSIM NDLQNESPES RKRIATYCIK DGVLPLRLID KLLFIYNYVE
MARVTGTPFV YLLTRGQQIK VTSQLYRKCK ELNYVIPSTY MKVNTNEKYE GATVLEPIKG
YYIEPISTLD FASLYPSIMI AHNLCYSTLI KSNGKNNLKF VKKNVKKGIL PLIVEELIEA
RKKVKLLIKN EKNNITKMVL NGRQLALKIS ANSVYGYTGA SSGGQLPCLE VAVSITTLGR
SMIEKTKERV ESFYSKSNGY EHNSTVIYGD TDSVMVKFGT NNIEEAMTLG KDAAERISKE
FLSPIKLEFE KVYCPYLLLN KKRYAGLLYT NPNKHDKMDC KGIETVRRDF CILIQQMMET
VLNKLLMKKP FIQVAPMINV TNRHFRAMVR IITKRAQLWT EMIVDNTLLY NLNNLEEHLG
FDNNEHPIVC QLGGCDMNSM SEAAILVEQA GYDEININVG CPSTKVANKG AFGASLMKNP
EQVRNIVYEI KKKVQIPVTV KIRTGVDNYD SFDFLKTFIE TVSSVGCNHF IVHARKAWLK
GLDPKQNRKI PPLEYYKVYD LCKLYPHLKF TLNGGIQTIQ EAIALLLNGV MIGRACMENI
TVLSQTDKLV YNQDIPSTAY SRRTILEAYK KYLEKNSLFY NLFELLKPVL GILKGMPGHR
IFRFLKNFFF KNPVGHVGVV ALKNSSAKLI QPFTSNVDDI LSSILKERTA GLQGSPSLEE
GLQIAHDLLI DMPLYGTKEV LIMYGSIRTC DKKNILNVLE LLVKSNIYVN CISIAPEMHI
LKCGIHLISM HDLSHITNNL QGSPLFIEIM GSSSLPMSQQ MYFSTHNALR INENDVINTL
FYEINGSRHI SLLIFPFYDV QMLKRLLIKK LNLPGVKVND IIIFYKGIKL PNYRIISTYK
KINKLYWAIK DTNPNASIRV IDSKYPEFFE DILNEIKLSF KKNIAPKLTM DGTGGTYLLY
NAKKKICSVF KPLDEEAFAP FNPRGYEGKM YQEGFRAGVL SGEGASREIA AYLLDNCYNN
FSNVPCTIMV EACNPHFNNK SKLKYVDKET NLKWKCGSLQ EFIDSRESVG NYDYKQFSIR
DIHKIAILDI RVMNLDRNDG NILVSPTHLG IEQSRRDDIE ALGYVLMYFL RGSLPWQGLK
AISKKDKYDK IMEKKISTSV EVLCLLNVCD HIDSCIIIDR RVDMITPFCT PFTYEGLIDH
FFGIDNLQIE IPLYNDIKDL SQNEIGSFLH KKASDIQKTY EKDSLKDIEE INQYMRIFKE
KHYEHNSLST HVNIASFILN NIKKEYNFNK LKLEDEIIQL ETNTNKNILL SIVKQIQLLI
YTNEDIYEVY RLLCLFSILT NGKKDIIEQY GIKELTRLNK LYTCNILKFN NKQKFLWAQL
KNNFHLLSND ENDISYVCNG YAPLSVRLIE YKNNLQVFPE IFNLLNGPTL DIIQDTIEVR
SMCINCEKEG LNKIVKINIP YFKNVLIHSF ECEFCNYKNN VIQDLNQIKD KGVKISMKIN
NEELLDRQLI KSEYGVLKIP EIDFEIPKET QKGSINTIEG FLHTALNNLY IKMIENTVQN
LFTIQIIDPS GLSSLEYNRT KEELNELGFY SFASNCPCCN HMGMNNFCEI NIPGFKKCLI
LSFVCPNCNF KTSEIKSSGE INPKGKKITL TVNNKNDLNR FVIKSETASI NIPVVELTSD
YGTLGGTLTT VEGLIMKIIE SLEEKFKFLM YVLNRKGEEE DISFDQILKR IQRLSYGLHE
LVDPARVTQG VINGMYSGIK TCELDELAAQ TCAYMATTHP DFSILAARIT TDNLHKNTSD
DVAEVAEALY TYKDVRGRPA SLISKEVYDF ILLHKDRLNK EIDYTRDFNY DYFGFKTLER
SYLLRINNKI IERPQHLLMR VSIGIHIDDI DKALETYHLM SQKYFTHATP TLFNSGTPRP
QMSSCFLLSM KADSIEGIFE TLKQCALISK TAGGIGVAVQ DIRGQNSYIR GTNGISNGLV
PMLRVFNDTA RYVDQGGGKR KGSFAVYIEP WHSDIFEFLD LRKNHGKEEL RARDLFYAVW
VPDLFMKRVK ENKNWTLMCP NECPGLSETW GEEFEKLYTK YEEENMGKKT VLAQDLWFAI
LQSQIETGVP YMLYKDSCNA KSNQKNLGTI KCSNLCCEII EYTSPDEVAV CNLASIALCK
FVDLEKKEFN FKKLYEITKI ITRNLDKIIE RNYYPVKEAK TSNTRHRPIG IGVQGLADTF
MLLRYPYESD AAKELNKRIF ETMYYAALEM SVELASIHGP YESYQGSPAS QGILQFDMWN
AKVDNKYWDW DELKAKIRKH GLRNSLLLAP MPTASTSQIL GNNESFEPYT SNIYYRRVLS

GEFFVVNPHL LKDLFDRGLW DEDMKQQLIA HNGSIQYISE IPDDLKELYK TVWEIKQKNI
IDMAADRGIF IDQVPRLCFE EMRNEMNKYG VEINQSTLKN PSTEDIQGVY SICIKYILNK
DIQNIRIEEY TGDLKSILPN EGKNHLQAIG NLRFLRHCEK INKILNLDNI LSYIFKPVGS
HMTKLINAFI INETNELIFQ YSRYRQKKED LEDQIVPSPE KLQKYNEELK DHLYEHIAQF
DDDKKKNEDI KNKINIADIC IKKLVDLLTA LNDHIKLHIE KKNNLQTIEK QYKSLDWWTL
GIFIYEILVG CPPFYANEPL LIYQKILEGI IYFPKFLDNN CKHLMKKLLS HDLTKRYGNL
KKGAQNVKEH PWFSNIDWVN LLNKNVEVPY KPKYKNIFDS SNFQEDLTIA DKITNENDPF
YDWGISQFYI LSPRGDTIIN RDFRGDIIKG RGDAPPVFYL NGINFTYLKS NSLYFGVTSL
FNISPSYLIE LLHRLLKIFK DFWGQITEEL IRTNFILIYE IIDEIIDYGY LQNSNTEYIK
NLIHNETLPS NASQKPIQIN DKKNEIFIDI VEKIDGVIQI KSYLLGNPFI KIALNDDLYI
KNIHHDNSNN IIIDDCNFNH LVLSLYQPDG ECVLMNYRIN MIHIYKPDDI IACEIQRIST
DGTIILHTRS SIYGKLSNGV LIIVPQTLIH NQKKHIFVFP CVDHTTRKNI SIITNIIKLL
VKYHININYD TINKIYVQEW VAPKNKQIKA ASSNSSQIVI SLSGGELIYF EIDESHTLVE
IFRKNLNVEV LCLSIQQNRV RANFLAVGCL DNVVRLLSID KYFKQLSTHL LPNNSSPQDI
CIIFLNIGLN TGVLLRSIID PVGTLSNHYS KYLGAKSIKI CPVNPALLVL CEKTYLCYMH
QGKFLYSPLN YDMLEYASSF YSPQCSDGYV AISSNSLRIF RFYRLGEVFS QNILHLTFTP
RKIVPLPFPS MLAIIEADHN SYDENTQREI QKALKDIKLG TFKAGQGKWG SCIXIINPVN
LQILDKISLD MEEAALSVCA CELHALHCLI VGTTTNLSLK ASLRVYTYDI QYKLNLLHIT
PIEEQPYCFC SYNGKLIASI GNKLRIYALG KKKLLKKCEY KDIPEAIVSI KISNRIFACD
IRESVLIFFY DPNQNTLRLI SDDIIPRWIT CSEILDHHTI MAADKFDSVF ILRVLKKKPD
ITYNDIGGCK KQLEKFREVV EMPLLQPERF VTLRIDPPKG VLLYGPPGTG KTLTARAIAN
RTDACFICVI GSELVQKYVG EGARMVRELF QMAKSKKACI LFIDEVDAIG GSRGDESAHG
DHEVQRTMLE IVNQLDGFDN RGNIKVLMAT NRPDTLDSAL VRPGRIDRRI EFSLPDLEGR
THIFKIHANT MNMSRDVRFE LLARLCPNST GSDIRSVCTE AGMFAIRARR KTITEKDLLL
AINKVIHGCK QFSATGKYMV YNIKAKVEQN VPLIFSKINK LYSVTNDNIF NMPRYSRIPK
EKKKTKWQLF AENKLMKKNK SGLIYDKASK GWVRRFQKKQ IKLNEQKNNF VHEYKNKEDI
DPFEKEQEEK DIKKMKQKMR ELKNKVFERL NDKQFYTGVQ KNKFKKPCNY VVTPTKTIFL
FNNEKKYDKG VYFLVKSYIK NIKSLCYEIT KILQPSIGPT RKIYDQNNGG KYLCTSGDPP
APIRNLSHFD ARKYFKEGQK IITPPNGDGT RAFYESLLEE NPNSVIAIKY CIEHGILSGT
KHHEALYKYY VLKKNNAFKS NFGGVRIDFK KLLNVKFIQE KKLIGKFFEE IAQDTGKVVY
GIDDTLKALE IGAVELLILY EGLDIIRLTT KNPVTNQTKT MHISPCDEKQ ESLYKENNVE
LEVVEKISLT DWVIGNYKKY GASLDFVTNK SQEGAQFLQV IYEFIFLCIR IYDDISKLFA
LPYTIVSDTI CEEIISIIVL PFNYLGLSAL NARNMQTLLN SITEKHKKKL SLDIIDAIIE
CKKKYITYED VEKILKYISY IFNIEEKYNI CMLFYKYISN STYLVHLLPT IIFTLLHVVM
LEAKLNNASI LKKLFECIKD LVNDANVDAD ESGLKLQALD GNHVSLVSLH LLDSGFSHYR
CDRERVLGVN IASLNKVFKL CGANESVVIS SKDDEDNLNF VFENNKEDKV TNFSLKLMSI
ELDSLNIPEE GFDAEVELSS KELTNIFRNL SEFSDTVFIE IDSNCIKFTT KGIVGDAEVA
LKPRDSTDDI GVTIKSKKKI KQSFAIKYLN LFSKSNILAD VVVLGLSDSR PIEFKYEIKD
GFVKFFLAPK MDDDCPEKCD EEVGIVKGTF LYTYDSPICK SAIHSGVLNV AEDIVLSIAH
THNNFIGTKR NNIESHDFKG TSKSFTISIP TGFNGKENDY IDCTNLPNEK YIKSLSNFTF
IVYFGEGTWR TILSHSLCEG ISISINEDNE LIIEQNCNPH LLKSKFKPKF GQTYHISLVF
NKINKTLYLY INGKKVITEK NTYNFTLSGD LIIGRSNQTT KDYFIGNIHL VEIYKYTLSE
QEIKESFNSS LSQKTIDGRD CLTPCKSKNM INKDLQINTQ QINLKCQDNL LSEQFNGKGS
QFLVSCIENC TKSKYFIKGT NYYTPDSSIC KAAIHAGVIR IVEGLLEYKS SRGHFGILSK
SEKQSCSTDG QFILNLSVGE KRTINCPSNC GTNIYSPISV LCKAAIHSGA LSNQGGIVEI
IVGTGQQEFK GSTQNNVESF SSNNHSRSQK YIYDNADVGT QKKVFDLKLN MGPYTCTYTR
NGKYLLMTGV KGHVSLIDTH NLESLCEFQV DEMIRCNTTL HNYKLFAVSQ KKYMYIYDNT
GMEINCIKDI LYTYNMVFLP YHFLLTSIGE FGELVYQDIS VGNIVTRKKT KRGPCSIMKQ
NKKDAIIYLG HKNGHVTLWS PNIDKNYLIT ASVDSTYKLW DIRKMEYIKS YKSNIINNID
ISDTSLVAFS MNTHFRTYKD FFTNPYITHN IYGDQINSIS FQPFEDICSL GLKHSIKTLL
VPGAGIANID TFFNNPYETK KQNEVKLLLD KLPADTITND YKKIILVRKI KTKTQIKRII
TSPRDIIELT LKSYSNKNWP KWSDSESICT CLINNQVYIY KDNEQGSKGN PSIFKIFHID
NLNKHIYSKN FFNSDEIKLK WNKNGSSLLL QIHTDKEKQS YYGSSNLYFI DTVTLKDVNV
MTNKGLIYDT IWSNNQNKFY VCKGEIPAEI VLHDKNGNVS HSYGRHKFNT LKLNYNEKLL
LTGGFGNLSG DISIWNTSTK KEITKTKSSC AVICEFFNDG KHFLTATTHP RLRVDNNLKI
FKYNGLIVSR INFEELYNVI ILPPGCNFVN ENKNYKKKKK KREEDTIPLP NIKTPILKKI
IEYMEYHINN PADEIPKPLI TSNLQDVTVE DNSSKYKDLA QRMIQEIEMA VVLFKRKFIL
KKIPKLPPCY IINSGSLSIA RAKVKLPSTY SKLGNPLSFS KLPDFNYSYD MIEELQQFFM
KQRRCDYFSL LNNFINLLIS VSNLLAAEPD IDLRNELLRR FIYSLNSWMN MRRCIVACCE
NIFAMTGLCI PLQILHFNNE ECKIFFSKKR APYLLMFEVA DLDEDISHII HFLHQIYIYG
DFNRDTISSL LNNPLARSVL NELSNNPEML TNLVSNNPIL RNTFPLMQPV LENPNLLREF
MRPEILQAGL PPEERYASQL SSLQEMGFLD NAANIQALQE TGGDVNSAVT RLLERGSGDR
LTRAARVLEQ LTEQKPIFGK CRFTIRSFGV RRNEKISCFV TVRGKKALEI LEKGLKVKEY
ELRRKNFSDT GNFGFGIQEH IDLGIKYDPS TGIYGMDFYV HLSRSGYRVT RRTRERLGFF
ISLKMINQIK GLSSVDTNYQ WLPLLYMALA ADTSVSKITL SVIKPYSITL IRLLRDFFNV
VFLSQCVGIG YQNMSGLNCV AIACDLRLGA NTFTTVSTKF SKIFKMNNNV YVGLSGLATD
IQTLYEILRY RVNLYEVRQD AEMDVECFAN MLSSILYSNR FSPYFVNPIV VGPYLTAYDL
IGAKCETRDF VVNGVTSEQL FGMCESLYVK DILLVGAGGI GSEFLKNIIT IGCKNIDIID
IDTIDITNLN RQFLFKKKDV KEYKSHVAKE RALEHKKDLN INAYTFDVCT MKSSDIRKYD
YVINALDNIK ARKYVNKLCI MEKKVLIEAG STGYNGQVYP IYYNQTKCYS CEEKPKNKTY
AICTIRQTPS LPEHCVAWGR LIFETFFCKN DNETLIDIKN HIEEESKKRN MDKKEIIIFI
FNYLFNDTIK ELIYLKKDYT TIPIPNINKK EQQYLIFDKD DDECINFITS ISNIRMLNFS
INQKSKFDIQ SIAGNIIPAI SSTNAIVASL QFQRVYICKP QSSRNKSDKI LVLNFGSQYF
HLIVKRLNNI KIFSETKDYG VELKDIKNIK GVILSGGPYS VPHLKKEVFE YKIPIFGICY
GMQEIAIQMN GEVKKSKTSE YGCTVWMNHN DEVTKIPENF YLVSSSENCL IYNKEYNIYG
VQYHPEVYES LDGELMFYNF AICKCKKQFD PIRYHELELK NIEKYKHDHY VIAAMSGGID
STVAAAYTHK IFKERFFGIF IDNGLLRKNE AENVFLKSTF PDMNITKIDA SENFLSNLQG
VTDPEQKRKI IGKLFIQEFE KAVNNIDIDI NKTFLLQGTL YPDIIESKCS KNSDTIKTHH

NVGGLPKNLK FKLFEPFKYL FKDDVKTLSR ELNLPDEITN RHPFPGPGLA IRVIGEINKH
KLNILREVDD IFINDLKQYG LISQAFAVLL SSKSYDYVCV LRAVKTSSFM TANWYQIPYD
ILDKITTRIS EVKGVNRILY DVSSKPPATI EFEMPLPGFV SDKTLYLKKP LILYKDENDK
IEVDPILAQY LREHQREGVQ FVFECLMNIK DDKISGCILA DDMGLGKTLQ SITVLYTLLK
QGFHKKSAVR RCLILCPASL INNWNDEISK WIPNRCNVTC VNDNAKEKIV SKLEGFKYDI
QSTVLICSYE CFRINNEFLD KSSIDMIICD EAHRLKNDKT KTYTSIYNLT AKKRLLLSGT
PIQNDLGEFY ALISLCNPDL FDDINFFRKK FANPILIGRD KDATEKEQEI ASERLTELSN
INKFILRRTN NLLSKVLPVK YLINIFIKLN PIQEALYVLF LKDKKILKND NTNNKVNVLI
NIKKLEKICN HPLLLNVNDI KSCKFLLLHF LLKNIKQNTN DKVVIVSNYT QTLDYMEILC
KENMYKFVRL DGGINIKKRH KVINDFTHTA DIFIFLLSSK SGGCGINLIS SNRLILLDPD
WNPANDKQAL ARVWREGQKK ICYIYRLFCT GTIDEKVYQR QISKDGLSNM IVTTTNLSKD
QMSDENVKKL FNYKMNTVSE THDNIECNRC RKVENAGFMF YIKYEKGQFK KGSNLIKKCI
DQNIDVYDVL GVEETDDLET IKSCYKKLIL LFHPDKFLKI QDSYTILSDK ILRKQYDSSI
PWSAIKPVPD IGDENTEIKE VKYFYDFWYN FNNWRDFSYQ NEYDYEQAEC REERRWMERE
NKKIQKKASK TENLRIIKLV DLAYNNDPRI IAENKRIKLE KLKKKASVKL WKHHIKSFDD
LCEFIYDIYV ILWSAQEVSL LAKALKLYPG GTRNRWVLIS NSIKTKTVKE VIKKTKEMFE
NDTLWTHEEQ HLLEQALIKY PTSIPMPKKR RNGGRSKHNR GHVNPLRCSN CGRCVPKDKA
IKRFNIRNIV DTSAQRDIKE ASVYSTFQLP KLYIKQCYCV SCAIHSRFVR VRSREQRRVR
KETAKHVNPS QKKVSVSEIN FDSSYTVLDT SEGSIMLHVN HVLYHLDFNA LAVVKNVDMT
EEMQIDAIDC ANQALQKYNV EKDIAAHIKK EFDRKYDPTW HCVVGRNFGS YVTHETKNFI
YFYIGQVAIL LFKSGVCGWS KAIRKQGGRF CFVNLNDGSC HLNLQVVVNQ CIENYEKLLK
CGAGCCFRFT GELIISPVQN DIHNFEIYGE DPQKYPLSKK NHGKEFLREV AHLRPRSYFI
SSVIRIRNSL SIATHLFFQS RGFLYIHTPL ITTSDCEGGG EMFTVTTLDY KKDFFSKQAF
LTVSGQLSLE NLCSSMGDVY TFGPTFRAEN SHTSRHLAEF WMIEPEIAFA DLYDNMELAE
AYIKYCIEYV LNNNFHDIYY FEENVETNLI KRLKNILNED FAKITYTNAI EILKNYSDSF
EVKVEWGMDL QSEHERFIAE KIFKKPVIVY NYPKDLKAFY MKLNEDNKTV AAMDVLVPKI
GEVIGGSQRE DNLERLDKMI KEKKLNIDSY WWYRQLRQYG SHPHAGFGLG FERLIMLVTG
VDNIKDTIPF PRYPGHAEFI KDLLEMDLEN STNILKNLFL KDKKNYFLIC TLNNKIVDLK
NLSNILKTNN LRFVDENNLN NILNIQPGCL SPLAIKNDKE NVVKLYFDEE IKNMVIIHPL
HNYSSLYIKT EDVIKFCESF NHAPILGITS KKIENFSDWY TQVIVKSELI EYYDISGCYI
LRPAAYYIWE CVQAFFNKEI KKLNVENSYF PLFVTKNKLE KEKHHIEGFS PEVAWVTKYG
DSNLPEEIAI RPTSETIMYS VFPKWIRSYR DLPLKLNQWN TVVRWEFKQP TPFIRTMGKL
SKQQKKQMYI EKLSSLIQQY SKILIGHVDN GGSNQMARVR KSLRGKATIL MGKNTRIRTA
LKKNLQAVPQ IEKLLPLVKL NMGFVFCKDD LSEIRILENK SPAPARLGVI APIDVFIPPG
PTGMDPSHTS FFQSLGISTK IVKGQIEIQE HVHLIKQGEK VTASSATLLQ KFNMKPFSYG
VDVRTVYDDG VIYDAKVLDI TDEDILEKFS KGVSNVAALS RATGVITEAS YPHVFVEAFK
NIVALIIDSD YTFPLMENIK KMVENPEAFA AVAAPAEEEE EEDGFMGFGM FDQERDLARE
PCPDRIIEDM GGAFGMGCIG GYIWHFLKGA RNSPKGDVLS GALYSSRMRA PILGGNFAVW
GGTFSCFDCA FQYMRKKEDH WNAIGSGFCT GGVLAMRGGW RSASRNAIVG GVLLAIIEIV
SIVLTRKTTP TPRQQFQQQM ELEKMLVDNI GDVTITNDGA TILKQLEVQH PAAKILVNLS
ELQDQEVGDG TTSVVLLASE LLRRGNELIK MDIHPTTVIC GYKLAMKESV KYIKEKLSER
NLGKDVIINI AKTTLSSKFI SYESDYFAKM VANAIQSVKI INESGKTKYP VSSVNVIKVH
GMSSLDSKLI EGYAIMSGRA SQAMPTVIKN AKIAFLDFPL KQYRLHLGVQ VNINDPKELE
KIRQKEKDIT KERVNKILES GANVILTTQG IDDMPLKYFV EAGAIAVRRV NKDDLRRIAK
LTNGQIRLTL SSIDGTEKFE ASSLGYCDEV YEDKVGDWDL MFFKGCRTSK SNTILLRGAN
DFVLDEMQRS IHDALCSVSR ALESNYVVVG GGCVEVALSV YLEDFAKTLG SREQLAIAEF
AESLLVIPKI LALKASYDSI DLVCKLRGFY TKFPGNFEDL KGFKRYGFGL VKGKVANHFK
NGVFEALISK NKFFRFGSGA TITILGKDVF FKVVPERNPP VIKLKSHTSS IEFVHVNPAY
SEILASCSED MSIRIWLNGH KKKVNILSWN PMNYFILSST SFDSSVNIWD IENEKKAFEI
NMPKKLSSLQ WDIGGNLLSG TCQNKKIHII DPRKQEICNS FLIHDGGKST KCIWIDGFGG
EDILTTGFSK NNMRELKLWS LKNTELPLTT ITLDNAAAPL LPHYDESVGM IYLIGKGDGN
CRYYQYSQGS IRKVDEYKSC LPFRSFGFLP KRMCDVYKCE IGRVYKNENN TDIRPISFYV
PRKNFQEDLY PPIIGINQDI KRISIFNKLK ICGQFNKGFV ISKLFIIDQH AADEKSNFEK
YNKIFTMKSQ KLVYLLSLPV FNGKILEVVD FMSLLHHLWF NYNFPRPQKV WRILASKMKE
LVIIFLKKIS DTYTEDQTKW LEQMKSSQEQ QNDKKLNEWN ENVENKCFIY PASSAPCGAC
TSAGAIIHHR RYKEPRRKKE YLGTDILCQA KSGMGKTAVF VLSILQQLVR CLGLAHTREL
AYQIKNEFDR FSKYLKNVRC EVVYGGISMN KHIKLFKIPH IIIGTPGRIL ALIREKYLIT
DKIQHFVLDE CDKCLEKLDM RSDVQKIFIS TPLKKQVMFF SATMAKEMRD VCKKFLQNPV
EIFIDDEAKL KLHGLLQHYV KLQEKDKTRK LIEILDALEF NQVIIFVKSV TRAITLDKLL
TECNFPSIAI HGGLEQQERI ERYDKFKKFE NRILVSTDLF GRGIDIERVN IVINYDMPEN
SDSYLHRVGR AGRFGTKGLA VTFVSSQEDT LALNEVQTRF EVAISEMPNK IDCNEYINQR
LSVSAEAYGD WNKKINFIPK VYKKDEKEKA KIREALNESF LFNHLNKKEF EIIVNAFFDK
NVEKGVNIIN EGDYGDLLYV IDQGEVEIYK TKNNKKEVLT VLKSKDVFGE LALLYNSKRA
ATATALTKCH LWALDRESFT YIIKDMVAKK RKMYEDILSH VNILKDMDPY ERCKVADCLK
SKSYNDEIII KEGEEGDTFF ILIDGNAVAS KDNKVIKTYT KGDYFGELAL LKNKPRAATI
KAQNFCQVVY LDRKSFKRLL GPIEDILHRN VENYKKVLNE LMHLKIVCLS DEVREMYKNH
KTHHEGDSGL DLFIVKDEVL KPKSTTFVKL GIKAIANTSF LLFPRSSISK TPLRLANSIG
LIDAGYRGEI IAALDNTSDQ EYHIKKNDKL VQLVSFTGEP LSFELVEELD ETSRGEGGFG
STLGAAFGTA KSGVGVCSVG VMRPDLIMKS ILPVVMAGVL GIYGIIMSIL IYGDYIKAYL
LGFTLEDSLA LLRIEDLYIE SFQIQDVKIL KGDHLSRCIG RICGSNGSTK YAIENATKTR
IVIANDKIHI LGSFNNIKMA RHSICSLILG STQGKIFNKL NILAKRMKER KKKKKIYMHI
FSNIDGMKAA CSYTYDDIIC MPGYIDFALS DIDLTNNMTD NITLKTPVIS SPMDTVTGHK
MSIALALSGG LGVIHNNMSI EKQIEEVKKV KRFENGNKVL CDEKKSVLPI VNKNNEFPHA
SKSQNKQLIV GASISTDLER ANQLIKNMID VICIDSSQGN SIYQIDTIKK IKSAPIIAGN
VVTSQQAKNL IDAGADVLRI GMGSGSICTT QDVCAVGRAQ GTAVYHVSKY AHNVKTIADG
GIKNSGNIVK ALSLGADFVM LGNLLAATEE SCSEYYFENN VRLKIYRGMG SMEAMVSQGV
SASLVDKGSV LNLIPHLFKA VKHGFQSMGI RKIPELHSKL YSGMKENKQY QEALKELKKL
KKRIEENIDF FQKLKEKLIL AQESAWDKFG SKLKDMPFLN NFFENPILGK LFGETELAAA

LREMKMIDKN FKLSELMYLF EYVISKHIVE SYLIGDEETL RLHCGSSAFN SLNASITERK
KKKVFLDTNV LIYKNHELKG AQRMEESSPW FIFTFHTQQI NCLKNKNDEI IEGKIDDIRE
VVYTIALSKH PEPEGLLYPY IVREFAIIGN TPSWMVLYII GLGLGDEKDI TIKGKELIEK
SDVVYLETYT SILFVSKDVL EETYKKSIEE VDRDFAEENC DKILDEAKNK KVSFLVVGDP
LCATTHHDII LRAKKKNIDV EIIHNTSIIS AIGECGMQLY NFGPIVSIPY FEDNYKPTSY
YDKIYINLKN NFHTLCLLDI KVKERTVENI MRNKKIYEPP RFMTINDSIE QLLYCEHKKN
IITKNTLGIA IIQIGTDNQQ IISGDLLTLK DISYNKPLHS LIICAPTLHD IEKEYFDLYP
NSAYRKCVRV QLIKNGKKIT AFVPGDGCLN FIDENDEVLV SGFGRSGHSV GDLPGVKFKV
VKVARVSLLA LFKEKKEKPR SMGIKGLTKF IADAAPNAIK EIKIESLMGR IIAIDASMSL
YQFIIAIRDS EQYGNLTNES GETTSHISGL MSRSIRLMEN GLKPIYVFDG APPELKGSEL
EKRGEKRQKA EELLKKAKEE NLEEIKKQSG RTVRVTRKQN EEAKKLLTLM GIPVIEAPCE
AESQCAFLTK YNLAHATATE DADALVFGTK ILIRNLINLE QVLKGLNLTM DEFIDFCILC
GCDYCDTIKG IGSKTAYNLI KEYNCIEKII ENIDFQEARK SFINPNVKED IKIDWNEPQI
EELKHFLIKD YNFNELRVTN YINRLLKARK VTTQRRLDNF FVNNNKFYEV LNLKKNCTTD
EVKKAYRKLA IIHHPDKGGD PQKFKEISRA YEVLSDEEKR KLYDEYGEEG LENGEQPADA
TDLFDFILNA GKGKKKRGED IVSEVKVTLE QLYNGATKKL AISKDIICTN CEGHGGPKDA
KVDCKQCNGR GTKTYMRYHS SVLHQTEVTC NTCRGKGKIF NEKDKCANCK GMCVLKTRKI
IEVYIPKGAP NKHKIVFNGE ADEKPNVITG NLVVILNEKQ HPVFRREGID LFMNYKISLY
ESLTGFVAEV THLDERKILV NCTNSGFIRH GDIREVLDEG MPTYKDPFKK GNLYITFEVE
YPLIITNENK EVLKILKKQN EVEDLENSEL EVVSCSPVDK EYIKVRLKRL KNYLPYLCKI
LIDNTVYTKW DYLTMDESHF QNDNADEMSS RTWGNDWTVR KGAALCLDYL SNVYNDEILE
FVLPHIEEKL MSDKWNIRES AVLTLGAIAK GCMYSLSPFI PKVLEYLIKL LNDEKPLARS
ISCWCVTRFS SWICHPDKWF EPVLLNLLKR ILDSNKRVQE AACSSFANLE EDALELLNNY
LHEIVHTIQQ AFQIYQAKNY FILFDVVGTL IDSVNIVKEN NELAHEIVYA ILSKWVNIRI
SSPYIIALME CMSCITSAYG KEFLKYAKDV IRTCIKFLVI LYIDDLIECS FDLLSRIILQ
SNFALIGDIS RFCPQYLILN DIIPFLIAHI THPSTPVSNN ASWAIGEISI HINSEYMEIY
VDEITKQLIY ICNSKYHGCL LQNICITFGR LTSTYPKKLI FYFPQFLKTW LKIMAHGTQE
NEKINFFHQF LATMKTCIYM PHIYIYIFIG LLKSIIVNKL TSNGCTFIFW IADWFAHLNN
KMSGDLKKIK KVGSYFIEVW KSCGMNMENV QFLWASEEIN KKPNEYWSLV LDISRSFNIN
RMKRCLKIMG RSEGEENYCS QILYPCMQCA DIFFLNVDIC QLGIDQRKVN MLAREYCDIK
KIKKKPVILS HGMLPGLLEG QEKMSKSDEN SAIFMDDSES DVNRKIKKAF CPPNVIENNP
IYAYAKSIIF PSYNQFNLVR KEKNGGVKMY VKEEIVEEEK LSDIINKKKE NIKYMKGMKI
PENILAISNL KEVIDDADLL IFVLPHQMER ILSSIGKLSV VAGGLSLIPY TFIYDVDGGE
RCVMFNRFGG VSENTFGEGS HFYVPWFQTP YIYDIKMKPK VINTTTGTRD LQIVTISLRL
LFRPHTQHLP YLHSTLGPDY DERVLPSIGN EVLKAVVAKY NAESLLTQRD KISKEIRENI
TTRAKHFNIL LDDVAITHLS YGKEFAKAIE DKQVAQQESE RVKFIVAKTE QEKIAAVIKA
QGEAEAAKLI SSAVKEYGKS LIEIRKLEAA KEIAENLSKS KNVTYFPSNS NILYINILKE
ENGGYNFDNL KRNEILKEKG PQFRKTGTTI CGLVCQNAVI LGADTRATEG PIVADKNCSK
LHYISKNIWC AGAGVAGDLE HTTLWLQHNV ELHRLNTNTQ PRVSMCVSRL TQELFKYQGY
KVCAIVLGGV DVNGPQLYGI HPHGSSCLLP FTALGSGSLN AMAVLEAKYR DNMTIEEGKN
LVCEAICAGI FNDLGSGGNV DICVITKDSY QHIRPYKEPN MRLYHLPKGT TPILSEKIEY
IKKFISLNDL GEARGTVLSV KLDELIDNVE GQTVIDPKGY LTNLNANDAD IADINKARSL
LKSVISTNPK HGPGWIAAAR IEELAQRKDK AKEIIMKGCV VCSKNEDIWL EAVRLEKLSE
VKIILAKAIK HIPTSVKLWL EAYKKEKNVD DKRKVLRKAI ECIPNSVKLW KEAISLENEN
NAYILIMVKI NRNCITHNKN VNPIISEALK ECPSSGILWS KAIELENKNL QNSKSVSAFN
HCGNNAYVIL TVAKLFWVNF KIQKARKWFY RVINLNPHFG DGWATFLAFE IDQQNEINQK
DIINKCIKAE PNRGWVRGRI HDIRSKGSIA FIILRHKLYS LQCILDIKNN NDKNMMKWVS
NLSLECIVDI YGEIKKPEIP IDSTNIKYEI HINKIFCLSK TMKELPFLLK DANMKETNDE
ITIKVNQDNR LNNRCFDLRT YANYSIFSLQ SVICHIFRTF LLQHNFVEIH TPKLLGESSE
GGANAFKINY FNQNGYLAQS PQLYKQMCIN SGFDKVFEVG PVFRAENSNT YRHLCEYVSL
DIEMTYKFDY MENVHFYDCM FKHIFKELKN QYPSDDFVWL DKTPIFTYEE AIKILIEDIL
TYDLTTDLEK ELGKLIKLSH NTDYYIIINF PSSLRPFYTM YKEDDPKISN SYDFFMRGEE
ILSGSQRISD MKLLLENIKL FNLDPNKLNF YIDSFAYSSY PHSGLLFENL NKEYKFITTQ
DNFDGFRFEV DKNINKFLQS THTLFLGTRE VGYLYQFGAN FTNLDNTLLM ISRINIDGSV
NGRFCKKINN DCKLNFNTYA KSDTRNMYEM SLEVNKPLYT YNFKSIWQGG VDLTYIASNC
ASIGSFGLRY NHKNNVLTMQ IVRQPNFKSP EFMLNQTHLY KIQYAKKISD RLSLGTELEI
TPQTKESAMR LGWDYSFRHA KVQGSIDTSG KISVFTQDYS GFGVSGYIDY LNNDYKFGMM
HISPSQEQNN KMGIYLYVYI YIYIFFFFFF LATLGVEVHP LKFQTNFGKT QFNVWDTAGQ
EKFGGLRDGY YIKSDCAIIM FDVSSRITYK NVPNWYRDIT RVCETIPMVL VGNKVDVKDR
QVKSRQIQFH RKRNLQYYDL SARSNYNFEK PFLWLARRLS NQPNLVFVGE HAKAPEFQID
LNIVREAEKE LEQAAAVAID EEDGVITVKS ILSEPTIHQY DIKKLIKNKL QECVPFYNYN
MNRSFAEKIY GDCIYDNYGL SKEIEINLII LEEWNINCNK NRVLKNTGLI KEITINQFKY
STNKESLEVH FAVSPKYTDI LPKNKVLPPS GINYNKLIKE FGCSKITENH IKRIEKLTAH
HFIRRGIFFS HRDLDFLLNY YEQHKCFYIY TGRGPSSLSM HLGHLIPFYF CKYLQEAFNV
PLVIQLSDDE KYLFNQNYSL INITKLTKDE LDFTLYNSNS GIANALRRIM LSEIPTLAID
VVNVYENTSA FHDEFIAHRL GLIPIDSRNV NNYEFREKCK CKETCSKCTI QYIIEVKCNN
KIDVSHYDIL EHEPNVPMPI PIPIVTLSKN QTLHMKLIAT KGIGKMHAKW IPANVSYRID
HKVLIKHNLI DKLSNEHKLL LANNLNNDCY ILLKLKENMS VVMAESSIDL LSELGYKDII
KIVYDETMFH FHVESVGSIP PEQIVQMAID ILENKLKVLE PQIKSSFYSI DEVAKQLKLP
DPEKAKNEEE RCFLTLLKNS ILKNPKKWTN IAKKIIGVSE ETTTGVLRLK KMDKQNELLF
TAINVNDAVT KQKYDNVYGC RHSLPDGLMR ATDFLISGKI VVICGYGDVG KGCASSMKGL
GARVYVTEID PICAIQAVME GFNVVTLDEI VDKGDFFITC TGNVDVIKLE HLLKMKNNAV
VGNIGHFDDE IQVNELFNYK GIHIENVKPQ VDRITLPNGN KIIVLARGRL LNLGCATGHP
AFVMSFSFCN QTFAQLDLWQ NKKYENKVYL LPKHLDEKVA LYHLKKLNMG SYGMDVDDAY
LHQGQYAAPY DNQYDTPSPR GENHTPFVGY FSSHLLRTGF FLQCVSLVLM FVFYWAFGGT
GIFIFDLYAG PECVKVSSTF HLTISILMAL YLLGTLYIAM FQVFVADNSK WCRGFRAGSK
LLSAAVTLDL LSSILRLVQY LYAYFYMSMR WWARYQQTKS DWTLLHFGSI VHSFALFIYG
AAFFYMEAYH DEGTYEELAW SNLTLFKLAG LAGANKPKKR TFRTFQYRGV DLDKLLDLSQ

DELIKLFKAR QRRKFQRGIS KKAKSLLKKI RKSKKNCEPG EKPNPVPTHL RNMTIIPEMV
GSIVAVHNGK QYTNVEIKPE MIGYYLGEFS ITYKHTRHGK PGIGATHSSR FIPLKLMVNV
FLGEAPEQFE LKNLGNEENT PTAPHFLENQ YAGEAPFDEW GFLQIIKDHH ETMYELKQKI
RPRDQVVGWF CSGSELSELS CAVHGWFKEH NSISKFYPHT PLNEPIHLLV DASLESGFLN
IKAYVQLPIS LVKDYFVHFH EIQTELLPCN VERADVSLKK LLIMLKQCKS YVQDVIDQKK
KGNISVGRYL HKVLSNDTFL TLEKFDSINE SVLQDNLMIS YLSNLANLQF LIAEKLNWLN
HLPIKEDDAE GRRVHKNLID LVSQNHPLLF GKDNSNTSKI IEIFLTIYET DFSDTDCNKK
ISTLINSLDK SYLNNLALSH KQAKKLNNIN GMNLMDFYSK NLNLKPYLKI IKDFDKYPII
VDSSEQILSL PPIINCDHTK ISLNTKNVFI ECTAIDRNKA QIALNILCSM LSEYCVPKYS
IQSFVVIYPI FENKSLTCNI DYVRKLSGIS HITVHEVNNL LKRMMLDNNT FKVTIPFYRS
DIMHCCDIIE DIAIAYGYGN IKYEPPQICK KHSLNNCSEL FRNVLVECGY TEVMTNALLS
RDENYNCMLR PIQIKNSKTS EYEIIRTSLI VNLLKFVSAN KHRELPLRFF EIGDVSYNKT
DTNAVNKKYL SIIFSDKTAG LEELHGVLEA ILKEYQLFSD YKIEEKKKEN ISIRVLSVGN
IRGFGGSDFG SFRMSNEFLG WKNKKTNNVY QYKCSDIDEG CWIKTSYNNN RLHLKLGESK
ENIIIYFDGF PDRNVNEITQ HFQKYFNIRL NNRKIATKGW NWGEFKLENS NLCFDIDNKY
AFNLPTNNIN QLNVQIKTDI AMEFKNEENE DFLAEIRFYY PHENDENQNF QNLKNDLLEK
VNIGDTKSES IASLSNIPLL VPRGRYDIEM YSSTFKLHGK SYDFNIQYTN INKMILVPKS
NSNQYVLIFS LSNKMKQGQT EYPFILIQLN NDDDMELDIS ASDEVMRKYK LEKTISGKAH
DVVTKLFTAL VNKNVIVPGD YRTSKNQHGI TCSYRAASGQ LYPLNKYFLF IVKPVILISF
DDIVTLSFQR TGNNQHRFFS LIIKHKRGMS YEYTNIDKSE YNPLLTFLKS KNINIKDISI
YQKEEYPQKI IIKCEEKFSK SKKVHQTVRH VAKEHGITVE ELNEKAIWPL YERYGHALDA
LKEATMNPEN VFKGLDIFEE IKNSLLKDIK LRLTPQALKL RGRIDVWCFG YEGIDAVKEA
LKKGKVSINI KLIAPPQYVI VTSCHDKDLG MAKIQEAMKV ISDKIKEYKG GDFKQQGEIL
VILLDKHDGI SSDDDGERGI FEFLLCDVGV GLSLSVRDVL PIEYDSIFIG VLPYYTFHHE
YIIYDNSQIL PRYLIQFECD PNDDEHFSLP LCDYCSDAPA ILYCESDEVK LCEKCDTLIH
SNKIVKKHIR KALNEAQGKC KRHMTNDVNM FCTICHIPIC NLCISSHVHI SLNMAYKAIL
HHSNNPSNFI KQKKKNLNDL LAKIDTLHEQ VRINMNDTEK SVYTILEDLV KQLHIITDQK
MCSVLSEEYE LKRQFNEIIW NESFLYYLQT ILPPADFMNA WLKHCQFREQ IEKNSLIFPD
MRIKGNINIV TESSMHHFFF LSLLGKSTTF NVLTKLNIPA ENYPFCTIDP HEAKVTVEDE
RFEWLVKHFN PKSNVHAYLS IFDIAGLVKN AHLGEGLGNN FLSNISAVDG IYHVVRAFEN
EDIIHTEGNI NPVRDLEIIN SELIYKDISH CEKNLEEVTK VNRNKKDKVK QNEHDVLTSV
LNYLKEHKWI KDGTWKSNEI EVLNEYNFLT AKPVVYLVNM SEADFIRQKN KYLAKIYNWV
QEKNKGTIIP YSAEVEQKIL SMDEEEKKQY FQTNNIKQSM LNKIIKTGYY EINLIHFFTC
GHDEVKCWTI RKGTKAPQAA GVIHTDFEKG FICAEVVYKYT DLVEYKSEGE VKANGKYLQK
GKDYVVEDGD IIFFKFNVSS GGRINKFVNQ LRISYSTLEE FVDNFVYELK KGLEAHRKHP
NLWIPHECSF KMLDSCIANI PTGQEKGTYY AIDFGGTNFR AVRASLDGKG KIKRDQETYS
LKFGSYSHEK GLLDKHATAS QLFDHFAERI KYIMGEFNDL DNKEVKSVGF TFSFPCTSPS
INCSILIDWT KGFETGRATN DPVEGRDVCK LMNDAFVRAA IPAKVCCVLN DAVGTLMSCA
YQKGRGTPPC YIGIILGTGS NGCYYEPEWK KYKYAGKIIN IEFGNFDKDL PTSPIDLVMD
WYSANRSRQL FEKMISGAYL GEIVRRFMVN VLQSACSKKM WISDSFNSES GSVVLNDTSK
NFEDSRKVAK AAWDMDFTDE QIYALRKICE AVYNRSAALA AGTIAAIAKR IKICGVDGSL
FVKNAWYCKR LQEHLKVILA DKAENLIIIP ADDGSGKGAA ITAAYFLIRW LCKVIVKSVF
RDVNVINPEN VPLYGSVIFV GNHNNQFIDA CVLIANIPRQ VKFIVAEKSM RRAVIGKLAS
VIGCISVKRP QDLKFKGIGH ICWNEGDVKI TGINTRFRLD VQMGDKLLIQ NKMFPVVKIE
SETELLIQEV INIECEDNGV PFKILPKINQ TEVYNLVTNS LKNGDTIGIF PEGGSHDRTN
LLPLKPGVAI MTLCALADGD VSIIPVGLSY SKLYQLQGCA TLFYGNAIII SQDLCKEYNN
NNREAISKLL SKIEEGMRSC MLTSKDHETS RCIELCVSLY TPERMTISKN KIYNNLQLFC
KMFWKFGNSK VIENLSYELK CYEKLLQANK IKDDEVWMLK QSTSAATLKF IEHICTFIFC
VIFGMTFSLL WLPLVLISIY LAERHRKDAL RNSTIKIQGG DVVSSYKVLV LIVLLPTFNI
VYGLLFSIYR LMKKKDIDGL QKLLNEHLKQ YNLHAIFSKE DVAHWFTPID QVIYTYVNEE
NGEIKDLISF YSLPSKVLGN NKYNILHAAF SFYNISTTTT FKNLIQDAIC LAKRNNFDVF
NALEVMDNYS VFQDLKFGEG DGMDIDSVLG LQAILISANY KEKEFIRIAY YMNSFYKDME
LRENPPVVPQ YDKICRHIVE NPRIVKFSIG WDDAYRVYPI LKELKQKDYF RVFKVNLHLS
CKFLKGNEKC KEIKKCSVCE CEQDEIPYNF RTNEIEVDLV YNSPSFTAYE GRNIWELRGS
SNYKYFGAAK NLKGVRELLF KENEDKKQKK KDARNFEKVI NIHYFGYCDE TNEHLLQQEV
KIQKKLMKHG TLVLPSDRAR EYLDCLGKEV DIQFIDMNEK TMKRQYKKYI QRIDDMERIL
RFLEENINKL PNVKIKKSKI DNFLEHDNIY ELDQVEESLN RLHVQFVRFC NNNKDLIDEK
NNAIEEKHVI LTALNQLSPS LSTHIMKDGI NMMFTNISGV IKTKDQESFS RTIFRAFRGN
TYTYFQNIDE KSVFVVYCQG SAQSNIYDKI MKICKAYDVK TYDWPRTYEH AKKRLKELRE
IINDKEKALK AYEEYFINEI FVLINVVEPN KNSLIEEWKL FCKKERHIYN NLNYFEGSDI
TLRCDCWYSA NDEEKIRHIL INKSSNDLVS ALLLSDKLRP NVSPPTYIKT NEFTKSYQSM
VDTYGVPRYG EINPAISTII TFPFLFGIMY GDVGHGLCIF LFALFLIIMN NKVKNNEMVT
MLFDGRYMLL LMGFFAVYAG FLYNDFFSMP LNLFSSPYIF GFDCKWLGAE NELTYINSFK
MKFSIIIGFI HMTFGVLMKG FNALHFKRKM DFFFEFLPQL IMMLSMIGYL VFLIIYKWVT
PGGFQKQGII NTIINMYLMK EINSTNQFYP YQNIIQILLL SLFVLCIPFM FICKPAIRTY
HIMKEIWIEQ LIETIEFILG LISNTASYLR LWALSLAHQQ LSFVFFEQTI LNSLKRNSFM
SVLINLILFS QLFSILTIAV ILCMDTLECF LHSLRLQWVE FQNKFYKGDG IPFKPFNIKK
LLPENYSAIL ARALSERPLT YLPTIERVCY EVLNDEDEHL NYIQINLLNT IRPTPIRGLL
AATQERFVVV PGIIVQASKP QHKMRKITLQ CRYCDHKMSI DVPLWRDKPQ LPPYCRYVLE
PYVILPNECT FVDIQSLKMQ ELPEAVPTGD MPRHLQLNVT RYLCEKMIPG DRVYVHGVLT
SYNPNPTRAD GTNFSYLHVL GFQKYDGNDL NFDVEERNEL TLLAAEHNIH EKIFKSIAPE
LYGMDEVKKA CACLLFGGTR KRIGEETKIR GDINMLMLGD PSVAKSQILK FVNRCAPVSV
YTSGKGSSAA GMVRQGLLGE NEEKLDYVLG LTLPKLLERR LQTKVFKLGL AKSVHHARVL
IRQRHIRVGK QMVDIPSFLV RVDSEKHIDF ATTSPFGGAR PGRVKRKSLV QIIIVIIISY
YNYYPFLKFL EYAIKAVKNT NITSVGVKGE NCAVIISQKK MATQYISQDK LLDYNNITNI
YNITDEIGCS MVGMPGDCLS MVYKARSEAS EFLYSNGYNV NAETLCRNIC DKIQVYTQHA
YMRLHACSNK NITEDNIKEM FSPYGTVEEV FIMKDNTGLG KGCSFVKFSY KEQALYAIKS
LNGKKTLEGC TRPVEVRFAE PKSSKQTLKV CSRDLPGHLK MKTRDLSDHV EDKNMIETIN

LAVKYAKEAV VEDEKKNYKE ALNLYIQSLQ YFNFFCKYEK NSNIRDLILK KMEVYMTRAE
NLKEMLNILN KNNNIKWPDV CGLETAKEVL KEAIIFPLKF PKLFNSSTLP YKGILLYGPP
GTGKTFLALA CSNECNMNFF NVSSSDLVSK YQGESEKYIK CLFETAKEHS PAIIFIDEID
SLCGSRTDGE NESTRRIKTE FLINMSGLTN YKNNIIVMGA TNTPWSLDSG FRRRFEKRIY
IPLPNIYARD IKQFATLTEN YTGADIDILC RDAVYMPVKK GLYVKCGSRY EGMSVMLENM
AFHSTAHLSH LRTIKSLEKI GATVSCNAFR EHMVYSCECL KEYLPIVTNL IIGNVLFPRF
LSWEMKNNVN RLNLMREKLF ENNELYITEL LHNTAWYNNT LGNKLYVYES SIENYTSENL
RNFMLKHFSP KNMTLIGVNV EHDELTKWTS RAFQDYVPIP YTNQKEVTPK YTGGFISVED
NVKKTNIAIA YETQGWKSSD MITLTVLQTL MGGGGSFSTG GPGKGMYSRL FLNVLNSYNF
IESCMAFSTQ HSDTGLFGLY FTGEPSNTSD IINAMALEFQ KMNRVTDEEL NRAKKSLKSF
MWMSLEYKSI LMEDLARQMM ILNRILTGKQ LSDAIDSITK EDIQRVVHNF LKTKPTVVVY
GNINYSPHYD EICNILDAIK WIIYLMGYPE LASVNFGNST VVRCKKCRTY INPFVRFEAG
GKKWNCNMCY NINDTPQFYK RKDLFQRPEL CTGSVEFIAP SDYMIRPPQP PVYLFLIDVT
VTSVNSGLLD VVCSTIKSLL DSRTLIGIMT FDSTIHFYNL NSNLKQTQMM VVPDIQDIFI
PLPEDILVNV HECQNVIDVL LDNLPGMWRN NKISDCCAGN ALKAAFMVLK KVGGKLLFFL
SSVPNIGDNL YAELAQNVTQ YQIAVDLFAC PYNLDLASIY PLIKNSGGSL YYYPFNVHQY
NDKLRQELLF ALTTETAWES VMRIRISRGW TITNWYGNYQ FRGADLLALP NCHSGQNFSI
IVDLEENVVQ DSIVYVQSAL LYTNSNGERR IRLHTYALPI TQNIKTITDS INPQVVVSLL
AHQAIDISKK GKIADGRNLI QNLCSQVLSS QLLQSECARL LSLYILGMLK SIAFRDPDLR
IYHWYRLENI PVESVEANFY PRMFSLHNPD ALNLTCENMT QDGCYIVEDG ETIVMWIGRY
ISLAVVNVAA GLDGCDDQLL PASFRALEAD LNLHPSLLGY ITLAQTLMLS LFSPIWGFLS
DKYSRKWMLV FGTALWGVAT ILLANINDFA HILFFRAING LALGSIGPIS QSILADAAKN
ELGLSFGLVQ LSSSLGRLIG GVVTTTVALK YFGGIRGWRL CFIVVGILSV LLSIIVALFV
EDAPSLSKKS IIIILLEGFT GTIPWLALSF NTMFFQYCGL SDLQAAIITG FLLIGSAIGG
VVGGHFGDIM HDISNKHGRP LLGQLAMFGR VPLVLLIYLV IPKRKESFEL FLSCFCLGLS
SIAGVAVNRP IVSDIIRPDY RGTVFSLTIA IEGVGSSLIG APLFGYLAEK IFKYQNNNLL
IADMPEDIRI NNAQALSKTL FYLTIIPWIL SFIFYSLLHF TYGKEYLKMN EIIQNEMLMF
GLRSLSDFCN PTSKAYKENA SDALDRGVVV SIKNAVINYK DDDDILFCSS RVLLSMSDYC
MSEKDTNALK KLITDGGGIV EIVKCFPSDQ DTLKNCMAFI KNVGIALLNV FTSKTYTNKL
TNGIVLALCI ISKSTSGSKG LNDEGAHHKL LDYCLDDTAE IVESVFDIIK NMSSNGYVDP
TIIEKSVIIL DKFKSYPRVI SKGSDAMKCA VGPEELTKCL NVLKKSAQGS KEQDAALELL
SSLSYISSIT DKVVESGGIP VLIELINSGL QQYESNPEKI SRLVAGASRM LGRISNNPPH
AAIVVEYGGI ATLCTAISYF PNDVECSKAI CNALTPFVSR SYSLFASLLP ILYGSLESVE
LAKASMECIA SASMINEFHE QMVNNQAIEI LSTCVQYHLT EMDYLLNCFT AYFRLSDYIT
TVEPINQYGG VDGIANALLA VDSVLTVMLE NENKEVIIQE GTKIMEKLAT ESDCQRHITN
LETISETNQE EAYKTLAAIS GLSRIESLKN ILESKGADTS IFNGIKIWIE SARFIEQTKL
IKAGLKTIKI VDLMCLSQVK RIAEDEPDEN ILITSSECIN YLTEVNKIHS AEIVEASLEN
IFKLMKKYSE SRLTQINLIS AMNNILLSSN GVDILINKGY IKHIITYLQK VPMYVDVQII
GFTVLANLVK ISPDSVESIK KLNALIPLQT ALRTHAKNMK LKTTCAPLLS VLMPLDSLTN
EIQDIIKLCN KSMNDKNLSK LHEYLVALNE LLLTPEACKI ASRSNIISEI AHASTNISQT
RLGLVHLTKC NMVSSLVQLH DILKLPGDNY TEEAVSNILE ALSLLLKYDI TNAEIGFNSG
LIKKLCAGIN YFSHSDSVIN KTFGCLACMC TSKNRVGQLI SCPEYEGLIK LIVELIGDSK
LSRGSAIKAV YELLKTEDED IIKDISCKTS IVNLYKIMGE YQADLSIVQD CSRCLAIIVD
YVNIEEDKYT PMKVLLECLN KSKNDELTVL EMLTVLVKLC NSDDKMKLKE LGAIDVISDI
TMIHSENEEI SRLGGVMYSY MGADEQVKKL MKLILNVKKE DSDAVQKIDN FTSKLEMFLR
APLENPSDAL QYTEATLQVL NSYLASEVDN SSLQTNIALV TKRLVDRVKH DSEDPLGSWA
VASAGTLNQY IDMISNKIGL SNFKFVSPVY GVLAACVMNM DKKAREYAQD ALKFIQRSGS
NFLACKNLKE RLENNGFINL SEWNLNKNEG YVLCKENRNI CGFFVGKNFN IDTGSILISI
GHIDSCALKI SPNNNVIKKK IHQINVLVEK LIQINKSVLF LPSLAIHLQN RTRSVKINYE
NHIKPIISTT LFNQLPLLYL LSKELNCKEE DILDFELCLM DTQEPCFTGV YEEFIEGARF
DNLLGSFCVF EGFIEFVLFF ITIYEIKCQL RFASLGDWGK TKGQILNAKY FKQFIKNERV
TFIVSPGSNF IDGVWKNLYE DVYSEEDMYM PFFTVLGTRD WTGNYNAQLL KGQGIYPKWI
MPNYWYHYFT HFTVSSGPTG HKDLAAAFLF IDTWVLSSNF PYKKIHEKAW NDLKSQLSVA
KKIIVVGDQP IYSSYLLPLL KDAEVDLYIS GHDNNMEIIE DNDMAHITCG SFCVHELSNN
GIVTKFVSSK KGALQHFAAL PNVELTDVPS SGPMGNKDTF VRVVGTIGIL ILYVYILINY
NDIIMNLNKT WCMNPDQGLI KPDVVFYLVC IYDNDICDIS DVGVNFYINE KDVEDKSDAV
LKELQELNNY VKRIAFLSCN IYGLCGYIFV DFNKEFICYD SNGEQVKSCN VSKISKELEG
KVSFDFDKTS PFEEGDYVQF SNVEGMEINN KIYKIKNLKK YTFEIGDTST YSDYIKGGIC
TQVKKHLKLN FYPYEDYAKF DMSNHLHVEQ LKKDVVYNVC RYSKSHIAPI ASFFGGLLAQ
EVIKFTGKYM PIYQLLYLDF FEKNDNIITV FGKSFQKKLN DLNVFLVGSG ALGCEYAKLF
SLLDGKLTIT DNDNIEVSNL NRQFLFRREH VGKSKSLVSS EIIKKKNNNM HVQSLETKVG
AENEHIFNEA FWTKQNIIVN ALDNIQARQY VDNKCVWYSK PLFESGTLGT KGNVQVIIPY
LTQSYNDSYD PPEDSIPLCT LKHFPYDIVH TIEYARDIFQ GLFYNTPLSI KQFENLQNVI
NSLKISSECN FDFCIKKSVE LFHNNFINQI NQLLYSFPLD YKLSSGEYFW VGQKKPPQPI
VFDVNNEMIQ EFLLSTSNLL AQVYNIPPCF DINYIINVIE VKPFEPKKVK INMDPIEFDK
DEQTNLHVNF IYAFANLRAI NYKINTCDKL KAKIVAGKII PALATTTSII TGLVGIELLK
YVNYLSYFKN AFINSALPLF LFSEPMPPLR MMDKEYDELM KGPVKAIPNG FSSWDKIVIS
IKDYDTQRDK RFSGTVRLSN EVRKKLKVCI LGDAVHVEEA QKLELDYMDI EAMKKLNKDK
TLVKKLAKKY DAFLASQVIL PQIPKLLGPG LNKAGKFPSL ITHNDKIFLV YNIASFCFEL
QFRFIEDTTF DWLPALGYLL PYEKIKFLRT FFPIVFFISI CASAYSYTDK NATLIFLMRS
ILSTNRIIVE RSNDVNYLKK NGEKLVTKLQ QIRKFALTLD SQQISLLEKK NKKKSWFEFF
SIQVIFGVIF VYIWLTSKKP ATYEVPLSLS LEELYSGCKK KLKITRKRFM GTKSYEDDNY
VTIDVKAGWK DGTKITFYGE GDQLSPMAQP GDLVFKVKTK THDRFLRDAN HLIYKCPVPL
DKALTGFQFI VKSLDNRDIN VRVDDIVTPK SRKIVAKEGM PSSKYPSMKG DLIVEFDIVF
PKSLTSEKKK IIRETLGLYH ICISCPSTNW FKSTAIKWSL SIEVGGSDID PENLAKKSEL
SETLTILHVD TGKTKLLDKL RHTNVQDNEA GGITQQIGAT FFPKDVLDKE IKKIDIKCLS
KGIMIIDTPG HESFYNLRKR GSSLCDIAIL VIDLMHGLEQ QTKESIQILK QRNCPFVIAL
NKIDRLYMWN KNDWSPFNNT FKKQKPDTQE EFHDRLKNII NELSEQGLNC QLYWENMNPR

KYVSIVPTSA ITGEGIADLI MVLVKLTQTF MLKNIQYHDK LECTVLEVKN IEGLGTTIDV
ILTNGILRES DTIVLCGING PIVTVIRALL TPQPLKELRI KNEYIHHKYI KACIGVKISA
NNLEEVLCGT SLFVVNNIEE EEEYKKKVMT DVSDVFNHVD KTGVGLYVMA STLGSLEALL
IFLNDSKIPV FGVNIGTIQK KDVKKASIMR EKGRPEYAVI LAFDVKIDPE AEKEAALLGV
EIMQRDIIYH LFDSFTAYLK KIEEEKKQSK ITDAIFPCEL SVINDCVFNK KDPIVIGVRV
ECGQLKIGTP LFVPEKNIKI GNVVSVQSNK KNFDKARKGD EVCIKICGEP HVTYGKHFDS
TQKIYSKITR ESIDVLKEYF RSELTMEDWK LVVQLKKIFN IVMNINEKDK LAEQNLETLD
VTKLTPLSED VISRQATINL GTIGHVAHGK STLVHAISGV HTVRFKHEKE RNITIKLGYA
NAKIYKCTNP DCLPPECYKS YESSKEDNPI CPRKDCNHEM KLLRHVSFVD CPGHDILMAT
MLNGAAVMDA ALLLVAGNES CPQPQTSEHL AAVEIMRLKH ILILQNKVEL IKEEQALKQQ
EEIRNFVSGT AADSAPIIPI SAVLKYNIDV VCEYIVTQIS IPKKGIXXST XMXLVIYIFI
YLFIYLFIYI FYFGRYASLF FIACIDKGDN ELITLEIIHH YVEILDKYFG NVCELDLIFN
FHKAYYLLDE ILVTGEMQES SKKTILRIVA AQDSLMEDNK FAKSLLDVAD NLSLAIKNIN
EESLKTNENI YKGIEMTETI LHNIFNKYGI DKYNPINEKF NPQLHEAIFE INDSTKKGTV
ATVIQHGYKI KDRILRSLDI ESVQALIVAL NMYKGGLIII SHDTYLIKHV ADEIYHINNI
TKLVKIDYEF DKYTQLFLNN KIMPREIITL QCGQCGNQIG VEFWKQLCNE HNIDQEGILK
NNFLNEDRKD IFFYQADDEH FIPRALLFDL EPRVINSIQT SEYRNLYNPE NMFISKEGGG
AGNNWGCGYS QGHKVEEEII DMIDREVDNS DNLEGFILSH SIAGGTGSGM GSYLLELLND
NYSKKMIQTF SVFPLLNESS DVVVQPYNSI LTLKRLILST DSVVVIDNTS LNRIFVERLK
LNNPTFQQTN TIISNVMSAS TTTLRYPGSM NNDMISLISS LIINPKCHFL ITSSNVQKTT
VLDVMKRLLH TKNIMVSAPV RRGMYISILN IIRGETDPTQ VHKGLQRIRD RKLVNFIKWN
PASIQVTLAK QSPHSQHKVC GLMMANHTSI SALFERCVTQ FDRLYKRRAF LENYKKESMF
QGNFEEMESS KEITQNLIDE YKSAERDDYL NISRAFGIPV RKYTHEVVTL WYRAPDVLMG
SKKYSTTIDI WSVGCIFAEM VNGTPLFPGV SEADQLMRIF RILGTPNSKN WPNVTELPKY
DPNFTVYEPL PWESFVKKNK IVHIYIYIYL YLYLYLCAYT EMILIFVMGK EKTHINLVVI
GHVDSGKSTT TGHIIYKLGG IDRRTIEKFE KESAEMGKGS FKYAWVLDKL KAERERGITI
DIALWKFETP RYFFTVIDAP GHKDFIKNMI TGTSQADVAL LVVPAEVFEG AFSKEGQTKE
HALLAFTLGV KQIVVGVNMD TVKYSEDRYE EIKKEVKDYL KKVGYQADKV DFIPISGFEG
DNLIEKSDKT PWYKGRTLIE ALDTMEPPKR PYDKPLRIPL QGVYKIGGIG TVPVGRVETG
ILKAGMVLNF APSAVVSECK SVEMHKEVEE ARPGDNIGFN VKNVSVKEIK RGYVASDTKN
EPAKGCSKFT AQVIILNHPG EIKNGYTPVL DCHTSHISCK FLNIDSKIDK RSGKVVEENP
KAIKSGDSAL VSLEPKKPMV VETFTEYPPL GRFAIRDMRQ TIAVGIIKSV EKKEPGLPIV
LLKEGTDTAQ GRSQIIKNIN ACQIIVDIVK TTLGPRGMDK LIYTERDVTI TNDGATVMNL
NISHPAASIL VDIAKSQDDE VGDGTTSVVV VAGELLNEAK GLLNDGIEPN MIIDGFRNAC
NVAINKLNEL SLNFSNKNEE EKRSILLKCA QTALNSKLVS NHKEFFGELV VNAVYKLGDN
LDKSNIGIKK VTGGSCLDTQ LIYGVAFKKT FSYAGFEQQP KKFINPKILL LNVELELKAE
KENAEVRIEN PNEYNSIVQA EWDIIFKKLN LIKDSGANIV LSKLPIGDIA TQFFADHDIF
CAGRVEDADL KRTANATGAI VQTSLFNLNN DVLGTCGVFE EVQIGNERYN IFKECLKTKS
VTIILRGGAK QFIEEVERSI NDAIMIVLRC ITNSEIVPGA GSIEMQLSKY LRIYSRSICN
KEQIVLFSFA KALESIPRHL SHNAGYDSTD ILNKLRKKHS EQTSDIWYGV DCMEGDIINA
YDNCIFEVTK IKRNVIYSAT EAACLILSID ETIKNPSRNL GLPDCFKELL KTDKIKHVLC
TGNVGCNENL ELLKNIADSV HITKGDMDDN FDFPEDITLC IGDFKISLIH GHQIIPWGDM
NALLQWQKKY DSDIIISGHT HKNSIVQYEG KYFINPGSVT GAFQPWLSEP TPTFILMKSN
IVLYVYEEKN GKTNVEMSEL HKYSVKISEP RDKNSTGIYR HPEYKDKLCE NFDDCMGVRE
KEDNKRGAYQ WKNFGEIKEL IIKVGSGLGL YMPNCPEWNI CDLGCNAYNI ITVPLYDSLG
PQSSRFILDQ TQMETIVCNK TCARNLFKSL QGSLNNIFSI CYTSGTTGYP KGVIMTNRNF
IGILAAAYIG PSKFPNENDI HISYLPLAHI YERLMMYLFI AHGVKVGYYS GNIQTLLEDI
QELKPTLFIS VPRLYNRIHE RIFNSLKKKS GIVQSLFNKG LQNKNSSGST THVLWDKLLF
NKAKKILGGR IRAMLNGSAP ISVDVVKKLR TIFCVNILEG YGMTESLGFI THSRDRNIGH
IGGPVPCIEF KLVSVPEMNY LVTDNPPKGE LYLRGPSTGY FKLEKETNED GFIRTGDIVV
LNPNGSLTII DRKKNIFKLA QGEYVAVEKV EASYKQSLFI SQIFVFGYSY ESVLVCVICP
STDSIDIWRT QKKIKATDEE VIKLPEFKAD VINDLTSLGK KDGLKGFEQI KDIHFTLEAF
TIENDLMTPT GKIKRHEAKK RFKKEIDEMY GTKTTKTINE GQTILVVFNE GYAPDGVWLG
GTKYQFINIE RDLEFEGYNF DVATCAKLKG GLHLVKVPGG NILVVLYDEE KEQDRGNNLN
MLAVVYLACR EAGHIKSIKE LITFDRSYKE KDLGKTINKL KKVLPSRAFV YNENISHLIY
SLQLSIDLIE AIEYVVKKAS TLIWSDIERY FKDPELLTAE ILFVALTLCN VFVMYRLFLD
VIPYPIFVTW WQLAQGLLVA YVCGLKVLFV PSIFYCLMLV LSNYLLYKTP CIASYPVLVS
FTVVFHHLTR FIGCGEEYML RWKSIVFLLA AFIIGCFDSK TTGKGVIVWA LLYALFSAIF
RAGFMQKIMH LVDGKGNTLH NNQHLLGVLI LPVLILLTGE LSVFVHMPYD ITSLHTGCLI
TVGTLPFIKN VISNRLVRRT GQGPWRFLEI ISIILVFFIG MTYNAPSFKG YLAILCVIIG
RSLGAFDVLL NKTKVGEEMR NASFSLAKSV WAAGDFKGQI IEGIKRPVVT LSLSTNNVAG
VKLPIFQVNI DPTVDVLGNL GVAAGGQVIN NTRENYLQCL NMLVKLASMQ YVYFFFFFFF
FFFFFYAKLN EDLTATKINL MRDSSTSSKD DNPYCSINDG KVIIKNNELL SGIICKRTVG
SSSGSLIHVL WHEMGPDKTK DFLSALQKVT NNWLEYVGFT VSCSDIIASN KVLGKVREIL
DKSKSEVSKL VEKAQKGELE CQPGKSLYES FETRVNNELN CAREMAGKVA SESLDERNNI
FSMVASGSKG SIINISQIIS CVGQQNVEGK RIPFGFNHRS LPHFIKFDYG PESRGFVSNS
YLSGLTPQEV FFHAMGGREG IIDTACKTSE TGYIQRRLIK AMEDVMVQYD RTVRNSYGDI
IQFLYGEDGM AGEYIEDQII DLMKLDNKEI NKLYKYNFDE EPFGDYNKQN ILNQEFEELY
KCKNYLCKEI FPDGDIRQHL PINMNRLIEY AKSQFPNPID VVHKVNNFLE KLVIIKQINS
NDTLSVEAQN NATILLKAHL RTYLNSKLLT QTHKVSVKGL DWLLQEIEKI FYKSLCHPGE
CVGALAAQSI GEPATQMTLN TFHFAGVGSK NVTLGVPRLK ELINIVKNVK TPSTTIYLDD
MVSNDQQKAK DILTKLEYTT LKQLTSHAQI IYDPNTTTTI LEEDKSWVNE FYEFPDEDQY
SLGEWVLRIQ LTNIHVNEKK LTMKEIVYII YSVFSSDELD IIYTDDNSED LVLRIRVKYL
EDTFLKKLME QCLSTLKLRG IENITKVYMR EESKITYDSD NGKFVRSSHW VLDTDGCNLE
NIFCAPQVDF KKTVSNDIVE IFEVLGIEAV RRALLKELRT VISFDSSYVN YRHLSILCDV
MTQKGYLMSI TRHGINRVDK GPLIKCSFEE TVEILLEAAA FAQVDNLKGI TENIMLGQLC
KIGTGSFDII IDNQKLNDAN QNETIQDLTS AGFTTPDSSP LPFSPTYNAN IRNVVIPGNI
RKSEHFLNLM RIVVVYLKKY INIYDITSEG PLSFLYKFEK DTKLDTSFFK YCFDRLKSLL

NNLQIVEDYS SLNIVCNFCT LLGNYFKGFI IICEPYPEAT IYDPLIQFAC LDSSIAMKTV
INKYKSIILT SGTITPLELY PKLLNFKTVL TASFPMSFDR NCVCPLIVTK GSDLIPLSSQ
FSLRNDLSVI KNYGILLVDM CKCIPDGIVA YFPSYIYMEQ VISSWYELGV IANILDYKLI
FIETKDIVST TIALHNFKKA CDLGKGAVFL SICRGKIAEG IDFDKHYGKC VILFGIPYQY
TLSKILKSRL DFLKETYNIQ ENEFLTFDAM RQASQCVGRI IRNKKDYGIM IFSDIRYAKH
DKKNKLPPWI IKCMDISNIN LTVTTAVDIS KQFLLNMSQE YRETGQTKNQ PITRVTELSN
KLKVATVHTN CEIPTIGLWI SSGSKYENKK NNGVAHFLEH MIFKGTKKRN RIQLEKEIEN
MGAHLNAYTA REQTGYYCKC FKSDIKWCIE LLSDILSNSI FDDNLIELEK HVILREMEEV
EKCKDEVIFD KLHMTAFRDH PLGFTILGPE ENIKNMKRKD IIDYIDKNYT SDRMVLCAVG
DVQHEEIVKL AELNFKPFFC GSEIIIRDDD SGPNAHVAVA FEGVPWNSPD SITFMLMQCI
IGTYKKNEEG ILPGTVNNIC NKMTVGCADY FTSFNTCYNN TGLFSKYLWK ARIFFIWQRL
FTSSNDYYYS LKINIIDPIE IFKTIKIIST QFKIYVLSNF SMYLAFYNYT SAYQRILDLL
SQISHFQYTF TGRMGIKRMY QKIPATILVL LKDFDPDTDI LEEPHFVDSQ NNLSFQEQIC
LINYCFSMIR FNPHYDEIKF EKLNAVISRC LKYQNWLLHS CMLWFKCKGE TFRFKTVDRA
AAQLNELHKE CYDIKPESVE RLKFIYDVYY PTTWEMKKEI GSVMIKIGSV VTAFNIFKDL
KLWEEAISCL IQADRKEEAR ELLDDLLKKK KSPCLLCLYG LNYYIDAWEV SNFKYAKAAR
LIGKYYYEKE MYEKCSEYLE KALELSPLFP EIWFILGCSY MKIQNFDESI KAFTRMISMN
LAYLYMKKGT YKAAKICINQ AVKMNNNEWK YWDTYLKLSI IQNDIDSFCL ALRMICQVNQ
VKQIQPWVFD YISDVIVKDK ELDTFWNAHS FFLFIKGKFP DSFEAKIKEI RSIEIIIIWM
EMVIVGREAP YFKAEAVFAD NTFGEVNLHD FIGKYVLLYF YPLDFTFVCP SEIIALDKAL
DAFKERNVEL IGCSVDSKYT HLAWKKTPLS KGGIGNIQHT LISDITKSIS RSYNVLFGDS
VSLRAFVLID KQGVVQHLLN NLAIGRSVEE VLRIIDAVQH HEQHGDVCPA NWKKGKVAMK
PSEEGVSEYL SKLMDDNTAN IISHDNILTD PRITQDCSAE TNELLNRAEE AIEIIYEISN
ILNVNLDKET IVILIQLCEY G

> ***Babesia bovis***
NPGGDFKKTK KKLHWLPKDD DKLVKCTLKL YSDLLEPEDM RKFLEPVTEW TTECLADKQL
ASLSKGTIVQ LERRGYYIID MVSIPDGKMK KAGLSDQAEP LLHCPSIYYY GNECQQRMSY
MSTTNVVDHG HIFDYDIAAG LWKYAFDTMD VEPKGASVLM IEPVLCSAEH HRKTGEILLE
GMGVEEIHTS MSGVLSLYGV GKSSGMVVDI GDGMIQVVPM EEGHLEKKAI RRIDFGGLEL
TMYLQKLLCE LGYPMTSRDD FDTCRKIKEE LCFTSLDPRA DENNAGLEKQ YVLPDGQTLR
DGENVVGLSV ERFYVCEILF NPTIIGFSHP GLSQLLWQSI QDSGLTQRKL LMENIYLCGA
SSKFENLGER LSFELQNLAP AAARSNIMVV HREQATMFAN EWNLHLFEAS AVSGYNVKDV
FEFLLQMEQP KLAKVNKVLG RTGSRGGVTQ VRVDFMGEGG WEGRVLIRNV KGPVREGDIL
ALLETEREAR RLRGTQSLLL RDSHCYCHLS TGDILRSAIR SGDPIGMEAK TYMDQGKLVP
DDVVVKLIEG NINSPRCSRG FILDGFPRTE TQADRLKTLL SNLGKRLNAV FLFECPDDEI
QRRITGRLVH EPSGRVYHMT SKPPKVPMRD DITNEPLTQR KDDTLEVIRT RLDAYHKQTA
PLIKYYENMH LLCGSCYNQL PQPEVVLSPE DSELNYYLWM PGVQYQPFNN AASNGMMDLK
TAAEFIRQLG ATPSQAELVE YSATCGNSLN FDQLKELLAL SMYPKEDAAY LQKVLVSLSK
DQEKIEFPHF EYIMANYGEP MTAEELAAVK SIVFGNNNVR MVKLFNIIDE NKDELEKFNS
RNLQRVQNMQ LEQEMQMMDK NKDGFVDFEE ISISFPPEAG TPEDFMEGLQ RRFNVADKDG
NGKLNKTEVY ILLNPAHDES MLDLEVKDIM LTHDKNGDGL ISEQDDEFLE AEFKFDLNND
GLLSILEIID FATWNKHALE LSTSSITDHG ELLRHPEDYD ILCLCNCLGS GIIMGMAYLH
ILPEYFLAMI AFCIMLLIER VLSSGRTPCS DSSSDDDENR VVKSARAKAL EAIQNIGKAL
DHHKTISDYS EVLKDYDNLA RLVEKQSRGR IPKLIVQIIV ELQEFIDEKQ KDKDSLKKMS
KARSISFNTL KSRLRKFNEQ YAAVVDEYNK DPSSYWSDSD ASSISDADAG DKHKSALAKW
GVKKVQKPKA SKKPKEKADL GAADNPFSLS TPEINAVISQ VMVSADSVRS YVKSIVERRG
KRGGNTAETI RHLKVLPYIA KGISHSLYIF VVETLLHIEF DSYSNAYGAM TPKQWIDSYR
IVSHLIDELR RHPHALLSSE TNERDPANAG VSREERIQRS MTILESVVRK LNDELYKGLL
YIEVGSDDYN TMLVYNVNML YLLHKTLLYC LAANIAIIML EHLHYKDDVV SGKIWELVEK
PPEQERAEKR RLLPYHMHLN IELIETVNNI CACLLESANL AKSSLNSREI ISRQFRRMYE
MHEKQVFMGP PENNRDVVMS TFRHLQNGNW KECYDLLAGL TIWNRLPDEV LQILKDRIKV
EAFNTYIFKY VPVYDSFSVD QLSSMFDLDN NVIHSLLSKM IITGDIHATW DDSSKYCLIN
HTEPTDLQKC AIKLAENLTT AVEQNEMTLN MKNPKLGLSP HQTVVVGTRA REFLLNEKTD
IRTAEQLLER VLKRSVIDSR GNMLPLHLLE LFSLSIQAKE SGDFIANKAE MKRQIAMGQR
EVEDLIKTSF ESKEGMTVVE RLLQMFSING FLTTLDSKYQ TLVANTFRNL EKLVRKKMEL
ERTQSLRESI ALFIRQFVEV VQKMMTGNYT IMKLPPEEFL KYYGGSLRDN LEDGVKPGRY
VRYFTSKINA HMFGLIEPPI SNDYKSDEMI NVEFQINNGQ ENSLHKNIDR SRIPLPSVSD
QMQVPWHKTQ SSTGWILVRP IVIDRLDKGA KTDHDKSGRH AEAHLLNQLA VTQFWATKML
LASPLATKIY THFTHQKLKP AIVTALGDIA MASGSGFNNF VEPSMRLLLQ AAGTSFDLGP
VDNEEWIWYI NDLREGTLLS FTGILYGQKD INQVDSLRGY VSSILYFIQQ VVETPAEYFS
AGNFRLAVAL TGDLITAFGG DLSVHLVNSP LVEKIYERQK ALEAEEHPCR EKVNWLYKLL
NMYMLQLRFT SNAQIDKAGT LLLKAISGGD TFVELRLKLM QMLYNSVDVT LPLYVEILEF
ASRHDLFHTL LPVIQKLDEW MKDWVIDKKT RINIYRIISE QLDKMGNSEL SFHYWAKCIE
CCDDALYTSD NLKVIADFCV RSINADGVLY FDRLRHKPAI DHLSKTEFAL VVDILDLLIQ
GSEDDFDVFI SPETVRAKLK LLTIASICQN EPEVPIARIQ ECLKLSKDES EELVVTAITK
GVLDGLIDQR SEKVIIRSVM QRQFRKEQLQ QLHSNLLQWK SCVANEFYTF KDARATSTRA
FQIGASYWCS AGNHASTDFV SWTGELWDVA KISQIDVIWE YAPNEVEIST SMAQDSFNVV
MPFRKTFESK PSYKEVFKLD APVEAKFVRL TLRGPINEYF GIREVHIVGA GNPLFVIKSG
ISSPLEMCLQ DLWRHDSRQR LVSAVTKPPK CLTSVNPNKI GDDQCRWEFL GNGQISLKLC
MTQSDVYSDK AGMGDLKKKV KLIASTADRH KVESIMDENV KTYWASNLFL DSVHTVTITI
DFGEITRAAK VRIDWEYQPV TYTVEGSTDK ELARNMSNAD HVTIDTLEDR DFKQLRLVML
RPHHTYGKVE GGYVYGIRQL QVLSSNLETV VGDCRAAANT PDARDKYFVS YVSAFEPALA
HEIKNMENEI HGITGEVIDD MATLGDTLDE TDTCMQEKKE YDKTLEQIHT RETAMIQPQC
SKHPLRVYCD MGSQTSMLIW DGLTSPQAIR YQCAAYGLEP LIIKSKHQVD GLREALFLMG
FERKADHYIP LAYKFGNFRD LMNIYTFMTE SSITPPEQPA MQNAAARNRG LEPTTLGHVL
RRREKLWAKI QCIDKSARSD FEKHLDLYQQ QFEHYSKGWR YNWNNVIFTP NIGDSSDLIR
RYGKKRLYKN LDCLVHGWVH PGLVVCEVKD ATHPIRFATP PDQDCYTVIY AGPTILDTTQ

RVVFGEYTGV VREESLEDSL FEYAFELNFV LLPNTSKYVL DSSRACNELS LVNHYQSIYG
EKWPFPNCEW QQVFLDGWPH VVLTSKLGVS IKPGDELVAD FGALWFSKVE ETAHKAIRNE
IIQYRLDIGL KSNTLAAAAA VCSICYISSD NDGDYSVSCD GCDRFFHIRC IRSLNSDTYK
FYCSYCRHLA KKIANVYVCR LLKRHLGGNE LVLMAYEQHV QMLLRLLVPG FSYIYRQFKD
GYYKGIITNL VDGSEYFQVG YSDGDDEVIT HDDLIVEEYK EEGNKLYKQK RFEEALEMYK
KAIEHDPDNL LLENNKAAVY LEMGDYAKCI ATCNAAIDRR YEVKADFLVI SKIYNRLGSC
YTKMEDYDAA LAAYQKSLLE DNNRNTRCAM NEVERLKEKK EREAYIDPQK AEEHREKGNA
FFKKFQFPEA KKEYDEAIRR NPSDIKLYTN RAAALTKLGE YPSALADCNK AVEMDPTFVK
AWARKGNLHV LLKEYSKALE AYDKGLALDP NNQECITGKY DCMAKIQAMS QGTVDEEQYR
QAMADPEVQQ MLGDPQFQII LKRLSENPAA MNEYLSDPKI AKGIQKLMAC GILFRPGEER
PDSYGPNRDW NVDLIPKFVL AGGTLVKILK ATETSHYLEW QVLDGSYVYQ HQKATLFYDE
KFIHKVPATD KEALQSPLMG FFEKTRCHNF YRFVAQFDHT NPETWKGLNP FKDSIRAYYD
KYGLEENTVD FLGHAVALHT CDDYMNEPAY FSIMKMKLYM NSLMRFGSSP FIYPVYGLGG
IPEAFSRRCA IYNGTYMLNK PINGFEFDDK VCAVKTAEGE ARCSMVVCDP TYVTLPHKVK
SVGKVIRCIC ILSQPIPGTN DASSCQVIIP QKQLNRKHDI YITLVSHSHG VAAKGKYDVR
IDTTLNRYIW SNGIRNLPRR VRVRVSRKRN DDDDAKEPMF TLVQHVPVED FTGLQTEQMD
GDRRRTLDCT TANFSGLLSI MDPQKSWAAR YNSLVDVIPG CYAISVVGEL GCGSGYISTY
FIKLLQGSHR TIPFVITVDI NPAANKVVEA DTMTADMFLP LLPQLDMVMF NPPYVPSEEI
GNPKSIDRAW EGGFMGREII DRFLETVQQY LSNNGIFYLL LEKRNDIPDV EKSEFPTLCE
TCLGPNPLIR MLKERCGKEC KICERPFTMF RWKPGPKARY KQTIVCQSCS KMKNVCQTCL
FDLEYGLPVQ VRDEYLKNGL ELSEVKANLN HQLGKLELEK LARVAPYYRR NKPRICTFWL
RNACNRGEEC PYSHDNKHRV VIFNLPNVTE SAIRELCRPF GPITEVYCYV TWVFPSDAIK
FRDAKNRSIF CGRIIHVDLA SYKRQLQKKR QSTSGESSIW NTLHIDINAT VAAVSRELEL
QKKEVMENSA AVNVALTETL VLNELTRWLD EQGIRSDDTI IIKNLSKESC EHELVELFSQ
YGTLMRLSIS PYQVMGIVQY IDPKCASTAF KKMAYRPYKG LPIYVEWAPV KLFDPSLSNT
SVYIKNLHFK TRNDALQHHF GSCKGYITSK VSRGFGFVEF DSLVNAKAAI RAKTGLIIDG
KVIEMSIAKI IVKNLAFQAT KQDLYKLFSF YGNVKSVRIP KSLKSNNRGF AFVEYSSKQE
SARAVESLQH SHLYGRHLVL EFAPADAIIL EPIVRERIIE VERPEIQERI VDVPEIQYVQ
RVTEVPEPVI QENIVRVAKP VLQERIKKVV KPIIQEKVVE VPVVEIVEKV VEVPQYVYQE
KVIEIPKVVV QERVVNIPKK PQYRNVPTPV EVPVKQIRNV PVTKIIERDV PVPVEIDVVQ
EFTCRNIEAR YHEIPVPVHV QRIIEHPLPQ EAFQNTNLVP MYYLGSTALA IATKEGVIFA
SEHRVNSPLM ESVSLEKIME IDTHIGCTMS GLIADARTLI DHGRLECANH RFVYNEPLAI
RSCVESIADM ALDFSDIFDT RKKKTMSRPF GVALLVGGID IDGPSIWCVD PSGTSIKYKA
SAIGSAQEGA ESVLQERYRD DMSFRDAELL VLEVLRQVMK DQMTTKNIEM ARIKDRQFRE
YTEDEIGEII QDLPLLNCIL KEYNMISLTY VNDLSRCEEK LSEIANSIKD LEDRLGIYLD
EQRFICPRYP PMIITPLTET CLISISEAMD NCLIPNPQGP AGTGKTESIK VLAELCGHPF
WIFNCSEGFD SISMERIFAG LCQMGAWGIF DEFNRLIDGV LSSIAEKIIT LVNRKILLDK
NVGIFITINP GYISRRQFPY GVGFKMAFAR TAFGCAVMSR TIDSIGIGML SMELMSQCES
REMSVPLCMW RLPSKELINK EGSRMVDQRH HQRLLMTYSP FNSATLLAEQ INKLGTAPGT
RIMFFPLWKS STYSMDYCLP VYLYWLHLRT SCAITVQDYD LRGQVVVIAA TNRQNSIDPA
LRRFGRFDKE IDIGVPDDTG RLEILKIHTR NMKLAPEVKL EELAANSHGF VGADLAQLCT
EAALGCIREK MGAIDLEEDT IDTAILDSMA VTQEHFNAAI ATCNPSSLRE TVVEIPNVKW
DDIGGLESVK NSLREMILYP IEHIRRNLYG ANMTSPTVIQ RLVFKPSLTA GMHIVACAET
GSGKTLAFSL PIALSLLAIL PTRELALQVK DTMSMLIKGT QHSVFAIIGG MSIQKQERVL
KHQPEVIVAT PGRLQYLVLD EADRLVSEQS FKELGNIVDK VRTQCFIYSA TILQALFKLL
KLSNPMVCTA SHLPPNLRFR IIKCLDDERE LKLIAYMMEH HIIIFVNSIS YAYRLEPLLS
LIFWRDKHEL RIAKKITSIH SRLKQKQRLK RLEQYKNKKA VLICTDVAAR GVDIPNITEV
IHFQVPRNAS TFVHRSGRTA RCEAAGETTV STEVCGLSFF LSSDAIRALS DSALGPSDLC
NICSTCQDAC DGHLGHVNFP VPIYHPLLMP RLVKLLKSTC LYCRKLKLTF DLTFWNNLRN
RFLAEAGAVN QCPHCERKDL FNIAAASDLS SIQVQLQAFQ VVPYLKELFN EVILGHVFPQ
TRKMGWKMFV MFCMGVPANR FRPVLLGLHA RRETLCVLLK VGDNSNLKKC MRLQRKVSLY
TDIKQSLEHK EGAVRHNMLG KRVNYAARTV IAPDCFLDSN QMGIPLMFAT ELTVPENVTS
YNVNMLRKLS PQDFILTSEA DYRHLSLIAD FMTGELCMDI LKSNWSPAWT LQYLCKGITY
ILDDPNADSP LNCDAGNLIR GIKVKSAAEV GKRAVEYTRG DEISKWIEAN KETVFKLCPN
ILRDLDITHD DNVLDICNVL IEAGFIYRAQ YQPSSGSFRR PMWPKRLIKT AKQEFNPVGF
YIISYEGNQR WNYLMLTAMI VGIFSICMFW YASVVLLSIL FTLIILRLIL FLLFWFFGYD
FWLFPNLFDE DLGVIDSFKP LHSICYRNDA TYMLCCRVLC SIVLAASINE LRKTHDLKDV
GDFAKQSFMD IIEWGHNKLT DDDYKCLLAC GYKSLHQLMK ECMLSCECMS LVDSFLDDLE
ELEREHFVFS KKVNDIEECN QAVQEIDNEI INIYNYVRDI YSKRFPKLES IVYSPLDYIA
VVRRAQNEMD FTKVTLSDIL PNTMVMAITV AATSSGSYLS SHVLKEVLAA CNEGMILADF
RNDILVYLET RMALLAPNVS AIIGTALAAR LITQAGGLTT LAKMPSQNIM LVGGNRKGVI
YSCDIIQNAP SAVKHRAVKL VSGKLSLAAK IDMFTMDRPT LRTKVLESPE VSQTYDLILR
DKYMSRHCKY ILRQARLPAY RQFLRPYKSV TIENMSHAFQ LPPDFIEVKS AAQLIHDMAE
KIDNVDELLD AFDTMEQFYE HPGNASSVHR TGMLDAIAQH INTKILPAAL SLLITTISNN
EKVQEEATKG PLMQNLLDLR EKVDNTNLEP KLITAIAAVT RHCTTAEKHF VKVGGMRYIA
QCTAKHNLKV KEKAALLIYH FVNLQKLDKR EANNVQLLTT VRNLMPHGIQ YAEVCVNLFA
AIVSKYPNSI NKNDALTLWN QLAKAIENTF KDLGVPEWLI QLASAVSIKA PTKIQELCLP
AAFSGHNVIG CAQTGTGKTI CFCWPILVAL AKNPYGVFGL VLTGSRELAF QIGDQFNVFG
VQMNIRICVC VGGDDFVEQP HVVIATPGRF KKVKYLVFDE ADKLLHSEFE EPLQQILKCL
PRITYLFSAT ITKAIQELSK SFKGTQFHMF DVTKGQEEQL DLKQNYGIIF TATKKKCQLT
AVALEYLFKV TCIHSLMKQR KRNACLAKFR TGVSKILVAT DLIARGIDIQ AVSFVVNLDF
PRTTEDYIHR VGRTARSKST GIAISFVDEF DIEKLKTVEK AANIKLEKLE VDDAQAVKLL
NKVSMATQRA QIYLQEVVLF PSNLYITKAL ECFDKQFKIG VQNFSQAKCG AFTGEIAIPM
LNELGLEWTL IGHSERRTLF GETDAIVAQK VNTAQQGKLR AAVCIGENLT ERESGRVEAV
LTTQLDAFMG MVTDWDIVVI AYEPVWAIGT GKVATCEEVR EAHQMIRDYM TSKLGAETIR
IVYGGSVNEQ NCQELLHVPN MDGFLVGGKS ITPGFADIME AARDLGKDTT HRFGPNTFKL
HRLPVPRPGQ ILGLVGTNGI GKSTALKILS GKLKPNLGRF DNEPDWPEII QYFRGSELQS
YFTRILENSM KTAVKPQYVD NIPRQVSGRV GEILEAKDKK GRAEDLIITL ELAHLLNRQV

SELSGGELQR FAICVAVLCD SDVTMFDEPS SYLDIRQRII AARVIRETVE HDKYVIVVEH
DLSVLDYMSD YICCLWGRPS VYGVVTSPFS VREGINIFLD GFVPTENLRF REESLCFRME
EIERLHNYKY PEMKKTLGTF SLTVSAGDFC DSEILVMLGE NGTGKTTFIK MLAGILAADN
AEAMPKLSIS YKPQIITAKF DGTLRQLLMM KIKEAFGSPM FQTDVIKPLQ IEDLYDQQLK
NLSGGELQRV ALILVLGKPA DIYLIMSLIC SISGVQPEEP CISKTGYVFE RKLIEKHLQE
SQTCPATGKP LTVDDLIPIQ CDKTVIPRPA TAMSIPGLLS LMQSEWDALA LETYNLRKHT
NTVRKQLCQS LYEHDAATRV IARLIKERDA ALQQVESLEK LLLEFRTNYN AGAIEVGLDD
SSVSRIEDLA KALMAERKKR DVARYSTTES IAKYTLKGDY RAHSSTSPGI LSVTLDFTGG
ADGAVVYFDL DAGRTVTRMT SHLKPVNTVV SHPYANVVIS GSDDKTVRVW KGPSSKAPVV
SMSLHPSNEY FLAGASDGLW HLVDLESGQI IKICRDIPSC SKVQFHPDGL LAAGSGTDGA
VHIWDIRTQS LASTLFSENG YHLATVSQEG HLRLWDLRKS VVFANADCNM SPTAVSFYTG
QVKDGLFHGV GIFYYGDNER YEGNFVYGKR EGKGKFFYTD GAVYDGDWVD DKIKGHGVAH
FASGNVYEGH WDNGRINGYG TLKYVNGDVY EGEWMDGAMH GQGTYKYAEG DIYVGEWRND
KRHGKGILNY MSPKGEVLES YDGDWVDNAM SGKGKYQYSD GAVYEGDWYN GKMHGSGQYV
FPNGNKYDGE WVNDHKEGYG TLTYATGEKY DGYWVNDKAH GHGSFIYPSN DKYIGEWQNS
KKHGTGELIY VNGDRFKGTW VDDDATGFGV FYANGNRYEG EWLMNKRHGR ATFYCQEDGS
TYNGEYANNR KEGFGTLKLG LGHVIHGLWT LGSLATIEKF EISPSSPWSD PDLDMETLYD
VGHKLIEALR KESVTAGDVI RIDKSTGSVR KLGRVYSRAR DYDAVGPHIK YVQCPSGELQ
KRQKVVHTVT LHDVDVVNSR SEGFLALFAG DTGEIDNNIR KQIDEKVREW QADNRAELLP
GVLFIDEAHM LDVECFSFLC RHLETEMCPF LILATNRGIT NVRGTFYKSP HGIPLDLLDR
LLIIPTYPFQ PEDTEKIIQE RCNEEDVELD EESLHLLCKV ASETSLRYAL QLINAADLIR
RRRGTKVVTS LDIRRAFGLF LDTRRSTKYL VEFQHDFMFS ELYDNISDFW TSDEEGTEEY
IRTKWSVVDD SSTPELEDLV IEYPFELDDF QKRAIYHLHK MKHVFVAAHT SSGKTVVAEY
AIALALSRGK KAVYTSPIKA LSNQKFREFT KRYETVGIIT GDVSCNPNAP CLIVTTEILR
NLLYRGDPII GQLGVVIFDE VHYINDFQRG VVWEEVFIML PKSIQLVMLS ATVPNYAEFA
DWIGAIMERE VITIVTTRRP VPLVHFMYIY NRIFLLLDNK GFNKDAYHNM YRTTFKGQVQ
KLQRLIRHLE MTQKLPVVLF CFSRAKCESY AREMPNLNLN SNHQRSKIHI FLKESLSSIS
EDDRDLMQVK SIIKLLYRGI GVHHSGLLPL MKEIVEILFS RGLIKVLFAT ETFAMGVNMP
ARSVIFTSIH KHDGQKTRHL TASEYTQMAG RAGRRGLDSF GSVYIFCPDD PPDLQDLTTM
MFEKSTKLES KFRITYNMLL QVHSREHMNI TEMMLKSFKE TYKMKNIPIF KRDNIRKRQE
LSTIPKVDCI YIEEYHKLDG CSRTIADNLN ESLVRISNIS FIYNELHKLL LTSKIELLAF
NKQLKQTSIQ FYDVMHYSIQ ERVESCRNEL ERITSLLKEE SLSSYDEMVA KVEVLKQLDF
LDENGKPTVK GRIATYLTTG DEITLTETIT QNVLNDLEPE ECAAILSAFV HNDRPEKEVP
SPTAAIQKAR DMVLDLHSKV DVVQRALNVV VSREDHSALC NFSLSYVIYQ WAIGTPFSEI
MQYTDLQEGH IVRAITRLDE LCRKIGQVAN INGDQALQSK IEKVSNSIKR GIVFMPSLYL
DAIALAEFFA KVENMKQDGT LFTADELILG SMSSQCRADM PDNISFHPIS SIGSNCAVVH
YRATEEIKAK IEPKIYLLDS GGQYPGGTTD VTRTIHFGTP SDEEKEAYTQ VLKGHLALGH
AIFPEHTSGA TLDILARQYL WASGRNYYHG TGHGVGSYLN VHEGPMSISP RMGDYLEPGM
VLSNEPGFYK EGHYGIRIEN MIYVKPVESK DKTEFLTFET LTLVPYCKEL MNIAMLSQQE
IDWINQYHAR IADILLPRME ASPKASFQLQ KNPEFIKSRL AIFEELYNRI NLEIPDKHVE
TGKSWLTCPA DLLKHLSKSE SQGIIVAEIR PSTKLVEHVA DMWDLYRPLE CDCTVRFFWH
SSAHLLGSAL ETLYGAYITI GPALSQGFYY DCYLGNNTFK PDDMALITEQ VSTVTKTKSP
FQRLVCSKEE ALELMKHNPF KVELIRKKVP DGSSTVCYRC GDFVDLCRGP HLPFTTSVKA
MTVTKFSSCY WLSKNTADSL QRVYGISFPK EAQLKAYERR IEDAKANDHR LLGTTLNLFH
FDNIHSPGSC FWLPNGAKIY NRLVDFMREN YRLRGYQEVI TPNIYSCELW KTSGHYDNYK
ENMYMFTVDE AEWGMKGMNC PGHCLMFKHM NPSYRQLPLR FADFGVLHRN ELTGSLSGLT
RVRRFQQDDA HIFCTPEQIS AEVLSMLKFI DDVYSLFNFD IQSIKHRGYD SCGIGTMIEV
TKCSSYAPAN CFDRLRERLV DRHLGSTIGI AHTRWATMGP PTDENAHPHC DPKGRVALVH
NGTVTNTVEL FNELRENGLE PHPDSDSSAI AYLIGLNLDL GADPFTAMKT VVSRLEGSWA
ICLITANNPH SLYVARSGCP LLLMKDDDSV YIASEAVAFM HRTDYFIVLE DGDVMELNYP
VVEKLFTTRE VCRITKQIIR STPEPHQFWI QKEVFDQPLV ARVALQHFKL VNASQKVRLL
KQELEGLMDV HKINLVAAGS SHNAAKYVAS LFQRSAMFDH IETDDPTELK MYRYTNPDST
FIYVSQSGET LDTVNACNQL AELNPSKIAL LNNLNTLLDR SCDLTMLVNI GREISLASTK
TFTVQIMLLM ALIGYIVQAQ HHEELRQMKK SIIGYGLALK KVLCTEDICQ RVAERLCNDS
MYVIGSGEGY AIAQEAALKF KEITYIHAEG IASGTMKHGS LASIDPKKHT PVICIMTPDP
EVTVNATKQL KARGAFIIML ASNPAMGEDE FIQIPECGML TAACAVVPVQ MMAYKIAMLR
GWNPDTPRGL AKTVTVWLVD IEKYSSANIS KLLIGNKVDL EDSRAVTYDE AREFAEQNNM
DYIEASAKTA QNVEKAFESI ARALKDKATR CTTPAAANTF NLSTTRVSIR TLNSRPPPEG
WDVISDTLDS FDERMKAAER ESGEGKRRSE VQWPIFRIHH QRSRYIYDLF YVQKAISYDL
AVIGGGCSGL AAAKEAARLG AKTVLFDYVR PSPRGTKWGL GGTCVNVGCI PKKLMHYAGI
LGEHDREMLG WSDASKHDWS KMIQTIQNYV KMLNFSYRSG LLTGVKYINA FATLEKDHQV
SYLGPNGPER IKAKHILIAI GTRPIIPEEV KGAYEYSITS DDLMSLSHPV GKTLIVGGSF
VALECAGFLT ALGYDVTVAV RSIILRGFDR QCAEKVGDLM ENMGTRFSPT SVTKADGKLE
VTFDNGHVET YDTLMYATGR KLHGIYKYLG MTGYPNVYAV GDVAEGNPAL ATVAVKDGEM
LARRLFGNSN KLMDLNYVPM CVFTPIEYGK CGLSEEEAVK KYGDVDIYLK EFTSLEFSAV
HRHKQTDEMD VDMPPTCLSK MICKKDGTIV GIHFVGPNAG EIIQGLCVAV RLGAKKSDFD
DTIGVHPTDA ESFMNLTVTK ASGESWVQSA GCGGGRCGDT YASGLQVVEC LLSKGFLQRD
IDVLKAAGYV TLDSIAQVAS KTLLEVKGLS EQKVAKIKEI VKELCPPDIC TAAEYLECRL
NLIKFTTGST ALDALLQGGI ESGSITEIIG DFSTGKTQLC HTLAITSQLP IEQNGGEGKC
LWIDTQNSFR PERLGPIANR FGLSHAECVA NIVYVKVSNT EQQFDMLVEA AHYMAQSRFA
MLIVDSATAL YRTDYTGRGE LAARQMSLGK YFRALKRLAD IYGVAVVVTN QVMARVDNMS
FMGNDKVPVG GHVVAQNTQT RLFLRKARGN SRVCKVYNSP SLPEGEAVFA IAEGGIVDYM
AIRTQYENSN EVGVFATLTN SYALVSLGSS CNFASVFEAE LMPQIPVVQT TIGGTRVVGS
VTVGNRKGLL VSSICTDTEL RHLRNSLPDS VEIRRIDDRL SALGNVITCN DYVGLIHVDI
DRETEEIVED VLGIEVFRAS IAGNVLIGSY CRFQNKGGLV HVKTTTDEME ELSQLLQIPL
TSGTVNRGSD VIGGGLIAND WVAFCGMSTT ATEIATIERI FQLSRPKDPS VSNSHTLRNA
LIDTLIVVDT YWQTENGGIV VAPLAGVTPM KPGSATLPFF GIDVALVDSA TGKEILQNDG
GLLVVRRPWP GIFRTLYNSH KRGIVTYFSK VPGSYLTGDA AYRDKDGYIW INGRVDDTLN

ISGHRIGSAD IEHALVEVSY VAEAAAVAFP HPIKGNGIFC FVSLKLKLAV RRIVGPFATP
DIIISSPNLP KTRSGKIMRR ILRKLVSQAK DLGDTSTLAD QTVLEGLPFP IPCTIGDALR
YYLDLTGLPD EETLRNLGTF LQSNHSKELH LTLQEFIEIF MHDAIFNIGG FLQIIPKKVT
KAYTVSSHPK RMYKGACTHY LCSLQKGDVV KLYKRPSAFE QHILYKEEIE GLKNMPNYSV
NIALSRTGPW VEKYRPASLD DIVFHTNAMT TMRHIVESYD MPHMIFHGPP GTGKTSAALA
IARQIYGPEG MKERVLELNA SDERGINVVR ERIKTYTRLN ISSNRVNTQT GRVMPNFKMI
ILDEADMITP DAQAALRRII ENFSNISRFI LICNYVHKII GPIYSRCSAF HFKPISQDAQ
IERLRYICTA ESLEYEDFLT QVSQGDMRRS VTILQSTASL FNKVTEEAVR NVSGYPPKEI
VNEIFATCKG TTQDVEELCK KIIYDGWEVA TLFQQISEYV VDVQKATITI ELSGRELALI
QGGLQYFQLA SLCFHIRSII VKEVMYRSSD SKHIQNVFRA IKDLIKQVKQ RETDDANRVI
AEQEKLMLKK EGRRIVLKDL MVRPNVHGAR RIIGFLEAHH NGLRYVVIDI TYANIRHAIF
QPCERELIVL LHFHLKSPIM VGKRKSMDVQ FYCEVGTQID DLDNRRGRSY NDPDETLEEM
RDREMKRRLN AEFKQFVTQL QEMSNLVFDM PYRELMFSGV PSKSNVEILP TAHCLVNLVE
WPPFVLTLED VEMVSLERVQ HGLRNFDMVL VNKDYSKAVR RIDLIPVEYL DVLKSWLNEL
DMVLKTILDD VDAFVENGGF DGFLGESEGE EELDDEDEEY EHDSEEEVEE VRTLANIEFD
YIRRCSSAII AFPRIILFCK GGDNVMLNKL KNKSEMDKDT ISHCEQYCRD GLRTLVFAKR
ELTTQEFNDW NQKYIEAQGN ILNREESVAR CATLIEQQLE LQGITGIEDK LQDGVGEAIE
LLGYAGINVW MLTGDNLETA INIGIATNLL RNFSIDGIAT NELTKEHIVD DFIELCTHCH
SAICCRMTPA HKGLFVSLFK KKLGTVMMAI GDGGNDCNMI QTADVGIGIK GKEGLQAYNV
SDYGIGQFRF LVPLILDHGR NCYRRIAKTV AYMFYKNITL IMPIFFYGYL SLFSGQRILL
EVLVALYNVL FTGISVILVG SIDRDIDRSL SYQYPHVYQL GQRNYYLNPK VFLGWLFNSF
IHAVVIFFVV TFGLLPGGSG MPLNSQQLGV GMMIVMVIVS SKLIMETWFY TRLTTATYLF
SFFNFLLCIY VVSLSSKLGS ALLGGAMILM SNGRFWIVLL TSTMALYRDY LTKVIHYSFM
PRFYSPEVKD HFYKPRNVGS FDKNDPNVGT AVVGKAACGD VIKFQVRVED GVIKDACFKT
FGCGSAIASS SYVTELIKGK TCAEAESIKN TDISEVLKLP PVRIVVLGTG WSSLFFVKNL
DLSKFDLQVV SPRNYFTFTP LLPKLVSGRI STKTCTVPFS SFVQKHRGSF NFVHASCVNV
DPHSKLVYCV SNTRVNLPYD RLVIAVGAES NTFIPGVAEH AYFMKEVEHA NIIYQKIISN
FEQASLPGIS EEEKRRLLHL VIVGGGPTGV ETTGEIAILL NKAQSFPAVA SYVKVTIVEG
GQRLLGTFSL GNSQYADRVL SAKDVNILLG KQVCAVGEND CTVKDVTMPC GIVLWASGLK
QLELVDKVRA HFKVQNNPRA LLVDQHLALR GTSIFAVGDC CKILPVQITA EQILRDAVEW
QSRENKQNRT FVDQDELVYY KAQRRKEFED KLRRQRHHMG TWIKYALWEA NQQDFRRARS
VFERALQVDP NNVNLWLRYI ETEMKNKNVN AARNLFDRVV SLLPRVDQFW FKYAHFEELL
GNYAGARTVF ERWMEWNPDD RSWMLYIKFE ERCGELDRCR QIFERFLESR PSCASFLKFA
KFEQRQKNYP LAVKCLEIIP PELLTEEFFL KFAAFETQQG NLSGAEKVYE QGLGILPRES
SEQLYRSFVS FQKQHETIDN LVVTKRRNEY EEQLIDSPCN YDIWFDYIRM EERVCELYER
AISLLHLAMA NGCLLRGIHQ VTKAIEAKNA RACVVSTQTS EEAYLKLIRA LCKEHGVPCI
ETDSEKIGEW AGLCKYDIEG VARKIVGATS VAITNFDEKS ESAEEMGYYQ ATIGELLGDR
YKVISESAGK GVFASVARCL DIDTNTTVAV KVIRNHDIMV RAAEKEISIL QRLNRRHVVR
LLGRFDYRGH VCMVFPWLWG NLRNALRQQR IMTPFFEKVR EHHSTHNSRV DVQRKYLSDI
KDLYIGGLTE AILPAVGFRG ATKRNRPNIN EGDVIFCQIT KQYPNELVEV SCLDVDDMKQ
WTTKETYFGS LEGGFMFSVP IPYSYCLSGA RCHVLEKCRF KYEIAVGLNG GALGFSQQLP
DEAMLFSREA LLDKVAVMLG GRAAEDIFIG RITTGATDDL NKVTRMCYAF VSQWGMNPAL
GLVSYQRGSG DEPEFYRTYS ENTAQLIDTE VRTMIESQYA RVKSMLREKA ELVHKLSKLL
YQRETITYHD IASCIGLSHQ RVQQHQKSAL DEPVLRGVTS RAVRACENCG ASTHDVKSCV
ERPRKKGYEA VRDRWSGFDA STHQLVLNAH QVVEDELYRR RMEDLIDTNL PVVSHTTEKE
TDVMLLGHTS VWGSYYDRST GLWGYKCCLS TSNTSRCIVP LMDPYYASYY TKEVMNILVK
EFATPDEEMK HIVLKVVRQC ISTEGIQADY IRHELLDPFF KSFWIVRNSM DKKNLDLIIE
TTVEISNKVG VINRLVDDLK DPSESFRVMV AQCIEAVISS LQKVELDQRM EELLIDGMIY
AFQQQASDDC TVLLDAFGTL LHYLGDRSLP YLTQIVGVIR WRLGTQSPRT RQQAADMIAK
IAPIMRLCGR QDMLASLGQH LYEYLGEEYP EVLGSILGAL KAIVSAIGPA AMSPPIKDLL
PRLTPILKNR HEKVQENVIE LVGRIADRGG DLVSPKEWDR ICFDLLELLK ANKKAIRRAT
VNTFGYIART IGPNDVVATL LNHLRVQERQ LRLCTTIAIA IVAETCLPYS VLPALMTEYR
VPEINVQTGV LKALCFLFEY IGEMAKDYIY AITPLLENAL MDRNLVHRTA AWTCKHLALG
VAGLNCEDAL LHLLNYVWPN IFETSPHLTQ SCFDAIDGFR VALGPGVIFN YILQGLFHPA
TKVREVYWRL YNNLYVGNQD ALVPLFPLVR EGVENCHQAT ELLYMGRMYG KGKGISSSSI
PYRRRPASWV KTKPLDVEQQ IVKLAKKGMT PSQIGVVLRD SNAIPLVKTI TNNKILRILR
GHGWAPDIPE DLYFLVKKAV VMRKHMEHNL NDKDSKFRLI LVESRIHRLA RYFKKKRRLP
ANWKYKSETA SALRHVERAP GVQFAHVPND FFQYDDDEDE AAQLEVFDEG GGVAPAIVPH
RKTVNHRLRR KDYKNDYEFI EENVISTLHM LEFLYLSGVS LEEQDSYGRT ALMLASRRGC
QFVVQWLLSR GANLAHRDHL GNSVLHHACH SSDLDTLRFL CRHGAIGLIH AKSLAVTSSS
AFGICCVKRR FLQYSVLKLW SWQYGLTGRI ITFSSPYPVY YWLLAFINLL LYYKMYATMG
QMTPLQASVD IWILGWLMNQ CFWFITFASD PGERQMHLLQ KQQELINLQT DMHALYPQVG
VERRDHCVFG YADAVMSIRV LQRLIAPFIL RRKKRTVMHE LPEKRTLLVR CKMTGIQLDM
YMTEVETKIK QNRDTLVKSM VFLMRRICNH PLLVRGYYKE DLLQKLIKYY WSKVEGYKGN
PLERVEKELR SWSDFEIHRS LQSLVPVEPR LERFLIPKEQ FLESAKVQEM FKIIDRVEQA
GKKALIFSQF TMYLDLLETC LYLRLDGGHN PSTRTDIVER FTNITLLLIS TKAGGTGLNL
TVASTVILMD LWAKLLEQEE TIFNMCQNII QTGCNVCEKG VSDLAQHFLS KAGISCIRRI
RKTDANRLAR VTGATIVNRT EELLPSDVGT GCGLFEVKKI GDEYFSYFIN CKDPKACSIV
LRGSSKDVLN EIERNLQDAL NVCRNIMLEG KLLPGGGATE IEVSCRLAEK LDSKSGLKRW
SYRAAAKAFE VIPKTLAQNC GANPVRVITE LTSLHGNVNA GLDGETGEVC DTMKRKIFDT
FAVKAQIFKS AIEAACMLLR IDMLAVLTLY EACLENPMAK VTHVSNRSTK RYPPLPLDTV
EMHKDAAMHL KISSHESMRL AESLYNKGYV SYPRTETNVF PSSMNLRDLV GMFSSHPLFG
EYANRLLQGE PRKGNKNDEA HPPIHPVKPL SWDLYVYITR RFLACCSPAA VGDESIVFLD
ISGEGFHAKG LVVRERNWLD IYPYASWNGT PIPAMEEGQE LMPSRILLSD GCTRPPGLLT
ETDLIDLMNK HGIGTDATMH EHIQKVQDRR YVKKESNFTL IPTNLGKALY NAFKSYAHQQ
IDLTKPNLRA LMESDICAIA EGICMIMTGK TEYTDETKIV ITSYDLCVSN RRLRGFETII
CDESHYLKNQ NAKRTQFITP LLKEATRVIL LSGTPSLNNP AELYEQLSCL IPSFCSSSTF
VERYCEKRLH WFTKRMTYSG SQHASELHMF LVKTVMIRRL KENVLNELPP KIRSKVPIYI

APDILRGLSV FRKTGEAKVK GVCDYVLHLI KSSVKFIIFA HHMFMMDAIE QVLKAQHCCY
MRIDGSTNAQ QRESRVTEFQ NNSKCRVALL SLTACGVGLN LTSSSTVVFA ELHWVPGQMI
QAEDRAHRMG TKHRIINIHY LIAEGSIEET MWRVVSRKWE TVTATLNGEV SNLLIVDGDV
VCIGVSGGKD SSVLAHVLAT LKERYHRKWT LYLLAADEGI KGYRDDSLGV VFKERFGFDM
DEVVSLIGQK NNCTVCGTFR RQILEIGARM LGANKLCTGH NIDDNAETVL LNICRNDLFK
LFSTECIYAP EAYRGYMRTF IKQLECKKCG IEGINVEKLT ALRSNDKTSH CSIEVSMDYK
DLDPMPIEGE WQSIGPIRTL SFDIECVKFT GPGFPNANND PVIQIASVLH THGDSAQKFV
FTLDTCDNLH GAHVLSFANE AALLMAWKQF FIAVDPDFLT GYNIINFDIP YLINRANALK
LPEFTQLPRI TRSKSSVRDI ISSNNTMGTF ENKEINIEGR ILFDVYDLVR RDHKLKSYTL
NYVSFEFLKQ QKEDVHHSTM SKLQLGSAAD RRRIASYCLK DSVLPLLLID KLLLLFNYVE
MARVTSTPIK MLISRGQQIR VTMQIYRQCK LMGYAVPSVN RAANSEASYE GATVLEPKKG
YHRNAIAVLD FQSLYPSIMI AHNLCYSTLV PPAGHPGLYF IKAHVRKGML PLIVERLIEA
RKKAKEEMKV CTDPMLRSVL DGRQLALKIT TNSVYGYTGA STSGFLPCVE VATAITSFGR
YMIVDTKEKI ESHFTKANGY DTDASVVYGD TDSVMINFGT KDISRAIELG VQAAAMITNQ
AIKPITLLFE KVYLPLLLLA KKRYAGLYYT KADKYDKIDC KGIETVRRDF CLLVQQMMER
ILHMLLVDLP LVQIAPMLDI TYYEFRQFMR LITKRAQLWT EMMADGSLIY DSDKVGSMLH
CDANEHPIVL QLGGNNLDSL SKAGNIALGY GYTEFNLNVG CPSTRVSGKG CFGAALMNDA
PLVGRIVKHL RSELGTPVTV KHRLGVDHND SYEFVRDFIS TVADQGCTDF IVHARKAWLN
GINPRKNRSI PPLDHERVYR LCQEFPNLNI SINGGIKSLE EIKYALVYGV MVGRMAYENP
CGLVRVDTDI YGATNPATCK TRRILLETYA NFIDSQASRT SDCMLVKPIL GTFHGEVGTK
IFRLIQGLFH QGPMTQLAII TMRNKRSTMV SKLGTPPSEH IALLDEKIKE GTDGVPSLQN
GLEMAISILT DMPPYTTREI LVIFGATKTF DPGNILTTLQ KLKDEHVSVS AVSIAPEMYI
LKCRLYLVSP PDISRMFHQL IPPKPFINVM SSGGLRISQQ NYFSTHNALR VSGDKGVRYV
LHELHGERHG ALRLYPFYDV QMVKRMLIKK LNLSGVSVAD LRILYKGVEL PNYRTMETYK
TDHRLFWSLR EKNPSAGIRR TGIKTTARMD AIINEIALSL KSNIKPKLTM DGTGGTYLMY
NKHRKCCAVF KPIDEEAFAP CNPRGYEGTL NHQGFRSGVL SGEGASREVA AYLLDSAYGG
VCGVPDTTMV EASHPCFKNS CDERFVKDAS GPKWKPGSLQ EFIDCKESSG NYNPALFSVG
DVHRIGIFDI RVVNLDRNDG NILVMDTHLG IEQSRRDDLE ALGYVLLYFM RGSLPWQGLK
ANSKKDKYDK ISEKKIAIPV DLLCLLKQSG TIDEAVIIDR RVDMVTPMCL NVTYEGLLDN
VFGITNGSLQ CPLYREIRWL NYSEVGKHLH QRALQVHKGY ERGDLATLDE MGAFVKKFKN
LQKEHSELSI HVNMMSWMNS LISGDCMQLL HQLEDSILQS ATDIKPSKIA SLTAKILDLI
YWNVDVTQVY RLLILLSQTR DGKRAIVDQY GFCQLMTLHK LETMGLIRIN DPDGLRWARL
CKKLNLLVDR EADYASIFGG YAPISVRLFQ LARNVSSVEA DLRLLDCPVA VLRQDRASVE
SLCVQCGAMG ITMVLMHMIP HFKEVILMSF ECPSCGYRNS ELQDAAPLQD YGLRLKAHVA
YEGALRNQVV LSGTTSCRIE EIDFEFQPTM EKGTVTTIEG YLMRLAGGLY LYSLNSIKQS
LFTLILDDPA GNTYIEYNRS PEQQEMLGYI SLPVDCPHCG KVGNNKICEV LVPGFGPCVI
MAFTCENCGA KSNEIKPGGG YKEHARKWTL KVQDVTDLNR DVIISETATI HIPLLELDMT
AGTIGAVYTT VEGMLIKHAD SLETAYPFLM FVINRKGESE EVHFDRILER IRKLSFGLHS
LVDAPRVTQS VINGMYSGIR TSQLDELAAQ TCAYMAATHP DYSKLAARIV VDNLHKNTLD
NYADVITALF KYKYIYDSNA SLISEEVYDF IMANIDRINA EIDYSRDFQY DYFGLKTLER
SYLLRINDKI VERPQHMLMR VSAGIHTGDI ERTIQTYHLM SQKYFTHATP TLFFSGTPRP
QMSSCFLLDM KDDSLAGIFE TLTQCAFISK CAGGIGLACH KIRASGAYIR GTNGKSNGLV
PMLRIFNSTA RYVDQGGGKR KGSFAIYLEP WHADIMDFLD LRKNHGAEEA RARDLFYALW
IPDLFMKRVE SNGDWTLMCP DECRGLYDVW GDEFEALYTK YEMEGLGRKT IPAQTLWFAI
LQSQIETGNP FMLYKDACNA KSNQQNLGTI KSSNLCCEIV QYTSPEEVAV CNLASIALPM
YVDKENKTYD FKKLYDVARV ITYNLNKVID RNYYPVPEAK VSNHRHRPIG VGVQGLADTF
MLMRYPFESE EAKELNRRIF ETIYYGCLDE SISLAEKYGT YESYPGSPAS QGKLQFDLWG
ATVDNKLWDW DGLKQRMAKH GLRNSLFLAP MPTASTSQIL GNNESFEPYT SNIYYRRVLS
GEFFVVNPHL LRDLVDLGLW DDAMKEKLIA YNGSLRHIEG IPQHIKDLYK TVWEIKQKSI
IDMAADRGIF IDQSRKVRVD EVVEDLKELG VDVREETLKN PTPEAALCFY GLAIQVVFGK
TRTDIRPEDV YSHIHVITSD NLEFLKRGIG NLRFWRYCQK LHETLGLQKI ERYIFNPTPE
SFHRFISAFI KLSINEVQLK KTKSRHLNET LSEQVVVEPD ALYNQKNDLE AEDSVATATL
NNLEERFEDV IGRIKELEES SKFLVDMKTK LSQHLKPLLE NNSTSQLLQN RNETLDWWTL
GILIYEMLVG YPPFYNEDPM GIYKKILDCR LVFPRNYDPD AKALTKQLLT FDPSKRIGNL
HKGAKDIIAS QWFAKMDFDK LLQKKLTPPY VPELKGKNDT TKFPDSVEVP RAVEGKADPF
DNWALSRFFV ISSGGDRILL RCLRGEGEGG SEGNLPLIRI GDVFYYSLKR NGLYFVATTS
FAVPPSYMLE LLNRIIGTFK DFCGILTEES LRQNFILAYE LLDELLDFGY VQCTNTSQLK
QKVYNVTVPS SVSQRPIKEG ARSNEIFVDV LEKVEGQIRM KSFLSGNPMV RVALNEDIVI
NNRRCKVPNV AVLDFCNFHE CVLSLTPLEG EFTLMSYRIS MRNLFGIGHV ISCEVQRVSP
NGTVLLQTRT SKYGRLNNGI LVKVKPNLML RQSKHMQELS CIHAQVYHDI CTFRRIILSL
AGLSICINFA LLCEIFVREW KTPTAKRVKV AASNQHQLVL ALSGGELVYF ELDESHTLVE
VAKRSLNVEI TCLSLQPGRL MANFMSVGAL DNLVRVLSLD RQLKQYSTQL LPNNSTPESV
CILMLVVGLN TGVMIRATID AVGALSDQYT RFLGSRAVKF KYIKNQVIGT SDRPWLLYEY
QGVVQCLPLS YDTLESVASF SSPLCNDGYV AISGSNLRIF RCCRLGETFS EHRLPLDYTP
RKLVMMPNPN MVAVVESDHN AYGPENVAEI SKALGDIKLA NYKAGTGRWA SCVRIVNPLN
LTTAAKLLFE TNEAATAAAV VVLDGMQCLC IGTTVGYDLK SYIRVYCYGA NFEIRLLHVT
RVGGVVRAFT GYEGRLLASV GKRIRLYALG KKQLLLKAEH RTCSDGFIWL NAVSRIFAGD
IREGIQILFY SEEAAEFEWV GGATGPRWLT SCAQLDYSTV IAGDKFDSIF VTRVVEEKPD
ITYNDVGGCK VQLEKLREVV EMPLLYPERF VELGIDPPKG VLLYGPPGTG KTLTARAVAN
RTDACFICVI GSELVQKYVG EGARLVRELF QMARSKKACI LFIDEVDAIG GSRGDESAHG
DHEVQRTMLE IVNQLDGFDA RGNIKVIMAT NRPDTLDPAL LRPGRIDRKI EFGLPDLDGR
KHIFKIHAKT MSVEKNIRYE LLARLCPNST GADLRSVCTE AGMFAIRSRR KTITEKDFID
AITKVIQGYK KFSATGRYMV YNLTNLSRDN TQLLINRIFS LATLPKENGI VMPRMYPLPK
PKPKTRWQIF AEARGKKHKR SRLVFDKSVN DWVPRWGYKS IKKGPLHAPP IVEVTGSKVP
DPFEAASRKK SERKTRQKIR EIRNKVFERL TDYRFFTGSH RERFRGPPKT VVPRPKLMWL
YRNGDKYHDG IAFYVRPFIK NMDILYQHIS RDLELIAGPV RRIYDQNDGA KYLCTSGEPP
APAHRLQKFD ISIFAKLGQK HVTPPKGDGT RGFYESLLEA HPNSVLAVVY CVEYGLFGGQ
KHHELYERYQ EMRQQGLLKG AAGGVRPAAI QVLNVKFIQE KKLITRFFEE ISTDTGKYIF

GAKETIEALE NGTVETLLVY EALEVVRVVL HNPVTGEDSV IFLNDANERR DEEFKKNNVD
LETVERKLLT EWIVENYSNY GAALHFVSNK SQEGAQFLEL QASFVEFTST VYPGLTHVVN
VLSSCGTPEA CESIVKLLTL PLHTLGLRSL DMQHNEPLLG FLPKHLHRNV ARAMIDALID
SKLKIESCEV FESICRYLKS LFDPKDQFDI YQRLSKRMIS PMHYRYSIST LICRSLMLVM
LELKLNHAVV LRRIFDCMRD IISDGNIDFD ATGMSLQALD GNHVALVHLK LHESGFSLYR
CDRPRALGIN LNSVTKAFKS CSNHDSVLIQ SEEEKDYISF IFENNVDDRV MSFSLKLMSI
EQDALSIPTE GYDAEITLGS KELANICKQM NEFSDTLKLD VSINSVSFAT QGDLGFGEIV
LKNRPPTSDC GVSVKVRRPI KQSYATKYLL MFAKSCCLSD VVTLGLCQNR PIEVKYDVKD
GELKFYLAPK VDDACPSGCK GDPSSLKGTY IYTEDSSICK AAIHVGVIDN GGEIVVIRAI
TQPTFYGSVM NGVTSLGAHK EAGAFLVSRA TGFNGSALDF VSLANFPNAE KMKTLRDFTF
SVQMLSGKWS TIFSFQGCGG LTCAIDNTGE VIIEENCRPE LFKTSYFPKI GRRTNLTIVY
YSLTRDLGFF ADGNLIASRN TDFNFNLQGD LILGKSAETD SDFFVGQILS VEVFDYLMSP
NQVQHHNRML QSRMTVEGNL CLSKCVKMRS PGRESGPTNP AIHLGCHDTL EDERFNGAGD
QFLVSCSRSC TDPLLTLKGS KVYTSDSSIC KAAIHAGIVT IMHGKESYDY CMGHYDRDCK
YKVEPFNMYG YWYIHAENAH AKVSSWLPGT LKGIYNPISS VCQAAIHSGH LGELGGEVEI
EILGSQDSFN GSTAHGMTSL ESGQYLKSQS ELLKSVDVGT KRKVFNLQLS LGPYFANYTR
DGRHMLLGGS KGQLALFDII DMKPFFDISV KQTIRAVQFL DNHELMAVAQ KKYVHIYDNH
GMEVYVLRDL GLTYQLDYMA PFWLLTSIGE FGELAWQDVS SGQVVARYKT RKGPCRLMRH
NKDNGVVHLG HGNGVVTLWT PNQGRNYMAT SGFDGYWSTW DLRSKSIHVN FIGTPPQAMT
VSQTGILGMA LGGRVEFYRD VFTAPYLRHM YHGDQVNDIQ FQPFEDICAV GTGTGFSTML
VPGAGIANFD AYEPNPYESS SKRQVQRLLD KIPYDTMTHG GDRICINRKL PPDCVISGIL
FSTKDVLAIP YKSYTVKRYP IWTPCETYCT IRVNNDLFVF KDGTPEQKGT SGLVRIYNLQ
DATKPCYENV FKYAEEGEFF WSTRGTTAVL RTFIVKGLSS YYGGNGLFLL QPHKANHETI
METEGQAHEV SWSRTANDIL IIKGTRPAEL DMYDGNGNKI LTFGRHHRNT IRRDAFDRLV
LIGGFGNLSG DIDIWDLKKR CKIAQTKSDC AVFCDFAPDG RYFVTATTCP RMRVNNCFKV
FSYSGKLVSQ IDFDELYHLY ICSPGADPTL LAQAARISRK KKETEPIELK NIPTRTLGKI
LDYCKYHYNN PAKPIPKPLK STRLADVVIS DQASKIGHLA QKMTQETEMA VVLLIRRAAI
TAIPTMPKQM IYNATTLLCP FSKVKVPTPY SKIGDPLDLE TPPYVECPDE IQYELERLMM
KQRRLDYFNM LSNFVKLCME VSKLLTTIAK RDARLPLLTL FAKSMNEWML VQRCVVAAYE
ESFAYRGLSL PFQFLRIVED EIKIFLSKKR APFVLYFEIA NLDEDVRVIA TPENQRLIFD
DFNPQTAAAL FNNPMIQDMM QQIANNPQLF KDIVSSNPML QPMVPMLSYM MNNPELLRNM
MRPGVLQAGL PPEERYAFQL QTLQEMGFTN RDENISVLNL TNGDISAAIS RLLEARSGDR
LTRAGKVLEQ LTDQKPVFSK CRFTIRSLGV RRNEKIACHV TVRGKKALEL LERGLKVKEY
ELKSENFSNT GNFGFGIQEH IDLGLKYDPS TGIYGMDFYV QLIRPGYRVT KRRKEAIGAS
VARRLLGEIQ LKGVFDTTHQ HLPLIFMAMS GDYQISVLRM GRMNDYSVQM LRKIKQFLGV
TFLLKCVGSS FHNMVGNGCV AIACDKRLGM NGQHTISSNF PKAFKVTDTA YFAACGLATD
IQTMKSEIEF KTNMYALRCE KNMGVKTLAH MVGSLLYSRR FGPWFVSSVV AGPHIYCFDL
IGAPCNAKDF VVVGTCSEQL YGICESLYRP NLLVVGAGGI GCELIKNLVL CGVRNLVIVD
IDTIDVSNLN RQFLYRAEDV GRYKAEVARD ALLKWVPKCK VTAEVCDVLK WRPIDLSKYD
VVLNALDNIR ARSHINYCCM RAGIPLIEAG STGYNGQVYP IVHGITACYD CHEKPRNKDI
PVCSVRQIPE KAEHCVAWAR QLYELIFGPD NDNNMLDLDP QIPDVDSITD STAQKWVRDI
FEYLFDTQIT QLLTLDKVWA ERQPPSHRKN ILGSAIFDKE DPICVDFVSS AANLRMINFN
IPHLSTWDVQ SIAGSITPAI AATNAIVAAT QFDDFSIHKP AASRLTSNII FIFDFGSHQS
GSMVRFLRGL GITCELFPVE KAVETLSVPA AVILSGGSES VIKIDKKILE IHVPVLALAY
AMYALCETLG GKVTKLGPQE SEYRAHGCHL DKIDSIPTGF KVSMKDAHGN VVNSQSNVRA
FCFHPQEVEE GKADMIMKKF CLSRCEKTWD MRSYHEQTKK DVIKQGSKKF VVAGLSGGVD
STVCAAIVHE AIKERFHGIM INTGLMRYQE TQKCRLKAEI PGIQLTIRDS SAVFFKELKG
VHDPENKRKV IGRVYIEEFE KAMKELGYNH DNCLLLQGTI YPDILESDLN RRKLPVKSHH
NVGGLPENLK FELIEPVRLL FKEEVRDLGR LLGLSETTFK RHPFPGPGLG ARVLGELTPE
RVEIARQADR YMFEELEARG LVSQCACVLL PTRSYGMVVV VRIIMTVDFV TAKFAHIDME
CLAAISRKIE NIPEVNRVCY DITDKPPATI EWEMPVEGHL PGVSLAKRKP LVLYTSPEDK
IEVDPMLSRF LRDHQRQGVQ FVFDCLMGLK EFNGQGCILA DDMGLGKTLQ SITVMWTLLN
NGLNGKPAAR KCAIVCPASL VNNWESEIKK WLKGKCPCTA VAEGVKEKVV SKFTGFKYDR
QSNVIISSYE TFRLHAKKLE GVPIDLVICD EAHRLKNDKT LTSVAIQNLP AKMRLMLSGT
PIQNDLNEFY ALVSLCNPNV LGDISNFRKH YANPILLGRE PDATKAQQEI AAERLADLSY
INQFVLRRTN TLLSKVLPPK INMNVFCNLT ETQKIIYTSY TNSASCRKLI NSGEMTKSLG
VILSLMKVCN HPGLIKPSPS KSAKTLVLFR LLHNIRRTTS DRIVIISNYT QTLDVFERMC
KQCNYPCVRL DGTLSIKKRH KLVTTFNDNS HSFAFLLSSK AGGCGINLIG ANRLVLFDPD
WNPANDKQAL ARVWRDGQRK TCYIYRFFST GTIEEKIYQR QICKDGLSAM LVTDENEIKD
SLSGEYLRNL FEFKEDTISD THDSLSCTRC RKIEPAGFAF FLTKEKANSK VCLGLVRDFV
SKNQNAYELL DVCDSDDLSK IKANYKRIVL LLHPDKFILL QDAFTIMSDP QLRHEYDCSL
PWSRTKPVPS LGAANSSDDD IDFFYDFWRN FETTRTFSHA APHLLDDAES REEKRWMERE
NLKVQRKLIK KELVRIQKLV DLAQAFDPRL KARAERRLQE KVERKQIVKK LRQHVRAYAF
MKETCESIYA LLWTTEELSR LSKGVEMHVA GVTDRWSLIA KHVKTKTAAQ CIQMAREIAS
GKRLWSVEQQ SEFEAALVKY PSSLDMPTKR RNGGRSKHSR GRVRPIRCSN CGRSVPKDKA
IKRFNVRNIV DASSQRDIKE ACAFSTFNLP KLYIKQCYCV SCAIHSRVVR VRSVEARKIR
TFVQPRSMPM RKKVSVTEIS FDSSYTILDT SQGAVIIHVG HVAFHLDFSA LTVVKHVDMD
EPTKKFALEL ASDAIEKFKI EKDIAAYMKR EFDKRFDPTW HCVVGRNFGS YVTHEKHCFI
YFYIGSIAIL LFKNGVAGWC KTVRMQAGKL GFVILNDGSC KADLQVVVHE GAIGHQAVDK
CTSGTTIAIR GTLVERPSKL EGHYLEVIGE DAAKYPIAKK YITQEFLREV AHLRPRSYFI
SSVMRVRSAL AMATHIYFQR LGCIYLHTPL ITTTDCEGAG ELFQVTTMDY KKDFFKRKAY
LTCSGQLSAE NYACSMGKVY TFGPTFRAEH SHTSRHLAEF WMVEPEIDFC DLAKNMDIAE
EYVQFAIKYA LDHCLDDLLY LESIGDKGLV DSLRSFATEK FVRITYTDVI DILMQHETAF
ENHVEWGIDL SSEHERFIAE AVFKKPTIIY NYPAKIKAFY MRRNDDGKTC AAMDVIVPRF
GELVGGSQRE ERRDVLEDSI KQNGLCMDDY TWYMDLRTYG SIPHSGFGLG FERLVMLVTG
VSNIRDVIPF PRYVGKADFV KQSLEIDFKG KYDIVKNLWL KDEKRYYLLV ALHDTKIDFK
YLSKQVKVKH LRMGPEECME AMVGMKRGHL NPFAVVNDKN NEVKVLLDER IKEQIMAHAL
HNTTSVCIST EDLLRFLREN NHEPILGITV KKDTNFPEWY TQAIIRGGMV EYYDISGCYI

YLPSSYFIWE VFQQWFNENI KKEGVENCYF PMFVSKQKLE TEKNHIEGFS PEVAWVTKYG
DSDFVEPIAI RPTSETIMYP EFSKWIRSHR DLPLKLNQWC SVVRWEFKQP TPFLRSMARM
SKQEKKKAYF ERLTHLVKTY PQILIVSVDY VGSRQMAHVR HSLRGKAEIL IGKNTMIRMV
LNTSFPNSEA ISKLLSCVKL NVGFVFCMGD PLEVRILDNK VPAPAKQGVI APCDVFISAG
ATGMDPSQTS FFQALGISTK IVKGQIEIQN DVHLIKVNDR VTASSATLLQ KLNMKPFAYG
LKIEKFYDSG HLVEASALDI TEDDSLDSVK TAVTNVNAFA LAIGFPTSLS ITHSLIGAFK
NCVALALEND YCFKEMQGIK DRLDNPELFA AAAPTAEEPE EEEDDMGFSL FDEGRDVSRE
PCPDRIVEDM GGAFGMGCVG GFLWHFVKGA RNAPRGIIMQ NAFYNARSRA PVLGGNFAVW
GGTFSTFDCT FQYLRGKEDH WNAIASGFAT GGTLALRGGM GHAARNAVIG GLLLSIIEVV
SVVVNRRMTP TPRQQFERQM EYERMLVDDV GDVTISNDGA TILKQLEIQH PAAKLLVDLS
ELQDQEVGDG TTSVVLLAVE LLRRANDLAN SGIHATSIIA GYKMAIKECV KYIKDNLSKR
DLGDEMAVNI AKTTLSSKMV CVNLEYFASM VVKAIKAIET CDDMGNRKFP VEAVNILKTH
GKSLKDSFLV NGYSIMMGRA AQGMPIDISN AKIAFLDFPL KHYRLHFGVQ VQITDPVELE
QIRLKEKDVT KERVQKILAT GANVVLTSQG IDDMSLKYFT EAGVMAYRRV PRKDLRRIAR
LTGGKLVLTL STFEGEEAFP EDSLGTCGKV YEQRVGDVDF TFFEQCSSSR AATIILRGAN
DYMVEEADRS IHDALCAVSR ALEKDSLVPG GGCVETALSL HLEAYSRTLA SREQMAIAEF
AESLLVIPKT LALNAALDAT ELVSKLRALH AKAQSATPED KECKWYGISL ADGELRNNLK
AGILEATVSK IKSIKFATEA AVTILRIDEL VTLEPERNSG AEKLRGHAGS LQDMVFNDFD
YSVLATGSDD CSVRVWLAGH TKKTTNVVWN ASTDYVLLSG SMDNTVKVWD VKHGSAVSTI
PIEGNYSYCN WSYDGNTVLV STKESYVAFA DPRDGKVKLA FKAHDSNKAT SVQWLGGNYG
GDLATTGYVG NQTRQIRVWD ARNTDKPVVS KDIDSAPGPL IPYWDSDTGL LTVVGKGDLT
VRIFQYLEGD LNRAGEFKCN GTIKSFCFLP NSACDKSRCE LGRLLYNCTS KEINPISIVV
LRRNAMGEIY GNVEGCDLGA PQKSIFLRMQ VCGQFNNGFI IAKVYLIDPH AADEKTKFEK
YNSSVKIQRQ PLIYLSSFPQ VLGQILGEED FVSFVHDLWG ANTIPRPKRI WNILANRMAT
LAIVWLKVLS DVYKQEVSMM CGTFSSSTVK QNANSPSELQ SQTAEKCFVY SSEMTPNGGC
ISAGAALPQR RERTQGRRKP TTGVDILCQA KSGMGKTAVF VLSVLQQLVA CLGISHTREL
AYQIKNEFDR FSKYMNGVRC EVVYGGVPIS RDIEMLKCPH ILVGTPGRLL ALIKGKHLNM
DGIRHFVLDE CDKCLEKLDM RADVQSIFMS TPKKKQVMFF SATMNNDVRD VCKRFVRSPV
EVFVDDESKL TLHGLLQYYV KLSESDKNRK LNDLLDNLEF NQVIIFVKSV SRAQTLDNLL
NECNFPSIAI HAGLDQDERI ARYTQFKNFD KRIMVSTDLF GRGIDVERVN IVINYDMPDS
TDSYLHRVGR AGRFGTKGLA ITFVATEADS TALADVQKRF EVDIPEMPES IDTSLYVNQR
VSISAEVFGA YNNPDLFVAP VHEKTPEQAT RIKETVLKCF LFSSVDAKDI DTLVKAFESS
DVSAGTKVIQ QGDPGDKLYL IESGTARFTK TSATEQHDLG TAGEGGCFGE LALMYNAPRA
CSVVAETDMK LWSLDRSTFN HIVRNAVIKK REKYDSLLQS VALLSNLDPY DRCRLADALT
EKTFVDEDII VEGDKGTSVF MILEGNAEAY CQGKLVKSYS EGGYFGEIAL IAQTPRASTV
KAKGKCVVAE LERESCVTLL GPMEECFRDN LKEYQKVLAE LMHIKILPRS PEVAERYKTH
KSYYPGDCGL DLFCPDTITL APKKTTDVVL GVKIAANVGW ILAPRSSISK TPLRLANSIG
IIDAAYRGDI KVAFDNISDE PYTIQSGDRL VQVISYDGEE ISYELVNELD QTERGEKGFG
STLGAAYGTA RSGVGISSMG VMRPDLVMRS IIPVIMAGVL GIYGLIMAVI IVLDYLRAFM
LGFELKDAEA LIRLEDIFIE SFDIKDVKRL NGDHLSRCIG RISGKDGKTK HAIENMTRTR
IVLAQDRIHI MGSFNSIKMA RHSISSLILG NQPGKVYNNL CNISKRLREK IDPFTCQIHQ
WSSITGMSGA CGYTYDDLIL LPGYISGSCN DVDVSSRLTR TLRLNTPVVS SPMDTVTEAK
MAIEIALQGG IGIIHNNLTM EESVEEVRKV KRYENGNNIL YKSRKGILPI VNASGEFPKA
SHNENMQLLV GVAISTSIEK AKKLMDAGAD VLVIDSSQGN SVYQIDLIKQ LRQSQIIGGN
VVTGSQAKNL IDAGVDALRV GMGSGSICST QGVVGVGRPQ ATAVYHVAKY AYGCPIIADG
GIRSSGDIMK ALALGASCCM LGGAIAGTNE SPGDFFYHNG IRVKQYRGMG SKAAFVSQGV
AGYTADKGSI HVLIPTMMQA VKHGMQNIGC NDIKSLHSGL YNGLEKDEKF KEAMKSLERL
GEIYTRSKDA CSHYVNKLVL AKESVWERFG TRLRDMPFLT NFFENPVFDQ LFGNSTLAKA
VKEMKRLDSS FDLPEFIESV EHVVAPHIVQ CYLDGDSKSL EAHCGELAFN VLNASIRERD
LQKLYLDPNI LILKDVELKG GMTMEEGYPW FIFNFKTQQI NCLRDSRGHV VAGEIDDIRQ
VVYSMAVSRH PDIDGLEYPY MVHEVAIIGN TQCWMTLTLV GLGLGAVEDI TLRGLKAIQN
ADAVLLEIYT SALIDSLHDL ESFIGKSIEQ ADRISVEESA DKILEEARAK NVVLLVAGDP
LSATTHCDLC LRAENAGVDV EVIHNASIIN AIGRTGMQLY RFGEIVSIPF FETNWSPDSF
YDKIVKNMEA NLHTLCLLDI KVRERSIENL MNNRMIFEPP RYMSVNIAID QIFRIDHTKH
RLPSNTRAIG VARLGSKTAK IAAGTLKELK DIDFGEPLHS MVICAPQLHD IEEEYFKHYP
NSAYRKSVRV QLIKNGKKIT AFVPRDGCLN YIDENDEVLV AGFGRSGHSV GDLPGVRFKV
VKVAGVSLLA LYKEKKEKPR SMGIKGLIGF LSDAAPGCIS EVTLESLSGT SIAIDASTAL
YQFTIAIREG SYLSSLTNSK GESTSHIAGL LNRCIRLLEL GIRPVFVFDS TPPEAKSQTL
AKRKLLREEA ESSLEKAIEE DKEAIRKYVG RTVRITQKEN ESAKKLLRLV GVPVIEAAEE
AEAQCAYLCQ RGFVTAVGSE DADALVFRCG VLLKNLVDLA KALELLELTH EQFTDFCILC
GCDYCGTLKG VGPKTAYNLI KKHGSISRIL EVRSYEAAQE YFRDPKVRDI TTIDRCEANI
DGLREFLISE NDFSEERVDK LIERLQKARS KKTQLSLKSF FVDNEKFYKV LGLSRDCSES
EIKKAYRKLA IKHHPDKGGD SEMFKEITRA YEVLSDPEKR RIYDEAGEDG LEGNMPHGDP
SDIFDLFFGG GRKGKKRGED VVTQLKVTLE QIYNGAMRKL AINKDVVCDT CDGLGGPSDA
FVSCDLCNGR GIRVQIRQMG AMIQQSQSMC HACNGQGRSI NESKKCKSCS GKGVKQMKKI
LEVNIDRGVP DQHKVTFHGE ADERPNEIPG NVVFIICQAP HDQFKRSGSD LIIVKQIQLY
EALTGAVFYI KHLDGRVLRI QTPANEVIRP SSIFVIENEG MPVYQSAFSK GNLYVNFEVQ
FPRKFSAAEK DQLKLFPYKP ESKPSGTTAA EDVDAREVDP QEIHDRISQL RTHLPQLIPV
LIEHTRYSSW DYMSMDESHF EEDNAEDEES ATWGNNWTPR KGAALALDYI SQVYGQEIVQ
FLLEHIEKRL ADSDWEMKES AVLVLGAIAS GCMLAMAPYL PKVVEYLIEL TRHPKPLMRS
IACWCLARYA GWACHENNWL YRVLTAVLAR VLDRSKRVQE AACSALASFI EEGGSQLKPH
LEPIVETIVK AFSSYQARNL MFLYDTVGTM GQVFGESLVQ TPCCEYLLQS VLQRLGSTET
HAPQYLALMD CISYLVQSWQ QLYARYAEVT IARAMNAVFE VLYDDIIGCS LDMIATVVLQ
NVFALLGDVA WQCADLVATE TVIASLNLNL LNPSKIVSNN VCWALGVISH TDHKKRIESV
VHEFYPKLVS ILVTETESMI LQNVCITIGY FAAGYPAYVG ANLQQFLEPW LRNISRSSSE
HDKANTLHAL AQRLSECIKQ EELEELLAQG ICKAYKVNQL KRMGISSVMW IADWFAMLND
KLNGNMENIR LVGEYFKHVW RASGMDMNAV KFLWASEEIN KNPDLYWRLV MDISRSFNIT
RLKRCSEALG RADGDNRPGA SLLYPAMQCA DIFYIGADIC QLGLDQRKIN MLAREYCELK

SVGARPIILS HHMMPSLAQQ GGKMSKSIPN SAIFMEDSAE EVNAKIKSAW CPEKEVCDNP
CIAYFEHIVF PMFNTVTIPR KEKNGGVIIY VLEEVFEGRN LSEIINTDHE NKKYLPGIKL
PHNILAVPDL KQCIQDSDIF IIVIPHQMEK LVRRLGKLSV LAGSVALVPS TCLVDVDGGQ
RVVMFNRFGG VSEKTLGEGS HFYLPWFQMP HIYDIRTKPK VINTTTGTRD LQMVSISLRL
LYRPITENLP RIHQKLGPDY DERVLPSISN EVLKAVVARY NAESLLTQRD QVSSDIRMAI
TARAKQFDIK LDDVAITHLS YGKDFSKAIE QKQVAQQESE RVKFIVQKSE QEKIAAIVKA
EGEAEAANLI SRAIQEHGTG MLEIRKLEAA KEIAETLASS KNIAYVPNTT NILYVREYMS
HQGGWDFSNH SRNARIKDNI FKVLKTGTTI CGVLVKDGVV LAADTRATEG PIVADKNCSK
LHRISDFIYC AGAGVAADLE HTTLWLENNI ELLRLNLKQK PKVQMCVSML VHELFKYQGY
KQCALILGGY DSKGPHLFSI SPRGSSDSLP FCTMGSGSLN AMSVLESEYR DGMSISEAVA
LATKAISAGI LNDLGSGGNV DVCVINRDGA THTRAHAVVG TRTYAPPPGA SCVLREKILS
MKEYISLNDL GEARGAVLSI TLDKVMDNIS GQTVVDPKGY LTDLNSSDSD IADIKKARKL
LKSVIATNPN HAPGWIAAAR IEELAGKISS AREIIAQACE KCGDREDVWL EAARLEKPEY
AKAVLAKAVR MVPQSVKIWV EAARRESNVN DKRRILRKAL EFIPNSVRLW KDAISLEDET
DAYVMLLERS RKQCGTNAGN AYFMMSSSLQ EFPDSGNLWA RAIFLEERNA QNSKAVDALN
QCSNSPLVVM AAAKLFWRDG KVLKTRKWFK RALAIEESNG VIWGTFLAFE LDSGDNDAIK
DVINGCTKAE PSTGWLRGRI NDVRGKGTMT FIILRQQHES LQCIVDSRQM VSKDMAKWAA
SLSMESIVDV LGKVVRPEVP VTSTTAKCEL QVHKLICISK ANPTMPFLIR DANNTDDKNP
SVIKVNQDTR LDNRPLDLRA TLNLAIFKIQ SEICQQFRNF LLKRDFIEIH SPKLLGGTSE
GGCNVFKLKY FDNDACLAQS PQLYKQLAVV GDFRRVFEIG PVFRAENSNT HRHLCEFVGL
DMEMEIRDNY MEVVDLIDDM LKHVFHGIQK HQPVEPFKWL DQTPRIRFTE AVEMLRDDIS
SYDFSTEQEK RLGRLVKEKY NTDYYIVYEY PLNARPFYTM PKEDGIMFTH SYDFFMRGEE
ILSGAQRIHN PDLLLQRAKE CGIDPKTISC YIEAFKMGAS PHAGLVFENL TREFKNVITQ
DNYDGFRLEA DRQLTNNLQL SHSLYLGTSE TGYLYQIGTN YASSDGNCLA MGKIGLDGML
TGRVFAKYAD EFKVSGNSFL RYDTRNAYEA GVDYFGKGWT ASLKGAWQGG SELTYIVANG
TSIGAIAARY VRGDHVFTGQ WSKQPNFKGM DYNLQDTDTC RLQYVRRVNE RLSLCTELEV
TPATKESALR VGWDYLFRHA RVQGNIDTAG RISMQAQDYS GFGISGCIDY WSNIYRFGMM
HLLPPPEQDG GVGKTTLVKR HLTGEFEKKY IPTLGVEVHP LKFRTNCGGI QFNAWDTAGQ
EKYGGLRDGY YIKGECAIIM FDVTSRITYR NVPNWHRDIV RVCDNIPMVL CGNKADVKER
QVKAGHIQFH RKRNLQYYDL SARSNFNFER PFLWLARRLL NQPQLVFVGE CAKEPEFRID
PQLAQECERN LEAAANVAID DDGGVITLTS VLNQPTDSQY TFKQLIAQKI DENSKFRAFL
VDRQIAESVY GESIYDARVV PIDVRLRLVA LEEWNINAST RAVVRTTGQV GHIDIEKLTY
KHDSQTLLIS FVIQPETDQC PPKFMVLPPG EIDYDKLIRE FGCQRITQEQ IERMERLIVH
PYLKRGLFFS HRGLDQLLDA YEQGVPFFIY TGRGPSTGAL HLGHLVPFLF TKWLQEVFQV
PVVIMMSDDE KFLFREQLEY IDIVSLTSTK MIFILLNADV ATANSLRRIM LSEIPSLAIE
VVTVLENTSV LHDEYIAHRL GLLPIESSSA RNFEYRDKCT CSDKCSRCTV EYSLDVKCMD
TRVVTHYDIL DSNVNAPMPV PIPIVKLKRG QCLSLKMTAT KGLGKFHAKW MVANVAYKME
PVITINRQVA EKMNLNEKTQ IVQSCPRKVF KLIVIDNNLN CIYCDECLNQ AREMGYRDLI
RVQPDETKFH FTIESNGAIS PEKILEICLD TLEEKLGSLH QHFEAQARSI GAKVHTTSLL
NPKDFDDRDI QCLVTFLNTL VTKEGYFWEK LANQVVGLSE ETTSGVTHFR KFWRAEGLLF
PVMSTNDCVT KQKYDNIYGC RHSGIHGFFN GGDFLIGGKT VVVIGYGNVG KGVAQGFRGQ
GAKVKITEID PICALQAAME GFDVVLLEDV LETADIFISC TGGIDNITVE HMKRMKNNAI
LGNIGQGDQE IQMAELHKVP GLEITNIKPG SDYFKFPDTG KVIILAFGRL YNLGCANGHP
AFVMSASFTD QALCLLELWK NRTYGKCINK LPKTLDETVA RYHLKALNMY PYVGEHQDHY
GMNYNHTGQM DADYEVISPR GEQHTPMVGF FSSKLLRSGL TFQCASLTLM FIFYWAFGGN
GIFVFDLYAA PESVKISRPF HLTVSSLQAI YLLGTFYVAM FQVFVSDNSK AVRGFRAGSK
ILSAAVTLDL ISSMLRLVQY IYAYFYMSMK WWTRYQQTKA DWIFFQFGSF TNSFALVMYG
AAFFYLEAYH DEGTSEEVAW TNLVLFCLAG LTELQGNRKR TFRTFKFRGY ELEKLLEMPM
ENFVGLLKSR QRRRFSRGIK TRIRTLLKKL KAAKKDLAYG EKPEPVKTHL RDTIIIPEMI
GSIVGVYNGK QYINVEIKPE MVGHYVGEFS ITYKPVMHGK PGIGSTHSSR FIPLKIMVDT
FFWRDAEQME LKAEQKEAMS SLRPPMDWNQ MTGARANADW GLLQIIKDHH ENMYDLKQKV
NPKEQVVGWF STGGEMTELT CAVHGWFKQF NTVSKFYPQP NLYEPIHLLV DATCDSGSMS
IKAYVQLPLN LTKEACFQFH EVDLELMISP SESVGVSLSK LSELIDLCIE RVEAAIAGNK
DADPELGRFL LKATTTDSII SQAKLEKLSA LALQDNLMIA YLSNLANLQF ALAEHLNWLK
SLPLKADEEE AKRVHKNLMD LIIANNPTIL GPDNCNMAQI AKIFITIYET DFSTEELNKQ
IIQLMKHLGE AFLQQLALPK RLQAQLKNIS ASELMRIYEG NSHLKPYLKI LENCDRYPVL
RDSSGKVCSW PPITNSDATK VTLDTRNILI EVTATDRVKG SICLNMVVYC FSQYCEHPFT
VEPFYTKYPD LTERVLHGDM SYFRSLVGVP SLDGQQVCSL LKRMMVETDS VEAVVSINRP
DIQHACDLGE DIAIAYGYNR IAKESFNCGG LRPLTYLKRT VRDVLSSCGY KETLMPILDS
LVNMYDRMGW PVLLKNAKVS DLEACRVSLL PGILNTISNL RSASLPIQVF EVGDVVWDES
DVQAVNNCNI ACGYANSTAG LEEVQGASET LLNELGFYSE YQRWELGESK IPIPVLVYAG
IRRPDSIDSG AFKVSTELFG WKNKRTGEVI QHKRSDLNSI SIINIGGGMY QVRFDMNASK
GYEILRFSGF SEKAVDELKQ HFDEHFKISP EVGSVAHTGW HWGVYGFEND TFKLTIDDNA
GIDIDAKDVT QVTVPTKTDL AVEFKQNKNG DELMEIRFCI PNKDDNELAL EDLKQTFLLK
AGLDELKSET LAFLTDVPLI VPRGRFEIEF SRKHIKYHGK SYDYTMFFTN ISRMFLVPKP
NSPHINFIIG LHQPMRQGQT RYPFVVMQFD AEEDIELEIN MPEEDLESMK LEKVMTGKTF
NVVTKLFGTL VNKPIVVPGE FKSEKEEAGF SCTYKATSGY MFPLNRSLLF IVKPVIFIRF
DEIISVEFSR TGVTQNRFFA FSISTKNGQE YEFTNVDRAE FEPLSKYLAS RDVKIKGYID
LSKRRVSPED IVKCEEKFSK AKKVHQTVRH IAQKHGMSVE ELNRICIWPL YQRYPNALDA
LKEAAINKTN IFKDLPIAPE VIDSLIADIQ LRLTPQALKL RCMIDVWCFG PDGIEAVKSA
LMLAKHNIQV KLIAPPQYEI MTTCHDKDKG MDIITTALEE ISNKIRSYAG GEFKQRGDII
IVLLEGQESS EEEDSGDKRI YEYFVCDVAL GKSISVRLSM PVEYDSVYIG VLPQYAFRKD
YIVYGASQIL PKYLIQFECD PSAEETFALP LCDSCQNDAA TLYCASDTAK ICKKCDEKLH
SHKVVSRHIR VPLNKMPAKC RLHPSKVYTM YCTVCHLPVC QLCTSGHIHI PIANAYDSAI
EMQQHSSETV TRRKEYLNKL LEQLKSIEKT VEDNCERVEV SCYENLEASL NDLHTSIQSS
VEIIVAEQTE NQRHLNQLKW AEHFAAYLKN TLLPADYLRA WLRHCRFRAE VSSTAEVFPN
IDLKGELSIM HEQSVDHLVG LPNVGKSTTF NLLSKQMVPA ENFPFCTINP HEAVINVPDE
RFKHLCKVFQ PKKEIAASLS IFDIAGLVRG AHKGEGLGNA FLSHIDAVDG IYHVVRGFED

DDIIHTDGEV NPINDLETIN QELILKDLDK CTKALVEINK VQRNMKIKSK KEELDTMTKA
KEVLEKNQWI SQASWKASEV PILNEYNFLT AKPVVYLVNL SEKDFVRQKN KWLPKIAKWV
ADNNPGPIVP YSAQFESALE AFTDDAREAY LKDKNGATSK IDKIIASGYN CLNLIHYFTC
GPDEVRCWTI RKGTKAPQAA GVIHTDFERG FICAETYNYT DIVEFGSESD VKGNGRYLQK
GKDYVVQDGD IIFFKFNVTN KKRLDQIIGQ LDIPIRYLKD VAQAFYAELV NGLMAHRRHR
NLWLPNECSF KMLDSYITHL PTGNEKGCYY AIDFGGSNLR AVRINVTGTG TMERMQSTFS
LRHATALRPK GLLDRTATAT ELFDHFAKNI GNLMEEAGDV SDPGHYPVGF TFSFPCTMLS
RRNAILLDWT KGFETGRDTT EQVEGRDIGM LMDEAFKRNH VNARVSIILN DTVGTLMSVA
YQKPPGYPEC RMGLILGTGF NICYVEHDYL HYGYIGKVVN IECGNFDKLL PTTPVDFEID
WYTSNSGRGM MEKLIAGAYL GDIIRRNMLL YLREKAPAKM WNIGTFTSID AAEILNDQSE
TFEKAKEIVK ENWDAVLEHH HLAGLRRICE AAFSRSAGLA AAAITATARK TRSCAVDGSL
YVKNQWYRDR LAYYLKVSRS DLIGSVVMYA CDDGSGKGAA IAAAYMFIKW LTIVVIRTFF
MKVTVINEER LPLYGPVILV GNHNNQFIDA ATLIYAVPRQ ISFLMAAKSL ARRMIGSLAR
LAGCIPVHRQ EDLKYAGIGK ITWEDNSTTI RGVDTHFTMD VGVGDKLFFI DEKIGVENVT
SDTELTLQRP ISRPCKDNGE EFVILPKVDL SDTYDAVSTA LRFGNSIAIF PEGGSHDRTN
LLPLKPGVVL MAIYSLLDGD VVILPVGLAY GDSHGLQSNA TVYYGTGITI SKRDVEEFQV
DRHTVVNRIL GIIEKGLSSC MITAPNKDIK GWIDLCGSLY PPERSMVPTN KAFELRKILA
RIFWDHGEDH KTKELIKKLA SYKQMLKNSF LHDDEVWLLR QSLHSATLLF VEQGIMFFCY
CFLALSFFPL WFPMYIISKI LAEQHRQKAL KASVVKLEGA DVVASYKILV LMGITPLFNL
GYGLLLGLYR PMEPRDIEGV TKLLNSYLTS YKIHQVFTDE EVNHAFLPKK DIVYTYVKSE
EGMVTDILSF YCLESSVINN PRVSHIRAAY SYYNVATTVS FKNLMQKALH FAHEHSFDVF
NALDLMENSS ILEDLKFGEG DGLDPNGILG MQAVLVTASY CDQEFIRIGY YTNNCYDDPE
LRECPPDTPI IEKMVRCIID QPRVTRFPIK WDDAELVHSK LAPLMRSLYF RILKVNLDTP
CPLRERNDIC SNIPKCYVGR CQPQEVSPEP TLDGLEVDLM HNPPSYTGYR GGEDWELKGG
GGYRYFGAAK NLPGVQELFE KQEMDERRTY VTRAELYRKI NPDYYGFRDD EDGMLSAAEA
ELERSLMSHG TLVIPQERAR DCIDLLCRNT NIQFVDMNER RLDRPYKKYI QRILNMERMI
RVLTEEVTSL PGTMIVKDRI DDFLRYDKVY RLDQVEESLV KLYEQFEKFK QNDLMLKTEL
EEVMNEYSVM LVALKQLNAS PSPESDSSGS TLAFSNIAGV ISAEDKDAFS RAIFRAMRGN
VYTFFQDIKE KIVFVIYCQS ASGSSTFQKL QKLCNGFQAK TFAWSKSHSH INQRLQELEE
IIRDRQKALN AFKRYFREEI ACLLECPRPD GNSVIEEWSL FCRKEKYIYY ILNHFEGSDI
TLRADCWFPE EEEETIRTCL QAEKSEGRVS ALLLIDHFKP ATMPPTYNKN DVFTSAFQGV
VDTYGVPRYK EMNPTPFTIV TFPFLFGIMF GDIGHGMCVI LAGLFLIIRY PQLRKDEMAL
MILNGRYMIL LMGIFATYTG FIYNDFLSLP NNFFGSPVSF GLDVAWIHAV NEQPMLHSFK
MKLSIIVGFL QMMMGILLKG MNAIYFRQPL DFFFEFIPQL VLMCCFVGYI TFLIFYKWLT
PADYPKPSII ITLIDMCLFK ELAEHDVMYP GQRHVQKVLV SMMMLCIPLM LLPKPLYMWY
QQRRDIFIHQ LIETIEFSLG IISNTASYLR LWALSLSHQQ LSAVFFNQTV LRTLSGESVV
GTTISLFFTS TLFAVITAAV MLGMDTLECY LHAMRLQWVE FQNKFYKADG KPFKPFNVKV
LLPASYAGDL VRVLTERPLI YLSTIERACF DVFDEVDESF NFIQINLLNT CRPTPIRALL
AAKQERFVVV PGIVVQAYRP QHKMKIMTIQ CRYCEHKMKL DVPLWISKPQ IPRTCRYAHN
PYVVLPNECQ FVDVQTLKLQ ELAEDVPTGD MPRHLQLNVT RYLCDKMIPG DRVMVHGVLT
NYNVNGNDST AIGSSYLHVL GIEKLTGEAI SFDLEETNDL VLLATQPDIH DKIFRSIAPA
IYGMENVKKA VACALFGGSR KEVGKDNRVR GDINILMLGD PSVAKSQILK FVDHVAPISV
YTSGKGSSAA GMVRYGLMGE NERKLDFVLG LTLNKMMERR LQTKVFKLGL AKSIHHARCM
IRQRHIRVGK QIVDIPSFMV RVDSEKHIDF ALTSPFGGGR PGRVHRKALS NAAYDRHITI
FSPEGKLFQL EYALKAVKNS NITGLAIKDN DAIAVVCQKK LSVQQGNQDV LLDQTCVTHL
YHITDDIMAL LIGLPGDCMS ILYKSREIAL EYQYKYGCSI PAKVLCEKIA DINQVHTQHA
YMRLRACTGK NAEEDLIREI FGPYGTLEDI FIMKDQNGAG KGCAFVKMAY KEQGLYAIRS
LDGMKQLEGC PRPMEVRFAE SKANKQTFKV SSRDLPSHSE LKRRDSTETA DHKRKLERQQ
RAVTLSQEAI ELDKAGRYSE AFDRYLRALD QWTIVCKYQQ NPVLQDRFYA KMREYVERAE
ALKQMLKLEV KRPHVKWSDI AGLETAKQSL QEAVVFPMRF PNLFTGSLKP WRGILLYGPP
GTGKTYLAKA CATELDASFI AISSSDVLSK WLGESEKFVK SLFQAARERA PCVIFIDEID
SLCSSRSESD SECGRRVKTE FLVQMQGVSE DSDGVLVLAA TNLPWALDSA IIRRFDRRIY
IPLPDLQARD LDELAQCTEG YSGSDVNVVV RDARMQPLRK GLYVGAGSRY EGVSSMIENM
AFHSTAHLSH LRTIKTVETL GGNASCNAFR EHIAYHGECL RRDVPIMVNL LIGNVLFPRF
LPWEMKASKS RLDDRRKQIM SSPDQYITEL LHSVAWHNNT LGLPNYCSES SVSNFKPEVM
RNFMLRHFAP NNCIIVGVNT DIAELSKWVM RAYNEYNAIE PVARNVEKPV YTGGVRYHED
NSPMLHLAVA YQIPGWDSSE LVVFTVLQSL LGGGGAFSTG GPGKGMHSRL FLNVLNKHEF
VESCMAFSTV YSDAGMFGMY MVVAPQASRG AIDVMSNEFR NMLSVTPKEL ERAKNSLKSF
LHMSLEHKAV QMEDIARQLL LCDRVLTVPE LERAIDSVTA LDIQRCVQSM LKGKPSVVAL
GNLAFMPHPE ELLKHFDTGR YVVLFMSDPE IPFVNTNSET ISRCKRCRTY INPFITLDGS
RRYWTCNICG VSNELPNRYI GMDDSVPVEL RKGLIEYMAS ADYMARSPQA PTIMFVIDVS
VSAVNSGMLE VVCQTISDLI GPRTLVGIMT FDTSVHIYQM NSGGSSPNIL MLSDLNDLFL
PLPNGILLNL YESESEILDL LSLLPSTWRN TNVAGSCMGS AMRVAHFAMQ KIGGKMCIFM
ATPSYFGDEK CKDFTTVLGK DNVSVELFVC PQSVNLKQLY HLASLTAANV HHIPLRTHVG
NAKLSDELTR VLTRETGWES VMRVRASKGW KITNWYGHCH VRGSDLMVLA NCHADQTYTV
TFEHEENVVT DKIAYIQSAL LHTTSNGERR IRVCTYAIPI SDNVSQVLCS VDPEAVVLTV
AHLGINSVLG GKLSDARAQV QTHCSRIANS SLNAQSALSQ IVVYTLGLLK SPCFSEDDTR
VYHCMRLMSL PLDHLAVYCY PRLMCISDPP SLKLTHSTLS QDSAYLIENG ECMILWVGKG
VAKILYHLLA FMDGYDIQSL SVCMRAFEIS LGLSPSSLAW MASVEIVSLV ACGTIWGYLA
DFYKIRYLLC IAMNLVGLSA IGIGCASNYA LIMFLRVVHG AAMGCTAPAI QQIVTGATDK
DYGTAFGIIH AVSCFGRLVS AILITSVAIK VFGKIYGWRI CYIAVGIVWI LLGALMAIYL
DSADIFRTWT SIILLFAIFI SDAPFFAAFTY MILYLQYLGL SDLEAGVACA LTLLGGLLGG
GFGGFAVDMC HKKSTRYGRL IAGNAIMLLR LSVTLAFFLP LPQNGLSWYH YVEIILLGSS
LMTVSAIDRP IMGAVVEKKY QASATGINRC IAGILSSLTF LPLAGLLTEM AFGYQKSQLP
IDQLEENVRS TNSDALRKAM MFIIGIGTVI NTMCYIAFFF TYPKDSAETE ERENEVMLMF
GMRSLSDFCN PQLQLYVENA LDALNRNVLP SLVTALSNFG DDEDIIFSCS QILQAMACGC
VEEENQEISQ KFAKDGGAVE LILNQCPQDD VVLAYCYTVL ECGLAGTISV NGAPSMGADV
AVKALACLSS ICATSSGPAK MKQHNGINVM LQLCLKSKVA SVESAMKAGA SLAKAGILDK

TCLDPVIRVI EKYKTSKAVV GYGSDIVKSV VSTAALKQSL NDIQKCQAGT PEHQAAVDTL
RSLSYISSVG EQLAKDGALG LIVDLVKNAT AQLETHAEVM LSVIAGAARI LGTVAVTKSY
CDEVVQKGGV DNLVAALPLC TSDGACVASI ADALAQLLQS GTDAVATSLP ILYQMADQEN
VAISMLGFVS AASQQSELQP AFVANKVVEI LCTCSQYHLE SLAIHMNIMH VFNRFSSHVT
DLSVVYEYGG LQGIAASMSA IDTILELMLE HQKNDAVNGA AMKVLELLAT EDDVRRTMKD
LSRASKEDVD GAYSKIAAVT GLARIARLRP IIVKSDMLKE IMQTVSTWVE GSNFTGRSKL
TKAAMQCAQI CDLACMPQVR RVIDEGGEDN FLLHCTGALA HLCSVDRGYS SEQCKAVVEH
INRVMRKHMD IKNAETQCID ALSHFLQVTG GMDALLSTNT IVAVVGYLSK IPMYLPCQIV
GVGFLLACAK MDYRALECLK QCNTYQLLRA LNRTHKKSRK LKTMVGELLS MIMPPDAFEA
ELIQLLADLE KGMEVNDILA VHTALSSINQ LLVSNECIRI AVRLHVNLEI AQSIYNMGQN
RSGLIAMTKL GTYATALKVW ESVQGPVTPI LEDEACASLD AMTQLFVHDM ANVDAALKND
VLSKVCRGFT LFQSSPNVIR SICRCLAAMC TTDARAKALV ESPEFNKLTA MLVQLLDGDE
NVSLGSVKAI AELLKCNNKL VIDHFAKKTN LVGLLRNIEM YPENSKLVAL SATCLNYFGK
GVVAGRDIPA VLTNITKALN ENKNVATTVL PVLKLLLNMC TPETKQALKS SGVMEVVSDV
MMIHIDDEAI TSVGGELFGY LGAEAQIRAL MRQVIEVQMR QDTMAQEVDS LCTRLAMFLL
SPLENRAEAL ADTEEFLGAL NTCMAYSADN KNLIANATLV SRRLGDAVFN DFEDQFGAWA
IANSSNLQQI IAILNSDYGA SNVKFLCHAY RVFSCCAVNM SNSAQRFVRS ILPYLDNTGS
PAHSVVELEK FLKDNFAKKL NRWSLVKGGT YYLYDHNATM MAFHVGKGYN VENKGIVIAA
GHTDSPALKL EYKSENVAHA FNQPGVLEER LIRIEKPIIV VPNLSVHLQT SEEVLKLNKE
KHLRGVVATE AVHNLPVLGF IAKELGVKVE DIVDMDLCMF DITKSSLSGL YEEFLSSARL
DNLASCFSVL GGFVDFAILA SHVAIVKAQL RFASVGNWGT SKYQKRVAET LKKSIANDRV
TFIVSPGSNF EYGVWDTHFQ SVYRSESMEI PMFTVLGAGD WLGDFNSQIN RNQQAYPRWT
MPNWWYHYYT HFATTASMSG HKDMSVGFIF IDTWILSTAF PYKDVSNAAW ADLKKVLEIA
PKIIVVGDKP IQSSYLLPLL RDAQVDAYIA GYDHNMEVID SNGIAMIVTG NFCIHELGAD
GMETKFINGE TGEVQHVSAL PTVSLYPIGE MASPTQMDAF VKIVGTIGLI IRYAFSGVAY
SVGAENLSYE WCITADDGLL SPDLVVYLIC ITDDNIVERR DLGVNFFIRS SDVEVKSDAC
LHHLQDLNRN VKRVGFIVAD TFGMVGAVFV DFGNEFVCVD PSGKEINTAI VSGISNEEAG
LVYIHTEGSM PFQSGDFVTF SEVEGMELNN GPIEITIKDK ESFTIGDTRG FGQYVTGGIV
KEIRRSKQID FISLEDLSLI GRAEQLHVEK IDEDVLNSFV KNARYRISPI CSFVGGVVAH
EVVKFTGKYH PIDQWLYCDF TLRYSDHIAI WGREIQSKIQ SAKIFTVGSG ALGCEFMKHF
ALLGGIVKIT DNDRIEVSNI SRQFLFRKKH VGMSKSKVAA ISAKEINEHM KIDALELAVG
ADSENMFNDS FWEELTVVVN ALDNIKARTY VDGRCVWYEK PLLESGTLGT MGNVQVIIPH
MTQCYSESQD PQENSIPLCT LKHFPYQVDH TIQWARDLFE GIFTQTAHDL KRIEKISLIA
KLLKINDTNV KTELLQIAAE LVNKYFINDI NQLLYSFPKD HRTSDGHKFW SPPKRMPTPL
TFNPSEKYVS MFLIATANIL ATVIGKKVLV NQDDVAMMMQ FEPFKPKILK LSQDSVEFEK
DDDTNYHIEF IWATANLRCQ NYDIDQCDRM KAKMISGKII PAIATTTSMI AGLVMLEFVK
TICYIEHFRN SFCCLATPLW LQSEPMPPTT TSDKEYDPVV GGAIRALPPN FTVWDKVKIN
IKDYDTQRDK RFSGTVVLQN VPRERMKICV FGDQVHCDQA KSLGIDYIDL EGLKKFNRNK
TLVKKLANKY GAFLASQTLL PQIPRFLGPG LNKAGKFPTQ LTHNDNVMML YSSIAFALEM
ECRFVTNSIF KNIFYVNKLL PFQQIALLRG AAPAIALFLN AICARLFVDI ERLSYFLWHS
SVTTNRVILE KMVDVNYIRS MGPKSTAHVK LNKSIVKILE SMDVSQRKKT EEAASWFERL
KTPAIYFALF VLFKYYWKKS VNYELDLPVT LEELYTGTTK KMKITRKRFS GNTEYKEEQI
LKVDVKAGWK DGTKLTFAHE GDQASPTSPP GDLIFIIRSK PHPRFTRDGN NLIYKFTVPL
VKALTGFQAT LTTLDNRRVT TRIVDVVSPS YRKVIPNEGM PISKSPSHRG DLILEFDITF
PRTLTPEQKK QMIAVFGTYY ICIDCPGQLW YTAQMAKIAL SIEIADDDVD RETTAKKDEM
KNLSEEMHVD TGKTKLLDKI RHSNVQNAEA GGITQQIGAT FFPKKMLDKH CELINFKLKS
PGLLIIDTPG HESFNNLRAR GSSLCDIAIL VVDIMHGLEP QTIESIGLLR GRKCYFVIAL
NKIDRLYKWK TTPWATFHKT FENQLEDTRG EFFERARNIM TELSEQGLNS ELYWENDDIR
RNISICPTSA ITGEGISDLI CLILQLTQKI MVKNITHKEE FRCSVLEVKA IEGLGTTVDV
ILLSGTINEG DKIVLCGLSG PIVTTIRTLL TPQPLAELRV KGEYVKHTSI KAAMGVKLVA
QGLEETVAGT ELLLVEDDDD IEQLCEDVMQ DMSSIFGNVN RTGVGVYVMA STLGSLEALL
QFLTDKKIPV FSVNIGTVQK KDVKKASIMR EKGYPEYSVI LAFDVKCATE AEKEAQVLGV
KIMSADIIYH LLDSFVKYLE ETQEQKKQSR ISEVVFPCEL TILPHCVFNK KDPFVFGVHV
DNGILKPNTP LVAMAKGLML GRVASMEHNK KPVDKAVKGQ EICIKVVGEP NIAYGRHFDC
NDRVYSRITR DSIDVLKEYF RDEMTNDAWK VVIHLKKVFG IILSTKDTSH LREQDLSKLD
VSTLTSLSPE VISRQATINI GTIGHVAHGK STVVHALSGV HTVRFKHEKE RNITIKLGYA
NAKIYKCTNP ECPPPECYKS YGSSKEDDPL CLRPGCGHKM ELKRHVSFVD CPGHDILMAT
MLNGAAVMDA ALLLIAGNEP CPQPQTSEHL AAVEIMRLRN IIILQNKVEL IKESQALQRQ
EEIKKFVSGT AADSAPIIPI SAVLNYNIDV ICEYLVTQVA VPKRDFKLAP QMIVIRNSKQ
CNFIEWRDDK LVFRRYASLY FVLCVDRDAN ELLMLEIIQH YVELLDRYFC NVCELDMVFN
VTKAYHILDE MLIDGNLYEC SKKAVLRNVS AQDALCEKTK FAKDMLDVAD AFELAFKALG
SQHNVDLDKF IEGIKMTESQ LHKTFEKYGI KRFESLNQMF NPEVHEAMYE IQDDSVKNTI
LQVVFNGYTI KDRILRHLDV YMQATLSTLL NNVKGGIIVA THDMELIKNL VTGVVYIHER
DRMYTFNGDF EAYMKLRSEN PHMPKEIITL QVGQCGNQIG IEFWKQLCAE HGIDQEGHVI
NQNYHHDRKD VFFYQADDEH YIPRAVLFDL EPRVVHGIMT SEYQRLYNPE NVFLSKDGGG
AGNNWARGYA TADRVQDELF DIIDREADGS DSLEGFVLCH SISGGTGSGM GSYLLESLNE
KYPKRLIQTY SVFPHLTETS DVVVQPYNSI LTLKRLTLNA DSVVVLDNAA LNRILVEKLK
VTTPSIQETN TLVSNVMAAS TATLRYPGPI NNDLLGLMAS LIAVPRCHFL ITSSSIQKTT
VLDVMRRLFQ TQNVMMSAPM KDGKYMSALN VIMGDVDPTE IHKSLQRIRE RKLVEFIKWN
PASIQVALSK HSPYQQHKVS GLLLANHTSI AGLFQRCIQQ FDKLYSRRAF LDNYKKEAMF
QGNFEEMEHS RDISQLLIDE YKRAEQDDFF GLARAFAIPV RSYTHEVVTL WYRAPDVLMG
SKKYSTEVDI WSVGCIFAEM INGVPLFPGV SEQDQLKRIF KVLGSPNVGT WPGVVDLPAY
NPDMDQFEKQ PWNVIVPKLG VDLISKMLQL DPFQRISARD ALCHEYFMPK EKTHINLVVI
GHVDSGKSTT TGHLIYKLGG IDKRTIEKFE KESTDMGKGS FKYAWVLDKL KSERERGITI
DITLWKFETT KYYYTVIDAP GHRDFIKNMI TGTSQADVAM LVVPAEAFEA AFSKEGQTRE
HALLAFTLGV KQIICAINMD KCDYKEDRYS EIQKEVQGYL KKVGYNIEKV PFVAISGFMG
DNMVERSTNM PWYKGKTLVE ALDQMEPPKR PVDKPLRLPL QGVYKIGGIG TVPVGRVETG
MLKAGMILTF APNPITTECK SVEMHHETEV AYPGDNVGFN VKNVSTSDIR SGHVASDSKN

DPAKAAVSFT AQVIVLNHPG TIKAGYCPVV DCHTAHISCK FEEITSRMDK RTGKSLEENP
KTIKNGDAAM VVLKPMKPMV VESFTEYAPL GRFAVRDMKQ TVAVGVIKSV EKKEPGLPVL
LLKEGTDTSQ GRAQIISNIN ACQVVVDCIK TTLGPRGMDK LIHSANGVTI TNDGATVLKL
DVAHPAAAVL VDIAKSQDDE VGDGTTSVTI LAGELLTEAK QFIIDGISPQ VIIKYFRIAC
ERALERIESL SIDIDSKDEA TKRSLLIKCA ETSLNSKLLS GHKNFFAQMV VDAVMLLDSD
LDQDMIGIKK VTGGSCSDSM LIKGVAFKKT FTYAGAEQQP KKFIDPKILL INIELELKAE
KENAEILIKD PNQYQSIIDA EWTILHDKLE KIAKMGTNVV LSKLPIGDIA TQFFADRNIF
AAGRVEQADM IRTSKATGAL IQNTVNGIST DVLGTCGIFE ERQIGNDRFN IFEGCPKTTT
ATLILRGGAQ QFVEESERSL NDAICIVRRT TRTQKIVGGG GAIEMELSKA LREYSLSVAG
KQQLIISAFA RALEVIPKTL AQNAGFNATD VISKLRRDHA LSKDVNWFGV NCLNGDIVDA
FQDCIWEPAM VKKNAIYAAT EAACQVLSID ETVKHASRAL DLPQCFRDLL NTDKIKQVLC
TGNVGSQQMK DLLLGISPNL HMVKGDFDQD TTLPEELIIH VGNFKIGLIN GYQLPSWGDK
NAVYEYAKNR DVDVLVYGHT HISDVSKISG KILVNPGSAT GAFQPWAPNA IPTFMLMGSK
IVIYVYEEHE GQANVVMSEV DQVSQSYPNS CGSGYSPIYR CPKYMDKLLK DVHDCLGSRE
KADGSLGEYV FKSYKEVESL VQRFGSGLGI YASNCVEWLI CEQTCNGYGY TIVPIYDTIG
EESIIHILEN SDINIVVCDP ACAEKLARVL PATPAMINTI SYTSGTSGIP KGVILTQGQM
ASLIVVVNHV VGELNDAVKC YLSYLPLAHM YERLYIDSSL FVGGKIGVYS GDVRNILDDL
ETLKPTVFVS VPRLFFRIHD KVFANVSRKP WFIRWLFNTA LNSKRNTGNC KHRFWDKIVF
KRFPALFGGN VRWMMTGSAP LAPRTYDRIR AIFGTELLSG YGLTETAAVM NRQGETDTTH
VGGIIPTLEF RLKSLPEFEY SVKDENPRGE IMFRGEHVGY FRNPEATAED GWLLTGDIAE
LLPNGAIKII DRRKNLFKLV QGEYISPEKL EAVLIGCALI SQAFVTGKST EVYPVAIVVP
DETEAQYWAE SQGHDMTLQD ICQHPVFKEA IMEQMAQAYD EANVKGFERC KQIYIEAEPF
SIGNNMLTTT NKLRRHHAKK RYEDIIDSLY GQPIKHQITE KATIMEVFEK RRSSIGIFIG
GNKYTFANYD DDCPVGDYTF KCVSAAKNKG GAHLVKTPGG YIVICVFDEN RGQNKTNMLN
VLSIVYMASR EVGVCRSLDE LTIYEAKISQ RDLSRAIGRM KKLLPQRGNA TVEDSAQIIP
SEQSSNATKD ITPYIASRRY HIFWDQVKPY FTNPSLITVE ALFVLLTLCN VYVLHTLFVT
VIAAPVFITW WQLTQGLVTA YLLGLKTLAM PTVAYVAMLC STNIMLCKAP STAAFPILAS
GAVAAHHAAR FIACGEEYMM RWKAIGFLLL AFVIGATDKH IAPGNIITVA FIYAFLAAVF
RAGFMERALH IVGGRGNALH NHQHFLGAMI LPFVFLVNGE LKVLNTLPWD ITAARTGCFV
AVGALPFVKN VVSNRLIRQT GQAPWRMLEL IAVALLFIIG SVKQSPSWQV VLATIFVIVG
RFLGAVDVIR NKEKVIEGFN EASYALSNAV WSAGDFKSLV VESVGRSAVT LRVRTENVAG
VIIPHFELKI DPTVDVIANI GLTTGGHVIH SVKTAHLEFL ETLAELASLQ YQSMFDWLNV
YAETFYMLFD GQEPVTRINL MRNSAVALEN DNPYCSANDS KVIISKNEHL SGIICKKTVG
TSSGSLIHVL WHEAGPDRCK DFLTTLQKVV NNWLVYNGFT VSCSDIMASE STLTQVAEIL
ERSKKEVQRL VGLAQRGKLK CQPGKSLFES FEARVNKELN EAREQSGTIA AKSLDERNNI
LAMVNSGSKG STINISQIIA CVGQQNVEGK RVPFGFRDRS LPHFIKHDYG PESRGFVSNS
YLSGLTPQEM FFHAMGGREG VIDTACKTSE TGYVQRRLMK AMEDIMVHYD KTVRSGGGDI
LQFLYGEDGM GAEYVEDQTL DLMKLDFASL NRLYAHDFRN ENYGDFSKQV ILVEEYQRIL
DMKAILCKQV FPDGEARQHL PINISRLLEY AKTQFPNPVD IAQRVQQLLD SLTIVVTSGP
HDILAAEAQE NATILIKAHL STALNSRRLM EREKIGNLAF DWLLGEVKRI FYKSICHPGE
CVGAIAAQSI GEPATQMTLN TFHFAGVSSK NVTLGLPRLK ELINVVRNVK TPSLTIHLDR
GVAHDQERAK DMQTRLEYTT LDKVVALSQV IYDPNVSQTI VPKDYAWVRE YYEFPDEDMN
RLGPWVLRIQ LSNKVMTDKR LTMKEIVDRI YQEFSNDEID CIHTDDNNDE LVLRIRVKYS
EGEFLQRFMS QVLVNVKLRG VSKITKVYMR EEARTKYNET NGRFERVSQW VLDTDGCNLE
DVLSIPCVDA QKSISNDISE IFHVLGIEAA RMALLRELRA VISFDGSYVN YRHLSLLCDV
MTQKGHIMSI TRHGLNRADR GPLVKCSFEE TLEALVDAAV FAELDLLKGV TENVMLGQLC
PMGTGSFDIM IDDEKLRDAN QNQIMPDSAL SGFTSPESSP LPFSPSYAAL VIQKAIPGNI
RRAEHFISFL RTVVGYLKQY LKVQEPRSEG PLMFLHRFEQ ETGIAYSTLQ YTYNRMKSLL
NTLSITGDLS SIQLVADFCT LVGTYTTGFI VIVEPYPQGS LYEPVIQFSC LDASIAMQPV
VENFQSVILT SGTISPLEMY PKILNFTPVL TQSLPMSLDR DCLCPLIVAK GANQLQMSTR
YELRNDVTVL RNYGTLLIEL CKHIPDGVVC FFPSYAYMEL IVSHWYECGI IASIMEHKLI
FMETKDVVTT TMALHNYRKA CDVGRGALFL SICRGKVAEG IDFDRHYGRC VILIGVPFQY
TLSRVLKARL DFMRTKYGIM ENEFLTFDAM RQAAQCVGRI IRNKSDFGLM VFADSRYSRA
DKRSKLPPWI LKNLEPGNMS LTTESAVTAA KVLLRNIAQD YVSSRLTRNQ PPCEITTLKN
GLRVASVWMP GNSTTVGVWI DSGSRFETKE TNGAAHFLEH MIFKGTKNRS RLELEEEIEQ
KGAHLNAYTA REQTGYYARC FNKDVPWCTE LLSDILQNSL IEPSQMEAEK HVILREMEEV
EKSTEEVIFD RLHMTAFRDS SLGFTILGPV ENIQNMKREY LVDYIQKNYT ADRMVFCCVG
NVEHDKVVEL AEKHLKPYFV GSELLNRNDD MGPHAYLAVA FEGVSWTNPD SVCFMLMQSI
IGSYKKNQEG IVPGTVHAIA NRMTVGCAEA FSAFNTCYKD TGLFTVGIWQ GRVAFIWQRI
VRNSTLNPCP FRTCVIDFGE SLKSCGILPG YLRPLLILEL GVRLPYYNMS RLFDSLLNIA
SSTLNFGYTF TGKLGIRRKH QVRETAQLVL LISVNDDSDI LERPRLSEEA DELSLAEQCL
LLCHALHMLK STPESDELNL EFLNAIVVRC LATTSWLLTS VALWVRCKTE YHRTKTVERA
TLQLYKLSDA YYEPSAAPGA RLEYIWNVWY PSAWGIKREI ARRMSSIGSF LTAFEIYKQL
HMWEDAIQCL IIVGRKKDAL ELVNQQLKTA PSALLWCFLG ISHYKTAWEV SKHRCARAQR
TLGSYYFNKG DLDQAIASLE LALSINPMRE SSQFMLGCCY LKKGSLERAI SVFARVVSMN
MCSAHLNIGN MKEATICIEQ AVKHNGNKWE FWDIRMRIAL RSRDIQNVCF AMEKLISLGK
KSAIDPLMVA FLVDASTKFD DNGDIWSQCA RYFGFQKCYL EALECTFREY RALEVKHLWK
NKCFVGQPAP NFRCEAVMPD NSFKEISLSD YAGKYVCLFF YPLDFTFVCP TEIVAFNDAM
AQFEARNVQI LACSVDSKFA HVTWRNTPRD KGGIGNVMFP VLTDITKTVC DAYEVLIEEG
VALRGLFLID KKGIVQHLQN NLPLGRSVTE VLRIIDALQF YEKHGEVCPA NWKAGDKGMA
ATTEGVIAHL TSKMEDNTAE FLSTEATFRD EPMVEDLSDL SSKTLAIVEE GLEIIYEISK
ILNTGLDRET LAILVGLCEK G

> ***Plasmodium gallinaceum***
NFQGDFKTTK KKIHWLPYII EKLITCTLYE YDHLIDKNDW KNFINYNSKF ETTVYADPAI
TSLKVSDKLQ FERRGYFIVD LIKIPDGKSK KAGLNAYEEP SIIFPTLIFV GDDAIYHESE
LSIYRPIDHG HISDWDMAQK IWDYTLNCID KNKNAKDILL TEPPLCSTSH RTKMGEMFFE
YFDFLNLNIS VSGLMSIYAA GLTTGLVLDV GEGVTQCLPI FDGYIEKNSV IRSDFGGEEL

SMFLQKLICD IGYSMTTRKN FEYVKSIKEN LCFCSLNPSQ DQLRDDLSVT YTLPDGDVLR
DGYDTIEISH ERFYVPEALF NPQLCHRDSL SIVDIIWKSI LLCPIENRKS LSSCIVLSGG
STLFPNLVER IETEVKNNAP ANARSAVKVV TYEQGANFAK ENNLFFAETS AVSKLNVKHI
FESLLQMEKS KLAKVEKVLG RTGSRGSVIQ VRAQFMGDTE LSGRFLIRNV KGPVREGDIL
ALLETEREAR RLRGTQSLNL KKSHCYCHLS TGDLLREAIE KKSEIGNKVK DIMSEGKLVD
DDIVLSLVDE KLKSPQCKKG FILDGYPRNV KQAEDLNKLL QKNQTKLNGV FYFNVPDDVL
VKRISGRLIH KPSGRIYHKI FNPPKTPFKD DLTDEPLIQR EDDNEEVLKK RLHIFKNETT
PLVNYYENKN LLCPVCYFNL PDPESTLAPY DTELNYFMWG PGFEWQPFNE KSKNGKISVE
NASVNARKLG LAPSSTDEEK IKEKYGDNLT YDQYLEYLSL CIHDRDNTEE LVKMFAHFDN
ATGYLSKKQM KNILTTWGDA LTEEEATDAL NAFSKEDKIR LEKIFHIIDK NNDELEIWSN
FVKNEVFLKQ VQVEMKQIDS DKDGFISLAE LNDAFSQNLK EVEKHSEGLL KRFQIVDKDK
DSKLNINEVG LLIDPVKDEE LKELEISEIL DHHDINKDGK ISKKDDDVAL DDFNFDSNKD
GFIDKEEIIS YDLWNEKALK IAVTSLTDYG DIIRFPEDFK LLSNLNCFGS GFIFSIIMFH
LLPEFFFVFI GFCMQLALEY VLPTDRHICC DSSSSEEEER VIKSYEGKRL DFYENMGNSI
NENMNNNEFN QLLKDYENLY KFMIKESSEN IPKFAIVYLD KLSKYVEKIF QNNVEKKNLS
KNKAQTLNKL RAKIRKCSEF YQERLNAYNE NPDDFWSYSD EEEYASNEED DKTKSAISKW
GLKTSEKVEK KKTIKKEEKT VHIDEVQSSK NKGYLDLLNT KNLTEDVIRK RVKLVIEKRG
RKGLDKHEHI NILSKLCEIA KTISTQSYIE VLEHLINLEF DVVSSVYTYM SFNIWNKAFK
YIELILDLLI QNDNFYLVSI NLAEEITEES TNEKEKIIKS CKTLISFLAK LDDELLKALL
YIDVQTEEYR KRLGKTIHVI GLLHKGYNYV KAIYISSRIL EHMYYKSETI FKQIWNFIEK
TLEQERAEKR RLLSFHMHIS IELIECVNNI CAMLLEVPNL AKNSYESKDI ISRQFRRFLD
MYDKQVFNSP PENNKEIIIL ATKYLQKGNW KLCCEKIFGL SIWSKFTDKV QDILKEKIKQ
EAMRTYIFRY IFIYDSFSID QLCLMFDLSQ NTVHSILSKM MINQEIPACW NESSQFILIN
KINPTILQSM ALKLAENVND VMEQNELALN MKNPKLGLQP KQCIVVGTRA LEFLNNELNN
IKTLSELYER VKKRSIMDNN DNILSLYLLE LFSIPIEQKE KNDFMTNRIA MYSKILNGRK
NVLDLLLNKF ENDCNDSIKK ELIDCFDVEK FKQEVNNKFM NILIQQLRKV EKLEKKKAKM
EFYLSIREQI KLYIRELVNI ISNLLTGNYP ILNLNGESFL KKYGGTLMDN LRDGLVEGQL
VRFILAKEES NMFGIIQNSS TDVKSKNILV TFYFRNSNTE EQVQIKSVEK DRLPVETLNC
DASFLWYKMV RDDGWVGFDK AEIIKVFNNN SKIKDVLIRH SDLKILNQLA ITQFWAIKML
ISVPLATKIY AHFVHDSIKI SILTVLGDIA TALNRCFSRY LNFFANILTE TSKITISSGP
PESDDWVNYV FELRDAILLT YSNIIYALID GNEIAKLKTY IPNILDLIEL ILVKEINHFN
AQNFQNAVSL LGDLVHAYGY ELIENSKLTD LIISVYGKID ILSSQGDECV LKIKWLKRIC
NISILQLEFK SVEDLNNATN NFIKAIKNYN VFPELRLKIL QLLYNSFSVN FSFFIAILQF
SSQNNIFHNI LPYIKFIDQW IKEWNISSRE KRQIYLIIAQ ELKKLKKYED SFKHLKKHIY
YFQEILNHPN TINASVELIV DSINLNSNIY FHEILNLDAI QNLQNIEHQP LFELLVIFYK
YGIHEFLTFI NLEACENKIY LLSIISLFKD IKIQNIQYIS EKLNITPLKI EQILVAAIGS
GVIDAKIDQI NKTIQMKTTI LRHFDDTHWE LLNNQINKYI NNVATSFYKF IDSTASSTRA
IQNNPNYWCS SGNHSKDEEI TWIGYLNTKG FIKGVKISWE YCPELVKISV SADGENFKTV
IPYKRISGNE ASFEEIYFFK KLEEVISIKI GLKNPIHKYF GIREVKIIGG GNPYFLLLSG
ITSENEMCLQ ELWKTNSNNQ IISAFSDPPK CLSVINLDNL GDGKSNWIFE SNSQIRLQLC
ISQKNIYGNV PGIHDIDVSI DSNSILDNDH NPDNTIDGNL NSYWASATFA DNEHLVYLNL
DLNKLVEISR IKIYWEYPPL HYNIEVSSDK VIVENLANPS HITIDTLKNI ETRYIKISMI
KPHPNHGKME DQFLYGIRSI EVQANNLETV IEYCRDAANS DDARDKYFVE YITEFDKDLT
NKLINIEDDV SKNVNSISNN LSKLEELLPS IETCLQEKKG YDEELKEFKE KANELIKAKC
SSEPLRVYCD MTSSTSIYIW NGINSVDDIR YHCAEVGLEP LILRSRSQLN SLILALKKIG
FVLNGKINIP LAYDYSCFHD LLNGNIDLTT LIYESPDSTK IRQTALEEKK MVFCKIEDIA
KKRNKRYVKT QVLSEDSKIE RHKMKKFYNS KSGYFGCGWK MEWTPFIHAP FFDNQHNAIY
KNRNKKLYEE IDTLLHGRIH PDIRIVELKD HKHPVRLCTP HNEDCYSIVY IGKKINATDD
RIIFGEYTGF VNNRELSQEK HQYMFALTFI ILPDNYTYAV DSSYMFNEMS LVNHYKTCFN
NYDFRINAEW QLVYLDGWPH IILTSIPGIE IYPGEEIFAD FGFEWFERIN DTCLNEFIKN
NFEHRLDDIV EKYNLLKNNT TCNICMHNVN IDGNNFITCS GCNNIYHLKC VHKLNNENYD
WFCSSCIQFS LNLAKAIICR ITKMHFHSNE DLFKSSSECI QSVIRELALG KTKFQREFTN
GTFVGTVTKH IDNNNFFVIT YEDGDVEWIT PCFLFQEEHK LKGNEFYKQK KFEEALKEYD
EAIRVNPNDI MYYYNKAAVY IEMKNYEKSI ETCLYAIENR YNFKADFNKV AKLYNRLAIC
YTNLKNYDKA IEAYQKSLVE DNNRVTRNAL KELERIKEKE EKEAYIDPVK AEEHKNKGNE
YFKNGDFPNA KKEYDEAIRR NPNDAKLYSN RAAALTKLIE YPSALEDVMK AIELDPKFVK
AYSRKGNLHF FMKDYYKALQ AYNKGLEIDP NNKECMEGYQ RCVYKIDEMS KEKVDEEQFK
KSMSDPEIQQ IISDPQFQII LQRLNENPNS ISEYLKDPKI FNGLQKLIAA GILFFLDEKI
PSKYGENRHW NVDLIPKFIL VGGNLVKILK KTRVTNYLEW LVVEGSYVYQ HQKKGLLYSE
KFIHKVPATD MEALVSPLLS LMEKNRCKNF YQYVSEWDSN NKSTWKNLDP FKLTMMDIYK
YFNLCQLTID FLGHAVALYL NDDYLKEPAY VTLERIKLYM QSISAFGKSP FIYPLYGLGG
IPEGFSRMCA INGGTFMLNK NAVDFIFNDK VCGIKSSDGE AYCDKVICDP SYVHLENKIK
KIGQVIRCIC ILSNPIPETN QANSCQIIIP QNQLNRKSDI YINLVSFQHG VTLKGSIDVR
LDVKLNKFIW SKGIRNPPKR VRVKLERKRN EDEDSKEKMY TIVQHVMVDS FKGLVNEQMT
GDRHRIHDCT TENFHGFIAI TNPNKSWMAQ YNDLSKYVPG FYALQIVGEL RAGTGYLILS
LYEMLLKRNK KLELLYCIDI NEAANKVSNV EILNNNLFNN MRKCFDLVLF NPPYVPTEED
EMNKTIVASY SGGKLGREII LKFLLNVYDY VSNKGVIYLL LEKNNISHEI ENSDLPILCE
TCLGENPYVR LIREENGKEC KICKNAFTLF RWKPGQKARY KQTVICGMCA KVKNVCQTCL
FDLEYNLPVQ VRDKFIENAI TLPENETNRN FFLEQMELSK LKRYDPYFKR NMARVCSFWR
NNSCNRGSEC PYLHKEIHKI IIFNLPPTNE HDIKDLCEKY GPVVDVYAFV SFMFPSACEK
AKENLNNAIY KGKILYVKYA SYKKILEIQR KRNCQNENIW NILYTDINSN IYNFCKENKC
SRESLLDKNI AVNVSLTETY IINKMKEWIK KEGIRSNDII IIKNLSMNTN QNDIINLFKK
YGTLKKVSFS PYNNLAIIQF ENPENAKKAF ISNSYIRYKK LPLYLEWAPI NLYDEEITHA
SIYIKNINFN TKEEDLKNLF KNLEGFLTCN ISLGYGFAEF KSKEFAIEAI KKLTGTSLDD
HILELSLSKL VVKNLAFQVT KEELRKLFSA FGNVKSVRIP KNAYNRSRGY AFIEFMSKNE
CLSAIDSLQH THLYGRHLII DFAPKNARIL KPLIQEKIVE IMKPEIEEKI IEVPQVQYVE
KVVEVPHVIL QEKLIHIPKP VIHERIKKCP KTIFQEKIVE VPQIKVVDKI VEVPQYVYQE
KIIEVPKVMV QERIIPVPKK PQYRHIPKPV EVPMAHYRTF PVEKLVDRNV PVPVELQIVQ

EFLCPKIEAR YKEIPVPVHV QRIIEHPIPK DAMNNPHLLP LYYLGSTAVG ICVNDGVILA
SERRISSALI EKDSVEKLLA IDDHIGCAMS GLMADARTLI DYARVECNHY KFIYNENINI
KSCVELISEL ALDFSNLSDN KRKKIMSRPF GVALLIGGVD KNGPCLWYTE PSGTNTRFLA
ASIGSAQEGA ELLLQENYNK NMSFQEAEIL ALTVLRQVME DKLSSTNVEI AAIKEKTFYK
YNSEDISRII DILPRFKIID KDFISIKLLE LFQIDGFQKQ LDRLSDSLSK IQKALGEYLE
KKEANFQDFR KVSQTQLTDA CFLTLTQALK MKLGGNPFGP AGTGKTESVK ALGAQLGRYV
LVFNCDESFD FTAMGRIFVG LCQVGAWGCF DEFNRLEERI LSAVSEQIIE LLNKKIGLNK
NVGIFVTMNP GYAGRSNLPY GVGFKMAYAR ISSSCAIMSR TVNTIGIGLL SLELMNHCDA
KELATPLCMW KLPNKELINR NIANKSEHRH HQKLLMSYTP FNSPSLLAEQ INILGTYSGT
RLLYFPLWDH PKDSIDYCLS TYLYWLYLRR NTNIFLQNTL LRGQVVVIAA TNRQNSIDPA
LRRFGRFDRE IDIGVPDDNG RFEILRIHTK NMKLSPDVKL EELASNTHGF VGADLAQLCT
EAALTCIREK MDVIDLEDEI IDKEVLESMC VTQDHFNMAL GTCNPSSLRE TVVEVPNVKW
DDIGGLDEVK NTLREMILYP IDHIMKSLYD SNFFKPTEIQ FKTLEHSINY KKDVIVISKT
GTGKTLTFCL AILNNILILV PTRELALQIL NHFSFINKYI NLYITTIIGG LNVNKQKRII
SKKPEVIICT PGRLRYLACD EVDKMIESSF INDINFIVKR LYVQTFLLSA TLLSKLLNSI
IIRKEKSFFI NLLPDGLTLN IVKCEKKMVL IKLFYLLKLY KIIIFVNTIN STKELKMIFR
FLFFDPSLES AIPKKIYSIH SKLKLKERIA NITKFSNNKS VLFCTDVLSR GIDLDKCDLI
VQLNCPITDI TFVHRSGRTA RNFKKGNNAR CVEIKSAELV LNSDELKRIS DPRYGTIDNK
QICSVCFENC FGHVGHLEFV LPLFNPLFYK ELQELLNLVC YNCYNFCCSY NYEEYLILSK
TIKMHMKKSS KCYYCKFHRN ISAKISQKRD TITIRLFSFQ VIDILKKIFN KEIIDLLYPF
TKKDGYKIFF LYDMGISANR FRSRYRGMHK RNNFLNLCLN AKKEIDFDSL LELQIGVNTF
YDIREILDKK EGILRKNIMG KRVNHCARTV ISPDTFIETN QVGMPLEFAK VLTVDEYVTH
HNLEYIKKLS LYDFILNKDA DFRHLSFISD FMTGELCMDI LKANWSPAWT IQSLCRAILF
LFTEPNAESP LNCDAGLKLQ GVKVKSAAEV GRRAVEYFRG DDFFNFINTN KDMLKKKFPK
LFVNRNLIDS KDIEDFADIF IQKGYIYKAQ YKPENGIYKR PKWPKRLIMT SKQNFDKTSF
YILVHERNKK LQYVMLIGLI TIVLICCMFW HLSVVFITII SVIIVGRLCT FVFFWFFGVD
YWIFPNLFDE DCNVIESFYP IQSWVYRKDT WLLFVARIFT AILLSIGIHQ LGKTHSISDI
RNFATQSFID ILEWGNKKLA EENYDCLKKC GFQTFEELVR RCFLKCECMT LADSFLKDLE
DLELEERKIS ELLYNIEKCI ELIIKIDTEI LNIHKYVKDI YSTKFPELDS IVYSPLEYIS
VVSKIKNEID LTNIDFSDIL PNTTVMAITV ASSTTGIKLS DHLLKNCLSF CSEALELNEN
RKMILLYLES KMFLLAPNLT MLLGSALTAR LISSVGSLKN LSVTSSQNII VVGGSKKGIL
SSSEIVQSVP DSYKKKAIKL LAGKCSLAAR IELLTEERTV LNKKILNNSV ILQIAIRVKR
DKYIGRHYRY FIRNTRVRAY KQFLEPFKNV TLKNMAYAFG VSEEFIEVED PYEAINEAVL
NFENKDEILA SAKIVERLVE YPEVARNLDK IKAIDPLLKL LNNHILESVL QILSLALSNN
PDLQESVFKK NALKTLLIKL QESQKTLIDK KLITAISALI RHHDEAENKF IDYGGVGFLV
YGMQTNIYKY QEKSALLLKH LVHQNKITFE IFIKNEIMKG LICLANTGIQ YGETTAELFL
AIIQNHRHKL AKSGLHNLKK LIEDRLIYLF ENLGIEDWLI KISKSVQINY PTKIQQLCLP
LIIQGKNVIG TSETGSGKTI CYCWSILQEL NKNFFAIFAL ILLPTRELVF QIIEQFLLYG
SKIGVKVLSC IGGYSLIEQP HIVIGTPGRF KRLKFLVLDE ADLLLQKCFE SKLKIILDNL
QRRTLFFSST ITHSIQLLIK SFPNDNLMLV NANKNQKPVK NLDQRYGIIF TANSYKCQLI
YSVLNILFSV ECIHSSKEQK KRISSLSKFK NGLCKIMVAT DIISRGIDIP RVAFVINFDF
PNDTIQYIHR IGRTARANRK GLAISFIDKK DIKNFKDVKK IMKDKLKPYI LDKNEVLKDM
FKIGKAIKKA EIMLEEVVIF PVSVHYEYTR KLLDKKFSMG IQNTSKYGNG SYTGEISAEI
AKDMNVEYVI IGHFERRKYF FEKDEDVREK LQQCLKNNLK AVVCFGESLE EREQNKTIDV
IKTQVKAFID LIQNFDNVIL AYEPLWAIGT GKTATPEQAQ NVHKEIRNIV KENCGSKQIR
ILYGGSVNTE NCASLIKQED IDGFLVGNAS LKDTFVDIIK SAKDINKDVV HRYGPNTFKL
HRLPIPKLGQ ILGLVGTNGI GKSTALKILS SKLKPNLGKF NNPPEWRDIL SFFRGNELQI
FFTKLLEEKL SPIIKPQNVD LIPKQIKGNI LDIIKKKDKL NQKDKYISEL DLEHLLDRNV
EDLSGGELQR FALLISIIQS TNVYMFDEPS SYLDIKQRIS MAKIIHKLVK HDNYIIVVEH
DLSILDYLSD YVCCLWGKAG AYGVVTCPFS VREGINIFLD GFVPTDNLRI REESLNFKLE
EKKRLHFYNY PSIVKTLNSF TLTIEKGIFS ESEIFVLLGQ NGSGKSTFIR LFAGLIKPDN
LEFLESLSVS YKPQQIQAKF TGTVRQLLMS KLKGLYTDPY FNNEIIKPLK IDSILDNQVL
TLSGGELQKV AIIVTLAKNT NIYLIMSILC TISGQTPEEP VVSKTGYIFE KRLIEKHIIN
YGICPVSGEL LTLQDLYPIK NEKIVKPRPI SASSIPGLLS IFQTEWDSII SEMFSLRTHV
NDIRNQLSHS LYQYDAATRV IAKLLKEKNS YKEEIDNLRN QIFQLKSGND INEFEIGITD
DLLNEMQTTA KNLLMKRKKR KIENVCSVDL WKEFKNSNEF NIHSSVVAGV TCLSLDFSGG
KDGNIYYVSL SDNKILSKLQ GHLKKINSIV SHPSNFICIS GSNDKTIRIW KGDKHKDNIT
SLSLHPLENC FISSSKDNTW ILHDLETSKT IKICKNNPSF KNVSIHPDGM MLGVASDDSN
IHIYDIKSQE YKASLFSENG YYLASCSKDN TVKLWDLRKA LSFQTLELEH TPKHITFYHG
NIKDGLFHGY GVLIYSKNEK YEGDFVYGKR EGKGKFTYAD GATYEGEWID DKIHGKGIAK
FISGNVYEGE WENGKINGFG ILSYNNGDIY EGEWLDGKMH GRGTYTYEDG DVYIGEWKND
KRHGKGCVKY KGNENKIAET YEGDWVEGKM QGKGIYTFAD GGIYEGDWID GKMEGKGIYK
YLNGNVYEGE WSNDMKNGYG VLTYVNGEKY EGYWKNDKVH GKGTLTYSKG DKYIGGWKYA
KKCGEGELIY ASGDRFKGEW KNDQANGFGI LYANGNKYEG EWIDDQRHGY GTFSCKEDGS
VYSGYFAYNR REGKGTLTFV NGNILEGIWN LGVLTKVTNF KLSPSSPWND ADLEMETLYD
LGNKMIDALQ KENITAGDVI SIDKSTGKIT KIGKSFARSK DYDAMDPNTN FVQCPEGELQ
KRKEVVHTVT LHDIDAINSR TQGFLALFSG DTGEIKNEIR EHIDMKISEW QEDEKAEIVP
GVLFIDEVHM LDIECFSYLN RALESEQSPI VIMATNRGIT HIRGTDYKAP HGIPLDLLDR
TLIIPTYPYK HQDILKILEQ RAEEEDVEID EYAKELLCKI ASESSLRYAL HLITLANLVS
KKRKATEVTV QDVRRVYNLF IDVKRSTQYL IEYQNEFMFS ELYSNLSDFW TSDEDDEDEY
IRKKWVIEDD VSNFSKNDML LNYDFELDDF QKRSINHLNN FKHVFVAAHT SAGKTLIAEH
AIALSIKLQK KAIYTSPIKA LSNQKYNEFK NIFKNVGIIT GDVKMNVNAN CIIMTTEILR
NLLYLNDKII NNIHCVIFDE VHYVNDEDRG VIWEESIIML PDHVQILLLS ATVPNYLEFA
DWVGFTKKKE VISISTKKRP VPLLHYIYAY DSIFLIMDEK NFYSSSFKEI YEASMKTEIQ
KLQTLIKKLE QDNKLPVVLF CFSRIKCETY AKCMPHLNFL NNKEKSKVHL FIKESISKLC
TRDRELNQIK NLSKLLEKGI GIHHSGLLPI LKEIVEILFS KGLIKVLFAT ETFAMGINMP
TKSVVFTSIY KHDHLKKRIL TSSEYTQMSG RAGRRSSDKY GYVYIFCCDN IPDQVQLTEM
MMQKAVSLKS KFKVTYNMIL KLLINKQINI EKMLFSSFLE SCRALQIPLF KKDLKRKKRL

LQNIKEVECI YIENYVQIDY KLKLIGLDLH KKLICIENIS IITNELDRLI EKENFEPIVL
SKVLKALKCE FYSVLHYELV CKKNDCLNDI ENIEKNINAK SLNLYEDMEG KLNVLRHFSF
IDDQNNLTIK GKIASYITLT DEITLTQVIF ENLLNKLNPP EIAAVLSCFV APEKKIEESP
DLTVNLQDVK ASLTNIHSKF EEFYKVIRLR VSSEDHWKLC NFKIMFIAYK WALGASFAEL
LEQTELEEGL IVRSILRLDD LCRKVKIAFL YLGNIELAQK VEETSNLLRR DIIFTTSLYL
DALALLQFFH WVNEKKKSKE LFNETEISLK NKIDYFRSTK KNFPSFATIS AIGPNAAIIH
YESTESSNTK ISPNIYLLDS GGQYLHGTTD VTRTTHFGEP TEEEKKAYTL VLKGHLHLRK
VIFASYTNSL ALDFMARSYL FKNYMDYSHG TGHGVGLSLN VHEGGCSISP VSGTPLKENM
VLSNEPGYYL ENKFGIRIEN MQYVIVKKKK DNTEYLSFND LTVYPYEKKL LDFSLLNQEE
IRDINEYHDN IRKTLLPLLK ENPKKKVVIE KDPSFIKERL EKYNELKEKI NIELLDGSIR
NGECYVTTPF QIASSISKKL AEDSIVSKVT YKEKVELELC DLWDMNEPLI GNCKIEFFWH
SSAHILGSSL EKLFGGYLTI GPALNEGFYY DIFLGDFSIS NEHYKKIEEE FNNLVKQNVE
FEKLICTKDE VLELFKYNPF KLELIKSKIP DNKKTSVYKC GNFIDLCLGP HIKNAGKVKA
FKVLKNSAAY WLGNKNNESL QRVYGITFQK KNELMEYIKF LEEAKKRDHR NLGKKLNFFF
FEKDTSPGSC FWLPHGAKIY NKLIDFIKKE YRLRKYDEVI SPNVYSCDLW KTSGHYQNYK
ECMFIFNVEN KEWGIKPMNC PGHCLMFKQL NLSYRSLPIR LADFGVLHRN EITGSLSGLT
RVRRFQQDDS HIFCSYEHIK KEVLDTLNFI FFVYIHLVLG IEILQNRGYD SCGMSTILKT
TKFSSTSTSD AIDKLKCNYM TNHVNDNIGI AHTRWATHGS KTDENAHPHT DYGERISLVH
NGIIENYREL KTFLLKNNIP FKSNTDTEVV ANLIGYFLDK NEKFEDAVLS AIRQLEGTWS
FCIIHKNYPD QIILAANGSP LHIGFKENEI FIASEHSALF MFTNEYISLK NGEILVVNKE
KINDLKILKK LESIPEIAIQ KTPHPFPHWT IKEIHEQSQS LSKSLNNFSI ANNCVKLGGL
DPYSEDLSKI DNLILIGCGT SYYAALYGKY IMNYLNCFNT VQVMDPVDFN ISVIPKEKEG
VIFVSQSGET RDVIKACKLA HDLNLKKMSV VNSVGSTIAN MTGCGVYLNA GREVAVASTK
CFTSQVSVLI LIALWFFQNK TSNKISSLIY SLHRLPLYAD MTIKCESLCK LLSHKLSNKS
MLIIGNGLSY PIALEGALKI KELTYIHCEG FTGSSLKHGP YALLGGDDNI PVIMLVFNDK
NVMINTGEQI KSRGAHIICL TDDENLVKDD IILIPNNGLL TPLLAVIPLQ MLAYYISVSK
GINPDKPRCL AKTVTVFVFI FKRYASEDVQ KILIGNKIDL KNDRNVSYEE GKELADSCNI
QFLETSAKIA HNVEQAFKTM AYEIKNKSHL ENQQKGKANI NLNAKPVKIR TMNSRKPPEG
WDKVESFLDE MNKKMRSLEN EDTSKKRKNE ILWPIFQINH QTSRYIYELY YKRKEISYDY
IVIGGGPGGM ASAKEAAANG AKVLLFDFVK PSIQGTKWGI GGTCVNVGCV PKKLMHYAGN
MGNLDSNEYG WKFDNKHDWN KLVTTVQSHI RSLNFSYMTS LRAKVKYING LAKLKNENTV
SYYLKGKEET VTGKYILIAT GCRPYIPEDV EGAKELSITS DDIFSLKRDP GKTLVVGASY
VALECAGFLN SLGYDVSVSV RSIVLRGFDQ QCANKVKTYM EEQGVVFLPK KLTKQNEKIL
VEFSNQTKEL YDTVLYATGR KGDTKHLNLS CTNIPNIFAV GDVVENIPEL APVAIKAGEI
LARRLFKQSD EIMDYSYIPT SIYTPIEYGS CGYSEEKAYE IFGNVEVFLQ EFNNLEISAV
HRKKRKDEYD IDISSTCFAK LVCLKDNRVI GFHYVGPNAG EVTQGMALAL KLKAKKKDFD
NCIGIHPTDA ESFMNLHITL SSGLSYAAKG GCGGGKCGEQ LYTGPLKIEQ LLAKGFVKRD
LELLKEGGLQ TVECVAYAPM RTLCSIKGIS EQKAEKLKKA CKELCNSGFC NAIDYHDARQ
NLIKFTTGSK QLDALLKGGI ETGGITELFG EFRTGKSQLC HTLAITCQLP IEQSGGEGKC
LWIDTEGTFR PERIVAIAKR YGLHPTDCLN NIAYAKAYNC DHQTELLIDA SAMMADTRFA
LLIVDSATAL YRSEYIGRGE LANRQSHLCR FLRGLQRIAD IYGVAVIITN QVVAKVDAMS
MFGHEKIPIG GNIIAHASQT RLYLRKGRGE SRICKIYDSP VLPEGEAVFA ITEGGIADYM
AIRVQFENSN EVGVFSRLTN SYALIALGSS ENFSSVFESE LSQHIPLVYT TIGGTKVIGR
VCVGNRKGLL VSSICTDQEL LHLRNSLPEN VKIKRIEERL SALGNCITAN DYVGLIHTDI
DRETEEIIQD VLDIEVFRTS IAGNLLVGTY SYFTNNGGLL HAMTSSQEIE ELSELLQIPL
ITGTINRGSD LIGSGLVAND WSAFCGMDTT AIELSIIEKV FKLNNINDNN ITNNFKYKSS
IIKTMIIVDT YWQTETGGIV IAPIPNLFKL KPGCATLPFF GVELEILDSK TLNPLKGPNC
GLLCIKSPWP GMLRTVYGNH NRLIKTYFAT CPNYYFTGDG AYRDEDGYYW ISGRIDDTLN
VSGHRLGAAE IEHALVQHFY IAEAAVVSFF HKVKGEGILC FVVKKLKLQV RQVIGPIATP
DLICIVPDLP KTRSGKIIRR ILRCIANGLN DYGDITTVAN YEIIDVIPFP TPCTVEKALE
YYCDLTTIPR VNVLKKFKCF IKDKEEKESD MTLIEFVDIY MSSSQFEISP FLQLIPKNNP
KNYTISSSPK RWFKGSCSFY LTELNIHDTI MFNLKPSKFE VDFLYEKEID AKEKKYIDEV
YFAFSRDQPW VEKYRPKKLD DIVHQSNAVR MLKEVVKTKN MPHLIFHGPP GTGKTSAINA
LAHELFGKDN IKDRVLELNA SDDRGINIVR EKIKAYTRIS ISKNKINNET NETLPSWKLV
VLDEADMMTD DAQSALRRII EIYSNVTRFI LICNYIHKIS DPIFSRCSCY RFQSIPINIK
KEKLLYICKS ENINITDKII ETTQGDLRRA VSILQLCSCI NPQITLESVL DVSGLPNDNI
ILKIIDACKM KDKNVEKTVQ DIIEDGFDVS YIFKAFNNYF VDSLKYQILL ELSRHDYRLH
SGATQYIQLL SFAVKIHSLL IKELIFKSKD EKHFQMVVKQ VKELIKQVKQ KEVEDVNDSK
HTQDKLILNK SGRRIILRDL MTRPNIFTGR KILGTLELHT NGLRYSAIDI LFDDIKHAFY
QPCDGQLIIL IHFHLKRYIM VGKKKTLDVQ FYCEAGTQID DLDRAKARNV YDPDEMHDEM
KEREQKNKLN LIFKNFVQQM QDASKIEFEI PYPELTFSGV PNKSNVEIFV TANTINHLVE
WPPFILSVED IEIASLERVH HGLRNFDMIF VFKDYTKPVK RIDVIPTEYI DTIKKWLTTI
DIVXKLXLSD IDSFVNSKGF EGFLGEDDDE EEDEDEDDEY ELDESELVII IDTLASIEFT
SQRKMSSIIC RIPKIMLFCK GAGSVILKKL AKKTKVDDIT IEHMEAYADE GLRTLCIAQR
ELSEETFAKW YELYKEASLS IKDRERNLEH VASFIENDLI LQGVTGIEDK LQEGVSSTIE
DLRIAGIHIW MLTGDKIETA MNIGIAANLI DNYSVDGNVI DILLSKKFEK KFFYLADKCT
SVICGRVSPY QKGAIVSSAN RLLKKITLAI GDGANDRNMI NRANIGIGIR GQEGVQAFNS
SDYGISQFRF LKNLLLVHGR LSYRRISKLV VYMFYKNIVL IFPLFIFGSL SLYSGQKIYY
EFLLQVYNVF FTSIPVIIFA ILDQDINLNT ALEKPVLYKL GIYHYYFNIN TFVSWVMNSL
FHGSVVFLIP LYFLIPSLDG IPYDIWTVGC VTFLTVLVVN LKILLETYYL NMLPIIAVSL
SIFSFLLLVT SFSFMYFGSS HILGTVIYLV RSLRFWLVSL LSLFALSRDY IFKVYKRNFN
PRNYSDNVKD HFNKPRNVGS FDKNEKNIGT SIVGKASCGD VIKLQLKIED NVIKDARFMA
FGCGSAIASS SYATELIKGK TIDEALKIKN DDIASHLNLP PVKVVILGSG WGGMHFLINI
DFKKYDVTLI SPRNYFTFTP LLPCLCSGTL SINVCTESVR NFLKKNGSVG NYLQLECTDI
IYKDNYITCK DNNEMKINYD YLIIAVGAKT NSFIKGVDKF AFYVKDIDDV LKIRRKFFEN
LEKSTLPNTT NEEKKNLLHI VIVGGGPTGV EVAGEFADFV NRKQNYEDIF NFISISIIEG
GKNLLPTFTQ NISDFTKYNF RNLNINVLTN YYVTEVNENY FYIQSKKFPY GVLIWASGLA
QTTLVNNLLK KIPQQVNNKI LNVDSQLKVI GVNVYAIGDC KKIFPIQITA EQLINEALDL

EEVEKKVNYN LIDEDELNEY KISKRKEFED KIRKRRYLIS TYIKYALWEI KQKDIRRGRS
IFERALNIDY TNKNLWLKYI EVELTNKNIN SARNLLERVV LLLPLENIFW KKYAHLEEIL
NNFINARNIY ERWIKWNIDE TSFLCYINFE ERCKEINKCR EIFEKLIVKI PKLECFYKFI
KFEKKYKNIE RAEKCIELLP SSFIDENFYI YFCHFEEENN EYERCRKIYI EALKRLPKNK
STLLYKNFLQ FQKKYDELDQ TLLLKERIYY EDELKKTPND YDIWFNYIXL EERNRELYER
AISVIKNAHV HDGLKVGIRE VIKAIESKEA KVCFLSSVCS EPAYKKLITA LCTEKNIPLF
LVDSKDLGHW AGLYKLDKEG NARKIIGASS VAIVDFGEDS AEREFLLFLK AIVGEVIDNR
YSVVCELVGK GVFSNVLKCY DMVNKMPVAI KVIRDNDMMK KAAEKEISIL KKLNKRHIVR
LLRSIKYKNH LCLVFEWMWG NLRIALKIEK CFNSLIIKTR ELEDVYISRR KLQSKYLMQI
KNLYINCNCD CIIHKIGFKY ATKSSFPNLV NGTLLYMIVE KINLDNNVVA SCINSSDVKS
WINYENYLGE LIDGFLFSVN ISFSKSLIGD RCYILDLIDI NYEIAIGHNG GALGYSQHLS
EEIMLFSREA ILDKIAVILG GRAAEELFIG KITTGAIDDL NKVTQLSYSF VSQYGMNKEI
GLVSFQPNSN SEYNLYRPHS ECLAHLIDNE VRNLIETQYK RVKSILLKNE QHVHNLANLL
YQKETISYHD IVKCVGLKHQ RYKGTDKVKI EEERNKKIFL KNNKFCKNCG SAAHKEKYCL
ERTRKKGYDG NRDRWVGYNS DNFEQIYREY EKIVEEKKKR KAEELIDPKR KKIKVLSKYE
EDIYAFDHSS VFGSYYDKEK KKWGYKCCLS TNKFEKCIIP LMDSYHANYY TREVMIILIN
EFSSPDEEMK KIVLKCVKQC IQTEGIEKDY INQEIVNPFF EKFWVMRNCN DKKNFTLIVE
TTVEIANKIG VISRIVDDLK DPSEQYRKMV MQTIQNIINN LGVDDIDQKL EEQLIDGILY
AFQEQVSDDY YVLLNAFDVI VNKLQLRMKP YLPQIAGIIR WRLNTPLPKI RQQSADLISR
IAMLIKICDE QQMLGHLALY LYEYLGEEYP EVLGNIIRAL KSIVVVLGVQ NMTPPIKDLL
PRITPILKNR HEKVQENVID LIGIIADKGG DLVSPKEWDR ICFDLIELLK SNKKLIRRAT
IQTFGYIART IGPFEVLTVL LNNLRVQERQ LRVCTTVAIA IVADTCLPYS VLAALMNEYR
TQDLNVQNGV LKALSFMFEY IGEIAKDYVY SVVSLLEHAL IDRDLVHRIA TWACKHLALG
CFGLNREDAL IHLLNYVWPN IFETSPHLIQ AVIDSIDGFR VALGPAIIFQ YLIQGLFHPS
RKVREIYWRI YNNVYIGHQD SLVPIYPPFE KLNDSNFSRD ELKYNGSYVR KRKRYIKFNC
SIKRKQPSWL KQKPSEIEDT IIKLAKKGQT PSQIGATLRD NYGIPQVKSV TGNKILRILR
APGVATTIPE DLYFLIKKAV SMGKHFEKNK KDKDCKFRLI LTESKIHRIS RYYKRKKFFP
SNWKYQSSPA SAFRNIEHAP GVQFSYVPPD FFDSEDDESD KNQYELDDDG GGRAAGTRIK
EHSSSHHLRR KNYEDDFEFN EDKILETLHI LELLYMNGIS LEEQNEHGQT ALFLSVKKNN
ISTLQWLLSK NVNINHRDFY GNTILHIAVR YSDIDIIRLL CDYGCLNLVY CSSIENKNTN
VFQLCIKNRY FLIYILLKKW ILQNKICKNL KICKTIYAFY FWFFSLLNLI IYFNISYSFS
LITKYQTLSI VWLSLWIFQK FLWCLLYFRS PGEYHLNSIE KEIYQINLEY QKLSLYSHVS
QERINSLDIN YRNAILEIIL LQLIIEPYIL RRSKKHVFID MPKKHSVIIK LPLNNTQLNL
YKNEIFSKLQ HTHKHLINAS IFILRRICNH PLLHKYYYSI EDIKNISKYF YNNTDQYLDL
DLKTVETEFM KISDFDIHLS IKHLISQDNN LNKYLITKEH ILNSTKIHHM ISLIKEIRQK
KEKVLIFSQF TTFLDIIEEA LYVRLDGTTN TIERQKIIKR FSNIFIFLLS TKAGGVGLNL
IAANHVILMD QWNQLLLQEE IVKKLCEYII DSKCDVVEKG VSDLAQHFLV KKNISVIRRV
RKTDLNRLER ISGATIVNRC DEIVESDIGT KCGLFEVKKI GDDYYSFFID CEDPHACTIL
LRGSTKDVLN EIERNLHDGM NVAKNIILEG KLLCGGGCTE MRVGQNLIKQ AKNFDDSRKS
ITEAVASALE IIPKILAQNC GVNVVKTINE LRIKHGGEKY GIDGITGEII DVSTKNIWDL
LSVKKQIYKS AIEAAAMILR IDDLYVVLIY EKLLKNPLCK VTNIYEKETK KYRPLPLNTL
QMTKQVSKFF HISSKECMNI AEKLYNKGYI SYPRTETNYF TDSMDLQKIV NELSKNNLFG
WYASKLTKNN PRKGKLNDKA HPPIHPVKNM EWILYEFICR HFLAVCSDDA IGFNSKVIVR
IGDEQFFCKG LKIIKKNYLE IYTYEKWNDK ILPTFHINDE FFPYSLLIEE GITQPPKFLS
ESNLLSLMDK FSIGTDATMH EHIENIQKRN YVTKNSKSLF IPTNLGIALI LSYKKFKDIG
VDLTEPSLRA KMEKDMSLVA SGICVVKSGK TDIPSNCKMI IISYELITKN DKYQKYKSII
CDESHYLKNS FSKRTKAITP IIKSAKRCVL LSGTPALNKP SELYEQVSSV IPNLFNYHEF
CDRYCFKDKN IYTRKIEYVG CKHTEELHLF LTNTIMIRRL KKDVLKELPE KLRSKIPVEI
PPKELSEILL FKMTGYAKVK AIKEYITYLI DADIKFLLFC HHKLVMDEIE EFLKEKKTQF
IRVDGLTSMD KRELYIKNFQ ENENIKIALL SLTACGIGLN LTAANTVVFG ELYWVPGQII
QAEDRAHRIG TTHENVNIHY LIAQNTIDEI VWKIINRKWN TLTTALNGME DTLMFEANDK
ICIAVSGGKD SSVLTHVLVN IKKKYNYKWD LFLLAIDEGI KGYRDDSLKV VFNDIFSYTM
DDVVKFIGKK NNCTVCGVFR RQAMEKGALL FNATKLVTGH NADDMAETIL MNLCRGDIDK
LFSTECTYSP NSFRGNLRSF IKDIECIKCG AYTSNVTGLN NYKDNRKISN CTFEIDASYE
SIEPLLIENE YQQIPKLRIL SFDIECIKLD GKGFPDAQND PIIQISSILY LQGDPSIKFI
FTLQECASIP GSNVIWFNDE KSLLNAWNEF LVRLDPDFLT GYNIINFDLP YILNRGTALN
LKKLKYLGRI KNISSSVKDS SFSSKQFGMH ETKEINITGR IQFDVYDLIK RDYKLKSYTL
NYVSFEFLKE QKEDVHYSIM NDLQRESPES RKRIATYCIK DGVLPLRLID KLLFIYNYVE
MARVTGTPFV YLLTRGQQIK VTSQLYRKCK ELNYVIPSTY IKVNTNEKYE GATVLEPIKG
YYIEPISTLD FASLYPSIMI AHNLCYSTLI KNNGKGNLKF VKKNVKKGIL PLIVEELIEA
RKKVKLLMKK EENRITQMVL NGRQLALKIS ANSVYGYTGA SSGGQLPCLE VAVSITTLGR
CMIEKTKERV ENYYNKNNGF EYNSTVIYGD TDSVMVKFGT NNIEEAMRLG KDAAERISKE
FLPPIKLEFE KVYCPYLLLN KKRYAGLLYT NPLKHDKMDC KGIETVRRDF CILIQQMMET
VLNKLLIEKP FIQVAPMINV TNRHFRALVR IFTKKAQLWT EMIVDNTLLY NLNNLEEYLG
FNDNEHPIVC QLGGCDITSM SEAAILVEQA GYDEININVG CPSTKVANKG AFGASLMKKP
EHVRNIVYEI KKKVQIPVTV KLRTGVDDYD SFSFLKSFIE TVSSAGCNHF IVHARKAWLK
GLDPKKNRKI PPLEYKKVYD LCKLYPNLKF TLNGGVKTIE EATALLLNGV MIGRACMENT
TILYQTDKLI YNEKVPDTAY SRRTALNAYK SYLEENSIFY NVFELLKPIL GILKGMPGHR
LFRFLKNFFF KNPVGHIGIV ALKNSSAKLI QPLTSSIDDI LNSLLKERSL GLQGSPSLQE
GLEIAHDLLI DMPLYGTKEI LIMYGSIRTC DKKNILKILE LLIKSNIYVN CISIAPEMHI
LKCGIHLISM HDLSHITNNL QGSPLFTEIM GRSSLAMAQQ MYFSTHNALR INENDVISTL
FYEINGNRHI SLLIFPFYDV QMLKRLLIKK LNLPGIKVND IIIFYKGIKL PNYRIISTYK
TVNKLYWAIK DTNPNASIRV IDNKYPPFFE NILNEIKLAF KKNISPKLTM DGTGGTYLLF
NGKKKVCSVF KPLDEEAFAP FNPRGYEGKM YQEGFRSGVL SGEGASREIA AYILDNNYNN
FSNVPCTIMV EACHPHFNNR SKLKYIDNES NLKWKCGSLQ EFIDSKESVG NYDYKQFSIR
DIHKIAILDI RVMNLDRNDG NILVSPSHLG IEQSRRDDIE ALGYVLMYFL RGSLPWQGLK
AISKKDKYDK IMEKKISTSV EVLCLLNVCY NIDSCIIIDR KIDMITPFCT PFTYEGLIDH
LFGIDNLQIE IPLYNDIKDL SQNEIGRFLH KKASEIQKTY EKDKLKDIEQ INEYMRKFKA

KHYEHNSLST HVNIASFILN TIKREKNFDK LKLEDEIIQL NSNTNKSKLL NILKKIQLLI
YTGEDIYEVY RILCLFSIIT NGKKDIVEQY GINELTRLNK LHTCNILKYQ SKQKFIWNNL
KNHFNLLSNE INDISYVCNG YAPLSVRLIE YKNNLQIIPE VLNFLNGPTL DVIQDTIEVK
SMCINCEKEG INKIVKIEIP YFKNVLIHSF ECEFCNYKNN VIQDLNTIKD KGIKIIFKVT
KDEHLDRQLI KSEYGILKIP EIDFEIPKET QKGSINTIEG FIQTALNNLY IKMIESTIYN
LFTIEIIDPS GLSSLEYERT KKELNELGFY SFTSNCPCCN YLGSNNFCEI VIPGFKKCLI
LSYVCANCNF KTSEIKSSGE INPKGKKITL TVKNKSDLDR FVIKSETASI HIPIIDLTSD
YGTLGGSLTT VEGLIIKIIE SLEDKFKFLM YVLNRKGEEE DISFDQILKR IQRLSYGLHE
LVDPARVTQG VINGMYSGIK TCELDELAAQ TCAYMATTHP DFSILAARIT TDNLHKNTSD
DIAEVAEALY SYKDIRGRPA SLISKEVYDF IMLHKDRLNK EIDYTRDFNY DYFGFKTLER
SYLLRINKKI IERPQHLLMR VSIGIHIDDI EKALETYHLM SQKYFTHATP TLFNSGTPRP
QMSSCFLLSI KADSIEGIFE TLKQCALISK TAGGIGVAVQ DVRGQNSYIR GTNGISNGLV
PMLRVFNDTA RYVDQGGGKR KGSFAVYIEP WHSDIFEFLD LRKNHGKEEL RARDLFYAIW
VPDLFMRRVK ENKNWTLMCP NECPGLSDTW GEEFEKLYTK YEEQNLGKKT VLAQDLWFAI
LQSQIETGVP YMLYKDSCNA KSNQKNLGTI KCSNLCCEII EYTSPDEVAV CNLASIALCK
FVDVEKKQFD FKKLYEITKI ITRNLDKIIE RNYYPVKEAR TSNIRHRPIG IGVQGLADTF
MLLRYPYESE PAKELNKRIF ETMYYAALEM SVELAQIYGP YESFQGSPAS QGILQFDMWN
AKVDNKYWNW DELKAKIRKH GLRNSLLLAP MPTASTSQIL GNNESFEPYT SNIYYRRVLS
GEFFVVNPHL LKDLFDRGLW DEDMKQQLIA HNGSVQYISE IPDDLKELYK TVWEIKQKNI
IDMAADRGIF IDQVPKYGFE EMKNEISKYG VEITQSTLKN PTTEDVQGVY SICIKYILNK
DINNIRIEEF TGDLKSILPN EGKNHLQAIG NLRFIRHCEK INKILNLDNI LSYIFKPVSS
HITKLISAFI INETNELIFQ FSRLRQKKED LEDQIVPSPE KLQQYNEELK DLLSEHMSYF
ESDKKKNEEI KSKINVADLS IKKLVDLLTI LTNHIKQHID KKNELKNLEK NLKSLDWWTL
GIFIYEILVG CPPFYANEPL LIYQKILEGI IYFPKFLDSN CKHLMKKLLS HDLTKRYGNL
KKGAENVKEH PWFRNIDWNN LLNKNVEVPY KPKYKNMFDA SNFEEDLTTA DKITNESDPF
VEWVISQFYI LSPRGDTIIN RDFRGDITKS SGDAPPLFYL NGINFTYLKN NSLYFVLTSL
FNISPSYLIE LLNRLLKIFK DFCGQITEEI IRMNFILIYE IIDEVIDYGY LQNSNTEYIR
NLIHNETLPS NASQKPIQIN DKKNEIFIDI VEKIDGVIQI KSYLLGNPYI KIAFNDDLYI
KNIHNDTSNN IIIDDCNFNH LVLSLYQPDG ECVLMNYRIN MINIYKPNDI IACEVQRILT
DGCIILHTRS SIYGKLSNGI LITVPQTLIQ NQKKHIFVFP FVDDTTRRNI SIISNIIKLL
AKYHININYD IITKIYVQEW VTPKNKQIKA ASSNNAQIVI ALSGGELIYF EIDESHTLVE
IFRKNLNVEV LCLSMQQNRL RANFLAVGCL DNVVRLLSID KYFKQLSTHL LPNNSSPQDI
CILFLNIGLN TGVLLRSIID PIGTLSNHYS KYLGAKSVKI CHVNPALLVL CEKTYLCYVH
QGKYVYSPLN YDMLEYASSF YSEQCSDGYV AISGNSLRIF RFYRLGEVFS QNILHLTFTP
RKIVPLPFPS MLAIIEADHN SYDENTQKEI QKALKDIKLG TFKAGAGKWG SCIKIINPIN
LQIVDKISLE MEEAALSVCA CELEALHCLI VGTTSNMSLK AALRVYTYDI KYKLNLLHIT
PIEDQPFCFC PFNGRVIASI GNKLRIYALG KKKLLKKCEY KDIPEAIVSI KVSDRIFASD
IRESVLIFFY DSNQNTIRLV SDDIIPRWIT CSEILDHHTI IAADKFDSVF ILRVVEEKPD
ITYNDIGGCK EQLEKLREVV EMPLLQPERF VTLGIDPPKG VLLYGPPGTG KTLTARAIAN
RTDACFICVI GSELVQKYVG EGARMVRELF QMAKSKKACI LFIDEVDAIG GSRGDESAHG
DHEVQRTMLE IVNQLDGFDN RGNIKVLMAT NRPDTLDSAL VRPGRIDRKI EFSLPDLEGR
THIFKIHANT MNMSRDVRFE LLARLCPNST GSDIRSVCTE AGMFAIRARR KTITEKDLLL
AINKVIHGCK QFSATGKYMV YNIKIKVEEN LSSIIKKINE LYFLEKNNFF NIPRYSKIPK
TKKSTKWEMF AEKKLKKKNK SGLIFDKNSK GWVRRYQKKH IKENEEKLKF VHEYKDNENL
DPFEKKEEEK EIKKMKQKMR EMKNKVFDRL TDTNFYTGIH KERFKKSKKP VVTPPKNIWI
FRNGDKHHNG ILFLVKPHIN NLKTLFFEIT KVLSPTIGPI RKIYDQNEGA KYLCTSGDPP
APIDRLQLFD ARKYFKEGQK IITPPNGDGT RAFYESLFEE NPNSVIAIKY CIEHGVLSGT
KHHEALYKYF ILKKNNAFKS NFGGIKSEFL KLLMLNFFKK KNLLESFLKK LPKILGKVVY
GIDDPLKALE IGAVEFLIVF EGLDFFGLTP GNNFSNPAKT LHFSPNGEKQ EFFFKENNVE
FEVFEKIFLT DWVFNNYKKF GASLDFVTNK SQEGAQFLQV IYEFIFLCIR IYNDISKLFV
LPYTVVSDLI CEQVISIIVL PFNYLGLNAL NSKDMQKLLS SVNEKHKKKL SLDIIDAIIE
CKNKSIVYED VEEVLKYISS IFDIEQKYNI CMLFYKYIAN SGYLIHLLPT IIFSMLNLVM
LEAKLNNASI LKKLFECIKD LVNDANVDAD ETGLKLQALD GNHVSLVSLH LLDSGFSHYR
CDRERVLGVN IASLNKVFKL CAANESVVIS SKDDEDNLNF VFENNKEDKV TNFSLKLMSI
ELDSLNIPEE GFDAEVELSS KELTNIFRNL SEFSDTVFIE IDSNSIKFTT KGLVGDAEVA
LKPRDSTDDI GVNIKSKKKI KQSFAIKYLN LFSKSSILSD VVTLGLSDSR PIEFKYEIKD
GFVKFFLAPK MDDDCPEKCD EEMGIIKGTF LYTFDSPICK AAIHAGVLNV TEDIVVIIAH
KHKNFIGTRR NNIESHTFSG ISKSYSVSIP TGFNGKENDF IDCSNLPNEK YIKGLYNFSF
IIYFGEGTWR TILSHSLCEG ISISIDEENE LIIEQNCNPY IIKSRFKPKF GETYHIAIIF
NKTNKTIALY INGKKAIFEK AKYDFTLNGD LIIGRSNQST TDYFIGNIHL VEVYKYVLSD
EEIKESLNSV LSRKTIDGRE CITPCKSRSI INKDLQVNAE KINLKCEDDL LSEQFNGKGS
QFLVNCLEDC TKSKFLVKGS NFYTPDSSIC KAAIHAGIVR IVEGLLEYKS SRGHFGIVSK
SEKQSCSTNG QFILNLSVGE KIIISCPSNC GTNIYSPLSS VCKAAIHSGA LSNQGGQVEI
IVGPKQEEFK GSKQNNIESY DSTSQNLSQN YIYNNADIGT QKKVFDLSLN LGPYSCNYSR
NGKYLLATGE KGHISLIDTQ NLESLCEFEV DETVRCNTIL HNHKLFAVSQ KKYIYIYDNT
GIEINCIKDI LYTYKMEFLP YHFLLVSIGE FGELVYQDIS VGNIVTRKKT KRGSCSIMKQ
NKSDAIIYLG HKNGHVTLWS PNVDKNYLIT ASIDCTYKLW DMRKLQFIKS YRSNVINNIE
ISDTSIVGFS MNSHFRTYKN FFTYPYLTHN TYGDKINSLS FQPFEDICCA GLKYSIKTLL
VPGAGLANID TFVNNPYETK KQNEIRLLLD KLPPDTITND YKNIILVRKI KTKTQIKRIM
TSPRDVIELT LKSYSNHNWP KWSDSESICT CLINNQINIY KGNEKGSKGN PSIFKIFNLD
NLNKHIYSKS FFNSDEIKIK WNKNGTALLL QIHTDKEKHS YYGSSSLYFI DTVTLKEVNI
MTNKGLIYDT IWSNNQNKFY VCKGEIPAEI VLHDKNGNVS HSFGRHKYNT LKLNCNEKLL
LTGGFGNLSG DISIWNTVSK KEITKTKSSC AVICEFFNDG KHFLTATTNP RLRVDNNLKI
YKYNGIIVSR INFEELYNVI ILPPGSNFVN ENKSYKKKKK KKEEDTIPLP NIKTQILKKI
IEYMEYHIQN PADEIPKPLS TSNLQDVIVE DNSSKYKDLA QKMIQEIEMA VVLFKRKFIL
IKIPKLPPCY IINSGSLSIA RAKVKLPSTY SKLGNPLSLS KLPDLNYSFD MIEELQQFFM
KQRRCDYFSL LNNFINLIIS VSNLLSSEPD IDLRNELLKR FIYSLNSWMN MRRCIVASCE
NTFAMTGLCI PLQILHFNHE ECKIFFSKKR APYLLVFEVA DLDEDISDII IFFYLFFFFG

EFNRDTISSL LNNPLARSLL NELSNNPEML TNLVSNNPIL RNTFPIMQPV LENPNLLREF
MRPEVLQAGL SPEEKYASQL LSLQEMGFID NAANIQALQE TGGDVNSAVT RLLEKGSGDR
LTRAARVLEQ LTEQKPIFGK CRFTIRSFGV RRNEKISCYV TVRGKKALEI LEKGLKVKEY
ELRRKNFSDT GNFGFGIQEH IDLGIKYDPS TGIYGMDFYV HLSRPGYRVT RRRRERLGFF
ISLKMINEIK GLSSIDTNYQ WLPILYMALA NDTSVSKITL NILKPYSIVL IRLLSDFFSV
AFIIKCVGVG YRNMSGTDCV AIACDLRLGS NGFTTVSTNF TKIFKMNDYV YVGLSGLATD
IQTLYELLRF RVNLYQIRQE KLMDVDCFSN MLSSILYSNR FSPYFVNPIV VGPYLTAFDL
IGAKCETNDF VINGITSEQL YGMCESLYIK DIFLVGAGGI GSEFLKNIIT IGCKNIDIID
IDTIDITNLN RQFLFKKKDV KKYKSVVAKE RALKYNKNLN INAYTFDVCI MKSSDIKKYD
YIINALDNIK ARKYVNKLCV MEKKVLIEAG STGYNGQVYP IYPNETKCYN CEEKPKNKTY
AICTIRQTPT LPEHCVAWGK LIFEMFFCKN DNETLIDIKN HIEEESKKRN MEKMEIIIFI
FNYLFHDTIC ELISLKKDFA IMPIPNINKN VEEYLIFDKD DDDCINFITA ISNLRMMNFS
INQKSKFDIQ SIAGNIIPAI SSTNAIVASL QFDEVVINDK ESHINDTDKI LVINFGTQYF
HLIIKRLNNI KIFSETKDFN IDLKDIINIK GVILSGGPKS TPHLKDEVFE YKIPILGICY
GMQEIALKMK GEVKKSKNCE YGSTVWMNHT EEVSKIPDSF YLTNSSENCL IYDKEHNIHG
VQYHPEVYES LVGEQLFFNF AVCKCKKQFD PIRYHELEFK KIEKHAHDHY VIAAMSGGID
STVAAAITYK IFKNRFFGIF IDNGLLRKNE GENVFLKKLF PDMNLTRINA SDNFLKNLKG
VTEPEKKRKI IGKLFIEEFE KAVTNMNIDV NYTYLLQGTL YPDVIESKCS KNSDTIKTHH
NVGGLPTNLK FKLFEPFRYL FKDDVKKLAQ ELNLPKEIIN RHPFPGPGLA IRVVGEIDEH
KLNILREIDD IFIKDLIHYG LINQAFAVLL TTKSYEYVCV LRAVKTSSFM TANWFKIPYD
ILEKISTRIS EVKGINRVLY DISSKPPSTI EFEMPLPGFV SDKTLYLKKP LILYEDENDK
IQVDPILAQH LREHQREGVQ FIFECLMNLR DEKISGCILA DDMGLGKTLQ SISVLYTLLK
QGFHNKSAIR RCLILCPASL INNWNDEISK WLPGRCNVTC VNDNVKEKIV SKLEGFKYDL
KSNVLICSYE CFRINNEFLD KSSIDMIICD EAHRLKNDKT KTYTSIYNLS AKKRLLLSGT
PIQNDLGEFF ALISLCNPDL FDDTNLFRKK YANPILIGRD KDATEKEQEI ASERLSELSN
INKFILRRTN NLLSKVLPVK YLINIFIKLN PLQEALYLLF LKDKKILKND QSNNKVNVLI
NIKKLEKICN HPLLLNANDI KSCKFLLLHF LLKNIKQNTT DKVVIVSNYT QTLDYMEILC
KENFYKFVRL DGGISIKKRH KVISDFTHSP DIFIFLLSSK SGGCGINLIS SNRLILLDPD
WNPANDKQAL ARVWREGQKK ICYIYRLFCT GTIDEKVYQR QISKDGLSSM IVTNTNLSKD
QLSDENVKKL FNYKINTICE THDNIECNRC KKIESAGFMF YIKYEKASYK KSSNLVKKCI
DENINVYEIL GVDETDDIEA IKASYKKLIL IFHPDKFLKI QDSYTILSDK TLRKQYDSSV
PWSAKKPVPD IGDENTNIKN VKYFYDFWYN FVNWRDFSYH NAYDYEQAEC REERRWMERE
NKKIQKKVSK LENLRILKLV DLAYNNDPRI IAENKRIKTE KQKKKAAVKI WKHHIKSFET
ICDFIYDIYV LLWTAHEVSL LAKALKFYPG GTKNRWVLIS SYIKTKSIKE VIKKTKEMFE
NETLWTQEEQ NLLEKALMKY PSSIPMPKKR RNGGRSKHNR GHVNPLRCSN CGRCVPKDKA
IKRFNIRNIV DASAQRDIKE ASVYSTFQLP KLYIKQCYCV SCAIHSRFVR VRSREQRRVR
KETAKHINPS QKDAQAVRIF MQMIYALMDG FLKKFLFLVQ HIYFFILFIS FAVVKNVDMT
EEMQIDAIDC ANQALQKYNV EKDIAAHIKK EFDRKYDPTW HCVVGRNFGS YVTHETKNFI
YFYIGQVAIL LFKSGVCGWS KAVRKQGGRF CFVNLNDGSC HLNLQVIVDQ HIDNYDKLLK
CGVGCCFRFT GKLILSPVQN SAHSFEIYGE DPQKYPLSKK NHSKEFLREV AHLRPRSYFI
SSVMRIRNAL SIATHLFFQS RGFLYIHTPL ITASDCEGGG EMFTVTTLDF KKDFFNRQAF
LTVSGQLSVE NFCSSMGDVY TFGPTFRAEN SHTSRHLAEF WMIEPEIAFS DLYDNMELAE
SYIKYCIEYV LNNNYHDIVY FEENVEKDLI KRLKNVLEED FAKITYTNAI DLLMKYSESF
EVPVHWGMDL QSEHERFIAE RIFKKPVIVY NYPKDLKAFY MKLNEDKKTV AAMDVLAPKI
GEIVGGSQRE DNLELLDKMI QEKKLNLDNY WWYRQLRKYG SHPHSGFGLG FERLIMLVTG
VDNIKDTIPF PRYSGHAEFI KDLLEMNLDN STNIIKNLFL KDKKKFFFIC TLNSKTVDLK
YLANALNTSN LRFVDEKNLK NILNLLPGCL TPLAMKYDKD NVVKLYFDAE LKNMIIIHPL
HNYSSLYMKK IDVIKFCDIH NHSPLLGITS KKLENFSDWY TQVIVKSELI EYYDISGCYI
LRPGAYYIWE CVQSFFDKEI KKRGVENSYF PLFVTKGKLE KEKNHIEGFS PEVAWVTKYG
DSNLPEEIAI RPTSETIMYS AFSKWIRSHR DLPLKLNQWN TVVRWEFKQP TPFIRTMAKL
SKAQKKQMYI DKLSTLIQQY TKILIVHVDN VGSDQMATVR QSLRGKAVIL MGKNTRIRTA
LKKNLQAVPQ IEKLLPLVKL NMGFVFCNDD LSEVRILENR SPAPARLGVI APIDVFIPPG
PTGMDPSHTS FFQSLGISTK IVKGQIEIQE KVHLIKQGEK VTASSATLLQ KFNMKPFSYG
VEVKTVYDDG VIYDANVLDI TEEDILKKFS KGVSNVAALS RAVGIITEAS YPHVFVEAFK
NIVSLVIDSD YTFPLMQNIK NMVENPQAYV AAAPAAEEED DEDGFVGFGM FDQERDLARE
PCPDRIIEDM GGAFGMGCVG GYIWHFLKGA RNSPKGDILS GALYSSRMRA PILGGNFAVW
GGTFSCFDCA FQYVRKKEDH WNAIGSGFCT GGVLAMRGGW RSASRNAIVG GVLLAIIEVV
SIVLTRKTTP TPRQQFQQQM EMEKMLVDNI GDVTITNDGA TILKQLEVQH PAAKILVNLS
ELQDQEVGDG TTSVVLLASE LLRRGNELIK MDIHPTTVIC GYKLAMKESV KYIKEKLSEK
NLGKDVIINI AKTTLSSKFI SYESEYFAKM VANAIQSVKI INEAGKVKYP VSSVNVIKVH
GLSSLDSKLI DGYAIMSGRA SQSMPSAIKN AKIAFLDFPL KQYRLHLGVQ VNINDPTELE
KIRQREKDIT KERVNKILES GANVILTTQG IDDMPLKYFV EAGAIAVRRV NKDDLRRIAK
LTNGQIRLTL SSIDGTEKFE ASSLGYCDEV YEEKVGDWDL MFFKGCKTSK CNTILLRGAN
DFVLDEMKRS IHDSLCSVSR ALESNYVVVG GGCVEVALSV YLEDFAKTLG SREQLAIAEF
AESLLVIPKI LALNASYDSI DLVCKLRAYH TKSQVNTEDS KDYKWYGLDL VNGKVANNLK
NGVLEAMISK IKSIRFATEA TITILRIDDL IKLSPEKNPP VIKMKGHTAN ILDLSFNPCY
SKIIASSSED LTVRIWLKGH KKKVSIIDWN PMSYYILAST AFDSFLNIWD IENEKKAFQI
NMPKKLTSLK WNIRGNLLSG ACLNKNLHII DPRKKEICFS FGVHNGGKSV KSTWIDGLCG
DDVLSTGFSK DNMREIKLWD LKNTSTPLTS ISLDNASAPL LPFYDETLGI VYLIGKGDGN
CRYYQYLQGN IHKVEEYKSN LPFRSFGFLP KQMCDIYKCE LGRVYKNENN NSIRPISFIV
PRKNFQKDLY PPIIGVDLDI KRIGIFDKLK ICGQFNRGFV ISKLFIIDQH AADEKSNFEK
YNKTFTMKSQ KLVYLLSLPV FNGKILEIVD FMSLLHHLWF NYNFPRPQKV WRILASKMKA
LVIIFLKKIS DTYIEDQAKW MQNMKVSQEE QNNKKLGEWN DTIENKCFVY PASSAPCGAC
TSAGAVLPYR RYKEPRQKKE YLGTDILCQA KSGMGKTAVF VLSILQQLVR CLGLAHTREL
AYQIKNEFDR FSKYLKNVRC EVVYGGISMS KHIKLFKIPH IIIGTPGRIL ALIREKYLVT
DKIQHFVLDE CDKCLEKLDM RSDVQKIFIS TPLKKQVMFF SATMAKEMRD VCKKFLQNPV
EIFIDDEAKL KLHGLLQHYV KLQEKDKTRK LIEILDALEF NQVIIFVKSV TRAITLDKLL
TECNFPSIAI HGGLDQQERI ERYDKFKKFE NRILVSTDLF GRGIDIERVN IVINYDMPEN

SDSYLHRVGR AGRFGTKGLA ITFVSSQEDT LALNEVQTRF EVAISEMPNK IDCNEYINQR
LSVSAEAYGE WNKKKNFVAK IYEKDEEEKL KIREALNESF LFNHLNEKEF ETIVDAFFDK
NVNEGMNIIN EGDDGDLLYV IDEGEVEIYK NKGDKKEILT VLKSKDVFGE LALLYNSKRA
ATATALTNCH LWALDRESFT YIIKDNVAKK RQMYEDILKQ VTILQDMDPY ERSKVADCLK
SKNYTDEIII KEGEEGDTFY MLIDGNAIAL KNDKIIKTYK KGDYFGELAL LKNQPRAATV
KCQKFCQVVY LDRKSFKRLL GPIEEILYRN VENYKDVLKQ LMHLKIKCLS DEVRELYKNH
KTYHEGDSGL DLFIIKDEVI KAKSTTFVKL GIKVVANTSF LLFPRSSISK TPLRLANSIG
LIDAGYRGEI IAALDNISED DYHIKKNDKL VQLVSFTGEP LSFELVDELD ETSRGEGGFG
STLGAAFGTA KSGVGVCSVG VMRPDLVMKS ILPVIMSGVL GIYGIIMSIL IKGDYIKAYL
LGFSIEDALA LLRIEDLYIE SFQIQDVKML KGDHLSRCIG RICGSNGSTK YAIENATKTR
IVIAGDKIHI LGSYNNIKMA RYSICSLILG STQGKIFNKL NILAKRLKER IEKLEDRMHP
WNKIYIKKSK CSYTYDDIIC MPGYIDFSLS EIDLCNNLTS NISLKTPIIS SPMDTVTEHK
MSISLALSGG LGLIHNNMSI ENQIEEVKKV KRFENGNKVL CEEKKSVLPI VNNNYEFPYA
SKSKNKQLIV GAAISTDLER ANQLIKNMID VICIDSSQGN SIYQIDTIKK IKSAPIIGGN
VVTCDQAKNL IDAGADILRI GMGSGSICTT QDVCAVGRAQ GTAVYHVSNY AHNIKTIADG
GIKNSGNIVK ALSLGADFVM LGNLLAATEE SCSDYYFENN VRLKIYRGMG SMEAMVSQGV
SASLVDKGSV LNLIPHLVKA VKHGFQSMGI KNIQELHKKL YSGMKENKQY QEALKELKKL
KKKIEENINF YKNIKEKLIL AYESAWDKFG SKLKDMPFLN NFFENPFIGK LFGETELAAA
LREMKMYDKN FKLSELMYLF EYVISKHIVE SYLIGDEETL RLHCGSSAFN SLNASINERK
KKKVYLDTNV LIYKNHELKG AQRMEESSPW FIFTFHTQQI NCLKNKNDEI IEGKIDDIRE
VVYTIALSKH PEPEGLLYPY IVREFAIIGN TPSWMVLYII GLGLGDEKDI TVKGKELIEK
SDIVYLESYT SILFISKEDL EKFYKKKIYE VDRNFAEENC EEILNEAKNK SVSFLVVGDP
LCATTHHDII LRAKKENIDV HIIHNTSIIS AIGESGMQLY NFGQIVSIPF FEKDYKPTSF
YDKIKINLDN NFHTLCLLDI KIKERTIENI MKNRNIFEPP RYMTINEAID QLLYCEHKKN
VITKNTLGIA MVRIGSNNQQ IISGNLLTLK SLNYDKPLHS LIICASTLHD IEKEYFNIYP
NSAYRKCVRV QLIKNGKKIT AFVPGDGCLN FIDENDEVLV SGFGRSGHSV GDLPGVKFKV
VKVARVSLLA LFKEKKEKPR SMGIKGLTKF IADAAPNAIK EIKIENLMGR IIAIDASMSL
YQFIIAIRDS DQYGNLTNES GETTSHISGL MSRSIKLMEN GLKPIYVFDG APPELKGSEL
EKRGEKRQKA EELLQKAKEE NLEEIKKQSG RTVRVTKKQN EEAKKLLTLM GIPVVEAPCE
AESQCAFLTK YNIAHATATE DADALVFGTK ILIRNLINLQ QVLEGLNLTM NQFIDFCILC
GCDYCDTIKG IGSKTAYNLI KEYNCIENII QNIDFEEARN SFLNPKVKEE IKIDWNEPKI
EELKDLLIKE YNFNEVRVTN YINRLLKARK ATTQRRLDNF FVNNSKYYEV LNLKKNCTTE
EVKKAYRKLA IIHHPDKGGD PEKFKEISRA YEVLADEEKR KLYDEYGEEG LENGEQPADA
TDLFDFILNA GKGKKKRGED IVSEVKVTLE QLYNGATKKL AISKDVICSN CEGHGGPKDA
KVDCKQCNGR GTKTYMRYHS SVLHQTEVTC NGCRGKGKIF NEKDKCTNCK GGCVLKTRKI
IEVYIPKGAP NKHKIVFNGE ADEKPNVITG NLVVVLNEKQ HPVFRREGVD LFMNYKISLY
QSLTGFVAEV THLDERKILV NCTNTGFIRH GDIREIKEEG MPTYKDPFKK GNLYITFEVE
YPLVITNEKK EVLKFLKKQN ESEDLENSEF EVVTCQAVDK EYLKQRLKTL KNYLPYLCKI
LVDNTVYTKW DYLTMDESHF QNDNADDMTA RTWGNDWTVR KGAALCLDYL SNVYNDDILE
YILPHIEEKL MSDKWNIRES AVLTLGAIAK GCMYSLSPFI PKVLEYLIKL LNDEKPLARS
ISCWCVTRFS SWICHPDKWF EPVLLNLLKR ILDSNKRVQE AACSSFANLE EDALELLNNY
LHEIVHTIQQ AFQIYQAKNY FILFDVVGTL IDSVNIVKEN NELAHQIVNS ILSKWNNIRI
SSPYIIALME CMSCITSAYG KEFLKYSQNV IRTCIKFLVL LYIDDLIECS FDLLSRIILQ
SNFALIGDIS RFCTQYLILN DIIPFLIAHI THPSTPVSNN ASWAIGEISI HINSQYMEMY
VDEIIKQLIF ICNSKYHGCL LQNICITLGR LSSTYPKKII FYFPQFLKTW LKIMSHGTQE
NEKINFFHQF LSTMKYFLFF FIFYYFLFIG LLKCLIVNKL TSNGCKFIFW IADWFAQLNN
KMSGDLKKIR KMGNYFIEVW KSCGMNMENV EFLWASDEIN KKPNDYWSLV IDISRSFNIN
RIKRCLKIMG RSEGEDNYCS QILYPCMQCA DIFFLNVDIC QLGIDQRKVN MLAREYCDIK
KMKKKPIILS HEMLPGLLEG QEKMSKSDEN SAIFMDDLEA DVNRKIKKAY CPPNIIENNP
IFSYAKSIIY PYYNKFHLVR KEKNGGVKMF VKEEIIDTEK LSDIINKKKE NVKYMKGMKI
PDNIVAVSDL NEATEEADLL VFVLPHQMEK VLSSIGKLSV VAGGLSLIPY TFIYDVDGGE
RCVMFNRFGG VSEKTYGEGS HFYIPWFQTP YIYDIKMKPK VINTTTGTRD LQIVTISLRL
LFRPHTQHLP YLHSTLGPDY DERVLPSIGN EVLKAVVAKY NAESLLTQRD KISKEIRESI
TARAKHFNIM LDDVAITHLS YGKEFAKAIE DKQVAQQESE RVKFIVAKTE QEKIAAVIKA
QGEAEAAKLI STAVKEYGNS LLEIRKLEAA KEIAENLSKS KNITYFPSSS NILYINVLKE
ENGGYNFENL NRNEILKEKG PQFRKTGTTI CGLVCQNAVI LGADTRATEG PIVADKNCSK
LHYISKNIYC AGAGVAGDLE HTTLWLQHNV ELHRLNTNSQ PRVAMCVSRL TQELFKYQGY
KVCAIVLGGV DVTGPQLYGI HPHGSSCLLP FTALGSGSLN AMAVLEAKYR DNMTIEEGKN
LVCEAICAGI FNDLGSGGNV DICVITKDGT QHIRSYKQPN VRLYHLPKGT TPVLCEKIEN
IKKYISLNDL GEARGTVLSV KLDELIDNVE GQTVIDPKGY LTNLNANDAD VADINKARSL
LKSVISTNPK HGPGWIAAAR VEELSQRKDK AKEIIMKGCI ECSKNEDVWL EAVRLEKISE
AKIIIAKAIK HIPTSVKLWL EAYKKEKNID DKRKVLRKAI ECIPNSVRLW KEAISLENEN
NAYILLLKKS RVQCNTNNKN INPFISEALK ECPSSGILWS KAIELENKNL QNSKSVSAFN
NCGNNAYVVL TVAKLFWFNF KIHKARKWFY RVITLNPYFG DGWATFLAFE IDQQNEINQK
DIINKCIKAE PNRGWIRGRI HDIRGKGSLS FIILRHKIYS LQCILDINNN NDKNMIKWVN
NLSLESIVDI YGKLIKPEIP IDSTNIKYEI HINKIFCISK TVKELPFLLK DANMKETSEE
STIKVNQDNR LNNRCIDLRT YANFSIFYLQ SQICQIFRNF LIQNNFIEIH TPKLLGESSE
GGANAFQINY FNQNGYLAQS PQLYKQMCIN SGFDRVFEIG PVFRAENSNT YRHLCEYISL
DIEMTYKYDY MENVYFYDSL FKHIFKELKN QYPSEDFKWL DVTPIFTYEE AIRLLIEDIL
NYDLTTDMEK ELGKIIKDSH NTHYYIIINF PSSLRPFYTM YDEKNPKISN SYDFFMRGEE
VLSGAQRIND VNLLLNNIKS LNLDSQKLNF YIDSFSYSSY PHSGLLFENL NKEYKFITTQ
DNFDGFRFEV DKNVNKYLQS THTLFLGTRD IGYLYQFGAN FTNSDNSLLM ISRINIDGSV
NGRFCKKINP DCKLNFNTFA KSDTRNMYEM SLEVNKPVYT YNIKTIWQGG VDLTYIASNC
ASIGSFGLRY NHKNNVITMQ VIRQPNFKSP EFMLNQTHLY KIQYAKKISD RLSLGTELEL
TPQTKESAMR LGWDYSFRHA KVQGTIDTSG KISVFTQDYS GFGVSGYIDY LNNEYKFGMM
HISPSQEQNT SIRIYIYIYI FVYIYFFFFF LATLGVEVHP LKFQTNFGKT QFNVWDTAGQ
EKFGGLRDGY YIKSDCAIIM FDVSSRITYK NVPNWYRDIT RVCETIPMVL VGNKVDVKDR
QVKSRQIQFH RKRNLQYYDL SARSNYNFEK PFLWLARRLS NQPNLVFVGE HAKAPEFQID

LNVVREAEKE LEQAAAVAID EEDGIITVKS IFKEPTISQY NIKQLIKTKI EENCPFYNYQ
INRTIAEKIY GDTIYDNYGL SKEIEVNLII LEEWNINCNR NRVLKHSGLI KNIEINKFKY
LNNKESLEVH FLVNPKYTDI LPKNKVLPPS GINYNKLIKE FGCSKISDEH IRKIEKLTAH
HFIRRGIFFS HRDLDFLLNY YEQNGYFYIY TGRGPSSLSM HLGHLIPFYF CKYLQDAFNV
PLIIQLSDDE KFLFNQNYSL IEITKLTKDE MSFILYNSNS GLANALRRII LSEIPTLAID
VVNVYENTSP FHDEYLAHRL GLIPIDSRNV NNYEFREKCK CKETCSKCTI QYVIEVKCNN
KIDISHYDIL DHEPNIPMPI PIPIVTLSKN QTLHMKLIAT KGIGKMHAKW IPANVSYRID
HKIKIKHHLI DKLPQSHKLM LANNLNKDCY VLLKLNENMS VVMAENCIEI LNELGYKDVI
KIIYDDTKFH FHIESVGSLP PEQIVEMALE VLENKLKNLE PQIKSSFYSI DEVAKQLKFT
RSSVSKKREE KCFLNLLKKS ILKNPKKWTN ISKKIIGVSE ETTTGVLRLK KMDKNKELLF
PAINVNDSVT KQKYDNVYGC RHSLPDGLMR STDFLISGKI VVICGYGDVG KGCASSMKGL
GARVYVTEID PICAIQAIME GFNVVTLDEI VDKGDFFITC TGNVDVIKLE HLLKMKNNAV
VGNIGHFDDE IQVNELFNYE GVHIENVKPQ VDRVTLPNGN KIIVLAKGRL LNLGCATGHP
AFVMSFSFCN QVFAQLNLWE NRKYENKVYL LPKELDEKVA LYHLKKLNMY TYGVEGDDGY
LQQAQYPSPY ENQYESPSPR GESHTPFIGY FSSHLLRTGF FLQCVSLILM FIFYWAFGGT
GIFVFDLYAG PECVKVSSAF HLTISILMAI YLLGTLYIAM FQVFVADNSK WCRGFRAGSK
LLSAAVTLDL LSSILRLVQY LYAYFYMNMR WWARYQQTKS DWTLLHFGSI VHSFALFIYG
AAFFYMEAYH DEGTYEELAW SNLTLFKLAG LAGINKPKKR TFRTFQYRGV DLDKLLDISQ
DELIKLFRAR QRRKFKRGIS KKAKSLLKKL RKAKKECEPG EKPKPIPTHL RNMTIIPEMV
GSIVAVHNGK QYTNVEIKPE MIGYYLGEFS ITYKHTRHGK PGIGATHSSR FIPLKVMVDM
FLWRDPEQFE LKNLANEENA ATAPHLVENQ FATEAPYEEW GFLQIIKDHH ETMYELKQKI
RPRDQVVGWF CSGSELSELS CAVHGWFKEH NSISKFYPHS PLNEPIHLLV DAALESGFLN
IKAFVQLPIS LVKEYFVHFH EIQTELLPCN VERAEVSLKK LLIMLKQCKS YVQDVIDKKK
KGNLSVGRYL HKVLSNDTFL SLEKFDSLNE NILQDNLMIS YLSNLANLQF LIAEKLNWVK
NFPIKKNYPK GRGVFKNLIG LVSQNLPLFF GKGGLKPSKK IEIFFTIFET DFLDRGCKKK
IFSQKNFLDQ FFLKNLALTH KQGKKLDFFN GSNLLNFYEN NLNLKPYLKI IKDFERYPII
VDADKKIISL PPIINCDHTK ITLNTKNIFI ECTAIDKNKA EIALNILCSM LSEYCTPKYS
IYSFLVKYPL FQKKMLKCDI DYVRKLSGIS DITIDSIEKL LKKMMIDEFC FNIEVPFYRS
DIMHCCDIIE DIAIAYGYGN ILPQNSKFSK KNILNTCSDL FKNALVECGY TEVMTNTLVS
KKENYDYMLR PVQILNSKTS EYEIVRTSLI VNLLKFVSTN KHRELPLRFF EIGDVAYNMT
DTNAVNKRHL SVIFADKTAG LEELHGILEA ILKEFNLFSD YKIEEKKKEN IYVRVISIGN
IRGFGGGDYG SFRMSNEFLG WKNKKTNNVY QYKFSDISEG EWIKTSYNNN RLHLKLIESK
DNLIVYFDGF PDRNINEITQ HFQKYFNIRL SNRKITTKGW NWGEFKFENS NLLFDIDKKY
AFNIPTNNIN QLNVQIKTDI AMELKNEENE DSLAEIRFYY PHENDENQTF QNLKNDLLEK
VNIGDSKSES IASLTNIPLL VPRGRYEIEM YIRTFKLHGK SYDFTIQYTN INKMILVPKS
NSNQYVLIFS LNNKMKQGQT EYPFILIQLN NDDDMDININ ANEEIIKKYK LEKTISGKPY
DVVTRLFTSL VKKSVIIPGD FRTAKNQYGI TCSYRAASGQ LYPLNKYFLF IIKPVILISF
EDIVTLSFQR TGNNQHRFFS LIIKHKRGIS YEYTNIDKNE YVPLLNFLKS KNLNIKGYID
LSKRRVSPKD IIKCEEKFSK SKKVHQTVRH VAQKHGMTVE ELNRRAIWPL YKKYDHALDA
LKEATMNPEG VFKGLDIDEE IKKSLLADIQ LRLTPQALKL RGRIDVWCFS YEGIDAVKEA
LKKGKVSINI KLIAPPQYVI VTSCHDKELG MAKIQEAMKV ISDKIKEYKG GDFKQQGDIL
VILLDKHDEL SSEEEGERSL FEFFLCDVGV GLSLAVRDTL PIEYDSIFIG VLPYYTFHHE
YIIYDNSQLL PRYLIQFECD PSADEHFAIP LCDYCQDAPS AFYCESDEVR LCEKCDNIIH
SNKLVMKHIR KSLNEAQGKC KIHLHNDINM FCTVCHVPIC ELCMSIHSHM SLNMAYKAII
QYSSKPSKLI KERKRNLNDL LNKIEKLYEQ VKLNIHDAEE NVYTILEDLV KQLHMITEKK
MCSVLSEEYE LKRQFNEIIW NESFLHYLQT ILPPADFMNA WLKHCKRREE IEKNSLVFPD
MRIKGNINIV TEGSIEHLVG LPNVGKSTTF NVLTKLNIPA ENYPFCTIDP HEAKVTVEDD
RFDWLVNHFK PKSSVHAYLS IFDIAGLVKN AHLGEGLGNN FLSNIAAVDG IYHVVRAFEN
EDIIHTEGNI NPVRDMEIIN SELIYKDISH CEKNLEEINK VNRNKKDKIK QNEHDVLTGV
LKHLKENKWI KDGNWKNSEV EVINDFNFLT AKPVVYLVNM SEEDFIRQKN KYLAKIYNWV
QENNKGTIIP YCAEFEQKIL NMTEEEKIDY FKQKNIKQSM LNKIIKTGYY EINLIHFFTC
GQDEVKCWTI RKGTKAPQAA GVIHTDFEKG FICAEVVKYT DLVEHKSESE VKANGKYLQK
GKDYIVEDGD IVFFKFNVSS SGRINKFVDH LRLPYYLLEE FVDNFVYELK KGLEAHRKHP
NLWIPHECSF KMLDSCISEI PSGKEKGTYY AIDFGGTNFR AVRASLDGNG KIKRDQETYS
LKFGTFSHEK GLLDKNATAS QLFDHFAERI KYIMGEFNDL DSKEVKSVGF TFSFPCTSPS
INCAILIDWT KGFETGRATN DPVEGRDVCK LMNEAFTRSS VPAKVCCVVN DAVGTLMSCA
YQKGKSTPPC YIGIILGTGS NGCYYEPEWK KYKYSGKIIN IEFGNFDKDL PLAPIDLVMD
WYSSNRSRQL FEKMISGAYL GEMVRRYMVN VLQSASSKKM WITDTFNSES GSVVLNDTSK
NFEETRKVAK DAWDIDLTDE QVYALRKICE AVYNRSAGLA AGAIAAIAKR IKICGVDGSL
FVKNAWYCKR LQEHLKVILA DKSENLIIIP ADDGSGKGAA ITAAYFVIRW LCKAIVRSLF
RDVNVINPEN VPLYGSVIFV GNHNNQFIDA CVLVANIPRQ VKFIVAEKSM KRAVIGKLAS
IIGCISVKRP QDLKFKGIGN IFWEIGDIRI KGVNTRFRLD VQIGDKLMTQ NKIFCVTKIE
SETELLIQEA INIECDDNGV PFKIIPKVNQ SDVYNLVTNS LKNGDTIGIF PEGGSHDRTN
LLPLKPGVAI MTLCALADGD VSIIPVGLSY SKLYQLQGCV TLFYGNAIMI SQDLCKDYNN
NHRETISKLL TKIEEGMRSC MLTSKDHETS RCIELCVSLY TPERMTISKN KIYNNLQLFC
KMFWKFGNTK VIKNLCYQLQ CYEKLLQANK IKDDEVWMLK QSTSAATLKF IEHICSVIFC
VIFGMTFSLL WLPLVLISIH LAEKHREDAL KNSTVKIQGC DVVSSYKVLV LIVLLPTFNL
FYGLIFSLYR KMKKKDIDGL HNLLNNYLEQ FKLHAVFSKE DIAHWFLPID NVIYTYVNEE
NGEIKDLISF YSLPSQILGN EKYNILNAAY SFYNIATTTS IKNLMQDAIY LAKKNSFDVF
NALEIMHNKS IFEDLKFGEG DGMDIDSVLG LQAILISANY KEKEFIRIAY YMNAFYKDIE
LREKPPLIPQ YDKICRHIVE NPRIVKFNIG WDDAYKVYPI LKELKQKDYF RIFKVNLHIS
CKFLKISEKC KEMKKCSVCE CDDDKIPYNF RTNEIEVDLV YNSPCFTAYE GKNIWELKGN
NNYKYFGAAK NLKGVKELLF RENEERKKKK KDKRNLDKFV NIHYFGYCDD ENEILLNEEL
KIQKKLMKHG TLVLPSDRAR EYLDCLGKEV DIQFVDMNEK TMKRQYKKYI QRIDDMERIL
RFLEENINKL PNVKIKKSKI DNFLEHDNIY ELDQVEESLN RLYVQFVRFC NNNKDLVDER
NNAIEEKHVI LAAINQLKPS LSTHIMKDGI NMMFTNISGV IKTKDQESFS RTIFRALRGN
AYTYFQNIDD KSVFVVYCQG SAQSSIYEKI MKICKAYDVK TYDWPKTYEQ SKKRLRELKE
IINDKDKALK AYEEYFINEI FVLINVVEPN KNSLIEEWKL FCKKERHIYN NLNYFEGSDI

TLRCDCWYSA SDEEKIRHIL INKSSNDLVS ALLLSDKLTP NISPPTYIKT NEFTKSYQVI
IDTYGVPRYG EINPAISTII TFPFLFGIMY GDVGHGFCIF LFAIFMILIH NKVKNNEMIA
MFFDGRYMLL LMGFFSVYAG FLYNDFFSMP LNLFSSPYIF GFDSNWLGAE NELIFINSFK
MKFSVIIGFL HMTFGTIIKG FNTLYFNKKL DFFFEFIPQL VMMICIIGYL VFLIIYKWVT
PGGYEKQGII NTIINMYLHK DIDKTNQFYS HQSIIETLLL SLFIISIPVM LICKPAIRTY
KIMKEIWIEQ LIETIEFVLG LISNTASYLR LWALSLAHQQ LSYVFFEQTI LNSLKQDKFI
TVLISLIIFV QLFSILTILI ILCMDTLECF LHSLRLQWVE FQNKFYKGDG IPFRPFNIKK
ILPENYSAIL ARALSERPLT YLPTIERVCY EVLNDEDEHL NYIQINLLNT IRPTPIRGLL
AATQERFVVV PGIIVQASKP QHKMRKITLQ CRYCDHKMSI DVPLWKDKPQ LPPYCRYVLE
PYVILPNECT FVDIQSLKMQ ELPEAVPTGD MPRHLQLNAT RYLCEKMIPG DRVYVHGVLT
SYNPNPTRAD GTNFSYLHVL GFQKYDGNDL NFDVEERNEL TLLAAEHDIH EKIFKSVAPE
LYGMDEVKKA CACLLFGGTR KRIGEETKIR GDINMLMLGD PSVAKSQILK FVNRCAPVSV
YTSGKGSSAA GMVRQGLLGE NEEKLDYVLG LTLPKLLERR LQTKVFKLGL AKSVHHARVL
IRQRHIRVGK QMVDIPSFLV RVDSEKHIDF ATTSPFGGAK PGRVKRKTLH NIFLFYMKDF
PHFFIFFFSS EYAIKAVKNT NITSIGVKGE NCAVIISQKK MATQYISQDK LLDYNNITNI
YNITDEIGCS MVGMPGDCLS MVYKARSEAS EFLYLNGYNV NVETLCRNIC DKIQVYTQHA
YMRLHACSKK NITEDNIKEM FSPYGSVEEV FIMKDNTGLG KGCSFVKFSY KEQALYAINS
LNGKKTLEGC TRPIEVRFAE PKSAKQTVKV CSRDLPSHTK MKTRDLSDYV EDKTLIETIN
LAVKYAKDAV IEDEKKNYKE ALNLYIQSLQ YFHFFCKYEK NANIRELILK KMEIYMARAE
SLKKIINIMN KNNNVKWSDV CGLELAKEVL KEAIIFPLKF PKLFNSSSLP YKGILLYGPP
GTGKTFLALA CANECSMNFF NVSSSDLISK YQGESEKYIK CLFETAKENS PSIIFIDEID
SLCGSRTDGE NESTRRIKTE FLINMSGLQN YENNIIVMGA TNTPWSLDSG FRRRFEKRIY
IPLPNLYARD IKYFATLTEN YTGADIDIIC RDAVYMPIKK GLYVKCGSRY EGMSVMIENM
AFHSTAHLSH LRTIKSLEKI GATVSCNAFR EHIVYSCECL KEYLPIVINL LIGNVLFPRF
LSWEMKNNVN RLNLLREKLF ENNELYITEL LHNTAWYNNT LGNKLYVCES SIENYTSENL
RNFMLKHFSP KNMALVGVNV DHDELTKWTA RAFQDYVSIP FTNQKEVTPK YTGGFVSVED
NVKKTNIAIA YETKGWKSSD MITLTVLQTL MGGGGSFSTG GPGKGMYSRL FLNVLNNYNF
IESCMAFSTQ HTDTGLFGLY FTGEPTNTVD IINAMALEFH KMNKVTEEEL NRAKKSLKSF
MWMSLEYKSI LMEDLARQMM ILNRILSGKQ LCDAIDAITK EDIYKVVNKF LKSKPTVVVY
GNINHSPHYD EICKILFLVN KIHFFMGYPE LSSVNFGNST VVRCKKCRTY INPFVRFESG
GKKWNCNMCY HINDTPQFYK RKDLFQRPEL CTGSVEFIAP SDYMIRPPQP PVYLFLIDVT
VTSVNSGLLD VICNTIKKLL DSRTLIGIIT FDSTIHFYNL NSNLKQTQMM IVPDIQDIFI
PLPEDILVNA HECQNIIDNL LDNLPTMWRN NKVSDCCAGN ALKAAFMVLK KIGGKLLFFL
SSVPNIGENL YAELAQNITQ YQIAVDLFAC PYNLDLATIY PLIKNSGGSL YYYPFNVHQY
NEKLREELLF ALTTETAWES VMRIRISRGW KITNWYGNFQ FRGVDLLALP NCHSSQSFSI
IVDLEENVVQ DSVVYVQSAL LYTNSNGERR IRLHTYALPV TQNIKTITDS INPQVVVSLL
SHQAIDISKK GKIADGRNLI QTLCSQVLSS QLLPSESAKL LSIYILGMLK SVSFRDSDLR
IYHWSRIENI PVESREVYFY PRMFSLHNPD TLNLTCDNMT QDGCYIIEDG ETITMWIGRY
YSLAVVNIAA GLDGCDDQLL PASFRALEAD LDLHPSLLGY ITLVQTLMLS LFSPIWGFLS
DKYSRKWMLV FGTALWGIAT ILLANINDFA HILFFRAING LALGSIGPIS QSILADAAKN
ELGLSFGLVQ LSSSTGRLIG GVITTTVALK YFGGIRGWRL CFIVVGILSI LLSITVALFV
DDAPSLSKKS IIIILLEGFT GTIPWLALSF NTMFFQYCGL TDLQAAVITG FLLVGAALGG
VIGGLFGDVM HDISNDHGRP FLGQLAMFGR VPLVILTYIV IPKRQESFEL FLSCFCLGMS
SIAGVAVNRP IVSDIIRPDY RGTVFSLTIA IEGVGSSLIG APLFGYLAEN VFNYQNNNLL
ISDMSEDLRK NNAEALSKTL LYLTLIPWIL SFVFYSLLHF TYGKEYRKMN KIIENEMLMF
GLRSLSDFCN PTSKIYKENA YDALDRGAVG SIKNAVVNYK DDDDILFCSS KILCAMSDYC
CSEKDQEALQ KLITDGGAIV EIIKSSPSDQ ETVKNCMSFI QNLGIGLLNV FTSNSYNTKL
GKEIANALSV AAKSSSGAKA LNDEGAHHKL IDQCLDDVAS MVEGAFNTIK NMASNGYIDA
TIIEKSVLIL DKFKSYPRVV SKGSDAMKCA VGPEQLTDCL NVLKKSEQGS KEQDSALELL
SSLSYISSIT DKVVQSGGIP VLIELINSGL QQYESNPEKI SRLVAGASRM LGRISANPPH
AAIVDEYGGI ATLCTALSYF PNDPECASAI CNALTPFVSR SYTLFASLLP ILYGSLESIE
LAKASMECIA SASMINEFHE HMVNNQAIEI LSTCIQYHLT ETDYLLNCLT AYFRLSDYIT
SIESINQYGG VTGIADALSA VDAVLTIMLE NENEEVIIQE GTKIMEKLAT ESDCQRHISN
LESVAQSNPE SAYKTLAAIS GLSRIESLRK ILESKKADYS IFNGIKVWIE APRFNDQTKL
IKAGLKTIKI VDIMCSANVK RIIEEEPDEN ILITSVVCIN DLTKVSKISS TEIVEANIDS
ILKLMKKYSE SRLTQTNLLS AINNILLSSD GADVLVNKGY IKQITNYLQK VPMYVDVQII
GFTVLANLIK INPESIEAIR KANALNPLQI ALRTHVRNNK LKMTCAPLLA LLMPLDSLFK
EIEELLKLCD NSMKEKNIAK LHECLVSLNE LLLTPEASKI SSRCNIISEI GHACINISQT
RIGLVHLTKN KMASALIEAY DLLQLPGNDY TEEAVSNILE SLSLLLKHDI INADTAFNSG
LVKKLCVGIN YFAESDVVIK STCGCLACMC TNGKRVNQLI SHPEYEELIS LIVKLIGNLK
HSRANAIKAL YELLKTQKEE IIVHLSNKTH VINLFKIMEE YQDYLPIVQE SSKCLAIIAD
YVTLEEDKHS AIKILIESLN KNKNDEETAL EILRVLVKLC NANDKMQLKE LGAIDVVSDV
TMIHNDNEEI SKLGGVLFSY MGADEQVKKL MKLILNVKRD DPDSVQKIDN LTGKLEMFLR
APLENPQDAL QYTEATLQVL NEYLASEVDN VSLQANIALV TKRLVDRVKY DYEDQLGSWV
VASAGTLNQY MDMISNKIGL SNAKFVSPVY SVLAGCVLNM DRKAREYAQD LLEFIRNSGS
SFLACKNLRE KLKKNNFIHL KEWNLKTNEA YVLSKENRNI CSFFIGKNFN MENGSILISI
GHIDSCSLKI SPNNNIIKSK INQINVLIEK LIQINKSLFF LPSLAIHLQK RTRSPNINYE
DHLKPILSTT LFDNMPLLYI LSKELNCKEE DILDFELCLI DAQDPCFTGA YEEFIEGPRF
DNLVGSYCIF EAFIEFVLFF ITIYQIQCQL RFASLGDWGK SKSQLLNAKY LKQYIKNERV
TFIVSPGSNF LDGVWKNLYE DVYAEENMYM PFFTVLGARD WTGNYTSEVL KGQGIYPKWI
IPNYWDHYFT HFTGSTGPTG HKDMAAAFIF IDTWILSPNF PYKNIPERAW DDLKFSINCL
QKLQISGDQP IYSSYLLPLL KDAQVDLYIA GHDNNMEVIE DNDITHVTCG SFCLHELNNN
GIITKFISSK SGSLQYVASL PKVELMDIPA SGPMGNKDTF IKIVGTVGLL IRYAYSGVAY
SAGALNLTKN WCMNPDQGLI KPDIVFYLVC IYDNDICEIS DVGVNFYISE SNVHNKSDAV
LKHLQELNNY VKKIAFLCCN IYGLCGYIFV DFNNEFVCYD QTGENVKSCN VSKISKESEG
KVSFDFDKSA PFQNGDYVKF TDVEGMEINN KIYKINNLQK YTFTIGDTSN FSNYIKGGIC
TQLKIPLKLN FHSYEDYAKI DMSNQLHVEE LKKEIVYNVS RYCKSHIAPV ASFFGGLLAQ
EVIKFTGKFM PIYQILYLDF FEKNDNIISI FGKSFQKKLN ELNVFLVGSG ALGCEYSKLF

CLLDGVLTIT DNDNIEISNL NRQFLFRREH VGKSKSHIAS EIIKNKNKNI NVQSLETKIC
AENEHIFNES FWERQNIIVN ALDNIQARQY VDNKCVWYSK PLFESGTLGT KGNVQIIIPF
LTQSYNDSYD PPEDSIPLCT LKHFPYDIVH TIEYARDIFQ GLFYNTPLNI QQFETLQNVI
NTLKVSNQCN FEFCIRKSVE LFHTNFINQI NQLLHSFPID YKLSSGEYFW IGQKKPPQPI
SFDLNNEYVT EFLLSTSNLY AQVYNIPSCY DINFIKEVIE VKPFQPKSVK VNIDPIEFDK
DEETNLHVNF IYSFANLRAK NYKIETCDKL KAKIVAGKII PALATTTSMI TGLVGIELLK
YVNYLSYFKN AFINSALPLF LFSEPMPPLK TVDKEYDELM KGPIRAIPNG FTSWDKIQIS
IKDYDTQRDK RFSGTVKLSN EVRKKLKVCI LGDAVHVEEA QKLQLDYMDI EAMKKLNKDK
TLVKKLAKKY DAFLASQVIL PQIPKLLGPG LNKAGKFPSL ITHNDKIFLV YNIASFCFEL
QFRFIEDTTF DWLPFLGYLL PYEKIKLLRM LFPIVFFISI CASAHIYTDK NATMIFLMRS
ILSTNRIIVE RSNDVNYLKK HGTDIISKLQ HIRKFSLTLD SQQMNLLEKK NKKKSWFEFF
SLQMIFGIIF VYIWLTSKKP ATYEVPLSLT LEELYSGCKK KLKITRKRFM GTKSYEDDNF
VTIDVKAGWK DGTKITFYGE GDQLSPMAQP GDLVFKVKTK AHDRFLRDSN HLIYKCPVPL
DKALTGFQFI VKSLDNRDIN VRIDDIVTPK TRKIVANEGM PSSKHPGMKG DLIVEFDIVF
PKNLTSEKKR IIREVLGLYY ICISCPSTNW FKSTSIKWTL SIDVGGTDID LENLAKKSEL
SETLSILHVD TGKTKLLDKL RHTNVQDNEA GGITQQIGAT FFPKDVLEKE IKKIDIKCLS
KGIMIIDTPG HESFYNLRKR GSSLCDIAIL VIDLMHGLEQ QTKESIQILK QRNCPFVIAL
NKIDRLYMWN KNDWSPFNNT FKKQKQDTQD EFYDRLRQIL NQLAEQGLNC QLYWENTNPR
KTVSIVPTSA ITGEGIADLI MILVKLTQSF MLKNIQYHDK LECTVLEVKN IEGLGTTIDV
ILTNGILRES DTIVLCGING PIVTVIRALL TPQPLKELRI KNEYIHHKYI KACIGVKISA
NNLEEVLCGT SLFVANNTEE IEEYKKKVMT DISDVFSHVD KTGVGLYVMA STLGSLEALL
IFLNDSKIPV FAVNIGTVQK KDVKKASIMR EKGRPEYSVI LAFDVKIDPE AEKEAAILGV
EIMQKDIIYH LFDAFTNYLK KIEEEKKQSK MTDAIFPCEL SIINDCVFNK KDPIVMGVKI
ESGILKIGTP LYIPEKNLKI GNVVSLESNK KSCDKARKGE EVCIKICGEP HVTYGRHFDF
NQKIYSKITR ESIDVLKEYF RNELTMEDWK LVVHLKKIFN IIMNINEKDK LAEQNLENLD
VTKLTPLSED VISRQATINL GTIGHVAHGK STLVHAISGV HTVRFKHEKE RNITIKLGYA
NAKIYKCTNP ECRPPECYKS YESSKEDDPM CPRENCNHKM KLLRHVSFVD CPGHDILMAT
MLNGAAVMDA ALLLVAGNES CPQPQTSEHL AAVEIMRLKH ILILQNKVEL IKEEQALKQQ
EEIRNFVSGT AADSAPIIPI SAVLKYNIDV VCEYIVTQIS IPKRDFISPP HMIVINFMRI
NNFIGIFIYI TFSFRYASLF FITCIDKNDN ELITLEIIHH YVEILDKYFG NVCELDLIFN
FHKAYYLLDE ILVTGELQES SKKTVLRVVA AQDALMEDNK FAKSYXDVAD NLSLAIQNIN
EESLKHNENI HKGIQMTETI LHNIFNKYGI NKYNPINEKF NPSLHEAIFE INDNTKKGTV
ATVIQQGYKI KDRILRHLDI ESVQALIVAL NLYKGGIIII SHDTYLIKHV ADEIYHINNC
TKLVKINYDF DKYTKLILEN KIMPREIITL QCGQCGNQIG VEFWKQLCNE HNIDQEGILK
NNFLNEDRKD IFFYQADDEH FIPRALLFDL EPRVINSIQT SEYRNLYNPE NMFISKEGGG
AGNNWGCGYS QGHKVEEEII DMIDREVDNS DNLEGFILSH SIAGGTGSGM GSYLLELLND
NYSKKMIQTF SVFPLLNESS DVVVQPYNSI LTLKRLILST DSVVVIDNTS LNRIFVDKLK
LNNPTFQQTN TIISNVMSAS TTTLRYPGSM NNDMISLISS LIINPKCHFL VTSSNVQKTT
VLDVMKRLLH TKNIMVSVPV RRGMYISILN IIRGETDPTQ VHKGLQRIRD RKLVNFIKWN
PASIQVTLAK QSPHSTHKVC GLMMANHTSI STLFERCVTQ FDRLFKRRAF LENYKKEPMF
QGNFEEMESS KEITQNLIDE YKSAERDDYN CIYRAFGIPV RKYTHEVVTL WYRAPDVLMG
SKKYSTTIDI WSVGCIFAEM VNGRPLFPGV SETDQLMRIF KILGTPNSQN WPSVTELPKY
DPNFTVYEPL PWESFVTKYV LSIVYNSIFF SFFIYVLNNS IFIIFLVMGK EKTHINLVVI
GHVDSGKSTT TGHIIYKLGG IDRRTIEKFE KESAEMGKGS FKYAWVLDKL KAERERGITI
DIALWKFETP RYYFTVIDAP GHKDFIKNMI TGTSQADVAL LIVPAEVFEG AFSKEGQTKE
HALLAFTLGV KQIVVGVNMD TVKYSEERYE EIKKEVRDYL KKVGYAADKV DFIPISGFEG
DNLIEKSDKT PWYKGRTLIE ALDTMEPPKR PYDKPLRIPL QGVYKIGGIG TVPVGRVETG
ILKAGMVLNF APSAVVSECK SVEMHKEVEE ARPGDNIGFN VKNVSVKEIK RGYVASDTKN
EPAKGCAKFT AQVIILNHPG EIKNGYTPVL DCHTSHISCK FVNIDSKIDK RSGKVVEENP
KAIKSGDSAL VSLEPKKPMV VETFTEYPPL GRFAIRDMRQ TIAVGIIKSV EKKEPGLPIV
LLKEGTDKSQ GKSQIIRNIN ACQIIVDIVK TTLGPRGMDK LIYTDKEVTI TNDGATVMNL
NITHPAASIL VDIAKSQDDE VGDGTTSVVI VAGELLNEAK GLLNDGIEPN MIIDGFRNAC
NVAINKLNEL SLNFSSKNEE EKRNILLKCA QTALNSKLVS NHKAFFAELV VNAAYKLGDN
LDKSNIGIKK VTGGSCLDTQ LIYGVAFKKT FSYAGFEQQP KKFNNPKILL LNVELELKAE
KENAEVRIEN PNEYNSIVQA EWDIIFKKLN LIKDSGANIV LSKLPIGDIA TQFFADHNIF
CAGRVEDADL KRTANATGAV IQTSLFNLNE NILGNCGIFE EVQIGNERYN IFKECLKTKS
VTIILRGGAK QFIEEVERSI NDAIMIVLRC MSNSEIVPGA GSIEMQLSKY LRIYSRSICN
KEQIVLYSFA KALESIPRHL SHNAGYDSTD ILNKLRKKHS EGTNDIWYGV DCLEGDIINA
YEHCIFEVTK IKRNVIYSAT EAACLILSID ETIKNPSRNL GLPDCFKDLL KTDKIKHVLC
TGNVGSRENL ELLKNIADSV HITKGDMDDN YDFPEEISLN IGDFKISLIH GHQIIPWGNM
NSLLQWQKKY DSDIIISGHT HKNSIVQYEG KYFINPGSAT GAFQPWLAET VPSFILMKNS
IVVYVYEEKY GKTNVEMSEL RKYSVKISED VGNECTGVYR SPMCKDRLME NFEDCLGSRI
KENNKLGEYK WKTYGEVKEL VMLIGSGLGF YMPNNPEWTI CDLSCNAYNI ITVPLYDSLG
IESSKFILEQ TLMKTIICNK SCALNLFKSL PANPDAIASI CYTSGTTGFP KGVIMVNRNM
MATLNASMLE LLKMSNDKDT HISYLPLAHI YERMILFLCF VFGIRIGYYS GNIQALIEDI
QVLKPSIFIS VPRLYNRIHE RIYNSLKKKS HLIQALFNKG IDHKNGSGSY THFLWDKFLF
NKAKKIMGGN IKVMLNASAP ISCDVVKKLK SIFCAPILEG YGMTETMGFL SHMYDPLMGH
IGGPIACMEF KIVSVPEMNY FINDKPPKGE LYLRGPSIGY FKLEKETKED GWVCTGDIVV
LNDNGSVSII DRKKNIFKLS QGEYVAVEKI ESSYRQSLFI NQIFVFGYSS ESFLVSVIFP
STDTIEIWKK NKKIDKNDEE IIILPDFKND VINDLIKIGK NDGLKGYEQI KDIYFTLEPF
TIENDLLTPT GKIKRHVALK KFKSQIDQMY GDKIKKTINE GQTLLTVFKE GYAPDGVWLG
GTKFQFINID RDLDFEGYTF DVATCAKLKG GLHLIKIPGG NILVALYDEE KEHDRGNNLN
MLAVVYLACR EAGHIKSIKE LITFDRSYKE KDLGKTINKL KKVLPTRAFV YNENISHLIY
TLQLSTDLIE AIEYVVKKAT TLIWSDVERY FKDPELITAE ILFVALTLCN VFVMYRLFLD
VIPFPIFVTW WQLAQGLLVA YVCGLKVLFI PSIFYCLMLV LSNYLLYKTP CIASYPVLVS
FTVVFHHLTR FVGCGEEYML RWQSIFFLLV AFIIGCFDSK IMGKGVILWA LLYALSSAIF
RAGFMQKIMH LVDGRGNTLH NNQHLLGVLI LPILIILSGE WTVFQYMPYD FTSLHTGCLI
TVGTMPFIKN VISNRLVRRT GQGPWRFLEI LSIILVFLIG MTFNAPTYKG YIAILCVIIG

RSIGAFDVLL NKVKVGEEMR NASFALAKAV WAAGDFKGQI IEGIKRPAVT LSLSTNNVAG
VKLPIFQVQI DPTVDVLGNL GVASGGQVIN NTRENYLQCL NMLVKLASMQ YINIFDWLNI
HSETFYTKKK KKKKKKKINL IRDSSTSCKD DNPYCSVNDG KVIIKNNELL SGIICKRTVG
SSSGSLIHIL WHEMGPDKTK DFISALQKVT NNWLEYIGFT VSCSDIIASN KILDKVKEIL
NKSKKEVTKL VKKAQRGELE CQPGKSLYES FETRVNNELN CAREMAGKVA SESLDEKNNI
FSMVASGSKG SIINISQIIS CVGQQNVEGK RIPFGFNQRS LPHFIKFDYG PESRGFVSNS
YLSGLSPQEV FFHAMGGREG IIDTACKTSE TGYIQRRLIK AMEDVMVQYD RTVRNSYGDI
IQFLYGEDGM AGEYVEDQII DLMKLDNKEI NKLYKYNFDE ESYGDYNKQN ILNQEFEELY
KCKNYLCKEI FPDGDIRQHL PINMNRLIEY AKSQFPNPID IVHKVNNFLE NLVIIKQINS
NDTLSLEAQN NATILLKAHL RTYLNSKLLT QTHKISLKGI DWLLQEIEKN FYKSLCHPGE
CVGALAAQSI GEPATQMTLN TFHFAGVGSK NVTLGVPRLK ELINIVKNVK TPSTTIYLDD
LVSNDQQKAK DILTKLEYTT LKQLTSHAQI IYDPYTTTTI LEEDKSWVNE FYEFPDEDQY
TLGEWVLRIQ LTNIHVNEKK LTMKEIVYII YSVFSSDELD IIYTDDNSED LILRIRVKYL
EDTFLKKLME QCLSTLKLRG IENITKVYMR EEAKINYDTE SGKFIRSSHW VLDTDGCNLE
NIFCAPLVDY KKTISNDIVE IFEVLGIEAV RRALLKELRT VISFDSSYVN YRHLSILCDV
MTQKGYLMSI TRHGINRVDK GPLIKCSFEE TVEILLEAAA FAQVDHLKGI TENIMLGQLC
KIGTGAFDII IDNQKLNDAN QNETIQDITS AGFTTPDTSP LPFSPTYNSN IKNIIIPGNI
RKSEHFLNLM RIVVVYLKKY INIYEITSEG PLSFLYKCEK DTKLDTSFFK YCFDRLKSLL
NTLQIVDDYS SLNIVCNFCT LLGNYFKGFI IICEPYPEAT IYDPVIQFAC LDSSIAMKSV
INKYKSIILT SGTITPLELY PKLLNFKTVL TASFPMSFDR NCVCPLIVTK SSDLVPLSSQ
FSLRNDINVI KNYGILLVEM CKNIPDGIVA YFPSYIYMEQ VISTWYELGV IANILEYKLI
FIETKDIVST TIALHNFKKA CDFGKGAVFL SICRGKIAEG IDFDKHYGKC VILFGIPYQY
TLSKILKSRL DFLKETYNIQ ENEFLTFDAM RQASQCVGRI IRNKKDYGIM IFSDIRYSRN
DKKNKLPPWI IKCMDISNVN LTIGTAVNIS KNFLLNMSQQ YKETGQTKNQ PITRISELSN
KLKIATVKNN CEIPTIGIWI SSGSKYENKF NNGVAHFLEH MIFKGTKKRN RIQLEKEIEN
MGAHLNAYTA REQTGYYCKC FKEDVKWCIE LLSDILTNSV FDENLIEMEK HVILREMEEV
EKSKDEVIFD KLHMTAFRDH PLGYTILGPV ENIKNMKRQD IIKYIQKNYT SDRMVLCAVG
DVDHEEIVKL AEKNFKPFFC GSEIIVRDDD SGPNAHVAVA FEGVEWKSPD SITFMLMQCI
IGSYKKNEEG VLPGTVNNIC NKMTVGCADY FTSFNTCYNN TGLFSKYLWK ARIYFIWQRL
FVSSNDYFYF LKIHIIDPFN IFKNIKLLSE KLKIYLLSNF SIYLAFYNYT SGYKKIMDII
SELSQFHYSF TGRMGIKRKY QKIPATILVL LKDLDPDTDI LEEPFFVDSQ NNLSFQEQII
LINYCFSLIR FNPHYDEIKF EKLSAVISRC LKYQNWLLHS CILWFKCKYE TFKFKTVDRA
AAQLNELLKE CYELEPKSIE RLKFIYDVYY PTTWEMKKEI GKVMIKIGSM ISAFNIFKDL
KLWEEAITCL IEAGRKEEAK ELLDDIIQKK RTPALLCLYG LNYYEDAWNL SNFKYSKAAR
FIGKYYYNKE MYKECCEYLE KSLEISPLFS DIWFILGCSY MKIDKFDEAI KSFTRMISMN
LAYLYMKKGT YKAAKICINQ AVKVNNNEWK FWDTYLKLSI LQNDVDSFCL SIRTLCQLNQ
VKQIQPWVFD YISDLIVKDK EYDSFWNAYS FFLFIKGKFT DSFDTKIKEI RSIEVNYIWK
NKIFVGKEAP FFKAEAVFGD NSFGEVSLTD FIGKYVLLYF YPLDFTFVCP SEIIALDRAL
HKFKERDVEL LGCSVDSKFS HLAWKKTSLS NGGIGNIKHT LLSDITKSIS KDYNVLFDDS
VSLRAFFLID KKGIIQHALN NLAIGRSVDE ILRIIDALQH HEKYGDVCPA NWAKGKEAMK
PSAEGVSDYL SKIEEDNTAK IISHDNLLTD PRIAQDFSNE TDELLKKAEE AIEIIYEISN
ILNVNLDKET IVILIQLCEY G

> ***Plasmodium falciparum***
NFHGDFKTTK KKIHWLPYLP QQLITCTLYE YDHLINKDDW TNFINFNSKH ETLVYAEPSI
SSLKVSDKFQ FERRGYFILD LIKIPDGKSK KAGINSSEEP TIVFPTIVYT GDEAFFHESN
LNIYRPIDHG HISDWDKAQK VWDYTLNCVD PSKSIKDILL TEPPLCSISH RKKMGEIFFE
YFDTLNLNLS VSGLMSLYAS GLTTGLVLDI GEGVTQCLPV FDGYIEKNSI IRSDFGGEEL
SMFLQKLICD IGYSMTTTKS LEYVKNIKET ICFCSLNPSE DQLRNDLAAT YTLPDGDVLR
DGYDSIEIAH ERFYVAEALF NPQLCHRDNL SIIDIIWKSI LSCPMENRKI LSSSIVLSGG
SSLFPNLVER IETEVRNNAP ESARSMVKVV TYEQGANFAR ENNLFFAEAS AVSKLNVKHI
FENLLQMEKS KLAKVEKVLG RTGSRGGVIQ VRAQFMGDSE LAGRFLIRNV KGPVREGDIL
ALLETEREAR RLRGTQSLNL KKSHCYCHLS TGDLLREAAE KKTELGLKIK NIINEGKLVD
DQMVLSLVDE KLKTPQCKKG FILDGYPRNV KQAEDLNKLL QKNQTKLDGV FYFNVPDEVL
VNRISGRLIH KPSGRIYHKI FNPPKVPFRD DVTNEPLIQR EDDNEDVLKK RLTVFKSETS
PLISYYKNKN LLCNVCYFNL PDPESTLGPY DNELNYFTWG PGFEYEPFNE KSSGGKISID
NASYNARKLG LAPSSIDEKK IKELYGDNLT YEQYLEYLSI CVHDKDNVEE LIKMFAHFDN
CTGYLTKSQM KNILTTWGDA LTDQEAIDAL NAFSSEDNIR IEKLFHLIDK NNDELNTWSS
FLKNEIFLKQ VQAEMGQIDS DKDGFISLNE LNDAFAQNLK EVEKHSEGLL KRFQIVDKDK
DGKLSINEVG LLIDPMKDEE LKELEINEIL EHHDVNKDGK ISKKDDEMAL DDFNFDANKD
GFIDKEEIIT YDLWNEKALK IAVTSLTDYG DVIRYPEDFK LLSNLNCFGS GFIFSIIMFH
LLPEFFFVFI GFCMQLGLEY VLPVDTNICC DSSSSEEEER VIKTYEGKRL HFYETTGNSL
NENMYNNDFN LLLKDYENLY KFMIKESADC IPNFAIIYLD KLSKYVEKTF QNNVEKKNLS
KNKAQTLNKL RAKIRKCSEF YQEKLNLYNE NPDDFWSYSE DDNYASDEED DKTKNAMSKW
GLKTSTKVEK KKTVKKEEKA NRVEDSQSAK NKGYAELLST KNLTEDVIRE RVKLVIEKRG
RKGLDKHEHI NILSKLCEIA KTISTQSYIE VLEHLINLEF DVVSSVYTYM SFNIWNKVFK
YVELILDILI QNENFYLVSI NIAEEITEET TNEKEKIIKS CKTLISFLAK LDDELLKALL
YIDVQTEEYR KRLGKTIHMI GLLKKGYNYV KAIHISSRIL EHMYYKPEML FKQIWTYLEK
TLEQERAEKR RLLSYHMHIS IELIECVNNI CAMLLEVPNL AKNTYESKDI ISRQFRRFLD
IYDKQIFNNP PENNKEIIIL ATKHLQKGNW KLCCEKIFSL SIWPKFPDKV QNILKEKIKQ
EAMRTYIFRY ISIYESFSID QLCVMFDLNQ NVVHSILSKM MINQEIPAFW NESSKFILIS
KVNPTTLQNL ALKLAENVNE VMEQNELALN MKNPKLGLQP KQCIVVGTRA LEFLNNELTN
IKTLSELHER VKKRSITDNN DNVLPLYLLE LFSIPIEQKE KNDFLTNRIS MYSKILNGRK
NVLDLLLNKF ENDCNDSIKK ELIDCFDVEK FKQEVNSKFM NILIQQLRKV EKLEKKKAKM
DFYLSVREQV KLYIRELVNI VSNLLTGNYP ILNLNGENFL KKYGGTLMDN LKDGLVEGVP
VRFMLAKEES NMFGVVQNAQ SDAKGKNILV NFYFRNNNTD EQVQVKSVEK DRLAVETLNG
DPSFLWYKMV RDDGWVGFDK AEIIKVFNNN SKIKDVLIRH TDLKILNQLA ITQFWAIKML
ISVPLATKIY AHFVHDSIKI SILTVLGDIA TALNRSFSKY LNFFANILSE TSKITITSGS

PDSDDWVNYV FELRDAILLT YSNIIYALID GNEINKLKPY IPNILDFIEL ILIKEINHFN
AQNFQNSVSL LGDLVHAYGY ELIENSKLTD LIISVYGKID ILSSQGDECV SKIKWLKRIC
NISILQLEFK SVEELNNATN NFIKAIKNYN EFPELRLKIL QLLYNSFNVN FSFFIAILQF
CSQNNIFHNM LPYIKYIDDW IKEWNISTRE KRHIYLIISQ ELKKLKKYEE SYEHLKKHIY
YYDDILNHPN SINASIELIV DAININNNIY FHEIINLHAI QNLQFIQHQP LFDLLLIFYK
YTINEFLVFI DIQTCESKIY LLSIISLFND HKVQNIQFIS KQLNISVVQI ENILVAAIGS
GVIDAKIDQI NQTVHMKTTI LRNFDDENWK QLNNQITKYI NNVATNFYKF IDSFASSTRA
IQNNPNYWCS SGNHSNDEEI TWTGYLNTKG FIKGVKVSWA YSPEFVKISV SSDGEKYRTI
IPYKKISSNE ASFDEIYFFK RLEEAMSIKI GLKNARHKYF GIREVKLIGG GNPYFLLLSG
ISSEEEMCLQ ELWKTNSNNQ VISAFSDPPK CLSVVNLDDL GDGKSNWIFE SNSQIRLQFC
ISQKNIYGNI PGIHDIDVSI YSNSTLDDDH NPDNTIDGNL NSYWASATFT DNDHLVYLVL
DLNKITDLSR IKIYWEYPPL HYNISVSTDT VVSENLANPS YITVDSLKNM ETRYIKISMI
KTHPKHGELG DNFLYGIRSI EVQANNLETV INHCRDAANS DDARDKYFVE YITEFDKDLT
NKLINLEDDV TKNVSSISDN LSKLEELLPN IETCLEEKKT YDEELKESKE KANDLIKPKC
SPEPLRVYCD MDSSTSIYIW NGINSVNDIR QHCAEVGLQP LILRSKNQLN SLIISLKKIG
YSLNGKVNIP LAYDYSCFHD LLNGNIDIST LIYESPDSTK VRQTALDEKQ MVICKIEDIA
KKRNKKYVKA QVLSKDSKIE RHKMKKFYNS KSSYFGCGWK KEWTPFIHAP FFENQHNAIY
KNRNKKLYEE IDTLLHGRIH PDIKVVELKD HKHPIRLCTP YNEDCYSVVY TGKKINATDD
RVIFGEYTGY VNNKELSQEK HQYMFALTFI ILPDNYTYAV DSSYMFNEMS LVNHYKTCFN
NFDFRINSEW QLVYLDGWPH IILTSIPGVE INPGEEIFAD FGFEWFEKVN DICLNEFIKN
NYFYRLEDIV EKYNLLKNHI TCNICMHNVN TDGNNFILCS GCNHVYHLKC VHKFNNENYE
WFCSSCLQFC FNIAKAIICR VTKMHFEANE DLFKTSSEFI QSVIRELALG KTKFQREFTN
GTFVGRVTEQ INNNNFFVVT YEDGDVEWIT PSFLFQEEHK LKGNEFYKQK KFDEALKEYE
EAIQINPNDI MYHYNKAAVH IEMKNYDKAV ETCLYAIENR YNFKAEFIQV AKLYNRLAIS
YINMKKYDLA IEAYRKSLVE DNNRATRNAL KELERRKEKE EKEAYIDPDK AEEHKNKGNE
YFKNNDFPNA KKEYDEAIRR NPNDAKLYSN RAAALTKLIE YPSALEDVMK AIELDPTFVK
AYSRKGNLHF FMKDYYKALQ AYNKGLELDP NNKECLEGYQ RCAFKIDEMS KEKVDEEQFK
KSMADPEIQQ IISDPQFQII LQKLNENPNS ISEYIKDPKI FNGLQKLIAA GILFKPKENI
PSKYGENRHW NVDLIPKFIL VGGNLVKILK KTRVTNYLEW LVVEGSYVYQ HQKKGFLTSE
KFIHKVPATD MEALVSPLLS LMEKNRCKNF YQYVSEWDAN KRNTWDNLDP YKLTMLEIYK
HFNLCQLTID FLGHAVALYL NDDYLKQPAY LTLERIKLYM QSISAFGKSP FIYPLYGLGG
IPEGFSRMCA INGGTFMLNK NVVDFVFDDK VCGIKSSDGE AYCDKVICDP SYVHLKNKIK
KIGQVIRCIC ILSNPIPETN QTNSCQIIIP QNQLNRKSDI YINLVSFQHG VTLKGKYDVR
LDVKLNKFIW SKGVRNPPKR VRVKLERKRN EDEDSKEKMY TIVEHVMVDS YKGLVNEQLA
GDRHRIHDCT TENFNGFMAI TTPNKSWMAQ YNDLSKYAPG FYALQVIGEL GSGSGYIILS
LYEMLLSRNK KIDMLYCVDI NKKANKIFNV EIIRNNLFNN IRRCFDIVLF NPPYVITGPD
EMNKTLTASY AGGKYGREII MKFLLDIHNY LSNKGVIYLL LEKSNIPQEI EDSNLPILCE
TCLGENPYVR IIREENGKEC KICKNVFTHF RWKPGENSRY KQTVICMKCA KVKNVCQTCL
FDLQYNLPVQ VRDKFLENSI VLPENETNRN FFLEQMELSK LKRRDPYFKR NMARVCSFWR
KNSCNRGDEC PYLHKEIHKL IIFNLPPINE QDIKSLCERY GPIVDVYAFV NFMFPSSCEK
AKIHLDNKIY RGKILIAKYS SYKKIVEIQK KKSCQNENMW NILYTDINTS INNFCKENNC
SVESVLDKNI AVNVSLTETY IINKIKTWIK NEGIRSDDTI IIKNLSIYTN QNDIINLFKQ
YGILKRVSFS PYNNICIIQY ENADNAKKAF ISNSYIRYKK LPLYLEWAPL NLFDDEGTHA
SIYIKNINFN TKEEDLKNLF DKMEGFITCN ISSGYGFAEF KNKELAMEAI KRLTGTRLND
HLLEMSLSKL VVKNLAFQVN KEELRKLFSA FGNVKSVRIP KNVYNRSRGY AFIEFMSKKE
SCNAIESLQH THLYGRHLII DFAPQNARIL KPLIQEKIVE IMKPEIEEKI IEVPQVQYIE
KLVEVPHVIL QEKLIHIPKP VIHERIKKCS KTIFQEKIVE VPQIKVVDKI VEVPQYVYQE
KIIEVPKIMV QERIIPVPKK PQYRHIPKPV EIPMAHYRTF PVEKIVDRNV PVPVELQIVQ
EFLCPKIEAR YKEIPVPVHV QRIIEHPIPK DAMNNPHLLP LYYLGSTAVG ICVNDGVILA
SERRISSTLI EKDSVEKLLS IDDHIGCAMS GLMADARTLI DYARVECNHY KFIYNENINI
KSCVELISEL ALDFSNLSDS KRKKIMSRPF GVALLIGGVD KNGPCLWYTE PSGTNTRFSA
ASIGSAQEGA ELLLQENYKK DMTFEQAEIL ALTVLRQVME DKLSTSNVEI CAIKDQTFYK
YNTDDISRII DVLPRFKIID SDFINIKLLE LFQMEGFQKQ LDRLSDSLSK IQKALGEYLE
KQRNKFPRFE KLVQTELTDA CFLTLTQALK MKLGGNPFGP AGTGKTESVK ALGAQLGRYV
LVFNCDESFD FTAMGRIFVG LCQVGAWGCF DEFNRLEERI LSAVSEQIIE ILNKKIGLNK
NVGIFVTMNP GYAGRSNLPY GVGFKMSFAR ISSSCAIMSR TFNTIGIGLL SLELMNHCEA
KELATPLCMW KLPNKELINR NIANKSEHRH HQKLLMSYTP FNSPSLLAEQ INILGTYSGT
RLLYFPLWDH PKDSIDYCLS TYLYWLYLRR NTNIFLQNTL LRGQVVVIAA TNRQNSIDPA
LRRFGRFDRE IDIGVPDDNG RFEILRIHTK NMKLSPDVKL EELASNTHGF VGADLAQLCT
EAALTCIREK MDVIDLEDEI IDKEVLESMC VTQDHFNMAL GTCNPSSLRE TVVEVPNVKW
DDIGGLDEVK STLREMILYP IDHVMKSLFD NAFFKPTEIQ SKTLEKSIND KNDIVVISKT
GTGKTLTFCL PILNNILILV PTRELALQIL KHFNYINKYI NLFISTIIGG LNLNKQKRIL
MKKPEILICT PGRLRYLVCD EIDKMIEISF MKDISYIAKH IYIQTFLLSA TLMTKLLNSI
IIRKDKSFII NLLPELLTLY IVKLNERDIV CKLFYLIKSY KIIIFVNTIK SAKQLNAIFK
HLFLHNNLES SIPKKIYSIH SKQKLKERLE NINKFQNHKA ILFCTDVLSR GIDLDKCDLI
IQLNCPISDI TFVHRSGRTA RNFKKGNKSI CAEIKSAELV LNSGELRRIS DPKYGTIDHN
QICSVCFENC TGHIGHIEFV LPLFNPLFYK ELQELLNLIC YHCYNFCCSY NYEEYLILSK
TIKLHMKKSS KCCFCKFQRN ISAKVSQRRD TITIRLFSFQ VIDILKKIFN KDIINLLYPF
TKRDGYKKFF LYDMGVSGNR FRSQSKGIHV RNNFIKLCIN AKKKVDFDYL LELQLGVNTF
YDIRQILDKK EGILRKNIMG KRVNNCARTV ISPDTFIETN QIGMPIEFAK VLTIDEYITQ
NNFTYIKKLS LYDFILNKNA DFRHLSFISD FMTGELCMDI LKANWSPAWT IQSLCRAILF
LFNEPNADSP LNCDAGNLIR GVKVKSAAEV GKRAVEYFRG DDFVNFLSTN GEMLKKKFPN
LFINRNLSDM KEIEEFADMF IQKGYIYKAQ YKPENGVYKR PKWPKRLIMT SKQNFDKTSF
YILVHERNKK LQYFMLISLI SIVLICCMFW HISVAFLTLI SIIIVGRLVA FIFFWFFGVD
YWIFPNLFDE ECNVVESFLP LQSWVYRNDT WFLFVARMCT AVLLAIAIQQ LGKTHSIADI
RNFATQSYID IIEWGNKKLA EENYDCLKKC GFTSFEELVR KCFLKCECMT LADSFLKDLE
DLEFEERKIS ELLYDIEKCM ETILKIDTEI LNIHKYVKDI YSTKFPELDS IVYSPVEYIS
VVNKIRNEVD LKNIDFSDIL PNTTVMAITV ASSTTGICLP DNLLKNCISF CNEGIQLNEY

RNIILLYLES KMFYLAPNVT MLLGSSLTAR LISAVGSLKN LSIISSQNLI VIASTKKGIL
CCSEIVQSVP DAYKKKAISL LASKCSLAAR IDYFTEERTV LDKKILNSSV ILQIAMRVKR
DRYLGRHYRY FIRNTRVRAY KQFLEPFKNV TLKNMAFAFG VSEEFIEVED PYEAINEAVR
NFENKDEILA SAKIVERLVE YPEVARNLDK IKAIDPLLKL LNNHILESVL QILSLALSNN
PELQDSVFKK NALKTLLIKL QESQKTIIDK KLITAISALI RHHDQGENKF IDYGGVGFLV
YGMQTNIFKY QEKSALLLKH LIHQNKITFD IFIKNDIMKG LVALVNTGIQ YGETTAELFL
ALIQNHRHKL AKSGLHTIKK LIEDRLSYLF EELGVEDWLI KISKSVHILY PTKIQQLCLP
LIIQGKNVIG SSETGSGKTI CYCWSILQEL NKNVYGIFSL ILLPTRELVF QIIEQFHLYG
SKIGVMILSC IGGFSLIEQP HIIVGTPGRF KRLRFLVLDE ADLLLQKCFE DKLQNILNNL
PRKTLFFSST ITNSLQLLID TFPYNNLILV NVNKKQKPPK NLDQRYGIIF TANSYKCELV
YTVLNMLDNV DAMHSSKDQK NRFATLAKFK NGLCKILVAT DIISRGIDIP KISFVINFDF
PNDTVQYIHR VGRTARANRK GLAISFIDKK DVNSFNQVKN IMKDKLKPYT LNKKEVLENM
FKIGRVIKKA EIMLEEVVVF PVSVHYDHTR KLLQSKFSTG IQNVSKFGNG SYTGEVSAEI
AKDLNIEYVI IGHFERRKYF HETDEDVREK LQASLKNNLK AVVCFGESLE QREQNKTIEV
ITKQVKAFVD LIDNFDNVIL AYEPLWAIGT GKTATPEQAQ LVHKEIRKIV KDTCGANQIR
ILYGGSVNTE NCSSLIQQED IDGFLVGNAS LKESFVDIIK SAKDINKDVV HRYGPNTFKL
HRLPIPKLGQ ILGLVGTNGI GKSTALKILS SKLKPNLGKF NNPPEWRDIL SFFRGNELQI
FFTKLLEEKL SPIIKPQNVD LIPKQIKGNI LEIINKKDKF NQKDKYIAEL DLEHLLDRNV
EDLSGGELQR FALLMSIIQS TNVYMFDEPS SYLDIKQRIS MAKIIHKLVK HDNYIIVVEH
DLSILDYLSD YVCCLWGKAG AYGVVTCPFS VREGINIFLD GFVPTDNLRI REESLNFKLE
DKKRLHFYNY PTMVKTLNSF SLTIDKGHFS ESEIFVLLGQ NGSGKSTFIR LFAGLIKPDN
LEFLESLSVS YKPQQIQAKF TGTVRQLLMS KLKGLYNDPY FNNEIIKPLK IESILDNQVL
TLSGGELQKV AIIVTLAKNT NIYLIMSIIC TISGQTPEEP VISKTGYIFE KRLIEKHIIN
YGICPVSGEV LTLEDLYPIK NEKIVKPRPI TASSIPGLLS IFQTEWDSII SEMFSLRTHV
NDIRNELSHS LYQYDAATRV IAKLLKEKNG YKEEIENLKK QIFQLKSSND LDIYEIGLNE
ELLEKMQNVA KDLLINRKKR KIDNVCSVEQ WKDFKNTNEF NIHSSTIPGV TCITLDFSGG
NDGNVYYVSL NDNKILSKLQ GHLKKVNSII SHPSNFICIT ASNDKTIRIW KGDKHKDHVT
SLALHPLENY FISSSKDSMW ILHDLETAKT IKTSKDNPSF KHLAIHPDGM MFGIAAQDSN
IHIYDIKSQE YKATLFSENG YYLASSSKDN TVKLWDLRKA QSFQTITLNE TPNFISFYNG
NIKDGLFHGF GILIYSQHEK YEGDFVYGKR EGRGKFTYAD GATYEGEWVD DKIHGKGIAN
FVSGNIYEGE WENGKINGFG MLCYNNGDKY EGEWLDGKMH GRGTYTYEDG DVYIGEWKND
KRHGKGCVKY KGNENKIAET YEGDWVDGKM QGRGTYFFAD GGIYEGDWVD GKMEGKGVYK
YLNGNKYEGE WINDMKNGYG TLAYVNGELY EGYWKNDKVH GKGTLTYSKG DKYIGEWKYA
KKCGEGELIY ASGDKFKGQW KNDKANGYGI LYNNGNKYEG EWLDDHRHGM GTFTCKEDGT
IYSGHFQFNR KHGKGTLTFV NGHILQGIWN SGLLEKVINY ELTPSSPWND PDLEMETLYD
LGSKMIEALQ KENITAGDVI CIDKGTGKIT KIGKSFARSK DYDAMDPNTL FVQCPEGELQ
KRKEVVHTVT LHDIDAINSR TQGFLALFSG DTGEIKNEIR EHIDMKINEW QEDEKAEIVP
GVLFIDEVHM LDIECFSYLN RALESEQSPI VIMATNRGIT HIRGTDYKAP HGIPLDLLDR
TLIIPTYPYK HQDILKILEQ RAEEEDVDID EYAKELLCKI ASESSLRYAL HLITLANLVS
KKRKATEVTV QDVRRVYNLF IDVKRSTQYL IEYQNEFMFS ELYSNLSDFW TSDDDEGDEY
IRKKWVIEDD ISNFNKNDLL LNYDFELDDF QKRSIKHLNN FKHVFVAAHT SAGKTLIAEH
AIALSIKLQK KAIYTSPIKA LSNQKYYEFK NIFKDVGIIT GDVKMNVNAN CIIMTTEILR
NLLYLNDNII NNIHCVIFDE VHYVNDEDRG VIWEESIIML PHHVQILLLS ATVPNYLEFA
DWVGFTKQKE VISISTKKRP VPLLHYIYVY DSVYLVMDEK NFYSSAFKEI YEASMKTEIQ
KLQTLIKKLD QDNKLPVVLF CFSRIKCETY AKCMPHLNFL DTNKKSKVHL FIKESISKLP
KQDRELNQIQ SLSKLLEKGI GVHHSGLLPI LKEIVEILFS KGLIKVLFAT ETFAMGINMP
TKSVVFTSIY KHDHLRKRIL TSSEYTQMSG RAGRRSSDKY GYVYICCCDN IPDQVQLTEM
MMQKAVSLKS KFKVTYNMIL KLLINKQINI EKMLFSSFLE SCRALQIPLF KKDLKRKRKL
LQNIKEVQCI YIEQYVQINY RLKYIGLNLH KKLVCIENIS IITNELDRLI EKNNFEPFVL
TKMLKSLKCE FYSVLHYQLI CKKNDCLDDI ENIERNINAK SLNLYEDLEG KLNVLKHFGF
IDDQNNLTVK GKIASYITLT DEITLTQVIF ENVLNKLNPA EIAAVLSCFV APEKKVEESP
DLTVNLQEVK AALTNIHSSF EEFYKVIRLR ISSEDHWKLC NFKIMFIAYK WTLGVSFAEL
LEQCELEEGL IVRSILRLDD LCRKVKIAFL YLGNIDLAQK VEKTSHLLRR DIIFTTSLYL
DGLALLQFFH WCEQKRKTKE LFNETEMSLR HKVDYFRSTK KNFPSFSTIS ASGPNAAVIH
YECTDKTNAT IKPAIYLLDS GGQYLHGTTD VTRTTHFGEP TAEEKRIYTL VLKGHLRLRK
VIFASYTNSS ALDFIARENL FNNFMDYNHG TGHGVGLTLN VHEGGCSIGP VGGAPLKKNM
VLSNEPGYYM KDKFGVRIEN MQYVISKEIT DTTEYLSFDD LTMYPYEKKL LDFSLLTNQE
IKELNEYHTT IRNTLLPLVK QSPKKKLVVG ENPEFIKKRL EKFNEIKEKI TIELLDGSIK
SGESYVTTPF DIALSISKRL AEDSIVCKVT YLEKVDVELC DLWDLNVPLL GNCRVEFFWH
SSAHILGSSL EKLFGGFLTI GPALKEGFYY DIFLNNFSIN NEDYKRIEDE FNKLVKDNVP
FEKVICTKEE ALELFDYNPF KLELIRSKIP DNKKTSVYRC GNFIDLCLGP HIKNTGKVKT
FKVLKNSSAY WLGQKENDSL QRVYGISFQK KSELVEYLKF LEEAKKRDHR NVGKILNLFF
FEKETSPGSC FWLPHGSKIY NKLIEFIRKE YRIRKYEEVI SPNVFSCDLW KTSGHYQNYK
DCMFLFNVEN KEWGMKPMNC PGHCLMFKQL NVSYRSLPVR LADFGVLHRN EISGSLSGLT
RVRRFQQDDS HIFCSMEHIK QEVLNTLNFL FYVYNLFGFG IEILQNRGYD SCGMSTILKT
TKYASNTTCD AIEKLKSNYL NSHKNDHIGI AHTRWATHGC KTDENAHPHV DYGERISIVH
NGIIENYREI KTFLLKNNIP FKSNTDTEVV ANLIGYFLDK KQSFQDAVLS AITQLEGTWS
FCIIHKNHPD EMILASNGSP LHIGFKDDEI FIASEHTALF MFTNEYISLK NGEILSISKD
KINDLKLLKK VENIPEIAIQ KTPHPYPHWT IKEIHEQSAT LSKSLNNFSS GDHLVKLGGL
DPYIQDLNKI ENLVLVGCGT SYYAALFAKY LMNYLNCFNT VQVMDPIDFN ISVIPKEKEG
VIFISQSGET RDVIKACKLA EDLNVRKLSV VNSVGSTIAN MTGRGVYLNA GREVGVASTK
CFTSEVSVLT LIALWFFQHK SSNKATSLIN SLHRLPLYTG VTIKCENTCK TLSEKFKNKS
MLIIGNGLSY PIAQEGALKI KELAYIHCEG FTGASLKHGP YALLGGEDNI PVIMLLFNDK
NAMINTGEQI KSRGAHIVCL TDDENLVKDD IILIPNNGIL TPLLAVIPLQ MLAYYTSVNK
GINPDKPRCL AKTVTVWIIE IEKYASEDVQ KILIGNKIDL KNDRNVSYEE GKELADSCNI
QFLETSAKIA HNVEQAFKTM AYEIKNKSQH ETINKGKTNI NLNARPIKIR TMNSRKPPEG
WHKVESFLEE MNKKMRSLEN EDTSKKRKNE ILWPIFQINH KTSRYIYELY YKRKEISYDY
VVIGGGPGGM ASAKEAAAHG ARVLLFDYVK PSSQGTKWGI GGTCVNVGCV PKKLMHYAGH

MGKLDSKAYG WKFDNKHDWK KLVTTVQSHI RSLNFSYMTG LRSKVKYING LAKLKDKNTV
SYYLKGKEET VTGKYILIAT GCRPHIPDDV EGAKELSITS DDIFSLKKDP GKTLVVGASY
VALECSGFLN SLGYDVTVAV RSIVLRGFDQ QCAVKVKLYM EEQGVMFLPK KLTKMDDKIL
VEFSDKTSEL YDTVLYAIGR KGDIDGLNLS CTNIPSIFAV GDVAENVPEL APVAIKAGEI
LARRLFKDSD EIMDYSYIPT SIYTPIEYGA CGYSEEKAYE LYGNVEVFLQ EFNNLEISAV
HRQKQKDEYD LDVSSTCLAK LVCLKDNRVI GFHYVGPNAG EVTQGMALAL RLKVKKKDFD
NCIGIHPTDA ESFMNLFVTI SSGLSYAAKG GCGGGKCGEQ LYTGPLKIEQ LLAKGFVKRD
LELLKEGGLQ TVECVAYAPM RTLCAIKGIS EQKAEKLKKA CKELCNSGFC NAIDYHDARQ
NLIKFTTGSK QLDALLKGGI ETGGITELFG EFRTGKSQLC HTLAITCQLP IEQSGGEGKC
LWIDTEGTFR PERIVAIAKR YGLHPTDCLN NIAYAKAYNC DHQTELLIDA SAMMADARFA
LLIVDSATAL YRSEYIGRGE LANRQSHLCR FLRGLQRIAD IYGVAVIITN QVVAKVDAMS
MFGHEKIPIG GNIIAHASQT RLYLRKGRGE SRICKIYDSP VLPEGEAVFA ITEGGIADYM
AIRVQFENSN EVGVFSRLTN SYALVALGGS ENFSSVFESE LSQHIPLVYT TIGGTRVIGR
VCVGNRKGLL VSSICTDQEL LHLRNCLPEN VKIKRIEERL SALGNCITTN DYVGLIHTDI
DKETEEIIQD VLDIEVFRTS IAGNLLVGTY SYFTNNGGLL HAMTSSQEIE ELSELLQIPL
ITGTINRGSD LIGSGLVAND WSAFCGMDTT AIELSIIEKV FKLNNITDNN MEDTFKYKSS
IIQTMIIVDT YWQTETGGIV IAPIPHLFSM KPGCASLPFL GVQLEILDSK TLQPLSGNNC
GLLCIKSPWP GMLRTVYGNH QRLIKTYFTM CPNYYFTGDG AFRDEDGYYW ISGRIDDTLN
VAGHRLGAAE IEHALVQHFY IAEAAVVSFH HNVKGEGILC FVVKKLKLYV RQVIGPIATP
DLICVVPDLP KTRSGKIVRR ILRCIANGIT DFGDISTVSN YEVIETIPFP TPCSVEDALS
YYCDLTTIPR LNILKKFKCF IKDIEEKECD MTFIEFVDMF MQSAVFELSP FLQLIPRNTP
KSYTISSSPK RWFKGSSSYY LTELNVNDIV KFNIKPSKFE VDFLYEMEID ALDKKHIDET
YFAFSRDQPW VEKYRPKRLD DIVHQNNAVM MLKEVVRTKN MPHLIFHGPP GTGKTSAINA
LAHELFGKEN ISERVLELNA SDDRGINVVR EKIKAYTRIS ISKNKIHSET KEVLPSWKLV
VLDEADMMTE DAQSALRRII EIYSNVTRFI LICNYIHKIS DPIFSRCSCY RFQSIPINIK
KEKLLYICQN ENIDIVDKII ETTEGDLRRA VSILQLCSCI NTKITLNSVL DVSGLPSDNI
VYKIIDACKM KDKLVEKTVQ DIIEDGFDVA YIFKSFNNYF VDSLKYQILL ELSRHDYRLH
CGATQYIQLL SFASSVHSLL IRELIFKSPN EKHFQMVVKQ VKELIKQVKQ KEVEDVNESK
TSQDRLVLNK SGRRIVLRDL MTRPNIFTGR KILGTLELHM NGLRYAAIDI LFDDIKHAFY
QPCDGQLIIL IHFHLKRYIM VGKKKTLDVQ FYCEAGTQID DLDRAKARNV YDPDEMHDEM
KEREQKNKLN LIFKNFVQQM QDISKIEFEI PYPELTFSGV PNKSNVEIFV TANTINHLVE
WPPFILSVED IEIASLERVH HGLRNFDMIF VFKDYTKPVK RIDVIPTEYI DTIKKWLTTI
DIVLKTILSD IDSFVNSKGF DGFLGEDDDE EEDEDEDDEY EVDESELSAE IDTLATIEFT
SKRKMSSVIC RIPKIMLFCK GAGSIILKKL AKRTDVDEIT IEHMETYADE GLRTLCIAQR
ELSEESFAEW YHLYKEASLS IKDREEKLES VAEYIENDLI LQGITGIEDK LQEGVSSTIE
DLRMAGIHIW MLTGDKIETA MNIGIAANLI DNYSVDGSVI DLLLSEKMER KFFYLADKCS
SVICGRVSPY QKGAIVSSAN RLLNKITLAI GDGANDRNMI NTANIGIGIR GQEGVQAFNS
SDYGISQFRF LKNLLLVHGR LSYRRISKLV VYMFYKNMVL IFPLFIFGSI SLYSGQKIYF
EFLLHLFNVL FTAIPVVIHA VLDQDISLNT AMEKPNLYKL GIHHYYFNIR TFISWVMNSL
FHGSVVFLIP LYFLIPTSDG IPYDIWTVGC ATYLTVLIVN FKILFETYYL NILPISGIAL
SIFSFVLLVT AFSFMCVGSI HLLGTIVYLV QSLRFWLVVI LGLFALLRDY VFKVYKRNFN
PRNYSDHVKD HFNKPRNVGS FDKNEKNIGT SIVGKASCGD VIKLQLKIEN DVIKDARFMA
FGCGSAIASS SYATELIKGK TIDEALKIKN NDIASHLSLP PVKIIILGSG WGGFNFLLNI
DFKKYDVTLI SPRNYFTFTP LLPCLCSGTL SVNVCTESIR NFLRKKNYCG NYLQLECTDV
FYEDKYINCI DNNKVKLFYD YLIIAVGAKT NTFINGVDKY AYFVKDIDDA LKIRKKFLDI
LEKCTLPNIS NEEKKKMLHV AVVGGGPTGV EVTAEFADFI NKKINYKDIF NFISISIIEG
GNNLLPTFTQ NISDFTKENF HNLNINVLTN YYVIDVDKHS FHIQSKKLSY GLLIWASGLA
QTTLIQKFLK TIPVQANNAI LKVDEKLRVI GINIYAIGDC KKIQPVQITA EQLINEALEL
EEVEHKVNYN LIDEEELNEY RINKRKEYED KIRKRRYMIS TYIKYGLWEI KQKDIERCRS
IFERALNIDY TNKNLWLKYI EVELINKNIN SARNLLERVV LLLPLENIFW KKYAHLEEIL
NNYVNARNIY ERWIKFKIDE SSFLCYIYFE ERCNEINKCR EIFERLIVSI PKLECFYKFI
KFEKKYKNIV RAEKCIELLP SCYIDENFYI HFCNFEEEQN EYERCKKIYI EALKILPKNK
SELLYKNFLQ FQKKYDELHE SLLIKERIFY EDELKKNKND YDIWFNYIKL EERIRDLYER
AISVIKNAHV HDGLKIGIRE VIKSIESQEA KVCFLSDVCS EPAYKKLITT LCAEKNIPLF
MVDSKDLGHW AGLFKLDNEG NARKIIGASS VAVVDFGEDS AEKDFLGYYK AMVGEVIDKR
YSVVCELVGK GVFSNVLKCY DMVNKIPVAV KVIRDNDMMK KAAEKEISIL KKLNKRHIIR
LLSSIKYKNH LCLVFEWMWG NLRIALKIQS YFNDLISKTE ELEDVYISRR KLQSKYLTQI
KNLYINCNCE CIIHKISFKY ATKSSFPNLL NGTLLYMVVE KMNLENNVVT SCINSSDVKS
WINYENYLGE LVDGFVFSVN IASAKSLIGD RCYILDLIDI KYEIAVGHNG GALGYSQHLS
EEIMLFSRDA ILDKIAVILG GRAAEELFIG KITTGAIDDL NKVTQLAYSY VSQYGMNQEI
GLVSFQPNSN SEYNLYRPHS ECLAHLIDNE VRSLIETQYK RVKSILMKNE KHVHNLANLL
YEKETISYHD IVKCVGLKHQ RYKGSDKIKI EEERNKKLFL NNNKFCKNCG SVAHKEKDCL
ERTRKKGYDG NRDRWVGYNV NNFDYIYKEY EKIVEEKKKR KAEDLIHNMK KNIKVPSKYE
EDIYLFDHSS VFGSYYDKHT KKWGYKCCSS TNKYDKCIIP LMDSYHANYY TKEVMIILIN
EFNSPDDEMK KIVLKCVKQC IQTEGVDKDY INEEIVNPFF EKFWVMRNSN DKKSFNLIVD
TTVEIAKKIG VIYRIVDDLK DPSEQYRKMV MQTIQNVVNE LGVDDIDQKL EEQLIDGMLY
AFQEQTSEDY YILLNSFDII CNKLNIRMKP YLPQIAGILR WRLNTPLPKV RQQSADLISR
ITNLIKICDE KQMLGHLSLY LYEYLGEEYP EVLANIIRAL KSILLVLGVQ NMTPPIKDLL
PRITPILKNR HEKVQENVID LIGIIADKGG DLVSPKEWDR ICFDLIELLK SNKKLIRRAT
IQTFGYIART IGPFEVLTVL LNNLKVQERQ LRVCTTVAIA IVADTCLPYS VLAALMNEYK
TQDMNVQNGV LKALSFMFEY IGEIAKDYVY SVVTLLEHAL MDRDLVHRIA TWACKHLALG
CFGLNRQDAL IHLLNYVWPN IFETSPHLIQ AVIDSIDGFR VALGPAIIFQ YLVQGIFHPS
RKVREIYWKI YNNVYIGHQD SLVPIYPPFE LLNDSTFVRD ELRYMGRMYG KGKGISSSTL
PYKRKQPSWL KQKPSEIEDA IIKLAKKGQT PSQIGATLRD NYGIPQVKSV TGNKILRILR
AQGIATTIPE DLYFLIKKAV SMRKHLEKNK KDKDCKFRLI LTESKIHRIS RYYKRKKLLP
SNWKYQSSTA SALRHIEHAP GVQFSYVPPD FFNSDDDESD KNQYELKDDG GGRAPGTRAK
EHSTTHHLRR KNYDDDFEFN EDKILEALHI LELLYMNGVS LEEQNEHGQT ALFLSVKKNN
ISTLQWLLTK EVNINHRDFY GNTVLHIAVR HCDIDILRLL CDYGCLNMVY YSSIENKNTN

VFQLCIKNRY FLVYILLKKW VLQNKICSKL KICKTIYAFY FWFFAILNLI VYFNIAHSFS
IINKYHFKSL IWITIWFFQQ FLWCMLYFKS PGEYQLNNIE REIFQINLEY QKLSLYSQVS
QERINSLDED YRNAILEIIL LQLIIEPYIL RRSKKHVFID MPKKHSLIIK LPLNNTQLNL
YKDEIFSKMQ KTFKHLINAS IFILRRICNH PLLHKYYYTV EDIKKISKYF YYNTDQYVDL
DLKTVENEFM KISDFDIHLS IKHLISQDNK LNKYLITKEH ILNSSKINHM LSLIKDIRKK
KEKVLIFSQF TTFLDIIEEA LYVRLDGSTN TIERQKIIKR FSNIFVFLLS TKAGGVGLNL
IAANHVILMD QWNELLLQEE IVKKMCEYII DSKCDIVEKG VSDLAQHFLV KKNISVIRRV
RKTDLNRLER ISGATIVNRC EEIVEGDIGT KCGLFEIKKI GDDYYSFFVE CKDPHACTIL
LRGSTKDVLN EIERNLHDGM NVAKNILMEG KLLYGGGCTE IRVGQYLIKE AAKFNDSRKS
ITEAVASALE IIPKILAQNS GVNVVKTMNE LRIKHGGQEF GIDGITGDII KVTTKNIWDL
LSVKKQIYKS AIEAASMILR IDDLAVILIY EELLKNPLCK ITNVYESETR KYKPYPLNTL
QMTKLVSIYF KISSKECMML AEKLYNKGYI SYPRTETNYF PDSMNLHKII NELRKNDNFG
WYANKLCEEH PRKGKMNDKA HPPIHPVKNM EWKLYEFICK HFLAVCSNDA IGYNTKVTAK
IQEEQFFCKG LKIKEKNYLE IYTYEKWNDK IIPSFQVDDE FYPTSLLIEE GITQPPKYLS
ESNLLTLMDK FSIGTDATMH EHIENIQKRN YVIKNSKSLF IPTNLGIALV QSYKKFKDIG
IDLTDPSLRA KMEKDMSLVA SGICVVKNGK TDIPSNTKMI IISYELITKN DKYQKYKSII
CDESHYLKNS LSKRTKVITP IIKNAKRCVL LSGTPALNKP SELYEQISSI MPNFFNYHEF
CDRYCFKDKN LYTKKIEYVG CKHTEELHLF LTNTIMIRRL KKDVLKELPD KLRSKIPVEI
PQKELSEILL FKITGYAKVK AIKEYISYLI DADIKFLLFC HHKLVMDEVE TFLKEQKCSY
IRVDGLTPME KREIYIKNFQ NDDNVKIALL SITACGMGLN LTAANTVVFG ELFWVPGQII
QAEDRAHRIG TAHDVVNIHY LIAQNTIDEI VWKIINRKWN TLTTALNGIE DSLMFEDNDK
ICIAVSGGKD SSVLAHVLVN LKKKYNYNWE LFLLAIDEGI KGYRDDSLKV VFENLFSYTM
DDVVKFIGKK NNCTVCGVFR RQSFEKGALL FNATKLVTGH NADDLAETIL MNMCRGDIDK
LFSTECTYSP NSFRGNLRCF IKDIECIKCG AYTSNIDGLN NYTDNKKISN CTFEIDISYE
HVEPITLENE YQQIPKLRIL SFDIECIKLD GKGFPEAKND PIIQISSILY FQGEPCTKFI
FTLLECASIP GSNVIWFNDE KTLLEAWNEF IIRIDPDFLT GYNIINFDLP YILNRGTALN
LKKLKFLGRI KNVASTVKDS SFSSKQFGTH ETKEINIFGR IQFDVYDLIK RDYKLKSYTL
NYVSFEFLKE QKEDVHYSIM NDLQNESPES RKRIATYCIK DGVLPLRLID KLLFIYNYVE
MARVTGTPFV YLLTRGQQIK VTSQLYRKCK ELNYVIPSTY MKVNTNEKYE GATVLEPIKG
YYIEPISTLD FASLYPSIMI AHNLCYSTLI KSNGKNNLKF VKKNVKKGIL PLIVEELIEA
RKKVKLLIKN EKNNITKMVL NGRQLALKIS ANSVYGYTGA SSGGQLPCLE VAVSITTLGR
SMIEKTKERV ESFYCKSNGY EHNSTVIYGD TDSVMVKFGT NNIEEAMTLG KDAAERISKE
FLSPIKLEFE KVYCPYLLLN KKRYAGLLYT NPNKHDKMDC KGIETVRRDF CILIQQMMET
VLNKLLIEKP FIQVAPMINV TNRHFRAMVR IITKRAQLWT EMIVDNTLLY NLNNLEEHLG
FDNNEHPIVC QLGGCDMNSM SEAAILVEQA GYDEININVG CPSTKVANKG AFGASLMKNP
EQVRNIVYEI KKKVQIPVTV KIRTGVDNYD SFDFLKTFIE TVSSVGCNHF IVHARKAWLK
GLDPKQNRKI PPLEYYKVYD LCKLYPHLKF TLNGGIQTIQ EAIALLLNGI MIGRACMENI
TVLSQTDKLV YNQDIPSTAY SRRTILEAYK KYLEKNSLFY NLFELLKPVL GILKGMPGHR
IFRFLKNFFF KNPVGHVGVV ALKNSSAKLI QPFTSNVDDI LNSILKERTA GLQGSPSLEE
GLQIAHDLLI DMPLYGTKEV LIMYGSIRTC DKKNILNVLE LLVKSNIYVN CISIAPEMHI
LKCGIHLISM HDLSHITNNL QGSPLFIEIM GSSSLPMSQQ MYFSTHNALR INENDVINTL
FYEINGSRHI SLLIFPFYDV QMLKRLLIKK LNLPGVKVND IIIFYKGIKL PNYRIISTYK
KINKLYWAIK DTNPNASIRV IDSKYPEFFE DILNEIKLSF KKNIAPKLTM DGTGGTYLLY
NAKKKICSVF KPLDEEAFAP FNPRGYEGKM YQEGFRAGVL SGEGASREIA AYLLDNCYNN
FSNVPCTIMV EACNPHFNNK SKLKYVDKET NLKWKCGSLQ EFIDSRESVG NYDYKQFSIR
DIHKIAILDI RVMNLDRNDG NILVSPTHLG IEQSRRDDIE ALGYVLMYFL RGSLPWQGLK
AISKKDKYDK IMEKKISTSV EVLCLLNVCD HIDSCIIIDR RVDMITPFCT PFTYEGLIDH
FFGIDNLQIE IPLYNDIKDL SQNEIGSFLH KKASDIQKTY EKDSLKDIEE INQYMRIFKE
KHYEHNSLST HVNIASFILN NIKKEYNFNK LKLEDEIIQL EINTNKNILL SIVKQIQLLI
YTNDDIYEVY RLLCLFSILT NGKKDIIEQY GIKELTRLNK LYTCNILKFN NKQKFLWTQL
KNNFHLLSND ENDISYVCNG YAPLSVRLIE YKNNLQVFPE IFNLLNGPTL DIIQDTIEVR
SMCINCEKEG LNKIVKINIP YFKNVLIHSF ECEFCNYKNN VIQDLNQIKD KGVKISMKIN
NKELLDRQLI KSEYGVLKIP EIDFEIPKET QKGSINTIEG FLHTALNNLY IKMIENTVQN
LFTIQIIDPS GLSSLEYNRT KEELNELGFY SFASNCPCCN HMGMNNFCEI NIPGFKKCLI
LSFVCPNCNF KTSEIKSSGE INPKGKKITL TVNNKNDLNR FVIKSETASI NIPVVELTSD
YGTLGGTLTT VEGLIMKIIE SLEEKFKFLM YVLNRKGEEE DISFDQILKR IQRLSYGLHE
LVDPARVTQG VINGMYSGIK TCELDELAAQ TCAYMATTHP DFSILAARIT TDNLHKNTSD
DVAEVAEALY TYKDVRGRPA SLISKEVYDF ILLHKDRLNK EIDYTRDFNY DYFGFKTLER
SYLLRINNKI IERPQHLLMR VSIGIHIDDI DKALETYHLM SQKYFTHATP TLFNSGTPRP
QMSSCFLLSM KADSIEGIFE TLKQCALISK TAGGIGVAVQ DIRGQNSYIR GTNGISNGLV
PMLRVFNDTA RYVDQGGGKR KGSFAVYIEP WHSDIFEFLD LRKNHGKEEL RARDLFYAVW
VPDLFMKRVK ENKNWTLMCP NECPGLSETW GEEFEKLYTK YEEENMGKKT VLAQDLWFAI
LQSQIETGVP YMLYKDSCNA KSNQKNLGTI KCSNLCCEII EYTSPDEVAV CNLASIALCK
FVDLEKKEFN FKKLYEITKI ITRNLDKIIE RNYYPVKEAK TSNTRHRPIG IGVQGLADTF
MLLRYPYESD AAKELNKRIF ETMYYAALEM SVELASIHGP YESYQGSPAS QGILQFDMWN
AKVDNKYWDW DELKAKIRKH GLRNSLLLAP MPTASTSQIL GNNESFEPYT SNIYYRRVLS
GEFFVVNPHL LKDLFDRGLW DEDMKQQLIA HNGSIQYISE IPDDLKELYK TVWEIKQKNI
IDMAADRGIF IDQSPRLGFE EMRNEMNKYG VEINQSTLKN PSTEDIQGIY SLCIKYILNK
DIQNIRIEEY TGDLKSILPN EGKNHLQAIG NLRFLRHCEK INKILNLDNI LSYIFKPVGS
HMTKLINAFI VNETNELIFQ YSRYRQKKED LEDQIVPSPE KLQKYNEELK DHLYEHIAQF
EDDRKKNEDI KNKINIADIC IKKLVDLLTA LNEHIKLHIE KKNNLQTIEK QYKSLDWWTL
GIFIYEILVG CPPFYANEPL LIYQKILEGI IYFPKFLDNN CKHLMKKLLS HDLTKRYGNL
KKGAQNVKEH PWFSNIDWVN LLNKNVEVPY KPKYKNIFDS SNFQEDLTIA DKITNENDPF
YDWVISQFYI LSPRGDTIIN RDFRGDIIKG SGDAPPVFYL NGINFTYLKS NSLYFVVTSL
FNISPSYLIE LLHRLLKIFK DFCGQITEEL IRTNFILIYE IIDEIIDYGY LQNSNTEYIK
NLIHNETLPS NASQKPIQIN DKKNEIFIDI VEKIDGVIQI KSYLLGNPFI KIALNDDLYI
KNIHHDNSNN IIIDDCNFNH LVLSLYQPDG ECVLMNYRIN MIHIYKPDDI IACEIQRIST
DGTIILHTRS SIYGKLSNGV LIIVPQTLIH NQKKHIFVFP CVDHTTRKNI TIITNIIKLL

VKYHININYD TINKIYVQEW VAPKNKQIKA ASSNSSQIVI SLSGGELIYF EIDESHTLVE
IFRKNLNVEV LCLSIQQNRV RANFLAVGCL DNVVRLLSID KYFKQLSTHL LPNNSSPQDI
CIIFLNIGLN TGVLLRSIID PVGTLSNHYS KYLGAKSIKI CPVNPALLVL CEKTYLCYMH
QGKFLYSPLN YDMLEYASSF YSPQCSDGYV AISSNSLRIF RFYRLGEVFS QNILHLTFTP
RKIVPLPFPS MLAIIEADHN SYDENTQREI QKALKDIKLG TFKAGQGKWG SCIKIINPVN
LQILDKISLD MEEAALSVCA CELEALHCLI VGTTTNLSLK ASLRVYTYDI QYKLNLLHIT
PIEEQPYCFC SYNGKLIASI GNKLRIYALG KKKLLKKCEY KDIPEAIVSI KISNRIFACD
IRESVLIFFY DPNQNTLRLI SDDIIPRWIT CSEILDHHTI MAADKFDSVF ILRVVEEKPD
ITYNDIGGCK EQLEKLREVV EMPLLQPERF VTLGIDPPKG VLLYGPPGTG KTLTARAIAN
RTDACFICVI GSELVQKYVG EGARMVRELF QMAKSKKACI LFIDEVDAIG GSRGDESAHG
DHEVQRTMLE IVNQLDGFDN RGNIKVLMAT NRPDTLDSAL VRPGRIDRRI EFSLPDLEGR
THIFKIHANT MNMSRDVRFE LLARLCPNST GSDIRSVCTE AGMFAIRARR KTITEKDLLL
AINKVIHGCK QFSATGKYMV YNIKAKVEQN LPLIFSKINK LYSVTNDNIF NMPRYSRIPK
EKKKTKWQLF AENKLMKKNK SGLIYDKVSK GWVRRFQKKQ IKLNEQKNNF VHEYKNKEDI
DPFEKEQEEK DIKKMKQKMR ELKNKVFERL NDKQFYTGVQ KTKFKKPCNY VVTPPKTIFL
FNNEKKYDKG VYFLVKSYIK NIKSLCYEIT KILQPSIGPT RKIYDQNNGG KYLCTSGDPP
APIRNLSHFD ARKYFKEGQK IITPPNGDGT RAFYESLLEE NPNSIIAIKY CIEHGILSGT
KHHEALYKYY VLKKNNAFKS NFGGVRIDFK KLLNVKFIQE KKLIGKFFEE IAQDTGKVVY
GIDDTLKALE IGAVELLILY EGLDIIRLTT KNPVTNQTKT MHISPCDEKQ ESLYKENNVE
LEVVEKISLT DWVIGNYKKY GASLDFVTNK SQEGAQFLQV IYEFIFLCIR IYDDISKLFG
LPYTIVSDTI CEEIISIIVL PFNYLGLSAL NARNMQTLLN SITEKHKKKL SLDIIDAIIE
CKKKYITYED VEKILKYISY IFNIEEKYNI CMLFYKYISN STYLVHLLPT IIFTLLHVVM
LEAKLNNASI LKKLFECIKD LVNDANVDAD ESGLKLQALD GNHVSLVSLH LLDSGFSHYR
CDRERVLGVN IASLNKVFKL CGANESVVIS SKDDEDNLNF VFENNKEDKV TNFSLKLMSI
ELDSLNIPEE GFDAEVELSS KELTNIFRNL SEFSDTVFIE IDSNCIKFTT KGIVGDAEVA
LKPRDSTDDI GVTIKSKKKI KQSFAIKYLN LFSKSNILAD VVVLGLSDSR PIEFKYEIKD
GFVKFFLAPK MDDDCPEKCD EEVGIVKGTF LYTYDSPICK SAIHSGVLNI AEDIVLSIAH
THNNFIGTKR NNIESHDFKG TSKSFTISIP TGFNGKENDY IDCTNLPNEK YIRSLSNFTF
IVYFGEGTWR TILSHSLCEG ISISINEDNE LIIEQNCNPH LLKSKFKPKF GQTYHISLVF
NKINKTLYLY INGKKVITEK NTYNFTLSGD LIIGRSNQTT KDYFIGNIHL VEIYKYTLSE
QEIKESLNSS LSQKTIDGRD CVTPCKSKNM INKDLQINTQ QINLKCQDNL LSEQFNGKGS
QFLVSCIENC TKSKYFIKGT NYYTPDSSIC KAAIHAGVIR IVEGLLEYKS SRGHFGILSK
SEKQSCSTDG QFILNLSVGE KRTINCPSNC GTNIYSPTSV LCKAAIHSGA LSNQGGLVEI
IVGTGQEEFK GSTQNNVESF SSNNHSRSQK YIYDNADVGT QKKVFDLKLN MGPYSCTYTR
NGKYLLMTGV KGHVSLIDTH NLESLCEFQV DEMIRCNTTL HNYKLFAVSQ KKYMYIYDNT
GMEINCIKDI LYTYNMVFLP YHFLLTSIGE FGELVYQDIS VGNIVTRKKT KRGPCSIMKQ
NKKDAIIYLG HKNGHVTLWS PNIDKNYLIT ASVDSTYKLW DIRKMEYIKS YKSNIINNID
ISDTSLVAFS MNTHFRTYKN LFTNPYITHN IYGDQINSIS FQPFEDICSL GLKHSIKTLL
VPGAGIANID TFFNNPYETK KQNEVKLLLD KLPADTITND YKKIILVRKI KTKTQIKRII
TSPRDIIELT LKSYSNKNWP KWSDSESICT CLINNQVYIY KDNEQGSKGN PSIFKIFHID
NLNKHIYSKN FFNSDEIKLK WNKNGSSLLL QIHTDKEKQS YYGSSNLYFI DTVTLKDVNV
MTNKGLIYDT IWSNNQNKFY VCKGEIPAEI VLHDKNGNVS HSYGRHKFNT LKLNYNEKLL
LTGGFGNLSG DISIWNTSTK KEITKTKSSC AVICEFFNDG KHFLTATTHP RLRVDNNLKI
FKYNGLIVSR INFEELYNVI ILPPGCNFVN ENKNYKKKKK KREEDTIPLP NIKTPILKKI
IEYMEYHINN PADEIPKPLI TSNLQDVVVE DNSSKYKDLA QRMIQEIEMA VVLFKRKFIL
KKIPKLPPCY IINSGSLSIA RAKVKLPSTY SKLGNPLSFS KLPDFNYSYD MIEELQQFFM
KQRRCDYFSL LNNFINLLIS VSNLLAAEPD IDLRNELLRR FIYSLNSWMN MRRCIVACCE
NIFAMTGLCI PLQILHFNNE ECKIFFSKKR APYLLMFEVA DLDEDISHII PVEAQRIIFG
DFNRDTISSL LNNPLARSVL NELSNNPEML TNLVSNNPIL RNTFPLMQPV LENPNLLREF
MRPEILQAGL PPEERYASQL SSLQEMGFLD NAANIQALQE TGGDVNSAVT RLLERGSGDR
LTRAARVLEQ LTEQKPIFGK CRFTIRSFGV RRNEKISCFV TVRGKKALEI LEKGLKVKEY
ELRRKNFSDT GNFGFGIQEH IDLGIKYDPS TGIYGMDFYV HLSRSGYRVT RRTRERLGFF
ISLKMINQIK GLSSVDTNYQ WLPLLYMALA TDTSVSKITL SIIKPYSITL IRLLRDFFNV
VFLIQCVGIG YQNMSGSNCV AIACDLRLGA NTFTTVSTKF SKIFKMNNNV YVGLSGLATD
IQTLYEILRY RVNLYEVRQD AEMDVECFAN MLSSILYSNR FSPYFVNPIV VGPYLTAYDL
IGAKCETRDF VVNGVTSEQL FGMCESLYVK DILLVGAGGI GSEFLKNIIT IGCKNIDIID
IDTIDITNLN RQFLFKKKDV KKYKSLVAKE RALMHKKDLN INAYTFDVCT MKSSDIKKYD
YVINALDNIK ARKYVNKLCI MEKKVLIEAG STGYNGQVYP IYYNHTKCYS CEEKPKNKTY
AICTIRQTPS LPEHCVAWGR LIFETFFCKN DNETLIDIKN HIEEESKKRN MDKKEIIIFI
FNYLFNDTIK ELIYLKKDYT TIPIPNINKK EEEYLIFDKD DDECINFITS ISNIRMLNFC
ISQKSKFDIQ SIAGNIIPAI SSTNAIVASL QFQRVYICKP QSSRNKSDKI LVLNFGSQYF
HLIVKRLNNI KIFSETKDYG VELKDIKNIK GVILSGGPYS VPHLKKEVFE YKIPIFGICY
GMQEIAVQMN GEVKKSKTSE YGCTVWMNHN DEVTKIPENF YLVSSSENCL IYNKEYNIYG
VQYHPEVYES LDGELMFYNF AICKCKKQFD PIRYHELELK NIEKYKHDHY VIAAMSGGID
STVAAAYTHK IFKERFFGIF IDNGLLRKNE AENVFLKSTF PDMNITKIDA SENFLSNLQG
VTDPEQKRKI IGKLFIEEFE KAVNNIDIDI NKTFLLQGTL YPDIIESKCS KNSDTIKTHH
NVGGLPKNLK FKLFEPFKYL FKDDVKTLSR ELNLPEEITN RHPFPGPGLA IRVIGEINKH
KLNILREVDD IFINDLKQYG LISQAFAVLL SSKSYDYVCV LRAVKTSSFM TANWYQIPYD
ILDKITTRIS EVKGVNRILY DVSSKPPATI EFEMPLPGFV SDKTLYLKKP LILYKDENDK
IEVDPILAQY LREHQREGVQ FVFECLMNIK DDKISGCILA DDMGLGKTLQ SITVLYTLLK
QGFHKKCAVR RCLILCPASL INNWNDEISK WIPNRCNVTC VNDNAKEKIV SKLEGFKYDI
QSTVLICSYE CFRINNEFLD KSSIDMIICD EAHRLKNDKT KTYTSIYNLT AKKRLLLSGT
PIQNDLGEFY ALISLCNPDL FDDINLFRKK FANPILIGRD KDATEKEQEI ASERLTELSN
INKFILRRTN NLLSKVLPVK YLINIFIKLN PIQEALYVLF LKDKKILKND NTNNKVNVLI
NIKKLEKICN HPLLLNVNDI KSCKFLLLHF LLKNIKQNTN DKVVIVSNYT QTLDYMEILC
KENMYKFVRL DGGINIKKRH KVINDFTHSA DIFIFLLSSK SGGCGINLIS SNRLILLDPD
WNPANDKQAL ARVWREGQKK ICYIYRLFCT GTIDEKVYQR QISKDGLSNM IVTTTNLSKD
QMSDENVKKL FNYKMNTVSE THDNIECNRC KKVENAGFMF YIKYEKGQFK KGSNLIKKCI

DQNIDVYDVL GVEETDDLET IKSCYKKLIL LFHPDKFLKI QDSYTILSDK ILRKQYDSSI
PWSAIKPVPD IGDENTDIKE VKYFYDFWYN FNNWRDFSYQ NEYDYEQAEC REERRWMERE
NKKIQKKASK TENLRIIKLV DLAYNNDPRI IAENKRIKLE KLKKKASVKL WKHHIKSFDD
LCEFIYDIYV ILWSAQEVSL LAKALKLYPG GTRNRWVLIS NSIKTKTVKE VIKKTKEMFE
NDTLWTHEEQ HLLEQALIKY PTSIPMPKKR RNGGRSKHNR GHVNPLRCSN CGRCVPKDKA
IKRFNIRNIV DTSAQRDIKE ASVYSTFQLP KLYIKQCYCV SCAIHSRFVR VRSREQRRVR
KETAKHVNPS QKKVSVSEIN FDSSYTVLDT SEGSIMLHVN HVLYHLDFNA LAVVKNVDMT
EEMQIDAIDC ANQALQKYNV EKDIAAHIKK EFDRKYDPTW HCVVGRNFGS YVTHETKNFI
YFYIGQVAIL LFKSGVCGWS KAIRKQGGRF CFVNLNDGSC HLNLQIVVNQ CIENYEKLLK
CGAGCCFRFT GELIISPVQN DIHNFEIYGE DPQKYPLSKK NHGKEFLREV AHLRPRSYFI
SSVIRIRNSL SIATHLFFQS RGFLYIHTPL ITTSDCEGGG EMFTVTTLDY KKDFFSKQAF
LTVSGQLSLE NLCSSMGDVY TFGPTFRAEN SHTSRHLAEF WMIEPEIAFA DLYDNMELAE
AYIKYCIDYV LNNNFHDIYY FEENVETNLI KRLKNILNED FAKITYTNAI EILQNYSDSF
EVKVEWGMDL QSEHERFIAE KIFKKPVIVY NYPKDLKAFY MKLNEDNKTV AAMDVLVPKI
GEVIGGSQRE DNLERLDKMI KEKKLNIDSY WWYRQLRQYG SHPHAGFGLG FERLIMLVTG
VDNIKDTIPF PRYPGHAEFI KDLLEMNLEN STNILKNLFL KDKKNYFLIC TLNNKTVDLK
NLSNILKTNN LRFVDENNLN NILNIQPGCL SPLAIKNDKE NIVKLYFDEE IKNMVIIHPL
HNYSSLYIKT QDVIKFCESF NHAPILGITS KKIENFSDWY TQVIVKSELI EYYDISGCYI
LRPAAYYIWE CVQAFFNKEI KKLNVENSYF PLFVTKNKLE KEKNHIEGFS PEVAWVTKYG
DSNLPEEIAI RPTSETIMYS VFPKWIRSYR DLPLKLNQWN TVVRWEFKQP TPFIRTMAKL
SKQQKKQMYI EKLSSLIQQY SKILIVHVDN VGSNQMASVR KSLRGKATIL MGKNTRIRTA
LKKNLQAVPQ IEKLLPLVKL NMGFVFCKDD LSEIRILDNK SPAPARLGVI APIDVFIPPG
PTGMDPSHTS FFQSLGISTK IVKGQIEIQE HVHLIKQGEK VTASSATLLQ KFNMKPFSYG
VDVRTVYDDG VIYDAKVLDI TDEDILEKFS KGVSNVAALS RATGVITEAS YPHVFVEAFK
NIVALIIDSD YTFPLMENIK KMVENPEAFA AVAAPAEEEE EEDGFMGFGM FDQERDLARE
PCPDRIIEDM GGAFGMGCIG GYIWHFLKGA RNSPKGDVLS GALYSSRMRA PILGGNFAVW
GGTFSCFDCA FQYMRKKEDH WNAIGSGFCT GGVLAMRGGW RSASRNAIVG GVLLAIIEIV
SIVLTRKTTP TPRQQFQQQM ELEKMLVDNI GDVTITNDGA TILKQLEVQH PAAKILVNLS
ELQDQEVGDG TTSVVLLASE LLRRGNELIK MDIHPTTVIC GYKLAMKESV KYIKEKLSER
NLGKDVIINI AKTTLSSKFI SYESDYFAKM VANAIQSVKI INESGKTKYP VSSVNVIKVH
GMSSLDSKLI EGYAIMSGRA SQAMPTVIKN AKIAFLDFPL KQYRLHLGVQ VNINDPKELE
KIRQKEKDIT KERVNKILES GANVILTTQG IDDMPLKYFV EAGAIAVRRV NKDDLRRIAK
LTNGQIRLTL SSIDGTEKFE ASSLGYCDEV YEDKVGDWDL MFFKGCRTSK SNTILLRGAN
DFVLDEMQRS IHDALCSVSR ALESNYVVVG GGCVEVALSV YLEDFAKTLG SREQLAIAEF
AESLLVIPKI LALNASYDSI DLVCKLRAYH TKSQVNIEDS KDYKWYGLDL VNGKVANNLK
NGVLEAMISK IKSIRFATEA TITILRIDDL IKLVPERNPP VIKLKSHTSP ILDLSFNPCY
SEILASCSED MSIRIWLNGH KKKVNILSWN PMNYFILSST SFDSSVNIWD IENEKKAFEI
NMPKKLSSLQ WDIGGNLLSG TCQNKQIHII DPRKQEICNS FLIHDGGKST KCIWIDGFGG
EDILTTGFSK NNMRELKLWS LKNTTSPLTT ITLDNAASPL LPHYDESVGM IYLIGKGDGN
CRYYQYSQGS IRKVDEYKSC LPFRSFGFLP KRMCDVYKCE IGRVYKNENN TDIRPISFYV
PRKNFQEDLY PPIIGINLDI KRVSIFNKLK ICGQFNKGFV ISKLFIIDQH AADEKSNFEK
YNKIFTMKSQ KLVYLLSLPV FNGKILEVVD FMSLLHHLWF NYNFPRPQKV WRILASKMKE
LVIIFLKKIS DTYTEDQTKW LEQMKSSQEQ QNDKKLNEWN ENVENKCFIY PASSAPCGAC
TSAGAIIHHR RYKEKRRKKE YTGTDILCQA KSGMGKTAVF VLSILQQLVR CLGLAHTREL
AYQIKNEFDR FSKYLKNVRC EVVYGGISMN KHIKLFKIPH IIIGTPGRIL ALIREKYLIT
DKIQHFVLDE CDKCLEKLDM RSDVQKIFIS TPLKKQVMFF SATMAKEMRD VCKKFLQNPV
EIFIDDEAKL KLHGLLQHYV KLQEKDKTRK LIEILDALEF NQVIIFVKSV TRAITLDKLL
TECNFPSIAI HGGLEQQERI ERYDKFKKFE NRILVSTDLF GRGIDIERVN IVINYDMPEN
SDSYLHRVGR AGRFGTKGLA VTFVSSQEDT LALNEVQTRF EVAISEMPNK IDCNEYINQR
LSVSAEAYGD WNKKINFIPK VYKKDEKEKA KIREALNESF LFNHLNKKEF EIIVNAFFDK
NVEKGVNIIN EGDYGDLLYV IDQGEVEIYK TKNNKKEVLT VLKSKDVFGE LALLYNSKRA
ATATALTKCH LWALDRESFT YIIKDMVAKK RKMYEDILSH VNILKDMDPY ERCKVADCLK
SKSYNDEIII KEGEEGDTFF ILIDGNAVAS KDNKVIKTYT KGDYFGELAL LKNKPRAATI
KAQNFCQVVY LDRKSFKRLL GPIEDILHRN VENYKKVLNE LMHLKIVCLS DEVREMYKNH
KTHHEGDSGL DLFIVKDEVL KPKSTTFVKL GIKAIANTSF LLFPRSSISK TPLRLANSIG
LIDAGYRGEI IAALDNTSDQ EYHIKKNDKL VQLVSFTGEP LSFELVEELD ETSRGEGGFG
STLGAAFGTA KSGVGVCSVG VMRPDLIMKS ILPVVMAGVL GIYGIIMSIL IYGDYIKAYL
LGFTLEDSLA LLRIEDLYIE SFQIQDVKIL KGDHLSRCIG RICGSNGSTK YAIENATKTR
IVIANDKIHI LGSFNNIKMA RHSICSLILG STQGKIFNKL NILAKRMKER IEKLEDRMHP
WSNIDGMKAA CSYTYDDIIC MPGYIDFALS DIDLTNNMTD NITLKTPVIS SPMDTVTGHK
MSIALALSGG LGVIHNNMSI EKQIEEVKKV KRFENGNKVL CDEKKSVLPI VNKNNEFPHA
SKSQNKQLIV GASISTDLER ANQLIKNMID VICIDSSQGN SIYQIDTIKK IKSAPIIGGN
VVTSQQAKNL IDAGADVLRI GMGSGSICTT QDVCAVGRAQ GTAVYHVSKY AHNIKTIADG
GIKNSGNIVK ALSLGADFVM LGNLLAATEE SCSEYYFENN VRLKIYRGMG SMEAMVSQGV
SASLVDKGSV LNLIPHLFKA VKHGFQSMGI RNIPELHSKL YSGMKENKQY QEALKELKKL
KKRIEENIDF FQKLKEKLIL AQESAWDKFG SKLKDMPFLN NFFENPILGK LFGETELAAA
LREMKMIDKN FKLSELMYLF EYVISKHIVE SYLIGDEETL RLHCGSSAFN SLNASITERK
KKKVFLDTNV LIYKNHELKG AQRMEESSPW FIFTFHTQQI NCLKNKNDEI IEGKIDDIRE
VVYTIALSKH PEPEGLLYPY IVREFAIIGN TPSWMVLYII GLGLGDEKDI TIKGKELIEK
SDVVYLETYT SILFVSKDVL EETYKKSIEE VDRDFAEENC DKILDEAKNK KVSFLVVGDP
LCATTHHDII LRAKKKNIDV EIIHNTSIIS AIGECGMQLY NFGQIVSIPY FEDNYKPTSY
YDKIYINLKN NFHTLCLLDI KVKERTVENI MRNKKIYEPP RFMTINDSIE QLLYCEHKKN
IITKNTLGIA IIQIGTDNQQ IISGDLLTLK DISYNKPLHS LIICAPTLHD IEKEYFDLYP
NSAYRKCVRV QLIKNGKKIT AFVPGDGCLN FIDENDEVLV SGFGRSGHSV GDLPGVKFKV
VKVARVSLLA LFKEKKEKPR SMGIKGLTKF IADAAPNAIK EIKIESLMGR IIAIDASMSL
YQFIIAIRDS EQYGNLTNES GETTSHISGL MSRSIRLMEN GLKPIYVFDG APPELKGSEL
EKRGEKRQKA EELLKKAKEE NLEEIKKQSG RTVRVTRKQN EEAKKLLTLM GIPIIEAPCE
AESQCAFLTK YNLAHATATE DADALVFGTK ILIRNLINLE QVLKGLNLTM DEFIDFCILC

GCDYCDTIKG IGSKTAYNLI KEYNCIEKII ENIDFQEARK SFINPNVKED IKIDWNEPQI
EELKHFLIKD YNFNELRVTN YINRLLKARK VTTQRRLDNF FVNNNKFYEV LNLKKNCTTD
EVKKAYRKLA IIHHPDKGGD PEKFKEISRA YEVLSDEEKR KLYDEYGEEG LENGEQPADA
TDLFDFILNA GKGKKKRGED IVSEVKVTLE QLYNGATKKL AISKDIICTN CEGHGGPKDA
KVDCKQCNGR GTKTYMRYHS SVLHQTEVTC NTCRGKGKIF NEKDKCANCK GMCVLKTRKI
IEVYIPKGAP NKHKIVFNGE ADEKPNVITG NLVVILNEKQ HPVFRREGID LFMNYKISLY
ESLTGFVAEV THLDERKILV NCTNSGFIRH GDIREVLDEG MPTYKDPFKK GNLYITFEVE
YPLIITNENK EVLKILKKQN EVEDLENSEL EVVSCSPVDK EYIKVRLKRL KNYLPYLCKI
LIDNTVYTKW DYLAMDESHF QNDNADEMSS RTWGNDWTVR KGAALCLDYL SNVYNDEILE
FVLPHIEEKL MSDKWNIRES AVLTLGAIAK GCMYSLSPFI PKVLEYLIKL LNDEKPLARS
ISCWCVTRFS SWICHPDKWF EPVLLNLLKR ILDSNKRVQE AACSSFANLE EDALELLNNY
LHEIVHTIQQ AFQIYQAKNY FILFDVVGTL IDSVNIVKEN NELAHEVVYA ILSKWVNIRI
SSPYIIALME CMSCITSAYG KEFLKYAKDV IRTCIKFLVI LYIDDLIECS FDLLSRIILQ
SNFALIGDIS RFCPQYLILN DIIPFLIAHI THPSTPVSNN ASWAIGEISI HINSEYMEIY
VDEITKQLIY ICNSKYHGCL LQNICITFGR LTSTYPKKLI FYFPQFLKTW LKIMAHGTQE
NEKINFFHQF LATMKECIQP DELRVKLAQG LLKSIIVNKL TSNGCTFIFW IADWFAHLNN
KMSGDLKKIK KVGSYFIEVW KSCGMNMENV QFLWASEEIN KKPNEYWSLV LDISRSFNIN
RMKRCLKIMG RSEGEENYCS QILYPCMQCA DIFFLNVDIC QLGIDQRKVN MLAREYCDIK
KIKKKPVILS HGMLPGLLEG QEKMSKSDEN SAIFMDDSES DVNRKIKKAY CPPNVIENNP
IYAYAKSIIF PSYNEFNLVR KEKNGGVKMY VKEEIVEEEK LSDIINKKKE NIKYMKGMKI
PENILAISNL KEVIDDADLL IFVLPHQMER ILSSIGKLSV VAGGLSLIPY TFIYDVDGGE
RCVMFNRFGG VSENTFGEGS HFYVPWFQTP YIYDIKMKPK VINTTTGTRD LQIVTISLRL
LFRPHTQHLP YLHSTLGPDY DERVLPSIGN EVLKAVVAKY NAESLLTQRD KISKEIRESI
TARAKHFNIL LDDVAITHLS YGKEFAKAIE DKQVAQQESE RVKFIVAKTE QEKIAAVIKA
QGEAEAAKLI SSAVKEYGKS LIEIRKLEAA KEIAENLSKS KNVTYFPSNS NILYINILKE
ENGGYNFDNL KRNEILKEKG PQFRKTGTTI CGLVCQNAVI LGADTRATEG PIVADKNCSK
LHYISKNIWC AGAGVAGDLE HTTLWLQHNV ELHRLNTNTQ PRVSMCVSRL TQELFKYQGY
KVCAIVLGGV DVNGPQLYGI HPHGSSCLLP FTALGSGSLN AMAVLEAKYR DNMTIEEGKN
LVCEAICAGI FNDLGSGGNV DICVITKDSY QHIRPYKEPN MRLYHLPKGT TPILSEKIEY
IKKFISLNDL GEARGTVLSV KLDELIDNVE GQTVIDPKGY LTNLNANDAD IADINKARSL
LKSVISTNPK HGPGWIAAAR IEELAQRKDK AKEIIMKGCV VCSKNEDIWL EAVRLEKLSE
VKIILAKAIK HIPTSVKLWL EAYKKEKNVD DKRKVLRKAI ECIPNSVKLW KEAISLENEN
NAYILLLKKS RVQCNTNNKN LNPIISEALK ECPSSGILWS KAIELENKNL QNSKSVSAFN
HCGNNAYVIL TVAKLFWVNF KIQKARKWFY RVINLNPHFG DGWATFLAFE IDQQNEINQK
DIINKCIKAE PNRGWVRGRI HDIRSKGSIA FIILRHKLYS LQCILDIKNN NDKNMMKWVS
NLSLECIVDI YGEIKKPEIP IDSTNIKYEI HINKIFCLSK TMKELPFLLK DANMKETNDE
ITIKVNQDNR LNNRCFDLRT YANYSIFSLQ SVICHIFRTF LLQHNFVEIH TPKLLGESSE
GGANAFKINY FNQNGYLAQS PQLYKQMCIN SGFDKVFEVG PVFRAENSNT YRHLCEYVSL
DIEMTYKFDY MENVHFYDSM FKHIFKELKN QYPSDDFVWL DKTPIFTYEE AIKILIEDIL
TYDLTTDLEK ELGKLIKLSH NTDYYIIINF PSSLRPFYTM YKEDDPKISN SYDFFMRGEE
ILSGSQRISD MKLLLENIKL FNLDPNKLNF YIDSFAYSSY PHSGLLFENL NKEYKFITTQ
DNFDGFRFEV DKNINKFLQS THTLFLGTRE VGYLYQFGAN FTNLDNSLLM ISRINIDGSV
NGRFCKKINN DCKLNFNTYA KNDTRNMYEM SLEVNKPLYT YNFKSIWQGG VDLTYIASNC
ASIGSFGLRY NHKNNVLTMQ IVRQPNFKSP EFMLNQTHLY KIQYAKKISD RLSLGTELEI
TPQTKESAMR LGWDYSFRHA KVQGSIDTSG KISVFTQDYS GFGVSGYIDY LNNDYKFGMM
HISPSQEQDG GVGKTTFVKR HLTGEFEKKY IPTLGVEVHP LKFQTNFGKT QFNVWDTAGQ
EKFGGLRDGY YIKSDCAIIM FDVSSRITYK NVPNWYRDIT RVCETIPMVL VGNKVDVKDR
QVKSRQIQFH RKRNLQYYDL SARSNYNFEK PFLWLARRLS NQPNLVFVGE HAKAPEFQID
LNIVREAEKE LEQAAAVAID EEDGVITVKS ILSEPTIHQY DIKKLIKNKL QECVPFYNYN
MNRSFAEKIY GDCIYDNYGL SKEIEINLII LEEWNINCNK NRVLKNTGLI KEITINQFKY
STNKESLEVH FAVSPKYTDI LPKNKVLPPS GINYNKLIKE FGCSKITENH IKRIEKLTAH
HFIRRGIFFS HRDLDFLLNY YEQHKCFYIY TGRGPSSLSM HLGHLIPFYF CKYLQEAFNV
PLVIQLSDDE KYLFNQNYSL INITKLTKDE LYFTLYNSNS GIANALRRIM LSEIPTLAID
VVNVYENTSA FHDEFIAHRL GLIPIDSRNV NNYEFREKCK CKETCSKCTI QYIIEVKCNN
KIDVSHYDIL EHEPNVPMPI PIPIVTLSKN QTLHMKLIAT KGIGKMHAKW IPANVSYRID
HKVLIKHNLI DKLSNEHKLL LANNLNKDCY ILLKLKENMS VVMAESSIDL LSELGYKDII
KIVYDETMFH FHVESVGSIP PEQIVQMAID ILENKLKVLE PQIKSSFYSI DEVAKQLKLP
DPEKAKNEEE RCFLTLLKNS ILKNPKKWTN IAKKIIGVSE ETTTGVLRLK KMDKQNELLF
TAINVNDAVT KQKYDNVYGC RHSLPDGLMR ATDFLISGKI VVICGYGDVG KGCASSMKGL
GARVYITEID PICAIQAVME GFNVVTLDEI VDKGDFFITC TGNVDVIKLE HLLKMKNNAV
VGNIGHFDDE IQVNELFNYK GIHIENVKPQ VDRITLPNGN KIIVLARGRL LNLGCATGHP
AFVMSFSFCN QTFAQLDLWQ NKKYENKVYL LPKHLDEKVA LYHLKKLNMG SYGMDVDDAY
LHQGQYAAPY DNQYDTPSPR GENHTPFVGY FSSHLLRTGF FLQCVSLVLM FVFYWAFGGT
GIFIFDLYAG PECVKVSSTF HLTISILMAL YLLGTLYIAM FQVFVADNSK WCRGFRAGSK
LLSAAVTLDL LSSILRLVQY LYAYFYMSMR WWARYQQTKS DWTLLHFGSI VHSFALFIYG
AAFFYMEAYH DEGTYEELAW SNLTLFKLAG LAELNKPKKR TFRTFQYRGV DLDKLLDLSQ
DELIKLFKAR QRRKFQRGIS KKAKSLLKKI RKSKKNCEPG EKPNPVPTHL RNMTIIPEMV
GSIVAVHNGK QYTNVEIKPE MIGYYLGEFS ITYKHTRHGK PGIGATHSSR FIPLKVMVDM
FLWRDPEQFE LKNLANEENT PTAPHLIENQ YAAEAPYDEW GFLQIIKDHH ETMYELKQKI
RPRDQVVGWF CSGSELSELS CAVHGWFKEH NSISKFYPHT PLNEPIHLLV DASLESGFLN
IKAYVQLPIS LVKDYFVHFH EIQTELLPCN VERADVSLKK LLIMLKQCKS YVQDVIDQKK
KGNISVGRYL HKVLSNDTFL TLEKFDSINE SVLQDNLMIS YLSNLANLQF LIAEKLNWLN
HLPIKEDDAE GRRVHKNLID LVSQNHPLLF GKDNSNTAKI IEIFLTIYET DFSDTDCNKK
ISTLINSLDK SYLNNLALSH KQAKKLNNIN GMNLIDFYSK NLNLKPYLKI IKDFDKYPII
VDSNEQILSL PPIINCDHTK ISLNTKNVFI ECTAIDRNKA QIALNILCSM LSEYCVPKYS
IQSFVVIYPI FENKSLTCNI DYVRKLSGIS HITVHEVNNL LKRMMLDNNT FKVTIPFYRS
DIMHCCDIIE DIAIAYGYGN IKYEPPQICK KHSLNNCSEL FRNVLVECGY TEVMTNALLS
RDENYNCMLR PIQIKNSKTS EYEIIRTSLI VNLLKFVSAN KHRELPLRFF EIGDVSYNQT

DTNAVNKKYL SIIFSDKTAG LEELHGVLEA ILKEYQLFSD YKIEEKKKEN ISIRVLSVGN
IRGFGGSDFG SFRMSNEFLG WKNKKTNNVY QYKCSDIDEG CWIKTSYNNN RLHLKLGESK
ENIIIYFDGF PDRNVNEITQ HFQKYFNIRL NNRKIATKGW NWGEFKLENS NLCFDIDNKY
AFNLPTNNIN QLNVQIKTDI AMEFKNDENE DFLAEIRFYY PHENDENQNF QNLKNDLLEK
VNIGDTKSES IASLSNIPLL VPRGRYDIEM YSSTFKLHGK SYDFNIQYTN INKMILVPKS
NSNQYVLIFS LSNKMKQGQT EYPFILIQLN NDDDMELDIS ASDEVMTKYK LEKTISGKAH
DVVTKLFTAL VNKNVIVPGD YRTSKNQHGI TCSYRAASGQ LYPLNKYFLF IVKPVILISF
DDIVTLSFQR TGNNQHRFFS LIIKHKRGMS YEYTNIDKSE YNPLLTFLKS KNINIKGYID
LSKRRVSPKD IIKCEEKFSK SKKVHQTVRH VAKEHGITVE ELNEKAIWPL YERYGHALDA
LKEATMNPEN VFKGLDISEE IKNSLLKDIK LRLTPQALKL RGRIDVWCFG YEGIDAVKEA
LKKGKVSINI KLIAPPQYVI VTSCHDKDLG MAKIQEAMKV ISDKIKEYKG GDFKQQGEIL
VILLDKHDGI SSDDDGERGI FEFLLCDVGV GLSLSVRDVL PIEYDSIFIG VLPYYTFHHE
YIIYDNSQIL PRYLIQFECD PNDDEHFSLP LCDYCSDAPA ILYCESDEVK LCEKCDTLIH
SNKIVKKHIR KALNEAQGKC KRHMTNDVNM FCTICHIPIC NLCISSHVHI SLNMAYKAIL
HHSNNPSNFI KQKKKNLNDL LAKIDTLHEQ VRINMNDAEK SVYTILEDLV KQLHIITDQK
MCSVLSEEYE LKRQFNEIIW NESFLYYLQT ILPPADFMNA WLKHCQFREQ IEKNSLIFPD
MRIKGNINIV TESSIQHLVG LPNVGKSTTF NVLTKLNIPA ENYPFCTIDP HEAKVTVEDE
RFEWLVKHFN PKSNVHAYLS IFDIAGLVKN AHLGEGLGNN FLSNIAAVDG IYHVVRAFEN
EDIIHTEGNI NPVRDLEIIN SELIYKDISH CEKNLEEVTK VNRNKKDKVK QNEHDVLTSV
LNYLKEHKWI KDGTWKSNEI EVLNEYNFLT AKPVVYLVNM SEADFIRQKN KYLAKIYNWV
QEKNKGTIIP YSAEVEQKIL SMDEEEKKQY FETNNIKQSM LNKIIKTGYY EINLIHFFTC
GHDEVKCWTI RKGTKAPQAA GVIHTDFEKG FICAEVYKYT DLVEYKSEGE VKANGKYLQK
GKDYVVEDGD IIFFKFNVSS GGRINKFVNQ LRISYSTLEE FVDNFVYELK KGLEAHRKHP
NLWIPHECSF KMLDSCIANI PTGQEKGTYY AIDFGGTNFR AVRASLDGKG KIKRDQETYS
LKFGSYSHEK GLLDKHATAS QLFDHFAERI KYIMGEFNDL DNKEVKSVGF TFSFPCTSPS
INCSILIDWT KGFETGRATN DPVEGRDVCK LMNDAFVRAA IPAKVCCVLN DAVGTLMSCA
YQKGRGTPPC YIGIILGTGS NGCYYEPEWK KYKYAGKIIN IEFGNFDKDL PTSPIDLVMD
WYSANRSRQL FEKMISGAYL GEIVRRFMVN VLQSACSKKM WISDSFNSES GSVVLNDTSK
NFEDSRKVAK AAWDMDFTDE QIYVLRKICE AVYNRSAALA AGTIAAIAKR IKICGVDGSL
FVKNAWYCKR LQEHLKVILA DKAENLIIIP ADDGSGKGAA ITAAYFLIRW LCKVIVKSVF
RDVNVINPEN VPLYGSVIFV GNHNNQFIDA CVLIANIPRQ VKFIVAEKSM RRAVIGKLAS
VIGCISVKRP QDLKFKGIGH ICWNEGDVKI TGINTRFRLD VQIGDKLLIQ NKMFPVVKIE
SETELLIQEV INIECEDNGV PFKIIPKINQ TEVYNLVTNS LKNGDTIGIF PEGGSHDRTN
LLPLKPGVAI MTLCALADGD VSIIPVGLSY SKLYQLQGCA TLFYGNAIII SQDLCKEYNN
NNREAISKLL SKIEEGMRSC MLTSKDHETS RCIELCVSLY TPERMTISKN KIYNNLQLFC
KMFWKFGNSK VIENLSYELK CYEKLLQANK IKDDEVWMLK QSTSAATLKF IEHICTFIFC
VIFGMTFSLL WLPLVLISIY LAERHRKAAL RNSTIKIQGG DVVSSYKVLV LIVLLPTFNI
VYGLLFSIYR LMKKKDIDGL QKLLNEHLKQ YNLHAIFSKE DVAHWFTPID QVIYTYVNEE
NGEIKDLISF YSLPSKVLGN NKYNILNAAF SFYNITTTTT FKNLIQDAIC LAKRNNFDVF
NALEVMDNYS VFQDLKFGEG DGMDIDSVLG LQAILISANY KEKEFIRIAY YMNSFYKDME
LRENPPVVPQ YDKICRHIVE NPRIVKFSIG WDDAYRAYPI LKELKQKDYF RIFKVNLHLS
CKFLKGNEKC KEIKKCSVCE CEQDEIPYNF RTNEIEVDLV YNSPSFTAYE GRNIWELRGS
SNYKYFGAAK NLKGVRELLF KENEDKKQKK KDARNFEKVI NIHYFGYCDE ANEHLLQQEV
KIQKKLMKHG TLVLPSDRAR EYLDCLGKEV DIQFIDMNEK TMKRQYKKYI QRIDDMERIL
RFLEENINKL PNVKIKKSKI DNFLEHDNIY ELDQVEESLN RLHVQFVRFC NNNKDLIDEK
NNAIEEKHVI LTALNQLSPS LSTHIMKDGI NMMFTNISGV IKTKDQESFS RTIFRAFRGN
TYTYFQNIDE KSVFVVYCQG SAQSNIYDKI MKICKAYDVK TYDWPRTYEH AKKRLKELRE
IINDKEKALK AYEEYFINEI FVLINVVEPN KNSLIEEWKL FCKKERHIYN NLNYFEGSDI
TLRCDCWYSA NDEEKIRHIL INKSSNDLVS ALLLSDKLRP NVSPPTYIKT NEFTKSYQSM
VDTYGVPRYG EINPAISTII TFPFLFGIMY GDVGHGLCIF LFALFLIIMN NKVKNNEMVT
MLFDGRYMLL LMGFFAVYAG FLYNDFFSMP LNLFSSPYIF GFDCKWLGAE NELTYINSFK
MKFSIIIGFI HMTFGVLMKG FNALHFKRKM DFFFEFLPQL VMMLSMIGYL VFLIIYKWVT
PGGFQKQGII NTIINMYLMK EINSTNQFYP YQSIIQILLL SLFVLCIPFM FICKPAIRTY
HIMKEIWIEQ LIETIEFILG LISNTASYLR LWALSLAHQQ LSFVFFEQTI LNSLKRNSFM
SVLINLILFS QLFSILTIAV ILCMDTLECF LHSLRLQWVE FQNKFYKGDG IPFKPFNIKK
LLPENYSAIL ARALSERPLT YLPTIERVCY EVLNDEDEHL NYIQINLLNT IRPTPIRGLL
AATQERFVVV PGIIVQASKP QHKMRKITLQ CRYCDHKMSI DVPLWRDKPQ LPPYCRYVLE
PYVILPNECT FVDIQSLKMQ ELPEAVPTGD MPRHLQLNVT RYLCEKMIPG DRVYVHGVLT
SYNPNPTRAD GTNFSYLHVL GFQKYDGNDL NFDVEERNEL TLLAAEHNIH EKIFKSIAPE
LYGMDEVKKA CACLLFGGTR KRIGEETKIR GDINMLMLGD PSVAKSQILK FVNRCAPVSV
YTSGKGSSAA GMVRQGLLGE NEEKLDYVLG LTLPKLLERR LQTKVFKLGL AKSVHHARVL
IRQRHIRVGK QMVDIPSFLV RVDSEKHIDF ATTSPFGGAR PGRVKRKSLS QSMYDRHLTI
FSPDGNLYQI EYAIKAVKNT NITSVGVKGE NCAVIISQKK MATQYISQDK LLDYNNITNI
YNITDEIGCS MVGMPGDCLS MVYKARSEAS EFLYSNGYNV NAETLCRNIC DKIQVYTQHA
YMRLHACSGK NITEDNIKEM FSPYGTVEEV FIMKDNTGLG KGCSFVKFSY KEQALYAIKS
LNGKKTLEGC TRPVEVRFAE PKSSKQTLKV CSRDLPGHLK MKTRDLSDYV EDKNMIETIN
LAVKYAKEAV VEDEKKNYKE ALNLYIQSLQ YFNFFCKYEK NSNIRDLILK KMEVYMTRAE
NLKEMLNILN KNNNIKWSDV CGLETAKEVL KEAIIFPLKF PKLFNSSTLP YKGILLYGPP
GTGKTFLALA CSNECNMNFF NVSSSDLVSK YQGESEKYIK CLFETAKEHS PAIIFIDEID
SLCGSRTDGE NESTRRIKTE FLINMSGLTN YKNNIIVMGA TNTPWSLDSG FRRRFEKRIY
IPLPNIYARD IKQFATLTEN YTGADIDILC RDAVYMPVKK GLYVKCGSRY EGMSVMLENM
AFHSTAHLSH LRTIKSLEKI GATVSCNAFR EHMVYSCECL KEYLPIVTNL IIGNVLFPRF
LSWEMKNNVN RLNLMREKLF ENNELYITEL LHNTAWYNNT LGNKLYVYES SIENYTSENL
RNFMLKHFSP KNMTLIGVNV EHDELTKWTS RAFQDYVPIP YTNQKEVTPK YTGGFISVED
NVKKTNIAIA YETQGWKSSD MITLTVLQTL MGGGGSFSTG GPGKGMYSRL FLNVLNSYNF
IESCMAFSTQ HSDTGLFGLY FTGEPSNTSD IIKAMALEFQ KMNRVTDEEL NRAKKSLKSF
MWMSLEYKSI LMEDLARQMM ILNRILTGKQ LSDAIDSITK EDIQRVVHNF LKTKPTVVVY
GNINYSPHYD EICNILDAVS KIHFFMGYPE LASVNFGNST VVRCKKCRTY INPFVRFEAG

GKKWNCNMCY NINDTPQFYK RKDLFQRPEL CTGSVEFIAP SDYMIRPPQP SVYLFLIDVT
VTSVNSGLLD VVCSTIKSLL DSRTLIGIMT FDSTIHFYNL NSNLKQTQMM VVPDIQDIFI
PLPEDILVNV HECQNVIDVL LDNLPGMWRN NKISDCCAGN ALKAAFMVLK KVGGKLLFFL
SSVPNIGDNL YAELAQNITQ YQIAVDLFAC PYNLDLASIY PLIKNSGGSL YYYPFNVHQY
NDKLRQELLF ALTTETAWES VMRIRISRGW KITNWYGNYQ FRGADLLALP NCHSGQNFSI
IVDLEENVVQ DSIVYVQSAL LYTNSNGERR IRLHTYALPI TQNIKTITDS INPQVVVSLL
AHQSIDISKK GKIADGRNLI QNLCSQVLSS QLLQSECARL LSLYILGMLK SIAFRDPDLR
IYHWYRLENI PVESVEANFY PRMFSLHNPD ALNLTCENMT QDGCYIVEDG ETIVMWIGRS
ISLAVVNVAA GLDGCDDQLL PASFRALEAD LNLHPSLLGY ITLAQTLMLS LFSPIWGFLS
DKYSRKWMLV FGTALWGVAT ILLANINDFA HILFFRAING LALGSIGPIS QSILADAAKN
ELGLSFGLVQ LSSSLGRLIG GVVTTTVALK YFGGIRGWRL CFIVVGILSV LLSIIVALFV
EDAPSLSKKS IIIILLEGFT GTIPWLALSF NTMFFQYCGL SDLQAAIITG FLLIGSAIGG
VVGGHFGDIM HDISNKHGRP LLGQLAMFGR VPLVLLIYLV IPKRKESFEL FLSCFCIGLS
SIAGVAVNRP IVSDIIRPDY RGTVFSLTIA IEGVGSSLIG APLFGYLAEK IFKYQNNNLL
ISDMPEDIRI NNAQALSKTL FYLTIIPWIL SFIFYSLLHF TYGKEYLKMN EIIQNEMLMF
GLRSLSDFCN PTSKAYKENA SDALDRGVVV SIKNAVINYK DDDDILFCSS RVLLSMSDYC
MSEKDTNALK KLITDGGGIV EIVKSFPSDP DTLKNCMAFI KNVGIALLNV FTSKTYTNKL
TNGIVLALCI ISKSTSGSKG LNDEGAHHKL LDYCLDDTAE IVESVFDIIK NMSSNGYVDP
TIIEKSVIIL DKFKSYPRVI SKGSDAMKCA VGPEELSKCL NVLKKSAQGS KEQDAALELL
SSLSYISSIT DKVVESGGIP VLIELINSGL QQYESNPEKI SRLVAGASRM LGRISNNPPH
AAIVVEYGGI ATLCTAISYF PNDVECSKAI CNALTPFVSR SYSLFASLLP ILYASLESVE
LAKASMECIA SASMINEFHE QMVNNQAIEI LSTCVQYHLT EMDYLLNCFT AYFRLSDYIT
TVEPINQYGG VDGIANALLA VDSVLTVMLE NENKEVIIQE GTKIMEKLAT ESDCQRHITN
LETISETNQE EAYKTLAAIS GLSRIESLKN ILESKGADTS IFNGIKIWIE SARFIEQTKL
IKAGLKTIKI VDLMCLSQVK RIAEDEPDEN ILITSAECIN YLTEVNKINS AEIVEACLEN
IFKLMKKYSE SRLTQINLIS AMNNILLSSN GVDILINKGY IKHIITYLQK VPMYVDVQII
GFTVLANLVK ISPDSVEGIK KLNALIPLQT ALRTHAKNMK LKTTCAPLLS VLMPLDSLTN
EIQDIIKLCN KSMNDKNLSK LHEYLVALNE LLLTPEACKI ASRSNIISEI AHASTNISQT
RLGLVHLTKC NMVSSLVQLH DILKLPGDNY TEEAVSNILE ALSLLLKYDI TNAEIGFNSG
LIKKLCAGIN YFSHSDSVIN KTFGCLACMC TSKNRVGQLI SCPEYEGLIK LIVELIGDSK
LSRGSAIKAV YELLKTEDED IIKDISCKTS IVNLYKIMGE YQADLSIVQD CSRCLAIIVD
YVNIEEDKYT PMKVLLECLN KSKNDELTVL EMLTVLVKLC NSDDKMKLKE LGAIDVISDI
TMIHSENEEI SRLGGVMYSY MGADEQVKKL MKLILNVKKE DSDAVQKIDN FTSKLEMFLR
APLENPSDAL QYTEVTLQVL NSYLASEVDN SSLQTNIALV TKRLVDRVKH DSEDPLGSWA
VASAGTLNQY IDMISNKIGL SNFKFVSPVY GVLAACVMNM DKKAREYAQD ALKFIQRSGS
NFLACKNLKE RLENNGFINL SEWNLNKNEG YVLCKENRNI CGFFVGKNFN IDTGSILISI
GHIDSCALKI SPNNNVIKKK IHQINVLVEK LIQINKSVLF LPSLAIHLQN RTRSVKINYE
NHIKPIISTT LFNQLPLLYL LSKELNCKEE DILDFELCLM DTQEPCFTGV YEEFIEGARF
DNLLGSFCVF EGFIEFVLFF ITIYEIKCQL RFASLGDWGK TKGQILNAKY FKQFIKNERV
TFIVSPGSNF IDGVWKNLYE DVYSEEDMYM PFFTVLGTRD WTGNYNAQLL KGQGIYPKWI
MPNYWYHYFT HFTVSSGPTG HKDLAAAFIF IDTWVLSSNF PYKKIHEKAW NDLKSQLSVA
KKIIVVGDQP IYSSYLLPLL KDAEVDLYIS GHDNNMEVIE DNDMAHITCG SFCVHELSNN
GIVTKFVSSK KGALQHFAAL PNVELTDVPS SGPMGNKDTF VRVVGTIGIL IRYAYSGVAY
SSGALNLNKT WCMNPDQGLI KPDVVFYLVC IYDNDICDIS DIGVNFYINE KDVEDKSDAV
LKELQELNNY VKRIAFLSCN IYGLCGYIFV DFNKEFICYD SNGEQVKSCN VSKISKELEG
KVSFDFDKTS PFEEGDYVQF SNVEGMEINN KIYKIKNLKK YTFEIGDTSL YSEYIKGGIC
TQVKKHLKLN FYPYEDYAKF DMSNHLHVEQ LKKDVVYNVC RYSKSHIAPV ASFFGGLLAQ
EVIKFTGKYM PIYQLLYLDF FEKNDNIITV FGKSFQKKLN NLNVFLVGSG ALGCEYAKLF
SLLDGKLTIT DNDNIEVSNL NRQFLFRREH VGKSKSLVSS EIIKKKNNNM HVQSLETKVG
AENEHIFNEE FWTKQNIIVN ALDNIQARQY VDNKCVWYSK PLFESGTLGT KGNVQVIIPY
LTQSYNDSYD PPEDSIPLCT LKHFPYDIVH TIEYARDIFQ GLFYNTPLSI KQFENLQNVI
NSLKISSQCN FDFCIKKSVE LFHNNFINQI NQLLYSFPLD YKLSSGEYFW VGQKKPPQPI
VFDVNNEMIQ EFLLSTSNLL AQVYNIPPCF DINYIINVIE VKPFEPKKVK INMDPIEFDK
DEQTNLHVNF IYAFSNLRAI NYKINTCDKL KAKIVAGKII PALATTTSII TGLVGIELLK
YVNYLSYFKN AFINSALPLF LFSEPMPPLR MMDKEYDELM KGPVKAIPNG FSSWDKIVIS
IKDYDTQRDK RFSGTVRLSN EVRKKLKVCI LGDAVHVEEA QKLELDYMDI EAMKKLNKDK
TLVKKLAKKY DAFLASQVIL PQIPKLLGPG LNKAGKFPSL ITHNDKIFLV YNIASFCFEL
QFRFIEDTTF DWLPALGYLL PYEKIKFLRT FFPIVFFISI CASAYSYTDK NATLIFLMRS
ILSTNRIIVE RSNDVNYLKK NGEKLVTKLQ QIRKFALTLD SQQISLLEKK NKKKSWFEFF
SIQVIFGVIF VYIWLTSKKP ATYEVPLSLS LEELYSGCKK KLKITRKRFM GTKSYEDDNY
VTIDVKAGWK DGTKITFYGE GDQLSPMAQP GDLVFKVKTK THDRFLRDAN HLIYKCPVPL
DKALTGFQFI VKSLDNRDIN VRVDDIVTPK SRKIVAKEGM PSSKYPSMKG DLIVEFDIVF
PKSLTSEKKK IIRETLGLYH ICISCPSTNW FKSTAIKWSL SIEVGGSDID PENLAKKSEL
SETLTILHVD TGKTKLLDKL RHTNVQDNEA GGITQQIGAT FFPKDVLDKE IKKIDIKCLS
KGIMIIDTPG HESFYNLRKR GSSLCDIAIL VIDLMHGLEQ QTKESIQILK QRNCPFVIAL
NKIDRLYMWN KNDWSPFNNT FKKQKPDTQE EFHDRLKNII NELSEQGLNC QLYWENMNPR
KYVSIVPTSA ITGEGIADLI MVLVKLTQTF MLKNIQYHDK LECTVLEVKN IEGLGTTIDV
ILTNGILRES DTIVLCGING PIVTVIRALL TPQPLKELRI KNEYIHHKYI KACIGVKISA
NNLEEVLCGT SLFVVNNIEE EEEYKKKVMT DVSDVFNHVD KTGVGLYVMA STLGSLEALL
IFLNDSKIPV FGVNIGTIQK KDVKKASIMR EKGRPEYAVI LAFDVKIDPE AEKEAALLGV
EIMQRDIIYH LFDSFTAYLK KIEEEKKQSK ITDAIFPCEL SVINDCVFNK KDPIVIGVRV
ECGQLKIGTP LFVPEKNIKI GNVVSVQSNK KNFDKARKGD EVCIKICGEP HVTYGKHFDS
TQKIYSKITR ESIDVLKEYF RSELTMEDWK LVVQLKKIFN IVMNINEKDK LAEQNLETLD
VTKLTPLSED VISRQATINL GTIGHVAHGK STLVHAISGV HTVRFKHEKE RNITIKLGYA
NAKIYKCTNP DCLPPECYKS YESSKEDNPI CPRKDCNHEM KLLRHVSFVD CPGHDILMAT
MLNGAAVMDA ALLLVAGNES CPQPQTSEHL AAVEIMRLKH ILILQNKVEL IKEEQALKQQ
EEIRNFVSGT AADSAPIIPI SAVLKYNIDV VCEYIVTQIS IPKRDFISSP HMIVIRTPKL
CNFVEWREYK LVFKRYASLF FIACIDKGDN ELITLEIIHH YVEILDKYFG NVCELDLIFN

FHKAYYLLDE ILVTGEMQES SKKTILRIVA AQDSLMEDNK FAKSLLDVAD NLSLAIKNIN
EESLKTNENI YKGIEMTETI LHNIFNKYGI DKYNPINEKF NPQLHEAIFE INDSTKKGTV
ATVIQHGYKI KDRILRHLDI ESVQALIVAL NMYKGGLIII SHDTYLIKHV ADEIYHINNI
TKLVKIDYEF DKYTQLLLNN KIMPREIITL QCGQCGNQIG VEFWKQLCNE HNIDQEGILK
NNFLNEDRKD IFFYQADDEH FIPRALLFDL EPRVINSIQT SEYRNLYNPE NMFISKEGGG
AGNNWGCGYS QGHKVEEEII DMIDREVDNS DNLEGFILSH SIAGGTGSGM GSYLLELLND
NYSKKMIQTF SVFPLLNESS DVVVQPYNSI LTLKRLILST DSVVVIDNTS LNRIFVERLK
LNNPTFQQTN TIISNVMSAS TTTLRYPGSM NNDMISLISS LIINPKCHFL ITSSNVQKTT
VLDVMKRLLH TKNIMVSAPV RRGMYISILN IIRGETDPTQ VHKGLQRIRD RKLVNFIKWN
PASIQVTLAK QSPHSQHKVC GLMMANHTSI STLFERCVTQ FDRLYKRRAF LENYKKESMF
QGNFEEMESS KEITQNLIDE YKSAERDDYF GLARAFGIPV RKYTHEVVTL WYRAPDVLMG
SKKYSTTIDI WSVGCIFAEM VNGTPLFPGV SEADQLMRIF RILGTPNSKN WPNVTELPKY
DPNFTVYEPL PWESFLKGLG IDLLSKMLKL DPNQRITAKQ ALEHAYFMGK EKTHINLVVI
GHVDSGKSTT TGHIIYKLGG IDRRTIEKFE KESAEMGKGS FKYAWVLDKL KAERERGITI
DIALWKFETP RYFFTVIDAP GHKDFIKNMI TGTSQADVAL LVVPAEVFEG AFSKEGQTKE
HALLAFTLGV KQIVVGVNMD TVKYSEDRYE EIKKEVKDYL KKVGYQADKV DFIPISGFEG
DNLIEKSDKT PWYKGRTLIE ALDTMEPPKR PYDKPLRIPL QGVYKIGGIG TVPVGRVETG
ILKAGMVLNF APSAVVSECK SVEMHKEVEE ARPGDNIGFN VKNVSVKEIK RGYVASDTKN
EPAKGCSKFT AQVIILNHPG EIKNGYTPVL DCHTSHISCK FLNIDSKIDK RSGKVVEENP
KAIKSGDSAL VSLEPKKPMV VETFTEYPPL GRFAIRDMRQ TIAVGIIKSV EKKEPGLPIV
LLKEGTDTAQ GRSQIIRNIN ACQIIVDIVK TTLGPRGMDK LIYTERDVTI TNDGATVMNL
NISHPAASIL VDIAKSQDDE VGDGTTSVVV VAGELLNEAK GLLNDGIEPN MIIDGFRNAC
NVAINKLNEL SLNFSNKNEE EKRSILLKCA QTALNSKLVS NHKEFFGELV VNAVYKLGDN
LDKSNIGIKK VTGGSCLDTQ LIYGVAFKKT FSYAGFEQQP KKFINPKILL LNVELELKAE
KENAEVRIEN PNEYNSIVQA EWDIIFKKLN LIKDCGANIV LSKLPIGDIA TQFFADHDIF
CAGRVEDADL KRTANATGAL VQTSLFNLND DVLGTCGVFE EVQIGNERYN IFKECLKTKS
VTIILRGGAK QFIEEVERSI NDAIMIVLRC ITNSEIVPGA GSIEMQLSKY LRIYSRSICN
KEQIVLFSFA KALESIPRHL SHNAGYDSTD ILNKLRKKHS EQTSDIWYGV DCMEGDIINA
YDNCIFEVTK IKRNVIYSAT EAACLILSID ETIKNPSRNL GLPDCFKELL KTDKIKHVLC
TGNVGCNENL ELLKNIADSV HITKGDMDDN FDFPEDITLC IGDFKISLIH GHQIIPWGDM
NALLQWQKKY DSDIIISGHT HKNSIVQYEG KYFINPGSVT GAFQPWLSEP TPTFILMKSN
IVLYVYEEKN GKTNVEMSEL HKYSVKISEP RDKNSTGIYR HPEYKDKLCE NFDDCMGVRE
KEDNKRGAYQ WKNFGEVKEL IMKVGSGLGL YMPNCPEWNI CDLGCNAYNI ITVPLYDSLG
PQSSRFILDQ TQMETIVCDK TCARNLFKSL QGALNNIFSI CYTSGTTGYP KGVIMTNRNF
IGILAAAYIG PSRLPNENDI HISYLPLAHI YERLMIYLFM AHGVKVGYYS GNVQTLLEDI
QELKPTLFIS VPRLYNRIHE RIFNSLKKKS GLVQSLFNKG LQNKSSSGST THVLWDKLLF
NKAKKILGGH VRAMLNGSAP ISVDVVKKLR TIFCVPMFEG YGMTESLGFI THSQDRNIGH
IGGPVPCIEF KLVSVPEMNY LVTDNPPKGE LYLRGPSTGY FKLEKETNED GFIRTGDIAL
LSPNGSLTII DRKKNIFKLA QGEYVAVEKV EASYKQSLFI SQIFVFGYSY ESVLVCVICP
STDSIDIWRT QKKIKATDEE VIKLPEFKAD VINDLTSIGK KDGLKGFEQI KDIHFTLEAF
TIENDLMTPT GKIKRHEAKK RFKKEIDEMY GTKTTKTINE GQTILVVFNE GYAPDGVWLG
GTKYQFINIE RDLEFEGYNF DVATCAKLKG GLHLVKVPGG NILVVLYDEE KEQDRGNNLN
MLAVVYLACR EAGHIKSIKE LITFDRSYKE KDLGKTINKL KKVLPSRAFV YNENISHLIY
SLQLSIDLIE AIEYVVKKAS TLIWSDIERY FKDPELLTAE ILFVALTLCN VFVMYRLFLD
VIPYPIFVTW WQLAQGLLVA YVCGLKVLFV PSIFYCLMLV LSNYLLYKTP CIASYPVLVS
FTVVFHHLTR FIGCGEEYML RWKSIVFLLA AFIIGCFDSK TTGKGVIVWA LLYALFSAIF
RAGFMQKIMH LVDGKGNTLH NNQHLLGVLI LPVLILLSGE LSVFVHMPYD ITSLHTGCLI
TVGTLPFIKN VISNRLVRRT GQGPWRFLEI ISIILVFFIG MTYNAPSFKG YLAILCVIIG
RSLGAFDVLL NKTKVGEEMR NASFSLAKSV WAAGDFKGQI IEGIKRPVVT LSLSTNNVAG
VKLPIFQVNI DPTVDVLGNL GVAAGGQVIN NTRENYLQCL NMLVKLASMQ YINIFDWLNV
YSETFYAKLN EDLTATKINL MRDSSTSSKD DNPYCSINDG KVIIKNNELL SGIICKRTVG
SSSGSLIHVL WHEMGPDKTK DFLSALQKVT NNWLEYVGFT VSCSDIIASN KVLGKVREIL
DKSKSEVSKL VEKAQKGELE CQPGKSLYES FETRVNNELN CAREMAGKVA SESLDERNNI
FSMVASGSKG SIINISQIIS CVGQQNVEGK RIPFGFNHRS LPHFIKFDYG PESRGFVSNS
YLSGLTPQEV FFHAMGGREG IIDTACKTSE TGYIQRRLIK AMEDVMVQYD RTVRNSYGDI
IQFLYGEDGM AGEYIEDQII DLMKLDNKEI NKLYKYNFDE EPFGDYNKQN ILNQEFEELY
KCKNYLCKEI FPDGDIRQHL PINMNRLIEY AKSQFPNPID VVHKVNNFLE KLVIIKQINS
NDTLSVEAQN NATILLKAHL RTYLNSKLLT QTHKVSVKGL DWLLQEIEKI FYKSLCHPGE
CVGALAAQSI GEPATQMTLN TFHFAGVGSK NVTLGVPRLK ELINIVKNVK TPSTTIYLDD
MVSNDQQKAK DILTKLEYTT LKQLTSHAQI IYDPNTTTTI LEEDKSWVNE FYEFPDEDQY
SLGEWVLRIQ LTNIHVNEKK LTMKEIVYII YSVFSSDELD IIYTDDNSED LVLRIRVKYL
EDTFLKKLME QCLSTLKLRG IENITKVYMR EESKITYDSD NGKFVRSSHW VLDTDGCNLE
NIFCAPQVDF KKTVSNDIVE IFEVLGIEAV RRALLKELRT VISFDSSYVN YRHLSILCDV
MTQKGYLMSI TRHGINRVDK GPLIKCSFEE TVEILLEAAA FAQVDNLRGI TENIMLGQLC
KIGTGSFDII IDNQKLNDAN QNETIQDLTS AGFTTPDSSP LPFSPTYNAN IKNVVIPGNI
RKSEHFLNLM RIVVVYLKKY INIYDITSEG PLSFLYKFEK DTKLDTSFFK YCFDRLKSLL
NNLQIVEDYS SLNIVCNFCT LLGNYFKGFI IICEPYPEAT IYDPLIQFAC LDSSIAMKTV
INKYKSIILT SGTITPLELY PKLLNFKTVL TASFPMSFDR NCVCPLIVTK GSDLIPLSSQ
FSLRNDLSVI KNYGILLVDM CKCIPDGIVA YFPSYIYMEQ VISSWYELGV IANILEYKLI
FIETKDIVST TIALHNFKKA CDLGKGAVFL SICRGKIAEG IDFDKHYGKC VILFGIPYQY
TLSKILKSRL DFLKETYNIQ ENEFLTFDAM RQASQCVGRI IRNKKDYGIM IFSDIRYAKH
DKKNKLPPWI IKCMDISNIN LTVTTAVDIS KQFLLNMSQE YRETGQTKNQ PITRVTELSN
KLKVATVHTN CEIPTIGLWI SSGSKYENKK NNGVAHFLEH MIFKGTKKRN RIQLEKEIEN
MGAHLNAYTA REQTGYYCKC FKNDIKWCIE LLSDILSNSI FDDNLIELEK HVILREMEEV
EKCKDEVIFD KLHMTAFRDH PLGFTILGPE ENIKNMKRKD IIDYINKNYT SDRMVLCAVG
DVQHEEIVKL AELNFKPFFC GSEIIIRDDD SGPNAHVAVA FEGVPWNSPD SITFMLMQCI
IGTYKKNEEG ILPGTVNNIC NKMTVGCADY FTSFNTCYNN TGLFSKYLWK ARIFFIWQRL
FTSSNDYYYS LKINIIDPIH IFKNIKIIST QFKIYVLSNF SIYLAFYNYT SAYQRILDLL

SQISHFQYFF TGRMGIKRMY QKIPATILVL LKDFDPDTDI LEEPHFVDSQ NNLSFQEQIC
LINYCFSMIR FNPHYDEIKF EKLNAVISRC LKYQNWLLHS CMLWFKCKGE TFRFKTVDRA
AAQLNELHKE CYDIKPESVE RLKFIYDVYY PTTWEMKKEI GSVMIKIGSV VTAFNIFKDL
KLWEEAISCL IQADRKEEAR ELLDDLLKKK KSPCLLCLYG LNYYIDAWEI SNFKYAKAAR
LIGKYYYEKE MYEKCSEYLE KALELSPLFP EIWFILGCSY MKIQNFDESI KAFTRMISMN
LAYLYMKKGT YKAAKICINQ AVKMNNNEWK YWDTYLKLSI IQNDIDSFCL ALRMICQVNQ
VKQIQPWVFD YISDVIVKDK ELDTFWNAHS FFLFIKGKFQ DSFEAKIKEI RSIEIMYIWK
NKIFVGREAP YFKAEAVFAD NTFGEVNLHD FIGKYVLLYF YPLDFTFVCP SEIIALDKAL
DAFKERNVEL IGCSVDSKYT HLAWKKTPLT KGGIGNIQHT LISDITKSIS RSYNVLFGDS
VSLRAFVLID KQGVVQHLLN NLAIGRSVEE VLRIIDAVQH HEQHGDVCPA NWKKGKVAMK
PSEEGVSEYL SKLMDDNTAN IISHDNILTD PRIAQDCSAE TNELLNKAEE AIEIIYEISN
ILNVNLDKET IVILIQLCEY G

> ***Plasmodium yoelii***
NFDGDFKTTK KKIHWLPYIP DKLIKCTLYE YDHLINKEDW TNCINQNSKF ETVGYAEPAL
INLKVSDKFQ FERRGYFIVD LIKIPDGKSK KVGLNTNTLP TIVFPTVVYV GDEAFFRERE
LSIYRPFDHG HISDWDLANN IWDYAINCVD PNKSVKSVLL TEPPLCSISH RKNMGEIFFE
NFGFENINIS VSGLMSIYAA GLTTGLVLDI GEGVTQCIPI FDGYIEKNSV IRSDFGGEEL
TMFMQKLICD IGYNMTTRKS YEYVKIMKET LCFCSLNPPK DQLREDLTTT YTLPDGDVLR
DGYNTIEISH ERFYVPEALF NPLLCHRDNL SISDIVCKSI LSCPIENRKT LSSYIILSGG
CSLFPNLAER LEREIKNNSP ENARSAVKVV TYEQGASFAR ENNLFFSEAS AVSKLNVKHI
FENLLQMEKS KLAKVEKVLG RTGSRGGVIQ VRAQFMGDSE LSGRFLIRNV KGPVREGDIL
ALLETEREAR RLRGTQSLNL KKSHCYCHLS TGDLLREAAE KQNELGNKIR SIINEGKLVD
NELVLSLVDD KLKSPQCKKG FILDGYPRNV KQAEDLNKLL DKNKIKLNGV FYFNVPDEVL
VERICGRLIH KPSGRIYHKT LNPPKTPFKD DITNEPLIQR DDDNEAVLKK RLGVFKNETT
PLINYYKNKN LLCHACYFEL PDPKTTIGPY DSELNYFMWG PGFEWKPFDE KSSGGKISIE
NASHNARRLG LAPSSKDEEK IRELYGDNLT YDQYLEYLSM SIHDKDNAEQ LVKMFAYFDN
TTGYLTKNQM KNILITWGDA LTEDEAMNAL NAFSNDDKIR LTKLFGVIDK NQDEITSWFE
YVKNEVFLKQ VQIEMKQIDS DKDGFISLPE LNDAFSQNLK EVEKHADGLL KRFQIVDKDK
DNKLNINEVG LLIDPMKDND LKELEINEIL EHHDTNKDGK ISKKDDELAL DDFNFDANRD
GFIDRDEIIT FDLWNEKALK FAVTSLTDYG DVIRYPQDFK LLSNLNCFGA GFIFSIVMFH
LLPEFFFVFV GFCMQLALEY VLPTDAKLCC DSSSSEEEER VIKSHEGKRL DFYENIEDNL
NDSMENDDFS QLLKEYDNLY KFMVKEDPDS IPNFVIIYLD KLTKYVDTTF QNNVEKKNLS
KNKAQTLNKL RAKIRKCSEL YQNKLNQYLE NPDKFWSYSE DAEYASDEED DKTKKAMSKW
GLKTSEKVDK KKVAKKDDKG SHIDDNQSSK KKTYAELLNT KNLSEDVIRN RVKSVIEKRG
RKGLDKHEHI NILSKLCEIA KTISTQSYIE VLEHLINLEF DVVSSVYTYM SFNIWNKTFK
YIELILDLLI QNEHFYLVSI NITEEIAEEE SNEKEKISKS CKTLISFLAK LDDELLKALL
YIDVQTEEYR KRLGKTVHMI SLLYKGYKYV KAIYISTRIL DHLYYKPELP FKQIWGFVEK
TIEQERTEKR RLLSFHMHIS IELIECVNNI CAMLLEVPNL AKHSYESKDI ISRQFRRFLD
IYDKQIFNSP PENNREIIIL ATKYLQKGNW KMCCEKIFSL SIWPKFTDKV QAILKEKIKQ
EAMRTYIFRY ISVYDSFSID QLCVMFDLPQ NTVHSILSKM MVNHEIPACW NESSKYILIN
TINPTPLQTM ALKLAENINE VMEQNELALN MKNPKLGLQT KQCIVVGTRA LEFLNNELST
IKTLTELHDR VKKRGISDNN DNMLSLYLLE LFSIPIEQKE KNDFLTNRIS MYSKILNGRK
NVLDLLLNKF ENDCSDSIKK ELIDCFDVEK FKQEVNSKFM NILIQQLRKV EKLEKKKAKM
EIYLSIREQV KLYIRELVNI ISNLLTGNYP ILNLNGDDFL KKYGGTLMEN LKDGLKVGQT
VRFLLAKEES SMFGIIQNIS NDVRSKNILV NFFFRSSNIE EQIQVKSVEK ERLPVETLSG
DLFFLWYKMT RDDGWVGFDK AEIIKVFNNN SKIKDVLIRH TDLKILNQLA ITQFWAIKML
ISVPFATKIY AHFVHDSIKI SILTVLGDIA TALNKSFSKY LNFFANILLE TSKITIASGS
PENDDWVSYI FELRDAILLT YSNIIYALID GNEINKLKMY IPNILDFIEL ILIKEINHFN
AQNFQNSVSL LGDLVHAYGY ELIENSKLTD LIISVYGKID ILSSQRDECV SKIKWLKKIC
NISILQLEFK SVEDLNNATN NFIKAIKNYN VFPELRLKIL QLLYNSFNVN FSFFIAILQF
SSQNNLFHSI LPYIKFIDEW IKEWNISNNE KRQIYLIVAE ELKKLKKYED SYKHLKKHIY
YFQEILNHAS TIKASVELIV DSINLNNNIF FHEIINLDAI QNLQYIEYKP IYELLNIFYK
YNIQEFLTFI DLENSENKMY LLSIISLFKD NKVQNIQYIA DQLNITMLKV EKILVSAIGS
DVIDAKIDQI NKTVHMKTTI LRHFDEQQWE HLNNQIEKYI KNVATNFYKF IDSFASSTRA
IQNNPSYWCS AGNHLKDEEI TWTGYLNTKG FVKGVKISWE YSPELVSIFV SSDGEHYKNV
IPYKKISSTE SSFDEIYFFK KLEEVISIKI GLKNAIHKYF GIREVKIIGG GNPYFLLLSG
ITSEQEMCLQ ELWKTNSNNQ IISALSDPPK CLAVINVDNL GDGKSNWVFE SNSQIRLQLC
ISQKNVHGNI PGIHDIDASV DATSILDDDH NADNTIDGNL NSFWASSIFG DSEHLVYFII
DLNKFVEISR IKVFWEYPPL HYIISFSTEK IVAENLANPS FITIDSLKNI ETRYIKISMI
KPHPKHGEMD GQFLYGIRSI EVQANNLESV LNFCRDAANS DDARDKYFIE YISEFDKNLS
NKLINLEDDV SKNVSSISDK LSKLEEVLPN IETCLNEKKE YETKLKVSME QVIELIKPKC
SPEPLRVYCD MDSSTSLYVW NGINSVDDIR KHCAEVGLEP LVLKSTDQLN SLIFALKKMG
FILNGKINIP LAYDYSCFHD LLNGNVDLTT LIYESPNSTS IRQTALEEKK MLLCRVEDIA
KKRNRIHVKT QILSEDSKIK RHKMKKVYNY KFGYFGCGWK TEWTPFIHAP FFDNQHNTIY
KNRNKKLYEE IDTILHGRIH PHIKVVELKD HMHPIRLCTP SNEDCYSVIY TGEKINSTDE
RVIFGEYTGF VNNKELPQEK HQYIFALTFI ILPDNYTYAV DSSYMFNEMS LVNHYKTCFN
NYDFRINAEW QIVYLDGWPH IILTSIPGVE IETGEEIFAD FGFEWFDRVN DICLNDFIKN
NYEHRLDDIV DKYNLLKNYT TCNICIHSVN TDCNNYILCS GCNHVYHLKC VNRLNNENYD
WFCSSCIQFS MNIAKAVICR VTKLHFETNE ELHKMSSECI QSVIKELALG KTKFQREFTN
GTYVGTVTKQ INDNHFFVVT YEDGDVEWIT PFFLFQEEHK LKGNDFYKQK KFEEALKEYD
EAIKVNPNDI MYYYNKAAVY LEMKSYEKCI ETCIYAIENR YNFKAEFSQV AKVYNRLAIG
YINIKNYDKA IEAYRKSLVE DNNRATRNAL KELERKKEKE EREAYIDPVK AEEHKNKGNE
FFKNNDFPNA KKEYDEAIRR NPNDAKLYSN RAAALTKLIE YPSALEDVMK AIELDPKFVK
AYSRKGNLHF FMKDYYKAIQ AYNKGLELDP NNKECIEGYQ RCVYKIDEMS KEKVDEEQIK
KSMADPEIQQ IISDPQFQII LQKINENPNS ISEYIKDPKI FNGLQKLIAA GILFKPNEKI
PSKYGENRHW NVDLIPKFIL VGGNLVKILK KTRVTNYLEW LVVEGSYVYQ HQKKSLLFSE
KFIHKVPSTD MEALVSPLLS LMEKNRCKNF YQYVSEWNAN DRNTWDNLDP YRLTMMDIYK

YFNLCQLTID FLGHAVALYL NDDYLKQPAY ITLERIKLYM HSISAFGKSP FIYPLYGLGG
IPEGFSRMCA INGGTFMLNK NVTDFIYNDQ VCGIKSSDGE AYCDKVICDP SYVHLENKIQ
KIGQVIRCIC ILSNPIPETN DINSCQIIIP QNQLNRKSDI YVNLVSFQHG VSYKGKYDVR
LDVKLNKFIW SKGIRNPPKR VRVKIERVRN EDEDSKERMY TLVQHVMVDS YKGLVNEQMT
GDRHRIQDCT TENFSGFIAI TTPTKSWIAQ YNDLSKYNPG FYALQVVGEL GTGSGYLILF
LYELLLKKNK RIDLLYCIDI NKDANGISNV EIINSDLFNN LRKCFDIILF NPPYVETEQD
EMNKTIVASY AGGKHGREVI LKFLHTVYDH LSNNGILYLL LEKSNIPHEI ENSDLPILCE
TCLGENPYVR IIREENGKEC KICKNAFTLF RWKPGHNARY KQTIICNKCA KVKNVCQTCL
FDLEYNLPVQ VRDKFLETSI TLPENETNRN FFLEQLELSK LKRRDPYFKR NMARVCSFWR
KNACNRGDEC PYLHKEIHKL IIFNLPPVDE QDVKSLCERY GPVIDVYAFV SFVFPSSCEK
AKNNLNETIY RGKVLSVKYA SYKKILEIQK KRNCQNENIW NILYTDINSN IYNFCKETNC
DPQSILDKNI AVNVSLTETF IINKMKEWIR KEGIRSDDTI IVKNLSMHTN ENDIINLFKK
HGILKKISFS PYKNIAILQY EKPEDAKKAL ISNSYIRYKK LPLYLEWAPV NLFDEEITHS
SIYIKNINFN TKEEDFKKLF EKLDGFITCN ISLGYGFAEF KSKELAIEAI KKLTATKLDG
HVLELSLSKL LVKNLAFQVT KEELKKLFSA FGNIKNVRIP KNAYNRSRGY GFVEFMSKNE
CLAAINALQH THLYGRHLII DFAPENARML KPLIQEKIIE IMKPEIEEKI IEVPQVQYIE
KLVEVPHVIL QEKLIHVPKP VIHERIKKCP KTIFQEKIVE VPQIKIVDKI IEVPQYVYQE
KIIQVPKVMV QERIIPVPKK PQYRHIPKPV EVPMAHYRTF PVEKLVDRNV PVPVEIQIVQ
EFLCPKIEAR YKEIPVPVHV QRIIEHPIPK DAMNNPFLLP LYYLGSTAIG ICVNDGVILA
SERRIASPLI EKDSVEKLLP IDDHIGCAMS GLMADARTLI DHARVECNHY KFIYNENINI
KSCVELISEL ALDFSNLSDS KRKKIMSRPF GVALLIGGVD KNGPCLWYTE PSGTNTRFLA
ASIGSAQEGA ELLLQENYNK NMSFEEAEIL ALTVLRQVME DKLSSSNVEI AAIKDQMFYK
YKPDDITKII DSLPRFKIID ADFINIKLLE LFQINGFQKQ LDRLSDSLSK IQKALGEYLE
KQRNQFPRFE KLVQTKLTDA CFLTLTQALK MKLGGNPFGP AGTGKTESVK ALGAQLGRYV
LVFNCDESFD FTAMGRIFVG LCQVGAWGCF DEFNRLEERI LSAVSEQIVE ILNKKVELNK
NVGIFVTMNP GYAGRSNLPY GVGFKTAFAR IASSCAIMSR TINTIGIGLL SLELMNHCDA
KELATPLCMW KLPNKELINR NIANKSEHRH HQKLLMSYTP FNSPSLLAEQ INILGTYSGT
RLLYFPLWDH PKDSIDYCLS TYLYWLYLRR STNIFLQNTL LRGQVVVIAA TNRQNSIDPA
LRRFGRFDRE IDIGVPDDNG RFEILRIHTK NMKLSPDVKL EELASNTHGF VGADLAQLCT
EAALTCIREK MDVIDLEDEI IDKEVLESMC VTQDHFNMAL GTCNPSSLRE TVVEVPNVKW
DDIGGLDEVK NTLREMILYP IDHIIKSLYD HNFYSPREIQ SKTLQHSINE KKDIIVVSKT
GTGKTLTFCL PILSNILVLV PTRELAVQIL NHFNYVNKYI NIYIITIIGG LNINKQLRLI
SKKPEIIICT PGRLRYFVCD EVDKMIETSF INDIHFISKH LYIQTFLLSA TLLAKLLNYI
SIRKNKSYII DLLPDHLTLN IIKCEKKIIL HKLYYLLKLY KIIIFLNTIK LVKDVSTIFK
YLFFESGLES SLPNKIYSIH SKQKLKERIQ SVSKFSNNSS ILFCTDVMSR GIDLNKCDLI
IQLNCPISDI TFIHRSGRTA RNLKSGNSAN CSEIKSAELV LSSQELKRIS DPRFGTIDYT
QICSVCFESC LGHIGHIEFV LPVFNPLFYK DLQELLNLVC YNCYALCYSY NYEEYVILNK
AIKLHMKKGA NCSNCKFRRS IVAKTSPKKD TITVRLFSFQ IIDLLKKIFN KEIINLVYPF
TKKDGYQVFF LYYMGISSNR FRTQSRGIHK RNNYVKLCIN SKKNVDFDHM IELQLSVNTF
LDIREILDKK EGILRKNIMG KRVNNCARTV ISPDTFIETN QIGVPIEFAK TLTIDEHITE
NNFEYVKKLS LYDFILKKKA DFRHLSFISD FMTGALCMDI LKTNWSPAWT IQSLCRAILF
LLNEPNAESP LNCDAGNLIR GVKVKSAAEV GKRAVEYFRG DDFVSFLSSK QDILKKKFPK
LIGNRNLSEM KEVEEFADLF IQKGFIYKAQ YKPENGVYKR PKWPKRLIMT SKQNFDKAGF
YILVYERNKK LQYFMLMILI SIVLICCMFW HLSVVFIGLL SAIIVGRIIT FVFFWFFGVD
YWIFPNLFDE DCSIVESFIP FHSWERRNDS WFLVFARAVT AVLVAIGIHQ LGKTHSISDI
QNFAKQSFID IIEWGNKKLS EENYDCLKKC GFPPFEELVR RCFLKCDCMT LADTLLKDLE
DLEEEERKIS ELLYDIEKCI ELIIKIDTEI LNIHKYLKDI YSTKFPELDS IVYTPLEYIS
VVSRIKNESD IKNIDFSDIL PNTTVMAIVV ASSTTGIKLP DHLLKSCMSF CNEALELNEN
RQKVLIYLEN KMFLLAPNLT MLLGSALTAR LISCVGSLKN LSVTSSQNLI VVGNSKKGIL
STSEIVQSVP DAYKKKAISL LAGKCSLASR IDYFTEERTV LDKNILNSSV ILQIALRVKK
DRYLGRHYRY FIRNTRVRAY KQFLEPFKSV TLKNMAYAFG VSEDFIEVED PYEAIGEAVR
NFESKDEILA SAKIIERLVE YPEVAKNLDK INALDPLLKL LNNHILESVL QIFSLALSNN
PVLQDCVFKK NGLKILLLKL QESKQTTVDK KLITAISALI RHHDEGENKF IDYGGIAFLV
YGMQTNIYKY QEKSALLLKH LIHQNKITFE TFEKNKVMNG LIALANTGIQ YGETTAELFL
ALMQSHRHKL AKAGLKQLKE LIEGRLNYLF EELGIDDWLI KISKSVQIIK PTKIQKLCLP
LIIEGKNVIG SSETGTGKTI CYCWGMLQEL NKNFYGIFGL IILPTRELVF QIVEQFHLYG
NKIGIKILSC IGGFSLIEQP HIVVGTPGRF KRLRFLVLDE ADLLLQKSFE DKLKIILSNI
PRRTLFFSST ITDSINLLNK TFPNDKLILV DANKKQKPLK NLDQRYGIIF TANSYKCELI
YNVLNSLFSV ESIHSSKDQR KRMSSLLRFK NGFCKILIAT DIISRGIDIP KVAFVINFDF
PNEIIQYIHR IGRTARANRK GLSISFIDKK DLQSFNNVKI AMKNKLKPYI LNKNEVLADM
LKIGKVVKKV EMMLQEVVVF PVSVHYDLTR KLLNKKIGTG IQNVSKFGNG SYTGEVSAEI
AKNLNIEYVL IGHFERRKYF NETDEDVRQK LQQAIKNNLK AVVCFGESLE QRESNKTIDV
ITKQVKAFVD LIENFDNVIL AYEPIWAIGT GKTATPEQAQ EVHKEIRKIV KEMCGANKIR
ILYGGSVSVE NCTSLIKQED IDGFLVGTSS LKTSFTEIIK SAKDINKDVV HRYGPNTFKL
HRLPVPKLGQ ILGLVGTNGI GKSTALKILS SKLKPNLGKF DNPPEWRDIL SFFRGSELQI
FFTKLLEEQL SPIIKPQNVD LIPKQVKGNI LEIINKKDKL NQKDKYMKVL ELDHLLDRNV
EDLSGGELQR FALLISIIQT TNVYMFDEPS SYLDIKQRIS MAKIIHGLVR HDNYIIVVEH
DLSILDYLSD YVCCLWGKAG AYGVVTSPFS VREGINVFLD GFIPTDNLRI REESLNFKLE
DKKRLHFYTY PKIVKTLNSF TLTIDKGNFS ESEIFVLLGQ NGSGKSTFIR LFAGLIKPDN
VDFLESLSVS YKPQQIQAKF TGTVRQLLMS KLKGLYTDPY FNNEIIKPLK IDGILDNQVL
TLSGGELQKV AIIITLAKNT NIYLIMSILC TISGQTPEEP VVSKTGYIFE KRLIEKHIKN
YGICPISGEI LTLEDLYPIK NEKYVKPRPI TATSIPGLLS IFQTEWDSMI SEMFNLRTHV
NDVRNQLSHS LYQYDAATRV IAKLLKEKNN YQEEINNLRN QILQLKNGND INDLEIGINE
DLLNEMQNIA KELLMNRKKR KVENVNSPNE WKKITATNEF NIHSSIIPGV TCLTIDFSGG
KDGNIYYVSL NNNKIISKLQ GHLKKVNSII SHPSNSICIS GSNDKTIRIW KGDKHKDKIN
SLSLHPLENY FISSSNDSIW ILHDMETGKT IKTCKSSPSF KNLSIHPDGM MFGIGSEDSN
IYIYDIKSQE YKASLFSENG YYLASISKDN TLKLWDLRKA TTFQTIELEN TPKHITFYNG
NIKDGLFHGY GILIYSKNEK YEGDFAYGRR EGKGKFTYAD GATYEGEWVD DKIHGKGVAH

FVSGNVYEGE WENGKISGFG ILNYNNGDKY EGEWSEGKMH GRGTYIYADG DVYVGEWKND
KRHGKGCVKY KGNKDKIAET YEGDWYEGKM QGKGVYSFAD GGIYEGDWVD GKMEGKGIYK
YLNGNKYDGD WSNDMKNGYG ILTYANGEMY EGYWKDDKVH GKGTLTYSKG DKYIGDWEFA
KKSGEGELIY SSGDKFKGKW KNDKANGFGV LYSNGNKYKG DWVNDQRHGF GVFTCKEDGS
IYSGQFSYNR KEGQGTLTFS NGTIVEGIWN SGVLTKVTKF QLYPSSPWND PDLEMETLYD
LGNKMIEALQ KENITAGDVI CIDKGTGKIT KIGKSFARSK DYDAMDPNTH FVQCPEGELQ
KRKEVVHTVT LHDIDAINSR TQGFLALFSG DTGEIKNEIR EHIDMKINEW QEDEKAEIVP
GVLFIDEVHM LDIECFSYLN RALESEQSPI VIMATNRGIT HIRGTDYKAP HGIPLDLLDR
TLIIPTYPYK HEDIRKILEQ RAEEEDVEID EFAKELLCKI ASESSLRYSL HLITLANLVA
KKRKATEVTV QDVRRVYNLF IDVKRSTQYL IEYQNEFMFS ELYSNLSDFW TSDDEEDGEY
IRKKWVIEDD VSNFSKNDLL LSYNFELDDF QKRSVKHINN FKHVFVAAHT SAGKTLIAEH
AIALSIKLNK KAIYTSPIKA LSNQKYYEFK NIFKNVGIIT GDVKMNVNAN CLIMTTEILR
NLLYLNDNII NNIHCVIFDE VHYVNDEFRG VIWEESIIML PPHVQIVLLS ATVPNYLQFA
DWVGFTKQKE VIAISTKKRP VPLLHYIYAH DSLFLIMDEK NFYSSAFKEI YEANMKTEIQ
KLQALIKKLD EDNKLPVVLF CFSRIKCETY AKSMPHLNFL DNKKKSKVHL FIKESASKLC
DQDRELNQIK ILSKLLENGI GVHHSGLLPI LKEIVEILFS KGLIKVLFAT ETFAMGINMP
AKSVIFTSIY KHDHLKKRIL TSSEYTQMSG RAGRRSSDSY GYVYIYCSDN IPDQVQLTEM
MMQKAVSLKS KFKVTYNMIL KLLINKQINI EKMLFSSFLE SCRALQIPLF KKDLKRKKKM
LQNIKQVECV YIENYVYIDH KLKNIGLNLH KKLVGIENIS LITNELDRLI KKETFEPFTL
TKMLKSLKCE FYSVLHYELV CKKNDCINDI ENIERNINAK SLNLYEDLEG KLNVLKHFSF
IDDDNNLTIK GKIASYITLT DEITLTQIIF ENVLNNLNPP EIAAVLSCFV SPEKKVEESP
DLTLNLQDVK LALTNIHSKF EEFYKVIRLK ISTEEHWKLC NFKLMFIAYK WALGVSFSEL
LEQSEFEEGL IVRSIQRLDN LCRKVRIAFL YLGNVDLAEK TEKASLLLRR DIVFTTSLYL
DGLALLQFFH WCEEKRKTKE LFKETEISLR NKIDYFRSTK KNFLSFSTIS AIGPNSAIIH
YESTEDTNAK ITPSIYLLDS GGQYLHGTTD VTRTTHFGEP TADEKKLYTL VLKGHLSLRK
VIFASYTNSM ALDFLARQAL FNNFLDYNHG TGHGVGICLN VHEGGYSISP AAGTPLKENM
VLSNEPGYYW ADHFGIRIEN MQYVVTKKQT DNAKFLTFND LTLYPYEKKL LDYSLLTPEE
IADINEYHQT IRNTLLPRIK ENPKKNFVIQ EDPDFIQYRL NKYNELKEKI NIELLDGSIK
TGQKNITTPY QIASQISKKL SENSIVAEII YLDNVNLNLC DLWDMNVPLI GNCKIKFFWH
SSAHILGSSL EKLYGGYLTI GPALNEGFYY DIYLGNNSII SENYNKIENE YNKLVKENVE
FEKMICTKEE VLELFKYNPF KIELIKSKIN DNEKTSVYKC GNFIDLCLGP HIKNTGKVKA
FKVLKNSSAY WLGNKNNDSL QRVYGISFQK KTELTDYIKF IEEAKKRDHR NVGKNLNLFF
FEKETSPGSG FWFSHGAKIY NKLIEFMRKE YRIRKYDEVI TPNIFSCDLW KTSGHYQNYK
NCMFIFNIEN KEWGMKPMNC PGHCLIFKQL NASYKSLPIR LADFGVLHRN EITGSLSGLT
RVRRFQQDDA HIFCSLDHIK TEVVNVLQFI FFVYNLFGFG IEILQNRGYD SCGMSTILKT
TKYASSSTAN AIEKLRGNYL TSHKNDNIGI AHTRWATHGS KTDENAHPHV DYKERISLVH
NGMIENYREL KKFLVQKNIP FKSNTDTEVV ANLIGYFLDQ KQSFQDAVVS SIKQLEGTWS
FCIIHKDFPD EMVLAANGSP LHIGIKDNEM FVASEHSALF AFTNEYISLK NGEIMLINKN
NINNLKMIKK FDNIPEIVIQ KTPDPYPHWT IKEIHEQSIS LSKSLNNFNL KNNTVKLGGL
DPYVEELKNI ENIILIGCGT SYYAALFCKY IMNYLHCFNT VQVMDPIEFN ISAIPKEKEG
IIFISQSGET RDIIKACKLA EYFNLKKLSV INSVGSTIAN MTGRGVYLNA GREVGVASTK
CFTSEVSVLT LIALWFFQNK SNNKVSSLIN SLYRLPLYAD TTIKCEDTCK ALSHKLSKKS
MFIIGNGLSY PIALEGALKI KEIAYIHCEG STGNALKHGP YALLGGDDNI PVIMLIFNDK
NSMISIGEQI KSRGAHIICL TDDVNLCNDD IILIPNNGLL TSLLAVIPLQ MLAYYMSVNL
GHDPDKPRSL AKTVTVWIIE IEKYASEDVQ KILIGNKIDL KNDRSVSYEE GKELAESCNI
QFLETSAKIS HNVEQAFKTM AYEIKNKSQL ENQQKGRANI NLNAKPIKIR TMNSKKPPEG
WNKVETFLNE MNQKMRSLEN EDTSKKRKNE ILWPIFQINH QTARYIYELY YKRKEISYDY
IVIGGGPGGM ASAKEAASHG AKVLLFDFVK PSSQGTKWGI GGTCVNVGCV PKKLMHYAGN
MGKSDSDKYG WECDNKHDWN KLVSTVQSHI RSLNFSYMVG LKSKVKYING LAKLKDKNTV
SYYLKGKEDC VTGKYILIAT GCRPNIPDDV IGAKELSITS DDIFSLKKNP GKTLVVGASY
VALECAGFLN SLGCDTTISV RSIILRGFDQ QCANKIKLYM EEQGVTFLPK KLTKENDKIL
VHFNNNTTEL FDTVLYAIGR KGDIDGLNLS CTNIPNIFAV GDIAENVPEL APVAIKAGEI
LARRLFKNSN EIMKYNLIPT SIYTPIEYGS CGYSEEQAYE QFGNIEIFLQ EFNNLEISAV
HRTKQKDEYD VDISSTCLSK LVCLKDNRVI GFHYVGPNAG EVTQGMALAL KLNAKKSDFD
NCIGIHPTDA ESFMNLSITL SSGLSYAAKG GCGGGKCGEH LYTGPLKIEQ LLAKGFVKRD
LELLKEGGLQ TVECVAYAPM RTLCSIKGIS EQKAEKLKKA CKELCNSGFC NAIDYHDARQ
NLIKFTTGSK QLDALLKGGI ETGGITELFG EFRTGKSQLC HTLAITCQLP IEQSGGEGKC
LWIDTEGTFR PERIVAIAKR YGLHPTDCLN NIAYAKAYNC DHQTELLIDA SAMMADTRFA
LLIVDSATAL YRSEYTGRGE LANRQSHLCR FLRGLQRIAD IYGVAVIITN QVVAKVDAMS
MFGHEKIPIG GNIIAHASQT RLYLRKGRGE SRICKIYDSP VLPEGEAVFA ITEGGIADYM
AIRVQFENSN EVGVFSRLTN SYGLIALGGS ENFSSVFEAE LSQHIPIIYA TIGGTRVIGR
VCVGNRKGLL VSSICTDQEL LHLRNSLPDN VKIKRVEERL SALGNCITCN DYVGLIHTDI
DRETEEIVQD VLDIEVFRTS IAGNLLVGTY SYFTNNGGLL HAMTTSQEIE ELSELLQIPL
VTGTINRGSD LIGSGLVAND WSAFCGMDTT AIELNIIEKI FKLNNIEDTN IEDTFKYKSS
IVQTMIIVDT YWQTETGGIV IAPIPHLFKM KPGSASLPFF GIQLEILNSK TLEPLKGPNC
GILCIKSSWP GMLRTVYGNH NRLIKTYFEA CPSYYFTGDG AYRDEDGYYW ISGRIDDTLN
VSGHRLGAAE IEHALVQHSC ISESAVVSFS HKVKGEGILC FVVKKLKLYV RKVIGPIATP
DIICIVPDLP KTRSGKIIRR ILRAIAIGLN DYGDISTVSN YDVIEIIPFP TPCTIEEALQ
CYCDLSTIPR VNVLKNFKCF IKDIEEKEFE MTFIEFVDIF MQSAIFELTP FLQLIPKIAP
KSYTISSSPK RWYKGSSSYY LTELYPNDII KFNVKTSIFG IDFLYEKEID SLENKYIDEV
YLAFSRDQPW VEKYRPKKLD DIVHQTNAIS MLKEVIKTKN MPHLIFHGPP GTGKTSAINA
LAHELFGKEN ISERVLELNA SDDRGINVVR EKIKAYTRIS ISKNKINSEN NEPLPPWKLV
VLDEADMMTE DAQSALRRII EIYSNVTRFI LICNYIHKIS DPIYSRCSCY RFQGIPINIK
KEKLLYICKN ENIDISDKII ETTQGDLRRA VSVLQLCSCI DSKITVDSVL DVSGLPDNDV
ILKIVDSCKM KDKILEKTVQ DIIEDGFDVS YIFKSLNEYF VDSIKYQILM ELSRHDFRLH
NGATKYIQLM SFASSVHSLL IKELIFKSND EKHLQIVVKQ VKELIKQVKQ KEVEDVNDSK
TSNEKLALNK TGRRIVLRDL MTRPNIFTGR KILGTLELHT NGLRYSAIDI LFDDIKHAFY
QPCDGQLIIL IHFHLKRYIM VGKKKTLDVQ FYCEVGTQID DLDRAKARNV YDPDEMHDEM

KEREQKNKLN LIFKNFVQQM QDISKIEFEI PYPELTFSGV PNKSNVEIFV TANTINHLIE
WPPFILSVED IEIASLERVH HGLRNFDMIF VFKDYTKPVK RIDVIPIEYI DTIKKWLTTI
DIVLKTILAD IESFVNSKGF DGFLGEDDDE EEDEDEDDEY EVDESEISAE IDILATVEFT
SKRKMSTIVC RIPKIVVFCK GAGCVIIKKL AKKTDVDDLT IEHMETYADE GLRTLCIAYK
ELSQEEFAVW YNSYKEASLS LNGREENIEK IAEDIEKDLI LQGVTGIEDK LQEGVGATIE
DLRLSGIHVW MLTGDKIETA INIGIATNLI DNGSVDGNVL DILLSKPFER KFFYLADKCS
SVICGRVSPY QKGSIVSSAN RLLKKNTLAI GDGANDCNMI KMANIGVGIR GQEGVQAFNS
SDYGISQFRF LRNLILIHGR LSYRRISKLV VYMFYKNIVF IFPLFIYGSI SLYSGQKIYY
EFLLHLYNVM FTSLPIVILA ILDKDVSLNT ALNNPCLYKL GIHNFYFNIN KFISWVLNSL
FQGLLVFIIP LYFLIPSSTG EPFDIWSIGC VTYLAVLIVN IKILLETYYL NTSPIVAVSM
SIISFIIMSI AFSFIGIGNK SFLGVAILLV KSLRFWLVLL LVLFALTRDY VYKVYKKNFY
PRNYSDNVKD HFNKPRNVGS FDKNEKNVGT SIVGKASCGD VIKLQLKIED NVIKDARFMA
FGCGSAIASS SYATELIKGK TIDEALKIKN NDIASHLNLP PVKVVILGSG WGGIHFLLNI
DFQKYDVTLI SPRNYFTFTP LLPCLCSGTL NVDACSENIE TLLKKNKISG KYLKLECTDI
VYKDKYIKCK DNNEIKINYD YLVISVGAKT NSFIKGVDKY AFYIKDIIDA LKIRTKFISN
LEACSSSSIS DDLAKKMLHI VVVGGGPTGV EVAAELADFV NKKNKYKEIY KYISISIIEG
GNNLLPTFTQ NISKFTEKIF KKLNINVYTN YHVIEIDENN FYIKSKKIPY GIIIWASGLA
QTPLINNFIK KIPEQENNKN LKVNQYLQII GINIYAIGDC KQISPVQITA EQLIKEALDF
EEVEKKVNYN LIDEDELNEY KISKRKEFED SIRKRRYLIN TYIKYALWEI KQKDIKRCRS
IFERALNIDY TNKNLWLKYI EVELTNKNIN SARNLLERVV LLLPLENIFW KKYAHLEEIL
NNFVNARNIY ERWVKWKIDE TAFLCYINFE ERCKEINKCR EIFEKLIVNI PKLECFYRFI
KFEKKYKNIS RAEKCIELLP SQFLDQHFYI HFSKFEEENN EYERCRKIYI EALKRLPREN
SDILYKNFLQ FQKKYEELDQ TLLYNERIHF EEALKKTPND YDIWFNYIKL EERIRELYER
AISVIKNALV HDGLKIGIRE VIKSIESKEA KVCFLSNVCS EPAYKKLVTA LCAEKQIPLF
MIDSKDLGQW SGLFKVDKEG NARKIIGASS VAVIDFGEES AERDFLGYYK AIVGEVIDNR
YSVVCELVGK GVFSNVLKCY DMTNKMHVAI KVIRDNHMMH KAAEKEIFIL KKLNKKHIIR
LLRSVKYKNH LCLIFEWMWG NLRIALKIQD SFNTLILKAK ELEDVYISRR KLQSKYLTQI
KNLYINSNCE CIIHKISFKY ASKSSFPNLL NGTLLYMIIE KINLDNNVVA SCINSADVKS
WINYENYLGE LVDGFLFAVN ISYAKSLIGD KCYILDLIDV PYEIAIGHNG GALGYSQHLS
EEIMLFSKEA IHDKIAVILG GRAAEELFIG KITTGAIDDL NKVTQLAYSY VSQYGMNKEI
GLVSFQQNGS GEYAFYRPHS ECLAHLIDNE ARSLIESQYN RVKAILKKNE KHVHNLANLL
YEKETISYHD IVKCVGLKHQ RYREADKVKI EEERNRKIYV KNIKFCKNCG SAAHTEKYCL
ERTRKKGYDG NRDRWVGYDP NNFDHIYREY EKIVDEQKKR KAEKLIDKKN NKIKILSKYE
EDIYISDHTS IFGSYYDRET KKWGYKCCKC TDKFQHCIIP LMDSYHANYY TKEVMIILIN
EFNSPDEEMK KIVLKCVKQC IQTEGIEKDY INKEILNPFF EQFWIIRNSN DKRNFNLIVD
TTVEIANKIG VISKIVDDLK DPSESYRKMV MQTIQNIINN LGVDYIDQKL EERLIDGILY
SFQEQTSDDY YVLLNSFDII VNKLKLRMKP YLPQIAGIIR WRLNTPLPKV RQQSAELIAR
ISKLIKTCDE QQMLGHLALY LYEYLGEEYP EVLANILKAL RSIVIVLGVN NMTPPIKDLL
PRITPILKNR HEKVQENVIN LIGIIADKGG DMVSPKEWDR ICFDLIELLK SNKKLIRRAT
IQTFGYIART IGPFEVLTVL LNNLRVQERQ LRVCTTVAIA IVADTCLPYS VLAALMNEYK
TQDLNVQNGV LKALSFMFEY IGEIAKDYIY SVVSLLEHAL TDRDLVHRIA TWACKHLALG
CFGLNREDAL IHLLNHVWPN IFETSPHLIQ AVIDSIDGFR VALGPAIIFQ YLVQGIFHPS
KKVREIYWKI YNNVYIGHQD SLVPIYPPFE MIGDSNFSRD ELRYMGRMYG KGKGISSSTI
PYKRKQPSWL KQKPSEIEDA IIKLAKKGQT PSQIGATLRD NYGIPQVKAV TGNKILRILR
AHGVATTIPE DLYFLIKKAV SMRKHLEKNK KDKDCKFRLI LTESKIHRIS RYYKRKRLLP
SNWKYQSSTA SALRNIEHAP GVQFSYVPPD FFDSDDDKSD KNQYELKDDG GGRAAGTRGK
EHSSTHHLRR KNYEDDFEFN EDKILEALHI LEFLYLNGIS LEEQNEYGQT ALFLGVKKNN
ISILQWLLSK NVNINHVDFY GNTILHIAVR YTDIDILRLL CDYGCLNLVY YSTFENNNTN
VFQLCINNRY FLVYILLKKW LIQNKICKGL KICKTIYAFY FWFFALLNLF VYINITQSFL
EIKKHHNKSV IWISLWLFQQ LLWCILYFKN PGEYQLNNIE KEIFQINLKY SKLSLYSQVS
QERINSLDVN YRNAILEIIL LQLIIEPYIL RRSKKHVFID MPKKHSIIIK LPLNNTQLNL
YKDEILSKLQ HTHKHLINAS IFILRRICNH PLLHKYYYSI DDIKKISKYF YNNTDQYLDL
DLKTVENEFM KISDFDIHLS IKHLISQDNN LNKYLIDKKH ILNSTKIHHM LTLIKNIKQK
KEKVLIFSQF TTFLDIIEES LYVRLDGSTN TIERQQIIKE FSNVFIFLLS TKAGGVGLNL
IAANHVILMD QWNQLLLQEE IVKKMCEYII DSKCDIVEKG VSDLAQHFLV KKNISVIRRV
RKTDLNRLER ITGATIVNRC DEIVEKDIGT KCGLFEVKKI GDDYYSFFVE CENPRACTIL
LRGATKDVLN EIERNLHDGM NVAKNIMLEG KLLYGGGCTE MRVSQYLIKE AANFDDSRKS
VIESVASAFE IIPKILAQNS GVNVVKCINE LRTKHGSEKL GIDGVTGEII DVSSKNIWDL
LSVKKQIYKS AIEAASMILR IDDLGVVLIY EDLLKNPLCR ISNIFEKEVK KYRPFPLNTL
QMTKLVSKYF HISSKECMNI AEKLYNKGYI SYPRTETNYF VDSMNLRKII HELKKNNIFG
SYATKLAEKN PRKGKLNDKA HPPIHPVKNM EWKIYEFICR HFLAVCSDDA IGFDTKVIAN
IGEEQFYCKG LKIKNKNYLE IYIYEKWNDK ILPPFQINDE FYPYSLVVEE XITQPPKYLS
ESDLLSLMDK YGIGTDATMH EHIENIQKRN YVYKNSKNLF IPTKLGIALI LSYKKFKDIG
VDLTEPSLRA KMERDMFLVA SGICVIKSGK MDIPRNTKMI IISYELITKN DKYQKYKCIV
CDESHYLKNS FSKRTKAIVP IIKSAKRCVL LSGTPALNKP SELYEQVSSI IPNLFNYNEF
CDRYCYKDKN IYTRKIEYVG CKHTEELHLF LTNTIMIRRL KKDVLKELPD KLRSKIPIEI
PPNELSEILL FKMTGYAKVK AIKEYITYLI DADIKFLLFC HHKLVMDEID EFLKEKKLGF
IRVDGLTPID KREIYIKNFQ SDEKIRIALL SITACGVGLN LTAANTVVFG ELYWVPGQMI
QAEDRAHRIG TTHDTVNIHY LVAQNTIDEV VWKIINRKWN TLTTALNGTE DSLMFEDNDK
ICIAVSGGKD SSVLTHVLVN IKKKYNYNWN LFLLAIDEGI KGYRDDSLKV VFQDIFSYTM
DDVVSYIGKK NNCTVCGVFR RQAMEKGALL FNATKLVTGH NADDLAETIL MNMCRGDIDK
LFSTECTYSP NSFRGNLRSF IKDLECIKCG AYTSNVDGLN NYTDNKQSSN CTFEIDIHYE
NIEPMALENE YQKIPKLRIL SFDIECIKLD GKGFPEAKTD PIIQISSILY LQGDPCAKFI
FTLLECASIP GSNVIWFNDE KTMLEAWNEF VIRIDPDFLT GYNIINFDIP YILNRGTALN
LKKLKYIGRI KNIPSLVKDA NFSSKQFGTH ETKEININGR IQFDVYDLIK RDYRLKSYTL
NYVSFEFLKE QKEDVHYSIM NDLQNENSES RKRIATYCIK DGLLPLRLID KLLFIYNYVE
MARVTGTPFV YLLTRGQQIK VTSQLYRKCK ELNYIIPSTY IKVNNNDKFE GATVLEPIKG
YYIEPISTLD FASLYPSIMI AHNLCYSTLI KNNGKNNFKF VKGNVKRGVL PLIVEELIRA

RKNVKAMMKN EQNPITKMVL NGRQLALKIS ANSVYGYTGA AAGGQLPCLE IATSITTFGR
SMIEKTKETV EAYYCKNNGF EHNATVVYGD TDSVMVKFGT NDVGEAMRLG KDAAERISKE
FLHPIKLEFE KVYCPYLLLN KKRYAGLLYT NPNKHDKMDC KGIETVRRDF CILIQQMMET
VLNKLLIEKP LIQVAPMINV TNRHFRALVR IISKKVQLWT EMIVDNTLLY NINNLEEHLG
FNKNEHPIVC QLGGSDPTSL SEAAILVEQA GYDEININVG CPSTKVANKG AFGAYLMKNP
QLVKNIVYEI KKKVQIPVTV KIRTGVDNLD SFSFLRSFIE TVSSAGCDHF IIHSRKAWLK
GLDPKQNRSI PPLEYNKVFD LCKLYPHIKF TLNGGIKSIE QGVALLLYGV MIGRACMENI
TVLAKTDKLV YNYDSPSTAH SRRTVLDAYK FYLEQNSSFY TLFELLKPIL GILKGMPGHR
LFRFLKNFFF KNPVGHVGVV ALKNSSAKLI QQLTSNIDDV LNSLVKEQKE GLNGSPSLQE
GLEIAHNLLM DMPLYGTKEI LIMYGSIRTC DKKNILKYLD LLIKNNMYVN CISIAPEMHI
LKCGIHLISM HDLSHITNNL QASPLFVEIM GSNSIAISQQ MYFSTHNALR INENDVISTL
FYEINGHRHI SLLIFPFYDV QMLKRLLIKK LDLPDIKVND ILIFYKGIKL PNYRIISTYK
KVNKLYWAIK DPNPNASIRV IDNKYPPFFE NILNDIKLAF KKNIAPKLTM DGTGGTYLLF
NSKKKVCSVF KPADEEAFSP FNPRGYEGKI YQEGFRAGVL SGEGASREIA AYILDNTYNN
FSNVPCTIMV EACNPHFNNK SNLKYIYNEN TLKWKCGSLQ EFIDSRESVG NYDHKQFSIR
DVHKIGILDI RVMNLDRNDG NILVSPTHLG IEQSRRDDIE ALGYVLMYFL RGSLPWQGLK
AISKKDKYDK IMEKKISTSV EVLCLLNTCT KIDSCIIIDR RIDMVTPFCT PFTYEGLIDH
IFCIENLQIE IPLYNDIKNL NQNEVGIFLH KKASDIQQTY EKDSLKDISQ INKFMIKFKE
KHYEHNSLSR HVNIASYILN EIKTEHTFNK LKLEDEIIQL NTNTNKNILS SIIKKIQTLI
YTGENIYEIY RLASLFSSIT NGKKDIIEQY GINELTRLNK LHISNILKYQ PKQKFIWNNL
KNHFNLLSND ENDISYVCNG YAPLSTRLIE YKNNMQVFPE VFSLINGPTF DIIQDTIEVK
SMCINCEQEG INKILKFEIP YFKNILIHSF ECVLCNYRNN TIQDLNPIKE KGVKILFSVT
KIEHLDRQLI XSEYGVLKXP EINFEIPKET QKGSINTIEG FIQTALSNLY MSMIEKTIHK
LFTVEIIDPS GLSSLEYQRS KQELNELGFY SFTSNCPCCN YLGDNNFCEI NIPGFKKCLI
LSYVCPNCNY KTSEIKSSGE INPKGKKITL TVKNKSDLNR FVIKSETASI QIPIIDLTSD
YGTLGGSLTT VEGIIIQIIE SLEDKFKFLM YVLNRKGEEE DISFDQILKR IQRLSYGLHE
LVDPARVTQG VINGMYSGIK TCELDELAAQ TCAYMATTHP DFSILAARIT TDNLHKNTSD
DIAKVAEALY SYKDIRGRSA SLISKEVYDF IMEHKDRLNK EIDYTRDFNY DYFGFKTLER
SYLLRINNKI IERPQHLLMR VSIGIHIDDL EKALETYHLM SQKYFTHATP TLFNSGTPRP
QMSSCFLLSM KSDSIEGIFE TLKQCALISK TAGGIGVAVQ DIRGQNSYIR GTNGISNGLV
PMLRVFNDTA RYVDQGGGKR KGSFAVYIEP WHSDIFEFLD LRKNHGKEEL RARDLFYAIW
VPDLFMKRVK ENRNWTLMCP NECPGLSESW GEEFEKLYTK YEEENLGKKT VLAQDLWFAI
LQSQIETGVP YMLYKDACNS KSNQKNLGTI KCSNLCCEII EYTSPDEVAV CNLASIALCK
FVDREKKEFD FKKLYDITKI ITRNLDKIIE RNYYPIKEAE KSNKRHRPIG IGVQGLADTF
MLLRYPYESD EAKELNKRIF ETMYYAALEM SVELAQIHGP YESYKGSPAS QGILQFDMWN
VKVDNKYWDW DLLKKKISMH GLRNSLLLAP MPTASTSQIL GNNESFEPYT SNIYYRRVLS
GEFFVVNPHL LKDLFDRGLW DEDMKQQLIA HNGSVQYISE IPADLKELYK TVWEIKQKNI
IDMAADRGVF IDQSPKLPFD EIRNEMNKYG VAITPSTLKH PTTEDVQGVY SICIKYILNK
DINNIRIEEF TGDLKSILPN EGKNHLQAIG NLRFFRHCEK INKILNMDNT LSYIFKPTSG
HITKLINAFI VNETNELIFQ FSRYRQKKED LEDQIVPSPE KLQQYNDELK DLLYEHMSHC
ETSKKKNEDI KNKINIADLC IKKLVNLLTI LTSHLKVHID KKNKLKDLGT NLKSLDWWTL
GIFIYEILVG YPPFYANEPL LIYQKILEGI IYFPKFLDNN CKHLMKKLLS HDLTKRYGNL
KKGAQSVKEH PWFANIEWNN LLNKKVDVPY KPKYKNIFDA SNFQEDLSIA DKVINENDPF
FDWVISQFYI LSPRGDTIIN RDFRGDVLKG SGDPPPLFYL NGINFCFLKN NNLYFVLTSL
FNISPSYLVE LLYRLLKIFK DFCGQLTEEI IRTNFILIYE IIDEVIDYGY LQNSNTEYIR
YLIHNETLPS NASQKPIQAD NKKNEIFIDI IEKIDGVIQI KSYLLGNPYI KIALNDDLYI
KNIHKDNTNN IIIDDCNFNH LVLSLYQPDG ECVLMNYRIN MINIYKPNDV IACEVQRILT
DGCIILHTRS SIYGKLSNGI LITVPQTLVQ NQKKHIFVFP CVDDTTRKNI SIISNIIKLL
AKYHININYD IITKIYVQEW IPPKNKQINA ATSNGSQIVV SLSGGELIYF EIDESHTLTE
IFRKNINVEI LCLSIQQNKL RASFLAVGCL DNVVRLLSID QYFKQLSTYI LPNNSSPQDI
CILYLNIGLN TGVLLRSVID PIGTLSNHYS KYLGAKSVKI CHVNPALLVL SEKTYLCYVY
QGKYIYSPLN YDVLEYASSF YSEQCSDGYV AISGNSLRIF RFYRLGEVFS QNILHLTFTP
RKIVPLPFPS MLAIIEADHN AYDENTQQEI QKALRDIKLG TPKAGLGKWG SCIKIINPIN
LQIIDKISLE LEEAALSVCA CELEALHCLI VGTTTNMTLK ASLRVYTYDI NYKLNLLHIT
PIEDQPYCFC PFNGRVIVSV GNKLRIYALG KKKLLKKCEY KDIPEAIVSI KVSDRIFASD
IRESVLIFFY DSNQNLIRLI SDDIIPRWIT CSEILDHHTI IAADKFDSVF ILRVVEEKPD
ITYNDIGGCK EQLEKLREVV EMPLLQPERF VTLGIDPPKG VLLYGPPGTG KTLTARAIAN
RTDACFICVI GSELVQKYVG EGARLVRELF QMAKSKKACI LFIDEVDAIG GSRGDESAHG
DHEVQRTMLE IVNQLDGFDN RGNIKVIMAT NRPDTLDSAL VRPGRIDRKI EFSLPDLEGR
THIFKIHANT MNMSRDVRFE LLARLCPNST GSDIRSVCTE AGMFAIRARR KTITEKDLLL
AINKVIHGCK QFSATGKYMV YHIKTKTEEN FALVLKKINE LYTLTQDNVF NLPRYSKFPE
AKKPTRWELF SQTKLKKKNK HGLIYDENSK GWVRRFQKKQ IKINKEKSDF VHEYKPSDNI
DPFEKMEEEK DIKKMKQKMR EMKNKVFERL TDQTFYTGTH KKKFKKEKKL VVTPPKSIWL
YRNGDKHHNG LLFFIKSHIN NLKLLLFEIT KVLNPIIGPI RKIYDQNDGS KYLCTSGDPP
APIDHLGKFD ITKYFKEGQK VITPPNGDGT RAFYESLLDE NPNSIIAIKY CIEHGVLSGT
KHHETLNKYY MLKKNNAFRN NFGGIKCEFV EMLNVKFIQE KKLIGKFFEE IAQDTGKVVY
GIEDTLKALE IGAVELLIVY EGLDIIRLTT KNNVTNQIKT MHIFPHDEKQ ESLYKENNVE
LEVVEKILLT DWIINNYKKY GASLDFVTNK SQEGAQFLQV LYEFIFLCIC MYDSINGLFE
LAYKIVSDEI SEQVINIIVL PFNHLGINAL KAKNIKNLLN SINDKYKKKL SLNIIDAIIE
CKNKEIIYQN VEEMLKFISC IFDIDQKYNT SMLFYNYIYD SIYFSQLLPS IIFTFLNLVM
LEAKLNNASI LKKLFECIKD LVNDANIDAD ENGLKLQALD GNHVSLVSLH LVDSGFSHYR
CDRERVLGVN IASLNKVFKL CGINESVVIS SKDDEDNLNF VFENNKEDKV TNFSLKLMSI
ELDSLNIPDE GFDAEVELSS KELTNIFRNL SEFSDTVFIE IDSNSIKFTT KGLVGDAEVA
LKPRESTDDV GVTIKSKKKI KQSFAIKYLN LFSKSSILSD VVILGLSDSR PIEFKYEIKD
GFIKFFLAPK MDDDCPEKCD EDIGAIKGTF VYTFDSYICK AAIHAGVLNV TDDVVLIITH
SRNKFIGTKR NNIESKEFIG ESKSFSLSIP TGFNGDENQY INANNLPNEK YIRTLSNFTF
IIHFGKNKWR TILSHSLCEG ISISIDEENE LIIEQNCNPH LVKTKFIPKF EHPYHLVLIY
NKSNKSISLY MNQKKINLEN TKFDFTLNGD LTIGRSNKQA TDYFIGDINF VKIYKYILTE

HEIKESYNSV ISRKTIDGRD CITPCKSKTN VNKNIQINTE EFYLNCSDNL LSERFNGKGA
QFLASCLEDC TNSKYIVKGS NYYTPDTSIC KAVMHSGIIK IVEGLTEYKS SRGHFGIVSK
SEKQSCFTDA AFLLELPIGT TKNVICPENC GTNTYSPLSS VCKAAIHAGV ISIKGGHIQI
VVGKGQQEFK SSTQNNIQSY IAEKQNRSQK YIYEHADVGT QKKVFDLHLN LGPYKCNYSR
NGKYLLVTGE KGHISLLDTH NMESLCELNV NETVRCNTTF HNHKLFAIGQ KKYIYIYDNT
GIEVNCIKDI LYPCQLEFLP YHFLLASIGD LGELVYQDIS VGNIITRKKT KRGPCSIMKQ
NKQNAIIYLG HRNGHVTLWS PNMDKNYLIT ASIDCTYKLW DIRKLEYIKS FKSNIINNID
ISDTSMVAFS MNSHFRTYKN FFTKPYLTHN TGGDKINSIA FQPFEDICCA GLKYSIKSFI
VPGSGLANID TFVNNPYETK KQNEIRQLLD KLPPETITRD YKKIILVRKI KTKTQIKRII
TSPRDVYEMG LNSYSNSNWP KWTESESNCL CLINNQIYIY KDNERGTKGN SSVFKIFNLD
NLNKHIYSKN FFNSDEIKLK WNKNGTSLLL QIHTDKEKQS YYGSSNLYFI DTIKIKDVNI
MTNKGLIYDT IWSYNQNKFY VCKGEIPADI VLHDKNGNII HSYGKHKFNT LKLNNNEKLL
LTGGFGNLSG DISIWNTINK KEITKTKSSC AVICEFFNDD NHFLTATTHP RLRVDNNIKI
FKYNGLIVSK LDFDELYNVI ILPPGCNFVV EEKTNXKKKK KKEEDTIPLP NIKTQILKKI
IEYMEYHIHN PPDEIPKPLI TSNLQDVVVE DNIPKYKEIS QKMIQEIEMA VVLFKRKFII
KKIPQLPSCY IINSGALSVA SAKIKLPSTY AKLGDPLSFS KLPDCNYSFD MIEELQQFFM
KQRRCDYFSL LNNFINILIT TSNLLANEPD IDIRNTLLNK FIYSLNTWMV MRRCIVASCE
NIFSMTGLCI PLQILHFNND ECKIFFSKKR APYLLVFEVA DLDEDISHII PVESQRIIFG
EFNRESISSL LNNPLARSLM NELSNNPEML TNLISNNPLL RNTFPLMQPM LDNPNLLREF
MRPEVLQAGL PPEERYASQL VSLQEMGFID NDANIQALQE TGGDVNSAVT RLLERGSGDR
LTRAARVLEQ LTEQKPIFGK CRFTIRSFGV RRNEKISCFV TVRGKKALEI LEKGLKVKEY
ELRRKNFSDT GNFGFGIQEH IDLGIKYDPS TGIYGMDFYV HLSRPGYRVT RRRRERLGFF
ISLKLMNEIK GLSSIDSSYQ WLPLLYMALA NDTAVSKISL SALKPYSIVL IRLLRDFFSV
VFLIKCVGIG YRNMSGSQCV AIACDLRLGS NSFTTVSTNF TKIFKINDHI YVGLSGLATD
IQSLYELLRY RVNLYQIRQE TDMNIDCFSN MLSNILYSNR FSPYFVNPIV VGPYLNAYDL
IGAKCETNDF VVNGVSNEQL YGMCESMYIK DILLVGAGGI GSEFLKSIIT IGCKNIDIID
IDTIDITNLN RQFLFKKKDV KKHKSIVARE RALKHRKDLN INAYTFDVCT MKGSDISKYD
YVINALDNIK ARKYVNKLCI TEKKVLIEAG STGYNGQVYP IFSNETKCYN CEEKPKNKTY
AICTIRQTPS LPEHCVAWGK LIFETFFCKN DNETLIDIKK HIEEESKKRN MDKEEIIRFI
FNYLFHDTIN ELISLKKDYT IMPKPNIKKT TEEYLIFDKD DDDCINFITC LSNLRMINFS
IKQKSKFDIQ SIAGNIIPAI SSTNAIVAAF QFEKIYVCKP QSSRNKSDMI LVLNFGSQYF
HLIVKRLNNI KIYSETKDYN VDLKDIKNIK GVILSGSPHS VPHIKKEVLN YKIPIFGICY
GMQEIAFHMN GKVGKSKNSE HGSTVWMNHT EEVIEIPENY YLVNSSENCF IYNKENNIYG
VQYHPEVYET VDGDQMFYNF AICKCTKTFD PIKYHEVEFN NIKKHAHDHY VIAAMSGGID
STVAAAMTHK IFKDRFYGIF IDNGLLRKNE GEKVFIKSTF PDMNITKIDA SENFLNQLKG
VTDPEQKRKI IGKLFIEEFE KAVYSMDIDI EKTYLLQGTL YPDIIESKCS KNSDTIKTHH
NVGGLPKNLK FKLFEPFKFL FKDDVKKLSQ ELNLPKELTN RHPFPGPGLA IRVIGEIDKH
KLDILREVDD IFINSLKEYN LIGQAFAVIF SSKSYDHICA LRAVKTTSFM TASYYKIPHD
ILEKITTRIS NVKGVNRILY DISSKPPSTI EFEMLYKGDI NNNHRRNIKP LVLYEDENSK
IEVDPILAQY LREHQREGVQ FVFECLMNLK DEKISGCILA DDMGLGKTLQ SISVLYTLLK
QGYNKKAAVR RCLILCPASL INNWNDEINK WLPNRCTVTC VNDSAKEKIV SKLEGFKYDL
KSTILICSYE CFRINNDSID KSAIDMIICD EAHRLKNDKT KTYTSIYKLS AKKRLLLSGT
PIQNDLGEFF ALISLCNPDL FDDTNSFRKK FANPILIGRD KDATEKEQQI ASERLAELST
INKFILRRTN NLLSKVLPVK YLINIFIKLN PIQEALYVLF LKDKKLLKPD NSNNKVNVLI
NIKKLEKICN HPLLLNANDI KSSKFQLLHF LLKTIKQETN DKVVIVSNYT QTLDYMEILC
RENYYKFVRL DGGISIKKRH KVISDFTNTD DIFIFLLSSK SGGCGINLIS SNRLILLDPD
WNPANDKQAL ARVWREGQKK ICYIYRLFCT GTIDEKVYQR QISKDGLSSM IVTNTNLSKD
QLSDENVKKL FNYKNNTICE THDNIECNRC KKIEPAGFAF YTKYEKPSLK KNSNIVKKCI
NNNINVYEIL GVEESDDFET IKLAYKKLIL IFHPDKFLKI QDSYTVLSDK VLRKQYDSSI
PWSSKKPVPN IGDENTSIDK VKYFYDFWYE FTSWRDFSYQ NEYDYEDAEC REERRWMERE
NKKIQKKASK AEKLRINKLV DLAYNNDPRI IAENKRIELE KQRKKAAYKI WKHHIKSFDT
MCQFIYEIYL FLWTPQEISL LSKALKLYPG GTKDRWTVIA NSIKTKNVKE VIKKAKEMFE
NETLWTHEEQ MLLEKALMKH PATIPMPKKR RNGGRSKHNR GHVNPLRCSN CGRCVPKDKA
IKRFNIRNIV DTSAQRDIKE ASVYSTFQLP KLYIKQCYCV SCAIHSRFVR VRSRQQRRVR
KETTKHAHAS QKKVSVSEIN FDSSYTILDT SEGAIMLHVN HVLYHLDFNA LAVVKNVDMT
EEMQIDAIDC ANQALQKYNV EKDIAAHIKK EFDRKYDPTW HCVVGRNFGS YVTHETKNFI
YFYIGQVAIL LFKSGVCGWS KAVRKQGGRF CFVNLNDGSC HLNLQIVVNQ NIDNYDKLLK
CGIGCCFRFT GTLILSPVQN SIHSFEIYGE DPQKYPLSKK NHGKEFLREV AHLRPRSYFI
SSVMRIRNAL MLSTHLFFQS RGFICIQTPL ITTSDCEGGG EMFTVTTLDF KKDFFSKQAF
LTVSGQLSLE NLCSSMGDVY TFGPTFRAEN SHTSRHLAEF WMIEPEMAFA DIYDNMEVAE
AYIKYCIRYV LNNNFHDIYY FEENVEKGLI DRLKNILNDN FAKITYTNAI DLLTKYSNNF
EVPIKWGMDL QSEHERFISE QIFKKPVIVY NYPKDLKAFY MKLNDDNKTV AAMDVLVPKI
GEVIGGSQRE DNLELLDKMI NEKKLNIESY WWYRQLRKYG THPHSGFGLG FERLIMLVTG
VDNIKDTIPF PRYHGHAEFI KEILDLNLEK SENVIKNLFL KDKKNYFYLC VANWKKLDLK
NVSTQLKTSN LRFVDDENLK NILNVNPGSL TPFSIKSDKD NIVKLYFDED IKNMVIIHPM
HNYSCIYVKT TDVIKYCDLH NHTPILGITS KKEQNFSDWY TQVIVKSELI EYYDISGCYI
LRPASYYIWE CIQTFFNNEI KKLDVENSYF PLFVTKNKLE KEKNHIEGFS PEVAWVTKYG
DTNLPEEIAI RPTSETIMYS VFSKWIRSHR DLPLKLNQWN TVVRWEFKQP TPFIRTMAKL
SKAQKKQIYM DKLSSLIQQY NKILIVHVDN VGSDQMASVR QSLRGKATIL MGKNTRIRTA
LKKNLQAVPQ IEKLLPLVKL NMGFVFCKDD LSEVRILQNK SPAPARLGVI APIDVFIPPG
PTGMDPSHTS FFQSLGISTK IVKGQIEIQE NVHLIKQGEK VTASSATLLQ KFNMKPFSYG
VDVRTVYDDG VIYDANVLDI TEEDILKKFS KGVANVAALS RSVGIITEAS YPHVFVEAFK
NIVSLVIDTD YTFPLMKKIK DMVENPQAYA AAPVAAEEEE EEDGFMGFGM FDQERDLARE
PCPDRIIEDM GGAFGMGCIG GYIWHFLKGA RNSPKGDMLS GALYSSRMRA PILGGNFAVW
GGTFSCFDCT FQYLRKKEDH WNAIGSGFFT GGVLAMRGGW RSSSRNAIVG GVLLAIIEFV
SMVLTRKTTP TPRQQFQQQM EMEKMLVDNI GDVTITNDGA TILKQLEIQH PAAKILVNLS
ELQDQEVGDG TTSVVLLASE LLRRGNELIK MDIHPTTVIC GYKLAMKESV KYIKEKLSER
NLGKDVIINI AKTTLSSKFI SYESEYFAKM VANAIQSVKI INDSGKTKYP VSSVNILKVH

GLSSLDSKLI DGYAIMTGRA SQSMPSAIKN AKIAFLDFPL KQYRLHLGVQ VNINDPNELE
KIRQREKDIT KERVNKILES GANVILTTQG IDDMPLKYFV EAGAIAVRRV KKDDLKRIAK
LTNGQIRLTL SSIDGTEKFE PASLGYCDEV YEEKVGDWDV MFFKGCKNSK SNTILLRGAN
DFVLDEMERS IHDALCSVSR ALESNYVVVG GGCVEVALSV YLEDFAKTLG SREQLAIAEF
AESLLIIPKI LALNASYDSI DLVCKLRAYH TKSQVNTDEP KDYRWYGLDL VNGKVVNNLK
NGVLEAMISK IKSIRFATEA TITILRIDDL IKLVPERNPP VYKLKGHTST ILDIQFNPCY
SEVIASSSED MSIRIWLKGH KKKVTIIDWN PLNYYILSSS GFDSNVNIWD IENEKKAFNI
NMPQKLTSLK WNSIGTLLSA TCLNKKLHII DPRQEKICTT FNGHSGGKCA KNIWIDGYSG
NEILSTGFSK NYMREMKLWD LKNISEPIYT ISIDNASAPL LPHYDESIGI IYIIGKGDGN
CRYYQHSEGV LRKINEYKSC LPFKSFGFIP KQACNIYKCE IGRIYKNEND KNIKPISFYV
PRKNFQKDLY PPIIGNNLEI KRINIFKKLK ICGQFNKGFI LSKLFIIDQH AADEKSNFEK
YNKIFTMKSQ KLVYLLSLPV FNGKILEVVD FMSLLHHLWF NYNFPRPQKV WKILASKMKN
LVLVFLKKIS DTYIEDQSKW MEKMKSSQEE QNNKKLDEWN ECIENKCFVY PASSAPCGAC
TSAGAVTPHR RYKEPRQKKE YTGTDILCQA KSGMGKTAVF VLSILQQLVR CLGIAHTREL
AYQIKNEFDR FSKYLKNVRC EVVYGGISMN KHVVLFKIPH IIIGTPGRIL ALIREKYMLT
DKIQHFVLDE CDKCLERLDM RGDVQKIFIS TPLKKQVMFF SATMAKEMRD VCKKFLQNPV
EIFIDDEAKL KLHGLLQHYV KLQEKDKTRK LIEILDALEF NQVIIFVKSV TRAITLDKLL
TECNFPSISI HGGLNQEERI ERYDKFKKFE NRILVSTDLF GRGIDIERIN IVINYDMPEN
SDSYLHRVGR AGRFGTKGLA ITFVSSQEDT LALNEVQTRF EVAISEMPNK IDCNEYINQR
MSVSAEAYGE WNKKKNFVAK VHKKDNKEKK KIREALNESF LFNHLNNSEM ETIIDAFFDE
HVEKGVNIIN EGDEGDLLYV IDEGEIEIYK TKDNKKEVLT TLKSKDVFGE LALLYNSKRA
ATAKALTKCH LWALDRESFT YIIKDNIAKK RQMYEDILKQ VTVLKDMDPY ERSKVADCLK
SKTFNTDIII NEGEQGDTFY ILIDGKATAL KNGQVIKTYT KGDYFGELAL LRNQPRAATV
KAESTCQVVH LERKGFKRLL GPIEKILIRN VENYKKVLKE LMHLKIMCLS DEVREMYKNH
KTHHEGDSGF DVFIIKDEIL KPKTTTFVKL GIKATANTSF LLFPRSSISK TPLRLANSIG
LIDAGYRGEI ILALDNTSDQ EYTIKKNDKL AQIVSFTGEP LSFELVTELD ETSRGEGGVH
VILGAAFGTA KSGVGVCSVG VMRPDLIMKS ILPVVMAGVL GIYGIIMSII ISGDYIKAYL
LGFSIEDALA LLRIDDLYIX SFQIKDVKIL KGDHLSRCIG RICGSNGSTK YAIENATKTR
IVIAGDKIHI LGSFNNIKMA RYSICSLILG STQGKIFNKL NILAKRLKER IEKLEDRMHP
WSNIDGMKAA CSYTYDDIIC MPGYIDFPLS EIDLSNNLTK DISLKTPIIS SPMDTVTEHK
MAISMALCGG LGIIHNNMSI ENQIEEVKKV KRFENGNKVL CDEKKSILPI VNDNYEFPHA
SKRENKQLIV GASISTDLEK VNKLAQNMID IICIDSSQGN SIYQIDMIKK IKSAPIIAGN
VVTSNQAKNL IDAGADVLRI GMGSGSICTT QDVCAVGRAQ GTAVYHVSNY AHNIKTIADG
GIKNSGNIVK ALSLGADFVM LGNLLAATEE SCSEYYFENN VRLKMYRGMG SMEAMISQGV
SASLVDKGSV LNLIPHLVKA VKHGFQSIGI RNIQQLHSKL YSGMKENKQY QEALKELKKL
KKKIEENINI LKNIKEKLIL AHESAWDKFG SKLKDMPFLN SFFENPILGK LFGETELAAA
LRVMKMDDKN FKLSELMYLF EYVISKHIVE SYLIGDEDTL RLHCGEAAFN SLNLSITERK
KKKLFLDTNV LIYKNHELKG AQRMEESSPW FIFTFHTQQI NCLKNKDDEI IEGKIDDIRE
VVYTIALSKH PEPEGLLYPY IVREFAIIGN TPSWMVLYII GLGLGDEKDI SVKGKELIDQ
SDVIYLESYT SILFISKDKL EEYYKKKIYE VDRNFAEENC EQILDEAINK KVSFLVVGDP
LCATTHHDII LRAKKKNIDV QVIHNASIMS AIGESGMQLY NFGQTVSIPY FEGDYKPTSY
YNKIKINLDN NFHTLCLLDI KVKERTIENI MKNKNIYEPP KFMTVNEAIE QLIYCEHNEN
VITKNTLAIA IVRIGSKDQQ IVSGNLFTLK TQKYNDPLHS LIICAPNLHD IEKEYFDMYP
NSAYRKCVRV QLIKNGKKIT AFVPGDGCLN FIDENDEVLV SGFGRSGHSV GDLPGVKFKV
VKVARVSLLA LFKEKKEKPR SMGIKGLTKF IADTAPNAIK EIKIENLMGR VVAIDASMSL
YQFIIAIRDG DQYGNLMNES GETTSHISGL MSRTIKLMEN GLKPIYVFDG APPELKGSEL
EKRGEKRQKA EELLIKAKAE NLEEIKKQSG RTVRVTKKQN EEAKKLLTLM GIPVIESPCE
AEAQCAFLTK YEMAHATATE DADALVFGTK ILIRNLINLE QVLKGLKLTM DEFIDFCILC
GCDYCDTIKG IGSKTAYNLI KEYNCIENII KNIDYVEARQ SFINPKVKSE VKIDWCEPKI
EELKTFLIKE HNFNEVRVTN YITRLLKARK VTTQRRLDTF FVNNSKYYES LNLKKNCTTE
EVKKAYRKLA IIHHPDKGGD PEKFKEISRA YEVLSDEEKR KLYDEYGEEG LEGGEQPTDA
TDLFDFILNA GKGKKKRGED IVSEIKVTLE QLYNGATKKL AISKDVICTN CEGHGGPKDA
KVDCKQCNGR GTKTYMRYHS SVLHQTEVTC NGCRGKGKIF NEKDKCVNCK GLCVLKTRKI
IEVYIPKGAP NKHKIIFNGE ADEKPNVITG NLVVILNEKQ HTTFRREGVD LFMNYKISLY
ESLTGFIAEI THLDERKILI DCTNSGFIKH GDIREVLEEG MPTYKDPFKK GNLYITFEVE
YPLVITKEKK EVLKILKKQN EVEDIENTDC EVVTCKPVDK EYLKQRLKTL KNYLPYLCKI
LIDNTVYTKW DYLTMDESHF QNDNADEMTS RTWGNDWTVR KGAALCLDYL SNVYNDDILE
YILPHIEEKL MSDKWNIRES AVLSLGAIAK GCMYSLSPFI PKVLEYLIKL LNDEKPLARS
ISCWCVTRFS SWICHPDKWF EPVLLNLLKR VLDSNKRVQE AACSSFANLE EDALELLNNH
LHEIVHTIQQ AFQIYQAKNY FILFDVVGTL IDSVNIVKEN IDLAHEIVNS ILIKWNNIRI
SSPYIIALME CMSCITSAYG KEFLKYAKIV IRTCIKFLVL LYIDDLIECS FDLLSRIILQ
SNFALIGDIS RFCAQYLILN DIIPFLIAHI SHPSIPVSNN ASWAIGEISI HINSQYIEPY
VDEIVKQHIY ICNSKYHGCL LQNICITVGR LCSTYPKKII YYFPQFLKTW LKIMSHGTQE
NEKINFFHQF LSTMKECIQP DELKLKLAQG LLKSHIVNTL TNNGCTFIFW IADWFAQLNN
KMSGDLNKIK KVGQYFIEVW KSCGMNMENV QFMWASDEIN KNPDKYWSTV IDISRSFNIN
RIKRCLTIMG RTEGEDNYCS QILYPCMQCA DIFFLNVDIC QLGTDQRKVN MLAREYCDIK
KIKKKPVILS HGMLPGLLEG QEKMSKSDEN SAIFMDDNEA DVNRKIKKAY CPPNVIESNP
IFAYAKTIIY XHYKEFSLAR KEKNGGVRMY VNEEIIDNEK LSDIINKTKE NVKYMKGMKL
PNNIVAIPDI DKVIEDADLL VFVVPHQMER ILSSIGRLSV VAGGLSLIPY TFIYDVDGGE
RCVMFNRFGG VSEKTYGEGS HFYFPWFQTP YIYDIKMKPK VINTTTGTKD LQIVTLSLRL
LFRPHTKHLP YLHSTLGPDY DERVLPSIGN EVLXAVVARY NAESLLTQRD TISKEIRESI
TARAKQFNIV LDDVAITHLS YGKEFAKAIE DKQVAQQESE RVKFIVAKTE QEKIAAVIKA
QGEAEAAKLI SSAVKEYGNS LLEIRKLEAA KEIAENLSKS KNVTYFPSTS NILYINAIKE
ENGGYNFENL KRNEILKEKG PKFRKTGTTI CGIVCQNAVI LGADTRATEG PIVADKNCSK
LHYISKNIYC AGAGVAGDLE HTTLWLQHNV ELHRLNTKTQ PRVAMCVSRL TQELFKYQGY
KVCAIVLGGV DVTGPQLYGI HPHGSSCLLP FTALGSGSLN AMTVLEAKYR DNMTIEEGKE
LVCEAICAGI FNDLGSGGNV DICVITKDGT QHIRPYKQPN TRLYHLPKGT TPILYEKIEN
IKKHISLNDL GEARGTVLSV KLDELIDNVE GQTVIDPKGY LTNLNANDSD IADINKARSL

LKSVINTNRK HGPGWIAAAR VEELAQRKDK AKEIIMKGCI ECSKNEDVWL EAVRLEKLSE
SKIILTKAIK NIPTSVKLWL EAYKKEKNVQ DKRKVLRKAI ECIPNSVVLW KEAISLENEN
NAYILLLKKS RVQCNTNNIN INPIISEALK ECPTSGILWS KAIELENKNL QNSKSVTAFN
NCGNNSYVIL IVAIIFWNNY KIGKSRKWFY RAITLNPSFG DGWATFLAFE IDQENEINQK
DIINKCIKAE PNRGWVRGRI HDIRSKGSLA FIILRNKIYS LQCILDIKNN NDKNMIKWVS
NLSLESIVDI YGKLVKPEIS IDSTIIKYEI QILKIFCISK NSKELPFLLK DANMKETSDE
ATIRVNQDNR LNNRCIDLRT YANYSIFYLQ SEICKIFRNY LIDNNFTEIH TPKLLGESSE
GGANAFQINY FNQNGFLAQS PQLYKQMCIN SGFDRVFEIA PVFRAENSNT YRHLCEYVSL
DVEMTYKYDY MENVYFYDSM FKNIFNKLKN QYPSEDFKWL AVTPIFTYEQ AIKLLIEEIL
TYDMTTDMEK ELGKIIKQTH DTDYYIIINF PSGLRPFYTM YNEKDPKISN SYDFFMRGEE
ILSGSQRISD VKLLLDNIKK FNLDPKKLDF YIDSFAYSSY PHSGLLFENL NKEYKYITTQ
DNFDGFRFEV DKNVNKYLQS THTLFLGTRD VGYLYQFGAN FANSDNTLLM ISRINLDGSV
NGRFCKKINN DCKFNFNTYN KSDPRNMYEM SLEVNNPQNT YNIKTIWQGG VDLTYIGSNC
ASIGSFGLRY NHKNHVLTMQ CIRQPNFKSP EFMLNQAHSY KMQYARKISD RLSVGTELEV
TPETKESAMR LGWDYSFRHA KVQGSIDSSG KIAVFTQDYS GFGVSGYIDY LNNEYKFGMM
HIAPSQEQDG GVGKTTFVKR HLTGEFEKKY IPTLGVEVHP LKFQTNFGKT QFNVWDTAGQ
EKFGGLRDGY YIKSDCAIIM FDVSSRITYK NVPNWYRDIT RVCETIPMVL VGNKVDVKDR
QVKSRQIQFH RKRNLQYYDL SARSNYNFEK PFLWLARRLS NQPNLVFVGE HAKAPEFQID
LNIVREAEKE LEQAAAVAID EEDGVLTVNS IKSEPTISQY DIKKLVKNKV LENAPFYNYQ
IERSFADKFY GDCIYDNFGV PKNIEINLII LEEWNINCNR NRILKHTGLI KNIEINNFKY
INNKESLEVH FSVNPMYTDL LEKNKVLPPS GIDYNKLIKQ FGCSKIKEEH IKRIEMLTAH
HFIRRNIFFS HRDLDFLLNY YEKNKSFYIY TGRGPSSLSM HLGHLIPFYF CKYLQDAFNV
PLIIQISDDE KFLFNKNYSL IEIKSISKNE MEFTLYNSNS ALANALRRIM LSEVPTLAID
IVNVYENTSP FHDEFIAHRI GLIPIDSRNI KNYEFRERCK CKETCSRCTV QYIIEVKCNN
KIDVSHYDIL DHEPNIPMPI PIPILTLSKN QTIHMKLTAT KGIGKMHAKW IPANVSYIID
HKIIINNHEV DKMSKEHKLL IANNLNSDCY ILLRLSENMS VVMAESCRDT LNELGYKDIV
KIIYDETKFH FKIESVGSMP PEQIVEMAIE ILENKLKDLE PQIKASFYSI EEVAKQLKLP
DPESGKNEEE RCFLSLIKSS ILKNPKKWTN MAKKIIGMSE ETTTGVLRVK KIEKNNGLLF
TAINVNDSVT KQKYDNIYGC RHSLPDGLMR ATDFLISGKI VVICGYGDVG KGCASAMKGL
GARVYVTEID PICAIQAVME GFNVVTLEEI VEKGDFFITC TGNVDIIKLE HLLKMKNNAV
VGNIGHFDDE IQVSDLFNHE GIEIENVKPQ VDRVTLPNGN KIIVLAQGRL LNLSCATGHP
AFVMSFSFCN QIFAQLELWE NRKYENKSYI LPKELDEKVA FYHLKKLNMY SYGMEGDDTY
LPQVQYPSPY ENQYESPSPR GESHTPFIGY FSSHLLRTGF LLQCLSLILM FVFYWAFGGT
GIFIFDLYAG PECVKVSSAF HLTISVLMSI YLLGTLYIAM FQVFVADNSK WCRGFRAGSK
LLSAAVTLDL LSSILRLVQY LYAYFYMNMR WWARYQQTKS DWTLLHFGSI VHSFSLFIYG
AAFFYMEAYH DEGTYEELAW SNLTLFKLAG LAELNKPKKR TFRTFHYRGI ELDKLLELNQ
EELVKLLPAR QRRKFRRGID KKAKSLLKKL RKAKKECEVG EKPKPIPTHL RNMTIIPEMV
GSIVAVHNGK QYTNVEIKPE MIGYYLGEFS ITYKHTRHGK PGIGATHSSR FIPLKVMVDM
FLWRDPEQFE LKNLANEEAT PTAPHLADNQ YATEAPYDDW GFLQIIKDHH ETMYELKQKI
RPRDQVVGWF CSGSELSELS CAVHGWFKEH NSISKFYPHS PLNEPIHLLV DASLESGFLN
VKAYVQLPIN LVKEYFVHFH EIQIELLPCN IERADVSLKK LLIMLKNCKS YVQDIVDKKK
KGNVAIGRYL HKVFSNDPFI SVEKFDSINE SILQDNLMIS YLSNLAHLQF LIAEKLNWLN
NLPIKEDESE GRRVHKNLID LVSQNHPLLF GKDNSNIGKI IEIFLSIYET EFSDSDCNKK
IVSLISSLDQ SYLSNLALTN KQSKKLNHIN GCDLFNFYED NINLKPYLKI LKDFDKYPII
VDSKNNILSL PPIINCDHTK ITLDTKNIFI ECTGIDLNKL EISLNIICSM LSEYSQPKYT
IHSILVLYPN FKNKKLTCDI EYVRKLSGIK DITIDDVKKL LKKMMIDNTT FEVNVPFYRS
DIMHACDIVE DIAIAYGYDN IKYEPIEISK KHLLNTVSDM FRNSMTECSY TEVITNALLS
LKENYDWMLR PVQIMNSKTS EYEIVRTSLI VNLLKFVAAN KHRELPLRFF EIGDISYNKT
DTNAFNKRNL SIIFADKTAG LEEIHGVLES ILKDFQLFSH YKIEEKRKEK VEIRVISIGN
IRGLGGCDYG SFRMSNEFLG WKNKKTNSVY QYKCNDISEG EWIKLSYNNN RLHLKFNESK
DNLIVFFDGF PDRNIAEITQ HFQKYFNIKL GTRKLATKGW NWGEFKLENS NLFFDIDKKY
AFNINTNNIN QLNVQIKTDI AIELKNDENE DVLSEIRFYY PHENDENQNF QDLKNNLLEK
VNIGDSKSEC IASLSNIPLL VPRGRYEIEM YSKTFKLHGK SYDFTVQYSN INKMLLVPKT
NSNQYILIFS LNNKIKQGQT EYPFILIQLS NDDDMDLDIN ASEEDIQNYK LEKTLTGKAY
DVVTRLFTAL AKKNAIIPGD YRTAKNEHGI TCSYRAASGQ LYPLNKYFLF VVKPVILISF
DDIVTLSFQR TGNNQHRFFS LIIKHKRGIS YEYTNIDKSE YAPLLEFLKS KNLNIKGYID
LSKRRVSPKD IIKCEEHFSK SKKVHQTVRH VAQKHNMTVE ELNRKVIWPL YKKYGHALDA
LKEATMNPDV IFKEMDISDA VKESLLSDIK LRLTPQALKL RGRIDVWCFG YEGIDAVKEA
LKKGKVTINI KLIAPPQYVI VTSCHDKELG MQKIQEAMKV ISDKIKEYKG GDFKQQGEIL
VILLDKHDGL SSDDEGERGI FEFFVCDVGV GLSLSVRDIL PIEYDSIFIG VLPYYTFNHE
YIIYDSSQLL PRYLIQFECD PSAEELFSIP LCDYCGNAPS LYYCESDEVK LCEKCDNIIH
SNKLVKKHIR KTLNEAQGNC KIHLQNEVNM FCTVCHIPIC NLCMCSHAHI SLKMAYNAIM
QHSSKPSNFI KERKKNLNNL LEKIDKLHEQ VRLNMNETEK NVYNVLEDLV KQLHTTTDKK
MSSILSEEYE LKRQFNEIMW NENFLYYLQT ILPPADFMNA WLKHCQYREE IEKNSLIFPD
IRIKGNINVI TEGSINHLVG LPNVGKSTTF NVLTKLNIPA ENYPFCTIDP HEAKVTVEDE
RFDWLVDHFK PKSSVHAYLS IFDIAGLVKN AHLGEGLGNN FLSNIAAVDG IYHVVRAFEN
EDIIHTEGNI XPVRDMEIIN SELIYKDISN CERNLEEISK VNRNKKDKIK QNEHDVLTIV
LEHLKEHKWI KDKAWKSSEI EVINEYNFLT AKPVVYLVNM SENDFIRQKN KYLAKIYNWV
QEKNKGTIIP YCAEFEQKLL SMTENEKEEY FKANNIKTSM LNKIIKTGYY EINLIHFFTC
GQDEVKCWTV RKGTKAPQAA GVIHTDFEKG FICAEVYKYT DLVEFKSEGE VKANGKYLQK
GKDYVVEDGD IVFFKFNVSS SGRINKFVNQ LRISYGTLEE FVDNFVYELK KGLEAHRRHP
NLWIPHECSF KMLDSCISDI PTGQERGTYY AIDFGGTNFR AVRASLDGNG KIKRDQETYS
LKFGTFSHEK GLLDKHATAS QLFDHFAERI KYIMGEFNDL DDNNEKSVGF TFSFPCTSPS
INCSILIDWT KGFETGRATN DPVEGRDVCK LMNDAFIRSS VPAKVSCVVN DAVGTLMSCA
YQKGKSAPPC YIGIILGTGS NGCYYEPDWK KYKYSGKIIN IELGNFDKDL PLSPIDLVMD
WYSANRSRQL FEKMISGAYL GEIVRRFMVN VLQSASSKKM WQSDSFNSES GSVVLNDTTP
DFRECKKIAK QTWDMDFTDE QIYALRKICE AVYNRSAALA AAAIAAIAKR IKICGVDGSL
FVKNAWYCNR LKEHLRVILA DKAENLIIIP ADDGSGKGAA ITAAYILIRW LCKAIVSSLF

GDVNIINPEN VPLYGSVIFV GNHNNQFIDA CVLVASIPRQ VKFIVAEKSM KRAVIGDLAR
LAGCISVKRP EDLKFKGIGR IYWNTGDTKI KGINTRFKLD VQMGDKLMTQ NKIFSVTKIE
SEIELILQDP ININCEDNGV PFKIVPKINQ SEVYNLVTHS LKNGDTIGIF PEGGSHDRTN
LLPLKPGVAI MTLCALADGD VSIIPVGLSY SKLYQLQGCV TIFFGNAIIA SQDLCKDYNN
NNRETISKLL GKIEEGMRSC MLTSKNHETS RCIELCVSLY TPERMTISKN KIYNNLQLFS
EMFWKFGNSK EIENLCYELQ CYEKLLEANK IKDDEVWMLK QSTSAATLKF IEQICSLIFC
TIFGMTFSLL WLPLVAISVY LAENHRKTSL KNSLVKIQGG DVVASYKVLV LLVLLPTFNI
IYGLLFSLYR LMKKKDVDQV HKLLNNYLSK FNIYVKFTKE EIAHWFMPIQ NVIYTYVNEV
DGEIKDMISF YSLPSKILAN EKYDMIYAAY SFYNVATTTS LKNLMQDAIC LAKRNNFDVF
NALEVMDNKS VFADLKFGEG DGMDTDSVLG LQAILISANY KEKEFIRIAY YMNSFYKDIE
LREKPPVSPQ YDKICRHIVD NPRIVKFSIP WDDSRIAYKI LKELTQKNYF RIFKVNLHVP
CKLQKITEKC NEKTKCSVCE CTEDEIPYNF RTNEVEVDLI YNSPSYTAYE GKSIWELKGN
NNYKYFGAAK NLKGVKELLF KENDDRKKRR KEKRNLDKIV NIHYFGYCDE ENEILLNEEL
KIQKKLMKHG TLVLPADRAR EYMDCLGKQV DIQFIDMNEK TMKRQYKKYI QRIDDMERIL
RFLEENINKL PNVKIKKSKI ENFLEHDNIY ELDQVEESLN RLHVQFVRFC NNNKDLIDER
NSAIEEKHVI LTALNQLHPA LSTHIMREGI NMMFTNISGV IKTKDQESFS RTIFRALRGN
TYTYFQNIDE KSVFVVYCHG STHSSIYEKI MKICKAYDVR NYEWPKTYEQ ANKRLNELKE
IINDKEKALK AYEEYFINEI FVLINVVEPN KNSLIEEWKL FCKKERHIYN NLNYFEGSDI
TLRCDCWYSA NDEEKIRHIL MNKSSNDLVS ALLLSDKLTP NISPPTYIKT NEFTSTYQSM
VDTYGIPRYG EINPAISTIV TFPFLFGIMY GDVGHGICIF LFALFLIIVH NRMKNNEMLN
MLFNGRYMLL LMGFFAVYAG LLYNDFFSMP LNLFTSPYIF GFDSKWLGAD NELTYINSFK
MKFSIIIGFF HMTFGVIIKG FNALYFKKKM DFFFEFLPQL VMMLSMIGYL VFLIIYKWIT
PGGYKKQGII NTIINMYLLK EINQDNKFYE HQEVVQIIII TLFALCIPIM LICKPAIKTY
KIIKEIWIEQ LIETIEFILG LISNTASYLR LWALSLAHQQ LSLVFFEQTI LSSLEKNTFI
GVVISLIIFS QLFSILTIAV ILCMDTLECF LHSLRLQWVE FQNKFYKGDG IPFKPFNIKK
LLPDNYSAIL ARALSERPLT YLPTVERVCY EVLSDEDEHL NYIQINLLNT IRPTPIRGLL
AATQERFVVV PGIIVQASKP QHKMRKITLQ CRYCDHKMSI DVPLWKDKPQ LPPYCRYVLE
PYVILPNECT FVDIQSLKMQ ELPEAVPTGD MPRHLQLNAT RYLCEKMIPG DRVYVHGVLT
SYNPNPTRVD GTNFSYLHVL GFQKYDGNDL NFDVEERNEL TLLAAEHDIH NKIFKSVAPE
LYGMDEVKKA CACLLFGGTR KRIGEETKIR GDINMLMLGD PSVAKSQILK FVNRCAPVSV
YTSGKGSSAA GMVRQGLLGE NEEKLDYVLG LTLPKLLERR LQTKVFKLGL AKSVHHARVL
IRQRHIRVGK QMVDIPSFLV RIDSEKHIDF ATASPFGGSR PGRLKEMIIS QSMYDRHLTI
FSPDGNLYQI EYAIKAVKNT NITSLGVKGE NCAVIISQKK MATQYITQDK LLDYNNITNI
YNISDEIGCS MVGMPGDCLS MVYKARSEAS EYLYNNGYNL NVETLCRNIC DKIQVFTQHA
YMRLHACSGK NITEESIKDM FSVYGSVEEV FIMKDNTGLG KGCSFVKFAY KEQALYAISS
LNGKKTLEGC NRPVEVRFAE PKSSKQTAKV CSRDLPGHTK MKMRDLSDYT EDKEIIETIN
LAVKYAKDAV IEDEKKNYKE ALNLYIQSLQ YFNYFCKYEK NDNIRELILK KMEVYITRAA
DLKEMLNILN KDQNVKWSDV CGLETAKEIL KEAVIFPLKF PKLFNSSALP YKGILLYGPP
GTGKTFLALA CANECNMNFF NVSSSDLVSK YQGESEKYIR CLFDTAKEYS PAIIFIDEID
SLCGSRTDGE NESTRRIKTE FLISMSGLNN YKNNIIVMGA TNTPWSLDSG FRRRFEKRIY
IPLPNLYARD IKNFANITEN YTGADIDIIC RDAIYMPVKK GLYIKCGSRY EGMSVMIENM
AFHSTAHLSH LRAIKSLEKI GANVSCNAFR EHIVYTCECL NEYLPVVINL LIGNVLFPRF
LSWEMKNNVN RLNTMRAKLF ENNEMYITEL LHNTAWYNNT LGNKLYVSES NIENYTSENL
RNFMLKHFSP KNMTLVGINV DHNELTKWTS RAFQDYVPIP YTKQKEVTPN YTGGFISVED
NIKKTNIAIA YETKGWKTSD MITLTVLQTL MGGGGSFSTG GPGKGMYSRL FLNVLNNYNF
IESCMAFSTQ HSDTGLFGLY FTGDPANTKD IINSMALEFH KMNKCTDEEL NRAKKSLKSF
MWMSLEYKSI LMEDLARQMM ILNRILSGKQ LCDAIDAVTK EDINRVVSQF LKTKPTVVVY
GNISHSPHYD EICKMLDAVN KIHFFMGYPE LASVNFGNST VVRCKKCRTY INPFARFEAG
GKKWNCNMCY NINETPQFYK RKDLFQRPEL CTGSVEFIAP SDYMIRPPQP PVYLFLIDVT
VTSINSGLLD VVCNTIKKLL DSRTLIGIIT FDSTIHFYNL NSNLKQTQMM VVSDIQDIFI
PLPENILVNV HECQNVIDNL LDNLPNMWRN NKMSDCCAGN ALKAAVMLIK KVGGKILFFL
SSVPNIGDNE YGEFAQSITQ FQIAVDLFAC PYNIDLASIY PLIKNSGGSL YYYPFNVHQY
SDKLREELLF ALTTETAWES VMRIRISRGW KITNWYGNFQ FRGVDLLALP NCHSSQTFSI
VVDLEENVVQ DSVVYVQSAL LYTNSNGERR IRLHTYALPI TQNIKTITDS INPQVVVSLL
SHQAIEVIKK GKIADGRNLI QTLCSQVLST QLSSSENSRL LPIYILGMLK SVAFRDPDMR
IYQWSRVENI PIESIEAYFY PRMFSLHNPA TLNLTCENMT QDGCYLVEDG ENMVMWIGRS
ISLAVVNVAA GLDGCDDQLL PASFRALEAD LNLHPSLLGY ITLAQTLMLS LFSPIWGFLS
DKYSRKWMLV FGTALWGLAT IFLANINDFA HIIIFRAING LALGSIGPIS QSILADAAKN
ELGLSFGIVQ LSSSIGRLIG GVVTTTVSMK YFGTIRGWRL CFIIVGALSI LLSIIVALFV
EDAPSLSKKS IIIILLEGFT GTIPWLALSF NTMFFQYCDL SDLQAAVITG FLLIGSALGG
VLGGHFGDIM HNISNKHGRP FLGQLAMFGR VPLVILTYLV IPKRKESFEL FLSCFFLGLS
SIAGVAVNRP IVSDIIRPDY RGTIFSLTIA IEGVGSSLIG APLFGYLAEE VFNYRNNNLL
ISDMTTEFRS HNAEALSKTL LYLTAVPWIL SFIFYSLLHF TYGAEYLKMN QIIESEMLMF
GLRSLSDFCN PTSKAYKENA YDALNRDAIP SINKAVNNYK DDDDILYCSS KVLFAMSDYC
CSEKDNEALK KLVSDGGAIT EIIKTIPKDQ DTLKNCMLFI QNLSVALLNV FTSDTYTTKL
GSSIISALSV VSKSPSGSEA LNGENAHHKL IDHCLDETAE IIEGAFDVIK NLLSNGYVVP
TIIEKSVVIL DKFKAYPRVV SKGSDTMKSA VGPEQLTDCL NILKKEQQGS KEHDSALELL
SSLSYISSIT DKIVESGGIP VLIELINSGL QQYDSNPDKI ARLVAGASRM LGRISNNPSH
AGVVYEYGGI ATLCTALSYF PNDADCVSAI CIALIPFVSR SYLLFASLFP ILYASVESLD
LAKASMACVA SASMINEFHE QMVNNQVIEI LSTCIQYHLT DSDYLSNVFS VYFRLSDHIK
TIEPINQYGG ISGIANALSA VDSVLTVMLE NENKEVIIQE GTKIMENLAT ESDCQRHISN
LESIAQSNPD RAYKTLAAIS GLSRIQSLKL MLESKGADSS IYNGMKTWIE SPKFNEQTKL
IKAALKTIKI VELMCVPQVK RLAEDGPDDN ILITSAECIN YLTEVNKIST KEIVESSLES
IFKMMKKYSE SRLTQTNLLS AINNILLSSN CAEVLVNKGY VKQIVTYIHK VPMYVDVQII
GFSVLANMLK INSDSLDAIK KANTLIPLQN ALRTHVKNTK LKTTCAPLLA VLMPLDTLTR
EIEELLRLCN KSIQANNLPQ LHEYLVSLNE LLLTAESSKI SARCNIISEV AHVCSNVSQS
RIGIVHLTKS NMASSLMHLY NLLKLPGDEY TEEAVANILE SLSLLLKHDI INTDIAIDLG
LIEKLCTGIN HFSESDTVIK STFSCLACIC TTNKRINQLI THPEYDKLIS VIVNLIGNSK

DSRKNAIKAL YELLKIEKEE ISVNISTKTP IVNLFKIMGE YQMDLPIIQD SSKCLATIAE
HVTIEEDKYS AMKILIECLG KNKNDESTAQ EIMSVLVKLC NNNDKPQFKE LGAIDIISDV
TMIHGKNEEI SRLGGILFSY MGADEQVKKL MKMILSVKDS DADAVQKIDN LSGKLELFLR
APLENPLDAL QYTDATLQKL NAYVGSNLEN VSLQTNIALV NKRLVDRVKY DFEDQLGAWA
VASAGILNQY TDMITNKVGL KHDKTVAPIY SVLSGCYLNM DKKAREYAQE ALKFIQRSGS
NFMACKNLRE KLESHGLIHI KEWKLQKNQG YVLCKENRNI CSFFIGKNFN INNGSILISI
GHIDSCTLKI SPNNKVTKDQ ISQLNVLIEK IIQINKSVLF LPSLAIHLQN RTRSVKVNYE
NHLKPIISTL LYEKLPLLYL LANELKCKEE DILDFELCLM DTNKPCFTGV YEEFIEGARF
DNLLGTFGVF EAYVEFVLFF ISSYNVKCQL RFASLGNWGK SKSQLLNAKY LKQFIKSERV
TFIVSPGSNF MDGVWKSLYE DVYEEEDMYM PFFTVLGTGD WTGNYNSEVL KGQGIYPKWI
MPNYWYHYFT HFTVSSGPTG HKDMAAAFIF IDTWILSSNF PYKDIHDKAW DDLKLQLNVA
KKIVVVGDKP IYSSYLLPLL KEAQVDLYIS GHDNNMEVIE DNDIAFINCG SFCIHELTNN
GIVTKFISSK NGSLQYFATL PKVELIDIPA VGPMGNKDTF VRIVGTIGIL IRXXYSGVXY
SSGALVKKFM KSSKFKNEFM KHMNIFLMVC IYDNEICEMS DVGVNFYITE NHVENKSNAV
LSNLKELNNY VKNIAFLSCN IYGLCGYIFN DFGNNFICYD KDGENVKSCN ISKIVKDVEG
IVSFDFDKSL PFQNGDYVKF TNVEGMEING KIYQIKNLKK YSFTIGDTSK FGDYIKGGEC
TQIKTNLKLN FKPYEDYSKL ESSNYLHVEK LDKNIIINVA KYSKSHISPI ASFFGGLLAQ
EIIKFTGKYM PIHQLLYMDF FEKNDNIISI FGKKFQDKLN KLNIFLVGSG ALGCEFAKLF
SLLDGSLVIT DNDNIEVSNL NRQFLFRREH IEKSKSLVAS NAIKNKNKNI NVISYVTKVG
QENEHIFNEQ FWSKQDFIIN ALDNIIARQY VDNKCVWYSK PLFESGTLGT KGNVQIIIPH
MTQSYNDSYD PPEDSIPLCT LKHFPYDIVH TIEYARDIFQ GLFYNVPLSI QQFENLENVL
NTLKIIKENN FNFCIKKAVH LFHSNFINQI SQLLYSFPLD YKLSTGEFFW VGQKKPPQII
NFDINNIYVQ EYLVSTSNLY AQVYNIPTCY DIKYILDVIK VEPFSPKNVK VNIDPIEFDK
DEISGLHVNF IYAFANLRAM NYKITTCDKL KTKMVAGKII PALSTTTSII TGLVGIEILK
YVNYLSYFKN AFINTALPLF IFSEPMPPLK IKDKEYDELM KGPVKAIPNG FTTWDKIEIS
IKDYDTQRDK RFSGTVKLSN EVRKKLKVCI LGDAVHSEEA QKLKLDYMDI EAMKKLNKDK
TLVKKLAKKY DAFLASQVIL PQIPKLLGPG LNKAGKFPSL ITHNDKVLFL YNIASFCFEL
QFRFIEDTTF DWLPVIGYLL PYEKIKLLRM IFPIVFFISI CISAYAYTDR NASLIYLMRS
ILSTNRIIVE RSNDVNYLKK NGDQLVSKLQ QTRKFSLTLD SQQMNLLEKK GKKKSWFEFF
SLQMVFGVIF IYMWFTSKKP ATYEVPLPLS LEELYKGCKK KLKITRKRFM GTKSYEDDNF
VTIDVKAGWK DGTKITFYGE GDQISPMSQP GDLVFKVQTK PHDRFTRDSN NLIYKCPVPL
DKALTGFQFV VKSLDNRDIN VRIDEIVYPK FRKIVANEGM PSSKTPNMKG DLIVEFDIIF
PKNLTSEKKR IIREALGLHY ICILCPSNNW FKDSSIKWNL SIEVGGIDID MNDTAKKSEI
SETLNTLHVD TGKTKLLDKL RHTNVQDNEA GGITQQIGAT FFPKDVLDKE IKKIDIKCLS
KGIMIIDTPG HESFYNLRKR GSSLCDIAIL VIDLMHGLEQ QTKESIQILK QRNCPFVIAL
NKIDRLYMWE KNDWEPFNDT FKNQKEYVKE EFKNRLQTIL NELSEQGLNC QLYWENKNPR
KFVSIVPTSA ITGEGIADLI MVLVKLTQSF MLKNIEYNNK LECTVLEVKN IEGLGTTIDV
ILTNGVLKES DTLVLCGMNG PIVTVARALL TPQPLKELRI KNEYIHHKSI KACIGVKISA
NGLEEVLCGT SLFVANNNDE IEEYKKKVMT DVSDVFNHVD KSGVGLYVMA STLGSLEALL
IFLNDSKIPV FSVNIGTVQK KDVKKASIMR EKGKPEYAVI LAFDVKIDPE AEKEAQILGV
EIMQKDIIYH LFDAFTAYLK KIEDEKKQSK MADAIFPCEV SIINDCVFNK KDPIVVGVKI
EAGILKIGTP LYIPEKNLKI GNVVSIESNK KSCNKAKKGE EVCVKIAGEP NVTYGRHFDF
NQKIYSKITR ESIDVLKQYF RNELTMDDWR LVVHLKKILN ILMIINEKDK LAEQNLETLD
VTKLTPLSED VISRQATINL GTIGHVAHGK STLVHAISGV HTVRFKHEKE RNITIKLGYA
NAKIYKCTNP DCPPPECYKS YESSKEDDPM CPRENCNSKM KLLRHVSFVD CPGHDILMAT
MLNGAAVMDA ALLLVAGNES CPQPQTSEHL AAVEIMRLKH ILILQNKVEL IKEEQALKQQ
EEIRNFVSGT AADSAPIIPI SAVLKYNIDV VCEYIVTQIS IPRRDFISSP HMIVIRTPKL
CNFVEWKEYK LVFKRYASLF FILCIDKSDN ELITLEIIHH YVEVLDKYFG NVCELDLIFN
FHKAYYLLDE ILVSGELQES SKKNILRVVT SQDSLMEDNK FAKSLLDVAD NLSLAIKNIS
EESLKSNENI YKGIEMTETI LHNIFNKYGI DKYNPINEKF NPMFHEAIFE VSDTTKKGTV
ATVIQPGYKI NDRILRHLDI ESVQALIVAL SLYKGGIVLI SHDTYLIKHV ADEIYHINNI
TKVVKIDYEF EKYTKLLLEN KIMPREIITL QCGQCGNQIG VEFWKQLCNE HNIDQEGILK
NNFINEDRKD IFFYQADDEH FIPRALLFDL EPRVINSIQT SEYRNLYNPE NMFISKEGGG
AGNNWGSGYS QGHKVEEEII DMIDREVDNS DNLEGFILSH SIAGGTGSGM GSYLLELLND
NYSKKMIQTF SVFPLLNESS DVVVQPYNSI LTLKRLILST DSVVVIDNTS LNRIFVEKLK
LNNPTFQQTN NLISNVMSAS TTTLRYPGSM NNDMISLISS LIINPKCHFL VTSSNVQKTT
VLDVMKRLLH TKNIMVSVPV RRGMYISILN IIRGETDPTQ VHKGLQRIRD RKLVNFIKWN
PASIQVTLAK QSPHSPHKVC GLMMANHTSI STLFERCVTQ FDRLFKRRAF LENYKKEPMF
QGNFEEMESS KEITQNLIDE YKSAERDDYF GLARAFGIPA RRYTHEVVTL WYRAPDILMG
SKKYSTPIDI WSVGCIFAEM VNGRPLFPGV SETDQLMRIF KILGTPNSQN WPDVFKLPKY
DPNFPVYEPL PWETFIKGLG IDLLSKMLKL DPNQRITAKQ AIEHPYFMGK EKTHINLVVI
GHVDSGKSTT TGHIIYKLGG IDRRTIEKFE KESAEMGKGS FKYAWVLDKL KAERERGITI
DIALWKFETP RYFFTVIDAP GHKDFIKNMI TGTSQADVAL LVVPAEVFEG AFSKEGQTKE
HALLAFTLGV KQIVVGVNMD TVKYSEDRYE EIKKEVKDYL KKVGYQADKV DFIPISGFEG
DNLIEKSDKT PWYKGRTLIE ALDTMEPPKR PYDKPLRIPL QGVYKIGGIG TVPVGRVETG
ILKAGMVLNF APSAVVSECK SVEMHKEVEE ARPGDNIGFN VKNVSVKEIK RGYVASDTKN
EPAKGCSKFT AQVIILNHPG EIKNGYTPVL DCHTSHISCK FLNIDSKIDK RSGKVVEENP
KAIKSGDSAL VTLEPKKPMV VETFTEYPPL GRFAIRDMRQ TIAVGIIKSV EKKEPGLPIV
LLKDGTDKAQ GKSQIIRNIN ACQVIVDIVK TTLGPRGMDK LIYTERDVTI TNDGATVMNL
NISHPAASIL VDIAKSQDDE VGDGTTSVVV VAGELLNEAK VLLNDGIEPN MIIDGFRNAC
NVSINKLNDL SLSFVNKSEE EKKNILLKCA QTALNSKLIS NHKNFFAELV VNAAYQLGDN
LDKTNIGIKK VTGGSCLDTQ LIYGVAFKKT FSYAGFEQQP KKFNNPKILL LNVELELKAE
KENAEVRIDN PNEYNSIVQA EWDIIFQKLN LIKDCGANIV LSRLPIGDIA TQFFADHDIF
CAGRVEDADL KRTATATGAV IQTSLFNLNE SILGNCGLFE EVQIGNERYN IFKDCLKTKS
VTIILRGGAK QFIEEVERSI NDAIMIVLRC IGNSEIVPGA GSIEMQLSKH LRIYSRSICN
KEQIVLYAFA KALESIPRYL SHNAGYDSTD ILNKLRKKHS EETNDIWYGV DCLEGDIINA
YSNCIFEVTK IKRNVIYSAT EACLILSIDE ETVKNPSRNL GLPDCFKDLL KTDKIKHVLC
TGNVGCNENL ELLKNIADSV HITKGDMDNN FDFPEKINIK IGDFKISLVH GHQIIPWGDL

NALLQWQKEY DSDIIISGHT HKNSINNFEG KYFINPGSAT GAFQPWISNP TPSFILMKSS
IVVYVYEEKN GKMNVEMSEL RKYAVEHSPS TSKDSTAVYR NPQFNDKLLD NFEDCFGTRV
RKNDKLGEYK WKTFKEVQEL IILIGSGLGL YLPNCEEWNI CDFSCNAFNI ITVPLYDSLG
IESSKFILDQ TMMQTIMCNR ACGLKLIKSL PGKLSDVCSL CYTSGTTGYP KGVIMTNQNF
IAQIASSCLG PIKFPNEKDT HLSYLPLAHV YERIMMCIFL HVGVRIGYYS GNILALTDDV
QELKPSLFLS VPRLYNRIHE RICNSLKKKS SVIQSLFHKG LDQKNSTGNP WSLFWDTLLF
NKAKKVLGGN LKGMLNGSAP LGVEVAKKLK CIFCVPLMEG FGMTEGLGFI TNPIDPDVGH
IGGPLPAVEY KLVSVPEMNY LVTDNPPRGE LLLRGPTIGY FKLEKETSED GWLRTGDIAS
FSQNQSLTII DRKKNIFKLS QGEYVAVEKI ESVYKQSLFI GQIFVFGYSY ESFLVCIIFP
SVDTMSIWAK ENKINLPNEE IIKLEKFKND VMQDLITIGK TDGLNGYEQI KDIHFIMEGF
TIENDLMTPT GKLKRHAVQK KYKQEIDQMY GNKTTKTITE GQTILTMFNE GYAPDGIWLG
GTKYQFINME KGLEYEGYSF DVATCAKLKG GMHIIKVGGG HILIVLYDEE KEQDRGNNLN
MLGVVYLACR EAGHIKSIKE LITFDRSYKE KDLGKTINKL KKVLPSRAFV YNENISHLIY
SLQLSTDLIE AIEYVVKKAT TLIWTDIERY FKDPELITSE ILFVGLTLCN VFVMYRLFLD
VIPFPIFVTW WQLAQGLLVA YVCGLKVLFV PSIFYCLMLV LSNYLLFKTP CISSYPVLVS
FTVVFHHIIR FIGCGEEYML RWKSIAFLLS AFILGCFDSQ TSGKGVIIWA LLYALFSAVF
RAGFMQKIMH LVEGKGNTLH NNQHMLGVLI LPIFILLSGE WKILGHMPYN ITSLYTGCLV
TVGALPFVKN VVSNRLVRRT GQGPWRFLEI ISIALVFMIG LGYNRPSFMG YLAIVCVIIG
RSLGAFDVLL NKNKVGEDMR NASFALAKSV WAAGDFKGQI IEGIKRPVVT LSLSTNNVAG
VKLPIFQVHI DPTVDVLGNL GVAAGGQVIN NTRENYLQCL NMLVKLASMQ FINMFDWLNI
HSETFYAKIN DDLIATKINL IRDSSTSCKD DNPYCSNNDG KVIIKNNELL SGIICKRVVG
SSSGSLIHIL WHEMGPDKTK DFISALQKVT NNWLEYVGFT VSCSDIIASN KVLDKVKEIL
NKSKNEVTKL VKKAQRGELE CQPGKSLYES FETRVNNELN CAREMAGKVA SESLDEKNNI
FSMVASGSKG SIINISQIIS CVGQQNVEGK RIPFGFNHRS LPHFIKFDYG PESRGFVSNS
YLSGLTPQEV FFHAMGGREG IIDTACKTSE TGYIQRRLIK AMEDVMVQYD RTVRNSYGDI
IQFLYGEDGM AGEYIEDQII DLMKLDNKEI KKLYKYNFDD DSYGDYNKQN ILNQEFEELY
RCKNYLCKEI FSDGDIRQHL PINMNRLIEH AKSQFPNPVE IVQKVNKFLD KLVIIKQINN
SDTLSLEAQN NATILLKAHL RTYLNSKLLI HTHKISLKGI DWLLQEIEKI FYKSLCHPGE
CVGALAAQSI GEPATQMTLN TFHFAGVGSK NVTLGVPRLK ELINIVKNVK TPSTTIYLDD
MISNDQQKAK DILTKLEYTT LKQLTSHAQI IYDPNTTSTI LEEDKLWVNE FYEFPDEDQY
TLGEWVLRVQ LTNIHVNEKK LTMKEIVYII YSVFSSDELD IIYTDDNSED LVLRIRVKYL
EDTFLKKLME ECLSSLKLRG VENITKVYMR EESKITYDSE NGKFVRSSHW VLDTDGCNLE
SIFCAPSVDF KKTISNDIVE IFEVLGIEAV RRALLKELRT VISFDSSYVN YRHLSILCDV
MTQKGYLMSI TRHGINRVDK GPLIKCSFEE TVEILLEAAA FAQVDNLRGI TENIMLGQLC
KIGTGVFDII IDNQKLNDAN QNETIQDITS AGFTTPDSSP LPFSPTYNSN IKNIVMPGNI
RKSEHFLNLM RIVVVYLKKY INIYEVTSEG PLSFLYKCEK DTKLDTSFFK YSFDRLKNLL
NTLQVVDDYA ALNIVCNFCT LIGNYFKGFI IICEPYPEAT IYDPVIQFAC LDSSIAMKSV
LNRYKSIILT SGTITPLELY PKLLNFSTVL TASFPMSFDR NCVCPLIVTK SSDLIPLSSQ
YSLRNDLNVI KNYGFLLVEM CKNIPDGIIS YFPSYIYMEH VMSTWYELGI ISNILEYKLI
FIETKDIVST TIALHNFKKA CDLGKGAVFL SICRGKIAEG IDFDKHYGKC VILFGIPYQY
TLSRILKARL DFLKETYNIQ ENEFLTFDAM RQASQCVGRI IRNKKDYGIM IFSDIRYTRN
DKKGKLPPWI IKCMDVSNTN LTIGAGVNIS KKFLLNMSQE YKETDQTKNQ PLTKITELSN
KMKVATIQNN CEVPTIGLWI SSGSKYENKM NNGVAHFLEH MIFKGTHKRN RIQLEKEIEN
MGAHLNAYTA REQTGYYFKC FKDDVKWCIE LLSDILTNSI FDEQLIEMEK HVILREMEEV
EKSTDEIIFD KLHMTAFRDH PLGYTILGPI ENIKNMKKND ILNYIQKNYT SDRMVLCAVG
NVNHDNIVKL AEQHFKPFFC GSEIIMRDDD SGPNAHVAVA FEGVPWTSSD SITFMLMQCI
IGTYRKNEEG IVPGTINNIS NKMTVGCADY FTSFNTCYNN TGLFSKYLWK ARIYFIWQRL
FSSSSNFMYV LKVHIIDPFN IFKNMNILSE KFKIYILSNY AVYLSFYNYT YAYDKLLDIL
SESSKFYYTF TGRMGIKRKY QKNPATILVL LKDFDPDTDI LEEPYFFDSQ NNLSFDEQIA
LINYCFSIIR FNPHYDEIKF EKLNAIISRC LKYQNYLLHS CILWFKCKCE SFRLKTVDRS
QAQLNELLKE YCNDEPLNKE RLKFIYDIYY PTTWEMKKEV GNIMTKTGSV VSAFNIFKDL
KLWEEAIQCL IEADRKVEAK ELLDTLLEKK KSPPLVCLYG LKYFIKAWDL SNYKYSKAAR
LIGKHYYNKE MYSECCDYLE KALEISPLLP EIWFILGCAY MKIDKFDQAI KAFTRMISMN
LAYLYMKNNV YKAAKICINQ AVKINNNEWK YWDTYLKLSI VQNDVDSFCL ALTTLCQLNQ
VKQIQPWVFD YISDLIVNDK EYDSFWNAYS FFLFVKGEFE DSFEAKIKEI RSIEVSYIWK
NKIFVGNQAP SFKAEAVFGD NTFGEVSLSD FIGKYVLLYF YPLDFTFVCP SEIIALDKAL
DSFKERNVEL LGCSVDSKFT HLAWKKTPLS QGGIGNIKHT LISDISKSIA RSYDVLFNES
VALRAFVLID KQGVVQHLLN NLALGRSVDE ILRLIDALQH HEKYGDVCPA NWQKGKESMK
PSEEGVAKYL SNLMEDTAAK IISHDGLLTD PRIAQDFSAE TNELLTKAEE AIEIIYEIAN
ILNVNLDKET IVILIQLCEY G

> ***Plasmodium chabaudi***
NFDGDFKTTK KKIHWVPYIP DKLIKCTLYE YDHLINKEDW TNCVNQNSKF ETVAYAEPAL
ANLKVSDKLQ FERRGYFIVD LIKIPDGKSK KVGLNTNTLP TIVFPTVVYV GDEAFYHESE
LSIYRPFDHG HISDWDLVNS IWDYAINCVD PNRSVKNILL TEPPLCSISH RKNMGEIFFE
NFGFENINIS VSGLMSIYAA GLTTGLVLDI GEGVTQCIPV FDGYIEKNSV IRSDFGGEEL
TMFMQKLICD IGYNMTTRKC YEYVKIMKET LCFCSLNPPK DQLREDLTAT YTLPDGDVLR
DGYNTIEISH ERFYVPEALF NPLLCHRDNL SIPDIVCKSI LSCPIENRKI LSSNIILSGG
CSLFPNLAER LEREIKNNSP ENARSAVKVV TYEQGASFAR ENNLFFSEAS AVSKLNVKHI
FENLLQMEKS KLAKVEKVLG RTGSRGGVIQ VRAQFMGDSE LSGRFLIRNV KGPVREGDIL
ALLETEREAR RLRGTQSLNL KNSHCYCHLS TGDLLREAAE KQNELGNKIR GIINEGKLVD
NEIVLSLVDD KLKSPQCKKG FILDGYPRNV KQAEDLNKLL DKNKMKLNGV FYFNVPDEVL
VERICGRLIH KPSGRIYHKT LNPPKTPFRD DITNEPLTQR DDDNEAVLKK RLGVFKNETT
PLINYYKNKN LLCYTCYFDL PDPKSTIGPY DNELSYFMWG PGFEWKPFDE KCIGGKISIA
DASYNARKLG LAPSSKDEEK IRDLYGDNLT YDQYLEYLSM SIHDKDNAEQ LVKMFAYFDN
TTGFLTKNQM KNILVTWGDA LTEDEAMNAL NAFSNDDRIR LTKLFGVIDK NQDEISAWFE
YVKNEVFLKQ VQIEMKQIDA DKDGFISLPE LNDAFSQNLK EVEKHADGLL KRFQIVDKDK
DNKLNLNEVG LLIDPMKDND LKELEINEIL EHHDTNKDGK ISKKDDELAL DDFNFDVNKD

GFIDREEIIT FDLWNEKSLK FAVTSLTDYG DVIRYPQDFK LLSNLNCFGA GFIFSIVMFH
LLPEFFFVFL GFCMQLALEY VLPTDTSLCC DSSSSEEEER VIKSSEGKRL HFYETIEDNL
NDSMENDDFN QLLKEYENLY KFMAKEGADR IPNFVIIYLD KLTKYVDTTF QNNVEKKELS
KNKAQTLNKL RAKIRKCSEL YQNKLNQYHE NPDKFWSYSD DAEYASDEED DKTKKAMSKW
GLKTSEKVEK KKVAKKDEKG THADDNQSSK KKTYAELLNT KNLSEDVIRN RVKSVIEKRG
RKGLDKHEHI NILSKLCELA KTISTQSYIE VLEHLINLEF DVVSSVYTYM SFNVWNKTFK
YIELILDLLI QNDHFYLVSI NITEEIAEDE TNEKEKITKS CKTLISFLAK LDDELLKALL
YIDVQTEEYR KRLGKTVHMI SLLYKGYKYV KAIYISTRIL DHLYYKPELP FKQIWGFVEK
TIEQERTEKR RLLSFHMHIS IELIECVNNI CAMLLEVPNL AKHSYESKDI ISRQFRRFLD
IYDKQIFNSP PENNREIIIL ATKYLQKGNW KMCCEKIFSL SIWPKFTDKV QAILTEKIKQ
EAMRTYIFRY ISVYDSFSID QLCVMFDLPQ NTVHSILSKM MVNHEIPACW NESSKYILIN
KVNPTPLQTM ALKLAENINE VMEQNELALN MKNPKLGLQN KQCIVVGTRA LEFLNNELST
IKTLTELHDR VKKRGITDNN DNMLSLYLLE LFSIPIEQKE KNDFLTNRIS MYSKILNGRK
NVLDLLLNKF ENDCSDSIKK ELIDCFDVEK FKQEVNSKFM NILIQQLRKV EKIEKKKAKM
EIYLSIREQV KLYIRELVNI ISNLLTGNYP ILNLNGDDFL KKYGGTLMEN LKDGLKVGQA
VRFLLAKEES SMFGVVQNIP NDARSKNILV NFFFRSNNVE EQIQVKSVEK ERLPVESLNS
DLFFLWYKMT RDDGWVGFDK AEIIKVFNNN SKIKDVLIRH TDLKILNQLA ITQFWAIKML
ISVPFATKIY AHFVHDSIKI SILTVLGDIA TALNKSFSKY LNFFANILLE TSKITIASGS
PENDDWVSYV FELRDAILLT YSNIIYALID GNEINKLKMY IPNILDFIEL ILIKEINHFN
AQNFQNSVSL LGDLVHAYGY ELIENSKLTD LIISVYGKID ILSSQRDECV SKIKWLKKIC
NISILQLEFK SVEDLNNATN NFIKAIKNYN VFPELRLKIL QLLYNSFNVN FSFFIAILQF
SSQNNIFHAI LPYIKSIDDW IKEWNISNHE KRQIYLIVAE ELKKLKKFEE SYKHLKKHVY
YFQEILNHAS TIKASVELVV DAINLNNNIY FHEIINLDAI QNLQYVEHKP IYELLTIFYK
YNIHEFLEFI DLESSENKIY LLSIISLFKD NKVQNIQYIS EQLNISMLKV EQILVSAIGS
DIIDAKIDQI NKTVHMKTTI LRHFDEQQWE HLNTQIAKYI KNVATNFYKF IDSFASSTRA
IQNNPSYWCS AGNHLKDEEI TWTGYLNTKG FVKGVKISWE YSPELVSIFV SSDGEHYKNV
IPYKKISSTE SSFDEIYFFK KLEEVTSIKI GLKNAIHKYF GIREVKIIGG GNPYFLLLSG
ITSEQEMCLQ ELWKTNSNNQ IISALSDPPK CLAVINLDNL GDGKSNWVFE SNSQIRLQLC
ISQKNIHGNI PGIHDIDASV DATSILDDDH SADNTIDGDL NSFWASSIFG DNEHLVYFVI
DLNKFVEISR VKVFWEYPPL HYIISFSTEK IVAENLANPS FTTIDSLKNI ETRYIKISMV
KPHPKHGEMD GQFLYGIRSI EVQANNLESV LNFCRDAANS DDARDKYFIE YISEFDKNLS
NKLINLEDDV SKNVSSISDK LSKLEEVLPN IETCLNEKKE YDTKLKASMD QVIELIKPKC
SPEPLRVYCD MDSSTSLYVW NGINSVDDIR KHCAEVGLEP LVLKSTDQLN SLIVALKKMG
FILNGKINIP LAYDYSCFHD LLNGNVDLTT LIYESPNTTT IRQTALEEKK MLLCRVEDIA
KKRNRIHVKT QILSEDSKIK RHKMKKVYNY KFGYFGCGWK TEWTPFIHAP FFDNQHNTIY
KSRNKKLYEE IDTILHGRIH PHIKVVELKD HMHPIRLCTP SNEDCYSVIY TGEKINATDE
RVIFGEYTGF VNNKELPQEK HQYIFALTFI ILPDNYTYAV DSSHMFNEMS LVNHYKTCFN
NYDFRINAEW QIVYLDGWPH IILTSIPGVE IETGEEIFAD FGFEWFDRVN DICLNDFIKN
NYEHRLDDIV DKYNLLKNYT TCNICMHSVN TDCNNYILCS GCNHVYHLKC VNRLNNENYD
WFCSSCIQFS MNIAKAVICR VTKLHFETNE ELHKMSSECI QSVIKELALG KTKFQREFTN
GTYVGTVTKQ INDNNFFVVT YEDGDVEWIT PFFLFQEEHK LKGNELYKQK KFEEALKEYD
EAIKVNPNDI MYYYNKAAVY LEMKSYEKSI ETCIYAIENR YNFKADFSQV AKVYNRLAIG
YINIKDYDKA LEAYRKSLVE DNNRATRNAL KELERKKEKE EREAYIDPVK AEEHKNKGNE
YFKNNDFPNA KKEYDEAIRR NPNDAKLYSN RAAALTKLIE YPSALEDVMK AIELDPKFVK
AYTRKGNLHF FMKDYYKAIQ AYNKGLELDP NNKECTEGYQ RCVYKIDEMS KEKVDEEQIK
KSMADPEIQQ IISDPQFQII LQKINENPNS ISEYIKDPKI FNGLQKLIAA GILFKPREMI
PSKYGENRHW NVDLIPKFIL VGGNLVKILK KTRVTNYLEW LVVEGSYVYQ HQKKSLLFSE
KFIHKVPSTD MEALVSPLLS LMEKNRCKNF YQYVSEWNAN DKSTWDNLDP YRLSMMDIYK
YFNLCQLTID FLGHAVALYL NDDYLKQPAY ITLERIKLYM HSISAFGKSP FIYPLYGLGG
IPEGFSRMCA INGGTFMLNK NVTDFIYNDQ VCGIKSSDGE AYCDKVICDP SYVHLENKIQ
KIGQVIRCIC ILSNPIPETN DINSCQIIIP QNQLNRKSDI YVNLVSFQHG VSYKGKYDVR
LDVKLNKFIW SKGIRNPPKR VRVKIERVRN EDEDSKERMY TLVQHVMVDS FKGLVNEQMT
GDRHRIQDCT TENFSGFIAI TTPTKSWIAQ YNDLSKFNPG FYALQVVGEL GTGSGYLILF
LYELLLKKNK KVDLLYCIDI NKDANAISNV EIINNNLFNN LRTCFDIILF NPPYVETEQD
EMNKTIVASY AGGKHGREVI LKFLHTVYDY LSNNGILYLL LEKNNIPHEI ENSDLPILCE
TCLGENPYVR IIREENGKEC QICKNAFTLF RWKPGHNARY KQTIICNKCA KVKNVCQTCL
FDLEYNLPVQ VRDKFLETSI ALPENETNRN FFLEQLELSK LKRKDPYFKR NMARVCSFWR
KNACNRGDEC PYLHKEIHKL IIFNLPPVDE QDVKSLCERY GPVIDVYAFV SFVFPSSCEK
AKNNLNETIY KGKILSVKYA SYKKILEIQK KRNCQNENIW NILYTDINSN IYNFCKETNC
DPQSILDKNI AVNVSLTETF IINKMKEWIR KEGIRSDDTI IVKNLSMHTN ENDIINLFKK
HGILKKISFS PYKNIAILQY EKPEDAKKAL ISNSYIRYKK LPLYLEWAPV NLFDEEITHS
SIYIKNINFN TKEEDFKKLF EKLDGFITCN ISLGYGFAEF KSKELAIEAI KKLTATKLDG
HVLELSLSKL LVKNLAFQVT KEELRKLFSA FGNIKNVRIP KNAYNRSRGY GFVEFMSKNE
CLAAINALQH THLYGRHLII DFAPENAKML KPLVQEKIIE VMKPEIEEKI IEVPQVQYIE
KLVEVPHVIL QEKLIHVPKP VIHERIKKCP KTIFQEKIVE VPQIKIVDKI IEVPQYVYQE
KIIQVPKVMV QERIIPVPKK PQYRHIPKPV EVPMAHYRTF PVEKLVDRNV PVPVEIQIVQ
EFLCPKIEAR YKEIPVPVHV QRIIEHPIPK DAMNNPLLLP LYYLGSTAIG ICVDDGVILA
SERRIASPLI EKDSIEKLLP IDDHIGCAMS GLMADARTLI DHARVECNHY KFVYNENINI
KSCVELISEL ALDFSNLSDS KRKKIMSRPF GVALLIGGVD KNGPCLWYTE PSGTNTRFLA
ASIGSAQEGA ELLLQENYNK NMSFQEAEIL ALTVLRQVME DKLSSSNVEI AAIKDQTFYK
YKPDDITKII DSLPRFKIID ADFINIKLLE LFQINGFQKQ LDRLSDSLSK IQKALGEYLE
KQRNQFPRFE KLVQTKLTDS CFLTLTQALK MKLGGNPFGP AGTGKTESVK ALGAQLGRYV
LVFNCDESFD FTAMGRIFVG LCQVGAWGCF DEFNRLEERI LSAVSEQIVE ILNKKVELNK
NVGIFVTMNP GYAGRSNLPY GVGFKTAFAR IASSCAIMSR TINTIGIGLL SLELMNHCDA
KELATPLCMW KLPNKELINR NIANKSEHRH HQKLLMSYTP FNSPSLLAEQ INILGTYSGT
RLLYFPLWDH PKDSIDYCLS TYLYWLYLRR STNIFLQNTL LRGQVVVIAA TNRQNSIDPA
LRRFGRFDRE IDIGVPDDNG RFEILRIHTK NMKLSPDVKL EELASNTHGF VGADLAQLCT
EAALTCIREK MDVIDLEDEI IDKEVLESMC VTQDHFNMAL GTCNPSSLRE TVVEVPNVKW

DDIGGLDEVK NTLREMILYP IDHIMKSLYD HNFFSPREIQ SKTLEHSING KKDIIVVSKT
GTGKTLTFCL PILTNILILV PTRELAVQIL NHFNYVNKYV NIYIITIIGG LNINKQLRLI
SKKPEIVICT PGRLRYFVCD EVDKMIETSF INDIHFISKH LYIQTFLLSA TLLAKLLNYV
SIRKDKSYII DLLPDHLKLN IIKCEKKIIL HKLYYLLKLY KIIIFLNTIK LVKDVSTIFK
YLFFEPGLES SIPNKIYSIH SKQTLKERIQ SVSKFSNNSS ILFCTDVLSR GIDLNKCDLI
IQLNCPISDI TFIHRSGRTA RNLKAGNSAK CSEIKSAELV LSSQELKRIS DPRFGTIDYS
QICSVCFENC LGHVGHIEFM LPVFNPLFYK DLQELLNLVC YNCYALCYSY NYEEYLILNK
AIKIHMKKGA NCSYCKFRRS ITVKTSPKKD TITVRLFSFQ IVDLLKKIFN KDIINLLYPF
TKKDGCQVFF LYDMGISANR FRTQFRGIHK RNNFVKLCIN SKKNIDFDYM IELQLAVNTF
FDIKEILDKK EGVVRKNIMG KRVNNCARTV ISPDTFIETN QIGVPIEFAK TLTIDEHITE
NNFEYIKKLS LYDFILKKKA DFRHLSFISD FMTGALCMDI LKTNWSPAWT IQSLCRAILF
LLNEPNAESP LNCDAGNLIR GVKVKSAAEV GKRAVEYFRG DDFAAFLSSN QDVLKKKFPK
LIGNRNLSEI KDIEEFGDLF IQRGFIYKAQ YKPENGVYKR PKWPKRLIMT AKQNFDKAGF
YILVYERNRK LQYFMLMTLI SIVLICCMFW HLSVVFITFL SAIIVGRLVA FLYFWFFGID
YWIFPNLFDE ECSIVESFIP FHSWERRNDS WILVFARMVT AVLVAIGIHQ LGKTHSISDI
QNFAKQSFID IIEWGNKKLS EENYDCLKKC GFPPFEELVR RCFLKCDCMT LADTLLKDLE
DLEEEERKIS ELLYDIEKCI ELIIKIDTEI LNIHKYLKDI YSTKFPELDS IVYTPLEYIS
VVSRIKNESD IKNIDFSDIL PNTTVMAIVV ASSTTGIKLP DHLLKSCMSF CDEALELNEN
RQKILIYLEN KMFLLAPNLT MLLGSALTAR LISCVGSLKN LSVTSSQNLI VVGNSKKGIL
STSEIVQSVP DAYKKKAISL LAGKCSLASR IDYFTEERTV LDKNILNSSV ILQIALRVKK
DRYLGRHYRY FIRNTRVRAY KQFLEPFKSV TLKNMAYAFG VSEDFIEVED PYEAIGEAVR
NFENKDEILA SAKIIERLVE YPEVAKNLDK INALDPLLKL LNNHILESVL QIFSLALSNN
PVLQDCVFKK NGLKILLLKL QESKQTSVDK KLITAISALI RHHDEGENKF IDYGGIAFLV
YGMQTNIYKY QEKSALLLKH LIHQNKITFE IFEKNKVMNG LIALTNTGIQ YGETTAELFL
ALMQNHRHKL AKGGLKQLKE LIEGRLNYLF EDLGVDDWLI KISKSVQILK PTKIQKLCLP
LIIEGKNVIG SSETGTGKTI CYCWSMLQEL NKNFYAIFGL ILLPTRELVF QIVEQFQLYG
NKIGIKILSC IGGFSLIDQP HIVVGTPGRF KRLKFLVLDE ADLLLQKSFE DKLKIILNNI
PRRTLFFSST ITDSINLLTK AFPNDKLVLV NANKKQKPVK NLDQRYGIIF TANSYKCELI
YTVLNELFSI ESIHSSKDQR KRMSSLLKFK NGLCKILVAT DIISRGIDIP KVAFVINFDF
PNDTIQYIHR VGRTARANRK GISISFVDKR DLKSFNNVKL IMKEKLKPYI LNKNEVLSDM
LKIGKVVKKA EMMLQEVVVF PVSVHYDLAR NLLNKKIGTG IQNVSKFGNG SYTGEVSAEI
AKNLDIEYVL IGHFERRKYF HETDEDVREK LQQAIKHNLK AVVCFGESLE QRESNKTIEV
ITKQVKAFVD LIENFDNVIL AYEPIWAIGT GKTATPEQAQ EVHKEIRKIV KEICGANKIR
ILYGGSVSVE NCTSLIKQED IDGFLVGTSS LKTSFTEIIK SAKDINKDVV HRYGPNTFKL
HRLPVPKLGQ ILGLVGTNGI GKSTALKILS SKLKPNLGKF DSPPEWRDIL AFFRGSELQI
FFTKLLEEQL SPIIKPQNVD LIPKQVKGNI LEIINKKDKL NQKDKYIKVL ELDHLLDRNV
EDLSGGELQR FALLISIIQT TNVYMFDEPS SYLDIKQRIS MAKIIHGLVR HDNYIIVVEH
DLSILDYLSD YVCCLWGKAG AYGVVTSPFS VREGINVFLD GFIPTDNLRI REESLNFKLE
DKKRLHFYTY PKMVKTLSSF TLTVDKGNFS ESEIFVLLGQ NGSGKSTFIR LFAGLIKPDN
VEFLESLSVS YKPQQIQAKF TGTVRQLLMS KLKGLYTDPY FNNEIIKPLK IDGILDNQVL
TLSGGELQKV AIIITLAKNT NIYLIMSILC TISGQTPEEP VVSKTGYIFE KRLIEKHIKN
YGICPVSGEV LTLDDLYPIK IEKFVKPRPI TATSIPGLLS IFQTEWDSMI SEMFTLRTHV
NDVRNQLSHC LYQYDAATRV IAKLLKEKNN YQEEINNLRN QILQLKNGND IDDLEMGISE
DLLNEMQNIA KDLLMNRKKR KVENVNSPND WKKITSTNEF NIHSSVIPGV TCLAIDFSGG
KDGNIYYVSL NNNKIISKLQ GHLKKVNSII SHPSNSICIS GSNDKTIRVW KGDKHKDNVH
SLSLHPLENY FISSSKDSIW ILHDMETGKT IKTCKSSPSF KNLSIHPDGM MFGIGSEDSN
IYIYDIKSQE YKASLFSENG YYLASISKDN TLKLWDLRKA TSFQTIELED TPKHITFYNG
NIKDGLFHGY GVLVYSKNEK YEGDFAYGRR EGKGKFTYAD GATYEGEWMD DKIHGKGVAH
FVSGNIYEGE WENGKISGFG ILNYSNGDKY EGEWSEGKMH GRGTYIYADG DIYVGEWKND
KRHGKGCVKY KGSKDKIAET YDGDWYEGKM QGKGVYSFAD GGIYEGDWVD GKMEGKGIYK
YLNGNKYDGD WSNDMKNGYG VLTYANGEMY EGYWKDDKVH GKGTLTYSKG DKYIGDWEFA
KKCGEGELIY SSGDKFKGKW KNDKANGFGV LYSNGNKYKG EWVNDQRHGF GVFTCKEDGS
VYSGQFSHNR KEGHGTLTFS NGTIVEGIWN LGVLTKVTKF QLYPSSPWND PDLEMETLYD
LGNKMIEALQ KENITAGDVI CIDKSTGKIT KIGKSFARSK DYDAMDPNTH FVQCPEGELQ
KRKEVVHTVT LHDIDAINSR TQGFLALFSG DTGEIKNEIR EHIDMKINEW QEDEKAEIVP
GVLFIDEVHM LDIECFSYLN RALESEQSPI VIMATNRGIT HIRGTDYKAP HGIPLDLLDR
TLIIPTYPYK HEDIMKILEQ RAEEEDVEID EFAKELLCKI ASESSLRYSL HLITLANLVA
KKRKATEVTV QDVRRVYNLF IDVKRSTQYL IEYQNEFMFS ELYSNLSDFW TSDDEEDGEY
IRKKWVIEDD VSNFSKNDLL LSYDFELDNF QKRAVKHINN FKHVFIAAHT SAGKTLIAEH
AIALSIKLNK KAIYTSPIKA LSNQKYYEFK NIFKNVGIIT GDVKMNVNAN CLIMTTEILR
NLLYLNDNII NNIHCVIFDE VHYVNDEFRG VIWEESIIML PPHVQIVLLS ATVPNYLQFA
DWVGFTKQKE VIAISTKKRP IPLLHYIYAH DSLFLIMDEK NFYSSAFKEI YEANMKTEIQ
KLQALIKKLD EDNKLPVVLF CFSRIKCETY AKSMPHLNFL DNKKKSKVHL FIKESASKLC
DQDRDLNQIK ILSKLLENGI GVHHSGLLPI LKEIVEILFS KGLIKVLFAT ETFAMGINMP
AKSVIFTSIY KHDHLKKRIL TSSEYTQMSG RAGRRSSDTH GYVYIYCSDN IPDQVQLTEM
MMQKAVSLKS KFKVTYNMIL KLLINKQINI EKMLFSSFLE SCRALQIPLF KKDLKRKKKM
LQNIKQVECV YIENYVYIDH KLKNIGLNLH KKLVGIENIS LITNELDRLI KKETFEPFTL
TKMLKSLKCE FYSVLHYELV CKKNDCINDI ENIERNINAK SLNLYEDLEG KLNVLKHFSF
IDDDNNLTIK GKIASYITLT DEITLTQVIF ENVLNNLNPP EIAAVLSCFV SPEKKVEEAP
DLTLNLQDVK LALTNIHSKF EEFYRVIRLK ISTEEHWKLC NFKLMFIAYK WALGVSFSEL
LEQSEFEEGL IVRSIQRLDN LCRKVRIAFL YLGNVDLAEK TEKASLLLRR DIVFTTSLYL
DGLALLQFFH WCDEKRKTKE LFKETEISLR NKIDYFRSTK KNFPSFSTIS AIGPNSAVIH
YESTEETNAK ITPSIYLLDS GGQYLYGTTD VTRTTHFGEP NADEKKLYTL VLKGHLSLRK
VIFASYTNSM ALDFLARQPL YNHFLDYNHG TGHGVGICLN VHEGGCSISP AAGTPLKESM
VLSNEPGYYW ADHFGIRIEN MQFVVTKKQT DDTTFLTFND LTLYPYEKKL LDYSLLTPQE
IADINEYHLT IRNTLLPRIK ENPKKNFVIK ESPDFIKYRL DKFNELKEKI NIELLDGSIK
IGQKNVTTPY QIASQISKKL SENSIVAKVI YLDDVNLNLC DLWDLNVPLI GNCKINFFWH
SSAHILGSSL EKIYGGYLTI GPALNEGFYY DIYLGNNSIV SDDYTKIENE YNKLVKENVE

FEKMVCTKEE VLELFKYNPF KIELIKSKIN DNEKTSVYKC GNFIDLCLGP HIKNTGKSKA
FQVLKNSSAY WLGDKNNDSL QRVYGISFQK KTELTDYIKF IEEAKKRDHR NVGKNLNLFF
FEKETSPGSG FWFTHGAKIY NKLIEFMRKE YRIRKYEEVI TPNIFSCDLW KTSGHYQNYK
NCMFIFNIEN KEWGMKPMNC PGHCLIFKQL NASYKSLPIR LADFGVLHRN EITGSLSGLT
RVRRFQQDDA HIFCSLDHIK TEVINVLQFI FYVYNLFGFG IEILQNRGYD SCGMSTILKT
TKYASSSATN AIEKLRGNYM TSHKNDNIGI AHTRWATHGS KTDENAHPHV DYKERISLVH
NGMIENYREL KKFLVQKNIP FKSNTDTEVV ANLIGYFLDQ KQSFQDAVVS SIKQLEGTWS
FCIIHKDFPD EMILAANGSP LHIGIKDNEM FVASEHSALF AFTNEYISLK NGEIMSINKN
NINNLKLIKK FDNIPEIVIQ KTPDPYPHWT IKEIHEQSIS LSKSLNNFNL QNNTVKLGGL
DPYVDELKNI ENIILIGCGT SYYAALFCKY IMNYLHCFNT VQVMDPSDFN ISSIPKEKEG
IIFISQSGET RDIIKACKLA EYFNLKKLSV INSVGSTIAN MTGRGVYLNA GREVGVASTK
CFTSEVSVLT LIALWFFQNK SNNKVSSLIN SLYRLPLYAD TTIKCEDTCK ALSHKLSNKS
MFIIGNGLSY PIALEGALKI KEIAYIHCEG STGNALKHGP YALLGGNDNI PVIMLIFNDK
NSMISIGEQI KSRGAHIICL TDDANLCNDD IILIPNNGML TSLLAVIPLQ MLAYYMSVNL
GHDPDKPRSL AKTVTVWIIE IEKYASEDVQ KILIGNKIDL KNDRNVSYEE GKELAESCNI
QFLETSAKIS HNVEQAFKTM AYEIKNKSQL ENQQKGRTNI NLNAKPIKIR TMNSKKPPEG
WNKVEAFLNE MNQKMRSLEN EDTSKKRKNE ILWPIFQINH QTARYIYELY YKRKEISYDY
IVIGGGPGGM ASAKEAASHG AKVLLFDFVK PSTQGTKWGI GGTCVNVGCV PKKLMHYAGN
MGKNDSEKYG WDCDNKHDWN KLVSTVQSHI RSLNFSYMVG LKSKVKYING LAKLKDKNTV
SYYLKGKEEC VTGKYILVAT GCRPNIPDDV IGAKELSITS DDIFSLKRCP GKTLVVGASY
VALECAGFLN SLGYDVTISV RSIILRGFDQ QCANKIKLYM EEQGVTFLPK KLTKENDKIL
VHFNNDTTEV FDTVLYAIGR KGDIDGLNLS CTNIPNIFAV GDIAENVPEL APVAIKAGEI
LARRLFKNSN EIMKYDFIPT SIYTPIEYGS CGYSEEKAYE IFGNIEVFLQ EFNNLEISAV
HRIKQKDEYD VDISSTCFSK LVCLKDNRVV GFHYVGPNAG EVTQGMALAL KLNAKKSDFD
NCIGIHPTDA ESFMNLTITL SSGLSYAAKG GCGGGKCGEH LYAGPLKIEQ LLAKGFVKRD
LELLKEGGLQ TVECVAYAPM RTLCSIKGIS EQKAEKLKKA CKELCNSGFC NAIDYHDARQ
NLIKFTTGSK QLDALLKGGI ETGGITELFG EFRTGKSQLC HTLAITCQLP IEQSGGEGKC
LWIDTEGTFR PERIVAIAKR YGLHPTDCLN NIAYAKAYNC DHQTELLIDA SAMMADTRFA
LLIVDSATAL YRSEYTGRGE LANRQSHLCR FLRGLQRIAD IYGVAVIITN QVVAKVDAMS
MFGHEKLPIG GNIIAHASQT RLYLRKGRGE SRICKIYDSP VLPEGEAVFA ITEGGIADYM
AIRVQFENSN EVGVFSRLTN SYGLIALGGS ENFSSVFEAE LSQHIPIVYA TIGGTRVIGR
VCVGNRKGLL VSSICTDQEL LHLRNSLPDD VKIKRVEERL SALGNCITCN DYVGLIHTDI
DRETEEIVQD VLDIEVFRTS IAGNLLVGTY SYFTNNGGLL HAMTTSQEIE ELSELLQIPL
ITGTINRGSD LIGSGLVAND WSAFCGMDTT AIELNIIEKI FKLNNIEDTN IEDTFKYKSS
IVQTMIIVDT YWQTETGGIV IAPIPHLFKM KPGSASLPFF GIQLEILNSK TLEPLNGPNC
GILCIKSSWP GMLRTVYGNH NRLIKTYFEP CPNYYFTGDG AYRDEDGYYW ISGRIDDTLN
VSGHRLGAAE IEHALVQHSC ISESAVVSFS HKVKGEGILC FVVKKLKQYV RKVIGPIATP
DIICIVPDLP KTRSGKIIRR ILRAIAIGVN DYGDISTVSN YDVIEIIPFP TPCTIEEALQ
CYCDLSTIPR VNVLNNFKCF IKDIEEKEFD MTFIEFVDIF MQSAIFELVP FLQLIPKIAP
KSYTISSSPK RWYKGSSSYY LTELYPNDIL KFNVKTSIFG IDFLYEKEID ALENKYIDEV
YLAFSRDQPW VEKYRPKKLD DIVHQTNAIS MLKEVIKTKN MPHLIFHGPP GTGKTSAINA
LAHELFGKEN ISERVLELNA SDDRGITVVR EKIKAYTRIS ISKNKINNET NEPLPPWKLV
VLDEADMMTE DAQSALRRII EIYSNVTRFI LICNYIHKIS DPIYSRCSCY RFQGIPLNIK
KEKLLYICNN ENINISDKII ETTQGDLRRA VSVLQLCSCI DSKITVESVL DISGLPDNDI
ILKIVDSCKV KDKILEKTIQ DIIEDGFDVS YIFKALNEYF VDSIKYQILM ELSRHDFRLH
NGATKYIQLM SFASSVHSLL IKELIFKSND EKHLQFVVKQ VKELIKQVKQ KEVEDVNDSK
TSNEKLALNK SGRRIVLRDL MTRPNIFTGR KILGTLELHT NGLRYSAIDI LFDDIKHAFY
QPCDGQLIIL IHFHLKRYIM VGKKKTLDVQ FYCEVGTQID DLDRAKARNV YDPDEMHDEM
KEREQKNKLN LIFKNFVQQM QDISKIEFEI PYPELTFSGV PNKSNVEIFV TANTINHLIE
WPPFILSVED IEIASLERVH HGLRNFDMIF VFKDYTKPVK RIDVIPIEYI DTIKKWLTTI
DIVLKTILAD IESFVNSKGF DGFLGEDDDE EEDEDEDDEY EVDESELSAE IDILATIEFT
SKRKMSTIVC RIPKIVVFCK GAGCVIIKKL ANKTDVDDLT IEHMETYADE GLRTLCIAYK
ELSPKEFAIW YNLYKEASLS LNGREENIEK IAGGIENDLI LQGVTGIEDK LQEGVGSTIE
DLRLAGIHVW MLTGDKIETA INIGIATNLI DNGSVDGSVL DILLSKPFER KFFYLADKCS
SVICGRVSPY QKGFIVSSAN RLLRKNTLAI GDGANDCNMI KMANIGIGIR GQEGVQAFNS
SDYGISQFRF LRNLILVHGR LSYRRISKLV VYMFYKNIVF IFPLFIYGAI SLYSGQKIYF
EILLHSYNVL FTSLPIIILA ILDKDVSINT ALKNPCLYKL GIHNFYFNIN KFISWVLNSL
FHGLLVFSIP LYFLIPSSTG EPFDLWSIGC VTYLSVVIVN IKILLETYYL NTSPIIGVSF
SIISFIVTAI AFSFTGIGNK SFLGVATLLA TSLRFWLVFV LGLFALTRDY VYKVYKKNFC
PRNYSDNVKD HFNKPRNVGS FDKNEKNVGT SIVGKASCGD VIKLQLKIED DVIKDARFMA
FGCGSAIASS SYATELIKGK TIDEALKIKN NDIASHLNLP PVKVVILGSG WGGIHFLLNI
DFQKYDVTLV SPRNYFTFTP LLPCLCSGTL NVDACSERID ILLKKNNISG KYLKLECTDI
VYKDKYIKCK ENNEIKIKYD YLIISVGAKT NSFIKGVDKY AFYIKDVIDA LKIRKKFISN
LEACNGNTTN DEFVKNMLHV VVVGGGPTGV EVAAELADFV NNKNKYKQIY KYISITIVEG
GNNLLPTFTQ NISNFTKDNF KKLNINVYTN YHVTEIDENH FYIKSKKIPY GMIIWASGLA
QTPLINNFIK KIPEQVNNRI LNVNQHLKVI GIDVYAIGDC KKIEPVQITA EQLIKEALDF
EEVEKKVNYN LIDEDELNEY KISKRKEFED SIRKRRYLIN TYIKYALWEI KQKDIKRCRS
IFERALNIDY TNKNLWLKYI EVELTNKNIN SARNLLERVV LLLPLENIFW KKYAHLEEIL
NNFVNARNIY ERWVKWKIDE TAFLCYINFE ERCKEINKCR EIFERLIVSI PKLECFYRFI
KFEKKYKNIS RAEKCIELLP SQFLDQHFYI HFSKFEEENN EYERCRKIYI EALKRLPREN
SDILYKNFLQ FQKKYEELDQ TLLYNERINF EEALKKTPND YDIWFNYIKL EERIRELYER
AISVIKNALV HDGLKIGIRE VIKSIESKEA KVCFLSNVCS EPAYKKLVTA LCAEKQIPLF
MIDSKDLGQW SGLFKVDKEG NARKIIGASS VAVIDFGEES AERDFLGYYK AIVGEVIDNR
YSVVCELVGK GVFSNVLKCY DMTNKMHVAI KVIRDNDMMH KAAEKEISIL KKLNKKHIIR
LLRSVKYKNH LCLIFEWMWG NLRIALKIKD SFSSLILKAK ELEDVYISRR KLQSKYLTQI
KNLYINSNCE CIIHKISFKY ASKSSFPNLL NGTLLYMIIE KINLDNNVVA SCINSADVKS
WINYENYLGE LVDGFLFSVN ISYAKSLIGD KCYILDLIDI AYEIAIGHNG GALGYSQHLS
EEIMLFSKEA IHDKIAVILG GRAAEELFIG KITTGAIDDL NKVTQLAYSY VSQYGMNKEI

GLVSFQQNGG SEYSFYRPHS ECLAHLIDNE ARNLIESQYN RVKAILKKNE KHVHNLANLL
YEKETISYHD IVKCVGLKHQ RYREADKVKI EEERNRKVFV KNTSFCKNCG SAAHTEKYCL
ERTRKKGYDG NRDRWVGYDP NNFDHIYREY EKIVEEQKKR KAEKLIDKKN NKIKVLSKYE
EDIYISDHTS VFGSYYDRKT NKWGYKCCQR TDKFQNCIIP LMDAYHANYY TKEVMIILIN
EFNSPDEEMK KIVLKCVKQC IQTEGIEKDY INQEIVNPFF EKFWVIRNSS DKKNFNLIVD
TTVEIANKIG VIAKIVDDLK DPSEPYRKMV MQTIQNIINN LGVDDIDQKL EEQLIDGILY
SFQEQTSDDY YVLLNSFDVI VNKLKLRMKP YLPQIAGIIR WRLNTPLPKV RQQSAELIAR
IAKLIKICDE QQMLGHLALY LYEYLGEEYP EVLGNILKAL KSIVVVLGVN NMTPPIKDLL
PRITPILKNR HEKVQENVID LIGIIADKGG DMVSPKEWDR ICFDLIELLK SNKKLIRRAT
IQTFGYIART IGPFEVLTVL LNNLRVQERQ LRVCTTVAIA IVADTCLPYS VLAALMNEYK
TQDLNVQNGV LKALSFMFEY IGEIAKDYVY SVISLLEHAL TDRDLVHRIA TWACKHLALG
CFGLNREDAL IHLLNHVWPN IFETSPHLIQ AVIDSIDGFR VALGPAIIFQ YLVQGIFHPS
KKVREIYWKI YNNVYIGHQD SLVPIYPPFE TMGDSNFARD ELRYMGRMYG KGKGISCSTI
PYKRKQPSWL KQKPSEIEDA IIKLAKKGQT PSQIGATLRD NYGVPQVKAV TGNKILRILR
AHGVATTIPE DLYFLIKKAV SMRKHLEKNK KDKDCKFRLI LTESKIHRIS RYYKRKKLLP
SNWKYQSSTA SALRNIEHAP GVQFSYVPPD FFDSDDDKSD KNQYELKDDG GGRAAGTRGK
EHSSSHHLRR KNYEDDFEFN EDKILEALHI LEFLYLNGVS LEEQNEYGQT ALFLGVKKNN
ISILQWLLSK NVNINHVDFY GNTILHIAVR YTDIDILRLL CDYGCLNLVY YSTFENNNTN
VFQLCINNRY FLVYILLKKW LLQNKICKGL KICKTIYAFY FWFFALLNLI VYINIAHSFL
QIQTHHNKSV IWISLWLFQQ LLWCVLYFKN PGEYQLNNIE RELFQINLRY SKLSLYSQVS
QERINSLDVN YRNAILEIIL LQLIIEPYIL RRSKKHVFID MPKKHSIIIK LPLNNTQLDL
YKDEILSKLQ HTHKHLINAS IFILRRICNH PLLHKYYYSV DDIKKISKYF YNNTDQYLDL
DLKTVENEFM KISDFDIHLS IKHLISQDNN LNKYLIDKKH ILNSTKIHHM LTLIKNIKEK
KEKVLIFSQY TTFLDIIEES LYVRLDGSTN TIERQQIIKE FSNVFIFLLS TKAGGVGLNL
IAANHVILMD QWNQLLLQEE IVKKLCEYII DSRCDIVEKG VSDLAQHFLV KKNISVIRRV
RKTDLNRLER ITGATIVNRC DEIVEKDIGT KCGLFEIKKI GDDYYSFFVE CENPRACTIL
LRGATKDVLN EVERNLHDGM NVAKNIMLEG KLLYGGGCTE MRVSQHLIKQ AANFDDSRKS
IIESVASAFE IIPKILAQNS GVNVVKCINE LRTKHESEKL GIDGVTGEII DVSSKNIWDL
LSVKKQIYKS AIEAASMILR IDDLGVVLIY EDLLKNPLCR ISNIFEKEVK KYRPFPLNTL
QMTKLVSKYF HISSKECMNI AEKLYNKGYI SYPRTETNYF VDSMNLRKFI HELKKNNIFG
SYAAKLAENG PRKGKLNDKA HPPIHPVKNM EWKIYEFICR HFLAVCSDDA IGFDTKVVAN
IGAEQFYCKG LKIKNKNYLE IYIYEKWNDK ILPPFQINDE FYPYSLIVEE GITQPPKYLS
ESDLLSLMDK YGIGTDATMH EHIENIQKRN YVYKNSKNLF IPTKLGIALI LSYKKFKDIG
VDLTEPSLRA KMERDMFLVA SGICVIKSGK MDIPRNTKMI IISYELITKN DKYQKYKCIV
CDESHYLKNS FSKRTKAIVP IIKSAKRCVL LSGTPALNKP SELYEQVSSI IPNLFNYNEF
CDRYCYKDKN IYTRKIEYVG CKHTEELHLF LTNTIMIRRL KKDVLKELPD KLRSKIPIEI
PQNELSEILL FKMTGYAKVK AIKEYITYLI DADIKFLLFC HHKLVMDEID EFLKEKKLGF
IRVDGLTPID KREVYIKNFQ SDEKIRIAIL SITACGVGLN LTAANTVVFG ELYWVPGQMI
QAEDRAHRIG TTHDTINIHY LVAQNTIDEV VWKIINRKWN TLTTALNGAE DSLMFEDNDK
ICIAVSGGKD SSVLTHVLVN IKKKYNYNWN LFLLAIDEGI KGYRDDSLKV VFQDIFSYTM
DDVVSYIGKK NNCTVCGVFR RQAMEKGALL FNATKLVTGH NADDLAETIL MNMCRGDIDK
LFSTECTYSP NSFRGNLRSF IKDLECIKCG AYTSNVDGLN NYTDNKQSSN CTFEIDIHYE
NIEPMPLENE YQKIPKLRIL SFDIECIKLD GKGFPEAKAD PIIQISSILY LQGDPCAKFI
FTLLECASIP GSNVIWFNDE KTMLEAWNEF IIRIDPDFLT GYNIINFDIP YILNRGTALN
LKKLKYIGRI KNVPSIVKDA NFSSKQFGTH ETKEININGR IQFDVYDLIK RDYRLKSYTL
NYVSFEFLKE QKEDVHYSIM NDLQNENSES RKRIATYCIK DGLLPLRLID KLLFIYNYVE
MARVTGTPFV YLLTRGQQIK VTSQLYRKCK ELNYIIPSTY MKVNSNDKFE GATVLEPIKG
YYIEPISTLD FASLYPSIMI AHNLCYSTLI KNNGKNNFKF VKGNVKRGVL PLIVEELIKA
RKNVKAMMKN EQNPITKMVL NGRQLALKIS ANSVYGYTGA AAGGQLPCLE IATSITTFGR
SMIEKTKETV EAYYCKKNGF EHNATVVYGD TDSVMVKFGT NDVGEAMRLG KDAADRISKE
FLNPIKLEFE KVYCPYLLLN KKRYAGLLYT NPNKHDKMDC KGIETVRRDF CILIQQMMET
VLNKLLIEKP LIQVAPMINV TNRHFRALVR IISKKVQLWT EMIVDNTLLY NINNLEEHLG
FNKNEHPIVC QLGGSDPTSL SEAAVLIEQA GYDEININVG CPSTKVANKG AFGAYLMKKP
ELVKNIVYEI KKKVQIPVTV KIRTGVDDLD SFSFLKSFIE TVSSVGCEHF IIHSRKAWLK
GLDPKQNRSV PPLEYNKVFD LCKLYPNLKF TLNGGIKSIE QGVALLLYGV MIGRACMDNI
TVLAKTDKLV YNHDTLATAY SRRTVLEAYK SYLEQNSSFY SLFELLKPIL GVLKGMPGHR
LFRFLKNFFF KNPVGHVGVV ALKNSSAKLI QQLTSNIDDV LNSLVKEQKE GLQGSPSLQE
GLEIAHNLLM DMPLYGTKEI LIMYGSIRTC DKKNILKYLD LLIKNNMYVN CISIAPEMHI
LKCGIHLISM HDLSHITNNL QASPLFIEIM GSNSIAISQQ MYFSTHNALR INENDVISTL
FYEINGHRHI SLLIFPFYDV QMLKRLLIKK LDLPDIKVND ILIFYKGIKL PNYRIISTYK
KVNKLYWAIK DPNPNASIRV IDNKYPPFFE NILNDIKLAF KKNIAPKLTM DGTGGTYLLF
NSKKKVCSVF KPADEEAFSP FNPRGYEGKI YQEGFRAGVL SGEGASREIA AYILDNTYNN
FSNVPCTIMV EACNPHFNNK SNLKYIYNEN TLKWKCGSLQ EFIDSRESVG NYDHKQFSIR
DIHKIAILDI RVMNLDRNDG NILVSPTHLG IEQSRRDDIE ALGYVLMYFL RGSLPWQGLK
AISKKDKYDK IMEKKISTSV EVLCLLNACT KVDSCIIIDR RIDMVTPFCT PFTYEGLIDH
IFCIENLQIE IPLYNDIKDL NQNEVGLFLH KKASDIQQTY EKDSLKDIGQ INKFMIKFKE
KHYEHNSLSR HVNIASYILK EIKTEHTFNK LKLEDEIIQL NTNTNKTILS NIVKQIQILI
YTGENLYEIY RLISLFSVIT NGKKDIIEQY GINELTRLNK LHISNILRYQ PKQKFIWNTL
KDHFNLLSND ENDISYVCNG YAPLSTRLIE YKNNMQVFPE VFSLINGPTF DIIQDTIEVK
SMCINCEQEG VNKILKFEIP YFKNILIHSF ECTLCNYRNN TIQDLNPIKE KGVKIIFSVT
KNEHLDRQLI KSEYGVLKIP EINFEIPKET QKGSINTIEG FIQTALSNLY MSMIEKTIHE
LFTIEIIDPS GLSSLEYQRS KQELNELGFY SFTSNCPCCN YLGDNNFCEI NIPGFKKCLI
LSYVCPNCNY KTSEIKSSGE INPKGKKITL TVKNKSDLNR FVIKSETASI QIPIIDLTSD
YGTLGGSLTT VEGIIMQIIE SLEDKFKFLM YVLNRRGEEE DISFDQILKR IQRLSYGLHE
LVDPARVTQG VINGMYSGIK TCELDELAAQ TCAYMATTHP DFSILAARIT TDNLHKNTSD
DIGKVAEALY TYKDVRGRPA SLISKEVYDF IMEHKDRLNK EIDYTRDFNY DYFGFKTLER
SYLLRINNKI IERPQHLLMR VSIGIHIDDL EKALETYHLM SQKYFTHATP TLFNSGTPRP
QMSSCFLLSM KSDSIEGIFE TLKQCALISK TAGGIGVAVQ DIRAQNSYIR GTNGISNGLV

PMLRVFNDTA RYVDQGGGKR KGSFAVYYVEP WHSDIFEFLD LRKNHGKEEL RARDLFYAIW
VPDLFMKRVK ENKNWTLMCP NECPGLSETW GEEFEKLYTK YEEENLGKKT ILAQDLWFAI
LQSQIETGVP YMLYKDACNS KSNQKNLGTI KCSNLCCEII EYTSPDEVAV CNLASIALCK
FVDREKKEFN FKKLYDITKI ITRNLDKIIE RNYYPVKEAE RSNKRHRPIG IGVQGLADTF
MLLRYPYESE EAKELNRRIF ETMYYAALEM SVELAQVSGP YESYQGSPAS QGILQFDMWN
AKVDNKYWDW DLLKKKISKH GLRNSLLLAP MPTASTSQIL GNNESFEPYT SNIYYRRVLS
GEFFVVNPHL LKDLFDRGLW DEDMKQQLIA HNGSVQYISE IPSDLKELYK TVWEIKQKNI
IDMAADRGIF IDQSPKLPFD EIRNEMHKYG VAITPATLKH PTTEDVQGVY SICIKYILNK
DINNIRIEEF TGDLKSILPN EGKNHLQAIG NLRFFRHCEK INKILNMENT LSYIFKPTSG
HITKLINAFI VNETNELIFQ FSRYRQKKED LEDQIVPSPE KLQQYNDELK DLLYEHMSHC
ETSKKKNEDI KNKINVADLC IKKLVNLLTT LTSHLKVHID KKNRLKDLGT NLKSLDWWTL
GIFIYEILVG YPPFYANEPL LIYQKILEGI IYFPKFLDNN CKHLMKKLLS HDLTKRYGNL
KKGAQSVKEH PWFANIEWNR LLNKRVDVPY KPKYKNILDA SNFQEDLSIA DKVINENDPF
YDWVISQFYI LSPRGDTIIN RDFRGDVLKG SGDPPPLFYL NGINFCFLKN NNLYYVLTSL
FNISPSYLIE LLYRLLKIFK DFCGQLTEEI IRTNFILIYE IVDEVIDYGY LQNSNTEYIR
YLIHNETLPS NASQKPIQVD NKKNEIFIDI VEKIDGVIQI KSYLLGNPYI KIALNDDLYI
KNIHKDNTNN IIIDDCNFNH LVLSLYQPDG ECVLMNYRIN MINIYKPNDV IACEVQRILT
DGCIVLHTRS SIYGKLSNGI LITVPQTLVQ NQKKHIFVFP CVDDTTRKNI SIISNIIKLL
AKYHININYD IITKIYVQEW VPPKNKQINA ATSNGSQIVV SLSGGELIYF EIDESHTLTE
IFRKNINVEI LCLSIQQNKL RASFLAVGCL DNVVRLLSID QYFKQLSTYI LPNNSSPQDI
CILYLNIGLN TGVLLRSVID PIGTLSNHYS KYLGAKSVKI CHVNPALLVL SEKTYLCYVY
QGKYIYSPLN YDVLEYASSF YSEQCSDGYV AISGNSLRIF RFYRLGEVFS QNILPLTFTP
RKIVPLPFPS MLAVIEADHN AYDENTQREI QKALRDIKLG TPKAGAGKWG SCIKIINPIN
LQVIDKVSLE LEEAALSVCA CELEALHCLI VGTTTNMTLK ASLRVYTYDI NYKLNLLHIT
PIEDQPYCFC PFNGRVIVSV GNKLRIYALG KKKLLKKCEY KDIPEAIVSI KVSDRIFASD
IRESVLIFFY DSNQNVIRLI SDDIIPRWIT CSEILDHHTI MAADKFDSVF ILRVVEEKPD
ITYNDIGGCK EQLEKLREVV EMPLLQPERF VTLGIDPPKG VLLYGPPGTG KTLTARAIAN
RTDACFICVI GSELVQKYVG EGARLVRELF QMAKSKKACI LFIDEVDAIG GSRGDESAHG
DHEVQRTMLE IVNQLDGFDN RGNIKVIMAT NRPDTLDSAL VRPGRIDRKI EFSLPDLEGR
THIFKIHANT MNMSRDVRFE LLARLCPNST GSDIRSVCTE AGMFAIRARR KTITEKDLLL
AINKVIHGCK QFSATGKYMV YNIKAKAEEN FAIVLKKINE LYNLTQDNVF NLPRYSKIPQ
AKKPTRWELF AQTKLKKRNK HGLIYDENSK GWVRRFQKKQ IKINKEKADF VHEYKPSDNI
DPFERMEEEK DIKKMKQKMR EMKNKVFDRL TDQTFYTGTH KKKFKKEKNL VVTPPKSIWL
YRNGDKHHNG LLFFIKPHIN NLKLLLFEIT KVLDPIIGPI RKMYDQNDGA KYLCTSGDPP
ASIDNLGKFD ITKYFKEGQK VITPPNGDGT RAFYESLLDE NPNSIIAIKY CIEHGVLSGT
KHHETLNKYY MLKKNNAFRN NFGGIKCEFV EMLNVKFIQE KKLIGKFFEE IAQDTGKVVY
GIEDTLKALE IGAVELLIVY EGLDIIRLTT KNNVTNQTKT MHIFPHDEKQ ESLYKENNVE
LEVVEKILLT DWIINNYKKY GASLDFVTNK SQEGAQFLQV LYEFIFLCIC MYDSINALFE
LAYKIVSGEI YEQVISIIVL PFNHLGLDAL KAKNIQNLLN SINDKYKKKL SLNIIDAIIE
CKHKEMVYEN VEEILKFISC IFNIEQKYNT TMLFYNYIYD SIYFSQLLPT IIFTLLNIVM
LEAKLNNASI LKKLFECIKD LVNDANIDAD ENGLKLQALD GNHVSLVSLH LVDSGFSHYR
CDRERVLGVN IASLNKVFKL CGINESVVIS SKDDEDNLNF VFENNKEDKV TNFSLKLMSI
ELDSLNIPDE GFDAEVELSS KELTNIFRNL SEFSDTVFIE IDSNSIKFTT KGLVGDAEVA
LKPRESTDDV GVTIKSKKKI KQSFAIKYLN LFSKSSILSD VVILGLSDSR PIEFKYEIKD
GFIKFFLAPK MDDDCPEKCD EDSGVIKGTF VYTFDSYICK AAIHAGVLNV ADDVVLIITH
SRNKFIGTKR NNVESKEFNG ESKSFSLSIP TGFNGNENEY INASNLPNEK YIRTLSNFTF
ITHFGKNTWR TILSHSLCEG ISISIDEENE LVIEQNCNPH LIKTKFIPKF EHPYHLVLIY
NKPNKSISLY INQKKINLEN MKIDFTLNGD LTIGRSNKQA TDYFIGDINF VKIYKYILTE
QEIKESFDSV LSRKTIDGRD CISSCKSKTN VNKNIQINTE EFYLNCSDNL LSERFSGKGT
QFLASCSEDC TSSKYIVKGS NYYTPDTSIC KAVMHSGIIK IVEGLTEYKS SRGHFGIISK
AEKQSCFTDG SFLFELPIGS TKNIICPENC GTNTYSPLSS VCKAAIHAGV ISIKGGQIQV
VVGKGQQEFK PSTQNNVQSY IAEKQDRSQK YIYENADVGT QKKVFDLHLN LGPYKCNYSR
NGKYLLVTGE KGHISLLDTH NMESLCELDV NETVRCNTIF HNHKLFAVGQ KKYIYIYDNT
GIEVNCIKDI LYPSQLEFLP YHFLLASIGD LGELVYQDIS VGNIVTRKKT KRGPCSIMKQ
NKHDAIIYLG HKNGHVTLWS PNMDKNYLIT SSLDCTYKLW DIRKLEYINT FKSNIINNID
ISDTSLVAFT MNSHFRTYKN FFTKPYLTHN TYGDKINSIA FQPFEDICCA GLKYSIKSFI
VPGAGLANID TFVNNPYETK KQNEIRQLLD KLPPETITRD YKKIILVRKI KTKTQIKRIV
TSPRDVHEMA LNSYSNSNWP KWTDSESNCL CLINNQIYIY KDNERGTKGN SSVFKIFNLD
NLSKHIYSKN FFNSDEIKLK WNKNGTSLLL QIHTDKEKQS YYGSSNLYFI DTIQLKDVNI
MTNKGLIYDT IWSYNQNKFY VCKGEIPADI VLHDKNGNII HSYGKHKFNT LKLNYNEKLL
LTGGFGNLSG DISIWNTINK KEITKTKSSC AVICEFFNDD KHFLTATTHP RLRVDNNIKI
YKYNGLIVSK LDFDELYNVI ILPPGCNFVV EEKTNKKKKK KKEEDTIPLP NIKTQILKKI
IEYMEYHIHN PPDEIPKPLI TSNLQDVVVE DNIPKYKEIS QKMIQEIEMA VVLFKRKFII
KKIPQMPSCY IINIGPLTAA SGKIKLPSTY AKLGDPLSFS KLPDCNYSFD MIEELQQFFM
KQRRCDYFSL LNNFINLLIT TSNLLANETD IDARNTLLNK FLYSLNTWMI MRRCIVASCE
NIFSMTGLCI PLQILHFNND ECKIFFSKKR APYLLVFEVA DLDEDISHII PVESQRIIFG
EFNRESISSL LNNPLARSLM NELSNNPEML TNLISNNPLL RNTFPLMQPM LDNPNLLREF
MRPEVLQAGL PPEERYASQL VSLQEMGFID NDANIQALQE TGGDVNSAVT RLLERGSGDR
LTRAARVLEQ LTEQKPIFGK CRFTIRSFGV RRNEKISCFV TVRGKKALEI LEKGLKVKEY
ELRRKNFSDT GNFGFGIQEH IDLGIKYDPS TGIYGMDFYV HLSRPGYRVT RRRRERLGFF
ISLKLMNEIK GLSSIDSSYQ WLPLLYMALA NDTAVSKISL SALKPYSIVL IRLLRDFFSV
VFLIKCVGIG YRNMSGSQCV AIACDLRLGS NSFTTVSTNF TKIFKINDHI YVGLSGLATD
IQSLYELLRY RVNLYQIRQE TEMNIDCFSN MLSNILYSNR FSPYFVNPIV VGPYLNAYDL
IGAKCETNDF VVNGVSNEQL YGMCESMYIK DILLVGAGGI GSEFLKTIIT IGCKNIDIID
IDTIDITNLN RQFLFKKKDV KKHKSLVAKE RALKHRKDLN INAYTFDVCT MKGSDISKYD
YVINALDNIK ARKYVNKLCV TEKKVLIEAG STGYNGQVYP IFSNETKCYN CEEKPKNKTY
AICTIRQTPS LPEHCVAWGK LIFETFFCKN DNETLIDIKK HIEEESKKRD MDKEEIIRFI
FNYLFHDTIN ELIALKKDYT IMPKPNIKKE TEEYLIFDKD DDDCINFITS LSNLRMINFS

IKQKSKFDIQ SIAGNIIPAI SSTNAIVAAF QFEKIYVCKP QSSRNKSDMI LVLNFGSQYF
HLIVKRLNNI KIYSETKDYN VDLKDIQNIK GVIFSGSPHS VPHVKKEVLN YKIPIFGICY
GMQELAFQMN GKVGKSKNSE HGSTVWMNHT EEVIEIPENY YLVNSSENCF IYNKEYNIYG
VQFHPEVYET IDGDQMFYNF AICKCTKTFD PIKYHEVEFN NIKKYAHDHY VIAAMSGGID
STVAAAMTHK IFKDRFFGIF IDNGLLRKNE GEKVFIKNTF PDMNITKIDA SEIFLNQLKG
VTDPEQKRKI IGKLFIEEFE KAVYSMDIDI EKTYLLQGTL YPDIIESKCS KNSDTIKTHH
NVGGLPKNLK FKLFEPFKFL FKDDVKKLSQ ELNLPKELTN RHPFPGPGLA IRVIGEIDKH
KLDILREVDD IFINSLKEFN LIGQAFAVIF SSKSYDHICA LRAVKTTSYM TASYYKIPHD
ILEKITTRIS NVKGVNRILY DISSKPPSTI EFEMPLPGFV SNKSLYLKKP LVLYEDENSK
IEVDPILAQY LREHQREGVQ FVFECLMNLK DEKISGCILA DDMGLGKTLQ SISVLYTLLK
QGYNKKPAVR RCLILCPASL INNWNDEINK WLPNRCTVTC VNDNAKEKIV SKLEGFKYDL
KSTILICSYE CFRINNESID KSAIDMIICD EAHRLKNDKT KTYTSIYKLS AKKRLLLSGT
PIQNDLGEFF ALISLCNPDL FDDTNSFRKK FANPILIGRD KDATEKEQQI ASERLAELST
INKFILRRTN NLLSKVLPVK YLINIFVKLN PIQEALYVLF LKDKKLLKSD NSNNKVNVLI
NIKKLEKICN HPLLLNANDI KSSKFQLLHF LLKTIKQDTN DKVVIVSNYT QTLDYMEILC
KENHYKFVRL DGGISIKKRH KVISDFTNTD DIFIFLLSSK SGGCGINLIS SNRLILLDPD
WNPANDKQAL ARVWREGQKK ICYIYRLFCT GTIDEKVYQR QISKDGLSSM IVTNTNLSKD
QLSDENVKKL FNYKQNTICE THDNIECNRC KKVEPAGFAF YIKYEKASLK KNTNLVKKCI
NNNINVYEIL DVEESDDLET IKSAYKKLIL IFHPDKFLKI QDSYAVLSDK ILRKQYDSSI
PWSSIKPVPN IGDENTSIDK VKYFYDFWYE FTSWRDFSYQ NEYDYEEAEC REERRWMERE
NKKIQKKASK AEKLRINKLV DLAYNNDPRI IAENKRIEME KQKKKTTSKI WKHHIKSFDT
LCQFVYEIYY FLWTPQEISL LSKALKLYPG GTKNRWTVIA NSIKTKNVKE VIKKTKEMFE
NETLWTHEEQ MLLEKALMKH PATIPMPKKR RNGGRSKHNR GHVNPLRCSN CGRCVPKDKA
IKRFNIRNIV DTSAQRDIKE ASVYSTFQLP KLYIKQCYCV SCAIHSRFVR VRSRQQRRVR
KETTKHAHAS QKKVSVSEIN FDSSYTILDT SEGAIMLHVN HVLYHLDFNA LAVVKNVDMT
EEMQIDAIDC ANQALQKYNV EKDIAAHIKK EFDRKYDPTW HCVVGRNFGS YVTHETKNFI
YFYIGQVAIL LFKSGVCGWS KAVRKQGGRF CFVNLNDGSC HLNLQIVVNQ NIENYDKLLK
CGIGCCFRFT GTLILSPVQN SIHSFEIYGE DPQKYPLSKK NHGKEFLREV AHLRPRSYFI
SSVMRIRNAL MVSTHLFFQS RGFICIQTPL ITTSDCEGGG EMFTVTTLDY KKDFFSKQAF
LTVSGQLSLE NLCSSMGDVY TFGPTFRAES SHTSRHLAEF WMIEPEMAFA DIYDNMEVAE
AYIKYCIRYV LDNHFHDIYY FEENVEKGLI DRLKNILNDD FAKITYTNAI DLLTKYSTNF
DVPVKWGMDL QSEHERFIAE QIFKKPVIVY NYPKDLKAFY MKLNDDQKTV AAMDVLVPKI
GEVIGGSQRE DNLELLDKMI VEKKLNIESY WWYRQLRKFG THPHSGFGLG FERLIMLVTG
VDNIKDTIPF PRYHGHAEFI KEILDLNLEK SENVIKNLFL KDKKNYFYLC VANWKKLDLK
NVSTQLKTSN LRFVDDENLK NILNVNPGSL TPFSIKSDKD NIVKLYFDED IKNMVIIHPM
HNYSCIYVKT SDVIKYCDLH NHTPILGITS KKEQNFSDWY TQVIVKSELI EYYDISGCYI
LRPASYYIWE CIQTFFNNEI KKLDVENSYF PLFVTKNKLE KEKNHIEGFS PEVAWVTKYG
DTNLPEEIAI RPTSETIMYS VFSKWIRSHR DLPLKLNQWN TVVRWEFKQP TPFIRTMAKL
SKAQKKQIYM DKLSSLIQQY NKILIVHVDN VGSDQMASVR QSLRGKATIL MGKNTRIRTA
LKKNLQAVPQ IEKLLPLVKL NMGFVFCKDD LSEVRILQNK SPAPARLGVI APIDVFIPPG
PTGMDPSHTS FFQSLGISTK IVKGQIEIQE NVHLIKQGEK VTASSATLLQ KFNMKPFSYG
VDVRTVYDDG VIYDAKVLDI TEEDILAKFS KGVANVAALS RSVGIITEAS YPHVFVEAFK
NIVALVIDTD YTFPMMQKIK DMVENPQAYA AAPAAAEEED EEDGFMGFGM FDQERDLARE
PCPDRIIEDM GGAFGMGCIG GYIWHFLKGA RNSPKGDMLS GALYSSRMRA PILGGNFAVW
GGTFSCFDCT FQYIRKKEDH WNAIGSGFFT GGVLAMRGGW RSSSRNAIVG GVLLAIIEFV
SMVLTRKTTP TPRQQFQQQM EMEKMLVDNI GDVTITNDGA TILKQLEIQH PAAKILVNLS
ELQDQEVGDG TTSVVLLASE LLRRGNELIK MDIHPTTVIC GYKLAMKESV KYIKEKLSER
NLGKDVIINI AKTTLSSKFI SYESEYFAKM VANAIQSVKI INDAGKTKYP VSSVNIIKVH
GLSSLDSKLI DGYAIMSGRA SQAMPSAIKN AKIAFLDFPL KQYRLHLGVQ VNINDPNELE
KIRQREKDIT KERVNKILES GANVILTTQG IDDMPLKYFV EAGAIAVRRV KKDDLKRIAK
LTNGQIRLTL SSIDGTEKFE PASLGYCDEV YEEKVGDWDV MFFKGCRNSK SNTILLRGAN
DFVLDEMERS IHDALCSVSR ALESNYVVVG GGCVEVALSV YLEDFAKTLG SREQLAIAEF
AESLLVIPKI LALNASYDSI DLVCKLRAYH TKSQVNTDDP KDYRWYGLDL VNGKVVNNLK
NGVLEAMISK IKSIRFATEA TITILRIDDL IKLVPERNPP VYKLKGHTSN ILDIQFNPCY
NEVIASSSED MSIRIWLNGH KKKVTIIDWN PLNYYILSSS SFDSTVNIWD IENEKKAFNI
SMPQKLTSLK WNNTGTLLTA TCLNKKLHII DPRQEKICTS FVGHTGGKCA KNIWIDGYSG
NEILSTGFSK NYMREIKLWD LKNISDPIST ISIDNASAPL LPHYDESIGI IYVIGKGDGN
CRYYQHSEGV LRKINEYKSC LPFKSFGFLP KQVCNIYKCE IGRIYKNENN KSIKPISFYV
PRKNFQEDLY PPIIGNNLEI TRINIFNKLK ICGQFNKGFI ISKLFIIDQH AADEKSNFEK
YNKIFTMKSQ KLVYLLSLPV FNGKILEVVD FMSLLYHLWF NYNFPRPQKV WRILASKMKN
LVLVFLKKIS DTYLEDQSKW MEKMKSSQEQ QNNRKLDEWN ECIENKCFVY PASSAPCGAC
TSAGAVTPHR RYKEPRQKKE YTGTDILCQA KSGMGKTAVF VLSILQQLVR CLGIAHTREL
AYQIKNEFDR FSKYLKNVRC EVVYGGISMN KHVALFKVPH IIIGTPGRIL ALIREKYLLT
DKIQHFVLDE CDKCLEKLDM RGDVQKIFIS TPLKKQVMFF SATMAKEMRD VCKKFLQNPV
EIFIDDEAKL KLHGLLQHYV KLQEKDKTRK LIEILDALEF NQVIIFVKSV TRAITLDKLL
TECNFPSIAI HGGLNQEERI ERYDKFKKFE NRILVSTDLF GRGIDIERIN IVINYDMPEN
SDSYLHRVGR AGRFGTKGLA VTFVSSQEDT LALNEVQTRF EVAISEMPNK IDCNEYINQR
MSVSAEAYGE WNKKKNFVPT VHKKDDKEKE KIRKALNESF LFNHLNHLEM ETIIDAFFDE
HVEEGDNIIN EGDEGDLLYV IDEGEIEIYK TKKNKKEVLT ILKSKDVFGE LALLYNSKRA
ATAKALTKCH LWALDRESFT YIIKDNIAKK RQMYENILKQ VTILKDMDPY ERSRVADCLK
SKTYNADIII NEGERGDTFY ILTYGNATAL KSDQVIKTYT KGDYFGELAL LRNKPRAATV
KADGVCQVVY LERKGFKRLL GPIEKILIRN VENYKQVLKE LMHLKIMCLS DEVREMYKNH
KTHHEGDSGL DVFIIKDEVL KPKTTTFVKL GIKATANTSF LLFPRSSISK TPLRLANSIG
LIDAGYRGEI ILALDNTSDQ EYTIKKNDKL AQIVSFSGEP LSFELVTELD ETSRGEGGFG
STLGAAFGTA KSGVGVCSVG VMRPDLIMKS ILPVVMAGVL GIYGIIMSII ISGDYIKAYL
LGFSIEDALA LLRIDDLYIE SFQIKDVKIL KGDHLSRCIG RICGSNGSTK YAIENATKTR
IVIAGDKIHI LGSFNNIKMA RYSICSLILG STQGKIFNKL NILAKRLKER IEKLEDRMHP
WSNIDGMKAA CSYTYDDIIC MPGYIDFPLS EIDLSNNMTK DICLKTPIIS SPMDTVTEHK

MAISMALCGG LGIIHNNLSI EKQVEEVKKV KRFENGNKVL CDEKKSILPI VNDNYEFPHA
SKRENKQLIV GASISTDLEK VNKLAQNMID IICIDSSQGN SIYQIDMIKK IKSAPIIAGN
VVTSNQAKNL IDAGADVLRI GMGSGSICTT QDVCAVGRAQ GTAVYHVSNY AHGVKTIADG
GIKNSGNIVK ALSLGADFVM LGNLLAATEE SCSEYYFENN VRLKIYRGMG SMEAMISQGV
SASLVDKGSV LNLIPHLVKA VKHGFQSIGS KSIQELHSKL YSGMKENKQY QEALKELKKL
KKKIEENINF LKNIKEKLIL AYESAWDKFG SKLKDMPFLN SFFENPLLGK LFGETELAAA
LRVMKMEDKN FKLSELMYLF EFVISKHIVE SYLIGDEETL RLHCGQAAFN SLNSSINERK
KKKLFLDTNV LIYKDHELKG AQRMEESSPW FIFTFHTQQI NCLKNKNDEI VEGKIDDIRE
VVYTMALSKH PEPEGLLYPY IVREFAIIGN TPSWMVLYII GLGLGDEKDI SVKGKELIDQ
SDVVYLESYT SILFISKDKL EDYYKKKIYE VDRNFAEENC EQILDEAINK KVSFLVVGDP
LCATTHHDII LRAKKKNIDV QVIHNASIMS AIGESGMQLY NFGQTISIPY FEGDYKPTSY
YNKIKINLDN NFHTLCLLDI KVKERTIENI MKNKNIYEPP KFMTVNEAIE QLIYCEHNQN
VITKNTLAIA IVRIGSKDQQ IVSGNIFTLK TQKYNDPLHS LIICAPNLHD IEKEYFDMYP
NSAYRKCVRV QLIKNGKKIT AFVPGDGCLN FIDENDEVLV SGFGRSGHSV GDLPGVKFKV
VKVARVSLLA LFKEKKEKPR SMGIKGLTKF IADTAPNAIK EIKIENLMGR VVAIDASMSL
YQFIIAIRDG DQYGNLMNEA GETTSHISGL MSRTIKLMEN GLKPIYVFDG APPELKGSEL
EKRGEKRQKA EELLLKAKEE NLEEIKKQSG RTVRVTKKQN EEAKKLLTLM GIPVIESPCE
AEAQCAFLTK YDMAHATATE DADALVFGTK ILIRNLINLE QVLKGLKLTM DEFIDFCILC
GCDYCDTIKG IGSKTAYNLI KEYNCIENII KNIDYVEARQ SFINPKVKSE VKIDWCEPKI
EELKTFLIKE HNFNEVRVTN YITRLLKARK VTTQRRLDTF FVNNSKYYES LNLKKNCTTD
EVKKAYRKLA IIHHPDKGGD PEKFKEISRA YEVLSDEEKR KLYDEYGEEG LEGGEQPTDA
TDLFDFILNA GKGKKKRGED IVSEVKVTLE QLYNGATKKL AISKDVICTN CEGHGGPKDA
KVDCKQCNGR GTKTYMRYHS SVLHQTEVTC NGCRGKGKIF NEKDKCVNCK GLCVLKTRKI
IEVYIPKGAP NKHKIVFNGE ADEKPNVITG NLVVILNEKQ HTTFRREGVD LFMSYKISLY
ESLTGFVAEI THLDERKILI DCTNAGFIKH GDIREILEEG MPTYKDPFKK GNLYITFEVE
YPLVITNEKK EILKILKKQN EVEDIENTDC EVVTCKAVDK EYLKQRLKTL KNYLPYLCKI
LIDNTVYTKW DYLTMDESHF QNDNADEMTS RTWGNDWTVR KGAALCLDYL SNVYNDDILE
YILPHIEEKL MSDKWNIRES AVLSLGAIAK GCMYSLSPFI PKVLEYLIKL LNDEKPLARS
ISCWCVTRFS SWICHPDKWF EPVLLNLLKR VLDSNKRVQE AACSSFANLE EDALELLNNH
LHEIVHTIQQ AFQIYQAKNY FILFDVVGTL IDSVNIVKEN IDLAHEIVNS ILIKWNSIRI
SSPYIIALME CMSCITSAYG KEFLKYAKIV IRTCIKFLVL LYIDDLIECS FDLLSRIILQ
SNFALIGDIS RFCAQYLILS DIIPFLIAHI SHPSIPVSNN ASWAIGEISI HINSQYIEAY
VDEIVKQHIY ICNSKYHGCL LQNICITIGR LCSTYPKKII YYFPQFLKTW LKIMAHGTQE
NEKINFFHQF LSTMKECIQP EELKLKLAQG LLKSHIVNTL TNNGCTFIFW IADWFAQLNN
KMSGDLNKIK KVGQYFIEVW KSCGMNMENV QFMWASDEIN KNPDKYWSTV IDISRSFNIN
RIKRCLTIMG RTEGEDNYCS QILYPCMQCA DIFFLNVDIC QLGTDQRKVN MLAREYCDIK
KIKKKPVILS HGMLPGLLEG QEKMSKSDEN SAIFMDDNEA DVNRKIKKGY CPPNVIENNP
IFAYAKTIIF PHYKEFNLTR KEKNGGVRMY VNEEIVDNEK LSDIINKTKE NVKYMKGMKL
PNNIVAIPDI NKVIEDADLL IFVVPHQMER ILSSIGRLSV FAGGLSLIPY TFVYDVDGGE
RCVMFNRFGG VSEKTYGEGS HFYFPWFQTP YIYDIKMKPK VINTTTGTKD LQIVTLSLRL
LFRPHTKHLP YLHSTLGPDY DERVLPSIGN EVLKAVVARY NAESLLTQRD TISKEIRESI
TARAKQFNIV LDDVAITHLS YGKEFAKAIE DKQVAQQESE RVKFIVAKTE QEKIAAVIKA
EGEAEAAKLI STAVKQYGNS LLEIRKLEAA KEIAENLSKS KNVTYFPSTS NILYINAIKE
ENGGYNFENL KRNEILKEKG PQFRKTGTTI CGIVCQNAVI LGADTRATEG PIVADKNCSK
LHYISKNIYC AGAGVAGDLE HTTLWLQHNV ELHRLNTKTQ PRVAMCVSRL TQELFKYQGY
KVCAIVLGGV DVTGPQLYGI HPHGSSCLLP FTALGSGSLN AMTVLEAKYR DNMTIEEGKE
LVCEAICAGI FNDLGSGGNV DICVITKDGT QHIRPYKQPN TRLYHLPKGT TPILYEKIES
IKKHISLNDL GEARGTVLSV KLDELIDNVE GQTVIDPKGY LTNLNASDSD IADINKARSL
LKSVINTNRK HGPGWIAAAR VEELAQRKDK AKEIIMKGCI ECSKNEDVWL EAVRLEKLSE
AKIILAKAIK NIPTSVKLWL EAYKKEKNVQ DKRKVLRKAI ECIPNSVVLW KEAISLENEN
NAYILLLKKS RVQCNTNNKN INPIISEALK ECPTSGILWS KAIELENKNL QNSKSVTAFN
NCGNNSYVIL IVAIIFWNNY KIVKARKWFH RAITLNPSFG DGWATFLAFE IDQENEINQK
DIINKCIKAE PNRGWVRGRI HDIRSKGSLA FIILRNKIYS LQCILDIKNN NDKNMIKWVS
NLSLESIVDI YGKLVKPEIS IDSTIIKYEI HIQKIFCISK NSKELPFLLK DANMKETNDE
ATIRVNQDNR LNNRCIDLRT YANYSIFYLQ SEICKIFRNY LIDNNFTEIH TPKLLGESSE
GGANAFQINY FNQNGFLAQS PQLYKQMCIN SGFDRVFEIA PVFRAENSNT YRHLCEYVSL
DVEMTYKFDY MENVYFYDSM FKNIFNKLKN QYPSEDFKWL AVTPIFTYEQ AIKILIEEIL
TYDMTTDMEK ELGKIIKQTH NTDYYIIINF PSGLRPFYTM YNEEDPKISN SYDFFMRGEE
ILSGSQRISD VKVLLDNIKK FNLNPKKLDF YIDSFAYSSY PHSGLLFENL NKEYKYITTQ
DNFDGFRFEV DKNVNKYLQS THTLFLGTRD VGYLYQFGAN FASSDNTLLM ISRVNLDGSV
NGRFCKKINN DCKFNFNTYN KNDPRNMYEM SLEVNNPQNT YNFKTIWQGG VDLTYIGSNC
ASIGSFGLRY NHNNHVLTMQ CVRQPNFKSP EFMLNQAHSY KMQYARKISD RLSVGTELEL
TPETKESAMR LGWDYSFRHA KVQGSIDTSG KIAVFTQDYS GFGVSGYIDY LNNEYKFGMM
HIAPSQEQDG GVGKTTFVKR HLTGEFEKKY IPTLGVEVHP LKFQTNFGKT QFNVWDTAGQ
EKFGGLRDGY YIKSDCAIIM FDVSSRITYK NVPNWYRDIT RVCETIPMVL VGNKVDVKDR
QVKSRQIQFH RKKRNLQYYDL SARSNYNFEK PFLWLARRLS NQPNLVFVGE HAKAPEFQID
LNIVREAEKE LEQAAAVAID EEDGVLTVNS IKSEPTISQY DIKKLVKNKI LENAPFYNYQ
IERSFADKFY GECMYDNFGL PKNIEINLII LEEWNINCNR NRILKHTGLI KNIEINNFKY
LNNKESLEVH FSVNPMYTDL LEKNKVLPPS GIDYNKLIKQ FGCSKIKEEH IKRIEMLTAH
HFIRRDIFFS HRDLDFLLNY YEKNKSFYIY TGRGPSSLSM HLGHLIPFYF CKYLQDAFNV
PLIIQISDDE KFLFNKNYSL IEIKSISKNE MSFILYNSNT ALANALRRIM LSEVPTLAVD
IVNVYENTSP FHDEFIAHRI GLIPIDSRNI KSYEFRERCK CKETCSRCTV QYIIEVKCNN
KIDVSHYDIL DHEPNIPMPI PIPIVTLSKN QTLHMKLTAT KGIGKMHAKW IPANVSYIID
HKIVINHPEV DKMPKEHKLI IANNLNPDCY ILLQLSENMS VVMAESCRDT LTELGYKDIV
KVIYDETKFH FKVESVGSMP PEQIVEMAIE ILESKLKDLE PQIKASFYSI EEVAKQLKLP
DPETGKNEEE RCFLSLIKSS ILKNPKKWTN MSKKIIGMSE ETTTGVLRVK KIEKNNGLLF
TAINVNDSVT KQKYDNIYGC RHSLPDGLMR ATDFMISGKI VVICGYGDVG KGCASAMKGL
GARVYVTEVD PICAIQAVME GFNVVTLEEI VEKGDFFVTC TGNVDIIKLE HLLKMKNNAV

VGNIGHFDDE IQIADLFSHE GIEIENVKPQ VDRVTLPNGN KIIVLAQGRL LNLACATGHP
AFVMSFSFCN QVFAQLELWE NRKYENKSYI LPKELDEKVA YYHLKKLNMY SYGMEGDDTY
LPQVQYPSPY ENQYESPSPR GENHTPFIGY FSSHLLRTGF LLQCVSLILM FVFYWAFGGT
GIFIFDLYAG PECVKVSSAF HLTISVLMSI YLLGTLYIAM FQVFVADNSK WCRGFRAGSK
LLSAAVTLDL LSSILRLVQY LYAYFYMNMR WWARYQQTKS DWTLLHFGSI VHSFALFMYG
AAFFYMEAYH DEGTYEELAW SNLTLFKLAG LAELNKPKKR TFRTFHYRGI ELDKLLELNQ
EELVKLLPAR QRRKFRRGID KKAKSLLKKL RKAKKECEVG EKPRPIPTHL RNMTIIPEMV
GSIVAVHNGK QYTNVEIKPE MIGYYLGEFS ITYKHTRHGK PGIGATHSSR FIPLKVMVDM
FLWRDPEQFE LKNLANEEAT PTAPHLADNQ YATDAPYDDW GFLQIIKDHH ETMYELKQKI
RPRDQVVGWF CSGSELSELS CAVHGWFKEH NSISKFYPHS PLNEPIHLLV DASLESGFLN
IKAYVQLPIN LVKEYFVHFH EIQIELLPCN VERADVSLKK LLIMLKNCKS YVQDIVDKKR
KGNVAIGRYL HKVFSNDPFV SIEKFDSINE SILQDNLMIS YLSNLAHLQF LIAEKLNWLN
HLPIKEDESE GRRVHKNLID LVSQNHPLLF GKDNSNIGKI IEIFLSVYET DFSDSDCNKK
IVSLISSLDQ SYLSNLALTN KQSKKLNHIN GCDLFKFYDD NINLKPYLKI LKDFDKYPLI
VDSENNILSL PPIINGDHTK ITINTKNLFV ECTGIDLNKL EICLNIISSM LSEYCQPKYT
IHSVLVSYPN FKNKKLTCDI EYVRKLSGIT DITIEDIKKL LKKMMINNTT FEVDVPFYRS
DIMHACDIVE DIAIAYGYGN IKHEPIEISK KHLLNTVSDM FRNSMTECGY IEVLTNALLS
MKENYDCMFR PVQIMNSKTS EYEIVRTSLI VNLLKFVAAN KHRELPLRFF EIGDISYNKT
DTNAFNKRNL SIIFADKTAG LEEIHGVLES ILKDFQLFSH YKIDEKRKEN IHIRVISIGN
IRGLGGCDYG SFRMSNEFLG WKNKKTNSVH QYKCSDISEG EWIKLSYNNN RLHLKFNESK
DHLIVFFDGF PDRNLAEITQ HFQKYFNIKL GNRKLATKGW NWGEFKLENS NLIFDIDKKY
AFNINTNNIN QLNVQIKTDI AIELKNDENE DVLSEIRFYY PHENDENQNF QDLKNNLLDK
VNIGDSKSEC IASLSNIPLL VPRGRYEIEL YSKTFKLHGK SYDFTVQYSN INKMLLVPKT
NSNQYILIFS LNNKIKQGQT EYPFILIQLN NDDDMDLDIN APEEDIKNYK LEKTLTGKAY
DVVTRLFTAL AKKNAIIPGD YRTAKNEHGI TCSYRAASGQ LYPLNKYFLF VVKPVILISF
DDIVTLSFQR TGNNQHRFFS LIIKHKRGMS YEYTNIDKSE YAPLLEFLKS KNLNIKGYID
LSKRRVSPKD IMKCEEHFSK SKKVHQTVRH VAQKHNMTVE ELNRKVIWPL YKKYGHALDA
LKEATMNPDI IFKEMDISDA VKESLLSDIK LRLTPQALKL RGRIDVWCFG YEGIDAVKEA
LKKGKVTINI KLIAPPQYVI VTSCHDKELG MQKIQEAMKV ISDKIKEYKG GDFKQQGEIL
VILLDKHDGL SSDDEGERGI FEFFVCDVGV GLSLSVRDIL PMEYDSIFIG VLPYYTFNHE
YVIYDSSQLL PRYLIQFECD PSAEEVFSIP LCDYCGNAPS LYYCESDEVK LCEKCDNIIH
SNKLVKKHIR KTLNEAQGNC KIHLQNEVNM FCTVCHIPIC NLCMCSHAHI SLKMAYNAIM
QHSSKPSNFI KERKKNLNNL LEKIDKLHEQ VSLNMNEAEK NVYTALEDLV KQLHTTTDKK
MSSILSEEYE LKRQFNEIMW NENFLYYLQT ILPPADFMNA WLKHCQYREE IEQNSLIFPD
IRIKGNINVI TEGSINHLVG LPNVGKSTTF NVLTKLNIPA ENYPFCTIDP HEAKVNVVDE
RFDWLVDHFK PKSSVHAYLS IFDIAGLVKN AHLGEGLGNN FLSNIAAVDG IYHVVRAFEN
EDIIHTEGNI NPVRDMEIIN SELIYKDISN CERNLEEISK VNRNKKDKIK QNEHDVLTIV
LEHLKEHKWI KDRAWKSSEI EVINEFNFLT AKPVVYLVNM SEHDFIRQKN KYLAKIYNWV
QEKNKGTIIP YCAEFEQKIL SMTETEKEEY FKANNIKNSM LNKIIKTGYY EINLIHFFTC
GQDEVKCWTI RKGTKAPQAA GVIHTDFEKG FICAEVYKYT DLVEFKSEGE VKANGKYLQK
GKDYVVEDGD IIFFKFNVSS SGRIDKFVNQ LRISYGTLEE FVDNFVYELK KGLEAHRRHP
NLWIPHECSF KMLDSCISDI PTGQERGTYY AIDFGGTNFR AVRASLDGNG KIKRDQETYS
LKFGTFSHEK GLLDKHATAS QLFDHFAERI KYIMGEFNDI DDSSEKSVGF TFSFPCTSPS
INCSILIDWT KGFETGRATN DPVEGRDVCK LMNDAFARSS VPARVSCVVN DAVGTLMSCA
YQKGKSAPPC YIGIILGTGS NGCYYEPDWK KYKYSGKIIN IELGNFDKDL PLSPIDLVMD
WYSANRSRQL FEKMISGAYL GEIVRRFMVN VLQSASSKKM WQSDSFNSES GSVVLNDTTP
DFSECKKIAK QTWDMDFTDE QIYALRKICE AVYNRSAALA AAAIAAIAKR IKICGVDGSL
FVKNAWYCNR LKEHLKVILA DKAENLIIIP ADDGSGKGAA ITAAYIVLRW LCKAIVSSLF
GDVNIINPEN VPLYGSVIFV GNHNNQFIDA CVLVASIPRQ IKFIVAEKSM KRPVIGELAR
LAGCISVKRP EDLKFKGIGR IYWNTGDTKI KGINTRFKLD VQIGDKLMTQ NKMFTVTKIE
SEIELILQNP ININCEDNGV PFKIVPKINQ TEVYNLVTHS LKNGDPIGIF PEGGSHDRTN
LLPLKPGVAI MTLCALADGD VSIIPVGLSY SKLYQLQGCV TIFVGNAIIA SQDLCNDYNN
NNRETISKLL AKIEEGMRSC MLTSKNHETS RCIELCVSLY TPERMTISKN KIYNILQLFS
EMFWKFGNSK EIENLCYELQ CYEKLLQANK IKDDEVWMLK QSTSAATLKF IEHICSLIFC
IIFGMTFSLL WLPLVAISVY LAENHRKMSL KNSLVKIQGG DVVASYKVLV LLVLLPTFNI
IYGLLFSLYR LMKKKDVDQV HKLLNNYLSK FNIYVKFTKE EIAHWLMPIQ NVIYTYVNEE
NGEIKDLISF YSLPSKILAN EKYDMIYAAY SFYNVATTTS LKNLMQDAIC LAKRNNFDVF
NALEVMDNKS VFADLKFGEG DGMDADSVLG LQAILISANY KEKEFIRIAY YMNSFYKDIE
LREKPPVVPQ YDKICRHIVD NPRIVKFSIP WDDSRVAYEI LKELTQKNYF RIFKVNLHIP
CKLQKISEKC NEKTKCSVCE CTEDEIPYNF RTNEVEVDLI YNSPSYTAYE GKSIWELKGN
NNYKYFGAAK NLKGVKELLF KENDDRKKRK KEKRNLDKIV NIHYFGYCDE ENEILLNEEL
KIQKKLMKHG TLVLPADRAR EYMDCLGKQV DIQFIDMNEK TMKRQYKKYI QRIDDMERIL
RFLEENINKL PNVKIKKSKI ENFLEHDHIY ELDQVEESLN RLHVQFVRFC NNNKDLVDER
NSAIEEKHVI LTALNQLHPS LSTHIMREGI NMMFTNISGV IKTKDQESFS RTIFRALRGN
TYTYFQNIDE KSVFVVYCHG STHSSIYEKI MKICKAYDVR NYEWPKTYEQ ATKRLSELKE
IINDKEKALK AYEEYFINEI FVLINVVEPN KNSLIEEWKL FCKKERHIYN NLNYFEGSDI
TLRCDCWYSA NDEEKIRHIL MNKSSNDLVS ALLLSDKLTP NISPPTYIKT NEFTSTYQSM
VDTYGIPRYG EINPAISTIV TFPFLFGIMY GDVGHGICIF LFALFLIIVH NRMKNNEMLS
MLFNGRYMLL LMGFFAVYAG FLYNDFFSMP LNLFTSPYIF GFDSKWLGAD NELTYINSFK
MKFSIIIGFC HMTFGVIIKG FNALHFKKKM DFFFEFLPQF VMMLSMIGYL VFLIIYKWVT
PGGYKKQGII NTIINMYLMK EINQDNQFYE HQEIVQAIII TLFALCIPVM LFCKPAIKTY
KMMKEIWIEQ LIETIEFILG LISNTASYLR LWALSLAHQQ LSLVFFEQTI LSSLEKDSFM
GVLISLIIFS QLFSILTIAV ILCMDTLECF LHSLRLQWVE FQNKFYKGDG IPFKPFNIKK
LLPDNYSAIL ARALSERPLT YLPTIERVCY EVLSDEDEHL NYIQINLLNT IRPTPIRGLL
AATQERFVVV PGIIVQASKP QHKMRKITLQ CRYCDHKMSI DVPLWKDKPQ LPPYCRYVLE
PYVILPNECT FVDIQSLKMQ ELPEAVPTGD MPRHLQLNAT RYLCEKMIPG DRVYVHGVLT
SYNPNPTRVD GTNFSYLHVL GFQKYDGNDL NFDVEERNEL TLLAAEHDIH DKIFKSIAPE
LYGMDEVKKA CACLLFGGTR KRIGEETKIR GDINMLMLGD PSVAKSQILK FVNRCAPVSV

YTSGKGSSAA GMVRQGLLGE NEEKLDYVLG LTLPKLLERR LQTKVFKLGL AKSVHHARVL
IRQRHIRVGK QMVDIPSFLV RIDSEKHIDF ATASPFGGSR PGRVKRKTLS QSMYDRHLTI
FSPDGNLYQI EYAIKAVKNT NITSLGVKGE NCAVIISQKK MATQYITQDK LLDYNNITNI
YNISDEIGCS MVGMPGDCLS MVYKARSEAS EYLYNNGYNL NVETLCRNIC DKIQVFTQHA
YMRLHACSGK NITEESIKDM FSVYGSVEEV FIMKDNTGLG KGCSFVKFAY KEQALYAISS
LNGKKTLEGC NRPVEVRFAE PKSSKQTAKV CSRDLPGHTK MKMRDLSDYT EDKEIIETIN
LAVKYAKDAV VEDEKKNYKE ALNLYIQSLQ YFNYFCKYEK NDNIRELILK KMEVYITRAA
DLKEMLNILN KDQSIKWSDV CGLETAKEIL KEAVIFPLKF PKLFNSSALP YKGILLYGPP
GTGKTFLASA CANECNMNFF NVSSSDLVSK YQGESEKYIR CLFDTAKEYS PAIIFIDEID
SLCGSRTDGE NESTRRIKTE FLISMSGLNN YKNNIIVMGA TNTPWSLDSG FRRRFEKRIY
IPLPNLYARD IKNFANITEN YTGADIDIIC RDAVYMPVKK GLYIKCGSRY EGMSVMIENM
AFHSTAHLSH LRAIKSLEKI GANVSCNAFR EHIVYTCECL NEYLPVVINL LIGNVLFPRF
LSWEMKNNVN RLNTMRAKLF ENNEMYITEL LHNTAWYNNT LGNKLYVSES NIENYTSENL
RNFMLKHFSP KNMTLVGVNV DHNELTKWTS RAFQDYVPIP YVKQNEVTPN YTGGFVSVED
NIKKTNIAIA YETKGWKTSD MITLTVLQTL MGGGGSFSTG GPGKGMYSRL FLNVLNNYNF
IESCMAFSTQ HSDTGLFGLY FTGDPANTKD IINSMALEFH KMNKCTDEEL NRAKKSLKSF
MWMSLEYKSI LMEDIARQMM ILNRILSGKQ LCDAIDAVTK EDINRVVSQF LKTKPTVVVY
GNISHSPHYD EICKMLDAVN KIHFFMGYPE LASVNFGNST VVRCKKCRTY INPFARFEAG
GKKWNCNMCY NINETPQFYK RKDLFQRPEL CTGSVEFIAP SDYMIRPPQP PVYLFLIDVT
VTSINSGLLD VVCNTIKKLL DSRTLIGIIT FDSTIHFYNL NSNLKQNQMM VVSDIQDIFI
PLPENILVNV HECQNAIDNL LDNLPNMWRN NKMSDCCAGN ALKAAVMLIK KVGGKILFFL
SSVPNIGDNE YGEFAQSITQ FQIAVDLFAC PYNLDLASIY PLIKNSGGSL YYYPFNVHQY
SDKLREELLF ALTTETAWES VMRIRISRGW KITNWYGNFQ FRGVDLLALP NCHSSQTFSI
VVDLEENVVQ DSVVYVQSAL LYTNSNGERR IRLHTYALPV TQNIKTITDS INPQVAVSLL
SHQAIDVIKK GKIADGRNLI QTLCSQVLST QLSSSENSRL LPIYILGMLK SVAFRDPDMR
IFQWSRVENI PVESIEAYFY PKMFSLHNPP TLNLTCENMT QDGCYLVEDG ENIVMWIGRS
ISLAVVNVAA GLDGCDDQLL PASFRALEAD LNLHPSLLGY ITLAQTLMLS LFSPIWGFLS
DKYSRKWMLV FGTALWGIAT IFLANINDFT HIIIFRAING LALGSIGPIS QSILADAAKN
ELGLSFGIVQ LSSSIGRLIG GVVTTTVSMK YFGTIRGWRL CFIVVGALSI LLSIIVAFFV
EDAPSLSKKS IIIILLEGFT GTIPWLALSF NTMFFQYCDL SDLQAAVITG FLLIGSALGG
VLGGHFGDIM HNISNKHGRP FLGQLAMFGR VPLVILTYLV IPKRKESFEL FLSCFFLGLS
SIAGVAVNRP IVSDIIRPDY RGTIFSLTIA IEGVGSSLIG APLFGYLAEE VFHYRNNNLL
ISDMTTEFRS HNAEALSKTL LYLTAVPWML SFVFYSLLHF TYGAEYSKMN QIIESEMLMF
GLRSLSDFCN PNSKAYKENA YDALNRDAIP SINKAVSNYK DDDDILYCAS RVLFAMSDYC
CSEKDNAALN KLVNDGGAIT EIIKTIPKDQ DTLKNCMLFI QNLSVALLNV LTSDTYNAKL
GSAIVSALFV VSKSPSGSKA LNDENAHHKL IDHCLDETAE VIEGVFDIIK NLSSNGYVVP
TIIEKSVVIL DKFKSYPRVV SKGSDTMKSA VGPEQLTNCL NILKKEQRGS KEHDSALELL
SSLSYISSIT DKIVQSGGIP VLIELINSGL QQYDSNPDKI GRLVAGASRM LGRISNNPSH
AGVVYDYGGI ATLCTALSYF PNDADCVSAI SIALIPFVSR SYLLFASLFP ILYASVESID
LAKASMSCVA AASMINEFHE QMVNNQVIEI LSTCIQYHLT DIDYLSNVFS VYFRLSDYIK
TIEPINQYGG IIGIANALSA VDSVLTVMLE NENKEVIIQE GTKIMENLAT ESDCQRHISN
LESIAQSNPD CAYKTLAAIS GLSRIQSLKL MLESKGADSS IYNGMKTWIE SPKFNEQTKL
IKAALKTIKI VELMCIAQVK RLAEEGPDDN ILITSAECIN YLTEVNKIST KEIVESSLES
IFKMMKKYSE SRLTQTNLLS AINNILLSSN CAEVLVNKGY VKQIVTYIHK VPMYVDVQII
GFSVLANMLK INSDSLDAIK KANTLIPLQN ALRTHVKNMK LKTTCAPLLA VLMPLDTLTR
EIEDLLNLCN KCIQGNNLSQ LHANLVSLNE LLLTAESSKI SARCNIISEV AHMCTNISQS
RIGLVHLTKS NMASSLIQLY NLLKIPGDEY TEEAVANILE ALSFLLKHDI VNADLAIELG
LIEKLCSGIN HFSESDIVIK STFSCLACMC TNEKRINQLI THPEYDKLIS VIVNLVGNSK
DSRMNAIKAL HELLKTEKEE IAIDISSKTP IVNLFKIMGE YQMDLPIIQN SSKCLATIAD
HATIEEDKYS AMKILIECLG KNKNDESTAK EIMSVLVKLC NSSDKPHFKE LGAIDIISDV
TMIHGKNEEI SKLGGTLFSY MGADEQVKKL MKLILSVKDS DADAVQKIDN LTGKLELFLR
APLENPSDAL QYTEATLQQL NAYLASNLEN VSLQTNIALV NKRLVDRVKY EFEDQLGAWA
VASAGILNQY TDMISNKVGL KHDKVVAPIY SVLAGCYINM DKKAREYAQE AIKFIQRSGS
SFMACKNLRE KLESHGLIHI KEWKLQKNQG YVLCKENRNI CSFFVGKNFN INNGSILISI
GHIDSCALKI SPNNKVTKDQ ISQLNVLVEK IIQINKSVLF LPSLAIHLQN RTRSVKVNYE
NHLKPILSTV LYEKLPLLYL LANELKCKED DILDFELCLM DTNQPCFTGV YEEFIEGARF
DNLLGTFSVF EAYIEFVLFF ISSYNVKCQL RFASLGNWGK SKSQLLNAKY LKQFIKSERV
TFIVSPGSNF VDGVWKSLYE DVYEEEDMYM PFFTVLGTGD WTGNYNSEVL KGQGIYPKWI
MPNYWYHYFT HFTVSSGPTG HKDMAAAFIF IDTWILSANF PYKDIHNKAW EDLKLQLNVA
KKIIVVGDKP IYSSYLLPLL KEAQVDLYIS GHDNNMEVIE DSDIAFVNCG SFCIHELTNN
GLVTKFISSN TGSLQYFATL PKVELIDVPA AGPMGNKDTF VRIVGTIGIL IRYAYSGVAY
SSGALKLPKE WCMNPDKGLI KPDAVCYLVC IYDNDICEIS DVGVNFYITE KHVENKSNAV
LSNLQELNNY VKNIAFLSCN VYGLCGYMFN DFGSNFICYD KDGENVKSCS ISQISKETEG
KVSFDFDKSS PFQNGDFVKF TNVEGMEING QIYQIQNLKK YTFTIGDTTK FSDYIKGGEC
TQVKTNLKID FKPYEDYSKL NASNYLHVEE LDKNIIINVA KYSKAHISPI TSFFGGLLAQ
EIVKFTGKYM PIHQLLYMDF FEKNDNIISI FGKNFQDKLN KLNIFLVGSG ALGCEFAKLF
SLLDGSLIIT DNDNIEVSNL NRQFLFRREH IEKSKSLVAS NAIKNKNKNI NVISHVTKVG
QENEHIFDEK FWTKQDFIIN ALDNIVARQY VDNKCVWYSK PLFESGTLGT KGNVQIIIPH
MTQSYNDSYD PPEDSIPLCT LKHFPYDIVH TIEYARDIFQ GLFYNVPLSI QQFENLENVL
NTLKIIKENN FNFCIKKAVH LFHSNFINQI SQLLYSFPLD YKLSTGEFFW VGQKKPPQVI
EFDLNNTYVQ EYLVSTSNLY AQVYNIPTCY DIKYIIDVIK VEPFSPKTVK VNIDPIEFDK
DEESGLHVNF IYAFANLRAM NYKITTCDKL KTKMVAGKII PALATTTSII TGLVGIEILK
YVNYLSYFKN AFINTALPLF IFSEPMPPLR MKDKEYDELM KGPVKAIPNG FTTWDKIEIS
IKDYDTQRDK RFSGTVKLSN EVRKKLKVCI LGDAVHSEEA QKLKLDYMDI EAMKKLNKDK
TLVKKLAKKY DAFLASQVIL PQIPKLLGPG LNKAGKFPSL ITHNDKIFLV YNIASFCFEL
QFRFIEDTTF DWLPVIGYLL PYEKIKLLRM IFPIVFFISI CISAYAYTDR NASLIYLMRS
ILSTNRIIVE RSNDVNYLKK NGDQIVSKLQ QMRKFSLSLD SQQMNLLQKK GKKKSWFEFF
SMQMIFGIIF VYIWFTSKKP ATYEVPLPLS LEELYKGCKK KLKITRKRFM GTKSYEDDNF

VTIDVKAGWK DGTKITFYGE GDQVSPMAQP GDLVFKVQTK PHDRFTRDSN NLIYKCPVPL
DKALTGFQFI VKSLDNRDIN VRIDEIVNPK FRKIVANEGM PSSKTPNMKG DLIVEFDIIF
PKNLTSEKKR IIREALGPHY VCILCPSNNW FKDTSVKWKL SIEAGGIDID LNDAAKKSEL
SKTISALHVD TGKTKLLDKL RHTNVQDNEA GGITQQIGAT FFPKDILDKE IKKIDIKCLS
KGIMIIDTPG HESFYNLRKR GSSLCDIAIL VIDLMHGLEQ QTKESIQILK QRNCPFVIAL
NKIDRLYMWE QSAWEPFNNT FKKQKEYVKE EFNNRLQTIL NELSEQGLNC QLYWENKNPR
KYVSIVPTSA ITGEGIADLI MVLVKLTQSF MLKNIEYNKK LECTVLEVKN IEGLGTTIDV
ILTNGVLKES DTLVLCGMNG PIVTVARALL TPQPLKELRI KNEYIHHKSI KACIGVKISA
NGLEEVLCGT SLFVANNNDE IEEYKKKVMT DVSDVFNHVD KSGVGLYVMA STLGSLEALL
IFLNDSKIPV FSVNIGTVQK KDVKKASIMR EKGKPEYSVI LAFDVKIDPE AEKEAQILGV
EIMQKDIIYH LFDAFTAYLK KIEDEKKQSK MADAIFPCEV SIISDCVFNK KDPIVVGVKI
EAGILKIGTP LYIPEKNLKI GSVVSIESNK KSCSKAKKGE EVCVKIAGEP NVTYGRHFDF
NQKIYSKITR ESIDVLKQYF RNELTMDDWR LVVQLKKILN ILMIINEKDK LAEQNLETLD
VTKLTPLSED VISRQATINL GTIGHVAHGK STLVHAISGV HTVRFKHEKE RNITIKLGYA
NAKIYKCTNP DCPPPECYKS YESSKEDDPM CPRENCNSKM KLLRHVSFVD CPGHDILMAT
MLNGAAVMDA ALLLVAGNES CPQPQTSEHL AAVEIMRLKH ILILQNKVEL IKEEQALKQQ
EEIRNFVSGT AADSAPIIPI SAVLKYNIDV VCEYIVTQIS IPRRDFISSP HMIVIRTPKL
CNFVEWKEYK LVFKKRYASLF FILCIDKGDN ELITLEIIHH YVEVLDKYFG NVCELDLIFN
FHKAYYLLDE ILVSGELQES SKKIILRVVA AQDSLMEDNK FAKSLLDVAD NLSLAIKNIS
EESLKSNENI YKGIEMTETI LHNIFSKYGI DKYNPINEKF NPMLHEAIFE VNDTTKKGTV
ATVIQPGYKI NDRILRHLDI ESVQALIVAL SLYKGGIVLI SHDTYLIKHV ADEIYHINNI
TKVVKIDYEF EKYTKLLLEN KIMPREIITL QCGQCGNQIG VEFWKQLCNE HNIDQEGILK
NNFLNEDRKD IFFYQADDEH FIPRALLFDL EPRVINSIQT SEYRNLYNPE NMFISKEGGG
AGNNWGSGYS QGHKVEEEII DMIDREVDNS DNLEGFILSH SIAGGTGSGM GSYLLELLND
NYSKKMIQTF SVFPLLNESS DVVVQPYNSI LTLKRLILST DSVVVIDNTS LNRIFVEKLK
LNNPTFQQTN NLISNVMSAS TTTLRYPGSM NNDMISLISS LIINPKCHFL VTSSNVQKTT
VLDVMKRLLH TKNIMVSVPV RRGMYISILN IIRGETDPTQ VHKGLQRIRD RKLVNFIKWN
PASIQVTLAK QSPHSPHKVC GLMMANHTSI STLFERCVTQ FDRLFKRRAF LENYKKEPMF
QGNFEEMESS KEITQNLIDE YKSAERDDYF GLARAFGIPA RRYTHEVVTL WYRAPDILMG
SKKYSTPIDI WSVGCIFAEM VNGRPLFPGV SDTDQLMRIF KILGTPNSQN WPDVFKLPKY
DPNFPVYEPL PWETFIKGLG IDLLSKMLKL DPNQRITAKQ AIEHPYFMGK EKTHINLVVI
GHVDSGKSTT TGHIIYKLGG IDRRTIEKFE KESAEMGKGS FKYAWVLDKL KAERERGITI
DIALWKFETP RYFFTVIDAP GHKDFIKNMI TGTSQADVAL LVVPAEVFEG AFSKEGQTKE
HALLAFTLGV KQIVVGVNMD TVKYSEDRYE EIKKEVKDYL KKVGYQADKV DFIPISGFEG
DNLIEKSDKT PWYKGRTLIE ALDTMEPPKR PYDKPLRIPL QGVYKIGGIG TVPVGRVETG
ILKAGMVLNF APSAVVSECK SVEMHKEVEE ARPGDNIGFN VKNVSVKEIK RGYVASDTKN
EPAKGCSKFT AQVIILNHPG EIKNGYTPVL DCHTSHISCK FLNIDSKIDK RSGKVVEENP
KAIKSGDSAL VTLEPKKPMV VETFTEYPPL GRFAIRDMRQ TIAVGIIKSV EKKEPGLPIV
LLKDGTDKAQ GKSQIIRNIN ACQVIVDIVK TTLGPRGMDK LIYTERDVTI TNDGATVMNL
NISHPAASIL VDIAKSQDDE VGDGTTSVVV VAGELLNEAK VLLNDGIEPN MIIDGFRNAC
NVSINKLNDL SLSFVNKNEE EKKNILLKCA QTALNSKLIS NHKSFFAELV VNAAYQLGDN
LDKSNIGIKK VTGGSCLDTQ LIYGVAFKKT FSYAGFEQQP KKFNNPKILL LNVELELKAE
KENAEVRIDN PNEYNSIVQA EWDIIFQKLN LIKNSGANIV LSRLPIGDIA TQFFADHDIF
CAGRVEDADL KRTATATGAV IQTSLFNLNE SILGNCGLFE EVQIGNERYN IFKECLKTKA
VTIILRGGAK QFIEEVERSI NDAIMIVLRC IGNSEIVPGA GSIEMQLSKH LRIYSRSICN
KEQIVLYAFA KALESIPRYL SHNAGYDSTD ILNKLRKKHS EETNDIWYGV DCLEGDIINA
YSNCIFEVTK IKRNVIYSAT EAACLILSID ETIRNPSRNL GLPDCFKKELL KTDKIKHVLC
TGNVGCNENL ELLKNIADSV HITKGDMDSN FDFPEKINIK IGDFKISLVH GHQIIPWGDL
NALLQWQKEY DSDIIISGHT HKNSINNFEG KYFINPGSAT GAFQPWVSNP IPSFILMKSS
IVVYVYEEKD GKMNVEMSEL RKYSVEYSPS KSKDSTGVFR NPKFKDKLVD NFDECFGTRV
RKDGKLGEYQ WKTFQEVREL IILVGSGLGL YLPNCEEWNI CDFSCNAFNI ITVPLYDSLG
IESSKFILDQ TMMQTITCNK PCALKLLKSL PGKLNDICSI CYTSGTTGYP KGVIMTNHNF
VAQMASSCYG PIKLPDEKDT HLSYLPLAHI YERIMLSIFI YLGIRIGYYS GNILALTDDI
QALKPTLFLS VPRLYNRIHE KICNSLKKKP SLLQSLFNKG LDQKNSTGNP SSFFWDTLLF
NKAKKILGGN VRGMLNGSAP LGVEVAKRLK CIFSVPLMEG FGMTEGLGFI TNTYDKDVGH
IGGPLPATEF RLVSVPEMNY LVTDNPPRGE LLLRGPTIGY FKLEKETSEE GWMRTGDIAC
FSSNNSLTII DRKKNIFKLS QGEYVAVEKI ESVYKQSLFI GQIFVFGYSH ESFLVCIVFP
SFDTMEIWAK ENKINKPNDE IIKLEKFKND VIQDLVKIGK NNGLNGYEQI KDIHFIMDGF
TIENDLMTPT GKIKRHAVQI KFKQDIDKMY GNKSTKTISE GQTILTMFNE GYAPDGIWLG
GTKYQFINME KGLDYEGYSF DVATCAKLKG GMHIIKVGGG HILIVLYDEE KEQDRGNNLN
MLAVVYLACR EAGHIKSIKE LITFDRSYKE KDLGKTINKL KKVLPSRAFV YNENISHLIY
SLQLSTDLIE AIEYVVKKAT TLIWTDIERY FKDPELITSE LLFVGLTLCN VFVMYRLFLD
VIPFPIFVTW WQLAQGLLVA YVCGLKVLFV PSIFYCLMLV LSNYLLFKTP CISSYPVLVS
FTVVFHHIIR FVGCGEEYML RWKSIAFLLS AFVFGCFDSQ TSGKGVIIWA LLYALFSAIF
RAGFMQKIMH LVEGKGNTLH NNQHMLGVLI LPVLILLSGE WRVLGHMPYN ITSLYTGCLV
TVGALPFVKN VVSNRLVRRT GQGPWRFLEI ISIALVFMIG LGYNKPSFMG YLAIICVIIG
RSLGAFDVLL NKNKVGEDMR NASFALAKSV WAAGDFKGQI IEGIKRPVVT LSLSTNNVAG
VKLPIFQVHI DPTVDVLGNL GVAAGGQVIN NTRENYLQCL NMLVKLASMQ FINMFDWLNI
HSETFYATIN EDLIATKINL IRDSSTSCKD DNPYCSNNDG KVIIKNNELL SGIICKRVVG
SSSGSLIHIL WHEMGPDKTK DFISALQKVT NNWLEYVGFT VSCSDIIASN KVLDKVKEIL
NKSKNEVTKL VKKAQSGELE CQPGKSLYES FETRVNNELN CAREMAGKVA SESLDEKNNI
FSMVASGSKG SIINISQIIS CVGQQNVEGK RIPFGFNHRS LPHFIKFDYG PESRGFVSNS
YLSGLTPQEV FFHAMGGREG IIDTACKTSE TGYIQRRLIK AMEDVMVQYD RTVRNSYGDI
IQFLYGEDGM AGEYIEDQII DLMKLDNKEI KKLYKYNFDD ESYGNYNKQN ILNQEFEELY
KCKNYLCKEI FSDGDIRQHL PINMNRLIEH AKSQFPNPVE IVQKVNKFLD KLVIIKQINN
SDTLSLEAQN NATILLKAHL RTYLNSKLLI HTHKISLKGI DWLLQEIEKI FYKSLCHPGE
CVGALAAQSI GEPATQMTLN TFHFAGVGSK NVTLGVPRLK ELINIVKNVK TPSTTIYLDD
MISNDQQKAK DILTKLEYTT LKQLTSHAQI IYDPNTTSTI LEEDKLWVNE FYEFPDEDQY

TLGEWVLRVQ LTNIHVNEKK LTMKEIVYII YSVFSSDELD IIYTDDNSED LILRIRVKYL
EDTFLKKLME QCLSSLKLRG VENITKVYMR EESKITYDSE NGKFVRSSHW VLDTDGCNLE
SIFCAPSVDF KKTISNDIVE IFEVLGIEAV RRALLKELRT VISFDSSYVN YRHLSILCDV
MTQKGYLMSI TRHGINRVDK GPLIKCSFEE TVEILLEAAA FAQVDNLRGI TENIMLGQLC
RIGTGVFDII IDNQKLNDAN QNETIQDITS AGFTTPDSSP LPFSPTYNSN IKNVVMPGNI
RKSEHFLNLM RIVVVYLKKY INIYEVTSEG PLSFLYKCEK DTKLDTSFFK YSFDRLKSLL
NALQVVDDYS ALNVVCNFCT LIGNYFKGFI IICEPYPEAT IYDPVIQFAC LDSSIAMKSV
LNRYKSVVLT SGTITPLELY PKLLNFSTVL TASFPMSFDR NCVCPLIVTK SSDLIPLSSQ
YSLRNDLNVI KNYGFLLVEM CKNIPDGIIS YFPSYIYMEH VMSTWYELGI ISNILEYKLI
FIETKDIVST TIALHNFKKA CDLGKGAVFL SICRGKIAEG IDFDKHYGKC VILFGIPYQY
TLSRILKARL DFLKETYNIQ ENEFLTFDAM RQASQCVGRI IRNKKDYGIM IFSDIRYARN
DKKGKLPPWI IKCMDVSNIN LTIGAGVSIS KKFLLNMSQE YKETDQTKNQ PLSKITELPN
KMKVATIKNN CEVPTIGLWI SSGSKYENKA NNGVAHFLEH MIFKGTNKRN RVQLEKEIEN
MGAHLNAYTA REQTGYYFKC FKDDVKWCIE LLSDILTNSV FDEKLIEMEK HVILREMEEV
EKSADEVIFD KLHMTAFRDH PLGYTILGPV ENIKNMKKND ILNYIQKNYT SDRMVLCAVG
DVEHDNIVKL VEQNFKPFFC GSEIIIRDDD SGPNAHVAVA FEGVPWTSSD SITFMLMQCI
IGTYKKNEEG IVPGTINNIS NKMTIGCADY FTSFNTCYNN TGLFSKYLWK ARIYFIWQRL
FSSSSSFMYI LKRHIIDPFN IFKNMNILSE KFKIYILSNY SVYLSFYNYT YAYDKLLEIL
SECSKFYYTF TGRMGIKRKY QKNPATILVL LKDFDPDTDI LEEPYFFDSQ NNLSFDEQIA
LINYCFSIMR FNPHYDEIKF EKLNAIISRC LKYQNNLLHS CILWFKCKCE SFRLKTVDRS
QAQLNELLKE YYNDEPQNKE RLKFIYDIYY PTTWEMKKEV GNIMIKTGSV VSAFNIFKDL
KLWEEAIECL IEADRKAEAK ELLDTLLEKK KSPSLVCLYG LKYFIQAWDL SNYKYSKAAR
LIGKHYYNKE MYSECCDYLE KALEISPLLP DIWFILGCAY MKIDKFDQAI KAFTRMISMN
LAYLYMKNNG YKAAKICINQ AVKINNNEWK YWDTYLKLSI VQNDVDSFCL ALTTLCQLNQ
VKQIQPWVFD YISDLIVKDK EYDSFWNAYS FFLFVKGEFE DSFEAKIKEI RSIEVSYIWK
NKIFVGSQAP HFKAEAVFGD NSFGEVSLSD YIGKYILLYF YPLDFTFVCP SEIIALDKAL
ESFKERNVEL LGCSVDSKFT HLAWKKTPLQ QGGIGNIRHT LLSDISKSIA RDYDVLFNES
VALRAFVLID KQGIVQHLLN NLALGRSVDE ILRLIDALQH HEKVGDVCPA NWQKGKESMK
PSEEGVAKYL SNLMEDTAAK IISSDGLLTD PRIAQDFSAE TNELLAKAEE AIEIIYEIAN
ILNVNLDKET IVILIQLCEY G

> ***Plasmodium vivax***
NFDGDFKTTK KKIHWLPYIP EKLITCTLYE YDHLINKDDW TKFINPSSKY ETVVYSEPAI
SSLKVCDKFQ FERRGYFIVD LIKIPDGKSK KAGLNAYDEP SIVFPTIVYV GDEAIFRESE
LSFYRPIDHG HISDWDLAQI AWEYAIKCVD QNRSVENILL TEPPLCSTSH RTKMGEIFFE
DFNFQNINIS VSGLMSIYAT GLTTGLVLDI GDGVTQCIPV FDGYIEKNSI IRSDFGGEEL
SMFLQKLICD IGYSMTTRKN FEYVKTIKET LCFCSLNPPK DQLRDDLNVT YTLPDGDVLR
DGYDSVEISH ERFYVPEALF NPLICHRDSL SIVDIVWKSL LLCPIENRKT LTSYIVLSGG
SSLFPNLVER LEKEVKNNAP ESARSAVKVV TYEQGASFAR ENDLFFAEAS AVSKLNVKHV
FENLLQMEKS KLAKVEKVLG RTGSRGGVIQ VRAQFMGDTE LSGRFLIRNV KGPVREGDIL
ALLETEREAR RLRGTQSLNL KKSHCYCHLS TGDLLREAAE KKNDLGNKIR NIINEGKLVD
DDVVLTLVDD KLKSPQCKKG FILDGYPRNV KQAEDLNKLL QTNQMKLNGV FYFNVPDDVL
VKRISGRLIH KPSGRIYHKI FNPPKTPFKD DITNEPLIQR EDDNEEVLKK RLNVFKSETT
PLINYYKNKN LLCPVCYFNL PDPESAIAPY DTELNYFMWG PGFEWQPFNE KSSNGKVSVE
DASQNARKLG LAPSSLDEKK VRDLYGDSLT YEQYLEYLAM CVHDRDNMEE LIKMFSHFDN
ASGFLTKNQM RNILTTWGDA LTEQEANDAL NAFSSEDRIR ISKLFAVIDK NNDELTAWSN
YVKNEVFLKQ VQVEMKQIDA DKDGFISLPE LNEAFSQNLK EVEKHAEGLL KRFQIVDKDK
DNKLNINEVG LLIDPMKDEE LKELEINEIL EHHDVNKDGR ISKKDDDVAL DDFNFDTNKD
GFIDKEEIIT LDLWNEKALK LAVTSLTDYG DILRYPEDFK LLSNLNCFGS GFIFSIVMFH
LLPEFFFVFV GFAMQLALEY VLPVDGNMCC DSSSSEEEER VIKSYEGKRI DFYKNIGNSL
NESMGSSDFN QLLKDYESVY KFMVKESSER IPNFAIVYLD KLTKYVETTF QNNVEKKVLS
KNKAQTLNKL KAKIRKCSEF YQNKLNLYHE NPEEFWSNSE EEGYVSDDAD DKTKSAMSKW
GLKTSEKVEK KKAVKKEEKA THVDENQSAK NKAYAELLST KNLSEEVIRN RVKFVIEKRG
RKGLDKHEHI NILSKLCELA KTISTQSYIE VLEQMINLEF DVVSSVYTYM SFNIWNKAFK
YIELILDLLI QNENFYLVSI NITEEITEEV INEKEKISRS CKTLISFLAK LDDELLKALL
YIDAQTEEYR RRLGKTVHMI GLLYKGYNYV KAIFISTRIL EHMYYKPEAL FMQIWNFVEK
TLEQERAEKR RLLSFHMHIS IELIECVNNI CAMLLEVPNL ARHTYESKDI ISRQFRRFLD
IYDKQIFNSP PENNKEIILL ATKYLQKGNW KLCCEKIFSL SIWSKFNEKV QNILQEKIKQ
EAMRTYIFRY ISIYDSFSVE QLCIMFDLNQ NVVHSILSKM MINHEIPACW NESSSHILIN
KVNPTALQTV AIKLAENINE IMEQNELTLN MRNPKLGLQN KQCIVVGTRA LEFLNNELST
IKTLTELHDR VKKRGITDHN DNILSLYLLE LFSIPIEQKE KNDFLTNRIS MYSKILNGRK
NVLDLLLNKF ENDCNDSIKK ELLDCFDVEK FKQEVNSKFM NILIQQLRKV EKLEKKKSKM
EFYLSIREQV KLYIRELVNI VSNLLTGNYP ILNLNGEDFL KKYGGTLMEN LKDGLVEGQP
VRFILAKEES SMFGVVQNTP TDAKSKNILV SFYFRNSNTE EQVQIKSVEK DRLAVETLNG
DPSFLWYKMI RDDGWVGFDK AEIIKVFNNN SKIKDVLIRH TDLKILNQLA ITQFWAIKML
ISVPFATKIY AHFVHDSIKI SILTVLGDIA TALNRSFSRY LNFFANILAE TSKITITSGP
PESDDWVNYV FELRDAILLT YSNIIYALID GNEIVKLKVY ITNILDLIEL ILIKEINHFN
AQNFQNAVSL LGDLVHAYGY ELIENSKLTD LIISVYGKID ILSSQGDECV SKIKWLKRIC
NISILQLEFK TVEDLNNATN NFIMAIKNFN VFPELRLKIL QLLYNSFSVN FSFFIAILQF
SSQNNIFHFI LPYIKFIDQW IKEWNISSRE KRQIYLIIAQ ELKKLKKYEE SFKHLNKHVY
YFQEVLNHPT TVNASVELIA DAINLNNNIY FHQLLTLDAI QNLQNIEHQP IFHLLTIFYQ
YSIHEFLAFI DLEVAENKIY LLSIISLFKE TKVQNIQYIS EKLNISTLKI EKILVAAIGS
GVIDAKIDQI NKSVQMKTTI LRHFDEAHWE ILNAQITKYI NNVATNFYKF VDSSASSTRA
IQNNPSYWCS SGNHSKDEEI NWTGYLNTKG FIKGVKISWE YSPELVSISV SSDGENYKNV
IPYRRISGNE ASFDEIYFFK KLEEVVSVKI GLKNAIHKYF GIREVKIIGG GNPYFLLLSG
ITSDNEMCLQ ELWKTNSNNQ IISAFSDPPK CLSVINLDDL GDGKSNWIFE SNSQIRLQLC
ISQKNIYGNV PGIHDIDVTV DANSTLDDDH NADNTVDGNL NSYWASATFA DNEHLVYLIL
DLNKYVEISR VKVSWEYPPL HYSISASVDK VIAENLANPS FVTMDSLKNV ETRYIKIVMM

KPHPKHGEMG DQFLYGIRSI EVQANNLESI ISYCRDAANS DDARDKYFVE YITEFDQDLT
NKLINLEDDV SKNVNSISDN LSKLEELLPN IETCVEEKKE YDEELKESKE KANELIKPKC
APEPLRVYCD MVSSTSLYVW NGINSVDDIR QHCAEIGLEP LILRSKSQLN SLILSLKKMG
YTLNGKNNIP LAYDYSCFHD LVNGNIDLTT LIYESPDSTK VRQTALEEKK MIFCKIEEVA
KRRNKRHVKT QVLSEDSKIE RHKMKKFYNS KSGYFGCGWR TQWTPFIHAP FFDNHHNTIY
KNRNKKMYEE IDTLLHGRIH PSVRIVELKD HKHPVRLCTP YYEDCYSVVY TGKKILASDD
RVIFGEYTGY VNNRELSQEK HQYTFALSFI ILPDNYTYAV DSSYMFNEMS LVNHYKTCFN
NYDFRINAEW QLVYLDGWPH IILTSIPGVE INTGEEIFAD FGFEWFEKVN DICLNEFIKN
SYAYRLDDIV EKYNLIKNHL TCNICMYSVN TDSSHFIACS GCNHIYHLAC VQKLNNENYD
WFCASCVKFC INVAKAIICR VTKMHFEANE DMFKTSSECI QSVIRELALG STKIQREFTN
GTYVGTVTEQ IHGNPFFVVT YEDGDAEWMT PCFLFQEEHK QKGNEFYKQK KFEEALNEYD
QAIQINPNDI MYHYNKAAVY IEMKQLDKAI ETCLYAIENR YNFKADFAQV AKVYNRLAIS
YANLKNYDKA IEAYRKSLVE DNNRATRNAL KELERKKEKE EREAYIDPEK AEEHKNKGNE
YFKNNDYPNA KKEYDEAIRR NPNDAKLYSN RAAALTKLIE YPSALEDVMK ALELDPNFVK
AYSRKGNLHF FMKDYYKALQ AYNKGLELDP NNKECLEGYQ RCVYKIDEMS KEKVDEEQFK
KSMADPEIQQ IISDPQFQII LQRLNENPNS ISEYIKDPKI FNGLQKLIAA GILFKPNEKI
PSKYGENRHW NVDLIPKFIL VGGNLVKILK KTRVTNYLEW LVVEGSYVYQ HQKKGLLYSE
KFIHKVPATD MEALVSPLLS LMEKNRCKNF YKYVSEWDAN NRNTWDDLDP YRLTMMDIYK
YFNLCQLTID FLGHAVALYL NDDYLNEPAY KTLERIKLYM QSISAFGKSP FIYPLYGLGG
IPEGFSRMCA INGGTFMLNK NVVDFVFNEK VCGIKSSDGE AYCDKVICDP SYVHLKNKVK
KIGQVIRCIC ILSNPIPETN DINSCQIIIP QNQLNRKSDI YVNLVSFQHG VSLKGKYDVR
LDVKLNKFIW SKGIRNPPNR VRVKIERKRN EDEDSKEKMY TIVQHVMVDT YKGLLNEQMA
GDRHRIHDCT TENFNGFMAI TTPTKSWMAQ YNDLSKYAPG FYALQVVGEL GTGSGYLILS
LRELLLKKKK SLDLLYCLDI NEKANKISNV EIINTDLFSN LRQCFDLVLF NPPYVVTEED
EMNKTIVASY AGGKLGREII LKFLLSVYDY VSDEGVIYLL MEKNNRPDEI EGAELPILCE
TCLGENPYVR LIKEENGKEC KICNNPFTLF RWKPGQKSRY KQTIICNMCA KVKNVCQTCL
FDLQYNLPVQ VRDKFLETSI SMPENETNRN FFLEQVELSK LRRRDPYFKR NMARVCSFWR
KNECNRGAEC PYLHKEIHKI IIFNMPPVNE QDVKSLCERF GPVVDVYAFV NFMFPSACER
AKNILNNAIF RGKVLSVKYA SYKKILEIQK KRNCQNENIW NLLYTDINSS IHSFCKENKC
SPQSILDRNI AVNVSLTETY IINKMKEWIR KEGIRSDDTI IVKNLSVQTN QKEVISLFKK
FGVLSKVSFS PYNNIAILQF EKAENAKKAF ISNSYIRYKK LPLYLEWAPM NLFDEEITHA
SIYIKNLNFN TKEEDLKKLF EKLDGFITCN ISQGYGFVEF KSKELAVEAI KKLTATTLDG
HVLELSLSKL LVKNLAFQVT KEELRKLFSA FGNIKSVRIP KNAYNRSRGY AFVEFMSKNE
CLTAIESLQH THLYGRHLII DFAPQNARIL KPLVQEKVVE IMKPEIEEKI IEVPQVQYIE
KLVEVPHVIL QEKLIHVPKP VIHERIKKCP KTIFQEKIVE VPQIKVVDKI VEVPQYVYQE
KIIQVPKIMV QERIIPVPKK PQYRHIPKPV EVPMAHYRTF PIEKLVDRNV PVPVELQIVQ
EFLCPKIEAR YKEIPVPVHV QRIIEHPIPK DAMNNPFLLP LYYLGSTAVG ICVNDGVILA
SERRIASALI EKDSVEKLLP IDDHIGCAMS GLMADARTLI DYARVECNHY KFIYNENINI
KSCVELISEL ALDFSNLSDN KRKKIMSRPF GVALLIGGVD KNGPCLWYTE PSGTNTRFLA
ASIGSAQEGA ELLLQENYNK DMTFEEAEIL ALTVLRQVME DKLSSSNVEI AAVKDQTFYK
YKTEDISRII EALPRFKIID ADFINIKLME VFQMEGFQKQ LDRLSDSLSK IQKALGEYLE
KQRNQFPRFE KLVQTKLTDA CFLTLTQALK MKLGGNPFGP AGTGKTESVK ALGAQLGRYV
LVFNCDESFD FTAMGRIFVG LCQVGAWGCF DEFNRLEERI LSAVSEQIIE ILNKKIGLNK
NVGIFVTMNP GYAGRSNLPY GVGFKMSFAR ISSSCAIMSR TINTIGIGLL SLELMNHCDA
KELATPLCMW KLPNKELINR NIANKSEHRH HQKLLMSYTP FNSPSLLAEQ INILGTYSGT
RLLYFPLWDH PKDSIDYCLS TYLYWLYLRR STNIFLQNTL LRGQVVVIAA TNRQNSIDPA
LRRFGRFDRE IDIGVPDDNG RFEILRIHTK NMKLSPDVKL EELASSTHGF VGADLAQLCT
EAALTCIREK MDVIDLEDEI IDKEVLESMC VTQDHFNMAL GTCNPSSLRE TVVEVPNVKW
DDIGGLDEVK NTLREMILYP IDHIMKSLHD ANFWKPTEIQ KQTLEHSINF KKDVIVVSKT
GTGKTLTFCI PILNNIIILV PTRELAIQIL SHFSNINKYT HIYIATIIGG LNLNKQKRII
SNKPEILVCT PGRLRYFACD EIDKMIETSF MRDINLIAKH LYIQTFLLSA TLLAKLLNCV
TIRKEQSCII NLLPDGLSLS MVKCERKKVL QKLFYLLKLY KIVIFVNTIK ETKELNSIFR
FLFFDQGVES SVPKKIFSIH SKQSLKERMQ SISKFSINHS VLFCTDVLSR GIDLDKCDVI
IQLNCPVSDI TFVHRSGRTA RNFKTGNKAV SVEVRSAELV LTSEELRRVS DPKYGTIDYR
QICSVCFENC VGHIGHLEFS LPLFNPLFYK DLCELLGLVC LDCFQVCCSY NYEEYLQLTK
TLKLLMKKSG RCPACKGRRN ISARMSQKRD TITVRLFSFQ VIDLLEKVFD REVMHLLYPF
TKRDGHKVFF LYDMGISANR FRVKLRGVHK KNALLNLCIS AKKEIDFEEL LELQVALNTF
FDVREILDKK EGILRKNIMG KRVNNCARTV ISPDTFIETN QIGVPLEFAK KLTMDECITE
NNFDYVKRLS LYDFILKKDA DFRHLSFISD FMTGELCMDI LKANWSPAWT IQSLCRAILF
LFTEPNADSP LNCDAGWFCG GVKVKGAVEV GKRAVEYFRG DEFVHFLYTR RDMLKKKFPT
LLQNRTLADL KDIEEFSDTF IQKGFIYKAQ YKPENGMYRR PKWPRRLIMS SKQNFDRTSF
YILVHERNKK LQYLMLITLI SIVLICCMFW HLSVVFISLM SVIVLIRLFL FIFFWFFGVD
YWLFPNLFDE ECNVVESFTP VHQWIYRNDT WLLVIARMFT AVLLAIGIHQ LGKTHSISDI
GNFATQSFID IIEWGNKKLA EENYDCLKGC GFQTFEELVR KCFLKCECMT LADTFLQDLE
DLEFEERKVS ELLYDIEKCI ELIIQIDTEI LNIHKYVRDI YSTKFPELDS IVYTPLEYIS
VVSKIKNESD LKSIDFSDIL PNTTVMAITV ASSTTGINLS DHSLKNCLSF CNEALELNEN
RRMILLYLES KMFLLAPNLT MLLGSALTAR LISSVGSLKN LSITSSQNLI VVGSSKKGIL
STSEIVQSVP DAFKKKAISL LAGKCSLASR VDYFTEERTV LDKKILNSSV ILQIAMRVKR
DKYLGRHYRY FIRNTRVRAY RQFLEPFKSV TLKNMAYAFG VSEEFIEVED PYEAINEAVK
NFEHADEVLA SAKIVERLVE YPEVSRNLHK INAIDPLLKL LNHHILESVL QIFSLALSNN
PELQESVFKK NALKTLLLKL QESQKSVIDK KLITAISALI RHHDEAENKF IDYGGVGFLV
YGMQTNMYQY QEKSALLLKH LVLQNKITFE IFLKNEIMKG LICLANTGIQ YGETTAELFL
ALMQNHRHRL AKSGLHNLKL LIEDRLAYLF EELGVEDWLV KISKTVQISH PTKIQQLCLP
LIMRGHNVIG TSETGTGKTI CYCWGILQEL NKNPFGVFAL VLLPTRELVV QVVEQFLLYG
HKIGIKIISC IGGSSLIEQP HVVVGTPGRF RRLRFLVLDE ADLLLRSCYE AKLEGILRGV
RRRTLLFSST ITDSLELLAN SFPQEDLLLV NVNRKQKPLK NLDQRYGIIF TDNSYRCELV
YTVLSMLFSV ESIHSSKEPK KRLAALSKIK NGGCNILVAT DIISRGIDIP KTSFVINFDF
PNDSILYVHR VGRTARANRK GVAISFVDKR DVNSFNSVMQ TMRGALKPWR LRRREVLQDM

FHVGRVLKKA ELLLEEVVVF PVSVHYELAK SLLKPKFHTG IQNVSKFGSG SYTGEISAEI
AKDLNIEYVI IGHFERRKYF NETDEDVKDK LQQCLKNNLK VVVCFGESLE QREKNQTIDV
ITKQVNSFVH LIDNFDNVVL AYEPIWAIGT GKTATPEQAQ EVHKEIRNIV KEKCGANQIR
ILYGGSVNTD NCASLIKQED IDGFLVGNAS LKSSFVEIIK SAKDINKDVV HRYGPNTFKL
HRLPIPKLGQ ILGLVGTNGI GKSTALKILS SKLKPNLGKF SNPPEWRDIL SFFRGNELQI
FFTKLLEEQL TPIIKPQNVD LIPKQIKGNI LEIINKKDKL NKKDQYMSAL ELDHLLDRNV
EDLSGGELQR FALLISIIQT TNVYMFDEPS SYLDIKQRIS MAKIIHSLVR HDNYIIVVEH
DLSILDYLSD YVCCLWGKAG AYGVVTSPFS VREGINVFLD GFVPTDNLRI REESLNFKLE
DKKRLHFYNY PTIKKTLNSF TLTINKGIFS ESEIFVLLGQ NGSGKSTFIR LFAGLIKPDN
VEFLESLSVS YKPQQIQAKY TGTVRQLLMS KLKGLYTDPY FNNEIIKPLK IEAILDNQVL
TLSGGELQKV AIIITLAKNT NIYLIMSILC TISGQTPEEP VVSKTGYVFE KRLIEKHILN
YGICPVSGEV LTLQDLYPLK NEQVVKPRPI TASSIPGLLS ILQTEWDAII SEMFTLRTHV
NDIRNQLTHS LYQYDAATRV IAKLLKEKND YKEEVKKLRN QILSIKSGND LNEFEVGLSE
DLLNEMQEVA KNLLMTRKKR NIEHVSSAEQ WKEVTNTNEF DVHSAAIPGV TCLSLDFSGG
MDGNVYYVSL EQSKVLAKLQ GHLKKVTAIV AHPKYSLCIS GANDKTVRIW KGDKHKDQIT
SLALHPMENY FVSSSKDNVW ILNDLETSRS IKICKDTPSF RQLAIHPDGI MLGIGCEDSN
IHIFDMKSQE YKASLFSENG YYLASCSKDK TVKLWDLRKA QCFQTIDVEE LPRSICFYNG
NIKDGLFHGH GILMYSRNEK YEGDFVYGKR EGKGKFTYAD GATYEGDWVD DKIHGKGTAK
FVSGNVYEGE WDNGKINGFG ILKYNNGDIY EGEWLDGKMH GRGTYTYEDG DIYVGEWKND
KRHGKGCVKY KGSENKIAET YEGDWFEGKM QGKGTYFFAD GGIYEGDWID GKMEGKGVYK
FLNGNKYDGD WSNDMKNGYG ILTYVNGEMY EGYWKDDKVH GKGTLTYSRG DKYIGEWKFA
KKSGQGELIY ASGDKFKGEW KNDKANGFGV LYSNGNKYEG EWVDDQRHGF GTFTCKEDGS
VYAGHFAFNR KEGRGTLTFV DGNVLEGLWT MGVLTKVSKF QLAPTSPWHD PDLEMETLYD
LGNKMIEALQ KENITAGDVI CIDKSTGKIT KIGKSFARSK DYDAMDPNTN FVQCPEGELQ
KRKEVVHTVT LHDIDAINSR TQGFLALFSG DTGEIKNEIR EHIDMKINEW QEDEKAEIVP
GVLFIDEVHM LDIECFSYLN RALESEQSPI VIMATNRGIT HIRGTDYKAP HGIPLDLLDR
TLIIPTYPYK HQDIMKILEQ RAEEEDVEID EYAKELLCKI ASESSLRYAL HLITLANLVS
KKRKATEVTV QDVRRVYNLF IDVKRSTQYL IEYQNEFMFS ELYSNLSDFW TSDDEDDEEY
IRKKWVIEDD VSNFSQNDLL LSYDFELDDF QKRSIKHLNN FKHVFVAAHT SAGKTLIAEH
AIAMSIKLNK KAIYTSPIKA LSNQKYHEFK NLFKSVGIIT GDIKMNVHAN CIIMTTEILR
NLLYINDNII NNIHCVIFDE VHYVNDNDRG FIWEESIIML PPHVQILLLS ATVPNYLEFA
DWVGFTKKKE IISISTKKRP VPLLHYIYAY DTIFQIMDEK NIYSSAFKEI YEANMKTEIQ
KLQALIKKLE QDNKLPVVLF CFSRIKCETY AKSMPHLNFL DNKHKSKVHL FIKESIAKLC
TQDRELNQIK ILTKLLEKGI GIHHSGLLPI LKEIVEILFS KGLIKILFAT ETFAMGINMP
AKSVVFTSIY KHDQLKKRIL TSSEYTQMSG RAGRRSSDKY GYVYIYCADK IPDQVQLTEM
LMQKAVSLKS KFKVTYNMIL KLLINKQINI EKMLFSSFLE SCRAVQIPLF KKDLKRKKKL
LQNIKQVQCI YIESYVQIDC KLKHIGLSLH RKLVPIEHIS IITNELDRLI EKGNFEPFVL
TKMLKSLKCE FYSVLHYELV CKRNNCINDI ENIERNINAK SLNLYEDLEG RLDVLRHFSF
IDDEHNLTVK GKIASYITLT DEITLTQVIF ENLLNNLNPP EIAAVLSCFV APEKKIEESP
DLTVNLQDVK MALTNIHSQF EEFYKIIRLK ISSEEHWKLC SFKIMFIAYK WALGVSFAEL
LEQSELEEGL IVRSILRLDD LCRKVKIAFL YLGNVDLAER LETTCTLLRR DIIFMTSLYL
DALALLQFFH WCDEKRKTKE LFNETEMSLK NKVDYFRSTK PNFPSFATIS ASGPNAAVIH
YEVTDSTNAK ITPGIYLLDS GGQYLHGTTD VTRTTHFGEP TAEEKKIYTL VLKGHLRLRK
VIFASYTNSM ALDFIARESL FKHFLDYNHG TGHGVGLFLN VHEGGCSIGP TAGTPLKPAM
VLSNEPGYYL ENKFGVRIEN MQFVISKKNT DNTEFYSFED LTLYPYEKKL LDFSILTAEE
IRDINEYHET IRKTLLPRLK QNPKQKLVVG ENPAFIQERL AKYNALKEKI TVQLLDGSVR
EGQCNVTTPF QIASSISKRL AEDSLVARVT YVERVDLELC DLWDMGAPLL GSCKIEFFWH
SSAHILGSSL EKLFGGYLTI GPPLKEGFYY DIYLGDFSIT NEHYKKVEDE FSKLIKENAE
FEKLICTKEE VMDLFQYNPF KLELIRTKIP EGKKTSVYKC GNFIDLCLGP HIKSTGKAKA
FKVLKNSAAY WLGNKNNESL QRVYGITFQK KTELNDYLNF LEEAKKRDHR NVGKKLHFFF
FDKDTSPGSC FWLPHGAKIY NKLIDFVRRE YRIRLYDEVI TPNVFSCDLW RTSGHYQNYK
DCMFIFNVEQ KEWGMKPMNC PGHCVMFKQL NASYRSLPIR LADFGVLHRN EISGSLSGLT
RVRRFQQDDS HIFCTFEQIK EEVLGTLHFI FFIYDLFGFG IEVLQNRGYD SCGMSTILKT
TKYASSSTSD AIEKLRGNYS ASHKNDNIGI AHTRWATHGC KVDENAHPHM DYNERISIVH
NGIIENYREL KTFLLGKKIP FRSNTDTEVV ANLIGYFLDQ RESFQNAVLS AIRQLEGTWS
FCIIHRDHPD EMILAANGSP LHIGFKDNEI FVASEHSALF MFTNEYISLK NGEILSINKD
KINDLKMEKK VGSIPEVVIQ KTPHPFPHWT LKEIHEQSIT LSKTLNNFSI LNSSVKLGGL
DPYVEELSKI ENIILIGCGT SYYAALFAKY VMQYLSCFNT VQVMDPIDFN VSVVPKEKEG
VIFISQSGET RDVIKACKLA DDLNLKKLSV VNSVGSTIAN MTGRGVYLNA GREVGVASTK
CFTAEVSVLT LIAIWFFQNK SHSKVSSLIN SMHRLPLYAG TTVKCEAKCK QLATKLTNKS
MLIVGNGLSY PIALEGALKI KELTYIHCEG FTGSALKHGP YALLGGEDNL PVIMLVFNDK
NVMMNTGEQI KSRGAHIICL TDDEDLCKDD IILIPNNGLL TPLLAVIPLQ MLAYYTAVAR
GNNPDRPRCL AKTVTVWIIE IEKYASEDVQ KVLIGNKIDL KNDRNVSYEE GKELADSCNI
QFLETSAKIA HNVEQAFKTM AHEIKNKSQL ENQQKGRVNI NLNAKPIKIR TMNSRKPPEG
WSKVESFLDE MNKKMRSLEN EDTSKKRKSE ILWPIFQINH QTSRYIYELY YKRKEISYDY
VVIGGGPGGM ASAKEAAAHG AKVLLFDYVK PSSKGTKWGI GGTCVNVGCV PKKLMHYAGN
MGKLDSTQYG WTCKDSHNWG KLVSTVQSHI RSLNFSYLTG LRSQVKYING LASLKDEHTV
AYYLKGQEET ITAKYILIAT GCRPHIPEDV EGARELSITS DDIFSMKKVP GKTLIVGASY
VALECAGFLN SLGFDVTVAV RSIVLRGFDS QCALKVKNYM EEQGVLFLPK KLSKEEDKVA
VLFTDGTTEL YDTVLYATGR KGDIAMLHLS CTNVPNIFAV GDVAVDVPEL APVAIKAGEI
LARRLFKQSQ EIMDYTFIPT AIYTPIEYGA CGYSEEKAYE AFGNVEVFLQ EFNNLEISAV
HREKQKDQYD TDVSSTCLSK LVCLKDNRVV GFHYVGPNAG EVTQGMALAL RLKARKSDFD
SCVGIHPTDA ESFMNLSVTR ASGLSFAAKG GCGGGKCGEH LYSGPLKIEQ LLAKGFVKRD
LELLKEGGLQ TVECVAYAPM RTLCAIKGIS EQKAEKLKKA CKELCNSGFC NAIDYHDARQ
NLIKFTTGSK QLDSLLKGGI ETGGITELFG EFRTGKSQLC HTLAITCQLP IEQSGGEGKC
LWIDTEGTFR PERIVAIAKR YGLHPTDCLN NIAYAKAYNC DHQTELLIDA SAMMADARFA
LLIVDSATAL YRSEYIGRGE LASRQSHLCR FLRGLQRIAD IYGVAVIITN QVVAKVDAMS
MFGHEKIPIG GNIIAHASQT RLYLRKGRGE SRICKIYDSP VLPEGEAVFA ITEGGIADYM

AIRVQFENSN EVGVFSRLTN SYALIAMGGS ENFSSVFESE LSQHIPLVYT TIGGTKVIGR
VCVGNRKGLL VSSICTDQEL LHLRNALPEN VKIKRIEERL SALGNCITAN DYVGLIHTDI
DRETEEIVQD VLDIEVFRTS IAGNLLVGTY SYFTNNGGLL HAMTSSQEIE ELSELLQIPL
ITGTVNRGSD LIGAGLVAND WSAFCGMDTT AIELSIIEKV FKLNSIEDAN IEDNFKYKSS
IIQTMIIVDT YWQTETGGIV IAPIPNLFKM KPGCASLPFF GVQLEILDSK TLEPLNGPNC
GLLCIKSPWP GMLRTVYGNH NRLVKTYFSS CPNYYFTGDG AYRDEDGYYW ISGRIDDTLN
VSGHRLGAAE IEHALVQHSC IAEAAVVSFR HVVKGEGILC FVVKKLKLYV RRAIGPIATP
DLICIVPDLP KTRSGKIIRR ILRCIANELN DFGDITTVAN HEVIDIIPFP TPCSVEDALA
YYCDLTTIPR VNILKKFKCF IKDVEEKEAD MTFIEFVDIF MAKAEFELTP FLQLIPRNVP
KSYTISSSPK RWFKGASSFY LTELSPNDSV KFNLKSSKFE IDFLYEREID AEQGKHIDQV
FLAFSRDQPW VEKYRPKKLD DIVHQTNAVS MLKEVVRTKN MPHLIFHGPP GTGKTSAINA
LAHELFGRDN ISERVLELNA SDDRGINVVR EKIKAYTRIS ISKNKINSET NETLPPWKLV
VLDEADMMTE DAQSALRRII EIYSNVTRFI LICNYIHKIS DPIYSRCSCY RFQGIPINIK
KDKLLYICKS EGINILDKII ETTQGDLRRA VSILQLCSCI DPMITLESVL DVSGLPADDI
ISKIIDACKM KDKNVEKAVQ DVIEDGYDVA YIFKSLNNYF VDSVKSQILL ELSRHDYRLH
SGATKYIQLM SFASSVHSLL IKELIFKSND ERHFQNVVKQ VKDLIKHVKQ KEVEDVNDPK
HAQEKLVLNK SGRRIILRDL MTRPNIFTGR KILGTLELHT NGVRYSAIDI LFDDIKYAFY
QPSDGQLIIL IHFHLKRYIM VGKKKTLDVQ FYCEAGTQID DLDRAKARNV YDPDEMHDEM
KEREQKNRLN LIFKNFVQQM QDISKIEFEI PYPELTFSGV PNKSNVEIFV TANTINHLVE
WPPFILSVED IEIASLERIH HGLRNFDMIF VFKDYTKPVK RIDVIPTEYI DTIKKWLTTI
DIVLKTILAD IDSFVNSKGF DGFLGDDEEE EEDEDEDDEY EVDESELSAE IETLAIVEFT
SKRKMSSVIC RIPKIMLFCK GAGSVILNKL AKKTEVDDIT IEHMETYADE GLRTLCIAQR
ELTEEEFAEW YRLYKEATVS IKDREENLEN VAEFIENKLT LQGVTGIEDK LQEGVSSTIE
DLRLAGVHIW MLTGDKIETA MNIGIAANLI DNYSVDGGLL DILLSKKFER KFFYLADKCS
SVICGRVSPY QKGAIVSSAN RLLKKITLAI GDGANDRNMI NTANIGVGIR GQEGVQAFNS
SDYGISQFRF LKNLLLVHGR LSYTRISKLV VYMFYKNIVL IFPLFIFGSL SLYSGQKIYY
EFLLHLHNIL FTAVPVVAHA ILDKDVSLNT ALVTPSLYKL GIYHYYFNIS TFVSWVINSL
FHGLVVFLIP LYFLIPSSDG TPYDMWTVGS VTYLTVLVVN LKVLLETYCL NVLPLTAVFM
SILAFVILVV SCSFMCFGSN NFLGTAVILA KSLRFWLVVL LGLFALSRDF IFKVFKRNFN
PRSYSDHVKD HFNKPRNVGS FDKNEKNIGT SIVGKASCGD VIKLQLKIED NVIKDARFMA
FGCGSAIASS SYATELIKGK TIDEALKIKN DDIASHLSLP PVKVVILGSG WGGIHFFISI
DFKKYDVTLI SPRSYFTFTP LLPCLCSGTL SAKVCTENIS TFLRKKGSSG SYLQMECTDI
VPEERQVICR DNNEVKISYD YLIISVGAKT NSFIKGVEKY AFFVKDIQGV INIRRRFLDI
LSICSTERIS NEEKKKLLHI VVVGGGPTGV EVAGEFADFI NKKRKYKSIF PFISVSIIEG
GNNLLPTFTQ NISDFTRKTF RRSNINVLTN YYVTEVDEHN ICVQSKHIPY GILIWASGLA
QTPLITNFLK KIPEQVNNKI LNVNGHLAVI GINIYAIGDC KKIQPVQITA EQLINEALDL
EEVEQKVNYN LIDEDELNEY KISKRKEYED KIRKRRYLIS TYIKYALWEV KQKDMRRARS
ILERALNIDY TNVNLWLKYI EVELTNKNIN SARNLFERAV LLLPMENIFW KKYAHLEEIL
NNFLNCRNIY ERWVKWKIDE TAFLCYINFE ERCREINKCR DIFERLIVTL PKMECFYRFI
KFERKYKNVD RAEKCIQLLP PSFLDEHFYI HFCNFEEENN EYERCRKIYI EALKILPKSK
SEFLYKSFLQ FQKKYDELDE TLMIKERITY EEEIKKNPSD YDTWFNYIKL EERIRELYER
AISVLKNALV HDGLKIGIRE VIKSIESKEA KACFLSDVCS EPAYKKLITA LCTEKNIPLF
MVDSKDLGQW VGLFKLDKEG NARKIIGASS VSIIDFGEES AERDYLGYYK AIVGEVIDNR
YSVVCELVGK GVFSNVLKCY DKVGKIPVAI KVIRDNDMMR KAAEKEISIL KKLNKRHIVR
LLRSLKYKNH LCLVFEWMWG NLRIALKVQE SFSSLVQKTK ELEDVYISRR KLQSKYLTQI
KNLYINCNCE CIIHKISFKY ATKNSFPNLA NGTLLYMIVE KINLDNNVVA SCINSSDVKS
WINYENYLGE LVDGYMFPVS ISYAKSLIGD KCYLLDLIDV SYEVAVGHNG GALGYSQHLS
EEVFLFSREA LLDKVAVILG GRAAEELFIG KITTGAIDDL NKVTQLSYSY VSQYGMNKEI
GLVSFQPNSS SDYSFYRPHS ECLAHLIDNE VRCLIETQYN RVKSILRTHE EQVHKLADLL
FRKETISYQD IVQCIGLKHQ RYRGTEKVKI EEERNKKVYV KNLKFCKNCG SATHKEKDCL
ERTRKKGYDG NRDRWVGYDP ENFEQVYREY EKIVEEKKKR KAEELIDSKV KRVKILSRYE
EDVHLFEHSS VFGSYYDRER GKWGYRCCRS TDRFQKCIIP LMDPYHASYY TREVMVILIN
EFNSPDEEMK KVVLKCVKQC IQTEGIERDY INQEVVNPFF EKFWVLRSSH DKRNLHLIVE
TTVEISNKIG VIARIVDDLK DPSEQFRKMV MQTIQSIVNN QGVDDIDQTL EEQLIDGILY
AFQEQASEDY YVLLNSFDAI VNKLQIRMKP YLPQIAGIIR WRLNTPLPKI RQQSAELIAR
IANLMHLCEE HQMLGHLALY LYEYLGEEYP EVLGNIIGAL KSIVVVLGVQ NMTPPIKDLL
PRVTPILKNR HEKVQENVID LIGIIADKGG DLVSPKEWDR ICFDLIELLK SNKKLIRRAT
IQTFGYIART IGPFEVLTVL LNNLRVQERQ LRVCTTVAIA IVADTCLPYS VLAALMNEYR
TQDLNVQNGV LKALSFMFEY IGEIAKDYVY AVVPLLEHAL MDRDLVHRIA TWACKHLALG
CFGLNREDAL IHLLNYVWPN IFETSPHLIQ AVIDSIDGFR VALGPAIIFQ YLVQGIFHPS
RKVREIYWKI YNNVYIGHQD SLVPVYPPFE RLADSNFARD ELRYMGRMYG KGKGISSSTI
PYKRKQPSWL KQKPSEIEDA IIKLAKKGQT PSQIGATLRD NYGIPQVKAV TGNKILRILR
AHGVATTIPE DLYFLIKKAV SMRKHLEKNK KDKDCKFRLI LTESKIHRIS RYYKRKKLLP
SNWKYQSSTA SALRNIEHAP GVQFAYVPPD FFDSEDDECE KNQYELKDDG GGRAAGTRAK
DHATSHHLRR KNYEDDFEFN EGKILEALHI LELLYLNGAS LEEQNEHGQT ALFLSVKRNN
ISTLQWLLSK GVNINHRDFY GNTILHIAVK HCDVDILRLL CDYGSLPLVH QTSLQNDNTN
VLQLCLRNRY FLVFILLNKW MLQDKLCKGI KICKTVYAFY FWFFAILNLI VFANISRSFW
AFRKYHALSI TWLAIWLFQQ FLWCLLYFKS PGEYQLNGLE MELYKLNLEG QKMPLYAQVS
QERVNSLDAD YRNAILEIIL LQLIIEPYIL RRSKKHVFID MPKKHSIIIK LPLNSTQLNL
YKDEIMSKMQ HTHKHLINAS IFILRRICNH PLLHKYYYSV EDIKKISKYF YANTDQYLDL
DLKTVENEFM KISDFDIHLS IKHLISQDEN LNKYLISKEH ILNSSKIHHM ISLIKEIRKK
KEKVLIFSQF TTFLDIIEEA LYVRLDGSTN TIERQKIIKR FSNVFIFLLS TKAGGVGLNL
IAANHVILMD QWNQLLLQEE IVKKLCEHII NSQCDVVEKG VSDLAQHFLV KRNISVIRRV
RKTDLNRLER ISGATIVSRC DEIVESDIGT KCGLFDVKKI GDDYYAHFVE CENPRACTIL
LRGSTKDVLN EVERNLHDGM NVAKNIIMEG KLLYGGGCTE MRVGQHLISQ ANQYDDSRKS
IMEAVGSALE IIPKILAQNS GANVVKTINE LRIRHGGEKF GVNGVTGEII DVSTENIWDL
LAVKKQIYKS AIEAAAMILR IDDLAVVLIY EELLKNPLCR ITNIYENQVK KYRPLPLNTL
QMTKLVSRHF HISSKQCMNI AEKLYNRGFI SYPRTETNYF PSSMNLRSIV AELKKSKVFG

SYAKKLCEGN PRKGRLNDQA HPPIHPVKNM EWAIYELICR HFLAVCSDDA IGFNSKVVAT
IGKEEFFCKG LKIVKKNYLE IYIYEKWNDK ILPPFQVNQE FYPYSLLVDE GMTQPPKYLS
ECDLLSLMDK YGIGTDATMH EHIENIQKRN YVFKNKKNLF IPTNLGIALV LSYKKFKDIG
VDLTEPSLRA KMEKDMTLVA SGICVVKSGK TDVPRNCKMI IISYELMTKN DKYQKYKSIV
CDESHYLKNS FSKRTKAITP IIRSAKRCVL LSGTPALNKP SELYEQVSSI IPNLFNYHEF
CERYCFKDKN IYTRKIEYVG CKHTEELHLF LTNTIMIRRL KKDVLKELPE KLRSKIPVEI
PPKELSEIIL FKMTGYAKVK AIKEYITYLI DADIKFLLFC HHKLVMDEID DFLREKKTMF
IRVDGLTPIE KREVYIKSFQ NDDHVKIALL SLTACGIGLN LTAANTVVFG ELYWVPGQII
QAEDRAHRIG TTHEVVNIHY LIAQNTIDEI VWKIINRKWN TLTTALNGME DSLMFEEKDK
ICIAVSGGKD SSVLAHVLVQ IRRKHNYKWD LFLLAIDEGI KGYRDDSLKI VFEDIFTYTM
DAVVSFIGKK NNCTVCGVFR RQAMERGALL FNATKLVTGH NADDLAETIL MNMCRGDLEK
LFSTECTYSP NSFRGNLRSF IKDLECASCG AYTSNVEGLR NYKDNKKVSN CSLEIDISYE
HVEPMLLEGE YQQIPRLRVL SFDIECIKLD GKGFPEAKND PIIQISSILY FQGDPCSKFI
FTLKECASIP GSNVIWFHDE KTLLDAWSEF ITRLDPDFLT GYNIINFDLP YILNRGTALN
LKKLKMLGRI KSISSVVKES SFSSKQFGTH ETKEININGR IQFDVYDLIK RDYKLKSYTL
NYVSFEFLKE QKEDVHYSIM NDLQNESPES RKRIATYCIK DGILPLRLID KLLFIYNYVE
MARVTGTPFV YLLTRGQQIK VTSQLYRKCK ELNYVIPSTY IKSGSNEKYE GATVLEPIKG
YYIEPISTLD FASLYPSIMI AHNLCYSTLV KNNGKSNIKF VKRSVKKGIL PLIVEELIDA
RKKVKLLIKN EQNKITKMVL NGRQLALKIS ANSVYGYTGA ASGGQLPCLE VAVSITTLGR
CMIDKTKETV EKYYSKSNGF EHNSTVVYGD TDSVMVKFGT NNIAEAMALG KDAAQRISKE
FLHPIKLEFE KVYCPYLLLN KKRYAGLLYT TPERHDKMDC KGIETVRRDF CILIQQMMET
VLNKLLIEKP FIQVAPMINV TNRHFRALVR TITRRAQVWT EMIVDNTLLY NLNNLEEHLG
FNSNEHPIVC QLGGSDATSL AEAAVLVEQA GYDEINLNVG CPSTKVANKG AFGAYLMKKP
EHVRNIVYEI KRKVHIPVSV KIRTGVDDCD SFPFLRSFVE CISSVGCSHF IVHARKAWLK
GLDPKQNRSV PPLEYPKVYS LCQLYPHLKF TLNGGVKTVE EAVALLLHGV MLGRACMENT
TVLSQTDQLV YNEKPPHTAF SRRTVLDAYK SYLEENSSLC SLFELLKPVL GILKGMPGHR
IFRFLTHFFF KNPVGHVGVV ALKNSSAKLI QPLTSNMEDI TNALLKERSM GLQGSPSLQQ
GLEIAHDLLI DIPLYGTKEI LIMYGSIRTC DKKNILNILN LIVKNNMHVN CVSIAPEMHI
LKCGMHLISM HDLSHITNNL QGSPLFLEIM GSNSLPMSQQ MYFSTHNALR INENDVISTL
FYEINGNRHI SLLIFPFYDV QMLKRLLIKK LNLPGVKVND IIIFYKGIKL PNYRIISTYK
KLNKLYWAIK DTNPNASIRV IDQKYPPFFE NILHDIKLAF KKNISPKLTM DGTGGTYLLF
NSKKKVCSVF KPLDEEAFAP FNPRGYEGKM YQEGFRSGVL SGEGASREIA AYILDNSYNN
FSSVPCTIMV EACNPHFNNK SKLKYVDNEA TLKWKCGSLQ EFVDSRESVG NYDYKQFSIR
DIHKIAILDI RVMNLDRNDG NILVSPTHLG IEQSRRDDIE ALGYVLMYFL RGSLPWQGLK
AISKKDKYDK IMEKKISTSV EVLCLLNVSP KIDCCVIIDR RIDMVTPFCT PFTYEGLLDH
LFGISNLQIE IPLYNDIKDL NPNQVGLYLH NKASEIKETY EKDTLKDIEE INTFLKKIKV
KHYEHNSLST HVNLASFILT TMKKEANFNK LKLEDEIIQL NSTSDRTTLL NIVQQIKLLI
YTNEDIYEVY RLLCLFSVVT NGKKDILESY GIRELSRINK LHLCNIIKHQ PKQKFIWGNL
RNHFNLLSND HNDISYVCNG YAPLSVRLIE YKNNMQAFPE IFNLLSGPTL DIVQETIQVR
SMCINCEQEG INQIAKLHIP YFKNVLIHSF ECGFCNYRNN VIQDLNTIKE KGVKIIFNIN
KREHMDRQLI KSEYGVLKIP QIDFEIPKET QKGSINTIEG FMQTALSSLY IKLIESTVHK
LFTIEIVDPS GLSSLEYKRS KQELNEMGFY SFTSNCPCCN YMGANNFCEI NIPGFKKCLI
MSYVCGNCNF KTSEIKSSGE INPKGKKITL TVRSKSDLDR FVIKSDTASI HIPIVDLTSD
YGTLGGSLTT VEGLILKIIE SLEDKFKFLM YVLNRKGEEE DISFDQILKR IQRLSYGLHE
LVDPARVTQG VINGMYSGIK TCELDELAAQ TCAYMATTHP DFSILAARIT TDNLHKNTSD
DIGEVAEALY KYTDVRGRPA SLISKEVYEF MIEHKDRLNK EIDYTRDFNY DYFGFKTLER
SYLLRINGKI IERPQHLLMR VSIGIHIGDL EKALETYHLM SQKYFTHATP TLFNSGTPRP
QMSSCFLLCM KSDSIEGIFE TLKQCALISK TAGGIGVAVQ DIRGQNSYIR GTNGISNGLV
PMLRVFNDTA RYVDQGGGKR KGSFAVYIEP WHSDIFEFLD LRKNHGKEEL RARDLFYAVW
VPDLFMKRVK ENKNWTLMCP NECPGLSESW GEEFEKLYTK YEEENMGKKT VLAQDLWFAI
LQSQIETGVP YMLYKDSCNA KSNQKNLGTI KCSNLCCEII EYTSPDEVAV CNLASIALCK
FVDVEKREFN FKKLYEITKI ITRNLDQIIE RNYYPVEEAK RSNKRHRPIG IGVQGLADTF
MLLRYPYESD SAKELNKRIF ETMYYGALEM SMELAQLYGP YETYQGSPAS QGILQFDMWN
VKVDKKYWDW DELKAKIKKH GLRNSLLLAP MPTASTSQIL GNNESFEPYT SNIYYRRVLS
GEFFVVNPHL LKDLFDRGLW DEDMKQQLIA HNGSVQYISE IPADLKELYK TVWEIKQKNI
IDMAADRGAF IDQSPRLSFE EMRGEMSKYG VEITQGTLKN PTTEDMQGVY SMCIKHILNK
DINNIRIEEF TGDLKSILPN EGKNHLQAIG NLRFIRHCEQ VNKILCVENT LSYLFKPVSS
HMARLISAFI VNETNEIIFQ FSRFRQKKED LEDQIVPSPE KLQEYNQELK NLLLEHVSYF
EKDKKKNEEI KNKINISDLC LKKLVDLVTI LTGHIKLHIG KKEELKGLEK HLKNLDWWTL
GIFIYEILVG CPPFYANEPL LIYQKILEGI IYFPKFLDAN CKHLMKKLLS HDLTKRYGNL
KKGAQNVKEH PWFGNIDWVS LLHKNVDVPY KPKYKNVFDS SNFQEDLTIA DKITNENDPF
FDWVVSQFYI LSPRGDTIIN RDFRGDVSKG SGDAPPLFYL NGIHFTYLKN NSLYFVFTSL
LNSSPSYVLE LLYRVVKIVK DFCGQINEEV IRANFILIYE IVDEVIDYGY IQNSSTESIR
HLIHNETLPS NASQKPIQLN EKKNEIFLDI VERIDGVILI KSYLQGNPFI KIALNEDLYI
KNVHSDSTNN IIIDDCNFNH LVLSLYQPDG ECVLMNYRIN MINIYKPNDI IACEVQRILA
DGCIVLHTRS SIYGKLSNGI LITVPQTLIQ NQKKHIFVFP CVDDTTRRNI SIISNIIKLL
AKYHININYD IVTKIYIHEW VPPKNKQIKA ATSNCAQIVI SLSGGELLYF EIDESHTLVE
TFRKNLNVET LCLSIQQNKL RANFLAVGCL DNVVRLLSIE KYFNQLSTFI LPNNSSAQDI
CILFLNIGLN NGVLLRSVVD PIGTLTNHYS KYLGAKNVKI CPVNAALLVL CEKTYLCYVH
QGKYIYSPLN YDILEYASSF HSEQCSDGYV AISGSSLRIF RFYRLGEVFS QNILHLTFTP
RKIVPLPFPS MLAIIEADHN SYDENTLSEI QRALKGIQLG TVKAGPGKWG SCIKIIHPVN
LQTIDKISLE MEEAALSVCA CELEALHCLI VGTTTSLSLK AALRVYTYDI NYKLNLLHIT
PVEDQPFCFC PFNGRLLASI GNKLRIYALG KKKLLKKCEY KDIPEAIISI KVSDRIFASD
IRESVLIFFY DANMNTLRLI SDDIIPRWIT CSEILDHHTI MAADKFDSVF VLRVVEEKPD
ITYNDIGGCK EQLEKLREVV EMPLLQPERF VTLGIDPPKG VLLYGPPGTG KTLTARAIAN
RTDACFICVI GSELVQKYVG EGARMVRELF QMAKSKKACI LFIDEVDAIG GSRGDESAHG
DHEVQRTMLE IVNQLDGFDN RGNIKVLMAT NRPDTLDSAL VRPGRIDRKI EFSLPDLEGR
THIFKIHANT MNMSRNVRFE LLARLCPNST GSDIRSVCTE AGMFAIRARR KTITEKDLLL

AINKVIHGCK QFSATGKYMV YNIKSKAEEN LRRIIQCMDA LYQMTKTNHF NLPRYSRIPK
EKKNTRWEIF AKKKLMKKNK SGLIYDQNSK GWVRRFQKKQ MKINEDKANF VHEYKPSDNI
DPFERMEEEK EIKKMKQKMR EMKNKVFDRL TDANFYTGMH KQKFKKGKTP CVVPPKNIWL
FRNGDEHHNG LLFLVKPHVH NWTSLLSEIT KVLGPTIGPV RTIYNANDGE KYLCTSGEPP
ARVDRLSRFD ARKYFKEGQK CITPPNGDGT RAFYESLLEE NPNSVIAIKY CIEHGVLSGT
KHHQTIYKYN VLKKNNAFRN NFGGLRGEFI KLLNVKFIQE KKLIGKFFEE IAQDTGKVVY
GIEDTLKALE IGAVELLILY EGLDVIRLTT RNAITNTTRT MHISPQDEKQ ESLYKENNVE
LEVVEKISLT DWVINNYKKF GASLDFVTNK SQEGAQFLQV LYEFIFLCIR IYDDISKLFE
LPYMIASDVL CEQIISIIVL PFNYLGLSAL KGKNMQALLA SVSQKHKKKL SLDIIDAIIE
CKNKAIVYRD VEEILSYISP IFDIDERYNI CMLFYKHIEN GPYLVHLLPT IVFTMLHLVM
LEAKLNNASI LKKLFECIKD LVNDANVDAD ESGLKLQALD GNHVSLVSLH LVDSGFSHYR
CDRERVLGVN IASLNKVFKL CGANESVVIS SKDDEDNLNF VFENNKEDKV TNFSLKLMSI
ELDSLNIPEE GFDAEVELSS KELTNIFRNL SEFSDTVFIE IDSNIIKFTT KGLVGDAEVA
LKPRESTDDV GVTIKSRKKI KQSFAIKYLN LFSKSTILSD VVTLGLSDSR PIEFKYEIKD
GFVKFFLAPK MDDECPEKCN EEVGVIKGTF LYTFDSPICK AAIHAGVLNV ADDLVLIISH
KHHSFLGTKR NDVESHGFTG TSKSFSVSIP TGFNGKETDF VNCTNLPNEK YIKSLSNFTF
IIYFGGGNWR TLLSHSLCDG ISISVNEENE LIIEQNCNPH LIKTKFKPVL GQTYHLAVAF
NKTNKGVTLY INGKELTLEK AKYDFTLNGD LIIGRSNQTT TDYFIGSIHL VEVYKFVLAD
DEIRQSASAA LSRKTVDGRQ CTTPCKPKSI INKELQINGE QINLSCQDDL LSHQFDGKGS
QFLVHCSDDC TKSKLIVKGS NYYTPDSSIC KAAIHAGIMR IVNGLFEYKA ARGHLGIVSK
SERQSCSSNG HLFLNLPVGT KRTIMCPSGC GTNVYAPSST LCKAAIHSGV LSNQGGLVDL
SLGSAVDEFT GSTQNGVESH SSARRSRSQK ELYARADVGT KKKVLDLRLN LGPYRCSYSR
NGKYLLATGE KGHITLVDTH NLEPLCELEV EESVRCSTIL HNHKLFAVGQ KKYTYIYDNT
GMEINCIKDI PYTYQLEFLP FHFLLTSIGE FGELVYQDIS MGSIITRKRT KRGPCKVMKQ
NKQDATIYLG HQNGHVTVWT PNIDKNYLIT SSVDCTYKLW DMRKLQLMED ARSNVINQME
ISTTGLVAMA INSHFRTYAN FFTKSYLTHN MHGDRINSLS FQPFEDICCV GARHSIKSLL
IPGAGLANID TYVNNPYETK KQNEVRSLLD KLPPDTITND YKKIILVRKI RAKTPIKAIC
SSPKDVRVDT LKSYSSKTWP KWSDSESVCA LRMNSSIFVY KDNERGSKGK PSVFKIFNSS
NLQSHLYTKS FFNSDEMKLS WNKNATAVLL NVHTDKEKQY YYGLSNLFFI ETEKYSEVNI
MTDRGQIYDC IWSYNQNKFY VCKGDIPAEI VSYDRCANVA HSFGRHKFNT LKLNGSEKLL
LTGGFGNLSG DITLWNTSTK KEVTKTKSSC AVVCEFFNDG KHFLTATTHP RLRVDNHLKI
FTHNGFIVSR INFEELYNVI ILPPGANFVQ EQKKKAPQKK KKEEDTIPLP NIKTPILKKI
IEYMEYHINN PAEEIPKPLI TSNLQDVVVE DNSSKYKDLA HKMTQEIEMA VVLFKRKYLI
KRIPKLPQCY IVNSGALSIA RARVKLPSTY AKLGNPLSAS KLPEFNCTFD MIGELQQFFM
KQRRCDYFSL LNNFVNLTIS VSNLLSTEPD VEIRNELLNR FIYSLNSWML MRRCIVAACT
NVFSMTGLCI PLQILHFNYD ECKIFFSKKR APYLLMFEVA DLDEDISHII PVECQRIIFG
EINRDSISSL LNNPLARSLM NEISNNPEML ANIVSNNPLL RNTFPIMQPV LENPNLLREL
MRPEFLQAGL PPEERYASQL LSLQEMGFID NDANIQALQE TGGDVNSAVT RLLEKGSGDR
LTRAARVLEQ LTEQKPIFGK CRFTIRSFGV RRNEKISCFV TVRGKKALEI LEKGLKVKEY
ELRRKNFSET GNFGFGIQEH IDLGIKYDPS TGIYGMDFYV HLSRPGYRVT RRRRERLGFF
IALKMMNEIK GLPSVDSNYQ WLPLLYMALG NDLAVSKISL SMVKPYSIAL IRLLRDFFSV
VFLIKCVGIG YRNMSGAQCV AIACDLRLGA NNFTTVSTNF TKIFKMNDYV YVGLSGLATD
IQTLYELLRY RVNLYEIRQE TPMDIDCFAN MLSSILYANR FSPYFVNPIV VGPYLTAYDL
IGAKCETKDF VVNGVTSEQL YGMCESLYIK DILLVGAGGI GSEFLKNIIT IGCKNVDIVD
IDTIDITNLN RQFLFKKEDV KKYKSFVAKE RALQHSKGLN INAYTFDVCT MKSSDIAKYD
YVVNALDNIK ARKYVNKLCV MERKVLIEAG STGYNGQVYP ILANETKCYN CEEKPKNKTY
AICTIRQTPS LPEHCVAWGR LIFETFFCKS DNETLMDIKN HVEEESKKRN MDQHEIITFI
FNYLFYDTIK ELAALKKDYV TEPIPNINKQ KEEYLVFDKD DDDCINFITA ISNLRMINFS
IKQKSKFDVQ SIAGNIIPAI SSTNAIVASL QFERVYVCKP QSSRNKSDKI LVLNFGSQYF
HLIVKRLNNI KIFSETRDYG IDVKEVENIK GVILSGGPHS VPHLKKEVLE YKIPIFAICY
GMQEIAVQMN GEVKKSKNSE YGCTVWMNHT DEVTKIPDNF FLVNSTDDCL IYNEEHNIYG
VQYHPEVYES VDGDQMFYNF AICECTKKFD PIRYHEIELN NIKKYAQDHY VIAAMSGGID
STVAAAFTHK IFKERFYGIF IDNGLLRKNE GEKVFLKGIF PDMNLTKIDA SEIFLNNLKG
VTDPEQKRKI IGKLFIEEFE KAVKNINIDI EKTYLLQGTL YPDIIESKCS KRSDTIKTHH
NVGGLPENLK FKLFEPFKYL FKDDVKKLSQ ELNLPEEITN RHPFPGPGLA IRVIGEIDKH
KLSILREVDD IFINDLKAYN LISQAFAVLL PTKSYDYVCS LRAVKTSSFM TASWYKIPYD
ILEKISTRIS EVKGVNRILY DISSKPPATI EFEMPLPGFV SDRRLYVQKP LILYESENSK
VEVDPILAQF LREHQREGVT FVFECLMNLR DDRISGCILA DDMGLGKTLQ SISVLYTLLK
QGIDKKPAVR RCLILCPASL INNWNDEINK WLPGRCNVTC VNDNAKEKIV SKLEGFKYDQ
KSTVIICSYE CFRINNDSLD KSSIDMIICD EAHRLKNDKT KTYMSIYKLA ARKRLLLSGT
PIQNDLSEFF ALISLCNPDL FDDTNSFRKK YANPILIGRD KDATEKEQEV ASERLAELST
INKFILRRTN NLLSKVLPVK YLINIFIKLN PIQEALYVLF LKDKRILKNE QSTNKVNVLI
NIKKLEKICN HPLLLNPNDM KSCKFQLLHF LLKTIKQNTT DKVVIVSNYT QTLDYMEILC
KENFYKFVRL DGGISIKKRH KVISDFTHSS DIFIFLLSSK SGGCGINLIS SNRLILLDPD
WNPANDKQAL ARVWREGQKK ICYIYRLFCT GTIDEKVYQR QISKDGLSSM IVTNTNFSKD
QLSDENVKKL FNYKMNTLCE THDNIECTRC KKVEPAGFMF YVKYEKANNK KSSNLVKKCI
NNNINVYEVL SVEEADDLET IKASYKKLIL LFHPDKFLKI QDSYAVLSDK TLRKQYDSSI
PWSAKKPVPD IGDEHTDIKN VKYFYDFWYN FINWRDFSYQ NEYNYEEAEC REERRWMERE
NKKIQKKASK AENLRIIKLV DLAYNNDPRI IAENKRLKLE KQKKKAAVKI WRHHIKSFET
LCEFINDIYV FLWTAQEVSL LAKALKSYPG GTKNRWNLIS NFIKTKSVKE VIKKTKEMFE
NETLWTHQEQ HLLEQALMKH PASLPMPKKR RNGGRSKHNR GHVNPLRCSN CGRCVPKDKA
IKRFNIRNIV DTSAQRDIKE ASVYSTFQLP KLYIKQCYCV SCAIHSRFVR VRSREQRRVR
KETSKHVNPS QKKVSVSEIN FDSSYTVLDT SEGAIMLHVN HVLYHLDFNA LAVVKNVDMT
EEMQIDAIDC ANQALQKYNV EKDIAAHIKK EFDRKYDPTW HCVVGRNFGS YVTHETKNFI
YFYIGQVAIL LFKSGVCGWS KAVRKQGGRF CFVNLNDGSC HLNLQVIVDQ SIDNYEKLLK
CGVGCCFRFT GKLIISPVQN SIHNFEIYGE DPQKYPLSKK NHGKEFLREV AHLRPRSYFI
SSVIRVRNAL ATATHLFFQS RGFLYVHTPL ITTSDCEGGG EMFTVTTLDY KKDFFNKQAF
LTVSGQLSLE NLCSSMGDVY TFGPTFRAEN SHTSRHLAEF WMIEPEIAFA DIYDNMELAE

SYIKYCIGYV LKNNFDDIYY FEENVEKGLI SRLRNILDEN FAKITYTNVI DLLMPFSNQF
EVPVKWGMDL QSEHERFVAE QIFKKPVVVY NYPKDLKAFY MKLNEDQKTV AAMDVLVPKI
GEVIGGSQRE DNLELLDKMI VEKKLNMESY WWYRQLRKFG SHPHAGFGLG FERLIMLVTG
VDNIKDTIPF PRYPGHAEFI KDLLDMNLEN SKNIIKNLFL KDKKNYFFLC TVNWKTVDLK
YLSSLFKTSN LRFVDEGNLK SMLNLLPGSL TPLALKFDQE NLVKLYFDDE LKSMMIVHPM
HNYSSLYMKQ EDVVKFCQLH NHAPILGITA KKTTSFSEWY TQVIVKSELI EYYDISGCYI
LRPASYYIWE CVQAFFNKEI KKLDVENSYF PLFVTKNKLE REKNHIEGFS PEVAWVTKYG
DSTLPEEIAI RPTSETIMYS VFSKWIRSHR DLPLKLNQWN TVVRWEFKQP TPFIRTMAKL
SKAQKKQIYI EKLSSLIQQY TKILIVHVDN VGSNQMASVR QSLRGKATIL MGKNTRIRTA
LKKNLQAVPQ IEKLLPLVKL NMGFVFCKED LTEVRILQNK SPAPARLGVI APIDVFIPPG
PTGMDPSHTS FFQSLGISTK IVKGQIEIQE HVHLIKQGEK VTASSATLLQ KFNMKPFSYG
VDVRTVYDDG VIYDAKVLDI TDEDILAKFG KGVANIAALS RSIGVLTEAS YPHVFVEAFK
NIVALVIDTD YTFPLMKKIK DMVENPEAYA AAAPVAEEEE EEDGFMGFGM FDQERDLARE
PCPDRIIEDM GGAFGMGCIG GYIWHFLKGA RNSPKGDVLS GALYSGRMRA PILGGNFAVW
GGTFSCFDCT FQYIRKKEDH WNAIGSGFFT GGVLAMRGGW RSASRNAIVG GVLLAIIEIV
SLVLTRKTTP TPRQQFQQQM ELEKMLVDNI GDVTITNDGA TILKQLEVQH PAAKILVNLS
ELQDQEVGDG TTSVVLLASE LLRRGNELIK MDIHPTTVIC GYKLAMKESV KYIKEKLSER
NLGKDVIMNI AKTTLSSKFI GYESDYFAKM VANAIQSVKI VNDAGKTKYP VSSVNVIKVH
GMSSLDSKLI EGYAIMSGRA SQSMPTGVKN AKIAFLDFPL KQYRLHLGVQ VNINEPTELE
KIRQREKDIT KERVNKILES GANVILTTQG IDDMPLKYFV ESGAIAVRRV NKDDLRRIAK
LTNGQIRLTM SSLDGTEKFE PASLGYCDEV YEERVGDWDV MFFKGCKTSK SNTILLRGAN
DFVLDEMQRS IHDALCSVSR ALESNYVVVG GGCVEVALSV YLEDFAKTLG SREQLAIAEF
AESLLVIPKI LALNASYDSI DLVCKLRAYH TKSQVNTEDP KDYRWYGLDL VNGKVANNLK
NGVLEAMISK IKSIRFATEA TITILRIDDL IKLTPERKPP VIKLKGHTSS ILDLQFNPCF
SEILASGSED LTIRVWLKGH KKKISIIDWN PMNYYIMCSS GFDSFVNIWD IENEKRAFQI
IMPKKLSSLK WNVKGSLLSG TCVGKHMHII DPRKKEIASS FHIHNGGKNT KNIWVDGLGG
DEILSTGFSK NNLREMKLWD LKNTSSALVT MSIDNASAPL IPHYDESTGL IYVIGKGDGN
CRYYQHSLGS IRKVNEYKSC SPFRSFGFLP KQICDVYKCE IGRVYKNENN SSIRPISFYV
PRKNFQEDLY PPILGKDNKM ERINVFEKLK ICGQFNKGFV ISKLFIIDQH AADEKSNFEK
YNKVFTMKSQ RLVYLLSLPV FNGKILEVED FMSLLHHLWF NYNFPRPQRV WRILASKMKA
LVLMFLKKIS DTYVDDQAKW MNKMRSSQEE QNNKKISEWN ETIENKCFVY PASSAPCGAC
TSAGAIAPYR RFKEPRRKKQ YTGTDILCQA KSGMGKTAVF VLSILQQLVR CLGLAHTREL
AYQIKNEFDR FSKYLKGVRC EVVYGGISMS KHIKMFKIPH IIIGTPGRIL ALIREKYLLT
DKIQHFVLDE CDKCLEKLDM RSDVQKIFIS TPLKKQVMFF SATMAKEMRD VCKKFLQNPV
EIFIDDEAKL KLHGLLQHYV KLQEKDKTRK LIEILDALEF NQVIIFVKSV TRAITLDKLL
TECNFPSIAI HGGLDQQERI ERYDKFKKFE NRILVSTDLF GRGIDIERVN IVINYDMPEN
SDSYLHRVGR AGRFGTKGLA VTFVSSQEDT LALNEVQTRF EVAISEMPNK IDCNEYINQR
MSVSAEAYGE WNKKKNFVPK VYKKEESEKE KIREALNDSF LFNHLNKNEM ETIVDAFFDE
HVEKNVNIIN EGEEGDLLYV IDQGEVEIFK TKENKKEVLT VLKSKDVFGE LALLYNSKRA
ATAKALTKCH LWALDRESFT YIIKDNVAKK RKMYEDFLTQ ISILKDMDPY ERSKVADSLK
TKTFADEDII KEGEPGDTFY IIVEGNALAI KDKTVIKTYG KGDYFGELAL LKNKPRAATV
KAKDTCQVVY LDRKSFKRLL GPIEEILHRN VENYRQVLKQ LMHLQIVCLT DEVREMYKHH
KTHHEGDSGL DLFIIKDEIL KPKSTTFVKL GIKAIANTSY LLFPRSSISK TPLRLANSIG
LIDAGYRGEL IAALDNTSEE EYLIKKNDKL VQLVSFTGEP LSFELVDELD ETSRGEGGFG
STLGAAYGTA KSGVGVCSVG VMRPDLIMKS ILPVVMAGVL GIYGIIMSIL IYGDYIKAYL
LGFTIEDSLA LLRIDDLYVE SFQVKDVKIL KGDHLSRCIG RICGSNGATK YAIENATKTR
IVIAGDKIHI LGSFNNIKMA RYSICSLILG STQGKIFNKL NILAKRMKER IEKLEDRMHP
WSNIDGMKAA CSYTYDDIIC LPGYINFPMS EIDLCNNLTP EISLKTPIIS SPMDTVTEHK
MSISLALCGG LGIIHNNMSI ENQIEEVKKV KRFENGNKVL CEEKKSVLPI VNSNYEFPHA
SKSQNKQLIV GASISTDLER ANQLIKNMID IICIDSSQGN SIYQIDTIKK IKGAPIIGGN
VVTCDQAKNL IDAGADVLRI GMGSGSICTT QDVCAIGRAQ GTAVYHVSNY AHNIKTIADG
GIKNSGNIVK ALSIGADFVM MGNLLAATEE SCSDYYFENN VRLKIYRGMG SMEAMVSQGV
SASLVDKGSV LNLIPHLVKA VKHGFQSMGI RNIPELHSRL YSGMKENKQY QEALKELKKL
KKKIEENINL LKHIKGKLVL AHESAWDKFG SKLKDMPFLN NFFENPILGK LFGETELAAA
LREMKMQDKN FKLCELMYLF EFVISKHIVE SYLIGDEETL RLHCGQSAFN SLNASINERK
KKKVYLDTNV LIYKNHELKG AQRMEESSPW FIFTFHTQQI NCLKNANDEI VEGHIDDIRE
VVYTIALSKH PEPEGLLYPY LVREFAIIGN TPSWMALYII GLGLGDERDV SVKGKELIEM
SDVVYLESYT SVLFVSKNTL EEFYKKNIKE VDRNLAEENC EEILKEAINK KVSFLVVGDP
LCATTHHDII LRAKKKNINV QVIHNASVMS AIGESGMQLY NFGQTVSIPY FEGNYKPTSF
YDKIKVNLDN NFHTLCLLDI KVKERTIENM MKNKNIYEPS RFMTVNEAIE QLLYCELKKN
VITDNTRGIA IVRIGSDSQQ IVSGSLLALK SVSYNDPLHS LIICAPTLHD VEREYFEMYP
NSAYRKCVRV QLIKNGKKIT AFVPGDGCLN FIDENDEVLV SGFGRSGHSV GDLPGVKFKV
VKVARVSLLA LFKEKKEKPR SMGIKGLTKF IADAAPNAIK EIKIENLMGR VVAIDASMSL
YQFIIAIRDS EQYGNLTNES GETTSHISGL MSRSIKLMEN GLKPIYVFDG APPELKGSEL
EKRGEKRQKA EELLKKAKEE NLEEIKKQSG RTVRVTRKQN EEAKKLLTLM GIPVVEAPCE
AESQCAFLTK YNLAHATATE DADALVFGTK ILIRNLINLE QVLKGLNLSM NEFIDFCILC
GCDYCDTIKG IGSKTAYNLI KEYNSIEKII ENIDFVEARD SFINPKVKEE VKIDWCEPKI
EELKNFLIKD YNFNEVRVTN YINRLLKARK VTTQRRLDNF FVNNSKYYEV LNLKKNCTTD
EVKKAYRKLA IIHHPDKGGD PEKFKEISRA YEVLSDEEKR KLYDEYGEEG LENGEQPADA
TDLFDFILNA GKGKKKRGED IVSEVKVTLE QLYNGATKKL AISKDVICAN CEGHGGPKDA
KVDCKQCNGR GTKTYMRYHS SVLHQTEVTC NGCRGKGKIF NEKDKCANCK GGCVLKTRKI
IEVYIPKGAP NKHKIVFNGE ADEKPNVITG NLVVILNEKP HQLFRREGVD LFITHKISLY
ESLTGFVAEI MHLDERKILV DCTNSGFVRH GDIREIAEEG MPTYKDPFKK GNLYITFEVE
YPLVITNEKK EILKVLKKQN EIEDLENSEC EVVTCQAVDK EYLKQRLKIL KNYLPYLCKI
LVDNTVVYTKW DYLTMDESHF QNDNADEMTA RTWGNDWTVR KGAALCLDYL SNVYNDEILE
FILPHIEEKL MSDKWNIRES AVLTLGAIAK GCMYSLSPFI PKVLEYLIKL LNDEKPLARS
ISCWCVTRFS SWICHPDKWF EPVLLNLLKR ILDTNKRVQE AACSSFANLE EDALDLLNNY
LHEIVHTIQQ AFQIYQAKNY FILFDVVGTL IDSVNIVKEN NDLAHEIVNS ILSKWNNIRI

SSPYIIALME CMSCITSAYG KDFLKYAKNV IRTCIKFLVL LYIDDLIECS FDLLSRIILQ
SNFALIGDIS RFCAQYLILN DIIPFLIAHI THPSTPVSNN ASWAIGEISI HINPQYMEVY
VDEIIKQLIY ICNSKYHGCL LQNICITLGR LSSTYPKKII FYFPQFLKTW LKIMSHGTQE
NEKINFFHQF LATMKECIQP EELKARLAQG LLKCQIVNKL TSNGCRFIFW IADWFAQLNN
KMSGDLKKIK KVGMYFIEVW KSCGMNMQNV EFLWASEEIN KKPNEYWSLV IDISKSFNIN
RIKRCLKIMG RSEGEENYCS QILYPCMQCA DIFFLNVDIC QLGIDQRKVN MLAREYCEIK
KMKKKPIILS HQMLPGLLEG QEKMSKSDEN SAIFMDDSEA DVNRKIKKGY CPPGVIESNP
IFAYARSIVF PHYNEFALQR KEKNGGVRMY VKEEIVDNEK LSNIINTKKE NVKYMKGMKV
PDNVVAISNL KDAVEDADLL IFVVPHQMEK LLSSIGRLSV VAGGLSLIPY TFIYDVDGGE
RCVMFNRFGG VSENTYGEGS HFYIPWFQTP YIYDIKMKPK VINTTTGTRD LQIVTLSLRL
LFRPHTKQLP YLHSTLGPDY DERVLPSIGN EVLKAVVAKY NAESLLTQRD KISKEIRESI
TARAKHFNIL LDDVAITHLS YGKEFAKAIE DKQVAQQESE RVKFIVAKTE QEKIAAVIKA
QGEAEAAKLI SSAVKEYGNS LLEIRKLEAA KEIAENLSKS KNVTYLPASS NILYMNALKE
ESGGFNFENL KRNEILKEKG PNFRKTGTTI CGLVCQNAVI LGADTRATEG PIVADKNCSK
LHYISKNIYC AGAGVAGDLE HTTLWLQHNV ELHRLNTNTQ PRVAMCVSRL TQELFKYQGY
KVCAIVLGGV DVTGPQLYGI HPHGSSCLLP FTALGSGSLS AMAVLEAKYR DNMTIEEGKE
LVCEAICAGI FNDLGSGGNV DICVITKDGS QHIRPYKQPN VRLYHLPKGT TPVLCQKIEN
IKKYISLNDV GEARGTVLSV KLDELIDSVE GQTVIDPKGY LTNLNANDAD IADINKARSL
LKSVISTNPK HGPGWIAAAR VEELAQRKDK AKEIITKGCI ECSKNEDVWL EAVRLEKLSE
VKIILTKAIK EIPTSVKLWL EAYRKENHID DKRKVLRKAI ECIPNSVRLW KEAISLESEN
NAYILLLKKS RVQCNTNRNN VSPVISEALK ECPSSGILWS KAIEFENKNL QNSKSVTAFN
NCGNNAYVIL TVAKLFWQHF KTQKARKWFY RVISLNPHFG DGWATFLAFE IDQQNEVNQK
DIINKCTKAE PNRGWVRGRI HDIRSKGSLA FIILRHKLYS MQCILDIKHD NDKNMMKWVS
NLPLESIVDI KGKLSKPEVP IDSTNIKYEA HIRKIFCISK TAKELPFLLK DANMKETNEE
GSIKVNQDNR LNNRCVDLRT YANYSIFCLQ SQICTIFKNF LLENNFIEIH TPKLLGESSE
GGANAFQINY FNQKGFLAQS PQLYKQMCIN SGFDRVFEVA PVFRAENSNT YRHLCEYVSL
DVEMTYKYDY LENVHFYDSM FKHIFTELKG QYPCEDFQWL EETPIFTYEE AIKMLIEEIL
AYDMSTDMEK ELGKIVKASH HTDYYIIINF PSALRPFYTM YKEDEPAISN SYDFFMRGEE
ILSGSQRISD VNLLLENIKR FNLDANKLNF YIDSFAYSSY PHSGLLFENL NKEYKFITTQ
DNFDGFRFEV DKSVNKYLQS THTLFLGTRD VGYLYQFGAN FTNSDNSLLM ISRVNIDGSV
NGRFCKRIDN DCKLNFNTYA KNDQRNMYEM AVEVNKPIYT YSVKTIWQGG VDLTYIASNC
ASIGSFGLRY NHKNNVVSMQ VVRQPNFKSP EFMLNQTHLY KIQYAKKVSD RLSLGTELEV
TPETKESAMR LGWDYSFRHA KVQGSIDTSG KIAVFTQDYS GFGVSGYIDY PNNEYKFGMM
HIAPSQEQDG GVGKTTFVKR HLTGEFEKKY IPTLGVEVHP LKFQTNFGKT QFNVWDTAGQ
EKFGGLRDGY YIKSDCAIIM FDVSSRITYK NVPNWYRDIT RVCETIPMVL VGNKVDVKDR
QVKSRQIQFH RKRNLQYYDL SARSNYNFEK PFLWLARRLS NQPNLVFVGE HAKAPEFQID
LNIVREAEKE LEQAAAVAID EEDGVLTVRS ILMEPTISQY DIKKLIRNKI QEEVPFYNYQ
MKRTLAETIY GDCIYDNFGL SKDIEVNLIA LEEWNINCNR NRVLQNTGLI KDIQINEFKY
LTTKESLEVH FAVNPKYSDI LQKNKVLPPS GINYNKLIKE FGCSKITESH IKRIEQLTAH
HFIRREIFFS HRDLDFLLNS YEQHKCFYIY TGRGPSSLSM HLGHLIPFYF CKYLQDAFNV
PLVIQMSDDE KFLFNQNYSL VEITKLTKDE MTFVLHNSNT GMANALRRIM LSEIPTLAID
VVNVYENTSP FHDEFLAHRL GLIPIDSTNV KNFEFREKCK CKETCSKCTI QYLIQVKCNS
KIEITHHDIV EHEPNVPMPV PIPIVTLSKN QTLHMKLIAT KGIGKMHAKW IPANVSYRID
HKVAIKHHLI NSLSREHKLL LANSLNKNCY VLLRLNENMS VVMAESCIEF LNELGYKDVV
KIIYDETKFH FKVESVGSMP PEQVVEMAIE ILENKLKTLE PQIKASYYSI DEVAKQLKLP
DPESAKNEEE KCFLSLLKKS ILKNPKKWTN IAKKIIGVSE ETTTGVLRLK KMDKNEELLF
TAINVNDAVT KQKYDNIYGC RHSLPDGLMR ATDFLISGKI VVICGFGDVG KGCASSMKGL
GARVCVTEID PICAIQAVME GFNVVTLDEI VEKGDFFITC TGNVDVIKLE HLLKMKNNAV
VGNIGHFDDE IQVNELFNCE GIHIENVKPQ VDRVTLPNGN KIIVLAKGRL LNLGCATGHP
AFVMSFSFCN QVFAQLDLWE SKKYQNKVYL LPKHLDEKVA LYHLKKLNMY TYGVEGDDTY
LPQPQYPSPY ENQYESPSPR GENHTPFIGY FSSHLLRTGF FLQCVSLMLM FIFYWAFGGT
GIFVFDLYAG PECVKVSSAF HLTISILMAI YLLGTLYIAM FQVFVADNSK WCRGFRAGSK
LLSAAVTLDL LSSILRLVQY LYAYFYMSMR WWARYQQTKS DWTLLHFGSI VHSFALFIYG
AAFFYMEAYH DEGTYEELAW SNLTLFKLAG LAELNKPKKR TFRTFHYRGV ELDKLLDLSQ
DELVKLFRAR QRRKFKRGIS KKEKSLLKKL RKAKKECEVG EKPRAIPTHL RNMTIIPEMV
GSIVAVHNGK QYNNVEIKPE MIGYYLGEFS ITYKHTRHGK PGIGATHSSR FIPLKVMVDM
FLWRDPEQFE LKNLAIEETA PAAPHLAENQ FATEAPYEEW GFLQIIKDHH ETMYELKQKI
RPRDQVVGWF CSGSELSELS CAVHGWFKEH NSISKFYPHS PLNEPIHLLV DAALESGFLN
IKAYVQLPIT LVKEYFVHFH EIQTELLPSN VERAEVSLKK LLIMLKQCKS YVQDVIDKKK
KGNLDVGRYL HKVFSNDSFS TLEKFDSINE SILQDNLMIS YLSNLAHLQF LIAEKLNWLN
NLPIKEDDAE GRRVHKNLID LVSQNHPLLF GKDNSNTAKI IEIFLTIYET DFSDADCNKK
IASLINSLDQ AYLSNLALTN KQAKKLNHIN GNNFINFYQD NINLKSYLKI ISDFEKFPVI
VDAGGQILSL PPIINCDYTK ITYDTRNLFI ECTAIDRNKA EIAVNIICSM LSEYCTPKYS
IHSFFVQYPV FKNKTLTCHM DYVRKLSGIL NLSVKDVEPL LKKMMIDSST FTVDVPFYRS
DIMHFCDIVE DIAIAYGYGN IVSEKIEIAK KNSLSACTEL FRNVLAECTY TEVMTNALLS
KRENYDCMLR PVQIMNSKTS EYEIVRTSLI VNMLKFVSAN KHRELPLRFF EIGDVSYDRT
DTNAVNKRYL SVIFADKTAG LEEAHGMLET VLKEFQLFSD YKIEEKSKEN VAIRVISIGN
IRGFGGCDYG SFRMSNEFLG WKNKKTNSVY QYKCSDISEA EWIKTSYNNN RLHLKFSEQK
DNLIIFFDGF PDRNTSEITQ HFQKYFNLRL ASRKLATRGW NWGEFKLENS NITFDIDNKY
AFTIPTNSIS QLNVQIKTDI AMELKNEENE DFLSEIRFCY PHENDENQNF QNFKNDLLEK
VNIGDSKSEC IASLANIPLL VPRGRYEIEM YTKSFKLHGK SYDFTIQYTN INKMLLVPKS
NSNQYVLIFS LNNKMKQGQT EYPFILIQLN NDDDMELDIN ASEEDLKKYK LEKSLSGKAY
DVVTRLFTAL VKKNAIIPGD YRTAKNEHGI TCSYRAASGQ LYPLNKYFLF IIKPVILISF
DDIVTLSFQR TGNNQHRFFS LIIKHKRGMS YEYTNIDKSE YLPLLEFLKS KNIHIKGYID
LSKRRVSPKD IMKCEEHFSK SKKVHQTVRH VAQKHNMTVE ELNRIAIWPL YKKYGHALDA
LKEATINPEA VFKGIELNED VKNSLLADIQ LRLAAQALKL RGRIDVWCFS YEGIDAVKEA
LKKGKVSINI KLIAPPQYVI VTSCQDKDLG MSKIQEAMKL ISDKIKEYKG GDFKQQGEIL
VILLDKQDDL SSDNEGERSV FEFFLCDVGV GLSLSVRDTV PVEYDSLFIG VLPHYTFHHE

YIIFDNSQLL PRYLIQFECD PSGEEHFSLP LCDYCGNAPS VFFCESDEVK LCAKCDQMIH
SNKLVKKHIR KTLNEAQGKC KIHVQQRVSM FCTICHIPIC NICVSSHSHI SLNLAYKAII
KHSAIPSNVV KEEKKKLNDV LKKVDKLYEQ VRSNMKDAEK HVYTILEDVI KQLHETTDQK
MCAVLSEEYE LKRQFCEIAW NENFLYYLQT VLPPADFMNA WLKHCLVREE IERNSLVFPD
MCIRGNINVA TEESARHLVG LPNVGKSTTF NVLTKLNIPA ENYPFCTIEP HEAKVTVEDE
RFDWLVSHFK PKSNVHAYLS IFDIAGLVKN AHLGEGLGNN FLSNIAAVDG IYHVVRAFEN
EDIIHTEGNI NPVRDMEIIN SELIYKDITH CEKNLEEVTK VNRNKKDKIK QNEHDVLTVV
LNFLKEHKWI KDGNWKASEI EVINEFNFLT AKPVVYLVNM SEADFIRQKN KHLAKIYNWV
QEKNKGTIIP YCADMELKLL SMSEEEKNAY FKENNVKQSM LSKIIKTGYY EINLIHFFTC
GPDEVKCWTI RKGTKAPQAA GVIHTDFEKG FICAEVYKYA DLVEFKSEGE VKANGKYLQK
GKDYVVEDGD IVFFKFNVSS SGRINKHVNQ LRITYSTLEE FVDNFVYELK KGLEAHRRHP
NLWIPHECSF KMLDSCIADI PTGQEKGTYY AIDFGGTNFR AVRASLDGNG KIKRDQETYS
LKFGTFSHEK GLLDKHATAS QLFDHFAERI KYIMGEFKDL DNPEGKNVGF TFSFPCTSPS
INCSILIDWT KGFETGRATN DPVEGRDVCK LMNDAFVRSE VPAKVCCVVN DAVGTLMSCA
YQKGKTTPPC YIGIILGTGS NGCYYEPEWK KYKYSGKIIN IELGNFDKDL PLSPIDLVMD
WHSANRSRQL FEKMISGAYL GEIVRRFMVN VLQSASSEKM WKSDSFNSEL GSVVLNDTSP
NFEESRKVAK DAWDMDFTDE QIYALRKICE SVYNRSAALA AAAIAAIAKR IKICGVDGSL
FVKNAWYCKR LQEHLKVILA DKAENLIIIP ADDGSGKGAA ITAAYFLIRW LCKAIVSSLF
GDVNVINPEN VPLYGSVIFV GNHNNQFIDA CVLVANIPRQ VKFIVAEKSM RRAVIGKLAS
IIGCISVKRP EDLKFKGIGH ICWVKGDKKI KGINTRFRLD VQMGDKLLTQ NKIFLVAKIE
SETELIIQDA INIECEDNGV PFKIIPKINQ TEVYNLVTSS LKNGDTIGIF PEGGSHDRTN
LLPLKPGVAI MTLCALADGD VSIIPVGLSY SKLYQLQGCV TLFYGNAIII SQDLCKDYNN
NHRETISKVL SKIEEGMRSC MLTSKDHETS RCIELCVSLY TPERMTISKN KIYNNLQLFC
KMFWKFGNSK EIENLSYELK CYEKLLKANK IKDDEVWMLK QSTSAATLKF IEHICSLIFC
VIFGMTFSLL WLPLVAISIY LAEKHRESAL KNSTVKIQGC DVVASYKVLV LLVLLPTFNI
MYGLVFSLYR LMKKKDVEGV HKLLGSYLEQ FNLYAVFTKE EIAHWFLPIE NVIYTYVNEE
NGKIKDMISF YSLPSQILGN DKYSTLNAAY SFYNVTTTAT FKQLMQDAIL LAKRNNFDVF
NALEVMQNKS VFEDLKFGEG DGMDVDSVLG LQAILISANY KEKEFIRIAY YMNSFYKDTE
LREKPPAVPQ YDKICRHIVE NPRIVKFCIT WDDAYTAYPI LKELKQKDYF RIFKVNLHIP
CKLSKVNEKC KEIKKCSVCE CHDDEIPYNF RTNEIEVDLV YNSPSFTAYE GRNIWELKGN
NNYKYFGAAK NLKGVKELLL KESDDRKKKK KENRFFDKHV NIHYFGYCDD QNEMLLREEL
KMQNRLMKHG TLVLPSDRAR EYLDCLGKNI DIQFIDMNEK TMKRQYKKYI QRIDDMERIL
RFLEENIKKL PNVKIKKSKI DSFLEHDNVY ELDQVEESLN RLHVQFVRFC NNNKDLVDER
NNAVEEKHVI LTAMNQLNPS LQTNMMKDGM NMMFTNISGV IKTKDQESFS RTIFRALRGN
TYTYFQSIDE KSVFVVYCQG SSQSNIYHKI LKICKAYDVK TYDWPKTYEQ ARQRLKELKE
IITDKEKALK AYEEYFINEI FVLINVVEPN KNSLIEEWKL FCKKERYIYN SLNCFEGSDI
TLRCDCWFSA NDEEKIRHML ITKSSNDLVS ALLLSDKLTP NISPPTYIKT NSFTKSYQAM
VDTYGIPRYG EINPAISTII TFPFLFGIMY GDVGHGVCIF LFALFLIMIN SRVKNNEMVS
MLLDGRYMLL LMGFFAIYAG FLYNDFFSMP LNLFTSPYIF GFDAKWLGAE NELTYINSFK
MKFSIIIGFL HMTFGVLMKG LNALHYRRKM DFFFEFLPQL MMMLSIIGYL VFLIIYKWVT
PGGYQKQGII NTIINMYLMK DLTPQNQFYA HQGLVQAFLI AIFVLCIPLM FVCKPAIRTY
HIMKEIWIEQ LIETIEFILG LISNTASYLR LWALSLAHQQ LSFVFFEQTI LNSLKKDSFI
SVLVNLIVFS QLFSILTIAV ILCMDTLECF LHSLRLQWVE FQNKFYKGDG IPFRPFNIKK
LLPENYSAIL ARALSERPLT YLPTVERVCY EVLNDEDEHL NYIQINLLNT IRPTPIRGLL
AATQERFVVV PGIIVQASKP QHKMRKITLQ CRYCDHKMSI DVPLWKDKPQ LPPYCRYVLE
PYVILPNECT FVDIQSLKMQ ELPEAVPTGD MPRHLQLNAT RYLCEKMIPG DRVYVHGVLT
SYNPNPTRAD GTNFSYLHVL GFQKYDGNDL NFDVEERNEL TLLAAEHDIH EKIFKSVAPE
LYGMDEVKKA CACLLFGGTR KRIGEETKIR GDINMLMLGD PSVAKSQILK FVNRCAPVSV
YTSGKGSSAA GMVRQGLLGE NEEKLDYVLG LTLPKLLERR LQTKVFKLGL AKSVHHARVL
IRQRHIRVGK QMVDIPSFLV RVDSEKHIDF STTSPFGGSR PGRVKRKTLS QSMYDRHLTI
FSPDGNLYQI EYAIKAVKNT NITSIGVKGE HCAVIISQKK MATQYISQDK LLDYNNITNI
YNITDEIGCS MVGMPGDCLS MVYKARIEAA EFLYSNGHNV NVETLCRNIC DKIQIFTQHA
YMRLHACSGK SITEESVKEM FSPYGSVEEV FIMKDNTGLG KGCSFVKFAY KEQALYAINS
LNGKKTLEGC ARPVEVRFAE PKSAKQTAKV CSRDLPGHTK MKTRDLSDYV EDKEVIETIN
QAVKFAKDAV IEDEKKNYKA ALNLYIQSLQ YFNFFCKYEK NSNIRDLILK KMEIYMTRAE
NLKEIINILN KDKNVKWSDV CGLETAKEIL KEAIIFPLKF PKLFNSSALP YKGILLYGPP
GTGKTFLALA CSNECNMNFF NVSSSDLVSK YQGESEKYIK CLFETAKEHA PAIIFIDEID
SLCGSRTDGE NESTRRIKTE FLINMSGLNN YKNNIIVMGA TNTPWSLDSG FRRRFEKRIY
IPLPNVYARD IKYFAAVTEN YTGADIDIIC RDAVYMPVKK GLYVKCGSRY EGMSVMLENM
AFHSTAHLSH LRTIKSLEKI GANVSCNAFR EHIVYTCECL KEYLPVVTNL LIGNVLFPRF
LSWEMKNNVN RLNTMRTKLF ENNELYITEL LHNTAWYNNT LGNKLYVCES SVENYTANNL
RNFMLKHFSP KNMTLVGVNV DHEELTKWTS RAFQDYVSIP YTSQKEVTPK YTGGFVSVED
NVKKTNIAIA YETKGWKTSD MITLTVLQTL MGGGGSFSTG GPGKGMYSRL FLNVLNNYNF
IESCMAFSTQ HSDTGLFGLY FTGEPANTMD IINAMALEFQ KMNKVTDEEL NRAKKSLKSF
MWMSLEYKSI LMEDLARQMM ILNRVLSGKQ LCDAIDAVTK EDISRIVGHF LKTKPTVVVY
GNINHSPHYD EICKILDAVN KIHFFMGYPE LASVNFGNST VVRCKKCRTY INPFVRFEAG
GKKWNCNMCY HVNDTPQFYK RKDLFQRPEL CTGSVEFIAP SDYMIRPPQP PVYLFLIDVT
VTAVNSGLLD VVCSTIKKLL DSRTLIGIMT FDSTVHFYNL NSNLKQTQMM VVPDIQDIFI
PLPEDILVNV HECQNVIDVL LDNLPTMWRN NKMTDCCAGN ALKAAIMVLK KVGGKLVFFL
SSPPNIGDNL YTELAQSITQ YQIAVDLFAC PYNLDLATIY PLVKNSGGSL YYYPFNVHQY
SDKLREELLF ALTTETAWES VMRIRISRGW KITNWYGNFQ FRGVDLLALP NCHSSQNFSI
IVDLEENVVQ DSVVYVQSAL LYTNSNGERR IRLHTYALPV TQNIKTITDS INPQVVVSLL
SHQAIDICKK GKIADGRNLI QTLCSQVLSS QLLLSETARL LSIYILGMLK SVAFRDPDLR
IYHWSRVQNI PVESVEAYFY PRMFCLHNPD TMSLTCENMT QDGCYIVEDG ENIIMWIGRS
ISLAVVNVAA GLDGCDDQLL PASFRALEAD LNLHPSLLGY ITLAQTLMLS LFSPIWGFLS
DKYSRKWMLV FGTALWGVAT ILLANINDFA HIIFFRAING LALGSIGPIS QSILADAAKN
ELGLSFGLVQ LSSSVGRLIG GVVTTTVALK YFGGIRGWRL CFIVVGILSI LLSIVVALFV
DDAPSLSKKS IIIILLEGFT GTIPWLALSF NTMFFQYCGL SDLQAAIITG FLLIGSALGG

VIGGHFGDIM HDISNKHGRP FLGQLAMFGR VPLVILTYLV IPQRKESFEL FLSCFFLGLS
SIAGVAVNRP IVSDIIRPDY RGTVFSLTIA IEGVGASLIG APLFGYLAEK VFNYQNNNLL
IAEMPEELRR NNAEALSKTL LYLTLVPWLL SFVFYSLLHF TYGKEYQKMN EIIESEMLMF
GLRSLSDFCN PTSKTYKENA FDALDRGAVE SIKNAVINYK DDDDILFCSS RVLFAMSDYC
CSEKDTDALQ KLITDGGAVV EIIKTVPSDQ DTLKNCMLFI QNLGVALLNV FTSSSYNVKL
GNVIVSTLSV AAKSASGSQI LNAEGAHHKL IDHCLDDTAE IVEGVFDAIK NLSSNGYVVP
TIIEKSVVIL DKFKSYPRIV SKGSDAMKCA VGPEQLTDCL NILKTSEQGS KEQDSALELL
SSLSYISSIT DKVVQSGGIP VLVELINSGL QQYESNPEKI SRLVAGASRM LGRISNNPPH
AVIVYEYGGI ATLCTALSYF PNDVDCASAV CNALIPFVSR SYSLFASLFP ILYASLESIE
LAKASISCIA SASMINEFHE QMVNNQAIEI LSTCIQYHLN EREYLLNCFT AYFRLSEYIT
TVEPINQYGG VTGIANALAA VDSVLTVMLE NETKEVIIQE GTKIMEKLAT ESDCQRHISN
LESIAQSNPE AAYKTLAAIS GLSRIQSLKS ILESKGADNS IYNGMKVWIE SPRFTEQPKL
IKAALKTIKI VDVMCIPQVK RLAEEEPDDN ILITAAECIN YLTEINKINN KEIVESSLDS
IFKLMKKYSE SRLTQTNLLA AIDNILLSSN GADVLVNKGY VKQIVTYIHK VPMYVDVQII
GFSVLGNIVK ISSESVDSIK KANTLVPLQN ALRTHAKNAK LKTTCAPLLA MLMPLDSLTK
EIEDLLKLCN ESMSENDLIK LHEYLISLNE LLLTAEASKI SSRCNVISEV GHACTNISQT
RIGLVHLTKS DMTSALIEAY DLLQLPVKDY TEEAVSNILE SLSLLLKHDI TNADIGFNNG
LVKKLCAGIN HFSESDAVIK STFGCLACMC TSEKRVNQLI SHPEYDKLIA LIVDLIGNSK
NSRGNAIKAL YELLKTEDAQ IITNIASMTP IVNLFKIMGE YQADLPIVQD STKCLAIIAD
YVNIEEDQYS AIKILLESLG KNKNDELTAQ DILTVLIKLC DSNDKMKLRE LGAVDVISDV
TMIHSENEEI SKLGGVLFSY MGADEQVKKL MKLILNVKSS DSDSVQKIDN LSSKLEMFLR
APLENPLDAL EYTEATLQQL NGYLTSQLDN ASLQANIALV TKRLVDRVKY DHEDQLGAWA
VASAGVLNQY TDMIANKVGL ANCKFVSPVY SVLAGCVLNM EKKAREYAQG AVRFIQKSGS
NFLACKNLRE KLEERGFKRI HEWELRKNEG YVLSKQSRNI CGFFIGKDFT IEKGSILISI
GHIDSCCLKV SPNNNVVKSK LHQLNVLVER LIQINKSLLF LPSLAIHLQN RTRSVKINYE
AHLKPILSTL LYEHLPLLYT LAKELQCQEK DILDFELCLM DVNQPCFTGA YEEFIEGARF
DNLLGSFCVF EAFAEFVLLF ISSYEIKCQL RFASLGDWGK SKSQLLNAKY FKQYIKNERV
TFIVSPGSNF LDGVWKSLYE DVYAEEDMYM PFFTVLGTRD WAGNYNSELL KGQGMYPKWI
MPNYWYHYFT HFTVSSGPTG HKDMAAAFIF IDTWILSSNF PYKKIHQRAW SDLKAQLNVA
KKIIVVGDQP IYSSYLLPLL KDAQVDLYIS GHDHNMEVLE DSEIAHITCG SFCIHELSSN
GIITKFVSGK SGSLQYFASL PKVQHVDIPA SGPMGNKDSF VRIVGTIGIL IRYAYSGVAY
SAGALKLSKT WCMNPDQGLI KPDVVFYLVC IYDNEVCQMP DVGVNFFINE EDVAKQSDAV
IKHLQELNSY VKKIAFLCCN IYGLCGYLFV DFGKGFICYD KDGENTKVCS ISKISKAPEG
MVSFDFDKGA PFQKGDYVKF SNVEGMQINH KIFKIKDMHK YTFTIGDTSH FDEYLKGGEC
TQVKSHLRMD FQPYEDYAKW DMSNQLHVEE VKKEVVVNVA KYCTAHLAPV ASFFGGLLAQ
EVIKFTGKYM PIYQLLYVDF FEKNDNIICV FGEAFQKRLN ELHVFLVGSG ALGCEYAKLF
SLLDGKLTIT DNDSIEVSNL NRQFLFRREN VGKSKSLVAS GIIKKKNPNM NVESLETKVG
PENEHIFNES FWTKQHMVVN ALDNIQARQY VDNKCVWYSK PLFESGTLGT KGNVQIILPF
LTQSYNDSYD PPEDSIPLCT LKHFPYDIVH TIEYARDIFQ GLFYNTPLSL QEFETLQNVL
TTLKVSKETN FTFCVKKAVD LFYANFINQI NQLLYSFPLD YKLASGEFFW VGQKKPPQVI
PFDLNNEFVQ EFLFSTANLF AQVYNIPQCY DLKHILDVIE VKPFQPKRVK VKMDPIEFDK
DEETNMHVNF IYAFANLRAI NYKIETCDKL KAKLVAGKII PALATTTSII TGLVGIELLK
YVNYLSYFKN AFINSALPLF LFSEPMPPIK MRDKEYDELM KGPIRAIPNG FTSWDKIQVQ
IKDYDTQRDK RFSGTVKLSN EVRKKLKVCI LGDAVHVEEA QKLELDYMDI EAMKKLNKDK
TLVKKLAKKY DAFLASQVIL PQIPKLLGPG LNKAGKFPSL ITHNDKIFLV YNIASFCFEL
QFRFIEDTTF DWLPSIGYLL PYEKIKLLRM LFPIVFFISI CSSAYTYTDR NATLIYLMRS
ILATNRIVFE RSNDVNYLKK NGEQLVSKLQ EMRKFALTLD SHQMGLLEKK NQKKSWFEFF
SMQMIFGIIF VYIWFTSKKP ATFEVPLALT LEELYSGCKK KLKITRKRFM GSKSYEDDNY
VTIDVKAGWK DGTKITFYGE GDQLSPMSQP GDLVFKVKTK THDRFVRDSN NLIYKCPVPL
DKALTGFQFI VKSLDNRDIN VRVDEIVTPK TKKVVSKEGM PSSKMPNTKG DLIVEFDIIF
PKNLTGEKKK IIREALGLYY ICISCPSSNW FKSTAIKWSL SIEVGGSDVD IENVAKKSEL
SETLSILHVD TGKTKLLDKL RHTNVQDNEA GGITQQIGAT FFPKDILDKQ IKKVDIKCMS
KGIMIIDTPG HESFYNLRKR GSSLCDIAIL VIDLMHGLEQ QTKESIQILK QRNCPFVIAL
NKIDRLYMWS KSDWSPFNYT FQNQKENTQE EFQDRLKNIL NELAEQGLNC HLYWENPNPR
KYVSIVPTSA ITGEGIADLI MILVKLTQNF MLKNIEYHEK LECTVLEVKN IEGLGTTIDV
ILTNGVLKES DTIVLCGING PIVTVIRALL TPQPLKELRI KNEYVHHKSI KACIGVKISA
NGLEEVLCGT SLFVANNTNE IEDYKKKAMT DVSDVFNHVD KTGVGLYVMA STLGSLEALL
IFLKDSKIPV FAVNIGTVQK KDVKKASVMR EKGKPEYSVI LAFDVKIDPE AEKEAQILGV
EIMQKDIIYH LFDAFTSYIK KIEEEKKQSK LTDAIFPCEL SIVNDCVFNK KDPIVIGVKV
DCGILKIGTP LYIPEKSLKI GNVVSILLNK KTCEKAKKGD EVSIKICGEP HITFGRHFDF
NQKIYSKITR ESIDVLKEYF RSELTMEDWR LVVQLKKIFN IIMNINEKDK LAEQNLETLD
VTKLTPLSED VISRQATINL GTIGHVAHGK STLVHAISGV HTVRFKHEKE RNITIKLGYA
NAKIYKCTNP DCLPPECYRS YESSKEDDPI CPRENCQHKM KLLRHVSFVD CPGHDILMAT
MLNGAAVMDA ALLLVAGNES CPQPQTSEHL AAVEIMRLKH ILILQNKVEL IKEEQALKQQ
EEIRNFVSGT AADSAPIIPI SAVLKYNIDV VCEYIVTQIS IPKRDFISSP HMIVIRTPKL
CNFVEWREYK LVFKRYASLF FIACIDKGDN ELITLEIIHH YVEILDKYFG NVCELDLIFN
FHKAYYLLDE ILVTGELQES SKKNILRVVS AQDSLMEDTK FAKSLLDVAD NLSLAIKNIN
EESLKQNENI YKGIQMTETI LHNIFNKYGI DKYDPINEKF NPLFHEALFE INDDTKKGTV
ATVVQQGYKI KDRILRHLDI ESVQALIVAL NLYKGGLIII SHDTYLIKHV ADEIYHINNL
TKLIKIDYDF DKYAKLLLEN KIMPREIITL QCGQCGNQIG VEFWKQLCNE HNIDREGILK
NNHLNEDRKD IFFYQADDEH FIPRALLFDL EPRVINSIQA SEYRNLYNPE NMFISKEGGG
AGNNWGCGYS QGHKVEEEII DMIDREVDNS DNLEGFILSH SIAGGTGSGM GSYLLELLND
NYSKKVIQTF SVFPLLNESS DVVVQPYNSI LTLKRLILST DSVVVIDNTS LNRIFVDRLK
LNNPTFQQTN TIISNVMSAS TTTLRYPGSM NNDMISLISS LIINPKCHFL VTSSNVQKTT
VLDVMKRLLH TKNIMVSVPV RRGMYISILN IIRGETDPTQ VHKGLQRIRD RKLVNFIKWN
PASIQVTLAK PSPHSTHKVS GLMMANHTSI STLFERCVTQ FDRLFKRRAF LENYKKEPMF
QGNFEEMESS KEITQNLIDE YKSAERDDYF GLARAFGIPV RKYTHEVVTL WYRAPDILMG
SKKYSTPIDM WSVGCIFAEM VNGRPLFPGV SETDQLMRIF RILGTPNSEN WPNVTELPKY

DPDFMVYEPL PWETFLKGLG IDLLSKMLRL DPNQRITAKQ ALEHAYFMGK EKTHINLVVI
GHVDSGKSTT TGHIIYKLGG IDRRTIEKFE KESAEMGKGS FKYAWVLDKL KAERERGITI
DIALWKFETP RYFFTVIDAP GHKDFIKNMI TGTSQADVAL LVVPAEVFEG AFSKEGQTKE
HALLAFTLGV KQIVVGVNMD TVKYSEDRYE EIKKEVRDYL KKVGYQADKV DFIPISGFEG
DNLIEKSDKT PWYKGRTLIE ALDTMEPPKR PYDKPLRIPL QGVYKIGGIG TVPVGRVETG
ILKAGMVLNF APSAVVSECK SVEMHKEVEE ARPGDNIGFN VKNVSVKEIK RGYVASDTKN
EPAKGCSKFT AQVIILNHPG EIKNGYSPVL DCHTAHISCK FLNIDSKIDK RSGKVVEENP
KSIKSGDSAL VSLEPKKPMV VETFTEYPPL GRFAIRDMRQ TIAVGIIKAV EKKEPGLPIV
LLKEGTDKAQ GKSQIIRNIN ACQIIVDIIK TTLGPRGMDK LIYTEKDVTI TNDGATVMNL
SISHPAACIL VDIAKSQDEE VGDGTTSVVV VAGELLNEAK QLLNDGIEPN MIIDGFRNAC
TVAINKLNDL SLRFVSKSEE EKKEILIKCA QTALNSKLVS NHKAFFSELV VNAVFKLGDS
IDKSNVGIKK VTGGSCLDTQ LIYGVAFKKT FSYAGFEQQP KTFLNPKILL LNVELELKAE
KENAEVRIDN PSDYNSIVQA EWEIIFKKLN LIKESGANIV LSRLPIGDIA TQFFADNDIF
CAGRVEDADL KRTANATGAI VQTSLFNLNE GILGTCGVFE EVQIGNERYN IFKECLKTKS
VTIILRGGAS QFIEEVERSI NDAIMIVLRC MGNSEIVPGA GSIEMQLSKH LRIYSRSICN
KEQIVLYSFA KALESIPRHL SHNAGYDSTD ILNKLRKKHS EETSDIWYGV DCHEGDIINA
YEHCIFEVTK IKRNVIYSAT EAACLILSID ETIKNPARNL GLPDCFKDLL KTDKIKHVLC
TGNVGCRENL ELLKNIADSV HITKGDMDDE YDFPEDISLT IGDFKMSLIH GHQIIPWGDT
NALLQWQKKH DSDIVISGHT HKNSIVRYEG KYFINPGSAT GAFQPWLSQP TPSFILMKSS
IVVYVYEEKN GKTNVEMSEL QKYSVKVSEP TRKDATGAYR NPQYKDKLFD NFEDCLGTRV
KVNNKLGPYK WKSFAEVKEL IIAVGSGLGF YMPNCEEWNI CDLSCNAFNI VTVPLYDSLG
IESSKFILDQ TLMQTIICNK TCAMNLFKSL PGNLKDVSCI CYTSGTTGYP KGVIMTNGNF
VAQLTSSVTG PSRLPNDTDT HISYLPLAHI YERMMMLVFC AQGVRTGYYS GNVQNLVEDI
QELKPTLFIS VPRLYNRIHE RIFNSLKKKP AVVQSLFNKG LEHKNSNGIP FHFFWDKLVF
NKAKKILGGN IRVMLNGSAP ISPDVVKKLK AIFCAPIFEG YGMTETLGFI SHTTDVNIGH
IGGPVPCVEY RVVSVPEMNY LITDNPPRGE LHLRGPAIGY FKLEKETNED GWISTGDIVS
FSENGSITII DRKKNIFKLS QGEYIAVEKI ESVYRQSLYI SQIFVFGYSY ESVLVCIVCP
SLDTIEIWKN EKKITKTDEE VMQMPEFKKD VIDDLIKMGK KDGLKGYEQI KDVYFASEPF
TIENDLLTPT GKIKRHAVQK KYKEQIDKMY GNKSQKTINE GQTLLTVFKE GYAPDGVWLG
GTKYQFINIE RDLEFEGYTF DVATCAKLKG GLHLIKVPGG NILVVLYDEE KEHDRGNNLN
MLAVVYLACR EAGHIKSIKE LITFDRSFKE KDLGKTINKL KKILPSRAFV YNENISHLIF
TLQLSIDVIE AIEYVVKKAT TLIWNDIERY FKDPELITAE ILFVGLTLCN VFVMYRLFLD
VIPFPIFVTW WQLAQGLLVA YVCGLKVLFV PSIFYCLMLV LSNYLLFKTP CIASYPVLVS
FTVVFHHLTR FVGCGEEYML RWKSIVFLLA AFVIGCFDSK TTGKGVLIWA LLYALFSAIF
RAGFMQKIMH LVDGKGNTLH NNQHLLGVLL LPILIVLSGE WAVFGHMPYN IMSLHTGCLI
TVGALPFIKN VISNRLVRRT GQGPWRFLEI ISIVLVFFIG MTYNAPSFLG YVAIICVIIG
RSLGAFDVML NKTKVGEEMR NASFSLAKAV WAAGDFKGQI IEGIKRPVVT LSLSTNNVAG
VKLPIFQVHI DPTVDVLGNL GIASGGQVIN NTRENYLQCL NMLVKLASMQ FISMFDWLNV
HSETFYAKLN EDLVATKINL MRDSSTSCKD DNPYCSVNDG KVIIKNNELL CGIICKRTVG
SSSGSLIHIL WHEMGPDKTK DFISALQKVT NNWLEYIGFT VSCSDIIASN KVLDKVKDIL
SKSKKEVSKI VKKAQRGELE CQPGKSLYES FETRVNNELN CAREMAGKVA SESLDERNNI
FSMVASGSKG SIINISQIIS CVGQQNVEGK RIPFGFNHRS LPHFIKFDYG PESRGFVSNS
YLSGLTPQEV FFHAMGGREG IIDTACKTSE TGYIQRRLIK AMEDVMVQYD RTVRNSYGDI
IQFLYGEDGM AGEYIEDQII DLMKLDNKEV KKLYKYNFDE DSYGEYSKQN VLNQEFEELL
KCKNNICKEI FPDGDVRQHL PINMNRLIEF AKSQFPNPVD IVHKVNRFLE KLVIIKQINS
NDTLSLEAQN NATVLLKAHL RTYLNSKLLT QTHKISMKGI DWLLQEIEKN FYKSLCHPGE
CVGALAAQSI GEPATQMTLN TFHFAGVGSK NVTLGVPRLK ELINIVKNVK TPSTTIYLDD
VVSNDQQKAK DILTKLEYTT LKQLTSHAQI IYDPNTTSTI LEEDKLWVDE FYEFPDEDQY
TLGEWVLRIQ LTNIHVNEKK LTMKEIVYII YSVFSSDELD IIYTDDNSED LILRIRVKYL
EDTFLKKLME QCLSTLKLRG IENITKVYMR EESKITYDST NGKFVRSSHW VLDTDGCNLE
HIFCASHVDY KKTVSNDIVE IFEVLGIEAV RRALLKELRT VISFDSSYVN YRHLSILCDV
MTQRGYLMSI TRHGINRVDR GPLVKCSFEE TVEILLEAAA FAQVDNLKGI TENIMLGQLC
KIGTGVFDII IDNQKLSDAN QNETLMDITS AGFTTPDSSP LPFSPTYNVN VKNVVLPGNI
RKSEHFLNLM RIVVMYLRKY VNIYEVTSEG PLSFLYKCER ETKLDTSFFK FCFDRLKALL
NALQIVDDYS ALNVVCNFCT LLGSYFQGFI IICEPYPEAT IYDPVIQFAC LDSSIAMKAV
IGKYRSVILT SGTITPLELY PKLLNFKTVL TASFPMSFDR NCVCPLIVTN SSDLVPLSSQ
FSLRSDLTVI KNYGMLLVEM CKTIPDGIVA YFPSYIYMEE VISSWYELGI ITSILECKLI
FIETKDIVST TIALHNFKRA CDLGKGAVFL SICRGKIAEG IDFDKHYGKC VILFGIPYQY
TLSRILKSRL DFLKETYNIQ ENEFLTFDAM RQASQCVGRI IRNKKDYGIM IFADIRYARN
DKKSKLPPWI IKCMELSNVN LTVSTAVNIS RKFLLNMSQE YKETGQTKNQ PGTRVSELPN
KLKIATVKSS CEVPTIGIWV SSGSKYESKQ NNGVAHFLEH MIFKGTKKRS RIQLEKEIEN
MGAHLNAYTA REQTSYYCRC FKGDVKWCIE LLSDILSNSI FDEDLIEMEK HVILREMEEV
EKSKDEVIFD KLHMTAFRDH ALGYTILGPI ENIKNMNRQS IINYIHTNYT SDRMVLCAVG
DVEHEEIVKL AEQHFKPYFC GSEIIVRDDD SGPSAHVAVA FEGVDWKSPD SITFMLMQCI
IGTYKKSEEG ILPGTVNNIC NKMTVGCADY FSAFNTCYNN TGLFSKYLWK ARIYFIWQRL
FASSNDWFYS LKIHVVDPLR IFKNVKLLSP NFQICALSNF SIYLAFYNYT YAYQRVLDVL
AEMSQFSYSF TGRMGIKRKY QKIPATILVL LKDLDPDTDI LEEPYFMDSE NNLTFDEQIA
LINFCYSMIR FNPHYDEIKF EKLSAVIARC LKYQNWLLHS CILWYKCKCE TFRLKTVDRA
AAQLNELLKE TYDEKPQGGE RVKFLFDVYY PTTWELKKEI GNVMVKTGSV VSAFNLFKDL
KLWDEAITCL IQADRKGEAK ELLDDLLKKK RTPSLLCLYG LNYYIDAWKL SNCKYAKAAR
FIGNFYYRKE MYNPCCEYLE KALEISPLFP DIWFILGCSY MKIEKVDESV KAFTRMVSMN
LAYLYMKKGT YKAAKICINQ AVKVDNNEWK YWDTYLKLSI MQNDVDSFCL ALTTICQKNQ
VKQIQPWIYE YISDLIVNDK ECDSYWNAFS FFLFVKGRFV DSYEAKVKEI RSLEVNYIWK
NKVFVGKEAP FFKAEAVFGD NSFGEVNLTQ FIGKYVLLYF YPLDFTFVCP SEIIALDKAL
DAFHERNVEL LGCSVDSKYT HLAWKKTPLA KGGIGNIKHT LLSDITKSIS KDYNVLFDDS
VSLRAFVLID MNGIVQHLLN NLAIGRSVDE ILRIIDAIQH HEKYGDVCPA NWQKGKVSMK
PSEEGVAQYL STLMEDNAAK IICHDSLLTD PRIAQDFSAE TEELLAEAEE AIEIIYEISN
ILNVNLDKET IVILIQLCEY G

> ***Plasmodium knowlesi***

```
NFDGDFKTTK KKIHWVPYIP EKLITCTLYE YDHLINKEDW TKFINSNSKY ETVVYSEPAI
TSLKVSDRFQ FERRGYFIVD LIKIPDGKSK KAGLNAYDEP SIVFPTIVYV GDEAIFREAD
LSFYRPIDHG HISDWDLARI VWEYAIKCVD KNRSVESILL TEPPLCSTSH RTKMGEIFFE
DFDYKNINIS VSGLMSIYAT GLTTGLVLDI GDGVTQCIPV FDGYIEKNSI IRSDFGGEEL
SMFLQKLICD IGYSMTTRKN FEYIKTMKET LCFCSLNPPK DQLREDLSVT YTLPDGDVLR
DGYNSVELSH ERFYVPEALF NPLICQRDSL SIVDIVWKSI LLCPIENRKT LTSYIVLSGG
SSLFPNLVER LEREVKNNAP ESARSAVKVV TYEQGASFAR ENNLFFAEAS AVSKLNVKHV
FENLLQMEKS KLAKVEKVLG RTGSRGGVIQ VRAQFMGDTE LSGRFLIRNV KGPVREGDIL
ALLETEREAR RLRGTQSLNL KKSHCYCHLS TGDLLREAAE KKNDLGNKIR NIINEGKLVD
DDVVLTLVDD KLKSPQCKKG FILDGYPRNV KQAEDLNKLL QTNQMKLNGV FYFNVPDDVL
VKRISGRLIH KPSGRIYHKT FNPPKTPFKD DITNEPLIQR EDDNEEVLKK RLNVFKSETT
PLINYYKNKN LLCPVCYYNL PDPESAIAPY DTELNYFMWG PGFEWQPFNT KSSNGKLRIE
DASHNARKLG LAPSSTDEKK IRDLYGDSLT YEQYLEYLTM CVHDRDNMEE LIKMFSHFDN
SSGFLTKNQM KNILTTWGDA LTEQEANDAL NAFSSEDRIR IEKLFAVIDK NNDELNAWSI
YVKNEVFLKQ VQVEMKQIDA DKDGFISLPE LNEAFSQNLK EVEKHAEGLL KRFQIVDKDK
DNKLNINEVG LLIDPMKDED LKELEINEIL EHHDVNKDGR ISKKDDDIAL DDFNFDTNKD
GFIDKEEIIT YDLWNEKALK LAVTSLTDYG DILRYPEDFK LLSNLNCFGS GFIFSIVMFH
LLPEFLFVFI GFAMQLALEY VLPVDGNMCC DSSSSEEEER VLKSYEGKRI DFYKNIGSSL
NESMESSDFN QMLKDYESLY KFMVKESSER IPNFAIVYLD KLSKYVDTTF QNNVEKKVLS
KSKAQTLNKL KAKIRKCSAY YQSKLDLYHE NPEEFWSNSE EEEYVSDDGD DKTKSAMSKW
GLKTSEKVEK KKVVKKEEKV VHVDENQSAK NKAYAELLST KNLSEEVIRN RVKFVIEKRG
RKGLDKHEHI NILSKLCELA KTVSTQSYIE VLEQLINLEF DVVSSLYTYM SFNIWNKAFK
YIEIILDLLI QNESFYLVSI NITEEITEEV INEKEKISRS CKTLISFLAK LDDELLKALI
YIDAQTEEYR RRLGKTIHMI ALLYKGYNYV KAIFISTRIL EHMYYKPEAL FMQVWKFVEK
TLEQERAEKR RLLSFHMHIS IELIECVNNI CAMLLEVPNL ARHSFESKDI ISRQFRRFLD
IYDKQVFNSP PENNKEIILL ATKYLQKGNW KMCCEKIFSL SIWSKFNDKV QNILREKIKQ
EAMRTYIFRY ISIYDSFSVD QLCIMFDLNQ NVVHSILSKM MINHEIPACW NESSSHILIN
KVNPTALQTV AIKLAENINE IMEQNELTLN MRNPKLGLQN KQCIVVGTRA LEFLNNELST
IKTLTELYDR VKKRGIMDHN DNILSLYLLE LFSIPIEQKE KNDFLTNRIS MYSKILNGRK
NVLDLLLNKF ENDCNDSIKK ELLDCFDVEK FKQEVNSKFM NILIQQLRKV EKLEKKKSKM
EFYLSIREQV KLYIRELVNI VSNLLTGNYP ILNLNGEDFL KKYGGTLMEN LKDGLVEGQP
VRFILAKEES SMFGVVQNTP TDAKSKNILV SFYFRNSNTE EQVQIKSVEK DRLAVETLNG
DPSFLWYKMI RDDGWVGFDK AEIIKVFNNN SKIKDVLIRH TDLKILNQLA ITQFWAIKML
ISVPFATKIY AHFVHDSIKI SILTVLGDIA TALNRSFSRY LNFFANILAE TSKITIASGS
AESDDWVNYV FELRDAILLT YSNIIYALID GNEIAKLKIY ITNILDLIEL ILIKEINHFN
AQNFQNAVSL LGDLVHAYGY ELIENSKLTD LIISVYGKID ILSSQGDECV SKIKWLKRIC
NISILQLEFK SVEDLNNATN NFIMAIKNFN VFPELRLKIL QLLYNSFSVN FSFFIAILQF
SSQNNIFHFM LPYIKFIDEW IKEWNISSRE KRQIYLIIAQ ELKKLKKYEE SFKHLNKHVY
YFQEVLNHPT TVNATIELIA DAINLNNNIY FHQLLTLDAV QNLQNIEHKP IFHLLNIFYQ
YSIHEFLAFI DLQVAENKIY LLSIISLFKD TKVQNIQYIS EELNISALKI EQILVAAIGS
GVIDAKIDQI NKSVQMKTTI LRHFDEAHWE ILNAQINKYI NNVATNFYKF VDSSASSTRA
IQNNPGYWCS SGNHGKDEEI SWTGYLNTKG FIKGVKISWE YSPESVSISV SSDGENYKNV
IPYRRISGNE ASFDEIYFFK KLEEVISIRI GLKNAIHKYF GIREVKIIGG GNPYFLLLSG
ITSDNEMCLQ ELWKTNSNNQ IISAFSNPPK CLSVINLDNL GDGKSNWIFE SNSQIRLQLC
ISQKNIYGNV PGIHDIDITV DANSTLDDDH NADNTIDGNL NSYWASATFA DNEHVVNIIL
DLNKYVEISR VKISWEYPPL HYSISASIDK VIAENLANPS FVTIDSLKNL ETRYIKITMM
KPHPKHGEMG DQFLYGVRSI EVQANNLETT IGYCRDAANS DDARDKYFVE YITEFDEDLT
SKLINLEDDV SKNVNSISDN LSKLEELLPN IETCIQEKKE YDEELKESKE KANELIKPKC
APEPLRVYCD MESSTSLYVW NGINSVDDIR QHCAEIGLEP LILRSKSQLN SLILSLKKMG
YTLNGKNNIP LAYDYSCFHD LVNGNIDLTT LIYESPDSTK VRQTALEEKK MFFCKIEEVA
KRRNKRHVKT QVLSKDCKIE RHKMKKFYNS KSGYFGCGWK TQWTPFIHAP FFDNNYNTIY
KNRNKKMYEE IDTLLHGRIH PAVRIVELKD HKHPVRLCTP YSEDCYSVVY TGEKILASDD
RVIFGEYTGY VNNSELSQDK HQYMFALSFI ILPDNYTYAV DSSYMFNEMS LVNHYKTCFN
NYDFRINAEW QLVYLDGWPH IILTSIPGVE INTGEEIFAD FGFEWFERVN DICLNDFIKN
SYACRLDDIV EKYNLIKNHT TCNICMHSVN TDGSNFIACS GCNHIYHLTC VQKLNNENYD
WFCASCVKFC INMAKAIICR VTKMHFEPNE DMFKTSSECV QSIIRELALG KTKIQREFTN
GTYVGTVMEQ INGIPFFVVT YDDGDTEWMT PYFLFQEEHK QKGNEFYKQK KFQDALHEYD
EAIKINPNQI MYHYNKAAVY IEMKEFDKAV ETCLNAIENR YNFKADFSQV AKMYNRLAIS
YTNMKNYDKA IEAYRKSLVE DNNRATRNAL KELERKKEKE EREAYIDPEK AEEHKNKGNE
YFKNNDYPNA KKEYDEAIRR NPNDAKLYSN RAAALTKLLE YPSALEDVMK ALELDPNFVK
AYSRKGNLHF FMKDYYKALQ AYNKGLELDP NNKECTEGYQ RCVYKIDEMS KEKVDEEQLK
KSMADPEIQQ IISDPQFQII LQKLNENPNS ISEYIKDPKI FNGLQKLIAA GILFKPSEKI
PSKYGENRHW NVDLIPKFIL VGGNLVKILK KTRVTNYLEW LVVEGSYVYQ HQKKGLLYSE
KFIHKVPATD MEALVSPLLS LMEKNRCKNF YKYVSEWDAN NRNTWDGLDP YRLTMMDIYK
YFNLCQLTID FLGHAVALYL NDDYLNEPAY KTLERIKLYM QSISAFGKSP FIYPLYGLGG
IPEGFSRMCA INGGTFMLNK NVVDFVFNEK VCGIKSSDGE AYCDKVICDP SYVHLKDKVK
KIGQVIRCIC ILSNPIPETN DINSCQIIIP QNQLNRKSDI YINLVSFQHG VSLKGKYDVR
LDVKLNKFIW SKGIRNPPKR VRVKIERKRN EDEDSKEKMY TIVQHVMVDT YKGLLNEQMA
GDRHRIHDCT TENFNGFMAI TTPTKSWMAQ YNDLSKYAPG FYALQVVGEL GTGSGYLILS
LYELLLKRKK KINLLYCLDI NEKANKISNV EIINTDLFSN LKHCFDLVLF NPPYVVTEEE
EMNKTIVASY AGGKYGREII LKFLLSVYDY VSDKGVIYLL MEKNNRPNEI ESAELPILCE
TCLGENPYVR LIKEENGKEC KICNNPFTLF RWKPGQKSRY KQTIICNMCA KVKNVCQTCL
FDLQYNLPVQ VRDKFLETSI SMPENETNRN FFLEQVELSK LRRRDPYFKR NMARVCSFWR
KNECNRGAEC PYLHKEIHKI IIFNLPPVSE QDVKNLCERF GPVVDVYAFV NFMFPSACER
AKQFLNHAIF RGKVLSVKYA SYKKILEIQK KRNCQNENIW NILYTDINSS IHSFCKENKC
SPNSILDRNI AVNVSLTETY IINKMKEWIK KEGIRSDDTI IVKNLSIQTN QKEVISLFKK
```

YGVLSKVSFS PYNNIAILQF EKAENAKKAF ISNSYIRYKK LPLYLEWAPM NLFDEEITHA
SIYVKNLNFN TKEEDLKKLF EKLEGFITCN ISQGYGFVEF KSKELALEAI KKLTATTLDG
HVLELSLSKL LVKNLAFQVT KEELRKLFSA FGNIKSVRIP KNAYNRSRGY AFVEFMSKNE
CLTAIESLQH THLYGRHLII DFAPQNARIL KPLVQEKVVE IMKPEIEEKI IEVPQVQYIE
KLVEVPHVIL QEKLIHVPKP VIHERIKKCP KTIFQEKIVE VPQIKVVDKI VEVPQYVYQE
KIIQVPKIMV QERIIPVPKK PQYRHIPKPV EVPMAHYRTF PIEKLVDRNV PVPVELQIVQ
EFLCPKIEAR YKEIPVPVHV QRIIEHPIPK DAMNNPFLLP LYYLGSTAVG ICVNDGVILA
SERRIASTLI EKSSVEKLLP IDDHIGCAMS GLMADARTLI DYARVECNHY KFIYNENINI
KSCVELISEL ALDFSNLSDN KRKKIMSRPF GVALLIGGVD KNGPCLWYTE PSGTNTRFLA
ASIGSAQEGA ELLLQENYNK NMTFEEAEIL ALTVLRQVME DKLSSSNVEI AAIKDQTFYK
YNTDDISRII EALPRFKIID TDFINIKLME LFQMEGFQKQ LDRLSDSLSK IQKALGEYLE
KQRNQFPRFE KLVQTKLTDA CFLTLTQALK MKLGGNPFGP AGTGKTESVK ALGAQLGRYV
LVFNCDESFD FTAMGRIFVG LCQVGAWGCF DEFNRLEERI LSAVSEQIIE ILNKKIGLNK
NVGIFVTMNP GYAGRSNLPY GVGFKMSFAR ISHSCAIMSR TINTIGIGLL SLELMNHCDA
KELATPLCMW KLPNKELINR NIANKSEHRH HQKLLMSYTP FNSPSLLAEQ INILGTYSGT
RLLYFPLWDH PKDSIDYCLS TYLYWLYLRR STNIFLQNTL LRGQVVVIAA TNRQNSIDPA
LRRFGRFDRE IDIGVPDDNG RFEILRIHTK NMKLSPDVKL EELASSTHGF VGADLAQLCT
EAALTCIREK MDVIDLEDEI IDKEVLESMC VTQDHFNMAL GTCNPSSLRE TVVEVPNVKW
DDIGGLDEVK NTLREMILYP IDHIMKSLHD AKFWKPTEIQ KQTLEHSINL KNDIIVVSKT
GTGKTLTFCI PILNNILILV PTRELAIQIL SHFSYINKYT HIYIATIIGG LNLNKQKRII
SNKPEILVCT PGRLRYFACD EIDKMIETSF MRDINFIAKH LYIQTFLLSA TLLAKLLNYV
TIRKEKSCII NLLPDGLSLN VVKCERKNII HKLFYLLKMY KIVIFVNTIK ETKDLNSIFR
FLFFDQGLES SVPKKIFSIH SKQSLKERMQ SISKFSTNHS VMFCTDVLSR GIDLDKCDVI
IQLSCPVSDI TFVHRSGRTA RNFKTGNKVV SVEVRSAELV LNSEELRKIS DPKYGTIDYR
QICSVCFENC VGHIGHLEFT LPLFNPLFYK DVYDLLGLVC LNCYHVCCSY NYEEYLKLIK
TLKVLMKRNG RCPICKFKRN ISARMSQKRD TITVRLFSFQ VIDLLGKIFD KDIMNLLYPF
TKRDGHKVFF LHDMGISANR FRVKLRGIHK KNALLNLCIR AKKEIDFEEL MELQVAMNTF
FDLKEILDKK EGILRKNIMG KRVNNCARTV ISPDTFIETN QIGVPLEFAK KLTMDECITE
SNLDYVKRLS LFDFILKKDA DFRHLSFISD FMTGELCMDI LKANWSPAWT IQSLCRAILF
LFTEPNADSP LNCDAGNLLR GVKVKGAVEV GKRAVEYFRG DEFVHFLYTK RDMLKKKFPV
LLQNRTLEDL KDIEEFADTL IQKGFIYKAQ YKPENGMYRR PKWPRRLIMS SKQNFDRTSF
YILVHERNKK LQYLMLITLI SVVLICCMFW HLSVVFVSLM SVIVFVRLFL FIFFWFFGVD
YWLFPNLFDE ECSIIESFTP VNDWYYRNDS WVFVIARMFT AVLLAIGIHQ LGKTHSISDI
SNFATQSFID IIEWGNKKLA EENYDCLKGC GFQTFEDLVR KCFLKCECMT LADTFLQDLE
DLEFEERKIS ELLYDIEKCI ELIIQIDTEI LNIHKYVKDI YSTKFPELDS IVYTPLEYIS
VVSKIKNESD LKNIDFSDIL PNTTVMAITV ASSTTGINLS DHSLKNCLSF CNEALELNEN
RRMILLYLES KMFLLAPNLT MLLGSALTAR LISSVGSLKN LSITSSQNLI VVGSSKKGIL
STSEIVQSVP DAYKKKAISL LAGKCSLASR VDYFTEERTV LDKKILNSSV ILQIAMRVKR
DRYFGRHYRY FIRNTRVRAY RQFLEPFKSV TLKNMAYAFG VSEEFIEVED PYEAIKEAVR
NFENADEILA SAKIVERLVE YPEVSRNLHK INAIEPLLKL LNNHILESVL QIFSLALSNN
PDLQECVFKK NGLKTLLLKL QESQKTVIDK KLITAISALI RHHDEAENKF IDYGGVGFLV
YGMQTNIYQY QEKSALLLKH LVHQNKITFE IFLKNEIMKG LICLAKTGIQ YGETTAELFL
ALIQNHRHKL AKSGLHNMKI LIEDRLAYLF EDLGVEDWLI KISKTVQITH PTKIQQLCLP
LIMRGHNVIG TSETGTGKTI CYCWGILQEL NKNAFGVFAL VLLPTRELVV QVVEQFLLYG
YKIGIKILSC IGGFSIIEQP HVVVGTPGRF KRLSFFVLDE ADLLLQKCYE SKLEVILRSL
PRRTLLFSST ITDTLELLAN SFPHENLILV NVNKKQKPLK NLDQRYGIIF TDNSYRCELV
YTVLCMLFSV ESIHSSKDPK KRLAALSKIK NGGCKILVAT DIISRGIDIP KTSFVINYDF
PNDTVLYVHR VGRTARANRK GVAISFVDKR DVNSFNNVMA IMKSALKPLR LKKKEVLQDM
FQVGRVLKKA ELLLEEVVVF PVSVHYELAK SLLKPKFHTG IQNVSKYGNG SYTGEISAEI
AKDLNIEYVI IGHFERRKFF NETDEDVKEK LQQCLKNNLK VVVCFGESLE QREKNQTIDV
IKKQVNSFVH LINNFDNVVL AYEPIWAIGT GKTATPEQAQ EVHKEIRNIV KEKCGAQQIR
ILYGGSVNTE NCASLIKQED IDGFLVGNAS LKPSFVEIIK SAKDINKDVV HRYGPNTFKL
HRLPVPKLGQ ILGLVGTNGI GKSTALKILS SKLKPNLGKF SNPPEWRDIL SFFRGNELQI
FFTKLLEEQL TPIIKPQNVD LIPKQIKGNI LEIINKKDKL NRKDQYMSAL ELDHLLDRNV
EDLSGGELQR FALLLSIIQT TNVYMFDEPS SYLDIKQRIS MAKIIHSLVR HDNYIIVVEH
DLSILDYLSD YVCCLWGKAG AYGVVTSPFS VREGINVFLD GFVPTDNLRI REESLNFKLE
DKKRLHFYNY PTIKKTLNSF TLTIDKGIFS ESEIFVLLGQ NGSGKSTFIR LFAGLIKPDN
VEFLESLSVS YKPQQIQAKY TGTVRQLLMS KLKGLYTDPY FNNEIIKPLK IEGILDNQVL
TLSGGELQKV AIIITLAKNT NIYLIMSILC TISGQTPDEP VVSKTGYIFE KRLIEKHILN
YGICPVSGEV LTLQDLYPLK NEKVVKPRPI TASSIPGLLS ILQTEWDSLI AEMFTLRTHV
NDIRNQLTHS LYQYDAATRV IAKLLKEKNN YTEEIKNLRN QILSIRSGND LSELEVGLSE
ELLNQMQDVA KNLLITRKKR NIEHVTSTDK WKEVTNTNEF DVHSAALPGV TCLSLDFSGG
VDGNVYYISL EESKILAKLQ GHLKKVNAIV SHPTYPVCIS GANDKTVRIW KGDKHKDQIN
SLALHPMENY FVSSSKDNVW ILHDLETSRT IKICKTNPSF KHLAIHPDGI MIGIGSEDSN
IHIYDIKSEE YKASLFSENG YYLASCSKDK TAKLWDLRKA QCFQTIDVNE HPKSIEFYNG
NIKDGLFHGR GILMYSRNEK YEGDFVYGKR EGKGKFTYAD GATYEGDWVD DKIHGKGTAK
FVSGNVYEGE WDNGRINGFG ILKYNNGDIY EGEWLDGKMH GRGTYTYEDG DVYVGEWKND
KRHGKGCVKY KGSENKIAET YEGDWFEGKM QGKGTYFFAD GGIYEGDWVD GKMEGKGVYK
FLNGNKYDGD WSNDMKNGYG VLTYVNGEMY EGYWKDDKVH GKGTLTYSRG DKYIGEWKFA
KKSGQGELIY ASGDKFKGEW KNDKANGFGV LYSNGNKYEG EWVDDQRHGF GTFTCKEDGS
IYAGHFAFNR KEGRGTLTFV GGNVLEGLWT MGVLTKVSKF QLSPASPWHD PDLEMETLYD
LGNKMIEALQ KENITAGDVI CIDKGTGKIT KIGKSFARSK DYDAMDPNTN FVQCPEGELQ
KRKEVVHTVT LHDIDAINSR TQGFLALFSG DTGEIKNEIR EHIDMKINEW QEDEKAEIVP
GVLFIDEVHM LDIECFSYLN RALESEQSPI VIMATNRGIT HIRGTDYKAP HGIPLDLLDR
TLIIPTYPYK HQDIMKILEQ RAEEEDVEID QYAKELLCKI ASESSLRYAL HLITLANLVS
KKRKATEVTV QDVRRVYNLF IDVKRSTQYL IEYQNEFMFS ELYSNLSDFW TSDDEDDEEY
IRKKWVIEDD VTDFNQNDLL LSYDFELDDF QKRSIKHLNN FKHVFVAAHT SAGKTLIAEH
AIAMSIKLNK KAIYTSPIKA LSNQKYHEFK NIFKSVGIIT GDIKMNVNAN CLIMTTEILR

NLLYINDNII NNIHCVIFDE VHYVNDNERG FIWEESIIML PPHVQILLLS ATVPNYLEFA
DWVGFTKKKE IVSISTKKRP VPLLHYIYAY DSIFQIMDEN NIYSSAFKEI YEANMKTEIQ
KLQALIKKLE QDNKLPVVLF CFSRIKCETY AKSMPHLNFL DNKHKSKVHL FIKESIAKLC
TQDRELNQIK ILSKLLEKGI GIHHSGLLPI LKEIVEILFS KGLIKILFAT ETFAMGINMP
AKSVVFTSIY KHDQLKKRIL TSSEYTQMSG RAGRRSSDKY GYVYIYCPDK IPDQVQLTEM
LMQKAVSLKS KFKVTYNMIL KLLINKQINI EKMLFSSFLE SCRAVQIPLF KKDLKRKKKL
LQSIKQVQCI YIENYVQIDC KLKHIGLSLH RKLVPLDHIS IITNELDRLI EKGNFEPFVL
TKMLKSLKCE FYSVLHYEIV CKRNKCINDI ENIEQNINAK SLNLYEDLEG RLDVLRHFSF
IDEDHNLTVK GKIASYITMT DEITLTQVIF ENVLNNLNPP EIAAVLSCFV APEKKIEESP
DLTVNLQDVK MALTNIHSQF EEFYKIIRLK ISSEDHWKLC SFKIMFIAYK WALGVSFAEL
LEQCELEEGL IVRSILRLDD LCRKVKIAFL YLGNVDLAER LETTCTLLRR DIIFMTSLYL
DALALLQFFH WCDEKRKTKE LFNETEMSLK NKVDYFRSTK SNFPSFATIS ASGPNAAVIH
YEVTESTNSK ITPSIYLLDS GGQYLHGTTD VTRTTHFGEP TAEEKKIYTL VLKGHLHLRK
VIFASYTNSM ALDFIARENL FKHFLDYNHG TGHGVGLFLN VHEGGCSIGP TAGTPLKPYM
VLSNEPGFYL ENKFGVRIEN MQFVISKKKT DNTEFYSFED LTLYPYEKKL LDYSILTTEE
IKDINEYHDN IRKTLLPRLK KNPKKNLVIG ENPAFIQERL AKYNELKEKI NVQLLDGSVK
VGQCNVTTPF HIATSISKRL AEDSIVARVT YVEKVELELC DLWDMNVPLL GSCKIEFFWH
SSAHILGSSL EKLFGGYLTI GPPLKEGFYY DIYLGDFSIT NEDYKKIEEE FNKLIKQNAE
FEKLICTKDE VMELFQYNPF KLELIKSKIP ENKKTSVYKC GNFIDLCLGP HIKSTGKAKA
FKVLKNSAAY WLGNKNNDSL QRIYGITFQK KTELNDYLNF LEEAKKRDHR NVGKKLHFFF
FDKDTSPGSC FWLPHGAKIY NKLVDFIRRE YRIRMYDEVI TPNVFSCDLW RTSGHYQNYK
DCMFIFNVEQ KEWGMKPMNC PGHCIMFKQL NASYRSLPIR LADFGVLHRN EISGSLSGLT
RVRRFQQDDS HIFCTFDQIK EEVLNTLHFI FFIYDLFGFG IEVLQNRGYD SCGMSTILKT
TKYASTSTSD AIEKLRGNYS TSHKNDKIGI AHTRWATHGC KVDENAHPHM DYKERISIVH
NGIIENYREL KSFLLGKNIP FKSNTDTEVV ANLIGYFLDQ KENFQNAVLS AIRQLEGTWS
FCIIHKDHPD EMILAANGSP LHIGFKDNEI FVASEHSALF MFTNEYISLK NGEILSINKD
KINDLKTEKK VESIPEVVIQ KTPHPFPHWT LKEIHEQSIT LSKTLNNFSI LNSSVKLGGL
DPYVDELSQI ENIILIGCGT SYYAALFAKY VMHYLNCFNT VQVMDPMDFN VSVIPKEKEG
VIFISQSGET RDVIKACKLA DDLNLKKLSV VNSVGSTIAN MTGRGVYLNA GREVGVASTK
CFTAEVSVLT LIAIWFFQNK SHSKVSSLIN SMHRLPLYAG TTVKCEAKCK TLANKLINKS
MLIVGNGLSY PIALEGALKI KELSYIHCEG FTGSALKHGP YALLGGDENM PVIMLIFNDK
NVMINTGEQI KSRGAHIICL TDDEDLCKDD IILIPNNGLL TPLLAVIPLQ MLAYYTSVAR
GNNPDRPRCL AKTVTVWIIE IEKYASEDVQ KILIGNKIDL KNDRNVSYEE GKELADSCNI
QFLETSAKIA HNVEQAFKTM AHEIKNKSQL ENQQKGRANI NLNAKPIKIR TMNSRKPPEG
WSKVESFLDE MNKKMRSLEN EDTSKKRKSE ILWPIFQINH QTSRYIYELY YKRKEISYDY
VVIGGGPGGM ASAKEAASHG AKVLLFDYVK PSNKGTKWGI GGTCVNVGCV PKKLMHYAGN
MGKMDSNPYG WSYNNIHNWG KLVTTVQSHI RSLNFSYMTG LRSKVKYMNG LASLKDEHTV
SYYLKGKEEL VTAKYILIAT GCRPSIPDDV EGAKELSITS DDIFSMKKDP GKTLIVGASY
VALECAGFLN SLGYDVTVAV RSIVLRGFDS QCALKVKIYM EEQGVLFLPK KLSKEGEKIS
VLFNDGTTEL FDTVLYATGR KGDIDMLNLS CTNISNIFAV GDVAVDVPEL APVAIKAGEI
LARRLFNQSE EIMDYTFIPT SIYTPIEYGT CGYSEEKAYE IFGNVEVFLQ EFNNLEISAV
HREKQKDQYD TDVSSTCLSK LVCLKDNRVV GFHYVGPNAG EITQGMALAL RLKAKKSDFD
KCIGIHPTDA ESFMNLTITR SSGLSFAAKG GCGGGKCGEP LYSGPLKIEQ LLAKGFVKRD
LELLKEGGLQ TVECVAYAPM RTLCAIKGIS EQKAEKLKKA CKELCNSGFC NAVDYHDARQ
NLIKFTTGSK QLDSLLKGGI ETGGITELFG EFRTGKSQLC HTLAITCQLP IEQSGGEGKC
LWIDTEGTFR PERIVAIAKR YGLHPTDCLN NIAYAKAYNC DHQTELLIDA SAMMADARFA
LLIVDSATAL YRSEYIGRGE LANRQSHLCR FLRGLQRIAD IYGVAVIITN QVVAKVDAMN
VFGNDKIPIG GNIIAHASQT RLYLRKSRGE SRICKIYDSP VLPEAEAVFA ITEGGIADYM
AIRVQFENSN EVGVFARLTN SYALIAMGGS ENFSSVFESE LSQHIPLVYT TIGGTKVIGR
VCVGNRKGLL VSSICTDQEL LHLRNALPEN VKIKRIEERL SALGNCITAN DYVGLVHTDI
DRETEEIIQD VLDIEVFRTS IAGNLLVGTY SYFTNNGGLV HAMTSSQEIE ELSELLQIPL
ITGTVNRGSD LIGAGLVAND WSAFCGMDTT AIELNIIEKV FKLNSIEDAN IEDNFKYKSS
IIQTMIIVDT YWQTETGGIV IAPIPNLFKM KPGCATLPFF GVELEILDSK TLEPLKGPNC
GLLCIKSPWP GMLRTVYGNH SRLVKTYFSS CPNYYFTGDG AYRDEDGYYW ISGRIDDTLN
VSGHRLGAAE IEHALVQHSC IAEAAVVSFR HVVKGEGILC FVVKKLKLYV RRAIGPIATP
DLICIVPDLP KTRSGKIIRR ILRCIANGIN DFGDMTTVAN YEVIDIIPFP TPCTVEDALA
YYCDLTTIPR VNILKKFKCF IKDVEEKESD MTLIEFVDIF MPKAEFELTP FLQLIPRNVP
KSYTISSSPK RWFKGSSSFY LTELTPHDSV KFNLKSSKFE IDFLYEREIE AVEGKHIDEV
FLAFSRDQPW VEKYRPKKLD DIVHQTNAVS MLKEVVRTKN MPHLIFHGPP GTGKTSAINA
LAHELFGRDN ISERVLELNA SDDRGINVVR EKIKAYTRIS ISKNKINSET NETLPPWKLV
VLDEADMMTE DAQSALRRII EIYSNVTRFI LICNYIHKIS DPIYSRCSCY RFQGIPIDVK
KEKLLYICKS EGINILDKII ETTQGDLRRA VSILQLCSCI DPMITLESVL DVSGLPADDI
ISKIIDACKM KDKNVEKAVQ DIIEDGYDVA YIFKSLNNYF VDSVKSQILL ELSRHDYRLH
SGATKYIQLM SFASSVHSLL IKELIFKSND ERHFQNIVKQ VKDLIKHVRQ KEVEDVNDPE
HAQEKLVLNK SGRRIILRDL MTRPNIFTGR KILGTLELHT NGLRYSAIDI LFDDIKYAFY
QPSDGQLIIL IHFHLKRYIM VGKKKTLDVQ FYCEAGTQID DLDRAKARNV YDPDEMHDEM
KEREQKNRLN LIFKNFVQQM QDASKIEFEI PYPELTFSGV PNKSNVEIFV TANTINHLVE
WPPFILSVED IEIASLERIH HGLRNFDMIF VFKDYTKPVK RIDVIPTEYI DTIKKWLTTI
DIVLKTILAD IDSFVESKGF DGFLGDDDDE EEDEDDDDEY ELDESEMSAE IETLAIVEFT
SKRKMSSVIC RIPKIMLFCK GAGSVILNKL ANKTEIDDIT IEHMETYADE GLRTLCIAQR
ELTEEEFAEW YRLYKEATVS LKDREENLEN VAEFIENNLT LQGVTGIEDK LQEGVSSTIE
DLRLAGIHIW MLTGDKIETA MNIGIAANLI DNYSVDGGLL DTLLSKKFER KFFYLADKCS
SVICGRVSPY QKGAIVSSAN RLLKKITLAI GDGANDRNMI NTANIGVGIR GQEGVQAFNS
SDYGISQFRF LKNLLLVHGR LSYARISKLV VYMFYKNIVL IFPLFMFGSI SLYSGQKIYY
EFLLHLYNIV FTSIPVVAHA ILDKDVSLKT ALVTPSLYKL GIYHYYFNIS TFVSWVINSL
FHGLVVFLLP LYFLIPSADG TPFDMWTVGS VTYLTVLVVN FKVLLETYCL NVLPLTAVFM
SILSFVILVS SCSFMCFGTN NFLGTAVILA KSLRFWLVVL LGLFTLSRDF IFKVFKRNFN
PRSYSDHVKD HFNKPRNVGS FDKNEKNIGT SIVGKASCGD VIKLQLKIED NVIKDARFMA

FGCGSAIASS SYATELIKGK TIDEALKIKN NDIASHLSLP PVKVVILGSG WGGIHFFINI
DFKKYDVTLI SPRSYFTFTP LLPCLCSGTL SAKVCTENVS TFLKKKGSSG KYLQMECTDI
SPEERQVICR DNNEVKIAYD HLVISVGAKT NSFIKGVDKH AFFVKDIEGV INIRKRFLDV
LDICCTDKIS NEEKKKLLHV VVVGGGPTGV EVAGEFADFI NKKKKYKNIF PLISVSIIEG
GKNLLPTFTQ NISDFTKRTF HTANINVLTN YYVKEVDEDT ICVQSKQIPY GLLIWASGLA
QTPLITNFLK KIPEQVNNRI LNVNGHLAVI GINIYAIGDC KKIQPVQITA EQLINEALDL
EEVEQKVNYN LIDEDELNEY KISKRKEYED KIRKRRYLIS TYIKYALWEV KQKDIRRARS
IFERALNIDY TNINLWLKYI EVELLNKNIN SARNLFERAV LLLPMENIFW KKYAHLEEIL
NNFVNARNIY ERWIKWKIDE TSFLCYINFE ERCREIDNCR NIFERLIVTL PKMECFYRFI
KFERKYRNVD RAEKCIELLP PSFLDEHFYI NFCNFEEENN EYERCRKIYI EALKILPKNK
SEFLYKSFLQ FQKKYEELDE TLMIKERITY EEEIKKNPSD YDTWFNYIKL EERIRELYER
AISVLKNALV HDGLKIGIRE VIKSIESKEA KACFLSDVCS EPAYKKLITA LCTEKNIPLF
MVDSKDLGQW VGLFKLDKEG NARKIIGASS VSIIDFGEES PERDYLGYYK AIVGEVIDNR
YSVVCELVGK GVFSNVLKCY DKVGKIPVAI KVIRDNDMMR KAAEKEISIL KKLNKRHIVR
LLRSLKYKNH LCLVFEWMWG NLRIALKIQE SFSSLVQKTK ELEDVYISRR KLQSKYLTQI
KNLYINCNCE CIIHKISFKY ATKSSFPNLA NGTLLYMIVE KINLDNNVVA SCINSSDVKS
WINYENYLGE LVDGYMFPVS IAYAKSLIGD KCYILDLIDI SYEVAVGHNG GALGYSQHLS
EEVMLFSRDA ILDKVAVILG GRAAEELFIG KITTGAIDDL NKVTQLSYSY VSQYGMNKEI
GLVSFQPNTS SEYSFYRPHS ECLAHLIDNE VRCLIETQYN RVKSILLKHE KQVHKLADLL
FQKETISYQD IVECIGLKHQ RYKGTDKVKI EEERNKKVYV KNLKFCKNCG STAHKEKDCL
ERTRKKGYDG NRDRWVGYNP DNFEHVYKEY EKIVEEKKKR KAEELVDPKV KRIKIMSRYE
EDIHLFDHSS VFGSYYDRDK KKWGYRCCRS TNKFEKCIIP LMDPYHASYY TREVMVILIN
EFNSPDEEMK KVVLKCVKQC IQTEGIEKDY INEEVVNPFF EKFWVLRSSH DKRNLHLIVE
TTVEISNKIG VIARIVDDLK DPSEQFRKMV MQTIQSIVNN QGVDDIDQTL EEQLIDGILY
AFQEQASEDY YILLNSFDAI VNKLQVRMKP YLPQIAGIIR WRLNTPLPKI RQQSAELISR
IANLMHLCGE HQMLGHLALY LYEYLGEEYP EVLGNIIGAL KSIVVVLGVQ HMTPPIKDLL
PRITPILKNR HEKVQENVID LIGIIADKGG DLVSPKEWDR ICFDLIELLK SNKKLIRRAT
IQTFGYIART IGPFEVLTVL LNNLRVQERQ LRVCTTVAIA IVADTCLPYS VLAALMNEYR
TQDLNVQNGV LKALSFMFEY IGEIAKDYVY AVVPLLEHAL MDRDLVHRIA TWACKHLALG
CFGLNREDAL IHLLNYVWPN IFETSPHLIQ AVIDSIDGFR VALGPAIIFQ YLVQGIFHPS
RKVREIYWKI YNNVYIGHQD SLVPIYPPFE HLDDSNFARD ELRYMGRMYG KGKGISSSTI
PYKRKQPSWL KQKPSEIEDA IIKLAKKGQT PSQIGATLRD NYGIPQVKAV TGNKILRILR
AHGVATTIPE DLYFLIKKAV SMRKHLEKNK KDKDCKFRLI LTESKIHRIS RYYKRKKLLP
SNWKYQSSTA SALRNIEHAP GVQFAYVPPD FFDSEDDECD KNQYELKDDG GGRAPGTRSK
EHSTTHHLRR KNYEDDFEFN EDKILEALHI LELLYLNGAS LEEQNEYGQT ALFLCVKRNN
ISTLQWLLSK SVNINHRDFY GNTILHIAVK YCDIDILRLL CDYGSLNLVH HTSMQNDSIN
VFQLCLRNRY FLVYILLKKW IIQDKLCKGI KICKTIYAFY FWFFAMLNLI VYLNISRSFW
IHRKYHSLSI TWIVIWLFQQ FLWFILYFKS PGEYQLNSIE MELFKINLEC QKLALYEKVS
KERINSLDVD YRNAILEIIL LQLIIEPYIL RRSKKHVFID MPKKHSIIIK LPMNTTQLNL
YKDEIMSKIQ HTHKHLINAS IFILRRICNH PLLHKYYYSV EDIKKISKYF YTNTDQYLDL
DLKTVENEFM KISDFDIHLS IKHLISQDEN LNRYLISKDH ILNSSKIHHM ISLIKDIRKK
KEKVLIFSQF TTFLDIIEEA LYVRLDGSTN TIERQKIIKR FSNIFIFLLT TKAGGVGLNL
IAANHVILMD QWNQLLLQEE IVKKLCEHII DSRCDIVEKG VSDLAQHFLV KKNISVIRRV
RKTDLNRLER ISGATIVNRC DEIVESDIGT KCGLFEVKKI GDDYYSHFIE CENPRACTIL
LRGSTKDVLN EVERNLHDGM NVAKNIILEG KLLYGGGCTE MRVGQHLISQ ASQYDDSRKS
IMEAVGSALE IIPKILAQNS GANVVKTINE LRIKHGGEKF GVDGITGEII DVSTKNIWDL
LAVKKQIYKS AIEAAAMILR IDDLAVVLIY EELLKNPLCR ITNIYQNEVK KYRPLPLNTL
QMTKLVSKHF HISSKQCMNI AEKLYNKGFI SYPRTETNSF PISMNLRSIV SELKKNNIFG
SYANKLCQGN PRKGKLNDKA HPPIHPVKNM EWIIYEFICR HFLAVCSEDA IGFNSKVVAT
IGKEEFFCKG LKIVKKNYLE IYIYEKWNNK NIPPFEVNQE FHPYSLLVEE GITQPPKYLS
ESDLLSLMDK YGIGTDATMH EHIENIQKRN YVFKNSKNLF IPTNLGIALV LSYKKFKDIG
IDLTDPSLRA KMEKDMTLVA SGICVVKSGK TDIPRNCKMI IISYELMTKN DKYQKYKSIV
CDESHYLKNS FSKRTKAITP IIRSAKRCVL LSGTPALNKP SELYEQVSSI IPDLFNYNEF
CERYCFRDKN MYTRKFEYVG CKHTEELHLF LTNTIMIRRL KKDVLKELPE KLRSKIPVEI
PPKELSEILL FKITGYAKVK AIKEYITYLI DADIKFLLFC HHKLVMDEID KFLTEKKCMF
IRIDGLTPID KRELYIKSFQ NDDKIKIALL SLTACGLGLN LTAANTVVFG ELYWVPGQII
QAEDRAHRIG TTHEVINIHY LIAQKTIDET VWRIINRKWN TLTTALNGME DSLMFHDKDK
ICIAVSGGKD SSVLAHVLVH IKRKYNYKWD LFLLAIDEGI KGYRDDSLKI VFEDIFTYTM
DTVVSFIGKK NNCTVCGVFR RQAMERGALL FNATKLVTGH NADDLAETIL MNMCRGDLEK
LFSTECTYSP NSFRGNLRSF IKDLECTRCG VYTSNVEGLR NYKDNKKISN CSFEIDISFE
HLEPMPLENE FQQIPKLRIL SFDIECIKLD GKGFPEAKND PIIQISSILY FQGDPCSKFI
FTLKECASIP GSNVIWFHDE KTLLDAWNEF ITRLDPDFLT GYNIINFDLP YILNRGTALN
LKKLKMLGRI KNISSVVKDS NFSSKQFGNH ETKEININGR IQFDVYDLIR RDYKLKSYTL
NYVSFEFLKE QKEDVHYSIM NDLQNESPES RKRIATYCIK DGILPLRLID KLLFIYNYVE
MARVTGTPFV YLLTRGQQIK VTSQLYRKCK ELNYVIPSTY IKSGSNEKYE GATVLEPIKG
YYIEPISTLD FASLYPSIMI AHNLCYSTLV KNNGKSNIKF VKKSVKKGIL PMIVEELIDA
RKKVKLLIKN EKNKITKMVL NGRQLALKIS ANSVYGYTGA ASGGQLPCLE VAVSITTLGR
SMIDKTKESV EKYYNKSNGF EHNCTVVYGD TDSVMIKFGT SSIAEAMALG KDAAQRISKE
FLHPIKLEFE KVYCPYLLLN KKRYAGLLYT TPEKHDKMDC KGIETVRRDF CILIQQMMET
VLNKLLIEKP FIQVAPMINV TNRHFRAFVR TITRRAQLWT EMIVDNTLLY NLNNLEEYLG
FNSNEHPVVC QLGGSDPTSL AEAAVLVEQA GYDEINLNVG CPSTKVANKG AFGAYLMKKP
EHVRNIVYEI KRKVQIPVSV KIRTGVDDCD SFPFLRSFIE CVSSVGCTHF IVHARKAWLK
GLDPKQNRSV PPLQYSKVYT LCQLYPHLKF TLNGGIKTIE EAVALLLNGV MLGRACMENT
TVLSQTDQLV YNEKPPHTAF SRRTVLDAYK SYLEENSSLC SLFELLKPIL GILKGMPGHR
IFRFLTNFFF KNPVGHVGVV ALKNSSAKLI QPLTSNMEDI MNALVKERSM GLQGSPSLEQ
GLEIAHDLLV DIPLYGTKEI LIMYGSIRTC DKKNILNILN LIVKNNMHVN CVSISPEMHI
LKCGMHLISM HDLSHITNNL QGSPLFIEIM GSNSLPMSQQ MYFSTHNALR VNENDVISTL
FYEINGNRHI SLLIFPFYDV QMLKRLLIKK LNLPGVKVND IIIFYKGIKL PNYRIISTYK

KINKLYWAIK DINPNASIRV IDQKYPPFFE NILHDIKLAF KKNISPKLTM DGTGGTYLLF
NSKKKVCSVF KPLDEEAFAP FNPRGYEGKM YQEGFRSGVL SGEGASREIA AYILDNSYNN
FSSVPCTIMV EACNPHFNNK SKLKYVDHEN NLKWKCGSLQ EFVDSRESVG NYDHKQFSIR
DIHKIAILDI RVMNLDRNDG NILVSPTHLG IEQSRRDDIE ALGYVLMYFL RGSLPWQGLK
AISKKDKYDK IMEKKISTSV EVLCLLNVSN KIDSCIIIDR KIDMVTPFST PFTYEGLLDH
LFGISNLQIE VPLYNDIKDL SPNQVGLYLH NKASEIQKTY EKDTLKDIEE INKFLKKIKM
KHFEHNSLST HVNLASFILT TMKKEPNFNK LKLEDEIIQL NNTSNRTTLH NIVQQIQLLI
YSNEDIHEVY RLLCLFSVVT NGKKDILEHY GIDELSRINK LHFCNILKHQ PKQKFIWSHL
RNHFNLLSNE HNDISYVCNG YAPLSVRLIE YKNNMQAFPE IFNLLSGPTL DIVQETIQVK
SMCINCEQEG LNKIAKLHIP YFKNVLIHSF ECGFCNYRNN VIQDLNTIKE KGVKIIFQIS
QREHMDRQLI KSEYGVLKIP QIDFEIPKDT QKGSINTIEG FLQTALSNLY IKLIESTVHK
LFTIEIIDPS GLSSLEYKRS KQELNEMGFY SFTSNCPCCN YMGSNNFCEI NIPGFKKCLI
MSYVCANCNF KTSEIKSSGE INPKGKKITL TVRSKSDLNR FVIKSDTASI HIPIVDLTSD
YGTLGGTLTT IEGLILKIIE SLEDKFKFLM YVLNRKGEEE DISFDQILKR IQRLSYGLHE
LVDPARVTQG VINGMYSGIK TCELDELAAQ TCAYMATTHP DFSILAARIT TDNLHKNTSD
DIAEVAEALY MYKDVRGRPA SLISKEVYDF MIQHKDRLNK EIDYTRDFNY DYFGFKTLER
SYLLRINGKI IERPQHLLMR VSIGIHIGDL EKALETYHLM SQKYFTHATP TLFNSGTPRP
QMSSCFLLCM KSDSIEGIFE TLKQCALISK TAGGIGVAVQ DIRGQNSYIR GTNGISNGLV
PMLRVFNDTA RYVDQGGGKR KGSFAVYIEP WHSDIFEFLD LRKNHGKEEL RARDLFYAVW
VPDLFMKRVK ENQNWTLMCP NECPGLSESW GAEFEKLYLK YEEEGMGKKT VLAQDLWFAI
LQSQIETGVP YMLYKDSCNA KSNQKNLGTI KCSNLCCEII EYTSPDEVAV CNLASIALCK
FVDVDKREFN FKKLYEITKI ITRNLDQIIE RNYYPVQEAE RSNKRHRPIG IGVQGLADTF
MLLRYPYESD SAKELNKRIF ETMYYAALEM SMELAQIYGP YETYQGSPAS QGILQFDMWN
VKVDNKYWDW DKLKEKIKKH GLRNSLLLAP MPTASTSQIL GNNESFEPYT SNIYYRRVLS
GEFFVVNPHL LKDLFDRGLW DEDMKQQLIA HNGSVQYISE IPNDLKELYK TVWEIKQKNI
IDMAADRGAF IDQSPRLSFE EMRSEMSKYG VDITQGTLKN PTTEDMQGVY SMCIKHILNK
DINNIRIEEF TGDLKSILPN EGKNHLQAIG NLRFIRHCEQ INRILCVENT LSYLFKPVSS
HITRLINAFI VNETNELIFQ FSRFRQKKED LEDQIVPSPE KLQEYNHELK NLLLEHVSYY
ESDKKKNEEI KNKINVADLC LKKLVELLTC LTGHIKHHIE KKDQLKDLEK DLKTLDWWTL
GIFIYEMLVG CPPFYANEPL LIYQKILEGI IYFPKFLDTN CKHLMKKLLS HDLTKRYGNL
KKGAQNVKEH PWFGNIDWVS LLHKNVEVPY KPKYKNVFDT SNFQEDLTIA DKITNENDPF
FDWVVSQFYI LSPRGDTIIN RDFRGDVSKG SGDAPPLFYL NGIHFTYLKY NNLYFVFTSL
FNTSPSYILE LLYRLVKIVK DFCGHISEEV IRANFILIYE IVDEIIDYGY IQNSNTESIR
HLIHNETLPS NASQKPIQLN EKKNEIFIDI VEKIDGVIQV KSYLQGTPYI KIALNEDLYI
KNLHSDNTNN VIIDDCNFNH LVLSLYQPDG ECILMNYRIN MINIYKPNDI IACEVQRILP
DGCIILHTRS SIYGKLSNGI LITVPQTLIQ NQKKHIFVFP CVDDTTRRNI SIISNIIKLL
AKYHININYD IITKIYINEW VPPKNKQVKA ATSNATQIVI SLSGGELIYF EIDESHSLVE
IFRKSLNVEI LCLSIQENKV RANFLAVGCL DNVVRLLSIE KYFNQLSTFI LPNNSSAQDI
CILFLNIGLN NGVLLRSVVD PIGTLTNHYS KYLGAKNVKI CPVNPALLVL CEKTYLCYVH
QGKYIYSPLN YDILEYASSF HSEQCSDGYV AISGSSLRIF RFYRLGEVFS QNILHLSFTP
RKIVPLPFPS MLAIIEADHN AYDENTLREI QRALKGIQLG TVKAGPGKWG SCIKIIHPVS
LQTIDKISLE MEEAALSVCA CELEALHCLI VGTTTNLSLK AALRVYTYDI NYKLNLLHIT
PVEDQPFCFS PFNGRLLASV GNKLRIYALG KKKLLKKCEY KDIPEAIISI KVSDRIFASD
IRESVLVFFY DANMNALRLI SDDIIPRWIT CSEILDHHTI MAADKFDSVF VLRVVEEKPD
ITYNDIGGCK EQLERLREVV EMPLLQPERF VTLGIDPPKG VLLYGPPGTG KTLTARAIAN
RTDACFICVI GSELVQKYVG EGARMVRELF QMAKSKKACI LFIDEVDAIG GSRGDESAHG
DHEVQRTMLE IVNQLDGFDN RGNIKVLMAT NRPDTLDSAL VRPGRIDRKI EFSLPDLEGR
THIFKIHANT MNMSRDVRFE LLARLCPNST GSDIRSVCTE AGMFAIRARR KTITEKDLLL
AINKVIHGCK QFSATGKIYG VQIKSRAQEN LKYIVQCMEA LYQMTKINHF NIPRYSKIPK
EKKNTKWENF AKKKMMKKNK SGLIYDKNTK GWVRRFQKKQ IKINEENASF VHEYKPSDNI
DPFERMEEEK EIKKMKQKMR EMKNKVFDRL TDENFYTGMH KQKFKKCKTS VVTPPKNIWL
FRNGDEHHNG LLFLVKPHVN NWKSLLSEIT KVLSPTIGPV RIIYNANDGE KYLCTSGEPP
ARVDRLTRFD ARRYFKEGQK CITPPNGDGT RAFYESLLEE NPNSIIAIKY CIEHGVLNGT
KHHQAIYKYK VLKKNNAFRN NFGGIRGEFI KMLNVKFIQE KKLIGKFFEE IAQDTGKVVY
GIEDTLKALE VGAVELLILY EGLDIIRLTT RNAITNTTRT IHISPQDEKQ ESLYKENNVE
LEVVEKISLT DWVINNYKKY GASLDFVTNK SQEGAQFLQV IYEFIFLCIR IYDDISKLFD
LPYTIACDVL CEQIINIIVL PFNYLGISAL KGKNMQALLR SISQKHKKKL SLDIIDAIID
CKNKAIVYKD VEEILNYISP IFDIDERYNI CILFYKHIEN GPYLVHLLPT IVFTMLDLVM
LEAKLNNASI LKKLFECIKD LVNDANVDAD ESGLKLQALD GNHVSLVSLH LLDSGFSHYR
CDRERVLGVN IASLNKVFKL CGANESVVIS SKDDEDNLNF VFENNKEDKV TNFSLKLMSI
ELDSLNIPEE GFDAEVELSS KELTNIFRNL SEFSDTVFIE IDSNIIKFTT KGLVGDAEVA
LKPRESTDDV GVTIKSRKKI KQSFAIKYLN LFSKSTILCD VVTLGLSDNR PIEFKYEIKD
GFVKFFLAPK MDDECPEKCD EEIGIIKGTF LYTFDSPICK AAIHAGVLNV ADDIVLIISH
KHHNFVGTKR NNVESHEFAG TSKGFSISIP TGFNGKENDF VNCINLPNEK YIKSMSNFTF
IIYFGAGNWR TLLSHSLCDG ISISVNEENE LIIEQNCNPH LLKSKFKPVI GQTYHLAVAF
NKTNKSVTLY VNGKKLPTEK VKYDFTLNGD LVIGRSNQST TDYFIGSIHL VEVYKFVLAD
DEIKQLANAA LSRKTVDGRE CMTPCKPQSI INKELQINAE QINLSCKDDL LSHQFNSKGS
HFLVHCSDNC SKSNFIVKGS NYYTPDSSIC KAAIHAGILK IVNGLFEYKS ARGHMGIVSK
AERQSCSSNG HLFLNLPVGE KRTIICPSGC GTNVYTPSST LCKAAIHSGA LSNQGGLVDM
SIGSGVDKFT GATQNGIESH SSAQHSRSQK ELYAQADMGI KKKIIDLRLN LGAYQCRYSR
NGKYLLSTGE KGHITLMDTQ NLEPMCELQV EESVRCSTIL HNHKLFAVAQ KKYIYFYDNT
GIEVNCIKDI LYTYQLEFLP YHFLLTSIGE FGELVYQDIS MGNIITRKKT KRGPCNIMKQ
SKHDATIYLG HQNGHVTVWT PNIDKNYLIT SSVDCTYKLW DLRKLQFIES YRSNIINEMD
ISDTGIVGMA INSHFRTYKD FFNKPYLTHN NYGDKINSLA FQPFEDICCV GSRYSIKSLL
IPGAGLANID TYVNNPYETK KQNEIRSLLD KLPPDTITND YKKIILVRKI KAKTPIKAIC
SSPKDVRVDT LKSYSSKLWP KWSENESICV IRINNTIYIY KENERGSKGK PSVFKLFKSS
DFGNHVYSKT FFNSDEMKLK WNKNASAVLL NVHTDKEKQY YYGLSNLFFI ETNKFSEVNI
MTDRGQIYDY IWSPNQNKFY VCKGDIPAEI VSYDKCANVA HSFGRHKFNT LRLNCSEKLL

LTGGFGNLSG DITLWNTSTE KEVSKTKASC AVVCEFFNDG KHFLTATTHP RLRVDNHLKI
FTHDGFIVSR INFEELYKVI ILPPGANFVQ QKEKKSKKKK NKEEDTIPLP NIKTPILKKI
IEYMEYHINN PADEIPKPLI TSNLQDVVVE DNTSKYKDLA HKMTQEIEMA VVLFKRKYII
KRIPKLPHCY IINSGALSIA RAKVKLPSTY AKLGNPLSAS KLPEFSCSFD MIEDLQQFFM
KQRRCDYFSL LNNFVNLTIS VSNLLSTEPD IEIRNELLNR FIYSLNSWML MRRCIVAACT
NVFSMTGLCI PLQILHFNYD ECKIFFSKKR APYLLMFEVA DLDEDISHII PVECQRLIFG
EINRDSISSL LNNPLARSLM NEISNNPEML ANIVSNNPLL RNTFPIMQPV LENPNLLREL
MRPEFLQAGL PPEERYASQL LSLQEMGFID NDANIQALQE TGGDVNSAVT RLLEKGSGDR
LTRAARVLEQ LTEQKPIFGK CRFTIRSFGV RRNEKISCFV TVRGKKALEI LEKGLKVKEY
ELRKKNFSET GNFGFGIQEH IDLGIKYDPS TGIYGMDFYV HLSRPGYRVT RRRRERLGFF
IALKMMNEIK GLPSVDSNYQ WLPLFYMAMA NDMAVSKISL SMVKPYSIAL IRLLRDFFSV
VFLIKCVGIG YRNMSGAQCV AIACDLRLGA NNFTTISTNF TKVFKMNDYV YVGLSGLATD
IQTLYELLRY RVNLYEIRQE TPMDIDCFAN MLSSILYANR FSPYFVNPIV VGPYLTAYDL
IGAKCETKDF VVNGVTSEQL YGMCESLYIK DILLVGAGGI GSEFLKNIIT IGCRNIDIVD
IDTIDITNLN RQFLFKKDDV KKYKSFVAKQ RALQHKKDLN INAYTFDVCT MKGSDIAKYD
YVVNALDNIK ARKYVNKLCV MEKKVLIEAG STGYNGQVYP ILANETKCYN CEEKPKNKTY
AICTIRQTPS LPEHCVAWGR LIFETFFCKS DNETLIDIKN HIEEESKKRN MEQYEIITFI
FNYLFYDTIK ELATLKKDYG TEPIPNINKK TEEYLVFDKD DDDCINFITA ISNLRMMNFS
IKQKSKFDVQ SIAGNIIPAI SSTNAIVASL QFERVYVCKP QSSRNKSDKI LVLNFGSQYF
HLIVKRLNNI KIFSETRDYG IELKEVKNIK GVILSGGPHS VPHLNKEVLE YKIPIFAICY
GMQEIAVQMN GEVNKSKNSE YGCTVWMNHT DEVTKIPENF FLVNSTDDCL IYNEEYNIYG
VQYHPEVYES LDGEQMFYNF AICKCTKKFD PIRYHEIELN NIKKYAKDHY VIAAMSGGID
STVAAAFTHQ IFKERFYGIF IDNGLLRKNE GEQVFLKGLF PDMNLTKIDA SEIFLNNLKG
VTDPEQKRKI IGKLFIEEFE KAVKNINIDI EKTYLLQGTL YPDIIESKCS KKSDTIKTHH
NVGGLPENLK FKLFEPFKYL FKDDVKKLSK ELNLPDEITN RHPFPGPGLA IRVIGEIDKH
KLSILREVDD IFIKDLKAYN LISQAFAVLL PTKSYDYVCS LRAVKTSSFM TASWYKIPYD
ILEKISTRIS EVKGVNRILY DISSKPPATI EFEMPLPGFV SDKSLYVKKP LILYEDENNK
IEVDPILAQF LREHQREGVT FVFECLMNLR DEKISGCILA DDMGLGKTLQ SISVLYTLLK
QGINKKPAVR RCLILCPASL INNWNDEINK WLPGRCNVTC VNDNAKEKIL SKLEGFKYDY
KSTVIICSYE CFRINNESLD KSSIDMIICD EAHRLKNDKT KTYMSIYNLS ARKRLLLSGT
PIQNDLSEFF ALISLCNPDL FDDTILFRKK YANPILIGRD KDATEKEQQI ASERLAELST
INKFILRRTN NLLSKVLPVK YLINIFIKLN PIQEALYMLF LKDKRILKND QSTNRVNVLI
NIKKLEKICN HPLLLNANDI KSCKFQLLHF LLKTIKQNTT DKVVIVSNYT QTLDYMEILC
RENSYKFVRL DGGINIKKRH KVINDFTHSN DIFIFLLSSK SGGCGINLIS SNRLVLLDPD
WNPANDKQAL ARVWREGQKK ICYIYRFFCT GTIDEKVYQR QISKDGLSSM IVTNTNLCKD
QLSDENVKKL FNYRMNTLCE THDNIECTRC KKVEPAGFMF YVKYEKANNK KSSNLVKKCI
NNNINVYEVL SVEEADDLET IKASYKKLIL LFHPDKFLKI QDSYAVLSDK TLRKQYDSSI
PWSAKKPVPD IGDEHTDIKN VKYFYDFWYN FINWRDFSYQ NEYNYEEAEC REERRWMERE
NKKIQKKASK AENLRIIKLV DLAYNNDPRI IAENKRVKLE KQRKKAAVKI WRHHIKSFET
LCEFIDDIYV FLWTAQEVSL LAKALKSYPG GTRNRWEQIS NFIKTKSVKE VIKKTKEMFE
NETLWTHQEQ HLLEQALMKH PASLPMPKKR RNGGRSKHNR GHVNPLRCSN CGRCVPKDKA
IKRFNIRNIV DTSAQRDIKE ASVYSTFQLP KLYIKQCYCV SCAIHSRFVR VRSRQQRRVR
KETSKHVNPS QKKVSVSEIN FDSSYTVLDT SEGAIMLHVN HVLYHLDFNA LAVVKNVDMT
EEMQIDAIDC ANQALQKYNV EKDIAAHIKK EFDRKYDPTW HCVVGRNFGS YVTHETKNFI
YFYIGQVAIL LFKSGVCGWS KAVRKQGGRF CFVNLNDGSC HLNLQIIVDQ SIQNYEKLLK
CGVGCCFRFT GKLIVSPVQN SIHNFEIYGE DPQKYPLSKK NHGREFLREV AHLRPRSYFI
SSVIRIRNAL AIATHLFFQS RGFLYIHTPL ITTSDCEGGG EMFTVTTLDY KKDFFSKQAF
LTVSGQLSLE NLCSSMGDVY TFGPTFRAEN SHTSRHLAEF WMIEPEIAFA DLYDNMELAE
SYIKYCIGYV LSNHFDDIYY FEKNVENGLI SRLKNVLDEK FAKITYTNVI DLLLPYSEKF
EVPVKWGMDL QSEHERFVAE QIFKKPVIVY NYPKDLKAFY MKLNEDQKTV AAMDVLVPKI
GEVIGGSQRE DNLERLDKMI LEKKLNMESY WWYRQLRKFG SHPHAGFGLG FERLIMLVTG
VDNIKDTIPF PRYPGHAEFI KDLLDMNLEN SKNIIKNLFL KDKKNYFFLC TVNWKTVDLK
YLSTIFKTSN LRFVDEGNLK SMLNLLPGCL TPLALKCDQE NLVKLYFDEE LKNMIIVHPM
HNYSSLYMKQ EDVVKFCELH NHAPILGIVA KKTVNFSEWY TQVIVKSELI EYYDISGCYI
LRPASYYIWE CLQTFFNKEI KKLDVENSYF PLFVTKNKLE KEKNHIEGFS PEVAWVTKYG
DSTLPEEIAI RPTSETIMYS VFSKWIRSHR DLPLKLNQWN TVVRWEFKQP TPFIRTMAKL
SKAQKKQIYM DKLSSLIQQY TKILIVHVDN VGSNQMATVR QSLRGKAIIL MGKNTRIRTA
LKKNLQTVPQ IEKLLPLVKL NMGFVFCKDD LTEVRILQNK SPAPARLGVI APIDVFIPPG
PTGMDPSHTS FFQSLGISTK IVKGQIEIQE NVHLIKQGEK VTASSATLLQ KFNMKPFSYG
VDVRTVYDDG VIYDAKVLDI TEEDILAKFS KGVANVAALS RSIGVITEAS YPHVFVEAFK
NIVALVIDTD YTFPLMKKIK DMVENPEAYA ASAPVAEEEE EEDGFMGFGM FDQERDLARE
PCPDRIIEDM GGAFGMGCIG GYIWHFLKGA RNSPKGDVLS GALYSGRMRA PILGGNFAVW
GGTFSCFDCT FQYIRKKEDH WNAIGSGFFT GGVLAMRGGW RSASRNAIVG GVLLAIIEVV
SLVLTRKTTP TPRQQFQQQM ELEKMLVDNI GDVTITNDGA TILKQLEVQH PAAKILVNLS
ELQDQEVGDG TTSVVLLASE LLRRGNELIK MDIHPTTVIC GYKLAMKESV KYIKEKLSER
NLGKDVIINV AKTTLSSKFI GYESDYFAKM VANAIQSVKI VNDAGKTKYP VSSVNVIKVH
GMSSLDSKLI DGYAIMSGRA SQSMPTGVKN AKIAFLDFPL KQYRLHLGVQ VNINDPTELE
KIRQREKDIT KERVNKILES GANVILTTQG IDDMPLKYFV ESGAIAVRRV NKDDLRRIAK
LTNGQIRLTM SSLDGTEKFE PSSLGYCDEV YEERVGDWDV MFFKGCKTSK SNTILLRGAN
DFVLDEMQRS IHDALCSVSR ALESNYVVVG GGCVEVALSV YLEDFAKTLG SREQLAIAEF
AESLLVIPKI LALNASYDSI DLVCKLRAYH TKSQVNTEDP KDYRWYGLDL VNGKVANNLK
NGVLEAMISK IKSIRFATEA TITILRIDDL IKLTPERKPP VIKLKGHTSS ILDLQFNPCF
SEILASGSED LTVRVWLKGH KKKISIIDWN PMNYYIMCSS GFDSFVNIWD IENEKRAFQI
VMPKKLSSLK WNIKGNLLSG TCVGKHMHII DPRKREIASS FHIHSGGKNT KNIWIDGLGG
DDILSTGFSK NNFREMKLWD LRNTTSALVT MSIDNASAPL IPHYDESTGL IYIIGKGDGN
CRYYQHSLGS IRKVNEYKSC SPFRSFGFLP KQICDVYKCE IGRVYKNENN SSIRPISFYV
PRKNFQEDLY PPILGKDNKM SRINIFEKLQ ICGQFNKGFV ISKLFIIDQH AADEKSNFEK
YNKIFTMKSQ KLVYLLSLPV FNGKILEVED FMSLLHHLWF NHNFPRPQKV WRILASKMKA

LVLMFLKKIS DTYVDDQAKW MDKMRSSQEE QNNKKLTEWN ETIENKCFVY PASSAPCGAC
TSAGAIAPYR RFKEPRRKKQ YTGTDILCQA KSGMGKTAVF VLSILQQLVR CLGLAHTREL
AYQIKNEFDR FSKYLKGVRC EVVYGGISMS KHIKMFKIPH IIIGTPGRIL ALIREKYLLT
DKIQHFVLDE CDKCLEKLDM RSDVQKIFIS TPLKKQVMFF SATMAKEMRD VCKKFLQNPV
EIFIDDEAKL KLHGLLQHYV KLQEKDKTRK LIEILDALEF NQVIIFVKSV TRAITLDKLL
TECNFPSIAI HGGLDQQERI ERYDKFKKFE NRILVSTDLF GRGIDIERIN IVINYDMPEN
SDSYLHRVGR AGRFGTKGLA VTFVSSQEDT LALNEVQTRF EVAISEMPNK IDCNEYINQR
MSVSAEAYGE WNKKKNFVPK VYKKDENEKE KIREALNDSF LFNHLNKNEM ETIVNAFFDE
HVEKNVNIIN EGEEGDLLYV IDEGEVEIYK MKENKKEVLT ILKSKDVFGE LALLYNSKRA
ATAKALTKCH LWALDRESFT YIIKDNIAKK RKMYEDFLTH ISILKDMDPY ERSKVADSLK
TKTFSDEVII KEGEPGDTFY IIVDGSALAI KDKTVIKTYS KGDYFGELAL LKNQPRAATV
KAKDSCQVVY LDRKSFKRLL GPIEEILHRN VENYKKVLKE LMHLQILCLN DEVREMYKNH
KTHHEGDSGL DLFIIKDEVL KPKSTTFVKL GIKAIANTSY LLFPRSSISK TPLRLANSIG
LIDAGYRGEL IAALDNTSEE EYVIKKNDKL VQLVSFTGEP LSFELVDELD ETSRGEGGFG
STLGAAYGTA KSGVGVCSVG VMRPDLIMKS ILPVVMAGVL GIYGIIMSIL IYGDYIKAYL
LGFTIEDSLA LLRIDDLYIE SFQVKDVKIL KGDHLSRCIG RICGSNGATK YAIENATKTR
IVIAGDKIHI LGSFNNIKMA RYSICSLILG STQGKIFNKL NILAKRMKER IEKLEDRMHP
WSNIDGMKAA CSYTYDDIIC LPGYINFPMS EIDLSNNLTP NICLKTPIIS SPMDTVTEHK
MSISLALCGG LGIIHNNMSI ENQIEEVKKV KRFENGNKVL CEEKKSVLPI VNNNYEFPHA
SKSQNKQLIV GASISTDLER ADQLIKNMID IICIDSSQGN SIYQIDTIKK IKGAPIIAGN
VVTCDQAKNL IDAGADVLRI GMGSGSICTT QDVCAIGRAQ GTAVYHVSNY AHNIKTIADG
GIKNSGNIVK ALSIGADFVM MGNLLAATEE SCSDYYFENN VRLKIYRGMG SMEAMVSQGV
SASLVDKGSV LNLIPHLVKA VKHGFQSMGI KSIPELHSKL YSGMKENKQY QEALKELKKL
KKKIRENINL LKHIKEKLVL AQESAWDKFG SKLKDMPFLN NFFENPILGK LFGETELAAA
LREMKMYDQN FKLSELMYLF EFVISKHIVE SYLIGDEETL RVHCGQSAFN SLNASINERK
KKKVYLDTNV LIYKNHELKG AQRMEESSPW FIFTFHTQQI NCLKNANDEI IEGNIDDIRE
VVYTIALSKH PEPEGLLYPY IVREFAIIGN TPSWMALYII GLGLGDERDV SVRGKELIEM
SDVVYLESYT SVLFVSKNAL EEFYKKNIKE VDRNFAEENC EEILEEAVNK KVSFLVVGDP
LCATTHHDII LRAKKKNINV QVIHNASIMS AIGESGMQLY NFGQTVSIPY FEETYKPTSF
YDKIKVNLDN NFHTLCLLDI KVKERTIENL MKNKNIYEPP RYMTINEAIE QLLYCEHKKN
VITDNTRGIA IVRIGSNSQQ IVSGNLLTLK TIKYNDPLHS LIICAPTLHD VEREYFDMYP
NSAYRKCVRV QLIKNGKKIT AFVPGDGCLN FIDENDEVLV SGFGRSGHSV GDLPGVKFKV
VKVARVSLLA LFKEKKEKPR SMGIKGLTKF IADAAPNAIK EIKIENLMGR VVAIDASMSL
YQFIIAIRDS EQYGNLTNES GETTSHISGL MSRSIKLMEN GLKPIYVFDG APPELKGSEL
EKRGEKRQKA EELLKKAKEE NLEEIKKQSG RTVRVTKKQN EEAKKLLTLM GIPVVEAPCE
AESQCAFLTK YNLAHATATE DADALVFGTK ILIRNLINLE QVLKGLNLNM NEFIDFCILC
GCDYCDTIKG IGSKTAYNLI KEYNSIEKII ENIDFVEARD SFINPKVKEE IKIDWGEPKI
EELKNFLIKD YNFNEVRVTN YINRLLKARK VTTQRRLDNF FVNNSKYYEV LNLKKNCTTD
EVKKAYRKLA IIHHPDKGGD PEKFKEISRA YEVLSDEEKR KLYDEYGEEG LENGEQPTEA
TDLFDFILNA GKGKKKRGED IVSEVKVTLE QLYNGATKKL AISKDVICAN CEGHGGPKDA
KVDCKQCNGR GTKTYMRYHS SVLHQTEVTC NGCRGKGKIF NEKDKCANCK GGCVLKTRKI
IEVYIPKGAP NKHKIVFNGE ADEKPNVITG NLVVILNEKP HQLFRREGVD LFISHKISLY
ESLTGFVAEI VHLDERKILV DCTNSGFVRH GDIREIAEEG MPTYKDPFKK GNLYITFEVE
YPLIITNEKK EILKILKKQN EIEDLENSEC EVVTCQTVDK EYLKQRLKIL KNYLPYLCKI
LVDNTVYTKW DYLTMDESHF QNDNADEMTA RTWGNDWTVR KGAALCLDYL SNVYNDEILE
FILPHIEEKL MSDKWNIRES AVLTLGAIAK GCMYSLSPFI PKVLEYLIKL LNDEKPLARS
ISCWCVTRFS SWICHPDKWF EPVLLNLLKR ILDTNKRVQE AACSSFANLE EDALDLLNNY
LHEIVHTIQQ AFQIYQAKNY FILFDVVGTL IDSVNIVKEN NDLAHEIVNS ILSKWNTIRI
NSPYIIALME CMSCITSAYG KDFLKYAKNV IRTCIKFLVL LYIDDLIECS FDLLSRIILQ
SNFALIGDIS RFCAQYLILN DIIPFLIAHI AHPSTPVSNN ASWAIGEISI HINPQYMEVY
VDEIIKQLIY ICNSKYHGCL LQNICITLGR LSSIYPKKII FYFPQFLKTW LKIMSHGTQE
NEKINFFHQF LATMKECIQP EELKARLAQG LLKCQIVNKL TSNGCTFIFW IADWFAQLNN
KMSGDLKKIR KVGNYFIEVW KSCGMNMENV KFLWASEEIN KKPNEYWSLV IDISKSFNIN
RIKRCLKIMG RSEGEENYCS QIMYPCMQCA DIFFLNVDIC QLGIDQRKVN MLAREYCDIK
KIKKKPIILS HEMLPGLLEG QEKMSKSDEN SAIFMDDSEA DVNRKIKKGY CPPGVIENNP
IFAYARNIIF PHYNEFALLR KEKNGGVKMY VKEEIVDNDK LSNIINKKKE NVKYMKGMKV
PDNVLATSNL KDAVEGADLL IFVVPHQMEK LLSSVGRLSV VAGGLSLIPY TFIYDVDGGE
RCVMFNRFGG VSENTYGEGS HFYIPWFQTP YIYDIKMKPK VINTTTGTRD LQIVTLSLRL
LFRPHTKQLP YLHSTLGPDY DERVLPSIGN EVLKAVVAKY NAESLLTQRD KISKEIRESI
TARAKHFNIL LDDVAITHLS YGKEFAKAIE DKQVAQQESE RVKFIVAKTE QEKIAAVIKA
QGEAEAAKLI SSAVKEYGNS LLEIRKLEAA KEIAENLSKS KNVTYFPASS NILYMNALKE
ESGGFNFENI KRNEILKEKG PNFRKTGTTI CGLVCQNAVI LGADTRATEG PIVADKNCSK
LHYISKNIYC AGAGVAGDLE HTTLWLQHNV ELHRLNTNTQ PRVAMCVSRL TQELFKYQGY
KVCAIVLGGV DVTGPQLYGI HPHGSSCLLP FTALGSGSLS AMAVLEAKYR DNMTIEEGKE
LVCEAICAGI FNDLGSGGNV DICVITKDGS QHIRPYKQPN VRLYHLPKGT TPVLCQKIEN
IKKYISLNDV GEARGTVLSV KLDELIDSVE GQTVIDPKGY LTNLNANDAD IADINKARSL
LKSVISTNPK HGPGWIAAAR VEELAQRKDK AKEIITKGCI ECSKNEDIWL EAVRLEKLSE
VKIILTKGIK EIPTSVKLWL EAYRKESNID DKRKVLRKAI ECIPNSVRLW KEAISLESEN
NAYILLLKKS RVQCNTNSNN ITPVISEALK ECPSSGILWS KAIEFENKNL QNSKSVTAFN
NCGNNAYVIL TVAKLFWQHF KTQKARKWFY RVISLNPNFG DGWATFLAFE IDQQNEVNQK
DIINKCIKAE PNRGWVRGRI HDIRSKGSLA FIILRQKLYS MQCILDIKND NDKNMMKWVS
NLPLESIVDI YGKLTKPEVP IDSTNIKYEV HIKKIFCISK TTKELPFLLK DANMKETNEE
GSIKVNQDNR LNNRCIDLRT YANYSIFCLQ SQICTLFKNF LLKNNFIEIH TPKLLGESSE
GGANAFQINY FNQKGFLAQS PQLYKQMCIN SGFDRVFEVA PVFRAENSNT YRHLCEYVSL
DIEMTYKYDF LENVFFYDSL FKHIFTELKG QYPCEDFQWL EVTPIFTYEE AIKMLIEDIL
SYDMSTDMEK ELGKIVKASH HTDYYIIINF PSALRPFYTM YKEDNPAISN SYDFFMRGEE
ILSGSQRISD VNLLLENIKR FNLDASKLNF YIDSFAYSSY PHSGLLFENL NKEYKFITTQ
DNFDGFRFEV DKSVNKYLQS THTLFLGTRD VGYLYQFGAN FTNSDNSLLM ISRVNIDGSV

NGRFCKKIKN DCKLNFNTYA KSDTRNMYEM AIEVNKPIYT YNVKTIWQGG VDLTYIASNC
ASIGSFGLRY QHKNNVLSMQ VVRQPNFKSP EFMLNQTHLY KIQYAKKISD RLSLGTELEV
TPQTKESAMR LGWDYSFRHA KVQGSIDTSG KIAVFTQDYS GFGVSGYIDY PNNEYKFGMM
HIAPAQEQDG GVGKTTFVKR HLTGEFEKKY IPTLGVEVHP LKFQTNFGKT QFNVWDTAGQ
EKFGGLRDGY YIKSDCAIIM FDVSSRITYK NVPNWYRDIT RVCETIPMVL VGNKVDVKDR
QVKSRQIQFH RKRNLQYYDL SARSNYNFEK PFLWLARRLS NQPNLVFVGE HAKAPEFQID
LNIVREAEKE LEQAAAVAID EEDGVLTVKS ILMEPTISQY DIKKLIRNKI QEQVPFYNYQ
MKRALAEKIY GDCIYDNFGL SKDIEVNLIA LEEWNINCNR NRVLQNTGLI KDIQINEFKY
LTTKESLEVH FAVNPKYTDI LQKNKVLPPS GINYNKLIKE FGCSKITENH IKRIEQLTAH
HFIRREIFFS HRDLDFLLNY YEQNKCFYIY TGRGPSSLSM HLGHLIPFYF CKYLQDAFNV
PLVIQMSDDE KFLFNQNYSL VEITKLTKDE MTFVLHNSNT GMANALRRIM LSEIPTLAID
VVNVYENTSP FHDEFLAHRL GLIPIDSRNV KNFEFREKCK CKETCSKCTI QYLIQVKCNS
KIDVTHYDIV EHEPNVPMPV PIPIVTLSKN QTLHMKLIAT KGIGKMHAKW IPANVSYRID
HKVSIKHHLI NSLSPEHKLL LANSLNKDCY VLLRLNENMS VVMAESCIEV LNELGYKDVV
KIIYDETKFH FKVESVGSMP PEQVVEMAIE ILENKLKTLE PQIKSSFYSI DEVAKQLKLP
DPESAKNEEE KCFLSLLKKS ILKNPKKWTN IAKKIVGVSE ETTTGVLRLK KMEKNNELLF
SAINVNDAVT KQKYDNIYGC RHSLPDGLMR ATDFLISGKI VVICGYGDVG KGCASSMKGL
GARVYVTEID PICAIQAVME GFNVVTLEEI VEKGDFFITC TGNVDVIKLE HLMKMKNNAV
VGNIGHFDDE IQVNELFNSE GIHIENVKPQ VDRVTLPNGN KIIVLAKGRL LNLGCATGHP
AFVMSFSFCN QVFAQLDLWE NRKYQNKVYL LPKQLDEKVA LYHLKKLNMY TYGVEGDDTY
LPQPQYPSPY ENQYESPSPR GENHTPFVGY FSSHLLRTGF FLQCVSLMLM FIFYWAFGGT
GIFVFDLYAG PECVKVSSAF HLTISILMAI YLLGTLYIAM FQVFVADNSK WCRGFRAGSK
LLSAAVTLDL LSSILRLVQY LYAYFYMSMR WWARYQQTKS DWTLLHFGSI VHSFALFIYG
AAFFYMEAYH DEGTYEELAW SNLTLFKLAG LAELNKPKKR TFRTFHYRGV ELDKLLDLSQ
EELVKLFRAR QRRKFKRGIS KKEKSLLKKL RKAKKECEVG EKPRAIPTHL RNMTIIPEMV
GSIVAVHNGK QYNNVEIKPE MIGYYLGEFS ITYKHTRHGK PGIGATHSSR FIPLKVMVDM
FLWRDPEQFE LKNLAIEETA PAAPHLTENQ FATEAPYEEW GFLQIIKDHH ETMYELKQKV
RPRDQVVGWF CSGSELSELS CAVHGWFKEH NSISKFYPHS PLNEPIHLLV DAALESGFLN
IKAYVQLPIT LVKEYFVHFH EIQTELLPSN VERAEVSLKK LLIMLKQCKS YVQDVIDKKK
KGNLEVGRYL HKVFSNDSFS TLEKFDSINE SILQDNLMIS YLSNLAHLQF LIAEKLNWLN
NLPLKEDDAE GRRVHKNLID LVSQNHPLLF GKDNSNTAKI IEIFLTIYET DFSDADCNKK
IASLINSLDQ AYLSNLALTN KQAKKLNHIN GNNFLKFYEE NINLKSYLKI IKDFDKFPVI
VDADNQILSL PPIINCDHTK ITYDTKNLFI ECTAIDKNKA EIAVNIICSM LSEYCTPKYA
IHSFFVQYPV FKNKSLTCHI DYVRKLSGIL DLSVKDVEPL LKKMMIDSST FTVDVPFYRS
DIMHCCDIVE DIAIAYGYGN IVSEKIEIAK KNSLSAYTEL FRNVLSECTY TEVMTNALLS
KRENYDCMLR PVQIMNSKTS EYEIVRTSLI VNMLKFVSAN KHRELPLRFF EIGDVSYNKT
DTNAVNKRYL SVIFADKTAG LEEAHGMLET VLKEFQLFSD YKIEEKRKEN VAIRVISIGN
IRGFGGCDYG SFRMSNEFLG WKNKQTNSVY QYKCSDISEA EWIKTGYNNN RLHIKFNKQK
DNLIIFFDGF PDRNISEITQ HFQKYFNLRL ASRKIATKGW NWGEFKLENT NINFDIDNKY
AFSIPTNSIN QLNVQIKTDI AMELKNEDNE DFLSEIRFCY PHENDENKHF QNFKNDLLEK
VNIGDSKSEC IASLANIPLL VPRGRYEIEM YPKSFKLHGK SYDFTVQYTN INKMLLVPKS
NSNQYVLIFS LNNKMKQGQT EYPFILVQLN NDDDMELDIN ASEEDLKKYK LEKSLCGRAY
EVIPRLFSAL VKKNAIIPGD FRTAKNEHGI TCSYRAASGQ LYPLNKYFLF IVKPVILISF
DDIVTLTFQR TGNNQHRFFS VIIKHKRGMS YEYTNIDKSE YLPLLEFLKS KNIHIKGYID
LSKRRVSPKD ILKCEEHFSK SKKVHQTVRH VAQKHNMTVE ELNRIAIWPL YKKYGHALDA
LKEATVNPEA VFKGIELNED VKNSLMADIQ LRLAAQALKL RGRIDVWCFS YEGIDAVKEA
LKKGKVSINI KLIAPPQYVI VTSCQDKDLG MAKIQEAMKV ISDKIKEYKG GDFKQQGEIL
VILLDKQDDL SSDNEGERSV FEFFLCDVGV GLSLSVRDTV PIEYDSVFIG VLPHYTFHHE
YIIFDNSQIL PRYLIQFECD PNGEEHFSLP LCDYCGNAPS VFYCESDEVK LCAKCDHMIH
TNKLVRKHIR KTLNEAQGKC KIHVEERVSM FCTICHMPIC NKCISSHEHI SLNLAYKAII
KHSAIPSNLV KEEKKKLNDM LKKVDKLYEQ VRSNIKDAEK HVYTILEDVI KQLHVVTDQK
MCAVLSEEYE LKRQFCEIAW NENFLYYLQT VLPPADFMNA WLKHCLVREE IEKNSLVFPD
MCIRGNINVV TEESAHHLVG LPNVGKSTTF NVLTKLNIPA ENYPFCTIDP HEAKVTVEDE
RFDWLVSHFK PKSNVHAYLS IFDIAGLVKN AHLGEGLGNN FLSNIAAVDG IYHVVRAFEN
EDIIHTEGNI NPVRDMEIIN SELIYKDISH CEKNLEEVTK VNRNKKDKVK QNEHDVLTTV
LSFLKEHKWI KDGNWKSSEV EVINEYNFLT AKPVVYLVNM SETDFIRQKN KHLAKIYNWV
QEKNKGTIIP YCADMELKLL SMSEDEKKTY FEENKIKQSM LSKIVKTGYY EINLIHFFTC
GADEVKCWTI RKGTKAPQAA GVIHTDFEKG FICAEVYKYT DLVEFKSESE VKANGRYLQK
GKDYVVEDGD IIFFKFNVSS SGRINKHVNQ LRITYSTLEE FVDNFVYELK KGLEAHRRHP
NLWIPHECSF KMLDSCIADI PTGQEKGTYY AIDFGGTNFR AVRASLDGNG KIKRDQETYS
LKFGTFSHEK GLLDKHATAS QLFDHFAERI KYIMGEFKDL DNREGKNVGF TFSFPCTSPS
INCSILIDWT KGFETGRATN DPVEGRDVCK LMNDAFVRSE VPAKVCCVVN DAVGTLMSCA
YQKGKTTPPC YIGIILGTGS NGCYYEPEWK KYKYAGKIIN IELGNFDKDL PLTPIDLVMD
WYSANRSRQL FEKMISGAYL GEIVRRFMVN VLQSASSEKM WKSDSFNSES GSVVLNDTSP
NFEDSRKVAK AAWDMDFTDE QIYALRKICE SVYNRSAALA AAAIAAIAKR IKICGVDGSL
FVKNAWYCKR LQEHLKVILA DKAENLIIIP ADDGSGKGAA ITAAYFLIRW LCKAIVSSLF
GDVNVINPEN VPLYGSVIFV GNHNNQFIDA CVLVANIPRQ VKFIVAEKSM RRAVIGKLAS
IIGCISVKRP EDLKFKGIGH VCWVKGDNKI KGINTRFKLD VQIGDKLLTQ NKVFSVTKIE
SETELIIQDA INIECEDNGV PFKIIPKINQ TEVYNLVTSS LKNGDTIGIF PEGGSHDRTN
LLPLKPGVAI MTLCALADGD VSIIPVGLSY SKLYQLQGCV TLFYGNAIIV SQDHCKDYNN
NHRETISKVL SKIEEGMRSC MLTSKDHETS RCIELCVSLY TPERMTISKN KIYNNLQLFC
KMFWKFGNSK EIENLSYELK CYEKFLQANK IKDDEVWMLK QSTSAATLKF IEHICSLIFC
VIFGMTFSLL WLPLVAISIY LAEKHRESAL KNSTVKIQGC DVVASYKVLV LLVLLPTFNI
MYGLVFSLYR LMKKKDIEGV HKLLSSYLEQ FNLYAVFTKE EIAHWFLPIE NVIYTYVNEE
AGKIKDMISF YSLPSQILGN DKYSTLNAAY SFYNVTTTTT FKNLMQDAIL LAKRNNFDVF
NALEVMQNKS VFEDLKFGEG DGMDVDSVLG LQAILISANY KEKEFIRIAY YMNSFYKDME
LREKPPAVPQ YDKICRHIVE NPRIVKFCIT WDDAYTAFPI LKELKQKDYF RIFKVNLHIP
CKYLKVNEKC KEMKKCSVCE CHDDEIPYNF RTNEIEVDLV YNSPSFTAYE GRNIWELKGN

NNYKYFGAAK NLKGVKELLL KENDDRKKKK KENRFFDKYV NIHYFGYCDD QNEMLLREEL
KMQDQLMKHG TLVLPSDRAR EYLDCLGKNV DIQFIDMNEK TMKRQYKKYI QRIDDMERIL
RFLEENIRKL PNVKIKKSKI DSFLEHDNVY ELDQVEESLN RLHVQFVRFC NNNKELVDER
NNAIEEKHVI LTAMNQLNPS LQTNMMKDGI SMMFTNISGV IKTKDQESFS RTIFRALRGN
TYTYFQSIDE KSVFVVYCQG SSQSNIYQKI LKICKAYDVK TYDWPKTYEQ ARQRLKELKE
IITDKQKALK AYEEYFINEI FVLINVVEPN KNSLIEEWKL FCKKERYIYN SLNCFEGSDI
TLRCDCWFSA NDEEKIRHML ITKSSNDLVS ALLLSDKLTP NISPPTYIKT NKFTKSYQSM
VDTYGIPRYG EINPAISTII TFPFLFGIMY GDVGHGVCIF LFALFLILIN NRIKNNEMVS
MLLDGRYMLL LMGFFAIYAG VLYNDFFSMP LNLFTSPYIF GFDAKWLGAE NELTYINSFK
MKFSIIIGFL HMTFGVLMKG LNALHYRRKM DFFFEFLPQL MMMLSIIGYL VFLIIYKWVT
PGGYKKQGII NTVINMYLMK DLTPDNQFYA HQGLVQAFLI AIFVLCIPLM FVCKPAIRTY
HIMKEIWIEQ LIETIEFILG LISNTASYLR LWALSLAHQQ LSFVFFEQTI LNSLRKDSFM
SVLINLIVFS QLFSILTIAV ILCMDTLECF LHSLRLQWVE FQNKFYKGDG IPFRPFNIKK
VLPENYSAIL ARALSERPLT YLPTVERVCY EVLNDEDEHL NYIQINLLNT IRPTPIRGLL
AATQERFVVV PGIIVQASKP QHKMRKITLQ CRYCDHKMSI DVPLWKDKPQ LPPYCRYVLE
PYVILPNECT FVDIQSLKMQ ELPEAVPTGD MPRHLQLNAT RYLCEKMIPG DRVYVHGVLT
SYNPNPTRAD GTNFSYLHVL GFQKYDGNDL NFDVEERNEL TLLAAEHDIH EKIFKSIAPE
LYGMDEVKKA CACLLFGGTR KRIGEETKIR GDINMLMLGD PSVAKSQILK FVNRCAPVSV
YTSGKGSSAA GMVRQGLLGE NEEKLDYVLG LTLPKLLERR LQTKVFKLGL AKSVHHARVL
IRQRHIRVGK QMVDIPSFLV RVDSEKHIDF ATTSPFGGSR PGRVKRKSLS QSMYDRHLTI
FSPDGNLYQI EYAIKAVKNT NITSIGVKGE NCAVIISQKK MATQYISQDK LLDYNNITNI
YNITDEIGCS MVGMPGDCLS MVYKARMEAA EFLYSNGHNV NVETLCRNIC DKIQIFTQHA
YMRLHACSGK SISEESVKEM FSPYGSVEEV FIMKDNTGLG KGCSFVKFAY KEQALYAINS
LNGKKTLEGC ARPVEVRFAE PKSAKQTAKV CSRDLPGHMK MKTRDLSDYV EDKEEIETIN
QAVKLAKDAV IEDEKKNYKS ALNLYIQSLQ YFNFFCKYEK NSNIRDLILK KMEIYMTRAE
ILKELINILN KDKNVKWSDV CGLETAKEVL KEAIIFPLKF PKLFNSSALP YKGILLYGPP
GTGKTFLALA CSNECNMNFF NVSSSDLVSK YQGESEKYIK CLFDTAKEHA PAIIFIDEID
SLCGSRTDGE NESTRRIKTE FLINMSGLNN YKNNIIVMGA TNTPWSLDSG FRRRFEKRIY
IPLPNVYARD IKYFATVTEN YTGADIDIIC RDAVYMPVKK GLYVKCGSRY EGMSVMLENM
AFHSTAHLSH LRTIKSLEKI GANVSCNAFR EHIVYTCECL KEYLPVVTNL LIGNVLFPRF
LSWEMKNNVN RLNTMRSKLF ENNELYITEL LHNTAWYNNT LGNKLYVCES SVENYNATNL
RNFMLKHFSP KNMTLVGVNV DHEELTKWTS RAFQDYVSIP YTNQKEVTPK YTGGFVSVED
NVKKTNIAIA YETKGWKTSD MITLTVLQTL MGGGGSFSTG GPGKGMYSRL FLNVLNNYNF
IESCMAFSTQ HSDTGLFGLY FTGEPANTMD IINAMAVEFQ KMNKVTDEEL NRAKKSLKSF
MWMSLEYKSI LMEDLARQMM ILNRVLSGKQ LCDAIDAVTK EDINRIVGHF LKTKPTVVVY
GNINHSPHYD EICKILDAVN KIHFFMGYPE LASVNFGNST IVRCKKCRTY INPFVRFEGG
GKKWNCNMCY HVNDTPQFYK RKDLFQRPEL CTGSVEFIAP SDYMIRPPQP PVYLFLIDVT
VTSVNSGLLD VVCSTIKKLL DPRTLIGIMT FDSTVHFYNL NSNLKQTQMM VVPDIQDIFI
PLPEDILVNV HECQNVIDTL LDNLPTMWRN NKMADCCAGN ALKAAIMVLK KVGGKLVFVL
SSAPNIGDNS YTELAQSITQ YQIAVDLFAC PYSLDLATIY PLVKNSGGSL YYYPFNVHQY
SDKLREELLF ALTTETAWES VMRIRISRGW KITNWYGNFQ FRGVDLLALP NCHSSQNFSI
VVDLEENVVQ DSVVYVQSAL LYTNSNGERR IRLHTYALPV TQNIKTITDS INPQVVVSLL
SHQAIDICKK GKIADGRNLI QTLCSQVLSS QLLLSETARL LSIYVLGMLK SIAFRDPDLR
IYHWSRVQNI PVDCVEAYFY PRMFSLHNPD TISLTCENMT QDGCYIVEDG ENIIMWIGRS
ISLAVVNVAA GLDGCDDQLL PASFRALEAD LNLHPSLLGY ITLAQTLMLS LFSPIWGFLS
DKYSRKWMLV FGTALWGVAT ILLANINDFA HIIFFRAING LALGSIGPIS QSILADAAKN
ELGLSFGLVQ LSSSVGRLIG GVVTTTVALK YFGGIRGWRL CFIVVGILSI LLSIIVALFV
DDAPSLSKKS IIIILLEGFT GTIPWLALSF NTMFFQYCGL RDLQAAIITG FLLIGSALGG
VIGGHFGDIM HDISNKHGRP FLGQLAMFGR VPLVILTYMV IPQRKESFEL FLSCFFLGLS
SIAGVAVNRP IVSDIIRPDY RGTVFSLTIA IEGVGASLIG APLFGYLAEK VFNYQNNNLL
ISEMPEELRR NNAEALSKTL LYLTMVPWLL SFVFYSLLHF TYGSEYKKMN EIIESEMLMF
GLRSLSDFCN PTSKTYKENA FDALDRGAVE SIKNAVINYK DDDDILFCSS RVLCAMSDYC
SSEKDTDALQ KLITDGGAVV EIIKTVPSDQ DTLKNCMLFI QNLGVALLNV FTSNAYNVKL
GNIIVSALSV AAKSASGAKI LNTEGAHHKL IDHCLDDTAE IVEGVFDAIK NLSSNGYVVP
TIIEKSVVIL DKFKSYPRIV SKGSDAMKCA VGPEQLTNCL NILKTSKQGS KEQDSALELL
SSLSYISSIT DKVVESGGIP VLVELINSGL QQYESNPEKI SRLVAGASRM LGRISNNPPH
AVIVYEYGGI ATLCTALSYF PNDADCASAV CNALIPFVSR SYSLFASLFP ILYASLESIE
LAKASMSCIA SASMINEFHE QMVNNQAIEI LSTCIQYHLN ETKYLLNCFT AYFRLSDYIT
TVEPIHQYGG VTGIANALSA VDAVLTVMLE NETKEVIIQE GTKIMEKLAT ESDCQRHISN
LESIAQSNPE AAYKTLAAIS GLSRIQSLKS ILESKGADSS IYNGMKVWIE SPRFNEQPKL
IKAALKTIKI VDVMCISQVK RLAEEEPDDN ILITASECIN YLTEINKINN KEIVESSLDS
IFKLMKKYSE SRVTQTNLLA AINNILLSSN GADVLVNKGY VKQIVNYIHK VPMYVDVQII
GFSVLGNILK ISPESIDSIR KANALVPLQN SLRTHEKNAK LKTTCAPLLA MLMPLDSLTK
EIEDLLKLCN DAMTENDLIK LHEYLISLNE LLLNAEASKI SSRCNVISEI AHACTNISQT
RIGLVHLTKS DMTSALMEGY NLLELPGNDY TEEAASNTLE SLSLLLKHDI TNADIGFNKG
LVKKLCTGIN YFSESDPVIK NTFACLACMC TSEKRVNQLI KHPEYDKLIA LIVDLIGNSK
NSRGNAIKAL YELLKTEDEQ IITNIASMTP IVNLFKIMGE YQADLPIVQD SAKCLAIIAD
HINIQEDQYT AIKILLETLD KNKNDELTAE DILTVLIKLC DSNDKMKLRE LGAVDVISDV
TMIHSENENI SRLGGVLFSY MGADEQVKKL MKLILNVKSS DKDAVQKIDN LSSKLEMFLR
APLENPLDAL EYTEATLQQL NGYLTSQLDN ASVQANIALV TKRLVDRVKY DHEDQLGAWA
VASSGVLNQY TDMIANKVGL ANSKFVSPVY SVLAGCVLNM EKKVRDYAQG AVKFIKKSGS
NFLACKNLRE KLEEKGFKRI QEWDLRKNEG YVFSKQNRNI CGFFIGKDFN MEKGSILISI
GHVDTCCLKI SPNNNTVKSK VNQLNVLVEK LIQINRSIIF LPSLAIHLQN RTRSVKVNFE
NHLKPIISTV LYDQLPLLYT LAKELQCEEK DILDFELCLM DVNEPCFTGA YEEFIEGARF
DNLLGSYCVF EAFAEFVLLF ISSYQIKCQL RFASLGDWGK SKSQLLNAKY FKQYIKNERV
TFIVSPGSNF LDGVWKSLYE DVYAEEDMYM PFFTVLGTRD WAGNYNSELL KGQGMYPKWI
MPNYWYHYFT HFTVSTGPTG HKDMAAAFIF IDTWILSSNF PYKKIHERAW ADLKAQLNVA
KKIIVVGDQP IYSSYLLPLL KDAQVDLYIS GHDHNMEVLE DSNIAHITCG SFCIHELSSN

GIITKFISGK SGSLQYFASL PKVEQVDVPA SGPMGNKDSF VRIVGTIGIL IRYAYSGVAY
SSGALKLSKT WCMNPDQGLI KPDVVFYLVC IYDNELCQVS DVGVNFYIDE EDVANKSDAV
IKKLKELNRY VRKIAFLCCN IYGLCGYLFV DFGKGFVCYD KDGENTKSCN ISKISKATEG
VVSFDFDKGA PFQKGDYIKF ANVEGMQINN KIYKINDMQK YTFTIGDTSQ FDEYLKGGEC
TQVKSHLRMN FQPYDDYAKC DMSNQLHVDE VKKDIVLKVA KYCTAHLAPV ASFFGGLLAQ
EVIKYTGKYM PIYQLLYVDF FEKNDNVITV FGKAFQKRLN ELNVFLVGSG ALGCEYAKLF
SLLDGKLTIT DNDNIEVSNL NRQFLFRREN VGKSKSLVAS GIIKQKNPNI NVQSLETKVG
PENEHIFNET FWEKQHIIVN ALDNIQARQY VDNKCVWYSK PLFESGTLGT KGNVQVILPF
LTQSYNDSYD PPEDSIPLCT LKHFPYDIVH TIEYARDIFQ GLFYNTPLSL QEFETLENVL
STLRVSKECN FNFCVKKAVD LFHTNFINQI DQLLYSFPLD YKLASGEFFW VGQKKAPQVI
SFDINNEFVK EFLFCTSNLF AQVYNIPQCY DLKYILDVIE VKPFQPKRVK VNMDPIEFDK
DEETNMHVNF IYSFANLRAI NYKIETCDKL KAKLVAGKII PALATTTSII TGLVGIELLK
YVNYLSYFKN AFINTALPLI LFSEPMPPIK MRDKEYDDLM KGPIKAIPNG FTSWDKIEIH
IKDYDTQRDK RFSGTVKLSN EVRKKLKVCI LGDAVHVEEA QKLELDYMDI EAMKKLNKDK
TLVKKLAKKY DAFLASQVIL PQIPKLLGPG LNKAGKFPSL ITHNDKIFLV YNIASFCFEL
QFRFIEDTTF DWLPSIGYLL PYEKIKLLRM LFPIVFFISI CAAAYTYTDR NATLIYLMRS
ILATNRIVFE RSNDVNYLKK NGEQLVSKLQ EMRKFALTLD SHQMGLLEKK NQKKSWFEFI
SMQMIFGIIF VYIWFTSKKP ATFEVPLSLT LEELYSGCKK KLKITRKRFM GSKSYEEDNF
VTIDVKAGWK DGTKITFYGE GDQLSPMSQP GDLVFKVKTK THDRFVREAN NLIYKCPVPL
DKALTGFQFI VKTLDNREIN VRVDEIVTPQ TKKIVSKEGM PSSKIPNTKG DLIVEFDIIF
PKNLTSEKKK IIREALGLYY ICISCPSSNW FKSTAIKWSL SIEVGGSDVD IENVAKKSEL
SETLAILHVD TGKTKLLDKL RHTNVQDNEA GGITQQIGAT FFPKDVLDKE IKKVDIKCMS
KGIMIIDTPG HESFYNLRKR GSSLCDIAIL VIDLMHGLEQ QTKESIQILK QRNCPFVIAL
NKIDRLYMWN KNDWSPFNST FNKQKENTQD EFKDRLQNIL NELAEQGLNC QLYWENPNPR
KYVSIVPTSA ITGEGIADLI MILVKLTQNF MLKNIEYHDK LECTVLEVKN IEGLGTTIDV
ILTNGVLKES DTIVLCGMNG PIVTVIRALL TPQPLKELRI KNEYVHHKSI KACIGVKISA
NGLEEVLCGT SLFVANNTNE IEDYKKKAMT DVSDVFNHVD KTGVGLYVMA STLGSLEALL
IFLKDSKIPV FAVNIGTVQK KDVKKASVMR EKGKPEYSVI LAFDVKIDPE AEKEAQILGV
EIMQKDIIYH LFDAFTSYIK KIEEEKKQSK LTDAIFPCEL SIVNDCVFNK KDPIVIGVKV
DCGILKIGTP LYIPEKSLKI GNVVSILLNK KTCEKAKKGD EVSIKICGEP HITFGRHFDF
NQKIYSKITR ESIDVLKEYF RSELTMEDWK LVVQLKKIYN IIMNINEKDK LAEQNLETLD
VTKLTPLSED VISRQATINL GTIGHVAHGK STLVHAISGV HTVRFKHEKE RNITIKLGYA
NAKIYKCTNP DCLPPECYKS YESSKEDDPM CPRENCNHKM KLLRHVSFVD CPGHDILMAT
MLNGAAVMDA ALLLVAGNES CPQPQTSEHL AAVEIMRLKH ILILQNKVEL IKEEQALKQQ
EEIRNFVSGT AADSAPIIPI SAVLKYNIDV VCEYIVTQIS IPKRDFISSP HMIVIRTPKL
CNFVEWREYK LVFKRYASLF FIACIDKGDN ELITLEIIHH YVEILDKYFG NVCELDLIFN
FHKAYYLLDE ILVTGELQES SKKNILRVVS AQDSLMEDTK FAKSLLDVAD NLSLAIKNIN
EESLKQNENI YKGIQMTETI LHNIFNKYGI DKYDPINEKF NPLFHEALFE INDSTKKGTV
ATVVQQGYKI KDRILRHLDI ESVQALIVAL NLYKGGLIII SHDTYLIKHV ADEIYHINNL
TKLIKIDYDF DKYAKLLLEN KIMPREIITL QCGQCGNQIG VEFWKQLCNE HNIDKEGILK
NNYLNEDRKD IFFYQADDEH FIPRALLFDL EPRVINSIQA SEYRNLYNPE NMFISKEGGG
AGNNWGCGYS QGHKVEEEII DMIDREVDNS DNLEGFILSH SIAGGTGSGM GSYLLELLND
NYSKKVIQTF SVFPLLNESS DVVVQPYNSI LTLKRLILST DSVVVIDNTS LNRIFVDRLK
LNNPTFQQTN TIISNVMSAS TTTLRYPGSM NNDMISLISS LIINPKCHFL VTSSNVQKTT
VLDVMKRLLH TKNIMVSVPV RRGMYISILN IIRGETDPTQ VHKGLQRIRD RKLVNFIKWN
PASIQVTLAK PSPHSTHKVS GLMMANHTSI STLFERCVTQ FDRLFKRRAF LENYKKEPMF
QGNFEEMESS KEITQNLIDE YKSAERDDYF GLARAFGIPV RKYTHEVVTL WYRAPDILMG
SKKYSTPIDI WSVGCIFAEM VNGRPLFPGV SETDQLMRIF RILGTPNSAN WPSVTELPKY
DPDFIVYEPL PWETFLKGLG IDLLSKMLRL DPNQRITAKE ALQHAYFMGK EKTHINLVVI
GHVDSGKSTT TGHIIYKLGG IDRRTIEKFE KESAEMGKGS FKYAWVLDKL KAERERGITI
DIALWKFETP RYFFTVIDAP GHKDFIKNMI TGTSQADVAL LVVPAEVFEG AFSKEGQTKE
HALLAFTLGV KQIVVGVNMD TVKYSEDRYE EIKKEVKDYL KKVGYQADKV DFIPISGFEG
DNLIEKSDKT PWYKGRTLIE ALDTMEPPKR PYDKPLRIPL QGVYKIGGIG TVPVGRVETG
ILKAGMVLNF APSAVVSECK SVEMHKEVEE ARPGDNIGFN VKNVSVKEIK RGYVASDTKN
EPAKGCSKFT AQVIILNHPG EIKNGYSPVL DCHTAHISCK FLNIDSKIDK RSGKVVEENP
KSIKSGDSAL VSLEPKKPMV VETFTEYPPL GRFAIRDMRQ TIAVGIIKAV EKKEPGLPIV
LLKDGTDKAQ GKSQIIRNIN ACQIIVDIIK TTLGPRGMDK LIYTDKDVTI TNDGATVMNL
NISHPAACIL VDIAKSQDEE VGDGTTSVVV VAGELLNEAK QLINDGIEPN MIIDGFRNAC
TVAINKLNDL SLSFVSKSPE EKKEILIKCA QTALNSKLVS NHKAFFSELV VNAVYKLGEY
MDKSNIGIKK VTGGSCLDTQ LIYGVAFKKT FSYAGFEQQP KTFLNPKILL LNVELELKAE
KENAEVRIDN PSDYNSIVQA EWEIIFKKLN LIKESGANIV LSRLPIGDIA TQFFADNDIF
CAGRVEDADL KRTANATGAI VQTSLFNLNE SILGNCGVFE EVQIGNERYN IFKECLKTKS
VTIILRGGAN QFIEEVERSI NDAIMIVLRC MGNSEIVPGA GSIEMQLSKH LRIYSRSICN
KEQIVLYSFA KALESIPRHL SHNAGYDSTD ILNKLRKKHS EETSDIWYGV DCQQGDIINA
YEHCIFEVTK IKRNVIYSAT EAACLILSID ETIKNPSRNL GLPDCFKDLL KTDKIKHVLC
TGNVGCRENL ELLKNIADSV HITKGDMDDE YDFPEDTSLT IGDFKISLIH GHQIIPWGDT
NALLQWQKKY DSDIVISGHT HKNSIVRYEG KYFINPGSAT GAFQPWLSQP TPSFILMKSS
IVVYVYEEKN GKTNVEMSEL QKYSVKVSEP TREDATGAYR NPEYKDKLFD NFEDCLGTRV
KVDNKLGPYK WKSFTEIREL ILAVGSGLGF YMPNCEEWNI CDLSCSAFNI VTVPLYDSLG
VESSKFILEQ TLMQTIICNK ACAMNLFKSL PGNLKDIACI CYTSGTTGYP KGVIMTNGNF
VAQLASSITG PSRMTNENDT HISYLPLAHI YERIMVLVFC AQGVRTGYYS GNVQTLVEDI
QELKPTLFIS VPRLYNRIHE RIFNSLKKKS SVVQSLFNKG LEHKNNNGIP FHFFWDKLVF
NKAKKILGGN VRAMLNGSAP ISPDVVKKLK AVFSVPIFEG YGMTETLGFI SHSTDVNIGH
IGGPVPCVEY RVVSVPEMNY LITDNPPRGE LHLRGPAIGY FKLEKETNDE GWISTGDIVS
FSENGSITII DRKKNIFKLS QGEYIAVEKI ESVYRQSLYI SQIFVFGYSY ESVLVCIVCP
SLDTIEIWKN QKKINKTDEE VMQMPEYKRD VIDDLIKMGK KDGLKGYEQI KDVYFATEPF
TIENDLLTPT GKIKRHAVQK KYKEQIDEMY GNKSKKTINE GQTLLTVFKE GYAPDGVWLG
GTKYQFINIE RDLEFEGYTF DVATCAKLKG GLHLIKVPGG NILVVLYDEE KEHDRGNNLN

MLAVVYLACR EAGHIKSIKE LITFDRSFKE KDLGKTINKL KKILPSRAFV YNENISHLIF
TLQLSIDVIE SIEYVVKKAT TLIWNDIERY FKDPELITAE ILFVGLTLCN VFVMYRLFLD
VIPFPIFVTW WQLAQGLLVA YVCGLKVLFV PSIFYCLMLV LSNYLLFKTP CIASYPVLVS
FTVVFHHLTR FVGCGEEYML RWKSIVFLLA AFVIGCFDSK TTGKGVIVWA LLYALFSAIF
RAGFMQKIMH LVDGKGNTLH NNQHLLGVLL LPILIVLSGE WAVFGHMPYN IMSLHTGCLI
TVGALPFIKN VISNRLVRRT GQGPWRFLEI ISIVLVFFIG MTYNTPSFLG YVAIVCVIIG
RSLGAFDVML NKTKVGEEMG NASFSLAKAV WAAGDFKGQI IEGIKRPVVT LSLSTNNVAG
VKLPIFQVHI DPTVDVLGNL GIASGGQVIN NTRENYLQCL NMLVKLASMQ FISMFDWLNV
HSETFYAKMN EDLVATKINL MRDSSTSCKD DNPYCSVNDG KVIIKNNELL CGIICKRTVG
SSSGSLIHIL WHEMGPDKTK DFISALQKVT NNWLEYIGFT VSCSDIIASN KVLDKVKDIL
NKSKREVSKI VKKAQRGELE CQPGKSLYES FETRVNNELN CAREMAGKVA SESLDERNNI
FSMVASGSKG SIINISQIIS CVGQQNVEGK RIPFGFNHRS LPHFIKFDYG PESRGFVSNS
YLSGLTPQEV FFHAMGGREG IIDTACKTSE TGYIQRRLIK AMEDVMVQYD RTVRNSYGDI
IQFLYGEDGM AGEYIEDQII DLMKLDNKEV KKLYKYNFDE DSYGDYNKQN VLNQEFEELL
KCKNNICKEI FPDGDVRQHL PINMNRLIEF AKSQFPNPVD IVHKVNRFLE KLVIIKQINS
NDTLSLEAQN NATVLLKAHL RTYLNSKLLT QTHKISMKGI DWLLQEIEKN FYKSLCHPGE
CVGALAAQSI GEPATQMTLN TFHFAGVGSK NVTLGVPRLK ELINIVKNVK TPSTTIYLDD
IVSNDQQKAK DILTKLEYTT LKQLTSHAQI IYDPNTTSTI LEEDKTWVDE FYEFPDEDQY
TLGEWVLRIQ LTNIHVNEKK LTMKEIVCII YSVFSSDELD IIYTDDNSED LILRIRVKYL
EDTFLKKLME QCLSTLKLRG IENITKVYMR EESKITYDSN NGKFVRSSHW VLDTDGCNLE
HIFCAPHVDY KKTISNDIVE IFEVLGIEAV RRALLKELRT VISFDSSYVN YRHLSILCDV
MTQRGYLMSI TRHGINRVDR GPLVKCSFEE TVEILLEAAA FAQVDNLKGI TENIMLGQLC
KIGTGVFDII IDNQKLSDAN QNETMMDITS AGFTTPDSSP LPFSPTYNVN VRNAVIPGHI
RKSEHFLNLM RIVVMYLKKY VNIYEITSEG PLSFLYKCER ETKLDTSFFK FCFDRLKSIL
NSLQIVDEYS ALNIVCNFCT LLGSYFQGFI IICEPYPEAT IYDPVIQFAC LDSSIAMKSV
INKYKSVVLT SGTITPLELY PKLLNFKTVL TASFPISFDR NCVCPLIVTK SSDLVPLSSQ
FSLRSDITVI KNYGMLLVEM CKTIPDGIVA YFPSYIYMEE VISSWYELGI ITSILEYKLI
FIETKDIVST TIALHNFKRA CDLGKGAIFL SICRGKIAEG IDFDKHYGKC VILFGIPYQY
TLSRILKARL DFLKETYNIQ ENEFLTFDAM RQASQCVGRI IRNKKDYGIM IFADIRYARN
DKKSKLPPWI IKCMDISNVN LTVSTAVNIS RKFLLNMSQE YKETGQTKNQ PVTRVTELPN
KLKIATVKST CEVPTIGIWI SSGSKYENKH NNGVAHFLEH MIFKGTKKRN RIQLEKEIEN
MGAHLNAYTA REQTSYYCRC FKDDIKWCIE LLSDILSNSI FDENLINMEK HVILREMEEV
EKSKDEVIFD KLHMTAFRDH PLGYTILGPV ENIKNMNREN IINYINTNYT SDRMVLCAVG
DVEHEQVVKL AEQHFKPYFC GSEIIMRDDD SGPSAHVAVA FEGVDWKSPD SITFMLMQCI
IGTYKKSEEG ILPGTVNNIC NKMTIGCADY FSAFNTCYNN TGLFSKYLWK ARIYFIWQRL
FASSNDCFYS LKIHVVDPLR IFKNVKLLSQ NFQICALSNF SIYLAFYNYV YAYQRLLDVL
AEMSQFSYSF TGRMGIKRKY QKIPATILVL LKDLDPDTDI LEEPYFMDSQ NNLTFDEQIT
LINFCYSMIR FNPHYDEIKF EKISAVISRC LKYQNWLLHS CILWYKCKCE TFRLKTVDRA
AAQLNELLKE TYDMKPHGGE RVKFLFDVYY PTTWELKKEI GNVMVKTGSV VSAFNLFKDL
KLWEEAITCL IQADRKEEAK ELLDDLLEKK KTPSLLCLYG LNYYIDAWKL SNYKYAKAAR
FIGNFYYRKE MYNPCCEYLE KALEISPLFP DIWFILGCSY MKIEKIDESV KAFTRMVSMN
LAYLYMKKEM YRAAKICINQ AVKVDNNEWK YWDTYLKLSI MQNDVDSFCL ALITLCQKNK
VKQIQPWVYE YISDLIVNDK ECDSYWNAFS FFLFIKGRFV DSYEAKVKEI RSLEVNYIWK
NKVFVGKEAP FFRAEAVFGD NSFGEVNLSQ FIGKYVLLYF YPLDFTFVCP SEIIALDKAL
DAFHERNVEL LGCSVDSKYT HLAWKKTPRE KGGIGNIKHT LLSDISKSIS RDYNVLFDDS
VSLRAFVLID KNGIVQHLLN NLALGRSVDE ILRIIDALQH HEKYGDVCPA NWKKGKVSMK
PSEEGVAQYL STLMEDSAAK IICHDSLLTD PRIAQDFSAE TDDLLTEAEE AIEIIYEISN
ILNVNLDKET IVILIQLCEY G

> ***Theileria annulata***
NLQGDFKLTK KKIHWLPVKT KTAVDCDLVE YGHLLDDNNM KEFLEPQTEW VTKALGEWAL
SNLKKGTVLQ LERKGYYIVD LVQIPDGKAA KIGLSGSYSP SFIVRSVVYY SEDVYSSLEF
MSLDHLIDHG HISDFDKMEG LWQYCFSKLE VHSDCRPVLL TEAPFTSPKH RIKSSEIFFE
RFNIDDLNIS VAGLLSMYGL GKLTGTVVEI GDGVTQVLPV IEGYSERSSI KRVDFGGIEL
TMYLQKMLCT RGYCLTSRPD FELVRELKEK FSFCSLDPFT DENRSDLVEV YELPDGNVLR
DGENEIDLSL ERFYVCEPLF NPSIVNSDAP SIVNTVWNAI TSSPIQDRTT LTNSIFVCGG
TSLFPNFEKR LQMELQDIAP PGGRSKVRVV LMIEGMEFAN ENNLYFFEAS AVTGYNVKEI
FEFLIQMEQP KLAKVNKILG RTGSRGGVTQ VRVDFMGESG WEGRTLIRNV KGPVREGDIL
ALLETEREAR RLRGTQSHIL KNSHCYCHVS TGDLFREAIK SGTPLGLKAK EFIDKGLLVP
DDLTLSLVQE RINSPKCRRG FLLDGYPRNI SQAKDLGKLL KSVGKKLNGV FSFNASDEVI
EKRVVGRLVH PGSNRVYHKV FKPPKEEGKD DLTGEPLITR KDDSPDIIRK RLEVYKKETA
PLVEYYNNEN LHCKDCYNDL PQPEFVLSPE DSELNYFLWM PGFKYQPFKK RARGGILDVH
LAGVLARELG ASPSQADLLE FEATCGKSVN FENFKDFLAV SMYSNENREY LQDLLANFEN
MSGGISLSKF ENLMKNYGEP LTDSELNELY KLVRVEKNVH MLQLFDKIDL NSDELDTFST
TLSKVISDRQ LANEMETIDK DKDGKVSLEE LLAAFSIEVE DALNNKEPLI QRFKVADKNK
DGHLDLPELG DLINPSRSPE LLKLEVDDVL KAHDSDGDGK ISGEDETQSS NDFKFDKDGD
GYLTRSELLT RELWEKHSDE LSRSSVTDFQ EVLEHPADYG LMCLCNCLAA GFIMGMSFLH
MFPEFVIMLS SFSIMLFLER VLSFGRTPCC DSSDDEGESR IVKSAKAKAL ETIQNHVKTI
EHLKKINDYS ELLKDYDILA KFVEKQSSTR LPKLVVKLIV ELSQFMEAQQ KDKESYKKLS
KAKTISFNTL RSRLRKFNEQ HSEIIEEYNK DPDNFWSDSG SHPASEAEDG DKHNKAMDKW
GVKKAEMAEK TKKTKKEVKL GNDDNPFSVM IPEVQEMLNT VHLSEEALRL FVKSIIEKRG
KRGTNNFENL KILQSLPYIA KTISQSLYLE VLETLVHVLF DTYSHAYGAM TPTEWINTYR
IASHLVSELI SNPKSYLSSE MAEKVNNDKV KAFDDRVKTS LGVLSTIVQK LNDELYKGLL
YTEVHNPDYK TMLAYTIDML YLLHRTLVYY LAASTALMIL DHCHYKDDEI SAKIWELVEK
TPEQERAEKR RLLPYHMHIS IELIESVNYI CALLLESANY ARYPLKAKEV ISRQFRRMYD
AYERQVFVGP PESNREVILT AFKHLQNGDW KKCYNFILSL NTWNNMPDKV QETLKELIKV
EGFRTYIFKY VNIYDSFSVE QLSSMFSLDE NVVHSLISKM IVNGEILGSW DYSSKCCLIN
HSEPTELQKL AVKLAENLST AVEQNELTLN MKNSKLGLTP QQTMVVGTRA KEFLINEKTD

IKTHEELIER VYKRGVIDSK GQMLPLHLLE LFSLSIQAKE SGDFLSNRDE MKAQISSSQR
QIYDLLLNSF NGTEANRLRD ELFNIFSIDS FLKTLNYKFQ HLMNNQLNNL ERLVRKKIEL
ERQQTLRESI KQFIRQLIEV VHNMITGNYT IMKLPAEQFL KTYGGNLQDN LQDGIKPGRY
VRYFTSKINS HMFGLIEPPL NKDYSSDELI NVEFQINNGV ETSLHKNIDR NKLPLPSVSE
RLQVPWHKTQ ASTGWVLVRP IVIDRLEKKF KTKLDKIDRH ADSNLLNQLA FTQFWATKML
LSSPLTSKLY SHFGDKTLKP LIIVAIGDIA MAVGGAFSSY VQNTMTLLLQ AATTTYEMGP
VDNEDWIYFV NQLQEASLQC FTGIVYGLKE GGALHLLRPY VSSLLQFAQQ VVETPDPFFD
TNLYKLAVSL IGDLSSSFGS DLSRHLVDSN LIRGIENRLK QLELAQDPCR DRVCWLHSTL
VMYMLQLRFT SSGQIEKAGH LLMKAIQGGE SFLELRLRLL QMLYNSVEST LPLYIAILEF
ASKHNIFHTL VPIVKEVDEW MVDWSIDKKT KIKIYHIIAE ELDKLNRADL AYKFWKKRVE
CLNDLFTTDE NVMATVTFVV RSLRSEDILY FDQLLLMPAV AHLKETRYAP LISLLEIFIR
GNLEDLDLFL DRKPLVEKLT LLTISTMCQQ QSEIPIEMIE KNLQLPPEEA EQMIVNAINK
GVMEALIDQN SKKVIINHVV HREFGNEELK QLYNNLKQWR NCIANEFYTF KDSKATSVRA
FQIGSSYWSS QGHHSDTDFV SWTGELYEKA TISQINVFWE YAPKEVEISL SLTGDDFNVV
MPFRPTFENK RSYKENFKLE MPYETKFVRL TLRGAINTYF GIRHIHLVGY GTPLFMIKSG
ISSKEEMCLQ ELWRQNSRMQ IVSAVSYPEM CLTTVESSKV NEDSCTWEFM GNSQLSVKLC
LTQRDENGSA AGMGNLKSEL KVTSTSSEKH EEKYAVDLDK KTYWASLIFP DDYHVTSLTL
DFDKMVHASR VLIDWEYQPL SYTIEASSDK EISRNLSNSY HTTTDSFPGT DFKQLRIVMM
KPHYLHGKVS GGYVYGIRDL SVLTNNLLTV VGDCRAAANS ADARDKYFVF YVSVFEPLLS
KKVRGMESDL NSVITEVSNE LSHLKESLEE SANCMDEKKD YDNQLNLASK KSQDLIQPPC
SKAPLRVYCD MDSHTSMYIW NGIRTPTSLR FQCASYGLEP LVIKSRRQID HLKKAMKLMP
LSNKLDSYLP LAYKFGSFKD FMNIFSFISG NSGSSDESNH DKNAVLRRRG FEPKTLGDVL
RSRNKQWAKT QCIHSSAKED FERHLNMYKN HFRSYRKGWK LTWSNIIFCP NFRPGGEIIY
RFSRKKTYNT LDTINKGWVH PDLLVCLVRD ASHPVRFATP PEQDCYTAIY TGPEISENDT
KVIFGEYTGI VREDCVPDSI FEYAFELNFI FLPNNSKYVL DSTHAFNELS MVNHCQSIYG
EYFLQANCEW QQVIFDGWPH VILTNKQGVK IQTGDELIAD FGALWFQKVE ENCHRQLKRE
LISYRLDYFV VNNNTSYVCA ICSTQTLSSD EDDEDCIVCD GCDRIFHIKC LEKITFQAYK
WYCMYCRYLC KQIANVYVCR VLKRHLSGNE LILISFEQHI NYLLRLLVPG KTIIYRKFSD
GYYQGLVNKY TNSSTYFKIE YSDGDDELMD PFDLMNEKYK EEGNNLYKQK KFAEALEMYN
KAIELDPNNL LLENNKAAVY LEMGDYEKCI KTCNDAIDRR YDVMADFTLV SKIYNRLAAC
YTKMEKYDDA ISCYQKSLIE NNTRQTRSLL SDLERLKERK EKEAYINPEL AEQHREKGNE
YFKEFKFPEA KKEYDEAIKR NPSDAKLYSN RAAALLKLCE YPSALADCNK AIELDPTFVK
AWARKGNLHV LMKEYHKAMD AYDKGLKVDP NNNECLQGRY NCINKIQEMN KGNIDEEQYK
HAMSDPEVQE IICDPQFQLI LKKISENPTT MGEYLKDPKI SHGIQKLMAA GLLFKRESTP
PESFGVNRDW NVDLIPKFVL AGGKLVKILR ATETSQYLEW QVLDGSYVYQ HQKGNFLYSE
KFIHKVPASD KEALSSPLMG FLEKNRCHNF YKFVFNFNER DKSTWKNHNP FLESITAYYK
HYGLEENTID FLGHAVALYT NDDYLKLPAC EPIKKMKLYM ESLMRFGSSP FIYPVYGLGG
IPEAFSRKCA IHRGTFMLNK PVKEFKFDEK VCGVVTAEGE ARCSMVVCDP TYCLAPEKVK
STGKVIRCIC ILSNPIPETN NASSCQIIIP QKQLNRKHDV YVTLVSYSHG VTSKGKFDVR
LDTRLNEFLW SNGIKNLPRR VRVRVSRRRN DDEDAKEPMY TLVQHIPVDD FSGLQTEQMD
GDHRRILDCT SSNFNGFISI IDPQKSWSAR YNNLSDLIPG CYAISVNGTL GSGSGYISTY
ILNLFKAQEH NIPLCISTDI NKIGNKLESS ECVCMDLFNN LRPVFDFIFF NPPYVVGTDD
DTSDMIDKAW NGGINGSETI IRFINSVDKY ISSGGFVYLL VEKRNKINEI EKSEFPILCE
TCLGPNPLIR MMKQNMGKEC KVCERPFTIF RWKPGPKSRY KQTIICQICA KIKNLCQTCL
FDLQYGLPIQ VRDKFMNNPI ELPDSNKNLI YKLNNIQLKR ISRVAPYYRR NKPRLCTFWI
RGICNRGEEC PYSHEQDSRV VIFNLPNVTE EMIRKLVKPF GKVEQIMCFV TFCFESDALK
FIEEKNNSIF SGRIITISHA SYSEFKAKKK RSEIENRTIW NILHIDINSA IKSIASDLRI
SPEEILGEQA GVNAAISESF ILNKVKKWLS DQGIEEDDVL LIKNLPHDTE DRELIRLFSS
CGKIIKFTTS PFKLLGLVQF SSKTESEKAF RTLSYKMFQN LPLYLQKVPK SLLSDRIGHV
SVYVSNIDGN VSEEEFEKHF SSLKGFVISK IGVRYGFIEF DNVNNAKEAI KRLCGTVIGS
KIITLELSVI IVKNLPFQAT KKELLELFKY YANVKTVRIP KSAGNTHRGF GFVVFMSKND
AKLAMENLKN VHLYGRRLVL QYVPSDAIIL DPVVHENIIR IPKPEIKERI VEVPEIEYVE
KIIEVPDPVY EEKIVHIPKP VVCERVKQVK RPVIQEKIVE VPVVQVVEKV VEVPQYVYQE
KVVEVPKVVV QERIVSIPRK TQYRNVPQPV EVPVTHYRRV PVEQVIDRNV PVPVELEVVK
KIQCQKFEPR YREVPVPVHV QRVIDHPLPE KVLRNPETAP EYYLGSTAIA VATKEGVIFA
SERRSNSPLL EFVSLEKIME IDDHIACAMS GLIADAKTLV DHARSECVNH TFVYNERMGI
RSCVESIADL ALEFSDVFDT KKKKTMSRPF GVALLVGGID SEGPVIWCVD PSGTIIKYKA
AAIGSAQEGA ESILLQKYDE NMEFSDAEVL VLEILRQVME EKMSPKNVEM ARIRDVKYRE
YDEESLEKLI SSLPKFMDFK DHGNIIKLSE VNKLSNIEQE FQSMIKRVDD IEKVAKKRLN
DIRYKCPRLQ KMITTKVTEH FYSIASMTLS CNLLPSAQGP AGTGKTETIK SLSYIAGSNV
MVFNLSELYE VEDMEKILSG LYQLGFWGIF DEFNRLSECV LSSVTEKLIT LLDRNIQVNG
NSAIFITMNP GYSGRSELPY GLGFKLAYGR LGNSVAVMSR THDSIGIGML SLDLMCQCES
REMAAPMCMW KLPSKELISR DGPCLIDQRH HQRLLMSYSP FNSAALLAEQ INVLGVNPGT
RLLFFPLWKT AKYSIDYCLP VYLYWLHLHS SCTLSVQDVQ LRGQVVVIAA TNRQNSIDPA
LRRFGRFDKE IDIGVPDDQG RLEILKIHTR NMKLDPQVKL EELAANSHGF VGADLAQLCT
ESALSCIREK MGVIDLEDDT IDSSILDSLA VTQEHFNNAM NTCNPSSLRE TVVEIPNVKW
EDIGGLEQVK ASLREMILYP IEHILVNLYK NNFNSPTPIQ RLTLVPSIIK KTHVLISSET
GSGKTLCFVL PIVISLLVIL PTRELAVQVK KIFFMILEGI DIRVLSIIGG ISVQKQERLL
KKDPSIVVAT PGRLRHLVLD EVDKFFEDNS YKEVQLIVKY VKIQCFLSSA TILISLFKLL
NISNPTVCIC SKIPENLTFK LIDSEDKYKE VRLIGYLVDY KCIIFVNTIT YVYRLESLLS
LIFWKDVHEH RLKRKVSGIH SRLKQKQRLN RLEKFSNKKS ILICTDVASR GLDIPNIDIV
IHFHPPKDKS LFLHRSGRTA RLKSDGDSTV STEVNGLSLF LSSAEIKSLS DPSMGSTEIY
RLCSTCNENC DGHLGHISFS IPLFHPMMIN TLCKLLKTVC FYCGRFKFKH DSNMWNEIRN
RFFNKTSQKF FCNECSQKGK IMIKPSSDLS YITVHLQSFH LYPYLDHLFY SRLLNHVFPQ
SIVLGHKIFF MDCMGVSANR FRPPLVTLHS RNEFVKLFLQ FKDEANYNDN GSLQKKLSSY
MDVKQTMEHK TGTVRQNMLG KRVNYSARTV IAPDCFIDTN QMGMPLKFAL ELTVPEYVTK
YNVNFLRKLS PKDFFLTETA DYRHLTLIAD FMTGELCIDI LKSNWSPAWT IQYLCRGVIY
ILSSPNPDSP LNCDAGNLVR GVKVKSAAEV GKRAVQFTRG DEILKWLMNN KELVYNKCPS

FFQNSKLEDN QDVSNFVDNL IENGFMYRAQ YQPETGSYKR PMWPKRLIRT QKQRFDTVGF
YIISYEGSQK WNYLKLCGII FGIIVSNVYP SVATVFKALV MASIILRLIL FLIMWFCGYD
FWLFPNLFDE DLGVVDSFKP LYSFTYRKDN LTMMACRLLC SILIGVSIYQ LGKTHDINDI
YKFTKQSFLD VLDWGHQKLA EDDYNCLLGC GFKSLDDLVK NCMTDCDCMT LADSFLADLE
DLENEENSFS TLIKDIDECN KTVIKIDKEI INIFNYVRDI YSKRFPKLES IVYSPLDYIA
VVKRAQNESD FTKIDLTDLL PNSMIMAVTV ASTASGTCLS TQFLNKVVSA CNEGLLLAEF
RNDLLVYLEG RMILIAPNTS ALIGSALTAR IIARVGSVEN LSKIPSQNLM MIGADKNGIL
NNCDLVLNSE PSLRIKALRL VCSKVSLASR IDLFTMGRNV INKKVLLSSE VAQVSKLILK
DRYLGRHCRY FVRQARLPAY KQFLRPYKSV TLKNMADAFQ VSTEFIEVAN SVSEVNRISE
NGENKNKLLE SMQKLEEYFE HPSNATSLAR QGLLESFTLL LDMEVLSSTL SIISCSFSNN
ESVLEEASKT QLVPNLLKLK NKLKDTQLEP RLITAISSSI RNCRRAEQLF VTLGGLSYLK
DSLESTNLKT RERAILLFNH FISLDKASRL IMATLNPYKI LNLLLPNGIQ FTELSCTLVF
LILQKHSNAF TGEELNEVSK VLDRELQSLF EGLGVPNWII EICKSLQIKK PTKIQQLCLP
SAFKGKNLIG CSETGTGKTI CFCWPILTSL AKNPYGVYSL VLTPTRELAF QISDQFRIFG
VNMNILVLSC VGGVDIVSQP HVIIATPGRF SNVKYLVFDE SDRLLDITFQ EHLKEILKCI
PRITFMFSAT ITDAIRTLAS KISNTNFEFY DATEESSKVR KIEHEYGIIF TCTKKRCQLV
SLTLDQLFKV TCIHSLMKQS KRTDSLSKFR SGYSNLLVAT DLVSRGIDVP EVAFVINLDF
PTTPSDYIHR VGRTGRGGRQ GIAFSFIDEF DVDKVKNVEN SVEIRLKKYE IKDKEAVKLL
NKVTVATQKA HIFLQEVVLF PPSLYVEHTR NELKSEFELG VQNVSQSKSG AFTGELSLTM
FTDFGLKWSL VGHSERRQLF NEDDSYVCEK VMMLQENGVN AVVCFGETLS EREQGQTENV
LKRQLDAFVK HVKDWDKVVL AYEPVWAIGT GKVATVDQVK EAHKFVRDYV RGLVGADKVR
LVYGGSVNEK NCLELSKCSD VDGFLVGGAS LKKEFLDILK SLRDLGKDTT HRFGPNSFKL
HRLPVPRPGQ VLGLVGTNGI GKSTALKVLS GKLKPNLGKF DSPPEWSEIL QYFRGSELQG
YFTKMLEDNL TTAVKPQYVD NIPKQVGGLV GDILEAKDKR GIGQDLIVTL ELSHLLSRKV
SELSGGELQR FAICVAILCD ADVLMFDEPS SYLDIKQRII AARVIRQCIH HERYIIVVEH
DLSVLDYLSD YVCCLWGKPS VYGVVTSPFS VREGINIFLD GFVPTENLRF REDSLSFKVE
EVESIHCYKY PQLDKKLGSF SLTVMPGDFN DSEIIVLLGE NGTGKTTFIK MLAGKLQPDN
ADLMPKLSVS YKPQKLSVKF DGTLRQLFHS KIRESFLSPI FQADVVKPMQ IDNILDQQLK
NLSGGELQRA AIILVLGTPA DIYLIMTFLC TISGVQPQEP CLSKTGYIFE RRLIEKHLEE
SPVCPATGEP LTPQDLINIK TDVVTKPRPV TASSIPGLLS LLQSEWDALA LETHNMRSHV
DEVRKQLSYS LYQHDAATRV IARLIKQRDS ALQEVEALKQ QLLLFRTNYD VNSLETEFDK
DTMVRLQDLA KVLLSERKKR DLSGYLDAEA FSKFKCAGEF RLHSSTKPGV LCVALDFTGG
NDGSVVYFDL FNQKTVHTLN GHMKPVNTVV THPLDNIALS GSDDSTIRVW REFHHKTSIK
NLAMHPSGEY LLSLSSDGVW GLCNIDSGKV IKMHRNVPKC NALKIHPDGL VCIGAATNGT
LQVWDIRDST LKDPIFNENG YYLVSVSEAG ELVLWDLRKQ TVINTFSCNV NPTRVKFYAG
QVFDGLFHGS GTFYYNDFER YEGDFVLGKR EGRGKFYYAD GSIYDGEWLN DKINGHGVAY
FSSGNFYDGN WENGRINGYG TLKYANGDVY EGDWLDGAMH GHGTYKYSEG DIYVGQWRQD
KRHGKGTMTY VDKLGKPCEK YEGDWVDNIM NGKGIYKYSD GSYYDGDWCN GKMHGTGKYV
YADGNKYEGE WVEDTKQGFG ILIYSNGEKY EGFWQNDKCH GSGILFYSTN DKYNGEWVDG
KKNGPGEIIY VNGDRFRGNW EDDHANGHGI YYSNGNRYEG DWVMDKRHGT GTFFCKQDSS
TYRGGFVNGK KEGYGTLTLG CGHIVHGVWH YGSLVSIDNF EISPTSPWNN PDLDMETLYD
IGGKLIDALK RENVSVGDII QIDKSSGRVT KLGRAYSYSH DYDAMSPNVN FIPCPSGELQ
RRKEVVHTVT LHDVDVINSR SQGFLSLFTG DTGEIKSEIR DQIDLKVQEW QDDGRAELIQ
GVLFIDEVHM LDIECFSYLS RALEADNCPI VIMATNRGIT RIRGTDYKSP HGIPLDVLDR
VLIIPTFPYQ PEDTKLIITE RCTEEDVDVE EDSLELLVKV ATDISLRYAL QLITASSLIR
KRKGGGSVTC DDIKRSFNLF LDSKRSTKYL INFQHDYMFS ELYENPFEFW TSDEEDTEEY
IRKKWAIVDD KEAPELSDLI VKYPFELDDF QKKSIYHLIN GKHVFVSAHT SAGKTVVAEY
SIALAISRGQ KAIYTSPIKA LSNQKYREFK VKFENVGIIT GDVLCNPGAS CLIVTTEILR
NLLYRGDAVI GQISVVIFDE IHYINDLSRG VVWEEVIILL PRNIQLVMLS ATVPNYLEFA
EWIGNVMQKE VLIIMTNHRP VPLKHYLYIY DRFFLIHGAK GFNKEAYHIM YKSTFKGQVQ
KLQRLLKQLE SEDKMPVVLF CFSRQKCEQY AKDMPNLNLV YNKQASKIHL FLKESLDGLS
ESDRNLPQLR KMVNLLTRGI GVHHSGLLPI IKEMVEILFS RGLIKVLFAT ETFAMGVNMP
ARSVVFTSIY KHDGINYRYL TSSEYTQMAG RAGRRGLDTF GNVYIFCCDE PPDVQDLTNM
MIERSTRLES RFRITYNMLL QIQSRDHMNI TEMMLKSFRE REKMMKIPLL KKQINKKKHE
LMSLPPISCI YIENYYKTLN YSMNVSHELH QHLVNLSSVS FIFDELCKLI EKNDFKLMSF
SKKFKQISLQ FYEILHFKTQ DKIHNYELEI EDINKQLKDE SLYFYEDMSN KLEVLKQLDF
LDENNRPTLK GRIATFITTS DEITLTEVLT QGILSELTPP ECAAILSAFI YNDKPEKEVP
SPTLALQQAK NQVVSIHKKI DVVQRALGVR VSHEDFNSLC NFSLSYVIYQ WASGTPFQEI
MELTDLQEGH IVRVILRLDE LCRKLLQTAN IFGHQKLAEK IDLVCNAIRR DIVFKQSLYL
DGIAMAKFFA TVYEMKENGT LFDKDEYELG QLSSECRFEQ ENNLSFEPIS SISENGAVVH
YRALKESCSK IGPHMYLLDS GGQYLTGTTD VTRTVHFGTP TEEEKLAYTL VLKGHLALRH
AKFPEGTPGE SLDVLAKLPL WERGMNYYHG TGHGVGSYLN VHEGPCNITP RIGKPLKPGM
VLSNEPGFYE AGKFGVRIEN MFYVKELDSK DNRKFYEFDD LTLVPYCKDL MDHSLLTKQE
VEWVNEYHKR ISDTLVPLMS SRPKNSFNLQ TNPKFIKDRL ELFNALYQKL VLVTDDGEVS
SGKSFLTTPY NVLYSLDKKR AKKAVVAKVL YENRPSEVFA DLWDMHRPFE DSCKVEFYWH
SSAHILGSAL ETCFGGFLTI GPALSSGFYY DVYLGNNSVK PEDVKPLLSH VEALTNLNSP
FERLVCTKEE ALELMKYNPF KVQLIKNKVP DGENTVCYRC GDFVDLCRGP HIPTTSMVNS
FDVTKISSSY WLSNAKSDTL QRVYGISFPE KEQLKMYKNR IEEAKQRDHR TIGTDLKLFY
FDTVHSPGSC FWLPNGAKIY NRLVEFMRDN YRVRGYQEVI TPNIFSCDLW KQSGHYDNYK
ENMYLFNLED TEWGMKPMNC PGHCLMFKHL FLSYKQLPLR LADFGVLHRN ELTGSLSGLT
RVRRFQQDDA HIFCTREQIM DEVLNILTFI GKVYSLFDFG INAMKSRGYD SCGVCTLLKV
TKCCSVTPAD SFNILKDKVL SSHPPSTVGI GHTRWATVGK LSNKNSHPHI DLAKNIALVH
NGTISNIEDL YHDLISQKNK FSPDSDSESV AIFVGLEYEN TGDLLLAFKN TIKKLKGTWA
LCMMSQHYPN SLFVAAHEAP LLVARSERGV YVGSEPNVFM KYVKDCIVLN DGDILELSLE
NVESYYSQYN LLKLESEVVE ETCEPYPNWY TKEILEQIYI LSEQSNFFSF QNQNQVMNEE
ANELLSLRDK KKLLFVACGS SLHAATYVAK ILQKIHHFDL VEVDDASDLT LYRYHDKDVT
VVHISNSGET LDCILALNFI KRINPDSISI INTVHTSLER SSDATIHLRI GREKSVPSTK
AFTAQVTVLL IFSLYIISNN CHNYIASLYK SLSIFPSAIA KLLKNDEQYD SLAQWLLKKI

VYILGRGCGH VVALEASLKM KEVAYIQAEG VLSGAMKHGI YAMIKEEENT TTISIITSEK
EMTINSTLQI KARGGYIIVI TDLEDEVDDV LIRIPSIGAL TPALAIIPIQ IITSKIAILS
NRNPDIPRGL AKTVTTWLQD IDKYATSNVC KLLIGNKIDL VDSRVVLADE AKHVAEQNNM
NYIEASAKTD SNVEKAFTTI AKALKDKVTQ YPSNAPTSTV NLNASKVTVR TLNTKPPPEG
WELISETLES LDDKMKQAQL ESGEGKRKTE ILWPIFRIHH QRSRYIYDMF YQKKLISYDL
IVLGGGPAGM AAAKEASRLG KRTVLFDYVT PSARGTSWGV GGTCVNVGCI PKKLMHYASL
LRNYDKFQYG LTNTQTPNWN KLIQTIQNYI KMLNFSYRSS LLSGVDYINA FGILKHNKII
EYNLNNEIKY VSGDKIIIAI GERPYIPSDV EGANEYAITS DDLFQLNTNP GKTLIVGASY
VALECAGFLT GLGYNVDVSV RSILLRGFDR QCVKKVEELM EASGVLFLPI KIEKHNQQLK
VTFNDQSVNY YDTVLYAIGR IPSQYTQHLE ETNIKDIYAV GDIVSKVPKL APVAIKSSEL
LIQRLYSNNN TQMNYENVPK CVYTPFEYSS CGLTEEEAIE KYGNLEIYLK EYNNLEISPV
HRINKKTNDE FDYPMTCLSK VICLKDGKII GMHFVGPNAG EIMQGFSVLL TLNAKKSDLD
KTVGIHPTDA ESFVNLTVTK SSGKSWIATG GCAGGKCGES GSQNPQRLEC LLSKGLLQRD
LDLLREAGYS TLECVAYAPQ KNLLVIKGLS EQKVLKIKAA CRELCHLGFC SGQDYLEARG
NLIKFTTGSS QLDKLLQGGV ETGSITEIIG EFKTGKSQLC HTLAVTCQLP VEQSGGEGKC
LWVDSEGTFR PERIVSIAKR FGLSPSDCLD NVAYARAYNT DHQLELLVEA SAMMAQTRFA
LLIVDSATSL YRSDYSGRGE LASRQMHLCK FLRALQRIAD TFGVAVVITN QVVARVDAMS
FFGNDKLPVG GHIIAHASQT RLFLRQSKGE SRICKVYDSP VLPEGEAVFA ITDGGINDYM
AIRAQYENSN EVGVFSTLTN SYALVSLGSS TNFSSLFEAE LTPHIPVVHT TIGGTRVIGR
VSVGNKKGLL VSSICTDKEL RHLRNSLPDS VEIRRIDERL SALGNCISAN DYVGLIHVDM
DKETEEIVED VLGIEVFRAS IAGDVLIGSY TRFQNKGGLV HVKTTTSEME ELSQLLQIPL
TSGTINRGSD VIGAGIVVND WVAFCGMSTT ATEIATVERI FNLARPSNSS IIDSYSLKSA
LIDTLIVVDS YWQTETGGII ISPIPGVTFT KPGSATYPFF GIELAIIDAN TGEELEGNNS
GLLCIKKPWP GMFRTIFGDH SRIHETYFPT KYPYYFTGDG AFRDKHGYIW ISGRIDDTIN
VSGHRLCSAE IEYALTQVDI VSEAAVVGYP HALKGQGIFC FVSLKLKMSI RHYVGPFATP
DVVLITPNLP KTRSGKIMRR ILRRLASKFH DFGDVSTLAN PEVVHQLWFY GTSTSTTTVK
LYCDLTSLPN DEIILNFCTF IKDKKEREVK LLFNEFLILF FPNVKFNLSG FLQLIPKQIP
KAYTISSIPN RIYKGNCSNY LCNLIVGDTI KFFIRSSIFK NHILYHEELN HLQNLNNFHI
SYAFSREQPW VEKYRPKKIS DVIFQTQAVS IMEQIIETFN MPHMIFHGPP GTGKTSAALA
MARQIYGLEG MRERVLELNA SDERGIDVVR DRIKTYTRIN ISNNRVNPET NRVMPNYKMI
ILDEADMITA DAQAALRRVI ENYSSISRFI LICNYLHKII GPIYSRCSVF HFKPIETNSQ
IDRLKYICNQ EGITFDPFLT TISSGDMRKS ITILQSTACL YNEITENAIY SVSGKPPKRV
VESIFEVCRR PEGDVESVCK QIVHDGWDIS SIFQQICEYV VDIEKSKISL ELANRDFALL
QGGSQYFQLA SACFHIKNTI IKEVLYKSKD VKHLQNVFKS LKELIKQMKQ RENDDMGLTL
ADQEKLNLNR TGKRIVLKDL MIRPSVHGSR RVLGFLEAHH NGLRYLVVDI SYANVRHAIF
QPCQRELIVL LHFHLKSPIL VGKKKTLDVQ FFSEVGTQID DLDNRRGRSY NDPDETLEEM
RERELKRKFN TDFKQFVSQL KDLTSMKVDL PIRELMFTGV PLKSNVELLP TVNCLVHLVE
WPPFVLPLTD IEIVSLERVQ HGLRNFDIVF VNRDYSKPIK RVDLVPIEYL DTIKRWLNEL
DIVLKTILED VEAFVESGGF DGFLGEGEDE EDEDDEDEEY KDDESDEEEE IKIIAQADFD
YKRKCSTTII SIPRIILYCK GGDNIMIKKL KEIKEVDVVT LRNMKKYSVG GLRTLVFAKR
EIELKEFNEW LKEYNRIKLT IESRDEKLAE CVSKLECDLE LQGVTGIEDK LQTGVSECIE
QLLMAGIRIW MLTGDNLDTS INIGIATNLV NMLSTEGNNN TKETPLGVKE IFIEILKRVH
SVICCRMTPY LKGAVVTFVK NKLGGITLAV GDGANDCNMI QIAHVGIGIK GREGSQAFNA
SDFGIGEFRF LSPLILHHGR LCYRNLSKCI SYMFYKNVIL IIPLFFYAYI SLFSGQKIYY
SLFVAIYNVV FTSIPVGIFG IVDQDYNREF SVKYPHVYQL GQINHYFNVI KFSGWILNAI
IQSAVIFFMM TVGLIPFPYG LIADAPTLGI MLLSVFIIVS CKLVLETWYF TKITLLSHLI
SIFFFIITVC SFSSSPIYSA NSIGSAFVLF TSYRFWIVIL GTLMSMYRDY FYKVFKYSFC
PRRYSPEVND HFNNPRNVGS FDKDDPSVGT AIVGKAACGD VIKLQVKIKD EVIEDACFKT
FGCGSAIASS SYVTEMVKGK TCKEALAIKN TDISGTFLLY SYKVLFLGSG WSSVFFIKNL
NPKLFDLTVI SPRNYFTFTP LLPKILSGTV ETNTSTEPII EYMRRNFNNP QFIHAKCVDV
DSDAKSVTCD PEPSFSVPYD FLVIGVGAQT NTFTKGVEEY AYFLKEIEHA EVAFQKIVDN
FRAASMPSLS DSERRRLLHF LVVGGGPTGV ECTGELSVLM SRGKCYPELM PFVKVSIVEA
GQRLLPSLSQ STSKFVLNVF NKSNVNMYFG KVVSEVKQKS CVLKEEEIEC GLVLWASGLK
ETDLVTKLKR KWNIPESSRA LLVDQYLRLQ GLNIFCLGDC CKITPVQITA EQILRDAVEW
QTKEVKTKQT IADEEELNFY KAQKRKEFED TLRRQRHHIG TWIKYAVWEA NQQEFRRARS
IFERALLVDP NNPSLWLRYI ETEMKNKNIN SARNLFDRVV CLLPRIDQFW FKYAHFEELL
GNYAGARSIY ERWMEWNPED KAWMLYIKFE ERCGELDRCR SIFNRYIENR PSCMSFLKLV
KFEEKYKKVS RAVKCVEVLD PELLDEDFFI KFANFEQRQN NIEGANSVYE QGLKLLDKTK
SEKLYDNFIS FQKQFEFIDD LISVKKRNEY EGDIALNPDN YDTWFNYIKL EERIVQVYER
AIAVLLFSLS HGGLVRGLHE VAKALDSKTA QVCFLSKGCS EPAYVKLVQA LCKEHSIPLI
ETDSKTLGQW SGLCKYDIEG KPRKIVGATS VAVKDFGEES EALVFLGYYQ AMIGEVMNDR
YSVISELAGK GVFSSVLKCY DSVENRNVAI KVIRNNDMMI KAAEKEMDIL RRLNKKHIVQ
LLTSFRYRGH LCMVFNWYWG NLRSHLKNKK VLEDFFELVL EQQNNLNGRL NIQRKYIEDI
KQLYINNLFN GYVMCIGFRG TTKKYKPALN IGTFIFIQLD NVNYNNNIEL SCITTDDNKN
WSTNETYFGQ LSGGFLFPVP LNYIKILYGE DNLVTQLLNL KYEIVLGFNG GALGFNQQMP
DDSMLFTRDA LLDKIAVILG GRAAEDIFIG KITTGATDDL SKVTKMCYAF VSQWGMNKEI
GLVSFQRDNT DDPYFYRNYS ENTAQLIDQQ VRTIIEDQYL RVKKMLLGKA ELVHKLSKLL
YDKETITYQD IVQCVGLRHQ RVSEVQKAPI DYVTLRGVKN KALKACENCG AMTHDSKSCV
ERPRKKGYDA TRDRWSGFDP STHLQLVEEY RDLEQERALN KIMNIVTKED IITFKESEYE
DEAKMLGHSQ IWGSFYDVEK GLWGYKCCKI TNRSQRCIIP LMDPYYANYY TREVMLILIN
EFNTPDEEMK SIVLKVVRQC VSTEGVTAEY IKSDLLSPFF SKFWIVRNSL DKKNSDLLIE
TTVEIAQKVG ILEKLVEDLK DPSEPFRRMV AQCIEAIITN IDILEISTRL EELLIDGMLY
AFQEQVNEDS GVLLDSFGTL IHVLGARVKP YLPQITGLIR WRLGTQSART RQQAADLISK
IAPVMKVCDE LQMLNHLSLY LYEYLGEEYP EVLGSILCAL KSIVNVVGTT EITPPIKDLL
PRLTPILKNR HEKVQENVIE LIGRIADRGG DLVSPKEWDR ICFDLIDLLR ANKKSIRRAT
VNTFGYIARC IGPHDVLSTL LNHLKVQERQ LRICTTIAIA IVAETCLPYS VLPAMMNEYK
IPDQNIQTGI LKSLCFMFEY IGEMSKDYIY SIVPLLEDAL MCRDLVHRTA AWTCKYLALG
VFGLNCEDAL IHLLNYVWPN IFETSPHLTQ SVFDALDGFR VSLGPSIIFN YTLQGLFHPA

RRVREAYWRV YNNLYLGHQD ALVPLYPLIT EGVERKHQSN ELLYMGRMYG KGKGISSSSI
PYGRKPPSWL KTKPFEVEEQ IAKLAKKGQT PSQIGVSLRD SMAIPQVKAV TNNKILRILK
AQGLAPEIPE DLYFLIKKAV SMRKHMEQNL NDKDSKFRLI LVESRIHRLA RYYKKKRQLP
ATWKYQASTA GTLRYVEKSP GVQFAHVPAD FLTRDDDVDE DLQKQIFDEG GGITTLSTRK
RVSTTHRLRR RDNKGEFEFT CDNLVAVLHV LEFLYLSGVS VDEQDNNGTS ALMYACKRGQ
LFVVQWLLRR GADISHRDHY GATVLHYAAL SPNLDVLLFL AQNGLAPLTW IKSISNNDQT
PMDLCWEKAN HLRYMLLWLF WLQWKVFGKV FRFNMFYPIL YWIMNLFNLA LVIPMYRSLK
DSTNFPNSFY SCLAFFLATN ALWIINKLSD PGEGVLESLH RRQQMVNITT EMRSVYPQVA
DERCNKNSIN YNKAVLELKI LQKLTTPFIL RRLKKNVINE LPIKYSNFIP CHMINYQYYI
YQSFITTEVT NTKSTIINNK IYKLRRICNH PLLIRKIYED EMLPKISKII KKLHEEFHEY
NLNKIIEYLF TLSDFNIHQL LLQCLQYDSN LNEIKINKKL YLESTKIRKM LELISNIIKK
KEKILIFSQF TNYLDIIEYI MILRLDGTVT LIEREKIIKK FNDVYILLIS IKVGNVGLNL
SIANHVILMD QWNKLLLQEE TIKQMCQYII NSNCNLIEKG VSDLAQHYLV KANITCLRRV
RKSDTNRIAK ACGATIVNRP EEITESDIGY NCKLFHVDKI GDEYYSFFDQ CINTKACSIL
LRGSSKDVLN EIERNLYDAL SICRNIIYNC KLLPGGGATE VYISNYLNQQ ISKKVGLKRL
SYECASKAFQ VIPKTLAQNC GINPVKLMSE LLMLHGEIHM GINGETGEII NVINHNIYDI
YLVKSQVYKS SFESVSSITN TQYLAVLTLY ENCIQNPSGI IRKVLKKEVR KHPPLPLDTI
EMNKDVSRYL RISSHKCMQL AEGLYNKGFI SYPRTETNVF PSSIDLKSII GKLSSVPEFS
EYTNTLLNED PTKGNNNDEA HPPIHPVNSL HWLLYEYITR RFLACCSKDS IGHQSNVILE
ISGELFNLKG LIIQERNWLN IYKYTTWEAK LIPNFSENQE ILPNEIILKD GATQPPDLLS
ESNLIDLMNK NAIGTDATMH EHIQKIQDRF YCIKDDKLRF VPTNLGKAIY YGFKEYNYQN
IDLTKPILRA NMERDMSDIS IGILMVMSSK PDLLDMYKVV IISYDLMVRI KELKEFNAVI
CDESHYLKNK SSQRSKRVVP VLKSAKRAIL LSGTPALNFP SELFEQIAAI IPGFSSSHLF
IDRYCKKRTN WFTKRIEYVD SKHTNELHLF LISTVMIRRL KNDVLTQLPP KIRSKIPIEI
PEKLIKTTKL FQLTGESKTK GVCKYIEEIL ENNNKFIIFA HHMFMMDAIE DTLKSKKVGY
IRIDGSTKIN DRARLVNLFQ NNNGVRVALL SLTSCGVGLN LTSSSTVIFA ELYWVPGVLL
QAEDRVHRIG TKFNKININY LIAQNSVEEV MWKVINKKYK TVTSTLDGET GTLLISDGDS
VCIGVSGGKD SSVLAHVLSK IKEKYKMDWN LYLLGVDEGI KGYRDDSLKV VFKDRFGFSM
DQVVKLIGKR GNCTVCGSFR RQMLEIGARI FGANVLCTGH NADDMAETVL LNLFRGDLLK
LFSTECIYSP EAYRGHMRSF IKNLECQICG IDSINVQQLH NLETNQLVST CNIEVNCGYS
DIITLPLENE YETIGPIKIL SFDIECIKLN GTGFPNASND PVIQISSVIH THGNDTKNFV
FTLKECDSLA NCTILSFDNE EQLLLAWNDF VIFVDPDFLT GYNIILFDLP YLLTRSSVLN
IERFKKLTRI KSVNCNFKDS ISNNNILGLY ENKEINIEGR ILFDVYDLVR RDYKLKSYSL
NYVSFEFLKQ QKEDVHYSTI LKLFNGNNND RRRIASYCLK DSILPLLLIN KLLLLYNYIE
MSRVTTTPIK LLITRGQQIR VTMQIYKQCK KMNYVIPVIT KNSSNENSYE GATVLEPLKG
YHKNPISVLD FQSLYPSIMI AHNICYSTLL NYNGYNDICF ISVNKRKGIL PIIVENLINE
RKKAKKMMNE CKDEMLKKVY DGRQLALKIT TNSVYGYTGA TSGGFLPCID VATAITSFGR
NMIVNTKNLI EEHFTVKNGF KFDSKVIYGD TDSVMINFGT DDIQEAIDLG KNAADLITSK
SVKPITLLFE KVYKPLLLLS KKRYAGLYYV NSEKYEKIDC KGIESVRRDF CLLIQQMLEQ
ILYLLLVKLP SLQIAPMLDV TYLQFRQFMR LLTRKTQLWT EMFVASSLIN SEESVSRWLK
FEENEHPIVA QLGGNCPETL VEAGRILKKF GYDEINLNVG CPSPRVSGKG CFGASLMREK
ELVRDIVHNM IKELEIPVTV KTRLGVDESD SYEFVKDFVS TVSQSGCEHF IIHSRKAWLK
GINPKKNRTV PPLQYEKVFR LQRDFPHLKF TINGGFKTME SILDALLNGV MIGRLAYENP
CILSNVDKII YGVENPDTCY TRRILLEVYA NYIDENVEET ANSLIVKPIL GVFHGEQGNK
IFRFMNVLYG SGPITQVGII VMRNKICNVI TQFGTNPDEQ MELLSNILKD GPEGSSSLQN
GLEMCLKIMC ELPYYMTREI LIIFGSNKTL DPGNILITLD KLKQNFITVN CISLSPELYI
LKCGIFMVSP PDISRAFHHL IPPKSFHKIM SSGCTRISQQ NYYSTHNALV ANDDQSVRFV
LHELEGTRHG ALRIFPFYDI QMIKRLLIKK LSLPTTRVKD LRILYKGQEL PNYRTMDCYK
RDDKLYWCLK QSPTGSGIRP LGMKMNSKMQ DLFNEIAISM KNNIKPKLTL DGTGGTYEIH
NKNGKCVGIF KPCDEEAFTP YNPRGYTGTM NQQGFRPGVL SGEGATREVA AYLLDSTYKN
FSNVPTTIMV EIAHQSLNNC NTNNLTNNGS RLKWKIGSLQ EFVVSRGTSG NYNYNFFSVE
DVHKIAILDI RLLNLDRNDC NILVTNTHLG IEQSRRDDME ALGYVLLYFM RGSLPWQGLK
ATSKKDKYDK ISEKKIAIPV DLLCLLTNSA SFKSSIIIDR KVDLITPMCT NFTYEGLLDS
VFGLKCNTVN VDLYEEIRWL DFSKVGSYLH EKALRVKKGY EGGGMQTIGE MGEFVKKFKS
LQQEHVTLST HVNIMGYLNS FVKSERFHLV QNIEDCILQG STDGNKSKLA NISKLWLELI
FWNIQVFTIF RLLILLSQTM DGKKAIIYQY GFQYLKTIQN FIKSGLIKIN NVDSSRWQKI
YGKFNLLVDS ESDCSGIFGG YAPLSIRIVQ LSNDSAPLSS EFGLLNCQIV STKQDEAVVE
SVCMSCGENG TTRILARKIP HFNDILVMSF ECSFCDNKNN EILNISKLQN LGVSYNIHVN
NPEGLNNQIV ITNTSAVKLI DLEFEIPKLD RKGIVTTIEG LLTNIINNLY LQKLDKIKNR
LFTLFIDDPS GNSYVEYERT NEHLEKMGYA FFFVNCTNCG FKGKNQICEI AIPGFDKCII
MSFVCDNCNY RTNELKPGGG IKEYGKVWHL KINSVDDIKR DIILSNTCEI SINELELTIS
PGSLSSLFTT IEGLINKIIE NLHSTFPFLM YVINRHGEKE DVSFDKILNR IRKLSLGLHS
LVDAPRVTQS VINGMYTGIR TSELDELAAQ TCAYMAVTHP DYSRLAANIT IDNLHKNTLN
DFSEVIRVLH SYKHVYNTNA SLISDDVFEF VMENKDRLNA EIDYSRDFQY DYFGFKTLER
SYLLKTNGKI VERPQHMIMR VSAGIHCGDL ERTIQTYHLM SQRYFTHATP TLFNAGTKHP
QMSSCFLLDM QDDSLAGIFN TLSQCAFISK SAGGIGLAIH KIRASGSYIR GTNGISNGIV
PMLKIFNSTA KYVDQGGGKR KGSFAIYLEP WHADIFKLLD LRKNHGSEDQ RARDLFYALW
IPDLFMKRVE ANKNWTLMCP DECRGLYEVW GDEFERLYTQ YEQQGMGRKT IPAQKLWFAI
LQSQIETGTP YMLYKDACNS KSNQQNLGTI KSSNLCCEIV QFTSKDEVAV CNLASVALPK
FVNTQTRTFD FKKLYEICRV ITYNLNKVID RNYYPVKQAR ASNFRHRPMG VGVQGLADTF
MLMRYPFESD EARELNKRIF ETMYYACLSE SIDLARQYGT YESYEGSPAS KGLLQFDLWG
AKVDNSLWDW DKLKADLREH GLRNSLFIAP MPTASTSQIL GNNESFEPYT SNIYYRRVLS
GEFFVVNPHL LNDLIDLGLW NETMKQKLIA YNGSLKHIDE IPSHIKELYK TVWEIKQKHI
IDMAADRGIF IDQSKSVRPD EILEDLQKFG VSVSASAFRN PKPEESLGLY SLAIQVVFGK
TIHDIKPEEL SGKLYEFSSE NVDFLKNAIG NLRLWRYSQR LHKVLGLGEI ERYLFNPTAK
SFNELISAFI NSLVNDTMLS KSKARFTFDD LSQHFSTNIE TANRLYSELN AEYISQSQRL
EALVSDVERL NTLHGSLVET LETFELLKSN LEEHIIPHIK SLELSKRLSS QTESLDWWTL
GILIYEMLAG YPPFYDSNPQ SLYDKILKCK LKFPIHYDED AKYLTSRLLV TEPSQRFGNL

HKGIDDIKKC KWFESMDFDA LVEKELSPPH IPERKEDLPA EEFIDSYRMP KEVTGEDDPF
VDWTISQFFI ISHSGDVLLS RNFRNETTKN VSNIGPIFEL EGMLYFYIRR SNLYFVMSTR
YITSPSYVME LLNKITNYLK DFIGILNEET IKSNFVLAYE ILDEILDYGY IQCISINQLK
QKIYNTMLPS VVSNKSLINP NNKNEIFVDV IEKVEGQIQI KSYLKGSPSI QMYISNNVQF
SNNTSRTSNK IVIEDYNLES DVMKFIPKEG EYTILNYKIK MSNLFNVNDI VSCEIQRISS
TGTIMLQTRT SKYGKLENGI LVKIRPNLVI RKGKHIYDMV CEKQNFITRV CTIRSILLMF
SSKLIKVNFE VIEKAFIKEW KVQSTKRVKL ADNNDTQLIL VLTGGEIIYF QLTDTEVLVE
VGRRNLSTEI TCLAIQHSGT KAEFCCCGSI DNIVRIMKLD KNLKLCSSQI LGNNSLPESV
TLIYLYVGLN NGVLIRNTLD MIGNLIDQES RFMGTKPLKL KLLKQCLILM SIKTYIIYPN
NNNLDILPLY INTVDSIDTF NSLLCLNGFV CILGNNLKIF RCIINGDVFS EITIPLEYTP
RKLLLLPSPS LILIVESDYN SYNIEQVEEI NKEMMSIKLK NYRAGVGKWS SCIRIINPIN
LETIAKLLFT ENEAATTAYT CILNSIQLLI VGTIKNAHLY SCIRVYEYDS NYNIKLLHIT
NTKGWIRCFN NYENKLLCAI GSKLRMYSLG KKQMLLKGEH RSLTSGFMDI KVISRIYCGD
IRESVQLLFY GEDLGEFELT TTSTGPRWLS SMELLDYSTV IAGDKFDSIF VSRVVEEKPN
ITYNDIGGCK DQLEKLREVV EMPLLQPERF VQLGIDPPNG VLLYGPPGTG KTLTARAVAN
RTDACFICVI GSELVQRYVG EGARLVRELF QMARSKKACI LFIDEVDAIG GSRGEDASNG
DHEVQRTMLE IVNQLDGFDA RGNIKVLMAT NRPDTLDSAL LRPGRIDRRI EFGLPDLEGR
KHIFKIHSRT MSVDKNIRYE LLARLCPNST GADLRSVCTE AGMFAIRARR KSISEKDLID
AISKVIKGYK KFSATGRYMV YNLLKLSCEN AQLLVNKIFG LADLPSNDEI VMPRVFPLPK
PREKTRWEKF AEMKGKKRKR SRKVFDPTVN DWVPRWGYKS IKKNKVNRPP IMEVKPGDDD
NVLDVESAKR SLVKMKQKMR ELRNKVFERL TDHRFFTGSH RERFPRQRRP MVPPPKLVWL
YRNGDKHHDG VSFYIRPYIR TMKTLLIEIG KELTLIAGPV RKIYDQNDGA KYLCTSGEPP
ASPEKLEKFL NSSFSKPGQR YMTPPQGDGT RGFYESLYEE NPNSLIAIKF CVEYGIFGGS
KHDEVLHKYT QLQKHGHFKG TSGAIKPSAI TFLNVKFIHE KKLIKRFFDE IAHDTGKYVY
GVYDTINALE NGMIEVLIIY EQLEIMRVLV KNPSTNTESV LLLNQEQERD EANFKDNNVD
LEVLDKIPLI EWIINNYHNY GSTLDFVTNK SQEGSQFLDL QAAFLEFITT LYPGLNKVVE
VLSNILGGPA ANSIVKLLTV PIKALSLKAL ELSYNEKLIS FLSWEMRKEM SYNLIDELVT
TNILMDELSS FEIFFNLVSP LFDVCDQFSI YKDLTERILK SLRMKHTLPC LVNCSLSLLM
LELKLNNAVV LRRIFDCIRD LITDGNIDFD ATGMTLQALD GNHIALVHLK LHESGFVLYR
CDRPRALGIN INSVTKAFKS TTNNDSVLIQ SEEDKDLINF VFENNVEDRV SSFSLKLMTI
EQDALSIPTE GFDAEITLSS KEMTNICKQM NEFSDTIKMD ISGNSITFST EGDLGHGEIV
LRNRAPSGDC GVTIRVKNPI KQSYATKYLL MFTKSGCLSD SVTFGLSQNR PIEVKYEVRD
GELKFYLAPK IDDDCPMGCM DEPASIKGTY IYTRDSSICK AAIHSGVLNS GGEIVVIISY
AQEHFYGTSM NGVESLSLSR REKAFMVSKP TGFNGNHADY INTKDLPGSK LIKTFRDATF
IFEIGVGNWS TIFSFQSCGG FLCSIDNFGE LVLQENCKPE LVKTSYFPTM GKRAHISILY
YVLTKDISVF VDGVPVTNQL TTFDLNFQGD LILGKMADTD SDYFKGHILG FQAFDYVMSP
EQIRQQYASS QNRLTTEGNV CLTKCMKKFF GKYDVNATNP AIKLDCSDTI ESENFNGPGK
SFLISCTKSC IDPDLTLKGT KVYTSDSSIC KAALHSGILT VLNGLSEYGN CHGHFGITSQ
KSNVPCQDTA VFVLKMHIGT RVLLDCPPGC GTGVYSPTSS LCQSAIHSGK LDNDGGEVEI
EVEGERNTFD SNESNGILSQ SSGHYLKSQT KLLSLVDQGT KNKALKLNLP YGPYFVDFSA
NGRYLLLGGE KGQLSLICTQ TYKDFFDISV RSSEDLCSHL RQHGVRSVYL KRPTIIYLFL
SYSKIQITNF QLTYKLEYLY YHYLLVTVGE FGDLCYQDIS TGEVVAKHNT KKGPCKVMCQ
NKNNAVIHLG HNDGLVSLYV PNMEKSYDDC LGFDGYWKVW DLRKEAVIRQ YVGNPPTCAT
VSQTGILSLN IGSRVEFYNN VFDGPYLKHH FNSQEIKSVA YQPYEDVCAV GTTFGMSNLI
IPGSGYPNFD ALEHNPYETG KIREVQRLLE KLPADSISNK GNLVCLLKEL PNDSVLKEVV
FSPLDVLSLP YKSYVVSRFP TWTQDESFCI LRVMDEIKVW KNNASNQKFD RGVLRIYSTT
QLEKPIFNKE FDASEEGELF WNSKGTSVLF RTFAVKGLAS YYGANSLYLI NLNNSKFKTI
STNDGIIHDI SWSRNGKDFL LLKGPMPAEI DLYDGVGLKT LSFGKNNRNT VKRDPFDRLV
LMGGFGNLRG EIDIWDMKTK KKISQSKSEC SVSCEFSPDG MYFVTATTVP RMRVDCCFKI
FSYSGKLVQR VDFEELYHVY VRNPGADAAL LSAASKIKKK NRESEPIPLP NIKTNVLNKI
IEYCKHHYNN PPSQIPQPLK SAQLNEVVID DHIPSIDRLT QKMTQDTEMA VVLLDRRLLL
ESFAKIPTSL VHNTFMLSTP TSKVKLVGVH PKLGNAIVLE THPILDYTQD LHLELQKLMM
KQRRLNYFNT LNNFVELCMQ NSSLLTTESK RDARDPLLRV FASALNEWML LRRCIVAAYE
ESFAYTGLSL PLQILRILEN ETKIFFSRKR APFVFYVEMG NLDEDIELIA PADKQRLIFP
ELNPQSAAAL LNSPVVQEML TQISSNPELF RTLVESSPFL QPMMPMFGQM LNNPELLRTL
MRPGMLQAGL PPEERFSSQL QSLQEMGFTD QAANLQALVQ TNGDISAAIA RLLNRTSGDR
LTRAGKVLEQ LTDQKPVFSK CRFTIRSLGV RRNEKIACHV TVRGQKALDI LERGLKVKEY
ELRKKNFSDT GNFGFGIQEH IDLGLKYDPS TGIYGMDFYV QLVRPGYRVC KRRKERVGEK
CAYRLCSEIA LDSVIDTTHQ HLLLYFMALS GDHQVSQIRL AKLNRYSVHL LRLIKIHLGI
IFLVKCVGSN YQNMMGEGCV AIACDKRLGL NQQVTVSSNF PKAFKVTESC FFAASGLATD
VQTLKDEIMF KVNMYKLRGD KEMSVKTLSN MVGSMLYSRR FGPWFVDSVI AGPYITCFDL
VGAPCTPTDF VVAGTCSEQL YGVCEALFKP GILLVGAGGI GCEVIKNLML NGVKKLTIVD
MDTIDVSNLN RQFLYLPEHV NKYKAEVARM RALEINPKSE VKSLVCDVNS WEPNDLLQYD
VVLNALDNIK ARSHINYCCI QSGVPLIESG STGYNGQVYP IVKDMTKCYE CDPLPKTSSI
PVCSIRQIPE KPTHCIAWAR MLYQLLFGTP DNNNLLDLSP TLPDLNNLDE PVVVDYLNRI
FDFLFNSEVK SLLKMEEVWI NRDPPLNNSD MVGSLIFSKN DEVCVDFVSS AANLRMINFG
IKPLSTWDVQ SIAGSIVPAI ASTNAIVASF QFDYCTIFKP SASRSSSGWV LVLDFGCTFS
STLVRAVREL GVRCELESLE KFKGLDKLPS GVFLVGGNES VLSVEKSLLD TKVPVLSLSF
GMMSVAKSFG AKLRKTDNKD YVVTVYLNTL DMVESVPSGF EVIAKHSEVP VYNSDYNLFC
LYFHPESVDT EKGNTILHNF LVCKCDRTWT LSEYLERELK NIEEQGSDKY VVAALSGGVD
STVSAKMVQS VIGDRFHGVM VDTGLMRHNE IAECRVRKEV PGIKLTVRYS QNVFFKELAG
VVDPEMKRKI IGRVYIEEFE KAIKELGFNE KNCLLLQGTI YPDIIESELN RRKLPVKSHH
NVGGLPERMK FELIEPVRYL FKEEVRELGR LLKLSEETFK RQPFPGPGLG VRVLGALTPE
NVQKVRMADK IVREEVEKSK AVSQYFCIYL PLKSYGTTVV VRCVKTKDYV TAKWVHFDHE
VLDRISQRIS EVPGVNRVLL DITNKEPATI EWEMPLEGHQ PGVSESQRKP LVLYTSDPEE
IKVDSILSRF LRDHQRQGVQ FIFDCLMGLK GFNGRGCILA DDMGLGKTLQ SITVMWTLLN
QGLDNKPAAR KCAIICPASL VNNWESEIKK WLRGKCPCTA VAESSKEKVI SSFQGFKYDR
TSKVIISSYE TYRLHCSYLE GVNIDLLICD EAHRLKNDKT RTSQSISTSS AQMRLMLSGT

PIQNDLNEFY SLVSLCNPDV LGDVNNFRRN FANPILIGRE PYATPAEQQK ASERLAELSN
INQFVLRRTN ALLAKVLPPK IILNVFCNLT DVQKDIYKSF VNSKRWKNIM NQDRESRALS
AIQSLMKLCN HPYLIKRGGL MSGKFLVLFR LLYQIRKNSN DRVVIISNYT QTLDLFERLC
KECSYPFERL DGGTSIKKRH KLVTTFNDNS NSFVFLLSSK AGGCGINLIG ANRLVLFDPD
WNPANDKQAL ARVWRDGQTK VCYIYRFFST GTIEEKIYQR QICKDGLSSM LVTDINELKD
SLSGEYLKNL FEYKEEVLSD THDLIECKRC KTIEPAGFTF FLLKEKFKSI KTLGFVRNFI
AKEKNAYELL NCTDSDETAK IKANYRRLVL LLHPDKFLLL QDAFTILSDP DMRLEYDSNL
PWSRVKPVPL LGTNESDDDY VEEFYEFWRC FETLRTFSHA APHLLEDAES REEKRWMERE
NLKVQKKLIK KEQLRIQKLI DITQQYDPRL KRRQDRIRNE KLIKKQITKK LRQHMRMYDE
MKKFSTQLYD ILWTKEDLKR LSKGVEINPA GTPGRWNLIA KYVKTKTAPQ CIEMSKLIAN
NSDIWSESQQ TVMIRRLKLH LRNIPMPFKR RNAGRSKHGR GHVNPVRCSN CGRSVPKDKS
IKRFNVRNIV DASAQRDIRE ACAYSLFNVP KLYIKQCYCV SCAIHSRVVR VRSAQGRKVR
TYVQQRQMQV KKKVSVTEIS FDSSYTILDT SEGAVIIHVG HVVFHLDFAT LAVIKNVDMD
ETTKAFALKV AFDAITKFEV EKDIAGHIKK EFDKTYEPTW HCIVGKNFGS FVTHEKHCFI
YFYLGKMAFL IFKNGVFGWC KSMRSQGGRL LFVIVNDGSC KPNLQIVVHN DSKGYKEALK
CKSGTSIQAN GFLVNRVTQD DSNYIQVLGL CPGTYPIAKK DLTMEFLREN AHLRPRTYLI
SAVMRIRSSL SIAIHLFFQS KNFHYLNSPV ITTADCEGAG ELFQVTTMDF KKDFFKKKSF
LTCSGQLSAE NYCCSMGSVY TFGPTFRAEN SHTNRHLSEF WMIEPEMTLV DLPGLMELTE
EFIKFLVNYI LKHNYDDLVF FNNTVDKELL SRLYNIVNKE FVHLSYTGVI DILQEYIQPF
ENDVHWGIDL QSEHERFISE KVFNGPVIIY NYPKQIKAFY MRRNDDDKTV AAMDLIIPKI
GELIGGSQRE ERFDYLEKSI KENKLNMQDY WWYLDLRRYG TIVHSGFGLG FERLIMMVTG
VQNIKDVIPF PRYSGHSLFV KDMLEESLAG FRDCIRNLFM LDSKRFYLIS ALYDTEVNWK
VLRKGLNVNK LHMGDENDML SLLGVGRGNL TPFSAMNDTE NKVRVLFDIR LKSKVVAHPL
SNDQSVVMKM EDMLKFLTQI NHFPILGITV KKHENFAEWY SQVIIRSEMI EYYDISGCYI
FLPSSYFIWE TFNEWFNKQI KQRGVENCYF PMFVTKEKLE AEKTHLEGFS PEVAWVTRNG
DVDLPDPIAI RPTSETIMYP EFARWIRSHR DLPLKLNQWN SVVRWEFKQP TPFIRSMAKL
SKSEKKKLYF ERLTNLMKTY SKILIVSVDH VGSRQMASVR HSLRGMATIL MGKNTVIRTA
LQKNFPDSPD VEKVTQCVKL NTGFVFCEAD PMEVRILNNR VPAPARQGVI APSDVFIPAG
STGLDPSQTS FFQALGISTK IVKGQIEIQN EVHLIKKDDK VSASGATLLQ KLNIKPFSYG
LKVEKIYDSG AISDASVLDV TDEDILAVVK LGVSYANALS RQLGYPTTLS VDHAMLEGFK
NCVGLVLDSD YTFPQMAAVK QFLENPEAFA VATPSAVEEE EEDDDLGFSL FDQGRDISRQ
PCPDRIVEDM GGAFGMGSVG GFLWHFIAGA KNSPRGLILK NALYTASSKS PVLGGNFAIW
GGTFSTFDCT FQALRNKEDH WNAIFSGFVT GGVLALRGGL KNASRNAFIG GVLLSIIETV
SIVVNKRIAV TPRQHFQRQM EYEKMLVDDL GDVTITNDGA TMLKQLEVQH PAAKLLVDLS
ELQDQEVGDG TTSVVLIAAE LLKRANALAN SGIHPTSIIT GYKMALRESV KFIRDHMSLS
SMGTEVLMNI AKTTLSSKLV GFDSEYFAQL VVKAIKTVKT LSDDGDYKYP VGRINVIKVH
GKSAKESYVV NGYAVLMGRA SQGMPLAVKN AKIAFLDFPL KQYRLHLGIQ VNVTDPQELE
NIRLKEKDIT KERVKKILDS GCNVVLSSQG IDDMSMKYFV EAGVIAARRV PKKDLKNISK
ITNGKLLLTL SNLDGEESFS SEYLGTCESV EEKRIGDWDA LFFNTSNNSS SCTLVLRGAN
DFFINELERS VHDALCALST ALEQNSLVPG GGSVETSLSI HLHNYSKSMS SREQLAIDEF
AEALLVIPKT LSLNAALDAT EHVSLLKSYH AKYHSDREKY KEYKWYGLSL SNGKVGNNLE
MGVLEATMSK VKSVKFATEA AITILRIDDL ITLEPERNPP VYKLLGHTGS ILDIDFNGFN
ENILCSSSDD CSIKIWLQGH RMKVTNLKWN PTVDYALLTT SFDCTAKVWD ASNGKEIFST
SICEHPSSCS WTPNGDKILV STKEANVSLI DPRSGNCCTK FKAHDSNKLT NALWLGGHYG
DDLFTSGFVD NKTRQIRVWD TRKLDKHLIS NDIDSSPSPL IPHWDPQIGL IVLASKGDLT
VRIFQYIDKE LNRAGEFKAT GSMKSFCLVP TDICDRTKCE LGRFLFNTDC KQINLTSMFI
IRRNTMNELY GEEYGIEKNL GNLTIFEKME LIGQFNKSFI ITKLYVIDQH AADEKAKYSE
INRIDTIFNT SKVYVVNTLPQ ILGKVLGEDD FIDFLNELWG LGNIPRPHKI WSILASKMKL
LAIIWLKLLS DSYFSKQPNY LTHMTNNNTN NNNQFIHITT NTLTNKCYTY PASLIPNGSC
ASAGAIIPHR RLKNNKPIKN ITGVDILCQA KSGMGKTAVF VLSILQQLVS CVGISHTREL
AFQIKNEFDR FSKYLPQVRC EVVYGGVPIQ KDVAMLKTPH ILVGTPGRLL ALVKAKHLNM
DSVKHFVLDE CDKCLEKLDM RQDVQSIFLS TPKKKQVMFF SATMNNDIRE LCKRFMQSPV
EVFVDDESKL TLHGLLQYYV KLAESDKNRK LNDLLDTLEF NQVIIFVKSV SRAVTLNNLL
TECNFPSIAI HAGLDQSERI NRYTQFKNFD KRIMVATDLF GRGIDVERVN IVINYDMPDS
TDSYLHRVGR AGRFGTKGLA ITFVSSPEDS SQLEDVQKRF EVNISEIPAT IDTSLYLNQR
LSVSAEVFGD NHSKSKVVIP NYEKTPQEQA KIMEMIKRCF LFSGVNSSGL DLLVKAFDFK
TANPGDVLIK QGDDGDKLYL IESGTVEVTR KNTGQEEFLC NLTAGDYFGE LALMYNSPRA
ATVVAKTEMH LWTLDRTTFN HVVRMAVIKK REKYDSILSK LDLFKKVNPY DRCRLADALV
ERTFEDETVI KQGEPGSSLF MVLEGQAESF VENKLVKSYN PGDYFGEIGF ILKKPRASTV
KAKGKCLFVE LERENFINLL GPMEDVLNKN IKNYKKVLEE LMHVLLKPNN DEVKALYQNH
KSFYEGDCGL DLFCVEDQTV EAHDTSIINL GIRVSASVGW LMFPRSSMAK TPLRLSNSVG
VIDPQFRGEL RLSLDNIKDF PYTVKKGDRL VQMVSYDGEP ITFEVVDELD ETQRGSKGFG
STLGAAYGTA RSGVGISSMG VMRPDLVMKS IIPVIMAGVL GIYGLIISIV ITGDYIRAFM
LGFELKDAEA ILRLEDIFIE SFEINDVKRL HGDHLSRCIG RISGKDGRTK HAIENMTKTR
VILANNKIHI MGSFNSIKLA RHSICSLILG SQPGKVYNNL CNSAKRLRER IQKLENKLHT
WSSLDPFRAA CSLSYEDLII LPGYIRDSVD KVDLSSNVTR NIKLRIPILS SPMDTVTESK
MATAMALLGG LGVIHNNLSI DNLIKEVKAV KRFENGNELL FMSKKGVLPI VNEDYEYPYA
SKDDNKQLLV GAAISTGLEV AKKLIDAKVD VILVDSSQGN SVFQIDLIKQ LKSAQIIGGN
VVSAQQAKNV LEAGCDSIKV GMGIGSICTT QNICGVGRGQ ATSVYYVSRY THGVPVIADG
GIKTSGDIVK ALSLGASCVM GGSIFAGSKE APGEYYFNNG VRMKSYRGMG SKDAIISQGV
SGLVIDKGSV NNILPNLTQG VKHGLQNIGA FSVKELHEAL YSGLEKDKSL QEAMEKLERI
SEYFEKTRDF SKDCINKLVL AKESVWDRFG SKIRDMPFLY EFFENPIISK LFGDTSLASA
LREMKRLDPS FNLPDLVELV EHVIAPHVVE SYLKGDGEAL KLHCGEVAFN ILNTSIKERN
LQKLVLDPSI LILKNVELKG GMKVKEGDPW LIFNFTTQQI NCLRDTKGKV VLGQIDDIRE
VVYSIAISRH PNPENLEYPY MVIQYIYTKQ ITILMVLSIV GLGLGDVEDI TIKGFKAIKD
ADIVYLEIYT SFLINSKHKL EEYYGKEIKE VDRIFVEEQN DTLLNEAKDK NVALLIAGDP
FYATTHVEIY YKAMNSGINV NVIHNASILN SVGITGLQLY RFGETVSIPF FEENWKPFSF
YDKIMQNYNN NLHTLCLLDI KVRERSVENI MKNKLIFEEP SYLTVTHYHN YYYNFTLRFK

IGKIDFMVIG IARLSSEDQI IKSGKLEDLL NFDFGPPLHS LIVCSPHLHH YEQLFFNHYP
NSAYRKSVRV QLIKNGKKIT AFVPRDGCLN FIDENDEVLV SGFGRSGHSV GDLPGVRFKV
VKVSGVSLLA LYKEKKEKPR SMGIKGLIPF LSEKVPSSIS ELSLECLSGE SLAIDASAAL
YQFTIAIRDS SYFSSLVNSK GESTSHIYGL MNRCSKLLEY GIKPVFVFDS KPPELKSKTL
DKRRQKREEA KTDFKKAISE DKESAKKLVG RTVKVTKDMN DSAKKLLRLM GIPVIEALEE
AEAQCAYLVT KNLCHFVASE DTDTLVFGGW FLLRNVVDLQ KVLDGLEFNF DQFVDFCILC
GCDYCDTLEG VGPKTAYSLV KKYQSLEEIV RFKGFKEAKD YFLSPKVYDE NSVKMGTIDP
EGLTEFLVQE NNFSKERVEK FIEKLLKFKT KKIQTSLLSF LVDTEKLYKL LDLSKDCSDS
EIKKAYRKLA IKHHPDKGGD PEKFKEISKA YEILSDPDKR RIYDEHGEEG LDGSYTATDA
SDIFDLFFGG SRKGKKRGED IVSHLKVSLE QIYNGTMRKL AINKDIICNG CDGHGGPKDS
FVTCTSCNGQ GIRVQIRQMG SMIHQTQTTC SSCNGQGKSL PESKRCKNCN GKGVKQTKKI
LEVFVEKGVP DQHKITFHGE ADERPNEIPG SVIFIINQNP HDTFKRNGND LFMTKSIPLY
QALTGCTFYL THLDDRILKI NTPAGEVVKP GSCKVITGEG MPIYKSAYGK GNLYVTFDVI
FPRTFSPSEK EMLLLFPFTP ETPAKPDTQV DEYTAQHFDL DDYKSSLGKM ANEFGTLLKT
LVDNTKYSSW DYMSMDRTHL EDDNAEDEET NTWGNTWTVR KGSALLLDTI SQLYAQEVIK
ILLSYIQEKL DSTDWELKES GVLTLGAISK GSLYTLYPYL PKVIDYLIVV ATDPKPLLRI
ISCWCLSRFV EWMFLPNTYL SKTLSVILRG MLDRNKRVQE SACSSFTSFE ECGTTLLLPY
AGQILHVILS CIELYQSRNF MILYDVIGTL YQSLGESITQ QAEHNQLIDV LLNRLEIVGL
GDVQYIGLIE CLSSIISVLG SKLPQFVQKI TKHCVSSLCE LVGDDTISIL LTSTQGSILQ
SCIALMGDLS NSSIQLNQLK VLVPNEQSSI NSSSTGVVNN CVWVFGVLCD NQLGIYNSSN
IDLVFLLVVK VILCSNNFCI LQNCCVTLGK FSNHFPNVAI KYLNSFLNPL CKHLIHSKND
KEKFNLFKLY ISCTKECIKP DELLSLLAQG LCKVDTVNIM SKIGIKSVIW IADWHAMLNN
KFGGDLKKIR IVGEYFIHIW KAAGMNPDSV KFLWASDEVD KNPDLYWRLS MDISRSFNIT
RMKRCSQALG RTEGDDQPSA QLLYPAIQCA DIFFIGADIC QLGMDQRKIN VLAREYSELR
KIPRSPVVLS HRMLPGLVEG QEKMSKSNPN SAIFMDDSAE EVSSKIKKAF CPPGVVDGNP
VIAYFCFIVF KRFNSVTIER KEKDGGVRMW VLEEKVDGVN LSELINTTHE NKKYLPGIKL
PDNLLAVPDL NECVKDADLF IFVIPHQMSQ FMGRVSKLAG LGAASVVVPY LCLFDVDGGE
RAVMFNRFGG VSKKTFGEGS HFYLPWFQVP YLYDIRAKPK VINTTTGTQD LQMVSISLRL
LYRPLAEHLP RIHQKLGPDF DERVLPSIGN EVLKAVVAKY NAESLLTQRD KVSKDIREAI
TARAMQFDIK LDDVAITHLS YGKDFSKAIE EKQVAQQESE RVKFIVAKSE QEKIAAIIRA
EGEAEAANLI SKAVQTHGSG MLEVRKLEAA KEIAETLSNS KNVVYVPNNL NMLYLNTYMS
QKGGWDFSNY SRNSRIQNSI HNYLKTGTTI CGVMGRDSVV LAADTRATQG IIVADKNCSK
LHKISDNIYC AGAGVAADLE HTTLWLANNI ELHRLNTKKA PRVQMCISML VHELFKYQGY
KQCALILGGF DYTGPHLFSV SPHGSSDSLP FCTMGSGSLN AMTVLEQNYF DGMSVEEATE
LAVKAISAGI TNDLGSGGNV DVVVMDKNGS KHSRTFKKVC SRTYAATAGT TEYLREHIEH
IKKHISLNLL GEARGEVLSS TLDKVTDNLS GQTVVDPKGY LTDLNSTEFE EADVQKARTL
LKSLINTNQK HAQGWIAAAR MEELAGKIEA ARELIAQGCE NCPDKEDVWL EAARLEKPEY
AKSILAKAIK IIPTSVKLWL EAADKETSND NRKRVLRKAL EFIPNSIRLW KEAISLENET
NAYILLLEKA RTRCNTVREN AHFAMSKALQ ELPDSGLLWA HSIFLEEPNA QKTKAAEALK
RNQNSPHIVL AAAKIFWNCK MIDKARRWFQ TCITLDDSNG VSWGTFIAFE LDCGTEESMK
QAINKFIEAE PNRGWIRGRI HELRGKGGIC FLILRQQREL LQCVIDSKSN NTKDMVKWTS
TLSFESIVDV YGKVVVPNMP ILSTTSSSEL NVSKIFCISK SSTNLPFLLR DANNTDNNNP
NVIKVNQDTR LDNRALDLRC FMNNIIFKIQ SIVCQLYREF LLSNDFIEIH TPKLLSGSSE
GGSSVFKFKY FEQDACLAQS PQLYKQMAIC GDLKRVFEIG PVFRAENSNT HRHLCEYVGL
DLEMELKNNY MEVVNLIDEM LKFVFNGLYK LNPIQPFQFL NQTPKITFQQ AVEMLNKDLN
EYDFTTEHEK LLGKIIKNKY NTDYYIIYQY PLNVRPFYTM PLDERTIWSR SYDFFMRGEE
ILSGAQRIHD SEELEKRAKE CGIDVNTIKD YIQVFKYASF PHAGLVYENL AREYKNVITQ
DNYDGFRLEA DRQLTKNLQA SHSLYLGTKD VGYIYQIGAN YASDDGKKFL MFKVGLDGNI
ALKAFAKLGD ELKAVTNSRL KIDNQSTFEV GADYLSDYWT ATLKCCWQGG SEVTYIDANG
ASIGSLSARY VRGDNIFTCQ LTRQPDFKRM DFSKKEINCA RVQYTRKVND RLSLATELEL
SPSIKESALR VGWEYLFRHA RVQGNIDSCG RIAMQTQDYN GFGVSGCIDY WNNIYRFGMM
HLLPQPEQDG GVGKTTLVKR HLTGEFEKKY IPTLGVEVHP LKFRTNCGTV QFNAWDTAGQ
EKYGGLRDGY YIKGECAIIM FDVTSRITYR NVPNWHRDIV RVCENIPMVL VGNKADVKER
QVKAAHIQFH RKRNLQYYDL SARSNYNFER PFLWLARRLL NKPQLVFVGE CAKAPEIAID
PLLVQQSERE LEAAANVAID DDGGIMTLSK LNNIPSDGDY NFKKLIEDKI AENARFRVIN
VDRSMAESLY GESIYDDFVV PATVTLRLVV LDEWNINANI HNVLYSTGLI REICINKFKY
KPETNTLDIH FNVIPASEDC PLKENVLPPG VVDYNKLTFQ FGCSLITNHI LNRVKALVVH
PFLRRKMFFC HRDFDQLLDN YEKGNKFFIY TGRGPSSEAL HLGHLIPFIF TCWLQKSFNV
PVVIMLSDDE KFVFREELEL IEVVDLRKDR MDFILLNSDV STANAIRRVI LSEIPSLAIE
IVTVLENTSV LHDEYISHRL GLLPIDSTLA SEFEFRDRCQ CTDKCAKCTV DYTLDVSCNN
SRLVTHFDID DTGKNLPMPI PIPIVKLKRG QSINMKLTAS KGLGKFHAKW IVANVNYKME
PRFSFNSALM EQLSSDEKAG IAASCPRNVF KYLQVVNKLD CIYCDECINY CRELGHKDLI
RIQPDESKFH FTIESTGSIP PEKILEIALI VLEKKLQDLQ SNFEAQSRTL GSTTTAHHLY
DLSVLDDNDT RALLTFLNHL VTKKPNFFTN LVKRTVGLSE ETTSGLTEMR KLYRNHGLLF
PIYSTNDVVC KTKFDNNYGA RYSSVDGMHR GVEVLLGGRQ VVVIGYGNTG KGVCMGFRGA
GAIVKVCEAD PICALQCVMD GYQVVLLEDV VETADIFVTA TGCIEVIRLH HVRRMKEGAI
LGNIGQGDRE ILIHEILTDP NLEVTEVRKN VHYYKFKDLN KVIILSYGRL YNLGCANGHP
SLVMSMSFTT QFFALSQLLE NKKFDNKIHK MPKKLDEMVA RYHLEFINMY PYSSTPKTYD
SMQFNPSGPV DPDYEVASPR GESHTPFVGF FSSKLLRSGF TLQTVTLSLM FVFYWAFGGT
GIFVFDLYAA PESVKVSKPF HLTVSTLMAL YLLGTLYIAM FQVFVTDNSK SVRGFRAGSK
ILSAAVTLDL LSNMLRLVQY VYSYFFMSMK WWTRYQQTKA DWIFFQFGSF SNSFALVMYG
AAFFYLEAYH DEGTSEEVAW INLTLFSLAG LAELSILKKR TFRTFKYRGY ELEKLLEMPI
EKLTELLPAR QRRRFSRGVK RQSLTLLNKL RAAKKDLPYG QKPEPVKTHL RNMVVIPEMI
GSIVGVHNGK QYINVEIKPE MVGYYLGEFS ITYKPVRHGK PGIGATHSSR FIPLKVLVDT
FFWRDPEQFE QKPENTEDLM ASRPNVDWST VDPSHANSDW GLLQIIKDHH ENMYELKQKI
NPKEQVVGWF CTGSEMTELT CAVHGWFKQF NSVSKFYPNP NLYEPIHLLV DASCDSASMM
IKAYVQLPLT ITKDACFQFH EVDLELLVSP SDTAGISLLK LKDLIDKCIK LVENAMEGNS
KIYPEVGRFL LKTVSTEKLM NLKKIEKICE LSLQDNLMVT VKIHIQTWQT YNSRSLSWLK

NLPLKQDETE GKRVHKELME LVLSNNQTIL GADNSNLGQL AKIFISIYET DFSTEELNTL
ILHLMKHLGQ DFLKQLSLSK RLQMQLKVIN ALELMEVYDS HQQLKNYSKL LKGAPYYPVI
RDSDRNVCSL PPVINSHRTR ITVNTRNIFI EVTSTDFNKG SIVLNQLVSS FSKYCDEPYT
IEPVLVQYPD LSRRSLRASV SYLSKLVGIK ELTAEYSCDL LGRMMVDSET IEAMVPITRS
DIQHPCDLGE DIAIAYGYKN IKRNRFTMGN LLKKTLLADK VRFVFTSCSF KETLMPVLDS
FKSSYEMMCK PVVIKNGQLS ENETVRTSLL PGLLKTVHYK KGSNLPIRLF EVGEVVWEDS
DVGAKNNTNC GCVYANTSSG LEEVQGVSEL LLNNLGFISE YQVWEYNEFD KPIPVSSFGN
IKGPDVPDFG AFKVSNELFG WKNKRTGEVL QHRSSDVSSI TFVKTNSNLY QLRIELNESK
QFKVLRFDGF TEKNVLDLSK HFEENYKMSC DKDEVSCTGW HWGTYEFDNT TFRLRINNNS
GLEIDAQSII QATIPSKTDL AIELKNANNS DDLVEIRFCV PSKEDAEIKL EDLKQTFLVK
SGLDEMKSEK IALLMDIPLI VPRGRYEIEF TKRSIKLHGK SYDYTLLFTN IIRMFLLPKP
NSPYINFILG LSQSMRQGQT RYAYIVMQFE SDHETKVDLN LQDNDLKQYK LDKVLEGKTY
NVVSRLFGSL VNRSIVVPGD FKSEKGDSAI SCTYKATSGH LFPLNRSLLF IVKPVIFIRF
EDIVSVEFSR TGVTQNRFFA ILVSMRGGIE YEFTNIDKTE FKNLNEYLMT KDIKVKGYID
LSKRRVTPED IIKCEERFSK SKKVHQTVRH IAQKHGISVE ELNRECIWPL YKSYPHALDA
LKEAAANKDN VFKNISISQE VIDSLLQDIQ LRLVPQALKL KCSIDVWCFG PEGINAVKMS
LGKAKPQISI RLIAPPQYEI LTSCFDKEAG LALMNQTLEV IEKNIKSFIG GDFKQKCDVV
VILLELHETT DEEDEGEKRV FEYFVCDVGV GKSLEVRVSM PVEYDSYFLG VLPQNTFKHN
YIIYDSSQIL PRYLIQFEFD PSADESFALP LCDNCQSDVS TIYCPSDSAR ICTKCDVRLH
SNKVVSRHIR VPLSEMPTKC KIHQTKSYHL YCTVCETPIC QLCTVNHIHI PISTAYEAVV
NLNSTTDVSL RERKNQLLEI LNKVDEVKNK VSENCQNVEA QCYEKLESAL SDLNRMVECS
LEVVSTEQTE NKRQLNEINW SESFVDHMRS TLLPADYLRS WLRHCRLRDE FAQNSELFPD
ISLEAELSII PKDTHAHLVG LPNVGKSTTF NLLSKQCVPA ENFPFCTINP HEAVVSVPDD
RFDHLCKVFA PKKEISATVT IFDIAGLVRG AHKGEGLGNA FLSHIDAVDG ILHVVRAFED
DEIVHTDGEV NPVNDLDTIN QELILKDLDK CNKAISEVDK VQRNMKIKSK KEELDTLIKV
KEHLESNKWI SQGNWKSSEV PYINEYNFLT AKPIVYLVNL SENDFVRQKN KWLSKIAKWV
QENNPGPIIP YSAQFEQSLE AFTEEALKSY LKDKNNAVSK IGKIITSGYH ELQLIHYFTC
GPDEVRCWTI RNGTKAPQAA GMSNMNITRG FICAEVYNYQ DIVQFGTESK LELMGDICRR
GKDYVVNDGD IIFFKFNVTA SKRLQQIVDL LTVSLNDLKD VSHNFYSELM HGLKAHRRHR
NLWLPNECSF KMLDSFIPNI PTGKEKGSYF ALDFGGSNFR AVRIVIDGDG KMERNQSTFS
LRYSSALGPK GLLDQKATAT ELFDHFAKKI EHVMRESGVD PNPSVHKVGF TFSFPCTMLS
PCNAILLDWT KDFETGRATN DQVEGKDVGL LMNEAFKRNN INAEVSIVLN DTVGTLLSCA
YQKPKDYPPC RVGVILGTGF NICYEEDEFR RFGYVGRVIN IECGNFDTEL PLNPVDFEID
FYTSNRGRGK LEKLVAGAYL GEIIRRFMIL YLREQAPPKM WEVGTFTSVD ASEILNDNSD
DLSLSRQVAM RAWDVELPKK SLIALRKISE AAFGRSAGFA AASICATARK AKAVAIDGSL
YVKNEWYRNK LQYYINVTRP DLVGNVVLLS SDDGSGKGAA IAAAYLFIKW LCRVVISSIF
TNITIIHEER LPLYGPLIVV SNHNNQFVDA ALLIYAIPRQ MCFLVATKTL KRKLIGTLCS
LAGCISVYRP DDFKYTGVGK IHWKKGTNLI HGRDTKFTLD LNLGDKLQFD LEKIVITEII
SDTQLKLETP LQLECPDNGV QFSVIPKMDQ SETYEQVSAS LKHGNAIVIF PEGGSHDRTN
LLPLKPGVAL MAFFSILDGD VVILPVGLVY NNVKKDQSDA TIYYGNVISI TREECAEFER
DRRSVVTKLL GKIEQEINNC MITAPDIKIK KWIDLCASLY PPERSKVPVN IAFDLRKFIS
KIFWKYGDSP ETLELVDKLS VYKKRLDNGY LHDDEIWLLK QSMYSAFLSF LEDTIRLICY
SIIGLSFAPL WVPLYLLSNY LAERHRVKAL KNSSVKLVGT DVFVSYKCLV LIVVVPLLNF
SLGLFIGLYR PMEQKDVLSV HKLLRKYLQK YKLYQEFDVH EVEHQFMPRE DIIQTFVKTN
EDEVTDMVSY YSLPSTVINN RKVHTIRAAY SFYNIATTMP FKSLMEHAIF FAKSQGYDVY
NALDLMENSL VFKDLKFGMG DGLNPECILG MQAILISGNY CEQEFIRIGY YTNNVYDEES
LVENPPDLPI LDKIVRCIID QPRVTRFPIK WDEAETIHLK LSNILSDYYF RIFLVNLDSN
CKSKPRINSC PSVAKCHVDR CNSREVPLSI MGNRKEVDLL RNPPSYTGYS GQDDWELKGG
TGYKYFGAAK NLPGVRELFE KQQKEVEQKD VSRAELYQMI NPDYYGFKDE LDEDLLIQEY
NKEQELMVHG TLVIPHERAR SCIDLLSRHT NIQYIDMNER RMDRPYKKYV QRIDHMERMI
RVLYEEIAKL PNSKIVRHNI DNFLEHDNMY RLDQVEESLV KLYDQFQMFK ENDSLLRLER
DEALSEYYVL LVASKQLSPS PYDLSSRTSS SISFTNIAGL ISSQEKEAFS RAIFRAMRGN
VFTLLHDLRA KTVFVIYCQS SNNNATYNKI KKLCTGFQAK LFNWCKTQSE LAPRLKTLED
VIKDKKRALE AYKEYFRSEI ACLLEVIRPG GNSVIEEWFL FCKKEKYLYY ILNHFEGSDI
TLRADCWFPA DEEEKIREHL LAEKASGSVS ALLLVDIAPL SHIPPTYNKT NKISKSFQNV
VDTYGISRYK EVNPAPFTVM TFPFLFGLMF GDIAHGFCVI LFALFLILYY RKLKRGDIAN
MILEGRYMIL LMGIMATYAG FIYNDFLSLP NSFFGTPVVF GLDSAWIGAV NEQSVLHSFK
MKFSVIFGFF QMTLGIVLKG FNAIYFSSVL DFFFEFVPQL AMMCSFVGYM NFLIFHKWLT
PSGYAKPSII TTLIDMCMMK TLEPHEIMYE GQQTVQRVLM IILILSVPMM LIPKPLILYF
TIKKELFIHQ FIETIEFTLG TISNTASYLR LWALSLSHQQ LSLVLFKQLI LNCLDSSTLF
VMIFGLFIRS IFFSVFTFFI MLCMDSLECY LHALRLQWVE FQNKFFKADG RFFRPFNIKL
LLPPFYVHEL AKVLTQRPLI YLSTIEKVCY DVMDYEDEKF NFIQINLLNN ASPTPIRSLL
SDKQETFVVV PGIIVQANRT QHKMRVCTIQ CRYCGHKMII EVPLWISRPQ IPKTCRYVQN
PYVIIVNECQ FVDVQLLKMQ ELAEDVPTGD MPRHLQLNVT RYLCDKVIPG DRIYAHGVLT
SYNNSPNSHT DINSSYLHVL GIQKLNNETY EFDIDEGNDL LLLASQPDIH TKIFNSIAPS
IYGLDDVKKA CACALFGGTR KEVGNGAKIR GDINILILGD PSIAKSQILK FIDFIAPISI
YTSGKGSSAA GMVRYGLMNE NEQKLDFVLG LTLSKLMERR LQTKVFKLGL AKSIHHARCL
IRQRHICVHR QLVDIPSFLV RVDSEKHIEL ALTSPYAGAR PGRVRRKTLS HSGYDRHITI
FSPEGKLFQL EYALKAVKNC NLTGLAIKDD SAIAVVAQKK LPAQQGNQDV LLDTSSVTSL
YHITDEIFAL LVGLPGDCLS ILYKARQVAL DYSYKYGINI PASVLCQKIS DLNQVYTQHA
YMRLHACTGK SLTEEDLSSL FKEFGEAMEV FVLKDLCGGN KGCGFVKMKY KEQALHAIKE
LNGKKMLEGS VRPLEVRFAM NKTGLSTVSV SSKDLPSHTE LKRRTLSELS ETKKLLKEVE
KAIELTKEAI DLDNRKEYKR SLDMYIRALQ QWSMICKCET DTNLRDKYFN KMKQYLERAE
NIKSYLNINK NSNEIQWDDI VGHDGVKIIL KESILLPMKF PKLFSNNIIN YNCILLYGPP
GTGKTYLANA LSNEFKYHFL SISSSNILSK YYGESERYIR NLFNFCILKS PCVLFIDEID
SICNTRNVNQ HEATNRIKTE FMIQINRKMT ASNVVLLLGS TNLPWLLDNA IIRRFEKRIY
IPLPNQNNRD FLYMAENTNN FNCYDINILV KEIVLYALKK ALYVNAGSAH EGVTSMIENM
AFHSTAHLSH LRTIKTVETL GANVSCNAFR EHTVYQAEFL RQDLPFLVNL LVGNVLFPRF

LTWELAANKH RLSEKRNKVL ENPDQLVTEH LHSVAWHNNT LGNFNFCLEP SEDKYTPELM
RDFMLNHFYP QNCVLVSVNS GLDELSKWAM RAFSEYNPIP NPSGEVLEPK YTGGVKYVEG
NTPFTHVTVA YPVKGWDSKQ VVVTTLLQSI LGGGGSFSTG GPGKGLTTSL YNNVLNRYEF
VESCMAFNTV HSTSGLFGIY LVVNGAYASQ VFTLVKDEFE RMKRITNHEL SGGKNSLKSF
LHMSMEHKAV LCEDVGRQLL FCNRVLDASD LENLIDEVTL DDLKSVVNEL RVNNPSVVVY
GKLSKVPHPD TVLQLLDKGQ NLVLFMLVET VPLVNHGNES ITRCKQCRAY INPFVRTDAS
KRFWICNLCE TSNELQTRYP GSNQANELEL NCGVIEFMAS ADYTVRPPQP PSYLFLIDVS
SNAVNSRMLE VVCKTIKELI DNRTLVGLMT FDSSVHFYQI SRGSENYQLL VVADLEDLFL
PLPGEVLLNL QESSEDFLKL LDTLPSLWKN TTTTGSALGS AIRSAHYSMK HVGGKLIVFA
ASPCTFGDEK CKDFSCMMCQ TQTTLDLFVC PQSLNLDKLQ YMSTMTSGNI YYHPFKNHAE
NFKLVNELKH LVRRTTVWES VMRIRLSKGW KVTNWHGNCF VRGSDLMVLP TTSEDHSYTI
TFSNNTSSAG KKVMYIQTAL LHTNSSGERR IRVFNTAVGV SNDLGTVLNS VNVETLVFNM
LLGAVKVYQS GKMADARNHL TTHCSRVMNS ILGSADSARV LSLYVLGLLK STIFTDQDYR
VYLSTKFRSC KIDQVILYAY PQLYNLSNLQ GLPLSIESVL QECFYLLFNG EYLLLWVGKN
VIFSVFNIAT FLEYFNLQIL PSSMRGLEMS LYFTSKDNSM LSMAENLGLV SFIPIWGALC
DKIELKYILL FGVIATGLIN IWLSTISNYS LILILRIFNG GLIGSVTPSA QKFIATNMQN
KLPFGFGILH AVMCLGRMIS GLIATSFSTE VYRDIYGWRI VIFIFGATSL ALSPILLLVV
RRIDSLNNAT SLLLALLNFF SDGPFVAFNY VTLLIQYMRL SNVVSAVTTG LTILGGIIGG
IIGAFISGYF DRKWPKHGLL NFGILNVGIR ITTFMISFLV IDVTNIDYGL LVCLIINGMT
FMTVSCVDRT LLANVVMPSV HSSSISIIRC IGGVLSAVIF NPVLAKLNES VFHFQSSSLA
VKHMPFDLIK KNSDALRYSI SIISLGTTGV VLLLYIIIHF TYGKDCEKIK KRIEKEMLVF
GLRSLSDLCN PSNQLYVENA LEALERNVVK GILTAVENFG DDEDLVLCST TIMSTMSQGC
VEYEDEKLLK KLVNEGGVVQ KALERAPQDE DVLENCLKFL ENEFVPHVCK CMEVVKSYRV
ANRLVVCLSN LTDQNESCLA LKNSQGVQPL LDLCLDKSTG LVESCFKTLV ALSQLKLVDE
SNLQSVINLV ECCKDSKVVL SRASEVIKSV VDTEKLQNSL KVLEKNQYDS PEYKVAVNTL
RSLSYISTLS DELAKKGVIP ILLKLLSSGD AKPGASAEQL AEVVFGTSRM LASISSNSEY
GQQVLSNKGL DVLVRALSQS TQHPRSVVGL SFALVQLLKH EGSAVSVALP ILYQLSEDEA
VSQALVEFLL ACSQYSELES VFIQNKVLEI LSTCCQYHTS NLAYQFNVVS ILNRFSRFIE
NLKLIHEYGG LQGITFALEQ ADVILELMLH YRDNDAVIDL SVKILDLVLE EKDVEKYAKR
LNTASVKDPE GTFKALAALT GVHKISRLRP LLSKYNPLNG VLSAVTGWLD HNGVRQRTNL
TRAALSLVVV SELACVYQVK KVIDEQSDDN FLLHCTGAIV VLCGIDRTYD EAEMQESVEC
VSKVMKRHQD VRQAQASLLE ALNRLMEQSD FLSAMLNTGC LGLIVKYLST VPVYLNLQIL
GIGLIHKCSQ LDPQVVEFLK TSNCFQLLRT VNRTHTKNKK LKAIVGSLLS LLMPADALES
ELDQLLKDLV NYVAEKDAEG VNTCLVSINQ LLVSKEAVKA AVMLNILAEF SLLGLNMLVS
RMGLVYATKN SFTKFFLTLF TCLTANSKVM MEDGVVSNLD GLVLLFRHDV TNIDEALKED
LLAKLSGCFT RILNSPAVVS SVCRCLGSMC ATPGKLDKLL NNSNFSKFCN YLVDNLSSPE
KVVETTLVAL NELLSTKYQI LIDYFDKNTK IVSLVGLLEE YYKTYEIVVL ASNLLSYFDK
RVVLSRNLSS FLETVSKTLL YNKADVGTVI ALLTLLVHLL DDTNKEEFKK TSVVENISTV
MLMHLENDEV SRLGGILFSL LGAECQISAL MKNVIQVQAK EENMGQKVDK LCLMLAMYLS
SELKERSEAL KHTEPFLSSL NDCVSFLADN SNLLSSTCCV SRRLCDSAFE DHEDPFGAWA
VASSSNMAQI SSLVKTEYGY SNLKFLVHAF RVFTACVYNM SKEAQVLARK FVDFLNATGS
PFHTVKQLSL YLTNHLIKHL NEWKLENGQS YYVTNNNGTM MAFNIGKKFD PSKGGLILVC
SHTDSPCLKL DFKCHVNNKG FNQLSVLEEK LLHVQKPLIL LPNLAIHLQN STEALKLNKD
NHLKPLISTE VVHNLPLLKL VSSELKCEVE DLVDFELCLM DSNPSCLSGV YEEFVSSGRL
DNLGSCFGSI SAFTDPALIV IYSSSVKASL RFASLGNWGT SKTQKLVAEK LKEYVKNERL
TYLLSPGFNF NNGVWKKYFE SVYNDDLMDL PMFTVLGSED WLGDYNAQYN RYHQFYPRLI
MPNWWYHFFT SFSTNASVSG HKDLSVGFIF VDTWVLSNQF PYKDVTNDAW NELKKTLEIA
PKIVVVGDKP VLSSKLLPLL KQAQVDAYVA GYDQDMELLD YEGTALVVCG SFCVHELNAE
GFTTKFVNGN TGELKLINKL PDVMFHPVGD LDVASYSDAF TKIIGTLGLL IRYAFSGIAY
SVGAEGLDFN WCLIPDTGIV EPDVVFYMIK ILDNDVVQRR DLGVNYFVRA SSVGKEASAC
LHNLKDLNRN VKRIGFIACD TFGMIGSVFV DFGDNFISFD PTGTELKTGI IESITNDKEG
LVTLITDGVI DFQTGDYVRF SEIEGMELNN EPVQIKVNSK NSFLIGDLSH YTPHTSGGLV
TEVRYPKRIE FRSYEDYSLV NRAEQLHVES VDEELFKSFF SQVNFKVPPL ASFIGGIVAH
EVIKFTGKYH PINQWLYVDF SLRYFDQVSL WGSDLQNKLQ NSKIFIVGAG ALGCEFLKNF
ALLGGLLTIT DNDRIEVSNI SRQFLFRTRH VGLSKSSVAC ESALEINPSI KVKPLEIRVG
EETEDIFDEH FWSSLNVVVN ALDNIQARQY VDGICVWYEK PLVESGTLGT LGNVQVVVPH
MTQSYSESQD PPETSIPLCT LKHFPYQVEH TIEWARDVFE GLFTQIPLDI KKIERLELIS
KLLNCTPKNA KEQLLRISSE LYNLHFVNNI QQLLNSFPKD HVLSDGQKFW SPPKRPPTPL
TFDLSDKIVQ LFILSTTKIF ASMMNLDLDV VESDILSLLR LPEFQPRVLK LSQDAVEFEK
DDESNYHIEF IWSASVLRCR NYAIKECNKM KAKLISGKII PAIATTTAMI GGLVTIEFLK
ALCYISHFRN AFACLATPIW LQSEPLPPIP TKDKDYDPVT CGPVRALPPN FTVWNKLIVL
IQFWKVQRPK KETGTVVLQN APKKNMKVCV FGDAVHCDEA KALGVDYIDL EGLKKFNRNK
TLVKKLANKY SAFLASQSLL PQIPRFLGPG LNKAGKFPTQ LLHTDKITVL YNAVGFFLEV
ELRFLYACFM KNIFYLNKLL PYQHVEFLRA IPPFVMMVAS LLTHSFVTDM DKLNMLLWQS
AVATNGIILE KMANVDYLKS LGRDNLSLPE DLFKFGEFLD SLDESQKKKK TPKSRIIEFL
KPALIYIVMF LVIRVFLKKS TNYELELPLT LEELYTGTVK KMKVTRKRFN GNKQYKEEHT
LKIDIKPGWK DGTKLTFTGE GDQQSPMATP GDLIFIIKTK KHMRFVRDGN NLIYKFTVPL
VKALTGFNAV LTTLDNRRLT IRVTEVVSHK SRKVIAREGM PLSKNPNQRG DLILEFDVVF
PETLTNEQKA SISNIFDNYN ICVHCPGRLW YMSQMCKISL NFEIADSDVN YETTAKKHQV
ESLTNHLHVD TGKTKLLDKI RHSNVQNAEA GGITQQIGAT FFPKDLLDMH CHKIDMYVKS
PGLLIIDTPG HESFNNLRAR GSSLCDIAIL VVDIMHGLEP QTIESINLLK ARKCYFVIAL
NKIDRIYNWS STPWLTFRES LEKQPKESQL EFSDRTKQIM LELSENGLNS SLYWENDNIK
KNVSICPTSA ITGEGISDLL YLLVQLTQLL MSKRLTFSQK LKCTVLEVKT IEGLGVTIDV
ILLDGILREG DKIVLCGLSG PIVTTIRTLL TPQPLSELRV KGEYVKHSYI KAAMSVKIVA
NGLDDTVAGT ELFVVGEGDD VDELCNEVMT DISSIFDCID RTGIGVYVMA STLGSLEALL
HFLNDKKIKI YSVNIGPVQK KDVKKASIMR EKGHPEYSTI LAFDIKVTQD AEKEAEILGV
KLLSADIIYH LLDSFLAYMD QVQEERKQQQ IQNVVFPCEL TILPHCVFNK KDPFVFGVHV
DAGVLKSNTP LVAITKTLFL GRVASLEHNK KPVEQALKGQ EVCIKVVGEP NVAYGRHFDH

TNKVYSKITR ESIDILKEYF REEVAMDGWK LVAQLKKVFN IFMTTNAEDH LLKQDLSTLD
VAKLTSLTPE VISRQATINI GTIGHVAHGK STVVKALSGV HTVRFKHEKE RNITIKLGYA
NAKIYKCTNP EHEPPSCYKS YGSSKIDDPL CEKPGCGHKM ELKRHVSFVD CPGHDILMAT
MLNGAAVMDA ALLLIAGNES CPQPQTSEHL AAVEIMRLKN ILILQNKVEL IKESQALLRQ
QEIKKFISGT AADGAPIIPI SAVLNYNIDV ISEYLVTQIA VPKRNFTVPP QMIIIRSLKQ
CNFLEWREYK VVFKRFASLY FIACVDKDAN ELLILEMIQR YVEILDSYFC NVCELDLVFN
FTKAYHLLDE ILIDGDIYDT NKKGILRNMA AQDAMSEKTK FAKGLLEVAD TFELALKHLG
ESDPKKSTDF VDGIKMTEAM LHQTFEKFGI KKYESMMEDF DPQIHEAMFE VKDNDSHNKV
VQVVKNGYTI SGRVLRHLDL FMKKLLKLIV NGIKGGFMIA THDLDFIRDL VNSFIYIYSK
DKVFRFSDKF PEYLCFKSQF PDMTKEIVTL HVGQCGNNIG NEFWNQICLE HGINKDGFLL
DKTPIGDDKD VFFFQTGTNR YYPRALLIDL EPRVISSILN SENKNLFNPE NVFLSKDSMG
AGNNWGVGYT YGNQFNDELS EIVDREVDNA DNLEGFVLSH SIGGGTGSGL GSYLLEMINE
NYPKKLIKTF SVFPQLKKSS DVVVQPYNTI LSLKRLILNA DLVNVIDNNV VNNHSFNSLK
ENSTSFEENN IQIGNIMSSV TSCIRFPGPI NNDLISLVSS LVIIPRCHFL ISSMDHENIT
LLNLVKKLYY PNNFLVYTST KNGKYLSALN IIRGNNNPSD VYKCIEKIKE KRLVEFIKWN
PANIQVNLIK QSPHKQDKTN GILVANHTSI NQVFEDCILQ FDKLYSRRAF LDNYRKAEFF
KGDFEEMEHS REVVELVKEE YIRSQQDDYF GLARAFAIPV RSYTHEVVTL WYRAPDVLMG
SKKYSTAVDI WSVGCIFAEM INGVPLFPGI SEQDQLKRIF KILGTPNVDS WPQVVNLPAY
NPDFCYYEKQ AWSSIVPKLG IDLISRMLQL DPVQRISAKE ALKHDYFMGK EKTHINLVVI
GHVDSGKSTT TGHLIYKLGG IDKRTIEKFE KESADMGKGS FKYAWVLDKL KNERERGITI
DITLWKFETG KYYYTVIDAP GHRDFIKNMI TGTSQADVAM LVVPAESFEA AFSKEGQTRE
HALLAFTLGV KQMICAINMD KCDYKEDRYN EIQKEVCGYL KKIGYNVEKV PFVPISGFLG
DNMIDKSDKM PWYKGKILVE ALDLMEPPKR PVDKPLRLPI QAVYKIGGIG TVPVGRVETG
QLKPGMIVTF APSQITTECK SVEMHHESEV ASPGDNVGFN VKNVSTSDIR PGHVASDSKN
DPAKEANKFD AQVIVLNHPG TIKEGYSPVV DCHTAHISCK FEQIQSRMDK RTGKTLEENP
KTIKNGDAAM VTLKPNKPMV VETFTEYPPL GRFAVRDMKQ TVAVGVIKTV DKKEPGLPIL
VLKEGTDTSQ GQAQIISNIN ACQAIVDCVK TTLGPRGMDK LIHTENDVTI TNDGATVLKL
DITHPAASVL VDIAKSQDDE VGDGTTSVTV LAGELLNEAK AFILDGINPQ VIIKYYREAC
QVALNLIDKV AINLSNKSSS DKRELLVKCA ETTFNSKLLS GYKTFFAEMV VEAVATLDED
LDEDMIGVKK VTGGSCEDSL LVKGVAFKKT FSYAGAEQQP KKFINPKILL LNLELELKSE
KENAEIVINN PQEYQKIIDA EYQIIFEKLE NAVKLGANVV LSKLPIGDLA TQYFADKNVF
CAGRVDENDL IRTSKATGAS IQTTLNNLSV DVLGTCGVFE EVQIGSERYN MFTDCKSAST
CTIVLRGGGQ QFIDESERSL HDAIMIVRRA TKCNTILPGA GAIEMLLSTY LLHYSKSLVG
KRHIIMNGFA KALECIPRNL ATNSGYNSND LLSLLRNKYN KNNEENWYGI DCYKGSVCNA
YVECIWEPSL VKKNSIYSAT EAACLVLSVD ETVKNQSRSL FLPPCFKRLL KTDKIKRVIC
TGNVGSKEML EVLNDISPSL HIVQGDYDDD FDHPDTLTLS VGDLKIGVIN GYQIPTWNNK
DLLLKVAVDM NVDILVYGHS HVSDISKHGG KIFVNPGSAT GCYQPWQPNS IPTFMLMGSK
VVIYVYEEHD GEAQVIMTEL DNYSVPLEGS EEEGYTPVYR RPDYKDKLLE DYFDFLGKRV
KEDGTLGEFQ FLTYGEGETQ IKRFGSGLGI YSQNTVEWLI TEQVCNAYNL TLVPLYDTLG
EESLLYIVNV TKLNVIVCDY KCSLKLMELL PCTKDSIGTI SYTSGVSGIP KGVIIKHFQH
VSLIVIVNRI VCDEKENPKV HLSYLPLSHM FERLYIGTSI IDGSAIGLFS GDIKNVLEDI
KALKPNVFPS VPRVYMRIHD KIFSTVSQKS FLIKSLFGLG LHRKKKTGVV THRFWDKILF
SKFNMLLGGR VNWMLTGSAP LTPKIFDNIR ALFSIPLVSG YGLTETCAFH TERYEPDSTH
VGGPVPCMEF RLKSLPDYNY YTTDKIPKGE LLLRGHNISY FNDEVTNKEN KWFLTGDIAE
LLPNGAIRII DRRKNIFKLS QGEYISPEKI ESILNTVPII CQSYVTGKSH FLKPVAVVVP
DEFELELWSK KYGFNLDRKE QCNLKELQEY MSKEIEKVFH NSDVKGFEKI KNFYIEHEMF
TIENNLLTTT SKLKRYVLSQ VGQIGLILLL GNPLKVKVDE KFTIREVFEK KMSSEGIFLG
GEKYTFASYD PDMESGSFKF ECVCGAKNKG GCHLIKTPGN YIVVVVYDET RGQDKTNSLN
TLAVLYLACR ESSVSRSLRE LVIYDRSLTV KELGRAINRL KKVLPNRGNA PTEDVSQLMP
RFNLSNEFMT TCEAIAQKSF VLLWENVKTY FTDPTLLTLE VLFIVLTLSN VYVVHTLFVT
VLPAPLFVTW WQLAQGLWTA WVLGLKELFI PTVSYVAMLS SANVLLSKAP STAAFPILAS
GAVAAHHAAR FVACGEEYML RWKAVGFLLM AFVLGATDSK VAPGNVVTVA CLYALLAAVF
RAGCMERALH VVNGKGNALH NHQHLIGTLL LPLAIVFSGE LTVLRDLPFD PFSTRTGCFV
TVGALPFLKN VVSNRLIRQT GQAPWRCLEL LSVALLFVIG SAQLSPSWRS VLSTGFVLAG
RFLGAMDVVK NKERLVEGLK DATYSLANAV WSAEDFKSLV IESVGRPSVT LKLRGENIAG
VLLPVFSLHT DPTVDLFANL SLSSGGSAIQ SVKTTHLAAL DILVELASLQ YLPMFEWLNV
NAEAFYFLHD GNTSVTKINL IRDGTIKLDK DNLNCSINDS RVIIRKNEHL SGIICKKTVG
CSSGSLIHIL WHEAGPEKCR DFLTTLQKVV NNWFLQIGFT VSCSDIYCSE STLNKVYRIL
DKSKKEVQKL VLQAQKGKLK CQPGKSLFES FEARVNKELN DAREQSGSVV ASSLNLTNNI
LSMVNSGSKG STINISQIIA CVGQQNVEGK RIPFGFRDRS LPHFIKHDYG PESRGFVSNS
YLSGLTPQEM FFHAMGGREG IIDTACKTSE TGYVQRRLIK AMEDVMVQYD RTARNGNGEI
LQFLYGEDGM GAEYIEDQFI DLMRLDDEEI HRRFSHDFRS ESYGDFSQQV ILVEEFQKLL
DLKKMICEEI FPGGDYSQHL PINIKRILEY ATTQFPNPVE IAQRTSKLLE SLIIINTSGP
TDILSEEAQQ NATILIKAHL RCHLNSRYLM EHVQISSLAL DWVYGEVERC FFRAIANPGE
CVGAIAAQSI GEPATQMTLN TFHFAGVSSK NVTLGLPRLK ELINVVTNVR TPSLTIYLDP
SINKDQERAK EMQTLLEYTS FEKIVLGYTV VYDPVVDRTI IKEDYEWVRD YYEFPDEEMG
ALGQFVLRIV LNSKIMTDKR LTMKEVGEII YSEFSNGEID AIYTDDNSEL LVMRIRVKYG
EADFLNKFMT DVLCGIKLRG VKGITKVYMR EENCVRYNST VGSFDRVSQW VLDTDGCNLE
SVLPITCVDY TKTFSNDVSE IFHVFGIEAA RRALLREIRA VISFDGAYVN YRHLSLLCDI
MTQKGYLMSI TRHGINRADR GPLIKCSFEE TLETLLTAAV FGEVDHLKGV TENVIVGQLS
PYGTGAFDIM IDEVKLRDAN QTNAINVADT LGLVSPDSSP MAFSPTYTSL IVDRVMPGSI
RKAEHFISFL KVVIGYLKKY IKVKEPKSEG PLMFLYRFQN ETGIISDVFQ HTYNRFKSLL
NTLKMTGNLT ALHLVIDFCS LVGTYYKGFI IIVDPFPKSA AYDPVIQFSC LDASVAMKPV
LENFQSVILT SGTMSPLEFY PKILNFSPIL TQSLPMSLDR ECLCPIIVSK GDNQVHMTTK
FDLRKDITLL RNYGSLVIEL CKSIPDGVVC FFPSYAYMEL ILSHWYETGI LSSIMSHKLV
FIETKESIST SLALHNYRRA CDAGRGGLFL SVCRGKVAEG IDFDMHYGRC VILIGIPFQY
TLSRTLKARL DFMRCNYGIL ESEFITFDAM RQAAQCIGRV IRNKGDYGLM VLADSRYTRV
GKRSKLPVWI LKRLDLGNFY LTCESASSIG KAFIRRMSQE YISTKRTKNQ PPCHVSTLKN

GLRVATVWMP GSSSTVGVWI DSGSRFETPE TNGSAHFLEH MIFKGTKSRS RQQLEEQIEH
KGAHLNAYTS REQTAYYARC FNNDIPWCTE LLSDILQNSQ IDPDHMENEK HVILREMEEV
EKSHDEVIFD RLHMTAFRDC SLGFTILGPV ENIKNMQREY LLDYINHNYT ADRMVLCAVG
NFDHDKFVTL AEKHFKPYFV GSELLNRNDE MGPYAHMAVA FEGVPWNSPD SVAFMLMQSI
IGTYNKSNEG VVPGTIHAVA NRMTVGCAEF FSAFNTFYKD TGLFSKEIWR SRIGYIYQLM
IEDSGLILCP YHCSIINFLH YLRLSNVLDD YFKSLLLIEL LQRLPIYNLN RLYKPVSENL
TKYLNFSFEF TGKLGVRRKF QSFSIPQLVL LNEINEENDI YETPRFDEDE KTLSDLEQLF
LLSKGLNILS TTSKHDQLSL QFLNTISNTI LSKKRRLIML MSLWLRCKTE YNRTKTIERA
TIQLNNILNA YYVTTMQSGN RNEYFFQMSY PAIWNIKREI GKYMFMIGSI TTACNMYKEL
HMWEDVIKCL VLTQQKQQAN ELINERIKIM ATPSLYCYLG IQHYHTAWEM SNERCGRAVR
SIGVKYYNSG DFEKALEFLE KSIQLNPMNE NVQFIVGCCY LKLLKFENAI TPFSRVVSIN
ISSAHFKVSN YQSGKIAITQ ALKSNSTRWQ FWDILLRISA NLNDVKCACN CIQTLINLGM
KDKVEVWAVK YLVDSSINEL ESAGVWSEYS RYLSNKEDYV GALESKFKQY RKVEVLHLWR
NTLFVGLQAP NFKCEAVMPD GSFKEISLGD YLGKYVVLFF YPLDFTFVCP TEIVAFNDAV
AQFEQRNVQL LACSVDSKYC HLAWRNTPRD KAGVGQVKFP MLSDMTKEVA TSYGVLVDAG
LALRGLFLID KKGVLQHSLN NLPLGRSVNE VLRLVDALQV FETKGEVCPA NWKLGDKGMP
PTTEGVVAHL TTKMDDESYG IIDKDSYYKD PPREEDLSEL VNNTLESVKT DFDLICKLSD
YLDTGIDRKL LRILVNLCLL G

> ***Plasmodium berghei***
NFDGDFKTTK KKIHWLPYIP DKLINCTLYE YDHLINKEDW TNCINQNSKF ETVAYAEPAL
IDLKVSDKFQ FERRGYFIVD LIKIPDGKSK KVGLNTHNLP AIVFPTVVYV GDEAFFHESE
LSIYRPFDHG HISDWDLANN IWDYAISCVD PNKSVKSALL TEPPLCSISH RKNMGEIFFE
NFGFESINIS VSGLMSIYAA GLTTGLVLDI GEGVTQCIPI FDGYIEKNSV IRSDFGGEEL
TMFMQKLICD IGYNMTTRKS YEYVKIMKET LCFCSLNPPK DQLRDDLTVT YTLPDGDVLR
DGYSTIEISH ERFYVPEALF NPLLCHRDNL SISDIVCKSI LSCPIENRKI LSSYIILSGG
CSLFPNLVER LEREIKNNSP ENARSAVKVV TYEQGASFAK ENNLFFSEAS AVSKLNVKHI
FENLLQMEKS KLAKVEKVLG RTGSRGGVIQ VRAQFMGDSE LSGRFLIRNV KGPVREGDIL
ALLETEREAR RLRGTQSLNL KKSHCYCHLS TGDLLREAAE KQNELGNKIR SIINEGKLVD
NELVLSLVDD KLKSPQCKKG FILDGYPRNV KQAEDLNKLL DKNKIKLNGV FYFNVPDEVL
VERICGRLIH KPSGRIYHKT LNPPKIPFKD DITNEPLIQR DDDNEEVLKK RLGVFKNETT
PLINYYKNKN LLCHMCYFEL PDAKTTIGPY DNELNYFMWG PGFEWKPFDE KSSSGKISIE
NASYNARRLG LAPSSKDEEK IRDLYGDNLT YDQYLEYLSM TIHDKDNAEQ LVKMFAYFDN
TTGFLTKNQM KNILVTWGDA LTEDEAMNAL NAFSNDDKIR LTKLFGVIDK NQDEITAWFE
YVKNEVFLKQ VQIEMKQIDS DKDGFISLPE LNDAFSQNLK EVEKHADGLL KRFQIVDKDK
DNKLNINEVG LLIDPMKDND LKELEINEIL EHHDTNKDGK ISKKDDELAL DDFNFDANRD
GFIDREEIIT FDLWNEKALK FAVTSLTDYG DVIRYPQDFK LLSNLNCFGA GFIFSIVMFH
LLPEFFFVFV GFCMQLALEY VLPTDTNICC DSSSSEEEER VIKSSEGKRL DFYENIEDNL
NESMENDDFN QLLKEYENLY KFMVKEDSDR IPNFVIIYLD KLTKYVDTTF QNNVEKKNLS
KNKAQTLNKL RAKIRKCSEL YQNKLNQYLE NPEKFWSYSE DAEYASDEED DKTKKAMSKW
GLKTIEKVDK KKVAKKDDKG SHIDDNQSSK KKTYAELLNT KNLSEDVIRN RVKSVIEKRG
RKGLDKHEHI NILSKLCEIA KTISTQSYIE VLEHLINLEF DVVSSVYTYM SFNIWNKTFK
YIELILDLLI QNEHFYLVSI NITEEIAEEE SNEKEKISKS CKTLISFLAK LDDELLKALL
YIDVQAEEYR KRLGKTVHMI SLLYKGYKYV KAIYISTRIL DHLYYKPELP FKQIWGFVEK
TIEQERTEKR RLLSFHMHIS IELIECVNNI CAMLLEVPNL AKHSYESKDI ISRQFRRFLD
IYDKQIFNSP PENNREIIIL ATKYLQKGNW KMCCEKIFSL SIWPKFTDKV QAILKEKIKQ
EAMRTYIFRY ISVYDSFSID QLCVMFDLPQ NTVHSILSKM MVNHEIPACW NESSKYILIS
TIKPTPLQTM ALKLAENINE VMEQNELALN MKNPKLGLQT KQCIVVGTRA LEFLNNELST
IKTLTELHDR VKKRGISDNN DNMLSLYLLE LFSIPIEQKE KNDFLTNRIS MYSKILNGRK
NVLDLLLNKF ENDCSDSIKK ELIDCFDVEK FKQEVNSKFM NILIQQLRKV EKLEKKKAKM
EIYLSIREQV KLYIRELVNI ISNLLTGNYP ILNLNGDDFL KKYGGTLMEN LKDGLKIGQT
VRFLLAKEES SMFGIIQNIS NDARSKNILV NFFFRSSNIE EQIQVKSVEK ERLPVETLSG
DLFFLWYKMT RDDGWVGFDN AEIIKVFNNS SKIKDVLIRH TDLKILNQLA ITQFWAIKML
ISVPFATKIY AHFVHDSIKI SILTVLGDIA TALNKSFSKY LNFFANILLE TSKITIASGS
PENDDWISYI FELRDAILLT YSNIIYALID GKEINKLKMY IPNILDFIEL ILIKEINHFN
AQNFQNSVSL LGDLVHAYGY ELIENSKLTD LIISVYGKID ILSSQRDECV SKIKWLKKIC
NISILQLEFK SVDDLNNATN NFIKAIKNYN VFPELRLKIL QLLYNSFNVN FSFFIAILQF
SSQNNIFHAI LPYIKSIDDW IKEWNISNHE KRQIYLIVAE ELKKLKKYED SYKHLKKHIY
YFQEILNHAS TIKASVELVV DSINLNNNIF FHEIVNLDAI QNLQYIEHKP IYELLTIFYK
YNIQEFLTFI DLENSENKIY LLSIISLFKD NKVQNIQYIS EQLNINMLKV EKILVSAIGS
DIIDAKIDQI NKTVHMKTTI LRQFDEQQWE HLNNQIAKYI KNVATNFYKF IDSFASSTRA
IQNNPSYWCS AGNHLKDEEI TWTGYLNTKG FVKGVKVSWE YSPELVSIFV SSDGEHYKNV
IPYKKISSTE SSFDEIYFFK KLEEVISIKI GLKNAIHKYF GIREVKIIGG GNPYFLLLSG
ITSEQEMCLQ ELWKTNSNNQ IISALSDPPK CLSVINVDSL GDGKSNWVFE SNSQIRLQLC
ISQKNIHGNI PGIHDIDASV DATSILDDDH NADNTIDGNL NSFWASSIFG DNEHLVYFII
DLNKFVEVSR IKVFWEYPPL HYIISFSTEK IVAENLANPS FITIDSLKNI ETRYIKISMI
KPHPKHGEMD GQFLYGIRSI EVQANNLESV LNFCRDAANS DDARDKYFIE YVSEFDKNLS
NKLINLEDDV SKNVSSISDK LSKLEEVLPN IETCLNEKKE YETKLKASME QVIELIKPKC
SPEPLRVYCD MDSSTSLYVW NGINSVDDIR KHCAEVGLEP LVLKSTDQLN SLIFALKKMG
FILNGKINIP LAYDYSCFHD LLNGNVDLTT LIYESPNSTS VRQTALEEKK MLLCRVEDIA
KKRNRIHVKT QILSEDSKIK RHKMKKVYNY KFGYFGSGWK TEWTPFIHAP FFDNQHNTIY
KNRNKKLYEE IDTILHGRIH PHIKVVELKD HMHPIRLCTP SNEDCYSVIY TGEKIKSTDE
RVIFGEYTGF VNNKELPQEK HQYIFALTFI ILPDNYTYAV DSSYMFNEMS LVNHYKTCFN
NYDFRINAEW QIVYLDGWPH IILTSIPGVE IETGEEIFAD FGFEWFDRVN DICLNDFIKN
NYEHRLDDIV DKYNLLKNYT TCNICIHSVN TDCSNYIICS GCNHVYHLKC VNRLNNENYD
WFCSSCIQFS MNIAKAVICR VTKLHFETNE ELHKMSSECI QYVIKELALG KTKFQREFTN
GTYVGTVTKQ INDNHFFVVT YEDGDVEWIT PFFLFQEEHK LKGNDFYKQK KFEEALKEYD
EAIKVNPNDI MYYYNKAAVY LEMKSYEKCI ETCIYAIENR YNFKAEFSQV AKVYNRLAIG

YINIKNYDKA IEAYRKSLVE DNNRATRNAL KELERKKEKE EREAYIDPVK AEEHKNKGNE
FFKNNDFPNA KKEYDEAIRR NPNDAKLYSN RAAALTKLIE YPSALEDVMK AIELDPKFVK
AYSRKGNLHF FMKDYYKAIQ AYNKGLELDP NNKECIEGYQ RCVYKIDEMS KEKVDEEQIK
KSMADPEIQQ IISDPQFQII LQKINENPNS ISEYIKDPKI FNGLQKLIAA GILFKPNEKI
PSKYGENRHW NVDLIPKFIL VGGNLVKILK KTRVTNYLEW LVVEGSYVYQ HQKKSLLFSE
KFIHKVPSTD MEALVSPLLS LMEKNRCKNF YQYVSEWNAN DRNTWDNLDP YRLTMMDIYK
YFNLCQLTID FLGHAVALYL NDDYLKQPAY ITLERIKLYM HSISAFGKSP FIYPLYGLGG
IPEGFSRMCA INGGTFMLNK NVTDFIYNDQ VCGIKSSDGE AYCDKVICDP SYVHLENKIQ
KIGQVIRCIC ILSNPIPETN DINSCQIIIP QNQLNRKSDI YVNLVSFQHG VSYKGKYDVR
LDVKLNKFIW SKGIRNPPKR VRVKIERIRN EDEDSKERMY TLVQHVMVDS YKGLVNEQMT
GDRHRIQDCT TENFSGFIAI TTPTKSWIAQ YNDLSKYNPG FYALQVVGEL GTGSGYLILF
LYELLLKKNK KIDLLYCIDI NRDANGISNV EIINSNLFNN LRTCFDIILF NPPYVETEQD
ELNKTIVASY AGGKQGREVI LKFLHTVYDH LSNNGILYLL LEKSNIPHEI ENSDLPILCE
TCLGENPYVR IIREENGKEC KICKNAFTLF RWKPGHNARY KQTIICNKCA KVKNVCQTCL
FDLEYNLPVQ VRDKFLETSI TLPENETNRN FFLEQLELSK LKRRDPYFKR NMARVCSFWR
KNACNRGDEC PYLHKEIHKL IIFNLPPVDE QDVKSLCERY GPVIDVYAFV SFVFPSSCEK
AKNNLNETIY RGKILSVKYA SYKKILEIQK KRNCQNESIW NILYTDINSN IYNFCKETNC
DPQSILDKNI AVNVSLTETF IINKMKEWIR KEGIRSDDTI IVKNLSMHTN ENDIINLFKK
HGILKKISFS PYKNIAILQY EQPENAKKAL ISNSYIRYKK LPLYLEWAPI NLFDEEITHS
SIYIKNINFN TKEEDFKKLF EKLDGFITCN ISLGYGFAEF KSKELAIEAI KKLTATRLDG
HVLELSLSKL LVKNLAFQVT KEELKKLFSA FGNIKNVRIP KNAYNRSRGY GFVEFMSKNE
CLAAINALQH THLYGRHLII DFAPENARML KPLVQEKIIE IMKPEIEEKI IEVPQIQYVE
KLVEVPHVIL QEKLIHVPKP VIHERIKKCP KTIFQEKIVE VPQIKIVDKI IEVPQYVYQE
KIIQVPKVMV QERIIPVPKK PQYRHIPKPV EVPMAHYRTF PVEKLVDRNV PVPVEIQIVQ
EFLCPKIEAR YKEIPVPVHV QRIIEHPIPK DAMNNPFLLP LYYLGSTAIG ICVNDGVILA
SERRIASPLI EKDSVEKLLP IDDHIGCAMS GLMADARTLI DHARVECNHY KFIYNENINI
KSCVELISEL ALDFSNLSDS KRKKIMSRPF GVALLIGGVD KNGPCLWYTE PSGTNTRFLA
ASIGSAQEGA ELLLQENYNK NMSFEEAEIL ALTVLRQVME DKLSSSNVEI ASIKDQTFYK
YKPDDITKII NSLPRFKIID ADFINIKLLE LFQINGFQKQ LDRLSDSLSK IQKALGEYLE
KQRNQFPRFE KLVQTKLTDA CFLTLTQALK MKLGGNPFGP AGTGKTESVK ALGAQLGRYV
LVFNCDESFD FTAMGRIFVG LCQVGAWGCF DEFNRLEERI LSAVSEQIVE ILNKKVELNK
NVGIFVTMNP GYAGRSNLPY GVGFKTAFAR IASSCAIMSR TINTIGIGLL SLELMNHCDA
KELATPLCMW KLPNKELINR NIANKSEHRH HQKLLMSYTP FNSPSLLAEQ INILGTYSGT
RLLYFPLWDH PKDSIDYCLS TYLYWLYLRR STNIFLQNTL LRGQVVVIAA TNRQNSIDPA
LRRFGRFDRE IDIGVPDDNG RFEILRIHTK NMKLSPDVKL EELASNTHGF VGADLAQLCT
EAALTCIREK MDVIDLEDEI IDKEVLESMC VTQDHFNMAL GTCNPSSLRE TVVEVPNVKW
DDIGGLDEVK NTLREMIVYP IDHIIKSLYD HNFYSPREIQ SKTLKHSINE KKDIIVVSKT
GTGKTLTFCL PILSNILVLV PTRELAVQIL NHFNYVNKYI NIYIITIIGG LNINKQLRLI
SKKPEIIICT PGRLRYFVCD EVDKMIETSF INDIHFISKH LYIQTFLLSA TLLAKLLNYI
SIRKDKSYII DLLPDHLTLN IIKCEKKIIL HKLYYLLKLY KIIIFLNTIK LVKDVSTIFK
YLFFEPGLES SLPNKIYSIH SKQKLKERIQ SISKFSNNSS ILFCTDVMSR GIDLNKCDLI
IQLNCPISDI TFIHRSGRTA RNLKSGNSAK CAEIKSADLV LSSQELKRIS DPRFGTIDYS
QICSVCFEGC LGHIGHIEFV LPVFNPLFYK DLQELLNLVC YNCYGLCYSY NYEEYIILNK
AIKLHMKKGA NCSNCKFRRS ITVKTSQKKD TITVRLFSFQ IIDLLKKIFN KEIINLLYPF
TKKDGYQVFF LYYMGISSNR FRTQSRGIHK RNSFVKLCIN SKKNIDFDYL IELQLTINTF
LDIKDILDKK EGILRKNIMG KRVNNCARTV ISPDTFIETN QIGVPIEFAK TLTIDEHITE
NNFEYIKKLS LYDFILKKKA DFRHLSFISD FMTGALCMDI LKTNWSPAWT IQSLCRAILF
LLNEPNAESP LNCDAGNLIR GVKVKSAAEV GKRAVEYFRG DDFVNFLSSK QDILKKKFPN
LIGNRNLSEM KEIEEFADLF IQKGFIYKAQ YNPENGVYKR PKWPKRLIMT SKQNFDKAGF
YILVYERNKK LQYFMLMTLI SIVLICCMFW HLSVVFIGLL SAIIVGRIIA FVYFWFFGVD
YWIFPNLFDE ECSIVESFIP FHSWERRNDS WLLVFARVVT AVLVAIGIHQ LGKTHSISDI
QNFAKQSFID IIEWGNKKLS EENYDCLKKC GFPPFEELVR RCFLKCDCMT LADTLLKDLE
DLEEEERKIS ELLYDIEKCI ELIIKIDTEI LNIHKYLKDI YSTKFPELDS IVYTPLEYIS
VVSRIKNESD IKNIDFSDIL PNTTVMAIVV ASSTTGIKLP DHLLKSCMSF CNEALELNEN
RQNILIYLEN KMFLLAPNLT MLLGSALTAR LISCVGSLKN LSVTSSQNLI VVGNSKKGIL
STSEIVQSVP DAYKKKAINL LAGKCSLASR IDYFTEERTV LDKNILNSSV ILQIALRVKK
DRYLGRHYRY FIRNTRVRAY KQFLEPFKSV TLKNMAYAFG VSEDFIEVED PYEAIGEAVR
NFESKDEILA SAKIIERLVE YPEVAKNLDK INALDPLLKL LNNHILESVL QIFSLALSNN
PVLQDCVFKK NGLKILLLKL QESKQTTVDK KLITAISALI RHHDEGENKF IDYGGIAFLV
YGMQTNIYKY QEKSALLLKH LIHQNKITFE TFEKNKVMNG LIALANTGIQ YGETTAELFL
ALMQNHRHKL AKVGLKQLKE LIEGRLNYLF EELGVDDWLI KISKSVQIIK PTKIQKLCLP
LIIEGKNVIG SSETGTGKTI CYCWGMLQEL NKNLYAIFGL ILLPTRELVF QIVEQFQLYG
NKIGVKILSC IGGFSLIDQP HIVVGTPGRF KRLKFLVLDE ADLLLQKSFE EKLKIILNNI
PRRTLFFSST ITDSINLLIK TFPNDKLILV NANKKQKPLK NLDQRYGIIF TANSYKCELI
YTVLNSLFSI ESIHSSKDQR KRMSSLLKFK NGLCKILIAT DIISRGIDIP KVAFVINFDF
PNDTIQYIHR IGRTARANRK GLSISFVDKK DLKSFNNVKI IMKKKLKPYI LNKNEVLTDM
LKIGKVVKKA EMMLQEVVVF PVSVHYDLTR KLLNKKIGTG IQNVSKFGNG SYTGEVSAEI
AKNLNIEYVL IGHFERRKYF HETDEDVRQK LQQAIKNNLK AVVCFGESLE QRESNKTIDV
ITKQVKAFVD LIENFDNVIL AYEPIWAIGT GKTATPEQAQ EVHKEIRKIV KEICGANKIR
ILYGGSVSVE NCTSLIKQED IDGFLVGTSS LKTSFTEIIK SAKDINKDVV HRYGPNTFKL
HRLPVPKLRQ ILRLVGTNGI GKSTALKILS SKLKPNLRKF DNSPEWRDIL SFFRGSELQI
FFTKLLEEQL SPIIKPQNVD LIPKQVKGNI LEIINKKDKL NQKDKYIKVL ELDHLLDRNV
EDLSGGELQR FALLISIIQT TNVYMFDEPS SYLDIKQRIS MAKIIHGLVR HDNYIIVVEH
DLSILDYLSD YVCCLWGKAG AYGVVTSPFS VREGINVFLD GFIPTDNLRI REESLNFKLE
DKKRLHFYTY PKIVKTLNSF TLTIDKGNFS ESEIFVLLGQ NGSGKSTFIR LFAGLIKPDN
VDFLESLSVS YKPQQIQAKF TGTVRQLLMS KLKGLYTDPY FNNEIIKPLK IDGILDNQVL
TLSGGELQKV AIIITLAKNT NIYLIMSILC TISGQTPDEP VVSKTGYIFE KRLIEKHIKN
YGICPVSGEI LTLEDLYPIK IEKFVKPRPI TATSIPGLLS IFQTEWDSMI SEMFSLRTHV

NDVRNQLSHC LYQYDAATRV IAKLLKEKNN CQEEINNLRN QILQLKNGND IDDLEIGISE
DLLNEMQNIA KELLMNRKKR KVENVNSPNE WKKITSTNEF NIHSSIIPGV TCLSIDFSGG
KDGNIYYVSL NNNKIISKLQ GHLKKVNSII SHPSNSICIS GSNDKTIRIW KGDKHKDKIN
SLSLHPLENY FISSSNDSIW ILHDMETGKT IKTCKSSPSF KNLSIHPDGM MLGIGSEDSN
IYIYDIKSQE YKASLFSENG YYLASISKDN TLKLWDLRKA TSFQTIELEN TPKHITFYNG
NIKDGLFHGY GILIYSKNEK YEGDFAYGRR EGKGKFTYAD GATYEGEWMD DKIHGKGMAH
FVSGNIYEGE WENGKISGFG ILNYNNGDKY EGEWSEGKMH GRGTYIYADG DIYVGEWKND
KRHGKGCVKY KGSKDKIAET YEGDWYEGKM QGKGVYSFAD GGIYEGDWVD GKMEGKGIYK
YLNGNKYDGD WSNDMKNGYG ILTYANGEMY EGYWKDDKVH GKGTLTYSKG DKYIGDWEFA
KKSGEGELIY SSGDKFKGKW KNDKANGFGV LYSNGNKYKG EWVNDQRHGF GVFTCKEDGT
IYSGQFSYNR KEGQGTLTFS NGTIVEGIWN SGVLIKVTKF QLYPSSPWND PDLEMETLYD
LRNKMIEALQ KENITAGDVI CIDKSTGKIT KIGKSFARSK DYDAMDPNTH FVQCPEGELQ
KRKEVVHTVT LHDIDAINSR TQGFLALFSG DTGEIKNEIR EHIDMKINEW QEDEKAEIVP
GVLFIDEVHM LDIECFSYLN RALESEQSPI VIMATNRGIT HIRGTDYKAP HGIPLDLLDR
TLIIPTYPYM HEDIMKILEQ RAEEEDVEID EFAKELLCKI ASESSLRYSL HLITLANLVA
KKRKATEVTV QDVRRVYNLF IDVKRSTQYL IEYQNEFMFS ELYSNLSDFW TSDDEEDGDY
IRKKWVIEDD VSNFSKNDLL LSYDFELDDF QKRSVKHINN FKHVFVAAHT SAGKTLIAEH
AIALSIKLNK KAIYTSPIKA LSNQKYYEFK NIFKNVGIIT GDVKMNVNAN CLIMTTEILR
NLLYLNDNII NNIHCVIFDE VHYVNDEFRG VIWEESIIML PPHVQIVLLS ATVPNYLQFA
DWVGFTKQKE VIAISTKKRP VPLLHYIYAH DSLFLIMDEK NFYSSAFKEI YEANMKTEIQ
KLQALIKKLD EDNKLPVVLF CFSRIKCETY AKSMPHLNFL DNKKKSKVHL FIKESASKLC
DQDRELNQIK ILSKLLENGI GVHHSGLLPI LKEIVEILFS KGLIKVLFAT ETFAMGINMP
AKSVIFTSIY KHDHLKKRIL TSSEYTQMSG RAGRRSSDSY GYVYIYCSDN IPDQVQLTEM
MMQKAVSLKS KFKVTYNMIL KLLINKQINI EKMLFSSFLE SCRALQIPLF KKDLKRKKKI
LQNIKQVECV YIENYVYIDH KLKNVGLNLH KKLVGIENIS LITNELDRLI KKETFEPFTL
TKMLKSLKCE FYSVLHYELV CKKNDCINDI ENIERNINAK SLNLYEDLEG KLNVLKHFSF
IDDDNNLTIK GKIASYITLT DEITLTQIIF ENVLNNLNPP EIAAVLSCFV SPEKKVEESP
DLTLNLQDIK LALTNIHSKF EEFYRVIRLK ISTEEHWKLC NFKLMFIAYK WALGVSFSEL
LEQSEFEEGL IVRSIQRLDN LCRKVRIAFL YLGNADLAEK TEKASLLLRR DIVFTTSLYL
DGLALLQFFH WCEEKRKTKE LFKETEISLR DKIDYFRSTK KNFPSFSTIS AIGPNSAVIH
YESTEDTNAK ITPNIYLLDS GGQYLHGTTD VTRTTHFGEP TPEEKKLYTL VLKGHLSLRK
VIFASYTNSM ALDFLARQPL FNNFLDYNHG TGHGVGTCLN VHEGGCSISP ATGTPLKENM
VLSNEPGYYW ADHFGIRIEN MQYVVTKKQT NDAKFLTFND LTLYPYEKKL LDYYLLTPEE
IADINEYHQT IRNTLLPRIK ENPKKHFVIQ ENPDFIKYRL DKFNELKEKI NIELLDGSIK
IGQKNVTTPY QIASQISKKL SENSIVAKVI YLDNINLNLC DLWDMNVPLI GNCKIKFFWH
SSAHILGSSL EKLYGGYLTI GPALSEGFYY DIYLGNNSIL SENYNKIENE YNKLVKENVE
FEKMVCTKEE VLELFKYNPF KIELIKSKIN DNEKTSVYKC GDFIDLCLGP HIKNTGKVKA
FKVLKNSSAY WLGNKNNDSL QRVYGISFQK KTELTDYIKF IEEAKKRDHR NVGKNLNLFF
FEKETSPGSG FWFAHGAKIY NKLIEFMRKE YRIRKYEEVI TPNIFSCDLW KTSGHYQNYK
NCMFIFNIEN KEWGMKPMNC PGHCLIFKQL NASYKSLPIR LADFGVLHRN EITGSLSGLT
RVRRFQQDDA HIFCSLDHIK TEVVNVLQFI FFVYNLFGFG IEILQNRGYD SCGMSTILKT
TKYASSSASN AIEKLRGNYM TSHKNDNIGI AHTRWATHGS KTDENAHPHA DYKERISLVH
NGMIENYREL KKFLVQKNIP FKSNTDTEVV ANLIGYFLDQ KQSFQDAVVS SIKQLEGTWS
FCIIHKDFPD EMILAANGSP LHIGIKDNEM FVASEHSALF AFTNEYISLK NGEIMLINKN
NINNLKMIKK FDNIPEIVIQ KTPDPYPHWT IKEIHEQSIS LSKSLNNFNL KNNIVKLGGL
DPYVDELKNI ENIILIGCGT SYYAALFCKY IMNYLHCFNT VQVMDPSDFN ISSIPKEKEG
IIFISQSGET RDIIKACKLA EYFNLKKLSV INSVGSTIAN MTGRGVYLNA GREVGVASTK
CFTSEVSVLT LIALWFFQNK SNNKVSSLIN SLYRLPRYAD TTIKCEDTCK ALSHKLSNKS
MFIIGNGISY PIALEGALKI KEIAYIHCEG STGNALKHGP YALLGGNDNI PVIMLIFNDK
NSMISIGEQI KSRGAHIICL TDDVNLCNDD IILIPNNGLL TSLLAVIPLQ MLAYYMSVNL
GHNPDKPRSL AKTVTVWIIE IEKYASEDVQ KILIGNKIDL KNDRSVSYEE GKELAESCNI
QFLETSAKIS HNVEQAFKTM AYEIKNKSQL ENQQKGRTNI NLNAKPIKIR TMNSKKPPEG
WNKVETFLNE MNQKMRSLEN EDTSKKRKNE ILWPIFQINH QTARYIYELY YKRKEISYDY
IVIGGGPGGM ASAKEAASHG AKVLLFDFVK PSSQGTKWGI GGTCVNVGCV PKKLMHYAGN
MGKNDSDKYG WECNNKHDWN KLVSTVQSHI RSLNFSYMIG LKSKVKYING LAKLKNKNTV
SYYLKGKEDC VTGKYILIAT GCRPNIPDDV IGAKELSITS DDIFSLKNDP GKTLVVGASY
VALECAGFLN SLGYDTTVSV RSIILRGFDQ QCANKIKLYM EEQGVTFLPK KLTKENDKIL
VHFNNNTTEL FDTVLYAIGR KGDIDGLNLS CTNIPNIFAV GDIAENVPEL APVAIKAGEI
LARRLFKNSN EIMKYNFIPT SIYTPIEYGS CGYSEEKAYE LFGNIEIFLQ EFNNLEISAV
HRTKQKDEYD VDISSTCLSK LVCLKDNRVV GFHYVGPNAG EVTQGMALAL KLNAKKSDFD
NCIGIHPTDA ESFMNLSITL SSGLSYAAKG GCGGGKCGEH LYAGPLKIEQ LLAKGFVKRD
LELLKEGGLQ TVECVAYAPM RTLCSIKGIS EQKAEKLKKA CKELCNSGFC NAIDYHDARQ
NLIKFTTGSK QLDALLKGGI ETGGITELFG EFRTGKSQLC HTLAITCQLP IEQSGGEGKC
LWIDTEGTFR PERIVAIAKR YGLHPTDCLN NIAYAKAYNC DHQTELLIDA SAMMADTRFA
LLIVDSATAL YRSEYTGRGE LANRQSHLCR FLRGLQRIAD IYGVAVIITN QVVAKVDAMS
MFGHEKIPIG GNIIAHASQT RLYLRKGRGE SRICKIYDSP VLPEGEAVFA ITEGGIADYM
AIRVQFENSN EVGVFSRLTN SYGLIALGGS ENFSSVFEAE LSQHIPIVYA TIGGTRVIGR
VCVGNRKGLL VSSICTDQEL LHLRNSLPDN VKIKRIEERL SALGNCITCN DYVGLIHTDI
DRETEEIVQD VLDIEVFRTS IAGNLLVGTY SYFTNNGGLL HAMTTSQEIE ELSELLQIPL
ITGTVNRGSD LIGSGLVAND WSAFCGMDTT AIELNIIEKI FKLNNIEDTN IEDTFKYKSS
IVQTMIIVDT YWQTETGGIV IAPIPHLFKM KPGSASLPFF GIQLEILNSK TLEPLNGPNC
GLLCIKGSWP GMLRTVYGNH NRLVKTYFET CPNYYFTGDG AYRDEDGYYW ISGRIDDTLN
VSGHRLGAAE IEHALVQHPC ISESAVVSFS HKVKGEGILC FVVKKLKLYV RKVIGPIATP
DIICIVPDLP KTRSGKIIRR ILRAIAIGLN DYGDISTVSN YDVIEIIPFP TPCTIEEALQ
CYCDLSTIPR VNVLKNFKCF IKDIEEKEFE MTFIEFVDIF MQSAIFELTP FLQLIPKISP
KSYTISSSPK RWYKGSSSYY LTELYPNDIV KFNVKTSIFG IDFLYEKEID SLENKYIDEI
YLAFSRDQPW VEKYRPKKLD DIVHQTNAIS MLKEVIKTKN MPHLIFHGPP GTGKTSAINA
LAHELFGKEN INERVLELNA SDDRGINVVR EKIKAYTRIS ISKNKINTET NEQLPPWKLV

VLDEADMMTE DAQSALRRII EIYSNVTRFI LICNYIHKIS DPIYSRCSCY RFQGIPINIK
KEKLLYICKN ENIDISDKII ETTQGDLRRA VSVLQLCACI DSKITVDSVL DVSGLPGNDV
ILKIVNSCKM KNKILEKTIQ DIIEDGFDVS YIFKALNEYF VDSIKYQILM ELSRHDFRLH
NGATKYIQLM SFASSVHSLL IKELIFKSSD EKHLQILVKQ VKELIKQVKQ KEVEDVNDSK
TSNEKLALNK TGRRIVLRDL MTRPNIFTGR KILGTLELHT NGLRYSAIDI LFDDIKHAFY
QPCDGQLIIL IHFHLKRYIM VGKKKTLDVQ FYCEVGTQID DLDRAKARNV YDPDEMHDEM
KEREQKNKLN LIFKNFVQQM QDISKIEFEI PYPELTFSGV PNKSNVEIFV TANTINHLIE
WPPFILSVED IEIASLERVH HGLRNFDMIF VFKDYTKPVK RIDVIPVEYI DTIKKWLTTI
DIVLKTILAD IESFVNSKGF DGFLGEDDDE EEDEDEDDEY EVDESEMSAE IDILATVEFT
SKRKMSTVVC RIPKIVVFCK GAGCVIIKKL AKKTDVDDLT IEHMETYADE GLRTLCIAYK
ELSQEEFAIW YNSYKEASLS LNCREQNIEK IAENIEKDLI LQGVTGIEDK LQEGVGSTIE
DLRLSGIHVW MLTGDKIETA INIGIATNLI DNGSVDGSVL DILLSKPFER KFFYLADKCS
SVICGRVSPY QKGSIVSSAN RLLKKNTLAI GDGANDCNMI KMANIGVGIR GQEGVQAFNS
SDYGISQFRF LRNLILVHGR LSYRRISKLV VYMFYKNIVF IFPLFIYGSI SLYSGQKIYY
EFLLHLYNVM FTSLPIVILA ILDKDVSLNT ALKNPCLYKL GIHNFYFNIN KFISWVLNSL
FHGLLVFIIP LYFLIPSSTG EPFDIWSIGC VTYLTVFIVN IKILLETYYL NTSPIVAVSM
SVISFIIMSI AFSFIGIGNK SFLGVAILLA KSLRFWLVLL LVLFALTRDY VYKVYKKNFY
PRNYSDNVKD HFNKPRNVGS FDKNEKNVGT SIVGKASCGD VIKLQLKIED NVIKDARFMA
FGCGSAIASS SYATELIKGK TIDEALKIKN NDIASHLNLP PVKVVILGSG WGGIHFLLNI
DFQKYDVTLI SPRNYFTFTP LLPCLCSGTL NVDACSENVE TLLKKNKISG KYLKLECIDI
VYKDKYIKCK DNNEIKIYYD YLVISVGAKT NSFIKGVDKY AFYIKDIIDA LKIRRKFISN
LETCTNNNIN DDLAKNMLHV VIVGGGPTGV EVAAELADFV NKKNKYKEIY KYISISIIEG
GNNLLPTFTQ NISNFTKDTF KKLNINVYTN YYVIEIDENN FYIKSQKIPY GIIIWASGLA
QIPLINNFIK KIPEQVNNRI LNVNQYLRVI GINIYAIGDC KQINPVQITA EQLIKEALDF
EEVEKKVNYN LIDEDELNEY KISKRKEFED SIRKRRYLIN TYIKYALWEI KQKDIKRCRS
VFERALNIDY TNKNLWLKYI EVELTNKNIN SARNLLERVV LLLPLENIFW KKYAHLEEIL
NNFVNARNIY ERWVKWKIDE TAFLCYINFE ERCKEINKCR EIFEQLIVNI PKLECFYRFI
KFEKKYKNIS RAEKCIELLP SQFLDQHFYI HFSKFEEENN EYERCRKIYI EALKRLPREN
SDILYKNFLQ FQKKYEELDQ TLLYNERIHF EEALKKTPND YDIWFNYIKL EERIRELYER
AISVIKNALV HDGLKIGIRE VIKSIESKEA KVCFLSNVCS EPAYKKLVTA LCAEKQIPLF
MIDSKDLGQW SGLFKVDKEG NARKIIGASS VAIIDFGEES AERDFLGYYK AIVGEVIDNR
YSVVCELVGK GVFSNVLKCY DMTNKIHVAI KVIRDNHMMH KAAEKEISIL KKLNKKHIIR
LLRSVKYKNH LCLIFEWMWG NLRIALKIQN SFNSLILKAK ELEDVYISRR KLQSKYLTQI
KNLYINSNCE CIIHKISFKY ASKSSFPNLL NGTLLYMIIE KINLDNNVVA SCINSADVKS
WINYENYLGE LIDGFLFAVN ISYAKSLIGD KCYILDLIDV PYEIAIGHNG GALGYSQHLS
EEIMLFSKEA IHDKIAVILG GRAAEELFIG KITTGAIDDL NKVTQLAYSY VSQYGMNKEI
GLVSFQQNGS GEYAFYRPHS ECLAHLIDNE ARNLIESQYN RVKAILKKNE KHVHKLANLL
YEKETISYHD IVKCVGLKHQ RYREADKVKI EEERNRKIYV KNTKFCKNCG SAAHTEKYCL
ERTRKKGYDG NRDRWVGYDP NNFDHIYREY EKIVDEQKKR KAEKLIDKKN NKIKILSKYE
EDIYISDHTS VFGSYYDRET KKWGYKCCKC TDKFQNCIIP LMDGYHANYY TKEVMIILIN
EFNSPDEEMK KIVLKCVKQC IQTEGIEKDY INQEIVNPFF EQFWIIRNSN DKKNFNLIVD
TTVEIANKIG VIAKIVDDLK DPSESYRKMV MQTIQNIINN LGVDDIDQKL EEQLIDGILY
SFQEQTSDDY YVLLNAFDVI VNKLKLRMKP YLPQIAGIIR WRLNTPLPKV RQQSAELIAR
ISKLIKICDE QQMLGHLALY LYEYLGEEYP EVLGNILKAL KSIVIVLGVN NMTPPIKDLL
PRITPILKNR HEKVQENVID LIGIIADKGG DMVSPKEWDR ICFDLIELLK SNKKLIRRAT
IQTFGYIART IGPFEVLTVL LNNLRVQERQ LRVCTTVAIA IVADTCLPYS VLAALMNEYK
TQDLNVQNGV LKALSFMFEY IGEIAKDYVY SVVSLLEHAL TDRDLVHRIA TWACKHLALG
CFGLNREDAL IHLLNHVWPN IFETSPHLIQ AVIDSIDGFR VALGPAIIFQ YLVQGIFHPS
KKVREIYWKI YNNVYIGHQD SLVPIYPPFE TIGDSNFARD ELRYMGRMYG KGKGISSSTI
PYKRKQPSWL KQKPSEIEDA IIKLAKKGQT PSQIGATLRD NYGIPQVKAV TGNKILRILR
AHGVATTIPE DLYFLIKKAV SMRKHLEKNK KDKDCKFRLI LTESKIHRIS RYYKRKRLLP
SNWKYQSSTL VLLRNIEHAP GVQFSYVPPD FFDSDDDKSD KNQYELKDDG GGRAAGTRGK
EHSSTHHLRR KNYEDDFEFN EDKILEALHI LEFLYLNGIS LEEQNEYGQT ALFLGVKKNN
ISILQWLLSK NVNINHVDFY GNTILHMAVR YTDIDILRLL CDYGCLNLVY YSTFENNNTN
VFQLCINNRY FLVYILLKKW LIQNKICKGL KICKTIYAFY FWFFALLNLF VYINIAQSFL
EIQKHHNKSV IWISLWLFQQ LLWCILYFKN PGEYKLNNIE REIFQINLRY SKLSLYPQVS
QERINSLDVN YRNAILEIIL LQLIIEPYIL RRSKKHVFID MPKKHSIIIK LPLNNTQLNL
YKDEILSKLQ HTHKHLINAS IFILRRICNH PLLHKYYYSV NDIKKISKYF YNNTDQYLDL
DLKTVENEFM KISDFDIHLS IKHLISQDNN LNKYLIDKKH ILNSTKIQHM LTLIKNIKQK
KEKVLIFSQF TTFLDIIEES LYVRLDGSTN TIERQQIIKE FSNVFIFLLS TKAGGVGLNL
IAANHVILMD QWNQLLLQEE IVKKMCEYII DSRCDILEKG VSDLAQHFLV KKNISVIRRV
RKTDLNRLER ITGATIVNRC DEIVEKDIGT KCGLFEIKKI GDDYYSFFVE CENPRACTIL
LRGATKDVLN EIERNLHDGM NVAKNIMLEG KLLYGGGCTE MRVSQYLIKQ AANFDDSRKS
VIESVASAFE IIPKILAQNS GVNVVKCINE LRTKHESEKL GIDGVTGEII DVSSKNIWDL
LSVKKQIYKS AIEAASMILR IDDLGVVLIY EDLLKNPLCR ISNIFEKEVK KYRPFPLNTL
QMTKLVSKYF HISSKECMNI AEKLYSKGYI SYPRTETNYF VDSMNLRKII HELKKNNIFG
NYATKLAEKN PIKGKLNDKA HPPIHPVKNM EWKIYEFICR HFLAVCSDDA IGFDTKVVAN
IGEEQFYCKG LKIKNKNYLE IYIYEKWNDK ILPPFQINDE FYPYSLVVEE GITQPPKYLS
ESDLLSLMDK YGIGTDATMH EHIENIQKRN YVYKNSKNLF IPTKLGIALI LSYKKFKDIG
VDLTEPSLRA KMERDMFLVA SGICVIKSGK MDIPRNTKMI IISYELITKN DKYQKYKCIV
CDESHYLKNS FSKRTKAIVP IIKSAKRCVL LSGTPALNKP SELYEQVSSI IPNLFNYNEF
CDRYCYKDKN IYTRKIEYVG CKHTEELHLF LTNTIMIRRL KKDVLKELPD KLRSKIPIEI
PPNELSEILL FKMTGYAKVK AIKEYITYLI DADIKFLLFC HHKLVMDEID EFLKEKKLGF
IRVDGLTPID KREIYIKNFQ SDEKIRIALL SITACGVGLN LTAANTVVFG ELYWVPGQMI
QAEDRAHRIG TTHDTINIHY LVAQNTIDEV VWKIINRKWN TLTTALNGTE DSLMFEDNDK
ICIAVSGGKD SSVLTHVLVN IKKKYNYNWN LFLLAIDEGI KGYRDDSLKV VFQDIFSYTM
DDVVSYIGKK NNCTVCGVFR RQAMEKGALL FNATKLVTGH NADDLAETIL MNMCRGDIDK
LFSTECTYSP NSFRGNLRSF IKDLECIKCG AYTSNVDGLN NYTDNKQSSN CTFEIDIHYE

NIEPMALENE YQKIPKLRIL SFDIECIKLD GKGFPEAKTD PIIQISSILY LQGDPCAKFI
FTLLECASIP GSNVIWFNDE KTMLEAWNEF VIRIDPDFLT SYNIINFDIP YILNRGTALN
LKKLKYIGRI KNIPSLVKDA NFSSKQFGSH ETKEININGR IQFDVYDLIK RDYRLKSYTL
NYVSFEFLKE QKEDVHYSIM NDLQNENSES RKRIATYCIK DGLLPLRLID KLLFIYNYVE
MARVTGTPFV YLLTRGQQIK VTSQLYRKCK ELNYIIPSTY IKVSNNDKFE GATVLEPIKG
YYIEPISTLD FASLYPSIMI AHNLCYSTLI KNNGKNNFKF VKGNVKRGVL PLIVEELIRA
RKNVKAMMKN EQNPITKMVL NGRQLALKIS ANSVYGYTGA AAGGQLPCLE IATSITTFGR
SMIEKTKETV EAYYCKNNGF EHNATVVYGD TDSVMVKFGT NDVGEAMRLG KDAAERISKE
FLHPIKLEFE KVYCPYLLLN KKRYAGLLYT NPNKHDKMDC KGIETVRRDF CILIQQMMET
VLNKLLIEKP LIQVAPMINV TNRHFRALVR IISKKVQLWT EMIVDNTLLY NINNLEEHLG
FNKNEHPIVC QLGGSDPISL SEAAILVEQA GYDELNINVG CPSTKVANKG AFGAYLMKKP
QLVKNIVYEI KKKVQIPVTV KIRTGVDDLD SFSFLRSFIE TVSSAGCDHF IIHSRKAWLK
GLDPKQNRSI PPLEYNKVFD LCKLYPNIKF TLNGGIKSIE QGVALLLYGV MIGRSCMENI
TVLAKTDKLV YNYDIPTTAY NRRTVLDAYK SYLEQNSSFY SLFELLKPIL GILKGMPGHR
LFRFLKNFFF KNPVGHVGVV ALKNSSAKLI QQLTSNIDDV LNSLVKEQKE GLQGSPSLQE
GLEIAHNLLM DMPLYGTKEI LIMYGSIRTC DKKNILKYLD LLIKNNMYVN CISIAPEMHI
LKCGIHLISM HDLSHITNSL QASPLFVEIM GSNSIAISQQ MYFSTHNALR INENDVISTL
FYEINGHRHI SLLIFPFYDV QMLKRLLIKK LDLPDIKVND ILIFYKGIKL PNYRIISTYK
KVNKLYWAIK DPNPNASIRV IDNKYPPFFE NILNDIKLAF KKNIAPKLTM DGTGGTYLLF
NSKKKVCSVF KPADEEAFSP FNPRGYEGKI YQEGFRAGVL SGEGASREIA AYILDNTYNN
FSNVPCTIMV EACNPHFNNK SNLKYIYNEN TLKWKCGSLQ EFIDSRESVG NYDHKQFSIR
DIHKIGILDI RVMNLDRNDG NILVSPTHLG IEQSRRDDIE ALGYVLMYFL RGSLPWQGLK
AISKKDKYDK IMEKKISTSV EVLCLLNTCT KIDACIIIDR RIDMVTPFCT PFTYEGLIDH
IFCIENLQIE IPLYNDIKDL NQNEVGIFLH KKASDIQQTY EKDSLKDIGQ INKFMIKFKE
KHYEHNSLSR HVNIASYILN EIKTENTFNK LKLEDEIIQL NTNTNKTILS NIVKKIQTLI
YTGENIYEIY RLISLFSSIT NGKKDIIEQY GINELTRLNN LHISNILKYQ PKQKFIWNTL
KNHFNLLSND ENDISYVCNG YAPLSTRLIE YKNNMQVFPE VFSLINGPTF DIIQDTIEVK
SMCINCEQEG INKILKFEIP YFKNILIHSF ECVLCNYRNN TIQDLNPIKE KGVKILFSVT
KTEHLDRQLI KSEYGVLKIP EINFEIPKET QKGSINTIEG FIQTALSNLY MSMIEKTIHK
LFTVEIIDPS GLSSLEYQRS KQELNELGFY SFTSNCPCCN YLGDNNFCEI NIPGFKKCLI
LSYVCPNCNY KTSEIKSSGE INPKGKKITL TVKNKSDLNR FVIKSETASI QIPIIDLTSD
YGTLGGSLTT VEGIIIQIIE SLEDKFKFLM YVLNRKGEEE DVSFDQILKR IQRLSYGLHE
LVDPARVTQG VINGMYSGIK TCELDELAAQ TCAYMATTHP DFSILAARIT TDNLHKSTND
DIGKVAEALY TYKDIRGRSA SLISKEVYDF IMEHKDRLNK EIDYTRDFNY DYFGFKTLER
SYLLRINNKI IERPQHLLMR VSIGIHIDDL EKALETYHLM SQKYFTHATP TLFNSGTPRP
QMSSCFLLSM KSDSIEGIFE TLKQCALISK TAGGIGVAVQ DIRGQNSYIR GTNGISNGLV
PMLRVFNDTA RYVDQGGGKR KGSFAVYIEP WHSDIFEFLD LRKNHGKEEL RARDLFYAIW
VPDLFMKRVK ENKNWTLMCP NECPGLSESW GDEFEKLYTK YEEENLGKKT VLAQDLWFAI
LQSQIETGVP YMLYKDACNS KSNQKNLGTI KCSNLCCEII EYTSPDEVAV CNLASIALCK
FVDREKKEFN FKKLYDITKI ITRNLDKIIE RNYYPIKEAE KSNKRHRPIG IGVQGLADTF
MLLRYPYESD EAKELNKRIF ETMYYAALEM SVELAQVHGP YESYKGSPAS QGILQFDMWN
AKVDNKYWDW DLLKKKISIH GLRNSLLLAP MPTASTSQIL GNNESFEPYT SNIYYRRVLS
GEFFVVNPHL LKDLFDRGLW DEDMKQQLIA HNGSVQYISE IPNDLKELYK TVWEIKQKNI
IDMAADRGVF IDQSPKLPFD EIRNEMNKYG VVITPSTLKH PTTEDVQGVY SICIKYILNK
DINNIRIEEF TGDLKSILPN EGKNHLQAIG NLRFFRHCEK INKILNMDNT LSYIFKPTSG
HITKLINAFI VNETNELIFQ FSRYRQKKED LEDQIVPSPE KLQQYNDELK DLLYEHMSHC
ETSKKKNEDI KNKINIADLC IKKLVNLLTI LTSHLKVHID KKNKLKDLGT NLKSLDWWTL
GIFIYEILVG YPPFYANEPL LIYQKILEGI IYFPKFLDNN CKHLMKKLLS HDLTKRYGNL
KKGAQSVKEH PWFSNIEWNN LLNKRVDVPY KPKYKNIFDA SNFQEDLSIA DKVINENDPF
FDWVISQFYI LSPRGDTIIN RDFRGDVLKG SGDPPPLFYL NGINFCFLKN NNLYYVLTSL
FNISPSYLIE LLYRLLKIFK DFCGQLTEEI IRANFILIYE IVDEVIDYGY LQNSNTEYIR
YLIHNETLPS NASQKPIQVD NKKNEIFIDI VEKIDGVIQI KSYLLGNPYI KIALNDDLYI
KNIHKDNTNN IIIDDCNFNH LVLSLYQPDG ECVIMNYRIN MINIYKPNDV IACEVQRILT
DGCIILHTRS SIYGKLSNGI LITVPQTLVQ NQKKHIFVFP CVDDTTRKNI SIISNIIKLL
AKYHININYD IITKIYVQEW VPPKNKQINA ATSNGSQIVI SLSGGELIYF EIDESHTLTE
IFRKNINVEI LCLSIQQNKL RASFLAVGCL DNVVRLLSID QYFKQLSTYI LPNNSSPQDI
CILYLNIGLN TGVLLRSVID PIGTLSNHYS KYLGAKSVKI CHVNPALLVL SEKTYLCYVY
QGKYIYSPLN YDVLEYASSF YSEQCSDGYV AISGNSLRIF RFYRLGEVFS QNILHLTFTP
RKIVPLPFPS MLAVIEADHN AYDENTQQEI QKALRDIKLG TPKAGLGKWG SCIKIINPIN
LQVIDKISLE LEEAALSVCA CELEALHCLI VGTTTNMTLK ASLRVYTYDI NYKLNLLHIT
PIEDQPYCFC PFNGKVIVSV GNKLRIYALG KKKLLKKCEY KDIPEAIVSI KVSNRIFASD
IRESVLIFFY DSNQNVIRLI SDDIIPRWIT CSEILDHHTI IAADKFDSVF ILRVVEEKPD
ITYNDIGGCK EQLEKLREVV EMPLLQPERF VTLGIDPPKG VLLYGPPGTG KTLTARAIAN
RTDACFICVI GSELVQKYVG EGARLVRELF QMAKSKKACI LFIDEVDAIG GSRGDESAHG
DHEVQRTMLE IVNQLDGFDN RGNIKVIMAT NRPDTLDSAL VRPGRIDRKI EFSLPDLEGR
THIFKIHANT MNMSRDVRFE LLARLCPNST GSDIRSVCTE AGMFAIRARR KTITEKDLLL
AINKVIHGCK QFSATGKYMV YNIKSKTEEN FALVLKKINE LYNLTQDNVF NLPRYSKFPE
AKKPTKWELF SQKKLKKKNK HGLIYDENSK GWVRRFQKKQ IKINKEKSDF VHEYKPNDNI
DPFEKMEEEK DIKKMKQKMR EMKNKVFERL TDQNFYTGTH KKKFKKEKNL VVTPPKSIWL
YRNGDKHHNG LLFFIKSHIN NFKLLLFEIT KVLNPIIGPI RKIYDQNDGS KYLCTSGDPP
ASIDHLGKFD ITKYFKERQK VITPPNGDGT RAFYESLLDE NPNSIIAIKY CIEHGVLSGT
TYHETLNKYY ILKKNNAFRN NFGRIKCEFV DMLNVKFIQE KKLIGKFFEE IAQDTGKVVY
GIEDTLKALE IGAVELLIVY EGLDIIRLTT KNNVTNQTKT MHIFPHDEKQ ESLYKENNVE
LEVVEKILLT DWIINNYKKY GASLDFVTNK SQEGAQFLQV LYEFIFLCIC MYDSINELFE
LAYKIVSDEI SEQVINIIVL PFNHLGLNAL KAKNIKNLLN SINDKYKKKL SLNIIDAIIE
CKNKEMAYQN VEEILKFISC IFDIDKKYNT SMLFYNYIYD SIYFSQLLPS IIFTLLNIVM
LEAKLNNAYI LKKLFECIKD LVNDANIDAD ENGLKLQALD GNHVSLVSLH LVDSGFSHYR
CDRERVLGVN IASLNKVFKL CGINESVVIS SKDDEDNLNF VFENNKEDKV TNFSLKLMSI

ELDSLNIPDE GFDAEVELSS KELTNIFRNL SEFSDTVFIE IDSNSIKFTT KGLVGDAEVA
LKPRESTDDV GVTIKSKKKI KQSFAIKYLN LFSKSSILSD VVILGLSDSR PIEFKYEIKD
GFIKFFLAPK MDDDCPEKCD EDIGVIKGTF VYTFDSYICK AGIHAGVLSV TDDMILIITH
SRNKFIGTKR NNIESKEFNG ESKSFSLSIP TGFNGDENQY INANNLPNEK YIRTLSNFTF
IIHFGKNKWR TILSHSLCEG ISISIDEENE LVIEQNCNPH LVKTKFIPKF EHPCHLVLIY
NKPNKSISLY INQKKINLEK MKFDFTLNGD LTIGRSNKQA TDYFIGDINF VKIYKYILTE
QEIKESYDSV LSRKTIDGRD CITPCKSKTN VNKNVQINTE EFYLNCSDNL LSERFNGKGA
QFLVSCLEDC TNSKYIVKGS NYYTPDTSIC KAVMHSGIIK IVEGLTEYKS SRGHYGIVSK
PEKQSCFTDA AFLFELPIGT TKNIICPENC GTNTYSPLSS VCKAAIHAGV ISTKGGQIQI
VVGKGQQEFK SSTQNNIQSY IAEKQNRSQK YIYENADVGT QKKVFDLHLN MGPYTCNYSR
NGKYLLITGE KGHISFLDTH NMETLCELQV NETVKCNTIF HNHKLFAIGQ KKYIYIYDNT
GIEVNCIKDI LYPCQLEFLP YHFLLASIGD LGELVYQDVS VGNIITRKKT KRGPCSIMKQ
NKQNAIIYLG HKNGHVTLWS PNMDKNYLIT AGIDCTYKLW DIRKLEYINS FKSNIINNID
ISDTSMVAFS MNSHFRTYKN FFTKPYLTHN TWGDRINSIT FQPFEDICCA GLKYSIKSFI
VPGSGLANID TFVNNPYETK KQNEIRQLLD KLPPETITHD YKKIIIVRKI KTKTQIKRIV
TSPRDVYEMG LNSYSNSNWP KWTESESNCL CLINNQIYIY KDNERGTKGN SSVFKIFNLD
NLNKHIYSKK FFNSDEIKLK WNKNGTSLLL QIHTDKEKQS YYGSSNLYFI DTVKIKDVNI
MTNKGLIYDT IWSYNQNKFY VCKGEIPADI VLHDKNGNII HSYGKHKFNT LKLNYSEKLL
LTGGFGNLSG DISIWNTINK KEITKTKSSC AVICEFFNDD KHFLTATTHP RLRVDNNIKI
FKYNGLIVSK LDFDELYNVI ILPPGCNFVV EEKTNKKKKK KKEEDTIPLP NIKTQILKKV
IEYMEYHIHN PPDEIPKPLI TSNLQDVVVE DNISKYKEIS QKMIQEIEMA VVLFKRKFII
KKIPQLPSCY IINSGALSVA SAKIKLPSTY AKLGDPLSFS KLPDCNYSFD MIEELQQFFM
KQRRCDYFSL LNNFIDILIT TSNLLANEPD IDIRNTILNK FIYSLNTWMV MRRCIVASCE
NIFSMTGLCI PLQILHFNND ECKIFFSKKR APYLLVFEVA DLDEDISHII PVESQRIIFG
EFNRESISSL LNNPLARSLM NELSNNPEML TNLISNNPLL RNTFPLMQPM LDNPNLLREF
MRPEVLQAGL PPEERYASQL VSLQEMGFID NDANIQALQE TGGDVNSAVT RLLERGSGDR
LTRAARVLEQ LTEQKPIFGK CRFTIRSFGV RRNEKISCFV TVRGKKALEI LEKGLKVKEY
ELRRKNFSDT GNFGFGIQEH IDLGIKYDPS TGIYGMDFYV HLSRPGYRVT RRRRERLGFF
ISLKLMNEIK GLSSIDSSYQ WLPLLYMALA NDTAVSKISL SALKPYSIVL IRLLRDFFSV
VFLIKCVGID YRNMTGSQCV AIACDLRLGS NSFTTVSTNF TKIFKINDHI YVGLSGLATD
IQSLYELLRY RVNLYQIRQE TDMNIDCFSN MLSNILYSNR FSPYFVNPIV VGPYLNAYDL
IGAKCETSDF VVNGVSNEQL YGMCESMYIK DILLVGAGGI GSEFLKSIIT IGCKNIDIID
IDTIDITNLN RQFLFKKKDV KKHKSIVAKE RALKHRKDLN INAYTFDVCT MKGSDISKYD
YVINALDNIK ARKYVNKLCI TEKKVLIEAG STGYNGQVYP IFSSETKCYN CEEKPKNKTY
AICTIRQTPS LPEHCVAWGK LIFETFFCKN DNETLIDIKK HIEEESKKRN MDKEEIIRFI
FNYLFHDTIN ELISLKKDYA IMPKPNIKKT TEEYLIFDKD DDDCINFITC LSNLRMINFS
IKQKSKFDIQ SIAGNIIPAI SSTNAIVAAF QFEKIYVCKP QSSRNKSDII LVLNFGSQYF
HLIVKRLNNI KIYSETKDYN VDLKDIKNIK GVILSGSPHS VPHIKKEVLN YKIPIFGICY
GMQEIAFHMN GQVGKSKNSE HGSTVWMNHT EEVIEIPENY YLVNSSENCF IYNKENNIYG
VQYHPEVYET ADGDQMFYNF AICKCTKTFD PIKYHEVEFN NIKKHAHDHY VIAAMSGGID
STVAAAMTHK IFKDRFYGIF IDNGLLRKNE GEKVFIKSTF PDMNITKIDA SENFLNQLKG
VTDPEQKRKI IGKLFIEEFE KAVYSMDIDI EKTYLLQGTL YPDIIESKCS KNSDTIKTHH
NVGGLPKNLK FKLFEPFKFL FKDDVKKLSQ ELNLPKELTN RHPFPGPGLA IRVIGEIDKH
KLDILREVDD IFINSLKEYN LIGQAFAVIF SSKSYDHICA LRAVKTTSFM TASYYKIPHD
ILEKITTRIS NVKGVNRILY DISSKPPSTI EFEMPLPGFV SDKSLYLRKP LVLYEDENSK
IEVDPILAQY LREHQREGVQ FVFECLMNLK DEKISGCILA DDMGLGKTLQ SISVLYTLLK
QGYNKKAAVR RCLILCPASL INNWNDEINK WLPNRCTVTC VNDSVKEKIV SKLEGFKYDL
KSTILICSYE CFRINNDSID KSAIDMIICD EAHRLKNDKT KTYTSIYKLS AKKRLLLSGT
PIQNDLGEFF ALISLCNPDL FDDTNSFRKK FANPILIGRD KDATEKEQQI ASERLAELST
INKFILRRTN NLLSKVLPVK YLINIFIKLN PIQEGLYVLF LKDKKLLKSD NSNNKVNVLI
NIKKLEKICN HPLLLNANDI KSSKFQLLHF LLKTIKHETN DKVVIVSNYT QTLDYMEILC
KENHYKFVRL DGGISIKKRH KVISDFTNTD DIFIFLLSSK SGGCGINLIS SNRLILLDPD
WNPANDKQAL ARVWREGQKK ICYIYRLFCT GTIDEKVYQR QISKDGLSSM IVTNTNLSKD
QLSDENVKKL FNYKNNTICE THDNIECNRC KKIEPAGFAF YTKYEKASLK KNSNIVKKCI
NNNINVYKVL GVEESDDFET IKLAYKKLIL IFHPDKFLKV QDSYAVLSDK ILRKQYDSSI
PWSSKKPVPN IGDENTSIDK VKYFYDFWYE FTSWRDFSYQ NEYDYEDAEC REERRWMERE
NKKIQKKASK AEKLRINKLV DLAYNNDPRI IAENKRIELE KQRKKAASKI WKHHIKSFDT
LCQFIYEIYL FLWTPQEVSL LSKALKLYPG GTKNRWNVIA NSIKTKNVKE VIKKAKEMFE
NETLWTHEEQ MLLEKALMKH PATIPMPKKR RNGGRSKHNR GHVNPLRCSN CGRCVPKDKA
IKRFNIRNIV DTSAQRDIKE ASVYSTFQLP KLYIKQCYCV SCAIHSRFVR VRSRQQRRVR
KETTKHAHVS QKKVSVSEIN FDSSYTILDT SEGAIMLHVN HVLYHLDFNT LAVVKNVDMT
EEMQIDAIDC ANQALQKYNV EKDIAAHIKK EFDRKYDPTW HCVVGRNFGS YVTHETKNFI
YFYIGQVAIL LFKSGVCGWS KAVRKQGGRF CFVNLNDGSC HLNLQIVVNQ NIDNYDKLLK
CGIGCCFRFT GTLILSPVQN SIHSFEIYGE DPQKYPLSKK NHGKEFLREV AHLRPRSYFI
SSVMRIRNAL MLSTHLFFQS RGFICIHTPL ITASDCEGGG EMFTVTTLDF KKDFFSKQAF
LTVSGQLSLE NLCSSMGDVY TFGPTFRAEN SHTSRHLAEF WMIEPEMAFA DIYDNMELAE
AYIKYCIRYV LNNNFHDIYY FEENVEKGLI ERLKNILNDD FAKITYTNAI ELLTKYSNNF
DIPVKWGMDL QSEHERFISE QIFKKPVIVY NYPKDLKAFY MKLNDDNKTV AAMDVLVPKI
GEVIGGSQRE DNLELLDKMI IEKKLNIESY WWYRQLRKYG THPHSGFGLG FERLIMLVTG
VDNIKDTIPF PRYHGHAEFI KDILDLNLEK SENVIKNLFL KDKKKYFYLC VANWKKLDLK
NVSSQLKTSN LRFVDDENLK NILNVNPGSL TPFSIKSDKD NIVKLYFDED IKNMVIIHPM
HNYSCIYVKT TDVIKYCDLH NHTPILGITS KKEQNFSDWY TQVIVKSELI EYYDISGCYI
LRPASYYIWE CIQTFFNNEI KKLDVENSYF PLFVTKNKLE KEKNHIEGFS PEVAWVTKYG
DTNLPEEIAI RLTSETIMYS VFSKWIRSHR DLPLKLNQWN TVVRWEFKQP TPFIRTMAKL
SKAQKKQIYM DKLSSLIQQY NKILIVHVDN VGSDQMASVR QSLRGKATIL MGKNTRIRTA
LKKNLQAVPQ IEKLLPLVKL NMGFVFCKDD LSEVRILQNK SPAPARLGVI APIDVFIPPG
PTGMDPSHTS FFQSLGISTK IVKGQIEIQE NVHLIKQGEK VSASSATLLQ KFNMKPFSYG
VDVRTVYDDG VIYDAKVLDI TEEDILEKFS KGVANVAALS RSVGIITEAS YPHVFVEAFK

NIVSLVIDTD YTFPLMKKIK DMVENPQAYA AAPVAAEEEE EEDGFMGFGM FDQERDLARE
PCPDRIIEDM GGAFGMGCIG GYIWHFLKGA RNSPKGDMLS GALYSSRMRA PILGGNFAVW
GGTFSCFDCT FQYLRKKEDH WNAIGSGFFT GGVLAMRGGW RSSSRNAVVG GVLLAIIEFV
SMVLTRKTTP TPRQQFQQQM EMEKMLVDNI GDVTITNDGA TILKQLEIQH PAAKILVNLS
ELQDQEVGDG TTSVVLLASE LLRRGNELIK MDIHPTTVIC GYKLAMKESV KYIKEKLSER
NLGKDVITNI AKTTLSSKFI SYESEYFAKM VSNAIQSVKI INDSGKTKYP VSSVNILKVH
GLSSLDSKLI DGYAIMSGRA SQSMPSAIKN AKIAFLDFPL KQYRLHLGVQ VNINDPNELE
KIRQREKDIT KERVNKILES GANVILTTQG IDDMPLKYFV EAGAIAVRRI KKDDLKRIAK
LTNGQIRLTL SSIDGTEKFE PASLGYCDEV YEEKVGDWDV MFFKGCKNSK SNTILLRGAN
DFVLDEMERS IHDALCSVSR ALESNYVVVG GGCVEVALSV YLEDFAKTLG SREQLAIAEF
AESLLIIPKI LALNASYDSI DLVCKLRAYH TKSQVNTDEP KDYRWYGLDL VNGKVANNLK
NGVLEAMISK IKSIRFATEA TITILRIDDL IKLVPERNPP VYKLKGHTSN ILDIQFNPCY
SEVIASSSED MSIRIWLKGH KKKVTIIDWN PLSYYILCSS SFDSTVNIWD IENEKKAFSI
NMPQKLTSLK WNSSGALLSA TCLNKKLHII DPRQEKICTT FNAHSGSKCA KNIWIDGYSG
NEILSTGFSK NYMREMKLWD LKNISDPLCT ISIDNASAPL LPHYDESIGI IYIIGKGDGN
CRYYQNSEGV LRKINEYKSC LPFKSFGFLP KQVCNIYKCE IGRIYKNEND KSIKPISFYV
PRKNFQEDLY PPIIGNNLEI KRIYIFKKLK ICGQFNKGFI ISKLFIIDQH AADEKSNFEK
YNKIFTMKSQ KLVYLLSLPV FNGKILEVVD FMSLLYHLWF NYNFPRPQKV WKILASKMKN
LVLVFLKKIS DTYLEDQSKW MEKMKSSQEE QNNKKLDEWN ECIENKCFVY PASSAPCGAC
TSAGAVTPHR RYKEPRQKKE YTGTDILCQA KSGMGKTAVF VLSILQQLVR CLGIAHTREL
AYQIKNEFDR FSKYLKNVRC EVVYGGISMN KHVVLFKIPH IIIGTPGRIL ALIREKYMLT
DKIQHFVLDE CDKCLERLDM RGDVQKIFIS TPLKKQVMFF SATMAKEMRD VCKKFLQNPV
EIFIDDEAKL KLHGLLQHYV KLQEKDKTRK LIEILDALEF NQVIIFVKSV TRAITLDKLL
TECNFPSISI HGGLNQEERI ERYDKFKKFE NRILVSTDLF GRGIDIERIN IVINYDMPEN
SDSYLHRVGR AGRFGTKGLA ITFVSSQEDT LALNEVQTRF EVAISEMPNK IDCNEYINQR
MSVSAEAYGE WNKKKNFVAK VHKKDNKEKK KIREALNESF LFNHLNNSEM ETIIDAFFDD
HVEEGANIIN EGDEGDLLYV IDEGEIEIYK TKDNKKEVLT TLKSKDVFGE LALLYNSKRA
ATAKALTKCH LWALDRESFT YIIKDNIAKK RQMYEDILKH VTILKDMDPY ERSKVADCLK
SKTFNADVII NEGEQGDTFY ILTDGNATAL KNCQIIKTYT KGDYFGELAL LRNQPRAATV
KAESTCQVVY LERKGFKRLL GPIEKILIRN VENYKKVLKE LMHLKIICLS DEVREMYKNH
KTHHEGDSGL DVFIIKDEIL KPKTTTFVKL GIKATANTSF LLFPRSSISK TPLRLANSIG
LIDAGYRGEI ILALDNTSDQ EYTIKKNDKL AQIVSFSGEP LSFELVTELD ETSRGEGGFG
STLGAAFGTA KSGVGVCSVG VMRPDLIMKS ILPVVMAGVL GIYGIIMSII ISGDYIKAYL
LGFSIEDALA LLRIDDLYIE SFQIKDVKIL KGDHLSRCIG RICGSNGSTK YAIENATKTR
IVIAGDKIHI LGSFNNIKMA RYSICSLILG STQGKIFNKL NILAKRLKER IEKLEDRMHP
WSNIDGMKAA CSYTYDDIIC MPGYIDFPLS EIDLSNNMTK DICLKTPIIS SPMDTVTEHK
MAISMALCGG LGIIHNNMSI ENQIEEVKKV KRFENGNKVL CNEKKSILPI VNDNYEFPHA
SKRENKQLIV GASISTDLEK VNKLVQNMID IICIDSSQGN SIYQIDMIKK IKSAPIIAGN
VVTSNQAKNL IDAGADVLRI GMGSGSICTT QDVCAVGRAQ GTAVYHVSNY AHNIKTIADG
GIKNSGNIVK ALSLGADFVM LGNLLAATEE SCSEYYFENN VRLKMYRGMG SMEAMISQGV
SASLVDKGSV LNLIPHLVKA VKHGFQSIGI KNIQQLHSKL YSGMKENKQY QEALKELKKL
KKKIEENINI LKNIKGKLIL AHESAWDKFG SKLKDMPFLN NFFENPILGK LFGETELAAA
LRIMKMDDKN FKLSELMYLF EFVISKHIVE SYLIGDEDTL RLHCGEAAFN SLNSSINERK
KKKLFLDTNV LIYKNHELKG AQRMEESSPW FIFTFHTQQI NCLKNKDDEI VEGKIDDIRE
VVYTIALSKH PEPEGLLPYY IIREFAIIGN TPSWMVLYII GLGLGDEKDI SVKGKELIDQ
SDVIYLESYT SILFISKDKL EAYYKKKIYE VDRNFAEENC EQILDEAINK KVSFLVVGDP
LCATTHHDII LRAKKKNIDV HVIHNASIMS AIGESGMQLY NFGQTVSIPY FEGEYKPTSY
YNKIKINLDN NFHTLCLLDI KVKERTIENI MKNKNIYEPP KFMTVNEAIE QLIYCEHNEN
VITKNTLAIA IVRIGSNDQQ IVSGNLFTLK TQEYNDPLHS LIICAPNLHD IEKEYFDMYP
NSAYRKCVRV QLIKNGKKIT AFVPGDGCLN FIDENDEVLV SGFGRSGHSV GDLPGVKFKV
VKVARVSLLA LFKEKKEKPR SMGIKGLTKF IADTAPNAIK EIKIENLMGR VVAIDASMSL
YQFIIAIRDG DQYGNLMNES GETTSHISGL MSRTIKLMEN GLKPIYVFDG APPELKGSEL
EKRGEKRQKA EELLIKAKEE NLEEIKKQSG RTVRVTKKQN EEAKKLLTLM GIPVIESPCE
AEAQCAFLTK YEMAHATATE DADALVFGTK ILIRNLINLE QVLKGLKLTM DEFIDFCILC
GCDYCDTIKG IGSKTAYNLI KEYNCIENII KNIDYVEARQ SFIKPKVKSE VKIDWCEPKI
EELKKFLIKE HNFNEVRVTN YITRLLKARK VTTQRRLDTF FVNNSKYYES LNLKKNCTID
EIKKAYRKLA IIHHPDKGGD PEKFKEISRA YEVLSDEEKR KLYDEYGEEG LEGGEQPTDA
TDLFDFILNA GKGKKKRGED IVSEVKVTLE QLYNGATKKL AISKDVICTN CEGHGGPKDA
KVDCKQCNGR GTKTYMRYHS SVLHQTEVTC NGCRGKGKIF NEKDKCVNCK GLCVLKTRKI
IEVYIPKGAP NKHKIIFNGE ADEKPNVITG NLVVILNEKP HTTFKREGVD LFMSYKISLY
ESLTGFIAEI THLDERKILI DCTNAGLIKH GDIREVLEEG MPTYKDPFKK GNLYITFEVE
YPLVITKEKK EMLKILKKQN EVEDIENTDC EVVTCKLVDK EYLKQRLKTL KNYLPYLCKI
LIDNTVYTKW DYLTMDESHF QNDNADEMTS RTWGNDWTVR KGAALCLDYL SNVYNDDILE
YILPHIEEKL MSDKWNIRES AVLSLGAIAK GCMYSLSPFI PKVLEYLIKL LNDEKPLARS
ISCWCVTRFS SWICHPDKWF EPVLLNLLKR VLDSNKRVQE AACSSFANLE EDALELLNNH
LHEIVHTIQQ AFQIYQAKNY FILFDVVGTL IDSVNIVKEN IDLAHEIVNS ILIKWNNIRI
SSPYIIALME CMSCITSAYG KEFLKYAKIV IRTCIKFLVL LYIDDLIECS FDLLSRIILQ
SNFALIGDIS RFCAQYLILN DIIPFLIAHI SHPSIPVSNN ASWAIGEISI HINSQCIEPY
VDEIVKQHIY ICNSKYHGCL LQNICITVGR LCSTYPKKII YYFPQFLKTW LKIMSHGTQE
NEKINFFHQF LSTMKECIQP EELKLKLAQG LLKSHIVNTL TNNGCTFIFW IADWFAQLNN
KMSGDLNKIK KVGQYFIEVW KSCGMNMENV QFMWASDEIN KNPDKYWSTV IDISRSFNIN
RIKRCLTIMG RTEGEDNYCS QILYPCMQCA DIFFLNVDIC QLGIDQRKVN MLAREYCDIK
KIKKKPVILS HGMLPGLLEG QEKMSKSDEN SAIFMDDNEA DVNRKIKKGY CPPNIIENNP
IFAYAKIIIY PHYKEFNLIR KEKNGGVRMY VNEEIIDNEK LSDIINKTKE NVKYMKGMKL
PNNIVAIPDI DKVIEGADLL IFVVPHQMER ILSSIGRLSV VAGGLSLIPY TFIYDVDGGE
RCVMFNRFGG VSEKTYGEGS HFYFPWFQTP YIYDIKMKPK VINTTTGTKD LQIVTLSLRL
LFRPHTKHLP YLHSTLGPDY DERVLPSIGN EVLKAVVARY NAESLLTQRD TISKEIRESI
TARAKQFNIV LDDVAITHLS YGKEFAKAIE DKQVAQQESE RVKFIVAKTE QEKIAAVIKA

QGEAEAAKLI SSAVKEYGNS LLEIRKLEAA KEIAENLSKS KNVTYFPSTS NILYINAIKE
ENGGYNFENL KRNEILKEKG PKFRKTGTTI CGIVCQNAVI LGADTRATEG PIVADKNCSK
LHYISKNIYC AGAGVAGDLE HTTLWLQHNV ELHRLNTKTQ PRVAMCVSRL TQELFKYQGY
KVCAIVLGGV DVTGPQLYGI HPHGSSCLLP FTALGSGSLN AMTVLEAKYR DNMTIEEGKE
LVCEAICAGI FNDLGSGGNV DICVITKDGT QHIRPYKQPN TRLYHLPKGT TPILYEKIEN
IKKHISLNDL GEARGTVLSV KLDELIDNVE GQTVIDPKGY LTNLNANDSD IADINKARAL
LKSVINTNRK HGPGWIAAAR VEELAQRKDK AKEIIIKGCI ECSKNEDVWL EAIRLEKLSE
SKIILAKAIK NIPTSVKLWL EAYKKEKNVQ DKRKVLRKAI ECIPNSVVLW KEAISLENEN
NAYILLLKKS RVQCNTNNLN INPIISEALK ECPTSGILWS KAIELENKNL QNSKSVTAFN
NCGNNCYVIL IVAIIFWNNY KIGKSRKWFY RAITLNPSFG DGWATFLAFE IDQENEINQK
DIINKCIKAE PNRGWVRGRI HDIRSKGSLA FIILRNKIYS LQCILDIKNN NDKNMIKWVN
NLSLESIVDI YGKLVKPEIS IDSTIIKYEI HILKIFCISK NSKELPFLLK DANMKEIDDE
ATIRVNQDNR LNNRCIDLRT YANYSIFYLQ SEICKIFRNY LIDNNFTEIH TPKLLGESSE
GGANAFQINY FNQNGFLAQS PQLYKQMCIN SGFDRVFEIA PVFRAENSNT YRHLCEYVSL
DVEMTYKYDY MENVYFYDSM FKNIFNKLKN QYPSEDFKWL AVTPIFTYEQ AIKLLIEEIL
TYDMTTDMEK ELGKIIKQTH DTDYYIIINF PSALRPFYTM YNEEDPKISN SYDFFMRGEE
ILSGSQRISD VKLLLDNIKK FNLDPKKLDF YIDSFAYSSY PHSGLLFENL NKEYKYITTQ
DNFDGFRFEV DKNVNKYLQS THTLFLGTRD VGYLYQFGAN FANSDNTLLM ISRINLDGSV
NGRFCKKINH DCKFNFNTYN KSDPRNMYEM SLEVNNPQNT YNFKTIWQGG VDLTYIGSNC
ASIGSFGLRY NHKNHVLTMQ CVRQPNFKSP EFMLNQAHSY KMQYARKISD RLSVGTELEV
TPETKESAMR LGWDYSFRHA KVQGSIDSSG KIAVFTQDYS GFGVSGYIDY LNNEYKFGMM
HIAPSQEQDG GVGKTTFVKR HLTGEFEKKY IPTLGVEVHP LKFQTNFGKT QFNVWDTAGQ
EKFGGLRDGY YIKSDCAIIM FDVSSRITYK NVPNWYRDIT RVCETIPMVL VGNKVDVKDR
QVKSRQIQFH RKRNLQYYDL SARSNYNFEK PFLWLARRLS NQPNLVFVGE HAKAPEFQID
LNIVREAEKE LEQAAAVAID EEDGILTVNS IKSEPTISQY DIKKLVKNKI LENAPFYNYQ
IERSFADKFY GDCIYDNFGL PKNIEINLII LEEWNINCNR NRILKHTGLI KNIEINNFKY
INNKESLEVH FSVNPMYTDL LEKNKVLPPS GIDYNKLIKQ FGCSKIKEEH IKRIEMLTAH
HFIRRNIFFS HRDLDFLLNY YEKNKSFYIY TGRGPSSLSM HLGHLIPFYF CKYLQDVFNV
PLIIQISDDE KFLFNKNYSL IEIKSISKNE MEFTLYNSNA ALANALRRIM LSEVPTLAID
IVNVYENTSP FHDEFIAHRI GLIPIDSRNI KSYEFRERCK CKETCSRCTV QYIIEVKCNN
KINVSHYDIL DHEPNIPMPI PIPIVTLSKN QTIHMKLTAT KGIGKMHAKW IPANVSYIID
HKIVINNHEV DKMSKEHKLL IANNLNPDFY ILLRLSENMS VVMAESCRDA LNELGYKDIV
KIIYDETKFH FKIESVGSMP PEQIVEMAIE ILESKLKDLE PQIKASFYSI EEVAKQLKLP
DPESGKNEEE RCFLSLIKSS ILKNPKKWTN MAKKIIGMSE ETTTGVLRVK KIEKNNGLLF
TAINVNDSVT KQKYDNIYGC RHSLPDGLMR ATDFLISGKI VVICGYGDVG KGCASAMKGL
GARVYVTEID PICAIQAVME GFNVVTLDEI VEKGDFFVTC TGNVDIIKLE HLLKMKNNAV
VGNIGHFDDE IQVTDLFNHE GIHIENVKPQ VDRVTLPNGN KIIVLAQGRL LNLSCATGHP
AFVMSFSFCN QIFAQLELWE NRKYANKSYI LPKELDEKVA FYHLKKLNMY SYGMEGDDTY
LPQVQYPSPY ENQYESPSPR GENHTPFIGY FSSHLLRTGF LLQCLSLILM FVFYWAFGGT
GIFIFDLYAG PECVKVSSAF HLTISVLMSI YLLGTLYIAM FQVFVADNSK WCRGFRAGSK
LLSAAVTLDL LSSILRLVQY LYAYFYMNMR WWARYQQTKS DWTLLHFGSI VHSFSLFIYG
AAFFYMEAYH DEGTYEELAW SNLTLFKLAG LAELNKPKKR TFRTFHYRGI ELDKLLELNQ
EELVKLLPAR QRRKFRRGID KKAKSLLKKL RKAKKECEIG EKPKPIPTHL RNMTIIPEMV
GSIVAVHNGK QYTNVEIKPE MIGYYLGEFS ITYKHTRHGK PGIGATHSSR FIPLKVMVDM
FLWRDPEQFE LKNLANEEAA PTAPHLADNQ YATEAPYDDW GFLQIIKDHH ETMYELKQKI
RPRDQVVGWF CSGSELSELS CAVHGWFKEH NSISKFYPHS PLNEPIHLLV DASLESGFLN
IKAYVQLPIN LVKEYFVHFH EIQIEILPCN VERSDVSLKK LLIMLKNCKS YVQDVVDKKK
KGNVAIGRYL HKVFSNDPFI SIEKFDSINE SILQDNLMIS YLSNLAHLQF LIAEKLNWLN
NLPIKEDESE GRRVHKNLID LVSQNHPLLF GKDNSNIGKI IEIFLSIYET EFSDSDCNKK
IVSLISSLDQ SYLSNLALTN KQSKKLNHIN GCDLFKFYED NINLKPYLKI LKDFDKYPII
VDSENNILSL PPIINCDYTK ITLNTKNIFV ECTGTDLNKL EISLNIICSM LSEYSQPKYT
IHSVLVLYPN FKNKKLTCDI EYVRKLSGIK DITIDNVKKL LKKMMINNTI FEVNVPFYRS
DIMHACDIVE DIAIAYGYDN IKYEPIEISK KHLLNTISDM FRNSMTECSY TEVITNALLS
LKENYDYMLR PVQIMNSKTS EYEIVRTSLI VNLLKFVAAN KHRELPLRFF EIGDISYNKT
DTNAFNKRNL SIIFADKTAG LEEIHGVLES ILKDFQLFSH YKIEEKRKEK IEIRVISIGN
IRGLGGCDYG PFRMSNEFLG WKNKKTNSVY QYKCNDISEG EWIKLSYNNN RLHLKFNESK
DNLIVFFDGF PDRNLSEITQ HFQKYFNIKL GTRKLATKGW NWGEFKLENS NLIFDIDKKY
AFNINTNNIN QLNVQIKTDI AIELKNDENE DVLSEIRFYY PHENDENQNF QDLKNNLLEK
VNIGDSKSEC IASLSNIPLL VPRGRYEIEM YSKTFKLHGK SYDFTVQYSN INKMLLVPKT
NSNQYILIFS LNNKIKQGQT EYPFILIQLS NDDDMDLDIN ASEEDIQNYK LEKTLTGKAY
DVVTRLFTAL AKKNAIIPGD YRTAKNEHGI TCSYRAASGQ LYPLNKYFLF VVKPVILISF
DDIVTLSFQR TGNNQHRFFS LIIKHKRGIS YEYTNIDKSE YAPLLEFLKS KNLNIKGYID
LSKRRVSPKD IIKCEEHFSK SKKVHQTVRH VAQKHNMTVE ELNRKVIWPL YKKYGHALDA
LKEATMNPDI IFKEMDISDA VKESLLSDIK LRLTPQALKL RGRIDVWCFG YEGIDAVKEA
LKKGKVTINI KLIAPPQYVI VTSCHDKELG MQKIQEAMKV ISDKIKEYKG GDFKQQGEIL
VILLDKHDGL SSDDEGERGI FEFFVCDVGV GLSLSVRDIL PIEYDSIFIG VLPYYTFNHE
YIIYDSSQLL PRYLIQFECD PSAEELFSIP LCDYCGNAPS LYYCESDEVK LCEKCDDIIH
SNKLVKKHIR KTLNEARGNC KIHLQNEVNM FCTVCHIPIC NLCMCSHAHI SLKMAYNAIM
QRSSKPSNFI KERKKNLNNL LEKIDKLHEQ VSLNMNETEK NVYNVLEDLV KQLHTTTDKK
MSSILSEEYE LKRQFNEIMW NENFLYYLQT ILPPADFMNA WLKHCQYREE IEKNSLIFPD
IRIKGNINVI TEESINHLVG LPNVGKSTTF NVLTKLNIPA ENYPFCTIDP HEAKVTVEDE
RFDWLVDHFK PKSSVHAYLS IFDIAGLVKN AHLGEGLGNN FLSNIAAVDG IYHVVRAFEN
EDIIHTEGNI NPVRDMEIIN SELIYKDISN CERNLEEISK VNRNKKDKIK QNEHDVLTIV
LEHLKEHKWI KDRTWKSSEI EVINEFNFLT AKPVVYLVNM SENDFIRQKN KYLAKIYNWV
QEKNKGTIIP YCAEFEQKIL FMTENEKEEY FKANNIKTSM LNKIIKTGYY EINLIHFFTC
GQDEVKCWTI RKGTKAPQAA GVIHTDFEKG FICAEVYKYT DLVEFKSEGE VKANGKYLQK
GKDYVIEDGD IVFFKFNVSS SGRINKFVNQ LRISYGTLEE FVDNFVYELK KGLEAHRRHP
NLWIPHECSF KMLDSCISDI PTGQERGTYY AIDFGGTNFR AVRASLDGNG KIKRDQETYS

LKFGTFSHEK GLLDKHATAS QLFDHFAERI KYIMGEFNDL DDNNEKSVGF TFSFPCTSPS
INCSILIDWT KGFETGRATN DPVEGRDVCK LMNDAFVRSN VPAKVSCVVN DAVGTLMSCA
YQKGKSAPPC YIGIILGTGS NGCYYEPDWK KYKYSGKIIN IELGNFDKDL PLSPIDLVMD
WYSANRSRQL FEKMISGAYL GEIVRRFMVN VLQSASSKKM WQSDSFNSES GSVVLNDTTP
DFSECKKIAK QTWDMDFTDE QIYALRKICE AVYNRSAALA AAAIAAIAKR IKICGVDGSL
FVKNAWYCKR LKEHLRVILA DKAENLIIIP ADDGSGKGAA ITAAYILIRW LCKAIVSSLF
GDVNIINPEN VPLYGSVIFV GNHNNQFIDA CVLVASIPRQ VKFIVAEKSM KRAVIGELAR
VAGCISVKRP EDLKFKGIGR IYWNTGDTKI KGINTRFKLD VQIGDKLMTQ NKIFSVTKIE
SEIELILQDP ININCEDNGV PFKIVPKINQ SEVYNLVTHS LKNGDTIGIF PEGGSHDRTN
LLPLKPGVAI MTLCALADGD VSIIPVGLSY SKLYQLQGCV TIFFGNAIIA SQDLCKDYNN
NNRETISKLL AKIEEGMRSC MLTSKNHETS RCIELCVSLY TPERMTISKN KIYNNLQLFS
EMFWKFGNSK EIENLCYELQ CYEKLLEANK IKDDEVWMLK QSTSAATLKF IEQICSLIFC
TIFGMTFSLL WLPLVAISVY LAEKHRKTSL KNSLVKIQGG DVVASYKVLV LLVLLPTFNI
IYGLLFSLYR LMKKKDVDQV HKLLNNYLSK FNIYVKFTKE EISHWFMPIQ NVIYTYVNEE
NGEIKDMISF YSLPSKILAN EKYDMIYAAY SFYNVATTTS LKNLMQDAIC LAKRNNFDVF
NALEVMDNKS VFADLKFGEG DGMDTDSVLG LQAILISANY KEKEFIRIAY YMNSFYKDIE
LREKPPMSPQ YDKICRHIVD NPRIVKFTIP WDDSRVAYEI LKELTQKNYF RIFKVNLHVP
CKLQKISEKC NEKTKCSVCE CTEDEIPYNF RTNEVEVDLI YNSPSYTAYE GKSIWELKGN
NNYKYFGAAK NLKGVKELLF KENDDRKKRR KEKRNLDKIV NIHYFGYCDE ENEILLNEEL
KIQKKLMKHG TLVLPADRAR EYMDCLGKQV DIQFIDMNEK TMKRQYKKYI QRIDDMERIL
RFLEENINKL PNVKIKKSKI ENFLEHDNIY ELDQVEESLN RLHVQFVRFC NNNKDLVDER
NSAIEEKHVI LTALNQLHPS LSTHIMREGI NMMFTNISGV IKTKDQESFS RTIFRALRGN
TYTYFQNIDE KSVFVVYCHG STHSSIYEKI MKICKAYDVR NYEWPKTYEQ ANKRLNELKE
IINDKEKALK AYEEYFINEI FVLINVVEPN KNSLIEEWKL FCKKERHIYN NLNYFEGSDI
TLRCDCWYSV NDEEKIRHIL MNKSTNDLVS ALLLSDKLTP NISPPTYIKT NEFTNTYQSM
VDTYGIPRYG EINPAISTIV TFPFLFGIMY GDVGHGICIF LFALFLIIVH NRMKNNEMLN
MLFNGRYMLL LMGFFAIYAG FLYNDFFSMP LNLFTSPYIF GFDSKWLGAD NELTYINSFK
MKFSIIIGFF HMTFGVIIKG FNALYFKKKM DFFFEFLPQL VMMLSMIGYL VFLIIYKWIT
PGGYKKQGII NTIINMYLLK EINKDNQFYE HQEIVQIIII TLFALCIPIM LICKPAIKTY
KMIKEIWIEQ LIETIEFILG LISNTASYLR LWALSLAHQQ LSLVFFEQTI LSSLEKNTFM
GVLISLIIFS QLFSILTIAV ILCMDTLECF LHSLRLQWVE FQNKFYKGDG IPFKPFNIKK
LLPDNYSAIL ARALSERPLT YLPTVERVCY EVLSDEDEHL NYIQINLLNT IRPTPIRGLL
AATQERFVVV PGIIVQASKP QHKMRKITLQ CRYCDHKMSI DVPLWKDKPQ LPPYCRYVLE
PYVILPNECT FVDIQSLKMQ ELPEAVPTGD MPRHLQLNAT RYLCEKMIPG DRVYVHGVLT
SYNPNPTRVD GTNFSYLHVL GFQKYDGNDL NFDVEERNEL TLLAAEHDIH DKIFKSVAPE
LYGMDEVKKA CACLLFGGTR KRIGEETKIR GDINMLMLGD PSVAKSQILK FVNRCAPVSV
YTSGKGSSAA GMVRQGLLGE NEEKLDYVLG LTLPKLLERR LQTKVFKLGL AKSVHHARVL
IRQRHIRVGK QMVDIPSFLV RIDSEKHIDF ATASPFGGSR PGRVKRRTLS QSMYDRHLTI
FSPDGNLYQI EYAIKAVKNT NITSLGVKGE NCAVIISQKK MATQYITQDK LLDYNNITNI
YNISDEIGCS MVGMPGDCLS MVYKARSEAS EYLYNNGYNL NVETLCRNIC DKIQVFTQHA
YMRLHACKGK NITEESIKDM FSVYGSVEEV FIMKDNTGLG KGCSFVKFAY KEQALYAISS
LNGKKTLEGC NRPVEVRFAE PKSSKQTAKV CSRDLPGHTK MKMRDLNDYT EDKEIIETIN
LAVKYAKDAV VEDEKKNYKE ALNLYIQSLQ YFNYFCKYEK NDNIRELILK KMEVYITRAA
DLKEIINILN KDQNVKWSDV CGLETAKEIL KEAVIFPLKF PKLFNSSALP YKGILLYGPP
GTGKTFLALA CANECNMNFF NVSSSDLVSK YQGESEKYIR CLFDTAKEYS PAIIFIDEID
SLCGSRTDGE NESTRRIKTE FLISMSGLNN YKNNIIVMGA TNTPWSLDSG FRRRFEKRIY
IPLPNLYARD IKNFANITEN YTGADIDIIC RDAIYMPVKK GLYIKCGSRY EGMSVMIENM
AFHSTAHLSH LRAIKSLEKI GANVSCNAFR EHIVYTCECL NEYLPIVINL LIGNVLFPRF
LSWEMKNNVN RLNTMRAKLF ENNEMYITEL LHNTAWYNNT LGNKLYVSES NIENYTSENL
RNFMLKHFSP KNMTLVGINV DHNELTKWTS RAFQDYVPIP YIKQKEVTPN YTGGFISVED
NIKKTNIAIA YETKGWKTSD MITLTVLQTL MGGGGSFSTG GPGKGMYSRL FLNVLNNYNF
IESCMAFSTQ HSDTGLFGLY FTGDPANTKD IINSMALEFH KMNKCTDEEL NRAKKSLKSF
MWMSLEYKSI LMEDLARQMM ILNRILSGKQ LCDAIDAVTK EDINRVVSQF LKTKPTVVVY
GNISHSPHYD EICKMLDAVN KIHFFMGYPE LASVNFGNST VVRCKKCRTY INPFARFESG
GKKWNCNMCY NINETPQFYK RKDLFQRPEL CTGSVEFIAP SDYMIRPPQP PVYLFLIDVT
VTSINSGLLD VVCNTIKKLL DPRTLIGIIT FDSTIHFYNL NSNLKQTQMM VVSDIQDIFI
PLPENILVNV HECQNVIDNL LDNLPSMWRN NKMSDCCAGN ALKAAVMLIK KVGGKILFFL
SSVPNIGDNE YGEFAQSITQ FQIAVDLFAC PYNIDLASIY PLIKNSGGSL YYYPFNVHQY
SDKLREELLF ALTTETAWES VMRIRISRGW KITNWYGNFQ FRGVDLLALP NCHSSQTFSI
VVDLEENVVQ DSVVYVQSAL LYTNSNGERR IRLHTYALPI TQNIKTITDS INPQVVVSLL
SHQAIEVIKK GKIADGRNLI QTLCSQVLST QLSSSENLQL LPIYILGMLK SVAFRDPDMR
IYQWSRLENI PVESIEAYFY PRMFSLHNPA TLNLTCENMT QDGCYLVEDG ENIVMWIGRS
ISLAVVNVAA GLDGCDDQLL PASFRALEAD LNLHPSLLGY ITLAQTLMLS LFSPIWGFLS
DKYSRKWMLV FGTALWGLAT IFLANINDFA HIIIFRAING LALGSIGPIS QSILADAAKN
ELGLSFGIVQ LSSSIGRLIG GVVTTTVSMK YFGTIRGWRL CFIIVGALSI LLSIIVALFV
EDAPSLSKKS IIIILLEGFT GTIPWLALSF NTMFFQYCDL SDLQAAVITG FLLIGSALGG
VLGGHFGDIM HNISNKHGRP FLGQLAMFGR VPLVILTYLV IPKKKESFEL FLSCFFLGLS
SIAGVAVNRP IVSDIIRPDY RGTIFSLTIA IEGVGSSLIG APLFGYLAEE VFNYRNNNLL
ISDMTTEFRS HNAEALSKTL LYLTAVPWVL SFIFYSLLHF TYGAEYSKMN QIIESEMLMF
GLRSLSDFCN PTSKAYKENA YDALNRDAIP SINKAVNNYK DDDDILYCSS KVLFAMSDYC
CSEKDNDALK KLVNDGGAIT EIIKTIPKDQ DTLKNCMLFI QNLSIALLNV FTSDTHTAKL
GSSIISALSV VSKSPSGSKA LNDENAHHKL IDHCLDETAE IIEGAFDVIK NLLSNGYVVP
TIIEKSVVIL DKFKSYPRVV SKGSDTMKSA VGPEQLTDCL NILKKEQQGS KEHDSALELL
SSLSYISSIT DKIVESGGIP VLIELINSGL QQYDSNPEKI ARLVAGASRM LGRISNNPSH
AGVVYEYGGI ATLCTALSYF PNDVDCVSAI CIALIPFVSR SYLLFASLFP ILYASVESIE
LSKASMACIA SASMINEFHE QMVNSQVIEI LSTCIQYHLT DIDYLSNVFS VYFRLSDYIK
TIEPINQYGG ISGIANALSA IDSVLTVMLE NENKEVIIQE GTKIMENLAT ENDCQRHISN
LESIAQSNPD CAYKTLAAIS GLSRIQSLKL MLESKGADSS IYNGMKTWIE SPKFNEQTKL

IKAALKTIKI VELMCIAQVK RLAEEGPDDN ILITSAECIN YLTEVNKINT KEIVESSLES
IFKMMKKYSE SRLTQTNLLS AINNILLSSN CADVLVNKGY VKQIVTYIHK VPMYVDVQII
GFSVLANMLK INSDSLDAIK KANTLIPLQN ALRTHVKNTK LKTTCAPLLA VLMPLDTLTR
EIEELLKLCN KSIQGNNLSQ LHEYLVSLNE LLLTAESSKI SARCNIISET AHVCSNVSQS
RIGIVHLTKN NMASSLMQLY NLLKMPGDEY TEEAVVNILE SLCLLLKHDI VNADIAIDLG
LIEKLCSGIN HFSEFDTVIK STFSCLACIC ATNKRINQLI THPEYDKLIS VIVNLIGNSK
DSRRNAIKAL YELLKVEKEE ISIDISTKTP IVNLFKIMGE YQMDLPIIQD SSKCLSNIAD
HVTIEEDKYS AMKILLECLG KNKNDESTAK EIMSVLVKLC NNNDKPQFKE LGAIDVISDV
TMIHGKNEEI SKLGGILFSY MGADEQVKKL MKLILSVKDN DADAVQEIDN LTGKLELFLR
APLEIHSDAL QYTDATLQKL NGYLGSNLEN VSFQTNIALV NKRLVDRVKY DFEDHIGAWA
VASAGILNQY TDMISNKVGL KHDRIVAHIY SVLAGCYINM DKKAREYAQE ALKFIQRSGS
NFMACKNLRE KLESHGLIHI KEWTLQKNQG YVLCKENRNI CSFFVGKNFN INNGSILISI
GHIDSCTLKI SPNNRVTKDQ ISQLNVLIEK IIQINKSVIF LPSLAIHLQN RTRSVKVNYE
NHLKPILSTL LYEKLPLLYI LANELKCKEE DILDFELCLM DTNKPCFTGV YGEFIEGARF
DNLLGTFGVF EAYVEFVLFF ISSYNVKCQL RFASLGNWGK SKSQLLNAKY LKQFIKSERV
TFIVSPGSNF VDGVWKSLYE DVYEEEDMYM PFFTVLGTGD WTGNYNSEVL KGQGIYPKWI
MPNYWYHYFT HFTVSSGPTG HKDMAAAFIF IDTWILSSNF PYKDIHDKAW EDLKLQLNVA
KKIVVVGDKP IYSSYLLPLL KEAQVDLYVS GHDNNMEVIE DNDIAFVNCG SFCIHELTNN
GIVTKFISSK TGSLQYFATL PKVELIDVPA AGPMGNKDTF VRIVGTIGIL IRYAYSGVAY
SSGALKLPKE WCMNPDKGLI KPDAVCYLVC IYDNDICEIS DIGVNFYINE NHVENKSNAV
LSNLQELNNY VKNIAFLSCN VYGLCGYIFN DFGNNFICYD KDGENIKSCN ISKISKDVNG
IVSFDFDKSS PFQNGDFVKF TNVEGMEING KIYQIKNLKK YTFTIGDTSK FSDYIKGGEC
TQIKTNLKLN FKPYEDYSKL NLSNYLHVEE LDKNVIINVA KYSKAHISPI TSFFGGLLAQ
EIVKFTGKYM PIHQLLYMDF FEKNDNIISI FGKKFQDKLN KLNIFLVGSG ALGCEFAKLV
SLLDGSLIIT DNDNIEVSNL NRQFLFRKEH IEKSKSLVAS NAIKNKNKNI NVISYVTKVG
QENEHIFDEQ FWSKQDFIIN ALDNIIARQY VDNKCVWYSK PLFESGTLGT KGNVQVIIPH
MTQSYNDSYD PPEDSIPLCT LKHFPYDIVH TIEYARDIFQ GLFYNVPLSI QQFENLENVL
NTLKIIKENN FNFCIKKAVH LFHSNFINQI SQLLYSFPLD YKLSTGEFFW VGQKKPPQVI
DFDINNIYVQ EYLVSTSNLY AQVYNIPTCY DIKYILDVIK VEPFSPKSVK VNIDPIEFDK
DEESGLHVNF IYAFANLRAM NYKISTCDKL KTKMVAGKII PALSTTTSII TGLVGIEILK
YVNYLSYFKN AFINTALPLF IFSEPMPPFK IKDKEYDELM KGPIKAIPNG FTTWDKIEIS
IKDYDTQRDK RFSGTVKLSN EVRKKLKVCI LGDAVHSEEA QKLKLDYMDI EAMKKLNKDK
TLVKKLAKKY DAFLASQVIL PQIPKLLGPG LNKAGKFPSL ITHNDKIFLV YNIASFCFEL
QFRFIEDTTF DWLPVIGYLL PYEKIKLLRM IFPIVFFISI CISAYVYTDR NANLIYLMRS
ILSTNRIIVE RSNDVNYLKK NGDQLISKLQ QTRKFSLTLD SQQMNLLEKK GKKKSWFEFF
SLQMVFGVIF VYMWLTSKKP TTYEVPLPLS LEELYKGCKK KLKITRKRFM GTKSYEDDNF
VTIDVKAGWK DGTKITFYGE GDQISPMAQP GDLVFKVQTK PHDRFIRDSN NLIYKCPVPL
DKALTGFQFI VKSLDNRDIN VRIDEIVNPK FRKIVANEGM PSSKTANMKG DLIVEFDIIF
PKNLTSEKKR IIREALGLHY ICILCPSNNW FKDTSIKWNF SIEVGGADID INNTAKKSEL
SATLNTLHVD TGKTKLLDKL RHTNVQDNEA GGITQQIGAT FFPKDILDKE IKKIDIKCLS
KGIMIIDTPG HESFYNLRKR GSSLCDIAIL VIDLMHGLEQ QTKESIQILK QRNCPFVIAL
NKIDRLYMWE KNDWEPFNNT FKNQKEYVKE EFNNRLQTIL NELSEQGLNC QLYWENKNPR
KYVSIVPTSA ITGEGIADLI MVLVKLTQSF MLKNIEYNKK LECTVLEVKN IEGLGTTIDV
ILTNGILKES DTLVLCGMNG PIVTVARALL TPQPLKELRI KNEYIHHKSI KACIGVKISA
NGLEEVLCGT SLFVANNNDE IEEYKKKVMT DVSDVFNHVD KSGVGLYVMA STLGSLEALL
IFLNDSKIPV FSVNIGTVQK KDVKKASIMR EKGKPEYSVI LAFDVKIDPE AEKEAQILGV
EIMQKDIIYH LFDAFTAYLK KIEDEKKQSK MTDAIFPCEV SIINDCVFNK KDPIVVGVKI
EAGILKIGTP LYIPEKNLKI GNVVSIESNK KSCNKAKKGE EVCVKIAGEP NVTYGRHFDF
NQKIYSKITR ESIDVLKQYF RNELTMDDWR LVVQLKKILN ILMIINEKDK LAEQNLETLD
VTKLTPLSED VISRQATINL GTIGHVAHGK STLVHAISGV HTVRFKHEKE RNITIKLGYA
NAKIYKCTNP DCPPPECYKS YESSKEDDPI CPRENCNSKM KLLRHVSFVD CPGHDILMAT
MLNGAAVMDA ALLLVAGNES CPQPQTSEHL AAVEIMRLKH ILILQNKVEL IKEEQALKQQ
EEIRNFVSGT AADSAPIIPI SAVLKYNIDV VCEYIVTQIS IPRRDFISSP HMIVIRTPKL
CNFVEWKEYK LVFKRYASLF FILCIDKSDN ELITLEIIHH YVEVLDKYFG NVCELDLIFN
FHKAYYLLDE ILVSGELQES SKKIILRVVA SQDSLMEVNK FAKSLLDVAD NLSLAIKNIS
EESLKSNENI YKGIEMTETI LHNIFNKYGI DKYNPINEKF NPMFHEAIFE VSDTTKKGTV
ATVIQPGYKI NDRILRHLDI ESVQALIVAL SLYKGGIVLI SHDTYLIKHV ADEIYHINNI
TKVVKIDYEF EKYTKLLLEN KIMPREIITL QCGQCGNQIG VEFWKQLCNE HNIDQEGILK
NNFLNEDRKD IFFYQADDEH FIPRALLFDL EPRVINSIQT SEYRNLYNPE NMFISKEGGG
AGNNWGSGYS QGHKVEEEII DMIDREVDNS DNLEGFILSH SIAGGTGSGM GSYLLELLND
NYSKKMIQTF SVFPLLNESS DVVVQPYNSI LTLKRLILST DSVVVIDNTS LNRIFVEKLK
LNNPTFQQTN NLISNVMSAS TTTLRYPGSM NNDMISLISS LIINPKCHFL VTSSNVQKTT
VLDVMKRLLH TKNIMVSVPV RRGMYISILN IIRGETDPTQ VHKGLQRIRD RKLVNFIKWN
PASIQVTLAK QSPHSPHKVC GLMMANHTSI STLFERCVTQ FDRLFKRRAF LENYKKEPMF
QGNFEEMESS KEITQNLIDE YKSAERDDYF GLARAFGIPA RRYTHEVVTL WYRAPDILMG
SKKYSTPIDI WSVGCIFAEM VNGRPLFPGA SETDQLMRIF KILGTPNSQN WPDVFKLPKY
DPNFPVYNPL PWETFIKGLG IDLLSKMLKL DPNQRITAKQ AIEHPYFMGK EKTHINLVVI
GHVDSGKSTT TGHIIYKLGG IDRRTIEKFE KESAEMGKGS FKYAWVLDKL KAERERGITI
DIALWKFETP RYFFTDIDAP GHKDFIKNMI TGTYQADVAL LVVPAEVFEG AFSKEGQTKE
HALLAFTLGV KQIVVGVNMD TVKYSEDRYE EIKKEVKDYL KKVGYQADKV DFIPISGFEG
DNLIEKSDKT PWYKGRTLIE ALDTMEPPKR PYDKPLRIPL QGVYKIGGIG TVPVGRVETG
ILKAGMVLNF APSAVVSECK SVEMHKEVEE ARPGDNIGFN VKNVSVKEIK RGYVASDTKN
EPAKGCSKFT AQVIILNHPG EIKNGYTPVL DCHTSHISCK FLNIDSKIDK RSGKVVEENP
KAIKSGDSAL VTLEPKKPMV VETFTEYPPL GRFAIRDMRQ TIAVGIIKSV EKKEPGLPIV
LLKDGTDKAQ GKSQIIRNIN ACQVIVDIVK TTLGPRGMDK LIYTERNVTI TNDGATVMNL
NISHPAASIL VDIAKSQDDE VGDGTTSVVV VAGELLNEAK VLLNDGIEPN MIIDGFRNAC
NVSINKLNDL SLSFVNKSEE EKKNILLKCA QTALNSKLIS NHKSFFAELV VSAAYQLGDN
LDKSNIGIKK VTGGSCLDTQ LIYGVAFKKT FSYAGFEQQP KKFNNPKILL LNVELELKAE

KENAEVRIDN PNEYNSIVQA EWDIIFQKLN LIKNSGANIV LSRLPIGDIA TQFFADHDIF
CAGRVEDADL KRTATATGAV IQTSLFNLNE SILGNCGLFE EVQIGNERYN IFKDCLKTKA
VTIILRGGAK QFIEEVERSI NDAIMIVLRC IGNSEIVPGA GSIEMQLSKH LRIYSRSICN
KEQIVLYAFA KALESIPRYL SHNAGYDSTD ILNKLRKKHS EETNDIWYGV DCLEGDIINA
YSNCIYEVTK IKRNVIYSAT EAACLILSID ETIRNPSRNL GLPDCFKDLL KTDKIKHVLC
TGNVGCNENL ELLKNIADSV HITKGDMDNN FDFPEKISIK IGDFKISLVH GHQIIPWGDL
NALLQWQKEY DSDIIISGHT HKNSINNFEG KYFINPGSAT GAFQPWISNP TPSFILMKSS
IVVYVYEEKN GKMNVEMSEL RKYAVEHTPS TSKDSTAVYR NPKFNDKLLD NFEDCFGTRI
RKNDKLGEYK WKTFKEVQEL IILIGSGLGL YLPNCEEWNI CDFSCNAFNI ITVPLYDSLG
IESSKFILDQ TMMQTIMCNK TCGLKLIKSL PGKLSDVSSI CYTSGTTGYP KGVIMTNQNF
IAQIASSCLG PSKFPNEKDT HLSYLPLAHV YERIMMCLFL YLGIRVGYYS GNILALTDDI
QELKPTLFLS VPRLYNRIHE RICNSLKKKS SVVQSLFHKG LDHKNNTGNP WSLFWDTLLF
NKAKKILGGN LRGMLNGSAP LGVEVAKKLK CIFCVPLMEG FGMTEGLGFI TNPIDPDVGH
IGGPLPSVEY KLVSVPEMNY LVTDNPPRGE LLLRGPTIGY FKLEKETNED GWMKTGDIAS
FSQNQSITII DRKKNIFKLS QGEYVAVEKI ESVYKQSLFI AQIFVFGYSY ESFLVCIVFP
SVDTMRIWAK ENKINKSNEE IIKLEKFKND VMKDLIKIGK TDGLNGYEQI KDIHFIMEGF
TIENDLMTPT GKIKRHAVQN KFKQEIDKMY GSKTTKTITE GQSILTMFNE GYASDGIWLG
GTKYQFINMD KGLEYEGHSF DVATCAKSKG GMHIIKVGGG HILIVLYDEE KEQDRGNNLN
MLAVVYLACR EAGHIKSIKE LITFDRSYKE KDLGKTINKL KKVLPSRAFV YNENISHLIY
SLQLSTDLIE AIEYVVKKAT TLIWTDIERY FKDPELITSE ILFVGLTLCN VFVMYRLFLD
VIPFPIFVTW WQLAQGLLVA YVCGLKVLFV PSIFYCLMLV LSNYLLFKTP CISSYPVLVS
FTVVFHHIIR FIGCGEEYML RWKSIAFLLS AFVLGCFDSQ TSGKGVIIWA LLYALFSAVF
RAGFMQKIMH LVEGKGNTLH NNQHMLGVLI LPILILLSGE WKVLGHMPYN ITSLYTGCLV
TVGALPFVKN VVSNRLVRRT GQGPWRFLEI ISIALVFMIG LGYNRPSFMG YLAIVCVIIG
RSLGAFDVLL NKNKVGEDMR NASFALAKSV WAAGDFKGQI IEGIKRPVVT LSLSTNNVAG
VKLPIFQVHI DPTVDVLGNL GVAAGGQVIN NTRENYLQCL NMLVKLASMQ FINMFDWLNI
HSETFYAKIN DDLLATKINL IRDSSTSCKD DNPYCSNNDG KVIIKNNELL SGIICKRVVG
SSSGSLIHIL WHEMGPDKTK DFISALQKVT NNWLEYVGFT VSCSDIIASN KVLDKVKEIL
NKSKNEVTKL VKKAQRGELE CQPGKSLYES FETRVNNELN CAREMAGKVA SESLDEKNNI
FSMVASGSKG SIINISQIIS CVGQQNVEGK RIPFGFNHRS LPHFIKFDYG PESRGFVSNS
YLSGLTPQEV FFHAMGGREG IIDTACKTSE TGYIQRRLIK AMEDVMVQYD RTVRNSYGDI
IQFLYGEDGM AGEYIEDQII DLMKLDNKEI KKLYKYNFDD ESYGDYDKQN ILNQEFEELY
KCKNYLCKEI FSDGDIRQHL PINMNRLIEH AKSQFPNPVE IAQKVNNFLD KLVIIKQINN
SDTLSLEAQN NATILLKAHL RTYLNSKLLI HTHKISLKGI DWLLQEIEKI FYKSLCHPGE
CVGALAAQSI GEPATQMTLN TFHFAGVGSK NVTLGVPRLK ELINIVKNVK TPSTTIYLDD
MISNDQQKAK DILTKLEHTT LKQLTSHAQI IYDPNTTSTI LEEDKLWVNE FYEFPDEDQY
TLGEWVLRVQ LTNIHVNEKK LTMKEIVYII YSVFSSDELD IIYTDDNSED LVLRIRVKYL
EDTFLKKLME ECLSSLKLRG IENITKVYMR EESKITYDSE NGKFVRSSHW VLDTDGCNLE
SIFCAPSVDF KKTISNDIVE IFEVLGIEAV RRALLKELRT VISFDSSYVN YRHLSILCDV
MTQKGYLMSI TRHGINRVDK GPLIKCSFEE TVEILLEAAA FAQVDNLRGI TENIMLGQLC
KIGTGVFDII IDNQKLNDAN QNETIQDITS AGFTTPDSSP LPFSPTYNSN IKNVVMPGNI
RKSEHFLNLM RIIVVYLKKY INIYEVTSEG PLSFLYKCEK DTKLDTSFFK YSFDRLKSLL
NTLQVVDDYS ALNIVCNFCT LIGNYFKGFI IICEPYPEAT IYDPVIQFAC LDSSIAMKSV
LNRYKSIVLT SGTITPLELY PKLLNFSTVL TASFPMSFDR TCVCPLIVTK SSDLIPLSSQ
YSLRNDLNVI KNYGFLLVEM CKNIPDGIIS YFPSYIYMEH VMSTWYELGI ISNILEYKLI
FIETKDIVST TIALHNFKKA CDLGKGAVFL SICRGKIAEG IDFDKHYGKC VILFGIPYQY
TLSRILKARL DFLKETYNIQ ENEFLTFDAM RQASQCVGRI IRNKKDYGIM IFSDIRYTRN
DKKSKLPPWI IKCMDISNTN LTIGASVNIS KKFLLNMSQE YKETDQTKNQ PLTQITELSN
KMKVATIQNN CEVPTIGLWI SSGSKYENKT NNGVAHFLEH MIFKGTNKRN RVQLEKEIEN
MGAHLNAYTA REQTGYYFKC FKDDVKWCIE LLSDILTNSI FDEKLIEMEK HVILREMEEV
EKSIDEVIFD KLHMTAFRDH PLGYTILGPI ENIKNMKKND ILNYIQKNYT SDRMVLCAVG
DVDHANIVKL AEQYFKPFFC GSEIIIRDDD SGPNAHVAVA FEGVPWASSD SITFMLMQCI
IGTYRKNEEG IVPGTINNIS NKMTVGCADY FTSFNTCYNN TGLFSKYLWK ARIYFIWQRL
FSSSSNFMYV LKVHIIDPFN IFKNMSILSE KFKIYILSNY AVYLSFYNYT YAYDKLLDIL
SESSKFYYTF TGRMGIKRKY QKNPATILVL LKDFDPDTDI LEEPYFFDSQ NNLSFDEQIA
LINYCFSIIR FNPHYDEIKF EKLNAIISRC LKYQNYLLHS CILWFKCKCE SFRLKTVDRS
QAQLNELLKE YCNDEPPNKE RIKFIYDIYY PTTWEMKKEV GNVMTKTGSV VSAFNIFKDL
KLWEEAIQCL IEADRKVEAK ELLDTLLEKK KSPPLVCLYG LKYFIEAWDL SNYKYSKAAR
FIGKHYYNKE MYSECCDYLE KALEISPLLP DIWFILGCAY MKIDKFDQAI KAFTRMISMN
LAYLYMKNNV YKAAKICINQ AVKINNNEWK YWDTYLKLSI VQNDVDSFCL ALTTLCQLNQ
VKQIQPWVFD YISDLIVNDK EYDSFWNAYS FFLFVKGEFE DSFEAKIKEI RSIEVSYIWK
NKIFVGNQAP SFKAEAVFGD NNFGEVSLSD FIGKYILLYF YPLDFTFVCP SEIIALDKAL
ESFKERNVEL LGCSVDSKFT HLAWKKTPLS QGGIGNIKHT LISDISKSIA RSYDVLFNES
VALRAFVLID KQGVVQHLLN NLALGRSVDE ILRLIDALQH HEKYGDVCPA NWKKGKESMK
PSEEGVAKYL SSLMEDTAAK IISHSDLLTD PRIAQDFSAE TDELLAKAEE AIEIIYEIAN
ILNVNLDKET IVILIQLCEY G

> ***Cryptosporidium parvum***
NLEGDFRSTK KKIHWLANLP SVLTKCILRE YDHLIDGDEI QDLVNTESLF ETPAFCDPLI
KDLKSGDRLQ LERRGYFIVD LIKIPDGKSK KAGTDSDKSP TCIFPSMVFV GEEAIAQRKK
LSLTFPIDHG HIDDWTKFEE LLNYLFRGLD IDPIDSSVII TKPPLCSNRH EEKITELMFE
MFQTQSLNIA LQGLMALYSA GRTTGVACDI GEGVTQVVPV YDGYCDSSSL RRADIGGQEI
TMYLQKLLSD KGYIATTRDD LEHVRIIKET LCYIAKDPAA ENEREEINEV YTLPDGLTLH
DEHNKIEIDK ERFYAPEVIF DPKLIMRDVQ PVHELIMESI MSSPMEVRKT LMGSILLSGG
TSLTTGIEER LEEELMYICP SQAKSNIRVV PFDIAANFAQ ENNLFFSEAS AVTRCNVKHI
FEHLLQDSAP KLAKVEKILG RTGSRGGVIQ VRVQFMGESE LAGRSLIRNV RGPVREGDIL
ALLETEREAR RLRGTQCEFI KKEYGLAHLS TGDMLREAIK NGTKIGLEAK SIIESGNFVG
DEIVLGLVKE KFDLGVCVNG FVLDGFPRTI PQAEGLAKIL SEIGDSLTSV IYFEIDDSEI

IERISGRCTH PASGRIYHVK YNPPKQPGID DVTGEPLVWR DDDNAEAVKV RLDVFHKQTA
PLVKFYEDLG ILCSGCKVVI DDYKTTGREY GVFVGYYSES EESDYDEFDN ASSGGECSLD
QTCHLAHRMG LAPSKSDLEQ LNEETGGKVT YEDFERWIMS ITHPEDHIDY MVSYFRKYDR
GNGKISRQQF IWLTSIGGDI LTREEAEAIL DKLSIGGDVR FGSLLEIIDL DKNELIKWIK
FVSERSSLKE VEAEFRILDK NKDGKLSNEE FINHFVSEKK EITELNNFYR ELFKEVDTDK
DGYLTVGEYY YLTNYYSLSK ELFVKINSFL SQNDKNGDGI IDITNDSGKL VVFGVDVSGE
KELSVKKIIP IDFVRSNYVI YIQSILTDYG DVFKYPHDYD LLQYINVFAG GTLLSLSLCH
LIPEAYLTLF GLTIILFFEK ALFSPQDVAC DSDSSEDDRR VVRGAKEKFH DELRMIIRRV
NNHVKVSDFS SLSDEYDKLL KCMQKSKNYG IPKFFISVLV ELEAFLDEKF RDKEAIKKLS
KAKATSFNTL RAKFRKSVEE YRDQMDDCKN NPLAYSSSSS SSEWSSDEYE DRHASALAKW
GTRTVKKKEG KKANRKDLQD TTVSGGANNN IPSGDLLSFE GEVTVDMIVE KVGEIVAARG
KKGTDRQEQI RLLKKAAEIS RPLSLQAYAD VLTHLISAQF DTITGAFHCI TSGIWSEICD
NINILLDLVL LNNKDKRVYI SNTDENSSDI VDLKEQDKST VTFLVSFIER LDGESLKALQ
LTDVHSSEYK DRLVQSLHLL ALLWRCYKIC EVSSLSVHLI NQLHFKNDSL AIKVWEFVER
TPEQERAEKR RLLPYHMHLS LEVIDCIYLI CSMLLEVPYL AYHNSHILRP ISKQFRRLLE
QYERQAITGP PESLRDTIIA ATRSLQLGKW RECRDYVFSL SIWDNQEDQT QERLELNIKQ
EALRTYLFTY GHLYSSYSVN NLIEMFQLPR EKIHSLLSKM MLKNELQALW DQTGEFVLLN
HKQASKIQND SLIVADKLLQ FVDCNESMIS SKGNALGLGK EQAIVVGTRA LEYLQEEVKE
ISNGKQLLER IKKRELNDAS GAPLPLFMLE LFSLSREERA IKKMSSRKNA MERRISAGEK
AIKEIITQLC DQDHETEVKE RLLSYFSRDL FEMELRSKFG RILLKQLNSL ERLFKNKSCS
SIYQLWREDI ILIIRKFAEI VYEIVTGNFK ILDFNGSQFL DNFGGNLWMN LNDALNSSSE
IIYLRQKRSP YLIGKCISLV SDSSSNSDLI KVEFKKKDGL TGDHIKYLSK SQLPVKKVQS
KVEQNWLVGF KEERWKKLIP VQVLSKVYYL ELCGEYALYM YDLLLLNEMS FRHFEKLKES
LSSSLCTKLY LSFVDRKVKP LAIIALGDIS MNLGEDFIPY AISVLQLFQQ ASITQYNDGP
VNSEDWIEYL GELREAVLQG YTGIVYGMKD AKRLEILGPY VPSIIQFIDN IVNDYSGEFP
NSNLKNATAL VGDLITAFNG QLIQYLLSKD KRSILENICT VGETSRDPII SNIKWVRKLC
NISMLLHPKF DKSRIDTCKK IFLEKLMSKG EIPKLRINVL QVYYDIFGQD LELLVNILKY
KAQYGCFNNF ETMESSLDKK ITNIDESKSE KRNLYLIISS ESKDNKNYKK AYDYLVGFFE
TFKSIKSNSR ILDLGVQFLI STILLPEILF FDSLLSMPIY QYIKESEYKV LLELFDICYQ
GTVGDFLQNI NESNIVNKLQ LLTISTLAKG KSSIKLDELE KEFRLSSFDT QDAVVNAISV
GLIDGNISEN SNTVNINCVT KRQFGKAEWE SLDKKLNQWM GHLESNYYFF KKAISSSTNA
LNMGNSEWRS ASNLSAEQQI VYTAFISATA KAIGLKIVWS SSPRETQIQV SPDGINYEIV
VPNRRATQNE KEFTEDFMFD HSRDVRAVKI QMKAKPSQYI GIRQLVALGS GSPLAMIISG
ITDEKDNCLQ ELWKMGPNGQ IISAASNPPK CLIVANGDFS EDGRSFWEFN ENSQLQVKAC
VNQINKAGFG VGTTKLELSG TASSTMDALH TVDAAIDSDI ATYWASAAFE STEHKVSINL
SANMTYQLKN LRIDWEFPPF TYSIAAKVDK QITKVEGNPS NTTLNEMNGV ISDFIQIVME
KPHPRYGKYS NKYLYGIREI ELMTNNLRSV VSFCRDAALS KDARDKYFLN YVEDFNSKAS
EMVKSAARDV LSRTKNLGVL NDRVNTLLPS IDECFTDKKE YVSRLEDYIS NAKQLILPRC
APEPIRVWCD IKFGASYYIW NGVTSVSDIR KYCAHVGMEP LVLRNIEQIS GIKSALGIMG
FKLSEGSGIP LAVDYGCYQD LRDGTTDLTS LVLASPESTA TTDAVLLERG ISYTQILDAH
AKKLKNEINT RCREFQSVDE LNSDQNRNDS NGLLSNFDWR SSYLPHIYSA TLGLKNGFID
PKLQGELYQS IKCINQGFVS GNIGIVRISD HSHPVRLATP IDKTCYSLVW RKKDLSETDI
PIVLGEYTGD VYVGDEEISD ESYSFQLTFL LLPNENEYIL SADKTCNEMA FLNHYECVFN
NFNFRINVQW HSVYVDGWPH IILTSIPGVG IKLGDELAAD FGENWFSRIR RISENNIKNE
LILFRLDENL SLSNLINFYE ICEVCQNSIT SKSKFYLLCN GCNRAFHWYC VNRPETSSKN
WFCFQCLSLT LRIGNVFLIK TIPITIYNEE LNEKSHSEIL NTIRFSAILK KQIYKKHLIN
NRHESSLNKS INSNETFVYS YQDKDTNTSS EESLLGNFYK NKGNELYKQK KFDEALVQYD
LAIEIDPNDI SFLTNKGAVY LEMGEYQKCL EVCMQALEKR FEVKADFTKV AKAYNRMASC
YIKMNELQKA KEMYEKSLLE DNNRHTRTSL KELERLIEKA EKEAYINPEL AEKHRIEGND
LFKQKNYPAA KKEYDEAIKR NPSDSRLYSN RAACYMQLLE YPSALIDVQK ALDLDPKFTK
AWSRKGNIHY FLKEYHKALH AYQEGLKCDP DNKECNEGLK NTMAKIQQVS SDQIDEEQVA
HALADPEIQS LLSDPQFRLV LEQLKQNPAT LTQVIQDPTI ANGIQKLMAA GILFRPGTSP
PANYGANRDW NVDLIPKFVM ASGDLVKILL KTKVTRYLEW QVIEGTYVYQ FQKGGLLFNP
KFIHKVPATE MEALKSPLLG IMEKNRCRSF FSFVANWSDD DVSKQMGFNR DKNTMKDIYD
HFGLSSTTID FVGHALALYT NDDYINKPCG ETLDKIRLYM MSLSRYGKSP FIYPVYGLGG
LPEGFSRLCA IHGGTFMLNT NIEKFLYDEK VSGVVTSQGK AECKMVICDP SYVDSKPKVK
CIGKVLRCIC ILNSPINDTN DVSSCQIIIP QNELGRKNDI YVMMVSCTHG VALKGKYDVR
IDAKLNKFIF SKGVRNLPTR VRVRISRKRS ESEDSKDSLY TLVQYIPVAT FAGLQTEAME
FDRQKVNSCT SMSFKGMISM LKPNESWVAR YNKLLNIVPG CYAVSVSGDM GCGSGYLTAC
LLKIIKDAEF SLPISYLVDV NTKANKINSI ELIKMSLFTC LNRNFEIIIF NPPYVPSSNK
ELNQSIDSAW SGGVNGLFFV SYFLPSLVDV LAPKGVCYLL LEENNCPEQI EDCNFPIVCD
RCLGESKFLR MTKSNQERSC KICNRPCTMF RWKLHNSKKY NQTTICYSCG KIKNVCQSCV
SDLNFGLSLY ARDDYIKSKI NIPDSVDNRD HFMENKLRKE IKKILAKDDK IEAKEVTNYS
KNTTECGSSS KTTIKSISRL MVVNISSTTE EDLNKFFSKW GEVKSVYGFV QYEFVEHAVS
ALSQAHLSLL HGRVLRVSPA DYKLKALNKR KESSIDKKTW NLLYISGNSA VNAFIDNEDV
KKHDIVAPDL ASRVSLMETH VISATKEWLK KEGIRSKDTI IIKHLSDQVT LSDLQKICSP
FGRINRLCLS PSKTIAIVQF LEESSAESAF KRLAFKRFKS VPLYIEWAPV NLFDINTNAV
HVFVKNLSFD TTNKALENLF SKVEGFRKAT ISMGYGFLEF KTSENAKECI KRMQSVTLDG
HTLELKISKL LIKNLPFQAT KSDIMSLFNS VGTVTSIRIP KKSDGTNKGY CFIEFLGKLE
AISALEQFQH THLYGRHLII EVAKNNGGQT DRDSESGLTD RQSNKVESNI VEVPELHFHE
KYSAVNIPVV QEKIRYETKE VIQEREVHVV KPVVKEKIVE VPKYKVVEKV VEVPQVVVQE
KLIEVERVEV TSRSRNQSRP TQYRQVPKPV EVPIAFYSAI PVPILIDRAV PVPMELQIIQ
DILCPKIEAI YKDIPIPIPV KRTIEKPVPI GLYDQPELLQ KYLLGSTAVG IKTKEGVILA
SEKRISSPLL EPRNLEKIMI IDRHVGCCMS GLVADAKTMI DHARVESQNY FFTYNENIPT
QSVVQSISDL ALDFSDIKEK GKKKSMSRPF GVAMLIAGAD SDGSSLWMTD PSGTYTQYSA
AAIGTAQEGA EAILLENYNS NMSLKEAEDL ALIVLRQVME EKINSVNVEV AAVKEKKFII
YDDKEIQRVL DRLPRFQTVD AEVQGLKVID LLTFEGLTKS LERISDYLNK IQKALGEYLE
KQRSMFPRFE KLVQTPLTDR TYLTLTQALH MRLGGNPFGP AGTGKTETVK ALGNQLGRFV

LVFNCDEQFD FTAMGRIFVG LCQVGAWGCF DEFNRLQARI LSAVSEQIVE LLNKTIPMSQ
DVGIFVTMNP GYAGRSELPY GLGFKHAFSR LSDTCMVFTK TSNYIGVGLL CKSIMKSENL
IEYWTPICYW YSDTMKPLIP KGSSISEYEE NQRLILKYGF VKDPSLFCDH FNSIDSCSGT
KMLFSPFWKS ERYSIDYSLS TYLSWLYLNK TQKIFCQGRL IRGQVVVIAA TNRPNSIDPA
LRRFGRFDRE IDIGVPDDNG RLEIIRIHTR NMKLAKDVKI DDIAANTHGF VGADLAQLCT
EAALCCIREK MDVIDMEDET IDAVILDSMA VSQDHFNSAL GVCNPSSLRE TVVEVPNIKW
DDIGGLEEVK RNLQEMILYP IEHILKGLSE LGFLNPTPIQ AACLVPAIRD RKDIVGAAET
GSGKTLAYGI PIIANILIVL PSRELAIQVR DHLRALGKYT GLGIHAFVGG LSLEKQERLI
ATRVQVAVGT PGRLRFLVLD EADRLIEQGH FRELKQILQL IYIQNYLFSA TLMQSIMQYV
KLRENQLFII DLLPKGLKIS MIKCESDELE MRLVLYLLKY KILVFVNSIS YVYRLAPLLS
LVLCKDTHEK ELPRKIIGIH GNLSQKQRIQ AIESFSSNSA ILICTDVLAR GLDIPEVDVV
VHLQAPRNIS LMIHRSGRTA RASRQGSAPI CSDIHSASFL LRASEVKKLS DLRMGPLSNR
DFCGTCGSEC PGHIGHIELE LPVFHPLFLG NLVRILKCCC WHCCCIRLSY SLEIWEGIKR
EFLDKASKHS SCSSCGNAFK GRVKESQLGI GITQHFQAFQ IVPIIKNIWN RKIFEFIFPI
CKSLGWEAFF MFTVPVSANK FRPLGMALHP RNDRLKFYVD LQKENTNESS IELQEKVNMY
LDIRQWLEKK AGVIRQKMMG KRVNYSARTV IGPDPYLDTN EIGVPLMFAL NLTIPERVGT
HNVSLMANLS ISDMLLTPQA NFTQLTLIRL NSSGEVCIDV IKDNWTPAWT LHAVCRAIIS
ILCDPNPNSP LNCDAGNILR GVRTKGAIEV GRRAVDYFRG IEFIRWMRKN EDHIKESYPE
VVKNISFESI NDLNQVGTEL IRRSFIVRAE YKPNTGDLKI PKWPKRLCVT SNQNFDEHSF
YIITFDSNKT MSNILMISIL VSVVALFMFW YISVVFLSAM LIVFFGRLIV FAFLWFFGFD
FWILPNLFDE DTGVIDSFKP LYSIIRRGDD WFMLSIRVFC AIFLAGAIYQ LSKTHTASDV
GQFARQSFLD VLDWGHRKIS NEEHICITRC RFESKDAILD ECATDCHCLS LQELLLKDLE
DLDYSNDRLF GKMIDFQEII DIVGIIDSKV SEKYECVKAL YKDHFGELAS IIINKIDYLE
VVKRIITHEN FEDVKLNDIL PNSTIMTITI SSSTSKRNIP TLEKKESILK CIDFVQINES
KSKILEFLQF QIEAITPNLS ALIGPEIAAN LLCVSGGLKN LAEMPSQNIM VLGSKKNSII
SQSDIVRNIQ DKYKKKAIRL VSLKCGLCAR IDFSSIDRRT IKSKLLTSPD ILKITYILQK
DYYFNRHHKY YLRIVRSKAY IQYLEPYESV SILSMAESFG ITQEFLEVVD PNKLILEAKD
KLSLSNDVLA SLAVIDRCVE LPDCALNFEK LGIVQPLLSC LSEEVRSITY QILSKSMQNN
LPVQNSFAKL GALSLLKQSV QGEDSETNKS KGITAISSLV RHNKTLEGSF ISDNGIPLIA
LWLHSENVGV RERALSLLRH LLIQGVVKSE YIIGNNIIDT ILTLSKQNIQ YGETISETLL
ELINAFNPKL SSSSKDKIRE EVNKRMIFLF LNLGLHKWVQ DTCDSLKIRT PTAIQSKSIP
YILKGRNVVG NAPTGSGKTL CYCLPILQIL AEDPFSVFGL VLVPSRELSY QVLDQFQVFG
NKVNANCQVL TGGFDESEQP HILIGTPGRL RNLRFLVLDE ADRLLSESLE DDMLPILSIL
PRQTLLFSAT LTNAIKEIVN NYSTAPMIIV NENPDDSPVE KIRQMYGIIF TATKQQCQML
TSCLEIMYSV TGLHSLMNQR RRLASLGKFR SKTSKLLVAT GVAARGLDIP DVEFVINYDF
PRSFEDYIHR IGRVGRANKT GISLTFVTEQ DVPYVYEFES KMKKEMELLK LDEDEVLKNM
NRVTVAQQKA LLMLEEVYVF PTSLHISLVK EFFGNDFKIG SQNISCTGNG AFTGEVSCEM
LKDMDVDCSL VGHSERRQYY SETDQIVNNK VKKGLENGLK IVLCIGESLS ERETGKTNDV
IQKQLTEALK DVSDLSNLVI AYEPIWAIGT GVVATPGQAQ EAHAFIREYV TRMYNSSNLR
IIYGGSVTPD NCNELIKCAD IDGFLVGGAS LKPTFAKIIE SAKNMSKDTT HRYGKNSFKL
HRLPVPRPGQ VLGLVGTNGI GKSTALQILS GKLRPNLGDY TKELEWKEVI AYFRGSELQT
YFNKMQNGEL KTVIKPQYVD HIPKRVKGRV GDIINAKDEK SIAETLIDQL ELRHLLDRQV
GELSGGELQR FALCVSTSVA SMVYMYDEPS SYLDIKQRIN AAKVIRNVLN HENYVIVVEH
DLSVLDYLSD LVCCLWGSPG VYGVVTTPFS VREGINIFLD GFVPTENMRF REEGLNFKII
AMQRSNFVKY PGFTKTMGSF KLSAEAGDFG NSEIIVMLGQ NGCGKTTFIK ILAGVSKPDD
SDQMPEFNVS YKPQTISPKF EGSVRDLFLM KIRDSFMDVQ FTSEVVKPFN IERISDQQVK
LLSGGELQRV ALILALGKKA DIYLIMSLIC SISGTTPEDP VISKTGYIFE KRLIEEYIRC
NNSCPITKSE LSLDDLIQVK SKSNLKPRLI KNTSIPGILD SLRTEWDAMA MEMFQLRSEL
EQTKSQLTHS LYQHDAACRV IARITREKDQ AISRLAEIQN SILEKDEDNI TSLQSVGIPE
DVIDLFTEYS DKMRPIRKKQ SFPDLIPAEQ VKEFSLKSEI KLKENCNIEV GVAFNDVSGM
ENGEILVSSI ENCEQAVSIF AIEKYSGSQY SSKNTSQLLS FQNSNKIHIF SRNESNIIIN
SLEKHPLGMH IIANSSNGLF SMFDLESRKQ LFLHQEQNNY SQLKPHPDGL ILGGIVPNGN
IDIWDIRNLE KISSLFSNNG YYLLSTSLDN KIHLWDLRKS AILDSLELST LPNNLQIYSG
DIKGGLFHGR GVLIYSKNEK YEGDFVMGKR EGFGKFTYAD GASYEGEWVD DKIHGQGKAS
FSSGNTYEGQ WENGKINGYG KLTFSNGDVY EGEWVDGKMH GRGVYKYVDG DIYSGEWRDD
KRHGKGTVTY VSSTGQIIEK YEGDWVNGKM HGHGKYVYVD SAVYEGDWFE GSMHGKGTYI
FPCGNVYEGE WVNDVKEGYG VLTYQNGEKY EGYWKDGKVN GKGTLTYSRG DKYVGDWLDA
KKHGEGELFY SNNDRFKGNW VADKACGFGV YYANGNRYEG YWENDRRHGK GIFYCAEDNN
VYEGEWANGR KDGKGILRFA MGHSIQGVWK DGVLSQFHSL QFPPESQWSN PNFDMETVYD
IGSKMIESLQ AENIVAGDVI SINKSSGKIT KLGRSFTRSK DYDAVGYQTR FIACPEGELQ
KKREVVHNVT LHDIDVINSR TQGFLALFAG DTGEIKPEVR AQIDEKVAEW KEESRAEIVH
GVLFIDEVHM LDVECFSFLN KALEEETSPI LIMASNRGIT KIRGTDYKSP HGIPIDLLDR
CLIIPTVPYS EEDVKKIIQE RASEEDLKLT DSAYQILTRI AMDTSLRYSL HLLTVSQVLA
NRKKKEEIDL DEIKKAYSLF IDVKRSTQYL IDYQQEYLFS EIFANQDDFW TSDEEEEEEF
IRKKWSIEDD VSDFRLENVV LKYPYELDDF QKRAVINIHN GDHVLVAAHT SAGKTAVAEY
AIELANKNGR KAIYTSPIKA LSSQKYREFL NRFNRIGIIT GDVSINPDAQ CVIMTTEILR
TMLYRNDPCI EQIQTVIFDE VHYINDLERG VVWEEVLILL DPKVQLVLLS ATIPNYIEFA
NWIGRIKQNT VYCIRTLHRP VPLKHYLYIY EKCFQIMDEN NFNINGYKEM LETKFKTEVY
RLQVFLKLLE KNDQLPVILF GFSRRKVEQL ATNLPNLNFL YNHEKSNIIT FIKESTSKLN
ELDQKIPQLL QCKELALRGI GIHHSGMLPI IKEMTEILFT RGLIKVLFAT ETISMGINCP
ARSIVFTSIK KYDGRKNRIL LSSEYTQMSG RAGRRGIDTF GNVFIFNSET IPECIDIVKM
MLNTYLPVQS KFRLTYQMIL QLSCRHSLKI EDMMTKSFKE MFRSINLPIF HRNLNRKLKR
HQIISKLHSI LISNYNQRNT NQKLISGQLF QKLINFKDIS ILHNSSSSNI PENWPKLLPI
SKTLKSIEIE YYELLNIQLI VELNKLDHEI SIYKHFINDE SLDDYPEMKL KIQLLIEKGF
LNENLTITTK GRIASELLTS DELTLIEILL NGMLHKLNNH EITAILSCFV FPEKGIDRPS
LPSVELLNAH DELINIHTDY EKTHYKHQIN LDTEHFWSLC NDKFMLIAYK WSNKESLKEI
MEEMNLHEGT IVRTILRLDE LVRKLIIAAK MMGDKILEEK LCLIHENIAR DIIFMTSLYF
DGLALTKFLY YLYKAGRDKT LFNVSEWDLS QKLLEFRKQQ PKFPSFDTIS SIGENGAIIH

YRPEKENSSI IKPDLYLCDS GGQYHTGTTD VTRTLFFGRP TIEQIESFTR VLIGFIRLHK
LVFPIGTNAT AIDVLARASL WEAGLDYLHG TGHGVGSFLS VHEEPWSICV GRDGALAAGA
VVSIEPGYYE EGKYGIRIEN LAEIIEVDGY RKNKFLKFSP LTFAPIQKEM IDISILSDDE
LDWLNWYHSK TLENLEPLVD DDPKGEFKVE KKPEFINRRL SLFKTLYEKI KITLQNGDQR
DGVKFQTSPM EIARQISKKL AESCIVAKIR YNKSLFIEDD ELWDATRPLE GDCELEIFWH
SSSHILGQCL ENEYGAQVTI GPALNPGFYY DSYMGTHSVS NTEYSDIENC AKTIISEKQQ
FERLQCNKEE ALELFKDNPF KVSLIMSKIP DGAQTTIYRC GSFVDLCTGP HIPHTGIVKA
FKVTKNSGCN WLGNTENDAL QRVYGVSFPD KKRLDEYLNM LEEAKKRDHR LLGSNLQLFF
FDSNVSPGSC FWLPAGARLY NKLMDFIRNE YRIREFTEVI TPNIFSCDLW KTSGHYFAYK
ENMFIFDVEE KEWGLKPMNC PGHCVMFKHM NPSYRQLPIR LADFGVLHRN EFSGALNGLT
RVRRFQQDDA HIFCTPEQIQ EEVFKALDFL FFIYGQLGFG IEILQNRGYD SCGMSTILIT
TKYSSKESGD SIERLKNDSE LLHGNHHIGI AHTRWATHGG KTDFNAHPHQ DYKKRISIVH
NGTIDNYCSL KSELMEKGIK FQSETDTEVI ANLIGSYLDD GEDFQNAVQK ALSRLQGTWG
IAVLHKDYKD LMILARHGSP LLVGVQSGHI YIASETSALA NYTNQYVALQ DGEIALLSHE
GINKLITPSR LLSIDHEKVE SSPSPYLHWT LKEIYDQPHA LARSLNFISP YNNMVKLGGL
DQRLDELKNV QNMILLGCGT SFHAALFAQL LMEHISGFNT VSAKDASEIF VTGFPREHAG
AIAISQSGET ADTVKAINIA DKLGIPKISV VNVVGSMLAR TTGCGVYLNA GREVAVASTK
AFSTQVLVLS LIAAWFAQNR ISQRCQELLE AIHRVPISVG VSLQAKDQCE QIAEMIKDNS
IFVLGKGYGY PVALEGALKI KEISYIHSEG YSAGALKHGP FALIDKDSQT PVILVILSDQ
SLMMNVAQQV KARGARVICI TDDENLCKCE KVLIPSNGPL TALNAVIPLQ LIAYYLAIKR
GINPDKPRGL AKAVTVWIQE IDRYAMENVN KLLVGNKCDL VSKRVVTSDE GRELADSHGI
KFIETSAKNA YNVEQAFHTM AGEIKKRVQV NSQNRGSAQQ KLGAQPIRVK QFTNVPIPQG
WDQIKDELEK YNEMMREAEN SSTKGKKKNE YLWPIYRINH LRSRFIYTKY YLDKEISYDL
VVIGGGSGGM AAAKEAAKYG KKVALFDFVK PSTQGTKWGL GGTCVNVGCV PKKLMHYSAL
IAHHDAQMFG HKTSSSFEWG KLVETLRNHI RMLNFSYRTG LRGNVEYINA LAKLIDPHSV
EYEDNGQKKT ITSRYILLAT GGRPSIPETV PGAIQYSITS DDIFFLSKSP GKTLVIGASY
IGLETAGFLN ELGFDTTVAM RSIPLRGFDR QCSEKIVEYM KATGTKFVPI NIEKVNENIK
VSFSDGSVEE FETVLYATGR NPDVKGLNLD ATSVPSIFAV GDIVEGRPEL TPVAVKAGIL
LARRLFAGSN EFIDYDFVPT TVFTPIEYGH VGLSSEAAIA KYGDIEEYLS EFSTLEIAAA
HREKRENEMD FALPLNCLAK LVVVKGEKVV GFHFVGPNAG EITQGFSLAV KLGATKKDFD
DMIGIHPTDA EVFGILEVTK RSGESFVSSG GCGGGKCGET VYNGPLKLEH LLPSGLTKRD
LEILRENGYH TIECLAYAPK KALLSVKGIS EQKCDKIKSA CKELVAMGFC SGTEYLEART
NLIKFTTGSS QLDRLLQGGI ETGSITEIFG EFRTGKTQLC HTLAVTCQLP VEHKGGEGKC
LWIDTEGTFR PERIVQIADR FNLNASDCLD NIAYARGFNT EHQMDLLQSA VAMMTESRFA
LMIVDSATAL YRSEYNGRGE LATRQSHLGQ FLRALQKIAD TFGVAVVITN QVMSKVDAMA
MFQNDKVPIG GNIIAHASQT RLFLKKGRGE TRICKIYDSP NLPEGDATFS ITEGGINDPM
ALRVAYESSS EIGVFANLTN RYCLLAHGSS AAFTSVFEAE LMDHIPIINT LIGGTRLVGR
CTVGNRNGLL VSNMATDQEL QHLRNSLPDN VKVQRIEERL SALGNCIACN DYVALIHTDM
DKESEEIIQD VLGVEVFRTT IAGHVLVGSY AKFTNQGGIV HTLASEDEMN ELSALLQIPI
TSGTVNRGSD VVSAGCVVND WAGFVGMETT AAEMAVIERI FKLGTYNEEM REKDIKLKSS
IIDTLAIVDT YWQTETGGIV IAPIPGCFDT KPGSATFPFF GIEPAILDPD TGKEIDGPGS
GVLCIKNSWP GMFRGIFGAH YLHEDLYTKP FPGYYFTGDG VLRDQDGYLW ITGRIDDTIN
VSGHRLSSKE IEDALTNHFG IAEAAAVAID HDVKGNALVC FVVLKLRMCV RTQIGPVATP
DHIIVVENIP KTRSGKVVRR LLRKIATGCN DYGDISTVAN PECIKSIPFP NNRSLMHILK
YYFDLMTLPP HSVMLQFVPY LNSIEGKSYE FSFHLFINRF MKSIPIPIEK FVKFTIRQYP
RSYSISSSSL KIIKGLCSSF LFEFDLNLPV LGMIRSSSLE NEIIYKDELD FKRIGALTEV
FFALSKTQLW VEKYRPGNVL DISHHKDVVS MLSHVLKNGN MPHLLFHGPP GTGKTSAVLA
LSRELFGPNE YKNRILELNA SDERGISVVR DKIKSWTRQV VQCNKTHEIT GNLLPSWKIV
ILDEAEMMTA DAQSALRRII EVSSKNTRFV IICNYISKII EPLASRCAKF RFQPISANSQ
IERLKYICSQ EDVSYEDTIV NLSQGDLRRG INILQSASEL DKRISMSSIL DVSGVPPIKI
IERIINSCKI LGESILIETA KLINEGWSVE LIFKGLAEFI IDSKKAFLML RISEADASVI
DGSNEYLTLL NVCSSVQTIF IKELMIRSED GKTLNSIFRS IKELIKRFKQ KEEEAEQDMI
KNQQPIDFNR SKQRVVLKDV GIRPTIGQGR RQHGILEAHN NGFRFSSIDI LYTSIKHAIF
QPVENDLIVI LHLHLKHSIW LGKKKTQDIQ FYSEVGNQID DLEQRRGRNV YDPDEIMEEQ
RERETKKRYN LEYKKFIQGI EELSKFEAEI PYRDLGFYGV PGRSNVQLFP TASCLVHLLE
FPPFVLSLDE IEVVSFERVE QGLRNFDMIF VTKDYSKPVK RVDSIPIEYL DLIKRWLNEM
EIVLKTILSD IEDFVQNGGF NGFLGEDSDD EEDDDDDEDE EYSESDEDDE VEILAKIPFT
SDRKRSSTVV RIKRIMVFSK GADSAMIPLL RQSNEKFQKD LEIGNKMANQ ALRVLCITEK
EISEDEFADW NQKYQLAVNN VENRESSLQE AASMIEKDLL LQGVTGVEDR LQDEVPETIK
CLRDAGIKIW MLTGDKVETA REIAASAGLL SPGHIDGAVL GEIFSGSLAK LFVQMCCKCR
TVVFARFAPS QKGNIVRIVR KHMDEITLAV GDGANDCNMI QTANVGVGIR GLEGNQAFLT
ADYGITSFKD LKVLLLVHGR LAYRRICKLA LYMFYKNLTV GIPVFIYGFF TLWSGTRLYF
DYWYQVYNVI LSSVPIVVVS VFDFDVTKSE SLSKPHLYSF GPENKFLNTK ICLIYLLNSA
WHIFVVFTIP YILFVNSTNL FGSNQSIVGA AIYLVILVVN IKVLLMADHI HYLLGFAVAF
SLFSWICTLF VCAASFSIGS DVYTIWIPLY NNVLLWITLI SGLSSLWPDY FFKVLFSEWL
KLFYSDTVHD HFRNPRNVGS LPSDDKNVGT AVVGKASCGD VVKLQVDIRD GIIKDAKFKT
FGCGSAIAST SYATELIIGK TTEEALKINN KTIADHLNLP PIKVLILGTG WGFMKLAKGL
DVNSNDIKVI SPNKYFCFTP LLTQIVSNRL PREVCEIPIN ELTYRGNEVI KYIQGLALDI
DKENKEVIYF DKKQERIPYD YLIINVGNED SNIVPGIKEY ALYLRNVEDS IKMRDAVVNC
IKEVNWDKMS DDEKRKKLTF IVAGGGPTGV EVSGAFAELT KNKNEYKKLA PFINIKIIEM
ANKLLPTAGD KVSGYTKYVL SLAGIEVLLE TKLKSVSKDY VVIQKELVPY GVFVWASGAS
PNSLTKQICD KVEEQSFKKA IVVDERLQVH GINAYALGDC ALVRPIQITV EQILKESYSG
SANSVGGLSR LHESDELEDY RIRKRKEFED SIRRKRWKIS LYLSYAKWES LQNNIKNSRS
IFERGILVNY ENVRIWREYI KLEITNGNIN NARNLFERVT HLLPRIDEFW IKYIQMELIL
KNYINVRHIY RKWIDWKPDP SIYIQYSKFE EECGEIKSAR GVMKDLIISY PDESNFIEYI
KFEQRHKNLF SSEQIINILS ETLIDINFFS SISDIFVEEK KIEEAIKLCN EGIKILTNEH
KNLKDKLFQL FKMRIPDENI EWIKHKLQNY RDKLLHNPQD FDILFDYIIF ITSVLQEYEN
LIFVLKNSFV RGGLLRGLNE VVLALDSKKA QVCFLAESCE EDCYKQLVEA LCRERGIPLI

MVDSKELGEM AGLCKVDREG NPRKVVGAAS VAIVDYGVES EAYHYLGYYV SYIGEIIGNR
YKVSSNSTGK GMFSSVVKCV DMETQNEVAI KIIRINDMMR STGEKEYSFV KKFKAPNIVQ
VQGTFMHQNH LCIVFEWLHG SLKNCIHIVC SYNKLLSYSK LLYSKYYSKR NMQMKYLEQI
RDLYLNCSHD GILPDMAFEG ATKRNKPNLV PGNYVCSRIN YVDLESGIEL TCITPEEKKT
WSNNENYLGV LKDGMTVTVP QSVAQILLAD QSYVLEALYF AYEICVGQNG GALGFAQMVP
NELRLLSKEA LLDKIAVLLA GRASEELYSE SITTGAYDDL QKATMIANSM ITLYGMDPQI
GLTTFNSNNT SSYSLYKPYS EATSQAIDNC IRKMINDQYS RVKELLILKK EQVHKLSDLL
LNKETVTNQD INECIGLEHQ RLRADEAIDL DSGIKKGVLG EHVKACTNCG SMTHQARDCL
ERPRKVNLDE KRDRWRGFRP EDYKPIIEQF EAVEELARQK RAKKVMNTKT TKKFVSSIYV
EDEYISNHTQ VWGSYYDLEA KKWGFRCCKQ TCRFSKCMIS LMETNQACYY IKEISPVLVR
EFGSQDDEMK RIVLRVLEQC VSVEEIGSEF VKQKLLGPFF GQFWTSRNSL DKRTSKLVIN
TTVSLSKQVG ILDGLLIFLR DGSETFRIQA LETVRNVMEI VPVIHLEQRL EKLLVDGILY
IFQESSTDED SSVVENVGRI LTLLGTRSKQ YLPQISSIIR WRLNTPSPRA RQTAADLVAG
IIGVMKQCEE EQMIAHIGLF LYEYLGEEYP EVLGSIIGAL HAIVTQVRVE KLSPPIKELV
PRLTPILKNR HEKVQENIIQ LLGCCAKKGG DFVSPKEWDR ICFDLLDSLK ANKKSIRRAS
VKTFGHIAKT IGPQDVLVTL LNNLRVQERQ LRVCTTIAIA IISEICMPYT VLPAIMNEYR
IPDLNVQNGV LKTLSFMFEY IGTMSKDYIY ALTPLLEVAL TDRDQVHRTA AWACKHLALG
VAGTGCNDAL IHLLNFLWPN VFENSPHLVQ AVYEALDAFR VALGPGVILN YLLQGLFHPA
KKVRSVYWRI YNNLYIGSQD SLVPFFPPIP QIGNRNFDIN EFYYMGRVYG RGKGISSSAK
PYRRRPPTWI KLKPSEAEDL ITKLARKGLT PSQIGVTLRD SHGIPMVQSL TGSKILRILK
KNGLAPDIPE DLYFLIKRAV SIRKHLEKFR KDTAAKYRLI LVESRIHRLA RYFKRSKQLP
ANWKYQAATA STLRYLEHAP GVEFAYVPSD FFDRESNEDE EREEELSSWG GGRAAGSTES
QGNKSSRKLQ KEHASEFEVA EGRVQDLLRV MEWLYLQGYS LEEQDILGQT PIFWATKRGN
YGIIQWLISH GANIGHRDHK GNTLLHAACS DVDDETVSFL CDLGLIHLIS HSNYENPANT
AFQICWTRKN YWLALMLNFW YYHNKLFGRI SLLRNPYAVY YWFISILNLL VVFIMVSTLK
SYREYLDGFY TWMILFAAAQ LFWVLSNIGN DNIYQLMLKE NEQIQLNLSN EIIGLMPFVA
KERQSNNSKI YIDIVEEVLF LQQIIAPFVL RRSKQDVLSC LPKKHTFIEF CELTKIQKKQ
YDNEIDKDSN QESSDEVNSI IFRMRRICNH TLLHQGHYTS KQIEELVDYL SDNVDEFKGY
SRIKVEQYIT QLCDYEIHQL VSRLIVKSPL LERFRIDDEL IINSCKLKKM NEIIQSVIEN
KEKCLIFCHH TMLLDIIEEY IYLRLDGTTP ILERQNMIEK FQQVPLFLLS TKAAGQGLNL
TVASSVIMMD LWEALLRQEE EVEAMCKDII ATGCNVVEKG VSDLAQHFLV KAGISVIRRV
RKSDNNRIAR VTGATIASRT EELTPNDVGT CCGRFEVKKI GDEYFCFLTE SKTPKACSIL
LRGGSKDVLN ELERNLHDAL AVARNILLDP ALLPGGGGTE MAISCYLNEK SFSIDDTQVW
AYKAFAQALE IIPKTLAQNC GANVMKTLTL LKSHYRGQVH GINGCTGAIT NVTELGIWDT
LAVKQQVYKT AVEATLMLLR IDDLFTFIIY ETCIENSNAR IKEIVNNKIL KRKPLPLNTV
EMTKIASDKL KFSPIKCINI AENLYRKGYI SYPRTETNSF SDSIDISKYI KEQEKSSIFG
SFANELLYLN PRKGCKDDGS HPPIHPVKCL EWLLYELITR HFLSSCSEDA VIMETIVKVD
ISGETFKTKG TVIIEENWLK IYPFERIKTK ILPAFNLGNL AFPYKLFLRK SKTIPPTLLS
EAELIDLMDK NGIGTDATMH EHIEKIQVRQ YVKKNSKSLL SPTALGVALY NGFELISNPA
CNLMHFRIRQ IIERSIEKIT SGITLIRTSN DTYSRKSNII IISYDLITRN EHFRDFQVVI
ADESHFLKNS TAKRTQMIVP LLHKARRAIL LSGTPALNNP TELYEQINAI VPKFPSYLDF
AQRYSDTRIN KFSHRKEFYG SRNTEELHLF IRESVMIRRL KKQVLHELPP KQRSKIPLEI
KDKVGIKMIL HKLTCEIKIN PVQEYIEYLL ENDEKYVIFG HHHVMLDAIE SVLLKKRKTF
IRIDGKTPGN KREEYVKEFQ NNENCKVALL SITACGQGLN LTSAGTVIFA ELYWVPGTML
QAEDRCHRIG TQYSCINIHY LIAEETLDDK MWGTLCRKQK IMASTLDGID QRKMFKRGNR
VGVCISGGKD SSVLLNVLYE LNKRKDYGIE LELIAVDEGI KGYRDDSLEV VFKDMFNTTM
DEIQSKSSKS NSCTYCGVFR RKALDIGSYK VNADVICTGH SCDDTCETLL LNILRGDFNR
LFSTECTYSV DAYRGVSREF IRKIQCTICG YISSSINALK HDNPNQRISS CQIEMDLFYD
DLIVLGSSGK WATLPPIRIL SFDIECITES GVGFPEPHKD SVIQISSVVT LLDSPICNVI
FTLKECASVA EAFVFWFESE KDMLLAWKDF LLALDPDVIT GYNCINFDMN YLLERAKLFQ
LNDFFFFTRL AKTKITSKDT RFSSRAFGTH ESKLINIEGR ILWDILETIR REHKLKSYSL
NYVSTNFLKE QKEDVHYSMI RGLQDGNPET RKRIAIYCLK DSILPLRLMK HLKLFPNIIE
MARVTGTTID ILLSRGQQIK VTSQILRKCK STNFLMPTVK NQSDGDNQYE GATVLEPLKG
FYKDPISTLD FASLYPSIMI AHNICYSTLI PPLSPTGHSF VLSSVRKGIL PLIVEELIAA
RKKAKKEMEE ATDPTLISIL NGRQLALKTS ANSVYGYTGA VAGGQLPCLE LSTSITSYGR
AMIDITKNEV EKIYRKENGY SADAKVVYGD TDSVMIQFGV SDIGEAMKLG LEAATSISKL
FVKPIKLEFE KVYCPFLLMN KKRYAGVLFK NPQFHERIDC KGIETVRRDN CLLVQKVVDT
VLKKILVDKS IISVAPMLDV TNSHFRMLCR IISKKTELWT EMVVDDTIIH CYNDENRHFK
KNDIENPLVL QLGGNNPQKM EKAIEIAYKY GFQNFNLNVG CPSCKVASKG SFGASLFKNP
LRVAKIVDTC NKKVNKRISV KTRIGVDQYD TYQHLYNFIS LVSGCGANVF IIHARKAWLN
GINPSKNRTI PKLKYYWVYM LTLDFPNLTF ILNGGVTSIQ ECISILIKGV MIGREVMNNP
FILSKVDSMI YGGNNSSIHL TRRIVLERYV EYLSKDQGQF TINMYLKPVF GIFHGFSGTK
FWRFIKQLLE DNPLTQISVI SIYDGIGEVI ISYNSNFLEI MTSILNYLKK GCKGSMSIQN
GLEKAKYLLV SIPPYGTKEI IFFLGSMRSV DNSFLFNWVE GFSSNNIIIN AILFIPELYI
IKCLIYLISP NHLAKSFAYL FQHPDITPIM NQNNFPVAQQ VYFSTHNALR AGENEIQKFV
LYELDGTKRI LLSIYPFYDC QMVKRLVIKY LDLPGTSIRD IQLFYRGVEI PNGRFMHTFK
QRHPLHYSLR MNKSDFGIRS TGLKWSSKIQ KLVVEVKLAM QRNVHPKLTL DGTGATYRMY
NAKGQVVAMF KPLDEEAFSP NNPRGYQGKL GQQGFRSGVL SGEGASREVA TAIWDAYYHN
FAGVPDTTLL EACHQAFNYD SWNKITLEEI TVDWKLGAFQ EFISTTETVG NFNPSVFCIR
DVHRIGILDI CLFNLDRNDS NILVVPTHLG IEQSRRDDLE ALGYVLMYFN RGTLPWQGLK
ATSKKDKYDK IMERKIATPI ETLCTNEFPM GFDQVIIIDR RCDLVTPFST PFSYHALLDF
LFGVQKTYVD IPLFAILKDL KLKDVGIYLH QKANELQSLY EKEKLKDISA IGDFIRKLKG
KQREQGTLAK HVNIATYLNE YFTKDQTLRR LELEDSIMSD SHQSVTGVVK ELSTKFDLLD
QEDIQIEEIY RLLCLSCIIE NGKKHILSVF GFEELYRMNI LERVGLFKFD PNKKSYWQLI
KRLLNLFVDE SNDISCVYSG YAPISTRLIE IMNNSKSLKE ALNYVWGPSV ELTPDTTIIQ
SLCMNCHKEG ETKLLLTSIP QFRDVILMSF ECPHCGFKNN EIQSGGVLQD KGECIELVVT
NVSDLDRQIV KSEFATISIL EQELDIPPST QKGVISTIEG IITKTIQGLV GGKIEKIINN
LFTIKLDDPS GNSFIQYDRT KEQLEEMGYY KFDVPCPNCG NNGESDVCEI DIPGFRRCLI

MAFVCNFCGI KTNELKPSGA YGELAKKWIL TVESELDLNR DILKSDTASI EIPEIELEMG
MGSLGSLFTT VEGMIVKITD SLKDCFTFQM YVVNRKGEEE PVSFDQILSR ITKLSYGLHP
LVDPARVTQA VINGLYSGIK TSELDELASQ TCAYMAATHN DFSKLAARIS TSNLHKNTSS
DIGDVASQLY NFKDNQGCPA PLISKPVYDF IMENRERINS KIDFSKDFEY DYFAFKTLER
SYLLKIDNKV VERPQHLLMR VSCGIHCGDI EAALETYELL SQKYFTHATP TLFNSGTPRP
QMSSCFLLRI PEDSINGIFD TLTKCANISK TAGGLGVAVS NIRGTGSYIR GTNGRSNGLI
PMLRVYNDTA RYIDQGGGKR KGAIAIYLEP WHVDVVEFIE IRKNHGKEEM RCRDLFPALW
VPDLFMERVE KDQDWTLMCP DECRGLQDVW GDDFKKLYEE YEKQGRGRKT MKAQKLWFLI
LQAQIETGTP FICYKDAANS KSNQKNLGTI VSSNLCTEII EYTSTDEVAV CNLASIGLPK
FVDKNNKTFD FDKLKEVTKV ITRNLNKLID VGYYSLKECK KSNLRHRPLG IGIQGLADCF
MMLRMPYESE GAKKLNKQIF EVIYYAALDA SCELAEKYGP YETYSGSPAS KGILQFDMWG
VTPDSGLCDW DLLKDRISKH GIRNSLLISP MPTASTSQIL GNNESFEPFT SNIYHRRVLS
GEFFVVNPHL LNDLLELGLW DDRLKQNIIA NNGSIQNILT IPEDIRELYK TVWEIKQKTV
IDMAADRGPY VCQSPDLEMG ELMNELDMLG FEVGSNFWES INHEIAVELY MNCLSIALEI
DTEDIRPEEL IGQLPSIISE NGKSQIKPIG NLRFLRYCKI LWVMIGIDDF SMNIYRPTPD
RIYSFLCGFL KEQLKDVEFR ITTGNQDIQD LKDQVVQSPE RLRNTLEELN KSLENERKQI
DQISIKNNEL KERQNLLQKT EKRLGKAKTF LEQTIKDANN IKQSIKEIEH HIEKDDWWTL
GILIYEMVVG FPPFYDDEPM GIYQKILAGK IFFPKYFDKN CKSLVKRLLT PDLTKRYGNL
KGGVSDIKLH KWFYNYDFNS LISRKVDPPY IPKVNSYDDS SNFPDSHEQP TTVTGNADPF
VDWMISQFLI LNVRGDTIIF RDFRGEKSLS ESDEPTVIYF EEQIYIYLRQ SSLFFVLTSY
YDVSPTYIIE LLYRIIKLVR DFCGTVNEDS IRRNFILVYE LIDEIIDYGY PQIVSTNQLK
YCVYNETISS NASQRPITCV DRNNEVFVDI FERIEGGILM KSYLIGQPEL TLGFSNSIVL
KEDDELSPSS TIIDDCNFHE SVLTLKPPEG EIIVMNYRIS MKSILDEGDV ICTEVQRVQS
EGICHLHTRS AKYGKLANGM VVKVPNKLIQ RQAQHIVKLK YVNSNIRKSI VMLAFIIKLF
GLKNMQITPE RICKVFIYDW KTPDSRKIIA ADSNGRQVSL ALEGGLIVIL ELNVNGVLVE
VCRREITCEI ICIGIQQGQM RSDYVVVGST ENALRLYKIE KRLKQTCTQI LPNNSIPENV
QLLILFVGLT TGVILSCKVD ASGSISDPRS KYLGNRGVNI CRIEMSLVCM SSRPWLVDSQ
TSGVNFTPLQ YRCIDSIAPL NTHQVNNGYV AVSGSTLLIF QVTQFGESFS QSSINLSYTP
RKLLTLPSPQ MIAIVETDHN SFDFGTKKEI ILALQDVKMG GFFAGEGKWG GCVRIVNLKS
METIQLIPLD TNEGCISACV CKFDELDCLV LGTVYGMKLE AAIKIFKYDS NYNFELVHIT
PIENSATALT GWRGRLLVGI NKTLRVYSLG KKRLLRKSEY RNIPQGLTWI KVVDRIFAGD
ISNGVLVFKF NNTSNQFILV AKDPMPRWLT SAEVLDYHTI AVSDKFDNII VSRVVEDKPD
VTYNDIGGAK EQLERLREVV EMPLLHPERF VQLGIDPPKG VLLYGPPGTG KTLTARAVAN
RTDACFICVI GSELVQKYVG EGARMVRELF KLARSKKACI LFIDEVDAIG GARGDESAHG
DHEVQRTMLE IVNQLDGFDA RGNIKVLMAT NRPDTLDPAL LRPGRLDRKV EFGLPDLEGR
TQIFRIHAKV MSMERDIRFE LLSRLCPNCT GADIRSVCTE AGMFAIRARR KSISEKDLLD
AINKVVKGYK KFSATAKYMV YNINKSGANN LQLMLNSIWS LVSLPSNQEI LLPRAYPLPT
KKEKTRWEKF AELKGKKRKR SRMVYDPITD DFVPRWGRNS IKKQKKHNEA IIEIKGNMDE
DPREAINLKR DMMINKQRLR ELKNKVFDRL LDPKLYTGMH KYRFKRHSYA NISSAKVIWL
YKNGDKYDKG TIYYVKPYIR NMTQLLNEIN RNVRLLGGPV RALYDQGDGA KYLCTSGEQP
TSIEKLEKFD IKKQFRDGQK YITPPNGDAT RAFYESLYNE NPYSIIALKY CVENGILMGA
KHTSAYKRLE HLRESGLLKR SLGGIQNEAI ESLNVKFVQE KKLITKFFDE VAQDTGKYVY
GINETLQALE MGAIELLIVW ENLETKRMVV KNPSTGEEKV FLNSPTEQHD ESKFKETGAE
LDVIEILPLT EWLVNTYQNY GAQLEFVTNK SQEGNQFLEL HAAFLAFTLT LYPDLGSTVT
LLTNALGSRC IDTIVEILSL PFQSMPLSIM EMNHFPNLLY FLNKQAGKKV ALSLINTIVE
NNTPFDDADA LQRFCSFILP MLDVNQIFSM YGILFDLFSR SSRFKYTFPT LGYCAINLIM
FEARLSNGGI LRKVFEAITN LVSDVNLECN ESGVTIQAMD NSHVSLVGLY LKDTAFERYR
CDKNRTLGLN TQNVVKLLKL CSNDDQVLLR HDDDSESLIF IFETPNGDRV SEFELTLISI
DQDSLQIPET SFSSVVTMPS NEFQRLCRDM AQFSDSLSID VTSKNVRFST KGSLGSGSII
LRPKEGADSE AVALDVSEPC QLVFSLRYLN NFAKATPLSN SVKLSMSENQ PLELEYPLEG
GHLRFYLARK ITEDCPSKCS KASSHIKGSY IYSDDTPVCK AAIHAGIIEV GGEVVITFVS
GQKQYFGSFN NGIQSRSIET SDLASIRSFT IGFRGGKNDF IDATNLPGGD RISSLDDFTI
SARVGQGTWR TIMAHTECLG ISLYIDRDNT IHFDQLCNSD TVSTSYAPAI NEWLFLTVAY
NFNEKRVVMY INGNEVANKV TGFVFNLKTK LIIGRSSDSD SEYFLGQISN VNIWDKFFTP
SEVKSAMEKS WHRHTIDGRQ CITSCQSEFP VAYKMYATNP SIQIKCDTNL DNARFSSKAN
KFRVECPAGC SKSRYPVKGH KVYSPKSSIC QAAMHMGVVA LYPGLKQYTG AAGKNNIVST
DENTPCSDTA SFIFQLPVGE RELVSCPSNC GMDIYSPISS ICRAAIHASK LSSTAGEIQI
EVGPEQFHFE QSNRNGIQSQ STGYYIRSQN ELGENISIGA INKRFSLDLS SGPYSINYSR
NGNQLLMGGR KGHISMLDIS NGAPLLELPL AEQINDVHFL HDHTLFAIAQ KKYIYIYDRE
GIEIHCIRDV TSPYKLDFLP YHFLLCSIGE FGELRYQDVS TGQVAALHKT GRGPCHIMKH
NQFNGVIHLG HSDGVVSLWT PNVSTNSLIT AGNDNSWKVW DIRTPLYIYK SFGSSVRSVS
ISGTGLVALG FGSHIQIWKD LHSKPYITHN HPAVRVSEVE FQPWNDVLCI GHTHGIETII
APGAGYANFD SRECNIFETS KQREVRMLLE KLPASTIEPQ GRGYCFVPKI ENDFPVRKLQ
YSPLGLFSFP FKTNSASTWP RWSNNEIFVF KVQDTSVSIF KSDEEGSKLS SLNFTVVSAQ
NMDVYVYHAE MDSKKEIQLS WNNSGDSILV FAQIETLGKS YFGSSSLHLL RIQNSKHQVV
VPEEGPVNDV AWSPKSNEFL LCKGIIPPEL TLNSGIGSPK VSFGKSRRNT IRWNPSGKWF
AYGGFGNLAG DLDIWDLDKQ KLIGQTNASC CITLEWSVCG RFLLASTTSP RLRVDNAIRI
FRYNGDLLKR VNFDTLYSAY WSPPTLEASA AAVASRQKQK QKIDDPVPLP NVRGDVLRKV
LDYCEYHVDN PSKEIPKPLR SNSLSNIVNE DNIPLISEYA QNMLQEIEMA MVVFQQNMKL
NDIGFLPTSI ISGSSSFGTF SSKVSIPYFF SKLGSPFDLK IDIQFRSKDN YISEIEKYLL
KQHRYNYFNR VNQLVSQFLE ISNYLQSNIN KEHRCEILEF IIEELNLWLF YSRFSISISR
TTFLLNSISL PCQFLRIIPN ECKIYNSRKR VHFLLAIEIA DLDDQDYELI PSSQQRLIYN
FGNIPDLNSL MNSPIFQQSI NELANNPQLV RNILHSNPMF AQLSPMLDQM LNNPEMMRMM
LNPQMIQSVL PAPQMYATQL SQLRDMGFID TDASLSALQE SGGDINAAIN KLLERGSGDR
LTRAAKVLEQ LTDQKPVFGQ ARFTIRSFSI RRAEKISCYV TVRGDKAEEI LEKGLKVKEY
ELRKRNFSAT GNFGFGIDEH IDLGIKYDPS TGIYGMDFFV QLTRPGNRVS LRRKEKIGWL
TASRLLLEID SQSNVDTSHQ IYPLLFMSLS QDTATSKLKL SKLAPMTIQF MRDLKTFTNI
EFILSCTGIG FSNMKGKECV AFATDMRLGS NGYRTISTDF DKVIRPSSKT LMGFSGLATD

```
IHTLTNLIKF KTNLYHLREE REIGVKALSH MTASILYSKR FSPYFVEPIV AGPFIAGYDL
IGCLSVCSEF AISGTADNQL FGICESYYRP DILVVGAGGI GCELVKDLIL SGFSNITIID
MDGIDISNLN RQFFFRRKHV GMNKSTVVAL EAKKLFKVSN IVGIVGNIMD YNTEFFSQFD
VVLNALDNIS ARSYVNKICI ASNIELIDSG SAGYNGQVHP IIPRVSRCYE CYPPPTQKTF
PVCTIRSVPD KPQHSIAWSK YLFDIVFGVE DSDNILDISK VQIDLDSLKQ LEKNEYIVNM
FNFLFYSEIT LLANNQEMYI SNNKKRMNEI GTASLCFDKD NKDAMDFVSA ASNLRSYNFH
IPLQSRWSCQ SIAGSIVPAV ASTNAIVSGV QFELVDCCKP ESSRNSSSKI IILDFGSQYS
HLIAKRFRAL GYYSEIALPS TNLNTFNNAK GIVFSGGPSS VPEFNNDILN LNIPILGLCY
GHYIVNIGYG GQVHKAPIGE FGYAVWMSHQ DEIIIPGNDF QLIASTINCK YQNLEKKRFS
LQFHCEVNDT PCGNIIFNNF AYCNMEKNWS QDFVLNHILN DIKIQKNNKN VLLFLSGGVD
STVTFALLNK ALGDKVLGLH IDNGFMRKNE SKNILLYHKF GFKNFIIKDY SNSFLNIIKN
ITDPQKKRMA IGEHFINIKN IFIQEQNFDP NNWLLAQGTL YPDIIESGGT KNSNTIKTHH
NRVDMIYDLK GLIIEPLREL YKDEVRMIGK KIGLNDELIM RHPFPGPGLS INVIGDFDKY
RLDQLREVDN IVINELNNFG WIFQHLTIHL PASNEKASFV LRPVCSEDVM TARFAFFPKD
LLEIIIQKIS LLNFVDAIYF DVTNKPPATF GWEMPMSGHV PGVSESRKMP LILWISSEDV
IEVDSMLTKW LREHQRQGVT FIFECLMGLR DFDGNGCILA DDMGLGKTLQ SITILWTLLN
QGFDGKPSVR KAVVVCPASL VKNWASEIEK WLQGKCKCTP VAERDREKVV SAFAGFKYDT
MSRILIASYE TFRMHVEQLD GVPIDLVICD EAHRLKNDKT KTAMAINNLP AKKRLLLSGT
PIQNDLVEFY SLVSLANPQV LGDVSQFKKI YANPILEGRE PDASEYQQEL ATQRLQELSN
INHFILRRAN TLLAKVLPPK IILNIFCNLT PIQNYLYRRF LRSSACKKLL DSDSTGQVLS
SIQSLMKLCN HPTLIRPKGG KSGKLYLLSR LLFHIRSNTK DRVVLVSNYT QTLDVFECLC
RDLQVPCVRL DGSTSITRRH NLVKTFNDNS NSFAFLLSSK AGGCGINLIG ANRLVMFDPD
WNPANDKQAL ARVWRDGQKK NCYIYRLFST GTIEEKIYQR QLCKDGLSAM LVTSNNELKD
SISADLVRDL FTLKEDTISD THDMIQCNRC KKIEPVGEAF FYRYELAKGV LSLARLKELV
EEKETLYEKL GLDENVCVKE IKQAYRRLVL SHHPDKFLKI QEAYEILSDK NLRHAYDSAL
PWSIVKPVPE IGNIDDNIEV IESFYEFWRG FQSNRDFSIH EEHELNHAEC REEKRWMERQ
NFKIRSKYIR NEISRINRLV DLAYKNDPRI KQHNKRKEEE KRKKKSAIKS LRVSIRNFSV
CLKTIEDIHI FLWTVSEMSL LAKALQKYPG GYKNRWDMIS EYLKTKTKEQ ILTKVKELSE
SEKLWTRDQQ CSLERALKQY PSSLPMPSKR RNNGRSKKGR GHVVAIRCSN CGRCVSKDKA
IKRFVVRNIV DASSQRDIRD ASVYSTYALP KLYNKMCYCV SCAIHSRTVR VRSVTDRKIR
ANPNSQRFKA QKKVSVSEIV FESSYTILDT SEKTVMLHVN HVLYHLDFNT LAVIKNADMS
DEMQQDAISC AAAAIERHNV EKDIAAYIKK EFDRKYNPTW HCVVGRNFGS YVTHETRHFI
YFYMGQIAVL LFKSGVGGWA RTVRKQSDTL LFISLNDGST SSNLQCVVEK TVKGFEEGLK
ATAGCSFKIT GTIVKSPAQT GDDELKICGL DASKYPLAKK HHSKEFLREV AHLRPRSQFF
SSVMRIRNSL AIAIHEYFQK NGFMYIHTPI ITAADCEGAG EMFQVTTVDY KKDFFGKASY
LTVSGQLALE NFACSMSDVY TFGPTFRAEN SHTTRHLAEF WMIEPEMAFA DLSDNMKLGE
GLLKYTVEYV LINNMPDLLY LDKNIENGLV ERLKVICKEE FARISYTEAI EMLKPHDKEF
TVPVSWGMDL GSEHEKYITD VLEKRPCIIY NYPKDIKSFY MKLNEDGNTV AAMDILVPKI
GEVIGGSQRE DDIEKLENAI KSRNMDPAPY WWYNELRKYG SVPHSGFGLG FERLIMMVTG
VENVRDVIPF PRYPNHCEFM ESMVEVAEKH NTDFAKNLLI KSKEGLFFVL AHHETDTKMK
NLGQIFGVNK LRLADEDVLN DTLKVKRGCL TPLSLIFEKS GGIQVYFDEC LKDKVFVHPL
TNTESFSIHI NDIVKFAESC GKKVLLGITA DKITSFADWY SQVIVKSEMI EYYDISGCYI
LRPWSYFIWE TIQSVFDQKI KQHDVQNAYF PIFVTQKKLE TEKDHVEGFS PEVAWVTKSG
KSDLAEPIAI RPTSETIMYP YFAKWIRSHR DLPLKINQWT SIVRWEFKHP TPFIRTMPSP
EKAAKKKQYF ERLSEYATSY PRILVANADH VGSKQMADIR LALRGKAAVL MGKNTMIRTA
LKQMLGSHPE LEKLIELVRL NVGLIFCIDE PSEVRIEEYR VPAPARQGVI APCNVVVPAG
ATGLDPSQTS FFQALGIATK IVKGQVEIQS DVNLIDEGKK VTASQAVLLQ KLNIKPFSYG
LKVNNIYDHG SVYSSSVLDI TSEDLISRVA EATKYVAAFS KETAIPTLPS ARDGIISSFR
NCVALGLDVD FDFPEMQAIK NALANPSSFV AASTAAEEEE EEEGDLGFSL FDSNSDFTRE
PFPGRVFEDL GGAFSMGCIG GFITSFFKGV KSSTKREMFS SGMLFARKAA PSLGTSFAIW
GGTFSCFDCL FAKLRGKEDH WNAIFSGTAT GGLLAIRGGL KTSLKSAMVG GILLTIIESV
SIGINRKTIQ TPRQQFKEQM SEERMLVDEV GEVIVTNDGA TILSQLEVKH PAGRVLVDLS
ELQDKEVGDG TTSVVLLAAE LLRRGTSLVS NGSHPSNVIS GYKLALKECV RYISGSLSIN
TISEECCLNV AKTVLSSKLA GADSEFFGKL VVDSIMTVKA TDPTGAVKYP VKSLNILKTH
GKGLSESFLV EGYALHTGRA CQGMPTSVKN VKIACIDFPL KQYRMQMGIR VELEDPKELA
RIRLEEKEVI HKRIEKILAT GCNVVLTSGG IDDQCMKYFV SAGCIAVRRV EKVDLRRIAK
ATGATICLTM AQLDSEESFD PAYLGECQEV REERIGDTDY MMFLGCKTHR AASIVLRGAN
EVLLDELERS LNDALCSVSK LLESNGYVAG GGAVEAALSI YLEDFARTLG SREQLAIAEF
AEALLVIPKT LAINSAKDAT DLVARLRAYH ASAQQKSQDQ DKYKFFGLDL INGTVRDNVQ
AGVLEPTISK LKSLRFATEA AITLLRIDDY IKVKPVYKPN VVYFKGHKAA VSDVEFSPFY
SCLLVSASQD KTIKLWFRGH TKKVSLVKFN PSAEWILASA SRDNTIKIWN CETVQDEINI
GLPGLPTSIK WSYDGSLLAV SCMDKVTRII DPRSERITYQ WQAHNGSRKS RCEWMGGTMG
NPLLTTGFSD KGERQIGVWD VRYLDKSVGV EFVDIEQPSL IPFWDEGTGL FYLAGKGDTN
IKVFEYSDGC VRRLEEYRSV NSLKGYCLTP KQNVEVMKCE IDRILRLESG GVIQPVSFIV
PRKTFYSDLY PDAIGHTGEP MRMSLFNQLQ VIGQFNKGFI LTKIFIIDQH ASDEKARFEK
LNSDLNIQTQ KLIQLTQLPV ILGIPLKQID FLDLLSQIWC PSGIPRPRRI WSILASKFKN
FVVCWLDRLI EAQDKDVENL NSALNNEQEK QRDAKKVELD EVVADTQIAY SSELTPFGAP
VSAGASTSFK RNIPKARKST KTGVDILCQA KSGMGKTAVF VLSILQQLVE CICIGHTREL
AFQVKNEFDR FSKYLKNVKP QVVYGGIPIQ KDIDMLSTPN ILIGTPGRII ALIRQKKLVT
EGIAHFVLDE CDKCLESLDM RKDVQEIFMS TPRKKQVMMF SATMTKEIRD VCRKFMQNPV
EIFVDDETKL TLHGLLQYYV KLGESEKNRK LNDLLDQLEF NQVIIFVKSV SRAQALHKLL
TECSFPSICI HAALSQQERI SRYQQFKNFE KRIMVATDLF GRGIDIERVN IVINYDMPEN
TDSYLHRVGR AGRFGTKGLA ITMVSSQTDS QVLNDVQSRF EVNIAEMPNQ IDTSSYINQR
TSVSAEAYGA WNKMKDFTPP SYPKTKEQEK RIREKLLESF MFTSLDDDEL KTVILACVET
SVKKDTEIIT QGDNGDKLYI IDQGVVECYK KTTEPRKHLC DLNPGDAFGE LALLYNCPRA
ASVVAKTDCL LWALDRETFN HIVKGSASKR ISTYETFLKE VEILKTMDVY ELNKLTMVLK
SSIFEDQEII KQGEQGDTFY LIITGNAVAL KDNVEVMSYK RGDYFGELAL LRNAPRAATV
KARGRCKVAY LDRKAFKRVL GPIEDILKRN TDKYKTVIKK IMHLSLLPLC PIAEELYKNH
```

KTFHDGDSGL DLFIIEDQVI KAGETAYIKL GFKAAAPVSY LLFARSSISK SPIRLCNSVG
LIDAGYRGEL LAPVDNIKDF DFQVKKGERY FQLVSFNGEK ITFSIVDELD KTTRGEGGFG
STLGAAYGIA KSGVGISSMA VMRPDLIMRS IIPAVMAGIL GIYGLIGSLV IFFDFIKAFL
LGFELQDAIA LLRLDDLYIE SFEIKDVKRL NGAHLSRCIG RISGRDGKTK YAIENSTRTR
IVLAGQKLHL MGAFHNIKLA RDALCSLILG TPPGKVYNHL RIVSKRVQEK IDKLDEPMHP
WSEIDGLRAA SGLTFEDILL VPNYSEVLPR EVSLETKLTK NVSLKIPLIS SAMDTVTEHL
MAVGMARLGG IGIIHKNMDM ESQVNEVLKV KNWISNQNLD KESTDGKDTK SNNNIDYSNE
NLDNKGRLRV GAAIGVEIER AKLLVEAGVD VIVLDSAHGH SLNIIRTLKE IKSKDVIVGN
VVTEEATKEL IENGADGIKV GIGPGSICTT RIVAGVGVPQ ITAIEKCSSV AFGIPIIADG
GIRYSGDIGK ALAVGASSVM IGSILAGTEE SPGEKELIGD TVYKYYRGMG SVGAMVPEGI
EGRVKYKGEM EGVVYQLVGG LRSCMGYLGS ASIEELWKKS SYVIKSDKIL QDDIALLKNL
YFKFYSNLPF INFYSSVVVL HNYSLRNRLG VKLKDMLLLK NIFESEYFKT IYNGNKISKV
VKEMKAINPN FKLTDFMSFF EEYILPKFME SYLKCDEHKL RLHCGDVAYR QLCSNIEKLK
KMGLCLNTKI LQLGNVELIG AEKIQTNQPE FMFTFKTQQI NCLQDSNGRI ISGSIDDIKE
LQYSIKVTPH PNAPGLEYPY LITNLEIIGS IPIFMVLYLI GTGLNDERDM TLGGIELMKV
ADKIYLESYT SILSQRADLM KYTDGKPLIE ADRKMVEENC DEIIEEAKDK SIVLLVVGDP
FCATTHSDLV LRAHEKDVKV EVRHNASIIS AIGCTGLQVY RFGETVSIPF FDGSWQPSSF
YDKIKANIER GLHTLCLLDI KVKEQTIENM MRNRPIFEPP RFMTVNQAIS QLFILELKQN
VISPNSLAIG VARIGSSDQK IVSGTLSELS DTDFGNPLHS LVICHPDLHL IEQRFFEIYP
NSAVRKCVRV QLIKNGKKIT AFVPRDGCLN YIDENDEVLV AGFGRKGHSV GDIPGVRFKA
VKVSGVSLLA LFKEKKEKPR SMGIKGLTKF LADNAPKSIQ QQGIGSLLGK RVAIDASMWI
YQFLAAIREG SQWGNLTNSS GESTSHINGM LSRTTRLLEA GIKPVFVFDG APPEMKKDEL
TKRDERREKA LAELEKAQEI DEELIKKQSV RTIHVTKKQV EDVKKLLGFL GMPCIDAPSE
AEAQCAELCK DGLVYGVVTE DADSLTFGTP IQIKQLIKLS LILSELDINM DQFIDLCILS
GCDYCGTIRG IGTSTAYKLL KKYHNIESIL KNIDFSKVRE LFKNPLVNNQ IKIKWSNPKY
EELMEWLIKE QNFNEARVNS YCERIKKSKN KTSQTCLDGF FVDNKKLYEI LEVSQEATLS
EIKKAYRRLA IKHHPDKGGD QEKFKEVSRA YEVLSDPEKR KIYDEYGEEG LEGGGGGADP
VDLFDVIFGG GRGGKRRGED LVTHLKVTLE QIYNGAVRKM AINKDTICAD CEGVGGPKDA
IQYCELCQGQ GVRVQIRQIG PMVQQTQSPC NPCKGTGKTI PVTKQCKKCS GSGSVKERKV
LEVNIDKGIP NHHKVTFHGE ADEKQGEIPG DVVFVLDEQE HSVFKRKGGD LFIEKDITLV
EALTGFKFII THLDGRKLLV KSNPGDITKP SDIKCVNNEG MPTYKNPFVK GHLFVIINII
FPDKLDSKTQ DLVKLLPAPK ALNDEDDPSI EIHYTSNTKP SEVKDRINIL RPFLPRLLIC
LLENSIFTDF DYIEMDPSHF EDKTEDEVEL GAWGNQWTVR KASALALDHI SVIYGDEILG
ELLPKIEATL QDPNWEKQES AILVLGAIAR GCIKGLSPFL PRVLSYLVKL TSNSKPLIRS
ISCWCISRFT PWLALQQPIL NSAFGALLAR MLDPNKRVEE AACSATATFI EDSAQSLSPF
LDDIVNTISG ALTVYQYRNL LILCDTISTL CFSVGPNVFS QTFENNLVPL LITKWKSFPI
DHPCLVASMD AIAKIFAVVG NKASGFADSI LQHCIQNILM SALNDTAECA LDLVSSIIKQ
SVFACVGDIA KFGGDFLKLP TLLQLLVLNL SSPNIGIANN AAWAIGEIAM YGNPEFLEHI
IDMLVSRITT SCSSSHIDNL AINACITIGR VAVVAPSRVG GRLGVCADRF FLVLTNVRND
QEKMNFSENI LPILQECIQE AELLELLAQC ILKTINVNKL TECGCVFVFY VADWFALLNN
KMGGDLEKIK IVGEYFVHIW KAAGMDMTNV RFVWASDFIN EDSNEYWLRV FDISRKFNIT
RIKRCCQIMG RQENDEQPCA SVFYPCMQCA DIFQLKADIC QLGMDQRKVN MLAREYCDAA
GIKHKPVILS HKMLPGLLEG QEKMSKSDTS SAIFVEDTPE AVVKKIKKAF CPPGIIEGNP
CIEYINTLVF PKFGHFHVSR KEEYGGVKLW LYDERLEGEY LADVINRDHV NVKYLPDFKL
PNNIRAVTDL KEACEDCNLM IFVIPSQVER ILTRIARGGL LLGAIGTIPM SFMFNVDGGE
KAIMFNRFGG VSPKAISEGT HFFLPWFQVP FIYDVRVKPK VINTTTGTKD LQMVNLSLRL
LFKPCTEFLP RLHQNLGPDY DEKVLPSVGN EILKAVVAKY DAESLLTQRE KVSREIRESI
MQRTKQFDII MEDVAITHLT YGKEFEKAIE EKQVAQQDAE RVKFVVQKAE YEKQAAIIRA
SGEAQAAEMI SKAVSNSGWG IVDVRRLDGA RDIIENLSKS DRVTLIQGDQ QHLYLNAYFS
EKSGFLFENN IATAPCLSDS LKTVKTGTTI VGVACNDCVV LGADTRATNG PIVADKDCEK
IHRLSDNIFA AGAGTAADLD HVTSLIEGNL ELQKLQMNRK PRVAHAVSML SDHLYKYQGY
IGAHLIVAGS DSTGNFVFQV SANGCIMQLP FTSMGSGSLC ARSILEARYR DGLTESECVE
LVSDAIRAGI YNDLYSGSNV NILIIKNNYV KHFRHFTKAS ERIYRQPVGT TPIIAEKTED
LSSFVELNEL GTAKGNILSL KLDKAMGSVS GQSVIDPSKY LSSLNTLNGD LSDIKKARLL
LKSVVNTNPK HSPGWIAAAR FEEFVGRLSH AREIIAKGCE MCPKNEDIWL EAIRLGKPEQ
IDKIIVKSIK FIPNSTKVWM VAANRETNKN KKLLIIKKAL EFIPNSIKLW KEAISLVDNE
SEKALILERE RTNSPSNNEF IPQILSKALK ECPKSGLLYA ESIFTEQKQK QKSKFLIALE
QCGNDPYVLV AIAISFWKEN DFHKSRKWFK SALEIDNKIG DTWIHYIAFE LLNGDFQSQR
DALNDFINAT PNKGWLRGRI TESRCKGSLG FVLLRQTFYR LQLVVDANNG SSKEMIKWLG
CLPIESMIDV YGTIVVPETP VVSSTQDIEV LVERVYCVSS ACSELPFQLK DANRVETEDS
TIIKVLQDVR LDNRVLDLRT YLSQAIFRIQ SEVCRLLREF LIEREFIEIH TPKLLPGASE
SGATVFKVDY FSNTACLAQS PQLHKQMSIC GDLERVFEIG PVFRAENSNT HRHLCEFVGV
DIEMNIENTY HELVDVFDAM FRHIFQGISE YNPFTPFVIS EKTPRLTFEE GCNLLKEDLS
DFDISTEQER LLGSIVKEKY NSDFYMMLKY PLKVRPFYTM PDFEPEKWSN SFDFFMRGEE
ILSGAQRVHD YDLLVKRCQE CGVSEHSLRD YLNSFKLGAP PHGGLQYENF SLESQSIFQR
ESFDGFRAEI AKSVTSNLQT SYSLFLGTNI KGYSYQLGPS YQSSSKNTLI LARVNDEGTV
SGRFSRCFGN EGRISINSSL SDENKNMSEV SIDYNGEESS YSLKVAYQGG GELTWIAANN
TSIMSLGSRY CHGKNIFFNQ ITRQPDFTSP GRIFANIHSL RSSFYRKLSD RLSLASELEV
SIPNFESTLR FGYEYLFKTA RIQGMIDTCG KISLQCLDNK GFGISAAIDY LRNDYKFGMM
QFFPNEKDDG GVGKTTLVKR HLTGEFEKKY IPTIGVEVHP LKFNTDFGPL IFNVWDTAGQ
EKFGGLRDGY YIKGQCAIIM FDVTSRITYK NVPNWHRDIV RVCENIPIVL VGNKVDVKDR
QVKARQIQYH RKRNLQYYDI SARSNYNFEK PFLWLARRLT NQPALQLVGQ HAKAPEVNID
PTLVAQAERE LSEAANAPIE DDDFNYSLSK EIEMNDDTLE ELNNIISYKI KENSFFQVFK
ILSKEAASIY GEHLESDQAI PDDIELRIVT LRNFYLSATR NPVLRNTKDI GNVLIENISL
DHENSALLVN FKVESEENDV PSLEDSLPIS GIDYNKLIDK FGCKLITKDM IERMERLTAH
HFFRRNIFLS HRDFEKILDV YEKGELFYLY TGRGPSSESL HVGHLVPFLF TKYLQDTFKV
PLVIQLTDDE KFIFKSNLTL AEITELTPNS IRFTLSNTDL SMANTLRRII LAEIPTLAID
LVTMIDNTSV LHDEFIVHRM GLIPLDSTNI RDFNFKDRCE CQERCNKCSV EYLLDVTCEA

TRNVTHYDIV ATSTLVPMPV PILIAKLGPG QRIAMRMTAC KGIGKFHAKW IVSVATFTNE
ADVRINYSLS SNLTLKQRKE IVNCCPKDVF ALLEVVSSKS CIFCNECVNL TKNYGYINLI
RVDTKPDKFH FLVESTGALP VESIVELAFE ILQEKLNTLS RGVRSETAQS GLDTNGSGIP
EYLETESMDL KCMYKVLKME LLKNPFRWRG MLKDLYGVSE ETTTGVLRLK IMESEGKLLL
PAINVNDSVT KSKFDNTYGC RQSLLHGLFN GCIQMLAGKK IVVLGYGEVG KGCAQGLSGV
GARVIVTEID PICALQASME GYQVSVLEDV VSEADIFITA TGNKDVITVE HMRKMKENAY
IANIGHFDDE IDVYGLENYP GIKVIEVKQN VHKFTFPDTQ KVILLCKGRL VNLGCATGHP
PLVMSMSFTN QVLAQMDLWK SRNTRFFVKK LSKELDEYVA RLHLDVLGMY SQPYSGEEPM
LYQSRGPNKS SNQFEALNER GEEITPLISN FSCITLRTGT LLQCASLLVL IILYNVFGNK
GLFTFDLYGN GGTLAEDLSY YAGVVSMLCV YFIGVLFLAG FQEFIADNSK APLGFRAGSR
LLNTANIIQI IVVALRVTQF SFTYSYFNQK WYGKFSQTKG DWCLYNFGIV CEAVSLIMYG
ISFFYVESYA DVGVGEQYAY WMLTLFTLAG ISELTEQKKR TFRTYSYRGV DLDKLLTMKL
DEVVELLPAR KRRKIARGLN RRTAAFIAKL RKSKAECPMG EKPVAVRTHL RNMVILPEMV
GSVAGVYNGK TYVTVEIKPE MIGMYLGEFS ITYKPVRHGK PGVGSTSSSR FIPLKVMVDV
FFWRDPEEYE NSAIGAELND GMGVDIEGAA VDSAAAANEW GMLSIMIDTH ETMFELKQKT
NSRLQVIGWY STCSGINSVS CAVNNWFKTD IGSSKFQQTP LLSEPIHIVV DPSFSNGKLS
INGYIQIQST WTNSIVSIFR PIPLDIVASP CERLHILLTK LILLVQRCQN YVKKVLSGEV
SMDPMIGRQI DYAIHSIYDF EYNIYNIIKS NSTQDALIIG CLTELTRVQL SLSEKLQWIS
LLPIKYDTDE AQNSHTALMN LVDSKNPLIL GQNLENFPRI LAIFIDIYGT SMSNDSLNSR
IKLLIAQTGP NNLQSFAFSK KQIDKLQKLD GNQLLDLLNK HQQLKKYVSL VENEIFLPVV
TDSNGIVLSV PPLINGSQSK ITLNTKNVFI EVTATDYSRA SIVLNQIVSS FSMYCKNKFE
IEPVKVEYPN LERLEFVVKA SEASNLLGIN PLDPIVTQSL LRKMMIDSES LKCFVPINRS
DILHPVDIIE DIGISFGFNN IELKKSRFYE LDKSSLMAEQ VKRELSLLGI SESLNWALCK
HSDCFESLFR PVVVKDAKTS EFEILRTTLI QSLLKTIASN KSLPLPQKIF EVGDVVINNT
PSGSRNDKRV SIAYCNSGSG LEEIHGFLDQ LLSKLGLVAE YSLNEPNSIH PNIIVLSYGN
IRGLGYQDQG IFKASKELFG WKNRRTNATY HYKPEEVMGV EWIQTSCEDS SCQLRVFIRE
KKDCIHFTGF KTEDYSVIKS HFETYYGINL ETKELNTKGI NWGDLTIHND TICIGNEGKV
MMYVPSININ QIAMPSKSEL VLEFNEGVDC DELMEIRLFV PNQGNSLSSA EKLRSDLLKL
TGIGSSGSDK VCRWNDIHLL VPRGRYEIEV LVNCLKLHGK SFDYTILFQS ISRLFLLPRP
GTSLVNLVVA LETPMRQGNT KYPFVVMQFD TQQDIEMPLN LSEKEIQRFT LSPIMTGKFW
DIVTRILKSL TGHSIIVPGD FRSASMYHCI RCSYKAQDGL LYPLNRSFIF ITKPVILIRF
DDILNIEFSR MGGNQTRFFE LTITIRGGGD YSFTSIDKAE YNPLIKFLQE KNIRIKGYID
LSKRRVNPED VERCEEKYSK MKKVYQTVRH IAQKEGISVE ELSEKLIWPL HRKYGHALDA
LKEAAVNPEA VLGEFDLPAS VKEALIQDIK FRLSPQQLKL RARIHVCCCG YDGIDAVRHA
IIAGQVEIQV KFIAPPQYLV TAVSYGKAAG VEAIEKAMET IKNVITQYKG GDFKRQGEIE
VVDLNSASES NESDYATRRL FEFFLCKVGV GCSVVKRFPI PPEYDTVFLG VLPQHTFKHE
YIIYDSFQVV PEYLIQFEYD SSSPELFAVP WCDECQDSPA ILWCQADTAR LCDSCDERTH
RNKLVTRHIR IPINQMPGNC PVHTMDSLEE FCTLCHVPMC RLCRPSHTHM PISRAYRAQL
ERHNKPHPCL NSRKMELLEK LESQQLIFND VRANMFEVEQ RIYSILESSI TQLQIATESK
MNKILCEQLI LNQLLQQVEW SEGFLQYLQS ILPPADFLFA WLKHCHYREE VFQLCQFFPD
SRLVGRVDIL SESALKHLVG LPNVGKSTTF NLLCKQAVPA ENFPFCTIEP HEARMNVPDD
RFRALCKHFS PKSEVPATLT IFDIAGLVPG AHKGEGLGNA FLSNIQAVDG IYHVVRAFES
DEIVHTEGEV NPVKDLETIS NELRMKDLER VDKLISELNR LTKNGTDRTK KEQLQILKDV
YAHLEAGNWI SSKDWKSSEV ELLNEHQFLT AKPVVYLVNL SEKDYIRQKN KFLPKIAEWV
KEHIDGVIIP YSAEFEAALA ELTEEEKMAY IKEKGASKSM IDKIINTGYS ALNLLHYFTA
GEDEVKCWTI RSGTKAPQAA GIIHTDFERG FICAEVYKFT DLMELGSEAA IKAAGKYLQK
GKDYVVEDGD IIFFKFNVTN SGRLDLYEEY FFLSNLKLLE LVDDFHKSLE DGLENHRLKI
DKYHSNYKPF KMLDSCVDRL PTGKEKGVYY AIDMGGTNLR CVRVNLLGNG QSETKFKKVK
LNKSKVDQEV NIFDKTVSSE TMFNSIAVFF NEFLDECGDL NDLTNLEVGF TFSFPIVQSK
IASAKLVIWT KEIETGRLTD DPVEGKDIGD LLNLAFKRNG VPAQCKCVLN DTVGTLISAM
YDNNISENQP LIGIVVGTGV NACYLEPNSS NFGYKGVIIN TECGDFSTKL PITDCDYSMD
WFSDNRGEQI FEKMISGTYL GEISRLLIIN FLKNKTPEIF FQKNSLKTEH IAKIINDNHQ
DLKSIENYLK ETFSSNLDHN STYIIAKISQ MVLMRAASLV SAIIAAFFKR FNKIAIDGSV
WTKIPKFQKY VKDSLSSLIQ ESGGSIHFYE SDDGSGRGAA ILASYFLVKL LAQIITRTFF
KNVTVLGKER IPLYGPVLFV GNHMNQFVDA AMLIAIFPRQ IRFLIAEASF KRPIIGRLAQ
SAGCIPVQRP QDLRYRGIGG LIWSSSSVRI RGIKTRFKID VKFQDTLIID DLGVSVLEVV
SDDEIIIDKG IGTESATDGF PFFIMPKIDQ STVYEEVSDA MRNGHSIGIF PEGGSHDRTT
LLPLKPGVAV MALSSVLEGD LLIVPVGLNY YEPHKTLSSA VVEIGQPIPV TLELAQKYEE
SPSDAVKELL SMVEKGMNSV LLSARDYTTL TCIRLCVQLY PPDRTALSQD NYYLLHQLFS
QFFWALHDDP ELEHLRKDLC EYEDTINHYG VPDREVWQLK QPVYECIKLI ITKFLLLILV
SIVGGSFFPL WAPIRYIPAF MAERHRKKAL ANSKVKIRGT DVLASYRILV IIALLPMLTV
FYGIFFSLAR PSVDSDAEQI CKLFENYFMK YKIHQCFNVE DVKHYFTNID KVIVTYVRNK
NKEITDLFSF FIIESTVINN ERFPTINIAY SYFNIANTCS LKELFNEMLI TAKNNNCDAF
NTLDLMQNLQ VIQDSKFIIG TGIPPEDIHG MAVVLILGSY RNEEFIRIGY YVHNVYTDPL
LEDNPPDIPI LDKLQRIISE SPRLTRFNIG WDDAQILQEK MEKIRLMNSI RIFALDLSQN
CIFEEKRNSC GDTGKCQVMG NESDLSSTLF KANDINVDML KNPPSNTQYK GGEVWISMPN
SSLKIFGVAI SLPEYKELLN YDDKTELSKI VSEPPKVTIC DAYYEEINKK EEDRIKTLER
EKEIEFMSHG TLVLPNDRAR EYIDILGREV NLQFVDMNSI TMNRQYKKYI QRIDEMERIL
RVLFSEIEKL PDVKVLKGNY ENFLDHDHVY QLDKVEESLQ SLYGQFISFR DNNADLIHQK
SSAIEECAVA KAASLSFAPT PSSPLMNPGI MMMFSSIAGV VKHEDQEKFA RALFRATRGN
TFTHFQSIAE KVVFVIYFQG ATTSAVYDKI SRICDAFNVS IYPWPSSYEH AIQRISELNT
LIQDKEKALQ AYEQYITLEI ETLLQPVNSN GNSLIEEWRL FCIKEKSIYA TLNLFEGSDI
TLRADCWYPT EEEEKIRKIL IAESSTQHVG AFLLTDDANI SNTPPTYIKT NDFTVAFQDF
VNSYGIPRYQ EVNPALFTLV SFPFLFGIMY GDVGHGFIVF LIGLVLVLNY GKLKKDENMK
ILVSGRYMIT MMGFFATYCG LIYNDFFAAG LDIFGSPYPF GFDPVWKGAV NEMSFLNSFK
MKFSVIIAFF QMTLGVILKG FNNLYFKNYV DFFMEFIPQF IFMVGFIGYL NFLIFFKWLT
PEGYNKPSIL NALIGLLFGA DIPLSDRFYL SQPVVQKYIT LALLISVPWM FFPKPLYLIY
KSRKEIFIHQ LIETVEFLIG SISNTASYLR LWALSLAHNM LALVALQFTI MKALNSKLLI

VKVVQLFNLF FMFFAFTSFI MILMDSLECF LHGLRLQWVE FQNKFYKGDG ILFAPLNHMR
IITSNELCCL IQCFKNAPLK YIPICEGVLK EVVGKHKKEI SEIQLQIVSN QEPTLIRDLR
SNVMEKLVTV PGIVIQSSKP QKKASKLKIL CRQCKGTRNI NIPIWRQGTM LPRVCNTPLD
PYFTLCDESE YIDIQSMKFQ ELPEHVPTGD IPRNISLHMT RGLIDKVIPG NRLYVVGVLS
STDKESSRNG SLRTSYLYVI GVMNYGNSSI SNQYNEIEEF RRISSLPNIH ELIVNSIAPA
IYGNETIKQA IACLLFSGSS KCLPDGNRIR GDLNVLLLGD PSTAKSQLLK FVEQVAPICI
YTSGKGSSAA GMERLGLLSE EERKLDYVLG LTISKFLERR LQTRVFKLGL AKSIHHARVL
IRQNHIRVGR QIVDVPSFMV RLDSEKHIDF ALTSPFGGGR AGRVRRRTLS QSLHDRHITI
FSPEGKLYQI EYTFRAVKNS NITAIAIKGK DTVCIVCEKK VPNQQGQQDK LLDPAYVTSL
YKVRKHIGAV MLGLAPDCRS IISKCREIAG KFAFEKGVEI PVSYLSHKIA DVNQLYTQHA
SMRLLGASGR NIIEDEIREL FSPYGQINEI FIMREPSGVG KGCAFVKYAF KEQGLFAIKS
LHGALTLADV NRPIEVRFAS KNHQSSSRKL HKSDQRGHLT LKYRDNNETK KNESVIEMIN
NILDLIKQGT EKEKIGNLEE ALNIYISALQ KWDHICKYQN DERVKKVLLT RMEQLVSRAE
QIKNLINILM ESPNISWDDI IGLEQAKTSL KEAVILPAKF PELFQGKLKP WKGILLYGPP
GTGKTFLAKA CATEMKGTFL SISSADLTSK WQGESEKLIK ALFDVARERA PSIIFIDEID
SLCSSRNEQE NEATRRIKTE FLVQMDGVNS NSNNILVLGT TNIPWEIDSG IRRRFERRIY
IPLPDEESRD INYIAKMTHG YSSSDVSILI KDALFEPIRK GIIIKMGSRF EGSSRVLFNM
ILSQEGKTSQ NCLPNKLALN GLMLAGGFNR EYTSFLLEYL KDQIENTQEF FDGIFKFYKF
SDEELELAKK NIKEELLFEL ENPSIMLNEL LHSTAWKENS LGNNQSTSFD QVSDLNIQNL
TDFRNSNFLS RNTIIVGTGI SHDHLIKKIL NSSRKFNNLK NDEQTMKIPK YVGGLVKNKL
HYGFTDILIA FETNNWKGRE LVALSVLQAY LGGGSSFSVG GPGKGIHSKL FLDVLNKFDW
VESCNCFVNQ YSDTGLFGIH ITSYPGYSLE SIKVIAKQLG KMKNISEREL ERAKNLVLST
ICTAYENRSH YMEEISKQIL SYSEFIELDE IINCIKSIGI EDIKKVADLI LSKRPTVVAV
GDMNQVPNYN EIISIIDLGQ KLIFLMGYPE VPTVSFGSSG VVRCKPCRTY INPFVRWEAG
GRRWICNMCG YSNETLSFYR RTDRFERPEL SVGSTEFIAS GEYMVRPPQP PVYFIVLDVS
MPSVSSGLVE TVCLAVKNAI GGRAMIGIIT FDSSIHFYDL NSNLSQPHMF VVSDLNDLFL
PLSEGVLVNI ADSTEQITNL LDNLPNLWRN NRVSENCMGS AIKAAYMAIR HIGGKVLLFS
SGAPTVGDDG YSNFVHALVR AYISVDLFMC SPYVDLPTIS PIVKKTSGDL TYIFFNSYLH
GQKLREDIFS TLTRNSAWEA CIRFRVSRGW KISNWYGNFY FSGVDLLLAP NCHRDQAFSI
VIDMDENVAP DPFVYIQAAL LHTNSDGERR IRVHTMALPV TQNYVDLVSS MDVQATVSII
CQNAMELSLK SKLLDGRNYL QTICSQIILP QSNQIEAAKQ LPLYILGVLK CPAFRDSDTR
IYHWIRLSSL RLESQMILFY PRMFCLSSPP ALNLTAEKMT QADAYLLEDG ESMYLWLGRA
ISIAIVCLAA SLDGCDEQLL PASLRALEVD LKLSPKDLGN IILCQILCLA LSCPIWGLLA
DRYSRKYILA TGVTVWGFVT ILLAFSSSYW EVLITRAVNG AFLGSVGPLA QSVLADTLNS
KRGLGFGMIQ LSSCVGRVFG AVLTTSISQK LIVGFQGWRF AFLLVGVLSA ILGGIIVFLM
DEIPSLIVKS VILMILEGIS GTIPWSSLTF MTMYLQYCDL SNFQAALVVA TMLAGSMIGG
PMGGLLGDCL NRISADHGRP LVGQISMAIR IPIMCILFLV IPKESSSFYY FVLSFLMGFF
AIAGAAASRP ILSDVVRASH RATVFSIAVL FEGISAATFG APVVGILSEN VFGYKTTAEN
VSQMNADSRL INANALANAL VFLTVFPWCI SLLLYSLLHF TYGNDKRSLK IELSEIMLLF
SLRSLSDLCV PPTLLYKENS LDAMQRGILE IMSEALQQFP DDEDISTNCV RIMFGISEFM
KESQDAELTN MFISRGGTVA MAVNGLSNDS AILASICTTI ENAASCLGKF YESKYLGSSE
LVKVTGAICA IAHQESGIAA LVQAGIGSKT LNFAHNESAA LIENCLEIVR LCASYGDVPE
DALKAVVHII DLYRSRRGIT EKGGACLELL MNPQKLMECL VVIKSPNSDI TQKDDSLTML
SAMAYVSSFA DEIVRQGGIS MVVEALNQGV SEYEEHTSKS SRIIIGSLRV LARVASNPSN
VDNLIEIGGI DAICSAFTTC INNLEISTAV CQSLYPLFVR EASIMGDLLQ LFYSNVENKD
FTKCAADLLS IASQHAPLAE ALISVQSVEI LATCLNYYCD DAAYQSASLA ALNHLAPFIK
TLQPISEFGG ISGIEKSIIE VSAVLEAMLV HNSNDYIKEA GLSILEIIAT DKDVTLHTTN
LPKVALSDPN AAYKDLAAIA GLCKISSLAP LFSSLNICDD ILGYVRKWIS ESPFEGQEKL
ISAAFQTTSL LDISTLPQMK TFAEKNTNDN PVVENMKAVR SLIEVQRIGS DETVVNVVNS
LVSTIRKYIE NRVVQLQCIK SFCIIAETES GAKAEIDNGV IKVCLAFLQR TKVLLECQIA
GFTLLSLLVK YSPESIEILR KSGAVEIVQG AMRLHNRSSE LRLIIAPLAL ALVPVGEVKK
IISEKVGEIK ISLDSGDLKT ILQAIITCNE LAVTPEGCKF TISEGILSAC STLCSLLTAS
RPGKVAIVKN NVTVSLLNMY RNLNCSLNEE VETGLVDNLV AVANIVKFDV KAAETCFENG
AVQLICTSLD NFQDNEKILG SACSAIAAMA TSPKRVEILL AEPSFNNLLT RLVNTVNESA
EVRIKCLNAI NDLISSHDTS IIQITTDVGS IVAAFGVVDK YPSESSQIRA ACRVLNSIGN
YVDIRADLHR CVEVVLRALD AQKNNEEVVE DLLILLNNLT NSMDNTILRE CAIIELMQNV
MMVHNDNPAV ISKCGEVLSK CGADETIKSL MVSIINQQEN GPDCARDLDQ LCRQLAVFVA
SQPENPEDAL QYTEACLQSL VAAAAEYPTD IRLLSSIAVL TQRITDRAFD NSEDSFGSWA
VATSGMMQHI EDNLEDTSGV SSKRYVCSGI RVLSGCLNNV SKMTKEFSDK FLEFLNNTGS
PYHSVDETIK ILKGSGFVKL DDEEIKKGGK YYITLNYSTI LAFKVGEEFD LEDSMVVITG
SHTDSPCLRV RPSSITCNEG YTQLSVCNEY LIKIKRPICA IPNLAIHLTT SEESFVYDKE
KHLQPIISKT DTNDKSLLDE ICREINVQPQ NISSFDLCLM DSVDSRYVGI NDEFIDSPRL
DNLGGVFSCF TALIDVLCIF VSSNRVNGEL YFASLSNYGC SGNQKKVASV LKAQAEKTPF
SLLVSPGDNF PGGIFKHCFE NIYSEKSLQI PLFAAMGQAD WDNGNANLLL RNNVTYPRFS
FPNYFYHYVS HYTDTSNVLS RRDGTVLFVF IDTFILSSSF PDHKVSEQAF QNLNATLHYG
HKTVVVGNKY LASSKVQQLI LDTHVELVIS GQNRGTYNST IEGTTFLNCD SYCSFKVDGS
KMVPYIINGN QVSFQGIAEL PALEMIELKK GAKHLSREAF LKIVGTVGLM IRYSFSGIAY
SSGAINLDFE WCKSREQGLI SPDIVVFLIT LVDDEICSFS DMGANFYITE NDVKKGSDAC
LNKLASLNEY VPNIGFISAN SLGLCGSIFV DFGDSFNVFD GNGEEPKSAI IAKISRGKET
TITCLAEKLL PFQEGDYVMF REVQGMELNG GPHKIISTGK HQFTIQDSSM FREYEREGLV
TQVKVPINYS FRSLKDLNKF GRSEQLFVSS IDRDILEKVC KYSRCCISPM AAFLGGIAAQ
EIVKFVGKYT PLRQFFFFDA FERYDDQIII FGRSFQNRLS EKNIFIVGAG ALGCEFLKSM
ALLGGTVTIT DMDNIEVSNL NRQFLFRQEH VGSPKSAIAA QVIRTINKDI NIISLQTRVG
TDTEDVFDDI FWNKTSFVIN ALDNVPSRMY INDRCLWYEK PLLESGTLGT KANSETYLPH
KTQSYSDNRD PAEESIPLCT LKHFPHAIEH TIEWARDAFQ GIFTSDPQEA ITFEKSQKIF
ELINISEKDT HEDCIRMAIH LFHDYFYCQI KQLLTNFPPD HINSDGLPFW SGPKRCPTPI
KLNIQDKLHF DFILSASNLY SNMVRLPEIS DSSIIFKVTI LPEFNAKTTI IKIDPIEFEK
DDDSNFHIDF MNSCANLRAR NYSIKECDRH KCKMIAGRII PAMATTTAMI TGLVSFEALK

VSSLIELFKN SFINLSLPLY VITEPLPAPK TISKEFDPIV EGPLRARPEG FTAWDKLVIE
QKDYDTQRDK RFAGTVRLPN VPRPNARVCV MGDAADCERA QKLGFDVMDI EEMKKINKNK
KVVKKLCKKY DLFLASQVLL PQIPRLLGPG LNKAGKFPTV ITPSDKLFPV YSIAVFCAEL
HMRLSDPNPL SFLPVIGGLL PFEKIRVLRS LFTCVLIISF VYASNYYRDY DEILNGMLKG
NLITNKRIID LTQSLVQLQT HGIGSTSTSS GQNKLASTMN ESQKKQLQTK KEGGGFLSRI
FGSSFFWIIL VIIKNYLSAP RSHEVPLLVT LEELYLGKRK KIKVTRKRFI EHKVRNEENI
VEVEIKPGWK DGTKLTYSGE GDQESPGTSP GDLVLIIQTK THPRFTRDDC HLIMKVTIPL
VRALTGFTCP VTTLDNRNLQ IPIKEIVNPK TRKIVPNEGM PIKNQPGQKG DLILEFDICF
PKSLTPEQKK LIKEALGDHK VCIRCDSSNW FKSEQMKWIL SIDTGNEHLD VDSIASKDEA
NYVEEFIHVD TGKTKLLDKM RKTNVQDNEA GGITQQIGAT YFPPEMLSEQ VKKVEFELQI
PGLLFIDTPG HESFNNLRSR GSSLCDIAVL VVDIMHGLEP QTRESIGLLR SRKCPFIIAL
NKIDRLYGWI EQNWSSSRST LSIQNESTRD EFDTRLNRVL LELSEEGLNC DIYWKNDDFR
GNVSIVPTSA VTGEGVPDLI YLIAQLTQNY MGLHLQLNRE LSCTILEVKA IDGLGVTIDV
ILVSGILREG DTIIVCGLSA PIVTTIRALL TPQPMHEMRV KGEYIHHRFI KASMGVKICA
NGLDDAVAGT QLLVQSKNEE IESLKEEVMK DMGDIFSSVD RTGNGVYVMA STLGSLEALL
VFLKSSNIPV VALNIGTVHK SDVRRASIMH ERGFPEMAVI LAFDIKVDAE AEVEAKKLNV
RIMKANIIYH LCDMFTKYYS DVQEEKKKEK SQKVVFPCIL KIIPQYIFNA RDPIICGVYV
EEGILKPGTP LCIPEKDLMI GRVTSVEFNK KPVNEGKKGQ EVAVKIASDT NITYGRHFDH
NDKLVSRITR DSIDILKQHF RDDLSKDDWK LVIQLKKTFG IPFIKMSSDH LSKQDADNLD
LTKLTPITPE VISRQATINL GTIGHVAHGK STVVRAVSGV QTVRFKDEKE RNITIKLGYA
NAKIYKCTNP NCPPPKCYRS YGSNKEDEPM CEVPSCNHKM QLLRHVSFVD CPGHDILMST
MLNGAAVMDA ALLLVAGNET CPQPQTSEHL AAVEIMKLKH IIILQNKVEL IKEAQAQEQY
KQIKDFVAGT SAQDAPIIPI SAVLKFNIDV LCEYICTQIP IPIRDFTSSP RMIIIRQGKL
CNFIDWKGHT LVVKRYASLY FVACIDKNDN ELLALEIIHH YVEVLDRYFG NVCELDLIFN
FHKAYFILDE IILAGEIEES SKKAALRVIS TQDSMMDENK FAKSLLDVSD SLSRALLSVD
IENVDKNSSL YNGISMTYSS LEKVFEAHGI KRFQSLGKQF NPKEHEAVFE VKDTSKKGQV
CEELLPGYKI HDRVLRHLDL DSIQALIVAL NSFNGGVIIV SHDAHLISCV ANSIWHIDHV
NKLKEFKGDF DLYRKTVVRT SVMPRELVTL QIGQCGNQIG MEFWKQLCTE HGLDANGTVI
DSGPLEDRKD VFFYLSDDSH YVPRALLFDL EPRVLNSIKS SEFKHLYNPE NFFIGKEGGG
AGNNWGCGYD TGEEVYDEIL DMIDREVEGC ESMEGFVLCH SIAGGTGSGM GSYILELLNE
HYPRKLIKTF SVFPMLNESS DVVVQPYNSV LTLKRLALNA DCVVVLDNTA LSGIAEDRLN
LTNPTFAQTN ALVSTVMAAS TTTLRYPGYM HNNLVSLFST LVPTPRCHFL VTSQNVRKTT
TLDVMRRLLQ PSNIMVSTNM RKGTYISILN IIRGETDPME VHKSLQRIRD RNMVRFIQWG
PAAIQVALSK YNPYTQHKVN GLMMANNTAI AGLFQRCVAQ FDRLMKRGAF LDNYKKQSVF
QDGLDEFENA REVSEMLIEE YKRAERNDYF GLARAFGIPV RSYTHEVVTL WYRAPDVLMG
SKKYSTSVDI WSIGCIFAEM ITGKPLFPGV TDDDQLPKIF SILGTPNPRE WPQVQELPLW
QRTFQVFEKK PWSSIIPGFG IDLLSNMLCF DPNKRISARD AMNHPYFMGK EKTHINLVVI
GHVDSGKSTT TGHLIYKLGG IDKRTIEKFE KESSEMGKGS FKYAWVLDKL KAERERGITI
DIALWQFETP KYHYTVIDAP GHRDFIKNMI TGTSQADVAL LVVPADRFEG AFSKEGQTRE
HALLAFTLGV RQMIVGINMD TCEYKQSRFD EIFNEVDGYL KKVGYNTEKI PFVAISGFVG
DNMVERSDKM PWYKGKTLVE ALDTMEPPKR PTDKPLRLPL QDVYKIGGVG TVPVGRVETG
IIRPGMNVTF APAGVTTEVK SVEMHHEQPE AVPGDNVGFN VKNVSIKDIK RGFVASDAKN
DPAKGCEDFT AQVIVLNHPG EIKNGYSPVV DCHTAHISCK FQTITAKMDK RSGKVLEENP
KLIKSGDAAL VVMQPLKPLC VEAFTDYPPL GRFAVRDMKQ TVAVGVIKSV TKKEATAPII
LLKEGSDTSQ GKGQILSNIT ACQAIVDIVK TTLGPYGMDK LIHSGNDVTI TNDGATVLNL
GIVHPAAKLL VEIAKAQDDE VGDGTTSVVI LAGEFLKEAK GFIEDGMSPQ VIISGFRKAS
QIAIDKVNSM KISLSEETPE KRRSMLIKCA ETTLNSKLLA HYKTHFAEVV VDAVSYLDNE
MDKDLIGIKK INGGSVMDSF LVKGVAFKKT FSYAGFEQQP KHFVNPRILL LNLELELKAE
KDNAEVRISD PTAYQSIVDA EWKIIFEKLD LIANSGVNVV LSRLAIGDLA TQYFADRNIF
CAGRVEEQDL KRTSLATGAI VQTTVYGLNK DVFGTCSEFE EVQIGAERYN IFRDCAKTKS
STMILRGGAQ QFIDEAERSL NDAIMIVRRA MKSSYIVPGG GAIEMAVSKT IRDHARTILG
KEQLVMNSYA RALEAIPRSL ATNSGFDSID ILNRLRQKHS QNTEDSNYGV DCSNGGICDT
FNSFIWEPAV NKLSAYSSAT EAACSILSID ETVKNKSGAK ELPSNFRELL ATDKINYVLC
TGNVCSQEYV EMLKNITKNV YIVSGDLDSN GVFPEYVVVQ IGEFKIGLMH GNQVLPWDDP
GSLEQWQRRL DCDILVTGHT HKLRVFEKNG KLFLNPGTAT GAFSALTPDA PPSFMLMGNK
VVLYVYDLRD GKTNVAMSEF SKYSCPIEGT GDSNSTEIRR CKETINSELS NFHEYMGVRV
PKNGELSDYK FVKYSYVIRK AKEVGSGLAL FAKNSLNWSI TEQACNAYGI STIPLYDVLG
NSGLTYILNS TLPKTVFCSV SCCKKLIPLL PGNLESIHSI HYTSGTTGNP KGAVLTNRAW
VSCTAAFVYG QLGREGSNDR HISYLPLAHI FERIVHMVIT YLGGKIGFYS GDVQKIVDDI
QLFKPTIFIT VPRVLNRIHD EVMMSIEEKP KPLQMLFHFA LKQKERSNSP FHFIWDSIIF
NKTKKILGGN VKAILSGAAP LDETVLTRIR CFSCSYCFEG YGMTELLACM SEINDNSKNI
IGGPPGCYEF KLVSIPEMDY SVKNDPPTGE LLMRGPSSGY FRNEEETKAE GWIRTGDICQ
LLPNGSIRIV DRRKNIFKLS QGEYVAPEKL ENIFVCCELI SQALVIGRST ESFLVAIFVL
DEEFTMKYVR TNQLDLKFKD AVTHPKIIEK IKLDIEKAEL QHKILGYEKI RAFKCISTPF
SVENELLTPT FKVVRHKAIK FYEESINEMY GVSEAELIND QTTLCQAISE GKAPNGVWVG
GNKYKIIRVE KDFQQNDATV HVTFCNRPQG GCFLVDTQNG TVVVAVYDES KDQSSGGKKH
VLAIVYLACR EEGVSRTVKE LLSFDRTISE RELVRSINKL KKDLPRRGPT LSSSAAELMP
RFQLSHEIVG IAEYVCRTAE QYILGTLREK YENSAILSLS SLFIFITLCN MFIMYRLLLD
VIPYPIGVTF SQLLVGMMLA YVLGFSEYAV PVGVYLLMFT IANILLKSTP VIAVYPCILA
LAVVLHHIFR YLFCDQRNLL SFKTVLITAL SYIVACFDTN LVPPTLLGLS ILYAISSAIF
RAVCLEKALH VTERDANKLY NVQVFCGVFA LLFATIIEPE FFLSQSKSFS EFILSIGCLV
TVGTIPFVKN IIANKLVTIG QQAPWRFSEI VSVALFFVFG VIFFEITITI IISFILVISG
RTMSMVDIIN NKRSIGNDIK EASFALAKAT WAAGDFKDRI IESCKRPTVT MEVGTENIAG
VRLPIFEMNV DNNSSTTCHI GVASGGQVIQ STREIYMKVL RDLVKLASLQ FLPLFEFLNV
YAETFFTVLP RNQTANKLNL MRDGSIVSSN NNPWISELDN QVIIQKGEHI CGVLTKKIAG
SSSGSLIHIL WNEIGPEKTG LFLTYTQMVV NTWLLEHGFT VGCQDIQPQN YTLEKIMNSL
EGSKLQVQQI IRRAQKGKLD CQPGKSLIES FEAQVNQELN SARELSGTIA TENLDSKNNI
VAMVQCGSKG STINISQIMA CVGQQNVEGK RIPFGFRDRS LPHFLKYDYG PESRGFVSNS

YLSGLTPQEV FFHAMGGREG IIDTACKTSE TGYIQRRLIK AMEDCMVQYD RTVRNSNGDI
IQFLYGEDGM AGEFIEDQVI ELLQMEAAVF QHKYRHNLEH PRWGDLESQN ILNSEYKQLV
TDREQLGKDI FPDGETRQHL PININRIIQM AQQKFPGPVY IIKKVESLLD KLLLWKNVGS
NDSIIAEVQK NATTLLGIHI RSSLASKKIL EVDRLGPTAF EWILGEIEHQ FYRSLAHSGE
MVGTIAAQSI GEPATQMTLN TFHFAGVGSK NVTLGVPRLR ELINVAKNVR TPTLTVYLES
GIANDQEQAK DVLTLLEHTM LQNITTIAHI LYDPEQDKTI VEADKSWVSD YYEFPDDATS
RLGTWLLRIQ LNNKLVTDKK LSMKEIGDKI LMEFSKDELD CIWTDDNSDE LVLRLRIKNI
EHKFLEKLMT ECLSQITLRG INNIKKVYMK EEQVSRYDSV SQKMVRDNQW VLDTDGCNLE
AVLCHTAIDS TRTISNDITE VFSVLGIEAV RRALLRELRT VISFDGSYVN YRHLALLCDN
MTQKGHLMSI TRHGINRVDK GPLQKCSFEE TVEVLMDAAM FGETDYLNGV SENVMLGQLA
PYGTACFDVL IDPDKLRDTA THEIHGEFAK LGKFSTLSSP LPMSPLYQSY IKKGLIPGSI
RRAEHFITIM KKLILYLQEY IRVYSTRIEG PLTFVKHIEA SCYIQSGLLK FCDERLRSLL
NTLRIVDQYS SLELVCTFFT ILGSYSKGFI VIVDPYPEVS LYDPVIQLSC LDSSIAMRPI
LKRYQSIVLT SGTLSPLDLY PKLLGFIPVV SQSLTMTLDR TCICPLIVTR GSDQTPLSSK
FESRADVSIQ QNYGKLILEI TKKVPDGVVC FFSSYLYMEQ MLSQWYESGL LAQIMEHKLV
FVETKDIVST TLALHHYRKA CDIGRGAIFF SIARGKVAEG IDFDRHYGRC VVMVGIPYQY
TLSKILQSRL SFLKENYGIQ ENEFLTFDAM RQASQCVGRV IRSKADYGLM IFADLRYNKK
DKREKIPPWI LKHLKPEYST LSTDMAVSIS SNFLKQMSQP YAVSNSIKND PDLKISKLSN
GMRVATMKFG PNSLTFGLWV DSGSRNEDPG KNGIAHFLEH LIFKGTYNRS RKEIESQIED
LGAHLNAYTT REQTVYQIRC FNQDLPKCMD LLSDIIKNSK FCKSAIEQEK GVVLREMEEV
SKSEEEIIFD DLHKEMYKNH PLGNTILGPK ENILGFKRED LINYIRTNYI PEKMMILGVG
NIDHNSFKNI AETYFNSQYL NEVLVHKKNN SDGKTLLAMA YNGTSWNSKD FLKVMFLQSM
LGEYGTNNIN RVTGYKNQII ERILSGIKDF FETFNTCYKD TGKLFINFWK QRFYRIWQRS
LEGGIKFAAT LESTILEYSN WLKNWNIIDK NVSSIFLLDL SLSLSTFSRA NPCAILLKQI
SLMNGFKYSF TGALGQKRMN QRESTAQLVV LKSVDPNIDI YENVKLDDDK NSLAIIEQCV
LLIHGVAIYE TSPTNDVIAY EQLNALTNRI LKIHNWLCFS TALWYRCYAE HHRGRTADRA
CLQLQSLVDQ FNDAEPGPEE RLRLVFSVNY PNIWEAKKEL GIRMMRIGSV LSAYNMFVEM
CMWEDAVDCL IVADRKTEAI ELVKEQLKVR ETPRLYCSLG LSFYEKSWEL SRHRFARAQR
SLGNAYFKKS QFELALDAYT KASLVNSTNV NCWFSLGCVA LRLERWEIAQ QAFSRVVSLN
LAAALSKKEL WDEAQSAINE GLKHSRDNWM MWDSSLKIAI KREDLSRIIE CLSGIMKLAS
YKERFPWSLP NIINLLKSEK SKPVVYYVLS EIQLLKNDFS SAYSSKMKEL RSMEVEHIWK
STCFVRKLAP NFTAEAVMAD GSFKKVSLSD YRGKYVVLFF YPLNFTFVCP SEILAFNQAQ
KDFEKLGVQL LAVSVDSQYS HAAWRRTPLE QGGIGPVNFP LISDSSHSIS KNYGVLLEEG
IALRGLFIID KEGVVRSEVY DLPLGRSVEE TLRVIDALQF TETYGEVCPA NWKKGQKGMS
ATHEGVSSYL KDSMQDETVN PIELESLVKD PKCTQDLSAE TDSLITKLEE VLETLADISS
LLNTGLDKET ILILIKLLEL G
