# Supplementary material for: Phylogenomic analyses of malaria parasites and evolution of their exported proteins
Source: BMC Evol Biol. 2011 Jun 15;11:167. doi: 10.1186/1471-2148-11-167 (PMC3146879; doi:10.1186/1471-2148-11-167)
Supplement: Additional file 4 — Likelihood ratio tests for 104 different tree topologies (with and without rate heterogeneity), including their expected likelihood weights (ELW) and probabilities in the approximately unbiased (AU) test. User specified test trees, as well as TREE-PUZZLE and CONSEL output files are given. [file 1471-2148-11-167-S4.PDF]

Additional file 4

Likelihood ratio tests for 104 different tree topologies (with and without rate heterogeneity), including their expected likelihood weights (ELW) and probabilities in the approximately unbiased (AU) test. User specified test trees, as well as TREE-PUZZLE and CONSEL output files are given.

■ 105 tree topologies were specified

```
USER DEFINED TREE # 1
((T. gondii:0.71051,C. parvum:1.17455):0.16883,(B. bovis:0.55109,
T. annulata:0.64817):0.59670,(((((P. reichenowi:0.03964,P. falciparum:0.00375)
:0.18665,(P. vivax:0.06187,P. knowlesi:0.05305):0.15897):0.18261,
(P. yoelii:0.02768,P. berghei:0.02404):0.02420):0.00001,P. chabaudi:0.03739)
:0.12577,P. gallinaceum:0.23734):0.76375);

USER DEFINED TREE # 2
((T. gondii:0.69798,C. parvum:1.15079):0.16652,(B. bovis:0.53948,
T. annulata:0.63604):0.58313,((((P. reichenowi:0.03863,P. falciparum:0.00420)
:0.17118,((P. vivax:0.06101,P. knowlesi:0.05263):0.15825,P. gallinaceum:0.18140)
:0.01749):0.19264,(P. yoelii:0.02729,P. berghei:0.02383):0.02161)
:0.00584,P. chabaudi:0.02913):0.85435);

USER DEFINED TREE # 3
((T. gondii:0.69735,C. parvum:1.14983):0.16617,(B. bovis:0.53879,
T. annulata:0.63582):0.58271,(((((P. reichenowi:0.03842,P. falciparum:0.00437)
:0.15704,P. gallinaceum:0.17332):0.04079,(P. vivax:0.06099,P. knowlesi:0.05260)
:0.15268):0.18570,(P. yoelii:0.02727,P. berghei:0.02381):0.02192)
:0.00569,P. chabaudi:0.02895):0.85391);

USER DEFINED TREE # 4
((T. gondii:0.71752,C. parvum:1.18462):0.17161,(B. bovis:0.55359,
T. annulata:0.65254):0.60092,((((P. reichenowi:0.03973,P. falciparum:0.00375)
:0.18736,(P. vivax:0.06200,P. knowlesi:0.05316):0.15960):0.18194,
((P. yoelii:0.02769,P. berghei:0.02406):0.02457,P. gallinaceum:0.36306)
:0.00050):0.00443,P. chabaudi:0.03373):0.86610);

USER DEFINED TREE # 5
((T. gondii:0.69827,C. parvum:1.15115):0.16656,(B. bovis:0.53954,
T. annulata:0.63642):0.58355,(((((P. reichenowi:0.03867,P. falciparum:0.00416)
:0.17346,(P. vivax:0.06102,P. knowlesi:0.05263):0.16286):0.01469,
P. gallinaceum:0.18110):0.19024,(P. yoelii:0.02728,P. berghei:0.02383)
:0.02160):0.00594,P. chabaudi:0.02905):0.85460);

USER DEFINED TREE # 6
 ((T. gondii:0.71759,C. parvum:1.18426):0.17156,(B. bovis:0.55360,
T. annulata:0.65239):0.60086,((((P. reichenowi:0.03973,P. falciparum:0.00375)
:0.18736,(P. vivax:0.06200,P. knowlesi:0.05315):0.15959):0.18182,
(P. yoelii:0.02769,P. berghei:0.02408):0.02519):0.00001,(P. chabaudi:0.03823,
P. gallinaceum:0.36303):0.00001):0.86743);

USER DEFINED TREE # 7
((T. gondii:0.71753,C. parvum:1.18453):0.17160,(B. bovis:0.55357,
T. annulata:0.65256):0.60092,(((((P. reichenowi:0.03973,P. falciparum:0.00375)
:0.18736,(P. vivax:0.06201,P. knowlesi:0.05315):0.15961):0.18195,
(P. yoelii:0.02770,P. berghei:0.02407):0.02492):0.00001,P. gallinaceum:0.36315)
:0.00454,P. chabaudi:0.03380):0.86606);

USER DEFINED TREE # 8
((T. gondii:0.73160,C. parvum:1.21058):0.17410,(B. bovis:0.56586,
T. annulata:0.66543):0.61474,((((P. reichenowi:0.04031,P. falciparum:0.00369)
:0.33956,((P. vivax:0.06187,P. knowlesi:0.05431):0.31217,P. chabaudi:0.04869)
:0.00023):0.00005,(P. yoelii:0.02787,P. berghei:0.02416):0.03579)
:0.09382,P. gallinaceum:0.25525):0.79371);

USER DEFINED TREE # 9
((T. gondii:0.73715,C. parvum:1.21768):0.17628,(B. bovis:0.56770,
T. annulata:0.66853):0.61808,(((P. reichenowi:0.04035,P. falciparum:0.00371)
:0.33762,(((P. vivax:0.06199,P. knowlesi:0.05439):0.31018,P. chabaudi:0.05055)
:0.00021,P. gallinaceum:0.34660):0.00004):0.00001,(P. yoelii:0.02784,
P. berghei:0.02419):0.03774):0.86472);

USER DEFINED TREE # 10
((T. gondii:0.71934,C. parvum:1.18642):0.17143,(B. bovis:0.55433,
T. annulata:0.65348):0.60185,((((P. reichenowi:0.03831,P. falciparum:0.00517)
:0.16033,P. gallinaceum:0.18026):0.19735,((P. vivax:0.06100,P. knowlesi:0.05358)
:0.32169,P. chabaudi:0.03853):0.00062):0.00001,(P. yoelii:0.02768,
P. berghei:0.02411):0.02586):0.86654);

USER DEFINED TREE # 11
((T. gondii:0.73718,C. parvum:1.21776):0.17628,(B. bovis:0.56771,
T. annulata:0.66857):0.61812,(((P. reichenowi:0.04035,P. falciparum:0.00371)
:0.33767,((P. vivax:0.06199,P. knowlesi:0.05439):0.31022,P. chabaudi:0.05051)
:0.00020):0.00012,((P. yoelii:0.02783,P. berghei:0.02419):0.03761,
P. gallinaceum:0.34655):0.00006):0.86471);

USER DEFINED TREE # 12
 ((T. gondii:0.73717,C. parvum:1.21773):0.17628,(B. bovis:0.56770,
T. annulata:0.66856):0.61810,((((P. reichenowi:0.04035,P. falciparum:0.00371)
:0.33766,((P. vivax:0.06199,P. knowlesi:0.05439):0.31021,P. chabaudi:0.05053)
:0.00020):0.00014,P. gallinaceum:0.34657):0.00001,(P. yoelii:0.02783,
```

P. berghei:0.02419):0.03764):0.86468);

USER DEFINED TREE # 13
((T. gondii:0.73714,C. parvum:1.21767):0.17628,(B. bovis:0.56770,
T. annulata:0.66852):0.61807,(((P. reichenowi:0.04035,P. falciparum:0.00371)
:0.33759,((P. vivax:0.06199,P. knowlesi:0.05438):0.31022,(P. chabaudi:0.05072,
P. gallinaceum:0.34656):0.00001):0.00005):0.00001,(P. yoelii:0.02784,
P. berghei:0.02418):0.03783):0.86470);

USER DEFINED TREE # 14
((T. gondii:0.72225,C. parvum:1.19128):0.17262,(B. bovis:0.55686,
T. annulata:0.65569):0.60413,(((P. reichenowi:0.04003,P. falciparum:0.00351)
:0.35033,(((P. vivax:0.06163,P. knowlesi:0.05372):0.15549,P. gallinaceum:0.20256)
:0.17694,P. chabaudi:0.03951):0.00001):0.00001,(P. yoelii:0.02775,
P. berghei:0.02419):0.02614):0.86952);

USER DEFINED TREE # 15
((T. gondii:0.73153,C. parvum:1.21061):0.17411,(B. bovis:0.56586,
T. annulata:0.66546):0.61475,(((((P. reichenowi:0.04031,P. falciparum:0.00369)
:0.33952,P. chabaudi:0.04888):0.00001,(P. vivax:0.06188,P. knowlesi:0.05430)
:0.31220):0.00006,(P. yoelii:0.02788,P. berghei:0.02416):0.03589)
:0.09378,P. gallinaceum:0.25525):0.79369);

USER DEFINED TREE # 16
((T. gondii:0.72222,C. parvum:1.19131):0.17263,(B. bovis:0.55689,
T. annulata:0.65568):0.60410,((((P. reichenowi:0.04003,P. falciparum:0.00351)
:0.35033,P. chabaudi:0.03951):0.00001,((P. vivax:0.06163,P. knowlesi:0.05372)
:0.15550,P. gallinaceum:0.20257):0.17694):0.00001,(P. yoelii:0.02775,
P. berghei:0.02419):0.02615):0.86954);

USER DEFINED TREE # 17
((T. gondii:0.73713,C. parvum:1.21766):0.17628,(B. bovis:0.56770,
T. annulata:0.66851):0.61807,(((((P. reichenowi:0.04035,P. falciparum:0.00371)
:0.33760,P. chabaudi:0.05075):0.00001,P. gallinaceum:0.34655):0.00001,
(P. vivax:0.06200,P. knowlesi:0.05438):0.31022):0.00001,(P. yoelii:0.02784,
P. berghei:0.02418):0.03787):0.86470);

USER DEFINED TREE # 18
((T. gondii:0.73718,C. parvum:1.21776):0.17627,(B. bovis:0.56772,
T. annulata:0.66857):0.61812,((((P. reichenowi:0.04035,P. falciparum:0.00371)
:0.33765,P. chabaudi:0.05068):0.00001,(P. vivax:0.06200,P. knowlesi:0.05438)
:0.31027):0.00013,((P. yoelii:0.02783,P. berghei:0.02419):0.03769,
P. gallinaceum:0.34652):0.00007):0.86469);

USER DEFINED TREE # 19
((T. gondii:0.73716,C. parvum:1.21773):0.17628,(B. bovis:0.56771,
T. annulata:0.66856):0.61810,(((((P. reichenowi:0.04035,P. falciparum:0.00371)
:0.33764,P. chabaudi:0.05069):0.00001,(P. vivax:0.06200,P. knowlesi:0.05438)
:0.31025):0.00015,P. gallinaceum:0.34653):0.00001,(P. yoelii:0.02784,
P. berghei:0.02419):0.03772):0.86465);

USER DEFINED TREE # 20
((T. gondii:0.73714,C. parvum:1.21767):0.17628,(B. bovis:0.56770,
T. annulata:0.66851):0.61807,((((P. reichenowi:0.04035,P. falciparum:0.00371)
:0.33760,(P. chabaudi:0.05075,P. gallinaceum:0.34655):0.00001):0.00001,
(P. vivax:0.06200,P. knowlesi:0.05438):0.31022):0.00001,(P. yoelii:0.02784,
P. berghei:0.02418):0.03787):0.86470);

USER DEFINED TREE # 21
((T. gondii:0.71938,C. parvum:1.18650):0.17147,(B. bovis:0.55434,
T. annulata:0.65352):0.60188,(((((P. reichenowi:0.03831,P. falciparum:0.00517)
:0.16033,P. gallinaceum:0.18027):0.19740,P. chabaudi:0.03892):0.00001,
(P. vivax:0.06101,P. knowlesi:0.05357):0.32180):0.00001,(P. yoelii:0.02769,
P. berghei:0.02411):0.02617):0.86654);

USER DEFINED TREE # 22
((T. gondii:0.68831,C. parvum:1.14036):0.16238,(B. bovis:0.53568,
T. annulata:0.63024):0.57947,((((P. reichenowi:0.03861,P. falciparum:0.00411)
:0.17328,(P. vivax:0.06076,P. knowlesi:0.05256):0.16123):0.01656,
((P. yoelii:0.02722,P. berghei:0.02371):0.02142,P. chabaudi:0.03444)
:0.18860):0.01805,P. gallinaceum:0.16378):0.75863);

USER DEFINED TREE # 23
((T. gondii:0.68756,C. parvum:1.13726):0.16245,(B. bovis:0.53547,
T. annulata:0.62864):0.57692,(((P. reichenowi:0.03854,P. falciparum:0.00415)
:0.17076,((P. vivax:0.06072,P. knowlesi:0.05252):0.15740,P. gallinaceum:0.18056)
:0.01656):0.05113,((P. yoelii:0.02721,P. berghei:0.02371):0.02153,
P. chabaudi:0.03431):0.14314):0.74758);

USER DEFINED TREE # 24
((T. gondii:0.68626,C. parvum:1.13654):0.16189,(B. bovis:0.53429,
T. annulata:0.62877):0.57658,((((P. reichenowi:0.03831,P. falciparum:0.00434)
:0.15670,P. gallinaceum:0.17257):0.04010,(P. vivax:0.06067,P. knowlesi:0.05251)
:0.15131):0.04729,((P. yoelii:0.02719,P. berghei:0.02369):0.02184,
P. chabaudi:0.03397):0.14026):0.74844);

USER DEFINED TREE # 25
((T. gondii:0.68835,C. parvum:1.14044):0.16262,(B. bovis:0.53556,
T. annulata:0.63020):0.57906,(((P. reichenowi:0.03861,P. falciparum:0.00411)
:0.17302,(P. vivax:0.06074,P. knowlesi:0.05256):0.16114):0.01392,
(((P. yoelii:0.02722,P. berghei:0.02370):0.02140,P. chabaudi:0.03446)
:0.18812,P. gallinaceum:0.18037):0.00425):0.77134);

USER DEFINED TREE # 26
((T. gondii:0.68772,C. parvum:1.13783):0.16237,(B. bovis:0.53564,
T. annulata:0.62912):0.57770,((((P. reichenowi:0.03857,P. falciparum:0.00413)
:0.17288,(P. vivax:0.06073,P. knowlesi:0.05254):0.16173):0.01382,
P. gallinaceum:0.18051):0.04859,((P. yoelii:0.02720,P. berghei:0.02372)
:0.02152,P. chabaudi:0.03434):0.14352):0.74801);

USER DEFINED TREE # 27
((T. gondii:0.70356,C. parvum:1.16930):0.16739,(B. bovis:0.54775,
T. annulata:0.64483):0.59228,(((P. reichenowi:0.03960,P. falciparum:0.00370)
:0.18664,(P. vivax:0.06159,P. knowlesi:0.05309):0.15698):0.04644,
((P. yoelii:0.02761,P. berghei:0.02396):0.02379,(P. chabaudi:0.03713,

P. gallinaceum:0.36244):0.00001):0.13905):0.77029);

USER DEFINED TREE # 28
((T. gondii:0.70357,C. parvum:1.16929):0.16739,(B. bovis:0.54772,
T. annulata:0.64487):0.59230,(((P. reichenowi:0.03960,P. falciparum:0.00370)
:0.18666,(P. vivax:0.06158,P. knowlesi:0.05309):0.15696):0.04644,
(((P. yoelii:0.02760,P. berghei:0.02396):0.02332,P. gallinaceum:0.36233)
:0.00072,P. chabaudi:0.03685):0.13902):0.77033);

USER DEFINED TREE # 29
((T. gondii:0.71053,C. parvum:1.17460):0.16884,(B. bovis:0.55111,
T. annulata:0.64819):0.59672,(((((P. reichenowi:0.03964,P. falciparum:0.00375)
:0.18666,(P. vivax:0.06187,P. knowlesi:0.05305):0.15896):0.18260,
P. chabaudi:0.03729):0.00025,(P. yoelii:0.02768,P. berghei:0.02404)
:0.02405):0.12574,P. gallinaceum:0.23733):0.76375);

USER DEFINED TREE # 30
((T. gondii:0.69807,C. parvum:1.15054):0.16648,(B. bovis:0.53951,
T. annulata:0.63586):0.58304,((((P. reichenowi:0.03863,P. falciparum:0.00420)
:0.17115,((P. vivax:0.06101,P. knowlesi:0.05263):0.15821,P. gallinaceum:0.18141)
:0.01752):0.19266,P. chabaudi:0.03460):0.00010,(P. yoelii:0.02728,
P. berghei:0.02384):0.02193):0.85592);

USER DEFINED TREE # 31
((T. gondii:0.69740,C. parvum:1.14958):0.16614,(B. bovis:0.53883,
T. annulata:0.63569):0.58264,(((((P. reichenowi:0.03842,P. falciparum:0.00438)
:0.15701,P. gallinaceum:0.17333):0.04082,(P. vivax:0.06099,P. knowlesi:0.05259)
:0.15265):0.18572,P. chabaudi:0.03427):0.00019,(P. yoelii:0.02727,
P. berghei:0.02382):0.02214):0.85536);

USER DEFINED TREE # 32
((T. gondii:0.71756,C. parvum:1.18426):0.17159,(B. bovis:0.55356,
T. annulata:0.65239):0.60085,((((P. reichenowi:0.03972,P. falciparum:0.00375)
:0.18736,(P. vivax:0.06200,P. knowlesi:0.05315):0.15957):0.18184,
P. chabaudi:0.03802):0.00001,((P. yoelii:0.02768,P. berghei:0.02407)
:0.02476,P. gallinaceum:0.36290):0.00060):0.86743);

USER DEFINED TREE # 33
((T. gondii:0.71760,C. parvum:1.18431):0.17157,(B. bovis:0.55360,
T. annulata:0.65241):0.60087,(((((P. reichenowi:0.03973,P. falciparum:0.00375)
:0.18737,(P. vivax:0.06201,P. knowlesi:0.05315):0.15959):0.18180,
P. chabaudi:0.03813):0.00024,P. gallinaceum:0.36302):0.00001,(P. yoelii:0.02769,
P. berghei:0.02408):0.02503):0.86745);

USER DEFINED TREE # 34
((T. gondii:0.71759,C. parvum:1.18425):0.17158,(B. bovis:0.55358,
T. annulata:0.65239):0.60085,((((P. reichenowi:0.03973,P. falciparum:0.00375)
:0.18736,(P. vivax:0.06200,P. knowlesi:0.05315):0.15959):0.18184,
(P. chabaudi:0.03824,P. gallinaceum:0.36303):0.00001):0.00001,(P. yoelii:0.02769,
P. berghei:0.02408):0.02518):0.86742);

USER DEFINED TREE # 35
((T. gondii:0.69834,C. parvum:1.15091):0.16654,(B. bovis:0.53959,
T. annulata:0.63630):0.58350,(((((P. reichenowi:0.03867,P. falciparum:0.00417)
:0.17347,(P. vivax:0.06102,P. knowlesi:0.05263):0.16286):0.01468,
P. gallinaceum:0.18113):0.19028,P. chabaudi:0.03462):0.00013,(P. yoelii:0.02727,
P. berghei:0.02384):0.02189):0.85617);

USER DEFINED TREE # 36
((T. gondii:0.73159,C. parvum:1.21056):0.17410,(B. bovis:0.56585,
T. annulata:0.66544):0.61473,((((P. reichenowi:0.04031,P. falciparum:0.00369)
:0.33951,((P. vivax:0.06188,P. knowlesi:0.05431):0.31219,(P. yoelii:0.02788,
P. berghei:0.02416):0.03594):0.00001):0.00001,P. chabaudi:0.04890)
:0.09382,P. gallinaceum:0.25522):0.79366);

USER DEFINED TREE # 37
((T. gondii:0.73712,C. parvum:1.21784):0.17633,(B. bovis:0.56773,
T. annulata:0.66861):0.61817,(((P. reichenowi:0.04036,P. falciparum:0.00371)
:0.33759,(((P. vivax:0.06199,P. knowlesi:0.05439):0.31022,(P. yoelii:0.02785,
P. berghei:0.02418):0.03779):0.00001,P. gallinaceum:0.34652):0.00003)
:0.00304,P. chabaudi:0.04774):0.86335);

USER DEFINED TREE # 38
((T. gondii:0.71926,C. parvum:1.18679):0.17149,(B. bovis:0.55431,
T. annulata:0.65369):0.60192,((((P. reichenowi:0.03831,P. falciparum:0.00517)
:0.16036,P. gallinaceum:0.18027):0.19749,((P. vivax:0.06101,P. knowlesi:0.05358)
:0.32194,(P. yoelii:0.02770,P. berghei:0.02411):0.02595):0.00001)
:0.00407,P. chabaudi:0.03490):0.86523);

USER DEFINED TREE # 39
((T. gondii:0.73714,C. parvum:1.21788):0.17633,(B. bovis:0.56774,
T. annulata:0.66863):0.61820,(((P. reichenowi:0.04036,P. falciparum:0.00371)
:0.33762,((P. vivax:0.06199,P. knowlesi:0.05439):0.31025,((P. yoelii:0.02784,
P. berghei:0.02418):0.03770,P. gallinaceum:0.34649):0.00010):0.00003)
:0.00303,P. chabaudi:0.04771):0.86339);

USER DEFINED TREE # 40
((T. gondii:0.72218,C. parvum:1.19154):0.17263,(B. bovis:0.55684,
T. annulata:0.65584):0.60422,(((P. reichenowi:0.04004,P. falciparum:0.00351)
:0.35049,(((P. vivax:0.06163,P. knowlesi:0.05374):0.15551,P. gallinaceum:0.20254)
:0.17708,(P. yoelii:0.02776,P. berghei:0.02419):0.02592):0.00001)
:0.00419,P. chabaudi:0.03536):0.86822);

USER DEFINED TREE # 41
((T. gondii:0.73713,C. parvum:1.21766):0.17628,(B. bovis:0.56770,
T. annulata:0.66851):0.61807,(((P. reichenowi:0.04035,P. falciparum:0.00371)
:0.33760,((P. vivax:0.06199,P. knowlesi:0.05438):0.31022,(P. yoelii:0.02784,
P. berghei:0.02419):0.03787):0.00001):0.00001,(P. chabaudi:0.05075,
P. gallinaceum:0.34655):0.00001):0.86470);

USER DEFINED TREE # 42
((T. gondii:0.73711,C. parvum:1.21795):0.17633,(B. bovis:0.56778,
T. annulata:0.66860):0.61817,((((P. reichenowi:0.04036,P. falciparum:0.00371)

:0.33759,((P. vivax:0.06199,P. knowlesi:0.05439):0.31021,(P. yoelii:0.02785,
P. berghei:0.02418):0.03781):0.00001):0.00001,P. gallinaceum:0.34651)
:0.00307,P. chabaudi:0.04773):0.86343);

USER DEFINED TREE # 43
((T. gondii:0.70934,C. parvum:1.17308):0.16744,(B. bovis:0.55101,
T. annulata:0.64657):0.59621,(((P. reichenowi:0.03868,P. falciparum:0.00469)
:0.16251,(((P. vivax:0.06081,P. knowlesi:0.05338):0.32215,(P. yoelii:0.02765,
P. berghei:0.02403):0.02461):0.00001,P. chabaudi:0.03770):0.19519)
:0.01724,P. gallinaceum:0.16482):0.77945);

USER DEFINED TREE # 44
((T. gondii:0.70953,C. parvum:1.17348):0.16783,(B. bovis:0.55102,
T. annulata:0.64659):0.59569,((P. reichenowi:0.03869,P. falciparum:0.00469)
:0.14361,((((P. vivax:0.06081,P. knowlesi:0.05339):0.32212,(P. yoelii:0.02765,
P. berghei:0.02402):0.02460):0.00001,P. chabaudi:0.03772):0.19397,
P. gallinaceum:0.17987):0.02163):0.78442);

USER DEFINED TREE # 45
((T. gondii:0.70824,C. parvum:1.17090):0.16766,(B. bovis:0.55026,
T. annulata:0.64576):0.59422,(((P. reichenowi:0.03850,P. falciparum:0.00484)
:0.15849,P. gallinaceum:0.17940):0.05786,(((P. vivax:0.06077,P. knowlesi:0.05335)
:0.32176,(P. yoelii:0.02762,P. berghei:0.02404):0.02467):0.00001,
P. chabaudi:0.03764):0.14265):0.76984);

USER DEFINED TREE # 46
((T. gondii:0.72725,C. parvum:1.20558):0.17331,(B. bovis:0.56415,
T. annulata:0.66253):0.61027,((P. reichenowi:0.04029,P. falciparum:0.00368)
:0.20686,(((P. vivax:0.06177,P. knowlesi:0.05422):0.31290,((P. yoelii:0.02785,
P. berghei:0.02412):0.03475,P. gallinaceum:0.34965):0.00015):0.00001,
P. chabaudi:0.04789):0.13359):0.78755);

USER DEFINED TREE # 47
((T. gondii:0.71145,C. parvum:1.17698):0.16910,(B. bovis:0.55284,
T. annulata:0.64856):0.59588,((P. reichenowi:0.03990,P. falciparum:0.00353)
:0.19404,((((P. vivax:0.06139,P. knowlesi:0.05355):0.15428,P. gallinaceum:0.20139)
:0.17813,(P. yoelii:0.02770,P. berghei:0.02411):0.02470):0.00001,
P. chabaudi:0.03830):0.15625):0.76800);

USER DEFINED TREE # 48
((T. gondii:0.72724,C. parvum:1.20554):0.17331,(B. bovis:0.56417,
T. annulata:0.66250):0.61025,((P. reichenowi:0.04029,P. falciparum:0.00368)
:0.20688,(((P. vivax:0.06177,P. knowlesi:0.05422):0.31285,(P. yoelii:0.02786,
P. berghei:0.02412):0.03489):0.00001,(P. chabaudi:0.04795,P. gallinaceum:0.34968)
:0.00001):0.13353):0.78747);

USER DEFINED TREE # 49
((T. gondii:0.72723,C. parvum:1.20553):0.17330,(B. bovis:0.56414,
T. annulata:0.66250):0.61024,((P. reichenowi:0.04029,P. falciparum:0.00368)
:0.20686,((((P. vivax:0.06177,P. knowlesi:0.05422):0.31286,(P. yoelii:0.02786,
P. berghei:0.02412):0.03489):0.00001,P. gallinaceum:0.34969):0.00001,
P. chabaudi:0.04794):0.13354):0.78752);

USER DEFINED TREE # 50
((T. gondii:0.73152,C. parvum:1.21057):0.17410,(B. bovis:0.56585,
T. annulata:0.66544):0.61473,((((P. reichenowi:0.04031,P. falciparum:0.00369)
:0.33951,P. chabaudi:0.04890):0.00001,((P. vivax:0.06188,P. knowlesi:0.05430)
:0.31219,(P. yoelii:0.02788,P. berghei:0.02416):0.03594):0.00001)
:0.09382,P. gallinaceum:0.25522):0.79367);

USER DEFINED TREE # 51
((T. gondii:0.73716,C. parvum:1.21770):0.17627,(B. bovis:0.56771,
T. annulata:0.66853):0.61809,(((P. reichenowi:0.04035,P. falciparum:0.00371)
:0.33759,P. chabaudi:0.05067):0.00001,(((P. vivax:0.06200,P. knowlesi:0.05438)
:0.31023,(P. yoelii:0.02784,P. berghei:0.02419):0.03782):0.00001,
P. gallinaceum:0.34658):0.00009):0.86469);

USER DEFINED TREE # 52
((T. gondii:0.73714,C. parvum:1.21766):0.17628,(B. bovis:0.56770,
T. annulata:0.66851):0.61807,((((P. reichenowi:0.04035,P. falciparum:0.00371)
:0.33760,P. chabaudi:0.05075):0.00001,P. gallinaceum:0.34655):0.00001,
((P. vivax:0.06200,P. knowlesi:0.05438):0.31022,(P. yoelii:0.02784,
P. berghei:0.02418):0.03787):0.00001):0.86470);

USER DEFINED TREE # 53
((T. gondii:0.73718,C. parvum:1.21774):0.17628,(B. bovis:0.56773,
T. annulata:0.66855):0.61811,(((P. reichenowi:0.04035,P. falciparum:0.00371)
:0.33762,P. chabaudi:0.05064):0.00001,((P. vivax:0.06200,P. knowlesi:0.05438)
:0.31026,((P. yoelii:0.02783,P. berghei:0.02419):0.03773,P. gallinaceum:0.34654)
:0.00011):0.00009):0.86473);

USER DEFINED TREE # 54
((T. gondii:0.72223,C. parvum:1.19132):0.17262,(B. bovis:0.55688,
T. annulata:0.65566):0.60411,(((P. reichenowi:0.04003,P. falciparum:0.00351)
:0.35035,P. chabaudi:0.03948):0.00001,(((P. vivax:0.06163,P. knowlesi:0.05373)
:0.15550,P. gallinaceum:0.20257):0.17693,(P. yoelii:0.02775,P. berghei:0.02419)
:0.02612):0.00006):0.86957);

USER DEFINED TREE # 55
((T. gondii:0.73714,C. parvum:1.21767):0.17628,(B. bovis:0.56770,
T. annulata:0.66852):0.61807,(((P. reichenowi:0.04035,P. falciparum:0.00371)
:0.33760,(P. chabaudi:0.05075,P. gallinaceum:0.34655):0.00001):0.00001,
((P. vivax:0.06200,P. knowlesi:0.05438):0.31022,(P. yoelii:0.02784,
P. berghei:0.02418):0.03787):0.00001):0.86471);

USER DEFINED TREE # 56
((T. gondii:0.71938,C. parvum:1.18651):0.17147,(B. bovis:0.55434,
T. annulata:0.65352):0.60188,((((P. reichenowi:0.03831,P. falciparum:0.00517)
:0.16033,P. gallinaceum:0.18027):0.19740,P. chabaudi:0.03891):0.00001,
((P. vivax:0.06101,P. knowlesi:0.05357):0.32180,(P. yoelii:0.02769,
P. berghei:0.02411):0.02618):0.00001):0.86654);

USER DEFINED TREE # 57
((T. gondii:0.68769,C. parvum:1.13910):0.16216,(B. bovis:0.53561,
T. annulata:0.62913):0.57857,(((P. reichenowi:0.03845,P. falciparum:0.00424)
:0.15892,((P. vivax:0.06074,P. knowlesi:0.05253):0.15197,((P. yoelii:0.02722,

P. berghei:0.02368):0.02169,P. chabaudi:0.03414):0.18524):0.03877)
:0.01637,P. gallinaceum:0.15858):0.76064);

USER DEFINED TREE # 58
((T. gondii:0.68788,C. parvum:1.13918):0.16239,(B. bovis:0.53561,
T. annulata:0.62908):0.57795,((P. reichenowi:0.03848,P. falciparum:0.00423)
:0.13865,(((P. vivax:0.06074,P. knowlesi:0.05253):0.15202,((P. yoelii:0.02722,
P. berghei:0.02368):0.02167,P. chabaudi:0.03416):0.18530):0.03767,
P. gallinaceum:0.17367):0.02216):0.76411);

USER DEFINED TREE # 59
((T. gondii:0.68675,C. parvum:1.13872):0.16216,(B. bovis:0.53455,
T. annulata:0.62929):0.57773,(((P. reichenowi:0.03837,P. falciparum:0.00430)
:0.15616,P. gallinaceum:0.17238):0.02370,((P. vivax:0.06069,P. knowlesi:0.05252)
:0.15109,((P. yoelii:0.02720,P. berghei:0.02367):0.02174,P. chabaudi:0.03407)
:0.18455):0.02020):0.76534);

USER DEFINED TREE # 60
((T. gondii:0.68819,C. parvum:1.14023):0.16267,(B. bovis:0.53567,
T. annulata:0.62994):0.57834,((P. reichenowi:0.03863,P. falciparum:0.00410)
:0.14457,((P. vivax:0.06074,P. knowlesi:0.05257):0.16159,(((P. yoelii:0.02722,
P. berghei:0.02369):0.02141,P. chabaudi:0.03445):0.18959,P. gallinaceum:0.18114)
:0.01372):0.03110):0.76196);

USER DEFINED TREE # 61
((T. gondii:0.68823,C. parvum:1.13971):0.16264,(B. bovis:0.53563,
T. annulata:0.62954):0.57794,((P. reichenowi:0.03861,P. falciparum:0.00412)
:0.14383,(((P. vivax:0.06075,P. knowlesi:0.05255):0.15705,P. gallinaceum:0.18120)
:0.01672,((P. yoelii:0.02723,P. berghei:0.02369):0.02141,P. chabaudi:0.03444)
:0.19174):0.02965):0.76153);

USER DEFINED TREE # 62
((T. gondii:0.70385,C. parvum:1.17133):0.16758,(B. bovis:0.54786,
T. annulata:0.64538):0.59283,((P. reichenowi:0.03963,P. falciparum:0.00369)
:0.15433,((P. vivax:0.06158,P. knowlesi:0.05313):0.15732,((P. yoelii:0.02764,
P. berghei:0.02393):0.02374,(P. chabaudi:0.03718,P. gallinaceum:0.36266)
:0.00001):0.18229):0.03560):0.77464);

USER DEFINED TREE # 63
((T. gondii:0.70392,C. parvum:1.17137):0.16759,(B. bovis:0.54786,
T. annulata:0.64539):0.59290,((P. reichenowi:0.03963,P. falciparum:0.00370)
:0.15438,((P. vivax:0.06157,P. knowlesi:0.05313):0.15730,(((P. yoelii:0.02763,
P. berghei:0.02393):0.02328,P. gallinaceum:0.36256):0.00070,P. chabaudi:0.03691)
:0.18225):0.03558):0.77464);

USER DEFINED TREE # 64
((T. gondii:0.70935,C. parvum:1.17308):0.16740,(B. bovis:0.55097,
T. annulata:0.64657):0.59622,(((P. reichenowi:0.03869,P. falciparum:0.00468)
:0.16250,(((P. vivax:0.06079,P. knowlesi:0.05340):0.32198,P. chabaudi:0.03719)
:0.00090,(P. yoelii:0.02764,P. berghei:0.02403):0.02416):0.19508)
:0.01721,P. gallinaceum:0.16484):0.77947);

USER DEFINED TREE # 65
((T. gondii:0.70955,C. parvum:1.17337):0.16781,(B. bovis:0.55101,
T. annulata:0.64654):0.59570,((P. reichenowi:0.03870,P. falciparum:0.00469)
:0.14362,((((P. vivax:0.06079,P. knowlesi:0.05340):0.32197,P. chabaudi:0.03721)
:0.00090,(P. yoelii:0.02765,P. berghei:0.02402):0.02414):0.19388,
P. gallinaceum:0.17987):0.02161):0.78436);

USER DEFINED TREE # 66
((T. gondii:0.70826,C. parvum:1.17089):0.16764,(B. bovis:0.55027,
T. annulata:0.64576):0.59422,(((P. reichenowi:0.03850,P. falciparum:0.00484)
:0.15848,P. gallinaceum:0.17940):0.05781,(((P. vivax:0.06075,P. knowlesi:0.05336)
:0.32163,P. chabaudi:0.03716):0.00084,(P. yoelii:0.02762,P. berghei:0.02404)
:0.02425):0.14261):0.76991);

USER DEFINED TREE # 67
((T. gondii:0.72729,C. parvum:1.20560):0.17332,(B. bovis:0.56419,
T. annulata:0.66256):0.61030,((P. reichenowi:0.04030,P. falciparum:0.00368)
:0.20687,(((P. vivax:0.06176,P. knowlesi:0.05423):0.31285,P. chabaudi:0.04769)
:0.00026,((P. yoelii:0.02785,P. berghei:0.02412):0.03464,P. gallinaceum:0.34970)
:0.00014):0.13361):0.78752);

USER DEFINED TREE # 68
((T. gondii:0.72724,C. parvum:1.20555):0.17330,(B. bovis:0.56416,
T. annulata:0.66251):0.61025,((P. reichenowi:0.04029,P. falciparum:0.00368)
:0.20688,((((P. vivax:0.06176,P. knowlesi:0.05423):0.31281,P. chabaudi:0.04772)
:0.00027,P. gallinaceum:0.34973):0.00001,(P. yoelii:0.02786,P. berghei:0.02412)
:0.03476):0.13356):0.78749);

USER DEFINED TREE # 69
((T. gondii:0.72723,C. parvum:1.20554):0.17331,(B. bovis:0.56416,
T. annulata:0.66250):0.61025,((P. reichenowi:0.04029,P. falciparum:0.00368)
:0.20688,(((P. vivax:0.06177,P. knowlesi:0.05422):0.31285,(P. chabaudi:0.04794,
P. gallinaceum:0.34968):0.00001):0.00001,(P. yoelii:0.02786,P. berghei:0.02412)
:0.03489):0.13353):0.78747);

USER DEFINED TREE # 70
((T. gondii:0.71147,C. parvum:1.17701):0.16911,(B. bovis:0.55287,
T. annulata:0.64859):0.59590,((P. reichenowi:0.03991,P. falciparum:0.00353)
:0.19404,((((P. vivax:0.06139,P. knowlesi:0.05355):0.15428,P. gallinaceum:0.20140)
:0.17812,P. chabaudi:0.03830):0.00001,(P. yoelii:0.02770,P. berghei:0.02411)
:0.02470):0.15625):0.76803);

USER DEFINED TREE # 71
((T. gondii:0.73153,C. parvum:1.21059):0.17411,(B. bovis:0.56585,
T. annulata:0.66545):0.61474,(((((P. reichenowi:0.04031,P. falciparum:0.00369)
:0.33951,(P. yoelii:0.02788,P. berghei:0.02416):0.03594):0.00001,
(P. vivax:0.06188,P. knowlesi:0.05431):0.31219):0.00001,P. chabaudi:0.04890)
:0.09382,P. gallinaceum:0.25522):0.79367);

USER DEFINED TREE # 72
((T. gondii:0.72216,C. parvum:1.19150):0.17263,(B. bovis:0.55684,
T. annulata:0.65581):0.60422,((((P. reichenowi:0.04004,P. falciparum:0.00351)
:0.35046,(P. yoelii:0.02776,P. berghei:0.02418):0.02590):0.00003,
((P. vivax:0.06162,P. knowlesi:0.05374):0.15550,P. gallinaceum:0.20255)

:0.17707):0.00419,P. chabaudi:0.03536):0.86818);

USER DEFINED TREE # 73
((T. gondii:0.73712,C. parvum:1.21797):0.17638,(B. bovis:0.56774,
T. annulata:0.66865):0.61818,(((((P. reichenowi:0.04036,P. falciparum:0.00371)
:0.33764,(P. yoelii:0.02785,P. berghei:0.02418):0.03762):0.00001,
P. gallinaceum:0.34659):0.00037,(P. vivax:0.06199,P. knowlesi:0.05439)
:0.31019):0.00284,P. chabaudi:0.04765):0.86341);

USER DEFINED TREE # 74
((T. gondii:0.73714,C. parvum:1.21801):0.17639,(B. bovis:0.56775,
T. annulata:0.66867):0.61820,((((P. reichenowi:0.04036,P. falciparum:0.00371)
:0.33767,((P. yoelii:0.02784,P. berghei:0.02418):0.03754,P. gallinaceum:0.34656)
:0.00009):0.00037,(P. vivax:0.06199,P. knowlesi:0.05439):0.31021)
:0.00283,P. chabaudi:0.04763):0.86345);

USER DEFINED TREE # 75
((T. gondii:0.71922,C. parvum:1.18684):0.17151,(B. bovis:0.55429,
T. annulata:0.65370):0.60195,(((((P. reichenowi:0.03831,P. falciparum:0.00517)
:0.16036,P. gallinaceum:0.18027):0.19742,(P. yoelii:0.02770,P. berghei:0.02411)
:0.02556):0.00072,(P. vivax:0.06101,P. knowlesi:0.05358):0.32186)
:0.00385,P. chabaudi:0.03472):0.86540);

USER DEFINED TREE # 76
((T. gondii:0.73714,C. parvum:1.21767):0.17627,(B. bovis:0.56770,
T. annulata:0.66852):0.61808,((((P. reichenowi:0.04035,P. falciparum:0.00371)
:0.33760,(P. yoelii:0.02784,P. berghei:0.02419):0.03787):0.00001,
(P. vivax:0.06200,P. knowlesi:0.05438):0.31022):0.00001,(P. chabaudi:0.05074,
P. gallinaceum:0.34656):0.00001):0.86471);

USER DEFINED TREE # 77
((T. gondii:0.73712,C. parvum:1.21784):0.17633,(B. bovis:0.56773,
T. annulata:0.66861):0.61818,(((((P. reichenowi:0.04036,P. falciparum:0.00371)
:0.33759,(P. yoelii:0.02785,P. berghei:0.02418):0.03780):0.00001,
(P. vivax:0.06199,P. knowlesi:0.05439):0.31022):0.00001,P. gallinaceum:0.34652)
:0.00307,P. chabaudi:0.04773):0.86335);

USER DEFINED TREE # 78
((T. gondii:0.73153,C. parvum:1.21058):0.17410,(B. bovis:0.56585,
T. annulata:0.66545):0.61475,((((P. reichenowi:0.04031,P. falciparum:0.00369)
:0.33954,(P. yoelii:0.02788,P. berghei:0.02416):0.03582):0.00001,
((P. vivax:0.06187,P. knowlesi:0.05431):0.31215,P. chabaudi:0.04870)
:0.00024):0.09386,P. gallinaceum:0.25523):0.79368);

USER DEFINED TREE # 79
((T. gondii:0.73717,C. parvum:1.21769):0.17626,(B. bovis:0.56771,
T. annulata:0.66853):0.61810,(((P. reichenowi:0.04035,P. falciparum:0.00371)
:0.33762,(P. yoelii:0.02784,P. berghei:0.02419):0.03775):0.00001,
(((P. vivax:0.06199,P. knowlesi:0.05439):0.31018,P. chabaudi:0.05055)
:0.00021,P. gallinaceum:0.34660):0.00004):0.86473);

USER DEFINED TREE # 80
((T. gondii:0.73713,C. parvum:1.21783):0.17635,(B. bovis:0.56770,
T. annulata:0.66858):0.61808,((((P. reichenowi:0.04036,P. falciparum:0.00371)
:0.33765,(P. yoelii:0.02784,P. berghei:0.02419):0.03764):0.00001,
P. gallinaceum:0.34664):0.00044,((P. vivax:0.06199,P. knowlesi:0.05439)
:0.31019,P. chabaudi:0.05039):0.00001):0.86465);

USER DEFINED TREE # 81
((T. gondii:0.73715,C. parvum:1.21787):0.17635,(B. bovis:0.56772,
T. annulata:0.66860):0.61810,(((P. reichenowi:0.04036,P. falciparum:0.00371)
:0.33768,((P. yoelii:0.02783,P. berghei:0.02419):0.03756,P. gallinaceum:0.34661)
:0.00010):0.00043,((P. vivax:0.06199,P. knowlesi:0.05439):0.31021,
P. chabaudi:0.05036):0.00001):0.86468);

USER DEFINED TREE # 82
((T. gondii:0.71932,C. parvum:1.18653):0.17149,(B. bovis:0.55432,
T. annulata:0.65352):0.60182,((((P. reichenowi:0.03831,P. falciparum:0.00517)
:0.16033,P. gallinaceum:0.18028):0.19729,(P. yoelii:0.02769,P. berghei:0.02411)
:0.02566):0.00067,((P. vivax:0.06099,P. knowlesi:0.05358):0.32173,
P. chabaudi:0.03839):0.00026):0.86651);

USER DEFINED TREE # 83
((T. gondii:0.73716,C. parvum:1.21768):0.17627,(B. bovis:0.56771,
T. annulata:0.66853):0.61809,(((P. reichenowi:0.04035,P. falciparum:0.00371)
:0.33760,(P. yoelii:0.02784,P. berghei:0.02419):0.03783):0.00001,
((P. vivax:0.06200,P. knowlesi:0.05438):0.31022,(P. chabaudi:0.05072,
P. gallinaceum:0.34657):0.00001):0.00005):0.86471);

USER DEFINED TREE # 84
((T. gondii:0.72221,C. parvum:1.19124):0.17263,(B. bovis:0.55683,
T. annulata:0.65566):0.60409,(((P. reichenowi:0.04003,P. falciparum:0.00351)
:0.35033,(P. yoelii:0.02775,P. berghei:0.02419):0.02608):0.00011,
(((P. vivax:0.06163,P. knowlesi:0.05373):0.15548,P. gallinaceum:0.20256)
:0.17694,P. chabaudi:0.03946):0.00001):0.86949);

USER DEFINED TREE # 85
((T. gondii:0.70609,C. parvum:1.17887):0.16717,(B. bovis:0.54978,
T. annulata:0.65001):0.59874,(((((P. reichenowi:0.03983,P. falciparum:0.00352)
:0.34997,(P. yoelii:0.02770,P. berghei:0.02408):0.02438):0.00021,
P. chabaudi:0.03809):0.17849,(P. vivax:0.06129,P. knowlesi:0.05363)
:0.15353):0.02356,P. gallinaceum:0.18077):0.76972);

USER DEFINED TREE # 86
((T. gondii:0.70648,C. parvum:1.17723):0.16734,(B. bovis:0.55013,
T. annulata:0.64920):0.59753,((((P. reichenowi:0.03984,P. falciparum:0.00351)
:0.34984,(P. yoelii:0.02768,P. berghei:0.02410):0.02442):0.00019,
P. chabaudi:0.03805):0.14329,((P. vivax:0.06130,P. knowlesi:0.05360)
:0.15102,P. gallinaceum:0.20188):0.03925):0.77322);

USER DEFINED TREE # 87
((T. gondii:0.70402,C. parvum:1.18041):0.16709,(B. bovis:0.54872,
T. annulata:0.65062):0.59872,(((((P. reichenowi:0.03983,P. falciparum:0.00352)
:0.34983,(P. yoelii:0.02770,P. berghei:0.02408):0.02438):0.00016,
P. chabaudi:0.03815):0.17549,P. gallinaceum:0.20139):0.03912,(P. vivax:0.06125,

P. knowlesi:0.05363):0.11914):0.77509);

USER DEFINED TREE # 88
((T. gondii:0.71901,C. parvum:1.20772):0.17158,(B. bovis:0.56066,
T. annulata:0.66347):0.61154,((((P. reichenowi:0.04020,P. falciparum:0.00369)
:0.34025,((P. yoelii:0.02784,P. berghei:0.02410):0.03421,P. gallinaceum:0.34989)
:0.00014):0.00033,P. chabaudi:0.04724):0.15169,(P. vivax:0.06163,
P. knowlesi:0.05428):0.16187):0.77956);

USER DEFINED TREE # 89
((T. gondii:0.70006,C. parvum:1.17478):0.16620,(B. bovis:0.54667,
T. annulata:0.64757):0.59527,(((((P. reichenowi:0.03810,P. falciparum:0.00519)
:0.15898,P. gallinaceum:0.17910):0.19788,(P. yoelii:0.02761,P. berghei:0.02400)
:0.02424):0.00088,P. chabaudi:0.03715):0.17044,(P. vivax:0.06062,
P. knowlesi:0.05347):0.15138):0.75764);

USER DEFINED TREE # 90
((T. gondii:0.71898,C. parvum:1.20760):0.17155,(B. bovis:0.56064,
T. annulata:0.66342):0.61150,((((P. reichenowi:0.04020,P. falciparum:0.00369)
:0.34016,(P. yoelii:0.02784,P. berghei:0.02410):0.03450):0.00001,
(P. chabaudi:0.04756,P. gallinaceum:0.34985):0.00001):0.15173,(P. vivax:0.06163,
P. knowlesi:0.05428):0.16183):0.77951);

USER DEFINED TREE # 91
((T. gondii:0.71899,C. parvum:1.20767):0.17157,(B. bovis:0.56064,
T. annulata:0.66345):0.61151,(((((P. reichenowi:0.04020,P. falciparum:0.00369)
:0.34021,(P. yoelii:0.02784,P. berghei:0.02410):0.03433):0.00001,
P. gallinaceum:0.34993):0.00034,P. chabaudi:0.04728):0.15165,(P. vivax:0.06163,
P. knowlesi:0.05428):0.16187):0.77954);

USER DEFINED TREE # 92
((T. gondii:0.68752,C. parvum:1.14013):0.16221,(B. bovis:0.53525,
T. annulata:0.63003):0.57919,((((P. reichenowi:0.03853,P. falciparum:0.00418)
:0.17105,((P. yoelii:0.02723,P. berghei:0.02369):0.02143,P. chabaudi:0.03442)
:0.19143):0.01878,(P. vivax:0.06075,P. knowlesi:0.05255):0.15665)
:0.01857,P. gallinaceum:0.16378):0.75904);

USER DEFINED TREE # 93
((T. gondii:0.68783,C. parvum:1.14009):0.16241,(B. bovis:0.53527,
T. annulata:0.62989):0.57863,(((P. reichenowi:0.03854,P. falciparum:0.00416)
:0.17072,((P. yoelii:0.02723,P. berghei:0.02369):0.02142,P. chabaudi:0.03442)
:0.19122):0.01238,((P. vivax:0.06074,P. knowlesi:0.05254):0.15591,
P. gallinaceum:0.18062):0.00848):0.77121);

USER DEFINED TREE # 94
((T. gondii:0.68389,C. parvum:1.14124):0.16177,(B. bovis:0.53351,
T. annulata:0.63042):0.57878,((((P. reichenowi:0.03849,P. falciparum:0.00420)
:0.17056,((P. yoelii:0.02722,P. berghei:0.02367):0.02138,P. chabaudi:0.03444)
:0.19183):0.01633,P. gallinaceum:0.18063):0.04557,(P. vivax:0.06067,
P. knowlesi:0.05255):0.11466):0.75472);

USER DEFINED TREE # 95
((T. gondii:0.68409,C. parvum:1.14150):0.16191,(B. bovis:0.53374,
T. annulata:0.63048):0.57878,(((P. reichenowi:0.03855,P. falciparum:0.00415)
:0.17279,(((P. yoelii:0.02722,P. berghei:0.02368):0.02138,P. chabaudi:0.03445)
:0.18980,P. gallinaceum:0.18047):0.01342):0.04928,(P. vivax:0.06065,
P. knowlesi:0.05257):0.11527):0.75462);

USER DEFINED TREE # 96
((T. gondii:0.68328,C. parvum:1.14009):0.16144,(B. bovis:0.53265,
T. annulata:0.63018):0.57789,((((P. reichenowi:0.03830,P. falciparum:0.00435)
:0.15658,P. gallinaceum:0.17258):0.03987,((P. yoelii:0.02720,P. berghei:0.02366)
:0.02168,P. chabaudi:0.03411):0.18450):0.04327,(P. vivax:0.06059,
P. knowlesi:0.05256):0.11129):0.75501);

USER DEFINED TREE # 97
((T. gondii:0.70062,C. parvum:1.17303):0.16683,(B. bovis:0.54633,
T. annulata:0.64629):0.59384,(((P. reichenowi:0.03957,P. falciparum:0.00374)
:0.18620,((P. yoelii:0.02763,P. berghei:0.02394):0.02372,(P. chabaudi:0.03720,
P. gallinaceum:0.36257):0.00001):0.18151):0.04662,(P. vivax:0.06151,
P. knowlesi:0.05315):0.11464):0.77320);

USER DEFINED TREE # 98
((T. gondii:0.70063,C. parvum:1.17308):0.16684,(B. bovis:0.54632,
T. annulata:0.64632):0.59387,(((P. reichenowi:0.03956,P. falciparum:0.00374)
:0.18621,(((P. yoelii:0.02762,P. berghei:0.02393):0.02328,P. gallinaceum:0.36245)
:0.00067,P. chabaudi:0.03694):0.18146):0.04658,(P. vivax:0.06150,
P. knowlesi:0.05315):0.11466):0.77321);

USER DEFINED TREE # 99
((T. gondii:0.70609,C. parvum:1.17886):0.16717,(B. bovis:0.54978,
T. annulata:0.65001):0.59873,(((((P. reichenowi:0.03983,P. falciparum:0.00352)
:0.35000,P. chabaudi:0.03818):0.00001,(P. yoelii:0.02770,P. berghei:0.02408)
:0.02450):0.17851,(P. vivax:0.06129,P. knowlesi:0.05363):0.15355)
:0.02356,P. gallinaceum:0.18076):0.76972);

USER DEFINED TREE # 100
((T. gondii:0.70649,C. parvum:1.17723):0.16734,(B. bovis:0.55013,
T. annulata:0.64920):0.59753,((((P. reichenowi:0.03984,P. falciparum:0.00351)
:0.34987,P. chabaudi:0.03814):0.00001,(P. yoelii:0.02768,P. berghei:0.02410)
:0.02453):0.14330,((P. vivax:0.06130,P. knowlesi:0.05359):0.15103,
P. gallinaceum:0.20187):0.03925):0.77323);

USER DEFINED TREE # 101
((T. gondii:0.70402,C. parvum:1.18053):0.16709,(B. bovis:0.54872,
T. annulata:0.65068):0.59872,(((((P. reichenowi:0.03983,P. falciparum:0.00352)
:0.34985,P. chabaudi:0.03822):0.00001,(P. yoelii:0.02770,P. berghei:0.02408)
:0.02448):0.17552,P. gallinaceum:0.20140):0.03914,(P. vivax:0.06125,
P. knowlesi:0.05363):0.11914):0.77516);

USER DEFINED TREE # 102
((T. gondii:0.71901,C. parvum:1.20766):0.17156,(B. bovis:0.56066,
T. annulata:0.66344):0.61153,((((P. reichenowi:0.04020,P. falciparum:0.00369)
:0.34021,P. chabaudi:0.04751):0.00001,((P. yoelii:0.02784,P. berghei:0.02410)
:0.03435,P. gallinaceum:0.34981):0.00016):0.15177,(P. vivax:0.06163,
P. knowlesi:0.05428):0.16183):0.77954);

USER DEFINED TREE # 103
((T. gondii:0.71898,C. parvum:1.20759):0.17155,(B. bovis:0.56064,
T. annulata:0.66341):0.61150,(((((P. reichenowi:0.04020,P. falciparum:0.00369)
:0.34016,P. chabaudi:0.04756):0.00001,P. gallinaceum:0.34984):0.00001,
(P. yoelii:0.02784,P. berghei:0.02410):0.03449):0.15174,(P. vivax:0.06163,
P. knowlesi:0.05428):0.16183):0.77950);

USER DEFINED TREE # 104
((T. gondii:0.71898,C. parvum:1.20760):0.17154,(B. bovis:0.56064,
T. annulata:0.66342):0.61150,((((P. reichenowi:0.04020,P. falciparum:0.00369)
:0.34016,(P. chabaudi:0.04757,P. gallinaceum:0.34985):0.00001):0.00001,
(P. yoelii:0.02784,P. berghei:0.02410):0.03450):0.15173,(P. vivax:0.06163,
P. knowlesi:0.05428):0.16183):0.77950);

USER DEFINED TREE # 105
((T. gondii:0.70008,C. parvum:1.17475):0.16617,(B. bovis:0.54670,
T. annulata:0.64755):0.59530,(((((P. reichenowi:0.03810,P. falciparum:0.00519)
:0.15899,P. gallinaceum:0.17910):0.19798,P. chabaudi:0.03765):0.00001,
(P. yoelii:0.02761,P. berghei:0.02401):0.02470):0.17054,(P. vivax:0.06063,
P. knowlesi:0.05346):0.15140):0.75762);

■ COMPARISON OF USER TREES

TREEPUZZLE-OUTPUT WITH RATE HETEROGENEITY

Tree	log L	difference	S.E.	p-1sKH	p-SH	c-ELW	2sKH
1	-1842524.45	22279.53	281.9222	0.0000 -	0.0000 -	0.0000 -	-
2	-1824345.37	4100.45	119.7048	0.0000 -	0.0000 -	0.0000 -	-
3	-1823358.19	3113.27	95.7522	0.0000 -	0.0000 -	0.0000 -	-
4	-1843705.55	23460.62	298.3982	0.0000 -	0.0000 -	0.0000 -	-
5	-1824436.59	4191.66	118.2266	0.0000 -	0.0000 -	0.0000 -	-
6	-1843730.24	23485.31	297.8565	0.0000 -	0.0000 -	0.0000 -	-
7	-1843709.50	23464.58	298.2286	0.0000 -	0.0000 -	0.0000 -	-
8	-1858894.25	38649.33	414.5865	0.0000 -	0.0000 -	0.0000 -	-
9	-1859507.78	39262.86	417.8315	0.0000 -	0.0000 -	0.0000 -	-
10	-1845749.82	25504.89	302.0184	0.0000 -	0.0000 -	0.0000 -	-
11	-1859506.22	39261.30	417.9196	0.0000 -	0.0000 -	0.0000 -	-
12	-1859506.64	39261.72	417.8920	0.0000 -	0.0000 -	0.0000 -	-
13	-1859508.76	39263.84	417.7210	0.0000 -	0.0000 -	0.0000 -	-
14	-1848630.96	28386.03	325.6143	0.0000 -	0.0000 -	0.0000 -	-
15	-1858895.22	38650.30	414.4716	0.0000 -	0.0000 -	0.0000 -	-
16	-1848630.98	28386.06	325.6139	0.0000 -	0.0000 -	0.0000 -	-
17	-1859508.98	39264.06	417.6881	0.0000 -	0.0000 -	0.0000 -	-
18	-1859507.00	39262.07	417.8146	0.0000 -	0.0000 -	0.0000 -	-
19	-1859507.43	39262.50	417.7859	0.0000 -	0.0000 -	0.0000 -	-
20	-1859509.10	39264.17	417.6865	0.0000 -	0.0000 -	0.0000 -	-
21	-1845753.44	25508.52	301.7819	0.0000 -	0.0000 -	0.0000 -	-
22	-1821629.87	1384.95	83.0617	0.0000 -	0.0000 -	0.0000 -	-
23	-1821193.53	948.61	72.0186	0.0000 -	0.0160 -	0.0000 -	-
24	-1820244.92	0.00	<---- best	1.0000 +	1.0000 +	0.9366 +	best
25	-1821722.48	1477.56	81.5495	0.0000 -	0.0000 -	0.0000 -	-
26	-1821274.52	1029.59	69.5386	0.0000 -	0.0080 -	0.0000 -	-
27	-1841320.33	21075.41	272.2079	0.0000 -	0.0000 -	0.0000 -	-
28	-1841315.06	21070.14	272.4310	0.0000 -	0.0000 -	0.0000 -	-
29	-1842523.61	22278.69	282.0019	0.0000 -	0.0000 -	0.0000 -	-
30	-1824373.06	4128.14	119.1759	0.0000 -	0.0000 -	0.0000 -	-
31	-1823384.90	3139.98	95.0677	0.0000 -	0.0000 -	0.0000 -	-
32	-1843725.23	23480.31	298.0639	0.0000 -	0.0000 -	0.0000 -	-
33	-1843729.12	23484.20	297.9453	0.0000 -	0.0000 -	0.0000 -	-
34	-1843730.15	23485.23	297.8476	0.0000 -	0.0000 -	0.0000 -	-
35	-1824465.34	4220.42	117.6595	0.0000 -	0.0000 -	0.0000 -	-
36	-1858895.61	38650.69	414.4390	0.0000 -	0.0000 -	0.0000 -	-
37	-1859497.05	39252.13	418.0423	0.0000 -	0.0000 -	0.0000 -	-
38	-1845735.63	25490.70	302.1607	0.0000 -	0.0000 -	0.0000 -	-
39	-1859496.08	39251.16	418.0999	0.0000 -	0.0000 -	0.0000 -	-
40	-1848612.48	28367.56	325.9474	0.0000 -	0.0000 -	0.0000 -	-
41	-1859509.24	39264.32	417.6920	0.0000 -	0.0000 -	0.0000 -	-
42	-1859497.32	39252.40	418.0279	0.0000 -	0.0000 -	0.0000 -	-
43	-1843905.17	23660.25	284.0103	0.0000 -	0.0000 -	0.0000 -	-
44	-1843888.49	23643.56	284.3327	0.0000 -	0.0000 -	0.0000 -	-
45	-1843547.90	23302.98	278.9197	0.0000 -	0.0000 -	0.0000 -	-
46	-1858259.45	38014.53	411.7416	0.0000 -	0.0000 -	0.0000 -	-
47	-1846686.29	26441.37	306.5781	0.0000 -	0.0000 -	0.0000 -	-
48	-1858261.05	38016.13	411.6586	0.0000 -	0.0000 -	0.0000 -	-
49	-1858260.88	38015.96	411.6611	0.0000 -	0.0000 -	0.0000 -	-
50	-1858895.53	38650.61	414.4400	0.0000 -	0.0000 -	0.0000 -	-
51	-1859508.56	39263.63	417.7473	0.0000 -	0.0000 -	0.0000 -	-
52	-1859509.08	39264.16	417.6921	0.0000 -	0.0000 -	0.0000 -	-
53	-1859507.55	39262.63	417.8079	0.0000 -	0.0000 -	0.0000 -	-
54	-1848630.92	28385.99	325.6314	0.0000 -	0.0000 -	0.0000 -	-
55	-1859509.20	39264.28	417.6906	0.0000 -	0.0000 -	0.0000 -	-
56	-1845753.47	25508.55	301.7841	0.0000 -	0.0000 -	0.0000 -	-
57	-1820667.16	422.24	62.0107	0.0000 -	0.2170 +	0.0000 -	-
58	-1820631.98	387.06	63.8125	0.0000 -	0.2510 +	0.0000 -	-
59	-1820530.20	285.27	44.9689	0.0000 -	0.3640 +	0.0000 -	-
60	-1821628.70	1383.78	85.3831	0.0000 -	0.0000 -	0.0000 -	-
61	-1821510.44	1265.52	87.3831	0.0000 -	0.0000 -	0.0000 -	-
62	-1841432.35	21187.43	276.0441	0.0000 -	0.0000 -	0.0000 -	-
63	-1841427.25	21182.33	276.2595	0.0000 -	0.0000 -	0.0000 -	-
64	-1843899.92	23655.00	284.3025	0.0000 -	0.0000 -	0.0000 -	-
65	-1843883.20	23638.28	284.6166	0.0000 -	0.0000 -	0.0000 -	-
66	-1843543.14	23298.22	279.1945	0.0000 -	0.0000 -	0.0000 -	-
67	-1858258.42	38013.49	411.8647	0.0000 -	0.0000 -	0.0000 -	-
68	-1858259.65	38014.72	411.7987	0.0000 -	0.0000 -	0.0000 -	-
69	-1858260.94	38016.01	411.6635	0.0000 -	0.0000 -	0.0000 -	-
70	-1846686.29	26441.36	306.5809	0.0000 -	0.0000 -	0.0000 -	-
71	-1858895.56	38650.64	414.4393	0.0000 -	0.0000 -	0.0000 -	-
72	-1848612.46	28367.54	325.9577	0.0000 -	0.0000 -	0.0000 -	-
73	-1859494.02	39249.10	418.2283	0.0000 -	0.0000 -	0.0000 -	-
74	-1859493.17	39248.25	418.2771	0.0000 -	0.0000 -	0.0000 -	-
75	-1845731.29	25486.36	302.4225	0.0000 -	0.0000 -	0.0000 -	-
76	-1859509.21	39264.29	417.6903	0.0000 -	0.0000 -	0.0000 -	-
77	-1859497.29	39252.36	418.0278	0.0000 -	0.0000 -	0.0000 -	-

78	-1858894.46	38649.54	414.5655	0.0000 -	0.0000 -	0.0000 -	-
79	-1859507.87	39262.95	417.8348	0.0000 -	0.0000 -	0.0000 -	-
80	-1859504.20	39259.28	417.9591	0.0000 -	0.0000 -	0.0000 -	-
81	-1859503.34	39258.41	418.0096	0.0000 -	0.0000 -	0.0000 -	-
82	-1845746.97	25502.05	302.1450	0.0000 -	0.0000 -	0.0000 -	-
83	-1859508.84	39263.92	417.7241	0.0000 -	0.0000 -	0.0000 -	-
84	-1848630.83	28385.91	325.6443	0.0000 -	0.0000 -	0.0000 -	-
85	-1846447.82	26202.90	304.4691	0.0000 -	0.0000 -	0.0000 -	-
86	-1846350.76	26105.84	301.8168	0.0000 -	0.0000 -	0.0000 -	-
87	-1846323.35	26078.43	306.0958	0.0000 -	0.0000 -	0.0000 -	-
88	-1857894.57	37649.64	412.1516	0.0000 -	0.0000 -	0.0000 -	-
89	-1843371.27	23126.35	281.5265	0.0000 -	0.0000 -	0.0000 -	-
90	-1857897.65	37652.73	411.9083	0.0000 -	0.0000 -	0.0000 -	-
91	-1857895.86	37650.94	412.0849	0.0000 -	0.0000 -	0.0000 -	-
92	-1821576.37	1331.45	87.1895	0.0000 -	0.0000 -	0.0000 -	-
93	-1821633.67	1388.74	85.6632	0.0000 -	0.0000 -	0.0000 -	-
94	-1821290.50	1045.58	89.4223	0.0000 -	0.0070 -	0.0000 -	-
95	-1821385.44	1140.52	86.9508	0.0000 -	0.0030 -	0.0000 -	-
96	-1820325.25	80.33	51.1859	0.0720 +	0.7920 +	0.0634 +	+
97	-1841289.91	21044.98	277.6424	0.0000 -	0.0000 -	0.0000 -	-
98	-1841285.07	21040.15	277.8520	0.0000 -	0.0000 -	0.0000 -	-
99	-1846448.18	26203.26	304.4100	0.0000 -	0.0000 -	0.0000 -	-
100	-1846351.06	26106.14	301.7627	0.0000 -	0.0000 -	0.0000 -	-
101	-1846323.59	26078.67	306.0495	0.0000 -	0.0000 -	0.0000 -	-
102	-1857896.14	37651.21	411.9910	0.0000 -	0.0000 -	0.0000 -	-
103	-1857897.64	37652.72	411.9101	0.0000 -	0.0000 -	0.0000 -	-
104	-1857897.75	37652.83	411.9098	0.0000 -	0.0000 -	0.0000 -	-
105	-1843376.29	23131.36	281.2282	0.0000 -	0.0000 -	0.0000 -	-

TREEPUZZLE-OUTPUT WITHOUT RATE HETEROGENEITY

Tree	log L	difference	S.E.	p-1sKH	p-SH	c-ELW	2sKH
1	-1891275.56	28767.88	338.9548	0.0000 -	0.0000 -	0.0000 -	-
2	-1870153.42	7645.74	165.0547	0.0000 -	0.0000 -	0.0000 -	-
3	-1868804.26	6296.58	134.7499	0.0000 -	0.0000 -	0.0000 -	-
4	-1895076.03	32568.35	369.7286	0.0000 -	0.0000 -	0.0000 -	-
5	-1870309.44	7801.76	162.8080	0.0000 -	0.0000 -	0.0000 -	-
6	-1895139.15	32631.47	368.5511	0.0000 -	0.0000 -	0.0000 -	-
7	-1895081.85	32574.17	369.4277	0.0000 -	0.0000 -	0.0000 -	-
8	-1912373.94	49866.26	482.3154	0.0000 -	0.0000 -	0.0000 -	-
9	-1914320.68	51813.00	480.8733	0.0000 -	0.0000 -	0.0000 -	-
10	-1897418.26	34910.58	369.7629	0.0000 -	0.0000 -	0.0000 -	-
11	-1914394.66	51886.98	481.6358	0.0000 -	0.0000 -	0.0000 -	-
12	-1914320.71	51813.03	480.8732	0.0000 -	0.0000 -	0.0000 -	-
13	-1914330.34	51822.66	480.3660	0.0000 -	0.0000 -	0.0000 -	-
14	-1901166.99	38659.31	401.7278	0.0000 -	0.0000 -	0.0000 -	-
15	-1912382.05	49874.37	481.7328	0.0000 -	0.0000 -	0.0000 -	-
16	-1901170.68	38663.00	401.5499	0.0000 -	0.0000 -	0.0000 -	-
17	-1914330.62	51822.94	480.2747	0.0000 -	0.0000 -	0.0000 -	-
18	-1914406.53	51898.85	481.0145	0.0000 -	0.0000 -	0.0000 -	-
19	-1914330.49	51822.81	480.2897	0.0000 -	0.0000 -	0.0000 -	-
20	-1914330.84	51823.16	480.2745	0.0000 -	0.0000 -	0.0000 -	-
21	-1897438.62	34930.94	369.0254	0.0000 -	0.0000 -	0.0000 -	-
22	-1864712.21	2204.53	118.6486	0.0000 -	0.0000 -	0.0000 -	-
23	-1863799.46	1291.78	95.3779	0.0000 -	0.0050 -	0.0000 -	-
24	-1862507.68	0.00	<---- best	1.0000 +	1.0000 +	0.9970 +	best
25	-1865013.13	2505.45	115.9000	0.0000 -	0.0000 -	0.0000 -	-
26	-1863971.79	1464.12	91.2408	0.0000 -	0.0010 -	0.0000 -	-
27	-1889363.24	26855.57	326.1503	0.0000 -	0.0000 -	0.0000 -	-
28	-1889349.80	26842.12	326.6535	0.0000 -	0.0000 -	0.0000 -	-
29	-1891270.52	28762.84	339.2010	0.0000 -	0.0000 -	0.0000 -	-
30	-1870209.67	7701.99	164.2145	0.0000 -	0.0000 -	0.0000 -	-
31	-1868856.18	6348.50	133.7605	0.0000 -	0.0000 -	0.0000 -	-
32	-1895129.25	32621.57	368.9733	0.0000 -	0.0000 -	0.0000 -	-
33	-1895127.06	32619.38	369.0036	0.0000 -	0.0000 -	0.0000 -	-
34	-1895130.21	32622.53	368.7723	0.0000 -	0.0000 -	0.0000 -	-
35	-1870367.05	7859.37	161.9436	0.0000 -	0.0000 -	0.0000 -	-
36	-1912382.16	49874.48	481.7326	0.0000 -	0.0000 -	0.0000 -	-
37	-1914263.79	51756.11	480.6006	0.0000 -	0.0000 -	0.0000 -	-
38	-1897394.32	34886.64	369.7808	0.0000 -	0.0000 -	0.0000 -	-
39	-1914263.67	51756.00	480.6208	0.0000 -	0.0000 -	0.0000 -	-
40	-1901123.83	38616.15	402.2372	0.0000 -	0.0000 -	0.0000 -	-
41	-1914408.81	51901.13	480.8952	0.0000 -	0.0000 -	0.0000 -	-
42	-1914264.01	51756.33	480.5853	0.0000 -	0.0000 -	0.0000 -	-
43	-1892787.83	30280.15	344.2275	0.0000 -	0.0000 -	0.0000 -	-
44	-1892788.10	30280.42	344.7680	0.0000 -	0.0000 -	0.0000 -	-
45	-1891937.16	29429.48	329.0659	0.0000 -	0.0000 -	0.0000 -	-
46	-1910966.83	48459.15	479.5391	0.0000 -	0.0000 -	0.0000 -	-
47	-1895723.59	33215.91	367.9015	0.0000 -	0.0000 -	0.0000 -	-
48	-1910967.47	48459.79	479.4723	0.0000 -	0.0000 -	0.0000 -	-
49	-1910967.25	48459.58	479.4775	0.0000 -	0.0000 -	0.0000 -	-
50	-1912382.05	49874.37	481.7288	0.0000 -	0.0000 -	0.0000 -	-
51	-1914406.60	51898.92	481.0293	0.0000 -	0.0000 -	0.0000 -	-
52	-1914408.71	51901.03	480.8747	0.0000 -	0.0000 -	0.0000 -	-
53	-1914406.46	51898.78	481.0413	0.0000 -	0.0000 -	0.0000 -	-
54	-1901177.48	38669.80	401.3717	0.0000 -	0.0000 -	0.0000 -	-
55	-1914408.95	51901.27	480.8715	0.0000 -	0.0000 -	0.0000 -	-
56	-1897446.89	34939.21	368.8046	0.0000 -	0.0000 -	0.0000 -	-
57	-1863412.38	904.70	97.1200	0.0000 -	0.0460 -	0.0000 -	-
58	-1863357.16	849.48	99.5492	0.0000 -	0.0560 +	0.0000 -	-
59	-1863227.61	719.93	69.6073	0.0000 -	0.1010 +	0.0000 -	-
60	-1864645.15	2137.47	125.5094	0.0000 -	0.0000 -	0.0000 -	-
61	-1864490.83	1983.15	125.8395	0.0000 -	0.0000 -	0.0000 -	-
62	-1889658.78	27151.10	338.3486	0.0000 -	0.0000 -	0.0000 -	-
63	-1889645.86	27138.18	338.8300	0.0000 -	0.0000 -	0.0000 -	-
64	-1892761.38	30253.70	344.9628	0.0000 -	0.0000 -	0.0000 -	-
65	-1892760.85	30253.17	345.5229	0.0000 -	0.0000 -	0.0000 -	-
66	-1891911.47	29403.79	329.8180	0.0000 -	0.0000 -	0.0000 -	-
67	-1910958.84	48451.16	480.0811	0.0000 -	0.0000 -	0.0000 -	-
68	-1910959.06	48451.38	480.0504	0.0000 -	0.0000 -	0.0000 -	-
69	-1910967.26	48459.58	479.5295	0.0000 -	0.0000 -	0.0000 -	-
70	-1895718.35	33210.67	368.0813	0.0000 -	0.0000 -	0.0000 -	-
71	-1912381.80	49874.12	481.8219	0.0000 -	0.0000 -	0.0000 -	-
72	-1901120.30	38612.62	402.4057	0.0000 -	0.0000 -	0.0000 -	-
73	-1914258.64	51750.96	481.0179	0.0000 -	0.0000 -	0.0000 -	-

74	-1914258.54	51750.86	481.0260	0.0000 -	0.0000 -	0.0000 -	-
75	-1897378.80	34871.12	370.4086	0.0000 -	0.0000 -	0.0000 -	-
76	-1914408.80	51901.12	480.9132	0.0000 -	0.0000 -	0.0000 -	-
77	-1914263.99	51756.32	480.5856	0.0000 -	0.0000 -	0.0000 -	-
78	-1912373.86	49866.18	482.3095	0.0000 -	0.0000 -	0.0000 -	-
79	-1914395.30	51887.62	481.6214	0.0000 -	0.0000 -	0.0000 -	-
80	-1914389.94	51882.26	481.7632	0.0000 -	0.0000 -	0.0000 -	-
81	-1914389.80	51882.12	481.7811	0.0000 -	0.0000 -	0.0000 -	-
82	-1897417.77	34910.09	369.7806	0.0000 -	0.0000 -	0.0000 -	-
83	-1914406.67	51898.99	481.0760	0.0000 -	0.0000 -	0.0000 -	-
84	-1901171.32	38663.64	401.6250	0.0000 -	0.0000 -	0.0000 -	-
85	-1895769.53	33261.86	369.7856	0.0000 -	0.0000 -	0.0000 -	-
86	-1895539.97	33032.29	360.6570	0.0000 -	0.0000 -	0.0000 -	-
87	-1895646.98	33139.30	372.7147	0.0000 -	0.0000 -	0.0000 -	-
88	-1910380.04	47872.36	480.9879	0.0000 -	0.0000 -	0.0000 -	-
89	-1891278.11	28770.43	333.7367	0.0000 -	0.0000 -	0.0000 -	-
90	-1910385.50	47877.82	480.5204	0.0000 -	0.0000 -	0.0000 -	-
91	-1910380.26	47872.59	480.9545	0.0000 -	0.0000 -	0.0000 -	-
92	-1864604.43	2096.75	126.8787	0.0000 -	0.0000 -	0.0000 -	-
93	-1864860.64	2352.96	122.5928	0.0000 -	0.0000 -	0.0000 -	-
94	-1864056.57	1548.89	127.5210	0.0000 -	0.0010 -	0.0000 -	-
95	-1864175.26	1667.58	125.2979	0.0000 -	0.0010 -	0.0000 -	-
96	-1862725.85	218.17	80.1430	0.0050 -	0.6270 +	0.0030 -	-
97	-1889487.69	26980.01	341.0992	0.0000 -	0.0000 -	0.0000 -	-
98	-1889476.09	26968.41	341.5468	0.0000 -	0.0000 -	0.0000 -	-
99	-1895779.43	33271.75	369.4815	0.0000 -	0.0000 -	0.0000 -	-
100	-1895549.88	33042.20	360.3595	0.0000 -	0.0000 -	0.0000 -	-
101	-1895655.25	33147.57	372.4518	0.0000 -	0.0000 -	0.0000 -	-
102	-1910385.08	47877.40	480.5240	0.0000 -	0.0000 -	0.0000 -	-
103	-1910385.56	47877.89	480.4701	0.0000 -	0.0000 -	0.0000 -	-
104	-1910385.78	47878.10	480.4672	0.0000 -	0.0000 -	0.0000 -	-
105	-1891304.96	28797.28	332.8834	0.0000 -	0.0000 -	0.0000 -	-

CONSEL-OUTPUT WITH RATE HETEROGENEITY

rank	item	obs	au	np	bp	pp	kh	sh	wkh	wsh
1	1	9917.3	1e-13	2e-08	0	0	0	0	0	0
2	2	2467.8	0.002	1e-05	0	0	0	0	0	0
3	3	1789.0	1e-15	2e-11	0	0	0	0	0	0
4	4	11605.9	3e-80	3e-23	0	0	0	0	0	0
5	5	2491.2	2e-06	1e-06	0	0	0	0	0	0
6	6	11639.6	1e-48	4e-17	0	0	0	0	0	0
7	7	11606.7	4e-45	1e-16	0	0	0	0	0	0
8	8	14345.9	3e-35	2e-14	0	0	0	0	0	0
9	9	14766.4	2e-28	3e-13	0	0	0	0	0	0
10	10	10270.4	1e-53	2e-17	0	0	0	0	0	0
11	11	14997.3	4e-08	7e-07	0	0	0	0	0	0
12	12	14765.6	9e-11	2e-08	0	0	0	0	0	0
13	13	14787.0	4e-33	3e-14	0	0	0	0	0	0
14	14	11669.0	2e-05	1e-06	0	0	0	0	0	0
15	15	14348.1	8e-83	8e-23	0	0	0	0	0	0
16	16	11664.3	2e-05	1e-06	0	0	0	0	0	0
17	17	14777.3	3e-45	5e-16	0	0	0	0	0	0
18	18	15009.9	1e-39	7e-16	0	0	0	0	0	0
19	19	14778.0	2e-13	8e-09	0	0	0	0	0	0
20	20	14785.0	2e-114	7e-28	0	0	0	0	0	0
21	21	10273.3	7e-66	1e-19	0	0	0	0	0	0
22	22	847.3	1e-89	2e-24	0	0	0	0.001	0	0
23	23	576.0	7e-48	3e-16	0	7e-251	0	0.031	0	0
24	24	-220.4	1.000	0.999	0.999	1.000	1.000	1.000	1.000	1.000
25	25	1071.6	8e-08	7e-07	0	0	0	2e-05	0	0
26	26	591.2	3e-06	3e-06	0	2e-257	0	0.027	0	0
27	27	10186.4	5e-10	1e-07	0	0	0	0	0	0
28	28	10181.5	2e-12	5e-08	0	0	0	0	0	0
29	29	9912.7	1e-70	1e-20	0	0	0	0	0	0
30	30	2515.6	1e-04	2e-05	0	0	0	0	0	0
31	31	1837.6	4e-05	2e-06	0	0	0	0	0	0
32	32	11635.8	1e-36	7e-15	0	0	0	0	0	0
33	33	11636.0	5e-58	5e-19	0	0	0	0	0	0
34	34	11639.8	9e-34	3e-14	0	0	0	0	0	0
35	35	2542.4	3e-06	5e-07	0	0	0	0	0	0
36	36	14352.3	9e-50	4e-17	0	0	0	0	0	0
37	37	14703.7	6e-06	7e-06	0	0	0	0	0	0
38	38	10240.9	6e-56	6e-18	0	0	0	0	0	0
39	39	14710.4	3e-04	3e-06	0	0	0	0	0	0
40	40	11635.6	7e-05	2e-06	0	0	0	0	0	0
41	41	15017.5	2e-08	6e-07	0	0	0	0	0	0
42	42	14705.8	6e-06	6e-06	0	0	0	0	0	0
43	43	8947.2	1e-50	2e-17	0	0	0	0	0	0
44	44	8970.8	4e-39	2e-15	0	0	0	0	0	0
45	45	8623.9	2e-44	8e-16	0	0	0	0	0	0
46	46	14371.2	7e-108	3e-26	0	0	0	0	0	0
47	47	9953.3	1e-86	5e-24	0	0	0	0	0	0
48	48	14372.6	5e-91	2e-23	0	0	0	0	0	0
49	49	14370.9	5e-64	5e-19	0	0	0	0	0	0
50	50	14348.2	9e-49	1e-16	0	0	0	0	0	0
51	51	15007.8	2e-08	6e-07	0	0	0	0	0	0
52	52	15008.0	6e-09	5e-07	0	0	0	0	0	0
53	53	15015.7	1e-07	8e-07	0	0	0	0	0	0
54	54	11664.0	3e-05	1e-06	0	0	0	0	0	0
55	55	15016.1	7e-09	5e-07	0	0	0	0	0	0
56	56	10273.8	2e-66	8e-20	0	0	0	0	0	0
57	57	220.4	0.001	0.001	0.001	2e-96	5e-04	0.407	5e-04	0.011
58	58	241.7	0.001	0.001	3e-04	1e-105	0	0.363	0	0.003
59	59	278.0	1e-57	8e-18	0	2e-121	0	0.289	0	0
60	60	916.2	0.002	5e-07	0	0	0	4e-04	0	0
61	61	854.7	7e-43	6e-16	0	0	0	0.001	0	0
62	62	10296.6	3e-48	8e-17	0	0	0	0	0	0
63	63	10289.3	4e-43	7e-16	0	0	0	0	0	0
64	64	8940.6	4e-42	7e-16	0	0	0	0	0	0
65	65	8962.7	5e-35	1e-14	0	0	0	0	0	0
66	66	8615.7	5e-136	2e-31	0	0	0	0	0	0
67	67	14366.7	1e-50	2e-16	0	0	0	0	0	0
68	68	14366.7	2e-71	4e-20	0	0	0	0	0	0
69	69	14372.9	3e-54	4e-17	0	0	0	0	0	0

70	70	9957.4	3e-59	4e-19	0	0	0	0	0	0
71	71	14352.8	1e-52	1e-17	0	0	0	0	0	0
72	72	11637.1	6e-05	2e-06	0	0	0	0	0	0
73	73	14706.7	7e-06	8e-06	0	0	0	0	0	0
74	74	14707.0	1e-05	8e-06	0	0	0	0	0	0
75	75	10238.6	1e-62	4e-19	0	0	0	0	0	0
76	76	15026.2	1e-07	8e-07	0	0	0	0	0	0
77	77	14712.4	3e-04	2e-06	0	0	0	0	0	0
78	78	14346.2	2e-72	1e-21	0	0	0	0	0	0
79	79	15003.9	2e-08	6e-07	0	0	0	0	0	0
80	80	15003.5	2e-08	6e-07	0	0	0	0	0	0
81	81	15003.8	2e-08	6e-07	0	0	0	0	0	0
82	82	10270.0	8e-82	2e-22	0	0	0	0	0	0
83	83	15026.1	3e-07	9e-07	0	0	0	0	0	0
84	84	11668.5	9e-40	4e-17	0	0	0	0	0	0
85	85	10279.4	2e-68	9e-21	0	0	0	0	0	0
86	86	10199.0	9e-66	2e-20	0	0	0	0	0	0
87	87	10392.1	1e-86	1e-23	0	0	0	0	0	0
88	88	14428.4	2e-04	2e-05	0	0	0	0	0	0
89	89	8596.9	1e-04	8e-06	0	0	0	0	0	0
90	90	14432.5	2e-36	1e-14	0	0	0	0	0	0
91	91	14428.3	2e-04	2e-05	0	0	0	0	0	0
92	92	850.5	6e-90	8e-23	0	0	0	0.001	0	0
93	93	1040.6	6e-46	2e-16	0	0	0	0	0	0
94	94	817.9	2e-78	2e-21	0	0	0	0.002	0	0
95	95	853.7	2e-51	2e-18	0	0	0	0.001	0	0
96	96	225.8	4e-05	3e-05	0	8e-99	8e-05	0.392	8e-05	1e-04
97	97	10373.0	1e-45	2e-16	0	0	0	0	0	0
98	98	10365.7	3e-49	4e-17	0	0	0	0	0	0
99	99	10272.1	6e-06	1e-06	0	0	0	0	0	0
100	100	10191.1	4e-43	5e-16	0	0	0	0	0	0
101	101	10384.1	7e-64	1e-19	0	0	0	0	0	0
102	102	14428.6	1e-37	5e-15	0	0	0	0	0	0
103	103	14427.8	1e-36	9e-15	0	0	0	0	0	0
104	104	14431.7	6e-106	4e-27	0	0	0	0	0	0
105	105	8601.9	8e-05	7e-06	0	0	0	0	0	0

CONSEL-OUTPUT WITHOUT RATE HETEROGENEITY

rank	item	obs	au	np	bp	pp	kh	sh	wkh	wsh
1	1	8629.7	2e-55	5e-18	0	0	0	0	0	0
2	2	1699.4	3e-51	1e-17	0	0	0	0	0	0
3	3	1166.5	0.001	4e-06	0	0	0	0	0	0
4	4	9509.6	1e-48	2e-16	0	0	0	0	0	0
5	5	1712.0	0.021	2e-06	0	0	0	0	0	0
6	6	9526.6	5e-93	3e-24	0	0	0	0	0	0
7	7	9510.0	2e-54	1e-17	0	0	0	0	0	0
8	8	12623.8	5e-52	1e-16	0	0	0	0	0	0
9	9	12857.4	1e-63	5e-18	0	0	0	0	0	0
10	10	8425.4	2e-05	9e-08	0	0	0	0	0	0
11	11	12919.1	2e-50	2e-16	0	0	0	0	0	0
12	12	12857.4	2e-67	1e-18	0	0	0	0	0	0
13	13	12858.2	1e-128	2e-28	0	0	0	0	0	0
14	14	9585.6	1e-63	3e-18	0	0	0	0	0	0
15	15	12623.8	3e-52	1e-16	0	0	0	0	0	0
16	16	9584.6	2e-53	2e-16	0	0	0	0	0	0
17	17	12858.1	5e-58	4e-17	0	0	0	0	0	0
18	18	12919.9	9e-73	2e-20	0	0	0	0	0	0
19	19	12858.0	6e-63	6e-18	0	0	0	0	0	0
20	20	12858.2	8e-63	6e-18	0	0	0	0	0	0
21	21	8426.0	1e-15	5e-12	0	0	0	0	0	0
22	22	622.8	5e-06	6e-07	0	3e-271	0	0.007	0	0
23	23	474.0	3e-07	3e-07	0	1e-206	0	0.040	0	0
24	24	-114.4	0.994	0.989	0.991	1.000	0.999	1.000	0.990	1.000
25	25	730.7	8e-70	1e-21	0	4e-318	0	0.001	0	0
26	26	477.7	1e-65	2e-19	0	4e-208	0	0.041	0	0
27	27	8686.4	2e-74	1e-20	0	0	0	0	0	0
28	28	8684.5	3e-67	2e-19	0	0	0	0	0	0
29	29	8627.4	7e-62	4e-19	0	0	0	0	0	0
30	30	1726.6	4e-39	6e-15	0	0	0	0	0	0
31	31	1193.6	0.168	4e-06	0	0	0	0	0	0
32	32	9524.5	8e-68	6e-20	0	0	0	0	0	0
33	33	9524.5	4e-78	1e-21	0	0	0	0	0	0
34	34	9526.6	1e-46	4e-16	0	0	0	0	0	0
35	35	1739.7	7e-06	3e-05	0	0	0	0	0	0
36	36	12624.0	9e-60	5e-18	0	0	0	0	0	0
37	37	12818.9	6e-71	2e-19	0	0	0	0	0	0
38	38	8409.5	5e-26	2e-13	0	0	0	0	0	0
39	39	12819.0	2e-66	1e-18	0	0	0	0	0	0
40	40	9568.3	1e-64	2e-18	0	0	0	0	0	0
41	41	12921.4	2e-50	2e-16	0	0	0	0	0	0
42	42	12819.1	1e-72	1e-19	0	0	0	0	0	0
43	43	7594.4	5e-06	6e-06	0	0	0	0	0	0
44	44	7616.0	1e-05	6e-06	0	0	0	0	0	0
45	45	7447.7	4e-56	4e-18	0	0	0	0	0	0
46	46	12644.5	2e-59	3e-18	0	0	0	0	0	0
47	47	8687.9	2e-102	3e-26	0	0	0	0	0	0
48	48	12644.6	3e-52	6e-17	0	0	0	0	0	0
49	49	12644.5	5e-49	2e-16	0	0	0	0	0	0
50	50	12623.9	4e-59	7e-18	0	0	0	0	0	0
51	51	12920.8	2e-60	3e-18	0	0	0	0	0	0
52	52	12921.1	3e-48	4e-16	0	0	0	0	0	0
53	53	12920.9	2e-55	2e-17	0	0	0	0	0	0
54	54	9584.3	9e-110	7e-26	0	0	0	0	0	0
55	55	12921.2	2e-50	2e-16	0	0	0	0	0	0
56	56	8425.9	9e-18	1e-12	0	0	0	0	0	0
57	57	118.6	0.013	0.008	0.008	3e-52	0.010	0.580	0.010	0.090
58	58	137.0	0.007	0.002	0.002	3e-60	0.003	0.527	0.003	0.031
59	59	128.1	8e-05	1e-05	0	2e-56	1e-04	0.553	1e-04	0.003
60	60	687.2	7e-41	1e-15	0	4e-299	0	0.003	0	0
61	61	638.5	6e-160	2e-34	0	5e-278	0	0.006	0	0
62	62	8738.6	7e-46	5e-16	0	0	0	0	0	0
63	63	8736.3	5e-59	2e-18	0	0	0	0	0	0
64	64	7592.4	5e-06	6e-06	0	0	0	0	0	0

65	65	7613.9	2e-05	5e-06	0	0	0	0	0	0
66	66	7445.7	6e-06	1e-05	0	0	0	0	0	0
67	67	12644.4	2e-100	3e-25	0	0	0	0	0	0
68	68	12644.4	2e-83	2e-22	0	0	0	0	0	0
69	69	12644.5	1e-74	7e-21	0	0	0	0	0	0
70	70	8689.4	4e-70	9e-21	0	0	0	0	0	0
71	71	12624.0	3e-62	2e-18	0	0	0	0	0	0
72	72	9568.9	3e-69	3e-19	0	0	0	0	0	0
73	73	12818.9	3e-114	2e-26	0	0	0	0	0	0
74	74	12818.9	1e-83	1e-21	0	0	0	0	0	0
75	75	8408.9	2e-30	2e-14	0	0	0	0	0	0
76	76	12921.5	2e-124	7e-29	0	0	0	0	0	0
77	77	12819.2	2e-77	2e-20	0	0	0	0	0	0
78	78	12623.9	5e-60	4e-18	0	0	0	0	0	0
79	79	12920.1	5e-48	5e-16	0	0	0	0	0	0
80	80	12920.1	4e-51	1e-16	0	0	0	0	0	0
81	81	12920.2	7e-50	2e-16	0	0	0	0	0	0
82	82	8425.0	2e-05	1e-07	0	0	0	0	0	0
83	83	12921.0	2e-66	3e-19	0	0	0	0	0	0
84	84	9585.3	1e-83	1e-21	0	0	0	0	0	0
85	85	8745.1	8e-07	5e-06	0	0	0	0	0	0
86	86	8717.8	2e-77	1e-20	0	0	0	0	0	0
87	87	8779.2	5e-63	3e-18	0	0	0	0	0	0
88	88	12636.6	6e-63	6e-19	0	0	0	0	0	0
89	89	7495.5	2e-07	3e-07	0	0	0	0	0	0
90	90	12636.7	9e-115	1e-27	0	0	0	0	0	0
91	91	12636.6	4e-79	9e-22	0	0	0	0	0	0
92	92	639.4	2e-05	6e-07	0	2e-278	0	0.006	0	0
93	93	716.0	3e-34	3e-14	0	1e-311	0	0.002	0	0
94	94	605.3	3e-07	3e-07	0	1e-263	0	0.009	0	0
95	95	631.8	2e-05	1e-06	0	4e-275	0	0.006	0	0
96	96	114.4	0.001	0.001	1e-04	2e-50	0.001	0.597	0.001	0.009
97	97	8747.6	4e-54	4e-17	0	0	0	0	0	0
98	98	8745.3	5e-50	1e-16	0	0	0	0	0	0
99	99	8743.5	8e-07	6e-06	0	0	0	0	0	0
100	100	8716.0	4e-92	4e-23	0	0	0	0	0	0
101	101	8777.4	5e-62	5e-18	0	0	0	0	0	0
102	102	12636.6	2e-70	3e-20	0	0	0	0	0	0
103	103	12636.6	4e-58	4e-18	0	0	0	0	0	0
104	104	12636.7	1e-61	9e-19	0	0	0	0	0	0
105	105	7496.9	1e-07	4e-07	0	0	0	0	0	0
