# Supplementary material for: Phylogenomic analyses of malaria parasites and evolution of their exported proteins
Source: BMC Evol Biol. 2011 Jun 15;11:167. doi: 10.1186/1471-2148-11-167 (PMC3146879; doi:10.1186/1471-2148-11-167)
Supplement: Additional file 5 — Sum of the expected substitutions per site for each of the 218 proteins used for phylogenetic inference. Note that only the proteins of primer taxa (P. falciparum, P. vivax, P. knowlesi, P. yoelii, P. berghei, P. chabaudi, T. gondii, and B. bovis) were considered. [file 1471-2148-11-167-S5.PDF]

Additional file 5

Sum of the expected substitutions per site for each of the 218 proteins used for phylogenetic inference. Note that only the proteins of primer taxa (*P. falciparum*, *P. vivax*, *P. knowlesi*, *P. yoelii*, *P. berghei*, *P. chabaudi*, *T. gondii*, and *B. bovis*) were considered.

MAL13P1.190	3.18943306078975
MAL13P1.205	1.73248408440143
MAL13P1.234	10.8042425973558
MAL13P1.279	0.84115266988692
MAL13P1.308	3.0353274391443
MAL13P1.337	1.47622186876619
MAL13P1.344	0.971557907565969
MAL13P1.36	3.18176173739441
MAL13P1.52	5.9668170461273
MAL13P1.76	3.02396325668684
MAL13P1.92	0.892838919618494
MAL7P1.122	1.14078666050757
MAL7P1.145	9.86718492764016
MAL7P1.162	6.82142348804727
MAL8P1.125	2.36729098654196
MAL8P1.128	1.63920791225437
MAL8P1.150	10.8132041711185
MAL8P1.65	8.08538993066271
MAL8P1.83	2.82957939615765
MAL8P1.96	4.26560665169785
PF07_0079	0.604933670362858
PF07_0091	4.99300615150718
PF07_0092	4.99300615150718
PF07_0112	1.18501165001585
PF07_0117	1.15926525599214
PF08_0006	0.792965452750112
PF08_0048	8.33527155857213
PF08_0069	2.70201850783883
PF08_0113	4.23563232589929
PF08_0125	0.710294140163439
PF08_0126	4.74159371440134
PF10_0086	2.28916573191201
PF10_0087	1.91023700644757
PF10_0123	2.64891069534622
PF10_0165	2.2029654847373
PF10_0174	2.86571448475591
PF10_0245	4.6301622186956
PF10_0264	1.1342439821942
PF10_0293	2.44210609945739
PF10_0294	2.95514963219718
PF10_0306	1.3659776923718
PF11_0051	3.68376781970565
PF11_0055	2.76774161353731
PF11_0087	1.19877381281875
PF11_0098	4.87901846761159
PF11_0108	3.60560826426042

PF11_0112	6.11328181705902
PF11_0114	2.72873352473893
PF11_0142	2.92339050162541
PF11_0156	3.8383049129697
PF11_0157	2.69267999535388
PF11_0183	0.414747991929411
PF11_0187	1.42998956977185
PF11_0202	2.77503663734661
PF11_0203	6.53136287507438
PF11_0225	4.92544558501081
PF11_0251	4.77567753437671
PF11_0258	10.03632657294
PF11_0259	4.02324369415074
PF11_0265	5.93093760867249
PF11_0270	3.80941707435322
PF11_0282	2.19975100220912
PF11_0303	2.20831230397468
PF11_0313	1.51804746185634
PF11_0331	1.02730005824433
PF11_0377	1.08681285827529
PF13_0016	4.03130344643587
PF13_0063	0.807052174057365
PF13_0156	1.48278125855693
PF13_0177	3.75867290151163
PF13_0178	0.856568074203576
PF13_0205	2.49681081711038
PF13_0217	2.76701479273378
PF13_0227	2.39545843243604
PF13_0251	3.30399510950485
PF13_0257	3.93448520818585
PF13_0305	0.571170352983591
PF13_0308	4.18767912716037
PF13_0313	5.54904922157483
PF13_0315	2.2474291187067
PF13_0316	0.991378880300674
PF13_0324	2.75611763930271
PF13_0328	1.36355022422706
PF13_0330	1.27301079130738
PF14_0064	1.74774528631177
PF14_0067	3.40775038601705
PF14_0104	0.627696292103833
PF14_0127	2.07477560223154
PF14_0193	5.94048450260224
PF14_0324	2.08691730405628
PF14_0328	1.3532996246806
PF14_0352	1.13075438451764
PF14_0359	1.94083062042588
PF14_0360	5.59210748610809
PF14_0361	2.62506942009394
PF14_0368	1.65313128267749
PF14_0370	6.24899595771665
PF14_0378	2.34084766353593
PF14_0391	0.61800142877888
PF14_0393	2.21116590229952
PF14_0429	6.04266277645647
PF14_0469	7.44853405345161

PF14_0493	2.02387653671165
PF14_0517	4.9122048083488
PF14_0518	2.98715371879482
PF14_0548	1.9380536102168
PF14_0585	0.58351897426319
PF14_0649	7.51564823330309
PF14_0661	3.69366532413514
PF14_0677	5.23651450640932
PF14_0688	3.77940050877936
PF14_0723	3.67238514164299
PFA0145c	4.02100392824127
PFA0400c	2.07288487131058
PFA0525w	3.16200328138852
PFB0275w	3.354336725854
PFB0445c	1.05443955239572
PFB0525w	3.11435920326622
PFB0550w	0.742359579974709
PFB0595w	1.54636828188204
PFB0640c	6.63489909909306
PFB0750w	4.39757068669801
PFB0830w	1.35143834208056
PFB0840w	1.99226834734985
PFC0160w	3.76092690715833
PFC0185w	6.89098293394943
PFC0290w	0.359796051209734
PFC0295c	2.0294652392107
PFC0350c	1.22451735061631
PFC0365w	2.54108506076199
PFC0375c	3.13450610198394
PFC0475c	9.06258918830265
PFC0720w	5.48877238072644
PFC0805w	3.45803864561113
PFD0180c	2.5776055106796
PFD0420c	5.32851975320954
PFD0450c	3.5712568566245
PFD0515w	3.64392681820519
PFD0525w	3.88728720968138
PFD0720w	1.85742525234155
PFD0725c	2.71669860798826
PFD0880w	3.2341075835742
PFD1110w	1.91622199776774
PFE0165w	2.46182527384085
PFE0185c	1.64913563736965
PFE0465c	7.21697805765309
PFE0485w	8.83148313034252
PFE0625w	1.09399450864722
PFE0785c	1.97898769331567
PFE0870w	2.48652192080079
PFE0890c	5.21618752114453
PFE0895c	3.47792508905919
PFE0965c	1.43375992014899
PFE1005w	0.795552295046913
PFE1050w	2.07331183959423
PFE1140c	1.15310712886961
PFE1155c	1.98604884100404
PFE1195w	2.64495174238914

PFE1250w	3.19531536348031
PFE1340w	3.85363750584704
PFF0185c	6.0440146980853
PFF0305c	1.38796482367309
PFF0345w	3.49451108572542
PFF0450c	4.13389713164715
PFF0500c	3.83662331091321
PFF0535c	6.94813053490874
PFF0610c	7.37238020159317
PFF0825c	4.68825524518771
PFF0940c	0.753657792169164
PFF1155w	2.42456404798981
PFF1345w	3.9121148317882
PFF1350c	4.56043831132287
PFI0200c	7.59661775067361
PFI0300w	3.307803905677
PFI0415c	11.8365460173976
PFI0480w	4.86215333027227
PFI0735c	4.06025797783232
PFI0880c	2.09130495724494
PFI0895c	2.11906108787858
PFI0920c	4.04994860231433
PFI0935w	3.81805075392904
PFI1020c	1.7579399720931
PFI1130c	2.8430374236818
PFI1140w	5.45973061491366
PFI1170c	2.92217145322438
PFI1260c	0.739006834683337
PFI1455c	4.00004517870669
PFI1565w	2.23192574990846
PFI1570c	3.82532370591472
PFI1625c	1.87146137275493
PFI1650w	4.67367541673702
PFI1685w	1.68701097424302
PFI1700c	9.10442704566932
PFL0095c	7.46875089699213
PFL0130c	7.94479107991774
PFL0310c	2.95945237181726
PFL0580w	1.32629504009982
PFL0620c	2.184783351907
PFL0660w	0.857148387551611
PFL0670c	2.61745774388941
PFL0815w	7.18562288193402
PFL0830w	6.3111464200302
PFL0895c	2.91906228151478
PFL0930w	3.03453407555706
PFL0950c	4.49261535359445
PFL1010c	10.6801686888531
PFL1110c	3.58284520607504
PFL1180w	6.98830024089473
PFL1245w	3.47407302850449
PFL1425w	1.50174246781549
PFL1680w	1.72719758606622
PFL1790w	4.81712422498357
PFL2005w	1.52770600509925
PFL2060c	1.23486923063658

PFL2225w	3.93405793707293
PFL2310w	3.23031407430532
PFL2460w	5.16678892939069
PFL2465c	3.37721647477248
