# Supplementary material for: Phylogenomic analyses of malaria parasites and evolution of their exported proteins
Source: BMC Evol Biol. 2011 Jun 15;11:167. doi: 10.1186/1471-2148-11-167 (PMC3146879; doi:10.1186/1471-2148-11-167)

Additional file 6

Split network of the plasmodium phylogeny reconstructed from the 65 slowest evolving genes in our data set. The network was calculated with SplitsTree v. 4.11.3 [71] considering only splits with a frequency of at least 10%. It displays alternative hypothesis concerning the relationships of the Plasmodium species that have at least some support by the data. Note that the topology placing *P. gallinaceum* as the sister of mammal *Plasmodium* species is not supported.

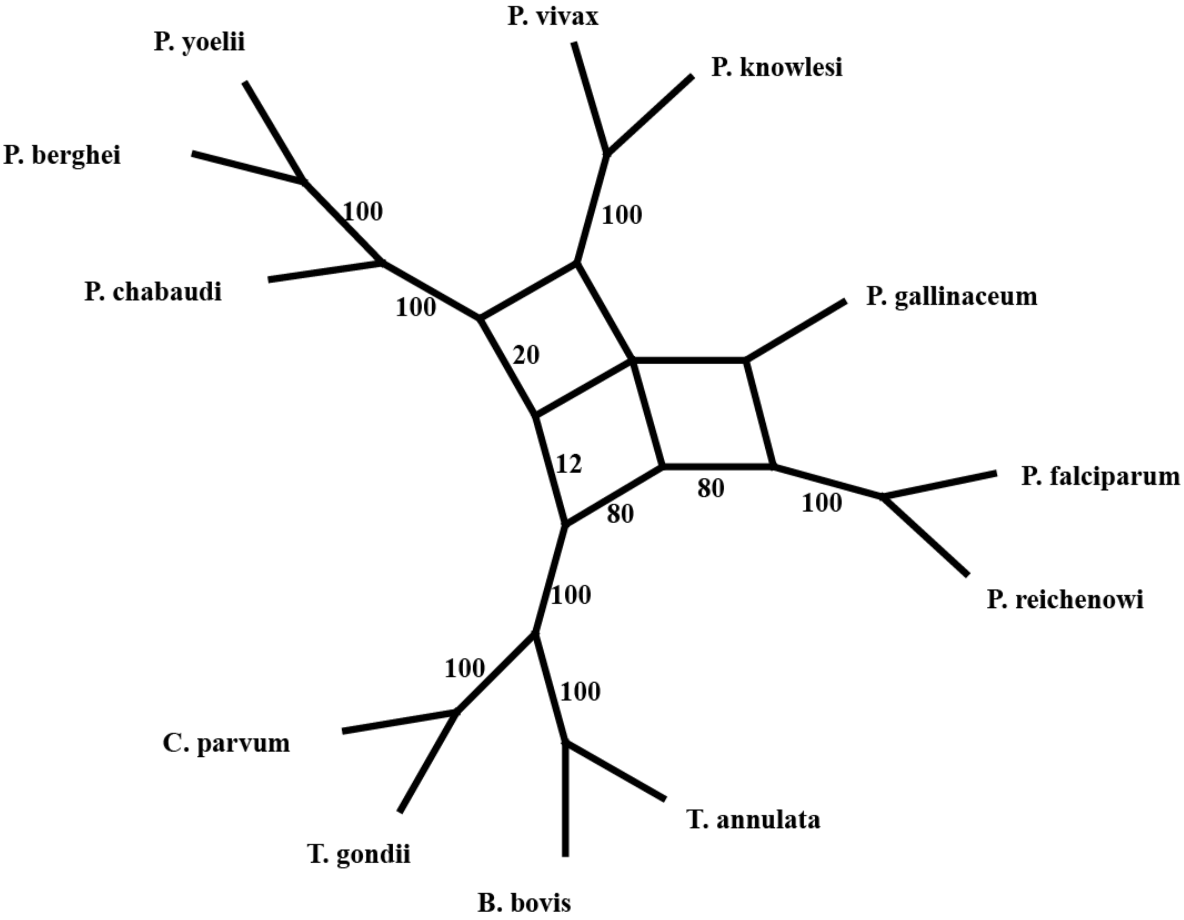

Supplement: Additional file 6 — Split network analyses of Plasmodium phylogeny, as reconstructed from the 65 slow-evolving proteins in our dataset. The network was calculated with SplitsTree v. 4.11.3 [71], considering only splits with frequencies of at least 10%. Notably, a sister group position of P. gallinaceum to all mammalian Plasmodium species is not supported. [file 1471-2148-11-167-S6.PDF]
