# Supplementary material for: Phylogenomic analyses of malaria parasites and evolution of their exported proteins
Source: BMC Evol Biol. 2011 Jun 15;11:167. doi: 10.1186/1471-2148-11-167 (PMC3146879; doi:10.1186/1471-2148-11-167)
Supplement: Additional file 7 — 102 paralog-free P. falciparum proteins that are exported into the host cell and information on the presence or absence of orthologs in other Plamodium species. [file 1471-2148-11-167-S7.PDF]

Additional file 7

Phylogeny of exported *P. falciparum* proteins with orthologs present in all *Plasmodium* species; corresponding amino acid alignments and information on the amount of missing data; *P. falciparum* PEXEL motifs and orthologous sequences of the other *Plasmodium* species.

■ PF13_0317

▪ PHYLOGENY AND PEXEL/VTS

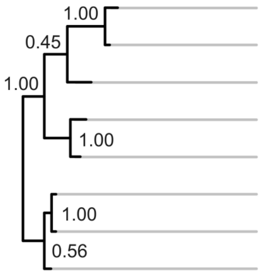

P. falciparum PF10_0134	RMPLWRRFFEGQYTN
P. reichenowi ortholog	RMPLWRRFFEGQYTN
P. gallinaceum ortholog	RMPLWRRFFEGQYTN
P. knowlesi ortholog	RMPLWRRFFEGQYTN
P. vivax ortholog	RMPLWRRFFEGQYTN
P. chabaudi ortholog	RMPLWRRFFEGQYTN
P. berghei ortholog	RMPLWRRFFEGQYTN
P .yoelii ortholog	RMPLWRRFFEGQYTN

▪ ALIGNMENT

```
>P_falciparum_PF13_0317
MNVSRVLLNNSKILKRNIEFKEIFTPRWFLECPNYS------RMPLWRRFFEGQYTN---
---GSFLFFGNAWTSMFAFAFMLWYSRIFDPPPLERIDKYWLNSPKFRILSAFYNQGKRP
GVKISLMTYEARYFYRGMDHPFTINEIKDLWFKLKENYLIESVPAIQYPYVFRQYNNISS
PSDLHVHLH

>P_reichenowi_ortholog
MNVSRVLLSNSKILKRNIEFKEIFTPRWFLECPNYS------RMPLWRRFFEGQYTN---
---GSFLFFGNAWTSMFAFAFMLWYSRIFDPPPLERIDKYWLNSPKFRILSAFYNQGKRP
GVKISLMTYEARYFYRGMDHPFTINEIKDLWFKLKENYLIESVPAIQYPYVFRQYNNVSS
PSDLHVHLH


>P_gallinaceum_ortholog
MNVTRI-LSNSKILKRNIEFKEVFTPRWFLESPNYS------RMPLWRRFFEGQYTN---
---GSFLFFGNTWTSMFAFAFVLWFSRIFDPPPLERVDKYWLNSPKFRILSAFYNEGKRP
GVKISLMTYEARYFYRGIDHPFTINEIKDLWFKLKENYLIESIPAIQYPYVFRQYNNVST
PSDLHVHLH▪


>P_knowlesi_ortholog
MNATRILLSSQKVLKRNVEFKEIFTPRWFLESPNYS------RMPLWRRFFEGQYTN---
---GSFLFFGNAWTSMFAFAFMLWFSRIFDPPPLERVDKYWLNSPKFRILSAFYNEGKRP
GVKISLMTYEARYFYRGIDHPFTINEIKDLWFKLRENYIIESIPAIQYPHVFRQYNNVST
PADLHVHLH

>P_vivax_ortholog
MNASRILLSSQKVLKRNVEFKEIFTPRWFLEAPNYS------RMPLWRRFFEGQYTN---
---GSFLFFGNAWTSMFAFAFMLWFSRIFDPPPLERVDRYWLNSPKFRILSAFYNEGKRP
GVKISLMTYEARYFYRGIDHPFTINEIKDLWFKLRENYLIESIPAIQYPHVFRQYNNVST
PADLHVHLH

>P_chabaudi_ortholog
MNVSRILLNSSKILKRNVEFKEIFTPRWFLESPNYS------RMPLWRRFFEGQYTN---
---GSFLFFGNAWTSMFAFAFFLWYSRIFDPPPLERVDRYWLNSPKFRILSAFYNEGKRP
GVKISLMTYEARYFYRGIDHPFTINEIKDLWFKLKENYLIESIPAIQYPHVFRQYNKVST
PADLHVHLH

>P_berghei_ortholog
MNVSRILLNNSKILKRNVEFKEIFTPRWFLESPNYS------RMPLWRRFFEGQYTN---
---GSFLFFGNAWTSMFAFAFFLWYSRIFDPPPLERVDRYWLNSPKFRILSAFYNEGKRP
GVKISLMTYEARYFYRGIDHPFTINEIKDLWFKLKENYLIESIPAIQYPHVFRQYNKVST
PADLHVHLH

>P_yoelii_ ortholog
MNVSRILLNSSKILKRNVEFKEIFTPRWFLESPNYS------RMPLWRRFFEGQYTN---
---GSFLFFGNAWTSMFAFAFFLWYSRIFDPPPLERVDRYWLNSPKFRILSAFYNEGKRP
GVKISLMTYEARYFYRGIDHPFTINEIKDLWFKLKENYLIESIPAIQYPHVFRQYNKVST
PADLHVHLH
```

▪ AMOUNT OF MISSING DATA

P. reichenowi	***P. gallinaceum***	***P. knowlesi***	***P. vivax***	***P. chabaudi***	***P. berghei***	***P. yoelii***
0%	0%	0%	0%	0%	0%	0%

■ PFC0935c

▪ PHYLOGENY AND PEXEL/VTS

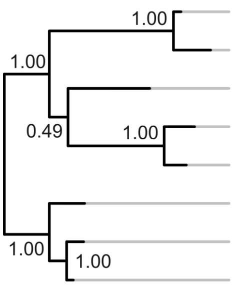

P. falciparum PFC0935c	YYNDHRILLEYNAGL
P. reichenowi ortholog	YYNDHRILLEYNAGL
P. gallinaceum ortholog	YFNDHKILLEYNAGL
P. knowlesi ortholog	YYNDHKILLEYNAGL
P. vivax ortholog	YYNDHKILLEYNAGL
P. chabaudi ortholog	YYNDHKILLEYNAGL
P. berghei ortholog	YYDDHKILLEYNAGL
P .yoelii ortholog	YYDDHKILLEYNAGL

▪ SUPPORT FOR ALTERNATIVE TOPOLOGY RESEMBLING THE SPECIES TREE:

Expected likelihood weights	0.1247 +
Shimodaira–Hasegawa test	0.9770 +
two-sided Kishino–Hasegawa test	+
one-sided KH test	0.2220 +

▪ ALIGNMENT

```
>P_falciparum_PFC0935c
MKNKILKNYVKPNKVIIYKPNISERCLFLILTIYLLFVLYVLKNTVYRNIILLYIAPCFI
LFKVTFICLPKFIHFLNEKGLCGIDLNKSGKEYVAEPIGLFPSILYFIFVLFYQLIYYND
HRILLEYNAGLLSIIFMTFLGFIDDILDLKWRYKVILPFFASLPLLLSYSGETHIRIPNF
LIFIFKHRIINIGFLYYVYIILLSVFCTNAINIYAGINGLEIGQSLIISFFITIHNLIEI
TLNIG----KSPIIENLILKQHFLSIIFTIPFLTINLATFSFNFYPSKGFVGNTLTYFCG
MFLAVVSIFGHFSKTLVLFLIPQFLNFFISLPQLFHIIPCPRHRLPIINYKTNKLMYSHN
YTLINLILYLFGPLSEYHLVLILLTFQFLTCSFGLFLRYYI---

>P_reichenowi_ortholog
XXXXXXXXXXXXXXXXXXXXXXXXXXXXXXXXXXXXXXXXXXXXXXXXXXXXXXXXXXXX
XXXXXXXXXXXXXXXXXXXXXXXXXXXXXXXXXXXXXXXXXXXXXXXXXXXXXXXXXYND
HRILLEYNAGLLSIIFMTFLGFIEDILDLKWRYKVFFPFFASLPLLLSYSGETHIRIPNF
LIFIFKHRIINIGFLYYVYIILLSVFCTNAINIYAGINGLEIGQNIIISFFITIHNLI-I
LINIG----KSPL-ENLILKQHFLSIIFTIPFLTINLATLSFNFYPSKGFVGNTLTYFCG
MFLAVVSIFGHFSKTLVLFLIPQFLNFFISLPQLFHIIPCPRHRLPIINYKTNKLIYSHN
YTLINLILYLFGPLSEYHLVLILFTFQFLTCSFGLFLRYYI---


>P_gallinaceum_ortholog
XXXXXXXXXXXXXXXXXXXXXXXXXXXXXXXXXXXXXXXXXXXXXXXXXXXXXXXXXXXX
XXXXXXXXXXXXXXXXXXXXXXXXXXXXXXXXXVAEPIGLFPAILYFIFVLFYQMIYFND
NKILLEYNAGLLSIIFMTFLGFIDDIFELKWRYKVALPFFACLPLLLSYSGETNIRIPNF
LYFIFKERIINIGFFYYLYIILLAVFCTNAINIYSGINGLKIGQSLIISFFISIHNLIEI
ILNID----KSSVESKLILKQHFLAIIFTLPFISINLVTFSFNFYPSKGFVGNTLTYFCG
IFLAVVSIFGHYSKTLILFLIPQFLNFFISLPQLFNFIPCPRHRLPNLNKKTNKLIYSHN
YTLINLILYLFGPLSEFHLVNVLLFFQIITCSFGLFLRYFI---



>P_knowlesi_ortholog
MKSKY-TTAPKHNKGYIYRESIPEQFLFFFLIFYLLIVLYVLRNTPYKNIILLYIVPCVL
LFKVSFICLPRFIHFLHEKGLYGIDLNKISKDKVAQPIGLFPSILYLIFTLFYQLLYYDD
HKILLEYNAGLLSIIFITFLGFIDDILELKWRYKVVLPFFASLPLLLSYSGNTNIRIPSF
LNFIFKERIIDIGFFYYLYIILLCVFCTNAINIYAGINGLEIGQSLIIAFFISIHNLIEI
ILNIGTGQSKGITEGAQILKQHFLSIIFILPFVSINLVTFSFNFYPSKGFVGNTLTYFCG
IFLAVLSIFGHFSKTLILFLIPQFLNFFLSLPQLLNFVPCPRHRLPVVNPKTNKLTYSHN
YTLINFILYLFGPLSEFHLVVLLLAFQFGTCSLGLFLRYFIDTT

>P_vivax_ortholog
MKSKY-TTTAKHNKGCIYRENIPEQFLFFFLIFYLLIVLYVLRNTPYKNIILLYIVPCVL
LFKVSFICLPRFIHFLHEKGLCGVDLNKTSKDKVAEPIGLFPSILYFIFTLFYQLLYYDD
HKILLEYNAGLLSIIFMTFLGFIDDILELKWRYKVILPFFASLPLLLSYSGKTIIRIPSF
LNFLFKERIIDIGFFYYLYIILLCVFCTNAINIYAGINGLEIGQSLIIAFFISIHNLIEI
ILNLGTGGGKGVIEGAQILKQHFLSIIFILPFVSINLVTFSFNFFPSKGFVGNTLTYFCG
IFLAVVSIFGHFSKTLILFLIPQFLNFFLSLPQLLNFVPCPRHRLPVVNPRTNKLTYSHN
YTLINLILYLFGPLSEFHLVVLLLAFQFGTCSLGLFLRYFIDTT

>P_chabaudi_ortholog
MKSKFANDKIKADATHIYKESIPERYLFSFLIFYLLIVLYALKDTIYKNIIIFYIGPCVL
LFKLSFICIPKFIQFLNQKGLCGIDLNKLSKDKVAEPIGLFPSILYFIFVLFYQILYYND
HKILLEYNAGLLSIIFMTFLGFIDDVLELKWRYKVILPFFASLPLLLCYSGETNIRIPNF
LIFIFKKKIINIGFFYYIYIILLSVFCTNTINIYAGMNGLEIGQTLIISIFISIHNLIEI
ILNIR----SSDIFGLRILKQHFLSIIFTLPFISINLATFAFNFYPSKGFVGNTLTYFCG
MFLAVVSIFGHYSKTLILFLVPQFLNFFLSLPQLFNFIPCPRHRLPIIDHKTNKLTHSYN
FTLINLILYIFGPLSEYHLVIVLLFFQFATCSIGLFLRYFIDT-

>P_yoelii_ ortholog
MKSKFANNKTKGNVTHIYKENIPEIYLFSFLIIYLLIVLYVLRDTIYKNIIIFYIGPCVL
LFKLSFICIPKFIQFLNQKGLCGTDLNKISKDKVAEPIGLFPSILYFIFVLFYQILYYND
HKILLEYNAGLLSIIFMTFLGFIDDVLELKWRYKVLLPFFASLPLLLCYSGETNIRIPNF
LIFIFKKKIINIGFFYYVYIILLSVFCTNTINIYAGINGLEIGQTLIISIFISIHNLIEI
```

```
VLNIG----SSDISGLLILKQHFLSVIFTLPFISINLATFAFNFYPSKGFVGNTLTYFCG
IFLAVVSIFGHYSKTLILFLIPQFLNFFLSLPQLFNFIPCPRHRLPIIDHKTNKLTYSYN
FTLINLILYIFGPLSEYHLVIILLFLQFVTCSIGLFLRYFIDTT

>P_berghei_ortholog
MKSKFANNKTKGNVTHIYKENIPERYLFSFLIIYLLIVLYVLRDTIYKNIIIFYIGPCVL
LFKLSFICMPKFIQFLNQKGLCGTDLNKISKDKVAEPIGLFPSILYFIFVLFYQILYYND
HKILLEYNAGLLSIISMTFLGFIDDVLELKWRYKVLLPFFASLPLLLCYSGETNIRIPNF
LIFIFKKKIINIGFFYYVYIILLSVFCTNTINIYAGINGLEIGQALIISIFISIHNLIEI
VLNIR----SFDVSGLLILKQHFLSVIFTLPFISINLATFAFNFYPSKGFVGNTLTYFCG
IFLAVVSIFGHYSKTLILFLIPQFLNFFLSLPQLFNFIPCPRHRLPIIDHKTNKLTYSYN
FTLINLILYIFGPLSEYHLVIVLLILQFVTCSIGLFLRYFIDT
```

- **AMOUNT OF MISSING DATA**

P. reichenowi	***P. gallinaceum***	***P. knowlesi***	***P. vivax***	***P. chabaudi***	***P. berghei***	***P. yoelii***
29%	23%	1%	1%	0%	0%	0%

■ PF14_0607

▪ PHYLOGENY AND PEXEL/VTS

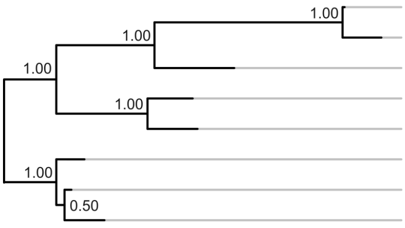

P. falciparum PF14_0607	EKLNNRILFEGSDDF
P. reichenowi ortholog	XXXXXXXXXXXXXXX
P. gallinaceum ortholog	-KLNYRQLEEEENDF
P. knowlesi ortholog	-ESRGRLLGEDNNEF
P. vivax ortholog	-ESRSRLLGEDNNEF
P. chabaudi ortholog	-KRYNRLLIEDNNEL
P. berghei ortholog	-KQYNRVLIEDNNEV
P .yoelii ortholog	-KWYNRVLIEDNNEV

▪ ALIGNMENT

>P_falciparum_PF14_0607

```
MLMLYLHIL------LCIFLNNPVLVTNENNFFIKILRSQEKKIENEVSLYEKLNNRILF
EGSDDFSNLDKLKSLLSFQKKIDNTIRKKQKEENKLKKFQQAIKNGNNIYPVGNNPQGFE
KKEREEKEQEKTYMNNVINKINEQKGNYYEPNKNINNKYTEDLTSNGLLYNKDKIKNQFN
NPHQEKFIRKHEHEREHEGKNEGKNEHKNEHKNEHKNEHVHVHEHNSKNYEGNPPKNEYP
NINFTNILNSIDFYKKIDLVKNYFGYNNNETLNQNMKEQKNMQNHHVENEKNNMIQANNQ
VDENQNNHQYNNNFHNQKNDNLKQDTKDNSSEQTYNNSNNNIEDNNHIKFFISKDIEKKK
NKQNDINESVYNA------IKNKNLHLRRNNIKEIFDVDDLVKNIKSFLGIKSNIHEAFE
NQKLIIKNCNYESFGPELCSVDEKAKEMLWKYEKKKNSAFLFIILFTLFFSLIIQNIVYF
IEKKVRNSKDQFRKDLLNTAFRQISLITIINLTIWGILQTNIAEALDEVIFNDILPRHRN
IDGVLHNVEPLLEVIFEKILFISMNFLICYSIFIVSIHFVTRTILKWFSESDNSDVSSVA
KEVKESQRKCFRNYFFFFRNVRNSKYLAHRYDFSENVDAISIPGLDPNGYYYYEYMRASL
LKYNVKLIKIPNAVILFLIFVCISLRPFYNIRLKAEVIFLNVLSLICIIGLISLFVYLYR
IDTKLLPRDISKYLLNKYHIETCDKNKRDVTPYYKLLKQESVYPSALNYFFYKTTFPNKH
EQLFLLWGNGPSLINFIFQTLCFCFLIILSCWIFLLRVDNITWFQLYSYGSLSICVCILV
FFFILKYIIYYNVMVTKTGYLIDTKLLEQ-------------------------------
----VWEYERSDNIKRISEFIDAIKIK-----STLHALKEGGEIFWRQLLIKSSTVPSNI
QEKMFSIWIGLDEENRGIIDSSKILKFLKSQGINLTSEHDIREFLEVFDRNNKNGLNQEE
FFVLIIIVKQILVELLDINAVQSLFEEVYGIPWKSLSSIDVNSLKKILTELNLKWPHGKI
RNLIDFVCENKKTKYVSAEYFIKQ---------LINIEEVTLQPFHSASDSK--------
-----------------------------------------------------
```

>P_reichenowi_ortholog

```
XXXXXXXXXXXXXXXXXXXXXXXXXXXXXXXXXXXXXXXXXXXXXXXXXXXXXXXXXXXX
XXXXXXXXXXXXXXXXXXXXXXXXXXXXXXXXXXXXXXXXXXXXXXXXXXXXXXXXXXXX
XXXXXXXXXXXXXXXXXXXXXXXXXXXXXXXXXXXXXXXXXXXXXXXXXXXXXXXXXXXX
XXXXXXXXXXXXXXXXXXXXXXXXXXXXXXXXXXXXXXXXXXXXXXXXXXXXXXXXXXXX
XXXXXXXXXXXXXXXXXXXXXXXXXXXXXXXXXXXXXXXXXXXXXXXXXXXXXXXXXXXX
XXXXXXXXXXXXXXXXXXXXNLKQDTKDNSSEQTYNNSNNNIEDNNHIKFFISKDIEEKK
NKQNDINESVYNA------IKNQNLHLRRNNIKEIFDVDDLVKNIKSFLGIKSNIHEAFE
NQKLIIKNCNYESFGPELCSVDEKAKEMLWKYEKKKNSAFLFIILFTLFFSLIIQNIVYF
IEKKVRNSKDQFRKDLLNTAFRQISLITIINLTIWGILQTNIAEAFDEVIFNDILPRHRN
IDGVLHNVEPLLEVIFEKILFISMNFLISYSIFIVSIHFVTRTILKWFSESDNSDVASVA
KEVKESQRKCFRNYFFFFRNVRNSKYLAHRYDFSENVDAISIPGLDPNGYYYYEYMRASL
LKYNVKLIKIPNAVILFLIFVCISLRPFYNIRLKARVIFLNVLSLICIIGLISLFVYLYR
IDTKLLPRDISKYLLNKYHIETCDKNKRDVTPYYKLLKQESVYPSALNYFFYKTTFPNKH
EQLFLLWGNGPSLINFIFQTLCFCFLIILSCWIFLLRVDNITWFQLYSYGSLSICVCILV
FFFILKYIIYYNVMVTKTGYLIDTKLLEQ-------------------------------
----VWEYERSDNIKRISEFIDAIKIK-----STLHALKEGGEIFWRQLLIKSSTVPSNI
QEKMFSIWIGLDEENRGIIDASKILKFLKSQGIYLTFEHDIREFLEVFDRNNKNGLNQEE
FFVLIIIVKQILVELLDINAVQSLFEEVYGIRWKSLSSIDVNSLKKILTEVNLKTPHTKI
NNLIDFVCKNKKYKYMRTCIYILL---------IISLRFHALNPFH--------------
-----------------------------------------------------
```

>P_gallinaceum_ortholog

```
XXXXXXXXXXXXXXXXXXXXXXXXXXXXXXXXXXXXXXXXXXKIKKDVNSY-KLNYRQLE
EEENDFYNIGKLKNLLFFQSNLDKTIXXXXXXXXXXXXXXXXXXXXXXXXXXXXXXXXXX
XXXXXXXXXXXXXXXXXXXXXXXXXXXXXXXXXXXXXXXXXXXXXXXXXXXXXXXXXXXX
XXXXXXXXXXXXXXXXXXXXXXXXXXXXXXXXXXXXXXXXXXXXXXXXXXXXXXXXXXXX
XXXXXXXXXXXXXXXXXXXXXXXXXXXXXXXXXXXXXXXXXXXXXXXXXXXXXXXXXXXX
XXXXXXXXXXXXXXXXXXXXXXXXXXXXXXXXXXXXXXXXXXXXXXXXXXLISKRNKENPK
---NNIHEYVYNI------IENKNFNLRRKDIKEFLDVHELVKNIKFVLGIKSNIHEKFE
HEKLILKDCNYESFGPDFCSVNDEAKETLWEFENKKNCVFLFIILFTLIFSLVIQNIVYF
IEKSVRNSKDQFRKDLLNTAFRQISLITIINLTIWGILQSNIAEALDEIIFNDVXXXXXX
XXXXXXXXXXXXXXXXXXXXXXXXXXXXXXXXXXXXXXXXXXXXXXXXXXXXXXXXXXXX
XXXXXXXXXXXXXXXXXXXXXXXXXXXXXXXXXXXXXXXXXXXXXXXXXXXXXXXXXXXX
XXXXXXXXXXXXXXXXXXXXXXXXXXXXXXXXXXXXXXXXXXXXXXXXXXXXXXXXXLYN
IDKKLLPKDISKYLLNRYHIESSEQSKKDIIPYYKLSKQKSIYPSTLNYFFFKTTFPNKH
EQLFYFWGNGPPLINFIFQTLCFCFLIILSCWIFLLRVDNITWFQLYSYGSIAICVCILV
FFFILKYIIYYNIMISKTGYLIDTKLLKK-------------------------------
----VWEFERSDNIKRISEFIDAIKIK-----STLHALKEGGEVFWRQLLIKSNTVPSNI
QEKMFSIWVGLDEENRGIIDSAKILKFLKSQGINLTSENDIKEFLEVFDRNNKNGLNQEE
FFVLIIIVKQILVELLDINAVQXXXXXXXXXXXXXXXXXXXXXXXXXXXXXXXXXXXXXX
XXXXXXXXXXXXXXXXXXXXXXXXXXXXXXXXXXXXXXXXXXXXXXXXXXXXXXXXXXXX
XXXXXXXXXXXXXXXXXXXXXXXXXXXXXXXXXXXXXXXXXXXXXXXXXXXXX
```

>P_knowlesi_ortholog

```
MFISFVGFFSPPLFFMQILATYAPFILKEENFDHTYYNKNESVKQRDTQDR-ESRGRLLG
EDNNEFFDIGKLKKLLYFQSNLDQTIKQKRREEKKLRKLYKAAKSGNFAAAIRNSQKDTQ
QNGAPPVENEHNTKGKSDMNDGGEMNQHEVLQRKEDNPVDMNQQTYGEEQMKNETGRRTG
EMQEEIHP-LDTKVNGPTEKFINKHDHVDNTRNVEKEKKEENVKHSD-------------
NAQLRNILQSVEFLKKTQSLKNYFGSSESQSRSSDVPP----------------GDQNKY
ADELKIQNENEQVTQLSGET---------------NYGTNNVEGNFQQEYPMSKNNEEKK
---NNYTEEPFYMFPKNDEAEKFNTHLRRSKIKEFFEVNDLVKYMKNLLGIKSNIHEKFE
HEKLIVKNCNYESFGPEFCSVDEEAKKMLWNYENKKNCAFLFIILFTLFFSLLIQNIVYY
IEKRVRNSKDQFRKDLLNTAFRQISLITIINLTIWGILQSNVAEALDEIIFNDILPQQRN
VDEVLHNVEPLLETIFEKILFISMNFLICYSLFIINIHFVTRRILKWFSEADNCDISNIA
KELKEARKGCFRNLFFFLRYSRNSKYLAHRYDFSENVDAISIPGLDPNGYYYYEYMRACL
LKYNVKLIKIPNAVILFLVFACISLRPFFNIRLKAEVIFLNFLSLICIMGLLLMLFYLYK
IDRKLLPRDIGKYLLNRYHIETCEQSKKDITPYYKLLKQQSVYPSAINYFLYKTTFPNKH
```

```
EQLFYFWGNGPRLINFIFQTLCFCFLIILSCWIFLLRVDNITWFQLYSYGSLAICVCIFL
FLIILKYIIYYNIMISKTGYLIDTKLLEKVSELAGGVCSVDPSWTQYAREQPHDKFFLGP
PSPQVWEFERSDNIKRISEFIDAIKIKVPLIYSTLHALKEGGEVFWRQLLIKSNTVPSNI
QEKMFSIWVGLDEENRGVIDSAKILKFLKSQGINLTSESDIKEFLEVFDRNKKNGLNQEE
FFVLIIIVKQILVELLDINAVQSLFEEVYGIPWNSLSSIDANSLKRILTELNLQWPHGKI
RNLIDFVCENKKTKYVSAEYFIKQ---------LINIEEVTLQPFHVRIPMCAQRSVCIL
LYDCAHTSNAYFTSNVKKCFVPPRIQSKDKSKDVTLYMFLFCFFYLPICLFLF
```

>P_vivax_ortholog

```
MFISSVRFL-PPAFALQISATYPLFILKEKNFDHIYCTKNESVRQRDTLDR-ESRSRLLG
EDNNEFFDIGKLKKLLYFQSNLDQTMKKKRREEKKLRKLYKAAKAGNFAERIRNAPKDTQ
QSGAPPGEKENGTKGKNDTNSAGEINQDGAVNRKEDNPADVNLQTYADEQMKNEKGKKAG
EIQEA--PTLQTAVSGPAEKFINKHDHVENTGNAEKEQKEGKTKHAD-------------
NAQLNNVLQSVEFLKKVQSLKSYFGSSESQSGSPGVPP----------------VEQNHS
ADGDKMKNENDQVAHLSGDT---------------NYGTNNAEANDKMGSPTSKDNEGKK
---NNYAEEPFSIFPEKEGAEKFNTHLRRNKIKEFSEVNDLVKYMKNLLGIKSNIHEKFE
HEKLIVKNCNYESFGPEFCSVDEEAKQMLWNYENKKNCAFLFIILFTLFFSLLIQNIVYY
IEKRVRNSKDQFRKDLLNTAFRQISLITIINLTIWGILQSNVAEALDEIIFNDILPRQRN
VDEVLHNVEPLLETIFEKILFISMNFLICYSLFIINIHFVTRRILKWFSEADNCDISNIA
KEMKEARKGCFKNFFFLLRYARNSKYLAHRYDFSENVDAISIPGLDPNGYYYYEYMRACL
LKYNVKLIKIPNAVILFLVFACISLRPFFNIRLKAEVIFLNFLSLICIVGLLLMFFYLYK
IDRKLLPRDISKYLLNRYHIETCEQSKKDITPYYKLLKQQSVYPSAINYFLYKTTFPNKH
EQLFYFWGNGPRLINFIFQTLCFCFLIILSCWIFLLRVDNITWFQLYSYGSLAICVCIFL
FLIILKYIIYYNIMISKTGYLIDTKLLEK-------------------------------
----VWEFERSDNIKRISEFIDAIKIK-----STLHALKEGGEVFWRQLLIKSNTVPSNI
QEKMFSIWVGLDEENRGVIDSTKILKFLKSQGINLASESDIKEFLEVFDRNNKKGLNQEE
FFVLIIIVKQILVELLDINAVQSLFEEVYGIPWNSLSSIDANSLKRILTELNLQWPHGKI
RNLIDFVCENKKTKYVSAEYFIKQ---------LINIEEVTLQPFHVRIPMCALRCVCIG
LYGTTHTSTAYFASNVKNVTLCLGYKVKIK---LRMYQHTFFFAYLTICLFLL
```

>P_chabaudi_ortholog

```
XXXXXXXXXXXFFFFFQILFKYPISILIENILGN-----NEKLIELDVHNH-KRYNRLLI
EDNNELFDIEHLKKLLYFQSKLDKKIXXXXXXXXXXXXXXXXXXXXXXXXXXXXXXXXXX
XXXXXXXXXXXXXXXXXXXXXXXXXXXXXXXXXXXXXXXXXXXXXXXXXXXXXXXXXXXX
XXXXXXXXXXXXXXXXXXXXXXXXXXXXXXXXXXXXXXXXXXXXXXXXXXXXXXXXXXXX
XXXXXXXXXXXXXXXXXXXXXXXXXXXXXXXXXXXXXXXXXXXXXXXXXXXXXXXXXXXX
XXXXXXXXXXXXXXXXXXXXXXXXXXXXXXXXXXXXXXXXXXXXXXXXXXXXXXXXXXXX
XXXXXXXXXXXXXXXXXXSMDNSNYSLRKSDIKELIGINDIVKYIKNLLGIKSNIHEKFE
QENLIIRDCNYDSFGPEFCSANEEAKQKLWKYENKKSFAFLFIILFTLFFGLFIQNIVYY
IEKKVRNSKDKFRKDLLNTAFRQISLITIINLTIWGILQSKVAEALDDIIFNDILPKQRN
VDEVLHNVEPLLEIIFEKILFISMNFLISYSLFIINVHFVTRGILKWFSEADNCDIKNII
KDMKERRKGCFRNIFSFIRYSRNSKYLAHRYDFSENVDAISIPXXXPNGYYYYEYMRACL
LKYNVKLIKIPNTVILFLVFASISLRPFFSIRLKEEVIFLNLLSLFCIFGFVSLFVYLYK
IDKKLLPENISKYLLNKYHIDISDQNAKEITPYYKLLKQQSLHPSSINYFIYKTTFPNKH
EQLFFFWGNGPSIINFIFQALCFCFLVILSCWIFLLKVDHITWFQLYAHGSVSICMCILI
FVFILKYIIYYYIMISKTGYLIDTKLLER-------------------------------
----VWEFERSDNIKRISELIDAIKIK-----STLHAVKEGGDLFWKQLLIKSSTVPSNI
QEKMFSIWVGLDEENRGVINSDKILKFLKSQGINLTSENDIKEFLEVFDRNNKNGLNQEE
FFVLIIIVKQILVELLDINAVQSLFEEVYGIPWNSLSSIDVNSLKRILSELNLHWPHGKI
KNLIDFVCESKKKTYVSAEYFIKQ---------LINIEEVTLQPFHV-------------
-------------------------------------------IN-------A
```

>P_berghei_ortholog

```
XXXXXXYFISYFFFFFQIFFKYPISILIENILEN-----NEKLIELDAHNH-KQYNRVLI
EDNNEVFNIEHLKKLLHFQSKLDKQIXXXXXXXXXXXXXXXXXXXXXXXXXXXXXXXXXX
XXXXXXXXXXXXXXXXXXXXXXXXXXXXXXXXXXXXXXXXXXXXXXXXXXXXXXXXXXXX
XXXXXXXXXXXXXXXXXXXXXXXXXXXXXXXXXXXXXXXXXXXXXXXXXXXXXXXXXXXX
XXXXXXXXXXXXXXXXXXXXXXXXXXXXXXXXXXXXXXXXXXXXXXXXXXXXXXXXXXXX
XXXXXXXXXXXXXXXXXXXXXXXXXXXXXXXXXXXXXXXXXXXXXXXXXXXXXXXXXXXX
XXXXXXXXXXXXXXXXXXSMDNINYSLRKSDIKELIGINDIVKYIKNLLGIKSNIHEKFE
HENLIIRDCNYDSFGPEFCSANEEAKQKLWKYENKKSFAFLFIILFTLFFGLLIQNVVYY
IEKKVRNSKDKFRKDLLNTAFRQISLITIINLTIWGILQSKAAEALDDIIFNDILPKQRN
VDEVLHNVEPLLEIIFEKILFISMNFLISYSLFILNVHFVTRSILKWFSEADNCDIKNII
KDMKDSRKGCFKNIFSFIKYTRNSKYLAHRYDFSENVDAISIPGLDPNGYYYYEYMRACL
LKYNVKLIKIPNAVILFLVFACISLRPFFSIRLKEEVIFLNLLSLFCILGFVLLFIYLYK
IDRKLLPGNISKYLLNKYHIDISDQNTKEIIPYYKLLKQQSLHPSSINYFIYKTTFPNKH
EQLFFFWGNGPSIINFIFQALCFCFLVILSCWIFLLRVDNITWFQLYAHGSVSICVCILV
FVFILKYIIYYYIMISKTGYLIDTKLLER-------------------------------
----VWEFERSDNIKRISELIDAIKIK-----STLHAVKEGGDLFWRQLLIKSSTVPSNI
QEKMFSIWVGLDEENRGVINSDKILKFLKSQGINLTSENDIKEFLEVFDRNNKNGLNQEE
FFVLIIIVKQILVELLDINAVQSLFEEVYGIPWNSLSSIDVNSLKRILSELNLHWPHGKI
KNLIDFVCESKKKTYVSAEYFIKQ---------LINIEEVTLQPFHV-------------
-------------------------------------------IN-------A
```

>P_yoelii_ortholog

```
XXXXXXXXXXXXXXXXXXXXXXXXXXXXXXXXXXXXXXXXXXXXXXDLHNH-KWYNRVLI
EDNNEVFDIEQLKKLLYFQSKLDKQIXXXXXXXXXXXXXXXXXXXXXXXXXXXXXXXXXX
XXXXXXXXXXXXXXXXXXXXXXXXXXXXXXXXXXXXXXXXXXXXXXXXXXXXXXXXXXXX
XXXXXXXXXXXXXXXXXXXXXXXXXXXXXXXXXXXXXXXXXXXXXXXXXXXXXXXXXXXX
XXXXXXXXXXXXXXXXXXXXXXXXXXXXXXXXXXXXXXXXXXXXXXXXXXXXXXXXXXXX
XXXXXXXXXXXXXXXXXXXXXXXXXXXXXXXXXXXXXXXXXXXXXXXXXXXXXXXXXXXX
XXXXXXXXXXXXXXXXXXXXXXXXXXXXXXXXXXXXXXNDIVKYIKNLLGIKSNIHEKFE
HENLIIRNCNYDSFGPEFCSANEEAKQKLWKYENKKSFAFLFIILFTLFFGLIIQNIVYY
IEKKVRNSKDKFRKDLLNTAFRQISLITIINLTIWGILQSKAAETLDDIIFNDILPKQRN
VDEVLHNVEPLLEIIFEKILFISMNFLICYSLFIINVHFVTRSILKWFSEADNCDIKNVI
KDMKERRKGCFKNIFSFIKYTRNSKYLAHXXXXXXXXXXXXXXXXXXXXXXXXXXXXXXX
XXXXXXXXXXXXXXXXXXXXXXXXXRPFFSIRLKEEVIFLNLLSLFCILGFVLLFIYLYK
IDRKLLPGNISKYLLNKYHIDISDQSTKEIIPYYKLLKQQSLHPSSINYFIYKTTFPNKH
EQLFFFWGNGPSIINFIFQXXXXXXXXXXXXXXXXXXXXXXXXXXXXXXXXXXXXXXXXX
XXXXXXXXXXXXXXXXXXXXXXXXXXXXXXXXXXXXXXXXXXXXXXXXXXXXXXXXXXXX
XXXXVWEFERSDNIKRISELIDAIKIK-----STLHAVKEGGDLFWRQLLIKSSTVPSNI
QEKMFSIWVGLDEENRGVINSDKILKFLKSQGINLTSENDIKXXXXXXXXXXXXXXXXXX
XXXXXXXXXXXXXXXXXXXXXXSLFEEVYGIPWNSLSSIDVNSLKRILSELNLHWPHGKI
KNLIDFVCESKKKTYVSAEYFIKHYLLGFVVIFILFMLNICVIYFFS-------------
-------------------------------------------LNFRLNFLFL
```

▪ AMOUNT OF MISSING DATA

P. reichenowi	***P. gallinaceum***	***P. knowlesi***	***P. vivax***	***P. chabaudi***	***P. berghei***	***P. yoelii***
29%	56%	13%	9%	34%	34%	52%

■ PF10_0070

▪ PHYLOGENY AND PEXEL/VTS

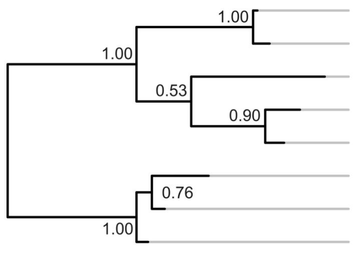

P. falciparum PF10_0070	INLNERILSVGLSWG
P. reichenowi ortholog	XXXXXXXXXXXXXXX
P. gallinaceum ortholog	XXXXXXXXXXXXXXX
P. knowlesi ortholog	INLKERILSVGLSWG
P. vivax ortholog	INLKERILSVGLSWG
P. berghei ortholog	FNIKSRVLSAGLSWG
P. chabaudi ortholog	FNIKSRILSTGLSWG
P .yoelii ortholog	FNIKSRILSTGLSWG

▪ SUPPORT FOR ALTERNATIVE TOPOLOGY RESEMBLING THE SPECIES TREE:

Expected likelihood weights	0.0570	+
Shimodaira-Hasegawa test	0.9990	+
two-sided Kishino-Hasegawa test		+
one-sided KH test	0.2860	+

▪ ALIGNMENT

```
>P_falciparum_PF10_0070
MALFHFVNCSLATFIPYYIIYDGFKLSKNVGATKLFFLVCFYYIISQI---LKLFALAFC
SIGLFQSMNLFNIIFQESANIIDLAGIYYILSHKHTNS-------INLNERILSVGLSWG
------------FYESLATNFFPFLI-GGKSLEFSLKYIYRSISANTFLFSNLSKTCLLF
MWMKNTSGKTKINAVNSLLVYFTFILPLVNRIILIKEESFRSDIFIQLLFEFALTFILSI
ITKFIFSSQLN-----------VSHEHKNATYSDDEIYQVNKDLKKKDKKKKKN----

>P_reichenowi_ortholog
MALFHFVNCSLATFIPYYIIYDGFKLSKNVGATKLFFLVCFYYIISQI---LKVXXXXXX
XXXXXXXXXXXXXXXXXXXXXXXXXXXXXXXXXXXXXX-------XXXXXXXXXXXXXXX
------------XXXXLANNFFPFLM-GGKSLEFSLKYIYRSISANTFLFSNLSKTCLLF
MWMKNTSGKTKINAVNSLLVYFTFILPFVNRIILINEESFRSDIFIQLLFEFALTFILSI
ITKFIFSSQLN-----------VSHEHKNATYSDDEIYQVNKDLKKKDKKKKKN----

>P_gallinaceum_ortholog
MSLFHFVNCSLTTFIPYYIIFDGFKLSKDVGSTKLFVIVCFYYVISQI---LKVFVLPFF
SIGLLQNMNLFNVCFXXXXXXXXXXXXXXXXXXXXXXX-------XXXXXXXXXXXXXXX
------------XXXXXXXXXXXXXXXXXXXXXXXXXXXXXXXXXXXXXXXXXXXXXXXX
XXXXXXXXXXXXXXXXXXXXXXXXXXXXXXXXXXXXXXXXXXXXXXXXXXXXXXXXXXXX
XXXXXXXXXXXXXXXXXXXXXXXXXXXXXXXXXXXXXXXXXXXXXXXXXXXXXXXXXX

>P_knowlesi_ortholog
MALFHFVNCSLTAFTPYYIIYDGFKLSKNAGSTKLFFLVCFYYIVSQI---LKLFTLAFF
SIGLLQNMNLFNIIFQECANFIDLAGLYYILSHKHTNT-------INLKERILSVGLSWG
------------FYESVATNFFPFFI-GGRSMDFSLKHIYRSISANTFMFSNLSKTCLLF
MWTRNTQSRKKINAVNFLLLYFTFILPLVNKIILIQEESFNKGIIIHLLVLLVCTFVLSF
ATKCIFNSKSNANVELYKESHTRSY-EKNDSNDDDGDGEMNKDQKKKKKKKKK-----

>P_vivax_ortholog
MALFHFVNCSLTAFTPYYIIYDGFKLSKNAGSTKLFLLVCFYYIVSQI---LKLFVLAFF
SIGLLQNMNLFNIIFQECANFIDLVGLYYILSHKHTNT-------INLKERILSVGLSWG
------------FYESVATNFFPFFI-GGRSMDFSLKHIYRSISANTFMFSNLSKTCLLF
MWLKNTQSRKKINVVNFLLFYFTFLLPLVNKIILIHEGSFNKRIIIHLIVLLLCTFVLSI
ATKCIFNSKSNANVEPYKELNTRSY-EKNDGHDDDGDGEMNKDQKKKNKKKKKGFFYK

>P_chabaudi_ortholog
MSLFHFVNCSISVFAPYYIISDGYKLSKNEGSTKLFFIAFFYYFTSQIVKVLQLFALAFL
SIGLMQSMTIFNIIFQEAGNFFDLIGLYYVLSHKQMHM-------FNIKSRVLSAGLSWG
------------FCESVATNFFPFLV-GGKSVDFSLKHIYRSISANTFLISNLSKTYLLY
LWIKNVQNKKKVNSVNSLLIYFTFILPLMNKIILINEDIISSVIFIKLVALFFITLILFC
CAKYIFNSQIE-KVEVVKSNTSYSYXXXXXXXXXXXXXXXXXXXXXXXXXXXXXXXXX

>P_yoelii_ ortholog
XXXXXXXXXXXXXXXXXXXXXXXXXXXXXXXXXXXXXXXXXXXXXXXXXXXXXXXXXXXX
SIGLIQSMTIFNIVFQEAWNFIDLIGLYYVLSHKQMHM-------FNIKSRILSTGLSWG
------------FCESVATNFFPFLIGGGKSVDFSLKHIYRSISANTFLVSNLSKTYLLY
LWIKNVQNKKKVNSVNSLLIYFTFXXXXXXXXXXXXXXXXXXXXXXXXXXXXXXXXXXXC
SVKYIFNSQTE-KVEIVKANSSHSYXXXXXXXXXXXXXXXXXXXXXXXXXXXXXXXXXF

>P_berghei_ortholog
MTLFHFVNCSISVFAPYYIIYDGYKLSKNEGFTKLFFITLFYYFASQIVKVLQLFALAFL
SIGLIQSMTIFNIVFQEAGNFFDLIGLYYVLSHKQMHM-------FNIKSRILSTGLSWG
------------FCESVATNFFPFLI-GGKSVDFSLKHIYRSISANTFLVSNLSKTYLLY
LWINNVQNKKKVNSVNSLLIYFTFILPLINKIILINEDIISSMIFIKLIALFLITFILFC
SVKYIFNSQNE-KVEIVKSNSSHSYVNDVNVKNEDNENDANKGLKKKYNRKKKS---R
```

▪ AMOUNT OF MISSING DATA

P. reichenowi	***P. gallinaceum***	***P. knowlesi***	***P. vivax***	***P. chabaudi***	***P. berghei***	***P. yoelii***
**24%**	**72%**	**5%**	**4%**	**15%**	**5%**	**48%**

■ PFL1630c

▪ PHYLOGENY AND PEXEL/VTS

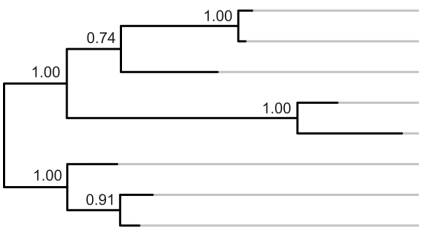

P. falciparum PFL1630c	VKIYNRCLCAWYCKY
P. reichenowi ortholog	VKIYNRCLCAWYCKY
P. gallinaceum ortholog	IKIYNRCLCAWFCKY
P. knowlesi ortholog	IATYNRCLCAWYCKN
P. vivax ortholog	IATYNRCLCAWYCKN
P. chabaudi ortholog	TKMYNRCLCAWYCKY
P. berghei ortholog	IKIYNRCLCAWYCKY
P .yoelii ortholog	IKIYNRCLCAWYCKY

▪ ALIGNMENT

```
>P_falciparum_PFL1630c
MSRKLNILKAKLPKAPTGRRPIGYR-----------------------------------
GNHQHGKSLYDPVYPTTKIPTSLIPRYPIDWRNGGRFLLCVGMRRIENRIIKKMKDSLNF
NMSECKICNDNCVKIYNRCLCAWYCKYKYRHVYKCDGLLIDYKGTIKTDKPIFTLKKEVG
IQNKKSMIEYIYNEDKHIFELNE-KEDIYKKFYKKIQTCASSEEK---------------
------------------------------------------------------------
-----RIPEE---FSDSENTDSDED--------

>P_reichenowi_ortholog
MSRKLNILKAKLPKAPTGRRPIGYR-----------------------------------
GNHQHGKSLYDPVYPTTKIPTSLIPRYPIDWRNGGRFLLCVGMRRIENRIIKKMKDSLNF
NMSECKICNDNCVKIYNRCLCAWYCKYKYRHVYKCDGLLIDYKGTIKTDKPIFTLKKEVG
IKNKKSMIEYIYNEDKHIFELNE-KEDIYKKFYKKIQTSAFSEEK---------------
------------------------------------------------------------
-----SVPEE---FSDSENTDSDED--------

>P_gallinaceum_ortholog
MSKKLDILKAKLPKAPTGRRPIGYR-----------------------------------
GNHQHGKSLYDPVYPTTKIPTSLIPRYPLDWRNGGRFLLCVGLRRIENRIINKMKESQDF
NICECNICNDNCIKIYNRCLCAWFCKYKYRHMYKCDNLLINYKGEIKTEKPIFTLKREVA
KKNLKKSVEYIYNNKTNLYEIKE-ENNIYKKFYQKIQ-----EEN---------------
------------------------------------------------------------
-----NNPKDNFYDSDSDITDSEEE--------

>P_knowlesi_ortholog
MARGLNIVKAKLPKAPTGRRPIGYR-----------------------------------
GNHQHGKSLYDPVYPTTKIPASLVPRYPIDWRNGGRFLLCVGLRRIENRIIKRMKESQDF
NRPECKTCNHDCIATYNRCLCAWYCKNKFKHLYQCDELLLQYKGETKTDKPLFTAPRWVA
NRNSDKGIQYEFNEKTRRFENVQGEEDMYKLFYKKLRYGADSTEHEGKEGNSIAGGTSGG
SAIGESAIGGSAIGGSAIGGSAIGGSAIGGSAIGGSAIGGSAIGGSAIGGSAIGGSAIGG
SAIGIPAEEEQLSGSDSDMTDSDEEG-------

>P_vivax_ortholog
MARGLDILKATLPKAPTGRRPIGYR-----------------------------------
GNHQHGKSLYDPVYPTTKIPASLVPRYPIDWRNGGRFLLCVGLRRIENRLIRRMKESQDF
NRPECKTCSHDCIATYNRCLCAWYCKNKFRHVYQCDQALLQFKGETPTDKPLFTVPRWVA
KRNPEQGVEYSFSEETGRFENGHGGEDMYSRFYRKLR-----------------------
-----------------------------------------------------CGRAVCG
GAIANPAEEGQLSGSDSDMTDSDEEG-------

>P_chabaudi_ortholog
MPKKLNILKAKLPKAPPGRRPIGYR------DVKSIIDLCSTVCFS-KQFSWLFHFSHFS
GNHKHGKALYDPVYPTTKIPSSLVPRYPLDWRNGGRFLLCVGLRRIENRIINKMKESLNY
NMPECSTCNDTCTKMYNRCLCAWYCKYKYRHVYNCDNILMEYKGEINTDKPIFTIKKQVA
KKNMSKAIEYVYNNNTSKFQTVE-KDDIYTKFYKKIQ----KKNQ---------------
------------------------------------------------------------
-----PSSQEETIDSDSDATDSDEEEMCSDS-

>P_yoelii_ ortholog
MPKKLNILKAKLPKAPTGRRPIGYR-----------------------------------
GNHKHGKALYDPVYPTTKIPSSLVPRYPLDWRNGGRFLLCVGLRRIENRIINKMKESLNY
NIPECSTCNNTCIKIYNRCLCAWYCKYKYKHVYDCDNILMEYKGEINTDKPIFTIKKQVA
KKNISRGIEYIYNKNISNFQTIE-KNDIYTKFYKKIQ----EKKQ---------------
------------------------------------------------------------
-----NSSKEEIIDSDSDATDSDEEEMHENVSN

>P_berghei_ortholog
MPKKLNILKAKLPKAPPGRRPIGYRGKHKKRNIKKIIDLYKIIYFTFRRKFYHFHIFHIS
GNHKHGKALYDPVYPTSKIPSSLVPRYPLDWRNGGRFLLCVGLRRIENRIINKMKESLNY
NIPECSTCNNTCIKIYNRCLCAWYCKYKYRHVYDCDNILMEYKGEINTDKPIFTIKKQVA
KKNISKGIEYVYNKNISKFQTIE-KNDIYAKFYKKIQ----EKKQ---------------
------------------------------------------------------------
-----TCSKEEAIYSDSDATDS-----------
```

▪ AMOUNT OF MISSING DATA

P. reichenowi	***P. gallinaceum***	***P. knowlesi***	***P. vivax***	***P. chabaudi***	***P. berghei***	***P. yoelii***
0%	3%	29%	11%	16%	18%	6%

■ MAL13P1.56

▪ PHYLOGENY AND PEXEL/VTS

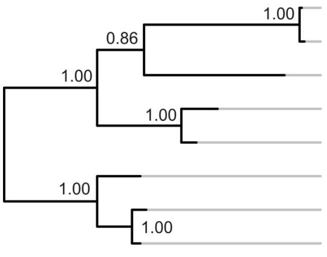

P. falciparum MAL13P1.56	CGIISRLLKS---NS
P. reichenowi ortholog	CGIISRLLKS---NS
P. gallinaceum ortholog	CGVISRLLKS---NS
P. knowlesi ortholog	CGITSRVLSGIHVNK
P. vivax ortholog	CGITSRVLSGIHVNK
P. chabaudi ortholog	CGIVNRVLRE----K
P. berghei ortholog	CCIVNRVLRE----K
P .yoelii ortholog	CGIVNRVLRE----K

▪ ALIGNMENT

```
>P_falciparum_MAL13P1.56
MKLTKGCAYKYIIFTVLI-LANIL-YDNKKRCMIKKNLRISSCGIISRLLKS---NSNYN
SFNKNYNFTSAISELQFSNFWNLDILQKDIFSNIHNNKNKPQ---SYIIHKRLMSEKGD-
--NNNNNHQNNNGNDNKKRLGSVVNNEEN------TCSDKRMKPFEEGHGITQVDKMNNN
SD-HLQQNGVMNLNSNNVENNNNNNSVVVKKNEPKIHYRKDYKPSGFIINNVTLNINIHD
NETIVRSVLDMDISKHNVGEDLVFDGVGLKINEISINNKKLVEGEEYTYDNEFLTIFSKF
VPKSKFAFSSEVIIHPETNYALTGLYKSKNIIVSQCEATGFRRITFFIDRPDMMAKYDVT
VTADKEKYPVLLSNGDKVNEFEIPGGRHGARFNDPHLKPCYLFAVVAGDLKHLSATYITK
YTKKKVELYVFSEEKYVSKLQWALECLKKSMAFDEDYFGLEYDLSRLNLVAVSDFNVGAM
ENKGLNIFNANSLLASKKNSIDFSYARILTVVGHEYFHNYTGNRVTLRDWFQLTLKEGLT
VHRENLFSEEMTKTVTTRLSHVDLLRSVQFLEDSSPLSHPIRPESYVSMENFYTTTVYDK
GSEVMRMYLTILGEEYYKKGFDIYIKKNDGNTATCEDFNYAMEQAYKMKKADNSANLNQY
LLWFSQSGTPHVSFKYNYDAEKKQYSIHVNQYTKPDENQKEKKPLFIPISVGLINPENGK
EMISQTTLELTKESDTFVFNNIAVKPIPSLFRGFSAPVYIEDNLTDEERILLLKYDSDAF
VRYNSCTNIYMKQILMNYNEFLKAKNEKLES----FNLTPVNAQFIDAIKYLLEDPHADA
GFKSYIVSLPQDRYIINFVSNLDTDVLADTKEYIYKQIGDKLNDVYYKMFKSLEAKADDL
TYFNDESHVDFDQMNMRTLRNTLLSLLSKAQYPNILNEIIEHSKSPYPSNWLTSLSVSAY
F--DKYFELYDKTYKLSKDDELLLQEWLKTVSRSDRKDIYEILKKLENEVLKDSKNPNDI
RAVYLPFTNNLRRFHDISGKGYKLIAEVITKTDKFNPMVATQLCEPFKLWNKLDTKRQEL
MLNEMNTMLQEPNISNNLKEYLLRLTNKL

>P_reichenowi_ortholog
MKLTKGCAYKYIIFTVLI-LANIL-YDNKKRCMIKKNLRISSCGIISRLLKS---NSNYN
SFNKNYNFTSAISELQFSNFWNLDILQKDIFSNIHNNKNKPQ---SYIIHKRLMSEKGDN
NNNNNNNHQNNNGNDNKKRLGSVVNNEEN------TCSDKRMKPFEEGHGITQVDKMNSN
SDHHLQQNVVMNLNTNNVENNNN--SAVVKKNEPKIHYRKDYKPSGFIINNVTLNINIHD
NETIVRSILDMDISKHNVGEDLVFDGVGLKINEISIDNKKLVEGEEYTYDNEFLTIFSKF
VPKSKFAFSSEVIIHPETNYALTGLYKSKNIIVSQCEATGFRRITFFIDRPDMMAKYDVT
VTADKEKYPVLLSNGDKVNEFEIAGGRHGARFNDPHLKPCYLFAVVAGDLKHLSATYITK
YTKKKVELYVFSEEKYVSKLQWALECLKKSMAFDEDYFGLEYDLSRLNLVAVSDFNVGAM
ENKGLNIFNANSLLASKKNSIDFSYARILTVVGHEYFHNYTGNRVTLRDWFQLTLKEGLT
VHRENLFSEEMTKTVTTRLSHVDLLRSVQFLEDSSPLSHPIRPESYVSMENFYTTTVYDK
GSEVMRMYLTILGEEYYKKGFDIYIKKNDGNTATCEDFNYAMEQAYKMKKADNSANLNQY
LLWFSQSGTPHVSFKYNYDAEKKQYSIHVNQYTKPDENQKEKKPLFIPISVGLINPENGK
EMISQTTLELTKESDTFVFNNIDVKPIPSLFRGFSAPVYIEDNLTDEERILLLKYDSDAF
VRYNSCTNIYMKQILMNYNEFLKAKNEKLES----FNLTPVNAQFIDAIKYLLEDPHADA
GFKSYIVSLPQDRYIINFVSNLDTDVLADTKEYIYKQIGEKLNDVYYKMFKSLEAKADDL
TYFNDESHVDFDQMNMRTLRNTLLSLLSKAQYPNILNEIIEHSKSPYPSNWLTSLSVSAY
F--DKYFELYDKTYKLSKDDELLLQEWLKTVSRSDRKDIYEILKKLENEVLKDSKNPNDI
RAVYLPFTNNLRRFHDISGKGYKLIAEVITKTDKFNPMVATQLCEPFKLWNKLDTKRQEL
MLNEMNTMLQESNISNNLKEYLLRLTNKL

>P_gallinaceum_ortholog
MNLKKVLNYNFFFISVLF-LANSL-NYHKNTCMINNIFRINSCGVISRLLKS---NSIKN
NINQKINFSSLIYDLEIFKIANLDLFQKDIFNSI-NKKKKPQ---SYIIQKRLMSEKGE-
--N-------NSSPDKDRKRGGPIGSGGN------TSQEKKLKLSSESLDPSAFDDITTR
ED-----------DSGNNESGDN-------KVVPKINYRTDYKPSGFKINNSTLHIEIYD
DHTTVKSTLDIELSEHYDGEDLVLDGVDLEIKEIAINETKLSEGEHYRYDNEYLTIFSSN
LPEGKFKFSSQVTIHPESNFALTGLYKSKNLIVSQCEATGFRRITFFIDRPDIMSKYDVT
LTADKKKYPVLLSNGDKVSEFDVNEGRHGARFVDPYLKPCYLFAVVAGDLKHLSDTYVTK
FSKRNVELYVFSEEKYVSKLKWALECLKKAMEFDENYFGLEYDLTRLNLVAVSDFNVGAM
ENKGLNIFNANSLLASKKKSIDFSFERILTVVGHEYFHNYTGNRVTLRDWFQLTLKEGLT
VHRENLFSEHTTKTATFRLDHVDLLRSVQFLEDSSPMSHPIRPESYVSMENFYTTTVYDK
GSEVMRMYMTILGEEYYKKGMTIYIKKNDGKTATCEDFNDAMNEAYKLKKGDNNANLDQF
LLWFSQSGTPHVTATYSYDENKKEFTIHVSQFTKEDNNQKEKKALFIPIKVGLVNPKNGE
EMIEETTLEFTKERDVFVFKNIEEKPIPSLFRGFSAPVYIKDNLTDEERILLFKYDKDSF
VRFNICTDIYMKQILTNYKELLQAKNEDIDE----PELTPVNKDFINAIRYLMNDSNADP
GFKSYIITLPRDRYIINFIDNLDTDVLYDTKMFIYKQIGNELNDDYFKIFKELKSEADDM
THFDDESYVDFDQINMRKLRNTLLSLLSRAKYPNILEYIMEQSNSPYPTNWLTSLAVSAY
Y--KTYYDLYEKTYILSKDDELLLQEWLKTVSRSDREDIYDIIKKLETDVLKDSKNPNEI
RAVYIPFTNNLKYFNDISGKGYKLIADVIIKVDSFNPQVATQLCDPFKLWNKLDLKRQKL
MLDEINRILKKDNISNNLKEYLLRLTNKL

>P_vivax_ortholog
MIRKKMLNFHFLFITVLVALANYTPVDYQNTCMISKSCRKNSCGITSRVLSGIHVNKTSA
RAKALISLSSLIYHLQLPKLVSLDLFRRDLFTGV-KQKGKRSPVPSYIIQNRLMSENGD-
--SGSTNMSVTANQEKKRPGTGDASEGNNQSGISAAAQDKRMKGGDQSEEVSNVSGSTNA
AMTNGASSTTEGGDNNNNGSGND------GKNEPKIHYRKDYKPSGFVIDNVTLNINIFD
NETSVRSTLDMKLSEHYGGEDLIFDGVSLEIKEISIDNNKLMEGEHYKYDNEFLTIYSKF
IPKGKFTFGSEVIIHPETNYALTGLYKSKNIIVSQCEATGFRRITFFIDRPDMMAKYDVT
ITADKEKYPVLLSNGDKLNEFEIPGGRHGARFNDPYLKPCYLFAVVAGDLKHLSDNYVTK
FSKKNVELYVFSEEKYVSKLKWALECLKKAMKFDEDYFGLEYDLSRLNLVAVSDFNVGAM
ENKGLNIFNANSLLASKKKSIDFSFERILTVVGHEYFHNYTGNRVTLRDWFQLTLKEGLT
VHRENLFSEQTTKTATFRLDHVDILRSVQFLEDSSPLAHPIRPESYVSMENFYTTTVYDK
```

```
GSEVMRMYQTILGDEYYKKGMDIYIKKNDGGTATCEDFNDAMNEAYKMKKGDKTANLDQY
LLWFSQSGTPHVTAEYSYDAGKKEFVIEVTQVTNPDPNQKEKKALFIPIRVGFINPKNGQ
DVIPEVTLEFKKDKEKFIFNNVNEKPIPSLFRGFSAPVYIKDNLTDSERILLLKYDTDAF
VRYNVCVDLYMKQILKNYQELLQAKSENKQESAEKPSLTPVSEDFINAIKYLMEDPHADA
GFKSYIITLPRDRFILNYIKNVDTDVLADTKDFIYKQLGDKLNDLYFQMFKSLQAKADDM
THFEDESYVDFEQLNMRKLRNTLLTLLSRAKYPNMLDQIMEHSKSPYPSNWLASLAVSAY
Y--DKYFDLYEKTYNQSKDDELLLQEWLKTVSRSDRKDIYDIIKKLETEVLKDSKNPNEI
RAVYLPFTYNLRYFNDISGKGYKMMADIIMKVDKFNPMVATQLCDPFKLWNKLDQKRQDM
MLNEMNRMLSMENISNNLKEYLLRLTNKL

>P_knowlesi_ortholog
MIRERMLNLYFLFITVLAALAIYTPVDYQNTCMISRSCRKNSCGITSRVLSGIHVNKAST
RARALISLSSLIYQLQLPKLVSLDLFRRDLFTGV-NQKGRRSPVPSYIIQSRLMSENID-
--SGNNNMSATGNQEKKRPVTGDASDAKNPDGSTISAQDKRMKGIDQNEGTSSVSGSTNA
AFTNGASSIMEGGENNNNGTGNE------GKNEPTIHYRKDYRPSGFIIDNVTLNINIFD
NETSVRSTLDMKLSDHYRGEDLIFDGVSLEIKEISIDGNKLMEGEHYKYDKEFLTIYSKF
IPKGKFTFGSEVIIHPETNYALTGLYKSKNIIVSQCEATGFRRITFFIDRPDMMAKYDVT
VTADKEKYPVLLSNGDKLNEFEIPGGRHGARFNDPYLKPCYLFAVVAGDLKHLSDNYVTK
FSKRNVELYVFSEEKYVSKLKWALECLKKAMKFDEDYFGLEYDLSRLNLVAVSDFNVGAM
ENKGLNIFNANSLLASKKKSIDFSFERILTVVGHEYFHNYTGNRVTLRDWFQLTLKEGLT
VHRENLFSEQTTKTATFRLDHVDLLRSVQFLEDSSPLAHPIRPESYVSMENFYTTTVYDK
GSEVMRMYQTILGDDYYKKGMDIYIKKNDGGTATCEDFNDAMNEAYKLKKGDKTANLDQY
LLWFAQSGTPHVTAEYSYDAGKKEFVIDITQVTHPDPNQKEKKALFIPIRVGFINPHNGK
EVIPEVTLEFKKDKEKFIFSNVNEKPIPSLFRGFSAPVYIKDNLTDSERIVLLKYDTDAF
VRYNVCVDLYMKQIMKNYQELLQAKAENKQESTEKPKLTPVSEDFISAIKYLMEDPHADA
GFKSYIITLPRDRFIINSIRNVDTDVLADTKDFIYKQLGDKLNDLYFQIFKSIQAKADDM
THFEDESYVDFEQLNMRKLRNTLLTLLSKAKYPNMLDHIMEHSKSPYPSNWLASLAVSAY
Y--DKYFDLYEKTYNQSKDDELLLQEWLKTVSRSDRKDIYDIIKKLENEVLKDSKNPNEI
RAVYLPFTNNLRYFNDISGKGYKMMADIIMKVDKFNPMVATQLCEPFKLWNKLDMKRQDM
MLNEMNRMLSMENISNNLKEYLLRLTNKL

>P_chabaudi_ortholog
MVTKKLLSFNLFLIIVLT-FENLS-FVKKNTCMINNTIRPNSCGIVNRVLRE-KSQQHSS
KIPKILPF---IRNFSLKKYITGDQLQKNILNHI-NKLSGAH---LFQGSKSQLSSITN-
--N---------------KFVGDAT--------------KSALGFVSNIGT----DISGN
KKTNLGRMLCEGHNNNGGEVTSTENAILKNSKDPQIHYRTDYKPSGFTIDTVTLNINIFD
NETTVRSSLSMCTNDNYANEDLVFDGVGLSIKEININDNKLTEGEDYTYDNEFLTVFAKN
VPKGNFVFVSEVIIHPETNYALTGLYKSKDIIVSQCEATGFRRITFFIDRPDMMAKYDVT
LTADKTKYPVLLSNGDKLNEFDIPGGRHGARFNDPHLKPCYLFAVVAGDLKFLSDKYVTK
FTKKPVELYVYSEEKYVSKLKWALECLKKAMKFDEDYFGLEYDLSRLNLVAVSDFNVGAM
ENKGLNIFNADSLLASKKTSIDFSFERILTVVGHEYFHNYTGNRVTLRDWFQLTLKEGLT
VHRENLFSEQTTKTATFRLTHIDILRSVQFLEDSSPLSHPIRPESYISMENFYTNTVYDK
GSEVMRMYQTILGDEYYKKGISIYLKKHDGGTATCEDFNEAMNEAYQMKNGNKEENLDQY
LLWFSQSGTPHVTAEYSYDANAKTFTIKLSQVTYPDDNQKEKFPLFIPVKVGLISPKDGK
DVIPEVVLEFKKDKDTFVFENIEEKPIPSLFREFSAPVYIKDNLTDEERIILLKYDSDAF
VRYNVCIDLYMKQIIKNYNEFLSQKTK--EANGLEHSLTPVSEDFINAIKHLLEDKHSDP
GFKAYIIALPRDRYIMNYIKEVDPIILADTKDYIYKQMGNRLNPILFSIFQDTESKANDM
THFKDESYVDFDQLNMRKLRNSIMVMLSKAQYPHMLKYVKDQAQSPYPSNWLASLSASAY
FTGDDYYNLYDKTYNLSKNDELLLQEWLKTVSRSDRSDIYNIIKKLETEILKDSKNPNNI
RAVYLPFTSNLRAFNDISGKGYKLMADVIMKVDKFNPMVATQLCDPFKLWNKLDLKRQAL
MHDEMNRMLSMDNISPNLKEYLLRLTNKM

>P_yoelii_ ortholog
MVTKKLLSFNLFLIIILT-FENLT-FDKKNTCMINNTIRPNSCGIVNRVLRE-KPYHYSS
KISKSIPF---IQNFSLEKNFTGESLQKNILNNI-NKLGGAH---LFHISKSHLAAKAG-
--N------------KNTEFIGEAT--------------ELFKGFKRNFGI----NMTEN
KQTNIGRMLCEDDNNNGGEVTSTEKTIFKNSKDPQIHYRTDYKPSGFTIDNVTLNINIFD
NETIVRSSLNMCTNENYADEDLVFDGVGLSIKEISINNNKLNEGEDYTYDNEFLTIFAKN
VPKENFVFLSEVVIHPETNYALTGLYKSKDIIVSQCEATGFRRITFFIDRPDMMAKYDVT
LTADKTKYPVLLSNGDKLNEFDIPGGRHGARFNDPHLKPCYLFAVVAGDLKHLSDNYVTK
YTKKPVELYVYSEAKYVSKLKWALECLKKAMKFDEDYFGLEYDLSRLNLVAVSDFNVGAM
ENKGLNIFNADSLLASKKTSIDFSFERILTVVGHEYFHNYTGNRVTLRDWFQLTLKEGLT
VHRENLFSEETTKTATFRLTHIDLLRSVQFLEDSSPLSHPIRPESYISMENFYTNTVYDK
GSEVMRMYQTILGDEYYKKGIDIYLKKHDGGTATCEDFNDAMNEAYQMKNGNTDENLDQY
LLWFSQSGTPHVTAEYIYDENEKTFTINLSQITYPDDNQKEKYPLFIPVKVGFISPKDGK
DVIPEVVLELKKDKESFVFQNVSEKPIPSLFREFSAPVYIKDNLTDEERIALLKYDSDAF
VRYNVCIDLYMKQIIKNYNELVSQKTK--ENDLVELSLTPVNDEFINAIKHLLEDKHADP
GFKAYIIALPRDRYIMNYIKEVDPIVLADTKDYIYKQIGSRLNPILFSIFQNTESKANDM
THFKDESYIDFDQLNMRKLRNSILVMLSKAQYPHMLKYIKEQSKSAYPSNWLASLSASAY
FSGDDYYDLYDKTYKLSKNDELLLQEWLKTVSRSDRSDIYSIIKKLEVEILKDSKNPNNI
RAVYLPFTANLRAFNDISGKGYKLMADVIMKVDKFNPMVATQLCDPFKLWNKLDLKRQAL
MHDEMNRMLSMENISPNLKEYLLRLTNKM

>P_berghei_ortholog
MVLKKLLCFNLFLIIILT-FENLS-FDKKNTCMINNTIRSNSCCIVNRVLRE-KTHHYSS
KISKSIPF---IQNFSLEKYFTGESLQKNILNNI-NKLGGAH---LFHISKSHLTAKSG-
--N------------KNTEFIGEAT--------------ELFKGFKRNFGI----NMTEN
KQTNIGRMLCENDNNNGGEDTSTEKAIFKKSKDSQIHYRTDYKPSGFTIDNVTLNINIFD
NETIVRSSLNMCTNENYADEDLVFDGVGLSIKEISINNNKLTEGEDYTYDNEFLTIFAKN
VPKENFVFLSEVVIHPETNYALTGLYKSKDIIVSQCEATGFRRITFFIDRPDMMAKYDVT
LTADKKKYPVLLSNGDKLNEFDIPGGRHGARFNDPHLKPCYLFAVVAGDLKHLSDNYVTK
YTKKPVELYVYSEAKYVSKLKWALECLKKAMKFDEDYFGLEYDLSRLNLVAVSDFNVGAM
ENKGLNIFNADSLLASKKTSIDFSFERILTVVGHEYFHNYTGNRVTLRDWFQLTLKEGLT
VHRENLFSEETTKTATFRLTHIDLLRSVQFLEDSSPLSHPIRPESYISMENFYTNTVYDK
GSEVMRMYQTILGDDYYKKGIDIYLKKHDGGTATCEDFNDAMNEAYQMKKGNTDENLDQY
LLWFSQSGTPHVTAEYIYDENEKTFTINLSQITYPDDNQKEKYPLFIPVKVGFISPKDGK
DVIPETVLELKKDKESFVFQNVSEKPIPSLFREFSAPVYIKDNLTDEERIALLKYDSDAF
VRYNVCIDLYMKQIIKNYNELISQKTK--ENNVLELSLTPVNDEFINAIKHLLEDKHADP
GFKSYIIALPRDRYIMNYIKEVDPIVLADTKDYIYKQIGSRLNPVLFSIFQNTESKANDM
THFKDESYIDFDQLNMRKLRNSILMMLSKAQYPHMLKYIKEQSNSPYPSNWLASLSASSY
FSGDDYYDLYDKTYKLSKNDELLLQEWLKTVSRSDRSDIYSIIKKLEVEILKDSKNPNNI
RAVYLPFTSNLRAFNDISGKGYKLMANVIMKVDKFNPMVATQLCDPFKLWNKLDLKRQAL
MHDEMNRMLNMENISPNLKEYLLRLTNKM
```

- **AMOUNT OF MISSING DATA**

P. reichenowi	***P. gallinaceum***	***P. knowlesi***	***P. vivax***	***P. chabaudi***	***P. berghei***	***P. yoelii***
0%	2%	2%	2%	3%	3%	3%

■ PF14_0281

▪ PHYLOGENY AND PEXEL/VTS

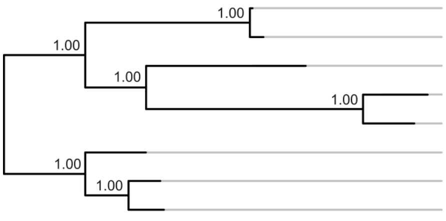

P. falciparum PF14_0281	IN-NSRILSDVDKHR
P. reichenowi ortholog	IN-NSRILSDVDKHR
P. gallinaceum ortholog	INNNSKPLKDVEFYI
P. knowlesi ortholog	XXXXXXXXXXXXXXX
P. vivax ortholog	XXXXXXXXXXXXXXX
P. chabaudi ortholog	LNKKSNNLPSLKNDQ
P. berghei ortholog	FNQNPNNLPSLKNNQ
P .yoelii ortholog	FNKNKYNLPSLKKNQ

▪ SUPPORT FOR ALTERNATIVE TOPOLOGY RESEMBLING THE SPECIES TREE:

Expected likelihood weights	0.1242	+
Shimodaira-Hasegawa test	0.9970	+
two-sided Kishino-Hasegawa test		+
one-sided KH test	0.1790	+

▪ ALIGNMENT

```
>P_falciparum_PF14_0281
MFFINFKKIKKKQFPIYLTQHRIITVFLIFIYFINLKDCFH-----IN-NSRILSDVDKH
R------GLY-YNIPKCNVCHKCSICTHENGEAQNVIPMVAIPSKRKHIQDINKEREENK
YPLHIFEEKDIYNNKDNVVKKEDIYKLRKKKKQKKNCLNFLEK-----------------
---DTMFLSPSHDKETFHINHMNK---IKDEKYKQEYEEEKEIYDNTNTSQEKNE-----
----TNNEQNLNINLINN--------------------DKVTLPLQQLEDSQYVGYIQIG
TPPQTIRPIFDTGSTNIWIVSTKCKDETCLKVHRYNHKLSSSFKYYEPHTNLDIMFGTGI
IQGVIGVETFKIGPFEIKNQSFGLVKREKASDNKSNVFERINFEGIVGLAFPEMLSTGKS
TLYENLMSSYKLQHNEFSIYIGKDSKYSALIFGGVDKNFFEGDIYMFPVVKEYYWEIHFD
GLYIDHQKFCCGVNSIVYDLKKKDQENN---KLFFTRKYFRK---NKFKTHLRKYLLKKI
KHQKKQ------------------------------------------------------
----------KHS-----NHKKKKLNKKKNYLIFDSGTSFNSVPKDEIEYFFRVVPSKKC
DDSNIDQVVSSYPNLTYVINKMPFTLTPSQYLVRKNDMCKPAFMEIEVSSEYGHAYILGN
ATFMRYYYTVYRRGNNNNSSYVGIAKAVHTEENEKYLSSLHNK-INNL-

>P_reichenowi_ortholog
MFLINFKKIKKKQFPIYLTQHRIITVFLIFIYFINLKDCFH-----IN-NSRILSDVDKH
R------GLY-YNIPKCNLCHKCSICTHQNGEARNVIPMVAIPSKRKHIQDINKEREENK
YPLHIFEEKDIYNNKDNVFKKEDIYKLRKKKKQEKNSLNFLEK-----------------
---DTMFLLPSHDKETFHINHMNK---IKDEKYKQEYEEEKEIYDNTNTPQEKNE-----
----TNNEQNLNINLINN--------------------DKVTLPLQQLEDSQFVGELLVG
TPPQTIYPIFDTGSTNIWIVSTKCKDETCLKVHRYNHKLSSSFKYYEPHTNLDIMFGTGI
IQGVIGVETFKIGPFEIKNQSFGLVKREKASDNKSNVFERINFEGIVGLAFPEMLSTGKS
TLYENLMSSYKLQHNEFSIYIGKDSKYSALIFGGVDKNFFEGDIYMFPVVKEYYWEIHFD
GLYIDHQKFCCGVNSIVYDLKKKDQENN---KLFFTRKYFRK---NKFKTHLRKYLLKKI
KHQKKQ------------------------------------------------------
----------KHF-----NHKKKKLNKKKNYLIFDSGTSFNSVPKDEIEYFFRVVPSKKC
DDSNIDQVVSSYPNLTYVINKMPFTLTPSQYLVRKNDMCKPAFMEIEVSSEYGHAYILGN
ATFMRYYYTVYRRGNNNNSXXXXXXXXXXXXXXXXXXXXXXXXXXXXXX

>P_gallinaceum_ortholog
MNFLIFKKLKKRIHSTILIQ-LILVLFLIHTSHIKLNNCIY-----INNNSKPLKDVEFY
I------DTPNYKIPKCNLCINCSVCIHENEVSENIIPLVAVASKRKYFSDKISKVKGDN
AEYGIKKE-DIIPSKYANDSEDNFFLLNKLNNGKKRSYNFLQN-----------------
---D----------KSYEISKNNKNLNIKKENDEQKENEYYISGKDFTNNYSHKNYRING
DINNKKKDQKVD-NKLSN--------------------TQVTLPLQQLQDSQYVGTVKIG
NPPQTIRPIFDTGSTNIWIVSTKCNDDTCLKVHRYNYKLSRSFRYYKPYTNLDIMFGTGI
IQGTIGIETFRIGPFKIENQSFGLVKKEKGNEKKSNVFERIKFEGIIGLAFPAMLSTGKT
TIYENLVSAYNFKHNEFSIYIGKDSKFSALIFGGVDTRFFHGDIYMFPVVKEYYWEINFD
GLYIDHQKFCCDSSSIVYDLRKKSKKK----KNSFIRKFFRXXXXXXXXXXXXXXXXXXI
SHFKNE------------------------------------------------------
----------NQM-EENDSMNEKKIKKDKNYLIFDSGTSFNSVPKSEIEYFLKIVPSKKC
DDNNIDEVVSGYPNLTYV-NKMPFTLTPAQYLVRNNDICKPAFMEIEVSPEYGHAYILGN
ATFMRYYYTVYRRGKKNESSYVGIAKAVHADENEVYLNSLHNE-INKM-

>P_knowlesi_ortholog
XXXXXXXXXXXXXXXXXXXXXXXXXXXXXXXXXXXXXXXXX-----XXXXXXXXXXXXXX
X------XXXXXXXXXXXXXXXXXXXXXXXXXXXNIIPMVAIPSKRKYLQEKIEKIKSEL
--------------------HQNLPEQKWKKKKKKESYSFFEGEDDDKGEVD--------
---EEEGDSTSHATMDNQIFHHNKGTHYEGEDKHPDEFQKCATSDCHMNKDASGIPDYLR
HFMDGSEEKAQT--------SWSSWSSAFKKKEVSSSTDQVTLPLQQLQDSQYVGYIQIG
NPPQTIRPIFDTGSTNIWVVSTKCKDDTCLKVHRYDYKLSKSFRYYKPRTNLDIMFGTGI
IQGVIGVENFRIGPFKLFNQPFGLVKREKRSEAKSNVFERINFEGIVGLAFPAMLSTGKT
TIYENLMDTYKLSHNEFSIYIGKDNKHSALIFGGVDRRFFEGDIYMFPVVKEYYWEIEFD
GLYIDHQKFCCDSSSIVYDMRKKEKKKGKVHRNSFVRKYLKK------KTDLMN--MSSV
WHHRREGAEVDSKEDQ------SGIDLSEEEKDGEHSIRGEVNTYGVHPGRHGKGVHSRQ
QRRHRRHGWRRHMRRVNHRGKDNKLKKNKNYLIFDSGTSYNSVPKSEIKYFFKILPSKKC
DDSNIEEVVASYPNLTYVINNMPFTLTPAQYLVRKSNMCKPAFMEIEVSPEYGHAYILGN
ATFMRYYYTVYRRGDGNKSSYVGIAKAVHAEDNEEYLTNLQRK-MNQME

>P_vivax_ortholog
XXXXXXXXXXXXXXXXXXXXXXXXXXXXXXXXXXXXXXXXX-----XXXXXXXXXXXXXX
X------XXXXXXXXXXXXXXXXXXXXXXXXXXXXXXXMMAIPSKRKYLQYKIGKLNSEL
--------------------LQNLPTKKLKKKKKKGSYSFFEGEDEDEGEEEQENEQDEQ
QQKEEEGDPPSDATMDNHISHHNKGTHYGGEDHHPDEFPNCVNSDCHMSNDASAEPDYLG
QFMNGSGEKARAQTQRSNWSNWNSWSGAFKKKEV-SSTDQVTLPLQQLQDSQYVGYIQIG
```

```
NPPQTIRPIFDTGSTNIWVVSTKCKDDTCLKVHRYNYKLSRSFRYYKPHTNLDIMFGTGI
IQGVIGVETFRIGPFKVFNQPFGLVKREKRSEAKSNVFERINFEGIVGLAFPAMLSTGKT
TIYENLMNTYKFSHNEFSIYIGKDNKHSALIFGGVERRFFEGDIYMFPVVREYYWEIQFD
GLYIDHQKFCCDSSSIVYDMRKK-KKKWKVQRNSFARKYLKK------KTDLRD--MSRV
WHHRREGAEEDSEEEDPSGENLSGESLSGEDKHGERSTGGEVNPYGAHPERRGKGAHRRR
RRRHR---WRRHRSRVNRRGKDEKLKKNQNYLIFDSGTSFNSVPKSEIGYFFKVVPPKKC
DDSNIDEVVASYPNLTYVINNMPFTLTPAQYLVRKSDMCKPAFMEIEVSPEYGHAYILGN
ATFMRYYYTVYRRGDGRKGSYVGIAKAVHAEDNEEYLTALQRK-MNPVG

>P_chabaudi_ortholog
MFFLNFKKLRKNYFLALLTHPTITVLFFIYIFNFVTSDYAH-----LNKKSNNLPSLKND
Q------EYNKQNIQPCNSCVNCSVCIHENAEPQDILPLVAVPSRRKYFYEQDRSKDDDL
NDFPV--E-DKINENDERTEYDNDYSQNELSKKKKKMYNFIEN-----------------
---------------HNEMPDMDNDVTD--EEYEENMEEENMEEENTTNLESNYE-----
NTFNSEKEDSTD--------------------------SKVILPLQQLKDSQYVGFIQIG
NPPQTIRPIFDTGSTDIWIVSTKCKDKTCLKVHRYNHKLSDTFKYYTPRTNLDIMFGTGI
IQGTIGIETFKIGPFKIENQSFGLVKREKGTDDKS-VFERINFEGIVGLAFPAMLSTGNI
PIYENLMSSYNFPHNEFSIYIGMDNKYSALIFGGVEKKFFEGDIYMFPVVREYYWEIKFD
GLYIDHQKFCCDSGSIVYDLKMKDENKHK--KKYSMRKYFHKHHFNHKKIWLRK--NHHT
KRWKRE------------------------------------------------------
----------KHF---------KPLNSDENYLIFDSGTSFNSVPKSEIKYFFKVVPPKEC
NDDNIDEVIDSYPNLTYVINNMPFTLTPSQYLIRKRNMCKPAFMEIEVSPEYGHAYILGN
AAFMKHYYTVYRRGKGNNDSYVGIAKAVHTKENAEYLNSLHKERMEDEE

>P_yoelii_ ortholog
MFFLTLKKLRKKYFLLFLTHPTITTLFFIYIFNLVKSDYPN-----FNKNKYNLPSLKKN
Q------KYFKQKIQPCNSCINCSICIHENGEPQNILPLVAIPSKRHYFYEQDMSKNSNL
NGSPV------KNKREDSIKFDRNYSQKELNKKNKKNYNFIEN-----------------
---------------HIAMSNINNDITDGDRETEDGLNQENIAKDNFNNLISSYE-----
NIYNQKTEHSID--------------------------NKVILPLQQLQDSQYVGSIQIG
NPPQTIKPIFDTGSTNIWVVSTKCRDKTCLKVHRYNHKLSNTFKYYSPRTNLDIMFGTGI
IQGTIGIDTFKIGPFKIENQSFGLVKREKGSNKKSNVFERINFEGIIGLAFPTMLSTGGN
PIYENLMASYNFPHNEFSIYIGMDNKYSALIFGGVEKKFFEGDIYMFPVVREYYWEIKFD
GLYIDHQKFCCDSGSIVYDLKMKDKNKNE--KNYFIRKYFNKHHFNHKKMWLRN--NHHT
KHWKRE------------------------------------------------------
----------KHF---------KPLSSNENYLIFDSGTSFNSVPKSEIKYFFKVVPPKKC
DANNIDEVIDSYPNLTYVINNMPFTLTPSQYLIRKHNMCKPAFMDIEVSPEYGHAYILGN
ATFMKHYYTVYRRGKGNNNSYVRKKKFXXXXXXXXXXXXXXXXXXXXXX

>P_berghei_ortholog
MFFLTLKKLRKKCFFVFLTHPTITALFFIYIFNFVKSYHVN-----FNQNPNNLPSLKNN
Q------EYYKQKIQPCNSCVNCFVCIHENGEPQNILPLVAIPSKRHYFYEQDMSKNSNL
NGFPV------KNKMDNST----NYFQKELNKK-KKNYNFIEN-----------------
---------------HTAISNIDNDITDEYKESESDLDEENIVKDNFNNLRSSYE-----
NIYNQKKEHSID--------------------------SKVILPLQQLQDSQYVGSIQIG
NPPQTIRPIFDTGSTNIWVVSTKCKDRTCLKVHRYNHKLSNTFKYYTPRTNLDIMFGTGI
IQGTIGIDTFKIGPFKIENQSFGLVKREKGTNKKSNVFKRINFEGIIGLAFSTMLSTGGN
PIYENLMSNYNFPHNEFSIYIGMDNKYSALIFGGVEKKFFEGNIYMFPVVREYYWEIKFD
GLYIDNQKFCCDNNSIVYDLKMKKKKNE--KKNFIRKYFNKHHINHKKMWLRN--NHHT
KHWKRE------------------------------------------------------
----------KHF---------NPLSSNENYLIFDSGTSFNSVPKSEIKYFFKVVPPKKC
DANNIDEVIDSYPNLTYVINNMPFTLTPSQYLIRKHNICKPAFMDIEVSPEYGHAYILGN
ATFMKHYYTVYRRGKGNNNSYVGIARAAHTKENAEYLNSLHKERMENEE
```

- **AMOUNT OF MISSING DATA**

P. reichenowi	***P. gallinaceum***	***P. knowlesi***	***P. vivax***	***P. chabaudi***	***P. berghei***	***P. yoelii***
4%	7%	19%	31%	7%	7%	9%

■ PFC0555c

▪ PHYLOGENY AND PEXEL/VTS

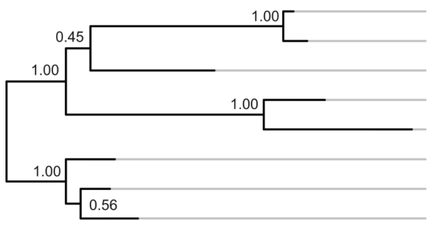

P. falciparum PFC0555c	KENMKRILYEWAKFL
P. reichenowi ortholog	KENMKHILYEWAKFL
P. gallinaceum ortholog	KENMKTILYEWAKFL
P. knowlesi ortholog	KDSMKTILNEWAEFL
P. vivax ortholog	KGSMKTILYEWAEFL
P. chabaudi ortholog	KDNMKTILYEWAKFL
P. berghei ortholog	KDNMKTILYEWAKFL
P .yoelii ortholog	KDNMKRILYEWAKFL

▪ ALI GNM ENT

>P_f alci paru

```
m_PFC0555c
MFRSKAHFLMLANLKYLELQDLLLKRFQVFKNEEIRILKKENMKRILYEWAKFLIKENDN
TNITYIPQKVLQNNKVQDILKNDIDCEWLVNKIHISNDEQI-----NIHSS-------NN
FEEFKKKKDALHFDEYPNLFYYEYPQFPYNYYDKKKMSNFYSLIKFPYQDILNISKNIKN
MSKEEEQKKE-----------NHQINNNVSN---------NEENKINQDIVHM----NNN
NVNYTDHSYVYAPCEMETSKENYNQTQID

>P_reichenowi_ortholog
MFRSKAHFLMLANLKYLELQDLLLKRFQVFKNEEIRILKKENMKHILYEWAKFLIKENHN
SNITYIPQEVLDNNKVQDILKNDMDCEWLVNKIHISNDEKI-----DIHSS-------NN
FEELKKKKDALHFDEYPNLFYYEYPQFPYNYYDKKKMSNFYSLIKFPYKDILNISKNIKN
MSYEEEQKKK-----------NHQTNNNVSN---------NEENKINQDIVHMNNNNNNN
NVNYTDHSYVYAPCEMETSKENYNQTQID

>P_gallinaceum_ortholog
MFRSKAHFLMLANLKYLELQKLLLNRFQIFKNEEIRILKKENMKTILYEWAKFLTEKNQD
TNITNIPPEVLKIKKVIEILKDDIDCKWLIDKINIENNNGN-----NINKV-------EN
DE--LKKIDNLHFDEYPQLFYYEYPQYPYNYYDKKKMTSLYSLIKFPYNDILNISKRVNV
DNLKKGNEIQXXXXXXXXXXXXXXXXXXXXXXXXXXXXXXXXXXXXXXXXXXXXXXXXXX
XXXXXXXXXXXXXXXXXXXXXXXXXXXXX

>P_knowlesi_ortholog
MFRSEAHFRMLTNLRFLELQKLLLKRFQIFKNEEITILKKDSMKTILNEWAEFLTKEQKG
TNITHIPDEVLQNKKVADILRDDIECRWLVGRINVGNNQESPVGTNDVNSSCSAALARKG
TA--LSKIDQLHFDEYPNLFHYEYPQFPYNYYDKKKMENFYSLLKFPYNDILKISKMIKR
DVISPSGNAEE---GEILQQGGKAQSEVLES---------THVDVVNDDF----------
-MNYSSNSYVYAPSEMETSKENYHE----

>P_vivax_ortholog
XXXXXXXXXMLANLRFLELQKLLLNRFQIFKNEEITVLKKGSMKTILYEWAEFLTKEQRA
TNMTHIPDEVLQSKRVADILRDDVECRWLVGRINVGAVSGV-SGVSPPSPLSGAHLARDD
AA--LSKVDQLHLDEYPDLFHYEYPQFPYDYYDKKKMSNFYSLMKFPYNDILNISKRIKR
EVSPPGGDAKE---GE-MRQMEEPRGGALES---------AGVDVVDDDF----------
-VSYSSNSYVYAPSEMETSKENYHE----

>P_chabaudi_ortholog
MFKSNSHFLMVTNLRFLELQKMLLNRFQVFKNEEIRILKKDNMKTILYEWAKFLTKQNIK
TNITNIPEQVLENKIVTKIINDDIDCKWLVDKINVKNKNEN-----NII---------QN
EE--LKKIDNLHFDEYPQLFYYEYPQFPYNYYDKKKMTNFYSLIKFPYNDIINISRKIKN
DKEDEQNDLSKVDDTKNMLNDKDHQDLYKLNKNCENIQKTEEEDSLNSE-----------
--NYSCDNYIYAPSEMETSKENYHQT---

>P_yoelii_ ortholog
MFRSDSHFLMVTNLRFLELQKMLLNRFQIFKNEEIRILKKDNMKRILYEWAKFLTKENIK
TNITNIPEKVLENKIVTKIINDDIDCKWLVNKINVKNQNEN-----NII---------KN
QE--LKKIDNLHFDDYPNLFYYEYPQFPYNYYDKKKMTNFYSLIKFPYNDIINISRKIKT
DEETEQNGQER---EKNGQETEQHEITDMLH---------PDKDILNCD-----------
--NYSCDNYIYAPSEMETSKENYHQT---

>P_berghei_ortholog
MFRSNSHFLMVTNLRFLELQKMLLNRFQIFKNEEIRILKKDNMKTILYEWAKFLTRENIE
TNITNIPEQVLENKIVTKIINDDIDCKWLVNKINVKNQSEN-----NII---------KN
EE--LKKIDNLHFDEYPQLFYYEYPQFPYNYYDKKKMTNFYSLIKFPYNDIINISRKIKN
DKGDEQNGKEN---KQN-----------------------EDKDSLNSD-----------
--NYGCDNYIYAPSEMETSKENYRQS---
```

▪ AMOUNT OF MISSING DATA

P. reichenowi	***P. gallinaceum***	***P. knowlesi***	***P. vivax***	***P. chabaudi***	***P. berghei***	***P. yoelii***
1%	24%	13%	15%	14%	12%	9%

■ PF10_0134

▪ PHYLOGENY AND PEXEL/VTS

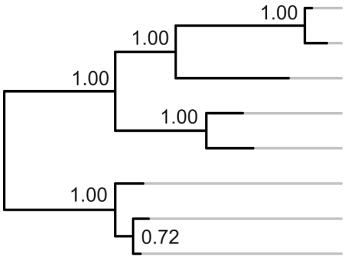

P. falciparum PF10_0134	FREVDRILTEHQIKL
P. reichenowi ortholog	FREVDRILTEHQIKL
P. gallinaceum ortholog	FREVARVLNENKIKL
P. knowlesi ortholog	FREVERIIAENKIKI
P. vivax ortholog	FREVARIIGENKIKI
P. chabaudi ortholog	FREVARIIEEKNLKI
P. berghei ortholog	FREVARIIEEKNLKI
P .yoelii ortholog	FREVARIIEEKNLKI

▪ ALIGNMENT

```
>P_falciparum_PF10_0134
M----FLLCLLLWRNIICDIKELNYEQFYKMLTTEKNNQKKNYILDKSINYKNFKSFQDF
LYIKTNEDFVLYVYAKWDTDSNNLISIFREVDRILTEHQIKLNFYIFNIDQAKDLCNFLN
ITSLPLILYVSSVHKKKYNTLLYKFLNSNKDIKISNAFRYNGDMYCYDYIVEWVEAHYYF
TKFVLLMKKLFSWKK-

>P_reichenowi_ortholog
MER------LLFWRNIIWDIKELNYEQFYKMVTTEKNNPKKNYILDKSINYKNFKSFQDF
LYIKTNEDFVLDVYAKWDTDSSNLISIFREVDRILTEHQIKLNFYIFNIDQAKDLCNFLN
ITSLPLILYVSSVHKKKYNTLLYKFLNSNKDIKISNAFRYNGDMYCYDYIVEWVEAHYYF
TKFVLLMKKLFSWKK-

>P_gallinaceum_ortholog
MKIFFLFFLCIFLKNVICYIKELSFQEFQKMLTVEKNNQKKNYVLDSSLNYKNVKFFQDY
LYIQTNNDFILYIYAKWDTDSSNLITVFREVARVLNENKIKLDFYIFNIDKVKDLCNFLN
ITTLPLILYVSSIHKKKYNSLLYKVFNSSKDVKISNAFRYNGDMYCYDHIAEWIEAHYYF
TKMITRIKYLFYGK--

>P_knowlesi_ortholog
MKLFFLLLFCALLKSAICEIKELSYHEFHRMLTTEKKDPKKTYAFDSKPKFKNVNYFQDF
LYLKTDVDFVLYVYAKWDTDSNNLITVFREVERIIAENKIKIDFYTFNIDNAKELCNFMN
IKTLPVILYVSSVHKKKYNSLLYKALSSSKDVRISNAFRYNGDMYCYEYIVEWIEAHHYF
TRGVMLVKKLFQWKKK

>P_vivax_ortholog
MKFVFPLLLYALLKSAFCEIKELSHHEFHRMLTTEKKDQKKIYALDSKPNFKNAHYFQDF
LHLKTDVDFVLYVYAKWDADSNNLITVFREVARIIGENKIKIDFYTFNIDNAKELCNFMN
IKTLPVILYVSSVHKKKYNSLLYKALSSSKDVKISNAFRYNGDMYCYDYIVEWIEAHHYF
TRAVMLAKKLFQWRKK

>P_chabaudi_ortholog
MKILIFVLFLIFIKNIICEIRELSFHEFERMLTTNNYHQNKNYILENKINYKKSHAINDY
LYLNTDNDFVLYLYAKWDADSNNLITVFREVARIIEEKNLKIPFYTFNVDNAKEFCNSIN
VTSLPLILYVSSVHKKKYGSLLQKTLISSKDIKIGNAFRYGGDMYCYDYIVEWVEVHHYF
SKALLFMKRIFMKRV-

>P_yoelii_ ortholog
MKISIFVLFLIFIKNIICEIRELSFHEFERMLTTKNYHQNKNYILENKINYKKSHTIKDY
LYLNTDNDFXLYLYAKWDADSSNLITVFREVARIIEEKNLKIPFYTFNVDNAKEFCNSIN
VTSLPLILYVSSVHKKKYDSLLQKIVNSSKDIKIGNAFRXXXXXXXXXXXXXXXXXXXXX
XXXXXXXXXXXXXXXX

>P_berghei_ortholog
MKISILVLFLIFIKNIICEIRELSFHEFERML-TKNYHQNKNYILENKINYKKSHTIKDY
LYLNTDNDFVLYLYAKWNADSNNLITVFREVARIIEEKNLKIPFYTFNVDNAKEFCNLIN
VTSLPLILYVSSVHKKKYDSLLQKIVNSSKDIKIGNAFRYSGDMYCYDYIVDWVEVHHYF
AKALLFMKRIFMKRI-
```

▪ AMOUNT OF MISSING DATA

P. reichenowi	***P. gallinaceum***	***P. knowlesi***	***P. vivax***	***P. chabaudi***	***P. berghei***	***P. yoelii***
3%	2%	2%	2%	2%	2%	20%

■ PF11_0212

▪ PHYLOGENY AND PEXEL/VTS

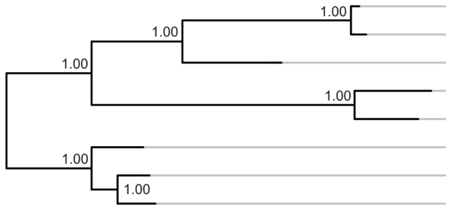

P. falciparum PF11_0212	NKFLKRQLCSNELKH
P. reichenowi ortholog	NKFIKRKLCDNELTH
P. gallinaceum ortholog	XXXXXXXXXXXXXXX
P. knowlesi ortholog	NALSTFKKVFDRLQM
P. vivax ortholog	NALSKFERVLDKLQM
P. berghei ortholog	NALSTFEKGFNKLQM
P. chabaudi ortholog	XXXXXXXXXXXXXXX
P .yoelii ortholog	CTLLATKKKHHHAKL

▪ ALI GNM ENT

>P_f alci paru

```
m_PF11_0212
MN--------------IFGLLLLNLLINVIFILCNIKRKNKFLKRQLCSNELKHLKYKND
NMIRKGILNYKYKYKYRFFYIIIKKNYSNKIKRKNKIKQIGVMENINNINDNNKWRCLSS
SNHISSNMMGN-------TFYENVYINYLKDYIIDEKE----------KAMEKNIVKDEE
QKKDKIYFPNSNNMLNAKDIENVIHERIRKEEENLFEFLKSVNERYNLNCTLRVVGGWVR
DKFLNINNDDIDITVDNMKGAEFCNYIKEYIKEKENKNFNFGIIKINSDQSKHLETSSFN
LFNFQVDIVNLRNEKYTEESRIPEIVIGTPEEDALRRDFTVNSLFYNLKNKKVEDYTEKG
IFHLKNHIISTPLEPLATFLDDPLRIVRCIRFCGFFNFFLEKSIFNVLKNEDIKKAFTKK
ISKSRLSSEIVKIFSAKCKNVILSLTLLNYSSYSSKIFQLPSNYFVKDEELFEKLKKKD-
-KINKGIVTPAQHYHTNDSVHDQHVDNLNDLNCVSSNNNINNKKIHLQNVENSNICNNSS
NIISSNDLIQNKVNNVS-----------------EQSDINKEDENNQCNNVMNVQNNDKQ
------------------------NFEQKWLFDGLSYLKFFKEIEKNNLLKETFNNLDYK
ENMNYIQLCLFLLPLKNHFLYIKNG-KTEYVVEYIIRESLKFPLKYSKFCVHIFEGFTHL
YNLYKTIDVLNFLKNKNYQEEHNIQIKGQTVLCLKKIGDKWNFVFLIFYIFHKFNELNKN
YITSITTNNICLSDFVAKLYRYIFKFNLQESYNMKPFLKWPDIKHNFPNISPNQINEVYE
QIIKFMAIHGENEKECIEYLKQHFT—K

>P_reichenowi_ortholog
XXXXXXXXXXXXXXXXXXXXXXXXXXXXXXXXXXXXXRLNKFIKRKLCDNELTHLKYEYD
NMIRKGILNYKYKYRYRFFYIIIKKNYSNKIKRKNKIKQIGMMENINNINDNNKWRCLSS
SNNISSNMMGN-------TFYENVYINYLKDYIIDEKE----------KAMEKNIAKDEV
QKKDRIYFPNSNNMLNVKDIENVIHERIRKEEENLFEFLKSVNERYNLNCTLRVVGGWVR
DKFLNITNDDIDITVDNMKGAEFCNYIKEYIKEKENKNFNFGIIKINSDQSKHLETSSFN
LFNFQVDIVNLRNEKYTEESRIPEIVIGTPEEDALRRDFTVNSLFYNLKNKKGEDYTGKG
IFHLKNHIISTPLEPLATFLDDPLRIVRCIRFCGFFNFFLEKSIFXXXKNEDIKKAFTKK
ISKSRLSSEIVKIFSSKCRNVILSLTLLNYSSYSSKIFQLPSNYFVKDEELFEKLKKKD-
-KINKAIVTPAQHYHTNDSVDDQHVDNLNDINYVSSNNNINNKKIHIQNVENSNICNNSS
NIISSNDLIQNKVNNVS-----------------EQSDINKEDKNNQCN---NVQNNHKQ
------------------------NFEEKWLFDGLSYLKFFKEIEKNNLLKETFNNLDYK
ENINYIQLCLFLLPLKNHFLYIKNG-KT--VVEYIIRESLKFPLKYSKFCVHIFEGFTHL
YNLYKTIDVLNFLKNKNYQEEHNIQIKGQTVLCLKKIGDKWNFVFLIFYIFHKFNELNKN
YITSITTNNICLSDFVAKLYQYIFKFNLQESYNMKPFLKWPDIKHNFPNISPNQINEVYE
QIVRXXXXXXXXXXXXXXXXXXXXXXXX

>P_gallinaceum_ortholog
XXXXXXXXXXXXXXXXXXXXXXXXXXXXXXXXXXXXXXXXXXXXXXXXXXXXXXXXXXXX
XXXXXXXXXXXXXXXXXXXXXXXXXXXXXXXXXXXXXXXXXXXXXXXXXXXXXXXXXXXX
XXXXXXXXXXXXXXXXXXXXXXXXXXXXXXXXXXXXXXXXXXXXXXXXXXXXXXXXXXXX
XXXXXXXXXXXXXXXNIKEIENLISTTIRKEEEDLFEFLTNVNEKYNLKSTLRVVGGWVR
DKFLRISNDDIDITVDNMKGADFCNYIKEYIKEKENKNFNFGIIKINSDQSKHLETSSFN
LFNFQVDIVNLRNEKYSEESRIPEIVIGTPEEDALRRDFTVNSLFYNLKNKKVEDYTKKG
IFHLKNKIICTPLDPLSTFLDDPLRIIRCIRFCGSFDFCLEKSIFNVLKNEDIKNSFSKK
ISKSRLSSEITKIFSEKCKNIVLCLTLLNYSSYSSKIFKLPLEYFIKDQENLKKNKKKD-
-KINKSIXXXXXXXXXXXXXXXXXXXXXXXXXXXXXXXXXXXXXXXXXXXXXXXXXXXXX
XXXXXXXXXXXXXXXXXXXXXXXXXXXXXXXXXXXXXXXXXXXXXXXXXXXXXXXXXXXX
XXXXXXXXXXXXXXXXXX-----------WLFYGLNYVKFFKEIE-HNLTKEVFHDLQYK
ENVNYIYICLFLLPLKNHFYYIKNS-KTEYVVEYIIRESLKLPLKYSKFCVNIFEGFIHL
YHLYKNIDLLNFLKN-NIDQKNNLHIKSDIVLFLKKVGERWNIIFFIFYIFHKYNEINKN
YLA-FTINDIYPSDFVKKLYQYILENNLQNSYKIKPFLKWPDIKNNFPRISSNQINEIYE
QIIRFMCIHGENKKECIEFLKEHFK--K

>P_knowlesi_ortholog
MSSLGAPAQIPPLTLLLFLALFLLLVETTTVKGVGRNTSCTLLATKKKHHHAKLFFGWKN
NHMNLLGEKKIINCTRGLFFINANRRSHHLGGRSNIKRF--------------NLHRYST
KEKMQ----------------DEAYVSYLKKYVVDDKKLGQVAREVVPNGQEGNLAPD--
---------------GVLQIEELIKERIRKEEEELFAFLQKVNETYKLQSTLRVVGGWVR
DKFLGISNDDIDVTVDNMKGSEFCNYIKEYIKEKEKKNFNFGIIKINSDQSKHLETSSFH
LFNFQVDIVNLRNEKYTEESRIPEIVIGTPEEDALRRDFTVNALFYNLSSRRVEDYTKRG
ILHLKNKVICTPLDPYTTFLDDPLRIIRCIRFCGVFNFLVEKSIFDVLKNEHIMKAFKKK
ISRSRLSTEILKIFSKRCENIVLSLTLLNYSSYAKEIFSLPQNIFVKNEEVFEQPRRKGD
-KLNRKADHLPA----------------GDALLPGSTVPGSTKKLHVGGDT---------
-----NETTNKDENGVAP----------------QNGGIHHEEGHGQGTFVNGSSRGDSK
MDRMTNTLETGAYSNPEESPQTNGDGETDWCAEGLQYVKFFKDMEKNNLLRESFPGLDYH
ENLNNVQLCLYLLSLKNHHVHVKKGGKTEPVVDFIIRESLKFPLKHGKFCVQIYEGFSSL
YKLYKNMDVFNFLKNSTCDGNKNAHITGDIVLFLKSVGEKWDLLLLIFFIFHKYKEVNKM
FVT-TITADVYLSDFAKKLYQYILKNKLQTAYNVKPFLKWPDIRHNFPQLSPNRIGEVYE
QIIRFSCIHGENEAKCIEFLKDHFK—E

>P_vivax_ortholog
XXXXXXXXXXXXXXXXXXXXXXXXXXXXXXXXXXXXXXXXXXXXXXXXXXXXXXXXXXXX
XXXXXXXXXXXXXXXXXXXXXXXXXXXXXXXXXXXXXXXXXXXXXXXXXXXXXXXXXXXX
XXXXXXXXXXXXXXXXXXXXXEEAYLSYLHKYVVDDRELARS--GGVSKGQQGHPPPD--
---------------GALQIEELITKTMRKEEEELFAFLQKVNETYKLQCTLRVVGGWVR
DKFLGISNDDIDLTVDNMKGSEFCNYIKEYIQEKEKKNFNFGIIKINSDQSKHLETSSFN
LFNFQVDVVNLRNEKYTEDSRIPEIIIGTPEEDALRRDFTVNALFYNLNNRRVEDYTRRG
ILHLKNKIICTPLDPLSTFLDDPLRIIRCIRFCGVFNFFLEKSIFDVLKNEQILKAFKKK
ISRSRLSSEIVKIFSQRCANIVLSLTLLNYSSYAKEIFSLPGNYFVKDEEVFERPRRKGD
-KVNKKGDP-PA----------------GGGLLPGSTAPGGKKKLHVGGGDPQEVREEIN
HEETHNAPINDEANGATPNGSLDSTGMQNGNVRPANGGIHRGEGLAQGALANSPPRGDSK
VGGMTNALEAGGHFQPGESAQLSADGETDWCAEGLQYVKFFKDIEKNNLLRETFPELDYQ
ENVNNVQLCLYLLPLKNHHVYVKKGGKTEPVVSFIIRESLKFPLKHGKFCVQIYEGFSSL
YKLYKNMDVFNFLKNSTCDGKRNAHIIGDIVLFLKSVGEKWDLLLLIFFIFHKYKEVNKM
FAT-TITAEVYLSDFAKKLYQYILKNKLQAAYNVKPFLKWPDIRHNFPQLSPNRIAEVYE
```

```
QIIRFSSVHGEDEAKCIEFLKDHFK--K

>P_chabaudi_ortholog
MN---------------FFSVFNFYTILIIIIVCHKNKGNALSTFKKVFDRLQMFVGAKR
NYNIL-KKKNFLYYTKKAFYISPKNISNNKHQNFNVIKF--KRWHSCEEKYNKQFKIFTT
NNLMNNEKMT----------YDKEYLSYLKGYIINGKDLDLSENGSSICQAENNKKDGE-
---------------PIWDIETVIDKAVTQRELELFEFLMKVNEQYKLDTTLRVVGGWVR
DKFLNISNDDIDITVDNMKGSDFCNYIKEYIKDKENKNFNFGIIKINCDQSKHLETSSFN
LFNFQVDIVNLRNETYTEESRIPEIAIGTPEEDALRRDFTINSLFYNLKNKKVEDYTKNG
IHHLKNQIISTPLNPLTTFLDDPLRIIRCIRFCGYFDFNLDQSIFDVLKNEDIKKSFNKK
ISKSRLASEIIKIFSHKCKNIVLSLTLLNYSNYSEEIFKLPSDYFIQNEEVFEKMKKKKN
HKIEDH-WY--------------------------ETNGVVNKRVHLFEDSKE---IDKN
LDKSIDEYVGHKDSSK------------------DDNKIESLENLK-----------DDR
------------------------NIENDWLFEGLNYVKFFKDIEKSELLKQILCDLDYK
ENINYIHMCLFLLPLKNYYIYIKKGVKTEYVIEYIIRESLKFPLKYSKFCVNIYEGFTHL
YKLYKNINVLEFLKDNN-DKKTNLNIKGDVVIFLKNVGDKWDLLILIFYIFHKYNELNKN
FIN-IITNDIYLSDFAIKLYQYILKNGIQKSYNIKPFLKWPNIKHHFPNIASNRINEVYE
QIIHFTCIHGEKEEECIEFLKKHFNEPE

>P_yoelii_ ortholog
MN---------------FFSVFILYIILIIIIICHKNKGNALSKFERVLDKLQMLIRVKR
TYNIF-KKNSFLNYTKKVFYISPKTI-NNKNINLNITKL--KKWNNFNGKYNKQFKIFTT
YNKMGDEKMGDEKMGDEKMIYDKEYMSYLKKYIINEKDLEQSETGNLIYQKEDDNKNDEQ
NNTNNNNN-------NIYDFEKMIDKAITHRELELFEFLIKINETYKLNTTLRVVGGWVR
DKFLNISNDDIDVTVDNMKGSDFCNYIKEYIKDKENKNFNFGIIKINCDQSKHLETSSFN
LFNFQVDIVNLRNEKYTEESRIPEISIGTVEEDALRRDFTINSLFYNLKNKKIEDYTKNG
IFHLRNXIISTPLNPLTTFLDDPLRIIRCIRFCGYFDFNLNKSIFDVLKNEDIKKSFNKK
ISKSRLASEIIKIFSHKCKNIVLSLTLLNYSNYSKEIFKLPTDYFVQNDEIFEKMKKKKN
HKIDNHSYY--------------------------DTNEMVNKRVHLFEDSKE---VHQN
VEKSISEYINDEDNNR---------------TYLNKNGDRNGENAKSQNDDKNDDKNDDK
------------------------NIENDWLFEGLNYVKFFKDVEKSELLKQIFYDLNYK
ENINYIHMCLFLLPLKNYYIYIKKNIKTEYVIEYIIRESLKFPLKYSKFCVNIYEGFTYL
YKLYKNINVLELLKDDN-DKKTDLNIKGDVVIFLKNVGDKWDLLILIFYIFHKYNELNKN
FIN-IITNDIYLSDFVIKLYQYILKNDIQKSYNIKPFLKWPNIKHHFPNINPNRINEIYE
KIIQFTCIHGEKEEECIEFLKNHFNNPE

>P_berghei_ortholog
MN---------------FFPVFILYITLIIIIICHKNKGNALSTFEKGFNKLQMFVRVKR
NHHILKKKKNFLNYPKNVFYISPKTI-SNKNRNLNIIKF--KRWIICDEKYNKQFKLFTA
YNKMA---------------YDNEYMFYLKKYIINEKELELSGTGNSIYQKEDDDDDDDN
NKNDEQNNTNK----YIYDIEKMIDKAITHRELELFEFLMKINETYKLNTTLRVVGGWVR
DKFLNISNDDIDITVDNMKGSDFCNYIKEYIKDKENKNFNFGIIKINCDQSKHLETSSFN
LFNFQVDIVNLRNEKYTEESRIPEISIGTVEEDALRRDFTINSLFYNLKNKKVEDYTKNG
IYHLRNKIISTPLNPLTTFLDDPLRIIRCIRVCGYFDFNLDQSIFDVLKNVDIKKSFNKK
ISKSRLASEIIKIFSHKCKNIVLSLTLLNYSNYSEEIFKLPRNYFVQNDEIFEKMKKKKS
HKIENH-WY--------------------------DIVEMENKRVHLLENSKE---VHKN
MEKSINEYVNNKDNSSI--------------TYLNKS-----ENLKNQ--------NDDK
------------------------NIENDWLFEGLNYVKFFKGIEKSKLLKQIFYDLNYK
ENINYIHMCLFLLPLKNYYIYIKKNIKTEYVIEYIIRESLKFPLKYSKFCVNIYEGFTYL
YKLYKNINVLKFLKDDN-DKKTDLNIKGDVVIFLKNVGDKWDLLILIFYIFHKYNELNKN
FIN-IITNDIYLSDFAIKLYQYILKNDIQKSYNIKPFLKWPNIKHHFPNITPNRINEIYE
KIIQFTCIHGEKEEECIEFLKNHFNNPE
```

- **AMOUNT OF MISSING DATA**

P. reichenowi	***P. gallinaceum***	***P. knowlesi***	***P. vivax***	***P. chabaudi***	***P. berghei***	***P. yoelii***
6%	33%	14%	24%	10%	9%	8%

■ PF13_0090

▪ PHYLOGENY AND PEXEL/VTS

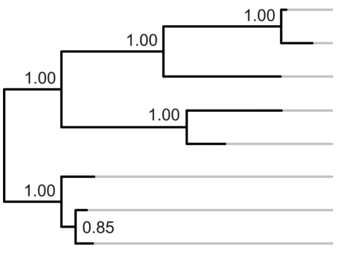

P. falciparum PF13_0090	RDDFIRILYEFRFIY
P. reichenowi ortholog	RDDFIRILYEFRFIY
P. gallinaceum ortholog	RDDFIRIIYDFRFIY
P. knowlesi ortholog	REEFIRIIYDFRFVY
P. vivax ortholog	REEFIRIIYDFRFVY
P. chabaudi ortholog	REDFIRILYEFRFIY
P. berghei ortholog	REDFIRIIYDFRFIY
P .yoelii ortholog	REDFIRIIYDFRFIY

▪ ALIGNMENT

```
>P_falciparum_PF13_0090
MN-------------VLDVLKRWFI------LVFFMLQRFFEFKSKITKKSFIIFGLPSS
GKTSIIYFFKLGYLITTVSTLFINEENFKINLKIEKDDTKEQNYDITFYEVGKNCSYNLI
KEYSDISNDVIYIVDSVQKGNLSEARDDFIRILYEFRFIYRKCKFLIFMNKQDSNGCLSS
QEIINFFALPKDLLIRCNFISCSTLSGQGLKEGLEWLL-YYNLPFYNNELNCID-RRTAT
KHLEL--

>P_reichenowi_ortholog
XXXXXXXXXXXXXXXXXXXXXXXXXXXXXXXXXXXXXXXXXXXXXXXXXXXXXXXXXXXX
XXXXIIYFFFIGYLITSIDTLFINEETFKINLKIEKDDTKDLNYDITFYEVGKNCSYNLI
KEYSDISNDVIYIVDSVQKGNLSEARDDFIRILYEFRFIYRKCKFLIFMNKQDSNGCLSS
QEIINFFALPKDLLIRCNFISCSTLSGQGLKEGLEWLL-YYNLPFYNNELNCID-RRTAT
KHLEL—

>P_gallinaceum_ortholog
MN-------------IFEVLKKCSQ------FVFFMFLRFFSLKP--TRKNLIIFGLPSS
GKTSIIYFFKLGYLITTVRTFLINEECFTITLKTDKDNFEEKKFKVKFFEVGSDCSFNLI
KEYSEISNDLIYIVDSSQKGSLREARDDFIRIIYDFRFIYRRCKFLIFLNKQDSNGCLKS
EEIINYFSLPKELLFRCKFISCSTLSGEGLKEGLDWLLFYYNLPVYSHELNTLDRRRTAR
KHLTL—

>P_knowlesi_ortholog
MYEQKYVPARLQGNEVFPYFTKKFIYLFIYLFFLFILYRLFSLKDP-TKKKFIIFGLPSS
GKTSIIYFFKLGYLITT--NTFHQRRKFQRENQNRQNTLGERNYEVTFFEVGTDCSYSLI
KEYADISNDMIYIVDSTHRSALSEAREEFIRIIYDFRFVYRKCKFLIFMNKQDSNGCLPS
DEIINYFALPKELHFRCKFFSCSTLSGQGLKEGLEWLV-STNV-FFDTNDDAVE--RSER
TFYNY—

>P_vivax_ortholog
MYEQKYLPAQMEGNELFPSFTKKXXXXXXXXXXXXMLYRLLSLKDP-TKKKFIIFGLPFS
GKTSIIYFFKLGYLITTVRTLFINEESFSVKIKTDENSLDERNYEVTFFEVGTDCSYSLI
KEYADVSNDVIYIVDSAHKSALSEAREEFIRIIYDFRFVYRKCKFLIFMNKQDSNGCLPS
EEIISYFALPNELRFRCKFFSCSTLSGQGLKEGLEWLV-NTNV-FVDKNDDAVE--RSGR
TFYNY—

>P_chabaudi_ortholog
MN-------------IFEFIKKHFM------FIFFMIYRFFNIKYP-IKKKFIIFGLPSS
GKTSXXXXXXXXXXXXXVKTYFINEEKFTLKINRDKNHNEEKNYEINFYEIGHNCSYNLI
KEYADISDDVIYIIDSIHKDKLCECREDFIRILYEFRFIYRKCKFLIFMNKQDSNGCLKP
EEIINYFALPNELQYRCKFIPSSTLSGQGLNEGLEWLL-NYNI--FSEKEDIMERRK---
KLYDY—

>P_yoelii_ ortholog
XXXXXXXXXXXXXXXXXXXXXXXXXXXXXXXXXXXXXXXXXXXXXXXXXXXXXXXXXXXX
XXXXXXXXXXXXXXXXXVKTYFINEEKFTLKIKNDKNHDEEKNYEINFYEIGHNCSYNLI
KEYADISDDIIYIVDSIRKDKLCECREDFIRIIYDFRFIYRKCKFLIFMNKQDSNGCIKP
EEIINYFALPNELQYRCKFISSSTLSGQGLNEGLEWLL-NYNV--FSEKEEIIERRK---
RLYDY—

>P_berghei_ortholog
XXXXXXXXXXXXXXXXXXXXXXXXXXXXXXXXXXXXXXXXXXXXXXXXXXXXXXXXXXXX
XXXXXXXXXXXXXXXXXVKTYFINEEKFTLKIKNDKNHNEEKNYEINFYEIGHNCSYNLV
KEYADISDDIIYIIDSIRKDKLCECREDFIRIIYDFRFIYRKCKFLIFMNKQDSNGCLKP
EEIINYFALPNELQYRCKFISSSTLSGQGLNEGLEWLL-NYNV--FSEKEEIIEQRK---
KLYDY—
```

▪ AMOUNT OF MISSING DATA

P. reichenowi	***P. gallinaceum***	***P. knowlesi***	***P. vivax***	***P. chabaudi***	***P. berghei***	***P. yoelii***
20%	1%	9%	9%	8%	28%	28%

■ PF10_0321

▪ PHYLOGENY AND PEXEL/VTS

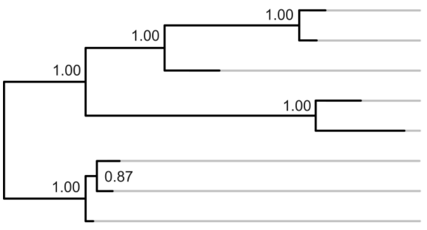

P. falciparum PF10_0321	RSQELRILSENCCVK
P. reichenowi ortholog	RSQELRILSENCCVK
P. gallinaceum ortholog	KTQELRLLSENCCVK
P. knowlesi ortholog	KSQELRVLSENCSNK
P. vivax ortholog	KSQQLRVLSENCCNK
P. chabaudi ortholog	KAQELRKLSENCCIK
P. berghei ortholog	KAQELRRLSESCCIK
P .yoelii ortholog	KAQELRKLSENCCIK

▪ ALIGNMENT

```
>P_falciparum_PF10_0321
MLKHVFFCY-----RWRAEYGWV--KSRLNFLCGASCYSSNINMDKIIDENKYEYIKNLR
SQELRILSENCCVKKVDDVIIWSEICRHSIEKYNSFKYFDALLLLSSFDKMNIVDKSLYK
TFSDVFIKQISYLKPEHFILLINLYCRVNIFPRVLFTEIFHGIIKYCNKLYPDEYVNLLT
CFANLKIANKDLIKTLCKSIIKNINLFDYIHLTNIVGALRSLEITDDIFYYVIDQKQLKE
LKFLTVQEIFDFIKKIKLLQYSWKLYEQDLMKEFLFKVYNFKNEKDVDQLDDPFVCLNFL
VSKGLLQGNNNTNIKKKNNNKKNNNNKKNNNNKKNNNNNNDNNNDNNNNDNNNNNDNNNN
NDNNNNNSSSDFIDDINCGGANFLVALSKWCANQVYHYPSRSTKRPTSYQLIKLYELMKE
FNIHNSDFIEKAIYRFVITRGGLENNRDKMFKPTSYQKGR-KYIFTKDPQ----------
-------IDHINY-----EKNKEHSYSHHYNLKDHTTDYEQQNMHYQTYDYNDEHNMNQQ
KTLSEKHKRIKLSMEKKTQNKK-ETHSNSRYCNFKLRQRPKRIKNSPAPIKV

>P_reichenowi_ortholog
MLKHVFFCY-----RWRAQYGCG--KSKLNFLCGALCYSSNINMDKIIHENKYEDIKNLR
SQELRILSENCCVKKVDDVIIWSEICRNSIEKYNSFKYFDALLLLSSFDKMNIVDKSLYK
TFSDVFIKQISYLQPEHFILLINLYCRVNIFPRVLFTEIFHGIIKYCNKLYPDEYVNLLT
CFANLKITNKDIIKTLCKSIIKNINLFDYIHLTSIVGALRSLEIADDIFYYVIDQKQLKE
LKFLTVQEIFDHIKKIKLLQYSWKLYEKDLMKEFLFKVYNFKNEKDVDQLDDPFVCLNFL
VSRGLLQGNNNTNGK--------------------NNNNNNNKKNNNNDDDNNNNDNNNN
DNNNNNSSSSDYIDDINCGGANFLVALSKWCANQVYHYPSRSTKRPTSYQLIKLYELMKE
FNIHNSDFIEKAIYRFVITRGGLENNRDKMFKPTSYQKGRKKYIFTKDPQ----------
-------IDHINY-----EKNKENSYSHYYNLTDHTTNYEQKNMHDQTNDYNDEHNMNQQ
KTLSEKLKXXXXXXXXXXXXXXXXXXXXXXXXXXXXXXXXXXXXXXXXXXXX

>P_gallinaceum_ortholog
XXXXXXXXXXXXXXXXXXXXXXXXXXXXXXXXXXXXXXXXXXXXXXXXXXXXXXXIKNLK
TQELRLLSENCCVKKINNVIIWSEISRNAIEKCNNFKYFDALLLLSSFDKMNILDKNLYK
SFSDVFIKQINYLEPRHFILLINLYCKADIFPRVLFIQVFHSIIKYSSKLYPDEYVDLLI
CFANLKIVNKDLIKTLCKSIIKNINLFDYLHLCSIVGCLRSLDISDDVFYYVLDEKQLKE
LKFLTVQELYDNLKKIKLLTYSWEIYEKDLIKEFLFKQKNFKNEKDXXXLDDNIKCLNXX
XXXXXXXXXXXXXXXXXXXXXXXXXXXXXXXXXXXXXXXXXXXXXXXXXXXXXXXXXXXX
DNNNNNSSSSDYIDDINCGGANFLIALSKWCANQVYEYPSRSAKRPLAFQLIKLYELMEE
KKVENYDFIEKAIYRFVISRGGLENNRDKMVKPVSYQKGR-KYIFTKDPXXXXXXXXXXX
XXXXXXXXXXXXXXXXXXXXXXXXXXXXXXXXXXXXXXXXXXXXXXXXXXXXXXXXXXXX
XXXXXXXXXXXXXXXXXXXXXXXXXXXXXXXXXXXXXXXXXXXXXXXXXXXX

>P_knowlesi_ortholog
MIRWAKSIVGVKPKGWRVSRKWVPLPTEQRRLFGSSTPPDEVT-RKIIIESNYGHIKQMK
SQELRVLSENCSNKKIRDVVLWSEISRNAINRCDEFKYFDALLLLSSFEKMNLADDNLYK
TFSHVFVKQINHMEPRDLILLINLYCRANLFPRVLFVQVFHAIVRYCSKFYPEEYVDLLI
CFASLNISNVDLIRTLCKSMVKNVNLFDYSNLCCIVGCLRRLDVNDDVIYYVFDEKQQKE
LKLLTTQELFDLMKKVKLLKYSWELYEKDLLEEFLNRIDNFQGVKDVNQLEDPFVCLNFL
VSK---------------------------------------------------------
----------------QCVRNNFLFALSKWCASHVYEYPSRSAKRPLAYQLIMLYQLMKE
HDVKNYNFIEKAIRRFVISRGGLAVNRDKMIKPVSYQKGR-KYIFAEDPL----------
EEARTAEVDASSD-----HTLDQPTGHDSYVQEGISHRMSQSDGY---TSPHSYADTEEP
NPMTEKKRSIMLSLGKTTECKKPSMYSRGRHFNFKLRQRPKCVKNAPVPVET

>P_vivax_ortholog
MIGWAKSIVGAKPNGWRAPRRWLPTGGERRRLFGSSSPTDELT-KKIIIESNYGHIKQLK
SQQLRVLSENCCNKKIRDVVLWSEISRSAINRCDEFKYFDAVLLLSSFEKMNMADDHLYK
TFAHVFVKQINHMEPRHLILLINLYCKANLFPRVLFVQVFHAIVRYCSKFYPEEYTDLLI
CFASLNISNGDLMKTLCKSMVQNVTLFDYNHLCCIVGCLRRLGVQDDVIYYVLDEKQQKE
LKLMTTQELFDCMKKVKLLKYSWELYEKDLLEEFKNRIAKFQGEKDVNQLEDPFVCLNFL
VSK---------------------------------------------------------
----------------QHVTKNFLIALSKWCASHVYEYPSRSAKRPLAYQLIMLYQLMKE
HDVNNYHFIEKAIHRFVVSRGGLAVNRDKMAKPVSYQRGR-KYVFARDPLEGGGTAEVGA
TEVGAAHVDASSD-----RLADQPTGEDFYLHEGIAHRMGEDDGYDGHTPPQRYAHPEEP
PPMTEKQRAIALSLGEGTERKKPSTHSHGRHFNFKLRQRPKRVKNAAVPVDM

>P_chabaudi_ortholog
MYAPFVTST---------------------------------TNDNIILKRNYEEIKNLK
AQELRKLSENCCIKKISDVIIWSEICRNAIKKSDEFKHFDALLLLSCFDKMNLLDKSLYT
HFSDIFIKHINNFEPRHLILLINLYCKVNIFPRILFIEVFHAIIRYSPKLYPNEYVDIFE
CFAKYEIANKDLISTLCKSIIKNINLFGYTDLCSIVGSLRSLKINDDVFFYCIDEKQLKE
LKMSTCQELFDYINKIKLLKYSWELYEKDLIKEFLNRINDFKNGNDINQLHDPFICLNYL
ISK---------------------------------------------------------
----------------NIIXNNFLLTLSMWCANQVYQYPSRSAKRPLSYQLIKLYQIMKE
HNVENFDFIEKAIHKFVISRGGLATNREKITKPVSYQKGR-KYIFAPDPL-------NAD
SEDTNKYIDSSNEEET--HQNEQLAYSNMYQGNDNDFDSIEQDSYNENANEHIKHNSN--
-ILTQKQRLINLSLEKKTENKK-ETYSNSRHCNFKLRQRPKRIKNQPISDQK

>P_yoelii_ ortholog
MCAPFVTCT---------------------------------TNDNIILKRNYEEIKNLK
AQELRKLSENCCIKKISDVIIWSEICRNAIKKSDEFKHFDALLLLSCFDKMNLLDKNLYT
HFSDVFIKNINNFEPRHLILLINLYCKVNIFPRILFIEVFHAIIRYSSKLYPSEYVDIFE
CFAKYEIANKDLISTLCKSIVKNINLFDYTDLCSIVGALRSLKINDDVFFYCIDEKQLKE
```

```
LKMSTCQELFDYINKIKLLKYSWELYEKDLIKEFLNRINDFKNGNDINQLHDPFICLNYL
ISK---------------------------------------------------------
----------------NIISNNFLLTLSMWCANQVYQYPSRSAKRPLSYQLIKLYQIMKE
HNVENFDFIEKAIHKFVISRGGLATNREKITKPVSYQKGR-KYIFTPDPL-------NTY
SEDTNKYIDSSNKTENIINKNEQLDYSNMYQENDKDLDIIETESYNDNTNEHIKYNSN--
-ILTQKQRLINLSLEKKTENKK-ETYSNSRHCNFKLRQRPKRIKNQPIPDQK

>P_berghei_ortholog
MYAHFVTCT---------------------------------TNDNIILKRNYEEIKNLK
AQELRRLSESCCIKKISDVIIWSEICRNAIKKSDEFKHFDALLLLSCFDKMNLLDKILYT
HFSDIFIKNINNFEPRHLILLINLYCKVNIFPRILF-EVFHAIIRYSSKLYPNEYVDIFE
CFAKYEIANKDLISTLCKSIVKNINLFGYTDLCSIVGALRSLKINDDVFFYCIDEKQLKE
LKMSTCQELFDYINKIKLLKYSWELYEKDLIKEFLNRINDFKNGNDVNQLHDPFICLNYL
ISK---------------------------------------------------------
----------------NIISNNFLLTLSMWCANQVYQYPSRSAKRPLSYQLIKLYQIMKE
HNVENYDFIEKAIHKFVISRGGLATNREKITKPVSYQKGR-KYIFTPDPL-------NAY
SEDTNKYIDSSNKTENIPNKNEQLAYSNMYQGNDKDFDLTETYSYNENANDHIKYNPN--
-ILTQKQKLINLSLEKKTENKK-ETYSNSRHCNFKLRQRPKRIKNQPIQDQK
```

- **AMOUNT OF MISSING DATA**

P. reichenowi	*P. gallinaceum*	*P. knowlesi*	*P. vivax*	*P. chabaudi*	*P. berghei*	*P. yoelii*
11%	37%	15%	16%	20%	20%	20%

■ PF14_0614

▪ PHYLOGENY AND PEXEL/VTS

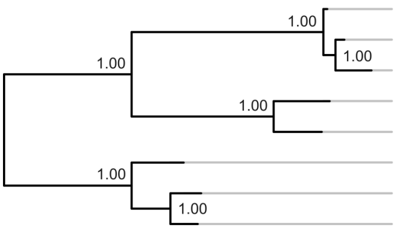

P. falciparum PF14_0614	IIKNERILDEYENIN
P. reichenowi ortholog	IIKNERILDEYENIN
P. gallinaceum ortholog	IIKNERILDEYENIN
P. knowlesi ortholog	LLR--RIIKEESGTK
P. vivax ortholog	LLR--RIIKEENGAK
P. chabaudi ortholog	LFK--RLMQEQNDNT
P. berghei ortholog	LFK--RVLQEQNEDI
P .yoelii ortholog	LFK--RVLQEQNEDI

▪ ALIGNMENT

```
>P_falciparum_PF14_0614
MNILNLFICCLSITLLKF-IEWEQYNY-DCVVAINKKNS-----IIKNERILDEYENIN-
-------NSENEEDE---------YEDYLDDKGSNEFEQVNYKYLDYVNFTIVKSQSTYQ
HAVNELFVFLGSQYSTDEKIVVHVKLIDILSLLFVHYRDNLSNFEHIINSFQDRNKLMNS
VEGEYFREFIDERDNYIFDVKNTYYNSQYVNKE-KETILNKKKTIYEIFQKNWKTDGYRF
YSVKNKKKYYYPRKINLDEPRKRKHKKKKKQKNIKCVNMVCKPLKIEYKSLNKPVNSPVD
DNTDVKTMKGEQHIKGQAEDKN-HVVQGIENVQQEKKEIMDRIDIQNEIQNQVQNDIENE
IEYELK-----------------------NDDNNNNIEESD----------------VMK
SYKNQICNADENRTSNV-GYVRENSIGPGIAYRMRKDFFLKDSSFNVITLINSITSNRDN
KVVTKLHEGLTKLGITTIEHLIRYTNILAIFFSYDIFDELYLQIKLVKEYFGLIKYKPNI
LDTEE--NEYVVGKKTFYGKYNMIDDEVFVSPNCLSAYCKLKSVWMQNRNITVKVEKSTT
NSLKIMMLGDIGQGFEEEKNFDVQNIYNFMGFNELKSTVQSMKKWHLENNADFVINLGDN
IPNDGSYNFIGNFQWHRLMRELFVFKKKSEKQVHKDLGTNTLSAESIANFYNDKIKEMNE
GNYEN----------HINYYNDKNLHKIKAQNENNEKENLNDNSIKMNNAKGTSSKINDL
QNVSNTNPYKIEVVDHTKLVESNTSINEKEEKSISSVE-ERANLYEDDEENDEEEDSNEF
ASEAIPFYSVLGEKDYFYFPSEQIQEHYSKRIPGYFMPNNYYCVNYDFTYNPVKKNVNGD
EDDDDDEEEEEGDSGTDRKVKTQEKFRASFIFIDTWALMVGFPIIRNYRAFREQFNWLSK
TLYESAKKSDWIFVVGHHPLISSGRRSDNYSYEEHSFHDIIRDFLFNYHVDAYFSAHDHL
MEYIKFGSVDLFINGSSSRVLFDNSSM-GRGYFGKIIGKLYPLSCYVLKTIHTGLKPKGC
NINRYSKWYNKSDIGFSTHKLTKDELVTQFISSRTGKPLSEKIITKNKKHERKKFYDLDG
FAEDRIKELEKKIIDFSVNNPDLINYKIQEFNENIEKLNLIIKKLKTKEEKEIFKELIQM
NNLIFDVSDHLDNVPIEKLKIMSELVSKYNIFFNKELAGFIVAALERAIQ-ME-------
-------RKKPHSSDENGSLND---EDKNLIELIESLGYQPEEFLQKYESMTSEQKVALK
NKIGRNISLEDYVNRIKFYVEKKKKEKE-KNGNEQEEAEEETEVVEEIDELKEMEKKRKE
SEGDITGEDENEIKEEQKDNEKEDETYEEYLDESQYNDEEIPLV----KQVHKDFKKLAN
QEKKLSEQKYILLMLASMRKFDIKKYALNLSTKKERIKDVTTSNYLSNIEPRKTFFQLCI
ELPPDIKRIINNFGGVGKRLPFFNFINKLYDEIIKLKDSLNRISR-

>P_reichenowi_ortholog
MNILNLFICCLSITLLKY-IEWEQYNY-DCVVAINKKNS-----IIKNERILDEYENIN-
-------NSENEEDE---------YEDYLDDKGSNEFEQVNYKYLDYVNFTIIKSQSTYQ
HAVNELFVFLGSQYSTDEKIVIHVKLIDILSLLFVHYRDNLSNFEHIINSFQDRNKLMNS
VEGEYFREFIDERDNYIFDVKNTYYNSQYVNKE-KETILNKKKTIYEIFQKNWKTDGYRF
YSVKNKKKYYYPRKINLDEPRKRKNKKKKKQKNIKCVNMVCKPLKIEYKSLNKPVNSPVD
DNTDVKTMKGEQHINGQAEDKN-DVVQGIENVQQEKKEIMDRFDIQNEIQNQVQNDIENE
IEYELK-----------------------NDDSNNNIEESD----------------VMX
SYXNXICNADENRX----XXXXXNSIGPRIAYRMRXDFFLXXISFNVITPYNSITSNRDN
KGVTKLHEGLTKLGITTIEHLIKYPTILAIFFSYDIFDEWYLQIKLVKEYFGLIKYNINI
LDTEE--NEYVVGKKTFMGKYNMIDDEVFVSPNCLSVYCKLKSVWMQNRNITVKVEKSTT
NSLKIMMLGDIGQGFEEEKNFDVQNIYNFMGFNELKSTVQSMKKWHLENNADFVINLGDN
IPNDGSYNFIGNFQWHQLMRELFVFKKKSEKQVHKDLGTNTLSAESIANFYNDKIKEMNE
GNYEN----------HINYYNDKNLHKIKAQNENNENKNLNDNSIKMNNAKGTSSKISDL
QNVSNTNPYKIEVVDHTKLVESNTSINEKEEKSISSVE-ERANLYEDDEESDEEEDSNEF
ASEAIPFYSVLGEKDYFYFPSEQIQEHYSKRIPGYFMPNNYYCVNYDFTYNPVKKNVNGD
EDDDDDEEEEERDSGKDRKVKTQEKFRASFIFIDTWALMVGFPIIRNYRAFREQFNWLSK
TLYESAKKSDWIFVVGHHPLISSGRRSDNYSYEEHSFHDIIRDFLFNYHVDAYFSAHDHL
MEYIKFGSVDLFINGSSSRVLFDNSSM-GRGYFGKIIGKLYPVSCYVLKTIHTGLKPKGC
NINRYSKWYNKSDIGFSTHKLTKDELVTQFISSRTGKPLSEKIITKNKKHERKKFYDLDG
FAEDRIKELEKKIIDFSVNNPDLINYKIQEFNENIEKLNLIIKKLKTKEEKEIFKELIQM
NNLIFDVSDHLDNVPIEKLKIMSQLVSKYNIFFNKELAGFIVAALERAIQ-MEG------
-------KKTHNSSDENGSLNE---EDKNLIELIESLGYQPEEFLQKYESMTSEQKVALK
NKIGRNISLEDYVNRIKFYVEKKKKEKEKKNGNEQ-EAEEETEVVEEIDELKEIEKKRKE
SEGDITDEDENEIKEDQKDNEKEDETYEEYLDESQYNDEEIPLV----KQVHKDFKKLAN
QEKKLSEQKYILLMLASMRKFDIKKYALNLSTKKERIKDVTTSNYLSNIEPRKTFFQLCI
ELPPDIKRIINNFSGVGKRLPFFNFINKLYDEIIKLKDSLNRISR-

>P_gallinaceum_ortholog
MNILNLFICCLSITLLKY-IEWEQYNY-DCVVAINKKNS-----IIKNERILDEYENIN-
-------NSENEEDE---------YEDYLDDKGSNEFEQVNYKYLDYVNFTIIKSQSTYQ
HAVNELFVFLGSQYSTDEKIVIHVKLIDILSLLFVHYRDNLSNFEHIINSFQDRNKLMNS
VEGEYFREFIDERDNYIFDVKNTYYNSQYVNKE-KETILNKKKTIYEIFQKNWKTDGYRF
YSVKNKKKYYYPRKINLDEPRKRKNKKKKKQKNIKCVNMVCKPLKIEYKSLNKPVNSPVD
DNTDVKTMKGEQHINGQAEDKN-DVVQGIENVQQEKKEIMDRFDIQNEIQNQVQNDIENE
IEYELK-----------------------NDDSNNNIEESD----------------VMX
SYXNXICNADENRAPNVXXXXXXNSIGPRIAYRMRXDFFLXXISFNVITPYNSITSNRDN
RVLLNYMKGLTKLGITTIEHLIKYPTILAIFFSYDIFDEWYLQIKLVKEYFGLIKYXXXX
XXXXXXXXXXXXXKKTFMGKYNMIDDEVFVSPNCLSVYCKLKSVWMQNRNITVKVEKSTT
NSLKIMMLGDIGQGFEEEKNFDVQNIYNFMGFNELKSTVQSMKKWHLENNADFVINLGDN
IPNDGSYNFIGNFQWHQLMRELFVFKKKSEKQVHKDLGTNTLSAESIANFYNDKIKEMNE
GNYEN----------HINYYNDKNLHKIKAQNENNENKNLNDNSIKMNNAKGTSSKISDL
QNVSNTNPYKIEVVDHTKLVESNTSINEKEEKSISSVE-ERANLYEDDEESDEEEDSNEF
ASEAIPFYSVLGEKDYFYFPSEQIQEHYSKRIPGYFMPNNYYCVNYDFTYNPVKKNVNGD
EDDDDDEEEEERDSGKDRKVKTQEKFTASFIFIDTWSLMMGFPIIRNYRSFREQYNWLGK
TLYESAKKSDWIFVFGHHPLFSSGRRSDSFSYEERSFHEIIRDFLFTYKVDGYFSGHDHL
MEYINFGKLNLFVNGSSSRIIFDDSKM-SRGYFGKFLGKIYPISCYVLKTVHRGLKPKGC
NINRYSKWHNKSDIGFSAHKLTKNEFITEFINGRNGKPLSHKIVIENKKQERIKFYDLEG
YANNRIKELEQRIIEFASKNPALVKYKIEEFKENTEKFNLIMEKLKTEEEKKIFKNLLFL
NNLVFDISEHVINVPIEKLKIMSQLVSKYNIFFNKELAGFIVAALERAIQ-MEXXXXXXX
XXXXXXXXXXXXXXDENGSLNE---EDKNLIELIESLGYQPEEFLQKYESMTSEQKVALK
```

```
NKIGRNISLEDYVNRIKFYVEKKKKEKEKKNGNEQ-EAEEETEVVEEIDELKEIEKKRKE
SEGDITDEDENEIKEDQKDNEKEDETYEEYLDESQYNDEEIPLV----KQVHKDFKKLAN
QEKKLSEQKYILLMLASMRKFDIKKYALNLSTKKERIKDVTTSNYLSNIEPRKTFFQLCI
ELPPDIKRIINNFSGVGKRLPFFNFINKLYDEIIKLKDSLNRISR-

>P_knowlesi_ortholog
MNITKYLLITLPLILLKYPLLKELSNHGNLFVRNNIRGA-----LLR--RIIKEESGTK-
-------EGEKEEDRE--------YEEYLENKGSNEFDKVKYKYMDYVNFTIYKSSKKYK
HLLNESLVFLGSQYSTDEKLTFHVKLIDILSLLFVHYKDNLSTFDHVLSSFQDRNKLMNS
IENEFFREFIDERDNYIFEVKNMYYKVDAQDEE-KERILRKKKTIYEIFLRNWKNDGYRF
YSMNNKKKDYIPKKID-----HKKKKKNETQYTLQCRSLVSSP---------------QD
SD------KGKGR-NATSSDNGLSIPSGVPKIRVKQNQ-----DSADKMLSHVEEDVDEE
TLEEIEQGEMGNKNRMEERTCVGQKEEQEEEQEENAEDKSDELYDQDVYEDDANQQALLE
SYNKQICHASEYNQGKSASMNTYRNGIPGVTYKMRKDFFLNGSSLNVITLINAITSNRDN
ATVTELHEALKKLGIKTFDHLVRYTNIIGIFFSYDIFDELYLQIKLVKEYFGLIQKKKND
FDIVEKVKKIKLKKRKAYGEYKMSDDEMFVPPNCLSAYCMLKSVWMRNRNVTMKIERTSS
NSMNFMMLGDIGQGFEEEKDFDEQNMLNLIGFNELKSTVQAMKDWHFANNADFVINLGDN
VPNDGTINYMENFQWHNLMKELFVFKKRSEQEISSMFGDNKLTKQSIKDFYNEKVKEMQD
N------------------YNSGQIGKEEKTDEKKDEK--KDEKKEEMKKKTKKKK----
--RSGTNEQNKENV-----VESPSN-----EEDLSVYDNDRKKLYQFD---DEEESDEDF
--EAIPFYSILGEKDYFFFPSEQIQEHYSYRIPGYFMPNNYYCVNYDFTYNNVGYK----
------------------GIINQEKFRASFIFIDTWSLMVGFPIIRNYRSFREQFNWLSK
TLYESAQSSDWIFVIGHHPLISSGRRADNYSYEEHSFHDILRDFLFNYNVDGYFSAHDHL
MEYIKFGNIDLFINGSSSRVMFDNSTM-GRGYFGKVIGKLYPVTCYILKTIHRGLKPKGC
SVNRYSKWSNKADIGFSTHKLSKDEFITEFINGRTGKPLSHKIVLKNKKSERKKFYNLDG
YVDDKIHELEKKIEEFKQKNPELIKYKIEEFNENIRKLNKIMKTLKTKEEVENFKELLFL
NNLIFDVSEHLSGMTVEKLRSMHALAVKYSIFFNKKIVNHIVVALEKAVQ-SENDESDNE
YEEIDEEVAEMRLKEKQKKQND---EKKSL-EIIETLGYNPEQFLEKYDSMTKEEKNLLK
EKVGKDVTLEDYVNRIRLYVEKKN------------LSESDLEAMQ--------EKLDEG
EGSD-----------EESTKESEEESS----QMSSEDIGEVALV----KDVHKTHQKFVF
MEKALSEQEYILMMLSALKIHDKNKYALNMSTKRENLKTIASSNFLYKIENHKTFFQLCI
ELAPDIKRIISNLGGVGVRLPFFKLMTKLYDEIMNLREGLDRVAK-

>P_vivax_ortholog
MNITKYLLIILPLILLKYPSRKGLISHDNVLLRSNIRGA-----LLR--RIIKEENGAK-
-------EKEKEEERE--------YEEYLQNKGSYEFDKVKYRYMDYVTFTIHKSSKKYK
HLLNESLVFLGSQYSTDEKLTFHVKLIDILSLLFVHYRDNLSTFDHVINSFQDRNKLMNS
IENEFFREFIDERDNYIFEVKNMYYKVDHKNEE-KEKILSKKKTIYEIFLKNWKNDGYRF
YSMNNKKKVYIPKKID-----HKKKRKNETQYSLQCRNLVSSP---------------QD
SE------EGKKEPNGKGPDKGSSTPSEETNSKEKQNQ-----GADAKKLSHVEDDVEEE
THEEVEQGKKGDTNKTEEAASAGKK----DAQEENAADESDELYDHDVYEDEANREALLE
SYNNQICHASEIDQGGSSPLSRYTNGIPGVTYKMRKDFFLNGSSFNVITLINAITSNRDN
ATVTKLYEALRKLGITTFDHLVRYTNIIGIFFSYDIFDELYLQIKLVKEYFGLTQKKKSD
FALVEKVERKKNKKRKAYGEYKMSDDEMFVPPNCLSAYCNLKSVWMRNRNVTVKIERTSS
NSMNFMMLGDIGQGFEEEKDFDVQNMLNLMGFNELKSTVQTMKDWHFKNNADFVINLGDN
VPNDGAMNYMENFQWHNLMKELFVFKKRSEQEINSMMGHNPLTKQSIKDFYNEKMKEMKD
D------------------YNNRQV---------------EDQEMKKKKKKSKKKK----
--KSANNEGKE--------VDSSSS-----QEDFSHYDNDRKKLYQFD---DEEESDENF
--EAIPFYSILGEKDYFFFPSEQIQEHYSYRIPGYYMPNNYYCVNYDFTYNNVGYK----
------------------DIISQEKFRASFIFIDTWSLMVGFPIIRNYRSFREQFNWLSK
TLYESAQNSDWIFVIGHHPLISSGRRADNYSYEEHSFHDILRDFLFNYNVDGYFSAHDHL
MEYIKFGNIDLFINGSSSRVMFDNSNM-GRGYFGKVIGKLYPVTCYILKTIHRGLKPKGC
SVNRYSKWSNKADIGFSTHRLTKDEFITEFINGRTGKPLSHKIVLKNKKSERKKFYNLDG
YADDRIDELEKKIKEFTLNNPDLIKYKIEEFNENMRKLNLIMKTLKTKEESENFKELLFL
NNLIFDVSKHLSGMTSKKLRSMHALAEKYSIFFNKELVNHLVVALEKAVQ-SENSQSDNL
NEEIDEEEAERRLKEKQKKQNE---EKKTL-EVIETLGYKPEQFLEKYDNMTKEEKDLLK
EKVGKDVTLEDYVNRIRIYVEKKN------------LSESDLEAMQ--------EKVDAE
EAGH---AEEANVQQGGSGERSSDGSS----DGSSESSGEVALV----KDVHKTHQYFMF
KEKALSEQNYILMMLSALKIHDKNKYALNMSTKKEDLKSIASSNFLYNIENHKTFFQLCI
ELAPDIKRIISNLGGVGLRLPFFKLMNKLYDEIMKLRDGLDRIAK-

>P_chabaudi_ortholog
MNISKFFLIFLPLVLFKYPANNELINY-NVILKYDVKD------LFK--RLMQEQNDNT-
-------EGDKDENHE--G-----YSIYIKSHGTNEFDKVKHKYADYVRFSIFKSKNIYT
HIFNELLVFLGSQYGTEEEIVVHVKLIDLLSLLFTHYRDNLSKFSHILNSFQDRSKLMNS
VESEFMNEFINERNNYIYEMKNAHSSSDNKDGEWIEAVVNKKNMLYDKFLKEWKNDGARF
YSIHNKKKAYIPKKVF-----LNKQNYIPDFESMEHVDLVCKPI--------------TD
SDTDENLIE-------EFEDKELNT--------NEENE----------------------
-----------------------------NSDSSNKTDGSD-------------------
-----VCKSESKYDKKTKNNKQNKKVKPGVIYKMRNDFFLSDASLNVISLISSITSNEDN
KVVKKLYTGLKKLGITTFDNLVRYTNIIGIFFSYDIFDELYLQIKLVKEYFGLIPKNNDE
LSIVS--KSKHLSKIKVYGKYNMTDDEMFVPPNCLSAYCKLRSVWMQNRDFNFKIEKDDS
NSINFMMLGDVGRGITKENSYDEEQMLKLIGFNELKSTSNAMKDWHETNNADFVINLGDN
VPDVDELDYLKNFEWHKIIKGLFTFKKKTEDEPEDDSYLYPDTNDSMHEFYKEVEKQMNN
ENKEKTNQQDDTSTTPVNNYTDKNESNNNSENNNGFNEHINDNTKDPNIDKNKTTK----
---------------------------------------------------KEGTD----
--DTIPFFSIFGEKDYFYFPSEQIQEHYTKRIPGYFFPNNYYRVNYDFVYNNKEKN----
------------------GV--QEKFRASFIFIDTWSLMIGFPIIRNYRSFREQYNWINT
VLLESAKESDWIFVVGHHPFISSGRRSDNYSFEELSFHNILRNFFFYYNIDGYFSAHDNL
MEYIKFGSLNLFVNGSSSRVLFDKSTMMGRGYFGKMVGSIYPVACYILTTLHSGLRPKGC
DISKYSKWSNKFDIGFSTHKLSKDEFVTEFINSRTGKPVSQKIVIKNKKYKRRHFYDLEG
YADNGIKQFEKQIHEFSSKHPNFIKYKIEEFKENDKKLNIIINNLKSQEEKDAFKSLMFL
NNLIFDISNHLSNISFKQLKLMCYLANKYHTFFNKKLVTFLVEELKNAIQNMDEDTANE-
------LLNE--NENEKIQPNDNMSEGMEIIELIEALGYKPDEFLEKYNAMTQEEKDALK
EQLGNDVPLEDYINRVKMYSHKKK------------LSAEELQEFEENEENIQIADAPDE
SK-D-----------DNTDSQTGDNNH-------QENKDEIDDIMNAPNEVHKNNKALVE
KEKNLNEHEYSLLLLSSLKSYDEMKYSLNILSKKEIIKNEAHPYGLYYIEKHKTFFQVSL
ELCPDIKRIIANLGQVGTKLAFYDYINNLYNKIMDLKNSLDKIAIF

>P_yoelii_ ortholog
MNISKYFLIFIPLVLFKYPANNELINY-NVILKYDVKD------LFK--RVLQEQNEDI-
-------KDDNDESGEEGGEESEEDSNNLENRGTNEFDKVKYKYADYVRFSIFKSKNIYT
HIFNELLVFLGAQYGTEEEIVVHVKLIDILSLLFIHYRDNLSKFGHILNSFQDRSKLMNS
VENEFMKEFVNERDNYIYEVKNAYNKSDDNDDEWIETVLNKKNMLYDKFLKEWRVDGSYF
YSIHNKKKTYLPKKVF-----QTKQNYIPDFENMEHVDLVCKPI--------------SD
SDTAEKGTE-------EYKDKELDT--------NEHNE-----QTNSEKEQNSNDNVSET
KMHKEE-----------------------SSDSSNKADESN-------------------
-----MCKSESKYIKKTNSNKQIKKGRPGVIYKMRNDFFLSDASLNVISLINSITSNEEN
KIVKKLYTGLKKLGIITFDNLVRYTNIIGIFFSYDIFDELYLQIKLIKEYFDLIPRNNDE
LSIVS--KAKNISKIKVYGKYNITDDETFVPPVCLSAYCKLRSVWMQNRDFNFKIEKSSS
NSINFMTLGDIGRGFKKENSYDEEQMLKLIGFNELKSTSNAMKDWHAANNADFIINLGDN
VPEVDELDYLKNFEWHKIIRELFTFRKQNEDEQKNVTDPYAITKDNIQEFYKEVEKQMNI
TNVEDTNQHNDISTTSLKNYTDDNEGNNDSENNNGFSEYISDNTKDFNIYKNESTE----
---------------------------------------------------KEETY----
--DSIPFFSIFGEKDYFYFPSEQIQEHYAKRIPGYFFPNNYYRINYDFVYNNKEKN----
```

```
------------------GV--QEKFRASFIFIDTWSLMIGFPIIRNYRSFREQFNWINK
ALLESAKESDWIFVVGHHPFISSGRRSDNYSFEELSFHNILRNFFFYYNIDGYFSAHDNL
MEYLKFGSLNLFVNGSSSRVLFDKSTMLGRGYFGKMIGSIYPVACYLLTTIHSGLRPKGC
DISKYSKWSNKYDIGFSTHKLSKDEFVTEFINSRSGKPVSQKIVLKNKKDKRRQFYDLDG
YANDRIKQFEKQIYEFSSKNPNFIKYKIEEFKENDKKLNIIMNNLKSEEEKDAFKSLMFL
NNLIFGISSHLSNISFEQLKLMCYLANKYRTFFNNKLITFLGEELKMAVQEMEEKGANEA
QLNSNDMVNTNENENENIQPNDQTLEAEQTMELIETLGYKPDEFLEKYNAMTQEEKDTLK
EKLGNDVSLEDYINRIKMYIHKKN------------LSAEELKEYEENEENIKIEEVPDE
SKED-----------DNTDSQPEDTID-------QENKDEINDIINAPNEVHKNYKELVE
KEKKLTENEHALLMLSSLKTYDEMKYSLNILSKKEVIKEESYPYGLHYIEKHKTFFQVSL
ELCPDIKRIISNLGKVGTKLAFYDYINNLYNKIMDLKNSIDKIAIF

>P_berghei_ortholog
MNISKFFLIFIPLVLFKYPANNELINY-NVILKYDVKD------LFK--RVLQEQNEDI-
-------KDDNDENDE--GDEEDEYYSYLKNRGTNEFDKVKYKYAEYVRFSIFKSKNIYT
HIFNELLVFLGAQYGTEEEIVVHVKLIDILSLLFTHYKDNLSKFGHILNSFQDRSKLMNS
VENEFMNEFINERDNYIYDVKNAYNKSDNNDDEWIETVLNKKNMLYDKFLKEWRIDGSNF
YSIHNKKKTYLPKKVF-----QNKQNYIPDFENMEHVDLVCKPI--------------SD
SAIDEKSAE-------KYEDKELNI--------NEQNE-----QSNSKKEQTGNDNVSET
KMHKEE-----------------------SSDSSNKTDESN-------------------
-----VCKSENKYIKKTNNNKKIKEERHGFIYKMRNDFFLSDASLNVISLINSITSNEEN
KIVKKLYTGLKKLKITTFDNLIRYTNIIGIFFSYDIFDELYLQIKLIKEYFGLIPRNNDE
LSIVS--KSKNVSKIKVYGKYNITDDEMFVPPVCLSAYCKLRSVWMQNRDFNFKIEKYSS
NSINFMTLGDIGRGFKKENSYDEEQMLKLIGFNELKSTSNAMKDWHASNNADFVINLGDN
VPEVDELDYLKNFEWHKIMRELFTFRKQDEDEEKNDADSYSITKDNIQEFYKEVEKQMNN
TNTDDKNQHNDISTTPINNYTDGNEGNNNSENNNGFREYINDNTKDYNIYKNETTE----
---------------------------------------------------KEETY----
--DSIPFFSIFGEKDYFYFPSEQIQEHYAKRIPGYFFPNNYYRINYDFVYNNKEKN----
------------------GV--QEKFKASFIFIDTWSLMIGFPIIRNYRSFREQFNWINK
ALLESAKESDWIFVVGHHPFISSGRRSDNYSFEELSFHNIIRNFFFYYNIDGYFSAHDNL
MEYLNFGPLNLFVNGSSSRVLFDKSTILGRGYFGKMVGSIYPVTCYLLTTIHSALRPKGC
DI-KYSKWSNKYDIGFSAHKLSKDELVTEFINSRSGKPVSQKIVIKNKKDKRRKFYDLDG
YTNDKIKQFENKIYEFSSKNPNFIKYKIEEFKENDKKLNIIMNNLKSEEEKDAFRSLMFL
NNLIFGISSHISNISFDQLKLMCYLANKYRTFFNKKLIKFLGEELKIAVQKMEVKTTNET
PHNSNEILNT--NENESIQPNDPTMKVEQIMELIDTLGYKPDEFLEKYDAMTQEEK-ALK
EKLGNDVSLEDYLSKVKMYIHKKK------------LSAEELKEYEENEENIKIAEVPDE
SKED-----------DNTNSQPEDTID-------QENKDDINDIINAPNEVHKNYKELVE
KEKKLTENEHALLMLSSLKTYDEMKYSLNILSKKEVIKEEAHPYGLYYIEKHKTFFQVSL
ELCPDIKRIISNLGKVGTKLAFYDYINNLYNKIMDLKNSIDKIAIF
```

▪ AMOUNT OF MISSING DATA

P. reichenowi	***P. gallinaceum***	***P. knowlesi***	***P. vivax***	***P. chabaudi***	***P. berghei***	***P. yoelii***
0%	0%	12%	12%	15%	14%	14%

■ MAL13P1.68

▪ PHYLOGENY AND PEXEL/VTS

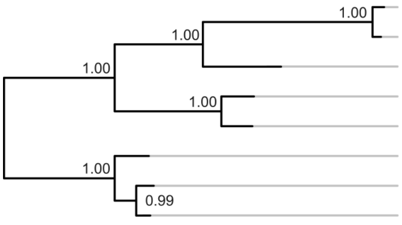

P. falciparum MAL13P1.68	-----RDLIDGVYDT
P. reichenowi ortholog	-----RDLIDGVYDT
P. gallinaceum ortholog	-----RDIIDGEDIT
P. knowlesi ortholog	SGILYKDLIDGEGDP
P. vivax ortholog	SGILYKDLIDGEGDP
P. chabaudi ortholog	SGILYKDLLDGEGDP
P. berghei ortholog	SGILYKDLLDGEGDP
P .yoelii ortholog	SGILYKDLLDGEGDP

▪ ALIGNMENT

```
>P_falciparum_MAL13P1.68
MVYFKR----FFFHKRHRILSIFNSYFFMKKQLLN----NLNKILFISLNSYFL-KLLFS
KNTYIYYLQEK-KNSYLEKKYRPDTPYKKTE-------------------RDLIDGVYDT
-------------VEEGDTVYIHYQGKTTNDFRIIESTFKSIIPPKIIAGHYDQKHIKAI
YEMVIGMKKNTRRECIIPPYLAHPNHFPTQPLIYEIDVVRIVKKNAQNKTTLQKLEVKLE
KIIDTICSYF

>P_reichenowi_ortholog
XXXXXXXXXXXXXXXXXXXXXXXXXXXXXXXXXXXXXXXXXXXXXXXXXXXXXXXXXXXX
XXXXXXXXXXXXXXXXXXXXXXXXXXXXXXX-------------------RDLIDGVYDT
-------------VEEGDTVYIHYQGKTTNDFRIIESTFKSIIPPKIIAGHYDQKHIKAI
YEMVIGMKKNTRRECIIPPYLAHPNHFPTQXXXXXXXXXXXXXXXXXXXXXXXXXXXXXX
XXXXXXXXXX

>P_gallinaceum_ortholog
XXXXXXXXXXXXXXXXXXXXXXXXXXXXXXXXXXXXXXXXXXXXXXXXXXXXXXXXXXXX
XXXXIFHLEEKR-STYLEKKYRSDTPYLKTE-------------------RDIIDGEDIT
-------------VEKGDIVYIHYQGKTTNDFRIIESTFKSIFPVKITAGFYDQKHIRAI
YEIVIGMKKNTRRQCIIPPHLAYPYHFPNQPLIYEIDVVKIIKKNSQKETFLQKMKMKIE
NIKIAISEYF

>P_knowlesi_ortholog
MRSLARRWGTFFKYYDRGKVSFRSFYFAGKKQFFI-------HLFFISLNSYFVKKYLFS
NTSTVFHLEGKGVDAYLEKKYKSDKPYIKTE--------------SGILYKDLIDGEGDP
-------------IEEGDIVYIHYQGKTTNDFRIIHSTFNSIIPPKIRAGQYDKKHIRAI
YEIVIGMKKHTRRQCIVPPHLAYPNHFPSQPLLYEIDVVKVVKKNSQGKTFIENAEKKIE
QIKSFISSFF

>P_vivax_ortholog
MRSFARR-GTFFKYYDRGKLPFRSFHFGDKKQFII-------HLFFISLNSYFVKKYLFS
NSSVVFHLEGKGADAYLEKKYKSDKPYVKTE--------------SGILYKDLIDGEGDP
-------------IEEGDIVYIHYQGKTTNDFRIIHSTFNSIIPPKIRAGQYDQKHIRAI
YEIVIGMKKHTRRQCVVPPHLAYPNHFPSQPLLYEIDVVKVVKKDSQGKTFIEKVEQKID
QIRSVISSYF

>P_chabaudi_ortholog
MINLINR-FIFFKLDNTIKYSHKICLFNNRKKQILTKFHDFCNIFFVSLNLYIVKKYILS
KNTRISYLEDRG-HAYLQKKYRTDTKF-KNR--------------SGILYKDLLDGEGDP
-------------IEEGDTVYIHYQGKTTNDFRIIQSTFTSIIPPKIKAGIYDKKHIKAI
YEIVIGMKKHTRRQCIVPPHLAYPNHFPNQPLIYEIDIVKVIKKGEEHDTLLERIKRNAN
YLKTAITSFF

>P_yoelii_ ortholog
XXXXXXXXXXXXXXXXXKKYSHKICLFNRRKKQIFTKFHNFCNIFFVSLNLYIVKKYILS
KNIRISYLEDRG-DAYLQKKYRTDTKFLRTR--------------SGILYKDLLDGEGDP
-------------IEEGDIVYIHYQGKTTNDFRIIQSTFKSIIPPKIKAGVYDKNHIKAI
YEIVIGMKKHTRRQCIVPPHLAYPIHFPNQXXXXEIDIVKVIKKGKENDTLLGRIKKNVN
YLKSAITSXX

>P_berghei_ortholog
XXXXXXXXXXXXXXXXXKKCSHKICLFNRRKKRIFTKFHDFCNIFFVSLNLYIVKKYILS
KNIRISYLEDRG-DAYLQKKYRTDTKFLRTK--------------SGILYKDLLDGEGDP
-------------IEEGDIVYIHYQGKTTNDFRIIQSTFKSIIPPKIKAGVYDKNHIKAM
YEIVIGMKKHTRRQCIVPPHLAYPIHFPNQPLIYEIDIVKVIKKGEENDTLLERIKKNVN
YLRTAITSFF
```

▪ AMOUNT OF MISSING DATA

P. reichenowi	*P. gallinaceum*	*P. knowlesi*	*P. vivax*	*P. chabaudi*	*P. berghei*	*P. yoelii*
58%	27%	6%	5%	7%	11%	13%

■ MAL8P1.93

▪ PHYLOGENY AND PEXEL/VTS

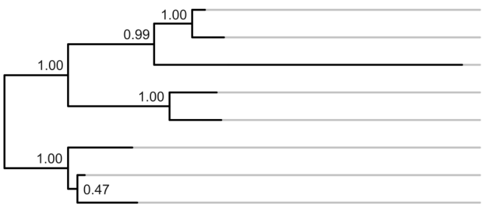

P. falciparum MAL8P1.93	LNKCKRFLTDLFIKL
P. reichenowi ortholog	LNKCKRFLTDLFIKL
P. gallinaceum ortholog	LNKCTHFIVNLFLKI
P. knowlesi ortholog	LLKCKRFLKNLFIEL
P. vivax ortholog	LLKCKRFLKNLFIQL
P. chabaudi ortholog	LNRCKNYIKELFVRL
P. berghei ortholog	LNKCKKYIKELFIRL
P .yoelii ortholog	LNRCKKYIKELFIRL

▪ ALIGNMENT

```
>P_falciparum_MAL8P1.93
MVKKDNIWVLCKLYLDEKNTQLNKLQEDDIFKIIQNSNTPLFVLIKEEFDKNALIFYGKI
FKTILFNPFSIIFANLELRIFIIKTSNKFKKEIEILTKFVTVCD-YLKVEILYIGVTLNK
CKRFLTDLFIKLKIEENIPCIMKEFENCS

>P_reichenowi_ortholog
MVKKDNIWVLCKLYLDEKNTQLNKLQEDDIFKIIQNSNTLLSVLIKEEFEKNALIFYGKI
FKTILFNPFSILFFPLKCRIFIISTNNIFKKEIEILTKFVTVCD-YLKVEILYIGVTLNK
CKRFLTDLFIKLKIEENIPCIMKEFENCS

>P_gallinaceum_ortholog
MMRKDNIFLLMKIYLNEASLNLKKNYEDDVYNYISGNNVPLKLNVNKEKIKNSLIFYGKI
KKILIFKIFTIIFNRKSLRIFIIRTYNKYKREVSLLTRFVTICDD-IRVKILYSGITLNK
CTHFIVNLFLKIGLIINIVCIFK------

>P_knowlesi_ortholog
MARKDYVWVLCKFYCGKKRGVVSKKQEEEIFKILESSNLPLTVMIKEEFDRKATMLYGKI
YKSLIFSRFLVIFANLRLRLFIIRTSNKFKQDISQLAQFVTLCDDYLDVQVLYIGVTLLK
CKRFLKNLFIELKIEENMPIIMKELER-S

>P_vivax_ortholog
MARKDHIWILCKFYCGNKRGVVSKKKEEEVFKILESSHIALPVLIKEEFERKATMLYGKI
YKSLIFSRFFVNFANLRLRLFIIRTSNKFRQEISLLTQFVTLCDDSLDVQILYIGVTLLK
CKRFLKNLFIQLKLEENIPLIMKELEH-L

>P_chabaudi_ortholog
MVKKDSVWILCRLHCSDNENFANKNEKNNIIKILQNSNIPLSSLIKEEYEKRGSILFGEI
FKSIIFSRFLVIFTHLNLRIFIIRTSNRYKNEIALLSQFVTMCDNYFSVQLLYIGVTLNR
CKNYIKELFVRLEIEEFIPSILK------

>P_yoelii_ ortholog
MVKKDSIWILCRFDYFDKENFANNNEQNKILKILQNVNIPLSSIIKEEYEKKGLILFGEI
FKSIIFSRFRVIFTNIKFRIFIIRASNKYKNEISLLSQFVTICDDRFSVQLLYIGVSLNR
CKKYIKELFIRLKIEESIPLILK------

>P_berghei_ortholog
MVKKDSIWILCRLHCSDKEDFANKNEQNNIFKILQNANIPLSSLIKEEYEKKGSILFGEI
FKSIIFSRFLVIFTNLKLRIFIIRASNKYKNEISLLSQFVTICDDYFSVQLLYIGVTLNK
CKKYIKELFIRLKIEESIPIILK------
```

▪ AMOUNT OF MISSING DATA

P. reichenowi	*P. gallinaceum*	*P. knowlesi*	*P. vivax*	*P. chabaudi*	*P. berghei*	*P. yoelii*
0%	5%	1%	1%	4%	4%	4%

■ PFL0805w

▪ PHYLOGENY AND PEXEL/VTS

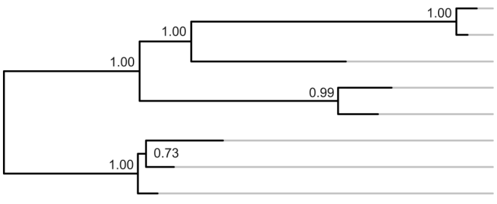

P. falciparum PFL0805w	NVEEIRNLEQYKLEL
P. reichenowi ortholog	NVEEIRNLEQYKLEL
P. gallinaceum ortholog	NVEEIRNLEQYKLEL
P. knowlesi ortholog	NVEEIQNLEQYKMEL
P. vivax ortholog	NVEEIQNLEQYKMEL
P. chabaudi ortholog	NVEEIKSLEEYTLEL
P. berghei ortholog	NVEEIKSLEEYTLEL
P .yoelii ortholog	NVEEIKSLEEYKLEL

▪ ALIGNMENT

```
>P_falciparum_PFL0805w
MNILLKYHLSYIFFFVHLIVINIKCKEINNNLVNDEKDSINNLEEPSPFHILKKKNSNNI
ENHSNKINNIFKNN---------YSYSYSYSLLDNSLYDNKYESDFFPQKKNNKINEEKY
KGTHGTNFAIINEHMNEIHNDMLGQNE---------------------------------
------ILSNELDTTKDIIKEEH--------VKNKKIKNHSFYMNKENEKKKFLDNTFPE
NK---------------------------------ENNKNKRNSKFIEENDLNSSGENYF
ENDKFIEIEYPIKNK---DIDEYEEGINRNHKEEDNKNRTYHSNGEDSIKSLNFIKYMNM
DKEKKGKKKSYNRKKKENYNNDNYNINDSD---LINVLKQGDYSPDCDTCNKNINGHMNK
GRGIRRRMYDRNYYKDFNLRDALPINKTHYSMLLKSDDDTIIKEKNENDVDDIITKEKRK
ENL-KEVYNDKKNNYLSLKYLGLGYDIIMGNPEGDPTLNVDPGFRGPVLQINLKEISLNS
YDNHKNDDNHMNDDIHKNDDNHMNSDNHKNDEYHKNDEYHKNDEDHNDDNYDNHADGDKR
SKERKSKIQTTNESMKPWVIPEHSCSQSK--------NVEEIRNLEQYKLE--L------
-------LSDVKVSTPSSFPYSFSASAEFKNALKKLKVQNNVIFLMKIYCLRYYTGIPIT
TTSYKFSENFKNALSKLPKYFDGLREDSKCSYEYYINKLNSPECEENVNKWMLFFKLHGT
HVAYEIYLGGKIIIKININKEEYNKMKENNINVKTFFNIYFHKMGLSSAFQKEAQKILNK
FRISKHIAILGGNPGLNVNNTSFFEKWVHSINTNSMPIRTKLLPFSFFMDDNDMIQAYKD
ALIFYGLTYGLQLFGQEKYNHNIISVGEYLEKCTQKLYAGPPPGLLTCPIGTTILMGFSI
NLNFYKNKYLSSTNGITLCEPMKESCSGNGFEKNYSDIRIFALCTNKPFDFITQVVQQGE
APKISASCPGELVILFGFALMKGIGSSSANKIDIYPCRTGQNSCEAVLQNHKFKQSMIYL
ACVDKTTNGLEYLQTYSKTKNLGDVISDKYKSDGYLNFSCPQNNTLVFGFSLEFHTNFQA
TRNNFLNCSKYTNICEISGIGINTHLSFFRSDKHSLAIIALCRSHGANNLVE

>P_reichenowi_ortholog
XXXXXXXXXXXXXXXXXXXXXXXXXXXXXXXXXXXXXXXXXXXXXXXXXXXXXXXXXXXX
XXXXXXXXXXXXXXXXXXXXXXXXXXXXXXXXXXXXXXXXXXXXXXXXXXXXXXXXXXXX
XXXXXXXXXXXXXXXXXXXXXXXXXXXXXXXXXXXXXXXXXXXXXXXXXXXXXXXXXXXX
XXXXXXXXXXXXXXXXXXXXXXXXXXXXXXXXXXXXXXXXXXXXXXXXXXXXXXXXXXXX
XXXXXXXXXXXXXXXXXXXXXXXXXXXXXXXXXXXXXXXXXXXXXXXXXXXXXXXXXXXX
XXXXXXXXXXXXXXXXXXXXXXXXXXXXXXXXXXXXXXXXXXXXXXXXXXXXXXXXXXXX
XXXXXXXXXXXXXXXXXXXXXXXXXXXXXXXXXXXXXXXXXXXXXXXXXXXXXXXXXXXX
XXXXXXXXXXXXXXXXXXXXXXXXXXXXXXXXXXXXXXXXXXXXXXXXXXXXXXXXXXXX
XXXXXXXXXXXXXXXXXXXXXXXXXXXXXXXXXXXXXXXXXXXXXXXXXXXXXXXXXXXX
XXXXXXXXXXXXXXXXXXXXXXXXXXXXXXXXXXXXXXXXXXXXXXXXXXXXXXXXXXXX
XXXXXXXXXXXNESMRPWVIPEHSCSQSK--------NVEEIRNLEQYKLE--L------
-------LSDVKVSTPSIFPYSFSASAEFKNALKKLKVQNNVIFLMKIYCLRYYTGIPIT
TRSYKFSENFKNALSKLPKYFDGLREDSKCSYEYYINKLNSPECEENVNKWMLFFKLHGT
HVAYEIYLGGKIIIKININKEEYNKMKENNINVKTFFNIYFHKMXXXXXXXXXXXXXXXX
XXXXXXXXXXXXXXXXXXXXXXXXXXXXXXXXXXXXXXXXXXXXXXXXXXXXXXXXXXXX
XXXXXXXXXXXXXXXXXXXXXXXXXXXXXXXXXXXXXXXXXXXXXXXXXXXXXXXXXXXX
XXXXXXXXXXXXXXXXXXXXXXXXXXXXXXXXXXXXXXXXXXXXXXXXXXXXXXXXXXXX
XXXXXXXXXXXXXXXXXXXXXXXXXXXXXXXXXXXXXXXXXXXXXXXXXXXXXXXXXXXX
XXXXXXXXXXXXXXXXXXXXXXXXXXXXXXXXXXXXXXXXXXXXXXXXXXXXXXXXXXXX
XXXXXXXXXXXXXXXXXXXXXXXXXXXXXXXXXXXXXXXXXXXXXXXXXXXX

>P_gallinaceum_ortholog
MRKLIEFFISHL---LYFFIKNINCEINSNLLNYQKDVTDVTPENFISSNIINEENSYII
DLNSVNKNKRLKNNYSKLLNNALYNYGNTETLRNVLDKGNNINEK---AKLHEERNFEKE
SRKIGSSFMLINKHNTENDLFKKNENEKYDKLYSEKITEKNCRKIQ--KEKSDKKGFIEK
NDNEVINNSKPNQLTNEASCKNI--------SKKKKKTVEPILYKYTDYNSNVGDKI---
NSNFKKTQEEKNISLHENYT-----INDENRIKSLKNSREKLNLLFLGNITNDYTGNNYF
EIDKYVKLEYPINNKKKRKINKYAHRDMNQINNKINKKSVFNN------NNLNFIKYTDD
E----------DNNSKENEDNENYVIDDSD---LLDILKDGYKPDNIVCTIK------KN
KNPERKKKIDEENYEDFDLKDALIDDIDNKNI-------KSIEDKEKNEHNKNKKPDNTN
NDIYSKLKLNNPDTHLSLKYLGLGYDIIMGNPEGDPTLNIDPGFRGPVLQINLEKINLNN
-----------------------NNININNNNNNNDSKR---------------------
--------------STPWVIPEHSCSQSR--------NVEEIRNLEQYKLE--L------
-------LSDVKVSTASIFPYSFSASVEFKNALEKLKVQNNVIFLMKIYCLRYYTGIP-T
TTLWKFTDHFKKALNNLPSTFDGLKEGSLCTYEDYINKINTPECEENVNKWIIFFKLHGT
HVAHEIYLGGKIILKMNIDKNEYNKLKENNLSMKPFFNFYFHKMGLSSSFSKQTQSILSK
FNISKNISILGGNPGLDVENSSFFERWVNSIGRNSMPIRTKLLPFSFFMKDPNMIQAYKD
ALTFYGLSYGVRIIDQEKYNNGVLSIGEYLEKCTQKLYAGPPPGLLTCPIGSSLLMGFSI
NLDFYKSKKLSNTIGMISCEQMKESCSGNGFENNYSDTRIWGLCSNNPLEFITQVVQQGE
SPKVTATCPRGLVILFGFALMKGMGSSSANNVDIYPCRTGQTSCSAVLQNSKFKQSMIYV
ACVDRTTNGLEHIQTFSQIKNMGYVVPSKFKEDGYLNFSCPSNNTLVFGFSLEFHTNFPS
TRNNFLYCSKFTNSCEIGGIGINTRLSFFRADMHSLAIIAVCRSHIEXXXXX

>P_knowlesi_ortholog
MRTTVERVLAHLLL-VYLSAHNVKCEISRYFLMHEKY-DDFNLEKFNSSDFLNEEKSYIA
DVNSVGKRKRLQNDNGIFAGNPLYGCFNKYALRRNGQYGQNVVNKDRSAKLNVKNEPAKC
NGDTCSSFMLIKAHLPARHTVKK--NEKNHTLCNLQREGRNCKKIPIGEEKMEQAVTINK
HN----YNSNLVDLTRELNKQHMPHGFPEGYSKEKKKNVHTEPSDIQSFTHSERDTASPS
NMKKNPKREEKQIRIHGNSK-----QNELPPNERSKNGRDNMVMLPPYNDSIGYSGGNYF
EKEKFINIKFPIKIS-----REVAEQNQEMDNEKQIMKKLHKHQAPRNNSDANFAEFTNK
EGEA-------DNTENRNKNIVNSKLGEGN---LIGIFKTPYILGRDSFFIKNEMEDLKQ
INSERG----NKQYKDFDLRDTLMGETGSITNTQPLGGGTQIMKNKNNVLTHNHNPNYGE
NHA-TYDKVHNPDIDLSLKYLGLGYDIIMGNPEGDPTINVDPGFRGPVLQINIEEMRVNK
--------------------------DISGDLNGTTSLYGSDIRGGGLPYRESTLMGDYT
IEEHKTK-------LTPWVIPEHSCSQSK--------NVEEIQNLEQYKME--L------
-------LSDVKVSTPSIFPYSFSASAEYKNAIKKLKVQNNVIFMMKIYCLRYYTGIP-T
TTSWRFTDNFRNALKKLPRTFDGLKEDSQCSYEYYINKIHTPECEENVNKWMMFFRLHGT
```

```
HVAHEIYLGGKIIIKMNMEKDEYNKLKDNNISIKTFFNLYFHKMGLSGGYSKHTQKLVKK
FRTSEDISILGGNPGLNIENSTFFEKWVNSINKNSMPIRTKLLPFSFFMDDPYMIQAYKD
ALTFYGLTYGVQIFDHEKYSHVVLSIGEYLEKCTQKLYAGPPPGLLTCPLGSSLLMGFSL
NLDFYKNKDLSSTNGMASCEQMKESCSGNGFGKKYSDIRIWGLCSEKPLDFITQVVQQGE
SPKITASCPGNLVILFGFALMKGKGSSSANKVDIYPCRTGQSSCSAVLQNNKIKQSMIYI
ACVDKSTNGLGNIQTYSKVKNMGYVNSDKDENDTSLSFACPKNSSLIFGFSLEFHTNFSK
TRNNFTGCSKASNTCHISGTRINTKLGFFKPDKYSLAIVAVCSTRRELSLRA

>P_vivax_ortholog
MRTTVERVLAHLLL-VYFSTHNVKCEISRYSLIHEND-DDYNLEKFNSSDFLNEEKSYIA
DVNSVEKSKRLQNGNGVFASNPLYGCFNRYALR-SGRYGKDVANQERSAKSHEKKETTKC
AGNTCSSFMLIKAHRAGGHAVKK--NEKNYNLCNLQREERSCEQIPSGKKKVGQAATIKR
HN----YNSNMMDLTRKTDKQHLLHGLPEDYPKEEKKNVHAEPSDLQSFSHFETDTVAPS
NLKKNPKREEKQIRIHGNSRRDEPPQNELPHNEPPQNGADDMVVPPSYNDSIGYSGGNYF
EKEKFIQIKFPIKIS-----SEAEEQDEEMNNEKEIMKELERQESLHTRSDANFEKYTNK
EGER-------DNAQKGGKNFVNSKLGEDN---IMGIFKNPYSLGSDNFFIKNEMEDLKR
INSERK----DEQYKDFDLRDALMGETGSITNTQPLSGGTQMKENENNALTNS--PNDGE
SHT-AYDKGHNPDIDLSLKYLGLGYDIIMGNPEGDPTINIDPGFRGPVLQINIEEMRVNK
--------------------------DSNVNANGTTSPYGSGFRGGGLPYRESTLLGDYT
LEEHKPK-------LTPWVIPEHSCSQSK--------NVEEIQNLEQYKME--L------
-------LSDVKVSTPSIFPYSFSASAEYKNAIKKLKVQNNVIFMMKIYCLRYYTGIP-T
TTSWRFTDNFRNAMKKLPPNFDGLKEDSQCSYEYYMNKIHTPQCEENVNKWMMFFKLHGT
HVAHEIYLGGKIIIKMNMEKDEYNKLKDNNISIKTFFNLYFHKMGLSAAYSKQTQKLVSK
FRTSKDVSILGGNPGLNIENSTFFEKWVNSINKNSMPIRTKLLPFSFFMDDPNMIQAYKD
ALTFYGLTYGVQIFDHEKYSHVVLSIGEYLEKCTQKLYAGPPPGLLTCPLGSSLLMGFSL
NLDFYKNKDLSNTNGIASCEQMKESCSGNGFGKKYSDIRIWALCSEKPLDFITQVVQQGE
SPKITASCPGNLVILFGFALMKGKGSSSANKVDIYPCRTGQTSCSAVLQNSKIKQSMIYI
ACVDKSTNGLENIQTFSKVKNMGHVNSDQHEDDTSLSFACPKNSSLVFGFSLEFHTNFSK
ARNNFTDCSKASNTCEIRGTQINTKLAFFKPDKYSLAIVAVCRARSEVPLRA

>P_chabaudi_ortholog
MTISVKGIFFHLIL-VQLVIYNIKCELSENSYTSKNG-DTHSIEEYSIANLINEEQPHIT
NTNLVNKNGRWKHIYKGLLNNHLYSLGKRPVLQQSP--G---NNNINTLEKLNKDNSLIK
HTKGGTNFISIKQNN-----------------------------------------VVRK
NETELNTNMNNAQFTN------------------TKNSIINSHDHLDEYNKNYIDNL---
-----------------------------------KSVKNHFNAQNLDNSNTDFDDNNYL
EPEKYVPIKYPFDNH-----------ETNNNSNNIKSNNIYHNEESSKKNDVTEEENYND
NIKP-------TPCTKNEKACINKTLDTGNEFYLRDVLLDGYKPDVSSYI--NTKKSFKN
KQKEKI----IEEFKDFDLIDPLTPNVNEHTSM------NDEEDDNKNSNFISMKDYDGD
DENPPDEPIPKEDVDMSLKYLGLGYDIIMGNPEGDPLLNVDPGFRAPVIQINITDTGINN
-----------------------NNDNVEEHEDSQDSHSNTGNIKHS------S------
---NKKK-------IIPWIIPEHSCNQSK--------NVEEIKSLEEYTLE--L------
-------LSDVKVSTPSIFPYSFSASAGYKNALKKLKIQNSIIFMMKIYCLRYYTGISTT
TNTWEFTNNFRNALNKLPNTFDGLKEDNECTYEYYITKSHSPQCEKNVNKWMTFFKLHGT
HVAHEMYLGGKIIIKVNIEKEEYNKMKETNLDMKTVFDFYFHKMGLSARKNRRIQKFINK
MHGSKTVSILGGHPGLNIDDPSFFEKWINSIDKNSMPIRTKLLPFSFFMDDPNMIKAYND
ALMFYGLTYGIQIFDQQQYNNVEIPIGDYLENSIQQIYHGSPPGLLTCPIGTTILMGFSL
NLDFYKNQSLNEIIGINPCEQLKESCSGNGFINKYSDIRIWGLCSEKTLYFIKQVVEQNE
TTKTTATCPGNYVILFGFALMKGIGRSSANAVDLYPCRTGQNSCSAILQNQKFKQSMIYI
ACVDKATIGLESLQTFTKVKNLGSVDSNNYQNDGYLDLECPENSTLVFGFAMEFHTNFQK
ARDNFIKCPKEENTCSIRGIGINTNLFFFKKDKHSLGIVALCRSTSSKMGRN

>P_yoelii_ ortholog
MTISVKDIFFHLIL-VQIIIYNIKCEISENLYTSKSD-DTHSIEEYNVANLINEKQSNIT
NTNLVNQNERLKNSYKGLLNNQLYSLGKRHVLQQSS--G---NNNINALEKFNKDNSLIK
NPKRGTDFIQIK-HN-----------------------------------------IINK
NETKLNTNTNNAQFTN------------------TKNSIISSHDHLDKYNKNYIDNL---
-----------------------------------KNVKNSFIVPILDDSNTDFDDDNYL
EPEKYIPIKYPFDNH-----------DMKYNSNNIDGNNIYHNEGTYKKSDVTEKGNYNR
NIKH-------TTCTKNKKASINKTLDTGNEFDLRNVLLDGYKPDVNNYT--NIKQSFKN
KKKKRV----PQKFKDFDLSDALTPNINEHNSI------NDGNRNNKNANFISVKDWDTD
DEISQNEALPKEDVDMSLKYLGLGYDIIMGNPEGDPLLNVDPGFRAPVLQINIKDVDISN
-----------------------NNDNIELNECSIDSHSNIDDIKHGDNNKTVS------
---SKKK-------IIPWIIPEHSCNQSK--------NVEEIKSLEEYKLE--L------
-------LSDVKVSTPSIFPYSFSASAGYKNALKKLKIQNSIIFMMKIYCLRYYTGIPTT
MTKWEFTDNFRNALNKLPHTFDGLKEDNECTYEYYISKSHTPQCEKNVNKWMTFFKLHGT
HVAHEMYLGGKIIIKVNIEKEEYNKIKENNLNMKTIFDFYFHKMGLSVKRNKQVQKFINK
IHGSKTVSILGGHPGLNIDDSSFFEKWIDSIDKNSMPIRTKLLPFSFFMDDPNMIKAYND
ALMFYGLTYGVQIFDQNQYNNNEISIGNYLEKSIQKIYHGSPPGLLTCPIGSTILMGFSL
NLDFYKNQNLNEIIGINACEQMKESCSGNGFTNKYSDIRIWGLCSEKQLYFIKQVVEQNE
STKTTATCPEDSVILFGFALMKGIGRSSANTVDLYPCRTGQNSCSAVLQNQKFKQSMIYI
ACVEKSTTGLDDLQTFTKIKNLGYVDPNNYQNDGYLDFECPQNSTLVFGFALEFHTNFQK
ARNNFIKCSKEENTCSIKGVGVNTNLYFFKKDKHSLGIVALCRSTVSKMGKN

>P_berghei_ortholog
MKISVKDIFFHLIL-VQIIIYNIKCEISESLYTSKSD-DTYSIDEYNVANLINEKQSHIT
NTNLVNQNERLNNSYKGLLNNQLDSLGKRHVLQHSP--GNNNNNNISVLEKLNKDNSLIK
HTKRGAYFIHIKQHN-----------------------------------------IINK
NETKLNTNTNNAQFTN------------------TKKSIISSHDHLDKYNKTYIDNL---
-----------------------------------KSVKNNFIVPILDDNNTDFDDNNYL
ESEKYIPIKYPFDGH-----------EMKHNNSNVDGNNIYHNEGTNKKSDVTEKENYNG
NIKP-------TTCTKNKKESINKTLDTGNEFDLRNALLDDYKPDVNNYT--NIKKSFKN
KKKQRV----TEKFKDFDLADALTPNINEHISI------NDGERNNENANFISMKDWGTN
DEISQKGTLYKEDVDMSLKYLGLGYDIIMGNPEGDPLLSVDPGFRAPVLQINIKDVGISN
-----------------------NNDNIELNEGNQDSHSNIDDIKDGDNKKNRS------
---NKKK-------IIPWIIPEHSCNQSK--------NVEEIKSLEEYTLE--L------
-------LSDVKVSTPSIFPYSFSASAGYKNALKKFKIQNSIIFMMKIYCLRYYTGIPTT
TTMWEFTHNFRNALNKLPHTFDGLKEDNECTYEYYISKSHSPQCEKNVNKWMTFFKLHGT
HVAHEMYLGGKIIIKVNIEKEEYNKIKENNLNMKTIFDFYFHKMGLSVRKNKQVQKFINK
IHGSKTVSILGGHPGLNIDDSSFFEKWINSIDKNSMPIRTKLLPFSFFMDDPNMIKAYSD
ALIFYGLTYGIQIFDEKQYNHNEISIGDYLEKSIQKIYHGAPPGLLICPIGSTILMGFSL
NLDFYKNQSLNEIVGINTCEQMKESCSGNGFTNKYSDIRIWGLCSEKPLYFIKQVVEQNE
ATKTTATCPENSVILFGFALMKGTGRSSANVVDLYPCRTGQNSCSAVLQNQKFKQSMIYI
ACVDKTTIGLDNLQTFTKVKNLGYVDPNNYQNDGYLDFECPQNSTLVFGFALEFHTNFQK
ARNNFIKCSKEENTCSIKGVGINTNLYLFKKDKHSLGIVALCRSAASKMGQN
```

- **AMOUNT OF MISSING DATA**

P. reichenowi	***P. gallinaceum***	***P. knowlesi***	***P. vivax***	***P. chabaudi***	***P. berghei***	***P. yoelii***
82%	15%	11%	12%	11%	10%	11%

■ PF14_0239

▪ PHYLOGENY AND PEXEL/VTS

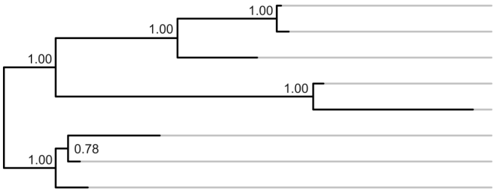

P. falciparum PF14_0239	KELTERNLIEILFDM
P. reichenowi ortholog	KELTERNLIEILFDM
P. gallinaceum ortholog	KELTERNLLEILFDM
P. knowlesi ortholog	KELIKRNIIEIVFDI
P. vivax ortholog	KELIRRNLIEIVFDL
P. chabaudi ortholog	TEVLERNVVEILFDL
P. berghei ortholog	TELIERNIVEILFDM
P .yoelii ortholog	TELIERNIVEILFDM

▪ ALIGNMENT

```
>P_falciparum_PF14_0239
MEEQNEEIEKTVEESEE-INEEVYEELFLFASSDNDLVKKESLRIILSLLEEKEFIDHII
KNNKKYLKTLISSLNSRCKLLTLECLLNLSAQLP------------KELTERNLIEILFD
M----------MKEEEKIED-NCIDLYIMIISNLTRCKEGVYKVLDINNDSNINIKE---
--DNFKVSFFLNKLLYFFFLPIKP-SINKNLSDKYIYVSHVLINISSIKESIVFFKNVAF
LNKISDQILNVERFRAILPFIINLCLNEVIHPYIFHDDCYLFPYVLSYLYTNDYNITKSG
SYNNSNNN--EEINTQNIHHIIMNKSSILVSCSVIKSRILIIILFYLCNRDYSREKLLSYG
ISDILKNWKSCEKNAEFINDIENVTNKLIEGSNSREAIAT

>P_reichenowi_ortholog
XXXXXXXXXXXXXXXXXXXXRKFYEELFLFASSDNDVVKKESLRIILSLLEEKEFIDHII
KNNKNFLKTXISSLNSRCKLLTLECLLNLSAQLP------------KELTERNLIEILFD
M----------MKEEEKIED-NCIDLYIMIISNLTRCKEGVYKVLDINNDSNINIKE---
--DNFKVSFFLNKLLYFFFLPIKP-SINKNLSDKYIYVSHVLINISSIKESIVFFKNVAF
LNKISDQILNVERFRAILPFIINLCLNEVIHAYIFHDDCYLFPYVLSYLYTNDYNITKSG
SYNNSNNN--EEINTQNIHHIIMNKSSILVPCSVIKSRILIIILFYLCNRDYSRXXXXXXX
XXXXXXXXXXXXXXXXXXXXXXXXXXXXXXXXXXXXXXXX

>P_gallinaceum_ortholog
MDMKKEE--DATGESNE-ANENIYEELFSYANSDNEIVKKESLRIILSLLEENELIEYIR
KNNKKCFKIIISNLNSRNKMLSLECLLNLSARLP------------KG-TERNLLEILFD
M----------VKEEEKYEE-NCIDLYIMIISNITRCKEGIYKILDLKEDTNIDINE---
--EKFFVSYYLNKLLYFFFLPIKP-SINKNINDKYIYVSHILINISSIKESSLFFKNVSF
LNKISEQILNKDRFRAILPFIINLCLNETTHNYLFHDSCILFPYILSYIYTNNYSLCSNN
IY-NSNNV--EK--SDNIHHIILQKATVLINCSIIKSRIIIILLYLCKKDYSRXXXXXXX
XXXXXXXXXXXXXXXXXXXXXXXXXXXXXXXXXXXXXXXX

>P_knowlesi_ortholog
MMLIAEEVENAKNAEREPIDASIYNELFSFAASDNEILKKESLKIILGLLDETDMVAYIQ
KNEKKCLKVLISGLNSNYEVVALQCLVNLSAHIP------------KELIKRNIIEIVFD
I----------LRDEEETEAKSHTELYIMLVANLSREKEGIYKILDLP-GEKVKEQEQEE
MKEELAVSYYLNKLLHLFSKPIVPSSINKQITDKYFFISHALINVSSVKECVHFFKSVFL
LNMLSNQMFQEERCAAVLQCVINLCVSELLHPYVFHEDCTLIPRVLSLVYTRQKGSEDNF
SKLSSVQTSQKETNKDPVHHLILNMSTTLTTSTDIKNRVMILLRNLFTRDQARQKLRNYG
IEHVLTNWLTYEKNTEIT----------------------

>P_vivax_ortholog
MEEEVDEGKKERDKRSSNHDDSLYDELFSLICADKEIVKKEAFKILLGLIDTESMVAYIQ
RNEKKCLKILISGLNSDYEVVALQCLVNLSAHIP------------KELIRRNLIEIVFD
L----------LRDEEEAQERSHTELYIMLVANLSREKAGLYKILDLPEGQKQEGEKQEE
TSRELAVSHYLNKLLHLFSKPIVTATINKQVTDKYFFIAHILINVSSVKECAHFFKSVIL
LNMLSKQMLQQERCAAVLQCVINLCMSETLHPYIFHEECNLMPRVLSLVYTREKGSNDEF
AALPSIATSQKGANKDSVHPLILDMSTVLTPSTDVKNRVMILLRNLFSRELARQKLRSYG
VEHVLRNWLLHEKNTGIT----------------------

>P_chabaudi_ortholog
-MLIAEELENSKNTPKEPIDVSVYNELFSFAASDNEVLKKESLKIILGLLDEPDLIAYIL
QNSQKSFRIIISSLNSQSKMVALECLLNLSAQIP------------TEVLERNVVEILFD
L----------IKDEEKYEE-NYIDMYIMIISNLTRCKEGVYKILDITDVNQVYHNT---
----FSVSYYLNKLLYFYFQPVQK-SLNKNMTDKYTHVSHILINISSMKESLPFFKNIAF
LNKLCDQILILERCRTFLPFIMNLSLHKEIHEFIFQADCNMFPYLLSYLYTPHTAIEDKN
SI-SGYTV--RP--NKTIHRTIVKKATSLVRCPVIKNRILVILLHLSKDNDTKERIKKYG
IIALLNNWKENENSPDIMXXXXXXXXXXXXXXXXXXXXXX

>P_yoelii_ ortholog
MMLIAEEAENAKNGGNEPIDISVYNELFSFAASDNDILKKESLKIILGLLDETDLIAYIL
QNSKKCFKIITSGLNSQCKMVALECLLNLSAQIP------------TELIERNIVEILFD
M----------IKEEEKNEE-NYIDMYIMIISNITRCKEGIYKILDISDEGQIYHNT---
----FSVSYYLNKLLFFYFQPITP-SINKNMSDKYMHISHILINISSIKESMPFFKNVAF
LNKLSEQILILERCRTFLPFIMNLSLHKDIHDFIFQSDCNMFPYLLSYVYSHDTPINEEN
IL-NGV-I--KP--DKSIHKLILKKSTSLVQCSIIKNRILVILLHLSKNNDTREKIKKFG
VIPLLNNWKANEKSTDIIXXXXXXXXXXXXXXXXXXXXXX

>P_berghei_ortholog
-MLIAEEVENAKNAEREPIDASIYNELFSFAASDNEILKKESLKIILGLLDETDLIAYIL
QNSKKCFKIIIS-LNSQCKMVALECLLNLSAQIP------------TELIERNIVEILFD
M----------IKDEEKIEE-NYIDMYIMIISNITRCKEGIYKILDINEEGQIYHNT---
----FSVSYYLNKLLFFYFQPITP-SINKNMSDKYMHVSHILINISSIKESMPFFKNVAF
LNKLSEQILILERCRTFLPFIMNLSLHKDIHDFIFQSDCNMFPYLLSYVYTPNTPIDDKK
SL-NGVTT--KP--YKSIHKVIVKKSTSLVRCSVIKNRILVILLHLSRNNDTREKIKKFG
VVALLNNWKANEKAPDIMXXXXXXXXXXXXXXXXXXXXXX
```

▪ AMOUNT OF MISSING DATA

P. reichenowi	***P. gallinaceum***	***P. knowlesi***	***P. vivax***	***P. chabaudi***	***P. berghei***	***P. yoelii***
17%	14%	8%	8%	7%	8%	7%

■ PF14_0553

▪ PHYLOGENY AND PEXEL/VTS

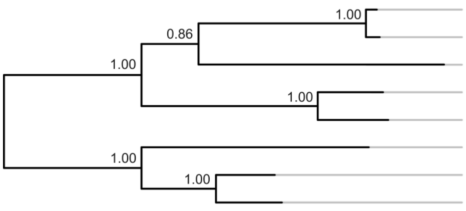

P. falciparum PF14_0553	EIELLRVLLEKYKKQ
P. reichenowi ortholog	EIELLRVLLEKYKNQ
P. gallinaceum ortholog	EIEIFKDLLKRYKEE
P. knowlesi ortholog	EIETLKFIFSKYPQG
P. vivax ortholog	EIETLRFIFSNYPHG
P. chabaudi ortholog	EIAHLREILKKYKAT
P. berghei ortholog	EIAYLREILKKYKTK
P .yoelii ortholog	EIAHLKEILKKYKTK

▪ ALIGNMENT

```
>P_falciparum_PF14_0553
MVAIKEMKEFAFARPSLVETLNKKKKFLKKKEKRTFVLSIYAFITFIIFCIGILYFTNKS
SAHNNNNNKNEHSL-------------------------KKEEIELLRVLLEKYKKQKDG
ILNES---------SNEEDEEKYTLNSETYNNKNNVSNIKNDSIKSKKEEYINLERILLE
KYKKFINENNEENRKELSNILHKLLEINKLILREEKDDKKVYLINDNYDEKGAL------
----EIGMNEEMKY----KKEDPIN---------NIKYASKFFKFMKEHNKVYKNIDEQM
RKFEIFKINYISIKNHNKL--NKNAMYKKKVNQFSDYSEEELKEYFKTLLHVPNHMIEKY
SKPFENHLKDNI---LISEFYTNGKRNEKDIFSKVPEILDYREKGIVHEPKDQGLCGSCW
AFASVGNIESVFAKK-NKNILSFSEQEVVDCSKDNFGCDGGHPFYSFLYVLQNELCLGDE
YKYKAKDDMFCLNYRCKRKVSLSSIGAVKENQLILALNEVGPLSVNVGVNNDFVAYSEGV
YNGTCSEELNHSVLLVGYGQVEKTKLNYNNKIQT-----YNTKENSNQP----DDNIIYY
WIIKNSWSKKWGENGFMRLSRNKNGDNVFCGIGEEVFYPIL

>P_reichenowi_ortholog
MVAIKEMKELAFARSSLIETLNKKKKFLKKKEKRTFVLCIYAFITFIIFCIGILYFTNKS
SAYNNNNNNNEHSL-------------------------KKEEIELLRVLLEKYKNQKDG
ISNES---------SNEEDEKKYALNSQAYNNNNNISNIKNDSIKSKKEEYINLERILLE
KYKKFINENNEENRKELSNILHKLLEINKLILSEEKDNKKVYLINDNYDEKGAF------
----EIGMNEEMKY----KKEDPIN---------NIKYASKFFKFMKEHNKVYKNIDEQM
RKFEIFKMNYISIKNHNKL--NKNAMYKKKVNQFSDYSEEELKQYFKTLLHVPNHMIQKY
SKPFENHLKDNI---LISEFYTNGKRNEKDIFTKVPEILDYREKGIVHEPNDQGLCGSCW
AFASVGNIESVFAKK-NKNILSFSEQEVVDCSKDNFGCDGGHPFYSFLYVLQNELCLGDE
YKYKAKDDMFCLNYRCKRKVSLSSIGAVKENQLILALNEVGPLSVNVGVNNDFVAYSEGV
YNGTCSEELNHSVLLVGYGQVEKTKLNYNNKIQT-----YNTKENSNQQ----DDNIIYY
WIIKNSWSKKWGENGFMRLSRNKNGDNVFCGIGEEVFFPIM

>P_gallinaceum_ortholog
MK--KEINNHTLSRRGL-ESLSKKKEHFKNEEKKVFRIYIYALITFIICSLILLYFTNNS
--------IESHDL-----------------------IFNSDEIEIFKDLLKRYKEENSL
KNDEKIENDYN---SNDLQKKK-----------------------------VNKNIIIDD
LYN-LIDEKNNISKERLFEILDDLLQKK----FSKKNEKEEYILLNGIAKK---------
--------NDNNLYREYDKNEELYDSKALVNLYSNLKYVSKFYEFMNEHNKNYNSMKEKI
EKYENFKINYLEIKKHNT----ENHLYKKKVNKFSDYSKKELENYFKKLVPVPSHLIEKH
VKPFSTILKSVKG--IEEK------EKNKDLFSTFPTNLDYREKGIVHEPKDQGVCGSCW
AFAGVGNIESMYAYK-NKKMISLSEQEVIDCSKKCFGCDGCHPFYAFLYALENKICFEEQ
YEYKIMENIFCLNYRCTNKVSLTSIGSVKENELIEALNKVGPLSVCVGASDDFVFYHEGI
FDGICTKEVNHAVLLVGYGQVEKNKNLQ------------KYKGSFSDE----DDNFIYY
WIIKNSWGSNWGENGFMRIIRNKNGDNLFCGIGKDVFYPVL

>P_knowlesi_ortholog
MA--QNMSIMNLTKSSL-EALNRNQMLSKRRNRKILKICIYIVLTFVMCGVVFVCFTTIS
--------RNDWSL------NRSDNLKDSSFNSGDGDILNKAEIETLKFIFSKYPQGKKD
LSGDEVEALADAAISNEED-EK------------------------IKIEEPGKHIKLMK
KYNEVVADLSQENREQLAKMLKELLKKK---MNEKKTGKKDPNSVNGEEGKEDINGGLSN
FNFDSVGANEDD-N----GGDAVLSEEHIEGLFFNLKYASKFFKFMNKYNRNYKDITEQM
EKYENFKINYLKIKKHNE----TSQMYRMRLNQFSDYSKKDFENYFRKLLPIPDHLKKKY
VVPFAS-LNNVKGK-MVDS------NKSADIFTDVPEILDYREKGIVHEPKDQGLCGSCW
AFASVGNVECIYAKEHDKTILTLSEQEIVDCSKLNFGCDGGHPFYSFIYAIENGICLGAD
YKYIAMDNLFCLNYRCKNKITLSSVGGVKENELIRALNEVGPVSVNVGVTDDFSFYDGGI
FNGTCTEELNHSVLLVGYGQVQTSKIFQQNNIYDDANGLTK-KGAIASPSKANDDGIQYY
WIIKNSWSKYWGENGFMRLSRNKEGDNVFCGIGVEVFYPIL

>P_vivax_ortholog
MA--QDIKIMNLTKSSL-EALNRNQMLSKKSSRKILKICMYAILTFAMCGVVLICLTAMS
--------NSDGSLTQSGSHNQSGSLKGLSSTPGDGEILNKAEIETLRFIFSNYPHGNRD
PTGDDVEKPADAALPNEED-QK------------------------VKIADAGKHIKLMK
QYNEIVADMSEDNKEQLAKMLRELLKKK---INERKKKREDPNG-NNEEGKEVINISVPS
FNYKRVSANQDD-S----DDEEEVSVAQIEGLFVNLKYASKFFNFMNKYKRSYKDINEQM
EKYKNFKMNYLKIKKHNE----TNQMYKMKVNQFSDYSKKDFESYFRKLLPIPDHLKKKY
VVPFSS-MNNGKGKNVVTS------SSGANLLADVPEILDYREKGIVHEPKDQGLCGSCW
AFASVGNVECMYAKEHNKTILTLSEQEVVDCSKLNFGCDGGHPFYSFIYAIENGICMGDD
YKYKAMDNLFCLNYRCKNKVTLSSVGGVKENELIRALNEVGPVSVNVGVTDDFSFYGGGI
FNGTCTEELNHSVLLVGYGQVQSSKIFQEKNAYDDASGVTK-KGALSYPSKA-DDGIQYY
WIIKNSWSKFWGENGFMRISRNKEGDNVFCGIGVEVFYPIL

>P_chabaudi_ortholog
MM--NDIRRINFTTTGI-DSLNENGTYSKGNHKKTIKICAYAITAIALFFIGGVYFKYQT
---------DRNAL-----------------NAIDEAELMNKEIAHLREILKKYKATTND
--------------DNEFEYPT--------------------------------------
--NDDMNEDSEDGEHQLLLMLHKLLKN-------NANKVNTFGIN---------------
--------NESNTN----TDNTYIFTQKLESMQDNIKYASKFFKYMKKYNKKYENMDEQL
ERFENFKISHMKVKKHNEMIGKNGVTYVQKVNQYSDFSKEEFNNYFKKFLSVPHDLKTKY
LVPLKEHLANNN---ITPA---------NDLVGDFPDSRDYRGKYTLLPPKDQGMCGSCW
AFATIANFEYLFAKIKGTMPTSFSEQQVVDCSTDNYGCDGGHPFYSFLYFINNGVCLGDE
YPYKGHDDFFCLNYRCSFLGSMHFIGDVKPNELIMALNYVGPVTIGVGASDEFVLYSGGV
FDGECASELNHAVLLVGYGQVKKSLAFDDSHSNVDSSLIKKYKENIKGD----DDETIYY
WIIRNSWGTEWGEGGYIRLKRNKEGDDGFCGVGTDVFFPIY
```

```
>P_yoelii_ ortholog
MV--NDIRRVNFATSGV-ESLNENSKYLNRNHKKTIKICTYAITTFALFFIVVVYFKNQT
----NVNDINSSTL-----------------SAIEETSLMNKEIAHLKEILKKYKTKINE
--------------DNEYGYEK--------------------------------------
--NDNINGDDEDGEQELLLMLHKYLKNK-----SNSNKIDSFDINNNESNK---------
--------NRSNEN----IDQTNILSQKLETMHDNIKYASKFFKYMKEYNKEYKNIDEQL
KRFENFKTTYMKVKKHNEMVGKNGITYVQKVNQFSDFSKEEFDNYFKKLLPISHDLKTKH
VVPLKTHLDDNK---IKPK---------EDVL-SYPEHRDYREWDILLPPKDQGMCGSCW
AFASVANYEALFAKKYAILPISFSEQQVVDCSSDNFGCDGGHPFLSFLYFLNNGVCFGDQ
YEYKAHDDFFCLSYRCGYKGRLKKIGNAYPYELIMALNEVGPITVNVGVSDEFVLYSGGI
FEGPCSPELNHSVLLVGYGKVKKSLAFEDSHTNVDSNLIKKYKENIKEN----DDDFLYY
WIIRNSWSSAWGEGGYIRIKRNKLGDDIFCGIGIDVFFPLL

>P_berghei_ortholog
MI--NDIRRINITTSSI-ESLNENSKYLKRNHKRTIKICAYAITTFALFFIVVVYFKNQT
----NVNDANRNTL-----------------AAIDETSLMNKEIAYLREILKKYKTKTNE
--------------NNEYAYEK--------------------------------------
--NDDINGDGED-EHELLLMLHKFLKNK-----GNPNKIDRFDINNNDSNK---------
--------NRGNEN----IDQINILSQKLESMHDNIKYASQFFQYMKE-NKKYKNIDEQL
VRFENFKTNYMKVKKHNEMVGKNGITYVQKVNQFSDFSKEELDSYFKKLLPIPHNLKTKH
VVPLKTHLDDNK---IKPK---------EGVL-DYPEQRDYREWNILLPPKDQGM-GSCW
AFASVGNYEALFAKKYSILPISFSEQQVVDCSSDNFGCDGGHPFLSFLYFLNNGVCFGDN
YEYKAHDDFFCLSYRCAYRSKLKKIGNAYPYELIMSLNEVGPITVNVGVSDEFVLYSGGI
FDGTCASELNHSVLLVGYGKVKRSLVFEDSHTNVDSNLIKNYKENIKDS----DDDYLYY
WIIRNSWSSTWGEGGYIRIKRNKLGDDVFCGIGIDVFFPIL
```

- **AMOUNT OF MISSING DATA**

P. reichenowi	*P. gallinaceum*	*P. knowlesi*	*P. vivax*	*P. chabaudi*	*P. berghei*	*P. yoelii*
0%	15%	17%	18%	18%	17%	16%

■ PF13_0218

▪ PHYLOGENY AND PEXEL/VTS

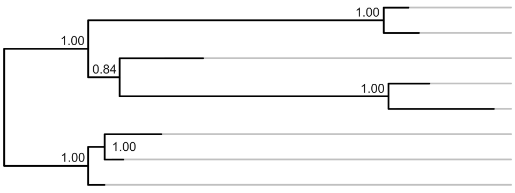

P. falciparum PF13_0218	ENYIFRAFLDMTKHE
P. reichenowi ortholog	ENYIIRAFLDMTKHE
P. gallinaceum ortholog	ENYIIRAICDMNKYE
P. knowlesi ortholog	NNFLLRAIRDMTKGE
P. vivax ortholog	KNFLLRAVADMTKGE
P. chabaudi ortholog	DNYIFRALYEMTSYE
P. berghei ortholog	DNYLFRALYEMTNYE
P .yoelii ortholog	DNYLFRALCEMTNYE

▪ SUPPORT FOR ALTERNATIVE TOPOLOGY RESEMBLING THE SPECIES TREE:

Expected likelihood weights	0.1539	+
Shimodaira-Hasegawa test	0.9990	+
two-sided Kishino-Hasegawa test		+
one-sided KH test	0.4030	+

▪ ALIGNMENT

```
>P_falciparum_PF13_0218
MGNSLSLCLLRETSKDVFRYFESLYIKYFKSYNYINYITLKYNKKDDKMHRKKVITCADI
NEGPKNNRIKK-----------ENYIFRAFLDMTKHE---------KTLLSLAMVFLIIN
AYTNINYPRIMGECIEASNHMNNNIFNNIHNNNNDNIFQSNDNVIYNTFCNINICN----
----NVINFFFDKISFIKNYFVD--RNIIKTIIFYFPYFICGGIASYMRIYFTNQCIKNI
EIRLKKQVHDKILMNNEEEFKEYKTSDYLVNCTFIEIKYSAKELITCITQTFRYMNSVVG
GLISMSCISLYLTKLCLFIIPIYGLSVLCLLKSLKRIKIETTTMEEKQIARFSDSLQKKN
IISLFGNESYEHRYFCKQLHVLNNLYDKYITCESIFYSFLNIGTNVVICTILCLGRLELT
TQHITHGQLVSFIVYSTMLGLGVGGMLKLKKDINLLQISLQKIYEILDLTKSETQTDINN
YEQGKLTIQDKQVNLSKNKQSGNSMGKYKPDNDDDNKEDNKEDSKDYNKDDNKDDNNDNN
NDNNNDNNNDNNNDNNNDNNNDNNNDNNNDHHNDHNNDNNNVVMNELPFKYPHKSNYIPN
NIKGSIRFENVSFSYNHYDKERKKSVLKNINLEIKASEKVAIIGKSGSGKSTLWKLLTCN
FSYEGNIFIDNLNIKNIHNHFLKRNILSVSEQECCIFNRTIYENLIYGLVPLYIKN----
-NNNNNNNN--YYYQYLSKCIFQNMN---------PGNNIHKKILNNIQSTKYNSQCYQK
NKHNITSNFITQSYRTQDEDPHNISNHKHIDNTSRNIYQNY-YTDFLNSYDEYKLNIINS
SINVLCEELDLKQFINSMPHNIHSNIQYNNMSSGQKQRLSIIRSLMKDTPIYIFDEITSF
LDEGNIHKLHKLIDLLIPNKTIIYITHSIHILNKMDKIIILDDGKIRAIGTYEQIKNDTL
FRDIFSLHKPA-V

>P_reichenowi_ortholog
MGNSLSLCLLRETSKDVFRYFESLYKKYFKSYNYINYIRLKYNKKDDKIHRKNVITCVDF
NEECKNNRIKK-----------ENYIIRAFLDMTKHE---------KALLSVAMVFLVIN
AYTNINYPRIMGECIEASNHMDNNIFNNNNNILKSNIFKSYDNVIYNYLCNINICN----
----NIINFFFDKIRFIKNGFLD--RNIMKTIIFYFPYFICGGIASYMRIYFTNQCIKNI
EIRLKKQVHDKILMNNEEEFKEYKTSDYLVNCTFIEIKYSSKELITCITQTFRYMNSVIG
GLISMSCISLYLTKLCLFIIPIYGLSVLFLLKSLKRIKMETITMEEKQIARFSDSLQKKN
IISLFGNESYEHKYFCKQLHALNNLYDKYITCESIFYSFLNIGTNVVICTILCLGRLELT
TQHITHGQLVSFIVYSTMLGLGVGGMLKLKKDINLLQISLQKIYEILDLTKRETQTNINN
YEKSKLFIQDKRVDHLKKEERENIMGK----NKSVNKEDNDDDNKDVNKDVNKDDDH-HH
NDNNNNNYNDNNNYNSNDNNNNNYNDNNNYNSNYNNYYYYNVVMNDLAFKYPHKCNYIPN
NIKGSIRFENVSFSYNHYDKERKKIVLQNINLEIKANEKVAIIGKSGSGKSTLWKLLTCN
FSYEGNIYIDNLNIKNIHNHFLKRNILSVSEQECCIFNRTIYENLIYGLVPLYIENHNNN
HNHNNNNNNYYYYYQYLSKCIFSNIN---------PSNIIHKKILNNIQTTKYNSQCYQN
NKHIITPNFITQSYQTQDEDAHNISNHKDIDNTNRNIYPNY-YTDFLNSYDEYKLNIINS
SINVLCEELDLKEFINSMPQNIHSNIQYNNMSSGQKQRLSIIRSLMKDTPIYIFDEITSF
LDEGNIHKLHKLIDLLIPNKTIIYITHSIHILNKMDKIIMLDDGKIRAVGTYEQIKNDTV
FMDIFSLHKPA-V

>P_gallinaceum_ortholog
XXXXXXXXXXXXXXXXXXXXXXXXXXXXXXXXXXXXXXXXXXXXXXXXXXXXXXXXXXXX
XXXXXXXXXXX-----------ENYIIRAICDMNKYE---------ITLLSMALIFLAIN
AYTNLSYPKIMGECIEASXXXXXXXXXXXXXXXXXXXXXXXXXXXXXXXXXXXXXXXXXX
XXXXXXXXXXXXXXXXXXXXXXXXXXXXXXXXXXXXPYFACGGLASYFRIYFTSKCIKNV
EYRLKKEVHNKIISENDEEFKKYKSSDYLVNCAFNEIKFASKELVTSITQMLRYVNSIIG
GMISMVLISSYLTKLCIFIVPAYGFFVLMILKKLKNIRIQTDNFDEKQMARFSDILQKKN
IVSIFGNEYYEKEYFSKKLKAVDKLHQKYLNCEATFYSFLNIGSNVVICSILCFGRMELK
NNNITHGQLVSFIAYSSMLGLGIVGLLKLKKDLNILHLSLKKIYEILDFSKXXXXXXXXX
XXXXXXXXXXXXXXXXXXXXXXXXXXXXXXXXXXXXXXXXXXXXXXXXXXXXXXXXXXXX
XXXXXXXXXXXXXXXXXXXXXXXXXXXXXXXXXXXXXXXXXXXXXXXXXXXXXXXXXXXX
NIKGSIKYENVSFSYNNFDENK-KEVLKNINFEINENEKVAIIGKSGSGKSTLWKLLTRE
YEYEGNIYIDNYNIKDINETYFKKNVLSISEQECSILNRSLYENLIYGLLPXXXXXXXXX
XXXXXXXXXXXXXXXXXXXXXXXXXXXXXXXXXXXXXXXXXXXXXXXXXXXXXXXXXXXX
XXXXXXXXXXXXXXXXXXXXXXXXXXXXXXXXXXXXXXXXXXXXXXXXXXXXXKLNTINS
SVNVLCEELDLINFINTLPQNFHTEIRHNSMSSGQKQRISIIRSLMKDTPIYVFDEITSF
LDESNTEKVYTLINTLIPNKTIIYITHSIQLLKEMDKIIIVDNGEIKSIGSYDEVKKDPL
FLDIFSMN-----

>P_knowlesi_ortholog
MGNAL-------------RKGKDAQGVFGKIPNNIYAVFSIHGEDSSLRNKPADGGVEEC
TSTKREDKKKK-----------NNFLLRAIRDMTKGE---------KTLLVIAFAFLAIN
AYTNLNYPKIMGECVETVNLAEGGVSAVNGVNGGDSVGGC----------------NIGA
PTKCNFLVTFLQKIKILNNLFLGTQQRGAHAVLCFLPYFICGGVASYFRIYFTNKCIKKV
ENRLKKRVHKTIITQNSEKFKSHKSPDYLINCVFNEIKFSAKHLITSITQMMRYGNSILG
GSISMICISPYLTKLCILVVPTYGFLVLLILRKLKSIKMKTSNSEEKQMARLSDILQKKN
VISFFGNDYYENSFFCKNLKYMNRLNDSYTNWESLFYSFLNIGSNIVICSILCFGRSELN
KKNMTHGQLVSFIAFSSMLGLGIVGMLKLKKDLGVLQLSLQKIYEIVDFTPMG-------
-----------------------------------------------NNQQDERMPLQRG
NVLIHMESPIERDPPPTIGDVNGGDDS------------------------PVLVDQCPR
```

```
NLRGSLKFENVNFAYNAFDPSKRKDVLKNINLEIKEKEKVAIIGKSGSGKSTLWKLLTME
YEYQGNIYIDTYNLKNINKTYFKKFIISVSEQDSSVLNRSLYENLIYALLPVKIED----
-------------QWELPKVI--TMGDDQLPSGNAKSICSDQSRYNDKSSSPKEGEDTSN
EKLDKAPHTQGQNCNTYNT---RLGEGKITPITEEAQLNDQVNYALLHEYGQDKLNLINE
TLNELCEQLDLTHFIRSLPVDIHSSIQNSAMSSGQRQRISIIRSLIKDTPIYVFDEITSF
LDESNEEKVYNLINTIIPNKTIIYVTHSVNILHQMDKIIIVDQGKICSIGTFSQIKNDPL
FLDIFSHSKMASR

>P_vivax_ortholog
MGNAL-------------RKAKEAQGVAGKILSSIYAAFSIHGQDSSRKGKPKDGGAEEG
ASAKGEAAKGR-----------KNFLLRAVADMTKGE---------KTLLTIAFAFLAIN
AYTNLSYPKIMGECVETVNLAEGGVPAVSGLNGGGSAGGSVGGSVGGSVGGSIDGSTIGT
PSKCNFLATLVQKTRILNKLFLGTPDRGAHAVLCFLPYFICGGVASYFRIYFTNKCIQKV
ENRLKRKVHKTIVTQNSERFKSLKSPDHLLNCVFHEIKFSAKQLITSITQMVRYGNSILG
GSISMVCISPYLTKLCVLVVPSYGLLVLLILRKLKSIKMKASNCEEKQMARLSDILQKKN
VISFFGNDYYENRFFCKNLKYMNRLNERYTNWESLFYSFLNIGSNIVICSILCFGRSELS
KKNITHGQLVSFIAFSSMLGLGIVGMLKLKKDLGVLQLSLQKIYEIVDFAPVG-------
------------------------------------------------------------
------------GAERDGGASAQGDDVSTEVHSQVGGT-------------PTVGAPWPR
NPRGSLKFENVHFAYNAFDASKRKEVLKNISFEIKEKEKVAIIGKSGAGKSTLWKLLTTE
FDYQGNIYIDEYNLKHMNKTYFKKCIISVSEQDCCLLNRSLYENLLYAVLPVKIEG----
-------------KREFPRVI--TMG--------------GREICTDQSTRPKEGEGTSN
GTPDEAAHSQRETCNTLNTSSTHPGEAKATPNSEAAQLKDHAHCALLQEYGQDKLILIND
TLNQLCEQLDLTQFIRSLPEHLHTSIQNSSMSSGQRQRISLIRSLIKDTPIYVFDEITSF
LDEANVERVYSLINTVLAKKTIIYVTHSVNILPQMDKIIIVDQGRICSIGTFSQIKNDPL
FLDIFSHSRVASV

>P_chabaudi_ortholog
XXXXXXXXXXXXXXXXXXXXXXXXXXXXXXXXXXXXXXXXXXXXXXXXXXXXXXXXXXXX
XXXXXXXXXXX-----------DNYIFRALYEMTSYE---------KTLLSISLIFLGIN
AITNLSYPKIMGECVEGENL---------------------------------------K
FCRSNIIVKLLQKLNILEKFKLNS-NKSISAMLYFLPYFICGGIASYFRIYFTNKCIKKI
EYRLKKQVHNKIINENDEKFKKYKSNDYLVNCLFNEIQFSSKELITSITQMLRYTNSIIG
GIMSMCLISSYLTKFCIFIVPTYGFCILIILKKLKNIKIEINNFEEKQMERFSDSLQKKN
IITIFGNEYYENKHFSKIINLTEKEHQKYINSESMFYSFLNIGTNLVICTILSFGKIELN
NNRITHGQLVSFIAYSSMLGLGIVGILKLKKDINLLKLSMKKIYEILDFTPET-------
-------------------------------SDGTVSTTKQLTDKPMDWGNDKNSDTSLA
SANLSN--------IAQLESSVQNDDK------------------------HEENNIICE
KIEGSIKFENVNFTYNKFDKDQ-KIILKNINFEIKKNEKVAIIGKSGSGKSTIWKLLTRQ
YEYEGNIYIDNYNIKNFDKTYLKKSILSITEQECCILNRSLYENIVYALLPTKVSD----
-------------ASGAKDLLLDSIGEXXXXXXXXXXXXXXXXXXXXXXXXXXXXXXXXX
XXXXXXXXXXXXXXXXXXXXXXXXXXXXXXXXXXXXXIQNY-DHMLLEKYG-NKINTINS
TIDILCKELNLDDFINSMPQNILTIVNNNSMSSGQKQRISIIRSLMKNSSIYIFDEITSF
LDESNLDKVYNLIHTLIPDKTIIHITHSLKHLKEMDKIIIIDQGTISAIGTYQEINKHPL
FLEIFSL------

>P_yoelii_ ortholog
XXXXXXXXXXXXXXXXXXXXXXXXXXXXXXXXXXXXXXXXXXXXXXXXXXXXXXXXXXXX
XXXXXXXXXXX-----------DNYLFRALCEMTNYE---------KTLLSISLVFLGIN
AITNLNYPKIMGECVEGENL---------------------------------------K
LCRPNIIVKILQKINILEKFKLNS-NKSLSAMLYFLPYFICGGIASYFRIYFTNKCVKKI
EYRLKKQVHNKIINENDEHFKKYKSNDYLVNCLFNEIKFSSKELITSITQMLRYINSIVG
GIISMCLISPYLTKFCIFIVPTYGFCILVILKKLKKIKIEINNFEEKQMERFSDSLQKKN
IITIFGNEYYENHYFSKIINLTEKEHQKYINSESMFYFFLNIGTNLVICTILSFGKIELN
NNRITHGQLVSFIAYSSMLGLGIVGILKLKKDVNLLKLSIKKIYDILDFSSEI-------
-------------------------------NNSITNSNNQLSNKMTNSICDQISDISLS
NTSLNNNDNNDNNDNMQLGNSIRKGDI------------------------IETNNTVCE
KIEGSIKFENVNFIYNKFDKDK-KLILKNINFEIKKNEKIAIIGKSGSGKSTIWKLLTRE
YEYEGNIYIDNFNIKNFDKTYLKKSILSITEQECCILNRSLYENIVYALLPTKISD----
-------------AKGMKDLLLENIGDLKRLDDELK----------------------VN
NSNNTNNYDKLNNCTNKFTVNENKLNEKKIINNKIEDIQNY-NYMLLEKYG-DKINTINS
TIDILCKELNLNDFINSMPQKILTNVNNNAMSSGQKQRISIIRSLMKNSSIYIFDEITSF
LDESNIDKVYNLIHTLIPDKTIIYITHSLKHLKEMDKIIIVDQGTISAIGTYQELNNHPL
FLEIFSL------

>P_berghei_ortholog
XXXXXXXXXXXXXXXXXXXXXXXXXXXXXXXXXXXXXXXXXXXXXXXXXXXXXXXXXXXX
XXXXXXXXXXX-----------DNYLFRALYEMTNYE---------KTLLSISLVFLGIN
AITNLNYPKIMGECLEGENF---------------------------------------K
FCRPNVIVKILQKLNILEKFKLNS-NKSLSAILYFLPYFICGGIASYFRLYFTNKCIKKI
EYRLKKQVHNKIISETDENFKKYKSNDYLVNCLFNEIQFSSKELITSITQMLRYINSIVG
GIMSMCLISPYLTKFCIFIVPTYGFCILIILKKLKKIKIEINNFEEKQMERFSDSLQKKN
IITIFGNEYYENQHFSKIINLTEKEHQKYINSESMFYSFLNIGTNLVICTILSFGKIELN
NNRITHGQLVSFIAYSSMLGLGIVGILKLKKDVNLLKLSIKKIYDILDFSPQI-------
-------------------------------NNNTTSFNNQLSNKTINSVCDKIDDISLS
NINLNN--------IIQLENSIQNDDI------------------------VEKNNIICE
QIQGSIKFENINFTYNKFDQDK-KLILKNINFEIKKNEKIAIIGKSGSGKSTIWKLLTRE
YEYEGNIYIDNFNIKNFDKTYLKKSILSITEQECCILNRSLYENIVYALLPTKVSD----
-------------PKGTKYLLLENIGDLGRLDNELKANISNN----------------TN
NANNKNHYDKLNNCTDKFTTIENKLNEKNFINNKLEDIQNY-NYMLLEKYG-DKINTINS
TIDILCKELNLNDFINSMPQNILTNVNNNSMSSGQKQRISIIRSLMKNSYIYIFDEITSF
LDESNIDKVYNLIHTLIPDKTIIYITHSLKHLKEMDKIIIIDQGTISAIGTYQELNNHPL
FLEIFSL------
```

- **AMOUNT OF MISSING DATA**

P. reichenowi	*P. gallinaceum*	*P. knowlesi*	*P. vivax*	*P. chabaudi*	*P. berghei*	*P. yoelii*
1%	38%	15%	14%	28%	23%	23%

■ PFL0535c

▪ PHYLOGENY AND PEXEL/VTS

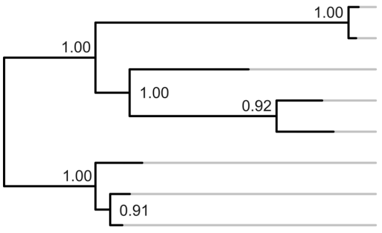

P. falciparum PFL0535c	ITPNRRILPSITYID
P. reichenowi ortholog	ITPNRRILPSITYID
P. gallinaceum ortholog	ISPNRKIVPMIRYIN
P. knowlesi ortholog	ISPNRKIIPSIRYIN
P. vivax ortholog	ISPNRKILPSITYIN
P. chabaudi ortholog	ITPNRKIMPSITYYN
P. berghei ortholog	ITPNRKIMPSITYYN
P .yoelii ortholog	ITPNKKVMPSITYYN

▪ SUPPORT FOR ALTERNATIVE TOPOLOGY RESEMBLING THE SPECIES TREE:

Expected likelihood weights	0.0750 +
Shimodaira-Hasegawa test	0.9880 +
two-sided Kishino-Hasegawa test	+
one-sided KH test	0.1400 +

▪ ALIGNMENT

```
>P_falciparum_PFL0535c
MLNSGGEHVESERRPCLKYLISLLLLLMLMLILRKQNDEEEEMNNRNKMNLRKNENY-RN
YTFGYMRDISYFKYSSVIY-----ITPNRRILPSITYID---------KYTIYVNGYDNI
LNNFKNISLSIDQNKDDIYISSSYDNNYNIYNDDNIYNDDNIYNNDNIFHNDDNIFHNDL
CKEGDDSNKDCAIINTILVDKYLFKNMKISNCNEEIDILCFLVFVQLNKKKCYNFDNLSY
SCHINNSIKNKNLFLLNNYCYVLLASSSIHKIYEFIKLELLNKEEINDNTKNEHVQIVTH
FCGNMINQIAMIDYVNTRILFIQRYIKHTDDDNNNNNNNTNDL------NTNDLNTNDLN
TNELNTNELNTNNHNNRMKGKSIELNLNEENKNNLYDTFNNDIKNTYETNLNFKLTYYLS
IINMNTQYKDNIEYVDYIKNKLTLNLFPPIYIGYDETSNFYYIIQYENKDFLTIIFISPI
TLTTYYTLKITDFYSGWLLIPKYYYENKGLILL--GEYPPYEDELHVWDITNIEHSKYFV
NVIKAYSNI

>P_reichenowi_ortholog
MLNSGGEHVESERRPCLKYLISLLLLLMLMLILRKQNDEEEEMNNRNKMNLRKNENY-RN
YTFGYMRDISYFKYSSVIY-----ITPNRRILPSITYID---------KYTIYVHGYDNI
LNNFXXXXXXXXXXXXXXXXXXXXXXXXXXXXXXXXXXXXXXXXXXXXXXXXXXXXXXXX
XXXXXXXXXXXXXXXXXXXXXXXXXXXXXXXXXXXXXXXXXXXXXXXXXXXXXXXXXXXX
XXXXXXXXXXXXXXXXXXXXXXXXXXXXXXXXXXXXXXXXXXXXXXXXXXXXXXXXXXXX
XXXXXXXXXXXXXXXXXXXXXXXXXXXXXXXXXXXXXXXXXXXXXXXXXXXXXXXXXXXX
XXXXXXXXXXXXXXXXXXXXXXXXXXXXXXXXXXXXXXXXXXXXXXXXXXXXXXXXXXXX
XXXXXXXXXXXXXXXXXXXXXXXXXXXXXXXXXXXXXXXXXXXXXXXXXXXXXXXXXXXX
XXXXXXXXXXXXXXXXXXXXXXXXXXXXXXXXXXXXXXXXXXXXXXXXXXXXXXXXXXXX
XXXXXXXXX

>P_gallinaceum_ortholog
MISQSGERLESERRSCCKCFITLLFLFILLLILRKQK-EEDEIKNRSKVNLRQNENYNKD
FSFGYIREISFLKYDSAIY-----ISPNRKILPSITYIN---------DYTLHVEGYDNI
LHNFENLSIKINEKNNNLYIFALSDNKNK----------------------------NSI
CINKSYSDDFCAKINTVLVDKSLFKNMKIQSCDEQ-NSFCFFVFVQLKKNKCYNFVNLSY
SCHENN-MKNPNIFLINNYCYVLLASSKIPSVYEFIQFNLLKNENFNENTKTKDVEIITH
FCGNLLNQIATIDSVNFRLLFVQQYIHINDKXXXXXXXXXXXXXXXXXXXXXXXXXXXXX
XXXXXXXXXXXXXXXXXXXXXXXXXXXXXXXXXXXXXXXXXXXXXXXXXXXXXXXXXXXX
XXXXXXXXXXXXXXXXXXXXXXXXXXXXXXXXXXXXXXXXXXXXXXXXXXXXXXXXXXXX
XXXXXXXXXXXXXXXXXXXXXXXXXXXXXXXXXXXXXXXXXXXXXXXXXXXXXXXXXXXX
XXXXXXXXX

>P_knowlesi_ortholog
MITASGERFDDDRRSCFKYFVTLMFLLVLMLILRKQKEEEEEMSNRAKMNLRQSENY-RN
FAFSYLREISFLKYDSAVY-----ISPNRKIIPSIRYIN---------NCTVYVQGYDNI
LYNFENVSLGINEEKNNFYIYAVANNRQG----------------------------NGN
CADNACGDVGDGIINTILVDTTLFRNMQIGSCQEKDTSMCFFIFVQLSKSKCYNFNNLSY
SCHANNTVKNSNVFLASTYCYVVLASSKVERIYEFIKEKLMGQDPINEKISTKEVEIVAH
FCGNLLNQVATIDAVNARLLFVQRYIRKFDKVEKNESNDSSEKDNPSAGNGNNPNEDDQK
NISMKFNAEEGQKEQYQGYTKSFVYNNNFKNENNM-------------NSLKLRISYQLT
VINLASENRDIIEYVEYAMDKPEFCLFPPIHIAFDEQSNFYYIVQHENNGFLNFIFISPF
PLMVYYTLKIRDFYSGWLLNSSRDYDNKGLILF--GEYPPYEDELHVWDIRNLQHRKYLV
NIIKTY---

>P_vivax_ortholog
MIAASGERFDDERRSCFKYFLTLMFLLVLMLILRKQKEEEEEISSRAKMNLRQNEHY-RN
FAFSYLREISFLKYDSAVY-----ISPNRKIVPMIRYIN---------NYTVYVQGYDNI
LHNFENVSVGISEEKNNLYIYAVANNRLE----------------------------NGT
CASAACGDADDGIINTVLVDTTLFRNMQIGSCLEKDTSMCFFVFVQLSKSKCYSFNNLSH
SCHANNTVKNPNVFLASSYCYVVLASSKVERIYEFVKEKLLGKDPFDEKTSTKEVEIVAH
FCGNLLNQIATIDAVNARLLFVQRYIRKFDKGGQEESNERRDSDSPSASNGNNPNADDQT
NSPVNFNTEQNQKEEYQRYTKSYLHNDSFKNENNS-------------NALKLQISYHFT
VINLASENRDIVEYVEYAKNKPEFCLFPPIHIAFDEQSNFYYIVQHENNGFLNFIFISPS
TLLVYYTLKIREFYSGWLLNSSRDYDSKGLILF--GEYPPYEDELHVWDIRTLQHRKYLV
NIIKTY---

>P_chabaudi_ortholog
MITPSADGIETERRTYFRYFIIFLFIFTLTSILQNEKEEEEAKAHQNKVNLRQNDNY-RN
PEFMYLRNINRLKYKSAIY-----ITPNKKVMPSITYYN---------NSTIYFEGYNNI
FNNFENVALGINKIHDDFHIYVTDKDNINTDK-------------------------NII
CMNNDTSKDSCAIINTILIDNYFFKNINISSCNDN-NELCFFVLVQLKKSKCYNFNNLSN
```

```
SCHINNAMKNHNIFLINNYCYVILASSDVNKIYEFIKLHLATDTNFDENTKMDNVQIVSH
FCGNVLNQVATLDAVNGRLLFIQQYIHKNDNYNETNKNEEHQKKSPN-------------
-----------------------VFLNNYSNQNNNND-----------DTLKFKINYYLT
IMDLKSENKDIIEYVQYMKNKPELSIYQPMHISFDEHSNLYYIVQHGSNGSLNFIFISPS
QLLVYYTLKIKNFYSGWLLNSDNEHENKGIVLF--GEYPPYEDELHIWDITNFQHRKYLV
NIIKTY---

>P_yoelii_ ortholog
MITPSADGIETERRTYFKYFIIFLFIFIFTSILQNEKEEEETKGQQNKVNLRQNDNY-RN
PEFIYLRNINHLKYKSAIY-----ITPNRKIMPSITYYN---------NCTIYLEGYGNI
FNNFENVSLGINKINNDFHIYVTDKDNINNDK-------------------------NII
CMNNDGSKDSCAIINTILIDNYFFKNINISSCNDN-NELCFFVFVQLKKNKCYNFNNLSN
SCHINNAMKNHNIFLIKNYCYVILASSDINKIYEFIKLHLASDKDFDENTKMDSVEIVTH
FCGNLLNQIATLDAINGRLLFIQQYIHKNDNYDETKKNEEYQKKSSG-------------
-----------------------VFFNNYSNKNNTDD-----------DTLKFKINYYLT
IMDLKSENKDIIEYVQYMKNKPELSMYQPMYVSFDEYSNFYYIVQHGNNGSLNFIFISPS
QLLVYYTLKINNFYSGWLLNPDSEYENKGIVLFGAGEYPPXXXXXXXXXXXXXXXXXXXX
XXXXXXXXX

>P_berghei_ortholog
MITPSADGIETERRTYFKYFIIFFFIFILTSLLQNEK-EEKTNAQQNKVNLRQTDNY-RN
PEFIYLRNINHLKYKSAIY-----ITPNRKIMPSITYYN---------NCTIYLEGYGNI
FNNFEDVSLGINKINNDFHIYVTDKDNINSDK-------------------------NII
CMNNDGSKDSCATINTILIDNYFFKNINISSCNDS-NELCFFVFVQLKKNKCYNFNNLSN
SCHINNAMKNHNIFLIKNYCYVILASSDVNKIYEFIKLHLASDKDFDENTKMDNVEIVTH
FCGNLLNQIATLDAINGRLLFIQQYIHKNDNYDETKKNEEYQKKSPS-------------
-----------------------VF-NNYSNKNNTDD-----------DTLKFKINYYLT
IMDLKSE-RDIIEYVQYMKNKPELSMYQPIYVSFDEYSNFYYIVQHGNNGSLNFIFISPS
QLLVYYTLKIRDFYCGWLLNSDSEYDNKGIVLF--GEYPPYEDELHIWDIINVQHRKYLV
NIIKTY---
```

- **AMOUNT OF MISSING DATA**

P. reichenowi	***P. gallinaceum***	***P. knowlesi***	***P. vivax***	***P. chabaudi***	***P. berghei***	***P. yoelii***
79%	45%	9%	9%	14%	15%	19%

■ MAL8P1.25

▪ PHYLOGENY AND PEXEL/VTS

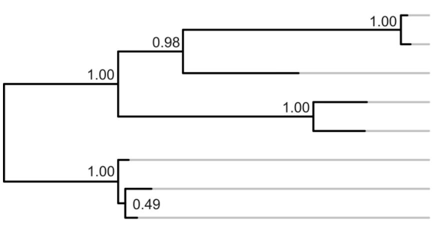

P. falciparum MAL8P1.25c	QAENKRMLSKKIKSS
P. reichenowi ortholog	QAENKRMLSKKIKSS
P. gallinaceum ortholog	QAKNKRTLSKKIKES
P. knowlesi ortholog	QAQNKRYLSKRIKDS
P. vivax ortholog	QAKNKRYLSKRIKDS
P. chabaudi ortholog	QSKNKRKLAKKIKES
P. berghei ortholog	QSKNKRKLAKKIKES
P .yoelii ortholog	QSKNKRKLAKKIKES

▪ ALIGNMENT

```
>P_falciparum_MAL8P1.25
MLKFFVKYLYTAIILDEEITTDIKIKEMLKGRQSLSFLLLSVPYFFLFL-W--NEDIKCQ
SLLPVSYASHQMYSFDKLTPHEIKSDLKKTFGNFISSKIQVLKNKFKLYDIKNREKYNNE
EGNDSDLIGDAYISSLEDEIKHLLEQAENKRMLSKKIKSSYERAHKNLKKRRNVE-----
-DDGKMKNVLKQNLQHIDYLNKKSDYLEKKAGELKNILERRGNENA---KEGNDNDEKNI
YNNEYHNNCSISKRGTLKFINSSNGLKHSIDNVNGMINENGFTIFYKNKKKMTYFWSVLE
LPIKMIGSIEQCFYFIYKNNNQIFCADNKIKAYSWMNSLSEASLCFHFGIKGVLINSNIN
NINKNDDMNNTNDENVKHSDFMNNISNGIKQGKKNKQKKIDENSLTVDIKPEDTGTRIFV
NDVEQKINKDVPGKDNVFNLNDIKKKMDDERKTPQSENDEDQ---------QGGYDKMEE
NEKEDTQMDN-------------------DDYEM

>P_reichenowi_ortholog
XXXXXXXXXXXXXXXXXXXXXXXXXXXXXKGRQSLAFLLLSVPYFFLFL-W--NEDIKCQ
SLLPVSYASHQMDSFDKLTPHEIKSDLKKTFSNFISSKIQVLKNKFKLYDIKNREKYNNE
EGNDSDLIGDAYISSLEDEIKHLLEQAENKRMLSKKIKSSYERAHKNLKKRRNVE-----
-DDGKMKNVLKQNLQHIDYLNKKSDYLEKKAGELKNILERRGNENV---KEGNNNDENNI
YNNEYHNNCSISKRGTLKFINSSNGLKHSIDNVKGMINENGFTIFYKNKKKMTYFWSVLE
LPIKMIGSIEQCFYFIYKNNNQIFCADNKIKAYSWMNSLSEASLCFHFGIK-VLINSNIN
NINKNDDMNNTNDENVNHSDFMNNXXXXXXXXXXXXXXXXXXXXXXXXXXXXXXXXXXXX
XXXXXXXXXXXXXXXXXXXXXXXXXXXXXXXXXXXXXXXXXXXXXXXXXXXXXXXXXXXX
XXXXXXXXXXXXXXXXXXXXXXXXXXXXXXXXXX

>P_gallinaceum_ortholog
XXXXXXXXXXXXXXXXXXXXXXXXXXXXXXXXXXXXXXLLNVYIFSLFFPL--NNKIKCQ
TFLPVSYPTHHLYSFEKLTPHDIRSNLKRTISNFITTKLENIKNKFRK----------ND
KNNDINDIKNEYGTSLEDQIKSLLQQAKNKRTLSKKIKESYDRALKNLKKKQSGV-----
-DEEKIIKILEHNLEQINYLNKKSHYLEKKAEYLKKNLENTNNENS---KQ---------
-----NNNCNISKTGKLKFVTSSNGLKHSINNVQGNINQNGFTIFHKNKKKMMYYWNIIE
LPIKVIGSIEQCFFFIYRNNNQILSADNKXXXXXXXXXXXXXXXXXXXXXXXXXXXXXXX
XXXXXXXXXXXXXXXXXXXXXXXXXXXXXXXXXXXXXXXXXXXXXXXXXXXXXXXXXXXX
XXXXXXXXXXXXXXXXXXXXXXXXXXXXXXXXXXXXXXXXXXXXXXXXXXXXXXXXXXXX
XXXXXXXXXXXXXXXXXXXXXXXXXXXXXXXXXX

>P_knowlesi_ortholog
XXXXXXXXXXXXXXXXXXXXXXXXXXXXXXXRSVKSRVTLSLAFFLLTC-LSPHGKANCQ
SLLPVSYATHQLYSFDKLTPVEVKSDLKKTLGSFITSKLKDLQKKFYDYKGYGSHGH-SS
SDEQGETLRSDYDASIEEEVKALLGQAQNKRYLSKRIKDSHDRALRNLKRNRNEE----G
RDEERTRKLLQHNLEQINYLNKKSHYLERKAEELRNHLKSSNSKNYNYQQE---------
-----GRNCDIYKIGKLKFVASSNGLKHSIDNVRGKIDNSGFTIFYKNKKKMTYLWNALE
LPIKLIGSVEQCFLFAYKNNNQILCTDNKLKAASWVNSLAEASACYHFGIKGILVNT---
----------------------NNVKNIQEKLGKSRKDDADGKMLVVDLKPEEDTTRVFV
NNHEQNVQD---GDDGVIDLNKIKRNMEEEEGKTKKLTTQEEVPSGEKADTEGVEEEMDE
TQVVDENGS---------------GAQTGDEDAE

>P._vivax_ortholog
XXXXXXXXXXXXXXXXXXXXXXXXXXXXXXXRSVKSKVTLSLAFFLLTC-LSPHGETNCQ
SLLPVSYATHQLYSFDKLTPVEVKSDLKKTLGSFIASKLKDLQRKFYDYKGYGTHGR-GS
SDGQGEVLKSDYDASLEEEVKALLGQAKNKRYLSKRIKDSHDRALKNLKRSSNGE----G
SDEEKTRKLLQHNLEQINYLNKKSHYLESKAEELKRHLKSGNNKNYNYEQQ---------
-----GRSCDIHKTGKLKFVTSSNGLKHSIDNVQGKINNSGFTIFYKNKKKMTYLWNALE
LPIKLIGSVEQCFLFAYKNSNQILCTDSRLKTASWVNSLAEASTCYHFGIRGMLVNT---
----------------------NNVKSVQEKIGKNRKKDAGDKLLVVDLKPEEGTTRVFV
NDREQNVQD---ANGGVIDLNKIKKNMEDEE--KKRRGTREEVPSGAEAG----EEEMGD
AEVVDEDASNDSGTTDDGDATDDGGAQAGDEDAE

>P_chabaudi_ortholog
XXXXXXXXXXXXXXXXXXXXXXXXXXXXXXXXKMKKSIRISYILFLSCL-LIRNG--ICQ
SFLPVSYATHKLYSFDKVSPNDIKSNVNNIVSNFITSNLKVIKNKIDDYNSY----Y-KN
NDEYDSLTQNEYDASLEDEVKSLLQQSKNKRKLAKKIKESYNRALNNLKNRKDKNNDNYQ
DDDELLQNMLEHNLEQIHSLNKKSEYLENKAMELKKYINKSQSSNY---ED---------
-----YKKCNISKTGKLKFVSSSNGLKHSIDNVQGIVNHNGFSIFYKKKKKMTYLLNIIE
LPIKIIGNVEQCFSFVYKNVDQIFCTNNKLKTLSWVNSLTEASFCANFKIKGILMNL---
----------------------DNIDK------ESFKNIKKDNLLRVHIKPDEKGTQVFV
NGKKQNAKE----NGGVINLNKIKKQAEDEEKISKKEMLKDD-------------DESEQ
NEEYENN----------------------DDDNL

>P_yoelii_ortholog
XXXXXXXXXXXXXXXXXXXXXXXXXXXXXXXRKMKSSIKISYILFVSCL-LIRNG--ICQ
SFLPVSYATHKLYSFDKVSPNDIKSNVHNIVSNFITSNLKVIKNKIEGYNSY----YNKN
NDEYDNLIQNEYDASLEDEVKSLLKQSKNKRKLAKKIKESYNRALNNLKNRKDKTNDNYQ
DDDEITKNMLEHNLEQINSLNKKSDYLENKAMELKKYINKNQLNNY---ED---------
-----YKKCNISKTGKLKFVSSSNGLKHSIDNVQGIVNHNGFSIFYKKKKKMTYLLNIIE
LPIKIIGNVEQCFSFVYKNVDQIFCTNNKLKTLSWVNSLTEASFCANFKIKGILINL---
----------------------DNVDN------ESYKNIKKDNLLRINIKPDEKGTQVFV
NGKKENAKE----N-GVINLNKIKKQAEDEEKISKKEILKED-------------DESEQ
NEESEDN----------------------DDDNL
```

```
>P_berghei_ortholog
XXXXXXXXXXXXXXXXXXXXXXXXXXXXXXXRKMKSSIKISYILFLFCL-LIRNG--ICQ
SFLPVSYATHKLYSFDKVSPNDIKSNVHNIVSNFITSNLKVIKNKIEGYNSY----Y-KN
NDEYDSLIQNEYDASLEDEVKSLLQQSKNKRKLAKKIKESYNRALNNLKNRKDKTNDNYQ
DDDELLQNFLENNLEQINSLNKKSDYLENKAMELKIY-KKNQSNNY---ED---------
-----YKKCNISKTGKLKFVTSSNGLKHSIDNVQGIINHNGFSIFYKKKKKMTYLLNIIE
LPIKIIGNVEQCFSFVYKNVDQIFCTNNKLKTLSWVNSLTEASFCANFKIKGILINL---
----------------------DNVDN------ESYKNIKKDNLLRIHIKPDEKGTQVFV
NGKKENAKE----NGGVINLNKIKKQAEDEEKISKKEILKED-------------DATEQ
NEEYEDN----------------------DDDNL
```

- **AMOUNT OF MISSING DATA**

P. reichenowi	***P. gallinaceum***	***P. knowlesi***	***P. vivax***	***P. chabaudi***	***P. berghei***	***P. yoelii***
27%	46%	19%	22%	21%	21%	21%

■ PF08_0024

▪ PHYLOGENY AND PEXEL/VTS

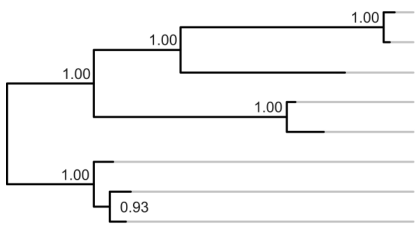

P. falciparum PF08_0024	HKKFNRNYSEDINEF
P. reichenowi ortholog	XXXXXXXXXXXXXXX
P. gallinaceum ortholog	YKKLNKNYSEDLNEF
P. knowlesi ortholog	YQKLNQNYSEDLNEF
P. vivax ortholog	FQKLNQNYSEDLNEF
P. berghei ortholog	LQNFNGNYSEDITEF
P. chabaudi ortholog	LTNFNGNYSEDITEF
P .yoelii ortholog	LTNFNGNYSEDITEF

▪ ALIGNMENT

```
>P_falciparum_PF08_0024
MRNIIFLFCLILFLVCFCQGHKNYLKRDNYLQYLRSQTFLQERSKHKKFNRNYSEDINEF
DEYEDEETDNLDNKISDEIKNKKKENYLS-QNINEQKNDSINNNNEKKLTFKNIENEINS
T-DTFMDDS-NVKSLGKTKECNVNEKGLLDVSLNSNDFFNMNKYNVEITSSNIIIKDKNN
IKIIKEIPFEHIKLPITTIEETRECWYIKTTNDKILLCDKEKEERDIWITNILKALFCYN
TNNLII---VNKEKKDTVSLPKESTVDKRINALKEKETIKSSDNNTSTNDNK--VNNITI
SNFSDHEPKIVFN

>P_reichenowi_ortholog
XXXXXXXXXXXXXXXXXXXXXXXXXXXXXXXXXXXXXXXXXXXXXXXXXXXXXXXXXXXX
XXXXXXXXXXXXXXXXXXXKNKKKENYLS-KNINEQENNRINNN-EKKLTFKNIENEINS
T-DTFMDDS-NVKSLGKTKECNVNEKGLLDVSLNSNDFFNMNKYNVEITSSNIIIKDKNN
IKIIKEIPFEHIKLPITTIEETRECWYIKTTNDKILLCDKEKEERDIWITNILKALFCYN
TNNLII---VNKEKNDTVSLPKESTVDKRINALKEKETIKSSDNNTSTNDNK--VNNITI
SNLSDHEPKIVFN

>P_gallinaceum_ortholog
MK--ILSIIIIVSFISLCKSHRSFSKKNNYLQFLRSQNFIQEKPKYKKLNKNYSEDLNEF
DDYEDEQT-----------YNENEVGHMSNKQLNNNNLENYNNNNENKLDFKDIENELNN
D-ELLRQDS-NINLVRGGKECTINEKGIINVSIDSNDIFNLNKYNVEITSSNILIKDIND
LKIIKSIPFKSIKLPIETIEETRECWNIKLNKEKILFCEQKKEDRDIWITNILKALFCYN
TNNLMI-EKSNTSNNESIHIPKESTVEERLKASKEGQDISNNNSPQYIKSSN--YNNINI
SNLRNDEPKILFD

>P_knowlesi_ortholog
MR--VISLLSVICLVSTCRGHRNLFARNNYLQYLRSQNFMQEKPKYQKLNQNYSEDLNEF
DNYEDDE------------LDAENLGAANKEKVTENSGTSSSST-ENKLEFKNIEKELNN
MGEVLHEEKNNLLLMGGGRECSVSDKGILDVSLNSSDIFNLTKYTVEMSSSGILIRDTQN
SNVVKEIPFDTVKLPIETIEETRECWKIRSHTETFLFCERSKEDRDRWITNILKALFCYN
TNNLTIDEGVHTGLSSKVDIPKESTVDARIAASLKNEPNDSGDNSSHGRAKRKGNNNITI
SNLKNDKPEIVVN

>P_vivax_ortholog
MR--VLSLLSALCLVSTCRGHRNLFARNNYLQYLRSQNFMQEKPKFQKLNQNYSEDLNEF
DNYEDDE------------LDAENVEATSKERVTQKSGTSSSSS-ENKLEFKNIEKELNN
MGEILHEEKDNLLLMGGGRECSVSDKGTLDVSLNSGDIFNLTKYTVEMSSSGILIRDTQN
SNVVKEIAFDTVKLPIETIEETRECWKIRSHKETFLFCERSKEARDRWITNMLKALFCYN
TNNLTIDEGVQTGLSSKVDIPKESTVDARIAASLKDEPNGSGDNSSHGRAKRKGNNNITI
SNLKNDKPEIVVN

>P_chabaudi_ortholog
MK--ILVSISFVCLVAFCRGHKNFSRRNNYLQYLRSQTFMQEKPKLQNFNGNYSEDITEF
DIHDDDEYNSF--------KND--------KDMKEN----------NQLEFKNIEKELNT
N-EVSNPDSINI--MGSGKECSVNEKGELDVSINSRDIFNLIKYMVEITSNSIIIKDIKN
SNVVKELTYEHIKLPIETIEETRECWSVKFNKEKIIFCEKNKQNRDRWVKDILKALFCYN
TNNLTI-ENNQKVYKQKSDIPKHSTVDERIQNLKETLTNDKNNLDKQKSTKH--NNNIVI
SNLKNSNPSISLK

>P_yoelii_ ortholog
MK--VFVSISFVCLIAFCRGHKNFSRRNNYLQYLRSQTFMQEKPKLTNFNGNYSEDITEF
DIHEDDEYNNL--------KNDDTKDTSNDKDMKEN----------NKLEFKNLDKELNT
I-EVNNPDSINI--MGSGKECSVNEKGELDVSINSQDIFNLIKYMVEITSNSIIIKDIKN
SNVVKELSYDHIKLPIETIEETRECWSIKFNKEKIIFCEKNKQNRDNWVKDILKALFCYN
TNNLTI-ENNQKVYKQKSDIPKHSTVNERIQNLKETLTNDKNNFDKQKSTKH--NNNIVI
SNLKNSNPNISLK

>P_berghei_ortholog
MK--IFVSISFVCLISFCKGHKNFSRRNNYLQYLRSQTFMQEKSKLTNFNGNYSEDITEF
DIHEDDEYNNF--------KNDKKKDTLNDKDMKEN----------NKLEFKNLEKELNT
N-DVNNSDSINI--MGSGKECSVNEKGELDVSINSQDIFNLIKYMVEITSNSIIIKDIKN
SNVVKELPYGNIKLPIETIEETRECWSIKFNKEKIIFCEKNKQNRDKWVKDILKALFCYN
TNNLTI-ENNQNVYKQKSDIPKHSTVNERIQNLKETLTNDKNNFDKQKSTKH--NNNIVI
SNLKSSNPNISLK
```

▪ AMOUNT OF MISSING DATA

P. reichenowi	***P. gallinaceum***	***P. knowlesi***	***P. vivax***	***P. chabaudi***	***P. berghei***	***P. yoelii***
26%	5%	7%	7%	10%	8%	8%

■ PFI1010w

▪ PHYLOGENY AND PEXEL/VTS

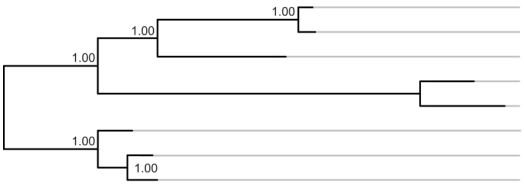

P. falciparum PFI1010w	SYDYDRILKKKKFLK
P. reichenowi ortholog	SYDYDRILKKKKFLK
P. gallinaceum ortholog	XXXXXXXXXXXXXXX
P. knowlesi ortholog	SYDYDRIFKNKKFLQ
P. vivax ortholog	SYDYDRIFKNKKFLQ
P. berghei ortholog	SYDYDRIFKNNKFLQ
P. chabaudi ortholog	SYDYERVLKGKEYLP
P .yoelii ortholog	SYDYERVLKGKEYLP

▪ ALIGNMENT

```
>P_falciparum_PFI1010w
M---IKCIQLYVITVCILLINIACKKYRDNSYAGYIRN-NIHN-AHRTRKKIYRRYAQKK
KE------KETYEEYFKELDKIFNECDYDAEQVEEEEKKKEEELNLITEKDLLEINFCNY
EKDPRSLANLVKEKS------------EEEIEKENNENNEKNIEHIENIENIENIENNEN
IKNNTLEYSYDYDRILKKKKFLK-EKIEYKDKFILNENHNKLVNNIDTSFMNDFLNNYNN
DFNKPFYKARRDQYFAREDLVKHEINNNNQSEYKEERNNSENNNNNNNHILYDEERKSDL
HYHKVSKDDQNNEDML-----KENLESVLFLKRIRNNFDIKRNIYINETINDIKEKY---
----------NNNEDKTNRWHNIKNEDSHKERDEE---------------N-RFHEYVEE
KIK-NANTDENVEKLILKKYIKMFSIDDEVLNINEKIKKCENVHSILEIFKDNKRINIIN
IMYSFIYIDRFKCLNITEYLYEKRFAYITYALEEIMKYYLYVLNKKGLKGLYQKKKINNL
NNINKEDTEKIKINKDYKNNNDNSDKDNNSYDPLFEHRTLQLNDKNLIFLIKYMNKLKLF
YINLNLYNMFFLILSKSFALCSANSFVILLSYINEYNYYNNNIHKKINISILNELFHIFK
RD--------------GMKSSGLNLAHFKILLPLLIKYKYVNN-----------------
-------NVFKLLYLHFEKDIKDMIHILTSNVKKKERKEICNVLFYDVKTKEQEKNKEKY
LSYLKYEEMKEKKENMEILSQLLNYLMMCKF-NEKDSMVLKLINIFIDYMHLLNVDQLLN
IFFNYHIFQNNNE--IILYRCKQELLNRKSLLKDYHINKILSYIIYNYRKAEIYRNYYRN
WFSYKNYTIFRDDVICDEIQRNQDLTQHEQEMYNIKNVSEDDKKKITTIDKIPRDNETEI
FNNNKKKNCNSPCYSFYDNYSEGRDMKKLPSHISLKKVLHKKKEIKELKKDYYINTKIFK
DVEFLYDLTHDIHIYVPFKNVHLLKFVYNIYLLSLLFYKNMDVIRIASEIAYDLTDTNIF
SFIDQYSYYLYDDIYIIIKTFKYISFFSIDLIDIWKKFFFLLNHFVNNLNLENIYDIFFF
VHISKITNLKNENYDVLNLLTSRMKTIVQLLFTHNVQNFNTYPHTYILNILNMINDQNNE
HVIDFVHFFFYSIYKRIQYLYSNGDNILGRSSYDESKMKMENTKMEDNIESDNNNTFGDN
KIVGDDQFFQDNKPLEDNKSFGDNIKPFSNNVKADKYFSYMSDTKNKKSNEIYKNVNNYI
YNFKDIRLFIKIFLRYYKKEQETFNLKIVDFILYNSEVFFFNMWTNRRYYQRHYFFGKEI
HEFLHLLKELIQNKLYNSNIMYRCVNILKYIIKMQSTFNIEDIPNVHCNLYSNFFLHFDK
INDINVWKYIRKVF------------------------------------

>P_reichenowi_ortholog
M---IKCIQLYVITVCILLINIVCKKYRDNSYAGYIRN-NIHN-THRTRKKIYRRYAQKK
KE------KETYEEYFKELDKIFNECDYDAEQVEEEEKKKEEELNLITEKDLLDINFCNY
EKDPRSLANLVKEKS------------EEEIKKENNENNE-NIENNKNNENIENIENNEN
IKNNTLEYSYDYDRILKKKKFLK-EKIEYKDKFILNENHNKLVNNIDTSFMNNVLNNY--
---------------------------------------------DNNHILYDEERKNDL
PYYKVSKDDQNNEDML-----KENLESVLFLKRIRNNFDIKRNIYINETINDIKDKY---
----------NKYEDKTNRWNNIKSEDIHKVRDDA---------------N-KFQVYVEE
KIK-NASIDENVEKLILKKYIKMFSIDDEVLNINEKIKKCENVHSILEIFKDNKRINIIN
IMYSFIYIDRFKCLNITEYLYEKRFAYITYALEEIMKYYLYVLNKKGLKGLYQKKKVNNL
NNINKEDREKTKINKDHKNNNDNCDKANNSYDPLFXXXXXXXXXXXXXXXXXXXXXXXXX
XXXXXXXXXXXXXXXXXXXXXXXXXXXXXXXXXXXXXXXXXXXXXXXXXXXXXXXXXXXX
RD--------------GMKSSGLNLAHFKILLPLLIKYKYVNN-----------------
-------NVFKLLYLYFEKDIKKMMHTLTSNVKKKERKEICNVLFYDVKTKEQEKNKEKY
LSYLKYEEMKEKKENMEILSQLLNYLMMCKF-NEKDSIVLKLVDIFIDYMHLLNVDQLLN
IFFNYHIFQYNNE--IILYRCKQELLNRKSLLKDYHINKILSYIIYNYRKAEIYRNYYRN
WFSYKNYTIFRNDVICDEIVRNEKLTQHEHEMYNIKNVSQDEKKKITXXXXXXXXXXXXX
XXXXXXXXXXXXXXXXXXXXXXXXXXXXXXXXXXXXXXXXXXXXXXXXXXXXXXXXXXXX
XXXXXXXXXXXXXXXXXXXXXXXXXXXXXXXXXXXXXXXXXXXXXXXXXXXXXXXXXXXF
SFIDQYSYYLYDDIYIIIKTFKYISFFSMDVIDIWKKFFFLLNHFVNNLNLENIYDIFFF
IHISKITNLKNENYDVLNLLTSRMKTILQLLFTHNVQNFNTYPHTYILNILNLINDQNNE
HVIDFVHFFFYSIYKRIQYLYSNGDNILARSSYDESKMKMENTKMEENIESD-NKTFGDN
KIVGDDKSFGDNKIVGDDKSFEDNIKSFSNNVKTDTYFSNMYDRKNKKSNEIYKNVNNYI
YNFKDIRLFIKIFLRYYKKEQETFNLKIVDFILYNSEVFFFNMWTNKRYYQRHYFFGKEI
HEFLHLLKELIQNKLYNSNIMYRCVNILKYIIKMQSTFNLEDIPNVHCNLYSNFFLHFDK
INDINVWKYIRKVF------------------------------------

>P_gallinaceum_ortholog
M---QKCINFSVFIVFLLLINIYCVKVKNNNYS-YVIN-KISNCKHKYKRKIIRKYAKKK
KT------KDDYEEYYKELDKIFNECDYDEKHFEEEEKK---XXXXXXXXXXXXXXXXXX
XXXXXXXXXXXXXXXXXXXXXXXXXXXXXXXXXXXXXXXXXXXXXXXXXXXXXXXXXXXX
XXXXXXXXXXXXXXXXXXXXXXX-XXXXXXXXXXXXXXXXXXXXXXXXXXXXXXXXXXXX
XXXXXXXXXXXXXXXXXXXXXXXXXXXXXXXXXXXXXXXXXXXXXXXXXXXXXXXXXXXX
XXXXXXXXXXXXXXXXXXXXXXXXXXXXXXXXXXXXXXXXXXXXXXXXXXXXXXXXXXXX
XXXXXXXXXXXXXXXXXXXXXXXXXXXXXXXXXXXXXXXXXXXXXXXXXXXXXXXXXXXX
XXXXXXXXXXXXXXXXXXXXXXXXXXXXXXXXXXXXXXXXENVSTILDIFKESKRINMIN
IMYAFMYIDRFKTLNINEYLYDKRFAHLTYALEELLKYYLYVL-----------------
-------------------------------------RTLILNDKNIIFLIKYMNRLKLF
YINLNIYNLLILILSKSFHLFNMNSFIILLAYLNEYTYFSNNIHKKINISLLQHIFNYFK
TY--------------TVHSSNINFYHFKILLPLLIKYKYIDN-----------------
-------DTFKMIFNYFQKDINKILEFL-KDENIPEQKEISDLLFYDVKTKEQKQNKENY
ISYIKYTNMMKKKEYLNFLSQLLHYLMISKF-NERDEIVLKIINIFINHFNFFDLEELLN
ILYNYDLFKNSNI--DIMICCKQELLXXXXXXXXXXXXXXXXXXXXXXXXXXXXXXXXXX
XXXXXXXXXXXXXXXXXXXXXXXXXXXXXXXXXXXXXXXXXXXXXXXXXXXXXXXXXXXX
XXXXXXXXXXXXXXXXXXXXXXXXXXXXXXXXXXXXXXXXXXXXXXXXXXXXXXXXXXXX
XXXXXXXXXXXXXXXXXXXXXXXXXXXXXXXXXXXXXXXXXXXXXXXXXXXXXXXXXXXX
XXXXXXXXXXXXXXXXXXXXXXXXXXXXXXXXXXXXXXXXXXXXXXXXXXXXXXXXXXXX
XXXXXXXXXXXXXXXXXXXXXXXXXXXXXXXXXXXXXXXXXXXXXXXXXXXXXXXXXXXX
XXXXXXXXXXXXXXXXXXXXXXXXXXXXXXXXXXXXXXXXXXXXXXXXXXXXXXXXXXXX
XXXXXXXXXXXXXXXXXXXXXXXXXXXXXXXXXXXXXXXXXXXXXXXXXXXXXXXXXXXX
XXXXXXXXXXXXXXXXXXXXXXXXXXXXXXXXXXXXXXXXXXXXXXXXXXXXXXXXXXXX
XXXXXXXXXXXXXXXXXXXXXXXXXXXXXXXXXXXXXXXXXXXXXXXXXXXXXXXXXXXX
```

```
XXXXXXXXXXXXXXXXXXXXXXXX--------------------------

>P_knowlesi_ortholog
M---LGTAFYVAQLLLLLTQIFECRK--RHTQQGYLNGIAHRTPRHEGRSRINKNYLRKK
KK---DKEKEYYENFFRELDKIFNDEDDEQKKVEEEERRKEEEMDAISEKDLLEVNFCNY
ERDPRSLANLVKNRT------------EHEEGNREDADRE---SSTKGE-PSSDEVSMHS
GKKETMPYSYDYERVLKGKEYLP-EKILLKEKFSVEEDEEKLVDRIDVNTMNDFLSNYQN
NFQEPFYKGRRDRYFGREDLQR------------------------------GNSASEVG
EEKMLRGAASAEVDIKDGDIGEDQLESVHFLRNVRREFDRERDCDMDEAVRQVEGQFVQG
NVT----GDGNRNYNKDWNSDGGEMEG-----------------------GLSFEKHVEE
EIKRRSTDDETVDKLIRNKFIDMFKVDDAVKKTNEQIKKSQSVSALLELFSEKKPKNIIN
IMYMFMYIYRFQNANMMEYIYDRRFAFLTDTFEQVLKFYLYMF-----------------
------------------------RKNMNCSGEKKKERTVVLNSRNIIFLLKYMNRLKLF
YVNLNIYNLLFLIMSQSLDMFSIDSLVVLLAYLNEYSYYGNEVHKKINLSILKRIFELVR
VD--------------RSNMSRLHFGHFKLLVPLLVKHRYVGS-----------------
--VDGVDDAASLLLDHFAKDTQRAIESVQSGKAYSRSKDICDILFYDSKTKEQKKNKENY
MAYLENQQMQQRKKDIKFLYTFLQYLMIGKF-AHEEETVQRLVDLFLGNLSLFQVEDVMT
IFFNSEQLQYGEQYSALVNRCKNELLQRKSLLKDFQINQVLSFFILGYRRSLLYRDYHRR
LFSHARYVLRRD------------------------QLNRGENPNVGIVDTVPR------
-------------------------------------LLSCRSEMRTMHRKHLLSNESFR
DPQFLYDFTHDIHIFVKFKNVHLLKFVYNMYLLSLLYYKNADVLRIVSEVSHDLIETNIF
SFIDQYAYYMYEDLYIIIKAFKYVSFFQIDLIEPWKKFFFLLEHFSSALNLENIYDIFFF
INICNLQKLKCENYNVFRVLTERMKRILEILAKHDLHQFSTHPHTYLLNILNLVREKQNE
HMCAFAQTFFYSVYTRLC---GDWTDNTSTGNISTDNNTSGNTCIDPR------------
--------------------------------------------------EAYRKVNRRP
YNFRDARLFLQSFLAYHNEKQQHMNLPIVHFILLNVEDFFSQLRESVQYYHSSYIYGAQM
EEFLSLIRQLVYRKLYNGHIMRTLVLIIKHMIHIQEIHNGNDYDS-GVNIFKPFFAHFPK
VTDANVWRFIRRHPSLSFYP------------------------------

>P_vivax_ortholog
M---WGATLYVAPLLLLLTQSLHCRK--RHPPQGYLNGIAPHAPRHESRSKISRTYTRKK
KKKKNEKEQDYYENFFRELDKIFNDEDDEQKQVEEEERRKEEEMDAISEKDLLEVNFCNY
ERDPRSLANLVRSRTDGGAADGGGADGEANANGEANANGE---ANTKGEAPPEGRAPTHG
GNKENMQYSYDYERVLKGKEYLP-EKIPLKEKFSLEEDEEKLVDRVDVNTLNDFLSNYQN
DFPGPFYKGRRDKHFGREDLQG------------------------------GESASKAG
EEKKLRGTPNEEADIHIGGTEKDQLESVHFLRRIRREFDRERGGDMDEAVRQVEGQFAQG
GVSGGGDGDGDGNTDGGSSTDGGYHRGCDHHRGGD---------------GHPFERHVEE
EIKRRVADDETVEKLIRSKFTSMFSVDDEVKKTNEEIKKSQSVSALLELFSENKRKNVIN
IMYMFMYICRFKNLNIMEYIYDRRFAYLTDALEQVLKFYLYMF-----------------
------------------------RKKMNCS-EEKRERTIILNSRNIIFLLKYMNRLKLF
YINLNIYNLLFLIMSQSLHLFSIDSLVVLLAYLNEYSYYGSEVHTKINLSILSRIFELVR
GD--------------EANLSRLHFGHFKLLVPLLVKHGYVGGGVGGAGGVDRVGGAGGV
NRLGGADEGAARLLAHFAKDTERAIESVRSGQALSRSSDVCDVLFYDSKTKEQKKNKENY
MAYLEIQHMQQRKKDIKFLFSFLQFLMIGKF-AHEEETVQRLVGLFLENLSLFQVEDVLA
IFFNSEQLQHGEQYSALVTRCKRELLQRKSLLKDFQINRVLSFIILGYRRSALFRDYHRR
LFAGARYVLRRGHPNSGSHPGESSHSIQSNQPSEGGHPNRGNHPN---IDALPR------
-------------------------------------LLSRRSHMRALQRKHLLSREAFR
DPQFLYDFTHDIHIFVKFKNLHLLKFLHSMYLLSLLYYQNADVLRIVSEASHELIETNIF
SFIDQYAYYMYEDLYIIIKAFKYVSFFQIDLITPWKKFFFLLDHFSNALNLENIYDIFFF
INICNLQNLKSENYDVFRVLTGRMQRILEVLSKHDLHQFSTHPHTYLLNILGLIREKRNE
HVCAFARAFFYSVYRRFCGGSGEKTPGETSGEMSGE-NTPGEPPAEPL------------
--------------------------------------------------E-----TPRP
YNFRDARLFLQAFLAYHNEKQEHLNLQIVHFVLLNVEAFFSQLREGVHYYHSGYIYGPQM
AELLSLLRQLVARKLYSGGIMRTIVCIVKHMLHIQGAHHGSAYAS-GANIFKPFFARFPG
VTDANVWRFIRRHAPLTLRGKRGV--------------------------

>P_chabaudi_ortholog
XXXXXXXXXXXXXXXXXXXXXXXXXXXXXXXXXXXXXXXXXXXXXXXXXXXXXXXXXXXX
XXXXXXXXKGNYDEYFKKLDKIFNECEYDEEQVEQEEKDNEDKIDLAYEQDLLNINFCNY
ENDPRSLSNLVKKKS------------QEEIEREKLQEKL-------------NSSDNKE
TINNTLPYSYDYDRIFKNKKFLQ-EKIQYKDKFSLADDKSKLIGTFDVNIMNDILNNHQN
DSDIPFYKARRDKYFGREDLNQDD----------------------------QPAAQNEH
E--------NAKENYL-----VNDLESINFLRKIKNDFHFERDEYIQASVKQIEQSYAKD
-------ENGDTYET--------------KRDKEE---------------E-EARQYISS
RIQ-NYEDKDVINKIVCKKFVSMFSVDDEVKKINEKIKISKNVNEILDIFSKEKRLNIIN
IMYIFIYMYRHKNINMNEYLYDKRLRYITDGLEELLKYYLYII-----------------
---------------------------NNNDCIKLEKKTLVLNDANIIMLIKYMNRLKLF
YINLNIYNMLFLILSKSYXXXXXXXXXXXXXXXXXXXXXXXXXXXXXXXXXXXXXXXXXX
XXXXXXXXXXXXXXXXXXXXXXXXXXXXXXXXXXXXXXXXXXXXXXXXXXXXXXXXXXXX
XXXXXXXXXXXXXXXXXXXXXXXXXXXXXXXXXXXXXXXXXXXXXXXXXXXXXXXXXXXX
XXXXXXXXXXXXXXXXXXXXXXXXXXXXXXXXXXXXXXXXXXXXXXXXXXXXXXXXXXXX
XXXXXXXXXXXXXXXXXXXXXXXXXXXXXXXXXXXXXXXXXXXXXXXXXXXXXXXXXXXX
XXXXXXXXXXXXXXXXXXXXXXXXXXXXXXXXXXXXXXXXXXXXXXXXXXXXXXXXXXXX
XXXXXXXXXXXXXXXXXXXXXXXXXXXXXXXXXXXXXXXXXXXXXXXXXXXXXXXXXXXX
XXXXXXXXXXXXXXXXXXXXXXXXXXXXXXXXXXXXXXXXXXXXXXXXXXXXXXXXXXXX
XXXXXXXXXXXXXXXXXXXXXXXXXXXXXXXXXXXXXXXXXXXXXXXXXXXXXXXXXXXX
XXXXXXXXXXXXXXXXXXXXXXXXXXXXXXXXXXXXXXXXXXXXXXXXXXXXXXXXXXXX
XXXXXXXXXXXXXXXXXXXXXXXXXXXXXXXXXXXXXXXXXXXXXXXXXXXXXXXXXXXX
XXXXXXXXXXXXXXXXXXXXXXXXXXXXXXXXXXXXXXXXXXXXXXXXXXXXXXXXXXXX
XXXXXXXXXXXXXXXXXXXXXXXXXXXXXXXXXXXXXXXXXXXXXXXXXXXXXXXXXXXX
XXXXXXXXXXXXXXXXXXXXXXXXXXXXXXXXXXXXXXXXXXXXXXXXXXXXXXXXXXXX
XXXXXXXXXXXXXXXXXXXXXXXXXXXXXXXXXXXXXXXXXXXXXXXXXXXXXXXXXXXX
XXXXXXXXXXXXXXXXXXXXXXXX--------------------------

>P_yoelii_ ortholog
MKKEIKCVKLVLCIFPFLWLYVFCIKLNKKEFYNYINVLLVKNNVKQRYKTINICYGRKK
TE------KENYDEYFKKLDKIFNECEYDEEQVEKEEKDNEDKIDLAYEQDLLNINFCNY
ENDPRSLSNLVKNKS------------KEEIEREKLEEKQ-------------NLKDNKE
TTNNTLPYSYDYDRIFKNKKFLQ-EKIHYKDKFSLKDDKTKLIDTYNVNIMNEILNNHQN
DSEIPFYKGRRDKYFGRED---------------------------------EPNCGE--
---------DVKENYL-----VDDLESVNFLRKIKSDFHFERDEYIQASIKQIEESYVKD
-------GNGNIYEN------GGEIRHGNKREIEEKEEAKEEGAKEEGAKEGEAKECISY
RIR-NYENKDVINKMLGIKFVKMFSVDDEVKKINEKIKTSKNINEILDIFSKEKRLNIIN
IMYIFIYMYRYKNININEYLYDKRLRYITDGLEELLKHYLYII-----------------
---------------------------NNSGCIKLEKKTIILNDVNIIMLIKYMNRLKLF
YINLNIYNMIFLILSKSLELFTLESMISLLAYINEYSYYDSEIHRKINLTILRNFFKKIK
KDFTPTFINKENEKFNKTDQKLISFSYFKLLIPILLKYKYVDK-----------------
-------IGISLLYSMFEENIKKILF---NDKNIVDPKEECDILFYNAKTKDQKKNKENY
FSYLKYEHLIEKKKNLHFLSQFLHYIIISKFYNDKDRLITDLVNIFINNFQIFNLNDILN
IYFNFHIFNKFGN--NFIFRCKQEILHRKSTLKDSQINKILSYIIYNYKKSKIFKDYHRH
IFVNRKYTLCRNXXXXXXXXXXXXXXXXXXXXXXXXXXXXXXXXXXXXXXXXXXXXXXXX
XXXXXXXXXXXXXXXXXXXXXXXKSDIKQTNIVSYSNLIKKRKELKSFKRLYITYKNNFE
DSQFVYDFTNNINMFVQFQNINLLRFIYNMYILSLLFYKNQDVIRIVSDLIYDMTETNIF
SFIDQYSYYIXXXXXXXXXXXXXXXXXXXXXXXXXXXXXXXXXXXXXXXXXXXXXXXXXX
XXXXXXXXXXXXXXXXXXXXXXXMKEIIHQVFNLDIKNLNTHPHTYILNILNLVTDHANQ
DFTSFVHFFFYSIYKKIEQKKNEQKNSEQKNSEQKNSEINDGKIADGKLH----------
```

```
--------------------------------------------------DVYKGVNNYV
YNFKDIRLFIKYFLLCFKENQEXXXXXXXXXXXXXXXXXXXXXXXXXXXXXXXXXXXXXX
XXXXXXXXXXXXXXXXXXXXXXXXXXXXXXXXXXXXXXXXXXXXXXXXXXXXXXXXXXXX
XXXXXXXXXXXXXXXXXXXXXXXX--------------------------

>P_berghei_ortholog
XXXXXXXXXXXXXXXXXXXXXXXXXXXXXXXXXXXXXXXXXXXXXXXXXXXXXXXXXXXX
XXXXXXXXXXXXXXXXXXXXXIFNECEYDEEQVEKEEKDNEDKIDLAYEQDLLNINFCNY
ENDPRSLSNLVKKKS------------KEEIEREKLEEKQ-------------NSSNNKE
TTNNTIPYSYDYDRIFKNNKFLQ-EKIHYKDKFSLKDDKTKLIDTYNVNIMNDILNNHQN
DSEIPFYKGRRDKYFGREDLNQED----------------------------GPNFGKEH
ETDIANGENNIKENYL-----VEDLESVNFLRKIKSDFHFERDEYIQASIKQIEENYVKG
-------EKDNIYENTKLNENRTKIEYGNKRDIEE---------------E-EVRQCISN
RIH-NYEDKDVINKILSRKFVNMFSVDDEVKKINEKIKTSKNINEILDIFSKEKRLNIIN
IMYIFIYMYRYKNININEYLYDKRLRYITNGLEELLKHYLYII-----------------
---------------------------NXXXXXXXXXXXXXXXXXXXXXXXXXXXXXXXX
XXXXXXXXXXXXXXXXXXXXXXXXXXXXXXXXXXXXXXXXXXXXXXXXXXXXXXXXXXXX
XXXXXXXXXXXXXXXXXXXXXXXXXXXXXXXXXXXXXXXXXXXXXXXXXXXXXXXXXXXX
XXXXXXXXXXXXXXXXXXXXXXXXXXXXXXXXXXXXXXXXXXXXXXXXXXXXXXXXXXXX
XXXXXXXXXXXXXXXXXXXXXXXXXXXXXXXXXXXXXXXXXXXXXXXXXXXXXXXXXXXX
XXXXXXXXXXXXXXXXXXXXXXXXXXXXXXXXXXXXXXXXXXXXXXXXXXXXXXXXXXXX
XXXXXXXXXXXXXXXXXXXXXXXXXXXXXXXXXXXXXXXXXXXXXXXXXXXXXXXXXXXX
XXXXXXXXXXXXXXXXXXXXXXXXXXXXXXXXXXXXXXXXXXXXXXXXXXXXXXXXXXXX
XXXXXXXXXXXXXXXXXXXXXXXXXXXXXXXXXXXXXXXXXXXXXXXXXXXXXXXXXXXX
XXXXXXXXXXXXXXXXXXXXXXXXXXXXXXXXXXXXXXXXXXXXXXXXXXXXXXXXXXXX
XXXXXXXXXXXXXXXXXXXXXXXXXXXXXXXXXXXXXXXXXXXXXXXXXXXXXXXXXXXX
XXXXXXXXXXXXXXXXXXXXXXXXXXXXXXXXXXXXXXXXXXXXXXXXXXXXXXXXXXXX
XXXXXXXXXXXXXXXXXXXXXXXXXXXXXXXXXXXXXXXXXXXXXXXXXXXXXXXXXXXX
XXXXXXXXXXXXXXXXXXXXXXXXXXXXXXXXXXXXXXXXXXXXXXXXXXXXXXXXXXXX
XXXXXXXXXXXXXXXXXXXXXXXXXXXXXXXXXXXXXXXXXXXXXXXXXXXXXXXXXXXX
XXXXXXXXXXXXXXXXXXXXXXXX--------------------------
```

- **AMOUNT OF MISSING DATA**

P. reichenowi	***P. gallinaceum***	***P. knowlesi***	***P. vivax***	***P. chabaudi***	***P. berghei***	***P. yoelii***
19%	70%	18%	18%	70%	73%	33%

■ PFC0435w

▪ PHYLOGENY AND PEXEL/VTS

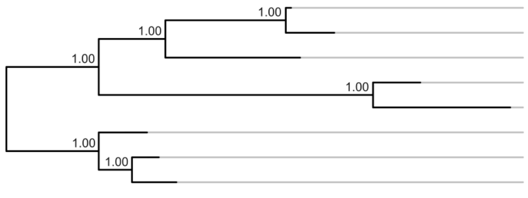

P. falciparum PFC0435w	YSSIFRLLVDTYKNI
P. reichenowi ortholog	YSSIFRLLVDTYKNI
P. gallinaceum ortholog	HSSIFRLLVDTYKKI
P. knowlesi ortholog	HTNIFHLLMDIYKQV
P. vivax ortholog	HTNVFHLLMDVYKQV
P. chabaudi ortholog	HSSIFKLLTNTYEQV
P. berghei ortholog	HSNIFKLLIETYETV
P .yoelii ortholog	HSSIFKLLIETYEQV

▪ ALIGNMENT

```
>P_falciparum_PFC0435w
MKV-GIIFFC-----LFFFVVLGACNNVKERIFKNIKKRTKFIILNEPIVDLSFSENLFH
TLLFDLDVD-KNLYTLDESLLNLENLNYSSIFRLLVDTYKNIKEN---EDDNKNIRYIFL
GTSFSRIHPLNFEYFLRKLNKYIYNGNIYEKGNVDIRGILEEYNKEIEEKKLEKQKLNKI
KDKNNNNNNNNNSKFSKDGDNEDFNNKNDLYNPSDKLYNNNDDIDVHELLEEIITKEKRF
FLNDDDDNDSNDKYILKT--------DEVNKYKGFFIGYGFNDDIPSVIHHYNFDKNFLF
PSLNSGIILDITLLKNIYEVSNILLSNNEKDQSIHIDYIYEVTKYIKENLRVRLTHSENV
CLNEEQNIHLLDND-PNNFEIYKYYQVLNLFKDYNKNTEEKQYEKIGHENVRHEETSSEG
NENLN-RNTKHNNDNNNDNNNYSEDAIAELLLSYFNVFYPISTCMCYSIRSKHESLMDYD
KYHMINLENDIKLKHYIKETEE--IHFNSIEEYKMKLNRINY-KYDTLLEEHENLVTHKN
ILIGIKTSINTEEERIPHIKNTYDNKENTQIIFNTFNYD------------------NKL
KEKNTFG-------------FYNNSLLQNALENDNIDLDIIYMSDKESQKYDNLYFNS--
------------KVTSKEGLCEKLKHMIYYYYEEYVMKNSEK---------KYFFIADDD
TFVNVKNLIDVTNLTLNTCSHSKKYMYDKYIKSYDFVKENEALFLQNFPKKTLFLYSYLK
DTFAKTIQTLKKYDYVPKYCQGGILSKKHKNNDSDDDHDHHVGNKQNNDSTNHQDIEKNQ
VNVINNNNNNNNNKAKSIPIYLGRRYSYNTFSTNSNEYFYDYLTGGAGILINDETAKRIY
ECK----------------------ECTCPSTNSSMDDMIFGKWAKELGILAINFEGYFQ
NSPLDYNKKYINTLVPITYHRLNKNRTTKESRDMYFNYLVN----YNRNDKEQNKDIYVD
YLDRNHKNMIDNVFHYFFYVNMYDE-----KNKVVTKIEHNADMNSKKNKSKNPQKLNNT
QGDKNVNDDENVNDDENVKGDENVKGDENVKGDEYMKGDENVKGDENVKDDENVKDDENI
KGDDNNYNVDNMENIDDIINMVESVDDDV-MERNKKGTGKEKKDDKNHNNKEKATDVKKS
SVPTNNIDKNEDTTKYVIKMNEKIYNRMQESGKYKQLFDINKFFKKEIEGHPYFQKIKKK
NEKAKKEKEKMNQLKKQKDYTNNYFHTS--NMQGNFNQQKMGNYQNQENEENDFFDQRPE
IEEDAINPMDYEEYMENLSNFE-----DDGEPYDEYD-DYDDFVNTINADKLKINDQNKH
LYEQIKD----------------------------------------------------I
AQPPVNFQNDQNSNTFDFDTDEL

>P_reichenowi_ortholog
MKV-GIIFFG-----LVFFVVLGACNNVKERIFKNIKRRTKFIILNEPIVDLSFSENLFH
TLLFDLDVD-KNLYTLDESLLNLQNLNYSSIFRLLVDTYKNIKEN---EDDNKNIRYIFL
GTSFSRIHPLNFEYFLRKLNKYIYNGNIYEKGNVDMRGILEEYNKEIEEKKLEKQKLNKI
KDK-NKNNNNNNSKISKDDNNNDFNNKNDLYNPSDKLYNNNDDIDVHQLLEEIITKEKRF
FLNDDDDNDSNDKYILKT--------DEDNKYKGFFIGYGFNDDIPSVIHHYNFDKNFLF
PSLNSGIILDITLLKNIYEVSNILLSNNEKDQSIHIDYIYEVTKYIKENLRVRLTHSENV
CLNEEQNIHLLDND-PNNFEIYKYYQVLNLFKDYNKNTQEQKYEKVEHENNKHEETSSEG
NENLN-RNTKHNNNNNDNNNNYSEDAIAELLLSYFNVFYPISTCMCYSIRXXXXXXXDTS
NYYLNPIDNDIS---HISQGREPFLNFNSFDDQKEDDYRNNVITYDHDIQENNNITTHNN
----------------NNNNNNNNNNNNNNNVYTTNDIAYNN---------------NLL
KDENYFKYNDNDLDCERIYHFFSKYFINKILYADNIDLDIIYMSDKESEKYDNLYFNS--
------------NVTSKEGLCEKLKHMIYYYYEEHVMKNSEK---------KY-FIADDD
TFVNVKN-IDVTNLTLNTCSHSKKYMYDKYIKSYDFVKENEALFLQNFPKKTLFLYSYLK
DTFAKTIQTLKKYDYVPKYCQGGIISKKHINNDGD---DHHMDNKQNNDSTNHRDIEKNQ
VNVVNNN------KAKSIPIYLGRRYSYNTFSTNSNEYFYDYLTGGAGILINDETAKRIY
ECK----------------------ECTCPSTNSSMDDMIFGKWAKELGILAINFEGYFQ
NSPLDYNKKYINTLVPITYHRLNKNRTTKESRDMYFNYLVN----YNRKDKEQNKDIYVD
YLDRNHKNMIDNVFHYFFYVNMYDEKNASQKNKVVTKIEHSADMNSKTNKSKNTQRLNNT
QGDK------------------NVKGDVNMKGDVNMKGDVNMKGDVNTI-------DVNT
NGDENNYNVDNMENIDDIINMVESVDDDNDMERNKKGTDKEKKDDKNHNNEEKATNVKKS
SVST-NIDKNEDTTKYVIKMNEKIYNRMQESDKYKQLFDINKFFKKEIEGHPYF------
-----------------------------------------XXXXXXXXXXXXXXXXXPE
IEEDAINPMDYEEYMENLSNFE-----DDGEPYDEYD-DYDDFVNTINADKLKINDQNKH
LYEQIKD----------------------------------------------------I
AQPPVNFQNDQNSNTFDFDTDEL

>P_gallinaceum_ortholog
MNLFSNSFFSFPSLIIHFFFIL-------SSLFLFIHRFFVFLLLNEPIVHLSFSENLIH
SLVFDLEND-KNLYTLDETLLNLENINHSSIFRLLVDTYKKIEED---KDDRENIRYIFL
LTSFSRIHPLNLEYFLRKLDKYIYNESIYKKGNINIKEILNEYNNEIKEKMEEKEEIKK-
--------------------------------------KNGEQININD-IEKIIRNEKIF
FLNNDD--KGSDKYVIK-----------DSIYNGLFIGYGLNDESPSIIHHYNLDNNFYF
PYFNSGVLIDITLLRNIYKKSLTLTNFE----IINIDYIYEVSQFLKENLNVKLTTFQNT
CLNDEKNIHLLDND-LNNFELYKYYQVLYLFKDLK---------------EKPQKDNNEN
NQNIEIKNDKENNTEKNYNQKNEDNELAELILSYFNIFYPISTCISYSIRSRNESLLGYD
KFHLMNIENDTKLENYIKETED--ITFSNIDEYKMKLNKVNN-KYDYLLDENENTLGYKN
ILIGVKTSINT-EERIPYIKNTYDNEENTKRIFNNFNYNYES----------------QL
KEKQHG--------------ILSNEFLNNVFHSERINIDVIYMSDKKSTKYDNLYMDT--
------------KITSKDGLCEKLKHMIFYFYEEYVKKNSEK---------KYFFIADDD
TYVNVKNLIDVMNLTLNECEHSKKYMYNKYIKSYEFLEENEPFFLQNFQNKSIILYQYLK
NNFLKTINSLKKYDYVPKYC----------------------------------------
------------------------------FSEKN---YYDYLTGGAGILINEETAKRIY
MCD----------------------SCICPTKNSFMDDMILGSWAKELEILAINFEGFFQ
NNPNEYNKIYLNTIVPITYHKLNKDKNVEETKKVYFDKLVN----YNK-NYTSTKSLQVD
YLDRNYKNMIDNIFHYLYYTNIY-------------------------------------
------------------------------------------------------------
------------------------------------------------------------
-----TIESNKN----IINMNKKIYNKMHESKNFNHLFDLNKFFKKQINDYEHLQTSGTY
KSKK----------KIKKGYSNNYIEKNNSNLSDLKWQKD--------------------
-ENESKSSYDSEEYLY--------------EDYEDYD-DYYEYLEKI-------------
------------------------------------------------------------
-----------------------
```

```
>P_knowlesi_ortholog
MSIMRRIFFA--LLVVS--GLMVHCIQVKDKLFKGIKRNTTFLVLNEPIVDLGLAEGLFH
SLLFDLEVG-DNLYTLDENLLNQKNLKHTNIFHLLMDIYKQVEN----SQGGHAIRYIFL
CTSFTRVHPLNLELLLRKFNKYIYNGHSHEKGDIDVGGILREYNEEVQAKRTALRGMSSV
SGS-NS--------------------------------DTTGRIPAKDIIERMMQDQVNF
LSSDDK--EGSDQFVNP-----------SGTYKGFFIGYGFSNQVPSQISGNPSPNGFFF
PSINSGIIMDIILLRRIYNH--LIKKSSEENLKVIKDNVHELSNYIGEHLQVNLTPFENA
CITDKDNVFLVEHEGGSNYDVYRYYLVMHLFRDYK------------KDNNVNEAD-NLG
GINTIMMNPP---GEE----GPRDAELAELIITYFNLAYPISTCASYSIRSKKEAFVDYD
NYHLMSIENEIKLEKYIKETED--VHYNSIDEYKTKLNQINQ-KYDMLFDETQRIWNYKN
ILLAVVTSAQT-EHRIAHVKNTYDNKKNIHQVFNSYHSHGGE--------DGVSGKGD--
-EQDTKG-N-----------LLNHDFLKSAFENEKINMEIIYFSDKESKEYDETLHL---
-EETDKKV----DSPNHGELWRKEKNIIYYLYEEYVRKNSPI---------NFFFICHDD
TFVNVKTLIDVINLTRNECTHSKRHMFRKYVKAFDHMDERESQFLENFDKKYLQLYRYLK
TNFVKTIDALKKYDYVPSYCKG--------------------------------------
--VGAGKSTFDGSPLTSVPLYIGKRYSHNSFDEDENS-FFHYLAGGAGIVLNAEAVKKNY
FCKGVCDVDLP-------SGIAPHGKC----DVSLLNDAAIGQWAKGLGIVPINFEGFYP
KAPEEYPAEYLNTITPVSFHKLNEGKTVEETKEAFFRHLVNPTQPRNKTNHKE-EDMCVD
YLDRNYKNTMDIIFHFFFYVNKVKECK---------------------------------
------------------------------------------------------------
------------------------------------------------------------
-----PDES-------IIKTTERIYDKMGSSKKFSHLFDMTTFFTRDVEDMLYFQKRSAS
KGNLNNKK------ESKGTYTNNYVERKDGTASGDTPDGHRQPGR------EDMNNFGGM
YEMDEADDLGEEDHLSSMDDFGSLDDLDEEEQFDDDDYDYEKYLDGIEAHKKGSGR----
-----------------------------------------------VPSDEPEWGDD--
---------GDDNLNLPPEAGEL

>P_vivax_ortholog
MNLMRRILLG--LLAVS--STVAHCREVKNKLFKGIKRNTKFLVLNEPIVHLGLSEGLLH
TLLFDLEIG-DSLYTLDEHLLNEQNLKHTNVFHLLMDVYKQVQN----SQGGDAIRYVFL
CSAFTRVHPLNLELLLRKLNKYIYDGRSHEKGDIDVGAILGEYSAEVRAKRAARSAIGSD
SGG-S---------------------------------------PAKDLVERMLKDEVDF
LSSEDQ--EGSDKFVKP-----------GGTYKGLFVGYGFGGQVPSGESGPPHKGGLLF
PSLNSGIIIDITLLRRLCNH--LAANPSEESVKLMKDNVQELSNYIGEHLHVKLTPFENA
CPTDRHNIFLVENEGGTNYDVYRYHLVLHLFGDYK------------KGSHAGETGAHLG
GPTDSALELPSDAAQE----RPTDAHLAQLIVAYFSLAYSISTCASYSVRSKQETFVDYD
SYHLMSIENEIKLHKYIKETED--VQYNSIEEYKSKLHQINK-KYDALFDEGQRNWTHKN
VLLAVVTSAQT-EHRIAHVKSTYDNKENTERVFNSYQPHGGEGNTKGFVGESASGRGDKP
KEQPRAGEN-----------LLNHDFLKSAFENENVDVEVVYFSDEESKRYDDMLHLKGG
GEEGDHPKGDAGDSPQGEELPKKEKHIIYYLYEEYVRKNSAI---------DFFFICHDD
TFVNVKTLIDVLNLTRNECTHARRHMFGKYVKAFDHMDEREDAFLKNFGKGYLHLYRYLK
TNFVQTIDALKRYDYLPSYCKG--------------------------------------
--GGAGKGASGGATLTGVPLYIGKRYSGNSSAGGDSG-LVHYLAGASGMVLNGEAIKRMY
FFKGACGVGAAVAADGATDGAAASGEC----DARLLNDAALGRWAKAVGILPIDFEGFFP
NAPEHYPPDYLSTIKPVSFHQLNEGRTANQTKEAFFHHLVGPTQRRNKTNHEE-ADTSVD
YLERNYKNTIDSIFHFFFYVNMAEECK---------------------------------
------------------------------------------------------------
------------------------------------------------------------
-----SADS-------IIKATERIYSKMGSSKRFNHLFDMATFFTGDVEDMLHFQKRSA-
---------------GKETYTNNYVQRK-----GGEPDGQRRPGRGEAGDFNKADDFAPL
DDLGEADDWGEEDHLGDM-DLGPLGDLDEEEQFDDDDYDYEKYLDGIEAHKRGSGSGSGS
VDDQPEDPEWPGGSDDSLDLPPHGDGGSDDSLDLPPHGDGGSDDSLDLPPDGPDYSDDSL
DLPPDGPDYSDDSFDLPPEGSEL

>P_chabaudi_ortholog
MKV---IIWLFPLLILS--IVIKCDNKIKDNIFQNLRKKTKFLVINEPIVDLDFSENLFH
SLLFDLEVYNNNIYALDENLLNLQNLNHSSIFKLLTNTYEQVSKENEKNDEENKIKYIII
ASSHTRVHPINLEYLLLKFDKYIYNGNIYQNGDIDIKGILNEYNNEIKESLEKKKKFKNI
S-------------------------------------NIIDEKYKDDIVSALLNKDI--
--------GSSDKFIKKGDNSNDSNSNYNNQYKGMFVGYGFNDDEPSILHSLNENKNFLF
PSMNSGIILDIILLKNIYENYINIQKQNEK--IIHKDYIYEISKFIYDNIHVELVHFENT
CLDAKQNVYLTNKE-NTEFDVYKYYITKNLFEDYG-------------------------
--SLSIKEVEEKNYEN-------------IVMSYFQLAYPITTCATYSGRSQ-ETFIGFD
KFNMISIENDEKLKYYIKETEE--ISFNDIEEYKRKFNDINK-RYDEILDNGENNLTHKD
IVLGIKSSINT-EDRISYIKKTFDNKKNNKHIFSNFKITP------------------VL
KNQKETS-------------IINTELLKGGFDSEDIDIQIFYMSDKESKLFNTVKYDI--
------------DDVSKNSSCEKMKQTIFHFYEEYVENDKNKNNNKNMKKQKYLFIGNDD
TYVNIKNLVDVMNVSSNKCVHIKKHMYDKYLKSFRFLKKNEPKFFKNFNEKPPLFYQYLK
QNLIDTIRNLKKYNYIPKYCKD--------------------------------------
------NNGIGNTPKSDVPILLGKRQSYNTFYNDNES--YDYLASEAGILINDSFAKKIY
FCK----------------------NCVCP-DNEQSDGMMLGEWANKLNLLTINFEGFFQ
SHPANYNKKYLNTLVPITYNNLNFNKTVDEIKKTYFQHLVN----YNKDEIDEKTDSYVD
YLDQNFKNTFDNIFHYFFYLKRYYNSP---------------------------------
------------------------------------------------------------
------------------------------------------------------------
-----NDEKNNDISKIISKINKKIYAKMEYNGSYKKLFNLSHFFKEEIENMIQFKHNKKN
GTR-----------KGKVNYANNYVVENSEIKTNDNYENE-------------------E
LEQDDYNDDSEED--------------DEDDLFSEDDFDYDEFIKDIEN-----------
------------------------------------------------------------
-----------------------

>P_yoelii_ ortholog
MKI---IIWLLPLLIIN--IVIKCNSKIKDNIFQNLRKKTKILVLNEPIIDIDFSENLFH
TLLFDLEIY-NNIYTLDETLLNLEKLNHSSIFKLLIETYEQVRKE---ASGENKINYIIL
ATSHTRLHPINLEYLLLKKLNYIYNQNTYKNGDIDIKGIIKEYXXXXXXXXXXXXXXXXX
XXXXXXXXXXXXXXXXXXXXXXXXXXXXXXXXXXXXXXXXXXXXXXXXXXXXXXXXXXXX
XXXXXXXXXXXXXXXXXXXXXXXXXXXXXXXXXXXXXXXXXXXXXXXXXXXXXXXXXXXX
XXXXXXXXXXXXXXXXXXXXXXXXXXXXXXXXXXXXXXXXXXXXXXXXXXXXXXXXXXXX
XXXXXXXXXXXXXXXXXXXXXXXXXXXXXXXXXXXXXXXXXXXXXXXXXXXXXXXXXXXX
XXXXXXXXXXXXXXXXXXXXXXXXXXXXXXXXXXXXXXXXXXXXXXXXXXXXXXXXXXXX
XXXXXXXXXXXXXXXXXXXXXXXXXXXXXXXXXXXXXXXXXXXXXXXXXXXXXXXXXXXX
XXXXXXXXXXXXXXXXXXXXXXXXXXXXXXXXXXXXXXXXXXXXXXXXXXXXXXXXXXXX
XXXXXXXXXXXXXXXXXXXXXXXXXXXXXXXXXXXXXXXXXXXXXXXXXXXXXXXXXXXX
XXXXXXXXXXXXXXXXXXXXXXXXXXXXXXXXXXXXXXXXXXXXXXXXXXXXXXXXXXXX
XXXXXXXXXXXXXXXXXXXXXXXXXXXXXXXXXXXXXXXXXXXXXXXXXXXXXXXXXXXX
XXXXXXXXXXXXXXXXXXXXXXXXXXXXXXXXXXXXXXXXXXXXXXXXXXXXXXXXXXXX
XXXXXXXXXXXXXXXXXXXXXXXXXXXXXXXXXXXXXXXXXXXXXXXXXXXXXXXXXXXX
XXXXXXXXXXXXXXXXXXXXXXXXXXXXXXXXXXXXXXXXXXXXXXXXXXXXXXXXXXXX
XXXXXXXXXXXXXXXXXXXXXXXXXXXXXXXXXXXXXXXXXXXXXXXXXXXXXXXXXXXX
XXXXXXXXXXXXXXXXXXXXXXXXXXXXXXXXXXXXXXXXXXXXXXXXXXXXXXXXXXXX
XXXXXXXXXXXXXXXXXXXXXXXXXXXXXXXXXXXXXXXXXXXXXXXXXXXXXXXXXXXX
XXXXXXXXXXXXXXXXXXXXXXXXXXXXXXXXXXXXXXXXXXXXXXXXXXXXXXXXXXXX
XXXXXXXXXXXXXXXXXXXXXXXXXXXXXXXXXXXXXXXXXXXXXXXXXXXXXXXXXXXX
XXXXXXXXXXXXXXXXXXXXXXXXXXXXXXXXXXXXXXXXXXXXXXXXXXXXXXXXXXXX
XXXXXXXXXXXXXXXXXXXXXXXXXXXXXXXXXXXXXXXXXXXXXXXXXXXXXXXXXXXX
```

```
XXXXXXXXXXXXXXXXXXXXXXXXXXXXXXXXXXXXXXXXXXXXXXXXXXXXXXXXXXXX
XXXXXXXXXXXXXXXXXXXXXXXXXXXXXXXXXXXXXXXXXXXXXXXXXXXXXXXXXXXX
XXXXXXXXXXXXXXXXXXXXXXX

>P_berghei_ortholog
MKI---ITWLLPLLVIN--IVIKCNNKIKDNIFQNLRKKTKFLVLNEPIIDLDFSENLFH
TLLFDLEIY-NNIYTLDKTLLNLEKSNHSNIFKLLIETYETVSKENEQANDENKIKYVIL
ATSHTRLHPINLEYLLLKFNKYIYNQNTYQNGDIDIKAILQEYNEEINESLEKKKKINNI
S-------------------------------------NNMDEIYKDNIISALLSQEK--
--------DSSDKFVKKNNISRNLN-NYNNQYKGMFVGYGFNDNESFTFHNLNENKKILF
PSINSGIILDIMLLKNIYENYINIHKKNEQ--IIHKDYIYEISKFIYDNINVELTHFENT
CLDTKQNIYIINKE-NTEFDVYKYYLTKALFQDYD-------------------------
--KSSIKEKEETHNEY-------------LIMSYFQLAYPITNCVTYSGRSQNETFIGFD
KFNMISIENDEKLKYYIKETEE--ISFNNIDEYKQKFNDINK-RYDEILEHSEHNLTHKD
VVFGIKTNINM-EYRINYIKNTFDNKQNNKQIFNNLKITK------------------TL
KNQKETS-------------VINTDLLKSGFDNENIDIQIFYISDIESKLFNTLKYDT--
------------YDISNNSSCEKMKRIIFHFYEEYVEKDKKKNNIKSLKKPKYLFIGNDN
TFVNIKNLVDVMNVASNKCMHIKKHMYDIYLKSFQFLKKNEPKFLKNFNKNPPLFYQYIK
QNFLDVIHNLKKYNYSPKYCKD--------------------------------------
----NNNNDVKSSFKYNVPIFLGKRYSYNTFYNNNET--YDYLASDAGILINDSFAKKIY
FCK----------------------NCVCLNNNDQSYDMILGKWVNKLNILTINFEGFFP
NHPDNYNKKYLNTLVPITYNNLNLNKTVEEIKKTYFQYLVN----FNKDEIEGKTDSYID
YLDQNFKNTFDNIFHYFFYIKNYYNSP---------------------------------
------------------------------------------------------------
------------------------------------------------------------
-----KDEKNNN----ISKINKKIYAKMEYNGSYKKLFNLSKFFKEEIENIIQFKYNKKN
GTR-----------KEKFNYVNNYVVENTEIKTNQNYENE-------------------E
LVQDYFPENSEED--------------DENDLFSEDDFDYDEYIKDIEN-----------
------------------------------------------------------------
-----------------------
```

- **AMOUNT OF MISSING DATA**

P. reichenowi	***P. gallinaceum***	***P. knowlesi***	***P. vivax***	***P. chabaudi***	***P. berghei***	***P. yoelii***
10%	30%	27%	29%	30%	30%	88%

■ PF08_0091

▪ PHYLOGENY AND PEXEL/VTS

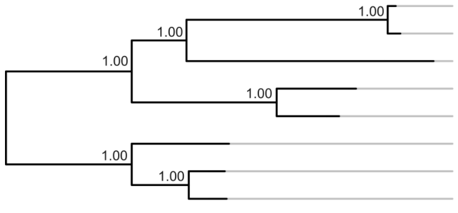

P. falciparum PF08_0091	NGHFIRSIDEYYVYE
P. reichenowi ortholog	NGHFIRSIDEYYVYE
P. gallinaceum ortholog	KGDFIRSIDEYYVYE
P. knowlesi ortholog	NGDFIRCIDEYYVYE
P. vivax ortholog	KGDFIRCIDEYYVYE
P. chabaudi ortholog	RGDFIRSINEYYVFE
P. berghei ortholog	KGDFVRSINEYYVFE
P .yoelii ortholog	KGDFVRSINEYYVFE

▪ ALIGNMENT

```
>P_falciparum_PF08_0091
MNNVIIFLLLLINFGTKVYGDDNGEHSANNING--KEEEVEDRWKMYALETNPFLDLKHP
NFNGHFIRSIDEYYVYETKTYNEYMKERYKNLENNNTFTKKQFHCALAVFNHGRPNPKQT
NDYKIWSAQGNILLNEVMHKKKYDKFIIYAPFVSLYNFLGKEGIHSHINFAYIIVSRLAL
TIKNALNDDSCHTYDLYLHGHCFGGNIIRIISSLSKDLWKIFQREVINFKIERETLAIID
SNFHLTLKDINIVTDSDSIEVRKDPYFKKDRFYLYKDFKYKESNKPISETFDDVFDDADI
GYH-----------------RLKDLDNPENTNNAHQV-----------------------
-------------------------SDEDDNLL-----------------RPIKRLHFYL
LNKKINLHSITVTGPPLSGTFSKMEDINVGHQGFFKQKYIVKYLPYYLKRATMHKFRDVE
EVLLIYNSELFCLLTVHEKFSYDYTNNLGSLMTFFKSVNFYSDLDNDEIVSMHASLALHS
PLHMRSLTYYLLNFRNEYYHRAFRIPSHLSDINVSNNYPFFEYLKNNNVCSSMSSRHIEQ
FISYVNEAINFNQKPKPLIPQRVIYKNPYLEHFVLPYIPDKSIYTPHTILGTGRTYLLLY
TFDIYKHIAGSAFSNANVLTNDKELLYDSDPFIYYNWTSHVGDQNNVQTNYFMQDIYVFS
DNVNMNIVDNLFNIFTSTHLVKFFIFNKNHSADVYKSLYRTVEKFALPMVDIVLRKQRKK
YIEYPFLKNDKFNDEENLVYEYINNYTNYLKNFSFNNSKVYISNDNFITNHETFKKYHTE
GINTLKEQLSSLHKFAQENKTFYEMKKKFESMHNMNK-----------------------
----INSSNNESFNEKELQEMYEEYDDFNNIEKPTQDETDNSNEENKSKLDV-----NYH
GIYLLIKRTLSLKEAFQSLKSYNSSYYNVKYIEFIIKNSYLEHVDDNISNIDDVSSCLFN
HDDDISLGYIAKYCNYNQRLYFNIKKHYISRDIFTVPKLKYCEKPKYKHLKICENVILLK
FYFRSDLETLLSNLNNKELKRMTEMKESVEAGIGKENIFSLTNDSLVTLFHHRNDDITNL
PVNACFKLSSISNMNDFFFKKNNVENSSS-------------------------------
------------------------------------------------------------
------------------------------------------------------------
-------------------TKNNGNNLSENIFCTQVTLPVILNKLTIKSINDIYLRAISN
VMKYKEFKNIFQLSEDEEPYDSFVYFFDKFTYIYNVRTFYQNYNEIETFSTPQNLKWNYF
YYLLKNGLNTDYNNENFLQNFFYGEHANLVKPLRENILKSFLKHFTVFFYLFKVDE----

>P_reichenowi_ortholog
XXXXXXXXXXXXXXXXXXXXXXXXXXXXXXING--KEEEVEDRWKMYALETNPFLDLKHP
NFNGHFIRSIDEYYVYETKTYNEYMKERYKNLENNNTFTRKQFHCALAVFNHGRPNPKQS
NDYKIWSAHGNILLNEVMHKKKYDKFIIYAPFVSLTNFLGKEGIHSHINFAYIIVSRLAL
TIKNALNDDSCHTYDLYLHGHCFGGNIIRIISSLSKDLWKIFQREVINFKIERETLAIID
SNFHLTLKDINIVTDTDSIEVRKDPYFKKDRFYLYKDFKYKESNKIISETFDDVFGDADI
GYH-----------------RLKDLDNPENANNAHEV-----------------------
-------------------------SDEDDNLL-----------------RPIKRLHFYL
LNKKINLLGITVTGPPLSGTFSKMEDINVGHQGFFKQKYIVKYLPYHLKRATMHKFRDVE
EVLLIYNSELFCLLTVHEKFSYDYTNNLGSLMTFFKSVNFYSDLDNDEIVSMHASLGLHS
PLHMRSLAYYLLNFRNEYYNRAFRIPSHLSDINVSNNYPFFEYLKNNNVCSSXXXXXXXX
XXXXXXXXXXXXXXXXXXXXXXXXXXXXXXXXXXXXXXXXXXXXXXXXXXXXXXXXXXXX
XXXXXXXXXXXXXXXXXXXXXXXXXXXXXXXXXXXXXXXXXXXXXXXXXXXXXXXXXXXX
XXXXXXXXXXXXXXXXXXXXXXXXXXXXXXXXXXXXXXXXXXXXXXXXXXXXXXXXXXXX
XXXXXXXXXXXXXXXXXXXXXXXXXXXXXXXXXXXXXXXXXXXXXXXXXXXXXXXXXXXX
XXXXXXXXXXXXXXXXXXXXXXXXXXXXXXXXXXXXXXXXXXXXXXXXXXXXXXXXXXXX
XXXXXXXXXXXXXXXXXXXXXXXXXXXXXXXXXXXXXXXXXXXXXXXXXXXXXXXXXXXX
XXXXXXXXXXXXXXXXXXXXXXXXXXXXXXXXXXXXXXXXXXXXXXXXXXXXXXXXXXXX
XXXXXXXXXXXXXXXXXXXXXXXXXXXXXXXXXXXXXXXXXXXXXXXXXXXXXXXXXXXX
XXXXXXXXXXXXXXXXXXXXXXXXXXXXXXXXXXXXXXXXXXXXXXXXXXXXXXXXXXXX
XXXXXXXXXXXXXXXXXXXXXXXXXXXXXXXXXXXXXXXXXXXXXXXXXXXXXXXXXXXX
XXXXXXXXXXXXXXXXXXXXXXXXXXXXXXXXXXXXXXXXXXXXXXXXXXXXXXXXXXXX
XXXXXXXXXXXXXXXXXXXXXXXXXXXXXXXXXXXXXXXXXXXXXXXXXXXXXXXXXXXX
XXXXXXXXXXXXXXXXXXXXXXXXXXXXXXXXXXXXXXXXXXXXXXXXXXXXXXXXXXXX
XXXXXXXXXXXXXXXXXXXXXXXXXXXXXXXXXXXXXXXXXXXXXXXXXXXXXXXXXXXX
XXXXXXXXXXXXXXXXXXXXXXXXXXXXXXXXXXXXXXXXXXXXXXXXXXXXXXXXXXXX

>P_gallinaceum_ortholog
XXXXXXXXXXXXXXXXXXXXXXXXXXXXXXXXXXXXXXXXXXRWMLYDLETNPYLDLKHP
NFKGDFIRSIDEYYVYETKEYNMYKQTRYANMMNNTSFERKQFNCVISVLLHGRPNSREF
YDYKIWSSQAAIIKDEVISKQKYGKFILYAPFISRLNFLGHGALHTHINFAYLVALRLTM
TLKNISSDDTCYNYDVFLHAHCFGANILRFILSLSNDIWKIKDTSLLDLNSERTALAILE
DNFKLTLRNIDIVTNKDSIKPNKDPYFRGPKYYLYKNFQVDLRHKELTESLDEIFNDENV
NYN-----------------NLSDDMLQDTNINYIK------------------------
-------------------------SEDTENQI----------------NRVIKRFHFYL
LNKKISLMNISATGPPLSGVVWNLEDIKIGHQKKLRYKSILKIVPSFV-KNNITRTRDVE
ELMYITNPKLLCHLAVEEKVDYNYNKNRGSLISYFKNVNYYADLDNDELVSMYTSLGLHS
PAHMRSLSYFMLNFRNEFYNRIFKIPVHLSNINVSDNNYFISYLEENDVCSYVKNKYLEY
FVSYVXELVNYNQKPSPYIPQRYAYKNPFLVHFKVPVDQKNNIYTSHSILGTGRTALLLY
SYELYKHLSSSGLNNNDVIRGESEYFYDTYPFVYYNWMTYIANQKDRKKENIMKDVLLYS
DNVNMDIVDNLFNVFTIFHYLKFFIFXXXXXXDAKKNIYKMINQFSLPNLNNIFKKKEKK
KIEFSMLKRNELNKQEKYLYDFFGIYTNFLKNFSNNNAFSYIPDDVFLIKHSTFKDHHNK
NIDSLKEQIKNIHDFVKENKTFYEMKKKLNLMYNID------------------------
------RTDNIVDDDEDIEELYKEFESYNDI-----QNLDIDNNNEDFNNSDKGTVNNYY
GMYFHIKRILNLKDAFVNIKQVNSSYKNVQYVEFVIKNAKYENIEDNLNNLENNNYCLFN
YDEEIDLSYIVHFCNYNQQIYYNLYKRYTSNSIYVLEKLSDCELDNYKYLKLCENAILLK
RFFHKELDVILSEVHKKELRKMYEMRTSIERGINARNIFSLSNDSLVAVLHNKNENMTNI
KINVCFKVSDVSKNKNLFFKDKLPEPEYD-------------------------------
------------------------------------------------------------
------------------------------------------------------------
-------------------FKDNVNSMNNSVFCTHVSLPVKINTRLIKSLNDIYLRAIRY
ISKDKHFQSIFNITEDEGSYKSFVYFFDKFSYVYDLRSTYKNSEEITSFTTPQNFTWKYF
```

YYLLKYDINTEFNNEEFLHNFFYEEDNETVXXXXXXXXXXXXXXXXXXXXXXXXXXXXXX

>P_knowlesi_ortholog
MNNVIICFLLLINYGKSVLGAEAQGKNPNNDNNPYEESEIDEKWRLHELETNPFIDLKHP
NFNGDFIRCIDEYYVYETKEYNAYKKERYKNIKNYKTFKKKQFNCAINLLVHGRPNSRNS
NDYKIWSAAANINKHQAMLQKKYSKYIIYTPFVSLSNVTSPEGLYTHLNYAYLLSNRMAI
TFKNMLEDDTCYNYDIFIHSHCYGANIVRLMFTLDNEIWKTLDKSLLNVTYEREVLAILE
DNFKLTLKDINIITDKNAIQIRKDPYFKIHKYYLYKTFQSKFNKNKIIEDFDDVFDDHSL
KYNFHPQGESNAIHGGGGKGSMENLVAQSGTSGARNAGAEESESEQGER-----------
-----------EEPEESEESEDSEDSDSDDEFIFFIKPKKVRKKGSHAPNRSLKRLHTYL
LNKKFTLLSIVSTAPPLIGIVSNMEDIRVGHQKLMKYKFMFQNVPSFL-RAKMLKTRDAQ
ELLHVVNPGLLCLLALHEKLNYNYENNTGSLISYFKNVNYYADLDNDEIMSLHTSLGLHS
TLHMRNLSYYLLNIRNEYHHRTYSLPAHLSDINVSNNYAFFEYIKENDVCSQIKHSELER
FVSYLSEIINFNQEPQPYIPHRQVYKNPFMSHYVIPYMQENNIYTPHSILGTGRTALLLF
SYDIYKHIAMNGFSERNVIKNDIDFLYDADPFVYYNWMTYIGNQNELDEDNFVKNFHIYS
DNINMNMVQNLFNLFVSTHYVKFFIFSKNNTADVYRSLHRTLKNFSLPINKVVLRRQREK
KINFPLLNNAQFDHEENVVYDYMCRYTNFLKTFSYLNSFQYITNEEFILNHKNFKKYQSE
RMAELKNEIKKAHNFIQQNSTFSDMKNKLTDMYNIE------------------------
------NTANSSERELEVNEEVGNLNTFESANYPLEESKKNSASNDSLQDIDKSYV-TYH
GIYVNIRRTMTLKEAFKSISMHNDIYDAMQYVEFVIKGSAYNDVEDHLNNMEDVNSCLFN
KNENIPLNFISKFCNYDQQAYFHIKKLYTSRNLFVLKSLKQCNYPKYRNLKLCENRNLLK
TFFQSDLSVTLSKLHEREKKRMATMRDGIERGMNEADILSISNDSLAAIFHNKNEDISTF
KMNACFIIPEKPLIQNLFSNDENMQSTKEDTEMGTVKKVGHGHITADTSDVITMTESSTN
WDSSLSNMEEVDSSRTSLHSLESLGGYEDVGSDVIEAAYELEREFGPVAKMAMSVVSANE
KEQEKE-----KEKEKETETEEEEEEEMERQKQKEKEREKKEKEKNYNIYAQSSAS---
-------------------RSGRDSGSGEKVFCTEVTLPVLLRKDILKSANDIYMRAIRS
VMQDPHFRSAFNLRNGDEPYESFIYFFDKFAYVYNQKNKYAHTGDINTFNTPQNIKWNYF
YYLLKYGSQTNYNSESFLKNFFYGKKSNLVKPQKDNILKDFLAHFTVFFYLFKLD-----

>P_vivax_ortholog
MNHVIIFFLLLFNYGKGVTGAEAQEGSPNSHNNPYEESEIDEQWRLYQLETNPFIDLKHP
NFKGDFIRCIDEYYVYETKEYNAYKKERYNSIKSYKAFEKKQFNCAINLFIHGRPNSRNA
NDYKVWSAAANINKHEAMLQKKYSKYIIYTPFISLSNFLGTEGLYTHLNYAYLLANRLAV
TFKNMQDDDTCHNYDIFIHSHCYGANIVRLMFTLDNDIWKLLDKSFLNMTYEREVLAILD
DNFKLTLKDINIITDKSTIQYRKDPYFKVDKYYLYKSFQAKINKKKITEHFDDVFDDHNV
KYDALPQAGSASDEGAGGSDSFENLMAQPGRSSALGGGEEEGEVEPEAA-----------
-------------------------SDNNEEFILFTKQKKVPKRSGHPANRSLKRLHTYL
LNKKFNLLSIVSTGPPLIGILSNMEDVRVGHQKLMRYKFMLQNVPSFL-RAKMLKSRDAQ
ELLHMVNPGLLCLLAVHEKINYNYEKDTGSLISLFKNVNYYADMDNDELMSLHTSLGLHS
TLHMRSLSYYLLNFRNEYYHRTYILPAHLSDINISNNYAFFEYIKRNNVCSQIKQSEMER
FVSYLNEVVNFDQKPQPYIPHRQIYKNPFLAHYIIPYMQENNVYTPHSILGTGRTALLLY
SYDIYKHIAMNGFSERNVLKNDIDFLYDADPFIYYNWMTYIGNQNNVEQDNFMEDLHVYS
DNINMNLVQNLFNLFVSTHYVKFFIFSKNNTADVYRSLYRTLRNFSLPINKVVLRRQKEK
KINFPLISNAKFDPDENVIYEYMGKYTNFLKTFSYLNSFQYITNEAFILNHKTFKRYQNE
RMEELKNEIKKAQNFIQENNTFSDMKNKISAMYNIE------------------------
------NTSNNNERELAVNEEVGDLAAFESANYALGEGKAASSSDGSVQNLDRTYV-TYH
GIYLNIRRNMTLMEAFRSISLYNSVYENIQYVEFVIKGSAYNDLEEHLSNPDDINSCLFD
KYEDIELSFISKFCNYDQQAYFHIKKLYTSRNLYVLKSLKQCNYPKYRHLKLCENLGLLK
TFFETDLSVTLSSLHEREKKRMTSMRDAIEREMHETDILSISNDSLAAVFHNKNEDISTF
KINACFIIPEKPLIQNLFSNEESMQSTEEDIEMGTLRKVGDGHLSASTSDLSAMTGSTTS
LDSSVSSSNE---SHGSMTSLESLEGFNDVDTEVIETGYELEHALGLSSHTGTGVEGESG
KEEDKEAKEKAKKEKKEKEKREKKERKEKEKKEKEMKEQEKKEQEKTDNNYAHSSSSSNR
SNNRSNNRSNNRSNNRSNNRSNNRNGSDHNVFCTQVEVPVLLRKNVLKSAHDIYMRAIRS
VMQSKHFRSAFNLREGEEPYESFIYFFDKFAYVYNKKNKYAHAGDVKAFNTPQNIKWNYF
YYLLKYGSGTNYNNEAFLENFFYGKESNLVKPQKDNILKEFLTHFTVFFYLFKLD-----

>P_chabaudi_ortholog
XXXXXXXXXXXXXXXXXXXXXXXXXXXXXXXXXXXXESEIDKKWKLHPLETNPFVDLQHP
NFRGDFIRSINEYYVFETEEYNAYKKERHNNIKNNIPFEKKQFHCAINVLLHGRPNSRHA
DDYRIWSPQMTLIKYEVMLKKKYHKYVVFAPFVVMTNFAGDEGVHAHANYAFLIANRMAM
TFKNLKDDDTCHTYDVLMHSHCVGGNIGRIAFTLDKEIWKTANISLINMENERTVLAILN
DNFKRILTDVNIITDKSTIKYRKDPYFKNNEYCLYKSFKYTPGKKKLTDDFEDIFDDYKV
DYDSLWSMENSLNKKKLSRRSKSKNSSGKNFGSSFKGHANLDSSDEEDAFE--------S
MNVPGPSNNTYDNFHYSYQHINSDLSDDDEDQI---------------SIKFVKRLHYHI
LNKKFNLLGATLTASPISGIYSNMEDLTIGYQHFSKYRHYLKWVPKYI-AHKLGKVRDYK
ELFYLVNPEMLCLLAVNEKLNYNFRQNTGSLMTLFKDVNYYSDLDTDEMVSFYSALGIHS
SMHMRSLSYFILNIRNEYYLRLYNIPAYLSDINVSNNFPFFNYIKNNPICKHVPDHNLGQ
FISFVNEIINYDQKPKPYIPNRYVYKNPKLSHFVLPTNMSDKTYTPHAVIGSGRTNLLLY
TYDVYRNVSRKQASEDNVLTSDVLFEYEGDPLIFYNWLSYIGDQNDMKRRNFMQKIYLYS
DNININVVDNLINAFSTTHYTKFFIFDKNHPVDAHKHLHRTLNNFSIPIQIVSFSVGNKK
KITFPILNTPKIDRDEAIAYEYINRYTNFLQNHVIRNSFYTTTDHNYILTHKTFKGYQQK
AVDRLRDQIKVVKNFINSHKTFNEMKKALRDSFNIH------------------------
-------GTAPINTDNYINHELGDLESFVEENYPNPIGLDEGVSNDDSSQYDLSYYDNYN
GTYLLVNSDLKLRSVYKYMLKYSKIYKNTKYIEFVMKNEMRGDVHDQLVNVENGSSCLFD
FNDNIRVSYIIDYCNYDKKSYFLFYKEYKSKNIYSVPSQDLCESAEYSYLKLCQNMSLLK
KFFTKTLDTQLSEIHKDEMKRMTKIKNAIEDNIDFKNILSISNDSLVSIIHDKNEGITTF
DINACFTVSAKLTLGNIFNVNSQIDPETA-------------------------------

-----------------------RTNINNSIFCTPVSVPVAVNRPIMRSINDVYIRAIFN
IMKDQQFREYMRIPVNSNPYHSFIYFFDKYAYVYKKRKWYKNMNHVKMFIPPQTIKWNMF
YYLLRNNSQTSYNNEMFLYDFFYGKKSADIKALSRNIMKPFLSHFTLFFYLYKVDESIGN

>P_yoelii_ ortholog
MYISISF-----------------------------ENDIDEKWMLHPLETNPFIDLQHP
NFKGDFVRSINEYYVFETKEYNAYKKERYNNIKNGIPFEKKQFHCAFNLLLHGRPNSRNA
DDYRIWSPQITLIKYDAMLKKKYHKYFLYAPYVVITNFVGNEGIHAHANYAFLITNRLSM
TFKHLLKDDTCHTYDVFVHAHCVGGNIGRIVFTLDRDIWKTVNLSLIDIQNERTTLAILN
HNFKRTLTDVNIITDKSTIKYRSDPYFKKHHHCLYKSFKFVPKKKRLTDYFEEMFSDHNV
EYDSLESVNKQLYKKKLSKLKKSKYSSSKRMRNPFKMDEDLTSNIKSDYSEDEDEEISDS
TSVFDPNNNSYDNFQYSYQTLDNDDSEDDEEVI---------------SLKFVKRLHYYI
LNKKFNLLGFTATGSPLSGLYANMEDVTIGYQNVSKYRHIIKYIPKIV-TSKLAKLRDFK
ELIYLINPELFCLLAVNEKLNYNYKKNIGSLMGFFKNVNFYSDLDNDEVVSLYSALGIHS
SMHMRSLSYYILNIRNEYYLRLYNIPVHLSDINVSNNYPFFNYIKHNPICKHVPSHNLGQ
FISFINEIVNFDQKPRPYIPNRYVYKSPKLTHFVIPMAVSSNIYTPHSILGSGRTNLLLY
TYDMYRHISHKLVSEDNVFTSDTEYSYEGDPFIYYNWLTYVGDQNNMKSRSFMKKIYLYS
DNLNINTVDNLINSFVNIHYTKVFIFDKNNPVDAYRHLYRTLNNFSIPIGRISFSVGNKK
KLSFLSLSTPKISRDEAIIYEYINRYTNFIQNYILMNSLYSIKYENHILTHKAFKEYHQK
AVERLKKQLKIVESFVKAHKTFNGMKQALRDSFNMYKNTYDERSSSLTEEIIYERNESSD
SRGSTSNDDAPINTDIYINKELGDLESFIESYYPDPDSVGENFSNENSKHDDSSSYDNYY
GTYLLVKSNTKIKSVYEYLLKYDKKYKNAKYIEFIMKNEMYGDINDQLNNIENGSSCLFS
VNDKVKISYIIDYCNYDKKSYFLFNKAYKSKNIYSVPSHDACETPEYSYFKLCQNASLLR
KFFTKRLDTEIGHIHRKELKRISTMKSSIESNMDSTHLFSISNDSLVSIIHDQNEDITTF
DVNACFTVSAKTTLGNIFNVNSRMDPETI-------------------------------

```
------------------------------------------------------------
-----------------------NTSINNSIFCTPVSVPVVSNRPIMKSINDIYIRAIVD
IIKNEQFRELMSIPEKLNPYYSFIYFFDKFAYVYKPKSWYKNMNRVKMFIPPQTIKWNMF
YYLLRNDISTVYNNEEFLYEFFYGNKSTSVKPLVKNILKPFLSHFTLFFYLYKVDESIGN

>P_berghei_ortholog
XXXXXXXXXXXXXXXXXXXXXXXXXXXXXXXXXXXXENDIDEKWMLHPLETNPFIDLQHP
NFKGDFVRSINEYYVFETKEYNEYKKERYNNIKNNIPFEKKQFHCAFNLLLHGRPNSRNA
DDYRIWSPQVALIKYDVMLKKKYHKYFLFAPYVVITNFVGNEGIHAHANYAFLITNRLAM
TFKNLLKDDTCHTYDVFVHAHCVGGNISRIVFTLDRDVWKTVNLSLINISNERTILAILN
DNFKRTLTDVNIIADKSTIKYRKDPYFKNHHHCLYKSFKFVPPKKKLTDYFEEMFSDHKV
EYDK-------------------------------------------------------S
TTVLDSNNNTYDNFQYSYQTLDSDDSEDDE-II---------------SLKFVKRFHYYM
LNKKINLLGYTSTGSPLSGLYANMEDVTIGYQNVSKYRHIIKYIPKFF-TSKLAKLRDFK
ELIYLINPELFCLLAVNEKLNYNYKKNVGSLMGFFKNVNFYSDLDNDEVVSLYSSLGIHS
SMHMRSLSYYILNIRNEYYLRIYNIPVYLSDINVSNNYPFFNYIKNNSICKHVPSHNLGQ
FISFINEIINFDQKPKPYIPNRYVYKNPKLSHFVIPMTVSNNIYTPHSILGSGRTNLLLY
TYDMYRHISHKLVSDDTVFTNNTEYSYERDPFIYYNWLTYIGDQNNMKIRGFMKKIYLYS
DNLNMNTVDSLINSFVNIHYTKFFIFDKNNPVDAYRHLYRTLNNFSIPIGRISFNVGNKK
KITFPSLSTPEISRDEAIIYEYINRYTNFIQNYVITNSVHSIKYENYISTHKSFKEYHQK
AFDRLKRQLRIVESFVNTYKTFNDMKQALRDSFNIH------------------------
-------DDAPINTDNYINKELGDLKSFVESYHSDPDSVGGNFLNENSKQDDSSSFDNYY
GTYLLAKSDIKLKYVYEYMLKYDKKYKNARYIEFIMKNEMHGDIHDQLNNIENSLSCLFS
LNDKIKISYIIDYCNYDKKSFFLFNKSYKSKNIFSVPSQDACTTPKYSYLKLCQNASLLR
KFFTNKIDTEIGHIHKKEIKRISTMKRSLESNIDIMNIFSISNDSLVSIIHDKNEDITTF
DVNACFIASAKPTLGNIFNVNSRMDPETM-------------------------------
------------------------------------------------------------
------------------------------------------------------------
-----------------------RTNINNSIFCTPVTVPVILNRPIMKSINDIYIRAIID
IIKNIEFRELMSIPEKSYPYHSFIYFFDKFAYVYKKKKWYKNINEVNMFIPPQTIKWNMF
YYLLRHDISTVYNNEEFLYDFFYGKKSTSVKPLVKNILKQFMSHITLFFYLYKVDESTGN
```

- **AMOUNT OF MISSING DATA**

P. reichenowi	***P. gallinaceum***	***P. knowlesi***	***P. vivax***	***P. chabaudi***	***P. berghei***	***P. yoelii***
60%	6%	15%	15%	8%	7%	10%

■ PF10_0177

▪ PHYLOGENY AND PEXEL/VTS

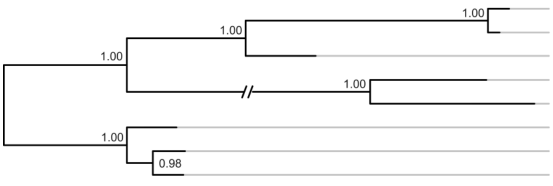

P. falciparum PF10_0177	LKNDKRKLKEFVIQE
P. reichenowi ortholog	LKNDKKKLKEFVIQE
P. gallinaceum ortholog	LKENKKKLKEYVINE
P. knowlesi ortholog	LKGNKKKMKKFVAVE
P. vivax ortholog	LKGNKKKMKKLVAVE
P. chabaudi ortholog	LKTDKNKLKNFVINE
P. berghei ortholog	LKTDKNKLKNFVINE
P .yoelii ortholog	LKTDKNKLKDFVINE

▪ ALIGNMENT

```
>P_falciparum_PF10_0177
MEKYSKVIKKNEGL----------------------------------------------
------------------LNSSSVEGLHYNKFSSDDSVYIKTNNLDKMYYAYNNNNNDRN
IVRKDFMPIEKYEDIKEDTNSNNYHNNSYYGRHHHNHILKNYKYDHYNKNCFKKYDDFNK
YYFLIPNNKINAHIDEHPNYRLSNKLKGTNINDHLNDPHIFKTSEYNYKNVIDKDTSYHM
YPVHNNNIPFIHNRKSTHTIKNKNCDFNNISQNLYVSSNPYISLDSYDNIIYSPKIGNYP
INLNENIFIGSRDMNSSSRQIYKYINNNNVDSHMNKMNEHIHASGTKNYTLQEYPHYIYE
SNVNIEKVCDINDNNICGGAKKKIISYDLEEKNHKMDDSKDNIKRNINNVNYVKINH---
------------------------------------------------------IDKEDK
NKKVNYDD-DKKV-----------------------------------------------
------S-DDDDKKVSDDDDNKKVSDDD-DKKVSDD-------DDKKVSNDDNKK--VYY
DDDKKIND-DDNNNISKDIRYD---------EKNNFMEWMSHNKLMKNIISSYNEDVNIE
NHGLFNLLFDIYGFNKDIF--------------------KEYLVHNK-------EETIKD
VIKNIVNELSNE--------KNNKISEKLIFLKYLFNEYGHTEYPNLISLKNFKLIFKNY
KNIFSSDKIVLFIFNCLDRGKRYHITQTDFIIGMLACSPQINNDISDDTGKLRHQLIFRA
YDLDRDGYLNDNEFLVFLYHIYELSKNLQYSK-------LKNDKRKLKEFVIQE------
--KNKIMKD-KKKISYDIFYHLVLTKQIQGTTNLLRSNYDVSSVVKKYFLYTYAKNFITD
TFIN-----MDTSFFITTKKNIKNE---------------HLKTQNLPNHENEVHIIHND
EVICNQEDDQKGNYVTNQDDNLAQEKKETHSEDHFHDSISNNNSYADVNACMGEMYAACD
ESEKNVEEHPYECVREVVHSENEQ--AEKCVEVKEEIKGDMNFIQSGDAASGDVEAADVE
GAGVEAADVEGEGVEAADVEGAGVEAADVEGADVEGADVEGADVEGVHVEC---------
-ADEQNNDHIPTKDKINNEDVFFDNNNDKEHIRSNSSNEESFTKQLKDEINSSNEKDDEE
KEDSLKKTENLNEEHIEKDGNNLSYLLNNNNNIVSCSGVDIKNNISERNIIYNMNDNLYD
NVAKDRYNYFEHDKKQPINNE-------------EDINKNNNNNDNMNCNTHISYSNINK
FVTNNKEYFMNNKNNENDKKMFPSYKEQKNSTNINKSL----------------------
----------------------------------PHFGKKED-IQDNVINKELHIEHIIE
DK-----------------IERIKKLVKIYREKYITDH-RLNTLNQDIAFKIFTMFYKVC
YGKKKHTYTSFFEKF--QICTYNDILLLCDEVAKLFKMENSLENVTLPCKVFGDVHGNLF
DLLDFFNLYNWPMHNNKNLLLTESDL-RKFEYIHNDKDIHIDKNKDMQYN----------
--------------------------DNDIKYVFLGNLINRGNYSLEVICLLFSLKILFP
KHIYLLRGNHEDRLFNYIYGFYKDIEIKMKTNMEYMGIINYQEQVISAHSYELFNRINDV
FEFFPLCVLLDKNILCIHGGIGDSVMTISDYQHIRKPILIPQNVDRYSNNKSEHVKKIII
DTLWSDPINYNDELDLQLLKNSST-YDIIPSRRGNITFKFGKHRLNDFLKNNQLKLIIRG
HECVQEGYKYSYKRKLLTLFSAKNYCNKYNNNASNAFIVRKNKNIIIFNQILKY------
------------------------------------------------------------
---------------------------------QHNCNTLINNENKQV------------
--TINQLQKYNFSNDKSNDKKTSPFFNNTHLTKK-------------------TFAVSHG
YLSKCDEEL-NDQLNTHPSMKNCSGNNKNFNKDADKNYMNGSGENKSFNKDVDKNYMNGS
GENKSFNKDVDKNYMNGSGDNKNFNNDVDKNYMNGSGDNKEDLNNSSSVPTTNINELNKN
GAISICENEVLMNNKYGVVSNMSEQSRNSTENVASNNDMDINTVNQISKSIIKKKWRDTR
TNQNLNSNKMNRNNNSQLINDMGSVNIYKEVEYEEDDKIEKE---IQREEQNDIQNNVQI
NLQN----------------DVLKQVENINVEISNDNMHNKNDVHYISMNEINSDKIKSS
HINDNVYDKNESSTFSFEHKETYDEKGDNEKEQRNLERADSEKKNIVNDDYDFYSYDEIY
INSLINSKIWEKNDDDYKIIERKDTNDELYKIEDKLQKQVNIHNVINEFFNNEEIYQEKT
DSNYNMSNENESISGDNENSKKNYYSKLSKINEMYTNKHNDGDKIDEQMSDINSVDLINE
FTSKSLDF-MMPPNLGLGNRTQLKKDISMKENNKDQRNNV-TTLKSLRGDNNIKDSSNKM
QMQCLPPDPKPQTKITFQKSL--------------------EKINDS

>P_reichenowi_ortholog
XXXXXXXXXXXXXXXXXXXXXXXXXXXXXXXXXXXXXXXXXXXXXXXXXXXXXXXXXXXX
XXXXXXXXXXXXXXXXXXXXXXXXXXXXXXXXXXXXXXXXXXXXXXXXXXXXXXXXXXXX
XXXXXXXXXXXXXXXXXXXXXXXXXXXXXXXXXXXXXXXXXXXXXXXXXXXXXXXXXXXX
XXXXXXXXXXXXXXXXXXXXXXXXXXXXXXXXXXXXXXXXXXXXXXXXXXXXXXXXXXXX
XXXXXXXXXXXXXXXXXXXXXXXXXXXXXXXXXXXXXXXXXXXXXXXXXXXXXXXXXXXX
XXXXXXXXXXXXXXXXXXXXXXXXXXXXXXXXXXXXXXXXXXXXXXXXXXXXXXXXXXXX
XXXXXXXXXXXXXXXXXXXXXXXXXXXXXXXXXXXXXXXXXXXXXXXXKVNY--------
---------------------------------------------------------DDN
NKKVNYDDNNKKV-----------------------------------------------
------NYDDNNKKVNDDDNNKKVNDDDNNKKVNDD-------DNNKKVNDND-------
DNNKKVNDNDDNNNISKDIRYD---------EKNNFMKWMSHNKLMKNIISSYNEDVNIE
NHGLFNLLFDIYGFNKDIF--------------------KEYLVHNK-------EETIKD
VIKNILNELSNE--------KNNKISEKLIFLKYLFNEYGHTEYPNLISLKNFKLIFKNY
KNIFSSDKIVLFIFNCLDRGKRYHITQTDFIIGMLACSPQINNDISDDTGKLRHQLIFRA
YDLDRDGYLNDNEFLVFLYHIYELSQNLQYSK-------LKNDKKKLKEFVIQE------
--KNKIMKDKKKKISYDIFYHLVLTKQIQGTTNLLRSNYDVSSVVKKYFLYTYAKNFITD
TYIN-----MDTSFFITTKKYIKNE---------------HLKTQNFPNHENEVHIIHND
EVICNQDDHHKANYVTNQYDNLAQEKKETHSEDHFHDSISNNNSYADVNACMGEMHVACD
ESEQNVEEHPYEGVREMVHSESEE--AEKCLEVKQEIKGDMDFIQSGDAASGGVECVDVE
CADVECIDVECADVECADVEGADVECADVECVDVECADVECVDVECVDVECVDVECADVE
GADEQSNNHIPIKDKINNEDVFFDNNNDKEHIRSNSSNEESFTNQLKDEINSSNEKDDEE
KEDSLKKTEKLNEEHIEKDGNNISYLLNNN-NIVNCNGVDIQNNISERNIIYNMNDNLYD
NVAKDRYNYFEYDKK-PINNE-------------EDMNKNN-NNDNINCNKNVSYTNINK
FVTNNKEYFINNKNNENDKKMFPSYKEQKNSTNMNKSL----------------------
----------------------------------PHFGKKEDDIQDNVINKELHIEHIIE
DK-----------------IERIKKLVKIYREKYITDH-RLNTLNQDIAFKIFTMFYKVC
YGKKKHTYTSFFEKF--QICTYNDILLLCDEVAKLFKMENSLENVTLPCKVFGDVHGNLY
DLLDFFNLYNWPMHNNKNLLLTETDL-RKFEYIHNDKDIHIDKNKDMQYN----------
--------------------------DNDIKYVFLGNLINRGNYSLEVICLLFSLKILFP
KHIYLLRGNHEDRLFNYIYGFYKDIEIKMKTNMEYMGIINYQEQVISAHSYELFNRINDV
FEFFPLCVLLDKNILCIHGGIGDSVMTISDYQHIHKPILIPQNVDRYSNNKSEHVKKIII
```

```
DTLWSDPINYNDELDLQLLKNSST-YDIIPSRRGNITFKFGKHRLNDFLKNNQLKLIIRG
HECVQEGYKYSYKRKLLTLFSAKNYCNKYNNNASNAFIVRKNKNIIIFNQILKC------
------------------------------------------------------------
---------------------------------QHNCNTLINNENKQL------------
--TINQLQKNNFSNDKSNDKKTSPFFNNRHLTKK--------------------TFVSHG
YLSKCDEELKNDQLNNHPSTKNCSGNNKNFNKDVDKNYMDGSGDNKNFNKDVDKNYMDGS
GDNKNFNKDVGKIYMDGSGDNKNFNKDVDKSYMNGSGDNKEDLNNSSSVPTTNINELNKN
GALSICENEVLMNNKYGVGSNISEQSRNSTENVASNNDMDINTVNQISKSIIKKKWRDTR
TNQNLNSNKMNRNNNSQLINDMGSVNMCKEVEYEEADKIEKE---VQREEQNDIQNNVQI
NLQN----------------DVLKQVENINGEISNDNVHNKTDVHYISMNEINSDKIKSS
HINDNVYDKNESSTFSFEHKETYDEKGDNEKEQRNLERADSEKKNIVNDDYDFYSYDEIY
INSLINSKIWEKKXXXXXXXXXXXXXXXXXXXXXXXXXXXXXXXXXXXXXXXXXXXXXXX
XXXXXXXXXXXXXXXXXXXXXXXXXXXXXXXXXXXXXXXXXXXXXXXXXXXXXXXXXXXX
XXXXXXXXXXXXXXXXXXXXXXXXXXXXXXXXXXXXXXXXXXXXXXXXXXXXXXXXXXXX
XXXXXXXXXXXXXXXXXXXXXXXXXXXXXXXXXXXXXXXXXXXXXXX

>P_gallinaceum_ortholog
XXXXXXXXXXXXXXXXXXXXXXXXXXXXXXXXXXXXXXXXXXXXXXXXXXXXXXXXXXXX
XXXXXXXXXXXXXXXXXXXXXXXXXXXXXXXXXXXXXXXXXXXXXXXXXXXXXXXXXXXX
XXXXXXXXXXXXXXXXXXXXXXXXXXXXXXXXXXXXXXXXXXXXXXXXXXXXXXXXXXXX
XXXXXXXXXXXXXXXXXXXXXXXXXXXXXXXXXXXXXXXXXXXXXXXXXXXXXXXXXXXX
XXXXXXXXXXXXXXXXXXXXXXXXXXXXXXXXXXXXXXXXXXXXXXXXXXXXXXXXXXXX
XXXXXXXXXXXXXXXXXXXXXXXXXXXXXXXXXXXXXXXXXXXXXXXXXXXXXXXXXXXX
XXXXXXXXXXXXXXXXXXXXXXXXXXXXXXXXXXXXXXXXXXXXXXXXXXXXXXXXXXXX
XXXXXXXXXXXXXXXXXXXXXXXXXXXXXXXXXXXXXXXXXXXXXXXXXXXXXXXXXXXX
XXXXXXXXXXXXXXXXXXXXXXXXXXXXXXXXXXXXXXXXXXXXXXXXXXXXXXXXXXXX
XXXXXXXXXXXXXXXXXXXXXXXXXXXXXXXXXXXXXXXXXXXXXXXXXXXXXXXXXXXX
XXXXXXXXXXXXXXXXXXXXXXXXXXXXXXXXXXXXXXWRSHNKLMKNIISSYNENIDME
NHGLFNLLFDIYGYNKDIF--------------------KNYLLNND-------DNLINE
VMKNIIYELSEE--------KNNKIIEKLIFLKYLFNEYGHTEHPNLISLKSFKLIFKNY
KDIFSSEKIVLFMFNCLDRGKRYHITETDFIIGMLACSPQMDNNIENDTGKLRHQLIFRA
YDLDRDGYLSDNELLVFLYHIYELSKNFKIVK-------LKENKKKLKEYVINE------
--KNRIMKY-CNKISYDYFYDLILNKEIEGTSNLFRSNQDMADIVKKYFLYTYARNINTE
KYIN-----NDKFFFITTRKGKNLEXXXXXXXXXXXXXXXXXXXXXXXXXXXXXXXXXXX
XXXXXXXXXXXXXXXXXXXXXXXXXXXXXXXXXXXXXXXXXXXXXXXXXXXXXXXXXXXX
XXXXXXXXXXXXXXXXXXXXXXXXXXXXXXXXXXXXXXXXXXXXXXXXXXXXXXXXXXXX
XXXXXXXXXXXXXXXXXXXXXXXXXXXXXXXXXXXXXXXXXXXXXXXXXXXXXXXXXXXX
XXXXXXXXXXXXXXXXXXXXXXXXXXXXXXXXXXXXXXXXXXXXXXXXXXXXXXXXXXXX
XXXXXXXXXXXXXXXXXXXXXXXXXXXXXXXXXXXXXXXXXXXXXXXXXXXXXXXXXXXX
XXXXXXXXXXXXXXXXXXXXXXXXXXXXXXXXXXXXXXXXXXXXXXXXXXXXXXXXXXXX
XXXXXXXXXXXXXXXXXXXXXXXXXXXXXXXXXXXXXXXXXXXXXXXXXXXXXXXXXXXX
XXXXXXXXXXXXXXXXXXXXXXXXXXXXXXXXXXXXXXXXXXXXXXXXXXXXXXXXXXXX
XXXXXXXXXXXXXXXXXXXXXXXXXXXXXXXXXXXXXXXXXXXXXXXXXXXXXXXXXXXX
XXXXXXXXXXXXXXXXXXXXXXXXXXXXXXXXXXXXXXXXXXXXXXXXXXXXXXXXXXXX
XXXXXXXXXXXXXXXXXXXXXXXXXXXXXXXXXXXXXXXXXXXXXXXXXXXXXXXXXXXX
XXXXXXXXXXXXXXXXXXXXXXXXXXXXXXXXXXXXXXXXXXXXXXXXXXXXXXXXXXXX
XXXXXXXXXXXXXXXXXXXXXXXXXXXXXXXXXXXXXXXXXXXXXXXXXXXXXXXXXXXX
XXXXXXXXXXXXXXXXXXXXXXXXXXXXXXXXXXXXXXXXXXXXXXXXXXXXXXXXXXXX
XXXXXXXXXXXXXXXXXXXXXXXXXXXXXXXXXXXXXXXXXXXXXXXXXXXXXXXXXXXX
XXXXXXXXXXXXXXXXXXXXXXXXXXXXXXXXXXXXXXXXXXXXXXXXXXXXXXXXXXXX
XXXXXXXXXXXXXXXXXXXXXXXXXXXXXXXXXXXXXXXXXXXXXXXXXXXXXXXXXXXX
XXXXXXXXXXXXXXXXXXXXXXXXXXXXXXXXXXXXXXXXXXXXXXXXXXXXXXXXXXXX
XXXXXXXXXXXXXXXXXXXXXXXXXXXXXXXXXXXXXXXXXXXXXXXXXXXXXXXXXXXX
XXXXXXXXXXXXXXXXXXXXXXXXXXXXXXXXXXXXXXXXXXXXXXXXXXXXXXXXXXXX
XXXXXXXXXXXXXXXXXXXXXXXXXXXXXXXXXXXXXXXXXXXXXXXXXXXXXXXXXXXX
XXXXXXXXXXXXXXXXXXXXXXXXXXXXXXXXXXXXXXXXXXXXXXXXXXXXXXXXXXXX
XXXXXXXXXXXXXXXXXXXXXXXXXXXXXXXXXXXXXXXXXXXXXXXXXXXXXXXXXXXX
XXXXXXXXXXXXXXXXXXXXXXXXXXXXXXXXXXXXXXXXXXXXXXXXXXXXXXXXXXXX
XXXXXXXXXXXXXXXXXXXXXXXXXXXXXXXXXXXXXXXXXXXXXXXXXXXXXXXXXXXX
XXXXXXXXXXXXXXXXXXXXXXXXXXXXXXXXXXXXXXXXXXXXXXXXXXXXXXXXXXXX
XXXXXXXXXXXXXXXXXXXXXXXXXXXXXXXXXXXXXXXXXXXXXXXXXXXXXXXXXXXX
XXXXXXXXXXXXXXXXXXXXXXXXXXXXXXXXXXXXXXXXXXXXXXX

>P_knowlesi_ortholog
MRNVRETGINRPYVMRKDNLYDALPKHLCMNQPDHHYGFNTYGGGYRASAGGALLKGGTP
NGAQGIPTAQINSLAAFRMGKIASHQVDWGGRSYDDSTKENLAGGSTSLLHLYDSAMNWS
NSRK----------FVNPSTGHSSGVEFPMLYHLRGEANSTMGGRRIGLNARGEHSSGRS
VSELSHHNQRVQKMKESH-------------GEYF---------------PLGMQHTKRT
TLLNSNFMGSPEQSIRGEALHSWWDQRGMGSPSREVSNGYPLPGVRYNKVESSPK-----
------------------------------------------------------------
----------------------GMMGVVLVPANGETKNGFTKWNQNGKEIDAGLIHK---
----------------------------------------------------CSNGQLSR
TSEYSYKDVEPIM-----YGSMAQGSTYPDDVSVESLPMNSSPWWSNEGFVQAKDNNHFT
ALTVEGRNYRSDGQSYDQSDIRRLSGENLQRGVMSKLEEGRYTDGLESLDSIPPVGAAMN
SLGKPIGGAASLSNEETVFEEDGSRLTQWNYNKGDFTEWISHNRLLKNIISSHKEDGLVE
GHVLYSLLFDAYGQNKETF--------------------RSYVKGDHDKSKMSMDEEIGE
VIHEIRTNKKKE-----ERKKSNRMVEKIIFLKYLFNQYAHTDYANFISFKCFKKMFEDY
KNVFSSEKIILFIFNCLDRCRRYYVSESDFLIGMLACSPQMENDITKDTGKLRHQLIFRA
YDLDRDGYWNKEEVFVFLYHLYELSKNMNQME-------LKGNKKKMKKFVAVE------
--RDKLMNT-CEKISYDKFYKLVLDGKIEGTENLLRSNCDVASVVKTYFLYTYAGGSSLT
MSIDTGASGNADSSNVQEDGQIDREKCEEGEETVNPVEKTQLEGRNTQQSSY---IEFSG
ESTTG-DPNSEEAPHISATSIKGEDEPEEGEAEEVVQKRSVDGTSSSNNTEGGK---SED
RIEVQSEEKPQS------------------------------------------------
------------------------------------------------------------
-----------DEVAVVGEEPPCGHGRESQKVSDLNSA-----DGKDEEVGLGE------
---------------------------------------------EEKVEKCDMPSGVSD
QVSNDVSKIVPDEMTKDVSNNVV-------------------------------------
------------EGREANADLSVPPEEKTDKAAIGDSI----------------------
----------------------------------PHLGEGQDEECA-------HKDNLVE
EEERANPIR--PERSWRSCVEKIKEIVKAYRKKYLTEKTRLFGLNQDIAFKIFTAFYKVS
YKRKREKYIRQFDHLCTGACTYNDIILLCDEAVKVFKGEDTIEYVDLPCKVFGDLHGSIF
DLLDFFNMYNWPMHGETNEWLNVEEV-SATNEVSLRSKNSTDPFPYVASH----------
--------------------------DKDIKYIFLGNYINRGDYSLEVICLLLSLKILFP
KHIYLLRGNHEERLFNYVHGFYGDIEKKMERNIKRGGLIRYQGEVIQAHAYELFNRINDA
LEFLPLSVLVGQSILCVHAGIGDSLENIEDFSDVHKPIVVPQCVDRTSKNEYERVQKIII
DTLWSDPINYEDEEDMLLLEKVKPGGDTIPSSRGEITLKFGQRRLSCFLKKNKLKMIIRG
HECVQEGYRYSYNRRLLTLFSAKNYCNSHGNDAANAFIVKRGKNIAIFNQILKCPRGRQL
E-----------------------------------------------------------
----------------------------GIHHGEEATSGPVDSTNKVIVNRNGFMDESDP
MMEEEEEEMEGQSCRKVDEGVTPFGPNEKNFISEWTDAVDKA------------------
-LCNSGVEGKDDLQSNHILRDSHSIVKRKLRDTRSNEDMKWGVSMQLEK---------RE
GAHRMGGHDGDGDRDGDYADGQFLIDSCNSIKDDHSNGDARGDNFEGG------TLRKSI
STNGTFSSEEGLYGGCSNGSNGRNYTGEMLSSKQYHFKGDGLEKGKIQNT-------EIE
RAHNRQSRKSHLKGD-------------------------------AYWEEKYLYDDYQG
```

```
YLNNVPDG------------DGLKNS-------PSDERASSDELPCEGQTEQDSRVNTNA
IIEKFFQQGRR------------DYASGEYIYTDHLEEKEVEMGGNLNHCQSAKLTPLSK
QTNDVNIRRGDEG-----------------------------------------------
---------------------------------------------GEWGDNGNCAKLMDE
LMSKPMDYMMMPPDLGLVNRPKLRRDTHSHQQKKEQGSATLRSLRSLRSENNTSEPLTKL
QMQYLPPDPKPQTKISLQKII-------------------DKFDDDA

>P_vivax_ortholog
XXXXXXXXXXXXXXXXXXXXXXXXXXXXXXXXXXXXXXXXXXXXXXXXXXXXXXXXXXXX
XXXXXXXXXXXXXXXXXXMGSMGSYQVDWARRSYDGSSQASLAGGSPSLLHQCEGGKRWS
DDRRGVNWSGVNSSWVNSSGVNSPVVNAPVVYHLGGEAN-TIG--------------GRS
VSEMAHHSQRVLKVKEAHAGCPPRGMEPIYAGDHYGEGTKQTNGQMKALPPVGMKHTKRA
PLMNANCVGSSNRSIRREVLHSWWGEERVGSPSSEASNCYTLPSVPYDQVGGSPK-----
------------------------------------------------------------
----------------------GMMGVVLVPANGETRNGFRKWDQNGKEVDAGLVHKWSS
GGLSGGGLSGGGLSGGGLSGGRLGSGGLSGGRLSSGRLSGGRLGSGGLSGGRLSSGRLSR
TSEYSYKDLDDSANVGADVGGVSRGKCYPDDASVESLPRNGFPWGSNETFLRAEDSNRCA
VVAVGGGRYESGEQWYGQAAGRRLSGESLQRGVMASWEEGSYKDGVAALASEEAV--SEE
AISEEAASEEAASGESVAREGDAGRPAQCTYNQSDFTQWVSHNRLVKNIISSHNEDGQVE
RHGLYSLLFDAYGHNREAFRGTGGGALVGRAVGKGEATCKEIAKETP-------KETPKE
TAKETGEETAKETANETDQRCSDSVVEKVIFLKYLFNQYAHTEYPNCISFKRFKAMFEDY
RGVFASEKIILFIFNCLDRCRRYYVSESDFLIGMLACSPQMQNDIRKDAGKLRHQLIFRA
YDLDRDGYLSRSEMFVFMYHLYELSKQFKHLE-------LKGNKKKMKKLVAVE------
--RDKLMRK-CHRVSYDQFYRLVLESKVQGTQNLLRSNCDVASVVKARFLDAYAGGRGLT
TWVDAGGAAN---------KAKNPS-------------SVQLTGGNELECSS---AQFSG
ESPTG-DANSEEDPQVSGTSVRGGDDPKEDGTEGVVQEGPAGGT------EGGKREQSEE
KQEEQSEEKPEE------------------------------------------------
------------------------------------------------------------
-KPEEQPQEQPQSDGDAAEEPPSGHSNEAPNGGGVNSAEEVTGDG-EEEVGEGE------
------------------------------------------------------------
------EEVSDGEEDKVEESDVV-------------------------------------
------------AGGEANAQLGAPPGESS-----TDSI----------------------
----------------------------------PHFGGGHQEESAPGEQRVHEGDHIHE
ESEQQHTSRAGPERSWSNCVEQMKEIVKAYRERHLTGKAKLFGLNQDVAFKIFTTFYRVS
YRRKREKYSRQVDDVCTAACSYNDVILLCDEAAKVLKGEDSIEQVELPCKVFGDLHGNVL
DLLDFFNMYGWPLHGGTNEWLSVEEV-----AMRGGRSVSVGKLKEAVPHTGGTSVSVEK
RRETVPHTDGTSVSVGREKEAAPHTGSNDVKYVFLGNYLNRGKHSLEVICLLLSLKVLFP
KHIYLLRGNHEERLLNYVNGFYADIEKKMKRNIKTAGLIKYQEEVIEAHAYELFNRVNDV
LEFLPLSVLVGGNILCVHAGIGDSLQNVGDFAAIPKPIVIPQFVNRSSNDAYEHVQKVII
DALWSDPINYEDEQDVFLLERLAHGEDVIPSSRGKITLKFGQRRLSSFLRRNKLKMLIRG
HECVQEGFRYSYGRRLLTLFSATNYCGRHGNDAANAFIVRRGSSIVIFNQIVKCPRSERF
ERLEPLEPLEPLHKVNWDGSLERPLPREQAAPYPPEPLHWGDFPHEVGQPQRGASLLSGH
RIMSSVCSPSEVPPLGVENWRSGDPSSRGDHIGEASTDGMGSQMNITTSKAKHSVGVLTG
LEEEEKAE--ELPHRRVNEEVTPFNPNEKELASD--------------------------
--------GGDDEQSNLILRDSHPIVKRKLRDTRSNEDVNWGAAVQLEKGEGVPSRFARG
GALRNDGHDGDRS------GDHSLNNSRNSMGDDHSGGDARDDPLEGG------PLRKSI
ITNGSCSSEEALYGGYTRGSSGRNYPGEMLPSKLSPFKGEGL---------------EIE
EAQNRQESKSPLEGDPHLGGD-------------------------PQWEGEYLYDDYQH
YLGNPPHG------------DALKKP-------PCDEPPGGDEPPCAGETKTNSPGNTNA
IIETFFQNGGR------------DHGGDEHIYRDHLEDSEVERRGHLNHRQCAKLTPLSK
RTNGVNVRRGEEG-----------------------------------------------
---------------------------------------------EQWEQNESSANLVEE
LMSKPMDYMMMPPDLGVANRPKLRRDAHSNQHKKEHGS---ATLRSLRSENNTSESLSKL
QMQCLPADPKPQTKISLQKIL-------------------DKLEDDA

>P_chabaudi_ortholog
XXXXXXXXXXXXXXXXXXXXXXXXXXXXXXXXXXXXXXXXXXXXXXXXXXXXXXXXXXXX
XXXXXXXXXXXXXXXXXXXXXXXXXXXXXXXXXXXXXXXXXXXXXXXXXXXXXXXXXXXX
XXXXXXXXXXXXXXXXXXXXXXXXXXXXXXXXXXXXXXXXXXXXXXXXXXXXXXXXXXXX
XXXXXXXXXXXXXXXXXXXXXXXXXXXXXXXXXXXXXXXXXXXXXXXXXXXXXXXXXXXX
XXXXXXXXXXXXXXXXXXXXXXXXXXXXXXXXXXXXXXXXXXXXXXXXXXXXXXXXXXXX
XXXXXXXXXXXXXXXXXXXXXXXXXXXXXXXXXXXXXXXXXXXXXXXXXXXXXXXXXXXX
XXXXXXXXXXXXXXXXXXXXXXXXXXXXXXXXXXXXXXXXXXXXXXXXEVPNGEAHH---
------------------------------------------------------DNTELS
SEIFSYNNINNFV-----------------------------------------------
------KGDTSLEYVNSYNNRKGVDKSEIEKDSGKN-------EN----NNIS-------
-----------MSNETIKINQK---------NRNEFLEWKSHNRLLKNIIASYNENTNIK
KHGLFNLLFDMYGYNKHIF--------------------KNYLSNND-------ENEIKN
VMKDIIYELFEK--------KNNKIIEKFIFLKYLFNEYGHTDYPNLISLKNFKQIFRNY
KNIFSSKKIILFIFNCLDRGRKYYITESDFIIGMLACSPQMGNDIFDDAGKLRHQLIFRA
YDLDRDGYLNSKEMLVFLYHIYELSNDLKHLE-------LKTDKNKLKNFVINE------
--RDKLMKN-NEKISYDYFYKLIANKQIEGTANLLRSNCDVASVVKKYFLYTYAKNLIPE
KYVD-----GDDFFFIYSKNKKNNEISKETNN----IIDSNLRNVSGYSHDT---IHFST
SSNNILDEDYNNFHMLRRMNAYEGSGIFTDITENASSEQIDIDTFSKVGT--GKVLANLK
EKETSMEKLPIETYSNTNKVKKNEFLASISQNFEKSKMNDIHFDGKG-------------
------------------------------------------------------------
-----------RNTFTDSQSVEF----------QTNSYETDYNNIYGDEKDKNE------
-------------------------IIKNKANIKNSKLGSMEHVVEESVGDMNELDTMGS
DLKKESNNFEKTNEETKINDD---------------------------------------
------------------------------------------------------------
---XXXXXXXXXXXXXXXXXXXXXXXXXXXXXXXXXXXXXXXXXXXXXXXXXXXXXXXX
XXXXXXXXXXXXXXXXXXXXXXXXXXXXXXXXXXXXXXXXXXXXXXXXXXXXXXXXXXXX
XXXXXXXXXXXXXXXXXXXXXXXXXXXXXXXXXXXXXXXXXXXXXXXXXXXXXXXXXXXX
XXXXXXXXXXXXXXXXXXXXXXXXXXXXXXXXXXXXXXXXXXXXXXXXXXXXXXXXXXXX
XXXXXXXXXXXXXXXXXXXXXXXXXXXXXXXXXXXXXXXXXXXXXXXXXXXXXXXXXXXX
XXXXXXXXXXXXXXXXXXXXXXXXXXXXXXXXXXXXXXXXXXXXXXXXXXXXXXXXXXXX
XXXXXXXXXXXXXXXXXXXXXXXXXXXXXXXXXXXXXXXXXXXXXXXXXXXXXXXXXXXX
XXXXXXXXXXXXXXXXXXXXXXXXXXXXXXXXXXXXXXXXXXXXXXXXXXXXXXXXXXXX
XXXXXXXXXXXXXXXXXXXXXXXXXXXXXXXXXXXXXXXXXXXXXXXXXXXXXXXXXXXX
XXXXXXXXXXXXXXXXXXXXXXXXXXXXXXXXXXXXXXXXXXXXXXXXXXXXXXXXXXXX
XXXXXXXXXXXXXXXXXXXXXXXXXXXXXXXXXXXXXXXXXXXXXXXXXXXXXXXXXXXX
XXXXXXXXXXXXXXXXXXXXXXXXXXXXXXXXXXXXXXXXXXXXXXXXXXXXXXXXXXXX
XXXXXXXXXXXXXXXXXXXXXXXXXXXXXXXXXXXXXXXXXXXXXXXXXXXXXXXXXXXX
XXXXXXXXXXXXXXXXXXXXXXXXXXXXXXXXXXXXXXXXXXXXXXXXXXXXXXXXXXXX
XXXXXXXXXXXXXXXXXXXXXXXXXXXXXXXXXXXXXXXXXXXXXXXXXXXXXXXXXXXX
XXXXXXXXXXXXXXXXXXXXXXXXXXXXXXXXXXXXXXXXXXXXXXXXXXXXXXXXXXXX
XXXXXXXXXXXXXXXXXXXXXXXXXXXXXXXXXXXXXXXXXXXXXXXXXXXXXXXXXXXX
XXXXXXXXXXXXXXXXXXXXXXXXXXXXXXXXXXXXXXXXXXXXXXXXXXXXXXXXXXXX
XXXXXXXXXXXXXXXXXXXXXXXXXXXXXXXXXXXXXXXXXXXXXXXXXXXXXXXXXXXX
XXXXXXXXXXXXXXXXXXXXXXXXXXXXXXXXXXXXXXXXXXXXXXXXXXXXXXXXXXXX
XXXXXXXXXXXXXXXXXXXXXXXXXXXXXXXXXXXXXXXXXXXXXXX

>P_yoelii_ ortholog
XXXXXXXXXXXXXXXXXXXXXXXXXXXXXXXXXXXXXXXXXXXXXXXXXXXXXXXXXXXX
```

```
XXXXXXXXXXXXXXXXXXXXXXXXXXXXXXXXXXXXXXXXXXXXXXXXXXXXXXXXXXXX
XXXXXXXXXXXXXXXXXXXXXXXXXXXXXXXXXXXXXXXXXXXXXXXXXXXXXXXXXXXX
XXXXXXXXXXXXXXXXXXXXXXXXXXXXXXXXXXXXXXXXXXXXXXXXXXXXXXXXXXXX
XXXXXXXXXXXXXXXXXXXXXXXXXXXXXXXXXXXXXXXXXXXXXXXXXXXXXXXXXXXX
XXXXXXXXXXXXXXXXXXXXXXXXXXXXXXXXXXXXXXXXXXXXXXXXXXXXXXXXXXXX
XXXXXXXXXXXXXXXXXXXXXXXXXXXXXXXXXXXXXXXXXXXXXXXXESPKGKIV----
------------------------------------------------------DPTKLS
SEIFSYNNINNFL-----------------------------------------------
------KEYGSLEYINDYSNMKKDDKKEKEKDIVKN-------DNINEDNNIS-------
-----------MSNESLKINKK---------NRNEFLEWKSHNRLLKNIIASYNENTNIK
KHGLFNLLFDIYGYNKTIF--------------------KNYLSNND-------ENEIKN
VIKDIIYELFEK--------KNNKILEKFIFLKYLFNEYGHTEYPNLISLKNFKQIFRNY
KNIFSSKKIVLFIFNCLDRGRKYYITESDFIIGMLACSPQMGNDIYDDSGKLRHQLIFRA
YDIDRDGYLNSKEMLVFLYHIYELSNDLKYLK-------LKTDKNLKDFVINE-------
--RDKLMKN-HEKISYDYFYNLIVNKQIEGTTNLLRSNCDVANVVKKYFLYTYAKNLIPE
KYID-----GDDFFFIHSKDKKNNQVDN--------IIDKKLKNVSRYSHDT---IPFNT
SSNHILDEDYSNSYMLRRMNAYEGSGIYTDATENISSEQIDRDTLSKVWT--GNIFPELK
ETETSMEKISIETYNNTKKVKENEFLSNIDHNFEKSKINDINFIDKG-------------
------------------------------------------------------------
-----------RDTFTDNQSVEFQKNNMNIESYQTNSCETGYNNIYVDEKDKNE------
-------------------------MVKNKANIKNVNLGSMEYVVDDSIGKINELDAMNS
DLKKESNTFEKSNEETKINGDISKKMENGDSFVNESLGVNDKDEIKYSGGINLNPIRLNI
SGFGEKCDNKCENKREN----HENLKKTSGQLDRKKPLYSNSDNLKTEKKIIDKDYVEYE
IRNDNILVENCNNINKGDNILLVNSDKTKNSEVLPHFGKEENCESNLRIKEVCDYDLIVN
DKEKME--------------NSIKEVVKEYREKYITDH-KLLTLNQDIAFKVFTTFYNIC
YKKKREDYKNYFDIF--RVCNYNDVLLLCDEVVKLFKLEDSLEFANLPCKVFGDIHGNLF
DIVDFFNMYNWPMHDNNNRIISLLKIERDFENVGNSENNMNNKC---VSN----------
--------------------------HSDLKYVFLGNYLNRGELSLEVICFLFSLKILFP
KHIYLIRGNHENRVFNYVYGFYKDIERKIKSNFNCLGKINYKDDVICAYSYELFNRINDV
LEFLPLSVLLDNEILCIHSGIGDSIQNVKDYLNIEKPIIIPEYVDRNSNNNSEILKKIII
DTLWSDPINYLDDNDMLLLKDCTK-YDIIPSSRGKITVKFGKHRLTKFLKNNKIKMIIRG
NECVSEGYKYEFNKKILTLFSATNYCNKYKNNASSALIIKKNKNITIFNQILKS------
------------------------------------------------------------
---------------------------------ECENMNLNESSNKEKVNIAYTNG----
--EEDKLNKSDKLQNGQNETKNGFYFDHTKHKDKPSKKYGNEYKEIENNNDLINLKTPQF
YVNKDSIKLSNDDNIENILGDDSASKNNLYDTDVCEKKNDNLNDSGVMNEMLKSLSYEKN
LDNCNDNKDKEDQ------NNDVLQNDRKRVNLLESDTDKNSAN------------LFEN
PCNEHIENEENVNKE---KKEKNEEKDESKETMTYDENGDGYKAKDDSSE-------KND
SGENYKHNKIFNNKENEIFNDFENCCNGKSIDNINKDLIKFNDFGIEQNEHNDIFLEEKK
YLENIEMMKEEMLKECDYKVDSNKYTDGIIEENIEENIEEKNNELYKSRNNSNSRNNTNS
IINNFFKNEKTIEQNCSNMFLNYDQVNNSKEHEDNLKNGNCSKISSLSNE------NIGA
INYMNEID----------------------------------------------------
----------------------------------------------------------NE
LVYKRINY-MMPPNLTLTNKTQMKNGSYTREHNS-------ATMRNIRSLK------CNI
CLQTLNHKQKYHSKKYIIIFIFFINFCVLLLFCQFLIFFHYRLFINV

>P_berghei_ortholog
XXXXXXXXXXXXXXXXXXXXXXXXXXXXXXXXXXXXXXXXXXXXXXXXXXXXXXXXXXXX
XXXXXXXXXXXXXXXXXXXXXXXXXXXXXXXXXXXXXXXXXXXXXXXXXXXXXXXXXXXX
XXXXXXXXXXXXXXXXXXXXXXXXXXXXXXXXXXXXXXXXXXXXXXXXXXXXXXXXXXXX
XXXXXXXXXXXXXXXXXXXXXXXXXXXXXXXXXXXXXXXXXXXXXXXXXXXXXXXXXXXX
XXXXXXXXXXXXXXXXXXXXXXXXXXXXXXXXXXXXXXXXXXXXXXXXXXXXXXXXXXXX
XXXXXXXXXXXXXXXXXXXXXXXXXXXXXXXXXXXXXXXXXXXXXXXXXXXXXXXXXXXX
XXXXXXXXXXXXXXXXXXXXXXXXXXXXXXXXXXXXXXXXXXXXXXXXESPKRRILH---
------------------------------------------------------DPIELS
SEILSYNNINNFL-----------------------------------------------
------KEYGSLEYINDYNNMKRIDAN--EKDIVKN-------ENINEDNNIS-------
-----------MSNESIKINKK---------NKSEFLEWKSHNRLLKNIIASYNENTNIK
KHGLFNLLFDIYGYNKTIF--------------------KNYLSNND-------ENEIKH
VMKDIIYELFEK--------KNNKILEKFIFLKYLFNEYGHTEYPNLISLKNFKQIFRNY
KNIFSSKKIVLFIFNCLDRGRKCYITESDFIIGMLACSPQMGNDIFDDSGKLRHQLIFRA
YDLDRDGYLNGKEMLVFLYHIYELSNNLKHLE-------LKTDKNLKNFVINE-------
--RNKLMKN-HEKISYDYFYNLIVNKQIEGTTNLLRSNCDVANVVKKYFLYTYAKNLIPE
NYID-----GDDFFFIYSKNKKNNKKNNETNKEINKEINKKLKNVSEYSHDT---IPFNT
SSNNILDDDYSNSYMLRRMNAYEGSGIYTDVTENISSELVDRDTFSKVWT--GNIFDELK
ETETSMENCSIETYNNARKMNKDEFLVNIGQNFKKSKINDINFIDKG-------------
------------------------------------------------------------
-----------RNTFMDNQSVNFQKHNMNIKSYQTSSCETGYNNIYVDEKDKNE------
-------------------------IIKNKANIKNSNLGSLEYVMEESIGKINELDAMYY
DLKTESNVFEKSNEETKINDNISKKMENDDSCVNEKLGVNDKNDINYSDSINLNPIRLNI
SGFDEKCDHILENKNENNTKCHDNLKKASGQLDKERLLYSNDENMKTEKSIIDKDYVEYE
----------------------------------PRFGKEENCESNLRIKEISGYDLIGN
DKEKME--------------NSIKEVVKEYREKYITDH-KLLTLNQDIAFKVFTTFYNIC
YKKKREDYKNYFDIF--RVCNYNDVLLLCDEVVKLFKLENSLEVANLPCKIFGDIHGNLF
DIVDFFNMYNWPMHDNNNRIISLLKIEKDFENI---ENNMNNKC---VTN----------
--------------------------HRDLKFVFLGNYLNRGELSLEVICFLFSLKILFP
KHIYLIRGNHENRVFNYVYGFYKDIERKIKSNFNCLGKINYKDDVICAYSYEIFNRINDV
LEFLPLSVLLDNEILCIHSGIGDSIQNVKDYLNIEKPIIIPEYVDRNSNNKSEILKKIII
DTLWSDPINYLDGNDMLLLKSCTK-YDIIPSSRGKITVKFGKHRLTKFIKNNKIKMIIRG
NECVPEGYKYDFNKKILTLFSATNYCNKYKNNASSALIIKKNKNITIFNQILKS------
------------------------------------------------------------
---------------------------------ECENMKLNESKNKEKANIVCTNG----
--EENKLNKSDKLENVKNETKIGFYFDHVKHKDKPSKKYGNKYKEIENNNDLINLKTPQF
YVNKDSIKMSNDDNIENILGDDSSDKNNLYDTDVCEKKIDDLDNSEVMNEMLKNLSYEKN
LDNCNDNKDEEDE------NNNVLQNDMEQVNLLKSNTDKNSDN-----------LLFEN
PCNEHIENEENADKEKKEKKEKNEEKDESKETMIHDENGDGYKAKDDNNE-------KND
SVENYKHNKFCNSKENEIFNDLENCCNGKSMDDINKDLIRLDDLGIEKNEHNDIFLDEKK
CLENIEMMKEEMLQECDYKIELIKYANGI----IEENFDEKNNELYKSRNNNNSRNNTNS
IINNFFKNEKTIEKNCSNIFLNYDQVNNSKEHEDNLKNGNCSKISSLSYENIGKCYNIGG
INSMYEID----------------------------------------------------
----------------------------------------------------------DE
LMYKXINY-MMPPNLTLTNKTQMKKGSYARKHNS-------TTMRNIRSESNTVDSLNKL
EMQHLPPDPQPQTK--------------------------------V
```

▪ AMOUNT OF MISSING DATA

P. reichenowi	*P. gallinaceum*	*P. knowlesi*	*P. vivax*	*P. chabaudi*	*P. berghei*	*P. yoelii*
26%	86%	30%	34%	73%	32%	33%

■ PFL0600w

▪ PHYLOGENY AND PEXEL/VTS

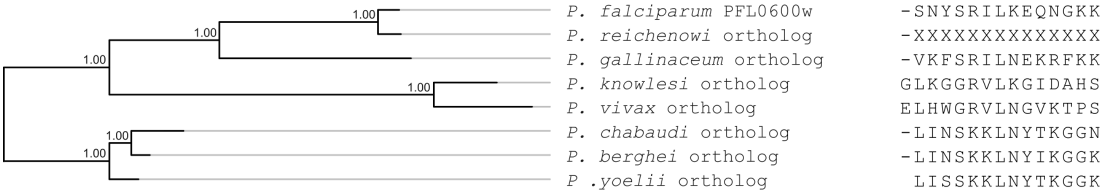

▪ ALIGNMENT

```
>P_falciparum_PFL0600w
MIYYIRKVAINFLFLYIINFCLLHYLKECNT-SNYSRILKEQNGKKYIY-YNLSKKKNNG
IIKGGSKHNGHLFINNYK-KKRNVKYKINK----------YKPCSIFSFFKKKDKNDKND
KNDKTHM--------KDNSNLENKEKGEIGKDKEQKEKEKEKIIILKDNKKLDEKKKKEL
QKEEKKTKLTNVLKQNNKNKNNNNKNNNNKNNNNNN---------NNNNNNNNMDDTLKK
DKQTELIKKDNLNNKNDVKKKKIDLNIN----NIDTNSRDILKNENKYKKHLENVKDKFI
ISLNQIFCSYMNSLDKKENFKYFFEKILQNVEKNFHTYTFIIDIEKMNNQQIKKTYMINN
INCFNEIMNIIQENLYNILVFKIQYLKVKAINDIKE-IISNKKNMNDYISYSNNINKIIQ
IYEEQLTKLCPLNYIFTLYNN------FME-N-----NNYLLNFSYQLPLYKYKNIIDSN
IEYLRTFSNDLTKHKKKSLYNEIEKNKNYQSILQIIDNQQKQIEVLQEQLETSIDGINGK
YSPFNCALAYRIPDTNLNISTQYVKGKFNVKVNCIPDDSLHLLGSYGFVKGLPYGNLGLS
LSLNF

>P_reichenowi_ortholog
------------------------------------------------------------
------------------------------------------------------------
------------------------------------------------------------
------------------------------------------------------------
------------------------------------------------------------
------------------------------------------------------------
------------------------------------------------------------
-----INKLCPLNYIFTLYNN------FME-N-----NNYLVNFSYELPLYKYKNIINSN
IEYLRTFSNDITKHKKKTLYNEIEKNKNYQSILQIIDNQQKQIEVLQEQLETSIEGINGK
YSPFNCALAYRIPDTNLNISTQYVKGKFNVKVNCIPDDSLHLLGSYGFVKGLPYGNLGLS
LSLNF

>P_yoelii_ortholog
MKLVVSKTVLNTLPLLLI----LLAICTCNI-LINSKKLNYIKGGKHAY-NYNAWQKNS-
-------INKYFYINNWK-KRKRINYKLKHYKLQSYKLNRYKLCSIFSIFKKQNDEKKKK
-----NV--------SLISNQTAIPKGNVQKD----------------------------
-GINEKSKPGNSGKIETEKRGKNETEKGGETDKGGE---------NDDRRN---------
------------------KIEGYPEIVL----ELNKNPKDALKNVQKYTEHLKNVMDKMI
KLLNNIFFYKTKFIEKDNDFKMFFEYIFKEFEKNYKKITYINEIEKMHYEDTKNEYYLAN
YKYFVKMLNIIEEHLLHILNLKLQFLKLKALNDIKD-TISKQTNNIDYISFYNNINNIFI
SYEKELNNLQPNKYIFTIYDN------IKEKK-----SNILINFNYDISIDKYQNAIKKI
IDYLKNFSQDLTKKKKKKIYKNIEKNEHYENILKIIDNQQKQIEVLQEQLESSIDGSNEK
YSPFYCSIAYRIPDTNLNISTQYLKKKFNIKLNCIPDDSMHLLGSYGFVKALPFGNLGLS
FSLNF

>P-berhei_ortholog
MKLVVSKTVLNALPLLLI----LLSICTCNI-LISSKKLNYTKGGKHAY-NYNAWQKNG-
-------INKYFYIHNWK-KRKRTNYKLHH----------YKLCSIFSIFKKQNDEKNAN
ETTKKNV--------PLITNQTTIPKGNIQKG----------------------------
-GINGKNNTGNIIKSETEKSKKI------DINKNDE---------NNKISH---------
------------------KIEEYPEIVL----DLNKNPQDALKNIQKYTEHLKNVMDKMI
NLLNNIFFYKTKFIEKDKDFKKFFEYIFKEFEKNYKKITYINEIEKMHYENTKNEYYLAN
YKYFVKIANIIEEHLLHILKLKLQLLKLKALNDIKD-IISKHTNNTEYVSFYNNINNIFV
SFEKELNNLQPNKYIFTIYDN------IKEKK-----SNMLINFNYDISIDKYQNVIKKI
IDYLKKFSQDLTKKKKKKFYKDIEKNEHYENILRVIDNQQKQIEVLQEQLESSIDGSNGK
YSPFYCSIAYRIPDTNLNISTQYLKKKFNIKLNCIPDDSMHLLGSYGFVKALPFGNLGLS
FSLNF

>P_chabaudi_ortholog
MKL-ASKTVLNTLPLLLI----LLTICTRNI-LINSKKLNYTKGGNHTY-NYNGWKKND-
-------INKYFYIHNWK-KRKRRNYKLQH----------YKLCSIFSIFKKQNDEKGEK
KT---NV--------SPISNQTATPKSDTQKG----------------------------
-GSDGKGKVENVIKGEAKGIKKN------DIKKRGE---------DSQTSH---------
------------------EGETYPEIVL----DMNKNPQDALKNIQKYTEHLKNVMDKMI
SLLNNIFFYKTKYIENNKDFKMFFEYIFKEFEKNYKKVTYINEIEKMHYEDTKNEYYLAN
YKYFVKIASIIEEHLLHILKLKLQLLKLKALNEIKD-IISKQTNNTDYVSFYNNINNIFI
SFEKELNNLQPDKYIFTIYDN------IKEKK-----SNMLVNFNYNISIDKYQNHIKKI
IDYLKKFSQDLTKKKKKKFYKDIEKNEHYENILRVIDNQQKQIEVLQEQLESSIDGGNGK
YSPFYCSIAYRIPDTNLNISTQYLKKKFNIKLNCIPDDSMHLLGSYGFVKALPFGNLGLS
LSLNF

>P_gallinaceum_ortholog
XXXXXXXXXXNILLIFLLNIYMFQGLIERNK-VKFSRILNEKRFKKSEY-FKFKQQYGI-
---SSRFKNYHLFINNWEGKKNKRSYKFKK----------HKVCSIFSNFMKKKNEENKK
ESKKILVNENENIDSKIINSKENIINNDEKKNNLVHDL----------------------
-HENKIDTKNNKLLRQEEKLNEENEKNSDNLNKENN---------RNVINNNSILKE---
------------------NVNSHNKNIMINFKDIDINSNKILKNINSYKKHLEILNEKSI
ISLNKIFCTYTKSLDENDDFKYFFENILKYLETNFHNYSLIGDIEKLNNEKTKKEYILNN
LNYFIEVINMVDEQLFNILVFKIQFLKLKAINEIRN-IISNKKYSTDYIEYSNNINNVVE
KYKEQLKNLYPKNYEFNLYNK------TLE-N-----YGLSLESCYKLPINKYKEFIDTN
IEYINKISQDLTQKKKKNLYTEIERNKNYQSIMQIIDNQQKQIEVLQEQLESSVEGINGK
YNPFHCAISYRIPDTNLNISTQYLKGKVNIKLNCIPDDSQHLLGSYGFVKSLPFGNLGLS
FSLNF

>P_vivax_ortholog
MLFLIKNLIGCALLLFVVNTHLHGRWHKWQEELHWGRVLNGVKTPSCVHPNRNKWPYNI-
--NRNCKPKSYLYVQNKK-RRKRQAYKLNR----------YKACSIFSIFDKSNEGKKKR
GGRPS-D--------GGVPQEGKQPSAEVPQE----------------------------
```

```
-GKQPSVEVPQEGKQPSVEVPQEGKQPSVEVPQEGKQPSAEVPQVGKQPSGGVPQVGKPP
TGGAPPGEVPPLSTPHGCESEG-----CIELSNLESNPKEALKNVATYKQHLENFKSRVI
LSLQTIFCSYTKGIDKKDNLRGFVLHILSDFERKHLEHTHLADVQNMRNEKVREEYLQSN
YNCFLDIVKTIEENLYNILLYKIQSVKLKALDDIKETVLSHRESNADYISYVNHVNKTFE
AYEKKLQSLLPASFKFSLYERMSRDSGVAEEEQRKRHPSSLSNFAYHFPMEKYEQLIKKN
INYVKKLSHDLTKKKKKRLIKEIERSKNYQNILHIIDNQQKQIEILQEQLEANVEGFPGK
YSPFHCAVAYRIPDTNLNISTQYVKGKFNVKLNCIPDDSLHLLGSYGFVKALPFGNLGLS
FSLNF

>P_knowlesi_ortholog
MLFLIRNLSGWILLLFVVNTQLHDGSHKWQKGLKGGRVLKGIDAHSCIHANKNKWQPNR-
--NKYCKPRSYLYIQNQR-RKKRQSYMLNR----------YNVCSIFSIFDKSNEAKKKN
IERESTS--------TGVPHKGNAPNKGMLQ-----------------------------
---------------------------------------------GEKTSTGVVAPGKV-
---LPSMETPPLITSPGCKLEG-----CIDLSNLESNPKGALKNEIEYRKHLENFKSKII
LSLQTIFCSYTKSIDTSDNLRNFVLNILSDFELKFRKYTHLTNVQNMHNHKMKEEYLQSN
YNCFLDIVKMIEENLYNILLYKIQSIKLKALDDIKETVLSHRESNADYISYVNHVNKIFE
VYEKKLQNLFPANFKFSLYNKMSKNCGVEEQQ-----PSSMSNFVYNFPIEKYEQLIKKN
INYVKKLSHDLTKKKKKKLIKEIERSKNYQNILHIIDNQQKQIEILQEQLETNVEGSPGK
YSPFHCAVAYRIPDTNLNVSTQYVKGKFNVKMNCIPDDSLHLLGSYGFVKALPFGNLGLS
FSMNF
```

- **AMOUNT OF MISSING DATA**

P. reichenowi	***P. gallinaceum***	***P. knowlesi***	***P. vivax***	***P. chabaudi***	***P. berghei***	***P. yoelii***
69%	11%	17%	9%	14%	14%	15%

■ PFA0200w

▪ PHYLOGENY AND PEXEL/VTS

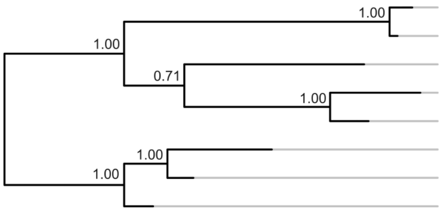

P. falciparum PFA0200w	SFINNRLLNE-HAH-
P. reichenowi ortholog	SFINNRLLNE-HAH-
P. gallinaceum ortholog	SVIKNRILKEDNPH-
P. knowlesi ortholog	PALRNRMLQEAKPY-
P. vivax ortholog	SALRNRMLQEEKPH-
P. chabaudi ortholog	QIFNRRILDGSELND
P. berghei ortholog	QTLNRRILNENELRN
P .yoelii ortholog	QALNNRVLNENVLNN

▪ SUPPORT FOR ALTERNATIVE TOPOLOGY RESEMBLING THE SPECIES TREE:

Expected likelihood weights	0.0157	-
Shimodaira-Hasegawa test	0.9730	+
two-sided Kishino-Hasegawa test		+
one-sided KH test	0.0660	+

▪ ALIGNMENT

```
>P_falciparum_PFA0200w
MLMKISRYFFLLYLIKAH-LDFFLRYRTGFIRSRLETYIGNSDVRYNKSFINNRLLNE-H
AH-C-DAWSEWSACSKTCDYGIKIRVKISTDQTKSKACSNITESTICHEHICPRTFEEAE
ETYLHNKEKEKKKKFRTTYILIFTIFSVVNIVVLLICVILSIKKKII

>P_reichenowi_ortholog
MLMKISRYFFLLYLIKAH-LDFFLRYRTGFIRSRLETYIGNSDVRYNKSFINNRLLNE-H
AH-CDDAWSEWSACSKTCDYGIKIRVKISTDPEKSKACSNITESTICHEHICPRTFEEAD
ETYFQNKENEKKRKFRTTYILIFTIFSVLNIVVLLICVILSIKKKII

>P_gallinaceum_ortholog
MLINISIYFFLLYLFKSH-LSKFXXXXXXXXXXXXXXXXXXXXXXYDDSVIKNRILKEDN
PH-C-EHWSEWTSCSKTCDVGVKMRVKTSSNKDYFEECSKISETSICLLRKCSESDYEGN
EDEKEQRENE-VRGTQKKYFLIFGIIFVVNILLLLICVIVS-KKKII

>P_knowlesi_ortholog
MWTNSLTSFFLLCLISAH-LNFFF----GQDNHSLGGHLTTDRIAHNEPALRNRMLQEAK
PY-C-DQWTEWSDCSKSCGMGVKMRVRTSSQKEHSRECSNIKETAVCFNDNCAELMGARE
RDEKEQRENE-IRNTRRKYLLIFGIFSAINIVILLVCVIMSIKRKVI

>P_vivax_ortholog
MWTNILKSFFLFCLINAH-LNFFF----GQDNHSLGGHLATDRTAHNDSALRNRMLQEEK
PH-C-DQWTEWSECSKSCDIGVKLRVRSSSQKEYSRECSNITETTVCFLNNCSDSKETRE
RDEKEQRENE-KNSAKRKYLLILSIFSAINVVILLVCVIMSIKKKVI

>P_chabaudi_ortholog
MFITISRYLFLLFLIKSCLLDLF-----PQHNDTFFERGPQPNKIGNNQIFNRRILDGSE
LNDC-DRWSDWSQCSKTCDIGIKIRARISNNPIHSLFCSKFTESSVCFMQECPESSERRS
RDEREKRKNK-KSKMLTKYLIIFCVFSAANIIILLICAIISIKKKLI

>P_yoelii_ ortholog
MFITISRYLFLLFLIKSC-LNIF-----PQYNDTFLEPGPHQNKIINNQALNNRVLNENV
LNNC-DGWTEWSQCSKTCDIGIKMRVRISSNPIYSLLCSKYTETSVCFIQECPDHSEMRN
RDEREKRKNK-KKKMLSKYVTIFCVFSVINXXXXXXXXXXXXXXXXX

>P_berghei_ortholog
MFITISRYLFLLFLIKSC-LNIF-----PQYNDTFLESGPQQNKIRNNQTLNRRILNENE
LRNC-DKWTEWSQCSKTCDIGIKIRARVSSNPIHSLFCSKFTETSVCFIQECPDNSEGRS
RDEKEKIKNK-KNRMLSKYVTIFCIFSVVNIIILLICAILSIKKKII
```

▪ AMOUNT OF MISSING DATA

P. reichenowi	***P. gallinaceum***	***P. knowlesi***	***P. vivax***	***P. chabaudi***	***P. berghei***	***P. yoelii***
0%	15%	3%	3%	5%	4%	15%

■ PFA0210c

▪ PHYLOGENY AND PEXEL/VTS

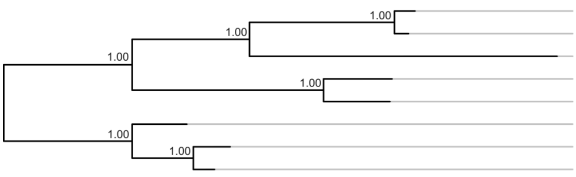

P. falciparum PFA0210c	-KIKSRILKENK-EE
P. reichenowi ortholog	-EIKSRMLKENK-EE
P. gallinaceum ortholog	-YLKKQLLKEKE-NK
P. knowlesi ortholog	DISRNRILHEQV-EE
P. vivax ortholog	DMPRFRTLHEKV-EV
P. chabaudi ortholog	-VTNYRSLEEAILDV
P. berghei ortholog	-VTNYRILEEALMDV
P .yoelii ortholog	-VTNYRILEAELMDV

▪ ALIGNMENT

```
>P_falciparum_PFA0210c
MSLKRRKFILLSFVFSVVELIFGYDNIINNYGGLSSVVVY-------NKNVGINKIGDNV
------------YKN-----------------------------------KIKSRILKEN
K-EES--------LETAAVNENTKDVKDSCNYSTPSSVFNNDKENTASFIKTGESYDGNN
VQAQAQAQAQAEAEGSLENAAVNLRTKSATLINDGMLDYYLNFCDQVNNKTFPFNECTNL
HTSDMVTLCKNNNDEKSKITYDLLGYGEMDDVSAYSLSYALNDVETIKDWNKNIYKLNYL
NLKKSDIQEKIDKNEKINVNNFILQSEKDVPEDERKYLYLINGLPWPFKSQDTIYEVYQK
YYNNKNMLLIINKSLNDVFDNNSSYARINNYENFFCIYPKSKNSYDKGVKYVMSIIYDVN
IPKFIQNNILNQIFPDLIFNLHNTSIAITNKTVGTVVDLSKNEQNAWHAHSLKNVKPEDT
PNTE-------HVQTDP--------ENSYGLGFIKMIFVDGPYNLWIINVNFFKKIFGIF
FNKD

>P_reichenowi_ortholog
MSLKRRKFILLSFVLLAVELIIGYDNIINNYGGLLNVVVY-------NKNVGINKKGDNI
------------FKN-----------------------------------EIKSRMLKEN
K-EES--------LETAAVNEKTKDVKDSCNYSTPSSVFNNDKENTSSFIKTGESYEGNN
VPSQ----AQADAEVSLENAPENLRTKSATLINDDMLNYYLNFCDQVNNKTFPFNECTNL
HTSDMVTLCKNNNDEKSKITYDLLGYGEMDDVSAYSLSYALNDVETIKDWNKNIYKLNYL
NLKKSDIQEKIDKNEKINVNNFILQNEKDLPEDERKYLYLINGLPWPFKSQDTIYEVYQK
YYNNKNMLLIINKSLNDVFDNNSSYARINNYENFFCICPKSKNSYDKGVKYVMSIIYDVN
IPKFIQNNILNQIFPDLIFNLHNTSIAITDKTVGTIVDLSKNEQNAWHAHSLKTLKSADT
PNDE-------LVQTDP--------ENSYGLGFIKMIFVDGPYNLWIINVNFFKKIFGMF
FNKE

>P_gallinaceum_ortholog
XXXXXXXXXXXXXXXXXXXXXXXXXXXXXXXXXXXXXXXXXXXXXXXXXXXXXXKKGDNI
------------FKN-----------------------------------YLKKQLLKEK
E-NKRET------LEKTEINEKKRRKKDRKLKK------NAEKEKGNGSVNE--------
-----------KGESVKEXXXXXXXXXXXXXXXXXXXXXXXXXXXXXXXXXXXLKNSSKV
HNSNILTLYKNLLEGKDK-KYDLLGYGEMKDVSAYVLNYSLNDIKSIKKWNTNIKRLNYL
NISKSTLLKKKSSREMLDVNKYIVNDEKDIENEKKNILYLVNSLPWPFKSHDVIYEVFQN
YDESENMFIIVNKSINDLFNKNENFSRINNYENFFCIYPKKKDIFQKGLDYVISLYYNAS
LPKFIQNNLFSNLFPSLIYNLYSYSQKET---------IKKKDIN--HEDKMKLLKHNDL
ENCEKNDNKKREIQDEKYNEKLDKRNNCYIKKLFKFVFTEKINTIWKAKKNFLKKLFFYL
YNXX

>P_knowlesi_ortholog
MILKKSKLLALSLLLALVE-YVCRDGRRKWMSLLTSWEVASFD----GSNVILNENRRGK
YVHIFPRKNASHFARNMTMYRQK------------ERRER-NVVCIVPSDISRNRILHEQ
V-EEENE------SVVREINGNDVNVSGNSDNA------NDIAEY-EETIKG--------
-----------QASTQEELPQQSVGTKVSPLIEKDTIDHYLDVCTRIRNKTFSLENCEIF
YKSPEVTLYKNILEDKSETRYDLIGYGTLDDVSLYGGSQALNNIDIIKEWNKNIYKINYL
KLNKTSILEKFKDGQKIDPSKYILQNEHLREN--RRYIYLINGLPWPFKSQDTVYEFYQK
YIEDQNILLVANKSVNNVFTDSSYYTRIRDYENFFCLYPKNKNSYEKGLDYVISVYYDVN
IPKFIRNNILGQIFPALIFDLHKSSKELTEKGLS----LSSEEIN--KNQLPFQM-DNFS
SEDGAGGLAGMDGQVAK--------SPFFGSTVLRVIFVDPFYFIWTTNVNFFKKIYVIV
TSIF

>P_vivax_ortholog
MILKKSKLLAVSLLLALVE-YLCRDGRRKWMGLLSRWEATPYGGSDVGSDVGLGQTGGGK
AAHIFALKSALQFANGYEEVRQKGRSGRSGRSERRERRERVNPVCILPSDMPRFRTLHEK
V-EVEEE------LLLQEKTADDMNVSGDADNT------NNKDEYGQPLIKG--------
-----------QTATEGVLPQQTVATKVSPLIEKSTIDHYLDVCTQIRKKTFSFKNCEIF
YESPEVTLYKNILEDKSETRYDLIGYGTLNDVSLYGASQALNNLDVIKEWNKNIYKINYL
KLNKASILEKYENDEKIDATKYILEKEHLREN--RRYIYLINGLPWPFRSHDTVYEFYQK
YIENQNMLLVANKSVNEVFSDNSYYTRIRDYENFFCIYPKSKNSYEKGLDYVISVYYDVN
IPKFIRNNILSQIFPSLIFNLHEVSKTITEKGLA----MSSDDIK--KNELPFQLKDNFS
PGEGGAGQDGKEGQDGK--------SPFFGATVLRVIFVDPFHFIWTTNVNFFKKIFVIV
TSIF

>P_chabaudi_ortholog
MNFKHNKYYLFLVSLFVLAKY--------------------------NNFVGSCKS----
-------------TN-----------------------------------VTNYRSLEEA
ILDVENVKENQKYLEQAKIEEAELNV-----EA------NTEKESTQTLVKT--------
-----------KSEILTE--EKIAINEKSVFINDELVTKYLNICDSVRNNTFSNENNEFF
NKSEDCTLFKNPVD-KNKPNYEIVGHGKLNDVSLYGMNYALRDLSAIREWNTHISYLNYL
DLTKDGIEDKMKKNESIDPSKHTIEDTEIFNN--NSYLYLVNGLPWPFRSHDTPYEYYQK
YFPDKNMLLVANRSVKKAFKDVSGYTRIRNYENFFCLYSKNKDIYSPGLDYVASIFYDVN
ISAFLQNSILNQLFPKLIFDLNATSRKYTQAGLS----MSEEEKT--TYQLLLQGKTHNX
XXXXXXXXXXXXXXXXXXXXXXXXXXXXXXXXXXXXXXXXXXXXXXXXXXXXXXXXXXXX
XXXX

>P_yoelii_ ortholog
MNFKQNKYYIFLVSLFVLAKY--------------------------DNNVGPCNLKNSL
NGESYISKNLIYFKN-----------------------------------VTNYRILEAE
LMDVENVKENLKYVQQAKMEENELNV-----EG------NAQKESPVSRVKS--------
-----------KSEILIE--EKTNLNETSTFITDELISKYLNICDSVHNNTFTYNNDESF
NKSEDCTLHKNLVDEKNNSNYEIIGHGKLKDVSIYGMNYALRNISAIKEWNTHISYLNYL
NLTKETIENKIKKHEQIDPSKHTLNDTELSNN--NSYLYLVNGLPWPFRSHDSPYEYYQK
YFPDKNMLLIVNRSIKNAFKDVSGYTRIRNYENFFCLYSKNNDMYTPGLDYVSSILYDVS
```

```
IPPFLKNSILNQLFPKLILDLNQCSRKYTKIGLS----LTDEEKA--TYLLLLQSKGHNX
XXXXXXXXXXXXXXXXXXXXXXXXXXXXXXXXXXXXXXXXXXXXXXXXXXXXXXXXXXXX
XXXX

>P_berghei_ortholog
MNFKQNKYYIFLVSLLVLDKY--------------------------NNNAGACKSKNSL
NEESYISTNLISFKN-----------------------------------VTNYRILEEA
LMDVENVKENLKYVQQAKMEENELNV-----EG------NAQKESPIPSVKS--------
-----------KSE-LIE--EKKKINETSAFITDELVTKYLNICDSVHNNTFIYNNDESF
NKSEDCTLHKNLIDEKNNSNYEIIGHGKLNDVSVYGMNYALSNISAIKEWNTHISYLNYL
NLTKETIEDKIKRHEKIDPSKYTLNDANMLNN--NSYLYLINGLPWPFRSHDAPYEYYQK
YFPDKNMILIVNRSIKNAFKDVSGYTRIRNYESFFCLYPKTNDMYTPGLDYVSSILYDVS
IPPFLKNSILNQLFPKLILDLNQSSRKYTKVGLS----LTDEEKA--SYLLSLQIKGDNX
XXXXXXXXXXXXXXXXXXXXXXXXXXXXXXXXXXXXXXXXXXXXXXXXXXXXXXXXXXXX
XXXX
```

- **AMOUNT OF MISSING DATA**

P. reichenowi	***P. gallinaceum***	***P. knowlesi***	***P. vivax***	***P. chabaudi***	***P. berghei***	***P. yoelii***
0%	26%	30%	16%	26%	26%	26%

■ PFL1660c

▪ PHYLOGENY AND PEXEL/VTS

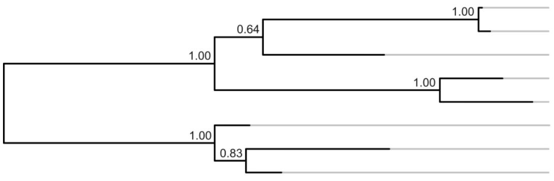

P. falciparum PFL1660c	KKIIKRILSSNSLHV
P. reichenowi ortholog	XXXXXXXXXXXXXXX
P. gallinaceum ortholog	XXXXXXXXXXXXXXX
P. knowlesi ortholog	---------------
P. vivax ortholog	---------------
P. chabaudi ortholog	---------------
P. berghei ortholog	XXXXXXXXXXXXXXX
P .yoelii ortholog	XXXXXXXXXXXXXXX

▪ ALIGNMENT

```
>P_falciparum_PFL1660c
MLFLLHIYIPQNSVAYVSNVFPMTYIYPSFTFVSFLLFSYLFSHFTYILKYEKKIIKRIL
SSNSLHVVHNFSFVKNDIERNIIKDDKNDENIDNINKQYMSDEEKIEKKEKIEKNEKIEK
NENGDHPQLFQHNYNNLNIRHNNNQMLDFLSSVQKKESRR--FYSINLKEKNFRWSMILS
IGSKKKDLELGILTNSNITGLYCSYN--YKDNDELKNLKYDITLSEDLKYVECKSIMCDN
VEHSSPCLPLENYFKLINDYKIRKRSCQNKFCDYVNNMNFLNMIDKLNDRNLSICSFNTT
INNEQIKGFYFKDSFFLYKKIKTSFLYFACITESDPLHFNNKLSGFIGLANYINDKEKYH
NQNKKIYSTILYSFIYKSISKKNIFSLCFKQGGGFITFGGYDQNVLTKKEAPLSKIQ---
--LYSIDKNTNKKYVQQLNSV--DVYDLLWIHYYAKNKSYYSLFLKNVTATMDQNKMNSV
INAEAKVDSYNYFLSFPADITIKLKNFVHDKCPDTDQFNDCSNLIEKGLIKLKNKNIKDF
PKIELLFEEGTVIVHPEDYIIHESDGVYRVLI-NSSGELKLGIPFFLNKYLIFDNENERL
GVSKSECALELNDNEL-FGVDLSSNDDPYEEKDDDSMKENLFQKYKTHIILISSGIFVSS
IIGSILYFS
```

```
>P_reichenowi_ortholog
XXXXXXXXXXXXXXXXXXXXXXXXXXXXXXXXXXXXXXXXXXXXXXXXXXXXXXXXXXXX
XXXXXXXXXXXXXXXXXXXXXXXXXXXXXXXXXXXXXXXXXXX------EKNEKNEKIEK
NENGDHPQLLQHNYDTLNIRHNNNHMLDFLSSVQKKESRK--FYSINLKEKNFRWSMILS
IGSKKKDLELGILTNSNITGLYCSYN--YKDNDELKNLKYDITVSEDLKYVECKSIMCDN
VEHSSPCLPLENYFKLINDYKIRKRSCQNKFCDYVNNMNFLNMIDRLNDRNLSICSFNTT
INNEQIIGFYFKDSFFLYKKIKTSFLYFACITESDPLHFNNKLSGFIGLANYINDKEKYH
NQNKKIYSTILYSFIYKSISKKNIFSLCFKQGGGFITFGGYDQNVLTKKEAPLSKIQ---
--LYSIDKNTNKKYVQQLNSF--DVYDLLWIHYYAKNKSYYSLFLKNVTATIDQNKMNSV
INAEAKVDSXXXXXXXXXXXXXXXXXXXXXXXXXXXXXXXXXXXXXXXXXXXXXXXXXXX
XXXXXXXXXXXXXXXXXXXXXXXXXXXXXXXXXXXXXXXXXXXXXXXXXXXXXXXXXXXX
XXXXXXXXXXXXXXXXXXXXXXXXXXXXXXXXXXXXXXXXXXXXXXXXXXXXXXXXXXXX
XXXXXXXXX
```

```
>P_gallinaceum_ortholog
XXXXXXXXXXXXXXXXXXXXXXXXXXXXXXXXXXXXXXXXXXXXXXXXXXXXXXXXXXXX
XXXXXXXXXXXXXXXXXXXXXXXXXXXXXXXXXXXXXXXXXXXXXXXXXXXXXXXXXXXX
XXXXXXXXXXXXXXXXXXXXXXXXXXXXXXXXXXXXXXXXXXXXXXXLNEFNFRWSIPLY
IGSKKTKIELCIITSSSITALYCTDS--FKEKDNLKKLKYNINESIDLRYVECRSKSCDN
IL-NNECLPLKNYFKMLHEYNIKKKNCKDRFCDYINNMNFLNMIDKLNDRNISVCSFNST
IDNDQIKGFYFKDSFYINNTVKYFYDYFGCISESDYLKFNEVLSGFIGLGN-------YN
ENNNKKYPTILNSFIHKSISKKNIFALCFVENSGLISFGGYEKSIL-KKKEHIPKLE---
INRRKIIRDREEEM--PVEDI--DQYEILWLHYSNPNKSIYNIFLKEANVNSKSKKLINT
IKRDAIVDSCNYFLSLPADITAKIKTFIYNECSDKT--NECSRLIEKGIFELKNKNINNF
PQIELVFKXXXXXXXXXXXXXXXXXXXXXXXXXXXXXXXXXXXXXXXXXXXXXXXXXXXX
XXXXXXXXXXXXXXXXXXXXXXXXXXXXXXXXXXXXXXXXXXXXXXXXXXXXXXXXXXXX
XXXXXXXXX
```

```
>P_knowlesi_ortholog
MALIIPLLFTLATLLLHTQNVTVC------------------------------------
---------------------------------------SADHFGEAIRRKAFKVQRSVR
GDPGVNSAITSKRVNGASTTSKGNSLLE----AK-KREQD--YFTLKINEQNFRWSLPLL
MGSKKTPLELGIVTSTPITALYCSYN--AKPNEEMNNIKYEVNASEGVKYISCKSRYCTA
IGQGNTCAPIEHFFKMIHDFGLRKKNCTNRFCTYINDINFLNVNTKLDKRNMSVCSFSSN
LGSEQVEGFYFRDSFYLYDTVKFDYKHFGCVTQSGVLTFNNVISGFIGLAY----NRADA
IANKKESSSILHTLVQKSVSKKNIFGLCFVEGGGFATFGGINNEAL-RKVLPVSKLQ---
MGFQHLGGEDPQAT----------SHEIVWLAYSDTSKSTYSLLLKEVNMVSTSNRVENA
IGRVAVIDSYNYFLSFPAEITAKLKTAIYNSCVGND--NKCSNIINKGVFTLKNGGVDDF
PTVELVFDDGKVLIHPKDYLIHEGDGVYRVLI-NSEETLKLGIPFFLNKYLTFDNENGKI
GVGPSDCTFEMKGVSPGVDLSATEADSNDDPEDKDFTIEDFFQENKLMILALTSLSVVGA
IVGGVFFFG
```

```
>P_vivax_ortholog
MGRIAPLLLTLAELLLYAHNATVC------------------------------------
---------------------------------------SAYHLGEAARKKACKY-----
-----------------GCTGKGNSLVE----AKTQSEQD--YFTLKLSEHNFRWSVRLL
MGSKKTPLQLGVVTSTPITALYCSYNASARESDQMKDLKYGVNESEDVKYVGCKSRQCTA
AQRGNSCAPINHFLKMIHEFGLRKKNCTSRFCSYINDMNFLNVDTQLDKRNMSVCSFSST
VGSEHIEGFYFKDSFYLHETVRCSYNYFGCLTQSDDLTFNNAISGFIGLAY----NRADD
MAHSKESPSMMHTLVQKSISKRNVFTLCFVEGGGFASFGGVNNEAL-RKTPAVSKLQ---
MSSQHLEADAPLELVADQQAA---PHQIVWLAYSDTSKDTYSLLLKEVNLVSGSKRVENS
-----------YFLSFPAEITAKLKTAVHSSCAGGA--NTCSEIINKGVFTLKNQGVADF
PALELVFDDGKVLIEPQDYLIHEGDGVYRVLL-NSEGTLKLGVPFFLNKYLIFDNERGKL
GVGPSDCTYKMKETFPGVDLSTPEADSKGDPEDEDCTRESFFQANKLTILALTSLSVVGG
LVGGVFFFC
```

```
>P_chabaudi_ortholog
XXXXXXXXXXXXXXXXXXXXXXXXXXXXXXXXXXXXXXXXXXXXXXXXXXXXXXXXXXXX
XXXXXXXXXXXXXXXXXXXXXXXXXXXXXXXXXXXXXXXXXXXXXXXXXXXXXXXXXXXX
XXXXXXXXXXXXXXXXXXXXXXXXXXXXXXXXVQDDEATD--FYNLKLHEKNFRWSLPLA
LGSEKSVVDLVLTLGNSSTAFYCHDE--TKPASETDVLAYDLSKSKDLKYVDCKSAECTE
ILGSNKCLVLDEYFKQLKGYTLRKKNCKSKLCDYVTSMNFLSMNDPSVDKNASVCPFDHK
IDSEQIKGFYFNDSFLINKDKKITYDYFGCITENKSLNINADTYGVIGLTN--------N
HQADKKYSSILNSFVSNSASKKNIFGLCLIEGGGFISFGGHDKAAL-GPVPPPKEAVDTA
LDTSDYDD-SDLTYKNTLLSV-NESDGLIWVDYAGPTTESYKIKVTKINFNVTDDSSEHK
IDKEFTLDTYDYFISLPKEVSTKLTEQIDKICKGLG--EKCKYTKESGSFQMASEHLGSF
```

```
PILEFSFGEHKVMVHPQDYIIDNGXXXXXXXXXXXXXXXXXXXXXXXXXXXXXXXXXXXX
XXXXXXXXXXXXXXXXXXXXXXXXXXXXXXXXXXXXXXXXXXXXXXXXXXXXXXXXXXXX
XXXXXXXXX

>P_yoelii_ ortholog
MSQKYLVIVHCVFLLLNLFKTNCY------------------------------------
--------------------------------------------------SVQSHSNLKT
NNSQCIGCMNGKTKTLRNIESKNNRINSSFLHVREDEEDDNTFYSLKLNEKNFRWSIPLA
IGSDKTVIDLVLTIANSSTAFYCYDE--KKPSPDTETLGYDLSKSTDLKYVDCKDETCTE
ILGSNKCLILEEYFKLLNGYVLRKKSCKSKFCDYVNKMNFLNMSNPGVDKNASVCPFDNK
VDSDKIKGFYFNDSFLISKEKKVTYNYFGCITENENLNINENTSGIIGLTN--------D
YKADKKYSSILNSFISNSESKKNIFGVCLIDGGGFISFGGYDKLAL-KPGVPPKKVK---
-GPEDSDDSVDTSYRNTLLSIGDDSDGLIWVDYSESTNELYKVKVTKINVNVTDDSSEHE
VNKDFILDTYDYFISLPREISAKLTEKINKICKDLN--DKCKQVENSGTFQMESEQVASF
PAIEFYFNENKVXVQPQDYIINDGDKNYKILVKHAESNEKLGVPFFLSKYIIFDNEQKKL
D--------------------------------------QIIKMKRIKDMRIILVLLFPF
LYFYLNLII

>P_berghei_ortholog
XXXXXXXXXXXXXXXXXXXXXXXXXXXXXXXXXXXXXXXXXXXXXXXXXXXXXXXXXXXX
XXXXXXXXXXXXXXXXXXXXXXXXXXXXXXXXXXXXXXXXXXXXXXXXXXXXXXXXXXXX
XXXXXXXXXXXXXXXXXXXXXXXXXXXXXXXXXXXXXXXXXXXXXXXXXXXXXXXXXXXX
XXXXXXXXXXXXXXXXXXXXXXXXXXXXXXXXXXXXXXXXXXXXXXXXXXXXXXXXXXXX
XXXSNKCGMGDEYFKLLKGYALRKKSCKSKFCDYVNKMNFLNMSNPGVDKNASVCPFDKK
VDNDKIKGFYFNDSFLISKEKKITYNYFGCITENENLNINENTSGIIGLAN--------D
YKADKKYSSILNAFISNSGSKKNIFGLCLIDGGGFISFGGYDKAAL-EPALPAKEIK---
-GSEEYED-LDTSYRNPLLSIGDDSDNLIWTNYSESTNELYKVKVTKINLNVTDDNSEYD
VNKDFILDTYDYFISLPREISTKLTEKINKICKDLN--DKCKNVENSGTFQMGNEQVGSF
PAIEFFFNDNKVVVPPHDYIINDGDNNYKILVKHTENSEKLGIPFFLSKYIIFDNEQKKL
G----------------------------------------------------YTYIHKY
IYIYIYIYM
```

▪ AMOUNT OF MISSING DATA

P. reichenowi	***P. gallinaceum***	***P. knowlesi***	***P. vivax***	***P. chabaudi***	***P. berghei***	***P. yoelii***
43%	45%	14%	18%	41%	46%	21%

■ PFE1190c

▪ PHYLOGENY AND PEXEL/VTS

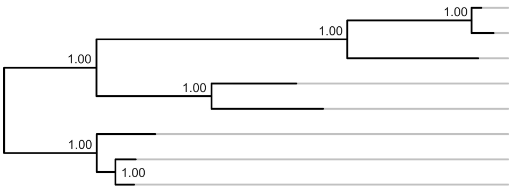

P. falciparum PFE1190c	KMIKKRNLDHCCSIK
P. reichenowi ortholog	KMIKKRNLDPCCSIK
P. gallinaceum ortholog	KILKKNNLDNCCFTK
P. knowlesi ortholog	KLLKSKNLDRCCKHK
P. vivax ortholog	QLLKSRNMDRCCKHK
P. chabaudi ortholog	KLLKEKNLDHCCKYD
P. berghei ortholog	KLLKEKNLDHCCKYD
P .yoelii ortholog	KLLKEKNLDHCCKYD

▪ ALIGNMENT

```
>P_falciparum_PFE1190c
MTPICSLCILFNFIQLNNVLLLTWGIFSVILFVISHGHFNI-GPKCNELLEKYHVFISIL
SIILLLSNNYTS-----QKMIKKRNLDHCCSIK-----RFGKNANNSSFTN--------H
HSSEHACNEDHHDHHNRTISASSNNGNKYYMNYDKNER------------------ELVS
FL---

>P_reichenowi_ortholog
XXXXXXXCILFNFIQLKNVLLLTWGIFSVILFVISHGHFNI-GPKCNELLEKYHVFISIL
SIILLLSNNYTS-----PKMIKKRNLDPCCSIK-----RFGKKANNSPFTN--------P
HFSEHACNEDHHDHHNRTNSASSNNGNKYYMNYDKNER------------------ELVS
FL---

>P_gallinaceum_ortholog
MAPICSLCIIFNYYQLKNVLLLLWGVFGIILFTISHGHFSI-NEKINIFLQKFHIIISLF
SILILLSNNYVS-----QKILKKNNLDNCCFTK-----NNEKNENGQKYA---------Y
HXXXXXXXXXXXXXXXXXXXXXXXXXXXXXXXXXXXXXXXXXXXXXXXXXXXXXXXXXXX
XX---

>P_knowlesi_ortholog
MTPIISFTTIYNFIQLKNISLFLMTLVGITLFVLTHAHIQVPNQNVMNFLRKMHIPLAIL
AAIFLIGTNRAA-----HKLLKSKNLDRCCKHK-----KITNNFDKHSCTEHQSCDHHHH
HHHDHHDHHHHHHHHHQDDLEMNEDNYDMHNNKGKNDNSASFERYYHIGFQQNGDQELVR
FL---

>P_vivax_ortholog
MTPIISFTTIYNFVQLKNFPLLLTTLIGITMFVLSHAHIEFSSPSVANIFKKMHIPMAIL
AAIFLVSTNYAA-----HQLLKSRNMDRCCKHK-----KISHHLEEQSCEQ--------H
HHHPQHPHHHHHY-----DLEMNDDTYGMHSHPHNNDNVVNLEKFYNIGFHQHNDHELVR
FL---

>P_chabaudi_ortholog
MMPIITLTTVINFYKLRNIPLLMSALTGMTLFIISHAHIEFSNDNINDILEVLHIPLALL
GAAFLLSTNYAS-----HKLLKEKNLDHCCKYD-----HIKSYQNNHPHCH--------H
HCHHH--RHHHATNNNRSNLTVNKNVLDSFGKPLDENS------------------DLLS
SL---

>P_yoelii_ ortholog
MMPIITLTTIINFYKLRNVPLLMSALTGMTLFIISHAHIEFSNDNINDIIETLHIPLALL
GATFLLSTNYAS-----HKLLKEKNLDHCCKYD-----HIKNYHNNNHHHH-----NHHH
HNHHHHNHHHHAANNNRSDLTGDKDEFDNFEKPSDENT------------------DLLS
YL---

>P_berghei_ortholog
M-PIITLTTIINFYKLRNVPLLMSALTGMTLFIISHAHIEFSNDNINDIIEILHIPLALL
GAAFLLSANYAS-----HKLLKEKNLDHCCKYD-----HIKNY----------------H
HNHHH---QQHADNNNRSDLTVDKDVFGNFEKPSDENT------------------DLLS
YL---
```

▪ AMOUNT OF MISSING DATA

P. reichenowi	***P. gallinaceum***	***P. knowlesi***	***P. vivax***	***P. chabaudi***	***P. berghei***	***P. yoelii***
4%	30%	15%	14%	2%	8%	2%

■ PFD0495c

▪ PHYLOGENY AND PEXEL/VTS

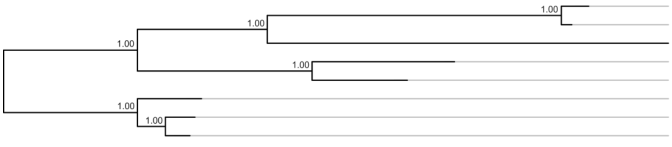

P. falciparum PFD0495c	QSSFSRIIAEYCDTK
P. reichenowi ortholog	QSSFSRIIAEYRDTK
P. gallinaceum ortholog	XXXXXXXXXXXXXXX
P. knowlesi ortholog	HSNHGRVLTEYVSSN
P. vivax ortholog	GSKESKKESETRKET
P. chabaudi ortholog	LNRVNRILSSTNSDT
P. berghei ortholog	LNRVNRILSNVKPDT
P .yoelii ortholog	LNRVNRILSNTKSDT

▪ ALIGNMENT

```
>P_falciparum_PFD0495c
MYKKCFILYPIFFPSLYIYIIKNDHLN-------SEQSSFSRIIAEYCDTKKNEFFLGES
ILGATSSRSTSLNIEQNKNTNIIKDKNE------------------------QSYDEHIV
MNPNTNRALSINTVFNYNKENKEKKIFSFSEFPKEFNILDVVWPYMKQPKELFKKSSVIT
FLMDHYFRHELYILESRIAMKPRRRTYEAPCFEHDDFELERDFFFLEDCDEDHQFFNKYK
SYFFSLNVLDHCKSLRTKKQKCNNMKDDEVSNINDDEVSNINDDEVSNINDDEVSNIKDD
VVRNINDDVVRNINDDEVHHTNDDKVNHTND--DKVNHTNDDKVNHTNDDKVNHTNDDKV
NHTNDDNVNHTNDDKVNHTNDDKVNHTNDDKVNHTNDDKVNHTNNYYNDKKNNAGDIKTN
NSIREEKKLEHPDRNIEKKIDLITYNKKR-------------------------------
---IEEYYDSIISYFFGLIILYHNKKETNLNYYTKFLTLDKYKNMYNCLNNDISKIYEKA
ILFSHEEFCIIQKKDLKP------HGLRGNIKYYYFFNRIVSTSLYLLHEILQKLDGKMY
TFQKLPLKIQNHLINLPDIRIKEIKKRMRQQKKKNQNSLLESSSYKDLYYVSSEYYDYVS
KCLIWSNYYFFNYMSTTIVYSVKKRS--YEYIQKEKSKINLFLEYAHNDIIEYIKDITYY
FKLIVNKIESKRLFSEPVMLCFQLFSDHYLYLLKNILSILLIHIEKPVTRKSNRDLKKIF
NCIKDQENITKNILDEFHSKNKIRYNPTEFLIYKFFSSIQYKQNIAHKYIIQSNINIISL
MLKIFNYFHILVISLIFYLNLHSMYTLFIDLDIDDTLKFQHDQEFLNYFKRYQDFNNQLF
DSFRSDDR--------

>P_reichenowi_ortholog
MYKKCFILYPIFFPSLYIYIIKNDHLN-------SEQSSFSRIIAEYRDTKKNEFFLGES
ILGATSSRSTSLNKEQNKNTNIIIDKNE------------------------QGYDEHIV
INPNINPAVSINTVFNYNKENKEKNIFSFSEFPKEFNILDVVWPYMKQPKEVFKKSSVIS
FLMDHYFRHELYILESRNTMKPRRRTYEAPCFQYDDFELERDFFFLEECDEDHQFFNKYK
SYFFSLNVLDHCKSLRTKKQKCNDMKDDEVSNMDGDDVSNMDGYGVSNMDGDDVSNMDGY
EVSNMDGDEVSNMDGYGVSNMDGDEVSNTND--DKVNHTNDDKVNHTNDDKVNHTNDDNV
NHTNDDNVNHTNDDNVNHRNDDNVNHTNDDNVNHTNDDNVNHRNNYYNDKKNNPGDIKTN
NSMREEKKIEQVDRKSENKNDLTTYNKKR-------------------------------
---IEEYYDSIISYFFGLIILYHNKKQTNLNYYTKFLTLDKYKNMYNSLNNDISKIYEKS
LLFSYEEFCIIQKKDIKP------HDLRGNIKYYYFFNRIVSTSLYLLHEILQKLDGKIY
TFQKLPLKIQNHLINLPDIRIKEIKKRMRHKKKKNQNSLLESSNYKDLYYISSEYYDYVS
KCLIWSNYYFFNYMSKTIVYSVKKRS--YEYIEKEKSKINLFLEYTHNDIIEYIKDITYY
FKLIVNKIESKSLFSEPVMLCFQLFFDHYLYLLKNILSILLIHIEKPVTRKSSRDLKNIF
NCIKDQENITKNILDEFHSKNKIRYNPTEFLIYKFFSSIQYKQNIAHKYIIQSNNNIISL
MLKIFNYFHILVIALIFYLNLHSMYTLFIDLDIDDTLKFQHDQEFLNYFKRYQDFNNQLF
DSFRSDDR--------

>P_gallinaceum_ortholog
XXXXXXXXXXXXXXXXXXXXXXXXXXXXXXXXXXXXXXXXXXXXXXXXXXXXXXXXXXXX
XXXXXXXXXXXXXXXXXXXXXXXXXXXXXXXXXXXXXXXXXXXXXXXXXXXXXXXXXXXX
XXXXXXXXXXXXXXXXXXXXXXXXXXXXXXXXXXXXXXXXXXXXXXXXXXXXXXXXXXXX
XXXXXXXXXXXXXXXXXXXXXXXXXXXXXXXXXXXXXXXXXXXXXXXXXXXXXXXXXXXX
XXXXXXXXXXXXXXXXXXXXXXXXXXXXXXXXXXXXXXXXXXXXXXXXXXXXXXXXXXXX
XXXXXXXXXXXXXXXXXXXXXXXXXXXXXXXXXXXXXXXXXXXXXXXXXXXXXXXXXXXX
XXXXXXXXXXXXXXXXXXXXXXXXXXXXXXXXXXXXXXXXXXXXXXXXXXXXXXXXXMSN
--VLE----------MDNPVDITVIN----------------------------------
---DKKYYEKMFARFIALILIFSNKP-NNIDDLAQILPEKYMLR-YVDVNGSLLRACKNE
FPGRIEEF--------RKDVGLNIVPTMGALKYYFLFDRQVKTLYHLLRDTIKRNRALIS
VIEPLSSTRE-----------------ADQERSLHKGSDIVQSTR-DLYDITLSRYNAAY
KNTSRVFSFYFYHWIDKAITAIKKKD--IPWIEGERRDLNVYLTIIENKLKYLTQDLALY
IRFLFHQAKKDNLFSSSVELCYTLFFEHYKNLINKTIQIFEIHIHSTPSKNIESTLRKVR
TIFQDHQKEVQTEIQKFYMNNVLILNTKE-LTENYIFKEKYRQSVANDYSREKQKKFISL
LPCIFYYSKVILASATLLISLQNVDVLFDDLM-HQRIKFADALEIVSFLEKHKKESNHVF
AXXXXXXXXXXXX---

>P_knowlesi_ortholog
MLNKVICFLAPFYLRAPYIIDLYDNFRPTVLHEGNRHSNHGRVLTEYVSSNFNE------
-----DRKSRQAS------------------------GGLRGIREECFRESDQGNEDR--
------------------------------------------------------------
------------------------------------------------------------
-------------------------GEGPFSTADAKD-----------------------
----------------------------GHEVCHGKYGRQNFHLHMPNGDRSDGA-----
------------------------------------------------------SRVDSN
--MFS--------------MDYSPYNSGRIFYRD--------------------------
---NWLYYERMMTHLLSLAILFYSNN-SYMEKMATQLPPNHYFL-YNFLQQDINGLYENN
ISQICEDFYEVEKKE-KT------IESKGIEKYHFAFNRMECTLNSFLDEILRKMDSLHL
SFESLPIEFS-----------------LRRNELIDEVIKSTPNEINDIYLITYKFFDSIC
SCLDMTSSFNFNYVSKKILSNLNGEGNFHRNVILEMKKINYYITNIHNNIVEYIKDLNSY
FKNFFDYAARENLFCEPVLYSYSLFLDYYLTTMDQLMIHLNLHLRYPVSKDSQTSLNHIY
KEIKTHANNLGCTIRRFYFKRNIKSDPEF-SIIPFFADFNFRLRLADMYNNEKYANFIPL
MINIIDYLRALVASIVSLVMLQTVYALLVDTE-NGHIKDVDAVKALTYLTEYMSHNSSLF
APPLKEKH--------

>P_vivax_ortholog
MARK----------------------------ESNKGSKESKKESETRKETKKE------
-----TRKETKKETKKETKKETKKTANRLPSGRLSSYGGGPPDARECRKQILKGGENHAC
KKKPKENVATPSTDEAVLNKNKFRQ-----------------------------------
-----DFLNFYYALIGKGINRSKADVKAAKNLDGGCEKK--------CKEKPKVYPKAK
SSLLDECCVKNKENSQNPLTVSKEEGGLRNGVFEPEE-----------------------
----------------------------KHA--RRKRGPQKSHLAMPNGGSVGGVGSVGG
VSGVGSVGSISSFSGLGSPNSVSSVGSISSLDSVSNLSSVSSVDSSGVSVGGSLSSVDSS
--VIS----------LGNSRDSSVISLGNSRDSSVISLGNSRDSSVLSLGSSRDSRYSSW
GTGNKTYHDKMLTHLFGLAILFYSNS-SYVEKMAKQLPQNYYLC-YRDLHQNIKRIYENN
FTQMYEDFYEMEKKM-KP------IEQKGVEKYYYAFNRVVYTLLLFLYEILQKEDALHL
```

```
SFESLPMEYR-----------------LRRDELVDTVTKATPSDMNDIYFTTYKFFDAMC
TCMDLSSCFNFNYIGKKLINNLHQKGSFHSKVTLEMKKIKEYMANMQNDIVEYVKDLNSY
FKHLLEYAARENLFCEPVLYCYSLFLDYYLATIDQLLDHLNIHLKYPVNKESQESLSHIY
RELKTHADNLGCTMRCFYFKKNIKSDPEL-SIISLFTDSKFRLQLADKYASKKYADFIAL
MLNILDYLRVLVASLTSLVTLQTVHALLMDTE-HGNIKYEDAMKVMRYFTRYISHNSSVL
AQRSGAPWSGA-----

>P_chabaudi_ortholog
XXHI------------------------------SKLNRVNRILSSTNSDTSDN------
-----TIKGTKSSNDSQKKGLGKKSHKK------------------------KGSSNGSI
EQEDSELKTSTEGENTY-------------------------------------------
----------------------------------------TGYLSSNTIGSTGSTGSTGP
DEFVYNINEGAIKKHRRHSSIFNCFN-----SNNDDD-----------------------
----------------------------GDS--YKQKDANESNLKIKSDDDPGEG-----
---------QSQAWGNNKGNKYEGNTNPHKLDDNYLTRRNSSTSNYEINLALVTKVIDSN
KNILK----------R--ENDRIIKDNMNKNSSTVRSRDMLFNTSARNI-----------
---DTFFYDKIITHLLGYAYIFYEHK-ININKLEKTLIKDYYYN-YKVLKGSINTI----
----PEEFKYNQNIN-KTDLVYSRLNHLGIEKYYYGFNRNLHTFLFLARECLQKEHALEK
VYESLPLDFE-----------------FDRKELKEKIQASTSDEM-DIYGFSYNMFDQIC
KFLHTYNCFNLNYISNKLINNLNSKNS-NSIITKELNNLTEYTQATYSEIIEYLKDLTYY
ISNLFEYGHKENLFNELVIFCYSLFFDYYIDTLDKLLDIMQIHLDHPFNERIKNELSTIN
IRVKAHRDFVENEVVSFYRKYNISENLSE-CVTNIFSNNEYRVIILEQYLDSRYSNYLSL
ILLIFRYLKVFVFSMSTYSNLQITYSLLSDLK-KKKVKYDEALNILSHHASFNIFDREFF
NKKNRNSKSAKNK---

>P_yoelii_ ortholog
XXYI------------------------------RKLNRVNRILSNTKSDTSDE------
-----KTKETKFSNDSHKNGKGEKSHKK------------------------KG-SNDHI
EGENSESKSSIECENKY-------------------------------------------
----------------------------------------KDDLLNE---------SNGS
GDFSHSVKEGAIKKYQCHSSIFNCFTKSRNDDDDDDD-----------------------
----------------------------EDS--YKQKDTNENNLNIKPDDDPDEG-----
---------HSKFWRNKKKNKHGIDTNPHKINISYLTRKKNPLPNYEFNLETVTKFIDSN
QKIID----------L--GNDTIIKDNMNRSSNSVKSRDMLLNVSLKNI-----------
---DNLFYDKIVTHLLGYAYIFYGHK-VNINKLEKELIKDYYYN-YKVLKDSINNI----
----PDEFKYTQNNS-KIDPVYNLLDYLGIEKYYYGFNRNLYTFLFLAREFWQKEHALTK
VYESLPVDFE-----------------FDRKELIEQIHDSTSDEK-DIYDFSYKIFDPIC
NFLHTYNCFNLNYISNKLINNLRAKKS-NGVIKKELSKLTEYIQATHFDIIEYLKDLTYY
ITNLFEYGHKENLYNELVIFCYSLFFDYYLDTLDKLLYIIQIHLKYPFNEQIKNDLSTIN
VRIKTHRDFVGNELTSFYHKYNISENLLE-CVTGVFSNNEYRVLILEQYLDSKYSNYLSL
ILLLFKYLKVFIFSMSTYSNLQITHSLLSDLE-KKKVKYDEALDILTHYASFNIFDSAFF
NKKKSNSTNPKTD---

>P_berghei_ortholog
XXYI------------------------------RKLNRVNRILSNVKPDTSND------
-----KKKETKSSNDSPKNYKGKKSHKK------------------------KGSSNDHI
EMENSELKPSIECENKY-------------------------------------------
----------------------------------------TDDLLNE---------SNGS
GDFLHSVKDVAVKKNQCHSYIFNCFAKSRN-DNDDDD-----------------------
----------------------------EDS--YKQKYTSENNLDIKSGDDPDEG-----
---------HSKFWRIKKGSKRGSDTNPFKLDMNYLTRKKSSLPNYEFNLAMVTKFIDSN
QKIID----------LENENDTVIKDNMNRNSNTVRSRDLLLSTSFKNI-----------
---DNFVYGKIITHLLGYAYIFYGHK-ININKLEKELIKDYYYN-YIVLKESINNI----
----PDEFKYSQNSS-KTDAIYNLLDYLGIEKYYYGFNRNLHTFLFLAREFLQKEYALKK
VYEVLPVDFE-----------------LDRKELIEQIHDSTSGEK-DIYEFSYKIFDPIC
SFLHAYNCFNLNYISNKLINNLRIKKS-NAVITKELSKLTEYVQTTHFDIIEYLKDLTYY
ITNLFEYGHKENLYNELVIFYYSLFFDYYLDTLGKLLYITQIHLKYPFNEQIKNDLSTIN
ARIKTHRDFVENEMIRFYHKYNISENLLE-CVTGIFSNNEYRVLILEQYLDSKNSNYLSL
ILLLFKYLKVFIFSMSTYSNLQITHSLLSDLE-KKNVKYDEALDILTHHASFNIFDSAFF
NKKK------------
```

- **AMOUNT OF MISSING DATA**

P. reichenowi	***P. gallinaceum***	***P. knowlesi***	***P. vivax***	***P. chabaudi***	***P. berghei***	***P. yoelii***
0%	51%	40%	26%	30%	30%	31%

■ PF11_0343

▪ PHYLOGENY AND PEXEL/VTS

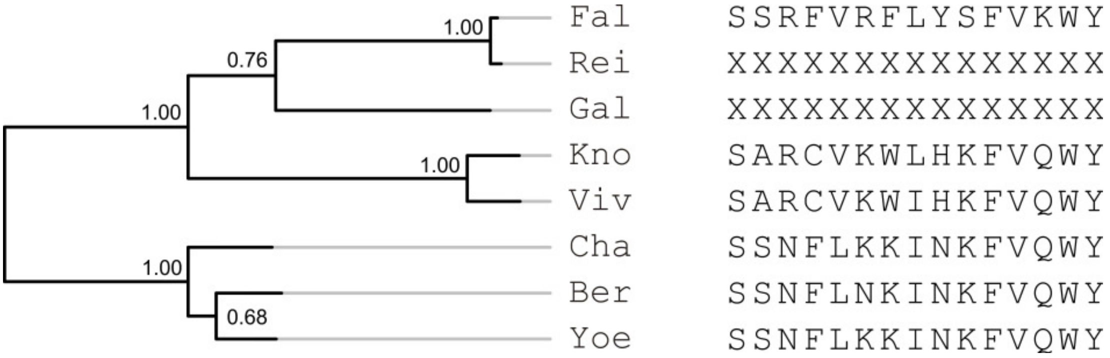

▪ ALIGNMENT

```
>P_falciparum_Pf11_0343
MSNLKNDMFISKKKRKFYYKKSSRFVRFLYSFVKWYKMERVVGPVWINKYSSMIYFLMFL
FILNLSVGILILILSSKYIECRIPYEYKGETFTKYSIVKVTPEQCKGQKNLKELNGNINV
HYEILGMQQNHYKFVSGMKKEQLNGNIFLKKEELEECYPLITFSEGKKKKKLLHPCGIFP
WNVFTDSYIFYDKEPDEVPFPTPLPLKQNVEEITIKYYRQFYKNPSPQNVQLYKDHIYFW
MEPDIQYERLQENKETNEKLLVLPQTLKYNQAGKAIENSHFINWMIPSALNYIKRLYGKL
YIPLKFPFYIYIENNFKINDTKIIVISTSQYYMRTFLIGFIFIIISIIALILCIFYLIRM
NKYENKXXXXX

>P_reichenowi_ortholog
XXXXXXXXXXXXXXXXXXXXXXXXXXXXXXXXXXXXXXXXXXXXXXXXXXXXXXXXXXXX
XXXXXXXXXXXXXXXXXXXXXXXXXXXXXXXXXXXXXXXXXXXXXXGQKNLKELNGNINV
HYEILGMQQNHYKFVSGMKKEQLNGNIFLKKEELEECYPLITFSEGKKKKKLLHPCGIFP
WNVFTDSYIFYDKEPDEIPFPTPLPLKQNVEEITIKYYRQFYKNPSPQNVQLYKDHIYFW
MEPDIQYERLQENKETNEKLLVLPQTLKYNQAGKAIENSHFINWMIPSALNYIKRLYGKL
YIPLKFPFYIYIENNFKINDTKIIVISTSQYYMRTFLIGFIFIIISIIALILCIFYLIRM
NXXXXXXXXXX

>P_gallinaceum_ortholog
XXXXXXXXXXXXXXXXXXXXXXXXXXXXXXXXXXXXXXXXXXXXXXXXXXXXXXXFLIFL
FIFNMIIGIIVLLLSNQYIECRIPYEYKGQSYTKYSIVRVTPEHCKGIGNLKELKGKLNV
HYELQGLQQNHYRFVSSFKKEQLSGQIFLKKEDVSECHPFITFSE-GGVNKILHPCGIFP
WNVFSDSYIFYDREPDEFPFPTPIPIQQKVEDITIKYYRKFFKNPSAELINSHKDHVYFW
MDEKLQSKSLQENKETNEKLVILPQTLKYDQAGKAIENSHFINWMIPSALNYIKRLYAKI
DGPLSFPFYIYIXXXXXXXXXXXXXXXXXXXXXXXXXXXXXXXXXXXXXXXXXXXXXXXX
XXXXXXXXXXX

>P_knowlesi_ortholog
MRIPRRDGDISKMRKQLSYKKSARCVKWLHKFVQWYRMEKVVGPIWVPTYCSIIVFLLFL
FLFNLLLGIAILIISSKYIECRIPYEYKSQAYTKYSIVKVTPEHCKGNENLKELKGPINI
HYEISGVEQNHYRFLTSFKKEQLHGDLFLQEKELSECFPLITYEQ-NGIRKILHPCGILQ
WNVFTDSYIFYDKEPDESPFPTPLPLKQMPEDITIKYYRKFFKNPTREIINLHKKNIYFW
MDEDVQLQILQEHAETNDKLVVLPQTLKYKKAGKAVENSHFMNWVIPSAFNYVKRLYAKF
DGPLVFPFYIYIENNFKISDTKIIVISNADFYLNTTLIGIFFIITAVFAFLLSLLYFIRM
KKHQFMXXXXX

>P_vivax_ortholog
MRSPRRDGGLSKKRKQLSYKKSARCVKWIHKFVQWYRMEKVIGPIWVPTYCSIIVFLLFL
FFFNLLVGVAILIISSNYIECRVPYEYKSQAYTKYSIVKVTPEHCKGNENLKQLKGPINI
HYEIYGVQQNHYRFLTSFKKEQLRGDLFLQEKELSECFPLITYEQ-SGTRKILHPCGILQ
WNVFTDSYIFYDKEPDESPFPTPLPLKQRAEDITIKYYRKFFKNPTRDIINLHKKRIYFW
MDEEVQLKILQEHAETNDKLVVLPQTLKYKKAGKAVENSHFMNWVIPSAFNYVKRLYAKL
DGPLVFPFYIYIENNFKISDTKIIVISNADFYFNTTLIGIFFIITAVFALLLSLLYFIRM
KKHQFKXXXXX

>P_chabaudi_ortholog
XXXXXXXXXXKFNNNELFYKKSSNFLKKINKFVQWYRMEKVFGPVFVYKYSTSIVFFIFL
FILNLSVGIAILYLSSQYIECKIPYEYKTQPYTKYSIIKVTPEHCKGHENLKELKGEINV
HYEIYGVQQNHYSFMKSFNTKQLGGKIFVSKDYLNHCYPLITYFK-DRINKILHPCGVLP
WHVFTDNYIFYDKEPEDAPFPDPLPLKERVEDITIKYFRKFFKNPHPETIDLYKDKVYFW
MDRTTQSEALHENIVANEKLLILPQALKYNIAGNAMENSHFINWMIPSPFRYIKRLYGKL
XXXXXXXXXXXXXXXXXXXXXXXXXXXXXXXXXXXXXXXXXXXXXXXXXXXXXXXXXXXX
XXXXXXXXXXX

>P_yoelii_ortholog
XXXXXXXXXXKRNNNKTFYKKSSNFLNKINKFVQWYRMEKVFGPAFVYKYSTLIAFFIFL
FILNLSIGIAILYLSSQYIECKIPYEYKSQPYTKYSIIKVTPKHCKGRENLKELKGEINI
HYEIYGVQQNHYSFMKSLNTEQLGGKIVVSKNDLNQCYPLITYFK-DRINKILHPCGILP
WNVFNDNYIFYDKEPDDPPFPSPLPLKERVEDITIKYFRKFFKNPHPENIKLYKDKVYFW
MDAETQSEALXXXXXXXXXXXXXXXXXXXXXXXXXXXXXXXXXXXXXXXXXXXXXXXXXX
XXXXXXXXXXXXXXXXXXXXXXXXXXXXXXXXXXXXXXXXXXXXXXXXXXXXXXXXXXXX
XXXXXXXXXXX

>P_berghei_ortholog
XXXXXXXXXXKLNNNKIFYKKSSNFLKKINKFVQWYRMEKIFGPVFVYKYSTLIAFFIFL
FILNLSIGIAILYLSSQYIECKIPYEYKSQPYTKYSIIKVTPEHCKGRENLKELKGKINV
HYEIYGVQQNHYSFMKSFNAEQIGGGIDVYKHDLNQCYPLITYFK-DRINKILHPCGILP
WSVFTDNYIFYDKEPDDAPFPDPLPLNERVEDITIKYFRKFFKNPHPENIKLYKDKVYFW
MDAKTQSEALHENIVANEKLLILSQALKYDIARNAMENSHFINWMIPSPFSYIKRLYGKL
NGPISFPFYIYIENNFRTAEAKAIIITEANFYINNNLIGIIFTIISIFSLILSILYYMRM
KKHKFMRQIDE
```

▪ **AMOUNT OF MISSING DATA**

P. reichenowi	*P. gallinaceum*	*P. knowlesi*	*P. vivax*	*P. chabaudi*	*P. berghei*	*P. yoelii*
30%	30%	0%	0%	21%	4%	34%
